# Lactic Acid Bacteria Convert Human Fibroblasts to Multipotent Cells

**DOI:** 10.1371/journal.pone.0051866

**Published:** 2012-12-26

**Authors:** Kunimasa Ohta, Rie Kawano, Naofumi Ito

**Affiliations:** Department of Developmental Neurobiology, Graduate School of Life Sciences, Kumamoto University, Kumamoto, Japan; University of Minnesota Medical School, United States of America

## Abstract

The human gastrointestinal tract is colonized by a vast community of symbionts and commensals. Lactic acid bacteria (LAB) form a group of related, low-GC-content, gram-positive bacteria that are considered to offer a number of probiotic benefits to general health. While the role of LAB in gastrointestinal microecology has been the subject of extensive study, little is known about how commensal prokaryotic organisms directly influence eukaryotic cells. Here, we demonstrate the generation of multipotential cells from adult human dermal fibroblast cells by incorporating LAB. LAB-incorporated cell clusters are similar to embryoid bodies derived from embryonic stem cells and can differentiate into endodermal, mesodermal, and ectodermal cells in vivo and in vitro. LAB-incorporated cell clusters express a set of genes associated with multipotency, and microarray analysis indicates a remarkable increase of *NANOG*, a multipotency marker, and a notable decrease in *HOX* gene expression in LAB-incorporated cells. During the cell culture, the LAB-incorporated cell clusters stop cell division and start to express early senescence markers without cell death. Thus, LAB-incorporated cell clusters have potentially wide-ranging implications for cell generation, reprogramming, and cell-based therapy.

## Introduction

Living organisms have been classified on the basis of cell structure into two groups: the eukaryotes and the prokaryotes. However, genomic analyses have shown that bacteria can be as widely divergent in their evolutionary history as any prokaryote is from any eukaryote. The prokaryotes comprise two distinct groups that are called the eubacteria and the archaebacteria. Thus, the living world has three major divisions: eubacteria, archaebacteria, and eukaryotes [1]. The generation of eukaryotic cells can be explained by the endosymbiotic theory, which was advanced and substantiated with microbiological evidence [2]. Now, it is widely believed that eubacteria infected archaebacteria, genomic DNA was transferred to the archaebacteria, and they evolved into eukaryotic cells [1].

Humans are in contact with components of the microflora from birth. Thus, a delicate balance exists in the symbiotic relationship between microorganisms and the human host during metabolic activities. When considering the positive influence of microorganisms on eukaryotic cells, lactic acid bacteria (LAB) deserve to be studied. The LAB form a group of related, low-GC-content, gram-positive bacteria and occupy important niches in the gastrointestinal tract of humans [3]. To exert beneficial effects on human physiology, probiotic LAB adhere to the surface of intestinal cells through mucin, extracellular matrix, and lectin [4], [5]. Although the role of LAB in gastrointestinal microecology has been the subject of extensive research, the effect of LAB incorporation into the cells is poorly understood because the gastrointestinal mucusal layer provides a protective barrier between the epithelium and the lumen, which contains noxious agents and microorganisms [6], [7]. Hooper et al. reported the global intestinal transcriptional responses to colonization, suggesting that commensals are able to modulate the expression of host genes that participate in diverse and fundamental physiological functions [8], [9]. It is likely that LAB contribute to the function of digestive cells in the gastrointestinal tract through epigenetic effects.

Thus, we studied the reaction of human fibroblasts, which are far different from digestive cells, when incorporated with LAB. To determine whether LAB can directly affect human gene expression in general, we incorporated LAB into adult human dermal fibroblasts (HDFs) and observed cell clusters similar to embryoid bodies derived from embryonic stem cells. The LAB-incorporated cell clusters expressed a set of genes associated with multipotency and differentiated into cell types of all three germ layers both in vivo and in vitro. Our quantitative RT-PCR analysis showed the higher expression of *NANOG* in LAB-incorporated cells than iPS cells. The LAB-incorporated cell clusters stopped proliferating and expressed early senescence markers during culture. Gene expression profiling indicated a remarkable remodeling of HDFs, especially increased *NANOG* and a notable decrease in *HOX* genes, showing that the normal skin cells acquired the multipotency. This study establishes LAB-incorporated cell clusters as an accessible human multipotential cell type for a better understanding of the molecular mechanism of cell origins and reprograming.

## Results

### Incorporation of lactic acid bacteria into human dermal cells

**Figure 1:**
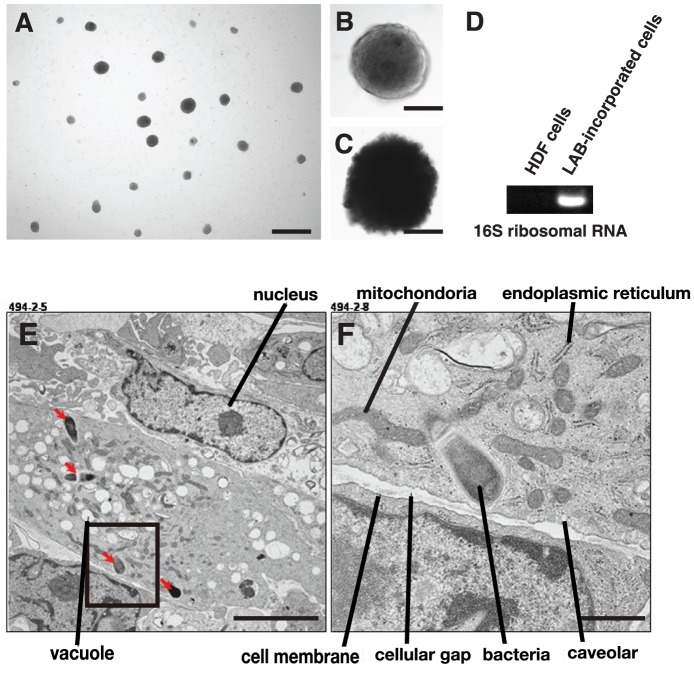
Characterization of LAB-incorporated cell clusters. (A) Typical LAB-incorporated cell clusters occurring on adherent culture dishes. (B) Characteristics of LAB-incorporated cell clusters. (C) ALP staining of LAB-incorporated cell clusters. (D) RT-PCR on LAB-incorporated cell clusters after 12 days of incorporation using a 16S ribosomal RNA primer set. (E) Ultrastructural picture of LAB-incorporated cells. (F) Hyper-magnification of a square in (E). Scale bars: 1 mm in (A), 100 µm in (B) and (C), 5 µm in (E), and 1 µm in (F).

To examine whether the incorporation of bacteria into a mammalian cell can reprogram the cell's characteristics, we incorporated a LAB (*Lactobacillus acidophilus*; JCM 1021) into adult HDFs that were pretreated with trypsin/EDTA to remove their cell surface proteins. After 2–3 days incubation, cell clusters (30–100 clusters; 500,000 cells) were generated similar to the embryoid bodies formed by human embryonic stem cells (hESCs) at an early stage (Fig. 1 A–B), while no cell clusters were observed from LAB-unincorporated adult HDFs (Fig. S1A). The pretreatment of HDFs with trypsin alone produced a similar number of clusters (Fig. S1B). At a certain size (50–300 µm), these cell clusters stopped growing and had a heterogeneous appearance. Direct application of LAB onto the HDFs cultured on the dish did not generate any cell clusters (data not shown). When we used three other strains (*Streptococcus salivarius* subsp. *thermophilus*, JCM 20026; *Lactobacillus* sp., JCM 20061; *Lactococcus lactis* subsp. *lactis*, JCM 20101) for the incorporation, they also demonstrated the ability to generate cell clusters (Fig. S1C), but *E. coli* (XL10-Gold, Stratagene) failed to form cell clusters (data not shown). Most of these cell clusters were attached to the dish and looked similar to those of hESC-derived embryoid bodies and/or Muse cells isolated from stress-tolerant mesenchymal cell populations [10], [11]. Moreover, these cells were not immortalized but could be maintained more than one month by replacing the fresh ES cell culture medium one week after bacterial incorporation. The LAB-incorporated cell clusters were positive for alkaline phosphatase (ALP) staining (Figs. 1C, S2A–B). We performed RT-PCR using a bacteria-specific 16S ribosomal RNA primer set and found this rRNA band in LAB-incorporated cell clusters (Fig. 1D). Further, the ultrastructural analysis indicated the localization of LAB in the cell cytoplasm (Fig. 1 E–F). Cell clusters were also generated from GFP-expressing mouse embryonic fibroblasts (MEFs) incorporated with LAB (*Lactobacillus acidophilus*; JCM 1021) (Fig. S3A–C). The LAB-incorporated GFP-MEF clusters were also ALP positive (Fig. S3D).

### Characterization of LAB-incorporated cell clusters

**Figure 2:**
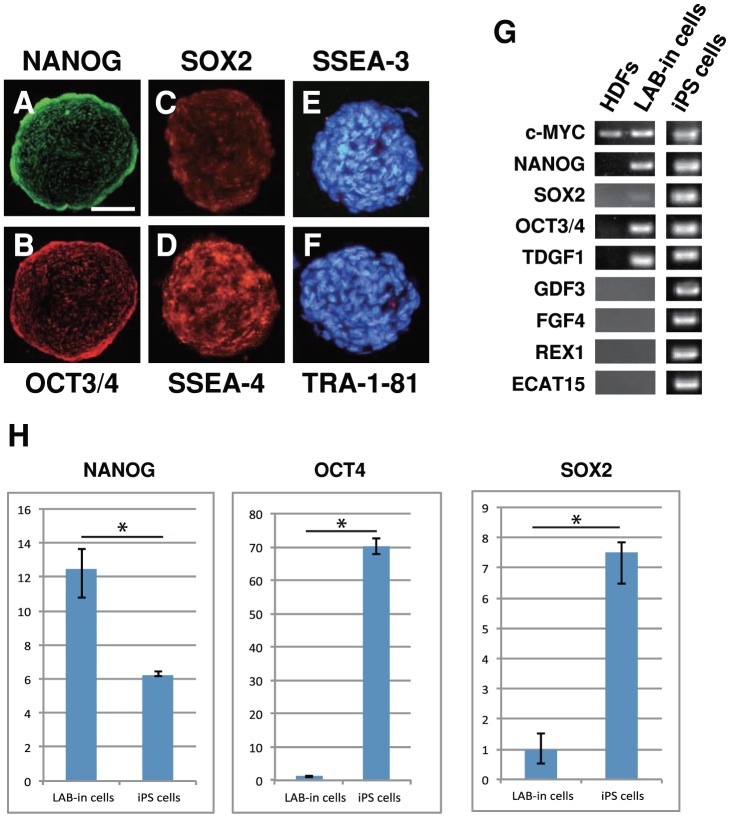
LAB-incorporated cell clusters express pluripotency markers. Immunocytochemistry for (A) NANOG, (B) OCT3/4, (C) SOX2, (D) SSEA-4, (E) SSEA-3, and (F) TRA-1-81 in LAB-incorporated cell clusters. (E–F) DAPI. (G) RT-PCR analysis on HDFs, LAB-incorporated cell clusters after 12 days of incorporation, and iPS cells. (H) The relative mRNA expression levels of *NANOG*, *OCT4*, and *SOX2* were quantified with LAB-incorporated cells and iPS cells by quantitative RT-PCR and normalized relative to the expression of endogenous human *GAPDH*. Error bars indicate the standard deviation. ^*^p<0.005, two-tailed Student *t* test. Three samples were used in each group. Scale bar, 100 µm.

Initially, we examined the expression of pluripotency markers by immunocytochemistry. The LAB-incorporated cell clusters after 14 days of incorporation were positive for NANOG (Fig. 2A), OCT3/4 (Fig. 2B), SOX2 (Fig. 2C), and SSEA-4 (Fig. 2D), but not for SSEA-3 (Fig. 2E) or TRA-1-81 (Fig. 2F). Double staining of LAB-incorporated cell clusters showed the heterogeneous expression pattern of pluripotency markers (Fig. S4). Next, we also assessed the expression of pluripotency markers by RT-PCR in LAB-incorporated cell clusters after 12 days of incorporation. As shown in Fig. 2G, LAB-incorporated cell clusters specifically expressed *NANOG*, *SOX2*, *OCT3*/*4*, and *TDGF1*, but not *GDF3*, *FGF4*, *REX1*, or *ECAT15*, while iPS cells expressed all of these markers. To compare the cell characteristic, the mRNA expression levels of *NANOG*, *OCT4*, and *SOX2* of LAB-incorporated cell clusters at 14 days after incorporation and iPS cells were examined by quantitative RT-PCR (Fig. 2H). The mRNA expression level of *NANOG* was upregulated in LAB-incorporated cell clusters, while *OCT4* and *SOX2* mRNA expression levels were low in LAB-incorporated cell clusters compared with iPS cells (Fig. 2H). These data suggest that the cell characteristic of LAB-incorporated cell clusters is different from that of human iPS cells.

**Figure 3:**
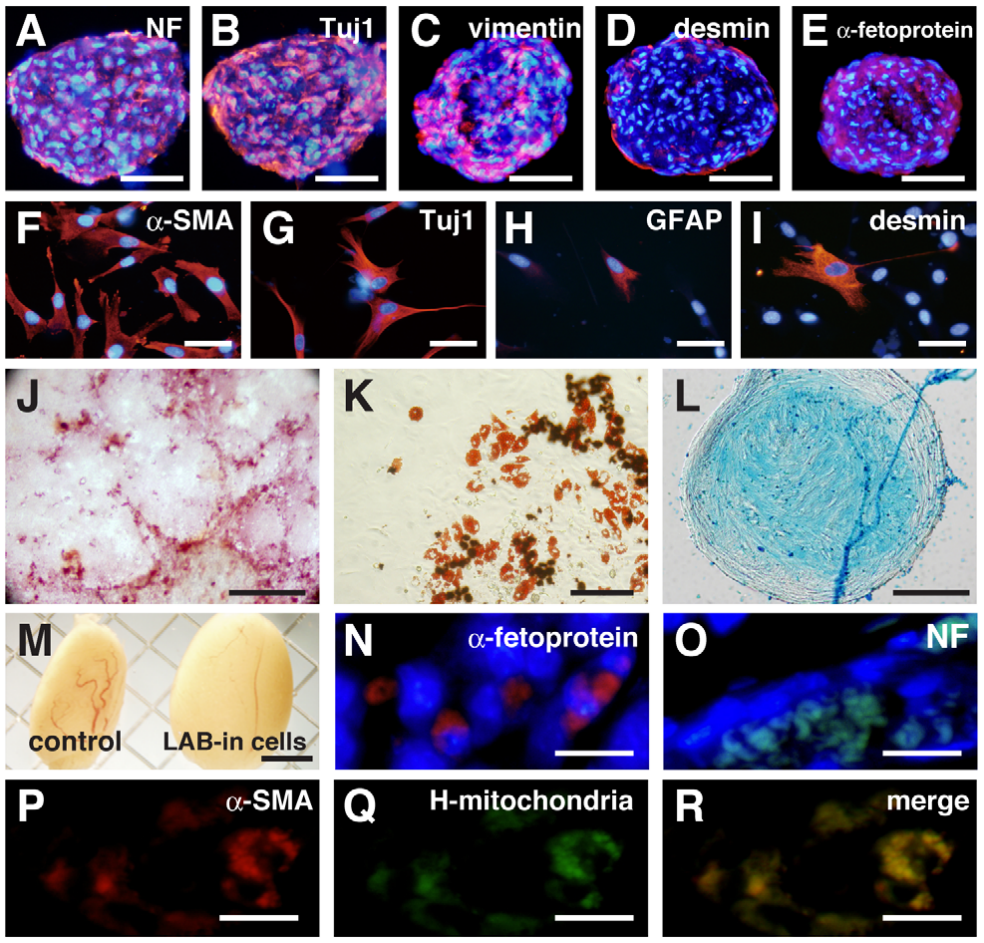
Multiple-lineage differentiation of LAB-incorporated cell clusters. (A, B) The floating culture for the neural induction produced clusters that expressed the neural markers neurofilament (NF, A) and Tuj1 (B). (C–I) Differentiating LAB-incorporated cell clusters cultured in the floating condition expressed vimentin (C), desmin (D), and α-fetoprotein (E). Differentiating LAB-incorporated cell clusters cultured in the attached condition expressed α-SMA (F), Tuj1 (G), GFAP (H), and desmin (I). (J) Induced osteocytes were stained with Alizarin Red S. (K) Induced adipocytes were stained with Oil Red O. (L) Induced chondrocytes were stained with Alcian Blue. (M) Uninjected testis and testis injected with LAB-incorporated cell clusters (12 weeks). (N, O) Immunocytochemistry of α-fetoprotein (N) and NF (O) in testis injected with LAB-incorporated cell clusters. (P–R) Double-staining of α-SMA (P) and human mitochondria (Q). Scale bars: 100 µm in (A–E), 50 µm in (F–I), 500 µm in (J), 100 µm in (K), 50 µm in (L), 200 µm in (M), and 20 µm in (N–R).

Next, to determine the differentiation ability of LAB-incorporated cell clusters in vitro, we cultured LAB-incorporated cell clusters after 14 days of incorporation and examined the cell differentiation by immunocytochemistry. When the LAB-incorporated cell clusters were cultured in the floating condition for additional 14 days, they showed positive immunostaining for the ectodermal markers neurofilament (Fig. 3A) and Tuj1 (Fig. 3B), the mesoendodermal marker vimentin (Fig. 3C), the mesodermal marker desmin (Fig. 3D), and the endodermal marker α-fetoprotein (Fig. 3E). RT-PCR analyses also showed the expression of α-fetoprotein, *GATA6* (endodermal), and microtubule-associated protein-2 (*MAP2*; ectodermal) in LAB-incorporated cell clusters that were cultured in the differentiation medium (Fig. S5). Furthermore, we cultured LAB-incorporated cell clusters on poly-L-lysine (PLL)-laminin-coated cover slips and continued cultivation for another 10 days; the attached cells showed various morphologies. Immunocytochemistry detected cells that were positive for α-smooth muscle actin (α-SMA, mesoderm, Fig. 3F), Tuj1 (Fig. 3G), glial fibrillary acidic protein (GFAP, ectoderm, Fig. 3H), and desmin (Fig. 3I). The LAB-incorporated GFP-MEF clusters were also positive for neurofilament, GFAP, α-SMA, and desmin (Fig. S6A–D).

Besides, we assessed whether the differentiation of LAB-incorporated cell clusters could be directed by certain induction media. For this purpose, the LAB-incorporated cell clusters were treated to the conditions that induced differentiation into adipocytes, osteocytes, or chondrocytes. We observed that osteocytes induction produced cells stained positively with Alizarin Red S (Fig. 3J; 3±1.2%), while adipocytes induction produced cells with lipid droplets that stained with Oil Red O (Fig. 3K; 5±3.3%) and chondrocytes induction produced cells stained with Alcian Blue (Fig. 3L). As shown in Figure S7, it is clear that the cell clusters were crumbled into the single cell and differentiate into adipocytes or osteocytes (attached condition). For the differentiation into chondrocytes (floating condition), the size of sphere is almost the same during the sphere culture (Fig. S7). The LAB-incorporated GFP-MEF clusters also could be differentiated into adipocytes and osteoblasts with the above induction media (Fig. S6E–F). To evaluate the multipotency of the LAB-incorporated cell clusters in vivo, we transplanted the LAB-incorporated cell clusters into the testicles of adult SCID mice (n = 6). The injection of LAB-incorporated cell clusters into immunodeficient mice failed to give rise to teratomas after 3 months (Fig. 3M and Fig. S8). In LAB-incorporated cell cluster-injected testes, cells positive for human mitochondria and for endodermal (α-fetoprotein), ectodermal (neurofilament), and mesodermal (α-SMA) lineage markers were detected (Fig. 3N–R). These data demonstrate that LAB-incorporated cell clusters can differentiate into cell types of all three germ layers both in vivo and in vitro.

### Cellular state of LAB-incorporated cell clusters during culture

**Figure 4:**
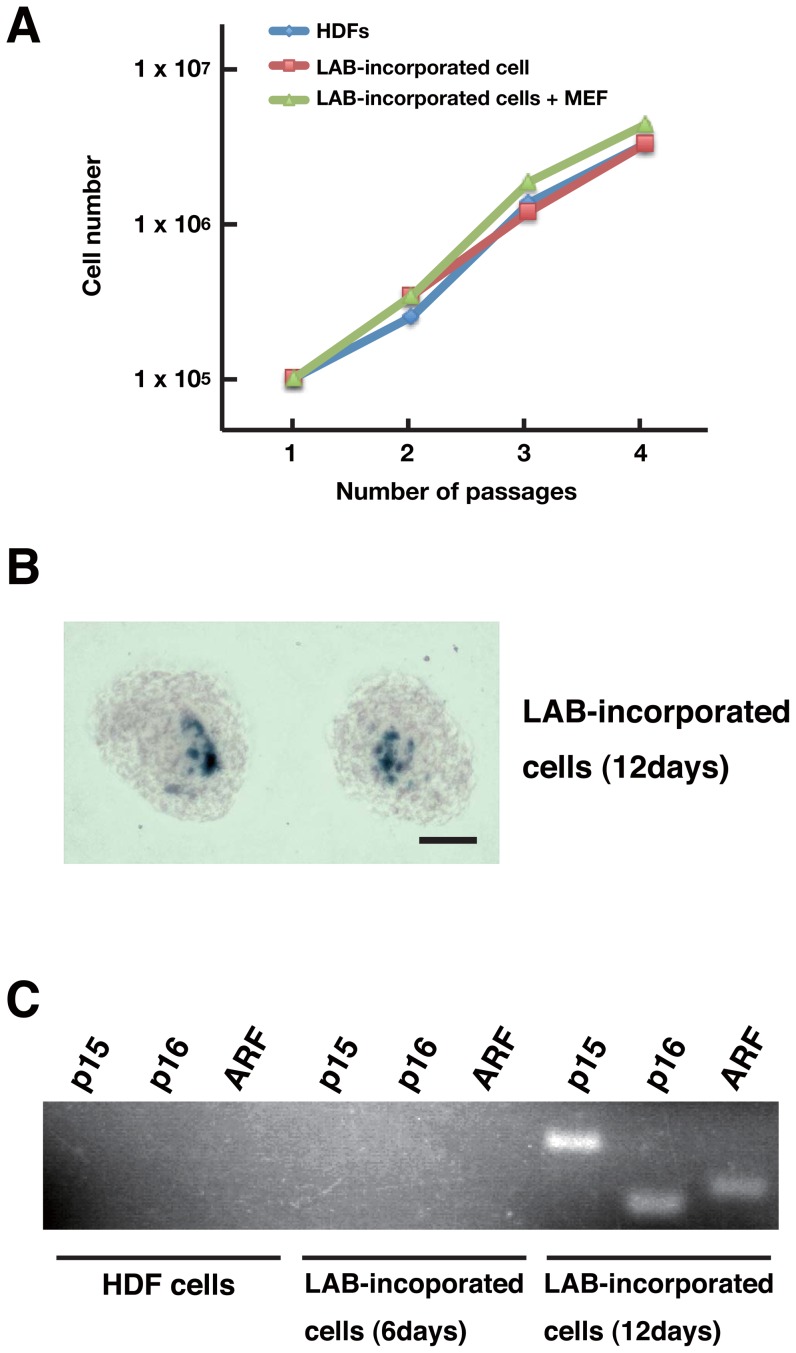
LAB-incorporated cell clusters express early senescence markers. (A) Growth curves of HDFs, LAB-incorporated cell clusters, and LAB-incorporated cell clusters cultured on MEFs. Cells (1×10^5^) were passaged every 4 days onto the wells of 6-well plates. (B) SA-β-galactosidase staining of LAB-incorporated cell clusters at 12 days after incorporation. Scale bar, 100 µm. (C) RT-PCR analyses on HDFs and LAB-incorporated cell clusters at 6 or 12 days after incorporation. Note that the expression of early senescence markers (p15, p16, and ARF) was induced in LAB-incorporated cell clusters at 12 days after incorporation.

We examined the cell growth curves of HDFs, LAB-incorporated cell clusters after 12 days of incorporation, and LAB-incorporated cell clusters after 12 days of incorporation cultured on the MEFs. As shown in Figure 4A, when cells were dissociated into a single cell, there was no difference in cell growth rate among the three groups. However, when LAB-incorporated cell clusters were cultured and kept as cell clusters in human ES cell culture medium, they stopped dividing and stayed as cell clusters for one month without any obvious cell death (Fig. S9). Next, to investigate the cellular status during culture, LAB-incorporated cell clusters after 12 days of incorporation were stained with SA-β-galactosidase (Fig. 4B). The cells positive for SA-β-galactosidase staining were localized in the central region of LAB-incorporated cell clusters (Fig. 4B; 1±0.7%). Further, the early senescence markers p15, p16, and ARF were upregulated in LAB-incorporated cell clusters after 12 days of incorporation, but not in HDFs or LAB-incorporated cell clusters after 6 days of incorporation (Fig. 4C). These data suggest that LAB-incorporated cell clusters stop dividing and growing and remain stationary and multipotent.

### Gene expression profiles in LAB-incorporated cell clusters

**Figure 5:**
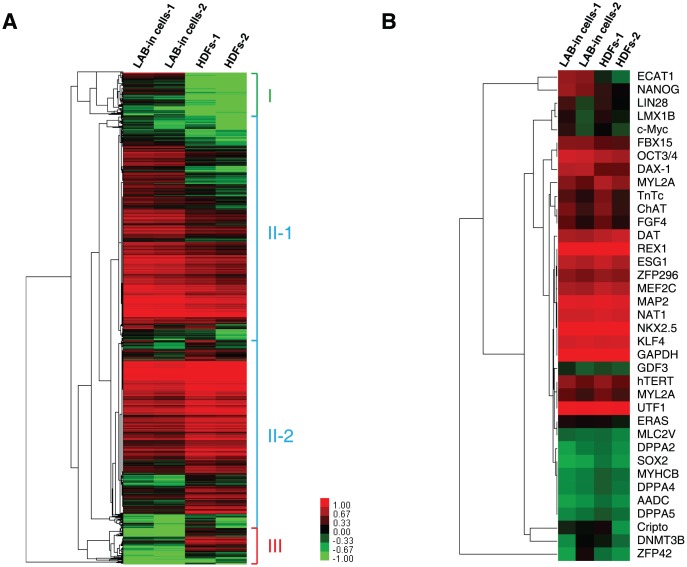
Gene expression profiles of the LAB-incorporated cell clusters. (A) Expression profile of whole genes in LAB-incorporated cells relative to the respective, untreated HDFs at 14 days after incorporation. The gene expression profiling was performed in two independent replicates. The guide at the right side of the figure indicates the expression difference between the samples with green (indicating lower expression) and red (indicating higher expression). (B) Expression profile of 37 selected pluripotency markers in the LAB-incorporated cell clusters and HDFs.

To identify any transcriptional effects of LAB incorporation during differentiation, genome-wide expression profiling was performed by comparing LAB-incorporated cell clusters after 14 days of incorporation to untreated HDFs (Table S1). Using a Whole Human Genome Microarray, we first considered those genes that were differentially expressed (genes with expression changes of at least twofold) between the HDFs and LAB-incorporated cell clusters at 14 days after incorporation (Fig. 5A and Table S2). Unsupervised clustering identified three major clusters of genes (Table S3). The largest cluster (cluster II) contained mostly genes with either higher or lower expression levels in the LAB-incorporated cell clusters versus the HDFs. Accordingly, the most significantly enriched gene oncology (GO) terms were associated with development in this cluster. The second largest cluster (cluster I) consisted of genes that were high in LAB-incorporated cell clusters but low in HDFs. The analysis also revealed a cluster of genes that were low in LAB-incorporated cell clusters but high in HDFs (cluster III).

We selected 37 genes related to pluripotency and compared them between LAB-incorporated cell clusters and HDFs (Fig. 5B and Table S4). The expression of *NANOG* was increased 7.6 times in the LAB-incorporated cell clusters, although the expression of other genes did not show significant differences. As *NANOG* is a highly divergent homeodomain-containing protein commonly afforded a certain position in the transcriptional network of pluripotency [12], [13] and thought to be the gateway to the multipotent ground state [14], we conclude that LAB-incorporated cell clusters attain multipotency.

We found approximately 800 genes differentially expressed by a factor of 10 or more in the LAB-incorporated cell clusters. The GO-based classification revealed that the differentially expressed genes were associated with early development, with a notable decrease in *HOX* gene expression. The 38 genes that are related to the Antennapedia gene of *Drosophila* species are subdivided into four clusters (*HOX* clusters), each containing 8–11 genes. Interestingly, 21 *HOX* genes were downregulated in LAB-incorporated cell clusters by a factor of 15 or more among 50 genes that had a GO term of “development” (Table S5). These results suggest that LAB-incorporated cell clusters lose their positional information and revert to multipotent cells.

## Discussion

This study describes the generation of multipotent cells by incorporating LAB into adult HDFs. The LAB-incorporated cell clusters can differentiate into endodermal, ectodermal, and mesodermal cells in vivo and in vitro. Furthermore, the LAB-incorporated cell clusters are positive for a set of genes associated with multipotency and ALP staining. Moreover, the LAB-incorporated cell clusters stop dividing, retaining the cluster structure, and express early senescence markers to exhibit cell differentiation ability. Finally, the microarray analysis demonstrates a notable decrease in *HOX* gene expression in the LAB-incorporated cell clusters, indicating the loss of positional information. These data suggest that the LAB or LAB-produced material(s) convert normal skin cells into multipotent cells.

The microbial colonization of the human gastrointestinal tract is an evolution-driven process that has beneficial effects on immune function, nutrient processing, and a broad range of the host activities. Mucus is secreted by the epithelial surfaces throughout the gastrointestinal tract and provides a protective barrier between the underlying epithelium and the lumen, which contains noxious agents, destructive hydrolases, and microorganisms [7], [15]. The probiotic LAB adhere to the mucosal surface and prevent the growth of putrefactive microorganisms responsible for disease by competitive inhibition, the generation of a non-conductive acidic environment and/or the production of antibiotic-like substances [16]. Previous studies with germ-free mice have revealed that the microbiota are not functionally insulated from the mucosa, but in contrast, gut bacteria can fundamentally influence epithelial metabolism, proliferation, and survival. In this study, we treated HDFs with trypsin/EDTA and exposed them to LAB, resulting the incorporation of LAB into the cytoplasmic region, as observed by electron microscopy. Depending on the efficiency of LAB-incorporation into the cells in each independent experiment, the number and diameter of spheres varied. Although the molecular mechanism of the LAB-incorporation is not clear, the cell surface proteins are supposed not to be involved in the incorporation of LAB into HDFs. It would be exciting to see whether the dead LAB or extracts of LAB also could generate cell clusters with multipotency.

Gene expression profiles in LAB-incorporated cell clusters demonstrated two interesting issues: the upregulation of *NANOG*, a gene related to pluripotency, and the downregulation of *HOX* genes, which are associated with early development. *NANOG* is one of the canonical quartets of transcription factors employed to reprogram human fibroblasts and dose-facilitate molecular reprogramming [17]–[19]. Furthermore, *NANOG* is expressed in pluripotent embryonic cells and derivative ES cells, and forced *NANOG* expression is sufficient to drive the cytokine-independent self-renewal of undifferentiated ES cells [20], [21]. *HOX* genes encode DNA-binding proteins that regulate gene expression and control various aspects of early embryonic development [22]. Mammalian *HOX* genes are divided into two broad categories: a family of 38 genes that are related to the Antennapedia gene of *Drosophila* species, and the other group comprises more distally related genes. These data suggest that LAB-incorporated cell clusters lose their positional information and revert to the pluripotent state. Although many questions regarding the actual molecular processes occurring in LAB-incorporated cell clusters remain unanswered, further epigenetic studies will be helpful to characterize LAB-incorporated cell clusters.

Pluripotent stem cells are able to differentiate into all types of cells in the body and have great potential for applications in regenerative medicine, developmental biology research, and drug development. ES cells, which are derived from the inner cell mass of the blastocyst, can differentiate into more than 200 types of cells. However, the fact that the isolation and establishment of human ES cell lines involve embryo destruction leads to extensive ethical concerns and debates. iPS cells, from fibroblasts with four Yamanaka factors, share a similar gene expression profile and differentiation potential with ES cells [18], [19]. Although the successful derivation of iPS cells solves the problems of ethical issues and immunological rejection, there are concerns about the safety of using iPS cells for therapeutic applications. The LAB-incorporated cell clusters can avoid the safety issue because we ingest items (e.g., milk, yogurt) that include LAB, which have beneficial effects on human health. Further, LAB-incorporated cell clusters can be readily generated with LAB from patients themselves, thus solving the immunological rejection problem and ethical issues. Although LAB-incorporated cell clusters failed to proliferate like ES cells and iPS cells, the area-specific transplantation of LAB-incorporated cell clusters would be possible for safe clinical application. It would be interesting to examine whether the incorporation of LAB or LAB-producing materials into cancer cells would induce cell cluster formation and change the cancer cell characteristics. After the identification of the factor(s) produced by LAB, the ultimate goal is treatment with patient-specific cancer cells and LAB-producing factors.

## Materials and Methods

### Cells and lactic acid bacteria cultures

HDFs from the facial dermis of a 34-year-old Caucasian female were purchased from Cell Applications, Inc. We used the HDFs for the generation of LAB-incorporated cell clusters within three passages after receipt. The HDFs were maintained in Fibroblast Growth Medium (Cell Applications, Inc) containing 0.1% gentamycin (Sigma). We purchased the following LAB strains from RIKEN BioResource Center: *Lactobacillus acidophilus* (JCM 1021), *Streptococcus salivarius* subsp. *thermophilus* (JCM 20026), *Lactobacillus* sp. (JCM 20061), and *Lactococcus lactis* subsp. *lactis* (JCM 20101). *E. coli* (XL10-Gold, Stratagene) were grown in LB medium and prepared for incorporation. LABs were grown in M.R.S. medium (OXOID). After treatment of HDFs with 0.1% trypsin and 1 mM EDTA for 5 min at 37°C, 5×10^5^ cells suspended in 2 ml of Fibroblast Growth Medium (without gentamycin) and approximately 1×10^8^ LAB suspended in 60 µl of Fibroblast Growth Medium (without gentamycin) were plated into a six-well dish (NUNC) containing 10 µg/ml lactoferrin (Sigma). After 5 days of incubation, half of the medium (1 ml) was replaced with Fibroblast Growth Medium containing 0.5×10^8^ LAB. The medium was changed every 3 days, and the clusters were collected at the indicated times. For longer culture, LAB-incorporated cell clusters after 8 days of incorporation were maintained in human ES cell culture medium [DMEM/F12 supplemented with 20% knockout serum replacement (GIBCO), 1% v/v non-essential amino acids (Sigma), 1 mM L-glutamine (GIBCO), 0.1 mM ß-mercaptoethanol, and 5 ng/ml basic fibroblast growth factor (bFGF, Sigma)].

The LAB-incorporated cell clusters at 12 days after incorporation were washed twice in PBS, fixed for 10 min in 4% PFA, and embedded in Optimal Cutting Temperature compound (Sakura). Sections (12 µm) were incubated for 12 h at 37°C with freshly prepared ß-gal staining solution containing 1 mg/ml 5-bromo-4-chloro-3indolyl-β-D-galactopyranoside (X-gal.), 5 mM potassium ferrocyanide, 150 mM NaCl, 2 mM MgCl_2_, and 40 mM citric acid and then titrated with NaH_2_PO_4_ to pH 6.0.

### Immunocytochemistry

For immunocytochemistry, LAB-incorporated cell clusters were fixed with 4% paraformaldehyde (PFA) for 15 min at room temperature (RT). After washing with PBS, the cells were treated with PBS containing 5% heat-inactivated goat serum and 0.1% Triton X-100 for 30 min at RT. The following primary antibodies were used for immunocytochemistry: rabbit anti-NANOG (ReproCELL), mouse anti-Oct-3/4 (Santa Cruz Biotechnology), rabbit anti-SOX2 (Millipore), rat anti-SSEA-3 (Millipore), mouse anti-SSEA-4 (Millipore), mouse anti-TRA-1-81 (Millipore), mouse anti-vimentin (Santa Cruz), rabbit anti-desmin (Thermo), mouse anti-Tuj1 (DSHB), and mouse anti-neurofilament (ZYMED). After three washes with PBS, the sections were incubated with anti-mouse IgG antibody conjugated with Cy3 or FITC (Jackson ImmunoResearch), anti-rabbit IgG antibody conjugated with Cy3 (Amersham), or anti-rat IgG or IgM antibody conjugated with Cy3 (Jackson ImmunoResearch) in the presence of DAPI in the antibody dilutions for 2 h at RT.

### In vitro cell differentiation assay

For neural induction with floating, LAB-incorporated cell clusters were cultured for 14 days in DMEM/F12 medium containing B-27 supplement (GIBCO), 1% FCS, and 20 ng/ml bFGF (Sigma). LAB-incorporated cell clusters were cultured in the floating condition for 14 days in DMEM/F12 medium containing 10% FCS to induce cell differentiation. For cell differentiation induction under the attachment condition, LAB-incorporated cell clusters were cultured on PLL-laminin-coated coverslips for 14 days in DMEM/F12 medium containing 1% FCS and 20 ng/ml bFGF (Sigma). The following primary antibodies were used for immunocytochemistry: mouse anti-vimentin (Santa Cruz), rabbit anti-desmin (Thermo), mouse anti-Tuj1 (DSHB), rabbit anti-α-SMA (Thermo), mouse anti-α-fetoprotein (DAKO), rabbit anti-GFAP (DAKO), and mouse anti-neurofilament (ZYMED). After three washes with PBS, the sections (10 µm) were incubated with anti-mouse IgG antibody conjugated with Cy3 (Jackson ImmunoResearch) or anti-rabbit IgG antibody conjugated with Cy3 (Amersham) in the presence of DAPI in the antibody dilutions for 2 h at RT.

For the lineage-specific differentiation, the LAB-incorporated cell clusters were cultured for 3 weeks in STEMPRO Adipogenesis, Chondrogenesis, and Osteogenesis Differentiation Medium (GIBCO). The culture media were changed every 3 days. Adipocytes were identified by the production of lipid droplets, detected by staining with Oil Red O. Cells were washed with PBS and overlaid with 0.18% Oil Red O dye/60% 2-propanol (Sigma) solution for 15 min, then rinsed in distilled water. Chondrocytes were revealed by Alcian Blue staining, which detects the synthesis of glycosaminoglycans. Cells were fixed with 4% PFA for 30 min and washed three times with PBS, then incubated with 1% Alcian Blue solution (Wako) in 3% acetic acid at pH 2.5 for 30 min. Osteogenic differentiation, indicated by calcium deposits, was revealed by Alizarin Red S staining. Cells were fixed with 4% PFA for 30 min. After rinsing in distilled water, cells were stained with 40 mM Alizarin Red S (Sigma) solution at pH 4.2 and rinsed in distilled water.

### RNA isolation and RT-PCR

We have purchased iPS (IPS-TIG120-3f7) from Center for iPS Cell Research and Application (Kyoto, Japan). Total RNA of iPS cells and LAB-incorporated cells at 6 or 12 days after incorporation was purified using Trizol reagent (Invitrogen) and then treated with DNase I (Invitrogen) to remove genomic DNA contamination. The first-strand cDNA was synthesized from 1 µg total RNA using an oligo d(T)12–18 primer and Superscript III reverse transcriptase (Invitrogen). The PCR was performed with the following primers:

16S ribosomal RNA-S (5′-TGATGCATAGCCGAGTTGAG-3′);

16S ribosomal RNA-AS (5′-CAATAAATCCGGACAACGCT-3′);

OCT3/4-S (5′-GACAGGGGGAGGGGAGGAGCTAGG-3′);

OCT3/4-AS (5′-CTTCCCTCCAACCAGTTGCCCCAAAC-3′);

SOX2-S (5′-GGGAAATGGGAGGGGTGCAAAAGAGG-3′);

SOX2-AS (5′-TTGCGTGAGTGTGGATGGGATTGGTG-3′);

NANOG-S (5′-CAGCCCCGATTCTTCCACCAGTCCC-3′);

NANOG-AS (5′-CGGAAGATTCCCAGTCGGGTTCACC-3′);

MYC-S (5′-GCGTCCTGGGAAGGGAGATCCGGAGC-3′);

MYC-AS (5′-TTGAGGGGCATCGTCGCGGGAGGCTG-3′);

TDGF1-S (5′-CTGCTGCCTGAATGGGGGAACCTGC-3′);

TDGF1-AS (5′-GCCACGAGGTGCTCATCCATCACAAGG-3′);

REX1-S (5′-CAGATC CTA AAC AGC TCG CAG AAT-3′);

REX1-AS (5′-GCGTACGCAAATTAAAGTCCAGA-3′);

FGF4-S (5′-CTACAACGCCTACGAGTCCTACA-3′):

FGF4-AS (5′-GTTGCACCAGAAAAGTCAGAGTTG-3′);

GDF3-S (5′-CTTATGCTACGTAAAGGAGCTGGG-3′);

GDF3-AS (5′-GTGCCAACCCAGGTCCCGGAAGTT-3′);

ECAT15-1-S (5′-GGAGCCGCCTGCCCTGGAAAATTC-3′);

ECAT15-1-AS (5′-TTTTTCCTGATATTCTATTCCCAT-3′);

P15-S (5′-GCGGGGACTAGTGGAGAAG-3′);

P15-AS (5′-CTGCCCATCATCATGACCT-3′);

P16-S (5′-CAACGCACCGAATAGTTACG-3′);

P16-AS (5′-CTGCCCATCATCATGACCT-3′);

ARF-S (5′-CTACTGAGGAGCCAGCGTCT-3′);

ARF-AS (5′-CTGCCCATCATCATGACCT-3′).

### Quantitative RT-PCR

The mRNA expression levels of *NANOG*, *OCT4*, and *SOX2* in LAB-incorporated cells and iPS cells were examined by quantitative RT-PCR assay using the ΔΔCt method [23] of ABI StepOne™ system (Applied Biosystems). The quantitative RT-PCR was performed using TaqMan Fast PCR reagent with the following sequence-specific primers and FAM and NFQ-MGB conjugated TaqMan probes; SOX2∶Hs01053049_s1, POU5F1∶Hs00999634_gH, NANOG∶Hs04260366_g1. Human Glyceraldehyde-3-phosphate dehydrogenase (GAPDH∶Hs02758991_g1) was used to normalize the mRNA levels. The relative levels of expression were determined to compare each target gene and housekeeping gene mRNA expression levels.

### Teratoma assay

LAB-incorporated cell clusters were treated with 0.05% trypsin/1 mM EDTA for 5 min, followed by collection, two washes with PBS, and suspension in PBS. A total of 500,000 cells per sample were injected intratesticularly into 8-week-old male SCID mice. The mice were killed 12 weeks after the initial injection. For immunohistochemistry, the testis sections (6 µm) were treated with PBS containing 5% heat-inactivated goat serum and 0.1% Triton X-100 for 30 min at RT. The following primary antibodies were used for immunohistochemistry: mouse anti-human mitochondria (abcam), rabbit anti-α-SMA (Thermo), mouse anti-α-fetoprotein (DAKO), and mouse anti-neurofilament (ZYMED). The sections were incubated with anti-mouse IgG antibody conjugated with Cy3 or FITC (Jackson ImmunoResearch) or anti-rabbit IgG antibody conjugated with Cy3 (Amersham) in the presence of DAPI in the antibody dilutions for 2 h at RT.

### DNA microarray

Total RNA from the HDFs and LAB-incorporated cell clusters was labeled with Cy3 (2 replicates). Each sample was hybridized once using a one-color protocol. Sample labeling and hybridization to Whole Human Genome Microarray 4×44K (Agilent) were performed by Oncomics (Japan). The arrays were scanned using a G2505B Microarray Scanner System (Agilent), and the Affymetrix data were analyzed by using GeneSpring GX11.5.1 software (Agilent).

## Supporting Information

Figure S1 **Typical LAB-incorporated cell clusters generated by HDFs and LAB.** (**A**) The cell clusters were not generated from HDFs without LAB that were dissociated with trypsin/EDTA. (**B**) *Lactobacillus acidophilus* (JCM 1021) is able to generate LAB-incorporated cell clusters from HDFs that were dissociated with trypsin. (**C**) *Lactococcus lactis* subsp. *lactis* (JCM 20101), *Streptococcus salivarius* subsp. *thermophilus* (JCM 20026), and *Lactobacillus* sp. (JCM 20061) lactic acid bacteria are also able to generate LAB-incorporated cell clusters from HDFs. Scale bars, 100 µm.
(PDF)

Figure S2 **LAB-incorporated cell clusters are ALP positive.** (**A**) LAB-incorporated cell clusters generated by *Lactobacillus acidophilus* (JCM 1021) from HDFs are ALP positive. (**B**) LAB-incorporated cell clusters generated by *Lactococcus lactis* subsp. *lactis* (JCM 20101), *Streptococcus salivarius* subsp. *thermophilus* (JCM 20026), or *Lactobacillus* sp. (JCM 20061) from HDFs are ALP positive. Scale bars, 100 µm.
(PDF)

Figure S3 **Generation of GFP-MEF-derived LAB-incorporated cell clusters.** Lactic acid bacteria (*Lactobacillus acidophilus*; JCM 1021) can generate cell clusters from LAB-incorporated GFP-MEFs. Lower (**A, B**) and higher (**C**) magnifications of LAB-incorporated GFP-MEF clusters after 6 days of incorporation. (**D**) LAB-incorporated GFP-MEF clusters are ALP positive. (**A, D**) Bright field. (**B, C**) GFP. Scale bars: 500 µm in (**A, B**), 100 µm in (**C, D**). For GFP-MEF preparation, the uteri isolated from 12.5-day-pregnant GFP-mice (C57BL/6-Tg(CAG-EGFP)C15-001-FJ001Osb mice) were washed with PBS. The head and visceral tissues were removed from the isolated embryos. The remaining bodies were washed, minced using scissors, transferred into a 0.1% trypsin/1 mM EDTA solution, and incubated at 37°C for 20 min. An equal amount of culture medium was added and pipetted up and down a few times to dissociate tissue. After passing through a cell strainer (BD Falcon), cells were collected by centrifugation (1,000 rpm for 5 min) and cultured in a 10 cm dish (one animal/dish). A total of 5×10^5^ cells (2 ml) and approximately 1×10^8^ lactic acid bacteria suspended in 60 µl Fibroblast Growth Medium containing 10 µg/ml lactoferrin (Sigma) were plated into the 6-well dish (NUNC).
(PDF)

Figure S4 **Double immunostaining.** To examine the heterogeneous expression pattern of pluripotency markers in LAB-incorporated cell clusters at 14 days after incorporation, double staining was performed with rabbit anti-NANOG (ReproCELL) and mouse anti-OCT3/4 (Santa Cruz Biotechnology) antibodies. After three washes with PBS, the sections (10 µm) were incubated with anti-mouse IgG antibody conjugated with Cy3 and anti-rabbit IgG antibody conjugated with FITC in the presence of DAPI in the antibody dilutions for 2 h at RT. The long white arrows indicate cells that express NANOG and OCT3/4 in the nucleus. The short arrow indicates the cell that expresses only NANOG. The large arrowhead indicates the cell that expresses OCT3/4 in the cytoplasm. The small arrowhead indicates the cell that expresses both NANOG and OCT3/4 in the cytoplasm. Scale bar: 100 µm in (**A–D**), 18 µm in (**E–H**).
(PDF)

Figure S5 **RT-PCR analysis.** RT-PCR analysis of native HDFs, LAB-incorporated cell clusters, and LAB-incorporated cell clusters induced to differentiate in culture medium (DMEM/F12 medium containing 10% FCS) for 12 days. Note that LAB-incorporated cell clusters induced to differentiate express α-fetoprotein and GATA6. The PCR was performed with the following primers: α-fetoprotein-S (5′-CCACTTGTTGCCAACTCAGTGA -3′); α-fetoprotein-AS (5′-TGCAGGAGGGACATATGTTTCA-3′); GATA6-S (5′-CCTGCGGGCTCTACAGCAAGATGAAC-3′); GATA6-AS (5′-CGCCCCTGAGGCTGTAGGTTGTGTT-3′); MAP-2-S (5′-ACTACCAGTTTCACACCCCCTTT-3′); MAP-2-AS (5′-AAGGGTGCAGGAGACACAGATAC-3′).
(PDF)

Figure S6 **Cell differentiation.** LAB-incorporated GFP-MEF clusters were cultured on PLL-laminin-coated coverslips for 14 days in DMEM/F12 medium containing 1% FCS and 20 ng/ml bFGF (Sigma). The following primary antibodies were used for immunocytochemistry: rabbit anti-desmin (Thermo), mouse anti-Tuj1 (DSHB), rabbit anti-α-SMA (Thermo), and rabbit anti-GFAP (DAKO). After three washes with PBS, the slides were incubated with anti-mouse IgG antibody conjugated with Cy3 (Jackson ImmunoResearch) or anti-rabbit IgG antibody conjugated with Cy3 (Amersham) in the presence of DAPI in the antibody dilutions for 2 h at RT. LAB-incorporated GFP-MEF clusters after 14 days of incorporation were cultured for 3 weeks in STEMPRO Adipogenesis and Osteogenesis Differentiation Medium (GIBCO). Adipocytes were identified by the production of lipid droplets, detected by staining with Oil Red O. Osteogenic differentiation, indicated by calcium deposits, was revealed by Alizarin Red S staining. Scale bars: 20 µm in (**A**–**D**), 20 µm in (**E**, **F**).
(PDF)

Figure S7 **Time course of the differentiation of LAB-incorporated cells.** LAB-incorporated cell clusters after 14 days of incorporation were treated to the conditions that induced differentiation into adipocytes, osteocytes, or chondrocytes. The cell clusters were crumbled into the single cell and differentiate into adipocytes or osteocytes (attached condition), whereas, for the differentiation into chondrocytes (floating condition), the size of sphere is almost the same during the sphere culture. Scale bars: 100 µm.
(PDF)

Figure S8 **Teratoma assay.** The control testes and the testes injected with LAB-incorporated cell clusters at 14 days after incorporation were extracted, embedded in paraffin and sectioned in 6-µm intervals followed by deparaffinization in xylene and processing through a graded series of alcohol concentrations. The samples were stained with hematoxylin and eosin. Scale bar, 100 µm.
(PDF)

Figure S9 **Cell death.** To examine cell death, LAB-incorporated cell clusters at 14 days after incorporation were immunostained with rabbit anti-single-stranded DNA antibody (DAKO). After three washes with PBS, the sections (10 µm) were incubated with anti-rabbit IgG antibody conjugated with FITC in the presence of DAPI in the antibody dilutions for 2 h at RT. Scale bar, 100 µm.
(PDF)

Table S1 Comparison between HDF-1,2 and LAB-incorporated cell clusters-1,2.
(PDF)

Table S2 List of 7580 genes that shows the difference by factor of two or more between LAB-incorporated cell clusters-1 vs. HDF-1 and LAB-incorporated cell clusters-2 vs. HDF-2.
(PDF)

Table S3 List of cluster genes that shows the difference by factor of two or more between LAB-incorporated cell clusters-1 vs. HDF-1 and LAB-incorporated cell clusters-2 vs. HDF-2.
(PDF)

Table S4 Selected 37 pluripotency maker genes.
(PDF)

Table S5 List of 50 genes that is decreased by a factor of fifteen or more at least between LAB-incorporated cell clusters-1 vs. HDF-1 and LAB-incorporated cell clusters-2 vs. HDF-2 and has a GO term [development].
(PDF)
