# Supplementary figures and images for: Lactic Acid Bacteria Convert Human Fibroblasts to Multipotent Cells
Source: PLoS One. 2012 Dec 26;7(12):e51866. doi: 10.1371/journal.pone.0051866 (PMC3530539; doi:10.1371/journal.pone.0051866)

## Supplementary Fig. 1

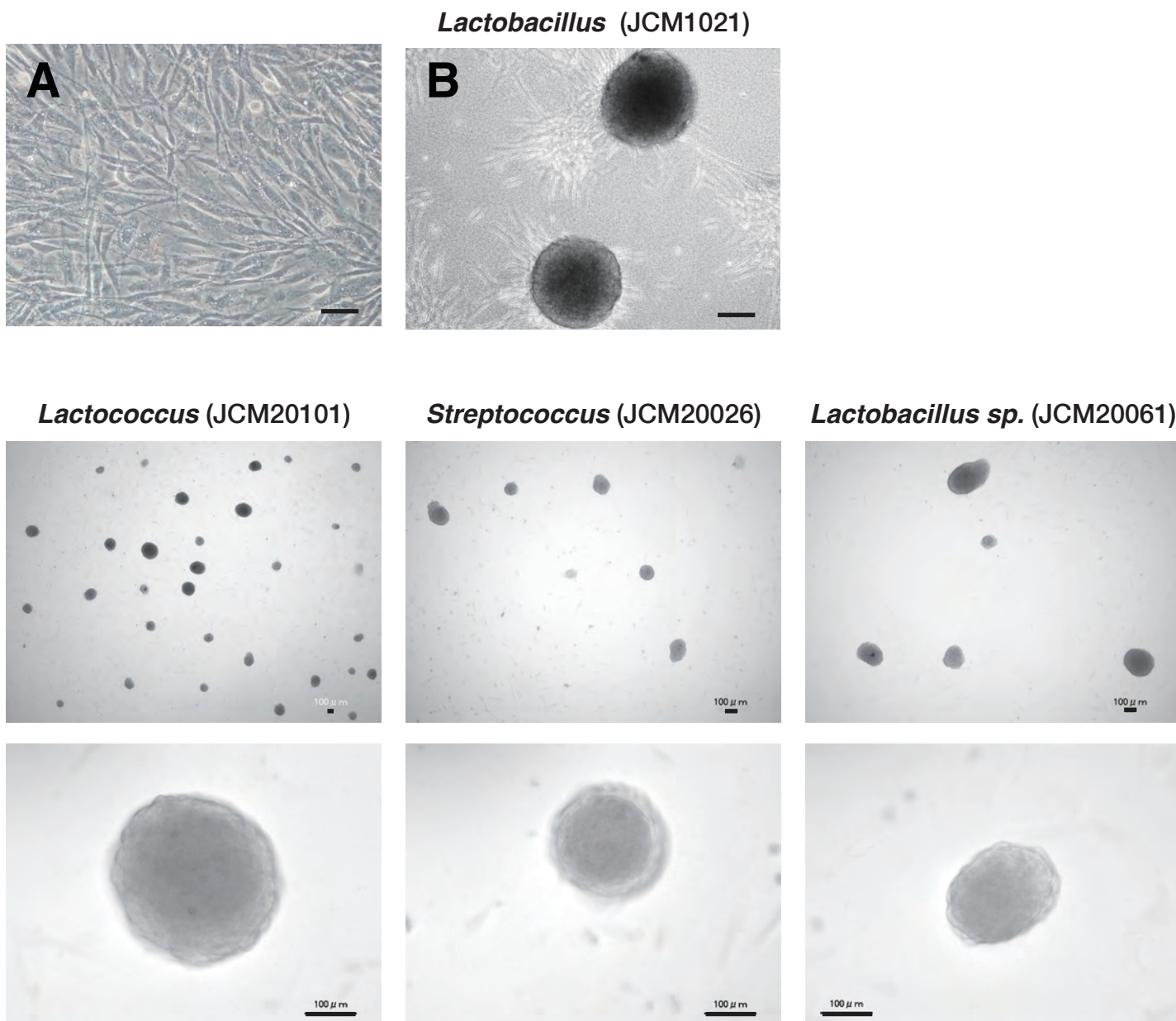

Supplement: Figure S1 — Typical LAB-incorporated cell clusters generated by HDFs and LAB. (A) The cell clusters were not generated from HDFs without LAB that were dissociated with trypsin/EDTA. (B) Lactobacillus acidophilus (JCM 1021) is able to generate LAB-incorporated cell clusters from HDFs that were dissociated with trypsin. (C) Lactococcus lactis subsp. lactis (JCM 20101), Streptococcus salivarius subsp. thermophilus (JCM 20026), and Lactobacillus sp. (JCM 20061) lactic acid bacteria are also able to generate LAB-incorporated cell clusters from HDFs. Scale bars, 100 µm. (PDF) [file pone.0051866.s001.pdf]

### Supplementary Fig. 3

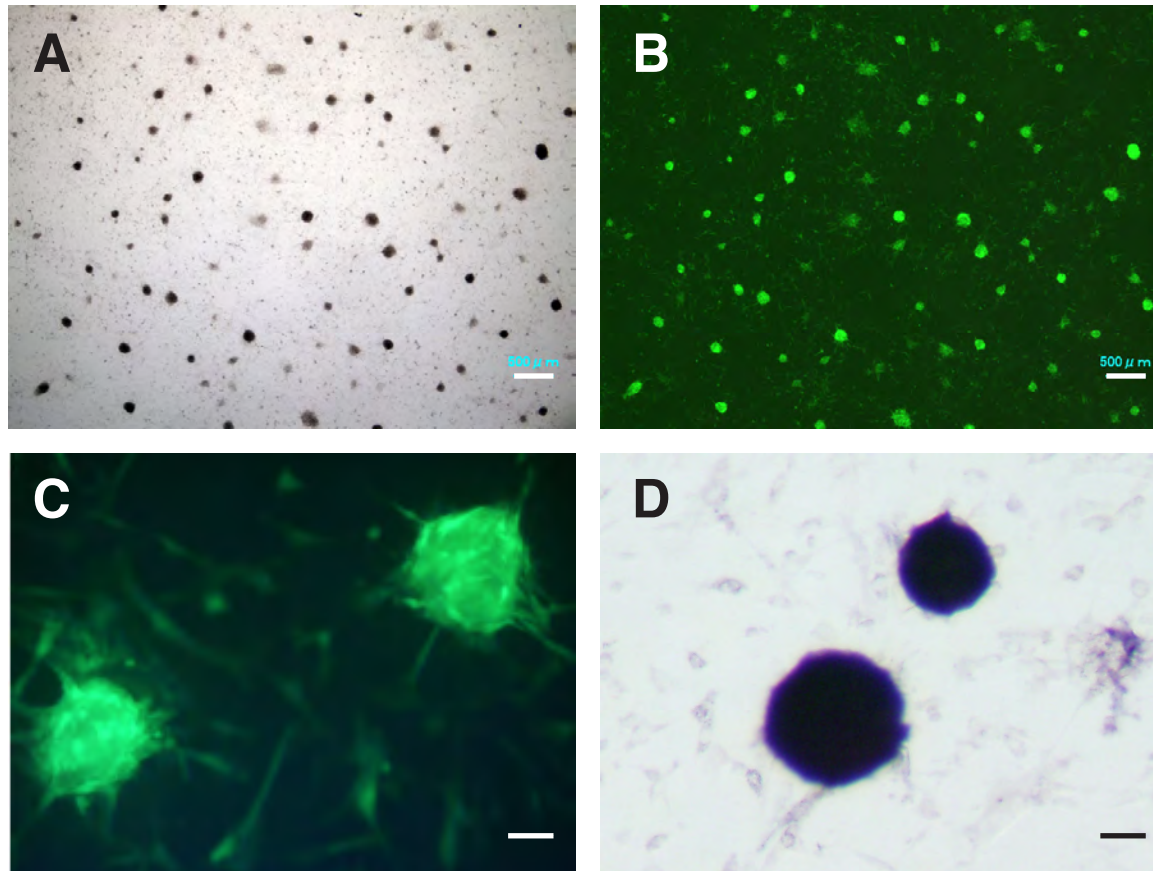

Supplement: Figure S3 — Generation of GFP-MEF-derived LAB-incorporated cell clusters. Lactic acid bacteria (Lactobacillus acidophilus; JCM 1021) can generate cell clusters from LAB-incorporated GFP-MEFs. Lower (A, B) and higher (C) magnifications of LAB-incorporated GFP-MEF clusters after 6 days of incorporation. (D) LAB-incorporated GFP-MEF clusters are ALP positive. (A, D) Bright field. (B, C) GFP. Scale bars: 500 µm in (A, B), 100 µm in (C, D). For GFP-MEF preparation, the uteri isolated from 12.5-day-pregnant GFP-mice (C57BL/6-Tg(CAG-EGFP)C15-001-FJ001Osb mice) were washed with PBS. The head and visceral tissues were removed from the isolated embryos. The remaining bodies were washed, minced using scissors, transferred into a 0.1% trypsin/1 mM EDTA solution, and incubated at 37°C for 20 min. An equal amount of culture medium was added and pipetted up and down a few times to dissociate tissue. After passing through a cell strainer (BD Falcon), cells were collected by centrifugation (1,000 rpm for 5 min) and cultured in a 10 cm dish (one animal/dish). A total of 5×10^5^ cells (2 ml) and approximately 1×10^8^ lactic acid bacteria suspended in 60 µl Fibroblast Growth Medium containing 10 µg/ml lactoferrin (Sigma) were plated into the 6-well dish (NUNC). (PDF) [file pone.0051866.s003.pdf]

Supplementary Fig. 4

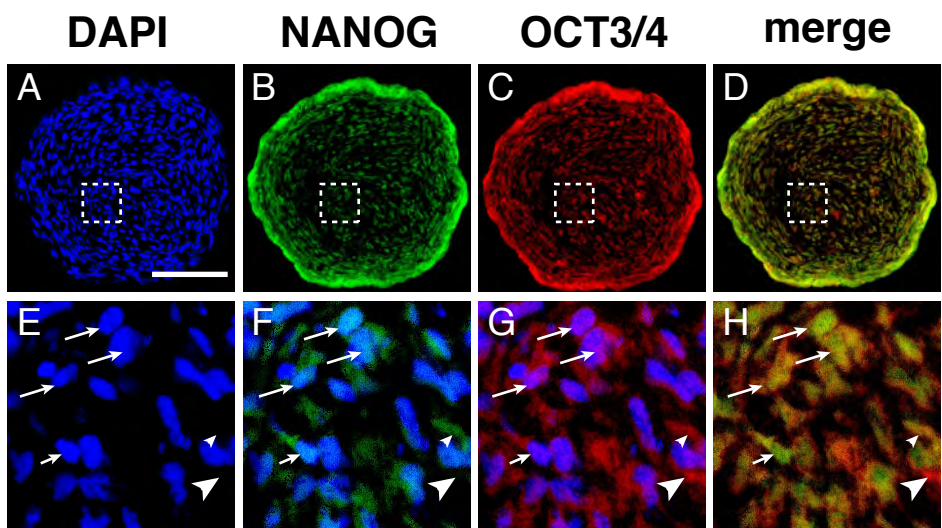

Supplement: Figure S4 — Double immunostaining. To examine the heterogeneous expression pattern of pluripotency markers in LAB-incorporated cell clusters at 14 days after incorporation, double staining was performed with rabbit anti-NANOG (ReproCELL) and mouse anti-OCT3/4 (Santa Cruz Biotechnology) antibodies. After three washes with PBS, the sections (10 µm) were incubated with anti-mouse IgG antibody conjugated with Cy3 and anti-rabbit IgG antibody conjugated with FITC in the presence of DAPI in the antibody dilutions for 2 h at RT. The long white arrows indicate cells that express NANOG and OCT3/4 in the nucleus. The short arrow indicates the cell that expresses only NANOG. The large arrowhead indicates the cell that expresses OCT3/4 in the cytoplasm. The small arrowhead indicates the cell that expresses both NANOG and OCT3/4 in the cytoplasm. Scale bar: 100 µm in (A–D), 18 µm in (E–H). (PDF) [file pone.0051866.s004.pdf]

## Supplementary Fig. 5

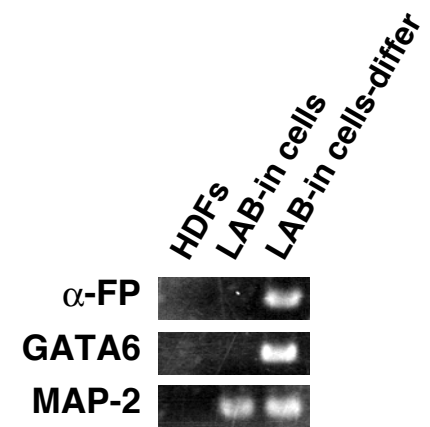

Supplement: Figure S5 — RT-PCR analysis. RT-PCR analysis of native HDFs, LAB-incorporated cell clusters, and LAB-incorporated cell clusters induced to differentiate in culture medium (DMEM/F12 medium containing 10% FCS) for 12 days. Note that LAB-incorporated cell clusters induced to differentiate express α-fetoprotein and GATA6. The PCR was performed with the following primers: α-fetoprotein-S (5′-CCACTTGTTGCCAACTCAGTGA -3′); α-fetoprotein-AS (5′-TGCAGGAGGGACATATGTTTCA-3′); GATA6-S (5′-CCTGCGGGCTCTACAGCAAGATGAAC-3′); GATA6-AS (5′-CGCCCCTGAGGCTGTAGGTTGTGTT-3′); MAP-2-S (5′-ACTACCAGTTTCACACCCCCTTT-3′); MAP-2-AS (5′-AAGGGTGCAGGAGACACAGATAC-3′). (PDF) [file pone.0051866.s005.pdf]

## Supplementary Fig. 6

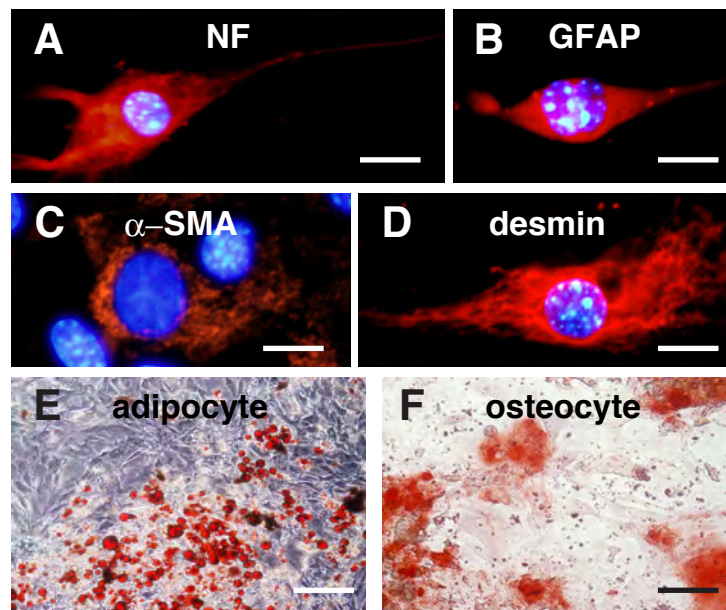

Supplement: Figure S6 — Cell differentiation. LAB-incorporated GFP-MEF clusters were cultured on PLL-laminin-coated coverslips for 14 days in DMEM/F12 medium containing 1% FCS and 20 ng/ml bFGF (Sigma). The following primary antibodies were used for immunocytochemistry: rabbit anti-desmin (Thermo), mouse anti-Tuj1 (DSHB), rabbit anti-α-SMA (Thermo), and rabbit anti-GFAP (DAKO). After three washes with PBS, the slides were incubated with anti-mouse IgG antibody conjugated with Cy3 (Jackson ImmunoResearch) or anti-rabbit IgG antibody conjugated with Cy3 (Amersham) in the presence of DAPI in the antibody dilutions for 2 h at RT. LAB-incorporated GFP-MEF clusters after 14 days of incorporation were cultured for 3 weeks in STEMPRO Adipogenesis and Osteogenesis Differentiation Medium (GIBCO). Adipocytes were identified by the production of lipid droplets, detected by staining with Oil Red O. Osteogenic differentiation, indicated by calcium deposits, was revealed by Alizarin Red S staining. Scale bars: 20 µm in (A–D), 20 µm in (E, F). (PDF) [file pone.0051866.s006.pdf]

Supplementary Fig. 7

Adiocyte

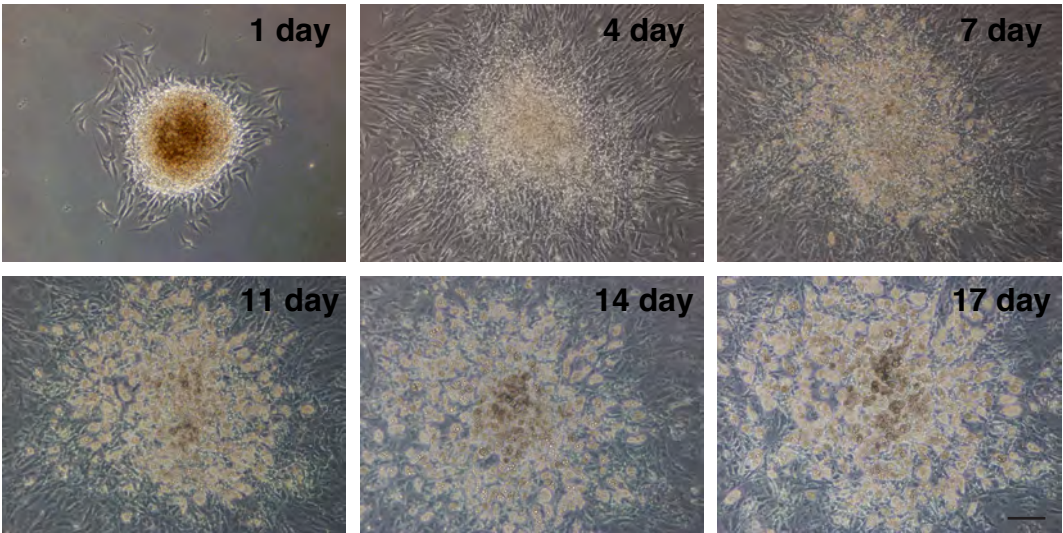

Osteocyte

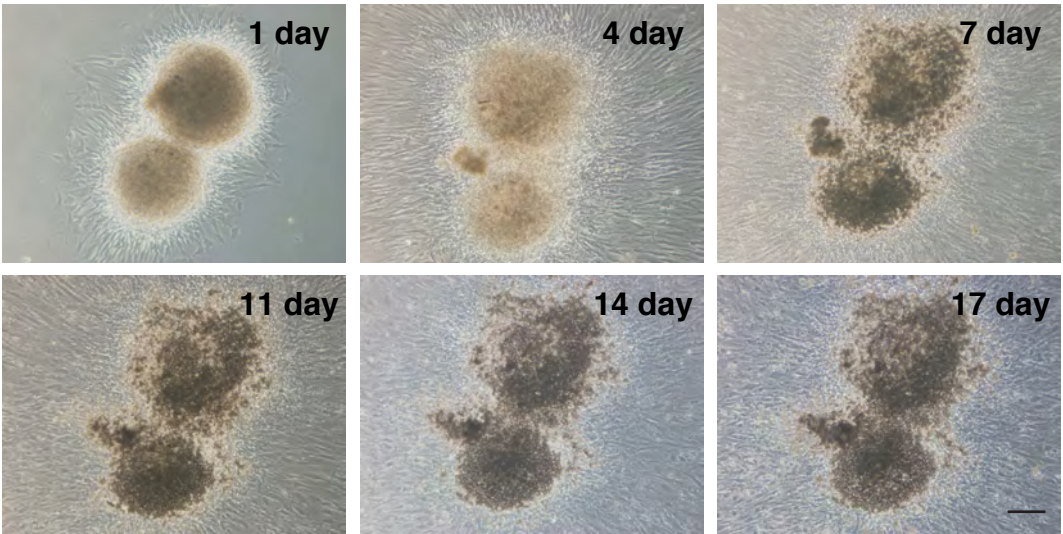

Chondrocyte

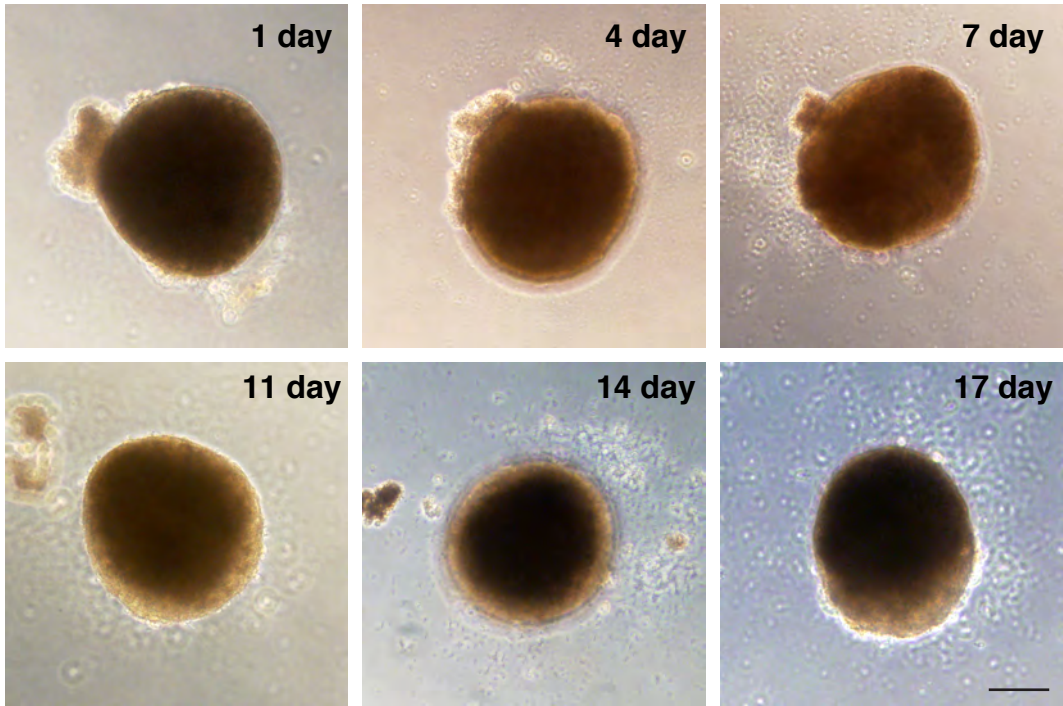

Supplement: Figure S7 — Time course of the differentiation of LAB-incorporated cells. LAB-incorporated cell clusters after 14 days of incorporation were treated to the conditions that induced differentiation into adipocytes, osteocytes, or chondrocytes. The cell clusters were crumbled into the single cell and differentiate into adipocytes or osteocytes (attached condition), whereas, for the differentiation into chondrocytes (floating condition), the size of sphere is almost the same during the sphere culture. Scale bars: 100 µm. (PDF) [file pone.0051866.s007.pdf]

## Supplementary Fig. 8

**control**

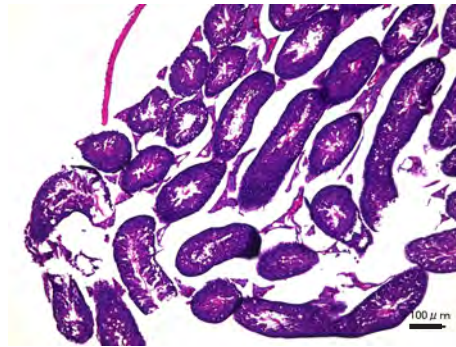

**LAB-incorporated cells**

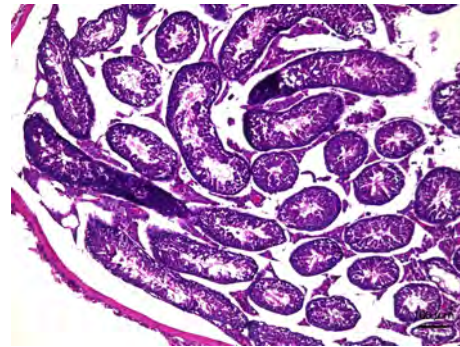

Supplement: Figure S8 — Teratoma assay. The control testes and the testes injected with LAB-incorporated cell clusters at 14 days after incorporation were extracted, embedded in paraffin and sectioned in 6-µm intervals followed by deparaffinization in xylene and processing through a graded series of alcohol concentrations. The samples were stained with hematoxylin and eosin. Scale bar, 100 µm. (PDF) [file pone.0051866.s008.pdf]

## Supplementary Fig. 9

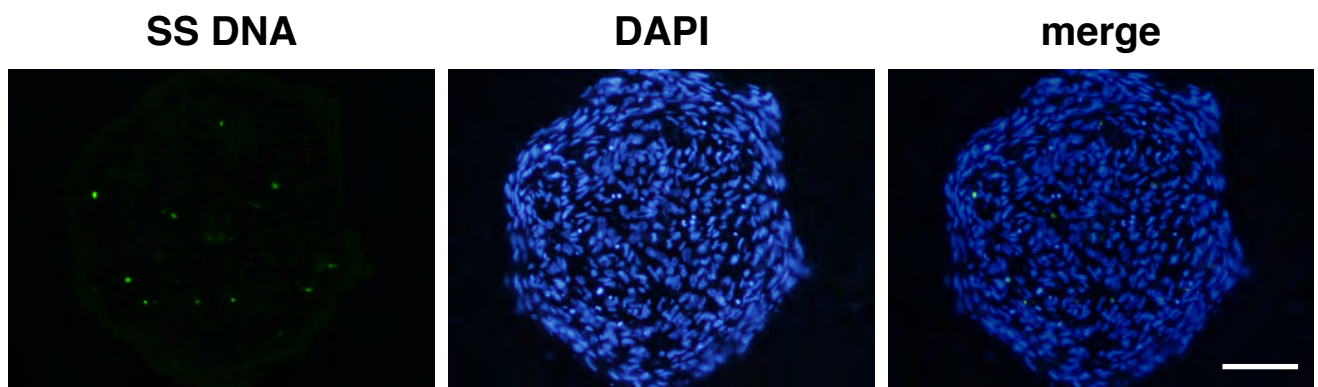

Supplement: Figure S9 — Cell death. To examine cell death, LAB-incorporated cell clusters at 14 days after incorporation were immunostained with rabbit anti-single-stranded DNA antibody (DAKO). After three washes with PBS, the sections (10 µm) were incubated with anti-rabbit IgG antibody conjugated with FITC in the presence of DAPI in the antibody dilutions for 2 h at RT. Scale bar, 100 µm. (PDF) [file pone.0051866.s009.pdf]
