# Supplementary material for: Lactic Acid Bacteria Convert Human Fibroblasts to Multipotent Cells
Source: PLoS One. 2012 Dec 26;7(12):e51866. doi: 10.1371/journal.pone.0051866 (PMC3530539; doi:10.1371/journal.pone.0051866)

Supplementary Fig. 2

A

Lactobacillus **(JCM1021)**

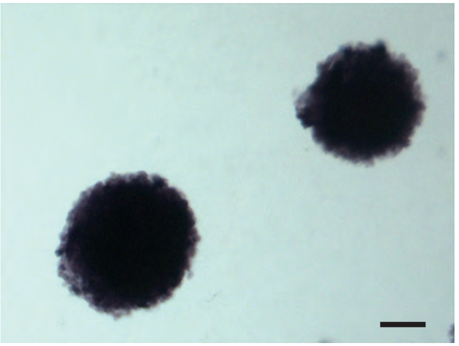

B

Lactococcus **(JCM20101)** ***Streptococcus*** **(JCM20026)** ***Lactobacillus sp.*** **(JCM20061)**

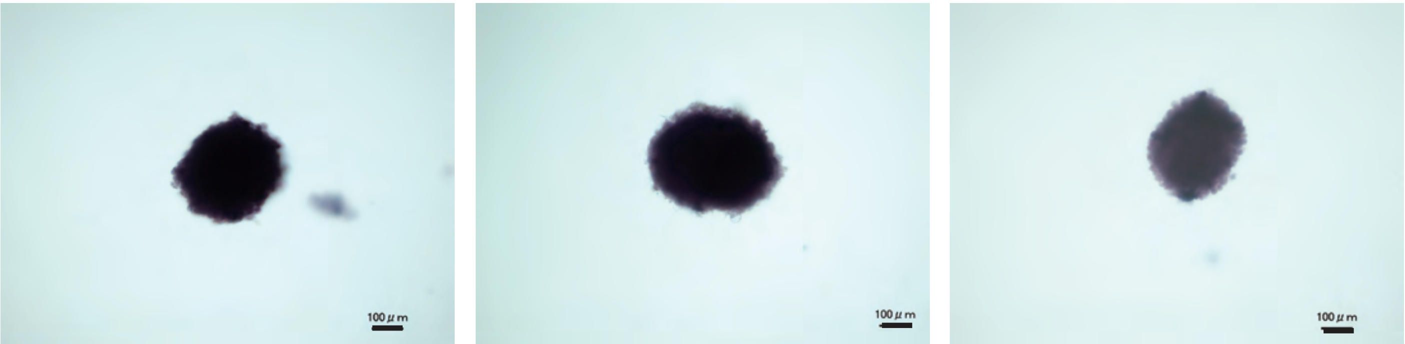

Supplement: Figure S2 — LAB-incorporated cell clusters are ALP positive. (A) LAB-incorporated cell clusters generated by Lactobacillus acidophilus (JCM 1021) from HDFs are ALP positive. (B) LAB-incorporated cell clusters generated by Lactococcus lactis subsp. lactis (JCM 20101), Streptococcus salivarius subsp. thermophilus (JCM 20026), or Lactobacillus sp. (JCM 20061) from HDFs are ALP positive. Scale bars, 100 µm. (PDF) [file pone.0051866.s002.pdf]
