# Supplementary material for: Lactic Acid Bacteria Convert Human Fibroblasts to Multipotent Cells
Source: PLoS One. 2012 Dec 26;7(12):e51866. doi: 10.1371/journal.pone.0051866 (PMC3530539; doi:10.1371/journal.pone.0051866)
Supplement: Table S2 — List of 7580 genes that shows the difference by factor of two or more between LAB-incorporated cell clusters-1 vs. HDF-1 and LAB-incorporated cell clusters-2 vs. HDF-2. (PDF) [file pone.0051866.s011.pdf]

List of 7580 genes that shows the difference by a factor of two or more between LAB-incorporated cell clusters-1vsHDF-1 & LAB-incorporated cell clusters-2vsHDF-2

no	ProbeName	GeneSymbol	UniGeneID	GeneName	Description	Fold change [LAB-inc. cells-1]vs[HDF-1]	Regulation [LAB-inc. cells-1]vs[HDF-2]	Fold change [LAB-inc. cells-2]vs[HDF-2]	Regulation [LAB-inc. cells-2]vs[HDF-2]	raw data [LAB-inc. cells-1]	raw data [HDF-1]	raw data [LAB-inc. cells-2]	raw data [HDF-2]	Flag [LAB-inc. cells-1]	Flag [HDF-1]	Flag [LAB-inc. cells-2]	Flag [HDF-2]
1	A_23_P259314	RPS4Y1	Hs.282376	ribosomal protein S4, Y-linked 1	Homo sapiens ribosomal protein S4, Y-linked 1 (RPS4Y1), mRNA [NM_001008]	18060.07600	up	14754.83200	up	54349.08200	3.11606	43508.62500	2.60020	Detected	Compromised	Detected	Compromised
2	A_23_P324384	RPS4Y2	Hs.367761	ribosomal protein S4, Y-linked 2	Homo sapiens ribosomal protein S4, Y-linked 2 (RPS4Y2), mRNA [NM_001039567]	4066.76780	up	13989.86800	up	44035.61000	11.21212	40853.48400	2.57502	Detected	Compromised	Detected	Compromised
3	A_33_P3224331	DDX3Y	Hs.99120	DEAD (Asp-Glu-Ala-Asp) box polypeptide 3, Y-linked	Homo sapiens DEAD (Asp-Glu-Ala-Asp) box polypeptide 3, Y-linked (DDX3Y), transcript variant 1, mRNA [NM_001122665]	1037.07930	up	615.24634	up	4161.63000	4.15513	2590.18330	3.71233	Detected	Compromised	Detected	Compromised
4	A_24_P38290	TAC1	Hs.2563	tachykinin, precursor 1	Homo sapiens tachykinin, precursor 1 (TAC1), transcript variant beta, mRNA [NM_003182]	686.36470	up	444.92096	up	2521.29250	3.80366	1705.67370	3.38048	Detected	Compromised	Detected	Compromised
5	A_24_P291658	ADH1A	Hs.654433	alcohol dehydrogenase 1A (class I), alpha polypeptide	Homo sapiens alcohol dehydrogenase 1A (class I), alpha polypeptide (ADH1A), mRNA [NM_000667]	571.48706	up	841.44680	up	5846.36330	10.59284	3907.21220	4.09455	Detected	Compromised	Detected	Compromised
6	A_24_P11506	KYNU	Hs.470126	kynureninase (L-kynurenine hydrolase)	Homo sapiens kynureninase (L-kynurenine hydrolase) (KYNU), transcript variant 2, mRNA [NM_001032998]	355.01260	up	591.24176	up	3616.03830	10.54684	3028.44920	4.51670	Detected	Compromised	Detected	Compromised
7	A_23_P81158	ADH1C	Hs.654537	alcohol dehydrogenase 1C (class I), gamma polypeptide	Homo sapiens alcohol dehydrogenase 1C (class I), gamma polypeptide (ADH1C), mRNA [NM_000669]	319.68158	up	771.92334	up	10654.07700	34.50893	12332.11600	14.08734	Detected	Detected	Detected	Detected
8	A_24_P400324	THSD7A	Hs.120855	thrombospondin, type I, domain containing 7A	Homo sapiens thrombospondin, type I, domain containing 7A (THSD7A), mRNA [NM_015204]	287.57077	up	180.97974	up	1179.51700	4.24710	781.19403	3.80622	Detected	Compromised	Detected	Compromised
9	A_33_P3353737	ADH1B	Hs.4	alcohol dehydrogenase 1B (class I), beta polypeptide	Homo sapiens alcohol dehydrogenase 1B (class I), beta polypeptide (ADH1B), mRNA [NM_000668]	212.37578	up	114.41269	up	665.44110	3.24442	351.00528	2.70523	Detected	Compromised	Detected	Compromised
10	A_33_P3232965	TDRD6	Hs.656983	tudor domain containing 6	Homo sapiens tudor domain containing 6 (TDRD6), mRNA [NM_001010870]	201.56079	up	137.27667	up	746.39404	3.83438	453.79120	2.91491	Detected	Compromised	Detected	Compromised
11	A_33_P3252809				Protein FAM118A [Source:UniProtKB/Swiss-Prot;Acc:Q9NWS6] [ENST00000405673]	197.02629	up	187.28546	up	1307.81470	6.87314	1139.50610	5.36510	Detected	Compromised	Detected	Compromised
12	A_23_P28334	IL18RAP	Hs.158315	interleukin 18 receptor accessory protein	Homo sapiens interleukin 18 receptor accessory protein (IL18RAP), mRNA [NM_003853]	181.63411	up	155.65323	up	1391.98900	7.93545	1164.60740	6.59761	Detected	Compromised	Detected	Compromised
13	A_24_P150580	RASL12	Hs.27018	RAS-like, family 12	Homo sapiens RAS-like, family 12 (RASL12), mRNA [NM_016563]	174.26625	up	96.89568	up	1016.60394	6.04048	965.77515	8.78895	Detected	Compromised	Detected	Compromised
14	A_33_P3379076					170.42334	up	59.04181	up	573.78625	3.48621	577.58410	8.62623	Detected	Compromised	Detected	Compromised
15	A_33_P3339246	LHX8	Hs.403934	LIM homeobox 8	Homo sapiens LIM homeobox 8 (LHX8), mRNA [NM_001001933]	164.66695	up	102.12812	up	503.34500	3.16514	308.50230	2.66366	Detected	Compromised	Detected	Compromised
16	A_23_P214208	CNR1	Hs.709067	cannabinoid receptor 1 (brain)	Homo sapiens cannabinoid receptor 1 (brain) (CNR1), transcript variant 2, mRNA [NM_033181]	153.46800	up	153.92642	up	493.54886	3.33001	440.57306	2.52389	Detected	Compromised	Detected	Compromised
17	A_23_P73848	NCRNA00185	Hs.138453	non-protein coding RNA 185	Homo sapiens non-protein coding RNA 185 (NCRNA00185), non-coding RNA [NR_001544]	148.06490	up	134.41275	up	427.09998	2.98683	374.96707	2.45991	Detected	Compromised	Detected	Compromised
18	A_32_P35947					143.33679	up	57.08913	up	581.07090	4.19764	703.69010	10.86910	Detected	Compromised	Detected	Compromised
19	A_23_P254944	GSTT1	Hs.268573	glutathione S-transferase theta 1	Homo sapiens glutathione S-transferase theta 1 (GSTT1), mRNA [NM_000853]	141.92702	up	163.10300	up	1264.40190	9.22472	1203.25930	6.50523	Detected	Compromised	Detected	Compromised
20	A_33_P3217700	USP9Y	Hs.598540	ubiquitin specific peptidase 9, Y-linked	Homo sapiens ubiquitin specific peptidase 9, Y-linked (USP9Y), mRNA [NM_004654]	136.82297	up	166.75487	up	836.06976	6.32727	605.62220	3.20250	Detected	Compromised	Detected	Compromised
21	A_24_P942743	ZFY	Hs.522845	zinc finger protein, Y-linked	Homo sapiens zinc finger protein, Y-linked (ZFY), transcript variant 1, mRNA [NM_003411]	123.99677	up	112.68932	up	386.42886	3.22695	344.56055	2.69618	Detected	Compromised	Detected	Compromised
22	A_24_P33982	C17orf60	Hs.631749	chromosome 17 open reading frame 60	Homo sapiens chromosome 17 open reading frame 60 (C17orf60), mRNA [NM_001085423]	112.69669	up	68.35141	up	798.46460	7.33631	603.78030	7.78927	Detected	Compromised	Detected	Compromised
23	A_32_P8546	C6orf176		chromosome 6 open reading frame 176	Homo sapiens chromosome 6 open reading frame 176 (C6orf176), transcript variant 1, non-coding RNA [NR_026860]	110.78636	up	104.23385	up	362.18805	3.38517	339.72490	2.87398	Detected	Compromised	Detected	Compromised
24	A_23_P106906	PPL	Hs.192233	periplakin	Homo sapiens periplakin (PPL), mRNA [NM_002705]	109.77239	up	169.79262	up	2648.28200	24.98068	2728.29690	14.16897	Detected	Detected	Detected	Detected
25	A_23_P96658	CYorf15B	Hs.592254	chromosome Y open reading frame 15B	Homo sapiens chromosome Y open reading frame 15B (CYorf15B), mRNA [NM_032576]	108.38292	up	73.16099	up	350.89370	3.35234	233.44202	2.81362	Detected	Compromised	Detected	Compromised
26	A_32_P53524	NTN1	Hs.660885	netrin 1	Homo sapiens netrin 1 (NTN1), mRNA [NM_004822]	105.13181	up	86.89350	up	4960.21200	48.85389	3970.34200	40.29082	Detected	Detected	Detected	Detected
27	A_33_P3234025				Putative uncharacterized protein C1orf196 [Source:UniProtKB/Swiss-Prot;Acc:B1AJZ1] [ENST00000401953]	98.42491	up	40.55052	up	306.80780	3.22771	297.64487	6.47243	Detected	Compromised	Detected	Compromised
28	A_24_P330263	EDNRB	Hs.82002	endothelin receptor type B	Homo sapiens endothelin receptor type B (EDNRB), transcript variant 2, mRNA [NM_003991]	96.93665	up	36.39593	up	455.31522	4.86359	388.11847	9.40323	Detected	Compromised	Detected	Compromised
29	A_32_P167471	CLMN	Hs.301478	calmin (calponin-like, transmembrane)	Homo sapiens calmin (calponin-like, transmembrane) (CLMN), mRNA [NM_024734]	92.82680	up	68.86951	up	323.90180	3.61304	234.17175	2.99829	Detected	Compromised	Detected	Compromised
30	A_33_P3308347	ADAMTS8	Hs.271605	ADAM metallopeptidase with thrombospondin type 1 motif, 8	Homo sapiens ADAM metallopeptidase with thrombospondin type 1 motif, 8 (ADAMTS8), mRNA [NM_007037]	84.65422	up	63.63633	up	292.92110	3.58290	238.65303	3.30695	Detected	Compromised	Detected	Compromised

31	A_33_P3252281	EYA4	Hs.596680	eyes absent homolog 4 (Drosophila)	Homo sapiens eyes absent homolog 4 (Drosophila) (EYA4), transcript variant 1, mRNA [NM_004100]	79.30304	up	80.15833	up	547.71387	7.15150	282.40942	3.10668	Detected	Compromised	Detected	Compromised
32	A_23_P136777	APOD	Hs.522555	apolipoprotein D	Homo sapiens apolipoprotein D (APOD), mRNA [NM_001647]	77.89092	up	51.44027	up	1574.38330	20.92940	1158.70530	19.86256	Detected	Detected	Detected	Detected
33	A_23_P217277	SLITRK2	Hs.320368	SLIT and NTRK-like family, member 2	Homo sapiens SLIT and NTRK-like family, member 2 (SLITRK2), transcript variant 1, mRNA [NM_032539]	77.83112	up	58.56053	up	296.35090	3.94263	232.92703	3.50736	Detected	Compromised	Detected	Compromised
34	A_23_P364792	CYorf15A	Hs.522863	chromosome Y open reading frame 15A	Homo sapiens chromosome Y open reading frame 15A (CYorf15A), mRNA [NM_001005852]	77.05311	up	21.94398	up	264.94186	3.56036	254.09647	10.21054	Detected	Compromised	Detected	Compromised
35	A_24_P237511	EIF1AY	Hs.461178	eukaryotic translation initiation factor 1A, Y-linked	Homo sapiens eukaryotic translation initiation factor 1A, Y-linked (EIF1AY), mRNA [NM_004681]	75.00179	up	58.85675	up	297.78046	4.11110	239.38290	3.58643	Detected	Compromised	Detected	Compromised
36	A_23_P119943	IGFBP2	Hs.438102	insulin-like growth factor binding protein 2, 36kDa	Homo sapiens insulin-like growth factor binding protein 2, 36kDa (IGFBP2), mRNA [NM_000597]	74.71652	up	72.95371	up	98704.35000	1367.89500	85870.87000	1037.92020	Detected	Detected	Detected	Detected
37	A_24_P174793	PCSK1	Hs.78977	proprotein convertase subtilisin/kexin type 1	Homo sapiens proprotein convertase subtilisin/kexin type 1 (PCSK1), mRNA [NM_000439]	69.55891	up	85.26331	up	2139.17900	31.84399	1470.16550	15.20441	Detected	Detected	Detected	Compromised
38	A_33_P3381666	ABLIM2	Hs.233404	actin binding LIM protein family, member 2	Homo sapiens actin binding LIM protein family, member 2 (ABLIM2), transcript variant 7, mRNA [NM_001130088]	68.62967	up	26.94885	up	281.47607	4.24681	265.85660	8.69907	Detected	Compromised	Detected	Compromised
39	A_23_P71880	SPINK4	Hs.555934	serine peptidase inhibitor, Kazal type 4	Homo sapiens serine peptidase inhibitor, Kazal type 4 (SPINK4), mRNA [NM_014471]	67.98492	up	62.12992	up	201.07626	3.06254	180.89660	2.56741	Detected	Compromised	Detected	Compromised
40	A_33_P3366540	CSGALNACT1	Hs.613729	chondroitin sulfate N-acetylgalactosaminyltransferase 1	Homo sapiens chondroitin sulfate N-acetylgalactosaminyltransferase 1 (CSGALNACT1), transcript variant 1, mRNA [NM_001130518]	66.61250	up	34.17123	up	213.92242	3.32532	366.23657	9.45076	Detected	Compromised	Detected	Compromised
41	A_23_P102000	CXCR4	Hs.593413	chemokine (C-X-C motif) receptor 4	Homo sapiens chemokine (C-X-C motif) receptor 4 (CXCR4), transcript variant 1, mRNA [NM_001008540]	66.13092	up	194.85002	up	1326.76600	20.77413	994.89056	4.50236	Detected	Not Detected	Detected	Compromised
42	A_23_P19333	TREM1	Hs.283022	triggering receptor expressed on myeloid cells 1	Homo sapiens triggering receptor expressed on myeloid cells 1 (TREM1), mRNA [NM_018643]	63.95784	up	24.53825	up	411.24466	6.65794	396.14578	14.23564	Detected	Compromised	Detected	Detected
43	A_33_P3258923	MKRN3	Hs.72964	makorin ring finger protein 3	Homo sapiens makorin ring finger protein 3 (MKRN3), mRNA [NM_005664]	63.06924	up	45.81807	up	227.17850	3.72978	164.29243	3.16189	Detected	Compromised	Detected	Compromised
44	A_33_P3402091	MERTK	Hs.306178	c-mer proto-oncogene tyrosine kinase	Homo sapiens c-mer proto-oncogene tyrosine kinase (MERTK), mRNA [NM_006343]	60.40837	up	24.94035	up	178.49197	3.05953	104.58090	3.69756	Detected	Compromised	Detected	Compromised
45	A_23_P382065	EMCN	Hs.152913	endomucin	Homo sapiens endomucin (EMCN), transcript variant 1, mRNA [NM_016242]	59.85459	up	79.65573	up	1237.75990	21.41273	994.08000	11.00449	Detected	Detected	Detected	Compromised
46	A_24_P6626	TMEM132B	Hs.524838	transmembrane protein 132B	Homo sapiens transmembrane protein 132B (TMEM132B), mRNA [NM_052907]	59.73206	up	27.89287	up	468.37958	8.11939	309.51443	9.78483	Detected	Compromised	Detected	Compromised
47	A_33_P3219572	LONRF3		LON peptidase N-terminal domain and ring finger 3	LON peptidase N-terminal domain and RING finger protein 3 (RING finger protein 127) [Source:UniProtKB/Swiss-Prot;Acc:Q496Y0] [ENST00000365713]	58.95693	up	44.92536	up	189.15330	3.32210	141.19566	2.77138	Detected	Compromised	Detected	Compromised
48	A_33_P3379106	LOC283867	Hs.444774	hypothetical LOC283867	Homo sapiens hypothetical LOC283867 (LOC283867), non-coding RNA [NR_027755]	57.61020	up	59.14799	up	311.24050	5.59410	204.82478	3.05357	Detected	Compromised	Detected	Compromised
49	A_23_P94103	SCARA5	Hs.591833	scavenger receptor class A, member 5 (putative)	Homo sapiens scavenger receptor class A, member 5 (putative) (SCARA5), mRNA [NM_173833]	55.25531	up	60.53935	up	390.93230	7.32589	337.01797	4.90886	Detected	Compromised	Detected	Compromised
50	A_23_P110837	IRX4	Hs.196927	iroquois homeobox 4	Homo sapiens iroquois homeobox 4 (IRX4), mRNA [NM_016358]	54.29953	up	49.93983	up	1106.68080	21.10374	989.28080	17.46779	Detected	Not Detected	Detected	Detected
51	A_32_P153812	TMEM132B	Hs.524838	transmembrane protein 132B	Homo sapiens transmembrane protein 132B (TMEM132B), mRNA [NM_052907]	53.91463	up	57.11242	up	2151.39010	41.31860	1649.45290	25.46683	Detected	Detected	Detected	Detected
52	A_33_P3252939	MYLK4	Hs.127830	myosin light chain kinase family, member 4	Homo sapiens myosin light chain kinase family, member 4 (MYLK4), mRNA [NM_001012418]	51.44025	up	36.85075	up	179.17750	3.60673	119.96481	2.87060	Detected	Compromised	Detected	Compromised
53	A_23_P203972	FZD10	Hs.31664	frizzled homolog 10 (Drosophila)	Homo sapiens frizzled homolog 10 (Drosophila) (FZD10), mRNA [NM_007197]	51.32589	up	130.12695	up	498.99650	10.06686	372.01550	2.52092	Detected	Compromised	Detected	Compromised
54	A_23_P51126	IL1RL1	Hs.66	interleukin 1 receptor-like 1	Homo sapiens interleukin 1 receptor-like 1 (IL1RL1), transcript variant 1, mRNA [NM_016232]	50.44381	up	64.04615	up	2127.36040	43.66830	1837.31930	25.29631	Detected	Detected	Detected	Detected
55	A_23_P255672	ABLIM2	Hs.233404	actin binding LIM protein family, member 2	Homo sapiens actin binding LIM protein family, member 2 (ABLIM2), transcript variant 6, mRNA [NM_032432]	49.60078	up	55.43421	up	493.69733	10.30636	355.38593	5.65312	Detected	Compromised	Detected	Compromised
56	A_24_P51909	CPLX1	Hs.478930	complexin 1	Homo sapiens complexin 1 (CPLX1), mRNA [NM_006651]	49.56390	up	42.50464	up	145.27829	3.03507	146.47609	3.03876	Detected	Compromised	Detected	Compromised
57	A_33_P3400578	HLF	Hs.196952	hepatic leukemia factor	Homo sapiens hepatic leukemia factor (HLF), mRNA [NM_002126]	48.00860	up	30.80689	up	156.09073	3.36660	100.69540	2.88222	Detected	Compromised	Detected	Compromised
58	A_23_P372308	RGMA	Hs.271277	RGM domain family, member A	Homo sapiens RGM domain family, member A (RGMA), transcript variant 4, mRNA [NM_020211]	47.81368	up	36.64224	up	1537.38490	33.29382	837.68400	20.15876	Detected	Detected	Detected	Detected
59	A_24_P32085	MOBKL2B	Hs.369022	MOB1, Mps One Binder kinase activator-like 2B (yeast)	Homo sapiens MOB1, Mps One Binder kinase activator-like 2B (yeast) (MOBKL2B), mRNA [NM_024761]	46.96169	up	48.88302	up	754.67940	16.63994	427.45444	7.71076	Detected	Compromised	Detected	Compromised
60	A_23_P121441	NLGN4Y	Hs.439199	neuroligin 4, Y-linked	Homo sapiens neuroligin 4, Y-linked (NLGN4Y), transcript variant 1, mRNA [NM_014893]	46.59098	up	38.02197	up	188.14577	4.18144	151.81349	3.52080	Detected	Compromised	Detected	Compromised

61	A_33_P3347291	INMT	Hs.632629	indolethylamine N-methyltransferase	Homo sapiens indolethylamine N-methyltransferase (INMT), mRNA [NM_006774]	46.57341	up	194.73220	up	1179.10220	26.21479	955.78310	4.32800	Detected	Detected	Detected	Compromised
62	A_23_P63660	C10orf58	Hs.500333	chromosome 10 open reading frame 58	Homo sapiens chromosome 10 open reading frame 58 (C10orf58), transcript variant 1, mRNA [NM_032333]	46.22512	up	44.27332	up	6211.44730	139.13866	5824.77300	116.01186	Detected	Detected	Detected	Detected
63	A_23_P41804	NKD2	Hs.240951	naked cuticle homolog 2 (Drosophila)	Homo sapiens naked cuticle homolog 2 (Drosophila) (NKD2), mRNA [NM_033120]	45.72899	up	50.84110	up	1645.56700	37.26122	1397.77970	24.24316	Detected	Detected	Detected	Detected
64	A_23_P55632	SERPINB3	Hs.227948	serpin peptidase inhibitor, clade B (ovalbumin), member 3	Homo sapiens serpin peptidase inhibitor, clade B (ovalbumin), member 3 (SERPINB3), mRNA [NM_006919]	44.67560	up	41.30827	up	162.06169	3.75615	155.98383	3.32972	Detected	Compromised	Detected	Compromised
65	A_33_P3368193	PNLIPRP3	Hs.276724	pancreatic lipase-related protein 3	Homo sapiens pancreatic lipase-related protein 3 (PNLIPRP3), mRNA [NM_001011709]	43.85113	up	36.37052	up	280.34708	6.61985	203.79060	4.94083	Detected	Compromised	Detected	Compromised
66	A_23_P32279	BARX1	Hs.164960	BARX homeobox 1	Homo sapiens BARX homeobox 1 (BARX1), mRNA [NM_021570]	43.17076	up	36.02054	up	128.62550	3.08511	103.85482	2.54239	Detected	Compromised	Detected	Compromised
67	A_23_P163216	ATP8B4	Hs.511311	ATPase, class I, type 8B, member 4	Homo sapiens ATPase, class I, type 8B, member 4 (ATP8B4), mRNA [NM_024837]	42.46031	up	33.17026	up	438.19717	10.68611	250.54417	6.66041	Detected	Compromised	Detected	Compromised
68	A_23_P45999	FBXO2	Hs.132753	F-box protein 2	Homo sapiens F-box protein 2 (FBXO2), mRNA [NM_012168]	41.53993	up	37.53362	up	4425.72500	110.31938	4584.95260	107.71592	Detected	Detected	Detected	Detected
69	A_24_P579356	ARHGAP28	Hs.183114	Rho GTPase activating protein 28	Homo sapiens Rho GTPase activating protein 28 (ARHGAP28), transcript variant 1, mRNA [NM_001010000]	41.13934	up	28.78119	up	152.18360	3.83039	105.18620	3.22267	Detected	Compromised	Detected	Compromised
70	A_33_P3276713	HGF	Hs.396530	hepatocyte growth factor (hepapoietin A; scatter factor)	Homo sapiens hepatocyte growth factor (hepapoietin A; scatter factor) (HGF), transcript variant 2, mRNA [NM_001010931]	40.99867	up	40.19474	up	1101.39330	27.81671	1136.79310	24.93893	Detected	Detected	Detected	Detected
71	A_24_P497318	LOC100129973	Hs.569494	hypothetical protein LOC100129973	Homo sapiens clone 23963 mRNA sequence [AF007131]	40.14883	up	52.14708	up	247.13031	6.37362	157.09143	2.65637	Detected	Compromised	Detected	Compromised
72	A_23_P146551	MOBKL2B	Hs.369022	MOB1, Mps One Binder kinase activator-like 2B (yeast)	Homo sapiens MOB1, Mps One Binder kinase activator-like 2B (yeast) (MOBKL2B), mRNA [NM_024761]	39.56636	up	26.27050	up	122.35542	3.20207	98.99301	3.32278	Detected	Compromised	Detected	Compromised
73	A_33_P3251876	IL18R1	Hs.469521	interleukin 18 receptor 1	Homo sapiens interleukin 18 receptor 1 (IL18R1), mRNA [NM_003855]	39.03991	up	40.03226	up	140.94081	3.73819	141.96802	3.12713	Detected	Compromised	Detected	Compromised
74	A_32_P100109	REPS2	Hs.186810	RALBP1 associated Eps domain containing 2	Homo sapiens RALBP1 associated Eps domain containing 2 (REPS2), transcript variant 1, mRNA [NM_004726]	38.63219	up	22.88877	up	133.74800	3.58485	77.76267	2.99581	Detected	Compromised	Detected	Compromised
75	A_23_P102391	SLC40A1	Hs.643005	solute carrier family 40 (iron-regulated transporter), member 1	Homo sapiens solute carrier family 40 (iron-regulated transporter), member 1 (SLC40A1), mRNA [NM_014585]	38.44627	up	29.83511	up	163.22023	4.39595	118.72677	3.50902	Detected	Compromised	Detected	Compromised
76	A_24_P208567	IL18R1	Hs.469521	interleukin 18 receptor 1	Homo sapiens interleukin 18 receptor 1 (IL18R1), mRNA [NM_003855]	38.35930	up	45.17770	up	2480.25510	66.95126	1807.45400	35.27838	Detected	Detected	Detected	Detected
77	A_33_P3228977				Histone demethylase UTY (EC 1.14.11.-)(Ubiquitously transcribed Y chromosome tetratricopeptide repeat protein)(Ubiquitously transcribed TPR protein on the Y chromosome) [Source:UniProtKB/Swiss-Prot;Acc:O14607] [ENST00000382893]	37.70444	up	26.93844	up	151.53046	4.16141	105.54459	3.45485	Detected	Compromised	Detected	Compromised
78	A_32_P131640	SLITRK4	Hs.272284	SLIT and NTRK-like family, member 4	Homo sapiens SLIT and NTRK-like family, member 4 (SLITRK4), mRNA [NM_173078]	37.36375	up	23.44451	up	1426.11610	39.52187	1046.09900	39.34568	Detected	Detected	Detected	Detected
79	A_23_P61406	SHC3	Hs.656806	SHC (Src homology 2 domain containing) transforming protein 3	Homo sapiens SHC (Src homology 2 domain containing) transforming protein 3 (SHC3), mRNA [NM_016848]	37.04697	up	31.46639	up	1888.98830	52.79705	1255.19140	35.17455	Detected	Detected	Detected	Detected
80	A_23_P329835	UTY	Hs.115277	ubiquitously transcribed tetratricopeptide repeat gene, Y-linked	Homo sapiens ubiquitously transcribed tetratricopeptide repeat gene, Y-linked (UTY), transcript variant 3, mRNA [NM_007125]	36.99965	up	17.47010	up	105.47289	2.95173	48.09137	2.42738	Detected	Compromised	Detected	Compromised
81	A_24_P383609	NANOS1	Hs.591918	nanos homolog 1 (Drosophila)	Homo sapiens nanos homolog 1 (Drosophila) (NANOS1), mRNA [NM_199461]	36.86504	up	36.11005	up	3418.01980	96.00481	2713.12130	66.25317	Detected	Detected	Detected	Detected
82	A_23_P41344	EREG	Hs.115263	epiregulin	Homo sapiens epiregulin (EREG), mRNA [NM_001432]	36.37867	up	40.60798	up	3908.62520	111.25263	3326.96340	72.24406	Detected	Detected	Detected	Detected
83	A_24_P183664	TRIL	Hs.21572	TLR4 interactor with leucine rich repeats	Homo sapiens TLR4 interactor with leucine rich repeats (TRIL), mRNA [NM_014817]	36.16660	up	58.18153	up	660.81910	18.91943	446.87784	6.77282	Detected	Compromised	Detected	Compromised
84	A_33_P3347697	NOVA1	Hs.31588	neuro-oncological ventral antigen 1	Homo sapiens neuro-oncological ventral antigen 1 (NOVA1), transcript variant 1, mRNA [NM_002515]	35.99863	up	33.48424	up	3895.28740	112.04350	2550.43460	67.16443	Detected	Detected	Detected	Detected
85	A_23_P5903	SLCO4A1	Hs.235782	solute carrier organic anion transporter family, member 4A1	Homo sapiens solute carrier organic anion transporter family, member 4A1 (SLCO4A1), mRNA [NM_016354]	35.69670	up	42.76345	up	328.30510	9.52320	306.34595	6.31691	Detected	Compromised	Detected	Compromised
86	A_23_P135381	SP5	Hs.368802	Sp5 transcription factor	Homo sapiens Sp5 transcription factor (SP5), mRNA [NM_001003845]	35.15185	up	31.24466	up	113.03018	3.32950	98.87079	2.79035	Detected	Compromised	Detected	Compromised
87	A_32_P15958	C21orf121	Hs.626078	chromosome 21 open reading frame 121	Homo sapiens chromosome 21 open reading frame 121 (C21orf121), non-coding RNA [NR_027273]	34.90670	up	36.79043	up	103.47908	3.06957	106.39017	2.54995	Detected	Compromised	Detected	Compromised
88	A_23_P253350	C8orf4	Hs.591849	chromosome 8 open reading frame 4	Homo sapiens chromosome 8 open reading frame 4 (C8orf4), mRNA [NM_020130]	34.03380	up	13.84523	up	257.75253	7.84198	173.23439	11.03315	Detected	Compromised	Detected	Compromised
89	A_23_P134835	CSGALNACT1	Hs.613729	chondroitin sulfate N-acetylgalactosaminyltransferase 1	Homo sapiens chondroitin sulfate N-acetylgalactosaminyltransferase 1 (CSGALNACT1), transcript variant 2, mRNA [NM_018371]	34.03196	up	35.23470	up	3581.97270	108.98540	2820.94480	70.59753	Detected	Detected	Detected	Detected
90	A_23_P17065	CCL20	Hs.75498	chemokine (C-C motif) ligand 20	Homo sapiens chemokine (C-C motif) ligand 20 (CCL20), transcript variant 1, mRNA [NM_004591]	33.94013	up	38.58477	up	891.41380	27.19563	593.61926	13.56618	Detected	Detected	Detected	Detected

91	A_24_P326491	MKX	Hs.128193	mohawk homeobox	Homo sapiens mohawk homeobox (MKX), mRNA [NM_173576]	33.22589	up	22.87169	up	155.40741	4.84315	110.28508	4.25191	Detected	Compromised	Detected	Compromised
92	A_23_P5342	LRP1B	Hs.656461	low density lipoprotein-related protein 1B (deleted in tumors)	Homo sapiens low density lipoprotein-related protein 1B (deleted in tumors) (LRP1B), mRNA [NM_018557]	33.13724	up	39.84983	up	167.17760	5.22390	121.01801	2.67787	Detected	Compromised	Detected	Compromised
93	A_23_P161458	OLAH	Hs.24309	oleoyl-ACP hydrolase	Homo sapiens oleoyl-ACP hydrolase (OLAH), transcript variant 2, mRNA [NM_001039702]	33.06666	up	33.34927	up	2227.14280	69.74142	1944.02030	51.40200	Detected	Detected	Detected	Detected
94	A_33_P3400763	PLIN4	Hs.591387	perilipin 4	Homo sapiens perilipin 4 (PLIN4), mRNA [NM_001080400]	33.01618	up	34.24303	up	363.90515	11.41286	283.48096	7.29991	Detected	Compromised	Detected	Compromised
95	A_23_P212105	DAZL	Hs.131179	deleted in azoospermia-like	Homo sapiens deleted in azoospermia-like (DAZL), mRNA [NM_001351]	33.00124	up	29.00014	up	110.35501	3.46254	96.09358	2.92186	Detected	Compromised	Detected	Compromised
96	A_32_P310335	JAM2	Hs.655065	junctional adhesion molecule 2	Junctional adhesion molecule B Precursor (JAM-B)(Junctional adhesion molecule 2)(Vascular endothelial junction-associated molecule)(VE-JAM)(CD322 antigen) [Source:UniProtKB/Swiss-Prot;Acc:P57087] [ENST00000480456]	32.73216	up	30.34047	up	202.03134	6.39112	157.40321	4.57464	Detected	Compromised	Detected	Compromised
97	A_23_P73571	MUM1L1	Hs.592221	melanoma associated antigen (mutated) 1-like 1	Homo sapiens melanoma associated antigen (mutated) 1-like 1 (MUM1L1), mRNA [NM_152423]	32.66191	up	30.81178	up	99.31375	3.14848	91.74905	2.62573	Detected	Compromised	Detected	Compromised
98	A_33_P3221458	ZNF204	Hs.8198	zinc finger protein 204 pseudogene	Homo sapiens zinc finger protein 204 pseudogene (ZNF204), non-coding RNA [NR_002722]	32.52627	up	89.68151	up	402.69946	12.81976	253.62761	2.49379	Detected	Compromised	Detected	Compromised
99	A_33_P3211634	PPIL6	Hs.32234	peptidylprolyl isomerase (cyclophilin)-like 6	Homo sapiens peptidylprolyl isomerase (cyclophilin)-like 6 (PPIL6), transcript variant 1, mRNA [NM_173672]	32.42908	up	40.07022	up	159.21518	5.08374	137.91795	3.03504	Detected	Compromised	Detected	Compromised
100	A_24_P220485	OLFML2A	Hs.357004	olfactomedin-like 2A	Homo sapiens olfactomedin-like 2A (OLFML2A), mRNA [NM_182487]	31.84938	up	34.26382	up	7593.59700	246.87650	4915.97800	126.51431	Detected	Detected	Detected	Detected
101	A_23_P131208	NR4A2	Hs.563344	nuclear receptor subfamily 4, group A, member 2	Homo sapiens nuclear receptor subfamily 4, group A, member 2 (NR4A2), mRNA [NM_006186]	31.49993	up	53.63770	up	1141.15920	37.51196	476.82245	7.83885	Detected	Detected	Detected	Compromised
102	A_33_P3248052	NLGN4Y	Hs.439199	neuroligin 4, Y-linked	Homo sapiens neuroligin 4, Y-linked (NLGN4Y), transcript variant 2, mRNA [NM_001164238]	31.42489	up	26.34670	up	114.60700	3.77633	98.18084	3.28599	Detected	Compromised	Detected	Compromised
103	A_23_P27229	MYO15A	Hs.462390	myosin XVA	Homo sapiens myosin XVA (MYO15A), mRNA [NM_016239]	31.28414	up	10.33449	up	107.32022	3.55214	72.77074	6.20916	Detected	Compromised	Detected	Compromised
104	A_33_P3479449	LOC151438	Hs.516245	hypothetical protein LOC151438	Homo sapiens cDNA FLJ31315 fis, clone LIVER1000303 [AK055877]	31.08377	up	19.17067	up	98.46256	3.27998	71.40328	3.28433	Detected	Compromised	Detected	Compromised
105	A_33_P3375566		Hs.126160		BX093647 Soares_testis_NHT Homo sapiens cDNA clone IMAGp998F023561 ; IMAGE:1409665, mRNA sequence [BX093647]	30.45936	up	14.55300	up	109.08609	3.70836	92.31986	5.59381	Detected	Compromised	Detected	Compromised
106	A_33_P3396214	KREMEN2	Hs.661128	kringle containing transmembrane protein 2	Homo sapiens kringle containing transmembrane protein 2 (KREMEN2), transcript variant 4, mRNA [NM_172229]	30.24967	up	7.42596	up	104.14642	3.56498	93.47362	11.09947	Detected	Compromised	Detected	Compromised
107	A_23_P66432	TTYH2	Hs.27935	tweety homolog 2 (Drosophila)	Homo sapiens tweety homolog 2 (Drosophila) (TTYH2), transcript variant 1, mRNA [NM_032646]	30.17794	up	30.11486	up	142.92722	4.90409	96.84503	2.83571	Detected	Compromised	Detected	Compromised
108	A_23_P85800	CD52	Hs.276770	CD52 molecule	Homo sapiens CD52 molecule (CD52), mRNA [NM_001803]	29.96984	up	46.27201	up	507.29092	17.52693	446.61680	8.51103	Detected	Compromised	Detected	Compromised
109	A_32_P133072	SPON1	Hs.643864	spondin 1, extracellular matrix protein	Homo sapiens spondin 1, extracellular matrix protein (SPON1), mRNA [NM_006108]	29.46121	up	26.38959	up	7575.24760	266.24368	5809.44530	194.11876	Detected	Detected	Detected	Detected
110	A_32_P197340		Hs.443729		Uncharacterized protein LOC285141 [Source:UniProtKB/Swiss-Prot;Acc:A1L162] [ENST00000375280]	29.08856	up	15.63092	up	670.04670	23.85151	622.16797	35.09851	Detected	Detected	Detected	Detected
111	A_33_P3226775	MUC20	Hs.308992	mucin 20, cell surface associated	Homo sapiens mucin 20, cell surface associated (MUC20), transcript variant S, mRNA [NM_001098516]	28.83724	up	19.27050	up	105.88018	3.80184	60.99501	2.79105	Detected	Compromised	Detected	Compromised
112	A_33_P3403053					28.82709	up	24.09965	up	114.81238	4.12402	101.79661	3.72467	Detected	Compromised	Detected	Compromised
113	A_23_P156907	SOBP	Hs.445244	sine oculis binding protein homolog (Drosophila)	Homo sapiens sine oculis binding protein homolog (Drosophila) (SOBP), mRNA [NM_018013]	28.62486	up	47.26331	up	289.61200	10.47626	219.32841	4.09201	Detected	Compromised	Detected	Compromised
114	A_23_P51587	RGS7	Hs.655739	regulator of G-protein signaling 7	Homo sapiens regulator of G-protein signaling 7 (RGS7), mRNA [NM_002924]	28.38625	up	20.62096	up	103.42441	3.77266	79.21559	3.38741	Detected	Compromised	Detected	Compromised
115	A_23_P160336	LEFTY1	Hs.720965	left-right determination factor 1	Homo sapiens left-right determination factor 1 (LEFTY1), mRNA [NM_020997]	27.25997	up	19.68110	up	94.15096	3.57629	80.80880	3.62055	Detected	Compromised	Detected	Compromised
116	A_32_P172141	CDON	Hs.38034	Cdon homolog (mouse)	Homo sapiens Cdon homolog (mouse) (CDON), mRNA [NM_016952]	27.23026	up	33.82092	up	159.92126	6.08118	112.84336	2.94209	Detected	Compromised	Detected	Compromised
117	A_33_P3246883	DMKN	Hs.417795	dermokine	Homo sapiens dermokine (DMKN), transcript variant 1, mRNA [NM_001035516]	26.97098	up	20.76584	up	4352.25200	167.08998	4441.97700	188.62196	Detected	Detected	Detected	Detected
118	A_33_P3363271	CACNB2	Hs.59093	calcium channel, voltage-dependent, beta 2 subunit	Homo sapiens calcium channel, voltage-dependent, beta 2 subunit (CACNB2), transcript variant 1, mRNA [NM_000724]	26.95618	up	38.19681	up	539.53580	20.72502	363.61682	8.39426	Detected	Detected	Detected	Compromised
119	A_23_P45025	MAPK10	Hs.125503	mitogen-activated protein kinase 10	Homo sapiens mitogen-activated protein kinase 10 (MAPK10), transcript variant 3, mRNA [NM_138980]	26.88256	up	20.18612	up	89.23782	3.43725	66.96814	2.92537	Detected	Compromised	Detected	Compromised
120	A_23_P320261	DMKN	Hs.417795	dermokine	Homo sapiens dermokine (DMKN), transcript variant 1, mRNA [NM_001035516]	26.58805	up	22.65370	up	21769.64600	847.80890	16328.26500	635.57446	Detected	Detected	Detected	Detected
121	A_23_P115261	AGT	Hs.19383	angiotensinogen (serpin peptidase inhibitor, clade A, member 8)	Homo sapiens angiotensinogen (serpin peptidase inhibitor, clade A, member 8) (AGT), mRNA [NM_000029]	26.29971	up	26.45119	up	1720.13070	67.72418	1436.19140	47.87763	Detected	Detected	Detected	Detected

122	A_23_P417261	EFHB	Hs.670883	EF-hand domain family, member B	Homo sapiens EF-hand domain family, member B (EFHB), mRNA [NM_144715]	25.67548	up	51.96707	up	219.54419	8.85394	158.34073	2.68677	Detected	Compromised	Detected	Compromised
123	A_33_P3226212	JAM2	Hs.517227	junctional adhesion molecule 2	Homo sapiens junctional adhesion molecule 2 (JAM2), mRNA [NM_021219]	25.66978	up	29.49885	up	1741.77920	70.25935	1490.58920	44.55725	Detected	Detected	Detected	Detected
124	A_33_P3312658				Kynureninase (EC 3.7.1.3)(L-kynurenine hydrolase) [Source:UniProtKB/Swiss-Prot;Acc:Q16719] [ENST00000410015]	25.64462	up	16.14607	up	92.85991	3.74943	60.55794	3.30727	Detected	Compromised	Detected	Compromised
125	A_23_P91334	HSPA12B	Hs.516854	heat shock 70kD protein 12B	Homo sapiens heat shock 70kD protein 12B (HSPA12B), mRNA [NM_052970]	25.64325	up	44.37856	up	458.91040	18.53053	383.27050	7.61549	Detected	Detected	Detected	Compromised
126	A_33_P3216277	LOC100131354	Hs.675317	hypothetical LOC100131354	PREDICTED: Homo sapiens hypothetical LOC100131354 (LOC100131354), mRNA [XM_001718006]	25.52585	up	21.35703	up	98.77198	4.00670	58.69536	2.42342	Detected	Compromised	Detected	Compromised
127	A_24_P224488	MAPT	Hs.101174	microtubule-associated protein tau	Homo sapiens microtubule-associated protein tau (MAPT), transcript variant 1, mRNA [NM_016835]	25.16290	up	30.70013	up	218.93666	9.00929	202.72017	5.82267	Detected	Compromised	Detected	Compromised
128	A_33_P3210492	C1QTNF4	Hs.662633	C1q and tumor necrosis factor related protein 4	Homo sapiens C1q and tumor necrosis factor related protein 4 (C1QTNF4), mRNA [NM_031909]	24.92442	up	22.31644	up	549.33105	22.82139	227.83722	9.00255	Detected	Detected	Detected	Compromised
129	A_23_P157766	C9orf68	Hs.179615	chromosome 9 open reading frame 68	Homo sapiens chromosome 9 open reading frame 68 (C9orf68), mRNA [NM_001039395]	24.71063	up	22.96372	up	136.48941	5.71937	74.74722	2.87024	Detected	Compromised	Detected	Compromised
130	A_33_P3216090	SHC3	Hs.656806	SHC (Src homology 2 domain containing) transforming protein 3	Homo sapiens SHC (Src homology 2 domain containing) transforming protein 3 (SHC3), mRNA [NM_016848]	24.43761	up	23.57672	up	606.78467	25.71041	739.49786	27.65789	Detected	Detected	Detected	Detected
131	A_33_P3259183	FAM78B	Hs.493518	family with sequence similarity 78, member B	Homo sapiens family with sequence similarity 78, member B (FAM78B), mRNA [NM_001017961]	24.41408	up	14.40718	up	318.97970	13.52869	244.20084	14.94630	Detected	Compromised	Detected	Compromised
132	A_23_P90453	KRTDAP	Hs.112457	keratinocyte differentiation-associated protein	Homo sapiens keratinocyte differentiation-associated protein (KRTDAP), mRNA [NM_207392]	24.25390	up	19.91099	up	76.82884	3.28001	62.71435	2.77741	Detected	Compromised	Detected	Compromised
133	A_23_P204847	LCP1	Hs.381099	lymphocyte cytosolic protein 1 (L-plastin)	Homo sapiens lymphocyte cytosolic protein 1 (L-plastin) (LCP1), mRNA [NM_002298]	23.96012	up	26.26848	up	2142.38280	92.58511	1806.19030	60.63090	Detected	Detected	Detected	Detected
134	A_23_P167920	DLL1	Hs.379912	delta-like 1 (Drosophila)	Homo sapiens delta-like 1 (Drosophila) (DLL1), mRNA [NM_005618]	23.86572	up	20.80848	up	536.94806	23.29651	409.12323	17.33721	Detected	Detected	Detected	Detected
135	A_33_P3315134	DIRC3	Hs.572788	disrupted in renal carcinoma 3	Homo sapiens disrupted in renal carcinoma 3 (DIRC3), non-coding RNA [NR_026597]	23.34748	up	17.01258	up	156.27208	6.93066	108.12130	5.60411	Detected	Compromised	Detected	Compromised
136	A_33_P3269110	GUCY1A3	Hs.24258	guanylate cyclase 1, soluble, alpha 3	Homo sapiens guanylate cyclase 1, soluble, alpha 3 (GUCY1A3), transcript variant 6, mRNA [NM_001130686]	23.25774	up	20.70064	up	97.22958	4.32877	75.02280	3.19577	Detected	Compromised	Detected	Compromised
137	A_23_P133293	MCTP1	Hs.655087	multiple C2 domains, transmembrane 1	Homo sapiens multiple C2 domains, transmembrane 1 (MCTP1), transcript variant L, mRNA [NM_024717]	23.02720	up	20.42284	up	1205.85190	54.22330	747.16660	32.26019	Detected	Detected	Detected	Detected
138	A_32_P167705	AGBL2	Hs.147377	ATP/GTP binding protein-like 2	Homo sapiens ATP/GTP binding protein-like 2 (AGBL2), mRNA [NM_024783]	22.77683	up	7.79309	up	70.56771	3.20808	42.20715	4.77575	Detected	Compromised	Detected	Compromised
139	A_23_P156890	TCF21	Hs.720025	transcription factor 21	Homo sapiens transcription factor 21 (TCF21), transcript variant 2, mRNA [NM_003206]	22.56896	up	16.27856	up	144.70180	6.63889	168.67722	9.13705	Detected	Compromised	Detected	Compromised
140	A_23_P319232	KIAA1045	Hs.7989	KIAA1045	Homo sapiens KIAA1045 (KIAA1045), mRNA [NM_015297]	22.52243	up	21.26069	up	70.04020	3.22007	64.85850	2.69002	Detected	Compromised	Detected	Compromised
141	A_23_P302207	ZNF853	Hs.592170	zinc finger protein 853	Homo sapiens zinc finger protein 853 (ZNF853), mRNA [NM_017560]	22.49574	up	9.58104	up	84.28135	3.87940	72.36288	6.65991	Detected	Compromised	Detected	Compromised
142	A_24_P63537	ERAP1	Hs.716426	endoplasmic reticulum aminopeptidase 1	Homo sapiens endoplasmic reticulum aminopeptidase 1 (ERAP1), transcript variant 1, mRNA [NM_016442]	22.38158	up	12.87026	up	205.45345	9.50508	51.66121	3.53951	Detected	Compromised	Detected	Compromised
143	A_33_P3409974	THRB	Hs.187861	thyroid hormone receptor, beta (erythroblastic leukemia viral (v-erb-a) oncogene homolog 2, avian)	Homo sapiens thyroid hormone receptor, beta (erythroblastic leukemia viral (v-erb-a) oncogene homolog 2, avian) (THRB), transcript variant 3, mRNA [NM_001128177]	22.18514	up	16.88650	up	81.50356	3.80406	78.22823	4.08498	Detected	Compromised	Detected	Compromised
144	A_23_P78782	CA11	Hs.428446	carbonic anhydrase XI	Homo sapiens carbonic anhydrase XI (CA11), mRNA [NM_001217]	22.16652	up	34.45066	up	252.82680	11.81024	288.16467	7.37579	Detected	Compromised	Detected	Compromised
145	A_33_P3350374	C10orf58	Hs.500333	chromosome 10 open reading frame 58	Homo sapiens chromosome 10 open reading frame 58 (C10orf58), transcript variant 1, mRNA [NM_032333]	22.04568	up	34.69864	up	298.76492	14.03263	361.52570	9.18739	Detected	Compromised	Detected	Compromised
146	A_23_P163238	STRC	Hs.657395	stereocilin	Homo sapiens stereocilin (STRC), mRNA [NM_153700]	21.97016	up	8.40048	up	70.51856	3.32356	72.56361	7.61694	Detected	Compromised	Detected	Compromised
147	A_33_P3419190	AREG	Hs.270833	amphiregulin	Homo sapiens amphiregulin (AREG), mRNA [NM_001657]	21.79760	up	22.24821	up	15615.27900	741.77840	9681.86800	383.73340	Detected	Detected	Detected	Detected
148	A_24_P48204	SECTM1	Hs.558009	secreted and transmembrane 1	Homo sapiens secreted and transmembrane 1 (SECTM1), mRNA [NM_003004]	21.65068	up	20.72855	up	19150.86000	915.90375	9143.48400	388.96295	Detected	Detected	Detected	Detected
149	A_24_P278747	CCND2	Hs.376071	cyclin D2	Homo sapiens cyclin D2 (CCND2), mRNA [NM_001759]	21.36604	up	16.80621	up	1283.24300	62.18964	1037.41260	54.43112	Detected	Detected	Detected	Detected
150	A_33_P3269109	GUCY1A3	Hs.24258	guanylate cyclase 1, soluble, alpha 3	Homo sapiens guanylate cyclase 1, soluble, alpha 3 (GUCY1A3), transcript variant 6, mRNA [NM_001130686]	21.33896	up	9.51538	up	63.07116	3.06049	39.90500	3.69800	Detected	Compromised	Detected	Compromised
151	A_24_P254850	SEC14L5	Hs.512856	SEC14-like 5 (S. cerevisiae)	Homo sapiens SEC14-like 5 (S. cerevisiae) (SEC14L5), mRNA [NM_014692]	21.22127	up	11.23725	up	60.90013	2.97153	31.17798	2.44655	Detected	Compromised	Detected	Compromised
152	A_23_P259071	AREG	Hs.270833	amphiregulin	Homo sapiens amphiregulin (AREG), mRNA [NM_001657]	21.10707	up	22.91913	up	51166.50000	2510.09900	45443.98400	1748.41210	Detected	Detected	Detected	Detected

153	A_33_P3403576	FCGR2A	Hs.352642	Fc fragment of IgG, low affinity IIa, receptor (CD32)	Homo sapiens Fc fragment of IgG, low affinity IIa, receptor (CD32) (FCGR2A), transcript variant 1, mRNA [NM_001136219]	20.96401	up	4.70850	up	88.30619	4.36164	74.44190	13.94122	Detected	Compromised	Detected	Compromised
154	A_32_P69849	BEX5	Hs.47209	brain expressed, X-linked 5	Homo sapiens brain expressed, X-linked 5 (BEX5), transcript variant 1, mRNA [NM_001012978]	20.89748	up	19.77356	up	500.22473	24.78589	406.50775	18.12798	Detected	Detected	Detected	Detected
155	A_33_P3387766	LOC284276	Hs.390287	hypothetical LOC284276	Homo sapiens hypothetical LOC284276 (LOC284276), non-coding RNA [NR_015417]	20.58447	up	19.46914	up	294.78238	14.82842	169.29780	7.66779	Detected	Compromised	Detected	Compromised
156	A_23_P354705	ST8SIA1	Hs.408614	ST8 alpha-N-acetyl-neuraminide alpha-2,8-sialyltransferase 1	Homo sapiens ST8 alpha-N-acetyl-neuraminide alpha-2,8-sialyltransferase 1 (ST8SIA1), mRNA [NM_003034]	20.50480	up	15.84092	up	68.03804	3.43581	52.09912	2.90012	Detected	Compromised	Detected	Compromised
157	A_24_P329487	FAM84B	Hs.124951	family with sequence similarity 84, member B	Homo sapiens family with sequence similarity 84, member B (FAM84B), mRNA [NM_174911]	20.48407	up	18.21981	up	202.27562	10.22493	61.89389	2.99550	Detected	Compromised	Detected	Compromised
158	A_33_P3883116	LOC253962	Hs.558704	hypothetical protein LOC253962	Homo sapiens hypothetical protein LOC253962, mRNA (cDNA clone IMAGE:5244335), partial cds [BC041476]	20.34616	up	12.95133	up	59.99200	3.05312	36.84636	2.50868	Detected	Compromised	Detected	Compromised
159	A_33_P3220919	ADRBK2	Hs.657494	adrenergic, beta, receptor kinase 2	Homo sapiens adrenergic, beta, receptor kinase 2 (ADRBK2), mRNA [NM_005160]	20.27522	up	19.78268	up	1639.76640	83.74319	1101.26150	49.08750	Detected	Detected	Detected	Detected
160	A_33_P3367636	C6orf221	Hs.128326	chromosome 6 open reading frame 221	Homo sapiens chromosome 6 open reading frame 221 (C6orf221), mRNA [NM_001017361]	20.27000	up	22.70337	up	109.27738	5.58226	82.21005	3.19301	Detected	Compromised	Detected	Compromised
161	A_33_P3330911	BCAS1	Hs.400556	breast carcinoma amplified sequence 1	Homo sapiens breast carcinoma amplified sequence 1 (BCAS1), mRNA [NM_003657]	20.04318	up	29.30792	up	471.96838	24.38256	236.39279	7.11238	Detected	Detected	Detected	Compromised
162	A_23_P392470	NR3C2	Hs.163924	nuclear receptor subfamily 3, group C, member 2	Homo sapiens nuclear receptor subfamily 3, group C, member 2 (NR3C2), transcript variant 1, mRNA [NM_000901]	19.98141	up	12.73011	up	59.13012	3.06419	44.21335	3.06258	Detected	Compromised	Detected	Compromised
163	A_33_P3220738	ASTN1	Hs.495897	astrotactin 1	Homo sapiens astrotactin 1 (ASTN1), transcript variant 2, mRNA [NM_207108]	19.93486	up	13.76883	up	59.78233	3.10522	40.28374	2.57987	Detected	Compromised	Detected	Compromised
164	A_23_P136671	UGT2B7	Hs.654424	UDP glucuronosyltransferase 2 family, polypeptide B7	Homo sapiens UDP glucuronosyltransferase 2 family, polypeptide B7 (UGT2B7), mRNA [NM_001074]	19.92933	up	8.56417	up	65.10293	3.38252	27.84269	2.86676	Detected	Compromised	Detected	Compromised
165	A_23_P207456	CCL8	Hs.271387	chemokine (C-C motif) ligand 8	Homo sapiens chemokine (C-C motif) ligand 8 (CCL8), mRNA [NM_005623]	19.68330	up	14.61421	up	61.14666	3.21668	44.24966	2.66993	Detected	Compromised	Detected	Compromised
166	A_33_P3398578	LOC100131208	Hs.658178	similar to hCG2045825	Homo sapiens cDNA FLJ42971 fis, clone BRSTN2018083 [AK124961]	19.67581	up	14.53590	up	66.97075	3.52440	49.46848	3.00090	Detected	Compromised	Detected	Compromised
167	A_32_P57810	RNF157	Hs.500643	ring finger protein 157	Homo sapiens ring finger protein 157 (RNF157), mRNA [NM_052916]	19.63899	up	17.21282	up	1199.13500	63.22402	878.70080	45.01472	Detected	Detected	Detected	Detected
168	A_24_P245838	MGAT3	Hs.276808	mannosyl (beta-1,4-)-glycoprotein beta-1,4-N-acetylglucosaminyltransferase	Homo sapiens mannosyl (beta-1,4-)-glycoprotein beta-1,4-N-acetylglucosaminyltransferase (MGAT3), transcript variant 1, mRNA [NM_002409]	19.52502	up	11.00780	up	71.27815	3.78005	39.87063	3.19388	Detected	Compromised	Detected	Compromised
169	A_33_P3222424	CSF3	Hs.2233	colony stimulating factor 3 (granulocyte)	Homo sapiens colony stimulating factor 3 (granulocyte) (CSF3), transcript variant 1, mRNA [NM_000759]	19.50861	up	19.62701	up	1023.51210	54.32501	587.20544	26.38162	Detected	Detected	Detected	Detected
170	A_24_P348861	TTTY15	Hs.433656	testis-specific transcript, Y-linked 15 (non-protein coding)	Homo sapiens testis-specific transcript, Y-linked 15 (non-protein coding) (TTTY15), non-coding RNA [NR_001545]	19.50555	up	9.74274	up	65.23033	3.46278	32.06854	2.90244	Detected	Compromised	Detected	Compromised
171	A_24_P383459	RNF157	Hs.500643	ring finger protein 157	Homo sapiens ring finger protein 157 (RNF157), mRNA [NM_052916]	19.50244	up	12.61229	up	63.39179	3.36572	40.37465	2.82280	Detected	Compromised	Detected	Compromised
172	A_24_P352952	FAM20A	Hs.268874	family with sequence similarity 20, member A	Homo sapiens family with sequence similarity 20, member A (FAM20A), transcript variant 1, mRNA [NM_017565]	19.50080	up	15.72512	up	199.26454	10.58061	213.21626	11.95616	Detected	Compromised	Detected	Compromised
173	A_33_P3309551	PTPRD	Hs.446083	protein tyrosine phosphatase, receptor type, D	Homo sapiens protein tyrosine phosphatase, receptor type, D (PTPRD), transcript variant 1, mRNA [NM_002839]	19.49539	up	13.40210	up	120.84581	6.41849	88.34084	5.81239	Detected	Compromised	Detected	Compromised
174	A_33_P3337134	ABLIM2	Hs.233404	actin binding LIM protein family, member 2	Homo sapiens actin binding LIM protein family, member 2 (ABLIM2), transcript variant 1, mRNA [NM_001130083]	19.32979	up	25.40682	up	118.96076	6.37250	97.44179	3.38190	Detected	Compromised	Detected	Compromised
175	A_33_P3523583	LOC642924	Hs.434882	hypothetical protein LOC642924	Homo sapiens cDNA clone IMAGE:5277089 [BC037909]	19.32162	up	16.05821	up	59.20582	3.17288	47.81736	2.62575	Detected	Compromised	Detected	Compromised
176	A_32_P119033	PLCXD3	Hs.145404	phosphatidylinositol-specific phospholipase C, X domain containing 3	Homo sapiens phosphatidylinositol-specific phospholipase C, X domain containing 3 (PLCXD3), mRNA [NM_001005473]	19.26149	up	23.59467	up	349.46060	18.78631	189.44217	7.07992	Detected	Detected	Detected	Compromised
177	A_33_P3234202	DNASE1L3	Hs.476453	deoxyribonuclease I-like 3	Homo sapiens deoxyribonuclease I-like 3 (DNASE1L3), mRNA [NM_004944]	19.23502	up	70.31754	up	323.46573	17.41280	223.38309	2.80125	Detected	Detected	Detected	Compromised
178	A_33_P3324675					19.16414	up	8.96456	up	4156.93650	224.60384	812.28656	79.89982	Detected	Detected	Detected	Detected
179	A_23_P60146	PDGFRL	Hs.458573	platelet-derived growth factor receptor-like	Homo sapiens platelet-derived growth factor receptor-like (PDGFRL), mRNA [NM_006207]	19.03447	up	19.35363	up	2403.17380	130.73070	1754.59250	79.94280	Detected	Detected	Detected	Detected
180	A_33_P3275702	FMO2	Hs.144912	flavin containing monooxygenase 2 (non-functional)	Homo sapiens flavin containing monooxygenase 2 (non-functional) (FMO2), mRNA [NM_001460]	18.92319	up	16.11199	up	74.85105	4.09578	63.28128	3.46331	Detected	Compromised	Detected	Compromised
181	A_33_P3775848	CLIC2	Hs.655445	chloride intracellular channel 2	Homo sapiens chloride intracellular channel 2 (CLIC2), mRNA [NM_001289]	18.90731	up	20.92745	up	1406.09060	77.00459	1114.58330	46.96363	Detected	Detected	Detected	Detected
182	A_33_P3221129	LRRN4CL	Hs.427449	LRRN4 C-terminal like	Homo sapiens LRRN4 C-terminal like (LRRN4CL), mRNA [NM_203422]	18.89436	up	21.51344	up	2375.74270	130.19678	1711.85230	70.16525	Detected	Detected	Detected	Detected

183	A_23_P137665	CHI3L1	Hs.382202	chitinase 3-like 1 (cartilage glycoprotein-39)	Homo sapiens chitinase 3-like 1 (cartilage glycoprotein-39) (CHI3L1), mRNA [NM_001276]	18.78310	up	17.45700	up	382.54202	21.08847	427.23523	21.58057	Detected	Detected	Detected	Detected
184	A_33_P3245290	AQP7P1		aquaporin 7 pseudogene 1	Homo sapiens aquaporin 7 pseudogene 1 (AQP7P1), non-coding RNA [NR_002817]	18.65274	up	31.35724	up	257.98795	14.32155	193.14859	5.43149	Detected	Compromised	Detected	Compromised
185	A_33_P3392829	ESPNL	Hs.127724	espin-like	Homo sapiens espin-like (ESPNL), mRNA [NM_194312]	18.63819	up	13.60274	up	100.17568	5.56534	94.01432	6.09443	Detected	Compromised	Detected	Compromised
186	A_33_P3328410	LOC100291851	Hs.710930	similar to Putative ubiquitin-like protein FUBI-like protein ENSP00000310146	PREDICTED: Homo sapiens similar to Putative ubiquitin-like protein FUBI-like protein ENSP00000310146 (LOC100291851), mRNA [XM_002344576]	18.52163	up	15.40838	up	309.13718	17.28244	217.92830	12.47160	Detected	Detected	Detected	Detected
187	A_33_P3328626					18.46876	up	6.50731	up	56.58904	3.17269	38.77446	5.25424	Detected	Compromised	Detected	Compromised
188	A_24_P397817	LEP	Hs.194236	leptin	Homo sapiens leptin (LEP), mRNA [NM_000230]	18.43113	up	10.13718	up	79.96177	4.49224	34.25934	2.98008	Detected	Compromised	Detected	Compromised
189	A_33_P3309491	PTPRU	Hs.19718	protein tyrosine phosphatase, receptor type, U	Homo sapiens protein tyrosine phosphatase, receptor type, U (PTPRU), transcript variant 3, mRNA [NM_005704]	18.24097	up	14.66093	up	23720.78300	1346.52400	16916.72500	1017.46650	Detected	Detected	Detected	Detected
190	A_32_P88880					18.09206	up	63.11618	up	261.83160	14.98536	222.54968	3.10922	Detected	Compromised	Detected	Compromised
191	A_23_P12082	CHI3L2	Hs.514840	chitinase 3-like 2	Homo sapiens chitinase 3-like 2 (CHI3L2), transcript variant 3, mRNA [NM_001025199]	18.05685	up	17.24178	up	495.44020	28.41073	384.22250	19.65017	Detected	Detected	Detected	Detected
192	A_32_P112910	UBL4B	Hs.374027	ubiquitin-like 4B	Homo sapiens ubiquitin-like 4B (UBL4B), mRNA [NM_203412]	18.04254	up	5.54037	up	105.18018	6.03628	112.14821	17.84922	Detected	Compromised	Detected	Detected
193	A_23_P25615	SOHLH2	Hs.124519	spermatogenesis and oogenesis specific basic helix-loop-helix 2	Homo sapiens spermatogenesis and oogenesis specific basic helix-loop-helix 2 (SOHLH2), mRNA [NM_017826]	18.01618	up	12.19154	up	51.85608	2.98037	33.81922	2.44608	Detected	Compromised	Detected	Compromised
194	A_24_P365679	RRP7B	Hs.534041	ribosomal RNA processing 7 homolog B (S. cerevisiae)	Homo sapiens cDNA FLJ40882 fis, clone UTERU2000424 [AK098201]	17.89545	up	8.14775	up	70.84905	4.09944	69.48125	7.51962	Detected	Compromised	Detected	Compromised
195	A_23_P418431	C6orf164	Hs.645177	chromosome 6 open reading frame 164	Homo sapiens chromosome 6 open reading frame 164 (C6orf164), non-coding RNA [NR_026784]	17.88613	up	7.72426	up	62.19009	3.60029	35.18921	4.01715	Detected	Compromised	Detected	Compromised
196	A_33_P3251552	RSPO4	Hs.444980	R-spondin family, member 4	Homo sapiens R-spondin family, member 4 (RSPO4), transcript variant 1, mRNA [NM_001029871]	17.86719	up	16.45114	up	888.90186	51.51464	711.03360	38.11181	Detected	Detected	Detected	Detected
197	A_23_P68031	STAT4	Hs.80642	signal transducer and activator of transcription 4	Homo sapiens signal transducer and activator of transcription 4 (STAT4), mRNA [NM_003151]	17.85223	up	13.42182	up	1149.00940	66.64448	871.92413	57.28394	Detected	Detected	Detected	Detected
198	A_33_P3317603	B4GALNT4	Hs.148074	beta-1,4-N-acetyl-galactosaminyl transferase 4	Homo sapiens beta-1,4-N-acetyl-galactosaminyl transferase 4 (B4GALNT4), mRNA [NM_178537]	17.81463	up	17.20743	up	336.60320	19.56476	374.65118	19.19891	Detected	Detected	Detected	Detected
199	A_33_P3374987	C7orf52	Hs.213044	chromosome 7 open reading frame 52	Homo sapiens chromosome 7 open reading frame 52 (C7orf52), mRNA [NM_198571]	17.78886	up	22.36739	up	279.63280	16.27696	216.27054	8.52604	Detected	Compromised	Detected	Compromised
200	A_33_P3341601	WDR86	Hs.647083	WD repeat domain 86	Homo sapiens WD repeat domain 86 (WDR86), mRNA [NM_198285]	17.74816	up	46.52995	up	166.58890	9.71910	155.19218	2.94105	Detected	Compromised	Detected	Compromised
201	A_33_P3288594	LOC100129055	Hs.148265	cyclin Y-like pseudogene	Homo sapiens cyclin Y-like pseudogene (LOC100129055), non-coding RNA [NR_024524]	17.66299	up	9.30161	up	82.89698	4.85968	35.88683	3.40206	Detected	Compromised	Detected	Compromised
202	A_23_P326893	CCDC151	Hs.124010	coiled-coil domain containing 151	Homo sapiens coiled-coil domain containing 151 (CCDC151), mRNA [NM_145045]	17.64206	up	18.17707	up	191.25830	11.22546	225.04561	10.91722	Detected	Compromised	Detected	Compromised
203	A_23_P136077	PARK2	Hs.132954	Parkinson disease (autosomal recessive, juvenile) 2, parkin	Homo sapiens Parkinson disease (autosomal recessive, juvenile) 2, parkin (PARK2), transcript variant 1, mRNA [NM_004562]	17.57617	up	9.96484	up	71.08962	4.18808	42.12795	3.72791	Detected	Compromised	Detected	Compromised
204	A_23_P214011	CDH6	Hs.171054	cadherin 6, type 2, K-cadherin (fetal kidney)	Homo sapiens cadherin 6, type 2, K-cadherin (fetal kidney) (CDH6), mRNA [NM_004932]	17.57215	up	12.25478	up	55.89781	3.29384	38.52780	2.77226	Detected	Compromised	Detected	Compromised
205	A_24_P195669					17.52130	up	7.05039	up	56.77738	3.35538	52.61725	6.58083	Detected	Compromised	Detected	Compromised
206	A_23_P502413	SERPINB4	Hs.123035	serpin peptidase inhibitor, clade B (ovalbumin), member 4	Homo sapiens serpin peptidase inhibitor, clade B (ovalbumin), member 4 (SERPINB4), mRNA [NM_002974]	17.51934	up	20.63824	up	130.46657	7.71107	122.02151	5.21350	Detected	Compromised	Detected	Compromised
207	A_23_P411761	CABP1	Hs.458482	calcium binding protein 1	Homo sapiens calcium binding protein 1 (CABP1), transcript variant 3, mRNA [NM_001033677]	17.49139	up	12.68175	up	50.30909	2.97821	37.61747	2.61563	Detected	Compromised	Detected	Compromised
208	A_33_P3627001	PEBP4	Hs.491242	phosphatidylethanolamine-binding protein 4	Homo sapiens phosphatidylethanolamine-binding protein 4 (PEBP4), mRNA [NM_144962]	17.40757	up	25.04212	up	2036.98270	121.16641	2251.50780	79.28079	Detected	Detected	Detected	Detected
209	A_23_P334173	LY75	Hs.153563	lymphocyte antigen 75	Homo sapiens lymphocyte antigen 75 (LY75), mRNA [NM_002349]	17.39153	up	20.53688	up	92.83244	5.52707	61.82533	2.65459	Detected	Compromised	Detected	Compromised
210	A_23_P76364	CD9	Hs.114286	CD9 molecule	Homo sapiens CD9 molecule (CD9), mRNA [NM_001769]	17.31517	up	15.03373	up	8418.92400	503.45750	8283.59800	485.86728	Detected	Detected	Detected	Detected
211	A_32_P74409	LOC387763	Hs.714890	hypothetical protein LOC387763	Homo sapiens hypothetical protein LOC387763 (LOC387763), mRNA [NM_001145033]	17.18526	up	16.48727	up	46052.15600	2774.76980	33126.72700	1771.72070	Detected	Detected	Detected	Detected
212	A_23_P31124	COL21A1	Hs.47629	collagen, type XXI, alpha 1	Homo sapiens collagen, type XXI, alpha 1 (COL21A1), mRNA [NM_030820]	16.99639	up	23.95970	up	230.03070	14.01400	192.30711	7.07749	Detected	Compromised	Detected	Compromised
213	A_32_P225816	PRDM16	Hs.99500	PR domain containing 16	Homo sapiens PR domain containing 16 (PRDM16), transcript variant 1, mRNA [NM_022114]	16.96804	up	19.55479	up	54.21896	3.30866	62.82385	2.83294	Detected	Compromised	Detected	Compromised
214	A_23_P320578	RGS16	Hs.413297	regulator of G-protein signaling 16	Homo sapiens regulator of G-protein signaling 16 (RGS16), mRNA [NM_002928]	16.86487	up	17.74958	up	1795.71150	110.25202	1776.87080	88.27409	Detected	Detected	Detected	Detected
215	A_23_P419150	VENTX	Hs.125231	VENT homeobox homolog (Xenopus laevis)	Homo sapiens VENT homeobox homolog (Xenopus laevis) (VENTX), mRNA [NM_014468]	16.73882	up	10.49100	up	56.10853	3.47086	35.47286	2.98157	Detected	Compromised	Detected	Compromised

216	A_32_P2452	TMTC1	Hs.401954	transmembrane and tetratricopeptide repeat containing 1	Homo sapiens transmembrane and tetratricopeptide repeat containing 1 (TMTC1), mRNA [NM_175861]	16.52263	up	16.74582	up	6265.59770	392.65980	4233.32600	222.91579	Detected	Detected	Detected	Detected
217	A_23_P107247	CACNA1G	Hs.591169	calcium channel, voltage-dependent, T type, alpha 1G subunit	Homo sapiens calcium channel, voltage-dependent, T type, alpha 1G subunit (CACNA1G), transcript variant 1, mRNA [NM_018896]	16.49892	up	16.28450	up	1343.89110	84.34154	1270.26770	68.78381	Detected	Detected	Detected	Detected
218	A_32_P24140	GAS2	Hs.632151	growth arrest-specific 2	Homo sapiens growth arrest-specific 2 (GAS2), transcript variant 1, mRNA [NM_005256]	16.49192	up	22.39991	up	92.59290	5.81353	78.18802	3.07793	Detected	Compromised	Detected	Compromised
219	A_23_P165414	KLHL23	Hs.655150	kelch-like 23 (Drosophila)	Homo sapiens kelch-like 23 (Drosophila) (KLHL23), mRNA [NM_144711]	16.48298	up	7.20114	up	47.26945	2.96946	37.71457	4.61821	Detected	Compromised	Detected	Compromised
220	A_23_P428129	CDKN1C	Hs.106070	cyclin-dependent kinase inhibitor 1C (p57, Kip2)	Homo sapiens cyclin-dependent kinase inhibitor 1C (p57, Kip2) (CDKN1C), transcript variant 1, mRNA [NM_000076]	16.46644	up	16.32702	up	9082.93000	571.16187	7648.93360	413.10422	Detected	Detected	Detected	Detected
221	A_23_P501754	CSF3	Hs.2233	colony stimulating factor 3 (granulocyte)	Homo sapiens colony stimulating factor 3 (granulocyte) (CSF3), transcript variant 1, mRNA [NM_000759]	16.44386	up	13.52571	up	5151.21830	324.36890	4555.96400	297.02005	Detected	Detected	Detected	Detected
222	A_23_P85693	GBP2	Hs.386567	guanylate binding protein 2, interferon-inducible	Homo sapiens guanylate binding protein 2, interferon-inducible (GBP2), mRNA [NM_004120]	16.30703	up	14.91568	up	1501.62720	95.34990	1054.83370	62.36007	Detected	Detected	Detected	Detected
223	A_23_P17420	BCAS1	Hs.400556	breast carcinoma amplified sequence 1	Homo sapiens breast carcinoma amplified sequence 1 (BCAS1), mRNA [NM_003657]	16.28021	up	8.99733	up	70.13487	4.46074	49.66695	4.86765	Detected	Compromised	Detected	Compromised
224	A_24_P460419	LRRC10B	Hs.441122	leucine rich repeat containing 10B	Homo sapiens leucine rich repeat containing 10B (LRRC10B), mRNA [NM_001145077]	16.19034	up	12.58499	up	150.32277	9.61395	51.78709	3.62856	Detected	Compromised	Detected	Compromised
225	A_23_P111361	PPIL6	Hs.32234	peptidylprolyl isomerase (cyclophilin)-like 6	Homo sapiens peptidylprolyl isomerase (cyclophilin)-like 6 (PPIL6), transcript variant 1, mRNA [NM_173672]	16.11174	up	27.13692	up	286.86790	18.43625	183.14062	5.95100	Detected	Detected	Detected	Compromised
226	A_24_P390928	TRAPPC6A	Hs.466929	trafficking protein particle complex 6A	Homo sapiens trafficking protein particle complex 6A (TRAPPC6A), mRNA [NM_024108]	16.05205	up	17.24298	up	3576.17380	230.68602	4485.03400	229.36072	Detected	Detected	Detected	Detected
227	A_24_P160680	CCDC40	Hs.202542	coiled-coil domain containing 40	Homo sapiens coiled-coil domain containing 40 (CCDC40), mRNA [NM_017950]	16.04955	up	17.46065	up	46.96877	3.03025	49.37777	2.49366	Detected	Compromised	Detected	Compromised
228	A_33_P3277527	LAMC3	Hs.201805	laminin, gamma 3	Homo sapiens laminin, gamma 3 (LAMC3), mRNA [NM_006059]	15.94822	up	11.95579	up	127.50391	8.27836	150.00008	11.06315	Detected	Compromised	Detected	Compromised
229	A_33_P3396537	CDH29	Hs.363312	cadherin-like 29	Homo sapiens cadherin-like 29 (CDH29), mRNA [NM_001007540]	15.91217	up	11.08265	up	49.31988	3.20942	34.05270	2.70940	Detected	Compromised	Detected	Compromised
230	A_24_P94402	MYCN	Hs.25960	v-myc myelocytomatosis viral related oncogene, neuroblastoma derived (avian)	Homo sapiens v-myc myelocytomatosis viral related oncogene, neuroblastoma derived (avian) (MYCN), mRNA [NM_005378]	15.90164	up	9.83994	up	49.64475	3.23269	30.36570	2.72118	Detected	Compromised	Detected	Compromised
231	A_23_P95231	CASC1	Hs.407771	cancer susceptibility candidate 1	Homo sapiens cancer susceptibility candidate 1 (CASC1), transcript variant 1, mRNA [NM_018272]	15.88912	up	12.17471	up	56.61573	3.68953	43.11943	3.12306	Detected	Compromised	Detected	Compromised
232	A_33_P3384078				Adenylate kinase domain-containing protein 2 [Source:UniProtKB/Swiss-Prot;Acc:Q5TCS8] [ENST00000355283]	15.83777	up	11.13639	up	81.36618	5.31965	72.64971	5.75248	Detected	Compromised	Detected	Compromised
233	A_32_P108254	FAM20A	Hs.268874	family with sequence similarity 20, member A	Homo sapiens family with sequence similarity 20, member A (FAM20A), transcript variant 1, mRNA [NM_017565]	15.80986	up	19.33295	up	2160.38400	141.49340	2084.30080	95.06654	Detected	Detected	Detected	Detected
234	A_24_P131646	MYL3	Hs.517939	myosin, light chain 3, alkali; ventricular, skeletal, slow	Homo sapiens myosin, light chain 3, alkali; ventricular, skeletal, slow (MYL3), mRNA [NM_000258]	15.78626	up	11.41208	up	144.23552	9.46076	136.31840	10.53308	Detected	Compromised	Detected	Compromised
235	A_33_P3291484	ST8SIA4	Hs.308628	ST8 alpha-N-acetyl-neuraminide alpha-2,8-sialyltransferase 4	Homo sapiens ST8 alpha-N-acetyl-neuraminide alpha-2,8-sialyltransferase 4 (ST8SIA4), transcript variant 1, mRNA [NM_005668]	15.77099	up	23.52642	up	235.49402	15.46159	155.52869	5.82935	Detected	Compromised	Detected	Compromised
236	A_33_P3320062	PLD1	Hs.382865	phospholipase D1, phosphatidylcholine-specific	Homo sapiens phospholipase D1, phosphatidylcholine-specific (PLD1), transcript variant 1, mRNA [NM_002662]	15.69643	up	9.63724	up	522.73880	34.48394	370.27994	33.87997	Detected	Detected	Detected	Detected
237	A_23_P340263	RNF175	Hs.388364	ring finger protein 175	Homo sapiens ring finger protein 175 (RNF175), mRNA [NM_173662]	15.65216	up	14.79071	up	1145.89500	75.80601	1278.16940	76.20176	Detected	Detected	Detected	Detected
238	A_33_P3854953	LOC440117	Hs.127100	hypothetical gene supported by BC037858	Homo sapiens hypothetical gene supported by BC037858, mRNA (cDNA clone IMAGE:5272477) [BC037858]	15.58360	up	2.45732	up	91.32793	6.06833	26.25056	9.41983	Detected	Compromised	Detected	Compromised
239	A_33_P3209960	RASGRP2	Hs.99491	RAS guanyl releasing protein 2 (calcium and DAG-regulated)	Homo sapiens RAS guanyl releasing protein 2 (calcium and DAG-regulated) (RASGRP2), transcript variant 2, mRNA [NM_153819]	15.55960	up	18.05850	up	1899.03060	126.37660	2270.63480	110.87439	Detected	Detected	Detected	Detected
240	A_23_P48740	DIO2	Hs.202354	deiodinase, iodothyronine, type II	Homo sapiens deiodinase, iodothyronine, type II (DIO2), transcript variant 1, mRNA [NM_013989]	15.54008	up	26.01893	up	2382.41240	158.74380	2019.19790	68.43132	Detected	Detected	Detected	Detected
241	A_24_P270496	NOVA1	Hs.31588	neuro-oncological ventral antigen 1	Homo sapiens neuro-oncological ventral antigen 1 (NOVA1), transcript variant 3, mRNA [NM_006491]	15.41141	up	29.73354	up	137.77759	9.25698	109.45982	3.24619	Detected	Compromised	Detected	Compromised
242	A_23_P252556	BAI3	Hs.13261	brain-specific angiogenesis inhibitor 3	Homo sapiens brain-specific angiogenesis inhibitor 3 (BAI3), mRNA [NM_001704]	15.32081	up	10.76405	up	54.80786	3.70420	37.94757	3.10866	Detected	Compromised	Detected	Compromised
243	A_24_P122137	LIF	Hs.2250	leukemia inhibitory factor (cholinergic differentiation factor)	Homo sapiens leukemia inhibitory factor (cholinergic differentiation factor) (LIF), mRNA [NM_002309]	15.31744	up	15.11951	up	1810.80360	122.41039	1770.29370	103.24599	Detected	Detected	Detected	Detected

244	A_24_P350437	THAP2	Hs.720665	THAP domain containing, apoptosis associated protein 2	Homo sapiens THAP domain containing, apoptosis associated protein 2 (THAP2), mRNA [NM_031435]	15.30183	up	7.74388	up	43.90980	2.97133	27.79999	3.16557	Detected	Compromised	Detected	Compromised
245	A_33_P3364869					15.24554	up	17.77186	up	7328.43100	497.73868	5648.88670	280.28220	Detected	Detected	Detected	Detected
246	A_33_P3227944	C17orf69	Hs.128813	chromosome 17 open reading frame 69	Homo sapiens chromosome 17 open reading frame 69 (C17orf69), transcript variant 2, non-coding RNA [NR_026906]	15.17439	up	13.54736	up	1349.81790	92.10795	1015.02660	66.06757	Detected	Detected	Detected	Detected
247	A_33_P3283824	SLC39A8	Hs.288034	solute carrier family 39 (zinc transporter), member 8	Homo sapiens solute carrier family 39 (zinc transporter), member 8 (SLC39A8), transcript variant 3, mRNA [NM_001135147]	15.17165	up	9.05527	up	50.50548	3.44698	36.34823	3.53954	Detected	Compromised	Detected	Compromised
248	A_33_P3308232	LOC401630		hypothetical LOC401630	Homo sapiens hypothetical LOC401630 (LOC401630), non-coding RNA [NR_002161]	15.10849	up	11.64846	up	124.01547	8.49939	91.63625	6.93688	Detected	Compromised	Detected	Compromised
249	A_23_P29096	PDE9A	Hs.473927	phosphodiesterase 9A	Homo sapiens phosphodiesterase 9A (PDE9A), transcript variant 1, mRNA [NM_002606]	15.09680	up	15.58961	up	433.75180	29.75018	438.25340	24.78880	Detected	Detected	Detected	Detected
250	A_23_P417853	MYO3B	Hs.671900	myosin IIIB	Homo sapiens myosin IIIB (MYO3B), transcript variant 2, mRNA [NM_138995]	15.09235	up	11.11161	up	50.28934	3.45026	36.54825	2.90038	Detected	Compromised	Detected	Compromised
251	A_23_P134854	CLDN23	Hs.183617	claudin 23	Homo sapiens claudin 23 (CLDN23), mRNA [NM_194284]	15.05430	up	14.75358	up	1764.39040	121.35770	1950.67290	116.58769	Detected	Detected	Detected	Detected
252	A_33_P3375934	NAMPT	Hs.489615	nicotinamide phosphoribosyltransferase	Homo sapiens nicotinamide phosphoribosyltransferase (NAMPT), mRNA [NM_005746]	15.04148	up	14.49525	up	26719.00800	1839.34390	19228.75800	1169.74430	Detected	Detected	Detected	Detected
253	A_24_P307653	SLC6A15	Hs.44424	solute carrier family 6 (neutral amino acid transporter), member 15	Homo sapiens solute carrier family 6 (neutral amino acid transporter), member 15 (SLC6A15), transcript variant 1, mRNA [NM_182767]	15.02631	up	17.51310	up	2143.47780	147.70651	1727.86880	86.99880	Detected	Detected	Detected	Detected
254	A_33_P3354847	C9orf171	Hs.201709	chromosome 9 open reading frame 171	Homo sapiens chromosome 9 open reading frame 171 (C9orf171), mRNA [NM_207417]	14.95810	up	22.35022	up	128.99500	8.92955	104.60102	4.12686	Detected	Compromised	Detected	Compromised
255	A_23_P43337	FREM1	Hs.709555	FRAS1 related extracellular matrix 1	Homo sapiens FRAS1 related extracellular matrix 1 (FREM1), mRNA [NM_144966]	14.78838	up	6.51907	up	58.50824	4.09666	43.25238	5.85045	Detected	Compromised	Detected	Compromised
256	A_32_P88231					14.74764	up	12.52694	up	221.12338	15.52549	132.96002	9.35926	Detected	Compromised	Detected	Compromised
257	A_23_P212469	ENTPD3	Hs.441145	ectonucleoside triphosphate diphosphohydrolase 3	Homo sapiens ectonucleoside triphosphate diphosphohydrolase 3 (ENTPD3), mRNA [NM_001248]	14.74358	up	7.69741	up	227.86139	16.00298	176.23895	20.18937	Detected	Compromised	Detected	Detected
258	A_23_P78037	CCL7	Hs.251526	chemokine (C-C motif) ligand 7	Homo sapiens chemokine (C-C motif) ligand 7 (CCL7), mRNA [NM_006273]	14.73203	up	15.28368	up	243.96648	17.14749	236.21362	13.62833	Detected	Detected	Detected	Compromised
259	A_23_P392476	AKD1	Hs.205144	adenylate kinase domain containing 1	Homo sapiens adenylate kinase domain containing 1 (AKD1), transcript variant 1, mRNA [NM_001145128]	14.69336	up	5.79418	up	45.02810	3.17319	26.56213	4.04237	Detected	Compromised	Detected	Compromised
260	A_33_P3223592	APOE	Hs.654439	apolipoprotein E	Homo sapiens apolipoprotein E (APOE), mRNA [NM_000041]	14.64105	up	12.61276	up	213789.52000	15119.84000	177031.81000	12376.75500	Detected	Detected	Detected	Detected
261	A_23_P108075	SLC7A10	Hs.58679	solute carrier family 7, (neutral amino acid transporter, y+ system) member 10	Homo sapiens solute carrier family 7, (neutral amino acid transporter, y+ system) member 10 (SLC7A10), mRNA [NM_019849]	14.50091	up	10.57297	up	50.11593	3.57860	32.91339	2.74499	Detected	Compromised	Detected	Compromised
262	A_33_P3382860	RND2	Hs.603111	Rho family GTPase 2	Homo sapiens Rho family GTPase 2 (RND2), mRNA [NM_005440]	14.49278	up	9.16711	up	149.69434	10.69515	132.49510	12.74480	Detected	Compromised	Detected	Compromised
263	A_33_P3871347	SNED1	Hs.471834	sushi, nidogen and EGF-like domains 1	Homo sapiens sushi, nidogen and EGF-like domains 1 (SNED1), mRNA [NM_001080437]	14.49096	up	14.95887	up	29000.48800	2072.24560	29473.91000	1737.41860	Detected	Detected	Detected	Detected
264	A_23_P41424	SLC39A8	Hs.288034	solute carrier family 39 (zinc transporter), member 8	Homo sapiens solute carrier family 39 (zinc transporter), member 8 (SLC39A8), transcript variant 1, mRNA [NM_022154]	14.48727	up	14.44510	up	8019.47270	573.18195	7354.79350	448.96796	Detected	Detected	Detected	Detected
265	A_23_P15450	TMEM100	Hs.173233	transmembrane protein 100	Homo sapiens transmembrane protein 100 (TMEM100), transcript variant 2, mRNA [NM_018286]	14.47442	up	44.45409	up	225.62056	16.14026	227.26352	4.50800	Detected	Compromised	Detected	Compromised
266	A_33_P3283964		Hs.722996		Putative uncharacterized protein ENSP00000355193 [Source:UniProtKB/TrEMBL;Acc:A6NIM1] [ENST00000442512]	14.40344	up	16.50703	up	445.12600	32.00000	265.32480	14.17341	Detected	Detected	Detected	Detected
267	A_33_P3365193	AMY1C	Hs.655232	amylase, alpha 1C (salivary)	Homo sapiens amylase, alpha 1C (salivary) (AMY1C), mRNA [NM_001008219]	14.29056	up	12.27161	up	852.28735	61.75469	670.78674	48.20017	Detected	Detected	Detected	Detected
268	A_24_P102293	SLITRK5	Hs.591208	SLIT and NTRK-like family, member 5	Homo sapiens SLIT and NTRK-like family, member 5 (SLITRK5), mRNA [NM_015567]	14.27035	up	9.13421	up	44.90061	3.25800	28.58114	2.75914	Detected	Compromised	Detected	Compromised
269	A_23_P414654	RAB37	Hs.351413	RAB37, member RAS oncogene family	Homo sapiens RAB37, member RAS oncogene family (RAB37), transcript variant 3, mRNA [NM_175738]	14.13913	up	14.77893	up	265.10898	19.41489	282.82678	16.87497	Detected	Detected	Detected	Detected
270	A_23_P65789	MCTP2	Hs.33368	multiple C2 domains, transmembrane 2	Homo sapiens multiple C2 domains, transmembrane 2 (MCTP2), transcript variant 1, mRNA [NM_018349]	14.09772	up	16.18181	up	78.35747	5.75526	49.24344	2.68341	Detected	Compromised	Detected	Compromised
271	A_23_P42882	CAMK2B	Hs.351887	calcium/calmodulin-dependent protein kinase II beta	Homo sapiens calcium/calmodulin-dependent protein kinase II beta (CAMK2B), transcript variant 6, mRNA [NM_172082]	14.07956	up	10.69363	up	548.92523	40.36987	601.17320	49.57243	Detected	Detected	Detected	Detected
272	A_23_P207632	ATP2A3	Hs.513870	ATPase, Ca++ transporting, ubiquitous	Homo sapiens ATPase, Ca++ transporting, ubiquitous (ATP2A3), transcript variant 5, mRNA [NM_174953]	14.07836	up	11.09807	up	582.04010	42.80889	392.92307	31.21948	Detected	Detected	Detected	Detected
273	A_23_P748	IRF6	Hs.591415	interferon regulatory factor 6	Homo sapiens interferon regulatory factor 6 (IRF6), mRNA [NM_006147]	14.00593	up	10.63017	up	42.24844	3.12343	31.67430	2.62744	Detected	Compromised	Detected	Compromised
274	A_33_P3378790	CASC1	Hs.407771	cancer susceptibility candidate 1	Homo sapiens cancer susceptibility candidate 1 (CASC1), transcript variant 3, mRNA [NM_001082972]	13.99620	up	9.70065	up	48.57039	3.59331	33.91716	3.08308	Detected	Compromised	Detected	Compromised

275	A_24_P592012	ZBTB46	Hs.585028	zinc finger and BTB domain containing 46	Zinc finger and BTB domain-containing protein 46 (BTB/POZ domain-containing protein 4)(Zinc finger protein 340) [Source:UniProtKB/Swiss-Prot;Acc:Q86UZ6] [ENST00000245663]	13.86121	up	17.09782	up	100.55039	7.51131	62.70635	3.23397	Detected	Compromised	Detected	Compromised
276	A_33_P3349761					13.84120	up	7.18725	up	43.08311	3.22305	56.32444	6.91035	Detected	Compromised	Detected	Compromised
277	A_32_P36143	LOC729088	Hs.416013	hypothetical protein LOC729088	PREDICTED: Homo sapiens hypothetical protein LOC729088 (LOC729088), miscRNA [XR_078341]	13.81794	up	24.92972	up	232.55809	17.42694	159.74730	5.65043	Detected	Detected	Detected	Compromised
278	A_24_P303145	ANKH	Hs.156727	ankylosis, progressive homolog (mouse)	Homo sapiens ankylosis, progressive homolog (mouse) (ANKH), mRNA [NM_054027]	13.81610	up	15.95897	up	8222.79500	616.26434	5899.31600	325.95840	Detected	Detected	Detected	Detected
279	A_23_P76749	GALNTL1	Hs.21035	UDP-N-acetyl-alpha-D-galactosamine:polypeptide N-acetylgalactosaminyltransferase-like 1	Homo sapiens UDP-N-acetyl-alpha-D-galactosamine:polypeptide N-acetylgalactosaminyltransferase-like 1 (GALNTL1), mRNA [NM_020692]	13.79476	up	14.68780	up	1317.66990	98.90668	1273.81790	76.47443	Detected	Detected	Detected	Detected
280	A_23_P23611	AMY1C	Hs.655232	amylase, alpha 1C (salivary)	Homo sapiens amylase, alpha 1C (salivary) (AMY1C), mRNA [NM_001008219]	13.76373	up	11.49081	up	1353.09200	101.79452	944.50604	72.48026	Detected	Detected	Detected	Detected
281	A_24_P390668	FMNL1	Hs.720131	formin-like 1	Homo sapiens formin-like 1 (FMNL1), mRNA [NM_005892]	13.70310	up	6.07807	up	107.11089	8.09372	170.83469	24.78425	Detected	Compromised	Detected	Detected
282	A_23_P13753	NFE2	Hs.75643	nuclear factor (erythroid-derived 2), 45kDa	Homo sapiens nuclear factor (erythroid-derived 2), 45kDa (NFE2), transcript variant 1, mRNA [NM_006163]	13.70127	up	18.28596	up	199.92067	15.10880	194.15591	9.36264	Detected	Compromised	Detected	Compromised
283	A_33_P3311750	CCDC144A	Hs.721149	coiled-coil domain containing 144A	Homo sapiens coiled-coil domain containing 144A (CCDC144A), mRNA [NM_014695]	13.65329	up	6.98650	up	99.35824	7.53528	44.80317	5.65477	Detected	Compromised	Detected	Compromised
284	A_33_P3347343	CCDC102B	Hs.280781	coiled-coil domain containing 102B	Homo sapiens coiled-coil domain containing 102B (CCDC102B), transcript variant 1, mRNA [NM_001093729]	13.65265	up	7.74551	up	77.50802	5.87845	68.13087	7.75639	Detected	Compromised	Detected	Compromised
285	A_33_P3218649	GPM6B	Hs.495710	glycoprotein M6B	Homo sapiens glycoprotein M6B (GPM6B), transcript variant 1, mRNA [NM_001001995]	13.59791	up	13.03356	up	28022.99400	2133.90670	22387.32600	1514.62400	Detected	Detected	Detected	Detected
286	A_23_P206280	GPR56	Hs.513633	G protein-coupled receptor 56	Homo sapiens G protein-coupled receptor 56 (GPR56), transcript variant 3, mRNA [NM_201525]	13.59210	up	11.97638	up	10218.61800	778.46450	7705.78900	567.35785	Detected	Detected	Detected	Detected
287	A_33_P3369058	LRRK2	Hs.187636	leucine-rich repeat kinase 2	Homo sapiens leucine-rich repeat kinase 2 (LRRK2), mRNA [NM_198578]	13.55443	up	15.82110	up	380.78506	29.08921	272.88240	15.20913	Detected	Detected	Detected	Detected
288	A_23_P386384	C1orf87	Hs.47385	chromosome 1 open reading frame 87	Homo sapiens chromosome 1 open reading frame 87 (C1orf87), mRNA [NM_152377]	13.52864	up	11.27687	up	55.31427	4.23366	48.83391	3.81855	Detected	Compromised	Detected	Compromised
289	A_23_P136936	SYP	Hs.632804	synaptophysin	Homo sapiens synaptophysin (SYP), mRNA [NM_003179]	13.49859	up	15.68671	up	51.06352	3.91702	59.05452	3.31961	Detected	Compromised	Detected	Compromised
290	A_33_P3364864	NAMPT	Hs.489615	nicotinamide phosphoribosyltransferase	Homo sapiens nicotinamide phosphoribosyltransferase (NAMPT), mRNA [NM_005746]	13.45482	up	13.70757	up	2450.68820	188.60071	2941.43900	189.21902	Detected	Detected	Detected	Detected
291	A_23_P149189	ZP4	Hs.136241	zona pellucida glycoprotein 4	Homo sapiens zona pellucida glycoprotein 4 (ZP4), mRNA [NM_021186]	13.45369	up	15.25292	up	196.96684	15.15949	187.11588	10.81741	Detected	Compromised	Detected	Compromised
292	A_23_P57155	CHGB	Hs.516874	chromogranin B (secretogranin 1)	Homo sapiens chromogranin B (secretogranin 1) (CHGB), mRNA [NM_001819]	13.32786	up	9.76589	up	100.27039	7.79014	101.51112	9.16573	Detected	Compromised	Detected	Compromised
293	A_33_P3397865	TNNT1	Hs.631558	troponin T type 1 (skeletal, slow)	Homo sapiens troponin T type 1 (skeletal, slow) (TNNT1), transcript variant 1, mRNA [NM_003283]	13.32465	up	11.63226	up	4095.03610	318.22552	4024.74560	305.09824	Detected	Detected	Detected	Detected
294	A_23_P347169	MTUS1	Hs.7946	microtubule associated tumor suppressor 1	Homo sapiens microtubule associated tumor suppressor 1 (MTUS1), transcript variant 1, mRNA [NM_001001924]	13.20848	up	7.99117	up	45.87645	3.59642	27.55657	3.04075	Detected	Compromised	Detected	Compromised
295	A_23_P216448	NFIB	Hs.644095	nuclear factor I/B	Homo sapiens nuclear factor I/B (NFIB), mRNA [NM_005596]	13.20284	up	12.47033	up	743.03174	58.27377	560.78570	39.65376	Detected	Detected	Detected	Detected
296	A_32_P50223	PLK5P	Hs.195352	polo-like kinase 5 pseudogene	Homo sapiens polo-like kinase 5 pseudogene (PLK5P), non-coding RNA [NR_026557]	13.16503	up	11.32671	up	167.70512	13.19038	121.56341	9.46378	Detected	Compromised	Detected	Compromised
297	A_33_P3725324	USP9Y	Hs.598540	ubiquitin specific peptidase 9, Y-linked	Homo sapiens ubiquitin specific peptidase 9, Y-linked (USP9Y), mRNA [NM_004654]	13.14798	up	12.81610	up	38.07638	2.99868	35.94472	2.47312	Detected	Compromised	Detected	Compromised
298	A_33_P3212172	SNX22	Hs.708268	sorting nexin 22	Homo sapiens sorting nexin 22 (SNX22), mRNA [NM_024798]	13.14327	up	11.05008	up	40.37183	3.18059	33.23529	2.65216	Detected	Compromised	Detected	Compromised
299	A_23_P25706	CLMN	Hs.301478	calmin (calponin-like, transmembrane)	Homo sapiens calmin (calponin-like, transmembrane) (CLMN), mRNA [NM_024734]	13.14211	up	9.79910	up	275.35977	21.69544	269.68890	24.26848	Detected	Detected	Detected	Detected
300	A_24_P224727	CEBPA	Hs.699463	CCAAT/enhancer binding protein (C/EBP), alpha	Homo sapiens CCAAT/enhancer binding protein (C/EBP), alpha (CEBPA), mRNA [NM_004364]	13.10156	up	7.96280	up	792.61420	62.64290	731.16410	80.96820	Detected	Detected	Detected	Detected
301	A_24_P170983	ESPNL	Hs.127724	espin-like	Homo sapiens espin-like (ESPNL), mRNA [NM_194312]	13.08612	up	11.43897	up	3088.87940	244.41240	3319.10820	255.85866	Detected	Detected	Detected	Detected
302	A_33_P3266674	ZBTB46	Hs.585028	zinc finger and BTB domain containing 46	Homo sapiens zinc finger and BTB domain containing 46 (ZBTB46), mRNA [NM_025224]	13.05072	up	12.13737	up	242.48145	19.23876	238.95975	17.36064	Detected	Detected	Detected	Detected
303	A_23_P334857	ZNF385B	Hs.655005	zinc finger protein 385B	Homo sapiens zinc finger protein 385B (ZNF385B), transcript variant 1, mRNA [NM_152520]	13.03578	up	9.44226	up	39.53168	3.14008	28.19162	2.63275	Detected	Compromised	Detected	Compromised
304	A_24_P7121	NSUN7	Hs.570821	NOL1/NOP2/Sun domain family, member 7	Homo sapiens NOL1/NOP2/Sun domain family, member 7 (NSUN7), mRNA [NM_024677]	12.90857	up	7.35239	up	37.31111	2.99291	20.51969	2.46098	Detected	Compromised	Detected	Compromised
305	A_23_P428298	UNC5CL	Hs.158357	unc-5 homolog C (C. elegans)-like	Homo sapiens unc-5 homolog C (C. elegans)-like (UNC5CL), mRNA [NM_173561]	12.90159	up	10.57598	up	90.28457	7.24609	61.76273	5.14957	Detected	Compromised	Detected	Compromised

306	A_33_P3226995	CD7	Hs.36972	CD7 molecule	Homo sapiens CD7 molecule (CD7), mRNA [NM_006137]	12.89459	up	69.95476	up	438.65160	35.22453	340.17343	4.28794	Detected	Detected	Detected	Compromised
307	A_33_P3317613	SYN2	Hs.445503	synapsin II	Homo sapiens synapsin II (SYN2), transcript variant IIa, mRNA [NM_133625]	12.85199	up	18.44367	up	131.70909	10.61154	102.00219	4.87672	Detected	Compromised	Detected	Compromised
308	A_23_P421811	C20orf132	Hs.349125	chromosome 20 open reading frame 132	Homo sapiens chromosome 20 open reading frame 132 (C20orf132), transcript variant 1, mRNA [NM_152503]	12.84612	up	9.23524	up	269.55347	21.72731	216.83226	20.70338	Detected	Detected	Detected	Detected
309	A_33_P3355946					12.80525	up	9.06953	up	38.25811	3.09363	31.96529	3.10784	Detected	Compromised	Detected	Compromised
310	A_23_P426663	MITF	Hs.166017	microphthalmia-associated transcription factor	Homo sapiens microphthalmia-associated transcription factor (MITF), transcript variant 1, mRNA [NM_198159]	12.80226	up	16.14208	up	549.13640	44.41470	367.20917	20.05947	Detected	Detected	Detected	Detected
311	A_23_P150609	IGF2	Hs.272259	insulin-like growth factor 2 (somatomedin A)	Homo sapiens insulin-like growth factor 2 (somatomedin A) (IGF2), transcript variant 1, mRNA [NM_000612]	12.70947	up	10.49984	up	64196.12500	5230.15230	42567.65600	3574.88800	Detected	Detected	Detected	Detected
312	A_23_P153964	INHBB	Hs.1735	inhibin, beta B	Homo sapiens inhibin, beta B (INHBB), mRNA [NM_002193]	12.63014	up	11.19111	up	1161.98900	95.26359	1057.78110	83.34669	Detected	Detected	Detected	Detected
313	A_33_P3374623	ABCA7	Hs.134514	ATP-binding cassette, sub-family A (ABC1), member 7	Homo sapiens ATP-binding cassette, sub-family A (ABC1), member 7 (ABCA7), mRNA [NM_019112]	12.58678	up	12.72510	up	312.98970	25.74829	378.68980	26.24146	Detected	Detected	Detected	Detected
314	A_33_P3209962	RASGRP2	Hs.99491	RAS guanyl releasing protein 2 (calcium and DAG-regulated)	Homo sapiens RAS guanyl releasing protein 2 (calcium and DAG-regulated) (RASGRP2), transcript variant 2, mRNA [NM_153819]	12.56661	up	17.65408	up	753.44965	62.08250	906.86570	45.29638	Detected	Detected	Detected	Detected
315	A_33_P3723580	LOC339975	Hs.639361	hypothetical protein LOC339975	Homo sapiens hypothetical protein LOC339975, mRNA (cDNA clone IMAGE:5548761), partial cds [BC042049]	12.51843	up	7.42321	up	47.22739	3.90641	27.87228	3.31090	Detected	Compromised	Detected	Compromised
316	A_24_P248185	C12orf53	Hs.44067	chromosome 12 open reading frame 53	Homo sapiens chromosome 12 open reading frame 53 (C12orf53), mRNA [NM_153685]	12.49081	up	11.59670	up	1478.97810	122.60394	787.68616	59.89415	Detected	Detected	Detected	Detected
317	A_24_P411121	TNFRSF18	Hs.212680	tumor necrosis factor receptor superfamily, member 18	Homo sapiens tumor necrosis factor receptor superfamily, member 18 (TNFRSF18), transcript variant 2, mRNA [NM_148901]	12.43651	up	19.00104	up	383.77170	31.95271	386.92190	17.95608	Detected	Detected	Detected	Detected
318	A_33_P3310189	ADRB1	Hs.99913	adrenergic, beta-1-, receptor	Homo sapiens adrenergic, beta-1-, receptor (ADRB1), mRNA [NM_000684]	12.42670	up	10.40225	up	37.56814	3.13038	30.74129	2.60592	Detected	Compromised	Detected	Compromised
319	A_33_P3239185	SYT7	Hs.502730	synaptotagmin VII	Homo sapiens synaptotagmin VII (SYT7), mRNA [NM_004200]	12.38018	up	13.01602	up	1937.53640	162.05264	1367.36980	92.63463	Detected	Detected	Detected	Detected
320	A_24_P321766	SERPINA5	Hs.159628	serpin peptidase inhibitor, clade A (alpha-1 antiproteinase, antitrypsin), member 5	Homo sapiens serpin peptidase inhibitor, clade A (alpha-1 antiproteinase, antitrypsin), member 5 (SERPINA5), mRNA [NM_000624]	12.35275	up	5.03303	up	38.37420	3.21669	34.30006	6.00940	Detected	Compromised	Detected	Compromised
321	A_32_P83784	ARAP2	Hs.479451	ArfGAP with RhoGAP domain, ankyrin repeat and PH domain 2	Homo sapiens ArfGAP with RhoGAP domain, ankyrin repeat and PH domain 2 (ARAP2), mRNA [NM_015230]	12.31989	up	9.16298	up	36.46962	3.06519	26.73872	2.57318	Detected	Compromised	Detected	Compromised
322	A_33_P3246885	DMKN	Hs.417795	dermokine	Homo sapiens dermokine (DMKN), transcript variant 3, mRNA [NM_001126056]	12.31044	up	24.94187	up	4826.60350	405.97660	5578.00240	197.20377	Detected	Detected	Detected	Detected
323	A_23_P501933	CACNG6	Hs.631560	calcium channel, voltage-dependent, gamma subunit 6	Homo sapiens calcium channel, voltage-dependent, gamma subunit 6 (CACNG6), transcript variant 1, mRNA [NM_145814]	12.28915	up	22.41440	up	358.90784	30.24085	334.77650	13.17023	Detected	Detected	Detected	Compromised
324	A_24_P6125	KCNJ4	Hs.32505	potassium inwardly-rectifying channel, subfamily J, member 4	Homo sapiens potassium inwardly-rectifying channel, subfamily J, member 4 (KCNJ4), transcript variant 1, mRNA [NM_152868]	12.25405	up	15.31211	up	54.32608	4.59052	42.75810	2.46235	Detected	Compromised	Detected	Compromised
325	A_24_P122921	BCL2L11	Hs.469658	BCL2-like 11 (apoptosis facilitator)	Homo sapiens BCL2-like 11 (apoptosis facilitator) (BCL2L11), transcript variant 1, mRNA [NM_138621]	12.20752	up	12.94587	up	1824.21410	154.73251	1361.43690	92.73245	Detected	Detected	Detected	Detected
326	A_33_P3365750	EML5	Hs.558671	echinoderm microtubule associated protein like 5	Homo sapiens echinoderm microtubule associated protein like 5 (EML5), mRNA [NM_183387]	12.18421	up	6.09295	up	42.74799	3.63289	22.00829	3.18511	Detected	Compromised	Detected	Compromised
327	A_23_P45185	FIGF	Hs.11392	c-fos induced growth factor (vascular endothelial growth factor D)	Homo sapiens c-fos induced growth factor (vascular endothelial growth factor D) (FIGF), mRNA [NM_004469]	12.11088	up	7.40567	up	37.73736	3.22648	22.50844	2.68007	Detected	Compromised	Detected	Compromised
328	A_23_P392738	DAZ2	Hs.592257	deleted in azoospermia 2	Homo sapiens deleted in azoospermia 2 (DAZ2), transcript variant 2, mRNA [NM_001005785]	12.10560	up	3.35183	up	40.79509	3.48943	12.92762	3.40097	Detected	Compromised	Compromised	Compromised
329	A_23_P125588	TNMD	Hs.132957	tenomodulin	Homo sapiens tenomodulin (TNMD), mRNA [NM_022144]	12.10195	up	10.22953	up	34.44680	2.94732	28.06541	2.41925	Detected	Compromised	Detected	Compromised
330	A_23_P145786	MLXIPL	Hs.647055	MLX interacting protein-like	Homo sapiens MLX interacting protein-like (MLXIPL), transcript variant 1, mRNA [NM_032951]	12.08586	up	15.49834	up	186.47588	15.97638	154.20868	8.77383	Detected	Detected	Detected	Compromised
331	A_33_P3531970	LOC643542	Hs.652901	hypothetical protein LOC643542	Homo sapiens hypothetical protein LOC643542, mRNA (cDNA clone IMAGE:5192916) [BC053410]	12.04224	up	6.94601	up	35.09199	3.01741	19.52180	2.47828	Detected	Compromised	Detected	Compromised
332	A_24_P515319	FAM90A7	Hs.715411	family with sequence similarity 90, member A7	Homo sapiens family with sequence similarity 90, member A7 (FAM90A7), mRNA [NM_001136572]	12.00210	up	17.24830	up	335.25464	28.92349	316.86030	16.19896	Detected	Detected	Detected	Detected
333	A_24_P109214	APOC1	Hs.110675	apolipoprotein C-I	Homo sapiens apolipoprotein C-I (APOC1), mRNA [NM_001645]	11.97815	up	12.25198	up	3813.63400	329.67236	2614.95170	188.20149	Detected	Detected	Detected	Detected
334	A_33_P3263497				Sushi, nidogen and EGF-like domain-containing protein 1 Precursor (Insulin-responsive sequence DNA-binding protein 1)(IRE-BP1) [Source:UniProtKB/Swiss-Prot;Acc:Q8TER0] [ENST00000401884]	11.96735	up	13.17509	up	779.20330	67.41958	841.68207	56.33265	Detected	Detected	Detected	Detected

335	A_23_P165788	PHOSPHO2	Hs.655150	phosphatase, orphan 2	Homo sapiens phosphatase, orphan 2 (PHOSPHO2), mRNA [NM_001008489]	11.94623	up	10.98770	up	2624.51440	227.48424	2228.56540	178.84800	Detected	Detected	Detected	Detected
336	A_23_P149858	ELOVL3	Hs.302130	elongation of very long chain fatty acids (FEN1/Elo2, SUR4/Elo3, yeast)-like 3	Homo sapiens elongation of very long chain fatty acids (FEN1/Elo2, SUR4/Elo3, yeast)-like 3 (ELOVL3), mRNA [NM_152310]	11.86257	up	10.54188	up	221.83790	19.36378	259.45157	21.70221	Detected	Detected	Detected	Detected
337	A_23_P149064	PTPRU	Hs.19718	protein tyrosine phosphatase, receptor type, U	Homo sapiens protein tyrosine phosphatase, receptor type, U (PTPRU), transcript variant 3, mRNA [NM_005704]	11.85507	up	12.43588	up	687.14777	60.01766	621.86780	44.09478	Detected	Detected	Detected	Detected
338	A_32_P94160	PRKAA2	Hs.437039	protein kinase, AMP-activated, alpha 2 catalytic subunit	Homo sapiens protein kinase, AMP-activated, alpha 2 catalytic subunit (PRKAA2), mRNA [NM_006252]	11.82062	up	8.44890	up	53.61281	4.69636	27.89293	2.91112	Detected	Compromised	Detected	Compromised
339	A_33_P3320159	GSDMC	Hs.133244	gasdermin C	Homo sapiens gasdermin C (GSDMC), mRNA [NM_031415]	11.81687	up	2.74925	up	35.77604	3.13489	9.35923	3.00187	Detected	Compromised	Compromised	Compromised
340	A_33_P3420862	PAGE2B	Hs.293317	P antigen family, member 2B	Homo sapiens P antigen family, member 2B (PAGE2B), mRNA [NM_001015038]	11.79107	up	13.03769	up	51.24704	4.50037	37.86754	2.56113	Detected	Compromised	Detected	Compromised
341	A_33_P3320079	NFIB	Hs.644095	nuclear factor I/B	Homo sapiens nuclear factor I/B (NFIB), mRNA [NM_005596]	11.77349	up	11.11541	up	295.99106	26.03193	284.66965	22.58297	Detected	Detected	Detected	Detected
342	A_24_P10226	SEMA6D	Hs.511265	sema domain, transmembrane domain (TM), and cytoplasmic domain, (semaphorin) 6D	Homo sapiens sema domain, transmembrane domain (TM), and cytoplasmic domain, (semaphorin) 6D (SEMA6D), transcript variant 6, mRNA [NM_024966]	11.75831	up	3.90316	up	41.98612	3.69738	19.02612	4.29832	Detected	Compromised	Detected	Compromised
343	A_33_P3210099	ALPK3	Hs.459183	alpha-kinase 3	Homo sapiens alpha-kinase 3 (ALPK3), mRNA [NM_020778]	11.73128	up	5.45540	up	70.29649	6.20471	31.45920	5.08495	Detected	Compromised	Detected	Compromised
344	A_33_P3251492	hCG_1994695		similar to hCG1994695	Homo sapiens similar to hCG1994695 (LOC100133669), non-coding RNA [NR_026913]	11.72778	up	13.62956	up	325.67563	28.75427	233.40407	15.10053	Detected	Detected	Detected	Compromised
345	A_33_P3211666	IL18R1	Hs.469521	interleukin 18 receptor 1	Homo sapiens interleukin 18 receptor 1 (IL18R1), mRNA [NM_003855]	11.72612	up	20.75935	up	72.12494	6.36890	59.36427	2.52161	Detected	Compromised	Detected	Compromised
346	A_23_P378416	GPM6B	Hs.495710	glycoprotein M6B	Homo sapiens glycoprotein M6B (GPM6B), transcript variant 2, mRNA [NM_001001996]	11.72039	up	12.03249	up	17453.87700	1541.99450	9502.02800	696.34796	Detected	Detected	Detected	Detected
347	A_33_P3299066	NR4A2	Hs.563344	nuclear receptor subfamily 4, group A, member 2	Homo sapiens nuclear receptor subfamily 4, group A, member 2 (NR4A2), mRNA [NM_006186]	11.71749	up	17.23327	up	703.23956	62.14436	714.76166	36.57287	Detected	Detected	Detected	Detected
348	A_33_P3319967	ARG1	Hs.440934	arginase, liver	Homo sapiens arginase, liver (ARG1), mRNA [NM_000045]	11.68063	up	11.18537	up	34.73758	3.07940	32.85294	2.58994	Detected	Compromised	Detected	Compromised
349	A_23_P143247	TSHZ2	Hs.473117	teashirt zinc finger homeobox 2	Homo sapiens teashirt zinc finger homeobox 2 (TSHZ2), mRNA [NM_173485]	11.60447	up	7.70209	up	237.28267	21.17259	189.07724	21.64692	Detected	Detected	Detected	Detected
350	A_33_P3320082	NFIB	Hs.644095	nuclear factor I/B	Homo sapiens nuclear factor I/B (NFIB), mRNA [NM_005596]	11.55030	up	14.37200	up	134.71878	12.07725	99.95924	6.13298	Detected	Compromised	Detected	Compromised
351	A_32_P112592	LOC339524		hypothetical LOC339524	Homo sapiens hypothetical LOC339524 (LOC339524), transcript variant 5, non-coding RNA [NR_026989]	11.53217	up	12.94134	up	1059.45430	95.12710	653.98390	44.56083	Detected	Detected	Detected	Detected
352	A_33_P3422289					11.52742	up	13.96167	up	1174.75870	105.52359	924.01980	58.35923	Detected	Detected	Detected	Detected
353	A_23_P40108	COL9A3	Hs.716639	collagen, type IX, alpha 3	Homo sapiens collagen, type IX, alpha 3 (COL9A3), mRNA [NM_001853]	11.52205	up	11.16829	up	1469.43380	132.05458	1365.08750	107.78027	Detected	Detected	Detected	Detected
354	A_23_P421306	SYT12	Hs.287636	synaptotagmin XII	Homo sapiens synaptotagmin XII (SYT12), mRNA [NM_177963]	11.49538	up	27.55599	up	141.13390	12.71280	106.00036	3.39201	Detected	Compromised	Detected	Compromised
355	A_33_P3240018	PDE3B	Hs.445711	phosphodiesterase 3B, cGMP-inhibited	Homo sapiens phosphodiesterase 3B, cGMP-inhibited (PDE3B), mRNA [NM_000922]	11.49484	up	1.94775	up	242.72777	21.86501	188.31978	85.25655	Detected	Detected	Detected	Detected
356	A_33_P3408042	OR2K2	Hs.381312	olfactory receptor, family 2, subfamily K, member 2	Homo sapiens olfactory receptor, family 2, subfamily K, member 2 (OR2K2), mRNA [NM_205859]	11.42644	up	9.28767	up	40.01284	3.62595	33.20751	3.15279	Detected	Compromised	Detected	Compromised
357	A_24_P79040	CAPN12	Hs.712632	calpain 12	Homo sapiens calpain 12 (CAPN12), mRNA [NM_144691]	11.34787	up	30.22135	up	80.27560	7.32491	91.66485	2.67457	Detected	Compromised	Detected	Compromised
358	A_23_P128323	SCNN1A	Hs.591047	sodium channel, nonvoltage-gated 1 alpha	Homo sapiens sodium channel, nonvoltage-gated 1 alpha (SCNN1A), transcript variant 1, mRNA [NM_001038]	11.33340	up	3.34622	up	38.91761	3.55565	29.74988	7.83964	Detected	Compromised	Detected	Compromised
359	A_23_P382705	TMTC2	Hs.577775	transmembrane and tetratricopeptide repeat containing 2	Homo sapiens transmembrane and tetratricopeptide repeat containing 2 (TMTC2), mRNA [NM_152588]	11.32137	up	23.86380	up	122.84638	11.23560	91.62411	3.38560	Detected	Compromised	Detected	Compromised
360	A_33_P3315814	LOC728208	Hs.647892	hypothetical protein LOC728208	PREDICTED: Homo sapiens hypothetical protein LOC728208 (LOC728208), miscRNA [XR_040782]	11.27692	up	5.47531	up	34.23108	3.14314	16.29635	2.62451	Detected	Compromised	Detected	Compromised
361	A_23_P88033	FGF14	Hs.508616	fibroblast growth factor 14	Homo sapiens fibroblast growth factor 14 (FGF14), transcript variant 2, mRNA [NM_175929]	11.27546	up	11.60610	up	39.68076	3.64400	39.58983	3.00790	Detected	Compromised	Detected	Compromised
362	A_33_P3413355	CHST1	Hs.104576	carbohydrate (keratan sulfate Gal-6) sulfotransferase 1	Homo sapiens carbohydrate (keratan sulfate Gal-6) sulfotransferase 1 (CHST1), mRNA [NM_003654]	11.25781	up	12.21776	up	703.24930	64.68276	387.51987	27.96841	Detected	Detected	Detected	Detected
363	A_24_P120251	TM4SF18	Hs.22026	transmembrane 4 L six family member 18	Homo sapiens transmembrane 4 L six family member 18 (TM4SF18), mRNA [NM_138786]	11.25759	up	10.90936	up	34.86007	3.20639	32.86276	2.65626	Detected	Compromised	Detected	Compromised
364	A_24_P923102	KLHL23	Hs.574626	kelch-like 23 (Drosophila)	Kelch-like protein 23 [Source:UniProtKB/Swiss-Prot;Acc:Q8NBE8] [ENST00000392647]	11.21242	up	11.64489	up	40.36454	3.72764	41.62518	3.15200	Detected	Compromised	Detected	Compromised
365	A_23_P142738	TMEM178	Hs.40808	transmembrane protein 178	Homo sapiens transmembrane protein 178 (TMEM178), mRNA [NM_152390]	11.20162	up	6.64857	up	65.16959	6.02417	54.29431	7.20098	Detected	Compromised	Detected	Compromised
366	A_23_P216108	ANK1	Hs.654438	ankyrin 1, erythrocytic	Homo sapiens ankyrin 1, erythrocytic (ANK1), transcript variant 3, mRNA [NM_000037]	11.18899	up	8.89323	up	353.00410	32.66795	326.95584	32.41865	Detected	Detected	Detected	Detected

367	A_23_P35564	SEC31B	Hs.18889	SEC31 homolog B (S. cerevisiae)	Homo sapiens SEC31 homolog B (S. cerevisiae) (SEC31B), mRNA [NM_015490]	11.16450	up	13.66384	up	1824.37930	169.20332	1382.33070	89.20821	Detected	Detected	Detected	Detected
368	A_33_P3405459	C20orf195	Hs.197755	chromosome 20 open reading frame 195	Homo sapiens chromosome 20 open reading frame 195 (C20orf195), mRNA [NM_024059]	11.14529	up	11.50988	up	1157.32290	107.52171	954.09510	73.09479	Detected	Detected	Detected	Detected
369	A_23_P163492	BAIAP3	Hs.458427	BAI1-associated protein 3	Homo sapiens BAI1-associated protein 3 (BAIAP3), mRNA [NM_003933]	11.08961	up	8.88073	up	41.67684	3.89145	33.76196	3.35231	Detected	Compromised	Detected	Compromised
370	A_33_P3355937	LOC284276	Hs.390287	hypothetical LOC284276	Homo sapiens hypothetical LOC284276 (LOC284276), non-coding RNA [NR_015417]	11.08263	up	3.02561	up	43.14889	4.03144	29.68110	8.65033	Detected	Compromised	Detected	Compromised
371	A_23_P29005	SAMSN1	Hs.473341	SAM domain, SH3 domain and nuclear localization signals 1	Homo sapiens SAM domain, SH3 domain and nuclear localization signals 1 (SAMSN1), mRNA [NM_022136]	11.06859	up	8.56369	up	32.63537	3.05302	24.83676	2.55741	Detected	Compromised	Detected	Compromised
372	A_33_P3243887	IL11	Hs.467304	interleukin 11	Homo sapiens interleukin 11 (IL11), mRNA [NM_000641]	11.04859	up	11.98718	up	12435.78600	1165.46530	8772.45800	645.31230	Detected	Detected	Detected	Detected
373	A_24_P658427	NFIB	Hs.644095	nuclear factor I/B	Homo sapiens nuclear factor I/B (NFIB), mRNA [NM_005596]	11.03872	up	13.51909	up	3430.19040	321.76053	1965.67790	128.21251	Detected	Detected	Detected	Detected
374	A_23_P94840	DYNLRB2	Hs.98849	dynein, light chain, roadblock-type 2	Homo sapiens dynein, light chain, roadblock-type 2 (DYNLRB2), mRNA [NM_130897]	11.00938	up	13.89792	up	253.64806	23.85622	220.48407	13.98920	Detected	Detected	Detected	Detected
375	A_33_P3318414	HMHA1	Hs.465521	histocompatibility (minor) HA-1	Homo sapiens histocompatibility (minor) HA-1 (HMHA1), mRNA [NM_012292]	10.99954	up	10.76441	up	9852.38500	927.47000	7999.04700	655.25960	Detected	Detected	Detected	Detected
376	A_24_P365349	CACNG7		calcium channel, voltage-dependent, gamma subunit 7	Voltage-dependent calcium channel gamma-7 subunit (Neuronal voltage-gated calcium channel gamma-7 subunit) [Source:UniProtKB/Swiss-Prot;Acc:P62955] [ENST00000391767]	10.99320	up	12.71972	up	7325.11400	689.95930	6722.64060	466.04500	Detected	Detected	Detected	Detected
377	A_24_P943566		Hs.436996		Phosphatase and actin regulator 1 [Source:UniProtKB/Swiss-Prot;Acc:Q9C0D0] [ENST00000379350]	10.96246	up	3.48536	up	51.18278	4.83447	14.03618	3.55113	Detected	Compromised	Compromised	Compromised
378	A_33_P3316539	SLC7A2	Hs.448520	solute carrier family 7 (cationic amino acid transporter, y+ system), member 2	Homo sapiens solute carrier family 7 (cationic amino acid transporter, y+ system), member 2 (SLC7A2), transcript variant 2, mRNA [NM_001008539]	10.95091	up	12.26519	up	2980.82370	281.85086	2685.27800	193.05470	Detected	Detected	Detected	Detected
379	A_23_P318904	SERTAD4	Hs.600545	SERTA domain containing 4	Homo sapiens SERTA domain containing 4 (SERTAD4), mRNA [NM_019605]	10.94622	up	10.33855	up	405.99750	38.40541	228.23148	19.46622	Detected	Detected	Detected	Detected
380	A_33_P3395008	ACOXL	Hs.253320	acyl-Coenzyme A oxidase-like	Homo sapiens acyl-Coenzyme A oxidase-like (ACOXL), transcript variant 1, mRNA [NM_001142807]	10.92841	up	13.28509	up	31.17739	2.95404	36.48599	2.42174	Detected	Compromised	Detected	Compromised
381	A_33_P3439713	LOC283713	Hs.4986	hypothetical protein LOC283713	Homo sapiens cDNA FLJ37663 fis, clone BRHIP2011120 [AK094982]	10.92461	up	7.23527	up	74.07384	7.02089	50.07333	6.10264	Detected	Compromised	Detected	Compromised
382	A_23_P308136	TRIM50	Hs.647053	tripartite motif-containing 50	Homo sapiens tripartite motif-containing 50 (TRIM50), mRNA [NM_178125]	10.92353	up	9.18446	up	68.23266	6.46789	83.60413	8.02674	Detected	Compromised	Detected	Compromised
383	A_33_P3376546	PTGFR	Hs.654365	prostaglandin F receptor (FP)	Homo sapiens prostaglandin F receptor (FP) (PTGFR), transcript variant 2, mRNA [NM_001039585]	10.88628	up	12.50082	up	1404.28850	133.57033	829.32960	58.49977	Detected	Detected	Detected	Detected
384	A_23_P48585	SALL2	Hs.719293	sal-like 2 (Drosophila)	Homo sapiens sal-like 2 (Drosophila) (SALL2), mRNA [NM_005407]	10.84966	up	9.53120	up	1871.77400	178.63657	1140.57320	105.52149	Detected	Detected	Detected	Detected
385	A_23_P129458	SDR42E1	Hs.87779	short chain dehydrogenase/reductase family 42E, member 1	Homo sapiens short chain dehydrogenase/reductase family 42E, member 1 (SDR42E1), mRNA [NM_145168]	10.76160	up	8.11631	up	32.71462	3.14774	24.53266	2.66533	Detected	Compromised	Detected	Compromised
386	A_33_P3394868	LOC388588		hypothetical protein LOC388588	Homo sapiens hypothetical protein LOC388588 (LOC388588), mRNA [NM_001163724]	10.73694	up	14.61793	up	535.71540	51.66384	659.22360	39.76603	Detected	Detected	Detected	Detected
387	A_23_P259442	CPE	Hs.75360	carboxypeptidase E	Homo sapiens carboxypeptidase E (CPE), mRNA [NM_001873]	10.73581	up	11.27468	up	11785.77700	1136.72770	8258.97200	645.93286	Detected	Detected	Detected	Detected
388	A_23_P357284	GPR4	Hs.17170	G protein-coupled receptor 4	Homo sapiens G protein-coupled receptor 4 (GPR4), mRNA [NM_005282]	10.68811	up	9.76092	up	1731.05040	167.70346	1539.17870	139.04767	Detected	Detected	Detected	Detected
389	A_23_P252471	PECAM1	Hs.514412	platelet/endothelial cell adhesion molecule	Homo sapiens platelet/endothelial cell adhesion molecule (PECAM1), mRNA [NM_000442]	10.68410	up	8.35486	up	357.17136	34.61559	346.58447	36.57932	Detected	Detected	Detected	Detected
390	A_33_P3340454				UPF0594 protein C13orf38 [Source:UniProtKB/Swiss-Prot;Acc:A6NNP5] [ENST00000340225]	10.67764	up	3.22275	up	32.82027	3.18273	22.20318	6.07510	Detected	Compromised	Detected	Compromised
391	A_23_P37410	CYP19A1	Hs.260074	cytochrome P450, family 19, subfamily A, polypeptide 1	Homo sapiens cytochrome P450, family 19, subfamily A, polypeptide 1 (CYP19A1), transcript variant 2, mRNA [NM_031226]	10.65677	up	11.98918	up	993.54330	96.53709	887.89000	65.30329	Detected	Detected	Detected	Detected
392	A_33_P3404257	LOC729885	Hs.685640	similar to pheromone receptor	PREDICTED: Homo sapiens similar to pheromone receptor (LOC729885), miscRNA [XR_015687]	10.64205	up	9.07362	up	58.53431	5.69532	54.92576	5.33778	Detected	Compromised	Detected	Compromised
393	A_33_P3274462					10.63180	up	9.07326	up	30.56850	2.97715	25.20293	2.44936	Detected	Compromised	Detected	Compromised
394	A_23_P212508	TF	Hs.518267	transferrin	Homo sapiens transferrin (TF), mRNA [NM_001063]	10.60173	up	5.01823	up	36.56189	3.57096	17.42358	3.06163	Detected	Compromised	Detected	Compromised
395	A_33_P3570193	FLJ36000	Hs.711145	hypothetical FLJ36000	Homo sapiens hypothetical FLJ36000 (FLJ36000), non-coding RNA [NR_027084]	10.58263	up	2.77897	up	32.86160	3.21535	26.17442	8.30537	Detected	Compromised	Detected	Compromised
396	A_33_P3330643	KIF6	Hs.588202	kinesin family member 6	Homo sapiens kinesin family member 6 (KIF6), mRNA [NM_145027]	10.57832	up	13.32620	up	116.06253	11.36079	136.87100	9.05671	Detected	Compromised	Detected	Compromised
397	A_33_P3238295	FAM66C	Hs.512257	family with sequence similarity 66, member C	Homo sapiens family with sequence similarity 66, member C (FAM66C), non-coding RNA [NR_026788]	10.55419	up	12.25409	up	187.90637	18.43527	81.00075	5.82873	Detected	Compromised	Detected	Compromised

398	A_24_P37589	ACPP	Hs.433060	acid phosphatase, prostate	Homo sapiens acid phosphatase, prostate (ACPP), transcript variant 1, mRNA [NM_001099]	10.54633	up	3.90621	up	32.75102	3.21556	23.29688	5.25906	Detected	Compromised	Detected	Compromised
399	A_23_P107116	RNF112	Hs.189482	ring finger protein 112	Homo sapiens ring finger protein 112 (RNF112), mRNA [NM_007148]	10.52743	up	10.50394	up	3522.35550	346.45276	2656.85990	223.03967	Detected	Detected	Detected	Detected
400	A_23_P94517	DBC1	Hs.532316	deleted in bladder cancer 1	Homo sapiens deleted in bladder cancer 1 (DBC1), mRNA [NM_014618]	10.52361	up	12.38724	up	377.23993	37.11812	273.39642	19.46183	Detected	Detected	Detected	Detected
401	A_23_P390148	RALGPS1	Hs.432842	Ral GEF with PH domain and SH3 binding motif 1	Homo sapiens Ral GEF with PH domain and SH3 binding motif 1 (RALGPS1), mRNA [NM_014636]	10.46444	up	10.57677	up	55.35303	5.47720	52.88453	4.40901	Detected	Compromised	Detected	Compromised
402	A_33_P3423941	IFITM1	Hs.458414	interferon induced transmembrane protein 1 (9-27)	Homo sapiens interferon induced transmembrane protein 1 (9-27) (IFITM1), mRNA [NM_003641]	10.44985	up	10.46953	up	27394.09200	2714.43800	21991.41800	1852.21620	Detected	Detected	Detected	Detected
403	A_23_P18447	PPARGC1A	Hs.527078	peroxisome proliferator-activated receptor gamma, coactivator 1 alpha	Homo sapiens peroxisome proliferator-activated receptor gamma, coactivator 1 alpha (PPARGC1A), mRNA [NM_013261]	10.44337	up	6.39084	up	136.79720	13.56344	92.20883	12.72272	Detected	Compromised	Detected	Compromised
404	A_33_P3389704	CES1	Hs.558865	carboxylesterase 1 (monocyte/macrophage serine esterase 1)	Homo sapiens carboxylesterase 1 (monocyte/macrophage serine esterase 1) (CES1), transcript variant 3, mRNA [NM_001266]	10.42760	up	8.89456	up	668.23180	66.35536	647.87980	64.22967	Detected	Detected	Detected	Detected
405	A_23_P129133	OCA2	Hs.654411	oculocutaneous albinism II	Homo sapiens oculocutaneous albinism II (OCA2), mRNA [NM_000275]	10.40769	up	21.44476	up	136.87029	13.61720	102.98220	4.23454	Detected	Compromised	Detected	Compromised
406	A_33_P3331916					10.40136	up	11.45069	up	537.71387	53.52961	421.38748	32.45006	Detected	Detected	Detected	Detected
407	A_23_P402936	PPFIA2	Hs.506216	protein tyrosine phosphatase, receptor type, f polypeptide (PTPRF), interacting protein (liprin), alpha 2	Homo sapiens protein tyrosine phosphatase, receptor type, f polypeptide (PTPRF), interacting protein (liprin), alpha 2 (PPFIA2), mRNA [NM_003625]	10.37351	up	17.30042	up	70.73332	7.06043	71.99606	3.66959	Detected	Compromised	Detected	Compromised
408	A_23_P257803	DMGDH	Hs.655653	dimethylglycine dehydrogenase	Homo sapiens dimethylglycine dehydrogenase (DMGDH), nuclear gene encoding mitochondrial protein, mRNA [NM_013391]	10.29127	up	4.15070	up	44.86228	4.51383	41.58643	8.83477	Detected	Compromised	Detected	Compromised
409	A_33_P3353030	UCN	Hs.534363	urocortin	Homo sapiens urocortin (UCN), mRNA [NM_003353]	10.27478	up	10.04579	up	2347.14840	236.53807	2149.90190	188.71219	Detected	Detected	Detected	Detected
410	A_23_P109269	LAMA5	Hs.473256	laminin, alpha 5	Homo sapiens laminin, alpha 5 (LAMA5), mRNA [NM_005560]	10.27451	up	9.93541	up	701.03300	70.64972	769.16620	68.26530	Detected	Detected	Detected	Detected
411	A_24_P215653	CLEC14A	Hs.525307	C-type lectin domain family 14, member A	Homo sapiens C-type lectin domain family 14, member A (CLEC14A), mRNA [NM_175060]	10.23899	up	8.24649	up	94.50938	9.55765	110.31227	11.79560	Detected	Compromised	Detected	Compromised
412	A_23_P73609	NDP	Hs.522615	Norrie disease (pseudoglioma)	Homo sapiens Norrie disease (pseudoglioma) (NDP), mRNA [NM_000266]	10.22683	up	9.88189	up	209.89755	21.25198	189.68622	16.92628	Detected	Detected	Detected	Detected
413	A_23_P86470	CH25H	Hs.47357	cholesterol 25-hydroxylase	Homo sapiens cholesterol 25-hydroxylase (CH25H), mRNA [NM_003956]	10.21213	up	9.81912	up	1060.55300	107.53478	877.67505	78.81826	Detected	Detected	Detected	Detected
414	A_23_P161135	LEPR	Hs.23581	leptin receptor	Homo sapiens leptin receptor (LEPR), transcript variant 1, mRNA [NM_002303]	10.16809	up	8.95892	up	36.10723	3.67695	31.60733	3.11098	Detected	Compromised	Detected	Compromised
415	A_24_P59667	JAK3	Hs.515247	Janus kinase 3	Homo sapiens Janus kinase 3 (JAK3), mRNA [NM_000215]	10.15420	up	8.77331	up	5083.65430	518.39813	4898.01760	492.29178	Detected	Detected	Detected	Detected
416	A_33_P3210203	SPIRE2	Hs.461786	spire homolog 2 (Drosophila)	Homo sapiens spire homolog 2 (Drosophila) (SPIRE2), mRNA [NM_032451]	10.13470	up	9.27045	up	1052.75790	107.55999	1078.50610	102.58582	Detected	Detected	Detected	Detected
417	A_33_P3356361	LOC100131089	Hs.386000	hypothetical protein LOC100131089	Homo sapiens cDNA FLJ45796 fis, clone NT2RI2012542. [AK127696]	10.08544	up	5.02432	up	113.84589	11.68842	108.81562	19.09762	Detected	Compromised	Detected	Detected
418	A_33_P3718269	LOC285628	Hs.604728	hypothetical protein LOC285628	Homo sapiens mRNA full length insert cDNA clone EUROIMAGE 2005635 [AL389942]	10.07387	up	12.36708	up	615.74430	63.29032	393.57580	28.06252	Detected	Detected	Detected	Detected
419	A_33_P3235677					10.07307	up	8.23990	up	36.93503	3.79673	31.93821	3.41786	Detected	Compromised	Detected	Compromised
420	A_33_P3271001	NIPSNAP3B	Hs.720820	nipsnap homolog 3B (C. elegans)	Homo sapiens nipsnap homolog 3B (C. elegans) (NIPSNAP3B), mRNA [NM_018376]	10.05388	up	6.49294	up	31.68244	3.26301	33.86998	4.59980	Detected	Compromised	Detected	Compromised
421	A_23_P202520	ABLIM1	Hs.438236	actin binding LIM protein 1	Homo sapiens actin binding LIM protein 1 (ABLIM1), transcript variant 3, mRNA [NM_001003408]	10.00016	up	10.19339	up	2657.81200	275.20130	1933.52270	167.26155	Detected	Detected	Detected	Detected
422	A_23_P151059	FAM90A1	Hs.196086	family with sequence similarity 90, member A1	Homo sapiens family with sequence similarity 90, member A1 (FAM90A1), mRNA [NM_018088]	9.98958	up	10.89579	up	269.46597	27.93120	302.20680	24.45744	Detected	Detected	Detected	Detected
423	A_24_P6674	WDR31	Hs.133331	WD repeat domain 31	Homo sapiens WD repeat domain 31 (WDR31), transcript variant 1, mRNA [NM_001012361]	9.93943	up	4.45878	up	29.11773	3.03340	12.67022	2.50573	Detected	Compromised	Detected	Compromised
424	A_24_P243749	PDK4	Hs.8364	pyruvate dehydrogenase kinase, isozyme 4	Homo sapiens pyruvate dehydrogenase kinase, isozyme 4 (PDK4), mRNA [NM_002612]	9.93847	up	38.06233	up	1036.09610	107.94779	867.62580	20.10030	Detected	Detected	Detected	Detected
425	A_24_P75917	CCDC144B	Hs.721149	coiled-coil domain containing 144B	Homo sapiens coiled-coil domain containing 144B (CCDC144B), mRNA [NM_182568]	9.93555	up	7.56204	up	39.48937	4.11549	32.32752	3.76963	Detected	Compromised	Detected	Compromised
426	A_23_P72737	IFITM1	Hs.458414	interferon induced transmembrane protein 1 (9-27)	Homo sapiens interferon induced transmembrane protein 1 (9-27) (IFITM1), mRNA [NM_003641]	9.93314	up	9.09901	up	52089.05500	5429.91700	54394.91000	5271.44630	Detected	Detected	Detected	Detected
427	A_24_P288915		Hs.649499		Coiled-coil domain-containing protein 144A [Source:UniProtKB/Swiss-Prot;Acc:A2RUR9] [ENST00000340621]	9.92664	up	8.90566	up	66.31436	6.91733	58.88777	5.83075	Detected	Compromised	Detected	Compromised
428	A_23_P217845	RGS16	Hs.413297	regulator of G-protein signaling 16	Homo sapiens regulator of G-protein signaling 16 (RGS16), mRNA [NM_002928]	9.90416	up	10.85331	up	273.68140	28.61284	325.16925	26.41878	Detected	Detected	Detected	Detected
429	A_23_P205900	NTRK3	Hs.410969	neurotrophic tyrosine kinase, receptor, type 3	Homo sapiens neurotrophic tyrosine kinase, receptor, type 3 (NTRK3), transcript variant 1, mRNA [NM_001012338]	9.88527	up	2.92004	up	58.84325	6.16370	57.21378	17.27734	Detected	Compromised	Detected	Detected

430	A_24_P203298	IQUB	Hs.159650	IQ motif and ubiquitin domain containing	Homo sapiens IQ motif and ubiquitin domain containing (IQUB), mRNA [NM_178827]	9.87373	up	4.50717	up	32.16048	3.37267	26.01299	5.08923	Detected	Compromised	Detected	Compromised
431	A_33_P3347869	C3	Hs.529053	complement component 3	Homo sapiens complement component 3 (C3), mRNA [NM_000064]	9.85969	up	7.86171	up	1440.64990	151.29625	1072.49220	120.29360	Detected	Detected	Detected	Detected
432	A_33_P3241269	CES1	Hs.558865	carboxylesterase 1 (monocyte/macrophage serine esterase 1)	Homo sapiens carboxylesterase 1 (monocyte/macrophage serine esterase 1) (CES1), transcript variant 1, mRNA [NM_001025195]	9.82588	up	9.32342	up	50269.65600	5297.46040	33968.60000	3212.68500	Detected	Detected	Detected	Detected
433	A_33_P3276718	HGF	Hs.396530	hepatocyte growth factor (hepapoietin A; scatter factor)	Homo sapiens hepatocyte growth factor (hepapoietin A; scatter factor) (HGF), transcript variant 5, mRNA [NM_001010934]	9.81608	up	10.21739	up	361.13947	38.09519	304.65887	26.29294	Detected	Detected	Detected	Detected
434	A_33_P3338733	MITF	Hs.166017	microphthalmia-associated transcription factor	Homo sapiens microphthalmia-associated transcription factor (MITF), transcript variant 1, mRNA [NM_198159]	9.81411	up	10.79317	up	3599.23500	379.74540	2816.83230	230.13230	Detected	Detected	Detected	Detected
435	A_24_P31627	KCNB1	Hs.84244	potassium voltage-gated channel, Shab-related subfamily, member 1	Homo sapiens potassium voltage-gated channel, Shab-related subfamily, member 1 (KCNB1), mRNA [NM_004975]	9.81067	up	1.13675	up	30.20052	3.18749	3.46456	2.68750	Detected	Compromised	Compromised	Compromised
436	A_24_P388528	ST6GAL1	Hs.207459	ST6 beta-galactosamide alpha-2,6-sialyltranferase 1	Homo sapiens ST6 beta-galactosamide alpha-2,6-sialyltranferase 1 (ST6GAL1), transcript variant 1, mRNA [NM_173216]	9.80316	up	7.35401	up	30.55300	3.22716	22.69565	2.72135	Detected	Compromised	Detected	Compromised
437	A_23_P363647	DDX26B	Hs.496829	DEAD/H (Asp-Glu-Ala-Asp/His) box polypeptide 26B	Homo sapiens DEAD/H (Asp-Glu-Ala-Asp/His) box polypeptide 26B (DDX26B), mRNA [NM_182540]	9.80048	up	7.39646	up	335.12012	35.40678	230.75648	27.51032	Detected	Detected	Detected	Detected
438	A_24_P49260	SPTLC3	Hs.425023	serine palmitoyltransferase, long chain base subunit 3	Homo sapiens serine palmitoyltransferase, long chain base subunit 3 (SPTLC3), mRNA [NM_018327]	9.79065	up	10.62372	up	54.48208	5.76203	31.29885	2.59787	Detected	Compromised	Detected	Compromised
439	A_23_P66881	RGS9	Hs.664380	regulator of G-protein signaling 9	Homo sapiens regulator of G-protein signaling 9 (RGS9), transcript variant 1, mRNA [NM_003835]	9.78291	up	15.03692	up	273.88373	28.98886	265.73007	15.58286	Detected	Detected	Detected	Detected
440	A_23_P218002	PRG2	Hs.512633	proteoglycan 2, bone marrow (natural killer cell activator, eosinophil granule major basic protein)	Homo sapiens proteoglycan 2, bone marrow (natural killer cell activator, eosinophil granule major basic protein) (PRG2), mRNA [NM_002728]	9.76122	up	10.91528	up	1356.21910	143.86629	1262.40490	101.98341	Detected	Detected	Detected	Detected
441	A_23_P110777	LECT2	Hs.512580	leukocyte cell-derived chemotaxin 2	Homo sapiens leukocyte cell-derived chemotaxin 2 (LECT2), mRNA [NM_002302]	9.72469	up	4.65029	up	94.45591	10.05742	74.50124	14.12698	Detected	Compromised	Detected	Detected
442	A_23_P417383	ASPRV1	Hs.516253	aspartic peptidase, retroviral-like 1	Homo sapiens aspartic peptidase, retroviral-like 1 (ASPRV1), mRNA [NM_152792]	9.71514	up	9.53292	up	4057.33230	432.43860	2917.69500	269.88544	Detected	Detected	Detected	Detected
443	A_24_P109838	SLC6A15	Hs.44424	solute carrier family 6 (neutral amino acid transporter), member 15	Homo sapiens solute carrier family 6 (neutral amino acid transporter), member 15 (SLC6A15), transcript variant 1, mRNA [NM_182767]	9.71492	up	9.57774	up	274.79120	29.28846	250.89487	23.09905	Detected	Detected	Detected	Detected
444	A_33_P3417695	ODF3B	Hs.531314	outer dense fiber of sperm tails 3B	Homo sapiens outer dense fiber of sperm tails 3B (ODF3B), mRNA [NM_001014440]	9.68329	up	9.60861	up	1713.26860	183.20420	2160.94970	198.31232	Detected	Detected	Detected	Detected
445	A_24_P940006	EFNB3	Hs.26988	ephrin-B3	Homo sapiens ephrin-B3 (EFNB3), mRNA [NM_001406]	9.65353	up	9.14287	up	956.46820	102.59290	745.17000	71.86846	Detected	Detected	Detected	Detected
446	A_33_P3330952	ATP8A1	Hs.435052	ATPase, aminophospholipid transporter (APLT), class I, type 8A, member 1	Homo sapiens ATPase, aminophospholipid transporter (APLT), class I, type 8A, member 1 (ATP8A1), transcript variant 1, mRNA [NM_006095]	9.63941	up	18.34085	up	244.64282	26.27939	185.91254	8.93830	Detected	Detected	Detected	Compromised
447	A_23_P381746	ASXL3	Hs.464876	additional sex combs like 3 (Drosophila)	Homo sapiens additional sex combs like 3 (Drosophila) (ASXL3), mRNA [NM_030632]	9.61154	up	4.67885	up	33.91995	3.65423	18.61565	3.50837	Detected	Compromised	Detected	Compromised
448	A_23_P156708	TNXB	Hs.485104	tenascin XB	Homo sapiens tenascin XB (TNXB), transcript variant XB-S, mRNA [NM_032470]	9.59115	up	9.58199	up	5805.15700	626.72424	5762.05130	530.25757	Detected	Detected	Detected	Detected
449	A_33_P3432135	LOC285965	Hs.642649	hypothetical protein LOC285965	Homo sapiens mRNA; cDNA DKFZp686O0656 (from clone DKFZp686O0656) [AL833583]	9.56009	up	1.84964	up	27.38919	2.96654	5.14404	2.45236	Detected	Compromised	Compromised	Compromised
450	A_33_P3390580	CCDC144A	Hs.721149	coiled-coil domain containing 144A	Homo sapiens coiled-coil domain containing 144A (CCDC144A), mRNA [NM_014695]	9.53925	up	8.10330	up	88.15624	9.56912	58.62548	6.37955	Detected	Compromised	Detected	Compromised
451	A_23_P34045	EDA	Hs.105407	ectodysplasin A	Homo sapiens ectodysplasin A (EDA), transcript variant 1, mRNA [NM_001399]	9.53450	up	12.73433	up	188.46124	20.46714	147.10527	10.18633	Detected	Detected	Detected	Compromised
452	A_33_P3389113					9.49794	up	7.54023	up	175.47730	19.13042	136.40890	15.95232	Detected	Detected	Detected	Detected
453	A_33_P3351371	CYP19A1	Hs.260074	cytochrome P450, family 19, subfamily A, polypeptide 1	Homo sapiens cytochrome P450, family 19, subfamily A, polypeptide 1 (CYP19A1), transcript variant 2, mRNA [NM_031226]	9.49635	up	9.30440	up	801.56730	87.40097	997.23000	94.50887	Detected	Detected	Detected	Detected
454	A_24_P759674	OBFC1	Hs.62314	oligonucleotide/oligosaccharide-binding fold containing 1	Homo sapiens oligonucleotide/oligosaccharide-binding fold containing 1 (OBFC1), mRNA [NM_024928]	9.49633	up	9.66432	up	1293.16530	141.00398	787.50500	71.85342	Detected	Detected	Detected	Detected
455	A_24_P290751	DTX1	Hs.372152	deltex homolog 1 (Drosophila)	Homo sapiens deltex homolog 1 (Drosophila) (DTX1), mRNA [NM_004416]	9.49618	up	11.88122	up	28.64580	3.12353	35.12608	2.60696	Detected	Compromised	Detected	Compromised
456	A_33_P3222762	HULC		highly up-regulated in liver cancer (non-protein coding)	Homo sapiens highly up-regulated in liver cancer (non-protein coding) (HULC), non-coding RNA [NR_004855]	9.47423	up	6.03604	up	37.15744	4.06102	23.54878	3.44019	Detected	Compromised	Detected	Compromised
457	A_23_P46025	ETNK2	Hs.497469	ethanolamine kinase 2	Homo sapiens ethanolamine kinase 2 (ETNK2), mRNA [NM_018208]	9.38866	up	8.26364	up	236.97293	26.13535	141.08897	15.05522	Detected	Detected	Detected	Compromised

458	A_24_P148796	MST1	Hs.512587	macrophage stimulating 1 (hepatocyte growth factor-like)	Homo sapiens macrophage stimulating 1 (hepatocyte growth factor-like) (MST1), mRNA [NM_020998]	9.36618	up	13.31739	up	4319.08060	477.48734	4542.70170	300.78818	Detected	Detected	Detected	Detected
459	A_23_P324813	BCL6B	Hs.22575	B-cell CLL/lymphoma 6, member B (zinc finger protein)	Homo sapiens B-cell CLL/lymphoma 6, member B (zinc finger protein) (BCL6B), mRNA [NM_181844]	9.35002	up	14.89408	up	98.39480	10.89664	70.21331	4.15692	Detected	Compromised	Detected	Compromised
460	A_33_P3351554	ETNK2	Hs.497469	ethanolamine kinase 2	Homo sapiens ethanolamine kinase 2 (ETNK2), mRNA [NM_018208]	9.33878	up	9.56199	up	5174.66850	573.75400	3572.62960	329.46190	Detected	Detected	Detected	Detected
461	A_33_P3284838		Hs.132442		Putative uncharacterized protein C10orf115 [Source:UniProtKB/Swiss-Prot;Acc:Q5QP74] [ENST00000399806]	9.33818	up	2.88170	up	30.72678	3.40712	32.55511	9.96175	Detected	Compromised	Detected	Compromised
462	A_33_P3257182	MYBPC2	Hs.85937	myosin binding protein C, fast type	Homo sapiens myosin binding protein C, fast type (MYBPC2), mRNA [NM_004533]	9.32309	up	9.88208	up	28.88374	3.20794	33.51798	2.99085	Detected	Compromised	Detected	Compromised
463	A_33_P3364348	JPH1	Hs.657367	junctophilin 1	Homo sapiens junctophilin 1 (JPH1), mRNA [NM_020647]	9.28563	up	6.64898	up	131.98358	14.71776	77.05670	10.21930	Detected	Compromised	Detected	Compromised
464	A_23_P146294	EFCAB1	Hs.23245	EF-hand calcium binding domain 1	Homo sapiens EF-hand calcium binding domain 1 (EFCAB1), transcript variant 1, mRNA [NM_024593]	9.25280	up	7.26284	up	411.62897	46.06443	346.81710	42.10753	Detected	Detected	Detected	Detected
465	A_23_P116898	A2M	Hs.212838	alpha-2-macroglobulin	Homo sapiens alpha-2-macroglobulin (A2M), mRNA [NM_000014]	9.24928	up	8.84767	up	8472.32900	948.47900	5920.55600	590.06384	Detected	Detected	Detected	Detected
466	A_23_P167030	PTH1R	Hs.1019	parathyroid hormone 1 receptor	Homo sapiens parathyroid hormone 1 receptor (PTH1R), mRNA [NM_000316]	9.23811	up	9.86247	up	142.52359	15.97485	170.30977	15.22718	Detected	Compromised	Detected	Detected
467	A_24_P928969	PTPN3	Hs.436429	protein tyrosine phosphatase, non-receptor type 3	Homo sapiens protein tyrosine phosphatase, non-receptor type 3 (PTPN3), transcript variant 3, mRNA [NM_001145369]	9.20607	up	9.84601	up	852.26874	95.85953	493.61120	44.20694	Detected	Detected	Detected	Detected
468	A_23_P125423	C1R	Hs.524224	complement component 1, r subcomponent	Homo sapiens complement component 1, r subcomponent (C1R), mRNA [NM_001733]	9.20476	up	7.41375	up	29453.14600	3313.23780	26236.24400	3120.53830	Detected	Detected	Detected	Detected
469	A_23_P125748	ZMAT1	Hs.496512	zinc finger, matrin type 1	Homo sapiens zinc finger, matrin type 1 (ZMAT1), transcript variant 3, mRNA [NM_032441]	9.16610	up	6.67031	up	88.71359	10.02164	49.78720	6.58169	Detected	Compromised	Detected	Compromised
470	A_33_P3275878	SFTPB	Hs.512690	surfactant protein B	Homo sapiens surfactant protein B (SFTPB), transcript variant 1, mRNA [NM_000542]	9.15881	up	5.14571	up	28.34736	3.20484	15.73904	2.69711	Detected	Compromised	Detected	Compromised
471	A_33_P3242543	MAOA	Hs.183109	monoamine oxidase A	Homo sapiens monoamine oxidase A (MAOA), nuclear gene encoding mitochondrial protein, mRNA [NM_000240]	9.13875	up	9.21871	up	8552.10600	968.99040	5194.00730	496.81860	Detected	Detected	Detected	Detected
472	A_23_P25155	GPR84	Hs.306199	G protein-coupled receptor 84	Homo sapiens G protein-coupled receptor 84 (GPR84), mRNA [NM_020370]	9.12849	up	5.23166	up	114.77057	13.01862	92.71485	15.62699	Detected	Compromised	Detected	Detected
473	A_23_P202071	CUGBP2	Hs.309288	CUG triplet repeat, RNA binding protein 2	Homo sapiens CUG triplet repeat, RNA binding protein 2 (CUGBP2), transcript variant 3, mRNA [NM_001025077]	9.12815	up	9.15363	up	11394.29200	1292.52170	7654.13800	737.34140	Detected	Detected	Detected	Detected
474	A_23_P115645	CUGBP2	Hs.309288	CUG triplet repeat, RNA binding protein 2	Homo sapiens CUG triplet repeat, RNA binding protein 2 (CUGBP2), transcript variant 1, mRNA [NM_001025076]	9.12721	up	9.31670	up	775.10376	87.93366	587.70910	55.62444	Detected	Detected	Detected	Detected
475	A_33_P3420816	GDF1	Hs.412355	growth differentiation factor 1	Homo sapiens growth differentiation factor 1 (GDF1), mRNA [NM_001492]	9.10775	up	9.12628	up	494.57013	56.22764	519.62946	50.20715	Detected	Detected	Detected	Detected
476	A_23_P134419	ZP3	Hs.656137	zona pellucida glycoprotein 3 (sperm receptor)	Homo sapiens zona pellucida glycoprotein 3 (sperm receptor) (ZP3), transcript variant 2, mRNA [NM_007155]	9.05221	up	6.48660	up	133.51172	15.27206	137.33998	18.67003	Detected	Detected	Detected	Detected
477	A_23_P215744	CTTNBP2	Hs.592285	cortactin binding protein 2	Homo sapiens cortactin binding protein 2 (CTTNBP2), mRNA [NM_033427]	9.03376	up	10.72283	up	167.83832	19.23782	116.03054	9.54176	Detected	Compromised	Detected	Compromised
478	A_23_P343398	CCR7	Hs.370036	chemokine (C-C motif) receptor 7	Homo sapiens chemokine (C-C motif) receptor 7 (CCR7), mRNA [NM_001838]	9.03262	up	9.44478	up	497.52982	57.03458	424.99814	39.67899	Detected	Detected	Detected	Detected
479	A_33_P3237927	ARHGAP4	Hs.701324	Rho GTPase activating protein 4	Homo sapiens Rho GTPase activating protein 4 (ARHGAP4), transcript variant 1, mRNA [NM_001164741]	9.03262	up	9.45522	up	904.85440	103.72851	805.22860	75.09538	Detected	Detected	Detected	Detected
480	A_23_P256047	ANKRD5	Hs.70903	ankyrin repeat domain 5	Homo sapiens ankyrin repeat domain 5 (ANKRD5), transcript variant 1, mRNA [NM_022096]	9.02772	up	4.14157	up	28.74980	3.29754	25.42098	5.41243	Detected	Compromised	Detected	Compromised
481	A_24_P295590	RASSF4	Hs.522895	Ras association (RalGDS/AF-6) domain family member 4	Homo sapiens Ras association (RalGDS/AF-6) domain family member 4 (RASSF4), mRNA [NM_032023]	8.99940	up	8.89714	up	1131.59290	130.19958	948.09283	93.96493	Detected	Detected	Detected	Detected
482	A_24_P217572	EDNRA	Hs.183713	endothelin receptor type A	Homo sapiens endothelin receptor type A (EDNRA), transcript variant 1, mRNA [NM_001957]	8.99458	up	9.41405	up	7132.73440	821.12360	5827.34900	545.83330	Detected	Detected	Detected	Detected
483	A_33_P3334220	ACACB	Hs.234898	acetyl-Coenzyme A carboxylase beta	Homo sapiens acetyl-Coenzyme A carboxylase beta (ACACB), mRNA [NM_001093]	8.98756	up	8.44658	up	564.99210	65.09295	436.61673	45.58112	Detected	Detected	Detected	Detected
484	A_32_P420520	FLJ39582	Hs.517430	hypothetical LOC439931	Homo sapiens hypothetical LOC439931 (FLJ39582), transcript variant 1, non-coding RNA [NR_027051]	8.98557	up	7.27263	up	79.71062	9.18552	78.34224	9.49883	Detected	Compromised	Detected	Compromised
485	A_33_P3219279	JPH4	Hs.597841	junctophilin 4	Homo sapiens junctophilin 4 (JPH4), transcript variant 1, mRNA [NM_032452]	8.97272	up	2.17488	up	28.55239	3.29497	32.48565	13.17107	Detected	Compromised	Detected	Compromised
486	A_23_P144020	CNTN4	Hs.298705	contactin 4	Homo sapiens contactin 4 (CNTN4), transcript variant 3, mRNA [NM_175613]	8.96547	up	4.61144	up	32.27991	3.72814	22.81206	4.36207	Detected	Compromised	Detected	Compromised
487	A_24_P97825	CCDC69	Hs.655336	coiled-coil domain containing 69	Homo sapiens coiled-coil domain containing 69 (CCDC69), mRNA [NM_015621]	8.94441	up	14.16009	up	296.36618	34.30915	189.38914	11.79382	Detected	Detected	Detected	Compromised

488	A_24_P260101	MME	Hs.307734	membrane metallo-endopeptidase	Homo sapiens membrane metallo-endopeptidase (MME), transcript variant 2b, mRNA [NM_007289]	8.92754	up	8.87739	up	18017.11300	2089.71140	14984.50100	1488.40930	Detected	Detected	Detected	Detected
489	A_33_P3391915	BTBD17	Hs.211870	BTB (POZ) domain containing 17	Homo sapiens BTB (POZ) domain containing 17 (BTBD17), mRNA [NM_001080466]	8.90654	up	5.61901	up	26.35087	3.06351	16.04398	2.51778	Detected	Compromised	Detected	Compromised
490	A_33_P3363933	FCRL6	Hs.196955	Fc receptor-like 6	Homo sapiens Fc receptor-like 6 (FCRL6), mRNA [NM_001004310]	8.90051	up	2.36260	up	43.49829	5.06047	33.61454	12.54593	Detected	Compromised	Detected	Compromised
491	A_23_P139682	PZP	Hs.707491	pregnancy-zone protein	Homo sapiens pregnancy-zone protein (PZP), mRNA [NM_002864]	8.89916	up	1.56918	up	338.51840	39.38823	5.94839	3.34267	Detected	Detected	Compromised	Compromised
492	A_23_P344421	ROBO4	Hs.524121	roundabout homolog 4, magic roundabout (Drosophila)	Homo sapiens roundabout homolog 4, magic roundabout (Drosophila) (ROBO4), mRNA [NM_019055]	8.89075	up	4.74146	up	154.22581	17.96187	107.01122	19.90135	Detected	Compromised	Detected	Detected
493	A_33_P3393821	C1R	Hs.524224	complement component 1, r subcomponent	Homo sapiens complement component 1, r subcomponent (C1R), mRNA [NM_001733]	8.83962	up	7.25900	up	97825.36000	11459.10800	89336.28000	10852.17700	Detected	Detected	Detected	Detected
494	A_33_P3275687	TBC1D3B	Hs.454713	TBC1 domain family, member 3B	Homo sapiens TBC1 domain family, member 3B (TBC1D3B), mRNA [NM_001001417]	8.83298	up	6.32259	up	51.13411	5.99428	44.80544	6.24887	Detected	Compromised	Detected	Compromised
495	A_24_P167642	GCH1	Hs.86724	GTP cyclohydrolase 1	Homo sapiens GTP cyclohydrolase 1 (GCH1), transcript variant 1, mRNA [NM_000161]	8.82830	up	8.99703	up	340.39905	39.92495	230.72102	22.61275	Detected	Detected	Detected	Detected
496	A_23_P80974	TDO2	Hs.183671	tryptophan 2,3-dioxygenase	Homo sapiens tryptophan 2,3-dioxygenase (TDO2), mRNA [NM_005651]	8.82215	up	7.28481	up	31.87280	3.74092	50.39909	6.10056	Detected	Compromised	Detected	Compromised
497	A_23_P21063	SCN5A	Hs.517898	sodium channel, voltage-gated, type V, alpha subunit	Homo sapiens sodium channel, voltage-gated, type V, alpha subunit (SCN5A), transcript variant 1, mRNA [NM_198056]	8.82108	up	9.57018	up	35.78612	4.20074	35.08748	3.23294	Detected	Compromised	Detected	Compromised
498	A_33_P3323692					8.81146	up	6.71130	up	34.61069	4.06720	26.11261	3.43091	Detected	Compromised	Detected	Compromised
499	A_24_P182696	MYO3B	Hs.671900	myosin IIIB	Homo sapiens myosin IIIB (MYO3B), transcript variant 2, mRNA [NM_138995]	8.78489	up	5.00851	up	26.40539	3.11236	14.61391	2.57290	Detected	Compromised	Detected	Compromised
500	A_23_P85082	RHOXF1	Hs.644617	Rhox homeobox family, member 1	Homo sapiens Rhox homeobox family, member 1 (RHOXF1), mRNA [NM_139282]	8.74278	up	1.88051	up	26.52185	3.14114	21.84637	10.24401	Detected	Compromised	Detected	Compromised
501	A_23_P77328	GCHFR	Hs.631717	GTP cyclohydrolase I feedback regulator	Homo sapiens GTP cyclohydrolase I feedback regulator (GCHFR), mRNA [NM_005258]	8.73454	up	9.57904	up	1000.03820	118.55219	1270.83040	116.98530	Detected	Detected	Detected	Detected
502	A_23_P11685	PLA2G4A	Hs.497200	phospholipase A2, group IVA (cytosolic, calcium-dependent)	Homo sapiens phospholipase A2, group IVA (cytosolic, calcium-dependent) (PLA2G4A), mRNA [NM_024420]	8.72787	up	9.07815	up	931.89905	110.55888	821.69480	79.81390	Detected	Detected	Detected	Detected
503	A_23_P63736	LOC84856	Hs.55977	hypothetical LOC84856	Homo sapiens hypothetical LOC84856 (LOC84856), non-coding RNA [NR_026827]	8.71147	up	9.24559	up	1181.67020	140.45530	983.54670	93.80493	Detected	Detected	Detected	Detected
504	A_23_P164814	C19orf57	Hs.143288	chromosome 19 open reading frame 57	Homo sapiens chromosome 19 open reading frame 57 (C19orf57), mRNA [NM_024323]	8.70453	up	8.64498	up	319.39746	37.99438	273.92140	27.94006	Detected	Detected	Detected	Detected
505	A_24_P179585	MARK1	Hs.497806	MAP/microtubule affinity-regulating kinase 1	Homo sapiens MAP/microtubule affinity-regulating kinase 1 (MARK1), mRNA [NM_018650]	8.68853	up	2.56389	up	35.55531	4.23732	7.49786	2.57871	Detected	Compromised	Compromised	Compromised
506	A_33_P3293164	CHGA	Hs.150793	chromogranin A (parathyroid secretory protein 1)	Homo sapiens chromogranin A (parathyroid secretory protein 1) (CHGA), mRNA [NM_001275]	8.68229	up	2.72491	up	28.87394	3.44354	22.29040	7.21327	Detected	Compromised	Detected	Compromised
507	A_33_P3276435	FILIP1	Hs.696158	filamin A interacting protein 1	Homo sapiens filamin A interacting protein 1 (FILIP1), mRNA [NM_015687]	8.67586	up	2.03350	up	25.60925	3.05645	13.43503	5.82586	Detected	Compromised	Detected	Compromised
508	A_23_P29939	SNCA	Hs.21374	synuclein, alpha (non A4 component of amyloid precursor)	Homo sapiens synuclein, alpha (non A4 component of amyloid precursor) (SNCA), transcript variant 4, mRNA [NM_007308]	8.65166	up	9.07805	up	532.17030	63.69193	519.76685	50.48723	Detected	Detected	Detected	Detected
509	A_33_P3257297	CCDC147	Hs.253576	coiled-coil domain containing 147	Homo sapiens coiled-coil domain containing 147 (CCDC147), mRNA [NM_001008723]	8.58491	up	9.19480	up	934.89276	112.76108	611.42633	58.63641	Detected	Detected	Detected	Detected
510	A_33_P3399480	LOC728052	Hs.535825	similar to hCG2031213	PREDICTED: Homo sapiens hypothetical LOC728052 (LOC728052), mRNA [XM_001717850]	8.58137	up	7.31462	up	41.43734	4.99998	20.49097	2.47022	Detected	Compromised	Detected	Compromised
511	A_32_P47157	LOC92973	Hs.720274	hypothetical LOC92973	Homo sapiens hypothetical LOC92973 (LOC92973), non-coding RNA [NR_024006]	8.54971	up	6.18356	up	25.20415	3.05249	17.59429	2.50899	Detected	Compromised	Detected	Compromised
512	A_23_P19816	RNF32	Hs.490715	ring finger protein 32	Homo sapiens ring finger protein 32 (RNF32), mRNA [NM_030936]	8.53891	up	4.74720	up	66.25548	8.03438	40.36942	7.49860	Detected	Compromised	Detected	Compromised
513	A_23_P44466	CCDC102B	Hs.280781	coiled-coil domain containing 102B	Homo sapiens coiled-coil domain containing 102B (CCDC102B), transcript variant 2, mRNA [NM_024781]	8.53441	up	11.58575	up	64.58260	7.83565	48.19966	3.66847	Detected	Compromised	Detected	Compromised
514	A_33_P3237379	KCNC1	Hs.552896	potassium voltage-gated channel, Shaw-related subfamily, member 1	Homo sapiens potassium voltage-gated channel, Shaw-related subfamily, member 1 (KCNC1), transcript variant B, mRNA [NM_004976]	8.53097	up	8.15560	up	29.11208	3.53352	27.44197	2.96705	Detected	Compromised	Detected	Compromised
515	A_23_P79978	SLC24A3	Hs.654790	solute carrier family 24 (sodium/potassium/calcium exchanger), member 3	Homo sapiens solute carrier family 24 (sodium/potassium/calcium exchanger), member 3 (SLC24A3), mRNA [NM_020689]	8.52277	up	9.47874	up	2154.42160	261.74786	1576.61830	146.67012	Detected	Detected	Detected	Detected
516	A_23_P27306	COLEC12	Hs.464422	collectin sub-family member 12	Homo sapiens collectin sub-family member 12 (COLEC12), mRNA [NM_130386]	8.52240	up	8.46793	up	3279.30300	398.43057	2427.54000	252.78703	Detected	Detected	Detected	Detected
517	A_23_P368996	LRRC56	Hs.567655	leucine rich repeat containing 56	Homo sapiens leucine rich repeat containing 56 (LRRC56), mRNA [NM_198075]	8.51819	up	6.86437	up	946.60120	115.06748	944.39990	121.31680	Detected	Detected	Detected	Detected
518	A_23_P209055	CD22	Hs.579691	CD22 molecule	Homo sapiens CD22 molecule (CD22), mRNA [NM_001771]	8.51413	up	4.90330	up	25.63793	3.11800	14.63540	2.63198	Detected	Compromised	Detected	Compromised

519	A_23_P77304	AP3B2	Hs.199593	adaptor-related protein complex 3, beta 2 subunit	Homo sapiens adaptor-related protein complex 3, beta 2 subunit (AP3B2), mRNA [NM_004644]	8.50659	up	6.47646	up	74.11170	9.02120	65.30547	8.89155	Detected	Compromised	Detected	Compromised
520	A_33_P3230663	LOC100130232	Hs.656046	LP2209	Homo sapiens LP2209 mRNA, complete cds. [AY203950]	8.48325	up	8.35175	up	50.07653	6.11231	24.74789	2.61292	Detected	Compromised	Detected	Compromised
521	A_33_P3247175	C4orf47	Hs.549398	chromosome 4 open reading frame 47	Homo sapiens chromosome 4 open reading frame 47 (C4orf47), mRNA [NM_001114357]	8.46483	up	9.28802	up	162.56609	19.88587	113.34570	10.76087	Detected	Detected	Detected	Compromised
522	A_23_P204640	NANOG	Hs.661360	Nanog homeobox	Homo sapiens Nanog homeobox (NANOG), mRNA [NM_024865]	8.45846	up	6.81780	up	129.54599	15.85863	67.55973	8.73794	Detected	Compromised	Detected	Compromised
523	A_23_P54144	BMP4	Hs.68879	bone morphogenetic protein 4	Homo sapiens bone morphogenetic protein 4 (BMP4), transcript variant 1, mRNA [NM_001202]	8.45809	up	8.30980	up	26.27405	3.21653	25.27938	2.68251	Detected	Compromised	Detected	Compromised
524	A_23_P152002	BCL2A1	Hs.227817	BCL2-related protein A1	Homo sapiens BCL2-related protein A1 (BCL2A1), transcript variant 1, mRNA [NM_004049]	8.45745	up	11.83541	up	167.29521	20.48223	149.31548	11.12467	Detected	Detected	Detected	Compromised
525	A_32_P70927	PAGE2	Hs.662489	P antigen family, member 2 (prostate associated)	Homo sapiens P antigen family, member 2 (prostate associated) (PAGE2), mRNA [NM_207339]	8.45491	up	2.82465	up	25.55557	3.12975	22.32546	6.96949	Detected	Compromised	Detected	Compromised
526	A_23_P200670	WDR78	Hs.49421	WD repeat domain 78	Homo sapiens WD repeat domain 78 (WDR78), transcript variant 2, mRNA [NM_207014]	8.45391	up	5.90577	up	502.26712	61.51915	438.36957	65.45293	Detected	Detected	Detected	Detected
527	A_24_P130936	DDX3Y	Hs.99120	DEAD (Asp-Glu-Ala-Asp) box polypeptide 3, Y-linked	Homo sapiens DEAD (Asp-Glu-Ala-Asp) box polypeptide 3, Y-linked (DDX3Y), transcript variant 2, mRNA [NM_004660]	8.44730	up	4.80505	up	276.60654	33.90607	219.05167	40.19888	Detected	Detected	Detected	Detected
528	A_23_P314755	STC1	Hs.25590	stanniocalcin 1	Homo sapiens stanniocalcin 1 (STC1), mRNA [NM_003155]	8.38850	up	8.04466	up	2781.67300	343.36410	2337.79270	256.25006	Detected	Detected	Detected	Detected
529	A_33_P3233436	PNMA6A	Hs.721963	paraneoplastic antigen like 6A	Homo sapiens paraneoplastic antigen like 6A (PNMA6A), mRNA [NM_032882]	8.38298	up	7.91726	up	1203.08510	148.60420	1632.12610	181.77916	Detected	Detected	Detected	Detected
530	A_33_P3351175	WNK2	Hs.654856	WNK lysine deficient protein kinase 2	Homo sapiens WNK lysine deficient protein kinase 2 (WNK2), mRNA [NM_006648]	8.38194	up	5.60014	up	28.41998	3.51085	20.12625	3.16905	Detected	Compromised	Detected	Compromised
531	A_33_P3371341	FAM66C	Hs.512257	family with sequence similarity 66, member C	Homo sapiens family with sequence similarity 66, member C (FAM66C), non-coding RNA [NR_026788]	8.33006	up	7.67553	up	104.23381	12.95668	56.71936	6.51611	Detected	Compromised	Detected	Compromised
532	A_33_P3400147	TLE6	Hs.334507	transducin-like enhancer of split 6 (E(sp1) homolog, Drosophila)	Homo sapiens transducin-like enhancer of split 6 (E(sp1) homolog, Drosophila) (TLE6), transcript variant 1, mRNA [NM_001143986]	8.30741	up	5.89706	up	54.14709	6.74904	53.50415	8.00051	Detected	Compromised	Detected	Compromised
533	A_24_P247026	FAM154B	Hs.459117	family with sequence similarity 154, member B	Homo sapiens family with sequence similarity 154, member B (FAM154B), mRNA [NM_001008226]	8.30256	up	5.08427	up	25.75222	3.21171	15.53116	2.69365	Detected	Compromised	Detected	Compromised
534	A_33_P3245120	LOC200772	Hs.647893	hypothetical protein LOC200772	Homo sapiens, clone IMAGE:5745636, mRNA [BC040629]	8.29697	up	23.85590	up	142.66220	17.80421	144.66664	5.34734	Detected	Detected	Detected	Compromised
535	A_24_P472007	UNC13A	Hs.164502	unc-13 homolog A (C. elegans)	Homo sapiens unc-13 homolog A (C. elegans) (UNC13A), mRNA [NM_001080421]	8.28609	up	5.85682	up	32.32014	4.03884	17.77806	2.67663	Detected	Compromised	Detected	Compromised
536	A_33_P3254431	LOC100128003		hypothetical protein LOC100128003	Homo sapiens hypothetical protein LOC100128003 (LOC100128003), non-coding RNA [NR_024445]	8.25627	up	2.76380	up	52.81763	6.62412	15.01266	4.78979	Detected	Compromised	Compromised	Compromised
537	A_33_P3330209	AUTS2	Hs.21631	autism susceptibility candidate 2	Homo sapiens autism susceptibility candidate 2 (AUTS2), transcript variant 3, mRNA [NM_001127232]	8.24780	up	6.89733	up	106.54691	13.37630	99.80445	12.75952	Detected	Compromised	Detected	Detected
538	A_23_P98686	ATHL1	Hs.353181	ATH1, acid trehalase-like 1 (yeast)	Homo sapiens ATH1, acid trehalase-like 1 (yeast) (ATHL1), mRNA [NM_025092]	8.23778	up	8.60605	up	1742.68200	219.04900	1547.57510	158.56718	Detected	Detected	Detected	Detected
539	A_33_P3333282	FGF11	Hs.655193	fibroblast growth factor 11	Homo sapiens fibroblast growth factor 11 (FGF11), mRNA [NM_004112]	8.21253	up	8.83962	up	113.39652	14.29735	91.81173	9.15862	Detected	Compromised	Detected	Compromised
540	A_24_P250922	PTGS2	Hs.196384	prostaglandin-endoperoxide synthase 2 (prostaglandin G/H synthase and cyclooxygenase)	Homo sapiens prostaglandin-endoperoxide synthase 2 (prostaglandin G/H synthase and cyclooxygenase) (PTGS2), mRNA [NM_000963]	8.19191	up	8.81709	up	1656.07690	209.32854	1191.17150	119.12826	Detected	Detected	Detected	Detected
541	A_33_P3292196	PNMA6A	Hs.721963	paraneoplastic antigen like 6A	Homo sapiens paraneoplastic antigen like 6A (PNMA6A), mRNA [NM_032882]	8.18830	up	8.28970	up	152.08212	19.23169	169.55922	18.03632	Detected	Detected	Detected	Detected
542	A_23_P166508	LOC100134361	Hs.335413	similar to hCG1811002	PREDICTED: Homo sapiens hypothetical LOC100134361 (LOC100134361), mRNA [XM_001726827]	8.15863	up	18.29105	up	705.41660	89.52860	702.20780	33.85265	Detected	Detected	Detected	Detected
543	A_23_P345692	IL17D	Hs.655142	interleukin 17D	Homo sapiens interleukin 17D (IL17D), mRNA [NM_138284]	8.15196	up	7.69389	up	1784.27080	226.63747	1443.01650	165.38304	Detected	Detected	Detected	Detected
544	A_23_P5983	PLTP	Hs.439312	phospholipid transfer protein	Homo sapiens phospholipid transfer protein (PLTP), transcript variant 1, mRNA [NM_006227]	8.14628	up	8.80226	up	3554.11060	451.75687	4003.48320	401.05980	Detected	Detected	Detected	Detected
545	A_23_P57474	OSBP2	Hs.517546	oxysterol binding protein 2	Homo sapiens oxysterol binding protein 2 (OSBP2), transcript variant 1, mRNA [NM_030758]	8.12138	up	9.23521	up	106.95686	13.63679	84.62169	8.07980	Detected	Compromised	Detected	Compromised
546	A_33_P3338186	HEXDC	Hs.444743	hexosaminidase (glycosyl hydrolase family 20, catalytic domain) containing	Homo sapiens hexosaminidase (glycosyl hydrolase family 20, catalytic domain) containing (HEXDC), mRNA [NM_173620]	8.09436	up	7.10755	up	8314.43500	1063.61190	6559.04830	813.74133	Detected	Detected	Detected	Detected
547	A_33_P3379091	SYNGR1	Hs.216226	synaptogyrin 1	Homo sapiens synaptogyrin 1 (SYNGR1), transcript variant 1b, mRNA [NM_145731]	8.07832	up	7.92747	up	880.93414	112.91608	891.82170	99.19933	Detected	Detected	Detected	Detected
548	A_33_P3290780	IL24	Hs.719320	interleukin 24	Homo sapiens interleukin 24 (IL24), transcript variant 1, mRNA [NM_006850]	8.04391	up	8.44953	up	649.64110	83.62560	836.68066	87.31576	Detected	Detected	Detected	Detected
549	A_33_P3319140	OVOS2	Hs.524331	ovostatin 2	Homo sapiens ovostatin 2, mRNA (cDNA clone IMAGE:4827636). [BC039117]	8.03633	up	4.74312	up	73.18561	9.42977	53.39385	9.92642	Detected	Compromised	Detected	Compromised

550	A_23_P216812	CDKN2B	Hs.72901	cyclin-dependent kinase inhibitor 2B (p15, inhibits CDK4)	Homo sapiens cyclin-dependent kinase inhibitor 2B (p15, inhibits CDK4) (CDKN2B), transcript variant 1, mRNA [NM_004936]	8.01473	up	6.31469	up	105.57578	13.63982	123.55238	17.25301	Detected	Compromised	Detected	Detected
551	A_32_P456318	SFRS13B	Hs.254414	splicing factor, arginine/serine-rich 13B	Homo sapiens splicing factor, arginine/serine-rich 13B (SFRS13B), mRNA [NM_080743]	8.00781	up	1.15321	up	37.40963	4.83730	24.75887	18.93168	Detected	Compromised	Detected	Detected
552	A_33_P3355921		Hs.678899		cDNA FLJ37701 fis, clone BRHIP2016990HCG1998535 ; [Source:UniProtKB/TrEMBL;Acc:Q8N9D1] [ENST00000398177]	7.99882	up	14.97550	up	90.64121	11.73364	45.11845	2.65667	Detected	Compromised	Detected	Compromised
553	A_23_P254181	MGC5566	Hs.255479	hypothetical protein MGC5566	Homo sapiens hypothetical protein MGC5566, mRNA (cDNA clone IMAGE:3458308). [BC000849]	7.97111	up	8.69939	up	188.44904	24.47981	212.33977	21.52325	Detected	Detected	Detected	Detected
554	A_23_P209288	CUL3	Hs.372286	cullin 3	Homo sapiens cullin 3 (CUL3), mRNA [NM_003590]	7.96038	up	9.63882	up	84.66550	11.01301	75.32278	6.89077	Detected	Compromised	Detected	Compromised
555	A_33_P3335366	MAML3	Hs.586165	mastermind-like 3 (Drosophila)	Homo sapiens mastermind-like 3 (Drosophila) (MAML3), mRNA [NM_018717]	7.95240	up	7.11715	up	43.66573	5.68559	44.18113	5.47389	Detected	Compromised	Detected	Compromised
556	A_23_P105963	AK7	Hs.667462	adenylate kinase 7	Homo sapiens adenylate kinase 7 (AK7), mRNA [NM_152327]	7.95174	up	6.89834	up	44.38477	5.77970	41.50077	5.30490	Detected	Compromised	Detected	Compromised
557	A_23_P101407	C3	Hs.529053	complement component 3	Homo sapiens complement component 3 (C3), mRNA [NM_000064]	7.94058	up	9.33844	up	4513.73240	588.59515	3410.00150	321.99243	Detected	Detected	Detected	Detected
558	A_32_P59678	C7orf46	Hs.592178	chromosome 7 open reading frame 46	Homo sapiens chromosome 7 open reading frame 46 (C7orf46), transcript variant 2, mRNA [NM_001127364]	7.93099	up	8.96531	up	73.48454	9.59404	71.73080	7.05514	Detected	Compromised	Detected	Compromised
559	A_23_P30666	TNFRSF21	Hs.443577	tumor necrosis factor receptor superfamily, member 21	Homo sapiens tumor necrosis factor receptor superfamily, member 21 (TNFRSF21), mRNA [NM_014452]	7.91594	up	7.76166	up	7500.66460	981.13820	6455.31150	733.37820	Detected	Detected	Detected	Detected
560	A_23_P5018	LOC80054	Hs.287521	hypothetical LOC80054	Homo sapiens hypothetical LOC80054 (LOC80054), non-coding RNA [NR_026887]	7.89870	up	4.97715	up	29.94474	3.92553	33.00670	5.84772	Detected	Compromised	Detected	Compromised
561	A_23_P64792	KCNMB4	Hs.525529	potassium large conductance calcium-activated channel, subfamily M, beta member 4	Homo sapiens potassium large conductance calcium-activated channel, subfamily M, beta member 4 (KCNMB4), mRNA [NM_014505]	7.88355	up	7.47561	up	2530.45070	332.36047	2179.88650	257.12985	Detected	Detected	Detected	Detected
562	A_33_P3232692	IL24	Hs.719320	interleukin 24	Homo sapiens interleukin 24 (IL24), transcript variant 1, mRNA [NM_006850]	7.85889	up	8.30030	up	5098.97800	671.82294	4074.78690	432.88904	Detected	Detected	Detected	Detected
563	A_33_P3219840	NCRNA00171	Hs.653168	non-protein coding RNA 171	Homo sapiens non-protein coding RNA 171 (NCRNA00171), non-coding RNA [NR_026751]	7.85330	up	5.59349	up	105.55917	13.91801	89.23410	14.06738	Detected	Compromised	Detected	Compromised
564	A_23_P53763	C13orf18	Hs.98117	chromosome 13 open reading frame 18	Homo sapiens chromosome 13 open reading frame 18 (C13orf18), mRNA [NM_025113]	7.84288	up	8.20862	up	765.34607	101.04516	529.63650	56.89488	Detected	Detected	Detected	Detected
565	A_23_P66017	PRRT2	Hs.655071	proline-rich transmembrane protein 2	Homo sapiens proline-rich transmembrane protein 2 (PRRT2), mRNA [NM_145239]	7.84249	up	7.79280	up	1851.86720	244.50580	1928.21040	218.18588	Detected	Detected	Detected	Detected
566	A_32_P125338	FAM43B	Hs.429179	family with sequence similarity 43, member B	Homo sapiens family with sequence similarity 43, member B (FAM43B), mRNA [NM_207334]	7.84149	up	6.38987	up	159.30914	21.03658	120.04231	16.56563	Detected	Detected	Detected	Detected
567	A_24_P366509	PIK3R6	Hs.255809	phosphoinositide-3-kinase, regulatory subunit 6	Homo sapiens phosphoinositide-3-kinase, regulatory subunit 6 (PIK3R6), mRNA [NM_001010855]	7.82701	up	4.08911	up	25.60742	3.38769	22.77060	4.91033	Detected	Compromised	Detected	Compromised
568	A_33_P3270767	TSC22D2	Hs.665220	TSC22 domain family, member 2	RST23433 Athersys RAGE Library Homo sapiens cDNA, mRNA sequence [BG204039]	7.82025	up	5.37405	up	23.16541	3.06727	15.49602	2.54264	Detected	Compromised	Detected	Compromised
569	A_33_P3260223	CYorf15B	Hs.592254	chromosome Y open reading frame 15B	Homo sapiens chromosome Y open reading frame 15B (CYorf15B), mRNA [NM_032576]	7.81634	up	24.08387	up	756.09600	100.16287	471.55392	17.26516	Detected	Detected	Detected	Compromised
570	A_24_P7790	WFDC10B	Hs.237392	WAP four-disulfide core domain 10B	Homo sapiens WAP four-disulfide core domain 10B (WFDC10B), transcript variant 1, mRNA [NM_172006]	7.79049	up	4.34465	up	31.88236	4.23759	18.86051	3.82794	Detected	Compromised	Detected	Compromised
571	A_23_P315772	KCND1	Hs.55276	potassium voltage-gated channel, Shal-related subfamily, member 1	Homo sapiens potassium voltage-gated channel, Shal-related subfamily, member 1 (KCND1), mRNA [NM_004979]	7.78910	up	3.75876	up	48.49635	6.44696	26.60850	6.24225	Detected	Compromised	Detected	Compromised
572	A_33_P3316273	CCL3	Hs.514107	chemokine (C-C motif) ligand 3	Homo sapiens chemokine (C-C motif) ligand 3 (CCL3), mRNA [NM_002983]	7.77769	up	8.54236	up	225.88904	30.07306	171.57172	17.71060	Detected	Detected	Detected	Detected
573	A_23_P203957	TMTC1	Hs.401954	transmembrane and tetratricopeptide repeat containing 1	Homo sapiens transmembrane and tetratricopeptide repeat containing 1 (TMTC1), mRNA [NM_175861]	7.77614	up	8.93069	up	2082.08080	277.24670	1652.38620	163.15189	Detected	Detected	Detected	Detected
574	A_24_P145019		Hs.720676		cDNA FLJ38235 fis, clone FCBBF2005428 [Source:UniProtKB/TrEMBL;Acc:Q8N984] [ENST00000356642]	7.76885	up	3.57712	up	23.59186	3.14441	10.63042	2.62049	Detected	Compromised	Compromised	Compromised
575	A_33_P3340324	hCG_38984	Hs.170876	hCG38984	PREDICTED: Homo sapiens hCG38984 (LOC345051), miscRNA [XR_040587]	7.76360	up	10.94891	up	156.81924	20.91556	128.38272	10.33954	Detected	Detected	Detected	Compromised
576	A_24_P298587	C8ORFK29	Hs.122231	hypothetical LOC340393	Homo sapiens hypothetical LOC340393 (C8ORFK29), non-coding RNA [NR_015428]	7.75522	up	6.22087	up	94.84693	12.66375	95.65533	13.55888	Detected	Compromised	Detected	Compromised
577	A_24_P305038	C17orf69		chromosome 17 open reading frame 69	Homo sapiens chromosome 17 open reading frame 69 (C17orf69), transcript variant 1, non-coding RNA [NR_026905]	7.74112	up	6.59887	up	64.70684	8.65525	76.28774	10.19415	Detected	Compromised	Detected	Compromised
578	A_33_P3477521	LOC284080	Hs.274864	hypothetical protein LOC284080	Homo sapiens cDNA FLJ39442 fis, clone PROST2005904 [AK096761]	7.73789	up	7.35760	up	189.52510	25.36165	105.45741	12.63881	Detected	Detected	Detected	Compromised
579	A_24_P289299	GEFT	Hs.61581	RhoA/RAC/CDC42 exchange factor	Homo sapiens RhoA/RAC/CDC42 exchange factor (GEFT), transcript variant 1, mRNA [NM_182947]	7.72623	up	4.02937	up	119.51981	16.01789	127.23877	27.84503	Detected	Detected	Detected	Detected
580	A_33_P3216945		Hs.640952		DB028998 TESTI2 Homo sapiens cDNA clone TESTI2012045 5', mRNA sequence [DB028998]	7.70252	up	8.16420	up	797.80725	107.25018	700.73096	75.68382	Detected	Detected	Detected	Detected

581	A_33_P3535649	FLYWCH1	Hs.655321	FLYWCH-type zinc finger 1	Homo sapiens FLYWCH-type zinc finger 1 (FLYWCH1), transcript variant 1, mRNA [NM_032296]	7.68000	up	8.05731	up	375.83410	50.67199	362.68710	39.69240	Detected	Detected	Detected	Detected
582	A_23_P205738	BCL11B	Hs.709690	B-cell CLL/lymphoma 11B (zinc finger protein)	Homo sapiens B-cell CLL/lymphoma 11B (zinc finger protein) (BCL11B), transcript variant 1, mRNA [NM_138576]	7.67555	up	11.47816	up	364.73890	49.20457	272.20856	20.91197	Detected	Detected	Detected	Detected
583	A_33_P3230478	C1S	Hs.458355	complement component 1, s subcomponent	Homo sapiens complement component 1, s subcomponent (C1S), transcript variant 1, mRNA [NM_201442]	7.67387	up	7.08031	up	3915.60230	528.34470	3311.75100	412.44962	Detected	Detected	Detected	Detected
584	A_33_P3740529	LOC645485	Hs.232332	hypothetical LOC645485	Homo sapiens, clone IMAGE:5741993, mRNA [BC039526]	7.65914	up	2.64696	up	23.93516	3.23586	16.16532	5.38520	Detected	Compromised	Detected	Compromised
585	A_23_P113613	CDCP1	Hs.476093	CUB domain containing protein 1	Homo sapiens CUB domain containing protein 1 (CDCP1), transcript variant 1, mRNA [NM_022842]	7.65075	up	7.94523	up	1550.28330	209.81682	1471.51890	163.31450	Detected	Detected	Detected	Detected
586	A_23_P102611	WISP2	Hs.592145	WNT1 inducible signaling pathway protein 2	Homo sapiens WNT1 inducible signaling pathway protein 2 (WISP2), mRNA [NM_003881]	7.63944	up	6.72916	up	13860.26300	1878.63890	13252.32000	1736.58600	Detected	Detected	Detected	Detected
587	A_24_P202319	ATP2A3	Hs.513870	ATPase, Ca++ transporting, ubiquitous	Homo sapiens ATPase, Ca++ transporting, ubiquitous (ATP2A3), transcript variant 7, mRNA [NM_174958]	7.63152	up	3.54322	up	24.27437	3.29360	32.57851	8.10773	Detected	Compromised	Detected	Compromised
588	A_33_P3671291	SNORA12	Hs.662165	small nucleolar RNA, H/ACA box 12	EST91069 Synovial sarcoma Homo sapiens cDNA 5' end, mRNA sequence [AA378382]	7.61973	up	7.82452	up	642.27500	87.27995	609.14620	68.64820	Detected	Detected	Detected	Detected
589	A_32_P96036	MEX3A	Hs.591496	mex-3 homolog A (C. elegans)	Homo sapiens mex-3 homolog A (C. elegans) (MEX3A), mRNA [NM_001093725]	7.61670	up	7.13788	up	725.40920	98.61645	455.00530	56.20992	Detected	Detected	Detected	Detected
590	A_33_P3356587	LOC100134040	Hs.552781	hypothetical LOC100134040	Homo sapiens mRNA; cDNA DKFZp434G1726 (from clone DKFZp434G1726) [AL162052]	7.59399	up	6.01596	up	149.45530	20.37862	85.51138	12.53385	Detected	Detected	Detected	Compromised
591	A_33_P3261298	SLC6A7	Hs.241597	solute carrier family 6 (neurotransmitter transporter, L-proline), member 7	Homo sapiens solute carrier family 6 (neurotransmitter transporter, L-proline), member 7 (SLC6A7), mRNA [NM_014228]	7.57180	up	14.71027	up	130.13478	17.79621	96.49406	5.78423	Detected	Detected	Detected	Compromised
592	A_33_P3302260	C11orf9	Hs.473109	chromosome 11 open reading frame 9	Homo sapiens chromosome 11 open reading frame 9 (C11orf9), transcript variant 1, mRNA [NM_013279]	7.56261	up	2.76341	up	50.79312	6.95451	14.61400	4.66326	Detected	Compromised	Detected	Compromised
593	A_24_P135748	GRTP1	Hs.170904	growth hormone regulated TBC protein 1	Homo sapiens growth hormone regulated TBC protein 1 (GRTP1), mRNA [NM_024719]	7.56150	up	10.19003	up	110.03301	15.06774	111.97868	9.69003	Detected	Compromised	Detected	Compromised
594	A_33_P3391120	LOC646870	Hs.674889	hypothetical protein LOC646870	PREDICTED: Homo sapiens hypothetical protein LOC646870 (LOC646870), miscRNA [XR_040282]	7.55422	up	5.19235	up	24.84197	3.40510	18.12376	3.07787	Detected	Compromised	Detected	Compromised
595	A_23_P252082	TMEM176A	Hs.647116	transmembrane protein 176A	Homo sapiens transmembrane protein 176A (TMEM176A), mRNA [NM_018487]	7.51730	up	9.63331	up	256.53650	35.33626	304.49338	27.87198	Detected	Detected	Detected	Detected
596	A_33_P3251562	RSPO1	Hs.135015	R-spondin homolog (Xenopus laevis)	Homo sapiens R-spondin homolog (Xenopus laevis) (RSPO1), mRNA [NM_001038633]	7.51680	up	8.03557	up	35.83232	4.93599	22.71169	2.49229	Detected	Compromised	Detected	Compromised
597	A_24_P403417	PTGES	Hs.146688	prostaglandin E synthase	Homo sapiens prostaglandin E synthase (PTGES), mRNA [NM_004878]	7.51213	up	7.39460	up	1509.08740	208.01013	1316.15000	156.94815	Detected	Detected	Detected	Detected
598	A_23_P218086	TPCN1	Hs.524763	two pore segment channel 1	Homo sapiens two pore segment channel 1 (TPCN1), transcript variant 1, mRNA [NM_001143819]	7.50966	up	6.86498	up	24734.60200	3410.49950	15893.71900	2041.51070	Detected	Detected	Detected	Detected
599	A_23_P356425	C1orf125	Hs.658505	chromosome 1 open reading frame 125	Homo sapiens chromosome 1 open reading frame 125 (C1orf125), transcript variant 1, mRNA [NM_144696]	7.50919	up	7.02896	up	51.96343	7.16536	26.74605	3.35532	Detected	Compromised	Detected	Compromised
600	A_23_P165624	TNFAIP6	Hs.437322	tumor necrosis factor, alpha-induced protein 6	Homo sapiens tumor necrosis factor, alpha-induced protein 6 (TNFAIP6), mRNA [NM_007115]	7.50887	up	7.44853	up	3267.00240	450.51390	3085.84670	365.31656	Detected	Detected	Detected	Detected
601	A_33_P3489675	PRR5-ARHGAP8	Hs.720401	PRR5-ARHGAP8 readthrough	Homo sapiens PRR5-ARHGAP8 fusion (LOC553158), mRNA [NM_181334]	7.50606	up	9.10672	up	35.05180	4.83539	33.34568	3.22881	Detected	Compromised	Detected	Compromised
602	A_23_P304897	BDKRB2	Hs.654542	bradykinin receptor B2	Homo sapiens bradykinin receptor B2 (BDKRB2), mRNA [NM_000623]	7.49730	up	7.00011	up	7759.26500	1071.63960	7206.35000	907.77020	Detected	Detected	Detected	Detected
603	A_33_P3877349	LOC440173		hypothetical LOC440173	Homo sapiens hypothetical LOC440173 (LOC440173), non-coding RNA [NR_027471]	7.46742	up	5.36045	up	27.75637	3.84880	19.76743	3.25173	Detected	Compromised	Detected	Compromised
604	A_33_P3266780	PODXL2	Hs.591290	podocalyxin-like 2	Homo sapiens podocalyxin-like 2 (PODXL2), mRNA [NM_015720]	7.40972	up	2.87423	up	24.97647	3.49030	11.10032	3.40549	Detected	Compromised	Compromised	Compromised
605	A_32_P453321	C1orf228	Hs.173679	chromosome 1 open reading frame 228	Homo sapiens chromosome 1 open reading frame 228 (C1orf228), mRNA [NM_001145636]	7.40199	up	6.39397	up	112.67412	15.76191	108.05853	14.90232	Detected	Detected	Detected	Detected
606	A_23_P79518	IL1B	Hs.126256	interleukin 1, beta	Homo sapiens interleukin 1, beta (IL1B), mRNA [NM_000576]	7.37966	up	7.91385	up	1613.51530	226.39677	1139.80290	127.00109	Detected	Detected	Detected	Detected
607	A_33_P3388006	CCDC52	Hs.477144	coiled-coil domain containing 52	Homo sapiens coiled-coil domain containing 52 (CCDC52), mRNA [NM_144718]	7.37023	up	3.79944	up	21.44811	3.01329	12.21372	2.83461	Detected	Compromised	Detected	Compromised
608	A_33_P3218925	STEAP2	Hs.489051	six transmembrane epithelial antigen of the prostate 2	Homo sapiens six transmembrane epithelial antigen of the prostate 2 (STEAP2), transcript variant 1, mRNA [NM_152999]	7.35935	up	8.26298	up	101.28952	14.25143	69.37874	7.40381	Detected	Compromised	Detected	Compromised
609	A_24_P28722	RSAD2	Hs.17518	radical S-adenosyl methionine domain containing 2	Homo sapiens radical S-adenosyl methionine domain containing 2 (RSAD2), mRNA [NM_080657]	7.33513	up	9.49808	up	25.38974	3.58413	26.39944	2.45089	Detected	Compromised	Detected	Compromised
610	A_24_P246841	SLC25A27	Hs.40510	solute carrier family 25, member 27	Homo sapiens solute carrier family 25, member 27 (SLC25A27), nuclear gene encoding mitochondrial protein, mRNA [NM_004277]	7.32929	up	7.75624	up	104.70060	14.79177	69.97617	7.95545	Detected	Compromised	Detected	Compromised

611	A_23_P403898	PTPN3	Hs.436429	protein tyrosine phosphatase, non-receptor type 3	Homo sapiens protein tyrosine phosphatase, non-receptor type 3 (PTPN3), transcript variant 1, mRNA [NM_002829]	7.31743	up	6.16256	up	332.30313	47.02288	203.04002	29.05266	Detected	Detected	Detected	Detected
612	A_33_P3381338	TNXB	Hs.485104	tenascin XB	Homo sapiens tenascin XB (TNXB), transcript variant XB, mRNA [NM_019105]	7.31668	up	7.48042	up	1737.41860	245.88000	1497.45800	176.52000	Detected	Detected	Detected	Detected
613	A_23_P131676	CXCR7	Hs.471751	chemokine (C-X-C motif) receptor 7	Homo sapiens chemokine (C-X-C motif) receptor 7 (CXCR7), mRNA [NM_020311]	7.30382	up	6.65820	up	11804.55300	1673.52610	9467.92000	1253.90190	Detected	Detected	Detected	Detected
614	A_33_P3212716		Hs.640952		DB028998 TESTI2 Homo sapiens cDNA clone TESTI2012045 5', mRNA sequence [DB028998]	7.29581	up	8.69042	up	656.72656	93.20602	610.00446	61.89534	Detected	Detected	Detected	Detected
615	A_24_P217234	SLC3A1	Hs.112916	solute carrier family 3 (cystine, dibasic and neutral amino acid transporters, activator of cystine, dibasic and neutral amino acid transport), member 1	Homo sapiens solute carrier family 3 (cystine, dibasic and neutral amino acid transporters, activator of cystine, dibasic and neutral amino acid transport), member 1 (SLC3A1), mRNA [NM_000341]	7.28255	up	3.49631	up	23.52974	3.34555	19.30765	4.86951	Detected	Compromised	Detected	Compromised
616	A_23_P79108	ATP8B3	Hs.306212	ATPase, class I, type 8B, member 3	Homo sapiens ATPase, class I, type 8B, member 3 (ATP8B3), mRNA [NM_138813]	7.27230	up	3.56536	up	72.12391	10.26929	50.27422	12.43389	Detected	Compromised	Detected	Compromised
617	A_33_P3287223	DPP4	Hs.368912	dipeptidyl-peptidase 4	Homo sapiens dipeptidyl-peptidase 4 (DPP4), mRNA [NM_001935]	7.26734	up	6.81552	up	5817.76170	828.92180	5390.58060	697.43190	Detected	Detected	Detected	Detected
618	A_33_P3216928				Putative uncharacterized protein C1orf191 Precursor [Source:UniProtKB/Swiss-Prot;Acc:Q7Z2R9] [ENST00000361350]	7.26379	up	2.07738	up	22.48648	3.20547	6.38011	2.70818	Detected	Compromised	Compromised	Compromised
619	A_23_P61057	IL16	Hs.459095	interleukin 16 (lymphocyte chemoattractant factor)	Homo sapiens interleukin 16 (lymphocyte chemoattractant factor) (IL16), transcript variant 1, mRNA [NM_004513]	7.26055	up	4.25595	up	63.03963	8.99036	88.42661	18.32111	Detected	Compromised	Detected	Detected
620	A_33_P3358042		Hs.675311		Liver-related low express protein 1 [Source:UniProtKB/TrEMBL;Acc:Q6SA06] [ENST00000431438]	7.23865	up	4.19700	up	42.75609	6.11608	31.04437	6.52243	Detected	Compromised	Detected	Compromised
621	A_23_P326931	TTC18	Hs.591367	tetratricopeptide repeat domain 18	Homo sapiens tetratricopeptide repeat domain 18 (TTC18), mRNA [NM_145170]	7.21864	up	8.33507	up	170.29208	24.42710	139.43523	14.75125	Detected	Detected	Detected	Compromised
622	A_33_P3324206	HR	Hs.272367	hairless homolog (mouse)	Homo sapiens hairless homolog (mouse) (HR), transcript variant 1, mRNA [NM_005144]	7.19136	up	6.37325	up	2981.85030	429.34644	2543.22390	351.87555	Detected	Detected	Detected	Detected
623	A_33_P3228355	ERV3	Hs.250693	endogenous retroviral sequence 3 (includes zinc finger protein H-plk/HPF9)	Homo sapiens endogenous retroviral sequence 3 (includes zinc finger protein H-plk/HPF9) (ERV3), mRNA [NM_001007253]	7.17834	up	7.97668	up	91.80429	13.24257	110.73068	12.24084	Detected	Compromised	Detected	Compromised
624	A_23_P79496	GTF2A1L	Hs.44385	general transcription factor IIA, 1-like	Homo sapiens general transcription factor IIA, 1-like (GTF2A1L), transcript variant 2, mRNA [NM_172196]	7.16416	up	6.33186	up	62.05046	8.96835	48.18505	6.71037	Detected	Compromised	Detected	Compromised
625	A_32_P146113	C6orf81	Hs.533066	chromosome 6 open reading frame 81	Homo sapiens chromosome 6 open reading frame 81 (C6orf81), mRNA [NM_145028]	7.16002	up	8.89184	up	70.83432	10.24383	61.30997	6.08002	Detected	Compromised	Detected	Compromised
626	A_23_P254863	MGC42105	Hs.25845	serine/threonine-protein kinase NIM1	Homo sapiens serine/threonine-protein kinase NIM1 (MGC42105), mRNA [NM_153361]	7.15758	up	6.56844	up	385.26578	55.73489	400.35233	53.74591	Detected	Detected	Detected	Detected
627	A_23_P158318	ROR2	Hs.98255	receptor tyrosine kinase-like orphan receptor 2	Homo sapiens receptor tyrosine kinase-like orphan receptor 2 (ROR2), mRNA [NM_004560]	7.14728	up	6.64486	up	773.56506	112.07002	751.34485	99.70541	Detected	Detected	Detected	Detected
628	A_32_P220750	PLGLB1	Hs.652174	plasminogen-like B1	Homo sapiens plasminogen-like B1 (PLGLB1), mRNA [NM_001032392]	7.10435	up	1.91861	up	31.64338	4.61202	19.40338	8.91777	Detected	Compromised	Detected	Compromised
629	A_24_P341674	DIO2	Hs.202354	deiodinase, iodothyronine, type II	Homo sapiens deiodinase, iodothyronine, type II (DIO2), transcript variant 1, mRNA [NM_013989]	7.09681	up	17.33626	up	72.66090	10.60158	68.62457	3.49052	Detected	Compromised	Detected	Compromised
630	A_32_P196263	ADAMTS9	Hs.656071	ADAM metallopeptidase with thrombospondin type 1 motif, 9	Homo sapiens ADAM metallopeptidase with thrombospondin type 1 motif, 9 (ADAMTS9), mRNA [NM_182920]	7.08794	up	10.90263	up	202.86755	29.63640	131.94072	10.67120	Detected	Detected	Detected	Compromised
631	A_33_P3245243					7.08375	up	2.60300	up	335.86508	49.09472	39.47469	13.37244	Detected	Detected	Detected	Compromised
632	A_23_P167818	FAM184A	Hs.443789	family with sequence similarity 184, member A	Homo sapiens family with sequence similarity 184, member A (FAM184A), transcript variant 1, mRNA [NM_024581]	7.07978	up	8.28033	up	108.42570	15.85789	99.45076	10.59073	Detected	Detected	Detected	Compromised
633	A_23_P113034	C10orf11	Hs.118161	chromosome 10 open reading frame 11	Homo sapiens chromosome 10 open reading frame 11 (C10orf11), mRNA [NM_032024]	7.07885	up	6.37025	up	42.39873	6.20188	36.82369	5.09725	Detected	Compromised	Detected	Compromised
634	A_33_P3290089					7.07459	up	5.14633	up	282.89093	41.40481	195.78583	33.54662	Detected	Detected	Detected	Detected
635	A_32_P104432	NCRNA00087	Hs.656427	non-protein coding RNA 87	Homo sapiens non-protein coding RNA 87 (NCRNA00087), non-coding RNA [NR_024493]	7.05366	up	7.35662	up	1827.52110	268.27536	1177.58340	141.14928	Detected	Detected	Detected	Detected
636	A_33_P3389658	PDE4D	Hs.117545	phosphodiesterase 4D, cAMP-specific (phosphodiesterase E3 dunce homolog, Drosophila)	Homo sapiens phosphodiesterase 4D, cAMP-specific (phosphodiesterase E3 dunce homolog, Drosophila) (PDE4D), transcript variant 3, mRNA [NM_001165899]	7.03810	up	3.95760	up	45.17578	6.64635	58.90025	13.12352	Detected	Compromised	Detected	Compromised
637	A_33_P3289227	DNALI1	Hs.406050	dynein, axonemal, light intermediate chain 1	Homo sapiens dynein, axonemal, light intermediate chain 1 (DNALI1), mRNA [NM_003462]	7.03807	up	6.60107	up	762.18000	112.13401	532.49790	71.13265	Detected	Detected	Detected	Detected
638	A_23_P383009	IGFBP5	Hs.607212	insulin-like growth factor binding protein 5	Homo sapiens insulin-like growth factor binding protein 5 (IGFBP5), mRNA [NM_000599]	7.03738	up	8.91316	up	131.08830	19.28795	80.21664	7.93594	Detected	Detected	Detected	Compromised
639	A_33_P3364062	FLJ31356	Hs.562970	hypothetical protein FLJ31356	Homo sapiens cDNA FLJ31356 fis, clone MESAN2000337 [AK055918]	7.03553	up	5.18600	up	22.31790	3.28466	16.45729	2.79828	Detected	Compromised	Detected	Compromised

640	A_23_P66011	NECAB2	Hs.140950	N-terminal EF-hand calcium binding protein 2	Homo sapiens N-terminal EF-hand calcium binding protein 2 (NECAB2), mRNA [NM_019065]	7.03538	up	6.61375	up	20.81387	3.06336	19.02355	2.53635	Detected	Compromised	Detected	Compromised
641	A_23_P6066	CPXM1	Hs.659346	carboxypeptidase X (M14 family), member 1	Homo sapiens carboxypeptidase X (M14 family), member 1 (CPXM1), mRNA [NM_019609]	7.01986	up	11.32421	up	208.82698	30.80286	215.79158	16.80320	Detected	Detected	Detected	Detected
642	A_23_P21485	PID1	Hs.715695	phosphotyrosine interaction domain containing 1	Homo sapiens phosphotyrosine interaction domain containing 1 (PID1), transcript variant 1, mRNA [NM_017933]	7.01960	up	7.43536	up	15322.27800	2260.18530	12243.05300	1451.95530	Detected	Detected	Detected	Detected
643	A_33_P3258617				Adipophilin (Adipose differentiation-related protein)(ADRP) [Source:UniProtKB/Swiss-Prot;Acc:Q99541] [ENST00000380464]	7.00668	up	7.87524	up	2671.99440	394.87200	2063.65530	231.06764	Detected	Detected	Detected	Detected
644	A_33_P3377364	ITGB4	Hs.632226	integrin, beta 4	Homo sapiens integrin, beta 4 (ITGB4), transcript variant 1, mRNA [NM_000213]	6.99413	up	5.76255	up	408.56836	60.48727	481.80383	73.72607	Detected	Detected	Detected	Detected
645	A_32_P2392	GOLGA8A	Hs.720151	golgi autoantigen, golgin subfamily a, 8A	Homo sapiens golgi autoantigen, golgin subfamily a, 8A (GOLGA8A), transcript variant 1, mRNA [NM_181077]	6.99208	up	8.75336	up	467.74530	69.26858	314.52844	31.68479	Detected	Detected	Detected	Detected
646	A_24_P626850	H6PD	Hs.463511	hexose-6-phosphate dehydrogenase (glucose 1-dehydrogenase)	Homo sapiens hexose-6-phosphate dehydrogenase (glucose 1-dehydrogenase) (H6PD), mRNA [NM_004285]	6.98240	up	7.64687	up	606.41656	89.92889	516.40515	59.54870	Detected	Detected	Detected	Detected
647	A_33_P3418716	EFHD1	Hs.516769	EF-hand domain family, member D1	Homo sapiens EF-hand domain family, member D1 (EFHD1), transcript variant 1, mRNA [NM_025202]	6.97931	up	7.59319	up	106.44068	15.79168	135.26530	15.70824	Detected	Compromised	Detected	Detected
648	A_33_P3251522	LVRN	Hs.98288	laeverin	Homo sapiens laeverin (LVRN), mRNA [NM_173800]	6.96637	up	6.79072	up	122.62955	18.22728	105.04511	13.64034	Detected	Detected	Detected	Compromised
649	A_24_P329795	C10orf10	Hs.93675	chromosome 10 open reading frame 10	Homo sapiens chromosome 10 open reading frame 10 (C10orf10), mRNA [NM_007021]	6.94372	up	6.63648	up	11099.41600	1655.16410	5629.94530	748.05273	Detected	Detected	Detected	Detected
650	A_23_P405110	CDC14A	Hs.127411	CDC14 cell division cycle 14 homolog A (S. cerevisiae)	Homo sapiens CDC14 cell division cycle 14 homolog A (S. cerevisiae) (CDC14A), transcript variant 3, mRNA [NM_033313]	6.94362	up	1.43120	up	19.97107	2.97816	3.97670	2.45014	Detected	Compromised	Compromised	Compromised
651	A_33_P3388651	ABLIM1	Hs.438236	actin binding LIM protein 1	Homo sapiens actin binding LIM protein 1 (ABLIM1), transcript variant 3, mRNA [NM_001003408]	6.93236	up	11.04492	up	85.66856	12.79598	40.75651	3.25387	Detected	Compromised	Detected	Compromised
652	A_33_P3352382	ARG1	Hs.440934	arginase, liver	Homo sapiens arginase, liver (ARG1), mRNA [NM_000045]	6.89363	up	4.57451	up	24.63676	3.70057	16.70089	3.21929	Detected	Compromised	Detected	Compromised
653	A_23_P206733	CES1	Hs.558865	carboxylesterase 1 (monocyte/macrophage serine esterase 1)	Homo sapiens carboxylesterase 1 (monocyte/macrophage serine esterase 1) (CES1), transcript variant 3, mRNA [NM_001266]	6.87506	up	8.08715	up	3190.61300	480.54108	3727.48050	406.42984	Detected	Detected	Detected	Detected
654	A_33_P3293858	MTUS2	Hs.22287	microtubule associated tumor suppressor candidate 2	Homo sapiens microtubule associated tumor suppressor candidate 2 (MTUS2), transcript variant 1, mRNA [NM_001033602]	6.87124	up	6.26198	up	30.03388	4.52594	22.33073	3.14454	Detected	Compromised	Detected	Compromised
655	A_33_P3276703	VGF	Hs.587325	VGF nerve growth factor inducible	Homo sapiens VGF nerve growth factor inducible (VGF), mRNA [NM_003378]	6.86888	up	6.74450	up	5413.69900	816.09600	4037.55500	527.87900	Detected	Detected	Detected	Detected
656	A_23_P28834	PHACTR3	Hs.473218	phosphatase and actin regulator 3	Homo sapiens phosphatase and actin regulator 3 (PHACTR3), transcript variant 1, mRNA [NM_080672]	6.85968	up	4.96664	up	21.47659	3.24186	15.20828	2.70012	Detected	Compromised	Detected	Compromised
657	A_23_P46482	IL20	Hs.272373	interleukin 20	Homo sapiens interleukin 20 (IL20), mRNA [NM_018724]	6.85233	up	3.99505	up	25.90193	3.91406	18.29435	4.03794	Detected	Compromised	Detected	Compromised
658	A_33_P3328375	TTC29	Hs.378893	tetratricopeptide repeat domain 29	Homo sapiens tetratricopeptide repeat domain 29 (TTC29), mRNA [NM_031956]	6.85105	up	2.38646	up	21.15531	3.19739	19.45469	7.18845	Detected	Compromised	Detected	Compromised
659	A_23_P79488	STON1-GTF2A1L	Hs.44385	STON1-GTF2A1L readthrough transcript	Homo sapiens STON1-GTF2A1L readthrough transcript (STON1-GTF2A1L), mRNA [NM_172311]	6.83954	up	6.63232	up	854.30500	129.33589	678.98060	90.27293	Detected	Detected	Detected	Detected
660	A_33_P3309075	TBC1D8	Hs.442657	TBC1 domain family, member 8 (with GRAM domain)	Homo sapiens TBC1 domain family, member 8 (with GRAM domain) (TBC1D8), mRNA [NM_001102426]	6.83868	up	6.44097	up	7926.22560	1200.12710	7572.35400	1036.68180	Detected	Detected	Detected	Detected
661	A_33_P3421923	CADM3	Hs.365689	cell adhesion molecule 3	Homo sapiens cell adhesion molecule 3 (CADM3), transcript variant 1, mRNA [NM_021189]	6.83582	up	12.03720	up	65.71242	9.95383	92.42572	6.77069	Detected	Compromised	Detected	Compromised
662	A_23_P133712	CYP39A1	Hs.387367	cytochrome P450, family 39, subfamily A, polypeptide 1	Homo sapiens cytochrome P450, family 39, subfamily A, polypeptide 1 (CYP39A1), mRNA [NM_016593]	6.82391	up	7.90140	up	21.64694	3.28470	24.61440	2.74695	Detected	Compromised	Detected	Compromised
663	A_23_P207245	TEKT3	Hs.414648	tektin 3	Homo sapiens tektin 3 (TEKT3), mRNA [NM_031898]	6.80932	up	1.98950	up	20.63918	3.13850	17.57672	7.79037	Detected	Compromised	Detected	Compromised
664	A_33_P3348086					6.80800	up	2.31116	up	21.88845	3.32912	12.97779	4.95149	Detected	Compromised	Compromised	Compromised
665	A_23_P96590	GPRASP1	Hs.710048	G protein-coupled receptor associated sorting protein 1	Homo sapiens G protein-coupled receptor associated sorting protein 1 (GPRASP1), transcript variant 1, mRNA [NM_014710]	6.80673	up	12.58670	up	1086.01300	165.20740	647.91840	45.39145	Detected	Detected	Detected	Detected
666	A_23_P208302	APOC2	Hs.75615	apolipoprotein C-II	Homo sapiens apolipoprotein C-II (APOC2), mRNA [NM_000483]	6.80348	up	6.86199	up	43.14624	6.56667	38.91995	5.00136	Detected	Compromised	Detected	Compromised
667	A_23_P434398	TXLNB	Hs.535820	taxilin beta	Homo sapiens taxilin beta (TXLNB), mRNA [NM_153235]	6.80140	up	5.89683	up	27.23257	4.14595	16.71405	2.49936	Detected	Compromised	Detected	Compromised
668	A_23_P201551	VAV3	Hs.267659	vav 3 guanine nucleotide exchange factor	Homo sapiens vav 3 guanine nucleotide exchange factor (VAV3), transcript variant 1, mRNA [NM_006113]	6.79582	up	7.11222	up	1982.75660	302.10712	1764.24340	218.73529	Detected	Detected	Detected	Detected
669	A_23_P71530	TNFRSF11B	Hs.81791	tumor necrosis factor receptor superfamily, member 11b	Homo sapiens tumor necrosis factor receptor superfamily, member 11b (TNFRSF11B), mRNA [NM_002546]	6.79450	up	6.28911	up	826.93080	126.02147	664.49290	93.16795	Detected	Detected	Detected	Detected

670	A_32_P70315	TIMP4	Hs.591665	TIMP metallopeptidase inhibitor 4	Homo sapiens TIMP metallopeptidase inhibitor 4 (TIMP4), mRNA [NM_003256]	6.79088	up	6.53749	up	1974.25680	301.03085	1567.95130	211.48856	Detected	Detected	Detected	Detected
671	A_33_P3423949	CBX2	Hs.368410	chromobox homolog 2 (Pc class homolog, Drosophila)	Homo sapiens chromobox homolog 2 (Pc class homolog, Drosophila) (CBX2), transcript variant 1, mRNA [NM_005189]	6.77804	up	5.82936	up	2502.97920	382.37213	1845.42590	279.15244	Detected	Detected	Detected	Detected
672	A_23_P364444					6.77780	up	1.73782	up	41.99950	6.41635	6.09994	3.09518	Detected	Compromised	Compromised	Compromised
673	A_33_P3861634	LOC285084	Hs.275398	hypothetical protein LOC285084	Homo sapiens mRNA; cDNA DKFZp667G2020 (from clone DKFZp667G2020) [AL833646]	6.76070	up	6.33152	up	753.68976	115.43408	618.84350	86.18633	Detected	Detected	Detected	Detected
674	A_23_P101642	PTPRH	Hs.179770	protein tyrosine phosphatase, receptor type, H	Homo sapiens protein tyrosine phosphatase, receptor type, H (PTPRH), transcript variant 1, mRNA [NM_002842]	6.74886	up	25.70233	up	248.55237	38.13473	223.58888	7.67085	Detected	Detected	Detected	Compromised
675	A_23_P130961	ELANE	Hs.99863	elastase, neutrophil expressed	Homo sapiens elastase, neutrophil expressed (ELANE), mRNA [NM_001972]	6.74519	up	62.67902	up	240.62944	36.93919	222.60243	3.13165	Detected	Detected	Detected	Compromised
676	A_23_P69573	GUCY1A3	Hs.24258	guanylate cyclase 1, soluble, alpha 3	Homo sapiens guanylate cyclase 1, soluble, alpha 3 (GUCY1A3), transcript variant 1, mRNA [NM_000856]	6.73449	up	9.31997	up	135.01192	20.75872	107.33898	10.15567	Detected	Detected	Detected	Compromised
677	A_23_P363968	C1RL	Hs.631730	complement component 1, r subcomponent-like	Homo sapiens complement component 1, r subcomponent-like (C1RL), mRNA [NM_016546]	6.72902	up	6.96225	up	1141.08900	175.59038	773.58350	97.97678	Detected	Detected	Detected	Detected
678	A_23_P377616	CIRBP	Hs.618145	cold inducible RNA binding protein	Homo sapiens cold inducible RNA binding protein (CIRBP), transcript variant 2, transcribed RNA [NR_023312]	6.71722	up	5.21659	up	710.36460	109.50278	620.83110	104.94281	Detected	Detected	Detected	Detected
679	A_33_P3398316	MMP27	Hs.534479	matrix metallopeptidase 27	Homo sapiens matrix metallopeptidase 27 (MMP27), mRNA [NM_022122]	6.71506	up	3.04064	up	24.91867	3.84245	11.03678	3.20069	Detected	Compromised	Compromised	Compromised
680	A_23_P381261	ADCY4	Hs.443428	adenylate cyclase 4	Homo sapiens adenylate cyclase 4 (ADCY4), mRNA [NM_139247]	6.69895	up	6.83609	up	1069.82910	165.36392	1320.51610	170.33397	Detected	Detected	Detected	Detected
681	A_23_P107775	TMEM190	Hs.590943	transmembrane protein 190	Homo sapiens transmembrane protein 190 (TMEM190), mRNA [NM_139172]	6.68831	up	7.93560	up	442.68550	68.53498	612.68620	68.08065	Detected	Detected	Detected	Detected
682	A_33_P3252588	LOC100130093		hypothetical LOC100130093	Homo sapiens hypothetical LOC100130093 (LOC100130093), transcript variant 1, non-coding RNA [NR_024485]	6.68445	up	4.53443	up	133.93530	20.74734	118.36622	23.01815	Detected	Detected	Detected	Detected
683	A_33_P3399208		Hs.77961		HLA class I histocompatibility antigen, B-18 alpha chain Precursor (MHC class I antigen B*18) [Source:UniProtKB/Swiss-Prot;Acc:P30466] [ENST00000359635]	6.64752	up	5.50218	up	13922.18100	2168.60550	17484.75600	2802.14580	Detected	Detected	Detected	Detected
684	A_33_P3883985	LOC650392	Hs.371980	hypothetical protein LOC650392	Homo sapiens cDNA clone IMAGE:5264670 [BC036550]	6.64218	up	6.32226	up	1250.59240	194.95682	1196.47230	166.87672	Detected	Detected	Detected	Detected
685	A_23_P306215	FAM84A	Hs.260855	family with sequence similarity 84, member A	Homo sapiens family with sequence similarity 84, member A (FAM84A), mRNA [NM_145175]	6.63029	up	8.32427	up	299.84778	46.82756	279.87286	29.64694	Detected	Detected	Detected	Detected
686	A_23_P167585	GDF9	Hs.25022	growth differentiation factor 9	Homo sapiens growth differentiation factor 9 (GDF9), mRNA [NM_005260]	6.62429	up	6.33227	up	22.61592	3.53515	21.61738	3.01030	Detected	Compromised	Detected	Compromised
687	A_23_P114903	HSPA6	Hs.654614	heat shock 70kDa protein 6 (HSP70B')	Homo sapiens heat shock 70kDa protein 6 (HSP70B') (HSPA6), mRNA [NM_002155]	6.61456	up	9.22900	up	404.18704	63.27242	459.65482	43.91800	Detected	Detected	Detected	Detected
688	A_33_P3243500	LOC728650	Hs.696467	hypothetical LOC728650	PREDICTED: Homo sapiens hypothetical LOC728650 (LOC728650), mRNA [XM_002343155]	6.60753	up	12.18407	up	160.91562	25.21694	196.54771	14.22463	Detected	Detected	Detected	Detected
689	A_23_P93658	ACCN3	Hs.647113	amiloride-sensitive cation channel 3	Homo sapiens amiloride-sensitive cation channel 3 (ACCN3), transcript variant 3, mRNA [NM_020322]	6.59570	up	7.18133	up	59.50608	9.34186	54.43339	6.68384	Detected	Compromised	Detected	Compromised
690	A_23_P425681	CCK	Hs.458426	cholecystokinin	Homo sapiens cholecystokinin (CCK), mRNA [NM_000729]	6.59245	up	7.24848	up	3663.55200	575.42450	2321.33400	282.39432	Detected	Detected	Detected	Detected
691	A_24_P169386	C2orf84	Hs.467868	chromosome 2 open reading frame 84	Homo sapiens chromosome 2 open reading frame 84 (C2orf84), mRNA [NM_001040710]	6.59107	up	5.32051	up	99.06247	15.56276	76.50373	12.67929	Detected	Compromised	Detected	Compromised
692	A_23_P157007	TMEM176B	Hs.647090	transmembrane protein 176B	Homo sapiens transmembrane protein 176B (TMEM176B), transcript variant 1, mRNA [NM_014020]	6.58039	up	22.98909	up	234.20009	36.85263	218.94563	8.39808	Detected	Detected	Detected	Compromised
693	A_33_P3313779	CCDC64	Hs.369763	coiled-coil domain containing 64	Homo sapiens coiled-coil domain containing 64 (CCDC64), mRNA [NM_207311]	6.57217	up	2.42958	up	36.16052	5.69717	22.14104	8.03587	Detected	Compromised	Detected	Compromised
694	A_33_P3261054	CCDC114	Hs.112645	coiled-coil domain containing 114	Homo sapiens coiled-coil domain containing 114 (CCDC114), transcript variant 2, mRNA [NM_144577]	6.56411	up	6.69359	up	37.90872	5.97994	34.55167	4.55172	Detected	Compromised	Detected	Compromised
695	A_24_P406060	RNF144B	Hs.148741	ring finger protein 144B	Homo sapiens ring finger protein 144B (RNF144B), mRNA [NM_182757]	6.56194	up	5.94384	up	240.10771	37.88845	170.62404	25.31272	Detected	Detected	Detected	Detected
696	A_33_P3413815	LAMB2L		laminin, beta 2-like	Homo sapiens laminin, beta 2-like (LAMB2L), non-coding RNA [NR_004405]	6.55874	up	5.93132	up	815.41060	128.73285	530.09143	78.80706	Detected	Detected	Detected	Detected
697	A_33_P3316278	ABCC6P1	Hs.13188	ATP-binding cassette, sub-family C, member 6 pseudogene 1	Homo sapiens ATP-binding cassette, sub-family C, member 6 pseudogene 1 (ABCC6P1), non-coding RNA [NR_003569]	6.55542	up	2.31262	up	19.72365	3.11544	9.61065	3.66449	Detected	Compromised	Compromised	Compromised
698	A_23_P155596	FMO3	Hs.445350	flavin containing monooxygenase 3	Homo sapiens flavin containing monooxygenase 3 (FMO3), transcript variant 2, mRNA [NM_001002294]	6.55514	up	7.00396	up	22.59917	3.56980	23.85950	3.00388	Detected	Compromised	Detected	Compromised
699	A_23_P387585	CXorf50	Hs.676982	chromosome X open reading frame 50	Homo sapiens chromosome X open reading frame 50 (CXorf50), non-coding RNA [NR_026595]	6.55148	up	3.78072	up	20.15196	3.18501	13.70104	3.19554	Detected	Compromised	Detected	Compromised
700	A_33_P3380762	LOC729467		hypothetical LOC729467	Homo sapiens hypothetical LOC729467 (LOC729467), non-coding RNA [NR_027120]	6.54469	up	3.06005	up	80.16272	12.68284	72.74611	20.96267	Detected	Compromised	Detected	Detected

701	A_23_P73632	NR0B1	Hs.268490	nuclear receptor subfamily 0, group B, member 1	Homo sapiens nuclear receptor subfamily 0, group B, member 1 (NR0B1), mRNA [NM_000475]	6.53813	up	6.99447	up	257.53506	40.78642	306.05145	38.58382	Detected	Detected	Detected	Detected
702	A_23_P218646	TNFRSF6B	Hs.434878	tumor necrosis factor receptor superfamily, member 6b, decoy	Homo sapiens tumor necrosis factor receptor superfamily, member 6b, decoy (TNFRSF6B), transcript variant M68C, mRNA [NM_032945]	6.53570	up	6.26144	up	3738.91600	592.36180	4989.08060	702.60580	Detected	Detected	Detected	Detected
703	A_32_P131143	CECR4		cat eye syndrome chromosome region, candidate 4 (non-protein coding)	Homo sapiens cat eye syndrome chromosome region, candidate 4 (non-protein coding) (CECR4), transcript variant 2, non-coding RNA [NR_024482]	6.53170	up	6.50357	up	177.56955	28.14981	201.00829	27.25383	Detected	Detected	Detected	Detected
704	A_33_P3305571	TNFRSF6B	Hs.434878	tumor necrosis factor receptor superfamily, member 6b, decoy	Homo sapiens tumor necrosis factor receptor superfamily, member 6b, decoy (TNFRSF6B), transcript variant M68C, mRNA [NM_032945]	6.52663	up	5.86240	up	1209.04930	191.81737	1358.43620	204.32875	Detected	Detected	Detected	Detected
705	A_23_P2492	C1S	Hs.458355	complement component 1, s subcomponent	Homo sapiens complement component 1, s subcomponent (C1S), transcript variant 2, mRNA [NM_001734]	6.52277	up	5.85421	up	99613.09000	15813.12000	66059.58600	9950.22800	Detected	Detected	Detected	Detected
706	A_23_P129425	TSNAXIP1	Hs.632212	translin-associated factor X interacting protein 1	Homo sapiens translin-associated factor X interacting protein 1 (TSNAXIP1), mRNA [NM_018430]	6.51881	up	3.96706	up	134.33679	21.33831	129.42801	28.76905	Detected	Detected	Detected	Detected
707	A_32_P58614	KIAA1377	Hs.156352	KIAA1377	Homo sapiens KIAA1377 (KIAA1377), mRNA [NM_020802]	6.50835	up	13.55465	up	128.32167	20.41560	48.60483	3.16196	Detected	Detected	Detected	Compromised
708	A_23_P313550	SLC25A41	Hs.375135	solute carrier family 25, member 41	Homo sapiens solute carrier family 25, member 41 (SLC25A41), mRNA [NM_173637]	6.50589	up	8.84281	up	73.00770	11.61971	126.56374	12.62074	Detected	Compromised	Detected	Compromised
709	A_33_P3267948	SLC25A23	Hs.356231	solute carrier family 25 (mitochondrial carrier; phosphate carrier), member 23	Homo sapiens solute carrier family 25 (mitochondrial carrier; phosphate carrier), member 23 (SLC25A23), nuclear gene encoding mitochondrial protein, mRNA [NM_024103]	6.49325	up	6.92547	up	655.41860	104.51759	565.00635	71.93987	Detected	Detected	Detected	Detected
710	A_23_P140725	IFT140	Hs.389438	intraflagellar transport 140 homolog (Chlamydomonas)	Homo sapiens intraflagellar transport 140 homolog (Chlamydomonas) (IFT140), mRNA [NM_014714]	6.47682	up	5.26228	up	516.51746	82.57649	486.54575	81.52964	Detected	Detected	Detected	Detected
711	A_33_P3377005	C11orf35	Hs.669395	chromosome 11 open reading frame 35	Homo sapiens chromosome 11 open reading frame 35 (C11orf35), mRNA [NM_173573]	6.47675	up	6.59977	up	1069.36820	170.96336	860.87420	115.02079	Detected	Detected	Detected	Detected
712	A_32_P24372	TBC1D3B	Hs.454713	TBC1 domain family, member 3B	Homo sapiens TBC1 domain family, member 3B (TBC1D3B), mRNA [NM_001001417]	6.47430	up	5.94574	up	217.75290	34.82607	40.65864	6.02993	Detected	Detected	Detected	Compromised
713	A_33_P3319905					6.47366	up	14.48513	up	110.68692	17.70433	97.31604	5.92417	Detected	Compromised	Detected	Compromised
714	A_23_P106024	JAG2	Hs.433445	jagged 2	Homo sapiens jagged 2 (JAG2), transcript variant 1, mRNA [NM_002226]	6.46737	up	5.96949	up	46.37534	7.42493	29.68383	4.38479	Detected	Compromised	Detected	Compromised
715	A_33_P3404601	C2	Hs.408903	complement component 2	Homo sapiens complement component 2 (C2), transcript variant 1, mRNA [NM_000063]	6.45326	up	5.29894	up	57.17582	9.17417	55.39499	9.21821	Detected	Compromised	Detected	Compromised
716	A_23_P39251	PLIN5	Hs.131034	perilipin 5	Homo sapiens perilipin 5 (PLIN5), mRNA [NM_001013706]	6.43596	up	5.08832	up	119.09570	19.16089	39.63485	6.86860	Detected	Detected	Detected	Compromised
717	A_24_P135769	VWA5A	Hs.152944	von Willebrand factor A domain containing 5A	Homo sapiens von Willebrand factor A domain containing 5A (VWA5A), transcript variant 2, mRNA [NM_198315]	6.43411	up	7.54227	up	110.08601	17.71646	107.84480	12.60848	Detected	Detected	Detected	Compromised
718	A_23_P100660	SERPINF1	Hs.532768	serpin peptidase inhibitor, clade F (alpha-2 antiplasmin, pigment epithelium derived factor), member 1	Homo sapiens serpin peptidase inhibitor, clade F (alpha-2 antiplasmin, pigment epithelium derived factor), member 1 (SERPINF1), mRNA [NM_002615]	6.43110	up	6.04053	up	18300.59800	2946.54570	17098.51600	2496.02500	Detected	Detected	Detected	Detected
719	A_24_P160401	CDCP1	Hs.476093	CUB domain containing protein 1	Homo sapiens CUB domain containing protein 1 (CDCP1), transcript variant 2, mRNA [NM_178181]	6.41645	up	6.85149	up	366.74655	59.18397	279.56445	35.98012	Detected	Detected	Detected	Detected
720	A_33_P3210880					6.41551	up	3.40216	up	24.81117	4.00451	27.90160	7.23168	Detected	Compromised	Detected	Compromised
721	A_32_P86289	CYP19A1	Hs.260074	cytochrome P450, family 19, subfamily A, polypeptide 1	Homo sapiens cytochrome P450, family 19, subfamily A, polypeptide 1 (CYP19A1), transcript variant 2, mRNA [NM_031226]	6.41310	up	6.02797	up	25.81845	4.16865	20.56400	3.00817	Detected	Compromised	Detected	Compromised
722	A_32_P139738	HERC2P4	Hs.531536	hect domain and RLD 2 pseudogene 4	Homo sapiens hect domain and RLD 2 pseudogene 4 (HERC2P4), non-coding RNA [NR_002827]	6.38919	up	2.83049	up	21.13700	3.42555	21.12554	6.58129	Detected	Compromised	Detected	Compromised
723	A_23_P73150	TTC25	Hs.201134	tetratricopeptide repeat domain 25	Homo sapiens tetratricopeptide repeat domain 25 (TTC25), mRNA [NM_031421]	6.38510	up	6.50933	up	555.06696	90.01417	487.57605	66.04984	Detected	Detected	Detected	Detected
724	A_33_P3249976	JAM2	Hs.517227	junctional adhesion molecule 2	Homo sapiens junctional adhesion molecule 2 (JAM2), mRNA [NM_021219]	6.37976	up	6.32122	up	161.29411	26.17866	159.98735	22.31775	Detected	Detected	Detected	Detected
725	A_23_P107173	MEOX1	Hs.438	mesenchyme homeobox 1	Homo sapiens mesenchyme homeobox 1 (MEOX1), transcript variant 1, mRNA [NM_004527]	6.37756	up	6.81689	up	32.32433	5.24817	27.00598	3.49333	Detected	Compromised	Detected	Compromised
726	A_23_P62890	GBP1	Hs.62661	guanylate binding protein 1, interferon-inducible, 67kDa	Homo sapiens guanylate binding protein 1, interferon-inducible, 67kDa (GBP1), mRNA [NM_002053]	6.37617	up	6.14213	up	375.55002	60.98749	337.86630	48.50555	Detected	Detected	Detected	Detected
727	A_23_P155057	CYTH4	Hs.170944	cytohesin 4	Homo sapiens cytohesin 4 (CYTH4), mRNA [NM_013385]	6.37421	up	4.59795	up	72.78553	11.82366	85.37134	16.37245	Detected	Compromised	Detected	Compromised
728	A_23_P106371	GOLGA6A	Hs.546408	golgi autoantigen, golgin subfamily a, 6A	Homo sapiens golgi autoantigen, golgin subfamily a, 6A (GOLGA6A), mRNA [NM_001038640]	6.36758	up	6.81067	up	285.72748	46.46338	137.64340	17.82095	Detected	Detected	Detected	Detected
729	A_33_P3680462	LOC339539	Hs.434301	hypothetical protein LOC339539	Homo sapiens cDNA clone IMAGE:5170739 [BC043541]	6.36703	up	2.50676	up	20.55779	3.34328	9.58752	3.37255	Detected	Compromised	Compromised	Compromised
730	A_23_P121926	SEPP1	Hs.275775	selenoprotein P, plasma, 1	Homo sapiens selenoprotein P, plasma, 1 (SEPP1), transcript variant 1, mRNA [NM_005410]	6.35521	up	7.03898	up	2963.17580	482.79280	1896.74230	237.60953	Detected	Detected	Detected	Detected

731	A_23_P212756	GRK4	Hs.32959	G protein-coupled receptor kinase 4	Homo sapiens G protein-coupled receptor kinase 4 (GRK4), transcript variant 3, mRNA [NM_001004057]	6.33623	up	5.50155	up	164.35370	26.85849	160.58409	25.73848	Detected	Detected	Detected	Detected
732	A_23_P127781	SCGB1D1	Hs.202686	secretoglobin, family 1D, member 1	Homo sapiens secretoglobin, family 1D, member 1 (SCGB1D1), mRNA [NM_006552]	6.33581	up	3.18241	up	23.60077	3.85707	12.36649	3.42654	Detected	Compromised	Compromised	Compromised
733	A_23_P321223	PMCH	Hs.707990	pro-melanin-concentrating hormone	Homo sapiens pro-melanin-concentrating hormone (PMCH), mRNA [NM_002674]	6.33402	up	1.98497	up	22.13522	3.61857	15.33052	6.81033	Detected	Compromised	Detected	Compromised
734	A_23_P412577	ANKRD29	Hs.374774	ankyrin repeat domain 29	Homo sapiens ankyrin repeat domain 29 (ANKRD29), mRNA [NM_173505]	6.32960	up	7.34935	up	1618.53710	264.77640	1342.29380	161.05120	Detected	Detected	Detected	Detected
735	A_23_P368681	GIMAP2	Hs.647071	GTPase, IMAP family member 2	Homo sapiens GTPase, IMAP family member 2 (GIMAP2), mRNA [NM_015660]	6.32581	up	6.45963	up	20.67360	3.38402	20.88170	2.85052	Detected	Compromised	Detected	Compromised
736	A_24_P167654	SLC8A3	Hs.337696	solute carrier family 8 (sodium/calcium exchanger), member 3	Homo sapiens solute carrier family 8 (sodium/calcium exchanger), member 3 (SLC8A3), transcript variant c, mRNA [NM_183002]	6.31854	up	2.47439	up	20.04742	3.28530	21.81239	7.77320	Detected	Compromised	Detected	Compromised
737	A_33_P3241984	PTPN22	Hs.535276	protein tyrosine phosphatase, non-receptor type 22 (lymphoid)	Homo sapiens protein tyrosine phosphatase, non-receptor type 22 (lymphoid) (PTPN22), transcript variant 1, mRNA [NM_015967]	6.31163	up	6.03207	up	944.46170	154.94449	555.36240	81.18499	Detected	Detected	Detected	Detected
738	A_33_P3407469	MYH7B	Hs.414122	myosin, heavy chain 7B, cardiac muscle, beta	Homo sapiens myosin, heavy chain 7B, cardiac muscle, beta (MYH7B), mRNA [NM_020884]	6.30143	up	5.90513	up	150.09232	24.66338	149.09140	22.26326	Detected	Detected	Detected	Detected
739	A_24_P23921	C17orf98	Hs.250182	chromosome 17 open reading frame 98	Homo sapiens chromosome 17 open reading frame 98 (C17orf98), mRNA [NM_001080465]	6.29694	up	7.89319	up	25.02121	4.11445	33.07570	3.69507	Detected	Compromised	Detected	Compromised
740	A_23_P28466	WDR69	Hs.424594	WD repeat domain 69	Homo sapiens WD repeat domain 69 (WDR69), mRNA [NM_178821]	6.29388	up	6.54712	up	436.32706	71.78383	418.93643	56.42390	Detected	Detected	Detected	Detected
741	A_23_P138352	WNT2B	Hs.258575	wingless-type MMTV integration site family, member 2B	Homo sapiens wingless-type MMTV integration site family, member 2B (WNT2B), transcript variant WNT-2B1, mRNA [NM_004185]	6.28515	up	2.25923	up	20.87522	3.43913	14.80531	5.77860	Detected	Compromised	Detected	Compromised
742	A_24_P56330	C13orf16	Hs.210677	chromosome 13 open reading frame 16	Homo sapiens chromosome 13 open reading frame 16 (C13orf16), mRNA [NM_152324]	6.27958	up	11.59735	up	647.93164	106.83939	615.80820	46.82224	Detected	Detected	Detected	Detected
743	A_23_P145054	FAM162B	Hs.126712	family with sequence similarity 162, member B	Homo sapiens family with sequence similarity 162, member B (FAM162B), mRNA [NM_001085480]	6.27587	up	6.84243	up	368.83080	60.85365	280.76584	36.18255	Detected	Detected	Detected	Detected
744	A_33_P3289386	LOC100130168	Hs.663697	hypothetical protein LOC100130168	Homo sapiens cDNA FLJ44638 fis, clone BRACE2027312. [AK126601]	6.27449	up	7.40669	up	18.09130	2.98555	20.58281	2.45045	Detected	Compromised	Detected	Compromised
745	A_24_P142503	SLC47A1	Hs.232054	solute carrier family 47, member 1	Homo sapiens solute carrier family 47, member 1 (SLC47A1), mRNA [NM_018242]	6.26584	up	12.37965	up	41.06432	6.78608	57.12029	4.06862	Detected	Compromised	Detected	Compromised
746	A_23_P37736	TNFRSF17	Hs.2556	tumor necrosis factor receptor superfamily, member 17	Homo sapiens tumor necrosis factor receptor superfamily, member 17 (TNFRSF17), mRNA [NM_001192]	6.25760	up	7.66126	up	62.76551	10.38595	42.85702	4.93273	Detected	Compromised	Detected	Compromised
747	A_33_P3220837	MAFB	Hs.169487	v-maf musculoaponeurotic fibrosarcoma oncogene homolog B (avian)	Homo sapiens v-maf musculoaponeurotic fibrosarcoma oncogene homolog B (avian) (MAFB), mRNA [NM_005461]	6.25740	up	5.70261	up	22195.72300	3672.89500	14432.80700	2231.73660	Detected	Detected	Detected	Detected
748	A_24_P22050	RAB20	Hs.508720	RAB20, member RAS oncogene family	Homo sapiens RAB20, member RAS oncogene family (RAB20), mRNA [NM_017817]	6.25273	up	4.82104	up	343.59024	56.89898	403.56980	73.81477	Detected	Detected	Detected	Detected
749	A_23_P385911	KIAA1712	Hs.555989	KIAA1712	Homo sapiens KIAA1712 (KIAA1712), transcript variant 2, mRNA [NM_001145314]	6.24044	up	9.00689	up	38.86466	6.44871	30.01562	2.93858	Detected	Compromised	Detected	Compromised
750	A_33_P3252068	JHDM1D	Hs.308710	jumonji C domain containing histone demethylase 1 homolog D (S. cerevisiae)	Homo sapiens jumonji C domain containing histone demethylase 1 homolog D (S. cerevisiae) (JHDM1D), mRNA [NM_030647]	6.24002	up	2.94227	up	18.57213	3.08183	27.59835	8.27115	Detected	Compromised	Detected	Compromised
751	A_33_P3269803	CLSTN3	Hs.535378	calsyntenin 3	Homo sapiens calsyntenin 3 (CLSTN3), mRNA [NM_014718]	6.23633	up	4.04409	up	3730.11550	619.33580	4428.73100	965.65900	Detected	Detected	Detected	Detected
752	A_24_P39195	OXGR1	Hs.352218	oxoglutarate (alpha-ketoglutarate) receptor 1	Homo sapiens oxoglutarate (alpha-ketoglutarate) receptor 1 (OXGR1), mRNA [NM_080818]	6.23139	up	3.43382	up	23.95931	3.98128	15.46829	3.97219	Detected	Compromised	Compromised	Compromised
753	A_33_P3412468	SLC25A27	Hs.40510	solute carrier family 25, member 27	Homo sapiens solute carrier family 25, member 27 (SLC25A27), nuclear gene encoding mitochondrial protein, mRNA [NM_004277]	6.22980	up	6.04627	up	1077.63880	179.11522	857.74146	125.09339	Detected	Detected	Detected	Detected
754	A_23_P409623	PPFIBP2	Hs.655714	PTPRF interacting protein, binding protein 2 (liprin beta 2)	Homo sapiens PTPRF interacting protein, binding protein 2 (liprin beta 2) (PPFIBP2), mRNA [NM_003621]	6.21911	up	5.65902	up	1399.33460	232.98431	982.64984	153.11699	Detected	Detected	Detected	Detected
755	A_32_P205624	SHC2	Hs.30965	SHC (Src homology 2 domain containing) transforming protein 2	Homo sapiens SHC (Src homology 2 domain containing) transforming protein 2 (SHC2), mRNA [NM_012435]	6.21733	up	11.32253	up	135.27054	22.52849	158.03465	12.30763	Detected	Detected	Detected	Compromised
756	A_23_P133359	ZFP2	Hs.654533	zinc finger protein 2 homolog (mouse)	Homo sapiens zinc finger protein 2 homolog (mouse) (ZFP2), mRNA [NM_030613]	6.21660	up	8.04942	up	196.07133	32.65834	163.67316	17.92992	Detected	Detected	Detected	Detected
757	A_23_P163697	SYT17	Hs.258326	synaptotagmin XVII	Homo sapiens synaptotagmin XVII (SYT17), mRNA [NM_016524]	6.21600	up	7.18347	up	18.09910	3.01494	20.26524	2.48762	Detected	Compromised	Detected	Compromised
758	A_33_P3284508	CD14	Hs.163867	CD14 molecule	Homo sapiens CD14 molecule (CD14), transcript variant 1, mRNA [NM_000591]	6.21590	up	6.43557	up	2234.99320	372.31020	2127.98050	291.57208	Detected	Detected	Detected	Detected
759	A_33_P3290709	EGFL6	Hs.12844	EGF-like-domain, multiple 6	Homo sapiens EGF-like-domain, multiple 6 (EGFL6), mRNA [NM_015507]	6.20943	up	4.01966	up	66.06165	11.01617	57.18763	12.54521	Detected	Compromised	Detected	Compromised
760	A_33_P3322999	C10orf105	Hs.568788	chromosome 10 open reading frame 105	Homo sapiens chromosome 10 open reading frame 105 (C10orf105), mRNA [NM_001164375]	6.20651	up	12.85524	up	19.06629	3.18091	72.14101	4.94843	Detected	Compromised	Detected	Compromised

761	A_23_P213699	NRG2	Hs.408515	neuregulin 2	Homo sapiens neuregulin 2 (NRG2), transcript variant 3, mRNA [NM_013982]	6.20311	up	5.71832	up	185.20956	30.91628	118.61247	18.29059	Detected	Detected	Detected	Detected
762	A_23_P76823	ADSSL1	Hs.592327	adenylosuccinate synthase like 1	Homo sapiens adenylosuccinate synthase like 1 (ADSSL1), transcript variant 1, mRNA [NM_199165]	6.19948	up	6.53409	up	1545.11170	258.07016	1630.57210	220.04967	Detected	Detected	Detected	Detected
763	A_23_P421379	IGF2	Hs.272259	insulin-like growth factor 2 (somatomedin A)	Homo sapiens insulin-like growth factor 2 (somatomedin A) (IGF2), transcript variant 1, mRNA [NM_000612]	6.19029	up	9.52472	up	97.98582	16.39024	134.85498	12.48476	Detected	Compromised	Detected	Compromised
764	A_23_P36795	SYT1	Hs.310545	synaptotagmin I	Homo sapiens synaptotagmin I (SYT1), transcript variant 1, mRNA [NM_005639]	6.18858	up	3.07687	up	25.03312	4.18848	10.26871	2.94288	Detected	Compromised	Compromised	Compromised
765	A_23_P116642	SLC26A10	Hs.159481	solute carrier family 26, member 10	Homo sapiens solute carrier family 26, member 10 (SLC26A10), mRNA [NM_133489]	6.17976	up	7.61056	up	346.27853	58.02129	409.56076	47.45342	Detected	Detected	Detected	Detected
766	A_33_P3405213	PECAM1	Hs.514412	platelet/endothelial cell adhesion molecule	Homo sapiens platelet/endothelial cell adhesion molecule (PECAM1), mRNA [NM_000442]	6.17553	up	7.17261	up	134.79103	22.60061	108.91990	13.39046	Detected	Detected	Detected	Compromised
767	A_33_P3293918	SH2D3C	Hs.306412	SH2 domain containing 3C	Homo sapiens SH2 domain containing 3C (SH2D3C), transcript variant 1, mRNA [NM_170600]	6.17456	up	9.94581	up	68.12656	11.42467	56.33297	4.99445	Detected	Compromised	Detected	Compromised
768	A_23_P204947	GJB2	Hs.524894	gap junction protein, beta 2, 26kDa	Homo sapiens gap junction protein, beta 2, 26kDa (GJB2), mRNA [NM_004004]	6.16611	up	5.10954	up	19.86098	3.33521	17.26534	2.97960	Detected	Compromised	Detected	Compromised
769	A_33_P3232354	BTBD8	Hs.676102	BTB (POZ) domain containing 8	Homo sapiens BTB (POZ) domain containing 8 (BTBD8), mRNA [NM_183242]	6.15981	up	1.88405	up	20.03293	3.36752	13.84200	6.47845	Detected	Compromised	Detected	Compromised
770	A_33_P3267230	TBC1D3G	Hs.617010	TBC1 domain family, member 3G	Homo sapiens TBC1 domain family, member 3G (TBC1D3G), mRNA [NM_001040282]	6.15488	up	5.37048	up	972.72296	163.64487	780.97460	128.23001	Detected	Detected	Detected	Detected
771	A_33_P3844650	ANGPTL2	Hs.653262	angiopoietin-like 2	Homo sapiens angiopoietin-like 2 (ANGPTL2), mRNA [NM_012098]	6.14893	up	5.33197	up	16207.33000	2729.26030	17331.30700	2866.21850	Detected	Detected	Detected	Detected
772	A_33_P3247117	CXorf1	Hs.106688	chromosome X open reading frame 1	Homo sapiens chromosome X open reading frame 1 (CXorf1), mRNA [NM_004709]	6.14153	up	6.05791	up	22.36280	3.77036	21.87972	3.18482	Detected	Compromised	Detected	Compromised
773	A_33_P3246997		Hs.130293		Cisplatin resistance-associated overexpressed protein (cAMP regulatory element-associated protein 1)(CRE-associated protein 1)(CREAP-1)(Luc7A)(Okadaic acid-inducible phosphoprotein OA48-18) [Source:UniProtKB/Swiss-Prot;Acc:O95232] [ENST00000311571]	6.14053	up	6.39213	up	618.27190	104.25731	299.26996	41.28412	Detected	Detected	Detected	Detected
774	A_33_P3315342	ARHGEF19	Hs.591532	Rho guanine nucleotide exchange factor (GEF) 19	Homo sapiens Rho guanine nucleotide exchange factor (GEF) 19 (ARHGEF19), mRNA [NM_153213]	6.13765	up	5.85844	up	82.18242	13.86469	125.20313	18.84511	Detected	Compromised	Detected	Detected
775	A_23_P500400	ABCA6	Hs.709514	ATP-binding cassette, sub-family A (ABC1), member 6	Homo sapiens ATP-binding cassette, sub-family A (ABC1), member 6 (ABCA6), mRNA [NM_080284]	6.12629	up	6.37888	up	3165.52730	535.03450	2400.01460	331.76846	Detected	Detected	Detected	Detected
776	A_23_P342398	C12orf33	Hs.408739	chromosome 12 open reading frame 33	PREDICTED: Homo sapiens chromosome 12 open reading frame 33 (C12orf33), miscRNA [XR_041988]	6.05879	up	2.37705	up	22.64219	3.86959	8.80809	3.26745	Detected	Compromised	Compromised	Compromised
777	A_33_P3389653	PDE4D	Hs.117545	phosphodiesterase 4D, cAMP-specific (phosphodiesterase E3 dunce homolog, Drosophila)	Homo sapiens phosphodiesterase 4D, cAMP-specific (phosphodiesterase E3 dunce homolog, Drosophila) (PDE4D), transcript variant 3, mRNA [NM_001165899]	6.04372	up	6.58585	up	3283.78250	562.60430	2216.23540	296.73550	Detected	Detected	Detected	Detected
778	A_23_P250245	CD72	Hs.116481	CD72 molecule	Homo sapiens CD72 molecule (CD72), mRNA [NM_001782]	6.03851	up	5.49951	up	109.94769	18.85338	103.73989	16.63365	Detected	Detected	Detected	Detected
779	A_24_P99046	STK38L	Hs.184523	serine/threonine kinase 38 like	Homo sapiens serine/threonine kinase 38 like (STK38L), mRNA [NM_015000]	6.03750	up	4.23339	up	125.80090	21.57544	73.38590	15.28585	Detected	Detected	Detected	Detected
780	A_33_P3367301	GJD3	Hs.444663	gap junction protein, delta 3, 31.9kDa	Homo sapiens gap junction protein, delta 3, 31.9kDa (GJD3), mRNA [NM_152219]	6.03524	up	5.90245	up	4682.28800	803.33530	2914.62430	435.42780	Detected	Detected	Detected	Detected
781	A_23_P420863	NOD2	Hs.592072	nucleotide-binding oligomerization domain containing 2	Homo sapiens nucleotide-binding oligomerization domain containing 2 (NOD2), mRNA [NM_022162]	6.03295	up	4.85235	up	18.15163	3.11544	14.23124	2.58616	Detected	Compromised	Detected	Compromised
782	A_23_P53724	CLSTN3	Hs.535378	calsyntenin 3	Homo sapiens calsyntenin 3 (CLSTN3), mRNA [NM_014718]	6.03191	up	2.80809	up	108.27712	18.58726	127.87801	40.15599	Detected	Detected	Detected	Detected
783	A_33_P3306110	CALCRL	Hs.470882	calcitonin receptor-like	Homo sapiens calcitonin receptor-like (CALCRL), mRNA [NM_005795]	6.03133	up	3.10278	up	32.64962	5.60529	20.93564	5.94978	Detected	Compromised	Detected	Compromised
784	A_33_P3237215	FBXO24	Hs.283764	F-box protein 24	Homo sapiens F-box protein 24 (FBXO24), transcript variant 1, mRNA [NM_033506]	6.01333	up	2.32955	up	22.18432	3.82001	8.49560	3.21579	Detected	Compromised	Compromised	Compromised
785	A_24_P389415	PNMA2	Hs.591838	paraneoplastic antigen MA2	Homo sapiens paraneoplastic antigen MA2 (PNMA2), mRNA [NM_007257]	6.00116	up	4.51192	up	18.10950	3.12467	15.36844	3.00355	Detected	Compromised	Detected	Compromised
786	A_24_P68079	TRANK1	Hs.170999	tetratricopeptide repeat and ankyrin repeat containing 1	Homo sapiens tetratricopeptide repeat and ankyrin repeat containing 1 (TRANK1), mRNA [NM_014831]	6.00027	up	10.08452	up	180.18192	31.09379	103.40311	9.04157	Detected	Detected	Detected	Compromised
787	A_23_P45365	COL4A5	Hs.369089	collagen, type IV, alpha 5	Homo sapiens collagen, type IV, alpha 5 (COL4A5), transcript variant 2, mRNA [NM_033380]	5.99631	up	5.00334	up	848.75995	146.56627	580.25070	102.26360	Detected	Detected	Detected	Detected
788	A_24_P122746	VWA1	Hs.449009	von Willebrand factor A domain containing 1	Homo sapiens von Willebrand factor A domain containing 1 (VWA1), transcript variant 1, mRNA [NM_022834]	5.98979	up	11.43424	up	49.22819	8.51012	35.62725	2.74752	Detected	Compromised	Detected	Compromised
789	A_23_P134176	SOD2	Hs.487046	superoxide dismutase 2, mitochondrial	Homo sapiens superoxide dismutase 2, mitochondrial (SOD2), nuclear gene encoding mitochondrial protein, transcript variant 2, mRNA [NM_001024465]	5.96872	up	5.26832	up	38512.18800	6681.13430	41074.55500	6874.89900	Detected	Detected	Detected	Detected

790	A_23_P74088	MMP23B	Hs.192316	matrix metallopeptidase 23B	Homo sapiens matrix metallopeptidase 23B (MMP23B), mRNA [NM_006983]	5.96488	up	4.25727	up	332.33850	57.69151	287.20184	59.48697	Detected	Detected	Detected	Detected
791	A_24_P185709	EPB41L1	Hs.704100	erythrocyte membrane protein band 4.1-like 1	Homo sapiens erythrocyte membrane protein band 4.1-like 1 (EPB41L1), transcript variant 1, mRNA [NM_012156]	5.95977	up	5.99127	up	405.51980	70.45564	326.80048	48.09829	Detected	Detected	Detected	Detected
792	A_23_P160214	TTC39A	Hs.112949	tetratricopeptide repeat domain 39A	Homo sapiens tetratricopeptide repeat domain 39A (TTC39A), transcript variant 2, mRNA [NM_001080494]	5.95285	up	6.63346	up	44.53681	7.74689	29.42247	3.91115	Detected	Compromised	Detected	Compromised
793	A_33_P3326904	LOC728650	Hs.696467	hypothetical LOC728650	PREDICTED: Homo sapiens hypothetical LOC728650 (LOC728650), mRNA [XM_002347291]	5.94082	up	6.81868	up	352.75470	61.48365	252.71835	32.68150	Detected	Detected	Detected	Detected
794	A_33_P3337267	NRARP	Hs.535075	NOTCH-regulated ankyrin repeat protein	Homo sapiens NOTCH-regulated ankyrin repeat protein (NRARP), mRNA [NM_001004354]	5.93998	up	3.21015	up	18.49615	3.22425	9.88387	2.71499	Detected	Compromised	Compromised	Compromised
795	A_23_P214360	IRF4	Hs.401013	interferon regulatory factor 4	Homo sapiens interferon regulatory factor 4 (IRF4), mRNA [NM_002460]	5.92326	up	1.23252	up	19.71884	3.44710	4.15737	2.97435	Detected	Compromised	Compromised	Compromised
796	A_33_P3283213	MGC57346	Hs.654930	hypothetical LOC401884	Homo sapiens hypothetical LOC401884 (MGC57346), transcript variant 2, non-coding RNA [NR_027295]	5.91347	up	6.22101	up	85.84335	15.03132	67.44554	9.55999	Detected	Compromised	Detected	Compromised
797	A_33_P3280157	SNORD116-19	Hs.680185	small nucleolar RNA, C/D box 116-19	Homo sapiens small nucleolar RNA, C/D box 116-19 (SNORD116-19), small nucleolar RNA [NR_001290]	5.91295	up	6.30276	up	538.43024	94.28845	507.06560	70.94123	Detected	Detected	Detected	Detected
798	A_23_P157963	CNTLN	Hs.435381	centlein, centrosomal protein	Homo sapiens centlein, centrosomal protein (CNTLN), transcript variant 1, mRNA [NM_017738]	5.90464	up	5.19585	up	237.04797	41.56959	148.71367	25.23831	Detected	Detected	Detected	Detected
799	A_33_P3326483	CRABP1	Hs.346950	cellular retinoic acid binding protein 1	Homo sapiens cellular retinoic acid binding protein 1 (CRABP1), mRNA [NM_004378]	5.89411	up	13.01186	up	74.64743	13.11383	55.41202	3.75517	Detected	Compromised	Detected	Compromised
800	A_24_P256380	GPR177	Hs.647659	G protein-coupled receptor 177	Homo sapiens G protein-coupled receptor 177 (GPR177), transcript variant 1, mRNA [NM_024911]	5.88334	up	5.71197	up	6315.05860	1111.44170	3967.65280	612.51050	Detected	Detected	Detected	Detected
801	A_33_P3252539	HIGD1C	Hs.702849	HIG1 hypoxia inducible domain family, member 1C	Homo sapiens HIG1 hypoxia inducible domain family, member 1C (HIGD1C), mRNA [NM_001109619]	5.87811	up	2.79158	up	21.88695	3.85550	10.45727	3.30319	Detected	Compromised	Compromised	Compromised
802	A_33_P3234804	CBR4	Hs.659311	carbonyl reductase 4	Homo sapiens carbonyl reductase 4 (CBR4), mRNA [NM_032783]	5.87159	up	6.31673	up	2664.11250	469.81808	1800.62900	251.36093	Detected	Detected	Detected	Detected
803	A_33_P3375710					5.86560	up	6.35124	up	558.00085	98.50436	544.37286	75.57945	Detected	Detected	Detected	Detected
804	A_33_P3249274	LOC100127967	Hs.689466	hypothetical protein LOC100127967	Homo sapiens cDNA FLJ46267 fis, clone TESTI4027170. [AK128146]	5.83183	up	5.08613	up	17.05671	3.02847	14.34302	2.48667	Detected	Compromised	Detected	Compromised
805	A_33_P3370094	MME	Hs.307734	membrane metallo-endopeptidase	Homo sapiens membrane metallo-endopeptidase (MME), transcript variant 2b, mRNA [NM_007289]	5.82983	up	5.34548	up	7869.74170	1397.77590	6427.11570	1060.21730	Detected	Detected	Detected	Detected
806	A_33_P3306202	LOC653602	Hs.657296	hypothetical LOC653602	Homo sapiens cDNA FLJ35392 fis, clone SKNSH2000716 [AK092711]	5.82338	up	5.38890	up	169.04843	30.05863	107.83158	17.64457	Detected	Detected	Detected	Detected
807	A_33_P3227400	COL4A4	Hs.591645	collagen, type IV, alpha 4	Homo sapiens collagen, type IV, alpha 4 (COL4A4), mRNA [NM_000092]	5.82134	up	6.36245	up	254.18837	45.21323	213.89528	29.64439	Detected	Detected	Detected	Detected
808	A_32_P88905					5.81273	up	6.29335	up	7422.15400	1322.15720	6680.93100	936.09680	Detected	Detected	Detected	Detected
809	A_33_P3307197	PTGFRN	Hs.418093	prostaglandin F2 receptor negative regulator	Homo sapiens prostaglandin F2 receptor negative regulator (PTGFRN), mRNA [NM_020440]	5.81183	up	6.18457	up	2294.32130	408.76584	1267.54250	180.72510	Detected	Detected	Detected	Detected
810	A_33_P3351249	CXCL16	Hs.513885	chemokine (C-X-C motif) ligand 16	Homo sapiens chemokine (C-X-C motif) ligand 16 (CXCL16), transcript variant 2, mRNA [NM_001100812]	5.80824	up	5.03708	up	214.68492	38.27278	215.68155	37.75723	Detected	Detected	Detected	Detected
811	A_23_P380379		Hs.407154		Calcyphosin-2 (Calcyphosine-2) [Source:UniProtKB/Swiss-Prot;Acc:Q9BXY5] [ENST00000328705]	5.80331	up	1.38362	up	17.81407	3.17849	17.01925	10.84651	Detected	Compromised	Detected	Compromised
812	A_23_P305198	STAT4	Hs.80642	signal transducer and activator of transcription 4	Homo sapiens signal transducer and activator of transcription 4 (STAT4), mRNA [NM_003151]	5.79822	up	7.17099	up	17.02406	3.04020	20.34003	2.50114	Detected	Compromised	Detected	Compromised
813	A_32_P396186	TRIM66	Hs.130836	tripartite motif-containing 66	Homo sapiens tripartite motif-containing 66 (TRIM66), mRNA [NM_014818]	5.79272	up	4.92168	up	524.36084	93.73052	239.64003	42.93500	Detected	Detected	Detected	Detected
814	A_24_P319113	P2RX7	Hs.507102	purinergic receptor P2X, ligand-gated ion channel, 7	Homo sapiens purinergic receptor P2X, ligand-gated ion channel, 7 (P2RX7), mRNA [NM_002562]	5.78814	up	3.38354	up	16.90800	3.02473	9.65661	2.51663	Detected	Compromised	Compromised	Compromised
815	A_33_P3409027				Putative helicase Mov10l1 (EC 3.6.1.-)(Moloney leukemia virus 10-like protein 1)(MOV10-like 1) [Source:UniProtKB/Swiss-Prot;Acc:Q9BXT6] [ENST00000354853]	5.77828	up	2.51641	up	18.43828	3.30411	10.07463	3.53032	Detected	Compromised	Compromised	Compromised
816	A_33_P3209361	LOC100130015	Hs.599784	hypothetical LOC100130015	Homo sapiens hypothetical LOC100130015 (LOC100130015), transcript variant 2, non-coding RNA [NR_027335]	5.76333	up	10.97750	up	196.01775	35.21719	247.27779	19.86311	Detected	Detected	Detected	Detected
817	A_33_P3356356	LOC100131089	Hs.386000	hypothetical protein LOC100131089	Homo sapiens cDNA FLJ45796 fis, clone NT2RI2012542. [AK127696]	5.75596	up	4.49596	up	17.09302	3.07493	12.98163	2.54608	Detected	Compromised	Detected	Compromised
818	A_24_P370156	MAN2B1	Hs.356769	mannosidase, alpha, class 2B, member 1	Homo sapiens mannosidase, alpha, class 2B, member 1 (MAN2B1), mRNA [NM_000528]	5.75441	up	4.45714	up	4426.14940	796.45040	3857.49950	763.15906	Detected	Detected	Detected	Detected
819	A_33_P3242863	NT5M	Hs.513977	5',3'-nucleotidase, mitochondrial	Homo sapiens 5',3'-nucleotidase, mitochondrial (NT5M), nuclear gene encoding mitochondrial protein, mRNA [NM_020201]	5.75228	up	5.57370	up	4248.41700	764.75183	3433.95200	543.27030	Detected	Detected	Detected	Detected
820	A_33_P3274562	SLC19A3	Hs.221597	solute carrier family 19, member 3	Homo sapiens solute carrier family 19, member 3 (SLC19A3), mRNA [NM_025243]	5.75014	up	22.75973	up	91.46468	16.47054	86.49270	3.35103	Detected	Compromised	Detected	Compromised
821	A_33_P3278455	ANKRD6	Hs.702213	ankyrin repeat domain 6	Homo sapiens ankyrin repeat domain 6 (ANKRD6), mRNA [NM_014942]	5.74298	up	8.15369	up	62.13267	11.20253	52.52755	5.68066	Detected	Compromised	Detected	Compromised

822	A_23_P502464	NOS2	Hs.709191	nitric oxide synthase 2, inducible	Homo sapiens nitric oxide synthase 2, inducible (NOS2), mRNA [NM_000625]	5.73767	up	5.57756	up	41.90181	7.56190	27.47658	4.34394	Detected	Compromised	Detected	Compromised
823	A_24_P264166	GOLGA8F	Hs.510812	golgi autoantigen, golgin subfamily a, 8F	Homo sapiens golgi autoantigen, golgin subfamily a, 8F (GOLGA8F), mRNA [NM_001012452]	5.73411	up	10.07385	up	216.56589	39.10727	95.13943	8.32781	Detected	Detected	Detected	Compromised
824	A_23_P109171	BFSP1	Hs.129702	beaded filament structural protein 1, filensin	Homo sapiens beaded filament structural protein 1, filensin (BFSP1), transcript variant 1, mRNA [NM_001195]	5.73304	up	5.39961	up	2199.23070	397.20908	1914.57340	312.66193	Detected	Detected	Detected	Detected
825	A_33_P3284315	LOC729076	Hs.609521	hypothetical LOC729076	PREDICTED: Homo sapiens hypothetical LOC729076 (LOC729076), mRNA [XM_001132474]	5.71491	up	4.60759	up	17.57098	3.18361	14.14493	2.70703	Detected	Compromised	Detected	Compromised
826	A_23_P119562	CFD	Hs.155597	complement factor D (adipsin)	Homo sapiens complement factor D (adipsin) (CFD), mRNA [NM_001928]	5.71213	up	4.96521	up	23835.96700	4320.83700	28125.65800	4994.94140	Detected	Detected	Detected	Detected
827	A_33_P3270489	C6orf97	Hs.660044	chromosome 6 open reading frame 97	Homo sapiens chromosome 6 open reading frame 97 (C6orf97), mRNA [NM_025059]	5.71092	up	8.21839	up	145.51329	26.38336	87.39236	9.37675	Detected	Detected	Detected	Compromised
828	A_23_P207850	TNS4	Hs.438292	tensin 4	Homo sapiens tensin 4 (TNS4), mRNA [NM_032865]	5.70280	up	6.07952	up	381.07263	69.19153	466.15408	67.61229	Detected	Detected	Detected	Detected
829	A_23_P212968	UGT2B11	Hs.339811	UDP glucuronosyltransferase 2 family, polypeptide B11	Homo sapiens UDP glucuronosyltransferase 2 family, polypeptide B11 (UGT2B11), mRNA [NM_001073]	5.69977	up	2.33372	up	20.71307	3.76288	8.85763	3.34683	Detected	Compromised	Compromised	Compromised
830	A_24_P101704	ROR2	Hs.98255	receptor tyrosine kinase-like orphan receptor 2	Homo sapiens receptor tyrosine kinase-like orphan receptor 2 (ROR2), mRNA [NM_004560]	5.69659	up	13.19707	up	36.80332	6.68968	43.71309	2.92079	Detected	Compromised	Detected	Compromised
831	A_32_P187704	LOC100127998	Hs.591869	hypothetical LOC100127998	PREDICTED: Homo sapiens hypothetical LOC100127998 (LOC100127998), mRNA [XM_001714676]	5.69240	up	5.32094	up	85.97659	15.63932	72.32417	11.98562	Detected	Compromised	Detected	Compromised
832	A_23_P428260	STEAP2	Hs.489051	six transmembrane epithelial antigen of the prostate 2	Homo sapiens six transmembrane epithelial antigen of the prostate 2 (STEAP2), transcript variant 1, mRNA [NM_152999]	5.69225	up	5.91260	up	3822.22750	695.28955	2244.31150	334.71100	Detected	Detected	Detected	Detected
833	A_32_P151263	SLC2A4	Hs.380691	solute carrier family 2 (facilitated glucose transporter), member 4	Homo sapiens solute carrier family 2 (facilitated glucose transporter), member 4 (SLC2A4), mRNA [NM_001042]	5.68747	up	2.61647	up	18.87401	3.43620	8.62833	2.90788	Detected	Compromised	Compromised	Compromised
834	A_32_P220463	FBLL1		fibrillarin-like 1	Homo sapiens fibrillarin-like 1 (FBLL1), non-coding RNA [NR_024356]	5.68176	up	13.53239	up	324.43690	59.12625	176.51984	11.50230	Detected	Detected	Detected	Compromised
835	A_23_P18684	CLGN	Hs.86368	calmegin	Homo sapiens calmegin (CLGN), transcript variant 1, mRNA [NM_004362]	5.67625	up	5.94501	up	1876.50040	342.31073	1546.86120	229.43753	Detected	Detected	Detected	Detected
836	A_33_P3336592	LOC283711	Hs.585100	hypothetical protein LOC283711	Homo sapiens cDNA FLJ45109 fis, clone BRAWH3034097. [AK127052]	5.67373	up	6.77588	up	212.14960	38.71746	182.26791	23.71974	Detected	Detected	Detected	Detected
837	A_23_P217528	KLF8	Hs.646614	Kruppel-like factor 8	Homo sapiens Kruppel-like factor 8 (KLF8), transcript variant 1, mRNA [NM_007250]	5.67139	up	4.76536	up	75.23125	13.73542	61.31319	11.34551	Detected	Compromised	Detected	Compromised
838	A_24_P42446	PURG	Hs.373778	purine-rich element binding protein G	Homo sapiens purine-rich element binding protein G (PURG), transcript variant B, mRNA [NM_001015508]	5.66121	up	3.23417	up	17.76934	3.25009	10.10114	2.75406	Detected	Compromised	Compromised	Compromised
839	A_23_P17345	MAFB	Hs.169487	v-maf musculoaponeurotic fibrosarcoma oncogene homolog B (avian)	Homo sapiens v-maf musculoaponeurotic fibrosarcoma oncogene homolog B (avian) (MAFB), mRNA [NM_005461]	5.66114	up	5.76740	up	5045.40230	922.83620	4115.01370	629.15340	Detected	Detected	Detected	Detected
840	A_33_P3375809	LOC729970	Hs.297988	similar to hCG2028352	PREDICTED: Homo sapiens similar to hCG2028352 (LOC729970), mRNA [XM_001129522]	5.65927	up	4.86005	up	995.84730	182.20726	814.28485	147.74089	Detected	Detected	Detected	Detected
841	A_23_P404606	C5orf41	Hs.484195	chromosome 5 open reading frame 41	Homo sapiens chromosome 5 open reading frame 41 (C5orf41), mRNA [NM_153607]	5.65175	up	5.66235	up	4124.22900	755.60140	1835.82480	285.89050	Detected	Detected	Detected	Detected
842	A_24_P245815	ASPHD2	Hs.567547	aspartate beta-hydroxylase domain containing 2	Homo sapiens aspartate beta-hydroxylase domain containing 2 (ASPHD2), mRNA [NM_020437]	5.64790	up	5.98546	up	306.69100	56.22733	317.02075	46.70420	Detected	Detected	Detected	Detected
843	A_24_P158718	DTX4	Hs.523696	deltex homolog 4 (Drosophila)	Homo sapiens deltex homolog 4 (Drosophila) (DTX4), mRNA [NM_015177]	5.64187	up	4.28570	up	41.44530	7.60650	34.24625	7.04623	Detected	Compromised	Detected	Compromised
844	A_33_P3290800	DDX26B	Hs.496829	DEAD/H (Asp-Glu-Ala-Asp/His) box polypeptide 26B	Homo sapiens DEAD/H (Asp-Glu-Ala-Asp/His) box polypeptide 26B (DDX26B), mRNA [NM_182540]	5.61804	up	6.90530	up	193.87960	35.73391	150.07372	19.16407	Detected	Detected	Detected	Detected
845	A_23_P36972	ZIC2	Hs.653700	Zic family member 2 (odd-paired homolog, Drosophila)	Homo sapiens Zic family member 2 (odd-paired homolog, Drosophila) (ZIC2), mRNA [NM_007129]	5.61189	up	2.29540	up	18.38676	3.39258	7.45333	2.86323	Detected	Compromised	Compromised	Compromised
846	A_32_P183904	SHF	Hs.310399	Src homology 2 domain containing F	Homo sapiens Src homology 2 domain containing F (SHF), mRNA [NM_138356]	5.60931	up	6.27405	up	381.23312	70.37430	315.02243	44.27502	Detected	Detected	Detected	Detected
847	A_24_P300777	ADAM8	Hs.720760	ADAM metallopeptidase domain 8	Homo sapiens ADAM metallopeptidase domain 8 (ADAM8), transcript variant 1, mRNA [NM_001109]	5.60411	up	5.33758	up	827.43384	152.88332	726.76080	120.06397	Detected	Detected	Detected	Detected
848	A_23_P202245	RET	Hs.350321	ret proto-oncogene	Homo sapiens ret proto-oncogene (RET), transcript variant 2, mRNA [NM_020975]	5.60159	up	3.62481	up	21.89975	4.04819	14.71816	3.58042	Detected	Compromised	Compromised	Compromised
849	A_33_P3329339	LOC644990	Hs.631912	hypothetical LOC644990	PREDICTED: Homo sapiens hypothetical LOC644990 (LOC644990), miscRNA [XR_040213]	5.59452	up	9.59812	up	123.95170	22.94157	113.72761	10.44829	Detected	Detected	Detected	Compromised
850	A_33_P3389649		Hs.117545		cAMP-specific 3',5'-cyclic phosphodiesterase 4D (EC 3.1.4.17)(DPDE3)(PDE43) [Source:UniProtKB/Swiss-Prot;Acc:Q08499] [ENST00000309641]	5.59388	up	5.79201	up	37.99368	7.03285	68.33392	10.40334	Detected	Compromised	Detected	Compromised
851	A_33_P3267160	LOC100132652	Hs.650662	similar to hCG2038304	PREDICTED: Homo sapiens similar to hCG2038304 (LOC100132652), miscRNA [XR_038224]	5.59218	up	7.48485	up	145.90959	27.01693	51.06142	6.01555	Detected	Detected	Detected	Compromised

852	A_33_P3380783	LOC100128184	Hs.678942	hypothetical protein LOC100128184	Homo sapiens cDNA FLJ46151 fis, clone TESTI4000957. [AK128032]	5.59001	up	5.60456	up	1489.44760	275.89618	1721.12240	270.79200	Detected	Detected	Detected	Detected
853	A_32_P523096	MGC12982	Hs.127762	hypothetical protein MGC12982	Homo sapiens hypothetical protein MGC12982 (MGC12982), non-coding RNA [NR_026878]	5.58756	up	4.91043	up	82.64507	15.31539	70.92104	12.73566	Detected	Compromised	Detected	Compromised
854	A_33_P3389872		Hs.187636		Leucine-rich repeat serine/threonine-protein kinase 2 (EC 2.7.11.1)(Dardarin) [Source:UniProtKB/Swiss-Prot;Acc:Q5S007] [ENST00000343742]	5.58514	up	3.80030	up	28.54237	5.29163	32.58225	7.56011	Detected	Compromised	Detected	Compromised
855	A_24_P400044	NUDT10	Hs.375178	nudix (nucleoside diphosphate linked moiety X)-type motif 10	Homo sapiens nudix (nucleoside diphosphate linked moiety X)-type motif 10 (NUDT10), mRNA [NM_153183]	5.57245	up	7.12687	up	32.85714	6.10544	25.17526	3.11488	Detected	Compromised	Detected	Compromised
856	A_32_P64200	GUCA1B	Hs.446529	guanylate cyclase activator 1B (retina)	Homo sapiens guanylate cyclase activator 1B (retina) (GUCA1B), mRNA [NM_002098]	5.56881	up	4.99506	up	265.88593	49.43856	201.63210	35.59463	Detected	Detected	Detected	Detected
857	A_23_P254353	NOXA1	Hs.495554	NADPH oxidase activator 1	Homo sapiens NADPH oxidase activator 1 (NOXA1), mRNA [NM_006647]	5.55896	up	3.72103	up	131.27791	24.45296	184.75035	43.78117	Detected	Detected	Detected	Detected
858	A_23_P159027	ZNF521	Hs.116935	zinc finger protein 521	Homo sapiens zinc finger protein 521 (ZNF521), mRNA [NM_015461]	5.55093	up	5.20679	up	4290.11900	800.27075	3140.01590	531.77400	Detected	Detected	Detected	Detected
859	A_23_P154338	EFHD1	Hs.516769	EF-hand domain family, member D1	Homo sapiens EF-hand domain family, member D1 (EFHD1), transcript variant 1, mRNA [NM_025202]	5.54824	up	4.82713	up	264.15594	49.29900	197.14328	36.01294	Detected	Detected	Detected	Detected
860	A_32_P37592	SCARNA17	Hs.668351	small Cajal body-specific RNA 17	Homo sapiens small Cajal body-specific RNA 17 (SCARNA17), guide RNA [NR_003003]	5.54535	up	5.27197	up	4342.96630	810.94310	4166.35060	696.86414	Detected	Detected	Detected	Detected
861	A_33_P3412900	CBLN3	Hs.207603	cerebellin 3 precursor	Homo sapiens cerebellin 3 precursor (CBLN3), mRNA [NM_001039771]	5.53887	up	5.28985	up	1569.42590	293.39510	985.58050	164.29108	Detected	Detected	Detected	Detected
862	A_23_P118392	RASD1	Hs.25829	RAS, dexamethasone-induced 1	Homo sapiens RAS, dexamethasone-induced 1 (RASD1), mRNA [NM_016084]	5.53128	up	5.11438	up	29303.37900	5485.62060	15891.10400	2739.84570	Detected	Detected	Detected	Detected
863	A_33_P3379967	HLA-F	Hs.519972	major histocompatibility complex, class I, F	Homo sapiens major histocompatibility complex, class I, F (HLA-F), transcript variant 3, mRNA [NM_001098478]	5.52895	up	3.41291	up	978.10693	183.17949	737.90650	190.65210	Detected	Detected	Detected	Detected
864	A_24_P14634	EMID1	Hs.289106	EMI domain containing 1	Homo sapiens EMI domain containing 1 (EMID1), mRNA [NM_133455]	5.52642	up	5.79592	up	57.34748	10.74492	64.32759	9.78679	Detected	Compromised	Detected	Compromised
865	A_23_P161297	OGDHL	Hs.17860	oxoglutarate dehydrogenase-like	Homo sapiens oxoglutarate dehydrogenase-like (OGDHL), transcript variant 1, mRNA [NM_018245]	5.49945	up	5.85741	up	54.69819	10.29880	64.38902	9.69331	Detected	Compromised	Detected	Compromised
866	A_23_P41390	SH3TC1	Hs.479116	SH3 domain and tetratricopeptide repeats 1	Homo sapiens SH3 domain and tetratricopeptide repeats 1 (SH3TC1), mRNA [NM_018986]	5.48823	up	6.79118	up	160.36287	30.25551	147.53238	19.15612	Detected	Detected	Detected	Detected
867	A_23_P425332	PPP4R4	Hs.259599	protein phosphatase 4, regulatory subunit 4	Homo sapiens protein phosphatase 4, regulatory subunit 4 (PPP4R4), transcript variant 1, mRNA [NM_058237]	5.47025	up	8.12577	up	443.00223	83.85555	273.56924	29.68715	Detected	Detected	Detected	Detected
868	A_23_P45304	XK	Hs.78919	X-linked Kx blood group (McLeod syndrome)	Homo sapiens X-linked Kx blood group (McLeod syndrome) (XK), mRNA [NM_021083]	5.46634	up	2.66425	up	20.49775	3.88278	10.38769	3.43803	Detected	Compromised	Compromised	Compromised
869	A_23_P3602	NUDT7	Hs.282665	nudix (nucleoside diphosphate linked moiety X)-type motif 7	Homo sapiens nudix (nucleoside diphosphate linked moiety X)-type motif 7 (NUDT7), transcript variant 1, mRNA [NM_001105663]	5.46080	up	6.36018	up	970.30634	183.98637	668.95490	92.74550	Detected	Detected	Detected	Detected
870	A_24_P231104	LEPR	Hs.23581	leptin receptor	Homo sapiens leptin receptor (LEPR), transcript variant 3, mRNA [NM_001003679]	5.45812	up	5.87146	up	1074.31650	203.80840	650.25037	97.65616	Detected	Detected	Detected	Detected
871	A_23_P59637	DOCK4	Hs.654652	dedicator of cytokinesis 4	Homo sapiens dedicator of cytokinesis 4 (DOCK4), mRNA [NM_014705]	5.45627	up	6.64799	up	922.59220	175.08434	492.09235	65.27118	Detected	Detected	Detected	Detected
872	A_33_P3283744	LOC339742	Hs.98178	hypothetical protein LOC339742	PREDICTED: Homo sapiens hypothetical protein LOC339742 (LOC339742), mRNA [XM_001714261]	5.44652	up	10.73018	up	223.84058	42.55518	177.21689	14.56343	Detected	Detected	Detected	Compromised
873	A_32_P75902	C16orf73	Hs.662737	chromosome 16 open reading frame 73	Homo sapiens chromosome 16 open reading frame 73 (C16orf73), transcript variant 2, mRNA [NM_152764]	5.44206	up	5.56898	up	237.07697	45.10858	204.68510	32.40978	Detected	Detected	Detected	Detected
874	A_33_P3418668	PAR-SN	Hs.564847	paternally expressed transcript PAR-SN	Homo sapiens paternally expressed transcript PAR-SN (PAR-SN), non-coding RNA [NR_022011]	5.43909	up	4.20977	up	480.06006	91.39077	658.66907	137.96678	Detected	Detected	Detected	Detected
875	A_33_P3249872	FBLN1	Hs.24601	fibulin 1	Homo sapiens fibulin 1 (FBLN1), transcript variant C, mRNA [NM_001996]	5.43235	up	4.71560	up	97043.20000	18497.39500	107709.17000	20141.00000	Detected	Detected	Detected	Detected
876	A_33_P3376551	PTGFR	Hs.654365	prostaglandin F receptor (FP)	Homo sapiens prostaglandin F receptor (FP) (PTGFR), transcript variant 2, mRNA [NM_001039585]	5.42717	up	5.90061	up	5007.08060	955.31060	4037.26120	603.33060	Detected	Detected	Detected	Detected
877	A_33_P3306103	CALCRL	Hs.470882	calcitonin receptor-like	Homo sapiens calcitonin receptor-like (CALCRL), mRNA [NM_005795]	5.42540	up	1.37703	up	19.27153	3.67805	18.57612	11.89538	Detected	Compromised	Detected	Compromised
878	A_23_P143512	HSF2BP	Hs.406157	heat shock transcription factor 2 binding protein	Homo sapiens heat shock transcription factor 2 binding protein (HSF2BP), mRNA [NM_007031]	5.42471	up	3.57855	up	66.72583	12.73651	76.42082	18.83087	Detected	Compromised	Detected	Detected
879	A_23_P152970	RAPGEFL1	Hs.632254	Rap guanine nucleotide exchange factor (GEF)-like 1	Homo sapiens Rap guanine nucleotide exchange factor (GEF)-like 1 (RAPGEFL1), mRNA [NM_016339]	5.42230	up	4.30178	up	75.32838	14.38494	65.30569	13.38655	Detected	Compromised	Detected	Compromised
880	A_23_P74290	GBP5	Hs.513726	guanylate binding protein 5	Homo sapiens guanylate binding protein 5 (GBP5), transcript variant 1, mRNA [NM_052942]	5.42057	up	4.92432	up	652.39667	124.62351	675.21375	120.90954	Detected	Detected	Detected	Detected
881	A_32_P222695	FLJ41603	Hs.256206	FLJ41603 protein	Homo sapiens FLJ41603 protein (FLJ41603), mRNA [NM_001001669]	5.41923	up	3.37600	up	71.09638	13.58448	64.62899	16.88072	Detected	Compromised	Detected	Compromised

882	A_23_P63209	HSD11B1	Hs.195040	hydroxysteroid (11-beta) dehydrogenase 1	Homo sapiens hydroxysteroid (11-beta) dehydrogenase 1 (HSD11B1), transcript variant 2, mRNA [NM_181755]	5.41714	up	5.86893	up	1451.68270	277.48212	1199.62430	180.24023	Detected	Detected	Detected	Detected
883	A_23_P333640	PAPLN	Hs.655583	papilin, proteoglycan-like sulfated glycoprotein	Homo sapiens papilin, proteoglycan-like sulfated glycoprotein (PAPLN), mRNA [NM_173462]	5.41395	up	5.85248	up	225.43503	43.11618	172.09070	25.92884	Detected	Detected	Detected	Detected
884	A_23_P160159	SLC2A5	Hs.530003	solute carrier family 2 (facilitated glucose/fructose transporter), member 5	Homo sapiens solute carrier family 2 (facilitated glucose/fructose transporter), member 5 (SLC2A5), transcript variant 1, mRNA [NM_003039]	5.41263	up	4.65819	up	146.28929	27.98577	110.88728	20.99083	Detected	Detected	Detected	Detected
885	A_23_P98571	C11orf66	Hs.502726	chromosome 11 open reading frame 66	Homo sapiens chromosome 11 open reading frame 66 (C11orf66), mRNA [NM_145017]	5.41246	up	3.27517	up	57.86992	11.07112	81.38003	21.91039	Detected	Compromised	Detected	Detected
886	A_24_P390060	IQCD	Hs.568276	IQ motif containing D	Homo sapiens IQ motif containing D (IQCD), mRNA [NM_138451]	5.40616	up	4.47995	up	603.65060	115.61916	533.98300	105.10408	Detected	Detected	Detected	Detected
887	A_23_P120428	TP53TG5	Hs.401982	TP53 target 5	Homo sapiens TP53 target 5 (TP53TG5), mRNA [NM_014477]	5.39491	up	1.15118	up	122.44292	23.50081	8.32186	6.37444	Not Detected	Not Detected	Compromised	Compromised
888	A_23_P344884	ATPGD1	Hs.502982	ATP-grasp domain containing 1	Homo sapiens ATP-grasp domain containing 1 (ATPGD1), transcript variant 1, mRNA [NM_001166222]	5.38964	up	4.79150	up	98.13254	18.85325	80.57062	14.82761	Detected	Compromised	Detected	Compromised
889	A_23_P144476	SPRY1	Hs.436944	sprouty homolog 1, antagonist of FGF signaling (Drosophila)	Homo sapiens sprouty homolog 1, antagonist of FGF signaling (Drosophila) (SPRY1), transcript variant 2, mRNA [NM_199327]	5.38631	up	5.49006	up	403.17890	77.50677	255.63626	41.05924	Detected	Detected	Detected	Detected
890	A_24_P100613	LAMA1	Hs.270364	laminin, alpha 1	Homo sapiens laminin, alpha 1 (LAMA1), mRNA [NM_005559]	5.38319	up	5.46462	up	447.18280	86.01578	319.64080	51.57841	Detected	Detected	Detected	Detected
891	A_24_P168925	CHRDL1	Hs.496587	chordin-like 1	Homo sapiens chordin-like 1 (CHRDL1), transcript variant 1, mRNA [NM_001143981]	5.37824	up	3.60071	up	18.17714	3.49960	12.29744	3.01156	Detected	Compromised	Compromised	Compromised
892	A_33_P3411628	CDKN2A	Hs.512599	cyclin-dependent kinase inhibitor 2A (melanoma, p16, inhibits CDK4)	Homo sapiens cyclin-dependent kinase inhibitor 2A (melanoma, p16, inhibits CDK4) (CDKN2A), transcript variant 3, mRNA [NM_058197]	5.37651	up	5.13388	up	5914.95460	1139.15730	8230.98400	1413.74600	Detected	Detected	Detected	Detected
893	A_23_P21092	CALB2	Hs.106857	calbindin 2	Homo sapiens calbindin 2 (CALB2), transcript variant CALB2, mRNA [NM_001740]	5.37372	up	5.55155	up	777.09430	149.73784	418.83868	66.52699	Detected	Detected	Detected	Detected
894	A_33_P3236906	C6orf217	Hs.510098	chromosome 6 open reading frame 217	Homo sapiens chromosome 6 open reading frame 217 (C6orf217), non-coding RNA [NR_026805]	5.36982	up	11.56005	up	77.77840	14.99797	42.23470	3.22162	Detected	Compromised	Detected	Compromised
895	A_24_P381505	GLIPR1L2	Hs.406728	GLI pathogenesis-related 1 like 2	Homo sapiens GLI pathogenesis-related 1 like 2 (GLIPR1L2), mRNA [NM_152436]	5.36769	up	4.48053	up	28.09599	5.41989	12.72841	2.50502	Detected	Compromised	Detected	Compromised
896	A_23_P304511	ZNF397	Hs.591061	zinc finger protein 397	Homo sapiens zinc finger protein 397 (ZNF397), transcript variant 2, mRNA [NM_032347]	5.36017	up	6.24456	up	74.03908	14.30263	69.64598	9.83467	Detected	Compromised	Detected	Compromised
897	A_33_P3685572	LOC157562	Hs.27371	hypothetical protein LOC157562	Homo sapiens mRNA; cDNA DKFZp686N0244 (from clone DKFZp686N0244) [BX649145]	5.35522	up	6.84146	up	682.59430	131.98306	415.00290	53.48939	Detected	Detected	Detected	Detected
898	A_32_P54503	LOC100134229	Hs.634333	hypothetical protein LOC100134229	Homo sapiens hypothetical protein LOC100134229 (LOC100134229), non-coding RNA [NR_024451]	5.35223	up	4.57320	up	619.58685	119.86723	484.02880	93.32899	Detected	Detected	Detected	Detected
899	A_24_P371628	ANKH	Hs.156727	ankylosis, progressive homolog (mouse)	Homo sapiens ankylosis, progressive homolog (mouse) (ANKH), mRNA [NM_054027]	5.35150	up	5.74803	up	1970.51100	381.27368	1514.61200	232.35280	Detected	Detected	Detected	Detected
900	A_24_P268993	LEAP2	Hs.337588	liver expressed antimicrobial peptide 2	Homo sapiens liver expressed antimicrobial peptide 2 (LEAP2), mRNA [NM_052971]	5.32956	up	4.12800	up	222.54770	43.23792	150.37643	32.12225	Detected	Detected	Detected	Detected
901	A_24_P678104	STMN3	Hs.639609	stathmin-like 3	Homo sapiens stathmin-like 3 (STMN3), mRNA [NM_015894]	5.32864	up	4.95670	up	957.99506	186.15714	914.94160	162.76710	Detected	Detected	Detected	Detected
902	A_33_P3229818	TBPL2	Hs.528278	TATA box binding protein like 2	Homo sapiens TATA box binding protein like 2 (TBPL2), mRNA [NM_199047]	5.32736	up	3.25263	up	20.33875	3.95317	12.92671	3.50444	Detected	Compromised	Compromised	Compromised
903	A_23_P407565	CX3CR1	Hs.78913	chemokine (C-X3-C motif) receptor 1	Homo sapiens chemokine (C-X3-C motif) receptor 1 (CX3CR1), mRNA [NM_001337]	5.32588	up	2.51030	up	21.83493	4.24515	16.45748	5.78100	Detected	Compromised	Compromised	Compromised
904	A_32_P108156	MIR155HG	Hs.697120	MIR155 host gene (non-protein coding)	Homo sapiens MIR155 host gene (non-protein coding) (MIR155HG), non-coding RNA [NR_001458]	5.32586	up	5.51035	up	320.03870	62.22223	318.92530	51.03583	Detected	Detected	Detected	Detected
905	A_32_P188825	TDRD12	Hs.646351	tudor domain containing 12	Homo sapiens tudor domain containing 12 (TDRD12), mRNA [NM_001110822]	5.32431	up	2.78282	up	21.97001	4.27268	22.56587	7.15044	Detected	Compromised	Detected	Compromised
906	A_33_P3398597	EPS8L1	Hs.438862	EPS8-like 1	Homo sapiens EPS8-like 1 (EPS8L1), transcript variant 1, mRNA [NM_133180]	5.32371	up	7.34048	up	48.29141	9.39267	56.12793	6.74249	Detected	Compromised	Detected	Compromised
907	A_32_P66035	TMEM86A	Hs.502100	transmembrane protein 86A	Homo sapiens transmembrane protein 86A (TMEM86A), mRNA [NM_153347]	5.32251	up	9.89532	up	47.58443	9.25724	36.57175	3.25898	Detected	Compromised	Detected	Compromised
908	A_33_P3363120	SCARNA10		small Cajal body-specific RNA 10	Homo sapiens small Cajal body-specific RNA 10 (SCARNA10), guide RNA [NR_004387]	5.31447	up	10.38952	up	95.02969	18.51537	64.32024	5.45905	Detected	Compromised	Detected	Compromised
909	A_23_P119464	RHPN2	Hs.466435	rhophilin, Rho GTPase binding protein 2	Homo sapiens rhophilin, Rho GTPase binding protein 2 (RHPN2), mRNA [NM_033103]	5.31437	up	3.25838	up	24.11280	4.69817	27.73145	7.50476	Detected	Compromised	Detected	Compromised
910	A_33_P3281985	CR2	Hs.445757	complement component (3d/Epstein Barr virus) receptor 2	Homo sapiens complement component (3d/Epstein Barr virus) receptor 2 (CR2), transcript variant 1, mRNA [NM_001006658]	5.31260	up	8.98688	up	55.89632	10.89455	27.37403	2.68593	Detected	Compromised	Detected	Compromised
911	A_33_P3383233	NDRG2	Hs.525205	NDRG family member 2	Homo sapiens NDRG family member 2 (NDRG2), transcript variant 1, mRNA [NM_201535]	5.31220	up	4.90125	up	534.21844	104.13041	450.45462	81.04186	Detected	Detected	Detected	Detected

912	A_23_P25503	FNDC3A	Hs.508010	fibronectin type III domain containing 3A	Homo sapiens fibronectin type III domain containing 3A (FNDC3A), transcript variant 1, mRNA [NM_001079673]	5.31211	up	5.21785	up	17337.04700	3379.41110	13145.40500	2221.50980	Detected	Detected	Detected	Detected
913	A_24_P49199	GLDN	Hs.526441	gliomedin	Homo sapiens gliomedin (GLDN), mRNA [NM_181789]	5.31108	up	5.20489	up	2829.93600	551.73000	1863.59850	315.72330	Detected	Detected	Detected	Detected
914	A_33_P3284404	SYNGR1	Hs.216226	synaptogyrin 1	Homo sapiens synaptogyrin 1 (SYNGR1), transcript variant 1b, mRNA [NM_145731]	5.31075	up	4.96814	up	1474.15540	287.42215	1682.16280	298.56552	Detected	Detected	Detected	Detected
915	A_23_P383679	HYDIN	Hs.461229	hydrocephalus inducing homolog (mouse)	Homo sapiens hydrocephalus inducing homolog (mouse) (HYDIN), transcript variant 1, mRNA [NM_032821]	5.31027	up	5.59890	up	27.99303	5.45842	35.48910	5.58931	Detected	Compromised	Detected	Compromised
916	A_23_P212119	GALNTL2	Hs.411308	UDP-N-acetyl-alpha-D-galactosamine:polypeptide N-acetylgalactosaminyltransferase-like 2	Homo sapiens UDP-N-acetyl-alpha-D-galactosamine:polypeptide N-acetylgalactosaminyltransferase-like 2 (GALNTL2), mRNA [NM_054110]	5.30881	up	5.60146	up	7680.41260	1498.03120	6226.96240	980.25780	Detected	Detected	Detected	Detected
917	A_23_P433016	FBLN1	Hs.24601	fibulin 1	Homo sapiens fibulin 1 (FBLN1), transcript variant C, mRNA [NM_001996]	5.29900	up	4.13912	up	21080.49000	4119.26460	18950.06400	4037.08520	Detected	Detected	Detected	Detected
918	A_24_P82466	GAS7	Hs.462214	growth arrest-specific 7	Homo sapiens growth arrest-specific 7 (GAS7), transcript variant c, mRNA [NM_201433]	5.29619	up	7.49040	up	123.92180	24.22800	73.06556	8.60148	Detected	Detected	Detected	Compromised
919	A_33_P3261530				Putative uncharacterized protein ENSG00000214546 [Source:UniProtKB/Swiss-Prot;Acc:A8MU10] [ENST00000398554]	5.29159	up	2.99535	up	15.11053	2.95684	9.23385	2.71832	Detected	Compromised	Compromised	Compromised
920	A_23_P34744	CTSK	Hs.632466	cathepsin K	Homo sapiens cathepsin K (CTSK), mRNA [NM_000396]	5.28199	up	5.04865	up	162622.66000	31879.86000	144722.05000	25276.95500	Detected	Detected	Detected	Detected
921	A_24_P242646	CTSS	Hs.181301	cathepsin S	Homo sapiens cathepsin S (CTSS), mRNA [NM_004079]	5.26659	up	7.33458	up	29.42997	5.78620	24.75462	2.97609	Detected	Compromised	Detected	Compromised
922	A_23_P6651	C3orf63	Hs.116877	chromosome 3 open reading frame 63	Homo sapiens chromosome 3 open reading frame 63 (C3orf63), transcript variant 2, mRNA [NM_015224]	5.26549	up	6.25166	up	932.78534	183.43243	707.00195	99.72189	Detected	Detected	Detected	Detected
923	A_33_P3861385	PGM5P1	Hs.650460	phosphoglucomutase 5 pseudogene 1	Homo sapiens cDNA FLJ38840 fis, clone MESAN2003490 [AK096159]	5.26441	up	3.23065	up	20.53688	4.03941	12.81287	3.49722	Detected	Compromised	Compromised	Compromised
924	A_23_P422766	KLHDC1	Hs.509258	kelch domain containing 1	Homo sapiens kelch domain containing 1 (KLHDC1), mRNA [NM_172193]	5.26283	up	5.79761	up	288.49840	56.76197	198.51042	30.19255	Detected	Detected	Detected	Detected
925	A_33_P3300495				Plasminogen-related protein B Precursor [Source:UniProtKB/Swiss-Prot;Acc:Q02325] [ENST00000409310]	5.26227	up	4.52480	up	355.92218	70.03501	153.10292	29.83661	Detected	Detected	Detected	Detected
926	A_23_P73345	MITF	Hs.166017	microphthalmia-associated transcription factor	Homo sapiens microphthalmia-associated transcription factor (MITF), transcript variant 1, mRNA [NM_198159]	5.25993	up	9.88404	up	76.27582	15.01550	75.48770	6.73453	Detected	Compromised	Detected	Compromised
927	A_23_P337729	TMEM180	Hs.309069	transmembrane protein 180	Homo sapiens transmembrane protein 180 (TMEM180), mRNA [NM_024789]	5.25724	up	4.69201	up	623.67960	122.83911	692.44214	130.13388	Detected	Detected	Detected	Detected
928	A_33_P3414912	NPL	Hs.496969	N-acetylneuraminate pyruvate lyase (dihydrodipicolinate synthase)	Homo sapiens N-acetylneuraminate pyruvate lyase (dihydrodipicolinate synthase) (NPL), mRNA [NM_030769]	5.25488	up	3.42956	up	48.08538	9.47510	43.04340	11.06710	Detected	Compromised	Detected	Compromised
929	A_23_P50517	ZNF541	Hs.14161	zinc finger protein 541	Homo sapiens zinc finger protein 541 (ZNF541), mRNA [NM_001101419]	5.25365	up	3.45633	up	22.83933	4.50148	10.50694	2.68057	Detected	Compromised	Compromised	Compromised
930	A_23_P384748	PLEKHH2	Hs.164162	pleckstrin homology domain containing, family H (with MyTH4 domain) member 2	Homo sapiens pleckstrin homology domain containing, family H (with MyTH4 domain) member 2 (PLEKHH2), mRNA [NM_172069]	5.25306	up	5.27821	up	401.65756	79.17293	250.45103	41.84094	Detected	Detected	Detected	Detected
931	A_23_P422911	HS6ST3	Hs.171001	heparan sulfate 6-O-sulfotransferase 3	Homo sapiens heparan sulfate 6-O-sulfotransferase 3 (HS6ST3), mRNA [NM_153456]	5.24280	up	7.32828	up	100.11653	19.77315	62.11768	7.47444	Detected	Detected	Detected	Compromised
932	A_23_P41114	CSTA	Hs.518198	cystatin A (stefin A)	Homo sapiens cystatin A (stefin A) (CSTA), mRNA [NM_005213]	5.24028	up	3.77240	up	214.67030	42.41802	128.85059	30.11859	Detected	Detected	Detected	Detected
933	A_33_P3339011	GOLGA2L1	Hs.524660	golgi autoantigen, golgin subfamily a, 2-like 1	Homo sapiens golgi autoantigen, golgin subfamily a, 2-like 1 (GOLGA2L1), transcript variant 2, transcribed RNA [NR_024261]	5.23925	up	5.33121	up	195.58221	38.65387	143.24832	23.69350	Detected	Detected	Detected	Detected
934	A_23_P120594	ACSS1	Hs.529353	acyl-CoA synthetase short-chain family member 1	Homo sapiens acyl-CoA synthetase short-chain family member 1 (ACSS1), nuclear gene encoding mitochondrial protein, mRNA [NM_032501]	5.23293	up	4.54798	up	783.54300	155.04270	575.39484	111.56105	Detected	Detected	Detected	Detected
935	A_23_P345460	PLEKHG4	Hs.188781	pleckstrin homology domain containing, family G (with RhoGef domain) member 4	Homo sapiens pleckstrin homology domain containing, family G (with RhoGef domain) member 4 (PLEKHG4), transcript variant 1, mRNA [NM_015432]	5.23132	up	4.88301	up	5489.18260	1086.49900	2597.03440	468.98093	Detected	Detected	Detected	Detected
936	A_33_P3322115	LOC728800		similar to FLJ00402 protein	PREDICTED: Homo sapiens hypothetical LOC728800 (LOC728800), mRNA [XM_001715974]	5.22946	up	3.35888	up	14.86533	2.94341	9.21653	2.41957	Detected	Compromised	Compromised	Compromised
937	A_23_P30098	ADH4	Hs.1219	alcohol dehydrogenase 4 (class II), pi polypeptide	Homo sapiens alcohol dehydrogenase 4 (class II), pi polypeptide (ADH4), mRNA [NM_000670]	5.22574	up	4.56657	up	16.78831	3.32654	14.34894	2.77073	Detected	Compromised	Detected	Compromised
938	A_23_P146849	APBA2	Hs.618112	amyloid beta (A4) precursor protein-binding, family A, member 2	Homo sapiens amyloid beta (A4) precursor protein-binding, family A, member 2 (APBA2), transcript variant 1, mRNA [NM_005503]	5.22560	up	8.01534	up	101.93027	20.19760	83.10006	9.14209	Detected	Detected	Detected	Compromised
939	A_23_P92042	ITPR1	Hs.567295	inositol 1,4,5-triphosphate receptor, type 1	Homo sapiens inositol 1,4,5-triphosphate receptor, type 1 (ITPR1), transcript variant 2, mRNA [NM_002222]	5.22509	up	4.96950	up	470.72705	93.28436	436.75528	77.49809	Detected	Detected	Detected	Detected
940	A_33_P3390773	TRIM66	Hs.130836	tripartite motif-containing 66	Homo sapiens tripartite motif-containing 66 (TRIM66), mRNA [NM_014818]	5.22028	up	7.00544	up	355.77423	70.56895	194.62930	24.49843	Detected	Detected	Detected	Detected

941	A_32_P83811	FAM47E	Hs.720509	family with sequence similarity 47, member E	Homo sapiens family with sequence similarity 47, member E (FAM47E), mRNA [NM_001136570]	5.21885	up	4.81702	up	15.27917	3.03151	13.82513	2.53079	Detected	Compromised	Detected	Compromised
942	A_33_P3414107	LOC729040	Hs.678658	hypothetical LOC729040	PREDICTED: Homo sapiens hypothetical LOC729040 (LOC729040), miscRNA [XR_078317]	5.21386	up	2.61004	up	53.62075	10.64894	47.26759	15.96916	Detected	Compromised	Detected	Detected
943	A_23_P501007	EFEMP1	Hs.76224	EGF-containing fibulin-like extracellular matrix protein 1	Homo sapiens EGF-containing fibulin-like extracellular matrix protein 1 (EFEMP1), transcript variant 1, mRNA [NM_004105]	5.21251	up	5.18391	up	25235.10000	5012.92900	19172.07600	3261.20040	Detected	Detected	Detected	Detected
944	A_33_P3336562	LOC100132701	Hs.132394	hypothetical LOC100132701	PREDICTED: Homo sapiens hypothetical LOC100132701 (LOC100132701), mRNA [XM_001715461]	5.20610	up	6.81002	up	370.88160	73.76598	309.49048	40.07418	Detected	Detected	Detected	Detected
945	A_33_P3220822	LOC441687	Hs.685242	similar to testis expressed gene 21	PREDICTED: Homo sapiens similar to testis expressed gene 21 (LOC441687), mRNA [XM_001717077]	5.20023	up	3.90127	up	100.22500	19.95661	79.45029	17.95789	Detected	Detected	Detected	Detected
946	A_23_P97283	PAQR6	Hs.235873	progestin and adipoQ receptor family member VI	Homo sapiens progestin and adipoQ receptor family member VI (PAQR6), transcript variant 1, mRNA [NM_024897]	5.19998	up	5.31183	up	1708.25320	340.16060	1398.29350	232.12390	Detected	Detected	Detected	Detected
947	A_23_P210109	CYP26B1	Hs.91546	cytochrome P450, family 26, subfamily B, polypeptide 1	Homo sapiens cytochrome P450, family 26, subfamily B, polypeptide 1 (CYP26B1), mRNA [NM_019885]	5.19271	up	2.45685	up	16.69956	3.33000	11.42504	4.10058	Detected	Compromised	Compromised	Compromised
948	A_33_P3353471	NCRNA00110		non-protein coding RNA 110	Homo sapiens non-protein coding RNA 110 (NCRNA00110), non-coding RNA [NR_027021]	5.18880	up	14.31691	up	99.17326	19.79068	73.17088	4.50666	Detected	Detected	Detected	Compromised
949	A_23_P77000	VASH1	Hs.525479	vasohibin 1	Homo sapiens vasohibin 1 (VASH1), mRNA [NM_014909]	5.18857	up	5.23342	up	1781.85220	355.59600	1720.44460	289.88130	Detected	Detected	Detected	Detected
950	A_23_P130677	LOC55908	Hs.534467	hepatocellular carcinoma-associated gene TD26	Homo sapiens hepatocellular carcinoma-associated gene TD26 (LOC55908), mRNA [NM_018687]	5.18578	up	13.19516	up	85.62395	17.09679	108.47509	7.24905	Detected	Detected	Detected	Compromised
951	A_33_P3322814	NTRK3	Hs.410969	neurotrophic tyrosine kinase, receptor, type 3	Homo sapiens neurotrophic tyrosine kinase, receptor, type 3 (NTRK3), transcript variant 3, mRNA [NM_001007156]	5.18520	up	5.11359	up	287.61640	57.43557	213.15840	36.75718	Detected	Detected	Detected	Detected
952	A_33_P3245178	BEX2	Hs.398989	brain expressed X-linked 2	Homo sapiens brain expressed X-linked 2 (BEX2), mRNA [NM_032621]	5.18012	up	5.88545	up	1429.86650	285.81757	1146.21240	171.73200	Detected	Detected	Detected	Detected
953	A_32_P189781	C14orf34		chromosome 14 open reading frame 34	Homo sapiens chromosome 14 open reading frame 34 (C14orf34), transcript variant 1, non-coding RNA [NR_026796]	5.17909	up	5.51170	up	279.14950	55.81053	154.39142	24.70036	Detected	Detected	Detected	Detected
954	A_23_P118042	LRRC36	Hs.125139	leucine rich repeat containing 36	Homo sapiens leucine rich repeat containing 36 (LRRC36), transcript variant 1, mRNA [NM_018296]	5.17889	up	3.73944	up	104.36546	20.86667	84.64722	19.96049	Detected	Detected	Detected	Not Detected
955	A_33_P3269650	LOC100128402	Hs.689574	hypothetical protein LOC100128402	Homo sapiens cDNA FLJ42583 fis, clone BRACE3009090. [AK124574]	5.17110	up	2.51513	up	44.45732	8.90212	20.54203	7.20193	Detected	Compromised	Detected	Compromised
956	A_23_P415611	NHEDC1	Hs.666728	Na+/H+ exchanger domain containing 1	Homo sapiens Na+/H+ exchanger domain containing 1 (NHEDC1), transcript variant 1, mRNA [NM_139173]	5.16976	up	3.71998	up	51.62310	10.33967	46.39995	10.99874	Detected	Compromised	Detected	Compromised
957	A_33_P3394272	C6orf176		chromosome 6 open reading frame 176	Homo sapiens chromosome 6 open reading frame 176 (C6orf176), transcript variant 2, non-coding RNA [NR_026861]	5.16554	up	2.34725	up	17.97071	3.60232	8.32217	3.12638	Detected	Compromised	Compromised	Compromised
958	A_32_P227605	RGS9BP	Hs.528491	regulator of G protein signaling 9 binding protein	Homo sapiens regulator of G protein signaling 9 binding protein (RGS9BP), mRNA [NM_207391]	5.16512	up	5.73688	up	61.75375	12.37988	68.15114	10.47522	Detected	Compromised	Detected	Compromised
959	A_33_P3210556	FLJ42289	Hs.136982	hypothetical LOC388182	Homo sapiens hypothetical LOC388182 (FLJ42289), transcript variant 1, non-coding RNA [NR_028139]	5.16464	up	7.76636	up	36.25965	7.26970	33.01471	3.74848	Detected	Compromised	Detected	Compromised
960	A_33_P3414389	SH2B2	Hs.489448	SH2B adaptor protein 2	Homo sapiens SH2B adaptor protein 2 (SH2B2), mRNA [NM_020979]	5.16325	up	4.65553	up	3480.98700	698.09190	3191.83450	604.55664	Detected	Detected	Detected	Detected
961	A_23_P160720	BATF3	Hs.62919	basic leucine zipper transcription factor, ATF-like 3	Homo sapiens basic leucine zipper transcription factor, ATF-like 3 (BATF3), mRNA [NM_018664]	5.15968	up	4.96409	up	1406.03020	282.16610	1440.00260	255.79346	Detected	Detected	Detected	Detected
962	A_33_P3402404	SCN3B	Hs.4865	sodium channel, voltage-gated, type III, beta	Homo sapiens sodium channel, voltage-gated, type III, beta (SCN3B), transcript variant 1, mRNA [NM_018400]	5.15948	up	2.64678	up	17.51334	3.51476	8.88044	2.95857	Detected	Compromised	Compromised	Compromised
963	A_33_P3331125	SLC2A12	Hs.486508	solute carrier family 2 (facilitated glucose transporter), member 12	Homo sapiens solute carrier family 2 (facilitated glucose transporter), member 12 (SLC2A12), mRNA [NM_145176]	5.15906	up	2.45152	up	16.06225	3.22381	7.60245	2.73454	Detected	Compromised	Compromised	Compromised
964	A_33_P3246833	IL1RN	Hs.81134	interleukin 1 receptor antagonist	Homo sapiens interleukin 1 receptor antagonist (IL1RN), transcript variant 1, mRNA [NM_173842]	5.15845	up	4.99084	up	15056.78500	3022.36160	13401.92500	2367.87670	Detected	Detected	Detected	Detected
965	A_23_P160004	UTY	Hs.115277	ubiquitously transcribed tetratricopeptide repeat gene, Y-linked	Homo sapiens ubiquitously transcribed tetratricopeptide repeat gene, Y-linked (UTY), transcript variant 1, mRNA [NM_182660]	5.14406	up	4.81160	up	17.99259	3.62177	16.44621	3.01399	Detected	Compromised	Detected	Compromised
966	A_33_P3232861	LOC728558	Hs.538374	hypothetical LOC728558	PREDICTED: Homo sapiens hypothetical LOC728558 (LOC728558), mRNA [XM_001130206]	5.14329	up	2.49954	up	16.42915	3.30755	7.85209	2.77007	Detected	Compromised	Compromised	Compromised
967	A_32_P183970	C15orf62	Hs.631715	chromosome 15 open reading frame 62	Homo sapiens chromosome 15 open reading frame 62 (C15orf62), mRNA [NM_001130448]	5.13973	up	2.39361	up	16.14035	3.25167	16.50773	6.08134	Detected	Compromised	Detected	Compromised
968	A_23_P128084	ITGA7	Hs.524484	integrin, alpha 7	Homo sapiens integrin, alpha 7 (ITGA7), transcript variant 2, mRNA [NM_002206]	5.13459	up	5.01097	up	9235.27000	1862.41870	11556.64600	2033.64880	Detected	Detected	Detected	Detected
969	A_33_P3413483	SORD	Hs.633539	sorbitol dehydrogenase	Homo sapiens cDNA FLJ33419 fis, clone BRACE2019877 [AK090738]	5.13335	up	5.39830	up	762.24854	153.75491	459.68338	75.08749	Detected	Detected	Detected	Detected

970	A_32_P313405	LAMA1	Hs.270364	laminin, alpha 1	Homo sapiens laminin, alpha 1 (LAMA1), mRNA [NM_005559]	5.13223	up	4.70291	up	1416.44190	285.77600	868.29300	162.80391	Detected	Detected	Detected	Detected
971	A_33_P3221925	C6orf170	Hs.121396	chromosome 6 open reading frame 170	Homo sapiens chromosome 6 open reading frame 170 (C6orf170), mRNA [NM_152730]	5.13187	up	1.10187	up	34.94195	7.05025	15.48297	12.39049	Detected	Compromised	Detected	Compromised
972	A_33_P3276364		Hs.492925		3-keto-steroid reductase (EC 1.1.1.270)(Estradiol 17-beta-dehydrogenase 7)(EC 1.1.1.62)(17-beta-hydroxysteroid dehydrogenase 7)(17-beta-HSD 7) [Source:UniProtKB/Swiss-Prot;Acc:P56937] [ENST00000367913]	5.13167	up	10.07742	up	34.33613	6.92828	30.28444	2.64994	Detected	Compromised	Detected	Compromised
973	A_23_P88278	RPGRIP1	Hs.126035	retinitis pigmentosa GTPase regulator interacting protein 1	Homo sapiens retinitis pigmentosa GTPase regulator interacting protein 1 (RPGRIP1), mRNA [NM_020366]	5.11678	up	5.24105	up	16.46728	3.33241	16.91269	2.84551	Detected	Compromised	Detected	Compromised
974	A_24_P595237	TMEM90A	Hs.12400	transmembrane protein 90A	Homo sapiens transmembrane protein 90A (TMEM90A), mRNA [NM_001105579]	5.11476	up	2.54073	up	16.55605	3.35170	8.09341	2.80892	Detected	Compromised	Compromised	Compromised
975	A_33_P3378634					5.11391	up	5.52070	up	34.05927	6.89628	29.58238	4.72503	Detected	Compromised	Detected	Compromised
976	A_33_P3434239	FLJ37638	Hs.131718	hypothetical gene supported by AK094957	Homo sapiens cDNA FLJ37638 fis, clone BRHIP1000058 [AK094957]	5.11265	up	2.87334	up	16.76293	3.39498	9.26654	2.84378	Detected	Compromised	Compromised	Compromised
977	A_24_P110141	C15orf51	Hs.567763	dynamin 1 pseudogene	Homo sapiens chromosome 15 open reading frame 51 (C15orf51), non-coding RNA [NR_003260]	5.10584	up	4.39807	up	121.87061	24.71523	120.24781	24.10910	Detected	Detected	Detected	Detected
978	A_23_P156390	JAKMIP2	Hs.184323	janus kinase and microtubule interacting protein 2	Homo sapiens janus kinase and microtubule interacting protein 2 (JAKMIP2), mRNA [NM_014790]	5.10560	up	3.98764	up	14.94080	3.03012	13.27854	2.93630	Detected	Compromised	Detected	Compromised
979	A_33_P3295523	RAC3	Hs.45002	ras-related C3 botulinum toxin substrate 3 (rho family, small GTP binding protein Rac3)	Homo sapiens ras-related C3 botulinum toxin substrate 3 (rho family, small GTP binding protein Rac3) (RAC3), mRNA [NM_005052]	5.10508	up	5.02369	up	11400.10800	2312.27370	11214.11900	1968.37460	Detected	Detected	Detected	Detected
980	A_33_P3244803	ACOX1	Hs.464137	acyl-Coenzyme A oxidase 1, palmitoyl	Homo sapiens acyl-Coenzyme A oxidase 1, palmitoyl (ACOX1), transcript variant 1, mRNA [NM_004035]	5.10499	up	3.85315	up	9369.03400	1900.34670	7639.90400	1748.38670	Detected	Detected	Detected	Detected
981	A_24_P73577	ALDH1A2	Hs.643455	aldehyde dehydrogenase 1 family, member A2	Homo sapiens aldehyde dehydrogenase 1 family, member A2 (ALDH1A2), transcript variant 3, mRNA [NM_170697]	5.10186	up	2.99748	up	18.49955	3.75462	10.58465	3.11377	Detected	Compromised	Compromised	Compromised
982	A_24_P696761	LEMD1	Hs.655520	LEM domain containing 1	Homo sapiens LEM domain containing 1 (LEMD1), mRNA [NM_001001552]	5.09883	up	4.15287	up	29.16211	5.92217	26.10342	5.54261	Detected	Compromised	Detected	Compromised
983	A_33_P3319625	NFIA	Hs.191911	nuclear factor I/A	Homo sapiens nuclear factor I/A (NFIA), transcript variant 1, mRNA [NM_001134673]	5.09397	up	2.36740	up	51.04963	10.37695	53.51714	19.93367	Detected	Compromised	Detected	Detected
984	A_24_P16559	FOXP2	Hs.656280	forkhead box P2	Homo sapiens forkhead box P2 (FOXP2), transcript variant 2, mRNA [NM_148898]	5.09006	up	2.39104	up	18.74792	3.81385	8.85488	3.26559	Detected	Compromised	Compromised	Compromised
985	A_33_P3357858	ZNF815	Hs.372707	zinc finger protein 815	Homo sapiens zinc finger protein 815 (ZNF815), non-coding RNA [NR_023382]	5.08797	up	5.11472	up	346.89000	70.59599	210.83147	36.34790	Detected	Detected	Detected	Detected
986	A_23_P397543	NCRNA00174	Hs.50755	non-protein coding RNA 174	Homo sapiens non-protein coding RNA 174 (NCRNA00174), non-coding RNA [NR_026873]	5.08590	up	3.24241	up	118.46078	24.11794	60.70594	16.50932	Detected	Detected	Detected	Detected
987	A_33_P3247988	PCSK4	Hs.46884	proprotein convertase subtilisin/kexin type 4	Homo sapiens proprotein convertase subtilisin/kexin type 4 (PCSK4), mRNA [NM_017573]	5.07671	up	7.70736	up	1169.35850	238.50572	1092.87670	125.03481	Detected	Detected	Detected	Detected
988	A_33_P3311917					5.06854	up	5.16130	up	202.12732	41.29287	151.32675	25.85368	Detected	Detected	Detected	Detected
989	A_33_P3651948	NEO1	Hs.388613	neogenin homolog 1 (chicken)	Homo sapiens neogenin homolog 1 (chicken) (NEO1), mRNA [NM_002499]	5.06713	up	12.46270	up	142.04532	29.02674	142.29526	10.06801	Detected	Detected	Detected	Compromised
990	A_33_P3295991	FLJ25694		hypothetical protein FLJ25694	Homo sapiens cDNA FLJ46084 fis, clone TESTI2006543. [AK127969]	5.06549	up	2.68774	up	25.64085	5.24137	17.85759	5.85869	Detected	Compromised	Detected	Compromised
991	A_24_P419276	ZNF248	Hs.528423	zinc finger protein 248	Homo sapiens zinc finger protein 248 (ZNF248), mRNA [NM_021045]	5.06483	up	2.45627	up	23.86897	4.87980	8.46436	3.03867	Detected	Compromised	Compromised	Compromised
992	A_24_P75157	TCP10	Hs.351	t-complex 10 homolog (mouse)	Homo sapiens t-complex 10 homolog (mouse) (TCP10), mRNA [NM_004610]	5.06196	up	4.85396	up	51.12970	10.45894	33.88594	6.15587	Detected	Compromised	Detected	Compromised
993	A_23_P156687	CFB	Hs.69771	complement factor B	Homo sapiens complement factor B (CFB), mRNA [NM_001710]	5.05489	up	4.16677	up	294.11030	60.24644	242.97676	51.41982	Detected	Detected	Detected	Detected
994	A_33_P3351101	TYSND1	Hs.720071	trypsin domain containing 1	Homo sapiens trypsin domain containing 1 (TYSND1), transcript variant 1, mRNA [NM_173555]	5.05093	up	6.50208	up	246.82085	50.59920	234.55255	31.80925	Detected	Detected	Detected	Detected
995	A_33_P3420204	CRTC1	Hs.371096	CREB regulated transcription coactivator 1	Homo sapiens CREB regulated transcription coactivator 1 (CRTC1), transcript variant 3, mRNA [NM_001098482]	5.05061	up	4.48363	up	4634.88570	950.22986	3083.12280	606.35450	Detected	Detected	Detected	Detected
996	A_24_P64167	PTGS1	Hs.201978	prostaglandin-endoperoxide synthase 1 (prostaglandin G/H synthase and cyclooxygenase)	Homo sapiens prostaglandin-endoperoxide synthase 1 (prostaglandin G/H synthase and cyclooxygenase) (PTGS1), transcript variant 1, mRNA [NM_000962]	5.04041	up	4.53811	up	32750.52300	6727.98900	25833.26600	5019.61100	Detected	Detected	Detected	Detected
997	A_23_P406986	MOSPD2	Hs.190043	motile sperm domain containing 2	Homo sapiens motile sperm domain containing 2 (MOSPD2), mRNA [NM_152581]	5.03975	up	5.08370	up	761.24646	156.40466	501.17200	86.93050	Detected	Detected	Detected	Detected
998	A_23_P217379	COL4A6	Hs.145586	collagen, type IV, alpha 6	Homo sapiens collagen, type IV, alpha 6 (COL4A6), transcript variant B, mRNA [NM_033641]	5.03439	up	3.68731	up	32.31705	6.64689	14.02160	3.35315	Detected	Compromised	Compromised	Compromised
999	A_24_P865226	LOC440356	Hs.448823	hypothetical LOC440356	Homo sapiens hypothetical LOC440356 (LOC440356), transcript variant 1, non-coding RNA [NR_015396]	5.03195	up	5.38969	up	69.95384	14.39489	88.38527	14.46046	Detected	Compromised	Detected	Detected
1000	A_33_P3357591	ATHL1	Hs.353181	ATH1, acid trehalase-like 1 (yeast)	Homo sapiens ATH1, acid trehalase-like 1 (yeast) (ATHL1), mRNA [NM_025092]	5.03186	up	3.92812	up	30.21907	6.21850	34.57362	7.76114	Detected	Compromised	Detected	Compromised

1001	A_23_P212126	COLQ	Hs.146735	collagen-like tail subunit (single strand of homotrimer) of asymmetric acetylcholinesterase	Homo sapiens collagen-like tail subunit (single strand of homotrimer) of asymmetric acetylcholinesterase (COLQ), transcript variant II, mRNA [NM_080538]	5.03006	up	5.99511	up	76.34126	15.71519	58.37831	8.58658	Detected	Detected	Detected	Compromised
1002	A_23_P348749	AKD1	Hs.205144	adenylate kinase domain containing 1	Homo sapiens adenylate kinase domain containing 1 (AKD1), transcript variant 2, mRNA [NM_145025]	5.02899	up	5.50079	up	71.85540	14.79488	62.75016	10.05901	Detected	Compromised	Detected	Compromised
1003	A_33_P3423874	GCOM1	Hs.437256	GRINL1A complex locus	Homo sapiens GRINL1A complex locus (GCOM1), transcript variant 12, mRNA [NM_001018100]	5.02462	up	5.26506	up	366.86996	75.60349	283.71643	47.51673	Detected	Detected	Detected	Detected
1004	A_24_P940086	NXPH3	Hs.55069	neurexophilin 3	Homo sapiens neurexophilin 3 (NXPH3), mRNA [NM_007225]	5.01687	up	4.14141	up	187.19920	38.63708	184.58086	39.30105	Detected	Detected	Detected	Detected
1005	A_23_P383118	ZSWIM5	Hs.656613	zinc finger, SWIM-type containing 5	Homo sapiens zinc finger, SWIM-type containing 5 (ZSWIM5), mRNA [NM_020883]	5.00723	up	8.27769	up	140.61896	29.07901	106.45200	11.33992	Detected	Detected	Detected	Compromised
1006	A_23_P201687				Transcription factor HES-2 (Hairy and enhancer of split 2) [Source:UniProtKB/Swiss-Prot;Acc:Q9Y543] [ENST00000377836]	5.00692	up	3.50056	up	15.40227	3.18528	17.51439	4.41188	Detected	Compromised	Detected	Compromised
1007	A_32_P129752	TMEM30B	Hs.146180	transmembrane protein 30B	Homo sapiens transmembrane protein 30B (TMEM30B), mRNA [NM_001017970]	5.00307	up	4.60998	up	182.10304	37.68894	89.49455	17.11839	Detected	Detected	Detected	Compromised
1008	A_33_P3300747	ADHFE1	Hs.720023	alcohol dehydrogenase, iron containing, 1	Homo sapiens alcohol dehydrogenase, iron containing, 1 (ADHFE1), nuclear gene encoding mitochondrial protein, mRNA [NM_144650]	5.00286	up	5.94647	up	18.83893	3.89916	18.65873	2.76687	Detected	Compromised	Detected	Compromised
1009	A_23_P137248	PRKY	Hs.632287	protein kinase, Y-linked	Homo sapiens protein kinase, Y-linked (PRKY), non-coding RNA [NR_028062]	4.99645	up	5.12049	up	601.05920	124.56294	412.87408	71.10033	Detected	Detected	Detected	Detected
1010	A_23_P201181	PTPN22	Hs.535276	protein tyrosine phosphatase, non-receptor type 22 (lymphoid)	Homo sapiens protein tyrosine phosphatase, non-receptor type 22 (lymphoid) (PTPN22), transcript variant 2, mRNA [NM_012411]	4.99318	up	2.48461	up	70.96196	14.71572	49.29162	17.49363	Detected	Compromised	Detected	Detected
1011	A_33_P3372099	DDIT4L	Hs.480378	DNA-damage-inducible transcript 4-like	Homo sapiens DNA-damage-inducible transcript 4-like (DDIT4L), mRNA [NM_145244]	4.99283	up	5.46561	up	2207.59100	457.83112	1506.00900	242.97113	Detected	Detected	Detected	Detected
1012	A_24_P6517	PLEKHG1	Hs.189781	pleckstrin homology domain containing, family G (with RhoGef domain) member 1	Homo sapiens pleckstrin homology domain containing, family G (with RhoGef domain) member 1 (PLEKHG1), mRNA [NM_001029884]	4.98781	up	5.91685	up	30.57878	6.34810	18.39860	2.74195	Detected	Compromised	Detected	Compromised
1013	A_33_P3748410	LOC654841	Hs.659982	hypothetical LOC654841	Homo sapiens cDNA clone IMAGE:5265552 [BC035052]	4.98763	up	2.11653	up	57.91830	12.02417	26.76186	11.14953	Detected	Compromised	Detected	Compromised
1014	A_23_P130359	ARHGAP28	Hs.183114	Rho GTPase activating protein 28	Homo sapiens Rho GTPase activating protein 28 (ARHGAP28), transcript variant 2, mRNA [NM_030672]	4.98375	up	2.05344	up	14.77368	3.06949	5.99593	2.57478	Detected	Compromised	Compromised	Compromised
1015	A_33_P3215635	CHRNB2	Hs.2306	cholinergic receptor, nicotinic, beta 2 (neuronal)	Homo sapiens cholinergic receptor, nicotinic, beta 2 (neuronal) (CHRNB2), mRNA [NM_000748]	4.98041	up	2.40634	up	18.44157	3.83412	15.58569	5.71129	Detected	Compromised	Detected	Compromised
1016	A_24_P408047	PLEKHA4	Hs.9469	pleckstrin homology domain containing, family A (phosphoinositide binding specific) member 4	Homo sapiens pleckstrin homology domain containing, family A (phosphoinositide binding specific) member 4 (PLEKHA4), transcript variant 1, mRNA [NM_020904]	4.97959	up	4.71008	up	32617.67400	6782.54740	23390.27700	4378.98050	Detected	Detected	Detected	Detected
1017	A_23_P144807	SEPT8	Hs.522057	septin 8	Homo sapiens septin 8 (SEPT8), transcript variant 1, mRNA [NM_001098811]	4.97926	up	3.92878	up	614.05994	127.69660	509.69327	114.39742	Detected	Detected	Detected	Detected
1018	A_24_P498854	LOC284998	Hs.410180	hypothetical protein LOC284998	Homo sapiens cDNA FLJ39302 fis, clone OCBBF2012990 [AK096621]	4.96440	up	5.02519	up	51.75044	10.79395	43.14697	7.57117	Detected	Compromised	Detected	Compromised
1019	A_33_P3263902	MXI1	Hs.501023	MAX interactor 1	Homo sapiens MAX interactor 1 (MXI1), transcript variant 1, mRNA [NM_005962]	4.96365	up	5.16837	up	68.63699	14.31826	85.42926	14.57533	Detected	Compromised	Detected	Detected
1020	A_23_P149529	TACSTD2	Hs.23582	tumor-associated calcium signal transducer 2	Homo sapiens tumor-associated calcium signal transducer 2 (TACSTD2), mRNA [NM_002353]	4.96109	up	4.78280	up	615.56080	128.47751	409.90692	75.57325	Detected	Detected	Detected	Detected
1021	A_23_P218358	FBXW10	Hs.592128	F-box and WD repeat domain containing 10	Homo sapiens F-box and WD repeat domain containing 10 (FBXW10), mRNA [NM_031456]	4.95898	up	3.28491	up	15.65503	3.26885	16.96856	4.55499	Detected	Compromised	Detected	Compromised
1022	A_24_P158314	GARNL3	Hs.29304	GTPase activating Rap/RanGAP domain-like 3	Homo sapiens GTPase activating Rap/RanGAP domain-like 3 (GARNL3), mRNA [NM_032293]	4.95722	up	5.38251	up	104.13960	21.75260	104.68707	17.15039	Detected	Detected	Detected	Detected
1023	A_23_P131089	KANK3	Hs.322473	KN motif and ankyrin repeat domains 3	Homo sapiens KN motif and ankyrin repeat domains 3 (KANK3), mRNA [NM_198471]	4.95385	up	5.27755	up	620.36920	129.67040	651.26260	108.81519	Detected	Detected	Detected	Detected
1024	A_23_P121082	GBE1	Hs.436062	glucan (1,4-alpha-), branching enzyme 1	Homo sapiens glucan (1,4-alpha-), branching enzyme 1 (GBE1), mRNA [NM_000158]	4.95374	up	4.81197	up	50205.56200	10494.25600	36161.38700	6626.56300	Detected	Detected	Detected	Detected
1025	A_23_P50368	OSCAR	Hs.347655	osteoclast associated, immunoglobulin-like receptor	Homo sapiens osteoclast associated, immunoglobulin-like receptor (OSCAR), transcript variant 1, mRNA [NM_206818]	4.95261	up	7.32111	up	2993.29880	625.82010	2376.73540	286.26590	Detected	Detected	Detected	Detected
1026	A_23_P73114	PROS1	Hs.64016	protein S (alpha)	Homo sapiens protein S (alpha) (PROS1), mRNA [NM_000313]	4.95118	up	5.11571	up	6521.92240	1363.95560	3663.78930	631.52466	Detected	Detected	Detected	Detected
1027	A_23_P371039	NTSR1	Hs.590869	neurotensin receptor 1 (high affinity)	Homo sapiens neurotensin receptor 1 (high affinity) (NTSR1), mRNA [NM_002531]	4.95019	up	18.02848	up	140.45374	29.37950	106.88300	5.22775	Detected	Detected	Detected	Compromised
1028	A_33_P3232637	LOC729417	Hs.378425	hypothetical LOC729417	PREDICTED: Homo sapiens hypothetical LOC729417 (LOC729417), mRNA [XM_001130175]	4.93102	up	3.47178	up	64.83331	13.61427	38.43239	9.76136	Detected	Compromised	Detected	Compromised
1029	A_23_P127557	KBTBD3	Hs.101949	kelch repeat and BTB (POZ) domain containing 3	Homo sapiens kelch repeat and BTB (POZ) domain containing 3 (KBTBD3), transcript variant 2, mRNA [NM_198439]	4.92969	up	8.99072	up	70.11935	14.72825	54.14061	5.30999	Detected	Compromised	Detected	Compromised

1030	A_23_P104188	ELF3	Hs.67928	E74-like factor 3 (ets domain transcription factor, epithelial-specific)	Homo sapiens E74-like factor 3 (ets domain transcription factor, epithelial-specific) (ELF3), transcript variant 1, mRNA [NM_004433]	4.92884	up	3.85855	up	16.28275	3.42071	13.55224	3.09708	Detected	Compromised	Compromised	Compromised
1031	A_24_P339416	ARSG	Hs.437249	arylsulfatase G	Homo sapiens arylsulfatase G (ARSG), mRNA [NM_014960]	4.92708	up	6.13373	up	608.62090	127.90578	719.41080	103.42311	Detected	Detected	Detected	Detected
1032	A_33_P3276100	LOC100130015	Hs.719553	hypothetical LOC100130015	Homo sapiens hypothetical LOC100130015 (LOC100130015), transcript variant 1, non-coding RNA [NR_027336]	4.92685	up	4.09913	up	294.14316	61.81912	201.16812	43.27463	Detected	Detected	Detected	Detected
1033	A_23_P40611	TCN2	Hs.417948	transcobalamin II; macrocytic anemia	Homo sapiens transcobalamin II; macrocytic anemia (TCN2), mRNA [NM_000355]	4.91426	up	5.48596	up	98.12585	20.67563	101.35101	16.29076	Detected	Detected	Detected	Detected
1034	A_23_P21560	FAM49A	Hs.467769	family with sequence similarity 49, member A	Homo sapiens family with sequence similarity 49, member A (FAM49A), mRNA [NM_030797]	4.91061	up	4.96244	up	90.51275	19.08567	84.09650	14.94334	Detected	Detected	Detected	Detected
1035	A_23_P340376	MSTP9	Hs.655432	macrophage stimulating, pseudogene 9	Homo sapiens macrophage stimulating, pseudogene 9 (MSTP9), non-coding RNA [NR_002729]	4.90796	up	4.72500	up	16.01123	3.37798	21.15229	3.94749	Detected	Compromised	Detected	Compromised
1036	A_23_P73413	SLC26A7	Hs.354013	solute carrier family 26, member 7	Homo sapiens solute carrier family 26, member 7 (SLC26A7), transcript variant 1, mRNA [NM_052832]	4.90746	up	4.41081	up	15.13291	3.19300	13.47427	2.69372	Detected	Compromised	Detected	Compromised
1037	A_23_P26386	TPPP3	Hs.534458	tubulin polymerization-promoting protein family member 3	Homo sapiens tubulin polymerization-promoting protein family member 3 (TPPP3), mRNA [NM_016140]	4.90607	up	4.88889	up	91.88711	19.39341	86.69645	15.63712	Detected	Detected	Detected	Detected
1038	A_33_P3302957	PLEKHG4	Hs.188781	pleckstrin homology domain containing, family G (with RhoGef domain) member 4	Homo sapiens pleckstrin homology domain containing, family G (with RhoGef domain) member 4 (PLEKHG4), transcript variant 1, mRNA [NM_015432]	4.90529	up	4.51352	up	2367.56520	499.76993	2145.76590	419.21124	Detected	Detected	Detected	Detected
1039	A_23_P19182	REEP2	Hs.416090	receptor accessory protein 2	Homo sapiens receptor accessory protein 2 (REEP2), mRNA [NM_016606]	4.90397	up	4.68518	up	10293.22000	2173.38450	9871.66900	1857.93050	Detected	Detected	Detected	Detected
1040	A_32_P221991	RALGPS1	Hs.432842	Ral GEF with PH domain and SH3 binding motif 1	Homo sapiens Ral GEF with PH domain and SH3 binding motif 1 (RALGPS1), mRNA [NM_014636]	4.90345	up	4.34820	up	97.67721	20.62646	95.78336	19.42431	Detected	Detected	Detected	Detected
1041	A_23_P64828	OAS1	Hs.524760	2',5'-oligoadenylate synthetase 1, 40/46kDa	Homo sapiens 2',5'-oligoadenylate synthetase 1, 40/46kDa (OAS1), transcript variant 2, mRNA [NM_002534]	4.89465	up	2.37652	up	18.03274	3.81481	8.80052	3.26537	Detected	Compromised	Compromised	Compromised
1042	A_33_P3352148	AGAP2	Hs.302435	ArfGAP with GTPase domain, ankyrin repeat and PH domain 2	Homo sapiens ArfGAP with GTPase domain, ankyrin repeat and PH domain 2 (AGAP2), transcript variant 1, mRNA [NM_001122772]	4.88578	up	12.25563	up	80.01869	16.95864	46.05290	3.31350	Detected	Compromised	Detected	Compromised
1043	A_33_P3394297				Threonine synthase-like 2 (TSH2)(EC 4.2.3.-) [Source:UniProtKB/Swiss-Prot;Acc:Q86YJ6] [ENST00000402102]	4.87958	up	5.29729	up	149.06760	31.63256	140.17593	23.33377	Detected	Detected	Detected	Detected
1044	A_33_P3282988	LOC100131582	Hs.671136	hypothetical protein LOC100131582	PREDICTED: Homo sapiens hypothetical protein LOC100131582 (LOC100131582), miscRNA [XR_078828]	4.87442	up	2.89547	up	66.58831	14.14516	70.79940	21.56137	Detected	Compromised	Detected	Detected
1045	A_33_P3384362					4.86357	up	3.40371	up	17.98289	3.82858	13.12855	3.40118	Detected	Compromised	Compromised	Compromised
1046	A_33_P3630129	FLJ46875	Hs.641142	hypothetical LOC440918	Homo sapiens cDNA FLJ46875 fis, clone UTERU3014446 [AK128708]	4.86268	up	6.53903	up	287.96625	61.31950	194.59586	26.24131	Detected	Detected	Detected	Detected
1047	A_23_P98844	GEFT	Hs.61581	RhoA/RAC/CDC42 exchange factor	Homo sapiens RhoA/RAC/CDC42 exchange factor (GEFT), transcript variant 1, mRNA [NM_182947]	4.85520	up	3.76392	up	327.26917	69.79605	640.58704	150.07327	Detected	Detected	Detected	Detected
1048	A_33_P3231613	CSAG3	Hs.721609	CSAG family, member 3	Homo sapiens CSAG family, member 3 (CSAG3), transcript variant 2, mRNA [NM_001129828]	4.85078	up	5.33233	up	335.31204	71.57658	166.93796	27.60601	Detected	Detected	Detected	Detected
1049	A_23_P37994	TP53TG3	Hs.592038	TP53 target 3	Homo sapiens TP53 target 3 (TP53TG3), mRNA [NM_016212]	4.84474	up	5.72223	up	190.25005	40.66186	115.41032	17.78462	Detected	Detected	Detected	Detected
1050	A_33_P3270445	BAI2	Hs.524138	brain-specific angiogenesis inhibitor 2	Homo sapiens brain-specific angiogenesis inhibitor 2 (BAI2), mRNA [NM_001703]	4.84301	up	4.16014	up	472.03265	100.92283	417.83597	88.56529	Detected	Detected	Detected	Detected
1051	A_24_P216421	DTNB	Hs.307720	dystrobrevin, beta	Homo sapiens dystrobrevin, beta (DTNB), transcript variant 3, mRNA [NM_033148]	4.83929	up	4.41555	up	205.77829	44.03020	106.35621	21.23948	Detected	Detected	Detected	Detected
1052	A_23_P92093	CELSR3	Hs.631926	cadherin, EGF LAG seven-pass G-type receptor 3 (flamingo homolog, Drosophila)	Homo sapiens cadherin, EGF LAG seven-pass G-type receptor 3 (flamingo homolog, Drosophila) (CELSR3), mRNA [NM_001407]	4.83875	up	6.13592	up	352.94574	75.52793	355.62470	51.10666	Detected	Detected	Detected	Detected
1053	A_33_P3392391	CPT1C	Hs.112195	carnitine palmitoyltransferase 1C	Homo sapiens carnitine palmitoyltransferase 1C (CPT1C), transcript variant 2, mRNA [NM_152359]	4.83805	up	4.48652	up	1989.02890	425.70023	1684.93620	331.16090	Detected	Detected	Detected	Detected
1054	A_33_P3362148	LOC284900		hypothetical LOC284900	Homo sapiens hypothetical LOC284900 (LOC284900), transcript variant 1, non-coding RNA [NR_026963]	4.83485	up	5.80876	up	236.40208	50.62920	235.46126	35.74389	Detected	Detected	Detected	Detected
1055	A_23_P134426	GPNMB	Hs.190495	glycoprotein (transmembrane) nmb	Homo sapiens glycoprotein (transmembrane) nmb (GPNMB), transcript variant 1, mRNA [NM_001005340]	4.83172	up	5.20551	up	28336.66600	6072.67000	23220.44500	3933.44560	Detected	Detected	Detected	Detected
1056	A_32_P128391	LOC728431	Hs.380738	hypothetical LOC728431	PREDICTED: Homo sapiens hypothetical LOC728431 (LOC728431), mRNA [XM_001716728]	4.83150	up	4.40908	up	685.19830	146.84780	866.53270	173.30140	Detected	Detected	Detected	Detected
1057	A_24_P630490	DFNB59	Hs.87734	deafness, autosomal recessive 59	Homo sapiens deafness, autosomal recessive 59 (DFNB59), mRNA [NM_001042702]	4.83027	up	6.09650	up	308.57205	66.14830	299.01940	43.24982	Detected	Detected	Detected	Detected
1058	A_33_P3733417	DRD2	Hs.73893	dopamine receptor D2	Homo sapiens dopamine receptor D2 (DRD2), transcript variant 1, mRNA [NM_000795]	4.81824	up	3.07761	up	13.94650	2.99716	8.68712	2.48902	Detected	Compromised	Compromised	Compromised

1059	A_33_P3349883				Galectin-8 (Gal-8)(Prostate carcinoma tumor antigen 1)(PCTA-1)(Po66 carbohydrate-binding protein)(Po66-CBP) [Source:UniProtKB/Swiss-Prot;Acc:O00214] [ENST00000366583]	4.81591	up	2.15016	up	26.88005	5.77942	26.08296	10.69672	Detected	Compromised	Detected	Compromised
1060	A_24_P213370		Hs.720324		cDNA FLJ26472 fis, clone KDN04506 [Source:UniProtKB/TrEMBL;Acc:Q6ZP57] [ENST00000357132]	4.81171	up	5.60432	up	796.91327	171.49223	388.07460	61.06018	Detected	Detected	Detected	Detected
1061	A_33_P3407895	RINL	Hs.446933	Ras and Rab interactor-like	Homo sapiens Ras and Rab interactor-like (RINL), mRNA [NM_198445]	4.80731	up	4.30713	up	780.31100	168.07335	897.40247	183.72337	Detected	Detected	Detected	Detected
1062	A_33_P3262376	OTUD7A	Hs.355236	OTU domain containing 7A	Homo sapiens OTU domain containing 7A (OTUD7A), mRNA [NM_130901]	4.80534	up	6.82898	up	120.16871	25.89410	106.65578	13.77191	Detected	Detected	Detected	Compromised
1063	A_33_P3297606		Hs.177968		Phospholipid scramblase 2 (PL scramblase 2)(Ca(2+)-dependent phospholipid scramblase 2) [Source:UniProtKB/Swiss-Prot;Acc:Q9NRY7] [ENST00000463633]	4.80252	up	5.08588	up	15.15626	3.26780	22.53095	3.90642	Detected	Compromised	Detected	Compromised
1064	A_23_P61487	LRRC20	Hs.7778	leucine rich repeat containing 20	Homo sapiens leucine rich repeat containing 20 (LRRC20), transcript variant 3, mRNA [NM_018205]	4.80184	up	4.70901	up	3053.15500	658.37610	3148.28170	589.53516	Detected	Detected	Detected	Detected
1065	A_32_P130788	SAMD13	Hs.591445	sterile alpha motif domain containing 13	Homo sapiens sterile alpha motif domain containing 13 (SAMD13), transcript variant 1, mRNA [NM_001010971]	4.79023	up	7.27905	up	141.98491	30.69155	121.84958	14.76097	Detected	Detected	Detected	Detected
1066	A_23_P324327	GPRC5B	Hs.148685	G protein-coupled receptor, family C, group 5, member B	Homo sapiens G protein-coupled receptor, family C, group 5, member B (GPRC5B), mRNA [NM_016235]	4.78839	up	5.77940	up	144.73958	31.29900	145.35898	22.17813	Detected	Detected	Detected	Detected
1067	A_24_P682579					4.78737	up	4.64458	up	20.08476	4.34413	17.73376	3.36682	Detected	Compromised	Detected	Compromised
1068	A_23_P7957	GNMT	Hs.144914	glycine N-methyltransferase	Homo sapiens glycine N-methyltransferase (GNMT), mRNA [NM_018960]	4.78702	up	6.28200	up	96.36959	20.84528	118.07719	16.57423	Detected	Detected	Detected	Detected
1069	A_23_P502343	ADAM33	Hs.173716	ADAM metallopeptidase domain 33	Homo sapiens ADAM metallopeptidase domain 33 (ADAM33), transcript variant 1, mRNA [NM_025220]	4.76658	up	5.50664	up	3425.10600	744.04626	3382.75460	541.68854	Detected	Detected	Detected	Detected
1070	A_23_P211522	SYNGR1	Hs.216226	synaptogyrin 1	Homo sapiens synaptogyrin 1 (SYNGR1), transcript variant 1c, mRNA [NM_145738]	4.76622	up	4.43900	up	889.98016	193.34769	1409.33070	279.95853	Detected	Detected	Detected	Detected
1071	A_24_P309645	TPCN1	Hs.524763	two pore segment channel 1	Homo sapiens two pore segment channel 1 (TPCN1), transcript variant 2, mRNA [NM_017901]	4.76478	up	4.12708	up	991.42030	215.45064	881.98350	188.44418	Detected	Detected	Detected	Detected
1072	A_24_P73075	TTC12	Hs.288772	tetratricopeptide repeat domain 12	Homo sapiens tetratricopeptide repeat domain 12 (TTC12), mRNA [NM_017868]	4.76058	up	5.13604	up	320.41846	69.69331	220.34850	37.83093	Detected	Detected	Detected	Detected
1073	A_23_P304110	ANKRD43	Hs.13308	ankyrin repeat domain 43	Homo sapiens ankyrin repeat domain 43 (ANKRD43), mRNA [NM_175873]	4.75926	up	2.94896	up	18.75188	4.07980	10.58516	3.16515	Detected	Compromised	Compromised	Compromised
1074	A_33_P3244274	RNF208	Hs.512767	ring finger protein 208	Homo sapiens ring finger protein 208 (RNF208), mRNA [NM_031297]	4.75610	up	4.07712	up	497.28550	108.26498	593.35016	128.32846	Detected	Detected	Detected	Detected
1075	A_33_P3281028	MACROD2	Hs.661576	MACRO domain containing 2	Homo sapiens MACRO domain containing 2 (MACROD2), transcript variant 1, mRNA [NM_080676]	4.75595	up	4.53521	up	14.55479	3.16885	13.68711	2.66121	Detected	Compromised	Detected	Compromised
1076	A_33_P3416787					4.75555	up	4.68585	up	19.65307	4.27920	19.58363	3.68528	Detected	Compromised	Detected	Compromised
1077	A_24_P73730	CCDC113	Hs.11614	coiled-coil domain containing 113	Homo sapiens coiled-coil domain containing 113 (CCDC113), transcript variant 1, mRNA [NM_014157]	4.75178	up	6.21937	up	191.24718	41.67465	116.12389	16.46420	Detected	Detected	Detected	Compromised
1078	A_33_P3475906	LOC100130331	Hs.661736	actin, gamma-like	Homo sapiens actin, gamma-like (LOC100130331), non-coding RNA [NR_027247]	4.75060	up	4.53641	up	14.43980	3.14736	13.58900	2.64144	Detected	Compromised	Detected	Compromised
1079	A_33_P3417305	LOC645225		similar to ATP synthase, H+ transporting, mitochondrial F0 complex, subunit F2	PREDICTED: Homo sapiens similar to ATP synthase, H+ transporting, mitochondrial F0 complex, subunit F2 (LOC645225), mRNA [XM_001720758]	4.74431	up	24.91087	up	433.88000	94.69554	309.63763	10.96050	Detected	Detected	Detected	Compromised
1080	A_33_P3265260					4.74401	up	2.75549	up	16.87539	3.68333	10.96474	3.50885	Detected	Compromised	Compromised	Compromised
1081	A_33_P3358745	SEPP1	Hs.275775	selenoprotein P, plasma, 1	Homo sapiens selenoprotein P, plasma, 1 (SEPP1), transcript variant 3, mRNA [NM_001093726]	4.73522	up	6.21029	up	2137.99700	467.51993	1985.97560	281.98600	Detected	Detected	Detected	Detected
1082	A_24_P12435	NCOA7	Hs.171426	nuclear receptor coactivator 7	Homo sapiens nuclear receptor coactivator 7 (NCOA7), transcript variant 1, mRNA [NM_181782]	4.72960	up	4.60080	up	3495.45390	765.26560	2739.63010	525.07886	Detected	Detected	Detected	Detected
1083	A_23_P65532	PELI2	Hs.657926	pellino homolog 2 (Drosophila)	Homo sapiens pellino homolog 2 (Drosophila) (PELI2), mRNA [NM_021255]	4.72567	up	4.42256	up	2086.19600	457.11472	2279.81370	454.55948	Detected	Detected	Detected	Detected
1084	A_23_P218817	CPT1B	Hs.439777	carnitine palmitoyltransferase 1B (muscle)	Homo sapiens carnitine palmitoyltransferase 1B (muscle) (CPT1B), nuclear gene encoding mitochondrial protein, transcript variant 3, mRNA [NM_152246]	4.72221	up	4.61717	up	496.39972	108.84767	452.31473	86.38345	Detected	Detected	Detected	Detected
1085	A_33_P3228132	LOC400891		chromosome 14 open reading frame 166B pseudogene	Homo sapiens chromosome 14 open reading frame 166B pseudogene (LOC400891), non-coding RNA [NR_027006]	4.72032	up	2.58015	up	43.54580	9.55231	8.22259	2.81015	Detected	Compromised	Compromised	Compromised
1086	A_33_P3262884	LOC440104	Hs.720564	hypothetical LOC440104	PREDICTED: Homo sapiens similar to RIKEN cDNA 1110012D08, transcript variant 1 (LOC440104), mRNA [XM_001721986]	4.72015	up	2.94697	up	20.16032	4.42257	18.72776	5.60370	Detected	Compromised	Detected	Compromised
1087	A_23_P99515	C13orf33	Hs.147880	chromosome 13 open reading frame 33	Homo sapiens chromosome 13 open reading frame 33 (C13orf33), mRNA [NM_032849]	4.71820	up	4.66863	up	32043.70700	7032.33940	30612.00000	5781.85740	Detected	Detected	Detected	Detected
1088	A_33_P3219398	ZNF521	Hs.116935	zinc finger protein 521	Homo sapiens zinc finger protein 521 (ZNF521), mRNA [NM_015461]	4.71485	up	4.70203	up	472.12003	103.68549	508.35406	95.33361	Detected	Detected	Detected	Detected

1089	A_33_P3221203	MMP13	Hs.2936	matrix metallopeptidase 13 (collagenase 3)	Homo sapiens matrix metallopeptidase 13 (collagenase 3) (MMP13), mRNA [NM_002427]	4.71329	up	2.16280	up	14.07820	3.09283	9.67851	3.94601	Detected	Compromised	Compromised	Compromised
1090	A_23_P302672	DDIT4L	Hs.480378	DNA-damage-inducible transcript 4-like	Homo sapiens DNA-damage-inducible transcript 4-like (DDIT4L), mRNA [NM_145244]	4.71277	up	4.33118	up	917.03240	201.48427	720.10740	146.60779	Detected	Detected	Detected	Detected
1091	A_23_P120863	GAL3ST1	Hs.17958	galactose-3-O-sulfotransferase 1	Homo sapiens galactose-3-O-sulfotransferase 1 (GAL3ST1), mRNA [NM_004861]	4.71012	up	2.99743	up	28.98957	6.37298	22.76339	6.69659	Detected	Compromised	Detected	Compromised
1092	A_23_P24433	CTSF	Hs.11590	cathepsin F	Homo sapiens cathepsin F (CTSF), mRNA [NM_003793]	4.70868	up	4.31483	up	30439.95700	6693.87900	28025.80900	5727.43360	Detected	Detected	Detected	Detected
1093	A_23_P251031	IL13	Hs.845	interleukin 13	Homo sapiens interleukin 13 (IL13), mRNA [NM_002188]	4.70451	up	3.69135	up	29.29162	6.44707	13.45505	3.21415	Detected	Compromised	Compromised	Compromised
1094	A_23_P501831	C5orf4	Hs.519694	chromosome 5 open reading frame 4	Homo sapiens chromosome 5 open reading frame 4 (C5orf4), mRNA [NM_032385]	4.70293	up	4.79990	up	2789.32930	614.13620	2174.94530	399.55997	Detected	Detected	Detected	Detected
1095	A_32_P126375	NHS	Hs.201623	Nance-Horan syndrome (congenital cataracts and dental anomalies)	Homo sapiens Nance-Horan syndrome (congenital cataracts and dental anomalies) (NHS), transcript variant 1, mRNA [NM_198270]	4.70256	up	5.40982	up	1340.76590	295.22406	981.63446	160.00449	Detected	Detected	Detected	Detected
1096	A_23_P160886	TAS1R1	Hs.124574	taste receptor, type 1, member 1	Homo sapiens taste receptor, type 1, member 1 (TAS1R1), transcript variant 2, mRNA [NM_138697]	4.70083	up	3.83593	up	361.56300	79.64212	360.13248	82.78603	Detected	Detected	Detected	Detected
1097	A_33_P3327842	ZNF436	Hs.293798	zinc finger protein 436	Homo sapiens zinc finger protein 436 (ZNF436), transcript variant 1, mRNA [NM_001077195]	4.69345	up	2.02925	up	18.07686	3.98808	17.51654	7.61163	Detected	Compromised	Detected	Compromised
1098	A_32_P178966	TMEM170B	Hs.146317	transmembrane protein 170B	Homo sapiens transmembrane protein 170B (TMEM170B), mRNA [NM_001100829]	4.69212	up	5.35830	up	331.79984	73.22176	260.85242	42.92730	Detected	Detected	Detected	Detected
1099	A_23_P203439	KCNC1	Hs.552896	potassium voltage-gated channel, Shaw-related subfamily, member 1	Homo sapiens potassium voltage-gated channel, Shaw-related subfamily, member 1 (KCNC1), transcript variant B, mRNA [NM_004976]	4.68346	up	5.21333	up	23.78365	5.25829	20.46592	3.46164	Detected	Compromised	Detected	Compromised
1100	A_33_P3229552	LRFN1	Hs.97860	leucine rich repeat and fibronectin type III domain containing 1	Homo sapiens leucine rich repeat and fibronectin type III domain containing 1 (LRFN1), mRNA [NM_020862]	4.68061	up	12.78048	up	54.53041	12.06339	59.94556	4.13595	Detected	Compromised	Detected	Compromised
1101	A_23_P161399	MXI1	Hs.501023	MAX interactor 1	Homo sapiens MAX interactor 1 (MXI1), transcript variant 2, mRNA [NM_130439]	4.67872	up	5.13212	up	3046.89670	674.31670	1828.73330	314.20944	Detected	Detected	Detected	Detected
1102	A_33_P3290040	CPT1C	Hs.112195	carnitine palmitoyltransferase 1C	Homo sapiens carnitine palmitoyltransferase 1C (CPT1C), transcript variant 2, mRNA [NM_152359]	4.67785	up	4.35979	up	3486.45870	771.74020	3172.10900	641.57526	Detected	Detected	Detected	Detected
1103	A_32_P88415	MYOZ3	Hs.91626	myozenin 3	Homo sapiens myozenin 3 (MYOZ3), transcript variant 2, mRNA [NM_133371]	4.66894	up	3.93725	up	378.48758	83.93958	363.55865	81.42294	Detected	Detected	Detected	Detected
1104	A_23_P122796	HDDC2	Hs.32826	HD domain containing 2	Homo sapiens HD domain containing 2 (HDDC2), mRNA [NM_016063]	4.65671	up	4.98417	up	403.69067	89.76413	352.45148	62.35508	Detected	Detected	Detected	Detected
1105	A_24_P310256	LGI4	Hs.65256	leucine-rich repeat LGI family, member 4	Homo sapiens leucine-rich repeat LGI family, member 4 (LGI4), mRNA [NM_139284]	4.65600	up	4.53103	up	136.11810	30.27167	138.62450	26.97792	Detected	Detected	Detected	Detected
1106	A_23_P78742	FLT3LG	Hs.428	fms-related tyrosine kinase 3 ligand	Homo sapiens fms-related tyrosine kinase 3 ligand (FLT3LG), mRNA [NM_001459]	4.65472	up	4.07337	up	224.41699	49.92235	287.29944	62.19375	Detected	Detected	Detected	Detected
1107	A_24_P133017	TMEM25	Hs.564188	transmembrane protein 25	Homo sapiens transmembrane protein 25 (TMEM25), transcript variant 1, mRNA [NM_032780]	4.65166	up	3.91529	up	250.04425	55.65982	256.41800	57.74971	Detected	Detected	Detected	Detected
1108	A_33_P3300837	LDB2	Hs.714330	LIM domain binding 2	Homo sapiens LIM domain binding 2 (LDB2), transcript variant 1, mRNA [NM_001290]	4.65163	up	3.16345	up	33.87443	7.54049	28.66180	7.98930	Detected	Compromised	Detected	Compromised
1109	A_24_P177279	CSDC2	Hs.310893	cold shock domain containing C2, RNA binding	Homo sapiens cold shock domain containing C2, RNA binding (CSDC2), mRNA [NM_014460]	4.64873	up	2.77170	up	44.47540	9.90647	43.42648	13.81576	Detected	Compromised	Detected	Compromised
1110	A_23_P159952	BEX1	Hs.334370	brain expressed, X-linked 1	Homo sapiens brain expressed, X-linked 1 (BEX1), mRNA [NM_018476]	4.64833	up	4.89203	up	8086.93100	1801.44100	4423.39400	797.31903	Detected	Detected	Detected	Detected
1111	A_23_P257583	DENND2A	Hs.6385	DENN/MADD domain containing 2A	Homo sapiens DENN/MADD domain containing 2A (DENND2A), mRNA [NM_015689]	4.64435	up	4.61039	up	219.46133	48.92904	285.48126	54.60156	Detected	Detected	Detected	Detected
1112	A_23_P212050	BCHE	Hs.420483	butyrylcholinesterase	Homo sapiens butyrylcholinesterase (BCHE), mRNA [NM_000055]	4.64322	up	6.76945	up	156.14903	34.82197	160.59910	20.91968	Detected	Detected	Detected	Detected
1113	A_33_P3248982	FAIM2	Hs.567424	Fas apoptotic inhibitory molecule 2	Homo sapiens Fas apoptotic inhibitory molecule 2 (FAIM2), mRNA [NM_012306]	4.63904	up	8.07217	up	1640.81920	366.23972	939.99090	102.68311	Detected	Detected	Detected	Detected
1114	A_23_P61466	CD163L1	Hs.631727	CD163 molecule-like 1	Homo sapiens CD163 molecule-like 1 (CD163L1), mRNA [NM_174941]	4.63676	up	5.00990	up	1274.04190	284.51294	1365.30410	240.30660	Detected	Detected	Detected	Detected
1115	A_23_P125505	PPEF1	Hs.211589	protein phosphatase, EF-hand calcium binding domain 1	Homo sapiens protein phosphatase, EF-hand calcium binding domain 1 (PPEF1), transcript variant 1, mRNA [NM_006240]	4.63422	up	1.21955	up	15.54859	3.47414	4.04404	2.92403	Detected	Compromised	Compromised	Compromised
1116	A_32_P31963					4.63019	up	4.79430	up	13.21870	2.95613	13.18354	2.42478	Detected	Compromised	Detected	Compromised
1117	A_23_P113471	FAAH2	Hs.720593	fatty acid amide hydrolase 2	Homo sapiens fatty acid amide hydrolase 2 (FAAH2), mRNA [NM_174912]	4.62988	up	5.28911	up	17.73367	3.96608	22.46531	3.74537	Not Detected	Compromised	Detected	Compromised
1118	A_33_P3366471					4.62468	up	1.34771	up	26.53721	5.94164	7.48528	4.89754	Detected	Compromised	Compromised	Compromised
1119	A_23_P168771	CCDC146	Hs.113940	coiled-coil domain containing 146	Homo sapiens coiled-coil domain containing 146 (CCDC146), mRNA [NM_020879]	4.62132	up	4.96281	up	265.67792	59.52819	212.14160	37.69326	Detected	Detected	Detected	Detected
1120	A_23_P369701	FAM108C1	Hs.459072	family with sequence similarity 108, member C1	Homo sapiens family with sequence similarity 108, member C1 (FAM108C1), mRNA [NM_021214]	4.61564	up	4.66499	up	6549.07900	1469.20080	4147.35500	783.94610	Detected	Detected	Detected	Detected

1121	A_33_P3308949	DBT	Hs.709187	dihydrolipoamide branched chain transacylase E2	Homo sapiens dihydrolipoamide branched chain transacylase E2 (DBT), nuclear gene encoding mitochondrial protein, mRNA [NM_001918]	4.61551	up	5.02506	up	906.63916	203.39856	724.56714	127.14599	Detected	Detected	Detected	Detected
1122	A_23_P167599	FAM134B	Hs.711125	family with sequence similarity 134, member B	Homo sapiens family with sequence similarity 134, member B (FAM134B), transcript variant 1, mRNA [NM_001034850]	4.61499	up	5.41101	up	767.93115	172.29991	568.76636	92.68749	Detected	Detected	Detected	Detected
1123	A_23_P31273	AMPH	Hs.592182	amphiphysin	Homo sapiens amphiphysin (AMPH), transcript variant 1, mRNA [NM_001635]	4.61476	up	3.80119	up	731.79850	164.20102	628.08250	145.70097	Detected	Detected	Detected	Detected
1124	A_24_P6030		Hs.656195		Protein FAM165B [Source:UniProtKB/Swiss-Prot;Acc:P58511] [ENST00000399299]	4.60928	up	5.23095	up	2048.03400	460.08408	1352.40710	227.97784	Detected	Detected	Detected	Detected
1125	A_23_P66719	DHRS13	Hs.631760	dehydrogenase/reductase (SDR family) member 13	Homo sapiens dehydrogenase/reductase (SDR family) member 13 (DHRS13), mRNA [NM_144683]	4.60799	up	3.89054	up	1320.20620	296.66312	1130.45040	256.21637	Detected	Detected	Detected	Detected
1126	A_24_P73599	IL16	Hs.459095	interleukin 16 (lymphocyte chemoattractant factor)	Homo sapiens interleukin 16 (lymphocyte chemoattractant factor) (IL16), transcript variant 2, mRNA [NM_172217]	4.60425	up	3.15394	up	14.26820	3.20881	9.48871	2.65289	Detected	Compromised	Compromised	Compromised
1127	A_23_P139260	SLC22A18	Hs.50868	solute carrier family 22, member 18	Homo sapiens solute carrier family 22, member 18 (SLC22A18), transcript variant 2, mRNA [NM_183233]	4.60253	up	5.42993	up	8157.17330	1835.17080	8984.94600	1459.10610	Detected	Detected	Detected	Detected
1128	A_33_P3299416	GRASP	Hs.407202	GRP1 (general receptor for phosphoinositides 1)-associated scaffold protein	Homo sapiens GRP1 (general receptor for phosphoinositides 1)-associated scaffold protein (GRASP), mRNA [NM_181711]	4.60110	up	4.46710	up	5112.23140	1150.48840	4425.42970	873.56604	Detected	Detected	Detected	Detected
1129	A_33_P3297020	PSORS1C3	Hs.670091	psoriasis susceptibility 1 candidate 3 (non-protein coding)	Homo sapiens psoriasis susceptibility 1 candidate 3 (non-protein coding) (PSORS1C3), non-coding RNA [NR_026816]	4.59941	up	7.43772	up	83.73975	18.85222	70.24699	8.32824	Detected	Detected	Detected	Compromised
1130	A_24_P393565		Hs.351005		Zinc finger protein 396 (Zinc finger and SCAN domain-containing protein 14) [Source:UniProtKB/Swiss-Prot;Acc:Q96N95] [ENST00000399057]	4.59892	up	3.21661	up	24.32697	5.47728	28.87855	7.91667	Detected	Compromised	Detected	Compromised
1131	A_33_P3349466	ANKRD12	Hs.464585	ankyrin repeat domain 12	Homo sapiens ankyrin repeat domain 12 (ANKRD12), transcript variant 1, mRNA [NM_015208]	4.59833	up	5.72895	up	133.94592	30.16217	82.55695	12.70703	Detected	Detected	Detected	Detected
1132	A_23_P46045	RGS5	Hs.24950	regulator of G-protein signaling 5	Homo sapiens regulator of G-protein signaling 5 (RGS5), mRNA [NM_003617]	4.59506	up	4.37608	up	287.01130	64.67562	199.01865	40.10272	Detected	Detected	Detected	Detected
1133	A_33_P3379341	LOC391322	Hs.711777	similar to D-dopachrome tautomerase	Homo sapiens similar to D-dopachrome tautomerase (LOC391322), mRNA [NM_001144931]	4.59495	up	5.58052	up	13.57341	3.05873	16.05119	2.53629	Detected	Compromised	Detected	Compromised
1134	A_32_P114028	C10orf68	Hs.585464	chromosome 10 open reading frame 68	Homo sapiens chromosome 10 open reading frame 68 (C10orf68), mRNA [NM_024688]	4.59052	up	2.67823	up	46.33748	10.45210	30.88742	10.16948	Detected	Compromised	Detected	Compromised
1135	A_33_P3243439	GPR162	Hs.631654	G protein-coupled receptor 162	Homo sapiens G protein-coupled receptor 162 (GPR162), transcript variant A-2, mRNA [NM_019858]	4.58992	up	4.96466	up	1031.80580	232.76956	1050.26260	186.54080	Detected	Detected	Detected	Detected
1136	A_23_P80048	FER1L4	Hs.72222	fer-1-like 4 (C. elegans)	Homo sapiens fer-1-like 4 (C. elegans) (FER1L4), non-coding RNA [NR_024377]	4.58946	up	5.80347	up	858.55945	193.70550	722.50960	109.77960	Detected	Detected	Detected	Detected
1137	A_24_P82032	RHOJ	Hs.656339	ras homolog gene family, member J	Homo sapiens ras homolog gene family, member J (RHOJ), mRNA [NM_020663]	4.58881	up	6.98154	up	15.60592	3.52146	23.39463	2.95482	Detected	Compromised	Detected	Compromised
1138	A_32_P223059	SLC45A1	Hs.463036	solute carrier family 45, member 1	Homo sapiens solute carrier family 45, member 1 (SLC45A1), mRNA [NM_001080397]	4.58396	up	5.55438	up	104.31739	23.56400	96.16173	15.26625	Detected	Detected	Detected	Compromised
1139	A_33_P3395314		Hs.656750		DB335107 SYNOV4 Homo sapiens cDNA clone SYNOV4009599 3', mRNA sequence [DB335107]	4.58354	up	5.04731	up	14.46354	3.26743	15.73334	2.74869	Detected	Compromised	Detected	Compromised
1140	A_24_P162244				S phase cyclin A-associated protein in the endoplasmic reticulum (S phase cyclin A-associated protein in the ER)(Zinc finger protein 291) [Source:UniProtKB/Swiss-Prot;Acc:Q9BY12] [ENST00000303521]	4.57768	up	5.43371	up	55.98806	12.66436	55.47908	9.00323	Detected	Compromised	Detected	Compromised
1141	A_23_P200260	PCNXL2	Hs.370605	pecanex-like 2 (Drosophila)	Homo sapiens pecanex-like 2 (Drosophila) (PCNXL2), transcript variant 1, mRNA [NM_014801]	4.57458	up	4.08553	up	3050.21970	690.41920	2652.55180	572.50720	Detected	Detected	Detected	Detected
1142	A_23_P167389	ARAP3	Hs.25277	ArfGAP with RhoGAP domain, ankyrin repeat and PH domain 3	Homo sapiens ArfGAP with RhoGAP domain, ankyrin repeat and PH domain 3 (ARAP3), mRNA [NM_022481]	4.57164	up	4.71162	up	2536.54170	574.51764	1746.43350	326.84910	Detected	Detected	Detected	Detected
1143	A_23_P374892	CES4		carboxylesterase 4-like	Homo sapiens carboxylesterase 4-like (CES4), non-coding RNA [NR_003276]	4.56123	up	4.29278	up	13.88885	3.15295	12.84631	2.63879	Detected	Compromised	Compromised	Compromised
1144	A_23_P208240	ZNF160	Hs.655967	zinc finger protein 160	Homo sapiens zinc finger protein 160, mRNA (cDNA clone IMAGE:3452857), complete cds. [BC000807]	4.56041	up	9.83779	up	15.21404	3.45441	33.29692	2.98450	Detected	Compromised	Detected	Compromised
1145	A_33_P3336422	LOC729013	Hs.649389	hypothetical protein LOC729013	Homo sapiens, clone IMAGE:3930408, mRNA [BC014360]	4.56005	up	4.05193	up	2951.30790	670.15920	2269.43900	493.88080	Detected	Detected	Detected	Detected
1146	A_23_P250619	ZDHHC14	Hs.187459	zinc finger, DHHC-type containing 14	Homo sapiens zinc finger, DHHC-type containing 14 (ZDHHC14), transcript variant 2, mRNA [NM_153746]	4.55699	up	4.16743	up	1812.47670	411.83936	1712.24170	362.29483	Detected	Detected	Detected	Detected
1147	A_24_P13024	SLC16A12	Hs.530338	solute carrier family 16, member 12 (monocarboxylic acid transporter 12)	Homo sapiens solute carrier family 16, member 12 (monocarboxylic acid transporter 12) (SLC16A12), mRNA [NM_213606]	4.55221	up	1.15761	up	16.48366	3.74942	4.14773	3.15947	Detected	Compromised	Compromised	Compromised

1148	A_23_P135548	DPYD	Hs.335034	dihydropyrimidine dehydrogenase	Homo sapiens dihydropyrimidine dehydrogenase (DPYD), transcript variant 1, mRNA [NM_000110]	4.54825	up	4.47335	up	1642.86500	374.01648	1029.18530	202.87373	Detected	Detected	Detected	Detected
1149	A_33_P3245484	LOC100131000	Hs.658271	hypothetical protein LOC100131000	Homo sapiens cDNA FLJ42370 fis, clone UTERU2030280. [AK124361]	4.54347	up	4.93922	up	127.67575	29.09739	129.65665	23.14739	Detected	Detected	Detected	Detected
1150	A_23_P307844	PHYHD1	Hs.709447	phytanoyl-CoA dioxygenase domain containing 1	Homo sapiens phytanoyl-CoA dioxygenase domain containing 1 (PHYHD1), transcript variant 2, mRNA [NM_174933]	4.54242	up	4.24269	up	705.89100	160.91031	482.79248	100.34256	Detected	Detected	Detected	Detected
1151	A_24_P226962	KIAA0368	Hs.368255	KIAA0368	Homo sapiens KIAA0368 (KIAA0368), mRNA [NM_001080398]	4.54014	up	5.01180	up	500.20325	114.08032	369.18720	64.95589	Detected	Detected	Detected	Detected
1152	A_23_P114689	ASAP3	Hs.437379	ArfGAP with SH3 domain, ankyrin repeat and PH domain 3	Homo sapiens ArfGAP with SH3 domain, ankyrin repeat and PH domain 3 (ASAP3), transcript variant 1, mRNA [NM_017707]	4.53819	up	4.08684	up	886.52000	202.27348	882.92330	190.50250	Detected	Detected	Detected	Detected
1153	A_23_P48713	PTGR2	Hs.632344	prostaglandin reductase 2	Homo sapiens prostaglandin reductase 2 (PTGR2), transcript variant 1, mRNA [NM_152444]	4.53726	up	4.55666	up	384.41153	87.72746	363.63992	70.37046	Detected	Detected	Detected	Detected
1154	A_24_P320604	ARMC2	Hs.645481	armadillo repeat containing 2	Homo sapiens armadillo repeat containing 2 (ARMC2), mRNA [NM_032131]	4.53599	up	5.43194	up	142.82497	32.60357	113.98851	18.50424	Detected	Detected	Detected	Detected
1155	A_33_P3372069	COX8C	Hs.666459	cytochrome c oxidase subunit 8C	Homo sapiens cytochrome c oxidase subunit 8C (COX8C), nuclear gene encoding mitochondrial protein, mRNA [NM_182971]	4.52442	up	2.20317	up	13.59755	3.11194	6.56927	2.62926	Detected	Compromised	Compromised	Compromised
1156	A_23_P63232	CREB3L4	Hs.372924	cAMP responsive element binding protein 3-like 4	Homo sapiens cAMP responsive element binding protein 3-like 4 (CREB3L4), mRNA [NM_130898]	4.52346	up	4.52622	up	3004.69000	687.79950	3296.42700	642.20410	Detected	Detected	Detected	Detected
1157	A_33_P3252394	GADD45G	Hs.9701	growth arrest and DNA-damage-inducible, gamma	Homo sapiens growth arrest and DNA-damage-inducible, gamma (GADD45G), mRNA [NM_006705]	4.52141	up	5.30153	up	1010.35004	231.38281	808.46600	134.47029	Detected	Detected	Detected	Detected
1158	A_23_P363255	CCDC68	Hs.120790	coiled-coil domain containing 68	Homo sapiens coiled-coil domain containing 68 (CCDC68), transcript variant 1, mRNA [NM_025214]	4.51390	up	3.73731	up	128.43408	29.46196	51.23665	12.08892	Detected	Detected	Detected	Compromised
1159	A_23_P321466	PEX11G	Hs.515100	peroxisomal biogenesis factor 11 gamma	Homo sapiens peroxisomal biogenesis factor 11 gamma (PEX11G), mRNA [NM_080662]	4.51340	up	4.05669	up	123.82841	28.40856	138.05542	30.00870	Detected	Detected	Detected	Detected
1160	A_23_P319583	RIMS3	Hs.654808	regulating synaptic membrane exocytosis 3	Homo sapiens regulating synaptic membrane exocytosis 3 (RIMS3), mRNA [NM_014747]	4.51271	up	4.52296	up	88.49886	20.30641	49.97285	9.74265	Detected	Detected	Detected	Compromised
1161	A_33_P3299220	ADAMTSL4	Hs.719991	ADAMTS-like 4	Homo sapiens ADAMTS-like 4 (ADAMTSL4), transcript variant 2, mRNA [NM_025008]	4.50749	up	4.29277	up	3408.09720	782.90674	4386.43160	901.03015	Detected	Detected	Detected	Detected
1162	A_33_P3308872	POM121L1P	Hs.318898	POM121 membrane glycoprotein-like 1 (rat) pseudogene	Homo sapiens POM121 membrane glycoprotein-like 1 (rat) pseudogene (POM121L1P), non-coding RNA [NR_024591]	4.50101	up	4.82413	up	284.25183	65.39221	257.48587	47.06519	Detected	Detected	Detected	Detected
1163	A_33_P3666884	PNPLA7	Hs.294147	patatin-like phospholipase domain containing 7	Homo sapiens patatin-like phospholipase domain containing 7 (PNPLA7), transcript variant 1, mRNA [NM_001098537]	4.49943	up	9.98095	up	3373.47400	776.34210	2216.74830	195.84381	Detected	Detected	Detected	Detected
1164	A_24_P375322	GGT8P	Hs.650223	gamma-glutamyltransferase 8 pseudogene	Homo sapiens gamma-glutamyltransferase 8 pseudogene (GGT8P), non-coding RNA [NR_003503]	4.49157	up	2.52102	up	16.26524	3.74969	15.05574	5.26612	Detected	Compromised	Compromised	Compromised
1165	A_33_P3244610	SIRPD	Hs.664861	signal-regulatory protein delta	Homo sapiens signal-regulatory protein delta (SIRPD), mRNA [NM_178460]	4.49093	up	11.70869	up	26.55673	6.12310	34.94795	2.63196	Detected	Compromised	Detected	Compromised
1166	A_33_P3283684	C7orf31	Hs.122055	chromosome 7 open reading frame 31	Homo sapiens chromosome 7 open reading frame 31 (C7orf31), mRNA [NM_138811]	4.48947	up	1.36484	up	13.55404	3.12612	7.41918	4.79336	Detected	Compromised	Compromised	Compromised
1167	A_33_P3626301	DPY19L2P1	Hs.594815	dpy-19-like 2 pseudogene 1 (C. elegans)	Homo sapiens dpy-19-like 2 pseudogene 1 (C. elegans), mRNA (cDNA clone IMAGE:4838596). [BC066987]	4.48487	up	2.47980	up	42.05948	9.71062	44.21146	15.72116	Detected	Compromised	Detected	Detected
1168	A_33_P3233440					4.48261	up	3.07988	up	66.54591	15.37176	50.42661	14.43746	Detected	Compromised	Detected	Detected
1169	A_23_P411806	SLC44A1	Hs.573495	solute carrier family 44, member 1	Homo sapiens solute carrier family 44, member 1 (SLC44A1), mRNA [NM_080546]	4.48095	up	4.22385	up	2725.93000	629.90826	2734.24050	570.81310	Detected	Detected	Detected	Detected
1170	A_23_P106806	PRSS27	Hs.332878	protease, serine 27	Homo sapiens protease, serine 27 (PRSS27), mRNA [NM_031948]	4.47995	up	5.36422	up	105.59972	24.40746	116.40633	19.13533	Detected	Detected	Detected	Detected
1171	A_23_P2920	SERPINA3	Hs.534293	serpin peptidase inhibitor, clade A (alpha-1 antiproteinase, antitrypsin), member 3	Homo sapiens serpin peptidase inhibitor, clade A (alpha-1 antiproteinase, antitrypsin), member 3 (SERPINA3), mRNA [NM_001085]	4.47337	up	5.27765	up	209.56696	48.50884	232.33983	38.81937	Detected	Detected	Detected	Detected
1172	A_23_P115064	CRABP2	Hs.405662	cellular retinoic acid binding protein 2	Homo sapiens cellular retinoic acid binding protein 2 (CRABP2), mRNA [NM_001878]	4.47245	up	3.91688	up	1767.93600	409.31204	1529.48240	344.32623	Detected	Detected	Detected	Detected
1173	A_23_P333038	C10orf79	Hs.288927	chromosome 10 open reading frame 79	Homo sapiens chromosome 10 open reading frame 79 (C10orf79), mRNA [NM_025145]	4.46829	up	4.00524	up	13.92773	3.22755	12.23256	2.69312	Detected	Compromised	Compromised	Compromised
1174	A_24_P191971	SAP30L	Hs.592566	SAP30-like	Homo sapiens SAP30-like (SAP30L), transcript variant 1, mRNA [NM_024632]	4.46575	up	4.62594	up	1838.99610	426.40234	1366.40560	260.46250	Detected	Detected	Detected	Detected
1175	A_33_P3421200	hCG_1990547	Hs.656318	family with sequence similarity 86, member A pseudogene	Homo sapiens family with sequence similarity 86, member A pseudogene (FLJ10661), transcript variant 2, non-coding RNA [NR_024361]	4.46268	up	4.35454	up	932.64290	216.39775	849.52783	172.02876	Detected	Detected	Detected	Detected
1176	A_23_P52227	GDF10	Hs.2171	growth differentiation factor 10	Homo sapiens growth differentiation factor 10 (GDF10), mRNA [NM_004962]	4.46201	up	14.17792	up	53.35391	12.38138	45.57743	2.83467	Detected	Compromised	Detected	Compromised
1177	A_33_P3238290	FAM65C	Hs.372578	family with sequence similarity 65, member C	Homo sapiens family with sequence similarity 65, member C (FAM65C), mRNA [NM_080829]	4.46131	up	4.36510	up	5840.50340	1355.56650	4975.03120	1005.00250	Detected	Detected	Detected	Detected

1178	A_23_P257834	ALB	Hs.418167	albumin	Homo sapiens albumin (ALB), mRNA [NM_000477]	4.45567	up	4.83850	up	38.35960	8.91443	38.92801	7.09442	Detected	Compromised	Detected	Compromised
1179	A_23_P31721	E2F5	Hs.445758	E2F transcription factor 5, p130-binding	Homo sapiens E2F transcription factor 5, p130-binding (E2F5), transcript variant 1, mRNA [NM_001951]	4.45418	up	3.32628	up	99.44153	23.11706	67.42403	17.87401	Detected	Detected	Detected	Compromised
1180	A_24_P826348	ZC3H6	Hs.190477	zinc finger CCCH-type containing 6	Homo sapiens zinc finger CCCH-type containing 6 (ZC3H6), mRNA [NM_198581]	4.44850	up	4.90789	up	1192.37680	277.54504	625.84990	112.44510	Detected	Detected	Detected	Detected
1181	A_23_P42909	TMEM139	Hs.17558	transmembrane protein 139	Homo sapiens transmembrane protein 139 (TMEM139), mRNA [NM_153345]	4.44751	up	1.80420	up	13.19684	3.07246	5.20544	2.54412	Detected	Compromised	Compromised	Compromised
1182	A_33_P3254082		Hs.179615		Uncharacterized protein C9orf68 [Source:UniProtKB/Swiss-Prot;Acc:Q8N4H0] [ENST00000381890]	4.44720	up	4.39126	up	13.67299	3.18354	23.88859	4.79697	Detected	Compromised	Detected	Compromised
1183	A_24_P144439	EFHB	Hs.670883	EF-hand domain family, member B	Homo sapiens EF-hand domain family, member B (EFHB), mRNA [NM_144715]	4.44714	up	4.12205	up	16.02420	3.73103	15.17499	3.24624	Detected	Compromised	Compromised	Compromised
1184	A_32_P66222	ANKRD20A2	Hs.632663	ankyrin repeat domain 20 family, member A2	Homo sapiens ankyrin repeat domain 20 family, member A2 (ANKRD20A2), mRNA [NM_001012421]	4.44635	up	1.41156	up	16.25730	3.78598	9.40287	5.87389	Detected	Compromised	Compromised	Compromised
1185	A_23_P303803	C19orf18	Hs.134209	chromosome 19 open reading frame 18	Homo sapiens chromosome 19 open reading frame 18 (C19orf18), mRNA [NM_152474]	4.44557	up	5.64619	up	275.15582	64.08912	216.96654	33.88465	Detected	Detected	Detected	Detected
1186	A_33_P3228591		Hs.632807		WD repeat domain phosphoinositide-interacting protein 4 (WIPI-4)(WD repeat-containing protein 45) [Source:UniProtKB/Swiss-Prot;Acc:Q9Y484] [ENST00000376358]	4.44493	up	3.11226	up	70.93971	16.52561	52.24096	14.80132	Detected	Compromised	Detected	Compromised
1187	A_33_P3231670					4.44409	up	4.45574	up	490.95578	114.39117	608.64026	120.44980	Detected	Detected	Detected	Detected
1188	A_33_P3305731	NINL	Hs.631508	ninein-like	Homo sapiens ninein-like (NINL), mRNA [NM_025176]	4.44301	up	3.47479	up	159.46797	37.16459	126.57566	32.12083	Detected	Detected	Detected	Detected
1189	A_23_P61580	NSUN6	Hs.396175	NOL1/NOP2/Sun domain family, member 6	Homo sapiens NOL1/NOP2/Sun domain family, member 6 (NSUN6), mRNA [NM_182543]	4.44232	up	3.96747	up	493.22990	114.96678	437.74265	97.29054	Detected	Detected	Detected	Detected
1190	A_23_P323094	PHC1	Hs.305985	polyhomeotic homolog 1 (Drosophila)	Homo sapiens polyhomeotic homolog 1 (Drosophila) (PHC1), mRNA [NM_004426]	4.44099	up	4.90303	up	188.86420	44.03547	144.88390	26.05678	Detected	Detected	Detected	Detected
1191	A_33_P3813128	SEMA3F	Hs.32981	sema domain, immunoglobulin domain (Ig), short basic domain, secreted, (semaphorin) 3F	Homo sapiens sema domain, immunoglobulin domain (Ig), short basic domain, secreted, (semaphorin) 3F (SEMA3F), mRNA [NM_004186]	4.43996	up	3.74454	up	987.31440	230.25525	767.37750	180.70735	Detected	Detected	Detected	Detected
1192	A_33_P3392787	CCDC147	Hs.253576	coiled-coil domain containing 147	Homo sapiens coiled-coil domain containing 147 (CCDC147), mRNA [NM_001008723]	4.43922	up	2.20240	up	86.56288	20.19100	61.93649	24.79793	Detected	Detected	Detected	Detected
1193	A_23_P258190	AKR1B1	Hs.521212	aldo-keto reductase family 1, member B1 (aldose reductase)	Homo sapiens aldo-keto reductase family 1, member B1 (aldose reductase) (AKR1B1), mRNA [NM_001628]	4.43563	up	3.87189	up	59975.41800	14000.75700	68133.56000	15516.85800	Detected	Detected	Detected	Detected
1194	A_24_P362881	IFT57	Hs.412196	intraflagellar transport 57 homolog (Chlamydomonas)	Homo sapiens intraflagellar transport 57 homolog (Chlamydomonas) (IFT57), mRNA [NM_018010]	4.42352	up	6.38181	up	54.98898	12.87185	55.07467	7.60981	Detected	Compromised	Detected	Compromised
1195	A_33_P3212630	FAM90A10	Hs.715411	family with sequence similarity 90, member A10	Homo sapiens family with sequence similarity 90, member A10 (FAM90A10), mRNA [NM_001164447]	4.42294	up	3.97358	up	94.24470	22.06375	100.14470	22.22345	Detected	Detected	Detected	Detected
1196	A_33_P3298810	FFAR3	Hs.248055	free fatty acid receptor 3	Homo sapiens free fatty acid receptor 3 (FFAR3), mRNA [NM_005304]	4.42044	up	2.92052	up	22.33439	5.23169	31.04200	9.37250	Detected	Compromised	Detected	Compromised
1197	A_33_P3313830	FLJ33630	Hs.340623	hypothetical LOC644873	Homo sapiens hypothetical LOC644873 (FLJ33630), non-coding RNA [NR_015360]	4.41738	up	4.52622	up	612.39510	143.54910	435.59793	84.86240	Detected	Detected	Detected	Detected
1198	A_23_P208400	ADCK4	Hs.130712	aarF domain containing kinase 4	Homo sapiens aarF domain containing kinase 4 (ADCK4), transcript variant 1, mRNA [NM_024876]	4.41649	up	3.21060	up	177.98210	41.72846	171.90611	47.21402	Detected	Detected	Detected	Detected
1199	A_32_P129419	NARG2	Hs.200943	NMDA receptor regulated 2	Homo sapiens NMDA receptor regulated 2 (NARG2), transcript variant 1, mRNA [NM_024611]	4.41490	up	3.77737	up	127.11490	29.81319	60.29860	14.07613	Detected	Detected	Detected	Compromised
1200	A_32_P101570	SUGT1L1	Hs.442781	SGT1, suppressor of G2 allele of SKP1 like 1 (S. cerevisiae)	Homo sapiens SGT1, suppressor of G2 allele of SKP1 like 1 (S. cerevisiae) (SUGT1L1), non-coding RNA [NR_003365]	4.41347	up	5.33544	up	21.74824	5.10243	15.97699	2.64052	Detected	Compromised	Detected	Compromised
1201	A_24_P106794	NUDCD1	Hs.380291	NudC domain containing 1	Homo sapiens NudC domain containing 1 (NUDCD1), transcript variant 1, mRNA [NM_032869]	4.41189	up	4.45024	up	347.13248	81.47108	192.71810	38.18600	Detected	Detected	Detected	Detected
1202	A_23_P64689	PAN2	Hs.273397	PAN2 poly(A) specific ribonuclease subunit homolog (S. cerevisiae)	Homo sapiens PAN2 poly(A) specific ribonuclease subunit homolog (S. cerevisiae) (PAN2), transcript variant 3, mRNA [NM_014871]	4.41070	up	4.58343	up	610.54224	143.33139	390.88320	75.20065	Detected	Detected	Detected	Detected
1203	A_33_P3248957	LOC441493	Hs.496083	hypothetical LOC441493	PREDICTED: Homo sapiens hypothetical LOC441493 (LOC441493), miscRNA [XR_017739]	4.40354	up	2.17923	up	15.01440	3.53053	7.41246	2.99934	Detected	Compromised	Compromised	Compromised
1204	A_24_P30141	C10orf4	Hs.658192	chromosome 10 open reading frame 4	Homo sapiens chromosome 10 open reading frame 4 (C10orf4), mRNA [NM_145246]	4.40277	up	3.58398	up	68.40400	16.08749	41.50301	10.21126	Detected	Compromised	Detected	Compromised
1205	A_33_P3741059	LOC284475	Hs.196026	hypothetical protein LOC284475	Homo sapiens mRNA; cDNA DKFZp667K053 (from clone DKFZp667K053) [AL832882]	4.40084	up	1.82108	up	39.15750	9.21325	20.03738	9.70236	Detected	Compromised	Detected	Compromised
1206	A_24_P396702	CD302	Hs.130014	CD302 molecule	Homo sapiens CD302 molecule (CD302), mRNA [NM_014880]	4.39995	up	4.57333	up	1640.19020	385.99304	1189.57090	229.36284	Detected	Detected	Detected	Detected
1207	A_23_P320862	C9orf43	Hs.632691	chromosome 9 open reading frame 43	Homo sapiens chromosome 9 open reading frame 43 (C9orf43), mRNA [NM_152786]	4.39602	up	2.56760	up	49.57161	11.67632	52.58179	18.05815	Detected	Compromised	Detected	Detected
1208	A_23_P165707	LYG1	Hs.164589	lysozyme G-like 1	Homo sapiens lysozyme G-like 1 (LYG1), mRNA [NM_174898]	4.39179	up	4.08880	up	201.61037	47.53405	205.81415	44.38588	Detected	Detected	Detected	Detected

1209	A_23_P25030	HSD17B6	Hs.524513	hydroxysteroid (17-beta) dehydrogenase 6 homolog (mouse)	Homo sapiens hydroxysteroid (17-beta) dehydrogenase 6 homolog (mouse) (HSD17B6), mRNA [NM_003725]	4.39041	up	4.50660	up	243.50665	57.42999	261.99920	51.26448	Detected	Detected	Detected	Detected
1210	A_23_P81825	GUCA1B	Hs.446529	guanylate cyclase activator 1B (retina)	Homo sapiens guanylate cyclase activator 1B (retina) (GUCA1B), mRNA [NM_002098]	4.38951	up	3.91763	up	37.69983	8.89317	21.89507	4.92820	Detected	Compromised	Detected	Compromised
1211	A_24_P400604	RBMY1B	Hs.536001	RNA binding motif protein, Y-linked, family 1, member B	Homo sapiens RNA binding motif protein, Y-linked, family 1, member B (RBMY1B), mRNA [NM_001006121]	4.38691	up	3.32146	up	16.17409	3.81763	12.24152	3.24992	Detected	Compromised	Compromised	Compromised
1212	A_33_P3298206	PPP4R4	Hs.259599	protein phosphatase 4, regulatory subunit 4	Homo sapiens protein phosphatase 4, regulatory subunit 4 (PPP4R4), transcript variant 2, mRNA [NM_020958]	4.37958	up	9.95245	up	41.84698	9.89384	35.81535	3.17325	Detected	Compromised	Detected	Compromised
1213	A_23_P419213	KIAA1407	Hs.477159	KIAA1407	Homo sapiens KIAA1407 (KIAA1407), mRNA [NM_020817]	4.37442	up	4.46658	up	176.56757	41.79492	175.95824	34.73760	Detected	Detected	Detected	Detected
1214	A_23_P43490	CDKN2A	Hs.512599	cyclin-dependent kinase inhibitor 2A (melanoma, p16, inhibits CDK4)	Homo sapiens cyclin-dependent kinase inhibitor 2A (melanoma, p16, inhibits CDK4) (CDKN2A), transcript variant 3, mRNA [NM_058197]	4.37423	up	4.62509	up	23289.71300	5513.09960	23008.87000	4386.72460	Detected	Detected	Detected	Detected
1215	A_23_P151046	KLRC1	Hs.512576	killer cell lectin-like receptor subfamily C, member 1	Homo sapiens killer cell lectin-like receptor subfamily C, member 1 (KLRC1), transcript variant 1, mRNA [NM_002259]	4.37060	up	5.27064	up	14.72006	3.48740	17.74364	2.96856	Detected	Compromised	Detected	Compromised
1216	A_33_P3272948	C17orf107	Hs.579243	chromosome 17 open reading frame 107	Homo sapiens chromosome 17 open reading frame 107 (C17orf107), mRNA [NM_001145536]	4.37005	up	4.55362	up	634.48846	150.33842	535.32460	103.66349	Detected	Detected	Detected	Detected
1217	A_32_P394951	NTN5	Hs.326217	netrin 5	Homo sapiens netrin 5 (NTN5), mRNA [NM_145807]	4.36515	up	4.00559	up	27.43268	6.50733	26.73481	5.88539	Detected	Compromised	Detected	Compromised
1218	A_33_P3407235	LOC100133180	Hs.709917	similar to DEAD (Asp-Glu-Ala-Asp) box polypeptide 3, Y-linked	PREDICTED: Homo sapiens similar to DEAD (Asp-Glu-Ala-Asp) box polypeptide 3, Y-linked (LOC100133180), miscRNA [XR_037576]	4.36188	up	4.77797	up	335.97278	79.75597	57.15229	10.54766	Detected	Detected	Detected	Compromised
1219	A_24_P941359	FAM65B	Hs.559459	family with sequence similarity 65, member B	Homo sapiens family with sequence similarity 65, member B (FAM65B), transcript variant 1, mRNA [NM_014722]	4.36111	up	4.99454	up	284.77618	67.61446	188.60900	33.29909	Detected	Detected	Detected	Detected
1220	A_33_P3415888	TEPP	Hs.132976	testis, prostate and placenta expressed	Homo sapiens testis, prostate and placenta expressed (TEPP), transcript variant 1, mRNA [NM_199046]	4.35862	up	1.71749	up	14.53949	3.45409	5.85059	3.00379	Detected	Compromised	Compromised	Compromised
1221	A_23_P98205	CHST1	Hs.104576	carbohydrate (keratan sulfate Gal-6) sulfotransferase 1	Homo sapiens carbohydrate (keratan sulfate Gal-6) sulfotransferase 1 (CHST1), mRNA [NM_003654]	4.35462	up	8.60330	up	208.01633	49.46301	142.28868	14.58380	Detected	Detected	Detected	Detected
1222	A_23_P106661	CMTM1	Hs.698621	CKLF-like MARVEL transmembrane domain containing 1	Homo sapiens CKLF-like MARVEL transmembrane domain containing 1 (CMTM1), transcript variant 17, mRNA [NM_052999]	4.35042	up	4.72062	up	799.86930	190.37970	729.16110	136.20396	Detected	Detected	Detected	Detected
1223	A_32_P118568	RFPL1S	Hs.167750	RFPL1 antisense RNA (non-protein coding)	Homo sapiens RFPL1 antisense RNA (non-protein coding) (RFPL1S), antisense RNA [NR_002727]	4.34595	up	9.34787	up	43.10982	10.27129	31.32931	2.95532	Detected	Compromised	Detected	Compromised
1224	A_32_P114003	LOC100192378	Hs.596420	hypothetical LOC100192378	Homo sapiens hypothetical LOC100192378 (LOC100192378), non-coding RNA [NR_024360]	4.34358	up	1.23009	up	13.69514	3.26477	3.77483	2.70599	Detected	Compromised	Compromised	Compromised
1225	A_33_P3222451		Hs.628396		Proline-rich AKT1 substrate 1 (40 kDa proline-rich AKT substrate) [Source:UniProtKB/Swiss-Prot;Acc:Q96B36] [ENST00000391830]	4.34012	up	2.87848	up	17.10599	4.08113	18.05234	5.53013	Detected	Compromised	Detected	Compromised
1226	A_24_P14932	WDR52	Hs.584936	WD repeat domain 52	Homo sapiens WD repeat domain 52 (WDR52), transcript variant 2, mRNA [NM_018338]	4.33225	up	3.61970	up	13.55816	3.24056	10.82542	2.63717	Detected	Compromised	Compromised	Compromised
1227	A_33_P3831566		Hs.667154		Homo sapiens HSFE-1 mRNA, partial cds. [AF072164]	4.32906	up	3.83749	up	204.75128	48.97406	115.23281	26.47856	Detected	Detected	Detected	Detected
1228	A_23_P419107	TCP11L2	Hs.696047	t-complex 11 (mouse)-like 2	Homo sapiens t-complex 11 (mouse)-like 2 (TCP11L2), mRNA [NM_152772]	4.32646	up	3.18550	up	155.06088	37.11098	147.00056	40.69178	Detected	Detected	Detected	Detected
1229	A_33_P3209537	EBF4	Hs.471955	early B-cell factor 4	Homo sapiens early B-cell factor 4 (EBF4), mRNA [NM_001110514]	4.32379	up	8.94597	up	377.44170	90.38952	345.10098	34.01606	Detected	Detected	Detected	Detected
1230	A_24_P45476	XCL1	Hs.546295	chemokine (C motif) ligand 1	Homo sapiens chemokine (C motif) ligand 1 (XCL1), mRNA [NM_002995]	4.32246	up	1.34091	up	17.33644	4.15300	5.57219	3.66432	Detected	Compromised	Compromised	Compromised
1231	A_23_P66827	FAM106A	Hs.674403	family with sequence similarity 106, member A	Homo sapiens family with sequence similarity 106, member A (FAM106A), non-coding RNA [NR_026809]	4.32029	up	2.54521	up	17.14978	4.11035	12.76762	4.42336	Detected	Compromised	Compromised	Compromised
1232	A_33_P3256952	EGLN3	Hs.135507	egl nine homolog 3 (C. elegans)	Homo sapiens egl nine homolog 3 (C. elegans) (EGLN3), mRNA [NM_022073]	4.31928	up	7.13851	up	32.55422	7.80420	25.16468	3.10849	Detected	Compromised	Detected	Compromised
1233	A_33_P3359084	TAS1R3		taste receptor, type 1, member 3	Taste receptor type 1 member 3 Precursor (Sweet taste receptor T1R3) [Source:UniProtKB/Swiss-Prot;Acc:Q7RTX0] [ENST00000339381]	4.31672	up	4.97339	up	142.18000	34.10497	131.86534	23.37994	Detected	Detected	Detected	Detected
1234	A_33_P3412087	C6orf97	Hs.660044	chromosome 6 open reading frame 97	Homo sapiens chromosome 6 open reading frame 97 (C6orf97), mRNA [NM_025059]	4.30243	up	3.96715	up	223.97046	53.90266	189.53763	42.12913	Detected	Detected	Detected	Detected
1235	A_23_P207003	SEPT4	Hs.287518	septin 4	Homo sapiens septin 4 (SEPT4), transcript variant 1, mRNA [NM_004574]	4.29982	up	3.07146	up	22.97934	5.53376	19.66872	5.64674	Detected	Compromised	Detected	Compromised
1236	A_23_P33583	DNAH7	Hs.97403	dynein, axonemal, heavy chain 7	Homo sapiens dynein, axonemal, heavy chain 7 (DNAH7), mRNA [NM_018897]	4.29871	up	4.08467	up	53.21900	12.81922	45.18938	9.75539	Detected	Compromised	Detected	Compromised
1237	A_33_P3217317	C14orf81	Hs.650220	chromosome 14 open reading frame 81	Homo sapiens cDNA FLJ32169 fis, clone PLACE6000523. [AK056731]	4.29232	up	2.99681	up	15.65985	3.77771	13.40368	3.94395	Detected	Compromised	Detected	Compromised
1238	A_33_P3344204	ZDHHC11	Hs.654874	zinc finger, DHHC-type containing 11	Homo sapiens zinc finger, DHHC-type containing 11 (ZDHHC11), mRNA [NM_024786]	4.29081	up	3.28568	up	297.60416	71.81793	211.07822	56.64791	Detected	Detected	Detected	Detected

1239	A_23_P103756	OVGP1	Hs.1154	oviductal glycoprotein 1, 120kDa	Homo sapiens oviductal glycoprotein 1, 120kDa (OVGP1), mRNA [NM_002557]	4.28728	up	4.06465	up	703.40180	169.88507	609.74320	132.27867	Detected	Detected	Detected	Detected
1240	A_24_P230938		Hs.217409		MORN repeat-containing protein 4 (Protein 44050) [Source:UniProtKB/Swiss-Prot;Acc:Q8NDC4] [ENST00000370635]	4.28257	up	4.60970	up	1809.74100	437.56790	1637.10850	313.16312	Detected	Detected	Detected	Detected
1241	A_24_P100830	AMN1	Hs.591146	antagonist of mitotic exit network 1 homolog (S. cerevisiae)	Homo sapiens antagonist of mitotic exit network 1 homolog (S. cerevisiae) (AMN1), transcript variant 1, mRNA [NM_001113402]	4.28182	up	5.83566	up	243.64899	58.92087	175.91446	26.58136	Detected	Detected	Detected	Detected
1242	A_23_P207345	ADAM11	Hs.6088	ADAM metallopeptidase domain 11	Homo sapiens ADAM metallopeptidase domain 11 (ADAM11), mRNA [NM_002390]	4.28102	up	2.31261	up	18.49724	4.47397	27.62122	10.53186	Detected	Compromised	Detected	Compromised
1243	A_33_P3232995	TDRD1	Hs.333132	tudor domain containing 1	Homo sapiens tudor domain containing 1 (TDRD1), mRNA [NM_198795]	4.27767	up	9.34267	up	52.20979	12.63799	37.70708	3.55891	Detected	Compromised	Detected	Compromised
1244	A_24_P520767	LOC149351	Hs.546492	hypothetical protein LOC149351	Homo sapiens cDNA FLJ12836 fis, clone NT2RP2003206 [AK022898]	4.27678	up	5.77382	up	304.43420	73.70721	288.58423	44.07320	Detected	Detected	Detected	Detected
1245	A_33_P3327063	STRC	Hs.657395	stereocilin	Homo sapiens stereocilin (STRC), mRNA [NM_153700]	4.27340	up	3.72085	up	13.81736	3.34800	11.87717	2.81472	Detected	Compromised	Compromised	Compromised
1246	A_33_P3332521	PCDHA3	Hs.199343	protocadherin alpha 3	Homo sapiens protocadherin alpha 3 (PCDHA3), transcript variant 2, mRNA [NM_031497]	4.27089	up	9.24623	up	69.75982	16.91298	54.74779	5.22117	Detected	Compromised	Detected	Compromised
1247	A_33_P3401701	EDNRA	Hs.183713	endothelin receptor type A	Homo sapiens endothelin receptor type A (EDNRA), transcript variant 1, mRNA [NM_001957]	4.26784	up	4.23394	up	302.80350	73.46599	232.20662	48.36105	Detected	Detected	Detected	Detected
1248	A_33_P3323218	ALS2CR8	Hs.444982	amyotrophic lateral sclerosis 2 (juvenile) chromosome region, candidate 8	Homo sapiens amyotrophic lateral sclerosis 2 (juvenile) chromosome region, candidate 8 (ALS2CR8), mRNA [NM_024744]	4.26391	up	1.72678	up	302.18063	73.38234	185.46985	94.71148	Detected	Detected	Detected	Detected
1249	A_24_P14260	CARD8	Hs.446146	caspase recruitment domain family, member 8	Homo sapiens caspase recruitment domain family, member 8 (CARD8), mRNA [NM_014959]	4.26280	up	3.94065	up	216.15434	52.50522	189.69969	42.44869	Detected	Detected	Detected	Detected
1250	A_24_P409800	KCTD16	Hs.661870	potassium channel tetramerisation domain containing 16	Homo sapiens potassium channel tetramerisation domain containing 16 (KCTD16), mRNA [NM_020768]	4.25931	up	1.77734	up	14.80215	3.59848	15.21657	7.54938	Detected	Compromised	Detected	Compromised
1251	A_33_P3365408	UNQ2963	Hs.246046	hypothetical LOC283314	Homo sapiens hypothetical LOC283314 (LOC283314), non-coding RNA [NR_026947]	4.25869	up	4.26358	up	51.41370	12.50075	25.30603	5.23378	Detected	Compromised	Detected	Compromised
1252	A_33_P3361546	TFAP2A	Hs.519880	transcription factor AP-2 alpha (activating enhancer binding protein 2 alpha)	Homo sapiens transcription factor AP-2 alpha (activating enhancer binding protein 2 alpha) (TFAP2A), transcript variant 2, mRNA [NM_001032280]	4.25285	up	3.35815	up	12.67772	3.08670	13.38969	3.51590	Detected	Compromised	Detected	Compromised
1253	A_23_P309619	KIAA1671	Hs.419171	KIAA1671	Homo sapiens KIAA1671 (KIAA1671), mRNA [NM_001145206]	4.24529	up	4.65462	up	152.44044	37.18138	103.03821	19.52000	Detected	Detected	Detected	Detected
1254	A_23_P368484	C17orf76	Hs.25425	chromosome 17 open reading frame 76	Homo sapiens chromosome 17 open reading frame 76 (C17orf76), transcript variant 2, mRNA [NM_207387]	4.24506	up	4.74111	up	123.88449	30.21804	111.58860	20.75418	Detected	Detected	Detected	Detected
1255	A_24_P22079	FOXO1	Hs.370666	forkhead box O1	Homo sapiens forkhead box O1 (FOXO1), mRNA [NM_002015]	4.23472	up	4.89830	up	1664.30000	406.94894	1473.73840	265.30220	Detected	Detected	Detected	Detected
1256	A_33_P3213362	CASC2	Hs.89387	cancer susceptibility candidate 2	Homo sapiens cancer susceptibility candidate 2 (CASC2), transcript variant 1, non-coding RNA [NR_026939]	4.23409	up	4.69759	up	226.79146	55.46254	180.87760	33.95279	Detected	Detected	Detected	Detected
1257	A_23_P69383	PARP9	Hs.518200	poly (ADP-ribose) polymerase family, member 9	Homo sapiens poly (ADP-ribose) polymerase family, member 9 (PARP9), transcript variant 1, mRNA [NM_031458]	4.23395	up	4.01104	up	901.21246	220.40175	801.56946	176.21770	Detected	Detected	Detected	Detected
1258	A_23_P96271	MYOM1	Hs.464469	myomesin 1, 185kDa	Homo sapiens myomesin 1, 185kDa (MYOM1), transcript variant 1, mRNA [NM_003803]	4.23158	up	2.77341	up	40.11065	9.81500	37.11859	11.80164	Detected	Compromised	Detected	Compromised
1259	A_33_P3346473	PHF21A	Hs.502458	PHD finger protein 21A	Homo sapiens PHD finger protein 21A (PHF21A), transcript variant 1, mRNA [NM_001101802]	4.23113	up	4.78180	up	1713.12930	419.24420	623.53040	114.98240	Detected	Detected	Detected	Detected
1260	A_33_P3329750					4.23058	up	2.93927	up	32.80483	8.02918	33.68646	10.10606	Detected	Compromised	Detected	Compromised
1261	A_23_P145935	EPHB6	Hs.380089	EPH receptor B6	Homo sapiens EPH receptor B6 (EPHB6), mRNA [NM_004445]	4.23037	up	3.76165	up	376.65836	92.19390	474.44800	111.21818	Detected	Detected	Detected	Detected
1262	A_33_P3324383	FIGNL2	Hs.648218	fidgetin-like 2	Homo sapiens fidgetin-like 2 (FIGNL2), mRNA [NM_001013690]	4.22780	up	4.14428	up	318.77966	78.07452	238.73192	50.79574	Detected	Detected	Detected	Detected
1263	A_32_P213661	CCDC122	Hs.170849	coiled-coil domain containing 122	Homo sapiens coiled-coil domain containing 122 (CCDC122), mRNA [NM_144974]	4.22606	up	3.90342	up	333.25146	81.65242	184.68288	41.72022	Detected	Detected	Detected	Detected
1264	A_23_P121106	HESX1	Hs.171980	HESX homeobox 1	Homo sapiens HESX homeobox 1 (HESX1), mRNA [NM_003865]	4.22427	up	8.36301	up	68.82225	16.86980	65.66126	6.92329	Detected	Detected	Detected	Compromised
1265	A_24_P212481	MCTP1	Hs.655087	multiple C2 domains, transmembrane 1	Homo sapiens multiple C2 domains, transmembrane 1 (MCTP1), transcript variant L, mRNA [NM_024717]	4.22266	up	3.51481	up	317.66730	77.89672	247.94930	62.20525	Detected	Detected	Detected	Detected
1266	A_23_P24535	TTC12	Hs.288772	tetratricopeptide repeat domain 12	Homo sapiens tetratricopeptide repeat domain 12 (TTC12), mRNA [NM_017868]	4.22221	up	4.07287	up	973.86630	238.83199	858.95120	185.96600	Detected	Detected	Detected	Detected
1267	A_33_P3388810		Hs.480085		Fucose-1-phosphate guanylyltransferase (EC 2.7.7.30)(GDP-L-fucose pyrophosphorylase)(GDP-L-fucose diphosphorylase) [Source:UniProtKB/Swiss-Prot;Acc:O14772] [ENST00000370895]	4.21929	up	4.15450	up	363.03560	89.09296	360.86264	76.59300	Detected	Detected	Detected	Detected
1268	A_24_P759477	ITGB8	Hs.592171	integrin, beta 8	Homo sapiens integrin, beta 8 (ITGB8), mRNA [NM_002214]	4.21200	up	3.25778	up	177.05466	43.52636	80.70503	21.84463	Detected	Detected	Detected	Detected

1269	A_23_P55356	VMO1	Hs.122561	vitelline membrane outer layer 1 homolog (chicken)	Homo sapiens vitelline membrane outer layer 1 homolog (chicken) (VMO1), transcript variant 1, mRNA [NM_182566]	4.20626	up	3.30434	up	150.18779	36.97193	222.73726	59.43932	Detected	Detected	Detected	Detected
1270	A_23_P77135	ATPBD4	Hs.107196	ATP binding domain 4	Homo sapiens ATP binding domain 4 (ATPBD4), transcript variant 1, mRNA [NM_080650]	4.20308	up	2.94112	up	52.67199	12.97613	49.46977	14.83178	Detected	Compromised	Detected	Detected
1271	A_23_P89431	CCL2	Hs.303649	chemokine (C-C motif) ligand 2	Homo sapiens chemokine (C-C motif) ligand 2 (CCL2), mRNA [NM_002982]	4.20265	up	4.26056	up	1433.46640	353.18146	1353.83640	280.19830	Detected	Detected	Detected	Detected
1272	A_33_P3413840	GK	Hs.1466	glycerol kinase	Homo sapiens glycerol kinase (GK), transcript variant 3, mRNA [NM_001128127]	4.20101	up	4.37331	up	1398.76310	344.76514	703.70966	141.88902	Detected	Detected	Detected	Detected
1273	A_23_P152047	SCAMP5	Hs.374180	secretory carrier membrane protein 5	Homo sapiens secretory carrier membrane protein 5 (SCAMP5), mRNA [NM_138967]	4.19923	up	4.04478	up	246.35417	60.74674	203.43346	44.34990	Detected	Detected	Detected	Detected
1274	A_23_P150555	SCGB1D2	Hs.204096	secretoglobin, family 1D, member 2	Homo sapiens secretoglobin, family 1D, member 2 (SCGB1D2), mRNA [NM_006551]	4.19636	up	4.36102	up	21.28573	5.25230	16.61231	3.35898	Detected	Compromised	Detected	Compromised
1275	A_24_P274814	TBXAS1	Hs.520757	thromboxane A synthase 1 (platelet)	Homo sapiens thromboxane A synthase 1 (platelet) (TBXAS1), transcript variant 2, mRNA [NM_030984]	4.19410	up	8.27205	up	449.25684	110.91463	419.25920	44.69256	Detected	Detected	Detected	Detected
1276	A_24_P418408	FAM89A	Hs.38516	family with sequence similarity 89, member A	Homo sapiens family with sequence similarity 89, member A (FAM89A), mRNA [NM_198552]	4.19108	up	4.32402	up	6070.43850	1499.78000	4641.85800	946.60740	Detected	Detected	Detected	Detected
1277	A_32_P309404	SLC22A3	Hs.567337	solute carrier family 22 (extraneuronal monoamine transporter), member 3	Homo sapiens solute carrier family 22 (extraneuronal monoamine transporter), member 3 (SLC22A3), mRNA [NM_021977]	4.19067	up	2.17335	up	14.26344	3.52431	7.30801	2.96508	Detected	Compromised	Compromised	Compromised
1278	A_23_P334328	LAMB4	Hs.62022	laminin, beta 4	Homo sapiens laminin, beta 4 (LAMB4), mRNA [NM_007356]	4.19035	up	2.94441	up	14.78204	3.65273	10.30638	3.08655	Detected	Compromised	Compromised	Compromised
1279	A_24_P131173	C1orf115	Hs.519839	chromosome 1 open reading frame 115	Homo sapiens chromosome 1 open reading frame 115 (C1orf115), mRNA [NM_024709]	4.18514	up	2.67618	up	145.61519	36.02716	116.87350	38.50933	Detected	Detected	Detected	Detected
1280	A_23_P327483	SPATA6	Hs.538103	spermatogenesis associated 6	Homo sapiens spermatogenesis associated 6 (SPATA6), mRNA [NM_019073]	4.18350	up	4.11182	up	407.93903	100.96921	373.56552	80.11219	Detected	Detected	Detected	Detected
1281	A_23_P70746	AHI1	Hs.386684	Abelson helper integration site 1	Homo sapiens Abelson helper integration site 1 (AHI1), transcript variant 2, mRNA [NM_017651]	4.18281	up	4.08390	up	465.91443	115.33756	303.56808	65.54598	Detected	Detected	Detected	Detected
1282	A_33_P3266429	SAMD13	Hs.591445	sterile alpha motif domain containing 13	Homo sapiens sterile alpha motif domain containing 13 (SAMD13), transcript variant 1, mRNA [NM_001010971]	4.18197	up	2.49187	up	12.74593	3.15591	7.53123	2.66505	Detected	Compromised	Compromised	Compromised
1283	A_23_P94533	CTSL1	Hs.418123	cathepsin L1	Homo sapiens cathepsin L1 (CTSL1), transcript variant 1, mRNA [NM_001912]	4.17921	up	3.98334	up	166162.97000	41169.31600	148621.38000	32900.24600	Detected	Detected	Detected	Detected
1284	A_24_P315921					4.17001	up	4.23989	up	948.86220	235.61275	1090.01460	226.69583	Detected	Detected	Detected	Detected
1285	A_23_P436048	LPAR3	Hs.674915	lysophosphatidic acid receptor 3	Homo sapiens lysophosphatidic acid receptor 3 (LPAR3), mRNA [NM_012152]	4.16962	up	6.15561	up	11.89851	2.95480	16.90189	2.42119	Detected	Compromised	Detected	Compromised
1286	A_33_P3331437	ZNF518A	Hs.600823	zinc finger protein 518A	Homo sapiens zinc finger protein 518A (ZNF518A), mRNA [NM_014803]	4.16530	up	2.39922	up	49.41702	12.28466	27.61905	10.15088	Detected	Compromised	Detected	Compromised
1287	A_23_P502336	EMR2	Hs.531619	egf-like module containing, mucin-like, hormone receptor-like 2	Homo sapiens egf-like module containing, mucin-like, hormone receptor-like 2 (EMR2), transcript variant 1, mRNA [NM_013447]	4.16358	up	6.96068	up	121.46176	30.20690	103.78359	13.14748	Detected	Detected	Detected	Compromised
1288	A_33_P3436646	LOC151657	Hs.587187	hypothetical protein LOC151657	Homo sapiens cDNA FLJ33795 fis, clone CTONG1000097 [AK091114]	4.16341	up	1.94435	up	35.25608	8.76835	19.69265	8.93090	Detected	Compromised	Detected	Compromised
1289	A_23_P65230	TMTC4	Hs.190983	transmembrane and tetratricopeptide repeat containing 4	Homo sapiens transmembrane and tetratricopeptide repeat containing 4 (TMTC4), transcript variant 1, mRNA [NM_032813]	4.15981	up	4.24639	up	1967.65900	489.78950	1605.23330	333.33740	Detected	Detected	Detected	Detected
1290	A_23_P32404	ISG20	Hs.459265	interferon stimulated exonuclease gene 20kDa	Homo sapiens interferon stimulated exonuclease gene 20kDa (ISG20), mRNA [NM_002201]	4.15756	up	4.38343	up	9246.77800	2302.95200	9035.14300	1817.55050	Detected	Detected	Detected	Detected
1291	A_23_P29153	RTDR1	Hs.526920	rhabdoid tumor deletion region gene 1	Homo sapiens rhabdoid tumor deletion region gene 1 (RTDR1), mRNA [NM_014433]	4.15382	up	5.27717	up	120.39317	30.01149	124.28970	20.76825	Detected	Detected	Detected	Detected
1292	A_33_P3280694				Protein pigeon homolog [Source:UniProtKB/Swiss-Prot;Acc:A4D1B5] [ENST00000334003]	4.15292	up	4.00454	up	254.03325	63.33889	154.05493	33.92256	Detected	Detected	Detected	Detected
1293	A_32_P19294	GLT1D1	Hs.655668	glycosyltransferase 1 domain containing 1	Homo sapiens glycosyltransferase 1 domain containing 1 (GLT1D1), mRNA [NM_144669]	4.15165	up	2.12770	up	15.58739	3.88763	7.90697	3.27692	Detected	Compromised	Compromised	Compromised
1294	A_33_P3263867	P2RX7	Hs.507102	purinergic receptor P2X, ligand-gated ion channel, 7	Homo sapiens purinergic receptor P2X, ligand-gated ion channel, 7 (P2RX7), mRNA [NM_002562]	4.14758	up	4.18541	up	156.49123	39.06867	132.26050	27.86493	Detected	Detected	Detected	Detected
1295	A_33_P3387621	RHPN2	Hs.466435	rhophilin, Rho GTPase binding protein 2	Homo sapiens rhophilin, Rho GTPase binding protein 2 (RHPN2), mRNA [NM_033103]	4.14416	up	4.04402	up	779.31090	194.71843	712.31840	155.31970	Detected	Detected	Detected	Detected
1296	A_32_P209230	CITED4	Hs.355820	Cbp/p300-interacting transactivator, with Glu/Asp-rich carboxy-terminal domain, 4	Homo sapiens Cbp/p300-interacting transactivator, with Glu/Asp-rich carboxy-terminal domain, 4 (CITED4), mRNA [NM_133467]	4.14223	up	4.14959	up	22435.80000	5608.42400	22865.22300	4858.88130	Detected	Detected	Detected	Detected
1297	A_33_P3414880	LOC339192	Hs.560498	hypothetical protein LOC339192	PREDICTED: Homo sapiens hypothetical protein LOC339192 (LOC339192), mRNA [XM_001726300]	4.14170	up	7.49634	up	160.01030	40.00394	170.36528	20.03998	Detected	Detected	Detected	Detected
1298	A_24_P944827	ATG7	Hs.38032	ATG7 autophagy related 7 homolog (S. cerevisiae)	Homo sapiens ATG7 autophagy related 7 homolog (S. cerevisiae) (ATG7), transcript variant 1, mRNA [NM_006395]	4.14148	up	3.94967	up	352.01746	88.01202	309.09262	69.00704	Detected	Detected	Detected	Detected

1299	A_33_P3229370	ID4	Hs.519601	inhibitor of DNA binding 4, dominant negative helix-loop-helix protein	Homo sapiens inhibitor of DNA binding 4, dominant negative helix-loop-helix protein (ID4), mRNA [NM_001546]	4.13577	up	4.56578	up	1238.90590	310.18090	990.65704	191.32608	Detected	Detected	Detected	Detected
1300	A_23_P502350	RFX2	Hs.465709	regulatory factor X, 2 (influences HLA class II expression)	Homo sapiens regulatory factor X, 2 (influences HLA class II expression) (RFX2), transcript variant 1, mRNA [NM_000635]	4.13359	up	4.08139	up	1625.21860	407.11523	1509.00260	326.02222	Detected	Detected	Detected	Detected
1301	A_33_P3254460	DLK2	Hs.337251	delta-like 2 homolog (Drosophila)	Homo sapiens delta-like 2 homolog (Drosophila) (DLK2), transcript variant 2, mRNA [NM_206539]	4.13284	up	3.82540	up	292.87988	73.37940	308.35610	71.07905	Detected	Detected	Detected	Detected
1302	A_24_P372223	MSR1	Hs.147635	macrophage scavenger receptor 1	Homo sapiens macrophage scavenger receptor 1 (MSR1), transcript variant SR-AI, mRNA [NM_138715]	4.12681	up	2.27988	up	12.20193	3.06159	9.12126	3.52783	Detected	Compromised	Compromised	Compromised
1303	A_32_P128701	USP53	Hs.431081	ubiquitin specific peptidase 53	Homo sapiens ubiquitin specific peptidase 53 (USP53), mRNA [NM_019050]	4.12625	up	4.01253	up	2448.41300	614.41600	1730.34560	380.25986	Detected	Detected	Detected	Detected
1304	A_33_P3221999	GSDMB	Hs.306777	gasdermin B	Homo sapiens gasdermin B (GSDMB), transcript variant 3, mRNA [NM_001165958]	4.12451	up	4.23170	up	827.25714	207.68341	649.26166	135.29141	Detected	Detected	Detected	Detected
1305	A_24_P364057	C11orf70	Hs.98328	chromosome 11 open reading frame 70	Homo sapiens chromosome 11 open reading frame 70 (C11orf70), mRNA [NM_032930]	4.12170	up	4.05795	up	668.40900	167.91855	543.42224	118.08538	Detected	Detected	Detected	Detected
1306	A_23_P150249	CCDC85B	Hs.66713	coiled-coil domain containing 85B	Homo sapiens coiled-coil domain containing 85B (CCDC85B), mRNA [NM_006848]	4.12125	up	3.58539	up	6420.67400	1613.18580	14881.96700	3660.07370	Detected	Detected	Detected	Detected
1307	A_33_P3323718	UACA	Hs.108049	uveal autoantigen with coiled-coil domains and ankyrin repeats	Homo sapiens uveal autoantigen with coiled-coil domains and ankyrin repeats (UACA), transcript variant 2, mRNA [NM_001008224]	4.12033	up	4.01461	up	2155.06670	541.57935	1601.09480	351.67346	Detected	Detected	Detected	Detected
1308	A_32_P188860	IL17RD	Hs.150725	interleukin 17 receptor D	Homo sapiens interleukin 17 receptor D (IL17RD), mRNA [NM_017563]	4.11834	up	4.81212	up	510.33313	128.31130	305.19950	55.92594	Detected	Detected	Detected	Detected
1309	A_33_P3421084	FLJ42289	Hs.136982	hypothetical LOC388182	Homo sapiens hypothetical LOC388182 (FLJ42289), transcript variant 1, non-coding RNA [NR_028139]	4.11570	up	4.46446	up	31.21387	7.85303	40.69748	8.03830	Detected	Compromised	Detected	Compromised
1310	A_23_P113393	APLN	Hs.303084	apelin	Homo sapiens apelin (APLN), mRNA [NM_017413]	4.11289	up	4.04194	up	148.97510	37.50589	118.28753	25.80565	Detected	Detected	Detected	Detected
1311	A_33_P3289810					4.11142	up	1.73361	up	19.60316	4.93705	17.76529	9.03620	Detected	Compromised	Detected	Compromised
1312	A_33_P3355040	MGC21881	Hs.459704	hypothetical locus MGC21881	zx56g04.r1 Soares_fetal_liver_spleen_1NFLS_S1 Homo sapiens cDNA clone IMAGE:446550 5' similar to contains element MER12 repetitive element ;, mRNA sequence [AA203336]	4.10819	up	4.87983	up	286.07500	72.10458	183.35406	33.13226	Detected	Detected	Detected	Detected
1313	A_32_P8551	NRN1L	Hs.435464	neuritin 1-like	Homo sapiens neuritin 1-like (NRN1L), mRNA [NM_198443]	4.10699	up	3.97228	up	142.99129	36.05116	113.14838	25.11734	Detected	Detected	Detected	Detected
1314	A_24_P277615	TRIM7	Hs.487412	tripartite motif-containing 7	Homo sapiens tripartite motif-containing 7 (TRIM7), transcript variant 2, mRNA [NM_203297]	4.10586	up	4.03536	up	98.68065	24.88635	112.85958	24.66164	Detected	Detected	Detected	Detected
1315	A_33_P3407941					4.10562	up	1.41118	up	31.80863	8.02232	8.85773	5.53483	Detected	Compromised	Compromised	Compromised
1316	A_24_P168398	ZNF177	Hs.172979	zinc finger protein 177	Homo sapiens zinc finger protein 177 (ZNF177), mRNA [NM_003451]	4.10288	up	3.62436	up	231.35315	58.38754	149.67000	36.41404	Detected	Detected	Detected	Detected
1317	A_33_P3308137	C7orf46	Hs.592178	chromosome 7 open reading frame 46	Homo sapiens chromosome 7 open reading frame 46 (C7orf46), transcript variant 1, mRNA [NM_199136]	4.10065	up	5.71734	up	125.83962	31.77587	122.19788	18.84670	Detected	Detected	Detected	Detected
1318	A_23_P141992	HSD11B1L	Hs.631840	hydroxysteroid (11-beta) dehydrogenase 1-like	Homo sapiens hydroxysteroid (11-beta) dehydrogenase 1-like (HSD11B1L), transcript variant b, mRNA [NM_198706]	4.10043	up	3.77967	up	2259.72800	570.63730	2025.16770	472.46844	Detected	Detected	Detected	Detected
1319	A_33_P3520835	FLJ41455	Hs.494023	hypothetical gene supported by AK123449; BX641014	Homo sapiens cDNA FLJ41455 fis, clone BRSTN2012284 [AK123449]	4.10035	up	3.87664	up	81.45468	20.56972	37.69471	8.57414	Detected	Detected	Detected	Compromised
1320	A_23_P83134	GAS1	Hs.65029	growth arrest-specific 1	Homo sapiens growth arrest-specific 1 (GAS1), mRNA [NM_002048]	4.09816	up	3.93629	up	15664.98700	3957.98410	14627.56400	3276.80000	Detected	Detected	Detected	Detected
1321	A_32_P68942	C21orf81	Hs.364456	ankyrin repeat domain 20 family, member A3 pseudogene	Homo sapiens chromosome 21 open reading frame 81 (C21orf81), non-coding RNA [NR_027270]	4.09706	up	1.77122	up	11.97424	3.02628	6.22913	3.10114	Detected	Compromised	Compromised	Compromised
1322	A_23_P62298	NLGN3	Hs.438877	neuroligin 3	Homo sapiens neuroligin 3 (NLGN3), transcript variant 2, mRNA [NM_018977]	4.09360	up	2.16533	up	12.61127	3.18997	6.56902	2.67511	Detected	Compromised	Compromised	Compromised
1323	A_23_P406785	C9orf50	Hs.124223	chromosome 9 open reading frame 50	Homo sapiens chromosome 9 open reading frame 50 (C9orf50), mRNA [NM_199350]	4.09283	up	4.37074	up	586.85736	148.47101	793.08380	160.00354	Detected	Detected	Detected	Detected
1324	A_33_P3222977	TEKT5	Hs.143519	tektin 5	Homo sapiens tektin 5 (TEKT5), mRNA [NM_144674]	4.08990	up	2.06141	up	32.39949	8.20273	23.08075	9.87305	Detected	Compromised	Detected	Compromised
1325	A_33_P3287631	CTSB	Hs.520898	cathepsin B	Homo sapiens cathepsin B (CTSB), transcript variant 2, mRNA [NM_147780]	4.08864	up	3.73594	up	43379.19000	10985.90100	35090.57400	8282.38800	Detected	Detected	Detected	Detected
1326	A_32_P150632	ANXA11		annexin A11	Annexin A11 (Annexin-11)(Annexin XI)(Calcyclin-associated annexin 50)(CAP-50)(56 kDa autoantigen) [Source:UniProtKB/Swiss-Prot;Acc:P50995] [ENST00000372234]	4.08387	up	4.29261	up	255.22713	64.71246	196.91203	40.44983	Detected	Detected	Detected	Detected
1327	A_33_P3259443		Hs.635230		Putative uncharacterized protein ENSP00000340488 Fragment [Source:UniProtKB/TrEMBL;Acc:A6NL95] [ENST00000338857]	4.08331	up	3.12634	up	102.07733	25.88511	15.45255	4.35843	Detected	Detected	Detected	Compromised
1328	A_33_P3301351	RBM44	Hs.720233	RNA binding motif protein 44	Homo sapiens RNA binding motif protein 44 (RBM44), mRNA [NM_001080504]	4.08319	up	3.94047	up	87.92421	22.29676	69.63701	15.58324	Detected	Detected	Detected	Detected
1329	A_24_P218688	ALDH3B1	Hs.523841	aldehyde dehydrogenase 3 family, member B1	Homo sapiens aldehyde dehydrogenase 3 family, member B1 (ALDH3B1), transcript variant 1, mRNA [NM_000694]	4.08207	up	3.26276	up	1859.22300	471.61070	1401.24890	378.70032	Detected	Detected	Detected	Detected

1330	A_32_P199884	HORMAD1	Hs.298312	HORMA domain containing 1	Homo sapiens HORMA domain containing 1 (HORMAD1), mRNA [NM_032132]	4.07748	up	4.72932	up	14.39697	3.65606	16.56413	3.08841	Detected	Compromised	Detected	Compromised
1331	A_23_P103398	PSEN2	Hs.25363	presenilin 2 (Alzheimer disease 4)	Homo sapiens presenilin 2 (Alzheimer disease 4) (PSEN2), transcript variant 1, mRNA [NM_000447]	4.07392	up	3.70755	up	9182.99600	2334.02200	6278.15140	1493.17300	Detected	Detected	Detected	Detected
1332	A_23_P214533	ZNF323	Hs.656413	zinc finger protein 323	Homo sapiens zinc finger protein 323 (ZNF323), transcript variant 1, mRNA [NM_030899]	4.07361	up	2.45691	up	16.14028	4.10265	10.10185	3.62558	Detected	Compromised	Compromised	Compromised
1333	A_33_P3361388	MYCBPAP	Hs.398196	MYCBP associated protein	Homo sapiens MYCBP associated protein (MYCBPAP), mRNA [NM_032133]	4.07168	up	3.47633	up	28.68038	7.29365	17.27950	4.38304	Detected	Compromised	Detected	Compromised
1334	A_24_P400457	CYB5RL	Hs.591426	cytochrome b5 reductase-like	Homo sapiens cytochrome b5 reductase-like (CYB5RL), mRNA [NM_001031672]	4.06960	up	4.96759	up	76.25545	19.40226	35.40360	6.28445	Detected	Detected	Detected	Compromised
1335	A_24_P36229	DTNA	Hs.643454	dystrobrevin, alpha	Homo sapiens dystrobrevin, alpha (DTNA), transcript variant 3, mRNA [NM_001391]	4.06691	up	2.88156	up	23.37894	5.95242	21.00159	6.42674	Detected	Compromised	Detected	Compromised
1336	A_33_P3384885				Zinc finger protein 511 [Source:UniProtKB/Swiss-Prot;Acc:Q8NB15] [ENST00000359035]	4.06382	up	4.24033	up	452.78350	115.36909	370.71100	77.09063	Detected	Detected	Detected	Detected
1337	A_23_P410110	C10orf25	Hs.655124	chromosome 10 open reading frame 25	Homo sapiens chromosome 10 open reading frame 25 (C10orf25), mRNA [NM_001039380]	4.06193	up	3.49894	up	142.40912	36.30266	141.09029	35.55708	Detected	Detected	Detected	Detected
1338	A_23_P353014	CACNA2D4	Hs.13768	calcium channel, voltage-dependent, alpha 2/delta subunit 4	Homo sapiens calcium channel, voltage-dependent, alpha 2/delta subunit 4 (CACNA2D4), mRNA [NM_172364]	4.05274	up	3.06964	up	31.74249	8.11009	27.10278	7.78559	Detected	Compromised	Detected	Compromised
1339	A_33_P3408137	LOC100130248	Hs.656333	hypothetical protein LOC100130248	Homo sapiens cDNA FLJ42222 fis, clone THYMU2039411. [AK124216]	4.05169	up	1.25403	up	16.73500	4.27684	8.58857	6.03917	Detected	Compromised	Compromised	Compromised
1340	A_23_P9280	LOC389765	Hs.601256	kinesin family member 27 pseudogene	Homo sapiens kinesin family member 27 pseudogene (LOC389765), non-coding RNA [NR_029410]	4.04717	up	4.83612	up	371.47488	95.04108	216.14198	39.41015	Detected	Detected	Detected	Detected
1341	A_23_P10542	HTRA3	Hs.479119	HtrA serine peptidase 3	Homo sapiens HtrA serine peptidase 3 (HTRA3), mRNA [NM_053044]	4.04434	up	3.12971	up	256.98322	65.79461	263.47687	74.23415	Detected	Detected	Detected	Detected
1342	A_23_P339119	ACSS3	Hs.259559	acyl-CoA synthetase short-chain family member 3	Homo sapiens acyl-CoA synthetase short-chain family member 3 (ACSS3), mRNA [NM_024560]	4.03627	up	4.00633	up	1371.28470	351.78787	1364.29370	300.28000	Detected	Detected	Detected	Detected
1343	A_23_P145074	PNRC1	Hs.75969	proline-rich nuclear receptor coactivator 1	Homo sapiens proline-rich nuclear receptor coactivator 1 (PNRC1), mRNA [NM_006813]	4.03392	up	3.98440	up	31909.37700	8190.75440	17985.02000	3980.28320	Detected	Detected	Detected	Detected
1344	A_33_P3376264				Glycerol kinase (EC 2.7.1.30)(ATP:glycerol 3-phosphotransferase)(Glycerokinase)(GK) [Source:UniProtKB/Swiss-Prot;Acc:P32189] [ENST00000378938]	4.02742	up	7.77081	up	70.70306	18.17792	38.59238	4.37926	Detected	Detected	Detected	Compromised
1345	A_33_P3336622	ALDH3A2	Hs.499886	aldehyde dehydrogenase 3 family, member A2	Homo sapiens aldehyde dehydrogenase 3 family, member A2 (ALDH3A2), transcript variant 1, mRNA [NM_001031806]	4.02699	up	4.16222	up	4356.19240	1120.10580	3473.77150	735.93850	Detected	Detected	Detected	Detected
1346	A_23_P69109	PLSCR1	Hs.130759	phospholipid scramblase 1	Homo sapiens phospholipid scramblase 1 (PLSCR1), mRNA [NM_021105]	4.02646	up	4.19947	up	4864.03560	1250.85280	3857.17550	809.91670	Detected	Detected	Detected	Detected
1347	A_33_P3249936	C3orf67	Hs.368434	chromosome 3 open reading frame 67	Homo sapiens chromosome 3 open reading frame 67 (C3orf67), mRNA [NM_198463]	4.01730	up	4.32566	up	485.95013	125.25383	328.04327	66.87203	Detected	Detected	Detected	Detected
1348	A_24_P26792	TRPM6	Hs.272225	transient receptor potential cation channel, subfamily M, member 6	Homo sapiens transient receptor potential cation channel, subfamily M, member 6 (TRPM6), mRNA [NM_017662]	4.01522	up	1.43359	up	11.90988	3.07136	4.17710	2.56930	Detected	Compromised	Compromised	Compromised
1349	A_33_P3304133	GLB1L2	Hs.436178	galactosidase, beta 1-like 2	Homo sapiens galactosidase, beta 1-like 2 (GLB1L2), mRNA [NM_138342]	4.01138	up	11.67637	up	44.24946	11.42213	48.24004	3.64305	Detected	Compromised	Detected	Compromised
1350	A_33_P3332970	CLEC2B	Hs.85201	C-type lectin domain family 2, member B	Homo sapiens C-type lectin domain family 2, member B (CLEC2B), mRNA [NM_005127]	4.00844	up	3.66369	up	6559.26900	1694.38760	6492.38000	1562.61010	Detected	Detected	Detected	Detected
1351	A_23_P417891	ARHGEF7	Hs.508738	Rho guanine nucleotide exchange factor (GEF) 7	Homo sapiens Rho guanine nucleotide exchange factor (GEF) 7 (ARHGEF7), transcript variant 2, mRNA [NM_145735]	4.00629	up	6.35238	up	36.89678	9.53628	19.34209	2.68493	Detected	Compromised	Detected	Compromised
1352	A_23_P160546	FAM63A	Hs.3346	family with sequence similarity 63, member A	Homo sapiens family with sequence similarity 63, member A (FAM63A), transcript variant 2, mRNA [NM_001040217]	4.00535	up	3.65973	up	1415.32710	365.88910	852.81430	205.48088	Detected	Detected	Detected	Detected
1353	A_24_P401830	LOC100129196	Hs.659751	similar to hCG2033298	Homo sapiens cDNA FLJ33163 fis, clone UTERU2000541. [AK057725]	4.00417	up	4.22292	up	228.31047	59.04005	140.88440	29.41816	Detected	Detected	Detected	Detected
1354	A_33_P3269919	C1orf129	Hs.591489	chromosome 1 open reading frame 129	Homo sapiens chromosome 1 open reading frame 129 (C1orf129), transcript variant 2, mRNA [NM_025063]	4.00251	up	3.98939	up	46.47594	12.02345	42.83126	9.46716	Detected	Compromised	Detected	Compromised
1355	A_33_P3285992				Vitamin K epoxide reductase complex subunit 1 (EC 1.1.4.1)(Vitamin K1 2,3-epoxide reductase subunit 1) [Source:UniProtKB/Swiss-Prot;Acc:Q9BQB6] [ENST00000498155]	4.00197	up	2.57884	up	39.51190	10.22320	49.21316	16.82763	Detected	Compromised	Detected	Detected
1356	A_32_P213881	C20orf202	Hs.148503	chromosome 20 open reading frame 202	Homo sapiens chromosome 20 open reading frame 202 (C20orf202), mRNA [NM_001009612]	4.00160	up	2.64357	up	14.37132	3.71874	9.80482	3.27050	Detected	Compromised	Compromised	Compromised
1357	A_23_P114185	TSPAN7	Hs.441664	tetraspanin 7	Homo sapiens tetraspanin 7 (TSPAN7), mRNA [NM_004615]	4.00157	up	3.75845	up	13.22887	3.42315	12.42338	2.91472	Detected	Compromised	Not Detected	Compromised
1358	A_23_P48295	CDADC1	Hs.388220	cytidine and dCMP deaminase domain containing 1	Homo sapiens cytidine and dCMP deaminase domain containing 1 (CDADC1), mRNA [NM_030911]	3.99992	up	4.75412	up	145.79910	37.74307	91.41530	16.95566	Detected	Detected	Detected	Detected

1359	A_23_P133386	RASGRF2	Hs.162129	Ras protein-specific guanine nucleotide-releasing factor 2	Homo sapiens Ras protein-specific guanine nucleotide-releasing factor 2 (RASGRF2), mRNA [NM_006909]	3.99407	up	12.18387	up	38.70342	10.03382	36.86875	2.66833	Detected	Compromised	Detected	Compromised
1360	A_33_P3354823	BTBD19	Hs.632400	BTB (POZ) domain containing 19	Homo sapiens BTB (POZ) domain containing 19 (BTBD19), mRNA [NM_001136537]	3.99304	up	1.16583	up	26.18178	6.78935	15.23083	11.52002	Detected	Compromised	Compromised	Compromised
1361	A_32_P223210	C7orf45	Hs.351816	chromosome 7 open reading frame 45	Homo sapiens chromosome 7 open reading frame 45 (C7orf45), mRNA [NM_145268]	3.98562	up	7.23435	up	25.53161	6.63309	30.59643	3.72938	Detected	Compromised	Detected	Compromised
1362	A_23_P385067	CLIC6	Hs.473695	chloride intracellular channel 6	Homo sapiens chloride intracellular channel 6 (CLIC6), mRNA [NM_053277]	3.98175	up	4.47525	up	300.41122	78.12225	226.71584	44.67143	Detected	Detected	Detected	Detected
1363	A_23_P205046	ANKRD10	Hs.525163	ankyrin repeat domain 10	Homo sapiens ankyrin repeat domain 10 (ANKRD10), mRNA [NM_017664]	3.97836	up	4.27421	up	2511.67580	653.72064	1506.67720	310.83487	Detected	Detected	Detected	Detected
1364	A_33_P3235053	TRIM47	Hs.293660	tripartite motif-containing 47	Homo sapiens tripartite motif-containing 47 (TRIM47), mRNA [NM_033452]	3.97826	up	4.52904	up	23924.12900	6226.96240	17745.28000	3454.95630	Detected	Detected	Detected	Detected
1365	A_32_P197621	LOC492303	Hs.550796	FAM51A1 pseudogene	Homo sapiens FAM51A1 pseudogene (LOC492303), non-coding RNA [NR_002830]	3.97411	up	2.31137	up	34.31964	8.94204	6.36858	2.42962	Detected	Compromised	Compromised	Compromised
1366	A_24_P148026	LOC100294391	Hs.638348	hypothetical protein LOC100294391	PREDICTED: Homo sapiens hypothetical protein LOC100294391 (LOC100294391), mRNA [XM_002346360]	3.97402	up	3.31534	up	131.99005	34.39092	123.85309	32.94152	Detected	Detected	Detected	Detected
1367	A_23_P259741	SATB1	Hs.517717	SATB homeobox 1	Homo sapiens SATB homeobox 1 (SATB1), transcript variant 1, mRNA [NM_002971]	3.97349	up	3.73541	up	1120.19470	291.91364	592.48596	139.86392	Detected	Detected	Detected	Detected
1368	A_33_P3732688	LOC646034	Hs.512237	hypothetical LOC646034	PREDICTED: Homo sapiens hypothetical LOC646034 (LOC646034), mRNA [XM_001724735]	3.97306	up	2.04548	up	16.21443	4.22581	21.89357	9.43815	Detected	Compromised	Detected	Compromised
1369	A_24_P945283	DLG3	Hs.649747	discs, large homolog 3 (Drosophila)	Homo sapiens discs, large homolog 3 (Drosophila) (DLG3), transcript variant 1, mRNA [NM_021120]	3.97100	up	3.62094	up	2060.24240	537.21936	1754.79200	427.33580	Detected	Detected	Detected	Detected
1370	A_32_P180315	KIAA1529	Hs.435629	KIAA1529	Homo sapiens KIAA1529 (KIAA1529), mRNA [NM_020893]	3.96854	up	4.35828	up	131.57239	34.32949	131.35678	26.57680	Detected	Detected	Detected	Detected
1371	A_23_P42397	PRSS35	Hs.98381	protease, serine, 35	Homo sapiens protease, serine, 35 (PRSS35), mRNA [NM_153362]	3.96791	up	1.82946	up	27.41526	7.15424	26.93970	12.98481	Detected	Compromised	Detected	Compromised
1372	A_32_P224522	SLC25A23	Hs.356231	solute carrier family 25 (mitochondrial carrier; phosphate carrier), member 23	Homo sapiens solute carrier family 25 (mitochondrial carrier; phosphate carrier), member 23 (SLC25A23), nuclear gene encoding mitochondrial protein, mRNA [NM_024103]	3.96470	up	4.46638	up	1127.95140	294.58700	944.26320	186.42442	Detected	Detected	Detected	Detected
1373	A_23_P324754	KIAA1199	Hs.459088	KIAA1199	Homo sapiens KIAA1199 (KIAA1199), mRNA [NM_018689]	3.96435	up	3.79877	up	28472.25000	7436.74950	16810.92200	3902.24340	Detected	Detected	Detected	Detected
1374	A_33_P3226542	SNORD3B-1	Hs.658815	small nucleolar RNA, C/D box 3B-1	Homo sapiens small nucleolar RNA, C/D box 3B-1 (SNORD3B-1), small nucleolar RNA [NR_003271]	3.96417	up	3.48037	up	7005.36870	1829.83480	5235.83250	1326.55810	Detected	Detected	Detected	Detected
1375	A_23_P38206	LSMD1	Hs.565094	LSM domain containing 1	Homo sapiens LSM domain containing 1 (LSMD1), mRNA [NM_032356]	3.96331	up	2.94147	up	126.21130	32.97415	100.40221	30.09846	Detected	Detected	Detected	Detected
1376	A_23_P256581	PRDM13	Hs.287386	PR domain containing 13	Homo sapiens PR domain containing 13 (PRDM13), mRNA [NM_021620]	3.96278	up	1.16065	up	15.65043	4.08940	4.55909	3.46371	Detected	Compromised	Compromised	Compromised
1377	A_33_P3376321	ANK1	Hs.654438	ankyrin 1, erythrocytic	Homo sapiens ankyrin 1, erythrocytic (ANK1), transcript variant 2, mRNA [NM_020477]	3.96163	up	5.24256	up	40.78545	10.66018	34.80586	5.85430	Detected	Compromised	Detected	Compromised
1378	A_23_P383986	CHST15	Hs.287537	carbohydrate (N-acetylgalactosamine 4-sulfate 6-O) sulfotransferase 15	Homo sapiens carbohydrate (N-acetylgalactosamine 4-sulfate 6-O) sulfotransferase 15 (CHST15), mRNA [NM_015892]	3.96153	up	3.99081	up	1147.63930	299.96872	823.15674	181.88097	Detected	Detected	Detected	Detected
1379	A_24_P140405	ADAMTS3	Hs.590919	ADAM metallopeptidase with thrombospondin type 1 motif, 3	Homo sapiens ADAM metallopeptidase with thrombospondin type 1 motif, 3 (ADAMTS3), mRNA [NM_014243]	3.95738	up	4.32049	up	123.25340	32.24962	92.68060	18.91567	Detected	Detected	Detected	Detected
1380	A_23_P90542	ZNF540	Hs.121283	zinc finger protein 540	Homo sapiens zinc finger protein 540 (ZNF540), mRNA [NM_152606]	3.95715	up	8.37599	up	50.76350	13.28317	29.92186	3.15006	Detected	Compromised	Detected	Compromised
1381	A_23_P105251	GLI1	Hs.632702	GLI family zinc finger 1	Homo sapiens GLI family zinc finger 1 (GLI1), transcript variant 1, mRNA [NM_005269]	3.95705	up	3.76429	up	534.24560	139.79857	246.28310	57.69225	Detected	Detected	Detected	Detected
1382	A_23_P86283	LAPTM5	Hs.371021	lysosomal protein transmembrane 5	Homo sapiens lysosomal protein transmembrane 5 (LAPTM5), mRNA [NM_006762]	3.95479	up	2.15939	up	66.41994	17.39035	59.10638	24.13623	Detected	Compromised	Detected	Detected
1383	A_33_P3242483	ARHGAP5	Hs.592313	Rho GTPase activating protein 5	Homo sapiens Rho GTPase activating protein 5 (ARHGAP5), transcript variant 1, mRNA [NM_001030055]	3.95420	up	3.63082	up	2297.71660	601.68810	1830.94800	444.66840	Detected	Detected	Detected	Detected
1384	A_23_P74870	C1orf129	Hs.591489	chromosome 1 open reading frame 129	Homo sapiens chromosome 1 open reading frame 129 (C1orf129), transcript variant 2, mRNA [NM_025063]	3.95415	up	3.65744	up	69.03223	18.07723	35.39471	8.53349	Detected	Detected	Detected	Compromised
1385	A_24_P6825	ZCCHC18	Hs.648338	zinc finger, CCHC domain containing 18	Homo sapiens zinc finger, CCHC domain containing 18 (ZCCHC18), transcript variant 1, mRNA [NM_001143978]	3.95337	up	1.67722	up	14.43170	3.77993	6.43068	3.38089	Detected	Compromised	Compromised	Compromised
1386	A_24_P118196	GLT8D4	Hs.710275	glycosyltransferase 8 domain containing 4	Homo sapiens glycosyltransferase 8 domain containing 4 (GLT8D4), mRNA [NM_001080393]	3.95301	up	4.15405	up	725.89080	190.14111	611.78613	129.86551	Detected	Detected	Detected	Detected
1387	A_32_P86245	EFHC1	Hs.403171	EF-hand domain (C-terminal) containing 1	Homo sapiens EF-hand domain (C-terminal) containing 1 (EFHC1), mRNA [NM_018100]	3.95060	up	3.51717	up	25.92156	6.79409	25.66364	6.43415	Detected	Compromised	Detected	Compromised
1388	A_32_P48466	LCA5L	Hs.517284	Leber congenital amaurosis 5-like	Homo sapiens Leber congenital amaurosis 5-like (LCA5L), mRNA [NM_152505]	3.94677	up	4.14512	up	97.81629	25.66273	83.47168	17.75692	Detected	Detected	Detected	Compromised
1389	A_23_P102364	NGEF	Hs.97316	neuronal guanine nucleotide exchange factor	Homo sapiens neuronal guanine nucleotide exchange factor (NGEF), transcript variant 1, mRNA [NM_019850]	3.94372	up	3.89704	up	1081.15360	283.86688	750.09326	169.72530	Detected	Detected	Detected	Detected

1390	A_24_P822704	TMEM198	Hs.446664	transmembrane protein 198	Homo sapiens transmembrane protein 198 (TMEM198), mRNA [NM_001005209]	3.94368	up	3.50985	up	457.51315	120.12550	441.14774	110.83108	Detected	Detected	Detected	Detected
1391	A_33_P3396404	CLLU1OS	Hs.551199	chronic lymphocytic leukemia up-regulated 1 opposite strand	Homo sapiens chronic lymphocytic leukemia up-regulated 1 opposite strand (CLLU1OS), mRNA [NM_001025232]	3.94298	up	2.87000	up	61.93248	16.26399	47.94620	14.73121	Detected	Detected	Detected	Detected
1392	A_24_P40551	BEX4	Hs.184736	brain expressed, X-linked 4	Homo sapiens brain expressed, X-linked 4 (BEX4), mRNA [NM_001127688]	3.94295	up	3.62412	up	2022.77820	531.20306	1583.26700	385.22784	Detected	Detected	Detected	Detected
1393	A_23_P91636	POM121L9P	Hs.318898	POM121 membrane glycoprotein-like 9 (rat) pseudogene	Homo sapiens POM121 membrane glycoprotein-like 9 (rat) pseudogene (POM121L9P), non-coding RNA [NR_003714]	3.94133	up	3.96913	up	257.61490	67.68008	229.35724	50.95446	Detected	Detected	Detected	Detected
1394	A_23_P204246	PHC1	Hs.305985	polyhomeotic homolog 1 (Drosophila)	Homo sapiens polyhomeotic homolog 1 (Drosophila) (PHC1), mRNA [NM_004426]	3.93980	up	3.33722	up	101.40911	26.65234	71.05072	18.77369	Detected	Detected	Detected	Detected
1395	A_23_P333022	PXT1	Hs.520097	peroxisomal, testis specific 1	Homo sapiens peroxisomal, testis specific 1 (PXT1), mRNA [NM_152990]	3.93623	up	1.60664	up	12.42804	3.26930	9.68831	5.31736	Detected	Compromised	Compromised	Compromised
1396	A_33_P3231727	LOC100134240	Hs.98178	similar to LOC339742 protein	PREDICTED: Homo sapiens hypothetical LOC100134240 (LOC100134240), mRNA [XM_001718062]	3.93564	up	10.13036	up	65.25548	17.16860	36.36771	3.16561	Detected	Compromised	Detected	Compromised
1397	A_33_P3390939	LOC100129950		similar to UCA1 protein	PREDICTED: Homo sapiens similar to UCA1 protein (LOC100129950), mRNA [XM_001718934]	3.93409	up	2.50587	up	13.08650	3.44439	7.76351	2.73191	Detected	Compromised	Compromised	Compromised
1398	A_33_P3601202	IFNAP22	Hs.545842	interferon, alpha pseudogene 22	Messenger RNA for human leukocyte (alpha) interferon [V00539]	3.93058	up	4.53525	up	60.90162	16.04371	49.27830	9.58121	Detected	Detected	Detected	Compromised
1399	A_33_P3315554	ANKMY1	Hs.656615	ankyrin repeat and MYND domain containing 1	Homo sapiens ankyrin repeat and MYND domain containing 1 (ANKMY1), transcript variant 1, mRNA [NM_016552]	3.92714	up	3.75475	up	498.42090	131.41740	478.09375	112.27869	Detected	Detected	Detected	Detected
1400	A_33_P3245415	N4BP2L1	Hs.161220	NEDD4 binding protein 2-like 1	Homo sapiens NEDD4 binding protein 2-like 1 (N4BP2L1), transcript variant 1, mRNA [NM_052818]	3.92706	up	5.69033	up	148.62596	39.18866	158.29610	24.53004	Detected	Detected	Detected	Detected
1401	A_23_P99405	ZMYM2	Hs.644041	zinc finger, MYM-type 2	Homo sapiens zinc finger, MYM-type 2 (ZMYM2), mRNA [NM_003453]	3.92627	up	4.26607	up	3270.34300	862.47534	1891.97250	391.06775	Detected	Detected	Detected	Detected
1402	A_32_P178499	C16orf86	Hs.632208	chromosome 16 open reading frame 86	Homo sapiens chromosome 16 open reading frame 86 (C16orf86), mRNA [NM_001012984]	3.92509	up	3.74171	up	667.11255	175.98761	835.20966	196.82967	Detected	Detected	Detected	Detected
1403	A_23_P392126	C17orf108	Hs.434386	chromosome 17 open reading frame 108	Homo sapiens chromosome 17 open reading frame 108 (C17orf108), mRNA [NM_001076680]	3.92488	up	3.47278	up	5686.34770	1500.16870	3847.45040	976.92505	Detected	Detected	Detected	Detected
1404	A_24_P419177	ZNF638	Hs.434401	zinc finger protein 638	Homo sapiens zinc finger protein 638 (ZNF638), transcript variant 1, mRNA [NM_014497]	3.92189	up	4.12572	up	478.07290	126.22093	231.67874	49.51670	Detected	Detected	Detected	Detected
1405	A_33_P3269218	BAI2	Hs.524138	brain-specific angiogenesis inhibitor 2	Homo sapiens brain-specific angiogenesis inhibitor 2 (BAI2), mRNA [NM_001703]	3.91935	up	4.24733	up	2341.00780	618.47424	2213.78150	459.60500	Detected	Detected	Detected	Detected
1406	A_23_P86195	SLC44A3	Hs.483423	solute carrier family 44, member 3	Homo sapiens solute carrier family 44, member 3 (SLC44A3), transcript variant 2, mRNA [NM_152369]	3.91420	up	2.67141	up	71.93552	19.02978	62.75941	20.71590	Detected	Detected	Detected	Detected
1407	A_33_P3395688	GCOM1	Hs.437256	GRINL1A complex locus	Homo sapiens GRINL1A complex locus (GCOM1), transcript variant 12, mRNA [NM_001018100]	3.91412	up	3.18867	up	29.32269	7.75716	24.79713	6.85737	Detected	Compromised	Detected	Compromised
1408	A_23_P358542	KIFC2	Hs.528713	kinesin family member C2	Homo sapiens kinesin family member C2 (KIFC2), mRNA [NM_145754]	3.90962	up	3.58986	up	556.58040	147.40990	577.44290	141.83932	Detected	Detected	Detected	Detected
1409	A_33_P3336282	TAF4B	Hs.369519	TAF4b RNA polymerase II, TATA box binding protein (TBP)-associated factor, 105kDa	Homo sapiens TAF4b RNA polymerase II, TATA box binding protein (TBP)-associated factor, 105kDa (TAF4B), mRNA [NM_005640]	3.90695	up	1.94559	up	19.03182	5.04400	12.72849	5.76888	Detected	Compromised	Compromised	Compromised
1410	A_33_P3264163	DDR1	Hs.631988	discoidin domain receptor tyrosine kinase 1	Homo sapiens discoidin domain receptor tyrosine kinase 1 (DDR1), transcript variant 2, mRNA [NM_001954]	3.90601	up	1.68943	up	15.23726	4.03931	14.01807	7.31668	Detected	Compromised	Compromised	Compromised
1411	A_33_P3221414	NTNG1		netrin G1	Netrin-G1 Precursor (Laminet-1) [Source:UniProtKB/Swiss-Prot;Acc:Q9Y2I2] [ENST00000370068]	3.90567	up	2.83556	up	71.04034	18.83402	53.79359	16.72850	Detected	Detected	Detected	Detected
1412	A_24_P944049	CEP68	Hs.709257	centrosomal protein 68kDa	Homo sapiens centrosomal protein 68kDa (CEP68), mRNA [NM_015147]	3.90061	up	3.04144	up	226.58691	60.14996	149.94128	43.47173	Detected	Detected	Detected	Detected
1413	A_32_P337442	C10orf67	Hs.522360	chromosome 10 open reading frame 67	Homo sapiens chromosome 10 open reading frame 67 (C10orf67), mRNA [NM_153714]	3.89984	up	2.54999	up	12.49425	3.31739	8.10686	2.80337	Detected	Compromised	Compromised	Compromised
1414	A_23_P254442	ZNF783		zinc finger family member 783	Homo sapiens zinc finger family member 783 (ZNF783), non-coding RNA [NR_015357]	3.89560	up	3.85740	up	924.48290	245.72963	715.10950	163.47182	Detected	Detected	Detected	Detected
1415	A_23_P404821	KIAA1147	Hs.521240	KIAA1147	Homo sapiens KIAA1147 (KIAA1147), mRNA [NM_001080392]	3.89476	up	3.73509	up	315.45940	83.86786	232.79349	54.95851	Detected	Detected	Detected	Detected
1416	A_23_P39910	C2orf64	Hs.596537	chromosome 2 open reading frame 64	Homo sapiens chromosome 2 open reading frame 64 (C2orf64), mRNA [NM_001008215]	3.89305	up	4.19437	up	2596.91940	690.71954	2323.07030	488.38410	Detected	Detected	Detected	Detected
1417	A_23_P98645	DCHS1	Hs.199850	dachsous 1 (Drosophila)	Homo sapiens dachsous 1 (Drosophila) (DCHS1), mRNA [NM_003737]	3.89171	up	3.88217	up	569.51090	151.52866	538.25726	122.25888	Detected	Detected	Detected	Detected
1418	A_33_P3245631	TTC39A	Hs.112949	tetratricopeptide repeat domain 39A	Homo sapiens tetratricopeptide repeat domain 39A (TTC39A), transcript variant 2, mRNA [NM_001080494]	3.88785	up	1.62452	up	14.85822	3.95722	19.78834	10.74111	Detected	Compromised	Detected	Compromised
1419	A_24_P918907	LOC154761		hypothetical LOC154761	Homo sapiens hypothetical LOC154761 (LOC154761), non-coding RNA [NR_015421]	3.88440	up	4.06668	up	78.62959	20.96021	48.99228	10.62314	Detected	Detected	Detected	Compromised
1420	A_24_P396650	RPS6KA1	Hs.149957	ribosomal protein S6 kinase, 90kDa, polypeptide 1	Homo sapiens ribosomal protein S6 kinase, 90kDa, polypeptide 1 (RPS6KA1), transcript variant 1, mRNA [NM_002953]	3.88361	up	3.92335	up	669.94570	178.62280	574.82227	129.19400	Detected	Detected	Detected	Detected

1421	A_33_P3262685	POM121L1P	Hs.318898	POM121 membrane glycoprotein-like 1 (rat) pseudogene	Homo sapiens POM121 membrane glycoprotein-like 1 (rat) pseudogene (POM121L1P), non-coding RNA [NR_024591]	3.88246	up	3.31579	up	93.91161	25.04640	92.79379	24.67730	Detected	Detected	Detected	Detected
1422	A_23_P332326	ARHGEF19	Hs.591532	Rho guanine nucleotide exchange factor (GEF) 19	Homo sapiens Rho guanine nucleotide exchange factor (GEF) 19 (ARHGEF19), mRNA [NM_153213]	3.87855	up	3.80429	up	3027.87480	808.35330	3155.46970	731.40140	Detected	Detected	Detected	Detected
1423	A_33_P3311165		Hs.451549		cDNA FLJ44313 fis, clone TRACH2025911FLJ44313 protein ; [Source:UniProtKB/TrEMBL;Acc:Q6ZTR6] [ENST00000405150]	3.87412	up	4.84442	up	123.72891	33.06974	66.33850	12.07507	Detected	Detected	Detected	Compromised
1424	A_23_P348063	SYNGR1	Hs.216226	synaptogyrin 1	Homo sapiens synaptogyrin 1 (SYNGR1), transcript variant 1a, mRNA [NM_004711]	3.87357	up	4.08558	up	283.81640	75.86820	312.69934	67.48994	Detected	Detected	Detected	Detected
1425	A_23_P39024	MORG1	Hs.657204	mitogen-activated protein kinase organizer 1	Homo sapiens mitogen-activated protein kinase organizer 1 (MORG1), transcript variant 2, mRNA [NM_032332]	3.87285	up	1.27212	up	2078.98220	555.84393	1022.96960	709.08670	Not Detected	Detected	Detected	Detected
1426	A_33_P3293913	BICC1	Hs.158745	bicaudal C homolog 1 (Drosophila)	Homo sapiens bicaudal C homolog 1 (Drosophila) (BICC1), mRNA [NM_001080512]	3.86809	up	4.11923	up	75.19399	20.12886	15.77041	3.37592	Detected	Detected	Detected	Compromised
1427	A_23_P67299	DOCK6	Hs.591002	dedicator of cytokinesis 6	Homo sapiens dedicator of cytokinesis 6 (DOCK6), mRNA [NM_020812]	3.86124	up	3.47972	up	1748.44000	468.87552	1698.29540	430.36313	Detected	Detected	Detected	Detected
1428	A_23_P210330	HSPC159	Hs.372208	galectin-related protein	Homo sapiens galectin-related protein (HSPC159), mRNA [NM_014181]	3.85685	up	3.94276	up	647.40800	173.81142	568.76790	127.20379	Detected	Detected	Detected	Detected
1429	A_23_P114670	ARHGEF16	Hs.87435	Rho guanine exchange factor (GEF) 16	Homo sapiens Rho guanine exchange factor (GEF) 16 (ARHGEF16), mRNA [NM_014448]	3.85308	up	3.71087	up	103.10102	27.70688	115.26511	27.38974	Detected	Detected	Detected	Detected
1430	A_23_P60180	ABL1	Hs.431048	c-abl oncogene 1, receptor tyrosine kinase	Homo sapiens c-abl oncogene 1, receptor tyrosine kinase (ABL1), transcript variant a, mRNA [NM_005157]	3.85129	up	3.37780	up	17743.97700	4770.65530	14995.34100	3914.60080	Detected	Detected	Detected	Detected
1431	A_33_P3254216	MAPK10	Hs.125503	mitogen-activated protein kinase 10	Homo sapiens mitogen-activated protein kinase 10 (MAPK10), transcript variant 3, mRNA [NM_138980]	3.84996	up	4.09597	up	13.96894	3.75699	14.78389	3.18272	Detected	Compromised	Compromised	Compromised
1432	A_33_P3422439	LOC100129555	Hs.533086	similar to High-mobility group nucleosome binding domain 1	PREDICTED: Homo sapiens similar to high-mobility group nucleosome binding domain 1 (LOC100129555), miscRNA [XR_038415]	3.84283	up	1.77416	up	24.31159	6.55082	12.93433	6.42859	Detected	Compromised	Detected	Compromised
1433	A_33_P3268726					3.84096	up	3.16871	up	15.40797	4.15374	18.31080	5.09554	Detected	Compromised	Detected	Compromised
1434	A_23_P40096	PROC	Hs.224698	protein C (inactivator of coagulation factors Va and VIIIa)	Homo sapiens protein C (inactivator of coagulation factors Va and VIIIa) (PROC), mRNA [NM_000312]	3.84053	up	1.22244	up	13.99061	3.77206	6.58614	4.75081	Detected	Compromised	Compromised	Compromised
1435	A_32_P172848	GK	Hs.1466	glycerol kinase	Homo sapiens glycerol kinase (GK), transcript variant 2, mRNA [NM_000167]	3.83873	up	5.90882	up	331.90317	89.52751	323.37872	48.25875	Detected	Detected	Detected	Detected
1436	A_33_P3221418	NTNG1	Hs.657434	netrin G1	Homo sapiens netrin G1 (NTNG1), transcript variant 2, mRNA [NM_001113228]	3.83787	up	2.17658	up	12.31479	3.32254	16.20202	6.56387	Compromised	Compromised	Detected	Compromised
1437	A_23_P367586	DNHD1	Hs.720080	dynein heavy chain domain 1	Homo sapiens dynein heavy chain domain 1 (DNHD1), transcript variant 2, mRNA [NM_173589]	3.83736	up	4.07426	up	12.33440	3.32827	15.08395	3.26462	Compromised	Compromised	Detected	Compromised
1438	A_23_P322854	LOC100288439	Hs.616684	hypothetical protein LOC100288439	Putative uncharacterized protein [Source:UniProtKB/TrEMBL;Acc:Q96FU4] [ENST00000328202]	3.83602	up	2.90665	up	124.54832	33.61945	145.91930	44.26753	Detected	Detected	Detected	Detected
1439	A_24_P37540	TTLL3	Hs.709359	tubulin tyrosine ligase-like family, member 3	Homo sapiens tubulin tyrosine ligase-like family, member 3 (TTLL3), mRNA [NM_001025930]	3.83219	up	3.62226	up	720.61456	194.71031	944.70080	229.97460	Detected	Detected	Detected	Detected
1440	A_33_P3242878	EFCAB6	Hs.658996	EF-hand calcium binding domain 6	Homo sapiens EF-hand calcium binding domain 6 (EFCAB6), transcript variant 1, mRNA [NM_022785]	3.82929	up	7.67010	up	29.95172	8.09910	26.79373	3.08033	Detected	Compromised	Detected	Compromised
1441	A_24_P333567	hCG_2022304	Hs.593896	similar to hCG2022304	Homo sapiens similar to hCG2022304 (LOC100289341), non-coding RNA [NR_027447]	3.82644	up	3.63314	up	975.81305	264.06116	689.26090	167.28897	Detected	Detected	Detected	Detected
1442	A_33_P3389291	TEX9	Hs.511476	testis expressed 9	Homo sapiens testis expressed 9 (TEX9), mRNA [NM_198524]	3.82288	up	1.72788	up	13.78618	3.73410	6.28083	3.20531	Detected	Compromised	Compromised	Compromised
1443	A_33_P3345586	HCN2	Hs.124161	hyperpolarization activated cyclic nucleotide-gated potassium channel 2	Homo sapiens hyperpolarization activated cyclic nucleotide-gated potassium channel 2 (HCN2), mRNA [NM_001194]	3.82234	up	2.27681	up	30.22003	8.18651	19.88368	7.70081	Detected	Compromised	Detected	Compromised
1444	A_23_P202810	OVOL1	Hs.134434	ovo-like 1(Drosophila)	Homo sapiens ovo-like 1(Drosophila) (OVOL1), mRNA [NM_004561]	3.82144	up	5.02161	up	12.07757	3.27255	15.60781	2.74072	Compromised	Compromised	Detected	Compromised
1445	A_23_P92672	OCLN	Hs.592605	occludin	Homo sapiens occludin (OCLN), mRNA [NM_002538]	3.82014	up	2.40648	up	16.91504	4.58487	9.13593	3.34762	Detected	Compromised	Compromised	Compromised
1446	A_33_P3576797	LOC158863	Hs.138411	hypothetical protein LOC158863	Homo sapiens mRNA; cDNA DKFZp586J1922 (from clone DKFZp586J1922) [AL110203]	3.82007	up	2.25387	up	26.75259	7.25151	27.86678	10.90243	Detected	Compromised	Detected	Compromised
1447	A_23_P58266	S100P	Hs.2962	S100 calcium binding protein P	Homo sapiens S100 calcium binding protein P (S100P), mRNA [NM_005980]	3.81446	up	4.20956	up	1624.49260	440.97894	1623.27730	340.03360	Detected	Detected	Detected	Detected
1448	A_23_P142447	MYO1F	Hs.465818	myosin IF	Homo sapiens myosin IF (MYO1F), mRNA [NM_012335]	3.81399	up	3.34297	up	600.53060	163.03789	621.51935	163.94110	Detected	Detected	Detected	Detected
1449	A_23_P4082	CCT6B	Hs.73072	chaperonin containing TCP1, subunit 6B (zeta 2)	Homo sapiens chaperonin containing TCP1, subunit 6B (zeta 2) (CCT6B), mRNA [NM_006584]	3.81367	up	3.81569	up	1664.71810	451.99194	1194.77540	276.10800	Detected	Detected	Detected	Detected
1450	A_23_P124044	DEAF1	Hs.243994	deformed epidermal autoregulatory factor 1 (Drosophila)	Homo sapiens deformed epidermal autoregulatory factor 1 (Drosophila) (DEAF1), mRNA [NM_021008]	3.81364	up	3.46368	up	7695.43500	2089.42290	5654.64000	1439.57040	Detected	Detected	Detected	Detected
1451	A_23_P119143	ICAM5	Hs.465862	intercellular adhesion molecule 5, telencephalin	Homo sapiens intercellular adhesion molecule 5, telencephalin (ICAM5), mRNA [NM_003259]	3.81283	up	3.50499	up	462.23810	125.53117	346.64862	87.21031	Detected	Detected	Detected	Detected

1452	A_23_P127068	SEMA4G	Hs.591923	sema domain, immunoglobulin domain (Ig), transmembrane domain (TM) and short cytoplasmic domain, (semaphorin) 4G	Homo sapiens sema domain, immunoglobulin domain (Ig), transmembrane domain (TM) and short cytoplasmic domain, (semaphorin) 4G (SEMA4G), mRNA [NM_017893]	3.81206	up	3.85565	up	141.31444	38.38482	118.24230	27.04213	Detected	Detected	Detected	Detected
1453	A_24_P219474	MGAT5B	Hs.144531	mannosyl (alpha-1,6-)-glycoprotein beta-1,6-N-acetyl-glucosaminyltransferase, isozyme B	Homo sapiens mannosyl (alpha-1,6-)-glycoprotein beta-1,6-N-acetyl-glucosaminyltransferase, isozyme B (MGAT5B), transcript variant 1, mRNA [NM_144677]	3.81193	up	3.21846	up	94.82007	25.75658	50.82652	13.92539	Detected	Detected	Detected	Compromised
1454	A_33_P3353345	SULT4A1	Hs.189810	sulfotransferase family 4A, member 1	Homo sapiens sulfotransferase family 4A, member 1 (SULT4A1), mRNA [NM_014351]	3.80869	up	3.30144	up	174.02548	47.31189	143.57425	38.34769	Detected	Detected	Detected	Detected
1455	A_24_P678418	FLJ45244		hypothetical locus FLJ45244	Homo sapiens hypothetical locus FLJ45244 (FLJ45244), non-coding RNA [NR_015415]	3.80582	up	3.72186	up	502.15625	136.62286	423.51877	100.34090	Detected	Detected	Detected	Detected
1456	A_33_P3308585	UNQ2963	Hs.246046	hypothetical LOC283314	Homo sapiens hypothetical LOC283314 (LOC283314), non-coding RNA [NR_026947]	3.80383	up	1.00935	up	13.81035	3.75938	8.53236	7.45404	Detected	Compromised	Compromised	Compromised
1457	A_24_P281988	PLCL2	Hs.202010	phospholipase C-like 2	Homo sapiens phospholipase C-like 2 (PLCL2), transcript variant 2, mRNA [NM_015184]	3.80355	up	4.04886	up	715.88900	194.89008	537.98160	117.16563	Detected	Detected	Detected	Detected
1458	A_23_P78108	ALDOC	Hs.155247	aldolase C, fructose-bisphosphate	Homo sapiens aldolase C, fructose-bisphosphate (ALDOC), mRNA [NM_005165]	3.80205	up	3.56683	up	527.72205	143.72101	798.11110	197.30899	Detected	Detected	Detected	Detected
1459	A_33_P3228305	ARHGAP26	Hs.654668	Rho GTPase activating protein 26	Homo sapiens Rho GTPase activating protein 26 (ARHGAP26), transcript variant 1, mRNA [NM_015071]	3.80155	up	4.21495	up	86.02752	23.43202	69.06851	14.44951	Detected	Detected	Detected	Detected
1460	A_33_P3213792	KCNK4	Hs.647233	potassium channel, subfamily K, member 4	Homo sapiens potassium channel, subfamily K, member 4 (KCNK4), mRNA [NM_033310]	3.79975	up	3.20581	up	114.83621	31.29369	102.70381	28.24970	Detected	Detected	Detected	Detected
1461	A_33_P3401156	ETV1	Hs.22634	ets variant 1	Homo sapiens ets variant 1 (ETV1), transcript variant 1, mRNA [NM_004956]	3.79950	up	3.85688	up	874.94086	238.44322	798.51160	182.56226	Detected	Detected	Detected	Detected
1462	A_23_P369899	TMEM158	Hs.716411	transmembrane protein 158	Homo sapiens transmembrane protein 158 (TMEM158), mRNA [NM_015444]	3.79885	up	4.01390	up	20499.51600	5587.59000	13591.27700	2985.78930	Detected	Detected	Detected	Detected
1463	A_24_P892472	EMX2OS	Hs.312592	EMX2 opposite strand (non-protein coding)	Homo sapiens EMX2 opposite strand (non-protein coding) (EMX2OS), non-coding RNA [NR_002791]	3.79486	up	3.79630	up	4735.68750	1292.17140	3871.12230	899.16900	Detected	Detected	Detected	Detected
1464	A_33_P3361758	C15orf33	Hs.712959	chromosome 15 open reading frame 33	Homo sapiens chromosome 15 open reading frame 33 (C15orf33), mRNA [NM_152647]	3.79408	up	3.51256	up	11.72691	3.20045	12.89860	3.23806	Compromised	Compromised	Detected	Compromised
1465	A_24_P932939	LOC401052	Hs.662766	hypothetical LOC401052	Homo sapiens hypothetical LOC401052 (LOC401052), mRNA [NM_001008737]	3.79278	up	1.93672	up	22.57457	6.16304	18.38608	8.37119	Detected	Compromised	Detected	Compromised
1466	A_23_P99226	SIRT4	Hs.50861	sirtuin (silent mating type information regulation 2 homolog) 4 (S. cerevisiae)	Homo sapiens sirtuin (silent mating type information regulation 2 homolog) 4 (S. cerevisiae) (SIRT4), mRNA [NM_012240]	3.79266	up	4.17605	up	194.12563	52.99957	178.01634	37.58888	Detected	Detected	Detected	Detected
1467	A_33_P3229402	PECAM1	Hs.514412	platelet/endothelial cell adhesion molecule	Homo sapiens platelet/endothelial cell adhesion molecule (PECAM1), mRNA [NM_000442]	3.79197	up	10.55478	up	42.67268	11.65248	42.51489	3.55187	Detected	Compromised	Detected	Compromised
1468	A_23_P370454	KCNAB3	Hs.435074	potassium voltage-gated channel, shaker-related subfamily, beta member 3	Homo sapiens potassium voltage-gated channel, shaker-related subfamily, beta member 3 (KCNAB3), mRNA [NM_004732]	3.79073	up	3.24222	up	125.41696	34.25839	81.00155	22.03007	Detected	Detected	Detected	Detected
1469	A_23_P167509	CYFIP2	Hs.519702	cytoplasmic FMR1 interacting protein 2	Homo sapiens cytoplasmic FMR1 interacting protein 2 (CYFIP2), transcript variant 2, mRNA [NM_001037332]	3.79071	up	3.03920	up	65.15610	17.79787	60.37597	17.51742	Detected	Detected	Detected	Detected
1470	A_23_P106405	NDN	Hs.50130	necdin homolog (mouse)	Homo sapiens necdin homolog (mouse) (NDN), mRNA [NM_002487]	3.78980	up	3.94751	up	4478.53120	1223.63540	3049.49000	681.19244	Detected	Detected	Detected	Detected
1471	A_23_P103617	ANXA9	Hs.720072	annexin A9	Homo sapiens annexin A9 (ANXA9), mRNA [NM_003568]	3.78940	up	6.94954	up	17.42940	4.76261	29.12772	3.69587	Not Detected	Compromised	Not Detected	Compromised
1472	A_23_P376449	LOC642413	Hs.558776	similar to Putative cathepsin L-like protein 6	PREDICTED: Homo sapiens similar to Putative cathepsin L-like protein 6 (LOC642413), miscRNA [XR_016155]	3.78738	up	3.93248	up	18216.83400	4980.42770	19787.43400	4436.98830	Detected	Detected	Detected	Detected
1473	A_23_P423427	FAM171B	Hs.28872	family with sequence similarity 171, member B	Homo sapiens family with sequence similarity 171, member B (FAM171B), mRNA [NM_177454]	3.78712	up	3.90628	up	31.23506	8.54017	28.24580	6.37612	Detected	Compromised	Detected	Compromised
1474	A_24_P179816	SLC27A3	Hs.438723	solute carrier family 27 (fatty acid transporter), member 3	Homo sapiens solute carrier family 27 (fatty acid transporter), member 3 (SLC27A3), mRNA [NM_024330]	3.78702	up	3.17150	up	1644.66550	449.69003	1772.72350	492.88116	Detected	Detected	Detected	Detected
1475	A_23_P347468	FZD3	Hs.40735	frizzled homolog 3 (Drosophila)	Homo sapiens frizzled homolog 3 (Drosophila) (FZD3), mRNA [NM_017412]	3.78656	up	3.48770	up	139.20070	38.06531	67.09385	16.96326	Detected	Detected	Detected	Detected
1476	A_33_P3424067	SUV420H1	Hs.632120	suppressor of variegation 4-20 homolog 1 (Drosophila)	Homo sapiens suppressor of variegation 4-20 homolog 1 (Drosophila) (SUV420H1), transcript variant 1, mRNA [NM_017635]	3.78501	up	5.44438	up	92.04596	25.18086	47.91030	7.75972	Detected	Detected	Detected	Compromised
1477	A_23_P362148	DNER	Hs.234074	delta/notch-like EGF repeat containing	Homo sapiens delta/notch-like EGF repeat containing (DNER), mRNA [NM_139072]	3.78299	up	3.67949	up	607.48260	166.27700	420.82130	100.84981	Detected	Detected	Detected	Detected
1478	A_33_P3419460	VAPA	Hs.699980	VAMP (vesicle-associated membrane protein)-associated protein A, 33kDa	Homo sapiens VAMP (vesicle-associated membrane protein)-associated protein A, 33kDa (VAPA), transcript variant 1, mRNA [NM_003574]	3.78281	up	3.88242	up	460.91266	126.16458	320.39642	72.76965	Detected	Detected	Detected	Detected
1479	A_23_P316472	DNHD1	Hs.720080	dynein heavy chain domain 1	Homo sapiens dynein heavy chain domain 1 (DNHD1), transcript variant 1, mRNA [NM_144666]	3.78053	up	4.90939	up	84.83690	23.23619	117.27170	21.06353	Detected	Detected	Detected	Detected

1480	A_23_P79231	CREB1	Hs.516646	cAMP responsive element binding protein 1	Homo sapiens cAMP responsive element binding protein 1 (CREB1), transcript variant B, mRNA [NM_134442]	3.77870	up	3.88405	up	445.98395	122.21092	267.75995	60.78926	Detected	Detected	Detected	Detected
1481	A_23_P214139	REV3L	Hs.232021	REV3-like, catalytic subunit of DNA polymerase zeta (yeast)	Homo sapiens REV3-like, catalytic subunit of DNA polymerase zeta (yeast) (REV3L), mRNA [NM_002912]	3.77566	up	3.83687	up	7977.15970	2187.70480	5887.04440	1352.96420	Detected	Detected	Detected	Detected
1482	A_23_P3956	C1QTNF1	Hs.201398	C1q and tumor necrosis factor related protein 1	Homo sapiens C1q and tumor necrosis factor related protein 1 (C1QTNF1), mRNA [NM_198594]	3.77415	up	3.76233	up	619.57990	169.98517	639.86290	149.96681	Detected	Detected	Detected	Detected
1483	A_23_P382835	P2RY1	Hs.654526	purinergic receptor P2Y, G-protein coupled, 1	Homo sapiens purinergic receptor P2Y, G-protein coupled, 1 (P2RY1), mRNA [NM_002563]	3.77395	up	1.83752	up	17.15684	4.70732	5.47658	2.62810	Detected	Compromised	Compromised	Compromised
1484	A_33_P3368695	HES7	Hs.434828	hairy and enhancer of split 7 (Drosophila)	Homo sapiens hairy and enhancer of split 7 (Drosophila) (HES7), transcript variant 1, mRNA [NM_001165967]	3.77028	up	2.88273	up	161.90022	44.46381	94.14091	28.79653	Detected	Detected	Detected	Detected
1485	A_23_P105619	TMEM116	Hs.506815	transmembrane protein 116	Homo sapiens transmembrane protein 116 (TMEM116), mRNA [NM_138341]	3.76817	up	3.72310	up	427.42422	117.45231	390.12170	92.39768	Detected	Detected	Detected	Detected
1486	A_23_P99642	SLC7A7	Hs.513147	solute carrier family 7 (cationic amino acid transporter, y+ system), member 7	Homo sapiens solute carrier family 7 (cationic amino acid transporter, y+ system), member 7 (SLC7A7), transcript variant 1, mRNA [NM_003982]	3.76810	up	3.91321	up	765.40125	210.32936	527.87520	118.94975	Detected	Detected	Detected	Detected
1487	A_23_P129358	SETD6	Hs.460923	SET domain containing 6	Homo sapiens SET domain containing 6 (SETD6), transcript variant 2, mRNA [NM_024860]	3.76806	up	3.89688	up	816.05230	224.25023	497.02030	112.46640	Detected	Detected	Detected	Detected
1488	A_33_P3394140	LOC283050		hypothetical LOC283050	Homo sapiens hypothetical LOC283050 (LOC283050), transcript variant 1, non-coding RNA [NR_024431]	3.76802	up	3.92239	up	5194.86230	1427.55730	3780.84230	849.96936	Detected	Detected	Detected	Detected
1489	A_23_P404965	GNL1	Hs.654655	guanine nucleotide binding protein-like 1	Guanine nucleotide-binding protein-like 1 (GTP-binding protein HSR1) [Source:UniProtKB/Swiss-Prot;Acc:P36915] [ENST00000376621]	3.76600	up	3.79170	up	6723.96040	1848.74950	4459.11430	1037.00330	Detected	Detected	Detected	Detected
1490	A_23_P422245	C5orf36	Hs.425123	chromosome 5 open reading frame 36	Homo sapiens chromosome 5 open reading frame 36 (C5orf36), transcript variant 2, mRNA [NM_173665]	3.76260	up	2.06609	up	12.64150	3.47892	7.01882	2.99558	Detected	Compromised	Compromised	Compromised
1491	A_24_P275585	IQCH	Hs.657894	IQ motif containing H	Homo sapiens IQ motif containing H (IQCH), transcript variant 2, mRNA [NM_022784]	3.76187	up	2.08700	up	40.62959	11.18335	35.52412	15.00952	Detected	Compromised	Detected	Compromised
1492	A_23_P7684	CCNJL	Hs.14070	cyclin J-like	Homo sapiens cyclin J-like (CCNJL), mRNA [NM_024565]	3.76053	up	3.26124	up	12.07663	3.32529	20.28200	5.48395	Compromised	Compromised	Detected	Compromised
1493	A_24_P481375	LOC100131564	Hs.648647	hypothetical protein LOC100131564	Homo sapiens mRNA; cDNA DKFZp667D2123 (from clone DKFZp667D2123) [AL832786]	3.75806	up	4.74721	up	362.46060	99.86876	189.64716	35.22683	Detected	Detected	Detected	Detected
1494	A_23_P99360	TRIM13	Hs.436922	tripartite motif-containing 13	Homo sapiens tripartite motif-containing 13 (TRIM13), transcript variant 3, mRNA [NM_213590]	3.75761	up	4.94596	up	1300.43030	358.35098	1649.46260	294.07470	Detected	Detected	Detected	Detected
1495	A_24_P413669	PFKFB2	Hs.282702	6-phosphofructo-2-kinase/fructose-2,6-biphosphatase 2	Homo sapiens 6-phosphofructo-2-kinase/fructose-2,6-biphosphatase 2 (PFKFB2), transcript variant 2, mRNA [NM_001018053]	3.75458	up	4.04421	up	30.80143	8.49458	15.60664	3.40284	Detected	Compromised	Compromised	Compromised
1496	A_33_P3240652					3.75387	up	4.13103	up	37.31189	10.29204	37.89582	8.08906	Detected	Compromised	Detected	Compromised
1497	A_24_P38143	AHI1	Hs.386684	Abelson helper integration site 1	Homo sapiens Abelson helper integration site 1 (AHI1), transcript variant 2, mRNA [NM_017651]	3.75284	up	3.98535	up	1246.55310	343.94090	782.93260	173.23033	Detected	Detected	Detected	Detected
1498	A_33_P3298159	PTGDS	Hs.446429	prostaglandin D2 synthase 21kDa (brain)	Homo sapiens prostaglandin D2 synthase 21kDa (brain) (PTGDS), mRNA [NM_000954]	3.75108	up	3.69373	up	12328.52900	3403.20300	13469.03200	3215.41100	Detected	Detected	Detected	Detected
1499	A_33_P3221408	NTNG1	Hs.657434	netrin G1	Homo sapiens netrin G1 (NTNG1), transcript variant 1, mRNA [NM_001113226]	3.74984	up	3.72312	up	3468.41600	957.74940	2579.15820	610.85266	Detected	Detected	Detected	Detected
1500	A_33_P3892608	KCNT2	Hs.657046	potassium channel, subfamily T, member 2	Homo sapiens potassium channel, subfamily T, member 2 (KCNT2), mRNA [NM_198503]	3.74967	up	1.89389	up	15.01386	4.14603	7.48989	3.48727	Detected	Compromised	Compromised	Compromised
1501	A_32_P130630	SLC16A7	Hs.439643	solute carrier family 16, member 7 (monocarboxylic acid transporter 2)	Homo sapiens solute carrier family 16, member 7 (monocarboxylic acid transporter 2) (SLC16A7), mRNA [NM_004731]	3.74918	up	2.45916	up	91.78311	25.34896	65.52706	23.49634	Detected	Detected	Detected	Detected
1502	A_23_P145424	KIAA1009	Hs.485865	KIAA1009	Homo sapiens KIAA1009 (KIAA1009), mRNA [NM_014895]	3.74719	up	4.59266	up	435.30176	120.28680	266.60367	51.18790	Detected	Detected	Detected	Detected
1503	A_32_P46840	LOC729680	Hs.130652	hypothetical protein LOC729680	PREDICTED: Homo sapiens hypothetical protein LOC729680 (LOC729680), miscRNA [XR_040503]	3.74645	up	3.94487	up	1055.80350	291.80753	991.79584	221.69432	Detected	Detected	Detected	Detected
1504	A_23_P50081	IMPA2	Hs.720058	inositol(myo)-1(or 4)-monophosphatase 2	Homo sapiens inositol(myo)-1(or 4)-monophosphatase 2 (IMPA2), mRNA [NM_014214]	3.74426	up	3.55023	up	7392.97460	2044.49670	6137.30760	1524.36000	Detected	Detected	Detected	Detected
1505	A_33_P3333777	LOC100129387	Hs.720669	hypothetical LOC100129387	Homo sapiens hypothetical LOC100129387 (LOC100129387), non-coding RNA [NR_024490]	3.74167	up	3.59364	up	404.24475	111.86959	393.32663	96.51258	Detected	Detected	Detected	Detected
1506	A_23_P398566	NR4A3	Hs.279522	nuclear receptor subfamily 4, group A, member 3	Homo sapiens nuclear receptor subfamily 4, group A, member 3 (NR4A3), transcript variant 2, mRNA [NM_173198]	3.74140	up	4.20297	up	644.86800	178.47185	622.18320	130.53502	Detected	Detected	Detected	Detected
1507	A_24_P174503	AMT	Hs.102	aminomethyltransferase	Homo sapiens aminomethyltransferase (AMT), nuclear gene encoding mitochondrial protein, transcript variant 1, mRNA [NM_000481]	3.73965	up	4.25946	up	196.88203	54.51399	152.47295	31.56484	Detected	Detected	Detected	Detected
1508	A_23_P216225	EGR3	Hs.534313	early growth response 3	Homo sapiens early growth response 3 (EGR3), mRNA [NM_004430]	3.73655	up	5.46198	up	279.88803	77.56148	216.44210	34.94274	Detected	Detected	Detected	Detected
1509	A_33_P3773168	LOC285768	Hs.209463	hypothetical LOC285768	Homo sapiens hypothetical LOC285768 (LOC285768), non-coding RNA [NR_027115]	3.73592	up	1.71572	up	14.21312	3.93935	6.81712	3.50364	Detected	Compromised	Compromised	Compromised

1510	A_33_P3273584	SCARNA2	Hs.550815	small Cajal body-specific RNA 2	Homo sapiens small Cajal body-specific RNA 2 (SCARNA2), guide RNA [NR_003023]	3.73534	up	3.43040	up	398.76890	110.54126	349.17383	89.75574	Detected	Detected	Detected	Detected
1511	A_24_P70117	DTWD1	Hs.127432	DTW domain containing 1	Homo sapiens DTW domain containing 1 (DTWD1), transcript variant 1, mRNA [NM_020234]	3.73495	up	3.80609	up	2518.18950	698.12976	2234.94300	517.78920	Detected	Detected	Detected	Detected
1512	A_33_P3390057	TM4SF1	Hs.351316	transmembrane 4 L six family member 1	Homo sapiens transmembrane 4 L six family member 1 (TM4SF1), mRNA [NM_014220]	3.73289	up	3.13430	up	87213.04000	24191.88900	55772.64500	15690.85500	Detected	Detected	Detected	Detected
1513	A_24_P80500	BDH2	Hs.124696	3-hydroxybutyrate dehydrogenase, type 2	Homo sapiens 3-hydroxybutyrate dehydrogenase, type 2 (BDH2), mRNA [NM_020139]	3.73060	up	3.39669	up	159.72878	44.33409	138.96709	36.07628	Detected	Detected	Detected	Detected
1514	A_33_P3854030	MBD5	Hs.458312	methyl-CpG binding domain protein 5	Homo sapiens methyl-CpG binding domain protein 5 (MBD5), mRNA [NM_018328]	3.72926	up	4.01712	up	451.67593	125.41161	422.66632	92.77867	Detected	Detected	Detected	Detected
1515	A_33_P3390187	NHLRC4	Hs.664267	NHL repeat containing 4	Homo sapiens NHL repeat containing 4 (NHLRC4), mRNA [NM_176677]	3.72559	up	2.26201	up	26.81049	7.45149	25.40453	9.90337	Detected	Compromised	Detected	Compromised
1516	A_23_P52676	CATSPER1	Hs.189105	cation channel, sperm associated 1	Homo sapiens cation channel, sperm associated 1 (CATSPER1), mRNA [NM_053054]	3.72455	up	3.72468	up	313.74683	87.22459	431.14017	102.06937	Detected	Detected	Detected	Detected
1517	A_33_P3396379				Putative uncharacterized protein ENSP00000380631 Fragment [Source:UniProtKB/TrEMBL;Acc:A8MZG5] [ENST00000397494]	3.72421	up	4.07395	up	37.09197	10.31286	51.21211	11.08465	Detected	Compromised	Detected	Compromised
1518	A_33_P3371115	AQP7P3	Hs.658215	aquaporin 7 pseudogene 3	Homo sapiens aquaporin 7 pseudogene 3 (AQP7P3), non-coding RNA [NR_026558]	3.72384	up	5.42389	up	463.08527	128.76651	370.10010	60.16909	Detected	Detected	Detected	Detected
1519	A_23_P91910	PLSCR4	Hs.477869	phospholipid scramblase 4	Homo sapiens phospholipid scramblase 4 (PLSCR4), transcript variant 2, mRNA [NM_020353]	3.72279	up	4.15875	up	3377.51700	939.42500	2422.62330	513.67535	Detected	Detected	Detected	Detected
1520	A_33_P3392320	SLC6A15	Hs.44424	solute carrier family 6 (neutral amino acid transporter), member 15	Homo sapiens solute carrier family 6 (neutral amino acid transporter), member 15 (SLC6A15), transcript variant 2, mRNA [NM_018057]	3.72251	up	5.01654	up	118.91280	33.07696	119.35487	20.97982	Detected	Detected	Detected	Detected
1521	A_33_P3334923	LOC338758		hypothetical LOC338758	Homo sapiens hypothetical LOC338758 (LOC338758), non-coding RNA [NR_028138]	3.72069	up	3.02574	up	78.34594	21.80352	63.77973	18.58729	Detected	Detected	Detected	Detected
1522	A_23_P83200	C9orf98	Hs.421340	chromosome 9 open reading frame 98	Homo sapiens chromosome 9 open reading frame 98 (C9orf98), mRNA [NM_152572]	3.72054	up	3.72767	up	116.43977	32.40622	114.06912	26.98334	Detected	Detected	Detected	Detected
1523	A_23_P320242	KIAA1324L	Hs.208093	KIAA1324-like	Homo sapiens KIAA1324-like (KIAA1324L), transcript variant 2, mRNA [NM_152748]	3.71749	up	3.75358	up	535.53830	149.16720	405.43512	95.24467	Detected	Detected	Detected	Detected
1524	A_33_P3663974	THRB	Hs.187861	thyroid hormone receptor, beta (erythroblastic leukemia viral (v-erb-a) oncogene homolog 2, avian)	Homo sapiens thyroid hormone receptor, beta (erythroblastic leukemia viral (v-erb-a) oncogene homolog 2, avian) (THRB), transcript variant 3, mRNA [NM_001128177]	3.71528	up	3.94270	up	106.54867	29.69543	96.40772	21.56174	Detected	Detected	Detected	Detected
1525	A_33_P3302165	ABHD8	Hs.515664	abhydrolase domain containing 8	Homo sapiens abhydrolase domain containing 8 (ABHD8), mRNA [NM_024527]	3.70954	up	3.55298	up	9800.42000	2735.63090	9515.56700	2361.60840	Detected	Detected	Detected	Detected
1526	A_33_P3782469	PYGO1	Hs.87194	pygopus homolog 1 (Drosophila)	Pygopus homolog 1 [Source:UniProtKB/Swiss-Prot;Acc:Q9Y3Y4] [ENST00000302000]	3.70729	up	3.42496	up	451.14478	126.00648	285.68118	73.55151	Detected	Detected	Detected	Detected
1527	A_23_P257164	AMT	Hs.102	aminomethyltransferase	Homo sapiens aminomethyltransferase (AMT), nuclear gene encoding mitochondrial protein, transcript variant 1, mRNA [NM_000481]	3.70192	up	4.14602	up	344.69950	96.41560	358.31924	76.20873	Detected	Detected	Detected	Detected
1528	A_23_P253791	CAMP	Hs.51120	cathelicidin antimicrobial peptide	Homo sapiens cathelicidin antimicrobial peptide (CAMP), mRNA [NM_004345]	3.70175	up	3.58406	up	15.02276	4.20219	10.54299	2.59391	Detected	Compromised	Compromised	Compromised
1529	A_33_P3560878	LOC283587	Hs.525513	hypothetical protein LOC283587	Homo sapiens cDNA clone IMAGE:4734409 [BC029479]	3.69999	up	14.89842	up	106.59161	29.83018	51.57369	3.05249	Detected	Detected	Detected	Compromised
1530	A_23_P140821	PARD6A	Hs.112933	par-6 partitioning defective 6 homolog alpha (C. elegans)	Homo sapiens par-6 partitioning defective 6 homolog alpha (C. elegans) (PARD6A), transcript variant 1, mRNA [NM_016948]	3.69848	up	2.43429	up	38.57728	10.80045	62.34737	22.58453	Detected	Compromised	Detected	Detected
1531	A_23_P158533	DEAF1	Hs.243994	deformed epidermal autoregulatory factor 1 (Drosophila)	Homo sapiens deformed epidermal autoregulatory factor 1 (Drosophila) (DEAF1), mRNA [NM_021008]	3.69600	up	3.31597	up	4040.31840	1131.92360	3767.96040	1001.98490	Detected	Detected	Detected	Detected
1532	A_33_P3220663	STAC3	Hs.417595	SH3 and cysteine rich domain 3	Homo sapiens SH3 and cysteine rich domain 3 (STAC3), mRNA [NM_145064]	3.69383	up	3.55879	up	568.72205	159.42508	479.86017	118.89889	Detected	Detected	Detected	Detected
1533	A_33_P3328559	TBC1D10C	Hs.534648	TBC1 domain family, member 10C	Homo sapiens TBC1 domain family, member 10C (TBC1D10C), mRNA [NM_198517]	3.69353	up	6.45684	up	128.47165	36.01627	139.85380	19.09940	Detected	Detected	Detected	Detected
1534	A_23_P64044	FERMT3	Hs.180535	fermitin family homolog 3 (Drosophila)	Homo sapiens fermitin family homolog 3 (Drosophila) (FERMT3), transcript variant URP2LF, mRNA [NM_178443]	3.69286	up	3.25611	up	125.23417	35.11507	151.92232	41.14222	Detected	Detected	Detected	Detected
1535	A_33_P3374087	RBM44	Hs.720233	RNA binding motif protein 44	Homo sapiens RNA binding motif protein 44 (RBM44), mRNA [NM_001080504]	3.69253	up	3.60037	up	19.65494	5.51164	18.68643	4.57662	Detected	Compromised	Detected	Compromised
1536	A_33_P3309556	PTPRE	Hs.127022	protein tyrosine phosphatase, receptor type, E	Homo sapiens protein tyrosine phosphatase, receptor type, E (PTPRE), transcript variant 1, mRNA [NM_006504]	3.69219	up	5.53012	up	354.99945	99.55811	188.11212	29.99493	Detected	Detected	Detected	Detected

1537	A_33_P3420392				Xaa-Pro aminopeptidase 2 Precursor (EC 3.4.11.9)(X-Pro aminopeptidase 2)(Membrane-bound aminopeptidase P)(Membrane-bound APP)(Membrane-bound AmP)(mAmP)(Aminoacylproline aminopeptidase) [Source:UniProtKB/Swiss-Prot;Acc:O43895] [ENST00000371105]	3.69002	up	5.86501	up	96.36507	27.04108	55.49580	8.34367	Detected	Detected	Detected	Compromised
1538	A_33_P3618429	PRKXP1	Hs.12250	protein kinase, X-linked, pseudogene 1	Homo sapiens cDNA FLJ38474 fis, clone FEBRA2022255 [AK095793]	3.68867	up	3.43208	up	1432.22000	402.04300	817.58410	210.05856	Detected	Detected	Detected	Detected
1539	A_23_P431268	PLEKHA6	Hs.253146	pleckstrin homology domain containing, family A member 6	Homo sapiens pleckstrin homology domain containing, family A member 6 (PLEKHA6), mRNA [NM_014935]	3.68816	up	1.34745	up	34.45024	9.67197	17.41927	11.39942	Detected	Compromised	Detected	Compromised
1540	A_33_P3421626	KIAA1147	Hs.521240	KIAA1147	Homo sapiens KIAA1147 (KIAA1147), mRNA [NM_001080392]	3.68709	up	4.04137	up	926.03400	260.06198	502.30463	109.59837	Detected	Detected	Detected	Detected
1541	A_24_P190877	C1orf25	Hs.107149	chromosome 1 open reading frame 25	Homo sapiens chromosome 1 open reading frame 25 (C1orf25), mRNA [NM_030934]	3.68474	up	4.09297	up	287.19424	80.70522	192.66612	41.50810	Detected	Detected	Detected	Detected
1542	A_23_P127584	NNMT	Hs.503911	nicotinamide N-methyltransferase	Homo sapiens nicotinamide N-methyltransferase (NNMT), mRNA [NM_006169]	3.68432	up	3.03852	up	163610.11000	45981.79700	119643.73400	34721.07000	Detected	Detected	Detected	Detected
1543	A_33_P3264042	EFCAB3	Hs.152670	EF-hand calcium binding domain 3	Homo sapiens EF-hand calcium binding domain 3 (EFCAB3), transcript variant 1, mRNA [NM_001144933]	3.68135	up	2.01426	up	14.19927	3.99385	11.43922	5.00779	Detected	Compromised	Compromised	Compromised
1544	A_23_P58443	ANKHD1-EIF4EBP3	Hs.594084	ANKHD1-EIF4EBP3 readthrough transcript	Homo sapiens ANKHD1-EIF4EBP3 readthrough transcript (ANKHD1-EIF4EBP3), mRNA [NM_020690]	3.67835	up	3.67855	up	1667.69200	469.45703	1574.97050	377.53854	Detected	Detected	Detected	Detected
1545	A_23_P45324	TMEM35	Hs.45140	transmembrane protein 35	Homo sapiens transmembrane protein 35 (TMEM35), mRNA [NM_021637]	3.67728	up	3.06981	up	3834.39260	1079.70020	2609.58500	749.59360	Detected	Detected	Detected	Detected
1546	A_23_P383590	LOC339524	Hs.306423	hypothetical LOC339524	Homo sapiens hypothetical LOC339524 (LOC339524), transcript variant 1, non-coding RNA [NR_026985]	3.67488	up	7.95340	up	13.06541	3.68140	28.09778	3.11519	Compromised	Compromised	Detected	Compromised
1547	A_24_P371053	ORMDL1	Hs.700632	ORM1-like 1 (S. cerevisiae)	Homo sapiens ORM1-like 1 (S. cerevisiae) (ORMDL1), transcript variant 1, mRNA [NM_016467]	3.67375	up	4.06675	up	789.61300	222.55550	594.88104	128.98763	Detected	Detected	Detected	Detected
1548	A_33_P3282688		Hs.640142		Putative uncharacterized protein ENSP00000366511 [Source:UniProtKB/TrEMBL;Acc:B7WP54] [ENST00000377296]	3.67129	up	3.98879	up	661.98737	186.70863	427.74700	94.56090	Detected	Detected	Detected	Detected
1549	A_33_P3229215	C1orf203	Hs.664752	chromosome 1 open reading frame 203	Homo sapiens chromosome 1 open reading frame 203 (C1orf203), transcript variant 3, non-coding RNA [NR_024126]	3.66858	up	2.49668	up	19.91217	5.62022	17.97032	6.34684	Detected	Compromised	Detected	Compromised
1550	A_32_P202759	FAM171B	Hs.28872	family with sequence similarity 171, member B	Homo sapiens family with sequence similarity 171, member B (FAM171B), mRNA [NM_177454]	3.66674	up	2.40821	up	82.00626	23.15795	70.66210	25.87360	Detected	Detected	Detected	Detected
1551	A_23_P416711	ST6GALNAC3	Hs.337040	ST6 (alpha-N-acetyl-neuraminyl-2,3-beta-galactosyl-1,3)-N-acetylgalactosaminide alpha-2,6-sialyltransferase 3	Homo sapiens ST6 (alpha-N-acetyl-neuraminyl-2,3-beta-galactosyl-1,3)-N-acetylgalactosaminide alpha-2,6-sialyltransferase 3 (ST6GALNAC3), transcript variant 1, mRNA [NM_152996]	3.66537	up	3.76077	up	75.69702	21.38424	52.05541	12.20548	Detected	Detected	Detected	Compromised
1552	A_33_P3225522	OAS2	Hs.414332	2'-5'-oligoadenylate synthetase 2, 69/71kDa	Homo sapiens 2'-5'-oligoadenylate synthetase 2, 69/71kDa (OAS2), transcript variant 3, mRNA [NM_001032731]	3.66501	up	1.62968	up	795.27090	224.68470	279.25027	151.09770	Detected	Detected	Detected	Detected
1553	A_23_P352950	PNMA5	Hs.573567	paraneoplastic antigen like 5	Homo sapiens paraneoplastic antigen like 5 (PNMA5), transcript variant 3, mRNA [NM_052926]	3.66459	up	6.36860	up	2440.80350	689.66930	2165.92100	299.89140	Detected	Detected	Detected	Detected
1554	A_23_P131899	SDCBP2	Hs.657015	syndecan binding protein (syntenin) 2	Homo sapiens syndecan binding protein (syntenin) 2 (SDCBP2), transcript variant 1, mRNA [NM_080489]	3.66257	up	3.35063	up	3894.30640	1100.97550	4416.45200	1162.28630	Detected	Detected	Detected	Detected
1555	A_33_P3615922	C7orf63	Hs.657403	chromosome 7 open reading frame 63	Homo sapiens chromosome 7 open reading frame 63 (C7orf63), transcript variant 1, mRNA [NM_001039706]	3.66080	up	6.91279	up	68.84095	19.47169	42.75375	5.45364	Detected	Compromised	Detected	Compromised
1556	A_23_P82979	LAMC3	Hs.201805	laminin, gamma 3	Homo sapiens laminin, gamma 3 (LAMC3), mRNA [NM_006059]	3.66019	up	6.85817	up	338.02580	95.62666	394.93204	50.77848	Detected	Detected	Detected	Detected
1557	A_24_P605081	LOC253039	Hs.594170	hypothetical LOC253039	Homo sapiens hypothetical LOC253039 (LOC253039), non-coding RNA [NR_024408]	3.65857	up	4.55239	up	25.43391	7.19838	16.36787	3.17043	Detected	Compromised	Detected	Compromised
1558	A_32_P197561	EBF1	Hs.573143	early B-cell factor 1	Homo sapiens early B-cell factor 1 (EBF1), mRNA [NM_024007]	3.65833	up	3.72375	up	1812.24180	512.94040	1323.90030	313.50220	Detected	Detected	Detected	Detected
1559	A_23_P420236	PCDH9	Hs.654709	protocadherin 9	Homo sapiens protocadherin 9 (PCDH9), transcript variant 1, mRNA [NM_203487]	3.65730	up	1.30611	up	17.59978	4.98287	9.23901	6.23751	Detected	Compromised	Compromised	Compromised
1560	A_33_P3440636	LOC286071	Hs.410678	hypothetical protein LOC286071	Homo sapiens cDNA FLJ34440 fis, clone HLUNG2001214 [AK091759]	3.65721	up	3.62960	up	98.07129	27.76676	41.96167	10.19434	Detected	Detected	Detected	Compromised
1561	A_23_P160240	ACP6	Hs.562154	acid phosphatase 6, lysophosphatidic	Homo sapiens acid phosphatase 6, lysophosphatidic (ACP6), mRNA [NM_016361]	3.65554	up	3.26002	up	285.65872	80.91502	333.53930	90.21769	Detected	Detected	Detected	Detected
1562	A_23_P14673	IGDCC4	Hs.458607	immunoglobulin superfamily, DCC subclass, member 4	Homo sapiens immunoglobulin superfamily, DCC subclass, member 4 (IGDCC4), mRNA [NM_020962]	3.65551	up	3.66911	up	406.11694	115.03668	249.56769	59.97808	Detected	Detected	Detected	Detected
1563	A_23_P384044	CNIH3	Hs.28659	cornichon homolog 3 (Drosophila)	Homo sapiens cornichon homolog 3 (Drosophila) (CNIH3), mRNA [NM_152495]	3.65547	up	3.59904	up	15682.16600	4442.17700	15978.98500	3914.97100	Detected	Detected	Detected	Detected
1564	A_23_P157569	ADHFE1	Hs.720023	alcohol dehydrogenase, iron containing, 1	Homo sapiens alcohol dehydrogenase, iron containing, 1 (ADHFE1), nuclear gene encoding mitochondrial protein, mRNA [NM_144650]	3.65369	up	4.13561	up	284.59665	80.65495	208.03200	44.35641	Detected	Detected	Detected	Detected

1565	A_24_P150466	SMOC1	Hs.497349	SPARC related modular calcium binding 1	Homo sapiens SPARC related modular calcium binding 1 (SMOC1), transcript variant 1, mRNA [NM_001034852]	3.65205	up	3.53678	up	1984.49010	562.65860	2292.87040	571.65857	Detected	Detected	Detected	Detected
1566	A_23_P3212	DTWD1	Hs.127432	DTW domain containing 1	Homo sapiens DTW domain containing 1 (DTWD1), transcript variant 1, mRNA [NM_020234]	3.65192	up	3.79882	up	7187.43460	2037.91470	5620.43750	1304.62770	Detected	Detected	Detected	Detected
1567	A_33_P3228460	FXYD3	Hs.301350	FXYD domain containing ion transport regulator 3	Homo sapiens FXYD domain containing ion transport regulator 3 (FXYD3), transcript variant 3, mRNA [NM_001136007]	3.65042	up	3.14284	up	93.05353	26.39510	87.39114	24.51946	Detected	Detected	Detected	Detected
1568	A_33_P3242369		Hs.689808		Homo sapiens PP10881 mRNA, complete cds. [AF370399]	3.64806	up	3.33725	up	77.86032	22.09975	77.11816	20.37666	Detected	Detected	Detected	Detected
1569	A_33_P3313302	PARP6	Hs.270244	poly (ADP-ribose) polymerase family, member 6	Homo sapiens poly (ADP-ribose) polymerase family, member 6 (PARP6), mRNA [NM_020214]	3.64801	up	3.42680	up	1529.34100	434.09200	1067.67150	274.73508	Detected	Detected	Detected	Detected
1570	A_23_P362191	C17orf44	Hs.121692	chromosome 17 open reading frame 44	Homo sapiens chromosome 17 open reading frame 44 (C17orf44), non-coding RNA [NR_026951]	3.64517	up	4.74005	up	53.05029	15.06963	60.87252	11.32410	Detected	Compromised	Detected	Compromised
1571	A_24_P186065	DHFRL1	Hs.718516	dihydrofolate reductase-like 1	Homo sapiens dihydrofolate reductase-like 1 (DHFRL1), mRNA [NM_176815]	3.64470	up	3.77444	up	566.89040	161.05354	307.04030	71.73125	Detected	Detected	Detected	Detected
1572	A_33_P3386429	SENP6	Hs.485784	SUMO1/sentrin specific peptidase 6	Homo sapiens SUMO1/sentrin specific peptidase 6 (SENP6), transcript variant 1, mRNA [NM_015571]	3.64452	up	2.07260	up	40.14382	11.40541	27.55107	11.72163	Detected	Compromised	Detected	Compromised
1573	A_23_P321935	OSBP2	Hs.517546	oxysterol binding protein 2	Homo sapiens oxysterol binding protein 2 (OSBP2), transcript variant 1, mRNA [NM_030758]	3.64275	up	6.74190	up	154.94116	44.04231	155.70500	20.36507	Detected	Detected	Detected	Detected
1574	A_24_P102920	WDR33		WD repeat domain 33	WD repeat-containing protein 33 (WD repeat-containing protein WDC146) [Source:UniProtKB/Swiss-Prot;Acc:Q9C0J8] [ENST00000409658]	3.63966	up	3.46171	up	56.17299	15.98086	41.71609	10.62620	Detected	Compromised	Detected	Compromised
1575	A_23_P171107	CCNB3	Hs.130310	cyclin B3	Homo sapiens cyclin B3 (CCNB3), transcript variant 3, mRNA [NM_033031]	3.63903	up	3.79738	up	26.94489	7.66697	22.54833	5.23595	Detected	Compromised	Detected	Compromised
1576	A_33_P3378665	TC2N	Hs.510262	tandem C2 domains, nuclear	Homo sapiens tandem C2 domains, nuclear (TC2N), transcript variant 1, mRNA [NM_152332]	3.63840	up	4.40217	up	161.10220	45.84837	123.10281	24.65850	Detected	Detected	Detected	Detected
1577	A_24_P286114	SLC1A3	Hs.481918	solute carrier family 1 (glial high affinity glutamate transporter), member 3	Homo sapiens solute carrier family 1 (glial high affinity glutamate transporter), member 3 (SLC1A3), transcript variant 1, mRNA [NM_004172]	3.63596	up	3.67887	up	3182.95340	906.45160	2142.53700	513.54630	Detected	Detected	Detected	Detected
1578	A_23_P66948	FAM59A	Hs.444314	family with sequence similarity 59, member A	Homo sapiens family with sequence similarity 59, member A (FAM59A), mRNA [NM_022751]	3.63315	up	3.46723	up	1752.63230	499.50638	1476.24430	375.44055	Detected	Detected	Detected	Detected
1579	A_24_P242361	HS2ST1	Hs.719144	heparan sulfate 2-O-sulfotransferase 1	Homo sapiens heparan sulfate 2-O-sulfotransferase 1 (HS2ST1), transcript variant 1, mRNA [NM_012262]	3.63004	up	3.99284	up	3179.04440	906.81480	1960.46250	432.95400	Detected	Detected	Detected	Detected
1580	A_24_P84396	KIAA1199	Hs.459088	KIAA1199	Homo sapiens KIAA1199 (KIAA1199), mRNA [NM_018689]	3.62596	up	3.79346	up	220.31958	62.91629	185.60396	43.14364	Detected	Detected	Detected	Detected
1581	A_23_P250735	CBX7	Hs.356416	chromobox homolog 7	Homo sapiens chromobox homolog 7 (CBX7), mRNA [NM_175709]	3.62589	up	3.51104	up	3417.73500	976.01495	3322.14840	834.35210	Detected	Detected	Detected	Detected
1582	A_23_P170978	ZNF692	Hs.377705	zinc finger protein 692	Homo sapiens zinc finger protein 692 (ZNF692), transcript variant 2, mRNA [NM_017865]	3.62586	up	3.11649	up	1319.79170	376.90143	1346.99580	381.12326	Detected	Detected	Detected	Detected
1583	A_32_P78491	ETV1	Hs.22634	ets variant 1	Homo sapiens ets variant 1 (ETV1), transcript variant 1, mRNA [NM_004956]	3.62580	up	3.74082	up	1174.53590	335.42465	818.79990	193.00845	Detected	Detected	Detected	Detected
1584	A_32_P98683	MLLT6	Hs.91531	myeloid/lymphoid or mixed-lineage leukemia (trithorax homolog, Drosophila); translocated to, 6	Homo sapiens myeloid/lymphoid or mixed-lineage leukemia (trithorax homolog, Drosophila); translocated to, 6 (MLLT6), mRNA [NM_005937]	3.62552	up	3.25349	up	5771.53960	1648.37050	5743.26200	1556.58940	Detected	Detected	Detected	Detected
1585	A_23_P396299		Hs.246769		LOC100129827 proteinHCG1991909 ; [Source:UniProtKB/TrEMBL;Acc:Q8N6V7] [ENST00000324928]	3.62485	up	6.22258	up	13.46622	3.84671	19.30531	2.73572	Detected	Compromised	Detected	Compromised
1586	A_23_P32253	NFIL3	Hs.79334	nuclear factor, interleukin 3 regulated	Homo sapiens nuclear factor, interleukin 3 regulated (NFIL3), mRNA [NM_005384]	3.62369	up	3.74424	up	31709.07400	9060.78900	21012.61100	4948.58840	Detected	Detected	Detected	Detected
1587	A_33_P3482466	LOC100271715	Hs.99841	DH and coiled-coil domain-containing protein	Homo sapiens DH and coiled-coil domain-containing protein (LOC100271715), mRNA [NM_001145451]	3.62137	up	1.42254	up	43.01858	12.30030	17.95940	11.13249	Detected	Compromised	Detected	Compromised
1588	A_23_P134125	MAP3K5	Hs.186486	mitogen-activated protein kinase kinase kinase 5	Homo sapiens mitogen-activated protein kinase kinase kinase 5 (MAP3K5), mRNA [NM_005923]	3.61988	up	3.72194	up	5312.23050	1519.55100	3606.47780	854.43660	Detected	Detected	Detected	Detected
1589	A_23_P217704	GYG2	Hs.567381	glycogenin 2	Homo sapiens glycogenin 2 (GYG2), transcript variant 2, mRNA [NM_003918]	3.61803	up	2.94297	up	113.63329	32.52123	115.74461	34.68008	Detected	Detected	Detected	Detected
1590	A_33_P3233125	PSD	Hs.154658	pleckstrin and Sec7 domain containing	Homo sapiens pleckstrin and Sec7 domain containing (PSD), mRNA [NM_002779]	3.61673	up	6.50423	up	2574.41260	737.04600	2469.64550	334.81433	Detected	Detected	Detected	Detected
1591	A_23_P81103	SFRP2	Hs.481022	secreted frizzled-related protein 2	Homo sapiens secreted frizzled-related protein 2 (SFRP2), mRNA [NM_003013]	3.61486	up	3.67848	up	1752.75280	502.06827	1930.71290	462.82330	Detected	Detected	Detected	Detected
1592	A_33_P3318053	C17orf69		chromosome 17 open reading frame 69	Homo sapiens chromosome 17 open reading frame 69 (C17orf69), transcript variant 2, non-coding RNA [NR_026906]	3.61485	up	2.85084	up	39.50703	11.31663	20.75252	6.41894	Detected	Compromised	Detected	Compromised
1593	A_23_P118854	KRT37	Hs.673852	keratin 37	Homo sapiens keratin 37 (KRT37), mRNA [NM_003770]	3.61294	up	1.38119	up	12.05978	3.45631	4.58338	2.92615	Not Detected	Compromised	Compromised	Compromised

1594	A_33_P3282840	RPS29	Hs.156367	ribosomal protein S29	Homo sapiens ribosomal protein S29 (RPS29), transcript variant 2, mRNA [NM_001030001]	3.61072	up	3.92463	up	2364.17460	677.98315	1611.71170	362.12150	Detected	Detected	Detected	Detected
1595	A_33_P3389376	MDM4	Hs.497492	Mdm4 p53 binding protein homolog (mouse)	Homo sapiens Mdm4 p53 binding protein homolog (mouse) (MDM4), transcript variant 1, mRNA [NM_002393]	3.60834	up	3.11786	up	139.39375	40.00084	116.69451	33.00345	Detected	Detected	Detected	Detected
1596	A_24_P366787	FLJ42875	Hs.531041	hypothetical LOC440556	Homo sapiens hypothetical LOC440556 (FLJ42875), transcript variant 2, non-coding RNA [NR_024371]	3.60306	up	3.80597	up	141.65352	40.70885	175.66660	40.69949	Detected	Detected	Detected	Detected
1597	A_32_P76853		Hs.525905		Golgin subfamily A member 8-like protein 1 [Source:UniProtKB/Swiss-Prot;Acc:A6NC78] [ENST00000450802]	3.60159	up	3.89410	up	828.08330	238.07475	713.12400	161.48187	Detected	Detected	Detected	Detected
1598	A_23_P50508	PLA2G4C	Hs.631562	phospholipase A2, group IVC (cytosolic, calcium-independent)	Homo sapiens phospholipase A2, group IVC (cytosolic, calcium-independent) (PLA2G4C), transcript variant 1, mRNA [NM_003706]	3.60115	up	3.12145	up	304.54370	87.56720	275.25370	77.75748	Detected	Detected	Detected	Detected
1599	A_33_P3255290	JAKMIP2	Hs.184323	janus kinase and microtubule interacting protein 2	Homo sapiens janus kinase and microtubule interacting protein 2 (JAKMIP2), mRNA [NM_014790]	3.59998	up	4.20435	up	11.50306	3.30861	13.51270	2.83406	Compromised	Compromised	Detected	Compromised
1600	A_32_P149546	CROCC	Hs.309403	ciliary rootlet coiled-coil, rootletin	Homo sapiens ciliary rootlet coiled-coil, rootletin (CROCC), mRNA [NM_014675]	3.59752	up	3.00261	up	50.55953	14.55234	43.12153	12.66369	Detected	Compromised	Detected	Compromised
1601	A_33_P3302518	CCDC40	Hs.202542	coiled-coil domain containing 40	Homo sapiens coiled-coil domain containing 40 (CCDC40), mRNA [NM_017950]	3.59598	up	2.38884	up	172.90828	49.78875	160.99239	59.42691	Detected	Detected	Detected	Detected
1602	A_33_P3314643	SPEF1	Hs.72620	sperm flagellar 1	Homo sapiens sperm flagellar 1 (SPEF1), mRNA [NM_015417]	3.59452	up	3.20838	up	435.99808	125.59629	405.89447	111.55589	Detected	Detected	Detected	Detected
1603	A_33_P3367447	ALDH3B1	Hs.523841	aldehyde dehydrogenase 3 family, member B1	Homo sapiens aldehyde dehydrogenase 3 family, member B1 (ALDH3B1), transcript variant 3, mRNA [NM_001161473]	3.59314	up	2.87858	up	1011.63520	291.53010	771.86926	236.44507	Detected	Detected	Detected	Detected
1604	A_24_P88801	NPHP1	Hs.280388	nephronophthisis 1 (juvenile)	Homo sapiens nephronophthisis 1 (juvenile) (NPHP1), transcript variant 1, mRNA [NM_000272]	3.59216	up	3.38714	up	1405.62020	405.17764	1274.20360	331.71980	Detected	Detected	Detected	Detected
1605	A_23_P23947	MAP3K8	Hs.432453	mitogen-activated protein kinase kinase kinase 8	Homo sapiens mitogen-activated protein kinase kinase kinase 8 (MAP3K8), mRNA [NM_005204]	3.59159	up	3.82053	up	384.24410	110.77798	360.49335	83.20309	Detected	Detected	Detected	Detected
1606	A_23_P42257	IER3	Hs.591785	immediate early response 3	Homo sapiens immediate early response 3 (IER3), mRNA [NM_003897]	3.59103	up	3.30767	up	67134.43000	19357.94000	53813.27300	14346.06700	Detected	Detected	Detected	Detected
1607	A_23_P163258	PARP6	Hs.270244	poly (ADP-ribose) polymerase family, member 6	Homo sapiens poly (ADP-ribose) polymerase family, member 6 (PARP6), mRNA [NM_020214]	3.58963	up	3.39774	up	5390.45600	1554.92440	5197.07400	1348.76010	Detected	Detected	Detected	Detected
1608	A_33_P3335077	LOC100130840	Hs.638946	hypothetical LOC100130840	PREDICTED: Homo sapiens hypothetical LOC100130840 (LOC100130840), mRNA [XM_001722076]	3.58931	up	3.90131	up	152.71529	44.05592	94.43475	21.34453	Detected	Detected	Detected	Detected
1609	A_23_P35349	SVIL	Hs.499209	supervillin	Homo sapiens supervillin (SVIL), transcript variant 2, mRNA [NM_021738]	3.58849	up	3.44995	up	4242.21800	1224.09250	3465.01540	885.64080	Detected	Detected	Detected	Detected
1610	A_24_P115700	COX15	Hs.28326	COX15 homolog, cytochrome c oxidase assembly protein (yeast)	Homo sapiens COX15 homolog, cytochrome c oxidase assembly protein (yeast) (COX15), nuclear gene encoding mitochondrial protein, transcript variant 1, mRNA [NM_078470]	3.58679	up	2.94805	up	145.35582	41.96235	99.63257	29.80107	Detected	Detected	Detected	Detected
1611	A_33_P3216898	LOC286367	Hs.720355	FP944	Homo sapiens FP944 (LOC286367), non-coding RNA [NR_024011]	3.58517	up	4.01068	up	305.39200	88.20250	214.72481	47.20954	Detected	Detected	Detected	Detected
1612	A_33_P3355252				Novel proteinPutative uncharacterized protein ENSP00000368839 ; [Source:UniProtKB/TrEMBL;Acc:Q5VXX5] [ENST00000379524]	3.58489	up	4.31669	up	108.92868	31.46297	132.45271	27.05674	Detected	Detected	Detected	Detected
1613	A_33_P3344504		Hs.618112		Amyloid beta A4 precursor protein-binding family A member 2 (Neuron-specific X11L protein)(Neuronal Munc18-1-interacting protein 2)(Mint-2)(Adapter protein X11beta) [Source:UniProtKB/Swiss-Prot;Acc:Q99767] [ENST00000382938]	3.58220	up	2.82836	up	33.92819	9.80719	8.54840	2.66511	Detected	Compromised	Compromised	Compromised
1614	A_33_P3390172	ADAMDEC1	Hs.521459	ADAM-like, decysin 1	Homo sapiens ADAM-like, decysin 1 (ADAMDEC1), transcript variant 2, mRNA [NM_001145271]	3.58198	up	7.35125	up	12.73327	3.68086	25.90791	3.10768	Compromised	Compromised	Detected	Compromised
1615	A_33_P3398727		Hs.650636		AGENCOURT_7904926 NIH_MGC_82 Homo sapiens cDNA clone IMAGE:6105930 5', mRNA sequence [BQ441254]	3.57776	up	3.21129	up	57.06456	16.51537	33.11118	9.09201	Detected	Detected	Detected	Compromised
1616	A_23_P168551	SLC29A4	Hs.4302	solute carrier family 29 (nucleoside transporters), member 4	Homo sapiens solute carrier family 29 (nucleoside transporters), member 4 (SLC29A4), transcript variant 1, mRNA [NM_001040661]	3.57508	up	4.74021	up	120.09771	34.78423	130.02846	24.18835	Detected	Detected	Detected	Detected
1617	A_23_P119196	KLF2	Hs.107740	Kruppel-like factor 2 (lung)	Homo sapiens Kruppel-like factor 2 (lung) (KLF2), mRNA [NM_016270]	3.57506	up	3.46525	up	6568.12060	1902.35140	5248.78960	1335.64230	Detected	Detected	Detected	Detected
1618	A_23_P4190	ACSF2	Hs.288959	acyl-CoA synthetase family member 2	Homo sapiens acyl-CoA synthetase family member 2 (ACSF2), mRNA [NM_025149]	3.57065	up	3.52768	up	864.44560	250.68234	731.38354	182.81906	Detected	Detected	Detected	Detected
1619	A_23_P209564	CYBRD1	Hs.221941	cytochrome b reductase 1	Homo sapiens cytochrome b reductase 1 (CYBRD1), transcript variant 1, mRNA [NM_024843]	3.56845	up	3.12987	up	22160.46300	6430.32000	24767.81600	6977.93850	Detected	Detected	Detected	Detected
1620	A_33_P3258581	LOC389791	Hs.632678	hypothetical LOC389791	Homo sapiens hypothetical LOC389791 (LOC389791), non-coding RNA [NR_024425]	3.56718	up	3.44778	up	173.79689	50.44876	139.96048	35.79572	Detected	Detected	Detected	Detected

1621	A_23_P417942	FNBP1L	Hs.134060	formin binding protein 1-like	Homo sapiens formin binding protein 1-like (FNBP1L), transcript variant 1, mRNA [NM_001024948]	3.56612	up	3.92056	up	2283.83960	663.13574	1493.06930	335.81310	Detected	Detected	Detected	Detected
1622	A_33_P3398111		Hs.120392		Putative uncharacterized protein C16orf85 [Source:UniProtKB/Swiss-Prot;Acc:Q6ZSH3] [ENST00000378416]	3.56530	up	6.00368	up	40.53354	11.77204	40.46209	5.94287	Detected	Compromised	Detected	Compromised
1623	A_33_P3345643	ZDHHC11B		zinc finger, DHHC-type containing 11B	PREDICTED: Homo sapiens zinc finger, DHHC-type containing 11B (ZDHHC11B), mRNA [XM_001716434]	3.56511	up	3.11255	up	331.82440	96.37583	241.80437	68.50353	Detected	Detected	Detected	Detected
1624	A_33_P3240144					3.56408	up	3.46930	up	97.15759	28.22687	91.06741	23.14658	Detected	Detected	Detected	Detected
1625	A_33_P3342111	ZNF169	Hs.387623	zinc finger protein 169	Homo sapiens zinc finger protein 169 (ZNF169), mRNA [NM_194320]	3.56200	up	3.04901	up	440.11954	127.94099	306.36032	88.60101	Detected	Detected	Detected	Detected
1626	A_23_P125253	LDB2	Hs.714330	LIM domain binding 2	Homo sapiens LIM domain binding 2 (LDB2), transcript variant 1, mRNA [NM_001290]	3.55983	up	3.54417	up	75.30250	21.90350	74.57307	18.55379	Detected	Detected	Detected	Compromised
1627	A_23_P200298	AGL	Hs.904	amylo-1, 6-glucosidase, 4-alpha-glucanotransferase	Homo sapiens amylo-1, 6-glucosidase, 4-alpha-glucanotransferase (AGL), transcript variant 4, mRNA [NM_000028]	3.55967	up	3.27552	up	1425.77870	414.73935	967.82410	260.54430	Detected	Detected	Detected	Detected
1628	A_23_P143374	NINL	Hs.631508	ninein-like	Homo sapiens ninein-like (NINL), mRNA [NM_025176]	3.55930	up	3.31997	up	2125.77170	618.42230	1310.51390	348.07526	Detected	Detected	Detected	Detected
1629	A_23_P153867	LASS4	Hs.515111	LAG1 homolog, ceramide synthase 4	Homo sapiens LAG1 homolog, ceramide synthase 4 (LASS4), mRNA [NM_024552]	3.55828	up	4.89378	up	62.79794	18.27421	63.07991	11.36612	Detected	Detected	Detected	Compromised
1630	A_23_P316150	IFT80	Hs.478095	intraflagellar transport 80 homolog (Chlamydomonas)	Homo sapiens intraflagellar transport 80 homolog (Chlamydomonas) (IFT80), mRNA [NM_020800]	3.55800	up	3.22053	up	1245.15640	362.36908	1048.37870	287.04944	Detected	Detected	Detected	Detected
1631	A_23_P67453	TNNI3	Hs.709179	troponin I type 3 (cardiac)	Homo sapiens troponin I type 3 (cardiac) (TNNI3), mRNA [NM_000363]	3.55771	up	8.10103	up	64.91136	18.89221	96.89681	10.54714	Detected	Detected	Detected	Compromised
1632	A_23_P147397	DYNC2H1	Hs.503721	dynein, cytoplasmic 2, heavy chain 1	Homo sapiens dynein, cytoplasmic 2, heavy chain 1 (DYNC2H1), mRNA [NM_001080463]	3.55719	up	3.51423	up	2354.29660	685.30970	1913.72940	480.19305	Detected	Detected	Detected	Detected
1633	A_33_P3296119	C13orf38	Hs.720832	chromosome 13 open reading frame 38	Homo sapiens chromosome 13 open reading frame 38 (C13orf38), transcript variant 1, mRNA [NM_001144981]	3.55138	up	3.39422	up	115.73061	33.74306	117.21819	30.45235	Detected	Detected	Detected	Detected
1634	A_24_P9346	SYNGAP1	Hs.586264	synaptic Ras GTPase activating protein 1 homolog (rat)	Homo sapiens synaptic Ras GTPase activating protein 1 homolog (rat) (SYNGAP1), mRNA [NM_006772]	3.54982	up	1.50162	up	17.86921	5.21233	22.19660	13.03442	Detected	Compromised	Detected	Compromised
1635	A_33_P3229512	LOC100130387	Hs.720681	similar to hCG2042422	PREDICTED: Homo sapiens similar to hCG2042422 (LOC100130387), mRNA [XM_001715803]	3.54847	up	3.14560	up	100.52268	29.33296	103.04519	28.88612	Detected	Detected	Detected	Detected
1636	A_33_P3356392	RGL3	Hs.720291	ral guanine nucleotide dissociation stimulator-like 3	Homo sapiens ral guanine nucleotide dissociation stimulator-like 3 (RGL3), transcript variant 1, mRNA [NM_001161616]	3.54837	up	6.28886	up	13.58671	3.96477	19.03892	2.66954	Detected	Compromised	Detected	Compromised
1637	A_33_P3296220					3.54748	up	4.32383	up	10.82510	3.15969	12.95025	2.64104	Compromised	Compromised	Detected	Compromised
1638	A_24_P171873	FBXO4	Hs.165575	F-box protein 4	Homo sapiens F-box protein 4 (FBXO4), transcript variant 1, mRNA [NM_012176]	3.54690	up	3.94588	up	1860.97770	543.28253	1464.78850	327.33840	Detected	Detected	Detected	Detected
1639	A_33_P3394710	SLC35A1	Hs.423163	solute carrier family 35 (CMP-sialic acid transporter), member A1	Homo sapiens solute carrier family 35 (CMP-sialic acid transporter), member A1 (SLC35A1), mRNA [NM_006416]	3.54588	up	3.41519	up	409.90256	119.69861	561.53460	144.98631	Detected	Detected	Detected	Detected
1640	A_33_P3290403	IMPA2	Hs.720058	inositol(myo)-1(or 4)-monophosphatase 2	Homo sapiens inositol(myo)-1(or 4)-monophosphatase 2 (IMPA2), mRNA [NM_014214]	3.54584	up	3.26568	up	646.22410	188.71120	462.48260	124.87829	Detected	Detected	Detected	Detected
1641	A_33_P3369920	LOC100128402	Hs.689574	hypothetical protein LOC100128402	Homo sapiens cDNA FLJ42583 fis, clone BRACE3009090. [AK124574]	3.54424	up	3.22057	up	21.31879	6.22834	9.91581	2.71495	Detected	Compromised	Compromised	Compromised
1642	A_33_P3227231				cDNA FLJ61016 [Source:UniProtKB/TrEMBL;Acc:B4E0R4] [ENST00000418158]	3.54086	up	3.71779	up	51.36776	15.02155	12.51492	2.96831	Detected	Compromised	Compromised	Compromised
1643	A_24_P612446	C6orf89	Hs.433381	chromosome 6 open reading frame 89	Homo sapiens chromosome 6 open reading frame 89 (C6orf89), mRNA [NM_152734]	3.53918	up	3.30985	up	1758.58120	514.50934	1263.04260	336.49265	Detected	Detected	Detected	Detected
1644	A_23_P129157	NEIL1	Hs.512732	nei endonuclease VIII-like 1 (E. coli)	Homo sapiens nei endonuclease VIII-like 1 (E. coli) (NEIL1), mRNA [NM_024608]	3.53743	up	3.37163	up	465.97850	136.39890	552.45330	144.48430	Detected	Detected	Detected	Detected
1645	A_33_P3377130	MAP3K5	Hs.186486	mitogen-activated protein kinase kinase kinase 5	Homo sapiens mitogen-activated protein kinase kinase kinase 5 (MAP3K5), mRNA [NM_005923]	3.53708	up	3.67891	up	849.81820	248.77913	638.20070	152.96898	Detected	Detected	Detected	Detected
1646	A_23_P170186	OPLAH	Hs.305882	5-oxoprolinase (ATP-hydrolysing)	Homo sapiens 5-oxoprolinase (ATP-hydrolysing) (OPLAH), mRNA [NM_017570]	3.53418	up	3.09577	up	1979.40030	579.93390	1974.36650	562.37270	Detected	Detected	Detected	Detected
1647	A_24_P191417	NAB1	Hs.107474	NGFI-A binding protein 1 (EGR1 binding protein 1)	Homo sapiens NGFI-A binding protein 1 (EGR1 binding protein 1) (NAB1), mRNA [NM_005966]	3.53207	up	3.70580	up	2311.19800	677.54900	1538.06290	365.98004	Detected	Detected	Detected	Detected
1648	A_32_P846696	LOC349196	Hs.722381	hypothetical LOC349196	Homo sapiens hypothetical LOC349196 (LOC349196), non-coding RNA [NR_027000]	3.53042	up	3.35395	up	577.95800	169.51305	376.68848	99.03562	Detected	Detected	Detected	Detected
1649	A_33_P3269924					3.52969	up	2.74722	up	129.37459	37.95295	110.42434	35.44351	Detected	Detected	Detected	Detected
1650	A_23_P434890	CARD10	Hs.57973	caspase recruitment domain family, member 10	Homo sapiens caspase recruitment domain family, member 10 (CARD10), mRNA [NM_014550]	3.52860	up	3.28053	up	10937.75800	3209.66020	11371.94400	3056.72530	Detected	Detected	Detected	Detected
1651	A_24_P160413	MTMR9L	Hs.471067	myotubularin related protein 9-like	Homo sapiens myotubularin related protein 9-like (MTMR9L), non-coding RNA [NR_026850]	3.52709	up	3.63214	up	533.08640	156.50012	389.90180	94.65812	Detected	Detected	Detected	Detected
1652	A_33_P3423551	IER3	Hs.591785	immediate early response 3	Homo sapiens immediate early response 3 (IER3), mRNA [NM_003897]	3.51849	up	3.29986	up	23844.66600	7017.26600	27244.95900	7280.40700	Detected	Detected	Detected	Detected

1653	A_23_P346912	SPATA17	Hs.171130	spermatogenesis associated 17	Homo sapiens spermatogenesis associated 17 (SPATA17), mRNA [NM_138796]	3.51794	up	3.79146	up	81.48010	23.98258	70.66014	16.43363	Detected	Detected	Detected	Detected
1654	A_32_P156373		Hs.666632		DA110513 BRACE3 Homo sapiens cDNA clone BRACE3027555 5', mRNA sequence [DA110513]	3.51633	up	1.51763	up	86.19940	25.38326	43.79826	25.44821	Detected	Detected	Detected	Detected
1655	A_23_P59616	GTF2IRD2	Hs.647017	GTF2I repeat domain containing 2	Homo sapiens GTF2I repeat domain containing 2 (GTF2IRD2), mRNA [NM_173537]	3.51555	up	3.35763	up	280.25424	82.54521	318.89220	83.74839	Detected	Detected	Detected	Detected
1656	A_23_P66038	ZDHHC1	Hs.658333	zinc finger, DHHC-type containing 1	Homo sapiens zinc finger, DHHC-type containing 1 (ZDHHC1), mRNA [NM_013304]	3.51459	up	3.37710	up	325.30707	95.84110	324.18497	84.64754	Detected	Detected	Detected	Detected
1657	A_33_P3252499	WDR52	Hs.584936	WD repeat domain 52	Homo sapiens WD repeat domain 52 (WDR52), transcript variant 1, mRNA [NM_001164496]	3.51396	up	1.41660	up	17.42707	5.13524	8.96346	5.57949	Detected	Compromised	Compromised	Compromised
1658	A_33_P3302185	HS3ST6	Hs.670144	heparan sulfate (glucosamine) 3-O-sulfotransferase 6	Homo sapiens heparan sulfate (glucosamine) 3-O-sulfotransferase 6 (HS3ST6), mRNA [NM_001009606]	3.50833	up	3.23090	up	10.91772	3.22229	13.30177	3.63038	Compromised	Compromised	Detected	Compromised
1659	A_33_P3305487	REM2	Hs.444911	RAS (RAD and GEM)-like GTP binding 2	Homo sapiens RAS (RAD and GEM)-like GTP binding 2 (REM2), mRNA [NM_173527]	3.50817	up	3.55839	up	518.81020	153.13045	456.49930	113.12332	Detected	Detected	Detected	Detected
1660	A_32_P41026	SC5DL	Hs.287749	sterol-C5-desaturase (ERG3 delta-5-desaturase homolog, S. cerevisiae)-like	Homo sapiens sterol-C5-desaturase (ERG3 delta-5-desaturase homolog, S. cerevisiae)-like (SC5DL), transcript variant 2, mRNA [NM_001024956]	3.50639	up	6.05258	up	66.26868	19.56955	116.33959	16.94933	Detected	Detected	Detected	Detected
1661	A_24_P396720	PPP1CB	Hs.702907	protein phosphatase 1, catalytic subunit, beta isoform	Homo sapiens protein phosphatase 1, catalytic subunit, beta isoform (PPP1CB), transcript variant 1, mRNA [NM_002709]	3.50570	up	3.30802	up	14584.61800	4307.78100	13471.40100	3590.95560	Detected	Detected	Detected	Detected
1662	A_23_P48029	CLEC4A	Hs.504657	C-type lectin domain family 4, member A	Homo sapiens C-type lectin domain family 4, member A (CLEC4A), transcript variant 1, mRNA [NM_016184]	3.49800	up	3.80101	up	261.87177	77.51788	182.94298	42.44064	Detected	Detected	Detected	Detected
1663	A_23_P35343	ZNF248	Hs.528423	zinc finger protein 248	Homo sapiens zinc finger protein 248 (ZNF248), mRNA [NM_021045]	3.49670	up	3.48105	up	244.93214	72.53056	176.16220	44.62397	Detected	Detected	Detected	Detected
1664	A_23_P254688	TMEM108	Hs.191616	transmembrane protein 108	Homo sapiens transmembrane protein 108 (TMEM108), transcript variant 1, mRNA [NM_023943]	3.49493	up	2.28341	up	16.29843	4.82882	7.60343	2.93624	Detected	Compromised	Compromised	Compromised
1665	A_33_P3422240	KIAA1530	Hs.380475	KIAA1530	Homo sapiens KIAA1530 (KIAA1530), mRNA [NM_020894]	3.49083	up	1.94352	up	60.49444	17.94405	52.01551	23.59981	Detected	Compromised	Detected	Detected
1666	A_33_P3354374				Putative uncharacterized protein ENSP00000380701 [Source:UniProtKB/Swiss-Prot;Acc:A8MTB1] [ENST00000397571]	3.48928	up	6.82353	up	104.87907	31.12331	76.15112	9.84085	Detected	Detected	Detected	Compromised
1667	A_33_P3215611					3.48912	up	2.51871	up	231.60783	68.73375	23.10415	8.08869	Detected	Detected	Detected	Compromised
1668	A_23_P123454	NUDT18	Hs.721833	nudix (nucleoside diphosphate linked moiety X)-type motif 18	Homo sapiens nudix (nucleoside diphosphate linked moiety X)-type motif 18 (NUDT18), mRNA [NM_024815]	3.48853	up	3.43320	up	1228.29110	364.57907	1351.67600	347.16757	Detected	Detected	Detected	Detected
1669	A_33_P3243093	RGS5	Hs.24950	regulator of G-protein signaling 5	Homo sapiens regulator of G-protein signaling 5 (RGS5), mRNA [NM_003617]	3.48742	up	3.84304	up	164.69100	48.89890	119.81332	27.49131	Detected	Detected	Detected	Detected
1670	A_33_P3314813	C11orf94	Hs.193741	chromosome 11 open reading frame 94	Homo sapiens chromosome 11 open reading frame 94 (C11orf94), mRNA [NM_001080446]	3.48578	up	3.24945	up	71.51778	21.24453	59.79224	16.22558	Detected	Detected	Detected	Detected
1671	A_23_P201939	PPM1J	Hs.655231	protein phosphatase 1J (PP2C domain containing)	Homo sapiens protein phosphatase 1J (PP2C domain containing) (PPM1J), mRNA [NM_005167]	3.48513	up	3.43103	up	578.89020	171.99289	745.83720	191.68364	Detected	Detected	Detected	Detected
1672	A_33_P3316456	NPL	Hs.496969	N-acetylneuraminate pyruvate lyase (dihydrodipicolinate synthase)	Homo sapiens N-acetylneuraminate pyruvate lyase (dihydrodipicolinate synthase) (NPL), mRNA [NM_030769]	3.48494	up	4.76134	up	66.36359	19.71821	54.51500	10.09606	Detected	Detected	Detected	Compromised
1673	A_23_P134542	FOXP2	Hs.656280	forkhead box P2	Homo sapiens forkhead box P2 (FOXP2), transcript variant 2, mRNA [NM_148898]	3.48230	up	4.66033	up	34.95438	10.39367	19.81325	3.74891	Detected	Compromised	Detected	Compromised
1674	A_32_P118250	C14orf167		chromosome 14 open reading frame 167	Homo sapiens chromosome 14 open reading frame 167 (C14orf167), transcript variant 1, non-coding RNA [NR_023921]	3.47629	up	3.61179	up	673.70056	200.67060	459.84750	112.26830	Detected	Detected	Detected	Detected
1675	A_33_P3329149	CDC2L5	Hs.233552	cell division cycle 2-like 5 (cholinesterase-related cell division controller)	Homo sapiens cell division cycle 2-like 5 (cholinesterase-related cell division controller) (CDC2L5), transcript variant 2, mRNA [NM_031267]	3.47153	up	2.27811	up	305.51297	91.12592	131.37502	50.85149	Detected	Detected	Detected	Detected
1676	A_33_P3399571	VNN1	Hs.12114	vanin 1	Homo sapiens vanin 1 (VNN1), mRNA [NM_004666]	3.46848	up	3.04297	up	96.48717	28.80472	64.80814	18.78009	Detected	Detected	Detected	Detected
1677	A_33_P3237775	NR1H3	Hs.438863	nuclear receptor subfamily 1, group H, member 3	Homo sapiens nuclear receptor subfamily 1, group H, member 3 (NR1H3), transcript variant 1, mRNA [NM_005693]	3.46450	up	3.43277	up	2694.90920	805.44730	2761.34810	709.31964	Detected	Detected	Detected	Detected
1678	A_33_P3218951	RPA1	Hs.461925	replication protein A1, 70kDa	Homo sapiens replication protein A1, 70kDa (RPA1), mRNA [NM_002945]	3.46163	up	3.73125	up	378.90866	113.34091	339.45755	80.22252	Detected	Detected	Detected	Detected
1679	A_23_P62588	CALML6	Hs.85902	calmodulin-like 6	Homo sapiens calmodulin-like 6 (CALML6), mRNA [NM_138705]	3.46162	up	2.91189	up	47.31645	14.15356	37.23115	11.27448	Detected	Compromised	Detected	Compromised
1680	A_33_P3419865	LOC646719	Hs.697792	hypothetical LOC646719	PREDICTED: Homo sapiens hypothetical LOC646719 (LOC646719), miscRNA [XR_041071]	3.46143	up	1.94651	up	81.24563	24.30400	39.03117	17.68151	Detected	Detected	Detected	Detected
1681	A_33_P3887081	LOC338817	Hs.524257	hypothetical protein LOC338817	Homo sapiens hypothetical protein LOC338817, mRNA (cDNA clone IMAGE:5267955) [BC041827]	3.46029	up	3.20898	up	54.44406	16.29188	29.77458	8.18172	Detected	Compromised	Detected	Compromised
1682	A_23_P30976	GRM1	Hs.32945	glutamate receptor, metabotropic 1	Homo sapiens glutamate receptor, metabotropic 1 (GRM1), transcript variant 1, mRNA [NM_000838]	3.46025	up	3.36211	up	22.09716	6.61245	12.97252	3.40234	Detected	Compromised	Compromised	Compromised

1683	A_33_P3242014	PHC1	Hs.305985	polyhomeotic homolog 1 (Drosophila)	Homo sapiens polyhomeotic homolog 1 (Drosophila) (PHC1), mRNA [NM_004426]	3.45983	up	3.55190	up	2592.50800	775.88760	1949.52170	483.98657	Detected	Detected	Detected	Detected
1684	A_23_P398172	FAM135A	Hs.211700	family with sequence similarity 135, member A	Homo sapiens family with sequence similarity 135, member A (FAM135A), transcript variant 2, mRNA [NM_020819]	3.45667	up	4.17388	up	504.74140	151.19731	308.59796	65.19569	Detected	Detected	Detected	Detected
1685	A_33_P3301980	LOC100132625	Hs.711757	hypothetical LOC100132625	PREDICTED: Homo sapiens hypothetical LOC100132625 (LOC100132625), mRNA [XM_001720431]	3.45553	up	1.95668	up	13.16082	3.94368	6.58631	2.96817	Detected	Compromised	Compromised	Compromised
1686	A_23_P67569	LPPR3	Hs.546439	lipid phosphate phosphatase-related protein type 3	Homo sapiens lipid phosphate phosphatase-related protein type 3 (LPPR3), mRNA [NM_024888]	3.45144	up	4.53070	up	75.44769	22.63489	127.61361	24.83690	Detected	Detected	Detected	Detected
1687	A_33_P3239242	SPATA6	Hs.538103	spermatogenesis associated 6	Homo sapiens spermatogenesis associated 6 (SPATA6), mRNA [NM_019073]	3.44937	up	3.91859	up	153.80193	46.16944	115.49961	25.99058	Detected	Detected	Detected	Detected
1688	A_32_P66364	PPP1R1C	Hs.10941	protein phosphatase 1, regulatory (inhibitor) subunit 1C	Homo sapiens protein phosphatase 1, regulatory (inhibitor) subunit 1C (PPP1R1C), mRNA [NM_001080545]	3.44406	up	3.65198	up	369.87850	111.20429	313.43857	75.68146	Detected	Detected	Detected	Detected
1689	A_23_P73809	LRCH2	Hs.65366	leucine-rich repeats and calponin homology (CH) domain containing 2	Homo sapiens leucine-rich repeats and calponin homology (CH) domain containing 2 (LRCH2), mRNA [NM_020871]	3.44277	up	3.28187	up	299.69025	90.13582	299.90585	80.58026	Detected	Detected	Detected	Detected
1690	A_24_P521994	KLHL24	Hs.407709	kelch-like 24 (Drosophila)	Homo sapiens kelch-like 24 (Drosophila) (KLHL24), mRNA [NM_017644]	3.43452	up	3.56704	up	1701.70040	513.03810	1084.29200	268.04242	Detected	Detected	Detected	Detected
1691	A_33_P3317460	LOC100130141	Hs.722967	hypothetical LOC100130141	PREDICTED: Homo sapiens similar to Kruppel-like factor (LOC100130141), miscRNA [XR_037929]	3.43361	up	3.17538	up	1069.53860	322.53674	413.09738	114.71539	Detected	Detected	Detected	Detected
1692	A_23_P163402	CYP1A1	Hs.72912	cytochrome P450, family 1, subfamily A, polypeptide 1	Homo sapiens cytochrome P450, family 1, subfamily A, polypeptide 1 (CYP1A1), mRNA [NM_000499]	3.42941	up	2.63223	up	103.43932	31.23194	105.67860	35.40213	Detected	Detected	Detected	Detected
1693	A_33_P3261520	FLJ41309	Hs.364045	hypothetical protein LOC645079	Homo sapiens cDNA FLJ41309 fis, clone BRAMY2042641. [AK123303]	3.42917	up	6.88871	up	76.15928	22.99678	47.47089	6.07652	Detected	Detected	Detected	Compromised
1694	A_23_P90359	NRTN	Hs.234775	neurturin	Homo sapiens neurturin (NRTN), mRNA [NM_004558]	3.42729	up	5.61734	up	49.59145	14.98267	104.18828	16.35512	Detected	Compromised	Detected	Detected
1695	A_33_P3350580				Protein kinase C delta type (EC 2.7.11.13)(nPKC-delta) [Source:UniProtKB/Swiss-Prot;Acc:Q05655] [ENST00000394727]	3.42528	up	1.51866	up	32.04597	9.68748	10.09262	5.86017	Detected	Compromised	Compromised	Compromised
1696	A_33_P3212640	NOTCH2NL	Hs.709526	Notch homolog 2 (Drosophila) N-terminal like	Homo sapiens Notch homolog 2 (Drosophila) N-terminal like (NOTCH2NL), mRNA [NM_203458]	3.42504	up	2.50405	up	217.71080	65.81836	154.98172	54.57623	Detected	Detected	Detected	Detected
1697	A_23_P147255	PCBP3	Hs.474049	poly(rC) binding protein 3	Homo sapiens poly(rC) binding protein 3 (PCBP3), transcript variant 1, mRNA [NM_020528]	3.42335	up	3.13726	up	401.60260	121.47260	349.18950	98.14671	Detected	Detected	Detected	Detected
1698	A_24_P16124	IFITM4P		interferon induced transmembrane protein 4 pseudogene	Homo sapiens interferon induced transmembrane protein 4 pseudogene (IFITM4P), non-coding RNA [NR_001590]	3.42221	up	3.48262	up	19027.96500	5757.29600	17262.69100	4370.86570	Detected	Detected	Detected	Detected
1699	A_23_P381017	WBSCR27	Hs.647042	Williams Beuren syndrome chromosome region 27	Homo sapiens Williams Beuren syndrome chromosome region 27 (WBSCR27), mRNA [NM_152559]	3.42096	up	3.10278	up	114.25768	34.58360	152.67113	43.38819	Detected	Detected	Detected	Detected
1700	A_32_P106933	BCAR4	Hs.24611	breast cancer anti-estrogen resistance 4	Homo sapiens breast cancer anti-estrogen resistance 4 (BCAR4), non-coding RNA [NR_024049]	3.41651	up	1.47511	up	30.29793	9.18255	22.96960	13.73075	Detected	Compromised	Detected	Compromised
1701	A_33_P3385266	ABCC6	Hs.442182	ATP-binding cassette, sub-family C (CFTR/MRP), member 6	Homo sapiens ATP-binding cassette, sub-family C (CFTR/MRP), member 6 (ABCC6), transcript variant 2, mRNA [NM_001079528]	3.41307	up	4.36598	up	214.72296	65.14271	185.94418	37.55487	Detected	Detected	Detected	Detected
1702	A_33_P3275943	PWWP2A	Hs.438851	PWWP domain containing 2A	Homo sapiens PWWP domain containing 2A (PWWP2A), transcript variant 1, mRNA [NM_052927]	3.41295	up	1.26572	up	10.79449	3.27496	14.68760	10.23242	Compromised	Compromised	Detected	Compromised
1703	A_32_P217750	IL3RA	Hs.632790	interleukin 3 receptor, alpha (low affinity)	Homo sapiens interleukin 3 receptor, alpha (low affinity) (IL3RA), mRNA [NM_002183]	3.41175	up	2.54756	up	24.63777	7.47751	24.33654	8.42365	Detected	Compromised	Detected	Compromised
1704	A_23_P250231	ANKRD7	Hs.657737	ankyrin repeat domain 7	Homo sapiens ankyrin repeat domain 7 (ANKRD7), mRNA [NM_019644]	3.41122	up	3.75617	up	133.59859	40.55326	135.77437	31.87409	Detected	Detected	Detected	Detected
1705	A_32_P67623	FAM120C	Hs.86045	family with sequence similarity 120C	Homo sapiens family with sequence similarity 120C (FAM120C), mRNA [NM_017848]	3.40896	up	3.52722	up	2931.53340	890.44270	2161.20830	540.29315	Detected	Detected	Detected	Detected
1706	A_33_P3556116	LOC145678	Hs.23777	hypothetical protein LOC145678	Homo sapiens mRNA full length insert cDNA clone EUROIMAGE 35394 [AL109682]	3.40866	up	1.82839	up	14.45228	4.39022	16.12279	7.77567	Detected	Compromised	Detected	Compromised
1707	A_32_P231617	TM4SF1	Hs.351316	transmembrane 4 L six family member 1	Homo sapiens transmembrane 4 L six family member 1 (TM4SF1), mRNA [NM_014220]	3.40665	up	3.08102	up	17096.97900	5196.66900	16455.67000	4709.62650	Detected	Detected	Detected	Detected
1708	A_23_P121564	GUCY1B3	Hs.77890	guanylate cyclase 1, soluble, beta 3	Homo sapiens guanylate cyclase 1, soluble, beta 3 (GUCY1B3), mRNA [NM_000857]	3.40324	up	3.84279	up	175.75934	53.47599	111.30045	25.53968	Detected	Detected	Detected	Detected
1709	A_23_P150979	MUCL1	Hs.348419	mucin-like 1	Homo sapiens mucin-like 1 (MUCL1), mRNA [NM_058173]	3.40226	up	5.16980	up	90.45718	27.53015	60.18936	10.26625	Detected	Detected	Detected	Compromised
1710	A_23_P4161	ARSG		arylsulfatase G	Arylsulfatase G Precursor (ASG)(EC 3.1.6.-) [Source:UniProtKB/Swiss-Prot;Acc:Q96EG1] [ENST00000355179]	3.40167	up	3.49782	up	269.70120	82.09647	221.88147	55.93572	Detected	Detected	Detected	Detected
1711	A_32_P232559	LOC439949	Hs.590987	hypothetical protein LOC439949	PREDICTED: Homo sapiens hypothetical protein LOC439949 (LOC439949), mRNA [XM_001128367]	3.40082	up	4.06075	up	65.27770	19.87532	49.82679	10.81987	Detected	Detected	Detected	Compromised
1712	A_33_P3390207	TMEM155	Hs.27524	transmembrane protein 155	Homo sapiens transmembrane protein 155 (TMEM155), mRNA [NM_152399]	3.40038	up	5.85635	up	26.57940	8.09377	25.20557	3.79520	Detected	Compromised	Detected	Compromised

1713	A_24_P356916	SLC13A3	Hs.655498	solute carrier family 13 (sodium-dependent dicarboxylate transporter), member 3	Homo sapiens solute carrier family 13 (sodium-dependent dicarboxylate transporter), member 3 (SLC13A3), transcript variant 2, mRNA [NM_001011554]	3.39894	up	2.77815	up	279.44970	85.13203	213.41995	67.73985	Detected	Detected	Detected	Detected
1714	A_23_P80594	PLCL2	Hs.202010	phospholipase C-like 2	Homo sapiens phospholipase C-like 2 (PLCL2), transcript variant 2, mRNA [NM_015184]	3.39820	up	2.83208	up	2247.69500	684.89040	1238.15590	385.50934	Detected	Detected	Detected	Detected
1715	A_24_P131589	CD86	Hs.171182	CD86 molecule	Homo sapiens CD86 molecule (CD86), transcript variant 2, mRNA [NM_006889]	3.39751	up	1.72718	up	1226.64230	373.84344	46.24458	23.60967	Detected	Detected	Detected	Detected
1716	A_24_P416961	ARVCF	Hs.713616	armadillo repeat gene deletes in velocardiofacial syndrome	Homo sapiens armadillo repeat gene deletes in velocardiofacial syndrome (ARVCF), mRNA [NM_001670]	3.39537	up	4.04400	up	639.01373	194.87482	236.49852	51.56830	Detected	Detected	Detected	Detected
1717	A_23_P428248	TTC21A	Hs.443935	tetratricopeptide repeat domain 21A	Homo sapiens tetratricopeptide repeat domain 21A (TTC21A), transcript variant 2, mRNA [NM_145755]	3.39198	up	3.50495	up	1205.40160	367.96973	1042.41490	262.25513	Detected	Detected	Detected	Detected
1718	A_33_P3220698	EPS8L1	Hs.438862	EPS8-like 1	Homo sapiens EPS8-like 1 (EPS8L1), transcript variant 1, mRNA [NM_133180]	3.39170	up	3.16580	up	1609.46200	491.35645	1443.56980	402.08658	Detected	Detected	Detected	Detected
1719	A_33_P3285540	CLDN5	Hs.505337	claudin 5	Homo sapiens claudin 5 (CLDN5), transcript variant 1, mRNA [NM_001130861]	3.39007	up	1.31431	up	19.81551	6.05243	11.14288	7.47590	Detected	Compromised	Compromised	Compromised
1720	A_33_P3382303	FMNL1	Hs.720131	formin-like 1	Homo sapiens formin-like 1 (FMNL1), mRNA [NM_005892]	3.38982	up	3.75514	up	127.98245	39.09370	129.19661	30.33828	Detected	Detected	Detected	Detected
1721	A_33_P3373203					3.38952	up	3.11154	up	168.67639	51.52873	99.56650	28.21648	Detected	Detected	Detected	Detected
1722	A_33_P3399363	GPR155	Hs.516604	G protein-coupled receptor 155	Homo sapiens G protein-coupled receptor 155 (GPR155), transcript variant 9, mRNA [NM_001033045]	3.38946	up	3.64750	up	159.63731	48.76824	123.96223	29.96816	Detected	Detected	Detected	Detected
1723	A_33_P3239634	NFYA	Hs.10441	nuclear transcription factor Y, alpha	Homo sapiens nuclear transcription factor Y, alpha (NFYA), transcript variant 1, mRNA [NM_002505]	3.38613	up	2.80674	up	431.47147	131.94164	292.97107	92.04243	Detected	Detected	Detected	Detected
1724	A_33_P3421275	NKX6-3	Hs.647132	NK6 homeobox 3	Homo sapiens NK6 homeobox 3 (NKX6-3), mRNA [NM_152568]	3.38414	up	4.50921	up	374.16632	114.48536	258.07562	50.46759	Detected	Detected	Detected	Detected
1725	A_33_P3421611					3.38404	up	3.08973	up	804.58154	246.18813	821.20500	234.36671	Detected	Detected	Detected	Detected
1726	A_33_P3329462		Hs.722230		ig32f10.y1 HR85 islet Homo sapiens cDNA 5', mRNA sequence [BM264234]	3.38395	up	2.93942	up	837.24445	256.18960	211.83520	63.54808	Detected	Detected	Detected	Detected
1727	A_23_P130158	WNT3	Hs.445884	wingless-type MMTV integration site family, member 3	Homo sapiens wingless-type MMTV integration site family, member 3 (WNT3), mRNA [NM_030753]	3.38270	up	3.66578	up	719.23870	220.16235	544.41500	130.95697	Detected	Detected	Detected	Detected
1728	A_33_P3397905	BET1	Hs.489132	blocked early in transport 1 homolog (S. cerevisiae)	Homo sapiens blocked early in transport 1 homolog (S. cerevisiae) (BET1), mRNA [NM_005868]	3.38234	up	3.75171	up	439.11900	134.43042	486.01727	114.23219	Detected	Detected	Detected	Detected
1729	A_23_P58912	SLC35A1	Hs.423163	solute carrier family 35 (CMP-sialic acid transporter), member A1	Homo sapiens solute carrier family 35 (CMP-sialic acid transporter), member A1 (SLC35A1), mRNA [NM_006416]	3.38105	up	3.95291	up	1278.35280	391.50073	1099.35580	245.23778	Detected	Detected	Detected	Detected
1730	A_33_P3605352	LOC440983	Hs.535002	hypothetical gene supported by BC066916	Homo sapiens hypothetical gene supported by BC066916, mRNA (cDNA clone IMAGE:4838452) [BC066916]	3.37972	up	1.67420	up	17.84400	5.46695	15.09054	7.94811	Detected	Compromised	Detected	Compromised
1731	A_23_P10194	SEZ6L2	Hs.6314	seizure related 6 homolog (mouse)-like 2	Homo sapiens seizure related 6 homolog (mouse)-like 2 (SEZ6L2), transcript variant 2, mRNA [NM_201575]	3.37918	up	3.10171	up	8300.26100	2543.39200	8044.77300	2287.06200	Detected	Detected	Detected	Detected
1732	A_23_P85682	NFIA	Hs.191911	nuclear factor I/A	Homo sapiens nuclear factor I/A (NFIA), transcript variant 2, mRNA [NM_005595]	3.37602	up	3.04742	up	1977.55480	606.53610	1197.00230	346.36002	Detected	Detected	Detected	Detected
1733	A_33_P3271111	NINJ1	Hs.494457	ninjurin 1	Homo sapiens ninjurin 1 (NINJ1), mRNA [NM_004148]	3.37435	up	3.62090	up	31492.26600	9663.78600	28584.60700	6961.14550	Detected	Detected	Detected	Detected
1734	A_33_P3264662	CYP27C1	Hs.407639	cytochrome P450, family 27, subfamily C, polypeptide 1	Homo sapiens cytochrome P450, family 27, subfamily C, polypeptide 1 (CYP27C1), mRNA [NM_001001665]	3.37305	up	2.71830	up	499.73987	153.41045	315.68512	102.40508	Detected	Detected	Detected	Detected
1735	A_23_P40174	MMP9	Hs.297413	matrix metallopeptidase 9 (gelatinase B, 92kDa gelatinase, 92kDa type IV collagenase)	Homo sapiens matrix metallopeptidase 9 (gelatinase B, 92kDa gelatinase, 92kDa type IV collagenase) (MMP9), mRNA [NM_004994]	3.36932	up	4.68286	up	579.02760	177.94675	464.11280	87.39334	Detected	Detected	Detected	Detected
1736	A_23_P136964	RPGR	Hs.61438	retinitis pigmentosa GTPase regulator	Homo sapiens retinitis pigmentosa GTPase regulator (RPGR), transcript variant A, mRNA [NM_000328]	3.36878	up	3.25767	up	2732.72830	839.95630	2471.28170	668.93054	Detected	Detected	Detected	Detected
1737	A_33_P3266444	MRPS25	Hs.715617	mitochondrial ribosomal protein S25	Homo sapiens mitochondrial ribosomal protein S25 (MRPS25), nuclear gene encoding mitochondrial protein, mRNA [NM_022497]	3.36762	up	3.17167	up	1602.99580	492.88116	1236.05750	343.64975	Detected	Detected	Detected	Detected
1738	A_23_P210048	HDAC4	Hs.20516	histone deacetylase 4	Homo sapiens histone deacetylase 4 (HDAC4), mRNA [NM_006037]	3.36728	up	4.89179	up	1338.12560	411.48150	691.21790	124.59839	Detected	Detected	Detected	Detected
1739	A_33_P3387110	LOC145783	Hs.620525	hypothetical LOC145783	Homo sapiens hypothetical LOC145783 (LOC145783), non-coding RNA [NR_015419]	3.36695	up	5.91598	up	38.19764	11.74716	43.03569	6.41457	Detected	Compromised	Detected	Compromised
1740	A_24_P156267	SOX12	Hs.43627	SRY (sex determining region Y)-box 12	Homo sapiens SRY (sex determining region Y)-box 12 (SOX12), mRNA [NM_006943]	3.36651	up	4.81029	up	72.84500	22.40542	55.37658	10.15128	Detected	Detected	Detected	Compromised
1741	A_23_P90601	STEAP3	Hs.647822	STEAP family member 3	Homo sapiens STEAP family member 3 (STEAP3), transcript variant 1, mRNA [NM_182915]	3.36638	up	3.50103	up	15551.47400	4783.46000	19297.06000	4860.27540	Detected	Detected	Detected	Detected
1742	A_33_P3238335		Hs.535239		Mucolipin-3 [Source:UniProtKB/Swiss-Prot;Acc:Q8TDD5] [ENST00000370587]	3.36515	up	1.75982	up	80.26930	24.69891	23.95441	12.00282	Detected	Detected	Detected	Compromised
1743	A_23_P114947	RGS2	Hs.78944	regulator of G-protein signaling 2, 24kDa	Homo sapiens regulator of G-protein signaling 2, 24kDa (RGS2), mRNA [NM_002923]	3.36457	up	3.31915	up	6021.32500	1853.08690	4250.02930	1129.09350	Detected	Detected	Detected	Detected

1744	A_33_P3357949	ETV1	Hs.22634	ets variant 1	Homo sapiens ets variant 1 (ETV1), transcript variant 1, mRNA [NM_004956]	3.36277	up	3.24076	up	2218.69500	683.17706	1149.69780	312.82526	Detected	Detected	Detected	Detected
1745	A_23_P431330	CRIPAK	Hs.26410	cysteine-rich PAK1 inhibitor	Homo sapiens cysteine-rich PAK1 inhibitor (CRIPAK), mRNA [NM_175918]	3.36065	up	3.19568	up	1937.77210	597.05210	1074.60560	296.51843	Detected	Detected	Detected	Detected
1746	A_33_P3313640					3.35789	up	1.15604	up	346.62686	106.88788	33.98067	25.91929	Detected	Detected	Detected	Detected
1747	A_23_P104804	ZBTB16	Hs.591945	zinc finger and BTB domain containing 16	Homo sapiens zinc finger and BTB domain containing 16 (ZBTB16), transcript variant 1, mRNA [NM_006006]	3.35599	up	3.20976	up	4052.93870	1250.49820	4179.66600	1148.24630	Detected	Detected	Detected	Detected
1748	A_23_P306956	ZNF566	Hs.533939	zinc finger protein 566	Homo sapiens zinc finger protein 566 (ZNF566), transcript variant 3, mRNA [NM_032838]	3.35362	up	3.29340	up	24.33081	7.51235	18.33152	4.90817	Detected	Compromised	Detected	Compromised
1749	A_33_P3273480	BRSK2	Hs.170819	BR serine/threonine kinase 2	Homo sapiens BR serine/threonine kinase 2 (BRSK2), mRNA [NM_003957]	3.35322	up	4.18816	up	32.42313	10.01212	25.81219	5.43460	Detected	Compromised	Detected	Compromised
1750	A_23_P382043	NT5DC1	Hs.719198	5'-nucleotidase domain containing 1	Homo sapiens 5'-nucleotidase domain containing 1 (NT5DC1), mRNA [NM_152729]	3.35258	up	3.76160	up	1665.52160	514.40424	1035.24300	242.68092	Detected	Detected	Detected	Detected
1751	A_32_P5276	SGEF	Hs.240845	Src homology 3 domain-containing guanine nucleotide exchange factor	Homo sapiens Src homology 3 domain-containing guanine nucleotide exchange factor (SGEF), mRNA [NM_015595]	3.35067	up	3.35058	up	696.46990	215.23064	625.61490	164.64664	Detected	Detected	Detected	Detected
1752	A_23_P24948	KCNE3	Hs.523899	potassium voltage-gated channel, Isk-related family, member 3	Homo sapiens potassium voltage-gated channel, Isk-related family, member 3 (KCNE3), mRNA [NM_005472]	3.34961	up	3.45120	up	27.69973	8.56278	18.19641	4.64923	Detected	Compromised	Detected	Compromised
1753	A_23_P29663	ZMYND10	Hs.526735	zinc finger, MYND-type containing 10	Homo sapiens zinc finger, MYND-type containing 10 (ZMYND10), mRNA [NM_015896]	3.34895	up	3.07910	up	112.50269	34.78461	114.30830	32.73550	Detected	Detected	Detected	Detected
1754	A_23_P305877	C9orf123	Hs.7517	chromosome 9 open reading frame 123	Homo sapiens chromosome 9 open reading frame 123 (C9orf123), mRNA [NM_033428]	3.34610	up	4.25329	up	216.78940	67.08602	153.27502	31.77691	Detected	Detected	Detected	Detected
1755	A_24_P808100	MCTS1	Hs.102696	malignant T cell amplified sequence 1	Homo sapiens malignant T cell amplified sequence 1 (MCTS1), transcript variant 1, mRNA [NM_014060]	3.34420	up	3.26019	up	205.85536	63.73870	123.76613	33.47527	Detected	Detected	Detected	Detected
1756	A_24_P269129	ZDHHC14	Hs.187459	zinc finger, DHHC-type containing 14	Homo sapiens zinc finger, DHHC-type containing 14 (ZDHHC14), transcript variant 2, mRNA [NM_153746]	3.34347	up	3.41259	up	107.59398	33.32147	84.31381	21.78611	Detected	Detected	Detected	Detected
1757	A_33_P3269208	STX19	Hs.679768	syntaxin 19	Homo sapiens syntaxin 19 (STX19), mRNA [NM_001001850]	3.34270	up	8.44525	up	46.72910	14.47515	28.43114	2.96857	Detected	Compromised	Detected	Compromised
1758	A_33_P3309662	GPX4	Hs.433951	glutathione peroxidase 4 (phospholipid hydroperoxidase)	Homo sapiens glutathione peroxidase 4 (phospholipid hydroperoxidase) (GPX4), transcript variant 3, mRNA [NM_001039848]	3.34178	up	3.03888	up	38499.97000	11929.30200	33813.95300	9811.78300	Detected	Detected	Detected	Detected
1759	A_23_P354074	LYST	Hs.532411	lysosomal trafficking regulator	Homo sapiens lysosomal trafficking regulator (LYST), mRNA [NM_000081]	3.33780	up	3.50033	up	1707.49670	529.70337	914.33340	230.33540	Detected	Detected	Detected	Detected
1760	A_33_P3287228		Hs.568276		IQ domain-containing protein D [Source:UniProtKB/Swiss-Prot;Acc:Q96DY2] [ENST00000392574]	3.33619	up	7.44932	up	37.31191	11.58055	27.00180	3.19626	Detected	Compromised	Detected	Compromised
1761	A_33_P3456233	LOC286254	Hs.635171	hypothetical protein LOC286254	Homo sapiens cDNA FLJ35432 fis, clone SMINT2002311 [AK092751]	3.32730	up	4.93090	up	606.14166	188.63219	472.33295	84.46707	Detected	Detected	Detected	Detected
1762	A_33_P3388376	TBC1D3B	Hs.454713	TBC1 domain family, member 3B	Homo sapiens TBC1 domain family, member 3B (TBC1D3B), mRNA [NM_001001417]	3.32647	up	2.65039	up	18.65774	5.80775	13.53422	4.50287	Detected	Compromised	Compromised	Compromised
1763	A_33_P3376806	C10orf67	Hs.522360	chromosome 10 open reading frame 67	Homo sapiens chromosome 10 open reading frame 67 (C10orf67), mRNA [NM_153714]	3.32542	up	3.86883	up	149.93806	46.68728	126.95039	28.93476	Detected	Detected	Detected	Detected
1764	A_32_P191441	C10orf90	Hs.587663	chromosome 10 open reading frame 90	Homo sapiens chromosome 10 open reading frame 90 (C10orf90), mRNA [NM_001004298]	3.32210	up	3.41843	up	503.95825	157.07810	507.93720	131.02342	Detected	Detected	Detected	Detected
1765	A_33_P3240538	TUBE1	Hs.34851	tubulin, epsilon 1	Homo sapiens tubulin, epsilon 1 (TUBE1), mRNA [NM_016262]	3.32193	up	3.33073	up	1822.66150	568.13135	1274.64090	337.45337	Detected	Detected	Detected	Detected
1766	A_33_P3351664	CCDC57	Hs.631724	coiled-coil domain containing 57	Homo sapiens coiled-coil domain containing 57 (CCDC57), mRNA [NM_198082]	3.32182	up	3.71923	up	1948.60060	607.40630	1683.95740	399.24920	Detected	Detected	Detected	Detected
1767	A_33_P3266719	TET2	Hs.367639	tet oncogene family member 2	Homo sapiens tet oncogene family member 2 (TET2), transcript variant 2, mRNA [NM_017628]	3.32021	up	3.20611	up	108.72416	33.90730	75.47787	20.75903	Detected	Detected	Detected	Detected
1768	A_23_P434430	ZNF439	Hs.528731	zinc finger protein 439	Homo sapiens zinc finger protein 439 (ZNF439), mRNA [NM_152262]	3.31406	up	3.06807	up	478.76898	149.58860	287.89963	82.74487	Detected	Detected	Detected	Detected
1769	A_23_P123086	KIAA1908	Hs.436146	hypothetical LOC114796	Homo sapiens hypothetical LOC114796 (KIAA1908), transcript variant 1, non-coding RNA [NR_027329]	3.31108	up	2.67927	up	455.75275	142.52562	388.34558	127.81082	Detected	Detected	Detected	Detected
1770	A_23_P39131	GLTSCR2	Hs.421907	glioma tumor suppressor candidate region gene 2	Homo sapiens glioma tumor suppressor candidate region gene 2 (GLTSCR2), mRNA [NM_015710]	3.31005	up	2.85403	up	7135.15400	2232.04170	9520.51300	2941.48780	Detected	Detected	Detected	Detected
1771	A_23_P68487	BMP7	Hs.473163	bone morphogenetic protein 7	Homo sapiens bone morphogenetic protein 7 (BMP7), mRNA [NM_001719]	3.30932	up	1.43095	up	34.69455	10.85565	28.08055	17.30404	Detected	Compromised	Detected	Detected
1772	A_33_P3369461	AMIGO1	Hs.655656	adhesion molecule with Ig-like domain 1	Homo sapiens adhesion molecule with Ig-like domain 1 (AMIGO1), mRNA [NM_020703]	3.30928	up	2.76182	up	48.82508	15.27714	42.60979	13.60440	Detected	Compromised	Detected	Compromised
1773	A_23_P171388	TMSB4Y	Hs.159201	thymosin beta 4, Y-linked	Homo sapiens thymosin beta 4, Y-linked (TMSB4Y), mRNA [NM_004202]	3.30717	up	6.01317	up	168.74435	52.83315	63.03204	9.24322	Detected	Detected	Detected	Compromised
1774	A_23_P23639	MCOLN2	Hs.591446	mucolipin 2	Homo sapiens mucolipin 2 (MCOLN2), mRNA [NM_153259]	3.30703	up	2.65507	up	31.19053	9.76603	34.10172	11.32572	Detected	Compromised	Detected	Compromised
1775	A_33_P3404899	LPHN2	Hs.24212	latrophilin 2	Homo sapiens latrophilin 2 (LPHN2), mRNA [NM_012302]	3.30524	up	2.22107	up	49.21060	15.41660	32.42074	12.87143	Detected	Compromised	Detected	Compromised

1776	A_23_P110234	CSN1S1	Hs.3155	casein alpha s1	Homo sapiens casein alpha s1 (CSN1S1), transcript variant 1, mRNA [NM_001890]	3.30414	up	2.66120	up	69.34351	21.73105	53.49363	17.72514	Detected	Detected	Detected	Detected
1777	A_24_P74508	SLC25A18	Hs.282982	solute carrier family 25 (mitochondrial carrier), member 18	Homo sapiens solute carrier family 25 (mitochondrial carrier), member 18 (SLC25A18), nuclear gene encoding mitochondrial protein, mRNA [NM_031481]	3.30256	up	6.69899	up	24.55994	7.70033	19.89780	2.61916	Detected	Compromised	Detected	Compromised
1778	A_24_P49183	EXD3	Hs.495553	exonuclease 3'-5' domain containing 3	Homo sapiens exonuclease 3'-5' domain containing 3 (EXD3), mRNA [NM_017820]	3.30256	up	11.31794	up	34.96183	10.96166	41.65928	3.24571	Detected	Compromised	Detected	Compromised
1779	A_23_P128201	NCKAP1L	Hs.182014	NCK-associated protein 1-like	Homo sapiens NCK-associated protein 1-like (NCKAP1L), mRNA [NM_005337]	3.30046	up	3.77996	up	23.98062	7.52348	21.25066	4.95736	Detected	Compromised	Detected	Compromised
1780	A_23_P152620	TNFSF13	Hs.54673	tumor necrosis factor (ligand) superfamily, member 13	Homo sapiens tumor necrosis factor (ligand) superfamily, member 13 (TNFSF13), transcript variant gamma, mRNA [NM_172088]	3.29152	up	3.38086	up	241.37310	75.93219	250.86080	65.42909	Detected	Detected	Detected	Detected
1781	A_23_P106773	SULT1A2	Hs.546304	sulfotransferase family, cytosolic, 1A, phenol-preferring, member 2	Homo sapiens sulfotransferase family, cytosolic, 1A, phenol-preferring, member 2 (SULT1A2), transcript variant 2, mRNA [NM_177528]	3.29043	up	3.15481	up	2024.97100	637.23364	622.47650	173.98610	Detected	Detected	Detected	Detected
1782	A_32_P212058	C3orf34	Hs.282800	chromosome 3 open reading frame 34	Homo sapiens chromosome 3 open reading frame 34 (C3orf34), mRNA [NM_032898]	3.28655	up	3.26176	up	417.85086	131.64812	248.25688	67.11423	Detected	Detected	Detected	Detected
1783	A_24_P184445	MMP19	Hs.591033	matrix metallopeptidase 19	Homo sapiens matrix metallopeptidase 19 (MMP19), transcript variant 1, mRNA [NM_002429]	3.28633	up	3.33740	up	1573.85160	495.89005	1443.50400	381.39545	Detected	Detected	Detected	Detected
1784	A_23_P120062	C2orf3	Hs.303808	chromosome 2 open reading frame 3	Homo sapiens chromosome 2 open reading frame 3 (C2orf3), mRNA [NM_003203]	3.28617	up	3.27116	up	2689.47950	847.44360	1702.17190	458.84640	Detected	Detected	Detected	Detected
1785	A_33_P3217649	C9orf116	Hs.414028	chromosome 9 open reading frame 116	Homo sapiens chromosome 9 open reading frame 116 (C9orf116), transcript variant 1, mRNA [NM_001048265]	3.28421	up	2.87939	up	784.76636	247.42477	753.50745	230.75587	Detected	Detected	Detected	Detected
1786	A_23_P161156	ZNF438	Hs.660642	zinc finger protein 438	Homo sapiens zinc finger protein 438 (ZNF438), transcript variant 2, mRNA [NM_182755]	3.28395	up	3.05094	up	8951.29700	2822.42580	6589.07230	1904.39220	Detected	Detected	Detected	Detected
1787	A_33_P3345708	CREB3L4	Hs.372924	cAMP responsive element binding protein 3-like 4	Homo sapiens cAMP responsive element binding protein 3-like 4 (CREB3L4), mRNA [NM_130898]	3.28355	up	4.32702	up	763.54610	240.78235	704.89734	143.64902	Detected	Detected	Detected	Detected
1788	A_23_P130735	SLC6A16	Hs.130949	solute carrier family 6, member 16	Homo sapiens solute carrier family 6, member 16 (SLC6A16), mRNA [NM_014037]	3.28263	up	4.07650	up	429.04285	135.33570	239.76053	51.86281	Detected	Detected	Detected	Detected
1789	A_33_P3317543	GTF2IRD2	Hs.647017	GTF2I repeat domain containing 2	Homo sapiens GTF2I repeat domain containing 2 (GTF2IRD2), mRNA [NM_173537]	3.28066	up	2.86278	up	120.66916	38.08629	174.16417	53.64592	Detected	Detected	Detected	Detected
1790	A_33_P3649236	FLJ39534	Hs.570631	hypothetical FLJ39534	Homo sapiens cDNA FLJ33945 fis, clone CTONG2018279 [AK091264]	3.27914	up	2.57471	up	138.70801	43.80007	111.93742	38.33657	Detected	Detected	Detected	Detected
1791	A_23_P153583	PPP5C	Hs.654604	protein phosphatase 5, catalytic subunit	Homo sapiens protein phosphatase 5, catalytic subunit (PPP5C), mRNA [NM_006247]	3.27889	up	1.28325	up	1766.54940	557.86957	799.48020	549.36630	Detected	Detected	Detected	Detected
1792	A_23_P363275	WDR66	Hs.709837	WD repeat domain 66	Homo sapiens WD repeat domain 66 (WDR66), mRNA [NM_144668]	3.27776	up	3.39891	up	95.61202	30.20429	97.14960	25.20381	Detected	Detected	Detected	Detected
1793	A_24_P109071	KIF27	Hs.697514	kinesin family member 27	Homo sapiens kinesin family member 27 (KIF27), mRNA [NM_017576]	3.27561	up	3.66008	up	71.66179	22.65316	53.24745	12.82845	Detected	Detected	Detected	Compromised
1794	A_32_P489130	BRWD3	Hs.170667	bromodomain and WD repeat domain containing 3	Homo sapiens bromodomain and WD repeat domain containing 3 (BRWD3), mRNA [NM_153252]	3.27433	up	2.77034	up	194.48450	61.50298	123.97522	39.46098	Detected	Detected	Detected	Detected
1795	A_23_P302018	TXK	Hs.479669	TXK tyrosine kinase	Homo sapiens TXK tyrosine kinase (TXK), mRNA [NM_003328]	3.27417	up	3.78992	up	12.63491	3.99581	16.68772	3.88269	Compromised	Compromised	Detected	Compromised
1796	A_33_P3265301	GJD3	Hs.444663	gap junction protein, delta 3, 31.9kDa	Homo sapiens gap junction protein, delta 3, 31.9kDa (GJD3), mRNA [NM_152219]	3.27158	up	3.73841	up	415.18372	131.40623	436.67474	102.99990	Detected	Detected	Detected	Detected
1797	A_23_P111860	RADIL	Hs.667336	Ras association and DIL domains	Homo sapiens Ras association and DIL domains (RADIL), mRNA [NM_018059]	3.27080	up	3.98136	up	2471.22950	782.33405	1736.54140	384.60852	Detected	Detected	Detected	Detected
1798	A_23_P211167	C21orf2	Hs.517331	chromosome 21 open reading frame 2	Homo sapiens chromosome 21 open reading frame 2 (C21orf2), mRNA [NM_004928]	3.27018	up	2.89205	up	371.11703	117.50931	379.00534	115.55949	Detected	Detected	Detected	Detected
1799	A_23_P158925	GPR125	Hs.99195	G protein-coupled receptor 125	Homo sapiens G protein-coupled receptor 125 (GPR125), mRNA [NM_145290]	3.26799	up	3.42790	up	9099.14900	2883.05470	6871.06450	1767.50840	Detected	Detected	Detected	Detected
1800	A_33_P3380063	ADAMTSL4	Hs.719991	ADAMTS-like 4	Homo sapiens ADAMTS-like 4 (ADAMTSL4), transcript variant 2, mRNA [NM_025008]	3.26771	up	4.07263	up	2146.69950	680.23895	2664.96880	577.00867	Detected	Detected	Detected	Detected
1801	A_33_P3285132	ZNF599	Hs.590961	zinc finger protein 599	Homo sapiens zinc finger protein 599 (ZNF599), mRNA [NM_001007248]	3.26757	up	1.56556	up	34.52332	10.94009	25.91915	14.59877	Detected	Compromised	Detected	Detected
1802	A_24_P481783	LOC390595	Hs.659256	similar to ubiquitin-associated protein 1 (predicted)	Homo sapiens similar to ubiquitin-associated protein 1 (predicted) (LOC390595), mRNA [NM_001163692]	3.26738	up	3.10985	up	679.22797	215.25311	585.92430	166.13730	Detected	Detected	Detected	Detected
1803	A_24_P456490	C1orf204	Hs.647718	chromosome 1 open reading frame 204	Homo sapiens chromosome 1 open reading frame 204 (C1orf204), mRNA [NM_001134233]	3.26489	up	4.58459	up	56.72392	17.98997	48.61577	9.35065	Detected	Detected	Detected	Compromised
1804	A_23_P202496	NOC3L	Hs.74899	nucleolar complex associated 3 homolog (S. cerevisiae)	Homo sapiens nucleolar complex associated 3 homolog (S. cerevisiae) (NOC3L), mRNA [NM_022451]	3.26212	up	3.26686	up	2909.44630	923.51540	2156.93950	582.20013	Detected	Detected	Detected	Detected
1805	A_23_P200637	FBXO44	Hs.556006	F-box protein 44	Homo sapiens F-box protein 44 (FBXO44), transcript variant 4, mRNA [NM_001014765]	3.26043	up	2.45825	up	224.58595	71.32488	231.45303	83.02385	Detected	Detected	Detected	Detected
1806	A_33_P3353996	PPP1R3G	Hs.653089	protein phosphatase 1, regulatory (inhibitor) subunit 3G	Homo sapiens protein phosphatase 1, regulatory (inhibitor) subunit 3G (PPP1R3G), mRNA [NM_001145115]	3.26005	up	2.93385	up	489.36780	155.43356	587.98346	176.72258	Detected	Detected	Detected	Detected

1807	A_23_P43317	PRDM12	Hs.495311	PR domain containing 12	Homo sapiens PR domain containing 12 (PRDM12), mRNA [NM_021619]	3.25705	up	2.63694	up	77.02335	24.48676	66.33418	22.18207	Detected	Detected	Detected	Detected
1808	A_23_P133648	FAM8A1	Hs.95260	family with sequence similarity 8, member A1	Homo sapiens family with sequence similarity 8, member A1 (FAM8A1), mRNA [NM_016255]	3.25553	up	3.31803	up	802.26600	255.16994	444.42126	118.10822	Detected	Detected	Detected	Detected
1809	A_23_P156861	RGS17	Hs.166313	regulator of G-protein signaling 17	Homo sapiens regulator of G-protein signaling 17 (RGS17), mRNA [NM_012419]	3.24953	up	3.45866	up	574.71590	183.13281	349.17980	89.02399	Detected	Detected	Detected	Detected
1810	A_23_P364437	CDH23	Hs.656032	cadherin-like 23	Homo sapiens cadherin-like 23 (CDH23), transcript variant 1, mRNA [NM_022124]	3.24931	up	2.61979	up	37.57147	11.97293	14.81460	4.98642	Detected	Compromised	Detected	Compromised
1811	A_33_P3279708	RNU2-2	Hs.640133	RNA, U2 small nuclear 2	Homo sapiens RNA, U2 small nuclear 2 (RNU2-2), small nuclear RNA [NR_002761]	3.24894	up	2.74596	up	1051.34890	335.07250	1027.35620	329.90760	Detected	Detected	Detected	Detected
1812	A_33_P3368750	PAQR5	Hs.591096	progestin and adipoQ receptor family member V	Homo sapiens progestin and adipoQ receptor family member V (PAQR5), transcript variant 1, mRNA [NM_001104554]	3.24798	up	4.25104	up	1535.94560	489.66130	1506.67420	312.52844	Detected	Detected	Detected	Detected
1813	A_23_P312851	SILV	Hs.95972	silver homolog (mouse)	Homo sapiens silver homolog (mouse) (SILV), mRNA [NM_006928]	3.24616	up	2.47211	up	70.37856	22.44931	81.25911	28.98480	Detected	Detected	Detected	Detected
1814	A_23_P12241	MCOLN3	Hs.535239	mucolipin 3	Homo sapiens mucolipin 3 (MCOLN3), mRNA [NM_018298]	3.24608	up	2.62447	up	28.86564	9.20777	13.98728	4.69957	Detected	Compromised	Detected	Compromised
1815	A_23_P217098	VPS13A	Hs.459790	vacuolar protein sorting 13 homolog A (S. cerevisiae)	Homo sapiens vacuolar protein sorting 13 homolog A (S. cerevisiae) (VPS13A), transcript variant A, mRNA [NM_033305]	3.24242	up	3.25346	up	1091.27700	348.49725	638.02350	172.92480	Detected	Detected	Detected	Detected
1816	A_33_P3314166		Hs.689460		Homo sapiens cDNA FLJ46531 fis, clone THYMU3036310. [AK128388]	3.23628	up	3.39539	up	216.91240	69.40195	133.82097	34.75362	Detected	Detected	Detected	Detected
1817	A_33_P3397073		Hs.549204		Proline-rich transmembrane protein 1 [Source:UniProtKB/Swiss-Prot;Acc:Q99946] [ENST00000427462]	3.23552	up	3.09197	up	562.12177	179.89493	548.24457	156.35257	Detected	Detected	Detected	Detected
1818	A_32_P52018	PHACTR1	Hs.436996	phosphatase and actin regulator 1	Homo sapiens phosphatase and actin regulator 1 (PHACTR1), mRNA [NM_030948]	3.23366	up	2.52427	up	52.35361	16.76431	84.57846	29.54536	Detected	Compromised	Detected	Detected
1819	A_32_P134290	ZCCHC2	Hs.114191	zinc finger, CCHC domain containing 2	Homo sapiens zinc finger, CCHC domain containing 2 (ZCCHC2), mRNA [NM_017742]	3.23338	up	3.11755	up	1173.92880	375.93920	901.53064	254.99518	Detected	Detected	Detected	Detected
1820	A_23_P904	BEND5	Hs.475348	BEN domain containing 5	Homo sapiens BEN domain containing 5 (BEND5), mRNA [NM_024603]	3.23003	up	4.44474	up	116.40548	37.31645	78.48320	15.57027	Detected	Detected	Detected	Compromised
1821	A_32_P85999	CDH13	Hs.654386	cadherin 13, H-cadherin (heart)	Homo sapiens cadherin 13, H-cadherin (heart) (CDH13), mRNA [NM_001257]	3.22907	up	3.30198	up	10328.62200	3312.05960	7102.83840	1896.80870	Detected	Detected	Detected	Detected
1822	A_24_P943301	PEAR1	Hs.142003	platelet endothelial aggregation receptor 1	Homo sapiens platelet endothelial aggregation receptor 1 (PEAR1), mRNA [NM_001080471]	3.22842	up	2.58773	up	163.78783	52.53207	142.25570	48.47480	Detected	Detected	Detected	Detected
1823	A_24_P941922		Hs.490224		CCR4-NOT transcription complex subunit 4 (EC 6.3.2.-)(E3 ubiquitin-protein ligase CNOT4)(CCR4-associated factor 4)(Potential transcriptional repressor NOT4Hp) [Source:UniProtKB/Swiss-Prot;Acc:O95628] [ENST00000315544]	3.22487	up	3.06329	up	129.02762	41.42889	95.79286	27.57472	Detected	Detected	Detected	Detected
1824	A_33_P3888485	BCO2	Hs.647227	beta-carotene oxygenase 2	Homo sapiens beta-carotene oxygenase 2 (BCO2), transcript variant 1, mRNA [NM_031938]	3.21931	up	4.05313	up	292.88007	94.20188	155.74474	33.88350	Detected	Detected	Detected	Detected
1825	A_33_P3609033	LOC401022	Hs.98661	hypothetical LOC401022	Homo sapiens hypothetical LOC401022, mRNA (cDNA clone IMAGE:5296725) [BC047481]	3.21698	up	2.51477	up	367.97190	118.44038	270.66370	94.90667	Detected	Detected	Detected	Detected
1826	A_24_P192727		Hs.632079		Kazal-type serine protease inhibitor domain-containing protein 1 Precursor [Source:UniProtKB/Swiss-Prot;Acc:Q96I82] [ENST00000313664]	3.21601	up	2.60849	up	4094.32150	1318.24760	3417.45730	1155.25830	Detected	Detected	Detected	Detected
1827	A_23_P416894	PION	Hs.186649	pigeon homolog (Drosophila)	Homo sapiens pigeon homolog (Drosophila) (PION), mRNA [NM_017439]	3.21595	up	3.81272	up	233.35948	75.13628	219.79060	50.83225	Detected	Detected	Detected	Detected
1828	A_33_P3352103	LYPLAL1	Hs.657617	lysophospholipase-like 1	Homo sapiens lysophospholipase-like 1 (LYPLAL1), mRNA [NM_138794]	3.21459	up	3.19356	up	1960.50660	631.50290	1517.36580	418.96770	Detected	Detected	Detected	Detected
1829	A_23_P93311	DDR1	Hs.631988	discoidin domain receptor tyrosine kinase 1	Homo sapiens discoidin domain receptor tyrosine kinase 1 (DDR1), transcript variant 1, mRNA [NM_013993]	3.21362	up	2.73218	up	1082.10450	348.66455	1119.00240	361.14926	Detected	Detected	Detected	Detected
1830	A_33_P3427239	LOC100134937	Hs.659762	hypothetical LOC100134937	Homo sapiens cDNA clone IMAGE:5263734 [BC035129]	3.21241	up	3.32004	up	2536.55520	817.61170	1584.55690	420.85272	Detected	Detected	Detected	Detected
1831	A_33_P3362900	LOC375190	Hs.710370	hypothetical protein LOC375190	Homo sapiens hypothetical protein LOC375190 (LOC375190), mRNA [NM_001145710]	3.21125	up	3.39598	up	2917.37500	940.69950	2516.14770	653.33620	Detected	Detected	Detected	Detected
1832	A_33_P3344564		Hs.586199		Uncharacterized protein C5orf42 [Source:UniProtKB/Swiss-Prot;Acc:Q9H799] [ENST00000388739]	3.20983	up	2.65740	up	15.78332	5.09154	13.72050	4.55281	Detected	Compromised	Detected	Compromised
1833	A_23_P344578	FAM154A	Hs.720871	family with sequence similarity 154, member A	Homo sapiens family with sequence similarity 154, member A (FAM154A), mRNA [NM_153707]	3.20621	up	1.32835	up	9.94275	3.21106	15.86384	10.53082	Compromised	Compromised	Detected	Compromised
1834	A_33_P3331491	LOC728392	Hs.104305	hypothetical protein LOC728392	Homo sapiens hypothetical protein LOC728392 (LOC728392), mRNA [NM_001162371]	3.20611	up	3.33937	up	8745.28000	2824.41160	5729.08840	1512.81880	Detected	Detected	Detected	Detected
1835	A_33_P3334155	LOC730495	Hs.369771	hypothetical protein LOC730495	Homo sapiens cDNA FLJ45934 fis, clone PLACE7003684 [AK127831]	3.20605	up	3.50368	up	127.75951	41.26251	52.35310	13.17601	Detected	Detected	Detected	Compromised
1836	A_33_P3286621	SCARNA16	Hs.722642	small Cajal body-specific RNA 16	Homo sapiens small Cajal body-specific RNA 16 (SCARNA16), guide RNA [NR_003013]	3.20473	up	2.94030	up	792.75360	256.14117	563.69430	169.05106	Detected	Detected	Detected	Detected

1837	A_33_P3265855	PRR5-ARHGAP8	Hs.720401	PRR5-ARHGAP8 readthrough	Homo sapiens PRR5-ARHGAP8 fusion (LOC553158), mRNA [NM_181334]	3.20364	up	3.92503	up	608.85230	196.78946	583.90640	131.17946	Detected	Detected	Detected	Detected
1838	A_32_P79483	LOC283481	Hs.646604	hypothetical protein LOC283481	PREDICTED: Homo sapiens hypothetical protein LOC283481 (LOC283481), mRNA [XM_001128325]	3.20102	up	2.95682	up	846.57910	273.84988	642.83014	191.70665	Detected	Detected	Detected	Detected
1839	A_32_P68533	FAM161A	Hs.440466	family with sequence similarity 161, member A	Homo sapiens family with sequence similarity 161, member A (FAM161A), mRNA [NM_032180]	3.19860	up	2.46817	up	325.04373	105.22417	227.84587	81.40136	Detected	Detected	Detected	Detected
1840	A_24_P110558	C5orf53	Hs.696360	chromosome 5 open reading frame 53	Homo sapiens chromosome 5 open reading frame 53 (C5orf53), mRNA [NM_001007189]	3.19684	up	3.74135	up	873.86580	283.04617	522.34670	123.11062	Detected	Detected	Detected	Detected
1841	A_23_P306890	POLI	Hs.438533	polymerase (DNA directed) iota	Homo sapiens polymerase (DNA directed) iota (POLI), mRNA [NM_007195]	3.19606	up	3.28953	up	3923.29980	1271.07150	2167.64380	581.05750	Detected	Detected	Detected	Detected
1842	A_23_P145984	TSPAN12	Hs.16529	tetraspanin 12	Homo sapiens tetraspanin 12 (TSPAN12), mRNA [NM_012338]	3.19404	up	4.23272	up	164.56580	53.34979	87.04568	18.13400	Detected	Detected	Detected	Detected
1843	A_23_P324523	IQCK	Hs.460217	IQ motif containing K	Homo sapiens IQ motif containing K (IQCK), mRNA [NM_153208]	3.19286	up	3.08683	up	3506.24490	1137.09140	2589.39430	739.69147	Detected	Detected	Detected	Detected
1844	A_23_P90722	PTPRN	Hs.89655	protein tyrosine phosphatase, receptor type, N	Homo sapiens protein tyrosine phosphatase, receptor type, N (PTPRN), mRNA [NM_002846]	3.19272	up	3.12222	up	855.39900	277.42215	692.73193	195.64409	Detected	Detected	Detected	Detected
1845	A_33_P3394243	C1orf101	Hs.459534	chromosome 1 open reading frame 101	Homo sapiens chromosome 1 open reading frame 101 (C1orf101), transcript variant 2, mRNA [NM_173807]	3.19190	up	3.89946	up	186.21548	60.40869	159.18535	35.99682	Detected	Detected	Detected	Detected
1846	A_23_P46412	SCNN1D	Hs.512681	sodium channel, nonvoltage-gated 1, delta	Homo sapiens sodium channel, nonvoltage-gated 1, delta (SCNN1D), transcript variant 2, mRNA [NM_002978]	3.19053	up	4.12122	up	233.71454	75.85007	170.85457	36.55663	Detected	Detected	Detected	Detected
1847	A_33_P3368879	LMOD1	Hs.519075	leiomodin 1 (smooth muscle)	Homo sapiens leiomodin 1 (smooth muscle) (LMOD1), mRNA [NM_012134]	3.18963	up	3.78473	up	37.84213	12.28481	32.98058	7.68402	Detected	Compromised	Detected	Compromised
1848	A_23_P389102	MYO1D	Hs.658000	myosin ID	Homo sapiens myosin ID (MYO1D), mRNA [NM_015194]	3.18852	up	3.27488	up	7918.14000	2571.38530	4539.04900	1222.18100	Detected	Detected	Detected	Detected
1849	A_23_P147641	TCEA2	Hs.505004	transcription elongation factor A (SII), 2	Homo sapiens transcription elongation factor A (SII), 2 (TCEA2), transcript variant 1, mRNA [NM_003195]	3.18818	up	2.97140	up	3080.45600	1000.47394	3455.08420	1025.32690	Detected	Detected	Detected	Detected
1850	A_24_P921683	FOXP2	Hs.656280	forkhead box P2	Homo sapiens forkhead box P2 (FOXP2), transcript variant 1, mRNA [NM_014491]	3.18684	up	2.63705	up	97.62041	31.71851	100.64842	33.65540	Detected	Detected	Detected	Detected
1851	A_33_P3405384	C8orf45	Hs.437006	chromosome 8 open reading frame 45	Homo sapiens chromosome 8 open reading frame 45 (C8orf45), transcript variant 1, mRNA [NM_173518]	3.18586	up	2.21039	up	14.78272	4.80463	9.76846	3.89692	Detected	Compromised	Compromised	Compromised
1852	A_33_P3422679	FAM149B1	Hs.408577	family with sequence similarity 149, member B1	Homo sapiens family with sequence similarity 149, member B1 (FAM149B1), mRNA [NM_173348]	3.18531	up	3.08314	up	1732.32900	563.13450	1198.33860	342.72983	Detected	Detected	Detected	Detected
1853	A_33_P3238533	C18orf34	Hs.115461	chromosome 18 open reading frame 34	Homo sapiens chromosome 18 open reading frame 34 (C18orf34), transcript variant 1, mRNA [NM_001105528]	3.18268	up	1.99742	up	16.48797	5.36424	22.86861	10.09570	Detected	Compromised	Detected	Compromised
1854	A_24_P344961	AMOT	Hs.528051	angiomotin	Homo sapiens angiomotin (AMOT), transcript variant 2, mRNA [NM_133265]	3.18239	up	3.79340	up	110.54550	35.96836	75.13824	17.46619	Detected	Detected	Detected	Detected
1855	A_24_P261259	PFKFB3	Hs.195471	6-phosphofructo-2-kinase/fructose-2,6-biphosphatase 3	Homo sapiens 6-phosphofructo-2-kinase/fructose-2,6-biphosphatase 3 (PFKFB3), transcript variant 1, mRNA [NM_004566]	3.17826	up	2.76253	up	4227.55700	1377.31410	3518.72730	1123.16580	Detected	Detected	Detected	Detected
1856	A_33_P3279831	PNRC1	Hs.75969	proline-rich nuclear receptor coactivator 1	Homo sapiens proline-rich nuclear receptor coactivator 1 (PNRC1), mRNA [NM_006813]	3.17816	up	3.54741	up	4833.97270	1574.92870	4292.03120	1066.88490	Detected	Detected	Detected	Detected
1857	A_24_P314597				Uncharacterized protein KIAA0319-like [Source:UniProtKB/Swiss-Prot;Acc:Q8IZA0] [ENST00000335883]	3.17691	up	2.21622	up	226.68571	73.88444	166.48575	66.24146	Detected	Detected	Detected	Detected
1858	A_33_P3294901	SULT1A4	Hs.460558	sulfotransferase family, cytosolic, 1A, phenol-preferring, member 4	Homo sapiens sulfotransferase family, cytosolic, 1A, phenol-preferring, member 4 (SULT1A4), transcript variant 1, mRNA [NM_001017389]	3.17593	up	3.06160	up	853.39910	278.23703	981.85706	282.79090	Detected	Detected	Detected	Detected
1859	A_32_P132317	GPR155	Hs.516604	G protein-coupled receptor 155	Homo sapiens G protein-coupled receptor 155 (GPR155), transcript variant 9, mRNA [NM_001033045]	3.17493	up	3.01188	up	442.03516	144.16379	340.52194	99.69482	Detected	Detected	Detected	Detected
1860	A_33_P3306413	INCA1	Hs.658926	inhibitor of CDK, cyclin A1 interacting protein 1	Homo sapiens inhibitor of CDK, cyclin A1 interacting protein 1 (INCA1), mRNA [NM_213726]	3.17366	up	2.26333	up	77.87960	25.40955	107.72173	41.96820	Detected	Detected	Detected	Detected
1861	A_23_P157793	CA9	Hs.63287	carbonic anhydrase IX	Homo sapiens carbonic anhydrase IX (CA9), mRNA [NM_001216]	3.17109	up	4.85881	up	56.50852	18.45177	46.69330	8.47404	Detected	Detected	Detected	Compromised
1862	A_23_P216361	COL14A1	Hs.409662	collagen, type XIV, alpha 1	Homo sapiens collagen, type XIV, alpha 1 (COL14A1), mRNA [NM_021110]	3.17057	up	4.27010	up	27.85370	9.09658	26.13008	5.39596	Detected	Compromised	Detected	Compromised
1863	A_23_P64617	FZD4	Hs.591968	frizzled homolog 4 (Drosophila)	Homo sapiens frizzled homolog 4 (Drosophila) (FZD4), mRNA [NM_012193]	3.16831	up	3.25170	up	3876.91750	1267.04490	2830.62820	767.60583	Detected	Detected	Detected	Detected
1864	A_33_P3299656	RPGR	Hs.61438	retinitis pigmentosa GTPase regulator	Homo sapiens retinitis pigmentosa GTPase regulator (RPGR), transcript variant A, mRNA [NM_000328]	3.16618	up	3.27434	up	897.44760	293.49908	372.08435	100.20367	Detected	Detected	Detected	Detected
1865	A_33_P3222635	XGPY2		Xg pseudogene, Y-linked 2	Homo sapiens Xg pseudogene, Y-linked 2 (XGPY2), non-coding RNA [NR_003254]	3.16491	up	4.67914	up	21.75178	7.11650	14.97871	2.82276	Detected	Compromised	Detected	Compromised
1866	A_33_P3255509	FCHO1	Hs.96485	FCH domain only 1	Homo sapiens FCH domain only 1 (FCHO1), transcript variant 1, mRNA [NM_001161357]	3.16293	up	3.15349	up	2036.98400	666.85504	1496.57320	418.47750	Detected	Detected	Detected	Detected
1867	A_33_P3413098	LOC100129550	Hs.61558	hypothetical LOC100129550	Homo sapiens hypothetical LOC100129550 (LOC100129550), non-coding RNA [NR_024618]	3.16284	up	3.10889	up	7576.69000	2480.47800	4264.29400	1209.50260	Detected	Detected	Detected	Detected

1868	A_33_P3237135	MMP2	Hs.513617	matrix metallopeptidase 2 (gelatinase A, 72kDa gelatinase, 72kDa type IV collagenase)	Homo sapiens matrix metallopeptidase 2 (gelatinase A, 72kDa gelatinase, 72kDa type IV collagenase) (MMP2), transcript variant 1, mRNA [NM_004530]	3.16127	up	3.00790	up	23241.45300	7612.63040	17475.42000	5123.05660	Detected	Detected	Detected	Detected
1869	A_33_P3408514	SCNN1D	Hs.512681	sodium channel, nonvoltage-gated 1, delta	Homo sapiens sodium channel, nonvoltage-gated 1, delta (SCNN1D), transcript variant 2, mRNA [NM_002978]	3.15976	up	2.76239	up	17.88340	5.86043	21.79800	6.95821	Detected	Compromised	Detected	Compromised
1870	A_33_P3235330	MTMR3	Hs.474536	myotubularin related protein 3	Homo sapiens myotubularin related protein 3 (MTMR3), transcript variant 3, mRNA [NM_021090]	3.15795	up	3.76015	up	825.26196	270.59534	504.27518	118.25720	Detected	Detected	Detected	Detected
1871	A_33_P3363485	SOBP	Hs.445244	sine oculis binding protein homolog (Drosophila)	Homo sapiens sine oculis binding protein homolog (Drosophila) (SOBP), mRNA [NM_018013]	3.15180	up	5.52492	up	39.57497	13.00156	25.01229	3.99202	Detected	Compromised	Detected	Compromised
1872	A_24_P89891	TRAF1	Hs.531251	TNF receptor-associated factor 1	Homo sapiens TNF receptor-associated factor 1 (TRAF1), mRNA [NM_005658]	3.14990	up	3.54761	up	299.98737	98.61404	181.89702	45.21218	Detected	Detected	Detected	Detected
1873	A_33_P3214988	EFCAB1	Hs.23245	EF-hand calcium binding domain 1	Homo sapiens EF-hand calcium binding domain 1 (EFCAB1), transcript variant 2, mRNA [NM_001142857]	3.14781	up	5.74003	up	177.49733	58.38709	138.04059	21.20596	Detected	Detected	Detected	Detected
1874	A_23_P412562	C1orf162	Hs.288010	chromosome 1 open reading frame 162	Homo sapiens chromosome 1 open reading frame 162 (C1orf162), mRNA [NM_174896]	3.14708	up	2.74358	up	25.21941	8.29774	31.98726	10.28074	Detected	Compromised	Detected	Compromised
1875	A_33_P3301221	LOC100129110	Hs.642701	hypothetical protein LOC100129110	Homo sapiens cDNA FLJ46574 fis, clone THYMU3041736. [AK128431]	3.14608	up	3.45581	up	395.17020	130.06122	318.39996	81.24361	Detected	Detected	Detected	Detected
1876	A_24_P126139	RAB9B	Hs.522736	RAB9B, member RAS oncogene family	Ras-related protein Rab-9B (Rab-9L)(RAB9-like protein) [Source:UniProtKB/Swiss-Prot;Acc:Q9NP90] [ENST00000243298]	3.14055	up	5.05309	up	41.43655	13.66188	17.73630	3.09508	Detected	Compromised	Detected	Compromised
1877	A_23_P37623	GOLGA8A	Hs.720151	golgi autoantigen, golgin subfamily a, 8A	Homo sapiens golgi autoantigen, golgin subfamily a, 8A (GOLGA8A), transcript variant 1, mRNA [NM_181077]	3.14025	up	3.32771	up	1228.10120	404.95163	694.89670	184.13658	Detected	Detected	Detected	Detected
1878	A_23_P85716	FCGR2A	Hs.352642	Fc fragment of IgG, low affinity IIa, receptor (CD32)	Homo sapiens Fc fragment of IgG, low affinity IIa, receptor (CD32) (FCGR2A), transcript variant 2, mRNA [NM_021642]	3.13698	up	1.60141	up	62.22174	20.53822	73.48319	40.46230	Detected	Detected	Detected	Detected
1879	A_23_P56734	HNMT	Hs.42151	histamine N-methyltransferase	Homo sapiens histamine N-methyltransferase (HNMT), transcript variant 1, mRNA [NM_006895]	3.13659	up	3.88200	up	427.34088	141.07480	414.78076	94.21686	Detected	Detected	Detected	Detected
1880	A_23_P60259	TMEM38B	Hs.411925	transmembrane protein 38B	Homo sapiens transmembrane protein 38B (TMEM38B), mRNA [NM_018112]	3.13619	up	3.51590	up	2997.45700	989.65430	2365.52370	593.27484	Detected	Detected	Detected	Detected
1881	A_33_P3383029	MXI1	Hs.501023	MAX interactor 1	Homo sapiens MAX interactor 1 (MXI1), transcript variant 2, mRNA [NM_130439]	3.13593	up	3.23566	up	534.98706	176.64840	539.31830	146.97638	Detected	Detected	Detected	Detected
1882	A_23_P208334	PDE4A	Hs.89901	phosphodiesterase 4A, cAMP-specific (phosphodiesterase E2 dunce homolog, Drosophila)	Homo sapiens phosphodiesterase 4A, cAMP-specific (phosphodiesterase E2 dunce homolog, Drosophila) (PDE4A), transcript variant 4, mRNA [NM_006202]	3.13344	up	2.85476	up	155.30626	51.32163	171.96982	53.11884	Detected	Detected	Detected	Detected
1883	A_23_P344568	FAM124A	Hs.71913	family with sequence similarity 124A	Homo sapiens family with sequence similarity 124A (FAM124A), mRNA [NM_145019]	3.13284	up	2.15314	up	52.94270	17.49849	40.19567	16.46163	Detected	Compromised	Detected	Detected
1884	A_23_P408675	BTAF1	Hs.500526	BTAF1 RNA polymerase II, B-TFIID transcription factor-associated, 170kDa (Mot1 homolog, S. cerevisiae)	Homo sapiens BTAF1 RNA polymerase II, B-TFIID transcription factor-associated, 170kDa (Mot1 homolog, S. cerevisiae) (BTAF1), mRNA [NM_003972]	3.13265	up	3.33716	up	924.36370	305.53738	933.43176	246.64421	Detected	Detected	Detected	Detected
1885	A_33_P3762918	LOC100216546	Hs.369356	hypothetical LOC100216546	Homo sapiens cDNA FLJ41601 fis, clone CTONG2027327 [AK123595]	3.13150	up	3.21297	up	2685.75220	888.06840	1694.06620	464.93207	Detected	Detected	Detected	Detected
1886	A_23_P144096	CISH	Hs.655334	cytokine inducible SH2-containing protein	Homo sapiens cytokine inducible SH2-containing protein (CISH), transcript variant 2, mRNA [NM_145071]	3.13141	up	3.93848	up	611.42670	202.17950	372.81656	83.47033	Detected	Detected	Detected	Detected
1887	A_33_P3409337	C14orf45	Hs.644621	chromosome 14 open reading frame 45	Homo sapiens chromosome 14 open reading frame 45 (C14orf45), mRNA [NM_025057]	3.12996	up	2.55546	up	117.78099	38.96448	114.56307	39.53124	Detected	Detected	Detected	Detected
1888	A_23_P75800	RAB3IL1	Hs.13759	RAB3A interacting protein (rabin3)-like 1	Homo sapiens RAB3A interacting protein (rabin3)-like 1 (RAB3IL1), mRNA [NM_013401]	3.12996	up	2.78527	up	648.67303	214.59497	803.25195	254.30240	Detected	Detected	Detected	Detected
1889	A_33_P3224307	SCARNA12	Hs.689636	small Cajal body-specific RNA 12	Homo sapiens small Cajal body-specific RNA 12 (SCARNA12), guide RNA [NR_003010]	3.12958	up	2.82364	up	859.88800	284.50446	787.28845	245.86115	Detected	Detected	Detected	Detected
1890	A_33_P3217609		Hs.435044		TBC1 domain family member 22A [Source:UniProtKB/Swiss-Prot;Acc:Q8WUA7] [ENST00000380995]	3.12823	up	2.99467	up	91.85586	30.40470	73.24652	21.56771	Detected	Detected	Detected	Detected
1891	A_23_P217428	ARHGAP6	Hs.435291	Rho GTPase activating protein 6	Homo sapiens Rho GTPase activating protein 6 (ARHGAP6), transcript variant 1, mRNA [NM_013427]	3.12746	up	4.61496	up	193.71094	64.13506	100.44762	19.19276	Detected	Detected	Detected	Detected
1892	A_33_P3390823					3.12719	up	2.34970	up	2317.59300	767.38970	1903.72630	714.42535	Detected	Detected	Detected	Detected
1893	A_23_P67785	SPAG16	Hs.602792	sperm associated antigen 16	Homo sapiens sperm associated antigen 16 (SPAG16), transcript variant 2, mRNA [NM_001025436]	3.12511	up	3.40848	up	839.14770	278.03918	828.13916	214.24380	Detected	Detected	Detected	Detected
1894	A_33_P3326713	FAM188B	Hs.660192	family with sequence similarity 188, member B	Homo sapiens family with sequence similarity 188, member B (FAM188B), mRNA [NM_032222]	3.12495	up	2.75080	up	180.31573	59.74796	119.33266	38.25306	Detected	Detected	Detected	Detected
1895	A_33_P3307363	LPHN2	Hs.24212	latrophilin 2	Homo sapiens latrophilin 2 (LPHN2), mRNA [NM_012302]	3.12357	up	3.10102	up	3728.10380	1235.86060	2523.89720	717.68335	Detected	Detected	Detected	Detected
1896	A_23_P203115	TMEM25	Hs.564188	transmembrane protein 25	Homo sapiens transmembrane protein 25 (TMEM25), transcript variant 1, mRNA [NM_032780]	3.11875	up	2.97520	up	1422.11150	472.15656	1182.72740	350.53668	Detected	Detected	Detected	Detected
1897	A_33_P3342802	LOC649294	Hs.588395	hypothetical LOC649294	Homo sapiens cDNA FLJ33940 fis, clone CTONG2018069 [AK091259]	3.11873	up	1.62306	up	21.87080	7.26140	6.50987	3.53674	Detected	Compromised	Compromised	Compromised

1898	A_23_P160567	ZMYND12	Hs.294009	zinc finger, MYND-type containing 12	Homo sapiens zinc finger, MYND-type containing 12 (ZMYND12), transcript variant 1, mRNA [NM_032257]	3.11831	up	3.28305	up	26.71981	8.87254	27.62315	7.41927	Detected	Compromised	Detected	Compromised
1899	A_33_P3407746	PPM1B	Hs.416769	protein phosphatase 1B (formerly 2C), magnesium-dependent, beta isoform	Homo sapiens protein phosphatase 1B (formerly 2C), magnesium-dependent, beta isoform (PPM1B), transcript variant 2, mRNA [NM_177968]	3.11657	up	1.94805	up	17.91791	5.95311	11.92083	5.39601	Detected	Compromised	Compromised	Compromised
1900	A_33_P3257187	PRKAA1	Hs.43322	protein kinase, AMP-activated, alpha 1 catalytic subunit	Homo sapiens protein kinase, AMP-activated, alpha 1 catalytic subunit (PRKAA1), transcript variant 2, mRNA [NM_206907]	3.11414	up	3.39111	up	1046.12800	347.84064	661.96720	172.13160	Detected	Detected	Detected	Detected
1901	A_23_P90099	TMEM205	Hs.8036	transmembrane protein 205	Homo sapiens transmembrane protein 205 (TMEM205), transcript variant 1, mRNA [NM_198536]	3.11371	up	3.05882	up	47074.19500	15654.46000	50075.70000	14435.72700	Detected	Detected	Detected	Detected
1902	A_33_P3260733	GHR	Hs.125180	growth hormone receptor	Homo sapiens growth hormone receptor (GHR), mRNA [NM_000163]	3.11368	up	1.96998	up	29.98511	9.97161	21.49529	9.62162	Detected	Compromised	Detected	Compromised
1903	A_23_P309207	ZNF577	Hs.148322	zinc finger protein 577	Homo sapiens zinc finger protein 577 (ZNF577), transcript variant 1, mRNA [NM_032679]	3.11307	up	3.44411	up	295.31113	98.22547	160.04716	40.97667	Detected	Detected	Detected	Detected
1904	A_33_P3221960	IL18RAP	Hs.158315	interleukin 18 receptor accessory protein	Homo sapiens interleukin 18 receptor accessory protein (IL18RAP), mRNA [NM_003853]	3.11252	up	12.43740	up	1680.15580	558.94635	689.36346	48.87470	Detected	Detected	Detected	Detected
1905	A_23_P79931	ATRN	Hs.276252	attractin	Homo sapiens attractin (ATRN), transcript variant 2, mRNA [NM_139322]	3.11189	up	2.84631	up	5378.37200	1789.61820	5075.10940	1572.27420	Detected	Detected	Detected	Detected
1906	A_33_P3318696	CCBL2	Hs.481898	cysteine conjugate-beta lyase 2	Homo sapiens cysteine conjugate-beta lyase 2 (CCBL2), transcript variant 1, mRNA [NM_001008661]	3.10756	up	3.53023	up	1345.83180	448.43970	1063.09390	265.54290	Detected	Detected	Detected	Detected
1907	A_23_P257003	PCSK5	Hs.368542	proprotein convertase subtilisin/kexin type 5	Homo sapiens proprotein convertase subtilisin/kexin type 5 (PCSK5), mRNA [NM_006200]	3.10711	up	3.15117	up	3113.60700	1037.62380	2368.50600	662.77856	Detected	Detected	Detected	Detected
1908	A_24_P252364	NRCAM	Hs.21422	neuronal cell adhesion molecule	Homo sapiens neuronal cell adhesion molecule (NRCAM), transcript variant 1, mRNA [NM_001037132]	3.10625	up	3.03230	up	2559.17260	853.09230	1556.44530	452.61325	Detected	Detected	Detected	Detected
1909	A_23_P126836	TNFSF4	Hs.181097	tumor necrosis factor (ligand) superfamily, member 4	Homo sapiens tumor necrosis factor (ligand) superfamily, member 4 (TNFSF4), mRNA [NM_003326]	3.10589	up	3.38855	up	87.57895	29.19755	50.33538	13.09862	Detected	Detected	Detected	Compromised
1910	A_23_P343261	C1orf211	Hs.185688	chromosome 1 open reading frame 211	Homo sapiens chromosome 1 open reading frame 211, mRNA (cDNA clone MGC:40168 IMAGE:5141008), complete cds [BC030279]	3.10345	up	3.65721	up	105.40451	35.16799	142.92451	34.46053	Detected	Detected	Detected	Detected
1911	A_24_P541919	DENND5B	Hs.118166	DENN/MADD domain containing 5B	Homo sapiens DENN/MADD domain containing 5B (DENND5B), mRNA [NM_144973]	3.10040	up	3.17076	up	760.60920	254.02534	527.85670	146.79733	Detected	Detected	Detected	Detected
1912	A_33_P3230594				GB	3.09829	up	3.61667	up	868.46870	290.24560	547.20870	133.41669	Detected	Detected	Detected	Detected
1913	A_23_P403424	JMJD7-PLA2G4B	Hs.198161	JMJD7-PLA2G4B readthrough transcript	Homo sapiens JMJD7-PLA2G4B readthrough transcript (JMJD7-PLA2G4B), transcript variant 1, mRNA [NM_005090]	3.09802	up	3.51634	up	1220.54770	407.94740	1173.06000	294.16785	Detected	Detected	Detected	Detected
1914	A_24_P409126	FNDC3A	Hs.508010	fibronectin type III domain containing 3A	Homo sapiens cDNA FLJ31509 fis, clone NT2RI1000016. [AK056071]	3.09766	up	6.04483	up	39.30961	13.14010	21.45673	3.13000	Detected	Compromised	Detected	Compromised
1915	A_23_P118615	ABCA8	Hs.58351	ATP-binding cassette, sub-family A (ABC1), member 8	Homo sapiens ATP-binding cassette, sub-family A (ABC1), member 8 (ABCA8), mRNA [NM_007168]	3.09634	up	2.14435	up	65.97136	22.06179	66.81093	27.47368	Detected	Detected	Detected	Detected
1916	A_24_P379820	ITM2C	Hs.111577	integral membrane protein 2C	Homo sapiens integral membrane protein 2C (ITM2C), transcript variant 1, mRNA [NM_030926]	3.09436	up	2.10207	up	4030.14200	1348.59690	4211.08740	1766.49800	Detected	Detected	Detected	Detected
1917	A_23_P81973	HSD17B8	Hs.415058	hydroxysteroid (17-beta) dehydrogenase 8	Homo sapiens hydroxysteroid (17-beta) dehydrogenase 8 (HSD17B8), mRNA [NM_014234]	3.09401	up	3.39091	up	448.73563	150.17627	512.92040	133.38261	Detected	Detected	Detected	Detected
1918	A_33_P3269636	SBSN	Hs.433484	suprabasin	Homo sapiens suprabasin (SBSN), transcript variant 1, mRNA [NM_001166034]	3.09319	up	2.72999	up	25099.49600	8402.18400	20362.87900	6577.24460	Detected	Detected	Detected	Detected
1919	A_33_P3424800	HLA-B	Hs.77961	major histocompatibility complex, class I, B	Homo sapiens major histocompatibility complex, class I, B (HLA-B), mRNA [NM_005514]	3.09248	up	2.53489	up	159327.45000	53347.90600	55503.57000	19307.57400	Detected	Detected	Detected	Detected
1920	A_33_P3326225	SAMD12	Hs.359393	sterile alpha motif domain containing 12	Homo sapiens sterile alpha motif domain containing 12 (SAMD12), transcript variant 1, mRNA [NM_001101676]	3.08890	up	4.50178	up	32.08853	10.75671	26.68708	5.22736	Detected	Compromised	Detected	Compromised
1921	A_33_P3390778	TRIM46	Hs.287735	tripartite motif-containing 46	Homo sapiens tripartite motif-containing 46 (TRIM46), mRNA [NM_025058]	3.08631	up	3.13705	up	396.77448	133.11818	370.97623	104.27741	Detected	Detected	Detected	Detected
1922	A_23_P78980	B3GNT3	Hs.69009	UDP-GlcNAc:betaGal beta-1,3-N-acetylglucosaminyltransferase 3	Homo sapiens UDP-GlcNAc:betaGal beta-1,3-N-acetylglucosaminyltransferase 3 (B3GNT3), mRNA [NM_014256]	3.08402	up	7.82021	up	149.43481	50.17279	127.58443	14.38616	Detected	Detected	Detected	Compromised
1923	A_23_P377141	VPS13A	Hs.459790	vacuolar protein sorting 13 homolog A (S. cerevisiae)	Homo sapiens vacuolar protein sorting 13 homolog A (S. cerevisiae) (VPS13A), transcript variant B, mRNA [NM_015186]	3.08204	up	2.92573	up	339.35254	114.01082	156.21278	47.08122	Detected	Detected	Detected	Detected
1924	A_24_P235783	SF1	Hs.502829	splicing factor 1	Homo sapiens splicing factor 1 (SF1), transcript variant 4, mRNA [NM_201997]	3.08179	up	3.22868	up	84.96043	28.54606	54.26468	14.82034	Detected	Detected	Detected	Detected
1925	A_33_P3211929	RCOR2	Hs.98788	REST corepressor 2	Homo sapiens REST corepressor 2 (RCOR2), mRNA [NM_173587]	3.08084	up	2.89297	up	2262.04470	760.26434	1265.19950	385.63880	Detected	Detected	Detected	Detected
1926	A_33_P3665553	FLJ44253	Hs.595418	hypothetical gene supported by AK096952; AK126241; BC068588	Homo sapiens cDNA FLJ44253 fis, clone TKIDN2009092 [AK126241]	3.07994	up	5.51299	up	47.49411	15.96725	29.68685	4.74835	Detected	Compromised	Detected	Compromised
1927	A_23_P54918	LDHD	Hs.380929	lactate dehydrogenase D	Homo sapiens lactate dehydrogenase D (LDHD), nuclear gene encoding mitochondrial protein, transcript variant 1, mRNA [NM_153486]	3.07794	up	3.29567	up	52.11274	17.53140	49.97242	13.37062	Detected	Compromised	Detected	Compromised

1928	A_33_P3340490	FLJ46906	Hs.92290	hypothetical protein LOC441172	PREDICTED: Homo sapiens hypothetical protein LOC441172 (FLJ46906), mRNA [XM_001717237]	3.07553	up	3.31028	up	1584.79680	533.56445	1720.54000	458.31620	Detected	Detected	Detected	Detected
1929	A_23_P307536	C1orf113	Hs.524496	chromosome 1 open reading frame 113	SH3 domain-containing protein C1orf113 [Source:UniProtKB/Swiss-Prot;Acc:A4FU49] [ENST00000453908]	3.07436	up	3.25304	up	279.54670	94.15263	189.11418	51.26257	Detected	Detected	Detected	Detected
1930	A_23_P132718	SEMA3B	Hs.82222	sema domain, immunoglobulin domain (Ig), short basic domain, secreted, (semaphorin) 3B	Homo sapiens sema domain, immunoglobulin domain (Ig), short basic domain, secreted, (semaphorin) 3B (SEMA3B), transcript variant 1, mRNA [NM_004636]	3.07431	up	3.08225	up	1335.92570	449.95407	1290.79610	369.27994	Detected	Detected	Detected	Detected
1931	A_24_P943193	PREPL	Hs.444349	prolyl endopeptidase-like	Homo sapiens prolyl endopeptidase-like (PREPL), transcript variant C, mRNA [NM_006036]	3.07430	up	3.41274	up	1423.68690	479.51392	747.68835	193.18945	Detected	Detected	Detected	Detected
1932	A_33_P3289296	TMEM37	Hs.26216	transmembrane protein 37	Homo sapiens transmembrane protein 37 (TMEM37), mRNA [NM_183240]	3.07377	up	6.22575	up	196.58163	66.22242	140.02962	19.83323	Detected	Detected	Detected	Detected
1933	A_24_P137522	USP53	Hs.431081	ubiquitin specific peptidase 53	Homo sapiens ubiquitin specific peptidase 53 (USP53), mRNA [NM_019050]	3.07296	up	3.11116	up	1197.33700	403.45297	1145.90860	324.78256	Detected	Detected	Detected	Detected
1934	A_23_P74278	PDE4B	Hs.198072	phosphodiesterase 4B, cAMP-specific (phosphodiesterase E4 dunce homolog, Drosophila)	Homo sapiens phosphodiesterase 4B, cAMP-specific (phosphodiesterase E4 dunce homolog, Drosophila) (PDE4B), transcript variant d, mRNA [NM_001037341]	3.07171	up	3.13935	up	2477.39900	835.11860	2080.30760	584.32416	Detected	Detected	Detected	Detected
1935	A_33_P3309414	LOC643770	Hs.129589	hypothetical LOC643770	Homo sapiens cDNA FLJ44867 fis, clone BRALZ2017607 [AK126817]	3.07019	up	3.03724	up	23.86752	8.04962	16.72893	4.85684	Detected	Compromised	Detected	Compromised
1936	A_24_P93703	LOC440104	Hs.616500	hypothetical LOC440104	Homo sapiens cDNA FLJ32617 fis, clone STOMA2000257. [AK057179]	3.07015	up	3.09807	up	1645.30820	554.90810	1609.28330	458.04422	Detected	Detected	Detected	Detected
1937	A_23_P151529	C14orf132	Hs.6434	chromosome 14 open reading frame 132	Homo sapiens chromosome 14 open reading frame 132 (C14orf132), non-coding RNA [NR_023938]	3.06968	up	2.84417	up	3387.01050	1142.49960	2517.54030	780.52510	Detected	Detected	Detected	Detected
1938	A_24_P540057	CA5A	Hs.177446	carbonic anhydrase VA, mitochondrial	Homo sapiens carbonic anhydrase VA, mitochondrial (CA5A), nuclear gene encoding mitochondrial protein, mRNA [NM_001739]	3.06934	up	2.44985	up	52.43564	17.68948	57.16416	20.57550	Detected	Detected	Detected	Detected
1939	A_24_P63019	IL1R2	Hs.25333	interleukin 1 receptor, type II	Homo sapiens interleukin 1 receptor, type II (IL1R2), transcript variant 1, mRNA [NM_004633]	3.06924	up	2.16310	up	53.48637	18.04451	26.17889	10.67185	Detected	Compromised	Detected	Compromised
1940	A_33_P3323842	BDNFOS	Hs.675323	BDNF opposite strand (non-protein coding)	Homo sapiens BDNF opposite strand (non-protein coding) (BDNFOS), non-coding RNA [NR_002832]	3.06885	up	3.78847	up	286.20914	96.56967	154.44821	35.94885	Detected	Detected	Detected	Detected
1941	A_23_P110412	TMEM150C	Hs.720835	transmembrane protein 150C	Homo sapiens transmembrane protein 150C (TMEM150C), mRNA [NM_001080506]	3.06618	up	5.74081	up	68.57480	23.15794	55.91653	8.58880	Detected	Not Detected	Detected	Compromised
1942	A_23_P357966	PCMTD1	Hs.671268	protein-L-isoaspartate (D-aspartate) O-methyltransferase domain containing 1	Homo sapiens protein-L-isoaspartate (D-aspartate) O-methyltransferase domain containing 1 (PCMTD1), mRNA [NM_052937]	3.06020	up	3.32597	up	285.84875	96.72089	28.82434	7.64200	Detected	Detected	Detected	Compromised
1943	A_23_P161686	RICS	Hs.440379	Rho GTPase-activating protein	Homo sapiens Rho GTPase-activating protein (RICS), transcript variant 2, mRNA [NM_014715]	3.05630	up	3.76163	up	380.65897	128.96529	255.25388	59.83596	Detected	Detected	Detected	Detected
1944	A_33_P3676515	C21orf122	Hs.309203	chromosome 21 open reading frame 122	Homo sapiens chromosome 21 open reading frame 122 (C21orf122), non-coding RNA [NR_027292]	3.05575	up	2.63242	up	984.71893	333.67800	696.38110	233.26941	Detected	Detected	Detected	Detected
1945	A_23_P85952	DENND2D	Hs.557850	DENN/MADD domain containing 2D	Homo sapiens DENN/MADD domain containing 2D (DENND2D), mRNA [NM_024901]	3.05549	up	2.58870	up	237.78024	80.58021	185.11035	63.05417	Detected	Detected	Detected	Detected
1946	A_33_P3342663	ADAM33	Hs.173716	ADAM metallopeptidase domain 33	Homo sapiens ADAM metallopeptidase domain 33 (ADAM33), transcript variant 2, mRNA [NM_153202]	3.05537	up	2.37439	up	80.84690	27.39890	74.99921	27.85285	Detected	Detected	Detected	Detected
1947	A_23_P200710	PIK3C2B	Hs.497487	phosphoinositide-3-kinase, class 2, beta polypeptide	Homo sapiens phosphoinositide-3-kinase, class 2, beta polypeptide (PIK3C2B), mRNA [NM_002646]	3.05523	up	3.83427	up	60.94561	20.65533	50.10544	11.52305	Detected	Detected	Detected	Compromised
1948	A_33_P3317618	SYN2	Hs.445503	synapsin II	Homo sapiens synapsin II (SYN2), transcript variant IIb, mRNA [NM_003178]	3.05502	up	3.12982	up	113.04173	38.31403	74.20045	20.90509	Detected	Detected	Detected	Detected
1949	A_32_P430695	C3orf35	Hs.475945	chromosome 3 open reading frame 35	Homo sapiens chromosome 3 open reading frame 35 (C3orf35), transcript variant B, mRNA [NM_178339]	3.05315	up	4.60254	up	67.93494	23.03980	48.45763	9.28389	Detected	Detected	Detected	Compromised
1950	A_23_P153676	TLE2	Hs.332173	transducin-like enhancer of split 2 (E(sp1) homolog, Drosophila)	Homo sapiens transducin-like enhancer of split 2 (E(sp1) homolog, Drosophila) (TLE2), transcript variant 1, mRNA [NM_003260]	3.05304	up	2.96488	up	645.62850	218.96901	633.44830	188.39542	Detected	Detected	Detected	Detected
1951	A_33_P3407529	PRRT4	Hs.381096	proline-rich transmembrane protein 4	Homo sapiens proline-rich transmembrane protein 4 (PRRT4), mRNA [NM_001114726]	3.05128	up	3.76249	up	63.65750	21.60231	45.81823	10.73814	Detected	Detected	Detected	Compromised
1952	A_33_P3393766	C17orf96	Hs.445574	chromosome 17 open reading frame 96	Homo sapiens chromosome 17 open reading frame 96 (C17orf96), mRNA [NM_001130677]	3.05124	up	3.19321	up	8132.76700	2759.91020	6416.18950	1771.80270	Detected	Detected	Detected	Detected
1953	A_33_P3327822	SH3BGR	Hs.473847	SH3 domain binding glutamic acid-rich protein	Homo sapiens SH3 domain binding glutamic acid-rich protein (SH3BGR), transcript variant 1, mRNA [NM_007341]	3.05068	up	3.00459	up	421.20980	142.96670	443.56952	130.17944	Detected	Detected	Detected	Detected
1954	A_33_P3330443	FAM110B	Hs.154652	family with sequence similarity 110, member B	Homo sapiens family with sequence similarity 110, member B (FAM110B), mRNA [NM_147189]	3.04690	up	3.59160	up	606.44620	206.09460	406.22354	99.73395	Detected	Detected	Detected	Detected
1955	A_24_P380679	C7orf53	Hs.396189	chromosome 7 open reading frame 53	Homo sapiens chromosome 7 open reading frame 53 (C7orf53), transcript variant 1, mRNA [NM_182597]	3.04634	up	3.22839	up	295.29608	100.37212	218.04488	59.55600	Detected	Detected	Detected	Detected
1956	A_23_P85180	TMEM187	Hs.23119	transmembrane protein 187	Homo sapiens transmembrane protein 187 (TMEM187), mRNA [NM_003492]	3.04620	up	3.00097	up	2192.94780	745.42220	1148.21600	337.38623	Detected	Detected	Detected	Detected

1957	A_33_P3275873	SFTPB	Hs.512690	surfactant protein B	Homo sapiens surfactant protein B (SFTPB), transcript variant 2, mRNA [NM_198843]	3.04618	up	4.23378	up	11.17468	3.79850	15.54381	3.23739	Compromised	Compromised	Detected	Compromised
1958	A_23_P359897	JHDM1D	Hs.308710	jumonji C domain containing histone demethylase 1 homolog D (S. cerevisiae)	Homo sapiens jumonji C domain containing histone demethylase 1 homolog D (S. cerevisiae) (JHDM1D), mRNA [NM_030647]	3.04476	up	2.98343	up	1511.70030	514.09863	1446.97490	427.67123	Detected	Detected	Detected	Detected
1959	A_33_P3377239	LOC100270804	Hs.598768	hypothetical LOC100270804	Homo sapiens hypothetical LOC100270804 (LOC100270804), non-coding RNA [NR_026885]	3.04325	up	3.35414	up	342.58307	116.56322	321.20905	84.44455	Detected	Detected	Detected	Detected
1960	A_24_P148094	LEPROT	Hs.23581	leptin receptor overlapping transcript	Homo sapiens leptin receptor overlapping transcript (LEPROT), mRNA [NM_017526]	3.04201	up	3.39559	up	5808.13600	1977.00980	3804.80350	988.05740	Detected	Detected	Detected	Detected
1961	A_32_P141969	NTRK3	Hs.185701	neurotrophic tyrosine kinase, receptor, type 3	NT-3 growth factor receptor Precursor (EC 2.7.10.1)(Neurotrophic tyrosine kinase receptor type 3)(TrkC tyrosine kinase)(GP145-TrkC)(Trk-C) [Source:UniProtKB/Swiss-Prot;Acc:Q16288] [ENST00000394480]	3.04195	up	2.90889	up	20.53707	6.99068	17.46781	5.29513	Detected	Compromised	Detected	Compromised
1962	A_33_P3266889					3.04060	up	3.27378	up	366.57760	124.83607	120.72449	32.51704	Detected	Detected	Detected	Detected
1963	A_24_P194881	SHANK3	Hs.149035	SH3 and multiple ankyrin repeat domains 3	Homo sapiens SH3 and multiple ankyrin repeat domains 3 (SHANK3), mRNA [NM_001080420]	3.04060	up	2.84426	up	1950.59170	664.26416	1894.82500	587.44226	Detected	Detected	Detected	Detected
1964	A_23_P114740	CFH	Hs.363396	complement factor H	Homo sapiens complement factor H (CFH), transcript variant 1, mRNA [NM_000186]	3.03943	up	2.86996	up	5491.11000	1870.68550	4243.48830	1303.80350	Detected	Detected	Detected	Detected
1965	A_24_P127075	TMEM135	Hs.188591	transmembrane protein 135	Homo sapiens transmembrane protein 135 (TMEM135), mRNA [NM_022918]	3.03699	up	2.54811	up	35.41678	12.07530	21.68153	7.50305	Detected	Compromised	Detected	Compromised
1966	A_23_P372467	AHSA2	Hs.655602	AHA1, activator of heat shock 90kDa protein ATPase homolog 2 (yeast)	Homo sapiens AHA1, activator of heat shock 90kDa protein ATPase homolog 2 (yeast) (AHSA2), mRNA [NM_152392]	3.03553	up	3.03152	up	5222.73140	1781.54480	4575.80270	1330.98340	Detected	Detected	Detected	Detected
1967	A_23_P143147	TCFL5	Hs.126248	transcription factor-like 5 (basic helix-loop-helix)	Homo sapiens transcription factor-like 5 (basic helix-loop-helix) (TCFL5), mRNA [NM_006602]	3.03498	up	3.06983	up	4070.68380	1388.81450	2600.19700	746.89056	Detected	Detected	Detected	Detected
1968	A_33_P3351027	PXMP3	Hs.437966	peroxisomal membrane protein 3, 35kDa	Homo sapiens peroxisomal membrane protein 3, 35kDa (PXMP3), transcript variant 1, mRNA [NM_000318]	3.03109	up	6.38722	up	75.50127	25.79222	60.17407	8.30736	Detected	Detected	Detected	Compromised
1969	A_24_P153456	ZDHHC11	Hs.654874	zinc finger, DHHC-type containing 11	Homo sapiens zinc finger, DHHC-type containing 11 (ZDHHC11), mRNA [NM_024786]	3.03080	up	2.62518	up	101.79839	34.77900	99.72676	33.49789	Detected	Detected	Detected	Detected
1970	A_24_P382026	MIF4GD	Hs.325631	MIF4G domain containing	Homo sapiens MIF4G domain containing (MIF4GD), mRNA [NM_020679]	3.02841	up	2.50107	up	492.82187	168.50314	572.13983	201.71667	Detected	Detected	Detected	Detected
1971	A_33_P3293213	RGL3	Hs.720291	ral guanine nucleotide dissociation stimulator-like 3	Homo sapiens ral guanine nucleotide dissociation stimulator-like 3 (RGL3), transcript variant 1, mRNA [NM_001161616]	3.02739	up	3.18240	up	63.59910	21.75284	94.55399	26.19935	Detected	Detected	Detected	Detected
1972	A_23_P399681	TSSK3	Hs.512763	testis-specific serine kinase 3	Homo sapiens testis-specific serine kinase 3 (TSSK3), mRNA [NM_052841]	3.02649	up	2.32472	up	83.65308	28.62038	72.10115	27.34877	Detected	Detected	Detected	Detected
1973	A_33_P3842770	LOC644450	Hs.652926	hypothetical protein LOC644450	Homo sapiens hypothetical protein LOC644450, mRNA (cDNA clone IMAGE:4606942), partial cds [BC022881]	3.02600	up	3.06750	up	629.92030	215.55092	544.23180	156.44640	Detected	Detected	Detected	Detected
1974	A_23_P169278	AGTPBP1	Hs.719980	ATP/GTP binding protein 1	Homo sapiens ATP/GTP binding protein 1 (AGTPBP1), mRNA [NM_015239]	3.02415	up	3.12577	up	1521.22640	520.86270	1091.82900	308.00854	Detected	Detected	Detected	Detected
1975	A_23_P100795	STAT3	Hs.463059	signal transducer and activator of transcription 3 (acute-phase response factor)	Homo sapiens signal transducer and activator of transcription 3 (acute-phase response factor) (STAT3), transcript variant 3, mRNA [NM_213662]	3.02345	up	2.76878	up	636.58484	218.01482	335.40182	106.81764	Detected	Detected	Detected	Detected
1976	A_24_P23258	GRAMD4	Hs.475150	GRAM domain containing 4	Homo sapiens GRAM domain containing 4 (GRAMD4), mRNA [NM_015124]	3.02080	up	3.48345	up	1365.56760	468.08524	949.24800	240.28992	Detected	Detected	Detected	Detected
1977	A_23_P202458	ZNF22	Hs.462693	zinc finger protein 22 (KOX 15)	Homo sapiens zinc finger protein 22 (KOX 15) (ZNF22), mRNA [NM_006963]	3.02066	up	2.93670	up	5688.99070	1950.14040	3826.23440	1148.88560	Detected	Detected	Detected	Detected
1978	A_23_P67367	DHDH	Hs.631555	dihydrodiol dehydrogenase (dimeric)	Homo sapiens dihydrodiol dehydrogenase (dimeric) (DHDH), mRNA [NM_014475]	3.01993	up	5.45467	up	134.07211	45.97004	115.67278	18.69942	Detected	Detected	Detected	Detected
1979	A_33_P3255304	GGT5	Hs.437156	gamma-glutamyltransferase 5	Homo sapiens gamma-glutamyltransferase 5 (GGT5), transcript variant 1, mRNA [NM_001099781]	3.01809	up	2.87783	up	11398.43600	3910.62040	10511.74400	3220.88010	Detected	Detected	Detected	Detected
1980	A_33_P3260722	UVRAG	Hs.719175	UV radiation resistance associated gene	Homo sapiens UV radiation resistance associated gene (UVRAG), mRNA [NM_003369]	3.01799	up	2.71667	up	339.56033	116.50182	243.02728	78.88307	Detected	Detected	Detected	Detected
1981	A_33_P3401782	C19orf54	Hs.585105	chromosome 19 open reading frame 54	Homo sapiens chromosome 19 open reading frame 54 (C19orf54), mRNA [NM_198476]	3.01571	up	3.72068	up	43.22903	14.84289	61.06197	14.47151	Detected	Compromised	Detected	Detected
1982	A_24_P114032	EFNA3	Hs.516656	ephrin-A3	Homo sapiens ephrin-A3 (EFNA3), mRNA [NM_004952]	3.01175	up	1.58570	up	21.10319	7.25542	17.46218	9.71052	Detected	Compromised	Detected	Compromised
1983	A_23_P400603	MGC21881	Hs.721449	hypothetical locus MGC21881	Homo sapiens hypothetical locus MGC21881 (MGC21881), non-coding RNA [NR_015363]	3.00818	up	4.81193	up	133.80460	46.05748	63.02007	11.54849	Detected	Detected	Detected	Compromised
1984	A_23_P40059	PMS1	Hs.111749	PMS1 postmeiotic segregation increased 1 (S. cerevisiae)	Homo sapiens PMS1 postmeiotic segregation increased 1 (S. cerevisiae) (PMS1), transcript variant 1, mRNA [NM_000534]	3.00743	up	3.15183	up	1165.18870	401.17540	851.95557	238.35237	Detected	Detected	Detected	Detected

1985	A_33_P3363720	C1orf213	Hs.61884	chromosome 1 open reading frame 213	Homo sapiens chromosome 1 open reading frame 213 (C1orf213), transcript variant 2, mRNA [NM_001008896]	3.00719	up	2.42749	up	193.36092	66.57955	248.85594	90.39758	Detected	Detected	Detected	Detected
1986	A_33_P3229953	EEF1A2	Hs.433839	eukaryotic translation elongation factor 1 alpha 2	Homo sapiens eukaryotic translation elongation factor 1 alpha 2 (EEF1A2), mRNA [NM_001958]	3.00700	up	3.36044	up	80.12863	27.59227	119.70182	31.41015	Detected	Detected	Detected	Detected
1987	A_23_P74778	C1orf54	Hs.91283	chromosome 1 open reading frame 54	Homo sapiens chromosome 1 open reading frame 54 (C1orf54), mRNA [NM_024579]	3.00566	up	3.20331	up	7380.55220	2542.62200	5828.62840	1604.47360	Detected	Detected	Detected	Detected
1988	A_24_P343621	ECHDC3	Hs.22242	enoyl Coenzyme A hydratase domain containing 3	Homo sapiens enoyl Coenzyme A hydratase domain containing 3 (ECHDC3), nuclear gene encoding mitochondrial protein, mRNA [NM_024693]	3.00505	up	2.59341	up	744.48160	256.52835	860.37750	292.53854	Detected	Detected	Detected	Detected
1989	A_24_P177604	PPP1R3F	Hs.433652	protein phosphatase 1, regulatory (inhibitor) subunit 3F	Homo sapiens protein phosphatase 1, regulatory (inhibitor) subunit 3F (PPP1R3F), mRNA [NM_033215]	3.00445	up	2.83727	up	405.06677	139.60307	405.28558	125.95813	Detected	Detected	Detected	Detected
1990	A_23_P328621	UBQLNL	Hs.10688	ubiquilin-like	Homo sapiens ubiquilin-like (UBQLNL), mRNA [NM_145053]	3.00227	up	1.72009	up	18.69742	6.44859	10.91021	5.59305	Detected	Compromised	Compromised	Compromised
1991	A_33_P3334313	ACTR3B	Hs.647117	ARP3 actin-related protein 3 homolog B (yeast)	Homo sapiens ARP3 actin-related protein 3 homolog B (yeast) (ACTR3B), transcript variant 1, mRNA [NM_020445]	2.99978	up	3.11399	up	759.95886	262.32153	628.76086	178.04646	Detected	Detected	Detected	Detected
1992	A_24_P110914	LOC100190939	Hs.375766	hypothetical LOC100190939	Homo sapiens hypothetical LOC100190939 (LOC100190939), non-coding RNA [NR_024458]	2.99876	up	5.15297	up	4986.03760	1721.65840	4266.35800	730.07070	Detected	Detected	Detected	Detected
1993	A_33_P3246258		Hs.675058		cDNA FLJ26009 fis, clone HRT03250 [Source:UniProtKB/TrEMBL;Acc:Q6ZPD4] [ENST00000400449]	2.99322	up	1.82041	up	11.53324	3.98976	12.97457	6.28475	Detected	Compromised	Detected	Compromised
1994	A_23_P321354	TMEM71	Hs.293842	transmembrane protein 71	Homo sapiens transmembrane protein 71 (TMEM71), transcript variant 1, mRNA [NM_144649]	2.99319	up	2.94951	up	137.41730	47.53796	113.90599	34.05359	Detected	Detected	Detected	Detected
1995	A_24_P468810	RBBP4	Hs.16003	retinoblastoma binding protein 4	Homo sapiens retinoblastoma binding protein 4 (RBBP4), transcript variant 1, mRNA [NM_005610]	2.99286	up	3.20335	up	2148.65010	743.38354	1851.00320	509.52820	Detected	Detected	Detected	Detected
1996	A_23_P166616	AGTR1	Hs.477887	angiotensin II receptor, type 1	Homo sapiens angiotensin II receptor, type 1 (AGTR1), transcript variant 4, mRNA [NM_031850]	2.99282	up	3.32278	up	74.66245	25.83180	73.51761	19.50990	Detected	Detected	Detected	Detected
1997	A_32_P161913	C1orf70	Hs.668654	chromosome 1 open reading frame 70	Homo sapiens chromosome 1 open reading frame 70 (C1orf70), mRNA [NM_001114748]	2.99255	up	2.30950	up	380.63254	131.70338	515.75360	196.91965	Detected	Detected	Detected	Detected
1998	A_33_P3268618	WDR19	Hs.438482	WD repeat domain 19	Homo sapiens WD repeat domain 19 (WDR19), mRNA [NM_025132]	2.99154	up	3.07527	up	8196.03000	2836.88280	6540.60800	1875.42900	Detected	Detected	Detected	Detected
1999	A_23_P12554	PKD2L1	Hs.159241	polycystic kidney disease 2-like 1	Homo sapiens polycystic kidney disease 2-like 1 (PKD2L1), mRNA [NM_016112]	2.99047	up	29.51362	up	13.20559	4.57249	82.15697	2.45464	Detected	Compromised	Detected	Compromised
2000	A_33_P3359115	LMBRD1	Hs.271643	LMBR1 domain containing 1	Homo sapiens LMBR1 domain containing 1 (LMBRD1), mRNA [NM_018368]	2.98950	up	3.06211	up	19315.27300	6690.13670	12400.31500	3570.89900	Detected	Detected	Detected	Detected
2001	A_32_P40463	NUDT9P1		nudix (nucleoside diphosphate linked moiety X)-type motif 9 pseudogene 1	Homo sapiens nudix (nucleoside diphosphate linked moiety X)-type motif 9 pseudogene 1 (NUDT9P1), non-coding RNA [NR_002779]	2.98845	up	2.80380	up	372.01352	128.89795	229.39113	72.14327	Detected	Detected	Detected	Detected
2002	A_33_P3422822	GJC2	Hs.100072	gap junction protein, gamma 2, 47kDa	Homo sapiens gap junction protein, gamma 2, 47kDa (GJC2), mRNA [NM_020435]	2.98770	up	2.97294	up	1117.76040	387.38718	1043.61670	309.54245	Detected	Detected	Detected	Detected
2003	A_32_P131050	ZNF148	Hs.592591	zinc finger protein 148	Homo sapiens zinc finger protein 148 (ZNF148), mRNA [NM_021964]	2.98676	up	2.88570	up	711.98510	246.83333	613.58154	187.49352	Detected	Detected	Detected	Detected
2004	A_33_P3302736	ZNF397OS	Hs.596302	zinc finger protein 397 opposite strand	Homo sapiens zinc finger protein 397 opposite strand (ZNF397OS), mRNA [NM_001166012]	2.98673	up	2.33170	up	26.56560	9.20994	22.31098	8.43746	Detected	Compromised	Detected	Compromised
2005	A_33_P3423440	ZNF720	Hs.528826	zinc finger protein 720	Homo sapiens zinc finger protein 720 (ZNF720), mRNA [NM_001130913]	2.98669	up	2.66325	up	417.56840	144.76729	286.51730	94.86462	Detected	Detected	Detected	Detected
2006	A_23_P310460	MDGA1	Hs.437993	MAM domain containing glycosylphosphatidylinositol anchor 1	Homo sapiens MAM domain containing glycosylphosphatidylinositol anchor 1 (MDGA1), mRNA [NM_153487]	2.98619	up	3.28066	up	286.82030	99.45467	234.49550	63.02875	Detected	Detected	Detected	Detected
2007	A_33_P3323945	CEND1	Hs.22140	cell cycle exit and neuronal differentiation 1	Homo sapiens cell cycle exit and neuronal differentiation 1 (CEND1), mRNA [NM_016564]	2.98350	up	2.88468	up	1715.48860	595.38070	1699.07700	519.37500	Detected	Detected	Detected	Detected
2008	A_23_P76983	C14orf45	Hs.644621	chromosome 14 open reading frame 45	Homo sapiens chromosome 14 open reading frame 45 (C14orf45), mRNA [NM_025057]	2.98296	up	2.85462	up	806.80884	280.06293	736.07227	227.37245	Detected	Detected	Detected	Detected
2009	A_33_P3251640	LOC284440		hypothetical LOC284440	Homo sapiens hypothetical LOC284440 (LOC284440), non-coding RNA [NR_026956]	2.97765	up	2.32221	up	44.76602	15.56710	7.06456	2.68256	Detected	Compromised	Compromised	Compromised
2010	A_24_P239076	IGLL1	Hs.348935	immunoglobulin lambda-like polypeptide 1	Homo sapiens immunoglobulin lambda-like polypeptide 1 (IGLL1), transcript variant 1, mRNA [NM_020070]	2.97757	up	2.79335	up	570.68030	198.45590	528.39716	166.80167	Detected	Detected	Detected	Detected
2011	A_23_P153026	GAA	Hs.1437	glucosidase, alpha; acid	Homo sapiens glucosidase, alpha; acid (GAA), transcript variant 1, mRNA [NM_000152]	2.97734	up	2.80461	up	14493.13400	5040.41850	13046.86300	4102.03960	Detected	Detected	Detected	Detected
2012	A_33_P3298062	ABCC5	Hs.606340	ATP-binding cassette, sub-family C (CFTR/MRP), member 5	Homo sapiens ATP-binding cassette, sub-family C (CFTR/MRP), member 5 (ABCC5), transcript variant 2, mRNA [NM_001023587]	2.97631	up	2.95810	up	1481.47520	515.40530	1004.20960	299.34830	Detected	Detected	Detected	Detected
2013	A_33_P3408320	LASS1	Hs.412355	LAG1 homolog, ceramide synthase 1	Homo sapiens LAG1 homolog, ceramide synthase 1 (LASS1), transcript variant 2, mRNA [NM_198207]	2.97621	up	2.79456	up	892.38310	310.47076	678.11040	213.97015	Detected	Detected	Detected	Detected
2014	A_33_P3351197	MGC21881	Hs.721511	hypothetical locus MGC21881	Homo sapiens hypothetical locus MGC21881 (MGC21881), non-coding RNA [NR_015363]	2.97574	up	2.24049	up	215.36563	74.94025	193.17574	76.02843	Detected	Detected	Detected	Detected

2015	A_33_P3318746				Protein cornichon homolog 4 [Source:UniProtKB/Swiss-Prot;Acc:Q9P003] [ENST00000366856]	2.97441	up	2.97866	up	3766.94680	1311.35890	2875.61250	851.28564	Detected	Detected	Detected	Detected
2016	A_24_P411561	HAVCR2	Hs.710500	hepatitis A virus cellular receptor 2	Homo sapiens hepatitis A virus cellular receptor 2 (HAVCR2), mRNA [NM_032782]	2.97035	up	4.79614	up	26.30078	9.16842	17.67664	3.24993	Detected	Compromised	Detected	Compromised
2017	A_33_P3241521	EBF1	Hs.573143	early B-cell factor 1	Homo sapiens early B-cell factor 1 (EBF1), mRNA [NM_024007]	2.97012	up	1.24625	up	47.24593	16.47115	57.04293	40.36115	Detected	Compromised	Detected	Detected
2018	A_24_P414719	NFYA	Hs.10441	nuclear transcription factor Y, alpha	Homo sapiens nuclear transcription factor Y, alpha (NFYA), transcript variant 1, mRNA [NM_002505]	2.96949	up	2.90099	up	1035.96310	361.23975	1013.27290	307.99695	Detected	Detected	Detected	Detected
2019	A_33_P3351982	C2orf64	Hs.596537	chromosome 2 open reading frame 64	Homo sapiens chromosome 2 open reading frame 64 (C2orf64), mRNA [NM_001008215]	2.96531	up	3.18763	up	2605.54610	909.83320	2168.25000	599.80060	Detected	Detected	Detected	Detected
2020	A_23_P358410	SRGAP3	Hs.654743	SLIT-ROBO Rho GTPase activating protein 3	Homo sapiens SLIT-ROBO Rho GTPase activating protein 3 (SRGAP3), transcript variant 1, mRNA [NM_014850]	2.96281	up	2.94812	up	45.36979	15.85611	27.26046	8.15367	Detected	Compromised	Detected	Compromised
2021	A_23_P150147	SSH3	Hs.29173	slingshot homolog 3 (Drosophila)	Homo sapiens slingshot homolog 3 (Drosophila) (SSH3), mRNA [NM_017857]	2.96280	up	2.76108	up	402.31046	140.60223	395.28020	126.23850	Detected	Detected	Detected	Detected
2022	A_23_P101392	TMEM38A	Hs.436068	transmembrane protein 38A	Homo sapiens transmembrane protein 38A (TMEM38A), mRNA [NM_024074]	2.96232	up	4.45036	up	215.79321	75.42906	191.55850	37.95522	Detected	Detected	Detected	Detected
2023	A_33_P3321993					2.96140	up	1.86272	up	18.73614	6.55114	10.18288	4.82046	Detected	Compromised	Compromised	Compromised
2024	A_33_P3412184	LOC100133142		similar to zinc finger protein 208	PREDICTED: Homo sapiens similar to zinc finger protein 208 (LOC100133142), mRNA [XM_001718400]	2.95982	up	2.94408	up	46.42857	16.24253	43.33024	12.97797	Detected	Detected	Detected	Detected
2025	A_23_P431305	FAM69B	Hs.495480	family with sequence similarity 69, member B	Homo sapiens family with sequence similarity 69, member B (FAM69B), mRNA [NM_152421]	2.95958	up	5.99479	up	231.75310	81.08274	205.62580	30.24610	Detected	Detected	Detected	Detected
2026	A_23_P160025	IFI16	Hs.380250	interferon, gamma-inducible protein 16	Homo sapiens interferon, gamma-inducible protein 16 (IFI16), mRNA [NM_005531]	2.95903	up	3.04601	up	9834.12000	3441.27340	6287.23440	1820.09580	Detected	Detected	Detected	Detected
2027	A_33_P3319502					2.95889	up	2.58090	up	33038.22300	11561.68200	31721.59000	10837.98900	Detected	Detected	Detected	Detected
2028	A_23_P1691	MMP1	Hs.83169	matrix metallopeptidase 1 (interstitial collagenase)	Homo sapiens matrix metallopeptidase 1 (interstitial collagenase) (MMP1), transcript variant 1, mRNA [NM_002421]	2.95769	up	2.79074	up	468847.25000	164139.19000	383133.40000	121058.71000	Detected	Detected	Detected	Detected
2029	A_23_P105409	MAP3K12	Hs.713539	mitogen-activated protein kinase kinase kinase 12	Homo sapiens mitogen-activated protein kinase kinase kinase 12 (MAP3K12), mRNA [NM_006301]	2.95721	up	2.90150	up	553.57230	193.83224	596.00757	181.13179	Detected	Detected	Detected	Detected
2030	A_24_P494454	SPIN3	Hs.522672	spindlin family, member 3	Homo sapiens spindlin family, member 3 (SPIN3), transcript variant 1, mRNA [NM_001010862]	2.95623	up	2.97257	up	166.85257	58.44227	189.19601	56.12359	Detected	Detected	Detected	Detected
2031	A_23_P132341	C22orf46	Hs.517653	chromosome 22 open reading frame 46	Homo sapiens chromosome 22 open reading frame 46 (C22orf46), mRNA [NM_001142964]	2.95569	up	2.56367	up	495.12097	173.45440	525.61786	180.78955	Detected	Detected	Detected	Detected
2032	A_23_P92499	TLR2	Hs.519033	toll-like receptor 2	Homo sapiens toll-like receptor 2 (TLR2), mRNA [NM_003264]	2.95519	up	2.95155	up	3031.82840	1062.31350	2123.45430	634.39325	Detected	Detected	Detected	Detected
2033	A_23_P38167	GPRC5C	Hs.446438	G protein-coupled receptor, family C, group 5, member C	Homo sapiens G protein-coupled receptor, family C, group 5, member C (GPRC5C), transcript variant 1, mRNA [NM_022036]	2.95455	up	4.05598	up	233.73895	81.91668	184.60709	40.13455	Detected	Detected	Detected	Detected
2034	A_33_P3319542	SLC8A2	Hs.172884	solute carrier family 8 (sodium/calcium exchanger), member 2	Homo sapiens solute carrier family 8 (sodium/calcium exchanger), member 2 (SLC8A2), mRNA [NM_015063]	2.95346	up	9.62375	up	35.87996	12.57924	33.84625	3.10121	Detected	Compromised	Detected	Compromised
2035	A_33_P3300610	OR7G2	Hs.553774	olfactory receptor, family 7, subfamily G, member 2	Homo sapiens olfactory receptor, family 7, subfamily G, member 2 (OR7G2), mRNA [NM_001005193]	2.95191	up	1.24103	up	107.58154	37.73705	10.55803	7.50182	Detected	Detected	Compromised	Compromised
2036	A_24_P194931	SMEK2	Hs.516182	SMEK homolog 2, suppressor of mek1 (Dictyostelium)	Homo sapiens SMEK homolog 2, suppressor of mek1 (Dictyostelium) (SMEK2), transcript variant 2, mRNA [NM_020463]	2.95139	up	3.20871	up	1073.46400	376.61180	706.49554	194.15303	Detected	Detected	Detected	Detected
2037	A_23_P69100	SGEF	Hs.240845	Src homology 3 domain-containing guanine nucleotide exchange factor	Homo sapiens Src homology 3 domain-containing guanine nucleotide exchange factor (SGEF), mRNA [NM_015595]	2.94983	up	2.83023	up	106.25249	37.29717	100.07902	31.18078	Detected	Detected	Detected	Detected
2038	A_23_P155123	CYP2D6	Hs.648256	cytochrome P450, family 2, subfamily D, polypeptide 6	Homo sapiens cytochrome P450, family 2, subfamily D, polypeptide 6 (CYP2D6), transcript variant 1, mRNA [NM_000106]	2.94935	up	2.72445	up	100.15979	35.16417	99.32974	32.14886	Detected	Detected	Detected	Detected
2039	A_33_P3226439					2.94843	up	3.03758	up	1860.69970	653.45886	1494.11700	433.73320	Detected	Detected	Detected	Detected
2040	A_23_P130974	KIAA1683	Hs.313471	KIAA1683	Homo sapiens KIAA1683 (KIAA1683), transcript variant 2, mRNA [NM_025249]	2.94722	up	3.13897	up	228.67613	80.34187	220.36168	61.90340	Detected	Detected	Detected	Detected
2041	A_23_P153941	C2orf34	Hs.468349	chromosome 2 open reading frame 34	Homo sapiens chromosome 2 open reading frame 34 (C2orf34), mRNA [NM_024766]	2.94704	up	2.51088	up	21.22817	7.45865	15.49402	5.44132	Detected	Compromised	Detected	Compromised
2042	A_23_P59138	POU5F1	Hs.249184	POU class 5 homeobox 1	Homo sapiens POU class 5 homeobox 1 (POU5F1), transcript variant 1, mRNA [NM_002701]	2.94692	up	2.98864	up	665.32104	233.77365	520.54330	153.58511	Detected	Detected	Detected	Detected
2043	A_23_P110941	GSTA4	Hs.485557	glutathione S-transferase alpha 4	Homo sapiens glutathione S-transferase alpha 4 (GSTA4), mRNA [NM_001512]	2.94685	up	2.94953	up	1384.71950	486.55990	984.96700	294.46550	Detected	Detected	Detected	Detected
2044	A_33_P3308744	LAMA4	Hs.654572	laminin, alpha 4	Homo sapiens laminin, alpha 4 (LAMA4), transcript variant 1, mRNA [NM_001105206]	2.94646	up	3.03550	up	1066.23690	374.70218	1035.37990	300.77026	Detected	Detected	Detected	Detected
2045	A_23_P133691	RRAGD	Hs.31712	Ras-related GTP binding D	Homo sapiens Ras-related GTP binding D (RRAGD), mRNA [NM_021244]	2.94415	up	5.38316	up	72.32405	25.43640	45.94708	7.52638	Detected	Detected	Detected	Compromised

2046	A_24_P416997	APOL3	Hs.474737	apolipoprotein L, 3	Homo sapiens apolipoprotein L, 3 (APOL3), transcript variant beta/a, mRNA [NM_145641]	2.94313	up	2.37668	up	98.85288	34.77864	62.67815	23.25467	Detected	Detected	Detected	Detected
2047	A_33_P3294911	C12orf66		chromosome 12 open reading frame 66	UPF0536 protein C12orf66 [Source:UniProtKB/Swiss-Prot;Acc:Q96MD2] [ENST00000311915]	2.94164	up	2.41211	up	35.35727	12.44580	34.55707	12.63295	Detected	Compromised	Detected	Compromised
2048	A_23_P254165	RAI2	Hs.446680	retinoic acid induced 2	Homo sapiens retinoic acid induced 2 (RAI2), mRNA [NM_021785]	2.93924	up	2.85866	up	209.66472	73.86247	158.37370	48.85247	Detected	Detected	Detected	Detected
2049	A_33_P3401826	CMPK2	Hs.7155	cytidine monophosphate (UMP-CMP) kinase 2, mitochondrial	Homo sapiens cytidine monophosphate (UMP-CMP) kinase 2, mitochondrial (CMPK2), nuclear gene encoding mitochondrial protein, mRNA [NM_207315]	2.93912	up	2.72939	up	825.16974	290.70990	571.33026	184.58140	Detected	Detected	Detected	Detected
2050	A_33_P3241393	SLC4A5	Hs.594099	solute carrier family 4, sodium bicarbonate cotransporter, member 5	Homo sapiens solute carrier family 4, sodium bicarbonate cotransporter, member 5 (SLC4A5), transcript variant c, mRNA [NM_133478]	2.93902	up	2.85877	up	88.13061	31.04967	69.94727	21.57534	Detected	Detected	Detected	Detected
2051	A_32_P85539	HCFC2	Hs.506558	host cell factor C2	Homo sapiens host cell factor C2 (HCFC2), mRNA [NM_013320]	2.93844	up	3.14729	up	1616.85290	569.75415	1009.51820	282.84120	Detected	Detected	Detected	Detected
2052	A_33_P3272291	AKR1C4	Hs.567245	aldo-keto reductase family 1, member C4 (chlordecone reductase; 3-alpha hydroxysteroid dehydrogenase, type I; dihydrodiol dehydrogenase 4)	Homo sapiens aldo-keto reductase family 1, member C4 (chlordecone reductase; 3-alpha hydroxysteroid dehydrogenase, type I; dihydrodiol dehydrogenase 4) (AKR1C4), mRNA [NM_001818]	2.93551	up	3.61222	up	1686.14060	594.76227	312.36713	76.25301	Detected	Detected	Detected	Detected
2053	A_33_P3266928	LMTK3	Hs.207426	lemur tyrosine kinase 3	Homo sapiens lemur tyrosine kinase 3 (LMTK3), mRNA [NM_001080434]	2.93249	up	2.60112	up	382.56100	135.08199	376.16867	127.52279	Detected	Detected	Detected	Detected
2054	A_23_P136683	HLA-DQB1	Hs.409934	major histocompatibility complex, class II, DQ beta 1	Homo sapiens major histocompatibility complex, class II, DQ beta 1 (HLA-DQB1), mRNA [NM_002123]	2.92821	up	6.70233	up	10.89868	3.85394	20.64814	2.71657	Compromised	Compromised	Detected	Compromised
2055	A_23_P159974	KLHL13	Hs.348262	kelch-like 13 (Drosophila)	Homo sapiens kelch-like 13 (Drosophila) (KLHL13), mRNA [NM_033495]	2.92757	up	2.99623	up	66.84304	23.64190	63.65384	18.73332	Detected	Detected	Detected	Detected
2056	A_23_P130149	ENO3	Hs.224171	enolase 3 (beta, muscle)	Homo sapiens enolase 3 (beta, muscle) (ENO3), transcript variant 1, mRNA [NM_001976]	2.92326	up	3.01928	up	689.20950	244.12788	612.56433	178.90134	Detected	Detected	Detected	Detected
2057	A_24_P261005	C8orf40	Hs.655320	chromosome 8 open reading frame 40	Homo sapiens chromosome 8 open reading frame 40 (C8orf40), transcript variant 3, mRNA [NM_138436]	2.92248	up	2.98725	up	4703.88600	1666.62620	3282.40430	968.91550	Detected	Detected	Detected	Detected
2058	A_23_P27515	PLD3	Hs.257008	phospholipase D family, member 3	Homo sapiens phospholipase D family, member 3 (PLD3), transcript variant 2, mRNA [NM_012268]	2.92158	up	2.23594	up	2568.61940	910.36380	2497.27600	984.85370	Detected	Detected	Detected	Detected
2059	A_23_P328034	C20orf96	Hs.348112	chromosome 20 open reading frame 96	Homo sapiens chromosome 20 open reading frame 96 (C20orf96), mRNA [NM_153269]	2.92144	up	3.70952	up	75.09042	26.61464	82.52980	19.61817	Detected	Detected	Detected	Detected
2060	A_33_P3233666	RPP30	Hs.139120	ribonuclease P/MRP 30kDa subunit	Homo sapiens ribonuclease P/MRP 30kDa subunit (RPP30), transcript variant 1, mRNA [NM_001104546]	2.92006	up	3.12868	up	566.02540	200.71365	478.56424	134.87918	Detected	Detected	Detected	Detected
2061	A_24_P237586	ANKRD37	Hs.508154	ankyrin repeat domain 37	Homo sapiens ankyrin repeat domain 37 (ANKRD37), mRNA [NM_181726]	2.91993	up	2.83673	up	1622.78170	575.46747	1271.10850	395.12160	Detected	Detected	Detected	Detected
2062	A_23_P252062	PPARG	Hs.162646	peroxisome proliferator-activated receptor gamma	Homo sapiens peroxisome proliferator-activated receptor gamma (PPARG), transcript variant 3, mRNA [NM_138711]	2.91944	up	2.79086	up	3062.86800	1086.32960	2929.38920	925.55970	Detected	Detected	Detected	Detected
2063	A_33_P3411888				GB	2.91776	up	2.47162	up	81.24480	28.83233	62.48163	22.29135	Detected	Detected	Detected	Detected
2064	A_23_P123193	ACTR3B	Hs.647117	ARP3 actin-related protein 3 homolog B (yeast)	Homo sapiens ARP3 actin-related protein 3 homolog B (yeast) (ACTR3B), transcript variant 1, mRNA [NM_020445]	2.91633	up	3.11565	up	2398.17020	851.48430	1692.17580	478.91965	Detected	Detected	Detected	Detected
2065	A_32_P55241	SHISA2	Hs.433791	shisa homolog 2 (Xenopus laevis)	Homo sapiens shisa homolog 2 (Xenopus laevis) (SHISA2), mRNA [NM_001007538]	2.91518	up	2.39915	up	52.25579	18.56106	45.69510	16.79494	Detected	Detected	Detected	Detected
2066	A_23_P57961	PLXNB1	Hs.476209	plexin B1	Homo sapiens plexin B1 (PLXNB1), transcript variant 1, mRNA [NM_002673]	2.91411	up	2.36816	up	567.21533	201.54640	535.14410	199.26257	Detected	Detected	Detected	Detected
2067	A_23_P106835	BBS2	Hs.333738	Bardet-Biedl syndrome 2	Homo sapiens Bardet-Biedl syndrome 2 (BBS2), mRNA [NM_031885]	2.91406	up	2.95775	up	5034.76400	1789.01500	3699.98800	1103.07350	Detected	Detected	Detected	Detected
2068	A_33_P3260322					2.91193	up	2.66549	up	127.35831	45.28754	69.63222	23.03556	Detected	Detected	Detected	Detected
2069	A_23_P217475	IDS	Hs.460960	iduronate 2-sulfatase	Homo sapiens iduronate 2-sulfatase (IDS), transcript variant 2, mRNA [NM_006123]	2.91166	up	3.11697	up	12132.33300	4314.56640	9169.97200	2594.18120	Detected	Detected	Detected	Detected
2070	A_23_P126908	TNFRSF14	Hs.512898	tumor necrosis factor receptor superfamily, member 14 (herpesvirus entry mediator)	Homo sapiens tumor necrosis factor receptor superfamily, member 14 (herpesvirus entry mediator) (TNFRSF14), mRNA [NM_003820]	2.91105	up	2.98760	up	3789.02860	1347.75830	2289.28250	675.68300	Detected	Detected	Detected	Detected
2071	A_33_P3316073	INTS6	Hs.439440	integrator complex subunit 6	Homo sapiens integrator complex subunit 6 (INTS6), transcript variant 1, mRNA [NM_012141]	2.90584	up	2.90222	up	67.31969	23.98850	62.22517	18.90608	Detected	Detected	Detected	Detected
2072	A_33_P3388806				Fucose-1-phosphate guanylyltransferase (EC 2.7.7.30)(GDP-L-fucose pyrophosphorylase)(GDP-L-fucose diphosphorylase) [Source:UniProtKB/Swiss-Prot;Acc:O14772] [ENST00000370889]	2.90527	up	4.59342	up	33.63691	11.98844	19.23536	3.69258	Detected	Compromised	Detected	Compromised
2073	A_24_P941167	APOL6	Hs.257352	apolipoprotein L, 6	Homo sapiens apolipoprotein L, 6 (APOL6), mRNA [NM_030641]	2.90382	up	1.76568	up	71.45064	25.47823	143.11147	71.47081	Detected	Detected	Detected	Detected
2074	A_24_P62530	RHOU	Hs.647774	ras homolog gene family, member U	Homo sapiens ras homolog gene family, member U (RHOU), mRNA [NM_021205]	2.90355	up	3.30899	up	275.59726	98.28299	270.00394	71.95151	Detected	Detected	Detected	Detected

2075	A_23_P113351	SPARCL1	Hs.62886	SPARC-like 1 (hevin)	Homo sapiens SPARC-like 1 (hevin) (SPARCL1), transcript variant 2, mRNA [NM_004684]	2.90294	up	1.36039	up	12.89796	4.60063	4.49258	2.91204	Detected	Compromised	Compromised	Compromised
2076	A_33_P3352307	RPS6KA1	Hs.149957	ribosomal protein S6 kinase, 90kDa, polypeptide 1	Homo sapiens ribosomal protein S6 kinase, 90kDa, polypeptide 1 (RPS6KA1), transcript variant 1, mRNA [NM_002953]	2.90215	up	3.03785	up	213.66641	76.23403	166.99028	48.47187	Detected	Detected	Detected	Detected
2077	A_23_P117506	DHRS7	Hs.59719	dehydrogenase/reductase (SDR family) member 7	Homo sapiens dehydrogenase/reductase (SDR family) member 7 (DHRS7), mRNA [NM_016029]	2.90168	up	2.87034	up	10933.61500	3901.65010	9161.44700	2814.46850	Detected	Detected	Detected	Detected
2078	A_33_P3255914	MYLIP	Hs.484738	myosin regulatory light chain interacting protein	Homo sapiens myosin regulatory light chain interacting protein (MYLIP), mRNA [NM_013262]	2.89985	up	2.93423	up	3135.57710	1119.62990	2288.71830	687.80130	Detected	Detected	Detected	Detected
2079	A_33_P3379381	WDR78	Hs.49421	WD repeat domain 78	Homo sapiens WD repeat domain 78 (WDR78), transcript variant 2, mRNA [NM_207014]	2.89980	up	2.82029	up	206.67060	73.79790	170.77852	53.39558	Detected	Detected	Detected	Detected
2080	A_33_P3611762	FLJ37798	Hs.544586	hypothetical gene supported by AK095117	Homo sapiens cDNA FLJ37798 fis, clone BRSSN2000097 [AK095117]	2.89779	up	2.97507	up	6442.59900	2302.11650	5252.14900	1556.70360	Detected	Detected	Detected	Detected
2081	A_33_P3393543		Hs.590968		Putative uncharacterized protein LOC400692 [Source:UniProtKB/Swiss-Prot;Acc:Q8N3U1] [ENST00000316807]	2.89771	up	2.21072	up	57.80022	20.65415	23.89595	9.53139	Detected	Detected	Detected	Compromised
2082	A_23_P393777	PTGDR	Hs.306831	prostaglandin D2 receptor (DP)	Homo sapiens prostaglandin D2 receptor (DP) (PTGDR), mRNA [NM_000953]	2.89755	up	3.33757	up	497.72388	177.86531	466.53850	123.26009	Detected	Detected	Detected	Detected
2083	A_23_P250122	FAM20C	Hs.134742	family with sequence similarity 20, member C	Homo sapiens family with sequence similarity 20, member C (FAM20C), mRNA [NM_020223]	2.89488	up	2.72480	up	25324.03300	9058.07600	25336.24000	8199.23200	Detected	Detected	Detected	Detected
2084	A_32_P30831		Hs.586876		Homo sapiens cDNA FLJ43403 fis, clone OCBBF2016612 [AK125393]	2.89478	up	3.37425	up	71.10990	25.43589	95.09875	24.85211	Detected	Detected	Detected	Detected
2085	A_23_P46852	OBFC1	Hs.62314	oligonucleotide/oligosaccharide-binding fold containing 1	Homo sapiens oligonucleotide/oligosaccharide-binding fold containing 1 (OBFC1), mRNA [NM_024928]	2.89424	up	3.05379	up	7902.74000	2827.33130	8407.57300	2427.70850	Detected	Detected	Detected	Detected
2086	A_23_P129144	GCOM1	Hs.437256	GRINL1A complex locus	Homo sapiens GRINL1A complex locus (GCOM1), transcript variant 12, mRNA [NM_001018100]	2.89299	up	2.54211	up	34.49773	12.34743	30.71321	10.65361	Detected	Compromised	Detected	Compromised
2087	A_33_P3380126	MORN3	Hs.434154	MORN repeat containing 3	Homo sapiens MORN repeat containing 3 (MORN3), mRNA [NM_173855]	2.89224	up	7.46673	up	43.32909	15.51238	48.84450	5.76834	Detected	Compromised	Detected	Compromised
2088	A_33_P3351879	LOC100128905	Hs.456944	hypothetical LOC100128905	PREDICTED: Homo sapiens hypothetical LOC100128905 (LOC100128905), mRNA [XM_001721827]	2.89135	up	3.31061	up	150.14322	53.76984	102.15830	27.21020	Detected	Detected	Detected	Detected
2089	A_23_P3592	HSF4	Hs.512156	heat shock transcription factor 4	Homo sapiens heat shock transcription factor 4 (HSF4), transcript variant 2, mRNA [NM_001040667]	2.89051	up	3.19612	up	158.30745	56.71010	151.64409	41.83772	Detected	Detected	Detected	Detected
2090	A_23_P352717	TMEM31	Hs.98843	transmembrane protein 31	Homo sapiens transmembrane protein 31 (TMEM31), mRNA [NM_182541]	2.89026	up	1.86091	up	52.78613	18.91105	40.44062	19.16273	Detected	Detected	Detected	Detected
2091	A_33_P3241150					2.88862	up	3.19646	up	59.92439	21.48062	30.79430	8.49506	Detected	Detected	Detected	Compromised
2092	A_33_P3393694	GLTSCR2	Hs.421907	glioma tumor suppressor candidate region gene 2	Homo sapiens glioma tumor suppressor candidate region gene 2 (GLTSCR2), mRNA [NM_015710]	2.88716	up	2.48704	up	84522.08000	30313.23000	81016.13000	28724.60500	Detected	Detected	Detected	Detected
2093	A_32_P69166	ANKRD42	Hs.503438	ankyrin repeat domain 42	Homo sapiens ankyrin repeat domain 42 (ANKRD42), mRNA [NM_182603]	2.88661	up	2.85453	up	463.78006	166.36336	414.53104	128.05238	Detected	Detected	Detected	Detected
2094	A_33_P3209167	LOC100131053	Hs.697104	hypothetical LOC100131053	Homo sapiens cDNA FLJ38245 fis, clone FCBBF2007186. [AK095564]	2.88555	up	4.56798	up	10.51555	3.77343	17.46403	3.37121	Compromised	Compromised	Detected	Compromised
2095	A_33_P3308903	MIB2	Hs.135805	mindbomb homolog 2 (Drosophila)	Homo sapiens mindbomb homolog 2 (Drosophila) (MIB2), mRNA [NM_080875]	2.88503	up	2.27755	up	832.52200	298.79830	1062.63570	411.41678	Detected	Detected	Detected	Detected
2096	A_33_P3299898	LOC644021	Hs.690144	similar to Cathepsin L1	PREDICTED: Homo sapiens similar to Cathepsin L1 (LOC644021), mRNA [XM_001128559]	2.88460	up	3.71591	up	10856.85500	3897.18550	11138.44400	2643.16650	Detected	Detected	Detected	Detected
2097	A_32_P10936	CDH12	Hs.113684	cadherin 12, type 2 (N-cadherin 2)	Homo sapiens cadherin 12, type 2 (N-cadherin 2) (CDH12), mRNA [NM_004061]	2.88445	up	1.08774	up	21.86338	7.84852	12.62809	10.23711	Detected	Compromised	Detected	Compromised
2098	A_23_P44244	SMARCA1	Hs.152292	SWI/SNF related, matrix associated, actin dependent regulator of chromatin, subfamily a, member 1	Homo sapiens SWI/SNF related, matrix associated, actin dependent regulator of chromatin, subfamily a, member 1 (SMARCA1), transcript variant 1, mRNA [NM_003069]	2.88251	up	3.15339	up	6306.14400	2265.30080	4767.29800	1333.09400	Detected	Detected	Detected	Detected
2099	A_33_P3232173	PSPC1	Hs.721735	paraspeckle component 1	Paraspeckle component 1 (Paraspeckle protein 1) [Source:UniProtKB/Swiss-Prot;Acc:Q8WXF1] [ENST00000338910]	2.88206	up	2.15562	up	222.52740	79.94898	118.87029	48.62582	Detected	Detected	Detected	Detected
2100	A_23_P40880	CMTM8	Hs.154986	CKLF-like MARVEL transmembrane domain containing 8	Homo sapiens CKLF-like MARVEL transmembrane domain containing 8 (CMTM8), mRNA [NM_178868]	2.88194	up	2.73554	up	455.41907	163.62848	342.27386	110.33069	Detected	Detected	Detected	Detected
2101	A_24_P248240	SYT11	Hs.32984	synaptotagmin XI	Homo sapiens synaptotagmin XI (SYT11), mRNA [NM_152280]	2.88170	up	3.03856	up	1922.56970	690.82230	1295.06770	375.82864	Detected	Detected	Detected	Detected
2102	A_32_P45974	LOC100288755	Hs.635289	hypothetical LOC100288755	PREDICTED: Homo sapiens hypothetical LOC100288755 (LOC100288755), miscRNA [XR_078568]	2.88153	up	3.96767	up	392.59125	141.07527	15.24130	3.38728	Detected	Detected	Detected	Compromised
2103	A_33_P3291614	LOC100133039	Hs.667544	hypothetical protein LOC100133039	Homo sapiens cDNA FLJ25312 fis, clone SYN01070. [AK058041]	2.88142	up	2.90219	up	61.03328	21.93274	36.12417	10.97584	Detected	Detected	Detected	Compromised
2104	A_33_P3280721	GPR177	Hs.647659	G protein-coupled receptor 177	Homo sapiens G protein-coupled receptor 177 (GPR177), transcript variant 2, mRNA [NM_001002292]	2.87947	up	2.85478	up	12352.93750	4442.12400	10153.52700	3136.23850	Detected	Detected	Detected	Detected

2105	A_24_P778836	RSL1D1	Hs.401842	ribosomal L1 domain containing 1	Homo sapiens ribosomal L1 domain containing 1 (RSL1D1), mRNA [NM_015659]	2.87873	up	2.78658	up	3351.80150	1205.61910	3210.45800	1015.92340	Detected	Detected	Detected	Detected
2106	A_23_P67151	OLFM2	Hs.169743	olfactomedin 2	Homo sapiens olfactomedin 2 (OLFM2), mRNA [NM_058164]	2.87774	up	3.37005	up	234.24188	84.28424	241.07013	63.07721	Detected	Detected	Detected	Detected
2107	A_23_P79769	BIRC7	Hs.256126	baculoviral IAP repeat-containing 7	Homo sapiens baculoviral IAP repeat-containing 7 (BIRC7), transcript variant 2, mRNA [NM_022161]	2.87492	up	3.13553	up	42.24916	15.21686	42.22850	11.87571	Detected	Compromised	Detected	Compromised
2108	A_23_P64058	RASGRP2	Hs.99491	RAS guanyl releasing protein 2 (calcium and DAG-regulated)	Homo sapiens RAS guanyl releasing protein 2 (calcium and DAG-regulated) (RASGRP2), transcript variant 2, mRNA [NM_153819]	2.87388	up	9.13052	up	552.99225	199.24301	533.06354	51.48126	Detected	Detected	Detected	Detected
2109	A_33_P3234989	IFT81	Hs.528382	intraflagellar transport 81 homolog (Chlamydomonas)	Homo sapiens intraflagellar transport 81 homolog (Chlamydomonas) (IFT81), transcript variant 1, mRNA [NM_014055]	2.87191	up	2.94668	up	2924.80900	1054.53050	2390.37230	715.31610	Detected	Detected	Detected	Detected
2110	A_33_P3245449	LOC389834	Hs.720653	ankyrin repeat domain 57 pseudogene	Homo sapiens ankyrin repeat domain 57 pseudogene (LOC389834), non-coding RNA [NR_027420]	2.87037	up	4.16464	up	430.68870	155.36690	298.61328	63.22628	Detected	Detected	Detected	Detected
2111	A_32_P410272		Hs.467163		Putative G-protein coupled receptor GPCR39 (hGPRC39) [Source:UniProtKB/Swiss-Prot;Acc:Q8NGA4] [ENST00000376892]	2.86782	up	1.77166	up	10.05377	3.63002	14.85978	7.39600	Compromised	Compromised	Detected	Compromised
2112	A_33_P3327479	ZDHHC3	Hs.61430	zinc finger, DHHC-type containing 3	Homo sapiens zinc finger, DHHC-type containing 3 (ZDHHC3), transcript variant 2, mRNA [NM_016598]	2.86482	up	2.86761	up	4603.69730	1663.95680	4097.57860	1260.00720	Detected	Detected	Detected	Detected
2113	A_33_P3231297	CREG1	Hs.5710	cellular repressor of E1A-stimulated genes 1	Homo sapiens cellular repressor of E1A-stimulated genes 1 (CREG1), mRNA [NM_003851]	2.86447	up	2.70662	up	18655.48400	6743.64400	12225.44600	3982.93630	Detected	Detected	Detected	Detected
2114	A_33_P3361267	LOC100128386	Hs.635125	hypothetical protein LOC100128386	PREDICTED: Homo sapiens hypothetical protein LOC100128386 (LOC100128386), mRNA [XM_001725380]	2.86266	up	8.82983	up	79.53380	28.76835	53.01728	5.29457	Detected	Detected	Detected	Compromised
2115	A_24_P139901	GYPC	Hs.59138	glycophorin C (Gerbich blood group)	Homo sapiens glycophorin C (Gerbich blood group) (GYPC), transcript variant 1, mRNA [NM_002101]	2.86229	up	2.67324	up	5293.42100	1914.94170	8083.65530	2666.46460	Detected	Detected	Detected	Detected
2116	A_24_P119545				Inositol-trisphosphate 3-kinase B (EC 2.7.1.127)(Inositol 1,4,5-trisphosphate 3-kinase B)(IP3 3-kinase B)(IP3K-B)(IP3K B) [Source:UniProtKB/Swiss-Prot;Acc:P27987] [ENST00000366784]	2.86215	up	1.11404	up	18.39738	6.65573	10.97337	8.68570	Detected	Compromised	Compromised	Compromised
2117	A_23_P250800	ST3GAL6	Hs.148716	ST3 beta-galactoside alpha-2,3-sialyltransferase 6	Homo sapiens ST3 beta-galactoside alpha-2,3-sialyltransferase 6 (ST3GAL6), mRNA [NM_006100]	2.86122	up	4.82566	up	51.48545	18.63228	39.71555	7.25721	Detected	Detected	Detected	Compromised
2118	A_33_P3298775	PRSSL1	Hs.245146	protease, serine-like 1	Homo sapiens protease, serine-like 1 (PRSSL1), mRNA [NM_214710]	2.85964	up	4.05221	up	16.09948	5.82953	21.15192	4.60282	Detected	Compromised	Detected	Compromised
2119	A_33_P3245454	LOC389834	Hs.720653	ankyrin repeat domain 57 pseudogene	Homo sapiens ankyrin repeat domain 57 pseudogene (LOC389834), non-coding RNA [NR_027420]	2.85921	up	3.46406	up	88.69790	32.12179	62.19928	15.83307	Detected	Detected	Detected	Compromised
2120	A_33_P3234020	IGDCC3	Hs.128292	immunoglobulin superfamily, DCC subclass, member 3	Homo sapiens immunoglobulin superfamily, DCC subclass, member 3 (IGDCC3), mRNA [NM_004884]	2.85860	up	2.78299	up	25.31436	9.16953	16.54029	5.24079	Detected	Compromised	Detected	Compromised
2121	A_33_P3507270	EFCAB5	Hs.662411	EF-hand calcium binding domain 5	Homo sapiens EF-hand calcium binding domain 5 (EFCAB5), transcript variant 1, mRNA [NM_198529]	2.85757	up	2.22396	up	27.19609	9.85468	15.27364	6.05592	Detected	Compromised	Compromised	Compromised
2122	A_24_P356130	MAP2K5	Hs.114198	mitogen-activated protein kinase kinase 5	Homo sapiens mitogen-activated protein kinase kinase 5 (MAP2K5), transcript variant B, mRNA [NM_002757]	2.85718	up	2.92595	up	607.59670	220.19678	442.64365	133.39893	Detected	Detected	Detected	Detected
2123	A_23_P59179	RXRB	Hs.388034	retinoid X receptor, beta	Homo sapiens retinoid X receptor, beta (RXRB), mRNA [NM_021976]	2.85658	up	2.66175	up	1867.02640	676.76480	1553.91430	514.78520	Detected	Detected	Detected	Detected
2124	A_32_P137266	EFCAB7	Hs.652324	EF-hand calcium binding domain 7	Homo sapiens EF-hand calcium binding domain 7 (EFCAB7), mRNA [NM_032437]	2.85634	up	2.79346	up	1966.78260	712.98450	1449.03470	457.40550	Detected	Detected	Detected	Detected
2125	A_33_P3437283	LOC643551	Hs.513081	hypothetical LOC643551	Homo sapiens cDNA FLJ46123 fis, clone TESTI2039738 [AK128004]	2.85463	up	3.50278	up	132.71692	48.14031	61.36118	15.44709	Detected	Detected	Detected	Compromised
2126	A_24_P104538		Hs.559259		Nucleosome-remodeling factor subunit BPTF (Bromodomain and PHD finger-containing transcription factor)(Fetal Alzheimer antigen)(Fetal Alz-50 clone 1 protein) [Source:UniProtKB/Swiss-Prot;Acc:Q12830] [ENST00000342579]	2.85400	up	2.74528	up	103.08518	37.40027	72.01312	23.13080	Detected	Detected	Detected	Detected
2127	A_23_P45955	TEKT2	Hs.127111	tektin 2 (testicular)	Homo sapiens tektin 2 (testicular) (TEKT2), mRNA [NM_014466]	2.85326	up	2.87527	up	60.55796	21.97670	58.01646	17.79255	Detected	Detected	Detected	Detected
2128	A_23_P348121	FOSL2	Hs.220971	FOS-like antigen 2	Homo sapiens FOS-like antigen 2 (FOSL2), mRNA [NM_005253]	2.85323	up	2.74325	up	531.49774	192.88467	559.59960	179.87769	Detected	Detected	Detected	Detected
2129	A_23_P83798	ALX1	Hs.41683	ALX homeobox 1	Homo sapiens ALX homeobox 1 (ALX1), mRNA [NM_006982]	2.85273	up	2.78617	up	289.79892	105.18860	255.48160	80.85695	Detected	Detected	Detected	Detected
2130	A_23_P91552	FTCD	Hs.415846	formiminotransferase cyclodeaminase	Homo sapiens formiminotransferase cyclodeaminase (FTCD), transcript variant A, mRNA [NM_206965]	2.85271	up	1.68439	up	24.26622	8.80801	24.03099	12.58043	Detected	Compromised	Detected	Compromised
2131	A_24_P129277	NOD1	Hs.405153	nucleotide-binding oligomerization domain containing 1	Homo sapiens nucleotide-binding oligomerization domain containing 1 (NOD1), mRNA [NM_006092]	2.85170	up	2.81443	up	544.28796	197.63246	301.43228	94.44206	Detected	Detected	Detected	Detected
2132	A_23_P200138	SLAMF8	Hs.438683	SLAM family member 8	Homo sapiens SLAM family member 8 (SLAMF8), mRNA [NM_020125]	2.85155	up	2.72702	up	1115.08510	404.91095	1090.07080	352.47780	Detected	Detected	Detected	Detected
2133	A_33_P3295261	LMOD1	Hs.519075	leiomodin 1 (smooth muscle)	Homo sapiens leiomodin 1 (smooth muscle) (LMOD1), mRNA [NM_012134]	2.85017	up	1.49264	up	9.36807	3.40340	16.82253	9.93805	Compromised	Compromised	Detected	Compromised
2134	A_24_P262201	SULT1A4	Hs.460558	sulfotransferase family, cytosolic, 1A, phenol-preferring, member 4	Homo sapiens sulfotransferase family, cytosolic, 1A, phenol-preferring, member 4 (SULT1A4), transcript variant 1, mRNA [NM_001017389]	2.84893	up	2.86092	up	2390.93970	869.00050	2478.21460	763.83234	Detected	Detected	Detected	Detected

2135	A_33_P3298105	SPATA13	Hs.595391	spermatogenesis associated 13	Homo sapiens spermatogenesis associated 13 (SPATA13), transcript variant 1, mRNA [NM_001166271]	2.84874	up	2.00677	up	172.57828	62.72873	112.36742	49.37506	Detected	Detected	Detected	Detected
2136	A_23_P158277	TMCO4	Hs.656313	transmembrane and coiled-coil domains 4	Homo sapiens transmembrane and coiled-coil domains 4 (TMCO4), mRNA [NM_181719]	2.84738	up	2.46315	up	1554.97270	565.47040	1804.99320	646.17500	Detected	Detected	Detected	Detected
2137	A_24_P71468	QPCT	Hs.79033	glutaminyl-peptide cyclotransferase	Homo sapiens glutaminyl-peptide cyclotransferase (QPCT), mRNA [NM_012413]	2.84510	up	3.16925	up	6975.57760	2538.72240	5833.95560	1623.20230	Detected	Detected	Detected	Detected
2138	A_23_P250982	ISOC1	Hs.483296	isochorismatase domain containing 1	Homo sapiens isochorismatase domain containing 1 (ISOC1), mRNA [NM_016048]	2.84457	up	2.92520	up	1969.87540	717.06067	1481.04830	446.45620	Detected	Detected	Detected	Detected
2139	A_23_P119907	ANKZF1	Hs.437647	ankyrin repeat and zinc finger domain containing 1	Homo sapiens ankyrin repeat and zinc finger domain containing 1 (ANKZF1), transcript variant 1, mRNA [NM_018089]	2.84136	up	2.56693	up	736.00660	268.21878	589.90080	202.64246	Detected	Detected	Detected	Detected
2140	A_23_P52986	VWCE	Hs.60640	von Willebrand factor C and EGF domains	Homo sapiens von Willebrand factor C and EGF domains (VWCE), mRNA [NM_152718]	2.84016	up	2.64208	up	5303.60250	1933.57520	4788.86400	1598.27890	Detected	Detected	Detected	Detected
2141	A_23_P146077	ZNF395	Hs.435535	zinc finger protein 395	Homo sapiens zinc finger protein 395 (ZNF395), mRNA [NM_018660]	2.83982	up	2.68021	up	10988.08700	4006.48800	11013.39400	3623.41280	Detected	Detected	Detected	Detected
2142	A_24_P184305	BBS1	Hs.502915	Bardet-Biedl syndrome 1	Homo sapiens Bardet-Biedl syndrome 1 (BBS1), mRNA [NM_024649]	2.83964	up	3.44616	up	240.09296	87.54874	168.84958	43.20460	Detected	Detected	Detected	Detected
2143	A_33_P3290707	MME	Hs.307734	membrane metallo-endopeptidase	Homo sapiens membrane metallo-endopeptidase (MME), transcript variant 2b, mRNA [NM_007289]	2.83934	up	4.87579	up	2849.60500	1039.20120	3660.71480	662.04380	Detected	Detected	Detected	Detected
2144	A_24_P241815	JUNB	Hs.25292	jun B proto-oncogene	Homo sapiens jun B proto-oncogene (JUNB), mRNA [NM_002229]	2.83714	up	2.57049	up	654.95770	239.03748	681.14966	233.66388	Detected	Detected	Detected	Detected
2145	A_24_P373844	KCTD15	Hs.221873	potassium channel tetramerisation domain containing 15	Homo sapiens potassium channel tetramerisation domain containing 15 (KCTD15), transcript variant 1, mRNA [NM_024076]	2.83647	up	2.45212	up	387.38590	141.41602	241.98073	87.01706	Detected	Detected	Detected	Detected
2146	A_23_P137238	KDM5D	Hs.80358	lysine (K)-specific demethylase 5D	Homo sapiens lysine (K)-specific demethylase 5D (KDM5D), transcript variant 2, mRNA [NM_004653]	2.83643	up	6.33547	up	282.81165	103.24251	234.84654	32.68670	Detected	Detected	Detected	Detected
2147	A_23_P105002	ROM1	Hs.281564	retinal outer segment membrane protein 1	Homo sapiens retinal outer segment membrane protein 1 (ROM1), mRNA [NM_000327]	2.83576	up	2.78200	up	5963.19240	2177.42240	4960.14600	1572.18270	Detected	Detected	Detected	Detected
2148	A_23_P362228	C1orf213	Hs.61884	chromosome 1 open reading frame 213	Homo sapiens chromosome 1 open reading frame 213 (C1orf213), transcript variant 1, mRNA [NM_138479]	2.83385	up	2.35285	up	2012.35950	735.29560	1519.62040	569.51544	Detected	Detected	Detected	Detected
2149	A_33_P3245517	LOC441666		zinc finger protein 91 pseudogene	Homo sapiens zinc finger protein 91 pseudogene (LOC441666), non-coding RNA [NR_024380]	2.83230	up	3.01200	up	21.32151	7.79493	30.25097	8.85625	Detected	Compromised	Detected	Compromised
2150	A_33_P3364651	LOC389634	Hs.434403	hypothetical LOC389634	Homo sapiens cDNA FLJ90405 fis, clone NT2RP2006099 [AK074886]	2.83220	up	1.76312	up	56.83136	20.77771	57.79311	28.90414	Detected	Detected	Detected	Detected
2151	A_24_P46093	SLC6A6	Hs.529488	solute carrier family 6 (neurotransmitter transporter, taurine), member 6	Homo sapiens solute carrier family 6 (neurotransmitter transporter, taurine), member 6 (SLC6A6), transcript variant 1, mRNA [NM_003043]	2.83056	up	3.08243	up	6213.41400	2272.95650	5522.11960	1579.71250	Detected	Detected	Detected	Detected
2152	A_33_P3358731	PCSK5	Hs.368542	proprotein convertase subtilisin/kexin type 5	Homo sapiens proprotein convertase subtilisin/kexin type 5 (PCSK5), mRNA [NM_006200]	2.82958	up	2.38280	up	299.60547	109.63779	261.85168	96.90212	Detected	Detected	Detected	Detected
2153	A_23_P217009	C9orf24	Hs.50334	chromosome 9 open reading frame 24	Homo sapiens chromosome 9 open reading frame 24 (C9orf24), transcript variant 1, mRNA [NM_032596]	2.82904	up	3.24058	up	376.94827	137.96715	358.16437	97.45956	Detected	Detected	Detected	Detected
2154	A_23_P329375	POU6F1	Hs.555886	POU class 6 homeobox 1	Homo sapiens POU class 6 homeobox 1 (POU6F1), transcript variant 1, mRNA [NM_002702]	2.82796	up	2.62274	up	469.63860	171.95833	342.09560	115.01604	Detected	Detected	Detected	Detected
2155	A_24_P394368	WDR19	Hs.438482	WD repeat domain 19	Homo sapiens WD repeat domain 19 (WDR19), mRNA [NM_025132]	2.82750	up	2.90541	up	289.15155	105.89030	333.16425	101.11532	Detected	Detected	Detected	Detected
2156	A_23_P96369	CXorf57	Hs.274267	chromosome X open reading frame 57	Homo sapiens chromosome X open reading frame 57 (CXorf57), mRNA [NM_018015]	2.82657	up	2.93306	up	1024.83020	375.42640	806.11050	242.34772	Detected	Detected	Detected	Detected
2157	A_23_P1014	C1orf97		chromosome 1 open reading frame 97	Homo sapiens chromosome 1 open reading frame 97 (C1orf97), non-coding RNA [NR_026761]	2.82626	up	2.63448	up	3694.27400	1353.47310	3149.08100	1054.03220	Detected	Detected	Detected	Detected
2158	A_33_P3384287	PALM	Hs.631841	paralemmin	Homo sapiens paralemmin (PALM), transcript variant 1, mRNA [NM_002579]	2.82510	up	2.83828	up	1933.89600	708.81530	1793.61410	557.23640	Detected	Detected	Detected	Detected
2159	A_33_P3298850	SLC16A8	Hs.270285	solute carrier family 16, member 8 (monocarboxylic acid transporter 3)	Homo sapiens solute carrier family 16, member 8 (monocarboxylic acid transporter 3) (SLC16A8), mRNA [NM_013356]	2.82361	up	1.75698	up	49.02918	17.97970	49.19855	24.69163	Detected	Compromised	Detected	Detected
2160	A_33_P3262789	REEP6	Hs.76277	receptor accessory protein 6	Homo sapiens receptor accessory protein 6 (REEP6), mRNA [NM_138393]	2.82265	up	2.68258	up	1256.40090	460.89743	1352.45360	444.56552	Detected	Detected	Detected	Detected
2161	A_24_P47988	ELL3	Hs.706346	elongation factor RNA polymerase II-like 3	Homo sapiens elongation factor RNA polymerase II-like 3 (ELL3), mRNA [NM_025165]	2.82173	up	3.35288	up	103.93689	38.14064	124.68980	32.79282	Detected	Detected	Detected	Detected
2162	A_23_P424002	POU2F1	Hs.493649	POU class 2 homeobox 1	Homo sapiens POU class 2 homeobox 1 (POU2F1), mRNA [NM_002697]	2.81948	up	3.18485	up	3100.15900	1138.53860	1364.88090	377.89460	Detected	Detected	Detected	Detected
2163	A_24_P313993	CAPS	Hs.584744	calcyphosine	Homo sapiens calcyphosine (CAPS), transcript variant 1, mRNA [NM_004058]	2.81911	up	2.28949	up	447.17984	164.24948	454.80234	175.16560	Detected	Detected	Detected	Detected
2164	A_33_P3301782	LOC220930	Hs.372654	hypothetical LOC220930	Homo sapiens hypothetical LOC220930 (LOC220930), non-coding RNA [NR_024284]	2.81844	up	2.86892	up	168.16254	61.78086	105.26550	32.35434	Detected	Detected	Detected	Detected
2165	A_23_P259207	THNSL2	Hs.516179	threonine synthase-like 2 (S. cerevisiae)	Homo sapiens threonine synthase-like 2 (S. cerevisiae) (THNSL2), mRNA [NM_018271]	2.81832	up	2.39469	up	977.65640	359.19340	969.96200	357.16638	Detected	Detected	Detected	Detected

2166	A_24_P16856		Hs.522572		Splicing factor, arginine/serine-rich 17A (Protein XE7)(B-lymphocyte antigen)(721P) [Source:UniProtKB/Swiss-Prot;Acc:Q02040] [ENST00000381261]	2.81540	up	1.78949	up	18.40249	6.76814	18.31533	9.02510	Detected	Compromised	Detected	Compromised
2167	A_32_P8156	C10orf107	Hs.673160	chromosome 10 open reading frame 107	Homo sapiens chromosome 10 open reading frame 107 (C10orf107), mRNA [NM_173554]	2.81480	up	3.08553	up	147.18945	54.14541	152.01820	43.44410	Detected	Detected	Detected	Detected
2168	A_32_P213831	FAM40B	Hs.489988	family with sequence similarity 40, member B	Homo sapiens family with sequence similarity 40, member B (FAM40B), transcript variant 1, mRNA [NM_020704]	2.81409	up	3.30103	up	129.63658	47.70049	152.43607	40.71959	Detected	Detected	Detected	Detected
2169	A_23_P100386	IL34	Hs.461214	interleukin 34	Homo sapiens interleukin 34 (IL34), mRNA [NM_152456]	2.81361	up	3.22645	up	129.99280	47.83975	83.53969	22.83147	Detected	Detected	Detected	Detected
2170	A_33_P3310552		Hs.654630		Formin-like protein 2 (Formin homology 2 domain-containing protein 2) [Source:UniProtKB/Swiss-Prot;Acc:Q96PY5] [ENST00000421344]	2.81350	up	2.86084	up	141.73060	52.16145	128.23239	39.52482	Detected	Detected	Detected	Detected
2171	A_33_P3252099	ANKLE1	Hs.721610	ankyrin repeat and LEM domain containing 1	Homo sapiens ankyrin repeat and LEM domain containing 1 (ANKLE1), mRNA [NM_152363]	2.81219	up	4.38244	up	57.83297	21.29430	51.61974	10.38640	Detected	Detected	Detected	Compromised
2172	A_24_P120934	GADD45G	Hs.9701	growth arrest and DNA-damage-inducible, gamma	Homo sapiens growth arrest and DNA-damage-inducible, gamma (GADD45G), mRNA [NM_006705]	2.81173	up	3.37300	up	115.22611	42.43369	148.78069	38.89514	Detected	Detected	Detected	Detected
2173	A_32_P9468	COX19	Hs.121593	COX19 cytochrome c oxidase assembly homolog (S. cerevisiae)	Homo sapiens COX19 cytochrome c oxidase assembly homolog (S. cerevisiae) (COX19), mRNA [NM_001031617]	2.81153	up	2.59251	up	463.00964	170.52188	324.26257	110.29160	Detected	Detected	Detected	Detected
2174	A_23_P200143	DCAF8	Hs.632447	DDB1 and CUL4 associated factor 8	Homo sapiens DDB1 and CUL4 associated factor 8 (DCAF8), transcript variant 1, mRNA [NM_015726]	2.80947	up	2.48546	up	1555.59780	573.33276	1056.15730	374.70392	Detected	Detected	Detected	Detected
2175	A_33_P3393175	CAPN7	Hs.595234	calpain 7	Homo sapiens calpain 7 (CAPN7), mRNA [NM_014296]	2.80876	up	2.69000	up	1040.86010	383.71664	732.46250	240.10382	Detected	Detected	Detected	Detected
2176	A_24_P822692	LOC643669	Hs.355357	similar to mCG2980	PREDICTED: Homo sapiens similar to mCG2980, transcript variant 1 (LOC643669), mRNA [XM_933621]	2.80801	up	1.50011	up	27.68674	10.20954	26.04203	15.30801	Detected	Compromised	Detected	Detected
2177	A_23_P146855	MPPED1	Hs.159538	metallophosphoesterase domain containing 1	Homo sapiens metallophosphoesterase domain containing 1 (MPPED1), mRNA [NM_001044370]	2.80642	up	2.53648	up	95.71785	35.31615	16.73628	5.81826	Detected	Detected	Detected	Compromised
2178	A_23_P140830	ELMO3	Hs.377416	engulfment and cell motility 3	Homo sapiens engulfment and cell motility 3 (ELMO3), mRNA [NM_024712]	2.80527	up	2.90656	up	114.54050	42.27829	132.72249	40.26522	Detected	Detected	Detected	Detected
2179	A_23_P132237	C22orf31	Hs.50891	chromosome 22 open reading frame 31	Homo sapiens chromosome 22 open reading frame 31 (C22orf31), mRNA [NM_015370]	2.80390	up	3.80466	up	27.73865	10.24367	19.28583	4.46980	Detected	Compromised	Detected	Compromised
2180	A_24_P353103	ZNF599	Hs.590961	zinc finger protein 599	Homo sapiens zinc finger protein 599 (ZNF599), mRNA [NM_001007248]	2.80214	up	2.81265	up	76.48120	28.26169	51.81128	16.24329	Detected	Detected	Detected	Compromised
2181	A_33_P3291816	CLDN22	Hs.333179	claudin 22	Homo sapiens claudin 22 (CLDN22), mRNA [NM_001111319]	2.80145	up	2.97344	up	70.48438	26.05211	60.78074	18.02488	Detected	Detected	Detected	Detected
2182	A_32_P8221	GRM8	Hs.449625	glutamate receptor, metabotropic 8	Homo sapiens glutamate receptor, metabotropic 8 (GRM8), transcript variant 1, mRNA [NM_000845]	2.80007	up	1.53823	up	15.58556	5.76350	9.35802	5.36448	Detected	Compromised	Compromised	Compromised
2183	A_23_P213014	SLC2A9	Hs.656895	solute carrier family 2 (facilitated glucose transporter), member 9	Homo sapiens solute carrier family 2 (facilitated glucose transporter), member 9 (SLC2A9), transcript variant 2, mRNA [NM_001001290]	2.79836	up	2.49857	up	55.15659	20.40927	38.86599	13.71653	Detected	Detected	Detected	Compromised
2184	A_32_P415151	WDR27	Hs.131903	WD repeat domain 27	Homo sapiens WD repeat domain 27 (WDR27), mRNA [NM_182552]	2.79830	up	1.52642	up	38.49739	14.24524	38.15366	22.04081	Detected	Compromised	Detected	Detected
2185	A_33_P3241756		Hs.597664		Inhibitor of nuclear factor kappa-B kinase subunit beta (I-kappa-B-kinase beta)(IkBKB)(IKK-beta)(IKK-B)(EC 2.7.11.10)(I-kappa-B kinase 2)(IKK2)(Nuclear factor NF-kappa-B inhibitor kinase beta)(NFKBIKB) [Source:UniProtKB/Swiss-Prot;Acc:O14920] [ENST00000379708]	2.79713	up	1.70783	up	12.59193	4.66137	14.43501	7.45312	Detected	Compromised	Detected	Compromised
2186	A_33_P3225487	HSD17B7	Hs.492925	hydroxysteroid (17-beta) dehydrogenase 7	Homo sapiens hydroxysteroid (17-beta) dehydrogenase 7 (HSD17B7), mRNA [NM_016371]	2.79605	up	2.44941	up	290.94156	107.74419	289.11620	104.08241	Detected	Detected	Detected	Detected
2187	A_23_P142830	PLA2R1	Hs.410477	phospholipase A2 receptor 1, 180kDa	Homo sapiens phospholipase A2 receptor 1, 180kDa (PLA2R1), transcript variant 1, mRNA [NM_007366]	2.79600	up	3.49108	up	148.81900	55.11295	125.71582	31.75380	Detected	Detected	Detected	Detected
2188	A_23_P87545	IFITM3	Hs.374650	interferon induced transmembrane protein 3 (1-8U)	Homo sapiens interferon induced transmembrane protein 3 (1-8U) (IFITM3), mRNA [NM_021034]	2.79539	up	2.62922	up	54624.94500	20234.01400	64728.22000	21708.58800	Detected	Detected	Detected	Detected
2189	A_24_P48057	IRX5	Hs.435730	iroquois homeobox 5	Homo sapiens iroquois homeobox 5 (IRX5), mRNA [NM_005853]	2.79518	up	2.62417	up	1442.10110	534.21950	1232.52540	414.16046	Detected	Detected	Detected	Detected
2190	A_33_P3417452	ZGLP1	Hs.709296	zinc finger, GATA-like protein 1	Homo sapiens zinc finger, GATA-like protein 1 (ZGLP1), mRNA [NM_001103167]	2.79458	up	2.54793	up	756.55300	280.32110	674.41170	233.40129	Detected	Detected	Detected	Detected
2191	A_23_P500410	ATP6V1G2	Hs.249227	ATPase, H+ transporting, lysosomal 13kDa, V1 subunit G2	Homo sapiens ATPase, H+ transporting, lysosomal 13kDa, V1 subunit G2 (ATP6V1G2), transcript variant 1, mRNA [NM_130463]	2.79277	up	4.04675	up	369.15085	136.86820	388.91280	84.74439	Detected	Detected	Detected	Detected
2192	A_24_P330518	CA12	Hs.210995	carbonic anhydrase XII	Homo sapiens carbonic anhydrase XII (CA12), transcript variant 1, mRNA [NM_001218]	2.79082	up	2.46093	up	4428.40000	1643.03880	2400.52400	860.14606	Detected	Detected	Detected	Detected

2193	A_23_P361820	ATG2A	Hs.370671	ATG2 autophagy related 2 homolog A (S. cerevisiae)	Homo sapiens ATG2 autophagy related 2 homolog A (S. cerevisiae) (ATG2A), mRNA [NM_015104]	2.78862	up	2.23456	up	1221.41470	453.53030	1219.88320	481.38455	Detected	Detected	Detected	Detected
2194	A_24_P921155	C3orf17	Hs.591288	chromosome 3 open reading frame 17	Homo sapiens chromosome 3 open reading frame 17 (C3orf17), transcript variant 1, mRNA [NM_015412]	2.78820	up	2.73545	up	731.55910	271.68024	814.09863	262.42978	Detected	Detected	Detected	Detected
2195	A_32_P141768	AGPAT4	Hs.353175	1-acylglycerol-3-phosphate O-acyltransferase 4 (lysophosphatidic acid acyltransferase, delta)	Homo sapiens 1-acylglycerol-3-phosphate O-acyltransferase 4 (lysophosphatidic acid acyltransferase, delta) (AGPAT4), mRNA [NM_020133]	2.78668	up	2.52203	up	2407.74370	894.65594	2052.66110	717.68190	Detected	Detected	Detected	Detected
2196	A_33_P3298455		Hs.648259		Em:AC005003.4 protein [Source:UniProtKB/TrEMBL;Acc:Q6ICM0] [ENST00000436050]	2.78618	up	2.20778	up	43.11263	16.02244	37.16148	14.84233	Detected	Compromised	Detected	Compromised
2197	A_23_P70897	ZCWPW1	Hs.105191	zinc finger, CW type with PWWP domain 1	Homo sapiens zinc finger, CW type with PWWP domain 1 (ZCWPW1), mRNA [NM_017984]	2.78484	up	2.73343	up	1729.24790	642.96826	1232.76500	397.68330	Detected	Detected	Detected	Detected
2198	A_33_P3399267	IL15RA	Hs.524117	interleukin 15 receptor, alpha	Homo sapiens interleukin 15 receptor, alpha (IL15RA), transcript variant 2, mRNA [NM_172200]	2.78450	up	5.36169	up	27.45525	10.20966	68.09969	11.19977	Detected	Compromised	Detected	Compromised
2199	A_24_P319364	F11R	Hs.517293	F11 receptor	Homo sapiens F11 receptor (F11R), mRNA [NM_016946]	2.78410	up	2.54717	up	265.14560	98.61254	255.14026	88.32552	Detected	Detected	Detected	Detected
2200	A_23_P403588	USP47	Hs.577256	ubiquitin specific peptidase 47	Homo sapiens ubiquitin specific peptidase 47 (USP47), mRNA [NM_017944]	2.78359	up	2.74113	up	1381.58250	513.93146	943.29740	303.44812	Detected	Detected	Detected	Detected
2201	A_23_P422933	ARHGAP20	Hs.6136	Rho GTPase activating protein 20	Homo sapiens Rho GTPase activating protein 20 (ARHGAP20), mRNA [NM_020809]	2.78351	up	3.43687	up	65.14198	24.23267	48.93725	12.55574	Detected	Detected	Detected	Compromised
2202	A_23_P38346	DHX58	Hs.55918	DEXH (Asp-Glu-X-His) box polypeptide 58	Homo sapiens DEXH (Asp-Glu-X-His) box polypeptide 58 (DHX58), mRNA [NM_024119]	2.78349	up	2.69134	up	1611.72770	599.56354	1703.36390	558.09050	Detected	Detected	Detected	Detected
2203	A_33_P3341787				Iduronate 2-sulfatase Precursor (EC 3.1.6.13)(Alpha-L-iduronate sulfate sulfatase)(Idursulfase) [Contains Iduronate 2-sulfatase 42 kDa chain;Iduronate 2-sulfatase 14 kDa chain] [Source:UniProtKB/Swiss-Prot;Acc:P22304] [ENST00000428056]	2.78315	up	2.76741	up	606.83923	225.77245	532.47960	169.66612	Detected	Detected	Detected	Detected
2204	A_23_P163075	SLC35F4	Hs.28280	solute carrier family 35, member F4	Homo sapiens solute carrier family 35, member F4 (SLC35F4), mRNA [NM_001080455]	2.77887	up	3.11182	up	13.67247	5.09461	25.27107	7.16101	Detected	Compromised	Detected	Compromised
2205	A_23_P374322	C13orf31	Hs.210586	chromosome 13 open reading frame 31	Homo sapiens chromosome 13 open reading frame 31 (C13orf31), transcript variant 2, mRNA [NM_153218]	2.77831	up	2.42699	up	127.10078	47.36967	93.64394	34.02332	Detected	Detected	Detected	Detected
2206	A_33_P3246026	DEFB131	Hs.590200	defensin, beta 131	Homo sapiens defensin, beta 131 (DEFB131), mRNA [NM_001040448]	2.77757	up	3.06967	up	103.65009	38.64011	83.14529	23.88426	Detected	Detected	Detected	Detected
2207	A_23_P74252	HSPC157	Hs.279842	hypothetical LOC29092	Homo sapiens hypothetical LOC29092 (HSPC157), transcript variant 1, non-coding RNA [NR_023918]	2.77736	up	3.19576	up	1263.19960	470.94867	1104.07360	304.64136	Detected	Detected	Detected	Detected
2208	A_24_P16610	ZNRF1	Hs.427284	zinc and ring finger 1	Homo sapiens zinc and ring finger 1 (ZNRF1), mRNA [NM_032268]	2.77723	up	2.49279	up	1044.41240	389.39722	977.30680	345.70972	Detected	Detected	Detected	Detected
2209	A_33_P3323928	C10orf75	Hs.628088	chromosome 10 open reading frame 75	Homo sapiens chromosome 10 open reading frame 75 (C10orf75), non-coding RNA [NR_026762]	2.77686	up	2.35409	up	55.70394	20.77136	59.42593	22.25967	Detected	Detected	Detected	Detected
2210	A_23_P205200	DHRS12	Hs.266728	dehydrogenase/reductase (SDR family) member 12	Homo sapiens dehydrogenase/reductase (SDR family) member 12 (DHRS12), transcript variant 2, mRNA [NM_024705]	2.77644	up	2.85293	up	317.20398	118.29969	299.31012	92.51144	Detected	Detected	Detected	Detected
2211	A_33_P3797403	LOC283387	Hs.368755	hypothetical protein LOC283387	Homo sapiens hypothetical protein LOC283387, mRNA (cDNA clone IMAGE:4821676) [BC032840]	2.77586	up	3.81427	up	192.89774	71.95538	140.49553	32.48003	Detected	Detected	Detected	Detected
2212	A_33_P3271395	LOC100129534	Hs.655313	small nuclear ribonucleoprotein polypeptide N pseudogene	Homo sapiens small nuclear ribonucleoprotein polypeptide N pseudogene (LOC100129534), non-coding RNA [NR_024489]	2.77557	up	2.36832	up	95.21238	35.52013	75.60004	28.14799	Detected	Detected	Detected	Detected
2213	A_23_P57534	DDX17	Hs.528305	DEAD (Asp-Glu-Ala-Asp) box polypeptide 17	Homo sapiens DEAD (Asp-Glu-Ala-Asp) box polypeptide 17 (DDX17), transcript variant 1, mRNA [NM_006386]	2.77516	up	3.30270	up	23.39954	8.73076	38.75468	10.34713	Detected	Compromised	Detected	Compromised
2214	A_33_P3343493	GABBR1	Hs.167017	gamma-aminobutyric acid (GABA) B receptor, 1	Homo sapiens gamma-aminobutyric acid (GABA) B receptor, 1 (GABBR1), transcript variant 1, mRNA [NM_001470]	2.77503	up	3.77714	up	22.33238	8.33300	23.91073	5.58207	Detected	Compromised	Detected	Compromised
2215	A_23_P85598	CABC1	Hs.118241	chaperone, ABC1 activity of bc1 complex homolog (S. pombe)	Homo sapiens chaperone, ABC1 activity of bc1 complex homolog (S. pombe) (CABC1), nuclear gene encoding mitochondrial protein, mRNA [NM_020247]	2.77344	up	2.40094	up	1976.51720	737.92890	1316.16480	483.38610	Detected	Detected	Detected	Detected
2216	A_23_P344194	C12orf68	Hs.251699	chromosome 12 open reading frame 68	Homo sapiens chromosome 12 open reading frame 68 (C12orf68), mRNA [NM_001013635]	2.77256	up	1.91635	up	19.97324	7.45934	9.99934	4.60109	Detected	Compromised	Compromised	Compromised
2217	A_23_P134755	TRPS1	Hs.657018	trichorhinophalangeal syndrome I	Homo sapiens trichorhinophalangeal syndrome I (TRPS1), mRNA [NM_014112]	2.77223	up	2.42554	up	2567.84860	959.12230	1462.15040	531.55676	Detected	Detected	Detected	Detected
2218	A_23_P202860	CYP2R1	Hs.371427	cytochrome P450, family 2, subfamily R, polypeptide 1	Homo sapiens cytochrome P450, family 2, subfamily R, polypeptide 1 (CYP2R1), mRNA [NM_024514]	2.77147	up	2.75198	up	7128.93070	2663.46300	5839.52700	1871.10490	Detected	Detected	Detected	Detected
2219	A_24_P336137	C22orf23	Hs.517612	chromosome 22 open reading frame 23	Homo sapiens chromosome 22 open reading frame 23 (C22orf23), mRNA [NM_032561]	2.77076	up	1.90123	up	153.30167	57.29025	152.74997	70.84554	Detected	Detected	Detected	Detected

2220	A_33_P3691916	FAM13A	Hs.97270	family with sequence similarity 13, member A	Homo sapiens family with sequence similarity 13, member A (FAM13A), transcript variant 1, mRNA [NM_014883]	2.77056	up	2.40066	up	748.85620	279.87527	496.23404	182.27296	Detected	Detected	Detected	Detected
2221	A_23_P16915	QPCT	Hs.79033	glutaminyl-peptide cyclotransferase	Homo sapiens glutaminyl-peptide cyclotransferase (QPCT), mRNA [NM_012413]	2.76710	up	2.69226	up	4553.40200	1703.89990	4829.67400	1581.85250	Detected	Detected	Detected	Detected
2222	A_24_P659836	C22orf41	Hs.535660	chromosome 22 open reading frame 41	Homo sapiens chromosome 22 open reading frame 41 (C22orf41), mRNA [NM_001123225]	2.76695	up	1.96860	up	33.44235	12.51495	30.41700	13.62463	Detected	Compromised	Detected	Compromised
2223	A_32_P119569	GOLGA8E	Hs.454647	golgi autoantigen, golgin subfamily a, 8E	Homo sapiens golgi autoantigen, golgin subfamily a, 8E (GOLGA8E), mRNA [NM_001012423]	2.76670	up	2.31120	up	218.06116	81.61124	29.78781	11.36491	Detected	Detected	Detected	Compromised
2224	A_33_P3350853	LOC202781		hypothetical LOC202781	Homo sapiens hypothetical LOC202781 (LOC202781), non-coding RNA [NR_028090]	2.76662	up	3.18085	up	824.39390	308.54532	388.52250	107.70577	Detected	Detected	Detected	Detected
2225	A_23_P134953	PLIN2	Hs.3416	perilipin 2	Homo sapiens perilipin 2 (PLIN2), mRNA [NM_001122]	2.76087	up	2.60113	up	10996.10900	4124.06540	10729.60200	3637.36550	Detected	Detected	Detected	Detected
2226	A_33_P3401428	TMEM38B	Hs.411925	transmembrane protein 38B	Homo sapiens transmembrane protein 38B (TMEM38B), mRNA [NM_018112]	2.76037	up	2.83601	up	398.62234	149.52962	358.59387	111.49632	Detected	Detected	Detected	Detected
2227	A_24_P458479		Hs.567050		Protein FAM27E1 [Source:UniProtKB/Swiss-Prot;Acc:Q5T7N7] [ENST00000377525]	2.75972	up	4.06193	up	45.32588	17.00646	41.43237	8.99442	Detected	Compromised	Detected	Compromised
2228	A_24_P577694	ADCY1	Hs.192215	adenylate cyclase 1 (brain)	Homo sapiens adenylate cyclase 1 (brain) (ADCY1), mRNA [NM_021116]	2.75621	up	2.25716	up	20.22272	7.59733	8.27071	3.23106	Detected	Compromised	Compromised	Compromised
2229	A_32_P169735	TTC8	Hs.303055	tetratricopeptide repeat domain 8	Homo sapiens tetratricopeptide repeat domain 8 (TTC8), transcript variant 1, mRNA [NM_144596]	2.75553	up	2.82769	up	4555.19600	1711.72670	4294.59380	1339.23350	Detected	Detected	Detected	Detected
2230	A_33_P3351559	ETNK1	Hs.29464	ethanolamine kinase 1	Homo sapiens ethanolamine kinase 1 (ETNK1), transcript variant 1, mRNA [NM_018638]	2.75355	up	3.10440	up	1019.17020	383.25427	783.29443	222.49100	Detected	Detected	Detected	Detected
2231	A_23_P112061	HGSNAT	Hs.600384	heparan-alpha-glucosaminide N-acetyltransferase	Homo sapiens heparan-alpha-glucosaminide N-acetyltransferase (HGSNAT), mRNA [NM_152419]	2.75193	up	2.69635	up	11915.31300	4483.33250	9373.46300	3065.41820	Detected	Detected	Detected	Detected
2232	A_33_P3256914		Hs.513225		Protein ITFG3 [Source:UniProtKB/Swiss-Prot;Acc:Q9H0X4] [ENST00000301679]	2.75034	up	3.36739	up	44.86378	16.89049	31.12515	8.15048	Detected	Compromised	Detected	Compromised
2233	A_33_P3371718	SAT1	Hs.28491	spermidine/spermine N1-acetyltransferase 1	Homo sapiens spermidine/spermine N1-acetyltransferase 1 (SAT1), transcript variant 1, mRNA [NM_002970]	2.74941	up	2.83328	up	59177.38700	22286.92000	61022.78500	18991.91200	Detected	Detected	Detected	Detected
2234	A_33_P3290443	SCARNA9		small Cajal body-specific RNA 9	Homo sapiens small Cajal body-specific RNA 9 (SCARNA9), guide RNA [NR_002569]	2.74827	up	3.22067	up	3433.11430	1293.48750	2224.59000	609.07290	Detected	Detected	Detected	Detected
2235	A_33_P3334688	CPNE2	Hs.339809	copine II	Homo sapiens cDNA FLJ44150 fis, clone THYMU2031046. [AK126138]	2.74664	up	1.11909	up	10.86167	4.09475	12.82939	10.10898	Compromised	Compromised	Detected	Compromised
2236	A_23_P126486	CROCCL1	Hs.631865	ciliary rootlet coiled-coil, rootletin-like 1	Homo sapiens ciliary rootlet coiled-coil, rootletin-like 1 (CROCCL1), non-coding RNA [NR_026752]	2.74607	up	2.94759	up	7548.32500	2846.23900	6091.19600	1822.21830	Detected	Detected	Detected	Detected
2237	A_32_P58937	MBTD1	Hs.656803	mbt domain containing 1	Homo sapiens mbt domain containing 1 (MBTD1), mRNA [NM_017643]	2.74495	up	2.48773	up	575.32670	217.02635	445.87460	158.04301	Detected	Detected	Detected	Detected
2238	A_23_P424582	EGFL8	Hs.332138	EGF-like-domain, multiple 8	Homo sapiens EGF-like-domain, multiple 8 (EGFL8), mRNA [NM_030652]	2.74319	up	2.68181	up	2126.77400	802.78480	2198.38480	722.83826	Detected	Detected	Detected	Detected
2239	A_23_P104224	A1CF	Hs.499643	APOBEC1 complementation factor	Homo sapiens APOBEC1 complementation factor (A1CF), transcript variant 3, mRNA [NM_138933]	2.74294	up	1.46516	up	15.27172	5.76507	27.42098	16.50308	Detected	Compromised	Detected	Detected
2240	A_23_P104318	DDIT4	Hs.719970	DNA-damage-inducible transcript 4	Homo sapiens DNA-damage-inducible transcript 4 (DDIT4), mRNA [NM_019058]	2.74249	up	2.50768	up	8787.71100	3317.90600	6731.84130	2367.15360	Detected	Detected	Detected	Detected
2241	A_33_P3221859	SHOX2	Hs.55967	short stature homeobox 2	Homo sapiens short stature homeobox 2 (SHOX2), transcript variant 1, mRNA [NM_003030]	2.74215	up	2.48845	up	245.83199	92.82827	254.60356	90.21978	Detected	Detected	Detected	Detected
2242	A_24_P71661	CRTAP	Hs.719160	cartilage associated protein	Homo sapiens cartilage associated protein (CRTAP), mRNA [NM_006371]	2.74195	up	2.68751	up	677.63460	255.89934	601.12555	197.23361	Detected	Detected	Detected	Detected
2243	A_33_P3265429	LOC100132653	Hs.568145	similar to phosphorylase kinase gamma subunit 1	PREDICTED: Homo sapiens similar to phosphorylase kinase gamma subunit 1 (LOC100132653), mRNA [XM_001715253]	2.74177	up	4.26540	up	15.74395	5.94588	17.84281	3.68867	Detected	Compromised	Detected	Compromised
2244	A_23_P89780	LAMA3	Hs.436367	laminin, alpha 3	Homo sapiens laminin, alpha 3 (LAMA3), transcript variant 1, mRNA [NM_198129]	2.74168	up	2.99893	up	142.31633	53.74904	125.89790	37.01841	Detected	Detected	Detected	Detected
2245	A_24_P418044	HLA-J	Hs.720762	major histocompatibility complex, class I, J (pseudogene)	Homo sapiens major histocompatibility complex, class I, J (pseudogene) (HLA-J), non-coding RNA [NR_024240]	2.74136	up	3.14531	up	109189.96000	41243.02300	75351.33600	21124.85000	Detected	Detected	Detected	Detected
2246	A_24_P93309	CDNF	Hs.559067	cerebral dopamine neurotrophic factor	Homo sapiens cerebral dopamine neurotrophic factor (CDNF), mRNA [NM_001029954]	2.74130	up	2.70187	up	106.76208	40.32684	70.83563	23.11818	Detected	Detected	Detected	Detected
2247	A_23_P9662	IPP	Hs.699548	intracisternal A particle-promoted polypeptide	Homo sapiens intracisternal A particle-promoted polypeptide (IPP), transcript variant 1, mRNA [NM_005897]	2.74127	up	2.35659	up	672.14680	253.88971	881.59357	329.87552	Detected	Detected	Detected	Detected
2248	A_23_P350551	C12orf57	Hs.591045	chromosome 12 open reading frame 57	Homo sapiens chromosome 12 open reading frame 57 (C12orf57), mRNA [NM_138425]	2.74053	up	2.69612	up	26888.30500	10159.26800	33555.77000	10974.73700	Detected	Detected	Detected	Detected
2249	A_23_P396981	CCDC66	Hs.476399	coiled-coil domain containing 66	Homo sapiens coiled-coil domain containing 66 (CCDC66), transcript variant 2, mRNA [NM_001012506]	2.74043	up	2.23640	up	272.79346	103.07387	106.65109	42.05142	Detected	Detected	Detected	Detected

2250	A_23_P304489	FAM19A5	Hs.436854	family with sequence similarity 19 (chemokine (C-C motif)-like), member A5	Homo sapiens family with sequence similarity 19 (chemokine (C-C motif)-like), member A5 (FAM19A5), transcript variant 2, mRNA [NM_015381]	2.74026	up	2.07148	up	106.67150	40.30782	108.21547	46.06533	Detected	Detected	Detected	Detected
2251	A_33_P3327921	RHOQ	Hs.709193	ras homolog gene family, member Q	Homo sapiens ras homolog gene family, member Q (RHOQ), mRNA [NM_012249]	2.73760	up	2.94244	up	925.88710	350.20456	631.52510	189.25537	Detected	Detected	Detected	Detected
2252	A_23_P203900	SCARB1	Hs.709216	scavenger receptor class B, member 1	Homo sapiens scavenger receptor class B, member 1 (SCARB1), transcript variant 1, mRNA [NM_005505]	2.73751	up	2.54210	up	9151.40500	3461.51270	8312.78500	2883.49880	Detected	Detected	Detected	Detected
2253	A_23_P103371	ADC	Hs.101807	arginine decarboxylase	Homo sapiens arginine decarboxylase (ADC), mRNA [NM_052998]	2.73613	up	2.21314	up	482.97696	182.77731	413.13070	164.60535	Detected	Detected	Detected	Detected
2254	A_23_P426305	AOC3	Hs.198241	amine oxidase, copper containing 3 (vascular adhesion protein 1)	Homo sapiens amine oxidase, copper containing 3 (vascular adhesion protein 1) (AOC3), mRNA [NM_003734]	2.73563	up	3.21096	up	152.22842	57.61984	137.09679	37.64936	Detected	Detected	Detected	Detected
2255	A_24_P234732	MXD4	Hs.655020	MAX dimerization protein 4	Homo sapiens MAX dimerization protein 4 (MXD4), mRNA [NM_006454]	2.73452	up	2.16162	up	2188.94020	828.86975	2307.67300	941.37070	Detected	Detected	Detected	Detected
2256	A_33_P3404531	ZCWPW1	Hs.105191	zinc finger, CW type with PWWP domain 1	Homo sapiens zinc finger, CW type with PWWP domain 1 (ZCWPW1), mRNA [NM_017984]	2.73294	up	3.33840	up	223.51389	84.68529	189.96428	50.17631	Detected	Detected	Detected	Detected
2257	A_23_P66766	RPAIN	Hs.462086	RPA interacting protein	Homo sapiens RPA interacting protein (RPAIN), transcript variant 4, mRNA [NM_001160246]	2.73133	up	2.58567	up	5565.69700	2109.98140	5454.36200	1860.09700	Detected	Detected	Detected	Detected
2258	A_33_P3331601	THNSL1	Hs.645274	threonine synthase-like 1 (S. cerevisiae)	Homo sapiens threonine synthase-like 1 (S. cerevisiae) (THNSL1), mRNA [NM_024838]	2.72902	up	2.23738	up	65.70640	24.93071	48.71668	19.20007	Detected	Detected	Detected	Detected
2259	A_24_P352637	TSSK4	Hs.314432	testis-specific serine kinase 4	Homo sapiens testis-specific serine kinase 4 (TSSK4), mRNA [NM_174944]	2.72887	up	4.82971	up	10.93978	4.15106	21.11306	3.85474	Compromised	Compromised	Detected	Compromised
2260	A_33_P3335865	WDR35	Hs.205427	WD repeat domain 35	Homo sapiens WD repeat domain 35 (WDR35), transcript variant 1, mRNA [NM_001006657]	2.72864	up	2.97966	up	1400.68300	531.52830	1045.45680	309.38882	Detected	Detected	Detected	Detected
2261	A_23_P250629	PSMB8	Hs.180062	proteasome (prosome, macropain) subunit, beta type, 8 (large multifunctional peptidase 7)	Homo sapiens proteasome (prosome, macropain) subunit, beta type, 8 (large multifunctional peptidase 7) (PSMB8), transcript variant 1, mRNA [NM_004159]	2.72804	up	2.58023	up	3337.34890	1266.73000	3844.88770	1313.98380	Detected	Detected	Detected	Detected
2262	A_24_P165205	MORN1	Hs.642701	MORN repeat containing 1	Homo sapiens MORN repeat containing 1 (MORN1), mRNA [NM_024848]	2.72802	up	1.63692	up	20.52877	7.79199	21.77705	11.73105	Detected	Compromised	Detected	Compromised
2263	A_23_P371824	TUFT1	Hs.489922	tuftelin 1	Homo sapiens tuftelin 1 (TUFT1), transcript variant 1, mRNA [NM_020127]	2.72652	up	2.98720	up	911.69410	346.23734	886.69170	261.74250	Detected	Detected	Detected	Detected
2264	A_33_P3255544	RNF130	Hs.484363	ring finger protein 130	Homo sapiens ring finger protein 130 (RNF130), mRNA [NM_018434]	2.72480	up	2.28081	up	3022.85500	1148.72310	2527.85420	977.30230	Detected	Detected	Detected	Detected
2265	A_33_P3354414	AOX1	Hs.406238	aldehyde oxidase 1	Homo sapiens aldehyde oxidase 1 (AOX1), mRNA [NM_001159]	2.72471	up	2.52420	up	5323.11330	2022.91720	5324.66200	1860.08970	Detected	Detected	Detected	Detected
2266	A_33_P3382595	RN7SK	Hs.706653	RNA, 7SK small nuclear	Homo sapiens RNA, 7SK small nuclear (RN7SK), small nuclear RNA [NR_001445]	2.72448	up	2.39852	up	3720.59500	1414.04220	3145.01200	1156.23100	Detected	Detected	Detected	Detected
2267	A_33_P3243502	LOC389634	Hs.700070	hypothetical LOC389634	Homo sapiens hypothetical LOC389634 (LOC389634), non-coding RNA [NR_024420]	2.72371	up	4.74931	up	789.77740	300.24545	655.50130	121.70493	Detected	Detected	Detected	Detected
2268	A_33_P3393684	VSIG8	Hs.647718	V-set and immunoglobulin domain containing 8	Homo sapiens V-set and immunoglobulin domain containing 8 (VSIG8), mRNA [NM_001013661]	2.72363	up	2.75275	up	345.69006	131.42291	262.03317	83.93741	Detected	Detected	Detected	Detected
2269	A_23_P155755	CXCL6	Hs.164021	chemokine (C-X-C motif) ligand 6 (granulocyte chemotactic protein 2)	Homo sapiens chemokine (C-X-C motif) ligand 6 (granulocyte chemotactic protein 2) (CXCL6), mRNA [NM_002993]	2.72287	up	2.40006	up	186.20544	70.81077	261.69284	96.14695	Detected	Detected	Detected	Detected
2270	A_33_P3672756	LOC284561	Hs.504540	hypothetical protein LOC284561	Homo sapiens cDNA FLJ13747 fis, clone PLACE3000276 [AK023809]	2.72086	up	10.37302	up	44.58916	16.96899	29.81039	2.53412	Detected	Detected	Detected	Compromised
2271	A_24_P476086	KPNA5		karyopherin alpha 5 (importin alpha 6)	Importin subunit alpha-6 (Karyopherin subunit alpha-5) [Source:UniProtKB/Swiss-Prot;Acc:O15131] [ENST00000368564]	2.71990	up	3.02876	up	389.28653	148.20027	247.94650	72.18704	Detected	Detected	Detected	Detected
2272	A_23_P430902	MORN4	Hs.217409	MORN repeat containing 4	Homo sapiens MORN repeat containing 4 (MORN4), transcript variant 1, mRNA [NM_178832]	2.71953	up	2.33642	up	271.67020	103.43839	264.17780	99.70356	Detected	Detected	Detected	Detected
2273	A_33_P3361991	OR9A2	Hs.553594	olfactory receptor, family 9, subfamily A, member 2	Homo sapiens olfactory receptor, family 9, subfamily A, member 2 (OR9A2), mRNA [NM_001001658]	2.71925	up	1.43992	up	14.09131	5.36581	7.62735	4.67091	Detected	Compromised	Compromised	Compromised
2274	A_32_P56713	BCR	Hs.517461	breakpoint cluster region	Homo sapiens breakpoint cluster region (BCR), transcript variant 1, mRNA [NM_004327]	2.71884	up	2.38530	up	980.49115	373.41640	775.51580	286.69086	Detected	Detected	Detected	Detected
2275	A_23_P19322	C6orf64	Hs.58382	chromosome 6 open reading frame 64	Homo sapiens chromosome 6 open reading frame 64 (C6orf64), mRNA [NM_018322]	2.71871	up	2.73909	up	2718.09200	1035.22520	2479.50760	798.22240	Detected	Detected	Detected	Detected
2276	A_33_P3394213	GRIN3B	Hs.660378	glutamate receptor, ionotropic, N-methyl-D-aspartate 3B	Homo sapiens glutamate receptor, ionotropic, N-methyl-D-aspartate 3B (GRIN3B), mRNA [NM_138690]	2.71633	up	2.70513	up	512.19226	195.24643	480.25845	156.54953	Detected	Detected	Detected	Detected
2277	A_33_P3353372					2.71619	up	7.39717	up	83.43985	31.80871	29.37591	3.50180	Detected	Detected	Detected	Compromised
2278	A_33_P3285156	FLJ90757	Hs.448889	hypothetical LOC440465	Homo sapiens hypothetical LOC440465 (FLJ90757), non-coding RNA [NR_026857]	2.71609	up	2.56625	up	2567.01370	978.62560	2464.60600	846.86456	Detected	Detected	Detected	Detected
2279	A_24_P854492	MIAT	Hs.517502	myocardial infarction associated transcript (non-protein coding)	Homo sapiens myocardial infarction associated transcript (non-protein coding) (MIAT), non-coding RNA [NR_003491]	2.71533	up	1.74883	up	18.45537	7.03774	17.79155	8.97082	Detected	Compromised	Detected	Compromised

2280	A_32_P117170	NAPEPLD	Hs.324271	N-acyl phosphatidylethanolamine phospholipase D	Homo sapiens N-acyl phosphatidylethanolamine phospholipase D (NAPEPLD), transcript variant 1, mRNA [NM_001122838]	2.71370	up	2.46465	up	435.82000	166.29459	420.32336	150.38136	Detected	Detected	Detected	Detected
2281	A_33_P3239367				Obscurin (EC 2.7.11.1)(Obscurin-myosin light chain kinase)(Obscurin-MLCK)(Obscurin-RhoGEF) [Source:UniProtKB/Swiss-Prot;Acc:Q5VST9] [ENST00000366706]	2.71332	up	2.46797	up	99.30269	37.89597	59.26614	21.17544	Detected	Detected	Detected	Detected
2282	A_23_P154740	PLK1S1	Hs.187635	polo-like kinase 1 substrate 1	Homo sapiens polo-like kinase 1 substrate 1 (PLK1S1), transcript variant 1, mRNA [NM_018474]	2.71274	up	2.80582	up	3497.07180	1334.84000	3019.56180	948.96375	Detected	Detected	Detected	Detected
2283	A_33_P3326922		Hs.689400		CDNA FLJ26465 fis, clone KDN04238 [Source:UniProtKB/TrEMBL;Acc:Q6ZP60] [ENST00000377571]	2.71226	up	3.48239	up	16.59821	6.33671	15.58729	3.94692	Detected	Compromised	Detected	Compromised
2284	A_33_P3307990	MASP2	Hs.655645	mannan-binding lectin serine peptidase 2	Homo sapiens mannan-binding lectin serine peptidase 2 (MASP2), transcript variant 1, mRNA [NM_006610]	2.71088	up	2.11353	up	43.35148	16.55871	10.38281	4.33183	Detected	Compromised	Compromised	Compromised
2285	A_23_P422851	CABLES1	Hs.11108	Cdk5 and Abl enzyme substrate 1	Homo sapiens Cdk5 and Abl enzyme substrate 1 (CABLES1), transcript variant 1, mRNA [NM_138375]	2.70953	up	2.53192	up	4764.50630	1820.77820	4998.65700	1740.88350	Detected	Detected	Detected	Detected
2286	A_23_P7827	FAM26F	Hs.381220	family with sequence similarity 26, member F	Homo sapiens family with sequence similarity 26, member F (FAM26F), mRNA [NM_001010919]	2.70915	up	2.55861	up	226.72880	86.65763	223.49586	77.02497	Detected	Detected	Detected	Detected
2287	A_33_P3870906	LOC148987	Hs.158890	hypothetical protein LOC148987	Homo sapiens hypothetical protein LOC148987, mRNA (cDNA clone IMAGE:4830261) [BC040313]	2.70913	up	2.80399	up	48.53178	18.54938	48.41615	15.22578	Detected	Detected	Detected	Detected
2288	A_33_P3332362				POU domain, class 2, transcription factor 2 (Octamer-binding transcription factor 2)(Oct-2)(OTF-2)(Lymphoid-restricted immunoglobulin octamer-binding protein NF-A2) [Source:UniProtKB/Swiss-Prot;Acc:P09086] [ENST00000342301]	2.70703	up	3.08904	up	34.57213	13.22411	66.13220	18.87793	Detected	Compromised	Detected	Detected
2289	A_33_P3715177	MEGF8	Hs.132483	multiple EGF-like-domains 8	Homo sapiens multiple EGF-like-domains 8 (MEGF8), mRNA [NM_001410]	2.70483	up	2.88745	up	2074.76760	794.25850	1385.85960	423.22380	Detected	Detected	Detected	Detected
2290	A_23_P149695	C1orf203	Hs.664752	chromosome 1 open reading frame 203	Homo sapiens chromosome 1 open reading frame 203 (C1orf203), transcript variant 3, non-coding RNA [NR_024126]	2.70453	up	2.45802	up	96.68340	37.01635	92.72573	33.26439	Detected	Detected	Detected	Detected
2291	A_23_P68486	C20orf108	Hs.143736	chromosome 20 open reading frame 108	Homo sapiens chromosome 20 open reading frame 108 (C20orf108), mRNA [NM_080821]	2.70418	up	2.67942	up	12347.75300	4728.08700	12067.50500	3971.38430	Detected	Detected	Detected	Detected
2292	A_23_P256542	FAM162A	Hs.584881	family with sequence similarity 162, member A	Homo sapiens family with sequence similarity 162, member A (FAM162A), mRNA [NM_014367]	2.70333	up	2.74521	up	23549.29900	9020.10450	13823.70800	4440.32130	Detected	Detected	Detected	Detected
2293	A_33_P3333054	GOLSYN	Hs.390738	Golgi-localized protein	Homo sapiens Golgi-localized protein (GOLSYN), transcript variant 1, mRNA [NM_001099744]	2.70279	up	2.55352	up	160.25647	61.39557	145.88925	50.37893	Detected	Detected	Detected	Detected
2294	A_23_P16817	CLK1	Hs.433732	CDC-like kinase 1	Homo sapiens CDC-like kinase 1 (CLK1), transcript variant 1, mRNA [NM_004071]	2.70261	up	2.75917	up	7339.61100	2812.05100	6925.87260	2213.40530	Detected	Detected	Detected	Detected
2295	A_23_P382602	BCL9	Hs.415209	B-cell CLL/lymphoma 9	Homo sapiens B-cell CLL/lymphoma 9 (BCL9), mRNA [NM_004326]	2.70237	up	1.87085	up	21.38202	8.19289	18.49831	8.71885	Detected	Compromised	Detected	Compromised
2296	A_23_P144746	ZNF454	Hs.259441	zinc finger protein 454	Homo sapiens zinc finger protein 454 (ZNF454), mRNA [NM_182594]	2.70209	up	4.29596	up	35.44881	13.58422	16.33295	3.35251	Detected	Compromised	Detected	Compromised
2297	A_23_P48358	PCCA	Hs.80741	propionyl Coenzyme A carboxylase, alpha polypeptide	Homo sapiens propionyl Coenzyme A carboxylase, alpha polypeptide (PCCA), nuclear gene encoding mitochondrial protein, transcript variant 1, mRNA [NM_000282]	2.70201	up	2.89663	up	2382.41240	912.98230	2472.58740	752.70496	Detected	Detected	Detected	Detected
2298	A_33_P3311911					2.70198	up	2.43644	up	46.32223	17.75171	39.14465	14.16712	Detected	Compromised	Detected	Compromised
2299	A_23_P69908	GLRX	Hs.28988	glutaredoxin (thioltransferase)	Homo sapiens glutaredoxin (thioltransferase) (GLRX), transcript variant 1, mRNA [NM_002064]	2.69775	up	3.01359	up	27475.53700	10545.77100	32001.03100	9363.66000	Detected	Detected	Detected	Detected
2300	A_33_P3303250		Hs.650691		CDNA FLJ26691 fis, clone MPG07564 [Source:UniProtKB/TrEMBL;Acc:Q6ZP22] [ENST00000451205]	2.69703	up	9.32079	up	74.91072	28.76018	30.51932	2.88727	Detected	Detected	Detected	Compromised
2301	A_23_P128919	LGALS3	Hs.531081	lectin, galactoside-binding, soluble, 3	Homo sapiens lectin, galactoside-binding, soluble, 3 (LGALS3), transcript variant 1, mRNA [NM_002306]	2.69632	up	2.64176	up	80317.80500	30844.14800	65801.75000	21963.88900	Detected	Detected	Detected	Detected
2302	A_23_P171132	EDA2R	Hs.302017	ectodysplasin A2 receptor	Homo sapiens ectodysplasin A2 receptor (EDA2R), mRNA [NM_021783]	2.69515	up	1.05902	up	62.58739	24.04568	43.38207	36.12183	Detected	Detected	Detected	Detected
2303	A_23_P78018	ABCA5	Hs.421474	ATP-binding cassette, sub-family A (ABC1), member 5	Homo sapiens ATP-binding cassette, sub-family A (ABC1), member 5 (ABCA5), transcript variant 1, mRNA [NM_018672]	2.69455	up	2.98306	up	1227.13800	471.56424	783.20490	231.51515	Detected	Detected	Detected	Detected
2304	A_23_P85015	MAOB	Hs.654473	monoamine oxidase B	Homo sapiens monoamine oxidase B (MAOB), nuclear gene encoding mitochondrial protein, mRNA [NM_000898]	2.69411	up	5.03420	up	33.09285	12.71895	21.01397	3.68081	Detected	Compromised	Detected	Compromised
2305	A_23_P157361	WDR60	Hs.389945	WD repeat domain 60	Homo sapiens WD repeat domain 60 (WDR60), mRNA [NM_018051]	2.68834	up	2.83074	up	1735.24350	668.35730	1064.59530	331.62662	Detected	Detected	Detected	Detected
2306	A_23_P401076	SUSD3	Hs.88417	sushi domain containing 3	Homo sapiens sushi domain containing 3 (SUSD3), mRNA [NM_145006]	2.68798	up	3.92271	up	848.85254	326.99390	680.45526	152.96039	Detected	Detected	Detected	Detected

2307	A_33_P3404052	TNFAIP8	Hs.656274	tumor necrosis factor, alpha-induced protein 8	Homo sapiens tumor necrosis factor, alpha-induced protein 8 (TNFAIP8), transcript variant 1, mRNA [NM_014350]	2.68789	up	2.67531	up	151.25940	58.26980	106.60912	35.13874	Detected	Detected	Detected	Detected
2308	A_24_P406986	SLC43A3	Hs.99962	solute carrier family 43, member 3	Homo sapiens solute carrier family 43, member 3 (SLC43A3), mRNA [NM_199329]	2.68729	up	2.39497	up	2126.07960	819.21520	1828.93030	673.38446	Detected	Detected	Detected	Detected
2309	A_23_P110196	HERC5	Hs.26663	hect domain and RLD 5	Homo sapiens hect domain and RLD 5 (HERC5), mRNA [NM_016323]	2.68700	up	4.27036	up	71.27563	27.46673	40.60429	8.38442	Detected	Detected	Detected	Compromised
2310	A_24_P210082	C11orf71	Hs.715083	chromosome 11 open reading frame 71	Homo sapiens chromosome 11 open reading frame 71 (C11orf71), mRNA [NM_019021]	2.68400	up	2.77312	up	756.02106	291.66540	988.71140	314.38812	Detected	Detected	Detected	Detected
2311	A_33_P3416881	C2orf27A	Hs.655680	chromosome 2 open reading frame 27A	Homo sapiens chromosome 2 open reading frame 27A (C2orf27A), mRNA [NM_013310]	2.68272	up	2.75315	up	157.54677	60.80887	143.16309	45.85297	Detected	Detected	Detected	Detected
2312	A_23_P50735	ZNF181	Hs.720472	zinc finger protein 181	Homo sapiens zinc finger protein 181 (ZNF181), transcript variant 1, mRNA [NM_001029997]	2.68245	up	2.44450	up	217.11143	83.80767	133.10341	48.01355	Detected	Detected	Detected	Detected
2313	A_23_P76136	TSPAN11	Hs.505141	tetraspanin 11	Homo sapiens tetraspanin 11 (TSPAN11), mRNA [NM_001080509]	2.68132	up	2.48611	up	527.96594	203.88748	403.72270	143.19540	Detected	Detected	Detected	Detected
2314	A_24_P156501	EBF1	Hs.573143	early B-cell factor 1	Homo sapiens early B-cell factor 1 (EBF1), mRNA [NM_024007]	2.68064	up	2.35120	up	174.31447	67.33304	189.24274	70.97331	Detected	Detected	Detected	Detected
2315	A_24_P393372	PACS2	Hs.525626	phosphofurin acidic cluster sorting protein 2	Homo sapiens phosphofurin acidic cluster sorting protein 2 (PACS2), transcript variant 1, mRNA [NM_001100913]	2.67999	up	2.32175	up	2385.85900	921.81860	1793.84180	681.29285	Detected	Detected	Detected	Detected
2316	A_33_P3356631	ZNF192	Hs.57679	zinc finger protein 192	Homo sapiens zinc finger protein 192 (ZNF192), mRNA [NM_006298]	2.67985	up	1.25946	up	9.95997	3.84840	18.94888	13.26674	Compromised	Compromised	Detected	Compromised
2317	A_33_P3387971	FLJ42351	Hs.585221	hypothetical gene supported by AK124342	Homo sapiens cDNA FLJ42351 fis, clone UTERU2005664 [AK124342]	2.67907	up	1.50128	up	14.79518	5.71834	5.44075	3.19566	Detected	Compromised	Compromised	Compromised
2318	A_24_P187119				CDNA FLJ25625 fis, clone STM02974 [Source:UniProtKB/TrEMBL;Acc:Q8N1H0] [ENST00000399576]	2.67877	up	2.53466	up	121.76460	47.06722	85.36914	29.69933	Detected	Detected	Detected	Detected
2319	A_33_P3410859	LOC729603	Hs.674810	calcium binding protein P22 pseudogene	Homo sapiens calcium binding protein P22 pseudogene (LOC729603), non-coding RNA [NR_003288]	2.67708	up	3.15422	up	625.84534	242.06912	327.07083	91.43552	Detected	Detected	Detected	Detected
2320	A_33_P3293598	hCG_2038428	Hs.434120	hypothetical protein LOC144776	Homo sapiens hypothetical protein LOC144776 (LOC144776), non-coding RNA [NR_027039]	2.67542	up	1.11443	up	16.98447	6.57344	9.06844	7.17536	Detected	Compromised	Compromised	Compromised
2321	A_23_P143885	ARHGEF3	Hs.476402	Rho guanine nucleotide exchange factor (GEF) 3	Homo sapiens Rho guanine nucleotide exchange factor (GEF) 3 (ARHGEF3), transcript variant 3, mRNA [NM_019555]	2.67473	up	2.93686	up	263.36264	101.95470	342.90207	102.95630	Detected	Detected	Detected	Detected
2322	A_23_P54692				Putative uncharacterized protein ENSP00000330035 [Source:UniProtKB/TrEMBL;Acc:B7WNQ5] [ENST00000331346]	2.67461	up	1.29833	up	72.36359	28.01513	58.64199	39.82821	Detected	Detected	Detected	Detected
2323	A_23_P20852	AUH	Hs.175905	AU RNA binding protein/enoyl-Coenzyme A hydratase	Homo sapiens AU RNA binding protein/enoyl-Coenzyme A hydratase (AUH), nuclear gene encoding mitochondrial protein, mRNA [NM_001698]	2.67166	up	2.59494	up	2142.00050	830.17700	1548.35180	526.14795	Detected	Detected	Detected	Detected
2324	A_23_P209232	CLIP4	Hs.122927	CAP-GLY domain containing linker protein family, member 4	Homo sapiens CAP-GLY domain containing linker protein family, member 4 (CLIP4), mRNA [NM_024692]	2.66964	up	2.74715	up	4306.31150	1670.26480	2702.04250	867.31323	Detected	Detected	Detected	Detected
2325	A_23_P36724	FBXL14	Hs.367956	F-box and leucine-rich repeat protein 14	Homo sapiens F-box and leucine-rich repeat protein 14 (FBXL14), mRNA [NM_152441]	2.66855	up	3.90740	up	513.62646	199.29932	423.21848	95.50861	Detected	Detected	Detected	Detected
2326	A_24_P411851	C14orf49	Hs.354645	chromosome 14 open reading frame 49	Homo sapiens chromosome 14 open reading frame 49 (C14orf49), mRNA [NM_152592]	2.66828	up	2.38162	up	239.52228	92.94951	144.47006	53.48978	Detected	Detected	Detected	Detected
2327	A_33_P3343972	RSPH1	Hs.661069	radial spoke head 1 homolog (Chlamydomonas)	Homo sapiens radial spoke head 1 homolog (Chlamydomonas) (RSPH1), mRNA [NM_080860]	2.66812	up	2.17973	up	25.61244	9.93981	16.24456	6.57159	Detected	Compromised	Detected	Compromised
2328	A_33_P3322283	AZI2	Hs.706676	5-azacytidine induced 2	Homo sapiens 5-azacytidine induced 2 (AZI2), transcript variant 1, mRNA [NM_022461]	2.66808	up	2.76849	up	2200.28500	853.91205	1445.81980	460.50760	Detected	Detected	Detected	Detected
2329	A_24_P309594	SLC48A1	Hs.438867	solute carrier family 48 (heme transporter), member 1	Homo sapiens solute carrier family 48 (heme transporter), member 1 (SLC48A1), mRNA [NM_017842]	2.66547	up	2.43634	up	5426.91700	2108.20650	4635.96730	1677.90490	Detected	Detected	Detected	Detected
2330	A_23_P29638	NCKIPSD	Hs.655006	NCK interacting protein with SH3 domain	Homo sapiens NCK interacting protein with SH3 domain (NCKIPSD), transcript variant 2, mRNA [NM_184231]	2.66431	up	2.75669	up	415.94824	161.65468	449.89963	143.91057	Detected	Detected	Detected	Detected
2331	A_33_P3422732	LOC100131180	Hs.590740	hypothetical protein LOC100131180	PREDICTED: Homo sapiens hypothetical protein LOC100131180 (LOC100131180), mRNA [XM_001722021]	2.66429	up	4.52470	up	14.29618	5.55613	14.21573	2.77042	Detected	Compromised	Detected	Compromised
2332	A_33_P3620087	LOC400756	Hs.591444	hypothetical gene supported by BC030752	Homo sapiens hypothetical gene supported by BC030752, mRNA (cDNA clone IMAGE:4796358) [BC030752]	2.66409	up	1.46716	up	26.46292	10.28543	30.57357	18.37532	Detected	Compromised	Detected	Detected
2333	A_33_P3213317				Eppin Precursor (Epididymal protease inhibitor)(Serine protease inhibitor-like with Kunitz and WAP domains 1)(WAP four-disulfide core domain protein 7)(Protease inhibitor WAP7)(Cancer/testis antigen 71)(CT71) [Source:UniProtKB/Swiss-Prot;Acc:O95925] [ENST00000409554]	2.66308	up	2.46918	up	59.88384	23.28406	20.80347	7.42930	Detected	Detected	Detected	Compromised

2334	A_32_P104063	CRNDE	Hs.237396	colorectal neoplasia differentially expressed (non-protein coding)	PREDICTED: Homo sapiens hCG1815491 (LOC643911), miscRNA [XR_042100]	2.66282	up	2.89576	up	24081.86100	9364.43400	17524.34200	5336.36040	Detected	Detected	Detected	Detected
2335	A_24_P291401	TMEM150A	Hs.591559	transmembrane protein 150A	Homo sapiens transmembrane protein 150A (TMEM150A), transcript variant 1, mRNA [NM_001031738]	2.66124	up	2.52738	up	963.68600	374.95987	821.15890	286.49817	Detected	Detected	Detected	Detected
2336	A_33_P3382331	HSPA6	Hs.654614	heat shock 70kDa protein 6 (HSP70B')	Homo sapiens heat shock 70kDa protein 6 (HSP70B') (HSPA6), mRNA [NM_002155]	2.66110	up	4.26270	up	298.34850	116.09030	181.62470	37.57128	Detected	Detected	Detected	Detected
2337	A_33_P3257170				Proline-rich protein 3 (MHC class I region proline-rich protein CAT56) [Source:UniProtKB/Swiss-Prot;Acc:P79522] [ENST00000376555]	2.66088	up	2.23285	up	109.33479	42.54679	90.78534	35.85265	Detected	Detected	Detected	Detected
2338	A_24_P583225	LOC349114	Hs.661251	hypothetical LOC349114	Homo sapiens hypothetical LOC349114 (LOC349114), non-coding RNA [NR_026999]	2.66074	up	2.16978	up	37.91526	14.75520	28.80119	11.70471	Detected	Compromised	Detected	Compromised
2339	A_23_P43095	ZFHX4	Hs.458973	zinc finger homeobox 4	Homo sapiens zinc finger homeobox 4 (ZFHX4), mRNA [NM_024721]	2.66068	up	2.70822	up	396.07925	154.14275	158.96547	51.75873	Detected	Detected	Detected	Detected
2340	A_33_P3307875	C14orf147	Hs.269909	chromosome 14 open reading frame 147	Homo sapiens chromosome 14 open reading frame 147 (C14orf147), mRNA [NM_138288]	2.66059	up	2.89775	up	6130.82030	2386.02150	4405.52930	1340.60900	Detected	Detected	Detected	Detected
2341	A_23_P421401	PDGFRB	Hs.509067	platelet-derived growth factor receptor, beta polypeptide	Homo sapiens platelet-derived growth factor receptor, beta polypeptide (PDGFRB), mRNA [NM_002609]	2.66049	up	2.59819	up	52989.03000	20623.27000	35322.79000	11988.06800	Detected	Detected	Detected	Detected
2342	A_23_P131435	CD302	Hs.130014	CD302 molecule	Homo sapiens CD302 molecule (CD302), mRNA [NM_014880]	2.66032	up	2.84082	up	862.03470	335.52466	791.25525	245.60596	Detected	Detected	Detected	Detected
2343	A_24_P119609	MYO1D	Hs.658000	myosin ID	Homo sapiens myosin ID (MYO1D), mRNA [NM_015194]	2.65986	up	2.73130	up	101.33898	39.45036	95.53548	30.84335	Detected	Detected	Detected	Detected
2344	A_33_P3211793	LOC728650	Hs.696467	hypothetical LOC728650	PREDICTED: Homo sapiens hypothetical LOC728650 (LOC728650), mRNA [XM_002343155]	2.65914	up	5.24059	up	298.24066	116.13394	278.94510	46.93579	Detected	Detected	Detected	Detected
2345	A_33_P3385062	HAUS3	Hs.665869	HAUS augmin-like complex, subunit 3	Homo sapiens HAUS augmin-like complex, subunit 3 (HAUS3), mRNA [NM_024511]	2.65804	up	5.07206	up	12.89964	5.02516	15.17896	2.63890	Detected	Compromised	Detected	Compromised
2346	A_23_P26928		Hs.514222		Ig-like V-type domain-containing protein ENSP00000329499 Precursor [Source:UniProtKB/Swiss-Prot;Acc:A6NFU0] [ENST00000331733]	2.65649	up	2.43537	up	189.27588	73.77685	191.74980	69.42810	Detected	Detected	Detected	Detected
2347	A_33_P3215277	TTBK2	Hs.659846	tau tubulin kinase 2	Homo sapiens tau tubulin kinase 2 (TTBK2), mRNA [NM_173500]	2.65645	up	2.46279	up	1342.48820	523.28950	877.88160	314.32162	Detected	Detected	Detected	Detected
2348	A_33_P3379939	HLA-F	Hs.519972	major histocompatibility complex, class I, F	Homo sapiens major histocompatibility complex, class I, F (HLA-F), transcript variant 2, mRNA [NM_018950]	2.65620	up	2.66734	up	99214.07000	38676.38300	94341.70000	31188.24400	Detected	Detected	Detected	Detected
2349	A_24_P37264	RNF144A	Hs.22146	ring finger protein 144A	Homo sapiens ring finger protein 144A (RNF144A), mRNA [NM_014746]	2.65539	up	2.58464	up	234.84244	91.57585	150.96237	51.50310	Detected	Detected	Detected	Detected
2350	A_32_P174083	CYCS	Hs.437060	cytochrome c, somatic	Homo sapiens cytochrome c, somatic (CYCS), nuclear gene encoding mitochondrial protein, mRNA [NM_018947]	2.65505	up	2.56936	up	596.46630	232.61952	557.13140	191.20439	Detected	Detected	Detected	Detected
2351	A_23_P60565	ZNF354A	Hs.484324	zinc finger protein 354A	Homo sapiens zinc finger protein 354A (ZNF354A), mRNA [NM_005649]	2.65486	up	3.22345	up	1020.48010	398.01150	750.51636	205.30743	Detected	Detected	Detected	Detected
2352	A_23_P113111	AR	Hs.496240	androgen receptor	Homo sapiens androgen receptor (AR), transcript variant 1, mRNA [NM_000044]	2.65470	up	2.79181	up	2104.53610	820.86910	983.63250	310.67914	Detected	Detected	Detected	Detected
2353	A_33_P3343101				Arginine and glutamate-rich protein 1 [Source:UniProtKB/Swiss-Prot;Acc:Q9NWB6] [ENST00000426600]	2.65438	up	2.97194	up	40.75202	15.89717	33.49465	9.93805	Detected	Compromised	Detected	Compromised
2354	A_23_P376591	CLYBL	Hs.655642	citrate lyase beta like	Homo sapiens citrate lyase beta like (CLYBL), mRNA [NM_206808]	2.65272	up	2.23222	up	186.09581	72.64040	176.75195	69.82205	Detected	Detected	Detected	Detected
2355	A_23_P107166	ACBD4	Hs.110298	acyl-Coenzyme A binding domain containing 4	Homo sapiens acyl-Coenzyme A binding domain containing 4 (ACBD4), transcript variant 2, mRNA [NM_024722]	2.65204	up	2.71467	up	180.69081	70.54864	144.54988	46.95334	Detected	Detected	Detected	Detected
2356	A_24_P91094	RUNDC3A	Hs.500197	RUN domain containing 3A	Homo sapiens RUN domain containing 3A (RUNDC3A), transcript variant 2, mRNA [NM_006695]	2.65100	up	2.95669	up	75.60793	29.53190	72.62940	21.66069	Detected	Detected	Detected	Detected
2357	A_33_P3360675	PRKAR1B	Hs.520851	protein kinase, cAMP-dependent, regulatory, type I, beta	Homo sapiens protein kinase, cAMP-dependent, regulatory, type I, beta (PRKAR1B), transcript variant 1, mRNA [NM_001164761]	2.65053	up	2.57986	up	1440.59850	562.78510	1115.41690	381.24750	Detected	Detected	Detected	Detected
2358	A_23_P44195	MSI2	Hs.658922	musashi homolog 2 (Drosophila)	Homo sapiens musashi homolog 2 (Drosophila) (MSI2), transcript variant 1, mRNA [NM_138962]	2.65032	up	2.46608	up	958.55440	374.49940	852.47440	304.81740	Detected	Detected	Detected	Detected
2359	A_24_P161018	PARP14	Hs.518203	poly (ADP-ribose) polymerase family, member 14	Homo sapiens poly (ADP-ribose) polymerase family, member 14 (PARP14), mRNA [NM_017554]	2.64995	up	3.41538	up	449.63977	175.69504	296.76430	76.61930	Detected	Detected	Detected	Detected
2360	A_23_P257417	C2orf63	Hs.347014	chromosome 2 open reading frame 63	Homo sapiens chromosome 2 open reading frame 63 (C2orf63), transcript variant 1, mRNA [NM_152385]	2.64968	up	3.04330	up	122.79360	47.98616	80.46890	23.31573	Detected	Detected	Detected	Detected
2361	A_33_P3391339	LOC100287820	Hs.648247	hypothetical protein LOC100287820	Hypothetical gene supported by BC066547 [Source:UniProtKB/TrEMBL;Acc:Q6NYL1] [ENST00000383040]	2.64946	up	2.66484	up	96.22102	37.60498	68.31778	22.60620	Detected	Detected	Detected	Detected
2362	A_32_P50417	LOC100130107	Hs.710718	hypothetical LOC100130107	PREDICTED: Homo sapiens hypothetical LOC100130107 (LOC100130107), mRNA [XM_001716672]	2.64843	up	2.41043	up	2097.10100	819.90430	1340.98130	490.56195	Detected	Detected	Detected	Detected
2363	A_23_P412214	RAP1GAP2	Hs.499659	RAP1 GTPase activating protein 2	Homo sapiens RAP1 GTPase activating protein 2 (RAP1GAP2), transcript variant 1, mRNA [NM_015085]	2.64782	up	2.88580	up	37.15947	14.53162	35.00401	10.69590	Detected	Compromised	Detected	Compromised

2364	A_23_P137856	MUC1	Hs.89603	mucin 1, cell surface associated	Homo sapiens mucin 1, cell surface associated (MUC1), transcript variant 1, mRNA [NM_002456]	2.64641	up	2.26597	up	579.46295	226.72626	525.39060	204.45319	Detected	Detected	Detected	Detected
2365	A_33_P3391796	NOG	Hs.248201	noggin	Homo sapiens noggin (NOG), mRNA [NM_005450]	2.64519	up	2.84193	up	306.61194	120.02342	232.86661	72.25366	Detected	Detected	Detected	Detected
2366	A_24_P333857	SGIP1	Hs.132121	SH3-domain GRB2-like (endophilin) interacting protein 1	Homo sapiens SH3-domain GRB2-like (endophilin) interacting protein 1 (SGIP1), mRNA [NM_032291]	2.64444	up	2.68928	up	750.78827	293.97910	637.67060	209.08632	Detected	Detected	Detected	Detected
2367	A_24_P706752	PLA2G12A	Hs.389452	phospholipase A2, group XIIA	Homo sapiens phospholipase A2, group XIIA (PLA2G12A), mRNA [NM_030821]	2.64407	up	2.97161	up	1245.26260	487.66403	950.95000	282.18290	Detected	Detected	Detected	Detected
2368	A_32_P103837	FAM60A	Hs.505154	family with sequence similarity 60, member A	Homo sapiens family with sequence similarity 60, member A (FAM60A), transcript variant 2, mRNA [NM_021238]	2.64362	up	2.75208	up	1279.82400	501.28400	590.34430	189.15163	Detected	Detected	Detected	Detected
2369	A_23_P216630	SLC44A1	Hs.573495	solute carrier family 44, member 1	Homo sapiens solute carrier family 44, member 1 (SLC44A1), mRNA [NM_080546]	2.64066	up	2.54315	up	60259.83600	23629.21500	53149.87500	18428.78000	Detected	Detected	Detected	Detected
2370	A_23_P121885	ROPN1L	Hs.381089	ropporin 1-like	Homo sapiens ropporin 1-like (ROPN1L), mRNA [NM_031916]	2.63914	up	2.99138	up	189.76573	74.45407	171.42032	50.53081	Detected	Detected	Detected	Detected
2371	A_24_P385134	SCD5	Hs.379191	stearoyl-CoA desaturase 5	Homo sapiens stearoyl-CoA desaturase 5 (SCD5), transcript variant 1, mRNA [NM_001037582]	2.63807	up	2.71372	up	1867.68310	733.07720	1442.78090	468.81354	Detected	Detected	Detected	Detected
2372	A_23_P422115	C9orf116	Hs.414028	chromosome 9 open reading frame 116	Homo sapiens chromosome 9 open reading frame 116 (C9orf116), transcript variant 1, mRNA [NM_001048265]	2.63803	up	2.54321	up	2225.96240	873.71790	2368.95240	821.37006	Detected	Detected	Detected	Detected
2373	A_33_P3419567	RAD21L1	Hs.713451	RAD21-like 1 (S. pombe)	Homo sapiens RAD21-like 1 (S. pombe) (RAD21L1), mRNA [NM_001136566]	2.63791	up	1.68102	up	26.99780	10.59745	17.60497	9.23480	Detected	Compromised	Detected	Compromised
2374	A_33_P3247392	TPTE	Hs.122986	transmembrane phosphatase with tensin homology	Homo sapiens transmembrane phosphatase with tensin homology (TPTE), transcript variant 1, mRNA [NM_199261]	2.63600	up	3.37901	up	139.30809	54.72226	117.99721	30.79266	Detected	Detected	Detected	Detected
2375	A_23_P79155	GPR39	Hs.432395	G protein-coupled receptor 39	Homo sapiens G protein-coupled receptor 39 (GPR39), mRNA [NM_001508]	2.63589	up	2.80973	up	173.73503	68.24860	202.24092	63.47029	Detected	Detected	Detected	Detected
2376	A_23_P84872	SECISBP2	Hs.59804	SECIS binding protein 2	Homo sapiens SECIS binding protein 2 (SECISBP2), mRNA [NM_024077]	2.63444	up	2.65358	up	488.49902	192.00310	328.32812	109.10412	Detected	Detected	Detected	Detected
2377	A_24_P807031	ATP6AP1L	Hs.364045	ATPase, H+ transporting, lysosomal accessory protein 1-like	Homo sapiens ATPase, H+ transporting, lysosomal accessory protein 1-like (ATP6AP1L), mRNA [NM_001017971]	2.63325	up	2.58081	up	257.54730	101.27409	196.24289	67.05071	Detected	Detected	Detected	Detected
2378	A_23_P211244	PRMT2	Hs.154163	protein arginine methyltransferase 2	Homo sapiens protein arginine methyltransferase 2 (PRMT2), transcript variant 1, mRNA [NM_206962]	2.63305	up	2.46885	up	1390.70790	546.90234	1171.91060	418.56772	Detected	Detected	Detected	Detected
2379	A_23_P128728	ARG2	Hs.226007	arginase, type II	Homo sapiens arginase, type II (ARG2), nuclear gene encoding mitochondrial protein, mRNA [NM_001172]	2.63165	up	2.12293	up	405.57745	159.58018	414.65384	172.23233	Detected	Detected	Detected	Detected
2380	A_23_P57036	CD40	Hs.472860	CD40 molecule, TNF receptor superfamily member 5	Homo sapiens CD40 molecule, TNF receptor superfamily member 5 (CD40), transcript variant 1, mRNA [NM_001250]	2.63164	up	2.14054	up	399.17374	157.06107	486.50168	200.41289	Detected	Detected	Detected	Detected
2381	A_33_P3311083				Pleckstrin homology-like domain family B member 3 [Source:UniProtKB/Swiss-Prot;Acc:Q6NSJ2] [ENST00000314735]	2.63160	up	1.76198	up	8.04312	3.16474	16.88327	8.44929	Compromised	Compromised	Detected	Compromised
2382	A_33_P3295550	TET1	Hs.567594	tet oncogene 1	Homo sapiens tet oncogene 1 (TET1), mRNA [NM_030625]	2.63074	up	2.58637	up	386.89430	152.28192	346.90192	118.27197	Detected	Detected	Detected	Detected
2383	A_33_P3324505					2.63019	up	1.80521	up	41.20751	16.22265	24.68537	12.05809	Detected	Compromised	Detected	Compromised
2384	A_33_P3236102	IER5L	Hs.529857	immediate early response 5-like	Homo sapiens immediate early response 5-like (IER5L), mRNA [NM_203434]	2.62963	up	2.58589	up	2244.03100	883.62274	1469.55140	501.11768	Detected	Detected	Detected	Detected
2385	A_33_P3287907	LOC728723	Hs.161338	hypothetical LOC728723	Homo sapiens hypothetical LOC728723 (LOC728723), non-coding RNA [NR_024398]	2.62951	up	2.16734	up	31.82555	12.53240	8.27923	3.36843	Detected	Compromised	Compromised	Compromised
2386	A_33_P3367201	MMAB	Hs.12106	methylmalonic aciduria (cobalamin deficiency) cblB type	Homo sapiens methylmalonic aciduria (cobalamin deficiency) cblB type (MMAB), nuclear gene encoding mitochondrial protein, mRNA [NM_052845]	2.62933	up	2.60006	up	339.17648	133.57138	299.76157	101.66191	Detected	Detected	Detected	Detected
2387	A_33_P3209816	DPY19L3	Hs.194392	dpy-19-like 3 (C. elegans)	Homo sapiens dpy-19-like 3 (C. elegans) (DPY19L3), mRNA [NM_207325]	2.62661	up	3.24530	up	18.67290	7.36121	30.22174	8.21165	Detected	Compromised	Detected	Compromised
2388	A_33_P3783235	LOC286052	Hs.100691	hypothetical protein LOC286052	Homo sapiens cDNA FLJ37785 fis, clone BRHIP2028330 [AK095104]	2.62552	up	2.51462	up	988.82190	389.97397	721.54160	253.01965	Detected	Detected	Detected	Detected
2389	A_24_P303594	ACTR3B	Hs.647117	ARP3 actin-related protein 3 homolog B (yeast)	Homo sapiens ARP3 actin-related protein 3 homolog B (yeast) (ACTR3B), transcript variant 2, mRNA [NM_001040135]	2.62447	up	2.26151	up	194.17921	76.61145	184.54645	71.95707	Detected	Detected	Detected	Detected
2390	A_33_P3274129	TMEM151B	Hs.632851	transmembrane protein 151B	Homo sapiens transmembrane protein 151B (TMEM151B), mRNA [NM_001137560]	2.62332	up	3.32385	up	19.79187	7.81210	15.11412	4.00966	Detected	Compromised	Detected	Compromised
2391	A_23_P391228	MANEAL	Hs.534562	mannosidase, endo-alpha-like	Homo sapiens mannosidase, endo-alpha-like (MANEAL), transcript variant 1, mRNA [NM_001031740]	2.62227	up	2.68414	up	67.91811	26.81895	52.51436	17.25199	Detected	Detected	Detected	Detected
2392	A_33_P3354569	GPD2	Hs.512382	glycerol-3-phosphate dehydrogenase 2 (mitochondrial)	Homo sapiens glycerol-3-phosphate dehydrogenase 2 (mitochondrial) (GPD2), nuclear gene encoding mitochondrial protein, transcript variant 1, mRNA [NM_001083112]	2.61972	up	2.58521	up	4125.64100	1630.68430	2954.59500	1007.78326	Detected	Detected	Detected	Detected

2393	A_23_P12884	GRK5	Hs.524625	G protein-coupled receptor kinase 5	Homo sapiens G protein-coupled receptor kinase 5 (GRK5), mRNA [NM_005308]	2.61968	up	2.61426	up	4844.80130	1914.96700	4158.13770	1402.54250	Detected	Detected	Detected	Detected
2394	A_24_P379165	FOXO4	Hs.584654	forkhead box O4	Homo sapiens forkhead box O4 (FOXO4), mRNA [NM_005938]	2.61946	up	2.19949	up	207.34349	81.96180	156.73949	62.83793	Detected	Detected	Detected	Detected
2395	A_33_P3370310				UPF0599 protein C8orf83 [Source:UniProtKB/Swiss-Prot;Acc:Q629K1] [ENST00000378861]	2.61876	up	1.81346	up	73.54961	29.08158	72.92074	35.45758	Detected	Detected	Detected	Detected
2396	A_23_P211850	ABHD6	Hs.476454	abhydrolase domain containing 6	Homo sapiens abhydrolase domain containing 6 (ABHD6), mRNA [NM_020676]	2.61805	up	2.80193	up	2148.36450	849.69520	1780.49700	560.33810	Detected	Detected	Detected	Detected
2397	A_23_P86021	SELENBP1	Hs.632460	selenium binding protein 1	Homo sapiens selenium binding protein 1 (SELENBP1), mRNA [NM_003944]	2.61760	up	2.49365	up	4541.43460	1796.47970	3642.94560	1288.19710	Detected	Detected	Detected	Detected
2398	A_24_P135255		Hs.442781		SGT1, suppressor of G2 allele of SKP1 like 1 (S. cerevisiae)HCG2020254, isoform CRA_f ; [Source:UniProtKB/TrEMBL;Acc:Q5T9E9] [ENST00000304932]	2.61701	up	2.92399	up	38.62441	15.28231	21.92378	6.61157	Detected	Detected	Detected	Compromised
2399	A_23_P368794	TCERG1L	Hs.126575	transcription elongation regulator 1-like	Homo sapiens transcription elongation regulator 1-like (TCERG1L), mRNA [NM_174937]	2.61616	up	2.43389	up	8.76389	3.46870	16.24385	5.88510	Compromised	Compromised	Detected	Compromised
2400	A_23_P359655	ZNF664	Hs.524828	zinc finger protein 664	Homo sapiens zinc finger protein 664 (ZNF664), mRNA [NM_152437]	2.61608	up	2.56815	up	12359.41400	4891.92140	10681.62800	3667.60350	Detected	Detected	Detected	Detected
2401	A_32_P190461	LOC728534	Hs.679932	hypothetical LOC728534	PREDICTED: Homo sapiens hypothetical LOC728534, transcript variant 2 (LOC728534), mRNA [XM_001724486]	2.61605	up	2.10182	up	46.11272	18.25191	36.33460	15.24367	Detected	Detected	Detected	Detected
2402	A_33_P3773374	LOC286063	Hs.21550	hypothetical protein LOC286063	Homo sapiens cDNA FLJ33573 fis, clone BRAMY2010798 [AK090892]	2.61589	up	3.41384	up	118.21983	46.79547	43.67109	11.28020	Detected	Detected	Detected	Compromised
2403	A_33_P3379922	PROC	Hs.224698	protein C (inactivator of coagulation factors Va and VIIIa)	Homo sapiens protein C (inactivator of coagulation factors Va and VIIIa) (PROC), mRNA [NM_000312]	2.61319	up	4.35004	up	85.31436	33.80525	93.82890	19.01994	Detected	Detected	Detected	Detected
2404	A_32_P103669	GOLGA8E	Hs.454647	golgi autoantigen, golgin subfamily a, 8E	Homo sapiens golgi autoantigen, golgin subfamily a, 8E (GOLGA8E), mRNA [NM_001012423]	2.61263	up	3.06313	up	1608.15280	637.35660	144.90952	41.71546	Detected	Detected	Detected	Detected
2405	A_33_P3236993	ARVCF	Hs.713616	armadillo repeat gene deletes in velocardiofacial syndrome	Homo sapiens armadillo repeat gene deletes in velocardiofacial syndrome (ARVCF), mRNA [NM_001670]	2.61209	up	1.77297	up	59.78721	23.70028	47.86024	23.80345	Detected	Detected	Detected	Detected
2406	A_23_P41664	LRRC70	Hs.721348	leucine rich repeat containing 70	Homo sapiens leucine rich repeat containing 70 (LRRC70), mRNA [NM_181506]	2.61067	up	2.68330	up	134.31561	53.27308	93.84658	30.84002	Detected	Detected	Detected	Detected
2407	A_33_P3315719	PLEKHH2	Hs.164162	pleckstrin homology domain containing, family H (with MyTH4 domain) member 2	Homo sapiens pleckstrin homology domain containing, family H (with MyTH4 domain) member 2 (PLEKHH2), mRNA [NM_172069]	2.60970	up	2.58848	up	157.36633	62.43885	112.62922	38.36826	Detected	Detected	Detected	Detected
2408	A_33_P3380867	STAT5B	Hs.595276	signal transducer and activator of transcription 5B	Homo sapiens signal transducer and activator of transcription 5B (STAT5B), mRNA [NM_012448]	2.60928	up	2.67820	up	3150.59470	1250.27420	1811.62500	596.47260	Detected	Detected	Detected	Detected
2409	A_33_P3225313	FAM91A1	Hs.459174	family with sequence similarity 91, member A1	Homo sapiens family with sequence similarity 91, member A1 (FAM91A1), mRNA [NM_144963]	2.60913	up	1.74974	up	13.83474	5.49046	23.32592	11.75525	Detected	Compromised	Detected	Compromised
2410	A_33_P3250083	NFATC4	Hs.77810	nuclear factor of activated T-cells, cytoplasmic, calcineurin-dependent 4	Homo sapiens nuclear factor of activated T-cells, cytoplasmic, calcineurin-dependent 4 (NFATC4), transcript variant 1, mRNA [NM_001136022]	2.60912	up	2.51473	up	8602.42100	3413.97100	7543.43650	2645.10300	Detected	Detected	Detected	Detected
2411	A_24_P179183	ANKRD12	Hs.464585	ankyrin repeat domain 12	Homo sapiens ankyrin repeat domain 12 (ANKRD12), transcript variant 1, mRNA [NM_015208]	2.60856	up	3.06558	up	3054.66280	1212.53820	1294.06570	372.22754	Detected	Detected	Detected	Detected
2412	A_33_P3306146	PLAU	Hs.77274	plasminogen activator, urokinase	Homo sapiens plasminogen activator, urokinase (PLAU), transcript variant 2, mRNA [NM_001145031]	2.60846	up	2.37238	up	380.63810	151.09871	501.72690	186.48705	Detected	Detected	Detected	Detected
2413	A_32_P196021	FGF7	Hs.567268	fibroblast growth factor 7 (keratinocyte growth factor)	Homo sapiens fibroblast growth factor 7 (keratinocyte growth factor) (FGF7), mRNA [NM_002009]	2.60829	up	3.56643	up	269.14322	106.84673	202.96716	50.18317	Detected	Detected	Detected	Detected
2414	A_23_P73526	CITED1	Hs.40403	Cbp/p300-interacting transactivator, with Glu/Asp-rich carboxy-terminal domain, 1	Homo sapiens Cbp/p300-interacting transactivator, with Glu/Asp-rich carboxy-terminal domain, 1 (CITED1), transcript variant 1, mRNA [NM_004143]	2.60734	up	2.66254	up	52.00576	20.65315	57.90118	19.17595	Detected	Detected	Detected	Detected
2415	A_33_P3276585	IFT74	Hs.145402	intraflagellar transport 74 homolog (Chlamydomonas)	Homo sapiens intraflagellar transport 74 homolog (Chlamydomonas) (IFT74), transcript variant 4, mRNA [NM_001099224]	2.60719	up	1.96051	up	23.14281	9.19128	19.04984	8.56818	Detected	Compromised	Detected	Compromised
2416	A_33_P3244283	CAMK2N2	Hs.585003	calcium/calmodulin-dependent protein kinase II inhibitor 2	Homo sapiens calcium/calmodulin-dependent protein kinase II inhibitor 2 (CAMK2N2), mRNA [NM_033259]	2.60654	up	2.46525	up	1419.73600	563.99540	1537.76920	550.04140	Detected	Detected	Detected	Detected
2417	A_23_P380928	TTLL3	Hs.709359	tubulin tyrosine ligase-like family, member 3	Homo sapiens tubulin tyrosine ligase-like family, member 3 (TTLL3), mRNA [NM_001025930]	2.60617	up	3.32630	up	2198.25100	873.38934	1746.65620	463.03198	Detected	Detected	Detected	Detected
2418	A_23_P87082	ROBO3	Hs.435621	roundabout, axon guidance receptor, homolog 3 (Drosophila)	Homo sapiens roundabout, axon guidance receptor, homolog 3 (Drosophila) (ROBO3), mRNA [NM_022370]	2.60565	up	4.21614	up	462.81168	183.91700	503.43152	105.29097	Detected	Detected	Detected	Detected
2419	A_24_P146683	MSMB	Hs.255462	microseminoprotein, beta-	Homo sapiens microseminoprotein, beta- (MSMB), transcript variant PSP94, mRNA [NM_002443]	2.60556	up	3.10660	up	24.91783	9.90244	18.36275	5.21215	Detected	Compromised	Detected	Compromised
2420	A_24_P336759	MCL1	Hs.632486	myeloid cell leukemia sequence 1 (BCL2-related)	Homo sapiens myeloid cell leukemia sequence 1 (BCL2-related) (MCL1), transcript variant 1, mRNA [NM_021960]	2.60441	up	2.53843	up	14453.68750	5746.49000	9370.38400	3255.05370	Detected	Detected	Detected	Detected

2421	A_32_P49199	PGR	Hs.32405	progesterone receptor	Homo sapiens progesterone receptor (PGR), mRNA [NM_000926]	2.60267	up	5.11502	up	122.83887	48.87093	63.82708	11.00331	Detected	Detected	Detected	Compromised
2422	A_33_P3286066	PHLDB2	Hs.477114	pleckstrin homology-like domain, family B, member 2	Homo sapiens pleckstrin homology-like domain, family B, member 2 (PHLDB2), transcript variant 1, mRNA [NM_001134438]	2.60237	up	4.13551	up	222.14711	88.39044	63.80446	13.60464	Detected	Detected	Detected	Detected
2423	A_33_P3264364	DNM3	Hs.654775	dynamin 3	Homo sapiens dynamin 3 (DNM3), transcript variant 2, mRNA [NM_001136127]	2.60203	up	2.77493	up	373.09490	148.47072	362.93365	115.32948	Detected	Detected	Detected	Detected
2424	A_33_P3309468	PTPRS	Hs.644384	protein tyrosine phosphatase, receptor type, S	Homo sapiens protein tyrosine phosphatase, receptor type, S (PTPRS), transcript variant 1, mRNA [NM_002850]	2.60130	up	2.58480	up	24148.41600	9612.37100	17735.39300	6050.34380	Detected	Detected	Detected	Detected
2425	A_23_P137470	SIPA1L2	Hs.675721	signal-induced proliferation-associated 1 like 2	Homo sapiens signal-induced proliferation-associated 1 like 2 (SIPA1L2), mRNA [NM_020808]	2.60130	up	2.99520	up	260.11478	103.53970	207.45009	61.07366	Detected	Detected	Detected	Detected
2426	A_23_P342709	FBXO15	Hs.664011	F-box protein 15	Homo sapiens F-box protein 15 (FBXO15), transcript variant 1, mRNA [NM_152676]	2.60037	up	2.41878	up	79.46852	31.64416	75.16104	27.40072	Detected	Detected	Detected	Detected
2427	A_23_P210445	L3MBTL	Hs.709356	l(3)mbt-like (Drosophila)	Homo sapiens l(3)mbt-like (Drosophila) (L3MBTL), transcript variant II, mRNA [NM_032107]	2.60010	up	2.13868	up	28.56923	11.37737	12.45746	5.13629	Detected	Compromised	Compromised	Compromised
2428	A_24_P82155	ANKDD1A	Hs.207157	ankyrin repeat and death domain containing 1A	Homo sapiens ankyrin repeat and death domain containing 1A (ANKDD1A), mRNA [NM_182703]	2.59980	up	1.97129	up	89.05508	35.46917	52.62431	23.53967	Detected	Detected	Detected	Detected
2429	A_33_P3244112	UOX	Hs.647646	urate oxidase (pseudogene)	Homo sapiens urate oxidase (pseudogene) (UOX), non-coding RNA [NR_003927]	2.59932	up	4.23356	up	20.60939	8.20991	18.55851	3.86548	Detected	Compromised	Detected	Compromised
2430	A_24_P309317	PSAP	Hs.523004	prosaposin	Homo sapiens prosaposin (PSAP), transcript variant 2, mRNA [NM_001042465]	2.59798	up	2.21073	up	8785.77600	3501.68650	9851.92300	3929.62520	Detected	Detected	Detected	Detected
2431	A_23_P142872	TCF7L1	Hs.516297	transcription factor 7-like 1 (T-cell specific, HMG-box)	Homo sapiens transcription factor 7-like 1 (T-cell specific, HMG-box) (TCF7L1), mRNA [NM_031283]	2.59778	up	2.53273	up	416.58606	166.04880	215.01364	74.85880	Detected	Detected	Detected	Detected
2432	A_23_P372255	ITPKB	Hs.528087	inositol 1,4,5-trisphosphate 3-kinase B	Homo sapiens inositol 1,4,5-trisphosphate 3-kinase B (ITPKB), mRNA [NM_002221]	2.59773	up	2.75632	up	318.83636	127.08860	277.03710	88.62856	Detected	Detected	Detected	Detected
2433	A_24_P402690	ITM2C	Hs.111577	integral membrane protein 2C	Homo sapiens integral membrane protein 2C (ITM2C), transcript variant 1, mRNA [NM_030926]	2.59663	up	2.44587	up	23207.69300	9254.54400	12848.14100	4632.04640	Detected	Detected	Detected	Detected
2434	A_33_P3393650	PLEKHA5	Hs.188614	pleckstrin homology domain containing, family A member 5	Homo sapiens pleckstrin homology domain containing, family A member 5 (PLEKHA5), transcript variant 1, mRNA [NM_019012]	2.59614	up	1.85508	up	55.39465	22.09393	48.64742	23.12396	Detected	Detected	Detected	Detected
2435	A_33_P3217307		Hs.655921		Protein archease (Protein ZBTB8OS)(Zinc finger and BTB domain-containing opposite strand protein 8) [Source:UniProtKB/Swiss-Prot;Acc:Q8IWT0] [ENST00000341885]	2.59598	up	2.85906	up	21.43999	8.55176	19.22527	5.92945	Detected	Compromised	Detected	Compromised
2436	A_32_P22501	LOC645212	Hs.698054	hypothetical LOC645212	LOC645212 protein Fragment [Source:UniProtKB/TrEMBL;Acc:Q8TAS2] [ENST00000313807]	2.59479	up	2.37798	up	660.20874	263.45877	714.31200	264.87723	Detected	Detected	Detected	Detected
2437	A_33_P3266828	TMEM8B	Hs.493808	transmembrane protein 8B	Homo sapiens transmembrane protein 8B (TMEM8B), transcript variant 3, mRNA [NM_016446]	2.59330	up	2.65664	up	4234.77900	1690.87540	3262.92330	1083.02700	Detected	Detected	Detected	Detected
2438	A_23_P25964	GALC	Hs.513439	galactosylceramidase	Homo sapiens galactosylceramidase (GALC), transcript variant 1, mRNA [NM_000153]	2.59243	up	2.63131	up	7115.95000	2842.22680	4469.23300	1497.70840	Detected	Detected	Detected	Detected
2439	A_23_P42784	STK31	Hs.309767	serine/threonine kinase 31	Homo sapiens serine/threonine kinase 31 (STK31), transcript variant 2, mRNA [NM_032944]	2.59242	up	2.18433	up	20.39498	8.14612	6.64664	2.68318	Detected	Compromised	Compromised	Compromised
2440	A_24_P311604	PACRGL	Hs.479298	PARK2 co-regulated-like	Homo sapiens PARK2 co-regulated-like (PACRGL), transcript variant 1, mRNA [NM_145048]	2.59209	up	2.65764	up	868.48650	346.93402	844.30950	280.13700	Detected	Detected	Detected	Detected
2441	A_33_P3257903	GSTA4	Hs.485557	glutathione S-transferase alpha 4	Homo sapiens glutathione S-transferase alpha 4 (GSTA4), mRNA [NM_001512]	2.59149	up	2.52878	up	399.38144	159.57722	425.21033	148.27180	Detected	Detected	Detected	Detected
2442	A_33_P3681776	LOC255512	Hs.588291	hypothetical LOC255512	Homo sapiens hypothetical LOC255512 (LOC255512), non-coding RNA [NR_029409]	2.59089	up	2.63010	up	802.41980	320.69052	773.36060	259.28424	Detected	Detected	Detected	Detected
2443	A_23_P204269	USP15	Hs.434951	ubiquitin specific peptidase 15	Homo sapiens ubiquitin specific peptidase 15 (USP15), mRNA [NM_006313]	2.59056	up	3.01874	up	930.65186	371.98550	711.45020	207.81890	Detected	Detected	Detected	Detected
2444	A_33_P3304242	DNAI1	Hs.112667	dynein, axonemal, intermediate chain 1	Homo sapiens dynein, axonemal, intermediate chain 1 (DNAI1), mRNA [NM_012144]	2.58947	up	4.19335	up	13.60381	5.43979	11.74401	2.46957	Detected	Compromised	Compromised	Compromised
2445	A_24_P286054	ZFYVE16	Hs.482660	zinc finger, FYVE domain containing 16	Homo sapiens zinc finger, FYVE domain containing 16 (ZFYVE16), transcript variant 2, mRNA [NM_001105251]	2.58824	up	2.68341	up	1249.53800	499.89484	717.17017	235.66797	Detected	Detected	Detected	Detected
2446	A_23_P365614	NOTCH4	Hs.436100	Notch homolog 4 (Drosophila)	Homo sapiens Notch homolog 4 (Drosophila) (NOTCH4), mRNA [NM_004557]	2.58808	up	2.65937	up	725.85767	290.40662	582.00660	192.98141	Detected	Detected	Detected	Detected
2447	A_33_P3223002	GPR77	Hs.534412	G protein-coupled receptor 77	Homo sapiens G protein-coupled receptor 77 (GPR77), mRNA [NM_018485]	2.58806	up	1.31662	up	14.40925	5.76500	10.84595	7.26395	Detected	Compromised	Compromised	Compromised
2448	A_23_P80156	PRMT2	Hs.154163	protein arginine methyltransferase 2	Homo sapiens protein arginine methyltransferase 2 (PRMT2), transcript variant 1, mRNA [NM_206962]	2.58791	up	2.28188	up	1324.59620	529.98987	1296.25490	500.91367	Detected	Detected	Detected	Detected
2449	A_32_P420563	RNF215	Hs.592194	ring finger protein 215	Homo sapiens ring finger protein 215 (RNF215), mRNA [NM_001017981]	2.58607	up	2.38356	up	799.20917	320.00253	362.33005	134.04292	Detected	Detected	Detected	Detected

2450	A_24_P370670	LOC100130633	Hs.533986	hypothetical LOC100130633	ZMYM6 proteinZinc finger, MYM-type 6, isoform CRA_a ; [Source:UniProtKB/TrEMBL;Acc:Q8NCS4] [ENST00000373337]	2.58595	up	2.44763	up	2772.74170	1110.25350	3497.74680	1260.10800	Detected	Detected	Detected	Detected
2451	A_23_P330578	LRRC39	Hs.44277	leucine rich repeat containing 39	Homo sapiens leucine rich repeat containing 39 (LRRC39), mRNA [NM_144620]	2.58443	up	2.27984	up	294.63920	118.04792	196.84312	76.13449	Detected	Detected	Detected	Detected
2452	A_23_P331235	C5orf38	Hs.668017	chromosome 5 open reading frame 38	Homo sapiens chromosome 5 open reading frame 38 (C5orf38), mRNA [NM_178569]	2.58400	up	2.51198	up	322.67578	129.30272	338.18246	118.71370	Detected	Detected	Detected	Detected
2453	A_33_P3303464	C19orf39	Hs.631619	chromosome 19 open reading frame 39	Homo sapiens chromosome 19 open reading frame 39 (C19orf39), mRNA [NM_175871]	2.58382	up	1.74100	up	73.43026	29.42701	16.18536	8.19766	Detected	Detected	Detected	Compromised
2454	A_23_P360744	RAG1	Hs.73958	recombination activating gene 1	Homo sapiens recombination activating gene 1 (RAG1), mRNA [NM_000448]	2.58379	up	3.30036	up	148.56260	59.53677	121.81652	32.54691	Detected	Detected	Detected	Detected
2455	A_23_P217297	ZNF711	Hs.326801	zinc finger protein 711	Homo sapiens zinc finger protein 711 (ZNF711), mRNA [NM_021998]	2.58365	up	4.26820	up	10.09026	4.04391	16.58956	3.42732	Compromised	Compromised	Detected	Compromised
2456	A_24_P228026	CYB5D2	Hs.513871	cytochrome b5 domain containing 2	Homo sapiens cytochrome b5 domain containing 2 (CYB5D2), transcript variant 1, mRNA [NM_144611]	2.58351	up	2.44571	up	1846.20070	739.94870	2515.89820	907.09595	Detected	Detected	Detected	Detected
2457	A_24_P896205	LOC645722		hypothetical LOC645722	PREDICTED: Homo sapiens similar to hCG2037003 (LOC645722), mRNA [XM_944447]	2.58311	up	2.30473	up	8112.39600	3251.91580	6848.17430	2620.11160	Detected	Detected	Detected	Detected
2458	A_23_P13663	FAM60A	Hs.505154	family with sequence similarity 60, member A	Homo sapiens family with sequence similarity 60, member A (FAM60A), transcript variant 2, mRNA [NM_021238]	2.58262	up	2.50190	up	1955.51050	784.02954	1708.94540	602.31490	Detected	Detected	Detected	Detected
2459	A_33_P3213797	KCNK7	Hs.175218	potassium channel, subfamily K, member 7	Homo sapiens potassium channel, subfamily K, member 7 (KCNK7), transcript variant C, mRNA [NM_005714]	2.58247	up	1.88220	up	96.14352	38.54939	78.07604	36.57773	Detected	Detected	Detected	Detected
2460	A_33_P3218960	CACNA1H	Hs.459642	calcium channel, voltage-dependent, T type, alpha 1H subunit	Homo sapiens calcium channel, voltage-dependent, T type, alpha 1H subunit (CACNA1H), transcript variant 1, mRNA [NM_021098]	2.58157	up	2.86532	up	81.47275	32.67845	47.59345	14.64669	Detected	Detected	Detected	Compromised
2461	A_24_P182539	ATG4C	Hs.7353	ATG4 autophagy related 4 homolog C (S. cerevisiae)	Cysteine protease ATG4C (EC 3.4.22.-)(Autophagy-related protein 4 homolog C)(Autophagin-3)(Autophagy-related cysteine endopeptidase 3)(AUT-like 3 cysteine endopeptidase) [Source:UniProtKB/Swiss-Prot;Acc:Q96DT6] [ENST00000317868]	2.57985	up	2.35558	up	286.64612	115.04930	166.67702	62.39401	Detected	Detected	Detected	Detected
2462	A_33_P3411945	C19orf38	Hs.440535	chromosome 19 open reading frame 38	Homo sapiens chromosome 19 open reading frame 38 (C19orf38), mRNA [NM_001136482]	2.57956	up	1.94296	up	11.87056	4.76496	15.02921	6.82083	Detected	Compromised	Detected	Compromised
2463	A_33_P3261408	TMIE	Hs.185777	transmembrane inner ear	Homo sapiens transmembrane inner ear (TMIE), mRNA [NM_147196]	2.57931	up	1.29515	up	47.96891	19.25707	29.49900	20.08405	Detected	Detected	Detected	Detected
2464	A_24_P217365	ANKRD28	Hs.335239	ankyrin repeat domain 28	Homo sapiens ankyrin repeat domain 28 (ANKRD28), mRNA [NM_015199]	2.57930	up	2.16052	up	5978.13900	2399.91940	5291.85350	2159.80860	Detected	Detected	Detected	Detected
2465	A_32_P28223		Hs.656851		Homo sapiens full length insert cDNA YH73H08 [AF074986]	2.57784	up	2.42329	up	219.19884	88.04734	159.30766	57.96906	Detected	Detected	Detected	Detected
2466	A_23_P153616	MADCAM1	Hs.102598	mucosal vascular addressin cell adhesion molecule 1	Homo sapiens mucosal vascular addressin cell adhesion molecule 1 (MADCAM1), transcript variant 1, mRNA [NM_130760]	2.57709	up	5.17712	up	52.81176	21.21942	18.55060	3.15962	Detected	Detected	Detected	Compromised
2467	A_23_P5441	ABCB6	Hs.107911	ATP-binding cassette, sub-family B (MDR/TAP), member 6	Homo sapiens ATP-binding cassette, sub-family B (MDR/TAP), member 6 (ABCB6), nuclear gene encoding mitochondrial protein, mRNA [NM_005689]	2.57687	up	2.32885	up	1620.53700	651.17773	1270.89180	481.20670	Detected	Detected	Detected	Detected
2468	A_33_P3424364	CCDC152	Hs.718521	coiled-coil domain containing 152	Homo sapiens coiled-coil domain containing 152 (CCDC152), mRNA [NM_001134848]	2.57615	up	2.91744	up	105.69899	42.48478	105.29570	31.82538	Detected	Detected	Detected	Detected
2469	A_23_P376627	D2HGDH	Hs.516813	D-2-hydroxyglutarate dehydrogenase	Homo sapiens D-2-hydroxyglutarate dehydrogenase (D2HGDH), nuclear gene encoding mitochondrial protein, mRNA [NM_152783]	2.57278	up	2.47706	up	3348.77860	1347.77170	3834.78030	1365.11730	Detected	Detected	Detected	Detected
2470	A_23_P65983	CCDC102A	Hs.644611	coiled-coil domain containing 102A	Homo sapiens coiled-coil domain containing 102A (CCDC102A), mRNA [NM_033212]	2.57147	up	2.40137	up	2185.53400	880.05290	2180.18200	800.56860	Detected	Detected	Detected	Detected
2471	A_23_P134925	BNIP3L	Hs.131226	BCL2/adenovirus E1B 19kDa interacting protein 3-like	Homo sapiens BCL2/adenovirus E1B 19kDa interacting protein 3-like (BNIP3L), mRNA [NM_004331]	2.57142	up	2.58678	up	35592.54700	14332.41800	28244.13300	9627.95800	Detected	Detected	Detected	Detected
2472	A_24_P322474	PDE4A	Hs.89901	phosphodiesterase 4A, cAMP-specific (phosphodiesterase E2 dunce homolog, Drosophila)	Homo sapiens phosphodiesterase 4A, cAMP-specific (phosphodiesterase E2 dunce homolog, Drosophila) (PDE4A), transcript variant 4, mRNA [NM_006202]	2.57136	up	2.38718	up	19486.50600	7847.01000	15701.84400	5800.03270	Detected	Detected	Detected	Detected
2473	A_23_P74391	OPN3	Hs.409081	opsin 3	Homo sapiens opsin 3 (OPN3), mRNA [NM_014322]	2.57098	up	2.43247	up	715.10810	288.00928	624.65660	226.44370	Detected	Detected	Detected	Detected
2474	A_23_P332584	KIAA1107	Hs.21554	KIAA1107	Homo sapiens KIAA1107 (KIAA1107), mRNA [NM_015237]	2.57058	up	2.06793	up	48.87008	19.68544	34.45197	14.69076	Detected	Detected	Detected	Compromised
2475	A_23_P211136	BRWD1	Hs.654740	bromodomain and WD repeat domain containing 1	Homo sapiens bromodomain and WD repeat domain containing 1 (BRWD1), transcript variant 1, mRNA [NM_018963]	2.56971	up	2.54907	up	413.68164	166.69190	310.11346	107.27631	Detected	Detected	Detected	Detected
2476	A_33_P3227506	BPTF	Hs.444200	bromodomain PHD finger transcription factor	Homo sapiens bromodomain PHD finger transcription factor (BPTF), transcript variant 1, mRNA [NM_182641]	2.56887	up	2.45019	up	281.86447	113.61377	164.19461	59.09148	Detected	Detected	Detected	Detected
2477	A_23_P127840	ZNF214	Hs.445849	zinc finger protein 214	Homo sapiens zinc finger protein 214 (ZNF214), mRNA [NM_013249]	2.56827	up	2.52110	up	55.03595	22.18910	45.79773	16.01841	Detected	Detected	Detected	Detected

2478	A_33_P3263569	AKNAD1	Hs.720879	AKNA domain containing 1	Homo sapiens AKNA domain containing 1 (AKNAD1), mRNA [NM_152763]	2.56826	up	3.06632	up	113.92490	45.93173	52.07269	14.97472	Detected	Detected	Detected	Compromised
2479	A_23_P82929	NOV	Hs.235935	nephroblastoma overexpressed gene	Homo sapiens nephroblastoma overexpressed gene (NOV), mRNA [NM_002514]	2.56731	up	2.56602	up	1332.33900	537.36426	1363.50020	468.55453	Detected	Detected	Detected	Detected
2480	A_33_P3392952	FOXK2	Hs.591140	forkhead box K2	Homo sapiens forkhead box K2 (FOXK2), mRNA [NM_004514]	2.56658	up	2.87487	up	2116.57800	853.90980	1245.26830	381.95355	Detected	Detected	Detected	Detected
2481	A_33_P3364696	STAMBP	Hs.469018	STAM binding protein	Homo sapiens STAM binding protein (STAMBP), transcript variant 3, mRNA [NM_213622]	2.56559	up	2.51906	up	676.54870	273.05200	626.55610	219.32414	Detected	Detected	Detected	Detected
2482	A_23_P352799	NPW	Hs.233533	neuropeptide W	Homo sapiens neuropeptide W (NPW), mRNA [NM_001099456]	2.56495	up	2.14980	up	109.70782	44.28859	130.21352	53.41021	Detected	Detected	Detected	Detected
2483	A_33_P3331426	LOC100133086	Hs.631791	hypothetical LOC100133086	PREDICTED: Homo sapiens hypothetical LOC100133086 (LOC100133086), mRNA [XM_001723511]	2.56313	up	1.56197	up	337.43463	136.31741	29.14687	16.45449	Detected	Detected	Detected	Detected
2484	A_33_P3422581	C2orf86	Hs.414952	chromosome 2 open reading frame 86	Homo sapiens chromosome 2 open reading frame 86 (C2orf86), transcript variant 2, mRNA [NM_015910]	2.56234	up	2.27748	up	134.61362	54.39821	122.18694	47.30804	Detected	Detected	Detected	Detected
2485	A_33_P3252333	SNORD3B-1	Hs.658815	small nucleolar RNA, C/D box 3B-1	Homo sapiens small nucleolar RNA, C/D box 3B-1 (SNORD3B-1), small nucleolar RNA [NR_003271]	2.56215	up	2.45220	up	4319.25500	1745.57130	4004.44920	1439.96830	Detected	Detected	Detected	Detected
2486	A_23_P201368	CTBS	Hs.513557	chitobiase, di-N-acetyl-	Homo sapiens chitobiase, di-N-acetyl- (CTBS), mRNA [NM_004388]	2.56188	up	2.67730	up	2628.13260	1062.23830	2341.48600	771.18690	Detected	Detected	Detected	Detected
2487	A_23_P128574	ENOX1	Hs.128258	ecto-NOX disulfide-thiol exchanger 1	Homo sapiens ecto-NOX disulfide-thiol exchanger 1 (ENOX1), transcript variant 1, mRNA [NM_017993]	2.56176	up	2.49270	up	750.96000	303.53700	588.78780	208.28291	Detected	Detected	Detected	Detected
2488	A_24_P334640	PAQR8	Hs.239388	progestin and adipoQ receptor family member VIII	Homo sapiens progestin and adipoQ receptor family member VIII (PAQR8), mRNA [NM_133367]	2.56098	up	3.08063	up	101.20309	40.91867	121.41171	34.75251	Detected	Detected	Detected	Detected
2489	A_23_P315892	ST6GALNAC6	Hs.109672	ST6 (alpha-N-acetyl-neuraminyl-2,3-beta-galactosyl-1,3)-N-acetylgalactosaminide alpha-2,6-sialyltransferase 6	Homo sapiens ST6 (alpha-N-acetyl-neuraminyl-2,3-beta-galactosyl-1,3)-N-acetylgalactosaminide alpha-2,6-sialyltransferase 6 (ST6GALNAC6), mRNA [NM_013443]	2.56088	up	2.29051	up	16830.12500	6805.05200	12706.80900	4891.80500	Detected	Detected	Detected	Detected
2490	A_33_P3399988	HCP5P10	Hs.662200	HLA complex P5 pseudogene 10	Homo sapiens HLA complex P5 pseudogene 10, mRNA (cDNA clone IMAGE:5582570) [BC035828]	2.56078	up	2.47601	up	19.09507	7.72116	23.92854	8.52175	Detected	Compromised	Detected	Compromised
2491	A_24_P588897	SLCO3A1	Hs.311187	solute carrier organic anion transporter family, member 3A1	Homo sapiens solute carrier organic anion transporter family, member 3A1 (SLCO3A1), transcript variant 1, mRNA [NM_013272]	2.56047	up	2.55209	up	917.50476	371.04025	495.03983	171.04492	Detected	Detected	Detected	Detected
2492	A_24_P535219	PHF10	Hs.435933	PHD finger protein 10	Homo sapiens mRNA full length insert cDNA clone EUROIMAGE 2120537 [AJ420510]	2.55934	up	2.82864	up	347.87400	140.74320	225.31897	70.24012	Detected	Detected	Detected	Detected
2493	A_32_P353072	TMEM106B	Hs.396358	transmembrane protein 106B	Homo sapiens transmembrane protein 106B (TMEM106B), transcript variant 1, mRNA [NM_018374]	2.55811	up	2.73740	up	5253.55200	2126.51100	3789.75000	1220.78020	Detected	Detected	Detected	Detected
2494	A_33_P3385436	PLAC8L1	Hs.162369	PLAC8-like 1	Homo sapiens PLAC8-like 1 (PLAC8L1), mRNA [NM_001029869]	2.55790	up	2.27651	up	49.20490	19.91855	41.85890	16.21374	Detected	Detected	Detected	Detected
2495	A_23_P4144	COASY	Hs.709231	Coenzyme A synthase	Homo sapiens Coenzyme A synthase (COASY), nuclear gene encoding mitochondrial protein, transcript variant 1, mRNA [NM_025233]	2.55699	up	1.50277	up	4304.25700	1743.01970	4997.48440	2932.41720	Detected	Detected	Detected	Detected
2496	A_33_P3692756	LOC723809	Hs.124316	hypothetical LOC723809	Homo sapiens hypothetical LOC723809 (LOC723809), non-coding RNA [NR_027374]	2.55584	up	3.19100	up	50.07978	20.28904	30.61544	8.46016	Detected	Detected	Detected	Compromised
2497	A_32_P128209	C5orf27	Hs.8373	chromosome 5 open reading frame 27	Homo sapiens chromosome 5 open reading frame 27 (C5orf27), non-coding RNA [NR_026936]	2.55566	up	3.13791	up	343.63568	139.22878	281.45860	79.09323	Detected	Detected	Detected	Detected
2498	A_24_P586390	LOC100133331	Hs.646112	hypothetical LOC100133331	Homo sapiens hypothetical LOC100133331 (LOC100133331), non-coding RNA [NR_028327]	2.55533	up	2.57882	up	128.08100	51.90045	89.97714	30.76644	Detected	Detected	Detected	Detected
2499	A_33_P3422991	SLC36A4	Hs.148766	solute carrier family 36 (proton/amino acid symporter), member 4	Homo sapiens solute carrier family 36 (proton/amino acid symporter), member 4 (SLC36A4), mRNA [NM_152313]	2.55463	up	2.55179	up	1662.41760	673.82120	1322.35400	456.94946	Detected	Detected	Detected	Detected
2500	A_24_P3140	ZNF638	Hs.434401	zinc finger protein 638	Homo sapiens zinc finger protein 638 (ZNF638), transcript variant 1, mRNA [NM_014497]	2.55414	up	2.69446	up	1232.22310	499.54898	653.93414	214.00693	Detected	Detected	Detected	Detected
2501	A_24_P281439	OR2T5	Hs.553808	olfactory receptor, family 2, subfamily T, member 5	Homo sapiens olfactory receptor, family 2, subfamily T, member 5 (OR2T5), mRNA [NM_001004697]	2.55373	up	1.59558	up	14.86529	6.02743	8.09551	4.47396	Detected	Compromised	Compromised	Compromised
2502	A_33_P3434927	PR47	Hs.683780	platelet receptor for type III collagen, 47 kDa	Homo sapiens platelet receptor for type III collagen mRNA, partial cds [AF521131]	2.55275	up	3.07418	up	26.88312	10.90445	17.25789	4.95021	Detected	Compromised	Compromised	Compromised
2503	A_24_P702813	XPR1	Hs.227656	xenotropic and polytropic retrovirus receptor	Homo sapiens xenotropic and polytropic retrovirus receptor (XPR1), transcript variant 1, mRNA [NM_004736]	2.55273	up	2.84380	up	1053.15190	427.18893	618.54250	191.79468	Detected	Detected	Detected	Detected
2504	A_23_P53866	COG6	Hs.507805	component of oligomeric golgi complex 6	Homo sapiens component of oligomeric golgi complex 6 (COG6), transcript variant 1, mRNA [NM_020751]	2.55239	up	2.85614	up	2345.11230	951.36870	1641.21730	506.70108	Detected	Detected	Detected	Detected
2505	A_23_P255331	C4orf49	Hs.710036	chromosome 4 open reading frame 49	Homo sapiens chromosome 4 open reading frame 49 (C4orf49), mRNA [NM_032623]	2.55233	up	2.52796	up	424.66510	172.28300	345.81552	120.62577	Detected	Detected	Detected	Detected
2506	A_33_P3250963	TP53TG1	Hs.274329	TP53 target 1 (non-protein coding)	Homo sapiens TP53 target 1 (non-protein coding) (TP53TG1), non-coding RNA [NR_015381]	2.55193	up	2.60945	up	591.67380	240.07510	1034.71280	349.65170	Detected	Detected	Detected	Detected

2507	A_24_P244442	BSCL2	Hs.533709	Berardinelli-Seip congenital lipodystrophy 2 (seipin)	Homo sapiens Berardinelli-Seip congenital lipodystrophy 2 (seipin) (BSCL2), transcript variant 2, mRNA [NM_032667]	2.55073	up	2.28097	up	1259.10050	511.12753	1196.69920	462.62723	Detected	Detected	Detected	Detected
2508	A_33_P3366987	GPR125	Hs.99195	G protein-coupled receptor 125	Homo sapiens G protein-coupled receptor 125 (GPR125), mRNA [NM_145290]	2.54863	up	2.86022	up	280.18810	113.83508	231.64964	71.41628	Detected	Detected	Detected	Detected
2509	A_23_P111919	PURG	Hs.373778	purine-rich element binding protein G	Homo sapiens purine-rich element binding protein G (PURG), transcript variant A, mRNA [NM_013357]	2.54831	up	4.48056	up	50.30246	20.43952	22.47478	4.42312	Detected	Not Detected	Detected	Compromised
2510	A_23_P141893	FLJ40125	Hs.532872	protein phosphatase 1B-like	Homo sapiens protein phosphatase 1B-like (FLJ40125), mRNA [NM_001080401]	2.54808	up	2.71459	up	608.44556	247.25294	491.24830	159.57416	Detected	Detected	Detected	Detected
2511	A_33_P3351686	CCDC17	Hs.18912	coiled-coil domain containing 17	Homo sapiens coiled-coil domain containing 17 (CCDC17), mRNA [NM_001114938]	2.54800	up	4.60091	up	9.26591	3.76549	16.31429	3.12673	Compromised	Compromised	Detected	Compromised
2512	A_23_P502590	KIR2DS4	Hs.654608	killer cell immunoglobulin-like receptor, two domains, short cytoplasmic tail, 4	Homo sapiens killer cell immunoglobulin-like receptor, two domains, short cytoplasmic tail, 4 (KIR2DS4), mRNA [NM_012314]	2.54554	up	1.78163	up	36.19452	14.72299	8.09144	4.00474	Detected	Compromised	Compromised	Compromised
2513	A_33_P3252236	N6AMT1		N-6 adenine-specific DNA methyltransferase 1 (putative)	Homo sapiens N-6 adenine-specific DNA methyltransferase 1 (putative) (N6AMT1), transcript variant 1, mRNA [NM_013240]	2.54454	up	2.47341	up	675.44366	274.86060	569.93463	203.18623	Detected	Detected	Detected	Detected
2514	A_23_P134684	HMBOX1	Hs.591836	homeobox containing 1	Homo sapiens homeobox containing 1 (HMBOX1), transcript variant 1, mRNA [NM_024567]	2.54452	up	2.34208	up	2018.79080	821.52050	1518.69540	571.78630	Detected	Detected	Detected	Detected
2515	A_32_P100439	C7orf41	Hs.200100	chromosome 7 open reading frame 41	Homo sapiens chromosome 7 open reading frame 41 (C7orf41), mRNA [NM_152793]	2.54318	up	2.59148	up	5673.55960	2310.00050	5604.21400	1906.91660	Detected	Detected	Detected	Detected
2516	A_33_P3405424	IL4I1	Hs.574492	interleukin 4 induced 1	Homo sapiens interleukin 4 induced 1 (IL4I1), transcript variant 2, mRNA [NM_172374]	2.54275	up	2.42112	up	726.06550	295.66858	908.05230	330.71964	Detected	Detected	Detected	Detected
2517	A_23_P154962	RIMBP3	Hs.115429	RIMS binding protein 3	Homo sapiens RIMS binding protein 3 (RIMBP3), mRNA [NM_015672]	2.54226	up	2.45647	up	894.64703	364.38824	848.51650	304.58942	Detected	Detected	Detected	Detected
2518	A_23_P89902	RTN2	Hs.47517	reticulon 2	Homo sapiens reticulon 2 (RTN2), transcript variant 1, mRNA [NM_005619]	2.54169	up	2.33003	up	1591.60080	648.40295	1696.65720	642.09375	Detected	Detected	Detected	Detected
2519	A_23_P88522	NMB	Hs.386470	neuromedin B	Homo sapiens neuromedin B (NMB), transcript variant 1, mRNA [NM_021077]	2.54156	up	2.36949	up	27510.66800	11208.14550	25028.99800	9314.37700	Detected	Detected	Detected	Detected
2520	A_33_P3336878	TMEM180	Hs.309069	transmembrane protein 180	Homo sapiens transmembrane protein 180 (TMEM180), mRNA [NM_024789]	2.54115	up	1.63440	up	29.07715	11.84825	34.67252	18.70655	Detected	Compromised	Detected	Detected
2521	A_33_P3239455	GTF2IRD2	Hs.647017	GTF2I repeat domain containing 2	Homo sapiens GTF2I repeat domain containing 2 (GTF2IRD2), mRNA [NM_173537]	2.54103	up	3.36848	up	58.94321	24.01915	39.85822	10.43398	Detected	Detected	Detected	Compromised
2522	A_33_P3399045				Putative uncharacterized protein ENSP00000366043 [Source:UniProtKB/TrEMBL;Acc:Q8N5B5] [ENST00000376847]	2.54032	up	4.54818	up	18.01595	7.34349	13.11943	2.54357	Detected	Compromised	Detected	Compromised
2523	A_33_P3422298		Hs.547600		Putative uncharacterized protein ENSP00000374858 Fragment [Source:UniProtKB/TrEMBL;Acc:A6NKU1] [ENST00000390329]	2.53866	up	2.22408	up	75.83568	30.93161	49.64283	19.68213	Detected	Detected	Detected	Detected
2524	A_23_P328323	RAVER2	Hs.591443	ribonucleoprotein, PTB-binding 2	Homo sapiens ribonucleoprotein, PTB-binding 2 (RAVER2), mRNA [NM_018211]	2.53834	up	3.06399	up	155.08539	63.26355	147.29219	42.38940	Detected	Detected	Detected	Detected
2525	A_32_P84728	LOC100131727	Hs.563183	hypothetical LOC100131727	PREDICTED: Homo sapiens hypothetical LOC100131727 (LOC100131727), mRNA [XM_001723766]	2.53781	up	2.51189	up	255.97444	104.44083	145.09705	50.93578	Detected	Detected	Detected	Detected
2526	A_24_P381136	PACSIN3	Hs.334639	protein kinase C and casein kinase substrate in neurons 3	Homo sapiens protein kinase C and casein kinase substrate in neurons 3 (PACSIN3), mRNA [NM_016223]	2.53732	up	2.03964	up	1187.30600	484.53012	1297.02200	560.73750	Detected	Detected	Detected	Detected
2527	A_24_P131236	SV2A	Hs.516153	synaptic vesicle glycoprotein 2A	Homo sapiens synaptic vesicle glycoprotein 2A (SV2A), mRNA [NM_014849]	2.53675	up	2.78221	up	61.55216	25.12457	38.89967	12.32882	Detected	Detected	Detected	Compromised
2528	A_23_P130753	DBP	Hs.414480	D site of albumin promoter (albumin D-box) binding protein	Homo sapiens D site of albumin promoter (albumin D-box) binding protein (DBP), mRNA [NM_001352]	2.53651	up	2.20791	up	151.31460	61.77008	177.32323	70.81892	Detected	Detected	Detected	Detected
2529	A_33_P3792489	C21orf89	Hs.375832	chromosome 21 open reading frame 89	UI-E-CR0-adm-a-05-0-UI.r1 UI-E-CR0 Homo sapiens cDNA clone UI-E-CR0-adm-a-05-0-UI 5', mRNA sequence [BM686390]	2.53649	up	5.19961	up	41.06067	16.76201	45.48657	7.71397	Detected	Detected	Detected	Compromised
2530	A_24_P148750	SH3BP5	Hs.719150	SH3-domain binding protein 5 (BTK-associated)	Homo sapiens SH3-domain binding protein 5 (BTK-associated) (SH3BP5), transcript variant 1, mRNA [NM_004844]	2.53490	up	2.59639	up	1563.42740	638.63153	1188.71410	403.71307	Detected	Detected	Detected	Detected
2531	A_23_P81898	UBD	Hs.44532	ubiquitin D	Homo sapiens ubiquitin D (UBD), mRNA [NM_006398]	2.53333	up	20.46355	up	121.69443	49.74078	72.73988	3.13442	Detected	Detected	Detected	Compromised
2532	A_23_P303423	SLAIN2	Hs.479677	SLAIN motif family, member 2	Homo sapiens SLAIN motif family, member 2 (SLAIN2), mRNA [NM_020846]	2.53258	up	2.22885	up	109.06348	44.59124	81.25543	32.14678	Detected	Detected	Detected	Detected
2533	A_24_P386622	ARRB1	Hs.503284	arrestin, beta 1	Homo sapiens arrestin, beta 1 (ARRB1), transcript variant 1, mRNA [NM_004041]	2.53110	up	1.98862	up	243.61226	99.66040	184.05457	81.61316	Detected	Detected	Detected	Detected
2534	A_23_P115597	ANKRD26	Hs.361041	ankyrin repeat domain 26	Homo sapiens ankyrin repeat domain 26 (ANKRD26), mRNA [NM_014915]	2.53097	up	2.49812	up	317.10880	129.73405	268.57310	94.80158	Detected	Detected	Detected	Detected
2535	A_23_P132763	VGLL3	Hs.720159	vestigial like 3 (Drosophila)	Homo sapiens vestigial like 3 (Drosophila) (VGLL3), mRNA [NM_016206]	2.53085	up	2.54984	up	3788.87330	1550.15930	2621.36740	906.52710	Detected	Detected	Detected	Detected
2536	A_33_P3385842	CCDC7	Hs.585464	coiled-coil domain containing 7	Homo sapiens coiled-coil domain containing 7 (CCDC7), transcript variant 1, mRNA [NM_145023]	2.52976	up	2.44694	up	36.84833	15.08246	26.65815	9.60665	Detected	Detected	Detected	Compromised

2537	A_33_P3227225	LOC100130141	Hs.722967	hypothetical LOC100130141	PREDICTED: Homo sapiens similar to Kruppel-like factor (LOC100130141), miscRNA [XR_037929]	2.52902	up	2.43502	up	229.08170	93.79324	150.55179	54.51918	Detected	Detected	Detected	Detected
2538	A_33_P3569555	LOC253264	Hs.662111	hypothetical protein LOC253264	Homo sapiens cDNA FLJ13383 fis, clone PLACE1001024 [AK023445]	2.52611	up	3.54908	up	29.08017	11.92004	22.67742	5.63434	Detected	Compromised	Detected	Compromised
2539	A_33_P3409261	LOC729421	Hs.646665	hypothetical LOC729421	PREDICTED: Homo sapiens hypothetical LOC729421 (LOC729421), mRNA [XM_001133682]	2.52596	up	2.56633	up	173.21000	71.00346	192.46850	66.13221	Detected	Detected	Detected	Detected
2540	A_33_P3391791	MOG	Hs.141308	myelin oligodendrocyte glycoprotein	Homo sapiens myelin oligodendrocyte glycoprotein (MOG), transcript variant alpha4, mRNA [NM_206814]	2.52569	up	2.57038	up	19.41591	7.95996	11.50257	3.94605	Detected	Compromised	Compromised	Compromised
2541	A_23_P218597	NPAS2	Hs.156832	neuronal PAS domain protein 2	Homo sapiens neuronal PAS domain protein 2 (NPAS2), mRNA [NM_002518]	2.52561	up	2.33051	up	320.64136	131.45767	348.08838	131.70540	Detected	Detected	Detected	Detected
2542	A_24_P237757		Hs.644041		Zinc finger MYM-type protein 2 (Zinc finger protein 198)(Fused in myeloproliferative disorders protein)(Rearranged in atypical myeloproliferative disorder protein) [Source:UniProtKB/Swiss-Prot;Acc:Q9UBW7] [ENST00000382881]	2.52482	up	2.42816	up	794.61456	325.88170	455.68137	165.48135	Detected	Detected	Detected	Detected
2543	A_23_P61674	CLK4	Hs.406557	CDC-like kinase 4	Homo sapiens CDC-like kinase 4 (CLK4), mRNA [NM_020666]	2.52293	up	2.71019	up	1461.13000	599.67650	1211.53500	394.18660	Detected	Detected	Detected	Detected
2544	A_33_P3422233	KIAA1530	Hs.380475	KIAA1530	Homo sapiens KIAA1530 (KIAA1530), mRNA [NM_020894]	2.52032	up	2.60114	up	1540.61290	632.95105	1014.71936	343.99203	Detected	Detected	Detected	Detected
2545	A_24_P208909	TRIM2	Hs.435711	tripartite motif-containing 2	Homo sapiens tripartite motif-containing 2 (TRIM2), transcript variant 1, mRNA [NM_015271]	2.51913	up	2.57016	up	1864.69650	766.46344	1235.39750	423.85010	Detected	Detected	Detected	Detected
2546	A_33_P3546070	PHF20L1	Hs.304362	PHD finger protein 20-like 1	Homo sapiens PHD finger protein 20-like 1 (PHF20L1), transcript variant 1, mRNA [NM_016018]	2.51863	up	2.13093	up	1476.69310	607.09766	1269.07090	525.14935	Detected	Detected	Detected	Detected
2547	A_23_P26439	DBNDD1	Hs.301394	dysbindin (dystrobrevin binding protein 1) domain containing 1	Homo sapiens dysbindin (dystrobrevin binding protein 1) domain containing 1 (DBNDD1), transcript variant 1, mRNA [NM_001042610]	2.51806	up	1.57371	up	19.79746	8.14097	25.62077	14.35598	Detected	Compromised	Detected	Detected
2548	A_33_P3221403		Hs.710766		Putative uncharacterized protein ENSP00000386773 [Source:UniProtKB/TrEMBL;Acc:B9A039] [ENST00000409259]	2.51690	up	1.18266	up	8.22852	3.38524	17.73204	13.22099	Compromised	Compromised	Detected	Compromised
2549	A_24_P11462	ADC	Hs.101807	arginine decarboxylase	Homo sapiens arginine decarboxylase (ADC), mRNA [NM_052998]	2.51605	up	2.34406	up	1275.58650	524.95636	1247.45320	469.26810	Detected	Detected	Detected	Detected
2550	A_23_P314120	CHKB	Hs.654827	choline kinase beta	Homo sapiens choline kinase beta (CHKB), mRNA [NM_005198]	2.51436	up	2.65572	up	1945.75070	801.29610	1746.35270	579.85000	Detected	Detected	Detected	Detected
2551	A_23_P129085	SPESP1	Hs.657932	sperm equatorial segment protein 1	Homo sapiens sperm equatorial segment protein 1 (SPESP1), mRNA [NM_145658]	2.51333	up	2.62994	up	453.54560	186.85470	337.27563	113.08470	Detected	Detected	Detected	Detected
2552	A_23_P71300	CCDC25	Hs.445512	coiled-coil domain containing 25	Homo sapiens coiled-coil domain containing 25 (CCDC25), mRNA [NM_018246]	2.51302	up	2.93496	up	2156.07980	888.38790	1823.22910	547.77760	Detected	Detected	Detected	Detected
2553	A_23_P358662	KIAA1712	Hs.555989	KIAA1712	Homo sapiens KIAA1712 (KIAA1712), transcript variant 1, mRNA [NM_001040157]	2.51280	up	2.92917	up	699.72003	288.33597	444.88538	133.92720	Detected	Detected	Detected	Detected
2554	A_32_P28939	ALKBH2	Hs.374458	alkB, alkylation repair homolog 2 (E. coli)	Homo sapiens alkB, alkylation repair homolog 2 (E. coli) (ALKBH2), transcript variant 2, mRNA [NM_001001655]	2.51170	up	2.32059	up	7601.20070	3133.63350	9921.27000	3769.93870	Detected	Detected	Detected	Detected
2555	A_23_P46378	PIGV	Hs.259605	phosphatidylinositol glycan anchor biosynthesis, class V	Homo sapiens phosphatidylinositol glycan anchor biosynthesis, class V (PIGV), mRNA [NM_017837]	2.51092	up	2.86928	up	1035.71670	427.11154	783.38560	240.75099	Detected	Detected	Detected	Detected
2556	A_33_P3246133	HPCA	Hs.632391	hippocalcin	Homo sapiens hippocalcin (HPCA), mRNA [NM_002143]	2.50974	up	1.94236	up	17.34007	7.15411	15.09058	6.85082	Detected	Compromised	Compromised	Compromised
2557	A_23_P15272	ABCC6	Hs.442182	ATP-binding cassette, sub-family C (CFTR/MRP), member 6	Homo sapiens ATP-binding cassette, sub-family C (CFTR/MRP), member 6 (ABCC6), transcript variant 2, mRNA [NM_001079528]	2.50861	up	3.42489	up	742.62714	306.52844	403.74457	103.95039	Detected	Detected	Detected	Detected
2558	A_33_P3399980	TYRP1	Hs.270279	tyrosinase-related protein 1	Homo sapiens tyrosinase-related protein 1 (TYRP1), mRNA [NM_000550]	2.50812	up	4.77164	up	17.90754	7.39301	13.47027	2.48928	Detected	Compromised	Detected	Compromised
2559	A_23_P205531	RNASE4	Hs.283749	ribonuclease, RNase A family, 4	Homo sapiens ribonuclease, RNase A family, 4 (RNASE4), transcript variant 2, mRNA [NM_002937]	2.50771	up	2.41746	up	2726.94820	1125.98460	2920.86770	1065.41130	Detected	Detected	Detected	Detected
2560	A_24_P911676	SOX4	Hs.643910	SRY (sex determining region Y)-box 4	Homo sapiens SRY (sex determining region Y)-box 4 (SOX4), mRNA [NM_003107]	2.50706	up	2.24490	up	1557.54660	643.29395	1236.70000	485.77222	Detected	Detected	Detected	Detected
2561	A_23_P162746	CRYL1	Hs.370703	crystallin, lambda 1	Homo sapiens crystallin, lambda 1 (CRYL1), mRNA [NM_015974]	2.50692	up	2.55850	up	10214.54600	4219.01700	10386.29100	3579.64820	Detected	Detected	Detected	Detected
2562	A_23_P386268	C4orf26	Hs.24510	chromosome 4 open reading frame 26	Homo sapiens chromosome 4 open reading frame 26 (C4orf26), mRNA [NM_178497]	2.50668	up	1.46431	up	20.56818	8.49630	23.73701	14.29418	Detected	Compromised	Detected	Detected
2563	A_24_P19175	ZNF358	Hs.133475	zinc finger protein 358	Homo sapiens zinc finger protein 358 (ZNF358), mRNA [NM_018083]	2.50584	up	2.10776	up	944.63214	390.33994	1210.45280	506.39737	Detected	Detected	Detected	Detected
2564	A_32_P169179	MSX2P1	Hs.381329	msh homeobox 2 pseudogene 1	Homo sapiens msh homeobox 2 pseudogene 1 (MSX2P1), non-coding RNA [NR_002307]	2.50537	up	2.39453	up	417.28060	172.46013	365.86346	134.72974	Detected	Detected	Detected	Detected
2565	A_23_P408353	HLA-A	Hs.181244	major histocompatibility complex, class I, A	Homo sapiens major histocompatibility complex, class I, A (HLA-A), mRNA [NM_002116]	2.50422	up	2.00895	up	34335.16000	14197.12400	34391.88000	15095.70200	Detected	Detected	Detected	Detected
2566	A_33_P3590259	CXCL14	Hs.483444	chemokine (C-X-C motif) ligand 14	Homo sapiens chemokine (C-X-C motif) ligand 14 (CXCL14), mRNA [NM_004887]	2.50402	up	2.50450	up	910.33550	376.44080	696.19604	245.11865	Detected	Detected	Detected	Detected

2567	A_33_P3265394	WDR74	Hs.655372	WD repeat domain 74	Homo sapiens cDNA FLJ78749 complete cds, highly similar to Homo sapiens WD repeat domain 74 (WDR74), mRNA [AK292330]	2.50338	up	3.64188	up	163.39317	67.58331	141.28226	34.20799	Detected	Detected	Detected	Detected
2568	A_33_P3329740					2.50294	up	2.53521	up	21445.77500	8872.04700	19148.84600	6660.30760	Detected	Detected	Detected	Detected
2569	A_23_P133236	PCDHB14	Hs.658497	protocadherin beta 14	Homo sapiens protocadherin beta 14 (PCDHB14), mRNA [NM_018934]	2.50238	up	3.17614	up	80.37313	33.25761	61.64074	17.11332	Detected	Detected	Detected	Detected
2570	A_23_P42684		Hs.272195		cDNA FLJ20712 fis, clone HUV01027 [Source:UniProtKB/TrEMBL;Acc:Q9NWP0] [ENST00000311067]	2.50231	up	2.48846	up	46.57777	19.27396	19.86787	7.04022	Detected	Detected	Detected	Compromised
2571	A_23_P158053	C9orf16	Hs.522412	chromosome 9 open reading frame 16	Homo sapiens chromosome 9 open reading frame 16 (C9orf16), mRNA [NM_024112]	2.50177	up	2.23075	up	1194.02610	494.19592	1403.99050	554.98114	Detected	Detected	Detected	Detected
2572	A_32_P72110	PVR	Hs.171844	poliovirus receptor	Homo sapiens poliovirus receptor (PVR), transcript variant 1, mRNA [NM_006505]	2.50084	up	2.21669	up	53.30438	22.07043	78.25162	31.12825	Detected	Detected	Detected	Detected
2573	A_23_P53081	OSBPL5	Hs.436166	oxysterol binding protein-like 5	Homo sapiens oxysterol binding protein-like 5 (OSBPL5), transcript variant 1, mRNA [NM_020896]	2.49999	up	2.14301	up	17580.68600	7281.67800	18314.61100	7535.96600	Detected	Detected	Detected	Detected
2574	A_23_P251916	FAM82B	Hs.145386	family with sequence similarity 82, member B	Homo sapiens family with sequence similarity 82, member B (FAM82B), mRNA [NM_016033]	2.49927	up	2.29503	up	8983.55000	3721.92820	8850.39700	3400.47300	Detected	Detected	Detected	Detected
2575	A_33_P3335984				Uncharacterized protein FLJ39653 [Source:UniProtKB/Swiss-Prot;Acc:Q8N8D0] [ENST00000440429]	2.49872	up	2.25196	up	134.82810	55.87217	124.29179	48.66838	Detected	Detected	Detected	Detected
2576	A_33_P3350726	PPARG	Hs.162646	peroxisome proliferator-activated receptor gamma	Homo sapiens peroxisome proliferator-activated receptor gamma (PPARG), transcript variant 3, mRNA [NM_138711]	2.49758	up	2.45908	up	769.42706	318.99320	606.82440	217.59843	Detected	Detected	Detected	Detected
2577	A_23_P24104	PLAU	Hs.77274	plasminogen activator, urokinase	Homo sapiens plasminogen activator, urokinase (PLAU), transcript variant 1, mRNA [NM_002658]	2.49698	up	2.47550	up	7172.39840	2974.28520	6783.54300	2416.34740	Detected	Detected	Detected	Detected
2578	A_23_P216689	BRD3	Hs.522472	bromodomain containing 3	Homo sapiens bromodomain containing 3 (BRD3), mRNA [NM_007371]	2.49439	up	1.86543	up	689.19855	286.09677	1261.16800	596.15686	Detected	Detected	Detected	Detected
2579	A_33_P3298440	hCG_1986447	Hs.646781	hCG1986447	PREDICTED: Homo sapiens hCG1986447 (LOC729324), miscRNA [XR_041500]	2.49409	up	2.00091	up	45.69394	18.97054	24.90387	10.97501	Detected	Detected	Detected	Compromised
2580	A_23_P27606	IL27RA	Hs.132781	interleukin 27 receptor, alpha	Homo sapiens interleukin 27 receptor, alpha (IL27RA), mRNA [NM_004843]	2.49404	up	2.39949	up	653.52360	271.32578	627.74506	230.69022	Detected	Detected	Detected	Detected
2581	A_23_P31945	IL33	Hs.348390	interleukin 33	Homo sapiens interleukin 33 (IL33), mRNA [NM_033439]	2.49348	up	2.51772	up	409.89960	170.21780	387.54675	135.73192	Detected	Detected	Detected	Detected
2582	A_33_P3299565	LEKR1	Hs.478048	leucine, glutamate and lysine rich 1	Homo sapiens leucine, glutamate and lysine rich 1 (LEKR1), mRNA [NM_001004316]	2.49211	up	5.15428	up	7.97407	3.31318	16.51427	2.82525	Compromised	Compromised	Detected	Compromised
2583	A_33_P3394833	C3orf72	Hs.591303	chromosome 3 open reading frame 72	Homo sapiens chromosome 3 open reading frame 72 (C3orf72), mRNA [NM_001040061]	2.49194	up	2.32943	up	2294.50500	953.41925	1389.07340	525.82460	Detected	Detected	Detected	Detected
2584	A_32_P195401	FAM117B	Hs.471130	family with sequence similarity 117, member B	Homo sapiens family with sequence similarity 117, member B (FAM117B), mRNA [NM_173511]	2.49088	up	2.27071	up	1418.30830	589.59045	1077.65160	418.48750	Detected	Detected	Detected	Detected
2585	A_33_P3352877	SPG20	Hs.440414	spastic paraplegia 20 (Troyer syndrome)	Homo sapiens spastic paraplegia 20 (Troyer syndrome) (SPG20), transcript variant 1, mRNA [NM_015087]	2.49046	up	2.35052	up	648.40340	269.58730	411.50110	154.37320	Detected	Detected	Detected	Detected
2586	A_23_P82351	BBS9	Hs.372360	Bardet-Biedl syndrome 9	Homo sapiens Bardet-Biedl syndrome 9 (BBS9), transcript variant 2, mRNA [NM_198428]	2.48982	up	2.51268	up	1628.68920	677.33580	1462.48580	513.23920	Detected	Detected	Detected	Detected
2587	A_33_P3369371	GPX3	Hs.386793	glutathione peroxidase 3 (plasma)	Homo sapiens glutathione peroxidase 3 (plasma) (GPX3), mRNA [NM_002084]	2.48964	up	2.48071	up	85.25773	35.45927	58.32977	20.73385	Detected	Detected	Detected	Detected
2588	A_24_P64100	SLC25A37	Hs.596025	solute carrier family 25, member 37	Homo sapiens FP15737 mRNA, complete cds. [AF495725]	2.48903	up	2.66141	up	1194.87170	497.07680	1073.22690	355.58615	Detected	Detected	Detected	Detected
2589	A_23_P86838	SLC36A4	Hs.148766	solute carrier family 36 (proton/amino acid symporter), member 4	Homo sapiens solute carrier family 36 (proton/amino acid symporter), member 4 (SLC36A4), mRNA [NM_152313]	2.48609	up	2.47155	up	3367.66550	1402.63810	3402.16600	1213.81410	Detected	Detected	Detected	Detected
2590	A_33_P3327956	ZNF605	Hs.720299	zinc finger protein 605	Homo sapiens zinc finger protein 605 (ZNF605), transcript variant 1, mRNA [NM_183238]	2.48589	up	2.53138	up	1597.48350	665.40765	1029.25400	358.53485	Detected	Detected	Detected	Detected
2591	A_33_P3267263	RNU1-5	Hs.623806	RNA, U1 small nuclear 5	Homo sapiens RNA, U1 small nuclear 5 (RNU1-5), small nuclear RNA [NR_004400]	2.48304	up	2.95438	up	3608.08720	1504.62000	3732.75950	1114.11180	Detected	Detected	Detected	Detected
2592	A_33_P3383561		Hs.711755		DNA-directed RNA polymerases I, II, and III subunit RPABC2 (RNA polymerases I, II, and III subunit ABC2)(DNA-directed RNA polymerase II subunit F)(DNA-directed RNA polymerases I, II, and III 14.4 kDa polypeptide)(RPABC14.4)(RPB14.4)(RPB6)(RPC15) [Source:UniProtKB/Swiss-Prot;Acc:P61218] [ENST00000443002]	2.48270	up	1.80394	up	15.19413	6.33702	5.42324	2.65096	Detected	Compromised	Compromised	Compromised
2593	A_23_P103672	NES	Hs.527971	nestin	Homo sapiens nestin (NES), mRNA [NM_006617]	2.48138	up	2.14347	up	3163.19920	1319.97500	3497.96630	1439.01300	Detected	Detected	Detected	Detected
2594	A_23_P390116	SPATA13	Hs.595391	spermatogenesis associated 13	Homo sapiens spermatogenesis associated 13 (SPATA13), transcript variant 2, mRNA [NM_153023]	2.48016	up	2.15703	up	425.47320	177.63371	308.29355	126.02992	Detected	Detected	Detected	Detected
2595	A_23_P406448	RAB9B	Hs.522736	RAB9B, member RAS oncogene family	Homo sapiens RAB9B, member RAS oncogene family (RAB9B), mRNA [NM_016370]	2.47998	up	1.63310	up	14.06603	5.87295	20.41699	11.02412	Detected	Compromised	Detected	Compromised
2596	A_33_P3352767	MC1R	Hs.513829	melanocortin 1 receptor (alpha melanocyte stimulating hormone receptor)	Homo sapiens melanocortin 1 receptor (alpha melanocyte stimulating hormone receptor) (MC1R), mRNA [NM_002386]	2.47685	up	2.51510	up	6865.14840	2870.00660	5363.56700	1880.46280	Detected	Detected	Detected	Detected

2597	A_24_P481824	DPY19L4	Hs.567828	dpy-19-like 4 (C. elegans)	Homo sapiens dpy-19-like 4 (C. elegans) (DPY19L4), mRNA [NM_181787]	2.47630	up	3.03521	up	596.10016	249.25774	308.51492	89.62986	Detected	Detected	Detected	Detected
2598	A_33_P3228499					2.47604	up	3.81798	up	32.73024	13.68750	25.74217	5.94535	Detected	Compromised	Detected	Compromised
2599	A_23_P35316	ZNF695	Hs.669893	zinc finger protein 695	Homo sapiens zinc finger protein 695 (ZNF695), mRNA [NM_020394]	2.47451	up	2.49478	up	26.60894	11.13454	18.38606	6.49864	Detected	Compromised	Detected	Compromised
2600	A_23_P97860	LIPA	Hs.643030	lipase A, lysosomal acid, cholesterol esterase	Homo sapiens lipase A, lysosomal acid, cholesterol esterase (LIPA), transcript variant 2, mRNA [NM_000235]	2.47299	up	2.50411	up	11096.04900	4645.99900	9387.96800	3305.85840	Detected	Detected	Detected	Detected
2601	A_24_P190541	BRWD1	Hs.654740	bromodomain and WD repeat domain containing 1	Homo sapiens bromodomain and WD repeat domain containing 1 (BRWD1), transcript variant 1, mRNA [NM_018963]	2.47244	up	2.92858	up	581.29224	243.44540	402.90714	121.31480	Detected	Detected	Detected	Detected
2602	A_23_P55873	CC2D1A	Hs.269592	coiled-coil and C2 domain containing 1A	Homo sapiens coiled-coil and C2 domain containing 1A (CC2D1A), mRNA [NM_017721]	2.47193	up	2.19577	up	1194.55030	500.38150	1146.67770	460.49026	Detected	Detected	Detected	Detected
2603	A_23_P413641	PREX1	Hs.153310	phosphatidylinositol-3,4,5-trisphosphate-dependent Rac exchange factor 1	Homo sapiens phosphatidylinositol-3,4,5-trisphosphate-dependent Rac exchange factor 1 (PREX1), mRNA [NM_020820]	2.47123	up	2.65809	up	1698.27860	711.58984	1520.15480	504.29385	Detected	Detected	Detected	Detected
2604	A_33_P3409077	C6orf170	Hs.121396	chromosome 6 open reading frame 170	Homo sapiens chromosome 6 open reading frame 170 (C6orf170), mRNA [NM_152730]	2.47100	up	4.90091	up	22.31720	9.35191	20.28304	3.64940	Detected	Compromised	Detected	Compromised
2605	A_23_P29836	TMEM42	Hs.646856	transmembrane protein 42	Homo sapiens transmembrane protein 42 (TMEM42), mRNA [NM_144638]	2.46947	up	2.40651	up	13661.94200	5728.52000	12193.45300	4467.91260	Detected	Detected	Detected	Detected
2606	A_33_P3256957	LOC100128402	Hs.689574	hypothetical protein LOC100128402	Homo sapiens cDNA FLJ42583 fis, clone BRACE3009090. [AK124574]	2.46942	up	1.72866	up	13.65084	5.72396	7.38090	3.76501	Detected	Compromised	Compromised	Compromised
2607	A_23_P143935	PIGZ	Hs.518403	phosphatidylinositol glycan anchor biosynthesis, class Z	Homo sapiens phosphatidylinositol glycan anchor biosynthesis, class Z (PIGZ), mRNA [NM_025163]	2.46706	up	2.46548	up	5103.29050	2141.92260	4620.95700	1652.70960	Detected	Detected	Detected	Detected
2608	A_33_P3254660		Hs.712007		Putative uncharacterized protein ENSP00000329674 [Source:UniProtKB/TrEMBL;Acc:B7WPL7] [ENST00000330805]	2.46646	up	2.55471	up	464.04898	194.81511	403.30150	139.20462	Detected	Detected	Detected	Detected
2609	A_23_P35916	ATM	Hs.367437	ataxia telangiectasia mutated	Homo sapiens ataxia telangiectasia mutated (ATM), transcript variant 1, mRNA [NM_000051]	2.46478	up	2.76791	up	84.30158	35.41534	68.13844	21.70727	Detected	Detected	Detected	Detected
2610	A_23_P151307	RAPGEF3	Hs.8578	Rap guanine nucleotide exchange factor (GEF) 3	Homo sapiens Rap guanine nucleotide exchange factor (GEF) 3 (RAPGEF3), transcript variant 2, mRNA [NM_006105]	2.46458	up	2.06198	up	96.41627	40.50797	76.64784	32.77796	Detected	Detected	Detected	Detected
2611	A_24_P242299	ZRANB2	Hs.194718	zinc finger, RAN-binding domain containing 2	Homo sapiens zinc finger, RAN-binding domain containing 2 (ZRANB2), transcript variant 2, mRNA [NM_005455]	2.46453	up	2.55111	up	8524.73800	3581.62450	7611.28700	2630.83720	Detected	Detected	Detected	Detected
2612	A_32_P36942	C2orf60	Hs.204619	chromosome 2 open reading frame 60	Homo sapiens chromosome 2 open reading frame 60 (C2orf60), transcript variant 1, mRNA [NM_001039693]	2.46372	up	2.09060	up	270.02230	113.48579	190.58890	80.38809	Detected	Detected	Detected	Detected
2613	A_23_P202773	C11orf20	Hs.97174	chromosome 11 open reading frame 20	Homo sapiens chromosome 11 open reading frame 20 (C11orf20), mRNA [NM_001039496]	2.46363	up	3.28913	up	598.18670	251.41676	583.90250	156.53996	Detected	Detected	Detected	Detected
2614	A_24_P32887	BRD3	Hs.522472	bromodomain containing 3	Homo sapiens bromodomain containing 3 (BRD3), mRNA [NM_007371]	2.46336	up	1.76015	up	527.41200	221.69505	704.65830	353.01645	Detected	Detected	Detected	Detected
2615	A_23_P208143	ZNF397	Hs.591061	zinc finger protein 397	Homo sapiens zinc finger protein 397 (ZNF397), transcript variant 1, mRNA [NM_001135178]	2.46335	up	2.88931	up	425.07626	178.67896	333.65924	101.82972	Detected	Detected	Detected	Detected
2616	A_23_P162918	SERPINA3	Hs.534293	serpin peptidase inhibitor, clade A (alpha-1 antiproteinase, antitrypsin), member 3	Homo sapiens serpin peptidase inhibitor, clade A (alpha-1 antiproteinase, antitrypsin), member 3 (SERPINA3), mRNA [NM_001085]	2.46334	up	4.02014	up	130.63857	54.91352	79.91776	17.52944	Detected	Detected	Detected	Detected
2617	A_23_P135184	RALGDS	Hs.106185	ral guanine nucleotide dissociation stimulator	Homo sapiens ral guanine nucleotide dissociation stimulator (RALGDS), transcript variant 2, mRNA [NM_001042368]	2.46216	up	1.98372	up	5785.60800	2433.13720	3049.38300	1355.49570	Detected	Detected	Detected	Detected
2618	A_23_P123916	LRSAM1	Hs.495188	leucine rich repeat and sterile alpha motif containing 1	Homo sapiens leucine rich repeat and sterile alpha motif containing 1 (LRSAM1), transcript variant 1, mRNA [NM_138361]	2.45906	up	1.90436	up	192.53716	81.07355	169.83253	78.63876	Detected	Detected	Detected	Detected
2619	A_33_P3374504				Thioredoxin reductase 2, mitochondrial Precursor (EC 1.8.1.9)(Thioredoxin reductase TR3)(TR-beta)(Selenoprotein Z)(SelZ) [Source:UniProtKB/Swiss-Prot;Acc:Q9NNW7] [ENST00000334363]	2.45804	up	2.92765	up	362.18732	152.57301	504.50092	151.95255	Detected	Detected	Detected	Detected
2620	A_23_P404678	RAB3D	Hs.655274	RAB3D, member RAS oncogene family	Homo sapiens RAB3D, member RAS oncogene family (RAB3D), mRNA [NM_004283]	2.45746	up	3.06539	up	58.60295	24.69256	62.67073	18.02787	Detected	Detected	Detected	Detected
2621	A_24_P188164	SCAND3	Hs.176980	SCAN domain containing 3	Homo sapiens SCAN domain containing 3 (SCAND3), mRNA [NM_052923]	2.45727	up	1.69947	up	12.54010	5.28423	14.61694	7.58418	Detected	Compromised	Detected	Compromised
2622	A_32_P134209	ACVR2B	Hs.174273	activin A receptor, type IIB	Homo sapiens activin A receptor, type IIB (ACVR2B), mRNA [NM_001106]	2.45659	up	2.71678	up	216.89792	91.42310	167.57951	54.39159	Detected	Detected	Detected	Detected
2623	A_24_P29117	SLC26A1	Hs.658244	solute carrier family 26 (sulfate transporter), member 1	Homo sapiens solute carrier family 26 (sulfate transporter), member 1 (SLC26A1), transcript variant 2, mRNA [NM_134425]	2.45653	up	2.69879	up	56.04364	23.62315	56.65553	18.51136	Detected	Detected	Detected	Detected
2624	A_23_P104471	DUSP13	Hs.720464	dual specificity phosphatase 13	Homo sapiens dual specificity phosphatase 13 (DUSP13), transcript variant 1, mRNA [NM_001007271]	2.45642	up	2.85434	up	25.30144	10.66538	15.89626	4.91083	Detected	Compromised	Detected	Compromised
2625	A_33_P3335840	WDR33	Hs.620490	WD repeat domain 33	Homo sapiens WD repeat domain 33 (WDR33), transcript variant 3, mRNA [NM_001006623]	2.45613	up	2.39204	up	191.69594	80.81540	136.66740	50.38047	Detected	Detected	Detected	Detected

2626	A_23_P351734	NPHP4	Hs.462348	nephronophthisis 4	Homo sapiens nephronophthisis 4 (NPHP4), mRNA [NM_015102]	2.45528	up	2.80653	up	1529.90720	645.20526	800.98206	251.66286	Detected	Detected	Detected	Detected
2627	A_33_P3291569	LUC7L3	Hs.130293	LUC7-like 3 (S. cerevisiae)	Homo sapiens LUC7-like 3 (S. cerevisiae) (LUC7L3), transcript variant 1, mRNA [NM_016424]	2.45350	up	2.57141	up	19385.17600	8181.20360	16366.78900	5612.52540	Detected	Detected	Detected	Detected
2628	A_23_P218784	DDX17	Hs.528305	DEAD (Asp-Glu-Ala-Asp) box polypeptide 17	Homo sapiens DEAD (Asp-Glu-Ala-Asp) box polypeptide 17 (DDX17), transcript variant 1, mRNA [NM_006386]	2.45282	up	3.17677	up	804.29440	339.53340	557.73096	154.81206	Detected	Detected	Detected	Detected
2629	A_23_P161719	CWF19L2	Hs.212140	CWF19-like 2, cell cycle control (S. pombe)	Homo sapiens CWF19-like 2, cell cycle control (S. pombe) (CWF19L2), mRNA [NM_152434]	2.45205	up	2.58151	up	680.46790	287.35016	506.71915	173.08508	Detected	Detected	Detected	Detected
2630	A_33_P3278205	LOC100131102	Hs.654812	hypothetical protein LOC100131102	Homo sapiens cDNA, FLJ18231 [AK311189]	2.45021	up	2.02218	up	27.80112	11.74876	22.84285	9.96082	Detected	Compromised	Detected	Compromised
2631	A_24_P289471	RNASET2	Hs.529989	ribonuclease T2	Homo sapiens ribonuclease T2 (RNASET2), mRNA [NM_003730]	2.44787	up	2.01349	up	1790.45240	757.36957	1830.77610	801.77295	Detected	Detected	Detected	Detected
2632	A_23_P501805	LIPT1	Hs.719172	lipoyltransferase 1	Homo sapiens lipoyltransferase 1 (LIPT1), nuclear gene encoding mitochondrial protein, transcript variant 3, mRNA [NM_145197]	2.44785	up	2.55559	up	465.60068	196.95291	378.26648	130.51842	Detected	Detected	Detected	Detected
2633	A_32_P517749	RPS6KA3	Hs.445387	ribosomal protein S6 kinase, 90kDa, polypeptide 3	Homo sapiens ribosomal protein S6 kinase, 90kDa, polypeptide 3 (RPS6KA3), mRNA [NM_004586]	2.44717	up	2.30936	up	13783.41400	5832.10450	9224.87300	3522.36250	Detected	Detected	Detected	Detected
2634	A_33_P3571901	DKFZP586K1520	Hs.516864	DKFZP586K1520 protein	Homo sapiens mRNA; cDNA DKFZp586K1520 (from clone DKFZp586K1520) [AL050153]	2.44699	up	2.28339	up	6149.94630	2602.38940	6742.88600	2603.93630	Detected	Detected	Detected	Detected
2635	A_33_P3222689	H2BFM	Hs.376474	H2B histone family, member M	Homo sapiens H2B histone family, member M (H2BFM), mRNA [NM_001164416]	2.44545	up	2.12971	up	215.40335	91.20655	198.85130	82.33298	Detected	Detected	Detected	Detected
2636	A_23_P154065	TUBA4A	Hs.75318	tubulin, alpha 4a	Homo sapiens tubulin, alpha 4a (TUBA4A), mRNA [NM_006000]	2.44473	up	2.50268	up	14109.32000	5975.97360	15567.43700	5485.00050	Detected	Detected	Detected	Detected
2637	A_33_P3245439	CD40	Hs.472860	CD40 molecule, TNF receptor superfamily member 5	Homo sapiens CD40 molecule, TNF receptor superfamily member 5 (CD40), transcript variant 1, mRNA [NM_001250]	2.44448	up	2.24828	up	223.80263	94.80080	281.99493	110.60050	Detected	Detected	Detected	Detected
2638	A_23_P364837	VPS13D	Hs.439381	vacuolar protein sorting 13 homolog D (S. cerevisiae)	Homo sapiens vacuolar protein sorting 13 homolog D (S. cerevisiae) (VPS13D), transcript variant 1, mRNA [NM_015378]	2.44395	up	2.07457	up	164.84680	69.84261	58.99336	25.07494	Detected	Detected	Detected	Detected
2639	A_24_P50972	GOLGA6L6	Hs.125917	golgi autoantigen, golgin subfamily a, 6-like 6	Homo sapiens golgi autoantigen, golgin subfamily a, 6-like 6 (GOLGA6L6), mRNA [NM_001145004]	2.44375	up	2.32685	up	329.81033	139.74641	196.00479	74.27855	Detected	Detected	Detected	Detected
2640	A_23_P66608	KAT2A	Hs.463045	K(lysine) acetyltransferase 2A	Homo sapiens K(lysine) acetyltransferase 2A (KAT2A), mRNA [NM_021078]	2.44328	up	2.23717	up	7179.02500	3042.45870	6755.18600	2662.58620	Detected	Detected	Detected	Detected
2641	A_23_P212339	FYCO1	Hs.200227	FYVE and coiled-coil domain containing 1	Homo sapiens FYVE and coiled-coil domain containing 1 (FYCO1), mRNA [NM_024513]	2.44244	up	2.41681	up	2703.36990	1146.07980	2130.31270	777.25970	Detected	Detected	Detected	Detected
2642	A_24_P945113	ACVRL1	Hs.591026	activin A receptor type II-like 1	Homo sapiens activin A receptor type II-like 1 (ACVRL1), transcript variant 1, mRNA [NM_000020]	2.44070	up	2.22080	up	13233.51700	5614.27700	15768.31900	6260.98140	Detected	Detected	Detected	Detected
2643	A_23_P363406	ANGEL1	Hs.497448	angel homolog 1 (Drosophila)	Homo sapiens angel homolog 1 (Drosophila) (ANGEL1), mRNA [NM_015305]	2.44047	up	2.39716	up	246.57306	104.61752	177.48225	65.28661	Detected	Detected	Detected	Detected
2644	A_33_P3366221	NTNG1	Hs.657434	netrin G1	Homo sapiens netrin G1 (NTNG1), transcript variant 3, mRNA [NM_014917]	2.43943	up	2.24886	up	671.97620	285.23227	582.32910	228.33453	Detected	Detected	Detected	Detected
2645	A_23_P133133	ALPK1	Hs.652825	alpha-kinase 1	Homo sapiens alpha-kinase 1 (ALPK1), transcript variant 1, mRNA [NM_025144]	2.43898	up	2.55805	up	913.01610	387.61670	574.33080	197.97858	Detected	Detected	Detected	Detected
2646	A_24_P918175	LOC100128392	Hs.607889	hypothetical LOC100128392	PREDICTED: Homo sapiens hypothetical LOC100128392 (LOC100128392), mRNA [XM_001723572]	2.43891	up	2.12606	up	15.43492	6.55301	22.91605	9.50450	Detected	Compromised	Detected	Compromised
2647	A_23_P102607				Chromodomain-helicase-DNA-binding protein 6 (CHD-6)(EC 3.6.1.-)(ATP-dependent helicase CHD6)(Radiation-induced gene B protein) [Source:UniProtKB/Swiss-Prot;Acc:Q8TD26] [ENST00000373222]	2.43889	up	1.97702	up	40.64609	17.25676	31.60090	14.09466	Detected	Compromised	Detected	Compromised
2648	A_23_P405873	C9orf72	Hs.493639	chromosome 9 open reading frame 72	Homo sapiens chromosome 9 open reading frame 72 (C9orf72), transcript variant 2, mRNA [NM_145005]	2.43869	up	2.29506	up	709.64185	301.31195	656.29170	252.15576	Detected	Detected	Detected	Detected
2649	A_33_P3354539	CHURC1	Hs.325531	churchill domain containing 1	Homo sapiens churchill domain containing 1 (CHURC1), mRNA [NM_145165]	2.43808	up	2.58285	up	471.26663	200.14810	396.32178	135.30496	Detected	Detected	Detected	Detected
2650	A_33_P3262854					2.43648	up	2.04065	up	101.17997	42.99965	57.20247	24.71791	Detected	Detected	Detected	Detected
2651	A_23_P12680	PSAP	Hs.523004	prosaposin	Homo sapiens prosaposin (PSAP), transcript variant 2, mRNA [NM_001042465]	2.43645	up	2.30094	up	23059.81800	9800.14200	22953.10400	8796.32300	Detected	Detected	Detected	Detected
2652	A_33_P3281695	NLRP3	Hs.159483	NLR family, pyrin domain containing 3	Homo sapiens NLR family, pyrin domain containing 3 (NLRP3), transcript variant 1, mRNA [NM_004895]	2.43601	up	2.18336	up	30.17836	12.82775	23.77553	9.60219	Detected	Compromised	Detected	Compromised
2653	A_32_P207169	C1orf133	Hs.446946	chromosome 1 open reading frame 133	Homo sapiens chromosome 1 open reading frame 133 (C1orf133), non-coding RNA [NR_024337]	2.43386	up	2.74918	up	2171.39700	923.79750	1887.82740	605.51373	Detected	Detected	Detected	Detected
2654	A_33_P3221888	LOC100131426	Hs.546542	hypothetical LOC100131426	PREDICTED: Homo sapiens hypothetical LOC100131426, transcript variant 1 (LOC100131426), mRNA [XM_001724346]	2.43335	up	1.11187	up	39.47174	16.79631	32.45859	25.74188	Detected	Compromised	Detected	Detected

2655	A_23_P152115	NME3	Hs.514065	non-metastatic cells 3, protein expressed in	Homo sapiens non-metastatic cells 3, protein expressed in (NME3), mRNA [NM_002513]	2.43266	up	2.47232	up	11441.48700	4870.05570	12757.36600	4550.10100	Detected	Detected	Detected	Detected
2656	A_33_P3247372	LOC400927	Hs.720223	TPTE and PTEN homologous inositol lipid phosphatase pseudogene	Homo sapiens cDNA FLJ42070 fis, clone SYNOV2012326 [AK124064]	2.43266	up	1.54489	up	35.83493	15.25312	14.30008	8.16221	Detected	Detected	Detected	Compromised
2657	A_23_P210690	TRIB3	Hs.516826	tribbles homolog 3 (Drosophila)	Homo sapiens tribbles homolog 3 (Drosophila) (TRIB3), mRNA [NM_021158]	2.43175	up	2.21698	up	36691.48000	15623.50800	36604.41400	14559.21700	Detected	Detected	Detected	Detected
2658	A_33_P3358740	OSBPL7	Hs.463320	oxysterol binding protein-like 7	Homo sapiens oxysterol binding protein-like 7 (OSBPL7), transcript variant 1, mRNA [NM_145798]	2.43151	up	2.18676	up	2041.52950	869.38776	1520.55480	613.15094	Detected	Detected	Detected	Detected
2659	A_23_P408455	SLC25A36	Hs.144130	solute carrier family 25, member 36	Homo sapiens solute carrier family 25, member 36 (SLC25A36), transcript variant 1, mRNA [NM_001104647]	2.43129	up	2.72644	up	4647.45800	1979.29940	2823.44290	913.16340	Detected	Detected	Detected	Detected
2660	A_23_P154037	AOX1	Hs.406238	aldehyde oxidase 1	Homo sapiens aldehyde oxidase 1 (AOX1), mRNA [NM_001159]	2.43011	up	2.29765	up	8622.15300	3673.86620	8402.61700	3224.74680	Detected	Detected	Detected	Detected
2661	A_24_P201125	ZNF510	Hs.75264	zinc finger protein 510	Homo sapiens zinc finger protein 510 (ZNF510), mRNA [NM_014930]	2.43011	up	4.94606	up	33.06902	14.09059	15.03131	2.67980	Detected	Compromised	Detected	Compromised
2662	A_33_P3762913	LOC100216546	Hs.651341	hypothetical LOC100216546	Homo sapiens cDNA FLJ44142 fis, clone THYMU2016523 [AK126130]	2.43006	up	2.71932	up	169.92517	72.40585	161.84886	52.48252	Detected	Detected	Detected	Detected
2663	A_24_P265856	SENP7	Hs.529551	SUMO1/sentrin specific peptidase 7	Homo sapiens SUMO1/sentrin specific peptidase 7 (SENP7), transcript variant 1, mRNA [NM_020654]	2.42969	up	2.55494	up	1720.99050	733.43250	890.90430	307.47903	Detected	Detected	Detected	Detected
2664	A_23_P120860	NIPSNAP1	Hs.173878	nipsnap homolog 1 (C. elegans)	Homo sapiens nipsnap homolog 1 (C. elegans) (NIPSNAP1), mRNA [NM_003634]	2.42912	up	2.32726	up	1474.94010	628.72100	971.49370	368.09573	Detected	Detected	Detected	Detected
2665	A_23_P312150	EDN2	Hs.1407	endothelin 2	Homo sapiens endothelin 2 (EDN2), mRNA [NM_001956]	2.42870	up	2.13765	up	21.60421	9.21082	12.45530	5.13787	Detected	Compromised	Compromised	Compromised
2666	A_23_P500886	CLDN15	Hs.38738	claudin 15	Homo sapiens claudin 15 (CLDN15), mRNA [NM_014343]	2.42837	up	2.35146	up	151.00240	64.38749	120.14507	45.05403	Detected	Detected	Detected	Detected
2667	A_23_P3221	SQRDL	Hs.511251	sulfide quinone reductase-like (yeast)	Homo sapiens sulfide quinone reductase-like (yeast) (SQRDL), nuclear gene encoding mitochondrial protein, mRNA [NM_021199]	2.42719	up	2.48900	up	121426.74000	51801.71500	116282.48400	41195.93800	Detected	Detected	Detected	Detected
2668	A_33_P3236319	LOC100128869	Hs.694468	hypothetical LOC100128869	PREDICTED: Homo sapiens hypothetical LOC100128869 (LOC100128869), mRNA [XM_001719518]	2.42717	up	1.05322	up	32.61886	13.91561	10.75863	9.00752	Detected	Compromised	Compromised	Compromised
2669	A_23_P43079	INTS8	Hs.521693	integrator complex subunit 8	Homo sapiens integrator complex subunit 8 (INTS8), mRNA [NM_017864]	2.42699	up	2.62338	up	835.91170	356.63626	616.72200	207.29724	Detected	Detected	Detected	Detected
2670	A_23_P71037	IL6	Hs.654458	interleukin 6 (interferon, beta 2)	Homo sapiens interleukin 6 (interferon, beta 2) (IL6), mRNA [NM_000600]	2.42658	up	2.38097	up	2241.15360	956.33417	1901.17970	704.10190	Detected	Detected	Detected	Detected
2671	A_33_P3329108	MTAP	Hs.193268	methylthioadenosine phosphorylase	Homo sapiens methylthioadenosine phosphorylase (MTAP), mRNA [NM_002451]	2.42596	up	2.62456	up	2492.96120	1064.05770	1782.62020	598.91870	Detected	Detected	Detected	Detected
2672	A_32_P191895		Hs.454036		Homo sapiens cDNA clone IMAGE:4814292 [BC030617]	2.42462	up	2.63505	up	1222.35560	522.01984	1449.46190	485.04736	Detected	Detected	Detected	Detected
2673	A_33_P3212555	PROS1	Hs.64016	protein S (alpha)	Homo sapiens protein S (alpha) (PROS1), mRNA [NM_000313]	2.42414	up	2.77358	up	589.21760	251.68166	386.64860	122.92542	Detected	Detected	Detected	Detected
2674	A_33_P3288684					2.42370	up	4.13007	up	111.34014	47.56698	60.89954	13.00236	Detected	Detected	Detected	Compromised
2675	A_32_P132860	UNQ6494		hypothetical LOC100129066	Homo sapiens hypothetical LOC100129066 (LOC100129066), non-coding RNA [NR_024280]	2.42323	up	1.83558	up	18.13742	7.75024	6.78790	3.26083	Detected	Compromised	Compromised	Compromised
2676	A_33_P3289251					2.42312	up	2.77085	up	3451.20830	1474.78500	1465.29880	466.31467	Detected	Detected	Detected	Detected
2677	A_24_P258846	NFATC1	Hs.534074	nuclear factor of activated T-cells, cytoplasmic, calcineurin-dependent 1	Homo sapiens nuclear factor of activated T-cells, cytoplasmic, calcineurin-dependent 1 (NFATC1), transcript variant 1, mRNA [NM_172390]	2.42289	up	2.06483	up	968.79190	414.02853	1147.36010	489.98282	Detected	Detected	Detected	Detected
2678	A_23_P409168	NBEAL2	Hs.437043	neurobeachin-like 2	Homo sapiens neurobeachin-like 2 (NBEAL2), mRNA [NM_015175]	2.42270	up	2.53234	up	198.09344	84.66494	149.45175	52.04078	Detected	Detected	Detected	Detected
2679	A_33_P3311668	LOC100133598	Hs.577721	hypothetical protein LOC100133598	PREDICTED: Homo sapiens hypothetical LOC100133598 (LOC100133598), mRNA [XM_001721691]	2.42224	up	1.64181	up	19.60519	8.38085	10.79663	5.79869	Detected	Compromised	Compromised	Compromised
2680	A_33_P3672482	USP28	Hs.503891	ubiquitin specific peptidase 28	Homo sapiens ubiquitin specific peptidase 28 (USP28), mRNA [NM_020886]	2.42205	up	1.50049	up	31.70076	13.55251	15.27198	8.97485	Detected	Compromised	Detected	Compromised
2681	A_33_P3273068	ALX4	Hs.436055	ALX homeobox 4	Homeobox protein aristaless-like 4 [Source:UniProtKB/Swiss-Prot;Acc:Q9H161] [ENST00000329255]	2.42187	up	3.41335	up	29.70542	12.70040	33.75193	8.71934	Detected	Compromised	Detected	Compromised
2682	A_23_P157580	SDCBP	Hs.200804	syndecan binding protein (syntenin)	Homo sapiens syndecan binding protein (syntenin) (SDCBP), transcript variant 1, mRNA [NM_005625]	2.42172	up	2.38761	up	22007.96900	9409.99200	17086.66400	6310.43500	Detected	Detected	Detected	Detected
2683	A_33_P3405743	CRYZ	Hs.83114	crystallin, zeta (quinone reductase)	Homo sapiens crystallin, zeta (quinone reductase) (CRYZ), transcript variant 1, mRNA [NM_001130042]	2.41992	up	2.52390	up	2869.40330	1227.79110	2323.81670	811.88556	Detected	Detected	Detected	Detected
2684	A_23_P393686	C8orf42	Hs.289293	chromosome 8 open reading frame 42	Homo sapiens chromosome 8 open reading frame 42 (C8orf42), mRNA [NM_175075]	2.41911	up	2.19621	up	60.67338	25.97021	57.02685	22.89663	Detected	Detected	Detected	Detected
2685	A_23_P350555	TCP10L	Hs.42034	t-complex 10 (mouse)-like	Homo sapiens t-complex 10 (mouse)-like (TCP10L), mRNA [NM_144659]	2.41840	up	3.50402	up	361.97480	154.98264	269.50946	67.82232	Detected	Detected	Detected	Detected
2686	A_23_P79015	SCN1B	Hs.436646	sodium channel, voltage-gated, type I, beta	Homo sapiens sodium channel, voltage-gated, type I, beta (SCN1B), transcript variant b, mRNA [NM_199037]	2.41805	up	3.49664	up	67.54604	28.92458	55.52560	14.00258	Detected	Detected	Detected	Compromised
2687	A_23_P259090	NUDT12	Hs.434289	nudix (nucleoside diphosphate linked moiety X)-type motif 12	Homo sapiens nudix (nucleoside diphosphate linked moiety X)-type motif 12 (NUDT12), mRNA [NM_031438]	2.41756	up	2.38522	up	416.37134	178.33493	300.85977	111.22462	Detected	Detected	Detected	Detected

2688	A_33_P3588134	PANX2	Hs.440092	pannexin 2	Homo sapiens pannexin 2 (PANX2), transcript variant 1, mRNA [NM_052839]	2.41638	up	2.84495	up	124.87750	53.51217	98.97947	30.67860	Detected	Detected	Detected	Detected
2689	A_24_P382253	RPRD1A	Hs.464912	regulation of nuclear pre-mRNA domain containing 1A	Homo sapiens regulation of nuclear pre-mRNA domain containing 1A (RPRD1A), mRNA [NM_018170]	2.41610	up	2.25187	up	3233.75070	1385.87510	2306.38130	903.13635	Detected	Detected	Detected	Detected
2690	A_23_P420196	SOCS1	Hs.50640	suppressor of cytokine signaling 1	Homo sapiens suppressor of cytokine signaling 1 (SOCS1), mRNA [NM_003745]	2.41588	up	2.75806	up	578.10320	247.77815	487.96490	156.00934	Detected	Detected	Detected	Detected
2691	A_24_P9883	DKFZp761E198	Hs.591957	DKFZp761E198 protein	Homo sapiens DKFZp761E198 protein (DKFZp761E198), mRNA [NM_138368]	2.41575	up	1.98023	up	69.86681	29.94687	185.50381	82.60452	Detected	Detected	Detected	Detected
2692	A_33_P3402211		Hs.666398		RST27055 Athersys RAGE Library Homo sapiens cDNA, mRNA sequence [BG207573]	2.41550	up	5.88720	up	29.81048	12.77897	17.10826	2.56249	Detected	Compromised	Detected	Compromised
2693	A_23_P92517	TTC29	Hs.378893	tetratricopeptide repeat domain 29	Homo sapiens tetratricopeptide repeat domain 29 (TTC29), mRNA [NM_031956]	2.41427	up	2.28223	up	34.42820	14.76599	35.82701	13.84257	Detected	Compromised	Detected	Compromised
2694	A_32_P117313	C8orf83	Hs.440643	chromosome 8 open reading frame 83	Homo sapiens chromosome 8 open reading frame 83 (C8orf83), non-coding RNA [NR_015339]	2.41409	up	2.54452	up	1836.87610	787.87990	1397.76030	484.38730	Detected	Detected	Detected	Detected
2695	A_23_P312610		Hs.172847		DnaJ homolog subfamily C member 4 (Multiple endocrine neoplasia type 1 candidate protein number 18)(DnaJ-like protein HSPF2) [Source:UniProtKB/Swiss-Prot;Acc:Q9NNZ3] [ENST00000355040]	2.41386	up	2.33417	up	230.22108	98.75668	236.02367	89.16381	Detected	Detected	Detected	Detected
2696	A_23_P157607	INTS10	Hs.512627	integrator complex subunit 10	Homo sapiens integrator complex subunit 10 (INTS10), mRNA [NM_018142]	2.41373	up	2.40329	up	13873.02200	5951.35100	13738.12300	5040.64200	Detected	Detected	Detected	Detected
2697	A_33_P3379947	HLA-B	Hs.77961	major histocompatibility complex, class I, B	Homo sapiens major histocompatibility complex, class I, B (HLA-B), mRNA [NM_005514]	2.41367	up	2.25671	up	98676.94500	42332.19500	37149.52300	14515.84800	Detected	Detected	Detected	Detected
2698	A_33_P3404879	LPHN2	Hs.24212	latrophilin 2	Homo sapiens latrophilin 2 (LPHN2), mRNA [NM_012302]	2.41098	up	2.35147	up	68.56657	29.44773	61.18812	22.94526	Detected	Detected	Detected	Detected
2699	A_23_P11859	HSD17B7	Hs.492925	hydroxysteroid (17-beta) dehydrogenase 7	Homo sapiens hydroxysteroid (17-beta) dehydrogenase 7 (HSD17B7), mRNA [NM_016371]	2.40984	up	2.57902	up	303.81080	130.54123	229.04300	78.31182	Detected	Detected	Detected	Detected
2700	A_33_P3267330	HAR1B		highly accelerated region 1B (non-protein coding)	Homo sapiens highly accelerated region 1B (non-protein coding) (HAR1B), non-coding RNA [NR_003245]	2.40977	up	2.41401	up	35.58195	15.28928	42.57474	15.55173	Detected	Compromised	Detected	Detected
2701	A_23_P62881	SGIP1	Hs.132121	SH3-domain GRB2-like (endophilin) interacting protein 1	Homo sapiens SH3-domain GRB2-like (endophilin) interacting protein 1 (SGIP1), mRNA [NM_032291]	2.40973	up	2.26348	up	195.72934	84.10475	152.03152	59.22747	Detected	Detected	Detected	Detected
2702	A_32_P33083	VCX2	Hs.279737	variable charge, X-linked 2	Homo sapiens variable charge, X-linked 2 (VCX2), mRNA [NM_016378]	2.40930	up	3.97093	up	24.56661	10.55813	39.36513	8.74149	Detected	Compromised	Detected	Compromised
2703	A_33_P3286724	PLD2	Hs.104519	phospholipase D2	Homo sapiens phospholipase D2 (PLD2), mRNA [NM_002663]	2.40848	up	2.22269	up	12001.40500	5159.67000	10477.38400	4156.60700	Detected	Detected	Detected	Detected
2704	A_33_P3236642	MIA3	Hs.118474	melanoma inhibitory activity family, member 3	Homo sapiens melanoma inhibitory activity family, member 3 (MIA3), mRNA [NM_198551]	2.40801	up	2.28993	up	112.97820	48.58141	75.88051	29.21951	Detected	Detected	Detected	Detected
2705	A_33_P3308749	LAMA4	Hs.654572	laminin, alpha 4	Homo sapiens laminin, alpha 4 (LAMA4), transcript variant 3, mRNA [NM_001105207]	2.40762	up	3.01426	up	1733.11010	745.36900	1711.12040	500.57040	Detected	Detected	Detected	Detected
2706	A_33_P3422659	GDAP1	Hs.168950	ganglioside-induced differentiation-associated protein 1	Homo sapiens ganglioside-induced differentiation-associated protein 1 (GDAP1), transcript variant 1, mRNA [NM_018972]	2.40747	up	2.87999	up	75.46864	32.45929	39.01326	11.94502	Detected	Detected	Detected	Compromised
2707	A_24_P36097	RPAIN	Hs.462086	RPA interacting protein	Homo sapiens RPA interacting protein (RPAIN), transcript variant 5, mRNA [NM_001160266]	2.40664	up	2.50752	up	3953.25630	1700.89640	3774.77640	1327.43000	Detected	Detected	Detected	Detected
2708	A_23_P168669	CROT	Hs.125039	carnitine O-octanoyltransferase	Homo sapiens carnitine O-octanoyltransferase (CROT), transcript variant 2, mRNA [NM_021151]	2.40663	up	2.12521	up	120.67450	51.92051	94.46150	39.19382	Detected	Detected	Detected	Detected
2709	A_23_P149775	ARHGAP12	Hs.499264	Rho GTPase activating protein 12	Homo sapiens Rho GTPase activating protein 12 (ARHGAP12), mRNA [NM_018287]	2.40601	up	2.33183	up	14432.27200	6211.11470	10000.79100	3781.84450	Detected	Detected	Detected	Detected
2710	A_33_P3763846	MAPK12	Hs.432642	mitogen-activated protein kinase 12	Homo sapiens mitogen-activated protein kinase 12 (MAPK12), mRNA [NM_002969]	2.40546	up	2.05959	up	530.30560	228.27643	475.09170	203.40488	Detected	Detected	Detected	Detected
2711	A_23_P255714	IFT74	Hs.145402	intraflagellar transport 74 homolog (Chlamydomonas)	Homo sapiens intraflagellar transport 74 homolog (Chlamydomonas) (IFT74), transcript variant 1, mRNA [NM_025103]	2.40515	up	2.48426	up	1169.65310	503.55734	774.35040	274.85605	Detected	Detected	Detected	Detected
2712	A_23_P148015	AXIN2	Hs.156527	axin 2	Homo sapiens axin 2 (AXIN2), mRNA [NM_004655]	2.40506	up	2.22919	up	599.95130	258.29920	472.59308	186.94180	Detected	Detected	Detected	Detected
2713	A_33_P3299170	LUC7L	Hs.16803	LUC7-like (S. cerevisiae)	Homo sapiens LUC7-like (S. cerevisiae) (LUC7L), transcript variant 2, mRNA [NM_201412]	2.40462	up	2.25859	up	7433.45170	3200.94120	7307.82300	2853.09400	Detected	Detected	Detected	Detected
2714	A_23_P96383	SRPX	Hs.15154	sushi-repeat-containing protein, X-linked	Homo sapiens sushi-repeat-containing protein, X-linked (SRPX), mRNA [NM_006307]	2.40441	up	2.12604	up	61888.15000	26652.09800	41139.55000	17062.96000	Detected	Detected	Detected	Detected
2715	A_33_P3378126	FBXO32	Hs.403933	F-box protein 32	Homo sapiens F-box protein 32 (FBXO32), transcript variant 1, mRNA [NM_058229]	2.40298	up	2.46657	up	14752.09100	6356.78170	10623.82200	3797.98850	Detected	Detected	Detected	Detected
2716	A_23_P363778	FRZB	Hs.720164	frizzled-related protein	Homo sapiens frizzled-related protein (FRZB), mRNA [NM_001463]	2.40260	up	2.01971	up	98.89978	42.62336	72.66962	31.72701	Detected	Detected	Detected	Detected
2717	A_33_P3250953	SLC35B4	Hs.490181	solute carrier family 35, member B4	Homo sapiens solute carrier family 35, member B4 (SLC35B4), mRNA [NM_032826]	2.40233	up	2.52868	up	2078.29660	895.79510	1360.52200	474.43580	Detected	Detected	Detected	Detected
2718	A_33_P3386870					2.40187	up	2.37394	up	62.58938	26.98262	64.88757	24.10220	Detected	Detected	Detected	Detected

2719	A_23_P257176	SLC4A9	Hs.550313	solute carrier family 4, sodium bicarbonate cotransporter, member 9	Homo sapiens solute carrier family 4, sodium bicarbonate cotransporter, member 9 (SLC4A9), mRNA [NM_031467]	2.40123	up	2.70136	up	31.91404	13.76201	9.74067	3.17960	Detected	Compromised	Compromised	Compromised
2720	A_24_P286935	ARL3	Hs.182215	ADP-ribosylation factor-like 3	Homo sapiens ADP-ribosylation factor-like 3 (ARL3), mRNA [NM_004311]	2.39942	up	2.42293	up	565.07880	243.85716	517.30304	188.26495	Detected	Detected	Detected	Detected
2721	A_23_P15394	CD68	Hs.647419	CD68 molecule	Homo sapiens CD68 molecule (CD68), transcript variant 1, mRNA [NM_001251]	2.39864	up	2.09405	up	8877.93100	3832.47680	10008.60800	4214.55200	Detected	Detected	Detected	Detected
2722	A_24_P331830	RP1-21O18.1	Hs.368823	kazrin	Homo sapiens kazrin (KIAA1026), transcript variant A, mRNA [NM_015209]	2.39808	up	1.62480	up	86.75578	37.46002	86.24970	46.80832	Detected	Detected	Detected	Detected
2723	A_24_P225878	KIF9	Hs.373947	kinesin family member 9	Homo sapiens kinesin family member 9 (KIF9), transcript variant 1, mRNA [NM_022342]	2.39721	up	3.46715	up	62.38912	26.94854	48.96149	12.45222	Detected	Detected	Detected	Compromised
2724	A_23_P64888	TAS2R10	Hs.533756	taste receptor, type 2, member 10	Homo sapiens taste receptor, type 2, member 10 (TAS2R10), mRNA [NM_023921]	2.39703	up	2.40093	up	80.36944	34.71766	70.04536	25.72558	Detected	Detected	Detected	Detected
2725	A_33_P3350583	FAM122C	Hs.269127	family with sequence similarity 122C	Homo sapiens family with sequence similarity 122C (FAM122C), mRNA [NM_138819]	2.39663	up	2.41786	up	38.38320	16.58336	37.32691	13.61307	Detected	Compromised	Detected	Compromised
2726	A_23_P10442	OSBPL1A	Hs.370725	oxysterol binding protein-like 1A	Homo sapiens oxysterol binding protein-like 1A (OSBPL1A), transcript variant OSBPL1B, mRNA [NM_080597]	2.39428	up	2.32694	up	2378.80370	1028.76560	2031.50440	769.83510	Detected	Detected	Detected	Detected
2727	A_23_P9056	RB1CC1	Hs.196102	RB1-inducible coiled-coil 1	Homo sapiens RB1-inducible coiled-coil 1 (RB1CC1), transcript variant 1, mRNA [NM_014781]	2.39420	up	2.39906	up	6077.21600	2628.31500	5175.00730	1902.11120	Detected	Detected	Detected	Detected
2728	A_33_P3210223				Zinc finger CCHC domain-containing protein 6 [Source:UniProtKB/Swiss-Prot;Acc:Q5VYS8] [ENST00000375948]	2.39349	up	1.35296	up	16.06959	6.95193	11.13752	7.25885	Detected	Compromised	Compromised	Compromised
2729	A_24_P83787	DISC1	Hs.13318	disrupted in schizophrenia 1	Homo sapiens disrupted in schizophrenia 1 (DISC1), transcript variant S, mRNA [NM_001012959]	2.39314	up	4.16591	up	15.52889	6.71902	15.97240	3.38085	Detected	Compromised	Compromised	Compromised
2730	A_23_P122563	PFDN6	Hs.446374	prefoldin subunit 6	Homo sapiens prefoldin subunit 6 (PFDN6), mRNA [NM_014260]	2.39083	up	1.49289	up	3595.97000	1557.39730	5077.81050	2999.26270	Detected	Detected	Detected	Detected
2731	A_24_P139943	HS1BP3	Hs.531785	HCLS1 binding protein 3	Homo sapiens HCLS1 binding protein 3 (HS1BP3), mRNA [NM_022460]	2.39081	up	2.11785	up	3083.93600	1335.65110	3866.98410	1610.06120	Detected	Detected	Detected	Detected
2732	A_33_P3309924	HDAC2	Hs.3352	histone deacetylase 2	Homo sapiens histone deacetylase 2 (HDAC2), mRNA [NM_001527]	2.38688	up	2.46723	up	103.30002	44.81296	89.82162	32.10235	Detected	Detected	Detected	Detected
2733	A_23_P107322	CORO6	Hs.143046	coronin 6	Homo sapiens coronin 6 (CORO6), mRNA [NM_032854]	2.38682	up	2.60529	up	1165.93920	505.81223	827.40485	280.04460	Detected	Detected	Detected	Detected
2734	A_24_P13475	SPATA13	Hs.595391	spermatogenesis associated 13	Homo sapiens spermatogenesis associated 13 (SPATA13), transcript variant 1, mRNA [NM_001166271]	2.38589	up	2.14016	up	367.62497	159.54680	343.94590	141.71303	Detected	Detected	Detected	Detected
2735	A_32_P198303	DNAJC24	Hs.718544	DnaJ (Hsp40) homolog, subfamily C, member 24	Homo sapiens DnaJ (Hsp40) homolog, subfamily C, member 24 (DNAJC24), mRNA [NM_181706]	2.38485	up	2.48930	up	1077.84830	467.98220	617.44604	218.71924	Detected	Detected	Detected	Detected
2736	A_23_P139998	UBAC2	Hs.508545	UBA domain containing 2	Homo sapiens UBA domain containing 2 (UBAC2), transcript variant 2, mRNA [NM_177967]	2.38452	up	2.21028	up	4184.50100	1817.08680	3298.42380	1315.90300	Detected	Detected	Detected	Detected
2737	A_33_P3276153	LOC645752	Hs.574257	golgi autoantigen, golgin subfamily a, 6 pseudogene	Homo sapiens golgi autoantigen, golgin subfamily a, 6 pseudogene (LOC645752), non-coding RNA [NR_027024]	2.38435	up	2.50997	up	213.43448	92.68890	157.38075	55.29020	Detected	Detected	Detected	Detected
2738	A_23_P141680	BCAS3	Hs.655028	breast carcinoma amplified sequence 3	Homo sapiens breast carcinoma amplified sequence 3 (BCAS3), transcript variant 2, mRNA [NM_017679]	2.38362	up	2.03965	up	278.07153	120.79601	203.22055	87.85716	Detected	Detected	Detected	Detected
2739	A_33_P3288023	NCKIPSD	Hs.655006	NCK interacting protein with SH3 domain	Homo sapiens NCK interacting protein with SH3 domain (NCKIPSD), transcript variant 2, mRNA [NM_184231]	2.38350	up	2.29745	up	333.54526	144.90123	350.58466	134.55907	Detected	Detected	Detected	Detected
2740	A_33_P3221384					2.38325	up	1.11395	up	218.55946	94.95828	209.27245	165.65850	Detected	Detected	Detected	Detected
2741	A_23_P215997	CYP11B2	Hs.632054	cytochrome P450, family 11, subfamily B, polypeptide 2	Homo sapiens cytochrome P450, family 11, subfamily B, polypeptide 2 (CYP11B2), nuclear gene encoding mitochondrial protein, mRNA [NM_000498]	2.38290	up	2.31708	up	12.45421	5.41181	18.82797	7.16521	Detected	Compromised	Detected	Compromised
2742	A_33_P3394972	OSBPL5	Hs.436166	oxysterol binding protein-like 5	Homo sapiens oxysterol binding protein-like 5 (OSBPL5), transcript variant 1, mRNA [NM_020896]	2.38249	up	2.58999	up	156.49019	68.01246	140.49767	47.83389	Detected	Detected	Detected	Detected
2743	A_32_P104746	ZFYVE28	Hs.292056	zinc finger, FYVE domain containing 28	Homo sapiens zinc finger, FYVE domain containing 28 (ZFYVE28), mRNA [NM_020972]	2.38105	up	2.43791	up	491.07004	213.55408	471.85046	170.66832	Detected	Detected	Detected	Detected
2744	A_24_P944458	INSIG2	Hs.7089	insulin induced gene 2	Homo sapiens insulin induced gene 2 (INSIG2), mRNA [NM_016133]	2.38013	up	2.43528	up	911.71040	396.63315	643.32623	232.94212	Detected	Detected	Detected	Detected
2745	A_23_P43557	DENND1A	Hs.568340	DENN/MADD domain containing 1A	Homo sapiens DENN/MADD domain containing 1A (DENND1A), transcript variant 2, mRNA [NM_024820]	2.37983	up	2.25501	up	665.66370	289.62885	494.00290	193.17285	Detected	Detected	Detected	Detected
2746	A_23_P420256	C2orf43	Hs.187823	chromosome 2 open reading frame 43	Homo sapiens chromosome 2 open reading frame 43 (C2orf43), mRNA [NM_021925]	2.37696	up	2.15653	up	868.80460	378.47183	775.47220	317.08510	Detected	Detected	Detected	Detected
2747	A_33_P3221916	LOC100130138	Hs.560906	hypothetical LOC100130138	PREDICTED: Homo sapiens hypothetical LOC100130138 (LOC100130138), mRNA [XM_001725911]	2.37579	up	2.00054	up	12.57204	5.47938	5.62326	2.47860	Detected	Compromised	Compromised	Compromised
2748	A_33_P3277110	SLC5A3	Hs.302742	solute carrier family 5 (sodium/myo-inositol cotransporter), member 3	Homo sapiens solute carrier family 5 (sodium/myo-inositol cotransporter), member 3 (SLC5A3), mRNA [NM_006933]	2.37570	up	2.70954	up	3635.91820	1584.72970	2275.12870	740.41650	Detected	Detected	Detected	Detected

2749	A_23_P46017	CCBL2	Hs.481898	cysteine conjugate-beta lyase 2	Homo sapiens cysteine conjugate-beta lyase 2 (CCBL2), transcript variant 1, mRNA [NM_001008661]	2.37455	up	2.50477	up	2405.18750	1048.81870	2817.98320	992.05490	Detected	Detected	Detected	Detected
2750	A_33_P3309506	SEBOX	Hs.2257	SEBOX homeobox	Homo sapiens SEBOX homeobox (SEBOX), transcript variant 2, mRNA [NM_001083896]	2.37368	up	1.24215	up	69.18485	30.18020	11.95461	8.48648	Detected	Detected	Compromised	Compromised
2751	A_33_P3227551					2.37285	up	1.03706	up	13.10493	5.71870	10.40672	8.84863	Detected	Compromised	Compromised	Compromised
2752	A_32_P206104					2.37249	up	1.57268	up	17.51113	7.64262	15.11050	8.47235	Detected	Compromised	Detected	Compromised
2753	A_23_P9883	NLRP3	Hs.159483	NLR family, pyrin domain containing 3	Homo sapiens NLR family, pyrin domain containing 3 (NLRP3), transcript variant 3, mRNA [NM_001079821]	2.37151	up	2.49304	up	133.15118	58.13710	97.48431	34.48034	Detected	Detected	Detected	Detected
2754	A_23_P118722	ASGR1	Hs.12056	asialoglycoprotein receptor 1	Homo sapiens asialoglycoprotein receptor 1 (ASGR1), mRNA [NM_001671]	2.36913	up	2.32276	up	1028.25290	449.41156	843.52520	320.22787	Detected	Detected	Detected	Detected
2755	A_23_P349676	FBXO41	Hs.23158	F-box protein 41	Homo sapiens F-box protein 41 (FBXO41), mRNA [NM_001080410]	2.36878	up	2.42610	up	1214.21460	530.76680	800.96136	291.11700	Detected	Detected	Detected	Detected
2756	A_33_P3372727	SEMA5A	Hs.27621	sema domain, seven thrombospondin repeats (type 1 and type 1-like), transmembrane domain (TM) and short cytoplasmic domain, (semaphorin) 5A	Homo sapiens sema domain, seven thrombospondin repeats (type 1 and type 1-like), transmembrane domain (TM) and short cytoplasmic domain, (semaphorin) 5A (SEMA5A), mRNA [NM_003966]	2.36788	up	2.06385	up	30039.98800	13136.32800	25220.04100	10775.39100	Detected	Detected	Detected	Detected
2757	A_23_P90523	OCEL1	Hs.422676	occludin/ELL domain containing 1	Homo sapiens occludin/ELL domain containing 1 (OCEL1), mRNA [NM_024578]	2.36610	up	2.41639	up	1199.88510	525.09796	1433.88720	523.25525	Detected	Detected	Detected	Detected
2758	A_24_P247732	SLC5A6	Hs.435735	solute carrier family 5 (sodium-dependent vitamin transporter), member 6	Homo sapiens solute carrier family 5 (sodium-dependent vitamin transporter), member 6 (SLC5A6), transcript variant 1, mRNA [NM_021095]	2.36513	up	1.69957	up	11.55097	5.05704	15.24553	7.90988	Detected	Compromised	Detected	Compromised
2759	A_23_P151791	LTB4R	Hs.567248	leukotriene B4 receptor	Homo sapiens leukotriene B4 receptor (LTB4R), transcript variant 1, mRNA [NM_181657]	2.36405	up	2.13524	up	326.11703	142.83989	221.70648	91.55808	Detected	Detected	Detected	Detected
2760	A_23_P301328				Pyridoxal kinase (EC 2.7.1.35)(Pyridoxine kinase) [Source:UniProtKB/Swiss-Prot;Acc:O00764] [ENST00000327574]	2.36396	up	1.82776	up	66.58356	29.16489	35.37958	17.06861	Detected	Detected	Detected	Detected
2761	A_33_P3257714	RPS23	Hs.527193	ribosomal protein S23	Homo sapiens ribosomal protein S23 (RPS23), mRNA [NM_001025]	2.36329	up	2.20032	up	6501.25700	2848.48270	5814.66650	2330.26050	Detected	Detected	Detected	Detected
2762	A_23_P137016	SAT1	Hs.28491	spermidine/spermine N1-acetyltransferase 1	Homo sapiens spermidine/spermine N1-acetyltransferase 1 (SAT1), transcript variant 1, mRNA [NM_002970]	2.36308	up	2.49633	up	76012.30500	33307.28000	71746.69500	25343.42600	Detected	Detected	Detected	Detected
2763	A_23_P325887	TBC1D8B	Hs.351798	TBC1 domain family, member 8B (with GRAM domain)	Homo sapiens TBC1 domain family, member 8B (with GRAM domain) (TBC1D8B), transcript variant 1, mRNA [NM_017752]	2.36151	up	4.16199	up	103.12137	45.21602	49.63527	10.51610	Detected	Detected	Detected	Compromised
2764	A_24_P341222	C4orf52	Hs.479386	chromosome 4 open reading frame 52	Homo sapiens chromosome 4 open reading frame 52 (C4orf52), mRNA [NM_001145432]	2.36058	up	2.51449	up	4263.21730	1870.04140	4297.52730	1507.07260	Detected	Detected	Detected	Detected
2765	A_33_P3233754	ZNF154	Hs.646378	zinc finger protein 154	Homo sapiens zinc finger protein 154 (ZNF154), mRNA [NM_001085384]	2.36054	up	1.94772	up	29.79250	13.06857	17.96855	8.13489	Detected	Compromised	Detected	Compromised
2766	A_24_P15502				Putative uncharacterized protein IFITM8P [Source:UniProtKB/TrEMBL;Acc:A8MWN3] [ENST00000399573]	2.36033	up	1.93054	up	16764.60200	7354.50150	15668.59200	7156.77900	Detected	Detected	Detected	Detected
2767	A_23_P29124	SEPT5	Hs.283743	septin 5	Homo sapiens septin 5 (SEPT5), mRNA [NM_002688]	2.36015	up	2.07686	up	2646.31320	1161.00850	3115.13650	1322.61790	Detected	Detected	Detected	Detected
2768	A_33_P3333156	C11orf70	Hs.98328	chromosome 11 open reading frame 70	Homo sapiens chromosome 11 open reading frame 70 (C11orf70), mRNA [NM_032930]	2.35884	up	2.51342	up	317.96857	139.57830	220.50615	77.36099	Detected	Detected	Detected	Detected
2769	A_24_P96762	TRNAU1AP	Hs.533626	tRNA selenocysteine 1 associated protein 1	Homo sapiens tRNA selenocysteine 1 associated protein 1 (TRNAU1AP), transcript variant 1, mRNA [NM_017846]	2.35793	up	2.44256	up	959.55990	421.37997	779.50775	281.41022	Detected	Detected	Detected	Detected
2770	A_33_P3362143	LOC284900		hypothetical LOC284900	Homo sapiens hypothetical LOC284900 (LOC284900), transcript variant 2, non-coding RNA [NR_026962]	2.35703	up	2.60260	up	277.78660	122.03377	168.28268	57.01615	Detected	Detected	Detected	Detected
2771	A_33_P3237567		Hs.690701		CR987211 RZPD no.9017 Homo sapiens cDNA clone RZPDp9017M1210 5', mRNA sequence [CR987211]	2.35699	up	2.99045	up	1016.26935	446.46120	1155.10180	340.60320	Detected	Detected	Detected	Detected
2772	A_32_P163858	SCD	Hs.558396	stearoyl-CoA desaturase (delta-9-desaturase)	Homo sapiens stearoyl-CoA desaturase (delta-9-desaturase) (SCD), mRNA [NM_005063]	2.35656	up	2.52604	up	2810.46390	1234.90110	2130.86470	743.84265	Detected	Detected	Detected	Detected
2773	A_33_P3359856		Hs.721706		Protein FAM128A [Source:UniProtKB/Swiss-Prot;Acc:Q6P582] [ENST00000428332]	2.35653	up	2.53752	up	767.69464	337.32468	1008.73706	350.53738	Detected	Detected	Detected	Detected
2774	A_33_P3613516	LOC254057	Hs.586109	hypothetical protein LOC254057	Homo sapiens cDNA: FLJ21000 fis, clone CAE03359 [AK024653]	2.35561	up	2.27776	up	440.99380	193.84851	289.53830	112.08910	Detected	Detected	Detected	Detected
2775	A_33_P3335740	FLJ10038		hypothetical protein FLJ10038	Homo sapiens hypothetical protein FLJ10038 (FLJ10038), non-coding RNA [NR_026891]	2.35531	up	5.48010	up	111.27624	48.92008	65.02536	10.46308	Detected	Detected	Detected	Compromised
2776	A_33_P3226177	CYFIP2	Hs.519702	cytoplasmic FMR1 interacting protein 2	Homo sapiens cytoplasmic FMR1 interacting protein 2 (CYFIP2), transcript variant 2, mRNA [NM_001037332]	2.35153	up	2.90297	up	81.70776	35.97878	50.91779	15.46653	Detected	Detected	Detected	Compromised
2777	A_24_P68222					2.35005	up	2.42193	up	715.95430	315.45773	574.90170	209.31367	Detected	Detected	Detected	Detected

2778	A_33_P3409086	S100A1	Hs.515715	S100 calcium binding protein A1	Homo sapiens S100 calcium binding protein A1 (S100A1), mRNA [NM_006271]	2.34954	up	1.65963	up	211.72447	93.30845	33.45704	17.77634	Detected	Detected	Detected	Detected
2779	A_33_P3241299	LOC100128881	Hs.666282	similar to predicted protein	Homo sapiens cDNA FLJ38967 fis, clone NT2RI2002260 [AK096286]	2.34946	up	2.07457	up	97.31768	42.89000	119.72920	50.89067	Detected	Detected	Detected	Detected
2780	A_33_P3265494	C6orf138	Hs.659409	chromosome 6 open reading frame 138	Homo sapiens chromosome 6 open reading frame 138 (C6orf138), mRNA [NM_001013732]	2.34920	up	2.24561	up	19.64230	8.65778	22.74603	8.93176	Detected	Compromised	Detected	Compromised
2781	A_33_P3383326	LPAR1	Hs.126667	lysophosphatidic acid receptor 1	Homo sapiens lysophosphatidic acid receptor 1 (LPAR1), transcript variant 2, mRNA [NM_057159]	2.34897	up	2.46416	up	12336.42100	5438.08200	6781.10840	2426.59640	Detected	Detected	Detected	Detected
2782	A_32_P319858	NKPD1	Hs.299256	NTPase, KAP family P-loop domain containing 1	Homo sapiens NTPase, KAP family P-loop domain containing 1 (NKPD1), transcript variant 2, mRNA [NM_198478]	2.34812	up	1.17813	up	22.74639	10.03057	11.89693	8.90446	Detected	Compromised	Compromised	Compromised
2783	A_23_P206228	VPS13C	Hs.511668	vacuolar protein sorting 13 homolog C (S. cerevisiae)	Homo sapiens vacuolar protein sorting 13 homolog C (S. cerevisiae) (VPS13C), transcript variant 2A, mRNA [NM_020821]	2.34804	up	2.57400	up	2970.34380	1309.88600	1857.76350	636.42490	Detected	Detected	Detected	Detected
2784	A_33_P3309222	LOC441178	Hs.648083	hypothetical LOC441178	Homo sapiens mRNA; cDNA DKFZp686H0413 (from clone DKFZp686H0413) [AL832737]	2.34793	up	2.24538	up	465.61760	205.34212	312.76900	122.82831	Detected	Detected	Detected	Detected
2785	A_33_P3228762	SLC25A36	Hs.144130	solute carrier family 25, member 36	Homo sapiens solute carrier family 25, member 36 (SLC25A36), transcript variant 1, mRNA [NM_001104647]	2.34583	up	2.13162	up	3990.18300	1761.28480	2160.92820	893.91580	Detected	Detected	Detected	Detected
2786	A_33_P3220105	LOC644246	Hs.644600	hypothetical protein LOC644246	LOC644246 protein Fragment [Source:UniProtKB/TrEMBL;Acc:Q8WW58] [ENST00000398275]	2.34504	up	2.94493	up	153.64711	67.84344	235.44266	70.49780	Detected	Detected	Detected	Detected
2787	A_33_P3315239		Hs.493218		Zinc finger protein 7 (Zinc finger protein KOX4)(Zinc finger protein HF.16) [Source:UniProtKB/Swiss-Prot;Acc:P17097] [ENST00000325217]	2.34354	up	2.86902	up	38.93568	17.20318	60.54463	18.60835	Detected	Detected	Detected	Detected
2788	A_24_P64653	METTL7B	Hs.51483	methyltransferase like 7B	Homo sapiens methyltransferase like 7B (METTL7B), mRNA [NM_152637]	2.34229	up	2.97289	up	79.06632	34.95292	94.60976	28.06228	Detected	Detected	Detected	Detected
2789	A_24_P333421	ZNF862	Hs.301277	zinc finger protein 862	Homo sapiens zinc finger protein 862 (ZNF862), mRNA [NM_001099220]	2.34188	up	2.42542	up	967.14680	427.62290	636.14233	231.27760	Detected	Detected	Detected	Detected
2790	A_33_P3235262	PIP5KL1	Hs.445486	phosphatidylinositol-4-phosphate 5-kinase-like 1	Homo sapiens phosphatidylinositol-4-phosphate 5-kinase-like 1 (PIP5KL1), transcript variant 1, mRNA [NM_001135219]	2.34106	up	2.35361	up	406.55316	179.81961	350.87190	131.45598	Detected	Detected	Detected	Detected
2791	A_24_P365180	DSEL	Hs.124673	dermatan sulfate epimerase-like	Homo sapiens dermatan sulfate epimerase-like (DSEL), mRNA [NM_032160]	2.34034	up	2.51360	up	1420.88230	628.65360	675.55530	236.99023	Detected	Detected	Detected	Detected
2792	A_33_P3287710	LOC100128292		hypothetical LOC100128292	Homo sapiens hypothetical LOC100128292 (LOC100128292), non-coding RNA [NR_024585]	2.33994	up	2.49741	up	1077.96860	477.01855	816.79160	288.39450	Detected	Detected	Detected	Detected
2793	A_23_P21376	MAGI2	Hs.603842	membrane associated guanylate kinase, WW and PDZ domain containing 2	Homo sapiens membrane associated guanylate kinase, WW and PDZ domain containing 2 (MAGI2), mRNA [NM_012301]	2.33914	up	2.92175	up	293.67280	129.99911	174.71713	52.72998	Detected	Detected	Detected	Detected
2794	A_33_P3251369	GAB3	Hs.496982	GRB2-associated binding protein 3	Homo sapiens GRB2-associated binding protein 3 (GAB3), transcript variant 1, mRNA [NM_001081573]	2.33747	up	1.31246	up	34.36458	15.22294	24.17799	16.24427	Detected	Compromised	Detected	Compromised
2795	A_24_P391991	FAM183B	Hs.144075	acyloxyacyl hydrolase (neutrophil)	Homo sapiens family with sequence similarity 183, member B (FAM183B), non-coding RNA [NR_028347]	2.33703	up	2.10748	up	50.57869	22.40968	49.91826	20.88632	Detected	Detected	Detected	Detected
2796	A_33_P3277674	FBXL22	Hs.656997	F-box and leucine-rich repeat protein 22	Homo sapiens F-box and leucine-rich repeat protein 22 (FBXL22), mRNA [NM_203373]	2.33680	up	2.33731	up	35.19154	15.59376	20.35587	7.67961	Detected	Compromised	Detected	Compromised
2797	A_33_P3302861	RPGRIP1L	Hs.298382	RPGRIP1-like	Homo sapiens RPGRIP1-like (RPGRIP1L), transcript variant 1, mRNA [NM_015272]	2.33495	up	3.33732	up	67.14003	29.77402	65.51793	17.31122	Detected	Detected	Detected	Detected
2798	A_32_P154473	KIF5C	Hs.660699	kinesin family member 5C	Homo sapiens kinesin family member 5C (KIF5C), mRNA [NM_004522]	2.33480	up	4.15457	up	32.94513	14.61084	18.73256	3.97591	Detected	Compromised	Detected	Compromised
2799	A_23_P429461	FUK	Hs.7907	fucokinase	Homo sapiens fucokinase (FUK), mRNA [NM_145059]	2.33395	up	2.19714	up	979.59906	434.60077	879.63530	353.02975	Detected	Detected	Detected	Detected
2800	A_23_P206107	ULK3	Hs.513034	unc-51-like kinase 3 (C. elegans)	Homo sapiens unc-51-like kinase 3 (C. elegans) (ULK3), mRNA [NM_001099436]	2.33328	up	2.01522	up	1459.37130	647.63745	1221.96300	534.68976	Detected	Detected	Detected	Detected
2801	A_33_P3358943	GRM2	Hs.121510	glutamate receptor, metabotropic 2	Homo sapiens glutamate receptor, metabotropic 2 (GRM2), transcript variant 1, mRNA [NM_000839]	2.33313	up	2.20114	up	20.29636	9.00768	9.50821	3.80904	Detected	Compromised	Compromised	Compromised
2802	A_24_P144601	POU5F1	Hs.249184	POU class 5 homeobox 1	Homo sapiens POU class 5 homeobox 1 (POU5F1), transcript variant 1, mRNA [NM_002701]	2.33283	up	1.94667	up	34.96261	15.51868	40.08314	18.15659	Detected	Compromised	Detected	Detected
2803	A_33_P3220425	LOC100288667	Hs.720041	hypothetical protein LOC100288667	PREDICTED: Homo sapiens hypothetical protein LOC100288667 (LOC100288667), mRNA [XM_002344252]	2.33248	up	3.60038	up	18.25897	8.10571	17.99122	4.40634	Detected	Compromised	Detected	Compromised
2804	A_33_P3258383					2.33241	up	3.01843	up	55.43835	24.61155	63.49353	18.54869	Detected	Detected	Detected	Detected
2805	A_24_P456723	LOC728537	Hs.469369	hypothetical protein LOC728537	PREDICTED: Homo sapiens hypothetical protein LOC728537 (LOC728537), miscRNA [XR_041702]	2.33227	up	2.22359	up	346.23230	153.71710	304.51190	120.75785	Detected	Detected	Detected	Detected
2806	A_33_P3254331	CTRC	Hs.631869	chymotrypsin C (caldecrin)	Homo sapiens chymotrypsin C (caldecrin) (CTRC), mRNA [NM_007272]	2.33075	up	3.04160	up	105.74942	46.98032	116.66219	33.82156	Detected	Detected	Detected	Detected
2807	A_23_P29029	C21orf119	Hs.58149	chromosome 21 open reading frame 119	Homo sapiens chromosome 21 open reading frame 119 (C21orf119), non-coding RNA [NR_026845]	2.33009	up	2.18511	up	968.08130	430.20126	1164.46030	469.91345	Detected	Detected	Detected	Detected

2808	A_33_P3267865				major histocompatibility complex, class I, J (pseudogene) (HLA-J), non-coding RNA [Source:RefSeq DNA;Acc:NR_024240] [ENST00000376793]	2.32928	up	1.66471	up	2880.13260	1280.33420	1449.09310	767.57740	Detected	Detected	Detected	Detected
2809	A_23_P25348	ACAD10	Hs.331141	acyl-Coenzyme A dehydrogenase family, member 10	Homo sapiens acyl-Coenzyme A dehydrogenase family, member 10 (ACAD10), transcript variant 2, mRNA [NM_025247]	2.32928	up	2.12070	up	268.87170	119.52458	206.07866	85.68770	Detected	Detected	Detected	Detected
2810	A_23_P147025	RAB33A	Hs.654356	RAB33A, member RAS oncogene family	Homo sapiens RAB33A, member RAS oncogene family (RAB33A), mRNA [NM_004794]	2.32888	up	2.47846	up	1042.98770	463.72916	865.04297	307.76636	Detected	Detected	Detected	Detected
2811	A_23_P53198	DGAT2	Hs.129798	diacylglycerol O-acyltransferase homolog 2 (mouse)	Homo sapiens diacylglycerol O-acyltransferase homolog 2 (mouse) (DGAT2), mRNA [NM_032564]	2.32811	up	2.30272	up	377.54877	167.92021	338.92368	129.78552	Detected	Detected	Detected	Detected
2812	A_23_P56759	KRCC1	Hs.469254	lysine-rich coiled-coil 1	Homo sapiens lysine-rich coiled-coil 1 (KRCC1), mRNA [NM_016618]	2.32763	up	2.31382	up	6512.28500	2897.02370	4870.27440	1856.04990	Detected	Detected	Detected	Detected
2813	A_33_P3290296	LOC100130175	Hs.232543	hypothetical protein LOC100130175	Homo sapiens cDNA FLJ43841 fis, clone TESTI4006137. [AK125829]	2.32733	up	3.15363	up	1304.85530	580.54690	624.36145	174.57849	Detected	Detected	Detected	Detected
2814	A_33_P3234580	ASS1	Hs.160786	argininosuccinate synthetase 1	Homo sapiens argininosuccinate synthetase 1 (ASS1), transcript variant 1, mRNA [NM_000050]	2.32636	up	2.15684	up	25485.38500	11343.48900	15723.23900	6428.20360	Detected	Detected	Detected	Detected
2815	A_23_P346900	CACNA2D2	Hs.476273	calcium channel, voltage-dependent, alpha 2/delta subunit 2	Homo sapiens calcium channel, voltage-dependent, alpha 2/delta subunit 2 (CACNA2D2), transcript variant 1, mRNA [NM_001005505]	2.32627	up	1.99817	up	59.61094	26.53373	57.67135	25.45038	Detected	Detected	Detected	Detected
2816	A_32_P222684	PRDM6	Hs.135118	PR domain containing 6	Homo sapiens PR domain containing 6 (PRDM6), mRNA [NM_001136239]	2.32584	up	2.77220	up	174.47495	77.67580	170.55429	54.25047	Detected	Detected	Detected	Detected
2817	A_33_P3330503	ALDH7A1	Hs.483239	aldehyde dehydrogenase 7 family, member A1	Homo sapiens aldehyde dehydrogenase 7 family, member A1 (ALDH7A1), mRNA [NM_001182]	2.32577	up	2.67357	up	1016.87146	452.72336	675.88760	222.91962	Detected	Detected	Detected	Detected
2818	A_33_P3408203	TGFA	Hs.170009	transforming growth factor, alpha	Homo sapiens transforming growth factor, alpha (TGFA), transcript variant 1, mRNA [NM_003236]	2.32507	up	1.99030	up	8896.59600	3962.06250	1434.51870	635.55554	Detected	Detected	Detected	Detected
2819	A_32_P152437	AKAP12	Hs.371240	A kinase (PRKA) anchor protein 12	Homo sapiens A kinase (PRKA) anchor protein 12 (AKAP12), transcript variant 1, mRNA [NM_005100]	2.32467	up	2.30529	up	13265.36100	5908.69400	13115.11300	5016.63530	Detected	Detected	Detected	Detected
2820	A_23_P57760	ACPL2	Hs.657887	acid phosphatase-like 2	Homo sapiens acid phosphatase-like 2 (ACPL2), transcript variant 1, mRNA [NM_152282]	2.32264	up	2.38604	up	2976.98970	1327.17400	2150.30830	794.67340	Detected	Detected	Detected	Detected
2821	A_23_P356139	FAM178A	Hs.447458	family with sequence similarity 178, member A	Homo sapiens family with sequence similarity 178, member A (FAM178A), transcript variant 1, mRNA [NM_018121]	2.32254	up	1.98721	up	304.18620	135.61546	203.93115	90.49079	Detected	Detected	Detected	Detected
2822	A_33_P3284473	LOC100130798	Hs.527241	similar to hCG2045013	PREDICTED: Homo sapiens similar to hCG2045013 (LOC100130798), mRNA [XM_001718781]	2.32222	up	3.14538	up	16.55139	7.38014	10.56901	2.96296	Detected	Compromised	Compromised	Compromised
2823	A_23_P71972	WWOX	Hs.461453	WW domain containing oxidoreductase	Homo sapiens WW domain containing oxidoreductase (WWOX), transcript variant 1, mRNA [NM_016373]	2.32207	up	3.39895	up	177.84221	79.30357	167.45510	43.44295	Detected	Detected	Detected	Detected
2824	A_23_P1519	PITPNM1	Hs.372295	phosphatidylinositol transfer protein, membrane-associated 1	Homo sapiens phosphatidylinositol transfer protein, membrane-associated 1 (PITPNM1), transcript variant 1, mRNA [NM_004910]	2.32192	up	1.73966	up	562.36414	250.78635	575.59830	291.75583	Detected	Detected	Detected	Detected
2825	A_23_P160940	ABCA4	Hs.416707	ATP-binding cassette, sub-family A (ABC1), member 4	Homo sapiens ATP-binding cassette, sub-family A (ABC1), member 4 (ABCA4), mRNA [NM_000350]	2.32185	up	1.94074	up	28.90618	12.89106	32.47874	14.75695	Detected	Compromised	Detected	Detected
2826	A_24_P349196	CCDC30	Hs.473495	coiled-coil domain containing 30	Homo sapiens coiled-coil domain containing 30 (CCDC30), mRNA [NM_001080850]	2.32051	up	2.03115	up	83.26384	37.15405	68.28317	29.64396	Detected	Detected	Detected	Detected
2827	A_24_P74070	PARD6G	Hs.654920	par-6 partitioning defective 6 homolog gamma (C. elegans)	Homo sapiens par-6 partitioning defective 6 homolog gamma (C. elegans) (PARD6G), mRNA [NM_032510]	2.31916	up	2.24791	up	220.93913	98.64500	162.84933	63.88125	Detected	Detected	Detected	Detected
2828	A_23_P18798	PCDHB9	Hs.662726	protocadherin beta 9	Homo sapiens protocadherin beta 9 (PCDHB9), mRNA [NM_019119]	2.31871	up	1.47980	up	34.56656	15.43630	22.15333	13.20086	Detected	Detected	Detected	Detected
2829	A_23_P17242	ABHD1	Hs.375791	abhydrolase domain containing 1	Homo sapiens abhydrolase domain containing 1 (ABHD1), mRNA [NM_032604]	2.31800	up	2.21073	up	57.26365	25.57984	76.37738	30.46451	Detected	Detected	Detected	Detected
2830	A_23_P106922	CHST6	Hs.655622	carbohydrate (N-acetylglucosamine 6-O) sulfotransferase 6	Homo sapiens carbohydrate (N-acetylglucosamine 6-O) sulfotransferase 6 (CHST6), mRNA [NM_021615]	2.31561	up	2.86774	up	95.40712	42.66274	126.01718	38.74852	Detected	Detected	Detected	Detected
2831	A_23_P338534	HIF3A	Hs.420830	hypoxia inducible factor 3, alpha subunit	Homo sapiens hypoxia inducible factor 3, alpha subunit (HIF3A), transcript variant 2, mRNA [NM_022462]	2.31460	up	1.91545	up	25.31718	11.32589	24.02550	11.06030	Detected	Compromised	Detected	Compromised
2832	A_23_P67198	CPAMD8	Hs.631644	C3 and PZP-like, alpha-2-macroglobulin domain containing 8	Homo sapiens C3 and PZP-like, alpha-2-macroglobulin domain containing 8 (CPAMD8), mRNA [NM_015692]	2.31455	up	2.39897	up	458.34467	205.04947	467.16900	171.71740	Detected	Detected	Detected	Detected
2833	A_33_P3257312	ATG2A	Hs.370671	ATG2 autophagy related 2 homolog A (S. cerevisiae)	Homo sapiens ATG2 autophagy related 2 homolog A (S. cerevisiae) (ATG2A), mRNA [NM_015104]	2.31339	up	2.39149	up	16800.50200	7519.78800	11299.13300	4166.22100	Detected	Detected	Detected	Detected
2834	A_33_P3286146	LOC100132891	Hs.137674	hypothetical protein LOC100132891	Homo sapiens mRNA; cDNA DKFZp686E16147 (from clone DKFZp686E16147) [BX647470]	2.31326	up	1.89982	up	24.85642	11.12622	17.69148	8.21142	Detected	Compromised	Detected	Compromised
2835	A_23_P46396	PTBP2	Hs.269895	polypyrimidine tract binding protein 2	Homo sapiens polypyrimidine tract binding protein 2 (PTBP2), mRNA [NM_021190]	2.31092	up	1.99432	up	2844.90550	1274.72250	2396.92290	1059.80310	Detected	Detected	Detected	Detected
2836	A_23_P48988	SH3GL3	Hs.270055	SH3-domain GRB2-like 3	Homo sapiens SH3-domain GRB2-like 3 (SH3GL3), transcript variant 1, mRNA [NM_003027]	2.30984	up	3.83442	up	215.94376	96.80366	179.34459	41.24333	Detected	Detected	Detected	Detected

2837	A_24_P314451	F8	Hs.654450	coagulation factor VIII, procoagulant component	Homo sapiens coagulation factor VIII, procoagulant component (F8), transcript variant 1, mRNA [NM_000132]	2.30895	up	2.18187	up	142.65890	63.97610	148.23839	59.90983	Detected	Detected	Detected	Detected
2838	A_23_P103503	MDM4	Hs.497492	Mdm4 p53 binding protein homolog (mouse)	Homo sapiens Mdm4 p53 binding protein homolog (mouse) (MDM4), transcript variant 1, mRNA [NM_002393]	2.30715	up	2.33755	up	756.61145	339.57065	546.32025	206.08778	Detected	Detected	Detected	Detected
2839	A_32_P46214	SLC9A9	Hs.302257	solute carrier family 9 (sodium/hydrogen exchanger), member 9	Homo sapiens solute carrier family 9 (sodium/hydrogen exchanger), member 9 (SLC9A9), mRNA [NM_173653]	2.30705	up	2.22629	up	1814.53260	814.40760	1675.92520	663.80280	Detected	Detected	Detected	Detected
2840	A_24_P29277	COL4A3BP	Hs.270437	collagen, type IV, alpha 3 (Goodpasture antigen) binding protein	Homo sapiens collagen, type IV, alpha 3 (Goodpasture antigen) binding protein (COL4A3BP), transcript variant 3, mRNA [NM_001130105]	2.30696	up	2.21244	up	3519.91140	1579.87960	2687.12300	1070.97900	Detected	Detected	Detected	Detected
2841	A_23_P210763	JAG1	Hs.224012	jagged 1 (Alagille syndrome)	Homo sapiens jagged 1 (Alagille syndrome) (JAG1), mRNA [NM_000214]	2.30623	up	2.24314	up	506.22467	227.28627	381.18372	149.84561	Detected	Detected	Detected	Detected
2842	A_24_P79808	PBXIP1	Hs.505806	pre-B-cell leukemia homeobox interacting protein 1	Homo sapiens pre-B-cell leukemia homeobox interacting protein 1 (PBXIP1), mRNA [NM_020524]	2.30533	up	2.02605	up	877.11070	393.96155	828.46550	360.57007	Detected	Detected	Detected	Detected
2843	A_23_P207319	MAP3K14	Hs.404183	mitogen-activated protein kinase kinase kinase 14	Homo sapiens mitogen-activated protein kinase kinase kinase 14 (MAP3K14), mRNA [NM_003954]	2.30530	up	1.89458	up	659.59280	296.26627	780.44305	363.24063	Detected	Detected	Detected	Detected
2844	A_32_P43465	ZNF182	Hs.189690	zinc finger protein 182	Homo sapiens zinc finger protein 182 (ZNF182), transcript variant 1, mRNA [NM_006962]	2.30508	up	1.95825	up	1189.47110	534.34010	1156.96250	520.97500	Detected	Detected	Detected	Detected
2845	A_24_P737939	C6orf154	Hs.445552	chromosome 6 open reading frame 154	Homo sapiens chromosome 6 open reading frame 154 (C6orf154), mRNA [NM_001012974]	2.30474	up	2.53114	up	931.62370	418.55360	930.20715	324.06244	Detected	Detected	Detected	Detected
2846	A_24_P178444					2.30220	up	2.12078	up	7509.43100	3377.51700	7071.14600	2940.08810	Detected	Detected	Detected	Detected
2847	A_23_P201731	TRAF5	Hs.523930	TNF receptor-associated factor 5	Homo sapiens TNF receptor-associated factor 5 (TRAF5), transcript variant 1, mRNA [NM_004619]	2.30207	up	2.39001	up	883.25310	397.28363	688.33620	253.96054	Detected	Detected	Detected	Detected
2848	A_23_P414978	NUDT14	Hs.526432	nudix (nucleoside diphosphate linked moiety X)-type motif 14	Homo sapiens nudix (nucleoside diphosphate linked moiety X)-type motif 14 (NUDT14), mRNA [NM_177533]	2.30179	up	2.49665	up	77.89579	35.04144	94.93017	33.52837	Detected	Detected	Detected	Detected
2849	A_23_P121527	KLHL5	Hs.272251	kelch-like 5 (Drosophila)	Homo sapiens kelch-like 5 (Drosophila) (KLHL5), transcript variant 1, mRNA [NM_015990]	2.30177	up	2.44544	up	1230.58370	553.58220	987.60876	356.11777	Detected	Detected	Detected	Detected
2850	A_24_P170774	LRCH1	Hs.507971	leucine-rich repeats and calponin homology (CH) domain containing 1	Homo sapiens leucine-rich repeats and calponin homology (CH) domain containing 1 (LRCH1), transcript variant 2, mRNA [NM_015116]	2.30141	up	3.58235	up	27.38331	12.32039	18.23209	4.48780	Detected	Compromised	Detected	Compromised
2851	A_33_P3383184	SP9		Sp9 transcription factor homolog (mouse)	Sp9 transcription factor homolog [Source:RefSeq peptide;Acc:NP_001138722] [ENST00000394967]	2.30118	up	2.37177	up	1256.24300	565.27045	1066.03760	396.33698	Detected	Detected	Detected	Detected
2852	A_23_P10911	PLBD2	Hs.115896	phospholipase B domain containing 2	Homo sapiens phospholipase B domain containing 2 (PLBD2), transcript variant 1, mRNA [NM_173542]	2.30036	up	2.93600	up	766.39813	344.97806	771.40710	231.68225	Detected	Detected	Detected	Detected
2853	A_23_P436526	SLC25A42	Hs.303669	solute carrier family 25, member 42	Homo sapiens solute carrier family 25, member 42 (SLC25A42), mRNA [NM_178526]	2.30027	up	2.00497	up	450.39150	202.74222	405.11227	178.16902	Detected	Detected	Detected	Detected
2854	A_32_P225659	UTS2D	Hs.518492	urotensin 2 domain containing	Homo sapiens urotensin 2 domain containing (UTS2D), mRNA [NM_198152]	2.29964	up	2.03967	up	111.44338	50.17961	56.87801	24.58949	Detected	Detected	Detected	Detected
2855	A_33_P3362521	C3orf19	Hs.517820	chromosome 3 open reading frame 19	Homo sapiens chromosome 3 open reading frame 19 (C3orf19), mRNA [NM_016474]	2.29898	up	2.14456	up	5434.84770	2447.84990	4127.92200	1697.29880	Detected	Detected	Detected	Detected
2856	A_24_P311771	ZFR	Hs.435231	zinc finger RNA binding protein	Homo sapiens zinc finger RNA binding protein (ZFR), mRNA [NM_016107]	2.29874	up	1.85250	up	711.28090	320.39453	563.86346	268.39987	Detected	Detected	Detected	Detected
2857	A_23_P214627	AIF1	Hs.76364	allograft inflammatory factor 1	Homo sapiens allograft inflammatory factor 1 (AIF1), transcript variant 2, mRNA [NM_004847]	2.29797	up	2.78990	up	12.81018	5.77223	8.09217	2.55766	Detected	Compromised	Compromised	Compromised
2858	A_32_P150391	C17orf100	Hs.262480	chromosome 17 open reading frame 100	Homo sapiens chromosome 17 open reading frame 100 (C17orf100), mRNA [NM_001105520]	2.29789	up	2.20188	up	304.92603	137.40367	273.53470	109.54278	Detected	Detected	Detected	Detected
2859	A_33_P3291414		Hs.678863		Homo sapiens cDNA FLJ34930 fis, clone NT2RP7004961 [AK092249]	2.29773	up	2.45460	up	5282.55300	2380.55300	1166.80430	419.16217	Detected	Detected	Detected	Detected
2860	A_33_P3246829	IL1RN	Hs.81134	interleukin 1 receptor antagonist	Homo sapiens interleukin 1 receptor antagonist (IL1RN), transcript variant 1, mRNA [NM_173842]	2.29734	up	3.26650	up	261.05804	117.66419	212.56012	57.38056	Detected	Detected	Detected	Detected
2861	A_32_P42574	C1orf198	Hs.520494	chromosome 1 open reading frame 198	Homo sapiens chromosome 1 open reading frame 198 (C1orf198), transcript variant 1, mRNA [NM_032800]	2.29708	up	2.30443	up	39845.16000	17961.08000	34561.94000	13225.13900	Detected	Detected	Detected	Detected
2862	A_33_P3316978	RNF207	Hs.716549	ring finger protein 207	Homo sapiens ring finger protein 207 (RNF207), mRNA [NM_207396]	2.29626	up	4.41175	up	46.46422	20.95224	30.60785	6.11769	Detected	Detected	Detected	Compromised
2863	A_33_P3251163	LOC100131869	Hs.529989	hypothetical protein LOC100131869	Homo sapiens cDNA FLJ42372 fis, clone UTERU2031084. [AK124363]	2.29475	up	1.12487	up	18.87657	8.51767	10.86844	8.51985	Detected	Compromised	Compromised	Compromised
2864	A_23_P31921	ASS1	Hs.160786	argininosuccinate synthetase 1	Homo sapiens argininosuccinate synthetase 1 (ASS1), transcript variant 1, mRNA [NM_000050]	2.29379	up	2.10478	up	3960.42850	1787.80910	3800.42200	1592.17440	Detected	Detected	Detected	Detected
2865	A_33_P3283201		Hs.715264		Putative uncharacterized protein ENSP00000367676 [Source:UniProtKB/TrEMBL;Acc:B7WP47] [ENST00000378420]	2.29341	up	3.88352	up	24.06711	10.86615	12.12568	2.75325	Detected	Compromised	Compromised	Compromised
2866	A_23_P14708	ZNF280D	Hs.511477	zinc finger protein 280D	Homo sapiens zinc finger protein 280D (ZNF280D), transcript variant 1, mRNA [NM_017661]	2.29334	up	2.40493	up	542.96480	245.15300	477.73580	175.16635	Detected	Detected	Detected	Detected

2867	A_32_P125771	RGS22	Hs.120021	regulator of G-protein signaling 22	Homo sapiens regulator of G-protein signaling 22 (RGS22), mRNA [NM_015668]	2.29209	up	2.28812	up	20.21099	9.13038	9.85848	3.79924	Detected	Compromised	Compromised	Compromised
2868	A_33_P3293798	KRTAP10-1	Hs.688631	keratin associated protein 10-1	Homo sapiens keratin associated protein 10-1 (KRTAP10-1), mRNA [NM_198691]	2.29173	up	2.68833	up	1879.59290	849.24600	1016.12286	333.29507	Detected	Detected	Detected	Detected
2869	A_24_P287189	TOLLIP	Hs.719949	toll interacting protein	Homo sapiens toll interacting protein (TOLLIP), mRNA [NM_019009]	2.29168	up	5.45906	up	504.90024	228.13170	225.51454	36.42691	Detected	Detected	Detected	Detected
2870	A_32_P122940	LOC642852	Hs.710382	hypothetical LOC642852	Homo sapiens hypothetical LOC642852 (LOC642852), non-coding RNA [NR_026943]	2.29103	up	2.67759	up	766.01980	346.21237	723.54240	238.27888	Detected	Detected	Detected	Detected
2871	A_32_P90483	STXBP4	Hs.35199	syntaxin binding protein 4	Homo sapiens syntaxin binding protein 4 (STXBP4), mRNA [NM_178509]	2.28970	up	2.14294	up	101.43932	45.87333	109.50288	45.05888	Detected	Detected	Detected	Detected
2872	A_23_P154358	PROM2	Hs.469313	prominin 2	Homo sapiens prominin 2 (PROM2), transcript variant 3, mRNA [NM_144707]	2.28958	up	4.59937	up	26.23198	11.86339	15.08279	2.89167	Detected	Compromised	Detected	Compromised
2873	A_23_P112482	AQP3	Hs.234642	aquaporin 3 (Gill blood group)	Homo sapiens aquaporin 3 (Gill blood group) (AQP3), mRNA [NM_004925]	2.28935	up	1.90883	up	56.82177	25.70020	53.52462	24.72588	Detected	Detected	Detected	Detected
2874	A_23_P415401	KLF9	Hs.150557	Kruppel-like factor 9	Homo sapiens Kruppel-like factor 9 (KLF9), mRNA [NM_001206]	2.28878	up	2.28350	up	29898.14500	13526.10800	27647.41600	10676.27050	Detected	Detected	Detected	Detected
2875	A_33_P3233871	F12	Hs.1321	coagulation factor XII (Hageman factor)	Homo sapiens coagulation factor XII (Hageman factor) (F12), mRNA [NM_000505]	2.28839	up	2.09255	up	1038.35940	469.84015	907.89685	382.58400	Detected	Detected	Detected	Detected
2876	A_23_P68851	KREMEN1	Hs.229335	kringle containing transmembrane protein 1	Homo sapiens kringle containing transmembrane protein 1 (KREMEN1), transcript variant 3, mRNA [NM_001039570]	2.28835	up	2.24760	up	6949.47200	3144.57540	7645.68850	2999.59180	Detected	Detected	Detected	Detected
2877	A_33_P3223860					2.28737	up	2.27339	up	40.32455	18.25436	13.09129	5.07779	Detected	Detected	Detected	Compromised
2878	A_33_P3410589	FAM43A	Hs.708232	family with sequence similarity 43, member A	Homo sapiens family with sequence similarity 43, member A (FAM43A), mRNA [NM_153690]	2.28719	up	2.15202	up	2628.71460	1190.07670	2854.00320	1169.43040	Detected	Detected	Detected	Detected
2879	A_24_P350644	BAT2L	Hs.495349	HLA-B associated transcript 2-like	Homo sapiens HLA-B associated transcript 2-like (BAT2L), mRNA [NM_013318]	2.28684	up	2.35157	up	3456.32700	1564.99180	2754.58450	1032.91140	Detected	Detected	Detected	Detected
2880	A_23_P164057	MFAP4	Hs.296049	microfibrillar-associated protein 4	Homo sapiens microfibrillar-associated protein 4 (MFAP4), mRNA [NM_002404]	2.28671	up	2.17848	up	29031.48400	13145.95300	28135.60500	11388.54700	Detected	Detected	Detected	Detected
2881	A_23_P146367	C9orf89	Hs.434213	chromosome 9 open reading frame 89	Homo sapiens chromosome 9 open reading frame 89 (C9orf89), mRNA [NM_032310]	2.28666	up	2.05670	up	63335.78000	28680.07600	54753.35500	23475.03700	Detected	Detected	Detected	Detected
2882	A_24_P941912	DTX3L	Hs.518201	deltex 3-like (Drosophila)	Homo sapiens deltex 3-like (Drosophila) (DTX3L), mRNA [NM_138287]	2.28599	up	2.53797	up	175.87788	79.66542	182.67537	63.46859	Detected	Detected	Detected	Detected
2883	A_33_P3464555	LOC283070	Hs.600547	hypothetical LOC283070	Homo sapiens hypothetical LOC283070 (LOC283070), non-coding RNA [NR_027322]	2.28472	up	2.54870	up	168.94470	76.56745	132.18161	45.73171	Detected	Detected	Detected	Detected
2884	A_24_P132008	MSX2	Hs.89404	msh homeobox 2	Homo sapiens msh homeobox 2 (MSX2), mRNA [NM_002449]	2.28395	up	5.34033	up	43.29592	19.62881	44.62740	7.36884	Detected	Detected	Detected	Compromised
2885	A_23_P57417	MMP11	Hs.143751	matrix metallopeptidase 11 (stromelysin 3)	Homo sapiens matrix metallopeptidase 11 (stromelysin 3) (MMP11), mRNA [NM_005940]	2.28351	up	2.24067	up	1174.13220	532.41205	1062.87610	418.28280	Detected	Detected	Detected	Detected
2886	A_23_P218068	PLEKHA5	Hs.188614	pleckstrin homology domain containing, family A member 5	Homo sapiens pleckstrin homology domain containing, family A member 5 (PLEKHA5), transcript variant 1, mRNA [NM_019012]	2.28338	up	2.60868	up	1765.97270	800.82635	899.38184	304.01047	Detected	Detected	Detected	Detected
2887	A_33_P3327608	EML6	Hs.656692	echinoderm microtubule associated protein like 6	Homo sapiens echinoderm microtubule associated protein like 6 (EML6), mRNA [NM_001039753]	2.28242	up	2.47871	up	23.31490	10.57721	15.98530	5.68671	Detected	Compromised	Detected	Compromised
2888	A_33_P3254013				Cyclin G-associated kinase (EC 2.7.11.1) [Source:UniProtKB/Swiss-Prot;Acc:O14976] [ENST00000382997]	2.28228	up	2.89297	up	84.68058	38.41922	76.36857	23.27752	Detected	Detected	Detected	Detected
2889	A_32_P232214	LOC388630	Hs.576171	UPF0632 protein A	PREDICTED: Homo sapiens UPF0632 protein A (LOC388630), mRNA [XM_371250]	2.28197	up	2.05044	up	303.16843	137.56499	271.82680	116.89892	Detected	Detected	Detected	Detected
2890	A_33_P3493097	LOC648149	Hs.658840	hypothetical protein LOC648149	Homo sapiens cDNA FLJ41355 fis, clone BRAWH2016724. [AK123349]	2.28181	up	6.48182	up	53.79231	24.41031	32.41031	4.40912	Detected	Detected	Detected	Compromised
2891	A_33_P3305203	LOC283588	Hs.46519	hypothetical LOC283588	PREDICTED: Homo sapiens hypothetical LOC283588 (LOC283588), miscRNA [XR_041539]	2.28170	up	4.09758	up	109.82087	49.83778	49.59884	10.67357	Detected	Detected	Detected	Compromised
2892	A_33_P3288189	RHOBTB3	Hs.445030	Rho-related BTB domain containing 3	Homo sapiens Rho-related BTB domain containing 3 (RHOBTB3), mRNA [NM_014899]	2.28078	up	2.46559	up	2082.69900	945.53300	1852.28960	662.45090	Detected	Detected	Detected	Detected
2893	A_33_P3413989	SERPING1	Hs.384598	serpin peptidase inhibitor, clade G (C1 inhibitor), member 1	Homo sapiens serpin peptidase inhibitor, clade G (C1 inhibitor), member 1 (SERPING1), transcript variant 1, mRNA [NM_000062]	2.28065	up	1.94335	up	445.77234	202.38931	437.75568	198.63026	Detected	Detected	Detected	Detected
2894	A_24_P48495	LYPD3	Hs.631594	LY6/PLAUR domain containing 3	Homo sapiens LY6/PLAUR domain containing 3 (LYPD3), mRNA [NM_014400]	2.28026	up	1.71005	up	28.99650	13.16721	37.46451	19.31863	Detected	Compromised	Detected	Detected
2895	A_32_P88310	LOC730183	Hs.620916	hypothetical protein LOC730183	PREDICTED: Homo sapiens hypothetical LOC730183 (LOC730183), mRNA [XM_001714997]	2.27974	up	2.05525	up	653.70250	296.91150	602.06885	258.31354	Detected	Detected	Detected	Detected
2896	A_23_P111000	PSMB9	Hs.654585	proteasome (prosome, macropain) subunit, beta type, 9 (large multifunctional peptidase 2)	Homo sapiens proteasome (prosome, macropain) subunit, beta type, 9 (large multifunctional peptidase 2) (PSMB9), transcript variant 1, mRNA [NM_002800]	2.27952	up	3.30054	up	3037.46310	1379.74900	3415.92580	912.61786	Detected	Detected	Detected	Detected
2897	A_33_P3527721	LOC284219	Hs.657510	hypothetical protein LOC284219	Homo sapiens cDNA FLJ37117 fis, clone BRACE2022270 [AK094436]	2.27912	up	2.35162	up	1370.09680	622.46790	962.72327	360.99387	Detected	Detected	Detected	Detected
2898	A_23_P373054	C3orf23	Hs.55131	chromosome 3 open reading frame 23	Homo sapiens chromosome 3 open reading frame 23 (C3orf23), transcript variant 1, mRNA [NM_173826]	2.27822	up	2.21739	up	686.48770	312.01053	441.92178	175.73918	Detected	Detected	Detected	Detected

2899	A_24_P148717	CCR1	Hs.301921	chemokine (C-C motif) receptor 1	Homo sapiens chemokine (C-C motif) receptor 1 (CCR1), mRNA [NM_001295]	2.27805	up	3.17903	up	141.22101	64.19022	76.88025	21.32484	Detected	Detected	Detected	Detected
2900	A_24_P56363	CAB39L	Hs.87159	calcium binding protein 39-like	Homo sapiens calcium binding protein 39-like (CAB39L), transcript variant 1, mRNA [NM_030925]	2.27759	up	1.90541	up	621.12384	282.38162	502.12936	232.37637	Detected	Detected	Detected	Detected
2901	A_23_P207507	ABCC3	Hs.463421	ATP-binding cassette, sub-family C (CFTR/MRP), member 3	Homo sapiens ATP-binding cassette, sub-family C (CFTR/MRP), member 3 (ABCC3), transcript variant 1, mRNA [NM_003786]	2.27726	up	2.40103	up	3835.07930	1743.79280	3221.24220	1183.01670	Detected	Detected	Detected	Detected
2902	A_23_P36345	STARD10	Hs.188606	StAR-related lipid transfer (START) domain containing 10	Homo sapiens StAR-related lipid transfer (START) domain containing 10 (STARD10), mRNA [NM_006645]	2.27601	up	2.21549	up	758.61220	345.12747	833.33075	331.67526	Detected	Detected	Detected	Detected
2903	A_23_P55936	FCGRT	Hs.111903	Fc fragment of IgG, receptor, transporter, alpha	Homo sapiens Fc fragment of IgG, receptor, transporter, alpha (FCGRT), transcript variant 2, mRNA [NM_004107]	2.27546	up	2.25756	up	23787.28300	10824.50800	22535.41800	8802.19800	Detected	Detected	Detected	Detected
2904	A_33_P3402823	SPIN3	Hs.522672	spindlin family, member 3	Homo sapiens spindlin family, member 3 (SPIN3), transcript variant 1, mRNA [NM_001010862]	2.27506	up	1.30840	up	16.07388	7.31579	20.78083	14.00519	Detected	Compromised	Detected	Compromised
2905	A_23_P300076	IQCG	Hs.591675	IQ motif containing G	Homo sapiens IQ motif containing G (IQCG), transcript variant 1, mRNA [NM_032263]	2.27445	up	2.45931	up	100.42143	45.71759	89.66157	32.14840	Detected	Detected	Detected	Detected
2906	A_33_P3777207	MMEL1	Hs.591453	membrane metallo-endopeptidase-like 1	Homo sapiens membrane metallo-endopeptidase-like 1 (MMEL1), mRNA [NM_033467]	2.27412	up	1.20742	up	25.19880	11.47360	9.61180	7.01962	Detected	Compromised	Compromised	Compromised
2907	A_33_P3337977				Polyphosphoinositide phosphatase (EC 3.1.3.-)(Phosphatidylinositol 3,5-bisphosphate 5-phosphatase)(SAC domain-containing protein 3) [Source:UniProtKB/Swiss-Prot;Acc:Q92562] [ENST00000368941]	2.27387	up	2.15965	up	294.17154	133.95810	183.86224	75.07153	Detected	Detected	Detected	Detected
2908	A_23_P200780	TGFBR3	Hs.482390	transforming growth factor, beta receptor III	Homo sapiens transforming growth factor, beta receptor III (TGFBR3), mRNA [NM_003243]	2.27364	up	2.23966	up	1267.44950	577.22150	1232.22560	485.14740	Detected	Detected	Detected	Detected
2909	A_24_P942211	RP11-345P4.4	Hs.655255	similar to solute carrier family 35, member E2	Homo sapiens similar to solute carrier family 35, member E2 (LOC728661), mRNA [NM_001110781]	2.27357	up	2.30891	up	6403.00830	2916.14300	6235.51800	2381.39500	Detected	Detected	Detected	Detected
2910	A_23_P119857	TTC32	Hs.591547	tetratricopeptide repeat domain 32	Homo sapiens tetratricopeptide repeat domain 32 (TTC32), mRNA [NM_001008237]	2.27288	up	2.39441	up	4784.26300	2179.57100	3509.95750	1292.61660	Detected	Detected	Detected	Detected
2911	A_33_P3334515	NDRG2	Hs.525205	NDRG family member 2	Homo sapiens NDRG family member 2 (NDRG2), transcript variant 1, mRNA [NM_201535]	2.27274	up	2.66392	up	2472.27030	1126.36510	1646.85220	545.12800	Detected	Detected	Detected	Detected
2912	A_24_P116805	STAT3	Hs.463059	signal transducer and activator of transcription 3 (acute-phase response factor)	Homo sapiens signal transducer and activator of transcription 3 (acute-phase response factor) (STAT3), transcript variant 3, mRNA [NM_213662]	2.27244	up	1.77159	up	1370.47530	624.47090	847.36410	421.76620	Detected	Detected	Detected	Detected
2913	A_23_P81760	QKI	Hs.510324	quaking homolog, KH domain RNA binding (mouse)	Homo sapiens quaking homolog, KH domain RNA binding (mouse) (QKI), transcript variant 1, mRNA [NM_006775]	2.27178	up	2.26452	up	108.17091	49.30340	46.78173	18.21651	Detected	Detected	Detected	Detected
2914	A_23_P4489	NETO1	Hs.465407	neuropilin (NRP) and tolloid (TLL)-like 1	Homo sapiens neuropilin (NRP) and tolloid (TLL)-like 1 (NETO1), transcript variant 3, mRNA [NM_138966]	2.27120	up	3.91147	up	145.75449	66.45059	157.37918	35.47916	Detected	Detected	Detected	Detected
2915	A_33_P3283044	KIAA0802	Hs.650822	KIAA0802	Homo sapiens KIAA0802 (KIAA0802), mRNA [NM_015210]	2.27110	up	2.12552	up	406.41183	185.29498	282.10233	117.03249	Detected	Detected	Detected	Detected
2916	A_24_P189739	DUSP16	Hs.536535	dual specificity phosphatase 16	Homo sapiens dual specificity phosphatase 16 (DUSP16), mRNA [NM_030640]	2.27100	up	1.40204	up	57.12756	26.04725	64.82778	40.77245	Detected	Detected	Detected	Detected
2917	A_33_P3327592	RNF13	Hs.12333	ring finger protein 13	Homo sapiens ring finger protein 13 (RNF13), transcript variant 1, mRNA [NM_007282]	2.27061	up	2.20292	up	15010.57400	6845.23240	7842.13000	3139.07570	Detected	Detected	Detected	Detected
2918	A_33_P3413468	EDA2R	Hs.302017	ectodysplasin A2 receptor	Homo sapiens ectodysplasin A2 receptor (EDA2R), mRNA [NM_021783]	2.27035	up	2.36765	up	2954.96220	1347.69590	2415.00120	899.42460	Detected	Detected	Detected	Detected
2919	A_24_P42436	RICS	Hs.440379	Rho GTPase-activating protein	Homo sapiens Rho GTPase-activating protein (RICS), transcript variant 2, mRNA [NM_014715]	2.27030	up	1.90146	up	34.13775	15.56983	28.13359	13.04677	Detected	Detected	Detected	Detected
2920	A_23_P415633	FLJ25006	Hs.657973	uncharacterized serine/threonine-protein kinase SgK494	Homo sapiens uncharacterized serine/threonine-protein kinase SgK494 (FLJ25006), mRNA [NM_144610]	2.27028	up	3.21351	up	94.66159	43.17448	92.44334	25.36659	Detected	Detected	Detected	Detected
2921	A_23_P139123	SERPING1	Hs.384598	serpin peptidase inhibitor, clade G (C1 inhibitor), member 1	Homo sapiens serpin peptidase inhibitor, clade G (C1 inhibitor), member 1 (SERPING1), transcript variant 1, mRNA [NM_000062]	2.26975	up	2.01584	up	2228.46390	1016.62680	1566.13440	685.07510	Detected	Detected	Detected	Detected
2922	A_33_P3210468	MATN4	Hs.278489	matrilin 4	Homo sapiens matrilin 4 (MATN4), transcript variant 1, mRNA [NM_003833]	2.26882	up	1.48242	up	10.97351	5.00817	15.16150	9.01856	Compromised	Compromised	Detected	Compromised
2923	A_33_P3221055					2.26852	up	1.69482	up	32.18138	14.68908	14.12771	7.35045	Detected	Compromised	Compromised	Compromised
2924	A_23_P62967	DISC1	Hs.13318	disrupted in schizophrenia 1	Homo sapiens disrupted in schizophrenia 1 (DISC1), transcript variant L, mRNA [NM_018662]	2.26624	up	2.34540	up	494.50803	225.94347	386.41428	145.27892	Detected	Detected	Detected	Detected
2925	A_33_P3215739	LNP1	Hs.591294	leukemia NUP98 fusion partner 1	Homo sapiens leukemia NUP98 fusion partner 1 (LNP1), mRNA [NM_001085451]	2.26605	up	2.30439	up	494.85953	226.12366	454.12190	173.77293	Detected	Detected	Detected	Detected
2926	A_23_P108376	THADA	Hs.369592	thyroid adenoma associated	Homo sapiens thyroid adenoma associated (THADA), transcript variant 1, mRNA [NM_022065]	2.26593	up	2.24361	up	2281.72850	1042.67750	1880.75370	739.17820	Detected	Detected	Detected	Detected

2927	A_24_P233960	SNX27	Hs.192326	sorting nexin family member 27	Homo sapiens sorting nexin family member 27 (SNX27), mRNA [NM_030918]	2.26505	up	2.24386	up	2915.12920	1332.64110	2500.64580	982.70184	Detected	Detected	Detected	Detected
2928	A_23_P356004	KCNIP3	Hs.437376	Kv channel interacting protein 3, calsenilin	Homo sapiens Kv channel interacting protein 3, calsenilin (KCNIP3), transcript variant 1, mRNA [NM_013434]	2.26496	up	1.99777	up	8600.85700	3932.01320	7498.34470	3309.68290	Detected	Detected	Detected	Detected
2929	A_33_P3263538	NEAT1	Hs.523789	nuclear paraspeckle assembly transcript 1 (non-protein coding)	Human MEN1 region clone epsilon [AF001893]	2.26483	up	2.34536	up	2225.13840	1017.31180	1626.49000	611.51560	Detected	Detected	Detected	Detected
2930	A_23_P131825	TNNC2	Hs.182421	troponin C type 2 (fast)	Homo sapiens troponin C type 2 (fast) (TNNC2), mRNA [NM_003279]	2.26448	up	2.51029	up	262.35016	119.96271	222.99648	78.33204	Detected	Detected	Detected	Detected
2931	A_23_P418485	C11orf65	Hs.653180	chromosome 11 open reading frame 65	Homo sapiens chromosome 11 open reading frame 65 (C11orf65), mRNA [NM_152587]	2.26420	up	2.41272	up	95.74947	43.78801	97.70359	35.70824	Detected	Detected	Detected	Detected
2932	A_24_P18146	PSD3	Hs.434255	pleckstrin and Sec7 domain containing 3	Homo sapiens pleckstrin and Sec7 domain containing 3 (PSD3), transcript variant 1, mRNA [NM_015310]	2.26382	up	2.53132	up	1151.25830	526.57904	820.00680	285.65080	Detected	Detected	Detected	Detected
2933	A_23_P147711	NPR1	Hs.490330	natriuretic peptide receptor A/guanylate cyclase A (atrionatriuretic peptide receptor A)	Homo sapiens natriuretic peptide receptor A/guanylate cyclase A (atrionatriuretic peptide receptor A) (NPR1), mRNA [NM_000906]	2.26306	up	3.33545	up	174.77946	79.96999	87.42018	23.11125	Detected	Detected	Detected	Detected
2934	A_33_P3373765	DRD4	Hs.99922	dopamine receptor D4	Homo sapiens dopamine receptor D4 (DRD4), mRNA [NM_000797]	2.26262	up	2.35251	up	181.08372	82.87054	133.36740	49.99016	Detected	Detected	Detected	Detected
2935	A_23_P1492	AVPI1	Hs.23918	arginine vasopressin-induced 1	Homo sapiens arginine vasopressin-induced 1 (AVPI1), mRNA [NM_021732]	2.26224	up	2.08933	up	3427.74900	1568.93100	3907.66160	1649.20530	Detected	Detected	Detected	Detected
2936	A_23_P208961	MUM1	Hs.515016	melanoma associated antigen (mutated) 1	Homo sapiens melanoma associated antigen (mutated) 1 (MUM1), transcript variant 1, mRNA [NM_032853]	2.26221	up	2.14471	up	9262.38400	4239.58800	9552.49900	3927.48540	Detected	Detected	Detected	Detected
2937	A_23_P251412	SCGN	Hs.116428	secretagogin, EF-hand calcium binding protein	Homo sapiens secretagogin, EF-hand calcium binding protein (SCGN), mRNA [NM_006998]	2.26188	up	4.49240	up	56.60117	25.91132	36.47100	7.15872	Detected	Detected	Detected	Compromised
2938	A_33_P3248787	LOC729130	Hs.692489	hypothetical LOC729130	PREDICTED: Homo sapiens hypothetical LOC729130 (LOC729130), mRNA [XM_001133555]	2.26123	up	1.81253	up	15.20225	6.96141	15.62283	7.60045	Detected	Compromised	Detected	Compromised
2939	A_24_P842172	LOC349196	Hs.722381	hypothetical LOC349196	Homo sapiens hypothetical LOC349196 (LOC349196), non-coding RNA [NR_027000]	2.26072	up	2.02159	up	132.93391	60.88666	98.10594	42.79259	Detected	Detected	Detected	Detected
2940	A_33_P3339253	LOC646808	Hs.666639	similar to L antigen family, member 3	PREDICTED: Homo sapiens similar to L antigen family, member 3 (LOC646808), miscRNA [XR_017339]	2.25902	up	2.35417	up	4410.06000	2021.42020	6387.62840	2392.58400	Detected	Detected	Detected	Detected
2941	A_23_P148821	DPH5	Hs.440776	DPH5 homolog (S. cerevisiae)	Homo sapiens DPH5 homolog (S. cerevisiae) (DPH5), transcript variant 1, mRNA [NM_001077394]	2.25826	up	2.35777	up	2034.29690	932.76620	1756.72560	657.00530	Detected	Detected	Detected	Detected
2942	A_23_P123727	ZCCHC6	Hs.655162	zinc finger, CCHC domain containing 6	Homo sapiens zinc finger, CCHC domain containing 6 (ZCCHC6), mRNA [NM_024617]	2.25787	up	2.43306	up	1885.87790	864.86346	1123.71010	407.25546	Detected	Detected	Detected	Detected
2943	A_33_P3243230				Interleukin-8 Precursor (IL-8)(C-X-C motif chemokine 8)(Monocyte-derived neutrophil chemotactic factor)(MDNCF)(T-cell chemotactic factor)(Neutrophil-activating protein 1)(NAP-1)(Protein 3-10C)(Granulocyte chemotactic protein 1)(GCP-1)(Monocyte-derived neutrophil-activating peptide)(MONAP)(Emoctakin) [Contains MDNCF-a(IL8/NAP1 form I)(GCP/IL-8 protein IV);Interleukin-8(IL-8(1-77))(MDNCF-b)(IL8/NAP1 form II)(GCP/IL-8 protein II)((Ala-IL-8)77);IL-8(5-77);IL-8(6-77)(Lymphocyte-derived neutrophil-activating factor)(LYNAP)(Neutrophil-activating factor)(NAF)(MDNCF-c)(IL8/NAP1 form III)(GCP/IL-8 protein I)((Ser-IL-8)72);IL-8(7-77)(IL8/NAP1 form IV)(GCP/IL-8 protein V);IL-8(8-77)(IL8/NAP1 form V)(GCP/IL-8 protein VI);IL-8(9-77)(IL8/NAP1 form VI)(GCP/IL-8 protein III)] [Source:UniProtKB/Swiss-Prot;Acc:P10145] [ENST00000401931]	2.25777	up	1.71879	up	126.76268	58.13602	95.11688	48.79772	Detected	Detected	Detected	Detected
2944	A_33_P3240757	IGSF3	Hs.171057	immunoglobulin superfamily, member 3	Homo sapiens immunoglobulin superfamily, member 3 (IGSF3), transcript variant 1, mRNA [NM_001542]	2.25729	up	1.33064	up	31.93695	14.65003	17.02257	11.28056	Detected	Compromised	Detected	Compromised
2945	A_33_P3358233	NES	Hs.527971	nestin	Homo sapiens nestin (NES), mRNA [NM_006617]	2.25702	up	1.81471	up	710.15710	325.80093	811.58500	394.35944	Detected	Detected	Detected	Detected
2946	A_23_P115792	PLEKHA1	Hs.643512	pleckstrin homology domain containing, family A (phosphoinositide binding specific) member 1	Homo sapiens pleckstrin homology domain containing, family A (phosphoinositide binding specific) member 1 (PLEKHA1), transcript variant 2, mRNA [NM_001001974]	2.25696	up	2.04830	up	1977.07780	907.05220	1293.16190	556.70435	Detected	Detected	Detected	Detected
2947	A_33_P3240094				Ephexin-1 (Eph-interacting exchange protein)(Neuronal guanine nucleotide exchange factor) [Source:UniProtKB/Swiss-Prot;Acc:Q8N5V2] [ENST00000409079]	2.25682	up	1.97591	up	60.20647	27.62359	42.96730	19.17501	Detected	Detected	Detected	Detected
2948	A_33_P3294089					2.25671	up	2.67934	up	21.35827	9.79993	25.85777	8.50999	Detected	Compromised	Detected	Compromised
2949	A_33_P3354564	GPD2	Hs.512382	glycerol-3-phosphate dehydrogenase 2 (mitochondrial)	Homo sapiens glycerol-3-phosphate dehydrogenase 2 (mitochondrial) (GPD2), nuclear gene encoding mitochondrial protein, transcript variant 1, mRNA [NM_001083112]	2.25640	up	2.04859	up	244.26308	112.09188	212.81488	91.60376	Detected	Detected	Detected	Detected

2950	A_33_P3269779	ZNF3	Hs.435302	zinc finger protein 3	Homo sapiens zinc finger protein 3 (ZNF3), transcript variant 1, mRNA [NM_017715]	2.25617	up	1.50369	up	24.48342	11.23658	32.25094	18.91252	Detected	Compromised	Detected	Detected
2951	A_23_P360179	NEURL4	Hs.654794	neuralized homolog 4 (Drosophila)	Homo sapiens neuralized homolog 4 (Drosophila) (NEURL4), transcript variant 1, mRNA [NM_032442]	2.25601	up	1.90343	up	1403.54700	644.19730	1120.43620	519.05646	Detected	Detected	Detected	Detected
2952	A_23_P390384	ZNF630	Hs.650883	zinc finger protein 630	Homo sapiens zinc finger protein 630 (ZNF630), mRNA [NM_001037735]	2.25582	up	3.32103	up	430.44107	197.57956	391.71014	104.00582	Detected	Detected	Detected	Detected
2953	A_23_P41734	RNF130	Hs.484363	ring finger protein 130	Homo sapiens ring finger protein 130 (RNF130), mRNA [NM_018434]	2.25576	up	2.14930	up	7792.70200	3577.08400	5408.56300	2218.96630	Detected	Detected	Detected	Detected
2954	A_32_P25050	RDH10	Hs.244940	retinol dehydrogenase 10 (all-trans)	Homo sapiens retinol dehydrogenase 10 (all-trans) (RDH10), mRNA [NM_172037]	2.25527	up	2.54189	up	985.76230	452.59140	678.76117	235.46430	Detected	Detected	Detected	Detected
2955	A_23_P126103	CTH	Hs.19904	cystathionase (cystathionine gamma-lyase)	Homo sapiens cystathionase (cystathionine gamma-lyase) (CTH), transcript variant 1, mRNA [NM_001902]	2.25500	up	2.35423	up	1580.90760	725.92725	1221.60030	457.55790	Detected	Detected	Detected	Detected
2956	A_33_P3232552	GUCY1B3	Hs.77890	guanylate cyclase 1, soluble, beta 3	Homo sapiens guanylate cyclase 1, soluble, beta 3 (GUCY1B3), mRNA [NM_000857]	2.25480	up	3.57463	up	62.12696	28.53018	36.41146	8.98200	Detected	Detected	Detected	Compromised
2957	A_33_P3415052	NIPAL4	Hs.4285	NIPA-like domain containing 4	Homo sapiens NIPA-like domain containing 4 (NIPAL4), mRNA [NM_001099287]	2.25469	up	2.20638	up	150.43858	69.08849	129.04839	51.57492	Detected	Detected	Detected	Detected
2958	A_33_P3294524	ANKRD12	Hs.464585	ankyrin repeat domain 12	Homo sapiens ankyrin repeat domain 12 (ANKRD12), transcript variant 1, mRNA [NM_015208]	2.25442	up	2.20049	up	2269.50500	1042.39010	552.89940	221.56035	Detected	Detected	Detected	Detected
2959	A_33_P3279681	FLJ40125	Hs.532872	protein phosphatase 1B-like	Homo sapiens protein phosphatase 1B-like (FLJ40125), mRNA [NM_001080401]	2.25257	up	2.77246	up	84.04996	38.63606	113.19360	36.00167	Detected	Detected	Detected	Detected
2960	A_24_P32520	RNF214	Hs.591934	ring finger protein 214	Homo sapiens ring finger protein 214 (RNF214), transcript variant 2, mRNA [NM_001077239]	2.25254	up	2.35121	up	621.45276	285.67230	294.99738	110.63473	Detected	Detected	Detected	Detected
2961	A_24_P161463	C20orf199	Hs.356766	chromosome 20 open reading frame 199	Homo sapiens chromosome 20 open reading frame 199 (C20orf199), transcript variant 2, non-coding RNA [NR_003605]	2.25133	up	2.41096	up	62689.36700	28832.90800	40148.71000	14684.11300	Detected	Detected	Detected	Detected
2962	A_24_P113815	SLC35E2	Hs.719940	solute carrier family 35, member E2	Homo sapiens solute carrier family 35, member E2 (SLC35E2), mRNA [NM_182838]	2.25114	up	2.28286	up	625.13580	287.54434	502.44147	194.07591	Detected	Detected	Detected	Detected
2963	A_33_P3264089	ACTR3C	Hs.647117	ARP3 actin-related protein 3 homolog C (yeast)	Homo sapiens ARP3 actin-related protein 3 homolog C (yeast) (ACTR3C), transcript variant 1, mRNA [NM_001164458]	2.25056	up	1.47470	up	53.51188	24.62030	32.46536	19.41249	Detected	Detected	Detected	Detected
2964	A_33_P3221443	ZNF254	Hs.434406	zinc finger protein 254	Homo sapiens zinc finger protein 254 (ZNF254), mRNA [NM_203282]	2.25012	up	2.32462	up	411.61700	189.41751	339.35052	128.72488	Detected	Detected	Detected	Detected
2965	A_23_P124619	S100A14	Hs.288998	S100 calcium binding protein A14	Homo sapiens S100 calcium binding protein A14 (S100A14), mRNA [NM_020672]	2.24966	up	1.60653	up	55.32674	25.46544	33.82554	18.56610	Detected	Detected	Detected	Detected
2966	A_33_P3270337				Serine/threonine-protein kinase 32C (PKE)(EC 2.7.11.1)(YANK3) [Source:UniProtKB/Swiss-Prot;Acc:Q86UX6] [ENST00000368619]	2.24960	up	2.43693	up	676.98760	311.60860	669.91113	242.40355	Detected	Detected	Detected	Detected
2967	A_33_P3314436	FBXL17	Hs.721914	F-box and leucine-rich repeat protein 17	Homo sapiens F-box and leucine-rich repeat protein 17 (FBXL17), mRNA [NM_001163315]	2.24933	up	2.19633	up	136.69897	62.92830	83.79623	33.64286	Detected	Detected	Detected	Detected
2968	A_32_P219520	TNFAIP8	Hs.656274	tumor necrosis factor, alpha-induced protein 8	Homo sapiens tumor necrosis factor, alpha-induced protein 8 (TNFAIP8), transcript variant 1, mRNA [NM_014350]	2.24923	up	2.24177	up	2000.20590	920.81920	2081.44240	818.72687	Detected	Detected	Detected	Detected
2969	A_33_P3347413	PBXIP1	Hs.505806	pre-B-cell leukemia homeobox interacting protein 1	Homo sapiens pre-B-cell leukemia homeobox interacting protein 1 (PBXIP1), mRNA [NM_020524]	2.24838	up	2.03686	up	270.04980	124.36762	226.69194	98.13870	Detected	Detected	Detected	Detected
2970	A_23_P207399	NBR1	Hs.277721	neighbor of BRCA1 gene 1	Homo sapiens neighbor of BRCA1 gene 1 (NBR1), transcript variant 2, mRNA [NM_031858]	2.24762	up	2.20978	up	21008.00000	9678.22200	19788.38900	7896.35940	Detected	Detected	Detected	Detected
2971	A_23_P54891	C16orf58	Hs.9003	chromosome 16 open reading frame 58	Homo sapiens chromosome 16 open reading frame 58 (C16orf58), mRNA [NM_022744]	2.24703	up	2.00491	up	2231.13300	1028.13610	2074.00370	912.18120	Detected	Detected	Detected	Detected
2972	A_33_P3299796		Hs.279881		Endoplasmic reticulum mannosyl-oligosaccharide 1,2-alpha-mannosidase (EC 3.2.1.113)(ER alpha-1,2-mannosidase)(Mannosidase alpha class 1B member 1)(Man9GlcNAc2-specific-processing alpha-mannosidase) [Source:UniProtKB/Swiss-Prot;Acc:Q9UKM7] [ENST00000371587]	2.24695	up	1.45984	up	51.23203	23.60925	40.11793	24.23261	Detected	Detected	Detected	Detected
2973	A_33_P3376644	SENP6	Hs.485784	SUMO1/sentrin specific peptidase 6	Homo sapiens SUMO1/sentrin specific peptidase 6 (SENP6), transcript variant 1, mRNA [NM_015571]	2.24592	up	2.19625	up	235.20769	108.44029	137.85838	55.34997	Detected	Detected	Detected	Detected
2974	A_33_P3329652	ZFP90	Hs.461074	zinc finger protein 90 homolog (mouse)	Homo sapiens zinc finger protein 90 homolog (mouse) (ZFP90), mRNA [NM_133458]	2.24578	up	2.34222	up	4055.70730	1869.96350	2936.99560	1105.71100	Detected	Detected	Detected	Detected
2975	A_23_P106194	FOS	Hs.707896	FBJ murine osteosarcoma viral oncogene homolog	Homo sapiens FBJ murine osteosarcoma viral oncogene homolog (FOS), mRNA [NM_005252]	2.24550	up	2.18540	up	2280.67600	1051.67910	2165.02470	873.57120	Detected	Detected	Detected	Detected
2976	A_23_P98248	TRPT1	Hs.326586	tRNA phosphotransferase 1	Homo sapiens tRNA phosphotransferase 1 (TRPT1), transcript variant 1, mRNA [NM_001033678]	2.24550	up	2.08696	up	4759.54200	2194.75460	4312.33640	1822.06410	Detected	Detected	Detected	Detected

2977	A_24_P209171	SH3BGRL2	Hs.302772	SH3 domain binding glutamic acid-rich protein like 2	Homo sapiens SH3 domain binding glutamic acid-rich protein like 2 (SH3BGRL2), mRNA [NM_031469]	2.24536	up	2.65165	up	91.79768	42.33294	70.47483	23.43601	Detected	Detected	Detected	Detected
2978	A_23_P391926	LPHN1	Hs.94229	latrophilin 1	Homo sapiens latrophilin 1 (LPHN1), transcript variant 1, mRNA [NM_001008701]	2.24490	up	5.13229	up	320.01184	147.60540	257.91754	44.31344	Detected	Detected	Detected	Detected
2979	A_24_P194503	PHKA1	Hs.201379	phosphorylase kinase, alpha 1 (muscle)	Homo sapiens phosphorylase kinase, alpha 1 (muscle) (PHKA1), transcript variant 1, mRNA [NM_002637]	2.24476	up	2.34030	up	93.76606	43.25224	78.41022	29.54382	Detected	Detected	Detected	Detected
2980	A_33_P3217218	AGPAT5	Hs.624002	1-acylglycerol-3-phosphate O-acyltransferase 5 (lysophosphatidic acid acyltransferase, epsilon)	Homo sapiens 1-acylglycerol-3-phosphate O-acyltransferase 5 (lysophosphatidic acid acyltransferase, epsilon) (AGPAT5), mRNA [NM_018361]	2.24437	up	2.43287	up	508.60077	234.64754	417.62830	151.36873	Detected	Detected	Detected	Detected
2981	A_33_P3414907		Hs.496969		N-acetylneuraminate lyase (NALase)(EC 4.1.3.3)(N-acetylneuraminic acid aldolase)(N-acetylneuraminate pyruvate-lyase)(Sialic acid lyase)(Sialate lyase)(Sialate-pyruvate lyase)(Sialic acid aldolase) [Source:UniProtKB/Swiss-Prot;Acc:Q9BXD5] [ENST00000367555]	2.24409	up	1.40839	up	118.52078	54.68729	72.95692	45.67825	Detected	Detected	Detected	Detected
2982	A_33_P3357332		Hs.622548		Chromosome 9 open reading frame 147 Fragment [Source:UniProtKB/TrEMBL;Acc:A2A387] [ENST00000412934]	2.24364	up	1.49852	up	22.23703	10.26259	16.75543	9.85960	Detected	Compromised	Detected	Compromised
2983	A_33_P3780123	FLJ33065	Hs.660388	hypothetical gene supported by AK057627; BC031275; BC045736	Homo sapiens cDNA FLJ33065 fis, clone TRACH2000081 [AK057627]	2.24206	up	6.26439	up	31.22026	14.41857	30.98712	4.36182	Detected	Compromised	Detected	Compromised
2984	A_33_P3221293	DFNA5	Hs.520708	deafness, autosomal dominant 5	Homo sapiens deafness, autosomal dominant 5 (DFNA5), transcript variant 1, mRNA [NM_004403]	2.24185	up	2.12199	up	1045.69180	482.98080	1052.65770	437.43054	Detected	Detected	Detected	Detected
2985	A_33_P3618960	P2RX6	Hs.113275	purinergic receptor P2X, ligand-gated ion channel, 6	Homo sapiens purinergic receptor P2X, ligand-gated ion channel, 6 (P2RX6), transcript variant 1, mRNA [NM_005446]	2.24167	up	2.50177	up	13.52781	6.24869	7.15953	2.52350	Detected	Compromised	Compromised	Compromised
2986	A_33_P3321347	LOC388849	Hs.454905	similar to hCG1732289	PREDICTED: Homo sapiens similar to hCG1732289 (LOC388849), mRNA [XM_373945]	2.24144	up	2.56376	up	26.19149	12.09945	34.03787	11.70712	Detected	Compromised	Detected	Compromised
2987	A_33_P3659678	NR6A1	Hs.20131	nuclear receptor subfamily 6, group A, member 1	Nuclear receptor subfamily 6 group A member 1 (Germ cell nuclear factor)(GCNF)(hGCNF)(Retinoid receptor-related testis-specific receptor)(RTR)(hRTR) [Source:UniProtKB/Swiss-Prot;Acc:Q15406] [ENST00000487099]	2.24140	up	1.95913	up	141.00230	65.13892	106.16740	47.78513	Detected	Detected	Detected	Detected
2988	A_23_P103601	MAN1C1	Hs.197043	mannosidase, alpha, class 1C, member 1	Homo sapiens mannosidase, alpha, class 1C, member 1 (MAN1C1), mRNA [NM_020379]	2.24122	up	2.42996	up	534.25380	246.82867	354.27810	128.56140	Detected	Detected	Detected	Detected
2989	A_23_P73763	LAGE3	Hs.444619	L antigen family, member 3	Homo sapiens L antigen family, member 3 (LAGE3), mRNA [NM_006014]	2.24104	up	2.10311	up	13789.73800	6371.45560	15405.13100	6459.05470	Detected	Detected	Detected	Detected
2990	A_23_P377245		Hs.533986		Homo sapiens zinc finger, MYM-type 6, mRNA (cDNA clone IMAGE:5455214), [BC042172]	2.24095	up	2.77201	up	397.33510	183.59372	168.12486	53.48137	Detected	Detected	Detected	Detected
2991	A_33_P3402963	DPRX	Hs.579941	divergent-paired related homeobox	Homo sapiens divergent-paired related homeobox (DPRX), mRNA [NM_001012728]	2.24080	up	1.56618	up	370.57648	171.24069	37.38526	21.04859	Detected	Detected	Detected	Detected
2992	A_33_P3289561				Putative uncharacterized protein C1orf134 Precursor [Source:UniProtKB/Swiss-Prot;Acc:Q5TEV5] [ENST00000375605]	2.24008	up	2.29570	up	640.44855	296.04270	630.01904	241.99345	Detected	Detected	Detected	Detected
2993	A_24_P316257	NHLRC4	Hs.664267	NHL repeat containing 4	Homo sapiens NHL repeat containing 4 (NHLRC4), mRNA [NM_176677]	2.23996	up	2.19629	up	411.54626	190.24414	237.81436	95.48046	Detected	Detected	Detected	Detected
2994	A_33_P3336587	LOC283710	Hs.591097	hypothetical LOC283710	PREDICTED: Homo sapiens hypothetical LOC283710 (LOC283710), mRNA [XM_211174]	2.23921	up	1.80272	up	112.04903	51.81383	12.65105	6.18818	Detected	Detected	Compromised	Compromised
2995	A_24_P873659	MALAT1	Hs.621695	metastasis associated lung adenocarcinoma transcript 1 (non-protein coding)	Homo sapiens metastasis associated lung adenocarcinoma transcript 1 (non-protein coding) (MALAT1), non-coding RNA [NR_002819]	2.23810	up	1.76194	up	2071.52640	958.39350	1346.49560	673.87335	Detected	Detected	Detected	Detected
2996	A_23_P151614	PSME1	Hs.75348	proteasome (prosome, macropain) activator subunit 1 (PA28 alpha)	Homo sapiens proteasome (prosome, macropain) activator subunit 1 (PA28 alpha) (PSME1), transcript variant 1, mRNA [NM_006263]	2.23780	up	2.13571	up	1137.43270	526.30414	1033.87520	426.86627	Detected	Detected	Detected	Detected
2997	A_23_P106737	LUC7L	Hs.16803	LUC7-like (S. cerevisiae)	Homo sapiens LUC7-like (S. cerevisiae) (LUC7L), transcript variant 2, mRNA [NM_201412]	2.23691	up	2.15649	up	916.90875	424.43497	614.36890	251.21625	Detected	Detected	Detected	Detected
2998	A_33_P3303146	ATG4C	Hs.7353	ATG4 autophagy related 4 homolog C (S. cerevisiae)	Homo sapiens ATG4 autophagy related 4 homolog C (S. cerevisiae) (ATG4C), transcript variant 7, mRNA [NM_032852]	2.23610	up	2.41600	up	513.85535	237.94833	121.32635	44.28156	Detected	Detected	Detected	Detected
2999	A_23_P415021	METTL7A	Hs.655369	methyltransferase like 7A	Homo sapiens methyltransferase like 7A (METTL7A), mRNA [NM_014033]	2.23603	up	2.52943	up	4703.52250	2178.10620	4439.85200	1547.78800	Detected	Detected	Detected	Detected
3000	A_33_P3318971	C12orf72	Hs.585084	chromosome 12 open reading frame 72	UPF0516 protein C12orf72 [Source:UniProtKB/Swiss-Prot;Acc:Q8IXQ9] [ENST00000357721]	2.23586	up	3.93436	up	82.15444	38.04686	56.51485	12.66644	Detected	Detected	Detected	Compromised
3001	A_33_P3223097	C1orf97	Hs.523932	chromosome 1 open reading frame 97	Homo sapiens chromosome 1 open reading frame 97 (C1orf97), non-coding RNA [NR_026761]	2.23496	up	2.51177	up	798.95514	370.15735	1010.64660	354.80050	Detected	Detected	Detected	Detected

3002	A_24_P294851	TRIM38	Hs.584851	tripartite motif-containing 38	Homo sapiens tripartite motif-containing 38 (TRIM38), mRNA [NM_006355]	2.23464	up	2.65115	up	412.10513	190.95593	268.54150	89.31876	Detected	Detected	Detected	Detected
3003	A_32_P357301	PPHLN1	Hs.444157	periphilin 1	Homo sapiens periphilin 1 (PPHLN1), transcript variant 1, mRNA [NM_016488]	2.23454	up	1.94083	up	56.23978	26.06089	49.69147	22.57664	Detected	Detected	Detected	Detected
3004	A_24_P38754	CISD1	Hs.370102	CDGSH iron sulfur domain 1	Homo sapiens CDGSH iron sulfur domain 1 (CISD1), mRNA [NM_018464]	2.23414	up	2.40357	up	301.38885	139.68495	230.12263	84.42440	Detected	Detected	Detected	Detected
3005	A_23_P136909	HMGN5	Hs.282204	high-mobility group nucleosome binding domain 5	Homo sapiens high-mobility group nucleosome binding domain 5 (HMGN5), mRNA [NM_030763]	2.23411	up	2.24716	up	521.52590	241.71542	423.56525	166.20770	Detected	Detected	Detected	Detected
3006	A_33_P3544880	LOC142937	Hs.679824	hypothetical protein BC008131	Homo sapiens cDNA clone IMAGE:3454038. [BC008131]	2.23317	up	3.07184	up	38.48625	17.84503	38.84824	11.15162	Detected	Detected	Detected	Compromised
3007	A_33_P3231923		Hs.570209		Putative uncharacterized protein ENSP00000387129 [Source:UniProtKB/TrEMBL;Acc:B8ZZJ7] [ENST00000409590]	2.23245	up	2.86277	up	3553.63350	1648.25710	359.68760	110.79108	Detected	Detected	Detected	Detected
3008	A_23_P36753	ALDH2	Hs.632733	aldehyde dehydrogenase 2 family (mitochondrial)	Homo sapiens aldehyde dehydrogenase 2 family (mitochondrial) (ALDH2), nuclear gene encoding mitochondrial protein, mRNA [NM_000690]	2.23205	up	2.20203	up	3637.57230	1687.49210	2814.30300	1126.97280	Detected	Detected	Detected	Detected
3009	A_33_P3411090	ADAL	Hs.533913	adenosine deaminase-like	Homo sapiens adenosine deaminase-like (ADAL), transcript variant 2, mRNA [NM_001012969]	2.23178	up	2.36839	up	794.97076	368.83545	638.23950	237.62712	Detected	Detected	Detected	Detected
3010	A_24_P68819	KLRAQ1	Hs.654619	KLRAQ motif containing 1	Homo sapiens KLRAQ motif containing 1 (KLRAQ1), transcript variant 2, mRNA [NM_152994]	2.23174	up	2.49256	up	1947.13780	903.41455	1367.57500	483.80585	Detected	Detected	Detected	Detected
3011	A_32_P82189	FAM161A	Hs.440466	family with sequence similarity 161, member A	Homo sapiens family with sequence similarity 161, member A (FAM161A), mRNA [NM_032180]	2.23124	up	1.63227	up	74.66921	34.65197	22.47290	12.14042	Detected	Detected	Detected	Compromised
3012	A_24_P941051	CSTF2T	Hs.591358	cleavage stimulation factor, 3' pre-RNA, subunit 2, 64kDa, tau variant	Homo sapiens cleavage stimulation factor, 3' pre-RNA, subunit 2, 64kDa, tau variant (CSTF2T), mRNA [NM_015235]	2.23104	up	2.26311	up	2272.42300	1054.66500	1787.42980	696.44970	Detected	Detected	Detected	Detected
3013	A_32_P73452	ANO8	Hs.590990	anoctamin 8	Homo sapiens anoctamin 8 (ANO8), mRNA [NM_020959]	2.23098	up	2.42479	up	595.59674	276.43344	133.82800	48.66736	Detected	Detected	Detected	Detected
3014	A_23_P333498	EEPD1	Hs.487994	endonuclease/exonuclease/phosphatase family domain containing 1	Homo sapiens endonuclease/exonuclease/phosphatase family domain containing 1 (EEPD1), mRNA [NM_030636]	2.23058	up	2.18969	up	376.90717	174.96413	331.80774	133.61930	Detected	Detected	Detected	Detected
3015	A_33_P3279362	NCRNA00110		non-protein coding RNA 110	Homo sapiens non-protein coding RNA 110 (NCRNA00110), non-coding RNA [NR_027021]	2.23041	up	1.64426	up	23.91333	11.10170	24.60460	13.19507	Detected	Compromised	Detected	Detected
3016	A_23_P88559	LIPC	Hs.654472	lipase, hepatic	Homo sapiens lipase, hepatic (LIPC), mRNA [NM_000236]	2.22936	up	2.31325	up	101.33821	47.06812	63.28394	24.12325	Detected	Detected	Detected	Detected
3017	A_23_P122852	SMARCD3	Hs.647067	SWI/SNF related, matrix associated, actin dependent regulator of chromatin, subfamily d, member 3	Homo sapiens SWI/SNF related, matrix associated, actin dependent regulator of chromatin, subfamily d, member 3 (SMARCD3), transcript variant 2, mRNA [NM_003078]	2.22915	up	2.33462	up	10948.12300	5085.48970	13912.39300	5254.73970	Detected	Detected	Detected	Detected
3018	A_33_P3416473	LOC646999	Hs.72168	hypothetical LOC646999	Homo sapiens hypothetical LOC646999 (LOC646999), non-coding RNA [NR_024390]	2.22890	up	2.04877	up	342.35420	159.04466	257.79890	110.95687	Detected	Detected	Detected	Detected
3019	A_33_P3279526					2.22889	up	1.85715	up	587.14410	272.76532	294.43866	139.80171	Detected	Detected	Detected	Detected
3020	A_33_P3240647	C21orf2	Hs.517331	chromosome 21 open reading frame 2	Homo sapiens chromosome 21 open reading frame 2 (C21orf2), mRNA [NM_004928]	2.22854	up	2.30020	up	4746.81050	2205.54170	4340.98900	1664.13170	Detected	Detected	Detected	Detected
3021	A_23_P333138	MON2	Hs.389378	MON2 homolog (S. cerevisiae)	Homo sapiens MON2 homolog (S. cerevisiae) (MON2), mRNA [NM_015026]	2.22789	up	3.27205	up	72.59484	33.74004	43.92775	11.83816	Detected	Detected	Detected	Compromised
3022	A_33_P3393734				40S ribosomal protein S27 (Metallopan-stimulin 1)(MPS-1) [Source:UniProtKB/Swiss-Prot;Acc:P42677] [ENST00000392558]	2.22780	up	2.43839	up	166.93758	77.59078	143.55637	51.91401	Detected	Detected	Detected	Detected
3023	A_23_P386168	PRR24	Hs.128690	proline rich 24	Homo sapiens proline rich 24 (PRR24), mRNA [NM_178511]	2.22616	up	2.05745	up	4174.54930	1941.72010	4976.74500	2132.95340	Detected	Detected	Detected	Detected
3024	A_33_P3212109	DCDC2	Hs.61345	doublecortin domain containing 2	Homo sapiens doublecortin domain containing 2 (DCDC2), mRNA [NM_016356]	2.22589	up	4.10418	up	12.44481	5.78919	12.86791	2.76470	Detected	Compromised	Compromised	Compromised
3025	A_23_P423695	MXD4	Hs.655020	MAX dimerization protein 4	Homo sapiens MAX dimerization protein 4 (MXD4), mRNA [NM_006454]	2.22512	up	2.21919	up	7824.97950	3641.35640	3037.94300	1207.12070	Detected	Detected	Detected	Detected
3026	A_24_P126741					2.22443	up	2.59242	up	2112.93750	983.55900	3379.77510	1149.60500	Detected	Detected	Detected	Detected
3027	A_33_P3222228	FUT6	Hs.631846	fucosyltransferase 6 (alpha (1,3) fucosyltransferase)	Homo sapiens fucosyltransferase 6 (alpha (1,3) fucosyltransferase) (FUT6), transcript variant 1, mRNA [NM_000150]	2.22424	up	2.16113	up	32.51440	15.13658	33.43899	13.64388	Detected	Compromised	Detected	Detected
3028	A_23_P365086	ANKRD5	Hs.70903	ankyrin repeat domain 5	Homo sapiens ankyrin repeat domain 5 (ANKRD5), transcript variant 1, mRNA [NM_022096]	2.22409	up	4.34927	up	17.12206	7.97144	15.47770	3.13802	Detected	Compromised	Detected	Compromised
3029	A_24_P169896	LOC100289922		hypothetical protein LOC100289922	cDNA FLJ32655 fis, clone TESTI1000025, weakly similar to M.musculus testis-specific protein, DDC8 [Source:UniProtKB/TrEMBL;Acc:Q96MC4] [ENST00000322630]	2.22403	up	1.61609	up	17.60120	8.19474	11.43444	6.23901	Detected	Compromised	Compromised	Compromised
3030	A_23_P330537	SPSB3	Hs.592080	splA/ryanodine receptor domain and SOCS box containing 3	Homo sapiens splA/ryanodine receptor domain and SOCS box containing 3 (SPSB3), mRNA [NM_080861]	2.22402	up	1.97733	up	425.56067	198.13300	517.83984	230.93028	Detected	Detected	Detected	Detected
3031	A_33_P3324495	ZFP41	Hs.668016	zinc finger protein 41 homolog (mouse)	Homo sapiens zinc finger protein 41 homolog (mouse) (ZFP41), mRNA [NM_173832]	2.22309	up	2.33831	up	2808.01270	1307.90380	2471.86500	932.15410	Detected	Detected	Detected	Detected

3032	A_24_P329597	UBQLN1	Hs.9589	ubiquilin 1	Homo sapiens ubiquilin 1 (UBQLN1), transcript variant 1, mRNA [NM_013438]	2.22286	up	2.47773	up	590.53440	275.08505	188.27023	67.00285	Detected	Detected	Detected	Detected
3033	A_33_P3250887	C22orf41	Hs.535660	chromosome 22 open reading frame 41	Homo sapiens chromosome 22 open reading frame 41 (C22orf41), mRNA [NM_001123225]	2.22263	up	2.42790	up	34.68342	16.15800	32.61946	11.84706	Detected	Compromised	Detected	Compromised
3034	A_23_P381577	ZNF25	Hs.499429	zinc finger protein 25	Homo sapiens zinc finger protein 25 (ZNF25), mRNA [NM_145011]	2.22231	up	2.56651	up	1171.19900	545.70557	702.07556	241.21614	Detected	Detected	Detected	Detected
3035	A_33_P3245006	DAK	Hs.6278	dihydroxyacetone kinase 2 homolog (S. cerevisiae)	Homo sapiens dihydroxyacetone kinase 2 homolog (S. cerevisiae) (DAK), mRNA [NM_015533]	2.22148	up	3.27183	up	1026.40990	478.42350	742.99786	200.24557	Detected	Detected	Detected	Detected
3036	A_33_P3522511	KIAA0485	Hs.604754	hypothetical LOC57235	Homo sapiens mRNA, chromosome 1 specific transcript KIAA0485 [AB007954]	2.22129	up	2.63565	up	143.48929	66.88791	100.67370	33.68170	Detected	Detected	Detected	Detected
3037	A_33_P3221303	CCR10	Hs.278446	chemokine (C-C motif) receptor 10	Homo sapiens chemokine (C-C motif) receptor 10 (CCR10), mRNA [NM_016602]	2.22109	up	2.14478	up	2277.01100	1061.52800	1819.60740	748.10315	Detected	Detected	Detected	Detected
3038	A_32_P36046	C2orf68	Hs.516159	chromosome 2 open reading frame 68	Homo sapiens chromosome 2 open reading frame 68 (C2orf68), mRNA [NM_001013649]	2.22026	up	2.00594	up	921.02435	429.53690	761.71660	334.84244	Detected	Detected	Detected	Detected
3039	A_23_P171095	USP27X	Hs.143587	ubiquitin specific peptidase 27, X-linked	Homo sapiens ubiquitin specific peptidase 27, X-linked (USP27X), mRNA [NM_001145073]	2.21927	up	2.32938	up	39.39882	18.38253	13.24209	5.01283	Detected	Compromised	Compromised	Compromised
3040	A_32_P515088	KDSR	Hs.74050	3-ketodihydrosphingosine reductase	Homo sapiens 3-ketodihydrosphingosine reductase (KDSR), mRNA [NM_002035]	2.21901	up	2.24973	up	3151.88400	1470.76720	2044.82370	801.47565	Detected	Detected	Detected	Detected
3041	A_23_P93704	C7orf61	Hs.632306	chromosome 7 open reading frame 61	Homo sapiens chromosome 7 open reading frame 61 (C7orf61), mRNA [NM_001004323]	2.21797	up	2.31599	up	121.51906	56.73119	95.19649	36.24519	Detected	Detected	Detected	Detected
3042	A_33_P3265376	HOMER3	Hs.720208	homer homolog 3 (Drosophila)	Homo sapiens homer homolog 3 (Drosophila) (HOMER3), transcript variant 4, mRNA [NM_001145724]	2.21745	up	1.38018	up	3487.56270	1628.55350	5318.75200	3398.12040	Detected	Detected	Detected	Detected
3043	A_23_P116037	TM7SF2	Hs.31130	transmembrane 7 superfamily member 2	Homo sapiens transmembrane 7 superfamily member 2 (TM7SF2), mRNA [NM_003273]	2.21675	up	1.95058	up	56.69408	26.48223	41.57246	18.79347	Detected	Detected	Detected	Detected
3044	A_24_P201404	C11orf54	Hs.8360	chromosome 11 open reading frame 54	Homo sapiens chromosome 11 open reading frame 54 (C11orf54), mRNA [NM_014039]	2.21661	up	2.39950	up	869.39404	406.12610	753.98724	277.08200	Detected	Detected	Detected	Detected
3045	A_24_P7629	RPL32P3		ribosomal protein L32 pseudogene 3	Homo sapiens ribosomal protein L32 pseudogene 3 (RPL32P3), non-coding RNA [NR_003111]	2.21526	up	2.08038	up	24.22241	11.32208	16.68334	7.07140	Detected	Compromised	Detected	Compromised
3046	A_33_P3296240	CNTLN	Hs.435381	centlein, centrosomal protein	Homo sapiens centlein, centrosomal protein (CNTLN), transcript variant 1, mRNA [NM_017738]	2.21470	up	2.31434	up	96.60606	45.16704	53.23661	20.28381	Detected	Detected	Detected	Detected
3047	A_23_P145895	TP53TG1		TP53 target 1 (non-protein coding)	Homo sapiens TP53 target 1 (non-protein coding) (TP53TG1), non-coding RNA [NR_015381]	2.21297	up	2.24636	up	3586.01900	1677.91490	3139.65480	1232.44560	Detected	Detected	Detected	Detected
3048	A_33_P3242109					2.21287	up	2.78007	up	206.67018	96.70651	150.98778	47.89073	Detected	Detected	Detected	Detected
3049	A_33_P3753757	LOC158402	Hs.494822	hypothetical protein LOC158402	Homo sapiens cDNA FLJ38333 fis, clone FCBBF3025674 [AK095652]	2.21198	up	1.63739	up	19.12863	8.95440	16.96800	9.13783	Detected	Compromised	Detected	Compromised
3050	A_33_P3318534	ACPL2	Hs.657887	acid phosphatase-like 2	Homo sapiens acid phosphatase-like 2 (ACPL2), transcript variant 1, mRNA [NM_152282]	2.21122	up	1.43507	up	369.05618	172.81972	212.95341	130.85135	Detected	Detected	Detected	Detected
3051	A_23_P14892	IGFALS	Hs.839	insulin-like growth factor binding protein, acid labile subunit	Homo sapiens insulin-like growth factor binding protein, acid labile subunit (IGFALS), transcript variant 2, mRNA [NM_004970]	2.21112	up	1.72548	up	47.51930	22.25312	51.22060	26.17587	Detected	Detected	Detected	Detected
3052	A_24_P236091	ENO2	Hs.511915	enolase 2 (gamma, neuronal)	Homo sapiens enolase 2 (gamma, neuronal) (ENO2), mRNA [NM_001975]	2.21104	up	2.08510	up	823.66675	385.73364	662.95996	280.36688	Detected	Detected	Detected	Detected
3053	A_24_P200848	SNX13	Hs.487648	sorting nexin 13	Homo sapiens sorting nexin 13 (SNX13), mRNA [NM_015132]	2.21059	up	2.21868	up	1177.93840	551.75696	644.47034	256.13830	Detected	Detected	Detected	Detected
3054	A_23_P125643	ASB9	Hs.19404	ankyrin repeat and SOCS box-containing 9	Homo sapiens ankyrin repeat and SOCS box-containing 9 (ASB9), transcript variant 1, mRNA [NM_001031739]	2.21014	up	2.23901	up	135.25587	63.36802	88.68623	34.92737	Detected	Detected	Detected	Detected
3055	A_33_P3213962	OR13A1	Hs.532435	olfactory receptor, family 13, subfamily A, member 1	Homo sapiens olfactory receptor, family 13, subfamily A, member 1 (OR13A1), mRNA [NM_001004297]	2.21012	up	2.26897	up	69.06363	32.35684	64.60424	25.10714	Detected	Detected	Detected	Detected
3056	A_23_P152583	ENGASE	Hs.29288	endo-beta-N-acetylglucosaminidase	Homo sapiens endo-beta-N-acetylglucosaminidase (ENGASE), mRNA [NM_001042573]	2.20971	up	2.13178	up	1478.49040	692.81390	1176.57020	486.67828	Detected	Detected	Detected	Detected
3057	A_23_P377339	C4orf36	Hs.339646	chromosome 4 open reading frame 36	Homo sapiens chromosome 4 open reading frame 36 (C4orf36), mRNA [NM_144645]	2.20949	up	1.18439	up	19.89038	9.32149	23.73599	17.67168	Detected	Compromised	Detected	Detected
3058	A_23_P502047	CHRD	Hs.166186	chordin	Homo sapiens chordin (CHRD), mRNA [NM_003741]	2.20889	up	1.37595	up	525.83710	246.49597	627.15780	401.91970	Detected	Detected	Detected	Detected
3059	A_33_P3318668	C9orf169	Hs.512469	chromosome 9 open reading frame 169	Homo sapiens chromosome 9 open reading frame 169 (C9orf169), mRNA [NM_199001]	2.20797	up	2.14390	up	584.18823	273.96378	622.15770	255.89459	Detected	Detected	Detected	Detected
3060	A_23_P96761	ACADM	Hs.445040	acyl-Coenzyme A dehydrogenase, C-4 to C-12 straight chain	Homo sapiens acyl-Coenzyme A dehydrogenase, C-4 to C-12 straight chain (ACADM), nuclear gene encoding mitochondrial protein, transcript variant 1, mRNA [NM_000016]	2.20797	up	2.19304	up	1070.82100	502.17783	627.54720	252.32834	Detected	Detected	Detected	Detected
3061	A_24_P250650	RABL2A	Hs.446425	RAB, member of RAS oncogene family-like 2A	Homo sapiens RAB, member of RAS oncogene family-like 2A (RABL2A), transcript variant 1, mRNA [NM_013412]	2.20541	up	2.14050	up	8050.58200	3779.81270	6484.52640	2671.33060	Detected	Detected	Detected	Detected
3062	A_32_P151366	SIRT5	Hs.567431	sirtuin (silent mating type information regulation 2 homolog) 5 (S. cerevisiae)	Homo sapiens sirtuin (silent mating type information regulation 2 homolog) 5 (S. cerevisiae) (SIRT5), transcript variant 1, mRNA [NM_012241]	2.20537	up	2.13699	up	657.76360	308.83182	604.41516	249.40137	Detected	Detected	Detected	Detected

3063	A_33_P3243168	MZF1	Hs.399810	myeloid zinc finger 1	Homo sapiens myeloid zinc finger 1 (MZF1), transcript variant 2, mRNA [NM_198055]	2.20495	up	2.05579	up	3705.25020	1740.01380	3333.14450	1429.68490	Detected	Detected	Detected	Detected
3064	A_23_P259344	CECR6	Hs.209577	cat eye syndrome chromosome region, candidate 6	Homo sapiens cat eye syndrome chromosome region, candidate 6 (CECR6), transcript variant 1, mRNA [NM_031890]	2.20457	up	2.02739	up	103.64081	48.67877	84.73400	36.85411	Detected	Detected	Detected	Detected
3065	A_23_P60339	C9orf64	Hs.208914	chromosome 9 open reading frame 64	Homo sapiens chromosome 9 open reading frame 64 (C9orf64), mRNA [NM_032307]	2.20451	up	2.27825	up	262.53265	123.31159	244.53426	94.64636	Detected	Detected	Detected	Detected
3066	A_33_P3372297	ABR	Hs.159306	active BCR-related gene	Homo sapiens active BCR-related gene (ABR), transcript variant 3, mRNA [NM_001159746]	2.20332	up	2.03817	up	101.10068	47.51266	92.58960	40.05791	Detected	Detected	Detected	Detected
3067	A_32_P113584	ZNF292	Hs.485892	zinc finger protein 292	Homo sapiens zinc finger protein 292 (ZNF292), mRNA [NM_015021]	2.20331	up	1.98732	up	455.88202	214.24437	276.92285	122.87330	Detected	Detected	Detected	Detected
3068	A_32_P177539	IRX6	Hs.369907	iroquois homeobox 6	Homo sapiens iroquois homeobox 6 (IRX6), mRNA [NM_024335]	2.20309	up	2.14108	up	16.56641	7.78626	13.75806	5.66619	Detected	Compromised	Compromised	Compromised
3069	A_33_P3323822	GATAD2B	Hs.4779	GATA zinc finger domain containing 2B	Homo sapiens GATA zinc finger domain containing 2B (GATAD2B), mRNA [NM_020699]	2.20276	up	2.52925	up	447.56784	210.39014	326.16770	113.71430	Detected	Detected	Detected	Detected
3070	A_33_P3379456	SH3YL1	Hs.515951	SH3 domain containing, Ysc84-like 1 (S. cerevisiae)	Homo sapiens SH3 domain containing, Ysc84-like 1 (S. cerevisiae) (SH3YL1), transcript variant 1, mRNA [NM_015677]	2.20252	up	2.22439	up	3618.67100	1701.22800	3006.76250	1191.93950	Detected	Detected	Detected	Detected
3071	A_24_P89843	CYHR1	Hs.459379	cysteine/histidine-rich 1	Homo sapiens cysteine/histidine-rich 1 (CYHR1), transcript variant 2, mRNA [NM_032687]	2.20165	up	1.96526	up	1304.02930	613.29930	1424.48610	639.15270	Detected	Detected	Detected	Detected
3072	A_33_P3224362	PCBP3	Hs.474049	poly(rC) binding protein 3	Homo sapiens poly(rC) binding protein 3 (PCBP3), transcript variant 1, mRNA [NM_020528]	2.20141	up	2.83399	up	241.61534	113.64641	182.69499	56.84526	Detected	Detected	Detected	Detected
3073	A_23_P101297	EML2	Hs.24178	echinoderm microtubule associated protein like 2	Homo sapiens echinoderm microtubule associated protein like 2 (EML2), mRNA [NM_012155]	2.20052	up	1.83147	up	905.73285	426.19382	995.59980	479.34723	Detected	Detected	Detected	Detected
3074	A_32_P16315	RP11-631M21.2	Hs.532659	tubulin, beta 8	Homo sapiens tubulin, beta 8 (TUBB8), transcript variant 2, mRNA [NM_001164154]	2.20025	up	2.30732	up	74.24895	34.94233	29.85961	11.41146	Detected	Detected	Detected	Compromised
3075	A_33_P3333507	SENP7	Hs.529551	SUMO1/sentrin specific peptidase 7	Homo sapiens SUMO1/sentrin specific peptidase 7 (SENP7), transcript variant 1, mRNA [NM_020654]	2.20002	up	2.63484	up	51.49221	24.23524	55.42193	18.54781	Detected	Detected	Detected	Detected
3076	A_23_P114061	TRPM3	Hs.47288	transient receptor potential cation channel, subfamily M, member 3	Homo sapiens transient receptor potential cation channel, subfamily M, member 3 (TRPM3), transcript variant 9, mRNA [NM_001007471]	2.19998	up	2.01049	up	49.71554	23.39952	35.30045	15.48263	Detected	Detected	Detected	Detected
3077	A_24_P157087	CASP8	Hs.599762	caspase 8, apoptosis-related cysteine peptidase	Homo sapiens caspase 8, apoptosis-related cysteine peptidase (CASP8), transcript variant B, mRNA [NM_033355]	2.19997	up	2.75169	up	53.43244	25.14905	66.49148	21.30745	Detected	Detected	Detected	Detected
3078	A_33_P3248231	LOC283028	Hs.646699	hypothetical protein LOC283028	PREDICTED: Homo sapiens hypothetical protein LOC283028 (LOC283028), miscRNA [XR_017707]	2.19970	up	3.20002	up	43.56344	20.50653	22.81017	6.28551	Detected	Detected	Detected	Compromised
3079	A_23_P311010	SPRY3	Hs.381912	sprouty homolog 3 (Drosophila)	Homo sapiens sprouty homolog 3 (Drosophila) (SPRY3), mRNA [NM_005840]	2.19968	up	2.13678	up	268.54788	126.41378	197.07384	81.32700	Detected	Detected	Detected	Detected
3080	A_33_P3257367	TRIM33	Hs.26837	tripartite motif-containing 33	Homo sapiens tripartite motif-containing 33 (TRIM33), transcript variant a, mRNA [NM_015906]	2.19935	up	2.38970	up	2396.15620	1128.11770	1001.41016	369.51633	Detected	Detected	Detected	Detected
3081	A_23_P406385	FBXL16	Hs.513244	F-box and leucine-rich repeat protein 16	Homo sapiens F-box and leucine-rich repeat protein 16 (FBXL16), mRNA [NM_153350]	2.19849	up	2.22141	up	1099.33190	517.77140	675.88135	268.29184	Detected	Detected	Detected	Detected
3082	A_23_P23575	SLC39A1	Hs.7854	solute carrier family 39 (zinc transporter), member 1	Homo sapiens solute carrier family 39 (zinc transporter), member 1 (SLC39A1), mRNA [NM_014437]	2.19840	up	2.04758	up	4865.43460	2291.64920	5801.53600	2498.43000	Detected	Detected	Detected	Detected
3083	A_23_P137157	RENBP	Hs.158331	renin binding protein	Homo sapiens renin binding protein (RENBP), mRNA [NM_002910]	2.19734	up	2.13547	up	134.68500	63.46808	165.39267	68.29488	Detected	Detected	Detected	Detected
3084	A_23_P14493	C14orf104	Hs.231761	chromosome 14 open reading frame 104	Homo sapiens chromosome 14 open reading frame 104 (C14orf104), transcript variant 1, mRNA [NM_018139]	2.19707	up	2.21917	up	2810.37480	1324.50600	2195.48900	872.38245	Detected	Detected	Detected	Detected
3085	A_33_P3270084					2.19675	up	3.01066	up	228.33914	107.62980	101.22479	29.64773	Detected	Detected	Detected	Detected
3086	A_33_P3345354	C1orf66	Hs.512597	chromosome 1 open reading frame 66	Homo sapiens chromosome 1 open reading frame 66 (C1orf66), transcript variant 1, mRNA [NM_015997]	2.19584	up	2.85792	up	2562.64970	1208.43000	2757.65800	850.85583	Detected	Detected	Detected	Detected
3087	A_23_P251647	RFESD	Hs.399758	Rieske (Fe-S) domain containing	Homo sapiens Rieske (Fe-S) domain containing (RFESD), transcript variant 2, mRNA [NM_173362]	2.19465	up	2.43780	up	282.44810	133.26201	261.74698	94.67802	Detected	Detected	Detected	Detected
3088	A_23_P327361	DMXL2	Hs.511386	Dmx-like 2	Homo sapiens Dmx-like 2 (DMXL2), mRNA [NM_015263]	2.19463	up	2.63372	up	30.72939	14.49861	22.66277	7.58769	Detected	Compromised	Detected	Compromised
3089	A_23_P212511	TTC14	Hs.43213	tetratricopeptide repeat domain 14	Homo sapiens tetratricopeptide repeat domain 14 (TTC14), transcript variant 2, mRNA [NM_001042601]	2.19453	up	2.34123	up	574.75580	271.19040	509.56726	191.92099	Detected	Detected	Detected	Detected
3090	A_33_P3380693	ADAM15	Hs.312098	ADAM metallopeptidase domain 15	Homo sapiens ADAM metallopeptidase domain 15 (ADAM15), transcript variant 6, mRNA [NM_207197]	2.19399	up	1.66134	up	42563.49200	20087.97500	48000.73400	25477.31600	Detected	Detected	Detected	Detected
3091	A_33_P3396891	AVPI1	Hs.23918	arginine vasopressin-induced 1	Homo sapiens arginine vasopressin-induced 1 (AVPI1), mRNA [NM_021732]	2.19376	up	2.11827	up	5384.04600	2541.28300	3957.60200	1647.46300	Detected	Detected	Detected	Detected
3092	A_33_P3386117	RER1	Hs.525527	RER1 retention in endoplasmic reticulum 1 homolog (S. cerevisiae)	Homo sapiens RER1 retention in endoplasmic reticulum 1 homolog (S. cerevisiae) (RER1), mRNA [NM_007033]	2.19241	up	1.95115	up	3210.81450	1516.44340	3588.55880	1621.79520	Detected	Detected	Detected	Detected
3093	A_33_P3228023	SCPEP1	Hs.514950	serine carboxypeptidase 1	Homo sapiens serine carboxypeptidase 1 (SCPEP1), mRNA [NM_021626]	2.19231	up	1.67869	up	180.83676	85.41187	231.55954	121.63503	Detected	Detected	Detected	Detected

3094	A_33_P3248203	FUT5	Hs.631843	fucosyltransferase 5 (alpha (1,3) fucosyltransferase)	Homo sapiens fucosyltransferase 5 (alpha (1,3) fucosyltransferase) (FUT5), mRNA [NM_002034]	2.19215	up	1.55136	up	24.21105	11.43604	28.10486	15.97471	Detected	Compromised	Detected	Detected
3095	A_23_P14157	DZIP1	Hs.656580	DAZ interacting protein 1	Homo sapiens DAZ interacting protein 1 (DZIP1), transcript variant 2, mRNA [NM_198968]	2.19210	up	2.14183	up	2718.34550	1284.03550	2402.41630	989.07355	Detected	Detected	Detected	Detected
3096	A_24_P114249	GALNT3	Hs.170986	UDP-N-acetyl-alpha-D-galactosamine:polypeptide N-acetylgalactosaminyltransferase 3 (GalNAc-T3)	Homo sapiens UDP-N-acetyl-alpha-D-galactosamine:polypeptide N-acetylgalactosaminyltransferase 3 (GalNAc-T3) (GALNT3), mRNA [NM_004482]	2.19115	up	1.48657	up	45.18549	21.35303	20.21438	11.99059	Detected	Detected	Detected	Compromised
3097	A_33_P3380652	ADAM28	Hs.174030	ADAM metallopeptidase domain 28	Homo sapiens ADAM metallopeptidase domain 28 (ADAM28), transcript variant 3, mRNA [NM_021777]	2.19050	up	2.82807	up	46.64184	22.04786	28.90214	9.01167	Detected	Detected	Detected	Compromised
3098	A_23_P215037	WTAP	Hs.446091	Wilms tumor 1 associated protein	Homo sapiens Wilms tumor 1 associated protein (WTAP), transcript variant 3, mRNA [NM_152858]	2.19028	up	2.22113	up	9760.67800	4614.37900	6969.41940	2766.86250	Detected	Detected	Detected	Detected
3099	A_23_P17880	DNAL4	Hs.632766	dynein, axonemal, light chain 4	Homo sapiens dynein, axonemal, light chain 4 (DNAL4), mRNA [NM_005740]	2.19006	up	2.12454	up	424.15475	200.54045	446.51758	185.32689	Detected	Detected	Detected	Detected
3100	A_33_P3268129		Hs.575858		Putative uncharacterized protein C1orf98 [Source:UniProtKB/Swiss-Prot;Acc:A6NCI5] [ENST00000367356]	2.18997	up	2.99446	up	82.94727	39.21912	63.63493	18.73886	Detected	Detected	Detected	Detected
3101	A_23_P207058	SOCS3	Hs.527973	suppressor of cytokine signaling 3	Homo sapiens suppressor of cytokine signaling 3 (SOCS3), mRNA [NM_003955]	2.18981	up	1.97398	up	5844.79830	2763.73320	5653.16400	2525.30400	Detected	Detected	Detected	Detected
3102	A_24_P181149	BBS10	Hs.96322	Bardet-Biedl syndrome 10	Homo sapiens Bardet-Biedl syndrome 10 (BBS10), mRNA [NM_024685]	2.18946	up	2.31231	up	1288.75930	609.49176	770.27234	293.74094	Detected	Detected	Detected	Detected
3103	A_24_P135427	LOC100128398	Hs.721608	hypothetical protein LOC100128398	cDNA FLJ37429 fis, clone BRAWH2001666 [Source:UniProtKB/TrEMBL;Acc:Q8N9G5] [ENST00000313957]	2.18871	up	1.85334	up	91.35387	43.21880	53.48223	25.44599	Detected	Detected	Detected	Detected
3104	A_33_P3281258					2.18854	up	1.49128	up	20.75557	9.82003	4.94577	2.92442	Detected	Compromised	Compromised	Compromised
3105	A_24_P269814	PLEKHA1	Hs.643512	pleckstrin homology domain containing, family A (phosphoinositide binding specific) member 1	Homo sapiens pleckstrin homology domain containing, family A (phosphoinositide binding specific) member 1 (PLEKHA1), transcript variant 2, mRNA [NM_001001974]	2.18854	up	2.39877	up	5676.40670	2685.66670	3387.13570	1245.11350	Detected	Detected	Detected	Detected
3106	A_33_P3254946	PLEKHH3	Hs.632251	pleckstrin homology domain containing, family H (with MyTH4 domain) member 3	Homo sapiens pleckstrin homology domain containing, family H (with MyTH4 domain) member 3 (PLEKHH3), mRNA [NM_024927]	2.18816	up	1.93474	up	9381.95600	4439.64060	5067.00400	2309.37300	Detected	Detected	Detected	Detected
3107	A_33_P3292387	LRRIQ3	Hs.644625	leucine-rich repeats and IQ motif containing 3	Homo sapiens leucine-rich repeats and IQ motif containing 3 (LRRIQ3), mRNA [NM_001105659]	2.18787	up	2.34671	up	198.47008	93.93043	155.73573	58.51874	Detected	Detected	Detected	Detected
3108	A_33_P3638471	TRGV7	Hs.534032	T cell receptor gamma variable 7 pseudogene	Homo sapiens T cell receptor gamma variable 7 pseudogene, mRNA (cDNA clone IMAGE:5210958), [BC027954]	2.18724	up	2.28209	up	212.10983	100.41452	131.41263	50.77732	Detected	Detected	Detected	Detected
3109	A_24_P41371	CC2D1A	Hs.269592	coiled-coil and C2 domain containing 1A	Homo sapiens coiled-coil and C2 domain containing 1A (CC2D1A), mRNA [NM_017721]	2.18685	up	1.43238	up	44.81672	21.22040	42.12353	25.93178	Detected	Detected	Detected	Detected
3110	A_23_P31116	ACOT13	Hs.9676	acyl-CoA thioesterase 13	Homo sapiens acyl-CoA thioesterase 13 (ACOT13), transcript variant 1, mRNA [NM_018473]	2.18646	up	2.00605	up	8893.26200	4211.64400	8939.50000	3929.49020	Detected	Detected	Detected	Detected
3111	A_23_P214897	AKAP12	Hs.371240	A kinase (PRKA) anchor protein 12	Homo sapiens A kinase (PRKA) anchor protein 12 (AKAP12), transcript variant 2, mRNA [NM_144497]	2.18644	up	2.05608	up	7299.80860	3457.06270	3370.20650	1445.38320	Detected	Detected	Detected	Detected
3112	A_32_P42964	LOC389493	Hs.177948	hypothetical protein LOC389493	Homo sapiens hypothetical protein LOC389493 (LOC389493), mRNA [NM_001145712]	2.18513	up	2.21311	up	452.71768	214.52795	306.65088	122.18169	Detected	Detected	Detected	Detected
3113	A_23_P91640	ASPHD2	Hs.567547	aspartate beta-hydroxylase domain containing 2	Homo sapiens aspartate beta-hydroxylase domain containing 2 (ASPHD2), mRNA [NM_020437]	2.18502	up	4.00963	up	315.80950	149.65936	423.05765	93.03819	Detected	Detected	Detected	Detected
3114	A_23_P52531	FAM24B	Hs.114648	family with sequence similarity 24, member B	Homo sapiens family with sequence similarity 24, member B (FAM24B), mRNA [NM_152644]	2.18434	up	2.21001	up	1736.34280	823.09310	1515.78900	604.79690	Detected	Detected	Detected	Detected
3115	A_24_P248863	ZCCHC4	Hs.278945	zinc finger, CCHC domain containing 4	Homo sapiens zinc finger, CCHC domain containing 4 (ZCCHC4), mRNA [NM_024936]	2.18425	up	2.32952	up	1285.49890	609.39910	614.19390	232.48978	Detected	Detected	Detected	Detected
3116	A_32_P89827	LOC374491	Hs.406779	TPTE and PTEN homologous inositol lipid phosphatase pseudogene	Homo sapiens TPTE and PTEN homologous inositol lipid phosphatase pseudogene (LOC374491), non-coding RNA [NR_002815]	2.18372	up	2.65094	up	235.21585	111.53307	218.58627	72.70898	Detected	Detected	Detected	Detected
3117	A_33_P3285987	METT5D1	Hs.243326	methyltransferase 5 domain containing 1	Homo sapiens methyltransferase 5 domain containing 1 (METT5D1), transcript variant 2, mRNA [NM_152636]	2.18362	up	2.03536	up	1253.68470	594.49030	933.17880	404.28564	Detected	Detected	Detected	Detected
3118	A_33_P3338693	SNAP25	Hs.167317	synaptosomal-associated protein, 25kDa	Homo sapiens synaptosomal-associated protein, 25kDa (SNAP25), transcript variant 1, mRNA [NM_003081]	2.18273	up	10.33788	up	3170.61820	1504.09790	1991.59180	169.87694	Detected	Detected	Detected	Detected
3119	A_33_P3347457					2.18262	up	1.28927	up	12.42766	5.89582	5.20820	3.56212	Detected	Compromised	Compromised	Compromised
3120	A_23_P48747	DHRS1	Hs.348350	dehydrogenase/reductase (SDR family) member 1	Homo sapiens dehydrogenase/reductase (SDR family) member 1 (DHRS1), transcript variant 2, mRNA [NM_138452]	2.18256	up	2.17055	up	2077.05370	985.40594	2295.56230	932.57880	Detected	Detected	Detected	Detected
3121	A_33_P3361422	CYP27A1	Hs.516700	cytochrome P450, family 27, subfamily A, polypeptide 1	Homo sapiens cytochrome P450, family 27, subfamily A, polypeptide 1 (CYP27A1), nuclear gene encoding mitochondrial protein, mRNA [NM_000784]	2.18179	up	2.06936	up	4265.50050	2024.37540	4144.21140	1765.92360	Detected	Detected	Detected	Detected
3122	A_23_P255663	MANEA	Hs.533323	mannosidase, endo-alpha	Homo sapiens mannosidase, endo-alpha (MANEA), mRNA [NM_024641]	2.18177	up	2.16071	up	1389.76870	659.57930	1090.14800	444.89127	Detected	Detected	Detected	Detected

3123	A_33_P3361417	C6orf57	Hs.418495	chromosome 6 open reading frame 57	Homo sapiens chromosome 6 open reading frame 57 (C6orf57), mRNA [NM_145267]	2.18171	up	2.47527	up	1736.42880	824.12476	1263.12110	449.97488	Detected	Detected	Detected	Detected
3124	A_32_P175715	MEIG1	Hs.257249	meiosis expressed gene 1 homolog (mouse)	Homo sapiens meiosis expressed gene 1 homolog (mouse) (MEIG1), mRNA [NM_001080836]	2.18161	up	2.06776	up	77.52650	36.79655	61.36122	26.16729	Detected	Detected	Detected	Detected
3125	A_33_P3779229	GK5	Hs.135904	glycerol kinase 5 (putative)	Homo sapiens glycerol kinase 5 (putative) (GK5), mRNA [NM_001039547]	2.18160	up	2.20723	up	201.06330	95.43150	131.95859	52.71764	Detected	Detected	Detected	Detected
3126	A_24_P67806	MTR	Hs.498187	5-methyltetrahydrofolate-homocysteine methyltransferase	Homo sapiens 5-methyltetrahydrofolate-homocysteine methyltransferase (MTR), mRNA [NM_000254]	2.18086	up	2.16730	up	404.45297	192.03223	244.24532	99.37378	Detected	Detected	Detected	Detected
3127	A_33_P3364571	TNXB	Hs.485104	tenascin XB	Homo sapiens tenascin XB (TNXB), transcript variant XB, mRNA [NM_019105]	2.18070	up	1.49163	up	61.74926	29.32030	61.03250	36.07993	Detected	Detected	Detected	Detected
3128	A_33_P3341224	PCTK2	Hs.506415	PCTAIRE protein kinase 2	Homo sapiens PCTAIRE protein kinase 2 (PCTK2), mRNA [NM_002595]	2.17972	up	2.28319	up	744.30054	353.57413	535.92790	206.98094	Detected	Detected	Detected	Detected
3129	A_33_P3286929					2.17948	up	2.17156	up	272.62994	129.52531	231.31178	93.92732	Detected	Detected	Detected	Detected
3130	A_23_P110492	MARCH6	Hs.432862	membrane-associated ring finger (C3HC4) 6	Homo sapiens membrane-associated ring finger (C3HC4) 6 (MARCH6), mRNA [NM_005885]	2.17829	up	2.26190	up	2207.17380	1049.18980	1223.10000	476.82025	Detected	Detected	Detected	Detected
3131	A_23_P65022	ACADS	Hs.507076	acyl-Coenzyme A dehydrogenase, C-2 to C-3 short chain	Homo sapiens acyl-Coenzyme A dehydrogenase, C-2 to C-3 short chain (ACADS), nuclear gene encoding mitochondrial protein, mRNA [NM_000017]	2.17807	up	1.71388	up	419.87296	199.60863	287.04620	147.68489	Detected	Detected	Detected	Detected
3132	A_32_P486620	IGSF22	Hs.434152	immunoglobulin superfamily, member 22	Homo sapiens immunoglobulin superfamily, member 22 (IGSF22), mRNA [NM_173588]	2.17775	up	2.07249	up	20.89318	9.93411	17.45664	7.42735	Detected	Compromised	Detected	Compromised
3133	A_24_P287043	IFITM2	Hs.709321	interferon induced transmembrane protein 2 (1-8D)	Homo sapiens interferon induced transmembrane protein 2 (1-8D) (IFITM2), mRNA [NM_006435]	2.17741	up	2.07713	up	36095.73400	17165.23000	36411.90200	15457.72600	Detected	Detected	Detected	Detected
3134	A_23_P58676	C5orf23	Hs.13528	chromosome 5 open reading frame 23	Homo sapiens chromosome 5 open reading frame 23 (C5orf23), mRNA [NM_024563]	2.17727	up	2.17214	up	5236.76030	2490.47900	3325.21100	1349.88840	Detected	Detected	Detected	Detected
3135	A_23_P89570	ZMYND15	Hs.47223	zinc finger, MYND-type containing 15	Homo sapiens zinc finger, MYND-type containing 15 (ZMYND15), transcript variant 2, mRNA [NM_032265]	2.17699	up	2.47107	up	1772.48450	843.06024	735.01080	262.28497	Detected	Detected	Detected	Detected
3136	A_33_P3290562	GLI3	Hs.21509	GLI family zinc finger 3	Homo sapiens GLI family zinc finger 3 (GLI3), mRNA [NM_000168]	2.17686	up	2.18411	up	5636.14900	2680.92550	4363.65970	1761.74370	Detected	Detected	Detected	Detected
3137	A_33_P3268716	SNX1	Hs.188634	sorting nexin 1	Homo sapiens sorting nexin 1 (SNX1), transcript variant 1, mRNA [NM_003099]	2.17663	up	2.30866	up	1460.96400	695.00510	1248.79480	476.97565	Detected	Detected	Detected	Detected
3138	A_33_P3388061	KRTAP21-2	Hs.553699	keratin associated protein 21-2	Homo sapiens keratin associated protein 21-2 (KRTAP21-2), mRNA [NM_181617]	2.17655	up	4.12114	up	14.36426	6.83358	20.55763	4.39867	Detected	Compromised	Detected	Compromised
3139	A_23_P4628	ZNF606	Hs.654967	zinc finger protein 606	Homo sapiens zinc finger protein 606 (ZNF606), mRNA [NM_025027]	2.17435	up	2.24092	up	205.93724	98.07031	166.96725	65.70073	Detected	Detected	Detected	Detected
3140	A_23_P393645	ADAMTS13	Hs.131433	ADAM metallopeptidase with thrombospondin type 1 motif, 13	Homo sapiens ADAM metallopeptidase with thrombospondin type 1 motif, 13 (ADAMTS13), transcript variant 1, mRNA [NM_139025]	2.17425	up	2.38225	up	506.30330	241.12080	369.24774	136.67729	Detected	Detected	Detected	Detected
3141	A_23_P159125	SLC16A5	Hs.592095	solute carrier family 16, member 5 (monocarboxylic acid transporter 6)	Homo sapiens solute carrier family 16, member 5 (monocarboxylic acid transporter 6) (SLC16A5), mRNA [NM_004695]	2.17391	up	2.21672	up	13275.01100	6323.04930	11959.81200	4757.49760	Detected	Detected	Detected	Detected
3142	A_24_P188377	CD55	Hs.126517	CD55 molecule, decay accelerating factor for complement (Cromer blood group)	Homo sapiens CD55 molecule, decay accelerating factor for complement (Cromer blood group) (CD55), transcript variant 1, mRNA [NM_000574]	2.17315	up	2.07591	up	1973.88620	940.51420	1859.15170	789.71790	Detected	Detected	Detected	Detected
3143	A_23_P427122	ART5	Hs.125680	ADP-ribosyltransferase 5	Homo sapiens ADP-ribosyltransferase 5 (ART5), transcript variant 1, mRNA [NM_053017]	2.17248	up	1.45008	up	48.79375	23.25640	41.71713	25.36815	Detected	Detected	Detected	Detected
3144	A_23_P161424	PLXDC2	Hs.658134	plexin domain containing 2	Homo sapiens plexin domain containing 2 (PLXDC2), mRNA [NM_032812]	2.17146	up	2.37720	up	60.57085	28.88324	36.01918	13.36082	Detected	Detected	Detected	Detected
3145	A_33_P3387365	PXMP4	Hs.654857	peroxisomal membrane protein 4, 24kDa	Homo sapiens peroxisomal membrane protein 4, 24kDa (PXMP4), transcript variant 1, mRNA [NM_007238]	2.17107	up	2.37659	up	730.81330	348.55026	583.65326	216.55357	Detected	Detected	Detected	Detected
3146	A_24_P159837				Zinc finger protein 302 (ZNF135-like)(ZNF140-like) [Source:UniProtKB/Swiss-Prot;Acc:Q9NR11] [ENST00000221282]	2.17069	up	2.21023	up	2067.85570	986.40570	1543.27010	615.70000	Detected	Detected	Detected	Detected
3147	A_33_P3210358					2.17029	up	4.97848	up	97.83165	46.67620	81.06834	14.35888	Detected	Detected	Detected	Detected
3148	A_33_P3358898	SSC5D	Hs.554182	scavenger receptor cysteine-rich glycoprotein	Homo sapiens scavenger receptor cysteine-rich glycoprotein (SSC5D), mRNA [NM_001144950]	2.16932	up	2.03438	up	2906.18430	1387.17850	2920.43700	1265.84940	Detected	Detected	Detected	Detected
3149	A_33_P3243449	CD70	Hs.501497	CD70 molecule	Homo sapiens CD70 molecule (CD70), mRNA [NM_001252]	2.16892	up	2.81193	up	86.28574	41.19351	54.15869	16.98358	Detected	Detected	Detected	Detected
3150	A_33_P3405004	NFXL1	Hs.646325	nuclear transcription factor, X-box binding-like 1	Homo sapiens nuclear transcription factor, X-box binding-like 1 (NFXL1), mRNA [NM_152995]	2.16834	up	2.18730	up	3586.98170	1712.91190	2607.00020	1050.98900	Detected	Detected	Detected	Detected
3151	A_24_P29594	HBS1L	Hs.378532	HBS1-like (S. cerevisiae)	Homo sapiens HBS1-like (S. cerevisiae) (HBS1L), transcript variant 1, mRNA [NM_006620]	2.16731	up	1.94845	up	57.18043	27.31862	13.02524	5.89470	Detected	Detected	Compromised	Compromised
3152	A_23_P377214	HEXIM2	Hs.56382	hexamthylene bis-acetamide inducible 2	Homo sapiens hexamthylene bis-acetamide inducible 2 (HEXIM2), mRNA [NM_144608]	2.16667	up	2.08265	up	3774.06030	1803.63340	2692.84890	1140.14650	Detected	Detected	Detected	Detected
3153	A_24_P45005	NPEPL1	Hs.654868	aminopeptidase-like 1	Homo sapiens aminopeptidase-like 1 (NPEPL1), mRNA [NM_024663]	2.16477	up	1.94280	up	14713.02900	7037.57570	11568.40900	5250.63230	Detected	Detected	Detected	Detected

3154	A_23_P49009	LPCAT4	Hs.352614	lysophosphatidylcholine acyltransferase 4	Homo sapiens lysophosphatidylcholine acyltransferase 4 (LPCAT4), mRNA [NM_153613]	2.16435	up	2.32190	up	167.36717	80.07129	206.34229	78.36275	Detected	Detected	Detected	Detected
3155	A_24_P365975	COL8A2	Hs.353001	collagen, type VIII, alpha 2	Homo sapiens collagen, type VIII, alpha 2 (COL8A2), mRNA [NM_005202]	2.16393	up	2.08822	up	2195.47300	1050.55070	1470.97910	621.14886	Detected	Detected	Detected	Detected
3156	A_23_P143484	PIGP	Hs.656565	phosphatidylinositol glycan anchor biosynthesis, class P	Homo sapiens phosphatidylinositol glycan anchor biosynthesis, class P (PIGP), transcript variant 1, mRNA [NM_153681]	2.16323	up	2.32908	up	1301.41830	622.94040	1166.26680	441.54860	Detected	Detected	Detected	Detected
3157	A_32_P86905	KGFLP2		keratinocyte growth factor-like protein 2	Homo sapiens keratinocyte growth factor-like protein 2 (KGFLP2), non-coding RNA [NR_003670]	2.16297	up	2.15932	up	628.43054	300.84265	366.38525	149.61860	Detected	Detected	Detected	Detected
3158	A_23_P99811	MDP1	Hs.220963	magnesium-dependent phosphatase 1	Homo sapiens magnesium-dependent phosphatase 1 (MDP1), mRNA [NM_138476]	2.16074	up	2.27177	up	5811.06640	2784.75500	4632.14750	1797.97310	Detected	Detected	Detected	Detected
3159	A_23_P346006	CCPG1	Hs.285051	cell cycle progression 1	Homo sapiens cell cycle progression 1 (CCPG1), transcript variant 2, mRNA [NM_020739]	2.15986	up	2.34490	up	600.28296	287.78223	525.81525	197.73125	Detected	Detected	Detected	Detected
3160	A_33_P3286349	C19orf51	Hs.351582	chromosome 19 open reading frame 51	Homo sapiens chromosome 19 open reading frame 51 (C19orf51), mRNA [NM_178837]	2.15935	up	5.66484	up	50.80563	24.36252	60.23245	9.37581	Detected	Detected	Detected	Compromised
3161	A_23_P144622	GNPDA1	Hs.633853	glucosamine-6-phosphate deaminase 1	Homo sapiens glucosamine-6-phosphate deaminase 1 (GNPDA1), mRNA [NM_005471]	2.15911	up	2.16944	up	1547.27750	742.03830	1540.01110	625.95374	Detected	Detected	Detected	Detected
3162	A_32_P107493	C16orf55	Hs.655171	chromosome 16 open reading frame 55	Homo sapiens chromosome 16 open reading frame 55 (C16orf55), mRNA [NM_153025]	2.15832	up	2.36653	up	134.84169	64.69071	99.47999	37.06722	Detected	Detected	Detected	Detected
3163	A_23_P126528	KCNA10	Hs.248140	potassium voltage-gated channel, shaker-related subfamily, member 10	Homo sapiens potassium voltage-gated channel, shaker-related subfamily, member 10 (KCNA10), mRNA [NM_005549]	2.15808	up	1.22072	up	8.27743	3.97157	14.64676	10.58012	Compromised	Compromised	Detected	Compromised
3164	A_33_P3386237	LOC100132529	Hs.710296	hypothetical LOC100132529	PREDICTED: Homo sapiens hypothetical LOC100132529 (LOC100132529), mRNA [XM_001717495]	2.15744	up	2.33252	up	130.81581	62.78484	116.78588	44.14994	Detected	Detected	Detected	Detected
3165	A_23_P63289	SSU72	Hs.657061	SSU72 RNA polymerase II CTD phosphatase homolog (S. cerevisiae)	Homo sapiens SSU72 RNA polymerase II CTD phosphatase homolog (S. cerevisiae) (SSU72), mRNA [NM_014188]	2.15710	up	2.08429	up	17074.95500	8196.37100	12517.89900	5295.89160	Detected	Detected	Detected	Detected
3166	A_33_P3350413					2.15670	up	2.35466	up	399.90320	191.99834	271.98660	101.85540	Detected	Detected	Detected	Detected
3167	A_23_P137423	IGSF8	Hs.332012	immunoglobulin superfamily, member 8	Homo sapiens immunoglobulin superfamily, member 8 (IGSF8), mRNA [NM_052868]	2.15610	up	2.10491	up	558.54596	268.23972	640.46640	268.30515	Detected	Detected	Detected	Detected
3168	A_23_P111804	PARP12	Hs.12646	poly (ADP-ribose) polymerase family, member 12	Homo sapiens poly (ADP-ribose) polymerase family, member 12 (PARP12), mRNA [NM_022750]	2.15607	up	2.15180	up	778.31067	373.78537	599.01886	245.47343	Detected	Detected	Detected	Detected
3169	A_23_P5221	ZNF333	Hs.515215	zinc finger protein 333	Homo sapiens zinc finger protein 333 (ZNF333), mRNA [NM_032433]	2.15583	up	2.23619	up	417.73486	200.64056	368.29434	145.22841	Detected	Detected	Detected	Detected
3170	A_24_P225635	HNRPLL		heterogeneous nuclear ribonucleoprotein L-like	Heterogeneous nuclear ribonucleoprotein L-like (Stromal RNA-regulating factor) [Source:UniProtKB/Swiss-Prot;Acc:Q8WVV9] [ENST00000449105]	2.15522	up	2.37962	up	217.07750	104.29340	109.40680	40.54174	Detected	Detected	Detected	Detected
3171	A_33_P3352970	IRAK2	Hs.449207	interleukin-1 receptor-associated kinase 2	Homo sapiens interleukin-1 receptor-associated kinase 2 (IRAK2), mRNA [NM_001570]	2.15455	up	2.28294	up	3700.22020	1778.29380	2705.62260	1045.05110	Detected	Detected	Detected	Detected
3172	A_23_P162300	IRAK3	Hs.369265	interleukin-1 receptor-associated kinase 3	Homo sapiens interleukin-1 receptor-associated kinase 3 (IRAK3), transcript variant 1, mRNA [NM_007199]	2.15444	up	1.92696	up	573.99450	275.87173	467.30026	213.84021	Detected	Detected	Detected	Detected
3173	A_23_P209625	CYP1B1	Hs.154654	cytochrome P450, family 1, subfamily B, polypeptide 1	Homo sapiens cytochrome P450, family 1, subfamily B, polypeptide 1 (CYP1B1), mRNA [NM_000104]	2.15391	up	2.00275	up	9671.18000	4649.27440	6000.36500	2641.90530	Detected	Detected	Detected	Detected
3174	A_23_P98605	UBXN1	Hs.351296	UBX domain protein 1	Homo sapiens UBX domain protein 1 (UBXN1), mRNA [NM_015853]	2.15377	up	1.96445	up	74022.70000	35587.60500	58818.16400	26401.90600	Detected	Detected	Detected	Detected
3175	A_24_P373174	RAB27A	Hs.654978	RAB27A, member RAS oncogene family	Homo sapiens RAB27A, member RAS oncogene family (RAB27A), transcript variant 1, mRNA [NM_004580]	2.15261	up	2.21499	up	899.51200	432.68878	749.47030	298.36588	Detected	Detected	Detected	Detected
3176	A_24_P211565	C1QTNF6	Hs.720142	C1q and tumor necrosis factor related protein 6	Homo sapiens C1q and tumor necrosis factor related protein 6 (C1QTNF6), transcript variant 1, mRNA [NM_031910]	2.15180	up	2.06161	up	1158.20450	557.33590	1386.24350	592.92316	Detected	Detected	Detected	Detected
3177	A_24_P274615	ARRDC3	Hs.24684	arrestin domain containing 3	Homo sapiens arrestin domain containing 3 (ARRDC3), mRNA [NM_020801]	2.15098	up	2.28054	up	5402.46340	2600.69530	2439.44380	943.23130	Detected	Detected	Detected	Detected
3178	A_33_P3293668	DENND4C	Hs.249591	DENN/MADD domain containing 4C	Homo sapiens DENN/MADD domain containing 4C (DENND4C), mRNA [NM_017925]	2.15056	up	2.20934	up	4741.14400	2282.78760	3270.76880	1305.42820	Detected	Detected	Detected	Detected
3179	A_33_P3419945	LOC100128562	Hs.667110	hypothetical protein LOC100128562	Homo sapiens cDNA FLJ27214 fis, clone SYN03752. [AK130724]	2.15050	up	3.33693	up	1637.61610	788.50560	119.97112	31.70260	Detected	Detected	Detected	Detected
3180	A_33_P3358626	TAF4B	Hs.369519	TAF4b RNA polymerase II, TATA box binding protein (TBP)-associated factor, 105kDa	Homo sapiens TAF4b RNA polymerase II, TATA box binding protein (TBP)-associated factor, 105kDa (TAF4B), mRNA [NM_005640]	2.15021	up	2.52163	up	529.10736	254.79778	418.65515	146.39996	Detected	Detected	Detected	Detected
3181	A_24_P141214	STOM	Hs.253903	stomatin	Homo sapiens stomatin (STOM), transcript variant 2, mRNA [NM_198194]	2.14932	up	1.90468	up	14892.19100	7174.47070	10274.07700	4756.47560	Detected	Detected	Detected	Detected
3182	A_23_P14302	C14orf139	Hs.41502	chromosome 14 open reading frame 139	Homo sapiens chromosome 14 open reading frame 139 (C14orf139), non-coding RNA [NR_026779]	2.14889	up	2.44251	up	4851.15330	2337.56710	3064.74950	1106.42910	Detected	Detected	Detected	Detected
3183	A_33_P3299811	LQK1	Hs.552649	hypothetical LOC642946	Homo sapiens hypothetical LOC642946 (LQK1), transcript variant 2, non-coding RNA [NR_027286]	2.14882	up	2.31789	up	85.05660	40.98655	86.16750	32.78058	Detected	Detected	Detected	Detected

3184	A_23_P212089	NFKBIZ	Hs.319171	nuclear factor of kappa light polypeptide gene enhancer in B-cells inhibitor, zeta	Homo sapiens nuclear factor of kappa light polypeptide gene enhancer in B-cells inhibitor, zeta (NFKBIZ), transcript variant 1, mRNA [NM_031419]	2.14631	up	2.38612	up	604.77290	291.76453	512.55600	189.41525	Detected	Detected	Detected	Detected
3185	A_23_P112289	TMOD1	Hs.494595	tropomodulin 1	Homo sapiens tropomodulin 1 (TMOD1), transcript variant 1, mRNA [NM_003275]	2.14619	up	1.24839	up	19.13916	9.23398	18.46538	13.04287	Detected	Compromised	Detected	Detected
3186	A_24_P285032	IDS	Hs.460960	iduronate 2-sulfatase	Homo sapiens iduronate 2-sulfatase (IDS), transcript variant 2, mRNA [NM_006123]	2.14573	up	2.27560	up	452.59320	218.40645	517.00390	200.33792	Detected	Detected	Detected	Detected
3187	A_33_P3329686	SEPT5	Hs.283743	septin 5	Homo sapiens septin 5 (SEPT5), mRNA [NM_002688]	2.14523	up	1.61691	up	93.61037	45.18395	101.79849	55.51631	Detected	Detected	Detected	Detected
3188	A_33_P3332937	LOC650794	Hs.147563	similar to FRAS1 related extracellular matrix protein 2	PREDICTED: Homo sapiens similar to FRAS1 related extracellular matrix protein 2 (LOC650794), mRNA [XM_001716126]	2.14455	up	6.27206	up	36.33703	17.54474	22.09297	3.10606	Detected	Detected	Detected	Compromised
3189	A_33_P3386242	CNKSR1	Hs.16232	connector enhancer of kinase suppressor of Ras 1	Homo sapiens connector enhancer of kinase suppressor of Ras 1 (CNKSR1), transcript variant 1, mRNA [NM_006314]	2.14396	up	1.87552	up	35.24744	17.02335	23.37755	10.99112	Detected	Compromised	Detected	Compromised
3190	A_23_P50389	NAT14	Hs.719312	N-acetyltransferase 14 (GCN5-related, putative)	Homo sapiens N-acetyltransferase 14 (GCN5-related, putative) (NAT14), mRNA [NM_020378]	2.14394	up	2.36402	up	4181.36800	2019.47680	4534.95800	1691.56290	Detected	Detected	Detected	Detected
3191	A_23_P207125	NLGN2	Hs.26229	neuroligin 2	Homo sapiens neuroligin 2 (NLGN2), mRNA [NM_020795]	2.14371	up	2.06044	up	4248.26200	2052.00590	3368.05270	1441.40060	Detected	Detected	Detected	Detected
3192	A_23_P27744	FBXO46	Hs.128702	F-box protein 46	Homo sapiens F-box protein 46 (FBXO46), mRNA [NM_001080469]	2.14332	up	2.11077	up	2218.09520	1071.58230	1520.51660	635.20860	Detected	Detected	Detected	Detected
3193	A_23_P24716	TMEM132A	Hs.118552	transmembrane protein 132A	Homo sapiens transmembrane protein 132A (TMEM132A), transcript variant 1, mRNA [NM_017870]	2.14261	up	1.80357	up	12853.77200	6211.85350	10630.48800	5197.38670	Detected	Detected	Detected	Detected
3194	A_23_P421423	TNFAIP2	Hs.525607	tumor necrosis factor, alpha-induced protein 2	Homo sapiens tumor necrosis factor, alpha-induced protein 2 (TNFAIP2), mRNA [NM_006291]	2.14111	up	1.92974	up	19523.68000	9441.80800	15985.91600	7304.73730	Detected	Detected	Detected	Detected
3195	A_23_P92614	HELQ	Hs.480101	helicase, POLQ-like	Homo sapiens helicase, POLQ-like (HELQ), mRNA [NM_133636]	2.14062	up	2.10639	up	608.67285	294.42618	439.78220	184.10445	Detected	Detected	Detected	Detected
3196	A_24_P113131	BZRAP1	Hs.112499	benzodiazapine receptor (peripheral) associated protein 1	Homo sapiens benzodiazapine receptor (peripheral) associated protein 1 (BZRAP1), transcript variant 1, mRNA [NM_004758]	2.14047	up	5.31135	up	64.67962	31.28900	26.05792	4.32614	Detected	Detected	Detected	Compromised
3197	A_24_P487736	CXorf23	Hs.28896	chromosome X open reading frame 23	Homo sapiens chromosome X open reading frame 23 (CXorf23), mRNA [NM_198279]	2.14038	up	1.90982	up	429.28625	207.67703	268.02400	123.75030	Detected	Detected	Detected	Detected
3198	A_33_P3322230	FLJ31945	Hs.183953	hypothetical protein LOC440137	PREDICTED: Homo sapiens hypothetical protein LOC440137 (FLJ31945), mRNA [XM_001716811]	2.14036	up	2.14626	up	54.91789	26.56804	24.25895	9.96677	Detected	Detected	Detected	Compromised
3199	A_23_P88817	MLYCD	Hs.644610	malonyl-CoA decarboxylase	Homo sapiens malonyl-CoA decarboxylase (MLYCD), nuclear gene encoding mitochondrial protein, mRNA [NM_012213]	2.13983	up	1.98643	up	462.10703	223.61217	495.25223	219.84604	Detected	Detected	Detected	Detected
3200	A_23_P380881	ANKRD13B	Hs.662164	ankyrin repeat domain 13B	Homo sapiens ankyrin repeat domain 13B (ANKRD13B), mRNA [NM_152345]	2.13973	up	2.30111	up	75.89824	36.72879	80.31434	30.77660	Detected	Detected	Detected	Detected
3201	A_24_P122337	SYTL4	Hs.592224	synaptotagmin-like 4	Homo sapiens synaptotagmin-like 4 (SYTL4), transcript variant 1, mRNA [NM_080737]	2.13757	up	2.05116	up	192.51208	93.25483	137.57982	59.14547	Detected	Detected	Detected	Detected
3202	A_33_P3251480	MAPKSP1	Hs.433332	MAPK scaffold protein 1	Homo sapiens MAPK scaffold protein 1 (MAPKSP1), transcript variant 1, mRNA [NM_021970]	2.13672	up	1.71480	up	17.55518	8.50727	14.09272	7.24681	Detected	Compromised	Compromised	Compromised
3203	A_23_P204736	GPD1	Hs.524418	glycerol-3-phosphate dehydrogenase 1 (soluble)	Homo sapiens glycerol-3-phosphate dehydrogenase 1 (soluble) (GPD1), mRNA [NM_005276]	2.13629	up	1.12870	up	85.94099	41.65553	29.70165	23.20415	Detected	Detected	Detected	Detected
3204	A_33_P3216601	FHIT	Hs.655995	fragile histidine triad gene	Homo sapiens fragile histidine triad gene (FHIT), transcript variant 1, mRNA [NM_002012]	2.13624	up	2.64866	up	22.58867	10.94896	25.03205	8.33366	Detected	Compromised	Detected	Compromised
3205	A_24_P920646		Hs.260074		Cytochrome P450 19A1 (EC 1.14.14.1)(CYPXIX)(Aromatase)(Estrogen synthetase)(P-450AROM) [Source:UniProtKB/Swiss-Prot;Acc:P11511] [ENST00000405913]	2.13616	up	6.00521	up	35.04839	16.98896	20.52118	3.01328	Detected	Compromised	Detected	Compromised
3206	A_24_P280113	IL13RA1	Hs.496646	interleukin 13 receptor, alpha 1	Homo sapiens interleukin 13 receptor, alpha 1 (IL13RA1), mRNA [NM_001560]	2.13493	up	2.06055	up	48419.90200	23484.04000	33455.41400	14316.92700	Detected	Detected	Detected	Detected
3207	A_32_P110016	LOC727869	Hs.647210	hypothetical LOC727869	PREDICTED: Homo sapiens hypothetical LOC727869 (LOC727869), mRNA [XM_001128267]	2.13476	up	5.31240	up	26.83171	13.01468	14.70460	2.44078	Detected	Compromised	Detected	Compromised
3208	A_33_P3576853	LOC254128	Hs.712685	hypothetical protein LOC254128	Homo sapiens cDNA FLJ40945 fis, clone UTERU2008747 [AK098264]	2.13451	up	1.74805	up	90.14577	43.73006	110.16708	55.57293	Detected	Detected	Detected	Detected
3209	A_23_P139669	SLC2A3	Hs.419240	solute carrier family 2 (facilitated glucose transporter), member 3	Homo sapiens solute carrier family 2 (facilitated glucose transporter), member 3 (SLC2A3), mRNA [NM_006931]	2.13352	up	2.05521	up	305.25296	148.14822	183.16533	78.58719	Detected	Detected	Detected	Detected
3210	A_24_P46130	ACPP	Hs.433060	acid phosphatase, prostate	Homo sapiens acid phosphatase, prostate (ACPP), transcript variant 1, mRNA [NM_001099]	2.13339	up	1.86613	up	133.56331	64.82611	78.06742	36.88866	Detected	Detected	Detected	Detected
3211	A_23_P121898	PARP8	Hs.369581	poly (ADP-ribose) polymerase family, member 8	Homo sapiens poly (ADP-ribose) polymerase family, member 8 (PARP8), mRNA [NM_024615]	2.13304	up	1.96834	up	91.81804	44.57198	71.21668	31.90408	Detected	Detected	Detected	Detected
3212	A_24_P305570	RIN2	Hs.472270	Ras and Rab interactor 2	Homo sapiens Ras and Rab interactor 2 (RIN2), mRNA [NM_018993]	2.13185	up	2.26554	up	18160.27500	8820.61000	13562.50400	5278.78300	Detected	Detected	Detected	Detected
3213	A_23_P94095	ANKRD46	Hs.530199	ankyrin repeat domain 46	Homo sapiens ankyrin repeat domain 46 (ANKRD46), mRNA [NM_198401]	2.13126	up	2.36520	up	554.30210	269.30377	352.58594	131.45080	Detected	Detected	Detected	Detected

3214	A_33_P3364060	HR	Hs.272367	hairless homolog (mouse)	Homo sapiens hairless homolog (mouse) (HR), transcript variant 1, mRNA [NM_005144]	2.13076	up	3.56265	up	3319.86740	1613.31260	2510.93040	621.47906	Detected	Detected	Detected	Detected
3215	A_33_P3261167	GNS	Hs.334534	glucosamine (N-acetyl)-6-sulfatase	Homo sapiens glucosamine (N-acetyl)-6-sulfatase (GNS), mRNA [NM_002076]	2.13076	up	1.91835	up	46090.59000	22398.13300	38425.40000	17662.64500	Detected	Detected	Detected	Detected
3216	A_33_P3371954	IGSF8	Hs.332012	immunoglobulin superfamily, member 8	Homo sapiens immunoglobulin superfamily, member 8 (IGSF8), mRNA [NM_052868]	2.13067	up	1.84699	up	225.13606	109.41107	272.99155	130.33140	Detected	Detected	Detected	Detected
3217	A_33_P3243929					2.13051	up	1.37237	up	79.17230	38.47890	5.46459	3.51118	Detected	Detected	Compromised	Compromised
3218	A_33_P3340014	TRO	Hs.633653	trophinin	Homo sapiens trophinin (TRO), transcript variant 3, mRNA [NM_016157]	2.12974	up	2.44559	up	213.68541	103.89200	210.05148	75.73684	Detected	Detected	Detected	Detected
3219	A_24_P391230	CYYR1	Hs.37445	cysteine/tyrosine-rich 1	Homo sapiens cysteine/tyrosine-rich 1 (CYYR1), mRNA [NM_052954]	2.12908	up	2.65552	up	48.30590	23.49316	44.55092	14.79356	Detected	Detected	Detected	Detected
3220	A_33_P3371889	NUAK1	Hs.524692	NUAK family, SNF1-like kinase, 1	Homo sapiens NUAK family, SNF1-like kinase, 1 (NUAK1), mRNA [NM_014840]	2.12802	up	1.52520	up	24.93593	12.13341	12.90229	7.45943	Detected	Compromised	Compromised	Compromised
3221	A_23_P102842	NFS1	Hs.194692	NFS1 nitrogen fixation 1 homolog (S. cerevisiae)	Homo sapiens NFS1 nitrogen fixation 1 homolog (S. cerevisiae) (NFS1), nuclear gene encoding mitochondrial protein, mRNA [NM_021100]	2.12742	up	2.07165	up	2517.09600	1225.12290	2233.55620	950.70550	Detected	Detected	Detected	Detected
3222	A_23_P148584	DOCK11	Hs.368203	dedicator of cytokinesis 11	Homo sapiens dedicator of cytokinesis 11 (DOCK11), mRNA [NM_144658]	2.12702	up	2.33671	up	3784.85060	1842.51510	2198.25100	829.54120	Detected	Detected	Detected	Detected
3223	A_33_P3251841	DSEL	Hs.124673	dermatan sulfate epimerase-like	Homo sapiens dermatan sulfate epimerase-like (DSEL), mRNA [NM_032160]	2.12651	up	2.18357	up	4959.06450	2414.71240	2780.56270	1122.87330	Detected	Detected	Detected	Detected
3224	A_23_P131375	PQLC3	Hs.274415	PQ loop repeat containing 3	Homo sapiens PQ loop repeat containing 3 (PQLC3), mRNA [NM_152391]	2.12636	up	2.23661	up	2300.94340	1120.47680	1956.06770	771.18690	Detected	Detected	Detected	Detected
3225	A_33_P3508717	LOC729678	Hs.348292	hypothetical protein LOC729678	Homo sapiens hypothetical protein LOC729678 (LOC729678), non-coding RNA [NR_027183]	2.12555	up	2.02218	up	1390.70420	677.48140	1153.56800	503.02300	Detected	Detected	Detected	Detected
3226	A_23_P153098	HDHD2	Hs.465041	haloacid dehalogenase-like hydrolase domain containing 2	Homo sapiens haloacid dehalogenase-like hydrolase domain containing 2 (HDHD2), mRNA [NM_032124]	2.12530	up	2.26751	up	3714.59620	1809.77580	3528.47500	1372.15870	Detected	Detected	Detected	Detected
3227	A_23_P135742	C1orf170	Hs.271462	chromosome 1 open reading frame 170	Homo sapiens chromosome 1 open reading frame 170 (C1orf170), non-coding RNA [NR_027693]	2.12506	up	4.68264	up	35.56104	17.32753	20.51798	3.86375	Detected	Compromised	Detected	Compromised
3228	A_23_P34510	PHC2	Hs.524271	polyhomeotic homolog 2 (Drosophila)	Homo sapiens polyhomeotic homolog 2 (Drosophila) (PHC2), transcript variant 1, mRNA [NM_198040]	2.12405	up	1.65787	up	7617.77000	3713.61430	7480.42300	3978.70480	Detected	Detected	Detected	Detected
3229	A_24_P186342	ZFP14	Hs.35524	zinc finger protein 14 homolog (mouse)	Homo sapiens zinc finger protein 14 homolog (mouse) (ZFP14), mRNA [NM_020917]	2.12351	up	7.84806	up	37.18010	18.12965	22.31558	2.50733	Detected	Detected	Detected	Compromised
3230	A_32_P144596	TNKS	Hs.370267	tankyrase, TRF1-interacting ankyrin-related ADP-ribose polymerase	Homo sapiens tankyrase, TRF1-interacting ankyrin-related ADP-ribose polymerase (TNKS), mRNA [NM_003747]	2.12312	up	2.38484	up	627.73220	306.14856	523.99320	193.74545	Detected	Detected	Detected	Detected
3231	A_33_P3788772	LOC200609	Hs.635880	hypothetical protein LOC200609	Homo sapiens mRNA; cDNA DKFZp434E2221 (from clone DKFZp434E2221) [AL137458]	2.12296	up	1.86854	up	706.29150	344.48900	501.61115	236.71703	Detected	Detected	Detected	Detected
3232	A_24_P98613	TSPAN14	Hs.633177	tetraspanin 14	Homo sapiens tetraspanin 14 (TSPAN14), transcript variant 1, mRNA [NM_030927]	2.12130	up	2.16422	up	1398.45800	682.62366	1216.28870	495.56497	Detected	Detected	Detected	Detected
3233	A_23_P65278	NBEA	Hs.491172	neurobeachin	Homo sapiens neurobeachin (NBEA), mRNA [NM_015678]	2.12075	up	2.03336	up	847.98600	414.03125	574.71280	249.23145	Detected	Detected	Detected	Detected
3234	A_33_P3422260		Hs.148365		Putative uncharacterized protein ENSP00000363872 [Source:UniProtKB/TrEMBL;Acc:B7WPJ8] [ENST00000374740]	2.12074	up	1.79236	up	1450.88650	708.40076	180.22879	88.66742	Detected	Detected	Detected	Detected
3235	A_33_P3548860	SEC1		secretory blood group 1	Homo sapiens secretory blood group 1 (SEC1), non-coding RNA [NR_004401]	2.12018	up	1.50504	up	50.33355	24.58209	31.65206	18.54473	Detected	Detected	Detected	Detected
3236	A_24_P356338	GABARAPL2	Hs.461379	GABA(A) receptor-associated protein-like 2	Homo sapiens GABA(A) receptor-associated protein-like 2 (GABARAPL2), mRNA [NM_007285]	2.12004	up	2.14885	up	7008.46300	3423.04400	4467.54930	1833.28170	Detected	Detected	Detected	Detected
3237	A_33_P3292198	SPAG4L	Hs.375186	sperm associated antigen 4-like	Homo sapiens sperm associated antigen 4-like (SPAG4L), mRNA [NM_080675]	2.11994	up	1.82983	up	191.56349	93.56672	55.45942	26.72577	Detected	Detected	Detected	Detected
3238	A_23_P50646	LOC390940	Hs.22049	similar to R28379_1	Uncharacterized protein ENSP00000244321 Precursor [Source:UniProtKB/Swiss-Prot;Acc:A6NC86] [ENST00000244321]	2.11961	up	2.31546	up	536.68120	262.17697	465.61023	177.31725	Detected	Detected	Detected	Detected
3239	A_23_P216679	CDC14B	Hs.40582	CDC14 cell division cycle 14 homolog B (S. cerevisiae)	Homo sapiens CDC14 cell division cycle 14 homolog B (S. cerevisiae) (CDC14B), transcript variant 2, mRNA [NM_033331]	2.11951	up	2.36234	up	359.71826	175.73616	192.26530	71.76691	Detected	Detected	Detected	Detected
3240	A_33_P3338724	LENG9	Hs.590976	leukocyte receptor cluster (LRC) member 9	Homo sapiens leukocyte receptor cluster (LRC) member 9 (LENG9), mRNA [NM_198988]	2.11804	up	1.45986	up	411.04980	200.95248	176.01780	106.31888	Detected	Detected	Detected	Detected
3241	A_24_P224998				60S ribosomal protein L36a (60S ribosomal protein L44)(Cell migration-inducing gene 6 protein) [Source:UniProtKB/Swiss-Prot;Acc:P83881] [ENST00000392994]	2.11744	up	2.21126	up	677.19660	331.15933	456.86157	182.18431	Detected	Detected	Detected	Detected
3242	A_23_P127460	SIPA1	Hs.530477	signal-induced proliferation-associated 1	Homo sapiens signal-induced proliferation-associated 1 (SIPA1), transcript variant 1, mRNA [NM_153253]	2.11722	up	1.75194	up	1620.72770	792.64150	1803.80990	907.89560	Detected	Detected	Detected	Detected

3243	A_33_P3227284	OGT	Hs.405410	O-linked N-acetylglucosamine (GlcNAc) transferase (UDP-N-acetylglucosamine:polypeptide-N-acetylglucosaminyl transferase)	Homo sapiens O-linked N-acetylglucosamine (GlcNAc) transferase (UDP-N-acetylglucosamine:polypeptide-N-acetylglucosaminyl transferase) (OGT), transcript variant 1, mRNA [NM_181672]	2.11665	up	2.15029	up	9193.78900	4497.57760	7066.98700	2898.02900	Detected	Detected	Detected	Detected
3244	A_23_P347198	SP3	Hs.531587	Sp3 transcription factor	Homo sapiens Sp3 transcription factor (SP3), transcript variant 1, mRNA [NM_003111]	2.11603	up	2.14972	up	9267.34500	4534.89200	5312.06900	2178.95430	Detected	Detected	Detected	Detected
3245	A_33_P3297444	ABTB1	Hs.107812	ankyrin repeat and BTB (POZ) domain containing 1	Homo sapiens ankyrin repeat and BTB (POZ) domain containing 1 (ABTB1), transcript variant 2, mRNA [NM_172027]	2.11537	up	1.91441	up	9201.92400	4504.28120	8017.05570	3692.70400	Detected	Detected	Detected	Detected
3246	A_33_P3351615					2.11458	up	1.80931	up	60103.97000	29431.48600	36331.47700	17706.60000	Detected	Detected	Detected	Detected
3247	A_33_P3323368	FLJ39639	Hs.592092	hypothetical protein FLJ39639	PREDICTED: Homo sapiens hypothetical protein FLJ39639 (FLJ39639), mRNA [XM_001715751]	2.11345	up	1.98516	up	172.18156	84.35819	98.81339	43.89198	Detected	Detected	Detected	Detected
3248	A_23_P153853	ECH1	Hs.196176	enoyl Coenzyme A hydratase 1, peroxisomal	Homo sapiens enoyl Coenzyme A hydratase 1, peroxisomal (ECH1), mRNA [NM_001398]	2.11226	up	1.89778	up	3190.86820	1564.21000	3705.49270	1721.73010	Detected	Detected	Detected	Detected
3249	A_23_P13548	CHRDL2	Hs.432379	chordin-like 2	Homo sapiens chordin-like 2 (CHRDL2), mRNA [NM_015424]	2.11219	up	2.24905	up	25.25022	12.37841	13.76165	5.39557	Detected	Compromised	Detected	Compromised
3250	A_32_P507710	PI4KAP2	Hs.592456	phosphatidylinositol 4-kinase, catalytic, alpha pseudogene 2	Homo sapiens phosphatidylinositol 4-kinase, catalytic, alpha pseudogene 2 (PI4KAP2), non-coding RNA [NR_003700]	2.11209	up	1.34729	up	345.33215	169.29994	388.98096	254.58572	Detected	Detected	Detected	Detected
3251	A_23_P19348	CUL7	Hs.520136	cullin 7	Homo sapiens cullin 7 (CUL7), mRNA [NM_014780]	2.11193	up	1.92052	up	639.09440	313.34204	583.27795	267.80670	Detected	Detected	Detected	Detected
3252	A_32_P34552	POLB	Hs.654484	polymerase (DNA directed), beta	Homo sapiens polymerase (DNA directed), beta (POLB), mRNA [NM_002690]	2.11159	up	2.44030	up	290.09350	142.25322	186.35555	67.33865	Detected	Detected	Detected	Detected
3253	A_33_P3304794		Hs.130286		Homo sapiens cDNA FLJ39005 fis, clone NT2RI2024496. [AK096324]	2.10965	up	3.24788	up	145.62949	71.47785	99.06617	26.89624	Detected	Detected	Detected	Detected
3254	A_33_P3216955	C10orf104	Hs.426296	chromosome 10 open reading frame 104	Homo sapiens chromosome 10 open reading frame 104 (C10orf104), mRNA [NM_173473]	2.10938	up	2.16174	up	383.90213	188.45122	329.72750	134.49852	Detected	Detected	Detected	Detected
3255	A_23_P24004	IFIT2	Hs.437609	interferon-induced protein with tetratricopeptide repeats 2	Homo sapiens interferon-induced protein with tetratricopeptide repeats 2 (IFIT2), mRNA [NM_001547]	2.10905	up	1.94500	up	400.81253	196.78328	391.67624	177.57138	Detected	Detected	Detected	Detected
3256	A_33_P3351207	MGC21881	Hs.721511	hypothetical locus MGC21881	Homo sapiens hypothetical locus MGC21881 (MGC21881), non-coding RNA [NR_015363]	2.10872	up	2.07298	up	2234.59600	1097.26930	1592.76810	677.52260	Detected	Detected	Detected	Detected
3257	A_32_P219368	WTAP	Hs.446091	Wilms tumor 1 associated protein	Homo sapiens Wilms tumor 1 associated protein (WTAP), transcript variant 3, mRNA [NM_152858]	2.10823	up	2.26872	up	2944.54980	1446.21560	1680.60680	653.20660	Detected	Detected	Detected	Detected
3258	A_23_P64898	KLRG1	Hs.558446	killer cell lectin-like receptor subfamily G, member 1	Homo sapiens killer cell lectin-like receptor subfamily G, member 1 (KLRG1), mRNA [NM_005810]	2.10754	up	2.65571	up	42.03659	20.65307	26.95997	8.95167	Detected	Detected	Detected	Compromised
3259	A_23_P162378	CCDC41	Hs.279209	coiled-coil domain containing 41	Homo sapiens coiled-coil domain containing 41 (CCDC41), transcript variant 1, mRNA [NM_016122]	2.10739	up	2.10726	up	796.97390	391.59027	675.25570	282.56330	Detected	Detected	Detected	Detected
3260	A_23_P375354	TECTA	Hs.248162	tectorin alpha	Homo sapiens tectorin alpha (TECTA), mRNA [NM_005422]	2.10736	up	1.91614	up	13.04646	6.41044	5.47233	2.51831	Detected	Compromised	Compromised	Compromised
3261	A_23_P169887	GTF3C2	Hs.75782	general transcription factor IIIC, polypeptide 2, beta 110kDa	Homo sapiens general transcription factor IIIC, polypeptide 2, beta 110kDa (GTF3C2), transcript variant 1, mRNA [NM_001521]	2.10730	up	1.83518	up	4786.27200	2351.81710	4088.15280	1964.32560	Detected	Detected	Detected	Detected
3262	A_24_P268160	DRAM2	Hs.485606	DNA-damage regulated autophagy modulator 2	Homo sapiens DNA-damage regulated autophagy modulator 2 (DRAM2), mRNA [NM_178454]	2.10730	up	2.13402	up	3250.51460	1597.20060	2901.04610	1198.73240	Detected	Detected	Detected	Detected
3263	A_23_P356646	LOC100132288	Hs.487562	hypothetical protein LOC100132288	Homo sapiens hypothetical protein LOC100132288 (LOC100132288), mRNA [NM_001033515]	2.10666	up	2.33791	up	181.26780	89.09624	142.53410	53.75972	Detected	Detected	Detected	Detected
3264	A_23_P323180	HOXD3	Hs.93574	homeobox D3	Homo sapiens homeobox D3 (HOXD3), mRNA [NM_006898]	2.10624	up	2.05313	up	261.70737	128.65921	254.78667	109.42741	Detected	Detected	Detected	Detected
3265	A_33_P3815064	LOC283575	Hs.651736	hypothetical protein LOC283575	Homo sapiens cDNA FLJ35003 fis, clone OCBBF2011960 [AK092322]	2.10483	up	2.03369	up	62.93807	30.96203	66.20854	28.70741	Detected	Detected	Detected	Detected
3266	A_23_P42322	COL11A2	Hs.390171	collagen, type XI, alpha 2	Homo sapiens collagen, type XI, alpha 2 (COL11A2), transcript variant 1, mRNA [NM_080680]	2.10392	up	3.32671	up	148.73825	73.20255	117.34801	31.10476	Detected	Detected	Detected	Detected
3267	A_24_P368735	ARL10	Hs.424552	ADP-ribosylation factor-like 10	Homo sapiens ADP-ribosylation factor-like 10 (ARL10), mRNA [NM_173664]	2.10362	up	1.29281	up	14.19495	6.98716	19.09092	13.02139	Detected	Compromised	Detected	Compromised
3268	A_33_P3249364		Hs.401954		Transmembrane and TPR repeat-containing protein 1 [Source:UniProtKB/Swiss-Prot;Acc:Q8IUR5] [ENST00000381224]	2.10356	up	3.30269	up	81.02348	39.88319	71.09286	18.98118	Detected	Detected	Detected	Detected
3269	A_24_P142151	LRRC28	Hs.578684	leucine rich repeat containing 28	Homo sapiens leucine rich repeat containing 28 (LRRC28), mRNA [NM_144598]	2.10326	up	2.30267	up	430.52588	211.95271	512.29960	196.18115	Detected	Detected	Detected	Detected
3270	A_33_P3332865	NPEPL1	Hs.654868	aminopeptidase-like 1	Homo sapiens aminopeptidase-like 1 (NPEPL1), mRNA [NM_024663]	2.10315	up	1.85615	up	5005.97500	2464.63380	4344.52250	2063.92820	Detected	Detected	Detected	Detected
3271	A_23_P253012	GRAMD1C	Hs.24583	GRAM domain containing 1C	Homo sapiens GRAM domain containing 1C (GRAMD1C), mRNA [NM_017577]	2.10314	up	2.04687	up	3775.88180	1859.01660	3040.44820	1309.82280	Detected	Detected	Detected	Detected
3272	A_24_P41975	TDRKH	Hs.144439	tudor and KH domain containing	Homo sapiens tudor and KH domain containing (TDRKH), transcript variant 1, mRNA [NM_001083965]	2.10272	up	1.64037	up	65.60009	32.30399	52.24258	28.08325	Detected	Detected	Detected	Detected

3273	A_33_P3359953	LOC100131825	Hs.701271	hypothetical protein LOC100131825	PREDICTED: Homo sapiens hypothetical protein LOC100131825 (LOC100131825), miscRNA [XR_079485]	2.10262	up	1.33542	up	13.18120	6.49123	3.78093	2.49659	Detected	Compromised	Compromised	Compromised
3274	A_23_P154806	EPB41L1	Hs.704100	erythrocyte membrane protein band 4.1-like 1	Homo sapiens erythrocyte membrane protein band 4.1-like 1 (EPB41L1), transcript variant 1, mRNA [NM_012156]	2.10158	up	2.17350	up	8519.12200	4197.41940	6281.65530	2548.47460	Detected	Detected	Detected	Detected
3275	A_23_P371266	DNM3	Hs.654775	dynamin 3	Homo sapiens dynamin 3 (DNM3), transcript variant 1, mRNA [NM_015569]	2.10152	up	2.47757	up	382.21167	188.32277	300.72693	107.03173	Detected	Detected	Detected	Detected
3276	A_23_P210675	SYCP2	Hs.202676	synaptonemal complex protein 2	Homo sapiens synaptonemal complex protein 2 (SYCP2), mRNA [NM_014258]	2.10096	up	1.75975	up	55.64732	27.42587	28.83688	14.44981	Detected	Detected	Detected	Compromised
3277	A_23_P127186	TACC2	Hs.501252	transforming, acidic coiled-coil containing protein 2	Homo sapiens transforming, acidic coiled-coil containing protein 2 (TACC2), transcript variant 1, mRNA [NM_206862]	2.10073	up	2.07764	up	1574.31880	775.98860	1291.58130	548.17130	Detected	Detected	Detected	Detected
3278	A_33_P3329958	PYY	Hs.169249	peptide YY	Homo sapiens peptide YY (PYY), mRNA [NM_004160]	2.10051	up	6.28295	up	20.41986	10.06611	21.49755	3.01711	Detected	Compromised	Detected	Compromised
3279	A_23_P103932	FGR	Hs.1422	Gardner-Rasheed feline sarcoma viral (v-fgr) oncogene homolog	Homo sapiens Gardner-Rasheed feline sarcoma viral (v-fgr) oncogene homolog (FGR), transcript variant 2, mRNA [NM_001042747]	2.10045	up	2.79721	up	165.66338	81.66721	106.41331	33.54570	Detected	Detected	Detected	Detected
3280	A_33_P3257861	SARDH	Hs.198003	sarcosine dehydrogenase	Homo sapiens sarcosine dehydrogenase (SARDH), nuclear gene encoding mitochondrial protein, transcript variant 2, mRNA [NM_001134707]	2.10007	up	1.40920	up	79.09638	38.99924	82.24799	51.46558	Detected	Detected	Detected	Detected
3281	A_24_P873764	BCR	Hs.517461	breakpoint cluster region	Homo sapiens breakpoint cluster region (BCR), transcript variant 1, mRNA [NM_004327]	2.09980	up	1.86604	up	3972.70750	1959.03750	3344.45140	1580.40500	Detected	Detected	Detected	Detected
3282	A_33_P3390236	LOC338963	Hs.254464	hypothetical protein LOC338963	PREDICTED: Homo sapiens hypothetical protein LOC338963 (LOC338963), miscRNA [XR_040082]	2.09977	up	3.47263	up	127.66668	62.95638	89.69248	22.77526	Detected	Detected	Detected	Detected
3283	A_23_P391396	EBF3	Hs.591374	early B-cell factor 3	Homo sapiens early B-cell factor 3 (EBF3), mRNA [NM_001005463]	2.09936	up	2.16334	up	1128.68080	556.69556	885.29020	360.84976	Detected	Detected	Detected	Detected
3284	A_33_P3237580		Hs.434138		HCG2045601cDNA FLJ45513 fis, clone BRTHA2021450 ; [Source:UniProtKB/TrEMBL;Acc:Q6ZSH9] [ENST00000376609]	2.09910	up	1.11032	up	12.77991	6.30416	3.14430	2.49713	Detected	Compromised	Compromised	Compromised
3285	A_32_P192594	LOC400099	Hs.400432	hypothetical LOC400099	PREDICTED: Homo sapiens hypothetical LOC400099 (LOC400099), miscRNA [XR_040794]	2.09814	up	2.19304	up	10585.88300	5224.27150	8349.17100	3357.08570	Detected	Detected	Detected	Detected
3286	A_23_P200030	FPGT	Hs.480085	fucose-1-phosphate guanylyltransferase	Homo sapiens fucose-1-phosphate guanylyltransferase (FPGT), mRNA [NM_003838]	2.09770	up	1.90620	up	257.78476	127.24680	209.97437	97.13223	Detected	Detected	Detected	Detected
3287	A_33_P3330826	LOC100132077	Hs.679111	hypothetical protein LOC100132077	Homo sapiens cDNA FLJ37869 fis, clone BRSSN2017422 [AK095188]	2.09718	up	2.30196	up	26.70653	13.18604	25.66311	9.83053	Detected	Compromised	Detected	Compromised
3288	A_23_P138541	AKR1C3	Hs.78183	aldo-keto reductase family 1, member C3 (3-alpha hydroxysteroid dehydrogenase, type II)	Homo sapiens aldo-keto reductase family 1, member C3 (3-alpha hydroxysteroid dehydrogenase, type II) (AKR1C3), mRNA [NM_003739]	2.09711	up	3.50708	up	27028.15800	13345.30200	16328.14550	4105.40620	Detected	Detected	Detected	Detected
3289	A_32_P231493	LOC100129122	Hs.344872	hypothetical LOC100129122	PREDICTED: Homo sapiens hypothetical LOC100129122 (LOC100129122), mRNA [XM_001714405]	2.09697	up	1.90089	up	1242.42090	613.49350	1177.88610	546.40190	Detected	Detected	Detected	Detected
3290	A_23_P141092	TFAP4	Hs.513305	transcription factor AP-4 (activating enhancer binding protein 4)	Homo sapiens transcription factor AP-4 (activating enhancer binding protein 4) (TFAP4), mRNA [NM_003223]	2.09673	up	1.06114	up	20.41479	10.08173	12.45214	10.34755	Detected	Compromised	Compromised	Compromised
3291	A_24_P933418	ABI2	Hs.471156	abl-interactor 2	Homo sapiens abl-interactor 2 (ABI2), mRNA [NM_005759]	2.09610	up	2.01290	up	558.16284	275.72900	280.35745	122.81622	Detected	Detected	Detected	Detected
3292	A_33_P3221868	TMEM216	Hs.26745	transmembrane protein 216	Homo sapiens transmembrane protein 216 (TMEM216), mRNA [NM_016499]	2.09512	up	2.07842	up	999.85180	494.15182	1091.54170	463.09872	Detected	Detected	Detected	Detected
3293	A_23_P20022	C7orf68	Hs.710088	chromosome 7 open reading frame 68	Homo sapiens chromosome 7 open reading frame 68 (C7orf68), transcript variant 1, mRNA [NM_013332]	2.09464	up	2.11409	up	7732.06150	3822.25300	7576.07370	3159.98900	Detected	Detected	Detected	Detected
3294	A_23_P502170	DYNC2LI1	Hs.371597	dynein, cytoplasmic 2, light intermediate chain 1	Homo sapiens dynein, cytoplasmic 2, light intermediate chain 1 (DYNC2LI1), transcript variant 2, mRNA [NM_015522]	2.09451	up	2.13944	up	501.68180	248.01535	386.75058	159.40321	Detected	Detected	Detected	Detected
3295	A_33_P3360728	BLVRB	Hs.515785	biliverdin reductase B (flavin reductase (NADPH))	Homo sapiens biliverdin reductase B (flavin reductase (NADPH)) (BLVRB), mRNA [NM_000713]	2.09308	up	2.23772	up	14791.13000	7317.26860	14110.56100	5560.36470	Detected	Detected	Detected	Detected
3296	A_23_P167358	C4orf29	Hs.445817	chromosome 4 open reading frame 29	Homo sapiens chromosome 4 open reading frame 29 (C4orf29), mRNA [NM_001039717]	2.09108	up	1.81555	up	152.10526	75.31940	144.33481	70.10152	Detected	Detected	Detected	Detected
3297	A_33_P3238250	F11R	Hs.517293	F11 receptor	Homo sapiens F11 receptor (F11R), mRNA [NM_016946]	2.08992	up	1.44797	up	26.57830	13.16831	16.69701	10.16820	Detected	Compromised	Detected	Compromised
3298	A_33_P3287058		Hs.554395		Homo sapiens cDNA FLJ30719 fis, clone FCBBF2001718. [AK055281]	2.08965	up	2.09342	up	24.77448	12.27622	8.27130	3.48404	Detected	Compromised	Compromised	Compromised
3299	A_33_P3298634	NFS1	Hs.194692	NFS1 nitrogen fixation 1 homolog (S. cerevisiae)	Homo sapiens NFS1 nitrogen fixation 1 homolog (S. cerevisiae) (NFS1), nuclear gene encoding mitochondrial protein, mRNA [NM_021100]	2.08873	up	1.71908	up	107.45822	53.27097	100.38499	51.49176	Detected	Detected	Detected	Detected
3300	A_33_P3616021	LOC100292961	Hs.397403	hypothetical protein LOC100292961	PREDICTED: Homo sapiens hypothetical protein LOC100292961 (LOC100292961), mRNA [XM_002345453]	2.08817	up	7.82464	up	51.55629	25.56512	25.89193	2.91787	Detected	Detected	Detected	Compromised
3301	A_24_P228130	CCL3L3	Hs.512304	chemokine (C-C motif) ligand 3-like 3	Homo sapiens chemokine (C-C motif) ligand 3-like 3 (CCL3L3), mRNA [NM_001001437]	2.08813	up	4.03364	up	18.60374	9.22518	26.48538	5.78994	Detected	Compromised	Detected	Compromised

3302	A_33_P3403927	SAMD10	Hs.27189	sterile alpha motif domain containing 10	Homo sapiens sterile alpha motif domain containing 10 (SAMD10), mRNA [NM_080621]	2.08703	up	2.16252	up	382.39868	189.72308	345.49110	140.87752	Detected	Detected	Detected	Detected
3303	A_23_P350754	OR7E13P	Hs.684491	olfactory receptor, family 7, subfamily E, member 13 pseudogene	Homo sapiens olfactory-like receptor PJCG2 (PJCG2) mRNA, partial cds [AF238487]	2.08546	up	2.71764	up	321.41913	159.58899	223.20198	72.42220	Detected	Detected	Detected	Detected
3304	A_33_P3230588					2.08482	up	1.31709	up	15.43248	7.66479	11.22353	7.51414	Detected	Compromised	Compromised	Compromised
3305	A_23_P364766	C20orf94	Hs.668782	chromosome 20 open reading frame 94	Homo sapiens chromosome 20 open reading frame 94 (C20orf94), mRNA [NM_001009608]	2.08454	up	1.83128	up	211.12790	104.87419	119.44868	57.51648	Detected	Detected	Detected	Detected
3306	A_23_P37375	RPS6KA5	Hs.510225	ribosomal protein S6 kinase, 90kDa, polypeptide 5	Homo sapiens ribosomal protein S6 kinase, 90kDa, polypeptide 5 (RPS6KA5), transcript variant 1, mRNA [NM_004755]	2.08441	up	2.08993	up	1246.95360	619.44226	869.69293	366.94443	Detected	Detected	Detected	Detected
3307	A_33_P3254291	CNOT4	Hs.490224	CCR4-NOT transcription complex, subunit 4	Homo sapiens CCR4-NOT transcription complex, subunit 4 (CNOT4), transcript variant 2, mRNA [NM_001008225]	2.08397	up	2.24363	up	73.78885	36.66340	47.89127	18.82221	Detected	Detected	Detected	Detected
3308	A_33_P3396807	ATG4C	Hs.7353	ATG4 autophagy related 4 homolog C (S. cerevisiae)	Homo sapiens ATG4 autophagy related 4 homolog C (S. cerevisiae) (ATG4C), transcript variant 7, mRNA [NM_032852]	2.08336	up	1.97232	up	438.02023	217.70232	410.59763	183.57138	Detected	Detected	Detected	Detected
3309	A_23_P381102	CCDC74B	Hs.29383	coiled-coil domain containing 74B	Homo sapiens coiled-coil domain containing 74B (CCDC74B), mRNA [NM_207310]	2.08225	up	2.03211	up	3369.48930	1675.57400	2140.37550	928.76940	Detected	Detected	Detected	Detected
3310	A_23_P163161	SDR39U1	Hs.643552	short chain dehydrogenase/reductase family 39U, member 1	Homo sapiens short chain dehydrogenase/reductase family 39U, member 1 (SDR39U1), mRNA [NM_020195]	2.08151	up	1.88883	up	2174.32420	1081.63180	2497.01170	1165.71640	Detected	Detected	Detected	Detected
3311	A_23_P7253	LARP1B	Hs.657067	La ribonucleoprotein domain family, member 1B	Homo sapiens La ribonucleoprotein domain family, member 1B (LARP1B), transcript variant 1, mRNA [NM_018078]	2.08144	up	2.21035	up	274.13260	136.37361	212.97728	84.96464	Detected	Detected	Detected	Detected
3312	A_33_P3289406	LOC91450	Hs.406766	hypothetical protein LOC91450	Homo sapiens hypothetical protein LOC91450 (LOC91450), non-coding RNA [NR_026998]	2.08142	up	2.19734	up	378.09448	188.09303	264.57608	106.17438	Detected	Detected	Detected	Detected
3313	A_33_P3381943	DMTF1	Hs.196129	cyclin D binding myb-like transcription factor 1	Homo sapiens cyclin D binding myb-like transcription factor 1 (DMTF1), transcript variant 4, transcribed RNA [NR_024549]	2.08070	up	1.94929	up	107.38522	53.44023	103.71880	46.91865	Detected	Detected	Detected	Detected
3314	A_32_P131367	RNF13	Hs.12333	ring finger protein 13	Homo sapiens ring finger protein 13 (RNF13), transcript variant 1, mRNA [NM_007282]	2.08065	up	2.32722	up	1172.52820	583.52210	827.56290	313.56644	Detected	Detected	Detected	Detected
3315	A_24_P174755	SLC22A5	Hs.443572	solute carrier family 22 (organic cation/carnitine transporter), member 5	Homo sapiens solute carrier family 22 (organic cation/carnitine transporter), member 5 (SLC22A5), mRNA [NM_003060]	2.08042	up	2.20833	up	440.23040	219.11003	343.73315	137.25330	Detected	Detected	Detected	Detected
3316	A_24_P314179	ETS2	Hs.644231	v-ets erythroblastosis virus E26 oncogene homolog 2 (avian)	Homo sapiens v-ets erythroblastosis virus E26 oncogene homolog 2 (avian) (ETS2), mRNA [NM_005239]	2.07965	up	1.98134	up	3419.79830	1702.72220	2546.61380	1133.36330	Detected	Detected	Detected	Detected
3317	A_23_P105833	BIVM	Hs.288809	basic, immunoglobulin-like variable motif containing	Homo sapiens basic, immunoglobulin-like variable motif containing (BIVM), transcript variant 1, mRNA [NM_017693]	2.07842	up	2.09045	up	1253.26040	624.37010	914.37384	385.69950	Detected	Detected	Detected	Detected
3318	A_32_P201979	HECTD2	Hs.596096	HECT domain containing 2	Homo sapiens HECT domain containing 2 (HECTD2), transcript variant 1, mRNA [NM_182765]	2.07773	up	2.16196	up	674.49774	336.14290	453.95470	185.15290	Detected	Detected	Detected	Detected
3319	A_33_P3404671	LOC100130557		hypothetical LOC100130557	Homo sapiens hypothetical LOC100130557 (LOC100130557), non-coding RNA [NR_024567]	2.07767	up	2.18242	up	207.74046	103.53269	183.79085	74.25951	Detected	Detected	Detected	Detected
3320	A_23_P209499	PAX3	Hs.42146	paired box 3	Homo sapiens paired box 3 (PAX3), transcript variant PAX3D, mRNA [NM_181458]	2.07682	up	2.20007	up	1332.77940	664.49690	794.92505	318.60712	Detected	Detected	Detected	Detected
3321	A_33_P3249529	PCNX	Hs.446559	pecanex homolog (Drosophila)	Homo sapiens pecanex homolog (Drosophila) (PCNX), mRNA [NM_014982]	2.07660	up	5.47695	up	17399.60700	8676.00500	4817.22600	775.57477	Detected	Detected	Detected	Detected
3322	A_33_P3308740	RANBP2	Hs.199561	RAN binding protein 2	Homo sapiens RAN binding protein 2 (RANBP2), mRNA [NM_006267]	2.07643	up	2.45826	up	12.46262	6.21478	7.49281	2.68771	Detected	Compromised	Compromised	Compromised
3323	A_23_P77661	ZNF720	Hs.528826	zinc finger protein 720	Homo sapiens zinc finger protein 720 (ZNF720), mRNA [NM_001130913]	2.07568	up	2.36914	up	688.19830	343.30988	485.96234	180.87448	Detected	Detected	Detected	Detected
3324	A_33_P3411145	LOC285550	Hs.399980	hypothetical protein LOC285550	Homo sapiens hypothetical protein LOC285550 (LOC285550), mRNA [NM_001145191]	2.07494	up	1.86970	up	260.54843	130.02193	213.31825	100.60561	Detected	Detected	Detected	Detected
3325	A_33_P3407049					2.07438	up	2.06035	up	184.34400	92.01822	135.83720	58.13566	Detected	Detected	Detected	Detected
3326	A_33_P3233005	MBTD1	Hs.656803	mbt domain containing 1	Homo sapiens mbt domain containing 1 (MBTD1), mRNA [NM_017643]	2.07425	up	1.98260	up	55.26387	27.58754	40.83978	18.16411	Detected	Detected	Detected	Detected
3327	A_33_P3323544	LOC100130764		p150-like	PREDICTED: Homo sapiens p150-like (LOC100130764), mRNA [XM_001723337]	2.07413	up	2.21412	up	37.95797	18.94957	6.77445	2.69797	Detected	Detected	Compromised	Compromised
3328	A_23_P332399	GULP1	Hs.470887	GULP, engulfment adaptor PTB domain containing 1	Homo sapiens GULP, engulfment adaptor PTB domain containing 1 (GULP1), mRNA [NM_016315]	2.07363	up	2.17942	up	2335.25660	1166.09950	1951.23900	789.46950	Detected	Detected	Detected	Detected
3329	A_33_P3407042	B3GALT6	Hs.284284	UDP-Gal:betaGal beta 1,3-galactosyltransferase polypeptide 6	Homo sapiens UDP-Gal:betaGal beta 1,3-galactosyltransferase polypeptide 6 (B3GALT6), mRNA [NM_080605]	2.07335	up	2.14618	up	7329.06100	3660.23340	7649.73800	3143.00420	Detected	Detected	Detected	Detected
3330	A_24_P194886	EHBP1	Hs.271667	EH domain binding protein 1	Homo sapiens EH domain binding protein 1 (EHBP1), transcript variant 1, mRNA [NM_015252]	2.07327	up	1.41153	up	385.78693	192.67505	275.67578	172.21582	Detected	Detected	Detected	Detected
3331	A_32_P177955	LOC441461	Hs.163155	hypothetical LOC441461	PREDICTED: Homo sapiens hypothetical LOC441461 (LOC441461), miscRNA [XR_041357]	2.07273	up	1.80994	up	78.00308	38.96744	51.08580	24.88862	Detected	Detected	Detected	Detected

3332	A_24_P72518	AHCYL2	Hs.600789	adenosylhomocysteinase-like 2	Homo sapiens adenosylhomocysteinase-like 2 (AHCYL2), transcript variant 1, mRNA [NM_015328]	2.07255	up	2.26396	up	1300.92980	649.95260	757.03520	294.85858	Detected	Detected	Detected	Detected
3333	A_24_P234856	CAMLG	Hs.529846	calcium modulating ligand	Homo sapiens calcium modulating ligand (CAMLG), mRNA [NM_001745]	2.07224	up	2.23000	up	508.52646	254.10118	264.69626	104.66673	Detected	Detected	Detected	Detected
3334	A_33_P3620246		Hs.635230		Putative uncharacterized protein ENSP00000344626 Fragment [Source:UniProtKB/TrEMBL;Acc:A6NL00] [ENST00000395215]	2.07216	up	2.97065	up	87.09316	43.52060	38.84401	11.53025	Detected	Detected	Detected	Compromised
3335	A_23_P201979	CREM	Hs.200250	cAMP responsive element modulator	Homo sapiens cAMP responsive element modulator (CREM), transcript variant 19, mRNA [NM_183013]	2.07206	up	2.18405	up	1330.59720	664.93280	1169.48070	472.16810	Detected	Detected	Detected	Detected
3336	A_24_P712350	CHML	Hs.654545	choroideremia-like (Rab escort protein 2)	Homo sapiens choroideremia-like (Rab escort protein 2) (CHML), mRNA [NM_001821]	2.07131	up	2.03736	up	4630.38100	2314.75590	3044.36400	1317.63160	Detected	Detected	Detected	Detected
3337	A_23_P65262	N4BP2L2	Hs.507680	NEDD4 binding protein 2-like 2	Homo sapiens NEDD4 binding protein 2-like 2 (N4BP2L2), transcript variant 1, mRNA [NM_033111]	2.07109	up	2.10385	up	1810.32970	905.08950	1708.08480	715.91310	Detected	Detected	Detected	Detected
3338	A_33_P3263906				Tubulin epsilon chain (Epsilon-tubulin) [Source:UniProtKB/Swiss-Prot;Acc:Q9UJT0] [ENST00000368657]	2.07086	up	2.52171	up	10.98013	5.49022	14.61387	5.11018	Compromised	Compromised	Detected	Compromised
3339	A_33_P3387956	DPP7	Hs.37916	dipeptidyl-peptidase 7	Homo sapiens dipeptidyl-peptidase 7 (DPP7), mRNA [NM_013379]	2.07084	up	1.81407	up	8042.44900	4021.38670	8227.69300	3999.35700	Detected	Detected	Detected	Detected
3340	A_24_P274270	STAT1	Hs.642990	signal transducer and activator of transcription 1, 91kDa	Homo sapiens signal transducer and activator of transcription 1, 91kDa (STAT1), transcript variant beta, mRNA [NM_139266]	2.07037	up	1.96045	up	3114.68140	1557.75050	2593.71140	1166.62460	Detected	Detected	Detected	Detected
3341	A_24_P69691	ZNF25	Hs.499429	zinc finger protein 25	Homo sapiens zinc finger protein 25 (ZNF25), mRNA [NM_145011]	2.07015	up	2.10597	up	842.58780	421.45044	585.78990	245.27588	Detected	Detected	Detected	Detected
3342	A_33_P3333232				Coiled-coil domain-containing protein C6orf185 [Source:UniProtKB/Swiss-Prot;Acc:A2VCL2] [ENST00000440451]	2.06974	up	2.26664	up	41.58016	20.80196	27.30990	10.62436	Detected	Detected	Detected	Compromised
3343	A_23_P65068	EID3	Hs.659857	EP300 interacting inhibitor of differentiation 3	Homo sapiens EP300 interacting inhibitor of differentiation 3 (EID3), mRNA [NM_001008394]	2.06926	up	2.11861	up	477.25540	238.81890	523.64280	217.94588	Detected	Detected	Detected	Detected
3344	A_33_P3302305	BCKDHA	Hs.433307	branched chain keto acid dehydrogenase E1, alpha polypeptide	Homo sapiens branched chain keto acid dehydrogenase E1, alpha polypeptide (BCKDHA), nuclear gene encoding mitochondrial protein, transcript variant 1, mRNA [NM_000709]	2.06914	up	1.87581	up	3412.43430	1707.68640	4353.97270	2046.73860	Detected	Detected	Detected	Detected
3345	A_24_P72064	GHR	Hs.125180	growth hormone receptor	Homo sapiens growth hormone receptor (GHR), mRNA [NM_000163]	2.06870	up	2.10705	up	874.65060	437.79532	667.48440	279.33908	Detected	Detected	Detected	Detected
3346	A_32_P181527	C8orf85	Hs.437551	chromosome 8 open reading frame 85	Homo sapiens chromosome 8 open reading frame 85 (C8orf85), mRNA [NM_001025357]	2.06779	up	1.47872	up	131.02931	65.61386	109.05170	65.02959	Detected	Detected	Detected	Detected
3347	A_23_P341503	C3orf35	Hs.475945	chromosome 3 open reading frame 35	Homo sapiens chromosome 3 open reading frame 35 (C3orf35), transcript variant B, mRNA [NM_178339]	2.06763	up	2.14092	up	161.50237	80.87975	84.12185	34.64769	Detected	Detected	Detected	Detected
3348	A_33_P3423600	C17orf90	Hs.655241	chromosome 17 open reading frame 90	Homo sapiens chromosome 17 open reading frame 90 (C17orf90), mRNA [NM_001039842]	2.06747	up	1.91755	up	26356.01200	13199.96700	22424.10200	10311.79200	Detected	Detected	Detected	Detected
3349	A_33_P3409159	SLC22A23	Hs.713588	solute carrier family 22, member 23	Homo sapiens solute carrier family 22, member 23 (SLC22A23), transcript variant 1, mRNA [NM_015482]	2.06721	up	2.21371	up	1429.05850	715.81090	642.83990	256.06380	Detected	Detected	Detected	Detected
3350	A_23_P503182	ABR	Hs.159306	active BCR-related gene	Homo sapiens active BCR-related gene (ABR), transcript variant 1, mRNA [NM_021962]	2.06703	up	1.96486	up	22101.09800	11071.35800	23198.76600	10411.14700	Detected	Detected	Detected	Detected
3351	A_33_P3467872	LOC144920	Hs.567700	hypothetical protein LOC144920	Homo sapiens cDNA FLJ25324 fis, clone TST00330 [AK058053]	2.06657	up	2.61762	up	44.38470	22.23910	18.16539	6.11934	Detected	Detected	Detected	Compromised
3352	A_33_P3239569		Hs.190968		40S ribosomal protein S21 [Source:UniProtKB/Swiss-Prot;Acc:P63220] [ENST00000370562]	2.06597	up	2.07089	up	527.03040	264.14688	525.70920	223.84857	Detected	Detected	Detected	Detected
3353	A_23_P257043	GEM	Hs.654463	GTP binding protein overexpressed in skeletal muscle	Homo sapiens GTP binding protein overexpressed in skeletal muscle (GEM), transcript variant 1, mRNA [NM_005261]	2.06502	up	2.19543	up	1322.45800	663.11690	1283.06910	515.34260	Detected	Detected	Detected	Detected
3354	A_24_P238040	USP6	Hs.448851	ubiquitin specific peptidase 6 (Tre-2 oncogene)	Homo sapiens ubiquitin specific peptidase 6 (Tre-2 oncogene) (USP6), mRNA [NM_004505]	2.06476	up	1.29339	up	14.73115	7.38755	6.05095	4.12533	Detected	Compromised	Compromised	Compromised
3355	A_23_P147450	SPG21	Hs.242458	spastic paraplegia 21 (autosomal recessive, Mast syndrome)	Homo sapiens spastic paraplegia 21 (autosomal recessive, Mast syndrome) (SPG21), transcript variant 1, mRNA [NM_016630]	2.06470	up	2.11295	up	24415.37700	12244.45200	22739.69300	9489.88800	Detected	Detected	Detected	Detected
3356	A_33_P3331911	LOC100129767	Hs.639607	similar to rCG40189	PREDICTED: Homo sapiens similar to rCG40189 (LOC100129767), miscRNA [XR_039365]	2.06464	up	3.06139	up	18.31636	9.18605	10.60686	3.05515	Detected	Compromised	Compromised	Compromised
3357	A_23_P76102	GDF11	Hs.600883	growth differentiation factor 11	Homo sapiens growth differentiation factor 11 (GDF11), mRNA [NM_005811]	2.06442	up	1.91391	up	1293.39690	648.73520	1285.12160	592.09090	Detected	Detected	Detected	Detected
3358	A_23_P2283	TAC3	Hs.9730	tachykinin 3	Homo sapiens tachykinin 3 (TAC3), mRNA [NM_013251]	2.06439	up	1.56104	up	17.97314	9.01501	20.42554	11.53785	Detected	Compromised	Detected	Compromised
3359	A_23_P68072	WDR54	Hs.643480	WD repeat domain 54	Homo sapiens WD repeat domain 54 (WDR54), mRNA [NM_032118]	2.06381	up	2.02287	up	5700.47600	2860.05620	6317.20460	2753.73970	Detected	Detected	Detected	Detected
3360	A_23_P156289	OSMR	Hs.120658	oncostatin M receptor	Homo sapiens oncostatin M receptor (OSMR), mRNA [NM_003999]	2.06371	up	1.83192	up	627.20130	314.69647	622.91900	299.84006	Detected	Detected	Detected	Detected
3361	A_24_P268893	THAP6	Hs.479971	THAP domain containing 6	Homo sapiens THAP domain containing 6 (THAP6), mRNA [NM_144721]	2.06291	up	1.90673	up	686.15100	344.40820	573.78060	265.35130	Detected	Detected	Detected	Detected

3362	A_33_P3606465	LOC645158	Hs.712861	hypothetical protein LOC645158	PREDICTED: Homo sapiens hypothetical protein LOC645158 (LOC645158), miscRNA [XR_078662]	2.06269	up	2.42108	up	347.92157	174.65518	247.02962	89.97143	Detected	Detected	Detected	Detected
3363	A_23_P434289	GPR62	Hs.232213	G protein-coupled receptor 62	Homo sapiens G protein-coupled receptor 62 (GPR62), mRNA [NM_080865]	2.06238	up	2.25960	up	133.15550	66.85347	105.19013	41.04968	Detected	Detected	Detected	Detected
3364	A_32_P10396	WDFY3	Hs.480116	WD repeat and FYVE domain containing 3	Homo sapiens WD repeat and FYVE domain containing 3 (WDFY3), transcript variant 1, mRNA [NM_014991]	2.06179	up	1.85214	up	749.05920	376.18732	524.47840	249.70060	Detected	Detected	Detected	Detected
3365	A_23_P410312	C12orf76	Hs.44817	chromosome 12 open reading frame 76	Homo sapiens chromosome 12 open reading frame 76 (C12orf76), mRNA [NM_207435]	2.06156	up	2.24803	up	1268.19790	636.97675	1147.52090	450.11650	Detected	Detected	Detected	Detected
3366	A_23_P433229	PHYHIP	Hs.334688	phytanoyl-CoA 2-hydroxylase interacting protein	Homo sapiens phytanoyl-CoA 2-hydroxylase interacting protein (PHYHIP), transcript variant 2, mRNA [NM_014759]	2.06097	up	1.83133	up	56.49025	28.38153	57.59419	27.73176	Detected	Detected	Detected	Detected
3367	A_33_P3270776	HTRA3	Hs.479119	HtrA serine peptidase 3	Homo sapiens HtrA serine peptidase 3 (HTRA3), mRNA [NM_053044]	2.06066	up	1.56209	up	237.17336	119.17733	199.94624	112.86816	Detected	Detected	Detected	Detected
3368	A_33_P3252369	TMEM182	Hs.436203	transmembrane protein 182	Homo sapiens transmembrane protein 182 (TMEM182), mRNA [NM_144632]	2.06048	up	2.49352	up	155.72316	78.25609	129.45029	45.77780	Detected	Detected	Detected	Detected
3369	A_33_P3232504	C9orf169	Hs.512469	chromosome 9 open reading frame 169	Homo sapiens chromosome 9 open reading frame 169 (C9orf169), mRNA [NM_199001]	2.06007	up	2.10473	up	743.68480	373.79996	670.78723	281.03043	Detected	Detected	Detected	Detected
3370	A_23_P216038	PHF20L1	Hs.304362	PHD finger protein 20-like 1	Homo sapiens PHD finger protein 20-like 1 (PHF20L1), transcript variant 2, mRNA [NM_032205]	2.05912	up	2.01204	up	1933.83080	972.45685	1500.84020	657.75230	Detected	Detected	Detected	Detected
3371	A_33_P3336198	C16orf79	Hs.647399	chromosome 16 open reading frame 79	Homo sapiens chromosome 16 open reading frame 79 (C16orf79), mRNA [NM_182563]	2.05877	up	1.84967	up	236.97636	119.18749	249.79265	119.08342	Detected	Detected	Detected	Detected
3372	A_23_P93727	SDK1	Hs.653013	sidekick homolog 1, cell adhesion molecule (chicken)	Homo sapiens sidekick homolog 1, cell adhesion molecule (chicken) (SDK1), transcript variant 1, mRNA [NM_152744]	2.05782	up	9.52668	up	7.77630	3.91289	35.81147	3.31471	Compromised	Compromised	Detected	Compromised
3373	A_32_P206293	ZNF322A	Hs.126280	zinc finger protein 322A	Homo sapiens zinc finger protein 322A (ZNF322A), mRNA [NM_024639]	2.05712	up	1.96207	up	6251.20070	3146.57150	5245.45260	2357.40330	Detected	Detected	Detected	Detected
3374	A_24_P153576	SHPRH	Hs.124537	SNF2 histone linker PHD RING helicase	Homo sapiens SNF2 histone linker PHD RING helicase (SHPRH), transcript variant 2, mRNA [NM_173082]	2.05697	up	2.31727	up	516.04860	259.77438	360.71646	137.26318	Detected	Detected	Detected	Detected
3375	A_33_P3310366	LOC100289097	Hs.529357	hypothetical protein LOC100289097	PREDICTED: Homo sapiens hypothetical protein LOC100289097 (LOC100289097), mRNA [XM_002343891]	2.05668	up	2.25017	up	1214.21130	611.31036	1422.26090	557.35130	Detected	Detected	Detected	Detected
3376	A_33_P3362048				Serine hydroxymethyltransferase, cytosolic (SHMT)(Serine methylase)(EC 2.1.2.1)(Glycine hydroxymethyltransferase) [Source:UniProtKB/Swiss-Prot;Acc:P34896] [ENST00000395682]	2.05641	up	1.25732	up	153.98758	77.53721	26.00205	18.23591	Detected	Detected	Detected	Detected
3377	A_23_P158829	ARRB2	Hs.435811	arrestin, beta 2	Homo sapiens arrestin, beta 2 (ARRB2), transcript variant 1, mRNA [NM_004313]	2.05624	up	1.96786	up	999.16660	503.14874	1035.24570	463.88920	Detected	Detected	Detected	Detected
3378	A_33_P3372266		Hs.719643		V3-4 protein Fragment [Source:UniProtKB/TrEMBL;Acc:Q5NV62] [ENST00000390283]	2.05544	up	1.94321	up	17.73159	8.93256	6.95933	3.15801	Detected	Compromised	Compromised	Compromised
3379	A_33_P3354688	LOC730236	Hs.444913	hypothetical LOC730236	PREDICTED: Homo sapiens hypothetical LOC730236 (LOC730236), mRNA [XM_001714412]	2.05493	up	1.93564	up	243.22270	122.55770	176.38902	80.35494	Detected	Detected	Detected	Detected
3380	A_33_P3282213					2.05491	up	2.49007	up	56.28019	28.35936	59.08872	20.92463	Detected	Detected	Detected	Detected
3381	A_33_P3327140					2.05467	up	3.07602	up	2529.34030	1274.67200	201.80990	57.85199	Detected	Detected	Detected	Detected
3382	A_23_P330070	TFPI	Hs.516578	tissue factor pathway inhibitor (lipoprotein-associated coagulation inhibitor)	Homo sapiens tissue factor pathway inhibitor (lipoprotein-associated coagulation inhibitor) (TFPI), transcript variant 2, mRNA [NM_001032281]	2.05438	up	2.25226	up	8663.04800	4366.39800	7773.44970	3043.41240	Detected	Detected	Detected	Detected
3383	A_23_P51487	GBP3	Hs.720167	guanylate binding protein 3	Homo sapiens guanylate binding protein 3 (GBP3), mRNA [NM_018284]	2.05436	up	2.03052	up	1450.15670	730.92300	956.53015	415.39127	Detected	Detected	Detected	Detected
3384	A_24_P110354	C5orf50	Hs.591740	chromosome 5 open reading frame 50	Uncharacterized protein C5orf50 [Source:UniProtKB/Swiss-Prot;Acc:A6NLE4] [ENST00000330910]	2.05403	up	1.31073	up	12.36656	6.23413	7.95305	5.35041	Detected	Compromised	Compromised	Compromised
3385	A_23_P217737	ATP7A	Hs.496414	ATPase, Cu++ transporting, alpha polypeptide	Homo sapiens ATPase, Cu++ transporting, alpha polypeptide (ATP7A), mRNA [NM_000052]	2.05394	up	1.94425	up	603.74194	304.36680	388.37433	176.14241	Detected	Detected	Detected	Detected
3386	A_32_P71113	SCAI	Hs.59504	suppressor of cancer cell invasion	Homo sapiens suppressor of cancer cell invasion (SCAI), transcript variant 1, mRNA [NM_173690]	2.05390	up	2.27183	up	834.49280	420.70420	693.85754	269.31460	Detected	Detected	Detected	Detected
3387	A_23_P1782	CD82	Hs.527778	CD82 molecule	Homo sapiens CD82 molecule (CD82), transcript variant 1, mRNA [NM_002231]	2.05348	up	1.89783	up	5175.19870	2609.57960	4271.47950	1984.65810	Detected	Detected	Detected	Detected
3388	A_24_P649388	C21orf63	Hs.208358	chromosome 21 open reading frame 63	Homo sapiens chromosome 21 open reading frame 63 (C21orf63), mRNA [NM_058187]	2.05339	up	1.92852	up	180.68794	91.11498	229.37073	104.87658	Detected	Detected	Detected	Detected
3389	A_32_P83845	HEY1	Hs.234434	hairy/enhancer-of-split related with YRPW motif 1	Homo sapiens hairy/enhancer-of-split related with YRPW motif 1 (HEY1), transcript variant 2, mRNA [NM_001040708]	2.05310	up	2.08431	up	1826.20000	921.02435	1203.56470	509.18073	Detected	Detected	Detected	Detected
3390	A_23_P423864	PHC2	Hs.524271	polyhomeotic homolog 2 (Drosophila)	Homo sapiens polyhomeotic homolog 2 (Drosophila) (PHC2), transcript variant 1, mRNA [NM_198040]	2.05252	up	1.59595	up	107648.39000	54306.70700	100621.29000	55595.08000	Detected	Detected	Detected	Detected
3391	A_23_P203645	CREBZF	Hs.535319	CREB/ATF bZIP transcription factor	Homo sapiens CREB/ATF bZIP transcription factor (CREBZF), transcript variant 1, mRNA [NM_001039618]	2.05244	up	1.84864	up	720.37640	363.43085	517.71320	246.94660	Detected	Detected	Detected	Detected

3392	A_23_P399797	SMAD5OS	Hs.59666	SMAD family member 5 opposite strand	Homo sapiens SMAD family member 5 opposite strand (SMAD5OS), non-coding RNA [NR_026763]	2.05234	up	1.79029	up	108.36507	54.67307	120.71563	59.45725	Detected	Detected	Detected	Detected
3393	A_33_P3343616				PHD finger protein 12 (PHD factor 1)(Pf1) [Source:UniProtKB/Swiss-Prot;Acc:Q96QT6] [ENST00000378879]	2.05226	up	1.67497	up	75.29411	37.98934	59.39712	31.26974	Detected	Detected	Detected	Detected
3394	A_24_P275828	PGAP3	Hs.462971	post-GPI attachment to proteins 3	Homo sapiens post-GPI attachment to proteins 3 (PGAP3), mRNA [NM_033419]	2.05161	up	1.88530	up	1682.33190	849.08150	2074.93360	970.48596	Detected	Detected	Detected	Detected
3395	A_23_P436369	FILIP1	Hs.696158	filamin A interacting protein 1	Homo sapiens filamin A interacting protein 1 (FILIP1), mRNA [NM_015687]	2.05130	up	2.19558	up	24.33135	12.28200	18.19578	7.30780	Detected	Compromised	Detected	Compromised
3396	A_32_P790284	KATNAL2	Hs.404137	katanin p60 subunit A-like 2	Homo sapiens katanin p60 subunit A-like 2 (KATNAL2), mRNA [NM_031303]	2.05115	up	2.16987	up	34.35020	17.34065	22.95685	9.32918	Detected	Detected	Detected	Compromised
3397	A_33_P3225022	FTCD	Hs.415846	formiminotransferase cyclodeaminase	Homo sapiens formiminotransferase cyclodeaminase (FTCD), transcript variant A, mRNA [NM_206965]	2.05106	up	1.25670	up	25.94466	13.09793	17.52461	12.29656	Detected	Compromised	Detected	Compromised
3398	A_33_P3257451	SRRM3	Hs.511025	serine/arginine repetitive matrix 3	Homo sapiens serine/arginine repetitive matrix 3 (SRRM3), mRNA [NM_001110199]	2.04991	up	1.37959	up	79.56191	40.18864	63.33167	40.47966	Detected	Detected	Detected	Detected
3399	A_24_P942068	TANC2	Hs.410889	tetratricopeptide repeat, ankyrin repeat and coiled-coil containing 2	Homo sapiens tetratricopeptide repeat, ankyrin repeat and coiled-coil containing 2 (TANC2), mRNA [NM_025185]	2.04969	up	2.17518	up	3517.52950	1776.97780	2238.01370	907.26086	Detected	Detected	Detected	Detected
3400	A_33_P3227857	COX18	Hs.356697	COX18 cytochrome c oxidase assembly homolog (S. cerevisiae)	Homo sapiens COX18 cytochrome c oxidase assembly homolog (S. cerevisiae) (COX18), nuclear gene encoding mitochondrial protein, mRNA [NM_173827]	2.04956	up	1.87596	up	65.72430	33.20455	59.11636	27.78757	Detected	Detected	Detected	Detected
3401	A_24_P405981	TTC33	Hs.348915	tetratricopeptide repeat domain 33	Homo sapiens tetratricopeptide repeat domain 33 (TTC33), mRNA [NM_012382]	2.04952	up	2.54447	up	147.14912	74.34261	97.39520	33.75249	Detected	Detected	Detected	Detected
3402	A_23_P153320	ICAM1	Hs.643447	intercellular adhesion molecule 1	Homo sapiens intercellular adhesion molecule 1 (ICAM1), mRNA [NM_000201]	2.04758	up	1.84136	up	3194.19000	1615.29640	2412.40380	1155.25070	Detected	Detected	Detected	Detected
3403	A_33_P3313456	CXorf30	Hs.632791	chromosome X open reading frame 30	Homo sapiens chromosome X open reading frame 30 (CXorf30), mRNA [NM_001098843]	2.04711	up	1.02482	up	189.91864	96.06388	25.48234	21.92592	Detected	Detected	Detected	Detected
3404	A_23_P103864	TTC13	Hs.424788	tetratricopeptide repeat domain 13	Homo sapiens tetratricopeptide repeat domain 13 (TTC13), transcript variant 1, mRNA [NM_024525]	2.04693	up	2.06538	up	478.16202	241.88254	318.28357	135.88731	Detected	Detected	Detected	Detected
3405	A_23_P101829	LPAR2	Hs.122575	lysophosphatidic acid receptor 2	Homo sapiens lysophosphatidic acid receptor 2 (LPAR2), mRNA [NM_004720]	2.04622	up	2.01584	up	148.92572	75.36151	117.43903	51.37141	Detected	Detected	Detected	Detected
3406	A_33_P3406090					2.04608	up	1.81612	up	642.62665	325.21353	572.89750	278.16162	Detected	Detected	Detected	Detected
3407	A_23_P316974	SYNJ2	Hs.434494	synaptojanin 2	Homo sapiens synaptojanin 2 (SYNJ2), mRNA [NM_003898]	2.04598	up	2.12186	up	1011.67194	512.00070	973.11660	404.40220	Detected	Detected	Detected	Detected
3408	A_33_P3284883	MOBKL2B	Hs.369022	MOB1, Mps One Binder kinase activator-like 2B (yeast)	Homo sapiens MOB1, Mps One Binder kinase activator-like 2B (yeast) (MOBKL2B), mRNA [NM_024761]	2.04587	up	3.82452	up	16.59954	8.40139	10.70973	2.46926	Detected	Compromised	Compromised	Compromised
3409	A_32_P86118	PM20D2	Hs.356247	peptidase M20 domain containing 2	Homo sapiens peptidase M20 domain containing 2 (PM20D2), mRNA [NM_001010853]	2.04562	up	2.11688	up	400.22160	202.58540	294.15616	122.53121	Detected	Detected	Detected	Detected
3410	A_23_P94762	ZNF354B	Hs.591734	zinc finger protein 354B	Homo sapiens zinc finger protein 354B (ZNF354B), mRNA [NM_058230]	2.04526	up	1.84410	up	684.70250	346.64633	510.58620	244.14685	Detected	Detected	Detected	Detected
3411	A_32_P18547	C21orf57	Hs.474066	chromosome 21 open reading frame 57	Homo sapiens chromosome 21 open reading frame 57 (C21orf57), transcript variant 1, mRNA [NM_058181]	2.04426	up	2.04937	up	2755.58980	1395.76220	2809.18240	1208.71850	Detected	Detected	Detected	Detected
3412	A_23_P61960	ATP6V0E2	Hs.698060	ATPase, H+ transporting V0 subunit e2	Homo sapiens ATPase, H+ transporting V0 subunit e2 (ATP6V0E2), transcript variant 1, mRNA [NM_145230]	2.04410	up	1.82030	up	158.59523	80.33794	135.52759	65.65247	Detected	Detected	Detected	Detected
3413	A_24_P942030	VAMP4	Hs.605606	vesicle-associated membrane protein 4	Vesicle-associated membrane protein 4 (VAMP-4) [Source:UniProtKB/Swiss-Prot;Acc:O75379] [ENST00000401589]	2.04409	up	2.12404	up	1327.01950	672.21920	812.83330	337.44666	Detected	Detected	Detected	Detected
3414	A_24_P350200	LOC100288418	Hs.703561	similar to OK/SW-CL.16	PREDICTED: Homo sapiens similar to OK/SW-CL.16 (LOC100288418), mRNA [XM_002342023]	2.04367	up	2.14273	up	19471.42800	9865.50400	20811.10700	8564.31000	Detected	Detected	Detected	Detected
3415	A_33_P3319880	EVC2	Hs.87306	Ellis van Creveld syndrome 2	Homo sapiens Ellis van Creveld syndrome 2 (EVC2), transcript variant 2, mRNA [NM_001166136]	2.04350	up	1.76445	up	613.05164	310.63870	533.17560	266.45636	Detected	Detected	Detected	Detected
3416	A_23_P394377	KCNRG	Hs.660161	potassium channel regulator	Homo sapiens potassium channel regulator (KCNRG), transcript variant 2, mRNA [NM_199464]	2.04338	up	1.95998	up	47.57196	24.10659	37.27490	16.76990	Detected	Detected	Detected	Detected
3417	A_33_P3301034	LRRC28	Hs.578684	leucine rich repeat containing 28	Homo sapiens leucine rich repeat containing 28 (LRRC28), mRNA [NM_144598]	2.04333	up	2.24380	up	55.32303	28.03498	31.46162	12.36410	Detected	Detected	Detected	Compromised
3418	A_32_P3534	LOC145814	Hs.592021	pyroglutamyl-peptidase 1-like	Homo sapiens pyroglutamyl-peptidase 1-like (LOC145814), mRNA [NM_001102612]	2.04331	up	1.49709	up	27.79167	14.08358	18.44430	10.86374	Detected	Compromised	Compromised	Compromised
3419	A_33_P3371904	ASTN2	Hs.601562	astrotactin 2	Homo sapiens astrotactin 2 (ASTN2), transcript variant 4, mRNA [NM_198188]	2.04331	up	1.82919	up	92.23288	46.73956	87.10658	41.99103	Detected	Detected	Detected	Detected
3420	A_33_P3396129		Hs.275675		Katanin p80 WD40-containing subunit B1 (Katanin p80 subunit B1)(p80 katanin) [Source:UniProtKB/Swiss-Prot;Acc:Q9BVA0] [ENST00000394326]	2.04305	up	1.50580	up	727.39954	368.66116	138.86047	81.31601	Detected	Detected	Detected	Detected
3421	A_23_P411833	WDR90	Hs.511903	WD repeat domain 90	Homo sapiens WD repeat domain 90 (WDR90), mRNA [NM_145294]	2.04303	up	1.92761	up	6949.26070	3522.06450	5872.54300	2686.40550	Detected	Detected	Detected	Detected

3422	A_33_P3299882	UQCRB	Hs.131255	ubiquinol-cytochrome c reductase binding protein	Homo sapiens ubiquinol-cytochrome c reductase binding protein (UQCRB), nuclear gene encoding mitochondrial protein, mRNA [NM_006294]	2.04217	up	2.05760	up	206.06020	104.48071	152.63470	65.41221	Detected	Detected	Detected	Detected
3423	A_24_P196372	AGBL5	Hs.138207	ATP/GTP binding protein-like 5	Homo sapiens ATP/GTP binding protein-like 5 (AGBL5), transcript variant 3, mRNA [NM_001035507]	2.04209	up	1.61233	up	643.01843	326.04742	471.79900	258.02905	Detected	Detected	Detected	Detected
3424	A_33_P3379841	CG030	Hs.658751	hypothetical CG030	Homo sapiens hypothetical CG030 (CG030), non-coding RNA [NR_026928]	2.04208	up	2.47919	up	126.17167	63.97669	81.37260	28.94234	Detected	Detected	Detected	Detected
3425	A_33_P3403232	LOC100128336	Hs.570448	hypothetical LOC100128336	PREDICTED: Homo sapiens hypothetical LOC100128336 (LOC100128336), mRNA [XM_001715173]	2.04106	up	3.31889	up	17.69089	8.97486	9.57553	2.54411	Detected	Compromised	Compromised	Compromised
3426	A_33_P3764802	SIRT5	Hs.567431	sirtuin (silent mating type information regulation 2 homolog) 5 (S. cerevisiae)	Homo sapiens sirtuin (silent mating type information regulation 2 homolog) 5 (S. cerevisiae) (SIRT5), transcript variant 1, mRNA [NM_012241]	2.03964	up	2.12617	up	440.43008	223.59177	401.69473	166.59550	Detected	Detected	Detected	Detected
3427	A_24_P144773	RNF145	Hs.720757	ring finger protein 145	Homo sapiens ring finger protein 145 (RNF145), mRNA [NM_144726]	2.03958	up	2.00722	up	70.67612	35.88099	68.32006	30.01358	Detected	Detected	Detected	Detected
3428	A_33_P3320272	TMEM79	Hs.347408	transmembrane protein 79	Homo sapiens transmembrane protein 79 (TMEM79), transcript variant 1, mRNA [NM_032323]	2.03945	up	1.50319	up	171.55551	87.10136	163.55531	95.94391	Detected	Detected	Detected	Detected
3429	A_33_P3260572	CLIP4	Hs.122927	CAP-GLY domain containing linker protein family, member 4	Homo sapiens CAP-GLY domain containing linker protein family, member 4 (CLIP4), mRNA [NM_024692]	2.03936	up	2.33752	up	428.92914	217.78299	343.21387	129.47177	Detected	Detected	Detected	Detected
3430	A_23_P150950	ZFC3H1	Hs.527874	zinc finger, C3H1-type containing	Homo sapiens zinc finger, C3H1-type containing (ZFC3H1), mRNA [NM_144982]	2.03907	up	1.87789	up	1582.86830	803.79480	1354.26700	635.91440	Detected	Detected	Detected	Detected
3431	A_23_P28688	CPSF3	Hs.515972	cleavage and polyadenylation specific factor 3, 73kDa	Homo sapiens cleavage and polyadenylation specific factor 3, 73kDa (CPSF3), mRNA [NM_016207]	2.03842	up	2.07421	up	2878.60990	1462.25480	1886.91780	802.16736	Detected	Detected	Detected	Detected
3432	A_33_P3216803					2.03830	up	1.55467	up	29.44365	14.95739	31.49377	17.86284	Detected	Compromised	Detected	Detected
3433	A_33_P3276369	HSD17B7	Hs.492925	hydroxysteroid (17-beta) dehydrogenase 7	Homo sapiens hydroxysteroid (17-beta) dehydrogenase 7 (HSD17B7), mRNA [NM_016371]	2.03802	up	1.68856	up	111.05358	56.42326	118.17542	61.71288	Detected	Detected	Detected	Detected
3434	A_33_P3314441	FBXL17	Hs.721914	F-box and leucine-rich repeat protein 17	Homo sapiens F-box and leucine-rich repeat protein 17 (FBXL17), mRNA [NM_001163315]	2.03773	up	1.66442	up	156.44655	79.49745	108.74220	57.61035	Detected	Detected	Detected	Detected
3435	A_33_P3378644	PHC1	Hs.305985	polyhomeotic homolog 1 (Drosophila)	Homo sapiens polyhomeotic homolog 1 (Drosophila) (PHC1), mRNA [NM_004426]	2.03705	up	1.71508	up	1189.55530	604.66614	1267.16880	651.50024	Detected	Detected	Detected	Detected
3436	A_33_P3376341	LRRC24	Hs.715747	leucine rich repeat containing 24	Homo sapiens leucine rich repeat containing 24 (LRRC24), mRNA [NM_001024678]	2.03626	up	1.85053	up	563.64510	286.61996	642.87860	306.33542	Detected	Detected	Detected	Detected
3437	A_23_P411922	PRPF40B	Hs.706827	PRP40 pre-mRNA processing factor 40 homolog B (S. cerevisiae)	Homo sapiens PRP40 pre-mRNA processing factor 40 homolog B (S. cerevisiae) (PRPF40B), transcript variant 1, mRNA [NM_001031698]	2.03622	up	1.99739	up	335.70792	170.71419	304.64316	134.49140	Detected	Detected	Detected	Detected
3438	A_24_P379693	DNPEP	Hs.258551	aspartyl aminopeptidase	Homo sapiens aspartyl aminopeptidase (DNPEP), mRNA [NM_012100]	2.03527	up	1.57925	up	402.50412	204.77686	245.53162	137.09543	Detected	Detected	Detected	Detected
3439	A_32_P32905					2.03504	up	2.17460	up	74.30033	37.80515	78.44148	31.80772	Detected	Detected	Detected	Detected
3440	A_33_P3360867					2.03500	up	1.56356	up	489.32315	248.97968	224.25668	126.47250	Detected	Detected	Detected	Detected
3441	A_33_P3349455	LOC644525		hypothetical LOC644525	PREDICTED: Homo sapiens hypothetical LOC644525 (LOC644525), mRNA [XM_001727018]	2.03459	up	1.99469	up	322.67600	164.21909	303.76400	134.28445	Detected	Detected	Detected	Detected
3442	A_23_P57658	HRASLS	Hs.36761	HRAS-like suppressor	Homo sapiens HRAS-like suppressor (HRASLS), mRNA [NM_020386]	2.03420	up	2.18461	up	46.14607	23.48953	31.62794	12.76622	Detected	Detected	Detected	Detected
3443	A_33_P3415211	MPV17	Hs.75659	MpV17 mitochondrial inner membrane protein	Homo sapiens MpV17 mitochondrial inner membrane protein (MPV17), nuclear gene encoding mitochondrial protein, mRNA [NM_002437]	2.03416	up	1.92661	up	3994.58800	2033.38800	3847.93870	1761.16850	Detected	Detected	Detected	Detected
3444	A_32_P108826	ZBTB41	Hs.529439	zinc finger and BTB domain containing 41	Homo sapiens zinc finger and BTB domain containing 41 (ZBTB41), mRNA [NM_194314]	2.03403	up	2.62488	up	542.62000	276.23087	391.21097	131.42178	Detected	Detected	Detected	Detected
3445	A_23_P417282	IGF1R	Hs.643120	insulin-like growth factor 1 receptor	Homo sapiens insulin-like growth factor 1 receptor (IGF1R), mRNA [NM_000875]	2.03385	up	2.04250	up	3364.82150	1713.07500	3176.18550	1371.22660	Detected	Detected	Detected	Detected
3446	A_24_P53215	UNC13D	Hs.41045	unc-13 homolog D (C. elegans)	Homo sapiens unc-13 homolog D (C. elegans) (UNC13D), mRNA [NM_199242]	2.03369	up	1.76160	up	23.37373	11.90080	21.79528	10.90988	Detected	Compromised	Detected	Compromised
3447	A_33_P3239759	P2RY11	Hs.14468	purinergic receptor P2Y, G-protein coupled, 11	Homo sapiens purinergic receptor P2Y, G-protein coupled, 11 (P2RY11), mRNA [NM_002566]	2.03362	up	1.89404	up	291.85614	148.60430	156.61029	72.91163	Detected	Detected	Detected	Detected
3448	A_33_P3272090	PDE4DIP	Hs.568247	phosphodiesterase 4D interacting protein	Homo sapiens phosphodiesterase 4D interacting protein (PDE4DIP), transcript variant 1, mRNA [NM_014644]	2.03296	up	2.09810	up	1220.03310	621.40550	1041.96360	437.91745	Detected	Detected	Detected	Detected
3449	A_33_P3325502	ARHGAP29	Hs.483238	Rho GTPase activating protein 29	Homo sapiens Rho GTPase activating protein 29 (ARHGAP29), mRNA [NM_004815]	2.03222	up	2.31679	up	132.72836	67.62807	89.85413	34.19935	Detected	Detected	Detected	Detected
3450	A_33_P3347417	SPEN	Hs.558463	spen homolog, transcriptional regulator (Drosophila)	Homo sapiens spen homolog, transcriptional regulator (Drosophila) (SPEN), mRNA [NM_015001]	2.03213	up	1.01164	up	555.16270	282.87933	79.45810	69.25946	Detected	Detected	Detected	Detected
3451	A_33_P3271176	LOC100130057	Hs.689458	hypothetical protein LOC100130057	Homo sapiens cDNA FLJ46686 fis, clone TRACH3011454. [AK128528]	2.03195	up	4.22272	up	19.54599	9.96043	23.31935	4.86956	Detected	Compromised	Detected	Compromised
3452	A_33_P3414789	FSD1	Hs.28144	fibronectin type III and SPRY domain containing 1	Homo sapiens fibronectin type III and SPRY domain containing 1 (FSD1), mRNA [NM_024333]	2.03161	up	2.81214	up	2303.09200	1173.82410	1043.62500	327.24472	Detected	Detected	Detected	Detected

3453	A_33_P3380883	XPA	Hs.654364	xeroderma pigmentosum, complementation group A	Homo sapiens xeroderma pigmentosum, complementation group A (XPA), transcript variant 2, non-coding RNA [NR_027302]	2.03156	up	1.83503	up	119.18170	60.74545	115.25208	55.38242	Detected	Detected	Detected	Detected
3454	A_24_P315346	POM121L8P	Hs.645245	POM121 membrane glycoprotein-like 8 (rat) pseudogene	Homo sapiens POM121 membrane glycoprotein-like 8 (rat) pseudogene (POM121L8P), non-coding RNA [NR_024583]	2.03118	up	1.73828	up	35.23831	17.96387	17.35339	8.80298	Detected	Detected	Detected	Compromised
3455	A_23_P4611	SLC27A5	Hs.292177	solute carrier family 27 (fatty acid transporter), member 5	Homo sapiens solute carrier family 27 (fatty acid transporter), member 5 (SLC27A5), mRNA [NM_012254]	2.03117	up	2.05837	up	2012.86770	1026.13040	2095.60720	897.74304	Detected	Detected	Detected	Detected
3456	A_33_P3299781				DDRGK domain-containing protein 1 Precursor [Source:UniProtKB/Swiss-Prot;Acc:Q96HY6] [ENST00000380201]	2.03096	up	2.31188	up	409.82780	208.94539	279.29050	106.52638	Detected	Detected	Detected	Detected
3457	A_23_P46369	RAB13	Hs.151536	RAB13, member RAS oncogene family	Homo sapiens RAB13, member RAS oncogene family (RAB13), mRNA [NM_002870]	2.03025	up	2.11466	up	8092.15600	4127.13600	5463.58540	2278.25900	Detected	Detected	Detected	Detected
3458	A_24_P267293	SARM1	Hs.719354	sterile alpha and TIR motif containing 1	Homo sapiens sterile alpha and TIR motif containing 1 (SARM1), mRNA [NM_015077]	2.03000	up	1.78930	up	159.31409	81.26252	190.04422	93.65626	Detected	Detected	Detected	Detected
3459	A_23_P251717	WDR45	Hs.632807	WD repeat domain 45	Homo sapiens WD repeat domain 45 (WDR45), transcript variant 1, mRNA [NM_007075]	2.02945	up	1.88486	up	9483.54200	4838.65970	7956.72070	3722.37840	Detected	Detected	Detected	Detected
3460	A_33_P3291541	TMCO4	Hs.656313	transmembrane and coiled-coil domains 4	Homo sapiens transmembrane and coiled-coil domains 4 (TMCO4), mRNA [NM_181719]	2.02808	up	1.83849	up	197.04329	100.60291	165.69464	79.47163	Detected	Detected	Detected	Detected
3461	A_23_P327069	KIAA0232	Hs.79276	KIAA0232	Homo sapiens KIAA0232 (KIAA0232), transcript variant 1, mRNA [NM_014743]	2.02798	up	1.97479	up	5782.31600	2952.36940	4551.04830	2032.15310	Detected	Detected	Detected	Detected
3462	A_23_P64873	DCN	Hs.156316	decorin	Homo sapiens decorin (DCN), transcript variant A1, mRNA [NM_001920]	2.02796	up	1.84134	up	295721.12000	150992.44000	271926.72000	130222.02000	Detected	Detected	Detected	Detected
3463	A_24_P236522	CD2BP2	Hs.202677	CD2 (cytoplasmic tail) binding protein 2	Homo sapiens CD2 (cytoplasmic tail) binding protein 2 (CD2BP2), transcript variant 1, mRNA [NM_006110]	2.02782	up	1.66169	up	2107.30520	1076.04930	1812.92110	962.04580	Detected	Detected	Detected	Detected
3464	A_33_P3399101				Cathepsin L1 Precursor (EC 3.4.22.15)(Major excreted protein)(MEP) [Contains Cathepsin L1 heavy chain;Cathepsin L1 light chain] [Source:UniProtKB/Swiss-Prot;Acc:P07711] [ENST00000342020]	2.02747	up	2.37613	up	1029.46080	525.76100	467.65253	173.54770	Detected	Detected	Detected	Detected
3465	A_23_P77066	SNRPN	Hs.564847	small nuclear ribonucleoprotein polypeptide N	Homo sapiens small nuclear ribonucleoprotein polypeptide N (SNRPN), transcript variant 4, mRNA [NM_022807]	2.02738	up	1.95032	up	1557.26350	795.35500	1226.43810	554.50366	Detected	Detected	Detected	Detected
3466	A_32_P173058	TMEM41B	Hs.594563	transmembrane protein 41B	Homo sapiens transmembrane protein 41B (TMEM41B), transcript variant 3, non-coding RNA, 611 [NR_028491]	2.02697	up	2.00622	up	3709.04130	1894.72600	3209.68770	1410.74560	Detected	Detected	Detected	Detected
3467	A_23_P74042	LPHN2	Hs.24212	latrophilin 2	Homo sapiens latrophilin 2 (LPHN2), mRNA [NM_012302]	2.02681	up	2.21088	up	530.42377	270.98343	513.01330	204.61084	Detected	Detected	Detected	Detected
3468	A_23_P211631	FBLN1	Hs.24601	fibulin 1	Homo sapiens fibulin 1 (FBLN1), transcript variant D, mRNA [NM_006486]	2.02587	up	1.78050	up	82517.62500	42176.36700	75193.70000	37239.53500	Detected	Detected	Detected	Detected
3469	A_33_P3378051	SESTD1	Hs.30977	SEC14 and spectrin domains 1	Homo sapiens SEC14 and spectrin domains 1 (SESTD1), mRNA [NM_178123]	2.02569	up	2.01067	up	177.26598	90.61207	126.70780	55.56847	Detected	Detected	Detected	Detected
3470	A_24_P201381	FGFBP3	Hs.466120	fibroblast growth factor binding protein 3	Homo sapiens fibroblast growth factor binding protein 3 (FGFBP3), mRNA [NM_152429]	2.02470	up	1.71829	up	158.75125	81.18778	104.17248	53.45922	Detected	Detected	Detected	Detected
3471	A_33_P3235716	SPSB3	Hs.592080	splA/ryanodine receptor domain and SOCS box containing 3	Homo sapiens splA/ryanodine receptor domain and SOCS box containing 3 (SPSB3), mRNA [NM_080861]	2.02445	up	2.07555	up	27845.76200	14242.43200	23512.26400	9989.12300	Detected	Detected	Detected	Detected
3472	A_33_P3297305	ELMOD3	Hs.269990	ELMO/CED-12 domain containing 3	Homo sapiens ELMO/CED-12 domain containing 3 (ELMOD3), transcript variant 1, mRNA [NM_032213]	2.02420	up	1.82132	up	233.60356	119.49741	313.98727	152.01704	Detected	Detected	Detected	Detected
3473	A_33_P3221009		Hs.518622		Putative uncharacterized protein ENSP00000371928 [Source:UniProtKB/TrEMBL;Acc:B7WPA2] [ENST00000382488]	2.02396	up	4.63626	up	104.93871	53.68661	51.13940	9.72643	Detected	Detected	Detected	Compromised
3474	A_33_P3211864	ARMCX4	Hs.709762	armadillo repeat containing, X-linked 4	Homo sapiens armadillo repeat containing, X-linked 4 (ARMCX4), non-coding RNA [NR_028407]	2.02365	up	2.55067	up	53.58374	27.41762	48.41855	16.73875	Detected	Detected	Detected	Detected
3475	A_32_P16258	EXOC6B	Hs.303454	exocyst complex component 6B	Exocyst complex component 6B (Exocyst complex component Sec15B)(SEC15-like protein 2) [Source:UniProtKB/Swiss-Prot;Acc:Q9Y2D4] [ENST00000272427]	2.02357	up	2.14908	up	354.08075	181.18274	262.20260	107.58481	Detected	Detected	Detected	Detected
3476	A_33_P3404489	MAMLD1	Hs.20136	mastermind-like domain containing 1	Homo sapiens mastermind-like domain containing 1 (MAMLD1), mRNA [NM_005491]	2.02353	up	1.07256	up	17.55753	8.98434	15.65806	12.87312	Detected	Compromised	Compromised	Compromised
3477	A_23_P99625	FITM1	Hs.720731	fat storage-inducing transmembrane protein 1	Homo sapiens fat storage-inducing transmembrane protein 1 (FITM1), mRNA [NM_203402]	2.02339	up	3.58726	up	33.97564	17.38683	28.66168	7.04538	Detected	Detected	Detected	Compromised
3478	A_23_P127475	CCS	Hs.502917	copper chaperone for superoxide dismutase	Homo sapiens copper chaperone for superoxide dismutase (CCS), mRNA [NM_005125]	2.02315	up	1.88712	up	2320.91360	1187.85400	2950.43210	1378.64120	Detected	Detected	Detected	Detected
3479	A_23_P403398	DKFZP586I1420	Hs.112423	hypothetical protein DKFZp586I1420	Homo sapiens hypothetical protein DKFZp586I1420 (DKFZP586I1420), non-coding RNA [NR_002186]	2.02314	up	2.00147	up	574.26776	293.91516	382.26523	168.41551	Detected	Detected	Detected	Detected

3480	A_33_P3287428		Hs.663277		LOC652203 protein [Source:UniProtKB/TrEMBL;Acc:Q32Q44] [ENST00000398305]	2.02260	up	1.51634	up	114.07018	58.39755	70.22796	40.83948	Detected	Detected	Detected	Detected
3481	A_33_P3715909	LOC648556	Hs.203594	uncharacterized gastric protein ZA43P	BX118084 NCI_CGAP_Co16 Homo sapiens cDNA clone IMAGp998A105936, mRNA sequence [BX118084]	2.02253	up	2.29749	up	65.62284	33.59650	33.16374	12.72842	Detected	Detected	Detected	Compromised
3482	A_23_P68155	IFIH1	Hs.163173	interferon induced with helicase C domain 1	Homo sapiens interferon induced with helicase C domain 1 (IFIH1), mRNA [NM_022168]	2.02242	up	2.15125	up	210.88533	107.97138	173.78638	71.23462	Detected	Detected	Detected	Detected
3483	A_23_P385771	PAOX	Hs.532469	polyamine oxidase (exo-N4-amino)	Homo sapiens polyamine oxidase (exo-N4-amino) (PAOX), transcript variant 1, mRNA [NM_152911]	2.02226	up	1.91087	up	3074.66020	1574.31770	3012.40230	1390.10220	Detected	Detected	Detected	Detected
3484	A_33_P3841368	LOC286161	Hs.696258	hypothetical protein LOC286161	Homo sapiens cDNA FLJ32061 fis, clone OCBBF1000030 [AK056623]	2.02219	up	1.82381	up	1416.99820	725.57130	946.41406	457.57968	Detected	Detected	Detected	Detected
3485	A_33_P3252989	KIAA0913	Hs.65135	KIAA0913	Homo sapiens KIAA0913 (KIAA0913), mRNA [NM_015037]	2.02171	up	1.77425	up	17915.74600	9175.89600	13435.71200	6677.46900	Detected	Detected	Detected	Detected
3486	A_33_P3216532	EPHB4	Hs.437008	EPH receptor B4	Homo sapiens EPH receptor B4 (EPHB4), mRNA [NM_004444]	2.02146	up	1.79733	up	5929.46300	3037.27250	1365.94060	670.14680	Detected	Detected	Detected	Detected
3487	A_23_P134614	COG5	Hs.239631	component of oligomeric golgi complex 5	Homo sapiens component of oligomeric golgi complex 5 (COG5), transcript variant 1, mRNA [NM_006348]	2.02083	up	1.96493	up	1950.13530	999.23535	1546.08230	693.82635	Detected	Detected	Detected	Detected
3488	A_33_P3573141	LOC440149	Hs.41423	hypothetical LOC440149	Homo sapiens cDNA FLJ32523 fis, clone SMINT2000032 [AK057085]	2.02082	up	1.59688	up	13.51431	6.92469	6.77626	3.74182	Detected	Compromised	Compromised	Compromised
3489	A_23_P57697	DHX36	Hs.446270	DEAH (Asp-Glu-Ala-His) box polypeptide 36	Homo sapiens DEAH (Asp-Glu-Ala-His) box polypeptide 36 (DHX36), transcript variant 1, mRNA [NM_020865]	2.01957	up	2.20916	up	5171.26600	2651.37900	4454.00900	1777.82370	Detected	Detected	Detected	Detected
3490	A_33_P3252322	LOC286434	Hs.657302	hypothetical protein LOC286434	Homo sapiens cDNA FLJ20463 fis, clone KAT06143 [AK000470]	2.01920	up	4.10379	up	117.63922	60.32607	64.32689	13.82207	Detected	Detected	Detected	Compromised
3491	A_24_P376422	LOC100286937	Hs.649203	similar to HSPC047 protein	HSPC047 protein [Source:UniProtKB/TrEMBL;Acc:Q8IV37] [ENST00000344427]	2.01840	up	2.16594	up	379.28152	194.57547	393.49594	160.19900	Detected	Detected	Detected	Detected
3492	A_23_P250156	IGF2BP2	Hs.35354	insulin-like growth factor 2 mRNA binding protein 2	Homo sapiens insulin-like growth factor 2 mRNA binding protein 2 (IGF2BP2), transcript variant 1, mRNA [NM_006548]	2.01802	up	1.97412	up	1703.69710	874.17720	1140.71120	509.52650	Detected	Detected	Detected	Detected
3493	A_24_P412976	TMEM143	Hs.351335	transmembrane protein 143	Homo sapiens transmembrane protein 143 (TMEM143), mRNA [NM_018273]	2.01792	up	2.57654	up	1692.65010	868.55480	1457.16190	498.69708	Detected	Detected	Detected	Detected
3494	A_33_P3360942	SCAMP1	Hs.482587	secretory carrier membrane protein 1	Homo sapiens secretory carrier membrane protein 1 (SCAMP1), mRNA [NM_004866]	2.01771	up	2.11480	up	3079.67200	1580.44580	1858.23630	774.81226	Detected	Detected	Detected	Detected
3495	A_33_P3337026				Sodium- and chloride-dependent creatine transporter 1 (Creatine transporter 1)(CT1)(Solute carrier family 6 member 8) [Source:UniProtKB/Swiss-Prot;Acc:P48029] [ENST00000328897]	2.01622	up	1.78995	up	1784.18350	916.29376	674.38580	332.22540	Detected	Detected	Detected	Detected
3496	A_33_P3212037		Hs.567378		mRNA-capping enzyme (HCE)(HCAP1) [Includes Polynucleotide 5'-triphosphatase(EC 3.1.3.33)(mRNA 5'-triphosphatase)(TPase);mRNA guanylyltransferase(EC 2.7.7.50)(GTP--RNA guanylyltransferase)(GTase)] [Source:UniProtKB/Swiss-Prot;Acc:O60942] [ENST00000369475]	2.01596	up	1.54370	up	15.90878	8.17126	17.87254	10.20911	Detected	Compromised	Detected	Compromised
3497	A_23_P115011	ADAMTSL4	Hs.719991	ADAMTS-like 4	Homo sapiens ADAMTS-like 4 (ADAMTSL4), transcript variant 1, mRNA [NM_019032]	2.01560	up	2.26692	up	84.49645	43.40765	69.13534	26.89241	Detected	Detected	Detected	Detected
3498	A_33_P3362696	RIC8B	Hs.131306	resistance to inhibitors of cholinesterase 8 homolog B (C. elegans)	Homo sapiens resistance to inhibitors of cholinesterase 8 homolog B (C. elegans) (RIC8B), mRNA [NM_018157]	2.01558	up	2.06849	up	1215.74050	624.55835	806.94110	343.99728	Detected	Detected	Detected	Detected
3499	A_23_P360209		Hs.723065		F1262 Human cultured dermal papilla cell cDNA library(F) Homo sapiens cDNA, mRNA sequence [ES309389]	2.01525	up	2.30836	up	8614.19400	4426.06740	4911.44700	1876.16430	Detected	Detected	Detected	Detected
3500	A_23_P127911	PAMR1	Hs.55044	peptidase domain containing associated with muscle regeneration 1	Homo sapiens peptidase domain containing associated with muscle regeneration 1 (PAMR1), transcript variant 1, mRNA [NM_015430]	2.01400	up	1.82477	up	23156.69300	11905.55100	17066.68400	8247.22000	Detected	Detected	Detected	Detected
3501	A_33_P3331511	NCRNA00173	Hs.441601	non-protein coding RNA 173	Homo sapiens non-protein coding RNA 173 (NCRNA00173), transcript variant 1, non-coding RNA [NR_027345]	2.01340	up	1.74394	up	92.03972	47.33468	67.60577	34.18360	Detected	Detected	Detected	Detected
3502	A_24_P307854	FBXL18	Hs.623974	F-box and leucine-rich repeat protein 18	Homo sapiens F-box and leucine-rich repeat protein 18 (FBXL18), mRNA [NM_024963]	2.01318	up	1.80226	up	256.32004	131.83588	212.31584	103.87978	Detected	Detected	Detected	Detected
3503	A_23_P432573	MRGPRF	Hs.118513	MAS-related GPR, member F	Homo sapiens MAS-related GPR, member F (MRGPRF), transcript variant 2, mRNA [NM_145015]	2.01295	up	1.69596	up	4306.12940	2215.06370	3712.49240	1930.25350	Detected	Detected	Detected	Detected
3504	A_23_P53015	TUT1	Hs.144835	terminal uridylyl transferase 1, U6 snRNA-specific	Homo sapiens terminal uridylyl transferase 1, U6 snRNA-specific (TUT1), mRNA [NM_022830]	2.01239	up	1.65077	up	330.53714	170.07559	323.32764	172.71152	Detected	Detected	Detected	Detected
3505	A_33_P3361746	TRPS1	Hs.657018	trichorhinophalangeal syndrome I	Homo sapiens trichorhinophalangeal syndrome I (TRPS1), mRNA [NM_014112]	2.01125	up	2.30365	up	577.37760	297.25360	557.56650	213.42545	Detected	Detected	Detected	Detected
3506	A_23_P46812	CPEB3	Hs.131683	cytoplasmic polyadenylation element binding protein 3	Homo sapiens cytoplasmic polyadenylation element binding protein 3 (CPEB3), mRNA [NM_014912]	2.01060	up	3.23745	up	35.20803	18.13217	22.41621	6.10555	Detected	Detected	Detected	Compromised
3507	A_23_P101905	APC2	Hs.446376	adenomatosis polyposis coli 2	Homo sapiens adenomatosis polyposis coli 2 (APC2), mRNA [NM_005883]	2.01008	up	1.79466	up	341.53775	175.93759	353.33115	173.60617	Detected	Detected	Detected	Detected

3508	A_23_P39616	ORC2L	Hs.444870	origin recognition complex, subunit 2-like (yeast)	Homo sapiens origin recognition complex, subunit 2-like (yeast) (ORC2L), mRNA [NM_006190]	2.00988	up	2.03166	up	702.23940	361.78244	582.21210	252.69390	Detected	Detected	Detected	Detected
3509	A_24_P360269	RNASET2	Hs.529989	ribonuclease T2	Homo sapiens ribonuclease T2 (RNASET2), mRNA [NM_003730]	2.00985	up	1.90201	up	3395.47300	1749.32430	3163.65920	1466.70500	Detected	Detected	Detected	Detected
3510	A_23_P407614	PYDC1	Hs.58314	PYD (pyrin domain) containing 1	Homo sapiens PYD (pyrin domain) containing 1 (PYDC1), mRNA [NM_152901]	2.00969	up	1.70567	up	256.31952	132.06440	223.31600	115.44923	Detected	Detected	Detected	Detected
3511	A_24_P175059	ATG5	Hs.486063	ATG5 autophagy related 5 homolog (S. cerevisiae)	Homo sapiens ATG5 autophagy related 5 homolog (S. cerevisiae) (ATG5), mRNA [NM_004849]	2.00919	up	2.18893	up	1371.62920	706.88560	994.01890	400.43066	Detected	Detected	Detected	Detected
3512	A_33_P3379726	CCDC106	Hs.82482	coiled-coil domain containing 106	Homo sapiens coiled-coil domain containing 106 (CCDC106), mRNA [NM_013301]	2.00896	up	2.32107	up	4646.78860	2395.05250	5216.44500	1981.76600	Detected	Detected	Detected	Detected
3513	A_23_P215505	RAPGEF5	Hs.174768	Rap guanine nucleotide exchange factor (GEF) 5	Homo sapiens Rap guanine nucleotide exchange factor (GEF) 5 (RAPGEF5), mRNA [NM_012294]	2.00882	up	2.77025	up	30.84725	15.90043	12.38912	3.94355	Detected	Compromised	Compromised	Compromised
3514	A_33_P3337272	NRARP	Hs.535075	NOTCH-regulated ankyrin repeat protein	Homo sapiens NOTCH-regulated ankyrin repeat protein (NRARP), mRNA [NM_001004354]	2.00820	up	1.89463	up	161.29518	83.16628	125.26086	58.29841	Detected	Detected	Detected	Detected
3515	A_23_P49674	ARHGEF15	Hs.443109	Rho guanine nucleotide exchange factor (GEF) 15	Homo sapiens Rho guanine nucleotide exchange factor (GEF) 15 (ARHGEF15), mRNA [NM_173728]	2.00776	up	2.42261	up	4193.38130	2162.64300	425.91107	155.02490	Detected	Detected	Detected	Detected
3516	A_33_P3318069	LOC100129295	Hs.134183	hypothetical LOC100129295	PREDICTED: Homo sapiens similar to ribosomal protein L22 (LOC100129295), miscRNA [XR_039567]	2.00764	up	1.76283	up	43.36105	22.36383	36.83081	18.42329	Detected	Detected	Detected	Detected
3517	A_23_P28652	C2orf28	Hs.9527	chromosome 2 open reading frame 28	Homo sapiens chromosome 2 open reading frame 28 (C2orf28), transcript variant 1, mRNA [NM_016085]	2.00732	up	1.94443	up	9878.67300	5095.82700	8109.87060	3677.78560	Detected	Detected	Detected	Detected
3518	A_23_P86855	MACROD1	Hs.602898	MACRO domain containing 1	Homo sapiens MACRO domain containing 1 (MACROD1), mRNA [NM_014067]	2.00699	up	2.07395	up	739.23590	381.39166	721.68854	306.84390	Detected	Detected	Detected	Detected
3519	A_23_P48339	IFT88	Hs.187376	intraflagellar transport 88 homolog (Chlamydomonas)	Homo sapiens intraflagellar transport 88 homolog (Chlamydomonas) (IFT88), transcript variant 1, mRNA [NM_175605]	2.00615	up	1.98413	up	560.48810	289.29114	390.32523	173.46938	Detected	Detected	Detected	Detected
3520	A_23_P333592	ZNF547	Hs.446620	zinc finger protein 547	Homo sapiens zinc finger protein 547 (ZNF547), mRNA [NM_173631]	2.00573	up	1.95543	up	54.15966	27.95991	39.39948	17.76702	Detected	Detected	Detected	Detected
3521	A_24_P355626	ABCG4	Hs.126378	ATP-binding cassette, sub-family G (WHITE), member 4	Homo sapiens ATP-binding cassette, sub-family G (WHITE), member 4 (ABCG4), transcript variant 1, mRNA [NM_022169]	2.00547	up	1.66026	up	59.73692	30.84316	33.37523	17.72617	Detected	Detected	Detected	Detected
3522	A_23_P105545	VAMP1	Hs.20021	vesicle-associated membrane protein 1 (synaptobrevin 1)	Homo sapiens vesicle-associated membrane protein 1 (synaptobrevin 1) (VAMP1), transcript variant 3, mRNA [NM_016830]	2.00386	up	2.06086	up	141.68227	73.21196	135.64742	58.04005	Detected	Detected	Detected	Detected
3523	A_33_P3219434	MOSC2	Hs.369042	MOCO sulphurase C-terminal domain containing 2	Homo sapiens MOCO sulphurase C-terminal domain containing 2 (MOSC2), mRNA [NM_017898]	2.00331	up	1.93030	up	123.46970	63.81842	102.22758	46.69906	Detected	Detected	Detected	Detected
3524	A_24_P306594		Hs.585415		Uncharacterized protein C1orf167 [Source:UniProtKB/Swiss-Prot;Acc:Q5SNV9] [ENST00000444493]	2.00273	up	1.23448	up	36.06758	18.64783	27.14979	19.39312	Detected	Detected	Detected	Detected
3525	A_23_P19517	ITPR3	Hs.65758	inositol 1,4,5-triphosphate receptor, type 3	Homo sapiens inositol 1,4,5-triphosphate receptor, type 3 (ITPR3), mRNA [NM_002224]	2.00256	up	1.78451	up	21735.91200	11238.94200	15804.57100	7809.61670	Detected	Detected	Detected	Detected
3526	A_24_P213715	LOC90834	Hs.679408	hypothetical protein BC001742	Homo sapiens hypothetical protein BC001742 (LOC90834), non-coding RNA [NR_026993]	2.00239	up	1.69628	up	13.79330	7.13267	9.43854	4.90651	Detected	Compromised	Compromised	Compromised
3527	A_23_P330461	TMC4	Hs.355126	transmembrane channel-like 4	Homo sapiens transmembrane channel-like 4 (TMC4), transcript variant 2, mRNA [NM_144686]	2.00235	up	2.17469	up	114.83971	59.38625	103.53751	41.98234	Detected	Detected	Detected	Detected
3528	A_24_P243776	ZNF277	Hs.655904	zinc finger protein 277	Homo sapiens zinc finger protein 277 (ZNF277), mRNA [NM_021994]	2.00222	up	2.00314	up	3181.04370	1645.09500	2050.99270	902.85700	Detected	Detected	Detected	Detected
3529	A_23_P112846	MTHFD2L	Hs.479954	methylenetetrahydrofolate dehydrogenase (NADP+ dependent) 2-like	Homo sapiens methylenetetrahydrofolate dehydrogenase (NADP+ dependent) 2-like (MTHFD2L), mRNA [NM_001144978]	2.00194	up	1.78200	up	61.77427	31.95139	27.87302	13.79247	Detected	Detected	Detected	Detected
3530	A_24_P284523	MAP3K10	Hs.466743	mitogen-activated protein kinase kinase kinase 10	Homo sapiens mitogen-activated protein kinase kinase kinase 10 (MAP3K10), mRNA [NM_002446]	2.00192	up	1.90942	up	1545.06160	799.15686	1350.56040	623.70310	Detected	Detected	Detected	Detected
3531	A_23_P163227	CKMT1A	Hs.425633	creatine kinase, mitochondrial 1A	Homo sapiens creatine kinase, mitochondrial 1A (CKMT1A), nuclear gene encoding mitochondrial protein, mRNA [NM_001015001]	2.00149	up	2.35081	up	27.39458	14.17243	22.70342	8.51607	Detected	Compromised	Detected	Compromised
3532	A_33_P3382856	DCN	Hs.156316	decorin	Homo sapiens decorin (DCN), transcript variant E, mRNA [NM_133507]	2.00069	up	1.73183	up	51018.80500	26404.82000	45476.50000	23155.08800	Detected	Detected	Detected	Detected
3533	A_33_P3409447	AKAP11	Hs.105105	A kinase (PRKA) anchor protein 11	Homo sapiens A kinase (PRKA) anchor protein 11 (AKAP11), mRNA [NM_016248]	2.00040	up	1.18668	up	131.70996	68.17641	77.73790	57.76494	Detected	Detected	Detected	Detected
3534	A_33_P3424112	DHX40	Hs.29403	DEAH (Asp-Glu-Ala-His) box polypeptide 40	Homo sapiens DEAH (Asp-Glu-Ala-His) box polypeptide 40 (DHX40), transcript variant 1, mRNA [NM_024612]	2.00038	up	2.35684	up	198.12599	102.55642	187.77078	70.25285	Detected	Detected	Detected	Detected
3535	A_33_P3577142	LOC283861	Hs.192155	hypothetical locus LOC283861	Homo sapiens cDNA FLJ40824 fis, clone TRACH2011113 [AK098143]	1.99928	up	2.09501	up	821.93005	425.69067	671.15760	282.49030	Detected	Detected	Detected	Detected
3536	A_33_P3254811	C3orf70	Hs.380048	chromosome 3 open reading frame 70	Homo sapiens chromosome 3 open reading frame 70 (C3orf70), mRNA [NM_001025266]	1.99878	up	2.07665	up	430.29456	222.91217	348.92440	148.16124	Detected	Detected	Detected	Detected

3537	A_23_P413862		Hs.690460		Doublecortin domain-containing protein 5 [Source:UniProtKB/Swiss-Prot;Acc:Q6ZRR9] [ENST00000303697]	1.99728	up	6.70294	up	64.44672	33.41147	25.58884	3.36628	Detected	Detected	Detected	Compromised
3538	A_23_P10374	R3HDM2	Hs.443673	R3H domain containing 2	Homo sapiens R3H domain containing 2 (R3HDM2), mRNA [NM_014925]	1.99665	up	2.01566	up	2265.61380	1174.94350	1553.63180	679.66675	Detected	Detected	Detected	Detected
3539	A_33_P3341716	SP4	Hs.88013	Sp4 transcription factor	Homo sapiens Sp4 transcription factor (SP4), mRNA [NM_003112]	1.99623	up	2.04646	up	496.16876	257.36658	345.20460	148.74400	Detected	Detected	Detected	Detected
3540	A_33_P3253401	LOC100131774	Hs.641595	hypothetical LOC100131774	PREDICTED: Homo sapiens hypothetical LOC100131774 (LOC100131774), mRNA [XM_001717693]	1.99565	up	2.29853	up	137.46463	71.32478	111.34761	42.71650	Detected	Detected	Detected	Detected
3541	A_33_P3655775	LOC284630	Hs.502691	hypothetical protein LOC284630	Homo sapiens cDNA FLJ39065 fis, clone NT2RP7014721 [AK096384]	1.99532	up	2.55590	up	24.11570	12.51470	28.87646	9.96245	Detected	Compromised	Detected	Compromised
3542	A_24_P361457	FLJ35220	Hs.389678	hypothetical protein FLJ35220	Homo sapiens hypothetical protein FLJ35220 (FLJ35220), transcript variant 1, mRNA [NM_173627]	1.99498	up	3.08924	up	180.23248	93.54668	145.01985	41.39437	Detected	Detected	Detected	Detected
3543	A_23_P122615	SFRS18	Hs.520287	splicing factor, arginine/serine-rich 18	Homo sapiens splicing factor, arginine/serine-rich 18 (SFRS18), transcript variant 1, mRNA [NM_032870]	1.99450	up	2.02988	up	1306.21810	678.13330	754.11930	327.59340	Detected	Detected	Detected	Detected
3544	A_33_P3301514	NRCAM	Hs.21422	neuronal cell adhesion molecule	Homo sapiens neuronal cell adhesion molecule (NRCAM), transcript variant 1, mRNA [NM_001037132]	1.99449	up	2.12592	up	127.80258	66.35004	137.40176	56.99162	Detected	Detected	Detected	Detected
3545	A_24_P100761	BCAS3	Hs.655028	breast carcinoma amplified sequence 3	Homo sapiens breast carcinoma amplified sequence 3 (BCAS3), transcript variant 2, mRNA [NM_017679]	1.99392	up	2.10324	up	212.86700	110.54360	218.96994	91.80380	Detected	Detected	Detected	Detected
3546	A_33_P3237220	FBXO24	Hs.283764	F-box protein 24	Homo sapiens F-box protein 24 (FBXO24), transcript variant 1, mRNA [NM_033506]	1.99378	up	2.40426	up	651.24493	338.22113	404.04385	148.18774	Detected	Detected	Detected	Detected
3547	A_33_P3218013					1.99297	up	5.37157	up	28.58356	14.85074	25.09220	4.11910	Detected	Detected	Detected	Compromised
3548	A_23_P60499	ZNF462	Hs.370379	zinc finger protein 462	Homo sapiens zinc finger protein 462 (ZNF462), mRNA [NM_021224]	1.99261	up	2.00142	up	1093.59800	568.28796	944.47480	416.11984	Detected	Detected	Detected	Detected
3549	A_23_P20392	PSD3	Hs.434255	pleckstrin and Sec7 domain containing 3	Homo sapiens pleckstrin and Sec7 domain containing 3 (PSD3), transcript variant 1, mRNA [NM_015310]	1.99226	up	2.48627	up	2970.04900	1543.65380	1829.37520	648.81415	Detected	Detected	Detected	Detected
3550	A_33_P3417260	C9orf95	Hs.494186	chromosome 9 open reading frame 95	Homo sapiens chromosome 9 open reading frame 95 (C9orf95), transcript variant 1, mRNA [NM_017881]	1.99198	up	2.02481	up	1337.90980	695.46606	860.40200	374.69897	Detected	Detected	Detected	Detected
3551	A_23_P72503	KLHL2	Hs.388668	kelch-like 2, Mayven (Drosophila)	Homo sapiens kelch-like 2, Mayven (Drosophila) (KLHL2), transcript variant 1, mRNA [NM_007246]	1.99062	up	2.22785	up	860.52313	447.61667	676.17850	267.63327	Detected	Detected	Detected	Detected
3552	A_33_P3254141	LOC285696		hypothetical LOC285696	Homo sapiens hypothetical LOC285696 (LOC285696), non-coding RNA [NR_027253]	1.98958	up	4.34661	up	6.31693	3.28759	13.74076	2.78757	Compromised	Compromised	Detected	Compromised
3553	A_23_P4160	NBR2		neighbor of BRCA1 gene 2 (non-protein coding)	Homo sapiens neighbor of BRCA1 gene 2 (non-protein coding) (NBR2), non-coding RNA [NR_003108]	1.98877	up	2.23731	up	66.20338	34.46907	55.56208	21.89867	Detected	Detected	Detected	Detected
3554	A_23_P375147	RC3H2	Hs.709775	ring finger and CCCH-type zinc finger domains 2	Ring finger and CCCH-type zinc finger domain-containing protein 2 (Membrane-associated nucleic acid-binding protein)(RING finger protein 164) [Source:UniProtKB/Swiss-Prot;Acc:Q9HBD1] [ENST00000373670]	1.98875	up	2.11761	up	2160.22700	1124.74230	1513.71020	630.32227	Detected	Detected	Detected	Detected
3555	A_33_P3304107	KLHL28	Hs.550906	kelch-like 28 (Drosophila)	Homo sapiens kelch-like 28 (Drosophila) (KLHL28), mRNA [NM_017658]	1.98824	up	2.10363	up	1203.87110	626.96580	531.03940	222.59912	Detected	Detected	Detected	Detected
3556	A_23_P418199	LOC730098	Hs.459590	similar to chemokine (C-C motif) ligand 27	PREDICTED: Homo sapiens similar to chemokine (C-C motif) ligand 27 (LOC730098), miscRNA [XR_041278]	1.98730	up	2.00025	up	1388.05730	723.22970	1438.59350	634.19116	Detected	Detected	Detected	Detected
3557	A_33_P3677298	LOC152286	Hs.561180	hypothetical protein LOC152286	Homo sapiens mRNA; cDNA DKFZp434E036 (from clone DKFZp434E036) [AL117431]	1.98670	up	3.07007	up	44.36316	23.12185	16.97459	4.87547	Detected	Detected	Detected	Compromised
3558	A_23_P208937	TLE6	Hs.334507	transducin-like enhancer of split 6 (E(sp1) homolog, Drosophila)	Homo sapiens transducin-like enhancer of split 6 (E(sp1) homolog, Drosophila) (TLE6), transcript variant 2, mRNA [NM_024760]	1.98667	up	2.56986	up	71.28606	37.15451	74.84522	25.68150	Detected	Detected	Detected	Detected
3559	A_33_P3357087	MPV17	Hs.75659	MpV17 mitochondrial inner membrane protein	Homo sapiens MpV17 mitochondrial inner membrane protein (MPV17), nuclear gene encoding mitochondrial protein, mRNA [NM_002437]	1.98594	up	2.03899	up	16365.35000	8532.82300	13228.92500	5721.03120	Detected	Detected	Detected	Detected
3560	A_23_P331908		Hs.178715		cDNA FLJ40559 fis, clone THYMU2002910 [Source:UniProtKB/TrEMBL;Acc:Q8N1K6] [ENST00000399269]	1.98563	up	2.16863	up	508.66530	265.25730	419.05940	170.39433	Detected	Detected	Detected	Detected
3561	A_23_P314642	ZNF280C	Hs.308418	zinc finger protein 280C	Homo sapiens zinc finger protein 280C (ZNF280C), mRNA [NM_017666]	1.98387	up	2.01974	up	399.97113	208.76053	331.79590	144.85751	Detected	Detected	Detected	Detected
3562	A_24_P941787	PRPF4B	Hs.159014	PRP4 pre-mRNA processing factor 4 homolog B (yeast)	Homo sapiens PRP4 pre-mRNA processing factor 4 homolog B (yeast) (PRPF4B), mRNA [NM_003913]	1.98125	up	2.18792	up	4272.97900	2233.18850	2793.17820	1125.72420	Detected	Detected	Detected	Detected
3563	A_23_P20045	PEX1	Hs.164682	peroxisomal biogenesis factor 1	Homo sapiens peroxisomal biogenesis factor 1 (PEX1), mRNA [NM_000466]	1.98061	up	2.03292	up	402.75510	210.55946	491.49774	213.18997	Detected	Detected	Detected	Detected
3564	A_33_P3234197	TRIM45	Hs.301526	tripartite motif-containing 45	Homo sapiens tripartite motif-containing 45 (TRIM45), transcript variant 1, mRNA [NM_025188]	1.98037	up	2.15228	up	875.27930	457.65106	601.40620	246.39693	Detected	Detected	Detected	Detected
3565	A_23_P378427	STARD6	Hs.438779	StAR-related lipid transfer (START) domain containing 6	Homo sapiens StAR-related lipid transfer (START) domain containing 6 (STARD6), mRNA [NM_139171]	1.98029	up	2.56222	up	41.55079	21.72619	34.93115	12.02158	Detected	Detected	Detected	Compromised

3566	A_33_P3405168	MMAA	Hs.452864	methylmalonic aciduria (cobalamin deficiency) cblA type	Homo sapiens methylmalonic aciduria (cobalamin deficiency) cblA type (MMAA), nuclear gene encoding mitochondrial protein, mRNA [NM_172250]	1.97876	up	2.44105	up	256.49360	134.21950	178.96904	64.64983	Detected	Detected	Detected	Detected
3567	A_33_P3228325	SP100	Hs.369056	SP100 nuclear antigen	Homo sapiens SP100 nuclear antigen (SP100), transcript variant 1, mRNA [NM_001080391]	1.97821	up	2.20154	up	3722.38600	1948.42020	2244.82500	899.12860	Detected	Detected	Detected	Detected
3568	A_33_P3317880	ZNF252	Hs.330602	zinc finger protein 252	Homo sapiens zinc finger protein 252 (ZNF252), non-coding RNA [NR_023392]	1.97617	up	2.07626	up	2745.71100	1438.67910	2547.24000	1081.81500	Detected	Detected	Detected	Detected
3569	A_33_P3298043	ABCC6	Hs.442182	ATP-binding cassette, sub-family C (CFTR/MRP), member 6	Homo sapiens ATP-binding cassette, sub-family C (CFTR/MRP), member 6 (ABCC6), transcript variant 2, mRNA [NM_001079528]	1.97577	up	2.45721	up	93.21674	48.85289	92.33732	33.13603	Detected	Detected	Detected	Detected
3570	A_33_P3343976	NCRNA00171	Hs.653168	non-protein coding RNA 171	Homo sapiens non-protein coding RNA 171 (NCRNA00171), non-coding RNA [NR_026751]	1.97480	up	3.35481	up	18.99159	9.95797	17.08035	4.48946	Detected	Compromised	Detected	Compromised
3571	A_33_P3297907	FGF22	Hs.248087	fibroblast growth factor 22	Homo sapiens fibroblast growth factor 22 (FGF22), mRNA [NM_020637]	1.97411	up	2.52815	up	52.45741	27.51495	71.89887	25.07756	Detected	Detected	Detected	Detected
3572	A_33_P3232458	CLIP4	Hs.122927	CAP-GLY domain containing linker protein family, member 4	Homo sapiens CAP-GLY domain containing linker protein family, member 4 (CLIP4), mRNA [NM_024692]	1.97388	up	2.26502	up	440.60043	231.13051	341.31183	132.87527	Detected	Detected	Detected	Detected
3573	A_23_P53530	MTERFD3	Hs.5009	MTERF domain containing 3	Homo sapiens MTERF domain containing 3 (MTERFD3), transcript variant 1, mRNA [NM_001033050]	1.97388	up	2.13176	up	292.31818	153.34459	257.52298	106.52303	Detected	Detected	Detected	Detected
3574	A_24_P335901	ZNF266	Hs.656185	zinc finger protein 266	Homo sapiens zinc finger protein 266 (ZNF266), mRNA [NM_006631]	1.97178	up	2.04287	up	1748.79130	918.36150	1248.77160	539.02356	Detected	Detected	Detected	Detected
3575	A_24_P185604	NDRG3	Hs.437338	NDRG family member 3	Homo sapiens NDRG family member 3 (NDRG3), transcript variant 1, mRNA [NM_032013]	1.97055	up	2.01219	up	1279.07890	672.11300	800.05970	350.60504	Detected	Detected	Detected	Detected
3576	A_32_P209208	CAPS2	Hs.407154	calcyphosine 2	Homo sapiens calcyphosine 2 (CAPS2), mRNA [NM_032606]	1.97027	up	2.14640	up	161.32141	84.78112	131.10764	53.86200	Detected	Detected	Detected	Detected
3577	A_23_P47885	LRIG3	Hs.253736	leucine-rich repeats and immunoglobulin-like domains 3	Homo sapiens leucine-rich repeats and immunoglobulin-like domains 3 (LRIG3), transcript variant 2, mRNA [NM_153377]	1.97023	up	2.01970	up	977.49744	513.72730	681.83890	297.68716	Detected	Detected	Detected	Detected
3578	A_33_P3245606	BHLHA9		basic helix-loop-helix family, member a9	Homo sapiens basic helix-loop-helix family, member a9 (BHLHA9), mRNA [NM_001164405]	1.96997	up	3.46437	up	17.86018	9.38772	11.63444	2.96133	Detected	Compromised	Compromised	Compromised
3579	A_23_P202939	APLP2	Hs.370247	amyloid beta (A4) precursor-like protein 2	Homo sapiens amyloid beta (A4) precursor-like protein 2 (APLP2), transcript variant 1, mRNA [NM_001642]	1.96947	up	2.06267	up	54390.52300	28596.09800	40648.16400	17377.06800	Detected	Detected	Detected	Detected
3580	A_23_P9289	RFX3	Hs.136829	regulatory factor X, 3 (influences HLA class II expression)	Homo sapiens regulatory factor X, 3 (influences HLA class II expression) (RFX3), transcript variant 2, mRNA [NM_134428]	1.96880	up	2.09510	up	111.02668	58.39277	83.63274	35.19957	Detected	Detected	Detected	Detected
3581	A_23_P111402	RSPO3	Hs.135254	R-spondin 3 homolog (Xenopus laevis)	Homo sapiens R-spondin 3 homolog (Xenopus laevis) (RSPO3), mRNA [NM_032784]	1.96794	up	2.01285	up	69.72252	36.68558	37.23166	16.31046	Detected	Detected	Detected	Detected
3582	A_33_P3316379					1.96467	up	2.03232	up	20908.54100	11019.62700	8496.86100	3686.64330	Detected	Detected	Detected	Detected
3583	A_24_P555510	PCM1	Hs.491148	pericentriolar material 1	Homo sapiens pericentriolar material 1 (PCM1), mRNA [NM_006197]	1.96380	up	2.00912	up	1882.22880	992.45105	1115.03580	489.38270	Detected	Detected	Detected	Detected
3584	A_24_P289383	CHD7	Hs.20395	chromodomain helicase DNA binding protein 7	Homo sapiens chromodomain helicase DNA binding protein 7 (CHD7), mRNA [NM_017780]	1.96322	up	2.39438	up	202.66945	106.89371	127.86469	47.08938	Detected	Detected	Detected	Detected
3585	A_33_P3299025	LOC389834	Hs.720653	ankyrin repeat domain 57 pseudogene	Homo sapiens ankyrin repeat domain 57 pseudogene (LOC389834), non-coding RNA [NR_027420]	1.96262	up	3.22659	up	16.51271	8.71194	11.04182	3.01760	Detected	Compromised	Compromised	Compromised
3586	A_33_P3360087	BBS9	Hs.372360	Bardet-Biedl syndrome 9	Homo sapiens Bardet-Biedl syndrome 9 (BBS9), transcript variant 1, mRNA [NM_014451]	1.96088	up	2.79659	up	66.83823	35.29454	59.93847	18.89915	Detected	Detected	Detected	Detected
3587	A_33_P3399788	SERPINA3	Hs.534293	serpin peptidase inhibitor, clade A (alpha-1 antiproteinase, antitrypsin), member 3	Homo sapiens serpin peptidase inhibitor, clade A (alpha-1 antiproteinase, antitrypsin), member 3 (SERPINA3), mRNA [NM_001085]	1.96028	up	2.19322	up	468.69238	247.57309	688.07960	276.64478	Detected	Detected	Detected	Detected
3588	A_33_P3424867					1.96003	up	2.02554	up	495.41367	261.72086	274.30426	119.41446	Detected	Detected	Detected	Detected
3589	A_24_P191833	SFRS12	Hs.519347	splicing factor, arginine/serine-rich 12	Homo sapiens splicing factor, arginine/serine-rich 12 (SFRS12), transcript variant 2, mRNA [NM_139168]	1.95681	up	2.17618	up	2209.34000	1169.08870	1417.37290	574.32166	Detected	Detected	Detected	Detected
3590	A_23_P105923	DIO3	Hs.49322	deiodinase, iodothyronine, type III	Homo sapiens deiodinase, iodothyronine, type III (DIO3), mRNA [NM_001362]	1.95624	up	4.94579	up	34.90253	18.47426	23.13339	4.12448	Detected	Detected	Detected	Compromised
3591	A_23_P348257	NUAK1	Hs.524692	NUAK family, SNF1-like kinase, 1	Homo sapiens NUAK family, SNF1-like kinase, 1 (NUAK1), mRNA [NM_014840]	1.95577	up	2.04984	up	3859.91750	2043.58860	2831.93260	1218.22630	Detected	Detected	Detected	Detected
3592	A_33_P3862375	USP45	Hs.143410	ubiquitin specific peptidase 45	Homo sapiens ubiquitin specific peptidase 45 (USP45), mRNA [NM_001080481]	1.95320	up	2.10659	up	487.17075	258.26575	367.82280	153.96545	Detected	Detected	Detected	Detected
3593	A_24_P325176	KIAA1109	Hs.408142	KIAA1109	Homo sapiens KIAA1109 (KIAA1109), mRNA [NM_015312]	1.95286	up	2.15094	up	974.44650	516.67834	564.38150	231.37180	Detected	Detected	Detected	Detected
3594	A_24_P74064	ZFP161	Hs.592340	zinc finger protein 161 homolog (mouse)	Homo sapiens zinc finger protein 161 homolog (mouse) (ZFP161), transcript variant 2, mRNA [NM_003409]	1.95242	up	2.04635	up	742.17470	393.61066	549.32740	236.70975	Detected	Detected	Detected	Detected
3595	A_23_P88909	SYNGR3	Hs.435277	synaptogyrin 3	Homo sapiens synaptogyrin 3 (SYNGR3), mRNA [NM_004209]	1.95205	up	2.03137	up	172.61182	91.56157	183.05075	79.45996	Detected	Detected	Detected	Detected
3596	A_33_P3352822	ZNF547	Hs.446620	zinc finger protein 547	Homo sapiens zinc finger protein 547 (ZNF547), mRNA [NM_173631]	1.95059	up	2.35983	up	246.33936	130.76773	153.37865	57.31263	Detected	Detected	Detected	Detected

3597	A_33_P3389852	BCAS3	Hs.655028	breast carcinoma amplified sequence 3	Homo sapiens breast carcinoma amplified sequence 3 (BCAS3), transcript variant 2, mRNA [NM_017679]	1.94981	up	2.29034	up	89.49251	47.52570	83.55839	32.17040	Detected	Detected	Detected	Detected
3598	A_23_P33914	NUP62CL	Hs.163629	nucleoporin 62kDa C-terminal like	Homo sapiens nucleoporin 62kDa C-terminal like (NUP62CL), mRNA [NM_017681]	1.94964	up	2.20132	up	13.44632	7.14138	6.70081	2.68417	Detected	Compromised	Compromised	Compromised
3599	A_23_P142075	ACP5	Hs.1211	acid phosphatase 5, tartrate resistant	Homo sapiens acid phosphatase 5, tartrate resistant (ACP5), transcript variant 4, mRNA [NM_001611]	1.94931	up	2.08082	up	2288.90580	1215.85300	2310.70750	979.21124	Detected	Detected	Detected	Detected
3600	A_23_P149613	FMO1	Hs.1424	flavin containing monooxygenase 1	Homo sapiens flavin containing monooxygenase 1 (FMO1), mRNA [NM_002021]	1.94919	up	5.07418	up	191.70462	101.83814	120.43762	20.92966	Detected	Detected	Detected	Detected
3601	A_32_P114215	COMMD6	Hs.508266	COMM domain containing 6	Homo sapiens COMM domain containing 6 (COMMD6), transcript variant 1, mRNA [NM_203497]	1.94844	up	2.01250	up	35378.78000	18801.35700	31208.02300	13674.04000	Detected	Detected	Detected	Detected
3602	A_33_P3288074	LOC730202	Hs.647565	hypothetical protein LOC730202	PREDICTED: Homo sapiens hypothetical protein LOC730202 (LOC730202), miscRNA [XR_041804]	1.94813	up	2.36779	up	48.59230	25.82748	41.03780	15.28294	Detected	Detected	Detected	Detected
3603	A_33_P3379406				Protein GREB1 (Gene regulated in breast cancer 1 protein) [Source:UniProtKB/Swiss-Prot;Acc:Q4ZG55] [ENST00000389825]	1.94772	up	3.15305	up	44.66525	23.74519	30.83545	8.62353	Detected	Detected	Detected	Compromised
3604	A_24_P267522	ZNF585A	Hs.390568	zinc finger protein 585A	Homo sapiens zinc finger protein 585A (ZNF585A), transcript variant 1, mRNA [NM_152655]	1.94665	up	2.65558	up	19.30548	10.26894	17.28775	5.74043	Detected	Compromised	Detected	Compromised
3605	A_24_P945194	PDCD6IP	Hs.475896	programmed cell death 6 interacting protein	Homo sapiens programmed cell death 6 interacting protein (PDCD6IP), transcript variant 1, mRNA [NM_013374]	1.94354	up	2.11896	up	869.12340	463.04330	454.36090	189.07930	Detected	Detected	Detected	Detected
3606	A_33_P3388958	SNX32	Hs.591950	sorting nexin 32	Homo sapiens sorting nexin 32 (SNX32), mRNA [NM_152760]	1.94323	up	3.13674	up	402.90420	214.68896	303.47098	85.31072	Detected	Detected	Detected	Detected
3607	A_24_P236235	FLRT2	Hs.533710	fibronectin leucine rich transmembrane protein 2	Homo sapiens fibronectin leucine rich transmembrane protein 2 (FLRT2), mRNA [NM_013231]	1.94289	up	2.00799	up	454.28943	242.11241	260.16240	114.24820	Detected	Detected	Detected	Detected
3608	A_32_P475513	MYO15B	Hs.390817	myosin XVB pseudogene	Homo sapiens myosin XVB pseudogene (MYO15B), non-coding RNA [NR_003587]	1.94167	up	2.04561	up	20.01581	10.67407	11.82322	5.09658	Detected	Compromised	Compromised	Compromised
3609	A_24_P125283	HDAC5	Hs.438782	histone deacetylase 5	Homo sapiens histone deacetylase 5 (HDAC5), transcript variant 3, mRNA [NM_001015053]	1.94125	up	2.59723	up	2382.78860	1270.97830	1894.72970	643.28290	Detected	Detected	Detected	Detected
3610	A_33_P3239102					1.94053	up	2.63975	up	7676.01200	4095.89720	1228.99290	410.53683	Detected	Detected	Detected	Detected
3611	A_33_P3338417	WARS2	Hs.523506	tryptophanyl tRNA synthetase 2, mitochondrial	Homo sapiens tryptophanyl tRNA synthetase 2, mitochondrial (WARS2), nuclear gene encoding mitochondrial protein, transcript variant 2, mRNA [NM_201263]	1.93926	up	2.00955	up	1954.38230	1043.53530	1138.79980	499.70520	Detected	Detected	Detected	Detected
3612	A_23_P431569		Hs.655804		LOC100049716 protein Fragment [Source:UniProtKB/TrEMBL;Acc:Q8N503] [ENST00000318291]	1.93842	up	2.11263	up	402.96573	215.25511	256.92444	107.23760	Detected	Detected	Detected	Detected
3613	A_32_P355396	TECPR2	Hs.195667	tectonin beta-propeller repeat containing 2	Homo sapiens tectonin beta-propeller repeat containing 2 (TECPR2), mRNA [NM_014844]	1.93799	up	2.12611	up	2044.25210	1092.23400	1340.06680	555.78470	Detected	Detected	Detected	Detected
3614	A_24_P359322	C21orf49	Hs.54725	chromosome 21 open reading frame 49	Homo sapiens chromosome 21 open reading frame 49 (C21orf49), transcript variant 1, non-coding RNA [NR_024622]	1.93556	up	3.53055	up	11.80604	6.31582	23.10554	5.77085	Compromised	Compromised	Detected	Compromised
3615	A_33_P3216083	SHPRH	Hs.124537	SNF2 histone linker PHD RING helicase	Homo sapiens SNF2 histone linker PHD RING helicase (SHPRH), transcript variant 2, mRNA [NM_173082]	1.93408	up	2.07296	up	45.15646	24.17563	21.03370	8.94726	Detected	Detected	Detected	Compromised
3616	A_24_P388940	C6orf162	Hs.70769	chromosome 6 open reading frame 162	Homo sapiens chromosome 6 open reading frame 162 (C6orf162), transcript variant 1, mRNA [NM_001042493]	1.93405	up	2.25417	up	276.49905	148.03285	284.25803	111.19660	Detected	Detected	Detected	Detected
3617	A_23_P102950	RSPH1	Hs.661069	radial spoke head 1 homolog (Chlamydomonas)	Homo sapiens radial spoke head 1 homolog (Chlamydomonas) (RSPH1), mRNA [NM_080860]	1.93178	up	5.58034	up	39.55026	21.19944	22.31084	3.52550	Detected	Detected	Detected	Compromised
3618	A_23_P319013	ZNF383	Hs.590975	zinc finger protein 383	Homo sapiens zinc finger protein 383 (ZNF383), mRNA [NM_152604]	1.93174	up	2.15646	up	438.21832	234.89507	336.61990	137.64636	Detected	Detected	Detected	Detected
3619	A_33_P3270793	FAM84A	Hs.260855	family with sequence similarity 84, member A	Homo sapiens family with sequence similarity 84, member A (FAM84A), mRNA [NM_145175]	1.93117	up	2.61595	up	38.46104	20.62212	51.00356	17.19239	Detected	Detected	Detected	Detected
3620	A_33_P3415097	CLCN4	Hs.495674	chloride channel 4	Homo sapiens chloride channel 4 (CLCN4), mRNA [NM_001830]	1.92935	up	2.21027	up	578.52527	310.48780	478.53134	190.91081	Detected	Detected	Detected	Detected
3621	A_23_P204782	MDM1	Hs.655702	Mdm1 nuclear protein homolog (mouse)	Homo sapiens Mdm1 nuclear protein homolog (mouse) (MDM1), transcript variant 2, mRNA [NM_020128]	1.92874	up	2.01316	up	334.00262	179.31192	280.71756	122.95801	Detected	Detected	Detected	Detected
3622	A_24_P35169	GATAD1	Hs.21145	GATA zinc finger domain containing 1	Homo sapiens GATA zinc finger domain containing 1 (GATAD1), mRNA [NM_021167]	1.92833	up	2.00450	up	1446.44890	776.70350	1204.25480	529.75800	Detected	Detected	Detected	Detected
3623	A_23_P433132	KRTCAP3	Hs.59509	keratinocyte associated protein 3	Homo sapiens keratinocyte associated protein 3 (KRTCAP3), mRNA [NM_173853]	1.92637	up	2.06529	up	45.30082	24.35008	33.63108	14.35907	Detected	Detected	Detected	Compromised
3624	A_24_P367432	LOC100133862		similar to hCG1773549	Rheumatoid factor RF-IP9 Fragment [Source:UniProtKB/TrEMBL;Acc:A2J1M2] [ENST00000390633]	1.92506	up	2.79223	up	5.77586	3.10674	26.13817	8.25449	Compromised	Compromised	Detected	Compromised
3625	A_33_P3294598	PCDH10	Hs.192859	protocadherin 10	Homo sapiens protocadherin 10 (PCDH10), transcript variant 2, mRNA [NM_020815]	1.92315	up	3.61506	up	8.52342	4.58916	12.21554	2.97963	Compromised	Compromised	Detected	Compromised
3626	A_24_P845072	FAM91A1	Hs.459174	family with sequence similarity 91, member A1	Homo sapiens family with sequence similarity 91, member A1 (FAM91A1), mRNA [NM_144963]	1.92183	up	2.24668	up	966.61190	520.80035	676.36316	265.46286	Detected	Detected	Detected	Detected

3627	A_24_P196534	PKNOX1	Hs.431043	PBX/knotted 1 homeobox 1	Homo sapiens PBX/knotted 1 homeobox 1 (PKNOX1), mRNA [NM_004571]	1.92100	up	2.28377	up	1917.38000	1033.50900	1360.55700	525.32640	Detected	Detected	Detected	Detected
3628	A_23_P23279	RCSD1	Hs.493867	RCSD domain containing 1	Homo sapiens RCSD domain containing 1 (RCSD1), mRNA [NM_052862]	1.91990	up	2.43460	up	69.52772	37.49831	62.32583	22.57387	Detected	Detected	Detected	Detected
3629	A_32_P358115	LOC284648	Hs.253475	hypothetical protein LOC284648	PREDICTED: Homo sapiens hypothetical protein LOC284648 (LOC284648), mRNA [XM_001715964]	1.91952	up	2.58295	up	14.08319	7.59700	8.64931	2.95278	Detected	Compromised	Compromised	Compromised
3630	A_24_P116378	GCOM1	Hs.437256	GRINL1A complex locus	Homo sapiens GRINL1A complex locus (GCOM1), transcript variant 1, mRNA [NM_001018090]	1.91887	up	2.01558	up	2131.66920	1150.28760	1187.85400	519.67065	Detected	Detected	Detected	Detected
3631	A_24_P416159	RBL2	Hs.513609	retinoblastoma-like 2 (p130)	Homo sapiens retinoblastoma-like 2 (p130) (RBL2), mRNA [NM_005611]	1.91696	up	2.14528	up	1082.56750	584.75775	802.85065	330.00247	Detected	Detected	Detected	Detected
3632	A_33_P3311439	GCH1	Hs.86724	GTP cyclohydrolase 1	Homo sapiens GTP cyclohydrolase 1 (GCH1), transcript variant 4, mRNA [NM_001024071]	1.91407	up	3.46100	up	63.65474	34.43549	61.84089	15.75579	Detected	Detected	Detected	Detected
3633	A_23_P163408	SCAPER	Hs.458986	S-phase cyclin A-associated protein in the ER	Homo sapiens S-phase cyclin A-associated protein in the ER (SCAPER), transcript variant 1, mRNA [NM_020843]	1.91260	up	2.65442	up	113.12848	61.24646	97.63134	32.43288	Detected	Detected	Detected	Detected
3634	A_23_P348383	CC2D2A	Hs.590928	coiled-coil and C2 domain containing 2A	Homo sapiens coiled-coil and C2 domain containing 2A (CC2D2A), transcript variant 1, mRNA [NM_001080522]	1.91147	up	2.08874	up	240.12137	130.07591	165.59587	69.90871	Detected	Detected	Detected	Detected
3635	A_33_P3214466	MESP1	Hs.447531	mesoderm posterior 1 homolog (mouse)	Homo sapiens mesoderm posterior 1 homolog (mouse) (MESP1), mRNA [NM_018670]	1.90898	up	2.11017	up	189.52153	102.79931	156.76414	65.50794	Detected	Detected	Detected	Detected
3636	A_23_P157726	DENND4C	Hs.249591	DENN/MADD domain containing 4C	Homo sapiens DENN/MADD domain containing 4C (DENND4C), mRNA [NM_017925]	1.90830	up	2.03087	up	2634.84860	1429.68870	2352.46560	1021.42630	Detected	Detected	Detected	Detected
3637	A_23_P34433	ZCCHC11	Hs.655407	zinc finger, CCHC domain containing 11	Homo sapiens zinc finger, CCHC domain containing 11 (ZCCHC11), transcript variant 1, mRNA [NM_001009881]	1.90830	up	2.05636	up	1511.03780	819.90106	689.37213	295.61053	Detected	Detected	Detected	Detected
3638	A_33_P3254751	LOC100131355	Hs.660782	hypothetical protein LOC100131355	Homo sapiens cDNA FLJ42223 fis, clone THYMU2039989. [AK124217]	1.90665	up	2.51456	up	91.69806	49.79923	66.61211	23.35912	Detected	Detected	Detected	Detected
3639	A_33_P3341494	ZNF658	Hs.522147	zinc finger protein 658	Homo sapiens zinc finger protein 658 (ZNF658), mRNA [NM_033160]	1.90383	up	2.39002	up	180.87292	98.37334	152.45934	56.24946	Detected	Detected	Detected	Detected
3640	A_23_P361049	MYO1B	Hs.439620	myosin IB	Homo sapiens myosin IB (MYO1B), transcript variant 2, mRNA [NM_012223]	1.90310	up	2.14649	up	11705.36300	6368.77340	6133.96600	2519.86940	Detected	Detected	Detected	Detected
3641	A_23_P35645	RBM17	Hs.498548	RNA binding motif protein 17	Homo sapiens RNA binding motif protein 17 (RBM17), transcript variant 1, mRNA [NM_032905]	1.89794	up	2.08772	up	5679.22170	3098.40920	5591.55100	2361.70200	Detected	Detected	Detected	Detected
3642	A_33_P3352958	FLJ35220	Hs.389678	hypothetical protein FLJ35220	Homo sapiens hypothetical protein FLJ35220 (FLJ35220), transcript variant 3, mRNA [NM_001164638]	1.89631	up	2.04115	up	271.11243	148.03769	382.62598	165.29713	Detected	Detected	Detected	Detected
3643	A_23_P103486	CYP2J2	Hs.152096	cytochrome P450, family 2, subfamily J, polypeptide 2	Homo sapiens cytochrome P450, family 2, subfamily J, polypeptide 2 (CYP2J2), mRNA [NM_000775]	1.89550	up	2.04884	up	122.54324	66.94190	69.79430	30.03850	Detected	Detected	Detected	Detected
3644	A_23_P28485	GCA	Hs.377894	grancalcin, EF-hand calcium binding protein	Homo sapiens grancalcin, EF-hand calcium binding protein (GCA), mRNA [NM_012198]	1.89486	up	2.08578	up	543.39710	296.94330	378.93396	160.19928	Detected	Detected	Detected	Detected
3645	A_33_P3327587	LOC100127904	Hs.632266	hypothetical LOC100127904	PREDICTED: Homo sapiens hypothetical LOC100127904 (LOC100127904), miscRNA [XR_078564]	1.89380	up	2.58811	up	317.74900	173.73337	55.11639	18.77859	Detected	Detected	Detected	Detected
3646	A_23_P377965	C18orf18	Hs.657197	chromosome 18 open reading frame 18	Homo sapiens chromosome 18 open reading frame 18 (C18orf18), non-coding RNA [NR_026849]	1.89345	up	2.89130	up	352.27330	192.64587	219.24927	66.86680	Detected	Detected	Detected	Detected
3647	A_24_P161036	ACOT1	Hs.568046	acyl-CoA thioesterase 1	Homo sapiens acyl-CoA thioesterase 1 (ACOT1), mRNA [NM_001037161]	1.89304	up	2.07108	up	17085.04900	9345.22000	16663.53000	7094.71100	Detected	Detected	Detected	Detected
3648	A_33_P3348011	LOC222699	Hs.161441	transducer of ERBB2, 2 pseudogene	Homo sapiens transducer of ERBB2, 2 pseudogene (LOC222699), non-coding RNA [NR_002936]	1.88910	up	2.16438	up	306.09555	167.77763	253.94376	103.45911	Detected	Detected	Detected	Detected
3649	A_32_P210642	EGFL7	Hs.91481	EGF-like-domain, multiple 7	Homo sapiens EGF-like-domain, multiple 7 (EGFL7), transcript variant 2, mRNA [NM_201446]	1.87891	up	2.14133	up	1151.68600	634.69070	1178.89650	485.46405	Detected	Detected	Detected	Detected
3650	A_33_P3312807	IPW		imprinted in Prader-Willi syndrome (non-protein coding)	Homo sapiens imprinted in Prader-Willi syndrome (non-protein coding) (IPW), non-coding RNA [NR_023915]	1.87465	up	2.16767	up	83.59007	46.17073	56.35529	22.92488	Detected	Detected	Detected	Detected
3651	A_24_P305933	TMCC3	Hs.370410	transmembrane and coiled-coil domain family 3	Homo sapiens transmembrane and coiled-coil domain family 3 (TMCC3), mRNA [NM_020698]	1.87374	up	2.54699	up	283.08734	156.43910	251.12468	86.94157	Detected	Detected	Detected	Detected
3652	A_33_P3401990	VPREB3	Hs.136713	pre-B lymphocyte 3	Homo sapiens pre-B lymphocyte 3 (VPREB3), mRNA [NM_013378]	1.87310	up	2.50424	up	108.16008	59.79153	79.51505	27.99874	Detected	Detected	Detected	Detected
3653	A_23_P77529	MSLN	Hs.408488	mesothelin	Homo sapiens mesothelin (MSLN), transcript variant 1, mRNA [NM_005823]	1.87142	up	2.09734	up	23.33619	12.91192	8.48618	3.56787	Detected	Compromised	Compromised	Compromised
3654	A_24_P108291	IMPACT	Hs.515317	Impact homolog (mouse)	Homo sapiens Impact homolog (mouse) (IMPACT), mRNA [NM_018439]	1.87043	up	2.14477	up	840.16064	465.10696	525.59143	216.08942	Detected	Detected	Detected	Detected
3655	A_23_P363878	RFTN2	Hs.591615	raftlin family member 2	Homo sapiens raftlin family member 2 (RFTN2), mRNA [NM_144629]	1.86953	up	2.07171	up	92.76392	51.37819	92.49509	39.36906	Detected	Detected	Detected	Detected
3656	A_24_P370096	ZNF230	Hs.193583	zinc finger protein 230	Homo sapiens zinc finger protein 230 (ZNF230), mRNA [NM_006300]	1.86583	up	2.10162	up	224.00919	124.31620	162.65820	68.24775	Detected	Detected	Detected	Detected
3657	A_33_P3337627	TRPC6	Hs.159003	transient receptor potential cation channel, subfamily C, member 6	Homo sapiens transient receptor potential cation channel, subfamily C, member 6 (TRPC6), mRNA [NM_004621]	1.86313	up	2.07714	up	10329.43500	5740.72660	667.89000	283.53380	Detected	Detected	Detected	Detected

3658	A_33_P3339987	LOC100128655		similar to hCG1645245	PREDICTED: Homo sapiens similar to hCG1645245 (LOC100128655), mRNA [XM_001716495]	1.86287	up	2.09937	up	13.70002	7.61504	20.37450	8.55783	Detected	Compromised	Detected	Compromised
3659	A_23_P379746	DENND5B	Hs.118166	DENN/MADD domain containing 5B	Homo sapiens DENN/MADD domain containing 5B (DENND5B), mRNA [NM_144973]	1.86276	up	2.00071	up	380.99630	211.78563	318.52963	140.38838	Detected	Detected	Detected	Detected
3660	A_33_P3242508	ZNF365	Hs.22653	zinc finger protein 365	Homo sapiens zinc finger protein 365 (ZNF365), transcript variant B, mRNA [NM_199450]	1.86269	up	2.89938	up	21.38459	11.88760	21.51624	6.54375	Detected	Compromised	Detected	Compromised
3661	A_32_P25737	CHIC1	Hs.496323	cysteine-rich hydrophobic domain 1	Homo sapiens cysteine-rich hydrophobic domain 1 (CHIC1), mRNA [NM_001039840]	1.86210	up	2.10062	up	234.72844	130.52571	164.73367	69.15125	Detected	Detected	Detected	Detected
3662	A_23_P413923	DMRTA1	Hs.371976	DMRT-like family A1	Homo sapiens DMRT-like family A1 (DMRTA1), mRNA [NM_022160]	1.86192	up	3.33913	up	21.53626	11.97686	23.54715	6.21829	Detected	Compromised	Detected	Compromised
3663	A_23_P215318	VPS41	Hs.592184	vacuolar protein sorting 41 homolog (S. cerevisiae)	Homo sapiens vacuolar protein sorting 41 homolog (S. cerevisiae) (VPS41), transcript variant 1, mRNA [NM_014396]	1.85976	up	2.13759	up	593.43646	330.40717	527.13000	217.44990	Detected	Detected	Detected	Detected
3664	A_33_P3289338	LOC100131346	Hs.640125	hypothetical protein LOC100131346	Homo sapiens cDNA FLJ45704 fis, clone FEBRA2026977. [AK127606]	1.85894	up	2.29898	up	17.68627	9.85156	14.18271	5.43988	Detected	Compromised	Compromised	Compromised
3665	A_33_P3245480		Hs.656830		hypothetical LOC728855 (LOC728855), non-coding RNA [Source:RefSeq DNA;Acc:NR_024510] [ENST00000412305]	1.85862	up	3.55237	up	17.46534	9.73013	25.42032	6.30999	Detected	Compromised	Detected	Compromised
3666	A_33_P3241051	ADAMTS17	Hs.513200	ADAM metallopeptidase with thrombospondin type 1 motif, 17	Homo sapiens ADAM metallopeptidase with thrombospondin type 1 motif, 17 (ADAMTS17), mRNA [NM_139057]	1.85748	up	4.02284	up	14.46294	8.06244	12.66675	2.77651	Detected	Compromised	Detected	Compromised
3667	A_33_P3242493	PIBF1	Hs.441926	progesterone immunomodulatory binding factor 1	Homo sapiens progesterone immunomodulatory binding factor 1 (PIBF1), mRNA [NM_006346]	1.85562	up	2.30987	up	68.03877	37.96658	59.84474	22.84572	Detected	Detected	Detected	Detected
3668	A_23_P31739	STMN4	Hs.201058	stathmin-like 4	Homo sapiens stathmin-like 4 (STMN4), mRNA [NM_030795]	1.85484	up	8.24674	up	41.53862	23.18884	26.36396	2.81899	Detected	Detected	Detected	Compromised
3669	A_23_P428640	PPP1R3E	Hs.601513	protein phosphatase 1, regulatory (inhibitor) subunit 3E	Homo sapiens protein phosphatase 1, regulatory (inhibitor) subunit 3E (PPP1R3E), non-coding RNA [NR_026862]	1.85392	up	2.66505	up	84.25430	47.05807	92.37248	30.56352	Detected	Detected	Detected	Detected
3670	A_33_P3571254	CG012	Hs.687692	hypothetical gene CG012	Novel human gene mapping to chomosome 13 [AL049782]	1.85376	up	2.55187	up	462.74040	258.47446	268.33850	92.72365	Detected	Detected	Detected	Detected
3671	A_33_P3292235	PAQR3		progestin and adipoQ receptor family member III	Homo sapiens progestin and adipoQ receptor family member III, mRNA (cDNA clone MGC:39698 IMAGE:5271599), complete cds. [BC031256]	1.85369	up	2.11019	up	94.45179	52.76030	68.33144	28.55389	Detected	Detected	Detected	Detected
3672	A_23_P384329	DENND4A	Hs.654567	DENN/MADD domain containing 4A	Homo sapiens DENN/MADD domain containing 4A (DENND4A), transcript variant 1, mRNA [NM_001144823]	1.85355	up	2.36099	up	41.46604	23.16443	43.07253	16.08686	Detected	Detected	Detected	Compromised
3673	A_23_P34827	HCN3	Hs.706960	hyperpolarization activated cyclic nucleotide-gated potassium channel 3	Homo sapiens hyperpolarization activated cyclic nucleotide-gated potassium channel 3 (HCN3), mRNA [NM_020897]	1.85180	up	2.57895	up	159.80170	89.35528	113.56823	38.83109	Detected	Detected	Detected	Detected
3674	A_33_P3278515	LOC642340	Hs.655951	hypothetical LOC642340	PREDICTED: Homo sapiens hypothetical LOC642340 (LOC642340), mRNA [XM_001714255]	1.85083	up	2.09809	up	42.69819	23.88781	35.24102	14.81118	Detected	Detected	Detected	Detected
3675	A_32_P11230	LOC399744	Hs.534942	hypothetical LOC399744	Homo sapiens hypothetical LOC399744 (LOC399744), non-coding RNA [NR_024497]	1.85052	up	2.12715	up	1065.11900	595.98706	752.12250	311.78580	Detected	Detected	Detected	Detected
3676	A_33_P3276207	FAM55C	Hs.595933	family with sequence similarity 55, member C	Homo sapiens family with sequence similarity 55, member C (FAM55C), transcript variant 1, mRNA [NM_001134456]	1.84993	up	2.69171	up	150.68631	84.34351	189.42834	62.05583	Detected	Detected	Detected	Detected
3677	A_33_P3229527	LOC100134387		similar to DEAH (Asp-Glu-Ala-His) box polypeptide 40	PREDICTED: Homo sapiens similar to DEAH (Asp-Glu-Ala-His) box polypeptide 40 (LOC100134387), mRNA [XM_001717070]	1.84897	up	2.11564	up	265.18997	148.51180	229.39333	95.61038	Detected	Detected	Detected	Detected
3678	A_23_P373927	ERP44	Hs.154023	endoplasmic reticulum protein 44	Homo sapiens endoplasmic reticulum protein 44 (ERP44), mRNA [NM_015051]	1.84666	up	2.19343	up	184.82797	103.63680	121.40168	48.80535	Detected	Detected	Detected	Detected
3679	A_33_P3322589	FANCL	Hs.720331	Fanconi anemia, complementation group L	Homo sapiens Fanconi anemia, complementation group L (FANCL), transcript variant 1, mRNA [NM_001114636]	1.84645	up	2.08721	up	283.66486	159.07448	208.92177	88.26406	Detected	Detected	Detected	Detected
3680	A_33_P3446495	FRMD4B	Hs.709671	FERM domain containing 4B	Homo sapiens FERM domain containing 4B (FRMD4B), mRNA [NM_015123]	1.84449	up	2.95349	up	20.72036	11.63199	9.69492	2.89451	Detected	Compromised	Compromised	Compromised
3681	A_23_P157527	LRRCC1	Hs.193115	leucine rich repeat and coiled-coil domain containing 1	Homo sapiens leucine rich repeat and coiled-coil domain containing 1 (LRRCC1), transcript variant 1, mRNA [NM_033402]	1.84226	up	2.15777	up	219.86043	123.57446	146.89542	60.03005	Detected	Detected	Detected	Detected
3682	A_23_P140290	RTN1	Hs.368626	reticulon 1	Homo sapiens reticulon 1 (RTN1), transcript variant 1, mRNA [NM_021136]	1.84220	up	2.86871	up	17.32572	9.73843	13.27895	4.08172	Detected	Compromised	Detected	Compromised
3683	A_33_P3333627	PHACTR1	Hs.436996	phosphatase and actin regulator 1	Homo sapiens phosphatase and actin regulator 1 (PHACTR1), mRNA [NM_030948]	1.84194	up	4.14308	up	33.95574	19.08847	49.32464	10.49799	Detected	Detected	Detected	Compromised
3684	A_33_P3364443	LOC388279	Hs.681782	hypothetical gene supported by AF275804	PREDICTED: Homo sapiens hypothetical LOC388279 (LOC388279), mRNA [XM_373688]	1.84148	up	7.22290	up	41.89097	23.55514	33.57396	4.09880	Detected	Detected	Detected	Compromised
3685	A_23_P69154	FAM198A	Hs.720172	family with sequence similarity 198, member A	Homo sapiens family with sequence similarity 198, member A (FAM198A), mRNA [NM_001129908]	1.84136	up	2.23986	up	23.29176	13.09774	29.10685	11.45882	Detected	Compromised	Detected	Compromised
3686	A_33_P3333537	VPS13C	Hs.511668	vacuolar protein sorting 13 homolog C (S. cerevisiae)	Homo sapiens vacuolar protein sorting 13 homolog C (S. cerevisiae) (VPS13C), transcript variant 2B, mRNA [NM_001018088]	1.84130	up	2.06404	up	137.52003	77.33466	145.76201	62.27171	Detected	Detected	Detected	Detected

3687	A_33_P3333327					1.83984	up	2.65391	up	37.96538	21.36691	28.24818	9.38576	Detected	Detected	Detected	Compromised
3688	A_24_P115990	AMHR2	Hs.659889	anti-Mullerian hormone receptor, type II	Homo sapiens anti-Mullerian hormone receptor, type II (AMHR2), transcript variant 1, mRNA [NM_020547]	1.83957	up	8.62239	up	5.33054	3.00045	31.39744	3.21094	Compromised	Compromised	Detected	Compromised
3689	A_23_P169092	CYP7B1	Hs.667720	cytochrome P450, family 7, subfamily B, polypeptide 1	Homo sapiens cytochrome P450, family 7, subfamily B, polypeptide 1 (CYP7B1), mRNA [NM_004820]	1.83955	up	2.19712	up	101.53169	57.15080	121.74761	48.86221	Detected	Detected	Detected	Detected
3690	A_33_P3392460	LOC100128077	Hs.710111	hypothetical protein LOC100128077	PREDICTED: Homo sapiens hypothetical protein LOC100128077 (LOC100128077), miscRNA [XR_078411]	1.83889	up	2.17058	up	10.22851	5.75956	16.32040	6.63012	Compromised	Compromised	Detected	Compromised
3691	A_23_P345564	OPRL1	Hs.2859	opiate receptor-like 1	Homo sapiens opiate receptor-like 1 (OPRL1), transcript variant 1, mRNA [NM_182647]	1.83745	up	2.11890	up	106.92088	60.25311	94.40613	39.28757	Detected	Detected	Detected	Detected
3692	A_33_P3249743		Hs.370479		Putative uncharacterized protein ENSP00000305441 Fragment [Source:UniProtKB/TrEMBL;Acc:B5MDC0] [ENST00000304813]	1.83648	up	2.20821	up	15.92774	8.98049	9.11617	3.64030	Detected	Compromised	Compromised	Compromised
3693	A_23_P35970	SLC37A4	Hs.719203	solute carrier family 37 (glucose-6-phosphate transporter), member 4	Homo sapiens solute carrier family 37 (glucose-6-phosphate transporter), member 4 (SLC37A4), transcript variant 4, mRNA [NM_001467]	1.83565	up	2.21137	up	1397.58560	788.35450	1237.96850	493.64480	Detected	Detected	Detected	Detected
3694	A_33_P3303449	LEPR	Hs.23581	leptin receptor	Homo sapiens leptin receptor (LEPR), transcript variant 2, mRNA [NM_001003680]	1.83519	up	2.17437	up	121.97288	68.81999	113.61305	46.07445	Detected	Detected	Detected	Detected
3695	A_33_P3334185	AGTPBP1	Hs.719980	ATP/GTP binding protein 1	Homo sapiens ATP/GTP binding protein 1 (AGTPBP1), mRNA [NM_015239]	1.83319	up	2.68594	up	23.93548	13.51973	21.73516	7.13562	Detected	Compromised	Detected	Compromised
3696	A_23_P310086	BEND6	Hs.582993	BEN domain containing 6	Homo sapiens BEN domain containing 6 (BEND6), mRNA [NM_152731]	1.83295	up	2.08022	up	138.32306	78.14058	133.94261	56.77732	Detected	Detected	Detected	Detected
3697	A_23_P29773	LAMP3	Hs.518448	lysosomal-associated membrane protein 3	Homo sapiens lysosomal-associated membrane protein 3 (LAMP3), mRNA [NM_014398]	1.83188	up	3.67405	up	30.17534	17.05645	17.42260	4.18152	Detected	Compromised	Detected	Compromised
3698	A_24_P573533	CBWD5	Hs.645337	COBW domain containing 5	Homo sapiens COBW domain containing 5 (CBWD5), mRNA [NM_001024916]	1.83000	up	2.04573	up	504.75640	285.60397	130.44746	56.22807	Detected	Detected	Detected	Detected
3699	A_33_P3370521	LOC100133008	Hs.576457	hypothetical LOC100133008	PREDICTED: Homo sapiens hypothetical LOC100133008 (LOC100133008), mRNA [XM_001723587]	1.82959	up	2.05971	up	6159.20600	3485.82030	721.81836	309.02112	Detected	Detected	Detected	Detected
3700	A_33_P3236177	ANG	Hs.283749	angiogenin, ribonuclease, RNase A family, 5	Homo sapiens angiogenin, ribonuclease, RNase A family, 5 (ANG), transcript variant 1, mRNA [NM_001145]	1.82452	up	2.07483	up	2500.26070	1418.95530	2943.07280	1250.78910	Detected	Detected	Detected	Detected
3701	A_24_P69538	TLR4	Hs.174312	toll-like receptor 4	Homo sapiens toll-like receptor 4 (TLR4), transcript variant 1, mRNA [NM_138554]	1.82287	up	2.16676	up	203.27806	115.46950	209.31049	85.18146	Detected	Detected	Detected	Detected
3702	A_33_P3278220		Hs.19012		Rab9 effector protein with Kelch motifs (40 kDa Rab9 effector protein)(p40) [Source:UniProtKB/Swiss-Prot;Acc:Q7Z6M1] [ENST00000373544]	1.82210	up	2.15330	up	8955.37500	5089.15530	920.98540	377.14893	Detected	Detected	Detected	Detected
3703	A_33_P3378047	SESTD1	Hs.30977	SEC14 and spectrin domains 1	Homo sapiens SEC14 and spectrin domains 1 (SESTD1), mRNA [NM_178123]	1.82080	up	2.04299	up	1536.53660	873.80225	1113.96970	480.80798	Detected	Detected	Detected	Detected
3704	A_23_P432610	N4BP1	Hs.511839	NEDD4 binding protein 1	Homo sapiens NEDD4 binding protein 1 (N4BP1), mRNA [NM_153029]	1.81915	up	2.58148	up	521.91565	297.07404	455.26736	155.51193	Detected	Detected	Detected	Detected
3705	A_23_P55616	SLC14A1	Hs.101307	solute carrier family 14 (urea transporter), member 1 (Kidd blood group)	Homo sapiens solute carrier family 14 (urea transporter), member 1 (Kidd blood group) (SLC14A1), transcript variant 4, mRNA [NM_001146037]	1.81860	up	4.29687	up	16.12224	9.17954	15.61083	3.20360	Detected	Compromised	Detected	Compromised
3706	A_33_P3350703	ERO1LB	Hs.558519	ERO1-like beta (S. cerevisiae)	Homo sapiens ERO1-like beta (S. cerevisiae) (ERO1LB), mRNA [NM_019891]	1.81795	up	2.26649	up	228.95142	130.40518	175.89957	68.43462	Detected	Detected	Detected	Detected
3707	A_23_P105562	VWF	Hs.440848	von Willebrand factor	Homo sapiens von Willebrand factor (VWF), mRNA [NM_000552]	1.81764	up	2.32779	up	82.05867	46.74668	78.85735	29.87193	Detected	Detected	Detected	Detected
3708	A_33_P3424347	LOC100131271	Hs.624900	hypothetical protein LOC100131271	PREDICTED: Homo sapiens hypothetical LOC100131271 (LOC100131271), mRNA [XM_001716273]	1.81747	up	3.28077	up	44.51840	25.36332	39.87806	10.71824	Detected	Detected	Detected	Compromised
3709	A_23_P49865	YBX2	Hs.567494	Y box binding protein 2	Homo sapiens Y box binding protein 2 (YBX2), mRNA [NM_015982]	1.81726	up	2.68508	up	117.74308	67.08908	69.31644	22.76379	Detected	Detected	Detected	Detected
3710	A_33_P3395713	BRWD1	Hs.654740	bromodomain and WD repeat domain containing 1	Homo sapiens bromodomain and WD repeat domain containing 1 (BRWD1), transcript variant 1, mRNA [NM_018963]	1.81687	up	2.59552	up	198.85774	113.33181	218.22310	74.13821	Detected	Detected	Detected	Detected
3711	A_33_P3264835				Rap1 GTPase-activating protein 1 (Rap1GAP1)(Rap1GAP) [Source:UniProtKB/Swiss-Prot;Acc:P47736] [ENST00000374757]	1.81672	up	2.42318	up	21.20876	12.08817	8.96947	3.26397	Detected	Compromised	Compromised	Compromised
3712	A_23_P151565	RALGAPA1	Hs.113150	Ral GTPase activating protein, alpha subunit 1 (catalytic)	Homo sapiens Ral GTPase activating protein, alpha subunit 1 (catalytic) (RALGAPA1), transcript variant 1, mRNA [NM_014990]	1.81564	up	2.19797	up	1449.07590	826.40940	1039.91860	417.19925	Detected	Detected	Detected	Detected
3713	A_23_P360804	CPNE5	Hs.657869	copine V	Homo sapiens copine V (CPNE5), mRNA [NM_020939]	1.81454	up	2.07071	up	336.16782	191.83290	273.18668	116.33379	Detected	Detected	Detected	Detected
3714	A_33_P3409518	TUBBP5		tubulin, beta pseudogene 5	Homo sapiens tubulin, beta pseudogene 5 (TUBBP5), non-coding RNA [NR_027156]	1.81177	up	2.07314	up	6482.47360	3704.85600	889.81134	378.47333	Detected	Detected	Detected	Detected
3715	A_33_P3216442	COL11A2	Hs.390171	collagen, type XI, alpha 2	Homo sapiens collagen, type XI, alpha 2 (COL11A2), transcript variant 1, mRNA [NM_080680]	1.80888	up	2.33947	up	254.75609	145.83038	183.10619	69.01612	Detected	Detected	Detected	Detected

3716	A_33_P3380311	ATRX	Hs.533526	alpha thalassemia/mental retardation syndrome X-linked (RAD54 homolog, S. cerevisiae)	Homo sapiens alpha thalassemia/mental retardation syndrome X-linked (RAD54 homolog, S. cerevisiae) (ATRX), transcript variant 1, mRNA [NM_000489]	1.80572	up	3.57674	up	59.20575	33.95045	13.64940	3.36505	Detected	Detected	Detected	Compromised
3717	A_24_P148907	MAB21L2	Hs.584852	mab-21-like 2 (C. elegans)	Homo sapiens mab-21-like 2 (C. elegans) (MAB21L2), mRNA [NM_006439]	1.79942	up	2.04080	up	5011.31200	2883.71800	827.68604	357.62686	Detected	Detected	Detected	Detected
3718	A_33_P3237235	FBXO44	Hs.556006	F-box protein 44	Homo sapiens F-box protein 44 (FBXO44), transcript variant 4, mRNA [NM_001014765]	1.79842	up	2.44646	up	132.84291	76.48566	88.99882	32.07827	Detected	Detected	Detected	Detected
3719	A_33_P3388466	BTN3A1	Hs.191510	butyrophilin, subfamily 3, member A1	Homo sapiens butyrophilin, subfamily 3, member A1 (BTN3A1), transcript variant 1, mRNA [NM_007048]	1.79802	up	2.33954	up	20.39783	11.74689	14.30394	5.39127	Detected	Compromised	Detected	Compromised
3720	A_23_P500271	IRF5	Hs.521181	interferon regulatory factor 5	Homo sapiens interferon regulatory factor 5 (IRF5), transcript variant 1, mRNA [NM_002200]	1.79662	up	2.73609	up	64.92951	37.42121	50.05281	16.13107	Detected	Detected	Detected	Compromised
3721	A_23_P58862	FAM135A	Hs.211700	family with sequence similarity 135, member A	Homo sapiens family with sequence similarity 135, member A (FAM135A), transcript variant 2, mRNA [NM_020819]	1.79568	up	3.43994	up	47.05703	27.13497	36.56421	9.37284	Detected	Detected	Detected	Compromised
3722	A_24_P943815	PNPLA4	Hs.264	patatin-like phospholipase domain containing 4	Homo sapiens patatin-like phospholipase domain containing 4 (PNPLA4), transcript variant 1, mRNA [NM_004650]	1.79475	up	2.02392	up	97.26424	56.11547	55.23953	24.06701	Detected	Detected	Detected	Detected
3723	A_33_P3295056	PTPRCAP	Hs.155975	protein tyrosine phosphatase, receptor type, C-associated protein	Homo sapiens protein tyrosine phosphatase, receptor type, C-associated protein (PTPRCAP), mRNA [NM_005608]	1.79431	up	2.02412	up	18.56018	10.71070	19.49046	8.49084	Detected	Compromised	Detected	Compromised
3724	A_23_P18692	ADH5	Hs.78989	alcohol dehydrogenase 5 (class III), chi polypeptide	Homo sapiens alcohol dehydrogenase 5 (class III), chi polypeptide (ADH5), mRNA [NM_000671]	1.79297	up	2.00190	up	7084.92530	4091.61870	3727.28600	1641.78050	Detected	Detected	Detected	Detected
3725	A_33_P3391756	LOC100288271	Hs.445480	hypothetical protein LOC100288271	PREDICTED: Homo sapiens hypothetical protein LOC100288271 (LOC100288271), mRNA [XM_002342021]	1.78959	up	3.08729	up	59.16332	34.23195	46.79592	13.36585	Detected	Detected	Detected	Detected
3726	A_33_P3249066					1.78874	up	4.65612	up	42.95923	24.86806	29.13458	5.51760	Detected	Detected	Detected	Compromised
3727	A_33_P3418170	DDX58	Hs.190622	DEAD (Asp-Glu-Ala-Asp) box polypeptide 58	Homo sapiens DEAD (Asp-Glu-Ala-Asp) box polypeptide 58 (DDX58), mRNA [NM_014314]	1.78509	up	2.34415	up	242.55197	140.69469	154.42091	58.08788	Detected	Detected	Detected	Detected
3728	A_33_P3303542	SSC5D	Hs.554182	scavenger receptor cysteine-rich glycoprotein	Homo sapiens scavenger receptor cysteine-rich glycoprotein (SSC5D), mRNA [NM_001144950]	1.78494	up	2.04844	up	1612.87440	935.64270	1476.85420	635.74040	Detected	Detected	Detected	Detected
3729	A_33_P3391375	LANCL3	Hs.521932	LanC lantibiotic synthetase component C-like 3 (bacterial)	Homo sapiens LanC lantibiotic synthetase component C-like 3 (bacterial) (LANCL3), mRNA [NM_198511]	1.78298	up	2.34208	up	3509.34740	2038.03870	418.20210	157.45255	Detected	Detected	Detected	Detected
3730	A_33_P3324839	SYNGAP1	Hs.586264	synaptic Ras GTPase activating protein 1 homolog (rat)	Homo sapiens synaptic Ras GTPase activating protein 1 homolog (rat) (SYNGAP1), mRNA [NM_006772]	1.78259	up	4.21978	up	32.10061	18.64636	29.77667	6.22231	Detected	Detected	Detected	Compromised
3731	A_23_P147465	PARK2	Hs.132954	Parkinson disease (autosomal recessive, juvenile) 2, parkin	Homo sapiens Parkinson disease (autosomal recessive, juvenile) 2, parkin (PARK2), transcript variant 1, mRNA [NM_004562]	1.78254	up	3.63379	up	46.65476	27.10133	48.99404	11.88911	Detected	Detected	Detected	Compromised
3732	A_33_P3319231	AIFM2		apoptosis-inducing factor, mitochondrion-associated, 2	Apoptosis-inducing factor 2 (EC 1.-.-.-)(Apoptosis-inducing factor-like mitochondrion-associated inducer of death)(Apoptosis-inducing factor homologous mitochondrion-associated inducer of death)(p53-responsive gene 3 protein) [Source:UniProtKB/Swiss-Prot;Acc:Q9BRQ8] [ENST00000373248]	1.78246	up	3.61575	up	31.26964	18.16499	23.40287	5.70738	Detected	Detected	Detected	Compromised
3733	A_33_P3322082	LOC728806	Hs.646586	similar to N-ethylmaleimide-sensitive factor	PREDICTED: Homo sapiens similar to N-ethylmaleimide-sensitive factor (LOC728806), mRNA [XM_001128535]	1.78160	up	2.46499	up	19.85393	11.53901	25.75382	9.21280	Detected	Compromised	Detected	Compromised
3734	A_32_P117812		Hs.562568		Putative uncharacterized protein DKFZp779J2370 [Source:UniProtKB/TrEMBL;Acc:Q6MZR2] [ENST00000378904]	1.77807	up	2.14924	up	56.28162	32.77552	80.47266	33.01627	Detected	Detected	Detected	Detected
3735	A_24_P396753	TRIB2	Hs.467751	tribbles homolog 2 (Drosophila)	Homo sapiens tribbles homolog 2 (Drosophila) (TRIB2), transcript variant 1, mRNA [NM_021643]	1.77668	up	2.00358	up	146.57445	85.42449	92.53689	40.72614	Detected	Detected	Detected	Detected
3736	A_33_P3710442	FLJ11710	Hs.657294	hypothetical protein FLJ11710	Homo sapiens cDNA FLJ11710 fis, clone HEMBA1005149 [AK021772]	1.77652	up	2.92834	up	25.21724	14.69809	12.64960	3.80909	Detected	Compromised	Compromised	Compromised
3737	A_33_P3298128	ITPR2	Hs.512235	inositol 1,4,5-triphosphate receptor, type 2	Homo sapiens inositol 1,4,5-triphosphate receptor, type 2 (ITPR2), mRNA [NM_002223]	1.77620	up	2.06544	up	222.91933	129.95349	119.97032	51.21861	Detected	Detected	Detected	Detected
3738	A_23_P14986	HSD11B2	Hs.1376	hydroxysteroid (11-beta) dehydrogenase 2	Homo sapiens hydroxysteroid (11-beta) dehydrogenase 2 (HSD11B2), mRNA [NM_000196]	1.77613	up	3.39402	up	82.54202	48.12079	63.27200	16.43854	Detected	Detected	Detected	Detected
3739	A_33_P3474859	LOC203274	Hs.599821	hypothetical protein LOC203274	Homo sapiens cDNA clone IMAGE:5498355 [BC110369]	1.77606	up	2.18072	up	2056.24630	1198.81090	1063.64070	430.09152	Detected	Detected	Detected	Detected
3740	A_33_P3390753					1.77390	up	2.15337	up	817.70734	477.31125	177.66390	72.75214	Detected	Detected	Detected	Detected
3741	A_33_P3240229	CREBBP	Hs.459759	CREB binding protein	Homo sapiens CREB binding protein (CREBBP), transcript variant 1, mRNA [NM_004380]	1.77324	up	2.00170	up	1724.17320	1006.80510	888.24050	391.28806	Detected	Detected	Detected	Detected
3742	A_24_P21770	YPEL4	Hs.135978	yippee-like 4 (Drosophila)	Homo sapiens yippee-like 4 (Drosophila) (YPEL4), mRNA [NM_145008]	1.77313	up	2.25784	up	237.47269	138.67752	137.46909	53.68812	Detected	Detected	Detected	Detected

3743	A_23_P166280	ICOSLG		inducible T-cell co-stimulator ligand	ICOS ligand Precursor (B7 homolog 2)(B7-H2)(B7-like protein Gl50)(B7-related protein 1)(B7RP-1)(CD275 antigen) [Source:UniProtKB/Swiss-Prot;Acc:O75144] [ENST00000407780]	1.77117	up	3.97901	up	480.00104	280.61700	406.16098	90.00964	Detected	Detected	Detected	Detected
3744	A_33_P3392097	LOC344065	Hs.528395	similar to zinc finger protein 570	PREDICTED: Homo sapiens similar to zinc finger protein 570 (LOC344065), mRNA [XM_001714518]	1.77105	up	2.10116	up	30.64047	17.91423	22.54569	9.46172	Detected	Compromised	Detected	Compromised
3745	A_33_P3252594					1.76824	up	2.15463	up	320.64432	187.76520	206.05952	84.33048	Detected	Detected	Detected	Detected
3746	A_32_P315395	MGC16275	Hs.706954	hypothetical protein MGC16275	Homo sapiens hypothetical protein MGC16275 (MGC16275), non-coding RNA [NR_026914]	1.76758	up	2.86390	up	25.14880	14.73234	16.08369	4.95214	Detected	Compromised	Detected	Compromised
3747	A_24_P241183	CLEC2D	Hs.268326	C-type lectin domain family 2, member D	Homo sapiens C-type lectin domain family 2, member D (CLEC2D), transcript variant 1, mRNA [NM_013269]	1.76671	up	2.34295	up	93.89303	55.03017	60.44562	22.74927	Detected	Detected	Detected	Detected
3748	A_23_P28434	VAMP8	Hs.714302	vesicle-associated membrane protein 8 (endobrevin)	Homo sapiens vesicle-associated membrane protein 8 (endobrevin) (VAMP8), mRNA [NM_003761]	1.76654	up	2.17225	up	271.03610	158.86841	250.83228	101.82161	Detected	Detected	Detected	Detected
3749	A_23_P135248	CCL27	Hs.648124	chemokine (C-C motif) ligand 27	Homo sapiens chemokine (C-C motif) ligand 27 (CCL27), mRNA [NM_006664]	1.76223	up	2.20091	up	87.76936	51.57188	87.52036	35.06486	Detected	Detected	Detected	Detected
3750	A_23_P207582	CCL16	Hs.10458	chemokine (C-C motif) ligand 16	Homo sapiens chemokine (C-C motif) ligand 16 (CCL16), mRNA [NM_004590]	1.76222	up	6.80356	up	5.43966	3.19629	20.69105	2.68171	Compromised	Compromised	Detected	Compromised
3751	A_33_P3431595	C8orf31	Hs.660382	chromosome 8 open reading frame 31	Homo sapiens chromosome 8 open reading frame 31 (C8orf31), mRNA [NM_173687]	1.76158	up	2.01437	up	886.36884	521.00770	757.81226	331.73315	Detected	Detected	Detected	Detected
3752	A_24_P940135	CTBS	Hs.513557	chitobiase, di-N-acetyl-	Homo sapiens chitobiase, di-N-acetyl- (CTBS), mRNA [NM_004388]	1.76042	up	2.14641	up	1472.48220	866.09520	1095.50600	450.05728	Detected	Detected	Detected	Detected
3753	A_33_P3381781				Regulation of nuclear pre-mRNA domain-containing protein 2 [Source:UniProtKB/Swiss-Prot;Acc:Q5VT52] [ENST00000369067]	1.75706	up	6.23331	up	30.57419	18.01776	20.30864	2.87295	Detected	Detected	Detected	Compromised
3754	A_24_P818268				Putative uncharacterized protein DNM1P33 [Source:UniProtKB/TrEMBL;Acc:A6NN47] [ENST00000339692]	1.75660	up	2.02218	up	285.95100	168.55907	228.22884	99.52145	Detected	Detected	Detected	Detected
3755	A_24_P712562	C17orf67	Hs.658949	chromosome 17 open reading frame 67	Homo sapiens chromosome 17 open reading frame 67 (C17orf67), mRNA [NM_001085430]	1.75618	up	2.03361	up	70.44097	41.53251	45.73420	19.83071	Detected	Detected	Detected	Detected
3756	A_32_P62211		Hs.408455		Homo sapiens mRNA; cDNA DKFZp686J1595 (from clone DKFZp686J1595) [BX538057]	1.75466	up	2.42174	up	47.43689	27.99337	53.72361	19.56152	Detected	Detected	Detected	Detected
3757	A_23_P110624	CTNND2	Hs.314543	catenin (cadherin-associated protein), delta 2 (neural plakophilin-related arm-repeat protein)	Homo sapiens catenin (cadherin-associated protein), delta 2 (neural plakophilin-related arm-repeat protein) (CTNND2), mRNA [NM_001332]	1.75378	up	2.13378	up	61.01253	36.02285	42.13758	17.41351	Detected	Detected	Detected	Detected
3758	A_23_P166823	TNNC1	Hs.118845	troponin C type 1 (slow)	Homo sapiens troponin C type 1 (slow) (TNNC1), mRNA [NM_003280]	1.75371	up	2.70042	up	104.09418	61.46121	109.14289	35.63929	Detected	Detected	Detected	Detected
3759	A_33_P3307775	DENR	Hs.22393	density-regulated protein	Homo sapiens density-regulated protein (DENR), mRNA [NM_003677]	1.75286	up	2.10709	up	61.44254	36.29573	48.19349	20.16835	Detected	Detected	Detected	Detected
3760	A_33_P3217689	JMY	Hs.482605	junction mediating and regulatory protein, p53 cofactor	Homo sapiens junction mediating and regulatory protein, p53 cofactor (JMY), mRNA [NM_152405]	1.75181	up	2.49768	up	1333.44740	788.17580	1815.85360	641.07587	Detected	Detected	Detected	Detected
3761	A_23_P309720	GABRD	Hs.113882	gamma-aminobutyric acid (GABA) A receptor, delta	Homo sapiens gamma-aminobutyric acid (GABA) A receptor, delta (GABRD), mRNA [NM_000815]	1.75156	up	3.80506	up	19.41164	11.47546	12.39303	2.87198	Detected	Compromised	Compromised	Compromised
3762	A_33_P3463355	LOC286121	Hs.449418	hypothetical protein LOC286121	Homo sapiens hypothetical protein LOC286121, mRNA (cDNA clone IMAGE:4837614) [BC039302]	1.75128	up	3.81224	up	35.12813	20.76977	28.66014	6.62923	Detected	Detected	Detected	Compromised
3763	A_24_P363087	C5orf45	Hs.310781	chromosome 5 open reading frame 45	Homo sapiens chromosome 5 open reading frame 45 (C5orf45), transcript variant 1, mRNA [NM_016175]	1.75097	up	3.38284	up	2565.71240	1517.27050	2207.32280	575.37310	Detected	Detected	Detected	Detected
3764	A_33_P3287084	LOC100128009	Hs.474119	similar to hCG1642538	PREDICTED: Homo sapiens similar to hCG1642538 (LOC100128009), mRNA [XM_001720983]	1.74956	up	2.03951	up	32.17534	19.04269	35.13981	15.19281	Detected	Detected	Detected	Detected
3765	A_24_P791040	FBXW2	Hs.494985	F-box and WD repeat domain containing 2	Homo sapiens F-box and WD repeat domain containing 2 (FBXW2), mRNA [NM_012164]	1.74928	up	2.92574	up	21.89641	12.96121	24.10544	7.26514	Detected	Compromised	Detected	Compromised
3766	A_33_P3420446	LOC401387	Hs.417077	leucine-rich repeat and death domain-containing protein	Homo sapiens leucine-rich repeat and death domain-containing protein (LOC401387), mRNA [NM_001161528]	1.74873	up	2.16440	up	28764.96500	17032.37700	5142.26070	2094.98800	Detected	Detected	Detected	Detected
3767	A_33_P3762733	D21S2088E	Hs.145622	D21S2088E	Homo sapiens D21S2088E mRNA sequence [AY063451]	1.74815	up	2.00181	up	643.01970	380.87115	235.03299	103.53149	Detected	Detected	Detected	Detected
3768	A_24_P194688	EFHA2	Hs.403594	EF-hand domain family, member A2	Homo sapiens EF-hand domain family, member A2 (EFHA2), mRNA [NM_181723]	1.74760	up	2.08076	up	494.04797	292.72580	379.82123	160.96153	Detected	Detected	Detected	Detected
3769	A_33_P3343467	FLJ35390		hypothetical LOC255031	Homo sapiens hypothetical LOC255031 (FLJ35390), transcript variant 1, non-coding RNA [NR_024416]	1.74538	up	2.39588	up	121.47746	72.06722	97.39436	35.84551	Detected	Detected	Detected	Detected
3770	A_33_P3354975	MTMR9L	Hs.471067	myotubularin related protein 9-like	Homo sapiens myotubularin related protein 9-like (MTMR9L), non-coding RNA [NR_026850]	1.74192	up	2.17329	up	179.48122	106.69001	142.47010	57.80582	Detected	Detected	Detected	Detected
3771	A_23_P413285	CC2D2A	Hs.590928	coiled-coil and C2 domain containing 2A	Homo sapiens coiled-coil and C2 domain containing 2A (CC2D2A), transcript variant 1, mRNA [NM_001080522]	1.73967	up	2.08082	up	210.98009	125.57658	116.42557	49.33785	Detected	Detected	Detected	Detected

3772	A_33_P3243332	FLJ32810	Hs.269837	Rho-type GTPase-activating protein FLJ32810	Homo sapiens Rho-type GTPase-activating protein FLJ32810 (FLJ32810), mRNA [NM_152432]	1.73303	up	3.03212	up	25.30957	15.12208	13.05708	3.79721	Detected	Compromised	Compromised	Compromised
3773	A_33_P3297545				Putative uncharacterized protein ENSP00000374899 [Source:UniProtKB/TrEMBL;Acc:A6NME7] [ENST00000390376]	1.73235	up	2.63547	up	452.83290	270.66638	19.83942	6.63800	Detected	Detected	Detected	Compromised
3774	A_32_P199767	ZNF630	Hs.650883	zinc finger protein 630	Homo sapiens zinc finger protein 630 (ZNF630), mRNA [NM_001037735]	1.72953	up	2.51972	up	65.58949	39.26794	50.60721	17.71032	Detected	Detected	Detected	Detected
3775	A_24_P48408	RNMT	Hs.592347	RNA (guanine-7-) methyltransferase	Homo sapiens RNA (guanine-7-) methyltransferase (RNMT), mRNA [NM_003799]	1.72908	up	2.04250	up	650.58100	389.60062	266.60596	115.09921	Detected	Detected	Detected	Detected
3776	A_33_P3379669	RNF208	Hs.512767	ring finger protein 208	Homo sapiens ring finger protein 208 (RNF208), mRNA [NM_031297]	1.72306	up	2.46590	up	207.12746	124.47173	138.47227	49.51682	Detected	Detected	Detected	Detected
3777	A_33_P3358855					1.72030	up	2.00002	up	18.34070	11.03936	14.47940	6.38385	Detected	Compromised	Detected	Compromised
3778	A_23_P390621	PACRGL	Hs.479298	PARK2 co-regulated-like	Homo sapiens PARK2 co-regulated-like (PACRGL), transcript variant 1, mRNA [NM_145048]	1.71972	up	2.53857	up	170.51709	102.66970	100.92523	35.05710	Detected	Detected	Detected	Detected
3779	A_32_P89352	MACROD2	Hs.661576	MACRO domain containing 2	Homo sapiens MACRO domain containing 2 (MACROD2), transcript variant 1, mRNA [NM_080676]	1.71835	up	4.19747	up	117.07202	70.54652	68.29511	14.34720	Detected	Detected	Detected	Detected
3780	A_33_P3214463	MESP2	Hs.37311	mesoderm posterior 2 homolog (mouse)	Homo sapiens mesoderm posterior 2 homolog (mouse) (MESP2), mRNA [NM_001039958]	1.71779	up	2.77923	up	1118.79900	674.39740	583.84930	185.24268	Detected	Detected	Detected	Detected
3781	A_33_P3273230	HORMAD1	Hs.298312	HORMA domain containing 1	Homo sapiens HORMA domain containing 1 (HORMAD1), mRNA [NM_032132]	1.71611	up	2.60923	up	18.05679	10.89505	8.61857	2.91265	Detected	Compromised	Compromised	Compromised
3782	A_23_P420692	PPFIA4	Hs.153648	protein tyrosine phosphatase, receptor type, f polypeptide (PTPRF), interacting protein (liprin), alpha 4	Homo sapiens protein tyrosine phosphatase, receptor type, f polypeptide (PTPRF), interacting protein (liprin), alpha 4 (PPFIA4), mRNA [NM_015053]	1.71468	up	2.10166	up	113.75821	68.69614	97.69784	40.99103	Detected	Detected	Detected	Detected
3783	A_33_P3372910	DDX58	Hs.190622	DEAD (Asp-Glu-Ala-Asp) box polypeptide 58	Homo sapiens DEAD (Asp-Glu-Ala-Asp) box polypeptide 58 (DDX58), mRNA [NM_014314]	1.71399	up	2.24536	up	741.36755	447.87683	633.53754	248.80116	Detected	Detected	Detected	Detected
3784	A_33_P3240843	TMEM71	Hs.293842	transmembrane protein 71	Homo sapiens transmembrane protein 71 (TMEM71), transcript variant 1, mRNA [NM_144649]	1.71359	up	2.40176	up	51.26101	30.97508	39.53146	14.51369	Detected	Detected	Detected	Detected
3785	A_24_P16249	ZNF479	Hs.616660	zinc finger protein 479	Homo sapiens zinc finger protein 479 (ZNF479), mRNA [NM_033273]	1.71359	up	2.11863	up	201.65749	121.85429	7.31414	3.04421	Detected	Detected	Compromised	Compromised
3786	A_24_P93948		Hs.151219		Brain my050 protein [Source:UniProtKB/TrEMBL;Acc:Q9H3H7] [ENST00000455537]	1.71339	up	4.74900	up	26.21840	15.84463	17.36172	3.22371	Detected	Compromised	Detected	Compromised
3787	A_33_P3290959					1.71329	up	2.04762	up	17.21644	10.40508	16.95065	7.29967	Detected	Compromised	Detected	Compromised
3788	A_33_P3318946	HAPLN2	Hs.410719	hyaluronan and proteoglycan link protein 2	Homo sapiens hyaluronan and proteoglycan link protein 2 (HAPLN2), mRNA [NM_021817]	1.71295	up	4.41032	up	297.76727	179.99692	181.40578	36.26995	Detected	Detected	Detected	Detected
3789	A_23_P150325	TMEM133	Hs.44004	transmembrane protein 133	Homo sapiens transmembrane protein 133 (TMEM133), mRNA [NM_032021]	1.70967	up	2.03504	up	86.32243	52.28108	59.60612	25.82756	Detected	Detected	Detected	Detected
3790	A_24_P98161	KRIT1	Hs.531987	KRIT1, ankyrin repeat containing	Homo sapiens KRIT1, ankyrin repeat containing (KRIT1), transcript variant 4, mRNA [NM_194455]	1.70952	up	2.29023	up	63.98581	38.75643	32.40662	12.47729	Detected	Detected	Detected	Compromised
3791	A_32_P112623	LOC100293193	Hs.567050	hypothetical LOC100293193	PREDICTED: Homo sapiens hypothetical LOC100293193 (LOC100293193), miscRNA [XR_079078]	1.70901	up	2.21482	up	292.14856	177.00813	290.27840	115.56918	Detected	Detected	Detected	Detected
3792	A_32_P143000	FAM189A1	Hs.383564	family with sequence similarity 189, member A1	Homo sapiens family with sequence similarity 189, member A1 (FAM189A1), mRNA [NM_015307]	1.70871	up	2.24484	up	25.96095	15.73206	36.01842	14.14829	Detected	Detected	Detected	Detected
3793	A_23_P327140	RNF213	Hs.195642	ring finger protein 213	Homo sapiens ring finger protein 213 (RNF213), transcript variant 1, mRNA [NM_020914]	1.70791	up	2.90311	up	76.05743	46.11150	57.25914	17.39189	Detected	Detected	Detected	Detected
3794	A_24_P350576	TNIK	Hs.34024	TRAF2 and NCK interacting kinase	Homo sapiens TRAF2 and NCK interacting kinase (TNIK), transcript variant 1, mRNA [NM_015028]	1.70750	up	3.77760	up	27.35193	16.58675	31.07539	7.25381	Detected	Compromised	Detected	Compromised
3795	A_33_P3729375	LOC285181	Hs.434525	hypothetical protein LOC285181	Homo sapiens cDNA FLJ36538 fis, clone TRACH2005159 [AK093857]	1.70744	up	3.44308	up	25.51500	15.47332	21.30414	5.45610	Detected	Compromised	Detected	Compromised
3796	A_33_P3345936	CCDC9	Hs.227782	coiled-coil domain containing 9	Homo sapiens coiled-coil domain containing 9 (CCDC9), mRNA [NM_015603]	1.70692	up	2.09514	up	58668.54700	35589.77000	14873.05700	6259.69000	Detected	Detected	Detected	Detected
3797	A_23_P89973	BRSK1	Hs.182081	BR serine/threonine kinase 1	Homo sapiens BR serine/threonine kinase 1 (BRSK1), mRNA [NM_032430]	1.70331	up	2.44338	up	79.18384	48.13672	43.91546	15.84864	Detected	Detected	Detected	Detected
3798	A_33_P3231262	LOC257396	Hs.12326	hypothetical protein LOC257396	Homo sapiens hypothetical protein LOC257396, mRNA (cDNA clone IMAGE:5298658) [BC041894]	1.70290	up	2.48930	up	45.82232	27.86262	58.10454	20.58250	Detected	Detected	Detected	Detected
3799	A_33_P3602006	ADAT2	Hs.709561	adenosine deaminase, tRNA-specific 2, TAD2 homolog (S. cerevisiae)	Homo sapiens adenosine deaminase, tRNA-specific 2, TAD2 homolog (S. cerevisiae) (ADAT2), mRNA [NM_182503]	1.69857	up	2.19879	up	46855.20300	28563.17000	10441.96300	4187.58640	Detected	Detected	Detected	Detected
3800	A_24_P838448	FLJ22536	Hs.719939	hypothetical locus LOC401237	Homo sapiens hypothetical locus LOC401237 (FLJ22536), non-coding RNA [NR_015410]	1.69496	up	2.24761	up	64.44888	39.37206	31.48826	12.35361	Detected	Detected	Detected	Compromised
3801	A_33_P3320538		Hs.594723		Nucleoporin p58/p45 (Nucleoporin-like 1) [Source:UniProtKB/Swiss-Prot;Acc:Q9BVL2] [ENST00000381747]	1.69472	up	2.12613	up	1382.91650	844.94824	1052.80380	436.63867	Detected	Detected	Detected	Detected
3802	A_24_P522631	TMEM201	Hs.632365	transmembrane protein 201	Homo sapiens transmembrane protein 201 (TMEM201), transcript variant 1, mRNA [NM_001130924]	1.69371	up	2.06948	up	10622.69800	6494.24700	1706.45970	727.10960	Detected	Detected	Detected	Detected
3803	A_33_P3667484	LOC648740	Hs.721681	ACTB pseudogene	Homo sapiens ACTB pseudogene (LOC648740), non-coding RNA [NR_024438]	1.69211	up	7.75259	up	180685.05000	110567.23000	39458.83200	4488.10350	Detected	Detected	Detected	Detected

3804	A_24_P208998	TRIM23	Hs.792	tripartite motif-containing 23	Homo sapiens tripartite motif-containing 23 (TRIM23), transcript variant alpha, mRNA [NM_001656]	1.69178	up	2.02192	up	412.38010	252.39873	152.73370	66.60967	Detected	Detected	Detected	Detected
3805	A_24_P450285	CCDC153	Hs.114777	coiled-coil domain containing 153	Homo sapiens coiled-coil domain containing 153 (CCDC153), mRNA [NM_001145018]	1.68859	up	2.30247	up	57.86245	35.48187	110.02483	42.13683	Detected	Detected	Detected	Detected
3806	A_33_P3230583	LOC283788	Hs.657131	FSHD region gene 1 pseudogene	Homo sapiens FSHD region gene 1 pseudogene (LOC283788), non-coding RNA [NR_027436]	1.68742	up	2.28508	up	524.74260	321.99990	100.67909	38.85116	Detected	Detected	Detected	Detected
3807	A_32_P122226	AMDHD1	Hs.424907	amidohydrolase domain containing 1	Homo sapiens amidohydrolase domain containing 1 (AMDHD1), mRNA [NM_152435]	1.68504	up	3.47911	up	57.95345	35.61238	33.83199	8.57481	Detected	Detected	Detected	Compromised
3808	A_33_P3235454					1.68382	up	2.27957	up	11711.36900	7201.85940	508.86542	196.84091	Detected	Detected	Detected	Detected
3809	A_33_P3280993	HCG18		HLA complex group 18	Homo sapiens HLA complex group 18 (HCG18), non-coding RNA [NR_024052]	1.68343	up	2.35365	up	763.19690	469.43530	312.27237	116.99238	Detected	Detected	Detected	Detected
3810	A_33_P3400057	TCEANC	Hs.222855	transcription elongation factor A (SII) N-terminal and central domain containing	Homo sapiens transcription elongation factor A (SII) N-terminal and central domain containing (TCEANC), mRNA [NM_152634]	1.68315	up	2.05859	up	19.65783	12.09330	15.76408	6.75250	Detected	Compromised	Compromised	Compromised
3811	A_33_P3314212	PARG	Hs.536158	poly (ADP-ribose) glycohydrolase	Homo sapiens poly (ADP-ribose) glycohydrolase (PARG), mRNA [NM_003631]	1.68262	up	2.41064	up	41.63915	25.62411	41.40062	15.14398	Detected	Detected	Detected	Compromised
3812	A_24_P4877	ZCRB1	Hs.496279	zinc finger CCHC-type and RNA binding motif 1	Homo sapiens zinc finger CCHC-type and RNA binding motif 1 (ZCRB1), mRNA [NM_033114]	1.68076	up	2.07771	up	2561.57930	1578.09890	1349.87060	572.89330	Detected	Detected	Detected	Detected
3813	A_33_P3398401	CRBN	Hs.18925	cereblon	Homo sapiens cereblon (CRBN), mRNA [NM_016302]	1.67979	up	2.13860	up	6522.01700	4020.31300	6038.29300	2489.71500	Detected	Detected	Detected	Detected
3814	A_33_P3349002					1.67850	up	2.09838	up	40.36343	24.89996	31.01936	13.03507	Detected	Detected	Detected	Compromised
3815	A_33_P3300600	OR7G1	Hs.553576	olfactory receptor, family 7, subfamily G, member 1	Homo sapiens olfactory receptor, family 7, subfamily G, member 1 (OR7G1), mRNA [NM_001005192]	1.67688	up	2.46141	up	11.47180	7.08373	10.11555	3.62386	Detected	Compromised	Compromised	Compromised
3816	A_24_P233944	CEPT1	Hs.363572	choline/ethanolamine phosphotransferase 1	Homo sapiens choline/ethanolamine phosphotransferase 1 (CEPT1), transcript variant 2, mRNA [NM_001007794]	1.67530	up	2.02332	up	156.97133	97.02003	168.87173	73.59670	Detected	Detected	Detected	Detected
3817	A_24_P62783	FABP3	Hs.657242	fatty acid binding protein 3, muscle and heart (mammary-derived growth inhibitor)	Homo sapiens fatty acid binding protein 3, muscle and heart (mammary-derived growth inhibitor) (FABP3), mRNA [NM_004102]	1.67503	up	2.50447	up	53.91497	33.32879	56.31842	19.82895	Detected	Detected	Detected	Detected
3818	A_23_P72651	ECSCR	Hs.483538	endothelial cell-specific chemotaxis regulator	Homo sapiens endothelial cell-specific chemotaxis regulator (ECSCR), mRNA [NM_001077693]	1.67315	up	2.36779	up	76.42336	47.29586	64.63166	24.06958	Detected	Detected	Detected	Detected
3819	A_33_P3209162	APOC4	Hs.655423	apolipoprotein C-IV	Homo sapiens apolipoprotein C-IV (APOC4), mRNA [NM_001646]	1.67230	up	2.53413	up	84.61167	52.39004	71.81342	24.98857	Detected	Detected	Detected	Detected
3820	A_33_P3300740	LOC100128071	Hs.660539	similar to hCG41624	PREDICTED: Homo sapiens similar to hCG41624 (LOC100128071), mRNA [XM_001724939]	1.67144	up	3.60706	up	21.02881	13.02736	11.46088	2.80175	Detected	Compromised	Compromised	Compromised
3821	A_23_P321034	INADL	Hs.478125	InaD-like (Drosophila)	Homo sapiens InaD-like (Drosophila) (INADL), mRNA [NM_176877]	1.66919	up	4.33948	up	21.48222	13.32619	14.34168	2.91426	Detected	Compromised	Detected	Compromised
3822	A_24_P412734	PRSS36	Hs.256632	protease, serine, 36	Homo sapiens protease, serine, 36 (PRSS36), mRNA [NM_173502]	1.66838	up	2.09133	up	25013.10200	15524.10200	5460.73630	2302.46630	Detected	Detected	Detected	Detected
3823	A_33_P3265030	SEPT5	Hs.283743	septin 5	Homo sapiens septin 5 (SEPT5), mRNA [NM_002688]	1.66714	up	2.09701	up	7704.13400	4785.02540	6248.41200	2627.45500	Detected	Detected	Detected	Detected
3824	A_23_P43846	FABP6	Hs.519719	fatty acid binding protein 6, ileal	Homo sapiens fatty acid binding protein 6, ileal (FABP6), transcript variant 1, mRNA [NM_001040442]	1.66580	up	2.17809	up	26.45552	16.44474	21.28001	8.61514	Detected	Detected	Detected	Compromised
3825	A_24_P63347	PF4V1	Hs.72933	platelet factor 4 variant 1	Homo sapiens platelet factor 4 variant 1 (PF4V1), mRNA [NM_002620]	1.66529	up	2.01953	up	73.49332	45.69723	54.37374	23.74132	Detected	Detected	Detected	Detected
3826	A_23_P32175	LHX6	Hs.103137	LIM homeobox 6	Homo sapiens LIM homeobox 6 (LHX6), transcript variant 1, mRNA [NM_014368]	1.66492	up	2.21011	up	106.70690	66.36388	112.37116	44.83385	Detected	Detected	Detected	Detected
3827	A_23_P109072	SALL4	Hs.517113	sal-like 4 (Drosophila)	Homo sapiens sal-like 4 (Drosophila) (SALL4), mRNA [NM_020436]	1.66429	up	2.05913	up	57.44106	35.73766	44.71793	19.14973	Detected	Detected	Detected	Detected
3828	A_23_P501822	JUP	Hs.514174	junction plakoglobin	Homo sapiens junction plakoglobin (JUP), transcript variant 1, mRNA [NM_002230]	1.66286	up	2.08053	up	216.02950	134.52110	285.81058	121.13493	Detected	Detected	Detected	Detected
3829	A_33_P3881056	LOC100128567	Hs.676722	hypothetical protein LOC100128567	Homo sapiens cDNA FLJ27128 fis, clone SPL07659. [AK130638]	1.66266	up	2.30427	up	187.03110	116.47822	120.07949	45.95160	Detected	Detected	Detected	Detected
3830	A_23_P357546	PNKD	Hs.98475	paroxysmal nonkinesigenic dyskinesia	Homo sapiens paroxysmal nonkinesigenic dyskinesia (PNKD), transcript variant 1, mRNA [NM_015488]	1.66245	up	2.81169	up	74.47138	46.38449	66.78574	20.94506	Detected	Detected	Detected	Detected
3831	A_23_P110445	APBB3	Hs.529449	amyloid beta (A4) precursor protein-binding, family B, member 3	Homo sapiens amyloid beta (A4) precursor protein-binding, family B, member 3 (APBB3), transcript variant 4, mRNA [NM_006051]	1.66042	up	2.03634	up	477.55396	297.80942	394.38757	170.78038	Detected	Detected	Detected	Detected
3832	A_24_P316019		Hs.525905		Putative golgin subfamily A member 6-like protein 11 [Source:UniProtKB/Swiss-Prot;Acc:A6NCC3] [ENST00000333156]	1.65718	up	2.30762	up	79.30138	49.55001	38.29055	14.63163	Detected	Detected	Detected	Detected
3833	A_33_P3251771	CYLD	Hs.578973	cylindromatosis (turban tumor syndrome)	Homo sapiens cylindromatosis (turban tumor syndrome) (CYLD), transcript variant 1, mRNA [NM_015247]	1.65428	up	2.05384	up	2187.48070	1369.20760	933.60144	400.82950	Detected	Detected	Detected	Detected
3834	A_33_P3281905	LOC100144604	Hs.245476	hypothetical LOC100144604	Homo sapiens hypothetical LOC100144604 (LOC100144604), non-coding RNA [NR_021493]	1.65354	up	3.28862	up	2244.68380	1405.63800	1575.67850	422.49340	Detected	Detected	Detected	Detected
3835	A_23_P252462	GALNT9	Hs.301062	UDP-N-acetyl-alpha-D-galactosamine:polypeptide N-acetylgalactosaminyltransferase 9 (GalNAc-T9)	Homo sapiens UDP-N-acetyl-alpha-D-galactosamine:polypeptide N-acetylgalactosaminyltransferase 9 (GalNAc-T9) (GALNT9), transcript variant B, mRNA [NM_021808]	1.64796	up	2.45011	up	65.68143	41.26957	59.68341	21.47998	Detected	Detected	Detected	Detected

3836	A_33_P3231908	POU3F3	Hs.673855	POU class 3 homeobox 3	Homo sapiens POU class 3 homeobox 3 (POU3F3), mRNA [NM_006236]	1.64635	up	3.51000	up	11.94260	7.51123	20.20154	5.07508	Compromised	Compromised	Detected	Compromised
3837	A_33_P3580508	C14orf26	Hs.660501	chromosome 14 open reading frame 26	Homo sapiens cDNA FLJ43967 fis, clone TESTI4017254 [AK125955]	1.64626	up	2.19025	up	78.10838	49.12834	47.18171	18.99527	Detected	Detected	Detected	Detected
3838	A_33_P3330404	FAM180B	Hs.502529	family with sequence similarity 180, member B	Homo sapiens family with sequence similarity 180, member B (FAM180B), mRNA [NM_001164379]	1.64411	up	3.49904	up	611.92206	385.38780	375.26685	94.57062	Detected	Detected	Detected	Detected
3839	A_23_P99996	MAGEL2	Hs.141496	MAGE-like 2	Homo sapiens MAGE-like 2 (MAGEL2), mRNA [NM_019066]	1.64390	up	7.10904	up	414.38010	261.01044	240.16590	29.78970	Detected	Detected	Detected	Detected
3840	A_23_P416314	HRASLS5	Hs.410316	HRAS-like suppressor family, member 5	Homo sapiens HRAS-like suppressor family, member 5 (HRASLS5), transcript variant 1, mRNA [NM_054108]	1.64347	up	2.85093	up	31345.61500	19749.08000	3636.43260	1124.74460	Detected	Detected	Detected	Detected
3841	A_24_P254084	ZNF69	Hs.661955	zinc finger protein 69	Homo sapiens zinc finger protein 69 (ZNF69), mRNA [NM_021915]	1.64071	up	2.52322	up	36.58919	23.09166	27.05395	9.45454	Detected	Detected	Detected	Compromised
3842	A_23_P397455	ACVR1C	Hs.562901	activin A receptor, type IC	Homo sapiens activin A receptor, type IC (ACVR1C), transcript variant 1, mRNA [NM_145259]	1.63552	up	2.18674	up	28.49515	18.04053	24.13396	9.73190	Detected	Compromised	Detected	Compromised
3843	A_33_P3390868	SYNPO2	Hs.655519	synaptopodin 2	Homo sapiens synaptopodin 2 (SYNPO2), transcript variant 1, mRNA [NM_133477]	1.63456	up	2.08876	up	25.57292	16.19988	34.62911	14.61900	Detected	Compromised	Detected	Detected
3844	A_32_P162187	C2	Hs.408903	complement component 2	Homo sapiens complement component 2 (C2), transcript variant 1, mRNA [NM_000063]	1.62818	up	2.20433	up	467.02264	297.00885	202.34730	80.94431	Detected	Detected	Detected	Detected
3845	A_33_P3502311	HERC2P7	Hs.722631	hect domain and RLD 2 pseudogene 7	Homo sapiens cDNA FLJ43661 fis, clone SYNOV4004914 [AK125649]	1.62750	up	2.40862	up	185.14413	117.79369	152.24180	55.73543	Detected	Detected	Detected	Detected
3846	A_23_P84448	TUBA4A	Hs.75318	tubulin, alpha 4a	Homo sapiens tubulin, alpha 4a (TUBA4A), mRNA [NM_006000]	1.62697	up	2.18096	up	3445.81030	2193.02880	4222.82280	1707.34200	Detected	Detected	Detected	Detected
3847	A_33_P3262794	ZNF280D	Hs.511477	zinc finger protein 280D	Homo sapiens zinc finger protein 280D (ZNF280D), transcript variant 1, mRNA [NM_017661]	1.62661	up	2.32915	up	22.71745	14.46137	16.95596	6.41934	Detected	Compromised	Detected	Compromised
3848	A_33_P3382887	hCG_1659830		hCG1659830	PREDICTED: Homo sapiens hCG1659830 (LOC728111), mRNA [XM_001714799]	1.62262	up	2.21432	up	33102.01000	21123.70500	6651.83350	2648.90870	Detected	Detected	Detected	Detected
3849	A_23_P87982	ATP12A	Hs.147111	ATPase, H+/K+ transporting, nongastric, alpha polypeptide	Homo sapiens ATPase, H+/K+ transporting, nongastric, alpha polypeptide (ATP12A), mRNA [NM_001676]	1.61740	up	2.65776	up	16.11892	10.31931	7.84991	2.60445	Detected	Compromised	Compromised	Compromised
3850	A_33_P3354881					1.61620	up	2.31917	up	210.54735	134.89243	280.93030	106.81499	Detected	Detected	Detected	Detected
3851	A_24_P369898				Putative myosin-XVB (Unconventional myosin-15B)(Myosin XVBP) [Source:UniProtKB/Swiss-Prot;Acc:Q96JP2] [ENST00000293201]	1.61561	up	2.45288	up	97.56057	62.52729	73.18980	26.31119	Detected	Detected	Detected	Detected
3852	A_24_P15586	THAP5	Hs.707689	THAP domain containing 5	Homo sapiens THAP domain containing 5 (THAP5), transcript variant 2, mRNA [NM_182529]	1.61460	up	2.00376	up	115.08041	73.80212	31.44569	13.83827	Detected	Detected	Detected	Detected
3853	A_33_P3250750	LOC100130711	Hs.719173	similar to hCG1778032	PREDICTED: Homo sapiens similar to hCG1778032 (LOC100130711), mRNA [XM_001720669]	1.61399	up	2.11817	up	3303.07130	2119.09200	749.35730	311.95670	Detected	Detected	Detected	Detected
3854	A_33_P3418321					1.61062	up	2.99954	up	13.76204	8.84757	9.09398	2.67341	Detected	Compromised	Compromised	Compromised
3855	A_23_P429581	TMEM67	Hs.116240	transmembrane protein 67	Homo sapiens transmembrane protein 67 (TMEM67), transcript variant 1, mRNA [NM_153704]	1.60961	up	2.46555	up	47.64972	30.65307	37.87642	13.54631	Detected	Detected	Detected	Compromised
3856	A_33_P3324655	C8orf41	Hs.77135	chromosome 8 open reading frame 41	Homo sapiens chromosome 8 open reading frame 41 (C8orf41), transcript variant 2, mRNA [NM_025115]	1.60671	up	2.60463	up	86.65222	55.84383	48.84072	16.53488	Detected	Detected	Detected	Detected
3857	A_32_P144342	PARP4	Hs.117825	poly (ADP-ribose) polymerase family, member 4	Homo sapiens poly (ADP-ribose) polymerase family, member 4 (PARP4), mRNA [NM_006437]	1.60623	up	2.00534	up	1175.28060	757.64900	137.02680	60.25371	Detected	Detected	Detected	Detected
3858	A_33_P3343210	SLC25A37	Hs.716436	solute carrier family 25, member 37	Homo sapiens solute carrier family 25, member 37 (SLC25A37), nuclear gene encoding mitochondrial protein, mRNA [NM_016612]	1.60596	up	2.01946	up	1401.65730	903.73413	1073.36670	468.68280	Detected	Detected	Detected	Detected
3859	A_24_P153511	OSBPL8	Hs.430849	oxysterol binding protein-like 8	Homo sapiens oxysterol binding protein-like 8 (OSBPL8), transcript variant 1, mRNA [NM_020841]	1.60448	up	2.17028	up	465.02148	300.10437	375.45840	152.54987	Detected	Detected	Detected	Detected
3860	A_23_P103328	PTGER3	Hs.445000	prostaglandin E receptor 3 (subtype EP3)	Homo sapiens prostaglandin E receptor 3 (subtype EP3) (PTGER3), transcript variant 4, mRNA [NM_198714]	1.60343	up	2.04238	up	196.13907	126.66221	125.51521	54.19088	Detected	Detected	Detected	Detected
3861	A_33_P3260066	BEAN	Hs.97805	brain expressed, associated with Nedd4	Homo sapiens brain expressed, associated with Nedd4 (BEAN), mRNA [NM_001136106]	1.60091	up	2.47169	up	362.66074	234.56635	279.90543	99.85780	Detected	Detected	Detected	Detected
3862	A_32_P468743	ODF3L1	Hs.144348	outer dense fiber of sperm tails 3-like 1	Homo sapiens outer dense fiber of sperm tails 3-like 1 (ODF3L1), mRNA [NM_175881]	1.59887	up	2.79845	up	13.19396	8.54469	8.42982	2.65624	Detected	Compromised	Compromised	Compromised
3863	A_23_P55917	SYT3	Hs.515554	synaptotagmin III	Homo sapiens synaptotagmin III (SYT3), transcript variant 1, mRNA [NM_032298]	1.59821	up	2.33009	up	146.81276	95.11808	124.22932	47.01292	Detected	Detected	Detected	Detected
3864	A_23_P5002	MAP4K1	Hs.95424	mitogen-activated protein kinase kinase kinase kinase 1	Homo sapiens mitogen-activated protein kinase kinase kinase kinase 1 (MAP4K1), transcript variant 1, mRNA [NM_001042600]	1.59668	up	3.63038	up	79.58189	51.60943	40.78280	9.90581	Detected	Detected	Detected	Compromised
3865	A_33_P3305105	VWA5A	Hs.152944	von Willebrand factor A domain containing 5A	Homo sapiens von Willebrand factor A domain containing 5A (VWA5A), transcript variant 2, mRNA [NM_198315]	1.59649	up	4.37633	up	207.28683	134.44350	210.79391	42.47305	Detected	Detected	Detected	Detected
3866	A_33_P3374338	LOC100292758	Hs.414183	hypothetical protein LOC100292758	PREDICTED: Homo sapiens hypothetical protein LOC100292758 (LOC100292758), mRNA [XM_002345774]	1.59627	up	3.19282	up	11.39641	7.39258	15.88357	4.38671	Compromised	Compromised	Detected	Compromised
3867	A_33_P3399755					1.59309	up	2.10816	up	7594.20460	4935.99400	5587.33150	2337.04500	Detected	Detected	Detected	Detected

3868	A_33_P3408852	EPHA8	Hs.283613	EPH receptor A8	Homo sapiens EPH receptor A8 (EPHA8), transcript variant 2, mRNA [NM_001006943]	1.59305	up	2.12767	up	51.36903	33.38908	96.54698	40.01289	Detected	Detected	Detected	Detected
3869	A_33_P3414683	KIFC2	Hs.528713	kinesin family member C2	Homo sapiens kinesin family member C2 (KIFC2), mRNA [NM_145754]	1.58850	up	3.13362	up	87.21989	56.85388	47.76035	13.43961	Detected	Detected	Detected	Compromised
3870	A_24_P841677	CXorf23	Hs.28896	chromosome X open reading frame 23	Homo sapiens chromosome X open reading frame 23 (CXorf23), mRNA [NM_198279]	1.58608	up	2.07867	up	60.22088	39.31460	29.43163	12.48519	Detected	Detected	Detected	Compromised
3871	A_23_P76071	B3GNT4	Hs.363315	UDP-GlcNAc:betaGal beta-1,3-N-acetylglucosaminyltransferase 4	Homo sapiens UDP-GlcNAc:betaGal beta-1,3-N-acetylglucosaminyltransferase 4 (B3GNT4), mRNA [NM_030765]	1.58171	up	3.25759	up	26.36804	17.26171	22.88222	6.19394	Detected	Compromised	Detected	Compromised
3872	A_33_P3414477	LOC729739		hypothetical LOC729739	PREDICTED: Homo sapiens hypothetical LOC729739 (LOC729739), miscRNA [XR_016018]	1.57943	up	3.06894	up	26.10718	17.11563	11.63925	3.34427	Detected	Detected	Compromised	Compromised
3873	A_33_P3626709	LOC100129280	Hs.678032	hypothetical protein LOC100129280	Homo sapiens cDNA FLJ44650 fis, clone BRACE2039607. [AK126613]	1.57770	up	3.08588	up	72.66760	47.69230	34.98270	9.99633	Detected	Detected	Detected	Compromised
3874	A_23_P312132	ITGAX	Hs.248472	integrin, alpha X (complement component 3 receptor 4 subunit)	Homo sapiens integrin, alpha X (complement component 3 receptor 4 subunit) (ITGAX), mRNA [NM_000887]	1.57652	up	2.20027	up	45.72312	30.03092	22.56578	9.04357	Detected	Detected	Detected	Compromised
3875	A_32_P539599	FLJ40852	Hs.17589	hypothetical LOC285962	Homo sapiens hypothetical LOC285962 (FLJ40852), non-coding RNA [NR_015392]	1.57346	up	3.06145	up	14.65687	9.64538	10.73912	3.09320	Detected	Compromised	Compromised	Compromised
3876	A_24_P411186	BCL11A	Hs.370549	B-cell CLL/lymphoma 11A (zinc finger protein)	Homo sapiens B-cell CLL/lymphoma 11A (zinc finger protein) (BCL11A), transcript variant 1, mRNA [NM_022893]	1.57232	up	2.14879	up	116.56587	76.76495	80.73995	33.13287	Detected	Detected	Detected	Detected
3877	A_33_P3381361				Paraplegin (EC 3.4.24.-)(Spastic paraplegia protein 7) [Source:UniProtKB/Swiss-Prot;Acc:Q9UQ90] [ENST00000312632]	1.57187	up	2.13615	up	130.38264	85.88873	70.60164	29.14394	Detected	Detected	Detected	Detected
3878	A_24_P724153	LOC100130107	Hs.710718	hypothetical LOC100130107	PREDICTED: Homo sapiens hypothetical LOC100130107 (LOC100130107), mRNA [XM_001716672]	1.57155	up	2.09827	up	331.54530	218.44840	471.75443	198.25280	Detected	Detected	Detected	Detected
3879	A_33_P3365553	LOC389634	Hs.647958	hypothetical LOC389634	Homo sapiens hypothetical LOC389634 (LOC389634), non-coding RNA [NR_024420]	1.57065	up	3.79167	up	72.88973	48.05279	122.04389	28.38252	Detected	Detected	Detected	Detected
3880	A_24_P109887		Hs.375105		LOC554207 protein [Source:UniProtKB/TrEMBL;Acc:Q8N1B8] [ENST00000320322]	1.56496	up	2.21419	up	16.97433	11.23112	12.54624	4.99649	Detected	Compromised	Compromised	Compromised
3881	A_33_P3220674		Hs.636923		Novel proteinHCG1981372, isoform CRA_cPutative uncharacterized protein ENSP00000370498 ;; [Source:UniProtKB/TrEMBL;Acc:A8MSK0] [ENST00000425492]	1.56399	up	2.36105	up	95.78862	63.41810	126.44069	47.22224	Detected	Detected	Detected	Detected
3882	A_24_P85511	ANKRD26	Hs.361041	ankyrin repeat domain 26	Homo sapiens ankyrin repeat domain 26 (ANKRD26), mRNA [NM_014915]	1.56369	up	3.53232	up	26.29132	17.40988	10.54648	2.63277	Detected	Detected	Compromised	Compromised
3883	A_33_P3293524	NEURL	Hs.703959	neuralized homolog (Drosophila)	Homo sapiens neuralized homolog (Drosophila) (NEURL), mRNA [NM_004210]	1.56177	up	2.26686	up	43061.89000	28550.18200	10704.79900	4164.08740	Detected	Detected	Detected	Detected
3884	A_23_P338401	KDM1B	Hs.709336	lysine (K)-specific demethylase 1B	Homo sapiens lysine (K)-specific demethylase 1B (KDM1B), mRNA [NM_153042]	1.56093	up	2.65316	up	12.15473	8.06297	10.14892	3.37304	Detected	Compromised	Compromised	Compromised
3885	A_33_P3289113	COX11	Hs.591171	COX11 homolog, cytochrome c oxidase assembly protein (yeast)	Homo sapiens cDNA FLJ42819 fis, clone BRCAN2015464. [AK124809]	1.56049	up	2.61873	up	109.69422	72.78736	104.10512	35.05470	Detected	Detected	Detected	Detected
3886	A_23_P160992	FMO4	Hs.386502	flavin containing monooxygenase 4	Homo sapiens flavin containing monooxygenase 4 (FMO4), mRNA [NM_002022]	1.55956	up	2.25518	up	15.53553	10.31473	8.46942	3.31161	Detected	Compromised	Compromised	Compromised
3887	A_33_P3385376	SYPL2	Hs.528366	synaptophysin-like 2	Homo sapiens synaptophysin-like 2 (SYPL2), mRNA [NM_001040709]	1.55802	up	2.14210	up	32.13585	21.35747	14.35209	5.90800	Detected	Detected	Detected	Compromised
3888	A_23_P156993	CNOT4	Hs.490224	CCR4-NOT transcription complex, subunit 4	Homo sapiens CCR4-NOT transcription complex, subunit 4 (CNOT4), transcript variant 2, mRNA [NM_001008225]	1.55711	up	2.16884	up	41.63683	27.68796	39.73552	16.15536	Detected	Detected	Detected	Detected
3889	A_23_P50276	ANGPTL6	Hs.712850	angiopoietin-like 6	Homo sapiens angiopoietin-like 6 (ANGPTL6), mRNA [NM_031917]	1.55416	up	2.04293	up	120.57459	80.33311	151.01143	65.18100	Detected	Detected	Detected	Detected
3890	A_33_P3263274					1.55373	up	2.06251	up	17416.79000	11607.17600	2856.55300	1221.27260	Detected	Detected	Detected	Detected
3891	A_33_P3383696	SPEG	Hs.21639	SPEG complex locus	Homo sapiens SPEG complex locus (SPEG), mRNA [NM_005876]	1.55364	up	2.81254	up	945.03937	629.84326	683.88824	214.41304	Detected	Detected	Detected	Detected
3892	A_33_P3409635	LOC100129103	Hs.375763	similar to hCG2038970	PREDICTED: Homo sapiens similar to hCG2038970 (LOC100129103), mRNA [XM_001717390]	1.55286	up	2.82808	up	67.89369	45.27205	41.21406	12.85049	Detected	Detected	Detected	Compromised
3893	A_33_P3224730		Hs.32194		cDNA FLJ23816 fis, clone HSI02685HCG31249 ; [Source:UniProtKB/TrEMBL;Acc:Q8TCI8] [ENST00000302481]	1.55095	up	2.05757	up	180.25728	120.34463	110.04931	47.16255	Detected	Detected	Detected	Detected
3894	A_23_P152462	STX1B	Hs.542230	syntaxin 1B	Homo sapiens syntaxin 1B (STX1B), mRNA [NM_052874]	1.54891	up	2.72299	up	14.67962	9.81346	17.68655	5.72746	Detected	Compromised	Detected	Compromised
3895	A_23_P113572	CD19	Hs.652262	CD19 molecule	Homo sapiens CD19 molecule (CD19), mRNA [NM_001770]	1.54713	up	2.17454	up	67.70406	45.31294	54.63095	22.15318	Detected	Detected	Detected	Detected
3896	A_33_P3415032	KCNA3	Hs.169948	potassium voltage-gated channel, shaker-related subfamily, member 3	Homo sapiens potassium voltage-gated channel, shaker-related subfamily, member 3 (KCNA3), mRNA [NM_002232]	1.54202	up	6.64309	up	62.31734	41.84573	37.05456	4.91855	Detected	Detected	Detected	Compromised
3897	A_33_P3414127	ZNF260	Hs.18103	zinc finger protein 260	Homo sapiens zinc finger protein 260 (ZNF260), transcript variant 1, mRNA [NM_001012756]	1.54028	up	2.22013	up	16.34578	10.98853	13.26310	5.26785	Detected	Compromised	Detected	Compromised

3898	A_33_P3268532	PCK2	Hs.75812	phosphoenolpyruvate carboxykinase 2 (mitochondrial)	Homo sapiens phosphoenolpyruvate carboxykinase 2 (mitochondrial) (PCK2), nuclear gene encoding mitochondrial protein, transcript variant 2, mRNA [NM_001018073]	1.53839	up	2.05897	up	270.39276	181.99623	145.21228	62.18983	Detected	Detected	Detected	Detected
3899	A_24_P400997	SMCHD1	Hs.8118	structural maintenance of chromosomes flexible hinge domain containing 1	Homo sapiens structural maintenance of chromosomes flexible hinge domain containing 1 (SMCHD1), mRNA [NM_015295]	1.53200	up	2.39957	up	158.06845	106.83659	97.22261	35.72718	Detected	Detected	Detected	Detected
3900	A_33_P3391511	LOC100129395	Hs.91531	NS5ATP13TP1	PREDICTED: Homo sapiens NS5ATP13TP1 (LOC100129395), miscRNA [XR_078529]	1.52906	up	2.66079	up	44.22427	29.94817	33.13172	10.97993	Detected	Detected	Detected	Compromised
3901	A_33_P3240063	C14orf48	Hs.143845	chromosome 14 open reading frame 48	Homo sapiens chromosome 14 open reading frame 48 (C14orf48), transcript variant 1, non-coding RNA [NR_024182]	1.52845	up	7.64565	up	76.85667	52.06718	24.17394	2.78804	Detected	Detected	Detected	Compromised
3902	A_33_P3306452	LOC653510	Hs.635227	similar to COBW domain containing 1	COBW domain-containing protein 3 (Cobalamin synthetase W domain-containing protein 3) [Source:UniProtKB/Swiss-Prot;Acc:Q5JTY5] [ENST00000455061]	1.52005	up	2.01517	up	86.54411	58.95406	78.83817	34.49773	Detected	Detected	Detected	Detected
3903	A_33_P3358851					1.51623	up	4.73700	up	375.55646	256.47375	99.81415	18.58037	Detected	Detected	Detected	Detected
3904	A_32_P515431					1.51541	up	2.08932	up	14.06359	9.60949	21.10260	8.90628	Detected	Compromised	Detected	Compromised
3905	A_23_P354217	TMEM151A	Hs.399779	transmembrane protein 151A	Homo sapiens transmembrane protein 151A (TMEM151A), mRNA [NM_153266]	1.51490	up	2.23708	up	329.28772	225.07410	213.91386	84.31846	Detected	Detected	Detected	Detected
3906	A_33_P3228593	WDR45	Hs.632807	WD repeat domain 45	Homo sapiens WD repeat domain 45, mRNA (cDNA clone IMAGE:4657761), with apparent retained intron [BC035979]	1.50864	up	2.63007	up	338.74493	232.49922	178.60486	59.88124	Detected	Detected	Detected	Detected
3907	A_33_P3420496	SNORA53	Hs.693250	small nucleolar RNA, H/ACA box 53	Homo sapiens small nucleolar RNA, H/ACA box 53 (SNORA53), small nucleolar RNA [NR_003015]	1.50756	up	2.12053	up	27.10165	18.61462	30.54386	12.70118	Detected	Compromised	Detected	Compromised
3908	A_33_P3418394	ATG12	Hs.264482	ATG12 autophagy related 12 homolog (S. cerevisiae)	Homo sapiens ATG12 autophagy related 12 homolog (S. cerevisiae) (ATG12), mRNA [NM_004707]	1.49142	up	2.23525	up	48.82485	33.89796	98.99570	39.05317	Detected	Detected	Detected	Detected
3909	A_23_P49546	GRIN2C	Hs.436980	glutamate receptor, ionotropic, N-methyl D-aspartate 2C	Homo sapiens glutamate receptor, ionotropic, N-methyl D-aspartate 2C (GRIN2C), mRNA [NM_000835]	1.49099	up	2.05385	up	102.51369	71.19355	66.08572	28.37295	Detected	Detected	Detected	Detected
3910	A_33_P3283626					1.48735	up	11.40340	up	11.67043	8.12470	34.41100	2.66090	Compromised	Compromised	Detected	Compromised
3911	A_33_P3375527	MIER3	Hs.657594	mesoderm induction early response 1, family member 3	Homo sapiens mesoderm induction early response 1, family member 3 (MIER3), mRNA [NM_152622]	1.48358	up	2.81350	up	23.66355	16.51589	28.07819	8.80011	Detected	Detected	Detected	Compromised
3912	A_33_P3258265	SEMA6C	Hs.516316	sema domain, transmembrane domain (TM), and cytoplasmic domain, (semaphorin) 6C	Homo sapiens sema domain, transmembrane domain (TM), and cytoplasmic domain, (semaphorin) 6C (SEMA6C), mRNA [NM_030913]	1.48229	up	2.01583	up	61.76088	43.14322	41.53433	18.16845	Detected	Detected	Detected	Detected
3913	A_24_P209047	IL5	Hs.2247	interleukin 5 (colony-stimulating factor, eosinophil)	Homo sapiens interleukin 5 (colony-stimulating factor, eosinophil) (IL5), mRNA [NM_000879]	1.48001	up	11.23382	up	4.75747	3.32846	47.41226	3.72159	Compromised	Compromised	Detected	Compromised
3914	A_33_P3301095					1.47687	up	2.62615	up	1091.96180	765.59607	82.04286	27.54778	Detected	Detected	Detected	Detected
3915	A_33_P3400699	SLC26A5	Hs.585146	solute carrier family 26, member 5 (prestin)	Homo sapiens solute carrier family 26, member 5 (prestin) (SLC26A5), transcript variant b, mRNA [NM_206883]	1.47491	up	2.62587	up	26.87829	18.86993	27.91637	9.37458	Detected	Compromised	Detected	Compromised
3916	A_33_P3323535		Hs.508885		Putative uncharacterized protein ENSP00000374960 [Source:UniProtKB/TrEMBL;Acc:A6NJY9] [ENST00000390453]	1.47194	up	22.25909	up	5.51379	3.87877	62.57091	2.47874	Compromised	Compromised	Detected	Compromised
3917	A_33_P3398005					1.47111	up	2.03864	up	805.41223	566.90090	568.64307	245.95999	Detected	Detected	Detected	Detected
3918	A_33_P3297126	TSNARE1	Hs.370931	t-SNARE domain containing 1	Homo sapiens t-SNARE domain containing 1 (TSNARE1), mRNA [NM_145003]	1.46761	up	2.13525	up	49.10933	34.64867	56.54347	23.35066	Detected	Detected	Detected	Detected
3919	A_23_P412427	NS3BP	Hs.711081	NS3BP	PREDICTED: Homo sapiens NS3BP (NS3BP), miscRNA [XR_041088]	1.46655	up	2.14099	up	80.67822	56.96281	103.66239	42.69446	Detected	Detected	Detected	Detected
3920	A_33_P3407606					1.46543	up	2.13255	up	102.65633	72.53587	80.62126	33.33616	Detected	Detected	Detected	Detected
3921	A_33_P3328312	HNF1A	Hs.654455	HNF1 homeobox A	Homo sapiens HNF1 homeobox A (HNF1A), mRNA [NM_000545]	1.46342	up	2.23583	up	23.30296	16.48832	25.50851	10.06032	Detected	Detected	Detected	Compromised
3922	A_32_P123255	ANKRD58	Hs.355223	ankyrin repeat domain 58	Homo sapiens ankyrin repeat domain 58 (ANKRD58), mRNA [NM_001105576]	1.46145	up	2.70901	up	212.15044	150.31140	171.74454	55.90337	Detected	Detected	Detected	Detected
3923	A_33_P3261418	CACNA1G	Hs.591169	calcium channel, voltage-dependent, T type, alpha 1G subunit	Homo sapiens calcium channel, voltage-dependent, T type, alpha 1G subunit (CACNA1G), transcript variant 15, mRNA [NM_198397]	1.46137	up	3.29298	up	12.61722	8.93999	9.47794	2.53799	Detected	Compromised	Compromised	Compromised
3924	A_33_P3351790	LOC100133599		hypothetical protein LOC100133599	PREDICTED: Homo sapiens hypothetical LOC100133599 (LOC100133599), mRNA [XM_001715586]	1.45983	up	6.85620	up	4.35403	3.08832	45.68108	5.87515	Compromised	Compromised	Detected	Compromised
3925	A_33_P3315060	ZC3H11A	Hs.532399	zinc finger CCCH-type containing 11A	Homo sapiens mRNA; cDNA DKFZp781G2455 (from clone DKFZp781G2455) [CR627439]	1.45909	up	2.00300	up	84.48402	59.95505	64.69343	28.48030	Detected	Detected	Detected	Detected
3926	A_33_P3392087	IDUA	Hs.89560	iduronidase, alpha-L-	Homo sapiens iduronidase, alpha-L- (IDUA), mRNA [NM_000203]	1.45615	up	2.15741	up	1383.64340	983.89780	817.60680	334.17798	Detected	Detected	Detected	Detected
3927	A_33_P3285277	CACNB2	Hs.59093	calcium channel, voltage-dependent, beta 2 subunit	Homo sapiens calcium channel, voltage-dependent, beta 2 subunit (CACNB2), transcript variant 5, mRNA [NM_201593]	1.45614	up	4.24524	up	48.26880	34.32385	32.54459	6.75993	Detected	Detected	Detected	Compromised
3928	A_33_P3315906	PTP4A3	Hs.43666	protein tyrosine phosphatase type IVA, member 3	Homo sapiens protein tyrosine phosphatase type IVA, member 3 (PTP4A3), transcript variant 1, mRNA [NM_032611]	1.45505	up	2.33470	up	59.65834	42.45469	81.28342	30.69987	Detected	Detected	Detected	Detected

3929	A_33_P3357199	SH2D6	Hs.209542	SH2 domain containing 6	Homo sapiens SH2 domain containing 6 (SH2D6), mRNA [NM_198482]	1.44919	up	2.49185	up	28.19188	20.14340	31.75531	11.23727	Detected	Detected	Detected	Compromised
3930	A_33_P3335730		Hs.533036		Putative uncharacterized protein ENSP00000366515 [Source:UniProtKB/TrEMBL;Acc:B7WNS0] [ENST00000377300]	1.44837	up	2.17993	up	43.45096	31.06376	21.17938	8.56713	Detected	Detected	Detected	Compromised
3931	A_33_P3308219					1.44778	up	2.48065	up	110.40868	78.96509	112.92981	40.14286	Detected	Detected	Detected	Detected
3932	A_23_P101623	ZNF667	Hs.676605	zinc finger protein 667	Homo sapiens zinc finger protein 667 (ZNF667), mRNA [NM_022103]	1.44773	up	2.21588	up	156.87020	112.19800	53.37379	21.23962	Detected	Detected	Detected	Detected
3933	A_23_P137705	TBX19	Hs.716656	T-box 19	Homo sapiens T-box 19 (TBX19), mRNA [NM_005149]	1.44769	up	2.65921	up	643.15106	460.01474	326.19530	108.16612	Detected	Detected	Detected	Detected
3934	A_33_P3332156	C1orf95	Hs.116827	chromosome 1 open reading frame 95	Homo sapiens chromosome 1 open reading frame 95 (C1orf95), mRNA [NM_001003665]	1.44731	up	2.97718	up	33.62275	24.05494	33.32795	9.87117	Detected	Detected	Detected	Compromised
3935	A_33_P3312194				Tubulin folding cofactor B (Tubulin-specific chaperone B)(Cytoskeleton-associated protein 1)(Cytoskeleton-associated protein CKAPI) [Source:UniProtKB/Swiss-Prot;Acc:Q99426] [ENST00000392178]	1.44583	up	2.28019	up	28.82057	20.64041	36.21888	14.00650	Detected	Detected	Detected	Compromised
3936	A_32_P163147	VSIG1	Hs.177164	V-set and immunoglobulin domain containing 1	Homo sapiens V-set and immunoglobulin domain containing 1 (VSIG1), mRNA [NM_182607]	1.44361	up	2.38333	up	43.43386	31.15377	32.87548	12.16334	Detected	Detected	Detected	Compromised
3937	A_32_P82111	LRFN2	Hs.250015	leucine rich repeat and fibronectin type III domain containing 2	Homo sapiens leucine rich repeat and fibronectin type III domain containing 2 (LRFN2), mRNA [NM_020737]	1.44350	up	2.93666	up	14.57191	10.45277	10.38243	3.11754	Detected	Compromised	Compromised	Compromised
3938	A_23_P251232	TTTY14		testis-specific transcript, Y-linked 14 (non-protein coding)	Homo sapiens testis-specific transcript, Y-linked 14 (non-protein coding) (TTTY14), non-coding RNA [NR_001543]	1.44269	up	3.57590	up	908.98193	652.40314	321.14133	79.19099	Detected	Detected	Detected	Detected
3939	A_33_P3352098	MS4A7	Hs.530735	membrane-spanning 4-domains, subfamily A, member 7	Homo sapiens membrane-spanning 4-domains, subfamily A, member 7 (MS4A7), transcript variant 1, mRNA [NM_021201]	1.43884	up	4.02572	up	107.39803	77.28912	12.54706	2.74830	Detected	Detected	Compromised	Compromised
3940	A_32_P159535					1.43591	up	2.15220	up	90.39230	65.18322	14.40111	5.90036	Detected	Detected	Detected	Compromised
3941	A_33_P3383149	ZCWPW2	Hs.659030	zinc finger, CW type with PWWP domain 2	Homo sapiens zinc finger, CW type with PWWP domain 2 (ZCWPW2), mRNA [NM_001040432]	1.43565	up	2.05537	up	54.03496	38.97273	47.10782	20.21014	Detected	Detected	Detected	Detected
3942	A_33_P3350549					1.43221	up	3.48936	up	799.59863	578.09326	107.13720	27.07447	Detected	Detected	Detected	Detected
3943	A_33_P3410925	KLF1	Hs.37860	Kruppel-like factor 1 (erythroid)	Homo sapiens Kruppel-like factor 1 (erythroid) (KLF1), mRNA [NM_006563]	1.43169	up	2.32111	up	434.61456	314.33100	294.41060	111.84659	Detected	Detected	Detected	Detected
3944	A_33_P3301291	FOXB2	Hs.553843	forkhead box B2	Homo sapiens forkhead box B2 (FOXB2), mRNA [NM_001013735]	1.42755	up	2.37058	up	24.68746	17.90682	7.10983	2.64466	Detected	Detected	Compromised	Compromised
3945	A_23_P153855	ZNF492	Hs.232108	zinc finger protein 492	Homo sapiens zinc finger protein 492 (ZNF492), mRNA [NM_020855]	1.42712	up	5.00885	up	215.54400	156.38942	32.11036	5.65291	Detected	Detected	Detected	Compromised
3946	A_33_P3387616	RHPN1	Hs.521912	rhophilin, Rho GTPase binding protein 1	Homo sapiens rhophilin, Rho GTPase binding protein 1 (RHPN1), mRNA [NM_052924]	1.42603	up	2.34784	up	60.03308	43.59094	43.18220	16.21818	Detected	Detected	Detected	Detected
3947	A_33_P3376622	MLLT6	Hs.91531	myeloid/lymphoid or mixed-lineage leukemia (trithorax homolog, Drosophila); translocated to, 6	Homo sapiens myeloid/lymphoid or mixed-lineage leukemia (trithorax homolog, Drosophila); translocated to, 6 (MLLT6), mRNA [NM_005937]	1.42347	up	3.79397	up	37.55910	27.32118	46.45355	10.79670	Detected	Detected	Detected	Compromised
3948	A_23_P142125	HRC	Hs.436885	histidine rich calcium binding protein	Homo sapiens histidine rich calcium binding protein (HRC), mRNA [NM_002152]	1.42092	up	2.06749	up	144.42276	105.24421	97.27160	41.48659	Detected	Detected	Detected	Detected
3949	A_33_P3226788					1.41990	up	2.67150	up	1727.54100	1259.80570	122.48583	40.42934	Detected	Detected	Detected	Detected
3950	A_33_P3242703				DNA repair protein RAD52 homolog [Source:UniProtKB/Swiss-Prot;Acc:P43351] [ENST00000397230]	1.41465	up	2.28139	up	77.13312	56.45809	30.25491	11.69395	Detected	Detected	Detected	Compromised
3951	A_32_P116989	ZCCHC18	Hs.648338	zinc finger, CCHC domain containing 18	Homo sapiens zinc finger, CCHC domain containing 18 (ZCCHC18), transcript variant 1, mRNA [NM_001143978]	1.41416	up	2.87153	up	44.47654	32.56617	45.53941	13.98428	Detected	Detected	Detected	Compromised
3952	A_33_P3372074	LLGL2	Hs.514477	lethal giant larvae homolog 2 (Drosophila)	Homo sapiens lethal giant larvae homolog 2 (Drosophila) (LLGL2), transcript variant 3, mRNA [NM_001031803]	1.41091	up	2.03007	up	63.81958	46.83674	34.05516	14.79232	Detected	Detected	Detected	Detected
3953	A_24_P410610	DPYD	Hs.335034	dihydropyrimidine dehydrogenase	Homo sapiens dihydropyrimidine dehydrogenase (DPYD), transcript variant 2, mRNA [NM_001160301]	1.40466	up	2.24557	up	18.79734	13.85666	20.31011	7.97539	Detected	Compromised	Detected	Compromised
3954	A_33_P3416687				GB	1.40020	up	2.40617	up	361.41077	267.26694	119.65700	43.85080	Detected	Detected	Detected	Detected
3955	A_33_P3326733	LOC729668		golgi autoantigen, golgin subfamily a, 6 pseudogene	Homo sapiens golgi autoantigen, golgin subfamily a, 6 pseudogene (LOC729668), non-coding RNA [NR_003524]	1.39890	up	2.27896	up	2555.53440	1891.58980	1125.10230	435.33310	Detected	Detected	Detected	Detected
3956	A_33_P3648597	LOC283075	Hs.587657	hypothetical protein LOC283075	Homo sapiens cDNA FLJ39053 fis, clone NT2RP7012291 [AK096372]	1.39770	up	2.29161	up	105.30670	78.01463	59.65579	22.95503	Detected	Detected	Detected	Detected
3957	A_33_P3214869	KPNA7	Hs.253050	karyopherin alpha 7 (importin alpha 8)	Homo sapiens karyopherin alpha 7 (importin alpha 8) (KPNA7), mRNA [NM_001145715]	1.39554	up	2.04499	up	23.92537	17.75214	13.91151	5.99858	Detected	Compromised	Compromised	Compromised
3958	A_33_P3351356				Rho guanine nucleotide exchange factor 10 [Source:UniProtKB/Swiss-Prot;Acc:O15013] [ENST00000398560]	1.39319	up	2.15591	up	85.30164	63.39869	53.17164	21.74783	Detected	Detected	Detected	Detected
3959	A_33_P3352980					1.39272	up	2.57078	up	2132.55300	1585.51560	420.07617	144.08815	Detected	Detected	Detected	Detected
3960	A_33_P3280502					1.38626	up	2.02885	up	88.46894	66.08128	72.98690	31.72196	Detected	Detected	Detected	Detected
3961	A_24_P326739	GLS2	Hs.212606	glutaminase 2 (liver, mitochondrial)	Homo sapiens glutaminase 2 (liver, mitochondrial) (GLS2), nuclear gene encoding mitochondrial protein, mRNA [NM_013267]	1.38322	up	6.24036	up	27.46070	20.55669	25.84714	3.65232	Detected	Detected	Detected	Compromised

3962	A_33_P3349702	LOC400927		TPTE and PTEN homologous inositol lipid phosphatase pseudogene	Homo sapiens TPTE and PTEN homologous inositol lipid phosphatase pseudogene (LOC400927), non-coding RNA [NR_002821]	1.37658	up	2.43028	up	118.61581	89.22250	92.14616	33.43390	Detected	Detected	Detected	Detected
3963	A_33_P3314594	RAB37	Hs.351413	RAB37, member RAS oncogene family	Homo sapiens RAB37, member RAS oncogene family (RAB37), transcript variant 3, mRNA [NM_175738]	1.37588	up	2.03991	up	212.48490	159.91208	118.72873	51.32279	Detected	Detected	Detected	Detected
3964	A_23_P412029	PUS10	Hs.368348	pseudouridylate synthase 10	Homo sapiens pseudouridylate synthase 10 (PUS10), mRNA [NM_144709]	1.37484	up	2.10148	up	26.27853	19.79172	18.93067	7.94339	Detected	Detected	Detected	Compromised
3965	A_24_P50091	C15orf51	Hs.585319	dynamin 1 pseudogene	Homo sapiens chromosome 15 open reading frame 51 (C15orf51), non-coding RNA [NR_003260]	1.37145	up	2.05582	up	31.72077	23.94946	18.69385	8.01825	Detected	Detected	Detected	Compromised
3966	A_23_P129835	PPP1R1B	Hs.286192	protein phosphatase 1, regulatory (inhibitor) subunit 1B	Homo sapiens protein phosphatase 1, regulatory (inhibitor) subunit 1B (PPP1R1B), transcript variant 1, mRNA [NM_032192]	1.36838	up	2.70058	up	6.47051	4.89625	17.71794	5.78525	Compromised	Compromised	Detected	Compromised
3967	A_33_P3308167	POLR2J4	Hs.657028	polymerase (RNA) II (DNA directed) polypeptide J4, pseudogene	Homo sapiens polymerase (RNA) II (DNA directed) polypeptide J4, pseudogene (POLR2J4), non-coding RNA [NR_003655]	1.36550	up	2.03145	up	30.47665	23.11046	34.24526	14.86484	Detected	Detected	Detected	Detected
3968	A_33_P3316934					1.36261	up	3.32316	up	24.54410	18.65124	16.09832	4.27164	Detected	Detected	Detected	Compromised
3969	A_33_P3704280	FLJ38109	Hs.652083	hypothetical protein FLJ38109	Homo sapiens cDNA FLJ38109 fis, clone D3OST2001788 [AK095428]	1.36237	up	2.81429	up	30.08203	22.86355	16.16808	5.06588	Detected	Detected	Compromised	Compromised
3970	A_33_P3301469	NCRNA00164	Hs.380689	non-protein coding RNA 164	Homo sapiens non-protein coding RNA 164 (NCRNA00164), transcript variant 1, non-coding RNA [NR_027019]	1.36117	up	2.47989	up	131.40797	99.96354	22.34380	7.94493	Detected	Detected	Detected	Compromised
3971	A_33_P3332474	GOLGA6L1	Hs.531569	golgi autoantigen, golgin subfamily a, 6-like 1	Homo sapiens golgi autoantigen, golgin subfamily a, 6-like 1 (GOLGA6L1), mRNA [NM_001001413]	1.35861	up	2.44069	up	39.39558	30.02523	20.39983	7.37020	Detected	Detected	Detected	Compromised
3972	A_33_P3397603	LOC100130278	Hs.638469	hypothetical protein LOC100130278	Homo sapiens cDNA FLJ36795 fis, clone ADRGL2006767. [AK094114]	1.35181	up	2.47612	up	9.62989	7.37632	13.63893	4.85706	Compromised	Compromised	Detected	Compromised
3973	A_33_P3474250	LOC283270	Hs.629770	hypothetical protein LOC283270	Homo sapiens cDNA FLJ33733 fis, clone BRAWH2018267 [AK091052]	1.34332	up	2.00891	up	3026.37180	2332.79740	237.01600	104.03601	Detected	Detected	Detected	Detected
3974	A_33_P3262118		Hs.257900		Leucine zipper protein 1 [Source:UniProtKB/Swiss-Prot;Acc:Q86V48] [ENST00000314174]	1.34056	up	2.33167	up	17.28480	13.35092	10.92846	4.13293	Detected	Compromised	Compromised	Compromised
3975	A_33_P3405369	KRTAP5-4	Hs.539087	keratin associated protein 5-4	Homo sapiens keratin associated protein 5-4 (KRTAP5-4), mRNA [NM_001012709]	1.34035	up	2.40258	up	22.80168	17.61503	8.89320	3.26397	Detected	Compromised	Compromised	Compromised
3976	A_33_P3314857	KCNJ13	Hs.467338	potassium inwardly-rectifying channel, subfamily J, member 13	Homo sapiens potassium inwardly-rectifying channel, subfamily J, member 13 (KCNJ13), mRNA [NM_002242]	1.33638	up	2.88494	up	21.59785	16.73457	11.03369	3.37248	Detected	Compromised	Compromised	Compromised
3977	A_23_P104705	SLC29A2	Hs.569017	solute carrier family 29 (nucleoside transporters), member 2	Homo sapiens solute carrier family 29 (nucleoside transporters), member 2 (SLC29A2), mRNA [NM_001532]	1.33464	up	3.78798	up	51.82099	40.20459	22.52219	5.24286	Detected	Detected	Detected	Compromised
3978	A_23_P408232	MGC23270	Hs.352220	hypothetical LOC196872	Homo sapiens hypothetical LOC196872 (MGC23270), non-coding RNA [NR_024396]	1.33253	up	2.69705	up	16.35067	12.70547	12.59482	4.11783	Detected	Compromised	Compromised	Compromised
3979	A_33_P3336552	LOC100130701	Hs.711483	similar to hCG1657343	PREDICTED: Homo sapiens similar to hCG1657343 (LOC100130701), mRNA [XM_001715398]	1.33027	up	3.66945	up	13.29634	10.34963	21.47342	5.16019	Compromised	Compromised	Detected	Compromised
3980	A_33_P3454679	LOC100134702		hypothetical LOC100134702	PREDICTED: Homo sapiens similar to mucin (LOC100134702), mRNA [XM_002344283]	1.32946	up	2.17050	up	306.30768	238.57022	148.36280	60.27401	Detected	Detected	Detected	Detected
3981	A_33_P3714477	LOC285972	Hs.647110	hypothetical protein LOC285972	Homo sapiens cDNA FLJ39839 fis, clone SPLEN2014136 [AK097158]	1.32544	up	3.55458	up	30.11023	23.52273	12.31133	3.05410	Detected	Detected	Compromised	Compromised
3982	A_23_P29922	TLR3	Hs.657724	toll-like receptor 3	Homo sapiens toll-like receptor 3 (TLR3), mRNA [NM_003265]	1.31968	up	2.37392	up	14.24198	11.17471	12.45357	4.62588	Detected	Compromised	Detected	Compromised
3983	A_33_P3417547					1.31884	up	3.74995	up	45.70442	35.88381	19.43917	4.57107	Detected	Detected	Detected	Compromised
3984	A_33_P3237744	LOC100130827	Hs.712129	similar to Putative uncharacterized serine/threonine-protein kinase SgK110	PREDICTED: Homo sapiens similar to Putative uncharacterized serine/threonine-protein kinase SgK110 (LOC100130827), miscRNA [XR_042316]	1.31808	up	2.73442	up	32.76052	25.73616	25.67542	8.27977	Detected	Detected	Detected	Compromised
3985	A_23_P107981	SULT2B1	Hs.369331	sulfotransferase family, cytosolic, 2B, member 1	Homo sapiens sulfotransferase family, cytosolic, 2B, member 1 (SULT2B1), transcript variant 1, mRNA [NM_004605]	1.31805	up	3.80985	up	16.43002	12.90746	14.69255	3.40059	Detected	Compromised	Detected	Compromised
3986	A_33_P3658861	SNORA26	Hs.635034	small nucleolar RNA, H/ACA box 26	qi73d08.y5 NCI_CGAP_Ov26 Homo sapiens cDNA clone IMAGE:1862127 5', mRNA sequence [AI792523]	1.31154	up	2.90855	up	107.04500	84.51167	58.85277	17.84252	Detected	Detected	Detected	Detected
3987	A_33_P3271965	GRM1	Hs.32945	glutamate receptor, metabotropic 1	Homo sapiens glutamate receptor, metabotropic 1 (GRM1), transcript variant 1, mRNA [NM_000838]	1.30795	up	2.10729	up	37.78208	29.91083	13.17137	5.51152	Detected	Detected	Compromised	Compromised
3988	A_23_P161769	FXYD2	Hs.413137	FXYD domain containing ion transport regulator 2	Homo sapiens FXYD domain containing ion transport regulator 2 (FXYD2), transcript variant b, mRNA [NM_021603]	1.30592	up	2.93569	up	42.27184	33.51709	34.25326	10.28862	Detected	Detected	Detected	Compromised
3989	A_23_P30634	BACH2	Hs.269764	BTB and CNC homology 1, basic leucine zipper transcription factor 2	Homo sapiens BTB and CNC homology 1, basic leucine zipper transcription factor 2 (BACH2), mRNA [NM_021813]	1.30547	up	3.45878	up	42.06140	33.36184	31.40398	8.00621	Detected	Detected	Detected	Compromised
3990	A_33_P3218410		Hs.367437		Serine-protein kinase ATM (EC 2.7.11.1)(Ataxia telangiectasia mutated)(A-T, mutated) [Source:UniProtKB/Swiss-Prot;Acc:Q13315] [ENST00000389511]	1.29890	up	2.29266	up	22.29364	17.77213	8.93978	3.43838	Detected	Compromised	Compromised	Compromised

3991	A_33_P3295423	ZXDC	Hs.440049	ZXD family zinc finger C	Homo sapiens ZXD family zinc finger C (ZXDC), transcript variant 2, mRNA [NM_001040653]	1.29858	up	2.00618	up	86.53945	69.00472	59.36798	26.09438	Detected	Detected	Detected	Detected
3992	A_24_P941898	FAM22D	Hs.701044	family with sequence similarity 22, member D	Homo sapiens family with sequence similarity 22, member D (FAM22D), mRNA [NM_001009610]	1.29764	up	2.35244	up	53.55354	42.73327	26.86187	10.06892	Detected	Detected	Detected	Compromised
3993	A_33_P3280687	LENG8	Hs.720354	leukocyte receptor cluster (LRC) member 8	Homo sapiens leukocyte receptor cluster (LRC) member 8 (LENG8), mRNA [NM_052925]	1.29639	up	2.06797	up	13.79365	11.01731	18.27069	7.79071	Compromised	Compromised	Detected	Compromised
3994	A_33_P3279871		Hs.568075		Putative uncharacterized protein ENSP00000380325 Fragment [Source:UniProtKB/TrEMBL;Acc:A8MT48] [ENST00000397136]	1.29619	up	2.71008	up	33.69389	26.91633	19.35029	6.29608	Detected	Detected	Detected	Compromised
3995	A_33_P3404706	SPN	Hs.632188	sialophorin	Homo sapiens sialophorin (SPN), transcript variant 1, mRNA [NM_001030288]	1.29570	up	2.08815	up	25.29750	20.21648	17.65699	7.45623	Detected	Detected	Detected	Compromised
3996	A_33_P3402086				Proto-oncogene tyrosine-protein kinase MER Precursor (C-mer)(EC 2.7.10.1)(Receptor tyrosine kinase MerTK) [Source:UniProtKB/Swiss-Prot;Acc:Q12866] [ENST00000393237]	1.29440	up	3.39144	up	49.94673	39.95491	23.82013	6.19336	Detected	Detected	Detected	Compromised
3997	A_32_P119248	FOXD4	Hs.584759	forkhead box D4	Homo sapiens forkhead box D4 (FOXD4), mRNA [NM_207305]	1.29050	up	2.21114	up	22.48050	18.03764	18.57067	7.40590	Detected	Compromised	Detected	Compromised
3998	A_33_P3328087				PRO2532 [Source:UniProtKB/TrEMBL;Acc:Q9H399] [ENST00000391336]	1.28856	up	3.12656	up	25.92933	20.83626	9.56369	2.69727	Detected	Detected	Compromised	Compromised
3999	A_24_P935682	UNQ6228	Hs.661972	hypothetical LOC100131541	Homo sapiens clone DNA166629 MRSS6228 (UNQ6228) mRNA, complete cds. [AY358248]	1.27970	up	2.05435	up	74.27256	60.09716	39.07020	16.77013	Detected	Detected	Detected	Detected
4000	A_23_P123596	GLDC	Hs.584238	glycine dehydrogenase (decarboxylating)	Homo sapiens glycine dehydrogenase (decarboxylating) (GLDC), mRNA [NM_000170]	1.27797	up	2.34221	up	165.26158	133.90130	122.23808	46.02000	Detected	Detected	Detected	Detected
4001	A_33_P3277367	LOC732146		hypothetical protein LOC732146	PREDICTED: Homo sapiens hypothetical LOC732146 (LOC732146), mRNA [XM_001131995]	1.27486	up	4.59449	up	52.68789	42.79372	40.03375	7.68341	Detected	Detected	Detected	Compromised
4002	A_24_P942132	TRAPPC6B	Hs.13303	trafficking protein particle complex 6B	Homo sapiens trafficking protein particle complex 6B (TRAPPC6B), transcript variant 1, mRNA [NM_001079537]	1.27472	up	2.35653	up	65.89002	53.52268	13.96898	5.22707	Detected	Detected	Compromised	Compromised
4003	A_24_P337419	TIGD6	Hs.169333	tigger transposable element derived 6	Homo sapiens tigger transposable element derived 6 (TIGD6), mRNA [NM_030953]	1.27331	up	3.55820	up	62.75986	51.03650	42.15773	10.44751	Detected	Detected	Detected	Compromised
4004	A_33_P3298111				Spermatogenesis-associated protein 13 [Source:UniProtKB/Swiss-Prot;Acc:Q96N96] [ENST00000360220]	1.26706	up	2.83509	up	19.56452	15.98846	33.27119	10.34826	Detected	Detected	Detected	Compromised
4005	A_33_P3348973	PIKFYVE	Hs.720192	phosphoinositide kinase, FYVE finger containing	Homo sapiens phosphoinositide kinase, FYVE finger containing (PIKFYVE), transcript variant 3, mRNA [NM_152671]	1.26683	up	2.06788	up	31.13085	25.44510	33.24003	14.17428	Detected	Detected	Detected	Detected
4006	A_33_P3632866	C9orf96	Hs.720195	chromosome 9 open reading frame 96	Homo sapiens chromosome 9 open reading frame 96 (C9orf96), mRNA [NM_153710]	1.26661	up	3.25473	up	62.38347	50.99887	60.91553	16.50360	Detected	Detected	Detected	Detected
4007	A_33_P3294446	HMBOX1	Hs.591836	homeobox containing 1	Homo sapiens homeobox containing 1 (HMBOX1), transcript variant 1, mRNA [NM_024567]	1.26554	up	2.92078	up	33.33921	27.27810	21.81333	6.58550	Detected	Detected	Detected	Compromised
4008	A_33_P3229678	FLJ39739	Hs.636949	hypothetical FLJ39739	PREDICTED: Homo sapiens hypothetical FLJ39739 (FLJ39739), miscRNA [XR_079533]	1.26469	up	2.73683	up	20.09613	16.45361	8.51348	2.74299	Detected	Detected	Compromised	Compromised
4009	A_23_P47777	MARCH9	Hs.632709	membrane-associated ring finger (C3HC4) 9	Homo sapiens membrane-associated ring finger (C3HC4) 9 (MARCH9), mRNA [NM_138396]	1.25441	up	2.48888	up	1206.71860	996.08887	920.76135	326.21796	Detected	Detected	Detected	Detected
4010	A_23_P340308	TAS2R19	Hs.688196	taste receptor, type 2, member 19	Homo sapiens taste receptor, type 2, member 19 (TAS2R19), mRNA [NM_176888]	1.25264	up	2.82554	up	18.20434	15.04805	11.34689	3.54112	Detected	Compromised	Compromised	Compromised
4011	A_33_P3285456	C1orf68	Hs.601077	chromosome 1 open reading frame 68	Homo sapiens chromosome 1 open reading frame 68 (C1orf68), mRNA [NM_001024679]	1.24558	up	2.12257	up	88.84795	73.85980	29.51818	12.26289	Detected	Detected	Detected	Compromised
4012	A_24_P342944	CSPG5	Hs.45127	chondroitin sulfate proteoglycan 5 (neuroglycan C)	Homo sapiens chondroitin sulfate proteoglycan 5 (neuroglycan C) (CSPG5), mRNA [NM_006574]	1.24460	up	2.19365	up	3.93170	3.27103	17.97943	7.22728	Compromised	Compromised	Detected	Compromised
4013	A_33_P3282740		Hs.660463		hypothetical LOC728855 (LOC728855), non-coding RNA [Source:RefSeq DNA;Acc:NR_024510] [ENST00000412305]	1.24371	up	2.27379	up	13.03383	10.85138	17.60691	6.82808	Compromised	Compromised	Detected	Compromised
4014	A_23_P154643	BMP7	Hs.473163	bone morphogenetic protein 7	Homo sapiens bone morphogenetic protein 7 (BMP7), mRNA [NM_001719]	1.24323	up	2.19397	up	20.71513	17.25314	22.60521	9.08539	Detected	Detected	Detected	Compromised
4015	A_33_P3391419	SOBP	Hs.445244	sine oculis binding protein homolog (Drosophila)	Homo sapiens sine oculis binding protein homolog (Drosophila) (SOBP), mRNA [NM_018013]	1.24113	up	2.85560	up	16.67777	13.91401	66.77265	20.61893	Detected	Compromised	Detected	Detected
4016	A_23_P86599	DMBT1	Hs.279611	deleted in malignant brain tumors 1	Homo sapiens deleted in malignant brain tumors 1 (DMBT1), transcript variant 2, mRNA [NM_007329]	1.24076	up	2.41950	up	24.70851	20.62015	18.65137	6.79753	Detected	Detected	Detected	Compromised
4017	A_23_P251974	ZFYVE9	Hs.532345	zinc finger, FYVE domain containing 9	Homo sapiens zinc finger, FYVE domain containing 9 (ZFYVE9), transcript variant 2, mRNA [NM_007323]	1.23870	up	2.04510	up	27.90012	23.32234	19.98520	8.61705	Detected	Detected	Detected	Compromised
4018	A_32_P118397	HEPACAM2	Hs.443169	HEPACAM family member 2	Homo sapiens HEPACAM family member 2 (HEPACAM2), transcript variant 1, mRNA [NM_001039372]	1.23750	up	3.07223	up	17.98209	15.04626	10.17763	2.92118	Detected	Compromised	Compromised	Compromised
4019	A_23_P10647	CYTL1	Hs.13872	cytokine-like 1	Homo sapiens cytokine-like 1 (CYTL1), mRNA [NM_018659]	1.23367	up	6.93400	up	4.79888	4.02784	24.12216	3.06760	Compromised	Compromised	Not Detected	Compromised

4020	A_33_P3292994	LOC100131436		hypothetical LOC100131436	PREDICTED: Homo sapiens hypothetical LOC100131436 (LOC100131436), mRNA [XM_001720272]	1.23119	up	2.99752	up	4.04385	3.40097	33.98509	9.99752	Compromised	Compromised	Detected	Compromised
4021	A_33_P3423420	ZNF750	Hs.653124	zinc finger protein 750	Homo sapiens zinc finger protein 750 (ZNF750), mRNA [NM_024702]	1.22911	up	13.33363	up	6.00101	5.05555	49.01978	3.24182	Compromised	Compromised	Detected	Compromised
4022	A_23_P118571	SOST	Hs.349204	sclerosteosis	Homo sapiens sclerosteosis (SOST), mRNA [NM_025237]	1.22466	up	3.23268	up	21.52464	18.19920	18.17187	4.95681	Detected	Not Detected	Detected	Compromised
4023	A_33_P3357530	SLC12A7	Hs.172613	solute carrier family 12 (potassium/chloride transporters), member 7	Homo sapiens solute carrier family 12 (potassium/chloride transporters), member 7 (SLC12A7), mRNA [NM_006598]	1.22408	up	2.08645	up	2932.46200	2480.60280	1868.22020	789.56080	Detected	Detected	Detected	Detected
4024	A_23_P128598	TUBA3C	Hs.349695	tubulin, alpha 3c	Homo sapiens tubulin, alpha 3c (TUBA3C), mRNA [NM_006001]	1.21308	up	2.04083	up	1648.21250	1406.88170	892.21027	385.50220	Detected	Detected	Detected	Detected
4025	A_33_P3306823	ZNF846	Hs.665717	zinc finger protein 846	Homo sapiens zinc finger protein 846 (ZNF846), mRNA [NM_001077624]	1.20788	up	3.53874	up	14.85290	12.73272	11.89291	2.96350	Detected	Compromised	Compromised	Compromised
4026	A_33_P3279820					1.20767	up	2.29477	up	267.71410	229.53963	22.14985	8.51132	Detected	Detected	Detected	Compromised
4027	A_23_P150379	MPZL2	Hs.116651	myelin protein zero-like 2	Homo sapiens myelin protein zero-like 2 (MPZL2), transcript variant 2, mRNA [NM_144765]	1.20536	up	2.93529	up	16.10715	13.83676	9.02084	2.70995	Detected	Compromised	Compromised	Compromised
4028	A_33_P3885807	LOC399875	Hs.434602	hypothetical gene supported by BC040220	Homo sapiens cDNA clone IMAGE:4822188 [BC040220]	1.20334	up	2.95283	up	26.32151	22.64927	12.74148	3.80494	Detected	Detected	Compromised	Compromised
4029	A_33_P3375646	GTPBP6	Hs.437145	GTP binding protein 6 (putative)	Homo sapiens cDNA FLJ40553 fis, clone THYMU2002242, highly similar to Homo sapiens mRNA for GTP-binding protein [AK097872]	1.20147	up	2.05255	up	88.89113	76.60871	31.55012	13.55420	Detected	Detected	Detected	Compromised
4030	A_33_P3889862	LOC151475	Hs.528154	hypothetical protein LOC151475	Homo sapiens cDNA FLJ30454 fis, clone BRACE2009311 [AK055016]	1.20014	up	2.12194	up	25.53617	22.03210	19.62162	8.15393	Detected	Detected	Detected	Compromised
4031	A_33_P3251856					1.19876	up	3.27646	up	17.21193	14.86723	19.80901	5.33118	Detected	Compromised	Detected	Compromised
4032	A_24_P148499	CASP8	Hs.599762	caspase 8, apoptosis-related cysteine peptidase	Homo sapiens caspase 8, apoptosis-related cysteine peptidase (CASP8), transcript variant E, mRNA [NM_033358]	1.19845	up	2.42280	up	18.54948	16.02674	17.82007	6.48571	Detected	Detected	Detected	Compromised
4033	A_33_P3771899		Hs.632626		AGENCOURT_6773311 NIH_MGC_128 Homo sapiens cDNA clone IMAGE:5811573 5', mRNA sequence [BQ278507]	1.19691	up	2.11993	up	25.66982	22.20725	28.18316	11.72284	Detected	Detected	Detected	Compromised
4034	A_23_P255701	LRRC48	Hs.579264	leucine rich repeat containing 48	Homo sapiens leucine rich repeat containing 48 (LRRC48), transcript variant 2, mRNA [NM_031294]	1.19665	up	2.74855	up	13.27140	11.48371	11.00753	3.53144	Detected	Compromised	Compromised	Compromised
4035	A_33_P3301097					1.19160	up	3.15847	up	335.60208	291.62740	12.40597	3.46354	Detected	Detected	Compromised	Compromised
4036	A_33_P3346966	SPAG16	Hs.602792	sperm associated antigen 16	Homo sapiens sperm associated antigen 16 (SPAG16), transcript variant 1, mRNA [NM_024532]	1.19159	up	2.21048	up	23.50736	20.42728	55.72788	22.23059	Detected	Detected	Detected	Detected
4037	A_33_P3260116		Hs.550905		Putative uncharacterized protein ENSP00000374967 [Source:UniProtKB/TrEMBL;Acc:A6NGS7] [ENST00000390460]	1.19039	up	2.87175	up	13.13297	11.42369	15.22825	4.67594	Detected	Compromised	Detected	Compromised
4038	A_23_P390032	TMEM20	Hs.632085	transmembrane protein 20	Homo sapiens transmembrane protein 20 (TMEM20), transcript variant 2, mRNA [NM_153226]	1.18895	up	2.68716	up	95.73579	83.37634	128.48087	42.16102	Detected	Detected	Detected	Detected
4039	A_33_P3233165				Coiled-coil domain-containing protein 104 [Source:UniProtKB/Swiss-Prot;Acc:Q96G28] [ENST00000406691]	1.18784	up	4.28958	up	41.61646	36.27780	17.04373	3.50360	Detected	Detected	Detected	Compromised
4040	A_33_P3212350	CARD11	Hs.665701	caspase recruitment domain family, member 11	Homo sapiens cDNA FLJ39820 fis, clone SPLEN2010625. [AK097139]	1.18537	up	2.05626	up	57.13845	49.91240	67.23194	28.83119	Detected	Detected	Detected	Detected
4041	A_33_P3346327	KGFLP1	Hs.721495	keratinocyte growth factor-like protein 1	Homo sapiens keratinocyte growth factor-like protein 1 (KGFLP1), non-coding RNA [NR_003674]	1.18504	up	2.88261	up	2799.55930	2446.18380	1728.74960	528.82480	Detected	Detected	Detected	Detected
4042	A_33_P3697530	SEMA4D	Hs.494406	sema domain, immunoglobulin domain (Ig), transmembrane domain (TM) and short cytoplasmic domain, (semaphorin) 4D	Homo sapiens sema domain, immunoglobulin domain (Ig), transmembrane domain (TM) and short cytoplasmic domain, (semaphorin) 4D (SEMA4D), transcript variant 2, mRNA [NM_001142287]	1.18287	up	3.05946	up	38.79379	33.95916	12.15434	3.50310	Detected	Detected	Compromised	Compromised
4043	A_24_P357266	GRPR	Hs.567282	gastrin-releasing peptide receptor	Homo sapiens gastrin-releasing peptide receptor (GRPR), mRNA [NM_005314]	1.18134	up	3.95536	up	4.24077	3.71708	17.97457	4.00717	Compromised	Compromised	Detected	Compromised
4044	A_24_P92558	ZNF674	Hs.636105	zinc finger family member 674	Homo sapiens zinc finger family member 674 (ZNF674), transcript variant 1, mRNA [NM_001039891]	1.18111	up	2.32884	up	51.45010	45.10558	8.00332	3.03037	Detected	Detected	Compromised	Compromised
4045	A_24_P331704	KRT80	Hs.140978	keratin 80	Homo sapiens keratin 80 (KRT80), transcript variant 1, mRNA [NM_182507]	1.18069	up	3.54960	up	41.88565	36.73359	33.41480	8.30090	Detected	Detected	Detected	Compromised
4046	A_33_P3309626		Hs.513200		A disintegrin and metalloproteinase with thrombospondin motifs 17 Precursor (ADAMTS-17)(ADAM-TS 17)(ADAM-TS17)(EC 3.4.24.-) [Source:UniProtKB/Swiss-Prot;Acc:Q8TE56] [ENST00000378898]	1.17955	up	2.04724	up	16.62743	14.59630	13.18001	5.67692	Detected	Compromised	Compromised	Compromised
4047	A_23_P88880	ZNF423	Hs.530930	zinc finger protein 423	Homo sapiens zinc finger protein 423 (ZNF423), mRNA [NM_015069]	1.17805	up	2.46312	up	95.23167	83.70467	39.43089	14.11618	Detected	Detected	Detected	Compromised
4048	A_33_P3305088	LOC100129840	Hs.688480	hypothetical protein LOC100129840	Homo sapiens cDNA FLJ44675 fis, clone BRACE3007869. [AK126633]	1.17590	up	2.06227	up	39.85045	35.09106	11.23891	4.80556	Detected	Detected	Compromised	Compromised
4049	A_33_P3385837	C10orf68	Hs.585464	chromosome 10 open reading frame 68	Homo sapiens chromosome 10 open reading frame 68 (C10orf68), mRNA [NM_024688]	1.17437	up	3.16610	up	20.04126	17.67061	19.81384	5.51836	Detected	Compromised	Detected	Compromised
4050	A_33_P3407157		Hs.579287		DB050195 TESTI2 Homo sapiens cDNA clone TESTI2039685 5', mRNA sequence [DB050195]	1.16943	up	2.38344	up	14.06528	12.45397	6.76471	2.50271	Detected	Compromised	Compromised	Compromised

4051	A_33_P3246635	RP11-756A22.3		transmembrane phosphoinositide 3-phosphatase and tensin homolog 2 pseudogene	Homo sapiens transmembrane phosphoinositide 3-phosphatase and tensin homolog 2 pseudogene (LOC646405), non-coding RNA [NR_026730]	1.16833	up	2.44176	up	24.46156	21.67962	19.83631	7.16346	Detected	Detected	Detected	Compromised
4052	A_23_P413585	FOXD2	Hs.166188	forkhead box D2	Homo sapiens forkhead box D2 (FOXD2), mRNA [NM_004474]	1.16687	up	2.02196	up	71.13244	63.12175	35.13295	15.32175	Detected	Detected	Detected	Detected
4053	A_23_P66137	SOX8	Hs.243678	SRY (sex determining region Y)-box 8	Homo sapiens SRY (sex determining region Y)-box 8 (SOX8), mRNA [NM_014587]	1.16369	up	4.98778	up	4125.94400	3671.28320	1753.01230	309.91550	Detected	Detected	Detected	Detected
4054	A_23_P18017	CPA3	Hs.646	carboxypeptidase A3 (mast cell)	Homo sapiens carboxypeptidase A3 (mast cell) (CPA3), mRNA [NM_001870]	1.15339	up	2.02187	up	31.05916	27.88347	21.42946	9.34595	Detected	Detected	Detected	Compromised
4055	A_23_P69863	PCDHB5	Hs.119693	protocadherin beta 5	Homo sapiens protocadherin beta 5 (PCDHB5), mRNA [NM_015669]	1.15307	up	2.22740	up	28.55352	25.64111	20.77180	8.22323	Detected	Detected	Detected	Compromised
4056	A_33_P3259712				Putative uncharacterized protein C12orf33 [Source:UniProtKB/Swiss-Prot;Acc:Q8N6U2] [ENST00000399104]	1.15273	up	2.93408	up	36.30007	32.60730	19.28616	5.79614	Detected	Detected	Detected	Compromised
4057	A_23_P31407	AGR2	Hs.530009	anterior gradient homolog 2 (Xenopus laevis)	Homo sapiens anterior gradient homolog 2 (Xenopus laevis) (AGR2), mRNA [NM_006408]	1.14833	up	2.35892	up	10.62380	9.57962	18.14266	6.78193	Compromised	Compromised	Detected	Compromised
4058	A_23_P20484	FGL1	Hs.491143	fibrinogen-like 1	Homo sapiens fibrinogen-like 1 (FGL1), transcript variant 4, mRNA [NM_201553]	1.14790	up	2.45599	up	22.02630	19.86875	8.54289	3.06721	Detected	Detected	Compromised	Compromised
4059	A_24_P15658	LYSMD3	Hs.136235	LysM, putative peptidoglycan-binding, domain containing 3	Homo sapiens LysM, putative peptidoglycan-binding, domain containing 3 (LYSMD3), mRNA [NM_198273]	1.14461	up	2.65762	up	59.62058	53.93506	7.93394	2.63246	Detected	Detected	Compromised	Compromised
4060	A_33_P3313660	BRD7P3	Hs.648050	bromodomain containing 7 pseudogene 3	Homo sapiens bromodomain containing 7 pseudogene 3 (BRD7P3), non-coding RNA [NR_002730]	1.14399	up	2.17935	up	101.11929	91.52586	57.54833	23.28474	Detected	Detected	Detected	Detected
4061	A_33_P3253361	LOC644949	Hs.631716	hypothetical LOC644949	PREDICTED: Homo sapiens hypothetical LOC644949 (LOC644949), miscRNA [XR_040201]	1.14299	up	3.20611	up	16.23381	14.70653	12.48870	3.43483	Detected	Compromised	Compromised	Compromised
4062	A_23_P170574	SNAI3	Hs.673548	snail homolog 3 (Drosophila)	Homo sapiens snail homolog 3 (Drosophila) (SNAI3), mRNA [NM_178310]	1.13901	up	2.54125	up	22.02261	20.02051	10.15327	3.52310	Detected	Detected	Compromised	Compromised
4063	A_23_P207774	KSR1	Hs.133534	kinase suppressor of ras 1	Homo sapiens kinase suppressor of ras 1 (KSR1), mRNA [NM_014238]	1.13447	up	2.70013	up	8.77690	8.01092	13.83355	4.51767	Compromised	Compromised	Detected	Compromised
4064	A_24_P606538	GGNBP1	Hs.582973	gametogenetin binding protein 1	Homo sapiens gametogenetin binding protein 1 (GGNBP1), non-coding RNA [NR_028361]	1.13276	up	2.83923	up	13.01637	11.89831	8.02828	2.49337	Detected	Compromised	Compromised	Compromised
4065	A_33_P3264940	CXorf25	Hs.562045	chromosome X open reading frame 25	PREDICTED: Homo sapiens chromosome X open reading frame 25 (CXorf25), miscRNA [XR_078589]	1.13274	up	2.06481	up	21.20039	19.37977	7.55514	3.22647	Detected	Detected	Compromised	Compromised
4066	A_33_P3351499					1.12050	up	3.83535	up	28.61062	26.43931	12.36842	2.84364	Detected	Detected	Compromised	Compromised
4067	A_33_P3283054					1.11815	up	2.58124	up	29.13552	26.98086	20.68610	7.06668	Detected	Detected	Detected	Compromised
4068	A_24_P77082	KMO	Hs.720069	kynurenine 3-monooxygenase (kynurenine 3-hydroxylase)	Homo sapiens kynurenine 3-monooxygenase (kynurenine 3-hydroxylase) (KMO), mRNA [NM_003679]	1.11514	up	2.28575	up	19.81423	18.39842	7.93764	3.06216	Detected	Detected	Compromised	Compromised
4069	A_23_P401106	PDE2A	Hs.503163	phosphodiesterase 2A, cGMP-stimulated	Homo sapiens phosphodiesterase 2A, cGMP-stimulated (PDE2A), transcript variant 1, mRNA [NM_002599]	1.10910	up	2.99285	up	500.68225	467.43744	389.42963	114.73867	Detected	Detected	Detected	Detected
4070	A_33_P3334828	INSL3	Hs.37062	insulin-like 3 (Leydig cell)	Homo sapiens insulin-like 3 (Leydig cell) (INSL3), mRNA [NM_005543]	1.10760	up	3.90376	up	570.97840	533.79060	369.93180	83.56109	Detected	Detected	Detected	Detected
4071	A_33_P3379046	LOC729770	Hs.591612	hypothetical protein LOC729770	PREDICTED: Homo sapiens hypothetical protein LOC729770 (LOC729770), miscRNA [XR_041283]	1.10123	up	2.35802	up	1313.40100	1234.95900	134.67049	50.36055	Detected	Detected	Detected	Detected
4072	A_24_P221968	LOC643802	Hs.451336	similar to M-phase phosphoprotein 10 (U3 small nucleolar ribonucleoprotein)	PREDICTED: Homo sapiens similar to M-phase phosphoprotein 10 (U3 small nucleolar ribonucleoprotein) (LOC643802), mRNA [XM_001716860]	1.09726	up	3.24627	up	171.82118	162.14398	29.13746	7.91467	Detected	Detected	Detected	Compromised
4073	A_23_P346431	FAM194A	Hs.147128	family with sequence similarity 194, member A	Homo sapiens family with sequence similarity 194, member A (FAM194A), mRNA [NM_152394]	1.09557	up	3.17428	up	46.00853	43.48435	43.29216	12.02624	Detected	Detected	Detected	Compromised
4074	A_23_P402331	WFDC5	Hs.375031	WAP four-disulfide core domain 5	Homo sapiens WAP four-disulfide core domain 5 (WFDC5), mRNA [NM_145652]	1.08592	up	4.18215	up	14.24142	13.57964	25.79678	5.43916	Detected	Compromised	Detected	Compromised
4075	A_33_P3389261	TMEM194B	Hs.659824	transmembrane protein 194B	Homo sapiens transmembrane protein 194B (TMEM194B), mRNA [NM_001142645]	1.08440	up	3.19511	up	26.23440	25.05034	60.52998	16.70513	Detected	Detected	Detected	Detected
4076	A_33_P3424204	DLEU2L	Hs.547964	deleted in lymphocytic leukemia 2-like	Homo sapiens deleted in lymphocytic leukemia 2-like (DLEU2L), non-coding RNA [NR_002771]	1.06542	up	4.37822	up	25.46792	24.75173	16.21588	3.26594	Detected	Detected	Detected	Compromised
4077	A_33_P3239112					1.05781	up	3.13900	up	27.92028	27.33029	13.68500	3.84432	Detected	Detected	Detected	Compromised
4078	A_33_P3224078	LOC442421	Hs.585349	hypothetical LOC442421	Homo sapiens hypothetical LOC442421 (LOC442421), non-coding RNA [NR_024496]	1.05743	up	2.50896	up	17.34410	16.98378	12.05569	4.23705	Detected	Compromised	Compromised	Compromised
4079	A_23_P218369	CCL14	Hs.714858	chemokine (C-C motif) ligand 14	Homo sapiens chemokine (C-C motif) ligand 14 (CCL14), transcript variant 3, mRNA [NM_032963]	1.05714	up	2.54096	up	19.06380	18.67284	7.78236	2.70071	Detected	Detected	Compromised	Compromised
4080	A_32_P78681	GLP2R	Hs.248202	glucagon-like peptide 2 receptor	Glucagon-like peptide 2 receptor Precursor (GLP-2 receptor)(GLP-2-R)(GLP-2R) [Source:UniProtKB/Swiss-Prot;Acc:O95838] [ENST00000262441]	1.05714	up	2.23792	up	144.45564	141.49316	35.91961	14.15311	Detected	Detected	Detected	Compromised
4081	A_33_P3333364		Hs.572894		V1-20 protein Fragment [Source:UniProtKB/TrEMBL;Acc:Q5NV86] [ENST00000390287]	1.05372	up	3.49478	up	4.80212	4.71889	12.57025	3.17168	Compromised	Compromised	Detected	Compromised

4082	A_33_P3211432	NCF1	Hs.647047	neutrophil cytosolic factor 1	Homo sapiens neutrophil cytosolic factor 1 (NCF1), mRNA [NM_000265]	1.05046	up	2.39313	up	13.00003	12.81434	7.00201	2.58001	Detected	Compromised	Compromised	Compromised
4083	A_33_P3213169					1.04952	up	3.09447	up	9.98022	9.84647	18.63347	5.30973	Compromised	Compromised	Detected	Compromised
4084	A_24_P82074	CPSF2	Hs.657632	cleavage and polyadenylation specific factor 2, 100kDa	Homo sapiens cleavage and polyadenylation specific factor 2, 100kDa (CPSF2), mRNA [NM_017437]	1.04326	up	2.23604	up	65.92038	65.42741	33.87412	13.35839	Detected	Detected	Detected	Compromised
4085	A_23_P170901	PACRG	Hs.25791	PARK2 co-regulated	Homo sapiens PARK2 co-regulated (PACRG), transcript variant 1, mRNA [NM_152410]	1.04280	up	2.17008	up	171.63547	170.42796	97.84258	39.75740	Detected	Detected	Detected	Detected
4086	A_33_P3248394	INMT	Hs.632629	indolethylamine N-methyltransferase	Homo sapiens indolethylamine N-methyltransferase (INMT), mRNA [NM_006774]	1.04144	up	2.26476	up	46.21958	45.95400	16.62619	6.47346	Detected	Detected	Detected	Compromised
4087	A_23_P46215	TRIM46	Hs.287735	tripartite motif-containing 46	Homo sapiens tripartite motif-containing 46 (TRIM46), mRNA [NM_025058]	1.04120	up	2.02810	up	27.92592	27.77200	30.91382	13.44092	Detected	Detected	Detected	Compromised
4088	A_33_P3326634	GPC3	Hs.644108	glypican 3	Homo sapiens glypican 3 (GPC3), transcript variant 1, mRNA [NM_001164617]	1.04102	up	2.07995	up	21.94096	21.82385	18.75371	7.95061	Detected	Detected	Detected	Compromised
4089	A_33_P3239914	LOC100130741	Hs.661989	hypothetical protein LOC100130741	Homo sapiens cDNA FLJ45289 fis, clone BRHIP3002363. [AK127222]	1.03910	up	2.06601	up	16.56241	16.50435	13.50233	5.76292	Detected	Compromised	Compromised	Compromised
4090	A_33_P3409944	GNAT2	Hs.36973	guanine nucleotide binding protein (G protein), alpha transducing activity polypeptide 2	Homo sapiens guanine nucleotide binding protein (G protein), alpha transducing activity polypeptide 2 (GNAT2), mRNA [NM_005272]	1.03658	up	2.65162	up	15.46641	15.44964	16.66748	5.54274	Detected	Compromised	Detected	Compromised
4091	A_33_P3356577				Signal-regulatory protein beta-1 isoform 3 Precursor (SIRP-beta-1 isoform 3) [Source:UniProtKB/Swiss-Prot;Acc:Q5TFQ8] [ENST00000381596]	1.03043	up	2.11286	up	49.20210	49.44240	48.36412	20.18453	Detected	Detected	Detected	Detected
4092	A_33_P3248004		Hs.559970		Uncharacterized protein FLJ41423 [Source:UniProtKB/Swiss-Prot;Acc:A1L4Q6] [ENST00000442308]	1.02190	up	2.79936	up	28.50503	28.88320	17.06128	5.37426	Detected	Detected	Detected	Compromised
4093	A_33_P3406661	TMEM63C	Hs.22452	transmembrane protein 63C	Homo sapiens transmembrane protein 63C (TMEM63C), mRNA [NM_020431]	1.02160	up	2.73037	up	15.07922	15.28385	10.19408	3.29224	Detected	Compromised	Compromised	Compromised
4094	A_33_P3403913		Hs.633945		H.sapiens mRNA sequence (16p11.2) [X69637]	1.01413	up	2.73118	up	9.86891	10.07647	14.19159	4.58191	Compromised	Compromised	Detected	Compromised
4095	A_23_P24676	OR8U1	Hs.553618	olfactory receptor, family 8, subfamily U, member 1	Homo sapiens olfactory receptor, family 8, subfamily U, member 1 (OR8U1), mRNA [NM_001005204]	1.01195	up	2.38763	up	34.29009	35.08670	8.85800	3.27140	Detected	Detected	Compromised	Compromised
4096	A_23_P166566	CCDC48	Hs.134807	coiled-coil domain containing 48	Homo sapiens coiled-coil domain containing 48 (CCDC48), mRNA [NM_024768]	1.01004	up	5.12131	up	54.66911	56.04485	18.39978	3.16809	Detected	Detected	Detected	Compromised
4097	A_24_P392632	TRPM3	Hs.47288	transient receptor potential cation channel, subfamily M, member 3	Homo sapiens transient receptor potential cation channel, subfamily M, member 3 (TRPM3), transcript variant 7, mRNA [NM_206948]	1.00802	up	3.59459	up	3.19016	3.27700	19.58200	4.80367	Compromised	Compromised	Detected	Compromised
4098	A_23_P69310	CCRL2	Hs.535713	chemokine (C-C motif) receptor-like 2	Homo sapiens chemokine (C-C motif) receptor-like 2 (CCRL2), transcript variant 1, mRNA [NM_003965]	1.00623	up	2.72505	up	35.68720	36.72383	35.79386	11.58241	Detected	Detected	Detected	Compromised
4099	A_23_P313278		Hs.489354		Mucin-3B Fragment (MUC-3B)(Intestinal mucin-3B) [Source:UniProtKB/Swiss-Prot;Acc:Q9H195] [ENST00000319509]	1.00483	up	3.40874	up	17.64934	18.18739	10.32491	2.67090	Detected	Detected	Compromised	Compromised
4100	A_24_P124558	HOXC8	Hs.664500	homeobox C8	Homo sapiens homeobox C8 (HOXC8), mRNA [NM_022658]	594.18225	down	534.99910	down	4.66344	2869.18900	2.62351	1237.66160	Compromised	Detected	Compromised	Detected
4101	A_23_P500998	HOXA9	Hs.659350	homeobox A9	Homo sapiens homeobox A9 (HOXA9), mRNA [NM_152739]	592.35516	down	493.90430	down	3.55414	2179.96880	3.05108	1328.80490	Compromised	Detected	Compromised	Detected
4102	A_23_P70968	HOXA7	Hs.660918	homeobox A7	Homo sapiens homeobox A7 (HOXA7), mRNA [NM_006896]	547.44600	down	274.53156	down	6.21965	3525.65870	5.44903	1319.09750	Compromised	Detected	Compromised	Detected
4103	A_23_P363316	HOXB5	Hs.654456	homeobox B5	Homo sapiens homeobox B5 (HOXB5), mRNA [NM_002147]	489.89343	down	455.47385	down	3.73538	1894.82500	3.04086	1221.30730	Compromised	Detected	Compromised	Detected
4104	A_33_P3341686	XIST	Hs.529901	X (inactive)-specific transcript (non-protein coding)	Homo sapiens X (inactive)-specific transcript (non-protein coding) (XIST), non-coding RNA [NR_001564]	406.94977	down	1106.65300	down	17.06895	7192.51030	4.66421	4551.50000	Detected	Detected	Compromised	Detected
4105	A_23_P66682	HOXB6	Hs.98428	homeobox B6	Homo sapiens homeobox B6 (HOXB6), mRNA [NM_018952]	385.91574	down	1998.17210	down	44.43997	17758.20900	6.01446	10597.29500	Detected	Detected	Compromised	Detected
4106	A_23_P148541	CTAG1A	Hs.534310	cancer/testis antigen 1A	Homo sapiens cancer/testis antigen 1A (CTAG1A), mRNA [NM_139250]	249.25270	down	577.00867	down	93.85477	24223.07800	35.34504	17983.58200	Detected	Detected	Detected	Detected
4107	A_33_P3300965	HOXC6	Hs.549040	homeobox C6	Homo sapiens homeobox C6 (HOXC6), transcript variant 2, mRNA [NM_153693]	247.46118	down	651.67040	down	33.13974	8491.59500	10.06871	5785.85300	Detected	Detected	Compromised	Detected
4108	A_23_P7727	HAPLN1	Hs.2799	hyaluronan and proteoglycan link protein 1	Homo sapiens hyaluronan and proteoglycan link protein 1 (HAPLN1), mRNA [NM_001884]	241.03157	down	175.28838	down	2.67364	667.28190	2.98504	461.39066	Compromised	Detected	Compromised	Detected
4109	A_24_P77904	HOXA10	Hs.110637	homeobox A10	Homo sapiens homeobox A10 (HOXA10), transcript variant 1, mRNA [NM_018951]	212.57760	down	101.90968	down	2.51297	553.14400	2.69428	242.11569	Compromised	Detected	Compromised	Detected
4110	A_23_P7313	SPP1	Hs.313	secreted phosphoprotein 1	Homo sapiens secreted phosphoprotein 1 (SPP1), transcript variant 1, mRNA [NM_001040058]	170.12749	down	197.49605	down	5.62395	990.71533	4.06797	708.43726	Compromised	Detected	Compromised	Detected
4111	A_23_P374695	TEK	Hs.89640	TEK tyrosine kinase, endothelial	Homo sapiens TEK tyrosine kinase, endothelial (TEK), mRNA [NM_000459]	168.16338	down	99.82339	down	4.01029	698.29730	4.52445	398.25705	Compromised	Detected	Compromised	Detected
4112	A_33_P3264528	HOXA11	Hs.249171	homeobox A11	Homo sapiens homeobox A11 (HOXA11), mRNA [NM_005523]	135.72766	down	157.62318	down	2.86949	403.27990	2.75836	383.38577	Compromised	Detected	Compromised	Detected
4113	A_33_P3300975	HOXC4	Hs.549040	homeobox C4	Homo sapiens homeobox C4 (HOXC4), transcript variant 1, mRNA [NM_014620]	131.16129	down	304.63330	down	7.26983	987.33136	2.57020	690.41327	Compromised	Detected	Compromised	Detected
4114	A_24_P264943	COMP	Hs.1584	cartilage oligomeric matrix protein	Homo sapiens cartilage oligomeric matrix protein (COMP), mRNA [NM_000095]	126.96592	down	201.15356	down	308.84100	40602.72700	239.12202	42414.35000	Detected	Detected	Detected	Detected

4115	A_23_P256956	KIF20A	Hs.718626	kinesin family member 20A	Homo sapiens kinesin family member 20A (KIF20A), mRNA [NM_005733]	124.42455	down	110.00188	down	18.31738	2359.94800	16.03709	1555.57620	Detected	Detected	Detected	Detected
4116	A_23_P25150	HOXC9	Hs.658823	homeobox C9	Homo sapiens homeobox C9 (HOXC9), mRNA [NM_006897]	122.49983	down	222.03098	down	52.65937	6679.50440	23.26092	4554.13600	Detected	Detected	Detected	Detected
4117	A_24_P218805	HOXC10	Hs.44276	homeobox C10	Homo sapiens homeobox C10 (HOXC10), mRNA [NM_017409]	116.49500	down	100.85654	down	2.27947	274.96307	2.37521	211.23755	Compromised	Detected	Compromised	Detected
4118	A_23_P51085	SPC25	Hs.421956	SPC25, NDC80 kinetochore complex component, homolog (S. cerevisiae)	Homo sapiens SPC25, NDC80 kinetochore complex component, homolog (S. cerevisiae) (SPC25), mRNA [NM_020675]	111.57378	down	142.18135	down	98.13633	11337.70200	62.00933	7774.36960	Detected	Detected	Detected	Detected
4119	A_24_P319613	NEK2	Hs.153704	NIMA (never in mitosis gene a)-related kinase 2	Homo sapiens NIMA (never in mitosis gene a)-related kinase 2 (NEK2), mRNA [NM_002497]	107.40808	down	71.96903	down	3.01208	334.99344	3.40980	216.39153	Compromised	Detected	Compromised	Detected
4120	A_33_P3318343	CTAG2	Hs.87225	cancer/testis antigen 2	Homo sapiens cancer/testis antigen 2 (CTAG2), transcript variant 2, mRNA [NM_020994]	104.32980	down	144.68315	down	62.47739	6749.38800	50.49297	6441.90500	Detected	Detected	Detected	Detected
4121	A_23_P55281	HOXB7	Hs.436181	homeobox B7	Homo sapiens homeobox B7 (HOXB7), mRNA [NM_004502]	98.93823	down	44.19750	down	2.50469	256.59653	2.77442	108.12709	Compromised	Detected	Compromised	Detected
4122	A_23_P35219	NEK2	Hs.153704	NIMA (never in mitosis gene a)-related kinase 2	Homo sapiens NIMA (never in mitosis gene a)-related kinase 2 (NEK2), mRNA [NM_002497]	96.84769	down	146.36285	down	12.15671	1219.09720	8.98012	1158.98720	Detected	Detected	Compromised	Detected
4123	A_33_P3276918	FAM64A	Hs.592116	family with sequence similarity 64, member A	Homo sapiens family with sequence similarity 64, member A (FAM64A), mRNA [NM_019013]	95.78413	down	182.53444	down	21.28121	2110.68300	14.51748	2336.69260	Detected	Detected	Detected	Detected
4124	A_33_P3421243	AFP	Hs.518808	alpha-fetoprotein	Homo sapiens alpha-fetoprotein (AFP), mRNA [NM_001134]	91.34399	down	55.39948	down	4.93107	466.39575	6.94193	339.11865	Compromised	Detected	Compromised	Detected
4125	A_23_P118174	PLK1	Hs.592049	polo-like kinase 1 (Drosophila)	Homo sapiens polo-like kinase 1 (Drosophila) (PLK1), mRNA [NM_005030]	91.00964	down	131.35895	down	27.14974	2558.50440	23.68674	2743.66100	Detected	Detected	Detected	Detected
4126	A_23_P43164	SULF1	Hs.409602	sulfatase 1	Homo sapiens sulfatase 1 (SULF1), transcript variant 3, mRNA [NM_015170]	87.87735	down	98.15596	down	242.28316	22046.17600	155.72363	13478.34900	Detected	Detected	Detected	Detected
4127	A_23_P118815	BIRC5	Hs.514527	baculoviral IAP repeat-containing 5	Homo sapiens baculoviral IAP repeat-containing 5 (BIRC5), transcript variant 3, mRNA [NM_001012271]	84.09252	down	99.25370	down	897.69434	78166.19500	630.66320	55196.27000	Detected	Detected	Detected	Detected
4128	A_23_P118842	KRTAP1-5	Hs.534499	keratin associated protein 1-5	Homo sapiens keratin associated protein 1-5 (KRTAP1-5), mRNA [NM_031957]	82.79930	down	107.79884	down	69.21690	5934.33300	38.83360	3691.36670	Detected	Detected	Detected	Detected
4129	A_33_P3307495	STRA6	Hs.24553	stimulated by retinoic acid gene 6 homolog (mouse)	Homo sapiens stimulated by retinoic acid gene 6 homolog (mouse) (STRA6), transcript variant 1, mRNA [NM_001142617]	81.89140	down	93.67407	down	24.68651	2093.29790	17.08419	1411.16990	Detected	Detected	Detected	Detected
4130	A_23_P79302	LYPD6B	Hs.357567	LY6/PLAUR domain containing 6B	Homo sapiens LY6/PLAUR domain containing 6B (LYPD6B), mRNA [NM_177964]	81.38724	down	43.41795	down	2.33069	196.41484	6.15036	235.46982	Compromised	Detected	Compromised	Detected
4131	A_23_P88331	DLGAP5	Hs.77695	discs, large (Drosophila) homolog-associated protein 5	Homo sapiens discs, large (Drosophila) homolog-associated protein 5 (DLGAP5), transcript variant 1, mRNA [NM_014750]	80.77577	down	96.99825	down	74.09805	6197.56400	68.34229	5845.46300	Detected	Detected	Detected	Detected
4132	A_33_P3387524	DEPDC1	Hs.445098	DEP domain containing 1	Homo sapiens DEP domain containing 1 (DEPDC1), transcript variant 1, mRNA [NM_001114120]	78.75519	down	21.32339	down	2.93570	239.39984	3.27743	61.62465	Compromised	Detected	Compromised	Detected
4133	A_33_P3245218	ODZ2	Hs.631957	odz, odd Oz/ten-m homolog 2 (Drosophila)	Homo sapiens odz, odd Oz/ten-m homolog 2 (Drosophila) (ODZ2), mRNA [NM_001122679]	78.10015	down	136.65773	down	5.73433	463.73224	2.67215	322.00372	Compromised	Detected	Compromised	Detected
4134	A_23_P370588	HOXB8	Hs.514292	homeobox B8	Homo sapiens homeobox B8 (HOXB8), mRNA [NM_024016]	76.56639	down	23.89782	down	2.33384	185.03010	2.46445	51.93295	Compromised	Detected	Compromised	Detected
4135	A_32_P96719	SHCBP1	Hs.123253	SHC SH2-domain binding protein 1	Homo sapiens SHC SH2-domain binding protein 1 (SHCBP1), mRNA [NM_024745]	75.35630	down	91.03244	down	49.26647	3844.18380	33.25931	2669.77690	Detected	Detected	Detected	Detected
4136	A_23_P141624	KRTAP1-1	Hs.247934	keratin associated protein 1-1	Homo sapiens keratin associated protein 1-1 (KRTAP1-1), mRNA [NM_030967]	72.83463	down	52.05777	down	2.34718	177.01785	2.48257	113.95988	Compromised	Detected	Compromised	Detected
4137	A_23_P65757	CCNB2	Hs.194698	cyclin B2	Homo sapiens cyclin B2 (CCNB2), mRNA [NM_004701]	71.74918	down	79.26724	down	487.13727	36191.06200	315.03323	22019.91600	Detected	Detected	Detected	Detected
4138	A_23_P35871	E2F8	Hs.523526	E2F transcription factor 8	Homo sapiens E2F transcription factor 8 (E2F8), mRNA [NM_024680]	71.73170	down	34.99167	down	2.91475	216.49382	3.26183	100.64499	Compromised	Detected	Compromised	Detected
4139	A_33_P3288159	ASPM	Hs.121028	asp (abnormal spindle) homolog, microcephaly associated (Drosophila)	Homo sapiens asp (abnormal spindle) homolog, microcephaly associated (Drosophila) (ASPM), mRNA [NM_018136]	71.65447	down	143.60994	down	38.55512	2860.60820	11.57788	1466.15250	Detected	Detected	Compromised	Detected
4140	A_24_P323598	ESCO2	Hs.99480	establishment of cohesion 1 homolog 2 (S. cerevisiae)	Homo sapiens establishment of cohesion 1 homolog 2 (S. cerevisiae) (ESCO2), mRNA [NM_001017420]	69.80357	down	79.51831	down	4.74371	342.86966	3.38835	237.58604	Compromised	Detected	Compromised	Detected
4141	A_23_P107421	TK1	Hs.515122	thymidine kinase 1, soluble	Homo sapiens thymidine kinase 1, soluble (TK1), mRNA [NM_003258]	68.75995	down	79.18738	down	1940.31380	138146.73000	1558.03670	108792.55000	Detected	Detected	Detected	Detected
4142	A_33_P3291831	CEP55	Hs.14559	centrosomal protein 55kDa	Homo sapiens centrosomal protein 55kDa (CEP55), transcript variant 1, mRNA [NM_018131]	68.03040	down	91.99155	down	19.57336	1378.80080	10.99036	891.50810	Detected	Detected	Compromised	Detected
4143	A_23_P155711	NEIL3	Hs.405467	nei endonuclease VIII-like 3 (E. coli)	Homo sapiens nei endonuclease VIII-like 3 (E. coli) (NEIL3), mRNA [NM_018248]	67.63126	down	54.69765	down	3.82752	268.03870	3.23333	155.94969	Compromised	Detected	Compromised	Detected
4144	A_23_P215634	IGFBP3	Hs.450230	insulin-like growth factor binding protein 3	Homo sapiens insulin-like growth factor binding protein 3 (IGFBP3), transcript variant 1, mRNA [NM_001013398]	67.36516	down	66.17660	down	1824.75790	127283.97000	1505.56410	87855.55500	Detected	Detected	Detected	Detected
4145	A_23_P45011	PPP1R14C	Hs.486798	protein phosphatase 1, regulatory (inhibitor) subunit 14C	Homo sapiens protein phosphatase 1, regulatory (inhibitor) subunit 14C (PPP1R14C), mRNA [NM_030949]	66.81310	down	93.42963	down	29.65725	2051.75500	24.85876	2047.99870	Detected	Detected	Detected	Detected
4146	A_23_P316511	HOXB3	Hs.654560	homeobox B3	Homo sapiens homeobox B3 (HOXB3), mRNA [NM_002146]	65.99564	down	33.93036	down	2.28753	156.32037	4.18601	125.24307	Compromised	Detected	Compromised	Detected

4147	A_23_P52017	ASPM	Hs.121028	asp (abnormal spindle) homolog, microcephaly associated (Drosophila)	Homo sapiens asp (abnormal spindle) homolog, microcephaly associated (Drosophila) (ASPM), mRNA [NM_018136]	65.81075	down	70.01885	down	157.07414	10703.71700	108.06260	6671.99800	Detected	Detected	Detected	Detected
4148	A_23_P126212	CLSPN	Hs.175613	claspin homolog (Xenopus laevis)	Homo sapiens claspin homolog (Xenopus laevis) (CLSPN), mRNA [NM_022111]	65.52806	down	46.68759	down	2.25390	152.93062	2.33282	96.03928	Compromised	Detected	Compromised	Detected
4149	A_23_P138507	CDC2	Hs.334562	cell division cycle 2, G1 to S and G2 to M	Homo sapiens cell division cycle 2, G1 to S and G2 to M (CDC2), transcript variant 1, mRNA [NM_001786]	62.70113	down	68.01745	down	119.53158	7760.52200	82.25016	4933.13040	Detected	Detected	Detected	Detected
4150	A_23_P398854	DOK7	Hs.122110	docking protein 7	Homo sapiens docking protein 7 (DOK7), transcript variant 1, mRNA [NM_173660]	62.14818	down	34.53328	down	2.55532	164.43954	3.03850	92.52573	Compromised	Detected	Compromised	Detected
4151	A_24_P37253	LYPD6	Hs.21929	LY6/PLAUR domain containing 6	Homo sapiens LY6/PLAUR domain containing 6 (LYPD6), mRNA [NM_194317]	62.11988	down	32.23157	down	2.63021	169.18219	3.09921	88.08434	Compromised	Detected	Compromised	Detected
4152	A_33_P3271273	HOXB2	Hs.514289	homeobox B2	Homo sapiens homeobox B2 (HOXB2), mRNA [NM_002145]	60.37445	down	43.52597	down	23.25287	1453.65990	18.20533	698.73505	Detected	Detected	Detected	Detected
4153	A_33_P3313075	LOC100129619	Hs.714179	hypothetical LOC100129619	PREDICTED: Homo sapiens hypothetical LOC100129619 (LOC100129619), mRNA [XM_001717266]	59.50954	down	60.80922	down	5.94992	366.63214	11.18512	599.75740	Compromised	Detected	Compromised	Detected
4154	A_23_P34788	KIF2C	Hs.720061	kinesin family member 2C	Homo sapiens kinesin family member 2C (KIF2C), mRNA [NM_006845]	59.41870	down	69.13573	down	273.92862	16853.63900	207.39778	12643.64200	Detected	Detected	Detected	Detected
4155	A_24_P299474	ODZ2	Hs.631957	odz, odd Oz/ten-m homolog 2 (Drosophila)	Homo sapiens odz, odd Oz/ten-m homolog 2 (Drosophila) (ODZ2), mRNA [NM_001122679]	58.33110	down	88.31049	down	17.59517	1062.73900	8.75273	681.58655	Detected	Detected	Compromised	Detected
4156	A_23_P70249	CDC25C	Hs.656	cell division cycle 25 homolog C (S. pombe)	Homo sapiens cell division cycle 25 homolog C (S. pombe) (CDC25C), transcript variant 1, mRNA [NM_001790]	58.22949	down	70.52467	down	30.77745	1855.70400	18.09614	1125.36250	Detected	Detected	Detected	Detected
4157	A_23_P115872	CEP55	Hs.14559	centrosomal protein 55kDa	Homo sapiens centrosomal protein 55kDa (CEP55), transcript variant 1, mRNA [NM_018131]	57.81258	down	59.26899	down	178.33205	10675.41700	124.08308	6484.93360	Detected	Detected	Detected	Detected
4158	A_33_P3377529	HOXA4	Hs.533357	homeobox A4	Homo sapiens homeobox A4 (HOXA4), mRNA [NM_002141]	57.78450	down	98.33543	down	6.40620	383.30520	2.64844	229.64958	Compromised	Detected	Compromised	Detected
4159	A_33_P3807062	HJURP	Hs.532968	Holliday junction recognition protein	Homo sapiens Holliday junction recognition protein (HJURP), mRNA [NM_018410]	56.66154	down	56.32617	down	274.61868	16112.07700	196.39200	9754.37600	Detected	Detected	Detected	Detected
4160	A_23_P57588	GTSE1	Hs.386189	G-2 and S-phase expressed 1	Homo sapiens G-2 and S-phase expressed 1 (GTSE1), mRNA [NM_016426]	56.62428	down	52.05822	down	44.89710	2632.41300	41.87673	1922.32820	Detected	Detected	Detected	Detected
4161	A_24_P225616	RRM2	Hs.226390	ribonucleotide reductase M2	Homo sapiens ribonucleotide reductase M2 (RRM2), transcript variant 2, mRNA [NM_001034]	56.30852	down	87.60055	down	70.34309	4101.36700	30.43070	2350.62960	Detected	Detected	Detected	Detected
4162	A_23_P259586	TTK	Hs.169840	TTK protein kinase	Homo sapiens TTK protein kinase (TTK), transcript variant 1, mRNA [NM_003318]	56.15061	down	73.39811	down	25.58659	1487.64700	12.98803	840.60840	Detected	Detected	Compromised	Detected
4163	A_24_P297539	UBE2C	Hs.93002	ubiquitin-conjugating enzyme E2C	Homo sapiens ubiquitin-conjugating enzyme E2C (UBE2C), transcript variant 6, mRNA [NM_181803]	55.08201	down	59.43414	down	533.38605	30421.77100	342.11734	17929.84600	Detected	Detected	Detected	Detected
4164	A_23_P212844	TACC3	Hs.104019	transforming, acidic coiled-coil containing protein 3	Homo sapiens transforming, acidic coiled-coil containing protein 3 (TACC3), mRNA [NM_006342]	53.95437	down	50.58793	down	57.68809	3222.89280	66.68399	2974.63700	Detected	Detected	Detected	Detected
4165	A_24_P346855	MKI67	Hs.80976	antigen identified by monoclonal antibody Ki-67	Homo sapiens antigen identified by monoclonal antibody Ki-67 (MKI67), transcript variant 1, mRNA [NM_002417]	53.93493	down	90.15250	down	12.88017	719.32430	4.83019	383.97903	Compromised	Detected	Compromised	Detected
4166	A_23_P210176	ITGA6	Hs.133397	integrin, alpha 6	Homo sapiens integrin, alpha 6 (ITGA6), transcript variant 2, mRNA [NM_000210]	53.34168	down	45.93769	down	21.47094	1185.90750	17.42930	706.01610	Detected	Detected	Detected	Detected
4167	A_33_P3258627				Putative uncharacterized protein ENSP00000387024 [Source:UniProtKB/TrEMBL;Acc:B8ZZ63] [ENST00000409162]	52.59529	down	8.27522	down	2.31702	126.18560	6.65699	48.57613	Compromised	Detected	Compromised	Detected
4168	A_23_P10206	HAS2	Hs.159226	hyaluronan synthase 2	Homo sapiens hyaluronan synthase 2 (HAS2), mRNA [NM_005328]	51.20701	down	60.52638	down	50.60540	2683.23930	28.46118	1519.01870	Detected	Detected	Detected	Detected
4169	A_33_P3216008	SKA3	Hs.88523	spindle and kinetochore associated complex subunit 3	Homo sapiens spindle and kinetochore associated complex subunit 3 (SKA3), transcript variant 1, mRNA [NM_145061]	50.93999	down	48.42239	down	12.93789	682.42560	8.85430	378.06476	Detected	Detected	Compromised	Detected
4170	A_23_P423237	SGCG	Hs.37167	sarcoglycan, gamma (35kDa dystrophin-associated glycoprotein)	Homo sapiens sarcoglycan, gamma (35kDa dystrophin-associated glycoprotein) (SGCG), mRNA [NM_000231]	50.23103	down	13.21567	down	2.61723	136.12785	8.27940	96.48359	Compromised	Detected	Compromised	Detected
4171	A_23_P24129	DKK1	Hs.40499	dickkopf homolog 1 (Xenopus laevis)	Homo sapiens dickkopf homolog 1 (Xenopus laevis) (DKK1), mRNA [NM_012242]	49.39177	down	47.39847	down	971.48020	49684.58200	814.47595	34041.45300	Detected	Detected	Detected	Detected
4172	A_23_P163567	SMPD3	Hs.368421	sphingomyelin phosphodiesterase 3, neutral membrane (neutral sphingomyelinase II)	Homo sapiens sphingomyelin phosphodiesterase 3, neutral membrane (neutral sphingomyelinase II) (SMPD3), mRNA [NM_018667]	49.28304	down	25.49599	down	18.40829	939.38586	2.77939	62.48648	Detected	Detected	Compromised	Detected
4173	A_24_P416370	HOXB4	Hs.664706	homeobox B4	Homo sapiens homeobox B4 (HOXB4), mRNA [NM_024015]	48.99025	down	75.59501	down	21.78193	1104.94100	13.86268	924.07230	Detected	Detected	Detected	Detected
4174	A_24_P225970	SGOL1	Hs.105153	shugoshin-like 1 (S. pombe)	Homo sapiens shugoshin-like 1 (S. pombe) (SGOL1), transcript variant A1, mRNA [NM_001012409]	48.72279	down	32.40385	down	10.13061	511.09410	10.08757	288.23630	Compromised	Detected	Compromised	Detected
4175	A_24_P347378	ALOX5AP	Hs.507658	arachidonate 5-lipoxygenase-activating protein	Homo sapiens arachidonate 5-lipoxygenase-activating protein (ALOX5AP), mRNA [NM_001629]	48.64382	down	33.85954	down	2.26967	114.32042	2.35611	70.34632	Compromised	Detected	Compromised	Detected
4176	A_33_P3387831	CENPM	Hs.208912	centromere protein M	Homo sapiens centromere protein M (CENPM), transcript variant 1, mRNA [NM_024053]	47.62641	down	51.28153	down	238.83578	11778.23800	207.23128	9370.91100	Detected	Detected	Detected	Detected

4177	A_23_P356684	ANLN	Hs.62180	anillin, actin binding protein	Homo sapiens anillin, actin binding protein (ANLN), mRNA [NM_018685]	46.97221	down	53.60512	down	89.14799	4335.96440	61.57668	2910.63700	Detected	Detected	Detected	Detected
4178	A_33_P3330149	PAX6	Hs.270303	paired box 6	Homo sapiens paired box 6 (PAX6), transcript variant 1, mRNA [NM_000280]	46.52534	down	30.35840	down	2.62064	126.24968	3.08544	82.59633	Compromised	Detected	Compromised	Detected
4179	A_24_P30557	TBX5	Hs.381715	T-box 5	Homo sapiens T-box 5 (TBX5), transcript variant 1, mRNA [NM_000192]	45.04174	down	37.62053	down	4.14714	193.41812	3.04195	100.91200	Compromised	Detected	Compromised	Detected
4180	A_23_P146274	STMN2	Hs.521651	stathmin-like 2	Homo sapiens stathmin-like 2 (STMN2), mRNA [NM_007029]	43.26243	down	38.34229	down	251.80971	11280.19100	179.59544	6072.10000	Detected	Detected	Detected	Detected
4181	A_33_P3303956	LOC100133311	Hs.658061	similar to hCG1644697	PREDICTED: Homo sapiens similar to hCG1644697 (LOC100133311), mRNA [XM_002344295]	43.11292	down	26.67950	down	2.59771	115.96621	3.62127	85.19312	Compromised	Detected	Compromised	Detected
4182	A_23_P130182	AURKB	Hs.442658	aurora kinase B	Homo sapiens aurora kinase B (AURKB), mRNA [NM_004217]	42.91836	down	55.67188	down	24.55805	1091.36500	20.76012	1019.13385	Detected	Detected	Detected	Detected
4183	A_23_P56347	PSG3	Hs.654413	pregnancy specific beta-1-glycoprotein 3	Homo sapiens pregnancy specific beta-1-glycoprotein 3 (PSG3), mRNA [NM_021016]	42.86491	down	43.97244	down	93.47945	4149.07400	45.92394	1780.67700	Detected	Detected	Detected	Detected
4184	A_23_P122197	CCNB1	Hs.23960	cyclin B1	Homo sapiens cyclin B1 (CCNB1), mRNA [NM_031966]	42.74203	down	44.84546	down	517.57600	22906.70500	392.00906	15501.72600	Detected	Detected	Detected	Detected
4185	A_23_P100127	CASC5	Hs.181855	cancer susceptibility candidate 5	Homo sapiens cancer susceptibility candidate 5 (CASC5), transcript variant 1, mRNA [NM_170589]	42.70049	down	49.23346	down	13.65018	603.53723	7.98926	346.84253	Detected	Detected	Compromised	Detected
4186	A_24_P280983	HOXA11AS	Hs.587427	HOXA11 antisense RNA (non-protein coding)	Homo sapiens HOXA11 antisense RNA (non-protein coding) (HOXA11AS), antisense RNA [NR_002795]	41.97556	down	58.23160	down	34.17975	1485.58810	27.85350	1430.22380	Detected	Detected	Detected	Detected
4187	A_23_P74349	NUF2	Hs.651950	NUF2, NDC80 kinetochore complex component, homolog (S. cerevisiae)	Homo sapiens NUF2, NDC80 kinetochore complex component, homolog (S. cerevisiae) (NUF2), transcript variant 1, mRNA [NM_145697]	41.75897	down	42.07697	down	109.16320	4720.18600	109.68658	4069.71000	Detected	Detected	Detected	Detected
4188	A_23_P373521	HAND2	Hs.388245	heart and neural crest derivatives expressed 2	Homo sapiens heart and neural crest derivatives expressed 2 (HAND2), mRNA [NM_021973]	41.60090	down	32.86138	down	13.50888	581.90924	19.07774	552.81280	Detected	Detected	Detected	Detected
4189	A_33_P3311498	LOC283392	Hs.363603	hypothetical LOC283392	Homo sapiens hypothetical LOC283392 (LOC283392), transcript variant 1, non-coding RNA [NR_026837]	41.49494	down	40.66404	down	5.94969	255.63634	4.39060	157.43460	Compromised	Detected	Compromised	Detected
4190	A_23_P151150	FOXM1	Hs.239	forkhead box M1	Homo sapiens forkhead box M1 (FOXM1), transcript variant 1, mRNA [NM_202002]	41.37134	down	44.57644	down	121.89368	5221.72360	104.64352	4113.23200	Detected	Detected	Detected	Detected
4191	A_23_P148475	KIF4A	Hs.648326	kinesin family member 4A	Homo sapiens kinesin family member 4A (KIF4A), mRNA [NM_012310]	41.30457	down	52.96214	down	83.02992	3551.12330	51.60595	2410.07670	Detected	Detected	Detected	Detected
4192	A_23_P68610	TPX2	Hs.244580	TPX2, microtubule-associated, homolog (Xenopus laevis)	Homo sapiens TPX2, microtubule-associated, homolog (Xenopus laevis) (TPX2), mRNA [NM_012112]	41.25013	down	45.09609	down	243.59319	10404.55600	223.46461	8886.14000	Detected	Detected	Detected	Detected
4193	A_24_P140475	SORBS2	Hs.655143	sorbin and SH3 domain containing 2	Homo sapiens sorbin and SH3 domain containing 2 (SORBS2), transcript variant 2, mRNA [NM_021069]	41.04034	down	61.27197	down	6.06472	257.72375	2.78729	150.59439	Compromised	Detected	Compromised	Detected
4194	A_23_P124417	BUB1	Hs.469649	budding uninhibited by benzimidazoles 1 homolog (yeast)	Homo sapiens budding uninhibited by benzimidazoles 1 homolog (yeast) (BUB1), mRNA [NM_004336]	40.92328	down	48.56127	down	63.01636	2670.28000	43.82879	1876.78700	Detected	Detected	Detected	Detected
4195	A_23_P70007	HMMR	Hs.720052	hyaluronan-mediated motility receptor (RHAMM)	Homo sapiens hyaluronan-mediated motility receptor (RHAMM) (HMMR), transcript variant 2, mRNA [NM_012484]	40.85090	down	49.36233	down	116.10930	4911.35940	73.05530	3179.89580	Detected	Detected	Detected	Detected
4196	A_32_P150891	DIAPH3	Hs.283127	diaphanous homolog 3 (Drosophila)	Homo sapiens diaphanous homolog 3 (Drosophila) (DIAPH3), transcript variant 1, mRNA [NM_001042517]	40.27765	down	44.14895	down	13.45490	561.14830	11.06306	430.68640	Detected	Detected	Compromised	Detected
4197	A_33_P3708413	MFAP5	Hs.512842	microfibrillar associated protein 5	Homo sapiens microfibrillar associated protein 5 (MFAP5), mRNA [NM_003480]	39.62058	down	53.98369	down	356.31840	14618.14000	262.57050	12498.96400	Detected	Detected	Detected	Detected
4198	A_23_P96325	ERCC6L	Hs.47558	excision repair cross-complementing rodent repair deficiency, complementation group 6-like	Homo sapiens excision repair cross-complementing rodent repair deficiency, complementation group 6-like (ERCC6L), mRNA [NM_017669]	39.58863	down	64.94959	down	20.08920	823.50500	12.51515	716.76685	Detected	Detected	Detected	Detected
4199	A_23_P167159	SCRG1	Hs.7122	stimulator of chondrogenesis 1	Homo sapiens stimulator of chondrogenesis 1 (SCRG1), mRNA [NM_007281]	39.33724	down	24.93944	down	2.66140	108.40463	3.20510	70.48447	Compromised	Detected	Compromised	Detected
4200	A_23_P121795	SORBS2	Hs.655143	sorbin and SH3 domain containing 2	Homo sapiens sorbin and SH3 domain containing 2 (SORBS2), transcript variant 2, mRNA [NM_021069]	38.97343	down	33.97353	down	13.70106	552.91187	14.33083	429.31635	Detected	Detected	Detected	Detected
4201	A_23_P215454	ELN	Hs.647061	elastin	Homo sapiens elastin (ELN), transcript variant 1, mRNA [NM_000501]	37.86731	down	52.46331	down	51.50940	2019.68640	45.34365	2097.67200	Detected	Detected	Detected	Detected
4202	A_23_P117852	KIAA0101	Hs.81892	KIAA0101	Homo sapiens KIAA0101 (KIAA0101), transcript variant 1, mRNA [NM_014736]	36.88694	down	35.94107	down	540.27780	20635.85700	324.31445	10278.34300	Detected	Detected	Detected	Detected
4203	A_23_P72668	SDPR	Hs.26530	serum deprivation response	Homo sapiens serum deprivation response (SDPR), mRNA [NM_004657]	36.06926	down	30.90423	down	4.32851	161.66243	3.54686	96.65572	Compromised	Detected	Compromised	Detected
4204	A_23_P323751	FAM83D	Hs.472716	family with sequence similarity 83, member D	Homo sapiens family with sequence similarity 83, member D (FAM83D), mRNA [NM_030919]	35.86057	down	38.07982	down	68.57602	2546.37650	38.00895	1276.28080	Detected	Detected	Detected	Detected
4205	A_33_P3326210	ESCO2	Hs.99480	establishment of cohesion 1 homolog 2 (S. cerevisiae)	Homo sapiens establishment of cohesion 1 homolog 2 (S. cerevisiae) (ESCO2), mRNA [NM_001017420]	35.85314	down	25.91011	down	2.66392	98.89648	3.07945	70.35697	Compromised	Detected	Compromised	Detected
4206	A_23_P157136	SCIN	Hs.655515	scinderin	Homo sapiens scinderin (SCIN), transcript variant 2, mRNA [NM_033128]	35.78193	down	7.72451	down	2.38629	88.41367	5.01626	34.16774	Compromised	Detected	Compromised	Detected
4207	A_23_P375	CDCA8	Hs.524571	cell division cycle associated 8	Homo sapiens cell division cycle associated 8 (CDCA8), mRNA [NM_018101]	35.73556	down	45.04246	down	524.05695	19391.53300	375.53683	14915.58000	Detected	Detected	Detected	Detected

4208	A_23_P50108	NDC80	Hs.414407	NDC80 homolog, kinetochore complex component (S. cerevisiae)	Homo sapiens NDC80 homolog, kinetochore complex component (S. cerevisiae) (NDC80), mRNA [NM_006101]	35.48090	down	39.47569	down	492.26477	18085.33000	351.30728	12228.76100	Detected	Detected	Detected	Detected
4209	A_32_P62997	PBK	Hs.104741	PDZ binding kinase	Homo sapiens PDZ binding kinase (PBK), mRNA [NM_018492]	35.46259	down	35.71678	down	272.00460	9988.02700	235.21033	7407.88530	Detected	Detected	Detected	Detected
4210	A_32_P140489	GDF6	Hs.492277	growth differentiation factor 6	Homo sapiens growth differentiation factor 6 (GDF6), mRNA [NM_001001557]	34.81978	down	9.34949	down	3.73760	134.75726	6.48856	53.49357	Compromised	Detected	Compromised	Detected
4211	A_33_P3272957	LOC100133311	Hs.658061	similar to hCG1644697	PREDICTED: Homo sapiens similar to hCG1644697 (LOC100133311), mRNA [XM_002344295]	34.80172	down	23.70523	down	2.87419	103.57362	3.31359	69.26411	Compromised	Detected	Compromised	Detected
4212	A_23_P163481	BUB1B	Hs.513645	budding uninhibited by benzimidazoles 1 homolog beta (yeast)	Homo sapiens budding uninhibited by benzimidazoles 1 homolog beta (yeast) (BUB1B), mRNA [NM_001211]	34.57169	down	39.85715	down	88.89745	3182.31400	71.71636	2520.51930	Detected	Detected	Detected	Detected
4213	A_33_P3311755	KIF23	Hs.270845	kinesin family member 23	Homo sapiens kinesin family member 23 (KIF23), transcript variant 1, mRNA [NM_138555]	34.17050	down	25.71962	down	84.03610	2973.37900	79.24654	1797.25780	Detected	Detected	Detected	Detected
4214	A_23_P200310	DEPDC1	Hs.445098	DEP domain containing 1	Homo sapiens DEP domain containing 1 (DEPDC1), transcript variant 2, mRNA [NM_017779]	34.04056	down	51.95702	down	70.18784	2473.95400	28.20383	1292.16460	Detected	Detected	Detected	Detected
4215	A_23_P58321	CCNA2	Hs.58974	cyclin A2	Homo sapiens cyclin A2 (CCNA2), mRNA [NM_001237]	33.80436	down	39.61835	down	59.81775	2093.80300	43.77638	1529.33180	Detected	Detected	Detected	Detected
4216	A_23_P501538	HOXA3	Hs.659337	homeobox A3	Homo sapiens homeobox A3 (HOXA3), transcript variant 2, mRNA [NM_153631]	33.35369	down	18.09414	down	2.40679	83.12165	2.54286	40.57189	Compromised	Detected	Compromised	Detected
4217	A_33_P3423585	UNC13C	Hs.657273	unc-13 homolog C (C. elegans)	Homo sapiens unc-13 homolog C (C. elegans) (UNC13C), mRNA [NM_001080534]	33.21877	down	19.39248	down	2.73679	94.13646	3.03744	51.94047	Compromised	Detected	Compromised	Detected
4218	A_24_P399888	CENPM	Hs.208912	centromere protein M	Homo sapiens centromere protein M (CENPM), transcript variant 2, mRNA [NM_001002876]	32.49764	down	34.80446	down	30.25951	1018.23220	26.53851	814.47327	Detected	Detected	Detected	Detected
4219	A_24_P66027	APOBEC3B	Hs.226307	apolipoprotein B mRNA editing enzyme, catalytic polypeptide-like 3B	Homo sapiens apolipoprotein B mRNA editing enzyme, catalytic polypeptide-like 3B (APOBEC3B), mRNA [NM_004900]	31.39611	down	72.79544	down	82.84677	2693.29900	39.46157	2533.05150	Detected	Detected	Detected	Detected
4220	A_23_P107283	HOXB2	Hs.514289	homeobox B2	Homo sapiens homeobox B2 (HOXB2), mRNA [NM_002145]	31.19959	down	31.77469	down	256.95248	8301.08400	213.80014	5990.38600	Detected	Detected	Detected	Detected
4221	A_33_P3368358	NEDD9	Hs.37982	neural precursor cell expressed, developmentally down-regulated 9	Homo sapiens neural precursor cell expressed, developmentally down-regulated 9 (NEDD9), transcript variant 2, mRNA [NM_182966]	31.14883	down	19.13658	down	5.83776	188.28748	9.52556	160.73872	Compromised	Detected	Compromised	Detected
4222	A_33_P3255824					31.06368	down	9.36224	down	4.28859	137.94330	7.36967	60.84053	Compromised	Detected	Compromised	Detected
4223	A_23_P96158	KRT17	Hs.2785	keratin 17	Homo sapiens keratin 17 (KRT17), mRNA [NM_000422]	30.95973	down	18.17780	down	2.61022	83.67714	7.17710	115.04189	Compromised	Detected	Compromised	Detected
4224	A_33_P3242649	KIF18A	Hs.301052	kinesin family member 18A	Homo sapiens kinesin family member 18A (KIF18A), mRNA [NM_031217]	30.24299	down	50.83564	down	21.10955	661.05370	8.59912	385.46753	Detected	Detected	Compromised	Detected
4225	A_33_P3270514	NBLA00301	Hs.61435	Nbla00301	Homo sapiens Nbla00301 (NBLA00301), non-coding RNA [NR_003679]	30.17527	down	8.42936	down	4.17646	130.49452	10.87418	80.82700	Compromised	Detected	Compromised	Detected
4226	A_33_P3222600	LOC100132588		hypothetical protein LOC100132588	PREDICTED: Homo sapiens hypothetical LOC100132588 (LOC100132588), mRNA [XM_001724555]	29.99870	down	23.08425	down	2.46339	76.51893	2.80229	57.04184	Compromised	Detected	Compromised	Detected
4227	A_24_P276576	FCRLA	Hs.266331	Fc receptor-like A	Homo sapiens Fc receptor-like A (FCRLA), mRNA [NM_032738]	29.93015	down	23.49507	down	2.36212	73.20557	2.47585	51.29394	Compromised	Detected	Compromised	Detected
4228	A_24_P322354	SKA1	Hs.134726	spindle and kinetochore associated complex subunit 1	Homo sapiens spindle and kinetochore associated complex subunit 1 (SKA1), transcript variant 1, mRNA [NM_001039535]	29.75853	down	22.59668	down	33.14546	1021.33580	43.43451	865.45593	Detected	Detected	Detected	Detected
4229	A_23_P401	CENPF	Hs.497741	centromere protein F, 350/400ka (mitosin)	Homo sapiens centromere protein F, 350/400ka (mitosin) (CENPF), mRNA [NM_016343]	29.53957	down	29.72701	down	1128.54430	34518.81200	808.48630	21192.85400	Detected	Detected	Detected	Detected
4230	A_24_P296254	ARHGAP11A	Hs.591130	Rho GTPase activating protein 11A	Homo sapiens Rho GTPase activating protein 11A (ARHGAP11A), transcript variant 1, mRNA [NM_014783]	28.92835	down	14.87799	down	10.29851	308.48290	14.65840	192.30750	Compromised	Detected	Detected	Detected
4231	A_24_P96780	CENPF	Hs.497741	centromere protein F, 350/400ka (mitosin)	Homo sapiens centromere protein F, 350/400ka (mitosin) (CENPF), mRNA [NM_016343]	28.87474	down	24.69378	down	28.05992	838.95300	16.40652	357.24786	Detected	Detected	Detected	Detected
4232	A_24_P419132	CENPI	Hs.348920	centromere protein I	Homo sapiens centromere protein I (CENPI), mRNA [NM_006733]	28.78056	down	22.96875	down	2.85082	84.95754	3.28953	66.62502	Compromised	Detected	Compromised	Detected
4233	A_23_P149200	CDC20	Hs.524947	cell division cycle 20 homolog (S. cerevisiae)	Homo sapiens cell division cycle 20 homolog (S. cerevisiae) (CDC20), mRNA [NM_001255]	28.26161	down	36.94858	down	206.87822	6054.03200	168.00581	5473.78300	Detected	Detected	Detected	Detected
4234	A_23_P169097	WISP1	Hs.492974	WNT1 inducible signaling pathway protein 1	Homo sapiens WNT1 inducible signaling pathway protein 1 (WISP1), transcript variant 2, mRNA [NM_080838]	28.11829	down	23.15160	down	3.95122	115.04121	5.70281	116.42203	Compromised	Detected	Compromised	Detected
4235	A_24_P413884	CENPA	Hs.1594	centromere protein A	Homo sapiens centromere protein A (CENPA), transcript variant 1, mRNA [NM_001809]	28.00815	down	36.46183	down	64.97530	1884.36880	33.96244	1091.95030	Detected	Detected	Detected	Detected
4236	A_33_P3376116	SPC24	Hs.381225	SPC24, NDC80 kinetochore complex component, homolog (S. cerevisiae)	Homo sapiens SPC24, NDC80 kinetochore complex component, homolog (S. cerevisiae) (SPC24), mRNA [NM_182513]	27.98772	down	50.79770	down	6.32711	183.36061	3.35695	150.36780	Compromised	Detected	Compromised	Detected
4237	A_33_P3374205	MKI67	Hs.80976	antigen identified by monoclonal antibody Ki-67	Homo sapiens antigen identified by monoclonal antibody Ki-67 (MKI67), transcript variant 1, mRNA [NM_002417]	27.95661	down	132.04602	down	13.86352	401.32025	2.54857	296.74707	Detected	Detected	Compromised	Detected
4238	A_33_P3295333	CLEC1A	Hs.29549	C-type lectin domain family 1, member A	Homo sapiens C-type lectin domain family 1, member A (CLEC1A), mRNA [NM_016511]	27.91618	down	17.66912	down	2.85839	82.62470	3.45768	53.87229	Compromised	Detected	Compromised	Detected

4239	A_23_P48835	KIF23	Hs.270845	kinesin family member 23	Homo sapiens kinesin family member 23 (KIF23), transcript variant 1, mRNA [NM_138555]	27.51100	down	31.99163	down	474.74826	13523.92800	324.82614	9163.31800	Detected	Detected	Detected	Detected
4240	A_33_P3241334		Hs.709192		Pregnancy-specific beta-1-glycoprotein 10 Precursor (PSBG-10)(Pregnancy-specific beta-1-glycoprotein 12)(PSBG-12) [Source:UniProtKB/Swiss-Prot;Acc:Q15235] [ENST00000402456]	27.50140	down	1.17945	down	7.45382	212.25935	3.04265	3.16443	Compromised	Detected	Compromised	Compromised
4241	A_23_P89509	SPAG5	Hs.514033	sperm associated antigen 5	Homo sapiens sperm associated antigen 5 (SPAG5), mRNA [NM_006461]	27.46461	down	30.53343	down	536.76495	15264.78500	436.38416	11749.24800	Detected	Detected	Detected	Detected
4242	A_23_P393015	MYL10	Hs.247831	myosin, light chain 10, regulatory	Homo sapiens myosin, light chain 10, regulatory (MYL10), mRNA [NM_138403]	27.13719	down	17.72338	down	2.67354	75.12500	4.47056	69.86723	Compromised	Detected	Compromised	Detected
4243	A_33_P3230254	NCAPG	Hs.567567	non-SMC condensin I complex, subunit G	Homo sapiens non-SMC condensin I complex, subunit G (NCAPG), mRNA [NM_022346]	26.86137	down	13.70359	down	8.17935	227.49911	12.68985	153.34033	Compromised	Detected	Detected	Detected
4244	A_33_P3254844	CEACAM7	Hs.74466	carcinoembryonic antigen-related cell adhesion molecule 7	Homo sapiens carcinoembryonic antigen-related cell adhesion molecule 7 (CEACAM7), mRNA [NM_006890]	26.49349	down	6.39351	down	2.69588	73.95578	2.95227	16.64412	Compromised	Detected	Compromised	Detected
4245	A_23_P133956	KIFC1	Hs.436912	kinesin family member C1	Homo sapiens kinesin family member C1 (KIFC1), mRNA [NM_002263]	25.98211	down	30.89576	down	68.36352	1839.21250	67.40395	1836.32600	Detected	Detected	Detected	Detected
4246	A_23_P389281	HOXA13	Hs.592172	homeobox A13	Homo sapiens homeobox A13 (HOXA13), mRNA [NM_000522]	25.95339	down	14.20313	down	2.39783	64.43839	2.64672	33.14802	Compromised	Detected	Compromised	Detected
4247	A_23_P150935	TROAP	Hs.524399	trophinin associated protein (tastin)	Homo sapiens trophinin associated protein (tastin) (TROAP), transcript variant 1, mRNA [NM_005480]	25.85623	down	41.51222	down	198.92014	5325.70400	108.48868	3971.23700	Detected	Detected	Detected	Detected
4248	A_23_P50096	TYMS	Hs.592338	thymidylate synthetase	Homo sapiens thymidylate synthetase (TYMS), mRNA [NM_001071]	25.48037	down	24.16934	down	1101.61240	29064.81800	973.80450	20754.01200	Detected	Detected	Detected	Detected
4249	A_33_P3350488	NUSAP1	Hs.615092	nucleolar and spindle associated protein 1	Homo sapiens nucleolar and spindle associated protein 1 (NUSAP1), transcript variant 1, mRNA [NM_016359]	25.47006	down	24.61929	down	456.77460	12046.61500	337.07593	7317.60250	Detected	Detected	Detected	Detected
4250	A_32_P105549	ANXA8L2	Hs.705389	annexin A8-like 2	Homo sapiens annexin A8-like 2 (ANXA8L2), mRNA [NM_001630]	25.45810	down	21.92742	down	76.16319	2007.72500	90.35633	1747.07540	Detected	Detected	Detected	Detected
4251	A_33_P3383687	LOC100132588	Hs.689599	hypothetical protein LOC100132588	PREDICTED: Homo sapiens hypothetical LOC100132588 (LOC100132588), mRNA [XM_001724555]	25.20237	down	2.27886	down	2.30255	60.08739	23.29500	46.81074	Compromised	Detected	Detected	Detected
4252	A_33_P3232945	F2RL1	Hs.720399	coagulation factor II (thrombin) receptor-like 1	Homo sapiens coagulation factor II (thrombin) receptor-like 1 (F2RL1), mRNA [NM_005242]	25.13159	down	20.90183	down	91.22343	2373.88400	64.10367	1181.49760	Detected	Detected	Detected	Detected
4253	A_33_P3286372	C2orf48	Hs.676025	chromosome 2 open reading frame 48	Homo sapiens chromosome 2 open reading frame 48 (C2orf48), mRNA [NM_182626]	25.12050	down	18.76624	down	2.56037	66.59853	3.76110	62.23827	Compromised	Detected	Compromised	Detected
4254	A_33_P3237874	TROAP	Hs.524399	trophinin associated protein (tastin)	Homo sapiens trophinin associated protein (tastin) (TROAP), transcript variant 2, mRNA [NM_001100620]	24.72655	down	45.41724	down	92.35039	2364.47780	64.88549	2598.56620	Detected	Detected	Detected	Detected
4255	A_32_P51237	KANK4	Hs.283398	KN motif and ankyrin repeat domains 4	Homo sapiens KN motif and ankyrin repeat domains 4 (KANK4), mRNA [NM_181712]	24.48150	down	16.68460	down	2.36630	59.98484	2.53535	37.30093	Compromised	Detected	Compromised	Detected
4256	A_33_P3238215	COBLL1	Hs.470457	COBL-like 1	Homo sapiens COBL-like 1 (COBLL1), mRNA [NM_014900]	24.28693	down	27.82551	down	130.00362	3269.34860	79.11240	1941.12340	Detected	Detected	Detected	Detected
4257	A_32_P147078	SLC8A1	Hs.468274	solute carrier family 8 (sodium/calcium exchanger), member 1	Homo sapiens solute carrier family 8 (sodium/calcium exchanger), member 1 (SLC8A1), transcript variant A, mRNA [NM_021097]	24.25266	down	28.19601	down	86.19134	2164.49240	61.70829	1534.25200	Detected	Detected	Detected	Detected
4258	A_33_P3298387	PLK1	Hs.592049	polo-like kinase 1 (Drosophila)	Homo sapiens polo-like kinase 1 (Drosophila) (PLK1), mRNA [NM_005030]	24.01372	down	25.77529	down	16.77517	417.11844	17.10410	388.74908	Detected	Detected	Detected	Detected
4259	A_33_P3360216	HIST1H2AI	Hs.534035	histone cluster 1, H2ai	Homo sapiens histone cluster 1, H2ai (HIST1H2AI), mRNA [NM_003509]	23.89855	down	15.85401	down	2.35046	58.16457	4.54693	63.56579	Compromised	Detected	Compromised	Detected
4260	A_23_P385861	CDCA2	Hs.33366	cell division cycle associated 2	Homo sapiens cell division cycle associated 2 (CDCA2), mRNA [NM_152562]	23.84935	down	24.13222	down	521.70935	12883.63100	388.97968	8277.31900	Detected	Detected	Detected	Detected
4261	A_24_P206776	CRYAB	Hs.53454	crystallin, alpha B	Homo sapiens crystallin, alpha B (CRYAB), mRNA [NM_001885]	23.70014	down	23.36260	down	2041.03980	50088.21500	2418.37840	49820.84000	Detected	Detected	Detected	Detected
4262	A_33_P3239347	NKX3-1	Hs.55999	NK3 homeobox 1	Homo sapiens NK3 homeobox 1 (NKX3-1), mRNA [NM_006167]	23.59190	down	21.14121	down	36.95398	902.72864	33.94248	632.75990	Detected	Detected	Detected	Detected
4263	A_23_P388812	CKAP2L	Hs.434250	cytoskeleton associated protein 2-like	Homo sapiens cytoskeleton associated protein 2-like (CKAP2L), mRNA [NM_152515]	23.56590	down	26.92141	down	54.82716	1337.86650	66.33037	1574.61960	Detected	Detected	Detected	Detected
4264	A_23_P69030	COL8A1	Hs.654548	collagen, type VIII, alpha 1	Homo sapiens collagen, type VIII, alpha 1 (COL8A1), transcript variant 1, mRNA [NM_001850]	23.53891	down	28.27520	down	392.14618	9558.00700	288.84400	7201.69240	Detected	Detected	Detected	Detected
4265	A_33_P3212994	ZWINT	Hs.591363	ZW10 interactor	Homo sapiens ZW10 interactor (ZWINT), transcript variant 2, mRNA [NM_032997]	23.49981	down	23.20289	down	174.03929	4234.91600	126.22861	2582.64870	Detected	Detected	Detected	Detected
4266	A_24_P314571		Hs.621849		Kinetochore protein Spc24 (hSpc24) [Source:UniProtKB/Swiss-Prot;Acc:Q8NBT2] [ENST00000293743]	23.47924	down	26.93779	down	88.55961	2153.04350	98.14356	2331.25270	Detected	Detected	Detected	Detected
4267	A_33_P3395428	LOC100131594	Hs.689599	hypothetical protein LOC100131594	PREDICTED: Homo sapiens hypothetical protein LOC100131594 (LOC100131594), mRNA [XM_001713897]	23.47566	down	11.53013	down	2.57165	62.51181	4.25822	43.29396	Compromised	Detected	Compromised	Detected
4268	A_23_P379614	OIP5	Hs.661645	Opa interacting protein 5	Homo sapiens Opa interacting protein 5 (OIP5), mRNA [NM_007280]	23.44438	down	22.77427	down	280.73947	6815.14900	198.32660	3982.82300	Detected	Detected	Detected	Detected
4269	A_33_P3359047	LYPD6	Hs.21929	LY6/PLAUR domain containing 6	Homo sapiens LY6/PLAUR domain containing 6 (LYPD6), mRNA [NM_194317]	23.37007	down	39.18985	down	6.04121	146.18977	3.15517	109.03382	Compromised	Detected	Compromised	Detected

4270	A_33_P3253596	KIF4A	Hs.648326	kinesin family member 4A	Homo sapiens kinesin family member 4A (KIF4A), mRNA [NM_012310]	23.27730	down	39.24998	down	31.42260	757.36957	16.23617	561.93823	Detected	Detected	Detected	Detected
4271	A_23_P389897	NGFR	Hs.415768	nerve growth factor receptor (TNFR superfamily, member 16)	Homo sapiens nerve growth factor receptor (TNFR superfamily, member 16) (NGFR), mRNA [NM_002507]	23.03998	down	30.64351	down	13.95094	332.82703	9.57043	258.60416	Detected	Detected	Compromised	Detected
4272	A_24_P227091	KIF11	Hs.8878	kinesin family member 11	Homo sapiens kinesin family member 11 (KIF11), mRNA [NM_004523]	22.82629	down	21.38833	down	114.75316	2712.27150	102.49800	1933.11510	Detected	Detected	Detected	Detected
4273	A_24_P288890	FAM101A	Hs.432901	family with sequence similarity 101, member A	Homo sapiens family with sequence similarity 101, member A (FAM101A), mRNA [NM_181709]	22.72591	down	48.43768	down	19.58541	460.87915	10.31214	440.45105	Detected	Detected	Compromised	Detected
4274	A_23_P63789	ZWINT	Hs.591363	ZW10 interactor	Homo sapiens ZW10 interactor (ZWINT), transcript variant 2, mRNA [NM_032997]	22.61160	down	24.34384	down	358.83880	8401.63400	261.63540	5616.31100	Detected	Detected	Detected	Detected
4275	A_23_P154526	GRB14	Hs.411881	growth factor receptor-bound protein 14	Homo sapiens growth factor receptor-bound protein 14 (GRB14), mRNA [NM_004490]	22.55805	down	23.66319	down	82.34106	1923.31840	55.05007	1148.67370	Detected	Detected	Detected	Detected
4276	A_33_P3339375	ARHGAP11B	Hs.659621	Rho GTPase activating protein 11B	Homo sapiens Rho GTPase activating protein 11B (ARHGAP11B), mRNA [NM_001039841]	22.23459	down	20.62765	down	19.36673	445.88050	16.37650	297.87656	Detected	Detected	Detected	Detected
4277	A_24_P218979	CDCA3	Hs.524216	cell division cycle associated 3	Homo sapiens cell division cycle associated 3 (CDCA3), mRNA [NM_031299]	22.10996	down	27.92632	down	123.13178	2818.97440	116.81379	2876.55930	Detected	Detected	Detected	Detected
4278	A_33_P3372413	LOC390557		hypothetical LOC390557	PREDICTED: Homo sapiens similar to hCG2040392 (LOC390557), mRNA [XM_002347518]	22.09164	down	5.70985	down	2.82314	64.57932	3.47417	17.49210	Compromised	Detected	Compromised	Detected
4279	A_33_P3233906	RAMP1	Hs.471783	receptor (G protein-coupled) activity modifying protein 1	Homo sapiens receptor (G protein-coupled) activity modifying protein 1 (RAMP1), mRNA [NM_005855]	21.87682	down	20.80549	down	31.47056	712.88885	50.70985	930.32710	Detected	Detected	Detected	Detected
4280	A_33_P3318661	E2F7	Hs.416375	E2F transcription factor 7	Homo sapiens E2F transcription factor 7 (E2F7), mRNA [NM_203394]	21.46688	down	18.85418	down	2.46618	54.81842	2.76984	46.04974	Compromised	Detected	Compromised	Detected
4281	A_33_P3315385	MPPED2	Hs.289795	metallophosphoesterase domain containing 2	Homo sapiens metallophosphoesterase domain containing 2 (MPPED2), transcript variant 1, mRNA [NM_001584]	21.33950	down	10.78532	down	2.25900	49.91534	2.35537	22.40050	Compromised	Detected	Compromised	Detected
4282	A_23_P213336	FGF1	Hs.483635	fibroblast growth factor 1 (acidic)	Homo sapiens fibroblast growth factor 1 (acidic) (FGF1), transcript variant 1, mRNA [NM_000800]	21.22867	down	42.34012	down	23.06308	506.95938	10.85725	405.35687	Detected	Detected	Compromised	Detected
4283	A_23_P254733	MLF1IP	Hs.575032	MLF1 interacting protein	Homo sapiens MLF1 interacting protein (MLF1IP), mRNA [NM_024629]	21.19541	down	19.14272	down	491.06420	10777.37800	376.09010	6348.35400	Detected	Detected	Detected	Detected
4284	A_23_P369485	MGC45800		hypothetical LOC90768	Homo sapiens hypothetical LOC90768 (MGC45800), non-coding RNA [NR_027107]	21.11673	down	10.74253	down	3.61191	78.97618	7.26528	68.82154	Compromised	Detected	Compromised	Detected
4285	A_23_P83403	LIMCH1	Hs.335163	LIM and calponin homology domains 1	Homo sapiens LIM and calponin homology domains 1 (LIMCH1), transcript variant 1, mRNA [NM_014988]	21.08139	down	27.51155	down	3.52084	76.85609	2.77353	67.28425	Compromised	Detected	Compromised	Detected
4286	A_23_P46039	FCRLA	Hs.266331	Fc receptor-like A	Homo sapiens Fc receptor-like A (FCRLA), mRNA [NM_032738]	20.82261	down	36.28435	down	12.70706	273.97644	10.59259	338.91202	Compromised	Detected	Compromised	Detected
4287	A_23_P155969	PLK4	Hs.172052	polo-like kinase 4 (Drosophila)	Homo sapiens polo-like kinase 4 (Drosophila) (PLK4), mRNA [NM_014264]	20.70854	down	40.33532	down	26.50033	568.24250	9.90814	352.40582	Detected	Detected	Compromised	Detected
4288	A_33_P3348872					20.64854	down	1.16646	down	2.78004	59.43919	3.10964	3.19848	Compromised	Detected	Compromised	Compromised
4289	A_23_P333029	C8orf47	Hs.171455	chromosome 8 open reading frame 47	Homo sapiens chromosome 8 open reading frame 47 (C8orf47), mRNA [NM_173549]	20.55785	down	13.03977	down	2.47516	52.68829	2.76970	31.84703	Compromised	Detected	Compromised	Detected
4290	A_23_P103703	HSPB7	Hs.502612	heat shock 27kDa protein family, member 7 (cardiovascular)	Homo sapiens heat shock 27kDa protein family, member 7 (cardiovascular) (HSPB7), mRNA [NM_014424]	20.51337	down	23.21110	down	286.49078	6085.28370	206.45137	4225.50930	Detected	Detected	Detected	Detected
4291	A_23_P87011	TAGLN	Hs.410977	transgelin	Homo sapiens transgelin (TAGLN), transcript variant 1, mRNA [NM_001001522]	20.48880	down	23.24594	down	1784.49610	37858.66400	2158.99660	44255.23000	Detected	Detected	Detected	Detected
4292	A_23_P118246	GINS2	Hs.433180	GINS complex subunit 2 (Psf2 homolog)	Homo sapiens GINS complex subunit 2 (Psf2 homolog) (GINS2), mRNA [NM_016095]	20.36395	down	20.33517	down	185.42964	3909.97680	134.80475	2417.23240	Detected	Detected	Detected	Detected
4293	A_23_P87013	TAGLN	Hs.410977	transgelin	Homo sapiens transgelin (TAGLN), transcript variant 1, mRNA [NM_001001522]	20.32327	down	21.35219	down	865.50287	18213.57000	780.93330	14703.54000	Detected	Detected	Detected	Detected
4294	A_33_P3339361	ARHGAP11A	Hs.591130	Rho GTPase activating protein 11A	Homo sapiens Rho GTPase activating protein 11A (ARHGAP11A), transcript variant 2, mRNA [NM_199357]	20.30978	down	17.15621	down	46.56337	979.22546	27.35365	413.81128	Detected	Detected	Detected	Detected
4295	A_33_P3351999					20.29636	down	22.45215	down	7.29804	153.37602	3.82377	75.70328	Compromised	Detected	Compromised	Detected
4296	A_33_P3231447	ITGA6	Hs.133397	integrin, alpha 6	Homo sapiens integrin, alpha 6 (ITGA6), transcript variant 2, mRNA [NM_000210]	20.26199	down	18.95910	down	6.26121	131.36320	4.54921	76.05343	Compromised	Detected	Compromised	Detected
4297	A_32_P169406	LOC400043	Hs.622604	hypothetical LOC400043	Homo sapiens hypothetical LOC400043 (LOC400043), non-coding RNA [NR_026656]	20.26097	down	20.48495	down	150.84502	3164.64010	138.94614	2509.84520	Detected	Detected	Detected	Detected
4298	A_33_P3372217		Hs.656741		Pregnancy-specific beta-1-glycoprotein 5 Precursor (PSBG-5)(Fetal liver non-specific cross-reactive antigen 3)(FL-NCA-3) [Source:UniProtKB/Swiss-Prot;Acc:Q15238] [ENST00000401992]	20.25916	down	15.48512	down	2.86589	60.11926	3.22817	44.07943	Compromised	Detected	Compromised	Detected
4299	A_33_P3358208	PADI1	Hs.412941	peptidyl arginine deiminase, type I	Homo sapiens peptidyl arginine deiminase, type I (PADI1), mRNA [NM_013358]	20.08267	down	23.60190	down	4.16737	86.65952	3.39096	70.57236	Compromised	Detected	Compromised	Detected
4300	A_33_P3307903	CDKN3	Hs.84113	cyclin-dependent kinase inhibitor 3	Homo sapiens cyclin-dependent kinase inhibitor 3 (CDKN3), transcript variant 1, mRNA [NM_005192]	20.04709	down	16.54188	down	74.23734	1541.01530	98.50087	1436.78020	Detected	Detected	Detected	Detected
4301	A_33_P3233489					20.00280	down	9.71222	down	4.16698	86.30691	6.32585	54.17547	Compromised	Detected	Compromised	Detected

4302	A_33_P3632937	LOC100131262	Hs.657010	hypothetical LOC100131262	Homo sapiens cDNA FLJ35102 fis, clone PLACE6006474, weakly similar to ADHESIVE PLAQUE MATRIX PROTEIN PRECURSOR [AK092421]	19.99840	down	20.58349	down	443.55368	9184.90200	276.62120	5020.76560	Detected	Detected	Detected	Detected
4303	A_23_P150316	MMP12	Hs.1695	matrix metallopeptidase 12 (macrophage elastase)	Homo sapiens matrix metallopeptidase 12 (macrophage elastase) (MMP12), mRNA [NM_002426]	19.99392	down	39.26450	down	16.04060	332.08660	6.43880	222.93110	Detected	Detected	Compromised	Detected
4304	A_23_P69179	LEPREL1	Hs.374191	leprecan-like 1	Homo sapiens leprecan-like 1 (LEPREL1), transcript variant 1, mRNA [NM_018192]	19.84439	down	20.10372	down	151.32020	3109.33700	131.92650	2338.69700	Detected	Detected	Detected	Detected
4305	A_23_P258136	MXRA5	Hs.369422	matrix-remodelling associated 5	Homo sapiens matrix-remodelling associated 5 (MXRA5), mRNA [NM_015419]	19.74553	down	12.66393	down	20.71657	423.56470	24.52761	273.89822	Detected	Detected	Detected	Detected
4306	A_23_P50990	CENPO	Hs.718431	centromere protein O	Homo sapiens centromere protein O (CENPO), mRNA [NM_024322]	19.58921	down	32.51260	down	38.82269	787.47270	25.70120	736.83560	Detected	Detected	Detected	Detected
4307	A_33_P3271276	PSG5	Hs.654415	pregnancy specific beta-1-glycoprotein 5	Homo sapiens pregnancy specific beta-1-glycoprotein 5 (PSG5), transcript variant 2, mRNA [NM_001130014]	19.51882	down	23.10241	down	363.91020	7354.96830	304.04443	6193.83700	Detected	Detected	Detected	Detected
4308	A_23_P118834	TOP2A	Hs.156346	topoisomerase (DNA) II alpha 170kDa	Homo sapiens topoisomerase (DNA) II alpha 170kDa (TOP2A), mRNA [NM_001067]	19.44253	down	19.36505	down	227.44055	4578.82500	159.51663	2723.89280	Detected	Detected	Detected	Detected
4309	A_23_P93772	HOXA5	Hs.655218	homeobox A5	Homo sapiens homeobox A5 (HOXA5), mRNA [NM_019102]	19.32321	down	31.22134	down	12.14002	242.90233	5.89308	162.24042	Compromised	Detected	Compromised	Detected
4310	A_23_P71328	MATN2	Hs.189445	matrilin 2	Homo sapiens matrilin 2 (MATN2), transcript variant 2, mRNA [NM_030583]	19.15193	down	32.14108	down	6.90295	136.89272	4.29967	121.86009	Compromised	Detected	Compromised	Detected
4311	A_24_P355967	HTR2A	Hs.654586	5-hydroxytryptamine (serotonin) receptor 2A	Homo sapiens 5-hydroxytryptamine (serotonin) receptor 2A (HTR2A), transcript variant 1, mRNA [NM_000621]	19.14743	down	6.66174	down	9.45238	187.40652	20.17256	118.49892	Compromised	Detected	Detected	Detected
4312	A_33_P3397443	PKMYT1	Hs.77783	protein kinase, membrane associated tyrosine/threonine 1	Homo sapiens protein kinase, membrane associated tyrosine/threonine 1 (PKMYT1), transcript variant 2, mRNA [NM_182687]	19.13656	down	30.03717	down	845.12085	16746.18400	606.99930	16077.28700	Detected	Detected	Detected	Detected
4313	A_23_P8961	IL7	Hs.591873	interleukin 7	Homo sapiens interleukin 7 (IL7), mRNA [NM_000880]	19.05514	down	10.33761	down	3.94324	77.80329	3.08973	28.16472	Compromised	Detected	Compromised	Detected
4314	A_24_P355944	EFNB2	Hs.149239	ephrin-B2	Homo sapiens ephrin-B2 (EFNB2), mRNA [NM_004093]	18.91656	down	24.43176	down	59.01881	1156.02080	29.94339	645.09140	Detected	Detected	Detected	Detected
4315	A_24_P229234	MYO16	Hs.656587	myosin XVI	Homo sapiens myosin XVI (MYO16), mRNA [NM_015011]	18.65307	down	18.70774	down	22.75547	439.51047	18.95638	312.71030	Detected	Detected	Detected	Detected
4316	A_24_P62659	TSPAN2	Hs.310458	tetraspanin 2	Homo sapiens tetraspanin 2 (TSPAN2), mRNA [NM_005725]	18.51908	down	4.77308	down	2.87176	55.06810	8.26957	34.80544	Compromised	Detected	Compromised	Detected
4317	A_33_P3384942					18.46194	down	1.15349	down	2.38558	45.60415	2.64916	2.69456	Compromised	Detected	Compromised	Compromised
4318	A_23_P39799	LOXL3	Hs.720335	lysyl oxidase-like 3	Homo sapiens lysyl oxidase-like 3 (LOXL3), mRNA [NM_032603]	18.31738	down	14.01972	down	61.53202	1167.07190	58.72586	725.99590	Detected	Detected	Detected	Detected
4319	A_23_P8801	CYP3A5	Hs.695915	cytochrome P450, family 3, subfamily A, polypeptide 5	Homo sapiens cytochrome P450, family 3, subfamily A, polypeptide 5 (CYP3A5), mRNA [NM_000777]	18.31077	down	9.85480	down	2.44840	46.42182	2.62805	22.83744	Compromised	Detected	Compromised	Detected
4320	A_33_P3229017					18.30450	down	7.91693	down	2.34242	44.39714	2.58156	18.02206	Compromised	Detected	Compromised	Detected
4321	A_23_P388146		Hs.642598		Ubiquitin UBF-fl [Source:UniProtKB/TrEMBL;Acc:Q96JB9] [ENST00000416025]	18.06076	down	15.07035	down	176.48915	3300.55700	151.90073	2018.59190	Detected	Detected	Detected	Detected
4322	A_33_P3267003	LOC100132588	Hs.689599	hypothetical protein LOC100132588	PREDICTED: Homo sapiens hypothetical LOC100132588 (LOC100132588), mRNA [XM_001724555]	17.99580	down	25.72898	down	5.90499	110.03313	3.37833	76.64602	Compromised	Detected	Compromised	Detected
4323	A_23_P162719	DIAPH3	Hs.283127	diaphanous homolog 3 (Drosophila)	Homo sapiens diaphanous homolog 3 (Drosophila) (DIAPH3), transcript variant 2, mRNA [NM_030932]	17.95585	down	14.45076	down	32.37621	601.95660	47.86552	609.92865	Detected	Detected	Detected	Detected
4324	A_33_P3311493	LOC283392		hypothetical LOC283392	Homo sapiens hypothetical LOC283392 (LOC283392), transcript variant 2, non-coding RNA [NR_026836]	17.83773	down	18.79356	down	11.27594	208.26935	6.78669	112.46889	Compromised	Detected	Compromised	Detected
4325	A_23_P31073	MYB	Hs.606320	v-myb myeloblastosis viral oncogene homolog (avian)	Homo sapiens v-myb myeloblastosis viral oncogene homolog (avian) (MYB), transcript variant 2, mRNA [NM_005375]	17.55907	down	8.07867	down	3.90972	71.08532	6.33085	45.09901	Compromised	Detected	Compromised	Detected
4326	A_32_P117354	LIMCH1	Hs.335163	LIM and calponin homology domains 1	Homo sapiens LIM and calponin homology domains 1 (LIMCH1), transcript variant 1, mRNA [NM_014988]	17.50503	down	15.70622	down	37.94169	687.72125	28.95370	400.99698	Detected	Detected	Detected	Detected
4327	A_23_P80902	KIF15	Hs.658939	kinesin family member 15	Homo sapiens kinesin family member 15 (KIF15), mRNA [NM_020242]	17.49431	down	18.35486	down	54.24877	982.69727	51.76542	837.83075	Detected	Detected	Detected	Detected
4328	A_23_P48669	CDKN3	Hs.84113	cyclin-dependent kinase inhibitor 3	Homo sapiens cyclin-dependent kinase inhibitor 3 (CDKN3), transcript variant 1, mRNA [NM_005192]	17.43738	down	17.58140	down	540.94354	9767.11300	474.77963	7360.56150	Detected	Detected	Detected	Detected
4329	A_23_P161218	ANKRD1	Hs.448589	ankyrin repeat domain 1 (cardiac muscle)	Homo sapiens ankyrin repeat domain 1 (cardiac muscle) (ANKRD1), mRNA [NM_014391]	17.35818	down	19.33692	down	27.18278	488.57492	21.18681	361.25885	Detected	Detected	Detected	Detected
4330	A_32_P358887	SLC4A4	Hs.5462	solute carrier family 4, sodium bicarbonate cotransporter, member 4	Homo sapiens solute carrier family 4, sodium bicarbonate cotransporter, member 4 (SLC4A4), transcript variant 2, mRNA [NM_003759]	17.06936	down	19.00681	down	19.60631	346.53430	13.04699	218.66792	Detected	Detected	Compromised	Detected
4331	A_33_P3531206	PCOTH	Hs.721128	prostate collagen triple helix	Homo sapiens prostate collagen triple helix (PCOTH), transcript variant 1, mRNA [NM_001014442]	17.06187	down	11.44608	down	6.09393	107.66082	10.07329	101.67017	Compromised	Detected	Compromised	Detected
4332	A_23_P104651	CDCA5	Hs.434886	cell division cycle associated 5	Homo sapiens cell division cycle associated 5 (CDCA5), mRNA [NM_080668]	17.04429	down	17.55543	down	479.77963	8467.47000	399.53090	6184.82370	Detected	Detected	Detected	Detected

4333	A_23_P124642	RASGRP1	Hs.591127	RAS guanyl releasing protein 1 (calcium and DAG-regulated)	Homo sapiens RAS guanyl releasing protein 1 (calcium and DAG-regulated) (RASGRP1), transcript variant 1, mRNA [NM_005739]	16.94758	down	15.54540	down	3.27837	57.53051	2.68507	36.80635	Compromised	Detected	Compromised	Detected
4334	A_33_P3340468		Hs.348920		Centromere protein I (CENP-I)(Interphase centromere complex protein 19)(Follicle-stimulating hormone primary response protein)(FSH primary response protein 1)(Leucine-rich primary response protein 1) [Source:UniProtKB/Swiss-Prot;Acc:Q92674] [ENST00000403304]	16.82465	down	17.70007	down	9.16946	159.74335	10.91147	170.30363	Compromised	Detected	Compromised	Detected
4335	A_24_P792124	SCN9A	Hs.439145	sodium channel, voltage-gated, type IX, alpha subunit	Homo sapiens sodium channel, voltage-gated, type IX, alpha subunit (SCN9A), mRNA [NM_002977]	16.81000	down	2.92126	down	2.97357	51.75820	3.32227	8.55795	Compromised	Detected	Compromised	Compromised
4336	A_24_P174294	LRRTM4	Hs.285782	leucine rich repeat transmembrane neuronal 4	Homo sapiens leucine rich repeat transmembrane neuronal 4 (LRRTM4), transcript variant 2, mRNA [NM_024993]	16.79906	down	1.18548	down	2.35314	40.93218	2.54101	2.65623	Compromised	Detected	Compromised	Compromised
4337	A_23_P19663	CTGF	Hs.410037	connective tissue growth factor	Homo sapiens connective tissue growth factor (CTGF), mRNA [NM_001901]	16.63821	down	16.92576	down	1382.83520	23823.74400	1178.33010	17586.55500	Detected	Detected	Detected	Detected
4338	A_24_P923381	EPR1		effector cell peptidase receptor 1 (non-protein coding)	Homo sapiens effector cell peptidase receptor 1 (non-protein coding) (EPR1), non-coding RNA [NR_002219]	16.59728	down	39.36136	down	4.94781	85.03212	3.57990	124.25302	Compromised	Detected	Compromised	Detected
4339	A_33_P3303810	LAD1	Hs.519035	ladinin 1	Homo sapiens ladinin 1 (LAD1), mRNA [NM_005558]	16.51625	down	1.33313	down	2.35458	40.26772	2.54835	2.99570	Compromised	Detected	Compromised	Compromised
4340	A_32_P101031	LYPD1	Hs.719231	LY6/PLAUR domain containing 1	Homo sapiens LY6/PLAUR domain containing 1 (LYPD1), transcript variant 1, mRNA [NM_144586]	16.44540	down	12.44859	down	2.32768	39.63695	2.49361	27.37243	Compromised	Detected	Compromised	Detected
4341	A_33_P3392187	CCDC85A	Hs.117136	coiled-coil domain containing 85A	Homo sapiens coiled-coil domain containing 85A (CCDC85A), mRNA [NM_001080433]	16.38398	down	13.79487	down	11.20793	190.14186	11.74969	142.92550	Compromised	Detected	Compromised	Detected
4342	A_24_P88763	LOXL3	Hs.720335	lysyl oxidase-like 3	Homo sapiens lysyl oxidase-like 3 (LOXL3), mRNA [NM_032603]	16.36449	down	18.61668	down	321.03964	5439.94040	327.09680	5369.62600	Detected	Detected	Detected	Detected
4343	A_33_P3283944	LOC100133690	Hs.625330	hypothetical protein LOC100133690	PREDICTED: Homo sapiens hypothetical LOC100133690 (LOC100133690), mRNA [XM_001720217]	16.31190	down	15.69316	down	5.12165	86.50619	6.60534	91.40518	Compromised	Detected	Compromised	Detected
4344	A_23_P168368	C7orf69	Hs.287647	chromosome 7 open reading frame 69	Homo sapiens chromosome 7 open reading frame 69 (C7orf69), mRNA [NM_025031]	16.30508	down	1.18518	down	2.35173	39.70477	2.54654	2.66135	Compromised	Detected	Compromised	Compromised
4345	A_23_P59807	WNT2	Hs.567356	wingless-type MMTV integration site family member 2	Homo sapiens wingless-type MMTV integration site family member 2 (WNT2), transcript variant 1, mRNA [NM_003391]	16.28387	down	7.07583	down	2.35561	39.71857	2.53895	15.84151	Compromised	Detected	Compromised	Detected
4346	A_23_P361419	DEPDC1B	Hs.482233	DEP domain containing 1B	Homo sapiens DEP domain containing 1B (DEPDC1B), transcript variant 1, mRNA [NM_018369]	16.25246	down	16.90686	down	59.24345	996.99400	38.78222	578.17737	Detected	Detected	Detected	Detected
4347	A_23_P253752	FAM54A	Hs.121536	family with sequence similarity 54, member A	Homo sapiens family with sequence similarity 54, member A (FAM54A), transcript variant 2, mRNA [NM_138419]	15.96278	down	16.31740	down	213.55846	3529.86800	163.92441	2358.63160	Detected	Detected	Detected	Detected
4348	A_33_P3221284	CCDC80	Hs.477128	coiled-coil domain containing 80	Homo sapiens coiled-coil domain containing 80 (CCDC80), transcript variant 1, mRNA [NM_199511]	15.87260	down	17.41781	down	50.18488	824.81036	31.70135	486.89645	Detected	Detected	Detected	Detected
4349	A_23_P315815	NRG1	Hs.453951	neuregulin 1	Homo sapiens neuregulin 1 (NRG1), transcript variant HRG-gamma, mRNA [NM_004495]	15.87181	down	11.29507	down	16.08250	264.30966	22.70604	226.14952	Detected	Detected	Detected	Detected
4350	A_23_P74059	NPPA	Hs.75640	natriuretic peptide precursor A	Homo sapiens natriuretic peptide precursor A (NPPA), mRNA [NM_006172]	15.85257	down	1.18280	down	2.34556	38.50171	2.53344	2.64233	Compromised	Detected	Compromised	Compromised
4351	A_33_P3390367	LOC91149	Hs.711021	hypothetical protein LOC91149	Homo sapiens hypothetical protein LOC91149 (LOC91149), non-coding RNA [NR_026995]	15.82700	down	1.17735	down	2.25635	36.97755	2.34839	2.43805	Compromised	Detected	Compromised	Compromised
4352	A_23_P92441	MAD2L1	Hs.591697	MAD2 mitotic arrest deficient-like 1 (yeast)	Homo sapiens MAD2 mitotic arrest deficient-like 1 (yeast) (MAD2L1), mRNA [NM_002358]	15.58743	down	15.48211	down	400.73703	6467.95460	389.73584	5320.66500	Detected	Detected	Detected	Detected
4353	A_23_P303072	GRIA1	Hs.519693	glutamate receptor, ionotropic, AMPA 1	Homo sapiens glutamate receptor, ionotropic, AMPA 1 (GRIA1), transcript variant 1, mRNA [NM_000827]	15.56085	down	16.97557	down	3.36312	54.18872	2.92022	43.71250	Compromised	Detected	Compromised	Detected
4354	A_23_P206059	PRC1	Hs.567385	protein regulator of cytokinesis 1	Homo sapiens protein regulator of cytokinesis 1 (PRC1), transcript variant 1, mRNA [NM_003981]	15.48923	down	15.60178	down	1404.72850	22529.69700	1091.34190	15014.13700	Detected	Detected	Detected	Detected
4355	A_23_P349566	CCDC85A	Hs.117136	coiled-coil domain containing 85A	Homo sapiens coiled-coil domain containing 85A (CCDC85A), mRNA [NM_001080433]	15.48223	down	26.49447	down	23.81696	381.81494	8.78801	205.31049	Detected	Detected	Compromised	Detected
4356	A_23_P88731	RAD51	Hs.631709	RAD51 homolog (RecA homolog, E. coli) (S. cerevisiae)	Homo sapiens RAD51 homolog (RecA homolog, E. coli) (S. cerevisiae) (RAD51), transcript variant 1, mRNA [NM_002875]	15.45946	down	15.49924	down	307.68988	4925.38800	234.41080	3203.71220	Detected	Detected	Detected	Detected
4357	A_23_P106389	SEMA7A	Hs.24640	semaphorin 7A, GPI membrane anchor (John Milton Hagen blood group)	Homo sapiens semaphorin 7A, GPI membrane anchor (John Milton Hagen blood group) (SEMA7A), transcript variant 1, mRNA [NM_003612]	15.39454	down	18.77460	down	156.63383	2496.80880	101.89106	1686.83400	Detected	Detected	Detected	Detected
4358	A_24_P136866	SLC8A1	Hs.468274	solute carrier family 8 (sodium/calcium exchanger), member 1	Homo sapiens solute carrier family 8 (sodium/calcium exchanger), member 1 (SLC8A1), transcript variant A, mRNA [NM_021097]	15.35800	down	14.83782	down	37.23408	592.11790	33.32348	435.99924	Detected	Detected	Detected	Detected
4359	A_33_P3215640	PI16	Hs.720179	peptidase inhibitor 16	Homo sapiens peptidase inhibitor 16 (PI16), mRNA [NM_153370]	15.33325	down	16.68089	down	75.91495	1205.29880	80.89175	1189.84070	Detected	Detected	Detected	Detected

4360	A_33_P3403963	ST8SIA6	Hs.677766	ST8 alpha-N-acetyl-neuraminide alpha-2,8-sialyltransferase 6	Homo sapiens ST8 alpha-N-acetyl-neuraminide alpha-2,8-sialyltransferase 6 (ST8SIA6), mRNA [NM_001004470]	15.30703	down	1.17841	down	2.28488	36.21488	2.40743	2.50158	Compromised	Detected	Compromised	Compromised
4361	A_23_P112554	COL15A1	Hs.409034	collagen, type XV, alpha 1	Homo sapiens collagen, type XV, alpha 1 (COL15A1), mRNA [NM_001855]	15.17349	down	9.03242	down	12.94998	203.46391	12.76627	101.67959	Detected	Detected	Not Detected	Detected
4362	A_32_P152696	LOC729983	Hs.648133	hypothetical LOC729983	PREDICTED: Homo sapiens hypothetical LOC729983 (LOC729983), mRNA [XM_001718065]	15.13777	down	16.48681	down	122.57666	1921.33350	90.10722	1309.97080	Detected	Detected	Detected	Detected
4363	A_23_P216579	PALM2	Hs.591908	paralemmin 2	Homo sapiens paralemmin 2 (PALM2), transcript variant 1, mRNA [NM_053016]	15.12958	down	5.43421	down	2.40476	37.67316	4.88503	23.40827	Compromised	Detected	Compromised	Detected
4364	A_33_P3257678	HIST2H3A	Hs.706618	histone cluster 2, H3a	Homo sapiens histone cluster 2, H3a (HIST2H3A), mRNA [NM_001005464]	15.04909	down	17.76087	down	12.58176	196.05806	9.20432	144.15216	Detected	Detected	Compromised	Detected
4365	A_33_P3268555	SP140	Hs.632549	SP140 nuclear body protein	Homo sapiens SP140 nuclear body protein (SP140), transcript variant 2, mRNA [NM_001005176]	14.99314	down	19.09365	down	5.61608	87.18847	3.04745	51.30871	Compromised	Detected	Compromised	Detected
4366	A_23_P57379	CDC45L	Hs.474217	CDC45 cell division cycle 45-like (S. cerevisiae)	Homo sapiens CDC45 cell division cycle 45-like (S. cerevisiae) (CDC45L), mRNA [NM_003504]	14.98852	down	18.79490	down	438.00662	6797.86300	347.46466	5758.59000	Detected	Detected	Detected	Detected
4367	A_33_P3413741	OXTR	Hs.2820	oxytocin receptor	Homo sapiens oxytocin receptor (OXTR), mRNA [NM_000916]	14.92857	down	14.90245	down	122.57455	1894.74850	71.96461	945.67554	Detected	Detected	Detected	Detected
4368	A_23_P380318	EGR4	Hs.3052	early growth response 4	Homo sapiens early growth response 4 (EGR4), mRNA [NM_001965]	14.87685	down	18.41871	down	24.95375	384.39703	20.23956	328.71967	Detected	Detected	Detected	Detected
4369	A_23_P101671	ATCAY	Hs.418055	ataxia, cerebellar, Cayman type	Homo sapiens ataxia, cerebellar, Cayman type (ATCAY), mRNA [NM_033064]	14.85363	down	1.44656	down	2.46193	37.86528	2.68531	3.42527	Compromised	Not Detected	Compromised	Compromised
4370	A_33_P3230017	AURKAPS1		aurora kinase A pseudogene 1	Homo sapiens aurora kinase A pseudogene 1 (AURKAPS1), non-coding RNA [NR_001587]	14.84050	down	12.13505	down	151.15030	2322.68630	104.20519	1115.05490	Detected	Detected	Detected	Detected
4371	A_33_P3394883		Hs.579975		Putative uncharacterized protein ENSP00000386758 [Source:UniProtKB/TrEMBL;Acc:B8ZZS8] [ENST00000409910]	14.82705	down	7.81925	down	2.35522	36.15933	2.53591	17.48495	Compromised	Detected	Compromised	Detected
4372	A_24_P882732		Hs.579266		cDNA FLJ61294, highly similar to Keratin, type I cytoskeletal 17 [Source:UniProtKB/TrEMBL;Acc:B4DJM5] [ENST00000300992]	14.80811	down	14.77870	down	2.76101	42.33516	3.36381	43.83626	Compromised	Detected	Compromised	Detected
4373	A_23_P83818	COL5A1	Hs.210283	collagen, type V, alpha 1	Homo sapiens collagen, type V, alpha 1 (COL5A1), mRNA [NM_000093]	14.79478	down	16.72622	down	233.04053	3570.03980	226.51959	3340.94240	Detected	Detected	Detected	Detected
4374	A_23_P66732	GSG2	Hs.534059	germ cell associated 2 (haspin)	Homo sapiens germ cell associated 2 (haspin) (GSG2), mRNA [NM_031965]	14.73273	down	15.78214	down	19.45279	296.75510	15.50430	215.76633	Detected	Detected	Detected	Detected
4375	A_33_P3346331				Putative uncharacterized protein ENSP00000384476 [Source:UniProtKB/TrEMBL;Acc:B5MCL6] [ENST00000403628]	14.73241	down	12.38074	down	37.21508	567.70900	38.32880	418.44406	Detected	Detected	Detected	Detected
4376	A_32_P24376		Hs.406714		Keratin-associated protein 2-4 (Keratin-associated protein 2.4)(High sulfur keratin-associated protein 2.4) [Source:UniProtKB/Swiss-Prot;Acc:Q9BYR9] [ENST00000391418]	14.69707	down	23.33356	down	33.58148	511.05017	20.78677	427.69443	Detected	Detected	Detected	Detected
4377	A_23_P150343	SLN	Hs.334629	sarcolipin	Homo sapiens sarcolipin (SLN), mRNA [NM_003063]	14.68563	down	9.20060	down	2.53021	38.47531	2.71960	22.06412	Compromised	Detected	Compromised	Detected
4378	A_32_P224525	COL6A6	Hs.591282	collagen, type VI, alpha 6	Homo sapiens collagen type VI alpha 6 (COL6A6), mRNA [NM_001102608]	14.66730	down	10.34248	down	2.68150	40.72498	3.22207	29.38497	Compromised	Detected	Compromised	Detected
4379	A_33_P3322363	HMSD	Hs.721604	histocompatibility (minor) serpin domain containing	Homo sapiens histocompatibility (minor) serpin domain containing (HMSD), mRNA [NM_001123366]	14.58066	down	7.71276	down	14.86305	224.39763	25.78065	175.33514	Detected	Detected	Detected	Detected
4380	A_24_P3249	RARB	Hs.719896	retinoic acid receptor, beta	Homo sapiens retinoic acid receptor, beta (RARB), transcript variant 1, mRNA [NM_000965]	14.35181	down	15.74028	down	2.31652	34.42521	2.47098	34.29633	Compromised	Detected	Compromised	Detected
4381	A_23_P29723	SGOL1	Hs.105153	shugoshin-like 1 (S. pombe)	Homo sapiens shugoshin-like 1 (S. pombe) (SGOL1), transcript variant A2, mRNA [NM_001012410]	14.31705	down	22.82700	down	9.41642	139.59583	4.46658	89.90624	Compromised	Detected	Compromised	Detected
4382	A_33_P3327097				Myotubularin-related protein 1 (EC 3.1.3.-) [Source:UniProtKB/Swiss-Prot;Acc:Q13613] [ENST00000370387]	14.25575	down	4.07706	down	2.58600	38.17262	4.57051	16.43150	Compromised	Detected	Compromised	Detected
4383	A_23_P131866	AURKA	Hs.250822	aurora kinase A	Homo sapiens aurora kinase A (AURKA), transcript variant 1, mRNA [NM_198433]	14.13860	down	16.27255	down	567.97100	8315.06600	501.28497	7192.92870	Detected	Detected	Detected	Detected
4384	A_23_P18579	PTTG2	Hs.668806	pituitary tumor-transforming 2	Homo sapiens pituitary tumor-transforming 2 (PTTG2), mRNA [NM_006607]	14.11966	down	13.88121	down	712.10800	10411.25700	735.17993	8998.83700	Detected	Detected	Detected	Detected
4385	A_33_P3281651					14.07708	down	10.79814	down	2.44818	35.68527	2.60727	24.82567	Compromised	Detected	Compromised	Detected
4386	A_32_P210202	E2F7	Hs.416375	E2F transcription factor 7	Homo sapiens E2F transcription factor 7 (E2F7), mRNA [NM_203394]	14.04911	down	12.91462	down	412.02400	5993.82800	307.05228	3496.70870	Detected	Detected	Detected	Detected
4387	A_23_P106617	WFDC1	Hs.36688	WAP four-disulfide core domain 1	Homo sapiens WAP four-disulfide core domain 1 (WFDC1), mRNA [NM_021197]	14.04215	down	13.28701	down	14.54028	211.41685	13.83263	162.06807	Detected	Detected	Detected	Detected
4388	A_24_P200854	HOXA2	Hs.592177	homeobox A2	Homo sapiens homeobox A2 (HOXA2), mRNA [NM_006735]	13.90796	down	13.21049	down	2.32121	33.42809	2.48151	28.90680	Compromised	Detected	Compromised	Detected
4389	A_32_P148538	LPPR4	Hs.13245	lipid phosphate phosphatase-related protein type 4	Homo sapiens lipid phosphate phosphatase-related protein type 4 (LPPR4), transcript variant 1, mRNA [NM_014839]	13.89708	down	8.58384	down	8.97728	129.18188	12.65608	95.79578	Compromised	Detected	Compromised	Detected
4390	A_33_P3232080	CD177	Hs.232165	CD177 molecule	Homo sapiens CD177 molecule (CD177), mRNA [NM_020406]	13.89089	down	1.17740	down	2.25727	32.46732	2.35057	2.44042	Compromised	Detected	Compromised	Compromised
4391	A_23_P60016	PTTG3P	Hs.647156	pituitary tumor-transforming 3 (pseudogene)	Homo sapiens pituitary tumor-transforming 3 (pseudogene) (PTTG3P), non-coding RNA [NR_002734]	13.88853	down	14.47208	down	65.51600	942.18630	86.40104	1102.59380	Detected	Detected	Detected	Detected

4392	A_33_P3225397					13.71370	down	5.69887	down	2.42159	34.38652	6.62371	33.28554	Compromised	Detected	Compromised	Detected
4393	A_23_P74115	RAD54L	Hs.642042	RAD54-like (S. cerevisiae)	Homo sapiens RAD54-like (S. cerevisiae) (RAD54L), transcript variant 1, mRNA [NM_003579]	13.70042	down	12.96182	down	149.28099	2117.73360	135.04740	1543.53960	Detected	Detected	Detected	Detected
4394	A_23_P13094	MMP10	Hs.2258	matrix metallopeptidase 10 (stromelysin 2)	Homo sapiens matrix metallopeptidase 10 (stromelysin 2) (MMP10), mRNA [NM_002425]	13.63642	down	13.65127	down	46.99143	663.51680	29.52284	355.38312	Detected	Detected	Detected	Detected
4395	A_23_P408955	E2F2	Hs.194333	E2F transcription factor 2	Homo sapiens E2F transcription factor 2 (E2F2), mRNA [NM_004091]	13.62002	down	12.71674	down	36.62947	516.58480	39.07835	438.20502	Detected	Detected	Detected	Detected
4396	A_33_P3364263	LBH	Hs.567598	limb bud and heart development homolog (mouse)	Homo sapiens limb bud and heart development homolog (mouse) (LBH), mRNA [NM_030915]	13.55677	down	8.28714	down	15.72169	220.69296	22.99169	168.01231	Detected	Detected	Detected	Detected
4397	A_24_P943894	SCUBE3	Hs.12923	signal peptide, CUB domain, EGF-like 3	Homo sapiens signal peptide, CUB domain, EGF-like 3 (SCUBE3), mRNA [NM_152753]	13.50958	down	13.61309	down	53.05150	742.11725	42.05413	504.81348	Detected	Detected	Detected	Detected
4398	A_24_P254705	ZNF695	Hs.669893	zinc finger protein 695	Homo sapiens zinc finger protein 695 (ZNF695), mRNA [NM_020394]	13.49622	down	21.29071	down	12.26119	171.34747	5.81895	109.24460	Compromised	Detected	Compromised	Detected
4399	A_32_P142440	PCSK9	Hs.18844	proprotein convertase subtilisin/kexin type 9	Homo sapiens proprotein convertase subtilisin/kexin type 9 (PCSK9), mRNA [NM_174936]	13.46607	down	4.49118	down	2.92709	40.81410	5.23401	20.72815	Compromised	Detected	Compromised	Detected
4400	A_33_P3258339	C17orf76	Hs.25425	chromosome 17 open reading frame 76	Homo sapiens chromosome 17 open reading frame 76 (C17orf76), transcript variant 1, mRNA [NM_001113567]	13.46549	down	5.30512	down	47.81069	666.62280	34.30236	160.46663	Detected	Detected	Detected	Detected
4401	A_23_P40527	TBX1	Hs.173984	T-box 1	Homo sapiens T-box 1 (TBX1), transcript variant A, mRNA [NM_080646]	13.35310	down	15.45019	down	2.28643	31.61358	2.37873	32.40739	Compromised	Detected	Compromised	Detected
4402	A_23_P50946	RAMP1	Hs.471783	receptor (G protein-coupled) activity modifying protein 1	Homo sapiens receptor (G protein-coupled) activity modifying protein 1 (RAMP1), mRNA [NM_005855]	13.30559	down	18.52193	down	291.44992	4015.42330	233.02104	3805.80710	Detected	Detected	Detected	Detected
4403	A_33_P3286422	FANCA	Hs.719210	Fanconi anemia, complementation group A	Homo sapiens Fanconi anemia, complementation group A (FANCA), transcript variant 2, mRNA [NM_001018112]	13.06998	down	13.46745	down	34.67760	469.30685	27.57285	327.44055	Detected	Detected	Detected	Detected
4404	A_24_P100517	C9orf140	Hs.19322	chromosome 9 open reading frame 140	Homo sapiens chromosome 9 open reading frame 140 (C9orf140), mRNA [NM_178448]	13.06831	down	16.01504	down	68.86566	931.86950	70.17153	990.95636	Detected	Detected	Detected	Detected
4405	A_33_P3386262	CDT1	Hs.122908	chromatin licensing and DNA replication factor 1	Homo sapiens chromatin licensing and DNA replication factor 1 (CDT1), mRNA [NM_030928]	13.05675	down	13.26437	down	320.29288	4330.27540	284.40067	3326.46400	Detected	Detected	Detected	Detected
4406	A_24_P414658		Hs.51011		Histone H2A type 1 (H2A.1)(H2A/p) [Source:UniProtKB/Swiss-Prot;Acc:P0C0S8] [ENST00000359193]	13.05050	down	8.30552	down	2.31405	31.27040	2.50763	18.36517	Compromised	Detected	Compromised	Detected
4407	A_23_P58706	SPINK5L3	Hs.483771	serine PI Kazal type 5-like 3	Homo sapiens serine PI Kazal type 5-like 3 (SPINK5L3), mRNA [NM_001040129]	13.04836	down	1.84752	down	2.43811	32.94138	7.52278	12.25554	Compromised	Detected	Compromised	Compromised
4408	A_32_P99032	LOC100240734		hypothetical LOC100240734	Homo sapiens hypothetical LOC100240734 (LOC100240734), non-coding RNA [NR_026657]	12.96689	down	8.43801	down	3.28706	44.13436	3.32096	24.70979	Compromised	Detected	Compromised	Detected
4409	A_23_P58082	CCDC80	Hs.477128	coiled-coil domain containing 80	Homo sapiens coiled-coil domain containing 80 (CCDC80), transcript variant 1, mRNA [NM_199511]	12.95135	down	11.50418	down	2330.53760	31253.89500	3079.08180	31235.05900	Detected	Detected	Detected	Detected
4410	A_23_P50250	CKM	Hs.334347	creatine kinase, muscle	Homo sapiens creatine kinase, muscle (CKM), mRNA [NM_001824]	12.89479	down	10.53750	down	2.66216	35.54530	4.25211	39.51002	Compromised	Detected	Compromised	Detected
4411	A_23_P359214	LOC728449	Hs.463110	hypothetical protein LOC728449	Putative uncharacterized protein ENSP00000334090 [Source:UniProtKB/TrEMBL;Acc:A6NF06] [ENST00000335083]	12.83588	down	18.52038	down	18.79105	249.75244	17.23053	281.39346	Detected	Detected	Detected	Detected
4412	A_33_P3421985					12.75097	down	2.47442	down	2.86596	37.83955	6.36247	13.88242	Compromised	Detected	Compromised	Detected
4413	A_23_P7636	PTTG1	Hs.350966	pituitary tumor-transforming 1	Homo sapiens pituitary tumor-transforming 1 (PTTG1), mRNA [NM_004219]	12.71349	down	11.79510	down	5674.05100	74694.92000	4848.53100	50428.64500	Detected	Detected	Detected	Detected
4414	A_23_P251043	TMEM90B	Hs.124638	transmembrane protein 90B	Homo sapiens transmembrane protein 90B (TMEM90B), mRNA [NM_024893]	12.55207	down	10.09717	down	4.93395	64.12730	6.77633	60.33364	Compromised	Detected	Compromised	Detected
4415	A_33_P3274710					12.52025	down	3.42133	down	2.28297	29.59687	2.37813	7.17458	Compromised	Detected	Compromised	Compromised
4416	A_23_P250136	GRIK2	Hs.98262	glutamate receptor, ionotropic, kainate 2	Homo sapiens glutamate receptor, ionotropic, kainate 2 (GRIK2), transcript variant 2, mRNA [NM_175768]	12.48016	down	6.07048	down	2.41499	31.20818	2.93013	15.68465	Compromised	Detected	Compromised	Detected
4417	A_24_P175612	SFXN2	Hs.44070	sideroflexin 2	Homo sapiens sideroflexin 2 (SFXN2), mRNA [NM_178858]	12.47563	down	34.22838	down	9.20619	118.92561	2.55625	77.15347	Compromised	Detected	Compromised	Detected
4418	A_33_P3212305	LZTS1	Hs.521432	leucine zipper, putative tumor suppressor 1	Homo sapiens leucine zipper, putative tumor suppressor 1 (LZTS1), mRNA [NM_021020]	12.41443	down	1.19610	down	3.11246	40.00953	2.68269	2.82946	Compromised	Detected	Compromised	Compromised
4419	A_23_P121064	PTX3	Hs.591286	pentraxin-related gene, rapidly induced by IL-1 beta	Homo sapiens pentraxin-related gene, rapidly induced by IL-1 beta (PTX3), mRNA [NM_002852]	12.39700	down	12.15968	down	972.81750	12487.65800	677.31525	7262.37300	Detected	Detected	Detected	Detected
4420	A_33_P3298930	LOC389300	Hs.254516	similar to hCG1654959	PREDICTED: Homo sapiens similar to hCG1654959 (LOC389300), mRNA [XM_001714293]	12.37907	down	11.17368	down	8.30819	106.49459	4.20289	41.41038	Compromised	Detected	Compromised	Detected
4421	A_33_P3901921	C12orf48	Hs.330663	chromosome 12 open reading frame 48	Homo sapiens chromosome 12 open reading frame 48 (C12orf48), mRNA [NM_017915]	12.32767	down	7.74855	down	5.88287	75.09372	10.61924	72.55701	Compromised	Detected	Compromised	Detected
4422	A_32_P198731	NEURL1B	Hs.91521	neuralized homolog 1B (Drosophila)	Homo sapiens neuralized homolog 1B (Drosophila) (NEURL1B), mRNA [NM_001142651]	12.31738	down	10.96406	down	64.51976	822.89450	56.42166	545.48486	Detected	Detected	Detected	Detected
4423	A_24_P200219	UPK1B	Hs.271580	uroplakin 1B	Homo sapiens uroplakin 1B (UPK1B), mRNA [NM_006952]	12.29000	down	1.18538	down	2.51242	31.97251	2.86027	2.98971	Compromised	Detected	Compromised	Compromised
4424	A_23_P92730	HSPB3	Hs.41707	heat shock 27kDa protein 3	Homo sapiens heat shock 27kDa protein 3 (HSPB3), mRNA [NM_006308]	12.27595	down	12.65127	down	272.07672	3458.43400	274.89764	3066.69500	Detected	Detected	Detected	Detected

4425	A_23_P366983	TRHDE	Hs.199814	thyrotropin-releasing hormone degrading enzyme	Homo sapiens thyrotropin-releasing hormone degrading enzyme (TRHDE), mRNA [NM_013381]	12.26854	down	5.54106	down	4.89658	62.20409	3.66795	17.92180	Compromised	Detected	Compromised	Detected
4426	A_33_P3301876					12.14647	down	18.89383	down	10.75276	135.23929	3.94679	65.75504	Compromised	Detected	Compromised	Detected
4427	A_33_P3237359	HMGB3	Hs.19114	high-mobility group box 3	Homo sapiens high-mobility group box 3 (HMGB3), mRNA [NM_005342]	12.06067	down	12.10703	down	126.90768	1584.86600	85.42881	912.02686	Detected	Detected	Detected	Detected
4428	A_33_P3258732					12.03930	down	4.20350	down	11.33256	141.27399	2.53226	9.38610	Compromised	Detected	Compromised	Compromised
4429	A_23_P53390	PTPRB	Hs.434375	protein tyrosine phosphatase, receptor type, B	Homo sapiens protein tyrosine phosphatase, receptor type, B (PTPRB), transcript variant 2, mRNA [NM_002837]	11.97032	down	12.45743	down	2.28560	28.32945	2.39059	26.26020	Compromised	Detected	Compromised	Detected
4430	A_23_P133123	MND1	Hs.294088	meiotic nuclear divisions 1 homolog (S. cerevisiae)	Homo sapiens meiotic nuclear divisions 1 homolog (S. cerevisiae) (MND1), mRNA [NM_032117]	11.93079	down	12.53796	down	190.46000	2352.91330	132.15727	1461.11170	Detected	Detected	Detected	Detected
4431	A_33_P3703501	GALNT10	Hs.631797	UDP-N-acetyl-alpha-D-galactosamine:polypeptide N-acetylgalactosaminyltransferase 10 (GalNAc-T10)	Homo sapiens UDP-N-acetyl-alpha-D-galactosamine:polypeptide N-acetylgalactosaminyltransferase 10 (GalNAc-T10) (GALNT10), transcript variant 1, mRNA [NM_198321]	11.86230	down	12.02832	down	506.98587	6227.27150	386.07574	4094.89550	Detected	Detected	Detected	Detected
4432	A_23_P100642	PNMT	Hs.1892	phenylethanolamine N-methyltransferase	Homo sapiens phenylethanolamine N-methyltransferase (PNMT), mRNA [NM_002686]	11.81436	down	15.37287	down	6.27595	76.77553	6.60567	89.54417	Compromised	Detected	Compromised	Detected
4433	A_33_P3391290	C5orf13	Hs.36053	chromosome 5 open reading frame 13	Homo sapiens chromosome 5 open reading frame 13 (C5orf13), transcript variant 11, mRNA [NM_001142483]	11.80901	down	13.37855	down	86.16718	1053.63100	86.61972	1021.85986	Detected	Detected	Detected	Detected
4434	A_23_P134484	CALCR	Hs.489127	calcitonin receptor	Homo sapiens calcitonin receptor (CALCR), transcript variant 2, mRNA [NM_001742]	11.79050	down	5.58634	down	2.53666	30.96895	2.75759	13.58384	Compromised	Detected	Compromised	Compromised
4435	A_33_P3246920		Hs.656915		CUB and sushi domain-containing protein 2 (CUB and sushi multiple domains protein 2) [Source:UniProtKB/Swiss-Prot;Acc:Q7Z408] [ENST00000338325]	11.74381	down	9.12060	down	16.57642	201.57332	19.46281	156.52893	Detected	Detected	Detected	Detected
4436	A_33_P3238148		Hs.417077		Leucine-rich repeat and death domain-containing protein LOC401387 [Source:UniProtKB/Swiss-Prot;Acc:A4D1F6] [ENST00000343318]	11.70176	down	1.79525	down	3.32305	40.26442	8.40743	13.30925	Compromised	Detected	Compromised	Compromised
4437	A_23_P134204	FAM71F1	Hs.131098	family with sequence similarity 71, member F1	Homo sapiens family with sequence similarity 71, member F1 (FAM71F1), mRNA [NM_032599]	11.69357	down	2.86362	down	2.65462	32.14271	9.06420	22.88817	Compromised	Detected	Compromised	Detected
4438	A_33_P3254579	KRTAP1-5	Hs.534499	keratin associated protein 1-5	Homo sapiens keratin associated protein 1-5 (KRTAP1-5), mRNA [NM_031957]	11.68335	down	15.61468	down	15.00413	181.51439	11.67854	160.80042	Detected	Detected	Compromised	Detected
4439	A_24_P80204	MALL	Hs.720026	mal, T-cell differentiation protein-like	Homo sapiens mal, T-cell differentiation protein-like (MALL), mRNA [NM_005434]	11.61501	down	13.03403	down	60.29674	725.18097	64.13989	737.17810	Detected	Detected	Detected	Detected
4440	A_33_P3245126				E3 ubiquitin-protein ligase RNF144B (EC 6.3.2.-)(RING finger protein 144B)(IBR domain-containing protein 2)(p53-inducible RING finger protein) [Source:UniProtKB/Swiss-Prot;Acc:Q7Z419] [ENST00000397236]	11.60597	down	15.72656	down	12.30477	147.87270	10.44218	144.80730	Compromised	Detected	Compromised	Detected
4441	A_23_P301942	NPPC	Hs.247916	natriuretic peptide precursor C	Homo sapiens natriuretic peptide precursor C (NPPC), mRNA [NM_024409]	11.58496	down	42.84185	down	5.65335	67.81611	2.56138	96.76271	Compromised	Detected	Compromised	Detected
4442	A_24_P253780	DBF4B	Hs.720187	DBF4 homolog B (S. cerevisiae)	Homo sapiens DBF4 homolog B (S. cerevisiae) (DBF4B), transcript variant 1, mRNA [NM_145663]	11.49650	down	2.28256	down	2.35303	28.01084	5.01157	10.08700	Compromised	Detected	Compromised	Compromised
4443	A_33_P3307500	STRA6	Hs.24553	stimulated by retinoic acid gene 6 homolog (mouse)	Homo sapiens stimulated by retinoic acid gene 6 homolog (mouse) (STRA6), transcript variant 5, mRNA [NM_001142620]	11.48865	down	21.69311	down	9.30964	110.74777	4.63203	88.60502	Compromised	Detected	Compromised	Detected
4444	A_23_P253524	CENPE	Hs.75573	centromere protein E, 312kDa	Homo sapiens centromere protein E, 312kDa (CENPE), mRNA [NM_001813]	11.47482	down	12.78406	down	281.99063	3350.53000	274.09690	3089.85570	Detected	Detected	Detected	Detected
4445	A_24_P144784	HILS1	Hs.25934	histone linker H1 domain, spermatid-specific 1	Homo sapiens histone linker H1 domain, spermatid-specific 1 (HILS1), transcript variant 1, non-coding RNA [NR_024193]	11.46146	down	7.09797	down	7.21433	85.61871	13.07644	81.84448	Compromised	Detected	Compromised	Detected
4446	A_33_P3240328	PITX1	Hs.84136	paired-like homeodomain 1	Homo sapiens paired-like homeodomain 1 (PITX1), mRNA [NM_002653]	11.38954	down	11.81455	down	157.35880	1855.79600	117.62779	1225.44130	Detected	Detected	Detected	Detected
4447	A_23_P23303	EXO1	Hs.498248	exonuclease 1	Homo sapiens exonuclease 1 (EXO1), transcript variant 3, mRNA [NM_003686]	11.36499	down	10.91687	down	58.60251	689.63330	55.82058	537.35110	Detected	Detected	Detected	Detected
4448	A_23_P344555	NEDD9	Hs.37982	neural precursor cell expressed, developmentally down-regulated 9	Homo sapiens neural precursor cell expressed, developmentally down-regulated 9 (NEDD9), transcript variant 1, mRNA [NM_006403]	11.33920	down	8.07340	down	2.43618	28.60389	2.64807	18.85177	Compromised	Detected	Compromised	Detected
4449	A_23_P251132	SNTG2	Hs.595069	syntrophin, gamma 2	Homo sapiens syntrophin, gamma 2 (SNTG2), mRNA [NM_018968]	11.32209	down	4.16112	down	2.65865	31.16880	6.89224	25.28930	Compromised	Detected	Compromised	Detected
4450	A_24_P216014	GH2	Hs.406754	growth hormone 2	Homo sapiens growth hormone 2 (GH2), transcript variant 3, mRNA [NM_022558]	11.23941	down	1.18513	down	2.64374	30.76777	2.85337	2.98188	Compromised	Detected	Compromised	Compromised
4451	A_23_P65041	RACGAP1P	Hs.650500	Rac GTPase activating protein 1 pseudogene	Homo sapiens Rac GTPase activating protein 1 pseudogene (RACGAP1P), non-coding RNA [NR_026583]	11.23082	down	10.27700	down	26.28015	305.61334	32.67522	296.10828	Detected	Detected	Detected	Detected
4452	A_33_P3343250	GPR1	Hs.184907	G protein-coupled receptor 1	Homo sapiens G protein-coupled receptor 1 (GPR1), transcript variant 1, mRNA [NM_005279]	11.22817	down	19.42866	down	26.06809	303.07584	3.05885	52.40424	Detected	Detected	Compromised	Detected

4453	A_33_P3305250				26S proteasome non-ATPase regulatory subunit 5 (26S proteasome subunit S5B)(26S protease subunit S5 basic) [Source:UniProtKB/Swiss-Prot;Acc:Q16401] [ENST00000373920]	11.11289	down	6.01666	down	3.62711	41.73700	2.91894	15.48627	Compromised	Detected	Compromised	Detected
4454	A_33_P3281473	LOC100131726	Hs.660862	HCC-related HCC-C11_v3	Homo sapiens HCC-related HCC-C11_v3 (LOC100131726), non-coding RNA [NR_024479]	11.09951	down	7.89632	down	4.31624	49.60691	3.58392	24.95446	Compromised	Detected	Compromised	Detected
4455	A_23_P135990	SLCO2A1	Hs.518270	solute carrier organic anion transporter family, member 2A1	Homo sapiens solute carrier organic anion transporter family, member 2A1 (SLCO2A1), mRNA [NM_005630]	11.09150	down	26.54312	down	6.36072	73.05158	2.60263	60.91585	Compromised	Detected	Compromised	Detected
4456	A_33_P3407256	TRIP13	Hs.436187	thyroid hormone receptor interactor 13	Homo sapiens thyroid hormone receptor interactor 13 (TRIP13), transcript variant 2, mRNA [NM_001166260]	11.08604	down	14.87656	down	20.27239	232.70964	18.52462	243.00632	Detected	Detected	Detected	Detected
4457	A_23_P47704	UCP2	Hs.80658	uncoupling protein 2 (mitochondrial, proton carrier)	Homo sapiens uncoupling protein 2 (mitochondrial, proton carrier) (UCP2), nuclear gene encoding mitochondrial protein, mRNA [NM_003355]	11.07938	down	16.03194	down	2.86493	32.86715	2.36466	33.42870	Compromised	Detected	Compromised	Detected
4458	A_23_P151778	CMA1	Hs.135626	chymase 1, mast cell	Homo sapiens chymase 1, mast cell (CMA1), mRNA [NM_001836]	11.07161	down	1.37666	down	2.64003	30.26581	4.74813	5.76386	Compromised	Detected	Compromised	Compromised
4459	A_23_P218827	POLQ	Hs.241517	polymerase (DNA directed), theta	Homo sapiens polymerase (DNA directed), theta (POLQ), mRNA [NM_199420]	10.96682	down	14.68360	down	55.99027	635.80853	37.98750	491.85690	Detected	Detected	Detected	Detected
4460	A_23_P144843	ESM1	Hs.129944	endothelial cell-specific molecule 1	Homo sapiens endothelial cell-specific molecule 1 (ESM1), transcript variant 1, mRNA [NM_007036]	10.84666	down	11.05986	down	40.72820	457.42970	25.56133	249.28648	Detected	Detected	Detected	Detected
4461	A_33_P3238402					10.81443	down	2.44369	down	2.50390	28.03848	5.19282	11.18963	Compromised	Detected	Compromised	Compromised
4462	A_23_P404494	IL7R	Hs.591742	interleukin 7 receptor	Homo sapiens interleukin 7 receptor (IL7R), mRNA [NM_002185]	10.80711	down	14.07378	down	85.23578	953.81573	54.39650	675.06730	Detected	Detected	Detected	Detected
4463	A_33_P3230548	KIF14	Hs.3104	kinesin family member 14	Homo sapiens kinesin family member 14 (KIF14), mRNA [NM_014875]	10.70198	down	9.47822	down	107.26451	1188.64820	61.23342	511.77700	Detected	Detected	Detected	Detected
4464	A_33_P3242952	FAM72A	Hs.661924	family with sequence similarity 72, member A	Homo sapiens family with sequence similarity 72, member A (FAM72A), mRNA [NM_001123168]	10.69799	down	11.32691	down	26.63227	295.01456	30.12515	300.88900	Detected	Detected	Detected	Detected
4465	A_23_P167983	HIST1H2AC	Hs.622290	histone cluster 1, H2ac	Histone H2A type 1-C (H2A/l) [Source:UniProtKB/Swiss-Prot;Acc:Q93077] [ENST00000314088]	10.69018	down	9.89314	down	50.72849	561.52606	48.93801	426.91962	Detected	Detected	Detected	Detected
4466	A_33_P3328883	HGC6.3	Hs.448059	similar to HGC6.3	Homo sapiens similar to HGC6.3 (HGC6.3), mRNA [NM_001129895]	10.67553	down	3.53705	down	2.76951	30.61433	3.37365	10.52219	Compromised	Detected	Compromised	Compromised
4467	A_23_P64611	P2RY6	Hs.16362	pyrimidinergic receptor P2Y, G-protein coupled, 6	Homo sapiens pyrimidinergic receptor P2Y, G-protein coupled, 6 (P2RY6), transcript variant 2, mRNA [NM_176798]	10.65945	down	18.75149	down	4.36511	48.17956	3.08048	50.93531	Compromised	Detected	Compromised	Detected
4468	A_23_P64161	ANGPTL5	Hs.318370	angiopoietin-like 5	Homo sapiens angiopoietin-like 5 (ANGPTL5), mRNA [NM_178127]	10.62694	down	1.93604	down	2.84595	31.31619	12.18018	20.79379	Compromised	Detected	Compromised	Detected
4469	A_33_P3402020	CCDC8	Hs.97876	coiled-coil domain containing 8	Homo sapiens coiled-coil domain containing 8 (CCDC8), mRNA [NM_032040]	10.50293	down	18.44732	down	185.33307	2015.56400	97.20642	1581.22530	Detected	Detected	Detected	Detected
4470	A_23_P164958	CCDC8	Hs.97876	coiled-coil domain containing 8	Homo sapiens coiled-coil domain containing 8 (CCDC8), mRNA [NM_032040]	10.47582	down	11.63566	down	242.36613	2629.01510	243.71422	2500.55980	Detected	Detected	Detected	Detected
4471	A_23_P10385	DTL	Hs.656473	denticleless homolog (Drosophila)	Homo sapiens denticleless homolog (Drosophila) (DTL), mRNA [NM_016448]	10.43840	down	10.11828	down	81.02996	875.81525	61.50004	548.71590	Detected	Detected	Detected	Detected
4472	A_24_P125871	RIPK4	Hs.517310	receptor-interacting serine-threonine kinase 4	Homo sapiens receptor-interacting serine-threonine kinase 4 (RIPK4), mRNA [NM_020639]	10.39177	down	12.50697	down	19.48539	209.66777	13.19240	145.49270	Detected	Detected	Detected	Detected
4473	A_23_P129717	ERI2	Hs.248437	ERI1 exoribonuclease family member 2	Homo sapiens ERI1 exoribonuclease family member 2 (ERI2), transcript variant 2, mRNA [NM_080663]	10.38247	down	3.95443	down	2.31402	24.87717	5.17494	18.04489	Compromised	Detected	Compromised	Detected
4474	A_32_P211117	LOC401021	Hs.591636	hypothetical gene supported by BC047605	Homo sapiens hypothetical gene supported by BC047605, mRNA (cDNA clone IMAGE:5589309) [BC047605]	10.28565	down	2.62148	down	3.46688	36.92355	9.09632	21.02701	Compromised	Detected	Compromised	Detected
4475	A_33_P3227079				Probable lipid phosphate phosphatase PPAPDC3 (EC 3.1.3.-)(Phosphatidic acid phosphatase type 2 domain-containing protein 3) [Source:UniProtKB/Swiss-Prot;Acc:Q8NBV4] [ENST00000372261]	10.19616	down	10.35941	down	69.67550	735.61410	60.30766	550.90050	Detected	Detected	Detected	Detected
4476	A_33_P3406939	KIF24	Hs.710246	kinesin family member 24	Homo sapiens kinesin family member 24 (KIF24), mRNA [NM_194313]	10.19193	down	22.72142	down	25.42756	268.34540	5.73247	114.85307	Detected	Detected	Compromised	Detected
4477	A_33_P3339212	TRIP13	Hs.436187	thyroid hormone receptor interactor 13	Homo sapiens thyroid hormone receptor interactor 13 (TRIP13), transcript variant 1, mRNA [NM_004237]	10.13593	down	9.26729	down	831.93880	8731.48000	747.15424	6105.59960	Detected	Detected	Detected	Detected
4478	A_33_P3846653		Hs.527883		Keratin 19 Fragment [Source:UniProtKB/TrEMBL;Acc:A0A4R5] [ENST00000405395]	10.12079	down	7.44318	down	11.36448	119.09602	22.22349	145.86003	Compromised	Detected	Detected	Detected
4479	A_23_P88740	CENPN	Hs.55028	centromere protein N	Homo sapiens centromere protein N (CENPN), transcript variant 3, mRNA [NM_018455]	10.11463	down	9.65239	down	1710.36830	17913.17800	1506.79080	12824.87400	Detected	Detected	Detected	Detected
4480	A_33_P3304678	RP5-1022P6.6	Hs.123609	hypothetical LOC149837	Homo sapiens hypothetical LOC149837 (LOC149837), non-coding RNA [NR_015406]	10.09316	down	3.17443	down	2.82983	29.57470	2.94014	8.22998	Compromised	Detected	Compromised	Compromised
4481	A_33_P3441639	LOC145694	Hs.147229	hypothetical protein LOC145694	Homo sapiens cDNA FLJ32231 fis, clone PLACE6004491 [AK056793]	10.07201	down	8.69945	down	96.26368	1003.94870	101.53353	778.87370	Detected	Detected	Detected	Detected
4482	A_24_P142118	THBS1	Hs.164226	thrombospondin 1	Homo sapiens thrombospondin 1 (THBS1), mRNA [NM_003246]	10.06739	down	11.21227	down	8381.52000	87372.07000	6690.92400	66152.31000	Detected	Detected	Detected	Detected

4483	A_23_P149121	DIRAS3	Hs.194695	DIRAS family, GTP-binding RAS-like 3	Homo sapiens DIRAS family, GTP-binding RAS-like 3 (DIRAS3), mRNA [NM_004675]	10.04228	down	9.90943	down	62.51156	650.01830	54.66158	477.63540	Detected	Detected	Detected	Detected
4484	A_33_P3321293	IQGAP3	Hs.591495	IQ motif containing GTPase activating protein 3	Homo sapiens IQ motif containing GTPase activating protein 3 (IQGAP3), mRNA [NM_178229]	10.03782	down	19.00375	down	199.99000	2078.64700	102.83913	1723.30940	Detected	Detected	Detected	Detected
4485	A_33_P3415113	CLCN6	Hs.193043	chloride channel 6	Homo sapiens chloride channel 6 (CLCN6), transcript variant ClC-6b, mRNA [NM_021735]	10.02584	down	14.87768	down	15.92252	165.29736	13.10214	171.88696	Detected	Detected	Compromised	Detected
4486	A_23_P14083	AMIGO2	Hs.121520	adhesion molecule with Ig-like domain 2	Homo sapiens adhesion molecule with Ig-like domain 2 (AMIGO2), transcript variant 2, mRNA [NM_181847]	10.01934	down	9.88929	down	126.29672	1310.28090	76.09612	663.57996	Detected	Detected	Detected	Detected
4487	A_32_P183218	ZNF367	Hs.494557	zinc finger protein 367	Homo sapiens zinc finger protein 367 (ZNF367), mRNA [NM_153695]	10.00583	down	4.12613	down	14.49104	150.13632	10.17996	37.03856	Detected	Detected	Compromised	Detected
4488	A_23_P100344	ORC6L	Hs.49760	origin recognition complex, subunit 6 like (yeast)	Homo sapiens origin recognition complex, subunit 6 like (yeast) (ORC6L), mRNA [NM_014321]	9.98807	down	10.55351	down	193.56079	2001.85140	157.03879	1461.40050	Detected	Detected	Detected	Detected
4489	A_23_P134663	BAALC	Hs.533446	brain and acute leukemia, cytoplasmic	Homo sapiens brain and acute leukemia, cytoplasmic (BAALC), transcript variant 1, mRNA [NM_024812]	9.98213	down	9.94870	down	581.92426	6014.82100	391.14270	3431.36500	Detected	Detected	Detected	Detected
4490	A_32_P471485		Hs.58559		Rhotekin-2 (Pleckstrin homology domain-containing family K member 1) [Source:UniProtKB/Swiss-Prot;Acc:Q8IZC4] [ENST00000395260]	9.82234	down	8.06567	down	2.49951	25.42164	2.73433	19.44717	Compromised	Detected	Compromised	Detected
4491	A_24_P161764		Hs.721648		Putative uncharacterized protein ENSP00000375004 [Source:UniProtKB/TrEMBL;Acc:A6NMP9] [ENST00000390595]	9.74913	down	1.80463	down	3.99826	40.36176	2.69015	4.28084	Compromised	Detected	Compromised	Compromised
4492	A_24_P462899	C6orf173	Hs.486401	chromosome 6 open reading frame 173	Homo sapiens chromosome 6 open reading frame 173 (C6orf173), mRNA [NM_001012507]	9.74304	down	8.55238	down	1272.43850	12837.02000	753.98627	5686.11870	Detected	Detected	Detected	Detected
4493	A_24_P3045	CASP10	Hs.5353	caspase 10, apoptosis-related cysteine peptidase	Homo sapiens caspase 10, apoptosis-related cysteine peptidase (CASP10), transcript variant B, mRNA [NM_032974]	9.71254	down	5.39291	down	4.19933	42.23246	6.22266	29.59131	Compromised	Detected	Compromised	Detected
4494	A_23_P144959	VCAN	Hs.643801	versican	Homo sapiens versican (VCAN), transcript variant 1, mRNA [NM_004385]	9.70815	down	9.85742	down	700.70450	7043.75630	529.03290	4598.45070	Detected	Detected	Detected	Detected
4495	A_23_P114862	ANGPTL7	Hs.146559	angiopoietin-like 7	Homo sapiens angiopoietin-like 7 (ANGPTL7), mRNA [NM_021146]	9.68819	down	7.22973	down	2.51769	25.25676	2.79030	17.78848	Compromised	Not Detected	Compromised	Not Detected
4496	A_23_P258493	LMNB1	Hs.89497	lamin B1	Homo sapiens lamin B1 (LMNB1), mRNA [NM_005573]	9.66296	down	15.88211	down	33.51647	335.35260	77.72552	1088.52200	Detected	Detected	Detected	Detected
4497	A_33_P3300253	PTPN20B	Hs.499552	protein tyrosine phosphatase, non-receptor type 20B	Homo sapiens protein tyrosine phosphatase, non-receptor type 20B (PTPN20B), transcript variant 1, mRNA [NM_001042357]	9.66049	down	7.08076	down	3.86584	38.67013	4.44801	27.77223	Compromised	Detected	Compromised	Detected
4498	A_32_P192970	ALDH4A1	Hs.77448	aldehyde dehydrogenase 4 family, member A1	Homo sapiens aldehyde dehydrogenase 4 family, member A1 (ALDH4A1), nuclear gene encoding mitochondrial protein, transcript variant P5CDhS, mRNA [NM_170726]	9.47329	down	14.46507	down	33.76122	331.17096	28.35141	361.62680	Detected	Detected	Detected	Detected
4499	A_24_P270033	MPZL3	Hs.15396	myelin protein zero-like 3	Myelin protein zero-like protein 3 Precursor [Source:UniProtKB/Swiss-Prot;Acc:Q6UWV2] [ENST00000278949]	9.45431	down	9.79506	down	3.56797	34.92887	2.78325	24.03949	Compromised	Detected	Compromised	Detected
4500	A_33_P3343516	LOC100129617	Hs.657151	hypothetical protein LOC100129617	Homo sapiens cDNA FLJ41543 fis, clone CERVX2002006. [AK123537]	9.44973	down	4.13638	down	2.37330	23.22229	3.09636	11.29372	Compromised	Detected	Compromised	Compromised
4501	A_33_P3260634	PLCB4	Hs.472101	phospholipase C, beta 4	Homo sapiens phospholipase C, beta 4 (PLCB4), transcript variant 1, mRNA [NM_000933]	9.38440	down	6.74455	down	40.01251	388.80792	39.97358	237.73419	Detected	Detected	Detected	Detected
4502	A_23_P167468	PRLR	Hs.368587	prolactin receptor	Homo sapiens prolactin receptor (PRLR), mRNA [NM_000949]	9.34921	down	6.51474	down	3.64043	35.24203	3.68999	21.19769	Compromised	Detected	Compromised	Detected
4503	A_23_P206022	ITGA11	Hs.436416	integrin, alpha 11	Homo sapiens integrin, alpha 11 (ITGA11), mRNA [NM_001004439]	9.32716	down	9.91764	down	261.34277	2524.02220	275.41202	2408.55570	Detected	Detected	Detected	Detected
4504	A_23_P156218	GZMK	Hs.277937	granzyme K (granzyme 3; tryptase II)	Homo sapiens granzyme K (granzyme 3; tryptase II) (GZMK), mRNA [NM_002104]	9.30199	down	11.66739	down	5.09379	49.06254	4.55026	46.81402	Compromised	Detected	Compromised	Detected
4505	A_23_P323243	PNLDC1	Hs.349077	poly(A)-specific ribonuclease (PARN)-like domain containing 1	Homo sapiens poly(A)-specific ribonuclease (PARN)-like domain containing 1 (PNLDC1), mRNA [NM_173516]	9.30197	down	9.96252	down	2.64226	25.44977	2.88457	25.34057	Compromised	Detected	Compromised	Detected
4506	A_33_P3325135	ZSCAN23	Hs.721841	zinc finger and SCAN domain containing 23	Homo sapiens zinc finger and SCAN domain containing 23 (ZSCAN23), mRNA [NM_001012455]	9.20065	down	1.18399	down	2.50484	23.86334	2.83106	2.95572	Compromised	Detected	Compromised	Compromised
4507	A_33_P3387861	CENPN	Hs.55028	centromere protein N	Homo sapiens centromere protein N (CENPN), transcript variant 2, mRNA [NM_001100624]	9.18503	down	10.22693	down	319.12270	3035.08890	254.22676	2292.62040	Detected	Detected	Detected	Detected
4508	A_33_P3405911	TSIX		XIST antisense RNA (non-protein coding)	Homo sapiens XIST antisense RNA (non-protein coding) (TSIX), antisense RNA [NR_003255]	9.17552	down	4.59666	down	2.36100	22.43155	4.24215	17.19468	Compromised	Detected	Compromised	Detected
4509	A_23_P66798	KRT19	Hs.654568	keratin 19	Homo sapiens keratin 19 (KRT19), mRNA [NM_002276]	9.13790	down	11.51091	down	350.54920	3316.86900	264.09137	2680.58350	Detected	Detected	Detected	Detected
4510	A_33_P3716128	SMC4	Hs.58992	structural maintenance of chromosomes 4	Homo sapiens structural maintenance of chromosomes 4 (SMC4), transcript variant 1, mRNA [NM_005496]	9.11372	down	8.50121	down	948.89920	8954.65200	640.11720	4798.50000	Detected	Detected	Detected	Detected
4511	A_23_P321501	DHRS2	Hs.272499	dehydrogenase/reductase (SDR family) member 2	Homo sapiens dehydrogenase/reductase (SDR family) member 2 (DHRS2), transcript variant 1, mRNA [NM_182908]	9.10002	down	17.90205	down	65.39507	616.19836	33.44551	527.96630	Detected	Detected	Detected	Detected
4512	A_33_P3304668	COL1A1	Hs.172928	collagen, type I, alpha 1	Homo sapiens collagen, type I, alpha 1 (COL1A1), mRNA [NM_000088]	9.08634	down	8.83507	down	12927.84900	121632.08000	10123.93750	78872.40000	Detected	Detected	Detected	Detected

4513	A_33_P3415820	THBS1	Hs.164226	thrombospondin 1	Homo sapiens thrombospondin 1 (THBS1), mRNA [NM_003246]	9.08103	down	10.36795	down	415.86053	3910.35400	451.28854	4125.84330	Detected	Detected	Detected	Detected
4514	A_23_P143190	MYBL2	Hs.179718	v-myb myeloblastosis viral oncogene homolog (avian)-like 2	Homo sapiens v-myb myeloblastosis viral oncogene homolog (avian)-like 2 (MYBL2), mRNA [NM_002466]	9.08085	down	25.78563	down	247.45096	2326.74340	103.16248	2345.65940	Detected	Detected	Detected	Detected
4515	A_23_P121851	PCDHB15	Hs.130757	protocadherin beta 15	Homo sapiens protocadherin beta 15 (PCDHB15), mRNA [NM_018935]	9.03279	down	1.22671	down	3.14201	29.38744	2.94792	3.18877	Compromised	Not Detected	Compromised	Compromised
4516	A_23_P162355	HOXC5	Hs.549040	homeobox C5	Homo sapiens homeobox C5 (HOXC5), transcript variant 1, mRNA [NM_018953]	9.02139	down	7.62677	down	3.13995	29.33120	7.18272	48.30531	Compromised	Detected	Compromised	Detected
4517	A_23_P131263	MPP4	Hs.63085	membrane protein, palmitoylated 4 (MAGUK p55 subfamily member 4)	Homo sapiens membrane protein, palmitoylated 4 (MAGUK p55 subfamily member 4) (MPP4), mRNA [NM_033066]	9.01646	down	8.18834	down	44.18587	412.52707	19.90693	143.73611	Detected	Detected	Detected	Detected
4518	A_33_P3284345	NRG1	Hs.453951	neuregulin 1	Homo sapiens neuregulin 1 (NRG1), transcript variant HRG-gamma, mRNA [NM_004495]	8.97735	down	12.96108	down	32.91935	306.00793	20.54904	234.85416	Detected	Detected	Detected	Detected
4519	A_24_P52697	H19	Hs.533566	H19, imprinted maternally expressed transcript (non-protein coding)	Homo sapiens H19, imprinted maternally expressed transcript (non-protein coding) (H19), non-coding RNA [NR_002196]	8.97703	down	46.75808	down	9.89763	92.00195	3.18234	131.21063	Compromised	Detected	Compromised	Detected
4520	A_24_P384029	LOC100130171	Hs.711916	similar to hematological and neurological expressed 1	PREDICTED: Homo sapiens similar to hematological and neurological expressed 1 (LOC100130171), miscRNA [XR_038676]	8.95315	down	14.47509	down	131.62102	1220.20810	4.81989	61.52112	Detected	Detected	Compromised	Detected
4521	A_24_P214231	STIL	Hs.525198	SCL/TAL1 interrupting locus	Homo sapiens SCL/TAL1 interrupting locus (STIL), transcript variant 1, mRNA [NM_001048166]	8.88299	down	8.61571	down	21.11260	194.19313	15.16749	115.23121	Detected	Detected	Detected	Detected
4522	A_23_P137896	OXCT2	Hs.472491	3-oxoacid CoA transferase 2	Homo sapiens 3-oxoacid CoA transferase 2 (OXCT2), mRNA [NM_022120]	8.84161	down	11.69220	down	27.28027	249.75443	19.70523	203.16226	Detected	Detected	Detected	Detected
4523	A_32_P186474	RACGAP1	Hs.505469	Rac GTPase activating protein 1	Homo sapiens Rac GTPase activating protein 1 (RACGAP1), transcript variant 1, mRNA [NM_013277]	8.83306	down	7.72397	down	1142.04660	10445.47700	657.50240	4478.19970	Detected	Detected	Detected	Detected
4524	A_33_P3364268	LBH	Hs.567598	limb bud and heart development homolog (mouse)	Homo sapiens limb bud and heart development homolog (mouse) (LBH), mRNA [NM_030915]	8.80822	down	7.77108	down	77.30021	705.02090	80.12980	549.08673	Detected	Detected	Detected	Detected
4525	A_23_P80032	E2F1	Hs.654393	E2F transcription factor 1	Homo sapiens E2F transcription factor 1 (E2F1), mRNA [NM_005225]	8.76888	down	9.66277	down	512.26470	4651.27250	357.43234	3045.51730	Detected	Detected	Detected	Detected
4526	A_24_P941557	FAM125B	Hs.162659	family with sequence similarity 125, member B	Homo sapiens family with sequence similarity 125, member B (FAM125B), transcript variant 2, mRNA [NM_001011703]	8.75185	down	15.73737	down	14.19450	128.63310	4.88828	67.83497	Detected	Detected	Compromised	Detected
4527	A_24_P392110	PSG8	Hs.466843	pregnancy specific beta-1-glycoprotein 8	Homo sapiens pregnancy specific beta-1-glycoprotein 8 (PSG8), transcript variant 1, mRNA [NM_182707]	8.64318	down	9.10011	down	878.92096	7866.04900	927.49270	7442.56250	Detected	Detected	Detected	Detected
4528	A_33_P3325748	TRIOBP	Hs.533030	TRIO and F-actin binding protein	Homo sapiens TRIO and F-actin binding protein (TRIOBP), transcript variant 2, mRNA [NM_138632]	8.59614	down	1.17828	down	15.41921	137.24573	2.35087	2.44254	Detected	Detected	Compromised	Compromised
4529	A_33_P3401658	PSG2	Hs.709200	pregnancy specific beta-1-glycoprotein 2	Homo sapiens pregnancy specific beta-1-glycoprotein 2 (PSG2), mRNA [NM_031246]	8.54694	down	8.89981	down	334.67140	2961.84400	332.64578	2610.52690	Detected	Detected	Detected	Detected
4530	A_33_P3867534	MCM10	Hs.198363	minichromosome maintenance complex component 10	Homo sapiens minichromosome maintenance complex component 10 (MCM10), transcript variant 1, mRNA [NM_182751]	8.54474	down	17.74148	down	15.28417	135.23026	5.39638	84.42250	Detected	Detected	Compromised	Detected
4531	A_33_P3226761	SOX18	Hs.8619	SRY (sex determining region Y)-box 18	Homo sapiens SRY (sex determining region Y)-box 18 (SOX18), mRNA [NM_018419]	8.51783	down	1.38258	down	2.50588	22.10151	3.48455	4.24819	Compromised	Detected	Compromised	Compromised
4532	A_23_P94422	MELK	Hs.184339	maternal embryonic leucine zipper kinase	Homo sapiens maternal embryonic leucine zipper kinase (MELK), mRNA [NM_014791]	8.51146	down	8.09946	down	675.75670	5955.62900	609.38257	4352.22600	Detected	Detected	Detected	Detected
4533	A_33_P3415944	C6orf191	Hs.448372	chromosome 6 open reading frame 191	Homo sapiens chromosome 6 open reading frame 191 (C6orf191), mRNA [NM_001010876]	8.50365	down	6.42186	down	14.55207	128.13364	15.25652	86.39359	Detected	Detected	Detected	Detected
4534	A_33_P3340802				Dehydrodolichyl diphosphate synthase (Dedol-PP synthase)(EC 2.5.1.-) [Source:UniProtKB/Swiss-Prot;Acc:Q86SQ9] [ENST00000374186]	8.47346	down	3.75723	down	4.05853	35.60921	3.43037	11.36511	Compromised	Detected	Compromised	Compromised
4535	A_23_P420326	FNDC5	Hs.524234	fibronectin type III domain containing 5	Homo sapiens fibronectin type III domain containing 5 (FNDC5), mRNA [NM_153756]	8.43319	down	9.37426	down	3.62574	31.66074	3.32071	27.44941	Compromised	Detected	Compromised	Detected
4536	A_23_P415652	GALNT12	Hs.47099	UDP-N-acetyl-alpha-D-galactosamine:polypeptide N-acetylgalactosaminyltransferase 12 (GalNAc-T12)	Homo sapiens UDP-N-acetyl-alpha-D-galactosamine:polypeptide N-acetylgalactosaminyltransferase 12 (GalNAc-T12) (GALNT12), mRNA [NM_024642]	8.42560	down	1.70417	down	6.50325	56.73674	10.13794	15.23454	Compromised	Detected	Compromised	Detected
4537	A_23_P68167	CNGA3	Hs.234785	cyclic nucleotide gated channel alpha 3	Homo sapiens cyclic nucleotide gated channel alpha 3 (CNGA3), transcript variant 1, mRNA [NM_001298]	8.38726	down	8.92531	down	20.07795	174.37025	12.73791	100.25060	Detected	Detected	Compromised	Detected
4538	A_23_P99292	RAD51AP1	Hs.504550	RAD51 associated protein 1	Homo sapiens RAD51 associated protein 1 (RAD51AP1), transcript variant 2, mRNA [NM_006479]	8.37820	down	9.61286	down	124.78508	1082.54600	114.96819	974.53090	Detected	Detected	Detected	Detected
4539	A_24_P321581	SLC38A4	Hs.446077	solute carrier family 38, member 4	Homo sapiens solute carrier family 38, member 4 (SLC38A4), transcript variant 1, mRNA [NM_018018]	8.30192	down	4.32031	down	4.39538	37.78403	8.88294	33.84057	Compromised	Detected	Compromised	Detected
4540	A_32_P202013	FAM196A	Hs.613882	family with sequence similarity 196, member A	Homo sapiens family with sequence similarity 196, member A (FAM196A), mRNA [NM_001039762]	8.28199	down	4.47706	down	11.49315	98.56135	15.96267	63.01792	Compromised	Detected	Detected	Detected
4541	A_32_P116556	ZNF469	Hs.54925	zinc finger protein 469	Homo sapiens zinc finger protein 469 (ZNF469), mRNA [NM_001127464]	8.24956	down	8.33534	down	2682.95020	22917.97500	1912.84790	14059.48200	Detected	Detected	Detected	Detected

4542	A_33_P3709173	PHTF2	Hs.203965	putative homeodomain transcription factor 2	Homo sapiens putative homeodomain transcription factor 2 (PHTF2), transcript variant 5, mRNA [NM_001127360]	8.24325	down	4.98901	down	2.30800	19.70004	2.45491	10.79979	Compromised	Detected	Compromised	Compromised
4543	A_33_P3298216	MYO16	Hs.656587	myosin XVI	Homo sapiens myosin XVI (MYO16), mRNA [NM_015011]	8.23686	down	8.17679	down	31.71510	270.49610	26.65287	192.17305	Detected	Detected	Detected	Detected
4544	A_23_P57413	PPM1F	Hs.112728	protein phosphatase 1F (PP2C domain containing)	Homo sapiens protein phosphatase 1F (PP2C domain containing) (PPM1F), mRNA [NM_014634]	8.23210	down	11.92993	down	55.52918	473.33124	46.82087	492.54144	Detected	Detected	Detected	Detected
4545	A_24_P65098	TMEM87A	Hs.597178	transmembrane protein 87A	Homo sapiens transmembrane protein 87A (TMEM87A), transcript variant 2, mRNA [NM_001110503]	8.21011	down	3.15181	down	11.75098	99.89788	19.55616	54.35128	Compromised	Detected	Detected	Detected
4546	A_33_P3388771	LOC732272	Hs.572567	hypothetical LOC732272	PREDICTED: Homo sapiens hypothetical LOC732272 (LOC732272), miscRNA [XR_038449]	8.20544	down	5.16078	down	13.11345	111.41719	13.12062	59.70837	Detected	Detected	Detected	Detected
4547	A_23_P100240	CDH16	Hs.513660	cadherin 16, KSP-cadherin	Homo sapiens cadherin 16, KSP-cadherin (CDH16), mRNA [NM_004062]	8.20263	down	25.91708	down	6.51760	55.35724	2.44651	55.91123	Compromised	Detected	Compromised	Detected
4548	A_23_P374082	ADAM19	Hs.483944	ADAM metallopeptidase domain 19 (meltrin beta)	Homo sapiens ADAM metallopeptidase domain 19 (meltrin beta) (ADAM19), mRNA [NM_033274]	8.18717	down	8.91159	down	721.83026	6119.30500	622.56006	4892.17700	Detected	Detected	Detected	Detected
4549	A_23_P117437	IL25	Hs.302036	interleukin 25	Homo sapiens interleukin 25 (IL25), transcript variant 1, mRNA [NM_022789]	8.18346	down	1.18456	down	2.70902	22.95530	2.86861	2.99638	Compromised	Detected	Compromised	Compromised
4550	A_23_P125717	NAP1L3	Hs.21365	nucleosome assembly protein 1-like 3	Homo sapiens nucleosome assembly protein 1-like 3 (NAP1L3), mRNA [NM_004538]	8.17685	down	7.17852	down	6.91225	58.52456	4.83686	30.61713	Compromised	Detected	Compromised	Detected
4551	A_23_P429491	C11orf82	Hs.165607	chromosome 11 open reading frame 82	Homo sapiens chromosome 11 open reading frame 82 (C11orf82), mRNA [NM_145018]	8.15526	down	7.63438	down	158.07306	1334.83810	123.22599	829.54780	Detected	Detected	Detected	Detected
4552	A_33_P3401621	CCNB1	Hs.23960	cyclin B1	Homo sapiens cyclin B1 (CCNB1), mRNA [NM_031966]	8.14173	down	15.71186	down	422.79736	3564.36160	186.15253	2579.06270	Detected	Detected	Detected	Detected
4553	A_33_P3333187					8.13130	down	3.19737	down	14.85899	125.10724	16.00103	45.11354	Detected	Detected	Detected	Detected
4554	A_23_P100632	HN1	Hs.532803	hematological and neurological expressed 1	Homo sapiens hematological and neurological expressed 1 (HN1), transcript variant 3, mRNA [NM_001002033]	8.12470	down	7.20211	down	15509.49500	130478.28000	14559.64100	92464.78000	Detected	Detected	Detected	Detected
4555	A_23_P216023	ANGPT1	Hs.369675	angiopoietin 1	Homo sapiens angiopoietin 1 (ANGPT1), mRNA [NM_001146]	8.09189	down	8.49198	down	130.44817	1093.00240	114.93729	860.66705	Detected	Detected	Detected	Detected
4556	A_23_P93258	HIST1H3B	Hs.533292	histone cluster 1, H3b	Homo sapiens histone cluster 1, H3b (HIST1H3B), mRNA [NM_003537]	8.06998	down	9.86487	down	50.12993	418.89240	40.98689	356.53467	Detected	Detected	Detected	Detected
4557	A_33_P3242753					8.04604	down	1.19830	down	13.02594	108.52361	2.97160	3.13995	Compromised	Detected	Compromised	Compromised
4558	A_33_P3713357	ALCAM	Hs.591293	activated leukocyte cell adhesion molecule	Homo sapiens activated leukocyte cell adhesion molecule (ALCAM), mRNA [NM_001627]	8.04557	down	7.90001	down	1559.14640	12989.03500	1046.42200	7289.53760	Detected	Detected	Detected	Detected
4559	A_23_P101084	SPATA22	Hs.351068	spermatogenesis associated 22	Homo sapiens spermatogenesis associated 22 (SPATA22), mRNA [NM_032598]	8.04192	down	10.18348	down	5.01207	41.73591	2.89951	26.03677	Compromised	Detected	Compromised	Detected
4560	A_32_P80245	ZFP57	Hs.156326	zinc finger protein 57 homolog (mouse)	Homo sapiens zinc finger protein 57 homolog (mouse) (ZFP57), mRNA [NM_001109809]	8.03661	down	16.09754	down	9.20946	76.63728	4.44293	63.06582	Compromised	Detected	Compromised	Detected
4561	A_33_P3416009				Protein downstream neighbor of Son (B17) [Source:UniProtKB/Swiss-Prot;Acc:Q9NYP3] [ENST00000303113]	8.02522	down	1.78798	down	2.66017	22.10548	3.10114	4.88934	Compromised	Detected	Compromised	Compromised
4562	A_23_P104741	KIRREL3	Hs.657065	kin of IRRE like 3 (Drosophila)	Homo sapiens kin of IRRE like 3 (Drosophila) (KIRREL3), transcript variant 1, mRNA [NM_032531]	8.02436	down	8.32665	down	273.28130	2270.66630	296.64136	2178.04860	Detected	Detected	Detected	Detected
4563	A_33_P3221313	CENPI	Hs.348920	centromere protein I	Homo sapiens centromere protein I (CENPI), mRNA [NM_006733]	8.02247	down	7.61741	down	9.91329	82.34909	9.44057	63.41200	Compromised	Detected	Compromised	Detected
4564	A_23_P79803	VSTM2L	Hs.517029	V-set and transmembrane domain containing 2 like	Homo sapiens V-set and transmembrane domain containing 2 like (VSTM2L), mRNA [NM_080607]	8.01526	down	6.87369	down	44.37958	368.32720	49.42203	299.55457	Detected	Detected	Detected	Detected
4565	A_33_P3260530	KANK4	Hs.283398	KN motif and ankyrin repeat domains 4	Homo sapiens KN motif and ankyrin repeat domains 4 (KANK4), mRNA [NM_181712]	7.98969	down	7.02322	down	10.80301	89.37333	11.50272	71.23648	Compromised	Detected	Compromised	Detected
4566	A_33_P3406836	HTR1F	Hs.248136	5-hydroxytryptamine (serotonin) receptor 1F	Homo sapiens 5-hydroxytryptamine (serotonin) receptor 1F (HTR1F), mRNA [NM_000866]	7.98726	down	1.90591	down	2.45233	20.28198	2.65005	4.45370	Compromised	Detected	Compromised	Compromised
4567	A_33_P3340040	GINS4	Hs.656996	GINS complex subunit 4 (Sld5 homolog)	Homo sapiens GINS complex subunit 4 (Sld5 homolog) (GINS4), mRNA [NM_032336]	7.97494	down	7.91543	down	369.79416	3053.65750	243.39272	1698.82090	Detected	Detected	Detected	Detected
4568	A_33_P3377519	HOXA6	Hs.679517	homeobox A6	Homo sapiens homeobox A6 (HOXA6), mRNA [NM_024014]	7.97321	down	34.13733	down	15.34893	126.71986	4.61735	138.99133	Detected	Detected	Compromised	Detected
4569	A_23_P368154	PODN	Hs.586141	podocan	Homo sapiens podocan (PODN), mRNA [NM_153703]	7.95705	down	10.11153	down	6.83837	56.34272	7.41624	66.12500	Compromised	Detected	Compromised	Detected
4570	A_23_P85209	IL13RA2	Hs.336046	interleukin 13 receptor, alpha 2	Homo sapiens interleukin 13 receptor, alpha 2 (IL13RA2), mRNA [NM_000640]	7.94020	down	8.26163	down	744.42950	6120.51860	597.87040	4355.50150	Detected	Detected	Detected	Detected
4571	A_33_P3373259	CACNA2D3	Hs.656687	calcium channel, voltage-dependent, alpha 2/delta subunit 3	Homo sapiens calcium channel, voltage-dependent, alpha 2/delta subunit 3 (CACNA2D3), mRNA [NM_018398]	7.92884	down	4.79721	down	2.49471	20.48158	2.77605	11.74306	Compromised	Detected	Compromised	Compromised
4572	A_32_P213091	SHISA9	Hs.130661	shisa homolog 9 (Xenopus laevis)	Homo sapiens shisa homolog 9 (Xenopus laevis) (SHISA9), transcript variant 2, mRNA [NM_001145205]	7.92202	down	11.34854	down	12.20488	100.11581	6.75044	67.55193	Compromised	Detected	Compromised	Detected
4573	A_33_P3380346	WHSC1	Hs.113876	Wolf-Hirschhorn syndrome candidate 1	Homo sapiens Wolf-Hirschhorn syndrome candidate 1 (WHSC1), transcript variant 1, mRNA [NM_133330]	7.91765	down	8.25210	down	55.37885	454.01804	61.53497	447.76670	Detected	Detected	Detected	Detected
4574	A_24_P393958	DNAJB4	Hs.13852	DnaJ (Hsp40) homolog, subfamily B, member 4	Homo sapiens DnaJ (Hsp40) homolog, subfamily B, member 4 (DNAJB4), mRNA [NM_007034]	7.91703	down	7.44404	down	408.45602	3348.42430	290.73950	1908.43870	Detected	Detected	Detected	Detected

4575	A_24_P351466	KIAA1524	Hs.591308	KIAA1524	Homo sapiens KIAA1524 (KIAA1524), mRNA [NM_020890]	7.90081	down	1.16052	down	2.70181	22.10339	3.26288	3.33902	Compromised	Detected	Compromised	Compromised
4576	A_23_P211345	TBX1	Hs.173984	T-box 1	Homo sapiens T-box 1 (TBX1), transcript variant C, mRNA [NM_080647]	7.87664	down	3.06863	down	737.68555	6016.51860	3863.44040	10454.02800	Detected	Detected	Detected	Detected
4577	A_24_P827037	LRRC15	Hs.288467	leucine rich repeat containing 15	Homo sapiens leucine rich repeat containing 15 (LRRC15), transcript variant 2, mRNA [NM_130830]	7.86060	down	7.75884	down	394.72665	3212.81030	334.69840	2289.89700	Detected	Detected	Detected	Detected
4578	A_23_P51213	MYOM3	Hs.523413	myomesin family, member 3	Homo sapiens myomesin family, member 3 (MYOM3), mRNA [NM_152372]	7.85224	down	1.73678	down	2.35949	19.18428	11.41953	17.48877	Compromised	Detected	Compromised	Detected
4579	A_33_P3210363	LOC100128191	Hs.594042	hypothetical protein LOC100128191	Homo sapiens hypothetical protein LOC100128191 (LOC100128191), non-coding RNA [NR_027157]	7.84433	down	7.79175	down	24.37318	197.97092	21.18647	145.56563	Detected	Detected	Detected	Detected
4580	A_23_P414519	NRN1	Hs.103291	neuritin 1	Homo sapiens neuritin 1 (NRN1), mRNA [NM_016588]	7.83362	down	7.72414	down	41.57154	337.20316	32.53445	221.59447	Detected	Detected	Detected	Detected
4581	A_24_P411749	GPR126	Hs.318894	G protein-coupled receptor 126	Homo sapiens G protein-coupled receptor 126 (GPR126), transcript variant a1, mRNA [NM_020455]	7.82096	down	6.30008	down	2.92353	23.67560	3.25523	18.08394	Compromised	Detected	Compromised	Detected
4582	A_23_P163195	LRFN5	Hs.136893	leucine rich repeat and fibronectin type III domain containing 5	Homo sapiens leucine rich repeat and fibronectin type III domain containing 5 (LRFN5), mRNA [NM_152447]	7.82044	down	6.88402	down	11.98265	97.03247	14.42112	87.54005	Detected	Detected	Detected	Detected
4583	A_33_P3395321	HN1	Hs.532803	hematological and neurological expressed 1	Homo sapiens hematological and neurological expressed 1 (HN1), transcript variant 2, mRNA [NM_001002032]	7.79627	down	7.66898	down	1145.10600	9244.12600	1722.87100	11650.80900	Detected	Detected	Detected	Detected
4584	A_24_P62505	GLT25D2	Hs.387995	glycosyltransferase 25 domain containing 2	Homo sapiens glycosyltransferase 25 domain containing 2 (GLT25D2), mRNA [NM_015101]	7.78782	down	8.34892	down	14.61055	117.81903	11.79480	86.83330	Detected	Detected	Compromised	Detected
4585	A_24_P225534	RHBDL2	Hs.524626	rhomboid, veinlet-like 2 (Drosophila)	Homo sapiens rhomboid, veinlet-like 2 (Drosophila) (RHBDL2), mRNA [NM_017821]	7.76246	down	7.46802	down	19.36771	155.67212	18.00211	118.54804	Detected	Detected	Detected	Detected
4586	A_23_P9614	NDUFA4L2	Hs.720062	NADH dehydrogenase (ubiquinone) 1 alpha subcomplex, 4-like 2	Homo sapiens NADH dehydrogenase (ubiquinone) 1 alpha subcomplex, 4-like 2 (NDUFA4L2), mRNA [NM_020142]	7.74764	down	7.30257	down	22.48559	180.38762	19.47534	125.40830	Detected	Detected	Detected	Detected
4587	A_33_P3421595	C2orf27B	Hs.145567	chromosome 2 open reading frame 27B	Homo sapiens chromosome 2 open reading frame 27B (C2orf27B), mRNA [NM_214461]	7.74380	down	1.17934	down	2.31135	18.53332	2.45923	2.55742	Compromised	Detected	Compromised	Compromised
4588	A_23_P119254	ASF1B	Hs.26516	ASF1 anti-silencing function 1 homolog B (S. cerevisiae)	Homo sapiens ASF1 anti-silencing function 1 homolog B (S. cerevisiae) (ASF1B), mRNA [NM_018154]	7.73738	down	8.11242	down	230.42084	1846.07390	193.23960	1382.33170	Detected	Detected	Detected	Detected
4589	A_23_P162211	MANSC1	Hs.591145	MANSC domain containing 1	Homo sapiens MANSC domain containing 1 (MANSC1), mRNA [NM_018050]	7.73232	down	10.66141	down	11.77426	94.27062	6.32215	59.43534	Detected	Detected	Compromised	Detected
4590	A_33_P3229672	LOC642635	Hs.580122	similar to tubulin, alpha 1C	PREDICTED: Homo sapiens similar to tubulin, alpha 1C (LOC642635), miscRNA [XR_016188]	7.73068	down	4.86937	down	36.58497	292.85568	2.37607	10.20227	Detected	Detected	Compromised	Compromised
4591	A_24_P193592	CCNF	Hs.1973	cyclin F	Homo sapiens cyclin F (CCNF), mRNA [NM_001761]	7.72817	down	9.92364	down	27.45196	219.67622	19.30103	168.89502	Detected	Detected	Detected	Detected
4592	A_33_P3224324	NOX4	Hs.371036	NADPH oxidase 4	Homo sapiens NADPH oxidase 4 (NOX4), transcript variant 2, mRNA [NM_001143836]	7.72183	down	7.18524	down	22.75373	181.93068	26.26116	166.38739	Detected	Detected	Detected	Detected
4593	A_33_P3369885	HEATR7A	Hs.443139	HEAT repeat containing 7A	Homo sapiens HEAT repeat containing 7A (HEATR7A), transcript variant 2, mRNA [NM_001099280]	7.71394	down	4.63211	down	9.50098	75.88879	14.94341	61.03720	Compromised	Detected	Detected	Detected
4594	A_23_P389532	AMAC1	Hs.514814	acyl-malonyl condensing enzyme 1	Homo sapiens acyl-malonyl condensing enzyme 1 (AMAC1), mRNA [NM_152462]	7.68641	down	1.18453	down	2.49248	19.83752	2.81184	2.93700	Compromised	Detected	Compromised	Compromised
4595	A_23_P217755	SHROOM2	Hs.567236	shroom family member 2	Homo sapiens shroom family member 2 (SHROOM2), mRNA [NM_001649]	7.68279	down	1.18325	down	2.36952	18.85009	2.58399	2.69607	Compromised	Detected	Compromised	Compromised
4596	A_23_P325642	PDIA2	Hs.66581	protein disulfide isomerase family A, member 2	Homo sapiens protein disulfide isomerase family A, member 2 (PDIA2), mRNA [NM_006849]	7.67820	down	2.23141	down	2.34666	18.65706	4.77714	9.39969	Compromised	Detected	Compromised	Compromised
4597	A_23_P116387	INCENP	Hs.142179	inner centromere protein antigens 135/155kDa	Homo sapiens inner centromere protein antigens 135/155kDa (INCENP), transcript variant 1, mRNA [NM_001040694]	7.65182	down	7.48367	down	468.88020	3715.00630	230.12834	1518.62410	Detected	Detected	Detected	Detected
4598	A_33_P3292540	CDKN2C	Hs.525324	cyclin-dependent kinase inhibitor 2C (p18, inhibits CDK4)	Homo sapiens cyclin-dependent kinase inhibitor 2C (p18, inhibits CDK4) (CDKN2C), transcript variant 2, mRNA [NM_078626]	7.64785	down	7.56589	down	791.03455	6264.23540	677.02120	4516.76800	Detected	Detected	Detected	Detected
4599	A_23_P303978	LOC285943	Hs.714774	hypothetical protein LOC285943	PREDICTED: Homo sapiens hypothetical protein LOC285943 (LOC285943), miscRNA [XR_040113]	7.63318	down	5.56984	down	2.60220	20.56742	2.86364	14.06456	Compromised	Detected	Compromised	Compromised
4600	A_33_P3346891	MYBL1	Hs.445898	v-myb myeloblastosis viral oncogene homolog (avian)-like 1	Homo sapiens v-myb myeloblastosis viral oncogene homolog (avian)-like 1 (MYBL1), transcript variant 2, mRNA [NM_001144755]	7.60397	down	6.74603	down	2.39734	18.87573	2.56118	15.23542	Compromised	Detected	Compromised	Detected
4601	A_33_P3282489	GCNT1	Hs.521568	glucosaminyl (N-acetyl) transferase 1, core 2 (beta-1,6-N-acetylglucosaminyltransferase)	Homo sapiens glucosaminyl (N-acetyl) transferase 1, core 2 (beta-1,6-N-acetylglucosaminyltransferase) (GCNT1), transcript variant 1, mRNA [NM_001097634]	7.60052	down	7.40841	down	128.86017	1014.13390	87.73604	573.15015	Detected	Detected	Detected	Detected
4602	A_23_P345678	FANCD2	Hs.208388	Fanconi anemia, complementation group D2	Homo sapiens Fanconi anemia, complementation group D2 (FANCD2), transcript variant 1, mRNA [NM_033084]	7.59259	down	5.60040	down	5.75032	45.20794	3.79935	18.76265	Compromised	Detected	Compromised	Detected
4603	A_33_P3390097					7.58137	down	3.71555	down	2.29411	18.00925	2.39751	7.85505	Compromised	Detected	Compromised	Compromised
4604	A_23_P393766	WDR62	Hs.116244	WD repeat domain 62	Homo sapiens WD repeat domain 62 (WDR62), transcript variant 2, mRNA [NM_173636]	7.57702	down	6.04816	down	2.62190	20.57065	2.84593	15.17795	Compromised	Detected	Compromised	Detected

4605	A_23_P54622	KIF22	Hs.612151	kinesin family member 22	Homo sapiens kinesin family member 22 (KIF22), mRNA [NM_007317]	7.55828	down	8.31144	down	602.46700	4715.08400	611.75300	4483.50730	Detected	Detected	Detected	Detected
4606	A_24_P79403	PF4	Hs.81564	platelet factor 4	Homo sapiens platelet factor 4 (PF4), mRNA [NM_002619]	7.51988	down	8.05235	down	244.66570	1905.09730	185.89767	1319.96450	Detected	Detected	Detected	Detected
4607	A_33_P3306018		Hs.689480		Putative uncharacterized protein FLJ45999 [Source:UniProtKB/Swiss-Prot;Acc:Q6ZRX8] [ENST00000339808]	7.50804	down	1.18962	down	2.55258	19.84448	3.02145	3.16950	Compromised	Detected	Compromised	Compromised
4608	A_24_P343095	DHFR	Hs.648635	dihydrofolate reductase	Homo sapiens dihydrofolate reductase (DHFR), mRNA [NM_000791]	7.49027	down	6.86052	down	126.81158	983.53430	74.60490	451.32556	Detected	Detected	Detected	Detected
4609	A_33_P3271684	FAM23A	Hs.564139	family with sequence similarity 23, member A	Homo sapiens family with sequence similarity 23, member A (FAM23A), mRNA [NM_001098844]	7.48988	down	2.19507	down	2.35752	18.28365	9.31568	18.03135	Compromised	Detected	Compromised	Detected
4610	A_33_P3257503	LOC387647	Hs.660899	patched domain containing 3 pseudogene	Homo sapiens patched domain containing 3 pseudogene (LOC387647), non-coding RNA [NR_003930]	7.48944	down	6.77870	down	186.28159	1444.61610	169.56677	1013.56696	Detected	Detected	Detected	Detected
4611	A_23_P214821	EDN1	Hs.511899	endothelin 1	Homo sapiens endothelin 1 (EDN1), mRNA [NM_001955]	7.47316	down	7.16335	down	152.43022	1179.52870	146.98103	928.41644	Detected	Detected	Detected	Detected
4612	A_23_P411335	SGOL2	Hs.655182	shugoshin-like 2 (S. pombe)	Homo sapiens shugoshin-like 2 (S. pombe) (SGOL2), transcript variant 1, mRNA [NM_152524]	7.45942	down	7.04372	down	176.75575	1365.24820	146.04740	907.11280	Detected	Detected	Detected	Detected
4613	A_23_P47941	HOXC11	Hs.127562	homeobox C11	Homo sapiens homeobox C11 (HOXC11), mRNA [NM_014212]	7.45110	down	5.43341	down	2.86383	22.09534	3.22380	15.44565	Compromised	Detected	Compromised	Compromised
4614	A_33_P3335915				Nesprin-1 (Nuclear envelope spectrin repeat protein 1)(Synaptic nuclear envelope protein 1)(Syne-1)(Myocyte nuclear envelope protein 1)(Myne-1)(Enaptin) [Source:UniProtKB/Swiss-Prot;Acc:Q8NF91] [ENST00000367253]	7.44087	down	3.30723	down	11.74017	90.45476	9.63939	28.11122	Compromised	Detected	Compromised	Detected
4615	A_23_P23074	IFI44	Hs.82316	interferon-induced protein 44	Homo sapiens interferon-induced protein 44 (IFI44), mRNA [NM_006417]	7.43490	down	7.34857	down	331.72020	2553.76150	289.57140	1876.39370	Detected	Detected	Detected	Detected
4616	A_23_P257365	GFI1	Hs.73172	growth factor independent 1 transcription repressor	Homo sapiens growth factor independent 1 transcription repressor (GFI1), transcript variant 1, mRNA [NM_005263]	7.40008	down	8.15028	down	3.86174	29.59050	3.88114	27.89316	Compromised	Detected	Compromised	Detected
4617	A_33_P3398331	MMP24	Hs.715494	matrix metallopeptidase 24 (membrane-inserted)	Homo sapiens matrix metallopeptidase 24 (membrane-inserted) (MMP24), mRNA [NM_006690]	7.39601	down	5.22235	down	77.92255	596.75183	102.66319	472.76556	Detected	Detected	Detected	Detected
4618	A_23_P212284	WDR51A	Hs.476306	WD repeat domain 51A	Homo sapiens WD repeat domain 51A (WDR51A), transcript variant 1, mRNA [NM_015426]	7.37684	down	7.44564	down	565.71454	4321.16260	531.86505	3491.95970	Detected	Detected	Detected	Detected
4619	A_23_P15146	IL32	Hs.943	interleukin 32	Homo sapiens interleukin 32 (IL32), transcript variant 1, mRNA [NM_001012631]	7.37209	down	7.45856	down	75.85977	579.07544	91.34475	600.76404	Detected	Detected	Detected	Detected
4620	A_33_P3254606	WDR62	Hs.116244	WD repeat domain 62	Homo sapiens WD repeat domain 62 (WDR62), transcript variant 1, mRNA [NM_001083961]	7.35212	down	20.23320	down	1323.76900	10077.61200	303.36768	5412.51860	Detected	Detected	Detected	Detected
4621	A_23_P254507	HOPX	Hs.619396	HOP homeobox	Homo sapiens HOP homeobox (HOPX), transcript variant 2, mRNA [NM_139211]	7.33903	down	4.90331	down	8.58035	65.20436	12.33005	53.31134	Compromised	Detected	Compromised	Detected
4622	A_24_P315444	LOC644422	Hs.647418	similar to arginine/serine-rich splicing factor 6	PREDICTED: Homo sapiens similar to arginine/serine-rich splicing factor 6 (LOC644422), miscRNA [XR_019449]	7.32011	down	6.44472	down	19.13226	145.01631	12.38194	70.36520	Detected	Detected	Compromised	Detected
4623	A_33_P3348239	FBN1	Hs.591133	fibrillin 1	Homo sapiens fibrillin 1 (FBN1), mRNA [NM_000138]	7.29662	down	7.22852	down	449.62375	3397.06470	228.90285	1459.03560	Detected	Detected	Detected	Detected
4624	A_32_P151800	FAM72D	Hs.535577	family with sequence similarity 72, member D	Homo sapiens family with sequence similarity 72, member D (FAM72D), mRNA [NM_207418]	7.29141	down	7.81327	down	99.80597	753.53080	78.22633	538.95370	Detected	Detected	Detected	Detected
4625	A_23_P303548		Hs.516978		Uncharacterized protein C20orf112 [Source:UniProtKB/Swiss-Prot;Acc:Q96MY1] [ENST00000375673]	7.28696	down	3.82088	down	2.39316	18.05723	5.46754	18.42136	Compromised	Detected	Compromised	Detected
4626	A_24_P166613	EPDR1	Hs.710629	ependymin related protein 1 (zebrafish)	Homo sapiens ependymin related protein 1 (zebrafish) (EPDR1), mRNA [NM_017549]	7.27352	down	7.41466	down	1212.02860	9128.30800	854.52750	5587.05400	Detected	Detected	Detected	Detected
4627	A_23_P103720	AGMAT	Hs.567583	agmatine ureohydrolase (agmatinase)	Homo sapiens agmatine ureohydrolase (agmatinase) (AGMAT), mRNA [NM_024758]	7.24898	down	7.00531	down	65.23018	489.61954	54.77769	338.37344	Detected	Detected	Detected	Detected
4628	A_33_P3312119	LOC100130967	Hs.32804	similar to Putative uncharacterized protein C6orf99	Putative uncharacterized protein C6orf99 [Source:UniProtKB/Swiss-Prot;Acc:Q4VX62] [ENST00000367072]	7.21510	down	2.92233	down	2.34195	17.49656	2.47695	6.38280	Compromised	Detected	Compromised	Compromised
4629	A_24_P191312	SLC1A4	Hs.654352	solute carrier family 1 (glutamate/neutral amino acid transporter), member 4	Homo sapiens solute carrier family 1 (glutamate/neutral amino acid transporter), member 4 (SLC1A4), transcript variant 1, mRNA [NM_003038]	7.20153	down	7.70999	down	27.29878	203.56401	19.12932	130.05249	Detected	Detected	Detected	Detected
4630	A_24_P510377		Hs.647667		Homo sapiens cDNA clone IMAGE:5268928 [BC031250]	7.18389	down	7.82237	down	26.63440	198.12343	21.52508	148.47331	Detected	Detected	Detected	Detected
4631	A_23_P208880	UHRF1	Hs.108106	ubiquitin-like with PHD and ring finger domains 1	Homo sapiens ubiquitin-like with PHD and ring finger domains 1 (UHRF1), transcript variant 2, mRNA [NM_013282]	7.17819	down	7.18468	down	543.58500	4040.31570	392.17184	2484.55930	Detected	Detected	Detected	Detected
4632	A_33_P3350634	KIF22	Hs.612151	kinesin family member 22	Homo sapiens kinesin family member 22 (KIF22), mRNA [NM_007317]	7.16857	down	7.95159	down	549.70230	4080.30710	545.88920	3827.57760	Detected	Detected	Detected	Detected
4633	A_33_P3556532	DNAH17	Hs.713207	dynein, axonemal, heavy chain 17	full-length cDNA clone CS0DJ002YI14 of T cells (Jurkat cell line) Cot 10-normalized of Homo sapiens (human) [CR620279]	7.16679	down	3.99481	down	2.94526	21.85653	2.96272	10.43644	Compromised	Detected	Compromised	Compromised
4634	A_23_P125265	KPNA2	Hs.594238	karyopherin alpha 2 (RAG cohort 1, importin alpha 1)	Homo sapiens karyopherin alpha 2 (RAG cohort 1, importin alpha 1) (KPNA2), mRNA [NM_002266]	7.16243	down	7.26291	down	13168.67100	97664.22000	11489.94900	73585.86000	Detected	Detected	Detected	Detected

4635	A_23_P389919	WHSC1	Hs.113876	Wolf-Hirschhorn syndrome candidate 1	Homo sapiens Wolf-Hirschhorn syndrome candidate 1 (WHSC1), transcript variant 1, mRNA [NM_133330]	7.14438	down	7.39252	down	281.71732	2084.06370	170.28557	1110.03270	Detected	Detected	Detected	Detected
4636	A_33_P3846177	B4GALNT1	Hs.591019	beta-1,4-N-acetyl-galactosaminyl transferase 1	Homo sapiens beta-1,4-N-acetyl-galactosaminyl transferase 1 (B4GALNT1), mRNA [NM_001478]	7.11685	down	9.29604	down	66.17393	487.64984	53.93387	442.10440	Detected	Detected	Detected	Detected
4637	A_33_P3309607				Shugoshin-like 2 (Shugoshin-2)(Sgo2)(Tripin) [Source:UniProtKB/Swiss-Prot;Acc:Q562F6] [ENST00000409203]	7.11526	down	3.00319	down	2.81874	20.76727	3.15542	8.35612	Compromised	Detected	Compromised	Compromised
4638	A_24_P38895	H2AFX	Hs.477879	H2A histone family, member X	Homo sapiens H2A histone family, member X (H2AFX), mRNA [NM_002105]	7.10123	down	7.81943	down	5255.39300	38643.08600	3028.78760	20883.78700	Detected	Detected	Detected	Detected
4639	A_23_P416395	STC2	Hs.233160	stanniocalcin 2	Homo sapiens stanniocalcin 2 (STC2), mRNA [NM_003714]	7.09909	down	7.20956	down	760.69970	5591.76900	383.56146	2438.42300	Detected	Detected	Detected	Detected
4640	A_23_P137484	L1TD1	Hs.685462	LINE-1 type transposase domain containing 1	Homo sapiens LINE-1 type transposase domain containing 1 (L1TD1), transcript variant 2, mRNA [NM_019079]	7.09807	down	6.72630	down	2.36338	17.37025	2.53584	15.04053	Compromised	Detected	Compromised	Detected
4641	A_23_P345707	C15orf42	Hs.441708	chromosome 15 open reading frame 42	Homo sapiens chromosome 15 open reading frame 42 (C15orf42), mRNA [NM_152259]	7.09366	down	6.21526	down	41.35231	303.74072	41.48024	227.33504	Detected	Detected	Detected	Detected
4642	A_33_P3280729	SHISA9	Hs.130661	shisa homolog 9 (Xenopus laevis)	Homo sapiens shisa homolog 9 (Xenopus laevis) (SHISA9), transcript variant 1, mRNA [NM_001145204]	7.08224	down	6.57391	down	4.85130	35.57634	3.31543	19.21895	Compromised	Detected	Compromised	Detected
4643	A_24_P99090	CKAP2	Hs.444028	cytoskeleton associated protein 2	Homo sapiens cytoskeleton associated protein 2 (CKAP2), transcript variant 1, mRNA [NM_018204]	7.06006	down	8.58591	down	35.90656	262.49136	17.67922	133.84901	Detected	Detected	Detected	Detected
4644	A_23_P141802	SERPINB7	Hs.138202	serpin peptidase inhibitor, clade B (ovalbumin), member 7	Homo sapiens serpin peptidase inhibitor, clade B (ovalbumin), member 7 (SERPINB7), transcript variant 2, mRNA [NM_001040147]	7.04484	down	7.08274	down	66.62235	485.98645	74.93073	467.97940	Detected	Detected	Detected	Detected
4645	A_23_P26037	FRMD5	Hs.578544	FERM domain containing 5	Homo sapiens FERM domain containing 5 (FRMD5), transcript variant 2, mRNA [NM_032892]	7.03087	down	5.10698	down	30.18117	219.72450	20.69545	93.19758	Detected	Detected	Detected	Detected
4646	A_23_P216429	ASPN	Hs.435655	asporin	Homo sapiens asporin (ASPN), mRNA [NM_017680]	7.01575	down	5.65270	down	6.12644	44.50567	12.62557	62.93216	Compromised	Detected	Detected	Detected
4647	A_33_P3227360				Homeobox protein DLX-1 [Source:UniProtKB/Swiss-Prot;Acc:P56177] [ENST00000409492]	6.99283	down	1.92949	down	2.47228	17.90130	7.70356	13.10687	Compromised	Detected	Compromised	Compromised
4648	A_24_P330303	FRMD6	Hs.434914	FERM domain containing 6	Homo sapiens FERM domain containing 6 (FRMD6), transcript variant 1, mRNA [NM_001042481]	6.96897	down	6.33512	down	2243.21830	16187.25400	1651.06080	9223.23800	Detected	Detected	Detected	Detected
4649	A_33_P3423755	NCRNA00200	Hs.721191	non-protein coding RNA 200	Homo sapiens non-protein coding RNA 200 (NCRNA00200), non-coding RNA [NR_015376]	6.95478	down	3.78349	down	2.68139	19.30973	3.14298	10.48575	Compromised	Detected	Compromised	Compromised
4650	A_24_P680657	CCDC73	Hs.706808	coiled-coil domain containing 73	Homo sapiens coiled-coil domain containing 73 (CCDC73), mRNA [NM_001008391]	6.94328	down	4.52524	down	2.81349	20.22758	3.15073	12.57240	Compromised	Detected	Compromised	Compromised
4651	A_33_P3380502	GPR85	Hs.152009	G protein-coupled receptor 85	Homo sapiens G protein-coupled receptor 85 (GPR85), transcript variant 1, mRNA [NM_001146265]	6.93154	down	1.99166	down	2.54855	18.29177	2.94568	5.17329	Compromised	Detected	Compromised	Compromised
4652	A_24_P41850	MASP1	Hs.89983	mannan-binding lectin serine peptidase 1 (C4/C2 activating component of Ra-reactive factor)	Homo sapiens mannan-binding lectin serine peptidase 1 (C4/C2 activating component of Ra-reactive factor) (MASP1), transcript variant 3, mRNA [NM_001031849]	6.92804	down	8.09283	down	49.45198	354.75385	39.38162	281.03452	Detected	Detected	Detected	Detected
4653	A_23_P49060	SPINT1	Hs.233950	serine peptidase inhibitor, Kunitz type 1	Homo sapiens serine peptidase inhibitor, Kunitz type 1 (SPINT1), transcript variant 1, mRNA [NM_181642]	6.90691	down	1.58971	down	2.74321	19.61894	2.91923	4.09215	Compromised	Detected	Compromised	Compromised
4654	A_33_P3404480	MPP4	Hs.63085	membrane protein, palmitoylated 4 (MAGUK p55 subfamily member 4)	Homo sapiens membrane protein, palmitoylated 4 (MAGUK p55 subfamily member 4) (MPP4), mRNA [NM_033066]	6.90163	down	7.00214	down	36.59691	261.53442	26.78828	165.40224	Detected	Detected	Detected	Detected
4655	A_33_P3306894	C11orf41	Hs.502266	chromosome 11 open reading frame 41	Homo sapiens chromosome 11 open reading frame 41 (C11orf41), mRNA [NM_012194]	6.88529	down	9.30900	down	26.13967	186.36116	14.15435	116.18719	Detected	Detected	Compromised	Detected
4656	A_33_P3399593	CCDC134	Hs.474991	coiled-coil domain containing 134	Homo sapiens coiled-coil domain containing 134 (CCDC134), mRNA [NM_024821]	6.86174	down	9.48781	down	16.88953	120.00094	14.09410	117.91490	Detected	Detected	Compromised	Detected
4657	A_23_P38732	CDH2	Hs.464829	cadherin 2, type 1, N-cadherin (neuronal)	Homo sapiens cadherin 2, type 1, N-cadherin (neuronal) (CDH2), mRNA [NM_001792]	6.85809	down	7.23005	down	213.39350	1515.36610	191.76831	1222.59730	Detected	Detected	Detected	Detected
4658	A_24_P158089	SERPINE1	Hs.414795	serpin peptidase inhibitor, clade E (nexin, plasminogen activator inhibitor type 1), member 1	Homo sapiens serpin peptidase inhibitor, clade E (nexin, plasminogen activator inhibitor type 1), member 1 (SERPINE1), transcript variant 1, mRNA [NM_000602]	6.85678	down	6.87765	down	3734.19600	26512.47300	3013.17300	18273.82000	Detected	Detected	Detected	Detected
4659	A_24_P349965	TCF19	Hs.584807	transcription factor 19	Homo sapiens transcription factor 19 (TCF19), transcript variant 1, mRNA [NM_007109]	6.84845	down	5.67077	down	104.10759	738.25775	50.87435	254.39395	Detected	Detected	Detected	Detected
4660	A_33_P3267296	FKBP11	Hs.655103	FK506 binding protein 11, 19 kDa	Homo sapiens FK506 binding protein 11, 19 kDa (FKBP11), transcript variant 1, mRNA [NM_016594]	6.84568	down	6.44818	down	2607.15200	18480.60200	2472.24100	14057.02900	Detected	Detected	Detected	Detected
4661	A_33_P3230259	NCAPH	Hs.308045	non-SMC condensin I complex, subunit H	Homo sapiens non-SMC condensin I complex, subunit H (NCAPH), mRNA [NM_015341]	6.83272	down	32.82832	down	24.60024	174.04689	3.94879	114.30852	Detected	Detected	Compromised	Detected
4662	A_24_P53241	MYOCD	Hs.567641	myocardin	Homo sapiens myocardin (MYOCD), transcript variant 2, mRNA [NM_153604]	6.82716	down	1.18478	down	2.37836	16.81319	2.52439	2.63730	Compromised	Detected	Compromised	Compromised

4663	A_24_P149124	C5orf13	Hs.36053	chromosome 5 open reading frame 13	Homo sapiens chromosome 5 open reading frame 13 (C5orf13), transcript variant 1, mRNA [NM_004772]	6.82491	down	6.92562	down	3553.91550	25115.22900	2497.92970	15254.72900	Detected	Detected	Detected	Detected
4664	A_23_P363174	HIST1H2AL	Hs.233568	histone cluster 1, H2al	Homo sapiens histone cluster 1, H2al (HIST1H2AL), mRNA [NM_003511]	6.81042	down	13.66239	down	7.46676	52.65493	3.13748	37.79842	Compromised	Detected	Compromised	Detected
4665	A_33_P3415240	LOC730091	Hs.659905	hypothetical protein LOC730091	Homo sapiens cDNA FLJ31839 fis, clone NT2RP7000086 [AK056401]	6.81001	down	5.76382	down	3.03202	21.38025	3.53777	17.98065	Compromised	Detected	Compromised	Detected
4666	A_33_P3404989	HIST1H3H	Hs.591778	histone cluster 1, H3h	Homo sapiens histone cluster 1, H3h (HIST1H3H), mRNA [NM_003536]	6.80866	down	3.94233	down	5.74341	40.49154	6.64482	23.09946	Compromised	Detected	Compromised	Detected
4667	A_33_P3333238	HOXD9	Hs.236646	homeobox D9	Homo sapiens homeobox D9 (HOXD9), mRNA [NM_014213]	6.80310	down	9.28005	down	8.14635	57.38559	3.47431	28.43046	Compromised	Detected	Compromised	Detected
4668	A_33_P3278362	ANKRD2	Hs.73708	ankyrin repeat domain 2 (stretch responsive muscle)	Homo sapiens ankyrin repeat domain 2 (stretch responsive muscle) (ANKRD2), transcript variant 1, mRNA [NM_020349]	6.78587	down	7.13792	down	53.20981	373.87830	53.70923	338.05417	Detected	Detected	Detected	Detected
4669	A_33_P3247022	CCNE2	Hs.567387	cyclin E2	Homo sapiens cyclin E2 (CCNE2), mRNA [NM_057749]	6.77906	down	6.67016	down	73.33092	514.74200	45.47872	267.49158	Detected	Detected	Detected	Detected
4670	A_33_P3238305		Hs.222802		TAK1-like protein [Source:UniProtKB/Swiss-Prot;Acc:P57077] [ENST00000399926]	6.76676	down	7.55498	down	16.61694	116.43003	22.05594	146.93471	Detected	Detected	Detected	Detected
4671	A_23_P94338	ENPP2	Hs.190977	ectonucleotide pyrophosphatase/phosphodiesterase 2	Homo sapiens ectonucleotide pyrophosphatase/phosphodiesterase 2 (ENPP2), transcript variant 1, mRNA [NM_006209]	6.76665	down	7.40220	down	819.57050	5742.39650	646.95416	4222.79500	Detected	Detected	Detected	Detected
4672	A_33_P3275500	LOC400743	Hs.522935	FLJ45933 protein	Homo sapiens cDNA FLJ45933 fis, clone PLACE7003639. [AK127830]	6.74668	down	8.70307	down	14.36889	100.37981	9.80482	75.24503	Detected	Detected	Compromised	Detected
4673	A_24_P85557	GPR37	Hs.719904	G protein-coupled receptor 37 (endothelin receptor type B-like)	Homo sapiens G protein-coupled receptor 37 (endothelin receptor type B-like) (GPR37), mRNA [NM_005302]	6.71799	down	1.18178	down	2.45177	17.05505	2.63798	2.74898	Compromised	Detected	Compromised	Compromised
4674	A_33_P3579336	LOC285000	Hs.295012	hypothetical protein LOC285000	Homo sapiens hypothetical protein LOC285000, mRNA (cDNA clone IMAGE:3925223), partial cds [BC038566]	6.71730	down	1.18576	down	3.66306	25.47837	2.87368	3.00469	Compromised	Detected	Compromised	Compromised
4675	A_33_P3314574	hCG_2045048	Hs.617162	hCG2045048	PREDICTED: Homo sapiens hCG2045048 (LOC644058), mRNA [XM_001724795]	6.71338	down	4.95306	down	12.11299	84.20258	14.58711	63.71010	Compromised	Detected	Compromised	Detected
4676	A_32_P87697	HLA-DRA	Hs.520048	major histocompatibility complex, class II, DR alpha	Homo sapiens major histocompatibility complex, class II, DR alpha (HLA-DRA), mRNA [NM_019111]	6.71135	down	3.64946	down	2.51772	17.49646	5.39050	17.34696	Compromised	Compromised	Compromised	Detected
4677	A_33_P3342175	LOC100130580	Hs.662160	hypothetical protein LOC100130580	Homo sapiens cDNA FLJ44644 fis, clone BRACE2033128. [AK126607]	6.70626	down	3.79311	down	2.40093	16.67222	2.54488	8.51194	Compromised	Detected	Compromised	Compromised
4678	A_23_P25121	FKBP11	Hs.655103	FK506 binding protein 11, 19 kDa	Homo sapiens FK506 binding protein 11, 19 kDa (FKBP11), transcript variant 1, mRNA [NM_016594]	6.70528	down	6.83608	down	2337.28120	16227.85900	2160.92260	13026.02500	Detected	Detected	Detected	Detected
4679	A_23_P132644	NCEH1	Hs.444099	neutral cholesterol ester hydrolase 1	Homo sapiens neutral cholesterol ester hydrolase 1 (NCEH1), transcript variant 2, mRNA [NM_020792]	6.69268	down	6.59590	down	1132.31040	7846.91100	800.06177	4653.32030	Detected	Detected	Detected	Detected
4680	A_23_P154050	PFTK2	Hs.348711	PFTAIRE protein kinase 2	Homo sapiens PFTAIRE protein kinase 2 (PFTK2), mRNA [NM_139158]	6.64088	down	17.48687	down	9.36029	64.36475	3.20096	49.35809	Compromised	Detected	Compromised	Detected
4681	A_23_P111947	CDH17	Hs.591853	cadherin 17, LI cadherin (liver-intestine)	Homo sapiens cadherin 17, LI cadherin (liver-intestine) (CDH17), transcript variant 1, mRNA [NM_004063]	6.63649	down	1.30328	down	2.76736	19.01676	2.73337	3.14125	Compromised	Not Detected	Compromised	Compromised
4682	A_23_P27013	HOXB9	Hs.463350	homeobox B9	Homo sapiens homeobox B9 (HOXB9), mRNA [NM_024017]	6.62605	down	30.84917	down	48.61058	333.51770	12.12613	329.86115	Detected	Detected	Compromised	Detected
4683	A_32_P4595	SGCD	Hs.387207	sarcoglycan, delta (35kDa dystrophin-associated glycoprotein)	Homo sapiens sarcoglycan, delta (35kDa dystrophin-associated glycoprotein) (SGCD), transcript variant 1, mRNA [NM_000337]	6.62601	down	7.35240	down	344.43780	2363.17630	177.88036	1153.24710	Detected	Detected	Detected	Detected
4684	A_23_P333228	MARCH4	Hs.170388	membrane-associated ring finger (C3HC4) 4	Homo sapiens membrane-associated ring finger (C3HC4) 4 (MARCH4), mRNA [NM_020814]	6.59814	down	6.70920	down	263.17224	1798.02190	121.30525	717.65485	Detected	Detected	Detected	Detected
4685	A_23_P323102	PCDHA9	Hs.199343	protocadherin alpha 9	Homo sapiens protocadherin alpha 9 (PCDHA9), transcript variant 2, mRNA [NM_014005]	6.58658	down	1.06788	down	2.38889	16.29255	2.78823	2.62553	Compromised	Detected	Compromised	Compromised
4686	A_24_P402438	TGFB2	Hs.133379	transforming growth factor, beta 2	Homo sapiens transforming growth factor, beta 2 (TGFB2), transcript variant 2, mRNA [NM_003238]	6.58090	down	5.85699	down	4.42885	30.17932	3.12999	16.16530	Compromised	Detected	Compromised	Detected
4687	A_23_P135271	B4GALT1	Hs.272011	UDP-Gal:betaGlcNAc beta 1,4-galactosyltransferase, polypeptide 1	Homo sapiens UDP-Gal:betaGlcNAc beta 1,4- galactosyltransferase, polypeptide 1 (B4GALT1), mRNA [NM_001497]	6.57904	down	7.85385	down	119.75061	815.78046	107.91601	747.36670	Detected	Detected	Detected	Detected
4688	A_33_P3320493				Retinoic acid-induced protein 1 [Source:UniProtKB/Swiss-Prot;Acc:Q7Z5J4] [ENST00000395776]	6.57799	down	7.30047	down	40.42079	275.31573	35.76899	230.26225	Detected	Detected	Detected	Detected
4689	A_23_P315836	BAIAP2	Hs.128316	BAI1-associated protein 2	Homo sapiens BAI1-associated protein 2 (BAIAP2), transcript variant 2, mRNA [NM_017451]	6.57558	down	13.38980	down	13.31569	90.66317	7.23191	85.38717	Detected	Detected	Compromised	Detected
4690	A_23_P379034	BAIAP2L2	Hs.474822	BAI1-associated protein 2-like 2	Homo sapiens BAI1-associated protein 2-like 2 (BAIAP2L2), mRNA [NM_025045]	6.54253	down	6.68389	down	631.55510	4278.48800	472.11224	2782.53200	Detected	Detected	Detected	Detected
4691	A_23_P113777	ITGBL1	Hs.696554	integrin, beta-like 1 (with EGF-like repeat domains)	Homo sapiens integrin, beta-like 1 (with EGF-like repeat domains) (ITGBL1), mRNA [NM_004791]	6.53246	down	6.56886	down	965.24610	6529.00930	656.64970	3803.54860	Detected	Detected	Detected	Detected
4692	A_23_P152136	GINS3	Hs.47125	GINS complex subunit 3 (Psf3 homolog)	Homo sapiens GINS complex subunit 3 (Psf3 homolog) (GINS3), transcript variant 2, mRNA [NM_022770]	6.52575	down	6.62575	down	596.59766	4031.30100	504.73474	2948.92680	Detected	Detected	Detected	Detected
4693	A_33_P3346533					6.52480	down	6.35305	down	410.29510	2772.01980	435.57750	2440.13090	Detected	Detected	Detected	Detected

4694	A_24_P913227				Cell division cycle protein 23 homolog (Anaphase-promoting complex subunit 8)(APC8)(Cyclosome subunit 8) [Source:UniProtKB/Swiss-Prot;Acc:Q9UJX2] [ENST00000394884]	6.49663	down	6.60895	down	17.01369	114.45108	9.85829	57.45121	Detected	Detected	Compromised	Detected
4695	A_23_P84821	MRPL1	Hs.532019	mitochondrial ribosomal protein L1	Homo sapiens mitochondrial ribosomal protein L1 (MRPL1), nuclear gene encoding mitochondrial protein, mRNA [NM_020236]	6.49642	down	7.72029	down	94.57562	636.18896	74.86021	509.62450	Detected	Detected	Detected	Detected
4696	A_33_P3880302	EPHB2	Hs.523329	EPH receptor B2	Homo sapiens EPH receptor B2 (EPHB2), transcript variant 2, mRNA [NM_004442]	6.48655	down	6.93127	down	39.10874	262.67606	38.19669	233.45560	Detected	Detected	Detected	Detected
4697	A_23_P404481	S1PR1	Hs.154210	sphingosine-1-phosphate receptor 1	Homo sapiens sphingosine-1-phosphate receptor 1 (S1PR1), mRNA [NM_001400]	6.48174	down	6.43488	down	87.14776	584.89886	85.48776	485.07590	Detected	Detected	Detected	Detected
4698	A_33_P3511265	POSTN	Hs.136348	periostin, osteoblast specific factor	Homo sapiens periostin, osteoblast specific factor (POSTN), transcript variant 1, mRNA [NM_006475]	6.47247	down	5.67182	down	79.03504	529.69110	86.29932	431.61410	Detected	Detected	Detected	Detected
4699	A_24_P169634	MBL1P1	Hs.102310	mannose-binding lectin (protein A) 1, pseudogene 1	Homo sapiens mannose-binding lectin (protein A) 1, pseudogene 1 (MBL1P1), non-coding RNA [NR_002724]	6.46852	down	4.97035	down	3.97766	26.64194	8.55314	37.48676	Compromised	Detected	Compromised	Detected
4700	A_23_P411353	ZCCHC5	Hs.134873	zinc finger, CCHC domain containing 5	Homo sapiens zinc finger, CCHC domain containing 5 (ZCCHC5), mRNA [NM_152694]	6.46330	down	1.33284	down	3.32482	22.25131	14.20888	16.69952	Compromised	Detected	Detected	Detected
4701	A_33_P3379215	LOC402198		similar to hCG1641913	PREDICTED: Homo sapiens similar to hCG1641913 (LOC402198), mRNA [XM_001716525]	6.46290	down	1.18503	down	2.38070	15.93180	2.53013	2.64385	Compromised	Detected	Compromised	Compromised
4702	A_23_P84860	FAM107A	Hs.506357	family with sequence similarity 107, member A	Homo sapiens family with sequence similarity 107, member A (FAM107A), transcript variant 1, mRNA [NM_007177]	6.45651	down	8.28226	down	28.03433	187.42232	20.42995	149.20438	Detected	Detected	Detected	Detected
4703	A_33_P3337154					6.45579	down	3.51677	down	7.35498	49.16594	11.72220	36.35116	Compromised	Detected	Compromised	Detected
4704	A_33_P3217819	CCNE2	Hs.567387	cyclin E2	Homo sapiens cyclin E2 (CCNE2), mRNA [NM_057749]	6.45379	down	9.03854	down	7.36852	49.24119	3.61515	28.81312	Compromised	Detected	Compromised	Detected
4705	A_33_P3418209	ITGBL1	Hs.696554	integrin, beta-like 1 (with EGF-like repeat domains)	Homo sapiens integrin, beta-like 1 (with EGF-like repeat domains) (ITGBL1), mRNA [NM_004791]	6.44727	down	6.12141	down	1171.59300	7821.42140	952.68840	5142.42720	Detected	Detected	Detected	Detected
4706	A_24_P111996	HFE	Hs.233325	hemochromatosis	Homo sapiens hemochromatosis (HFE), transcript variant 1, mRNA [NM_000410]	6.43916	down	6.17508	down	14.01389	93.43751	12.81305	69.76872	Compromised	Detected	Compromised	Detected
4707	A_33_P3271652	HHIPL1	Hs.288522	HHIP-like 1	Homo sapiens HHIP-like 1 (HHIPL1), transcript variant 2, mRNA [NM_032425]	6.43487	down	5.92764	down	6.16106	41.05143	6.16175	32.20711	Compromised	Detected	Compromised	Detected
4708	A_23_P307502	C9orf53	Hs.721053	chromosome 9 open reading frame 53	Homo sapiens chromosome 9 open reading frame 53 (C9orf53), non-coding RNA [NR_024274]	6.42634	down	4.88934	down	80.93403	538.55200	33.38379	143.92998	Detected	Detected	Detected	Detected
4709	A_33_P3258392	EDN1	Hs.511899	endothelin 1	Homo sapiens endothelin 1 (EDN1), mRNA [NM_001955]	6.41764	down	6.37495	down	33.26720	221.06725	39.43983	221.70598	Detected	Detected	Detected	Detected
4710	A_33_P3423445	ZNF730	Hs.720743	zinc finger protein 730	Putative zinc finger protein 730 [Source:UniProtKB/Swiss-Prot;Acc:Q6ZMV8] [ENST00000327867]	6.41354	down	3.04603	down	4.77951	31.74057	5.36864	14.41995	Compromised	Detected	Compromised	Compromised
4711	A_23_P114314	CDX4	Hs.553488	caudal type homeobox 4	Homo sapiens caudal type homeobox 4 (CDX4), mRNA [NM_005193]	6.40196	down	1.29688	down	3.31210	21.95581	3.10158	3.54689	Compromised	Detected	Compromised	Compromised
4712	A_33_P3248519	SMC4	Hs.58992	structural maintenance of chromosomes 4	Homo sapiens structural maintenance of chromosomes 4 (SMC4), transcript variant 1, mRNA [NM_005496]	6.37328	down	5.67414	down	757.69760	5000.25000	260.09982	1301.38390	Detected	Detected	Detected	Detected
4713	A_23_P61778	NXN	Hs.527989	nucleoredoxin	Homo sapiens nucleoredoxin (NXN), mRNA [NM_022463]	6.37239	down	7.84752	down	87.37985	576.56256	73.49689	508.58865	Detected	Detected	Detected	Detected
4714	A_23_P75071	KIF20B	Hs.240	kinesin family member 20B	Homo sapiens kinesin family member 20B (KIF20B), mRNA [NM_016195]	6.35264	down	6.23597	down	83.81862	551.35046	54.67972	300.67386	Detected	Detected	Detected	Detected
4715	A_33_P3411975	ABI3BP	Hs.477015	ABI family, member 3 (NESH) binding protein	Homo sapiens ABI family, member 3 (NESH) binding protein (ABI3BP), mRNA [NM_015429]	6.34443	down	5.73243	down	8.09117	53.15412	10.41645	52.65311	Compromised	Detected	Compromised	Detected
4716	A_23_P80008	MYLK2	Hs.86092	myosin light chain kinase 2	Homo sapiens myosin light chain kinase 2 (MYLK2), mRNA [NM_033118]	6.33257	down	8.81233	down	34.62186	227.01952	25.14067	195.35875	Detected	Detected	Detected	Detected
4717	A_23_P102058	MATN3	Hs.656199	matrilin 3	Homo sapiens matrilin 3 (MATN3), mRNA [NM_002381]	6.33141	down	5.53635	down	15.85547	103.94723	5.30359	25.89163	Detected	Detected	Compromised	Detected
4718	A_23_P5392	TP53I3	Hs.50649	tumor protein p53 inducible protein 3	Homo sapiens tumor protein p53 inducible protein 3 (TP53I3), transcript variant 1, mRNA [NM_004881]	6.30961	down	6.43718	down	1282.72660	8380.50100	1145.63820	6502.92040	Detected	Detected	Detected	Detected
4719	A_32_P204722					6.29440	down	6.17052	down	40.74204	265.54025	36.00147	195.88770	Detected	Detected	Detected	Detected
4720	A_23_P131935	FERMT1	Hs.472054	fermitin family homolog 1 (Drosophila)	Homo sapiens fermitin family homolog 1 (Drosophila) (FERMT1), mRNA [NM_017671]	6.27021	down	6.41588	down	101.83689	661.18050	72.09717	407.88727	Detected	Detected	Detected	Detected
4721	A_24_P12626	CAV1	Hs.74034	caveolin 1, caveolae protein, 22kDa	Homo sapiens caveolin 1, caveolae protein, 22kDa (CAV1), mRNA [NM_001753]	6.26745	down	5.91353	down	4745.63700	30797.69700	4460.15200	23257.43800	Detected	Detected	Detected	Detected
4722	A_23_P70307	SMOC2	Hs.487200	SPARC related modular calcium binding 2	Homo sapiens SPARC related modular calcium binding 2 (SMOC2), transcript variant 1, mRNA [NM_022138]	6.25438	down	1.57505	down	5.30047	34.32664	2.85926	3.97113	Compromised	Detected	Compromised	Compromised
4723	A_24_P91991	NAT8L	Hs.318529	N-acetyltransferase 8-like (GCN5-related, putative)	Homo sapiens N-acetyltransferase 8-like (GCN5-related, putative) (NAT8L), mRNA [NM_178557]	6.24982	down	16.65014	down	10.40015	67.30389	2.79228	40.99614	Compromised	Detected	Compromised	Detected
4724	A_33_P3403075	PRR11	Hs.631750	proline rich 11	Homo sapiens proline rich 11 (PRR11), mRNA [NM_018304]	6.24341	down	8.06314	down	55.24514	357.14868	48.84374	347.27890	Detected	Detected	Detected	Detected
4725	A_33_P3362503	ISPD	Hs.636502	isoprenoid synthase domain containing	Homo sapiens isoprenoid synthase domain containing (ISPD), transcript variant 1, mRNA [NM_001101426]	6.23562	down	7.03611	down	4.70097	30.35291	4.87257	30.23124	Compromised	Detected	Compromised	Detected

4726	A_24_P381199	TRIM6	Hs.125300	tripartite motif-containing 6	Homo sapiens tripartite motif-containing 6 (TRIM6), transcript variant 1, mRNA [NM_001003818]	6.23017	down	5.24199	down	90.76167	585.51120	57.89333	267.60248	Detected	Detected	Detected	Detected
4727	A_24_P931443	GPR68	Hs.8882	G protein-coupled receptor 68	Homo sapiens G protein-coupled receptor 68 (GPR68), mRNA [NM_003485]	6.22946	down	5.97594	down	5417.59100	34945.36300	4146.32960	21849.20000	Detected	Detected	Detected	Detected
4728	A_33_P3365142	GAD1	Hs.420036	glutamate decarboxylase 1 (brain, 67kDa)	Homo sapiens glutamate decarboxylase 1 (brain, 67kDa) (GAD1), transcript variant GAD25, mRNA [NM_013445]	6.22926	down	5.26566	down	20.15288	129.98892	22.21679	103.15723	Detected	Detected	Detected	Detected
4729	A_33_P3629678	COL5A1	Hs.210283	collagen, type V, alpha 1	Homo sapiens collagen, type V, alpha 1 (COL5A1), mRNA [NM_000093]	6.22120	down	6.42459	down	5086.14840	32763.95000	4598.97950	26053.86000	Detected	Detected	Detected	Detected
4730	A_23_P111995	LOXL2	Hs.626637	lysyl oxidase-like 2	Homo sapiens lysyl oxidase-like 2 (LOXL2), mRNA [NM_002318]	6.20669	down	6.68828	down	9818.66100	63102.42600	6884.45360	40602.16400	Detected	Detected	Detected	Detected
4731	A_33_P3285260				ES1 protein homolog, mitochondrial Precursor (Protein KNP-I)(Protein GT335) [Source:UniProtKB/Swiss-Prot;Acc:P30042] [ENST00000389690]	6.20157	down	7.06232	down	310.96844	1996.87410	265.62155	1654.15450	Detected	Detected	Detected	Detected
4732	A_24_P146211	HIST1H2BD	Hs.591797	histone cluster 1, H2bd	Homo sapiens histone cluster 1, H2bd (HIST1H2BD), transcript variant 1, mRNA [NM_021063]	6.17541	down	4.28253	down	107.42239	686.89984	140.44548	530.36310	Detected	Detected	Detected	Detected
4733	A_33_P3244249				SH3 domain and tetratricopeptide repeats-containing protein 2 [Source:UniProtKB/Swiss-Prot;Acc:Q8TF17] [ENST00000394358]	6.17405	down	8.33459	down	6.70742	42.88037	5.22224	38.38015	Compromised	Detected	Compromised	Detected
4734	A_23_P252740	DSCC1	Hs.315167	defective in sister chromatid cohesion 1 homolog (S. cerevisiae)	Homo sapiens defective in sister chromatid cohesion 1 homolog (S. cerevisiae) (DSCC1), mRNA [NM_024094]	6.16602	down	6.28356	down	48.38689	308.93390	35.22436	195.17061	Detected	Detected	Detected	Detected
4735	A_33_P3415551	GPAT2	Hs.348629	glycerol-3-phosphate acyltransferase 2, mitochondrial	Homo sapiens glycerol-3-phosphate acyltransferase 2, mitochondrial (GPAT2), nuclear gene encoding mitochondrial protein, mRNA [NM_207328]	6.16546	down	6.58190	down	348.43805	2224.45750	329.21090	1910.69310	Detected	Detected	Detected	Detected
4736	A_24_P833129	ISPD	Hs.636502	isoprenoid synthase domain containing	Homo sapiens isoprenoid synthase domain containing (ISPD), transcript variant 1, mRNA [NM_001101426]	6.15976	down	3.01159	down	5.39254	34.39461	4.28294	11.37374	Compromised	Detected	Compromised	Compromised
4737	A_24_P339429	KCNJ12	Hs.200629	potassium inwardly-rectifying channel, subfamily J, member 12	Homo sapiens potassium inwardly-rectifying channel, subfamily J, member 12 (KCNJ12), mRNA [NM_021012]	6.14777	down	9.17641	down	20.63278	131.34341	9.33069	75.50088	Detected	Detected	Compromised	Detected
4738	A_23_P23669	PALMD	Hs.483993	palmdelphin	Homo sapiens palmdelphin (PALMD), mRNA [NM_017734]	6.13152	down	5.80493	down	26.10146	165.71666	21.52662	110.18900	Detected	Detected	Detected	Detected
4739	A_23_P118254	FOXF1	Hs.155591	forkhead box F1	Homo sapiens forkhead box F1 (FOXF1), mRNA [NM_001451]	6.12883	down	6.76577	down	79.43672	504.11752	46.29013	276.16678	Detected	Detected	Detected	Detected
4740	A_23_P101054	KRT34	Hs.296942	keratin 34	Homo sapiens keratin 34 (KRT34), mRNA [NM_021013]	6.10260	down	6.35309	down	149.22154	942.93024	145.92633	817.49384	Detected	Detected	Detected	Detected
4741	A_33_P3250438				UPF0608 protein C19orf42 Precursor [Source:UniProtKB/Swiss-Prot;Acc:Q9BQ49] [ENST00000358726]	6.07843	down	5.32833	down	152.76082	961.47170	159.04251	747.25653	Detected	Detected	Detected	Detected
4742	A_33_P3259092	FKTN	Hs.55777	fukutin	Homo sapiens fukutin (FKTN), transcript variant 1, mRNA [NM_001079802]	6.07569	down	2.92025	down	3.36455	21.16682	6.40561	16.49477	Compromised	Detected	Compromised	Compromised
4743	A_33_P3262635	CECR1	Hs.170310	cat eye syndrome chromosome region, candidate 1	Homo sapiens cat eye syndrome chromosome region, candidate 1 (CECR1), transcript variant 2, mRNA [NM_177405]	6.06742	down	6.22061	down	21.22471	133.34567	21.67738	118.90632	Detected	Detected	Detected	Detected
4744	A_24_P394075	NT5C1B	Hs.120319	5'-nucleotidase, cytosolic IB	Homo sapiens 5'-nucleotidase, cytosolic IB (NT5C1B), transcript variant 1, mRNA [NM_001002006]	6.06729	down	2.79241	down	2.71338	17.04660	2.72467	6.70899	Compromised	Detected	Compromised	Compromised
4745	A_23_P160537	C1orf135	Hs.149305	chromosome 1 open reading frame 135	Homo sapiens chromosome 1 open reading frame 135 (C1orf135), mRNA [NM_024037]	6.06211	down	6.05796	down	145.76831	914.99850	108.06578	577.27110	Detected	Detected	Detected	Detected
4746	A_23_P146134	DUSP26	Hs.8719	dual specificity phosphatase 26 (putative)	Homo sapiens dual specificity phosphatase 26 (putative) (DUSP26), mRNA [NM_024025]	6.05571	down	12.48702	down	14.09547	88.38480	9.97060	109.78570	Detected	Detected	Compromised	Detected
4747	A_23_P1819	OR8B8	Hs.351822	olfactory receptor, family 8, subfamily B, member 8	Homo sapiens olfactory receptor, family 8, subfamily B, member 8 (OR8B8), mRNA [NM_012378]	6.03055	down	8.06012	down	30.19206	188.53096	24.17651	171.83067	Detected	Detected	Detected	Detected
4748	A_23_P21976	CSPG4	Hs.513044	chondroitin sulfate proteoglycan 4	Homo sapiens chondroitin sulfate proteoglycan 4 (CSPG4), mRNA [NM_001897]	6.02973	down	6.44415	down	923.66376	5766.92870	815.41490	4633.50730	Detected	Detected	Detected	Detected
4749	A_33_P3300262		Hs.137415		Vitrin Precursor [Source:UniProtKB/Swiss-Prot;Acc:Q6UXI7] [ENST00000457137]	6.02854	down	7.03476	down	16.60020	103.62351	10.76577	66.78207	Detected	Detected	Compromised	Detected
4750	A_23_P71727	CKS2	Hs.83758	CDC28 protein kinase regulatory subunit 2	Homo sapiens CDC28 protein kinase regulatory subunit 2 (CKS2), mRNA [NM_001827]	6.00534	down	5.45346	down	5413.06600	33660.00400	4014.77030	19306.26000	Detected	Detected	Detected	Detected
4751	A_23_P141706	TCEB3B	Hs.375035	transcription elongation factor B polypeptide 3B (elongin A2)	Homo sapiens transcription elongation factor B polypeptide 3B (elongin A2) (TCEB3B), mRNA [NM_016427]	6.00044	down	1.18472	down	3.09817	19.24957	2.87890	3.00752	Compromised	Detected	Compromised	Compromised
4752	A_23_P57709	PCOLCE2	Hs.8944	procollagen C-endopeptidase enhancer 2	Homo sapiens procollagen C-endopeptidase enhancer 2 (PCOLCE2), mRNA [NM_013363]	5.99838	down	6.19582	down	1501.87540	9328.27200	1469.41050	8028.00150	Detected	Detected	Detected	Detected
4753	A_32_P212897	LOC643406	Hs.431161	hypothetical LOC643406	Homo sapiens hypothetical LOC643406 (LOC643406), non-coding RNA [NR_029405]	5.99736	down	9.68219	down	8.04846	49.98111	4.84814	41.39180	Compromised	Detected	Compromised	Detected
4754	A_23_P379945	CCDC88C	Hs.525536	coiled-coil domain containing 88C	Homo sapiens coiled-coil domain containing 88C (CCDC88C), mRNA [NM_001080414]	5.99674	down	8.00641	down	3.08286	19.14265	2.72660	19.24976	Compromised	Detected	Compromised	Detected

4755	A_33_P3392077	TP53I3	Hs.50649	tumor protein p53 inducible protein 3	Homo sapiens tumor protein p53 inducible protein 3 (TP53I3), transcript variant 1, mRNA [NM_004881]	5.98604	down	6.17101	down	2379.76640	14750.51200	2392.46040	13018.67400	Detected	Detected	Detected	Detected
4756	A_23_P15844	BRIP1	Hs.532799	BRCA1 interacting protein C-terminal helicase 1	Homo sapiens BRCA1 interacting protein C-terminal helicase 1 (BRIP1), mRNA [NM_032043]	5.98436	down	5.75183	down	26.35240	163.29420	12.15611	61.65468	Detected	Detected	Compromised	Detected
4757	A_33_P3222630	FBXO43	Hs.339577	F-box protein 43	Homo sapiens F-box protein 43 (FBXO43), transcript variant 1, mRNA [NM_001077528]	5.98191	down	4.62938	down	24.23979	150.14178	24.66990	100.70612	Detected	Detected	Detected	Detected
4758	A_33_P3344282				HCLS1-binding protein 3 (HS1-binding protein 3)(HSP1BP-3) [Source:UniProtKB/Swiss-Prot;Acc:Q53T59] [ENST00000406618]	5.97276	down	5.97041	down	41.84630	258.80066	30.82096	162.26150	Detected	Detected	Detected	Detected
4759	A_33_P3402565	DSP	Hs.519873	desmoplakin	Homo sapiens desmoplakin (DSP), transcript variant 1, mRNA [NM_004415]	5.97262	down	5.94804	down	1600.90330	9900.63900	1302.86270	6833.42330	Detected	Detected	Detected	Detected
4760	A_33_P3268472	CTSC	Hs.128065	cathepsin C	Homo sapiens cathepsin C (CTSC), transcript variant 3, mRNA [NM_001114173]	5.95346	down	5.44243	down	987.92615	6090.13670	969.57860	4653.08700	Detected	Detected	Detected	Detected
4761	A_23_P115482	UBE2T	Hs.5199	ubiquitin-conjugating enzyme E2T (putative)	Homo sapiens ubiquitin-conjugating enzyme E2T (putative) (UBE2T), mRNA [NM_014176]	5.94784	down	6.09248	down	1200.05410	7390.82900	825.05634	4432.43850	Detected	Detected	Detected	Detected
4762	A_33_P3220872	LOC1720	Hs.169235	dihydrofolate reductase pseudogene	Human dihydrofolate reductase pseudogene (psi-hd1). [J00146]	5.93584	down	1.89521	down	4.96579	30.52133	2.96354	4.95260	Compromised	Detected	Compromised	Compromised
4763	A_33_P3238856	FARP1	Hs.403917	FERM, RhoGEF (ARHGEF) and pleckstrin domain protein 1 (chondrocyte-derived)	Homo sapiens FERM, RhoGEF (ARHGEF) and pleckstrin domain protein 1 (chondrocyte-derived) (FARP1), transcript variant 2, mRNA [NM_001001715]	5.92383	down	8.24786	down	12.34271	75.70883	9.31471	67.74480	Compromised	Detected	Compromised	Detected
4764	A_23_P303671	ECM2	Hs.117060	extracellular matrix protein 2, female organ and adipocyte specific	Homo sapiens extracellular matrix protein 2, female organ and adipocyte specific (ECM2), mRNA [NM_001393]	5.91468	down	6.38802	down	28.01552	171.57869	19.85151	111.82150	Detected	Detected	Detected	Detected
4765	A_23_P213228	PHOX2B	Hs.87202	paired-like homeobox 2b	Homo sapiens paired-like homeobox 2b (PHOX2B), mRNA [NM_003924]	5.90662	down	1.18501	down	3.41934	20.91289	2.83012	2.95728	Compromised	Detected	Compromised	Compromised
4766	A_33_P3326989	RAGE	Hs.104119	renal tumor antigen	Homo sapiens renal tumor antigen (RAGE), mRNA [NM_014226]	5.89573	down	6.58951	down	46.27196	282.48074	43.58052	253.22765	Detected	Detected	Detected	Detected
4767	A_23_P356330	PPPDE2	Hs.570455	PPPDE peptidase domain containing 2	Homo sapiens PPPDE peptidase domain containing 2 (PPPDE2), mRNA [NM_015704]	5.89187	down	6.88832	down	153.68434	937.59546	83.17181	505.19037	Detected	Detected	Detected	Detected
4768	A_33_P3351851	HIST1H4L	Hs.533295	histone cluster 1, H4l	Homo sapiens histone cluster 1, H4l (HIST1H4L), mRNA [NM_003546]	5.88893	down	5.86637	down	6.44134	39.27768	5.11999	26.48525	Compromised	Detected	Compromised	Detected
4769	A_33_P3238815				FERM domain-containing protein 5 [Source:UniProtKB/Swiss-Prot;Acc:Q7Z6J6] [ENST00000402883]	5.87359	down	5.99213	down	14.56000	88.55196	10.16643	53.71743	Detected	Detected	Compromised	Detected
4770	A_23_P374844	GAL	Hs.278959	galanin prepropeptide	Homo sapiens galanin prepropeptide (GAL), mRNA [NM_015973]	5.86057	down	5.74262	down	1560.64470	9470.59300	1251.17200	6335.67240	Detected	Detected	Detected	Detected
4771	A_24_P69053	ODZ3	Hs.130438	odz, odd Oz/ten-m homolog 3 (Drosophila)	Homo sapiens odz, odd Oz/ten-m homolog 3 (Drosophila) (ODZ3), mRNA [NM_001080477]	5.85348	down	5.58371	down	50.41267	305.55334	30.27528	149.06538	Detected	Detected	Detected	Detected
4772	A_33_P3387856	CENPN	Hs.55028	centromere protein N	Homo sapiens centromere protein N (CENPN), transcript variant 1, mRNA [NM_001100625]	5.84901	down	5.03205	down	18.99727	115.05539	20.83892	92.46666	Detected	Detected	Detected	Detected
4773	A_33_P3330079	LOC100128242	Hs.651710	similar to GRAM domain containing 1B	PREDICTED: Homo sapiens similar to GRAM domain containing 1B (LOC100128242), miscRNA [XR_078445]	5.84869	down	6.71575	down	119.79380	725.48114	97.99329	580.30554	Detected	Detected	Detected	Detected
4774	A_33_P3215239	KIF20B	Hs.240	kinesin family member 20B	Homo sapiens kinesin family member 20B (KIF20B), mRNA [NM_016195]	5.84136	down	6.32239	down	101.26609	612.50684	79.09248	440.94220	Detected	Detected	Detected	Detected
4775	A_23_P58009	C3orf52	Hs.434247	chromosome 3 open reading frame 52	Homo sapiens chromosome 3 open reading frame 52 (C3orf52), mRNA [NM_024616]	5.84061	down	5.63324	down	161.91531	979.21770	117.84353	585.36920	Detected	Detected	Detected	Detected
4776	A_32_P160563	OPCML	Hs.4817	opioid binding protein/cell adhesion molecule-like	Homo sapiens opioid binding protein/cell adhesion molecule-like (OPCML), transcript variant 2, mRNA [NM_001012393]	5.82656	down	7.06456	down	2.37851	14.34993	2.56447	15.97528	Compromised	Compromised	Compromised	Detected
4777	A_33_P3400477	STIL	Hs.525198	SCL/TAL1 interrupting locus	Homo sapiens SCL/TAL1 interrupting locus (STIL), transcript variant 1, mRNA [NM_001048166]	5.80731	down	7.81219	down	73.38251	441.26645	36.21799	249.49544	Detected	Detected	Detected	Detected
4778	A_23_P309973	C14orf50	Hs.144696	chromosome 14 open reading frame 50	Homo sapiens chromosome 14 open reading frame 50 (C14orf50), mRNA [NM_172365]	5.80267	down	2.08938	down	3.39521	20.39990	5.79705	10.68047	Compromised	Detected	Compromised	Compromised
4779	A_23_P215060	PODXL	Hs.16426	podocalyxin-like	Homo sapiens podocalyxin-like (PODXL), transcript variant 1, mRNA [NM_001018111]	5.80112	down	5.34879	down	8632.86900	51856.15600	7474.39750	35253.09000	Detected	Detected	Detected	Detected
4780	A_23_P319859	EYA2	Hs.472877	eyes absent homolog 2 (Drosophila)	Homo sapiens eyes absent homolog 2 (Drosophila) (EYA2), transcript variant 1, mRNA [NM_005244]	5.80058	down	4.25633	down	31.42043	188.71936	22.63615	84.95781	Detected	Detected	Detected	Detected
4781	A_23_P104438	MYPN	Hs.55205	myopalladin	Homo sapiens myopalladin (MYPN), mRNA [NM_032578]	5.79883	down	5.68165	down	61.96690	372.07748	46.03132	230.61809	Detected	Detected	Detected	Detected
4782	A_23_P417113				RNA-binding protein 33 (RNA-binding motif protein 33)(Proline-rich protein 8) [Source:UniProtKB/Swiss-Prot;Acc:Q96EV2] [ENST00000287912]	5.79511	down	5.02111	down	128.72472	772.42560	100.11581	443.26965	Detected	Detected	Detected	Detected
4783	A_33_P3282018	MCF2L	Hs.170422	MCF.2 cell line derived transforming sequence-like	Homo sapiens MCF.2 cell line derived transforming sequence-like (MCF2L), transcript variant 2, mRNA [NM_024979]	5.79465	down	1.29674	down	12.28276	73.69808	4.83585	5.52957	Detected	Detected	Compromised	Compromised
4784	A_23_P53588	WNT5B	Hs.306051	wingless-type MMTV integration site family, member 5B	Homo sapiens wingless-type MMTV integration site family, member 5B (WNT5B), transcript variant 2, mRNA [NM_030775]	5.79008	down	6.48628	down	1831.00240	10977.57400	2779.46920	15897.28100	Detected	Detected	Detected	Detected

4785	A_33_P3334419	RPS6KL1	Hs.414481	ribosomal protein S6 kinase-like 1	Homo sapiens ribosomal protein S6 kinase-like 1 (RPS6KL1), mRNA [NM_031464]	5.78296	down	4.64186	down	13.14710	78.72505	11.52220	47.16211	Compromised	Detected	Compromised	Detected
4786	A_23_P250516					5.77991	down	14.91649	down	18.05566	108.06067	5.26174	69.20882	Detected	Detected	Compromised	Detected
4787	A_23_P397293	LY6K	Hs.69517	lymphocyte antigen 6 complex, locus K	Homo sapiens lymphocyte antigen 6 complex, locus K (LY6K), transcript variant 1, mRNA [NM_017527]	5.77675	down	5.75883	down	8178.64600	48921.34800	8288.28600	42088.58000	Detected	Detected	Detected	Detected
4788	A_33_P3283833	FOXS1	Hs.516971	forkhead box S1	Homo sapiens forkhead box S1 (FOXS1), mRNA [NM_004118]	5.73741	down	6.14674	down	36.19764	215.04498	27.02568	146.48302	Detected	Detected	Detected	Detected
4789	A_33_P3405103	FLYWCH1	Hs.655321	FLYWCH-type zinc finger 1	Homo sapiens FLYWCH-type zinc finger 1 (FLYWCH1), transcript variant 2, mRNA [NM_020912]	5.72716	down	5.75964	down	66.56827	394.76578	46.19363	234.60814	Detected	Detected	Detected	Detected
4790	A_24_P48723	PTGIS	Hs.302085	prostaglandin I2 (prostacyclin) synthase	Homo sapiens prostaglandin I2 (prostacyclin) synthase (PTGIS), mRNA [NM_000961]	5.72539	down	5.92772	down	225.35703	1336.00780	113.96143	595.67750	Detected	Detected	Detected	Detected
4791	A_33_P3584591	HIST2H2BA	Hs.585427	histone cluster 2, H2ba	AGENCOURT_8230913 Lupski_dorsal_root_ganglion Homo sapiens cDNA clone IMAGE:6182568 5', mRNA sequence [BQ880825]	5.71544	down	1.18419	down	4.37379	25.88455	2.86167	2.98817	Compromised	Detected	Compromised	Compromised
4792	A_23_P17593	CDH4	Hs.473231	cadherin 4, type 1, R-cadherin (retinal)	Homo sapiens cadherin 4, type 1, R-cadherin (retinal) (CDH4), mRNA [NM_001794]	5.69731	down	6.37063	down	134.48900	793.39480	119.07429	668.90670	Detected	Detected	Detected	Detected
4793	A_32_P170481	LOC100240735	Hs.635297	hypothetical LOC100240735	Homo sapiens hypothetical LOC100240735 (LOC100240735), non-coding RNA [NR_026658]	5.68053	down	5.19994	down	15.33198	90.18204	9.26442	42.47976	Detected	Detected	Compromised	Detected
4794	A_33_P3608210	LOC554202	Hs.458096	hypothetical LOC554202	Homo sapiens hypothetical LOC554202 (LOC554202), non-coding RNA [NR_027054]	5.67599	down	5.99245	down	360.81970	2120.62940	257.64470	1361.41800	Detected	Detected	Detected	Detected
4795	A_23_P156880	ENPP1	Hs.527295	ectonucleotide pyrophosphatase/phosphodiesterase 1	Homo sapiens ectonucleotide pyrophosphatase/phosphodiesterase 1 (ENPP1), mRNA [NM_006208]	5.66536	down	6.13000	down	520.16660	3051.42580	456.53460	2467.74100	Detected	Detected	Detected	Detected
4796	A_23_P156327	TGFBI	Hs.369397	transforming growth factor, beta-induced, 68kDa	Homo sapiens transforming growth factor, beta-induced, 68kDa (TGFBI), mRNA [NM_000358]	5.65901	down	4.78089	down	73874.69000	432881.84000	54878.18400	231352.42000	Detected	Detected	Detected	Detected
4797	A_23_P115200	FCRL4	Hs.120260	Fc receptor-like 4	Homo sapiens Fc receptor-like 4 (FCRL4), mRNA [NM_031282]	5.65804	down	8.52748	down	3.74802	21.95841	3.44157	25.87869	Compromised	Not Detected	Compromised	Not Detected
4798	A_23_P88630	BLM	Hs.169348	Bloom syndrome, RecQ helicase-like	Homo sapiens Bloom syndrome, RecQ helicase-like (BLM), mRNA [NM_000057]	5.65731	down	5.49223	down	270.38705	1583.90440	193.43558	936.80804	Detected	Detected	Detected	Detected
4799	A_23_P205789	GABPB1	Hs.654350	GA binding protein transcription factor, beta subunit 1	Homo sapiens GA binding protein transcription factor, beta subunit 1 (GABPB1), transcript variant gamma-1, mRNA [NM_002041]	5.64315	down	5.97990	down	259.79720	1518.06000	249.01811	1313.07730	Detected	Detected	Detected	Detected
4800	A_24_P945000	SKA2	Hs.463607	spindle and kinetochore associated complex subunit 2	Homo sapiens spindle and kinetochore associated complex subunit 2 (SKA2), transcript variant 1, mRNA [NM_182620]	5.64260	down	5.04924	down	222.10886	1297.71150	147.53517	656.88196	Detected	Detected	Detected	Detected
4801	A_33_P3271470	GREB1L	Hs.149020	growth regulation by estrogen in breast cancer-like	Homo sapiens growth regulation by estrogen in breast cancer-like (GREB1L), mRNA [NM_001142966]	5.64189	down	7.50632	down	4.37835	25.57806	3.25463	21.54242	Compromised	Detected	Compromised	Detected
4802	A_23_P214168	COL12A1	Hs.101302	collagen, type XII, alpha 1	Homo sapiens collagen, type XII, alpha 1 (COL12A1), transcript variant long, mRNA [NM_004370]	5.63714	down	5.28042	down	1915.52160	11180.96600	1511.68230	7038.72950	Detected	Detected	Detected	Detected
4803	A_23_P170213	NKAIN2	Hs.656604	Na+/K+ transporting ATPase interacting 2	Homo sapiens Na+/K+ transporting ATPase interacting 2 (NKAIN2), mRNA [NM_001040214]	5.63265	down	1.33815	down	5.56543	32.45965	4.27244	5.04132	Compromised	Detected	Compromised	Compromised
4804	A_24_P251599	CAV3	Hs.98303	caveolin 3	Homo sapiens caveolin 3 (CAV3), transcript variant 2, mRNA [NM_001234]	5.63260	down	4.01114	down	1368.57420	7981.97560	66.90283	236.63434	Detected	Detected	Detected	Detected
4805	A_23_P54996	TEX14	Hs.390221	testis expressed 14	Homo sapiens testis expressed 14 (TEX14), transcript variant 1, mRNA [NM_198393]	5.59169	down	6.08412	down	11.13445	64.46807	3.41842	18.33954	Compromised	Detected	Compromised	Detected
4806	A_24_P181295	C14orf37	Hs.535420	chromosome 14 open reading frame 37	Homo sapiens chromosome 14 open reading frame 37 (C14orf37), mRNA [NM_001001872]	5.58625	down	6.49878	down	62.33652	360.57507	47.69210	273.30246	Detected	Detected	Detected	Detected
4807	A_23_P122216	LOX	Hs.102267	lysyl oxidase	Homo sapiens lysyl oxidase (LOX), mRNA [NM_002317]	5.57305	down	5.64443	down	6056.68600	34951.09400	5062.68160	25198.01200	Detected	Detected	Detected	Detected
4808	A_33_P3423631	WHSC1	Hs.113876	Wolf-Hirschhorn syndrome candidate 1	Homo sapiens Wolf-Hirschhorn syndrome candidate 1 (WHSC1), transcript variant 8, mRNA [NM_007331]	5.55611	down	4.84574	down	85.14700	489.86136	123.90369	529.43170	Detected	Detected	Detected	Detected
4809	A_23_P12643	AS3MT	Hs.720370	arsenic (+3 oxidation state) methyltransferase	Homo sapiens arsenic (+3 oxidation state) methyltransferase (AS3MT), mRNA [NM_020682]	5.55596	down	6.45677	down	2.38905	13.74415	2.55687	14.55758	Compromised	Compromised	Compromised	Detected
4810	A_33_P3347971	TPD52	Hs.368433	tumor protein D52	Homo sapiens tumor protein D52 (TPD52), transcript variant 1, mRNA [NM_001025252]	5.54964	down	5.81387	down	6.19899	35.62203	3.33770	17.11112	Compromised	Detected	Compromised	Compromised
4811	A_23_P218858	ABI3BP	Hs.477015	ABI family, member 3 (NESH) binding protein	Homo sapiens ABI family, member 3 (NESH) binding protein (ABI3BP), mRNA [NM_015429]	5.54777	down	5.89446	down	238.96527	1372.73300	115.89896	602.40564	Detected	Detected	Detected	Detected
4812	A_23_P53276	TIMELESS	Hs.118631	timeless homolog (Drosophila)	Homo sapiens timeless homolog (Drosophila) (TIMELESS), mRNA [NM_003920]	5.54338	down	5.37356	down	1799.55690	10329.36600	1222.47600	5792.52700	Detected	Detected	Detected	Detected
4813	A_23_P342108	CCDC116	Hs.131615	coiled-coil domain containing 116	Homo sapiens coiled-coil domain containing 116 (CCDC116), mRNA [NM_152612]	5.54271	down	6.55883	down	8.67550	49.79090	9.25787	53.54308	Compromised	Detected	Compromised	Detected
4814	A_32_P34138	FAM25A	Hs.511787	family with sequence similarity 25, member A	Homo sapiens family with sequence similarity 25, member A (FAM25A), mRNA [NM_001146157]	5.53843	down	4.13207	down	11.56992	66.35140	13.27961	48.38582	Compromised	Detected	Compromised	Detected
4815	A_33_P3244643	FAM183A	Hs.657614	family with sequence similarity 183, member A	Homo sapiens family with sequence similarity 183, member A (FAM183A), mRNA [NM_001101376]	5.53384	down	5.87799	down	3.18328	18.24042	4.01155	20.79246	Compromised	Compromised	Compromised	Detected

4816	A_23_P305245	LOC401097	Hs.710781	Similar to LOC166075	LOC401097 protein Fragment [Source:UniProtKB/TrEMBL;Acc:Q8N5S4] [ENST00000326474]	5.51124	down	3.41983	down	3.18542	18.17814	2.79508	8.42875	Compromised	Detected	Compromised	Compromised
4817	A_33_P3247151	LOC100288900	Hs.711983	hypothetical protein LOC100288900	PREDICTED: Homo sapiens hypothetical protein LOC100288900 (LOC100288900), mRNA [XM_002343731]	5.50365	down	6.16395	down	2.89772	16.51358	3.25969	17.71742	Compromised	Compromised	Compromised	Detected
4818	A_24_P44462	TPM1	Hs.133892	tropomyosin 1 (alpha)	Homo sapiens tropomyosin 1 (alpha) (TPM1), transcript variant 5, mRNA [NM_000366]	5.47481	down	5.46019	down	4032.30830	22858.92200	3529.32890	16992.80300	Detected	Detected	Detected	Detected
4819	A_32_P84242	FAM169A	Hs.91662	family with sequence similarity 169, member A	Homo sapiens family with sequence similarity 169, member A (FAM169A), mRNA [NM_015566]	5.47185	down	4.79141	down	7.95284	45.05978	7.23377	30.56281	Compromised	Detected	Compromised	Detected
4820	A_33_P3325808	tcag7.1058		hypothetical protein LOC100130187	PREDICTED: Homo sapiens hypothetical protein LOC100130187 (LOC100130187), mRNA [XM_001723171]	5.46908	down	4.62343	down	411.18594	2328.55130	67.83411	276.55260	Detected	Detected	Detected	Detected
4821	A_32_P157945	DSP	Hs.519873	desmoplakin	Homo sapiens desmoplakin (DSP), transcript variant 1, mRNA [NM_004415]	5.45225	down	5.78343	down	7540.03660	42567.88000	4960.26400	25296.22700	Detected	Detected	Detected	Detected
4822	A_23_P16944	SDC1	Hs.224607	syndecan 1	Homo sapiens syndecan 1 (SDC1), transcript variant 1, mRNA [NM_001006946]	5.44746	down	6.25355	down	832.13790	4693.77600	780.44305	4303.61400	Detected	Detected	Detected	Detected
4823	A_23_P432598	CHMP4C	Hs.183861	chromatin modifying protein 4C	Homo sapiens chromatin modifying protein 4C (CHMP4C), mRNA [NM_152284]	5.44172	down	11.29031	down	2.39680	13.50519	4.13606	41.17736	Compromised	Compromised	Compromised	Detected
4824	A_33_P3229246	HIST2H2BE	Hs.2178	histone cluster 2, H2be	Homo sapiens histone cluster 2, H2be (HIST2H2BE), mRNA [NM_003528]	5.43989	down	3.34009	down	5.17327	29.13987	11.10427	32.70495	Compromised	Detected	Compromised	Detected
4825	A_23_P205713	STXBP6	Hs.508958	syntaxin binding protein 6 (amisyn)	Homo sapiens syntaxin binding protein 6 (amisyn) (STXBP6), mRNA [NM_014178]	5.43054	down	5.19837	down	327.07797	1839.19190	330.35210	1514.29160	Detected	Detected	Detected	Detected
4826	A_32_P197524	EIF2A	Hs.655782	eukaryotic translation initiation factor 2A, 65kDa	Homo sapiens eukaryotic translation initiation factor 2A, 65kDa (EIF2A), mRNA [NM_032025]	5.42694	down	8.16317	down	15.03625	84.49441	6.46038	46.50312	Detected	Detected	Compromised	Detected
4827	A_33_P3335966	TPM1	Hs.133892	tropomyosin 1 (alpha)	Homo sapiens tropomyosin 1 (alpha) (TPM1), transcript variant 1, mRNA [NM_001018005]	5.42583	down	5.74398	down	7805.83350	43854.98000	7258.56700	36764.53500	Detected	Detected	Detected	Detected
4828	A_32_P206698	CKS1B	Hs.374378	CDC28 protein kinase regulatory subunit 1B	Homo sapiens CDC28 protein kinase regulatory subunit 1B (CKS1B), transcript variant 1, mRNA [NM_001826]	5.42467	down	5.07312	down	710.91473	3993.22300	720.51780	3223.18460	Detected	Detected	Detected	Detected
4829	A_32_P181222	KCNMA1	Hs.144795	potassium large conductance calcium-activated channel, subfamily M, alpha member 1	Homo sapiens potassium large conductance calcium-activated channel, subfamily M, alpha member 1 (KCNMA1), transcript variant 2, mRNA [NM_002247]	5.40316	down	4.31213	down	35.13869	196.59216	43.77952	166.46692	Detected	Detected	Detected	Detected
4830	A_33_P3414964	PTPRS	Hs.644384	protein tyrosine phosphatase, receptor type, S	Homo sapiens protein tyrosine phosphatase, receptor type, S, mRNA (cDNA clone IMAGE:5272670), complete cds. [BC029496]	5.39559	down	7.99007	down	76.01405	424.68390	44.96117	316.77713	Detected	Detected	Detected	Detected
4831	A_23_P143994	FANCD2	Hs.208388	Fanconi anemia, complementation group D2	Homo sapiens Fanconi anemia, complementation group D2 (FANCD2), transcript variant 2, mRNA [NM_001018115]	5.39188	down	6.16581	down	53.44329	298.37790	41.35175	224.82770	Detected	Detected	Detected	Detected
4832	A_33_P3390017	C15orf54	Hs.376109	chromosome 15 open reading frame 54	Homo sapiens chromosome 15 open reading frame 54 (C15orf54), mRNA [NM_207445]	5.38802	down	9.20235	down	15.57670	86.90364	6.33467	51.40290	Detected	Detected	Compromised	Detected
4833	A_23_P47168	FLRT1	Hs.584876	fibronectin leucine rich transmembrane protein 1	Homo sapiens fibronectin leucine rich transmembrane protein 1 (FLRT1), mRNA [NM_013280]	5.37492	down	1.02112	down	11.87126	66.06969	2.89784	2.60927	Detected	Detected	Compromised	Compromised
4834	A_23_P214026	FBN2	Hs.519294	fibrillin 2	Homo sapiens fibrillin 2 (FBN2), mRNA [NM_001999]	5.35992	down	5.20221	down	581.03140	3224.71480	580.68427	2663.74800	Detected	Detected	Detected	Detected
4835	A_23_P407132	KIRREL3	Hs.657065	kin of IRRE like 3 (Drosophila)	Homo sapiens kin of IRRE like 3 (Drosophila) (KIRREL3), transcript variant 1, mRNA [NM_032531]	5.35714	down	3.29725	down	7.88733	43.75179	8.01686	23.30890	Compromised	Detected	Compromised	Detected
4836	A_33_P3336018					5.34611	down	2.49084	down	3.85981	21.36668	3.06622	6.73464	Compromised	Detected	Compromised	Compromised
4837	A_23_P207400	BRCA1	Hs.194143	breast cancer 1, early onset	Homo sapiens breast cancer 1, early onset (BRCA1), transcript variant 2, mRNA [NM_007300]	5.34418	down	5.40264	down	177.70929	983.38610	122.69441	584.51580	Detected	Detected	Detected	Detected
4838	A_33_P3618561	LOC730961	Hs.597933	hypothetical protein LOC730961	Homo sapiens cDNA clone IMAGE:4826905 [BC034282]	5.34379	down	4.83424	down	21.85534	120.93172	17.06258	72.73414	Detected	Detected	Detected	Detected
4839	A_33_P3407636					5.34225	down	5.20987	down	119.22460	659.51215	83.65967	384.33353	Detected	Detected	Detected	Detected
4840	A_24_P3005	SCN9A	Hs.439145	sodium channel, voltage-gated, type IX, alpha subunit	Homo sapiens sodium channel, voltage-gated, type IX, alpha subunit (SCN9A), mRNA [NM_002977]	5.34066	down	8.52840	down	17.04645	94.26747	8.24805	62.02753	Detected	Detected	Compromised	Detected
4841	A_23_P141555	TBX21	Hs.272409	T-box 21	Homo sapiens T-box 21 (TBX21), mRNA [NM_013351]	5.32979	down	5.31359	down	1477.72140	8155.22170	1039.61620	4871.09230	Detected	Detected	Detected	Detected
4842	A_33_P3333158	CPXM2	Hs.656887	carboxypeptidase X (M14 family), member 2	Homo sapiens carboxypeptidase X (M14 family), member 2 (CPXM2), mRNA [NM_198148]	5.32834	down	5.87874	down	44.43957	245.18541	41.77436	216.55050	Detected	Detected	Detected	Detected
4843	A_24_P195286	LOC203510		similar to hCG1644442	PREDICTED: Homo sapiens similar to hCG1644442 (LOC203510), mRNA [XM_001719132]	5.32135	down	5.18716	down	201.58711	1110.75420	16.75178	76.62241	Detected	Detected	Detected	Detected
4844	A_24_P287941	PSMC3IP	Hs.383019	PSMC3 interacting protein	Homo sapiens PSMC3 interacting protein (PSMC3IP), transcript variant 1, mRNA [NM_013290]	5.32069	down	5.29312	down	361.94320	1994.07370	276.43893	1290.25780	Detected	Detected	Detected	Detected
4845	A_33_P3211804	RUNX1	Hs.149261	runt-related transcription factor 1	Homo sapiens runt-related transcription factor 1 (RUNX1), transcript variant 2, mRNA [NM_001001890]	5.31406	down	5.34870	down	1593.63700	8768.98400	1287.10510	6070.55100	Detected	Detected	Detected	Detected
4846	A_32_P171328	UBE2S	Hs.396393	ubiquitin-conjugating enzyme E2S	Homo sapiens ubiquitin-conjugating enzyme E2S (UBE2S), mRNA [NM_014501]	5.30852	down	5.43527	down	6794.96040	37350.22700	6059.95800	29043.98000	Detected	Detected	Detected	Detected

4847	A_24_P276888	CENPO	Hs.718431	centromere protein O	Centromere protein O (CENP-O)(Interphase centromere complex protein 36) [Source:UniProtKB/Swiss-Prot;Acc:Q9BU64] [ENST00000380834]	5.28998	down	6.13074	down	406.66095	2227.50800	332.06950	1795.17630	Detected	Detected	Detected	Detected
4848	A_23_P388168	RAB3B	Hs.123072	RAB3B, member RAS oncogene family	Homo sapiens RAB3B, member RAS oncogene family (RAB3B), mRNA [NM_002867]	5.28882	down	5.06239	down	803.49520	4400.22950	557.61300	2489.16720	Detected	Detected	Detected	Detected
4849	A_23_P167129	HHIP	Hs.507991	hedgehog interacting protein	Homo sapiens hedgehog interacting protein (HHIP), mRNA [NM_022475]	5.28138	down	6.23850	down	68.96924	377.16910	54.35737	299.02258	Detected	Detected	Detected	Detected
4850	A_23_P44684	ECT2	Hs.518299	epithelial cell transforming sequence 2 oncogene	Homo sapiens epithelial cell transforming sequence 2 oncogene (ECT2), mRNA [NM_018098]	5.27814	down	4.55702	down	214.09161	1170.07530	265.90915	1068.51170	Detected	Detected	Detected	Detected
4851	A_23_P170491	TRAIP	Hs.517972	TRAF interacting protein	Homo sapiens TRAF interacting protein (TRAIP), mRNA [NM_005879]	5.27779	down	5.59030	down	681.31270	3723.32840	737.81085	3637.01680	Detected	Detected	Detected	Detected
4852	A_33_P3234222	TSPO2	Hs.357392	translocator protein 2	Homo sapiens translocator protein 2 (TSPO2), transcript variant 1, mRNA [NM_001010873]	5.26568	down	5.76133	down	1853.08690	10103.76000	609.54254	3096.64820	Detected	Detected	Detected	Detected
4853	A_24_P168726	NF2	Hs.187898	neurofibromin 2 (merlin)	Homo sapiens neurofibromin 2 (merlin) (NF2), transcript variant 13, mRNA [NM_181831]	5.24684	down	5.05431	down	343.62340	1866.86720	368.18164	1640.92660	Detected	Detected	Detected	Detected
4854	A_32_P52153					5.23919	down	4.78321	down	77.06253	418.06195	59.57576	251.27786	Detected	Detected	Detected	Detected
4855	A_33_P3340025	GINS1	Hs.658464	GINS complex subunit 1 (Psf1 homolog)	Homo sapiens GINS complex subunit 1 (Psf1 homolog) (GINS1), mRNA [NM_021067]	5.22995	down	5.82693	down	373.13315	2020.66400	230.54713	1184.58180	Detected	Detected	Detected	Detected
4856	A_23_P214459	PRPH2	Hs.654489	peripherin 2 (retinal degeneration, slow)	Homo sapiens peripherin 2 (retinal degeneration, slow) (PRPH2), mRNA [NM_000322]	5.21642	down	6.04036	down	35.85943	193.69070	23.47225	125.02110	Detected	Detected	Detected	Detected
4857	A_23_P76774	GSC	Hs.440438	goosecoid homeobox	Homo sapiens goosecoid homeobox (GSC), mRNA [NM_173849]	5.21498	down	4.61404	down	99.12335	535.25590	118.83870	483.50885	Detected	Detected	Detected	Detected
4858	A_23_P487	UCK2	Hs.458360	uridine-cytidine kinase 2	Homo sapiens uridine-cytidine kinase 2 (UCK2), mRNA [NM_012474]	5.21243	down	5.35292	down	3541.72500	19115.59800	3074.98730	14514.41800	Detected	Detected	Detected	Detected
4859	A_23_P105144	SCUBE2	Hs.523468	signal peptide, CUB domain, EGF-like 2	Homo sapiens signal peptide, CUB domain, EGF-like 2 (SCUBE2), mRNA [NM_020974]	5.20614	down	4.61871	down	38.50871	207.59084	22.64691	92.23489	Detected	Detected	Detected	Detected
4860	A_24_P516246	FLJ12825	Hs.350378	hypothetical LOC440101	Homo sapiens hypothetical LOC440101 (FLJ12825), non-coding RNA [NR_026655]	5.19401	down	3.37204	down	6.74914	36.29810	5.43618	16.16413	Compromised	Detected	Compromised	Compromised
4861	A_24_P335620	SLC7A5	Hs.513797	solute carrier family 7 (cationic amino acid transporter, y+ system), member 5	Homo sapiens solute carrier family 7 (cationic amino acid transporter, y+ system), member 5 (SLC7A5), mRNA [NM_003486]	5.18929	down	5.95787	down	855.25760	4595.55200	611.42145	3212.15820	Detected	Detected	Detected	Detected
4862	A_33_P3344603	ZNF280D	Hs.511477	zinc finger protein 280D	Homo sapiens zinc finger protein 280D (ZNF280D), transcript variant 3, mRNA [NM_001002844]	5.17996	down	6.03430	down	41.02706	220.05440	25.73241	136.92195	Detected	Detected	Detected	Detected
4863	A_23_P167997	HIST1H2BG	Hs.591809	histone cluster 1, H2bg	Homo sapiens histone cluster 1, H2bg (HIST1H2BG), mRNA [NM_003518]	5.17292	down	4.82838	down	80.57515	431.58870	95.09494	404.87830	Detected	Detected	Detected	Detected
4864	A_24_P212443	UBE3C	Hs.118351	ubiquitin protein ligase E3C	Homo sapiens ubiquitin protein ligase E3C (UBE3C), mRNA [NM_014671]	5.17227	down	4.26333	down	18.67925	100.04002	15.69420	59.00026	Detected	Detected	Detected	Detected
4865	A_23_P312863	TUBB2C	Hs.433615	tubulin, beta 2C	Homo sapiens tubulin, beta 2C (TUBB2C), mRNA [NM_006088]	5.16322	down	4.88925	down	6846.95200	36605.89000	6584.44000	28387.43600	Detected	Detected	Detected	Detected
4866	A_33_P3282634	ALDH1L2	Hs.42572	aldehyde dehydrogenase 1 family, member L2	Homo sapiens aldehyde dehydrogenase 1 family, member L2 (ALDH1L2), transcript variant 1, mRNA [NM_001034173]	5.16205	down	5.23953	down	27.16264	145.18670	23.58183	108.95201	Detected	Detected	Detected	Detected
4867	A_32_P55860	SKA2	Hs.463607	spindle and kinetochore associated complex subunit 2	Homo sapiens spindle and kinetochore associated complex subunit 2 (SKA2), transcript variant 1, mRNA [NM_182620]	5.15753	down	4.65911	down	113.54931	606.40015	101.11241	415.40610	Detected	Detected	Detected	Detected
4868	A_33_P3404221				DEP domain-containing protein 1A [Source:UniProtKB/Swiss-Prot;Acc:Q5TB30] [ENST00000370964]	5.15458	down	6.78709	down	18.65996	99.59500	6.31886	37.81705	Detected	Detected	Compromised	Detected
4869	A_32_P1381		Hs.510835		oo82d03.s1 NCI_CGAP_Kid5 Homo sapiens cDNA clone IMAGE:1572677 3' similar to gb:X13546_rna1 Human HMG-17 gene for non-histone chromosomal protein (HUMAN);, mRNA sequence [AA970917]	5.14235	down	5.05529	down	2686.72880	14306.01000	2493.68140	11116.09200	Detected	Detected	Detected	Detected
4870	A_33_P3251227				Glycoprotein-N-acetylgalactosamine 3-beta-galactosyltransferase 1 (EC 2.4.1.122)(Core 1 beta1,3-galactosyltransferase 1)(Core 1 beta3-Gal-T)(C1GalT1)(Core1 UDP-galactose:N-acetylgalactosamine-alpha-R beta 1,3-galactosyltransferase 1)(Beta 1,3-galactosyltransferase)(Core 1 O-glycan T-synthase)(B3Gal-T8) [Source:UniProtKB/Swiss-Prot;Acc:Q9NS00] [ENST00000402468]	5.13987	down	5.25184	down	185.99945	989.91340	136.37470	631.55430	Detected	Detected	Detected	Detected
4871	A_33_P3297930	COL8A2	Hs.353001	collagen, type VIII, alpha 2	Homo sapiens collagen, type VIII, alpha 2 (COL8A2), mRNA [NM_005202]	5.12497	down	1.50776	down	81.95367	434.90370	86.16969	114.56500	Detected	Detected	Detected	Detected
4872	A_33_P3213645	ERN2	Hs.592041	endoplasmic reticulum to nucleus signaling 2	Homo sapiens endoplasmic reticulum to nucleus signaling 2 (ERN2), mRNA [NM_033266]	5.12383	down	13.90805	down	9.48937	50.34607	2.41230	29.58440	Compromised	Detected	Compromised	Detected
4873	A_23_P93180	HIST1H2BC	Hs.658713	histone cluster 1, H2bc	Homo sapiens histone cluster 1, H2bc (HIST1H2BC), mRNA [NM_003526]	5.11242	down	4.68272	down	86.70694	459.00080	91.98714	379.83150	Detected	Detected	Detected	Detected
4874	A_33_P3369098	MYL10	Hs.247831	myosin, light chain 10, regulatory	Homo sapiens myosin, light chain 10, regulatory (MYL10), mRNA [NM_138403]	5.11036	down	10.31547	down	26.28557	139.09198	11.38359	103.54610	Detected	Detected	Compromised	Detected
4875	A_33_P3229122	HIST1H2BF	Hs.182137	histone cluster 1, H2bf	Homo sapiens histone cluster 1, H2bf (HIST1H2BF), mRNA [NM_003522]	5.10882	down	5.55097	down	3.49279	18.47677	3.07647	15.05870	Compromised	Detected	Compromised	Compromised

4876	A_33_P3317253	PTER	Hs.444321	phosphotriesterase related	Homo sapiens phosphotriesterase related (PTER), transcript variant 1, mRNA [NM_001001484]	5.09587	down	4.57032	down	94.17305	496.90990	67.41232	271.67593	Detected	Detected	Detected	Detected
4877	A_24_P355493	LHPP	Hs.527748	phospholysine phosphohistidine inorganic pyrophosphate phosphatase	Homo sapiens phospholysine phosphohistidine inorganic pyrophosphate phosphatase (LHPP), mRNA [NM_022126]	5.09295	down	4.74581	down	68.80198	362.83002	84.55750	353.85680	Detected	Detected	Detected	Detected
4878	A_23_P211110	SIM2	Hs.146186	single-minded homolog 2 (Drosophila)	Homo sapiens single-minded homolog 2 (Drosophila) (SIM2), transcript variant SIM2, mRNA [NM_005069]	5.08735	down	11.71559	down	8.86429	46.69481	2.32261	23.99417	Compromised	Detected	Compromised	Detected
4879	A_33_P3376925	LOC643371		similar to hCG1642839	PREDICTED: Homo sapiens similar to hCG1642839 (LOC643371), miscRNA [XR_078596]	5.07936	down	3.51635	down	1051.21480	5528.82960	146.95540	455.66208	Detected	Detected	Detected	Detected
4880	A_33_P3390122	GIPC2	Hs.659356	GIPC PDZ domain containing family, member 2	Homo sapiens GIPC PDZ domain containing family, member 2 (GIPC2), mRNA [NM_017655]	5.07078	down	6.71962	down	9.10933	47.82937	10.15155	60.15094	Compromised	Detected	Compromised	Detected
4881	A_23_P213153	HNRPDL	Hs.527105	heterogeneous nuclear ribonucleoprotein D-like	Homo sapiens heterogeneous nuclear ribonucleoprotein D-like (HNRPDL), transcript variant 2, mRNA [NM_031372]	5.06530	down	5.01680	down	3988.68530	20920.29100	2521.27900	11153.54200	Detected	Detected	Detected	Detected
4882	A_33_P3409477	UBASH3B	Hs.444075	ubiquitin associated and SH3 domain containing, B	Homo sapiens ubiquitin associated and SH3 domain containing, B (UBASH3B), mRNA [NM_032873]	5.05403	down	5.26956	down	128.88858	674.50574	87.85234	408.21930	Detected	Detected	Detected	Detected
4883	A_33_P3639068	GFRA1	Hs.388347	GDNF family receptor alpha 1	Homo sapiens GDNF family receptor alpha 1 (GFRA1), transcript variant 1, mRNA [NM_005264]	5.05258	down	3.65496	down	79.89974	418.01470	90.51180	291.71167	Detected	Detected	Detected	Detected
4884	A_33_P3280400	MGC72080	Hs.507862	MGC72080 pseudogene	Homo sapiens MGC72080 pseudogene (MGC72080), non-coding RNA [NR_002822]	5.05073	down	6.44908	down	69.33778	362.62450	45.86754	260.83658	Detected	Detected	Detected	Detected
4885	A_33_P3306898	C11orf41	Hs.502266	chromosome 11 open reading frame 41	Homo sapiens chromosome 11 open reading frame 41 (C11orf41), mRNA [NM_012194]	5.04089	down	3.85961	down	30.83955	160.97112	29.00075	98.70029	Detected	Detected	Detected	Detected
4886	A_24_P58529	TUBA1C	Hs.719091	tubulin, alpha 1c	Homo sapiens tubulin, alpha 1c (TUBA1C), mRNA [NM_032704]	5.04082	down	4.87195	down	12594.35100	65736.98000	17427.39600	74868.83600	Detected	Detected	Detected	Detected
4887	A_23_P120227	LBH	Hs.567598	limb bud and heart development homolog (mouse)	Homo sapiens limb bud and heart development homolog (mouse) (LBH), mRNA [NM_030915]	5.03746	down	5.18105	down	903.55070	4713.00240	603.57950	2757.51220	Detected	Detected	Detected	Detected
4888	A_33_P3384871	FBXO5	Hs.520506	F-box protein 5	Homo sapiens F-box protein 5 (FBXO5), transcript variant 2, mRNA [NM_001142522]	5.03545	down	4.51224	down	429.82947	2241.13300	323.89540	1288.73120	Detected	Detected	Detected	Detected
4889	A_23_P373119	HMGB3L1	Hs.558624	high-mobility group box 3-like 1	Homo sapiens high-mobility group box 3-like 1 (HMGB3L1), non-coding RNA [NR_002165]	5.03124	down	4.91625	down	1296.64650	6755.07030	837.34796	3629.98630	Detected	Detected	Detected	Detected
4890	A_24_P186943	ELN	Hs.647061	elastin	Homo sapiens elastin (ELN), transcript variant 1, mRNA [NM_000501]	5.02967	down	12.15771	down	228.40677	1189.54580	79.07068	847.68170	Detected	Detected	Detected	Detected
4891	A_33_P3237719	LOC100129652		hypothetical protein LOC100129652	PREDICTED: Homo sapiens hypothetical LOC100129652 (LOC100129652), mRNA [XM_001716612]	5.02272	down	1.87445	down	6.13799	31.92258	8.55560	14.14132	Compromised	Detected	Compromised	Compromised
4892	A_33_P3659876	NCAPG2	Hs.18616	non-SMC condensin II complex, subunit G2	Homo sapiens non-SMC condensin II complex, subunit G2 (NCAPG2), mRNA [NM_017760]	5.01665	down	4.95112	down	270.82816	1406.82780	178.78595	780.55347	Detected	Detected	Detected	Detected
4893	A_23_P122924	INHBA	Hs.583348	inhibin, beta A	Homo sapiens inhibin, beta A (INHBA), mRNA [NM_002192]	5.01462	down	4.70405	down	8814.29800	45767.67600	7573.83060	31416.12300	Detected	Detected	Detected	Detected
4894	A_23_P111672	TES	Hs.592286	testis derived transcript (3 LIM domains)	Homo sapiens testis derived transcript (3 LIM domains) (TES), transcript variant 2, mRNA [NM_152829]	5.01313	down	5.40680	down	65.47565	339.87695	36.94475	176.14014	Detected	Detected	Detected	Detected
4895	A_32_P66804	PTPRN2	Hs.490789	protein tyrosine phosphatase, receptor type, N polypeptide 2	Homo sapiens protein tyrosine phosphatase, receptor type, N polypeptide 2 (PTPRN2), transcript variant 1, mRNA [NM_002847]	5.01217	down	3.72176	down	5.99928	31.13566	4.30362	14.12366	Compromised	Detected	Compromised	Compromised
4896	A_23_P12079	KCNC4	Hs.153521	potassium voltage-gated channel, Shaw-related subfamily, member 4	Homo sapiens potassium voltage-gated channel, Shaw-related subfamily, member 4 (KCNC4), transcript variant 2, mRNA [NM_153763]	5.00864	down	5.90477	down	46.86871	243.07242	44.27237	230.51590	Detected	Detected	Detected	Detected
4897	A_33_P3304998	LOC100128960		similar to Putative paraneoplastic antigen-like protein 6B-like protein	PREDICTED: Homo sapiens similar to paraneoplastic antigen MA2 (LOC100128960), mRNA [XM_001714501]	5.00652	down	1.60616	down	2.39021	12.39096	17.92675	25.38967	Compromised	Compromised	Detected	Detected
4898	A_33_P3260146				Aminopeptidase O (AP-O)(EC 3.4.11.-) [Source:UniProtKB/Swiss-Prot;Acc:Q8N6M6] [ENST00000277198]	5.00144	down	5.43480	down	5.60996	29.05278	8.54823	40.96616	Compromised	Detected	Compromised	Detected
4899	A_33_P3323564	LOC646324	Hs.655713	hypothetical LOC646324	Homo sapiens cDNA FLJ42907 fis, clone BRHIP3016302 [AK124897]	5.00101	down	6.86238	down	27.79241	143.91875	21.90435	132.54730	Detected	Detected	Detected	Detected
4900	A_33_P3319765				Fanconi anemia group C protein (Protein FACC) [Source:UniProtKB/Swiss-Prot;Acc:Q00597] [ENST00000395345]	4.99626	down	5.41790	down	19.41148	100.42371	12.26209	58.58151	Detected	Detected	Compromised	Detected
4901	A_24_P413920	FAM84A	Hs.260855	family with sequence similarity 84, member A	Homo sapiens family with sequence similarity 84, member A (FAM84A), mRNA [NM_145175]	4.99565	down	2.90561	down	554.90330	2870.39940	207.21620	530.91730	Detected	Detected	Detected	Detected
4902	A_23_P91829	DCBLD2	Hs.203691	discoidin, CUB and LCCL domain containing 2	Homo sapiens discoidin, CUB and LCCL domain containing 2 (DCBLD2), mRNA [NM_080927]	4.99460	down	4.62646	down	285.92435	1478.71520	264.15753	1077.64810	Detected	Detected	Detected	Detected
4903	A_23_P11543	FUCA1	Hs.370858	fucosidase, alpha-L- 1, tissue	Homo sapiens fucosidase, alpha-L- 1, tissue (FUCA1), mRNA [NM_000147]	4.99369	down	5.30112	down	58.85664	304.33360	56.24582	262.91977	Detected	Detected	Detected	Detected
4904	A_23_P206018	TPM1	Hs.133892	tropomyosin 1 (alpha)	Homo sapiens tropomyosin 1 (alpha) (TPM1), transcript variant 3, mRNA [NM_001018004]	4.98365	down	5.04104	down	12588.56500	64961.57400	11102.85700	49353.77300	Detected	Detected	Detected	Detected

4905	A_33_P3224314				cDNA FLJ42076 fis, clone SYNOV2018921 [Source:UniProtKB/TrEMBL;Acc:Q6ZVU4] [ENST00000377652]	4.97741	down	9.31495	down	3.02778	15.60487	3.05173	25.06642	Compromised	Compromised	Compromised	Detected
4906	A_33_P3263157					4.96111	down	4.69317	down	873.11743	4485.23300	425.61893	1761.37680	Detected	Detected	Detected	Detected
4907	A_33_P3245321	CENPP	Hs.713775	centromere protein P	Homo sapiens centromere protein P (CENPP), mRNA [NM_001012267]	4.94776	down	4.64202	down	530.65063	2718.63280	605.38763	2478.02950	Detected	Detected	Detected	Detected
4908	A_33_P3280405	OR7E37P	Hs.507862	olfactory receptor, family 7, subfamily E, member 37 pseudogene	Homo sapiens olfactory receptor, family 7, subfamily E, member 37 pseudogene (OR7E37P), non-coding RNA [NR_002163]	4.94494	down	4.96780	down	196.59637	1006.62880	163.57385	716.54565	Detected	Detected	Detected	Detected
4909	A_23_P200001	NEXN	Hs.612385	nexilin (F actin binding protein)	Homo sapiens nexilin (F actin binding protein) (NEXN), mRNA [NM_144573]	4.93663	down	4.79937	down	331.07004	1692.32460	270.96927	1146.75280	Detected	Detected	Detected	Detected
4910	A_23_P329261	KCNJ2	Hs.1547	potassium inwardly-rectifying channel, subfamily J, member 2	Homo sapiens potassium inwardly-rectifying channel, subfamily J, member 2 (KCNJ2), mRNA [NM_000891]	4.92333	down	5.70215	down	69.63425	354.98868	46.99824	236.31209	Detected	Detected	Detected	Detected
4911	A_33_P3229083	HIST1H2BK	Hs.437275	histone cluster 1, H2bk	Homo sapiens histone cluster 1, H2bk (HIST1H2BK), mRNA [NM_080593]	4.92070	down	4.60755	down	149.72333	762.86755	246.97060	1003.41595	Detected	Detected	Detected	Detected
4912	A_24_P110780	C1orf118	Hs.632414	chromosome 1 open reading frame 118	PREDICTED: Homo sapiens chromosome 1 open reading frame 118 (C1orf118), miscRNA [XR_041258]	4.91811	down	5.15151	down	86.23591	439.15643	59.02673	268.13193	Detected	Detected	Detected	Detected
4913	A_33_P3419790		Hs.126409		Uncharacterized protein C6orf105 [Source:UniProtKB/Swiss-Prot;Acc:Q96IZ2] [ENST00000379415]	4.91759	down	7.27576	down	10.74763	54.72655	8.15376	52.31204	Compromised	Detected	Compromised	Detected
4914	A_23_P151405	CKAP2	Hs.444028	cytoskeleton associated protein 2	Homo sapiens cytoskeleton associated protein 2 (CKAP2), transcript variant 1, mRNA [NM_018204]	4.91515	down	4.77794	down	1918.27640	9762.94900	1245.34160	5246.80500	Detected	Detected	Detected	Detected
4915	A_23_P132277	MCM5	Hs.517582	minichromosome maintenance complex component 5	Homo sapiens minichromosome maintenance complex component 5 (MCM5), mRNA [NM_006739]	4.91332	down	5.28707	down	1318.36170	6707.22460	1529.83230	7132.21100	Detected	Detected	Detected	Detected
4916	A_24_P276102	RBL1	Hs.207745	retinoblastoma-like 1 (p107)	Homo sapiens retinoblastoma-like 1 (p107) (RBL1), transcript variant 2, mRNA [NM_183404]	4.90607	down	6.89707	down	11.03168	56.04131	5.49679	33.43024	Compromised	Detected	Compromised	Detected
4917	A_33_P3283083	INPP4B	Hs.658245	inositol polyphosphate-4-phosphatase, type II, 105kDa	Homo sapiens inositol polyphosphate-4-phosphatase, type II, 105kDa (INPP4B), transcript variant 1, mRNA [NM_003866]	4.90238	down	1.26523	down	7.55689	38.36041	6.10817	6.81470	Compromised	Detected	Compromised	Compromised
4918	A_23_P82775	SOX17	Hs.98367	SRY (sex determining region Y)-box 17	Homo sapiens SRY (sex determining region Y)-box 17 (SOX17), mRNA [NM_022454]	4.89976	down	3.37215	down	16.34719	82.93754	22.94141	68.21697	Detected	Detected	Detected	Detected
4919	A_33_P3323847	RECQL4	Hs.31442	RecQ protein-like 4	Homo sapiens RecQ protein-like 4 (RECQL4), mRNA [NM_004260]	4.88080	down	5.34507	down	2216.91380	11203.99800	1525.21280	7188.68900	Detected	Detected	Detected	Detected
4920	A_33_P3341429	NEXN	Hs.612385	nexilin (F actin binding protein)	Homo sapiens nexilin (F actin binding protein) (NEXN), mRNA [NM_144573]	4.87964	down	4.60348	down	500.97128	2531.24020	220.64992	895.68616	Detected	Detected	Detected	Detected
4921	A_33_P3310286				Lamin-B1 [Source:UniProtKB/Swiss-Prot;Acc:P20700] [ENST00000395354]	4.87909	down	6.67270	down	39.21571	198.12146	22.26442	131.00212	Detected	Detected	Detected	Detected
4922	A_33_P3402329	MGC16121		hypothetical protein MGC16121	Homo sapiens hypothetical protein MGC16121 (MGC16121), non-coding RNA [NR_024607]	4.86693	down	5.16666	down	328.72662	1656.62000	275.83310	1256.67250	Detected	Detected	Detected	Detected
4923	A_33_P3235905					4.85946	down	5.75249	down	147.08762	740.11210	23.53027	119.35708	Detected	Detected	Detected	Detected
4924	A_33_P3399061	ADARB1		adenosine deaminase, RNA-specific, B1 (RED1 homolog rat)	Homo sapiens adenosine deaminase, RNA-specific, B1 (RED1 homolog rat) (ADARB1), transcript variant 4, non-coding RNA [NR_027673]	4.84874	down	3.80261	down	33.89433	170.17253	48.96199	164.17480	Detected	Detected	Detected	Detected
4925	A_23_P410717	C1orf51	Hs.54680	chromosome 1 open reading frame 51	Homo sapiens chromosome 1 open reading frame 51 (C1orf51), mRNA [NM_144697]	4.84341	down	4.91848	down	135.76960	680.90520	159.60750	692.22840	Detected	Detected	Detected	Detected
4926	A_23_P116264	NRGN	Hs.524116	neurogranin (protein kinase C substrate, RC3)	Homo sapiens neurogranin (protein kinase C substrate, RC3) (NRGN), transcript variant 1, mRNA [NM_006176]	4.83638	down	4.69314	down	80.44029	402.83470	88.43649	365.98294	Detected	Detected	Detected	Detected
4927	A_23_P33759	DHRS3	Hs.289347	dehydrogenase/reductase (SDR family) member 3	Homo sapiens dehydrogenase/reductase (SDR family) member 3 (DHRS3), mRNA [NM_004753]	4.83401	down	5.17731	down	269.08078	1346.86330	333.75830	1523.70920	Detected	Detected	Detected	Detected
4928	A_33_P3361472				Proteasome activator complex subunit 4 (Proteasome activator PA200) [Source:UniProtKB/Swiss-Prot;Acc:Q14997] [ENST00000378234]	4.82886	down	11.55625	down	2.54125	12.70649	2.78029	28.33170	Compromised	Compromised	Compromised	Detected
4929	A_33_P3369760	GLIPR2	Hs.493819	GLI pathogenesis-related 2	Homo sapiens GLI pathogenesis-related 2 (GLIPR2), mRNA [NM_022343]	4.82528	down	4.10978	down	487.73367	2436.90200	471.18503	1707.55880	Detected	Detected	Detected	Detected
4930	A_33_P3259902	LOC100128372		similar to hCG2040192	PREDICTED: Homo sapiens similar to hCG2040192 (LOC100128372), mRNA [XM_001716747]	4.82382	down	4.95183	down	656.22534	3277.76200	766.50040	3346.90400	Detected	Detected	Detected	Detected
4931	A_24_P125469	LIPG	Hs.465102	lipase, endothelial	Homo sapiens lipase, endothelial (LIPG), mRNA [NM_006033]	4.82351	down	11.83337	down	6.46265	32.27798	2.44510	25.51355	Compromised	Detected	Compromised	Detected
4932	A_23_P91430	PXMP4	Hs.654857	peroxisomal membrane protein 4, 24kDa	Homo sapiens peroxisomal membrane protein 4, 24kDa (PXMP4), transcript variant 1, mRNA [NM_007238]	4.81926	down	4.97922	down	298.21344	1488.12790	318.45743	1398.22960	Detected	Detected	Detected	Detected
4933	A_24_P77968	FLNC	Hs.58414	filamin C, gamma	Homo sapiens filamin C, gamma (FLNC), transcript variant 1, mRNA [NM_001458]	4.81787	down	5.42464	down	1341.36340	6691.66700	1168.35100	5588.68700	Detected	Detected	Detected	Detected
4934	A_24_P813147	RP11-631M21.2	Hs.532659	tubulin, beta 8	Homo sapiens tubulin, beta 8 (TUBB8), transcript variant 1, mRNA [NM_177987]	4.81211	down	5.14839	down	1284.29530	6399.31840	1527.02450	6932.39500	Detected	Detected	Detected	Detected
4935	A_23_P99253	LIN7A	Hs.144333	lin-7 homolog A (C. elegans)	Homo sapiens lin-7 homolog A (C. elegans) (LIN7A), mRNA [NM_004664]	4.81096	down	4.19429	down	103.33338	514.76074	102.15040	377.80142	Detected	Detected	Detected	Detected
4936	A_33_P3336437	TTLL5	Hs.709609	tubulin tyrosine ligase-like family, member 5	Homo sapiens tubulin tyrosine ligase-like family, member 5 (TTLL5), mRNA [NM_015072]	4.80240	down	4.17000	down	35.93230	178.68008	31.67737	116.47976	Detected	Detected	Detected	Detected

4937	A_23_P138524	CPXM2	Hs.656887	carboxypeptidase X (M14 family), member 2	Homo sapiens carboxypeptidase X (M14 family), member 2 (CPXM2), mRNA [NM_198148]	4.79803	down	4.83914	down	588.70760	2924.79960	380.65695	1624.30360	Detected	Detected	Detected	Detected
4938	A_33_P3237729	PDLIM4	Hs.424312	PDZ and LIM domain 4	Homo sapiens PDZ and LIM domain 4 (PDLIM4), transcript variant 1, mRNA [NM_003687]	4.79563	down	4.95296	down	96.01392	476.77423	66.11311	288.74720	Detected	Detected	Detected	Detected
4939	A_23_P28733	RBL1	Hs.207745	retinoblastoma-like 1 (p107)	Homo sapiens retinoblastoma-like 1 (p107) (RBL1), transcript variant 1, mRNA [NM_002895]	4.78933	down	4.76244	down	59.32535	294.20360	59.22743	248.72429	Detected	Detected	Detected	Detected
4940	A_33_P3280801	LMO7	Hs.207631	LIM domain 7	Homo sapiens LIM domain 7 (LMO7), transcript variant 1, mRNA [NM_005358]	4.78344	down	4.75944	down	226.85376	1123.61880	254.31744	1067.32710	Detected	Detected	Detected	Detected
4941	A_33_P3420466	MATN3	Hs.656199	matrilin 3	Homo sapiens matrilin 3 (MATN3), mRNA [NM_002381]	4.77927	down	3.99631	down	17.29306	85.57879	19.28639	67.96354	Detected	Detected	Detected	Detected
4942	A_23_P43157	MYBL1	Hs.445898	v-myb myeloblastosis viral oncogene homolog (avian)-like 1	Homo sapiens v-myb myeloblastosis viral oncogene homolog (avian)-like 1 (MYBL1), transcript variant 1, mRNA [NM_001080416]	4.77451	down	4.61857	down	218.56741	1080.55700	185.30968	754.69385	Detected	Detected	Detected	Detected
4943	A_33_P3346483				Synaptopodin 2-like protein [Source:UniProtKB/Swiss-Prot;Acc:Q9H987] [ENST00000372872]	4.77343	down	8.67865	down	10.97932	54.26742	6.09552	46.64751	Compromised	Detected	Compromised	Detected
4944	A_23_P69586	FAT1	Hs.481371	FAT tumor suppressor homolog 1 (Drosophila)	Homo sapiens FAT tumor suppressor homolog 1 (Drosophila) (FAT1), mRNA [NM_005245]	4.76949	down	4.61213	down	1207.71370	5964.42630	889.89900	3619.16140	Detected	Detected	Detected	Detected
4945	A_23_P19030	ARSI	Hs.591252	arylsulfatase family, member I	Homo sapiens arylsulfatase family, member I (ARSI), mRNA [NM_001012301]	4.76940	down	5.13793	down	137.05452	676.84710	165.43192	749.50250	Detected	Detected	Detected	Detected
4946	A_24_P263653	DOCK2	Hs.586174	dedicator of cytokinesis 2	Homo sapiens dedicator of cytokinesis 2 (DOCK2), mRNA [NM_004946]	4.74544	down	5.47790	down	16.41420	80.65463	23.77668	114.85003	Detected	Detected	Detected	Detected
4947	A_24_P24685	LOC729595		similar to high mobility group box 3	PREDICTED: Homo sapiens similar to high mobility group box 3, transcript variant 1 (LOC729595), mRNA [XM_001130734]	4.74363	down	5.84415	down	108.68327	533.83470	77.04117	397.01743	Detected	Detected	Detected	Detected
4948	A_33_P3284951	MCM5	Hs.517582	minichromosome maintenance complex component 5	Homo sapiens minichromosome maintenance complex component 5 (MCM5), mRNA [NM_006739]	4.71559	down	5.59083	down	550.48260	2687.89770	594.46550	2930.68290	Detected	Detected	Detected	Detected
4949	A_33_P3319041	HMGB3	Hs.19114	high-mobility group box 3	Homo sapiens high-mobility group box 3 (HMGB3), mRNA [NM_005342]	4.70783	down	4.97913	down	1822.53380	8884.43600	1237.22490	5432.09600	Detected	Detected	Detected	Detected
4950	A_33_P3349840	LOC646049		hypothetical LOC646049	PREDICTED: Homo sapiens hypothetical LOC646049 (LOC646049), mRNA [XM_001722987]	4.69702	down	5.05447	down	1462.09910	7111.03120	900.85504	4015.09420	Detected	Detected	Detected	Detected
4951	A_33_P3401179					4.69508	down	4.25407	down	2.43390	11.83258	4.94412	18.54637	Compromised	Compromised	Compromised	Detected
4952	A_23_P148984	DARS2	Hs.647707	aspartyl-tRNA synthetase 2, mitochondrial	Homo sapiens aspartyl-tRNA synthetase 2, mitochondrial (DARS2), nuclear gene encoding mitochondrial protein, mRNA [NM_018122]	4.69403	down	5.00910	down	75.73225	368.09500	50.32705	222.29356	Detected	Detected	Detected	Detected
4953	A_23_P357207	MRAP2	Hs.370055	melanocortin 2 receptor accessory protein 2	Homo sapiens melanocortin 2 receptor accessory protein 2 (MRAP2), mRNA [NM_138409]	4.68824	down	4.17098	down	52.16884	253.25250	48.21416	177.32857	Detected	Detected	Detected	Detected
4954	A_23_P46781	ITGA8	Hs.171311	integrin, alpha 8	Homo sapiens integrin, alpha 8 (ITGA8), mRNA [NM_003638]	4.68750	down	2.83748	down	9.06729	44.01004	7.85035	19.64209	Compromised	Detected	Compromised	Detected
4955	A_23_P19291	TUBB2A	Hs.654543	tubulin, beta 2A	Homo sapiens tubulin, beta 2A (TUBB2A), mRNA [NM_001069]	4.68374	down	4.75856	down	14703.07300	71307.35000	13596.52500	57051.73000	Detected	Detected	Detected	Detected
4956	A_23_P134454	CAV1	Hs.74034	caveolin 1, caveolae protein, 22kDa	Homo sapiens caveolin 1, caveolae protein, 22kDa (CAV1), mRNA [NM_001753]	4.67558	down	4.56047	down	20129.15400	97452.82000	13978.40500	56212.50800	Detected	Detected	Detected	Detected
4957	A_24_P113144	ATAD5	Hs.528902	ATPase family, AAA domain containing 5	Homo sapiens ATPase family, AAA domain containing 5 (ATAD5), mRNA [NM_024857]	4.67547	down	4.55128	down	25.94260	125.59493	19.19552	77.03696	Detected	Detected	Detected	Detected
4958	A_23_P119040	GREB1L	Hs.149020	growth regulation by estrogen in breast cancer-like	Homo sapiens growth regulation by estrogen in breast cancer-like (GREB1L), mRNA [NM_001142966]	4.67520	down	3.28785	down	13.50212	65.36356	12.12886	35.16392	Detected	Detected	Not Detected	Detected
4959	A_23_P45917	CKS1B	Hs.374378	CDC28 protein kinase regulatory subunit 1B	Homo sapiens CDC28 protein kinase regulatory subunit 1B (CKS1B), transcript variant 1, mRNA [NM_001826]	4.67503	down	4.75047	down	8821.70600	42704.15000	9565.47200	40068.95700	Detected	Detected	Detected	Detected
4960	A_23_P209394	CFLAR	Hs.390736	CASP8 and FADD-like apoptosis regulator	Homo sapiens CASP8 and FADD-like apoptosis regulator (CFLAR), transcript variant 3, mRNA [NM_001127184]	4.67165	down	4.71388	down	733.48920	3548.11280	626.20910	2602.93480	Detected	Detected	Detected	Detected
4961	A_33_P3231953	COL12A1	Hs.101302	collagen, type XII, alpha 1	Homo sapiens collagen, type XII, alpha 1 (COL12A1), transcript variant long, mRNA [NM_004370]	4.66935	down	4.49362	down	45.62227	220.57993	39.70931	157.34549	Detected	Detected	Detected	Detected
4962	A_33_P3331307	CDC42EP3	Hs.369574	CDC42 effector protein (Rho GTPase binding) 3	Homo sapiens CDC42 effector protein (Rho GTPase binding) 3 (CDC42EP3), mRNA [NM_006449]	4.66804	down	5.64945	down	64.33516	310.96844	38.46300	191.60840	Detected	Detected	Detected	Detected
4963	A_24_P409250				Elongation factor 1-gamma (EF-1-gamma)(eEF-1B gamma) [Source:UniProtKB/Swiss-Prot;Acc:P26641] [ENST00000308436]	4.66024	down	5.99645	down	24.14008	116.48773	18.54161	98.04096	Detected	Detected	Detected	Detected
4964	A_23_P56578	VIT	Hs.137415	vitrin	Homo sapiens vitrin (VIT), mRNA [NM_053276]	4.65873	down	4.88177	down	75.32980	363.38544	66.07529	284.43450	Detected	Detected	Detected	Detected
4965	A_24_P67681	LOC441795		high mobility group protein B3-like protein-like	Putative high mobility group protein B3-like protein [Source:UniProtKB/Swiss-Prot;Acc:P0C6E5] [ENST00000447408]	4.62791	down	4.77405	down	1411.96500	6766.15330	1132.83900	4768.92400	Detected	Detected	Detected	Detected
4966	A_33_P3223495				Protein furry homolog [Source:UniProtKB/Swiss-Prot;Acc:Q5TBA9] [ENST00000380218]	4.62738	down	6.63436	down	25.85752	123.89551	9.05736	52.98659	Detected	Detected	Compromised	Detected

4967	A_33_P3797820	FAM22B	Hs.662805	family with sequence similarity 22, member B	PREDICTED: Homo sapiens family with sequence similarity 22, member B, transcript variant 1 (FAM22B), mRNA [XM_001133075]	4.62671	down	7.35893	down	36.15212	173.19661	13.93794	90.44382	Detected	Detected	Compromised	Detected
4968	A_33_P3265526	NARG1L	Hs.512914	NMDA receptor regulated 1-like	Homo sapiens NMDA receptor regulated 1-like (NARG1L), transcript variant 3, mRNA [NM_001110798]	4.62650	down	4.37924	down	10.14592	48.60461	3.14710	12.15277	Compromised	Detected	Compromised	Compromised
4969	A_24_P350245	DOCK5	Hs.195403	dedicator of cytokinesis 5	Homo sapiens dedicator of cytokinesis 5 (DOCK5), mRNA [NM_024940]	4.62643	down	4.32587	down	77.75825	372.49902	96.92073	369.70496	Detected	Detected	Detected	Detected
4970	A_33_P3560679	LOC651721	Hs.598754	hypothetical protein LOC651721	Homo sapiens hypothetical protein LOC651721, mRNA (cDNA clone IMAGE:4430430), partial cds [BC026225]	4.62614	down	7.95132	down	31.79598	152.30840	17.21336	120.68978	Detected	Detected	Detected	Detected
4971	A_33_P3301394	LOC729687		similar to high-mobility group nucleosomal binding domain 2	PREDICTED: Homo sapiens similar to high-mobility group nucleosomal binding domain 2 (LOC729687), mRNA [XM_001133530]	4.62578	down	4.92844	down	600.95233	2878.44430	758.60670	3296.79100	Detected	Detected	Detected	Detected
4972	A_23_P49459	LOC81691	Hs.177926	exonuclease NEF-sp	Homo sapiens exonuclease NEF-sp (LOC81691), transcript variant 1, mRNA [NM_030941]	4.62461	down	4.66151	down	85.03912	407.21832	80.32336	330.16720	Detected	Detected	Detected	Detected
4973	A_33_P3317523	STMN1	Hs.209983	stathmin 1	Homo sapiens stathmin 1 (STMN1), transcript variant 1, mRNA [NM_203401]	4.62218	down	4.65448	down	8335.58300	39894.79000	6252.74660	25663.01000	Detected	Detected	Detected	Detected
4974	A_32_P160561	DOK6	Hs.278285	docking protein 6	Homo sapiens docking protein 6 (DOK6), mRNA [NM_152721]	4.62094	down	2.64039	down	11.81122	56.51425	23.61119	54.97334	Compromised	Detected	Detected	Detected
4975	A_23_P250385	HIST1H1B	Hs.131956	histone cluster 1, H1b	Homo sapiens histone cluster 1, H1b (HIST1H1B), mRNA [NM_005322]	4.61948	down	3.62012	down	46.26167	221.28276	43.78721	139.77690	Detected	Detected	Detected	Detected
4976	A_32_P222383	HMGN2	Hs.181163	high-mobility group nucleosomal binding domain 2	Homo sapiens high-mobility group nucleosomal binding domain 2 (HMGN2), mRNA [NM_005517]	4.61657	down	4.55104	down	13079.31300	62522.70700	10102.02300	40540.05000	Detected	Detected	Detected	Detected
4977	A_24_P227927	IL21R	Hs.210546	interleukin 21 receptor	Homo sapiens interleukin 21 receptor (IL21R), transcript variant 2, mRNA [NM_181078]	4.61618	down	4.72694	down	29.49439	140.97920	38.96089	162.39569	Detected	Detected	Detected	Detected
4978	A_33_P3839897	RNU4ATAC	Hs.689638	RNA, U4atac small nuclear (U12-dependent splicing)	HHAGE001732 Human liver regeneration after partial hepatectomy Homo sapiens cDNA, mRNA sequence [DW419002]	4.61529	down	17.01053	down	36.73500	175.55450	7.73602	116.03821	Detected	Detected	Compromised	Detected
4979	A_23_P19673	SGK1	Hs.510078	serum/glucocorticoid regulated kinase 1	Homo sapiens serum/glucocorticoid regulated kinase 1 (SGK1), transcript variant 1, mRNA [NM_005627]	4.61040	down	4.79314	down	4232.22360	20204.15400	3493.31960	14764.68600	Detected	Detected	Detected	Detected
4980	A_23_P15876	ALPK2	Hs.628152	alpha-kinase 2	Homo sapiens alpha-kinase 2 (ALPK2), mRNA [NM_052947]	4.60869	down	4.78297	down	440.79620	2103.52780	288.80008	1218.03710	Detected	Detected	Detected	Detected
4981	A_23_P161615	POLA2	Hs.201897	polymerase (DNA directed), alpha 2 (70kD subunit)	Homo sapiens polymerase (DNA directed), alpha 2 (70kD subunit) (POLA2), mRNA [NM_002689]	4.60679	down	4.95227	down	415.58380	1982.39360	433.80743	1894.37920	Detected	Detected	Detected	Detected
4982	A_33_P3370305	TMEM106A	Hs.536474	transmembrane protein 106A	Homo sapiens transmembrane protein 106A (TMEM106A), mRNA [NM_145041]	4.59681	down	3.06683	down	5.57180	26.52067	7.45980	20.17356	Compromised	Detected	Compromised	Detected
4983	A_33_P3258117	HELLS	Hs.655830	helicase, lymphoid-specific	Homo sapiens helicase, lymphoid-specific (HELLS), mRNA [NM_018063]	4.59491	down	5.54712	down	79.99876	380.62173	58.06229	284.00577	Detected	Detected	Detected	Detected
4984	A_33_P3298102	hCG_1793639		hCG1793639	PREDICTED: Homo sapiens hCG1793639 (LOC645263), mRNA [XM_001726534]	4.59471	down	1.87103	down	13.92915	66.26987	3.62458	5.98003	Detected	Detected	Compromised	Compromised
4985	A_33_P3641427	MYEF2	Hs.6638	myelin expression factor 2	Homo sapiens myelin expression factor 2 (MYEF2), mRNA [NM_016132]	4.59378	down	3.27234	down	8.12736	38.65916	7.59356	21.91135	Compromised	Detected	Compromised	Detected
4986	A_24_P15877				FKSG41 [Source:UniProtKB/TrEMBL;Acc:Q9BZ77] [ENST00000413847]	4.59311	down	5.15039	down	27.37787	130.20848	3.48743	15.83837	Detected	Detected	Compromised	Compromised
4987	A_32_P120791	LOC727916	Hs.706850	hypothetical protein LOC727916	PREDICTED: Homo sapiens hypothetical protein LOC727916 (LOC727916), miscRNA [XR_039953]	4.59213	down	4.16899	down	53.48153	254.30273	43.69270	160.62193	Detected	Detected	Detected	Detected
4988	A_33_P3404729	LOC642350	Hs.632647	similar to Coagulation factor V precursor (Activated protein C cofactor)	PREDICTED: Homo sapiens similar to Coagulation factor V precursor (Activated protein C cofactor) (LOC642350), miscRNA [XR_041338]	4.59166	down	6.03695	down	14.27679	67.87863	8.64606	46.02578	Detected	Detected	Compromised	Detected
4989	A_23_P214798	SYNCRIP	Hs.571177	synaptotagmin binding, cytoplasmic RNA interacting protein	Homo sapiens synaptotagmin binding, cytoplasmic RNA interacting protein (SYNCRIP), transcript variant 1, mRNA [NM_006372]	4.58641	down	4.36381	down	3577.69650	16990.63500	2512.76510	9669.03800	Detected	Detected	Detected	Detected
4990	A_23_P204702	TMBIM6	Hs.708025	transmembrane BAX inhibitor motif containing 6	Homo sapiens transmembrane BAX inhibitor motif containing 6 (TMBIM6), transcript variant 1, mRNA [NM_003217]	4.58542	down	4.53334	down	4795.31350	22768.22700	4798.39200	19181.36700	Detected	Detected	Detected	Detected
4991	A_23_P410625	ZNF367	Hs.494557	zinc finger protein 367	Homo sapiens zinc finger protein 367 (ZNF367), mRNA [NM_153695]	4.58166	down	2.86888	down	2.89397	13.72936	7.01531	17.74702	Compromised	Compromised	Compromised	Detected
4992	A_24_P337239	FGF5	Hs.37055	fibroblast growth factor 5	Homo sapiens fibroblast growth factor 5 (FGF5), transcript variant 1, mRNA [NM_004464]	4.57302	down	1.19570	down	5.87650	27.82625	2.83158	2.98551	Compromised	Detected	Compromised	Compromised
4993	A_23_P350574	FCRLB	Hs.517422	Fc receptor-like B	Homo sapiens Fc receptor-like B (FCRLB), mRNA [NM_001002901]	4.56569	down	4.98208	down	352.37366	1665.87730	299.68570	1316.56510	Detected	Detected	Detected	Detected
4994	A_23_P45799	ORC1L	Hs.17908	origin recognition complex, subunit 1-like (yeast)	Homo sapiens origin recognition complex, subunit 1-like (yeast) (ORC1L), mRNA [NM_004153]	4.56285	down	6.84130	down	30.27161	143.02261	23.49678	141.74663	Detected	Detected	Detected	Detected
4995	A_33_P3280418	MGC72080	Hs.507862	MGC72080 pseudogene	Homo sapiens MGC72080 pseudogene (MGC72080), non-coding RNA [NR_002822]	4.56163	down	6.01834	down	193.90529	915.88920	172.86429	917.37616	Detected	Detected	Detected	Detected
4996	A_23_P389987	TLX2	Hs.168586	T-cell leukemia homeobox 2	Homo sapiens T-cell leukemia homeobox 2 (TLX2), mRNA [NM_016170]	4.56042	down	4.91624	down	44.07448	208.12553	40.50041	175.57297	Detected	Detected	Detected	Detected

4997	A_32_P380675	GRIP2	Hs.517819	glutamate receptor interacting protein 2	Glutamate receptor-interacting protein 2 (GRIP2 protein) [Source:UniProtKB/Swiss-Prot;Acc:Q9C0E4] [ENST00000443613]	4.55566	down	1.17303	down	4.34818	20.51126	2.49403	2.57974	Compromised	Detected	Compromised	Compromised
4998	A_33_P3229725	ARSA	Hs.88251	arylsulfatase A	Homo sapiens arylsulfatase A (ARSA), transcript variant 1, mRNA [NM_000487]	4.55455	down	4.97760	down	1960.64390	9246.49700	1680.44570	7375.81640	Detected	Detected	Detected	Detected
4999	A_33_P3544856	LOC100292680	Hs.391695	hypothetical LOC100292680	Homo sapiens cDNA FLJ33297 fis, clone BNGH42001406 [AK090616]	4.55398	down	7.34624	down	8.87600	41.85446	4.52442	29.30852	Compromised	Detected	Compromised	Detected
5000	A_32_P114284	IKZF2	Hs.604950	IKAROS family zinc finger 2 (Helios)	Homo sapiens IKAROS family zinc finger 2 (Helios) (IKZF2), transcript variant 2, mRNA [NM_001079526]	4.55096	down	4.15741	down	183.10570	862.85565	154.60490	566.77640	Detected	Detected	Detected	Detected
5001	A_33_P3315733				Trafficking protein particle complex subunit 10 (Transport protein particle subunit TMEM1)(TRAPP subunit TMEM1)(Trafficking protein particle complex subunit TMEM1)(Epilepsy holoprosencephaly candidate 1 protein)(EHOC-1)(Protein GT334) [Source:UniProtKB/Swiss-Prot;Acc:P48553] [ENST00000380221]	4.55096	down	4.42864	down	111.11552	523.61340	85.56742	334.15192	Detected	Detected	Detected	Detected
5002	A_32_P196142	LOC100130938	Hs.710069	hypothetical LOC100130938	PREDICTED: Homo sapiens hypothetical LOC100130938 (LOC100130938), mRNA [XM_001720237]	4.54489	down	4.65630	down	1107.07290	5209.94200	1258.51460	5167.31250	Detected	Detected	Detected	Detected
5003	A_33_P3225507	OR10G2	Hs.554580	olfactory receptor, family 10, subfamily G, member 2	Homo sapiens olfactory receptor, family 10, subfamily G, member 2 (OR10G2), mRNA [NM_001005466]	4.53174	down	2.20840	down	47.05341	220.79518	33.61902	65.46801	Detected	Detected	Detected	Detected
5004	A_23_P87351	RRM1	Hs.445705	ribonucleotide reductase M1	Homo sapiens ribonucleotide reductase M1 (RRM1), mRNA [NM_001033]	4.52815	down	4.21489	down	7833.72100	36730.11300	6160.22800	22895.39600	Detected	Detected	Detected	Detected
5005	A_23_P303210	IKBIP	Hs.252543	IKBKB interacting protein	Homo sapiens IKBKB interacting protein (IKBIP), transcript variant 1, mRNA [NM_153687]	4.52397	down	4.88737	down	480.37527	2250.26400	400.22476	1724.82410	Detected	Detected	Detected	Detected
5006	A_33_P3291092	THSD1	Hs.325667	thrombospondin, type I, domain containing 1	Homo sapiens thrombospondin, type I, domain containing 1 (THSD1), transcript variant 2, mRNA [NM_199263]	4.51972	down	2.06555	down	9.17102	42.92023	12.13744	22.10692	Compromised	Detected	Compromised	Detected
5007	A_32_P163089	C12orf75	Hs.368938	chromosome 12 open reading frame 75	Homo sapiens chromosome 12 open reading frame 75 (C12orf75), mRNA [NM_001145199]	4.51472	down	4.41816	down	5469.39200	25568.35500	4796.12800	18685.19000	Detected	Detected	Detected	Detected
5008	A_32_P7015	TSPAN15	Hs.499941	tetraspanin 15	Homo sapiens tetraspanin 15 (TSPAN15), mRNA [NM_012339]	4.51279	down	13.52915	down	11.02969	51.53963	2.93143	34.97155	Compromised	Detected	Compromised	Detected
5009	A_32_P918263					4.51276	down	4.04882	down	267.69394	1250.87340	187.08669	667.93900	Detected	Detected	Detected	Detected
5010	A_23_P93690	MCM7	Hs.438720	minichromosome maintenance complex component 7	Homo sapiens minichromosome maintenance complex component 7 (MCM7), transcript variant 2, mRNA [NM_182776]	4.50952	down	4.68434	down	6916.26460	32294.97500	8675.46000	35834.95300	Detected	Detected	Detected	Detected
5011	A_33_P3284129	LYPD1	Hs.719231	LY6/PLAUR domain containing 1	Homo sapiens LY6/PLAUR domain containing 1 (LYPD1), transcript variant 1, mRNA [NM_144586]	4.50684	down	1.85106	down	2.77338	12.94237	8.06536	13.16462	Compromised	Compromised	Compromised	Detected
5012	A_33_P3329352	LOC644992		hypothetical LOC644992	PREDICTED: Homo sapiens hypothetical LOC644992 (LOC644992), mRNA [XM_001717733]	4.50677	down	3.90407	down	76.94813	359.08417	37.69082	129.75331	Detected	Detected	Detected	Detected
5013	A_24_P3783	HIST1H2BM	Hs.182432	histone cluster 1, H2bm	Homo sapiens histone cluster 1, H2bm (HIST1H2BM), mRNA [NM_003521]	4.50042	down	4.40084	down	307.96744	1435.12950	363.77070	1411.65520	Detected	Detected	Detected	Detected
5014	A_33_P3257330	DCBLD1	Hs.658304	discoidin, CUB and LCCL domain containing 1	Homo sapiens discoidin, CUB and LCCL domain containing 1 (DCBLD1), mRNA [NM_173674]	4.50017	down	4.53250	down	67.11993	312.76157	32.62805	130.40501	Detected	Detected	Detected	Detected
5015	A_23_P166779	LOH3CR2A	Hs.591661	loss of heterozygosity, 3, chromosomal region 2, gene A	Homo sapiens loss of heterozygosity, 3, chromosomal region 2, gene A (LOH3CR2A), non-coding RNA [NR_024065]	4.49663	down	4.77085	down	167.67834	780.72424	100.77600	423.95407	Detected	Detected	Detected	Detected
5016	A_32_P12994	LOC440900	Hs.662205	hypothetical LOC440900	PREDICTED: Homo sapiens hypothetical LOC440900 (LOC440900), miscRNA [XR_041709]	4.49558	down	4.91045	down	31.67730	147.45746	24.28995	105.17532	Detected	Detected	Detected	Detected
5017	A_33_P3239322	LOC100132474	Hs.676024	hypothetical protein LOC100132474	Homo sapiens cDNA FLJ43170 fis, clone FCBBF3004847. [AK125160]	4.49213	down	1.17782	down	5.71118	26.56509	2.40374	2.49650	Compromised	Detected	Compromised	Compromised
5018	A_23_P168610	TSPAN13	Hs.364544	tetraspanin 13	Homo sapiens tetraspanin 13 (TSPAN13), mRNA [NM_014399]	4.48611	down	4.42517	down	358.68655	1666.16590	347.43594	1355.72070	Detected	Detected	Detected	Detected
5019	A_33_P3422888				Protein CLEC16A [Source:UniProtKB/Swiss-Prot;Acc:Q2KHT3] [ENST00000409552]	4.48513	down	4.63583	down	26.84907	124.69170	26.99679	110.35822	Detected	Detected	Detected	Detected
5020	A_24_P244699	NUDT15	Hs.144407	nudix (nucleoside diphosphate linked moiety X)-type motif 15	Homo sapiens nudix (nucleoside diphosphate linked moiety X)-type motif 15 (NUDT15), mRNA [NM_018283]	4.48313	down	4.42814	down	168.44261	781.92710	147.66917	576.60254	Detected	Detected	Detected	Detected
5021	A_33_P3280845	THY1	Hs.644697	Thy-1 cell surface antigen	Homo sapiens Thy-1 cell surface antigen (THY1), mRNA [NM_006288]	4.48070	down	3.68552	down	18913.14600	87749.11000	13449.04100	43707.47000	Detected	Detected	Detected	Detected
5022	A_24_P10214	STXBP6	Hs.508958	syntaxin binding protein 6 (amisyn)	Homo sapiens syntaxin binding protein 6 (amisyn) (STXBP6), mRNA [NM_014178]	4.47940	down	5.40021	down	182.04918	844.38770	172.94989	823.56274	Detected	Detected	Detected	Detected
5023	A_33_P3279730				D-dopachrome decarboxylase (EC 4.1.1.84)(D-dopachrome tautomerase)(Phenylpyruvate tautomerase II) [Source:UniProtKB/Swiss-Prot;Acc:P30046] [ENST00000430101]	4.47294	down	4.13579	down	50.48250	233.81213	68.81451	250.95956	Detected	Detected	Detected	Detected
5024	A_24_P397107	CDC25A	Hs.437705	cell division cycle 25 homolog A (S. pombe)	Homo sapiens cell division cycle 25 homolog A (S. pombe) (CDC25A), transcript variant 1, mRNA [NM_001789]	4.46766	down	5.26022	down	131.95741	610.44540	126.09592	584.88450	Detected	Detected	Detected	Detected

5025	A_33_P3267375	SGCD	Hs.387207	sarcoglycan, delta (35kDa dystrophin-associated glycoprotein)	Homo sapiens sarcoglycan, delta (35kDa dystrophin-associated glycoprotein) (SGCD), transcript variant 1, mRNA [NM_000337]	4.46409	down	4.79692	down	410.96070	1899.61700	226.99168	960.14810	Detected	Detected	Detected	Detected
5026	A_24_P49747	LOC646993	Hs.591080	similar to high mobility group box 3	PREDICTED: Homo sapiens similar to high mobility group box 3 (LOC646993), mRNA [XM_929965]	4.46362	down	4.73201	down	160.52905	741.94855	37.17392	155.11348	Detected	Detected	Detected	Detected
5027	A_33_P3387164	TCP11	Hs.435371	t-complex 11 homolog (mouse)	Homo sapiens t-complex 11 homolog (mouse) (TCP11), transcript variant 2, mRNA [NM_018679]	4.45426	down	1.35851	down	4.79983	22.13778	7.32468	8.77437	Compromised	Detected	Compromised	Compromised
5028	A_23_P111041	HIST1H2BI	Hs.553506	histone cluster 1, H2bi	Homo sapiens histone cluster 1, H2bi (HIST1H2BI), mRNA [NM_003525]	4.44803	down	4.53043	down	268.51025	1236.69180	310.05075	1238.61840	Detected	Detected	Detected	Detected
5029	A_33_P3296940	FNDC3B	Hs.159430	fibronectin type III domain containing 3B	Homo sapiens fibronectin type III domain containing 3B (FNDC3B), transcript variant 1, mRNA [NM_022763]	4.44759	down	4.03569	down	143.04219	658.75270	167.86288	597.36280	Detected	Detected	Detected	Detected
5030	A_33_P3275998	RABEP1	Hs.592121	rabaptin, RAB GTPase binding effector protein 1	Homo sapiens rabaptin, RAB GTPase binding effector protein 1 (RABEP1), transcript variant 1, mRNA [NM_004703]	4.44640	down	4.66180	down	260.47473	1199.24370	217.55586	894.31300	Detected	Detected	Detected	Detected
5031	A_33_P3294072	LOC100132878	Hs.710997	similar to Zinc finger protein 816A	PREDICTED: Homo sapiens similar to Zinc finger protein 816A (LOC100132878), miscRNA [XR_039398]	4.44048	down	13.26997	down	3.85614	17.73030	2.49958	29.24840	Compromised	Detected	Compromised	Detected
5032	A_24_P40626	GREM2	Hs.98206	gremlin 2, cysteine knot superfamily, homolog (Xenopus laevis)	Homo sapiens gremlin 2, cysteine knot superfamily, homolog (Xenopus laevis) (GREM2), mRNA [NM_022469]	4.43586	down	4.28679	down	3289.13300	15107.50200	2932.63840	11085.51400	Detected	Detected	Detected	Detected
5033	A_33_P3234697	LXN	Hs.478067	latexin	Homo sapiens latexin (LXN), mRNA [NM_020169]	4.43218	down	4.67260	down	204.84123	940.08720	173.78930	716.05554	Detected	Detected	Detected	Detected
5034	A_33_P3373364	CLIC4	Hs.440544	chloride intracellular channel 4	Homo sapiens chloride intracellular channel 4 (CLIC4), nuclear gene encoding mitochondrial protein, mRNA [NM_013943]	4.43053	down	4.14939	down	903.82074	4146.40100	833.44090	3049.46780	Detected	Detected	Detected	Detected
5035	A_23_P120002	SP110	Hs.145150	SP110 nuclear body protein	Homo sapiens SP110 nuclear body protein (SP110), transcript variant b, mRNA [NM_004510]	4.42805	down	4.44597	down	549.88110	2521.24240	387.31268	1518.42740	Detected	Detected	Detected	Detected
5036	A_33_P3263217	LRRC4	Hs.655003	leucine rich repeat containing 4	Homo sapiens leucine rich repeat containing 4 (LRRC4), mRNA [NM_022143]	4.42077	down	1.06305	down	4.56210	20.88315	11.34156	10.63141	Compromised	Detected	Compromised	Compromised
5037	A_32_P189204	GAS2L3	Hs.20575	growth arrest-specific 2 like 3	Homo sapiens growth arrest-specific 2 like 3 (GAS2L3), mRNA [NM_174942]	4.41818	down	3.97096	down	35.17792	160.93350	22.25418	77.92430	Detected	Detected	Detected	Detected
5038	A_32_P191527	SETD8	Hs.443735	SET domain containing (lysine methyltransferase) 8	Homo sapiens SET domain containing (lysine methyltransferase) 8 (SETD8), mRNA [NM_020382]	4.41753	down	4.52333	down	71.10564	325.24942	51.28594	204.56067	Detected	Detected	Detected	Detected
5039	A_24_P40094	ATCAY	Hs.418055	ataxia, cerebellar, Cayman type	Homo sapiens ataxia, cerebellar, Cayman type (ATCAY), mRNA [NM_033064]	4.41730	down	1.02055	down	9.59126	43.86983	6.63278	5.96894	Compromised	Detected	Compromised	Compromised
5040	A_33_P3223923	PDIA3	Hs.591095	protein disulfide isomerase family A, member 3	Homo sapiens protein disulfide isomerase family A, member 3 (PDIA3), mRNA [NM_005313]	4.40653	down	4.81978	down	286.47205	1307.11120	212.61816	903.63556	Detected	Detected	Detected	Detected
5041	A_23_P357929	SLC35D1	Hs.213642	solute carrier family 35 (UDP-glucuronic acid/UDP-N-acetylgalactosamine dual transporter), member D1	Homo sapiens solute carrier family 35 (UDP-glucuronic acid/UDP-N-acetylgalactosamine dual transporter), member D1 (SLC35D1), mRNA [NM_015139]	4.39346	down	4.39293	down	68.23728	310.42870	53.08312	205.62512	Detected	Detected	Detected	Detected
5042	A_32_P15799	HMGN2	Hs.181163	high-mobility group nucleosomal binding domain 2	Homo sapiens high-mobility group nucleosomal binding domain 2 (HMGN2), mRNA [NM_005517]	4.39215	down	3.93753	down	21575.49600	98123.01600	10546.88700	36619.56200	Detected	Detected	Detected	Detected
5043	A_32_P208350	TDRD9	Hs.21454	tudor domain containing 9	Homo sapiens tudor domain containing 9 (TDRD9), mRNA [NM_153046]	4.38833	down	2.60787	down	17.74113	80.61459	17.18819	39.52587	Detected	Detected	Detected	Detected
5044	A_24_P51201		Hs.35804		Probable E3 ubiquitin-protein ligase HERC3 (EC 6.3.2.-)(HECT domain and RCC1-like domain-containing protein 3) [Source:UniProtKB/Swiss-Prot;Acc:Q15034] [ENST00000407637]	4.37376	down	4.87225	down	21.17841	95.91395	5.73438	24.63660	Detected	Detected	Compromised	Detected
5045	A_33_P3368445	LOC648822	Hs.710955	similar to hCG2040565	PREDICTED: Homo sapiens similar to hCG2040565 (LOC648822), mRNA [XM_001716780]	4.37285	down	7.40903	down	430.96520	1951.36940	3.16628	20.68597	Detected	Detected	Compromised	Detected
5046	A_23_P39116	LIG1	Hs.1770	ligase I, DNA, ATP-dependent	Homo sapiens ligase I, DNA, ATP-dependent (LIG1), mRNA [NM_000234]	4.37236	down	4.90716	down	1743.32960	7892.74800	1793.52400	7760.73200	Detected	Detected	Detected	Detected
5047	A_23_P129075	WDR76	Hs.250154	WD repeat domain 76	Homo sapiens WD repeat domain 76 (WDR76), mRNA [NM_024908]	4.36562	down	3.89743	down	53.76137	243.02423	57.13434	196.35481	Detected	Detected	Detected	Detected
5048	A_33_P3436316	ASXL1	Hs.374043	additional sex combs like 1 (Drosophila)	Homo sapiens additional sex combs like 1 (Drosophila) (ASXL1), transcript variant 2, mRNA [NM_001164603]	4.36232	down	4.73408	down	251.65645	1136.73380	204.08989	851.96740	Detected	Detected	Detected	Detected
5049	A_32_P100430	LOC100128737	Hs.721809	hypothetical LOC100128737	PREDICTED: Homo sapiens hypothetical LOC100128737 (LOC100128737), mRNA [XM_001715932]	4.35949	down	4.78046	down	210.49495	950.18970	234.54007	988.67285	Detected	Detected	Detected	Detected
5050	A_23_P300056	CDC42	Hs.467637	cell division cycle 42 (GTP binding protein, 25kDa)	Homo sapiens cell division cycle 42 (GTP binding protein, 25kDa) (CDC42), transcript variant 2, mRNA [NM_044472]	4.35893	down	3.86047	down	109.17782	492.77332	60.26164	205.13826	Detected	Detected	Detected	Detected
5051	A_23_P205959	ALDH1A3	Hs.459538	aldehyde dehydrogenase 1 family, member A3	Homo sapiens aldehyde dehydrogenase 1 family, member A3 (ALDH1A3), mRNA [NM_000693]	4.34849	down	3.91021	down	208.54344	939.00590	173.49884	598.22186	Detected	Detected	Detected	Detected
5052	A_33_P3390092					4.34525	down	4.03782	down	7.77245	34.97081	7.64273	27.21205	Compromised	Detected	Compromised	Detected
5053	A_23_P216920	NEK6	Hs.197071	NIMA (never in mitosis gene a)-related kinase 6	Homo sapiens NIMA (never in mitosis gene a)-related kinase 6 (NEK6), transcript variant 2, mRNA [NM_014397]	4.34324	down	4.55403	down	2306.44850	10372.67200	2336.03780	9380.82700	Detected	Detected	Detected	Detected
5054	A_23_P395374	HIST1H4D	Hs.248179	histone cluster 1, H4d	Homo sapiens histone cluster 1, H4d (HIST1H4D), mRNA [NM_003539]	4.34009	down	4.25620	down	1161.45530	5219.56300	252.62883	948.13610	Detected	Detected	Detected	Detected

5055	A_33_P3390013	ADAMTS1	Hs.643357	ADAM metallopeptidase with thrombospondin type 1 motif, 1	Homo sapiens ADAM metallopeptidase with thrombospondin type 1 motif, 1 (ADAMTS1), mRNA [NM_006988]	4.33355	down	4.08942	down	320.86462	1439.79030	232.13419	837.07790	Detected	Detected	Detected	Detected
5056	A_24_P243396	TCOF1	Hs.519672	Treacher Collins-Franceschetti syndrome 1	Homo sapiens Treacher Collins-Franceschetti syndrome 1 (TCOF1), transcript variant 3, mRNA [NM_001008657]	4.32964	down	4.31941	down	117.56401	527.05860	107.63834	409.97464	Detected	Detected	Detected	Detected
5057	A_23_P152262	DPEP1	Hs.109	dipeptidase 1 (renal)	Homo sapiens dipeptidase 1 (renal) (DPEP1), transcript variant 1, mRNA [NM_004413]	4.32963	down	4.21869	down	7.82363	35.07455	7.00571	26.06125	Compromised	Detected	Compromised	Detected
5058	A_33_P3318763	LOC100128994	Hs.453788	hypothetical LOC100128994	PREDICTED: Homo sapiens hypothetical LOC100128994 (LOC100128994), mRNA [XM_001717144]	4.31309	down	3.70774	down	54.19221	242.02377	53.84109	176.03090	Detected	Detected	Detected	Detected
5059	A_23_P423074	FAM169A	Hs.91662	family with sequence similarity 169, member A	Homo sapiens family with sequence similarity 169, member A (FAM169A), mRNA [NM_015566]	4.31263	down	2.66643	down	4.55305	20.33189	2.34208	5.50678	Compromised	Detected	Compromised	Compromised
5060	A_24_P921321	PTPRJ	Hs.318547	protein tyrosine phosphatase, receptor type, J	Homo sapiens protein tyrosine phosphatase, receptor type, J (PTPRJ), transcript variant 1, mRNA [NM_002843]	4.30054	down	4.15283	down	331.86783	1477.81910	230.61170	844.48310	Detected	Detected	Detected	Detected
5061	A_23_P139547	TUBA1A	Hs.654422	tubulin, alpha 1a	Homo sapiens tubulin, alpha 1a (TUBA1A), mRNA [NM_006009]	4.29967	down	4.01356	down	13557.09900	60357.94500	18273.39800	64671.76000	Detected	Detected	Detected	Detected
5062	A_23_P69537	NMU	Hs.418367	neuromedin U	Homo sapiens neuromedin U (NMU), mRNA [NM_006681]	4.29906	down	8.46142	down	7.94597	35.37153	3.35202	25.01012	Compromised	Detected	Compromised	Detected
5063	A_33_P3271657	HHIPL1	Hs.288522	HHIP-like 1	Homo sapiens HHIP-like 1 (HHIPL1), transcript variant 1, mRNA [NM_001127258]	4.28159	down	5.28068	down	215.76305	956.56555	210.84135	981.77330	Detected	Detected	Detected	Detected
5064	A_33_P3328958					4.28052	down	3.76139	down	6.46318	28.64680	3.34601	11.09791	Compromised	Detected	Compromised	Compromised
5065	A_23_P113825	NACC2	Hs.112895	NACC family member 2, BEN and BTB (POZ) domain containing	Homo sapiens NACC family member 2, BEN and BTB (POZ) domain containing (NACC2), mRNA [NM_144653]	4.27787	down	4.17557	down	2208.61430	9783.18900	1584.71720	5834.90040	Detected	Detected	Detected	Detected
5066	A_32_P25357	CDH15	Hs.148090	cadherin 15, type 1, M-cadherin (myotubule)	Homo sapiens cadherin 15, type 1, M-cadherin (myotubule) (CDH15), mRNA [NM_004933]	4.27668	down	3.31864	down	22.61115	100.12960	20.82790	60.94959	Detected	Detected	Detected	Detected
5067	A_32_P41487	HMGN2	Hs.181163	high-mobility group nucleosomal binding domain 2	Homo sapiens high-mobility group nucleosomal binding domain 2 (HMGN2), mRNA [NM_005517]	4.27465	down	3.17169	down	42604.16800	188575.67000	38202.47300	106843.47000	Detected	Detected	Detected	Detected
5068	A_23_P35205	RCAN3	Hs.656799	RCAN family member 3	Calcipressin-3 (Regulator of calcineurin 3)(Down syndrome candidate region 1-like protein 2)(Myocyte-enriched calcineurin-interacting protein 3)(MCIP3) [Source:UniProtKB/Swiss-Prot;Acc:Q9UKA8] [ENST00000374395]	4.27272	down	4.12529	down	321.35263	1421.73830	260.73883	948.47400	Detected	Detected	Detected	Detected
5069	A_23_P259272	WSB2	Hs.719911	WD repeat and SOCS box-containing 2	Homo sapiens WD repeat and SOCS box-containing 2 (WSB2), mRNA [NM_018639]	4.26937	down	4.27102	down	6392.00830	28257.52300	4682.63130	17635.48400	Detected	Detected	Detected	Detected
5070	A_23_P212800	FGF5	Hs.37055	fibroblast growth factor 5	Homo sapiens fibroblast growth factor 5 (FGF5), transcript variant 1, mRNA [NM_004464]	4.26640	down	4.08437	down	371.24854	1640.06020	315.40643	1135.95400	Detected	Detected	Detected	Detected
5071	A_33_P3218356	TMEM167B	Hs.82933	transmembrane protein 167B	Homo sapiens transmembrane protein 167B (TMEM167B), mRNA [NM_020141]	4.26618	down	4.15481	down	337.39203	1490.41280	334.93080	1227.07710	Detected	Detected	Detected	Detected
5072	A_33_P3224105	C15orf23	Hs.525796	chromosome 15 open reading frame 23	Homo sapiens chromosome 15 open reading frame 23 (C15orf23), transcript variant 2, mRNA [NM_001142761]	4.26515	down	4.33094	down	4369.40100	19296.97300	4102.99700	15669.27500	Detected	Detected	Detected	Detected
5073	A_23_P206661	NQO1	Hs.406515	NAD(P)H dehydrogenase, quinone 1	Homo sapiens NAD(P)H dehydrogenase, quinone 1 (NQO1), transcript variant 1, mRNA [NM_000903]	4.26253	down	3.66023	down	303.08707	1337.72840	195.31755	630.39770	Detected	Detected	Detected	Detected
5074	A_23_P85783	PHGDH	Hs.487296	phosphoglycerate dehydrogenase	Homo sapiens phosphoglycerate dehydrogenase (PHGDH), mRNA [NM_006623]	4.26041	down	4.70117	down	1275.19620	5625.51100	1339.16260	5551.42530	Detected	Detected	Detected	Detected
5075	A_33_P3244728	LRP2	Hs.657729	low density lipoprotein-related protein 2	Homo sapiens low density lipoprotein-related protein 2 (LRP2), mRNA [NM_004525]	4.25888	down	5.90223	down	583.19740	2571.84080	190.76208	992.82770	Detected	Detected	Detected	Detected
5076	A_32_P831181	BRI3BP	Hs.596464	BRI3 binding protein	Homo sapiens BRI3 binding protein (BRI3BP), mRNA [NM_080626]	4.25817	down	5.02753	down	53.35552	235.25317	58.58858	259.73642	Detected	Detected	Detected	Detected
5077	A_33_P3280805	LMO7	Hs.207631	LIM domain 7	Homo sapiens LIM domain 7 (LMO7), transcript variant 2, mRNA [NM_015842]	4.25295	down	5.15996	down	41.05179	180.78215	52.77922	240.14589	Detected	Detected	Detected	Detected
5078	A_24_P259154	MGC16703	Hs.585006	tubulin, alpha pseudogene	Homo sapiens tubulin, alpha pseudogene (MGC16703), non-coding RNA [NR_003608]	4.24690	down	2.52806	down	482.56976	2122.09770	75.48191	168.26550	Detected	Detected	Detected	Detected
5079	A_23_P91590	RANBP1	Hs.24763	RAN binding protein 1	Homo sapiens RAN binding protein 1 (RANBP1), mRNA [NM_002882]	4.24606	down	4.78266	down	8336.71200	36653.42000	7187.89550	30313.53100	Detected	Detected	Detected	Detected
5080	A_23_P325040	TMPO	Hs.11355	thymopoietin	Homo sapiens thymopoietin (TMPO), transcript variant 1, mRNA [NM_003276]	4.24568	down	4.17001	down	102.42580	450.28763	80.07113	294.42773	Detected	Detected	Detected	Detected
5081	A_33_P3407937	PLCXD1	Hs.522568	phosphatidylinositol-specific phospholipase C, X domain containing 1	Homo sapiens phosphatidylinositol-specific phospholipase C, X domain containing 1 (PLCXD1), transcript variant 1, mRNA [NM_018390]	4.24394	down	5.95474	down	91.25652	401.02032	85.85397	450.80524	Detected	Detected	Detected	Detected
5082	A_23_P115519	LCE3D	Hs.244349	late cornified envelope 3D	Homo sapiens late cornified envelope 3D (LCE3D), mRNA [NM_032563]	4.23941	down	8.98598	down	28.68508	125.91992	17.07352	135.28632	Detected	Detected	Detected	Detected
5083	A_24_P319942	SSR3	Hs.518346	signal sequence receptor, gamma (translocon-associated protein gamma)	Homo sapiens signal sequence receptor, gamma (translocon-associated protein gamma) (SSR3), mRNA [NM_007107]	4.23918	down	5.64747	down	99.63514	437.34814	109.68317	546.20980	Detected	Detected	Detected	Detected

5084	A_33_P3382271				Homo sapiens cDNA clone IMAGE:5562656. [BC033643]	4.23458	down	3.77949	down	60.44880	265.05170	60.32363	201.04176	Detected	Detected	Detected	Detected
5085	A_33_P3293958	LOC100133479		hypothetical protein LOC100133479	PREDICTED: Homo sapiens hypothetical LOC100133479 (LOC100133479), mRNA [XM_001717909]	4.23134	down	3.28153	down	29.32807	128.49760	33.80766	97.82661	Detected	Detected	Detected	Detected
5086	A_23_P62607	IL22RA1	Hs.110915	interleukin 22 receptor, alpha 1	Homo sapiens interleukin 22 receptor, alpha 1 (IL22RA1), mRNA [NM_021258]	4.23083	down	4.11058	down	38.50855	168.70030	26.88339	97.44350	Detected	Detected	Detected	Detected
5087	A_23_P138706	ADRA2A	Hs.249159	adrenergic, alpha-2A-, receptor	Homo sapiens adrenergic, alpha-2A-, receptor (ADRA2A), mRNA [NM_000681]	4.22788	down	9.45882	down	9.42170	41.24627	3.56301	29.71798	Compromised	Detected	Compromised	Detected
5088	A_33_P3222109	XG	Hs.179675	Xg blood group	Homo sapiens Xg blood group (XG), transcript variant 2, mRNA [NM_001141919]	4.21595	down	4.02814	down	622.17664	2716.08030	442.00623	1569.99660	Detected	Detected	Detected	Detected
5089	A_32_P192823	PRPS1L1	Hs.169284	phosphoribosyl pyrophosphate synthetase 1-like 1	Homo sapiens phosphoribosyl pyrophosphate synthetase 1-like 1 (PRPS1L1), mRNA [NM_175886]	4.21344	down	3.91706	down	631.76807	2756.30570	623.39730	2153.22970	Detected	Detected	Detected	Detected
5090	A_23_P67771	BARD1	Hs.591642	BRCA1 associated RING domain 1	Homo sapiens BRCA1 associated RING domain 1 (BARD1), mRNA [NM_000465]	4.20839	down	4.28596	down	460.69882	2007.54970	412.85117	1560.29810	Detected	Detected	Detected	Detected
5091	A_33_P3309031	LOC728530	Hs.659020	hypothetical LOC728530	PREDICTED: Homo sapiens hypothetical LOC728530 (LOC728530), mRNA [XM_001127675]	4.20749	down	1.19764	down	4.95968	21.60779	2.86470	3.02531	Compromised	Detected	Compromised	Compromised
5092	A_23_P63521	LCE2C	Hs.553713	late cornified envelope 2C	Homo sapiens late cornified envelope 2C (LCE2C), mRNA [NM_178429]	4.20376	down	6.00603	down	19.64014	85.49013	11.85671	62.79385	Detected	Detected	Compromised	Detected
5093	A_23_P56505	ITGA4	Hs.440955	integrin, alpha 4 (antigen CD49D, alpha 4 subunit of VLA-4 receptor)	Homo sapiens integrin, alpha 4 (antigen CD49D, alpha 4 subunit of VLA-4 receptor) (ITGA4), mRNA [NM_000885]	4.20224	down	4.71250	down	61.73326	268.61676	46.28966	192.35397	Detected	Detected	Detected	Detected
5094	A_24_P376120	MYPN	Hs.55205	myopalladin	Homo sapiens myopalladin (MYPN), mRNA [NM_032578]	4.19850	down	6.35629	down	6.69271	29.09572	2.78283	15.59751	Compromised	Detected	Compromised	Detected
5095	A_24_P156490	KCNMA1	Hs.144795	potassium large conductance calcium-activated channel, subfamily M, alpha member 1	Homo sapiens potassium large conductance calcium-activated channel, subfamily M, alpha member 1 (KCNMA1), transcript variant 2, mRNA [NM_002247]	4.19337	down	3.96418	down	277.34824	1204.26500	185.66580	649.00890	Detected	Detected	Detected	Detected
5096	A_23_P355517	SYNPO2L	Hs.645273	synaptopodin 2-like	Homo sapiens synaptopodin 2-like (SYNPO2L), transcript variant 2, mRNA [NM_024875]	4.19199	down	2.34508	down	9.86973	42.84084	8.74944	18.09269	Compromised	Detected	Compromised	Detected
5097	A_33_P3346826	IL32	Hs.943	interleukin 32	Homo sapiens interleukin 32 (IL32), transcript variant 4, mRNA [NM_001012633]	4.19014	down	4.62824	down	60.08030	260.67145	55.96246	228.39061	Detected	Detected	Detected	Detected
5098	A_23_P167096	VEGFC	Hs.435215	vascular endothelial growth factor C	Homo sapiens vascular endothelial growth factor C (VEGFC), mRNA [NM_005429]	4.18984	down	4.15525	down	10963.04200	47562.11300	9239.17100	33852.84400	Detected	Detected	Detected	Detected
5099	A_33_P3351087				cGMP-dependent protein kinase 1, beta isozyme (cGK 1 beta)(cGKI-beta)(EC 2.7.11.12) [Source:UniProtKB/Swiss-Prot;Acc:P14619] [ENST00000373976]	4.18356	down	2.89636	down	26.90071	116.53132	14.61953	37.33798	Detected	Detected	Compromised	Detected
5100	A_33_P3392245	FAM178A	Hs.447458	family with sequence similarity 178, member A	Homo sapiens family with sequence similarity 178, member A (FAM178A), transcript variant 2, mRNA [NM_001136123]	4.17730	down	1.60359	down	9.40668	40.68789	5.21380	7.37247	Compromised	Detected	Compromised	Compromised
5101	A_24_P926960	MEGF6	Hs.593645	multiple EGF-like-domains 6	Homo sapiens multiple EGF-like-domains 6 (MEGF6), mRNA [NM_001409]	4.17486	down	4.24945	down	281.90063	1218.62740	325.07016	1218.07900	Detected	Detected	Detected	Detected
5102	A_33_P3407299	ANP32E	Hs.656466	acidic (leucine-rich) nuclear phosphoprotein 32 family, member E	Homo sapiens acidic (leucine-rich) nuclear phosphoprotein 32 family, member E (ANP32E), transcript variant 1, mRNA [NM_030920]	4.16875	down	3.65858	down	390.04410	1683.65250	277.51678	895.29663	Detected	Detected	Detected	Detected
5103	A_23_P132910	RBM47	Hs.518727	RNA binding motif protein 47	Homo sapiens RNA binding motif protein 47 (RBM47), transcript variant 2, mRNA [NM_019027]	4.16812	down	3.80970	down	33.11991	142.94289	19.92333	66.92962	Detected	Detected	Detected	Detected
5104	A_23_P433785	P2RX5	Hs.408615	purinergic receptor P2X, ligand-gated ion channel, 5	Homo sapiens purinergic receptor P2X, ligand-gated ion channel, 5 (P2RX5), transcript variant 1, mRNA [NM_002561]	4.16779	down	5.05332	down	108.52860	468.36410	115.11179	512.93430	Detected	Detected	Detected	Detected
5105	A_33_P3281785	NKX3-1	Hs.55999	NK3 homeobox 1	Homo sapiens NK3 homeobox 1 (NKX3-1), mRNA [NM_006167]	4.16630	down	6.59694	down	2.32629	10.03570	4.50406	26.20068	Compromised	Compromised	Compromised	Detected
5106	A_23_P366216	HIST1H2BH	Hs.247815	histone cluster 1, H2bh	Homo sapiens histone cluster 1, H2bh (HIST1H2BH), mRNA [NM_003524]	4.15571	down	4.08912	down	622.90010	2680.38000	741.87250	2675.00780	Detected	Detected	Detected	Detected
5107	A_24_P342632	AK5	Hs.559718	adenylate kinase 5	Homo sapiens adenylate kinase 5 (AK5), transcript variant 1, mRNA [NM_174858]	4.15522	down	3.21017	down	40.43916	173.99213	30.84461	87.31172	Detected	Detected	Detected	Detected
5108	A_24_P684186	EMB	Hs.561411	embigin homolog (mouse)	Homo sapiens embigin homolog (mouse) (EMB), mRNA [NM_198449]	4.15484	down	7.00172	down	11.82061	50.85430	5.01686	30.97437	Compromised	Detected	Compromised	Detected
5109	A_33_P3219651	BMPER	Hs.660998	BMP binding endothelial regulator	Homo sapiens BMP binding endothelial regulator (BMPER), mRNA [NM_133468]	4.15365	down	4.24079	down	701.65894	3017.79170	412.56277	1542.77550	Detected	Detected	Detected	Detected
5110	A_23_P110266	C4orf31	Hs.709520	chromosome 4 open reading frame 31	Homo sapiens chromosome 4 open reading frame 31 (C4orf31), mRNA [NM_024574]	4.14664	down	5.65484	down	21.75164	93.39444	8.78769	43.81885	Detected	Detected	Compromised	Detected
5111	A_23_P87769	C12orf48	Hs.330663	chromosome 12 open reading frame 48	Homo sapiens chromosome 12 open reading frame 48 (C12orf48), mRNA [NM_017915]	4.14654	down	4.00960	down	690.75930	2965.82520	521.31670	1843.18150	Detected	Detected	Detected	Detected
5112	A_23_P337270		Hs.506115		Serine/threonine-protein kinase Nek10 (EC 2.7.11.1)(NimA-related protein kinase 10) [Source:UniProtKB/Swiss-Prot;Acc:Q6ZWH5] [ENST00000383770]	4.14613	down	3.98962	down	12.39888	53.23028	13.00825	45.76311	Compromised	Detected	Compromised	Detected
5113	A_33_P3256293					4.14269	down	2.41673	down	5.36284	23.00438	7.01970	14.95934	Compromised	Detected	Compromised	Detected

5114	A_33_P3243524	RAPGEF6	Hs.483329	Rap guanine nucleotide exchange factor (GEF) 6	Homo sapiens Rap guanine nucleotide exchange factor (GEF) 6 (RAPGEF6), transcript variant 5, mRNA [NM_001164389]	4.14267	down	3.36642	down	12.84413	55.09579	11.18836	33.21243	Detected	Detected	Compromised	Detected
5115	A_23_P156826	C6orf105	Hs.126409	chromosome 6 open reading frame 105	Homo sapiens chromosome 6 open reading frame 105 (C6orf105), transcript variant 2, mRNA [NM_032744]	4.13812	down	3.96915	down	330.42178	1415.80820	223.17432	781.10060	Detected	Detected	Detected	Detected
5116	A_23_P70480	HIST1H4L	Hs.533295	histone cluster 1, H4l	Homo sapiens histone cluster 1, H4l (HIST1H4L), mRNA [NM_003546]	4.13334	down	4.16270	down	4069.09670	17415.36100	3905.88480	14337.06200	Detected	Detected	Detected	Detected
5117	A_33_P3299285	FCRLB	Hs.517422	Fc receptor-like B	Homo sapiens Fc receptor-like B (FCRLB), mRNA [NM_001002901]	4.13212	down	4.53147	down	38.55246	164.95206	54.47829	217.68490	Detected	Detected	Detected	Detected
5118	A_23_P342138	ADAMTSL1	Hs.522019	ADAMTS-like 1	Homo sapiens ADAMTS-like 1 (ADAMTSL1), transcript variant 4, mRNA [NM_001040272]	4.12763	down	4.82643	down	531.52550	2271.73680	465.98035	1983.16690	Detected	Detected	Detected	Detected
5119	A_33_P3215883	FLT1	Hs.594454	fms-related tyrosine kinase 1 (vascular endothelial growth factor/vascular permeability factor receptor)	Homo sapiens fms-related tyrosine kinase 1 (vascular endothelial growth factor/vascular permeability factor receptor) (FLT1), transcript variant 1, mRNA [NM_002019]	4.12179	down	7.45216	down	6.89830	29.44157	3.37186	22.15731	Compromised	Detected	Compromised	Detected
5120	A_23_P10305	CTPS2	Hs.227049	CTP synthase II	Homo sapiens CTP synthase II (CTPS2), transcript variant 2, mRNA [NM_175859]	4.11375	down	4.33865	down	54.16229	230.71088	44.44841	170.04990	Detected	Detected	Detected	Detected
5121	A_32_P95729	FANCI	Hs.513126	Fanconi anemia, complementation group I	Homo sapiens Fanconi anemia, complementation group I (FANCI), transcript variant 2, mRNA [NM_018193]	4.11238	down	3.65477	down	763.60620	3251.58900	795.07324	2562.31250	Detected	Detected	Detected	Detected
5122	A_23_P24903	P2RY2	Hs.339	purinergic receptor P2Y, G-protein coupled, 2	Homo sapiens purinergic receptor P2Y, G-protein coupled, 2 (P2RY2), transcript variant 1, mRNA [NM_176072]	4.11214	down	6.11951	down	4.72663	20.12577	2.54075	13.71020	Compromised	Detected	Compromised	Detected
5123	A_32_P72341	TRIM59	Hs.212957	tripartite motif-containing 59	Homo sapiens tripartite motif-containing 59 (TRIM59), mRNA [NM_173084]	4.11144	down	3.72007	down	477.01443	2030.75920	284.53458	933.36633	Detected	Detected	Detected	Detected
5124	A_23_P126727	NOL9	Hs.59425	nucleolar protein 9	Homo sapiens nucleolar protein 9 (NOL9), mRNA [NM_024654]	4.10907	down	3.87162	down	406.83920	1731.01030	364.22330	1243.44380	Detected	Detected	Detected	Detected
5125	A_23_P407840	FNDC1	Hs.520525	fibronectin type III domain containing 1	Homo sapiens fibronectin type III domain containing 1 (FNDC1), mRNA [NM_032532]	4.10761	down	3.54755	down	65.02814	276.58203	54.06905	169.13869	Detected	Detected	Detected	Detected
5126	A_33_P3288839	C14orf37	Hs.535420	chromosome 14 open reading frame 37	Homo sapiens chromosome 14 open reading frame 37 (C14orf37), mRNA [NM_001001872]	4.10182	down	4.00274	down	197.68538	839.62320	142.67120	503.56930	Detected	Detected	Detected	Detected
5127	A_24_P111242	SVIP	Hs.349096	small VCP/p97-interacting protein	Homo sapiens small VCP/p97-interacting protein (SVIP), mRNA [NM_148893]	4.10108	down	2.99680	down	20.07874	85.26444	14.26625	37.69934	Detected	Detected	Detected	Detected
5128	A_33_P3250895	PSMB11	Hs.508918	proteasome (prosome, macropain) subunit, beta type, 11	Homo sapiens proteasome (prosome, macropain) subunit, beta type, 11 (PSMB11), mRNA [NM_001099780]	4.10076	down	3.48257	down	15.26583	64.82123	3.09172	9.49436	Detected	Detected	Compromised	Compromised
5129	A_23_P80570	AADAC	Hs.506908	arylacetamide deacetylase (esterase)	Homo sapiens arylacetamide deacetylase (esterase) (AADAC), mRNA [NM_001086]	4.09567	down	2.74127	down	8.58929	36.42633	12.66254	30.60828	Compromised	Detected	Compromised	Detected
5130	A_23_P123974	DTYMK	Hs.471873	deoxythymidylate kinase (thymidylate kinase)	Homo sapiens deoxythymidylate kinase (thymidylate kinase) (DTYMK), mRNA [NM_012145]	4.09304	down	3.96534	down	10900.91700	46200.00800	7156.72700	25024.23200	Detected	Detected	Detected	Detected
5131	A_24_P55148	HIST1H2BJ	Hs.656567	histone cluster 1, H2bj	Homo sapiens histone cluster 1, H2bj (HIST1H2BJ), mRNA [NM_021058]	4.08783	down	5.60294	down	97.51760	412.77066	69.09530	341.37366	Detected	Detected	Detected	Detected
5132	A_23_P65506	SPTB	Hs.417303	spectrin, beta, erythrocytic	Homo sapiens spectrin, beta, erythrocytic (SPTB), transcript variant 2, mRNA [NM_000347]	4.08461	down	6.86540	down	7.19415	30.42732	3.11248	18.84246	Compromised	Detected	Compromised	Detected
5133	A_23_P203191	APOA1	Hs.93194	apolipoprotein A-I	Homo sapiens apolipoprotein A-I (APOA1), mRNA [NM_000039]	4.07973	down	3.90351	down	6.45793	27.28085	4.96122	17.07688	Compromised	Detected	Compromised	Detected
5134	A_33_P3339376		Hs.128067		WAS/WASL-interacting protein family member 1 (Wiskott-Aldrich syndrome protein-interacting protein)(WASP-interacting protein)(Protein PRPL-2) [Source:UniProtKB/Swiss-Prot;Acc:O43516] [ENST00000409415]	4.06854	down	4.25757	down	1367.90770	5762.73300	1049.89750	3941.61330	Detected	Detected	Detected	Detected
5135	A_23_P166087	RASSF2	Hs.631504	Ras association (RalGDS/AF-6) domain family member 2	Homo sapiens Ras association (RalGDS/AF-6) domain family member 2 (RASSF2), transcript variant 1, mRNA [NM_014737]	4.06574	down	3.41685	down	19.60497	82.53507	18.65502	56.20652	Detected	Detected	Detected	Detected
5136	A_24_P7965	ESRRG	Hs.444225	estrogen-related receptor gamma	Homo sapiens estrogen-related receptor gamma (ESRRG), transcript variant 2, mRNA [NM_206594]	4.06285	down	2.33449	down	8.91978	37.52477	15.82223	32.57055	Compromised	Detected	Detected	Detected
5137	A_23_P393025	SPATA4	Hs.481235	spermatogenesis associated 4	Homo sapiens spermatogenesis associated 4 (SPATA4), mRNA [NM_144644]	4.06095	down	3.12442	down	33.07120	139.06260	35.00371	96.43813	Detected	Detected	Detected	Detected
5138	A_33_P3211818	RUNX1	Hs.149261	runt-related transcription factor 1	Homo sapiens runt-related transcription factor 1 (RUNX1), transcript variant 2, mRNA [NM_001001890]	4.05252	down	4.98005	down	23.29842	97.76525	26.70613	117.27628	Detected	Detected	Detected	Detected
5139	A_33_P3739260	CAP2	Hs.132902	CAP, adenylate cyclase-associated protein, 2 (yeast)	Homo sapiens CAP, adenylate cyclase-associated protein, 2 (yeast) (CAP2), mRNA [NM_006366]	4.04999	down	4.30241	down	29.61644	124.19939	20.58231	78.08575	Detected	Detected	Detected	Detected
5140	A_23_P83098	ALDH1A1	Hs.76392	aldehyde dehydrogenase 1 family, member A1	Homo sapiens aldehyde dehydrogenase 1 family, member A1 (ALDH1A1), mRNA [NM_000689]	4.04910	down	4.17933	down	20.73626	86.94048	15.48779	57.07705	Detected	Detected	Detected	Detected
5141	A_33_P3225378	OR10G3	Hs.554581	olfactory receptor, family 10, subfamily G, member 3	Homo sapiens olfactory receptor, family 10, subfamily G, member 3 (OR10G3), mRNA [NM_001005465]	4.04757	down	1.47393	down	9.41191	39.44618	18.08694	23.50762	Compromised	Detected	Detected	Detected
5142	A_23_P401904	PHF19	Hs.460124	PHD finger protein 19	Homo sapiens PHD finger protein 19 (PHF19), transcript variant 2, mRNA [NM_001009936]	4.04559	down	4.20245	down	1946.19320	8152.69870	2070.30960	7671.90330	Detected	Detected	Detected	Detected

5143	A_33_P3210085	NET1	Hs.25155	neuroepithelial cell transforming 1	Homo sapiens neuroepithelial cell transforming 1 (NET1), transcript variant 1, mRNA [NM_001047160]	4.04558	down	4.06108	down	360.36182	1509.56630	268.14664	960.23883	Detected	Detected	Detected	Detected
5144	A_32_P211045	DHFR	Hs.648635	dihydrofolate reductase	Homo sapiens dihydrofolate reductase (DHFR), mRNA [NM_000791]	4.03933	down	3.73272	down	1067.30530	4464.06600	475.15230	1563.95250	Detected	Detected	Detected	Detected
5145	A_23_P25626	C13orf34	Hs.714340	chromosome 13 open reading frame 34	Homo sapiens chromosome 13 open reading frame 34 (C13orf34), mRNA [NM_024808]	4.02905	down	3.96190	down	351.51706	1466.49850	286.90356	1002.31750	Detected	Detected	Detected	Detected
5146	A_23_P390700	CNTN1	Hs.143434	contactin 1	Homo sapiens contactin 1 (CNTN1), transcript variant 1, mRNA [NM_001843]	4.02793	down	1.18602	down	4.43926	18.51506	2.86166	2.99279	Compromised	Detected	Compromised	Compromised
5147	A_23_P359540	HIST1H4F	Hs.247816	histone cluster 1, H4f	Homo sapiens histone cluster 1, H4f (HIST1H4F), mRNA [NM_003540]	4.02420	down	3.28756	down	42.68813	177.87689	55.66727	161.37617	Detected	Detected	Detected	Detected
5148	A_33_P3397733	FAM154B	Hs.459117	family with sequence similarity 154, member B	Homo sapiens family with sequence similarity 154, member B (FAM154B), mRNA [NM_001008226]	4.02201	down	3.49745	down	12.83743	53.46306	3.25652	10.04318	Compromised	Detected	Compromised	Compromised
5149	A_23_P162579	HSPB8	Hs.400095	heat shock 22kDa protein 8	Homo sapiens heat shock 22kDa protein 8 (HSPB8), mRNA [NM_014365]	4.01996	down	4.13115	down	93.56341	389.45822	71.08520	258.94970	Detected	Detected	Detected	Detected
5150	A_23_P47665	HBE1	Hs.655195	hemoglobin, epsilon 1	Homo sapiens hemoglobin, epsilon 1 (HBE1), mRNA [NM_005330]	4.01818	down	8.94765	down	4.46288	18.56851	2.38602	18.82561	Compromised	Detected	Compromised	Detected
5151	A_33_P3300332	hCG_2040054		hCG2040054	PREDICTED: Homo sapiens hCG2040054 (LOC644093), mRNA [XM_001725480]	4.01307	down	3.34178	down	129.19900	536.86960	32.55016	95.91708	Detected	Detected	Detected	Detected
5152	A_23_P211039	ADAMTS1	Hs.643357	ADAM metallopeptidase with thrombospondin type 1 motif, 1	Homo sapiens ADAM metallopeptidase with thrombospondin type 1 motif, 1 (ADAMTS1), mRNA [NM_006988]	4.01188	down	3.95781	down	558.26170	2319.09940	480.39910	1676.57230	Detected	Detected	Detected	Detected
5153	A_24_P332647	SSH1	Hs.199763	slingshot homolog 1 (Drosophila)	Homo sapiens slingshot homolog 1 (Drosophila) (SSH1), transcript variant 3, mRNA [NM_001161331]	4.01172	down	4.54461	down	206.51122	857.84296	149.85355	600.52185	Detected	Detected	Detected	Detected
5154	A_33_P3229241	HIST2H2BF	Hs.632451	histone cluster 2, H2bf	Homo sapiens histone cluster 2, H2bf (HIST2H2BF), transcript variant 1, mRNA [NM_001024599]	3.99755	down	3.71210	down	74.94844	310.23400	71.30449	233.40082	Detected	Detected	Detected	Detected
5155	A_24_P191781	PARM1	Hs.105460	prostate androgen-regulated mucin-like protein 1	Homo sapiens prostate androgen-regulated mucin-like protein 1 (PARM1), mRNA [NM_015393]	3.99105	down	3.00085	down	2.27328	9.39450	4.91601	13.00835	Compromised	Compromised	Compromised	Detected
5156	A_33_P3221898	LOC339240	Hs.420616	keratin pseudogene	Homo sapiens keratin pseudogene (LOC339240), non-coding RNA [NR_001443]	3.98927	down	6.39690	down	8.84978	36.55605	4.98367	28.11148	Compromised	Detected	Compromised	Detected
5157	A_33_P3404641	LOC150381		hypothetical LOC150381	Homo sapiens hypothetical LOC150381 (LOC150381), non-coding RNA [NR_027034]	3.98811	down	3.71283	down	54.05956	223.24013	47.56061	155.71060	Detected	Detected	Detected	Detected
5158	A_23_P122443	HIST1H1C	Hs.7644	histone cluster 1, H1c	Homo sapiens histone cluster 1, H1c (HIST1H1C), mRNA [NM_005319]	3.97740	down	3.82045	down	2330.71480	9598.89100	2705.52050	9114.45200	Detected	Detected	Detected	Detected
5159	A_33_P3257808	FANCD2	Hs.208388	Fanconi anemia, complementation group D2	Homo sapiens Fanconi anemia, complementation group D2 (FANCD2), transcript variant 2, mRNA [NM_001018115]	3.97608	down	3.95182	down	45.18990	186.04999	45.28367	157.79900	Detected	Detected	Detected	Detected
5160	A_33_P3259135	D4S234E	Hs.518595	DNA segment on chromosome 4 (unique) 234 expressed sequence	Homo sapiens DNA segment on chromosome 4 (unique) 234 expressed sequence (D4S234E), transcript variant 1, mRNA [NM_014392]	3.97525	down	2.62055	down	15.34606	63.16761	15.54790	35.92776	Detected	Detected	Detected	Detected
5161	A_24_P248741	ZNF501	Hs.401045	zinc finger protein 501	Homo sapiens zinc finger protein 501 (ZNF501), mRNA [NM_145044]	3.97349	down	1.00944	down	10.26006	42.21390	7.92068	7.05030	Compromised	Detected	Compromised	Compromised
5162	A_23_P425066	CRYBB2	Hs.373074	crystallin, beta B2	Homo sapiens crystallin, beta B2 (CRYBB2), mRNA [NM_000496]	3.96647	down	4.20490	down	264.54633	1086.52270	294.65106	1092.51920	Detected	Detected	Detected	Detected
5163	A_23_P27734	NPAS1	Hs.79564	neuronal PAS domain protein 1	Homo sapiens neuronal PAS domain protein 1 (NPAS1), mRNA [NM_002517]	3.96276	down	4.10195	down	157.13562	644.77075	246.37843	891.16520	Detected	Detected	Detected	Detected
5164	A_23_P59069	HIST1H2BO	Hs.673856	histone cluster 1, H2bo	Homo sapiens histone cluster 1, H2bo (HIST1H2BO), mRNA [NM_003527]	3.96150	down	3.81249	down	588.53990	2414.17600	739.42410	2485.81100	Detected	Detected	Detected	Detected
5165	A_24_P166407	HIST1H4B	Hs.143080	histone cluster 1, H4b	Homo sapiens histone cluster 1, H4b (HIST1H4B), mRNA [NM_003544]	3.96086	down	3.90042	down	3133.33330	12850.78200	1408.82300	4845.43550	Detected	Detected	Detected	Detected
5166	A_33_P3433873	ZWILCH	Hs.21331	Zwilch, kinetochore associated, homolog (Drosophila)	Homo sapiens Zwilch, kinetochore associated, homolog (Drosophila) (ZWILCH), transcript variant 1, mRNA [NM_017975]	3.95750	down	3.97691	down	685.95465	2810.92290	523.43690	1835.58810	Detected	Detected	Detected	Detected
5167	A_33_P3237393	ZNF140	Hs.181552	zinc finger protein 140	Homo sapiens zinc finger protein 140 (ZNF140), mRNA [NM_003440]	3.95236	down	1.24731	down	4.56436	18.67969	3.07390	3.38087	Compromised	Detected	Compromised	Compromised
5168	A_33_P3261640	LOC100134365		hypothetical protein LOC100134365	PREDICTED: Homo sapiens similar to ankyrin repeat domain 36 (LOC100134365), mRNA [XM_001715832]	3.94774	down	2.98486	down	40.21115	164.37189	19.78440	52.07305	Detected	Detected	Detected	Detected
5169	A_23_P143817	MYLK	Hs.477375	myosin light chain kinase	Homo sapiens myosin light chain kinase (MYLK), transcript variant 1, mRNA [NM_053025]	3.94632	down	3.99528	down	2449.65970	10009.93200	1911.55980	6734.42300	Detected	Detected	Detected	Detected
5170	A_24_P409661					3.94315	down	4.00473	down	1393.76150	5690.68700	1100.15770	3885.01930	Detected	Detected	Detected	Detected
5171	A_33_P3331376	EPHB2	Hs.523329	EPH receptor B2	Homo sapiens EPH receptor B2 (EPHB2), transcript variant 2, mRNA [NM_004442]	3.94230	down	4.35179	down	23.37711	95.42762	15.60890	59.89710	Detected	Detected	Detected	Detected
5172	A_33_P3286953	ADAMTS6	Hs.482291	ADAM metallopeptidase with thrombospondin type 1 motif, 6	Homo sapiens ADAM metallopeptidase with thrombospondin type 1 motif, 6 (ADAMTS6), mRNA [NM_197941]	3.94049	down	3.78766	down	181.99226	742.56824	146.56259	489.50787	Detected	Detected	Detected	Detected
5173	A_23_P28886	PCNA	Hs.147433	proliferating cell nuclear antigen	Homo sapiens proliferating cell nuclear antigen (PCNA), transcript variant 1, mRNA [NM_002592]	3.93942	down	3.66101	down	4267.33940	17406.92600	3028.38300	9776.35600	Detected	Detected	Detected	Detected
5174	A_23_P256158	ADRA2C	Hs.123022	adrenergic, alpha-2C-, receptor	Homo sapiens adrenergic, alpha-2C-, receptor (ADRA2C), mRNA [NM_000683]	3.93686	down	4.27035	down	476.37760	1941.93440	462.97263	1743.34690	Detected	Detected	Detected	Detected
5175	A_32_P122754	C9orf30	Hs.530272	chromosome 9 open reading frame 30	Homo sapiens chromosome 9 open reading frame 30 (C9orf30), mRNA [NM_080655]	3.93364	down	3.61552	down	1276.97020	5201.25340	1304.90280	4160.20500	Detected	Detected	Detected	Detected

5176	A_23_P116640	OR51B2	Hs.680163	olfactory receptor, family 51, subfamily B, member 2	Homo sapiens olfactory receptor, family 51, subfamily B, member 2 (OR51B2), mRNA [NM_033180]	3.93157	down	1.17786	down	4.53532	18.46321	2.79938	2.90751	Compromised	Not Detected	Compromised	Compromised
5177	A_23_P46539	PSRC1	Hs.405925	proline/serine-rich coiled-coil 1	Homo sapiens proline/serine-rich coiled-coil 1 (PSRC1), transcript variant 1, mRNA [NM_032636]	3.93042	down	4.04439	down	656.59110	2672.18530	577.50890	2059.57100	Detected	Detected	Detected	Detected
5178	A_23_P82651	NPTX2	Hs.3281	neuronal pentraxin II	Homo sapiens neuronal pentraxin II (NPTX2), mRNA [NM_002523]	3.92355	down	4.04043	down	3557.59400	14453.35300	2555.26250	9103.91400	Detected	Detected	Detected	Detected
5179	A_24_P6305	RPL10	Hs.534404	ribosomal protein L10	Homo sapiens ribosomal protein L10 (RPL10), transcript variant 1, mRNA [NM_006013]	3.92140	down	6.10690	down	12.14226	49.30301	7.22529	38.90824	Compromised	Detected	Compromised	Detected
5180	A_23_P11032	SLC9A7	Hs.496057	solute carrier family 9 (sodium/hydrogen exchanger), member 7	Homo sapiens solute carrier family 9 (sodium/hydrogen exchanger), member 7 (SLC9A7), mRNA [NM_032591]	3.92050	down	4.05986	down	129.97058	527.61774	146.92218	525.97340	Detected	Detected	Detected	Detected
5181	A_33_P3897734	LOC339894	Hs.634263	hypothetical protein LOC339894	Homo sapiens hypothetical protein LOC339894, mRNA (cDNA clone IMAGE:5269978) [BC038760]	3.91888	down	1.24012	down	5.54336	22.49410	7.35828	8.04647	Compromised	Detected	Compromised	Compromised
5182	A_33_P3404922	KIAA1217	Hs.445885	KIAA1217	Homo sapiens KIAA1217 (KIAA1217), transcript variant 2, mRNA [NM_001098500]	3.91885	down	1.78928	down	6.60209	26.79005	11.82033	18.64976	Compromised	Detected	Compromised	Detected
5183	A_23_P3302	MNS1	Hs.444483	meiosis-specific nuclear structural 1	Homo sapiens meiosis-specific nuclear structural 1 (MNS1), mRNA [NM_018365]	3.91862	down	3.93136	down	93.27447	378.46808	68.07374	235.98685	Detected	Detected	Detected	Detected
5184	A_33_P3407524	SUV39H1	Hs.522639	suppressor of variegation 3-9 homolog 1 (Drosophila)	Homo sapiens suppressor of variegation 3-9 homolog 1 (Drosophila) (SUV39H1), mRNA [NM_003173]	3.91108	down	4.52686	down	28.29967	114.60712	21.92879	87.53416	Detected	Detected	Detected	Detected
5185	A_33_P3412613	TMPO	Hs.11355	thymopoietin	Homo sapiens thymopoietin (TMPO), transcript variant 2, mRNA [NM_001032283]	3.90715	down	6.90736	down	6.31337	25.54195	3.01571	18.36822	Compromised	Detected	Compromised	Detected
5186	A_33_P3332596		Hs.722593		DKFZp686I24127_r1 686 (synonym: hlcc3) Homo sapiens cDNA clone DKFZp686I24127 5', mRNA sequence [BX471465]	3.89645	down	4.40806	down	6.47646	26.13003	6.38498	24.81833	Compromised	Detected	Compromised	Detected
5187	A_32_P4018	ROR1	Hs.128753	receptor tyrosine kinase-like orphan receptor 1	Tyrosine-protein kinase transmembrane receptor ROR1 Precursor (EC 2.7.10.1)(Neurotrophic tyrosine kinase, receptor-related 1) [Source:UniProtKB/Swiss-Prot;Acc:Q01973] [ENST00000371079]	3.89633	down	4.05488	down	300.31630	1211.62480	192.30661	687.60260	Detected	Detected	Detected	Detected
5188	A_23_P160934	ANP32E	Hs.656466	acidic (leucine-rich) nuclear phosphoprotein 32 family, member E	Homo sapiens acidic (leucine-rich) nuclear phosphoprotein 32 family, member E (ANP32E), transcript variant 1, mRNA [NM_030920]	3.88800	down	3.53078	down	189.92499	764.61285	129.73839	403.92905	Detected	Detected	Detected	Detected
5189	A_32_P109604	LOC100132733	Hs.709909	similar to FLJ00310 protein	PREDICTED: Homo sapiens similar to FLJ00310 protein (LOC100132733), mRNA [XM_001715342]	3.88218	down	1.95126	down	5.42426	21.80462	2.62435	4.51547	Compromised	Detected	Compromised	Compromised
5190	A_32_P75357		Hs.446164		Kelch-like protein 14 [Source:UniProtKB/Swiss-Prot;Acc:Q9P2G3] [ENST00000358095]	3.88155	down	3.04786	down	5.25134	21.10612	6.12600	16.46409	Compromised	Detected	Compromised	Detected
5191	A_23_P334845	ARHGAP19	Hs.80305	Rho GTPase activating protein 19	Homo sapiens Rho GTPase activating protein 19 (ARHGAP19), mRNA [NM_032900]	3.87842	down	2.20326	down	2.54511	10.22101	10.90299	21.18250	Compromised	Compromised	Compromised	Detected
5192	A_23_P202117	PCGF5	Hs.500512	polycomb group ring finger 5	Homo sapiens polycomb group ring finger 5, mRNA (cDNA clone IMAGE:3640258), complete cds. [BC007377]	3.87828	down	4.15640	down	190.79765	766.20560	179.38815	657.47150	Detected	Detected	Detected	Detected
5193	A_33_P3293446	KIAA1462	Hs.533953	KIAA1462	Homo sapiens KIAA1462 (KIAA1462), mRNA [NM_020848]	3.86874	down	3.94355	down	854.72015	3423.94460	757.26495	2633.29900	Detected	Detected	Detected	Detected
5194	A_23_P510	PLD5	Hs.672452	phospholipase D family, member 5	Homo sapiens phospholipase D family, member 5 (PLD5), mRNA [NM_152666]	3.86763	down	4.19045	down	5.17320	20.71751	5.18547	19.16083	Compromised	Detected	Compromised	Detected
5195	A_24_P84898	FEN1	Hs.409065	flap structure-specific endonuclease 1	Homo sapiens flap structure-specific endonuclease 1 (FEN1), mRNA [NM_004111]	3.86743	down	4.08794	down	615.32480	2464.11080	602.48730	2171.79170	Detected	Detected	Detected	Detected
5196	A_33_P3407339	C6orf204	Hs.656959	chromosome 6 open reading frame 204	Homo sapiens chromosome 6 open reading frame 204 (C6orf204), transcript variant 2, mRNA [NM_206921]	3.86320	down	4.44226	down	25.00476	100.02379	14.44568	56.58586	Detected	Detected	Detected	Detected
5197	A_33_P3250555				Uncharacterized protein C18orf2 [Source:UniProtKB/Swiss-Prot;Acc:Q9BZP3] [ENST00000269201]	3.86144	down	3.16921	down	19.92671	79.67424	12.27597	34.30616	Detected	Detected	Detected	Detected
5198	A_32_P148476	LOC389842		similar to RAN binding protein 1	PREDICTED: Homo sapiens similar to RAN binding protein 1 (LOC389842), mRNA [XM_372200]	3.85908	down	4.29177	down	301.46000	1204.60990	256.08310	969.13074	Detected	Detected	Detected	Detected
5199	A_23_P128351	EFCAB4B	Hs.504534	EF-hand calcium binding domain 4B	Homo sapiens EF-hand calcium binding domain 4B (EFCAB4B), transcript variant 3, mRNA [NM_032680]	3.85690	down	4.35018	down	68.50679	273.59310	62.95330	241.48537	Detected	Detected	Detected	Detected
5200	A_23_P356616	ABTB2	Hs.23361	ankyrin repeat and BTB (POZ) domain containing 2	Homo sapiens ankyrin repeat and BTB (POZ) domain containing 2 (ABTB2), mRNA [NM_145804]	3.85648	down	3.58953	down	436.00067	1741.04930	367.96410	1164.68520	Detected	Detected	Detected	Detected
5201	A_33_P3254650	LOC728353	Hs.621210	hypothetical protein LOC728353	PREDICTED: Homo sapiens hypothetical protein LOC728353 (LOC728353), miscRNA [XR_041035]	3.85567	down	3.84432	down	24.96817	99.68270	19.35469	65.61013	Detected	Detected	Detected	Detected
5202	A_23_P204417	ITFG2	Hs.446098	integrin alpha FG-GAP repeat containing 2	Homo sapiens integrin alpha FG-GAP repeat containing 2 (ITFG2), mRNA [NM_018463]	3.85499	down	4.58174	down	236.39012	943.59430	187.88979	759.10077	Detected	Detected	Detected	Detected
5203	A_33_P3271121					3.84858	down	3.77532	down	378.02026	1506.42920	226.53757	754.15375	Detected	Detected	Detected	Detected
5204	A_23_P163380	MTHFS	Hs.459049	5,10-methenyltetrahydrofolate synthetase (5-formyltetrahydrofolate cyclo-ligase)	Homo sapiens 5,10-methenyltetrahydrofolate synthetase (5-formyltetrahydrofolate cyclo-ligase) (MTHFS), mRNA [NM_006441]	3.84836	down	3.72155	down	1028.02870	4096.51170	1047.30640	3436.86550	Detected	Detected	Detected	Detected

5205	A_33_P3415843				Carnitine O-acetyltransferase (Carnitine acetylase)(EC 2.3.1.7)(Carnitine acetyltransferase)(CrAT)(CAT) [Source:UniProtKB/Swiss-Prot;Acc:P43155] [ENST00000393384]	3.84676	down	3.66171	down	321.47500	1280.48740	261.37576	843.94630	Detected	Detected	Detected	Detected
5206	A_23_P255653	TNFRSF10A	Hs.591834	tumor necrosis factor receptor superfamily, member 10a	Homo sapiens tumor necrosis factor receptor superfamily, member 10a (TNFRSF10A), mRNA [NM_003844]	3.84671	down	4.10795	down	88.61099	352.94740	91.94637	333.06207	Detected	Detected	Detected	Detected
5207	A_23_P19938	KDELR2	Hs.654552	KDEL (Lys-Asp-Glu-Leu) endoplasmic reticulum protein retention receptor 2	Homo sapiens KDEL (Lys-Asp-Glu-Leu) endoplasmic reticulum protein retention receptor 2 (KDELR2), transcript variant 1, mRNA [NM_006854]	3.84659	down	3.70201	down	8688.83300	34607.48400	5689.05470	18571.34800	Detected	Detected	Detected	Detected
5208	A_23_P320829	C14orf145	Hs.162889	chromosome 14 open reading frame 145	Homo sapiens chromosome 14 open reading frame 145 (C14orf145), mRNA [NM_152446]	3.84389	down	2.32335	down	12.44311	49.52603	12.80256	26.22872	Compromised	Detected	Compromised	Detected
5209	A_23_P353614	C8orf46	Hs.268869	chromosome 8 open reading frame 46	Homo sapiens chromosome 8 open reading frame 46 (C8orf46), mRNA [NM_152765]	3.84167	down	5.27271	down	13.99843	55.68430	7.76348	36.09570	Detected	Detected	Compromised	Detected
5210	A_32_P221799	HIST1H2AM	Hs.134999	histone cluster 1, H2am	Homo sapiens histone cluster 1, H2am (HIST1H2AM), mRNA [NM_003514]	3.84071	down	2.01269	down	20.67924	82.23918	36.91821	65.52141	Detected	Detected	Detected	Detected
5211	A_23_P399078	TIMP3	Hs.644633	TIMP metallopeptidase inhibitor 3	Homo sapiens TIMP metallopeptidase inhibitor 3 (TIMP3), mRNA [NM_000362]	3.83828	down	3.61801	down	11521.52800	45790.97700	10837.78900	34576.11000	Detected	Detected	Detected	Detected
5212	A_33_P3241661	LOC388780		hypothetical LOC388780	PREDICTED: Homo sapiens hypothetical LOC388780 (LOC388780), mRNA [XM_943910]	3.83580	down	4.14848	down	75.81969	301.14170	50.15599	183.47500	Detected	Detected	Detected	Detected
5213	A_33_P3273822				F-box only protein 11 (Vitiligo-associated protein 1)(VIT-1) [Source:UniProtKB/Swiss-Prot;Acc:Q86XK2] [ENST00000405808]	3.83049	down	1.19092	down	25.36062	100.58829	2.34863	2.46640	Detected	Detected	Compromised	Compromised
5214	A_23_P30363	P4HA2	Hs.519568	prolyl 4-hydroxylase, alpha polypeptide II	Homo sapiens prolyl 4-hydroxylase, alpha polypeptide II (P4HA2), transcript variant 1, mRNA [NM_004199]	3.82729	down	3.77913	down	12781.93000	50654.86300	8411.29200	28029.79300	Detected	Detected	Detected	Detected
5215	A_23_P96965	SYNC	Hs.712631	syncoilin, intermediate filament protein	Homo sapiens syncoilin, intermediate filament protein (SYNC), transcript variant 1, mRNA [NM_030786]	3.82636	down	3.46812	down	1679.43180	6653.97500	1497.15010	4578.52300	Detected	Detected	Detected	Detected
5216	A_24_P39639	EXOSC6	Hs.660633	exosome component 6	Homo sapiens exosome component 6 (EXOSC6), mRNA [NM_058219]	3.82052	down	4.17896	down	472.45566	1869.02930	445.46982	1641.54170	Detected	Detected	Detected	Detected
5217	A_23_P334870	TMEM217	Hs.520101	transmembrane protein 217	Homo sapiens transmembrane protein 217 (TMEM217), transcript variant 1, mRNA [NM_145316]	3.81872	down	1.31327	down	481.60257	1904.32040	246.80698	285.80908	Detected	Detected	Detected	Detected
5218	A_33_P3405500	SACS	Hs.159492	spastic ataxia of Charlevoix-Saguenay (sacsin)	Homo sapiens spastic ataxia of Charlevoix-Saguenay (sacsin) (SACS), mRNA [NM_014363]	3.81801	down	4.19625	down	283.24252	1119.77050	216.67580	801.74646	Detected	Detected	Detected	Detected
5219	A_23_P152082	SPTBN5	Hs.709819	spectrin, beta, non-erythrocytic 5	Homo sapiens spectrin, beta, non-erythrocytic 5 (SPTBN5), mRNA [NM_016642]	3.81438	down	15.70803	down	5.41050	21.36952	3.39958	47.08827	Compromised	Detected	Compromised	Detected
5220	A_23_P398530	WHSC1	Hs.113876	Wolf-Hirschhorn syndrome candidate 1	Homo sapiens Wolf-Hirschhorn syndrome candidate 1 (WHSC1), transcript variant 7, mRNA [NM_133334]	3.81391	down	3.02890	down	7.17742	28.34473	2.33800	6.24445	Compromised	Detected	Compromised	Compromised
5221	A_33_P3350758	RASAL2	Hs.549729	RAS protein activator like 2	Homo sapiens RAS protein activator like 2 (RASAL2), transcript variant 2, mRNA [NM_170692]	3.81278	down	3.95375	down	123.55180	487.78010	104.42902	364.07960	Detected	Detected	Detected	Detected
5222	A_23_P17130	C2orf88	Hs.720468	chromosome 2 open reading frame 88	Homo sapiens chromosome 2 open reading frame 88 (C2orf88), transcript variant 1, mRNA [NM_001042519]	3.80105	down	6.00192	down	13.79373	54.28985	6.57195	34.78159	Detected	Detected	Compromised	Detected
5223	A_23_P417918	PENK	Hs.339831	proenkephalin	Homo sapiens proenkephalin (PENK), transcript variant 2, mRNA [NM_006211]	3.79881	down	4.29323	down	445.45920	1752.22110	402.27747	1522.91470	Detected	Detected	Detected	Detected
5224	A_33_P3375899	LOC100133500		hypothetical protein LOC100133500	PREDICTED: Homo sapiens hypothetical LOC100133500 (LOC100133500), mRNA [XM_001720287]	3.79735	down	1.20925	down	4.75993	18.71606	2.89294	3.08475	Compromised	Detected	Compromised	Compromised
5225	A_23_P250347	CAMK4	Hs.591269	calcium/calmodulin-dependent protein kinase IV	Homo sapiens calcium/calmodulin-dependent protein kinase IV (CAMK4), mRNA [NM_001744]	3.79635	down	2.31339	down	14.27065	56.09746	19.44046	39.65708	Detected	Detected	Detected	Detected
5226	A_23_P163306	CGNL1	Hs.148989	cingulin-like 1	Homo sapiens cingulin-like 1 (CGNL1), mRNA [NM_032866]	3.79296	down	3.61588	down	14.33167	56.28698	8.46437	26.98820	Detected	Detected	Compromised	Detected
5227	A_33_P3407434		Hs.117259		BX117604 Soares_fetal_liver_spleen_1NFLS_S1 Homo sapiens cDNA clone IMAGp998E051013, mRNA sequence [BX117604]	3.78823	down	4.00163	down	16.92752	66.39913	7.46430	26.33849	Detected	Detected	Compromised	Detected
5228	A_23_P124122	PXMP2	Hs.430299	peroxisomal membrane protein 2, 22kDa	Homo sapiens peroxisomal membrane protein 2, 22kDa (PXMP2), mRNA [NM_018663]	3.78061	down	3.74940	down	966.07970	3781.88450	720.31880	2381.51000	Detected	Detected	Detected	Detected
5229	A_23_P252145	C1GALT1	Hs.592180	core 1 synthase, glycoprotein-N-acetylgalactosamine 3-beta-galactosyltransferase, 1	Homo sapiens core 1 synthase, glycoprotein-N-acetylgalactosamine 3-beta-galactosyltransferase, 1 (C1GALT1), mRNA [NM_020156]	3.77873	down	4.11757	down	327.45547	1281.24180	190.50627	691.69650	Detected	Detected	Detected	Detected
5230	A_33_P3238280	ESYT3	Hs.477711	extended synaptotagmin-like protein 3	Homo sapiens extended synaptotagmin-like protein 3 (ESYT3), mRNA [NM_031913]	3.77846	down	2.86865	down	18.38189	71.91812	23.33554	59.02829	Detected	Detected	Detected	Detected
5231	A_33_P3260575	CERCAM	Hs.495230	cerebral endothelial cell adhesion molecule	Homo sapiens cerebral endothelial cell adhesion molecule (CERCAM), mRNA [NM_016174]	3.77732	down	3.93905	down	5316.37940	20793.72500	5428.62840	18855.91000	Detected	Detected	Detected	Detected
5232	A_23_P124905	NPTX1	Hs.514556	neuronal pentraxin I	Homo sapiens neuronal pentraxin I (NPTX1), mRNA [NM_002522]	3.77588	down	3.54620	down	323.95770	1266.60020	224.94357	703.40090	Detected	Detected	Detected	Detected

5233	A_24_P734953	TRNP1	Hs.355747	TMF1-regulated nuclear protein 1	Homo sapiens TMF1-regulated nuclear protein 1 (TRNP1), mRNA [NM_001013642]	3.77497	down	4.09507	down	4016.02050	15697.93100	4386.20260	15838.56250	Detected	Detected	Detected	Detected
5234	A_23_P12816	HELLS	Hs.655830	helicase, lymphoid-specific	Homo sapiens helicase, lymphoid-specific (HELLS), mRNA [NM_018063]	3.77275	down	4.54889	down	90.17854	352.28516	60.02296	240.76237	Detected	Detected	Detected	Detected
5235	A_33_P3258612	PCNA	Hs.147433	proliferating cell nuclear antigen	Homo sapiens proliferating cell nuclear antigen (PCNA), transcript variant 1, mRNA [NM_002592]	3.77253	down	3.72687	down	3013.90010	11773.21000	2210.71340	7265.10000	Detected	Detected	Detected	Detected
5236	A_24_P205589	ACOT7	Hs.126137	acyl-CoA thioesterase 7	Homo sapiens acyl-CoA thioesterase 7 (ACOT7), transcript variant hBACHa, mRNA [NM_007274]	3.77080	down	4.45089	down	1732.32790	6763.89500	2208.02030	8665.93900	Detected	Detected	Detected	Detected
5237	A_33_P3235034		Hs.675838		Putative uncharacterized protein [Source:UniProtKB/TrEMBL;Acc:Q9HBN7] [ENST00000321930]	3.76618	down	2.69762	down	31.18600	121.61682	40.24796	95.73933	Detected	Detected	Detected	Detected
5238	A_32_P113812					3.76520	down	1.65100	down	6.16045	24.01778	7.54128	10.97889	Compromised	Detected	Compromised	Compromised
5239	A_23_P207911	TRPV2	Hs.279746	transient receptor potential cation channel, subfamily V, member 2	Homo sapiens transient receptor potential cation channel, subfamily V, member 2 (TRPV2), mRNA [NM_016113]	3.76487	down	3.89356	down	5539.44200	21594.77300	6876.06250	23607.59200	Detected	Detected	Detected	Detected
5240	A_23_P126075	KCNK1	Hs.208544	potassium channel, subfamily K, member 1	Homo sapiens potassium channel, subfamily K, member 1 (KCNK1), mRNA [NM_002245]	3.76349	down	3.68573	down	296.05307	1153.70000	213.70271	694.54310	Detected	Detected	Detected	Detected
5241	A_23_P104372	DNAJC9	Hs.654694	DnaJ (Hsp40) homolog, subfamily C, member 9	Homo sapiens DnaJ (Hsp40) homolog, subfamily C, member 9 (DNAJC9), mRNA [NM_015190]	3.76325	down	3.56651	down	6473.46830	25225.11000	4526.09900	14234.18700	Detected	Detected	Detected	Detected
5242	A_33_P3238166	PXDN	Hs.332197	peroxidasin homolog (Drosophila)	Homo sapiens peroxidasin homolog (Drosophila) (PXDN), mRNA [NM_012293]	3.75992	down	3.42673	down	19102.00600	74368.77000	13256.16600	40055.66400	Detected	Detected	Detected	Detected
5243	A_23_P22134	BNC1	Hs.459153	basonuclin 1	Homo sapiens basonuclin 1 (BNC1), mRNA [NM_001717]	3.75955	down	3.68716	down	164.02261	638.51715	125.52583	408.12270	Detected	Detected	Detected	Detected
5244	A_33_P3229918	PTCRA	Hs.169002	pre T-cell antigen receptor alpha	Homo sapiens pre T-cell antigen receptor alpha (PTCRA), mRNA [NM_138296]	3.75822	down	2.65455	down	6.28337	24.45166	9.99613	23.39850	Compromised	Detected	Compromised	Detected
5245	A_23_P34597	CDA	Hs.466910	cytidine deaminase	Homo sapiens cytidine deaminase (CDA), mRNA [NM_001785]	3.75707	down	5.44681	down	132.12090	513.98956	104.37382	501.30154	Detected	Detected	Detected	Detected
5246	A_23_P15123	UBFD1	Hs.3459	ubiquitin family domain containing 1	Homo sapiens ubiquitin family domain containing 1 (UBFD1), mRNA [NM_019116]	3.75688	down	3.33574	down	417.62885	1624.61600	408.02830	1200.18350	Detected	Detected	Detected	Detected
5247	A_33_P3342862	FAM101A	Hs.432901	family with sequence similarity 101, member A	Homo sapiens family with sequence similarity 101, member A (FAM101A), mRNA [NM_181709]	3.75668	down	9.31460	down	121.66808	473.27554	43.08754	353.90057	Detected	Detected	Detected	Detected
5248	A_23_P61180	PLCXD1	Hs.522568	phosphatidylinositol-specific phospholipase C, X domain containing 1	Homo sapiens phosphatidylinositol-specific phospholipase C, X domain containing 1 (PLCXD1), transcript variant 1, mRNA [NM_018390]	3.75622	down	3.86039	down	499.66818	1943.41640	495.06992	1685.24350	Detected	Detected	Detected	Detected
5249	A_33_P3275801	DES	Hs.594952	desmin	Homo sapiens desmin (DES), mRNA [NM_001927]	3.75461	down	4.70083	down	547.69434	2129.29900	388.88712	1611.99510	Detected	Detected	Detected	Detected
5250	A_23_P82738	RAD54B	Hs.30561	RAD54 homolog B (S. cerevisiae)	Homo sapiens RAD54 homolog B (S. cerevisiae) (RAD54B), mRNA [NM_012415]	3.75130	down	3.76019	down	142.36089	552.97473	109.51458	363.11770	Detected	Detected	Detected	Detected
5251	A_32_P56392	RBMX	Hs.380118	RNA binding motif protein, X-linked	Homo sapiens RNA binding motif protein, X-linked (RBMX), transcript variant 1, mRNA [NM_002139]	3.74887	down	2.97373	down	36.16409	140.38203	19.71775	51.70407	Detected	Detected	Detected	Detected
5252	A_33_P3407592					3.74669	down	3.06685	down	34.23169	132.80340	36.08723	97.59156	Detected	Detected	Detected	Detected
5253	A_24_P342086	WWP2	Hs.408458	WW domain containing E3 ubiquitin protein ligase 2	Homo sapiens WW domain containing E3 ubiquitin protein ligase 2 (WWP2), transcript variant 3, mRNA [NM_199423]	3.74508	down	3.43682	down	62.00339	240.44150	56.57099	171.44151	Detected	Detected	Detected	Detected
5254	A_23_P114983	TRIM63	Hs.279709	tripartite motif-containing 63	Homo sapiens tripartite motif-containing 63 (TRIM63), mRNA [NM_032588]	3.73993	down	5.62033	down	47.12495	182.49371	25.88433	128.28160	Detected	Detected	Detected	Detected
5255	A_32_P105083	LOC348751	Hs.471039	hypothetical protein LOC348751	PREDICTED: Homo sapiens hypothetical protein LOC348751 (LOC348751), mRNA [XM_001716816]	3.73935	down	2.80376	down	8.94348	34.62868	11.33699	28.02882	Compromised	Detected	Compromised	Detected
5256	A_23_P379789	ST8SIA5	Hs.465025	ST8 alpha-N-acetyl-neuraminide alpha-2,8-sialyltransferase 5	Homo sapiens ST8 alpha-N-acetyl-neuraminide alpha-2,8-sialyltransferase 5 (ST8SIA5), mRNA [NM_013305]	3.73860	down	3.93135	down	71.06592	275.10815	45.94891	159.28772	Detected	Detected	Detected	Detected
5257	A_23_P350005	TRIML2	Hs.276429	tripartite motif family-like 2	Homo sapiens tripartite motif family-like 2 (TRIML2), mRNA [NM_173553]	3.73656	down	2.72183	down	15.98617	61.85148	5.70030	13.68122	Detected	Detected	Compromised	Compromised
5258	A_33_P3394933	P4HA2	Hs.519568	prolyl 4-hydroxylase, alpha polypeptide II	Homo sapiens prolyl 4-hydroxylase, alpha polypeptide II (P4HA2), transcript variant 2, mRNA [NM_001017973]	3.73350	down	4.15462	down	698.97480	2702.16040	632.74480	2318.06180	Detected	Detected	Detected	Detected
5259	A_24_P405054	C1orf144	Hs.252967	chromosome 1 open reading frame 144	Homo sapiens chromosome 1 open reading frame 144 (C1orf144), transcript variant 2, mRNA [NM_015609]	3.73337	down	4.02531	down	261.73074	1011.78700	347.85260	1234.69530	Detected	Detected	Detected	Detected
5260	A_33_P3280597				Serine/threonine-protein phosphatase 4 regulatory subunit 1-like [Source:UniProtKB/Swiss-Prot;Acc:Q9P1A2] [ENST00000244070]	3.73196	down	3.20694	down	148.83548	575.14343	97.96050	277.01788	Detected	Detected	Detected	Detected

5261	A_33_P3231833				Acyl-coenzyme A thioesterase 8 (EC 3.1.2.27)(Choloyl-coenzyme A thioesterase)(Acyl-CoA thioesterase 8)(Peroxisomal acyl-coenzyme A thioester hydrolase 1)(PTE-1)(Peroxisomal long-chain acyl-CoA thioesterase 1)(HIV-Nef-associated acyl-CoA thioesterase)(Thioesterase II)(hTE)(hACTEIII)(hACTE-III)(PTE-2) [Source:UniProtKB/Swiss-Prot;Acc:O14734] [ENST00000372531]	3.72991	down	3.36454	down	95.53854	368.98618	91.19238	270.55136	Detected	Detected	Detected	Detected
5262	A_23_P116902	ART4	Hs.655792	ADP-ribosyltransferase 4 (Dombrock blood group)	Homo sapiens ADP-ribosyltransferase 4 (Dombrock blood group) (ART4), mRNA [NM_021071]	3.72962	down	1.54644	down	5.56126	21.47685	3.28363	4.47768	Compromised	Detected	Compromised	Compromised
5263	A_23_P17811	SEC14L2	Hs.335614	SEC14-like 2 (S. cerevisiae)	Homo sapiens SEC14-like 2 (S. cerevisiae) (SEC14L2), transcript variant 1, mRNA [NM_012429]	3.72881	down	4.43470	down	643.57935	2484.87960	546.94030	2138.79700	Detected	Detected	Detected	Detected
5264	A_33_P3263193	C12orf32	Hs.198853	chromosome 12 open reading frame 32	Homo sapiens chromosome 12 open reading frame 32 (C12orf32), transcript variant 1, non-coding RNA [NR_027363]	3.72823	down	3.43786	down	809.03326	3123.21900	438.11462	1328.13400	Detected	Detected	Detected	Detected
5265	A_23_P417415	ACOT11	Hs.234786	acyl-CoA thioesterase 11	Homo sapiens acyl-CoA thioesterase 11 (ACOT11), transcript variant 2, mRNA [NM_147161]	3.72370	down	4.28195	down	272.70764	1051.48860	246.57860	931.02655	Detected	Detected	Detected	Detected
5266	A_33_P3238976	TRMT5	Hs.380159	TRM5 tRNA methyltransferase 5 homolog (S. cerevisiae)	Homo sapiens TRM5 tRNA methyltransferase 5 homolog (S. cerevisiae) (TRMT5), mRNA [NM_020810]	3.72217	down	3.93484	down	915.45886	3528.32350	1061.80580	3684.14840	Detected	Detected	Detected	Detected
5267	A_33_P3258478	KIR3DP1	Hs.690615	killer-cell Ig-like receptor	Homo sapiens killer-cell Ig-like receptor (KIR3DP1), mRNA [NM_001015070]	3.72130	down	3.15913	down	23.74017	91.47687	14.91342	41.54417	Detected	Detected	Detected	Detected
5268	A_33_P3314531	LOC646853		similar to high-mobility group nucleosomal binding domain 2	PREDICTED: Homo sapiens similar to high-mobility group nucleosomal binding domain 2 (LOC646853), mRNA [XM_001719033]	3.71926	down	2.73868	down	37.07333	142.77480	12.77107	30.84141	Detected	Detected	Compromised	Detected
5269	A_24_P221485					3.71233	down	3.39973	down	43.39176	166.79643	20.70887	62.08211	Detected	Detected	Detected	Detected
5270	A_23_P394323	FAM98B	Hs.6799	family with sequence similarity 98, member B	Protein FAM98B [Source:UniProtKB/Swiss-Prot;Acc:Q52LJ0] [ENST00000305752]	3.71071	down	2.73959	down	6.30244	24.21579	3.28111	7.92631	Compromised	Detected	Compromised	Compromised
5271	A_33_P3235410				Protein-tyrosine phosphatase-like member A (Cementum attachment protein) [Source:UniProtKB/Swiss-Prot;Acc:B0YJ81] [ENST00000326961]	3.70999	down	3.66165	down	210.27188	807.76886	184.46120	595.59045	Detected	Detected	Detected	Detected
5272	A_33_P3371989	IGSF1	Hs.22111	immunoglobulin superfamily, member 1	Homo sapiens immunoglobulin superfamily, member 1 (IGSF1), transcript variant 2, mRNA [NM_205833]	3.70289	down	1.63882	down	4.96558	19.03900	2.99924	4.33418	Compromised	Detected	Compromised	Compromised
5273	A_33_P3531204	PCOTH	Hs.721128	prostate collagen triple helix	Homo sapiens prostate collagen triple helix (PCOTH), transcript variant 2, mRNA [NM_001135816]	3.70094	down	5.72179	down	36.40070	139.49385	22.44340	113.23648	Detected	Detected	Detected	Detected
5274	A_23_P163099	POLE2	Hs.162777	polymerase (DNA directed), epsilon 2 (p59 subunit)	Homo sapiens polymerase (DNA directed), epsilon 2 (p59 subunit) (POLE2), mRNA [NM_002692]	3.69761	down	3.25343	down	170.38876	652.37164	126.37004	362.53586	Detected	Detected	Detected	Detected
5275	A_23_P6771	LMCD1	Hs.475353	LIM and cysteine-rich domains 1	Homo sapiens LIM and cysteine-rich domains 1 (LMCD1), mRNA [NM_014583]	3.69693	down	3.92831	down	4452.59570	17044.63000	4454.90100	15431.52800	Detected	Detected	Detected	Detected
5276	A_23_P47247	TCP11L1	Hs.655341	t-complex 11 (mouse)-like 1	Homo sapiens t-complex 11 (mouse)-like 1 (TCP11L1), transcript variant 1, mRNA [NM_018393]	3.69319	down	3.71606	down	299.80020	1146.47910	294.51865	965.07380	Detected	Detected	Detected	Detected
5277	A_23_P81369	ADAM19	Hs.483944	ADAM metallopeptidase domain 19 (meltrin beta)	Homo sapiens ADAM metallopeptidase domain 19 (meltrin beta) (ADAM19), mRNA [NM_033274]	3.69254	down	5.33810	down	13.51513	51.67475	10.29465	48.45785	Compromised	Detected	Compromised	Detected
5278	A_23_P44436	GKN1	Hs.69319	gastrokine 1	Homo sapiens gastrokine 1 (GKN1), mRNA [NM_019617]	3.68694	down	2.54052	down	10.62038	40.54522	9.54230	21.37673	Compromised	Detected	Compromised	Detected
5279	A_24_P940275	FRMPD4	Hs.663678	FERM and PDZ domain containing 4	Homo sapiens FERM and PDZ domain containing 4 (FRMPD4), mRNA [NM_014728]	3.68547	down	1.92824	down	12.91231	49.27536	15.37892	26.14885	Compromised	Detected	Compromised	Detected
5280	A_23_P214487	HIST1H4C	Hs.46423	histone cluster 1, H4c	Homo sapiens histone cluster 1, H4c (HIST1H4C), mRNA [NM_003542]	3.68516	down	3.59859	down	21490.90200	82005.71000	16907.02000	53649.42600	Detected	Detected	Detected	Detected
5281	A_33_P3363637	BLNK	Hs.665244	B-cell linker	Homo sapiens B-cell linker (BLNK), transcript variant 1, mRNA [NM_013314]	3.68248	down	12.26093	down	2.57394	9.81458	3.20717	34.67455	Compromised	Compromised	Compromised	Detected
5282	A_33_P3302125	LCE2A	Hs.334054	late cornified envelope 2A	Homo sapiens late cornified envelope 2A (LCE2A), mRNA [NM_178428]	3.68122	down	3.39736	down	148.26230	565.13890	134.78899	403.79556	Detected	Detected	Detected	Detected
5283	A_23_P166526	RIBC2	Hs.475110	RIB43A domain with coiled-coils 2	Homo sapiens RIB43A domain with coiled-coils 2 (RIBC2), mRNA [NM_015653]	3.68058	down	3.55866	down	19.78241	75.39253	15.19062	47.66806	Detected	Detected	Compromised	Detected
5284	A_23_P429383	HOXD9	Hs.236646	homeobox D9	Homo sapiens homeobox D9 (HOXD9), mRNA [NM_014213]	3.67913	down	10.06876	down	67.95981	258.89886	25.21854	223.90378	Detected	Detected	Detected	Detected
5285	A_24_P109633	ITPK1	Hs.308122	inositol 1,3,4-triphosphate 5/6 kinase	Homo sapiens inositol 1,3,4-triphosphate 5/6 kinase (ITPK1), transcript variant 3, mRNA [NM_001142594]	3.67874	down	3.91942	down	22.39052	85.28960	13.96140	48.25214	Detected	Detected	Compromised	Detected
5286	A_23_P53126	LMO2	Hs.34560	LIM domain only 2 (rhombotin-like 1)	Homo sapiens LIM domain only 2 (rhombotin-like 1) (LMO2), transcript variant 1, mRNA [NM_005574]	3.67582	down	3.90573	down	93.84444	357.18740	61.91695	213.24445	Detected	Detected	Detected	Detected
5287	A_33_P3818787	LOC344967	Hs.534633	acyl-CoA thioesterase 7 pseudogene	Homo sapiens acyl-CoA thioesterase 7 pseudogene (LOC344967), non-coding RNA [NR_027277]	3.67134	down	3.65069	down	217.93270	828.47534	61.97788	199.51576	Detected	Detected	Detected	Detected

5288	A_23_P12363	ROR1	Hs.654491	receptor tyrosine kinase-like orphan receptor 1	Homo sapiens receptor tyrosine kinase-like orphan receptor 1 (ROR1), transcript variant 1, mRNA [NM_005012]	3.67092	down	4.02088	down	373.42627	1419.42630	264.36820	937.33710	Detected	Detected	Detected	Detected
5289	A_33_P3233459		Hs.452789		cDNA FLJ43791 fis, clone TESTI2053526 [Source:UniProtKB/TrEMBL;Acc:Q6ZUD9] [ENST00000339674]	3.66845	down	1.90419	down	275.01416	1044.64860	50.60420	84.96944	Detected	Detected	Detected	Detected
5290	A_23_P146946	CST6	Hs.139389	cystatin E/M	Homo sapiens cystatin E/M (CST6), mRNA [NM_001323]	3.66835	down	3.60980	down	153.73158	583.93884	201.52415	641.46880	Detected	Detected	Detected	Detected
5291	A_23_P68240	GPAT2	Hs.348629	glycerol-3-phosphate acyltransferase 2, mitochondrial	Homo sapiens glycerol-3-phosphate acyltransferase 2, mitochondrial (GPAT2), nuclear gene encoding mitochondrial protein, mRNA [NM_207328]	3.66567	down	4.93453	down	756.55396	2871.61400	505.43155	2199.24500	Detected	Detected	Detected	Detected
5292	A_32_P379467	ISLR2	Hs.254775	immunoglobulin superfamily containing leucine-rich repeat 2	Homo sapiens immunoglobulin superfamily containing leucine-rich repeat 2 (ISLR2), transcript variant 2, mRNA [NM_020851]	3.66564	down	3.80175	down	24.91619	94.57231	11.97065	40.12975	Detected	Detected	Compromised	Detected
5293	A_33_P3252286	CRLF1	Hs.114948	cytokine receptor-like factor 1	Homo sapiens cytokine receptor-like factor 1 (CRLF1), mRNA [NM_004750]	3.66271	down	3.82488	down	13849.45600	52525.27300	12980.70000	43780.62500	Detected	Detected	Detected	Detected
5294	A_32_P212373					3.66167	down	3.42420	down	193.54115	733.81415	156.42932	472.32764	Detected	Detected	Detected	Detected
5295	A_33_P3386344	FANCA	Hs.719210	Fanconi anemia, complementation group A	Homo sapiens Fanconi anemia, complementation group A (FANCA), transcript variant 2, mRNA [NM_001018112]	3.65940	down	2.54427	down	65.12068	246.75258	79.60854	178.60280	Detected	Detected	Detected	Detected
5296	A_33_P3321432	FAM198B	Hs.567498	family with sequence similarity 198, member B	Homo sapiens family with sequence similarity 198, member B (FAM198B), transcript variant 2, mRNA [NM_016613]	3.65178	down	4.38825	down	152.39822	576.25810	98.21914	380.06082	Detected	Detected	Detected	Detected
5297	A_23_P145238	HIST1H2BK	Hs.437275	histone cluster 1, H2bk	Homo sapiens histone cluster 1, H2bk (HIST1H2BK), mRNA [NM_080593]	3.65126	down	3.95895	down	1148.69890	4342.92630	911.53260	3182.13090	Detected	Detected	Detected	Detected
5298	A_33_P3217238	ATAD2	Hs.370834	ATPase family, AAA domain containing 2	Homo sapiens ATPase family, AAA domain containing 2 (ATAD2), mRNA [NM_014109]	3.65064	down	3.30123	down	1075.89710	4066.99100	892.74066	2598.76220	Detected	Detected	Detected	Detected
5299	A_23_P121586	HERC3	Hs.35804	hect domain and RLD 3	Homo sapiens hect domain and RLD 3 (HERC3), mRNA [NM_014606]	3.64814	down	2.84675	down	46.90350	177.17812	16.13589	40.50490	Detected	Detected	Detected	Detected
5300	A_33_P3271455	PXDN	Hs.332197	peroxidasin homolog (Drosophila)	Homo sapiens peroxidasin homolog (Drosophila) (PXDN), mRNA [NM_012293]	3.64584	down	3.56628	down	16410.87100	61953.02700	9350.12000	29403.44500	Detected	Detected	Detected	Detected
5301	A_23_P307544	PLXNA2	Hs.497626	plexin A2	Homo sapiens plexin A2 (PLXNA2), mRNA [NM_025179]	3.64236	down	5.06862	down	33.63466	126.85350	20.70332	92.53270	Detected	Detected	Detected	Detected
5302	A_24_P60680	OR10C1	Hs.631997	olfactory receptor, family 10, subfamily C, member 1	Homo sapiens olfactory receptor, family 10, subfamily C, member 1 (OR10C1), mRNA [NM_013941]	3.63912	down	2.13922	down	57.45465	216.49855	42.17722	79.56069	Detected	Detected	Detected	Detected
5303	A_32_P181103	GGCX	Hs.77719	gamma-glutamyl carboxylase	Homo sapiens gamma-glutamyl carboxylase (GGCX), transcript variant 1, mRNA [NM_000821]	3.63746	down	3.75849	down	327.34338	1232.92070	281.51086	932.98390	Detected	Detected	Detected	Detected
5304	A_32_P4199	RNF152	Hs.667457	ring finger protein 152	Homo sapiens ring finger protein 152 (RNF152), mRNA [NM_173557]	3.63175	down	4.50284	down	68.86626	258.97350	48.50383	192.58750	Detected	Detected	Detected	Detected
5305	A_33_P3327200	ATP2B4	Hs.343522	ATPase, Ca++ transporting, plasma membrane 4	Homo sapiens ATPase, Ca++ transporting, plasma membrane 4 (ATP2B4), transcript variant 1, mRNA [NM_001001396]	3.63161	down	3.50742	down	122.06923	459.02720	87.94022	271.98267	Detected	Detected	Detected	Detected
5306	A_23_P7172	PGM2	Hs.23363	phosphoglucomutase 2	Homo sapiens phosphoglucomutase 2 (PGM2), mRNA [NM_018290]	3.62926	down	2.96191	down	761.40045	2861.30620	511.06235	1334.78340	Detected	Detected	Detected	Detected
5307	A_23_P149545	HIST2H2BE	Hs.2178	histone cluster 2, H2be	Homo sapiens histone cluster 2, H2be (HIST2H2BE), mRNA [NM_003528]	3.62473	down	7.73174	down	14.49271	54.39488	2.77205	18.89919	Detected	Detected	Compromised	Detected
5308	A_33_P3392192		Hs.668810		MSTP131 [Source:UniProtKB/TrEMBL;Acc:Q7Z2R7] [ENST00000423322]	3.62171	down	3.49770	down	162.40160	609.02740	120.43778	371.45917	Detected	Detected	Detected	Detected
5309	A_33_P3310276	LOC652554		similar to piwi-like 2	PREDICTED: Homo sapiens similar to piwi like homolog 2 (LOC652554), mRNA [XM_942053]	3.62089	down	2.34175	down	24.23840	90.87666	24.04621	49.65377	Detected	Detected	Detected	Detected
5310	A_23_P8013	HIST1H2BL	Hs.137594	histone cluster 1, H2bl	Homo sapiens histone cluster 1, H2bl (HIST1H2BL), mRNA [NM_003519]	3.61351	down	3.95539	down	878.67523	3287.68770	1028.91190	3588.66550	Detected	Detected	Detected	Detected
5311	A_23_P150053	ACTA2	Hs.500483	actin, alpha 2, smooth muscle, aorta	Homo sapiens actin, alpha 2, smooth muscle, aorta (ACTA2), transcript variant 2, mRNA [NM_001613]	3.60917	down	3.76562	down	1443.42480	5394.28800	1353.69150	4494.91460	Detected	Detected	Detected	Detected
5312	A_33_P3376031		Hs.666223		Putative uncharacterized protein FLJ46214 [Source:UniProtKB/Swiss-Prot;Acc:Q6ZRN7] [ENST00000382225]	3.60599	down	2.37204	down	3.77485	14.09477	7.41909	15.51806	Compromised	Compromised	Compromised	Detected
5313	A_23_P151805	FBLN5	Hs.719986	fibulin 5	Homo sapiens fibulin 5 (FBLN5), mRNA [NM_006329]	3.60545	down	3.53951	down	13289.91100	49615.20000	11493.29200	35871.78000	Detected	Detected	Detected	Detected
5314	A_33_P3349536	CHEK1	Hs.24529	CHK1 checkpoint homolog (S. pombe)	Homo sapiens CHK1 checkpoint homolog (S. pombe) (CHEK1), transcript variant 2, mRNA [NM_001114121]	3.60125	down	3.30457	down	393.43924	1467.11610	390.91937	1139.11670	Detected	Detected	Detected	Detected
5315	A_24_P251969	FGF1	Hs.483635	fibroblast growth factor 1 (acidic)	Homo sapiens fibroblast growth factor 1 (acidic) (FGF1), transcript variant 1, mRNA [NM_000800]	3.59916	down	4.91719	down	9.94759	37.07256	7.56196	32.78812	Compromised	Detected	Compromised	Detected
5316	A_33_P3233764	LATS1	Hs.716697	LATS, large tumor suppressor, homolog 1 (Drosophila)	Homo sapiens LATS, large tumor suppressor, homolog 1 (Drosophila) (LATS1), mRNA [NM_004690]	3.59906	down	4.07261	down	11.82651	44.07368	10.48285	37.64586	Detected	Detected	Compromised	Detected
5317	A_33_P3212432	LOC100293406	Hs.553971	similar to hCG2002986	PREDICTED: Homo sapiens similar to hCG2002986 (LOC100293406), mRNA [XM_002346043]	3.59823	down	1.72822	down	4.94468	18.42301	5.80356	8.84422	Compromised	Detected	Compromised	Compromised
5318	A_24_P374675	UNKL	Hs.643536	unkempt homolog (Drosophila)-like	Homo sapiens unkempt homolog (Drosophila)-like (UNKL), transcript variant 2, mRNA [NM_001037125]	3.59549	down	4.08399	down	12.43607	46.29933	12.72793	45.83612	Compromised	Detected	Compromised	Detected

5319	A_24_P31235	EIF5A	Hs.534314	eukaryotic translation initiation factor 5A	Homo sapiens eukaryotic translation initiation factor 5A (EIF5A), transcript variant B, mRNA [NM_001970]	3.59170	down	3.65952	down	2230.47100	8295.25800	1824.00060	5885.92400	Detected	Detected	Detected	Detected
5320	A_33_P3258362	HBA2	Hs.654744	hemoglobin, alpha 2	Homo sapiens hemoglobin, alpha 2 (HBA2), mRNA [NM_000517]	3.59160	down	1.51256	down	28.27968	105.17101	11.54820	15.40257	Detected	Detected	Compromised	Compromised
5321	A_23_P409462	DCBLD1	Hs.658304	discoidin, CUB and LCCL domain containing 1	Homo sapiens discoidin, CUB and LCCL domain containing 1 (DCBLD1), mRNA [NM_173674]	3.58930	down	3.14684	down	72.92105	271.01675	50.13688	139.12242	Detected	Detected	Detected	Detected
5322	A_33_P3410409	LAMP2	Hs.496684	lysosomal-associated membrane protein 2	Homo sapiens lysosomal-associated membrane protein 2 (LAMP2), transcript variant C, mRNA [NM_001122606]	3.58897	down	3.74267	down	1713.55630	6367.96900	1486.29540	4905.14600	Detected	Detected	Detected	Detected
5323	A_33_P3226832	F3	Hs.62192	coagulation factor III (thromboplastin, tissue factor)	Homo sapiens coagulation factor III (thromboplastin, tissue factor) (F3), mRNA [NM_001993]	3.58681	down	3.52789	down	2749.87230	10213.02000	2131.62280	6631.18160	Detected	Detected	Detected	Detected
5324	A_32_P101860					3.58267	down	3.91925	down	347.75970	1290.08900	215.06146	743.24300	Detected	Detected	Detected	Detected
5325	A_33_P3363560	TMEM51	Hs.465305	transmembrane protein 51	Homo sapiens transmembrane protein 51 (TMEM51), transcript variant 1, mRNA [NM_001136216]	3.58237	down	3.46112	down	428.72476	1590.30940	403.14370	1230.38610	Detected	Detected	Detected	Detected
5326	A_23_P257936	TEX28	Hs.672606	testis expressed 28	Homo sapiens testis expressed 28 (TEX28), mRNA [NM_001586]	3.57870	down	1.10219	down	4.43554	16.43632	9.21441	8.95550	Compromised	Detected	Compromised	Compromised
5327	A_23_P85922	BMP8A	Hs.472497	bone morphogenetic protein 8a	Homo sapiens bone morphogenetic protein 8a (BMP8A), mRNA [NM_181809]	3.57717	down	3.69667	down	10.17271	37.67982	2.81777	9.18504	Compromised	Detected	Compromised	Compromised
5328	A_23_P19754	CPA4	Hs.93764	carboxypeptidase A4	Homo sapiens carboxypeptidase A4 (CPA4), transcript variant 1, mRNA [NM_016352]	3.57499	down	3.47603	down	14.26910	52.82070	12.46064	38.19352	Detected	Detected	Compromised	Detected
5329	A_24_P357726	PSG8	Hs.466843	pregnancy specific beta-1-glycoprotein 8	Homo sapiens pregnancy specific beta-1-glycoprotein 8 (PSG8), transcript variant 1, mRNA [NM_182707]	3.57210	down	1.35410	down	95.66386	353.83860	97.68231	116.63563	Detected	Detected	Detected	Detected
5330	A_24_P810290	PPAPDC1A	Hs.40479	phosphatidic acid phosphatase type 2 domain containing 1A	Homo sapiens phosphatidic acid phosphatase type 2 domain containing 1A (PPAPDC1A), mRNA [NM_001030059]	3.56904	down	3.74212	down	395.08350	1460.07090	343.31372	1132.85550	Detected	Detected	Detected	Detected
5331	A_33_P3265872	LOC100128994	Hs.453788	hypothetical LOC100128994	PREDICTED: Homo sapiens hypothetical LOC100128994 (LOC100128994), mRNA [XM_001717144]	3.56623	down	3.66058	down	139.54039	515.27820	164.21724	530.07150	Detected	Detected	Detected	Detected
5332	A_23_P435610	KIAA0090	Hs.439200	KIAA0090	Homo sapiens KIAA0090 (KIAA0090), mRNA [NM_015047]	3.56536	down	3.81056	down	354.83360	1309.96910	293.99094	987.84450	Detected	Detected	Detected	Detected
5333	A_23_P70843	BPGM	Hs.198365	2,3-bisphosphoglycerate mutase	Homo sapiens 2,3-bisphosphoglycerate mutase (BPGM), transcript variant 2, mRNA [NM_199186]	3.56381	down	3.25983	down	2182.77200	8054.82960	1954.05160	5616.89000	Detected	Detected	Detected	Detected
5334	A_24_P183150	CXCL3	Hs.89690	chemokine (C-X-C motif) ligand 3	Homo sapiens chemokine (C-X-C motif) ligand 3 (CXCL3), mRNA [NM_002090]	3.55833	down	3.66299	down	186.66193	687.75720	146.50864	473.22174	Detected	Detected	Detected	Detected
5335	A_33_P3280945	SNHG3		small nucleolar RNA host gene 3 (non-protein coding)	Homo sapiens small nucleolar RNA host gene 3 (non-protein coding) (SNHG3), non-coding RNA [NR_002909]	3.55523	down	3.73955	down	5548.47200	20425.54100	3471.29470	11446.60200	Detected	Detected	Detected	Detected
5336	A_33_P3281850				Cell growth regulator with EF hand domain protein 1 (Cell growth regulatory gene 11 protein) [Source:UniProtKB/Swiss-Prot;Acc:Q99674] [ENST00000402550]	3.55495	down	1.98076	down	42.03998	154.74959	32.63218	56.99597	Detected	Detected	Detected	Detected
5337	A_23_P128147	TUBA1B	Hs.524390	tubulin, alpha 1b	Homo sapiens tubulin, alpha 1b (TUBA1B), mRNA [NM_006082]	3.55446	down	3.78583	down	107757.17000	396600.30000	70294.20000	234663.94000	Detected	Detected	Detected	Detected
5338	A_23_P26511	GDPD3	Hs.289015	glycerophosphodiester phosphodiesterase domain containing 3	Homo sapiens glycerophosphodiester phosphodiesterase domain containing 3 (GDPD3), mRNA [NM_024307]	3.55303	down	4.05328	down	41.87480	154.05829	46.70894	166.94452	Detected	Detected	Detected	Detected
5339	A_23_P134601	WNT16	Hs.272375	wingless-type MMTV integration site family, member 16	Homo sapiens wingless-type MMTV integration site family, member 16 (WNT16), transcript variant 1, mRNA [NM_057168]	3.54859	down	3.23654	down	36.36884	133.63458	23.00234	65.64746	Detected	Detected	Detected	Detected
5340	A_23_P76731	RAGE	Hs.104119	renal tumor antigen	Homo sapiens renal tumor antigen (RAGE), mRNA [NM_014226]	3.54590	down	3.54097	down	1172.01210	4303.19600	1120.85770	3499.76340	Detected	Detected	Detected	Detected
5341	A_23_P33326	ADRA1B	Hs.368632	adrenergic, alpha-1B-, receptor	Homo sapiens adrenergic, alpha-1B-, receptor (ADRA1B), mRNA [NM_000679]	3.54319	down	3.97062	down	374.43750	1373.74700	347.30530	1216.00570	Detected	Detected	Detected	Detected
5342	A_23_P76705	TUBGCP3	Hs.224152	tubulin, gamma complex associated protein 3	Homo sapiens tubulin, gamma complex associated protein 3 (TUBGCP3), mRNA [NM_006322]	3.54304	down	3.63900	down	231.96722	851.01166	190.08424	609.94920	Detected	Detected	Detected	Detected
5343	A_33_P3264895	RHEBL1	Hs.159013	Ras homolog enriched in brain like 1	Homo sapiens Ras homolog enriched in brain like 1 (RHEBL1), mRNA [NM_144593]	3.54234	down	3.68896	down	1252.12350	4592.73050	970.29224	3156.25220	Detected	Detected	Detected	Detected
5344	A_23_P353717	C16orf75	Hs.347524	chromosome 16 open reading frame 75	Homo sapiens chromosome 16 open reading frame 75 (C16orf75), mRNA [NM_152308]	3.54040	down	4.26556	down	760.03820	2786.25170	643.79250	2421.51540	Detected	Detected	Detected	Detected
5345	A_33_P3280796				LIM domain only protein 7 (LOMP)(F-box only protein 20) [Source:UniProtKB/Swiss-Prot;Acc:Q8WWI1] [ENST00000377499]	3.54028	down	3.79997	down	28.58457	104.78590	20.30353	68.03269	Detected	Detected	Detected	Detected
5346	A_23_P120883	HMOX1	Hs.517581	heme oxygenase (decycling) 1	Homo sapiens heme oxygenase (decycling) 1 (HMOX1), mRNA [NM_002133]	3.53897	down	3.70083	down	45352.93400	166194.17000	33572.63000	109559.42000	Detected	Detected	Detected	Detected
5347	A_23_P155989	CENPK	Hs.529778	centromere protein K	Homo sapiens centromere protein K (CENPK), mRNA [NM_022145]	3.53495	down	2.89504	down	282.01190	1032.24870	118.56199	302.66720	Detected	Detected	Detected	Detected
5348	A_23_P134517	PURB	Hs.349150	purine-rich element binding protein B	Homo sapiens purine-rich element binding protein B (PURB), mRNA [NM_033224]	3.53174	down	3.61456	down	895.06525	3273.23240	691.15530	2202.91040	Detected	Detected	Detected	Detected

5349	A_33_P3423565	LOC100291714		hypothetical protein LOC100291714	PREDICTED: Homo sapiens hypothetical protein LOC100291714 (LOC100291714), mRNA [XM_002344976]	3.53127	down	2.28696	down	12.10876	44.27559	20.60895	41.56035	Detected	Detected	Detected	Detected
5350	A_23_P76435	GATC	Hs.369624	glutamyl-tRNA(Gln) amidotransferase, subunit C homolog (bacterial)	Homo sapiens glutamyl-tRNA(Gln) amidotransferase, subunit C homolog (bacterial) (GATC), mRNA [NM_176818]	3.53085	down	4.13800	down	52.15594	190.68456	48.54683	177.14010	Detected	Detected	Detected	Detected
5351	A_32_P160045	TCTEX1D1	Hs.479226	Tctex1 domain containing 1	Homo sapiens Tctex1 domain containing 1 (TCTEX1D1), mRNA [NM_152665]	3.52589	down	4.19416	down	9.89301	36.11855	5.05560	18.69746	Compromised	Detected	Compromised	Detected
5352	A_32_P416161	XKRX	Hs.364911	XK, Kell blood group complex subunit-related, X-linked	Homo sapiens XK, Kell blood group complex subunit-related, X-linked (XKRX), mRNA [NM_212559]	3.52292	down	3.66999	down	8.71600	31.79463	9.74857	31.54801	Compromised	Detected	Compromised	Detected
5353	A_23_P165201	PRODH2	Hs.515366	proline dehydrogenase (oxidase) 2	Homo sapiens proline dehydrogenase (oxidase) 2 (PRODH2), mRNA [NM_021232]	3.51936	down	1.72921	down	8.20413	29.89708	2.87968	4.39094	Compromised	Detected	Compromised	Compromised
5354	A_23_P29067	TMPRSS2	Hs.439309	transmembrane protease, serine 2	Homo sapiens transmembrane protease, serine 2 (TMPRSS2), transcript variant 2, mRNA [NM_005656]	3.51354	down	2.75185	down	58.68810	213.51477	10.03630	24.35359	Detected	Detected	Compromised	Detected
5355	A_23_P98350	BIRC3	Hs.127799	baculoviral IAP repeat-containing 3	Homo sapiens baculoviral IAP repeat-containing 3 (BIRC3), transcript variant 1, mRNA [NM_001165]	3.51217	down	3.31087	down	161.07797	585.79346	102.84661	300.26013	Detected	Detected	Detected	Detected
5356	A_33_P3325843	LOC100131686	Hs.689484	hypothetical protein LOC100131686	PREDICTED: Homo sapiens hypothetical LOC100131686, transcript variant 2 (LOC100131686), mRNA [XM_001722260]	3.51185	down	3.55226	down	261.11588	949.51720	133.40028	417.85583	Detected	Detected	Detected	Detected
5357	A_23_P111054	HIST1H2BB	Hs.553494	histone cluster 1, H2bb	Homo sapiens histone cluster 1, H2bb (HIST1H2BB), mRNA [NM_021062]	3.50981	down	3.49021	down	179.87553	653.71590	214.14377	659.05585	Detected	Detected	Detected	Detected
5358	A_33_P3344277				HCLS1-binding protein 3 (HS1-binding protein 3)(HSP1BP-3) [Source:UniProtKB/Swiss-Prot;Acc:Q53T59] [ENST00000402541]	3.50967	down	4.06850	down	60.86503	221.19077	41.65284	149.43239	Detected	Detected	Detected	Detected
5359	A_33_P3259775	DOCK5	Hs.195403	dedicator of cytokinesis 5	Homo sapiens dedicator of cytokinesis 5 (DOCK5), mRNA [NM_024940]	3.50323	down	3.24588	down	61.65580	223.65318	41.67636	119.28570	Detected	Detected	Detected	Detected
5360	A_23_P30254	PLK2	Hs.398157	polo-like kinase 2 (Drosophila)	Homo sapiens polo-like kinase 2 (Drosophila) (PLK2), mRNA [NM_006622]	3.49686	down	3.58726	down	957.32150	3466.32810	654.82007	2071.33740	Detected	Detected	Detected	Detected
5361	A_23_P3562	SLC7A6OS	Hs.709557	solute carrier family 7, member 6 opposite strand	Homo sapiens solute carrier family 7, member 6 opposite strand (SLC7A6OS), mRNA [NM_032178]	3.49680	down	3.04880	down	540.68190	1957.69630	502.00986	1349.60380	Detected	Detected	Detected	Detected
5362	A_33_P3372212		Hs.655399		Pregnancy-specific beta-1-glycoprotein 5 Precursor (PSBG-5)(Fetal liver non-specific cross-reactive antigen 3)(FL-NCA-3) [Source:UniProtKB/Swiss-Prot;Acc:Q15238] [ENST00000404580]	3.49676	down	3.94369	down	357.09415	1292.94900	270.83282	941.82324	Detected	Detected	Detected	Detected
5363	A_23_P63432	RHBDL2	Hs.524626	rhomboid, veinlet-like 2 (Drosophila)	Homo sapiens rhomboid, veinlet-like 2 (Drosophila) (RHBDL2), mRNA [NM_017821]	3.49088	down	2.98158	down	55.38981	200.21553	51.93965	136.55618	Detected	Detected	Detected	Detected
5364	A_24_P181585	LRRC59	Hs.370927	leucine rich repeat containing 59	Homo sapiens leucine rich repeat containing 59 (LRRC59), mRNA [NM_018509]	3.48785	down	3.24206	down	9999.81900	36114.66000	6029.58600	17237.49200	Detected	Detected	Detected	Detected
5365	A_23_P122815	CALU	Hs.718425	calumenin	Homo sapiens calumenin (CALU), transcript variant 1, mRNA [NM_001219]	3.48704	down	3.25888	down	3573.08300	12901.30700	2560.03200	7356.63130	Detected	Detected	Detected	Detected
5366	A_33_P3379454	UHRF1	Hs.108106	ubiquitin-like with PHD and ring finger domains 1	Homo sapiens ubiquitin-like with PHD and ring finger domains 1 (UHRF1), transcript variant 2, mRNA [NM_013282]	3.47909	down	4.58753	down	37.88173	136.46721	33.17343	134.19444	Detected	Detected	Detected	Detected
5367	A_23_P318039	RQCD1	Hs.148767	RCD1 required for cell differentiation1 homolog (S. pombe)	Homo sapiens RCD1 required for cell differentiation1 homolog (S. pombe) (RQCD1), mRNA [NM_005444]	3.47865	down	3.20806	down	117.86211	424.53992	84.84498	240.01234	Detected	Detected	Detected	Detected
5368	A_23_P144348	SLIT2	Hs.29802	slit homolog 2 (Drosophila)	Homo sapiens slit homolog 2 (Drosophila) (SLIT2), mRNA [NM_004787]	3.47757	down	3.57776	down	577.75750	2080.43400	629.35220	1985.50440	Detected	Detected	Detected	Detected
5369	A_33_P3308686	CC2D2A	Hs.590928	coiled-coil and C2 domain containing 2A	Homo sapiens coiled-coil and C2 domain containing 2A (CC2D2A), transcript variant 2, mRNA [NM_020785]	3.47552	down	2.94428	down	151.60490	545.58950	102.56459	266.28204	Detected	Detected	Detected	Detected
5370	A_33_P3259845	FAM53A	Hs.143314	family with sequence similarity 53, member A	Homo sapiens family with sequence similarity 53, member A (FAM53A), mRNA [NM_001013622]	3.47542	down	2.71118	down	26.95874	97.01531	21.68171	51.83439	Detected	Detected	Detected	Detected
5371	A_33_P3376971	CHAC1	Hs.155569	ChaC, cation transport regulator homolog 1 (E. coli)	Homo sapiens ChaC, cation transport regulator homolog 1 (E. coli) (CHAC1), transcript variant 1, mRNA [NM_024111]	3.47158	down	4.38778	down	500.00160	1797.34330	502.52450	1944.32100	Detected	Detected	Detected	Detected
5372	A_23_P419714	BTBD11	Hs.271272	BTB (POZ) domain containing 11	Homo sapiens BTB (POZ) domain containing 11 (BTBD11), transcript variant a, mRNA [NM_001018072]	3.46843	down	2.27990	down	13.52772	48.58366	16.14257	32.45290	Detected	Detected	Detected	Detected
5373	A_23_P1331	COL13A1	Hs.695934	collagen, type XIII, alpha 1	Homo sapiens collagen, type XIII, alpha 1 (COL13A1), transcript variant 1, mRNA [NM_005203]	3.46734	down	3.65189	down	1187.77160	4264.45170	736.68463	2372.27610	Detected	Detected	Detected	Detected
5374	A_33_P3316064		Hs.691047		DA567289 HEART2 Homo sapiens cDNA clone HEART2010630 5', mRNA sequence [DA567289]	3.46614	down	3.64841	down	21.99452	78.93935	22.14466	71.24229	Detected	Detected	Detected	Detected
5375	A_23_P52207	BAMBI	Hs.533336	BMP and activin membrane-bound inhibitor homolog (Xenopus laevis)	Homo sapiens BMP and activin membrane-bound inhibitor homolog (Xenopus laevis) (BAMBI), mRNA [NM_012342]	3.46492	down	3.69411	down	594.85390	2134.20580	509.33557	1659.12520	Detected	Detected	Detected	Detected
5376	A_24_P151121					3.46245	down	3.97912	down	15.67692	56.20525	8.77837	30.80112	Detected	Detected	Compromised	Detected

5377	A_33_P3283196				SFRS2-interacting protein (Splicing factor, arginine/serine-rich 2-interacting protein)(SC35-interacting protein 1)(CTD-associated SR protein 11)(Splicing regulatory protein 129)(SRrp129)(Renal carcinoma antigen NY-REN-40) [Source:UniProtKB/Swiss-Prot;Acc:Q99590] [ENST00000395453]	3.45788	down	3.15378	down	312.24120	1117.97910	260.88037	725.50220	Detected	Detected	Detected	Detected
5378	A_33_P3277097				lymphocyte antigen 6 complex, locus G6E (LY6G6E), transcript variant 2, non-coding RNA [Source:RefSeq DNA;Acc:NR_003673] [ENST00000383417]	3.44865	down	3.47182	down	132.29988	472.43472	151.45505	463.66724	Detected	Detected	Detected	Detected
5379	A_24_P133584	MFGE8	Hs.3745	milk fat globule-EGF factor 8 protein	Homo sapiens milk fat globule-EGF factor 8 protein (MFGE8), transcript variant 1, mRNA [NM_005928]	3.44577	down	4.26756	down	689.48517	2460.04860	638.23950	2401.75730	Detected	Detected	Detected	Detected
5380	A_33_P3352019	SCARA3	Hs.128856	scavenger receptor class A, member 3	Homo sapiens scavenger receptor class A, member 3 (SCARA3), transcript variant 2, mRNA [NM_182826]	3.44359	down	3.60844	down	168.41946	600.53260	182.38518	580.32930	Detected	Detected	Detected	Detected
5381	A_33_P3313065	OR5C1	Hs.534761	olfactory receptor, family 5, subfamily C, member 1	Homo sapiens olfactory receptor, family 5, subfamily C, member 1 (OR5C1), mRNA [NM_001001923]	3.44149	down	2.24851	down	71.75095	255.68637	21.24385	42.12041	Detected	Detected	Detected	Detected
5382	A_33_P3263232	LRRC3	Hs.326579	leucine rich repeat containing 3	Homo sapiens leucine rich repeat containing 3 (LRRC3), mRNA [NM_030891]	3.43806	down	3.56031	down	179.86240	640.30590	134.90565	423.52970	Detected	Detected	Detected	Detected
5383	A_23_P155765	HMGB2	Hs.434953	high-mobility group box 2	Homo sapiens high-mobility group box 2 (HMGB2), transcript variant 1, mRNA [NM_002129]	3.43732	down	3.28055	down	5733.48300	20406.62000	4430.14500	12815.32300	Detected	Detected	Detected	Detected
5384	A_23_P59602	MIOS	Hs.520215	missing oocyte, meiosis regulator, homolog (Drosophila)	Homo sapiens missing oocyte, meiosis regulator, homolog (Drosophila) (MIOS), mRNA [NM_019005]	3.43392	down	3.46502	down	1743.50350	6199.34230	1785.36450	5455.04700	Detected	Detected	Detected	Detected
5385	A_33_P3312258	TUBB	Hs.636480	tubulin, beta	Homo sapiens tubulin, beta (TUBB), mRNA [NM_178014]	3.43133	down	3.61192	down	6120.86300	21747.45300	5897.70560	18783.91600	Detected	Detected	Detected	Detected
5386	A_24_P282237	PRIM2	Hs.654580	primase, DNA, polypeptide 2 (58kDa)	Homo sapiens primase, DNA, polypeptide 2 (58kDa) (PRIM2), mRNA [NM_000947]	3.42646	down	3.38303	down	109.93461	390.04382	64.25757	191.68808	Detected	Detected	Detected	Detected
5387	A_33_P3214159	CDH2	Hs.464829	cadherin 2, type 1, N-cadherin (neuronal)	Homo sapiens cadherin 2, type 1, N-cadherin (neuronal) (CDH2), mRNA [NM_001792]	3.42621	down	3.64970	down	1946.82370	6906.75700	1861.10560	5989.53470	Detected	Detected	Detected	Detected
5388	A_23_P318604					3.42296	down	3.47489	down	91.08279	322.82830	77.14034	236.36763	Detected	Detected	Detected	Detected
5389	A_24_P22981	ZNF253	Hs.659321	zinc finger protein 253	Homo sapiens zinc finger protein 253 (ZNF253), mRNA [NM_021047]	3.42165	down	16.50574	down	2.36425	8.37651	4.00371	58.27250	Compromised	Compromised	Compromised	Detected
5390	A_23_P51376	NKAIN1	Hs.470259	Na+/K+ transporting ATPase interacting 1	Homo sapiens Na+/K+ transporting ATPase interacting 1 (NKAIN1), mRNA [NM_024522]	3.42062	down	5.05201	down	59.20457	209.69750	41.25796	183.79675	Detected	Detected	Detected	Detected
5391	A_23_P201636	LAMC2	Hs.591484	laminin, gamma 2	Homo sapiens laminin, gamma 2 (LAMC2), transcript variant 1, mRNA [NM_005562]	3.42014	down	3.59135	down	800.69934	2835.61180	720.53546	2281.80700	Detected	Detected	Detected	Detected
5392	A_33_P3335935	HN1L	Hs.513261	hematological and neurological expressed 1-like	Homo sapiens hematological and neurological expressed 1-like (HN1L), mRNA [NM_144570]	3.41902	down	3.31863	down	116.86543	413.73386	105.48631	308.68814	Detected	Detected	Detected	Detected
5393	A_23_P502747	RASAL2	Hs.549729	RAS protein activator like 2	Homo sapiens RAS protein activator like 2 (RASAL2), transcript variant 2, mRNA [NM_170692]	3.41673	down	4.27400	down	75.98702	268.83330	51.87065	195.48889	Detected	Detected	Detected	Detected
5394	A_24_P402825	CACNA2D3	Hs.656687	calcium channel, voltage-dependent, alpha 2/delta subunit 3	Homo sapiens calcium channel, voltage-dependent, alpha 2/delta subunit 3 (CACNA2D3), mRNA [NM_018398]	3.41612	down	8.16352	down	11.49971	40.67743	5.55219	39.96753	Compromised	Detected	Compromised	Detected
5395	A_33_P3253832					3.41605	down	3.61544	down	5836.16550	20643.56400	4327.42530	13796.10300	Detected	Detected	Detected	Detected
5396	A_33_P3402010		Hs.551210		Uncharacterized protein C14orf56 [Source:UniProtKB/Swiss-Prot;Acc:Q9H7N3] [ENST00000427346]	3.41427	down	1.77722	down	171.10538	604.91600	24.25271	38.00719	Detected	Detected	Detected	Detected
5397	A_32_P213459	DMRT2	Hs.59506	doublesex and mab-3 related transcription factor 2	Homo sapiens doublesex and mab-3 related transcription factor 2 (DMRT2), transcript variant 1, mRNA [NM_006557]	3.41415	down	4.34384	down	34.63318	122.43580	24.75905	94.83593	Detected	Detected	Detected	Detected
5398	A_23_P426989	SH3BP5L	Hs.298573	SH3-binding domain protein 5-like	Homo sapiens SH3-binding domain protein 5-like (SH3BP5L), mRNA [NM_030645]	3.41312	down	3.51348	down	3033.49600	10720.82500	3320.43140	10287.20700	Detected	Detected	Detected	Detected
5399	A_23_P80295	SYN3	Hs.608750	synapsin III	Homo sapiens synapsin III (SYN3), transcript variant IIIa, mRNA [NM_003490]	3.41138	down	3.10701	down	8.06113	28.47466	5.86243	16.06150	Compromised	Detected	Compromised	Detected
5400	A_33_P3365988	LOC728463	Hs.680183	hypothetical protein LOC728463	PREDICTED: Homo sapiens hypothetical protein LOC728463 (LOC728463), miscRNA [XR_041658]	3.41018	down	4.29386	down	6.40792	22.62699	3.35601	12.70683	Compromised	Detected	Compromised	Compromised
5401	A_33_P3341424	MYO10	Hs.481720	myosin X	Homo sapiens myosin X (MYO10), mRNA [NM_012334]	3.40986	down	3.09972	down	812.23444	2867.81620	695.86430	1902.00800	Detected	Detected	Detected	Detected
5402	A_23_P19624	BMP6	Hs.285671	bone morphogenetic protein 6	Homo sapiens bone morphogenetic protein 6 (BMP6), mRNA [NM_001718]	3.40700	down	3.36984	down	1000.64856	3530.09420	923.59540	2744.45730	Detected	Detected	Detected	Detected
5403	A_23_P81912	TUBB	Hs.636480	tubulin, beta	Homo sapiens tubulin, beta (TUBB), mRNA [NM_178014]	3.40157	down	3.67448	down	13773.70500	48513.58200	16979.76600	55016.57000	Detected	Detected	Detected	Detected
5404	A_23_P15348	MPRIP	Hs.462341	myosin phosphatase Rho interacting protein	Homo sapiens myosin phosphatase Rho interacting protein (MPRIP), transcript variant 1, mRNA [NM_015134]	3.39958	down	3.72961	down	6380.43300	22459.92800	5414.05370	17805.38000	Detected	Detected	Detected	Detected
5405	A_23_P387057	TUBB	Hs.636480	tubulin, beta	Homo sapiens tubulin, beta (TUBB), mRNA [NM_178014]	3.39656	down	3.34688	down	4480.39940	15757.58700	3812.83500	11252.63600	Detected	Detected	Detected	Detected
5406	A_33_P3214849	KDELC2	Hs.83286	KDEL (Lys-Asp-Glu-Leu) containing 2	Homo sapiens KDEL (Lys-Asp-Glu-Leu) containing 2 (KDELC2), mRNA [NM_153705]	3.39393	down	3.47129	down	1088.31910	3824.65140	649.86320	1989.19730	Detected	Detected	Detected	Detected

5407	A_32_P117464	C3orf59	Hs.151443	chromosome 3 open reading frame 59	Homo sapiens chromosome 3 open reading frame 59 (C3orf59), mRNA [NM_178496]	3.39218	down	3.38766	down	221.22879	777.05940	148.34941	443.14984	Detected	Detected	Detected	Detected
5408	A_24_P943997	ARL5B	Hs.25362	ADP-ribosylation factor-like 5B	Homo sapiens ADP-ribosylation factor-like 5B (ARL5B), mRNA [NM_178815]	3.38469	down	2.07373	down	20.73344	72.66457	23.64074	43.22933	Detected	Detected	Detected	Detected
5409	A_24_P184769	WDR20	Hs.36859	WD repeat domain 20	Homo sapiens WD repeat domain 20 (WDR20), transcript variant 1, mRNA [NM_181291]	3.38324	down	3.89757	down	235.56650	825.23956	166.60680	572.60030	Detected	Detected	Detected	Detected
5410	A_23_P358917	CYP3A7	Hs.111944	cytochrome P450, family 3, subfamily A, polypeptide 7	Homo sapiens cytochrome P450, family 3, subfamily A, polypeptide 7 (CYP3A7), mRNA [NM_000765]	3.38305	down	2.39064	down	15.91747	55.75911	14.74097	31.07464	Detected	Detected	Detected	Detected
5411	A_33_P3274397	CHM	Hs.496449	choroideremia (Rab escort protein 1)	Homo sapiens choroideremia (Rab escort protein 1) (CHM), transcript variant 2, mRNA [NM_001145414]	3.37952	down	3.26359	down	124.25421	434.80970	88.05138	253.39459	Detected	Detected	Detected	Detected
5412	A_24_P225468	ANP32E	Hs.656466	acidic (leucine-rich) nuclear phosphoprotein 32 family, member E	Homo sapiens acidic (leucine-rich) nuclear phosphoprotein 32 family, member E (ANP32E), transcript variant 1, mRNA [NM_030920]	3.37944	down	3.28218	down	866.63640	3032.59400	541.87620	1568.29440	Detected	Detected	Detected	Detected
5413	A_23_P26557	C16orf59	Hs.534491	chromosome 16 open reading frame 59	Homo sapiens chromosome 16 open reading frame 59 (C16orf59), mRNA [NM_025108]	3.37857	down	3.60126	down	397.71536	1391.35700	445.41122	1414.42750	Detected	Detected	Detected	Detected
5414	A_23_P31725	BLK	Hs.146591	B lymphoid tyrosine kinase	Homo sapiens B lymphoid tyrosine kinase (BLK), mRNA [NM_001715]	3.37837	down	3.78097	down	10.29856	36.02604	11.48014	38.27507	Compromised	Detected	Compromised	Detected
5415	A_24_P291826	SYTL3	Hs.436977	synaptotagmin-like 3	Homo sapiens synaptotagmin-like 3 (SYTL3), mRNA [NM_001009991]	3.37522	down	3.61795	down	107.96881	377.34042	119.45279	381.08698	Detected	Detected	Detected	Detected
5416	A_24_P69095	ENC1	Hs.104925	ectodermal-neural cortex (with BTB-like domain)	Homo sapiens ectodermal-neural cortex (with BTB-like domain) (ENC1), mRNA [NM_003633]	3.37495	down	3.39032	down	223.46048	780.91060	217.75276	650.98410	Detected	Detected	Detected	Detected
5417	A_33_P3316835				Uncharacterized protein C4orf21 [Source:UniProtKB/Swiss-Prot;Acc:Q86YA3] [ENST00000264370]	3.37477	down	3.83404	down	18.79868	65.69080	7.65548	25.88180	Detected	Detected	Compromised	Detected
5418	A_24_P124349	PDGFD	Hs.352298	platelet derived growth factor D	Homo sapiens platelet derived growth factor D (PDGFD), transcript variant 1, mRNA [NM_025208]	3.37415	down	3.84982	down	16.62358	58.07933	13.00552	44.15033	Detected	Detected	Compromised	Detected
5419	A_23_P218131	INF2	Hs.24956	inverted formin, FH2 and WH2 domain containing	Homo sapiens inverted formin, FH2 and WH2 domain containing (INF2), transcript variant 3, mRNA [NM_032714]	3.37374	down	3.51376	down	3484.78120	12173.64200	3088.35670	9568.97600	Detected	Detected	Detected	Detected
5420	A_32_P806841	ARL4A	Hs.245540	ADP-ribosylation factor-like 4A	Homo sapiens ADP-ribosylation factor-like 4A (ARL4A), transcript variant 1, mRNA [NM_005738]	3.37374	down	2.83190	down	214.72679	750.11957	118.97802	297.10507	Detected	Detected	Detected	Detected
5421	A_23_P91512	CLDN14	Hs.660278	claudin 14	Homo sapiens claudin 14 (CLDN14), transcript variant 1, mRNA [NM_144492]	3.37259	down	4.24126	down	23.47915	81.99339	10.71079	40.05732	Detected	Detected	Compromised	Detected
5422	A_33_P3344229	HIST1H4A	Hs.248178	histone cluster 1, H4a	Homo sapiens histone cluster 1, H4a (HIST1H4A), mRNA [NM_003538]	3.36936	down	2.78545	down	168.59038	588.18440	47.36457	116.33629	Detected	Detected	Detected	Detected
5423	A_23_P128532	C12orf45	Hs.295563	chromosome 12 open reading frame 45	Homo sapiens chromosome 12 open reading frame 45 (C12orf45), mRNA [NM_152318]	3.36765	down	3.53579	down	1417.38510	4942.51760	989.75440	3085.88350	Detected	Detected	Detected	Detected
5424	A_33_P3275495	LOC400743	Hs.522935	FLJ45933 protein	PREDICTED: Homo sapiens FLJ45933 protein (LOC400743), mRNA [XM_001719656]	3.36691	down	1.94756	down	334.55240	1166.35010	30.10249	51.69611	Detected	Detected	Detected	Detected
5425	A_24_P84608					3.36235	down	2.90525	down	367.15674	1278.28600	153.59040	393.47174	Detected	Detected	Detected	Detected
5426	A_33_P3244882	EIF2C3	Hs.657659	eukaryotic translation initiation factor 2C, 3	Homo sapiens eukaryotic translation initiation factor 2C, 3 (EIF2C3), transcript variant 1, mRNA [NM_024852]	3.36179	down	3.52148	down	358.69272	1248.60660	335.00763	1040.26930	Detected	Detected	Detected	Detected
5427	A_33_P3306024	SIGLEC16		sialic acid binding Ig-like lectin 16 (gene/pseudogene)	Homo sapiens sialic acid binding Ig-like lectin 16 (gene/pseudogene) (SIGLEC16), non-coding RNA [NR_002825]	3.36037	down	3.32248	down	1254.20850	4364.05500	561.81570	1645.97180	Detected	Detected	Detected	Detected
5428	A_23_P82379	CACNA2D1	Hs.282151	calcium channel, voltage-dependent, alpha 2/delta subunit 1	Homo sapiens calcium channel, voltage-dependent, alpha 2/delta subunit 1 (CACNA2D1), mRNA [NM_000722]	3.35881	down	2.79143	down	13.14338	45.71158	14.18157	34.90727	Compromised	Detected	Compromised	Detected
5429	A_33_P3245168	FAM71F2	Hs.445236	family with sequence similarity 71, member F2	Homo sapiens family with sequence similarity 71, member F2 (FAM71F2), transcript variant 2, mRNA [NM_001128926]	3.35537	down	4.71862	down	13.56868	47.14237	8.66985	36.07378	Compromised	Detected	Compromised	Detected
5430	A_23_P164826	RNASEH2A	Hs.532851	ribonuclease H2, subunit A	Homo sapiens ribonuclease H2, subunit A (RNASEH2A), mRNA [NM_006397]	3.35310	down	3.90665	down	2053.90160	7131.15040	2164.36350	7455.89750	Detected	Detected	Detected	Detected
5431	A_32_P116271	C4orf12		chromosome 4 open reading frame 12	Homo sapiens chromosome 4 open reading frame 12 (C4orf12), non-coding RNA [NR_015359]	3.35278	down	3.36016	down	96.27262	334.22702	98.83054	292.83070	Detected	Detected	Detected	Detected
5432	A_24_P258051	MASTL	Hs.276905	microtubule associated serine/threonine kinase-like	Homo sapiens microtubule associated serine/threonine kinase-like (MASTL), mRNA [NM_032844]	3.35198	down	3.13418	down	235.41441	817.08545	204.54713	565.30480	Detected	Detected	Detected	Detected
5433	A_23_P101380	B3GNT8	Hs.441681	UDP-GlcNAc:betaGal beta-1,3-N-acetylglucosaminyltransferase 8	Homo sapiens UDP-GlcNAc:betaGal beta-1,3-N-acetylglucosaminyltransferase 8 (B3GNT8), mRNA [NM_198540]	3.34853	down	3.53960	down	541.66956	1878.11120	432.96918	1351.37850	Detected	Detected	Detected	Detected
5434	A_33_P3305840	SFRS7	Hs.309090	splicing factor, arginine/serine-rich 7, 35kDa	Homo sapiens splicing factor, arginine/serine-rich 7, 35kDa (SFRS7), mRNA [NM_001031684]	3.34813	down	3.24653	down	5269.89600	18269.95700	4822.97700	13807.04200	Detected	Detected	Detected	Detected
5435	A_23_P140057	TNFRSF19	Hs.149168	tumor necrosis factor receptor superfamily, member 19	Homo sapiens tumor necrosis factor receptor superfamily, member 19 (TNFRSF19), transcript variant 1, mRNA [NM_018647]	3.34780	down	2.74817	down	6.51908	22.59849	4.57305	11.08194	Compromised	Detected	Compromised	Compromised
5436	A_23_P116414	PLA2G16	Hs.502775	phospholipase A2, group XVI	Homo sapiens phospholipase A2, group XVI (PLA2G16), transcript variant 1, mRNA [NM_007069]	3.34527	down	3.72630	down	1151.05590	3987.13000	813.60315	2673.35130	Detected	Detected	Detected	Detected

5437	A_23_P259692	PSAT1	Hs.494261	phosphoserine aminotransferase 1	Homo sapiens phosphoserine aminotransferase 1 (PSAT1), transcript variant 1, mRNA [NM_058179]	3.34414	down	3.19293	down	2168.18140	7507.80270	1129.59960	3180.38770	Detected	Detected	Detected	Detected
5438	A_23_P161727	HSPB2	Hs.709660	heat shock 27kDa protein 2	Homo sapiens heat shock 27kDa protein 2 (HSPB2), mRNA [NM_001541]	3.34362	down	3.32261	down	4778.94700	16545.60200	3946.50700	11562.66000	Detected	Detected	Detected	Detected
5439	A_33_P3284045	NEK10	Hs.506115	NIMA (never in mitosis gene a)-related kinase 10	Homo sapiens NIMA (never in mitosis gene a)- related kinase 10 (NEK10), transcript variant 3, mRNA [NM_199347]	3.33961	down	5.57757	down	6.68653	23.12226	3.30909	16.27492	Compromised	Detected	Compromised	Detected
5440	A_24_P270829	GP1BA	Hs.1472	glycoprotein Ib (platelet), alpha polypeptide	Homo sapiens glycoprotein Ib (platelet), alpha polypeptide (GP1BA), mRNA [NM_000173]	3.33202	down	1.83617	down	23.15328	79.88281	14.87222	24.07992	Detected	Detected	Detected	Detected
5441	A_33_P3242124	HAUS8	Hs.404088	HAUS augmin-like complex, subunit 8	Homo sapiens HAUS augmin-like complex, subunit 8 (HAUS8), transcript variant 1, mRNA [NM_033417]	3.33193	down	3.17718	down	117.90477	406.78058	110.64299	309.97860	Detected	Detected	Detected	Detected
5442	A_24_P319923	MYLK	Hs.477375	myosin light chain kinase	Homo sapiens myosin light chain kinase (MYLK), transcript variant 1, mRNA [NM_053025]	3.33130	down	3.51105	down	1076.15450	3712.11720	786.66473	2435.52510	Detected	Detected	Detected	Detected
5443	A_23_P215819	ZNF655	Hs.599798	zinc finger protein 655	Homo sapiens zinc finger protein 655 (ZNF655), transcript variant 2, mRNA [NM_024061]	3.32872	down	3.44040	down	521.18570	1796.40010	461.00497	1398.55690	Detected	Detected	Detected	Detected
5444	A_33_P3346348	WDR67	Hs.492716	WD repeat domain 67	Homo sapiens WD repeat domain 67 (WDR67), transcript variant 2, mRNA [NM_001145088]	3.32861	down	2.34793	down	6.36736	21.94600	4.71907	9.77026	Compromised	Detected	Compromised	Compromised
5445	A_32_P34920	FOXD1	Hs.519385	forkhead box D1	Homo sapiens forkhead box D1 (FOXD1), mRNA [NM_004472]	3.32843	down	3.08698	down	5160.05900	17783.90000	4136.47700	11259.78700	Detected	Detected	Detected	Detected
5446	A_24_P166663	CDK6	Hs.119882	cyclin-dependent kinase 6	Homo sapiens cyclin-dependent kinase 6 (CDK6), mRNA [NM_001259]	3.32586	down	3.03303	down	3692.00600	12714.48800	2710.45950	7249.11570	Detected	Detected	Detected	Detected
5447	A_23_P60488	ODF2	Hs.129055	outer dense fiber of sperm tails 2	Homo sapiens outer dense fiber of sperm tails 2 (ODF2), transcript variant 1, mRNA [NM_002540]	3.32533	down	3.15767	down	455.24330	1567.51180	440.88160	1227.59390	Detected	Detected	Detected	Detected
5448	A_23_P5785	BBS5	Hs.233398	Bardet-Biedl syndrome 5	Homo sapiens Bardet-Biedl syndrome 5 (BBS5), mRNA [NM_152384]	3.32285	down	3.25144	down	77.82369	267.76580	65.52163	187.85620	Detected	Detected	Detected	Detected
5449	A_33_P3409392	FZD6	Hs.591863	frizzled homolog 6 (Drosophila)	Homo sapiens frizzled homolog 6 (Drosophila) (FZD6), transcript variant 1, mRNA [NM_003506]	3.32278	down	3.05770	down	743.55010	2558.26250	432.54324	1166.24600	Detected	Detected	Detected	Detected
5450	A_23_P70670	CD83	Hs.595133	CD83 molecule	Homo sapiens CD83 molecule (CD83), transcript variant 1, mRNA [NM_004233]	3.32146	down	3.33656	down	109.50832	376.62500	99.16966	291.77164	Detected	Detected	Detected	Detected
5451	A_32_P150086	LOC284232	Hs.633153	ankyrin repeat domain 20 family, member A2 pseudogene	Homo sapiens ankyrin repeat domain 20 family, member A2 pseudogene (LOC284232), non-coding RNA [NR_027995]	3.32056	down	1.15992	down	6.08420	20.91937	7.41386	7.58294	Compromised	Detected	Compromised	Compromised
5452	A_33_P3362326	FHL2		four and a half LIM domains 2	Four and a half LIM domains protein 2 (FHL-2)(Skeletal muscle LIM-protein 3)(SLIM 3)(LIM domain protein DRAL) [Source:UniProtKB/Swiss-Prot;Acc:Q14192] [ENST00000409177]	3.32012	down	3.67956	down	1444.09670	4964.58100	798.77136	2591.69240	Detected	Detected	Detected	Detected
5453	A_33_P3416414					3.31945	down	3.55309	down	89.77190	308.56020	59.90820	187.69743	Detected	Detected	Detected	Detected
5454	A_23_P155103	ADSL	Hs.75527	adenylosuccinate lyase	Homo sapiens adenylosuccinate lyase (ADSL), transcript variant 1, mRNA [NM_000026]	3.31708	down	3.41019	down	1015.10710	3486.59470	1118.49280	3363.38940	Detected	Detected	Detected	Detected
5455	A_33_P3426943	LOC151484	Hs.559309	hypothetical protein LOC151484	Homo sapiens hypothetical protein LOC151484, mRNA (cDNA clone IMAGE:5180745), partial cds [BC043555]	3.31423	down	1.00262	down	8.63074	29.61856	18.66737	16.50391	Compromised	Detected	Detected	Detected
5456	A_23_P42868	IGFBP1	Hs.642938	insulin-like growth factor binding protein 1	Homo sapiens insulin-like growth factor binding protein 1 (IGFBP1), mRNA [NM_000596]	3.31350	down	3.18555	down	24.29037	83.34016	21.46993	60.30876	Detected	Detected	Detected	Detected
5457	A_23_P500130	KANK1	Hs.306764	KN motif and ankyrin repeat domains 1	Homo sapiens KN motif and ankyrin repeat domains 1 (KANK1), transcript variant 2, mRNA [NM_153186]	3.30805	down	3.30993	down	2456.86500	8415.63600	1905.91350	5562.71730	Detected	Detected	Detected	Detected
5458	A_23_P154208	NAGK	Hs.7036	N-acetylglucosamine kinase	Homo sapiens N-acetylglucosamine kinase (NAGK), mRNA [NM_017567]	3.30744	down	3.63006	down	2881.11900	9867.02300	3229.71310	10338.17100	Detected	Detected	Detected	Detected
5459	A_23_P69497	CLEC3B	Hs.476092	C-type lectin domain family 3, member B	Homo sapiens C-type lectin domain family 3, member B (CLEC3B), mRNA [NM_003278]	3.30717	down	3.81428	down	1889.17930	6469.37450	1760.17680	5920.17300	Detected	Detected	Detected	Detected
5460	A_23_P142096	GPR32	Hs.515555	G protein-coupled receptor 32	Homo sapiens G protein-coupled receptor 32 (GPR32), mRNA [NM_001506]	3.30509	down	3.79474	down	348.25983	1191.84570	22.37827	74.88146	Detected	Detected	Detected	Detected
5461	A_23_P321511	MARCH3	Hs.132441	membrane-associated ring finger (C3HC4) 3	Homo sapiens membrane-associated ring finger (C3HC4) 3 (MARCH3), mRNA [NM_178450]	3.30469	down	3.27205	down	273.87903	937.18030	309.50952	893.01570	Detected	Detected	Detected	Detected
5462	A_23_P150362	TEX12	Hs.524039	testis expressed 12	Homo sapiens testis expressed 12 (TEX12), mRNA [NM_031275]	3.30098	down	5.05924	down	9.92451	33.92230	5.93096	26.45913	Compromised	Detected	Compromised	Detected
5463	A_23_P255376	CCDC109B	Hs.234149	coiled-coil domain containing 109B	Homo sapiens coiled-coil domain containing 109B (CCDC109B), mRNA [NM_017918]	3.30003	down	3.55933	down	1293.94420	4421.46500	857.87756	2692.51760	Detected	Detected	Detected	Detected
5464	A_23_P161352	PTPLA	Hs.114062	protein tyrosine phosphatase-like (proline instead of catalytic arginine), member A	Homo sapiens protein tyrosine phosphatase-like (proline instead of catalytic arginine), member A (PTPLA), mRNA [NM_014241]	3.29994	down	2.95801	down	574.96970	1964.64640	568.94574	1484.00600	Detected	Detected	Detected	Detected
5465	A_23_P500433	CARD9	Hs.694071	caspase recruitment domain family, member 9	Homo sapiens caspase recruitment domain family, member 9 (CARD9), transcript variant 1, mRNA [NM_052813]	3.29775	down	3.71294	down	134.55565	459.46527	113.10810	370.31960	Detected	Detected	Detected	Detected
5466	A_23_P19590	EZR	Hs.487027	ezrin	Homo sapiens ezrin (EZR), transcript variant 1, mRNA [NM_003379]	3.29730	down	3.41749	down	2815.78980	9613.72500	2709.49930	8165.10900	Detected	Detected	Detected	Detected

5467	A_23_P214907	MTHFD1L	Hs.591343	methylenetetrahydrofolate dehydrogenase (NADP+ dependent) 1-like	Homo sapiens methylenetetrahydrofolate dehydrogenase (NADP+ dependent) 1-like (MTHFD1L), mRNA [NM_015440]	3.29447	down	3.27333	down	590.04315	2012.81040	437.22385	1261.99940	Detected	Detected	Detected	Detected
5468	A_24_P398500	MPHOSPH9	Hs.577404	M-phase phosphoprotein 9	Homo sapiens M-phase phosphoprotein 9 (MPHOSPH9), mRNA [NM_022782]	3.29437	down	3.59928	down	32.21681	109.89767	31.94882	101.39949	Detected	Detected	Detected	Detected
5469	A_33_P3403867	PMEPA1	Hs.517155	prostate transmembrane protein, androgen induced 1	Homo sapiens prostate transmembrane protein, androgen induced 1 (PMEPA1), transcript variant 1, mRNA [NM_020182]	3.29262	down	3.47231	down	776.55720	2647.57470	671.12210	2054.87800	Detected	Detected	Detected	Detected
5470	A_33_P3268974	RECQL5	Hs.632229	RecQ protein-like 5	Homo sapiens RecQ protein-like 5 (RECQL5), transcript variant 2, mRNA [NM_001003715]	3.29250	down	3.80106	down	41.53461	141.60170	27.91610	93.56759	Detected	Detected	Detected	Detected
5471	A_24_P229025		Hs.377070		Glutamate receptor 3 Precursor (GluR-3)(GluR-C)(GluR-K3)(Glutamate receptor ionotropic, AMPA 3)(AMPA-selective glutamate receptor 3) [Source:UniProtKB/Swiss-Prot;Acc:P42263] [ENST00000371264]	3.29040	down	3.31064	down	106.90501	364.23340	95.73006	279.46430	Detected	Detected	Detected	Detected
5472	A_33_P3210671	GPAT2	Hs.348629	glycerol-3-phosphate acyltransferase 2, mitochondrial	Homo sapiens glycerol-3-phosphate acyltransferase 2, mitochondrial (GPAT2), nuclear gene encoding mitochondrial protein, mRNA [NM_207328]	3.28798	down	5.63737	down	99.58111	339.03033	54.67298	271.77814	Detected	Detected	Detected	Detected
5473	A_33_P3388312	SKAP2	Hs.200770	src kinase associated phosphoprotein 2	Homo sapiens src kinase associated phosphoprotein 2 (SKAP2), mRNA [NM_003930]	3.28653	down	3.16492	down	1254.76500	4270.05660	924.84290	2581.05130	Detected	Detected	Detected	Detected
5474	A_23_P52121	PDZK1	Hs.444751	PDZ domain containing 1	Homo sapiens PDZ domain containing 1 (PDZK1), mRNA [NM_002614]	3.28453	down	4.23695	down	50.10766	170.41618	28.51170	106.52268	Detected	Detected	Detected	Detected
5475	A_23_P94998	LETM1	Hs.120165	leucine zipper-EF-hand containing transmembrane protein 1	Homo sapiens leucine zipper-EF-hand containing transmembrane protein 1 (LETM1), nuclear gene encoding mitochondrial protein, mRNA [NM_012318]	3.28387	down	3.36291	down	675.00530	2295.22680	774.48865	2296.65360	Detected	Detected	Detected	Detected
5476	A_24_P279489	VPS53	Hs.461819	vacuolar protein sorting 53 homolog (S. cerevisiae)	Homo sapiens vacuolar protein sorting 53 homolog (S. cerevisiae) (VPS53), transcript variant 1, mRNA [NM_001128159]	3.28249	down	2.73077	down	58.71769	199.57472	49.73733	119.76593	Detected	Detected	Detected	Detected
5477	A_33_P3356711	ING3	Hs.489811	inhibitor of growth family, member 3	Homo sapiens inhibitor of growth family, member 3 (ING3), transcript variant 3, mRNA [NM_198267]	3.28179	down	3.55419	down	344.33603	1170.11010	309.24940	969.20440	Detected	Detected	Detected	Detected
5478	A_23_P429998	FOSB	Hs.590958	FBJ murine osteosarcoma viral oncogene homolog B	Homo sapiens FBJ murine osteosarcoma viral oncogene homolog B (FOSB), transcript variant 1, mRNA [NM_006732]	3.28040	down	4.01334	down	246.31786	836.67290	154.63303	547.23505	Detected	Detected	Detected	Detected
5479	A_23_P256632	PGGT1B	Hs.254006	protein geranylgeranyltransferase type I, beta subunit	Homo sapiens protein geranylgeranyltransferase type I, beta subunit (PGGT1B), mRNA [NM_005023]	3.27865	down	2.43752	down	35.42237	120.25584	36.57559	78.61488	Detected	Detected	Detected	Detected
5480	A_33_P3267380	SGCD	Hs.387207	sarcoglycan, delta (35kDa dystrophin-associated glycoprotein)	Homo sapiens sarcoglycan, delta (35kDa dystrophin-associated glycoprotein) (SGCD), transcript variant 2, mRNA [NM_172244]	3.27793	down	2.67283	down	251.89944	854.98690	196.61067	463.38718	Detected	Detected	Detected	Detected
5481	A_33_P3619171	PMAIP1	Hs.96	phorbol-12-myristate-13-acetate-induced protein 1	Homo sapiens phorbol-12-myristate-13-acetate-induced protein 1 (PMAIP1), mRNA [NM_021127]	3.27635	down	3.14450	down	1125.90720	3819.66650	894.84045	2481.20780	Detected	Detected	Detected	Detected
5482	A_33_P3417339	SHROOM3	Hs.702168	shroom family member 3	Homo sapiens shroom family member 3 (SHROOM3), mRNA [NM_020859]	3.27601	down	3.31970	down	47.93352	162.59892	44.02505	128.87367	Detected	Detected	Detected	Detected
5483	A_23_P88234	FAM158A	Hs.271614	family with sequence similarity 158, member A	Homo sapiens family with sequence similarity 158, member A (FAM158A), mRNA [NM_016049]	3.27317	down	3.18411	down	553.35864	1875.46370	659.58844	1851.94070	Detected	Detected	Detected	Detected
5484	A_23_P210425	MYL9	Hs.504687	myosin, light chain 9, regulatory	Homo sapiens myosin, light chain 9, regulatory (MYL9), transcript variant 2, mRNA [NM_181526]	3.27241	down	3.72767	down	2269.80050	7691.10200	2354.34470	7738.79100	Detected	Detected	Detected	Detected
5485	A_24_P48248	C17orf53	Hs.437059	chromosome 17 open reading frame 53	Homo sapiens chromosome 17 open reading frame 53 (C17orf53), mRNA [NM_024032]	3.27212	down	3.09711	down	60.70640	205.68228	66.02950	180.32646	Detected	Detected	Detected	Detected
5486	A_24_P66545	KIAA0226	Hs.478868	KIAA0226	Homo sapiens KIAA0226 (KIAA0226), transcript variant 1, mRNA [NM_001145642]	3.26957	down	2.82138	down	8.19863	27.75651	11.03095	27.44350	Compromised	Detected	Compromised	Detected
5487	A_33_P3209866	KANK1	Hs.306764	KN motif and ankyrin repeat domains 1	Homo sapiens KN motif and ankyrin repeat domains 1 (KANK1), transcript variant 2, mRNA [NM_153186]	3.26931	down	3.40492	down	830.72320	2812.19780	811.87800	2437.60620	Detected	Detected	Detected	Detected
5488	A_24_P303497		Hs.279008		DNA replication licensing factor MCM9 (Mini-chromosome maintenance deficient 9)(hMCM9)(Mini-chromosome maintenance deficient domain-containing protein 1) [Source:UniProtKB/Swiss-Prot;Acc:Q9NXL9] [ENST00000316316]	3.26863	down	2.05182	down	6.78574	22.96652	9.11193	16.48603	Compromised	Detected	Compromised	Detected
5489	A_32_P29118	SEMA3D	Hs.201340	sema domain, immunoglobulin domain (Ig), short basic domain, secreted, (semaphorin) 3D	Homo sapiens sema domain, immunoglobulin domain (Ig), short basic domain, secreted, (semaphorin) 3D (SEMA3D), mRNA [NM_152754]	3.26625	down	2.89248	down	9.23622	31.23748	2.59148	6.60972	Compromised	Detected	Compromised	Compromised
5490	A_33_P3231750	ZNF738	Hs.124384	zinc finger protein 738	Homo sapiens zinc finger protein 738 (ZNF738), non-coding RNA [NR_027130]	3.26329	down	2.95532	down	60.09609	203.06493	55.84734	145.53680	Detected	Detected	Detected	Detected
5491	A_23_P431252	KBTBD8	Hs.116665	kelch repeat and BTB (POZ) domain containing 8	Homo sapiens kelch repeat and BTB (POZ) domain containing 8 (KBTBD8), mRNA [NM_032505]	3.26223	down	3.03922	down	81.72367	276.05527	56.79502	152.20795	Detected	Detected	Detected	Detected

5492	A_24_P672240	FRMPD4		FERM and PDZ domain containing 4	FERM and PDZ domain-containing protein 4 (PDZ domain-containing protein 10) [Source:UniProtKB/Swiss-Prot;Acc:Q14CM0] [ENST00000380682]	3.26178	down	4.91967	down	25.24073	85.24902	17.97349	77.97111	Detected	Detected	Detected	Detected
5493	A_33_P3296230					3.25643	down	4.97936	down	26.60233	89.70039	14.22454	62.45644	Detected	Detected	Detected	Detected
5494	A_32_P201496	POLR3H	Hs.712617	polymerase (RNA) III (DNA directed) polypeptide H (22.9kD)	Homo sapiens polymerase (RNA) III (DNA directed) polypeptide H (22.9kD) (POLR3H), transcript variant 2, mRNA [NM_001018052]	3.25540	down	3.26996	down	506.32710	1706.74510	597.65393	1723.28660	Detected	Detected	Detected	Detected
5495	A_33_P3390024				Exocyst complex component 7 (Exocyst complex component Exo70) [Source:UniProtKB/Swiss-Prot;Acc:Q9UPT5] [ENST00000406660]	3.25329	down	3.13203	down	113.89349	383.66670	99.27887	274.18750	Detected	Detected	Detected	Detected
5496	A_23_P159956	MID2	Hs.12256	midline 2	Homo sapiens midline 2 (MID2), transcript variant 1, mRNA [NM_012216]	3.25123	down	3.64521	down	205.57144	692.05960	135.97160	437.05478	Detected	Detected	Detected	Detected
5497	A_33_P3411907	FGF5	Hs.37055	fibroblast growth factor 5	Homo sapiens fibroblast growth factor 5 (FGF5), transcript variant 2, mRNA [NM_033143]	3.24854	down	3.86059	down	36.30327	122.11438	52.76553	179.62602	Detected	Detected	Detected	Detected
5498	A_23_P2423	MAGOHB	Hs.104650	mago-nashi homolog B (Drosophila)	Homo sapiens mago-nashi homolog B (Drosophila) (MAGOHB), mRNA [NM_018048]	3.24822	down	2.95450	down	1979.17260	6656.75100	1842.89270	4801.19730	Detected	Detected	Detected	Detected
5499	A_33_P3377691	C4orf46	Hs.380920	chromosome 4 open reading frame 46	Homo sapiens chromosome 4 open reading frame 46 (C4orf46), mRNA [NM_001008393]	3.24808	down	3.24011	down	291.06967	978.94006	223.33296	638.08420	Detected	Detected	Detected	Detected
5500	A_23_P409093	ANO4	Hs.58785	anoctamin 4	Homo sapiens anoctamin 4 (ANO4), mRNA [NM_178826]	3.24367	down	4.17703	down	90.61234	304.33844	52.78136	194.40788	Detected	Detected	Detected	Detected
5501	A_33_P3382565	KIF26B	Hs.368096	kinesin family member 26B	Homo sapiens kinesin family member 26B (KIF26B), mRNA [NM_018012]	3.24045	down	4.49135	down	32.59782	109.37719	28.17286	111.57668	Detected	Detected	Detected	Detected
5502	A_33_P3312642	CXorf64	Hs.130370	chromosome X open reading frame 64	Homo sapiens chromosome X open reading frame 64 (CXorf64), mRNA [NM_001122716]	3.23982	down	1.28130	down	33.81626	113.44353	18.77553	21.21330	Detected	Detected	Detected	Detected
5503	A_23_P137097	SLC16A2	Hs.75317	solute carrier family 16, member 2 (monocarboxylic acid transporter 8)	Homo sapiens solute carrier family 16, member 2 (monocarboxylic acid transporter 8) (SLC16A2), mRNA [NM_006517]	3.23673	down	3.28655	down	2224.80620	7456.44900	1511.55740	4380.56700	Detected	Detected	Detected	Detected
5504	A_33_P3243023	ADRA1D	Hs.557	adrenergic, alpha-1D-, receptor	Homo sapiens adrenergic, alpha-1D-, receptor (ADRA1D), mRNA [NM_000678]	3.23116	down	2.95062	down	8.43609	28.22494	11.79499	30.68854	Compromised	Detected	Compromised	Detected
5505	A_33_P3264926	SAMD4A	Hs.98259	sterile alpha motif domain containing 4A	Homo sapiens sterile alpha motif domain containing 4A (SAMD4A), transcript variant 1, mRNA [NM_015589]	3.22728	down	3.22882	down	200.65475	670.53143	233.63144	665.18250	Detected	Detected	Detected	Detected
5506	A_23_P49338	TNFRSF12A	Hs.355899	tumor necrosis factor receptor superfamily, member 12A	Homo sapiens tumor necrosis factor receptor superfamily, member 12A (TNFRSF12A), mRNA [NM_016639]	3.22499	down	3.35525	down	18841.79500	62919.28000	19337.05500	57211.10000	Detected	Detected	Detected	Detected
5507	A_33_P3358914	ARHGAP23	Hs.374446	Rho GTPase activating protein 23	Homo sapiens Rho GTPase activating protein 23 (ARHGAP23), mRNA [NM_020876]	3.22242	down	3.41034	down	58.70537	195.88110	61.10496	183.75502	Detected	Detected	Detected	Detected
5508	A_24_P334456	ADAMTS6	Hs.482291	ADAM metallopeptidase with thrombospondin type 1 motif, 6	Homo sapiens ADAM metallopeptidase with thrombospondin type 1 motif, 6 (ADAMTS6), mRNA [NM_197941]	3.22221	down	4.69581	down	9.31852	31.09095	5.58943	23.14423	Compromised	Detected	Compromised	Detected
5509	A_23_P78092	EVI2A	Hs.591198	ecotropic viral integration site 2A	Homo sapiens ecotropic viral integration site 2A (EVI2A), transcript variant 1, mRNA [NM_001003927]	3.22078	down	2.63514	down	45.00799	150.10085	52.93210	122.99532	Detected	Detected	Detected	Detected
5510	A_23_P326760	MYRIP	Hs.594535	myosin VIIA and Rab interacting protein	Homo sapiens myosin VIIA and Rab interacting protein (MYRIP), mRNA [NM_015460]	3.21841	down	3.12196	down	12.64973	42.15561	8.14695	22.42786	Compromised	Detected	Compromised	Detected
5511	A_32_P3572					3.21821	down	5.09577	down	12.95191	43.15993	5.57263	25.04007	Detected	Detected	Compromised	Detected
5512	A_23_P11841	ATP2B4	Hs.343522	ATPase, Ca++ transporting, plasma membrane 4	Homo sapiens ATPase, Ca++ transporting, plasma membrane 4 (ATP2B4), transcript variant 2, mRNA [NM_001684]	3.21704	down	3.00257	down	46.20483	153.91339	48.57132	128.59906	Detected	Detected	Detected	Detected
5513	A_33_P3334102	SH3D20	Hs.205326	SH3 domain containing 20	Homo sapiens SH3 domain containing 20 (SH3D20), mRNA [NM_174919]	3.21467	down	5.16139	down	675.71606	2249.23050	353.16980	1607.37070	Detected	Detected	Detected	Detected
5514	A_23_P156061	LNPEP	Hs.656905	leucyl/cystinyl aminopeptidase	Homo sapiens leucyl/cystinyl aminopeptidase (LNPEP), transcript variant 1, mRNA [NM_005575]	3.21194	down	3.24496	down	87.57545	291.26157	71.33482	204.11552	Detected	Detected	Detected	Detected
5515	A_23_P143958	RPL22L1	Hs.380933	ribosomal protein L22-like 1	Homo sapiens ribosomal protein L22-like 1 (RPL22L1), mRNA [NM_001099645]	3.21181	down	3.04128	down	36710.08600	122086.72000	27615.96300	74059.60000	Detected	Detected	Detected	Detected
5516	A_23_P123071	CAV2	Hs.212332	caveolin 2	Homo sapiens caveolin 2 (CAV2), transcript variant 1, mRNA [NM_001233]	3.21087	down	3.39831	down	2633.48170	8755.60450	2972.14090	8906.31600	Detected	Detected	Detected	Detected
5517	A_33_P3341524		Hs.541960		Putative uncharacterized protein MGC10955 [Source:UniProtKB/TrEMBL;Acc:Q53S31] [ENST00000401851]	3.20484	down	2.14478	down	17.95732	59.59111	20.02930	37.88041	Detected	Detected	Detected	Detected
5518	A_24_P192988	CCDC89	Hs.376241	coiled-coil domain containing 89	Homo sapiens coiled-coil domain containing 89 (CCDC89), mRNA [NM_152723]	3.20182	down	4.09185	down	44.48689	147.48987	38.00570	137.13028	Detected	Detected	Detected	Detected
5519	A_24_P10137	C13orf15	Hs.507866	chromosome 13 open reading frame 15	Homo sapiens chromosome 13 open reading frame 15 (C13orf15), mRNA [NM_014059]	3.20147	down	3.16461	down	1129.19130	3743.25880	977.29710	2727.17190	Detected	Detected	Detected	Detected
5520	A_23_P252681	PCYT1A	Hs.135997	phosphate cytidylyltransferase 1, choline, alpha	Homo sapiens phosphate cytidylyltransferase 1, choline, alpha (PCYT1A), mRNA [NM_005017]	3.19971	down	3.26293	down	162.24466	537.54330	178.06259	512.32470	Detected	Detected	Detected	Detected

5521	A_23_P165668				Solute carrier family 35 member F5 (Hepatitis C virus NS5A-transactivated protein 3)(HCV NS5A-transactivated protein 3) [Source:UniProtKB/Swiss-Prot;Acc:Q8WV83] [ENST00000409342]	3.19812	down	3.52929	down	69.46373	230.03108	55.39851	172.40558	Detected	Detected	Detected	Detected
5522	A_23_P37702	TPSAB1	Hs.405479	tryptase alpha/beta 1	Homo sapiens tryptase alpha/beta 1 (TPSAB1), mRNA [NM_003294]	3.19509	down	1.18317	down	5.43328	17.97537	2.89450	3.01986	Compromised	Detected	Compromised	Compromised
5523	A_24_P943358	DBF4	Hs.485380	DBF4 homolog (S. cerevisiae)	Homo sapiens DBF4 homolog (S. cerevisiae) (DBF4), mRNA [NM_006716]	3.19310	down	2.82759	down	6.91855	22.87499	4.25969	10.62089	Compromised	Detected	Compromised	Compromised
5524	A_24_P734720	CCNYL1	Hs.471234	cyclin Y-like 1	Homo sapiens cyclin Y-like 1 (CCNYL1), transcript variant 2, mRNA [NM_152523]	3.19125	down	2.53512	down	172.19437	569.00070	92.47002	206.71158	Detected	Detected	Detected	Detected
5525	A_24_P943613	TBC1D1	Hs.176503	TBC1 (tre-2/USP6, BUB2, cdc16) domain family, member 1	Homo sapiens TBC1 (tre-2/USP6, BUB2, cdc16) domain family, member 1 (TBC1D1), mRNA [NM_015173]	3.18618	down	3.17659	down	737.70166	2433.79830	498.70430	1396.91500	Detected	Detected	Detected	Detected
5526	A_33_P3325723	CHN1	Hs.654534	chimerin (chimaerin) 1	Homo sapiens chimerin (chimaerin) 1 (CHN1), transcript variant 1, mRNA [NM_001822]	3.18408	down	3.28371	down	778.31165	2566.08400	529.56190	1533.36880	Detected	Detected	Detected	Detected
5527	A_33_P3409746	C11orf91		chromosome 11 open reading frame 91	Homo sapiens chromosome 11 open reading frame 91 (C11orf91), mRNA [NM_001166692]	3.18392	down	4.07648	down	39.21393	129.28110	38.54809	138.56503	Detected	Detected	Detected	Detected
5528	A_23_P380208	VEPH1	Hs.658046	ventricular zone expressed PH domain homolog 1 (zebrafish)	Homo sapiens ventricular zone expressed PH domain homolog 1 (zebrafish) (VEPH1), mRNA [NM_024621]	3.18194	down	3.54381	down	66.67246	219.67033	50.51992	157.86963	Detected	Detected	Detected	Detected
5529	A_33_P3380076	LOC100131673		similar to hCG1995779	PREDICTED: Homo sapiens hypothetical LOC100131673 (LOC100131673), mRNA [XM_001723856]	3.17730	down	1.18403	down	5.85481	19.26212	2.86511	2.99137	Compromised	Detected	Compromised	Compromised
5530	A_24_P260639	HIST1H1D	Hs.136857	histone cluster 1, H1d	Homo sapiens histone cluster 1, H1d (HIST1H1D), mRNA [NM_005320]	3.17719	down	2.77023	down	408.88016	1345.15490	419.31656	1024.28980	Detected	Detected	Detected	Detected
5531	A_23_P32707	ESPL1	Hs.153479	extra spindle pole bodies homolog 1 (S. cerevisiae)	Homo sapiens extra spindle pole bodies homolog 1 (S. cerevisiae) (ESPL1), mRNA [NM_012291]	3.17617	down	3.49854	down	702.22830	2309.48580	594.55237	1834.17990	Detected	Detected	Detected	Detected
5532	A_23_P29303	RRP7A	Hs.720380	ribosomal RNA processing 7 homolog A (S. cerevisiae)	Homo sapiens ribosomal RNA processing 7 homolog A (S. cerevisiae) (RRP7A), mRNA [NM_015703]	3.16936	down	4.61394	down	149.90828	491.96094	134.40067	546.81274	Detected	Detected	Detected	Detected
5533	A_23_P48109	NINJ2	Hs.656450	ninjurin 2	Homo sapiens ninjurin 2 (NINJ2), mRNA [NM_016533]	3.16879	down	3.77729	down	46.87976	153.81956	39.25814	130.76013	Detected	Detected	Detected	Detected
5534	A_32_P192376	ENPP1	Hs.527295	ectonucleotide pyrophosphatase/phosphodiesterase 1	Homo sapiens ectonucleotide pyrophosphatase/phosphodiesterase 1 (ENPP1), mRNA [NM_006208]	3.16696	down	2.97743	down	774.22350	2538.88010	495.65933	1301.34010	Detected	Detected	Detected	Detected
5535	A_23_P254842	HDHD1A	Hs.185910	haloacid dehalogenase-like hydrolase domain containing 1A	Homo sapiens haloacid dehalogenase-like hydrolase domain containing 1A (HDHD1A), transcript variant 2, mRNA [NM_012080]	3.16554	down	3.05578	down	787.31340	2580.64870	677.89980	1826.63830	Detected	Detected	Detected	Detected
5536	A_33_P3372488				Tectonic-3 Precursor [Source:UniProtKB/Swiss-Prot;Acc:Q6NUS6] [ENST00000371209]	3.16503	down	1.55213	down	15.31944	50.20572	7.83487	10.72323	Detected	Detected	Compromised	Compromised
5537	A_23_P60227	CCIN	Hs.115460	calicin	Homo sapiens calicin (CCIN), mRNA [NM_005893]	3.16483	down	5.62170	down	241.68053	791.99884	135.92361	673.79584	Detected	Detected	Detected	Detected
5538	A_23_P350719	TMPRSS8	Hs.434388	transmembrane protease, serine 8 homolog (mouse) pseudogene	Homo sapiens transmembrane protease, serine 8 homolog (mouse) pseudogene (TMPRSS8), non-coding RNA [NR_026864]	3.16359	down	1.68976	down	10.66677	34.94184	8.64540	12.88179	Compromised	Detected	Compromised	Compromised
5539	A_33_P3344127	HIST1H2AC	Hs.484950	histone cluster 1, H2ac	Homo sapiens histone cluster 1, H2ac (HIST1H2AC), mRNA [NM_003512]	3.16263	down	2.72154	down	53.07601	173.81200	58.09112	139.40850	Detected	Detected	Detected	Detected
5540	A_33_P3363188	FLJ43315	Hs.632789	similar to Asparagine synthetase [glutamine-hydrolyzing] (Glutamine-dependent asparagine synthetase) (TS11 cell cycle control protein)	Homo sapiens similar to Asparagine synthetase [glutamine-hydrolyzing] (Glutamine-dependent asparagine synthetase) (TS11 cell cycle control protein), mRNA (cDNA clone IMAGE:30345211). [BC057848]	3.16255	down	3.09988	down	58.08824	190.22081	40.36947	110.34768	Detected	Detected	Detected	Detected
5541	A_33_P3311974				Prospero homeobox protein 2 (Homeobox prospero-like protein PROX2)(PROX-2) [Source:UniProtKB/Swiss-Prot;Acc:Q3B8N5] [ENST00000238583]	3.16219	down	2.03507	down	5.13720	16.82082	4.75180	8.52713	Compromised	Detected	Compromised	Compromised
5542	A_23_P121222	RAD18	Hs.375684	RAD18 homolog (S. cerevisiae)	Homo sapiens RAD18 homolog (S. cerevisiae) (RAD18), mRNA [NM_020165]	3.15445	down	2.80363	down	264.78900	864.88180	207.24443	512.35315	Detected	Detected	Detected	Detected
5543	A_23_P14193	RFC3	Hs.115474	replication factor C (activator 1) 3, 38kDa	Homo sapiens replication factor C (activator 1) 3, 38kDa (RFC3), transcript variant 1, mRNA [NM_002915]	3.15353	down	3.02196	down	162.44206	530.43005	158.75660	423.04422	Detected	Detected	Detected	Detected
5544	A_23_P162547	MYL2	Hs.75535	myosin, light chain 2, regulatory, cardiac, slow	Homo sapiens myosin, light chain 2, regulatory, cardiac, slow (MYL2), mRNA [NM_000432]	3.15344	down	2.83932	down	73.80911	241.00575	36.16277	90.54007	Detected	Detected	Detected	Detected
5545	A_23_P121533	SPON2	Hs.720064	spondin 2, extracellular matrix protein	Homo sapiens spondin 2, extracellular matrix protein (SPON2), transcript variant 1, mRNA [NM_012445]	3.15286	down	3.29741	down	57357.42000	187252.52000	38992.57400	113375.89000	Detected	Detected	Detected	Detected
5546	A_33_P3361202	C1orf96	Hs.585011	chromosome 1 open reading frame 96	Homo sapiens chromosome 1 open reading frame 96 (C1orf96), mRNA [NM_145257]	3.15266	down	2.90616	down	174.04498	568.16174	139.46707	357.40210	Detected	Detected	Detected	Detected
5547	A_23_P436281	HIST2H4B	Hs.706635	histone cluster 2, H4b	Homo sapiens histone cluster 2, H4b (HIST2H4B), mRNA [NM_001034077]	3.14919	down	2.74034	down	113.46137	369.98145	110.01450	265.83990	Detected	Detected	Detected	Detected

5548	A_23_P256724	TNFRSF10C	Hs.655801	tumor necrosis factor receptor superfamily, member 10c, decoy without an intracellular domain	Homo sapiens tumor necrosis factor receptor superfamily, member 10c, decoy without an intracellular domain (TNFRSF10C), mRNA [NM_003841]	3.14780	down	3.39738	down	35.48579	115.66289	26.96824	80.79075	Detected	Detected	Detected	Detected
5549	A_23_P219197	RGS3	Hs.494875	regulator of G-protein signaling 3	Homo sapiens regulator of G-protein signaling 3 (RGS3), transcript variant 4, mRNA [NM_134427]	3.14681	down	3.18681	down	4962.77340	16170.66600	4110.55220	11551.05600	Detected	Detected	Detected	Detected
5550	A_33_P3504659	CASP10	Hs.5353	caspase 10, apoptosis-related cysteine peptidase	Homo sapiens caspase 10, apoptosis-related cysteine peptidase (CASP10), transcript variant D, mRNA [NM_032977]	3.14676	down	3.25119	down	65.71383	214.11809	45.20422	129.59439	Detected	Detected	Detected	Detected
5551	A_24_P323114	ANXA2P3	Hs.591361	annexin A2 pseudogene 3	Homo sapiens annexin A2 pseudogene 3 (ANXA2P3), non-coding RNA [NR_001446]	3.14554	down	2.95864	down	5581.56900	18179.59000	1035.35520	2701.13900	Detected	Detected	Detected	Detected
5552	A_33_P3373200					3.14504	down	1.19699	down	10.26729	33.43602	2.76869	2.92233	Compromised	Detected	Compromised	Compromised
5553	A_23_P141965	HAUS8	Hs.404088	HAUS augmin-like complex, subunit 8	Homo sapiens HAUS augmin-like complex, subunit 8 (HAUS8), transcript variant 1, mRNA [NM_033417]	3.14356	down	3.11810	down	1395.77420	4543.28470	1386.16280	3811.26760	Detected	Detected	Detected	Detected
5554	A_23_P259189	CLIC4	Hs.440544	chloride intracellular channel 4	Homo sapiens chloride intracellular channel 4 (CLIC4), nuclear gene encoding mitochondrial protein, mRNA [NM_013943]	3.14298	down	3.13225	down	1334.81870	4344.06600	1020.13410	2817.59770	Detected	Detected	Detected	Detected
5555	A_23_P115105	C1orf144	Hs.252967	chromosome 1 open reading frame 144	Homo sapiens chromosome 1 open reading frame 144 (C1orf144), transcript variant 2, mRNA [NM_015609]	3.14053	down	3.17001	down	3118.76680	10141.88900	2434.52700	6805.19600	Detected	Detected	Detected	Detected
5556	A_23_P62932	ATP1B1	Hs.291196	ATPase, Na+/K+ transporting, beta 1 polypeptide	Homo sapiens ATPase, Na+/K+ transporting, beta 1 polypeptide (ATP1B1), transcript variant 1, mRNA [NM_001677]	3.13931	down	3.62003	down	1546.11900	5025.85250	1176.35170	3755.04320	Detected	Detected	Detected	Detected
5557	A_24_P46911	KCNS2	Hs.388045	potassium voltage-gated channel, delayed-rectifier, subfamily S, member 2	Homo sapiens potassium voltage-gated channel, delayed-rectifier, subfamily S, member 2 (KCNS2), mRNA [NM_020697]	3.13922	down	2.55367	down	29.73829	96.66528	32.70969	73.65569	Detected	Detected	Detected	Detected
5558	A_23_P86653	SRGN	Hs.1908	serglycin	Homo sapiens serglycin (SRGN), mRNA [NM_002727]	3.13800	down	3.14695	down	907.57700	2948.96200	689.72125	1913.94450	Detected	Detected	Detected	Detected
5559	A_24_P244706	SSTR1	Hs.248160	somatostatin receptor 1	Homo sapiens somatostatin receptor 1 (SSTR1), mRNA [NM_001049]	3.13713	down	3.54727	down	48.67087	158.10095	28.06864	87.79719	Detected	Detected	Detected	Detected
5560	A_23_P117494	MTHFD1	Hs.652308	methylenetetrahydrofolate dehydrogenase (NADP+ dependent) 1, methenyltetrahydrofolate cyclohydrolase, formyltetrahydrofolate synthetase	Homo sapiens methylenetetrahydrofolate dehydrogenase (NADP+ dependent) 1, methenyltetrahydrofolate cyclohydrolase, formyltetrahydrofolate synthetase (MTHFD1), mRNA [NM_005956]	3.13679	down	3.21752	down	3109.31840	10099.13300	2295.80800	6513.62400	Detected	Detected	Detected	Detected
5561	A_23_P35148	TAF13	Hs.632426	TAF13 RNA polymerase II, TATA box binding protein (TBP)-associated factor, 18kDa	Homo sapiens TAF13 RNA polymerase II, TATA box binding protein (TBP)-associated factor, 18kDa (TAF13), mRNA [NM_005645]	3.13553	down	3.23166	down	36.37207	118.08963	40.37082	115.04269	Detected	Detected	Detected	Detected
5562	A_23_P17663	MX1	Hs.517307	myxovirus (influenza virus) resistance 1, interferon-inducible protein p78 (mouse)	Homo sapiens myxovirus (influenza virus) resistance 1, interferon-inducible protein p78 (mouse) (MX1), transcript variant 2, mRNA [NM_002462]	3.13546	down	2.66173	down	196.99387	639.56946	226.36647	531.30280	Detected	Detected	Detected	Detected
5563	A_23_P391275	RCAN3	Hs.656799	RCAN family member 3	Homo sapiens RCAN family member 3 (RCAN3), mRNA [NM_013441]	3.13385	down	2.97680	down	142.12642	461.19640	111.41219	292.44730	Detected	Detected	Detected	Detected
5564	A_23_P408930	OR7E5P	Hs.350816	olfactory receptor, family 7, subfamily E, member 5 pseudogene	Homo sapiens olfactory receptor, family 7, subfamily E, member 5 pseudogene (OR7E5P), non-coding RNA [NR_027688]	3.12966	down	2.09140	down	49.24318	159.57928	18.91004	34.87352	Detected	Detected	Detected	Detected
5565	A_33_P3298960	KDELC2	Hs.83286	KDEL (Lys-Asp-Glu-Leu) containing 2	Homo sapiens KDEL (Lys-Asp-Glu-Leu) containing 2 (KDELC2), mRNA [NM_153705]	3.12880	down	3.94384	down	30.55508	98.99079	18.51525	64.38940	Detected	Detected	Detected	Detected
5566	A_23_P76761	VRK1	Hs.422662	vaccinia related kinase 1	Homo sapiens vaccinia related kinase 1 (VRK1), mRNA [NM_003384]	3.12691	down	3.17367	down	1575.39320	5100.78470	983.58307	2752.56760	Detected	Detected	Detected	Detected
5567	A_33_P3232828	SFRS3	Hs.405144	splicing factor, arginine/serine-rich 3	Homo sapiens splicing factor, arginine/serine-rich 3 (SFRS3), mRNA [NM_003017]	3.12679	down	3.31513	down	2374.86180	7688.99270	2459.61600	7190.06700	Detected	Detected	Detected	Detected
5568	A_33_P3406866	NFASC	Hs.13349	neurofascin homolog (chicken)	Homo sapiens neurofascin homolog (chicken) (NFASC), transcript variant 1, mRNA [NM_001005388]	3.12575	down	3.30579	down	2612.42240	8455.32600	2118.84840	6176.46880	Detected	Detected	Detected	Detected
5569	A_23_P209904	GPC1	Hs.328232	glypican 1	Homo sapiens glypican 1 (GPC1), mRNA [NM_002081]	3.12236	down	3.77655	down	9630.09700	31134.83200	9392.59200	31278.53100	Detected	Detected	Detected	Detected
5570	A_33_P3291939	CNGA3	Hs.234785	cyclic nucleotide gated channel alpha 3	Homo sapiens cyclic nucleotide gated channel alpha 3 (CNGA3), transcript variant 1, mRNA [NM_001298]	3.12142	down	5.35563	down	4.62787	14.95775	3.39421	16.02931	Compromised	Compromised	Compromised	Detected
5571	A_23_P79360	NOSTRIN	Hs.189780	nitric oxide synthase trafficker	Homo sapiens nitric oxide synthase trafficker (NOSTRIN), transcript variant 1, mRNA [NM_052946]	3.12081	down	8.33575	down	7.58485	24.51020	2.38415	17.52440	Compromised	Detected	Compromised	Detected
5572	A_33_P3384845	POFUT1	Hs.472409	protein O-fucosyltransferase 1	Homo sapiens protein O-fucosyltransferase 1 (POFUT1), transcript variant 2, mRNA [NM_172236]	3.12055	down	4.64619	down	23.18521	74.91607	9.41066	38.55512	Detected	Detected	Compromised	Detected
5573	A_24_P269779	CHST3	Hs.158304	carbohydrate (chondroitin 6) sulfotransferase 3	Homo sapiens carbohydrate (chondroitin 6) sulfotransferase 3 (CHST3), mRNA [NM_004273]	3.11906	down	3.47612	down	568.12160	1834.84200	347.33447	1064.65310	Detected	Detected	Detected	Detected
5574	A_23_P136355	HHAT	Hs.58650	hedgehog acyltransferase	Homo sapiens hedgehog acyltransferase (HHAT), transcript variant 1, mRNA [NM_018194]	3.11707	down	3.84842	down	29.14988	94.08417	23.16803	78.62073	Detected	Detected	Detected	Detected
5575	A_32_P46571	RHBDL2	Hs.524626	rhomboid, veinlet-like 2 (Drosophila)	Homo sapiens rhomboid, veinlet-like 2 (Drosophila) (RHBDL2), mRNA [NM_017821]	3.11673	down	2.81277	down	90.38510	291.69470	63.13185	156.58409	Detected	Detected	Detected	Detected

5576	A_24_P231829	BHMT2	Hs.114172	betaine-homocysteine methyltransferase 2	Homo sapiens betaine-homocysteine methyltransferase 2 (BHMT2), mRNA [NM_017614]	3.11606	down	3.39679	down	87.09492	281.01630	62.57184	187.41867	Detected	Detected	Detected	Detected
5577	A_33_P3283480	CTSC	Hs.128065	cathepsin C	Homo sapiens cathepsin C (CTSC), transcript variant 2, mRNA [NM_148170]	3.11383	down	3.33855	down	948.40250	3057.88330	685.53040	2018.13300	Detected	Detected	Detected	Detected
5578	A_23_P125233	CNN1	Hs.465929	calponin 1, basic, smooth muscle	Homo sapiens calponin 1, basic, smooth muscle (CNN1), mRNA [NM_001299]	3.11352	down	3.35658	down	2292.59450	7391.14700	2463.25420	7290.74270	Detected	Detected	Detected	Detected
5579	A_33_P3331188	ARHGAP23	Hs.374446	Rho GTPase activating protein 23	Homo sapiens Rho GTPase activating protein 23 (ARHGAP23), mRNA [NM_020876]	3.11247	down	3.31748	down	131.22339	422.91113	122.70849	358.96243	Detected	Detected	Detected	Detected
5580	A_23_P8571	SRCRB4D	Hs.567684	scavenger receptor cysteine rich domain containing, group B (4 domains)	Homo sapiens scavenger receptor cysteine rich domain containing, group B (4 domains) (SRCRB4D), mRNA [NM_080744]	3.11070	down	3.08539	down	202.05933	650.83307	166.77989	453.75342	Detected	Detected	Detected	Detected
5581	A_33_P3339436	LOC145845	Hs.302693	hypothetical LOC145845	Homo sapiens hypothetical LOC145845 (LOC145845), non-coding RNA [NR_024264]	3.11048	down	1.37076	down	434.93990	1400.84330	71.79256	86.77764	Detected	Detected	Detected	Detected
5582	A_23_P95764	PRPS1	Hs.56	phosphoribosyl pyrophosphate synthetase 1	Homo sapiens phosphoribosyl pyrophosphate synthetase 1 (PRPS1), mRNA [NM_002764]	3.10903	down	3.63238	down	3357.62060	10809.11300	5782.04500	18519.89500	Detected	Detected	Detected	Detected
5583	A_32_P193646	RBMX	Hs.380118	RNA binding motif protein, X-linked	Homo sapiens RNA binding motif protein, X-linked (RBMX), transcript variant 1, mRNA [NM_002139]	3.10660	down	2.72422	down	767.20660	2467.91720	511.97495	1229.86240	Detected	Detected	Detected	Detected
5584	A_23_P209778	POLR2D	Hs.554831	polymerase (RNA) II (DNA directed) polypeptide D	Homo sapiens polymerase (RNA) II (DNA directed) polypeptide D (POLR2D), mRNA [NM_004805]	3.10621	down	3.34165	down	1183.40390	3806.24490	1018.74790	3001.87700	Detected	Detected	Detected	Detected
5585	A_33_P3332126		Hs.709612		Selenocysteine lyase (hSCL)(EC 4.4.1.16) [Source:UniProtKB/Swiss-Prot;Acc:Q96I15] [ENST00000409736]	3.10605	down	4.00958	down	218.07831	701.38116	162.59698	574.87964	Detected	Detected	Detected	Detected
5586	A_33_P3287690	CNOT2	Hs.133350	CCR4-NOT transcription complex, subunit 2	Homo sapiens cDNA FLJ26456 fis, clone KDN03662. [AK129966]	3.10603	down	1.18513	down	5.35378	17.21865	2.53948	2.65384	Compromised	Detected	Compromised	Compromised
5587	A_23_P25293	NCAPD2	Hs.5719	non-SMC condensin I complex, subunit D2	Homo sapiens non-SMC condensin I complex, subunit D2 (NCAPD2), mRNA [NM_014865]	3.10084	down	3.18637	down	659.44366	2117.33470	729.57230	2049.88940	Detected	Detected	Detected	Detected
5588	A_23_P383819	TBX3	Hs.714737	T-box 3	Homo sapiens T-box 3 (TBX3), transcript variant 2, mRNA [NM_016569]	3.09961	down	3.18683	down	1834.99980	5889.47700	854.32250	2400.74930	Detected	Detected	Detected	Detected
5589	A_23_P141005	AMFR	Hs.295137	autocrine motility factor receptor	Homo sapiens autocrine motility factor receptor (AMFR), mRNA [NM_001144]	3.09914	down	4.03541	down	1555.67290	4992.20800	825.89716	2938.86000	Detected	Detected	Detected	Detected
5590	A_33_P3260342	NFASC	Hs.13349	neurofascin homolog (chicken)	Homo sapiens neurofascin homolog (chicken) (NFASC), transcript variant 1, mRNA [NM_001005388]	3.09853	down	2.73887	down	58.91195	189.01350	61.74173	149.11308	Detected	Detected	Detected	Detected
5591	A_23_P82523	ABCB1	Hs.489033	ATP-binding cassette, sub-family B (MDR/TAP), member 1	Homo sapiens ATP-binding cassette, sub-family B (MDR/TAP), member 1 (ABCB1), mRNA [NM_000927]	3.09646	down	2.03929	down	6.32705	20.28619	7.86219	14.13803	Compromised	Detected	Compromised	Detected
5592	A_24_P151498	PRMT2	Hs.154163	protein arginine methyltransferase 2	Homo sapiens protein arginine methyltransferase 2 (PRMT2), transcript variant 1, mRNA [NM_206962]	3.09619	down	3.29087	down	1086.87110	3484.48410	1271.60610	3690.02500	Detected	Detected	Detected	Detected
5593	A_33_P3225587	NAT13	Hs.596074	N-acetyltransferase 13 (GCN5-related)	Homo sapiens N-acetyltransferase 13 (GCN5-related) (NAT13), mRNA [NM_025146]	3.09558	down	2.80446	down	805.88460	2583.14380	777.68427	1923.17470	Detected	Detected	Detected	Detected
5594	A_32_P120043	FAM26E	Hs.660142	family with sequence similarity 26, member E	Homo sapiens family with sequence similarity 26, member E (FAM26E), mRNA [NM_153711]	3.09366	down	3.43724	down	46.13914	147.80028	27.43779	83.16188	Detected	Detected	Detected	Detected
5595	A_33_P3411357	DGCR11	Hs.720269	DiGeorge syndrome critical region gene 11	Homo sapiens DiGeorge syndrome critical region gene 11 (DGCR11), non-coding RNA [NR_024157]	3.09317	down	3.12435	down	108.01168	345.94516	94.95355	261.59910	Detected	Detected	Detected	Detected
5596	A_23_P145134	FGFR1OP	Hs.487175	FGFR1 oncogene partner	Homo sapiens FGFR1 oncogene partner (FGFR1OP), transcript variant 1, mRNA [NM_007045]	3.09269	down	3.47278	down	485.26736	1554.00060	359.61270	1101.22800	Detected	Detected	Detected	Detected
5597	A_23_P99452	BRCA2	Hs.34012	breast cancer 2, early onset	Homo sapiens breast cancer 2, early onset (BRCA2), mRNA [NM_000059]	3.08462	down	4.04832	down	17.06263	54.49803	8.90761	31.79808	Detected	Detected	Compromised	Detected
5598	A_23_P20823	WDR34	Hs.495240	WD repeat domain 34	Homo sapiens WD repeat domain 34 (WDR34), mRNA [NM_052844]	3.08350	down	3.21225	down	1397.11730	4460.76800	1505.87170	4265.43000	Detected	Detected	Detected	Detected
5599	A_33_P3228300	RCL1	Hs.194121	RNA terminal phosphate cyclase-like 1	Homo sapiens RNA terminal phosphate cyclase-like 1 (RCL1), mRNA [NM_005772]	3.08294	down	2.95784	down	924.96460	2952.72850	880.01800	2295.25730	Detected	Detected	Detected	Detected
5600	A_33_P3305472		Hs.288215		small nucleolar RNA, C/D box 1C (SNORD1C), non-coding RNA [Source:RefSeq DNA;Acc:NR_004397] [ENST00000375052]	3.08009	down	4.29798	down	39.18271	124.96590	18.12705	68.69997	Detected	Detected	Detected	Detected
5601	A_23_P379630	SLC38A10	Hs.352240	solute carrier family 38, member 10	Homo sapiens solute carrier family 38, member 10 (SLC38A10), transcript variant 2, mRNA [NM_138570]	3.07888	down	3.86654	down	118.41959	377.52832	117.92558	402.06454	Detected	Detected	Detected	Detected
5602	A_23_P309381	HIST2H2AA4	Hs.706714	histone cluster 2, H2aa4	Homo sapiens histone cluster 2, H2aa4 (HIST2H2AA4), mRNA [NM_001040874]	3.07490	down	3.01613	down	263.22757	838.09890	294.27090	782.64075	Detected	Detected	Detected	Detected
5603	A_23_P106761	CORO1A	Hs.415067	coronin, actin binding protein, 1A	Homo sapiens coronin, actin binding protein, 1A (CORO1A), mRNA [NM_007074]	3.07415	down	2.81142	down	55.34289	176.16493	66.37285	164.54393	Detected	Detected	Detected	Detected
5604	A_24_P168574				Guanine nucleotide-binding protein G(s) subunit alpha isoforms XLas (Adenylate cyclase-stimulating G alpha protein)(Extra large alphas protein)(XLalphas) [Source:UniProtKB/Swiss-Prot;Acc:Q5JWF2] [ENST00000371081]	3.07138	down	2.32541	down	27.38589	87.09503	33.84385	69.39768	Detected	Detected	Detected	Detected
5605	A_32_P215318	ACACA	Hs.160556	acetyl-Coenzyme A carboxylase alpha	Homo sapiens acetyl-Coenzyme A carboxylase alpha (ACACA), transcript variant 2, mRNA [NM_198839]	3.07102	down	2.48096	down	12.79545	40.68845	14.75733	32.28437	Detected	Detected	Detected	Detected

5606	A_33_P3342563	PITPNM3	Hs.183983	PITPNM family member 3	Homo sapiens PITPNM family member 3 (PITPNM3), transcript variant 1, mRNA [NM_031220]	3.06581	down	2.77746	down	127.91990	406.08502	94.89368	232.40817	Detected	Detected	Detected	Detected
5607	A_23_P361381	DMC1	Hs.339396	DMC1 dosage suppressor of mck1 homolog, meiosis-specific homologous recombination (yeast)	Homo sapiens DMC1 dosage suppressor of mck1 homolog, meiosis-specific homologous recombination (yeast) (DMC1), mRNA [NM_007068]	3.06563	down	2.27493	down	20.89205	66.31842	27.66866	55.50360	Detected	Detected	Detected	Detected
5608	A_33_P3378880				Histone H4 [Source:UniProtKB/Swiss-Prot;Acc:P62805] [ENST00000377803]	3.06445	down	2.78612	down	135.36536	429.53010	82.71588	203.21425	Detected	Detected	Detected	Detected
5609	A_23_P123228	SLC26A3	Hs.1650	solute carrier family 26, member 3	Homo sapiens solute carrier family 26, member 3 (SLC26A3), mRNA [NM_000111]	3.06307	down	1.40312	down	5.49373	17.42438	3.16996	3.92206	Compromised	Detected	Compromised	Compromised
5610	A_24_P71973	KDR	Hs.479756	kinase insert domain receptor (a type III receptor tyrosine kinase)	Homo sapiens kinase insert domain receptor (a type III receptor tyrosine kinase) (KDR), mRNA [NM_002253]	3.06144	down	3.43667	down	140.01378	443.84348	116.16223	352.02094	Detected	Detected	Detected	Detected
5611	A_33_P3382758		Hs.679193		BX404796 Homo sapiens FETAL LIVER Homo sapiens cDNA clone CS0DM011YO11 5-PRIME, mRNA sequence [BX404796]	3.06136	down	1.85589	down	9.23669	29.27957	8.40235	13.75051	Compromised	Detected	Compromised	Compromised
5612	A_33_P3415678				MKL/myocardin-like protein 1 (Myocardin-related transcription factor A)(MRTF-A)(Megakaryoblastic leukemia 1 protein)(Megakaryocytic acute leukemia protein) [Source:UniProtKB/Swiss-Prot;Acc:Q969V6] [ENST00000402630]	3.06004	down	2.89190	down	57.61629	182.55990	47.87204	122.07609	Detected	Detected	Detected	Detected
5613	A_24_P308029	HSPB6	Hs.534538	heat shock protein, alpha-crystallin-related, B6	Homo sapiens heat shock protein, alpha-crystallin-related, B6 (HSPB6), mRNA [NM_144617]	3.05943	down	4.67834	down	158.79968	503.06326	99.90445	412.13730	Detected	Detected	Detected	Detected
5614	A_24_P383523	SAMD4A	Hs.98259	sterile alpha motif domain containing 4A	Homo sapiens sterile alpha motif domain containing 4A (SAMD4A), transcript variant 1, mRNA [NM_015589]	3.05929	down	3.23846	down	443.15744	1403.81980	512.88617	1464.62200	Detected	Detected	Detected	Detected
5615	A_23_P140373	FLVCR2	Hs.509966	feline leukemia virus subgroup C cellular receptor family, member 2	Homo sapiens feline leukemia virus subgroup C cellular receptor family, member 2 (FLVCR2), mRNA [NM_017791]	3.05917	down	3.20855	down	29.11155	92.21517	17.89330	50.62498	Detected	Detected	Detected	Detected
5616	A_33_P3262156	SLC8A1	Hs.468274	solute carrier family 8 (sodium/calcium exchanger), member 1	Homo sapiens solute carrier family 8 (sodium/calcium exchanger), member 1 (SLC8A1), transcript variant A, mRNA [NM_021097]	3.05755	down	5.16874	down	61.28171	194.01587	25.11796	114.48138	Detected	Detected	Detected	Detected
5617	A_33_P3316522	DEFB124	Hs.381373	defensin, beta 124	Homo sapiens defensin, beta 124 (DEFB124), mRNA [NM_001037500]	3.05657	down	1.76417	down	205.09602	649.11870	58.34496	90.76322	Detected	Detected	Detected	Detected
5618	A_32_P352358	LOC650293	Hs.535167	seven transmembrane helix receptor	Homo sapiens seven transmembrane helix receptor (LOC650293), mRNA [NM_001040071]	3.05616	down	2.42163	down	24.44747	77.36475	19.35428	41.32858	Detected	Detected	Detected	Detected
5619	A_33_P3365924				Putative uncharacterized protein ENSP00000353084 Fragment [Source:UniProtKB/TrEMBL;Acc:A6NF24] [ENST00000391654]	3.05386	down	3.08996	down	31.33826	99.09613	37.50080	102.17822	Detected	Detected	Detected	Detected
5620	A_23_P59045	HIST1H2AE	Hs.121017	histone cluster 1, H2ae	Homo sapiens histone cluster 1, H2ae (HIST1H2AE), mRNA [NM_021052]	3.05154	down	2.59554	down	423.33880	1337.64180	237.01953	542.47296	Detected	Detected	Detected	Detected
5621	A_23_P100868	MYO19	Hs.302051	myosin XIX	Homo sapiens myosin XIX (MYO19), transcript variant 3, mRNA [NM_001033580]	3.05125	down	3.26739	down	907.86860	2868.36600	831.71230	2396.28930	Detected	Detected	Detected	Detected
5622	A_33_P3317073	MOBKL1A	Hs.700445	MOB1, Mps One Binder kinase activator-like 1A (yeast)	Homo sapiens MOB1, Mps One Binder kinase activator-like 1A (yeast) (MOBKL1A), mRNA [NM_173468]	3.05030	down	1.13996	down	12.22344	38.60724	21.09019	21.19990	Compromised	Detected	Detected	Detected
5623	A_33_P3411885	LOC100128851	Hs.679912	hypothetical protein LOC100128851	Homo sapiens cDNA FLJ45515 fis, clone BRTHA2022914. [AK127423]	3.05021	down	3.51950	down	1695.57520	5355.24850	1120.40200	3477.12800	Detected	Detected	Detected	Detected
5624	A_24_P339944	PDGFB	Hs.1976	platelet-derived growth factor beta polypeptide (simian sarcoma viral (v-sis) oncogene homolog)	Homo sapiens platelet-derived growth factor beta polypeptide (simian sarcoma viral (v-sis) oncogene homolog) (PDGFB), transcript variant 1, mRNA [NM_002608]	3.04940	down	2.40879	down	6.85375	21.64097	7.13727	15.15994	Compromised	Detected	Compromised	Detected
5625	A_23_P414308	FLCN	Hs.513975	folliculin	Homo sapiens folliculin (FLCN), transcript variant 2, mRNA [NM_144606]	3.04896	down	3.12549	down	461.19030	1456.01120	451.75537	1245.05180	Detected	Detected	Detected	Detected
5626	A_23_P25913	DNAL1	Hs.525445	dynein, axonemal, light chain 1	Homo sapiens dynein, axonemal, light chain 1 (DNAL1), mRNA [NM_031427]	3.04846	down	2.77518	down	242.22432	764.59410	195.18584	477.64487	Detected	Detected	Detected	Detected
5627	A_23_P91317	RAD21L1	Hs.713451	RAD21-like 1 (S. pombe)	Homo sapiens RAD21-like 1 (S. pombe) (RAD21L1), mRNA [NM_001136566]	3.04596	down	1.18368	down	6.79083	21.41805	2.88247	3.00859	Compromised	Detected	Compromised	Compromised
5628	A_33_P3253857					3.04576	down	2.27828	down	180.08672	567.94995	81.67855	164.08974	Detected	Detected	Detected	Detected
5629	A_33_P3289025	FAM176A	Hs.302346	family with sequence similarity 176, member A	Homo sapiens family with sequence similarity 176, member A (FAM176A), transcript variant 1, mRNA [NM_001135032]	3.04445	down	2.84429	down	284.20447	895.92834	429.49830	1077.21010	Detected	Detected	Detected	Detected
5630	A_33_P3333030	SUFU	Hs.404089	suppressor of fused homolog (Drosophila)	Homo sapiens suppressor of fused homolog (Drosophila) (SUFU), mRNA [NM_016169]	3.04428	down	2.72172	down	24.32019	76.66287	29.24629	70.19083	Detected	Detected	Detected	Detected
5631	A_23_P215525	OSBPL3	Hs.520259	oxysterol binding protein-like 3	Homo sapiens oxysterol binding protein-like 3 (OSBPL3), transcript variant 1, mRNA [NM_015550]	3.04349	down	3.30642	down	860.44464	2711.61180	657.90845	1918.17610	Detected	Detected	Detected	Detected
5632	A_23_P69810	AGPAT9	Hs.99196	1-acylglycerol-3-phosphate O-acyltransferase 9	Homo sapiens 1-acylglycerol-3-phosphate O-acyltransferase 9 (AGPAT9), mRNA [NM_032717]	3.04254	down	3.06132	down	363.46304	1145.06360	275.58212	743.91736	Detected	Detected	Detected	Detected

5633	A_33_P3272568	LOC100292021		similar to thioredoxin peroxidase	Peroxiredoxin-4 (EC 1.11.1.15)(Prx-IV)(Thioredoxin peroxidase AO372)(Thioredoxin-dependent peroxide reductase A0372)(Antioxidant enzyme AOE372)(AOE37-2) [Source:UniProtKB/Swiss-Prot;Acc:Q13162] [ENST00000379331]	3.04184	down	3.71843	down	12.50706	39.39356	3.35681	11.00656	Compromised	Detected	Compromised	Compromised
5634	A_33_P3291176	VMAC	Hs.620821	vimentin-type intermediate filament associated coiled-coil protein	Homo sapiens vimentin-type intermediate filament associated coiled-coil protein (VMAC), mRNA [NM_001017921]	3.04117	down	3.20077	down	226.97826	714.75745	215.07227	607.02140	Detected	Detected	Detected	Detected
5635	A_33_P3290532	LOC255480	Hs.720583	hypothetical protein LOC255480	Homo sapiens cDNA FLJ34447 fis, clone HLUNG2002059 [AK091766]	3.04080	down	10.12398	down	8.08405	25.45369	2.42988	21.69210	Compromised	Detected	Compromised	Detected
5636	A_32_P208403	GNG2	Hs.708109	guanine nucleotide binding protein (G protein), gamma 2	Homo sapiens guanine nucleotide binding protein (G protein), gamma 2 (GNG2), mRNA [NM_053064]	3.04060	down	2.66425	down	716.55600	2256.01760	395.25250	928.56990	Detected	Detected	Detected	Detected
5637	A_24_P671164	PRLR	Hs.368587	prolactin receptor	Homo sapiens prolactin receptor (PRLR), mRNA [NM_000949]	3.03957	down	2.86854	down	7.58914	23.88566	3.55654	8.99610	Compromised	Detected	Compromised	Compromised
5638	A_33_P3253175				WD repeat-containing protein 37 [Source:UniProtKB/Swiss-Prot;Acc:Q9Y2I8] [ENST00000381329]	3.03792	down	3.16379	down	32.21724	101.34390	24.57428	68.55726	Detected	Detected	Detected	Detected
5639	A_33_P3295203	HAS1	Hs.57697	hyaluronan synthase 1	Homo sapiens hyaluronan synthase 1 (HAS1), mRNA [NM_001523]	3.03548	down	3.36715	down	436.97534	1373.46640	392.35413	1164.94850	Detected	Detected	Detected	Detected
5640	A_23_P136232	IMPAD1	Hs.438689	inositol monophosphatase domain containing 1	Homo sapiens inositol monophosphatase domain containing 1 (IMPAD1), mRNA [NM_017813]	3.03134	down	3.11322	down	757.46870	2377.56670	457.53430	1256.02550	Detected	Detected	Detected	Detected
5641	A_23_P78664	DDX39	Hs.311609	DEAD (Asp-Glu-Ala-Asp) box polypeptide 39	Homo sapiens DEAD (Asp-Glu-Ala-Asp) box polypeptide 39 (DDX39), mRNA [NM_005804]	3.02939	down	3.06956	down	9388.38300	29449.62300	9159.12800	24791.05900	Detected	Detected	Detected	Detected
5642	A_23_P90470					3.02740	down	1.17387	down	7.15205	22.41985	2.47224	2.55905	Compromised	Detected	Compromised	Compromised
5643	A_33_P3368895					3.02728	down	3.09801	down	3199.80710	10030.20000	1203.02150	3286.40550	Detected	Detected	Detected	Detected
5644	A_23_P38795	FPR1	Hs.753	formyl peptide receptor 1	Homo sapiens formyl peptide receptor 1 (FPR1), mRNA [NM_002029]	3.02123	down	3.45878	down	145.88289	456.37500	121.58852	370.83575	Detected	Detected	Detected	Detected
5645	A_23_P203475	PRKCDBP	Hs.434044	protein kinase C, delta binding protein	Homo sapiens protein kinase C, delta binding protein (PRKCDBP), mRNA [NM_145040]	3.02060	down	3.21394	down	21024.57200	65758.79000	25662.04300	72726.73000	Detected	Detected	Detected	Detected
5646	A_32_P33434	ZNF812	Hs.626848	zinc finger protein 812	Putative zinc finger protein 812 [Source:UniProtKB/Swiss-Prot;Acc:P0C7V5] [ENST00000457674]	3.02010	down	2.59803	down	5.08619	15.90546	4.79885	10.99377	Compromised	Detected	Compromised	Compromised
5647	A_33_P3268734		Hs.571561		CR748640 Soares_NFL_T_GBC_S1 Homo sapiens cDNA clone IMAGp971L0898 ; IMAGE:2348699 5', mRNA sequence [CR748640]	3.01392	down	1.27358	down	8.52902	26.61729	15.13137	16.99302	Compromised	Detected	Compromised	Detected
5648	A_33_P3240787	LOC100131910	Hs.689306	hypothetical protein LOC100131910	Homo sapiens cDNA FLJ27192 fis, clone SYN02553. [AK130702]	3.01296	down	4.16777	down	70.84839	221.03299	27.68577	101.74807	Detected	Detected	Detected	Detected
5649	A_23_P152655	ICAM2	Hs.431460	intercellular adhesion molecule 2	Homo sapiens intercellular adhesion molecule 2 (ICAM2), transcript variant 5, mRNA [NM_000873]	3.01219	down	3.47897	down	108.77898	339.28143	103.91580	318.78473	Detected	Detected	Detected	Detected
5650	A_23_P145694	ASNS	Hs.489207	asparagine synthetase	Homo sapiens asparagine synthetase (ASNS), transcript variant 2, mRNA [NM_001673]	3.01104	down	3.09417	down	6760.25630	21077.17400	5756.45500	15705.99600	Detected	Detected	Detected	Detected
5651	A_23_P422193	SUV39H1	Hs.522639	suppressor of variegation 3-9 homolog 1 (Drosophila)	Homo sapiens suppressor of variegation 3-9 homolog 1 (Drosophila) (SUV39H1), mRNA [NM_003173]	3.01069	down	3.34616	down	160.72166	501.04050	213.61896	630.30690	Detected	Detected	Detected	Detected
5652	A_23_P132936	SPCS3	Hs.42194	signal peptidase complex subunit 3 homolog (S. cerevisiae)	Homo sapiens signal peptidase complex subunit 3 homolog (S. cerevisiae) (SPCS3), mRNA [NM_021928]	3.00993	down	3.01963	down	5045.07570	15723.79100	4488.09770	11950.36700	Detected	Detected	Detected	Detected
5653	A_33_P3380913	LOC100287245	Hs.650098	similar to nudix-type motif 15	PREDICTED: Homo sapiens similar to nudix-type motif 15 (LOC100287245), miscRNA [XR_078528]	3.00990	down	2.52504	down	43.25538	134.81104	20.64441	45.96591	Detected	Detected	Detected	Detected
5654	A_33_P3347320					3.00567	down	2.98429	down	275.64480	857.87640	200.90433	528.68290	Detected	Detected	Detected	Detected
5655	A_23_P152984	THOC4	Hs.534385	THO complex 4	Homo sapiens THO complex 4 (THOC4), mRNA [NM_005782]	3.00543	down	2.72688	down	7230.83350	22502.32600	5037.84030	12113.68500	Detected	Detected	Detected	Detected
5656	A_33_P3318581	PLOD2	Hs.477866	procollagen-lysine, 2-oxoglutarate 5-dioxygenase 2	Homo sapiens procollagen-lysine, 2-oxoglutarate 5-dioxygenase 2 (PLOD2), transcript variant 1, mRNA [NM_182943]	3.00457	down	2.84964	down	4076.25510	12681.68500	2914.74760	7324.13100	Detected	Detected	Detected	Detected
5657	A_24_P327181	WNK4	Hs.105448	WNK lysine deficient protein kinase 4	Homo sapiens WNK lysine deficient protein kinase 4 (WNK4), mRNA [NM_032387]	3.00359	down	2.69824	down	40.27748	125.26679	23.55896	56.05344	Detected	Detected	Detected	Detected
5658	A_23_P101093	COPZ2	Hs.408434	coatomer protein complex, subunit zeta 2	Homo sapiens coatomer protein complex, subunit zeta 2 (COPZ2), mRNA [NM_016429]	3.00228	down	3.06050	down	3416.54250	10621.14600	2415.69400	6519.27150	Detected	Detected	Detected	Detected
5659	A_23_P31765	PKIA	Hs.433700	protein kinase (cAMP-dependent, catalytic) inhibitor alpha	Homo sapiens protein kinase (cAMP-dependent, catalytic) inhibitor alpha (PKIA), transcript variant 6, mRNA [NM_006823]	3.00176	down	3.05832	down	788.10803	2449.60130	560.40656	1511.30160	Detected	Detected	Detected	Detected
5660	A_23_P127579	PTS	Hs.503860	6-pyruvoyltetrahydropterin synthase	Homo sapiens 6-pyruvoyltetrahydropterin synthase (PTS), mRNA [NM_000317]	2.99935	down	2.99315	down	3490.42500	10840.21800	3003.05790	7926.05800	Detected	Detected	Detected	Detected
5661	A_24_P51855	DUSP7	Hs.591664	dual specificity phosphatase 7	Homo sapiens dual specificity phosphatase 7 (DUSP7), mRNA [NM_001947]	2.99839	down	3.06222	down	29.15657	90.52259	39.54217	106.77337	Detected	Detected	Detected	Detected
5662	A_23_P405129	LTBP2	Hs.512776	latent transforming growth factor beta binding protein 2	Homo sapiens latent transforming growth factor beta binding protein 2 (LTBP2), mRNA [NM_000428]	2.99585	down	3.16660	down	824.17926	2556.67160	716.53570	2000.76660	Detected	Detected	Detected	Detected
5663	A_33_P3409347	C19orf53	Hs.231616	chromosome 19 open reading frame 53	Homo sapiens chromosome 19 open reading frame 53 (C19orf53), mRNA [NM_014047]	2.99080	down	2.50513	down	27.84484	86.23126	31.60478	69.81508	Detected	Detected	Detected	Detected

5664	A_23_P17053	IL1F9	Hs.211238	interleukin 1 family, member 9	Homo sapiens interleukin 1 family, member 9 (IL1F9), mRNA [NM_019618]	2.98881	down	1.52568	down	9.21036	28.50419	3.31594	4.46103	Compromised	Detected	Compromised	Compromised
5665	A_33_P3231367	ATXN10	Hs.475125	ataxin 10	Homo sapiens ataxin 10 (ATXN10), mRNA [NM_013236]	2.98693	down	2.81308	down	392.08320	1212.65200	307.86200	763.66570	Detected	Detected	Detected	Detected
5666	A_24_P701776	ARHGEF5L	Hs.534621	Rho guanine nucleotide exchange factor (GEF) 5-like	Homo sapiens Rho guanine nucleotide exchange factor (GEF) 5-like (ARHGEF5L), mRNA [NM_001003702]	2.98565	down	2.52318	down	22.13001	68.41540	24.18276	53.80462	Detected	Detected	Detected	Detected
5667	A_23_P336644	TOR1AIP2	Hs.655655	torsin A interacting protein 2	Homo sapiens torsin A interacting protein 2 (TOR1AIP2), transcript variant 2, mRNA [NM_145034]	2.98512	down	2.90584	down	108.62951	335.77072	81.21252	208.09396	Detected	Detected	Detected	Detected
5668	A_24_P149704	DAB2IP	Hs.522378	DAB2 interacting protein	Homo sapiens DAB2 interacting protein (DAB2IP), transcript variant 2, mRNA [NM_138709]	2.98190	down	2.89504	down	25.21265	77.84748	24.70412	63.06511	Detected	Detected	Detected	Detected
5669	A_33_P3240333	PITX1	Hs.84136	paired-like homeodomain 1	Homo sapiens paired-like homeodomain 1 (PITX1), mRNA [NM_002653]	2.98064	down	5.75448	down	91.91876	283.69223	42.20107	214.13847	Detected	Detected	Detected	Detected
5670	A_33_P3224745	PLA2G15	Hs.632199	phospholipase A2, group XV	Homo sapiens phospholipase A2, group XV (PLA2G15), mRNA [NM_012320]	2.97887	down	3.22974	down	8263.29100	25488.08000	6376.10700	18158.88500	Detected	Detected	Detected	Detected
5671	A_24_P206047	SLC25A4	Hs.246506	solute carrier family 25 (mitochondrial carrier; adenine nucleotide translocator), member 4	Homo sapiens solute carrier family 25 (mitochondrial carrier; adenine nucleotide translocator), member 4 (SLC25A4), nuclear gene encoding mitochondrial protein, mRNA [NM_001151]	2.97694	down	3.18034	down	590.48170	1820.15840	651.65790	1827.50550	Detected	Detected	Detected	Detected
5672	A_33_P3296772					2.97687	down	2.16678	down	28.53963	87.97130	32.48265	62.06285	Detected	Detected	Detected	Detected
5673	A_23_P83931	NET1	Hs.25155	neuroepithelial cell transforming 1	Homo sapiens neuroepithelial cell transforming 1 (NET1), transcript variant 1, mRNA [NM_001047160]	2.97624	down	3.17421	down	2421.09130	7461.27150	1569.30760	4392.47600	Detected	Detected	Detected	Detected
5674	A_23_P160582	HYI	Hs.709864	hydroxypyruvate isomerase homolog (E. coli)	Homo sapiens hydroxypyruvate isomerase homolog (E. coli) (HYI), mRNA [NM_031207]	2.97421	down	3.01106	down	1149.51610	3540.14040	1267.09680	3364.29440	Detected	Detected	Detected	Detected
5675	A_33_P3266958				Zinc finger protein 286A [Source:UniProtKB/Swiss-Prot;Acc:Q9HBT8] [ENST00000395893]	2.97401	down	1.54937	down	16.25680	50.06231	20.36998	27.82978	Detected	Detected	Detected	Detected
5676	A_24_P772488	PLXNA4	Hs.511454	plexin A4	Homo sapiens plexin A4 (PLXNA4), transcript variant 1, mRNA [NM_020911]	2.97264	down	5.37088	down	6.15336	18.94036	3.66594	17.36184	Compromised	Detected	Compromised	Detected
5677	A_24_P116669	CANT1	Hs.8859	calcium activated nucleotidase 1	Homo sapiens calcium activated nucleotidase 1 (CANT1), transcript variant 1, mRNA [NM_138793]	2.97187	down	3.23469	down	168.07591	517.21173	168.44190	480.44992	Detected	Detected	Detected	Detected
5678	A_33_P3336700	SHROOM3	Hs.702168	shroom family member 3	Homo sapiens shroom family member 3 (SHROOM3), mRNA [NM_020859]	2.96635	down	2.88006	down	991.32790	3044.89530	779.92114	1980.69470	Detected	Detected	Detected	Detected
5679	A_32_P54475		Hs.722066		AL566187 Homo sapiens FETAL BRAIN Homo sapiens cDNA clone CS0DF022YK06 3-PRIME, mRNA sequence [AL566187]	2.96124	down	2.06253	down	674.73410	2068.90040	452.71167	823.35480	Detected	Detected	Detected	Detected
5680	A_33_P3376273				Glycerol kinase (EC 2.7.1.30)(ATP:glycerol 3-phosphotransferase)(Glycerokinase)(GK) [Source:UniProtKB/Swiss-Prot;Acc:P32189] [ENST00000378941]	2.95450	down	4.20594	down	19.22633	58.81846	3.59255	13.32391	Detected	Detected	Compromised	Compromised
5681	A_33_P3217153	GRIA4	Hs.503743	glutamate receptor, ionotrophic, AMPA 4	Homo sapiens glutamate receptor, ionotrophic, AMPA 4 (GRIA4), transcript variant 2, mRNA [NM_001077243]	2.95283	down	1.57190	down	303.32556	927.42770	37.94178	52.59076	Detected	Detected	Detected	Detected
5682	A_24_P53519	CHAF1A	Hs.79018	chromatin assembly factor 1, subunit A (p150)	Homo sapiens chromatin assembly factor 1, subunit A (p150) (CHAF1A), mRNA [NM_005483]	2.95266	down	2.77340	down	1570.52590	4801.66360	1266.29300	3096.79570	Detected	Detected	Detected	Detected
5683	A_23_P30913	HLA-DPA1	Hs.347270	major histocompatibility complex, class II, DP alpha 1	Homo sapiens major histocompatibility complex, class II, DP alpha 1 (HLA-DPA1), mRNA [NM_033554]	2.95165	down	2.76049	down	184.20305	562.98267	192.37940	468.28452	Detected	Detected	Detected	Detected
5684	A_24_P925062	MXRA7	Hs.250723	matrix-remodelling associated 7	Homo sapiens matrix-remodelling associated 7 (MXRA7), transcript variant 1, mRNA [NM_001008528]	2.95046	down	2.69634	down	1202.87120	3674.86700	899.98550	2139.81540	Detected	Detected	Detected	Detected
5685	A_33_P3262537					2.94960	down	3.04715	down	25.82156	78.86404	26.80777	72.03114	Detected	Detected	Detected	Detected
5686	A_32_P300427	APCDD1L	Hs.119286	adenomatosis polyposis coli down-regulated 1-like	Homo sapiens adenomatosis polyposis coli down-regulated 1-like (APCDD1L), mRNA [NM_153360]	2.94873	down	2.88250	down	1917.96570	5856.10640	1273.29930	3236.42820	Detected	Detected	Detected	Detected
5687	A_23_P304450	GATA6	Hs.514746	GATA binding protein 6	Homo sapiens GATA binding protein 6 (GATA6), mRNA [NM_005257]	2.94847	down	3.21320	down	74.46387	227.33984	56.61322	160.40611	Detected	Detected	Detected	Detected
5688	A_23_P1387	ARHGAP19	Hs.80305	Rho GTPase activating protein 19	Homo sapiens Rho GTPase activating protein 19 (ARHGAP19), mRNA [NM_032900]	2.94842	down	3.18578	down	257.29938	785.52826	232.28217	652.52470	Detected	Detected	Detected	Detected
5689	A_24_P41570	H2AFZ	Hs.119192	H2A histone family, member Z	Homo sapiens H2A histone family, member Z (H2AFZ), mRNA [NM_002106]	2.94836	down	2.81480	down	16723.64800	51055.71500	17300.33400	42940.54300	Detected	Detected	Detected	Detected
5690	A_23_P68922	MICALL1	Hs.517610	MICAL-like 1	Homo sapiens MICAL-like 1 (MICALL1), mRNA [NM_033386]	2.94674	down	3.08602	down	231.45456	706.22190	179.47130	488.38086	Detected	Detected	Detected	Detected
5691	A_24_P325046		Hs.654700		Zinc finger CCHC domain-containing protein 7 [Source:UniProtKB/Swiss-Prot;Acc:Q8N3Z6] [ENST00000322831]	2.94539	down	3.02629	down	151.96289	463.46194	115.93329	309.37473	Detected	Detected	Detected	Detected
5692	A_33_P3257150	CDC42	Hs.467637	cell division cycle 42 (GTP binding protein, 25kDa)	Homo sapiens cell division cycle 42 (GTP binding protein, 25kDa) (CDC42), transcript variant 3, mRNA [NM_001039802]	2.94452	down	3.00471	down	2800.02000	8537.06000	2134.20300	5654.62500	Detected	Detected	Detected	Detected
5693	A_23_P74097	TCEB3	Hs.15535	transcription elongation factor B (SIII), polypeptide 3 (110kDa, elongin A)	Homo sapiens transcription elongation factor B (SIII), polypeptide 3 (110kDa, elongin A) (TCEB3), mRNA [NM_003198]	2.94332	down	2.89827	down	995.47210	3033.89330	874.38120	2234.62840	Detected	Detected	Detected	Detected

5694	A_23_P200976	HYI	Hs.709864	hydroxypyruvate isomerase homolog (E. coli)	Homo sapiens hydroxypyruvate isomerase homolog (E. coli) (HYI), mRNA [NM_031207]	2.93989	down	2.79095	down	2990.41750	9103.22900	2955.39200	7273.31200	Detected	Detected	Detected	Detected
5695	A_23_P143845	TIPARP	Hs.12813	TCDD-inducible poly(ADP-ribose) polymerase	Homo sapiens TCDD-inducible poly(ADP-ribose) polymerase (TIPARP), mRNA [NM_015508]	2.93818	down	3.20387	down	6523.20300	19845.97700	5583.55800	15774.32400	Detected	Detected	Detected	Detected
5696	A_23_P208310	CD3EAP	Hs.710495	CD3e molecule, epsilon associated protein	Homo sapiens CD3e molecule, epsilon associated protein (CD3EAP), mRNA [NM_012099]	2.93518	down	3.28927	down	335.18106	1018.70210	284.77014	825.96160	Detected	Detected	Detected	Detected
5697	A_32_P171313	GNB4	Hs.173030	guanine nucleotide binding protein (G protein), beta polypeptide 4	Homo sapiens guanine nucleotide binding protein (G protein), beta polypeptide 4 (GNB4), mRNA [NM_021629]	2.93409	down	2.70317	down	12750.47000	38737.61300	10215.64000	24350.30900	Detected	Detected	Detected	Detected
5698	A_33_P3339070	LOC100216001		hypothetical LOC100216001	Homo sapiens hypothetical LOC100216001 (LOC100216001), non-coding RNA [NR_024475]	2.93127	down	2.92962	down	80.41094	244.06439	55.68102	143.84119	Detected	Detected	Detected	Detected
5699	A_33_P3406899	TRAK1	Hs.535711	trafficking protein, kinesin binding 1	Homo sapiens trafficking protein, kinesin binding 1 (TRAK1), transcript variant 2, mRNA [NM_014965]	2.92971	down	2.68842	down	1165.44210	3535.48170	1013.55820	2402.76250	Detected	Detected	Detected	Detected
5700	A_23_P52761	MMP7	Hs.2256	matrix metallopeptidase 7 (matrilysin, uterine)	Homo sapiens matrix metallopeptidase 7 (matrilysin, uterine) (MMP7), mRNA [NM_002423]	2.92941	down	3.14511	down	15.54122	47.14092	13.93855	38.65622	Detected	Detected	Compromised	Detected
5701	A_33_P3254555		Hs.721800		DNA replication licensing factor MCM9 (Mini-chromosome maintenance deficient 9)(hMCM9)(Mini-chromosome maintenance deficient domain-containing protein 1) [Source:UniProtKB/Swiss-Prot;Acc:Q9NXL9] [ENST00000243218]	2.92889	down	1.78353	down	16.83345	51.05166	20.76773	32.66146	Detected	Detected	Detected	Detected
5702	A_24_P272225	LOC645676	Hs.568693	hypothetical LOC645676	Homo sapiens hypothetical LOC645676 (LOC645676), non-coding RNA [NR_027023]	2.92860	down	2.72217	down	435.37543	1320.25330	406.70908	976.25824	Detected	Detected	Detected	Detected
5703	A_33_P3369829	tcag7.1307		hypothetical LOC154822	Homo sapiens hypothetical LOC154822 (LOC154822), non-coding RNA [NR_024394]	2.92816	down	1.17935	down	6.03031	18.28381	3.08385	3.20701	Compromised	Detected	Compromised	Compromised
5704	A_24_P186274	IGFBPL1	Hs.349705	insulin-like growth factor binding protein-like 1	Homo sapiens insulin-like growth factor binding protein-like 1 (IGFBPL1), mRNA [NM_001007563]	2.92771	down	1.38712	down	16.92513	51.30895	22.91340	28.02642	Detected	Detected	Detected	Detected
5705	A_33_P3306267	PAX6	Hs.270303	paired box 6	Homo sapiens paired box 6 (PAX6), transcript variant 1, mRNA [NM_000280]	2.92371	down	4.01417	down	12.55757	38.01653	8.39845	29.72760	Compromised	Detected	Compromised	Detected
5706	A_24_P63380	BMPR1B	Hs.598475	bone morphogenetic protein receptor, type IB	Homo sapiens bone morphogenetic protein receptor, type IB (BMPR1B), mRNA [NM_001203]	2.92321	down	11.38564	down	4.49033	13.59159	3.05401	30.66152	Compromised	Compromised	Compromised	Detected
5707	A_33_P3253214				Histone H2A type 2-A (H2A.2)(H2A/o) [Source:UniProtKB/Swiss-Prot;Acc:Q6FI13] [ENST00000369159]	2.92117	down	3.09691	down	5751.40770	17396.60700	3995.19530	10910.20200	Detected	Detected	Detected	Detected
5708	A_24_P404033	C15orf38	Hs.6734	chromosome 15 open reading frame 38	Homo sapiens chromosome 15 open reading frame 38 (C15orf38), mRNA [NM_182616]	2.92004	down	2.93227	down	173.78426	525.45180	138.88650	359.11148	Detected	Detected	Detected	Detected
5709	A_33_P3339531	CHADL	Hs.344488	chondroadherin-like	Homo sapiens chondroadherin-like (CHADL), mRNA [NM_138481]	2.91736	down	3.43575	down	77.06607	232.80196	68.10331	206.32674	Detected	Detected	Detected	Detected
5710	A_32_P44568	LDHA	Hs.2795	lactate dehydrogenase A	Homo sapiens lactate dehydrogenase A (LDHA), transcript variant 1, mRNA [NM_005566]	2.91627	down	3.00010	down	17248.94700	52086.26600	13792.62900	36487.87000	Detected	Detected	Detected	Detected
5711	A_33_P3408918	SAA2	Hs.1955	serum amyloid A2	Homo sapiens serum amyloid A2 (SAA2), transcript variant 1, mRNA [NM_030754]	2.91487	down	2.43971	down	19.70596	59.47710	16.43667	35.36048	Detected	Detected	Detected	Detected
5712	A_23_P27400	HAS1	Hs.57697	hyaluronan synthase 1	Homo sapiens hyaluronan synthase 1 (HAS1), mRNA [NM_001523]	2.91396	down	3.34484	down	21.15866	63.84172	22.50650	66.38175	Detected	Detected	Detected	Detected
5713	A_33_P3237096	INPP5F		inositol polyphosphate-5-phosphatase F	Homo sapiens inositol polyphosphate-5-phosphatase F (INPP5F), transcript variant 3, transcribed RNA [NR_003251]	2.91321	down	2.95004	down	251.82303	759.62585	194.63162	506.29930	Detected	Detected	Detected	Detected
5714	A_33_P3312676		Hs.279562		Myelin transcription factor 1 (MyT1)(MyTI)(Proteolipid protein-binding protein)(PLPB1) [Source:UniProtKB/Swiss-Prot;Acc:Q01538] [ENST00000360149]	2.91135	down	4.22368	down	18.13263	54.66227	11.16169	41.57062	Detected	Detected	Compromised	Detected
5715	A_23_P109427	GSTT2	Hs.654462	glutathione S-transferase theta 2	Homo sapiens glutathione S-transferase theta 2 (GSTT2), mRNA [NM_000854]	2.90915	down	2.77277	down	1192.95310	3593.53900	1653.05050	4041.70730	Detected	Detected	Detected	Detected
5716	A_23_P3312	ISLR	Hs.699822	immunoglobulin superfamily containing leucine-rich repeat	Homo sapiens immunoglobulin superfamily containing leucine-rich repeat (ISLR), transcript variant 1, mRNA [NM_005545]	2.90689	down	3.20347	down	13129.58200	39519.65600	12272.19800	34666.41800	Detected	Detected	Detected	Detected
5717	A_24_P22800	PSG11	Hs.646353	pregnancy specific beta-1-glycoprotein 11	Homo sapiens pregnancy specific beta-1-glycoprotein 11 (PSG11), transcript variant 1, mRNA [NM_002785]	2.90641	down	2.75633	down	8.48527	25.53615	11.59793	28.18888	Compromised	Detected	Compromised	Detected
5718	A_33_P3223116				Homeodomain-interacting protein kinase 2 (hHIPk2)(EC 2.7.11.1) [Source:UniProtKB/Swiss-Prot;Acc:Q9H2X6] [ENST00000342645]	2.90559	down	2.96668	down	314.96994	947.62540	261.35504	683.70200	Detected	Detected	Detected	Detected
5719	A_33_P3361152	LOC100134259	Hs.114449	similar to hCG1987718	Homo sapiens similar to hCG1987718 (LOC100134259), non-coding RNA [NR_024452]	2.90042	down	3.53101	down	538.89636	1618.44840	252.78593	787.07770	Detected	Detected	Detected	Detected
5720	A_24_P33895	ATF3	Hs.460	activating transcription factor 3	Homo sapiens activating transcription factor 3 (ATF3), transcript variant 4, mRNA [NM_001040619]	2.89907	down	2.79579	down	46.31275	139.02478	48.77914	120.25544	Detected	Detected	Detected	Detected

5721	A_23_P101476	ZNF442	Hs.253193	zinc finger protein 442	Homo sapiens zinc finger protein 442 (ZNF442), mRNA [NM_030824]	2.89644	down	2.98820	down	89.26200	267.71005	40.17199	105.85170	Detected	Detected	Detected	Detected
5722	A_23_P156310	SKP2	Hs.23348	S-phase kinase-associated protein 2 (p45)	Homo sapiens S-phase kinase-associated protein 2 (p45) (SKP2), transcript variant 2, mRNA [NM_032637]	2.89593	down	3.10167	down	266.01180	797.66907	227.98456	623.54360	Detected	Detected	Detected	Detected
5723	A_23_P38567	CYTSB	Hs.431045	cytospin B	Homo sapiens cytospin B (CYTSB), transcript variant NSP5beta3alpha, mRNA [NM_152904]	2.89164	down	2.90011	down	449.70160	1346.48670	415.31137	1062.07320	Detected	Detected	Detected	Detected
5724	A_33_P3317979	MYO5B	Hs.720076	myosin VB	Homo sapiens myosin VB (MYO5B), mRNA [NM_001080467]	2.88831	down	2.45665	down	123.38212	369.00275	19.03550	41.23574	Detected	Detected	Detected	Detected
5725	A_23_P217570	CAPN6	Hs.496593	calpain 6	Homo sapiens calpain 6 (CAPN6), mRNA [NM_014289]	2.88765	down	13.76587	down	4.85299	14.51067	2.66936	32.40231	Compromised	Compromised	Compromised	Detected
5726	A_23_P216610	SUSD1	Hs.494827	sushi domain containing 1	Homo sapiens sushi domain containing 1 (SUSD1), mRNA [NM_022486]	2.88539	down	2.97186	down	381.25906	1139.09120	289.79547	759.42554	Detected	Detected	Detected	Detected
5727	A_24_P338187	HNRNPH3	Hs.643472	heterogeneous nuclear ribonucleoprotein H3 (2H9)	Homo sapiens heterogeneous nuclear ribonucleoprotein H3 (2H9) (HNRNPH3), transcript variant 2H9, mRNA [NM_012207]	2.88428	down	2.87791	down	2350.77420	7020.71340	1341.57890	3404.54170	Detected	Detected	Detected	Detected
5728	A_23_P145904	H2AFV	Hs.488189	H2A histone family, member V	Homo sapiens H2A histone family, member V (H2AFV), transcript variant 1, mRNA [NM_012412]	2.88378	down	2.61924	down	11790.97500	35208.33000	9640.27400	22265.37700	Detected	Detected	Detected	Detected
5729	A_23_P214300	GSTA2	Hs.94107	glutathione S-transferase alpha 2	Homo sapiens glutathione S-transferase alpha 2 (GSTA2), mRNA [NM_000846]	2.87882	down	3.95407	down	39.77264	118.55846	21.59130	75.28162	Detected	Detected	Detected	Detected
5730	A_24_P178503	ABCC9	Hs.446050	ATP-binding cassette, sub-family C (CFTR/MRP), member 9	Homo sapiens ATP-binding cassette, sub-family C (CFTR/MRP), member 9 (ABCC9), transcript variant SUR2A-delta-14, mRNA [NM_020298]	2.87856	down	2.97790	down	43.12259	128.53242	41.44601	108.83249	Detected	Detected	Detected	Detected
5731	A_33_P3376965	CHAC1	Hs.155569	ChaC, cation transport regulator homolog 1 (E. coli)	Homo sapiens ChaC, cation transport regulator homolog 1 (E. coli) (CHAC1), transcript variant 1, mRNA [NM_024111]	2.87856	down	4.07107	down	29.74660	88.66355	23.81865	85.50494	Detected	Detected	Detected	Detected
5732	A_33_P3347477		Hs.576925		DA375949 BRTHA2 Homo sapiens cDNA clone BRTHA2007423 5', mRNA sequence [DA375949]	2.87360	down	3.38460	down	41.24181	122.71481	28.28162	84.40677	Detected	Detected	Detected	Detected
5733	A_33_P3307133	GRIK2	Hs.98262	glutamate receptor, ionotropic, kainate 2	Homo sapiens glutamate receptor, ionotropic, kainate 2 (GRIK2), transcript variant 1, mRNA [NM_021956]	2.87345	down	3.22887	down	11.48295	34.16568	7.92374	22.56036	Compromised	Detected	Compromised	Detected
5734	A_23_P305140	C10orf32	Hs.34492	chromosome 10 open reading frame 32	Homo sapiens chromosome 10 open reading frame 32 (C10orf32), transcript variant 2, mRNA [NM_144591]	2.87250	down	2.66775	down	1398.56620	4159.83700	937.18835	2204.63530	Detected	Detected	Detected	Detected
5735	A_23_P92202	GMPPB	Hs.567488	GDP-mannose pyrophosphorylase B	Homo sapiens GDP-mannose pyrophosphorylase B (GMPPB), transcript variant 2, mRNA [NM_021971]	2.87243	down	3.10052	down	457.12076	1359.60940	455.60303	1245.62230	Detected	Detected	Detected	Detected
5736	A_23_P126120	CENPL	Hs.720473	centromere protein L	Homo sapiens centromere protein L (CENPL), transcript variant 2, mRNA [NM_033319]	2.87010	down	3.07375	down	141.24939	419.77582	93.57575	253.62836	Detected	Detected	Detected	Detected
5737	A_24_P21447	SURF6	Hs.274430	surfeit 6	Homo sapiens surfeit 6 (SURF6), mRNA [NM_006753]	2.87005	down	2.74524	down	231.85654	689.03680	172.62419	417.87628	Detected	Detected	Detected	Detected
5738	A_24_P349547					2.86924	down	2.42731	down	5768.00000	17136.63700	3106.61060	6649.32760	Detected	Detected	Detected	Detected
5739	A_23_P143535	WDR4	Hs.248815	WD repeat domain 4	Homo sapiens WD repeat domain 4 (WDR4), transcript variant 2, mRNA [NM_033661]	2.86675	down	3.27096	down	118.41016	351.48870	97.66119	281.68400	Detected	Detected	Detected	Detected
5740	A_33_P3249185				GTP-binding protein SAR1a (COPII-associated small GTPase) [Source:UniProtKB/Swiss-Prot;Acc:Q9NR31] [ENST00000373236]	2.86674	down	2.76199	down	1854.89580	5506.05960	1739.01220	4235.35250	Detected	Detected	Detected	Detected
5741	A_23_P22224	EIF4EBP1	Hs.411641	eukaryotic translation initiation factor 4E binding protein 1	Homo sapiens eukaryotic translation initiation factor 4E binding protein 1 (EIF4EBP1), mRNA [NM_004095]	2.86628	down	2.78357	down	2969.64480	8813.65100	3649.69210	8958.25300	Detected	Detected	Detected	Detected
5742	A_33_P3286804	ANO6	Hs.505339	anoctamin 6	Homo sapiens anoctamin 6 (ANO6), transcript variant 3, mRNA [NM_001142679]	2.86492	down	3.82158	down	7.36647	21.85271	5.29618	17.84726	Compromised	Detected	Compromised	Detected
5743	A_24_P221414	DYNC1I1	Hs.440364	dynein, cytoplasmic 1, intermediate chain 1	Homo sapiens dynein, cytoplasmic 1, intermediate chain 1 (DYNC1I1), transcript variant 1, mRNA [NM_004411]	2.86434	down	4.10648	down	36.74196	108.97340	26.92175	97.48515	Detected	Detected	Detected	Detected
5744	A_23_P121665	SORCS2	Hs.479099	sortilin-related VPS10 domain containing receptor 2	Homo sapiens sortilin-related VPS10 domain containing receptor 2 (SORCS2), mRNA [NM_020777]	2.86362	down	2.14979	down	15.44346	45.79244	13.65241	25.88040	Detected	Detected	Detected	Detected
5745	A_33_P3321120					2.86197	down	2.65583	down	580.11260	1719.13940	88.23506	206.63650	Detected	Detected	Detected	Detected
5746	A_23_P67339	RCN3	Hs.567550	reticulocalbin 3, EF-hand calcium binding domain	Homo sapiens reticulocalbin 3, EF-hand calcium binding domain (RCN3), mRNA [NM_020650]	2.85895	down	3.06314	down	3034.08180	8981.86400	2626.80200	7095.11430	Detected	Detected	Detected	Detected
5747	A_33_P3410836	HIST1H4D	Hs.248179	histone cluster 1, H4d	Homo sapiens histone cluster 1, H4d (HIST1H4D), mRNA [NM_003539]	2.85727	down	3.04797	down	65.81031	194.70543	71.92723	193.31682	Detected	Detected	Detected	Detected
5748	A_33_P3404336		Hs.676976		CDNA FLJ27256 fis, clone SYN09689 [Source:UniProtKB/TrEMBL;Acc:Q6ZNS0] [ENST00000426092]	2.85295	down	5.64382	down	58.86171	173.88405	28.69825	142.82162	Detected	Detected	Detected	Detected
5749	A_32_P934840	C8orf34	Hs.491941	chromosome 8 open reading frame 34	Homo sapiens chromosome 8 open reading frame 34 (C8orf34), mRNA [NM_052958]	2.85259	down	2.60545	down	27.51051	81.25890	20.41429	46.90112	Detected	Detected	Detected	Detected
5750	A_24_P136683	CA5BP	Hs.532326	carbonic anhydrase VB pseudogene	Homo sapiens carbonic anhydrase VB pseudogene (CA5BP), non-coding RNA [NR_026551]	2.85126	down	3.62421	down	53.27398	157.28429	47.90300	153.08800	Detected	Detected	Detected	Detected

5751	A_23_P256473	SEMA3C	Hs.269109	sema domain, immunoglobulin domain (Ig), short basic domain, secreted, (semaphorin) 3C	Homo sapiens sema domain, immunoglobulin domain (Ig), short basic domain, secreted, (semaphorin) 3C (SEMA3C), mRNA [NM_006379]	2.85076	down	2.57042	down	443.03766	1307.77930	348.35953	789.58325	Detected	Detected	Detected	Detected
5752	A_23_P166993	EPHB1	Hs.116092	EPH receptor B1	Homo sapiens EPH receptor B1 (EPHB1), mRNA [NM_004441]	2.85056	down	2.56736	down	103.46400	305.38837	88.78563	200.99918	Detected	Detected	Detected	Detected
5753	A_24_P20873	HIST1H4I	Hs.248172	histone cluster 1, H4i	Homo sapiens histone cluster 1, H4i (HIST1H4I), mRNA [NM_003495]	2.85010	down	2.79919	down	70.15601	207.04198	65.08009	160.63701	Detected	Detected	Detected	Detected
5754	A_23_P124300	BCMO1	Hs.212172	beta-carotene 15,15'-monooxygenase 1	Homo sapiens beta-carotene 15,15'-monooxygenase 1 (BCMO1), mRNA [NM_017429]	2.84954	down	2.32601	down	16.24094	47.92028	8.98558	18.42993	Detected	Detected	Compromised	Detected
5755	A_23_P349398	ZAR1	Hs.276457	zygote arrest 1	Homo sapiens zygote arrest 1 (ZAR1), mRNA [NM_175619]	2.84840	down	1.75472	down	7.72593	22.78685	14.09247	21.80524	Compromised	Detected	Compromised	Detected
5756	A_23_P357760	ARSD	Hs.528631	arylsulfatase D	Homo sapiens arylsulfatase D (ARSD), transcript variant 2, mRNA [NM_009589]	2.84809	down	2.87236	down	48.94005	144.32819	54.39506	137.77270	Detected	Detected	Detected	Detected
5757	A_23_P414913	GLIPR2	Hs.493819	GLI pathogenesis-related 2	Homo sapiens GLI pathogenesis-related 2 (GLIPR2), mRNA [NM_022343]	2.84742	down	2.89624	down	3772.81570	11123.73600	5311.05100	13563.75000	Detected	Detected	Detected	Detected
5758	A_23_P99906	HOMER2	Hs.578443	homer homolog 2 (Drosophila)	Homo sapiens homer homolog 2 (Drosophila) (HOMER2), transcript variant 2, mRNA [NM_199330]	2.84708	down	2.46108	down	63.56295	187.38577	62.03057	134.61620	Detected	Detected	Detected	Detected
5759	A_33_P3377209	ENSA	Hs.632456	endosulfine alpha	Homo sapiens endosulfine alpha (ENSA), transcript variant 8, mRNA [NM_207168]	2.84630	down	2.88374	down	428.56778	1263.08520	299.63043	761.91705	Detected	Detected	Detected	Detected
5760	A_33_P3372426	ADAMTS5	Hs.58324	ADAM metallopeptidase with thrombospondin type 1 motif, 5	Homo sapiens ADAM metallopeptidase with thrombospondin type 1 motif, 5 (ADAMTS5), mRNA [NM_007038]	2.84608	down	2.43879	down	3666.35030	10804.73300	2374.10520	5105.52440	Detected	Detected	Detected	Detected
5761	A_23_P145657	STAG3	Hs.592283	stromal antigen 3	Homo sapiens stromal antigen 3 (STAG3), mRNA [NM_012447]	2.84447	down	3.06051	down	148.51106	437.41492	128.02700	345.51025	Detected	Detected	Detected	Detected
5762	A_24_P65121	TTC23	Hs.513195	tetratricopeptide repeat domain 23	Homo sapiens tetratricopeptide repeat domain 23 (TTC23), transcript variant 1, mRNA [NM_001040655]	2.84443	down	1.64532	down	14.83880	43.70466	10.54224	15.29494	Detected	Detected	Compromised	Compromised
5763	A_33_P3380417	SLC25A30	Hs.591230	solute carrier family 25, member 30	Homo sapiens solute carrier family 25, member 30 (SLC25A30), mRNA [NM_001010875]	2.84300	down	2.95304	down	377.31598	1110.74680	281.06293	731.87740	Detected	Detected	Detected	Detected
5764	A_33_P3381313	FAM160A1	Hs.633810	family with sequence similarity 160, member A1	Homo sapiens family with sequence similarity 160, member A1 (FAM160A1), mRNA [NM_001109977]	2.84269	down	1.03519	down	8.80203	25.90867	18.60300	16.98121	Compromised	Detected	Detected	Detected
5765	A_33_P3251940	LOC100131608	Hs.650715	hypothetical LOC100131608	PREDICTED: Homo sapiens hypothetical LOC100131608 (LOC100131608), mRNA [XM_001715126]	2.84114	down	1.36759	down	6.93965	20.41564	4.59221	5.53787	Compromised	Detected	Compromised	Compromised
5766	A_23_P94141		Hs.30561		Fibrinogen silencer-binding protein [Source:UniProtKB/Swiss-Prot;Acc:O95073] [ENST00000481490]	2.84102	down	2.66058	down	44.87030	131.99780	33.64722	78.93891	Detected	Detected	Detected	Detected
5767	A_23_P217637	TIMM8A	Hs.447877	translocase of inner mitochondrial membrane 8 homolog A (yeast)	Homo sapiens translocase of inner mitochondrial membrane 8 homolog A (yeast) (TIMM8A), nuclear gene encoding mitochondrial protein, transcript variant 1, mRNA [NM_004085]	2.83829	down	2.72489	down	732.62760	2153.14450	778.02980	1869.43580	Detected	Detected	Detected	Detected
5768	A_33_P3236568	LCE2D	Hs.490225	late cornified envelope 2D	Homo sapiens late cornified envelope 2D (LCE2D), mRNA [NM_178430]	2.83491	down	2.16293	down	12.01387	35.26597	5.55010	10.58545	Detected	Detected	Compromised	Compromised
5769	A_24_P384200					2.83421	down	2.66940	down	853.39120	2504.45730	89.53719	210.75713	Detected	Detected	Detected	Detected
5770	A_33_P3227556					2.83347	down	3.73158	down	94.60057	277.55237	57.66731	189.75280	Detected	Detected	Detected	Detected
5771	A_24_P54131	DCLRE1B	Hs.591412	DNA cross-link repair 1B (PSO2 homolog, S. cerevisiae)	Homo sapiens DNA cross-link repair 1B (PSO2 homolog, S. cerevisiae) (DCLRE1B), mRNA [NM_022836]	2.83330	down	2.39591	down	217.30606	637.52435	154.04399	325.44750	Detected	Detected	Detected	Detected
5772	A_33_P3661631	PCBD2	Hs.710014	pterin-4 alpha-carbinolamine dehydratase/dimerization cofactor of hepatocyte nuclear factor 1 alpha (TCF1) 2	Homo sapiens pterin-4 alpha-carbinolamine dehydratase/dimerization cofactor of hepatocyte nuclear factor 1 alpha (TCF1) 2 (PCBD2), mRNA [NM_032151]	2.83230	down	2.24508	down	121.33881	355.85443	96.63998	191.31761	Detected	Detected	Detected	Detected
5773	A_24_P186216	PDS5A	Hs.331431	PDS5, regulator of cohesion maintenance, homolog A (S. cerevisiae)	Homo sapiens PDS5, regulator of cohesion maintenance, homolog A (S. cerevisiae) (PDS5A), transcript variant 3, mRNA [NM_001100400]	2.83195	down	2.73586	down	313.74120	920.00510	192.64104	464.73816	Detected	Detected	Detected	Detected
5774	A_23_P323685	HIST1H4H	Hs.591790	histone cluster 1, H4h	Homo sapiens histone cluster 1, H4h (HIST1H4H), mRNA [NM_003543]	2.82938	down	2.92027	down	99.91931	292.73413	82.56385	212.60733	Detected	Detected	Detected	Detected
5775	A_33_P3364989	C20orf134	Hs.592151	chromosome 20 open reading frame 134	Homo sapiens chromosome 20 open reading frame 134 (C20orf134), mRNA [NM_001024675]	2.82925	down	3.17200	down	184.42924	540.29890	169.56700	474.28494	Detected	Detected	Detected	Detected
5776	A_24_P739582					2.82909	down	2.58908	down	158.73729	465.00674	25.88620	59.09896	Detected	Detected	Detected	Detected
5777	A_24_P212024				Putative uncharacterized protein ENSP00000374793 [Source:UniProtKB/TrEMBL;Acc:A6NED6] [ENST00000498435]	2.82864	down	1.63730	down	47.94943	140.44121	39.05457	56.38527	Detected	Detected	Detected	Detected
5778	A_24_P277934	COL1A2	Hs.489142	collagen, type I, alpha 2	Homo sapiens collagen, type I, alpha 2 (COL1A2), mRNA [NM_000089]	2.82816	down	2.87648	down	68040.57000	199252.80000	52074.42000	132084.42000	Detected	Detected	Detected	Detected
5779	A_23_P36562	ITGA5	Hs.505654	integrin, alpha 5 (fibronectin receptor, alpha polypeptide)	Homo sapiens integrin, alpha 5 (fibronectin receptor, alpha polypeptide) (ITGA5), mRNA [NM_002205]	2.82788	down	3.21559	down	3545.26900	10381.09500	3820.78000	10833.74900	Detected	Detected	Detected	Detected
5780	A_24_P65616	PVR	Hs.171844	poliovirus receptor	Homo sapiens poliovirus receptor (PVR), transcript variant 1, mRNA [NM_006505]	2.82763	down	3.37357	down	976.36597	2858.69900	690.17554	2053.12100	Detected	Detected	Detected	Detected
5781	A_24_P177585	GEN1	Hs.467793	Gen homolog 1, endonuclease (Drosophila)	Homo sapiens Gen homolog 1, endonuclease (Drosophila) (GEN1), transcript variant 1, mRNA [NM_182625]	2.82626	down	2.85019	down	27.77951	81.29620	14.92911	37.52087	Detected	Detected	Detected	Detected

5782	A_23_P206960	SEC14L1	Hs.464184	SEC14-like 1 (S. cerevisiae)	Homo sapiens SEC14-like 1 (S. cerevisiae) (SEC14L1), transcript variant 1, mRNA [NM_003003]	2.82504	down	3.08211	down	1894.66710	5542.30600	1401.39800	3808.68120	Detected	Detected	Detected	Detected
5783	A_33_P3310274				Leucine-rich repeat-containing G-protein coupled receptor 6 Precursor [Source:UniProtKB/Swiss-Prot;Acc:Q9HBX8] [ENST00000420582]	2.82495	down	2.67037	down	13.79512	40.35233	13.31105	31.34366	Detected	Detected	Compromised	Detected
5784	A_33_P3356255				Annexin A11 (Annexin-11)(Annexin XI)(Calcyclin-associated annexin 50)(CAP-50)(56 kDa autoantigen) [Source:UniProtKB/Swiss-Prot;Acc:P50995] [ENST00000372219]	2.82488	down	4.21063	down	101.17352	295.93726	73.14206	271.56890	Detected	Detected	Detected	Detected
5785	A_23_P319565	PGBD3	Hs.654449	piggyBac transposable element derived 3	Homo sapiens piggyBac transposable element derived 3 (PGBD3), mRNA [NM_170753]	2.82231	down	2.88236	down	928.10410	2712.27500	801.00214	2035.85860	Detected	Detected	Detected	Detected
5786	A_23_P167479	IL17B	Hs.156979	interleukin 17B	Homo sapiens interleukin 17B (IL17B), mRNA [NM_014443]	2.82188	down	2.52967	down	17.24251	50.38161	17.19603	38.35809	Detected	Detected	Detected	Detected
5787	A_33_P3328666	CLTB	Hs.484241	clathrin, light chain (Lcb)	Homo sapiens clathrin, light chain (Lcb) (CLTB), transcript variant 2, mRNA [NM_007097]	2.81975	down	3.28659	down	838.24400	2447.45360	308.74893	894.77954	Detected	Detected	Detected	Detected
5788	A_23_P212696	FSTL1	Hs.269512	follistatin-like 1	Homo sapiens follistatin-like 1 (FSTL1), mRNA [NM_007085]	2.81930	down	2.93837	down	46115.00000	134622.40000	36858.75400	95501.88000	Detected	Detected	Detected	Detected
5789	A_33_P3368301	BOLA3	Hs.61472	bolA homolog 3 (E. coli)	Homo sapiens bolA homolog 3 (E. coli) (BOLA3), transcript variant 1, mRNA [NM_212552]	2.81827	down	2.57141	down	899.16250	2623.93850	407.87054	924.82580	Detected	Detected	Detected	Detected
5790	A_23_P205177	F10	Hs.361463	coagulation factor X	Homo sapiens coagulation factor X (F10), mRNA [NM_000504]	2.81744	down	2.99578	down	799.94440	2333.71200	950.68036	2511.36520	Detected	Detected	Detected	Detected
5791	A_23_P47148	NOX4	Hs.371036	NADPH oxidase 4	Homo sapiens NADPH oxidase 4 (NOX4), transcript variant 1, mRNA [NM_016931]	2.81733	down	2.72117	down	332.40770	969.71070	271.24277	650.84640	Detected	Detected	Detected	Detected
5792	A_33_P3397233		Hs.576854		DA992326 SYNOV4 Homo sapiens cDNA clone SYNOV4004518 5', mRNA sequence [DA992326]	2.81722	down	1.28605	down	14.50356	42.30858	21.80190	24.72399	Detected	Detected	Detected	Detected
5793	A_33_P3359753	C1orf96	Hs.585011	chromosome 1 open reading frame 96	Homo sapiens chromosome 1 open reading frame 96 (C1orf96), mRNA [NM_145257]	2.81607	down	2.80603	down	1218.26260	3552.36060	856.10220	2118.28120	Detected	Detected	Detected	Detected
5794	A_24_P36944	CEP170	Hs.533635	centrosomal protein 170kDa	Homo sapiens centrosomal protein 170kDa (CEP170), transcript variant alpha, mRNA [NM_014812]	2.81537	down	3.01124	down	91.06509	265.47340	65.83502	174.81049	Detected	Detected	Detected	Detected
5795	A_33_P3376527	CHST10	Hs.516370	carbohydrate sulfotransferase 10	Homo sapiens carbohydrate sulfotransferase 10 (CHST10), mRNA [NM_004854]	2.81124	down	3.08844	down	36.19944	105.37380	34.63421	94.32127	Detected	Detected	Detected	Detected
5796	A_23_P435521	TMEM106A	Hs.536474	transmembrane protein 106A	Homo sapiens transmembrane protein 106A (TMEM106A), mRNA [NM_145041]	2.81069	down	3.22372	down	13.62472	39.65275	10.31649	29.32615	Compromised	Detected	Compromised	Detected
5797	A_33_P3360382	MEG8		maternally expressed 8 (non-protein coding)	Homo sapiens maternally expressed 8 (non-protein coding) (MEG8), non-coding RNA [NR_024149]	2.81026	down	2.04787	down	35.93178	104.55823	33.20224	59.95642	Detected	Detected	Detected	Detected
5798	A_33_P3244021	MAVS	Hs.570362	mitochondrial antiviral signaling protein	Homo sapiens mitochondrial antiviral signaling protein (MAVS), nuclear gene encoding mitochondrial protein, mRNA [NM_020746]	2.80984	down	2.84718	down	1661.90890	4835.28960	1254.75920	3150.22200	Detected	Detected	Detected	Detected
5799	A_33_P3309289		Hs.591947		CMP-N-acetylneuraminate-beta-galactosamide-alpha-2,3-sialyltransferase (Beta-galactoside alpha-2,3-sialyltransferase)(EC 2.4.99.-)(Alpha 2,3-sialyltransferase IV)(Alpha 2,3-ST)(Gal-NAc6S)(STZ)(SIAT4-C)(ST3Gal III)(SAT-3)(ST-4) [Source:UniProtKB/Swiss-Prot;Acc:Q11206] [ENST00000239597]	2.80723	down	2.66477	down	24.08888	70.02098	21.26356	49.96451	Detected	Detected	Detected	Detected
5800	A_23_P34915	ATF3	Hs.460	activating transcription factor 3	Homo sapiens activating transcription factor 3 (ATF3), transcript variant 4, mRNA [NM_001040619]	2.80710	down	2.84920	down	1185.92400	3447.05420	912.66490	2292.97800	Detected	Detected	Detected	Detected
5801	A_23_P404730	SNX26	Hs.515364	sorting nexin 26	Homo sapiens sorting nexin 26 (SNX26), mRNA [NM_052948]	2.80701	down	3.77333	down	39.82050	115.73998	30.90159	102.81837	Detected	Detected	Detected	Detected
5802	A_33_P3307980				Homo sapiens cDNA FLJ16364 fis, clone THYMU2032976. [AK131345]	2.80601	down	2.98574	down	64.99880	188.85439	16.29204	42.89371	Detected	Detected	Detected	Detected
5803	A_33_P3397658	SYNPO		synaptopodin	Synaptopodin [Source:UniProtKB/Swiss-Prot;Acc:Q8N3V7] [ENST00000394243]	2.80589	down	3.32048	down	61.49945	178.67955	35.80001	104.82121	Detected	Detected	Detected	Detected
5804	A_24_P20383	ARPC4	Hs.323342	actin related protein 2/3 complex, subunit 4, 20kDa	Homo sapiens actin related protein 2/3 complex, subunit 4, 20kDa (ARPC4), transcript variant 1, mRNA [NM_005718]	2.80582	down	2.86767	down	296.60175	861.71967	285.19702	721.17303	Detected	Detected	Detected	Detected
5805	A_24_P307974				Transcription initiation factor TFIID subunit 8 (TBP-associated factor 8)(Transcription initiation factor TFIID 43 kDa subunit)(TBP-associated factor 43 kDa)(hTAFII43)(Protein taube nuss) [Source:UniProtKB/Swiss-Prot;Acc:Q7Z7C8] [ENST00000372978]	2.80480	down	2.60120	down	129.15000	375.08508	204.22640	468.43686	Detected	Detected	Detected	Detected
5806	A_24_P413126	PMEPA1	Hs.517155	prostate transmembrane protein, androgen induced 1	Homo sapiens prostate transmembrane protein, androgen induced 1 (PMEPA1), transcript variant 1, mRNA [NM_020182]	2.80414	down	2.94721	down	5515.95000	16015.94400	3364.78610	8744.47700	Detected	Detected	Detected	Detected
5807	A_23_P343837	PARP11	Hs.657268	poly (ADP-ribose) polymerase family, member 11	Homo sapiens poly (ADP-ribose) polymerase family, member 11 (PARP11), mRNA [NM_020367]	2.80400	down	2.00975	down	37.74524	109.59040	32.87626	58.26250	Detected	Detected	Detected	Detected

5808	A_33_P3230526	MPRIP	Hs.462341	myosin phosphatase Rho interacting protein	Homo sapiens myosin phosphatase Rho interacting protein (MPRIP), transcript variant 1, mRNA [NM_015134]	2.80174	down	3.64604	down	380.14500	1102.83510	360.09558	1157.72460	Detected	Detected	Detected	Detected
5809	A_23_P77401	CPPED1	Hs.460002	calcineurin-like phosphoesterase domain containing 1	Homo sapiens calcineurin-like phosphoesterase domain containing 1 (CPPED1), transcript variant 1, mRNA [NM_018340]	2.80106	down	2.59022	down	1294.42420	3754.33080	962.52026	2198.42680	Detected	Detected	Detected	Detected
5810	A_23_P58819	RANBP17	Hs.410810	RAN binding protein 17	Homo sapiens RAN binding protein 17 (RANBP17), mRNA [NM_022897]	2.80029	down	2.57896	down	171.39369	496.97168	149.00568	338.85400	Detected	Detected	Detected	Detected
5811	A_23_P370989	MCM4	Hs.460184	minichromosome maintenance complex component 4	Homo sapiens minichromosome maintenance complex component 4 (MCM4), transcript variant 1, mRNA [NM_005914]	2.79776	down	2.91650	down	391.27927	1133.52300	325.44320	836.95610	Detected	Detected	Detected	Detected
5812	A_23_P165608	SEMA4F	Hs.25887	sema domain, immunoglobulin domain (Ig), transmembrane domain (TM) and short cytoplasmic domain, (semaphorin) 4F	Homo sapiens sema domain, immunoglobulin domain (Ig), transmembrane domain (TM) and short cytoplasmic domain, (semaphorin) 4F (SEMA4F), mRNA [NM_004263]	2.79536	down	4.49420	down	104.54990	302.61780	44.46466	176.21089	Detected	Detected	Detected	Detected
5813	A_33_P3407356	LOC731656	Hs.702477	hypothetical LOC731656	Homo sapiens hypothetical LOC731656 (LOC731656), non-coding RNA [NR_027454]	2.79308	down	7.38485	down	10.73097	31.03528	3.46833	22.58541	Compromised	Detected	Compromised	Detected
5814	A_33_P3408221	LOC729799		SEC14-like 1 pseudogene	Homo sapiens SEC14-like 1 pseudogene (LOC729799), non-coding RNA [NR_026952]	2.79248	down	2.74570	down	426.58786	1233.47740	390.06010	944.38617	Detected	Detected	Detected	Detected
5815	A_33_P3327165	CCDC18	Hs.716682	coiled-coil domain containing 18	Homo sapiens coiled-coil domain containing 18 (CCDC18), mRNA [NM_206886]	2.79160	down	2.49488	down	242.87560	702.05280	194.23943	427.31998	Detected	Detected	Detected	Detected
5816	A_24_P204244	ANXA2P1	Hs.546235	annexin A2 pseudogene 1	Homo sapiens annexin A2 pseudogene 1 (ANXA2P1), non-coding RNA [NR_001562]	2.79143	down	2.89681	down	26224.94000	75800.86000	26965.82200	68880.94500	Detected	Detected	Detected	Detected
5817	A_23_P73208	GABPB2	Hs.654699	GA binding protein transcription factor, beta subunit 2	Homo sapiens GA binding protein transcription factor, beta subunit 2 (GABPB2), mRNA [NM_144618]	2.78768	down	3.94670	down	131.80171	380.44992	98.16433	341.62784	Detected	Detected	Detected	Detected
5818	A_33_P3343145	MAP1B	Hs.335079	microtubule-associated protein 1B	Homo sapiens microtubule-associated protein 1B (MAP1B), mRNA [NM_005909]	2.78747	down	2.51066	down	67.20338	193.97003	82.20322	181.98758	Detected	Detected	Detected	Detected
5819	A_24_P824592	RBMX	Hs.380118	RNA binding motif protein, X-linked	Homo sapiens RNA binding motif protein, X-linked (RBMX), transcript variant 1, mRNA [NM_002139]	2.78353	down	2.26550	down	142.08969	409.53586	72.17301	144.17990	Detected	Detected	Detected	Detected
5820	A_23_P403886	GLYAT	Hs.145384	glycine-N-acyltransferase	Homo sapiens glycine-N-acyltransferase (GLYAT), nuclear gene encoding mitochondrial protein, transcript variant 2, mRNA [NM_005838]	2.78224	down	1.66768	down	32.99304	95.04960	48.50984	71.33591	Detected	Detected	Detected	Detected
5821	A_33_P3282978				Brain and acute leukemia cytoplasmic protein [Source:UniProtKB/Swiss-Prot;Acc:Q8WXS3] [ENST00000306391]	2.78135	down	2.23064	down	927.42770	2670.97020	146.13687	287.44458	Detected	Detected	Detected	Detected
5822	A_33_P3288104				Disheveled-associated activator of morphogenesis 2 [Source:UniProtKB/Swiss-Prot;Acc:Q86T65] [ENST00000405961]	2.78130	down	2.95597	down	175.52922	505.51105	129.11739	336.55048	Detected	Detected	Detected	Detected
5823	A_23_P204484	RAB35	Hs.524788	RAB35, member RAS oncogene family	Homo sapiens RAB35, member RAS oncogene family (RAB35), transcript variant 1, mRNA [NM_006861]	2.77894	down	2.91155	down	3892.51270	11200.62100	3955.87060	10156.21200	Detected	Detected	Detected	Detected
5824	A_23_P48056	CKAP4	Hs.74368	cytoskeleton-associated protein 4	Homo sapiens cytoskeleton-associated protein 4 (CKAP4), mRNA [NM_006825]	2.77724	down	2.82307	down	5516.12900	15862.84000	2588.54370	6443.81300	Detected	Detected	Detected	Detected
5825	A_24_P943095	CYTSB	Hs.431045	cytospin B	Homo sapiens cytospin B (CYTSB), transcript variant NSP5beta3beta, mRNA [NM_001033553]	2.77506	down	2.35567	down	7.92652	22.77654	5.56396	11.55751	Compromised	Detected	Compromised	Compromised
5826	A_23_P211445	LIMK2	Hs.474596	LIM domain kinase 2	Homo sapiens LIM domain kinase 2 (LIMK2), transcript variant 2b, mRNA [NM_016733]	2.77078	down	2.97249	down	1460.39330	4189.91260	1018.28600	2669.04800	Detected	Detected	Detected	Detected
5827	A_33_P3255794	POLE	Hs.524871	polymerase (DNA directed), epsilon	Homo sapiens polymerase (DNA directed), epsilon (POLE), mRNA [NM_006231]	2.77059	down	2.08303	down	13.06518	37.48183	14.74840	27.08976	Compromised	Detected	Compromised	Detected
5828	A_24_P123408	ABLIM3	Hs.49688	actin binding LIM protein family, member 3	Homo sapiens actin binding LIM protein family, member 3 (ABLIM3), mRNA [NM_014945]	2.77014	down	3.13022	down	392.69610	1126.39860	423.11890	1167.89310	Detected	Detected	Detected	Detected
5829	A_24_P867342	AFMID		arylformamidase	Homo sapiens arylformamidase (AFMID), transcript variant 3, transcribed RNA [NR_027083]	2.76935	down	2.98407	down	41.14783	117.99358	41.07729	108.08778	Detected	Detected	Detected	Detected
5830	A_33_P3351120	TXNRD1	Hs.654922	thioredoxin reductase 1	Homo sapiens thioredoxin reductase 1 (TXNRD1), transcript variant 1, mRNA [NM_003330]	2.76764	down	2.60614	down	251.36260	720.34880	296.90070	682.29834	Detected	Detected	Detected	Detected
5831	A_23_P163455	MAP1A	Hs.194301	microtubule-associated protein 1A	Homo sapiens microtubule-associated protein 1A (MAP1A), mRNA [NM_002373]	2.76546	down	3.05788	down	1430.77220	4097.04800	1251.72130	3375.15360	Detected	Detected	Detected	Detected
5832	A_24_P937325				FYVE, RhoGEF and PH domain-containing protein 4 (Actin filament-binding protein frabin)(FGD1-related F-actin-binding protein)(Zinc finger FYVE domain-containing protein 6) [Source:UniProtKB/Swiss-Prot;Acc:Q96M96] [ENST00000395742]	2.76530	down	2.86912	down	20.78519	59.51542	14.10827	35.69336	Detected	Detected	Detected	Detected
5833	A_23_P36888	FAM113B	Hs.560100	family with sequence similarity 113, member B	Homo sapiens family with sequence similarity 113, member B (FAM113B), mRNA [NM_138371]	2.76480	down	2.56070	down	99.67381	285.35016	115.46590	260.72230	Detected	Detected	Detected	Detected
5834	A_33_P3405204	LOC729911	Hs.513931	hypothetical LOC729911	PREDICTED: Homo sapiens hypothetical LOC729911 (LOC729911), mRNA [XM_001134184]	2.76365	down	5.47977	down	8.25306	23.61732	2.60537	12.58916	Compromised	Detected	Compromised	Compromised

5835	A_23_P106127	KIAA0586	Hs.232532	KIAA0586	Homo sapiens KIAA0586 (KIAA0586), mRNA [NM_014749]	2.76223	down	2.78690	down	705.65870	2018.30770	478.54684	1176.01120	Detected	Detected	Detected	Detected
5836	A_33_P3337450				Putative uncharacterized protein FLJ34945 [Source:UniProtKB/Swiss-Prot;Acc:Q8NAQ8] [ENST00000432274]	2.76221	down	1.81172	down	22.67218	64.84600	15.24147	24.34909	Detected	Detected	Detected	Detected
5837	A_23_P203488	SMPD1	Hs.498173	sphingomyelin phosphodiesterase 1, acid lysosomal	Homo sapiens sphingomyelin phosphodiesterase 1, acid lysosomal (SMPD1), transcript variant ASM-1, mRNA [NM_000543]	2.76033	down	3.10795	down	1617.96600	4624.48050	1497.21150	4103.19500	Detected	Detected	Detected	Detected
5838	A_23_P46309	RCC1	Hs.469723	regulator of chromosome condensation 1	Homo sapiens regulator of chromosome condensation 1 (RCC1), transcript variant 1, mRNA [NM_001048194]	2.75993	down	2.90787	down	193.28334	552.36390	279.18826	715.87573	Detected	Detected	Detected	Detected
5839	A_33_P3230166	NALCN	Hs.525146	sodium leak channel, non-selective	Homo sapiens sodium leak channel, non-selective (NALCN), mRNA [NM_052867]	2.75838	down	2.31453	down	61.36000	175.25560	32.31105	65.94448	Detected	Detected	Detected	Detected
5840	A_23_P118158	HS3ST2	Hs.720257	heparan sulfate (glucosamine) 3-O-sulfotransferase 2	Homo sapiens heparan sulfate (glucosamine) 3-O-sulfotransferase 2 (HS3ST2), mRNA [NM_006043]	2.75793	down	2.84155	down	611.00050	1744.84790	512.09920	1283.14380	Detected	Detected	Detected	Detected
5841	A_23_P32135	C9orf9	Hs.62595	chromosome 9 open reading frame 9	Homo sapiens chromosome 9 open reading frame 9 (C9orf9), mRNA [NM_018956]	2.75741	down	2.60891	down	116.79298	333.46588	135.05087	310.68590	Detected	Detected	Detected	Detected
5842	A_23_P120125	COLEC11	Hs.32603	collectin sub-family member 11	Homo sapiens collectin sub-family member 11 (COLEC11), transcript variant 2, mRNA [NM_199235]	2.75507	down	2.56729	down	22.98119	65.55999	18.79908	42.55752	Detected	Detected	Detected	Detected
5843	A_24_P363100	RGMB	Hs.526902	RGM domain family, member B	Homo sapiens RGM domain family, member B (RGMB), mRNA [NM_001012761]	2.75313	down	2.89126	down	280.16403	798.67840	394.51117	1005.80200	Detected	Detected	Detected	Detected
5844	A_33_P3280603	PPP4R1L	Hs.473179	protein phosphatase 4, regulatory subunit 1-like	Homo sapiens protein phosphatase 4, regulatory subunit 1-like (PPP4R1L), non-coding RNA [NR_003505]	2.75280	down	3.04041	down	43.26352	123.31873	43.98033	117.91144	Detected	Detected	Detected	Detected
5845	A_33_P3295200	HAS1	Hs.57697	hyaluronan synthase 1	Homo sapiens hyaluronan synthase 1 (HAS1), mRNA [NM_001523]	2.74838	down	3.59578	down	22.04640	62.74035	27.31606	86.61165	Detected	Detected	Detected	Detected
5846	A_33_P3298612					2.74695	down	1.94244	down	9.03694	25.70428	8.93457	15.30335	Compromised	Detected	Compromised	Detected
5847	A_33_P3249439	NCAPH2	Hs.180903	non-SMC condensin II complex, subunit H2	Homo sapiens non-SMC condensin II complex, subunit H2 (NCAPH2), transcript variant 2, mRNA [NM_152299]	2.74255	down	2.88416	down	9450.85000	26838.54700	8418.79000	21410.91400	Detected	Detected	Detected	Detected
5848	A_23_P67725	LMNB2	Hs.538286	lamin B2	Homo sapiens lamin B2 (LMNB2), mRNA [NM_032737]	2.74158	down	2.59806	down	2246.70730	6377.94900	1009.52484	2312.76440	Detected	Detected	Detected	Detected
5849	A_23_P149975	FAM107B	Hs.446315	family with sequence similarity 107, member B	Homo sapiens family with sequence similarity 107, member B (FAM107B), mRNA [NM_031453]	2.74158	down	2.55354	down	2922.19820	8295.51100	2649.31500	5965.42400	Detected	Detected	Detected	Detected
5850	A_23_P165521	STRN	Hs.656726	striatin, calmodulin binding protein	Homo sapiens striatin, calmodulin binding protein (STRN), mRNA [NM_003162]	2.73976	down	2.37791	down	34.30307	97.31465	42.95839	90.07593	Detected	Detected	Detected	Detected
5851	A_32_P70203	FAM41C	Hs.449006	family with sequence similarity 41, member C	Homo sapiens family with sequence similarity 41, member C (FAM41C), non-coding RNA [NR_027055]	2.73953	down	1.17888	down	35.19427	99.83477	3.14977	3.27428	Detected	Detected	Compromised	Compromised
5852	A_33_P3218905	FAM13AOS	Hs.703446	FAM13A opposite strand (non-protein coding)	Homo sapiens FAM13A opposite strand (non-protein coding) (FAM13AOS), non-coding RNA [NR_002806]	2.73909	down	2.46612	down	37.77195	107.12952	23.80614	51.76888	Detected	Detected	Detected	Detected
5853	A_23_P341275	POP1	Hs.252828	processing of precursor 1, ribonuclease P/MRP subunit (S. cerevisiae)	Homo sapiens processing of precursor 1, ribonuclease P/MRP subunit (S. cerevisiae) (POP1), transcript variant 3, mRNA [NM_015029]	2.73666	down	2.83087	down	1654.27370	4687.72300	1311.80800	3274.57860	Detected	Detected	Detected	Detected
5854	A_24_P12413	TRAM2	Hs.520182	translocation associated membrane protein 2	Homo sapiens translocation associated membrane protein 2 (TRAM2), mRNA [NM_012288]	2.73623	down	2.57238	down	4790.47200	13572.64700	3906.67240	8861.51500	Detected	Detected	Detected	Detected
5855	A_24_P383598					2.73452	down	2.23353	down	443.29926	1255.19560	73.45451	144.66933	Detected	Detected	Detected	Detected
5856	A_24_P205213	ARSB	Hs.149103	arylsulfatase B	Homo sapiens arylsulfatase B (ARSB), transcript variant 2, mRNA [NM_198709]	2.73162	down	2.61913	down	111.82578	316.29755	139.22707	321.54830	Detected	Detected	Detected	Detected
5857	A_23_P153360	C19orf42	Hs.356467	chromosome 19 open reading frame 42	Homo sapiens chromosome 19 open reading frame 42 (C19orf42), mRNA [NM_024104]	2.73159	down	2.87773	down	1466.28810	4147.33000	1190.49680	3020.94920	Detected	Detected	Detected	Detected
5858	A_23_P88222	PLD4	Hs.407101	phospholipase D family, member 4	Homo sapiens phospholipase D family, member 4 (PLD4), mRNA [NM_138790]	2.73117	down	1.45187	down	7.57155	21.41245	4.13943	5.29950	Compromised	Detected	Compromised	Compromised
5859	A_33_P3272558	NCAPH2	Hs.180903	non-SMC condensin II complex, subunit H2	Homo sapiens non-SMC condensin II complex, subunit H2 (NCAPH2), transcript variant 2, mRNA [NM_152299]	2.73010	down	2.70022	down	9339.20500	26401.06800	9307.85900	22162.29500	Detected	Detected	Detected	Detected
5860	A_33_P3842551	IKZF2	Hs.604950	IKAROS family zinc finger 2 (Helios)	Homo sapiens IKAROS family zinc finger 2 (Helios) (IKZF2), transcript variant 2, mRNA [NM_001079526]	2.72992	down	3.73141	down	6.05244	17.10856	2.44329	8.03921	Compromised	Detected	Compromised	Compromised
5861	A_32_P809810	LOC100130433	Hs.638427	hypothetical protein LOC100130433	Homo sapiens cDNA FLJ38936 fis, clone NT2NE2015275. [AK096255]	2.72914	down	1.81372	down	73.74474	208.39592	40.71933	65.12342	Detected	Detected	Detected	Detected
5862	A_33_P3413523	DBF4	Hs.485380	DBF4 homolog (S. cerevisiae)	Homo sapiens DBF4 homolog (S. cerevisiae) (DBF4), mRNA [NM_006716]	2.72609	down	2.74662	down	241.25146	680.99347	128.35799	310.87576	Detected	Detected	Detected	Detected
5863	A_32_P148345	ANXA2	Hs.511605	annexin A2	Homo sapiens annexin A2 (ANXA2), transcript variant 2, mRNA [NM_001002857]	2.72543	down	2.27466	down	20438.35700	57678.43400	1060.35330	2126.82740	Detected	Detected	Detected	Detected
5864	A_33_P3238920	INPP5F	Hs.369755	inositol polyphosphate-5-phosphatase F	Homo sapiens inositol polyphosphate-5-phosphatase F (INPP5F), transcript variant 1, mRNA [NM_014937]	2.72499	down	2.52517	down	67.14090	189.44588	53.78373	119.75888	Detected	Detected	Detected	Detected
5865	A_23_P145761	ARL4A	Hs.245540	ADP-ribosylation factor-like 4A	Homo sapiens ADP-ribosylation factor-like 4A (ARL4A), transcript variant 1, mRNA [NM_005738]	2.72463	down	2.33214	down	283.53006	799.90686	201.56413	414.50912	Detected	Detected	Detected	Detected

5866	A_23_P142239	YIF1B	Hs.280741	Yip1 interacting factor homolog B (S. cerevisiae)	Homo sapiens Yip1 interacting factor homolog B (S. cerevisiae) (YIF1B), transcript variant 2, mRNA [NM_033557]	2.72344	down	3.83794	down	20.83361	58.75097	11.37190	38.48551	Detected	Detected	Compromised	Detected
5867	A_23_P391725	DR1	Hs.348418	down-regulator of transcription 1, TBP-binding (negative cofactor 2)	Homo sapiens down-regulator of transcription 1, TBP-binding (negative cofactor 2) (DR1), mRNA [NM_001938]	2.72033	down	2.54984	down	1734.64370	4886.12000	1154.77270	2596.41820	Detected	Detected	Detected	Detected
5868	A_23_P88626	ANPEP	Hs.1239	alanyl (membrane) aminopeptidase	Homo sapiens alanyl (membrane) aminopeptidase (ANPEP), mRNA [NM_001150]	2.71839	down	3.00064	down	7205.97360	20283.25400	7611.53860	20139.64300	Detected	Detected	Detected	Detected
5869	A_23_P100074	AVEN	Hs.555966	apoptosis, caspase activation inhibitor	Homo sapiens apoptosis, caspase activation inhibitor (AVEN), mRNA [NM_020371]	2.71674	down	2.74707	down	2960.03540	8326.81000	2313.94360	5605.15100	Detected	Detected	Detected	Detected
5870	A_23_P71644	FANCG	Hs.591084	Fanconi anemia, complementation group G	Homo sapiens Fanconi anemia, complementation group G (FANCG), mRNA [NM_004629]	2.71517	down	2.84118	down	1678.57390	4719.22500	1320.41400	3308.06420	Detected	Detected	Detected	Detected
5871	A_33_P3289045	GSTT2B	Hs.656498	glutathione S-transferase theta 2B (gene/pseudogene)	Homo sapiens glutathione S-transferase theta 2B (gene/pseudogene) (GSTT2B), mRNA [NM_001080843]	2.71213	down	2.53278	down	450.79840	1265.97580	691.95807	1545.40380	Detected	Detected	Detected	Detected
5872	A_23_P151820	RIN3	Hs.326822	Ras and Rab interactor 3	Homo sapiens Ras and Rab interactor 3 (RIN3), mRNA [NM_024832]	2.71206	down	3.28497	down	505.42352	1419.34660	440.24030	1275.22400	Detected	Detected	Detected	Detected
5873	A_24_P266285	IQCE	Hs.520627	IQ motif containing E	Homo sapiens IQ motif containing E (IQCE), transcript variant 1, mRNA [NM_152558]	2.71137	down	3.05621	down	138.20790	388.02097	174.45781	470.15268	Detected	Detected	Detected	Detected
5874	A_24_P302685	ARHGEF4	Hs.469935	Rho guanine nucleotide exchange factor (GEF) 4	Homo sapiens Rho guanine nucleotide exchange factor (GEF) 4 (ARHGEF4), transcript variant 1, mRNA [NM_015320]	2.70945	down	1.84893	down	3.91432	10.98175	8.35678	13.62465	Compromised	Compromised	Compromised	Detected
5875	A_23_P357316	UPP2	Hs.128427	uridine phosphorylase 2	Homo sapiens uridine phosphorylase 2 (UPP2), transcript variant 1, mRNA [NM_173355]	2.70832	down	1.14317	down	7.80181	21.87900	7.28632	7.34486	Compromised	Detected	Compromised	Compromised
5876	A_23_P55998	SLC1A5	Hs.631582	solute carrier family 1 (neutral amino acid transporter), member 5	Homo sapiens solute carrier family 1 (neutral amino acid transporter), member 5 (SLC1A5), transcript variant 1, mRNA [NM_005628]	2.70739	down	2.91471	down	994.70447	2788.54600	1100.99220	2829.72830	Detected	Detected	Detected	Detected
5877	A_33_P3413025		Hs.74647		Putative uncharacterized protein ENSP00000374964 [Source:UniProtKB/TrEMBL;Acc:A6NMI7] [ENST00000390457]	2.70633	down	2.72611	down	27.33961	76.61353	7.29396	17.53366	Detected	Detected	Compromised	Detected
5878	A_24_P133253	KITLG	Hs.1048	KIT ligand	Homo sapiens KIT ligand (KITLG), transcript variant b, mRNA [NM_000899]	2.70490	down	2.36191	down	2280.19500	6386.40400	1364.39650	2841.64620	Detected	Detected	Detected	Detected
5879	A_23_P209347	ANKRD44	Hs.654819	ankyrin repeat domain 44	Homo sapiens ankyrin repeat domain 44 (ANKRD44), mRNA [NM_153697]	2.70444	down	2.22571	down	21.50489	60.22086	21.18377	41.57541	Detected	Detected	Detected	Detected
5880	A_23_P41267	LOC401127	Hs.383197	WD repeat domain 5 pseudogene	Homo sapiens WD repeat domain 5 pseudogene (LOC401127), non-coding RNA [NR_026854]	2.70186	down	2.86633	down	334.72890	936.46070	336.61120	850.78670	Detected	Detected	Detected	Detected
5881	A_33_P3424864					2.69985	down	4.08761	down	12.31601	34.43050	6.09761	21.97832	Detected	Detected	Compromised	Detected
5882	A_23_P105873	TRPC4	Hs.262960	transient receptor potential cation channel, subfamily C, member 4	Homo sapiens transient receptor potential cation channel, subfamily C, member 4 (TRPC4), transcript variant alpha, mRNA [NM_016179]	2.69969	down	2.50528	down	86.88139	242.86981	78.24825	172.86086	Detected	Detected	Detected	Detected
5883	A_33_P3318122	C16orf62	Hs.654964	chromosome 16 open reading frame 62	Homo sapiens chromosome 16 open reading frame 62 (C16orf62), mRNA [NM_020314]	2.69875	down	2.81777	down	304.46762	850.81870	237.28877	589.58777	Detected	Detected	Detected	Detected
5884	A_33_P3357247	USP36	Hs.464243	ubiquitin specific peptidase 36	Homo sapiens ubiquitin specific peptidase 36 (USP36), mRNA [NM_025090]	2.69816	down	3.05170	down	2042.55550	5706.56150	1825.73410	4912.97300	Detected	Detected	Detected	Detected
5885	A_23_P362712	ABHD11	Hs.647045	abhydrolase domain containing 11	Homo sapiens abhydrolase domain containing 11 (ABHD11), transcript variant 6, mRNA [NM_148916]	2.69799	down	2.79960	down	311.63640	870.60520	409.26874	1010.34600	Detected	Detected	Detected	Detected
5886	A_33_P3340752					2.69784	down	3.28538	down	25.30535	70.69058	21.75145	63.01438	Detected	Detected	Detected	Detected
5887	A_23_P127175	SAR1A	Hs.499960	SAR1 homolog A (S. cerevisiae)	Homo sapiens SAR1 homolog A (S. cerevisiae) (SAR1A), transcript variant 2, mRNA [NM_020150]	2.69477	down	2.58337	down	12799.07600	35713.52000	11574.11500	26365.70000	Detected	Detected	Detected	Detected
5888	A_33_P3401647	PPP1R14A	Hs.631569	protein phosphatase 1, regulatory (inhibitor) subunit 14A	Homo sapiens protein phosphatase 1, regulatory (inhibitor) subunit 14A (PPP1R14A), mRNA [NM_033256]	2.69448	down	1.91201	down	35.51206	99.07933	28.80223	48.56027	Detected	Detected	Detected	Detected
5889	A_33_P3251985	MFSD1	Hs.58663	major facilitator superfamily domain containing 1	Homo sapiens major facilitator superfamily domain containing 1 (MFSD1), mRNA [NM_022736]	2.69425	down	2.62283	down	2021.91590	5640.70750	1849.33250	4277.10800	Detected	Detected	Detected	Detected
5890	A_23_P435636	DAND5	Hs.331981	DAN domain family, member 5	Homo sapiens DAN domain family, member 5 (DAND5), mRNA [NM_152654]	2.69356	down	4.58598	down	28.79373	80.30779	12.55294	50.76249	Detected	Detected	Compromised	Detected
5891	A_32_P52911	ADI1	Hs.502773	acireductone dioxygenase 1	Homo sapiens acireductone dioxygenase 1 (ADI1), mRNA [NM_018269]	2.69152	down	2.63400	down	1000.77290	2789.11720	714.53820	1659.61220	Detected	Detected	Detected	Detected
5892	A_23_P58002	TCTA	Hs.517962	T-cell leukemia translocation altered gene	Homo sapiens T-cell leukemia translocation altered gene (TCTA), mRNA [NM_022171]	2.69057	down	2.69221	down	514.23970	1432.65840	531.15250	1260.93630	Detected	Detected	Detected	Detected
5893	A_23_P85164	DNASE1L1	Hs.401929	deoxyribonuclease I-like 1	Homo sapiens deoxyribonuclease I-like 1 (DNASE1L1), transcript variant 1, mRNA [NM_006730]	2.69042	down	2.75214	down	833.20905	2321.17000	758.14100	1839.86550	Detected	Detected	Detected	Detected
5894	A_23_P40718	PARVB	Hs.475074	parvin, beta	Homo sapiens parvin, beta (PARVB), transcript variant 1, mRNA [NM_001003828]	2.69034	down	2.96786	down	733.58570	2043.57390	793.05600	2075.45480	Detected	Detected	Detected	Detected
5895	A_33_P3262475					2.68914	down	1.96806	down	9.39088	26.14885	8.60463	14.93264	Compromised	Detected	Compromised	Compromised
5896	A_33_P3243337	C19orf10	Hs.465645	chromosome 19 open reading frame 10	Homo sapiens chromosome 19 open reading frame 10 (C19orf10), mRNA [NM_019107]	2.68606	down	2.19481	down	60259.35500	167599.48000	51519.70700	99709.41400	Detected	Detected	Detected	Detected
5897	A_23_P99063	LUM	Hs.406475	lumican	Homo sapiens lumican (LUM), mRNA [NM_002345]	2.68582	down	2.67403	down	3847.00300	10698.72300	2716.49880	6405.33640	Detected	Detected	Detected	Detected

5898	A_23_P41166	B3GALNT1	Hs.418062	beta-1,3-N-acetylgalactosaminyltransferase 1 (globoside blood group)	Homo sapiens beta-1,3-N-acetylgalactosaminyltransferase 1 (globoside blood group) (B3GALNT1), transcript variant 5, mRNA [NM_001038628]	2.68576	down	2.97146	down	431.74652	1200.68570	358.65360	939.74740	Detected	Detected	Detected	Detected
5899	A_33_P3287680	C3orf43	Hs.631933	chromosome 3 open reading frame 43	Homo sapiens chromosome 3 open reading frame 43 (C3orf43), mRNA [NM_001077657]	2.68566	down	4.05640	down	24.22129	67.35677	14.36437	51.37987	Detected	Detected	Detected	Detected
5900	A_23_P39050	ZNF823	Hs.142167	zinc finger protein 823	Homo sapiens zinc finger protein 823 (ZNF823), mRNA [NM_001080493]	2.68554	down	2.52417	down	190.26483	529.08185	112.03851	249.37425	Detected	Detected	Detected	Detected
5901	A_24_P876522	GPX8	Hs.289044	glutathione peroxidase 8 (putative)	Homo sapiens glutathione peroxidase 8 (putative) (GPX8), mRNA [NM_001008397]	2.68237	down	2.65909	down	2686.06450	7460.49200	2674.32350	6270.64840	Detected	Detected	Detected	Detected
5902	A_33_P3242603	LOC645676	Hs.568693	hypothetical LOC645676	Homo sapiens hypothetical LOC645676 (LOC645676), non-coding RNA [NR_027023]	2.68230	down	3.03152	down	316.02106	877.72090	259.16720	692.79700	Detected	Detected	Detected	Detected
5903	A_33_P3402103		Hs.403187		UPF0490 protein C1orf201 [Source:UniProtKB/Swiss-Prot;Acc:Q5TH74] [ENST00000399974]	2.68160	down	2.37275	down	45.79633	127.16200	45.21000	94.59160	Detected	Detected	Detected	Detected
5904	A_23_P154605	SULF2	Hs.162016	sulfatase 2	Homo sapiens sulfatase 2 (SULF2), transcript variant 1, mRNA [NM_018837]	2.68092	down	3.21584	down	125.06792	347.18660	98.86739	280.35782	Detected	Detected	Detected	Detected
5905	A_33_P3336686	CLIC3	Hs.64746	chloride intracellular channel 3	Homo sapiens chloride intracellular channel 3 (CLIC3), mRNA [NM_004669]	2.67958	down	2.70641	down	549.24970	1523.94900	533.28420	1272.67660	Detected	Detected	Detected	Detected
5906	A_23_P76350	GNB3	Hs.631657	guanine nucleotide binding protein (G protein), beta polypeptide 3	Homo sapiens guanine nucleotide binding protein (G protein), beta polypeptide 3 (GNB3), mRNA [NM_002075]	2.67954	down	2.18385	down	282.53120	783.89740	64.16455	123.56165	Detected	Detected	Detected	Detected
5907	A_23_P136986	APOOL	Hs.706885	apolipoprotein O-like	Homo sapiens apolipoprotein O-like (APOOL), mRNA [NM_198450]	2.67786	down	2.53590	down	69.09710	191.59320	72.28335	161.63518	Detected	Detected	Detected	Detected
5908	A_23_P162171	MCAM	Hs.599039	melanoma cell adhesion molecule	Homo sapiens melanoma cell adhesion molecule (MCAM), mRNA [NM_006500]	2.67744	down	2.66438	down	797.15200	2210.00900	626.63226	1472.22620	Detected	Detected	Detected	Detected
5909	A_23_P430658	HEYL	Hs.472566	hairy/enhancer-of-split related with YRPW motif-like	Homo sapiens hairy/enhancer-of-split related with YRPW motif-like (HEYL), mRNA [NM_014571]	2.67656	down	2.38577	down	160.08390	443.66833	207.89192	437.35190	Detected	Detected	Detected	Detected
5910	A_23_P207766	ARHGDIA	Hs.159161	Rho GDP dissociation inhibitor (GDI) alpha	Homo sapiens Rho GDP dissociation inhibitor (GDI) alpha (ARHGDIA), mRNA [NM_004309]	2.67376	down	2.83762	down	2698.96480	7472.26860	3024.73000	7568.43550	Detected	Detected	Detected	Detected
5911	A_23_P93823	RFC2	Hs.647062	replication factor C (activator 1) 2, 40kDa	Homo sapiens replication factor C (activator 1) 2, 40kDa (RFC2), transcript variant 1, mRNA [NM_181471]	2.67289	down	2.60171	down	1035.79470	2866.73780	789.49396	1811.22990	Detected	Detected	Detected	Detected
5912	A_23_P128744	BDKRB1	Hs.525572	bradykinin receptor B1	Homo sapiens bradykinin receptor B1 (BDKRB1), mRNA [NM_000710]	2.67207	down	2.85950	down	336.64770	931.44210	293.69530	740.54620	Detected	Detected	Detected	Detected
5913	A_23_P203255	API5	Hs.435771	apoptosis inhibitor 5	Homo sapiens apoptosis inhibitor 5 (API5), transcript variant 2, mRNA [NM_006595]	2.67200	down	2.48771	down	198.85117	550.17194	205.20730	450.15073	Detected	Detected	Detected	Detected
5914	A_33_P3292660		Hs.377070		Glutamate receptor 3 Precursor (GluR-3)(GluR-C)(GluR-K3)(Glutamate receptor ionotropic, AMPA 3)(AMPA-selective glutamate receptor 3) [Source:UniProtKB/Swiss-Prot;Acc:P42263] [ENST00000335161]	2.66912	down	3.18850	down	96.61571	267.02325	103.32034	290.49475	Detected	Detected	Detected	Detected
5915	A_33_P3703637	LOC285300	Hs.638436	hypothetical protein LOC285300	Homo sapiens cDNA FLJ38336 fis, clone FCBBF3026678 [AK095655]	2.66779	down	2.31183	down	284.20280	785.07820	179.70844	366.34530	Detected	Detected	Detected	Detected
5916	A_32_P191859	SETD8	Hs.443735	SET domain containing (lysine methyltransferase) 8	Homo sapiens SET domain containing (lysine methyltransferase) 8 (SETD8), mRNA [NM_020382]	2.66736	down	3.68469	down	264.90700	731.65780	184.79523	600.42260	Detected	Detected	Detected	Detected
5917	A_33_P3395219	C1orf118	Hs.632414	chromosome 1 open reading frame 118	PREDICTED: Homo sapiens chromosome 1 open reading frame 118 (C1orf118), miscRNA [XR_041259]	2.66669	down	4.10713	down	51.74421	142.87856	32.09981	116.25349	Detected	Detected	Detected	Detected
5918	A_24_P647682	LOC442308		similar to tubulin, beta 5	Homo sapiens similar to tubulin, beta 5 (LOC442308), non-coding RNA [NR_003598]	2.66520	down	3.21971	down	3568.25600	9847.33200	1728.59420	4907.66260	Detected	Detected	Detected	Detected
5919	A_23_P319719	PGAM5	Hs.102558	phosphoglycerate mutase family member 5	Homo sapiens phosphoglycerate mutase family member 5 (PGAM5), mRNA [NM_138575]	2.66464	down	3.15127	down	160.68784	443.35846	205.26537	570.38275	Detected	Detected	Detected	Detected
5920	A_23_P89981	CYP2F1	Hs.558318	cytochrome P450, family 2, subfamily F, polypeptide 1	Homo sapiens cytochrome P450, family 2, subfamily F, polypeptide 1 (CYP2F1), mRNA [NM_000774]	2.66406	down	1.27286	down	5.51846	15.22285	20.69836	23.23175	Compromised	Compromised	Detected	Detected
5921	A_23_P205007	IPO5	Hs.712598	importin 5	Homo sapiens importin 5 (IPO5), mRNA [NM_002271]	2.66356	down	2.86958	down	955.10850	2634.19920	663.09015	1677.86390	Detected	Detected	Detected	Detected
5922	A_23_P140256	NP	Hs.75514	nucleoside phosphorylase	Homo sapiens nucleoside phosphorylase (NP), mRNA [NM_000270]	2.66194	down	2.72696	down	11246.79100	30999.82400	11443.06100	27516.10500	Detected	Detected	Detected	Detected
5923	A_33_P3218559					2.66073	down	2.82347	down	209.70630	577.75750	210.61563	524.37195	Detected	Detected	Detected	Detected
5924	A_33_P3268378	FAM167A	Hs.124299	family with sequence similarity 167, member A	Homo sapiens family with sequence similarity 167, member A (FAM167A), mRNA [NM_053279]	2.66018	down	3.30729	down	96.10974	264.73526	82.97532	241.98425	Detected	Detected	Detected	Detected
5925	A_23_P53663	PAWR	Hs.643130	PRKC, apoptosis, WT1, regulator	Homo sapiens PRKC, apoptosis, WT1, regulator (PAWR), mRNA [NM_002583]	2.65962	down	2.93462	down	322.41180	887.90060	206.29155	533.82520	Detected	Detected	Detected	Detected
5926	A_33_P3347132	EXOSC2	Hs.654643	exosome component 2	Homo sapiens exosome component 2 (EXOSC2), mRNA [NM_014285]	2.65867	down	2.78693	down	545.13306	1500.72020	468.58923	1151.55290	Detected	Detected	Detected	Detected
5927	A_23_P136817	KNTC1	Hs.300559	kinetochore associated 1	Homo sapiens kinetochore associated 1 (KNTC1), mRNA [NM_014708]	2.65720	down	2.60587	down	281.64804	774.93317	195.76562	449.83630	Detected	Detected	Detected	Detected
5928	A_32_P167904	ZNF681	Hs.399952	zinc finger protein 681	Homo sapiens zinc finger protein 681 (ZNF681), mRNA [NM_138286]	2.65628	down	3.59713	down	17.96584	49.41458	9.35550	29.67488	Detected	Detected	Compromised	Detected
5929	A_33_P3236986					2.65593	down	1.87418	down	161.91590	445.28610	95.32117	157.53099	Detected	Detected	Detected	Detected
5930	A_33_P3214052					2.65588	down	3.93641	down	81.36021	223.74516	18.90440	65.61891	Detected	Detected	Detected	Detected

5931	A_24_P75920	CCDC159	Hs.631636	coiled-coil domain containing 159	Homo sapiens coiled-coil domain containing 159 (CCDC159), mRNA [NM_001080503]	2.65497	down	2.65118	down	41.12437	113.05578	45.85074	107.18938	Detected	Detected	Detected	Detected
5932	A_33_P3214446	RFC3	Hs.115474	replication factor C (activator 1) 3, 38kDa	Homo sapiens replication factor C (activator 1) 3, 38kDa (RFC3), transcript variant 2, mRNA [NM_181558]	2.65489	down	2.81465	down	192.49675	529.17975	189.32380	469.88892	Detected	Detected	Detected	Detected
5933	A_33_P3307207	PRHOXNB	Hs.705356	parahox cluster neighbor	Homo sapiens parahox cluster neighbor (PRHOXNB), mRNA [NM_001105577]	2.65296	down	1.89534	down	7.13840	19.60943	4.52733	7.56650	Compromised	Detected	Compromised	Compromised
5934	A_24_P58620	GRAMD1B	Hs.144725	GRAM domain containing 1B	Homo sapiens GRAM domain containing 1B (GRAMD1B), mRNA [NM_020716]	2.64961	down	2.84049	down	58.04118	159.23955	46.23160	115.79726	Detected	Detected	Detected	Detected
5935	A_23_P372096		Hs.516978		Uncharacterized protein C20orf112 [Source:UniProtKB/Swiss-Prot;Acc:Q96MY1] [ENST00000375678]	2.64949	down	2.82026	down	82.57635	226.54326	67.42421	167.67569	Detected	Detected	Detected	Detected
5936	A_33_P3273255				Zinc finger protein 761 [Source:UniProtKB/Swiss-Prot;Acc:Q86XN6] [ENST00000396407]	2.64908	down	2.12562	down	30.57966	83.88039	20.52007	38.46174	Detected	Detected	Detected	Detected
5937	A_33_P3412945	SFRS2	Hs.713672	splicing factor, arginine/serine-rich 2	Homo sapiens splicing factor, arginine/serine-rich 2 (SFRS2), mRNA [NM_003016]	2.64841	down	2.59092	down	1252.03080	3433.46850	1524.02450	3481.86160	Detected	Detected	Detected	Detected
5938	A_23_P11025	ZNF185	Hs.16622	zinc finger protein 185 (LIM domain)	Homo sapiens zinc finger protein 185 (LIM domain) (ZNF185), mRNA [NM_007150]	2.64838	down	2.58630	down	440.92180	1209.13490	299.68650	683.45850	Detected	Detected	Detected	Detected
5939	A_33_P3297621	KIAA0090	Hs.439200	KIAA0090	Homo sapiens KIAA0090 (KIAA0090), mRNA [NM_015047]	2.64804	down	2.67767	down	870.75140	2387.55030	976.24530	2305.05700	Detected	Detected	Detected	Detected
5940	A_33_P3419835	NCRNA00095		non-protein coding RNA 95	Homo sapiens non-protein coding RNA 95 (NCRNA00095), non-coding RNA [NR_024348]	2.64703	down	2.64743	down	30.31763	83.09737	14.38931	33.59155	Detected	Detected	Detected	Detected
5941	A_33_P3312301	CIT	Hs.119594	citron (rho-interacting, serine/threonine kinase 21)	Homo sapiens citron (rho-interacting, serine/threonine kinase 21) (CIT), mRNA [NM_007174]	2.64621	down	3.09760	down	29.92613	81.99886	24.27054	66.29330	Detected	Detected	Detected	Detected
5942	A_33_P3247678					2.64555	down	3.08440	down	85.74915	234.89778	29.60079	80.50803	Detected	Detected	Detected	Detected
5943	A_32_P2634		Hs.661155		full-length cDNA clone CS0DI009YC01 of Placenta Cot 25-normalized of Homo sapiens (human) [CR604707]	2.64481	down	2.86427	down	167.67015	459.18076	166.35510	420.16086	Detected	Detected	Detected	Detected
5944	A_23_P92948	PRRC1	Hs.483259	proline-rich coiled-coil 1	Homo sapiens proline-rich coiled-coil 1 (PRRC1), mRNA [NM_130809]	2.64412	down	2.54792	down	392.12130	1073.58180	429.81100	965.67050	Detected	Detected	Detected	Detected
5945	A_33_P3409210		Hs.710582		cDNA FLJ44654 fis, clone BRACE2047232 [Source:UniProtKB/TrEMBL;Acc:Q6ZTH3] [ENST00000433265]	2.64286	down	3.18565	down	104.31122	285.45505	88.91026	249.75607	Detected	Detected	Detected	Detected
5946	A_24_P47547	RAN	Hs.10842	RAN, member RAS oncogene family	Homo sapiens RAN, member RAS oncogene family (RAN), mRNA [NM_006325]	2.64278	down	2.72293	down	7879.93400	21563.35000	6987.78000	16778.05000	Detected	Detected	Detected	Detected
5947	A_23_P57277	C21orf7	Hs.222802	chromosome 21 open reading frame 7	Homo sapiens chromosome 21 open reading frame 7 (C21orf7), mRNA [NM_020152]	2.64158	down	2.71501	down	166.53125	455.50458	130.44699	312.29984	Detected	Detected	Detected	Detected
5948	A_23_P121215	CAMK1	Hs.434875	calcium/calmodulin-dependent protein kinase I	Homo sapiens calcium/calmodulin-dependent protein kinase I (CAMK1), mRNA [NM_003656]	2.64103	down	2.86905	down	163.10564	446.04205	154.01813	389.65110	Detected	Detected	Detected	Detected
5949	A_24_P253003	WNT11	Hs.108219	wingless-type MMTV integration site family, member 11	Homo sapiens wingless-type MMTV integration site family, member 11 (WNT11), mRNA [NM_004626]	2.63940	down	3.29054	down	18.52752	50.63562	12.98368	37.67300	Detected	Detected	Compromised	Detected
5950	A_23_P206441	FANCA	Hs.719210	Fanconi anemia, complementation group A	Homo sapiens Fanconi anemia, complementation group A (FANCA), transcript variant 1, mRNA [NM_000135]	2.63804	down	2.60541	down	193.04425	527.31616	146.39120	336.32297	Detected	Detected	Detected	Detected
5951	A_23_P132378	CELSR1	Hs.252387	cadherin, EGF LAG seven-pass G-type receptor 1 (flamingo homolog, Drosophila)	Homo sapiens cadherin, EGF LAG seven-pass G-type receptor 1 (flamingo homolog, Drosophila) (CELSR1), mRNA [NM_014246]	2.63780	down	7.09151	down	2.48877	6.79767	3.18415	19.91119	Compromised	Compromised	Compromised	Detected
5952	A_33_P3413325		Hs.144795		Homo sapiens mRNA; cDNA DKFZp781N1049 (from clone DKFZp781N1049). [CR627384]	2.63662	down	5.00199	down	20.38964	55.66606	12.78259	56.38030	Detected	Detected	Compromised	Detected
5953	A_24_P305764	SMS	Hs.288487	spermine synthase	Homo sapiens spermine synthase (SMS), mRNA [NM_004595]	2.63511	down	2.52527	down	5591.95750	15257.95500	4541.58200	10113.02800	Detected	Detected	Detected	Detected
5954	A_23_P7976	HIST1H1E	Hs.248133	histone cluster 1, H1e	Homo sapiens histone cluster 1, H1e (HIST1H1E), mRNA [NM_005321]	2.63367	down	2.16303	down	412.17242	1124.01700	418.38818	798.00920	Detected	Detected	Detected	Detected
5955	A_33_P3228612	CACNA1E	Hs.437444	calcium channel, voltage-dependent, R type, alpha 1E subunit	Homo sapiens calcium channel, voltage-dependent, R type, alpha 1E subunit (CACNA1E), mRNA [NM_000721]	2.63257	down	4.63322	down	7.61690	20.76307	2.54779	10.40906	Compromised	Detected	Compromised	Compromised
5956	A_33_P3295358	ANGPTL4	Hs.9613	angiopoietin-like 4	Homo sapiens angiopoietin-like 4 (ANGPTL4), transcript variant 1, mRNA [NM_139314]	2.63048	down	2.60556	down	50708.93800	138118.80000	46730.95000	107367.25000	Detected	Detected	Detected	Detected
5957	A_24_P282309	MYOF	Hs.602086	myoferlin	Homo sapiens myoferlin (MYOF), transcript variant 2, mRNA [NM_133337]	2.62864	down	2.90892	down	156.00873	424.63310	152.63959	391.52920	Detected	Detected	Detected	Detected
5958	A_33_P3396607	UGDH	Hs.572518	UDP-glucose dehydrogenase	Homo sapiens UDP-glucose dehydrogenase (UGDH), mRNA [NM_003359]	2.62850	down	2.60108	down	4881.67140	13286.46800	3032.67820	6955.77300	Detected	Detected	Detected	Detected
5959	A_24_P107291	PPP2R1B	Hs.584790	protein phosphatase 2 (formerly 2A), regulatory subunit A, beta isoform	Homo sapiens protein phosphatase 2 (formerly 2A), regulatory subunit A, beta isoform (PPP2R1B), transcript variant 2, mRNA [NM_181699]	2.62670	down	2.46625	down	656.48610	1785.53920	549.33685	1194.65220	Detected	Detected	Detected	Detected
5960	A_33_P3233774	ZNF174	Hs.155204	zinc finger protein 174	Homo sapiens zinc finger protein 174 (ZNF174), transcript variant 2, mRNA [NM_001032292]	2.62663	down	2.55470	down	169.83708	461.91736	148.52484	334.58337	Detected	Detected	Detected	Detected

5961	A_24_P319715	PDIA6	Hs.212102	protein disulfide isomerase family A, member 6	Homo sapiens protein disulfide isomerase family A, member 6 (PDIA6), mRNA [NM_005742]	2.62543	down	2.81440	down	5827.30700	15841.68300	6830.06800	16950.27100	Detected	Detected	Detected	Detected
5962	A_33_P3234855	UTRN	Hs.133135	utrophin	Homo sapiens utrophin (UTRN), mRNA [NM_007124]	2.62263	down	1.30358	down	12.94645	35.15774	10.23947	11.77011	Compromised	Detected	Compromised	Compromised
5963	A_33_P3409289					2.62090	down	2.38950	down	153.57532	416.77777	93.20488	196.38590	Detected	Detected	Detected	Detected
5964	A_33_P3238415	GSG1	Hs.240053	germ cell associated 1	Homo sapiens germ cell associated 1 (GSG1), transcript variant 3, mRNA [NM_001080554]	2.62081	down	2.44940	down	91.68673	248.81383	79.88535	172.54080	Detected	Detected	Detected	Detected
5965	A_23_P46924	BUB3	Hs.418533	budding uninhibited by benzimidazoles 3 homolog (yeast)	Homo sapiens budding uninhibited by benzimidazoles 3 homolog (yeast) (BUB3), transcript variant 2, mRNA [NM_001007793]	2.62042	down	2.69069	down	3012.27900	8173.33900	2417.19730	5735.10160	Detected	Detected	Detected	Detected
5966	A_33_P3344276	HS1BP3	Hs.531785	HCLS1 binding protein 3	Homo sapiens HCLS1 binding protein 3 (HS1BP3), mRNA [NM_022460]	2.61951	down	3.62829	down	71.70268	194.48598	60.07006	192.18785	Detected	Detected	Detected	Detected
5967	A_23_P80040	PROCR	Hs.647450	protein C receptor, endothelial (EPCR)	Homo sapiens protein C receptor, endothelial (EPCR) (PROCR), mRNA [NM_006404]	2.61630	down	2.53215	down	3514.12940	9520.03400	3114.87920	6954.97750	Detected	Detected	Detected	Detected
5968	A_33_P3357580	MRTO4	Hs.463797	mRNA turnover 4 homolog (S. cerevisiae)	Homo sapiens mRNA turnover 4 homolog (S. cerevisiae) (MRTO4), mRNA [NM_016183]	2.61622	down	2.30562	down	661.24520	1791.30600	442.33154	899.29380	Detected	Detected	Detected	Detected
5969	A_23_P256244		Hs.148778		Oxidation resistance protein 1 [Source:UniProtKB/Swiss-Prot;Acc:Q8N573] [ENST00000395828]	2.61344	down	2.67399	down	65.98434	178.56082	55.19980	130.15552	Detected	Detected	Detected	Detected
5970	A_33_P3296198	FLJ44606	Hs.601507	glutaredoxin-like protein YDR286C homolog	Homo sapiens glutaredoxin-like protein YDR286C homolog (FLJ44606), transcript variant 1, mRNA [NM_001164479]	2.61250	down	4.31138	down	134729.94000	364463.38000	72000.98000	273728.40000	Detected	Detected	Detected	Detected
5971	A_33_P3389133	MINPP1	Hs.121260	multiple inositol polyphosphate histidine phosphatase, 1	Homo sapiens multiple inositol polyphosphate histidine phosphatase, 1 (MINPP1), mRNA [NM_004897]	2.61246	down	2.37971	down	116.85526	316.10483	123.97292	260.14520	Detected	Detected	Detected	Detected
5972	A_33_P3320888	CREB3L2	Hs.490273	cAMP responsive element binding protein 3-like 2	Homo sapiens cAMP responsive element binding protein 3-like 2 (CREB3L2), mRNA [NM_194071]	2.61145	down	2.94983	down	205.45256	555.55480	223.79634	582.12470	Detected	Detected	Detected	Detected
5973	A_23_P17275	DNAJC27	Hs.434993	DnaJ (Hsp40) homolog, subfamily C, member 27	Homo sapiens DnaJ (Hsp40) homolog, subfamily C, member 27 (DNAJC27), mRNA [NM_016544]	2.61063	down	2.86134	down	48.42781	130.90993	42.02477	106.03298	Detected	Detected	Detected	Detected
5974	A_33_P3238201	LOC100131315	Hs.610545	similar to hCG1796212	PREDICTED: Homo sapiens similar to hCG1796212 (LOC100131315), mRNA [XM_001716753]	2.61047	down	2.95184	down	41.27333	111.56348	32.55874	84.74739	Detected	Detected	Detected	Detected
5975	A_33_P3233947	FAM36A	Hs.657313	family with sequence similarity 36, member A	Homo sapiens family with sequence similarity 36, member A (FAM36A), mRNA [NM_198076]	2.60675	down	2.68522	down	2443.26680	6594.82400	1404.97840	3326.71260	Detected	Detected	Detected	Detected
5976	A_33_P3300395	APITD1	Hs.412311	apoptosis-inducing, TAF9-like domain 1	Homo sapiens apoptosis-inducing, TAF9-like domain 1 (APITD1), transcript variant B, mRNA [NM_199295]	2.60651	down	2.55178	down	1443.39430	3895.62230	1291.44980	2905.94210	Detected	Detected	Detected	Detected
5977	A_23_P204801	SLC41A2	Hs.577463	solute carrier family 41, member 2	Homo sapiens solute carrier family 41, member 2 (SLC41A2), mRNA [NM_032148]	2.60453	down	2.59265	down	117.18940	316.04675	100.55089	229.87710	Detected	Detected	Detected	Detected
5978	A_23_P136504	SLC25A21	Hs.693866	solute carrier family 25 (mitochondrial oxodicarboxylate carrier), member 21	Homo sapiens solute carrier family 25 (mitochondrial oxodicarboxylate carrier), member 21 (SLC25A21), nuclear gene encoding mitochondrial protein, mRNA [NM_030631]	2.60423	down	2.00279	down	9.90348	26.70546	10.47143	18.49300	Compromised	Detected	Compromised	Detected
5979	A_23_P30813	HIST1H4K	Hs.528055	histone cluster 1, H4k	Homo sapiens histone cluster 1, H4k (HIST1H4K), mRNA [NM_003541]	2.60420	down	2.78916	down	370.00443	997.73310	299.06192	735.52893	Detected	Detected	Detected	Detected
5980	A_33_P3343485		Hs.674397		Homo sapiens clone DNA108759 PIKR2786 (UNQ2786) mRNA, complete cds. [AY358103]	2.60251	down	3.17007	down	213.63188	575.69270	166.71701	466.03024	Detected	Detected	Detected	Detected
5981	A_33_P3407103	YTHDF1	Hs.11747	YTH domain family, member 1	Homo sapiens YTH domain family, member 1 (YTHDF1), mRNA [NM_017798]	2.60200	down	2.57553	down	32.37426	87.22479	37.54954	85.27808	Detected	Detected	Detected	Detected
5982	A_33_P3232516	KIF18B	Hs.135094	kinesin family member 18B	Homo sapiens kinesin family member 18B (KIF18B), mRNA [NM_001080443]	2.60163	down	2.19999	down	6.68345	18.00443	10.57910	20.52276	Compromised	Compromised	Compromised	Detected
5983	A_23_P119362	EMP3	Hs.9999	epithelial membrane protein 3	Homo sapiens epithelial membrane protein 3 (EMP3), mRNA [NM_001425]	2.59980	down	2.78355	down	6815.04100	18346.03100	10205.37400	25049.20700	Detected	Detected	Detected	Detected
5984	A_23_P100486	VKORC1	Hs.324844	vitamin K epoxide reductase complex, subunit 1	Homo sapiens vitamin K epoxide reductase complex, subunit 1 (VKORC1), transcript variant 2, mRNA [NM_206824]	2.59831	down	2.75368	down	14198.68300	38200.77700	16229.88700	39408.96500	Detected	Detected	Detected	Detected
5985	A_24_P133488	CDCA4	Hs.34045	cell division cycle associated 4	Homo sapiens cell division cycle associated 4 (CDCA4), transcript variant 1, mRNA [NM_017955]	2.59671	down	2.64167	down	153.17271	411.84833	111.15553	258.92590	Detected	Detected	Detected	Detected
5986	A_24_P358146	LOC100129478		hypothetical LOC100129478	PREDICTED: Homo sapiens hypothetical LOC100129478 (LOC100129478), mRNA [XM_001719774]	2.59661	down	2.75606	down	26.56233	71.41779	32.07050	77.93993	Detected	Detected	Detected	Detected
5987	A_23_P80839	MAP6D1	Hs.478465	MAP6 domain containing 1	Homo sapiens MAP6 domain containing 1 (MAP6D1), mRNA [NM_024871]	2.59631	down	2.45883	down	679.18260	1825.89820	636.15155	1379.28930	Detected	Detected	Detected	Detected
5988	A_23_P70318	ENPP4	Hs.643497	ectonucleotide pyrophosphatase/phosphodiesterase 4 (putative function)	Homo sapiens ectonucleotide pyrophosphatase/phosphodiesterase 4 (putative function) (ENPP4), mRNA [NM_014936]	2.59541	down	2.97615	down	303.78067	816.39435	200.24220	525.50420	Detected	Detected	Detected	Detected
5989	A_33_P3356092	BTBD9	Hs.603858	BTB (POZ) domain containing 9	Homo sapiens BTB (POZ) domain containing 9 (BTBD9), transcript variant 1, mRNA [NM_052893]	2.59357	down	2.19241	down	6.61973	17.77756	6.10343	11.79946	Compromised	Detected	Compromised	Compromised
5990	A_24_P73669	GSPT1	Hs.528780	G1 to S phase transition 1	Homo sapiens G1 to S phase transition 1 (GSPT1), transcript variant 1, mRNA [NM_002094]	2.59302	down	2.67098	down	807.27190	2167.50200	718.55110	1692.36490	Detected	Detected	Detected	Detected

5991	A_33_P3212804	C22orf42	Hs.426551	chromosome 22 open reading frame 42	Homo sapiens chromosome 22 open reading frame 42 (C22orf42), mRNA [NM_001010859]	2.59291	down	1.48906	down	16.78405	45.06262	10.48611	13.76863	Detected	Detected	Compromised	Compromised
5992	A_33_P3224705				Pecanex-like protein C14orf135 (Hepatitis C virus F protein-binding protein 2)(HCV F protein-binding protein 2) [Source:UniProtKB/Swiss-Prot;Acc:Q63HM2] [ENST00000391611]	2.59247	down	2.85051	down	2238.66480	6009.47560	1818.78970	4571.62450	Detected	Detected	Detected	Detected
5993	A_23_P150852	ESYT1	Hs.632729	extended synaptotagmin-like protein 1	Homo sapiens extended synaptotagmin-like protein 1 (ESYT1), mRNA [NM_015292]	2.59188	down	2.79078	down	2848.98730	7646.06740	2399.23270	5904.23540	Detected	Detected	Detected	Detected
5994	A_23_P160968	LAMC2	Hs.591484	laminin, gamma 2	Homo sapiens laminin, gamma 2 (LAMC2), transcript variant 2, mRNA [NM_018891]	2.59057	down	2.96437	down	246.36859	660.86540	192.93906	504.33353	Detected	Detected	Detected	Detected
5995	A_23_P218456	ILF3	Hs.465885	interleukin enhancer binding factor 3, 90kDa	Homo sapiens interleukin enhancer binding factor 3, 90kDa (ILF3), transcript variant 1, mRNA [NM_012218]	2.58977	down	2.65395	down	1930.40800	5176.58450	1607.09020	3760.96300	Detected	Detected	Detected	Detected
5996	A_23_P79259	SH3BP4	Hs.516777	SH3-domain binding protein 4	Homo sapiens SH3-domain binding protein 4 (SH3BP4), mRNA [NM_014521]	2.58702	down	2.76464	down	970.94543	2600.92750	762.42740	1858.67130	Detected	Detected	Detected	Detected
5997	A_32_P9575	MRPL45	Hs.462913	mitochondrial ribosomal protein L45	Homo sapiens mitochondrial ribosomal protein L45 (MRPL45), nuclear gene encoding mitochondrial protein, mRNA [NM_032351]	2.58639	down	2.60729	down	665.59460	1782.52950	734.00040	1687.52660	Detected	Detected	Detected	Detected
5998	A_23_P56404	EN1	Hs.271977	engrailed homeobox 1	Homo sapiens engrailed homeobox 1 (EN1), mRNA [NM_001426]	2.58500	down	2.56550	down	309.96796	829.67993	291.47690	659.38916	Detected	Detected	Detected	Detected
5999	A_23_P41948	CCDC99	Hs.368710	coiled-coil domain containing 99	Homo sapiens coiled-coil domain containing 99 (CCDC99), mRNA [NM_017785]	2.58461	down	2.43875	down	1656.22790	4432.48830	1145.57760	2463.52300	Detected	Detected	Detected	Detected
6000	A_23_P302787	LOC375295	Hs.659070	hypothetical protein LOC375295	PREDICTED: Homo sapiens hypothetical protein LOC375295 (LOC375295), mRNA [XM_001716150]	2.58284	down	2.29137	down	1065.05640	2848.41670	593.11487	1198.39260	Detected	Detected	Detected	Detected
6001	A_24_P194000	SAE1	Hs.515500	SUMO1 activating enzyme subunit 1	Homo sapiens SUMO1 activating enzyme subunit 1 (SAE1), transcript variant 1, mRNA [NM_005500]	2.58207	down	2.49707	down	17830.35000	47671.77700	13283.26300	29248.34200	Detected	Detected	Detected	Detected
6002	A_33_P3235562		Hs.432760		F-actin-capping protein subunit beta (CapZ beta) [Source:UniProtKB/Swiss-Prot;Acc:P47756] [ENST00000264202]	2.57942	down	2.30826	down	24.00909	64.12550	23.93008	48.70724	Detected	Detected	Detected	Detected
6003	A_33_P3334448	SNORA62	Hs.449909	small nucleolar RNA, H/ACA box 62	Homo sapiens small nucleolar RNA, H/ACA box 62 (SNORA62), small nucleolar RNA [NR_002324]	2.57805	down	2.65489	down	78.71155	210.11765	83.73945	196.03880	Detected	Detected	Detected	Detected
6004	A_23_P20443	LZTS1	Hs.521432	leucine zipper, putative tumor suppressor 1	Homo sapiens leucine zipper, putative tumor suppressor 1 (LZTS1), mRNA [NM_021020]	2.57669	down	2.97464	down	81.49607	217.43610	48.01293	125.93827	Detected	Detected	Detected	Detected
6005	A_33_P3833211	DNA2	Hs.532446	DNA replication helicase 2 homolog (yeast)	Homo sapiens DNA replication helicase 2 homolog (yeast) (DNA2), mRNA [NM_001080449]	2.57640	down	2.73005	down	41.13052	109.72628	31.38795	75.56111	Detected	Detected	Detected	Detected
6006	A_23_P77993	C1QL1	Hs.134012	complement component 1, q subcomponent-like 1	Homo sapiens complement component 1, q subcomponent-like 1 (C1QL1), mRNA [NM_006688]	2.57464	down	2.53150	down	16.33003	43.53473	20.28783	45.28758	Detected	Detected	Detected	Detected
6007	A_33_P3708309	LOC285638	Hs.532104	hypothetical protein LOC285638	Homo sapiens mRNA; cDNA DKFZp667J236 (from clone DKFZp667J236) [AL833206]	2.57330	down	1.86736	down	29.28161	78.02236	28.68316	47.23024	Detected	Detected	Detected	Detected
6008	A_23_P16469	PLAUR	Hs.466871	plasminogen activator, urokinase receptor	Homo sapiens plasminogen activator, urokinase receptor (PLAUR), transcript variant 3, mRNA [NM_001005377]	2.57317	down	2.70473	down	3130.33960	8340.53200	3909.75700	9324.80800	Detected	Detected	Detected	Detected
6009	A_24_P919452	LOC729839	Hs.661610	similar to DTW domain containing 2	PREDICTED: Homo sapiens similar to DTW domain containing 2 (LOC729839), miscRNA [XR_016021]	2.57229	down	2.12360	down	38.45179	102.41649	30.91932	57.89850	Detected	Detected	Detected	Detected
6010	A_33_P3235162					2.57213	down	2.37512	down	11.63942	30.99974	3.21604	6.73552	Compromised	Detected	Compromised	Compromised
6011	A_24_P325520	SORT1	Hs.485195	sortilin 1	Homo sapiens sortilin 1 (SORT1), mRNA [NM_002959]	2.57159	down	2.56935	down	440.65082	1173.35310	420.12308	951.84180	Detected	Detected	Detected	Detected
6012	A_33_P3362353				A-Raf proto-oncogene serine/threonine-protein kinase (EC 2.7.11.1)(A-raf-1)(Proto-oncogene Pks) [Source:UniProtKB/Swiss-Prot;Acc:P10398] [ENST00000377039]	2.56963	down	2.54010	down	335.57455	892.87933	292.41113	654.95386	Detected	Detected	Detected	Detected
6013	A_33_P3419733	DNAJC5	Hs.164419	DnaJ (Hsp40) homolog, subfamily C, member 5	Homo sapiens DnaJ (Hsp40) homolog, subfamily C, member 5 (DNAJC5), mRNA [NM_025219]	2.56935	down	3.85332	down	243.85095	648.75410	186.64607	634.18994	Detected	Detected	Detected	Detected
6014	A_23_P17855	TRIOBP	Hs.533030	TRIO and F-actin binding protein	Homo sapiens TRIO and F-actin binding protein (TRIOBP), transcript variant 6, mRNA [NM_001039141]	2.56903	down	2.95952	down	3372.02050	8969.99700	3991.62900	10416.87500	Detected	Detected	Detected	Detected
6015	A_33_P3295108	GANC	Hs.143261	glucosidase, alpha; neutral C	Homo sapiens glucosidase, alpha; neutral C (GANC), mRNA [NM_198141]	2.56755	down	2.64922	down	434.68410	1155.64780	293.03520	684.54724	Detected	Detected	Detected	Detected
6016	A_33_P3222783	SURF4	Hs.512465	surfeit 4	Homo sapiens surfeit 4 (SURF4), mRNA [NM_033161]	2.56754	down	2.97036	down	1676.78830	4457.88800	1317.89900	3451.89280	Detected	Detected	Detected	Detected
6017	A_24_P4334	RNF38	Hs.333503	ring finger protein 38	Homo sapiens ring finger protein 38 (RNF38), transcript variant 2, mRNA [NM_194328]	2.56438	down	2.01057	down	33.99410	90.26494	33.00624	58.51692	Detected	Detected	Detected	Detected
6018	A_33_P3409665	DEPDC5	Hs.435022	DEP domain containing 5	Homo sapiens DEP domain containing 5 (DEPDC5), transcript variant 2, mRNA [NM_001007188]	2.56395	down	2.45227	down	21.68472	57.56996	14.18932	30.68283	Detected	Detected	Detected	Detected
6019	A_23_P117851	CPLX3	Hs.187694	complexin 3	Homo sapiens complexin 3 (CPLX3), mRNA [NM_001030005]	2.56364	down	1.05283	down	7.70515	20.45369	18.53984	17.21188	Compromised	Detected	Detected	Detected
6020	A_23_P18806	YIPF5	Hs.372050	Yip1 domain family, member 5	Homo sapiens Yip1 domain family, member 5 (YIPF5), transcript variant 1, mRNA [NM_001024947]	2.56314	down	2.39738	down	1515.45630	4022.06200	1220.69140	2580.52760	Detected	Detected	Detected	Detected

6021	A_33_P3241884	SDC3	Hs.158287	syndecan 3	Homo sapiens syndecan 3 (SDC3), mRNA [NM_014654]	2.56258	down	2.89076	down	35.62002	94.51575	35.66364	90.90808	Detected	Detected	Detected	Detected
6022	A_32_P81768	TMEM167A	Hs.355606	transmembrane protein 167A	Homo sapiens transmembrane protein 167A (TMEM167A), mRNA [NM_174909]	2.56095	down	2.51248	down	1077.40810	2857.02320	853.53340	1890.98730	Detected	Detected	Detected	Detected
6023	A_24_P253251	SLC7A1	Hs.14846	solute carrier family 7 (cationic amino acid transporter, y+ system), member 1	Homo sapiens solute carrier family 7 (cationic amino acid transporter, y+ system), member 1 (SLC7A1), mRNA [NM_003045]	2.55839	down	2.54503	down	2154.40160	5707.24400	1371.49130	3077.87300	Detected	Detected	Detected	Detected
6024	A_33_P3396344	LOC728613		programmed cell death 6 pseudogene	Homo sapiens programmed cell death 6 pseudogene (LOC728613), non-coding RNA [NR_003713]	2.55612	down	2.71269	down	38.39051	101.61038	20.86679	49.91382	Detected	Detected	Detected	Detected
6025	A_33_P3307147	GRIK2	Hs.98262	glutamate receptor, ionotropic, kainate 2	Homo sapiens glutamate receptor, ionotropic, kainate 2 (GRIK2), transcript variant 3, mRNA [NM_001166247]	2.55569	down	4.08825	down	9.83601	26.02920	3.75112	13.52273	Compromised	Detected	Compromised	Compromised
6026	A_23_P127891	BDNF	Hs.502182	brain-derived neurotrophic factor	Homo sapiens brain-derived neurotrophic factor (BDNF), transcript variant 1, mRNA [NM_170735]	2.55460	down	2.45729	down	22.87861	60.51812	23.43706	50.78387	Detected	Detected	Detected	Detected
6027	A_33_P3345031	LOC645978	Hs.647847	similar to alkaline ceramidase 2	PREDICTED: Homo sapiens similar to alkaline ceramidase 2 (LOC645978), miscRNA [XR_017572]	2.55132	down	2.60126	down	7.65273	20.21688	9.23351	21.17952	Compromised	Detected	Compromised	Detected
6028	A_24_P937405	PRSS23	Hs.25338	protease, serine, 23	Homo sapiens protease, serine, 23 (PRSS23), mRNA [NM_007173]	2.54944	down	2.58708	down	2900.31540	7656.38040	2369.72440	5405.97100	Detected	Detected	Detected	Detected
6029	A_24_P290286	P4HA3	Hs.660541	prolyl 4-hydroxylase, alpha polypeptide III	Homo sapiens prolyl 4-hydroxylase, alpha polypeptide III (P4HA3), mRNA [NM_182904]	2.54749	down	2.68023	down	387.73460	1022.77420	428.14023	1011.86660	Detected	Detected	Detected	Detected
6030	A_23_P111888	CTHRC1	Hs.405614	collagen triple helix repeat containing 1	Homo sapiens collagen triple helix repeat containing 1 (CTHRC1), mRNA [NM_138455]	2.54723	down	2.52946	down	7924.49660	20901.31200	6844.78900	15267.00500	Detected	Detected	Detected	Detected
6031	A_33_P3297255	MXD3	Hs.702195	MAX dimerization protein 3	Homo sapiens MAX dimerization protein 3 (MXD3), transcript variant 1, mRNA [NM_031300]	2.54669	down	2.97217	down	3331.50120	8785.16100	2668.29320	6993.15400	Detected	Detected	Detected	Detected
6032	A_23_P83192	PHPT1	Hs.409834	phosphohistidine phosphatase 1	Homo sapiens phosphohistidine phosphatase 1 (PHPT1), transcript variant 3, mRNA [NM_014172]	2.54644	down	2.52842	down	8287.05400	21850.78700	9458.26200	21087.52700	Detected	Detected	Detected	Detected
6033	A_33_P3264910	NKX6-2	Hs.134013	NK6 homeobox 2	Homo sapiens NK6 homeobox 2 (NKX6-2), mRNA [NM_177400]	2.54634	down	1.02635	down	8.81191	23.23373	13.50177	12.21949	Compromised	Detected	Compromised	Compromised
6034	A_23_P120364	PPDPF	Hs.79625	pancreatic progenitor cell differentiation and proliferation factor homolog (zebrafish)	Homo sapiens pancreatic progenitor cell differentiation and proliferation factor homolog (zebrafish) (PPDPF), mRNA [NM_024299]	2.54229	down	2.75386	down	22242.42800	58551.73400	20681.11300	50220.59800	Detected	Detected	Detected	Detected
6035	A_33_P3766959	TDP1	Hs.209945	tyrosyl-DNA phosphodiesterase 1	Homo sapiens tyrosyl-DNA phosphodiesterase 1 (TDP1), transcript variant 1, mRNA [NM_018319]	2.54198	down	2.34706	down	2041.99390	5374.75800	1547.12000	3201.93800	Detected	Detected	Detected	Detected
6036	A_23_P15889	CBLN2	Hs.569851	cerebellin 2 precursor	Homo sapiens cerebellin 2 precursor (CBLN2), mRNA [NM_182511]	2.54193	down	2.44168	down	63.01534	165.86049	55.84948	120.24698	Detected	Detected	Detected	Detected
6037	A_23_P413761	SFRS3	Hs.405144	splicing factor, arginine/serine-rich 3	Homo sapiens splicing factor, arginine/serine-rich 3 (SFRS3), mRNA [NM_003017]	2.54157	down	2.53296	down	31272.47500	82299.51000	22369.75200	49963.74200	Detected	Detected	Detected	Detected
6038	A_23_P30630		Hs.279008		Homo sapiens cDNA FLJ20170 fis, clone COL09549. [AK000177]	2.54137	down	3.09525	down	29.71254	78.18810	22.37025	61.05659	Detected	Detected	Detected	Detected
6039	A_33_P3281807	PDDC1	Hs.218362	Parkinson disease 7 domain containing 1	Homo sapiens Parkinson disease 7 domain containing 1 (PDDC1), mRNA [NM_182612]	2.54124	down	3.30898	down	117.77679	309.91240	93.84003	273.80896	Detected	Detected	Detected	Detected
6040	A_33_P3272828	RAD1	Hs.38114	RAD1 homolog (S. pombe)	Homo sapiens RAD1 homolog (S. pombe) (RAD1), transcript variant 1, mRNA [NM_002853]	2.54028	down	2.60463	down	495.68490	1303.82850	313.61246	720.28450	Detected	Detected	Detected	Detected
6041	A_33_P3215953	MPZL1	Hs.493919	myelin protein zero-like 1	Homo sapiens myelin protein zero-like 1 (MPZL1), transcript variant 2, mRNA [NM_024569]	2.53983	down	2.78999	down	864.44965	2273.40720	938.67267	2309.30880	Detected	Detected	Detected	Detected
6042	A_33_P3266923	LMTK2	Hs.444179	lemur tyrosine kinase 2	Homo sapiens lemur tyrosine kinase 2 (LMTK2), mRNA [NM_014916]	2.53952	down	2.48420	down	285.02402	749.49160	184.87233	404.97170	Detected	Detected	Detected	Detected
6043	A_23_P22350	GRAMD3	Hs.363558	GRAM domain containing 3	Homo sapiens GRAM domain containing 3 (GRAMD3), transcript variant 2, mRNA [NM_023927]	2.53873	down	2.50708	down	510.79553	1342.75710	365.53033	808.08453	Detected	Detected	Detected	Detected
6044	A_23_P134935	DUSP4	Hs.417962	dual specificity phosphatase 4	Homo sapiens dual specificity phosphatase 4 (DUSP4), transcript variant 1, mRNA [NM_001394]	2.53792	down	3.36778	down	563.96454	1482.05210	312.40347	927.73850	Detected	Detected	Detected	Detected
6045	A_33_P3346057	ANKRD26P1	Hs.97414	ankyrin repeat domain 26 pseudogene 1	Homo sapiens ankyrin repeat domain 26 pseudogene 1 (ANKRD26P1), non-coding RNA [NR_026556]	2.53752	down	1.81601	down	12.89473	33.88094	7.96995	12.76261	Detected	Detected	Compromised	Compromised
6046	A_23_P2271	PTHLH	Hs.591159	parathyroid hormone-like hormone	Homo sapiens parathyroid hormone-like hormone (PTHLH), transcript variant 1, mRNA [NM_198965]	2.53666	down	3.22752	down	111.71067	293.42014	104.14428	296.39456	Detected	Detected	Detected	Detected
6047	A_24_P408736	GALNT5	Hs.269027	UDP-N-acetyl-alpha-D-galactosamine:polypeptide N-acetylgalactosaminyltransferase 5 (GalNAc-T5)	Homo sapiens UDP-N-acetyl-alpha-D-galactosamine:polypeptide N-acetylgalactosaminyltransferase 5 (GalNAc-T5) (GALNT5), mRNA [NM_014568]	2.53418	down	3.04319	down	230.25905	604.21027	148.92079	399.62228	Detected	Detected	Detected	Detected
6048	A_32_P178800	ITGA2	Hs.482077	integrin, alpha 2 (CD49B, alpha 2 subunit of VLA-2 receptor)	Homo sapiens integrin, alpha 2 (CD49B, alpha 2 subunit of VLA-2 receptor) (ITGA2), mRNA [NM_002203]	2.53387	down	2.22375	down	142.90501	374.94205	57.59190	112.93089	Detected	Detected	Detected	Detected
6049	A_23_P211627	NUP50	Hs.475103	nucleoporin 50kDa	Homo sapiens nucleoporin 50kDa (NUP50), transcript variant 2, mRNA [NM_007172]	2.53303	down	2.38239	down	189.14912	496.11038	194.19499	407.95926	Detected	Detected	Detected	Detected
6050	A_23_P12199	FAM46B	Hs.632378	family with sequence similarity 46, member B	Homo sapiens family with sequence similarity 46, member B (FAM46B), mRNA [NM_052943]	2.53221	down	2.81184	down	23.42695	61.42538	30.89461	76.60172	Detected	Detected	Detected	Detected

6051	A_23_P53345	ARNTL2	Hs.663740	aryl hydrocarbon receptor nuclear translocator-like 2	Homo sapiens aryl hydrocarbon receptor nuclear translocator-like 2 (ARNTL2), mRNA [NM_020183]	2.53179	down	3.12478	down	148.94034	390.45737	133.47813	367.78635	Detected	Detected	Detected	Detected
6052	A_33_P3420235				Actin-related protein 2/3 complex subunit 5 (Arp2/3 complex 16 kDa subunit)(p16-ARC) [Source:UniProtKB/Swiss-Prot;Acc:O15511] [ENST00000367534]	2.53171	down	3.00224	down	773.52110	2027.77590	666.90090	1765.52090	Detected	Detected	Detected	Detected
6053	A_23_P32078	SLC28A3	Hs.591877	solute carrier family 28 (sodium-coupled nucleoside transporter), member 3	Homo sapiens solute carrier family 28 (sodium-coupled nucleoside transporter), member 3 (SLC28A3), mRNA [NM_022127]	2.53168	down	8.08059	down	9.02851	23.66776	3.09259	22.03587	Compromised	Detected	Compromised	Detected
6054	A_23_P406424	RHOC	Hs.502659	ras homolog gene family, member C	Homo sapiens ras homolog gene family, member C (RHOC), transcript variant 1, mRNA [NM_175744]	2.53060	down	2.60652	down	5026.02540	13169.86300	6031.70260	13863.27700	Detected	Detected	Detected	Detected
6055	A_24_P394698	ORC5L	Hs.432948	origin recognition complex, subunit 5-like (yeast)	Homo sapiens origin recognition complex, subunit 5-like (yeast) (ORC5L), transcript variant 2, mRNA [NM_181747]	2.53000	down	2.19107	down	256.43274	671.77900	235.99830	455.96338	Detected	Detected	Detected	Detected
6056	A_32_P447001	FLJ27352	Hs.97300	hypothetical LOC145788	RST28817 Athersys RAGE Library Homo sapiens cDNA, mRNA sequence [BG209302]	2.52981	down	2.14532	down	63.44371	166.19188	48.17236	91.12878	Detected	Detected	Detected	Detected
6057	A_33_P3368452	MICA	Hs.130838	MHC class I polypeptide-related sequence A	Homo sapiens MHC class I polypeptide-related sequence A (MICA), mRNA [NM_000247]	2.52957	down	2.73209	down	1665.87730	4363.37100	2767.22300	6666.61500	Detected	Detected	Detected	Detected
6058	A_23_P38677	SLMO1	Hs.514718	slowmo homolog 1 (Drosophila)	Homo sapiens slowmo homolog 1 (Drosophila) (SLMO1), transcript variant 2, mRNA [NM_006553]	2.52936	down	2.67472	down	1760.95780	4612.03370	1383.03630	3261.94650	Detected	Detected	Detected	Detected
6059	A_33_P3361636	MGP	Hs.365706	matrix Gla protein	Homo sapiens matrix Gla protein (MGP), mRNA [NM_000900]	2.52689	down	2.33465	down	556.05340	1454.91060	381.32965	785.03357	Detected	Detected	Detected	Detected
6060	A_23_P149496	XKR8	Hs.55024	XK, Kell blood group complex subunit-related family, member 8	Homo sapiens XK, Kell blood group complex subunit-related family, member 8 (XKR8), mRNA [NM_018053]	2.52635	down	2.55800	down	5250.29200	13734.41800	5224.32000	11784.10300	Detected	Detected	Detected	Detected
6061	A_33_P3300267	VIT	Hs.137415	vitrin	Homo sapiens vitrin (VIT), mRNA [NM_053276]	2.52394	down	3.44368	down	85.68382	223.92923	50.45416	153.20938	Detected	Detected	Detected	Detected
6062	A_23_P1981	INS	Hs.272259	insulin	Homo sapiens insulin (INS), mRNA [NM_000207]	2.52175	down	2.18205	down	139.44559	364.11545	40.17003	77.29170	Detected	Detected	Detected	Detected
6063	A_33_P3254380	SLC9A7	Hs.620643	solute carrier family 9 (sodium/hydrogen exchanger), member 7	Sodium/hydrogen exchanger 7 (Na(+)/H(+) exchanger 7)(NHE-7)(Solute carrier family 9 member 7) [Source:UniProtKB/Swiss-Prot;Acc:Q96T83] [ENST00000328306]	2.52108	down	2.30829	down	151.78690	396.23560	124.76785	253.95550	Detected	Detected	Detected	Detected
6064	A_33_P3269578	PBRM1	Hs.189920	polybromo 1	Homo sapiens polybromo 1 (PBRM1), transcript variant 2, mRNA [NM_018313]	2.52092	down	2.36905	down	132.12141	344.87750	115.50369	241.28790	Detected	Detected	Detected	Detected
6065	A_23_P385690	WNT3A	Hs.336930	wingless-type MMTV integration site family, member 3A	Homo sapiens wingless-type MMTV integration site family, member 3A (WNT3A), mRNA [NM_033131]	2.52058	down	1.19988	down	7.54677	19.69674	2.97291	3.14547	Compromised	Detected	Compromised	Compromised
6066	A_33_P3294985	LOC554203	Hs.648316	alanyl-tRNA synthetase domain containing 1 pseudogene	Homo sapiens alanyl-tRNA synthetase domain containing 1 pseudogene (LOC554203), non-coding RNA [NR_024582]	2.51988	down	2.39459	down	66.83788	174.39539	46.88966	99.00875	Detected	Detected	Detected	Detected
6067	A_24_P262127	RRAD	Hs.1027	Ras-related associated with diabetes	Homo sapiens Ras-related associated with diabetes (RRAD), transcript variant 2, mRNA [NM_004165]	2.51985	down	3.06774	down	228.89052	597.22144	285.99597	773.64844	Detected	Detected	Detected	Detected
6068	A_23_P63190	NRAS	Hs.486502	neuroblastoma RAS viral (v-ras) oncogene homolog	Homo sapiens neuroblastoma RAS viral (v-ras) oncogene homolog (NRAS), mRNA [NM_002524]	2.51847	down	2.39325	down	508.72348	1326.63800	386.08365	814.76886	Detected	Detected	Detected	Detected
6069	A_24_P290770	OGDH	Hs.488181	oxoglutarate (alpha-ketoglutarate) dehydrogenase (lipoamide)	Homo sapiens oxoglutarate (alpha-ketoglutarate) dehydrogenase (lipoamide) (OGDH), nuclear gene encoding mitochondrial protein, transcript variant 2, mRNA [NM_001003941]	2.51771	down	2.90575	down	44.02111	114.76238	53.60998	137.36299	Detected	Detected	Detected	Detected
6070	A_32_P71447	NCAPD3	Hs.438550	non-SMC condensin II complex, subunit D3	Homo sapiens non-SMC condensin II complex, subunit D3 (NCAPD3), mRNA [NM_015261]	2.51747	down	2.26148	down	413.87637	1078.86700	272.70170	543.80820	Detected	Detected	Detected	Detected
6071	A_33_P3391496				Slit homolog 3 protein Precursor (Slit-3)(Multiple epidermal growth factor-like domains 5) [Source:UniProtKB/Swiss-Prot;Acc:O75094] [ENST00000404867]	2.51629	down	2.46751	down	674.61280	1757.71770	172.66180	375.68283	Detected	Detected	Detected	Detected
6072	A_23_P148194	ADI1	Hs.502773	acireductone dioxygenase 1	Homo sapiens acireductone dioxygenase 1 (ADI1), mRNA [NM_018269]	2.51575	down	2.63327	down	2069.96200	5392.15970	2306.94100	5356.70360	Detected	Detected	Detected	Detected
6073	A_32_P86763	TGM2	Hs.517033	transglutaminase 2 (C polypeptide, protein-glutamine-gamma-glutamyltransferase)	Homo sapiens transglutaminase 2 (C polypeptide, protein-glutamine-gamma-glutamyltransferase) (TGM2), transcript variant 1, mRNA [NM_004613]	2.51390	down	2.95012	down	1059.96140	2759.12200	1114.69900	2899.76730	Detected	Detected	Detected	Detected
6074	A_23_P256641	KCNE1L	Hs.522753	KCNE1-like	Homo sapiens KCNE1-like (KCNE1L), mRNA [NM_012282]	2.51375	down	3.45986	down	42.89677	111.65563	31.22060	95.25002	Detected	Detected	Detected	Detected
6075	A_33_P3325467	C1orf212	Hs.27160	chromosome 1 open reading frame 212	Homo sapiens chromosome 1 open reading frame 212 (C1orf212), transcript variant 1, mRNA [NM_001164824]	2.51368	down	2.57157	down	2771.84940	7214.60550	1743.39760	3953.29830	Detected	Detected	Detected	Detected
6076	A_23_P169003	SH2D4A	Hs.303208	SH2 domain containing 4A	Homo sapiens SH2 domain containing 4A (SH2D4A), mRNA [NM_022071]	2.50989	down	2.32534	down	143.82076	373.77426	142.13403	291.44107	Detected	Detected	Detected	Detected
6077	A_33_P3227880	BTN3A1	Hs.191510	butyrophilin, subfamily 3, member A1	Homo sapiens butyrophilin, subfamily 3, member A1 (BTN3A1), transcript variant 4, mRNA [NM_001145009]	2.50982	down	2.38759	down	97.49861	253.38077	82.59837	173.89894	Detected	Detected	Detected	Detected

6078	A_23_P377664	ALS2	Hs.471096	amyotrophic lateral sclerosis 2 (juvenile)	Homo sapiens amyotrophic lateral sclerosis 2 (juvenile) (ALS2), transcript variant 2, mRNA [NM_001135745]	2.50830	down	2.03062	down	175.92603	456.92320	125.99452	225.60312	Detected	Detected	Detected	Detected
6079	A_33_P3416366	CHAF1A	Hs.79018	chromatin assembly factor 1, subunit A (p150)	Homo sapiens chromatin assembly factor 1, subunit A (p150) (CHAF1A), mRNA [NM_005483]	2.50772	down	3.14510	down	151.97786	394.63272	149.46780	414.52173	Detected	Detected	Detected	Detected
6080	A_23_P77813	FN3KRP	Hs.31431	fructosamine 3 kinase related protein	Homo sapiens fructosamine 3 kinase related protein (FN3KRP), mRNA [NM_024619]	2.50687	down	2.63654	down	3332.87130	8651.34500	2808.80540	6530.11870	Detected	Detected	Detected	Detected
6081	A_23_P157736	PPAPDC3	Hs.134292	phosphatidic acid phosphatase type 2 domain containing 3	Homo sapiens phosphatidic acid phosphatase type 2 domain containing 3 (PPAPDC3), mRNA [NM_032728]	2.50612	down	2.56138	down	1563.39950	4056.99170	1377.32860	3110.83980	Detected	Detected	Detected	Detected
6082	A_23_P19020	SNCAIP	Hs.426463	synuclein, alpha interacting protein	Homo sapiens synuclein, alpha interacting protein (SNCAIP), mRNA [NM_005460]	2.50606	down	2.15893	down	123.14758	319.55880	93.24688	177.51604	Detected	Detected	Detected	Detected
6083	A_33_P3303385	NCAPD2	Hs.5719	non-SMC condensin I complex, subunit D2	Homo sapiens non-SMC condensin I complex, subunit D2 (NCAPD2), mRNA [NM_014865]	2.50551	down	2.51967	down	4493.94040	11658.85200	2878.19700	6394.83150	Detected	Detected	Detected	Detected
6084	A_23_P118300	FAHD1	Hs.513265	fumarylacetoacetate hydrolase domain containing 1	Homo sapiens fumarylacetoacetate hydrolase domain containing 1 (FAHD1), nuclear gene encoding mitochondrial protein, transcript variant 2, mRNA [NM_031208]	2.50527	down	2.45815	down	745.33990	1933.49340	669.80225	1451.84580	Detected	Detected	Detected	Detected
6085	A_24_P199655	VANGL1	Hs.515130	vang-like 1 (van gogh, Drosophila)	Homo sapiens vang-like 1 (van gogh, Drosophila) (VANGL1), mRNA [NM_138959]	2.50311	down	2.72575	down	1087.22750	2817.95170	627.55300	1508.35030	Detected	Detected	Detected	Detected
6086	A_33_P3789327	SNORD12B	Hs.711216	small nucleolar RNA, C/D box 12B	AV755695 BM Homo sapiens cDNA clone BMFBHD10 5', mRNA sequence [AV755695]	2.50297	down	1.07079	down	5.49398	14.23889	14.20500	13.41249	Compromised	Compromised	Detected	Detected
6087	A_23_P256504	AMBP	Hs.436911	alpha-1-microglobulin/bikunin precursor	Homo sapiens alpha-1-microglobulin/bikunin precursor (AMBP), mRNA [NM_001633]	2.50204	down	2.71173	down	2.89774	7.50736	7.41574	17.73235	Compromised	Compromised	Compromised	Detected
6088	A_33_P3543133	LOC283624	Hs.525210	hypothetical protein LOC283624	Homo sapiens cDNA clone IMAGE:5264647 [BC035145]	2.50167	down	2.05394	down	69.29273	179.49455	50.96552	92.30598	Detected	Detected	Detected	Detected
6089	A_33_P3346801	BMF	Hs.591104	Bcl2 modifying factor	Homo sapiens Bcl2 modifying factor (BMF), transcript variant 1, mRNA [NM_001003940]	2.50148	down	1.68808	down	7.11277	18.42336	3.08540	4.59273	Compromised	Detected	Compromised	Compromised
6090	A_23_P436138	MAX	Hs.285354	MYC associated factor X	Homo sapiens MYC associated factor X (MAX), transcript variant 4, mRNA [NM_145114]	2.50058	down	2.09089	down	98.50628	255.05763	108.89657	200.77528	Detected	Detected	Detected	Detected
6091	A_32_P212471	C1orf230	Hs.297967	chromosome 1 open reading frame 230	Homo sapiens chromosome 1 open reading frame 230 (C1orf230), mRNA [NM_001144956]	2.50013	down	2.48564	down	76.72168	198.61569	59.04790	129.42210	Detected	Detected	Detected	Detected
6092	A_33_P3394809	PCYT2	Hs.569843	phosphate cytidylyltransferase 2, ethanolamine	Homo sapiens phosphate cytidylyltransferase 2, ethanolamine (PCYT2), mRNA [NM_002861]	2.49891	down	2.65682	down	408.40662	1056.75930	488.21625	1143.77150	Detected	Detected	Detected	Detected
6093	A_24_P371425	ATRIP	Hs.694840	ATR interacting protein	Homo sapiens ATR interacting protein (ATRIP), transcript variant 2, mRNA [NM_032166]	2.49855	down	2.76371	down	207.66063	537.24880	186.33270	454.09518	Detected	Detected	Detected	Detected
6094	A_23_P29769	WWTR1	Hs.477921	WW domain containing transcription regulator 1	Homo sapiens WW domain containing transcription regulator 1 (WWTR1), mRNA [NM_015472]	2.49829	down	2.45124	down	300.61365	777.64980	292.62402	632.50226	Detected	Detected	Detected	Detected
6095	A_33_P3265104	PITRM1	Hs.528300	pitrilysin metallopeptidase 1	Homo sapiens pitrilysin metallopeptidase 1 (PITRM1), mRNA [NM_014889]	2.49799	down	2.59162	down	806.61370	2086.35740	712.91950	1629.21360	Detected	Detected	Detected	Detected
6096	A_24_P753476	LOC340508	Hs.680438	hypothetical protein LOC340508	Homo sapiens hypothetical protein LOC340508 (LOC340508), non-coding RNA [NR_002942]	2.49638	down	2.46027	down	2071.20460	5353.84670	801.01590	1737.76090	Detected	Detected	Detected	Detected
6097	A_24_P330112	MAGOHB	Hs.104650	mago-nashi homolog B (Drosophila)	Homo sapiens mago-nashi homolog B (Drosophila) (MAGOHB), mRNA [NM_018048]	2.49562	down	2.41069	down	56.82209	146.83475	45.77235	97.29925	Detected	Detected	Detected	Detected
6098	A_23_P59294	RREB1	Hs.298248	ras responsive element binding protein 1	Homo sapiens ras responsive element binding protein 1 (RREB1), transcript variant 1, mRNA [NM_001003699]	2.49536	down	2.89243	down	57.91427	149.64166	58.44019	149.05257	Detected	Detected	Detected	Detected
6099	A_23_P354297	CHTF18	Hs.153850	CTF18, chromosome transmission fidelity factor 18 homolog (S. cerevisiae)	Homo sapiens CTF18, chromosome transmission fidelity factor 18 homolog (S. cerevisiae) (CHTF18), mRNA [NM_022092]	2.49430	down	2.84411	down	1519.76300	3925.16500	1825.87410	4579.13430	Detected	Detected	Detected	Detected
6100	A_33_P3240946	DPF3		D4, zinc and double PHD fingers, family 3	Zinc finger protein DPF3 (cer-d4) [Source:UniProtKB/Swiss-Prot;Acc:Q92784] [ENST00000366353]	2.49360	down	2.25698	down	78.99602	203.96918	57.71237	114.85812	Detected	Detected	Detected	Detected
6101	A_33_P3404651	TTC7A	Hs.370603	tetratricopeptide repeat domain 7A	Homo sapiens tetratricopeptide repeat domain 7A (TTC7A), mRNA [NM_020458]	2.49325	down	2.87870	down	634.87573	1639.03380	613.56366	1557.47860	Detected	Detected	Detected	Detected
6102	A_33_P3309734	CCDC108	Hs.147762	coiled-coil domain containing 108	Homo sapiens coiled-coil domain containing 108 (CCDC108), transcript variant 2, mRNA [NM_152389]	2.49308	down	1.01566	down	3.41628	8.81907	17.17422	15.38123	Compromised	Compromised	Detected	Detected
6103	A_33_P3389573	CARD9	Hs.694071	caspase recruitment domain family, member 9	Homo sapiens caspase recruitment domain family, member 9 (CARD9), transcript variant 2, mRNA [NM_052814]	2.49307	down	3.07274	down	28.66742	74.00422	34.23233	92.75295	Detected	Detected	Detected	Detected
6104	A_23_P4944	CALM3	Hs.515487	calmodulin 3 (phosphorylase kinase, delta)	Homo sapiens calmodulin 3 (phosphorylase kinase, delta) (CALM3), mRNA [NM_005184]	2.49293	down	2.61785	down	2417.55200	6240.50340	2474.45460	5712.03000	Detected	Detected	Detected	Detected
6105	A_33_P3422897	ABCA1	Hs.429294	ATP-binding cassette, sub-family A (ABC1), member 1	Homo sapiens ATP-binding cassette, sub-family A (ABC1), member 1 (ABCA1), mRNA [NM_005502]	2.49273	down	2.56954	down	112.89477	291.39542	126.15270	285.83590	Detected	Detected	Detected	Detected
6106	A_33_P3371154	ZNF365	Hs.22653	zinc finger protein 365	Homo sapiens zinc finger protein 365 (ZNF365), transcript variant C, mRNA [NM_199451]	2.49224	down	2.04376	down	7.90200	20.39196	10.66245	19.21553	Compromised	Detected	Compromised	Detected

6107	A_24_P410605	ROR1	Hs.654491	receptor tyrosine kinase-like orphan receptor 1	Homo sapiens receptor tyrosine kinase-like orphan receptor 1 (ROR1), transcript variant 2, mRNA [NM_001083592]	2.49206	down	5.69445	down	29.28802	75.57572	13.11706	65.86491	Detected	Detected	Compromised	Detected
6108	A_23_P157170	DNAJC30	Hs.647046	DnaJ (Hsp40) homolog, subfamily C, member 30	Homo sapiens DnaJ (Hsp40) homolog, subfamily C, member 30 (DNAJC30), mRNA [NM_032317]	2.49017	down	2.62701	down	598.98004	1544.45200	877.11010	2031.80380	Detected	Detected	Detected	Detected
6109	A_24_P401174	KIAA0556	Hs.720307	KIAA0556	Homo sapiens KIAA0556 (KIAA0556), mRNA [NM_015202]	2.48968	down	1.77989	down	8.93182	23.02590	16.25209	25.50750	Compromised	Detected	Compromised	Detected
6110	A_24_P353300	LIMK2	Hs.474596	LIM domain kinase 2	Homo sapiens LIM domain kinase 2 (LIMK2), transcript variant 1, mRNA [NM_001031801]	2.48907	down	2.98029	down	138.75307	357.61243	112.42522	295.45288	Detected	Detected	Detected	Detected
6111	A_23_P27133	KRT15	Hs.654570	keratin 15	Homo sapiens keratin 15 (KRT15), mRNA [NM_002275]	2.48813	down	4.77803	down	179.01105	461.19702	85.27985	359.30334	Detected	Detected	Detected	Detected
6112	A_33_P3317548					2.48725	down	2.52355	down	9.50813	24.48768	11.18813	24.89634	Compromised	Detected	Compromised	Detected
6113	A_23_P40217	DOK5	Hs.656582	docking protein 5	Homo sapiens docking protein 5 (DOK5), mRNA [NM_018431]	2.48687	down	2.23888	down	39.52886	101.78900	32.38094	63.92725	Detected	Detected	Detected	Detected
6114	A_23_P47924	PTPRR	Hs.506076	protein tyrosine phosphatase, receptor type, R	Homo sapiens protein tyrosine phosphatase, receptor type, R (PTPRR), transcript variant 1, mRNA [NM_002849]	2.48652	down	2.76846	down	48.54148	124.97920	36.28098	88.56933	Detected	Detected	Detected	Detected
6115	A_23_P115149	WDR77	Hs.204773	WD repeat domain 77	Homo sapiens WD repeat domain 77 (WDR77), mRNA [NM_024102]	2.48650	down	2.72222	down	586.81230	1510.84510	483.81512	1161.36390	Detected	Detected	Detected	Detected
6116	A_23_P108673	FAM176A	Hs.302346	family with sequence similarity 176, member A	Homo sapiens family with sequence similarity 176, member A (FAM176A), transcript variant 2, mRNA [NM_032181]	2.48649	down	2.32517	down	1181.12110	3040.98500	926.00890	1898.60890	Detected	Detected	Detected	Detected
6117	A_33_P3349269	PIAS2	Hs.658013	protein inhibitor of activated STAT, 2	Homo sapiens protein inhibitor of activated STAT, 2 (PIAS2), transcript variant alpha, mRNA [NM_173206]	2.48625	down	2.47641	down	1026.33940	2642.21950	931.23150	2033.50620	Detected	Detected	Detected	Detected
6118	A_24_P100551	SH3RF1	Hs.301804	SH3 domain containing ring finger 1	Homo sapiens SH3 domain containing ring finger 1 (SH3RF1), mRNA [NM_020870]	2.48470	down	2.65593	down	2399.53700	6173.54200	1805.06120	4227.40330	Detected	Detected	Detected	Detected
6119	A_33_P3424207	RABEP2	Hs.555978	rabaptin, RAB GTPase binding effector protein 2	Homo sapiens rabaptin, RAB GTPase binding effector protein 2 (RABEP2), mRNA [NM_024816]	2.48329	down	2.83293	down	3258.06100	8377.60900	3548.11280	8863.38300	Detected	Detected	Detected	Detected
6120	A_24_P68631	HIST2H2AB	Hs.664173	histone cluster 2, H2ab	Homo sapiens histone cluster 2, H2ab (HIST2H2AB), mRNA [NM_175065]	2.48322	down	2.41808	down	1488.53820	3827.44360	1223.70410	2609.23460	Detected	Detected	Detected	Detected
6121	A_23_P52067	GRHL3	Hs.657920	grainyhead-like 3 (Drosophila)	Homo sapiens grainyhead-like 3 (Drosophila) (GRHL3), transcript variant 2, mRNA [NM_198173]	2.48116	down	1.92356	down	9.04561	23.23941	8.06883	13.68614	Compromised	Detected	Compromised	Compromised
6122	A_33_P3236798	SRMS	Hs.411061	src-related kinase lacking C-terminal regulatory tyrosine and N-terminal myristylation sites	Homo sapiens src-related kinase lacking C-terminal regulatory tyrosine and N-terminal myristylation sites (SRMS), mRNA [NM_080823]	2.48043	down	1.86080	down	167.50746	430.22403	51.95333	85.24708	Detected	Detected	Detected	Detected
6123	A_23_P251051	NF2	Hs.187898	neurofibromin 2 (merlin)	Homo sapiens neurofibromin 2 (merlin) (NF2), transcript variant 8, mRNA [NM_181832]	2.48016	down	2.50964	down	21018.50000	53977.72700	13933.72700	30834.99600	Detected	Detected	Detected	Detected
6124	A_33_P3832857	HAUS2	Hs.14347	HAUS augmin-like complex, subunit 2	Homo sapiens HAUS augmin-like complex, subunit 2 (HAUS2), transcript variant 1, mRNA [NM_018097]	2.47988	down	2.29437	down	289.12366	742.41460	231.88481	469.13907	Detected	Detected	Detected	Detected
6125	A_33_P3269899	RECK	Hs.388918	reversion-inducing-cysteine-rich protein with kazal motifs	Homo sapiens reversion-inducing-cysteine-rich protein with kazal motifs (RECK), mRNA [NM_021111]	2.47901	down	2.31027	down	473.27023	1214.84310	366.85492	747.34644	Detected	Detected	Detected	Detected
6126	A_33_P3382471	CYP4A22	Hs.720099	cytochrome P450, family 4, subfamily A, polypeptide 22	Homo sapiens cytochrome P450, family 4, subfamily A, polypeptide 22 (CYP4A22), mRNA [NM_001010969]	2.47876	down	2.01077	down	14.67549	37.66693	2.99643	5.31290	Detected	Detected	Compromised	Compromised
6127	A_24_P149036	DPYSL3	Hs.519659	dihydropyrimidinase-like 3	Homo sapiens dihydropyrimidinase-like 3 (DPYSL3), mRNA [NM_001387]	2.47867	down	2.69582	down	3321.12720	8523.89300	2517.92300	5985.48200	Detected	Detected	Detected	Detected
6128	A_24_P306720	LOC400236	Hs.585207	hypothetical LOC400236	PREDICTED: Homo sapiens hypothetical LOC400236 (LOC400236), mRNA [XM_001715158]	2.47848	down	2.55728	down	131.54169	337.58365	137.39941	309.83398	Detected	Detected	Detected	Detected
6129	A_32_P37360	LACE1	Hs.259666	lactation elevated 1	Homo sapiens lactation elevated 1 (LACE1), mRNA [NM_145315]	2.47841	down	2.22187	down	166.79219	428.03720	188.52467	369.36246	Detected	Detected	Detected	Detected
6130	A_33_P3311245	KRTAP19-7	Hs.553696	keratin associated protein 19-7	Homo sapiens keratin associated protein 19-7 (KRTAP19-7), mRNA [NM_181614]	2.47821	down	1.22272	down	258.95758	664.50745	72.93908	78.64144	Detected	Detected	Detected	Detected
6131	A_23_P258088	PACSIN1	Hs.520087	protein kinase C and casein kinase substrate in neurons 1	Homo sapiens protein kinase C and casein kinase substrate in neurons 1 (PACSIN1), mRNA [NM_020804]	2.47755	down	2.40133	down	10.45485	26.82089	9.09319	19.25454	Compromised	Detected	Compromised	Detected
6132	A_32_P473302	FLJ35024		hypothetical LOC401491	Homo sapiens hypothetical LOC401491 (FLJ35024), non-coding RNA [NR_015375]	2.47740	down	2.26662	down	19.76472	50.70144	13.08676	26.15627	Detected	Detected	Compromised	Detected
6133	A_23_P2355	CBX5	Hs.349283	chromobox homolog 5 (HP1 alpha homolog, Drosophila)	Homo sapiens chromobox homolog 5 (HP1 alpha homolog, Drosophila) (CBX5), transcript variant 3, mRNA [NM_012117]	2.47675	down	2.33323	down	434.09314	1113.26600	439.20462	903.62960	Detected	Detected	Detected	Detected
6134	A_33_P3287646	HSPB1	Hs.520973	heat shock 27kDa protein 1	Homo sapiens heat shock 27kDa protein 1 (HSPB1), mRNA [NM_001540]	2.47554	down	2.47905	down	28385.48400	72761.05500	15268.13600	33376.15600	Detected	Detected	Detected	Detected
6135	A_33_P3224505	LOC100131099	Hs.664462	hypothetical protein LOC100131099	Homo sapiens cDNA FLJ43943 fis, clone TESTI4014306. [AK125931]	2.47539	down	1.78872	down	20.03097	51.34262	11.63518	18.35186	Detected	Detected	Compromised	Compromised
6136	A_23_P216396	EXOSC2	Hs.654643	exosome component 2	Homo sapiens exosome component 2 (EXOSC2), mRNA [NM_014285]	2.47501	down	2.26699	down	204.91870	525.15906	160.91360	321.66820	Detected	Detected	Detected	Detected
6137	A_24_P2648	PTPN14	Hs.696573	protein tyrosine phosphatase, non-receptor type 14	Homo sapiens protein tyrosine phosphatase, non-receptor type 14 (PTPN14), mRNA [NM_005401]	2.47475	down	2.53917	down	149.74060	383.71133	103.39730	231.50789	Detected	Detected	Detected	Detected
6138	A_24_P20200	PLEKHB2	Hs.469944	pleckstrin homology domain containing, family B (evectins) member 2	Homo sapiens pleckstrin homology domain containing, family B (evectins) member 2 (PLEKHB2), transcript variant 2, mRNA [NM_017958]	2.47303	down	2.56647	down	359.18152	919.76434	471.39905	1066.82010	Detected	Detected	Detected	Detected

6139	A_23_P165937	DSN1	Hs.632268	DSN1, MIND kinetochore complex component, homolog (S. cerevisiae)	Homo sapiens DSN1, MIND kinetochore complex component, homolog (S. cerevisiae) (DSN1), transcript variant 3, mRNA [NM_024918]	2.47279	down	2.33463	down	1387.92600	3553.74630	1072.91850	2208.77050	Detected	Detected	Detected	Detected
6140	A_33_P3259042	MYOCD	Hs.567641	myocardin	Homo sapiens myocardin (MYOCD), transcript variant 3, mRNA [NM_001146313]	2.47275	down	1.88956	down	7.10948	18.20330	6.43418	10.72063	Compromised	Detected	Compromised	Compromised
6141	A_33_P3262416	ATPBD4	Hs.107196	ATP binding domain 4	Homo sapiens ATP binding domain 4 (ATPBD4), transcript variant 2, mRNA [NM_001141972]	2.47274	down	2.66263	down	10.49456	26.87047	3.42838	8.04942	Compromised	Detected	Compromised	Compromised
6142	A_23_P97394	BCAR3	Hs.36958	breast cancer anti-estrogen resistance 3	Homo sapiens breast cancer anti-estrogen resistance 3 (BCAR3), mRNA [NM_003567]	2.47124	down	2.43393	down	3851.13230	9854.52800	2828.53880	6070.65530	Detected	Detected	Detected	Detected
6143	A_23_P415882	TIMM50	Hs.590956	translocase of inner mitochondrial membrane 50 homolog (S. cerevisiae)	Homo sapiens translocase of inner mitochondrial membrane 50 homolog (S. cerevisiae) (TIMM50), nuclear gene encoding mitochondrial protein, mRNA [NM_001001563]	2.47029	down	2.52960	down	4650.21500	11894.73400	3940.98300	8790.66800	Detected	Detected	Detected	Detected
6144	A_23_P425502	DONSON	Hs.436341	downstream neighbor of SON	Homo sapiens downstream neighbor of SON (DONSON), mRNA [NM_017613]	2.46997	down	2.41261	down	547.40985	1400.02950	382.93063	814.65360	Detected	Detected	Detected	Detected
6145	A_33_P3299865	HIST1H4K	Hs.528055	histone cluster 1, H4k	Homo sapiens histone cluster 1, H4k (HIST1H4K), mRNA [NM_003541]	2.46931	down	2.60466	down	320.21942	818.75934	325.13916	746.76715	Detected	Detected	Detected	Detected
6146	A_23_P309361	C1orf59	Hs.7962	chromosome 1 open reading frame 59	Homo sapiens chromosome 1 open reading frame 59 (C1orf59), transcript variant 1, mRNA [NM_144584]	2.46858	down	2.53427	down	661.23160	1690.18140	513.56537	1147.66360	Detected	Detected	Detected	Detected
6147	A_33_P3239152	RABEP2	Hs.555978	rabaptin, RAB GTPase binding effector protein 2	Homo sapiens rabaptin, RAB GTPase binding effector protein 2 (RABEP2), mRNA [NM_024816]	2.46764	down	2.87826	down	4558.49760	11647.59800	3990.33620	10127.57200	Detected	Detected	Detected	Detected
6148	A_33_P3411025	ARHGAP19	Hs.80305	Rho GTPase activating protein 19	Homo sapiens Rho GTPase activating protein 19 (ARHGAP19), mRNA [NM_032900]	2.46761	down	2.54284	down	61.69612	157.64058	45.44716	101.90420	Detected	Detected	Detected	Detected
6149	A_33_P3267814	MICAL3	Hs.528024	microtubule associated monoxygenase, calponin and LIM domain containing 3	Homo sapiens microtubule associated monoxygenase, calponin and LIM domain containing 3 (MICAL3), transcript variant 2, mRNA [NM_001136004]	2.46758	down	2.97611	down	153.45004	392.07620	82.32313	216.04135	Detected	Detected	Detected	Detected
6150	A_33_P3315929	ROD1	Hs.269988	ROD1 regulator of differentiation 1 (S. pombe)	Homo sapiens ROD1 regulator of differentiation 1 (S. pombe) (ROD1), transcript variant 3, mRNA [NM_001163790]	2.46756	down	2.19899	down	81.58293	208.44910	69.26061	134.29942	Detected	Detected	Detected	Detected
6151	A_33_P3296333	NTM	Hs.504352	neurotrimin	Homo sapiens neurotrimin (NTM), transcript variant 3, mRNA [NM_001144058]	2.46276	down	2.84114	down	306.79260	782.34850	229.72618	575.52985	Detected	Detected	Detected	Detected
6152	A_23_P204286	MGP	Hs.365706	matrix Gla protein	Homo sapiens matrix Gla protein (MGP), mRNA [NM_000900]	2.46244	down	2.43947	down	306.85180	782.39820	254.50783	547.47314	Detected	Detected	Detected	Detected
6153	A_24_P34545	ING5	Hs.529172	inhibitor of growth family, member 5	Homo sapiens inhibitor of growth family, member 5 (ING5), mRNA [NM_032329]	2.46242	down	2.93772	down	109.24323	278.54086	106.71159	276.43106	Detected	Detected	Detected	Detected
6154	A_24_P40978	CYHR1	Hs.459379	cysteine/histidine-rich 1	Homo sapiens cysteine/histidine-rich 1 (CYHR1), transcript variant 1, mRNA [NM_138496]	2.46226	down	3.27665	down	164.65154	419.78998	162.61520	469.84760	Detected	Detected	Detected	Detected
6155	A_23_P125624	ACOT9	Hs.298885	acyl-CoA thioesterase 9	Homo sapiens acyl-CoA thioesterase 9 (ACOT9), transcript variant 1, mRNA [NM_001037171]	2.46205	down	2.33212	down	11937.86900	30433.82000	7223.85840	14855.45900	Detected	Detected	Detected	Detected
6156	A_23_P52219	ERLIN1	Hs.150087	ER lipid raft associated 1	Homo sapiens ER lipid raft associated 1 (ERLIN1), mRNA [NM_006459]	2.46181	down	2.60522	down	283.70856	723.20337	302.71564	695.41595	Detected	Detected	Detected	Detected
6157	A_32_P128656	MID1	Hs.27695	midline 1 (Opitz/BBB syndrome)	Homo sapiens midline 1 (Opitz/BBB syndrome) (MID1), transcript variant 1, mRNA [NM_000381]	2.46115	down	2.05541	down	37.14644	94.66491	29.05954	52.66860	Detected	Detected	Detected	Detected
6158	A_24_P219785	CALM3	Hs.515487	calmodulin 3 (phosphorylase kinase, delta)	Homo sapiens calmodulin 3 (phosphorylase kinase, delta) (CALM3), mRNA [NM_005184]	2.46104	down	2.61892	down	1654.72660	4216.75900	2210.56570	5104.95400	Detected	Detected	Detected	Detected
6159	A_23_P48561	EFS	Hs.24587	embryonal Fyn-associated substrate	Homo sapiens embryonal Fyn-associated substrate (EFS), transcript variant 1, mRNA [NM_005864]	2.46074	down	2.56792	down	1005.01110	2560.76500	913.70560	2068.96200	Detected	Detected	Detected	Detected
6160	A_33_P3354935	CSF1	Hs.591402	colony stimulating factor 1 (macrophage)	Homo sapiens colony stimulating factor 1 (macrophage) (CSF1), transcript variant 4, mRNA [NM_172212]	2.46054	down	2.00704	down	1645.12980	4191.43900	475.55264	841.62700	Detected	Detected	Detected	Detected
6161	A_23_P205074	SLC46A3	Hs.117167	solute carrier family 46, member 3	Homo sapiens solute carrier family 46, member 3 (SLC46A3), transcript variant 1, mRNA [NM_181785]	2.45964	down	2.80065	down	46.10125	117.41350	47.50271	117.31210	Detected	Detected	Detected	Detected
6162	A_33_P3589722	ATM	Hs.367437	ataxia telangiectasia mutated	Homo sapiens ataxia telangiectasia mutated (ATM), transcript variant 1, mRNA [NM_000051]	2.45949	down	2.23899	down	99.47134	253.32375	102.36593	202.10307	Detected	Detected	Detected	Detected
6163	A_23_P208293	PVRL2	Hs.655455	poliovirus receptor-related 2 (herpesvirus entry mediator B)	Homo sapiens poliovirus receptor-related 2 (herpesvirus entry mediator B) (PVRL2), transcript variant delta, mRNA [NM_001042724]	2.45907	down	2.40877	down	1186.22350	3020.43800	859.28796	1825.15490	Detected	Detected	Detected	Detected
6164	A_23_P40240	CTSZ	Hs.252549	cathepsin Z	Homo sapiens cathepsin Z (CTSZ), mRNA [NM_001336]	2.45895	down	3.07372	down	2796.76250	7120.95650	469.61874	1272.84570	Detected	Detected	Detected	Detected
6165	A_33_P3251347		Hs.188256		Mitogen-activated protein kinase kinase kinase 7-interacting protein 3 (TAK1-binding protein 3)(NF-kappa-B-activating protein 1) [Source:UniProtKB/Swiss-Prot;Acc:Q8N5C8] [ENST00000378928]	2.45885	down	1.48845	down	6.65649	16.94770	8.53700	11.20479	Compromised	Detected	Compromised	Compromised
6166	A_23_P104607	PSMC3	Hs.250758	proteasome (prosome, macropain) 26S subunit, ATPase, 3	Homo sapiens proteasome (prosome, macropain) 26S subunit, ATPase, 3 (PSMC3), mRNA [NM_002804]	2.45860	down	2.73951	down	5250.62900	13366.92400	5814.00930	14044.76000	Detected	Detected	Detected	Detected
6167	A_33_P3234849	UTRN	Hs.133135	utrophin	Homo sapiens utrophin (UTRN), mRNA [NM_007124]	2.45824	down	1.05040	down	9.02385	22.96936	17.02772	15.77162	Compromised	Detected	Detected	Detected

6168	A_33_P3382588	WNT1	Hs.248164	wingless-type MMTV integration site family, member 1	Homo sapiens wingless-type MMTV integration site family, member 1 (WNT1), mRNA [NM_005430]	2.45764	down	2.46902	down	7.53154	19.16614	5.79328	12.61290	Compromised	Detected	Compromised	Compromised
6169	A_23_P106299	SERF2	Hs.424126	small EDRK-rich factor 2	Homo sapiens small EDRK-rich factor 2 (SERF2), mRNA [NM_001018108]	2.45687	down	2.22477	down	13772.82100	35037.86300	15986.01100	31361.05700	Detected	Detected	Detected	Detected
6170	A_23_P354387	MYOF	Hs.602086	myoferlin	Homo sapiens myoferlin (MYOF), transcript variant 1, mRNA [NM_013451]	2.45657	down	2.42893	down	8086.66940	20569.89800	5400.19240	11566.19100	Detected	Detected	Detected	Detected
6171	A_23_P52311	TAF5	Hs.96103	TAF5 RNA polymerase II, TATA box binding protein (TBP)-associated factor, 100kDa	Homo sapiens TAF5 RNA polymerase II, TATA box binding protein (TBP)-associated factor, 100kDa (TAF5), mRNA [NM_006951]	2.45615	down	2.29989	down	97.32083	247.51009	66.78814	135.44788	Detected	Detected	Detected	Detected
6172	A_24_P314681		Hs.454452		BX281397 Soares_NFL_T_GBC_S1 Homo sapiens cDNA clone IMAGp998G075145 ; IMAGE:2087454, mRNA sequence [BX281397]	2.45537	down	1.92542	down	65.38776	166.24452	42.82311	72.70578	Detected	Detected	Detected	Detected
6173	A_23_P7655	BNIP1	Hs.145726	BCL2/adenovirus E1B 19kDa interacting protein 1	Homo sapiens BCL2/adenovirus E1B 19kDa interacting protein 1 (BNIP1), transcript variant BNIP1-a, mRNA [NM_013978]	2.45452	down	2.53291	down	540.90314	1374.73800	613.63184	1370.54330	Detected	Detected	Detected	Detected
6174	A_33_P3214625	INPP4B	Hs.658245	inositol polyphosphate-4-phosphatase, type II, 105kDa	Homo sapiens inositol polyphosphate-4-phosphatase, type II, 105kDa (INPP4B), transcript variant 1, mRNA [NM_003866]	2.45396	down	3.01445	down	30.56667	77.66917	17.96800	47.76100	Detected	Detected	Detected	Detected
6175	A_23_P401675	MARVELD2	Hs.657687	MARVEL domain containing 2	Homo sapiens MARVEL domain containing 2 (MARVELD2), transcript variant 2, mRNA [NM_144724]	2.45302	down	2.15381	down	171.31157	435.13257	128.06538	243.22343	Detected	Detected	Detected	Detected
6176	A_33_P3254412	ULK2	Hs.168762	unc-51-like kinase 2 (C. elegans)	Homo sapiens unc-51-like kinase 2 (C. elegans) (ULK2), transcript variant 1, mRNA [NM_014683]	2.45233	down	2.49249	down	115.71198	293.82560	70.14289	154.16388	Detected	Detected	Detected	Detected
6177	A_24_P317450	OSTCL	Hs.487035	oligosaccharyltransferase complex subunit-like	Homo sapiens oligosaccharyltransferase complex subunit-like (OSTCL), non-coding RNA [NR_028496]	2.45209	down	1.59725	down	4424.02900	11232.77800	1530.06500	2154.99980	Detected	Detected	Detected	Detected
6178	A_24_P377499	OSBPL3	Hs.520259	oxysterol binding protein-like 3	Homo sapiens oxysterol binding protein-like 3 (OSBPL3), transcript variant 1, mRNA [NM_015550]	2.45101	down	2.22136	down	609.49420	1546.85000	458.33075	897.76680	Detected	Detected	Detected	Detected
6179	A_24_P134235	KHSRP	Hs.91142	KH-type splicing regulatory protein	Homo sapiens KH-type splicing regulatory protein (KHSRP), mRNA [NM_003685]	2.45093	down	2.22758	down	72.53574	184.08406	94.75519	186.12355	Detected	Detected	Detected	Detected
6180	A_23_P26522	AQP8	Hs.176658	aquaporin 8	Homo sapiens aquaporin 8 (AQP8), mRNA [NM_001169]	2.45038	down	2.17691	down	74.23408	188.35146	48.04609	92.22843	Detected	Detected	Detected	Detected
6181	A_24_P389959	COPZ1	Hs.505652	coatomer protein complex, subunit zeta 1	Homo sapiens coatomer protein complex, subunit zeta 1 (COPZ1), mRNA [NM_016057]	2.45001	down	2.92838	down	2498.51590	6338.44530	2245.01030	5797.11230	Detected	Detected	Detected	Detected
6182	A_23_P20122	ZC3HAV1	Hs.133512	zinc finger CCCH-type, antiviral 1	Homo sapiens zinc finger CCCH-type, antiviral 1 (ZC3HAV1), transcript variant 2, mRNA [NM_024625]	2.44977	down	2.33915	down	104.76318	265.74606	75.86346	156.47876	Detected	Detected	Detected	Detected
6183	A_33_P3282973				Brain and acute leukemia cytoplasmic protein [Source:UniProtKB/Swiss-Prot;Acc:Q8WXS3] [ENST00000395861]	2.44959	down	3.51207	down	127.08301	322.33957	113.78412	352.37973	Detected	Detected	Detected	Detected
6184	A_33_P3214310	FOXP1	Hs.431498	forkhead box P1	Homo sapiens forkhead box P1 (FOXP1), transcript variant 2, mRNA [NM_001012505]	2.44877	down	4.86165	down	85.96473	217.97195	36.61164	156.95260	Detected	Detected	Detected	Detected
6185	A_33_P3397399		Hs.500067		Serine/threonine-protein phosphatase 2B catalytic subunit beta isoform (EC 3.1.3.16)(Calmodulin-dependent calcineurin A subunit beta isoform)(CAM-PRP catalytic subunit) [Source:UniProtKB/Swiss-Prot;Acc:P16298] [ENST00000394822]	2.44850	down	2.59471	down	256.80356	651.08010	268.57877	614.50616	Detected	Detected	Detected	Detected
6186	A_33_P3308050	EIF4E2	Hs.292026	eukaryotic translation initiation factor 4E family member 2	Homo sapiens eukaryotic translation initiation factor 4E family member 2 (EIF4E2), mRNA [NM_004846]	2.44765	down	2.40394	down	5805.62940	14714.00200	6912.24270	14652.37800	Detected	Detected	Detected	Detected
6187	A_32_P108889	DCLK1	Hs.507755	doublecortin-like kinase 1	Homo sapiens doublecortin-like kinase 1 (DCLK1), mRNA [NM_004734]	2.44764	down	1.18740	down	9.46958	24.00001	8.10572	8.48703	Compromised	Detected	Compromised	Compromised
6188	A_24_P565556	ALG1	Hs.592086	asparagine-linked glycosylation 1, beta-1,4-mannosyltransferase homolog (S. cerevisiae)	Homo sapiens asparagine-linked glycosylation 1, beta-1,4-mannosyltransferase homolog (S. cerevisiae) (ALG1), mRNA [NM_019109]	2.44546	down	2.73346	down	357.60180	905.50960	97.59224	235.23062	Detected	Detected	Detected	Detected
6189	A_23_P2041	MICALCL	Hs.128196	MICAL C-terminal like	Homo sapiens MICAL C-terminal like (MICALCL), mRNA [NM_032867]	2.44502	down	2.26584	down	113.37088	287.02362	94.48461	188.78000	Detected	Detected	Detected	Detected
6190	A_33_P3273552	KRT83	Hs.720768	keratin 83	Homo sapiens keratin 83 (KRT83), mRNA [NM_002282]	2.44217	down	3.12917	down	36.98336	93.52235	25.46344	70.26061	Detected	Detected	Detected	Detected
6191	A_33_P3365002	TUBB2A	Hs.654543	tubulin, beta 2A	Homo sapiens tubulin, beta 2A (TUBB2A), mRNA [NM_001069]	2.44205	down	2.73301	down	2988.80080	7557.59670	3583.82470	8636.80300	Detected	Detected	Detected	Detected
6192	A_33_P3884179	LOC150759	Hs.651352	hypothetical protein LOC150759	Homo sapiens cDNA FLJ33034 fis, clone THYMU2000236 [AK057596]	2.44203	down	2.13420	down	224.85059	568.56340	186.91480	351.75833	Detected	Detected	Detected	Detected
6193	A_33_P3345782				Coiled-coil domain-containing protein 88B (Brain leucine zipper domain-containing protein)(Hook-related protein 3)(HkRP3) [Source:UniProtKB/Swiss-Prot;Acc:A6NC98] [ENST00000318701]	2.44073	down	1.06520	down	9.68236	24.46997	4.07507	3.82765	Compromised	Detected	Compromised	Compromised
6194	A_23_P31444	ERVWE1		endogenous retroviral family W, env(C7), member 1	Homo sapiens endogenous retroviral family W, env(C7), member 1 (ERVWE1), transcript variant 1, mRNA [NM_014590]	2.44065	down	1.18321	down	6.86740	17.35525	2.88383	3.00881	Compromised	Detected	Compromised	Compromised
6195	A_33_P3209869	TCP11L1	Hs.655341	t-complex 11 (mouse)-like 1	Homo sapiens t-complex 11 (mouse)-like 1 (TCP11L1), transcript variant 1, mRNA [NM_018393]	2.44022	down	2.53542	down	942.49210	2381.43600	672.15300	1502.73730	Detected	Detected	Detected	Detected

6196	A_33_P3321611	PHACTR4	Hs.225641	phosphatase and actin regulator 4	Homo sapiens phosphatase and actin regulator 4 (PHACTR4), transcript variant 1, mRNA [NM_001048183]	2.43938	down	1.30154	down	42.61089	107.62991	70.58269	81.00669	Detected	Detected	Detected	Detected
6197	A_23_P166336	TMEM191A	Hs.546454	transmembrane protein 191A	Homo sapiens transmembrane protein 191A (TMEM191A), non-coding RNA [NR_026815]	2.43926	down	2.34592	down	432.18880	1091.60310	501.51700	1037.44410	Detected	Detected	Detected	Detected
6198	A_23_P47790	METTL1	Hs.42957	methyltransferase like 1	Homo sapiens methyltransferase like 1 (METTL1), transcript variant 1, mRNA [NM_005371]	2.43859	down	2.59265	down	911.14220	2300.69380	1117.72270	2555.30640	Detected	Detected	Detected	Detected
6199	A_33_P3321050	PHACTR2	Hs.102471	phosphatase and actin regulator 2	Homo sapiens phosphatase and actin regulator 2 (PHACTR2), transcript variant 2, mRNA [NM_001100165]	2.43760	down	2.53833	down	223.10963	563.13635	196.07315	438.86594	Detected	Detected	Detected	Detected
6200	A_23_P65240	COL4A1	Hs.17441	collagen, type IV, alpha 1	Homo sapiens collagen, type IV, alpha 1 (COL4A1), mRNA [NM_001845]	2.43742	down	2.49194	down	2371.95600	5986.46340	2028.57760	4457.52900	Detected	Detected	Detected	Detected
6201	A_23_P307328	WHSC1	Hs.113876	Wolf-Hirschhorn syndrome candidate 1	Homo sapiens Wolf-Hirschhorn syndrome candidate 1 (WHSC1), transcript variant 8, mRNA [NM_007331]	2.43615	down	2.24663	down	585.89140	1477.93330	478.07068	947.08563	Detected	Detected	Detected	Detected
6202	A_23_P350234	UBE2NL	Hs.585177	ubiquitin-conjugating enzyme E2N-like	Homo sapiens ubiquitin-conjugating enzyme E2N-like (UBE2NL), mRNA [NM_001012989]	2.43280	down	2.56071	down	324.17320	816.61346	380.47205	859.10900	Detected	Detected	Detected	Detected
6203	A_33_P3417620				U2 small nuclear ribonucleoprotein auxiliary factor 35 kDa subunit-related protein 2 (U2(RNU2) small nuclear RNA auxiliary factor 1-like 2)(CCCH type zinc finger, RNA-binding motif and serine/arginine rich protein 2)(Renal carcinoma antigen NY-REN-20) [Source:UniProtKB/Swiss-Prot;Acc:Q15696] [ENST00000380308]	2.43096	down	2.36823	down	126.49808	318.41574	97.35738	203.31012	Detected	Detected	Detected	Detected
6204	A_24_P193295	RAB15	Hs.512492	RAB15, member RAS onocogene family	Homo sapiens RAB15, member RAS onocogene family (RAB15), mRNA [NM_198686]	2.43028	down	2.49759	down	2195.66300	5525.28170	1646.90120	3627.06000	Detected	Detected	Detected	Detected
6205	A_23_P30805	HIST1H4J	Hs.278483	histone cluster 1, H4j	Homo sapiens histone cluster 1, H4j (HIST1H4J), mRNA [NM_021968]	2.42999	down	2.53875	down	363.35350	914.25300	387.43097	867.31960	Detected	Detected	Detected	Detected
6206	A_23_P112159	EIF2C2	Hs.449415	eukaryotic translation initiation factor 2C, 2	Homo sapiens eukaryotic translation initiation factor 2C, 2 (EIF2C2), transcript variant 1, mRNA [NM_012154]	2.42844	down	2.58673	down	838.95300	2109.58810	715.51850	1632.06570	Detected	Detected	Detected	Detected
6207	A_23_P394395	JPH2	Hs.441737	junctophilin 2	Homo sapiens junctophilin 2 (JPH2), transcript variant 1, mRNA [NM_020433]	2.42842	down	3.28445	down	60.57504	152.31790	60.70457	175.81221	Detected	Detected	Detected	Detected
6208	A_33_P3288589	LOC100128055	Hs.710623	hypothetical protein LOC100128055	PREDICTED: Homo sapiens hypothetical protein LOC100128055 (LOC100128055), mRNA [XM_001720585]	2.42831	down	4.02652	down	25.75042	64.74716	15.80330	56.11041	Detected	Detected	Detected	Detected
6209	A_23_P21033	GMPS	Hs.591314	guanine monphosphate synthetase	Homo sapiens guanine monphosphate synthetase (GMPS), mRNA [NM_003875]	2.42815	down	2.51394	down	5934.48600	14920.77700	4415.37800	9787.88000	Detected	Detected	Detected	Detected
6210	A_24_P277747	CNPY3	Hs.414099	canopy 3 homolog (zebrafish)	Homo sapiens canopy 3 homolog (zebrafish) (CNPY3), mRNA [NM_006586]	2.42727	down	2.33822	down	96.34708	242.15308	104.92540	216.33717	Detected	Detected	Detected	Detected
6211	A_33_P3267320	RFT1	Hs.720810	RFT1 homolog (S. cerevisiae)	Homo sapiens RFT1 homolog (S. cerevisiae) (RFT1), mRNA [NM_052859]	2.42664	down	2.39920	down	37.89586	95.22031	44.33249	93.78940	Detected	Detected	Detected	Detected
6212	A_23_P76690	UCHL3	Hs.162241	ubiquitin carboxyl-terminal esterase L3 (ubiquitin thiolesterase)	Homo sapiens ubiquitin carboxyl-terminal esterase L3 (ubiquitin thiolesterase) (UCHL3), mRNA [NM_006002]	2.42654	down	2.27245	down	3284.78100	8253.27900	2586.29100	5182.47700	Detected	Detected	Detected	Detected
6213	A_23_P333063	SMARCE1	Hs.643780	SWI/SNF related, matrix associated, actin dependent regulator of chromatin, subfamily e, member 1	Homo sapiens SWI/SNF related, matrix associated, actin dependent regulator of chromatin, subfamily e, member 1 (SMARCE1), mRNA [NM_003079]	2.42637	down	2.39365	down	885.01520	2223.51830	554.19660	1169.74120	Detected	Detected	Detected	Detected
6214	A_23_P70448	HIST1H1A	Hs.150206	histone cluster 1, H1a	Homo sapiens histone cluster 1, H1a (HIST1H1A), mRNA [NM_005325]	2.42598	down	2.85658	down	165.76979	416.41428	141.29810	355.91653	Detected	Detected	Detected	Detected
6215	A_24_P598406	RNF216L	Hs.520636	ring finger protein 216-like	Homo sapiens ring finger protein 216-like (RNF216L), transcript variant 1, non-coding RNA [NR_023384]	2.42518	down	2.49369	down	4408.47460	11070.47300	4525.04350	9950.18200	Detected	Detected	Detected	Detected
6216	A_24_P318967	PDXK	Hs.284491	pyridoxal (pyridoxine, vitamin B6) kinase	Homo sapiens pyridoxal (pyridoxine, vitamin B6) kinase (PDXK), mRNA [NM_003681]	2.42472	down	2.68120	down	2626.02080	6593.15900	3275.44020	7743.99500	Detected	Detected	Detected	Detected
6217	A_24_P406245	PMS2L2	Hs.720746	postmeiotic segregation increased 2-like 2 pseudogene	Homo sapiens cDNA clone IMAGE:3456494, partial cds [BC010535]	2.42246	down	2.55122	down	124.00018	311.03650	101.31708	227.92752	Detected	Detected	Detected	Detected
6218	A_24_P914513	BCKDHB	Hs.654441	branched chain keto acid dehydrogenase E1, beta polypeptide	Homo sapiens branched chain keto acid dehydrogenase E1, beta polypeptide (BCKDHB), nuclear gene encoding mitochondrial protein, transcript variant 1, mRNA [NM_183050]	2.42222	down	2.81725	down	33.29901	83.51744	10.99819	27.32194	Detected	Detected	Compromised	Detected
6219	A_24_P92163					2.41832	down	2.60000	down	91.78154	229.82756	70.62071	161.90894	Detected	Detected	Detected	Detected
6220	A_33_P3256685	TTF2	Hs.486818	transcription termination factor, RNA polymerase II	Homo sapiens transcription termination factor, RNA polymerase II (TTF2), mRNA [NM_003594]	2.41772	down	2.39616	down	521.99020	1306.77600	371.38846	784.70984	Detected	Detected	Detected	Detected
6221	A_23_P150693	FJX1	Hs.39384	four jointed box 1 (Drosophila)	Homo sapiens four jointed box 1 (Drosophila) (FJX1), mRNA [NM_014344]	2.41697	down	2.43571	down	5658.07600	14160.33700	4991.75600	10721.23500	Detected	Detected	Detected	Detected
6222	A_33_P3226955				Neuropilin-1 Precursor (Vascular endothelial cell growth factor 165 receptor)(CD304 antigen) [Source:UniProtKB/Swiss-Prot;Acc:O14786] [ENST00000455749]	2.41667	down	2.35727	down	377.16710	943.81070	323.96454	673.40000	Detected	Detected	Detected	Detected

6223	A_23_P163458	EHD4	Hs.143703	EH-domain containing 4	Homo sapiens EH-domain containing 4 (EHD4), mRNA [NM_139265]	2.41621	down	2.32343	down	978.82874	2448.91650	1185.44430	2428.71170	Detected	Detected	Detected	Detected
6224	A_23_P25019	PRIM1	Hs.534339	primase, DNA, polypeptide 1 (49kDa)	Homo sapiens primase, DNA, polypeptide 1 (49kDa) (PRIM1), mRNA [NM_000946]	2.41586	down	2.44769	down	295.59567	739.43870	311.15802	671.58673	Detected	Detected	Detected	Detected
6225	A_24_P275073	ADAMTS14	Hs.352156	ADAM metallopeptidase with thrombospondin type 1 motif, 14	Homo sapiens ADAM metallopeptidase with thrombospondin type 1 motif, 14 (ADAMTS14), transcript variant 1, mRNA [NM_139155]	2.41412	down	2.96642	down	68.63380	171.56543	21.49896	56.23612	Detected	Detected	Detected	Detected
6226	A_23_P166159	PDRG1	Hs.435755	p53 and DNA-damage regulated 1	Homo sapiens p53 and DNA-damage regulated 1 (PDRG1), mRNA [NM_030815]	2.41342	down	2.62313	down	1911.74560	4777.44630	2053.05050	4748.81640	Detected	Detected	Detected	Detected
6227	A_24_P219266	LRRC57	Hs.234681	leucine rich repeat containing 57	Homo sapiens leucine rich repeat containing 57 (LRRC57), mRNA [NM_153260]	2.41312	down	2.43587	down	526.00540	1314.32510	322.48877	692.68340	Detected	Detected	Detected	Detected
6228	A_33_P3273369	SH3BP4	Hs.516777	SH3-domain binding protein 4	Homo sapiens SH3-domain binding protein 4 (SH3BP4), mRNA [NM_014521]	2.41302	down	2.26227	down	79.81108	199.41457	107.49062	214.42757	Detected	Detected	Detected	Detected
6229	A_23_P138168	CNN3	Hs.483454	calponin 3, acidic	Homo sapiens calponin 3, acidic (CNN3), mRNA [NM_001839]	2.41146	down	2.44425	down	1373.70430	3430.09280	1432.24460	3086.94630	Detected	Detected	Detected	Detected
6230	A_23_P256470	NPY	Hs.1832	neuropeptide Y	Homo sapiens neuropeptide Y (NPY), mRNA [NM_000905]	2.41116	down	1.55344	down	19.79165	49.41306	22.13791	30.32477	Detected	Detected	Detected	Detected
6231	A_23_P84000	CACNG2	Hs.670146	calcium channel, voltage-dependent, gamma subunit 2	Homo sapiens calcium channel, voltage-dependent, gamma subunit 2 (CACNG2), mRNA [NM_006078]	2.40957	down	1.00489	down	17.05386	42.54961	7.66197	6.78929	Detected	Detected	Compromised	Compromised
6232	A_23_P208698	GYS1	Hs.386225	glycogen synthase 1 (muscle)	Homo sapiens glycogen synthase 1 (muscle) (GYS1), transcript variant 1, mRNA [NM_002103]	2.40957	down	2.41668	down	1551.61300	3871.28860	1303.96900	2778.76400	Detected	Detected	Detected	Detected
6233	A_24_P215765	ATP10A	Hs.659258	ATPase, class V, type 10A	Homo sapiens ATPase, class V, type 10A (ATP10A), mRNA [NM_024490]	2.40682	down	2.42874	down	1506.77780	3755.14230	1250.31460	2677.72880	Detected	Detected	Detected	Detected
6234	A_23_P210726	CDC25B	Hs.153752	cell division cycle 25 homolog B (S. pombe)	Homo sapiens cell division cycle 25 homolog B (S. pombe) (CDC25B), transcript variant 1, mRNA [NM_021873]	2.40583	down	2.95091	down	3435.34380	8557.93200	2537.57470	6602.99000	Detected	Detected	Detected	Detected
6235	A_32_P70818	PAX9	Hs.132576	paired box 9	Homo sapiens paired box 9 (PAX9), mRNA [NM_006194]	2.40500	down	1.83358	down	21.34461	53.15400	18.21684	29.45358	Detected	Detected	Detected	Detected
6236	A_23_P356021	FANCB	Hs.554740	Fanconi anemia, complementation group B	Homo sapiens Fanconi anemia, complementation group B (FANCB), transcript variant 1, mRNA [NM_001018113]	2.40246	down	1.75269	down	19.22428	47.82335	18.66384	28.84499	Detected	Detected	Detected	Detected
6237	A_33_P3219121					2.40111	down	1.46226	down	14.62771	36.36814	22.40896	28.89436	Detected	Detected	Detected	Detected
6238	A_23_P399897	KY	Hs.146730	kyphoscoliosis peptidase	Homo sapiens kyphoscoliosis peptidase (KY), mRNA [NM_178554]	2.40100	down	2.76878	down	9.27688	23.06360	6.75571	16.49399	Compromised	Detected	Compromised	Detected
6239	A_33_P3258997	TRMT2B	Hs.496501	TRM2 tRNA methyltransferase 2 homolog B (S. cerevisiae)	Homo sapiens TRM2 tRNA methyltransferase 2 homolog B (S. cerevisiae) (TRMT2B), mRNA [NM_024917]	2.40022	down	2.54100	down	48.14915	119.66649	47.86802	107.25458	Detected	Detected	Detected	Detected
6240	A_23_P250212	PRAGMIN	Hs.657673	homolog of rat pragma of Rnd2	Homo sapiens homolog of rat pragma of Rnd2 (PRAGMIN), mRNA [NM_001080826]	2.40010	down	2.48642	down	697.69700	1733.92370	574.19714	1258.92910	Detected	Detected	Detected	Detected
6241	A_23_P82959	FOXH1	Hs.708365	forkhead box H1	Homo sapiens forkhead box H1 (FOXH1), mRNA [NM_003923]	2.39986	down	2.42303	down	135.31789	336.25873	94.61042	202.14550	Detected	Detected	Detected	Detected
6242	A_23_P139600	RASAL1	Hs.528693	RAS protein activator like 1 (GAP1 like)	Homo sapiens RAS protein activator like 1 (GAP1 like) (RASAL1), mRNA [NM_004658]	2.39911	down	3.77247	down	110.68571	274.96307	90.93790	302.50778	Detected	Detected	Detected	Detected
6243	A_23_P420551	CIT	Hs.119594	citron (rho-interacting, serine/threonine kinase 21)	Homo sapiens citron (rho-interacting, serine/threonine kinase 21) (CIT), mRNA [NM_007174]	2.39898	down	2.40431	down	4550.50700	11303.68900	3538.14010	7501.21500	Detected	Detected	Detected	Detected
6244	A_24_P32215				Putative uncharacterized protein ARL4P [Source:UniProtKB/TrEMBL;Acc:A6NDQ6] [ENST00000242475]	2.39883	down	1.33513	down	87.32372	216.90236	11.01740	12.97082	Detected	Detected	Compromised	Detected
6245	A_24_P316234	LOC643475	Hs.675960	FLJ00409 protein	Homo sapiens mRNA for FLJ00409 protein [AK090485]	2.39845	down	2.02613	down	14.60104	36.26165	8.77799	15.68298	Detected	Detected	Compromised	Detected
6246	A_23_P214222	MARCKS	Hs.519909	myristoylated alanine-rich protein kinase C substrate	Homo sapiens myristoylated alanine-rich protein kinase C substrate (MARCKS), mRNA [NM_002356]	2.39786	down	2.25658	down	22550.87100	55991.14500	16289.39900	32413.22500	Detected	Detected	Detected	Detected
6247	A_32_P9382	C13orf37	Hs.28465	chromosome 13 open reading frame 37	Homo sapiens chromosome 13 open reading frame 37 (C13orf37), mRNA [NM_001071775]	2.39748	down	2.45947	down	2242.13300	5566.07200	1564.42300	3392.82540	Detected	Detected	Detected	Detected
6248	A_23_P73012	C9orf3	Hs.434253	chromosome 9 open reading frame 3	Homo sapiens chromosome 9 open reading frame 3 (C9orf3), mRNA [NM_032823]	2.39680	down	2.19971	down	673.55980	1671.63000	474.07650	919.55945	Detected	Detected	Detected	Detected
6249	A_24_P915007	NACC1	Hs.531614	nucleus accumbens associated 1, BEN and BTB (POZ) domain containing	Homo sapiens nucleus accumbens associated 1, BEN and BTB (POZ) domain containing (NACC1), mRNA [NM_052876]	2.39622	down	2.22219	down	6437.30760	15972.20500	4971.10300	9740.92900	Detected	Detected	Detected	Detected
6250	A_33_P3292337	KIF5B	Hs.327736	kinesin family member 5B	Homo sapiens kinesin family member 5B (KIF5B), mRNA [NM_004521]	2.39574	down	2.05328	down	302.04883	749.28845	271.56247	491.68005	Detected	Detected	Detected	Detected
6251	A_33_P3230161	GRIA2	Hs.32763	glutamate receptor, ionotropic, AMPA 2	Homo sapiens glutamate receptor, ionotropic, AMPA 2 (GRIA2), transcript variant 1, mRNA [NM_000826]	2.39549	down	1.43506	down	7.92119	19.64796	6.78169	8.58173	Compromised	Detected	Compromised	Compromised
6252	A_23_P349771	HAUS5	Hs.7426	HAUS augmin-like complex, subunit 5	Homo sapiens HAUS augmin-like complex, subunit 5 (HAUS5), mRNA [NM_015302]	2.39504	down	2.46992	down	19.07787	47.31258	13.87438	30.21779	Detected	Detected	Compromised	Detected
6253	A_23_P310532	C19orf52	Hs.164026	chromosome 19 open reading frame 52	Homo sapiens chromosome 19 open reading frame 52 (C19orf52), mRNA [NM_138358]	2.39435	down	2.50040	down	1270.71290	3150.41970	1056.75020	2329.95850	Detected	Detected	Detected	Detected
6254	A_23_P140434	MYO5C	Hs.487036	myosin VC	Homo sapiens myosin VC (MYO5C), mRNA [NM_018728]	2.39383	down	1.66252	down	14.27611	35.38635	10.46960	15.34837	Detected	Detected	Compromised	Detected

6255	A_33_P3268310	LIMS3	Hs.535619	LIM and senescent cell antigen-like domains 3	Homo sapiens LIM and senescent cell antigen-like domains 3 (LIMS3), transcript variant 1, mRNA [NM_033514]	2.39213	down	2.61996	down	293.52295	727.04333	319.16278	737.34863	Detected	Detected	Detected	Detected
6256	A_32_P150735				B-cell receptor-associated protein 29 (BCR-associated protein Bap29) [Source:UniProtKB/Swiss-Prot;Acc:Q9UHQ4] [ENST00000379121]	2.39200	down	2.52458	down	87.85406	217.59822	80.22090	178.58408	Detected	Detected	Detected	Detected
6257	A_23_P72961	PRPS1	Hs.56	phosphoribosyl pyrophosphate synthetase 1	Homo sapiens phosphoribosyl pyrophosphate synthetase 1 (PRPS1), mRNA [NM_002764]	2.39010	down	3.88238	down	542.25780	1342.00730	1291.66760	4421.95560	Detected	Detected	Detected	Detected
6258	A_33_P3344243	C4orf46	Hs.380920	chromosome 4 open reading frame 46	Homo sapiens chromosome 4 open reading frame 46 (C4orf46), mRNA [NM_001008393]	2.39009	down	2.37191	down	2867.72140	7097.15900	2252.21660	4710.57000	Detected	Detected	Detected	Detected
6259	A_24_P198844	MPDU1	Hs.246381	mannose-P-dolichol utilization defect 1	Homo sapiens mannose-P-dolichol utilization defect 1 (MPDU1), transcript variant 1, mRNA [NM_004870]	2.38948	down	2.58342	down	1538.85110	3807.44500	1276.66130	2908.27700	Detected	Detected	Detected	Detected
6260	A_32_P224234		Hs.536063		PREDICTED: Homo sapiens hypothetical LOC645195 (LOC645195), miscRNA [XR_040418]	2.38917	down	2.50690	down	65.37261	161.72432	52.73568	116.57539	Detected	Detected	Detected	Detected
6261	A_23_P7873	MCM3	Hs.179565	minichromosome maintenance complex component 3	Homo sapiens minichromosome maintenance complex component 3 (MCM3), mRNA [NM_002388]	2.38910	down	2.31777	down	6732.87800	16655.89800	6376.69730	13032.62300	Detected	Detected	Detected	Detected
6262	A_23_P33196	COL5A2	Hs.445827	collagen, type V, alpha 2	Homo sapiens collagen, type V, alpha 2 (COL5A2), mRNA [NM_000393]	2.38858	down	2.50586	down	9524.81800	23557.50400	6864.69040	15168.54300	Detected	Detected	Detected	Detected
6263	A_33_P3342628	HES4	Hs.154029	hairy and enhancer of split 4 (Drosophila)	Homo sapiens hairy and enhancer of split 4 (Drosophila) (HES4), transcript variant 2, mRNA [NM_021170]	2.38764	down	2.37045	down	5532.15620	13677.19500	5212.37060	10895.11700	Detected	Detected	Detected	Detected
6264	A_24_P296508	SLC43A2	Hs.160550	solute carrier family 43, member 2	Homo sapiens solute carrier family 43, member 2 (SLC43A2), mRNA [NM_152346]	2.38760	down	2.51008	down	956.45996	2364.62480	368.64752	815.95087	Detected	Detected	Detected	Detected
6265	A_33_P3317825	NRAS	Hs.486502	neuroblastoma RAS viral (v-ras) oncogene homolog	Homo sapiens neuroblastoma RAS viral (v-ras) oncogene homolog (NRAS), mRNA [NM_002524]	2.38677	down	2.26301	down	285.97577	706.76135	350.15155	698.72766	Detected	Detected	Detected	Detected
6266	A_24_P147398	VPS53	Hs.461819	vacuolar protein sorting 53 homolog (S. cerevisiae)	Homo sapiens vacuolar protein sorting 53 homolog (S. cerevisiae) (VPS53), transcript variant 2, mRNA [NM_018289]	2.38562	down	3.57164	down	26.40779	65.23292	10.51792	33.12555	Detected	Detected	Compromised	Detected
6267	A_24_P170753		Hs.122115		EP400 N-terminal-like protein [Source:UniProtKB/Swiss-Prot;Acc:Q6ZTU2] [ENST00000392352]	2.38513	down	2.50227	down	458.37195	1132.04410	363.75073	802.60810	Detected	Detected	Detected	Detected
6268	A_32_P506600	RAN	Hs.10842	RAN, member RAS oncogene family	Homo sapiens RAN, member RAS oncogene family (RAN), mRNA [NM_006325]	2.38506	down	2.19907	down	58519.81200	144522.44000	42442.34000	82300.92000	Detected	Detected	Detected	Detected
6269	A_23_P348298	SAC3D1	Hs.23642	SAC3 domain containing 1	Homo sapiens SAC3 domain containing 1 (SAC3D1), mRNA [NM_013299]	2.38460	down	2.38017	down	2461.94870	6078.92720	2271.81500	4768.10740	Detected	Detected	Detected	Detected
6270	A_23_P118150	ARL6IP1	Hs.634882	ADP-ribosylation factor-like 6 interacting protein 1	Homo sapiens ADP-ribosylation factor-like 6 interacting protein 1 (ARL6IP1), mRNA [NM_015161]	2.38448	down	2.32264	down	2155.11380	5321.04830	1496.81860	3065.61280	Detected	Detected	Detected	Detected
6271	A_23_P107412	P4HB	Hs.464336	prolyl 4-hydroxylase, beta polypeptide	Homo sapiens prolyl 4-hydroxylase, beta polypeptide (P4HB), mRNA [NM_000918]	2.38394	down	2.66079	down	28360.87700	70007.99000	20931.53700	49110.79700	Detected	Detected	Detected	Detected
6272	A_33_P3369029	LOC440300	Hs.546565	chondroitin sulfate proteoglycan 4 pseudogene	PREDICTED: Homo sapiens chondroitin sulfate proteoglycan 4 pseudogene (LOC440300), miscRNA [XR_078504]	2.38364	down	2.95469	down	27.54297	67.98065	23.23212	60.52940	Detected	Detected	Detected	Detected
6273	A_33_P3314192	PACSIN2	Hs.162877	protein kinase C and casein kinase substrate in neurons 2	Homo sapiens protein kinase C and casein kinase substrate in neurons 2 (PACSIN2), mRNA [NM_007229]	2.38339	down	2.49808	down	511.49560	1262.32150	642.05980	1414.32090	Detected	Detected	Detected	Detected
6274	A_24_P194420	CCDC134	Hs.474991	coiled-coil domain containing 134	Homo sapiens coiled-coil domain containing 134 (CCDC134), mRNA [NM_024821]	2.38299	down	3.98437	down	44.92689	110.85669	30.18556	106.05341	Detected	Detected	Detected	Detected
6275	A_23_P413788	FUT11	Hs.588854	fucosyltransferase 11 (alpha (1,3) fucosyltransferase)	Homo sapiens fucosyltransferase 11 (alpha (1,3) fucosyltransferase) (FUT11), mRNA [NM_173540]	2.38255	down	2.43215	down	179.66650	443.24270	168.40967	361.17910	Detected	Detected	Detected	Detected
6276	A_23_P43580	CEP110	Hs.653263	centrosomal protein 110kDa	Homo sapiens centrosomal protein 110kDa (CEP110), mRNA [NM_007018]	2.38243	down	1.63333	down	36.90078	91.03100	27.92016	40.21216	Detected	Detected	Detected	Detected
6277	A_23_P408768	DOT1L	Hs.713641	DOT1-like, histone H3 methyltransferase (S. cerevisiae)	Homo sapiens DOT1-like, histone H3 methyltransferase (S. cerevisiae) (DOT1L), mRNA [NM_032482]	2.38196	down	2.53330	down	342.23697	844.10190	336.23492	751.09656	Detected	Detected	Detected	Detected
6278	A_33_P3305855	LOC645967	Hs.604079	hypothetical LOC645967	PREDICTED: Homo sapiens hypothetical LOC645967 (LOC645967), miscRNA [XR_040575]	2.38196	down	4.60598	down	26.72227	65.90839	8.49016	34.48285	Detected	Detected	Compromised	Detected
6279	A_33_P3227209	PA2G4	Hs.524498	proliferation-associated 2G4, 38kDa	Homo sapiens proliferation-associated 2G4, 38kDa (PA2G4), mRNA [NM_006191]	2.38176	down	2.38053	down	12213.10450	30120.10000	12293.34100	25805.30900	Detected	Detected	Detected	Detected
6280	A_33_P3340792	DHDDS	Hs.369385	dehydrodolichyl diphosphate synthase	Homo sapiens dehydrodolichyl diphosphate synthase (DHDDS), transcript variant 2, mRNA [NM_205861]	2.38111	down	2.55784	down	148.87335	367.05432	158.81848	358.21220	Detected	Detected	Detected	Detected
6281	A_23_P312358	BEND7	Hs.498740	BEN domain containing 7	Homo sapiens BEN domain containing 7 (BEND7), transcript variant 1, mRNA [NM_152751]	2.38097	down	2.34528	down	60.32234	148.71849	47.69342	98.63226	Detected	Detected	Detected	Detected
6282	A_23_P134477	C7orf50	Hs.653258	chromosome 7 open reading frame 50	Homo sapiens chromosome 7 open reading frame 50 (C7orf50), transcript variant 1, mRNA [NM_032350]	2.38013	down	2.39667	down	1549.45960	3818.68200	2102.85200	4444.08300	Detected	Detected	Detected	Detected

6283	A_33_P3242798	CPS1	Hs.149252	carbamoyl-phosphate synthetase 1, mitochondrial	Homo sapiens carbamoyl-phosphate synthetase 1, mitochondrial (CPS1), nuclear gene encoding mitochondrial protein, transcript variant 2, mRNA [NM_001875]	2.37913	down	2.28962	down	124.52889	306.77658	78.26087	158.00630	Detected	Detected	Detected	Detected
6284	A_32_P49508	HMGCLL1	Hs.147054	3-hydroxymethyl-3-methylglutaryl-Coenzyme A lyase-like 1	Homo sapiens 3-hydroxymethyl-3-methylglutaryl-Coenzyme A lyase-like 1 (HMGCLL1), transcript variant 1, mRNA [NM_019036]	2.37874	down	1.43411	down	9.87206	24.31574	11.87820	15.02095	Compromised	Detected	Detected	Detected
6285	A_33_P3338341	PRODH	Hs.517352	proline dehydrogenase (oxidase) 1	Homo sapiens proline dehydrogenase (oxidase) 1 (PRODH), nuclear gene encoding mitochondrial protein, mRNA [NM_016335]	2.37773	down	1.19215	down	15.09261	37.15862	2.52538	2.65475	Detected	Detected	Compromised	Compromised
6286	A_23_P64837	SMAGP	Hs.652389	small cell adhesion glycoprotein	Homo sapiens small cell adhesion glycoprotein (SMAGP), transcript variant 1, mRNA [NM_001031628]	2.37621	down	2.49674	down	937.69220	2307.16500	951.29140	2094.36350	Detected	Detected	Detected	Detected
6287	A_23_P359245	MET	Hs.132966	met proto-oncogene (hepatocyte growth factor receptor)	Homo sapiens met proto-oncogene (hepatocyte growth factor receptor) (MET), transcript variant 2, mRNA [NM_000245]	2.37468	down	2.40219	down	3089.59200	7596.94630	1885.15870	3993.20430	Detected	Detected	Detected	Detected
6288	A_33_P3232339	LOC100129119	Hs.154829	hypothetical LOC100129119	PREDICTED: Homo sapiens hypothetical LOC100129119 (LOC100129119), mRNA [XM_001718523]	2.37465	down	1.29436	down	25.62673	63.01248	15.90509	18.15331	Detected	Detected	Detected	Detected
6289	A_32_P194264	CHAC2	Hs.585944	ChaC, cation transport regulator homolog 2 (E. coli)	Homo sapiens ChaC, cation transport regulator homolog 2 (E. coli) (CHAC2), mRNA [NM_001008708]	2.37441	down	2.92787	down	130.48581	320.81350	114.49580	295.60193	Detected	Detected	Detected	Detected
6290	A_33_P3321657	HSPG2	Hs.562227	heparan sulfate proteoglycan 2	Homo sapiens heparan sulfate proteoglycan 2 (HSPG2), mRNA [NM_005529]	2.37340	down	2.34435	down	21245.90200	52212.98400	16998.88000	35140.52700	Detected	Detected	Detected	Detected
6291	A_23_P330616	WIPF1	Hs.128067	WAS/WASL interacting protein family, member 1	Homo sapiens WAS/WASL interacting protein family, member 1 (WIPF1), transcript variant 2, mRNA [NM_001077269]	2.37257	down	2.57124	down	90.88859	223.28610	85.63651	194.16342	Detected	Detected	Detected	Detected
6292	A_24_P169148	HMGB1L1	Hs.568249	high-mobility group box 1-like 1	Homo sapiens high-mobility group box 1-like 1 (HMGB1L1), mRNA [NM_001008735]	2.37077	down	1.98444	down	9072.45200	22271.36700	7471.40400	13073.92000	Detected	Detected	Detected	Detected
6293	A_24_P410952	PEA15	Hs.517216	phosphoprotein enriched in astrocytes 15	Homo sapiens phosphoprotein enriched in astrocytes 15 (PEA15), mRNA [NM_003768]	2.36988	down	2.86838	down	1786.65490	4384.29540	2105.33900	5325.05960	Detected	Detected	Detected	Detected
6294	A_33_P3249354	CCDC99	Hs.368710	coiled-coil domain containing 99	Homo sapiens coiled-coil domain containing 99 (CCDC99), mRNA [NM_017785]	2.36716	down	2.41207	down	682.93640	1673.94430	550.39636	1170.66170	Detected	Detected	Detected	Detected
6295	A_33_P3218832	RIMS1	Hs.485729	regulating synaptic membrane exocytosis 1	Homo sapiens regulating synaptic membrane exocytosis 1 (RIMS1), mRNA [NM_014989]	2.36644	down	1.48816	down	21.48857	52.65455	14.00723	18.38090	Detected	Detected	Compromised	Detected
6296	A_23_P302654	CEP72	Hs.591741	centrosomal protein 72kDa	Homo sapiens centrosomal protein 72kDa (CEP72), mRNA [NM_018140]	2.36579	down	2.38356	down	236.12088	578.41974	233.85294	491.51138	Detected	Detected	Detected	Detected
6297	A_23_P169017	DEFB103A	Hs.283082	defensin, beta 103A	Homo sapiens defensin, beta 103A (DEFB103A), mRNA [NM_018661]	2.36523	down	5.19144	down	13.66321	33.46262	3.78317	17.31846	Compromised	Detected	Compromised	Compromised
6298	A_24_P193943	PIGN	Hs.157031	phosphatidylinositol glycan anchor biosynthesis, class N	Homo sapiens phosphatidylinositol glycan anchor biosynthesis, class N (PIGN), transcript variant 1, mRNA [NM_176787]	2.36324	down	1.47021	down	41.13775	100.66568	36.84714	47.76917	Detected	Detected	Detected	Detected
6299	A_33_P3269718	IVD	Hs.513646	isovaleryl Coenzyme A dehydrogenase	Homo sapiens isovaleryl Coenzyme A dehydrogenase (IVD), transcript variant 2, mRNA [NM_001159508]	2.36297	down	2.29750	down	655.92220	1604.88100	590.08170	1195.45640	Detected	Detected	Detected	Detected
6300	A_24_P370472	HLA-DRB4	Hs.696211	major histocompatibility complex, class II, DR beta 4	Homo sapiens major histocompatibility complex, class II, DR beta 4 (HLA-DRB4), mRNA [NM_021983]	2.36294	down	3.25969	down	6.73416	16.47669	4.93347	14.18061	Compromised	Detected	Compromised	Detected
6301	A_33_P3309859	LOC100132805	Hs.610785	hypothetical LOC100132805	PREDICTED: Homo sapiens similar to predicted protein (LOC100132805), mRNA [XM_001719188]	2.36220	down	2.13646	down	305.69235	747.71310	269.39350	507.51297	Detected	Detected	Detected	Detected
6302	A_23_P134058	C6orf114	Hs.674313	chromosome 6 open reading frame 114	Homo sapiens chromosome 6 open reading frame 114 (C6orf114), mRNA [NM_033069]	2.36057	down	2.01040	down	13.08679	31.98768	11.70166	20.74412	Compromised	Detected	Compromised	Detected
6303	A_33_P3310475	SEPT11	Hs.128199	septin 11	Homo sapiens septin 11 (SEPT11), mRNA [NM_018243]	2.35985	down	2.45260	down	19772.34600	48314.36000	14137.60900	30575.15200	Detected	Detected	Detected	Detected
6304	A_24_P75072	SMUG1	Hs.632721	single-strand-selective monofunctional uracil-DNA glycosylase 1	Homo sapiens single-strand-selective monofunctional uracil-DNA glycosylase 1 (SMUG1), mRNA [NM_014311]	2.35793	down	2.80624	down	617.95605	1508.76420	642.35100	1589.51060	Detected	Detected	Detected	Detected
6305	A_23_P139912	IGFBP6	Hs.274313	insulin-like growth factor binding protein 6	Homo sapiens insulin-like growth factor binding protein 6 (IGFBP6), mRNA [NM_002178]	2.35792	down	2.57043	down	3753.25630	9163.68800	6446.36100	14611.22300	Detected	Detected	Detected	Detected
6306	A_23_P426944	PAX9	Hs.132576	paired box 9	Homo sapiens paired box 9 (PAX9), mRNA [NM_006194]	2.35748	down	1.83196	down	26.66429	65.08959	25.22474	40.74806	Detected	Detected	Detected	Detected
6307	A_24_P367211	SOCS7	Hs.632261	suppressor of cytokine signaling 7	Homo sapiens suppressor of cytokine signaling 7 (SOCS7), mRNA [NM_014598]	2.35677	down	2.34208	down	11.68562	28.51689	11.77062	24.30900	Compromised	Detected	Compromised	Detected
6308	A_23_P74737	EYA3	Hs.185774	eyes absent homolog 3 (Drosophila)	Homo sapiens eyes absent homolog 3 (Drosophila) (EYA3), mRNA [NM_001990]	2.35626	down	2.35150	down	89.79858	219.09164	89.88936	186.38850	Detected	Detected	Detected	Detected
6309	A_24_P29975	ARPC5L	Hs.132499	actin related protein 2/3 complex, subunit 5-like	Homo sapiens actin related protein 2/3 complex, subunit 5-like (ARPC5L), mRNA [NM_030978]	2.35564	down	2.38880	down	2830.70870	6904.57960	3237.32030	6819.17330	Detected	Detected	Detected	Detected
6310	A_33_P3210909	SEC13	Hs.166924	SEC13 homolog (S. cerevisiae)	Homo sapiens SEC13 homolog (S. cerevisiae) (SEC13), transcript variant 1, mRNA [NM_183352]	2.35326	down	2.32439	down	6396.91160	15587.36100	5520.17100	11314.28300	Detected	Detected	Detected	Detected
6311	A_23_P146572	NPDC1	Hs.719906	neural proliferation, differentiation and control, 1	Homo sapiens neural proliferation, differentiation and control, 1 (NPDC1), mRNA [NM_015392]	2.35100	down	2.55530	down	4597.90040	11192.94500	4012.34550	9040.77050	Detected	Detected	Detected	Detected

6312	A_23_P161439	C10orf116	Hs.642660	chromosome 10 open reading frame 116	Homo sapiens chromosome 10 open reading frame 116 (C10orf116), mRNA [NM_006829]	2.35054	down	2.17923	down	5528.31800	13455.29200	6269.69900	12048.02400	Detected	Detected	Detected	Detected
6313	A_23_P138025	GPN2	Hs.14333	GPN-loop GTPase 2	Homo sapiens GPN-loop GTPase 2 (GPN2), mRNA [NM_018066]	2.34856	down	2.28674	down	937.90730	2280.83890	1277.48940	2575.95830	Detected	Detected	Detected	Detected
6314	A_33_P3401252	HSP90B3P	Hs.591435	heat shock protein 90kDa beta (Grp94), member 3 (pseudogene)	Homo sapiens heat shock protein 90kDa beta (Grp94), member 3 (pseudogene) (HSP90B3P), non-coding RNA [NR_003130]	2.34808	down	2.20274	down	4265.44970	10370.78600	4206.58000	8170.66500	Detected	Detected	Detected	Detected
6315	A_23_P61371	TMEM173	Hs.379754	transmembrane protein 173	Homo sapiens transmembrane protein 173 (TMEM173), nuclear gene encoding mitochondrial protein, mRNA [NM_198282]	2.34631	down	2.47218	down	4192.79100	10186.41400	3677.53520	8016.83840	Detected	Detected	Detected	Detected
6316	A_24_P89701	IMPDH1	Hs.654401	IMP (inosine monophosphate) dehydrogenase 1	Homo sapiens IMP (inosine monophosphate) dehydrogenase 1 (IMPDH1), transcript variant 1, mRNA [NM_000883]	2.34516	down	2.34783	down	19609.92600	47619.05500	17659.37500	36560.04000	Detected	Detected	Detected	Detected
6317	A_23_P127522	HYLS1	Hs.585071	hydrolethalus syndrome 1	Homo sapiens hydrolethalus syndrome 1 (HYLS1), transcript variant 1, mRNA [NM_145014]	2.34468	down	2.43323	down	660.31696	1603.12940	676.78490	1452.10690	Detected	Detected	Detected	Detected
6318	A_33_P3399718	LOC128322		similar to nuclear transport factor 2	PREDICTED: Homo sapiens similar to nuclear transport factor 2 (LOC128322), mRNA [XM_001716411]	2.34435	down	2.30505	down	478.85886	1162.41920	627.62683	1275.69620	Detected	Detected	Detected	Detected
6319	A_33_P3375451	NYNRIN	Hs.288348	NYN domain and retroviral integrase containing	Homo sapiens NYN domain and retroviral integrase containing (NYNRIN), mRNA [NM_025081]	2.34319	down	1.31541	down	27.04986	65.63056	22.58610	26.19789	Detected	Detected	Detected	Detected
6320	A_23_P67278	ZNF443	Hs.720755	zinc finger protein 443	Homo sapiens zinc finger protein 443 (ZNF443), mRNA [NM_005815]	2.34133	down	2.93591	down	46.28160	112.20278	11.78185	30.50156	Detected	Detected	Compromised	Detected
6321	A_33_P3354256	MTSS1	Hs.336994	metastasis suppressor 1	Homo sapiens metastasis suppressor 1 (MTSS1), mRNA [NM_014751]	2.34103	down	1.76063	down	25.01372	60.63431	21.30042	33.06904	Detected	Detected	Detected	Detected
6322	A_33_P3304908		Hs.722218		ie90h04.y1 Melton Normalized Human Islet 4 N4-HIS 1 Homo sapiens cDNA clone IMAGE:5674231 5', mRNA sequence [BM069901]	2.34058	down	2.24438	down	207.98209	504.06088	89.01482	176.16669	Detected	Detected	Detected	Detected
6323	A_33_P3402526	CSGALNACT2	Hs.657569	chondroitin sulfate N-acetylgalactosaminyltransferase 2	Homo sapiens chondroitin sulfate N-acetylgalactosaminyltransferase 2 (CSGALNACT2), mRNA [NM_018590]	2.33852	down	1.39430	down	211.26974	511.57780	380.51680	467.83713	Detected	Detected	Detected	Detected
6324	A_33_P3413701	ERAP1	Hs.716426	endoplasmic reticulum aminopeptidase 1	Homo sapiens endoplasmic reticulum aminopeptidase 1 (ERAP1), transcript variant 2, mRNA [NM_001040458]	2.33746	down	2.11009	down	333.07530	806.15740	223.59775	416.03980	Detected	Detected	Detected	Detected
6325	A_24_P162287	CEP250	Hs.443976	centrosomal protein 250kDa	Homo sapiens centrosomal protein 250kDa (CEP250), mRNA [NM_007186]	2.33572	down	2.20390	down	36.43417	88.11762	29.00269	56.36321	Detected	Detected	Detected	Detected
6326	A_24_P20630	LEF1	Hs.555947	lymphoid enhancer-binding factor 1	Homo sapiens lymphoid enhancer-binding factor 1 (LEF1), transcript variant 1, mRNA [NM_016269]	2.33350	down	1.92865	down	162.12495	391.73346	121.71104	206.98984	Detected	Detected	Detected	Detected
6327	A_24_P178065		Hs.477114		Pleckstrin homology-like domain family B member 2 (Protein LL5-beta) [Source:UniProtKB/Swiss-Prot;Acc:Q86SQ0] [ENST00000478922]	2.33321	down	1.32988	down	386.86642	934.64746	17.73886	20.80199	Detected	Detected	Detected	Detected
6328	A_23_P26223	ASL	Hs.632015	argininosuccinate lyase	Homo sapiens argininosuccinate lyase (ASL), transcript variant 1, mRNA [NM_001024943]	2.33234	down	2.25137	down	1411.58980	3409.04570	1954.22990	3879.60940	Detected	Detected	Detected	Detected
6329	A_24_P233786	FAM129A	Hs.518662	family with sequence similarity 129, member A	Homo sapiens family with sequence similarity 129, member A (FAM129A), transcript variant 2, mRNA [NM_052966]	2.33174	down	2.40136	down	1587.27000	3832.34500	1325.88490	2807.55620	Detected	Detected	Detected	Detected
6330	A_23_P165840	ODC1	Hs.467701	ornithine decarboxylase 1	Homo sapiens ornithine decarboxylase 1 (ODC1), mRNA [NM_002539]	2.33167	down	2.32514	down	13138.87200	31721.78000	10350.43200	21221.34000	Detected	Detected	Detected	Detected
6331	A_33_P3550894	GATA2	Hs.367725	GATA binding protein 2	Homo sapiens GATA binding protein 2 (GATA2), transcript variant 1, mRNA [NM_001145661]	2.33090	down	2.25758	down	43.18446	104.22799	41.48375	82.58216	Detected	Detected	Detected	Detected
6332	A_23_P431381	C14orf80	Hs.720306	chromosome 14 open reading frame 80	Homo sapiens chromosome 14 open reading frame 80 (C14orf80), transcript variant 1, mRNA [NM_001134875]	2.33074	down	2.62601	down	274.93780	663.53210	497.26020	1151.45310	Detected	Detected	Detected	Detected
6333	A_33_P3408212	HSP90B1	Hs.192374	heat shock protein 90kDa beta (Grp94), member 1	Homo sapiens heat shock protein 90kDa beta (Grp94), member 1 (HSP90B1), mRNA [NM_003299]	2.32980	down	2.22592	down	2519.94290	6079.15230	1664.37380	3266.82540	Detected	Detected	Detected	Detected
6334	A_33_P3284518	UHRF1BP1L	Hs.620701	UHRF1 binding protein 1-like	Homo sapiens UHRF1 binding protein 1-like (UHRF1BP1L), transcript variant 2, mRNA [NM_001006947]	2.32979	down	2.30091	down	149.22653	359.99470	114.90129	233.12581	Detected	Detected	Detected	Detected
6335	A_33_P3353360				Homo sapiens unknown mRNA, complete cds. [AF281279]	2.32957	down	2.49382	down	47.29825	114.09156	36.11374	79.41522	Detected	Detected	Detected	Detected
6336	A_33_P3221843				Hexaprenyldihydroxybenzoate methyltransferase, mitochondrial Precursor (EC 2.1.1.114)(Dihydroxyhexaprenylbenzoate methyltransferase)(3,4-dihydroxy-5-hexaprenylbenzoate methyltransferase)(DHHB methyltransferase)(DHHB-MT)(DHHB-MTase)(DHHB-MT) [Source:UniProtKB/Swiss-Prot;Acc:Q9NZJ6] [ENST00000369242]	2.32903	down	2.39355	down	526.17090	1268.92250	317.35876	669.81995	Detected	Detected	Detected	Detected
6337	A_33_P3367701	TMEM164	Hs.659535	transmembrane protein 164	Homo sapiens transmembrane protein 164 (TMEM164), transcript variant 2, mRNA [NM_032227]	2.32827	down	3.46092	down	11.33496	27.32665	9.05316	27.62849	Compromised	Detected	Compromised	Detected
6338	A_33_P3400278					2.32799	down	1.87838	down	13.02926	31.40756	4.36430	7.22875	Detected	Detected	Compromised	Compromised
6339	A_23_P145357	BAK1	Hs.485139	BCL2-antagonist/killer 1	Homo sapiens BCL2-antagonist/killer 1 (BAK1), mRNA [NM_001188]	2.32791	down	2.65414	down	1202.95950	2899.68100	1258.46310	2945.30710	Detected	Detected	Detected	Detected

6340	A_32_P48244	ZNF100	Hs.365142	zinc finger protein 100	Homo sapiens zinc finger protein 100, mRNA (cDNA clone MGC:45410 IMAGE:5576923), complete cds [BC035579]	2.32658	down	2.58641	down	6.75861	16.28205	2.51934	5.74579	Compromised	Detected	Compromised	Compromised
6341	A_33_P3252781	PLAC9	Hs.204947	placenta-specific 9	Homo sapiens placenta-specific 9 (PLAC9), mRNA [NM_001012973]	2.32372	down	2.25151	down	463.61545	1115.51120	833.56440	1654.92460	Detected	Detected	Detected	Detected
6342	A_23_P310972	PCDHGB1	Hs.368160	protocadherin gamma subfamily B, 1	Homo sapiens protocadherin gamma subfamily B, 1 (PCDHGB1), transcript variant 2, mRNA [NM_032095]	2.32229	down	2.76571	down	8.79056	21.13807	6.73388	16.42243	Compromised	Detected	Compromised	Detected
6343	A_32_P63848	OXCT1	Hs.278277	3-oxoacid CoA transferase 1	Homo sapiens 3-oxoacid CoA transferase 1 (OXCT1), nuclear gene encoding mitochondrial protein, mRNA [NM_000436]	2.32210	down	2.12275	down	1247.79800	3000.26000	803.16860	1503.38710	Detected	Detected	Detected	Detected
6344	A_23_P203267	TRIM29	Hs.504115	tripartite motif-containing 29	Homo sapiens tripartite motif-containing 29 (TRIM29), mRNA [NM_012101]	2.32154	down	2.39221	down	9.05919	21.77700	6.10862	12.88572	Compromised	Detected	Compromised	Compromised
6345	A_33_P3216150	PRB4	Hs.528651	proline-rich protein BstNI subfamily 4	Homo sapiens proline-rich protein BstNI subfamily 4 (PRB4), mRNA [NM_002723]	2.32132	down	2.36551	down	64.38258	154.75209	65.93984	137.54308	Detected	Detected	Detected	Detected
6346	A_33_P3320772	ATXN1	Hs.434961	ataxin 1	Homo sapiens ataxin 1 (ATXN1), transcript variant 1, mRNA [NM_000332]	2.32023	down	2.11026	down	45.63403	109.63612	48.18290	89.65896	Detected	Detected	Detected	Detected
6347	A_24_P250765	PRKG1	Hs.654556	protein kinase, cGMP-dependent, type I	Homo sapiens protein kinase, cGMP-dependent, type I (PRKG1), transcript variant 2, mRNA [NM_006258]	2.32003	down	2.17205	down	33.04324	79.37972	25.01828	47.91743	Detected	Detected	Detected	Detected
6348	A_24_P282416	ABL1	Hs.431048	c-abl oncogene 1, receptor tyrosine kinase	Homo sapiens c-abl oncogene 1, receptor tyrosine kinase (ABL1), transcript variant b, mRNA [NM_007313]	2.31979	down	2.44460	down	30.29367	72.76679	34.40602	74.16642	Detected	Detected	Detected	Detected
6349	A_33_P3410284				Dedicator of cytokinesis protein 9 (Cdc42 guanine nucleotide exchange factor zizimin-1) [Source:UniProtKB/Swiss-Prot;Acc:Q9BZ29] [ENST00000400224]	2.31974	down	1.42064	down	18.74068	45.01495	13.76184	17.23956	Detected	Detected	Compromised	Detected
6350	A_23_P28105	TSN	Hs.75066	translin	Homo sapiens translin (TSN), mRNA [NM_004622]	2.31941	down	2.23600	down	2349.64620	5643.04930	2457.87350	4846.15000	Detected	Detected	Detected	Detected
6351	A_33_P3330236				ATP-binding cassette sub-family D member 1 (Adrenoleukodystrophy protein)(ALDP) [Source:UniProtKB/Swiss-Prot;Acc:P33897] [ENST00000370129]	2.31920	down	2.92573	down	86.08221	206.72093	97.49584	251.52731	Detected	Detected	Detected	Detected
6352	A_23_P16722	DOCK10	Hs.46578	dedicator of cytokinesis 10	Homo sapiens dedicator of cytokinesis 10 (DOCK10), mRNA [NM_014689]	2.31785	down	2.38189	down	659.40796	1582.60460	579.55835	1217.26280	Detected	Detected	Detected	Detected
6353	A_33_P3294317	LOC100128913		hypothetical protein LOC100128913	PREDICTED: Homo sapiens hypothetical LOC100128913 (LOC100128913), mRNA [XM_001725730]	2.31771	down	2.64594	down	56.90114	136.55650	46.66309	108.87250	Detected	Detected	Detected	Detected
6354	A_32_P1173	C6orf150	Hs.658405	chromosome 6 open reading frame 150	Homo sapiens chromosome 6 open reading frame 150 (C6orf150), mRNA [NM_138441]	2.31694	down	2.68591	down	108.67078	260.71176	64.15840	151.95360	Detected	Detected	Detected	Detected
6355	A_23_P302470	SULT1B1	Hs.129742	sulfotransferase family, cytosolic, 1B, member 1	Homo sapiens sulfotransferase family, cytosolic, 1B, member 1 (SULT1B1), mRNA [NM_014465]	2.31677	down	2.30844	down	37.15598	89.13435	12.05587	24.54048	Detected	Detected	Compromised	Detected
6356	A_23_P47565	LDHA	Hs.2795	lactate dehydrogenase A	Homo sapiens lactate dehydrogenase A (LDHA), transcript variant 1, mRNA [NM_005566]	2.31662	down	2.41724	down	76183.23400	182746.06000	70225.93000	149686.80000	Detected	Detected	Detected	Detected
6357	A_33_P3419594	LOC100132338	Hs.473927	hypothetical protein LOC100132338	Homo sapiens cDNA FLJ90181 fis, clone MAMMA1000706. [AK074662]	2.31590	down	2.53791	down	1303.62940	3126.12740	753.68380	1686.67460	Detected	Detected	Detected	Detected
6358	A_23_P133438	FAM105A	Hs.155085	family with sequence similarity 105, member A	Homo sapiens family with sequence similarity 105, member A (FAM105A), mRNA [NM_019018]	2.31396	down	3.45310	down	21.21443	50.83005	17.16147	52.25510	Detected	Detected	Detected	Detected
6359	A_24_P140608	HBEGF	Hs.799	heparin-binding EGF-like growth factor	Homo sapiens heparin-binding EGF-like growth factor (HBEGF), mRNA [NM_001945]	2.31378	down	2.54991	down	99.59885	238.62132	81.78620	183.89543	Detected	Detected	Detected	Detected
6360	A_32_P233304	LIN9	Hs.120817	lin-9 homolog (C. elegans)	Homo sapiens lin-9 homolog (C. elegans) (LIN9), mRNA [NM_173083]	2.31374	down	2.23330	down	1239.48410	2969.53880	950.16266	1871.15430	Detected	Detected	Detected	Detected
6361	A_33_P3417626	ENHO	Hs.522085	energy homeostasis associated	Homo sapiens energy homeostasis associated (ENHO), mRNA [NM_198573]	2.31336	down	3.43628	down	11.86954	28.43218	9.00207	27.27695	Detected	Detected	Compromised	Detected
6362	A_23_P376488	TNF	Hs.241570	tumor necrosis factor (TNF superfamily, member 2)	Homo sapiens tumor necrosis factor (TNF superfamily, member 2) (TNF), mRNA [NM_000594]	2.31164	down	1.54580	down	18.88564	45.20486	20.70218	28.21864	Detected	Detected	Detected	Detected
6363	A_23_P394064	PTRF	Hs.437191	polymerase I and transcript release factor	Homo sapiens polymerase I and transcript release factor (PTRF), mRNA [NM_012232]	2.31148	down	2.51901	down	118298.15000	283139.72000	97201.65000	215908.19000	Detected	Detected	Detected	Detected
6364	A_33_P3388331	FARP1	Hs.403917	FERM, RhoGEF (ARHGEF) and pleckstrin domain protein 1 (chondrocyte-derived)	Homo sapiens FERM, RhoGEF (ARHGEF) and pleckstrin domain protein 1 (chondrocyte-derived) (FARP1), transcript variant 1, mRNA [NM_005766]	2.31123	down	2.69632	down	396.35690	948.55360	333.25278	792.33920	Detected	Detected	Detected	Detected
6365	A_23_P89327	ZNF286A	Hs.585799	zinc finger protein 286A	Homo sapiens zinc finger protein 286A (ZNF286A), transcript variant 1, mRNA [NM_020652]	2.31102	down	2.33160	down	45.34695	108.51379	27.06039	55.63560	Detected	Detected	Detected	Detected
6366	A_23_P72157	MFSD7	Hs.567612	major facilitator superfamily domain containing 7	Homo sapiens major facilitator superfamily domain containing 7 (MFSD7), mRNA [NM_032219]	2.31081	down	2.31473	down	1013.78070	2425.72400	1375.01260	2806.55400	Detected	Detected	Detected	Detected
6367	A_23_P111240	PHACTR2	Hs.102471	phosphatase and actin regulator 2	Homo sapiens phosphatase and actin regulator 2 (PHACTR2), transcript variant 3, mRNA [NM_014721]	2.30959	down	2.34968	down	765.06433	1829.64010	506.04733	1048.49230	Detected	Detected	Detected	Detected
6368	A_24_P404245	PCYT2	Hs.569843	phosphate cytidylyltransferase 2, ethanolamine	Homo sapiens phosphate cytidylyltransferase 2, ethanolamine (PCYT2), mRNA [NM_002861]	2.30941	down	2.48629	down	210.20413	502.66104	234.87923	514.94690	Detected	Detected	Detected	Detected

6369	A_23_P366394	ZAK	Hs.444451	sterile alpha motif and leucine zipper containing kinase AZK	Homo sapiens sterile alpha motif and leucine zipper containing kinase AZK (ZAK), transcript variant 1, mRNA [NM_016653]	2.30936	down	1.81645	down	334.13820	799.00840	215.54822	345.25020	Detected	Detected	Detected	Detected
6370	A_23_P100001	FAM174B	Hs.27373	family with sequence similarity 174, member B	Homo sapiens family with sequence similarity 174, member B (FAM174B), mRNA [NM_207446]	2.30662	down	2.40089	down	48.80207	116.55940	50.76467	107.47318	Detected	Detected	Detected	Detected
6371	A_33_P3277437	ERI2	Hs.248437	ERI1 exoribonuclease family member 2	Homo sapiens ERI1 exoribonuclease family member 2 (ERI2), transcript variant 1, mRNA [NM_001142725]	2.30659	down	2.48802	down	61.34220	146.50820	41.35730	90.73431	Detected	Detected	Detected	Detected
6372	A_23_P381449	SP2	Hs.514276	Sp2 transcription factor	Homo sapiens Sp2 transcription factor (SP2), mRNA [NM_003110]	2.30598	down	2.76874	down	165.98477	396.32965	87.86260	214.51236	Detected	Detected	Detected	Detected
6373	A_33_P3302210	LOC100129129		hypothetical protein LOC100129129	PREDICTED: Homo sapiens hypothetical protein LOC100129129 (LOC100129129), mRNA [XM_001715395]	2.30354	down	1.14771	down	525.75440	1254.04100	81.65868	82.64191	Detected	Detected	Detected	Detected
6374	A_33_P3284933	IL27	Hs.528111	interleukin 27	Homo sapiens interleukin 27 (IL27), mRNA [NM_145659]	2.30354	down	2.63621	down	51.34401	122.46668	55.38432	128.74553	Detected	Detected	Detected	Detected
6375	A_23_P422732	WDR63	Hs.97933	WD repeat domain 63	Homo sapiens WD repeat domain 63 (WDR63), mRNA [NM_145172]	2.30341	down	3.47573	down	34.82235	83.05445	18.24452	55.91696	Detected	Detected	Detected	Detected
6376	A_33_P3413114	ADAMTSL1	Hs.522019	ADAMTS-like 1	Homo sapiens ADAMTS-like 1 (ADAMTSL1), transcript variant 2, mRNA [NM_052866]	2.30268	down	2.57508	down	168.77023	402.40380	134.27980	304.90690	Detected	Detected	Detected	Detected
6377	A_23_P45099	HLA-DRB5	Hs.534322	major histocompatibility complex, class II, DR beta 5	Homo sapiens major histocompatibility complex, class II, DR beta 5 (HLA-DRB5), mRNA [NM_002125]	2.30263	down	1.51236	down	8.01390	19.10737	9.50742	12.67899	Compromised	Detected	Compromised	Compromised
6378	A_33_P3411925	WDR18	Hs.325321	WD repeat domain 18	Homo sapiens WD repeat domain 18 (WDR18), mRNA [NM_024100]	2.30261	down	2.76645	down	8627.75200	20570.77000	5317.62000	12971.97700	Detected	Detected	Detected	Detected
6379	A_33_P3268892		Hs.271721		Putative uncharacterized protein Fragment [Source:UniProtKB/TrEMBL;Acc:Q96HI3] [ENST00000391412]	2.30233	down	2.37754	down	253.43034	604.16950	225.38452	472.51657	Detected	Detected	Detected	Detected
6380	A_23_P207811	PAIP1	Hs.482038	poly(A) binding protein interacting protein 1	Homo sapiens poly(A) binding protein interacting protein 1 (PAIP1), transcript variant 1, mRNA [NM_006451]	2.30159	down	2.22990	down	2219.31250	5289.07300	1768.01090	3476.45240	Detected	Detected	Detected	Detected
6381	A_33_P3361393	EIF1AX	Hs.522590	eukaryotic translation initiation factor 1A, X-linked	Homo sapiens eukaryotic translation initiation factor 1A, X-linked (EIF1AX), mRNA [NM_001412]	2.30117	down	1.99397	down	12453.69400	29674.33000	9867.63700	17349.90000	Detected	Detected	Detected	Detected
6382	A_32_P218355	C6orf132	Hs.710148	chromosome 6 open reading frame 132	Homo sapiens chromosome 6 open reading frame 132 (C6orf132), mRNA [NM_001164446]	2.30083	down	2.18001	down	509.72830	1214.38300	358.12744	688.43220	Detected	Detected	Detected	Detected
6383	A_33_P3278211	LOC100133190	Hs.714477	hypothetical protein LOC100133190	Homo sapiens cDNA FLJ46161 fis, clone TESTI4002195. [AK128041]	2.29826	down	2.69450	down	76.06416	181.01395	76.02976	180.64578	Detected	Detected	Detected	Detected
6384	A_33_P3326025				KN motif and ankyrin repeat domain-containing protein 3 (Ankyrin repeat domain-containing protein 47) [Source:UniProtKB/Swiss-Prot;Acc:Q6NY19] [ENST00000381056]	2.29797	down	1.49777	down	2930.47360	6972.94040	830.19950	1096.46080	Detected	Detected	Detected	Detected
6385	A_33_P3386467	RPL23AP7	Hs.406135	ribosomal protein L23a pseudogene 7	Homo sapiens ribosomal protein L23a pseudogene 7 (RPL23AP7), transcript variant 4, non-coding RNA [NR_024530]	2.29746	down	2.24231	down	1648.42050	3921.47200	1426.96480	2821.46310	Detected	Detected	Detected	Detected
6386	A_24_P142024	CHMP4A	Hs.279761	chromatin modifying protein 4A	Homo sapiens chromatin modifying protein 4A (CHMP4A), mRNA [NM_014169]	2.29707	down	2.46835	down	1940.37930	4615.24200	2028.78690	4415.78370	Detected	Detected	Detected	Detected
6387	A_33_P3222105	EIF1AX	Hs.522590	eukaryotic translation initiation factor 1A, X-linked	Homo sapiens eukaryotic translation initiation factor 1A, X-linked (EIF1AX), mRNA [NM_001412]	2.29658	down	2.03174	down	20442.31600	48612.16800	13744.10000	24623.55500	Detected	Detected	Detected	Detected
6388	A_33_P3362891	HNRNPA2B1	Hs.487774	heterogeneous nuclear ribonucleoprotein A2/B1	Homo sapiens heterogeneous nuclear ribonucleoprotein A2/B1 (HNRNPA2B1), transcript variant A2, mRNA [NM_002137]	2.29656	down	2.56544	down	1017.28290	2419.08840	1197.04100	2707.92360	Detected	Detected	Detected	Detected
6389	A_33_P3270657	FAM111B	Hs.186579	family with sequence similarity 111, member B	Homo sapiens family with sequence similarity 111, member B (FAM111B), transcript variant 1, mRNA [NM_198947]	2.29519	down	3.24297	down	67.20326	159.71347	25.12072	71.83579	Detected	Detected	Detected	Detected
6390	A_24_P417935	hCG_2014417	Hs.656080	hypothetical LOC100130776	Homo sapiens hypothetical LOC100130776 (LOC100130776), non-coding RNA [NR_027032]	2.29474	down	2.71709	down	361.34985	858.60657	438.65830	1050.98410	Detected	Detected	Detected	Detected
6391	A_33_P3416588	RIT2	Hs.464985	Ras-like without CAAX 2	Homo sapiens Ras-like without CAAX 2 (RIT2), mRNA [NM_002930]	2.29401	down	1.66658	down	94.02600	223.34485	37.80473	55.55693	Detected	Detected	Detected	Detected
6392	A_23_P118888	PAFAH1B1	Hs.77318	platelet-activating factor acetylhydrolase, isoform Ib, subunit 1 (45kDa)	Homo sapiens platelet-activating factor acetylhydrolase, isoform Ib, subunit 1 (45kDa) (PAFAH1B1), mRNA [NM_000430]	2.29396	down	2.31016	down	2956.88100	7023.48440	1849.35200	3767.28050	Detected	Detected	Detected	Detected
6393	A_24_P281395	LOC642521		similar to nuclear DNA-binding protein	PREDICTED: Homo sapiens similar to nuclear DNA-binding protein (LOC642521), mRNA [XM_926017]	2.29327	down	1.71440	down	359.40550	853.44025	195.81113	296.01550	Detected	Detected	Detected	Detected
6394	A_33_P3284029	CSE1L	Hs.90073	CSE1 chromosome segregation 1-like (yeast)	Homo sapiens CSE1 chromosome segregation 1-like (yeast) (CSE1L), mRNA [NM_001316]	2.29322	down	2.13444	down	4353.30220	10337.05800	4588.51500	8636.16900	Detected	Detected	Detected	Detected
6395	A_33_P3230324	COPS8	Hs.531713	COP9 constitutive photomorphogenic homolog subunit 8 (Arabidopsis)	Homo sapiens COP9 constitutive photomorphogenic homolog subunit 8 (Arabidopsis) (COPS8), transcript variant 2, mRNA [NM_198189]	2.29314	down	2.18658	down	342.11392	812.33435	285.16534	549.82910	Detected	Detected	Detected	Detected
6396	A_23_P161563	RAB38	Hs.591975	RAB38, member RAS oncogene family	Homo sapiens RAB38, member RAS oncogene family (RAB38), mRNA [NM_022337]	2.29261	down	2.18690	down	56.28292	133.61050	56.52208	108.99661	Detected	Detected	Detected	Detected

6397	A_33_P3281710				Uncharacterized protein ENSP00000382042 [Source:UniProtKB/Swiss-Prot;Acc:A8MWP6] [ENST00000399093]	2.29239	down	2.73204	down	47.08192	111.75710	26.29483	63.34655	Detected	Detected	Detected	Detected
6398	A_23_P386764	DOLPP1	Hs.21701	dolichyl pyrophosphate phosphatase 1	Homo sapiens dolichyl pyrophosphate phosphatase 1 (DOLPP1), transcript variant 1, mRNA [NM_020438]	2.29203	down	2.30193	down	1068.53370	2535.95170	742.20510	1506.54080	Detected	Detected	Detected	Detected
6399	A_24_P213924	KIAA1609	Hs.288274	KIAA1609	Homo sapiens KIAA1609 (KIAA1609), mRNA [NM_020947]	2.29185	down	2.27519	down	4030.08600	9563.85900	3271.76370	6563.93650	Detected	Detected	Detected	Detected
6400	A_23_P109488	PIK3IP1	Hs.26670	phosphoinositide-3-kinase interacting protein 1	Homo sapiens phosphoinositide-3-kinase interacting protein 1 (PIK3IP1), transcript variant 1, mRNA [NM_052880]	2.29148	down	2.46090	down	144.37561	342.56560	148.12619	321.43335	Detected	Detected	Detected	Detected
6401	A_23_P110802	CENPH	Hs.631967	centromere protein H	Homo sapiens centromere protein H (CENPH), mRNA [NM_022909]	2.29139	down	2.22005	down	794.73270	1885.61240	521.67830	1021.24670	Detected	Detected	Detected	Detected
6402	A_33_P3351745	PVRIG	Hs.521075	poliovirus receptor related immunoglobulin domain containing	Homo sapiens poliovirus receptor related immunoglobulin domain containing (PVRIG), mRNA [NM_024070]	2.28930	down	2.04126	down	10.06876	23.86777	8.81948	15.87471	Compromised	Detected	Compromised	Detected
6403	A_23_P148785	SFT2D2	Hs.645435	SFT2 domain containing 2	Homo sapiens SFT2 domain containing 2 (SFT2D2), mRNA [NM_199344]	2.28903	down	2.49658	down	166.41273	394.43192	149.66891	329.49066	Detected	Detected	Detected	Detected
6404	A_24_P703462	LOC100133660	Hs.659695	hypothetical LOC100133660	PREDICTED: Homo sapiens similar to NEDD8-conjugating enzyme (LOC100133660), miscRNA [XR_036844]	2.28754	down	2.82345	down	377.30150	893.69720	102.09119	254.17584	Detected	Detected	Detected	Detected
6405	A_32_P192692	KCNMA1	Hs.144795	potassium large conductance calcium-activated channel, subfamily M, alpha member 1	Homo sapiens potassium large conductance calcium-activated channel, subfamily M, alpha member 1 (KCNMA1), transcript variant 1, mRNA [NM_001014797]	2.28657	down	1.89051	down	72.80983	172.38803	65.17525	108.64941	Detected	Detected	Detected	Detected
6406	A_23_P165722	EIF4E2	Hs.292026	eukaryotic translation initiation factor 4E family member 2	Homo sapiens eukaryotic translation initiation factor 4E family member 2 (EIF4E2), mRNA [NM_004846]	2.28640	down	2.32240	down	14696.79600	34794.29300	15347.95500	31430.66400	Detected	Detected	Detected	Detected
6407	A_24_P401090					2.28610	down	2.06575	down	661.18530	1565.13150	139.63788	254.35855	Detected	Detected	Detected	Detected
6408	A_33_P3374210	MKI67	Hs.80976	antigen identified by monoclonal antibody Ki-67	Homo sapiens antigen identified by monoclonal antibody Ki-67 (MKI67), transcript variant 1, mRNA [NM_002417]	2.28582	down	6.15307	down	294.90674	698.00570	74.25624	402.89355	Detected	Detected	Detected	Detected
6409	A_32_P113566	BTBD9	Hs.603858	BTB (POZ) domain containing 9	Homo sapiens BTB (POZ) domain containing 9 (BTBD9), transcript variant 3, mRNA [NM_152733]	2.28562	down	1.81748	down	9.86783	23.35382	6.20850	9.95000	Compromised	Detected	Compromised	Compromised
6410	A_23_P135474	MRPL37	Hs.584908	mitochondrial ribosomal protein L37	Homo sapiens mitochondrial ribosomal protein L37 (MRPL37), nuclear gene encoding mitochondrial protein, mRNA [NM_016491]	2.28425	down	2.16921	down	5312.01270	12564.21600	4528.39750	8661.85100	Detected	Detected	Detected	Detected
6411	A_23_P54517	TYRO3	Hs.381282	TYRO3 protein tyrosine kinase	Homo sapiens TYRO3 protein tyrosine kinase (TYRO3), mRNA [NM_006293]	2.28240	down	2.43025	down	683.90590	1616.30040	652.41300	1398.10490	Detected	Detected	Detected	Detected
6412	A_33_P3262575	BAIAP2L1	Hs.656063	BAI1-associated protein 2-like 1	Homo sapiens BAI1-associated protein 2-like 1 (BAIAP2L1), mRNA [NM_018842]	2.28235	down	2.26232	down	407.01782	961.89825	248.09094	494.91516	Detected	Detected	Detected	Detected
6413	A_33_P3424062	KCNF1	Hs.23735	potassium voltage-gated channel, subfamily F, member 1	Homo sapiens potassium voltage-gated channel, subfamily F, member 1 (KCNF1), mRNA [NM_002236]	2.28132	down	2.65050	down	13.46779	31.81377	14.71191	34.38448	Compromised	Detected	Compromised	Detected
6414	A_33_P3261132	CYCSP52		cytochrome c, somatic pseudogene 52	Homo sapiens cytochrome c, somatic pseudogene 52 (CYCSP52), non-coding RNA [NR_001560]	2.28072	down	1.72624	down	102.00030	240.88272	5.73450	8.72894	Detected	Detected	Compromised	Compromised
6415	A_24_P19828	TOE1	Hs.525091	target of EGR1, member 1 (nuclear)	Homo sapiens target of EGR1, member 1 (nuclear) (TOE1), mRNA [NM_025077]	2.28055	down	2.38961	down	260.31880	614.72190	268.21097	565.15650	Detected	Detected	Detected	Detected
6416	A_23_P162106	MRPL48	Hs.503239	mitochondrial ribosomal protein L48	Homo sapiens mitochondrial ribosomal protein L48 (MRPL48), nuclear gene encoding mitochondrial protein, mRNA [NM_016055]	2.28043	down	2.37801	down	2288.94970	5404.87100	1564.22240	3280.03340	Detected	Detected	Detected	Detected
6417	A_23_P202392	SUV39H2	Hs.554883	suppressor of variegation 3-9 homolog 2 (Drosophila)	Homo sapiens suppressor of variegation 3-9 homolog 2 (Drosophila) (SUV39H2), mRNA [NM_024670]	2.28033	down	2.43663	down	688.45540	1625.57310	622.60736	1337.73020	Detected	Detected	Detected	Detected
6418	A_33_P3348204	C4orf39	Hs.178648	chromosome 4 open reading frame 39	Homo sapiens chromosome 4 open reading frame 39 (C4orf39), mRNA [NM_153027]	2.27934	down	2.80630	down	55.01432	129.84311	50.10057	123.97739	Detected	Detected	Detected	Detected
6419	A_23_P13797	C12orf49	Hs.592011	chromosome 12 open reading frame 49	Homo sapiens chromosome 12 open reading frame 49 (C12orf49), mRNA [NM_024738]	2.27850	down	2.41432	down	325.08930	766.98250	239.42350	509.71480	Detected	Detected	Detected	Detected
6420	A_33_P3253792	CXorf67	Hs.97053	chromosome X open reading frame 67	Homo sapiens chromosome X open reading frame 67 (CXorf67), mRNA [NM_203407]	2.27747	down	4.13521	down	7.47837	17.63572	2.54193	9.26885	Compromised	Detected	Compromised	Compromised
6421	A_23_P17393	CSE1L	Hs.90073	CSE1 chromosome segregation 1-like (yeast)	Homo sapiens CSE1 chromosome segregation 1-like (yeast) (CSE1L), mRNA [NM_001316]	2.27718	down	2.28363	down	9280.00400	21881.56000	8756.91900	17633.64300	Detected	Detected	Detected	Detected
6422	A_23_P45699	FUBP1	Hs.567380	far upstream element (FUSE) binding protein 1	Homo sapiens far upstream element (FUSE) binding protein 1 (FUBP1), mRNA [NM_003902]	2.27693	down	2.12618	down	1492.37570	3518.52950	917.64606	1720.44570	Detected	Detected	Detected	Detected
6423	A_24_P89080	DCK	Hs.709	deoxycytidine kinase	Homo sapiens deoxycytidine kinase (DCK), mRNA [NM_000788]	2.27366	down	1.92916	down	255.18234	600.77160	151.52339	257.75850	Detected	Detected	Detected	Detected
6424	A_24_P109176	ZC3H12D	Hs.632618	zinc finger CCCH-type containing 12D	Homo sapiens zinc finger CCCH-type containing 12D (ZC3H12D), mRNA [NM_207360]	2.27351	down	1.43914	down	18.39056	43.29374	15.52489	19.70140	Detected	Detected	Detected	Detected
6425	A_33_P3362296				Potassium voltage-gated channel subfamily G member 1 (Voltage-gated potassium channel subunit Kv6.1)(kH2) [Source:UniProtKB/Swiss-Prot;Acc:Q9UIX4] [ENST00000396017]	2.27289	down	2.35791	down	38.40209	90.37862	31.04419	64.54649	Detected	Detected	Detected	Detected

6426	A_23_P211428	SMTN	Hs.149098	smoothelin	Homo sapiens smoothelin (SMTN), transcript variant 2, mRNA [NM_134269]	2.27275	down	2.64387	down	1545.50290	3637.10080	2150.85840	5014.38700	Detected	Detected	Detected	Detected
6427	A_23_P40295	C20orf103	Hs.22920	chromosome 20 open reading frame 103	Homo sapiens chromosome 20 open reading frame 103 (C20orf103), mRNA [NM_012261]	2.27245	down	2.30928	down	10.67436	25.11709	7.72828	15.73708	Compromised	Detected	Compromised	Detected
6428	A_33_P3271105	PABPC4	Hs.169900	poly(A) binding protein, cytoplasmic 4 (inducible form)	Homo sapiens poly(A) binding protein, cytoplasmic 4 (inducible form) (PABPC4), transcript variant 3, mRNA [NM_001135654]	2.27140	down	1.12478	down	16.80496	39.52430	35.25442	34.96610	Detected	Detected	Detected	Detected
6429	A_24_P289795				GB	2.27103	down	3.20147	down	98.39473	231.38152	51.13410	144.35289	Detected	Detected	Detected	Detected
6430	A_33_P3216232	ITGB1BP1	Hs.467662	integrin beta 1 binding protein 1	Homo sapiens integrin beta 1 binding protein 1 (ITGB1BP1), transcript variant 1, mRNA [NM_004763]	2.27080	down	2.16979	down	711.00916	1671.80790	432.04144	826.62360	Detected	Detected	Detected	Detected
6431	A_23_P258621	RTCD1	Hs.484222	RNA terminal phosphate cyclase domain 1	Homo sapiens RNA terminal phosphate cyclase domain 1 (RTCD1), transcript variant 2, mRNA [NM_003729]	2.27077	down	2.16746	down	5092.06250	11972.93500	3673.87350	7021.66000	Detected	Detected	Detected	Detected
6432	A_33_P3290602	LOC729580	Hs.356273	hypothetical LOC729580	PREDICTED: Homo sapiens hypothetical LOC729580 (LOC729580), mRNA [XM_001126751]	2.27073	down	2.23184	down	5950.62700	13991.38500	4820.89550	9487.61100	Detected	Detected	Detected	Detected
6433	A_33_P3279353	AZU1	Hs.72885	azurocidin 1	Homo sapiens azurocidin 1 (AZU1), mRNA [NM_001700]	2.26994	down	1.06089	down	10.55162	24.80081	14.15482	13.24163	Compromised	Detected	Compromised	Compromised
6434	A_24_P220058	MAPRE1	Hs.472437	microtubule-associated protein, RP/EB family, member 1	Homo sapiens microtubule-associated protein, RP/EB family, member 1 (MAPRE1), mRNA [NM_012325]	2.26981	down	2.17852	down	4491.42500	10556.19100	3229.02600	6202.94970	Detected	Detected	Detected	Detected
6435	A_23_P413760	P2RX5	Hs.408615	purinergic receptor P2X, ligand-gated ion channel, 5	Homo sapiens purinergic receptor P2X, ligand-gated ion channel, 5 (P2RX5), transcript variant 3, mRNA [NM_175081]	2.26831	down	3.24244	down	48.41437	113.71280	36.71761	104.98121	Detected	Detected	Detected	Detected
6436	A_23_P39561	UBE2F	Hs.471785	ubiquitin-conjugating enzyme E2F (putative)	Homo sapiens ubiquitin-conjugating enzyme E2F (putative) (UBE2F), mRNA [NM_080678]	2.26821	down	2.42859	down	1101.75010	2587.61470	898.92413	1925.05080	Detected	Detected	Detected	Detected
6437	A_23_P350059	C1orf152	Hs.632440	profilin 1 pseudogene	Homo sapiens chromosome 1 open reading frame 152 (C1orf152), non-coding RNA [NR_003242]	2.26788	down	2.51673	down	30770.16400	72257.58600	6922.24270	15362.04400	Detected	Detected	Detected	Detected
6438	A_33_P3301709	GNG4	Hs.159711	guanine nucleotide binding protein (G protein), gamma 4	Homo sapiens guanine nucleotide binding protein (G protein), gamma 4 (GNG4), transcript variant 1, mRNA [NM_001098722]	2.26743	down	2.43792	down	19.00255	44.61479	13.32931	28.65447	Detected	Detected	Compromised	Detected
6439	A_32_P164246	FOXQ1	Hs.591352	forkhead box Q1	Homo sapiens forkhead box Q1 (FOXQ1), mRNA [NM_033260]	2.26730	down	2.26098	down	2959.93200	6949.03120	2403.73460	4792.34700	Detected	Detected	Detected	Detected
6440	A_32_P234604	PFN1	Hs.494691	profilin 1	Homo sapiens profilin 1 (PFN1), mRNA [NM_005022]	2.26655	down	2.42630	down	6632.23830	15565.35800	8259.27700	17670.64500	Detected	Detected	Detected	Detected
6441	A_23_P84118	CDH18	Hs.317632	cadherin 18, type 2	Homo sapiens cadherin 18, type 2 (CDH18), mRNA [NM_004934]	2.26619	down	2.69877	down	13.89669	32.60922	8.69941	20.70241	Detected	Detected	Compromised	Detected
6442	A_23_P334798	LRRC2	Hs.657345	leucine rich repeat containing 2	Homo sapiens leucine rich repeat containing 2 (LRRC2), transcript variant 2, mRNA [NM_024750]	2.26562	down	2.27514	down	73.28420	171.92190	52.18354	104.69057	Detected	Detected	Detected	Detected
6443	A_33_P3363799	NCAM1	Hs.503878	neural cell adhesion molecule 1	Homo sapiens neural cell adhesion molecule 1 (NCAM1), transcript variant 2, mRNA [NM_181351]	2.26438	down	2.29033	down	100.27211	235.10501	69.17639	139.70822	Detected	Detected	Detected	Detected
6444	A_24_P405205	ATP2B4	Hs.343522	ATPase, Ca++ transporting, plasma membrane 4	Homo sapiens ATPase, Ca++ transporting, plasma membrane 4 (ATP2B4), transcript variant 1, mRNA [NM_001001396]	2.26392	down	2.27808	down	7633.94700	17895.50800	4279.26270	8596.15700	Detected	Detected	Detected	Detected
6445	A_23_P45087	ZNF107	Hs.50216	zinc finger protein 107	Homo sapiens zinc finger protein 107 (ZNF107), transcript variant 1, mRNA [NM_016220]	2.26380	down	2.36921	down	124.11240	290.92810	83.24802	173.91727	Detected	Detected	Detected	Detected
6446	A_33_P3378772	FAM98B	Hs.6799	family with sequence similarity 98, member B	Homo sapiens family with sequence similarity 98, member B (FAM98B), transcript variant 2, mRNA [NM_001042429]	2.26362	down	3.15224	down	10.81502	25.34919	10.23436	28.44759	Compromised	Detected	Compromised	Detected
6447	A_33_P3383079					2.26326	down	1.66847	down	16.15889	37.86865	7.75478	11.40914	Detected	Detected	Compromised	Compromised
6448	A_24_P254177	C19orf42	Hs.356467	chromosome 19 open reading frame 42	Homo sapiens chromosome 19 open reading frame 42 (C19orf42), mRNA [NM_024104]	2.26155	down	2.25837	down	2217.77420	5193.44780	1798.23690	3581.02080	Detected	Detected	Detected	Detected
6449	A_23_P301855	LSAMP	Hs.657246	limbic system-associated membrane protein	Homo sapiens limbic system-associated membrane protein (LSAMP), mRNA [NM_002338]	2.26141	down	1.88386	down	18.06320	42.29685	19.47525	32.35178	Detected	Detected	Detected	Detected
6450	A_24_P188071	TUBA1C	Hs.719091	tubulin, alpha 1c	Homo sapiens tubulin, alpha 1c (TUBA1C), mRNA [NM_032704]	2.26118	down	2.60208	down	126222.21000	295531.94000	69343.83600	159108.95000	Detected	Detected	Detected	Detected
6451	A_23_P91081	EPCAM	Hs.542050	epithelial cell adhesion molecule	Homo sapiens epithelial cell adhesion molecule (EPCAM), mRNA [NM_002354]	2.26071	down	4.31744	down	7.81336	18.29011	4.49730	17.12159	Compromised	Detected	Compromised	Detected
6452	A_24_P196019					2.26012	down	2.22124	down	237.38700	555.54895	167.72264	328.51288	Detected	Detected	Detected	Detected
6453	A_33_P3227793	CGREF1	Hs.159525	cell growth regulator with EF-hand domain 1	Homo sapiens cell growth regulator with EF-hand domain 1 (CGREF1), transcript variant 1, mRNA [NM_006569]	2.25998	down	2.74908	down	65.74155	153.84291	65.87000	159.67647	Detected	Detected	Detected	Detected
6454	A_23_P64630	RNF26	Hs.524084	ring finger protein 26	Homo sapiens ring finger protein 26 (RNF26), mRNA [NM_032015]	2.25984	down	2.31892	down	4371.03600	10228.08600	4151.04100	8488.04800	Detected	Detected	Detected	Detected
6455	A_24_P879740	MAP1B	Hs.335079	microtubule-associated protein 1B	Homo sapiens microtubule-associated protein 1B (MAP1B), mRNA [NM_005909]	2.25947	down	2.24691	down	1230.08940	2877.90600	827.96014	1640.44180	Detected	Detected	Detected	Detected
6456	A_23_P48977	MRPS11	Hs.111286	mitochondrial ribosomal protein S11	Homo sapiens mitochondrial ribosomal protein S11 (MRPS11), nuclear gene encoding mitochondrial protein, transcript variant 1, mRNA [NM_022839]	2.25856	down	2.29320	down	2840.63200	6643.24270	3574.40360	7227.88040	Detected	Detected	Detected	Detected
6457	A_23_P303238	VN1R5	Hs.553686	vomeronasal 1 receptor 5 (gene/pseudogene)	Homo sapiens vomeronasal 1 receptor 5 (gene/pseudogene) (VN1R5), mRNA [NM_173858]	2.25816	down	1.18559	down	13.41614	31.37005	19.72023	20.61628	Compromised	Detected	Detected	Detected

6458	A_33_P3376636	OPA3	Hs.719374	optic atrophy 3 (autosomal recessive, with chorea and spastic paraplegia)	Homo sapiens optic atrophy 3 (autosomal recessive, with chorea and spastic paraplegia) (OPA3), nuclear gene encoding mitochondrial protein, transcript variant 1, mRNA [NM_001017989]	2.25800	down	2.31795	down	355.12152	830.29610	262.60452	536.75040	Detected	Detected	Detected	Detected
6459	A_33_P3226377	PRH2	Hs.631731	proline-rich protein HaeIII subfamily 2	Homo sapiens proline-rich protein HaeIII subfamily 2 (PRH2), transcript variant 1, mRNA [NM_005042]	2.25749	down	2.55803	down	199.22035	465.68533	168.46227	379.99124	Detected	Detected	Detected	Detected
6460	A_23_P136347	EPS8	Hs.591160	epidermal growth factor receptor pathway substrate 8	Homo sapiens epidermal growth factor receptor pathway substrate 8 (EPS8), mRNA [NM_004447]	2.25728	down	1.89179	down	4739.51460	11077.75600	2184.29320	3643.75950	Detected	Detected	Detected	Detected
6461	A_23_P83328	ENG	Hs.76753	endoglin	Homo sapiens endoglin (ENG), transcript variant 2, mRNA [NM_000118]	2.25720	down	2.78881	down	1537.30470	3593.05200	1367.05350	3361.78030	Detected	Detected	Detected	Detected
6462	A_33_P3341509					2.25715	down	2.21252	down	937.45233	2191.00170	739.65920	1443.05850	Detected	Detected	Detected	Detected
6463	A_33_P3342957	STK17B	Hs.88297	serine/threonine kinase 17b	Homo sapiens serine/threonine kinase 17b (STK17B), mRNA [NM_004226]	2.25707	down	2.09234	down	604.75690	1413.37980	547.88440	1010.85110	Detected	Detected	Detected	Detected
6464	A_32_P92505	LCLAT1	Hs.468048	lysocardiolipin acyltransferase 1	Homo sapiens lysocardiolipin acyltransferase 1 (LCLAT1), transcript variant 1, mRNA [NM_182551]	2.25686	down	2.08403	down	2532.06030	5917.14840	1518.65830	2790.79930	Detected	Detected	Detected	Detected
6465	A_33_P3252196	EZH2	Hs.444082	enhancer of zeste homolog 2 (Drosophila)	Homo sapiens enhancer of zeste homolog 2 (Drosophila) (EZH2), transcript variant 1, mRNA [NM_004456]	2.25620	down	2.28927	down	1197.51430	2797.64090	813.46760	1642.11160	Detected	Detected	Detected	Detected
6466	A_33_P3275255	LOC100132966	Hs.632517	hypothetical LOC100132966	PREDICTED: Homo sapiens hypothetical LOC100132966 (LOC100132966), miscRNA [XR_078228]	2.25615	down	1.48618	down	26.58828	62.11427	24.91000	32.64459	Detected	Detected	Detected	Detected
6467	A_23_P75630	APOA5	Hs.283923	apolipoprotein A-V	Homo sapiens apolipoprotein A-V (APOA5), transcript variant 1, mRNA [NM_052968]	2.25600	down	1.87703	down	8.07216	18.85652	11.73876	19.42939	Compromised	Detected	Compromised	Detected
6468	A_33_P3383331	SLC35A3	Hs.448979	solute carrier family 35 (UDP-N-acetylglucosamine (UDP-GlcNAc) transporter), member A3	Homo sapiens solute carrier family 35 (UDP-N-acetylglucosamine (UDP-GlcNAc) transporter), member A3 (SLC35A3), mRNA [NM_012243]	2.25474	down	2.03941	down	199.55191	465.89316	76.82158	138.15091	Detected	Detected	Detected	Detected
6469	A_24_P934800	ERI2	Hs.248437	ERI1 exoribonuclease family member 2	Homo sapiens ERI1 exoribonuclease family member 2 (ERI2), transcript variant 1, mRNA [NM_001142725]	2.25466	down	2.23201	down	275.09033	642.22955	255.73276	503.32370	Detected	Detected	Detected	Detected
6470	A_33_P3393088	LOC100131320		hypothetical protein LOC100131320	PREDICTED: Homo sapiens hypothetical protein LOC100131320, transcript variant 1 (LOC100131320), mRNA [XM_001722766]	2.25412	down	2.48142	down	86.52884	201.96225	13.60549	29.77011	Detected	Detected	Detected	Detected
6471	A_23_P8380	C7orf49	Hs.720115	chromosome 7 open reading frame 49	Homo sapiens chromosome 7 open reading frame 49 (C7orf49), transcript variant 1, mRNA [NM_024033]	2.25337	down	2.33507	down	1755.15520	4095.25830	1249.94100	2573.68100	Detected	Detected	Detected	Detected
6472	A_23_P397308	KLC4	Hs.655123	kinesin light chain 4	Homo sapiens kinesin light chain 4 (KLC4), transcript variant 4, mRNA [NM_138343]	2.25290	down	1.21801	down	18.31725	42.73013	25.00060	26.85144	Detected	Detected	Detected	Detected
6473	A_23_P253221	ARHGEF4	Hs.469935	Rho guanine nucleotide exchange factor (GEF) 4	Homo sapiens Rho guanine nucleotide exchange factor (GEF) 4 (ARHGEF4), transcript variant 2, mRNA [NM_032995]	2.25222	down	2.66899	down	269.74258	629.06146	230.65292	542.83954	Detected	Detected	Detected	Detected
6474	A_23_P315571	RFTN1	Hs.98910	raftlin, lipid raft linker 1	Homo sapiens raftlin, lipid raft linker 1 (RFTN1), mRNA [NM_015150]	2.25101	down	2.24332	down	6515.51460	15186.57200	3921.42260	7757.10100	Detected	Detected	Detected	Detected
6475	A_24_P189533	ENDOD1	Hs.167115	endonuclease domain containing 1	Homo sapiens endonuclease domain containing 1 (ENDOD1), mRNA [NM_015036]	2.25051	down	2.58334	down	1167.45150	2720.52420	964.05237	2196.07540	Detected	Detected	Detected	Detected
6476	A_33_P3381870	EPB49	Hs.106124	erythrocyte membrane protein band 4.9 (dematin)	Homo sapiens erythrocyte membrane protein band 4.9 (dematin) (EPB49), transcript variant 1, mRNA [NM_001978]	2.25008	down	2.92808	down	52.08872	121.35983	27.72312	71.57974	Detected	Detected	Detected	Detected
6477	A_23_P146644	ANXA2	Hs.511605	annexin A2	Homo sapiens annexin A2 (ANXA2), transcript variant 2, mRNA [NM_001002857]	2.24951	down	2.12040	down	101888.86000	237327.17000	87421.41000	163456.17000	Detected	Detected	Detected	Detected
6478	A_33_P3286208	PPIL5	Hs.451090	peptidylprolyl isomerase (cyclophilin)-like 5	Homo sapiens peptidylprolyl isomerase (cyclophilin)-like 5 (PPIL5), transcript variant 3, mRNA [NM_203467]	2.24882	down	2.32711	down	191.65773	446.28595	263.79022	541.30340	Detected	Detected	Detected	Detected
6479	A_23_P384698	LOC100293611	Hs.666816	hypothetical protein LOC100293611	PREDICTED: Homo sapiens hypothetical protein LOC100293611 (LOC100293611), mRNA [XM_002345631]	2.24855	down	2.30359	down	101.52199	236.37146	69.98246	142.15442	Detected	Detected	Detected	Detected
6480	A_23_P27795	SPINT2	Hs.31439	serine peptidase inhibitor, Kunitz type, 2	Homo sapiens serine peptidase inhibitor, Kunitz type, 2 (SPINT2), transcript variant a, mRNA [NM_021102]	2.24731	down	3.06525	down	71.83084	167.15036	48.00599	129.75601	Detected	Detected	Detected	Detected
6481	A_23_P159688	TBC1D25	Hs.694353	TBC1 domain family, member 25	Homo sapiens TBC1 domain family, member 25 (TBC1D25), mRNA [NM_002536]	2.24688	down	2.13982	down	104.35929	242.79765	94.87559	179.01816	Detected	Detected	Detected	Detected
6482	A_33_P3220643	PTRH1	Hs.653265	peptidyl-tRNA hydrolase 1 homolog (S. cerevisiae)	Homo sapiens peptidyl-tRNA hydrolase 1 homolog (S. cerevisiae) (PTRH1), mRNA [NM_001002913]	2.24683	down	2.27719	down	2811.25370	6540.39200	2979.14330	5982.14360	Detected	Detected	Detected	Detected
6483	A_33_P3424367	TRIM14	Hs.575631	tripartite motif-containing 14	Homo sapiens tripartite motif-containing 14 (TRIM14), transcript variant 2, mRNA [NM_033219]	2.24644	down	1.33874	down	19.72015	45.87109	23.29956	27.50487	Detected	Detected	Detected	Detected
6484	A_23_P432360	PMM2	Hs.713724	phosphomannomutase 2	Homo sapiens phosphomannomutase 2 (PMM2), mRNA [NM_000303]	2.24598	down	1.86591	down	2056.67040	4783.04600	1989.41940	3273.26700	Detected	Detected	Detected	Detected
6485	A_23_P206532	PHKB	Hs.78060	phosphorylase kinase, beta	Homo sapiens phosphorylase kinase, beta (PHKB), transcript variant 2, mRNA [NM_001031835]	2.24550	down	2.31546	down	1954.04770	4543.39840	1644.12210	3356.88820	Detected	Detected	Detected	Detected
6486	A_33_P3258472	SPTBN1	Hs.503178	spectrin, beta, non-erythrocytic 1	Homo sapiens spectrin, beta, non-erythrocytic 1 (SPTBN1), transcript variant 1, mRNA [NM_003128]	2.24536	down	2.66961	down	18.54857	43.12497	10.72287	25.24206	Detected	Detected	Compromised	Detected

6487	A_23_P255503	PAFAH1B2	Hs.597488	platelet-activating factor acetylhydrolase, isoform Ib, subunit 2 (30kDa)	Homo sapiens platelet-activating factor acetylhydrolase, isoform Ib, subunit 2 (30kDa) (PAFAH1B2), mRNA [NM_002572]	2.24491	down	2.04381	down	422.98663	983.23850	341.62580	615.68207	Detected	Detected	Detected	Detected
6488	A_23_P259594	AKAP7	Hs.486483	A kinase (PRKA) anchor protein 7	Homo sapiens A kinase (PRKA) anchor protein 7 (AKAP7), transcript variant gamma, mRNA [NM_016377]	2.24474	down	2.28223	down	487.01650	1131.99010	562.43810	1131.87560	Detected	Detected	Detected	Detected
6489	A_33_P3257030	SC65	Hs.446459	synaptonemal complex protein SC65	Homo sapiens synaptonemal complex protein SC65 (SC65), mRNA [NM_006455]	2.24359	down	2.22522	down	4911.16460	11409.35100	6592.49270	12935.64500	Detected	Detected	Detected	Detected
6490	A_33_P3264846	SAMD9L	Hs.489118	sterile alpha motif domain containing 9-like	Homo sapiens sterile alpha motif domain containing 9-like (SAMD9L), mRNA [NM_152703]	2.24212	down	2.42834	down	49.94878	115.96233	31.94494	68.40323	Detected	Detected	Detected	Detected
6491	A_23_P47077	BAG3	Hs.523309	BCL2-associated athanogene 3	Homo sapiens BCL2-associated athanogene 3 (BAG3), mRNA [NM_004281]	2.24187	down	2.32424	down	1576.09840	3658.70460	1042.70170	2137.00930	Detected	Detected	Detected	Detected
6492	A_33_P3777165	FLJ31715	Hs.596176	hypothetical protein FLJ31715	Homo sapiens cDNA clone IMAGE:3831619 [BC022164]	2.24153	down	2.43462	down	231.31354	536.88275	200.74603	430.96692	Detected	Detected	Detected	Detected
6493	A_23_P122052	GPX8	Hs.289044	glutathione peroxidase 8 (putative)	Homo sapiens glutathione peroxidase 8 (putative) (GPX8), mRNA [NM_001008397]	2.24140	down	2.21135	down	2764.64300	6416.41400	2030.27200	3958.92100	Detected	Detected	Detected	Detected
6494	A_24_P196499				Homo sapiens mRNA for RBP1-like protein, complete cds. [AB030181]	2.24096	down	3.32788	down	8.55729	19.85654	3.14798	9.23774	Compromised	Detected	Compromised	Compromised
6495	A_33_P3888629	MECOM	Hs.721616	MDS1 and EVI1 complex locus	Homo sapiens MDS1 and EVI1 complex locus (MECOM), transcript variant 6, mRNA [NM_001164000]	2.23780	down	2.03295	down	251.04268	581.70310	163.39192	292.90292	Detected	Detected	Detected	Detected
6496	A_23_P83714	ZNF707	Hs.521922	zinc finger protein 707	Homo sapiens zinc finger protein 707 (ZNF707), transcript variant 1, mRNA [NM_173831]	2.23768	down	2.27923	down	683.53577	1583.77150	542.51166	1090.34030	Detected	Detected	Detected	Detected
6497	A_24_P391853	LOC100131482		similar to hCG1643669	PREDICTED: Homo sapiens similar to hCG1643669 (LOC100131482), mRNA [XM_001722583]	2.23766	down	2.16465	down	3798.16430	8800.37700	4768.40700	9101.77200	Detected	Detected	Detected	Detected
6498	A_23_P46936	EGR2	Hs.1395	early growth response 2	Homo sapiens early growth response 2 (EGR2), transcript variant 1, mRNA [NM_000399]	2.23670	down	2.41635	down	286.86203	664.37650	210.38345	448.26780	Detected	Detected	Detected	Detected
6499	A_23_P46606	LPGAT1	Hs.497674	lysophosphatidylglycerol acyltransferase 1	Homo sapiens lysophosphatidylglycerol acyltransferase 1 (LPGAT1), mRNA [NM_014873]	2.23626	down	1.66082	down	416.38260	964.15940	588.63600	862.05580	Detected	Detected	Detected	Detected
6500	A_23_P136978	SRPX2	Hs.306339	sushi-repeat-containing protein, X-linked 2	Homo sapiens sushi-repeat-containing protein, X-linked 2 (SRPX2), mRNA [NM_014467]	2.23616	down	2.26930	down	2682.15940	6210.41650	3072.39670	6148.01560	Detected	Detected	Detected	Detected
6501	A_23_P251548	TEX261	Hs.516087	testis expressed 261	Homo sapiens testis expressed 261 (TEX261), mRNA [NM_144582]	2.23606	down	2.19471	down	591.02800	1368.43580	458.26500	886.86870	Detected	Detected	Detected	Detected
6502	A_24_P384569	EWSR1	Hs.374477	Ewing sarcoma breakpoint region 1	Homo sapiens Ewing sarcoma breakpoint region 1 (EWSR1), transcript variant 5, mRNA [NM_001163287]	2.23598	down	1.98778	down	51.32769	118.83717	49.81579	87.31752	Detected	Detected	Detected	Detected
6503	A_33_P3308833	C21orf58	Hs.236572	chromosome 21 open reading frame 58	Homo sapiens chromosome 21 open reading frame 58 (C21orf58), mRNA [NM_058180]	2.23594	down	3.11743	down	35.19029	81.47326	26.96487	74.12437	Detected	Detected	Detected	Detected
6504	A_33_P3367106	C1orf152	Hs.632440	profilin 1 pseudogene	Homo sapiens chromosome 1 open reading frame 152 (C1orf152), non-coding RNA [NR_003242]	2.23542	down	2.51078	down	5463.38300	12646.03200	7038.53860	15583.18900	Detected	Detected	Detected	Detected
6505	A_24_P925040	CAV2	Hs.212332	caveolin 2	Homo sapiens caveolin 2 (CAV2), transcript variant 1, mRNA [NM_001233]	2.23538	down	2.05087	down	837.27875	1938.00010	452.67847	818.64246	Detected	Detected	Detected	Detected
6506	A_33_P3291776	NANS	Hs.522310	N-acetylneuraminic acid synthase	Homo sapiens N-acetylneuraminic acid synthase (NANS), mRNA [NM_018946]	2.23302	down	2.09147	down	12292.18700	28422.00400	11515.60450	21237.50600	Detected	Detected	Detected	Detected
6507	A_23_P13740	NAV3	Hs.655301	neuron navigator 3	Homo sapiens neuron navigator 3 (NAV3), mRNA [NM_014903]	2.23250	down	2.38614	down	1286.13290	2973.10570	1098.14370	2310.58080	Detected	Detected	Detected	Detected
6508	A_33_P3504002	LOC286186	Hs.449427	hypothetical protein LOC286186	Homo sapiens cDNA FLJ37641 fis, clone BRHIP2000087 [AK094960]	2.23248	down	1.25769	down	33.16493	76.66545	11.99236	13.29981	Detected	Detected	Compromised	Compromised
6509	A_23_P334955	FAM167A	Hs.124299	family with sequence similarity 167, member A	Homo sapiens family with sequence similarity 167, member A (FAM167A), mRNA [NM_053279]	2.23222	down	2.08637	down	803.27210	1856.65980	694.06670	1276.90340	Detected	Detected	Detected	Detected
6510	A_23_P131020	GATAD2A	Hs.118964	GATA zinc finger domain containing 2A	Homo sapiens GATA zinc finger domain containing 2A (GATAD2A), mRNA [NM_017660]	2.23131	down	2.33701	down	1768.25050	4085.41900	1520.00820	3132.35570	Detected	Detected	Detected	Detected
6511	A_24_P307572	ANKRD13A	Hs.528703	ankyrin repeat domain 13A	Homo sapiens ankyrin repeat domain 13A (ANKRD13A), mRNA [NM_033121]	2.23042	down	2.03624	down	340.39297	786.14230	266.14426	477.87100	Detected	Detected	Detected	Detected
6512	A_33_P3255404	CLDN11	Hs.31595	claudin 11	Homo sapiens claudin 11 (CLDN11), mRNA [NM_005602]	2.22907	down	2.12657	down	9547.06300	22035.67000	6177.75500	11584.47200	Detected	Detected	Detected	Detected
6513	A_23_P144005	C3orf32	Hs.561182	chromosome 3 open reading frame 32	Homo sapiens chromosome 3 open reading frame 32 (C3orf32), mRNA [NM_015931]	2.22840	down	2.42694	down	24.69904	56.99094	20.78343	44.47759	Detected	Detected	Detected	Detected
6514	A_23_P5654	IL1F7	Hs.166371	interleukin 1 family, member 7 (zeta)	Homo sapiens interleukin 1 family, member 7 (zeta) (IL1F7), transcript variant 1, mRNA [NM_014439]	2.22813	down	2.49745	down	138.51160	319.56580	48.38983	106.56529	Detected	Detected	Detected	Detected
6515	A_33_P3235831					2.22808	down	2.48713	down	1452.02920	3349.95870	177.98969	390.35464	Detected	Detected	Detected	Detected
6516	A_23_P56604	IL1RL2	Hs.659863	interleukin 1 receptor-like 2	Homo sapiens interleukin 1 receptor-like 2 (IL1RL2), mRNA [NM_003854]	2.22728	down	1.35225	down	12.67857	29.24002	15.74598	18.77548	Detected	Detected	Detected	Detected
6517	A_33_P3316800	AHR	Hs.171189	aryl hydrocarbon receptor	Homo sapiens aryl hydrocarbon receptor (AHR), mRNA [NM_001621]	2.22609	down	2.17197	down	95.68552	220.55725	110.14152	210.94585	Detected	Detected	Detected	Detected
6518	A_23_P19712	GMNN	Hs.234896	geminin, DNA replication inhibitor	Homo sapiens geminin, DNA replication inhibitor (GMNN), mRNA [NM_015895]	2.22581	down	2.21651	down	4159.04200	9585.48000	3683.24020	7198.88430	Detected	Detected	Detected	Detected
6519	A_23_P34460	SLC35A3	Hs.448979	solute carrier family 35 (UDP-N-acetylglucosamine (UDP-GlcNAc) transporter), member A3	Homo sapiens solute carrier family 35 (UDP-N-acetylglucosamine (UDP-GlcNAc) transporter), member A3 (SLC35A3), mRNA [NM_012243]	2.22485	down	2.25956	down	74.83668	172.40462	74.54286	148.52382	Detected	Detected	Detected	Detected

6520	A_33_P3396553	LOC732419		similar to AAA-ATPase TOB3	PREDICTED: Homo sapiens similar to belphegor (LOC732419), mRNA [XM_001718658]	2.22448	down	2.63361	down	807.77313	1860.59510	286.89597	666.25714	Detected	Detected	Detected	Detected
6521	A_23_P129856	HIC1	Hs.72956	hypermethylated in cancer 1	Homo sapiens hypermethylated in cancer 1 (HIC1), transcript variant 1, mRNA [NM_006497]	2.22410	down	2.43971	down	435.30234	1002.48780	432.44202	930.31910	Detected	Detected	Detected	Detected
6522	A_33_P3242659	KIF13A	Hs.189915	kinesin family member 13A	Homo sapiens kinesin family member 13A (KIF13A), transcript variant 1, mRNA [NM_022113]	2.22391	down	2.21564	down	204.78369	471.56890	226.06859	441.67792	Detected	Detected	Detected	Detected
6523	A_33_P3369158	KIF3C	Hs.21611	kinesin family member 3C	Homo sapiens kinesin family member 3C (KIF3C), mRNA [NM_002254]	2.22327	down	2.53916	down	120.94866	278.43665	115.49973	258.60486	Detected	Detected	Detected	Detected
6524	A_24_P143574	SIRPB1	Hs.709487	signal-regulatory protein beta 1	Homo sapiens signal-regulatory protein beta 1 (SIRPB1), transcript variant 1, mRNA [NM_006065]	2.22302	down	2.94987	down	27.46608	63.22277	24.31192	63.23936	Detected	Detected	Detected	Detected
6525	A_23_P250358	HERC6	Hs.529317	hect domain and RLD 6	Homo sapiens hect domain and RLD 6 (HERC6), transcript variant 1, mRNA [NM_017912]	2.22218	down	2.19011	down	14.22496	32.73130	3.54793	6.85185	Detected	Detected	Compromised	Compromised
6526	A_33_P3215392	EXOC3L2	Hs.721742	exocyst complex component 3-like 2	Homo sapiens exocyst complex component 3-like 2 (EXOC3L2), mRNA [NM_138568]	2.22138	down	1.20727	down	7.95078	18.28802	8.84290	9.41375	Compromised	Detected	Compromised	Compromised
6527	A_23_P94703	TOR1B	Hs.252682	torsin family 1, member B (torsin B)	Homo sapiens torsin family 1, member B (torsin B) (TOR1B), mRNA [NM_014506]	2.22039	down	1.98729	down	811.58344	1865.92830	862.59845	1511.59340	Detected	Detected	Detected	Detected
6528	A_33_P3329597	BLOC1S3	Hs.103902	biogenesis of lysosomal organelles complex-1, subunit 3	Homo sapiens biogenesis of lysosomal organelles complex-1, subunit 3 (BLOC1S3), mRNA [NM_212550]	2.22013	down	2.23607	down	2238.12600	5145.13400	1697.34940	3346.74490	Detected	Detected	Detected	Detected
6529	A_33_P3615955					2.21997	down	1.31640	down	99.63741	229.03528	52.90581	61.41233	Detected	Detected	Detected	Detected
6530	A_23_P254797	BPIL1	Hs.257045	bactericidal/permeability-increasing protein-like 1	Homo sapiens bactericidal/permeability-increasing protein-like 1 (BPIL1), mRNA [NM_025227]	2.21944	down	2.04648	down	488.12620	1121.78270	89.05166	160.70000	Detected	Detected	Detected	Detected
6531	A_33_P3712341	CXCL12	Hs.522891	chemokine (C-X-C motif) ligand 12 (stromal cell-derived factor 1)	Homo sapiens chemokine (C-X-C motif) ligand 12 (stromal cell-derived factor 1) (CXCL12), transcript variant 3, mRNA [NM_001033886]	2.21944	down	2.35518	down	372.28757	855.56920	208.04623	432.06543	Detected	Detected	Detected	Detected
6532	A_23_P416468	PIF1	Hs.112160	PIF1 5'-to-3' DNA helicase homolog (S. cerevisiae)	Homo sapiens PIF1 5'-to-3' DNA helicase homolog (S. cerevisiae) (PIF1), mRNA [NM_025049]	2.21903	down	2.14748	down	1322.01750	3037.61380	1087.43090	2059.19000	Detected	Detected	Detected	Detected
6533	A_24_P1255	BCCIP	Hs.715543	BRCA2 and CDKN1A interacting protein	Homo sapiens BRCA2 and CDKN1A interacting protein (BCCIP), transcript variant C, mRNA [NM_078469]	2.21876	down	2.38699	down	65.14663	149.67029	47.64932	100.29369	Detected	Detected	Detected	Detected
6534	A_23_P73982	TMEM48	Hs.476525	transmembrane protein 48	Homo sapiens transmembrane protein 48 (TMEM48), mRNA [NM_018087]	2.21765	down	2.25541	down	1190.70300	2734.19200	962.20795	1913.63450	Detected	Detected	Detected	Detected
6535	A_23_P77776	SFRS2	Hs.713672	splicing factor, arginine/serine-rich 2	Homo sapiens splicing factor, arginine/serine-rich 2 (SFRS2), mRNA [NM_003016]	2.21719	down	2.22584	down	7738.89300	17767.02700	6691.68000	13133.90500	Detected	Detected	Detected	Detected
6536	A_23_P103070	YWHAH	Hs.226755	tyrosine 3-monooxygenase/tryptophan 5-monooxygenase activation protein, eta polypeptide	Homo sapiens tyrosine 3-monooxygenase/tryptophan 5-monooxygenase activation protein, eta polypeptide (YWHAH), mRNA [NM_003405]	2.21696	down	2.15706	down	2437.30960	5595.00800	1686.43150	3207.72560	Detected	Detected	Detected	Detected
6537	A_33_P3399638	ZNF257	Hs.283900	zinc finger protein 257	Homo sapiens zinc finger protein 257 (ZNF257), mRNA [NM_033468]	2.21638	down	2.97554	down	32.16019	73.80660	17.58391	46.13667	Detected	Detected	Detected	Detected
6538	A_32_P198923	YWHAZ	Hs.492407	tyrosine 3-monooxygenase/tryptophan 5-monooxygenase activation protein, zeta polypeptide	Homo sapiens tyrosine 3-monooxygenase/tryptophan 5-monooxygenase activation protein, zeta polypeptide (YWHAZ), transcript variant 2, mRNA [NM_145690]	2.21584	down	2.32424	down	866.70630	1988.58460	974.33890	1996.90050	Detected	Detected	Detected	Detected
6539	A_23_P139864	GSG1	Hs.240053	germ cell associated 1	Homo sapiens germ cell associated 1 (GSG1), transcript variant 1, mRNA [NM_031289]	2.21561	down	2.18257	down	139.54771	320.14725	110.96970	213.56856	Detected	Detected	Detected	Detected
6540	A_32_P40288	TMEM200A	Hs.591341	transmembrane protein 200A	Homo sapiens transmembrane protein 200A (TMEM200A), mRNA [NM_052913]	2.21461	down	2.11590	down	2395.80620	5493.92720	1687.06040	3147.67820	Detected	Detected	Detected	Detected
6541	A_23_P32143	ALDOB	Hs.530274	aldolase B, fructose-bisphosphate	Homo sapiens aldolase B, fructose-bisphosphate (ALDOB), mRNA [NM_000035]	2.21429	down	1.70462	down	14.90303	34.16979	3.94471	5.92935	Detected	Detected	Compromised	Compromised
6542	A_23_P103877	LRRC38	Hs.657356	leucine rich repeat containing 38	Leucine-rich repeat-containing protein 38 Precursor [Source:UniProtKB/Swiss-Prot;Acc:Q5VT99] [ENST00000401092]	2.21400	down	5.20678	down	13.63118	31.24960	4.15942	19.09707	Detected	Detected	Compromised	Detected
6543	A_33_P3375636	FAM196B	Hs.721917	family with sequence similarity 196, member B	Homo sapiens family with sequence similarity 196, member B (FAM196B), mRNA [NM_001129891]	2.21390	down	2.78280	down	38.61979	88.53218	20.46457	50.21685	Detected	Detected	Detected	Detected
6544	A_33_P3219939	CUBN	Hs.166206	cubilin (intrinsic factor-cobalamin receptor)	Homo sapiens cubilin (intrinsic factor-cobalamin receptor) (CUBN), mRNA [NM_001081]	2.21340	down	1.82430	down	171.41138	392.85500	115.31191	185.49646	Detected	Detected	Detected	Detected
6545	A_33_P3241741	SNORA23	Hs.689720	small nucleolar RNA, H/ACA box 23	Homo sapiens small nucleolar RNA, H/ACA box 23 (SNORA23), small nucleolar RNA [NR_002962]	2.21338	down	1.58353	down	14.51758	33.27229	12.58132	17.56781	Detected	Detected	Detected	Detected
6546	A_33_P3256858	C14orf80	Hs.720306	chromosome 14 open reading frame 80	Homo sapiens chromosome 14 open reading frame 80 (C14orf80), transcript variant 1, mRNA [NM_001134875]	2.21232	down	2.21657	down	3430.14280	7857.66600	3467.88180	6778.13700	Detected	Detected	Detected	Detected
6547	A_33_P3331282	LOC648987	Hs.655947	hypothetical LOC648987	PREDICTED: Homo sapiens hypothetical LOC648987 (LOC648987), miscRNA [XR_039981]	2.21207	down	2.71948	down	383.98892	879.52830	239.10092	573.36730	Detected	Detected	Detected	Detected
6548	A_33_P3385775	ZNF703	Hs.288042	zinc finger protein 703	Homo sapiens zinc finger protein 703 (ZNF703), mRNA [NM_025069]	2.21201	down	2.38988	down	101.85399	233.29138	77.79172	163.93622	Detected	Detected	Detected	Detected
6549	A_23_P37598	NPTN	Hs.187866	neuroplastin	Homo sapiens neuroplastin (NPTN), transcript variant b, mRNA [NM_012428]	2.21192	down	2.22619	down	1284.58570	2942.15870	1297.20970	2546.46730	Detected	Detected	Detected	Detected

6550	A_24_P34155	RUNX1	Hs.149261	runt-related transcription factor 1	Homo sapiens runt-related transcription factor 1 (RUNX1), transcript variant 3, mRNA [NM_001122607]	2.21151	down	2.17766	down	449.18616	1028.60380	363.80627	698.59467	Detected	Detected	Detected	Detected
6551	A_23_P75310	ARHGAP22	Hs.655672	Rho GTPase activating protein 22	Homo sapiens Rho GTPase activating protein 22 (ARHGAP22), mRNA [NM_021226]	2.21131	down	2.40715	down	15794.79600	36165.71000	16419.27000	34851.61300	Detected	Detected	Detected	Detected
6552	A_33_P3409139	LOC143188	Hs.287723	hypothetical LOC143188	Homo sapiens hypothetical LOC143188 (LOC143188), non-coding RNA [NR_015409]	2.21119	down	2.48823	down	85.88192	196.63460	64.53490	141.59593	Detected	Detected	Detected	Detected
6553	A_32_P142028	HNRNPC	Hs.508848	heterogeneous nuclear ribonucleoprotein C (C1/C2)	Homo sapiens heterogeneous nuclear ribonucleoprotein C (C1/C2) (HNRNPC), transcript variant 1, mRNA [NM_031314]	2.21069	down	2.06053	down	10926.21700	25011.00200	9146.25900	16618.37300	Detected	Detected	Detected	Detected
6554	A_23_P433798	PODNL1	Hs.448497	podocan-like 1	Homo sapiens podocan-like 1 (PODNL1), transcript variant 1, mRNA [NM_024825]	2.21065	down	2.59425	down	224.55165	514.00616	182.32948	417.09473	Detected	Detected	Detected	Detected
6555	A_33_P3335391	FAM83G	Hs.710727	family with sequence similarity 83, member G	Homo sapiens family with sequence similarity 83, member G (FAM83G), mRNA [NM_001039999]	2.20944	down	2.74450	down	35.62516	81.50259	25.19721	60.97904	Detected	Detected	Detected	Detected
6556	A_24_P576506	SPRN	Hs.64968	shadow of prion protein homolog (zebrafish)	Homo sapiens shadow of prion protein homolog (zebrafish) (SPRN), mRNA [NM_001012508]	2.20894	down	2.16263	down	75.31041	172.25533	78.42929	149.56343	Detected	Detected	Detected	Detected
6557	A_23_P120931	APOBEC3C	Hs.441124	apolipoprotein B mRNA editing enzyme, catalytic polypeptide-like 3C	Homo sapiens apolipoprotein B mRNA editing enzyme, catalytic polypeptide-like 3C (APOBEC3C), mRNA [NM_014508]	2.20879	down	2.62642	down	279.26300	638.70630	260.96160	604.37366	Detected	Detected	Detected	Detected
6558	A_33_P3307253	AK5	Hs.559718	adenylate kinase 5	Homo sapiens adenylate kinase 5 (AK5), transcript variant 1, mRNA [NM_174858]	2.20775	down	2.15437	down	1281.09190	2928.61840	1071.80190	2036.10360	Detected	Detected	Detected	Detected
6559	A_23_P309396	RP1L1	Hs.33538	retinitis pigmentosa 1-like 1	Homo sapiens retinitis pigmentosa 1-like 1 (RP1L1), mRNA [NM_178857]	2.20645	down	1.38606	down	18.20810	41.59976	14.71859	17.98921	Detected	Detected	Detected	Detected
6560	A_33_P3232508	SMARCB1	Hs.534350	SWI/SNF related, matrix associated, actin dependent regulator of chromatin, subfamily b, member 1	Homo sapiens SWI/SNF related, matrix associated, actin dependent regulator of chromatin, subfamily b, member 1 (SMARCB1), transcript variant 1, mRNA [NM_003073]	2.20577	down	1.96705	down	6441.28300	14711.79400	3524.96660	6114.16200	Detected	Detected	Detected	Detected
6561	A_33_P3363061	ERLIN2	Hs.705490	ER lipid raft associated 2	Homo sapiens ER lipid raft associated 2 (ERLIN2), transcript variant 2, mRNA [NM_001003790]	2.20558	down	2.06523	down	116.49632	266.05243	138.86328	252.88371	Detected	Detected	Detected	Detected
6562	A_24_P95822	NPTN	Hs.187866	neuroplastin	Homo sapiens neuroplastin (NPTN), transcript variant b, mRNA [NM_012428]	2.20492	down	2.31994	down	434.33542	991.63390	572.98460	1172.15540	Detected	Detected	Detected	Detected
6563	A_32_P437004	XG	Hs.179675	Xg blood group	Homo sapiens Xg blood group (XG), transcript variant 1, mRNA [NM_175569]	2.20480	down	2.09547	down	590.04550	1347.06090	384.03537	709.60760	Detected	Detected	Detected	Detected
6564	A_33_P3283619	SH2D1A	Hs.349094	SH2 domain protein 1A	Homo sapiens SH2 domain protein 1A (SH2D1A), transcript variant 2, mRNA [NM_001114937]	2.20350	down	1.59366	down	28.77881	65.66265	18.72174	26.30914	Detected	Detected	Detected	Detected
6565	A_33_P3210278	SYNE2	Hs.525392	spectrin repeat containing, nuclear envelope 2	Homo sapiens spectrin repeat containing, nuclear envelope 2 (SYNE2), transcript variant 5, mRNA [NM_182914]	2.20264	down	2.06869	down	372.47934	849.52950	220.72856	402.64276	Detected	Detected	Detected	Detected
6566	A_33_P3264013				PRO2176 [Source:UniProtKB/TrEMBL;Acc:Q9P181] [ENST00000430036]	2.20253	down	1.65263	down	19.98295	45.57377	23.41162	34.11706	Detected	Detected	Detected	Detected
6567	A_24_P203056	BCL7A	Hs.530970	B-cell CLL/lymphoma 7A	Homo sapiens B-cell CLL/lymphoma 7A (BCL7A), transcript variant 1, mRNA [NM_020993]	2.20126	down	2.22491	down	432.36743	985.49976	329.79092	647.01917	Detected	Detected	Detected	Detected
6568	A_23_P3963	CDR2L	Hs.78358	cerebellar degeneration-related protein 2-like	Homo sapiens cerebellar degeneration-related protein 2-like (CDR2L), mRNA [NM_014603]	2.20052	down	2.38232	down	3247.87920	7400.44040	3290.75560	6912.90530	Detected	Detected	Detected	Detected
6569	A_23_P359043	AKAP2	Hs.591908	A kinase (PRKA) anchor protein 2	Homo sapiens A kinase (PRKA) anchor protein 2 (AKAP2), transcript variant 1, mRNA [NM_001004065]	2.19991	down	2.33923	down	453.33070	1032.64990	394.38235	813.49830	Detected	Detected	Detected	Detected
6570	A_33_P3285545	CLDN4	Hs.699253	claudin 4	Homo sapiens claudin 4 (CLDN4), mRNA [NM_001305]	2.19954	down	2.59778	down	1615.09680	3678.43970	1205.41520	2761.24600	Detected	Detected	Detected	Detected
6571	A_24_P72479	ARPC1A	Hs.124126	actin related protein 2/3 complex, subunit 1A, 41kDa	Homo sapiens actin related protein 2/3 complex, subunit 1A, 41kDa (ARPC1A), mRNA [NM_006409]	2.19934	down	2.34469	down	7425.28470	16909.78100	7775.52100	16076.08600	Detected	Detected	Detected	Detected
6572	A_33_P3382714	LOC144742	Hs.524782	hypothetical LOC144742	Homo sapiens hypothetical LOC144742 (LOC144742), non-coding RNA [NR_024246]	2.19924	down	7.03967	down	2.35740	5.36833	5.36713	33.31650	Compromised	Compromised	Compromised	Detected
6573	A_33_P3252043	P4HB	Hs.464336	prolyl 4-hydroxylase, beta polypeptide	Homo sapiens prolyl 4-hydroxylase, beta polypeptide (P4HB), mRNA [NM_000918]	2.19919	down	2.02303	down	82924.01600	188831.89000	70275.71000	125364.23000	Detected	Detected	Detected	Detected
6574	A_24_P726336	PHACTR2	Hs.102471	phosphatase and actin regulator 2	Homo sapiens phosphatase and actin regulator 2 (PHACTR2), transcript variant 1, mRNA [NM_001100164]	2.19893	down	1.91294	down	1769.84560	4029.77170	798.20496	1346.41910	Detected	Detected	Detected	Detected
6575	A_23_P11461	UBE2V1	Hs.719899	ubiquitin-conjugating enzyme E2 variant 1	Homo sapiens ubiquitin-conjugating enzyme E2 variant 1 (UBE2V1), transcript variant 4, mRNA [NM_001032288]	2.19858	down	2.15275	down	1541.40140	3509.06200	1217.36410	2310.89550	Detected	Detected	Detected	Detected
6576	A_32_P76720	NT5DC3	Hs.48428	5'-nucleotidase domain containing 3	Homo sapiens 5'-nucleotidase domain containing 3 (NT5DC3), mRNA [NM_001031701]	2.19780	down	1.99518	down	128.66261	292.80167	105.41978	185.46863	Detected	Detected	Detected	Detected
6577	A_23_P52402	PITRM1	Hs.528300	pitrilysin metallopeptidase 1	Homo sapiens pitrilysin metallopeptidase 1 (PITRM1), mRNA [NM_014889]	2.19776	down	2.16201	down	2844.84520	6473.98600	2715.27270	5176.50240	Detected	Detected	Detected	Detected
6578	A_23_P92860	CCNO	Hs.3041	cyclin O	Homo sapiens cyclin O (CCNO), mRNA [NM_021147]	2.19578	down	2.30498	down	82.25579	187.02054	74.60291	151.63116	Detected	Detected	Detected	Detected

6579	A_33_P3327762				Dedicator of cytokinesis protein 8 [Source:UniProtKB/Swiss-Prot;Acc:Q8NF50] [ENST00000382331]	2.19548	down	1.44539	down	39.62342	90.07700	19.49684	24.84941	Detected	Detected	Detected	Detected
6580	A_33_P3263851	FLJ46020	Hs.532855	FLJ46020 protein	PREDICTED: Homo sapiens FLJ46020 protein (FLJ46020), miscRNA [XR_041641]	2.19481	down	2.12869	down	173.36047	393.98602	29.31283	55.02189	Detected	Detected	Detected	Detected
6581	A_24_P123245	HNRNPD	Hs.480073	heterogeneous nuclear ribonucleoprotein D (AU-rich element RNA binding protein 1, 37kDa)	Homo sapiens heterogeneous nuclear ribonucleoprotein D (AU-rich element RNA binding protein 1, 37kDa) (HNRNPD), transcript variant 1, mRNA [NM_031370]	2.19480	down	2.02428	down	634.89460	1442.87630	581.71850	1038.36490	Detected	Detected	Detected	Detected
6582	A_33_P3297921	MRPL37	Hs.584908	mitochondrial ribosomal protein L37	Homo sapiens mitochondrial ribosomal protein L37 (MRPL37), nuclear gene encoding mitochondrial protein, mRNA [NM_016491]	2.19457	down	2.33190	down	3307.95040	7516.95500	4147.50050	8528.30100	Detected	Detected	Detected	Detected
6583	A_33_P3341586	SIL1	Hs.483521	SIL1 homolog, endoplasmic reticulum chaperone (S. cerevisiae)	Homo sapiens SIL1 homolog, endoplasmic reticulum chaperone (S. cerevisiae) (SIL1), transcript variant 1, mRNA [NM_001037633]	2.19275	down	2.65184	down	1256.88680	2853.77100	1107.59270	2589.95920	Detected	Detected	Detected	Detected
6584	A_23_P91814	SLC25A38	Hs.369615	solute carrier family 25, member 38	Homo sapiens solute carrier family 25, member 38 (SLC25A38), mRNA [NM_017875]	2.19252	down	2.24911	down	1091.84700	2478.78800	774.11500	1535.26230	Detected	Detected	Detected	Detected
6585	A_23_P54991	DYNLL2	Hs.720595	dynein, light chain, LC8-type 2	Homo sapiens dynein, light chain, LC8-type 2 (DYNLL2), mRNA [NM_080677]	2.19237	down	2.08443	down	7038.04100	15977.13700	5899.97070	10844.34700	Detected	Detected	Detected	Detected
6586	A_33_P3406493	GABBR2	Hs.198612	gamma-aminobutyric acid (GABA) B receptor, 2	Homo sapiens gamma-aminobutyric acid (GABA) B receptor, 2 (GABBR2), mRNA [NM_005458]	2.19175	down	2.10294	down	1325.41870	3007.99880	1160.16000	2151.34890	Detected	Detected	Detected	Detected
6587	A_23_P430181	ZBTB3	Hs.147554	zinc finger and BTB domain containing 3	Homo sapiens zinc finger and BTB domain containing 3 (ZBTB3), mRNA [NM_024784]	2.19151	down	2.34380	down	455.60938	1033.87870	369.34000	763.32860	Detected	Detected	Detected	Detected
6588	A_33_P3220857				UPF0553 protein C9orf64 [Source:UniProtKB/Swiss-Prot;Acc:Q5T6V5] [ENST00000376340]	2.19098	down	2.09181	down	56.42101	128.00050	69.20654	127.65440	Detected	Detected	Detected	Detected
6589	A_23_P62907	ATF6	Hs.492740	activating transcription factor 6	Homo sapiens activating transcription factor 6 (ATF6), mRNA [NM_007348]	2.19091	down	2.25484	down	1513.13700	3432.69240	1265.10240	2515.40260	Detected	Detected	Detected	Detected
6590	A_23_P428184	HIST1H2AD	Hs.679229	histone cluster 1, H2ad	Homo sapiens histone cluster 1, H2ad (HIST1H2AD), mRNA [NM_021065]	2.19023	down	2.92954	down	2691.97850	6105.12300	1796.40610	4640.54800	Detected	Detected	Detected	Detected
6591	A_24_P3973	HNRNPA2B1	Hs.487774	heterogeneous nuclear ribonucleoprotein A2/B1	Homo sapiens heterogeneous nuclear ribonucleoprotein A2/B1 (HNRNPA2B1), transcript variant A2, mRNA [NM_002137]	2.19003	down	2.13313	down	4330.20100	9819.52400	4228.98540	7954.62740	Detected	Detected	Detected	Detected
6592	A_32_P120895	LYSMD2	Hs.603629	LysM, putative peptidoglycan-binding, domain containing 2	Homo sapiens LysM, putative peptidoglycan-binding, domain containing 2 (LYSMD2), transcript variant 1, mRNA [NM_153374]	2.18994	down	2.39070	down	1514.78320	3434.91900	1077.56040	2271.60620	Detected	Detected	Detected	Detected
6593	A_33_P3302245	TMEM59L	Hs.329850	transmembrane protein 59-like	Homo sapiens transmembrane protein 59-like (TMEM59L), mRNA [NM_012109]	2.18806	down	2.36696	down	261.44644	592.34500	155.50412	324.56195	Detected	Detected	Detected	Detected
6594	A_33_P3353866	MOGAT2	Hs.288568	monoacylglycerol O-acyltransferase 2	Homo sapiens monoacylglycerol O-acyltransferase 2 (MOGAT2), mRNA [NM_025098]	2.18718	down	2.96239	down	2.67834	6.06573	9.13182	23.85420	Compromised	Compromised	Compromised	Detected
6595	A_33_P3325355	TOMM34	Hs.517066	translocase of outer mitochondrial membrane 34	Homo sapiens translocase of outer mitochondrial membrane 34 (TOMM34), nuclear gene encoding mitochondrial protein, mRNA [NM_006809]	2.18524	down	2.20352	down	18867.17000	42691.22300	22549.02300	43813.66000	Detected	Detected	Detected	Detected
6596	A_33_P3307408					2.18485	down	1.12742	down	11.33737	25.64879	9.59873	9.54254	Compromised	Detected	Compromised	Compromised
6597	A_33_P3261648	ANKRD36B	Hs.532921	ankyrin repeat domain 36B	Homo sapiens ankyrin repeat domain 36B (ANKRD36B), mRNA [NM_025190]	2.18474	down	2.66444	down	165.64880	374.73190	130.81260	307.34146	Detected	Detected	Detected	Detected
6598	A_23_P112187	FIBCD1	Hs.133205	fibrinogen C domain containing 1	Homo sapiens fibrinogen C domain containing 1 (FIBCD1), transcript variant 1, mRNA [NM_032843]	2.18366	down	2.00484	down	26.56942	60.07597	31.03868	54.87181	Detected	Detected	Detected	Detected
6599	A_33_P3399248	UFM1	Hs.643655	ubiquitin-fold modifier 1	Homo sapiens ubiquitin-fold modifier 1 (UFM1), mRNA [NM_016617]	2.18363	down	2.09212	down	1434.22570	3242.87430	1161.77090	2143.24540	Detected	Detected	Detected	Detected
6600	A_33_P3268622	LY6D	Hs.415762	lymphocyte antigen 6 complex, locus D	Homo sapiens lymphocyte antigen 6 complex, locus D (LY6D), mRNA [NM_003695]	2.18312	down	1.13561	down	8.17037	18.46939	8.87656	8.88870	Compromised	Detected	Compromised	Compromised
6601	A_33_P3420633	C16orf11	Hs.632220	chromosome 16 open reading frame 11	Homo sapiens chromosome 16 open reading frame 11 (C16orf11), mRNA [NM_145270]	2.18260	down	2.75092	down	12.35978	27.93304	8.52370	20.67623	Compromised	Detected	Compromised	Detected
6602	A_24_P42681	PSMD2	Hs.518464	proteasome (prosome, macropain) 26S subunit, non-ATPase, 2	Homo sapiens proteasome (prosome, macropain) 26S subunit, non-ATPase, 2 (PSMD2), mRNA [NM_002808]	2.18251	down	2.29864	down	15062.32000	34039.36300	12046.03400	24416.29700	Detected	Detected	Detected	Detected
6603	A_24_P382319	CEACAM1	Hs.512682	carcinoembryonic antigen-related cell adhesion molecule 1 (biliary glycoprotein)	Homo sapiens carcinoembryonic antigen-related cell adhesion molecule 1 (biliary glycoprotein) (CEACAM1), transcript variant 1, mRNA [NM_001712]	2.18203	down	4.21242	down	22.78962	51.49088	2.96325	11.00689	Detected	Detected	Compromised	Compromised
6604	A_33_P3385477	ATAD3B	Hs.23413	ATPase family, AAA domain containing 3B	Homo sapiens ATPase family, AAA domain containing 3B (ATAD3B), mRNA [NM_031921]	2.18112	down	2.22010	down	11669.33000	26354.74000	10203.61300	19975.23600	Detected	Detected	Detected	Detected
6605	A_33_P3235987	PIN4	Hs.655623	protein (peptidylprolyl cis/trans isomerase) NIMA-interacting, 4 (parvulin)	Homo sapiens protein (peptidylprolyl cis/trans isomerase) NIMA-interacting, 4 (parvulin) (PIN4), mRNA [NM_006223]	2.18080	down	2.12227	down	1988.29150	4489.82470	1473.16470	2756.87960	Detected	Detected	Detected	Detected
6606	A_33_P3407289	LOC283788		FSHD region gene 1 pseudogene	Homo sapiens FSHD region gene 1 pseudogene (LOC283788), non-coding RNA [NR_027436]	2.17931	down	1.72880	down	12.53490	28.28611	15.51859	23.65715	Detected	Detected	Detected	Detected
6607	A_23_P128974	BATF	Hs.509964	basic leucine zipper transcription factor, ATF-like	Homo sapiens basic leucine zipper transcription factor, ATF-like (BATF), mRNA [NM_006399]	2.17908	down	1.86728	down	57.07332	128.77748	65.73505	108.23628	Detected	Detected	Detected	Detected

6608	A_23_P101521	C19orf41	Hs.414175	chromosome 19 open reading frame 41	Homo sapiens chromosome 19 open reading frame 41 (C19orf41), mRNA [NM_152358]	2.17841	down	1.04051	down	9.57179	21.59066	4.23034	3.88139	Compromised	Detected	Compromised	Compromised
6609	A_32_P190416	MAP7	Hs.486548	microtubule-associated protein 7	Homo sapiens microtubule-associated protein 7 (MAP7), mRNA [NM_003980]	2.17806	down	1.37351	down	19.18441	43.26650	18.33527	22.20676	Detected	Detected	Detected	Detected
6610	A_23_P256773	TSSC1	Hs.502770	tumor suppressing subtransferable candidate 1	Homo sapiens tumor suppressing subtransferable candidate 1 (TSSC1), mRNA [NM_003310]	2.17803	down	2.19124	down	2439.29540	5501.24660	2752.17460	5317.79250	Detected	Detected	Detected	Detected
6611	A_23_P45864	TNR	Hs.659864	tenascin R (restrictin, janusin)	Homo sapiens tenascin R (restrictin, janusin) (TNR), mRNA [NM_003285]	2.17771	down	1.93524	down	262.72310	592.42130	74.08782	126.42899	Detected	Detected	Detected	Detected
6612	A_23_P112512	MCART1	Hs.645492	mitochondrial carrier triple repeat 1	Homo sapiens mitochondrial carrier triple repeat 1 (MCART1), nuclear gene encoding mitochondrial protein, transcript variant 1, mRNA [NM_033412]	2.17695	down	1.87604	down	243.80495	549.57150	234.20000	387.43045	Detected	Detected	Detected	Detected
6613	A_24_P42501	ACOT9	Hs.298885	acyl-CoA thioesterase 9	Homo sapiens acyl-CoA thioesterase 9 (ACOT9), transcript variant 1, mRNA [NM_001037171]	2.17695	down	2.29290	down	1966.19370	4432.07230	1740.85940	3519.77420	Detected	Detected	Detected	Detected
6614	A_23_P89343	SNX11	Hs.15827	sorting nexin 11	Homo sapiens sorting nexin 11 (SNX11), transcript variant 1, mRNA [NM_152244]	2.17586	down	2.07418	down	446.66904	1006.35333	440.48248	805.63904	Detected	Detected	Detected	Detected
6615	A_23_P130304	TXNL4A	Hs.465498	thioredoxin-like 4A	Homo sapiens thioredoxin-like 4A (TXNL4A), mRNA [NM_006701]	2.17584	down	2.19583	down	12271.70300	27648.11100	10339.89400	20020.75000	Detected	Detected	Detected	Detected
6616	A_33_P3700794	METTL1	Hs.42957	methyltransferase like 1	Homo sapiens methyltransferase like 1 (METTL1), transcript variant 1, mRNA [NM_005371]	2.17538	down	2.38640	down	839.50720	1891.00350	857.11346	1803.62630	Detected	Detected	Detected	Detected
6617	A_33_P3287785	NEFL	Hs.521461	neurofilament, light polypeptide	Homo sapiens neurofilament, light polypeptide (NEFL), mRNA [NM_006158]	2.17517	down	2.03799	down	14.31782	32.24802	12.74158	22.89769	Detected	Detected	Compromised	Detected
6618	A_23_P406591	LOC441046	Hs.135705	glucuronidase, beta pseudogene	Homo sapiens glucuronidase, beta pseudogene (LOC441046), non-coding RNA [NR_003675]	2.17508	down	1.73658	down	55.68340	125.41071	40.23959	61.61880	Detected	Detected	Detected	Detected
6619	A_24_P136161	HNRNPCL1	Hs.502617	heterogeneous nuclear ribonucleoprotein C-like 1	Homo sapiens heterogeneous nuclear ribonucleoprotein C-like 1 (HNRNPCL1), mRNA [NM_001013631]	2.17499	down	2.00443	down	238.24250	536.55000	73.76955	130.38678	Detected	Detected	Detected	Detected
6620	A_33_P3330413					2.17481	down	2.07962	down	2320.49340	5225.58740	2252.40400	4130.44400	Detected	Detected	Detected	Detected
6621	A_23_P70148	NNT	Hs.482043	nicotinamide nucleotide transhydrogenase	Homo sapiens nicotinamide nucleotide transhydrogenase (NNT), nuclear gene encoding mitochondrial protein, transcript variant 2, mRNA [NM_182977]	2.17417	down	2.15887	down	293.24970	660.18340	212.52597	404.57920	Detected	Detected	Detected	Detected
6622	A_33_P3235217	CTPS	Hs.473087	CTP synthase	Homo sapiens CTP synthase (CTPS), mRNA [NM_001905]	2.17317	down	2.38314	down	4127.44730	9287.70000	3256.01560	6842.28760	Detected	Detected	Detected	Detected
6623	A_23_P210708	SIRPA	Hs.581021	signal-regulatory protein alpha	Homo sapiens signal-regulatory protein alpha (SIRPA), transcript variant 1, mRNA [NM_001040022]	2.17299	down	1.96082	down	2217.69950	4989.91850	2030.40750	3510.64920	Detected	Detected	Detected	Detected
6624	A_32_P452655	LGALS9C	Hs.462402	lectin, galactoside-binding, soluble, 9C	Homo sapiens lectin, galactoside-binding, soluble, 9C (LGALS9C), mRNA [NM_001040078]	2.17266	down	2.91759	down	711.22070	1600.03260	484.56857	1246.65110	Detected	Detected	Detected	Detected
6625	A_23_P15542	HSD17B1	Hs.654385	hydroxysteroid (17-beta) dehydrogenase 1	Homo sapiens hydroxysteroid (17-beta) dehydrogenase 1 (HSD17B1), mRNA [NM_000413]	2.17252	down	2.24347	down	256.48900	576.98690	260.58770	515.51294	Detected	Detected	Detected	Detected
6626	A_24_P116871	TXNL4A	Hs.465498	thioredoxin-like 4A	Homo sapiens thioredoxin-like 4A (TXNL4A), mRNA [NM_006701]	2.17205	down	2.05948	down	3482.01590	7831.30300	2410.92260	4378.30030	Detected	Detected	Detected	Detected
6627	A_24_P340066	ELF4	Hs.271940	E74-like factor 4 (ets domain transcription factor)	Homo sapiens E74-like factor 4 (ets domain transcription factor) (ELF4), transcript variant 1, mRNA [NM_001421]	2.17182	down	2.07762	down	4249.67240	9556.79400	2904.52300	5321.16460	Detected	Detected	Detected	Detected
6628	A_23_P128246	FICD	Hs.661891	FIC domain containing	Homo sapiens FIC domain containing (FICD), mRNA [NM_007076]	2.17180	down	2.33777	down	289.04837	650.01400	336.16672	692.98250	Detected	Detected	Detected	Detected
6629	A_33_P3277328	LOC100131700	Hs.149540	similar to Quinone oxidoreductase-like protein 2	PREDICTED: Homo sapiens similar to mCG15963 (LOC100131700), miscRNA [XR_078881]	2.17148	down	1.22567	down	24.28950	54.61445	49.68618	53.69992	Detected	Detected	Detected	Detected
6630	A_24_P362540	ASAP2	Hs.555902	ArfGAP with SH3 domain, ankyrin repeat and PH domain 2	Homo sapiens ArfGAP with SH3 domain, ankyrin repeat and PH domain 2 (ASAP2), transcript variant 1, mRNA [NM_003887]	2.17020	down	2.07080	down	1355.60160	3046.24410	1115.06370	2036.11710	Detected	Detected	Detected	Detected
6631	A_23_P10232	BANK1	Hs.480400	B-cell scaffold protein with ankyrin repeats 1	Homo sapiens B-cell scaffold protein with ankyrin repeats 1 (BANK1), transcript variant 1, mRNA [NM_017935]	2.17008	down	2.62483	down	17.54608	39.42660	8.68094	20.09248	Detected	Detected	Compromised	Detected
6632	A_23_P168788	PLOD3	Hs.153357	procollagen-lysine, 2-oxoglutarate 5-dioxygenase 3	Homo sapiens procollagen-lysine, 2-oxoglutarate 5-dioxygenase 3 (PLOD3), mRNA [NM_001084]	2.16928	down	2.30571	down	17292.94700	38843.41000	16413.13900	33370.40000	Detected	Detected	Detected	Detected
6633	A_33_P3307144	GRIK2	Hs.98262	glutamate receptor, ionotropic, kainate 2	Homo sapiens glutamate receptor, ionotropic, kainate 2 (GRIK2), transcript variant 1, mRNA [NM_021956]	2.16919	down	1.98364	down	15.54582	34.91761	16.67583	29.16857	Detected	Detected	Detected	Detected
6634	A_33_P3264238	ZNF280B	Hs.43834	zinc finger protein 280B	Homo sapiens zinc finger protein 280B (ZNF280B), mRNA [NM_080764]	2.16913	down	2.03333	down	35.16718	78.98707	28.82525	51.68283	Detected	Detected	Detected	Detected
6635	A_33_P3262181	APOBEC3F	Hs.659809	apolipoprotein B mRNA editing enzyme, catalytic polypeptide-like 3F	Homo sapiens apolipoprotein B mRNA editing enzyme, catalytic polypeptide-like 3F (APOBEC3F), transcript variant 2, mRNA [NM_001006666]	2.16886	down	2.16711	down	255.86127	574.60460	261.54220	499.79044	Detected	Detected	Detected	Detected
6636	A_32_P1701	POLA1	Hs.567319	polymerase (DNA directed), alpha 1, catalytic subunit	Homo sapiens polymerase (DNA directed), alpha 1, catalytic subunit (POLA1), mRNA [NM_016937]	2.16778	down	2.20134	down	538.22205	1208.12100	427.85107	830.51184	Detected	Detected	Detected	Detected
6637	A_33_P3622472	ARD1A	Hs.433291	ARD1 homolog A, N-acetyltransferase (S. cerevisiae)	Homo sapiens ARD1 homolog A, N-acetyltransferase (S. cerevisiae) (ARD1A), mRNA [NM_003491]	2.16747	down	2.29609	down	1387.99390	3115.10960	1655.42020	3351.67970	Detected	Detected	Detected	Detected
6638	A_32_P50522	FKBP1A	Hs.471933	FK506 binding protein 1A, 12kDa	Homo sapiens FK506 binding protein 1A, 12kDa (FKBP1A), transcript variant 12B, mRNA [NM_000801]	2.16713	down	1.81621	down	3301.25300	7407.93900	2429.05830	3890.18210	Detected	Detected	Detected	Detected

6639	A_24_P127121	LOC644563	Hs.648342	similar to general transcription factor IIIC, polypeptide 6, alpha 35kDa	PREDICTED: Homo sapiens similar to general transcription factor IIIC, polypeptide 6, alpha 35kDa (LOC644563), miscRNA [XR_016572]	2.16687	down	1.98408	down	12969.14700	29098.90400	9248.16000	16180.07500	Detected	Detected	Detected	Detected
6640	A_23_P109345	PTTG1IP	Hs.474010	pituitary tumor-transforming 1 interacting protein	Homo sapiens pituitary tumor-transforming 1 interacting protein (PTTG1IP), mRNA [NM_004339]	2.16643	down	2.25716	down	10179.49500	22835.09000	9968.43750	19840.63700	Detected	Detected	Detected	Detected
6641	A_33_P3326898					2.16637	down	2.20252	down	497.45220	1115.87780	171.67195	333.41410	Detected	Detected	Detected	Detected
6642	A_32_P119197	TPM3	Hs.644306	tropomyosin 3	Homo sapiens tropomyosin 3 (TPM3), transcript variant 3, mRNA [NM_001043352]	2.16631	down	2.23650	down	138.41902	310.49130	96.61615	190.53928	Detected	Detected	Detected	Detected
6643	A_23_P118038	NUTF2	Hs.356630	nuclear transport factor 2	Homo sapiens nuclear transport factor 2 (NUTF2), mRNA [NM_005796]	2.16548	down	2.30862	down	3111.92360	6977.74950	3522.37650	7170.55900	Detected	Detected	Detected	Detected
6644	A_23_P9018	DEFB104B	Hs.559507	defensin, beta 104B	Homo sapiens defensin, beta 104B (DEFB104B), mRNA [NM_001040702]	2.16397	down	1.25285	down	9.49195	21.26865	9.11904	10.07428	Compromised	Detected	Compromised	Compromised
6645	A_33_P3294053	ANKRD39	Hs.709507	ankyrin repeat domain 39	Homo sapiens ankyrin repeat domain 39 (ANKRD39), mRNA [NM_016466]	2.16391	down	2.09737	down	3466.96730	7768.21530	2636.70100	4876.42720	Detected	Detected	Detected	Detected
6646	A_24_P860703	LOC388796	Hs.400876	hypothetical LOC388796	Homo sapiens hypothetical LOC388796 (LOC388796), transcript variant 1, non-coding RNA [NR_015366]	2.16342	down	2.18849	down	5176.01270	11594.97600	3396.51000	6554.54050	Detected	Detected	Detected	Detected
6647	A_23_P98898	CDK2	Hs.19192	cyclin-dependent kinase 2	Homo sapiens cyclin-dependent kinase 2 (CDK2), transcript variant 1, mRNA [NM_001798]	2.16317	down	2.55400	down	86.59659	193.96509	74.40871	167.57513	Detected	Detected	Detected	Detected
6648	A_33_P3403153	C1QTNF2	Hs.110062	C1q and tumor necrosis factor related protein 2	Homo sapiens C1q and tumor necrosis factor related protein 2 (C1QTNF2), mRNA [NM_031908]	2.16223	down	3.39319	down	20.84400	46.66761	9.08190	27.17382	Detected	Detected	Compromised	Detected
6649	A_23_P166686	AMOTL2	Hs.426312	angiomotin like 2	Homo sapiens angiomotin like 2 (AMOTL2), mRNA [NM_016201]	2.16181	down	2.14964	down	12692.48700	28411.76600	13985.12500	26509.26600	Detected	Detected	Detected	Detected
6650	A_23_P201655	MYCBP	Hs.591506	c-myc binding protein	Homo sapiens c-myc binding protein (MYCBP), mRNA [NM_012333]	2.16168	down	2.10234	down	1536.59750	3439.41330	1327.78820	2461.48970	Detected	Detected	Detected	Detected
6651	A_33_P3360392	NCRNA00092	Hs.434310	non-protein coding RNA 92	Homo sapiens non-protein coding RNA 92 (NCRNA00092), non-coding RNA [NR_024129]	2.16072	down	5.00630	down	94.03716	210.39278	35.62950	157.28668	Detected	Detected	Detected	Detected
6652	A_23_P73429	HCLS1	Hs.14601	hematopoietic cell-specific Lyn substrate 1	Homo sapiens hematopoietic cell-specific Lyn substrate 1 (HCLS1), mRNA [NM_005335]	2.16062	down	2.14163	down	374.30164	837.40216	378.12924	714.08590	Detected	Detected	Detected	Detected
6653	A_33_P3392177	CLIC5	Hs.485489	chloride intracellular channel 5	Homo sapiens chloride intracellular channel 5 (CLIC5), transcript variant 1, mRNA [NM_001114086]	2.16036	down	2.09268	down	11157.53100	24959.01000	11969.24700	22086.87300	Detected	Detected	Detected	Detected
6654	A_24_P141688	PCBP2	Hs.546271	poly(rC) binding protein 2	Homo sapiens poly(rC) binding protein 2 (PCBP2), transcript variant 1, mRNA [NM_005016]	2.16034	down	2.21126	down	829.47760	1855.49790	760.82690	1483.51060	Detected	Detected	Detected	Detected
6655	A_33_P3356517					2.16030	down	2.65623	down	151.25375	338.34055	115.90701	271.48150	Detected	Detected	Detected	Detected
6656	A_33_P3304097					2.16001	down	1.49229	down	57.15156	127.82531	18.15627	23.89157	Detected	Detected	Detected	Detected
6657	A_23_P77073	SPPL2A	Hs.401537	signal peptide peptidase-like 2A	Homo sapiens signal peptide peptidase-like 2A (SPPL2A), mRNA [NM_032802]	2.15961	down	2.21368	down	2504.74370	5601.07670	1908.45760	3725.30540	Detected	Detected	Detected	Detected
6658	A_23_P145965	TPST1	Hs.720092	tyrosylprotein sulfotransferase 1	Homo sapiens tyrosylprotein sulfotransferase 1 (TPST1), mRNA [NM_003596]	2.15958	down	2.07710	down	5381.19600	12033.18800	4498.23200	8238.80200	Detected	Detected	Detected	Detected
6659	A_33_P3331853	PDE1C	Hs.655694	phosphodiesterase 1C, calmodulin-dependent 70kDa	Homo sapiens phosphodiesterase 1C, calmodulin-dependent 70kDa (PDE1C), mRNA [NM_005020]	2.15957	down	33.37086	down	3.32886	7.44381	2.52417	74.27663	Compromised	Compromised	Compromised	Detected
6660	A_24_P272352	LOC284441		actin-related protein 2 pseudogene	Homo sapiens actin-related protein 2 pseudogene (LOC284441), non-coding RNA [NR_003128]	2.15923	down	2.17728	down	751.55840	1680.32910	566.60300	1087.82420	Detected	Detected	Detected	Detected
6661	A_33_P3273364	YIPF2	Hs.720136	Yip1 domain family, member 2	Homo sapiens Yip1 domain family, member 2 (YIPF2), mRNA [NM_024029]	2.15903	down	2.31023	down	2246.61820	5022.52150	1760.52000	3586.41970	Detected	Detected	Detected	Detected
6662	A_33_P3262625	CECR7		cat eye syndrome chromosome region, candidate 7 (non-protein coding)	Homo sapiens cat eye syndrome chromosome region, candidate 7 (non-protein coding) (CECR7), non-coding RNA [NR_015352]	2.15894	down	2.44786	down	95.35472	213.16524	52.40698	113.12040	Detected	Detected	Detected	Detected
6663	A_33_P3293254	GPSM1	Hs.239370	G-protein signaling modulator 1 (AGS3-like, C. elegans)	Homo sapiens G-protein signaling modulator 1 (AGS3-like, C. elegans) (GPSM1), transcript variant 2, mRNA [NM_015597]	2.15880	down	1.85692	down	24.38764	54.51491	36.98367	60.55776	Detected	Detected	Detected	Detected
6664	A_24_P377144	ANTXR2	Hs.162963	anthrax toxin receptor 2	Homo sapiens anthrax toxin receptor 2 (ANTXR2), transcript variant 1, mRNA [NM_058172]	2.15854	down	2.00106	down	209.28621	467.77063	179.63362	316.96650	Detected	Detected	Detected	Detected
6665	A_23_P202448	CXCL12	Hs.522891	chemokine (C-X-C motif) ligand 12 (stromal cell-derived factor 1)	Homo sapiens chemokine (C-X-C motif) ligand 12 (stromal cell-derived factor 1) (CXCL12), transcript variant 1, mRNA [NM_199168]	2.15847	down	2.13964	down	8794.89400	19656.62100	7958.87740	15016.15100	Detected	Detected	Detected	Detected
6666	A_33_P3209476	CCM2	Hs.148272	cerebral cavernous malformation 2	Homo sapiens cerebral cavernous malformation 2 (CCM2), transcript variant 1, mRNA [NM_001029835]	2.15787	down	1.70080	down	4053.04600	9056.06400	821.68396	1232.32340	Detected	Detected	Detected	Detected
6667	A_33_P3424462	CNST	Hs.368353	consortin, connexin sorting protein	Homo sapiens consortin, connexin sorting protein (CNST), transcript variant 2, mRNA [NM_001139459]	2.15493	down	2.31489	down	196.21461	437.82250	104.84539	214.01508	Detected	Detected	Detected	Detected
6668	A_24_P346587	MTFR1	Hs.584788	mitochondrial fission regulator 1	Homo sapiens mitochondrial fission regulator 1 (MTFR1), nuclear gene encoding mitochondrial protein, transcript variant 1, mRNA [NM_014637]	2.15468	down	2.03081	down	866.11350	1932.37280	793.81550	1421.52310	Detected	Detected	Detected	Detected
6669	A_23_P216501	TPM2	Hs.300772	tropomyosin 2 (beta)	Homo sapiens tropomyosin 2 (beta) (TPM2), transcript variant 2, mRNA [NM_213674]	2.15459	down	2.04676	down	91498.38000	204131.75000	97944.29000	176771.50000	Detected	Detected	Detected	Detected
6670	A_33_P3225096	MTMR10	Hs.30141	myotubularin related protein 10	Homo sapiens myotubularin related protein 10 (MTMR10), mRNA [NM_017762]	2.15329	down	1.98841	down	164.98499	367.85712	173.26881	303.80237	Detected	Detected	Detected	Detected

6671	A_23_P422724	PPIC	Hs.110364	peptidylprolyl isomerase C (cyclophilin C)	Homo sapiens peptidylprolyl isomerase C (cyclophilin C) (PPIC), mRNA [NM_000943]	2.15203	down	1.91254	down	2731.36650	6086.42240	2385.22500	4022.58060	Detected	Detected	Detected	Detected
6672	A_23_P136196	TBC1D19	Hs.479403	TBC1 domain family, member 19	Homo sapiens TBC1 domain family, member 19 (TBC1D19), mRNA [NM_018317]	2.15197	down	1.77951	down	289.01990	644.01685	187.26938	293.85492	Detected	Detected	Detected	Detected
6673	A_23_P8640	GPER	Hs.20961	G protein-coupled estrogen receptor 1	Homo sapiens G protein-coupled estrogen receptor 1 (GPER), transcript variant 3, mRNA [NM_001039966]	2.15190	down	2.42399	down	1097.87290	2446.29050	1032.70390	2207.35620	Detected	Detected	Detected	Detected
6674	A_23_P370097	ALS2CR4	Hs.12319	amyotrophic lateral sclerosis 2 (juvenile) chromosome region, candidate 4	Homo sapiens amyotrophic lateral sclerosis 2 (juvenile) chromosome region, candidate 4 (ALS2CR4), transcript variant 1, mRNA [NM_001044385]	2.15174	down	1.96188	down	1122.66320	2501.33840	1174.52500	2031.89250	Detected	Detected	Detected	Detected
6675	A_23_P313961	NME6	Hs.465558	non-metastatic cells 6, protein expressed in (nucleoside-diphosphate kinase)	Homo sapiens non-metastatic cells 6, protein expressed in (nucleoside-diphosphate kinase) (NME6), mRNA [NM_005793]	2.14994	down	2.33173	down	353.69757	787.39360	411.48993	846.06384	Detected	Detected	Detected	Detected
6676	A_32_P203786	FAM7A1	Hs.574255	family with sequence similarity 7, member A1	Homo sapiens family with sequence similarity 7, member A1 (FAM7A1), non-coding RNA [NR_026858]	2.14975	down	2.10108	down	10.91427	24.29497	9.81501	18.18439	Compromised	Detected	Compromised	Detected
6677	A_23_P141248	SRR	Hs.461954	serine racemase	Homo sapiens serine racemase (SRR), mRNA [NM_021947]	2.14909	down	2.01680	down	253.25903	563.57654	218.80774	389.12677	Detected	Detected	Detected	Detected
6678	A_32_P110390	TMEM171	Hs.162246	transmembrane protein 171	Homo sapiens transmembrane protein 171 (TMEM171), transcript variant 1, mRNA [NM_173490]	2.14879	down	1.85650	down	420.62940	935.89200	553.44100	906.00653	Detected	Detected	Detected	Detected
6679	A_33_P3707547	LOC541467	Hs.612192	hypothetical LOC541467	Homo sapiens hypothetical LOC541467, mRNA (cDNA clone IMAGE:4830703), partial cds [BC045815]	2.14858	down	2.21128	down	22.30807	49.63033	18.89605	36.84513	Detected	Detected	Detected	Detected
6680	A_33_P3269203	SERPINH1	Hs.596449	serpin peptidase inhibitor, clade H (heat shock protein 47), member 1, (collagen binding protein 1)	Homo sapiens serpin peptidase inhibitor, clade H (heat shock protein 47), member 1, (collagen binding protein 1) (SERPINH1), mRNA [NM_001235]	2.14846	down	2.32924	down	29063.24400	64655.47700	23792.29900	48866.95000	Detected	Detected	Detected	Detected
6681	A_33_P3229156	SLC17A9	Hs.512686	solute carrier family 17, member 9	Homo sapiens solute carrier family 17, member 9 (SLC17A9), mRNA [NM_022082]	2.14721	down	2.38270	down	2997.91280	6665.39450	1711.73800	3596.43820	Detected	Detected	Detected	Detected
6682	A_23_P155376	CRELD1	Hs.9383	cysteine-rich with EGF-like domains 1	Homo sapiens cysteine-rich with EGF-like domains 1 (CRELD1), transcript variant 2, mRNA [NM_015513]	2.14687	down	2.15217	down	541.73760	1204.28170	520.61176	987.99800	Detected	Detected	Detected	Detected
6683	A_33_P3370132	TNPO3	Hs.193613	transportin 3	Homo sapiens transportin 3 (TNPO3), mRNA [NM_012470]	2.14661	down	2.26146	down	1237.43590	2750.48880	1181.17420	2355.41580	Detected	Detected	Detected	Detected
6684	A_33_P3414949	LOC100129532		hypothetical LOC100129532	PXMP2 protein [Source:UniProtKB/TrEMBL;Acc:Q96GB0] [ENST00000439331]	2.14635	down	3.08436	down	7.35364	16.34317	5.84463	15.89599	Compromised	Detected	Compromised	Detected
6685	A_33_P3398513	hCG_1645220	Hs.602756	hCG1645220	Homo sapiens hCG1645220 (LOC728819), mRNA [NM_001101330]	2.14634	down	2.26639	down	47.31722	105.15989	28.41791	56.79264	Detected	Detected	Detected	Detected
6686	A_33_P3271166	OR6C74	Hs.554556	olfactory receptor, family 6, subfamily C, member 74	Homo sapiens olfactory receptor, family 6, subfamily C, member 74 (OR6C74), mRNA [NM_001005490]	2.14623	down	2.31595	down	60.71483	134.92890	17.05463	34.82874	Detected	Detected	Detected	Detected
6687	A_33_P3209025	ZNF623	Hs.694018	zinc finger protein 623	Homo sapiens zinc finger protein 623 (ZNF623), transcript variant 2, mRNA [NM_001082480]	2.14503	down	1.83762	down	11.65457	25.88588	12.61016	20.43342	Detected	Detected	Detected	Detected
6688	A_32_P720220	C12orf36	Hs.448717	chromosome 12 open reading frame 36	Homo sapiens chromosome 12 open reading frame 36 (C12orf36), mRNA [NM_182558]	2.14487	down	1.33590	down	29.12286	64.67966	6.22493	7.33287	Detected	Detected	Compromised	Compromised
6689	A_33_P3396692	SSR1	Hs.114033	signal sequence receptor, alpha	Homo sapiens signal sequence receptor, alpha (SSR1), mRNA [NM_003144]	2.14466	down	1.99471	down	2687.99880	5969.27400	2036.95310	3582.82980	Detected	Detected	Detected	Detected
6690	A_23_P89422	ABCA10	Hs.25377	ATP-binding cassette, sub-family A (ABC1), member 10	Homo sapiens ATP-binding cassette, sub-family A (ABC1), member 10 (ABCA10), mRNA [NM_080282]	2.14466	down	1.86908	down	20.97563	46.58079	10.63816	17.53319	Detected	Detected	Compromised	Detected
6691	A_33_P3229335	HIST3H2BB	Hs.376691	histone cluster 3, H2bb	Homo sapiens histone cluster 3, H2bb (HIST3H2BB), mRNA [NM_175055]	2.14349	down	1.13684	down	25.78246	57.22399	26.66236	26.72782	Detected	Detected	Detected	Detected
6692	A_23_P105730	MDM1	Hs.655702	Mdm1 nuclear protein homolog (mouse)	Homo sapiens Mdm1 nuclear protein homolog (mouse) (MDM1), transcript variant 2, mRNA [NM_020128]	2.14295	down	1.91986	down	7.61416	16.89531	8.14518	13.78905	Compromised	Detected	Compromised	Detected
6693	A_33_P3352522	LOC100128430	Hs.720848	hypothetical LOC100128430	PREDICTED: Homo sapiens similar to hCG2012058 (LOC100128430), mRNA [XM_002347413]	2.14287	down	2.66869	down	25.42752	56.42007	13.54976	31.88561	Detected	Detected	Compromised	Detected
6694	A_33_P3302423	TNRC6C	Hs.569831	trinucleotide repeat containing 6C	Homo sapiens trinucleotide repeat containing 6C (TNRC6C), transcript variant 1, mRNA [NM_001142640]	2.14209	down	2.27787	down	418.43850	928.11700	324.55637	651.90393	Detected	Detected	Detected	Detected
6695	A_33_P3280927	SNHG7		small nucleolar RNA host gene 7 (non-protein coding)	Homo sapiens small nucleolar RNA host gene 7 (non-protein coding) (SNHG7), transcript variant 2, non-coding RNA [NR_024542]	2.14146	down	2.02560	down	2252.31760	4994.27100	1330.15040	2375.85570	Detected	Detected	Detected	Detected
6696	A_33_P3398107	SYS1	Hs.655055	SYS1 Golgi-localized integral membrane protein homolog (S. cerevisiae)	Homo sapiens SYS1 Golgi-localized integral membrane protein homolog (S. cerevisiae) (SYS1), transcript variant 1, mRNA [NM_033542]	2.13967	down	2.19939	down	214.86708	476.04610	233.44078	452.73474	Detected	Detected	Detected	Detected
6697	A_24_P154948	GARS	Hs.404321	glycyl-tRNA synthetase	Homo sapiens glycyl-tRNA synthetase (GARS), mRNA [NM_002047]	2.13932	down	2.07495	down	4937.86430	10938.27100	2528.00460	4625.42800	Detected	Detected	Detected	Detected
6698	A_23_P349928	SP100	Hs.369056	SP100 nuclear antigen	Homo sapiens SP100 nuclear antigen (SP100), transcript variant 2, mRNA [NM_003113]	2.13917	down	2.02498	down	39478.00800	87444.76000	25006.67200	44652.16800	Detected	Detected	Detected	Detected
6699	A_23_P50591	KCNK6	Hs.240395	potassium channel, subfamily K, member 6	Homo sapiens potassium channel, subfamily K, member 6 (KCNK6), mRNA [NM_004823]	2.13908	down	2.38018	down	181.01460	400.93497	142.87407	299.86700	Detected	Detected	Detected	Detected

6700	A_33_P3225477	tcag7.1227	Hs.650534	hypothetical protein LOC731075	PREDICTED: Homo sapiens hypothetical protein LOC731075 (LOC731075), mRNA [XM_001714401]	2.13865	down	1.60256	down	53.81470	119.17197	24.40200	34.48311	Detected	Detected	Detected	Detected
6701	A_23_P34126	BGN	Hs.821	biglycan	Homo sapiens biglycan (BGN), mRNA [NM_001711]	2.13813	down	2.55975	down	383.01090	847.96470	440.74258	994.82680	Detected	Detected	Detected	Detected
6702	A_23_P784	LZIC	Hs.327252	leucine zipper and CTNNBIP1 domain containing	Homo sapiens leucine zipper and CTNNBIP1 domain containing (LZIC), mRNA [NM_032368]	2.13796	down	2.03667	down	1426.21410	3157.31640	1448.92740	2602.15900	Detected	Detected	Detected	Detected
6703	A_23_P359588	PCDHGB4	Hs.368160	protocadherin gamma subfamily B, 4	Homo sapiens protocadherin gamma subfamily B, 4 (PCDHGB4), transcript variant 2, mRNA [NM_032098]	2.13796	down	2.32746	down	76.29316	168.89574	70.24512	144.16612	Detected	Detected	Detected	Detected
6704	A_23_P164284	CLDN7	Hs.513915	claudin 7	Homo sapiens claudin 7 (CLDN7), mRNA [NM_001307]	2.13744	down	2.20138	down	615.63446	1362.54350	560.19880	1087.43530	Detected	Detected	Detected	Detected
6705	A_33_P3337019	LOC728975	Hs.647238	hypothetical protein LOC728975	PREDICTED: Homo sapiens hypothetical protein LOC728975 (LOC728975), miscRNA [XR_042005]	2.13733	down	1.93456	down	365.74182	809.42960	255.08148	435.13834	Detected	Detected	Detected	Detected
6706	A_33_P3240258	RPN2	Hs.370895	ribophorin II	Homo sapiens ribophorin II (RPN2), transcript variant 1, mRNA [NM_002951]	2.13731	down	2.22252	down	10777.37800	23851.43000	10439.94700	20460.20300	Detected	Detected	Detected	Detected
6707	A_23_P40354	MAPRE1	Hs.472437	microtubule-associated protein, RP/EB family, member 1	Homo sapiens microtubule-associated protein, RP/EB family, member 1 (MAPRE1), mRNA [NM_012325]	2.13617	down	2.20814	down	1271.74990	2813.00660	1364.57420	2656.99150	Detected	Detected	Detected	Detected
6708	A_23_P120566	RRBP1	Hs.472213	ribosome binding protein 1 homolog 180kDa (dog)	Homo sapiens ribosome binding protein 1 homolog 180kDa (dog) (RRBP1), transcript variant 1, mRNA [NM_001042576]	2.13574	down	2.36464	down	4643.10700	10268.12300	3533.72240	7368.22900	Detected	Detected	Detected	Detected
6709	A_33_P3361257	NOP16	Hs.696283	NOP16 nucleolar protein homolog (yeast)	Homo sapiens NOP16 nucleolar protein homolog (yeast) (NOP16), mRNA [NM_016391]	2.13551	down	1.98444	down	5930.65430	13114.06400	6031.30000	10553.95900	Detected	Detected	Detected	Detected
6710	A_33_P3215028	C6orf89	Hs.433381	chromosome 6 open reading frame 89	Homo sapiens chromosome 6 open reading frame 89 (C6orf89), mRNA [NM_152734]	2.13471	down	2.10900	down	2729.63920	6033.61300	1997.42860	3714.60620	Detected	Detected	Detected	Detected
6711	A_23_P105862	FRY	Hs.507669	furry homolog (Drosophila)	Homo sapiens furry homolog (Drosophila) (FRY), mRNA [NM_023037]	2.13453	down	2.02286	down	101.94138	225.31250	67.99375	121.28308	Detected	Detected	Detected	Detected
6712	A_32_P16489	MRPL35	Hs.433439	mitochondrial ribosomal protein L35	Homo sapiens mitochondrial ribosomal protein L35 (MRPL35), nuclear gene encoding mitochondrial protein, transcript variant 2, mRNA [NM_145644]	2.13434	down	1.87834	down	869.58830	1921.80860	407.18900	674.42816	Detected	Detected	Detected	Detected
6713	A_23_P66694	EVI2B	Hs.5509	ecotropic viral integration site 2B	Homo sapiens ecotropic viral integration site 2B (EVI2B), mRNA [NM_006495]	2.13365	down	2.13008	down	22.86298	50.51148	15.00267	28.17931	Detected	Detected	Detected	Detected
6714	A_24_P43092	SEPT2	Hs.712994	septin 2	Homo sapiens septin 2 (SEPT2), transcript variant 1, mRNA [NM_001008491]	2.13313	down	2.21955	down	230.83337	509.85736	197.36574	386.27970	Detected	Detected	Detected	Detected
6715	A_23_P141894	PVR	Hs.171844	poliovirus receptor	Homo sapiens poliovirus receptor (PVR), transcript variant 1, mRNA [NM_006505]	2.13302	down	2.57983	down	97.11194	214.48697	77.75445	176.88145	Detected	Detected	Detected	Detected
6716	A_23_P400465	GTF3C6	Hs.418520	general transcription factor IIIC, polypeptide 6, alpha 35kDa	Homo sapiens general transcription factor IIIC, polypeptide 6, alpha 35kDa (GTF3C6), mRNA [NM_138408]	2.13274	down	2.01200	down	20128.71500	44451.46500	17057.24400	30262.31600	Detected	Detected	Detected	Detected
6717	A_32_P219116	CENPJ	Hs.513379	centromere protein J	Homo sapiens centromere protein J (CENPJ), mRNA [NM_018451]	2.13060	down	2.08555	down	1045.16380	2305.78980	967.41986	1779.10050	Detected	Detected	Detected	Detected
6718	A_23_P17769	DDT	Hs.656723	D-dopachrome tautomerase	Homo sapiens D-dopachrome tautomerase (DDT), transcript variant 1, mRNA [NM_001355]	2.12970	down	2.18451	down	4815.29000	10618.73800	4986.30800	9605.03500	Detected	Detected	Detected	Detected
6719	A_33_P3270599	TPM2	Hs.300772	tropomyosin 2 (beta)	Homo sapiens tropomyosin 2 (beta) (TPM2), transcript variant 2, mRNA [NM_213674]	2.12959	down	2.17659	down	69898.86000	154134.22000	63249.09400	121393.99000	Detected	Detected	Detected	Detected
6720	A_33_P3389728	NR5A2	Hs.33446	nuclear receptor subfamily 5, group A, member 2	Homo sapiens nuclear receptor subfamily 5, group A, member 2 (NR5A2), transcript variant 1, mRNA [NM_205860]	2.12928	down	2.45468	down	298.60205	658.35345	206.43166	446.82407	Detected	Detected	Detected	Detected
6721	A_23_P101742	MRPL54	Hs.356578	mitochondrial ribosomal protein L54	Homo sapiens mitochondrial ribosomal protein L54 (MRPL54), nuclear gene encoding mitochondrial protein, mRNA [NM_172251]	2.12902	down	2.12374	down	1556.05960	3430.35820	1834.89180	3436.18630	Detected	Detected	Detected	Detected
6722	A_33_P3363310				Keratin-associated protein 5-10 (Keratin-associated protein 5.10)(Ultrahigh sulfur keratin-associated protein 5.10) [Source:UniProtKB/Swiss-Prot;Acc:Q6L8G5] [ENST00000376536]	2.12848	down	1.73219	down	1446.94320	3189.00000	270.05820	412.49542	Detected	Detected	Detected	Detected
6723	A_33_P3397298					2.12732	down	2.90458	down	429.15546	945.32240	350.95795	898.88434	Detected	Detected	Detected	Detected
6724	A_23_P365817	PPP1R14B	Hs.523760	protein phosphatase 1, regulatory (inhibitor) subunit 14B	Homo sapiens protein phosphatase 1, regulatory (inhibitor) subunit 14B (PPP1R14B), mRNA [NM_138689]	2.12663	down	2.05087	down	23511.65600	51773.67600	11957.66100	21624.71500	Detected	Detected	Detected	Detected
6725	A_32_P486693	NRIP3	Hs.523467	nuclear receptor interacting protein 3	Homo sapiens nuclear receptor interacting protein 3 (NRIP3), mRNA [NM_020645]	2.12619	down	2.40132	down	59.15313	130.23070	70.11028	148.45575	Detected	Detected	Detected	Detected
6726	A_32_P182511	KIAA1609	Hs.288274	KIAA1609	Homo sapiens KIAA1609 (KIAA1609), mRNA [NM_020947]	2.12566	down	2.29739	down	187.88048	413.53125	180.67159	366.00787	Detected	Detected	Detected	Detected
6727	A_23_P71241	SEC61G	Hs.488282	Sec61 gamma subunit	Homo sapiens Sec61 gamma subunit (SEC61G), transcript variant 1, mRNA [NM_014302]	2.12554	down	1.88909	down	9277.52600	20418.97900	7761.07100	12928.22500	Detected	Detected	Detected	Detected
6728	A_23_P201386	DDAH1	Hs.719968	dimethylarginine dimethylaminohydrolase 1	Homo sapiens dimethylarginine dimethylaminohydrolase 1 (DDAH1), transcript variant 1, mRNA [NM_012137]	2.12513	down	2.13246	down	7626.14400	16781.25600	6153.71200	11571.33100	Detected	Detected	Detected	Detected
6729	A_23_P96833	SLAMF9	Hs.661712	SLAM family member 9	Homo sapiens SLAM family member 9 (SLAMF9), transcript variant 1, mRNA [NM_033438]	2.12457	down	2.54721	down	439.70224	967.30347	492.34583	1105.86170	Detected	Detected	Detected	Detected

6730	A_33_P3418597	GAS2L1	Hs.322852	growth arrest-specific 2 like 1	Homo sapiens growth arrest-specific 2 like 1 (GAS2L1), transcript variant 3, mRNA [NM_152237]	2.12387	down	2.61805	down	1437.30160	3160.88100	1594.03940	3679.95000	Detected	Detected	Detected	Detected
6731	A_33_P3342992	UBE2QP2	Hs.641964	ubiquitin-conjugating enzyme E2Q family pseudogene 2	Homo sapiens ubiquitin-conjugating enzyme E2Q family pseudogene 2 (UBE2QP2), non-coding RNA [NR_004847]	2.12383	down	1.41850	down	105.66731	232.37761	78.91713	98.71082	Detected	Detected	Detected	Detected
6732	A_33_P3539223	C9orf100	Hs.534579	chromosome 9 open reading frame 100	Homo sapiens chromosome 9 open reading frame 100 (C9orf100), mRNA [NM_032818]	2.12339	down	2.59388	down	40.66789	89.41570	34.95396	79.94890	Detected	Detected	Detected	Detected
6733	A_23_P98631	HPS5	Hs.437599	Hermansky-Pudlak syndrome 5	Homo sapiens Hermansky-Pudlak syndrome 5 (HPS5), transcript variant 1, mRNA [NM_181507]	2.12331	down	2.23057	down	454.27560	998.76910	405.53590	797.64690	Detected	Detected	Detected	Detected
6734	A_23_P147729	SLC35E3	Hs.506011	solute carrier family 35, member E3	Homo sapiens solute carrier family 35, member E3 (SLC35E3), mRNA [NM_018656]	2.12305	down	1.95824	down	208.58810	458.54620	161.93335	279.62006	Detected	Detected	Detected	Detected
6735	A_24_P178423	HNRNPC	Hs.508848	heterogeneous nuclear ribonucleoprotein C (C1/C2)	Homo sapiens heterogeneous nuclear ribonucleoprotein C (C1/C2) (HNRNPC), transcript variant 1, mRNA [NM_031314]	2.12265	down	1.99525	down	3942.86940	8666.10600	3292.62480	5793.02830	Detected	Detected	Detected	Detected
6736	A_23_P5601	DOK1	Hs.103854	docking protein 1, 62kDa (downstream of tyrosine kinase 1)	Homo sapiens docking protein 1, 62kDa (downstream of tyrosine kinase 1) (DOK1), mRNA [NM_001381]	2.12206	down	2.22803	down	5768.48050	12675.12300	4269.10000	8387.31700	Detected	Detected	Detected	Detected
6737	A_24_P237613	USP12	Hs.42400	ubiquitin specific peptidase 12	Homo sapiens ubiquitin specific peptidase 12 (USP12), mRNA [NM_182488]	2.12156	down	1.92265	down	80.92330	177.77174	93.29781	158.17502	Detected	Detected	Detected	Detected
6738	A_23_P325726	ACOT11	Hs.234786	acyl-CoA thioesterase 11	Homo sapiens acyl-CoA thioesterase 11 (ACOT11), transcript variant 1, mRNA [NM_015547]	2.12125	down	2.39139	down	27.23157	59.81334	17.77024	37.47220	Detected	Detected	Detected	Detected
6739	A_33_P3225690	ZNF516	Hs.709890	zinc finger protein 516	Homo sapiens zinc finger protein 516 (ZNF516), mRNA [NM_014643]	2.12124	down	2.16493	down	897.14800	1970.54610	671.33636	1281.59000	Detected	Detected	Detected	Detected
6740	A_23_P819	ISG15	Hs.458485	ISG15 ubiquitin-like modifier	Homo sapiens ISG15 ubiquitin-like modifier (ISG15), mRNA [NM_005101]	2.12097	down	1.61635	down	9293.83500	20410.92400	6646.26900	9472.83400	Detected	Detected	Detected	Detected
6741	A_23_P215566	AHR	Hs.171189	aryl hydrocarbon receptor	Homo sapiens aryl hydrocarbon receptor (AHR), mRNA [NM_001621]	2.12014	down	2.10246	down	2162.68300	4747.77400	1764.58170	3271.41110	Detected	Detected	Detected	Detected
6742	A_33_P3405068	NAV1	Hs.585374	neuron navigator 1	Homo sapiens neuron navigator 1 (NAV1), mRNA [NM_020443]	2.11956	down	1.92521	down	707.75150	1553.31870	504.97500	857.26135	Detected	Detected	Detected	Detected
6743	A_23_P110253	KIT	Hs.479754	v-kit Hardy-Zuckerman 4 feline sarcoma viral oncogene homolog	Homo sapiens v-kit Hardy-Zuckerman 4 feline sarcoma viral oncogene homolog (KIT), transcript variant 1, mRNA [NM_000222]	2.11932	down	1.97216	down	26.55960	58.28436	17.32201	30.12346	Detected	Detected	Detected	Detected
6744	A_24_P131222	ATP13A2	Hs.128866	ATPase type 13A2	Homo sapiens ATPase type 13A2 (ATP13A2), transcript variant 1, mRNA [NM_022089]	2.11893	down	2.57305	down	207.87927	456.09995	188.49626	427.67830	Detected	Detected	Detected	Detected
6745	A_33_P3262452	PEX26	Hs.517400	peroxisomal biogenesis factor 26	Homo sapiens peroxisomal biogenesis factor 26 (PEX26), transcript variant 2, mRNA [NM_001127649]	2.11829	down	2.53371	down	205.72151	451.23068	150.73582	336.77414	Detected	Detected	Detected	Detected
6746	A_23_P145874	SAMD9L	Hs.489118	sterile alpha motif domain containing 9-like	Homo sapiens sterile alpha motif domain containing 9-like (SAMD9L), mRNA [NM_152703]	2.11813	down	2.28751	down	180.16281	395.13965	130.19489	262.61650	Detected	Detected	Detected	Detected
6747	A_33_P3213910	ARHGAP30	Hs.389374	Rho GTPase activating protein 30	Homo sapiens Rho GTPase activating protein 30 (ARHGAP30), transcript variant 2, mRNA [NM_181720]	2.11780	down	1.72159	down	80.82985	177.25104	32.33325	49.08453	Detected	Detected	Detected	Detected
6748	A_33_P3362321	FHL2	Hs.443687	four and a half LIM domains 2	Homo sapiens four and a half LIM domains 2 (FHL2), transcript variant 2, mRNA [NM_201555]	2.11773	down	2.27052	down	6334.86800	13891.23800	7268.36670	14552.13600	Detected	Detected	Detected	Detected
6749	A_23_P169909	DGKI	Hs.242947	diacylglycerol kinase, iota	Homo sapiens diacylglycerol kinase, iota (DGKI), mRNA [NM_004717]	2.11751	down	2.34045	down	127.27599	279.06445	119.32119	246.25339	Detected	Detected	Detected	Detected
6750	A_33_P3336557		Hs.721131		full-length cDNA clone CS0DL001YG15 of B cells (Ramos cell line) Cot 25-normalized of Homo sapiens (human) [CR591103]	2.11732	down	1.05923	down	8.35874	18.32567	9.71695	9.07582	Compromised	Detected	Compromised	Compromised
6751	A_32_P70158	LILRB3	Hs.631592	leukocyte immunoglobulin-like receptor, subfamily B (with TM and ITIM domains), member 3	Homo sapiens leukocyte immunoglobulin-like receptor, subfamily B (with TM and ITIM domains), member 3 (LILRB3), transcript variant 2, mRNA [NM_006864]	2.11698	down	1.53345	down	19.92083	43.66740	21.95743	29.69045	Detected	Detected	Detected	Detected
6752	A_24_P73389	STK24	Hs.508514	serine/threonine kinase 24 (STE20 homolog, yeast)	Homo sapiens serine/threonine kinase 24 (STE20 homolog, yeast) (STK24), transcript variant 2, mRNA [NM_001032296]	2.11668	down	2.33870	down	576.29010	1263.07350	469.68020	968.59595	Detected	Detected	Detected	Detected
6753	A_23_P63402	GPSM2	Hs.584901	G-protein signaling modulator 2 (AGS3-like, C. elegans)	Homo sapiens G-protein signaling modulator 2 (AGS3-like, C. elegans) (GPSM2), mRNA [NM_013296]	2.11596	down	2.03509	down	1775.73660	3890.62180	1770.76540	3177.68300	Detected	Detected	Detected	Detected
6754	A_33_P3331588	ATAD3B	Hs.23413	ATPase family, AAA domain containing 3B	Homo sapiens ATPase family, AAA domain containing 3B (ATAD3B), mRNA [NM_031921]	2.11511	down	2.34968	down	10746.49600	23536.00800	13221.15200	27393.22300	Detected	Detected	Detected	Detected
6755	A_24_P349151	SASS6	Hs.591447	spindle assembly 6 homolog (C. elegans)	Homo sapiens spindle assembly 6 homolog (C. elegans) (SASS6), mRNA [NM_194292]	2.11396	down	2.07268	down	93.53385	204.73788	58.67680	107.24178	Detected	Detected	Detected	Detected
6756	A_33_P3240602	IGFL2		IGF-like family member 2	Homo sapiens cDNA FLJ43766 fis, clone TESTI2049246. [AK125754]	2.11392	down	1.35663	down	19.67685	43.07025	8.73770	10.45258	Detected	Detected	Compromised	Compromised
6757	A_33_P3353170		Hs.491351		Clathrin heavy chain 1 (CLH-17) [Source:UniProtKB/Swiss-Prot;Acc:Q00610] [ENST00000393043]	2.11336	down	2.06796	down	210.07779	459.71347	141.11594	257.32642	Detected	Detected	Detected	Detected
6758	A_24_P412512	NIN	Hs.310429	ninein (GSK3B interacting protein)	Homo sapiens ninein (GSK3B interacting protein) (NIN), transcript variant 4, mRNA [NM_016350]	2.11335	down	2.03100	down	167.24850	365.98755	115.64679	207.11404	Detected	Detected	Detected	Detected
6759	A_23_P24616	SIAE	Hs.10056	sialic acid acetylesterase	Homo sapiens sialic acid acetylesterase (SIAE), mRNA [NM_170601]	2.11305	down	1.94112	down	116.93633	255.85408	98.95667	169.38042	Detected	Detected	Detected	Detected

6760	A_23_P153628	YIF1B	Hs.280741	Yip1 interacting factor homolog B (S. cerevisiae)	Homo sapiens Yip1 interacting factor homolog B (S. cerevisiae) (YIF1B), transcript variant 2, mRNA [NM_033557]	2.11304	down	2.44130	down	614.73206	1345.01440	604.98680	1302.36410	Detected	Detected	Detected	Detected
6761	A_24_P36745	CXorf38	Hs.495961	chromosome X open reading frame 38	Homo sapiens chromosome X open reading frame 38 (CXorf38), mRNA [NM_144970]	2.11278	down	2.21468	down	975.32935	2133.72600	869.52680	1698.08330	Detected	Detected	Detected	Detected
6762	A_23_P338890	PTPN1	Hs.417549	protein tyrosine phosphatase, non-receptor type 1	Homo sapiens protein tyrosine phosphatase, non-receptor type 1 (PTPN1), mRNA [NM_002827]	2.11219	down	1.87440	down	2374.95430	5194.22360	1166.46190	1927.96330	Detected	Detected	Detected	Detected
6763	A_33_P3281151	FUBP1	Hs.567380	far upstream element (FUSE) binding protein 1	Homo sapiens far upstream element (FUSE) binding protein 1 (FUBP1), mRNA [NM_003902]	2.11177	down	2.25709	down	730.42500	1597.18580	665.80850	1325.14360	Detected	Detected	Detected	Detected
6764	A_32_P18258	TALDO1	Hs.438678	transaldolase 1	Homo sapiens transaldolase 1 (TALDO1), mRNA [NM_006755]	2.11140	down	1.98640	down	24834.14600	54294.07000	13938.81200	24415.07000	Detected	Detected	Detected	Detected
6765	A_23_P28697	HAAO	Hs.368805	3-hydroxyanthranilate 3,4-dioxygenase	Homo sapiens 3-hydroxyanthranilate 3,4-dioxygenase (HAAO), mRNA [NM_012205]	2.11130	down	2.43569	down	245.23203	536.11710	51.42967	110.45883	Detected	Detected	Detected	Detected
6766	A_33_P3230723	KIF1B	Hs.97858	kinesin family member 1B	Homo sapiens kinesin family member 1B (KIF1B), transcript variant 2, mRNA [NM_183416]	2.11119	down	2.23050	down	134.69612	294.45306	124.35841	244.59277	Detected	Detected	Detected	Detected
6767	A_33_P3246418	MDFI	Hs.520119	MyoD family inhibitor	Homo sapiens MyoD family inhibitor (MDFI), mRNA [NM_005586]	2.11008	down	3.19873	down	31.51549	68.85824	26.23564	74.00040	Detected	Detected	Detected	Detected
6768	A_33_P3212274	F2R	Hs.679786	coagulation factor II (thrombin) receptor	602291527F1 NIH_MGC_86 Homo sapiens cDNA clone IMAGE:4386414 5', mRNA sequence [BG026194]	2.10989	down	2.07278	down	469.04385	1024.72180	433.64343	792.59644	Detected	Detected	Detected	Detected
6769	A_33_P3407400	SPEF2	Hs.298863	sperm flagellar 2	Homo sapiens sperm flagellar 2 (SPEF2), transcript variant 2, mRNA [NM_144722]	2.10966	down	1.69087	down	72.38953	158.13246	37.73844	56.26776	Detected	Detected	Detected	Detected
6770	A_23_P56590	C1D	Hs.602900	C1D nuclear receptor co-repressor	Homo sapiens C1D nuclear receptor co-repressor (C1D), transcript variant 1, mRNA [NM_006333]	2.10934	down	1.96526	down	392.30615	856.85120	319.42600	553.54850	Detected	Detected	Detected	Detected
6771	A_33_P3294297	RNF126	Hs.69554	ring finger protein 126	Homo sapiens ring finger protein 126 (RNF126), mRNA [NM_194460]	2.10879	down	2.24876	down	4841.84960	10572.48300	4770.55400	9459.70600	Detected	Detected	Detected	Detected
6772	A_24_P12281	AP4S1	Hs.293411	adaptor-related protein complex 4, sigma 1 subunit	Homo sapiens adaptor-related protein complex 4, sigma 1 subunit (AP4S1), transcript variant 1, mRNA [NM_007077]	2.10864	down	2.10911	down	74.46210	162.58118	53.91050	100.26260	Detected	Detected	Detected	Detected
6773	A_33_P3286218	DLEU2L	Hs.547964	deleted in lymphocytic leukemia 2-like	Homo sapiens deleted in lymphocytic leukemia 2-like (DLEU2L), non-coding RNA [NR_002771]	2.10815	down	2.19646	down	99.98975	218.26775	66.52727	128.85132	Detected	Detected	Detected	Detected
6774	A_33_P3323722	ARL4C	Hs.111554	ADP-ribosylation factor-like 4C	Homo sapiens ADP-ribosylation factor-like 4C (ARL4C), mRNA [NM_005737]	2.10775	down	2.03415	down	2411.62400	5263.33700	2570.67480	4611.01000	Detected	Detected	Detected	Detected
6775	A_23_P91657	EWSR1	Hs.374477	Ewing sarcoma breakpoint region 1	Homo sapiens Ewing sarcoma breakpoint region 1 (EWSR1), transcript variant 1, mRNA [NM_013986]	2.10758	down	2.51796	down	1331.60730	2905.99020	1147.41320	2547.61380	Detected	Detected	Detected	Detected
6776	A_33_P3357753	LOC728190		hypothetical LOC728190	Homo sapiens hypothetical LOC728190 (LOC728190), non-coding RNA [NR_024397]	2.10629	down	2.12179	down	1708.67430	3726.58860	1189.76120	2226.01250	Detected	Detected	Detected	Detected
6777	A_33_P3419621					2.10571	down	1.11019	down	1019.96014	2223.90190	54.45460	53.30845	Detected	Detected	Detected	Detected
6778	A_23_P46604	NIPAL3	Hs.523442	NIPA-like domain containing 3	Homo sapiens NIPA-like domain containing 3 (NIPAL3), mRNA [NM_020448]	2.10571	down	2.20097	down	265.23030	578.30120	241.78390	469.25256	Detected	Detected	Detected	Detected
6779	A_33_P3356701	ZDHHC12	Hs.133122	zinc finger, DHHC-type containing 12	Homo sapiens zinc finger, DHHC-type containing 12 (ZDHHC12), mRNA [NM_032799]	2.10546	down	2.40361	down	3431.86430	7481.85700	3140.85900	6656.99300	Detected	Detected	Detected	Detected
6780	A_24_P233915	MED8	Hs.301756	mediator complex subunit 8	Homo sapiens mediator complex subunit 8 (MED8), transcript variant 1, mRNA [NM_201542]	2.10494	down	2.21502	down	1248.09910	2720.32640	1158.85470	2263.45950	Detected	Detected	Detected	Detected
6781	A_23_P401084	ZNF575	Hs.213534	zinc finger protein 575	Homo sapiens zinc finger protein 575 (ZNF575), mRNA [NM_174945]	2.10437	down	2.34139	down	225.26558	490.85098	237.96855	491.31420	Detected	Detected	Detected	Detected
6782	A_33_P3220827	PEA15	Hs.517216	phosphoprotein enriched in astrocytes 15	Homo sapiens phosphoprotein enriched in astrocytes 15 (PEA15), mRNA [NM_003768]	2.10422	down	2.75013	down	3321.86060	7237.78700	3045.68040	7385.89160	Detected	Detected	Detected	Detected
6783	A_33_P3335147	MURC	Hs.99004	muscle-related coiled-coil protein	Homo sapiens muscle-related coiled-coil protein (MURC), mRNA [NM_001018116]	2.10394	down	2.22733	down	53.91845	117.46372	41.60881	81.72113	Detected	Detected	Detected	Detected
6784	A_24_P210675	NDE1	Hs.655378	nudE nuclear distribution gene E homolog 1 (A. nidulans)	Homo sapiens nudE nuclear distribution gene E homolog 1 (A. nidulans) (NDE1), transcript variant 2, mRNA [NM_017668]	2.10325	down	2.54568	down	97.12568	211.52309	51.54498	115.70583	Detected	Detected	Detected	Detected
6785	A_24_P156113	EHD2	Hs.631554	EH-domain containing 2	Homo sapiens EH-domain containing 2 (EHD2), mRNA [NM_014601]	2.10267	down	2.08747	down	4514.24900	9828.57000	3906.27730	7190.31450	Detected	Detected	Detected	Detected
6786	A_32_P930685	ZNF876P	Hs.673877	zinc finger protein 876 (pseudogene)	Homo sapiens zinc finger protein 876 (pseudogene) (ZNF876P), non-coding RNA [NR_027481]	2.10238	down	1.63979	down	40.80991	88.84038	40.10956	57.99666	Detected	Detected	Detected	Detected
6787	A_33_P3784253	PAK1	Hs.435714	p21 protein (Cdc42/Rac)-activated kinase 1	Homo sapiens p21 protein (Cdc42/Rac)-activated kinase 1 (PAK1), transcript variant 1, mRNA [NM_001128620]	2.10234	down	2.23023	down	1326.96070	2888.64010	1071.15170	2106.52370	Detected	Detected	Detected	Detected
6788	A_33_P3233649	C5orf33	Hs.81907	chromosome 5 open reading frame 33	Homo sapiens chromosome 5 open reading frame 33 (C5orf33), transcript variant 1, mRNA [NM_001085411]	2.10207	down	1.86168	down	74.30013	161.72240	87.84058	144.20001	Detected	Detected	Detected	Detected
6789	A_23_P502312	CD97	Hs.466039	CD97 molecule	Homo sapiens CD97 molecule (CD97), transcript variant 1, mRNA [NM_078481]	2.10201	down	2.42716	down	12405.13700	27000.37300	11619.81250	24869.30300	Detected	Detected	Detected	Detected
6790	A_33_P3335910	SYNE1	Hs.12967	spectrin repeat containing, nuclear envelope 1	Homo sapiens spectrin repeat containing, nuclear envelope 1 (SYNE1), transcript variant 2, mRNA [NM_033071]	2.10194	down	2.20583	down	38.98674	84.85370	19.61136	38.14566	Detected	Detected	Detected	Detected

6791	A_23_P80336	TOMM22	Hs.595072	translocase of outer mitochondrial membrane 22 homolog (yeast)	Homo sapiens translocase of outer mitochondrial membrane 22 homolog (yeast) (TOMM22), nuclear gene encoding mitochondrial protein, mRNA [NM_020243]	2.10121	down	2.07836	down	4727.34900	10285.37100	4178.33150	7657.52400	Detected	Detected	Detected	Detected
6792	A_24_P77364	BOLA3	Hs.61472	bolA homolog 3 (E. coli)	Homo sapiens bolA homolog 3 (E. coli) (BOLA3), transcript variant 1, mRNA [NM_212552]	2.10119	down	1.99932	down	11556.58500	25143.62000	10180.66600	17948.35700	Detected	Detected	Detected	Detected
6793	A_23_P369328	C10orf35	Hs.522992	chromosome 10 open reading frame 35	Homo sapiens chromosome 10 open reading frame 35 (C10orf35), mRNA [NM_145306]	2.10050	down	1.54015	down	31.52509	68.56648	31.58781	42.89898	Detected	Detected	Detected	Detected
6794	A_23_P13524	TMEM126A	Hs.533725	transmembrane protein 126A	Homo sapiens transmembrane protein 126A (TMEM126A), mRNA [NM_032273]	2.10021	down	2.03640	down	7383.06050	16055.84400	5234.75300	9399.92100	Detected	Detected	Detected	Detected
6795	A_33_P3257140	ROCK2	Hs.591600	Rho-associated, coiled-coil containing protein kinase 2	Homo sapiens Rho-associated, coiled-coil containing protein kinase 2 (ROCK2), mRNA [NM_004850]	2.10011	down	1.86840	down	129.91766	282.51605	102.93738	169.59329	Detected	Detected	Detected	Detected
6796	A_23_P100676	SMG6	Hs.448342	Smg-6 homolog, nonsense mediated mRNA decay factor (C. elegans)	Homo sapiens Smg-6 homolog, nonsense mediated mRNA decay factor (C. elegans) (SMG6), mRNA [NM_017575]	2.09987	down	2.32304	down	780.99884	1698.14950	688.79364	1410.94600	Detected	Detected	Detected	Detected
6797	A_23_P75978	CLPB	Hs.523877	ClpB caseinolytic peptidase B homolog (E. coli)	Homo sapiens ClpB caseinolytic peptidase B homolog (E. coli) (CLPB), mRNA [NM_030813]	2.09969	down	2.22419	down	735.78960	1599.71010	821.67255	1611.52420	Detected	Detected	Detected	Detected
6798	A_24_P13041	RTKN2	Hs.58559	rhotekin 2	Homo sapiens rhotekin 2 (RTKN2), mRNA [NM_145307]	2.09914	down	6.05376	down	2.44072	5.30510	2.74950	14.67723	Compromised	Compromised	Compromised	Detected
6799	A_32_P54553	USP41	Hs.581057	ubiquitin specific peptidase 41	Putative ubiquitin carboxyl-terminal hydrolase 41 (EC 3.1.2.15)(Ubiquitin thioesterase 41)(Ubiquitin-specific-processing protease 41)(Deubiquitinating enzyme 41) [Source:UniProtKB/Swiss-Prot;Acc:Q3LFD5] [ENST00000292729]	2.09826	down	2.96411	down	18.26599	39.68575	14.26793	37.29238	Detected	Detected	Detected	Detected
6800	A_23_P42975	PRKAR2B	Hs.433068	protein kinase, cAMP-dependent, regulatory, type II, beta	Homo sapiens protein kinase, cAMP-dependent, regulatory, type II, beta (PRKAR2B), mRNA [NM_002736]	2.09818	down	2.05250	down	71.98031	156.38275	39.34577	71.21104	Detected	Detected	Detected	Detected
6801	A_23_P57306	CHAF1B	Hs.75238	chromatin assembly factor 1, subunit B (p60)	Homo sapiens chromatin assembly factor 1, subunit B (p60) (CHAF1B), mRNA [NM_005441]	2.09791	down	2.11663	down	1814.68620	3942.04600	1618.94170	3021.62840	Detected	Detected	Detected	Detected
6802	A_33_P3252800	PTPRR	Hs.506076	protein tyrosine phosphatase, receptor type, R	Homo sapiens protein tyrosine phosphatase, receptor type, R (PTPRR), transcript variant 1, mRNA [NM_002849]	2.09757	down	3.12708	down	16.92621	36.76288	12.92952	35.65228	Detected	Detected	Compromised	Detected
6803	A_23_P81770	PTP4A1	Hs.227777	protein tyrosine phosphatase type IVA, member 1	Homo sapiens protein tyrosine phosphatase type IVA, member 1 (PTP4A1), mRNA [NM_003463]	2.09713	down	1.64981	down	7550.74560	16396.42800	5955.33740	8663.75200	Detected	Detected	Detected	Detected
6804	A_33_P3345608	C10orf137	Hs.468688	chromosome 10 open reading frame 137	Homo sapiens chromosome 10 open reading frame 137 (C10orf137), mRNA [NM_015608]	2.09630	down	2.41272	down	69.99095	151.92462	73.56426	156.50908	Detected	Detected	Detected	Detected
6805	A_23_P218892	EIF4G1	Hs.433750	eukaryotic translation initiation factor 4 gamma, 1	Homo sapiens eukaryotic translation initiation factor 4 gamma, 1 (EIF4G1), transcript variant 1, mRNA [NM_182917]	2.09630	down	2.23225	down	8865.28100	19243.26800	7265.86400	14301.94300	Detected	Detected	Detected	Detected
6806	A_32_P41405	THOC3	Hs.484227	THO complex 3	Homo sapiens THO complex 3 (THOC3), mRNA [NM_032361]	2.09610	down	2.04962	down	4343.02640	9426.23500	3870.17040	6994.70750	Detected	Detected	Detected	Detected
6807	A_33_P3345816	GPER	Hs.20961	G protein-coupled estrogen receptor 1	Homo sapiens G protein-coupled estrogen receptor 1 (GPER), transcript variant 3, mRNA [NM_001039966]	2.09570	down	2.44461	down	377.25363	818.64355	352.18180	759.17410	Detected	Detected	Detected	Detected
6808	A_23_P419624	BLCAP	Hs.718404	bladder cancer associated protein	Homo sapiens bladder cancer associated protein (BLCAP), mRNA [NM_006698]	2.09566	down	2.15394	down	1804.77000	3916.29860	1338.31930	2541.89940	Detected	Detected	Detected	Detected
6809	A_33_P3263307	RANGAP1	Hs.183800	Ran GTPase activating protein 1	Homo sapiens Ran GTPase activating protein 1 (RANGAP1), mRNA [NM_002883]	2.09562	down	2.22719	down	35303.34400	76605.72000	31365.24400	61598.69000	Detected	Detected	Detected	Detected
[truncated: 226,923 more chars]
