# Supplementary material for: Lactic Acid Bacteria Convert Human Fibroblasts to Multipotent Cells
Source: PLoS One. 2012 Dec 26;7(12):e51866. doi: 10.1371/journal.pone.0051866 (PMC3530539; doi:10.1371/journal.pone.0051866)
Supplement: Table S3 — List of cluster genes that shows the difference by factor of two or more between LAB-incorporated cell clusters-1 vs. HDF-1 and LAB-incorporated cell clusters-2 vs. HDF-2. (PDF) [file pone.0051866.s012.pdf]

Supplementary Table 3: List of cluster genes that shows the difference by a factor of two or more between LAB-incorporated cell clusters-1vs HDF-1& LAB-incorporated cell clusters-2 v sHDF-2

Genes included in Ⅰ	Genes included in Ⅱ-1	Genes included in Ⅱ-2	Genes included in Ⅲ
KCNB1	COL4A6	unknown	HOXC8
RPS4Y1	IL13	MMP25	HOXA9
RPS4Y2	TMEM108	unknown	HOXB5
TDRD6	PLIN5	SLC30A3	HAPLN1
ADH1B	UNQ2963	TGM7	HOXC10
LHX8	GRM1	SEC16B	HOXA11
DDX3Y	LOC100132652	SYT15	HOXA10
TAC1	PFKFB2	LOC401164	KRTAP1-1
CNR1	LOC100128402	BCL2	CLSPN
NCRNA00185	FBLL1	NEK10	ALOX5AP
ZFY	C7orf63	TMPRSS7	HOXB7
C6orf176	FLJ41309	OR10H4	HOXB8
SPINK4	FLJ44253	ZDHHC22	DEPDC1
KYNU	GOLGA8F	CDC6	MPPED2
ADH1A	FOXP2	CSAG1	unknown
USP9Y	unknown	OR2A1	WNT2
THSD7A	HAVCR2	RAP1GAP	CYP3A5
CYorf15B	SOBP	FSHR	unknown
ADAMTS8	MAOB	LOC100131165	HOXB3
CLMN	unknown	RNF125	LOC100133311
LONRF3	LOC100130798	RIMS3	HOXA7
BARX1	AIF1	unknown	NEIL3
MYLK4	MCOLN3	unknown	DOK7
HLF	CYB5RL	SLA	LYPD6
CPLX1	MOG	unknown	KANK4
C21orf121	SUV420H1	unknown	E2F8
MOBKL2B	unknown	LOC100129129	HOXA3
SLITRK2	RRAGD	SLC4A1	HOXA13
EIF1AY	LRCH1	unknown	NEK2
MKRN3	C6orf170	P2RX3	FCRLA
SP5	ANKRD5	LOC440028	TEK
MUM1L1	LOC100128386	CASP2	PAX6
SERPINB3	LOC645225	KRTAP19-7	ESCO2
IL18R1	ADAMTS17	LIN9	LOC100132588
unknown	FLJ33065	HAAO	SCRG1
KIAA1045	SLC14A1	unknown	UNC13C
DAZL	unknown	unknown	C8orf47
CCDC40	PYY	BPIL1	LOC100133311
MERTK	FNDC3A	unknown	LYPD1
UTY	RAB9B	OR4D10	unknown
FZD10	ZNF454	unknown	CLEC1A
ZNF204	unknown	unknown	unknown
EFHB	LOC100128336	unknown	SLN
DNASE1L3	P2RX6	INS	unknown
unknown	unknown	GNB3	NEDD9
INMT	MADCAM1	unknown	SPP1
WDR86	ZNF510	ANXA2	ESCO2
CXCR4	unknown	TNNT2	LIMCH1
IL18R1	LOC283587	LOC100129048	UCP2
NOVA1	LOC284561	DEFB124	CENPI
CAPN12	unknown	hCG_1817208	E2F7
SOBP	PROM2	HIST1H2AA	HOXA2
ABLIM2	MOBKL2B	FLJ46020	PTPRB
C6orf221	LOC650794	unknown	RARB
ABLIM2	unknown	PTPN20B	TBX1
GAS2	unknown	unknown	TBX5
CDON	LOC727869	FAM129C	SDPR
LY75	LOC100292961	LOC286186	RASGRP1
KCNJ4	UBD	LOC145845	LOC283392
PAGE2B	ZFP14	unknown	PADI1
C9orf68	STMN4	IGFL2	GRIA1
MCTP2	unknown	unknown	LYPD6B
LOC100129973	TMSB4Y	MGC16703	MYL10
EYA4	LOC284648	MAST4	C2orf48
LOC100131354	PURG	LOC285762	HIST1H2AI
LOC283867	LOC100129767	ERLIN2	LOC100131594
LRP1B	RANBP2	C5orf49	AFP
TTYH2	LOC648149	unknown	COL6A6
PPIL6	C1orf170	TRPC3	ANGPTL7
MUC20	KIF5C	FAM111B	unknown
REPS2	LOC389834	ABCA10	MYBL1
ASTN1	BHLHA9	HIST1H4A	L1TD1
LOC253962	unknown	ADAMTS14	LOC100129619
SOHLH2	DIO3	LOC100132672	CKM
SEC14L5	FLJ40852	HIST1H4D	WISP1
RASL12	LAMP3	unknown	unknown
THRB	LRFN2	LOC344967	PNLDC1
C17orf60	RSPH1	BTBD9	OPCML
unknown	GABRD	ANKRD26P1	AS3MT
IL18RAP	unknown	LOC100131099	HOXC4
NLGN4Y	INADL	GRIA2	ODZ2
unknown	CACNA1G	TTC23	XIST
LEFTY1	unknown	GP1BA	HOXA4
ARHGAP28	ODF3L1	MYO5C	SORBS2
MAPK10	NUP62CL	unknown	HOXC6
NLGN4Y	RAPGEF5	OR10G2	LOC100132588
KRTDAP	LOC152286	TMPRSS8	SPC24
LOC642924	unknown	PGA3	LYPD6
RGS7	FLJ11710	NCRNA00095	MKI67
SLC40A1	FLJ32810	LOC643475	SP140
ST8SIA1	FMO4	LOC100287245	HOXB6
unknown	BZRAP1	GEN1	MKI67
CABP1	LOC100128071	LOC644992	SLCO2A1
CCL8	FRMD4B	unknown	CDH16
LOC151438	HORMAD1	RBMX	SFXN2
NR3C2	ATP12A	BCMO1	H19
SCARA5	LOC729739	LOC100134365	EPR1
TM4SF18	KCNA3	unknown	NPPC
GSTT1	ZNF846	KCNN2	LOC100132878
ARG1	unknown	OR6C74	KRT17
USP9Y	TAS2R19	SULT1B1	unknown
MYCN	LOC644949	LOC643371	SCIN
VENTX	LOC388279	unknown	SGCG
SLITRK5	GLS2	OR5C1	PALM2

SLC7A10	SULT2B1	OR7E5P	MGC45800
MGAT3	unknown	ZNF443	NBLA00301
FIGF	P2RY1	unknown	TSIX
PNLIPRP3	LOC400891	BCKDHB	TSPAN2
JAM2	LOC492303	unknown	ERI2
FMO2	TP53TG5	LOC646993	SNTG2
BAI3	unknown	HERC3	unknown
CASC1	CD86	hCG_2040054	unknown
SDR42E1	OR7G2	tcag7.1058	HLA-DRA
ST6GAL1	LOC283710	PRHOXNB	SPINK5L3
RNF157	LOC284440	LCE2D	unknown
unknown	LOC100130764	LOC646853	unknown
CDH6	unknown	unknown	unknown
CDH29	unknown	unknown	DBF4B
MKX	unknown	unknown	PDIA2
CASC1	FLJ42351	DEFB104B	LOC401021
MYO3B	LOC649294	LY6D	C14orf50
SAMSN1	SPARCL1	EXOC3L2	LOC100132588
ZNF385B	unknown	COL8A2	MYOM3
ARAP2	USP6	RP1L1	FAM23A
LOC100131208	CDH23	ZC3H12D	FAM71F1
IRF6	GRM2	NYNRIN	ANGPTL5
GUCY1A3	RGS22	SNORA23	unknown
SNX22	TBC1D3B	unknown	ZCCHC5
ADRB1	LOC100130138	OR10C1	LYPD1
unknown	ADCY1	unknown	KIF4A
TNMD	BICC1	RIMS1	CCNA2
PRDM16	unknown	LOC100129119	ANLN
ACOXL	unknown	unknown	CDC2
DTX1	unknown	DCLK1	ANKRD1
SLCO4A1	STK31	UTRN	BUB1
KCNC1	LOC728723	C22orf42	MYO16
C1orf87	unknown	CXorf64	GSG2
unknown	SLC4A9	UPP2	EGR4
BAIAP3	TECTA	unknown	SULF1
OR2K2	PCMTD1	RTN4RL1	ASPM
LEPR	unknown	GIPC3	HMMR
NECAB2	KIR2DS4	unknown	CEP55
ADH1C	MPPED1	LOC100128328	GLT25D2
RSAD2	MASP2	DCLK2	NKX3-1
SYP	unknown	GNA15	CENPI
MYBPC2	LOC100131825	LOC96610	APOBEC3B
FGF14	C19orf39	unknown	CENPO
KLHL23	GOLGA8E	KRT33B	unknown
BMP4	HBS1L	NPHS1	PSG3
CYP39A1	UNQ6494	CLEC12B	FAM83D
STAT4	LOC100133180	KCNS1	CENPA
LOC100130168	unknown	APOA1	RIPK4
SYT17	unknown	unknown	EFNB2
GUCY1A3	USP27X	ENKUR	LOC400743
THAP2	unknown	SYNGR2	TACC3
SLC39A8	MSLN	PCDHGB2	COMP
C6orf164	ANKRD26	SNRNP48	CLCN6
AKD1	C14orf48	FAM151B	ELN
UGT2B7	ATRX	FOLR1	LOC728449
TTTY15	FOXB2	TRIM61	CCDC85A
NSUN7	ZNF492	unknown	GTSE1
LOC643542	KRTAP5-4	LOC100132188	CENPM
LEP	unknown	MYL4	DLGAP5
LOC728208	unknown	unknown	unknown
WDR31	LOC285972	ENGASE	KRTAP1-5
TF	unknown	HIST3H2A	ARHGAP11B
LOC100129055	FLJ39739	unknown	WFDC1
EML5	unknown	GPSM1	HHIPL1
SFTPB	unknown	SNAR-A3	DUSP26
CD22	SEMA4D	PER2	BAIAP2
MYO3B	CCL14	ADAMTSL1	CNGA3
FAM154B	FGL1	C16orf92	SHCBP1
PARK2	KMO	unknown	KRTAP1-5
MTUS1	CCDC48	LOC100133331	HIST2H3A
LOC339975	KCNJ13	LOC646890	SPC25
TSC22D2	SLC29A2	PRLHR	unknown
BTBD17	unknown	GLTPD1	HAS2
LOC92973	LOC399875	LOC644173	SLC4A4
unknown	SNAI3	unknown	RRM2
unknown	unknown	unknown	KIF4A
unknown	NCF1	MAP3K7IP3	LOC643406
CSGALNACT1	HEPACAM2	LOC100131646	DEPDC1
CYorf15A	MPZL2	SERPINA6	SHISA9
EDNRB	GGNBP1	LOC220077	MANSC1
ABLIM2	DLEU2L	MMRN2	CENPF
unknown	unknown	SMC1A	HIST1H4L
AGBL2	TMEM63C	F2RL3	KIF2C
MYO15A	unknown	LOC389033	CCNB2
KLHL23	NCRNA00164	ARMC6	HJURP
unknown	LOC100129840	C1orf152	FANCA
KREMEN2	TRAPPC6B	MRPL35	SLC8A1
TREM1	CXorf25	PHACTR2	OXCT2
ZNF853	LOC643802	PTPN1	KIF23
STRC	unknown	EPS8	SLC8A1
unknown	OR8U1	UBE2QP2	BIRC5
RRP7B	PZP	LOC642521	MFAP5
SERPINA5	unknown	NKX1-1	CDC20
TCF21	LOC100288755	CCDC132	PBK
NIPSNAP3B	unknown	RTN4R	HOXB2
unknown	MS4A7	unknown	KIF11
C8orf4	ZNF674	PRKAR2B	STMN2
FREM1	LYSMD3	HDAC9	UBE2C
CNTN4	ZNF479	unknown	SCUBE3
ACPP	unknown	DCAF12L2	LOC100128191
IQUB	unknown	C7orf58	NDUFA4L2
ANKRD5	unknown	PON3	RHBDL2
PIK3R6	unknown	GPR19	HFE
SLC3A1	unknown	NAV2	LOXL3
ERAP1	KSR1	MAML3	unknown
FAM84B	WFDC5	ABCA11P	CDCA3
LRRC10B	LOC100130701	SMURF2	ANXA8L2
LOC728052	LOC100130278	SOX11	E2F2

LOC100130232	PHACTR1	NDE1	unknown
ZBTB46	POU3F3	CECR7	FAM72A
SPTLC3	LOC100292758	C8orf60	RACGAP1P
RSPO1	unknown	CLTB	unknown
PRKAA2	CCL3L3	TPPP	TPX2
UNC13A	C21orf49	CLCN5	CENPM
TXLNB	IL15RA	PRLH	KIF15
GLIPR1L2	VCX2	ZNF259P	unknown
TMEM132B	unknown	SETD1B	ALDH4A1
BCAS1	SC5DL	LOC340508	CCDC134
ESPNL	DDX17	LOC732419	DHRS2
unknown	DPY19L3	SLC43A2	NRG1
PTPRD	NRTN	LOC441046	TROAP
CCDC144B	TCERG1L	tcag7.1213	CCNF
PNMA2	SLC35F4	unknown	POLQ
GJB2	DKFZp761E198	unknown	FGF1
HULC	ARL10	DLC1	C6orf191
LOC440173	SPIN3	CEP110	FOXM1
ARG1	APOL6	unknown	BUB1B
DIRC3	C4orf36	ICA1L	NUF2
unknown	EBF1	hCG_1645220	KIFC1
LOC729076	DBNDD1	ZNF286A	PLD5
WNK2	ZNF3	ARL4A	TRIP13
LOC646870	C4orf26	KIT	TROAP
ATP8B4	LOC400756	CCNYL1	FARP1
PHACTR3	FCGR2A	SDC2	unknown
FLJ31356	MATN4	NTF3	COBLL1
NOD2	RAB9B	unknown	GRB14
LOC100131089	FAM91A1	RBMX	DEPDC1B
LOC100127967	CPNE2	SYNE1	CCDC80
CXorf50	SYNGAP1	LOC100129794	F2RL1
MOBKL2B	LOC100128655	CENPK	DPEP1
TRIL	unknown	NALCN	MMP10
C9orf171	EEF1A2	LOC100130927	STIL
AQP7P1	RGL3	SPEF2	ESM1
PPFIA2	LOC100128077	ITGA2	unknown
LOC284276	unknown	SH2D1A	unknown
CCL20	CYP11B2	LOC100128059	LIMCH1
C7orf52	LOC100128392	LOC100130433	FAM169A
NFIB	SLC5A6	unknown	CNGA3
KCNC1	PARD6A	unknown	ARHGAP11A
CACNB2	PHACTR1	FAM84A	APOA1
SYN2	GEFT	unknown	SKA1
COL21A1	VMO1	C7orf29	NRG1
MEOX1	unknown	CLASP1	MXRA5
HSPA12B	TMOD1	AHSP	unknown
CD52	WDR27	SUPT3H	KANK4
ADHFE1	LOC643669	C21orf90	RAMP1
MTUS2	MORN1	unknown	DIAPH3
CYP19A1	FTCD	LOC728752	ABI3BP
UNC5CL	MIAT	unknown	LRFN5
WDR52	unknown	EPB49	CKAP2L
PCSK1	IQCH	unknown	CCDC116
ADH4	KIAA1530	TSPO2	GIPC2
SLC26A7	LAPTM5	LOC100128430	LBH
MACROD2	GPR77	EXOG	HMSD
LOC100130331	ZNF599	FAM196B	HOPX
CES4	BCAR4	LRP2	HEATR7A
UTY	EFNA3	WDR63	unknown
C10orf79	CCDC147	ZNF257	FAM196A
EMCN	BMP7	SEMA4F	TMEM106A
PRR5-ARHGAP8	C18orf34	hCG_2045830	hCG_2045048
PLIN4	unknown	ZNF442	unknown
FAM47E	LOC145678	EVI2B	LOC732272
PPL	C11orf66	FAM13AOS	KIRREL3
MAPT	C19orf38	unknown	MYEF2
SCN5A	SCAND3	DEPDC5	RPS6KL1
SERPINB4	unknown	FLJ35024	ZNF812
unknown	NOXA1	WNK4	COL15A1
FMO3	unknown	LOC728613	HIST1H3H
GIMAP2	CLSTN3	SMC4	ZNF730
GDF9	PRSS35	MYL2	LPPR4
C10orf58	NFIA	TPM3	C12orf48
CA11	unknown	CCM2	HILS1
unknown	MXI1	unknown	HTR2A
RALGPS1	LOC440356	OSTCL	SLC38A4
CXorf1	SCNN1D	unknown	FKTN
RPGRIP1	ARHGEF19	unknown	CEP55
LOC391322	CYTH4	CSF1	ODZ2
IRX4	TEKT5	unknown	SKA3
LOC729885	PTPN22	unknown	CASC5
APOD	LOC440983	LOC100132672	ITGA6
JAKMIP2	LOC401052	TMEM217	MPZL3
LOC401630	LOC158863	LOC401233	KIF20A
ELF3	DPY19L2P1	TNR	FNDC5
STRC	C9orf43	unknown	NEK2
DLL1	LOC100131582	C10orf82	MATN2
BEX5	NHLRC4	unknown	P2RY6
LOC100291851	CSDC2	ARHGAP30	FAM64A
PLK5P	LOC729040	tcag7.1227	CCDC88C
CHI3L2	LOC729467	RIT2	TTK
SHC3	unknown	SRMS	STRA6
GTF2A1L	HSF2BP	DAD1L	DIAPH3
IL18R1	GEFT	LOC100133660	HOXB4
ACCN3	ATPBD4	IL1F7	ERCC6L
CCL7	MYOM1	ALG1	CTAG1A
ZP4	FLJ41603	HNRNPCL1	HOXC9
C7orf46	IL3RA	unknown	GREB1L
CACNG6	CEBPA	CTSZ	CDC25C
ANKRD6	TSNAXIP1	SLC35A3	NGFR
MLXIPL	LOC100130093	ANXA2P3	HOXB2
CUL3	DDX3Y	NDST1	ITGA6
C6orf81	PDE3B	TMED9	HIST1H2BF
unknown	OR9A2	CABIN1	FAM101A
NFE2	LOC100128402	ELK4	FGF1
TMEM132B	C12orf68	unknown	LOC283392
NKD2	OR2T5	KIAA0564	KIF18A
IL1RL1	unknown	C10orf35	MMP12

CCDC114	unknown	ZNF876P	ASPM
APOC2	GRM8	ZNF683	SGOL1
C10orf11	UBQLNL	unknown	HOXA5
unknown	LOC151657	LOC554174	STRA6
NTN1	LOC284475	TRIM14	CCDC85A
AK7	LOC100129555	TBC1D28	PLK4
unknown	LOC654841	IL1RL2	ZNF695
EFHB	PPM1B	LILRB3	ZFP57
TSPAN7	HCN2	TNF	unknown
KIF6	C10orf68	NPY	unknown
unknown	ZNF518A	HIST3H2BB	SPATA22
MAPK10	NCRNA00174	FSD1L	ARHGAP11A
CCDC102B	C8orf45	LOC100132966	NCAPG
FAM78B	LOC100128402	PIGN	CASP10
TBC1D3B	MLLT6	MAP7	CTAG2
TMEM178	MIER3	PSG8	AURKB
FAM20A	STX1B	HHIPL2	HOXA11AS
MYL3	FBXW2	PHACTR4	PLK1
CHGB	LOC100131271	KLC4	ISPD
HGF	PARK2	VN1R5	unknown
MAML3	RASGRP2	unknown	PODN
CCDC151	GCH1	GLYAT	SORBS2
C15orf33	unknown	PAX3	PPP1R14C
LAMC3	LOC728650	GTF2A2	FCRLA
TRIM50	ZNF365	AEN	PNMT
TFAP2A	KIF9	FLJ44606	PLK1
LOC80054	SCN1B	GPI	GZMK
unknown	AGTPBP1	TUBA1C	GFI1
FBXW10	KDM5D	KLF6	FCRL4
RHOJ	FAM69B	SEC13	HAND2
LPAR3	ZNF585A	PTP4A1	TMEM90B
FAAH2	EFCAB1	ISG15	PCOTH
DNHD1	DHDH	DPH3	FAM183A
C17orf98	EGR3	P4HA1	NEDD9
KLRC1	TBXAS1	CFL2	LOC100133690
unknown	TNIK	BZW2	LOC100288900
OVOL1	DMRTA1	LOC644563	GDF6
HORMAD1	C19orf51	TBCE	unknown
JAKMIP2	unknown	TMEM126A	LOC100129617
SLC47A1	ANKLE1	MARCKS	unknown
unknown	EBF4	LHFP	PCSK9
RGL3	OSBP2	GLTPD1	unknown
CADM3	LOC100130057	MORF4L2	MYB
SFTPB	C21orf89	RAN	SGOL1
ANXA9	LOC100288667	EIF1AX	GPR126
SEMA6D	OR7G1	HMGB1L1	HOXC11
PODXL2	NCRNA00171	SP100	PTPN20B
LOC339539	B3GNT3	CORO1C	LOC730091
ABCC6P1	MSX2	EIF1AX	PRLR
unknown	unknown	SRRT	CCDC73
unknown	LOC253264	ARF4	LOC100240734
ASXL3	ST3GAL6	TALDO1	CACNA2D3
CCDC52	CHST1	RPN2	LOC285943
P2RX7	unknown	PPP1R14B	WDR62
LOC728800	TMEM150C	TBC1D22A	CALCR
NRARP	PXMP3	FEZ2	GRIK2
PURG	C3orf35	MYO9B	PHTF2
DRD2	MUCL1	ANKRD13A	IL7
unknown	DUSP13	MED10	unknown
IL20	PR47	MRPL12	LOC100131726
OXGR1	unknown	LOC388796	NCRNA00200
unknown	unknown	LPXN	DNAH17
CCDC144A	unknown	ELF4	LOC100130580
ZMAT1	LOC286071	TXNL4A	NAP1L3
LOC283713	EDN2	CLDN11	LOC389300
RGMA	CCDC68	TM7SF3	SHISA9
unknown	LOC643770	ABL2	TGFB2
RBMY1B	MSMB	MYOF	FANCD2
RET	ANKRD12	LOC728190	TPD52
TBPL2	IGDCC3	YWHAH	HIST1H2AL
PGM5P1	RIMS3	TMEM200A	PFTK2
LAMB4	GOLGA6A	ABCA1	LIPG
CCDC144A	C17orf69	MICB	HOXA6
IL16	LOC338817	MRPL12	HBE1
WFDC10B	C1orf129	CXCL2	HOXD9
CHRDL1	FLJ41455	RFTN1	FAM125B
ALPK3	EML6	ACOT9	CCNE2
SCGB1D1	SRGAP3	ATP2B4	P2RY2
FLJ37638	LOC100133039	HSPB1	KIF24
ALDH1A2	TNFSF4	FKBP1A	SPTB
SAMD13	FZD3	CLU	MYPN
ANKRD43	RSPH1	TMEM110	unknown
LOC100129950	MGAT5B	unknown	ADRA2A
C10orf67	unknown	WDR18	MCM10
RNF32	ZNF695	unknown	FLT1
KCND1	CSAG3	RRP1	NMU
unknown	C14orf34	CKAP5	TMPO
C20orf202	DTNB	SPPL2A	NAT8L
ZNF323	unknown	COL5A2	ERN2
FLJ25694	EFCAB5	TMEM47	SIM2
CALCRL	NARG2	FARP1	NCAPH
FAM106A	TMEM135	GLIS1	TSPAN15
unknown	unknown	BLCAP	NOSTRIN
CYP26B1	TMEM30B	PIN4	LOC255480
MMP13	PRRT4	GPX8	EIF2A
MSR1	CDADC1	BLOC1S3	RBL1
ELANE	unknown	SSR1	C15orf54
SYT12	TRIM66	C6orf89	EMB
TMTC2	SOX12	MAPRE1	LOC100292680
TMEM100	PPP4R4	RTCD1	NEK10
DIO2	GPRASP1	NF2	HOXB9
unknown	LOC154761	TSR2	CACNA2D3
VWA1	TP53TG3	NDE1	LOC339240
ROR2	CCDC113	HNRNPL	unknown
SIRPD	PIK3C2B	DOK1	PLXNA4
C1QTNF4	AMN1	SEPT11	RNU4ATAC
PLCXD3	LOC439949	SFRS3	LOC731656
NR4A2	FLJ46875	SRA1	SLC28A3

BCAS1	ST6GALNAC3	ANKRD39	HOXC5
SUGT1L1	KIF27	NACC1	unknown
ST8SIA4	KIAA1671	SUMF2	MBL1P1
CCDC102B	KLHDC1	FOXQ1	ZNF367
PPIL6	NFIB	KIAA1609	PARM1
LOC729088	CA9	AHR	LMNB1
BCL6B	C1orf204	GLIPR1	DOCK2
NUDT10	NPL	SAE1	unknown
CTSS	EMR2	BAT1	ASPN
OCA2	unknown	ZFP64	unknown
KIAA1712	LMOD1	MCM8	HIST2H2BE
FAM66C	IFNAP22	ASAP2	unknown
C1orf125	DDX26B	UBE2V1	ARHGEF4
PLEKHG1	SAMD13	UFM1	SMPD3
FAM66C	OTUD7A	UCHL3	SCN9A
TNFRSF17	LOC729417	PAIP1	unknown
JAG2	STK38L	PAK1	CEACAM7
NOS2	C2orf34	YIPF2	LOC390557
SCGB1D2	C10orf4	GMPS	unknown
TTC39A	LOC653602	SPON2	RP5-1022P6.6
CAMP	NRG2	TMEM48	LOC100130967
LOC200772	C12orf53	TMEM117	HGC6.3
FLJ42289	GOLGA8A	RPL23A	unknown
C7orf45	PTPN3	C19orf42	NT5C1B
ATP8A1	PTGFR	CIT	TRHDE
SH2D3C	RAD21L1	PIF1	unknown
SLC6A7	LPHN2	C4orf46	LOC401097
unknown	GHR	AK5	CMA1
KBTBD3	SENP6	SMS	SOX18
SCARNA10	LOC441666	GNB4	unknown
PCDHA3	C1orf162	H2AFV	HTR1F
PTPRH	HCP5P10	RPL22L1	SPINT1
TMEM176B	LPPR3	HNRNPD	GPR85
LOC100131180	C19orf54	FAM167A	ISPD
TMEM155	DNHD1	TRAM2	unknown
DCDC2	unknown	TUBB6	PTPRN2
LRFN1	unknown	PPP1R14B	LOC100128960
GLB1L2	MCOLN2	HSP90B1	ARHGAP19
PECAM1	ITGB4	PGAM1	AMBP
EXD3	C6orf138	TPM3	MOGAT2
HAUS3	DENND2A	PCTK1	CCDC108
MITF	PNMA6A	ATP6V0C	CYP2F1
LOC55908	RAB20	BCL2L12	IGFL1
LOC100132653	ARHGEF16	CLIC1	SULT1C2
CD7	TNS4	EFEMP2	C9orf117
SLC19A3	TRIM7	TPM2	D21S2091E
RASGRF2	PEX11G	RPSAP52	SERPINA4
GDF10	ODF3B	MYL6	DEFB108B
CRABP1	BCL9	PPIF	LOC642891
TMEM86A	SLC44A3	POP7	ST8SIA6
EGLN3	MMP23B	FHL2	LOC402160
PPP4R4	C12orf66	CALR	ZG16
EFCAB6	POM121L1P	P4HB	unknown
RFPL1S	IFT140	ANXA2	unknown
SLC25A18	NXPH3	CYR61	SPTBN5
LOC253039	LRRC56	CFL1	BMPR1B
XGPY2	CYFIP2	B4GALT2	CAPN6
NTSR1	TNNT1	LDHA	ZNF253
NCRNA00110	CD9	RANGAP1	CHMP4C
TDRD1	FAM90A10	SNRPF	NKX3-1
DNAI1	CXCL16	SNRPA	BLNK
unknown	ESPNL	CFL1	CELSR1
unknown	C15orf51	BZW2	RTKN2
TYRP1	MGC42105	MAP4	LOC642622
SLC8A2	SPIRE2	KLC1	LOC144742
unknown	WDR78	SERPINH1	CLEC7A
KIAA1377	CSF3	PTGIR	unknown
ABLIM1	SLC26A10	LRFN4	PDE1C
CR2	TMEM190	PTRF	STON2
C6orf217	IL24	RNF126	INSL4
AGAP2	GCHFR	SNRPA1	SLC22A2
ZNF540	NR0B1	DCBLD2	KCNK18
ARHGEF7	TRAPPC6A	BOLA3	IL12B
LOC100134240	CYP19A1	GTF3C6	LOC84740
STX19	NAMPT	DUS3L	LOC197350
TDO2	FHIT	FAM110A	C1QL3
MSTP9	C20orf96	DIXDC1	KRT2
unknown	LOC142937	POLE3	HPCAL4
unknown	PROC	CXCL12	RXFP2
CCNJL	LAMC3	IMPDH1	LOC283501
SLC25A41	IFT74	ATAD3B	MYEOV2
unknown	FAM124A	NANS	CLIC5
HYDIN	SLC16A7	MFSD1	LOC642587
HS3ST6	unknown	LIMS2	SLC27A2
TXK	C22orf41	THOC3	DEFB115
ZNF160	FAM171B	TOMM22	LUZP2
LOC339524	C1orf115	EHD2	LY6G6F
ADAMDEC1	LOC729130	SFRS2	LOC285629
HLA-DQB1	TCP11L2	PDIA3	NCRNA00159
TSSK4	ADCK4	TXNL4A	unknown
LOC100131053	CITED1	EIF4G1	C1orf106
CCDC17	TTC29	PSMD2	LOC441601
ZNF711	SYNGR1	MCFD2	LOC730242
LEKR1	CES1	SEC61G	KRT6C
LOC285696	DLK2	PSMD12	DGKB
PCDH10	ZMYND10	KDELR1	PRB4
DAZ2	ZNF521	HNRNPC	LOC387723
GSDMC	GBP5	C19orf10	unknown
LOC285965	KANK3	HNRNPC	LOC340074
CDC14A	ADSSL1	PPIC	LOC729426
MARK1	PLTP	PDIA4	unknown
unknown	ARSG	ODC1	unknown
LOC440117	KCNK4	TPST1	LAD1
C21orf81	LOC100294391	DYNLL2	unknown
SYT1	FXYD3	DDAH1	LRRTM4
ZNF541	CIRBP	HSPG2	C7orf69
unknown	HSD17B6	PDGFC	NPPA
C12orf33	LYG1	LOC729580	ST8SIA6

FBXO24
ZIC2
ARHGAP28
TMEM139
MMP27
SLC2A4
SLC2A12
LOC728558
TMEM90A
COX8C
XK
HIGD1C
SCN3B
UGT2B11
C6orf176
FOXP2
OAS1
LOC441493
SLC22A3
NLGN3
LOC100128003
C11orf9
TEPP
ZNF248
ZCCHC18
TEX9
KCNT2
LOC285768
GLT1D1
C5orf36
OCLN
LOC100132625
IRF4
SLC16A12
PPEF1
LOC100192378
TRPM6
unknown
XCL1
KRT37
PRDM13
FILIP1
SFRS13B
unknown
CALCRL
JPH4
RHOXF1
TEKT3
FCGR2A
PAGE2
FLJ36000
SCNN1A
LOC284276
CHGA
unknown
LOC645485
DMGDH
UBL4B
HERC2P4
C15orf62
unknown
unknown
SLC8A3
TTC29
WNT2B
PMCH
BTBD8
PLGLB1
PXT1
ATP2A3
JHDM1D
PDE4D
IL16
FFAR3
FMNL1
NTNG1
FCRL6
NTRK3
ZNF436
KCTD16
unknown
unknown
DDR1
TTC39A
ADAM11
LOC646034
unknown
C6orf170
BTBD19
UNQ2963
C7orf31
ANKRD20A2
LOC100130248
PROC
PCDH9
WDR52
PWWP2A
FAM154A
LMOD1
ZNF192
unknown
A1CF
KCNA10
unknown
C10orf105
PKD2L1
SDK1
CCL16

PRRT2
C10orf25
LOC100130387
TMEM25
JAK3
C13orf38
TNXB
SNED1
LOC650392
unknown
SHISA2
PRDM12
LSMD1
LOC148987
FMNL1
ANKRD7
WDR66
TEKT2
KLHL13
INTS6
SIRT4
HDDC2
ANKRD29
AGTR1
POM121L1P
MDM4
SULT4A1
LOC100134229
BDH2
TBC1D3G
POM121L9P
EDNRA
TIMP4
IL24
PTGR2
VAV3
CPT1B
ADAM8
GPM6B
KCNMB4
HMHA1
ITPR1
CARD8
STC1
TNXB
C6orf97
NAMPT
NDRG2
LOC387763
CSN1S1
LOC100131102
COL4A5
KIAA1199
LOC389791
unknown
PPFIBP2
unknown
FIGNL2
EDNRA
MAOA
SCAMP5
SLC25A27
COLEC12
ETNK2
unknown
C3
CPE
IL11
RAP1GAP2
LOC149351
SLC29A4
BIRC7
LDHD
DFNB59
LOC284900
KIAA1529
CELSR3
MST1
SERPINA3
GCOM1
AMIGO1
LOC338758
NINL
CFB
GPR56
PTPRU
LOC285084
TNFRSF11B
IL17D
LOC100133142
THRB
TTC25
SLC25A23
P2RX7
MITF
STON1-GTF2A1L
RNF112
SEMA4G
TMTC1
C11orf35
ASPRV1
unknown
ROR2
LDB2
MIR155HG
UCN
SLC39A8
KIAA1407

MRPL37
PICK1
P4HB
ATAD3B
FSTL1
RRBP1
CTPS
COL1A2
HP1BP3
PMM2
SIRPA
NCLN
TPM2
MFSD10
PEA15
SNRPB
TGFB1
BOK
SCARA3
SRM
FSTL3
FAM57A
FHL2
PMF1
C1orf152
ATAD3B
LOC100131482
EHD1
PHB
AMOTL2
CLIC5
FN1
TOMM34
C10orf116
SERF2
NRM
TMEM57
CYB561D2
SRPX2
MRPL54
EIF4E2
TSTA3
TFDP1
PHPT1
TSSC1
SNX8
NUTF2
VKORC1
MAPRE1
TGOLN2
ARPC5L
VPS24
COPG
PMS2L1
PTRH1
CDR2L
ANXA2P1
C11orf17
NOP16
MCM2
BOP1
GLTPD1
ARPC1A
ARHGAP22
unknown
POLD2
IFRD2
ZDHHC12
PPDPF
LRFN4
OLFML3
CD97
RPN2
PLOD3
PTTG1IP
TXNDC5
KCTD5
TPM3
RNF126
PA2G4
LRRC32
C14orf80
ARL4C
CSE1L
DDT
EIF4E2
CDC23
C20orf29
TOMM40L
ANKRD16
PRKCD
HRAS
C14orf153
BRD9
ARD1A
HNRNPA2B1
CSE1L
ANGPTL4
IDH2
RNF26
HES4
GPSM2
HSP90B3P
unknown
PITRM1
MCM3
CRISPLD2
DPH3B

CD177
LOC91149
ATCAY
LZTS1
unknown
UPK1B
GH2
C2orf27B
PCDHB15
ZSCAN23
SHROOM2
AMAC1
IL25
LOC402198
GPR37
MYOCD
PCDHA9
KIAA1524
unknown
CDH17
CDX4
TCEB3B

CYTL1
ZNF750
AMHR2
IL5
unknown
unknown
LOC100133862
CSPG5
LOC100131436
LOC100133599
TRPM3
GRPR
PPP1R1B
SOBP
unknown

unknown
SNORD116-19
ATHL1
unknown
CACNG7
PTGES
C9orf98
CDCP1
AREG
LASS4
BCHE
TBC1D10C
LOC339192
C7orf46
C13orf16
ALX4
SLC6A15
N4BP2L1
PRSS27
HSPA6
GPRC5B
RTDR1
DMKN
PHC1
CSTA
E2F5
LEAP2
unknown
TC2N
C19orf18
IRX6
TTC12
ZNF815
PLEKHH2
SPRY1
ADAMTS3
PHC1
LOC100132701
LOC100130015
RGS5
LAMB2L
CCDC146
NRN1L
APLN
SLC24A3
PTGS2
CASC2
TMEM170B
GCOM1
IL1B
LAMA1
C1RL
LCA5L
SPATA17
SHF
NTRK3
DNALI1
EFHD1
GUCA1B
C11orf94
ABLIM1
EPB41L1
ANKH
APOC1
HPCA
NTNG1
ZNF397OS
CLLU1OS
CROCC
C3
unknown
unknown
unknown
LRRC36
LOC441687
C17orf76
TAS1R3
TYSND1
LOC100131000
unknown
unknown
ARHGAP4
PLA2G4A
PRG2
H6PD
C16orf73
unknown
LOC84856
unknown
RASSF4
ASPHD2
LGI4
SYNGR1
WDR69
SLC7A2
SNORA12
GBP1
CDKN1C
GPR4
PHOSPHO2
DMKN
ARMC2
ARHGAP26
LOC728650
unknown
RBM44
C13orf18
PAPLN

TOR1B
TMEM18
LGMN
ALS2CR4
LZIC
PIGS
TNPO3
PDSS1
TSN
C21orf70
NPTN
unknown
XKR8
NCAPH2
H2AFZ
RNF216L
NP
ZNF536
GLT25D1
ZNF414
SIL1
ZDHHC12
ENG
COPZ1
CEP250
C17orf82
GPER
ISLR
ABHD12
YIF1B
KLHDC4
CNN3
CALM3
METTL1
TNFRSF12A
CHMP4A
ANPEP
TYRO3
CCRL1
HYI
HYLS1
SMAGP
RAB35
HCLS1
GPX8
DDX39
PLOD1
CDYL2
ALDH4A1
DPT
PTPN18
C19orf61
INF2
NFKBIL2
GALNS
LIPT2
SDSL
EWSR1
RABEP2
PTPLB
CENPJ
MTFR1
HSPA2
MYCBP
GABBR2
C21orf70
CHAF1B
SEC13
GMNN
CLN6
ACOT9
TMEM173
ZC3HC1
CTHRC1
FJX1
SAR1A
SMG6
FUBP1
PCBP2
CLDN7
F2R
PROCR
FEM1A
NAV3
FN3KRP
COL4A1
KCNG1
TIMM22
ATF6
PHKB
RPL23AP7
CXorf38
GATAD2A
TIMM50
LDHA
DUS2L
ACTN4
NPDC1
MED8
SAC3D1
RAN
NCAPH2
unknown
SKI
SH3RF1
SLMO1
AVEN
HIST2H2AB

FAM162B	POLA1
CDCP1	ZNF707
WDR33	YIPF5
GOLGA2L1	RAB15
CCDC147	IFI35
OBFC1	ATP10A
CALML6	GYS1
SALL2	PRSS23
MEX3A	FAM129A
SECTM1	UBE2F
CNTLN	BUB3
PTPN3	STK24
RNF144B	METTL2A
COL4A4	ILF3
LOC283711	C19orf52
SEC31B	PLA2G15
CUGBP2	MPDU1
BCL2L11	HMOX1
SYT7	CDC25B
ACACB	CLDN4
C20orf195	DPYSL3
EFNB3	TUBA1B
CH25H	CRELD1
SLC2A5	SNX11
TMTC1	IPPK
AREG	RSC1A1
MORN3	FAM107B
UOX	STK17B
DISC1	HMGN2
PDK4	SPSB1
BRSK2	TIPARP
SAMD12	ENDOD1
ZSWIM5	MXD3
LOC339742	IVD
COL14A1	C20orf196
unknown	unknown
LOC644990	APBB2
ANK1	FAHD1
APBA2	MAP4
PSORS1C3	PITRM1
unknown	NIPAL3
HESX1	PTS
NEO1	FBLN5
TMEM151B	TFPT
C22orf31	AKAP2
KCNE3	EIF2C2
HS6ST3	RGS3
GAS7	DOCK10
C6orf97	HIST1H4C
TRANK1	RCN3
CYorf15B	TMED7-TICAM2
LOC284998	MFSD1
CCNB3	PDSS1
MGC57346	PIAS2
DBC1	APITD1
NCKAP1L	EFS
COLQ	SUV39H2
BCL11B	SPCS3
ZNF566	HPS5
CHST1	PPAPDC3
LOC285628	ASNS
TBC1D3B	ESYT1
LOC100134040	WWOX
ETNK2	unknown
SERTAD4	KCTD5
LOC284080	HLA-DPA1
MYCBPAP	ATAD3C
GUCA1B	COL16A1
TCP10	PALLD
NANOG	WDR41
ADAMTS9	SMARCB1
IGFBP5	GARS
CCDC69	SLC35E3
JPH1	WEE1
SLC25A27	NAV1
CTTNBP2	CDGAP
STEAP2	KRTAP19-5
unknown	UBL3
OSBP2	TNFAIP3
FGF11	C9orf40
LRRK2	FUBP1
EDA	OXCT1
GUCY1A3	AEN
hCG_38984	LOC100130065
C4orf47	SNHG7
MITF	LCLAT1
hCG_1994695	GTF2H3
CSF3	CCDC85C
TAF4B	PTP4A1
EFCAB3	LOC728975
ATP8B3	XG
ROBO4	KITLG
CCDC64	ITGB1BP1
unknown	unknown
unknown	MAP4
ATHL1	PEG10
unknown	EPHB2
unknown	SLC13A4
RHPN2	LOC100132338
TDRD12	GDNF
GGT8P	SLFN12
CHRNB2	SMARCE1
CX3CR1	C14orf106
LOC440104	FAM36A
LECT2	C6orf132
ENTPD3	C9orf3
LOC100131089	CENPH
PRSSL1	ADAMTS5

IGF2	PHACTR2
KRTAP21-2	SLC7A1
TNNI3	DACT1
GABBR1	SLC17A9
LOC100134361	STX2
LOC100130015	ATP6V1H
LOC145783	unknown
IFT57	MET
CPXM1	PAFAH1B1
SHC2	HSP90B1
LOC728650	SLC25A38
C1orf203	ARL6IP1
NPL	BAG3
NHEDC1	NCAPD2
LDB2	DOLPP1
DTNA	MAP1B
EGFL6	EZH2
CAMK2B	UGDH
ANK1	C1orf212
CACNA1G	TRIM22
PECAM1	CALML3
DMKN	LOC100133638
RNF175	CAV2
C17orf44	CAPRIN1
GNMT	SYNE2
TMEM176A	LOC375295
ZMYND12	MECOM
TCN2	TBC1D19
GARNL3	ZAK
TPPP3	ADPRH
FAM49A	TARDBP
CYP19A1	LMNB2
FLYWCH1	LOC100129324
SNCA	unknown
JAM2	DCK
GALNTL1	unknown
MYH7B	CXCL12
LIF	TMEM59L
CD72	PRMT7
IGFBP2	TMEM87A
unknown	LOC100131490
RGS16	ARHGAP23
FAM20A	LOC442308
RALGPS1	PER3
COL9A3	TM7SF3
GRK4	C5orf42
CES1	ZNF714
INHBB	RANBP3
EFCAB1	CKAP4
GDF1	unknown
CECR4	ERAP1
CLDN23	NIN
LAMA5	NCAPD3
RASGRP2	BAIAP2L1
SLC45A1	unknown
SH3TC1	LRRC57
FAM84A	VANGL1
DNAH7	HNRNPH3
C1orf129	MGP
SLC6A15	MRTO4
CCR7	unknown
unknown	HMGN2
ATPGD1	ROCK2
AMY1C	SEC61A2
CACNA2D4	MAB21L1
RAPGEFL1	PLBD1
GCOM1	ALS2
KLF8	LEF1
MGC12982	CUBN
AMY1C	unknown
STAT4	unknown
C19orf57	MCM7
PTPRU	C17orf72
C17orf69	LOC100128163
SPON1	LOC648987
DDX26B	SLC12A8
NTRK3	unknown
GCH1	unknown
LOC339524	CERCAM
FAM171B	POLE
AKD1	APOBEC3F
TTC18	LGALS9C
PECAM1	FAM125B
CCL3	HIST1H2AD
LOC100127998	DNAH14
NFIB	MCART1
HGF	LOC400987
GBP2	SMC2
PDGFRL	USP10
unknown	USP13
LRRN4CL	TEF
OLFML2A	LYSMD2
ZFP2	ZNF516
DIO2	C7orf49
ALB	MRPL48
ZNF397	LOC284441
VWA5A	C13orf37
unknown	NAP1L5
NR4A2	BCAR3
unknown	C1D
PEBP4	unknown
MGC5566	LIN9
RGS9BP	TDP1
LOC388588	TEX261
RASGRP2	DSN1
EFHC1	FAM176A
C1orf228	THOC4
CCND2	LUM

SLC6A15	PAFAH1B2
unknown	THEM4
ATP2A3	ZNF544
C2orf84	MAPK6
FAM43B	BCL7A
LVRN	TCP11L1
CLIC2	PVRL2
unknown	CCDC99
RNF157	PMEPA1
RSPO4	CEP152
LCP1	C1orf55
ADRBK2	TNRC6C
DTX4	SRRT
SEPT4	unknown
C20orf132	ZBTB3
OVOS2	CCDC99
PPARGC1A	TMEM167A
GAL3ST1	SRR
NCRNA00171	ANTXR2
TSHZ2	WHSC1
PLD1	COPS8
GPR84	RUNX1
FLJ42289	SEPT6
EFHD1	PXDN
OGDHL	PLOD2
EMID1	LOC100132805
PNMA6A	KIF5B
FAM90A1	ORC5L
RGS16	SUSD2
NTN5	SURF4
NFIB	unknown
C10orf58	MTHFD1
C2	MCAM
AUTS2	ATF3
FBXO2	C1orf59
C8ORFK29	MAVS
CLMN	COPZ2
ERV3	SEPT2
PTH1R	HMGB2
ELOVL3	PRAGMIN
ABCA7	FANCG
CDKN2B	POP1
ZP3	CDC42
EPS8L1	FOXD1
FAM184A	C19orf42
BCL2A1	SEC14L1
RGS9	SH3BP4
GRTP1	C1orf144
TNFRSF18	unknown
FAM90A7	TRAK1
DYNLRB2	unknown
RBM44	PPP2R1B
JAM2	CHAF1A
NDP	TGFBI
AGT	UHRF1BP1L
NANOS1	GSG1
EREG	SEMA3C
CSGALNACT1	EXOSC2
ZBTB46	USP18
PDE9A	SAMD9L
OLAH	ZNF185
MCTP1	KIAA0586
NOVA1	GANC
C14orf81	VRK1
unknown	GDPD5
LEMD1	CRLF3
AP3B2	SIAE
SLITRK4	LOC150759
C17orf69	NT5DC3
CLEC14A	HAUS2
RAB37	SLC35E3
FLJ39582	LY86
B4GALNT4	ANKRD13A
CHI3L1	OSBPL3
SHC3	OPA3
TLE6	ADI1
RND2	TTF2
unknown	APCDD1L
PLEKHA6	GRAMD3
CLDN5	DONSON
CDH12	C10orf32
unknown	NNT
LOC100271715	DR1
hCG_2038428	LRRC59
unknown	PXDN
unknown	unknown
unknown	C9orf5
unknown	NR5A2
NKPD1	unknown
LOC100131869	SLC16A2
TFAP4	LOC727847
PTPN22	EGR2
KCNAB3	C12orf49
VNN1	NET1
MOSPD2	C3orf33
TACSTD2	C1orf96
GPM6B	IPO5
PRKY	SNHG3
NLRP3	LIMK2
KIAA0368	P4HA2
CLIC6	CALU
GPR155	DNAJC9
C3orf63	KDELR2
CLDN22	DTYMK
C10orf67	RECK
NCRNA00087	NRAS
ZDHHC14	MXRA7
ANXA11	CPPED1

AMT
NUDT7
IGSF22
ZNF177
LEPR
SORD
TET2
SLC2A9
RGS5
SPATA6
PDE4D
CBR4
NHS
PHYHD1
CBLN3
ZNF248
KIAA1147
CCK
SEPP1
FLJ33630
unknown
SLC4A5
ZNF214
C1orf25
PTGFRN
unknown
AHI1
PAN2
C3orf67
LOC286367
CLEC4A
MANEAL
FAM134B
ADHFE1
DEFB131
LAMA1
STEAP2
VAPA
C10orf10
PYGO1
FAM188B
BRWD3
APOL3
KIAA1107
THNSL1
unknown
COX15
LOC349114
CEP68
MAPKSP1
PCSK4
GK
ABCC6
C1orf101
FER1L4
C22orf41
LOC100132077
AQP7P3
TMEM38A
RPGRIP1L
GPRC5C
OSCAR
LOC286254
RAG1
WDR74
MMP9
unknown
ARMCX4
PLA2R1
TPTE
CYYR1
ATM
NETO1
FLJ25006
unknown
TRIM66
unknown
LOC157562
FAM65B
MGC21881
ARHGAP20
NUDCD1
CALB2
unknown
LOC100129196
CCDC7
LOC389765
GUCY1B3
DOCK4
LOC100130840
SF1
AMOT
LOC389834
LOC100131564
SV2A
unknown
RPL32P3
FAIM2
FILIP1
CCDC122
unknown
IL17RD
SYN2
ZNF599
HES7
LOC100132891
ZNF638
GLI1

RFC2
CDK6
THY1
FBLN7
C9orf86
TMEM171
LPGAT1
MSTO1
GPER
POFUT2
PPP1R15A
ARD1B
SEPW1
ASB1
SNX8
IFI30
SC65
unknown
MRPS11
PGAM1
C1orf152
MRPL37
PFN1
LYRM2
HSBP1L1
CIDECP
NR1D1
CLCC1
PDXK
C22orf25
GAS2L1
SARS2
TUBB2A
HYAL3
GLTPD1
HNRNPA2B1
TRIOBP
NCRNA00181
PLAUR
CLPB
YWHAZ
DDA1
PTTG1IP
PRKCDBP
PDIA6
ARD1A
RHOC
PSMC3
MARCKSL1
LOC729991
RABEP2
BAK1
DPYSL4
ADI1
PDRG1
ARHGDIA
EHD4
AKAP7
EIF4EBP1
SFRS2
WRAP53
TMEM191A
BGN
SLAMF9
BCAS4
FICD
CHTF18
METTL1
NME6
TUBB
PACSIN2
PEA15
FKRP
hCG_2014417
ATL1
GSTT2
PLEKHB2
C9orf114
FAM107B
MFSD7
C7orf50
GPN2
ASL
LOC128322
NPTN
CALM3
GLIPR2
SMTN
EMP3
HM13
CSTF3
IGFBP6
LOC100144603
UFSP1
DNAJC30
MICA
PRPS1
PPIL5
GSTT2B
PLAC9
CSGALNACT2
SLC6A3
HSF1
TMEM164
HN1L
CDK10
unknown
PTPRK

unknown
ZNF577
SLC6A16
C2orf63
ZC3H6
FAM135A
TRAF1
MCTS1
unknown
CREB1
CDNF
IGDCC4
LOC100287820
GK
KIAA1147
C5orf41
DHFRL1
LOC220930
GPX3
unknown
unknown
CNKSR1
KIAA1009
LOC100128905
IL34
C9orf123
NKX6-3
SCNN1D
PCDHB14
CKMT1A
PNPLA7
LOC283387
STARD6
unknown
PHF21A
PLXDC2
BCO2
CACNA1H
CYFIP2
BDNFOS
KATNAL2
unknown
CD70
HSPA6
unknown
TCEANC
IL1R2
LOC100133598
ITGB8
ZNF154
LOC90834
hCG_1986447
LOC646719
C5orf50
unknown
LOC100289922
OAS2
CDC2L5
unknown
unknown
PCDHB9
ANKDD1A
PSPC1
S100A14
LOC145814
F11R
ACTR3C
SEC1
IGSF3
MXI1
C14orf167
LOC349196
AHI1
PLEKHH2
FAM63A
PROS1
ANKRD10
KCNRG
PLEKHG4
ZNF439
unknown
TM7SF2
NUDT9P1
RNF144A
MBTD1
MIA3
RGS17
C3orf34
SATB1
TRPM3
unknown
C1orf113
SETD6
TNFAIP8
LOC100133331
LRRC70
unknown
C5orf53
MTMR3
PHF10
unknown
KPNA5
ZNF547
FAM110B
NLRP3
PARP14
KIAA0485
KRTCAP3

NXNL2
unknown
unknown
PGAM5
IQCE
SUV39H1
LYL1
SH3BP4
KHSRP
BLOC1S3
NRL
ABHD11
BGLAP
RCC1
RGMB
FAM176A
NRIP3
PRH2
ACOT7
RRAD
GPSM1
unknown
C5orf33
USP12
ERLIN2
BATF
LACE1
UBXN8
SRRT
ITGA5
AMACR
SMUG1
TGM2
ZNF575
MPZL1
GPC1
KIF13A
SYS1
MRPL45
KIAA0090
BNIP1
TMEM191A
ATRIP
FARP2
SUSD5
MCM7
TUBA1C
NRAS
IVL
TUBA1A
UFSP1
PCYT2
UBE2NL
F10
TRPV2
PCYT2
PRMT2
AFMID
PSMD5
PIK3IP1
CKS1B
SLC25A4
DHDDS
NCAPD2
ADSL
HIST1H1C
BTBD7
RNASEH2A
TRNP1
MYL9
TTC7A
TOE1
SFRS3
PRIM1
TIMM8A
TCTA
PRRC1
unknown
ERLIN1
HIST1H4K
LMCD1
CERCAM
MIOS
P4HA3
LIMS3
PARVB
NAGK
SLC1A5
CAV2
HIST1H4J
WDR34
HYI
SH3BP5L
CNN1
unknown
HEYL
AHR
TXNRD1
C10orf137
CREB3L2
ABLIM3
ABCA1
SAMD4A
POLR3H
FAM158A
LETM1
CDR2L
HIST1H2BL

unknown
PCBP3
C16orf55
LOC389634
IL1RN
FBXL14
RCSD1
SCAPER
SUSD3
PIBF1
ADAMTSL4
NBR2
TCP10L
LOC642340
CISH
RICS
BBS1
LOC389834
PANX2
SH3BGRL2
FGF7
LOC730202
SH3GL3
BBS9
COL11A2
ACVR1C
LYST
GOLGA8A
STAT3
NOD1
BPTF
ATG4C
FAM8A1
ZNF181
C14orf49
TCF7L1
LOC100131727
FBXL17
PREPL
ASB9
LOC284900
ARHGAP29
ETV1
GOLGA6L6
GK5
LOC441461
SENP6
TRGV7
LOC100128398
POU2F1
FAM60A
ANKRD12
RNF214
CDC14B
RNF215
ACPP
FLJ39639
TRIM38
POLB
OR7E13P
PAQR3
MMAA
VPREB3
HCN3
SALL4
DENR
SNX32
FLJ35390
CD19
ZCWPW2
LOC100130175
ZNF547
CHD7
TMEM133
PF4V1
ERP44
TRIB2
CG012
DDX58
RNF208
LOC100128567
BCL11A
C18orf18
FBXO44
unknown
HDAC4
HRASLS
LOC344065
LIPC
CNOT4
LOC729603
LOC202781
DPY19L4
ABCC6
IPW
MAGI2
CG030
TTC33
CTNND2
C17orf67
CLEC2D
FGR
LOC100131355
unknown
ZNF630
TMEM71
ICOSLG
FLJ35220

TRMT5
PLCB2
IPO11
VSIG10
HIST1H1E
SCHIP1
HIST1H4E
APOBEC3F
HSD17B1
MTMR10
ABL2
CBX5
HIC1
GPER
HYAL3
GATS
USP36
CRLF1
SMPD1
TUBB
CHSY3
KIAA1609
DOT1L
EZR
CEP72
CLIC3
HAUS8
WWTR1
SORT1
GIT2
unknown
SFRS7
PSMD9
TIMP3
ERI2
MAP6D1
GSPT1
TUBB
HSPB2
MAGOHB
unknown
RGS12
MAP1A
MPRIP
DNASE1L1
INF2
SMO
TRPV4
unknown
RIN3
DPP9
ATP13A2
APOBEC3C
DOCK2
ARHGEF4
FARP1
unknown
FLJ31715
EXOSC2
unknown
PGBD3
TCEB3
KIF1B
VEGFC
WDR77
NFASC
OR7E104P
SFT2D2
LTBP2
POLR2D
NRF1
PODNL1
PRH2
ATP1B1
B3GALNT1
unknown
PHACTR2
EIF5A
C13orf15
HS3ST2
HMGN2
HDHD1A
RRM1
HERC4
EN1
RCL1
NAT13
FUT11
LOC729799
BPGM
LOC645676
SYNC
MAX
SPRN
API5
NUP50
unknown
ATM
HIST1H1D
SH2D4A
PTPLA
MINPP1
HSPG2
CLEC3B
CDH2
NRL
DGKI
TUBB2A

ECSCR	MYO19
APOC4	TPM1
GALNT9	RANBP1
CNOT4	HIST1H4L
PNKD	C15orf23
COX11	KIF3C
unknown	LOC401127
PIKFYVE	GMPPB
unknown	KANK1
HNF1A	TMBIM6
SH2D6	SLC35A3
DPYD	EYA3
SLC26A5	C9orf30
C1orf95	ARPC4
VWA5A	RAGE
ZCCHC18	ACTA2
BMP7	FLCN
unknown	TUBB2C
POM121L8P	CCNO
VPS13D	ATRIP
MTHFD2L	PCDHGB4
QKI	PMEPA1
UBQLN1	ESPL1
ARVCF	SGK1
LRRC28	GREM2
CCR1	BDKRB1
NPR1	LAMP2
unknown	CYTSB
LOC648556	BMP6
RPGR	C12orf75
LOC100130141	INHBA
UTS2D	LOC100132774
VPS13A	C10orf47
CNTLN	CSMD2
SULT1A2	KLHL35
CCDC66	SART1
SYCP2	FCHSD2
ZFHX4	DTX3
RSPO3	SETD8
HNRPLL	LOC100131746
C3orf35	SEC61A2
PNPLA4	unknown
CYP2J2	MN1
ABCG4	FAM180A
unknown	DNAJC5
YBX2	LIMS2
C14orf26	KCNMA1
USP28	LOC100132832
NUAK1	MICALCL
GALNT3	SLC9A7
C1S	CCDC18
HR	MURC
LOC729970	CDCA4
SPATA6	SURF6
ATG7	unknown
IFITM1	unknown
TNFRSF21	unknown
SGEF	FANCA
NSUN6	ZNF703
CD14	CHN1
STMN3	ENSA
TNFAIP6	TBC1D1
unknown	ENPP4
IQCD	COL13A1
BAI2	LOC143188
MYOZ3	PLK2
HIF3A	ROD1
SEPT8	CCL25
unknown	BTN3A1
TSSK3	DNAL1
HSD11B1	RAD18
CLGN	C21orf7
C17orf107	INPP5F
DBT	AGPAT9
BEX2	MTHFD1L
unknown	PMS2L2
GADD45G	NACC2
ABCA6	SYNCRIP
ANKH	PXMP2
ARSG	unknown
TMEM71	unknown
CBX2	HNRPDL
MCTP1	TXNDC11
A2M	unknown
ACSS1	LIMK2
CUGBP2	unknown
PLAC8L1	NKX6-1
ZDHHC11	LOXL2
LOC728534	PVR
unknown	LAMC2
IGF2	HBEGF
FLJ39534	PURB
VGF	unknown
DDIT4L	unknown
PAQR6	B3GNT8
GSDMB	unknown
PPP1R1C	FGFR1OP
RPS6KA1	DSP
HSPC159	EPHB1
C11orf70	C1orf96
FLJ45244	CBLN2
RPA1	TRPC4
unknown	MASTL
GLT8D4	ABTB2
LPHN2	GSG1
CMTM1	unknown
MBD5	SLC41A2
MAP3K8	CKS2

MYO1D	MICALL1
SEPP1	C15orf38
HSF4	C4orf46
CCDC152	SRCRB4D
PION	ZWILCH
RUNDC3A	C12orf48
SLC26A1	FAT1
FAM122C	HMGB3
APOE	RECQL4
AMPH	DUSP22
TPCN1	PEX26
PLA2G4C	LOC100128851
PEAR1	LOC100126784
HEXDC	unknown
MME	CMKLR1
BFSP1	ANKRD36B
OVGP1	NTM
TMEM116	PVR
SCARNA2	NOX4
hCG_1990547	ATXN10
DCHS1	SLC25A30
RHPN2	SUSD1
FBXO15	C16orf62
BDKRB2	MYLK
DPP4	CTSC
VASH1	C12orf45
ANKMY1	PLA2G16
LOC100129387	PKIA
BATF3	CCDC109B
ZDHHC11	ADAMTSL1
CYP2D6	NPTX2
CD163L1	OSBPL3
TNFSF13	HIST1H4K
unknown	WSB2
C1R	PMAIP1
WISP2	SRGN
IFITM1	PCNA
KIFC2	LOC645676
C1QTNF1	RHEBL1
ASAP3	MCM4
TMEM198	MYLK
ZDHHC1	STMN1
LRCH2	TBC1D25
TBC1D8	KCNK6
FAM183B	MGP
C1orf203	SHROOM3
AMT	CLIC4
GPR162	CAV1
FUT6	KANK1
C14orf45	F3
ADAM33	SKAP2
MME	PCNA
CRABP2	EWSR1
unknown	RANBP17
ETV1	PBRM1
unknown	ZNF174
ADAM33	MYO10
CCL2	ATAD2
RFX2	SLC7A6OS
LOC729680	JPH2
TRIM46	RASAL1
GOLSYN	MDFI
SCARNA17	HS1BP3
PLEKHG4	RRP7A
SPEF1	STRN
SH2B2	YTHDF1
ZDHHC14	CORO1A
CACNA2D2	RFT1
ACSS3	ABL1
C10orf90	HIST1H2BB
CES1	LOC100128372
SEMA3F	CST6
SLC13A3	LOC729687
ZNF521	unknown
C5orf4	SLC38A10
ZNF783	MCM5
WDR78	SLC46A3
TPCN1	RREB1
DENND2D	CCDC159
unknown	ARSD
PTGFR	LOC100128994
NT5M	unknown
PID1	HIST1H4D
MAFB	MEGF6
STAC3	CRYBB2
REM2	HIST1H2BM
DHRS13	SYTL3
CPT1C	HIST1H2BI
PCBP3	POLA2
CXCR7	TRAIP
TTC12	SCARA3
PARP9	CENPP
C8orf42	ATF3
UNC13D	C17orf53
NCOA7	SDC3
GALNTL2	PPP4R1L
ACSF2	PXMP4
TMTC4	AFMID
ALX1	NF2
LOC729013	ARHGAP23
PPHLN1	LMO7
GJD3	SLC9A7
NR4A3	LOC100128737
KIAA1683	FNDC3B
OLFM2	ECT2
C10orf107	LOC100128517
PRR5-ARHGAP8	PTHLH
LOC100270804	C4orf39

RHOU	unknown
FOXO1	LOC100130131
unknown	CYHR1
C2orf27A	CGREF1
OR13A1	CHAC1
HNMT	CHAF1A
CREB3L4	MPRIP
ROPN1L	ING5
RAVER2	MFGE8
TRIM13	LOC100133190
BET1	LIG1
NOTCH2NL	PHGDH
DDIT4L	FIBCD1
GJD3	MAP1B
GLDN	unknown
PLCL2	MARCH3
KIAA1324L	C9orf9
DPYD	FAM113B
DNER	C16orf59
unknown	SAMD4A
SLC7A7	HIST1H2BH
C7orf53	LOC100130938
ORMDL1	ARSB
RPS6KA1	HIST1H2BO
MTMR9L	unknown
RAI2	IL27
GPR177	RP11-631M21.2
ARAP3	MCM5
CHST15	PCYT1A
NGEF	RAB38
hCG_2022304	ATXN1
IMPA2	PRB4
MAP3K5	HLA-DPA1
WNT3	FANCI
ACBD4	CNPY3
ETV1	MX1
ZNF169	CRKRS
C13orf31	LRRC8C
SAP30L	SLIT2
CD302	HIST2H2AA4
LOC100129110	LOC400236
MDGA1	PHF19
SLC25A23	OAZ3
ID4	RFC3
TAS2R10	unknown
HDAC2	MTHFS
IQCG	MYOF
ICAM5	unknown
ZDHHC11B	LY6K
LOC100129534	NEK6
RICS	TPM1
LAMA3	APOOL
AOC3	FAM174B
ZCWPW1	SNORA62
HLA-F	TMEM150B
LOC158402	CANT1
TMEM31	PLCXD1
ARVCF	FEN1
PLEKHA5	TRMT2B
PSD	TCP11L1
PNMA5	ENC1
WWOX	ADRA1B
ATP6V1G2	EXOSC6
unknown	COL5A1
CTRC	PENK
ROBO3	FLNC
TLE6	ADRA2C
DENND4A	TP53I3
PPP1R3E	CHAC2
unknown	ARNTL2
LOC100128009	C16orf75
PARG	SEC14L2
FABP3	CDK2
TNNC1	HIST1H1A
SNORA53	SKP2
ASPHD2	CD3EAP
LOC257396	HIST1H2BK
unknown	WIPF1
C1R	CAMK1
MGC21881	LAMC2
MAN2B1	HAS1
AQP3	UBE2S
PELI2	ODF2
MYO1F	PODXL
SLC44A1	unknown
CREB3L4	ZNF655
SH3BGR	BAMBI
LRRC20	ARHGAP19
C21orf2	KIAA1462
HSD17B8	LOX
GPR39	C20orf134
WDR19	P4HA2
LOC729421	EIF2C3
ANKHD1-EIF4EBP3	VMAC
NPHP1	unknown
GRASP	KPNA2
DLG3	ING3
HSD11B1L	ARSA
unknown	UCK2
KIAA1908	TPM1
PCNXL2	TMEM51
C2orf86	CHEK1
PCSK5	UBFD1
SERPINF1	RFC3
CPT1C	CLIC4
unknown	PRPS1L1
FAM26F	CAV1
SSH3	HN1

F11R	GATA2
PPP1R3F	PPAPDC1A
unknown	PSRC1
SGEF	ADAMTS1
BAI2	STAG3
DHRS12	LOC441795
S100P	GPR68
MXI1	unknown
C11orf65	HOMER2
LQK1	HIST2H4B
ANKRD13B	C9orf100
BTAF1	unknown
SPAG16	PDDC1
DTX3L	SULF2
unknown	LOC100128913
JMJD7-PLA2G4B	unknown
DNM3	BGLAP
RNF145	GPAT2
CLEC2B	unknown
DEAF1	DES
FBLN1	unknown
PLXNB1	CD83
OPLAH	HN1L
RNU2-2	LCE2A
DDR1	DNAJC27
CAPS	ITGBL1
SDCBP2	CARD9
JUNB	IKBIP
C3orf17	LOC389842
NPAS2	SACS
NCKIPSD	SLC7A5
ITGA7	ITFG2
ANGPTL2	HIST1H4I
EBF1	HAUS8
CFD	GLIPR2
SLC27A3	WDR4
SOD2	KDR
C22orf46	FPR1
SMOC1	NOL9
SULT1A4	KIAA0090
REEP2	GGCX
RAC3	RUNX1
TLE2	COL12A1
C9orf116	ARHGAP23
MORN4	BARD1
DOCK6	CFLAR
LMTK3	COL1A1
HSD17B7	SERPINE1
CLYBL	DGCR11
ACTR3B	FGF5
unknown	unknown
SHOX2	GAL
SFRP2	DSP
MAP3K12	TUBGCP3
NUDT18	C13orf34
STXBP4	C5orf13
ZNF692	FAM167A
PHYHIP	ACOT11
FLJ46906	ADAMTSL1
NR1H3	C4orf12
unknown	FBN2
GSTA4	ICAM2
ARHGEF19	PCOLCE2
C5orf38	TSPAN13
FOSL2	CTSC
F8	CKS1B
NCKIPSD	TMEM167B
SLC22A18	FKBP11
LOC283575	FKBP11
ACOX1	FCRLB
ADC	CHADL
LRSAM1	THBS1
IL1RN	CSPG4
IGLL1	PCGF5
EPS8L1	TP53I3
C14orf45	SDC1
MMP19	SEC14L2
GRIN3B	ANAPC1
SCARNA12	CNST
H2BFM	BOLA3
CBX7	MECOM
NFYA	FRY
NAPEPLD	GNG2
ISG20	LOC285300
SEMA3B	C10orf12
USP53	NOTCH1
LAMA4	TBX3
GJC2	HIST1H4B
LOC440104	SASS6
JHDM1D	CPS1
DMTF1	SUV420H1
RPGR	HIST1H2AE
CYCS	unknown
ASTN2	ZNF823
TUFT1	SIGLEC16
IL33	PDAP1
ADAMTSL4	MICAL3
NRCAM	C6orf150
CYP7B1	SP2
unknown	LOC100134259
ADAMTSL4	AQP8
PPP1R3G	ULK2
CDKN2A	DBF4
TTLL3	DHFR
PPM1J	SF3B4
NPW	BCCIP
LOC100128881	DEPDC6
ARHGEF3	GALNT5

FBXO44	CHST3
FAM19A5	TG
unknown	DUSP4
CLSTN3	AMFR
IGFALS	ANTXR2
unknown	PSMF1
C1orf70	TNS1
C1orf213	ADPRHL1
CXCL6	MGC13005
ECHDC3	PLCB4
MIF4GD	TPM3
RAB3IL1	RAD1
DBP	LOC100128130
PLAU	DLEU2L
CD40	LMTK2
C11orf71	PSAT1
RENBP	LRRC2
LPCAT4	ZC3HAV1
MIB2	AP4S1
IPP	LOC100293611
CD40	NCAM1
LOC254128	ZNF107
SEPT5	PDS5A
C22orf23	FZD6
RP1-21O18.1	FOXH1
TNXB	PTPN14
CC2D1A	ANP32E
unknown	KDELC2
unknown	KNTC1
TNFRSF6B	ENPP1
TMEM180	IMPAD1
TAS1R1	PAWR
GYG2	FHL2
LOC100128184	MARVELD2
unknown	ARL4A
CYP1A1	RBMX
HTRA3	SNCAIP
FOXP2	DCLRE1B
C10orf75	PGM2
TNFRSF6B	ZNF280B
ACP6	SGCD
RINL	PCBD2
SYNGR1	LOC283624
GTF2IRD2	DPF3
PDE4A	FLJ27352
unknown	TAF5
SPIN3	C12orf32
ELMO3	ABCG2
C16orf86	MTSS1
NEIL1	PAX9
C9orf50	PPP1R14A
CATSPER1	MID1
RNF208	LOC729839
LOC728431	PAX9
EPHB6	LOC285638
ABCA4	RNF38
POU5F1	FANCB
PAR-SN	MEG8
LOC100288439	PARP11
SILV	SAMD9L
WBSCR27	unknown
FLT3LG	PIWIL2
FERMT3	LZTS1
CA5A	ACOT11
ADCY4	LOC100131686
FLJ42875	unknown
C1orf211	LOC554203
unknown	PRDM15
LOC388849	ERI2
HAR1B	unknown
CCDC40	TRIM59
ABCA8	PRKG1
LOC389634	CC2D2A
SLC16A8	LOC100216001
NCRNA00110	BIRC3
ELL3	RHBDL2
GADD45G	NQO1
FAM40B	unknown
PAQR8	TNFSF10
METTL7B	LCTL
CASP8	SYNPO
FAM84A	ANO4
FAM198A	unknown
RAB3D	KBTBD8
unknown	FLJ43315
SENP7	CASP10
PTPRCAP	unknown
LOC284630	SGCD
LOC728806	TYRO3P
LOC644246	CDC42
NUDT14	GNG4
CHST6	HAUS5
FLJ40125	DOCK5
FGF22	C8orf34
FAM189A1	DCBLD1
SYNPO2	PRIM2
SYNGR1	NEXN
GTF2IRD2	TRIM6
PVR	KRT15
SLC35A1	unknown
ALDOC	unknown
ABHD1	VIT
INCA1	LOC100291610
LYPD3	unknown
TMEM180	FAM83G
FUT5	SEPT6
TAC3	NRP2
unknown	SELI

MORG1	SPINT2
PPP5C	SH3D20
ALS2CR8	C4orf39
TMIE	P2RX5
GAB3	C21orf58
FTCD	unknown
unknown	GABPB2
ART5	KCNE1L
unknown	HSPB6
EDA2R	CCIN
unknown	DOK5
LOC100131426	CEP250
MAMLD1	PITPNM3
ALDH3B1	RPS26
ALDH3B1	C1GALT1
FNDC3A	unknown
ARHGAP5	ARHGAP19
BEX4	C3orf59
LOC390595	CENPL
C2orf64	BMPER
TTC21A	unknown
LOC644450	CEP170
TMEM38B	MID2
ANKRD42	FOSB
IFT80	BEND7
FAM59A	C1orf230
FAM65C	MAGOHB
DTWD1	INPP5F
ABR	ANP32E
FAM89A	TOR1AIP2
ALDH3A2	NPTX1
PLSCR1	RCAN3
PTPRN	unknown
ACTR3B	HMGB3L1
NTNG1	KCNK1
ZCCHC2	unknown
MRPS25	CKAP2
ANKRD26	unknown
NIPAL4	XG
CCDC57	LOC646049
ENO3	DNA2
MMAB	BHMT2
EFEMP1	C14orf37
DYNC2H1	ATP2B4
ZNF148	unknown
COX18	LOC541467
unknown	unknown
TET1	PTPRJ
USP53	ADAMTS1
CCT6B	PTPRR
UACA	CPXM2
GPR155	ALPK2
CRTC1	ROR1
RAPGEF3	C6orf105
MAFB	POLE2
CCDC30	RQCD1
LOC100129295	ANP32E
EBF1	CHM
AGL	KCNMA1
SCARNA16	unknown
CMTM8	GATA6
PARP6	C3orf32
CROT	UBE2T
LOC283481	LNPEP
C6orf89	ADAMTS6
CECR6	BBS5
FOXO4	ALDH1A3
FBXO4	RAD54B
RPP30	BNC1
ITPKB	FBXO5
SLC35A1	ABCA3
OSBPL5	FRMD6
PLSCR4	RCAN3
POU5F1	CXCL3
CCBL2	unknown
unknown	unknown
LOC100132529	unknown
PTGDR	unknown
C9orf24	HIST1H4H
LOC100216546	TBX21
PAQR5	TIMELESS
PRDM6	unknown
LOC401022	GRAMD1B
FAM161A	RAB3B
unknown	NET1
KCNK7	LRRC3
UVRAG	LBH
NFYA	SP110
SLAIN2	ROR1
CYP27C1	WDR20
ZNF720	H2AFX
FRZB	NEFL
SPATA13	VPS53
CRIPAK	ANKRD44
KCTD15	PGGT1B
PRKXP1	unknown
ZMYM2	ZNF738
NT5DC1	unknown
PRKAA1	RHBDL2
BEX1	LOC440300
WDR33	CIT
VPS13A	LOC554202
LOC100130141	OR7E37P
FNBP1L	BAIAP2L2
KLHL24	VEPH1
LOC645752	LOC100131315
HS2ST1	SSH1
SMEK2	ITGBL1

DRD4	PDIA3
ANGEL1	NCEH1
SECISBP2	unknown
RPS29	EPDR1
NAB1	PSMC3IP
MAP2K5	ALCAM
FAM108C1	RASAL2
VSIG8	ASXL1
ATP6AP1L	RABEP1
CLDN15	LXN
DENND5B	MGC16121
unknown	unknown
MEIG1	NEXN
GPR125	ZNF469
C5orf27	NUDT15
SIPA1L2	MYBL1
PHKA1	IKZF2
NOG	MICAL3
ACVR2B	LOC100128239
LOC283070	LOC100287359
NBEAL2	C14orf80
C7orf61	PRPS1
RADIL	LOC100131700
GRAMD4	PABPC4
HSD17B7	PLAC8
ZNF397	TBX1
ZNF354A	KIAA0556
LOC100132288	ZAR1
LRRIQ3	APOA5
GPR62	ZNF365
LOC100131774	LOC100291714
TMEM182	HIST1H2AM
PLCL2	unknown
FAM178A	NPAS1
NDN	OGDH
SLC1A3	C1orf144
TUBE1	WNT5B
PMS1	DUSP7
BRWD1	FAM46B
POU6F1	HIST1H2BK
SGIP1	CARD9
GNL1	LOC100129478
MAP3K5	HN1
GSTA4	DHRS3
FAM149B1	HAS1
AGTPBP1	FGF5
CAPN7	FCRLB
CMPK2	LMO7
NR6A1	ITGA4
USP15	unknown
ETNK1	RBP1
ZNF606	LOC645967
NFIA	LRRC38
LRRC39	DEFB103A
RASD1	GRIK2
COX19	unknown
PARP8	unknown
NINL	TCTEX1D1
LOC349196	unknown
LOC100134937	C4orf31
RHOQ	unknown
SPESP1	LOC728463
PARD6G	unknown
CISD1	POFUT1
LOC100216546	CLDN14
C2orf3	BRCA2
ABCC5	unknown
NUDT12	MYRIP
USP47	CGNL1
SYT11	unknown
SPRY3	FAM22B
NRCAM	CYTSB
LTB4R	ISLR2
NCRNA00173	C9orf53
LOC441178	WNT1
SYTL4	SCUBE2
unknown	GRIK2
FAM13A	C6orf204
C2orf60	ITPK1
SPATA13	ODZ3
BCAS3	TRIM29
ACAD10	KIR3DP1
WDR35	RBM47
PLA2G12A	TCF19
ACTR3B	GAS2L3
CWF19L2	unknown
LPAR2	SVIP
PIGV	BTBD9
TTLL3	GKN1
IFIH1	EYA2
TAF4B	GREB1L
MESP1	EPCAM
CLIP4	ANO6
DNM3	HLA-DRB4
CAPS2	LOC651721
ZNF658	FAM71F2
TMCC3	LCE2C
PPFIA4	SLC1A4
OPRL1	ECM2
LOC100190939	MPP4
ZNF630	OXTR
VWF	C8orf46
C11orf20	CKAP2
LOC374491	unknown
ABCC6	ADAMTS6
BCAS3	TEX12
FBLN1	RPL10
TCEA2	MYL10

LOC645212	LOC642350
ARG2	C11orf41
CITED4	C2orf88
ZBTB16	SCN9A
MLLT6	KCNJ12
PTGDS	SYN3
LOC642413	FRMD5
DHX58	LOC100240735
REEP6	unknown
THNSL2	LOC644422
CARD10	BRIP1
CNIH3	MPP4
EGFL8	DBF4
SULT1A4	TNFRSF19
NOV	WDR67
DNAL4	FLJ12825
CDKN2A	unknown
C13orf33	ZNF367
SLAMF8	SYNPO2L
ABHD8	unknown
CTSF	ITGA8
PARP6	C17orf76
CEND1	unknown
SHANK3	DEF6
GAS1	unknown
XPA	JPH3
ZNRF1	FASLG
SPATA13	TRPM6
MUC1	CD24
NFS1	MKI67
CTSK	LOC100131366
C4orf29	NCRNA00092
IL27RA	FOXP1
PALM	PLEKHB1
C9orf72	unknown
RIMBP3	unknown
HOXD3	LOC645307
STAMBP	DHX57
PACRGL	CLDND2
LOC255512	LOC728145
ZFYVE28	CHRM3
CPAMD8	DENND2C
PCCA	ROR1
SYNGR3	HOXD9
BCAS3	LOC100128055
unknown	unknown
VPS13C	PITX1
unknown	FAM101A
PFKFB3	ELN
unknown	WDR62
ABL1	C20orf118
MAPK12	BANK1
SLC25A42	C1QTNF2
SEZ6L2	VPS53
PPP1CB	unknown
PPARG	SPTBN1
RSL1D1	LOC100131910
ATRN	ZNF681
BSCL2	DAND5
FLJ90757	YIF1B
ADC	SLC8A1
TM4SF1	unknown
CC2D1A	KIF18B
CCDC102A	ESRRG
IFIT2	DOK6
PRMT2	PTCRA
SERPING1	ABCB1
CTSB	unknown
AGPAT4	AADAC
C1orf97	LOC348751
SLC43A3	SOX17
C2orf43	KIAA1217
IFITM4P	unknown
ZDHHC3	HLA-DRB5
C6orf64	HMGCLL1
LOC644525	unknown
TTC8	IGFBPL1
NINJ1	unknown
ATG4C	POLE
FUK	SLC25A21
SIRT5	unknown
AHSA2	ARL5B
FAM60A	LOC652554
CHKB	unknown
PLK1S1	MYOCD
SLC6A6	GRHL3
CTBS	unknown
PRPF40B	unknown
BBS9	CYP3A7
LOC644021	D4S234E
C6orf154	C14orf145
unknown	TDRD9
unknown	FRMPD4
RNU1-5	SMC1A
LOC100286937	C6orf114
LPHN2	SORCS2
TRPS1	LOC650293
C1orf66	PVRIG
PSMB9	SAA2
CEPT1	FAM53A
VAMP8	AK5
LOC100293193	CDH18
PTGS1	DCBLD1
ZGLP1	C20orf103
GPNMB	WNT16
CRTAP	FLVCR2
EMX2OS	unknown
TMEM25	MARCH4

SVIL	INPP4B
MSX2P1	FAM26E
GBE1	SSTR1
DEAF1	C16orf11
SNORD3B-1	PDZK1
PLEKHA4	GSTA2
ANKZF1	KDELC2
ANKRD37	WNT11
DTWD1	PPPDE2
SGIP1	RECQL5
unknown	SGCD
PDE4B	ABI3BP
N6AMT1	PTGIS
C2orf64	FBN1
PIP5KL1	INCENP
PNRC1	C10orf47
ARL3	BMPER
IMPA2	C1orf118
KLF2	RBM33
GPD2	GPAT2
TMEM150A	CCDC134
OPN3	FAM105A
DGAT2	PTPRR
LOC728537	USP41
C17orf100	SNX26
LOC375190	DYNC1I1
RBBP4	NKAIN1
CD302	HIST1H2BJ
C9orf64	KRT83
VAMP1	FCHSD2
C1S	FAM198B
TMEM35	MNS1
PSEN2	NCAPG2
C17orf108	GINS1
MBTD1	unknown
C21orf122	RASAL2
unknown	RNF152
ARRB1	unknown
TDRKH	TNFRSF10C
unknown	BRCA1
LYPLAL1	unknown
PHC1	HELLS
LOC283050	FAM7A1
FAM120C	unknown
C4orf49	FNDC1
LASS1	TMPO
FCHO1	HSPB8
TSPAN11	BLM
REV3L	unknown
NOC3L	unknown
TMEM38B	SMC4
CHURC1	UHRF1
NOTCH4	ST8SIA5
CXorf57	LOC388780
FLJ40125	unknown
LIPT1	LMO2
SOCS1	LOH3CR2A
unknown	STC2
HSPC157	COLEC11
RFESD	SKA2
LOC100130557	RACGAP1
TNNC2	C6orf173
TMC4	PACSIN1
CABC1	IGFBP1
LOC728392	LOC727916
DCAF8	unknown
KIAA1199	SETD8
LOC646999	unknown
DENND1A	PDLIM4
TMEM158	UBASH3B
CXCL14	GINS4
MYLIP	SLC35D1
LPHN2	C3orf52
TLR2	DNAJB4
ZCWPW1	unknown
LOC286052	PTER
JAG1	DARS2
NRARP	C1orf118
DISC1	WHSC1
IQCK	unknown
PCSK5	MRAP2
ENOX1	LOC387647
FZD4	HHAT
ISOC1	HELLS
RGS2	LOC729595
EFCAB7	ITGA4
PPARG	C1orf135
TRIM47	BAALC
unknown	CTPS2
MOSC2	PTX3
ZNF254	E2F1
AGPAT5	PF4
NFIL3	MMP7
BBS2	GALNT10
ZNF22	KIF20B
C8orf40	COL12A1
CDH13	SGOL2
AUH	C11orf82
SMARCA1	LOC100128242
TRNAU1AP	ASF1B
RAB33A	PRC1
SCD5	KRT19
HMGN5	CENPN
SH3BP5	LOC283788
SLC35E2	SLC22A20
unknown	KCNS2
TRAF5	C19orf53
ADAL	unknown

PXMP4	LSAMP
NFKBIZ	ZNF623
TNKS	unknown
LOC100128292	FANCA
SLC22A5	GRIK2
C1orf213	NFASC
RXRB	HIST1H2AC
IRX5	EVI2A
PRMT2	DMC1
NTNG1	SUFU
MSI2	HIST1H4F
LOC388630	unknown
BCR	HIST1H2BD
PBXIP1	unknown
ATP6V0E2	ADARB1
C14orf132	unknown
GPR125	WHSC1
unknown	C1QL1
PRKAR1B	KCNF1
AXIN2	ARSI
C17orf96	unknown
C1orf54	TAF13
ASGR1	C1orf51
SLC36A4	CCDC80
CFH	DOCK5
ZNF438	P2RX5
TMCO4	C11orf91
FPGT	PLCXD1
LOC285550	GDPD3
WDR19	SSR3
FLJ37798	BRI3BP
QPCT	HAS1
C1orf133	THBS1
C11orf54	KIRREL3
SIRT5	NRGN
PREX1	GSC
SAMD10	LHPP
CLK4	HIST1H2BG
ABHD6	SIRPB1
C10orf104	CA5BP
TTC14	NGF
ZNF333	STXBP6
CCDC25	HHIPL1
LOC390940	CDA
MTERFD3	MGC72080
CCPG1	unknown
IFT81	TNFRSF10A
EEPD1	unknown
ZNF280D	CDC25A
SLC25A37	KIF22
LNP1	KIF22
GPD2	PSG8
TMEM106B	unknown
SCD	CCDC89
CTH	NINJ2
C14orf147	IL13RA2
PARP12	GATC
LMBRD1	EFCAB4B
VGLL3	unknown
ACPL2	ADAM19
NEAT1	MELK
GHR	GABPB1
C8orf83	GPAT2
C3orf70	WDR51A
KLHL5	BDNF
ZNF280C	ATP2B4
MDM1	STXBP6
SCAI	ABCC9
DENND5B	DCBLD2
unknown	HIST2H2BF
CLCN4	SHROOM3
KLHL2	TCOF1
TMEM143	CDKN2C
VPS41	CENPN
LOC222699	CHST10
LOC100134387	ENPP1
PLBD2	CENPO
LOC642852	GINS3
BEND6	ENPP2
LAMA4	PTTG1
C6orf162	MDM1
TLR4	ARHGEF5L
TRO	GFRA1
RFTN2	SOCS7
DHX40	unknown
LEPR	DAB2IP
CCL27	LIN7A
BRWD1	IL17B
LHX6	LOC100128994
C8orf85	unknown
HTRA3	LOC150381
SRRM3	SKA2
HCFC2	WWP2
ABCA5	SPATA4
DDX17	HIST1H1B
KIAA1712	FANCD2
PROS1	WDR76
C11orf70	unknown
BRWD1	HIST1H2BC
RFX3	MPHOSPH9
POLI	IL32
RCOR2	CDH4
SPG20	CDH2
LOC254057	RBL1
TMEM187	KRT34
unknown	PSG2
ZFYVE16	VIT
unknown	VCAN

XPR1	LOC81691
ALPK1	TRIP13
WDR60	IFI44
SESTD1	LRRC15
MAN1C1	ULBP3
unknown	unknown
ALDH7A1	LOC100129532
LOC389493	TMEM164
LOC91450	ENHO
GATAD2B	FRMPD4
LOC645158	LCE3D
FOXK2	MYBL2
ANKRD46	IQGAP3
CC2D2A	CCDC8
CHIC1	unknown
unknown	unknown
ZNF230	STIL
AR	PAX6
NPHP4	unknown
SLCO3A1	CDH15
ZNF638	RASSF2
NHLRC4	C11orf41
C20orf94	TTLL5
SENP7	C3orf43
KGFLP2	PLXNA2
DNAJC24	TRIM63
MTR	PCOTH
SLC2A3	PTPRS
KRTAP10-1	CCNB1
FBXO24	DMRT2
ZNF25	MGC72080
unknown	KY
LRRCC1	TES
COL11A2	PCDHGB1
unknown	TMEM106A
PNRC1	CDC42EP3
TTBK2	unknown
TCFL5	IL22RA1
KIAA0802	TRIM6
LOC100129550	RIBC2
MYO1D	CAP2
TNFRSF14	KIF20B
KIAA1530	DHFR
IFT74	LOC728353
ZNF605	FLYWCH1
FBXO41	MYPN
ZNF862	GCNT1
C3orf23	KCNJ2
IER5L	FERMT1
TRIM2	EPHB2
COG6	CCNE2
CORO6	FOXF1
AZI2	ZNF280D
INSIG2	PRPH2
LOC730236	ALDH1A1
MDM4	ATAD5
PCTK2	AMIGO2
PSD3	unknown
PM20D2	DSCC1
LEPROT	RAPGEF6
MEGF8	UBE3C
INTS8	LOC730961
LARP1B	AURKAPS1
SCARNA9	KIF14
ADAMTS13	CPA4
UQCRB	PDGFD
RDH10	LOC440900
EXOC6B	AGMAT
SHPRH	unknown
ZNF720	ALDH1L2
FANCL	MLF1IP
CLIP4	PITX1
LOC100130107	RAD51
CLIP4	CENPF
LUC7L	MND1
NIPSNAP1	FOXS1
SLC35B4	LOC100131262
IFI16	HMGB3
LOC284219	BTBD11
HELQ	CAMK4
MTAP	CACNA2D1
DYNC2LI1	ACACA
C6orf57	LOC100133479
KLRAQ1	ESYT3
USP45	KCNMA1
RNF13	MMP24
ZNF383	PSG11
CPNE5	ADRA1D
unknown	KIAA0226
ZCCHC6	LOC645978
BBS10	IL21R
ACADM	RUNX1
FBXL16	SERPINB7
STAT5B	IL32
TTC13	FAM98B
unknown	CHAC1
HECTD2	KIF26B
CA12	UHRF1
C3orf72	unknown
ATP7A	PTTG2
DKFZP586I1420	TLX2
NBEA	FUCA1
ZNF25	EDN1
IFT88	CTGF
NDRG2	S1PR1
SLC25A36	B4GALT1
VPS13C	POSTN
SLC5A3	ITGA11

SP4	CRYAB
TRIM45	TAGLN
FAM91A1	SUV39H1
GCA	FANCD2
TRPS1	C14orf37
FBXL17	FAM72D
ZNF292	E2F7
CXorf23	HHIP
FGFBP3	NXN
DAK	MRPL1
ZBTB41	ORC6L
ERO1LB	ANGPT1
MTMR9L	RAGE
HERC2P7	ANKRD2
SYT3	unknown
HR	MAD2L1
LOC653510	CPXM2
N4BP1	TAGLN
C5orf45	RAD51AP1
ACADS	CDCA5
STAT3	CDT1
RALGDS	CDKN3
unknown	TYMS
DNPEP	KCNC4
RNF13	AURKA
SP3	CCDC8
PLEKHH3	CENPE
CREG1	HSPB3
ZNF354B	ORC1L
RPRD1A	B4GALNT1
METT5D1	PRR11
SERPING1	RAMP1
MCL1	PKMYT1
MGC21881	CDC45L
NFXL1	FAM25A
TTC32	PDGFB
ZFP90	FBXO43
IRAK2	PLCB4
unknown	XKRX
PLEKHA1	C5orf13
WDFY3	BLK
LOC200609	EDN1
CREBZF	UNKL
FAM162A	WHSC1
COL8A2	COL5A1
FBXO46	LOXL3
KDSR	unknown
LRIG3	CENPN
GBP3	GAD1
HEY1	MATN3
GALC	VSTM2L
RPS6KA5	C15orf42
BIVM	LBH
ATG5	LOC145694
RBL2	PPM1F
ZFP161	ADAM19
GABARAPL2	MYLK2
CHML	OR8B8
ITM2C	FAM107A
LOC286161	unknown
C5orf23	LOC646324
CCDC74B	EPHB2
CPSF3	unknown
IGF2BP2	PSG5
R3HDM2	SPAG5
ZNF277	RAD54L
DENND4C	LATS1
RC3H2	ZWINT
ZNF266	HSPB7
TANC2	KIF23
PRPF4B	HIST1H3B
SESTD1	COL8A1
MARCH6	DIRAS3
PAX3	unknown
SFRS18	MASP1
DOCK11	CDCA2
WTAP	FAM54A
NDRG3	NUSAP1
VAMP4	unknown
PSD3	CECR1
SLC25A36	HIST1H2AC
LPAR1	EXO1
SCAMP1	LEPREL1
DSEL	LOC400043
SP100	DKK1
PCM1	IGFBP3
WARS2	TK1
unknown	CDCA8
C14orf139	NDC80
PLEKHA1	CCNB1
SFRS12	MYO16
TECPR2	unknown
RIC8B	ZWINT
C9orf95	NEURL1B
PKNOX1	PALMD
RALGAPA1	NRN1
LOC399744	DTL
HDAC5	TOP2A
APBB3	OIP5
DDX58	IL7R
EFHA2	SEMA7A
OSBPL8	LOC729983
CTBS	GINS2
unknown	KIAA0101
SLC25A37	unknown
unknown	NOX4
HLA-B	CDKN3
AKAP12	MALL

GCOM1	C9orf140
HLA-B	PI16
MYO1B	PTTG3P
ADH5	SNORD12B
unknown	CPLX3
MXD4	AZU1
ARRDC3	NKX6-2
LOC203274	unknown
CREBBP	MOBKL1A
ZCRB1	UTRN
IDUA	FAM160A1
AKR1C3	LOC151484
unknown	unknown
unknown	OR10G3
unknown	TEX28
ACPL2	LRRC4
AKAP11	LOC284232
AKR1B1	LOC339894
ATG2A	TCP11
CYBRD1	GALNT12
IER3	LOC100293406
PSMB8	unknown
STEAP3	LOC100129652
OCEL1	THSD1
IGSF8	unknown
OBFC1	TMEM87A
EID3	LOC100129900
QPCT	RREB1
C9orf116	MS4A5
C9orf169	unknown
STARD10	COL17A1
RTN2	HORMAD2
MLYCD	C17orf77
CAMK2N2	ZDHHC19
C16orf79	LOC100128230
D2HGDH	OAS2
CCBL2	unknown
CABLES1	LOC647343
RNASE4	unknown
APC2	unknown
C1orf97	ACAN
unknown	SPINK5L2
IDS	PLA2G1B
LRRC28	TAS2R19
LOC100289097	LOC441208
ITM2C	BECN1L1
PITPNM1	KIAA1644
MXD4	CXorf36
PACSIN3	GPR75
NFATC1	unknown
MAP3K14	REG1B
NES	unknown
C9orf16	C9orf47
LOC100130633	FUT1
C21orf119	unknown
PEX1	IL21
PLD3	OR51D1
TMEM79	NMS
unknown	unknown
TMCO4	unknown
HSD17B7	LOC642635
IL4I1	TRIOBP
SMAD5OS	unknown
GYPC	FLRT1
ELMOD3	LOC100132474
ZNF358	unknown
CYB5D2	unknown
GLTSCR2	unknown
IGSF8	FAM41C
SARM1	PRODH
SPSB3	unknown
C21orf63	LOC648822
unknown	LOC100132972
SERPINA3	unknown
FLJ35220	LOC285000
ALKBH2	PHOX2B
HS1BP3	HIST2H2BA
LOC646808	SMOC2
NES	FGF5
CYHR1	GRIP2
SEPT5	LOC728530
EML2	OR51B2
C1QTNF6	CNTN1
LRRC24	LOC100133500
PSAP	ZNF140
C12orf57	LOC1720
AVPI1	NKAIN2
IFITM3	LOC100132733
RER1	ART4
CD68	IGSF1
PHC1	SLC26A3
PRR24	hCG_1793639
ACVRL1	PRODH2
SDR39U1	IL1F9
SLC39A1	MCF2L
ECH1	TPSAB1
SIPA1	LOC100131673
SMARCD3	tcag7.1307
PGAP3	CNOT2
BCKDHA	RAD21L1
CCS	WNT3A
JMY	ERVWE1
LOC100130107	unknown
JUP	C19orf41
unknown	INPP4B
ANGPTL6	ATCAY
CTSL1	ZNF501
GGT5	HBA2

PBXIP1	unknown
VWCE	FAM178A
LOC730183	LOC100131608
CLK1	PLD4
TGFBR3	GPR1
unknown	FAM169A
ZNF395	LOC100130171
AOX1	unknown
SLC36A4	PSMB11
RPAIN	SEMA3D
DFNA5	FAM154B
PLIN2	BMP8A
C20orf108	WHSC1
TNFAIP8	TEX14
C7orf41	NARG1L
C4orf52	MATN3
GNPDA1	unknown
SCARB1	FAM98B
GPX4	HIST2H2BE
GAA	IKZF2
SNORD3B-1	LOC729911
SLC9A9	CACNA1E
CUL7	CXorf67
NFATC4	TRIML2
PIGZ	unknown
PLAU	PRLR
FOS	unknown
CD55	unknown
RPAIN	LOC100292021
MACROD1	ZNF100
GEM	ATPBD4
SYNJ2	tcag7.929
NNMT	CYCSP52
LOC645722	CYP4A22
PTBP2	LOC727918
SBSN	unknown
unknown	C12orf36
EVC2	FMO6P
NEURL4	unknown
PLD2	unknown
unknown	unknown
TMEM42	unknown
RPS23	REG4
MZF1	PPP2R2B
TRPT1	ZNF154
IGF1R	unknown
ZRANB2	LOC400743
ZNF664	GRIA4
DZIP1	ANKRD24
LIPA	unknown
TP53TG1	CACNG2
RB1CC1	GPR32
SNX27	CAV3
C21orf2	PGLYRP3
HDHD2	MYO5B
N4BP2L2	LOC100131320
PDE4A	LOC203510
ZFC3H1	TMPRSS2
LUC7L3	unknown
DRAM2	NCRNA00092
MAP3K10	unknown
NFS1	unknown
METTL7A	C19orf21
EGFL7	HLA-L
FAM20C	unknown
TMEM205	HAO1
ARRB2	CUX2
GLRX	ZNF569
DHRS1	MT1H
TMEM216	OR1L6
DKFZP586K1520	unknown
FAM43A	unknown
TUBA4A	GAD1
NME3	unknown
KREMEN1	ZSCAN1
ANKRD28	RPRML
C16orf58	SLC30A5
LOC100129122	C22orf34
unknown	LOC100131642
KAT2A	ZNF625
ASS1	CLDN16
AOX1	FLJ45482
LUC7L	unknown
FAM82B	LOC653712
GDF11	C12orf27
SSC5D	BMF
SAT1	ALDOB
C21orf57	unknown
AKAP12	HERC6
INTS10	SLC22A24
CYP27A1	ASPA
CRYL1	CEACAM1
NAT14	LOC100128006
SLC27A5	unknown
LOC730098	DSPP
RP11-345P4.4	OR2M2
ACP5	unknown
RNASET2	TAGLN3
ZNF182	LOC388906
OSMR	LOC100129363
TUT1	RGAG1
HMBOX1	unknown
FAM117B	MPV17L
PACS2	HAND1
SOX4	OR10J3
ABCB6	LOC100287919
FBXL18	GUCY2G
CROCCL1	TMEM72

SP9
IDS
TMEM8B
RAB27A
FYCO1
CRYZ
CCDC41
ENO2
IER3
SLC48A1
ULK3
RNF130
PHF20L1
RN7SK
PYDC1
ZFR
CAB39L
ROM1
DHRS7
GRK5
GPR177
OSBPL1A
CYP2R1
IL6
MMP11
F12
PSME1
MMP2
SELENBP1
C2orf68
IRAK3
DPH5
RHOBTB3
PIGP
C9orf169
SNX1
ZFP41
FAM24B
TSPAN14
CREM
C12orf76
TM4SF1
OSBPL7
DDIT4
HGSNAT
THAP6
THADA
UBAC2
LOC729678
CRNDE
C14orf104
MC1R
BAT2L
EDA2R
COL4A3BP
CCR10
TACC2
ABCC3
PDE4DIP
PQLC3
SH3YL1
ZNF462
GULP1
ENGASE
CSTF2T
MANEA
ORC2L
EBF3
LOC283861
C20orf199
ASS1
CYP1B1
RAB13
HLA-J
FBXO32
ARHGAP12
PDGFRB
ETS2
AVPI1
RNF130
RPS6KA3
HEXIM2
WTAP
EPB41L1
ATG2A
NUAK1
PTPRS
C3orf19
GLI3
COG5
ALDH2
KRCC1
PHF20L1
SNRPN
MDP1
GATAD1
ST6GALNAC6
SDCBP
RABL2A
BNIP3L
STAT1
NLGN2
GRAMD1C
TFPI
DENND4C
ZNF252
DHX36
SSC5D

ZFATAS
unknown
LOC150622
ADAMTSL1
LOC644925
KCTD7
ASCL2
UMODL1
FLJ30901
TRIM77
LOC100131195

SSU72
RIN2
OGT
LOC400099
KIAA0232
