# Supplementary material for: Lactic Acid Bacteria Convert Human Fibroblasts to Multipotent Cells
Source: PLoS One. 2012 Dec 26;7(12):e51866. doi: 10.1371/journal.pone.0051866 (PMC3530539; doi:10.1371/journal.pone.0051866)
Supplement: Table S4 — Selected 37 pluripotency maker genes. (PDF) [file pone.0051866.s013.pdf]

Selected 37 pluripotency marker genes

no	ProbeName	GeneSymbol	UniGeneID	GeneName	Description	Fold change [LAB-inc. cells-1]vs[HDF-1]	Regulation [LAB-inc. cells-1]vs[HDF-1]	Fold change [LAB-inc. cells-2]vs[HDF-2]	Regulation [LAB-inc. cells-2]vs[HDF-2]	raw data [LAB-inc. cells-1]	raw data [HDF-1]	raw data [LAB-inc. cells-2]	raw data [HDF-2]	Flag [LAB-inc. cells-1]	Flag [HDF-1]	Flag [LAB-inc. cells-2]	Flag [HDF-2]
1	A_23_P110851	TERT	Hs.492203	telomerase reverse transcriptase	Homo sapiens telomerase reverse transcriptase (TERT), transcript variant 1, mRNA [NM_198253]	1.02313	up	1.01886	up	95.27364	96.42165	41.57469	35.98146	Detected	Detected	Detected	Detected
2	A_23_P13899	GAPDH	Hs.544577	glyceraldehyde-3-phosphate dehydrogenase	Homo sapiens glyceraldehyde-3-phosphate dehydrogenase (GAPDH), mRNA [NM_002046]	1.10914	up	1.04260	down	135016.67000	126047.44500	135888.95000	124930.41000	Detected	Detected	Detected	Detected
3	A_23_P16058	ZNF296	Hs.192237	zinc finger protein 296	Homo sapiens zinc finger protein 296 (ZNF296), mRNA [NM_145288]	1.15618	down	1.50367	down	85.84721	102.77403	57.33899	76.02686	Detected	Detected	Detected	Detected
4	A_23_P162547	MYL2	Hs.75535	myosin, light chain 2, regulatory, cardiac, slow	Homo sapiens myosin, light chain 2, regulatory, cardiac, slow (MYL2), mRNA [NM_000432]	3.15344	down	2.83932	down	73.80911	241.00575	36.16277	90.54007	Detected	Detected	Detected	Detected
5	A_23_P204640	NANOG	Hs.661360	Nanog homeobox	Homo sapiens Nanog homeobox (NANOG), mRNA [NM_024865]	8.45846	up	6.81780	up	129.54599	15.85863	67.55973	8.73794	Detected	Compromised	Detected	Compromised
6	A_23_P320739	MEF2C	Hs.649965	myocyte enhancer factor 2C	Homo sapiens myocyte enhancer factor 2C (MEF2C), transcript variant 1, mRNA [NM_002397]	1.95959	down	1.77027	down	189.57979	384.67180	155.37459	242.54076	Detected	Detected	Detected	Detected
7	A_23_P32233	KLF4	Hs.376206	Kruppel-like factor 4 (gut)	Homo sapiens Kruppel-like factor 4 (gut) (KLF4), mRNA [NM_004235]	1.02345	down	1.00169	down	652.56530	691.54694	832.71185	735.51825	Detected	Detected	Detected	Detected
8	A_23_P342709	FBXO15	Hs.664011	F-box protein 15	Homo sapiens F-box protein 15 (FBXO15), transcript variant 1, mRNA [NM_152676]	2.60037	up	2.41878	up	79.46852	31.64416	75.16104	27.40072	Detected	Detected	Detected	Detected
9	A_23_P34700	TNNT2	Hs.533613	troponin T type 2 (cardiac)	Homo sapiens troponin T type 2 (cardiac) (TNNT2), transcript variant 1, mRNA [NM_000364]	2.09040	down	1.32848	down	28.24185	61.13021	12.93388	15.15124	Detected	Detected	Compromised	Compromised
10	A_23_P366376	TDGF1	Hs.385870	teratocarcinoma-derived growth factor 1	Homo sapiens teratocarcinoma-derived growth factor 1 (TDGF1), mRNA [NM_003212]	1.72332	down	2.56912	up	5.63538	10.05593	7.38659	2.53527	Compromised	Compromised	Compromised	Compromised
11	A_23_P380526	DPPA4	Hs.317659	developmental pluripotency associated 4	Homo sapiens developmental pluripotency associated 4 (DPPA4), mRNA [NM_018189]	1.30213	down	1.17999	down	2.62040	3.53308	2.85058	2.96604	Compromised	Compromised	Compromised	Compromised
12	A_23_P395582	ZFP42	Hs.335787	zinc finger protein 42 homolog (mouse)	Homo sapiens zinc finger protein 42 homolog (mouse) (ZFP42), mRNA [NM_174900]	1.26799	down	3.52599	up	2.37460	3.11772	10.32920	2.58316	Compromised	Compromised	Compromised	Compromised
13	A_23_P401055	SOX2	Hs.518438	SRY (sex determining region Y)-box 2	Homo sapiens SRY (sex determining region Y)-box 2 (SOX2), mRNA [NM_003106]	1.27169	down	1.17703	down	2.28901	3.01412	2.38275	2.47306	Compromised	Compromised	Compromised	Compromised
14	A_23_P405885	DPPA2	Hs.351113	developmental pluripotency associated 2	Homo sapiens developmental pluripotency associated 2 (DPPA2), mRNA [NM_138815]	1.27124	down	1.03344	down	2.37653	3.12827	2.87285	2.61798	Compromised	Compromised	Compromised	Compromised
15	A_23_P46894	CHAT	Hs.302002	choline acetyltransferase	Homo sapiens choline acetyltransferase (CHAT), transcript variant M, mRNA [NM_020549]	1.18237	down	1.03032	up	58.58926	71.73039	20.01846	17.13267	Detected	Detected	Detected	Detected
16	A_23_P59138	POU5F1	Hs.249184	POU class 5 homeobox 1	Homo sapiens POU class 5 homeobox 1 (POU5F1), transcript variant 1, mRNA [NM_002701]	2.94692	up	2.98864	up	665.32104	233.77365	520.54330	153.58511	Detected	Detected	Detected	Detected
17	A_23_P59738	MYL7	Hs.75636	myosin, light chain 7, regulatory	Homo sapiens myosin, light chain 7, regulatory (MYL7), mRNA [NM_021223]	1.27326	down	1.24645	down	36.03686	47.51137	20.32652	22.34098	Detected	Detected	Detected	Detected
18	A_23_P72817	GDF3	Hs.86232	growth differentiation factor 3	Homo sapiens growth differentiation factor 3 (GDF3), mRNA [NM_020634]	1.31792	up	1.15925	down	5.28466	4.15205	3.59586	3.67574	Compromised	Compromised	Compromised	Compromised
19	A_23_P73632	NR0B1	Hs.268490	nuclear receptor subfamily 0, group B, member 1	Homo sapiens nuclear receptor subfamily 0, group B, member 1 (NR0B1), mRNA [NM_000475]	6.53813	up	6.99447	up	257.53506	40.78642	306.05145	38.58382	Detected	Detected	Detected	Detected
20	A_23_P74895	LIN28	Hs.86154	lin-28 homolog (C. elegans)	Homo sapiens lin-28 homolog (C. elegans) (LIN28), mRNA [NM_024674]	1.52183	up	2.08789	down	22.24938	15.13858	4.23973	7.80570	Detected	Detected	Compromised	Compromised
21	A_23_P95594	NAT1	Hs.591847	N-acetyltransferase 1 (arylamine N-acetyltransferase)	Homo sapiens N-acetyltransferase 1 (arylamine N-acetyltransferase) (NAT1), transcript variant 5, mRNA [NM_000662]	1.31398	down	1.27421	down	591.03156	804.14450	529.49400	594.93370	Detected	Detected	Detected	Detected
22	A_24_P141227	LMX1B	Hs.129133	LIM homeobox transcription factor 1, beta	Homo sapiens LIM homeobox transcription factor 1, beta (LMX1B), mRNA [NM_002316]	1.02012	up	1.89265	down	12.01103	12.19164	3.88598	6.48542	Compromised	Compromised	Compromised	Compromised
23	A_24_P282383	MYH7	Hs.678918	myosin, heavy chain 7, cardiac muscle, beta	Homo sapiens myosin, heavy chain 7, cardiac muscle, beta (MYH7), mRNA [NM_000257]	1.31048	down	1.25126	down	2.59871	3.52632	2.84199	3.13572	Compromised	Compromised	Compromised	Compromised
24	A_24_P314585	ERAS	Hs.447330	ES cell expressed Ras	Homo sapiens ES cell expressed Ras (ERAS), mRNA [NM_181532]	1.14202	up	1.31809	up	11.08622	10.05175	9.40884	6.29443	Compromised	Compromised	Compromised	Compromised
25	A_24_P355720	FGF4	Hs.1755	fibroblast growth factor 4	Homo sapiens fibroblast growth factor 4 (FGF4), mRNA [NM_002007]	1.12486	down	1.17092	down	32.74595	38.14063	12.24211	12.64010	Detected	Detected	Compromised	Compromised
26	A_32_P233950	DPPA5	Hs.125331	developmental pluripotency associated 5	Homo sapiens developmental pluripotency associated 5 (DPPA5), mRNA [NM_001025290]	1.33378	down	1.17393	down	2.69710	3.72490	3.03895	3.14580	Compromised	Compromised	Compromised	Compromised
27	A_33_P3214665	MAP2	Hs.368281	microtubule-associated protein 2	Homo sapiens microtubule-associated protein 2 (MAP2), transcript variant 1, mRNA [NM_002374]	1.10565	down	1.07913	down	1285.73000	1471.96940	1054.59330	1003.51416	Detected	Detected	Detected	Detected
28	A_33_P3217719	TLE1	Hs.197320	transducin-like enhancer of split 1 (E(sp1) homolog, Drosophila)	Homo sapiens transducin-like enhancer of split 1 (E(sp1) homolog, Drosophila) (TLE1), mRNA [NM_005077]	1.35853	down	1.01156	up	325.97156	458.54380	222.32104	193.80008	Detected	Detected	Detected	Detected
29	A_33_P3294217	UTF1	Hs.458406	undifferentiated embryonic cell transcription factor 1	Homo sapiens undifferentiated embryonic cell transcription factor 1 (UTF1), mRNA [NM_003577]	1.36354	down	1.11815	down	13114.74100	18516.54100	2629.22200	2592.33670	Detected	Detected	Detected	Detected
30	A_33_P3299599	NKX2-5	Hs.54473	NK2 transcription factor related, locus 5 (Drosophila)	Homo sapiens NK2 transcription factor related, locus 5 (Drosophila) (NKX2-5), transcript variant 2, mRNA [NM_001166175]	1.11610	down	1.08543	down	83453.80000	96445.89000	106123.38000	101573.06000	Detected	Detected	Detected	Detected
31	A_33_P3300217	MYLK3	Hs.130465	myosin light chain kinase 3	Homo sapiens myosin light chain kinase 3 (MYLK3), mRNA [NM_182493]	1.06937	up	1.00590	up	3.33003	3.22442	3.13806	2.75089	Compromised	Compromised	Compromised	Compromised
32	A_33_P3303772	SLC6A3	Hs.406	solute carrier family 6 (neurotransmitter transporter, dopamine), member 3	Homo sapiens solute carrier family 6 (neurotransmitter transporter, dopamine), member 3 (SLC6A3), mRNA [NM_001044]	1.47399	down	3.54998	down	203.10928	309.99643	164.00641	513.39600	Detected	Detected	Detected	Detected
33	A_33_P3312682	REXO1	Hs.192477	REX1, RNA exonuclease 1 homolog (S. cerevisiae)	Homo sapiens REX1, RNA exonuclease 1 homolog (S. cerevisiae) (REXO1), mRNA [NM_020695]	1.38441	down	1.33395	down	58889.78500	84418.44500	23861.44300	28067.45000	Detected	Detected	Detected	Detected
34	A_33_P3355004	DDC	Hs.359698	dopa decarboxylase (aromatic L-amino acid decarboxylase)	Homo sapiens dopa decarboxylase (aromatic L-amino acid decarboxylase) (DDC), transcript variant 7, mRNA [NM_001242890]	1.27307	down	1.16312	down	2.38509	3.14406	2.56508	2.63082	Compromised	Compromised	Compromised	Compromised
35	A_33_P3359508	DNMT3B	Hs.713611	DNA (cytosine-5-)-methyltransferase 3 beta	DNA (cytosine-5-)-methyltransferase 3 beta [Source:HGNC Symbol;Acc:2979] [ENST00000537219]	2.34031	down	2.10028	up	2.66052	6.44722	7.67818	3.22364	Compromised	Compromised	Compromised	Compromised
36	A_33_P3367636	C6orf221	Hs.128326	chromosome 6 open reading frame 221	Homo sapiens chromosome 6 open reading frame 221 (C6orf221), mRNA [NM_001017361]	20.27000	up	22.70337	up	109.27738	5.58226	82.21005	3.19301	Detected	Compromised	Detected	Compromised
37	A_33_P3631022	LOC1002706	Hs.333720	hypothetical LOC100270680	Homo sapiens C-MYC proto-oncogene regulatory region [AJ315134]	1.74679	up	1.20201	down	14.60249	8.65605	3.97881	4.21723	Detected	Compromised	Compromised	Compromised
