# Supplementary material for: Lactic Acid Bacteria Convert Human Fibroblasts to Multipotent Cells
Source: PLoS One. 2012 Dec 26;7(12):e51866. doi: 10.1371/journal.pone.0051866 (PMC3530539; doi:10.1371/journal.pone.0051866)
Supplement: Table S1 — Comparison between HDF-1,2 and LAB-incorporated cell clusters-1,2. (PDF) [file pone.0051866.s010.pdf]

Comparison between HDF-1,2 and LAB-incorporated cell clusters-1,2

no	ProbeName	GeneSymbol	UniGeneID	GeneName	Description	raw data [LAB-inc. cells-1]	raw data [HDF-1]	raw data [LAB-inc. cells-2]	raw data [HDF-2]	Flag [LAB-inc. cells-1]	Flag [HDF-1]	Flag [LAB-inc. cells-2]	Flag [HDF-2]
1	A_23_P42935	BRAF	Hs.550061	v-raf murine sarcoma viral oncogene ho	Homo sapiens v-raf murine sarcoma vir	96.72971	163.01007	77.28133	115.48117	Detected	Detected	Detected	Detected
2	A_23_P117082	HEBP1	Hs.642618	heme binding protein 1	Homo sapiens heme binding protein 1 (H	29832.06600	26425.23400	19447.49000	14604.97500	Detected	Detected	Detected	Detected
3	A_23_P2683	RPAP3	Hs.437855	RNA polymerase II associated protein 3	Homo sapiens RNA polymerase II assoc	1301.96040	965.45667	804.10250	485.17947	Detected	Detected	Detected	Detected
4	A_24_P358131					38.94524	32.88369	10.21547	15.48231	Detected	Detected	Compromised	Compromised
5	A_23_P157316	C7orf34	Hs.239676	chromosome 7 open reading frame 34	Homo sapiens chromosome 7 open read	27.91531	38.73615	21.25393	11.25149	Detected	Detected	Detected	Compromised
6	A_32_P14850		Hs.448833		Nuclear pore complex-interacting prote	18239.03000	15742.11500	17450.29000	12535.06400	Detected	Detected	Detected	Detected
7	A_23_P158596	AGTRAP	Hs.464438	angiotensin II receptor-associated prote	Homo sapiens angiotensin II receptor-a	383.20514	317.61752	405.77480	308.25910	Detected	Detected	Detected	Detected
8	A_23_P350107	TRIM56	Hs.521092	tripartite motif-containing 56	Homo sapiens tripartite motif-containin	1870.74500	1520.12870	1724.65310	1275.22070	Detected	Detected	Detected	Detected
9	A_23_P388190	DIDO1	Hs.517172	death inducer-obliterator 1	Homo sapiens death inducer-obliterator	606.78107	746.48140	521.14780	532.82715	Detected	Detected	Detected	Detected
10	A_23_P106544	C16orf61	Hs.388255	chromosome 16 open reading frame 61	Homo sapiens chromosome 16 open rea	4613.91550	9466.83100	3846.75880	6727.16160	Detected	Detected	Detected	Detected
11	A_32_P85539	HCFC2	Hs.506558	host cell factor C2	Homo sapiens host cell factor C2 (HCF	1616.85290	569.75415	1009.51820	282.84120	Detected	Detected	Detected	Detected
12	A_23_P94998	LETM1	Hs.120165	leucine zipper-EF-hand containing tran	Homo sapiens leucine zipper-EF-hand c	675.00530	2295.22680	774.48865	2296.65360	Detected	Detected	Detected	Detected
13	A_33_P3235677					36.93503	3.79673	31.93821	3.41786	Detected	Compromised	Detected	Compromised
14	A_23_P103905	UFC1	Hs.301412	ubiquitin-fold modifier conjugating enzy	Homo sapiens ubiquitin-fold modifier co	1963.77260	3080.51900	1925.38160	2807.42600	Detected	Detected	Detected	Detected
15	A_24_P497186	IRF2BP2	Hs.350268	interferon regulatory factor 2 binding pr	Homo sapiens interferon regulatory fact	1455.00390	1155.36680	1053.70260	714.98944	Detected	Detected	Detected	Detected
16	A_23_P118536	SLFN12	Hs.663548	schlafen family member 12	Homo sapiens schlafen family member 1	166.22006	333.26028	116.15559	206.98546	Detected	Detected	Detected	Detected
17	A_23_P434289	GPR62	Hs.232213	G protein-coupled receptor 62	Homo sapiens G protein-coupled recep	133.15550	66.85347	105.19013	41.04968	Detected	Detected	Detected	Detected
18	A_33_P3326898					497.45220	1115.87780	171.67195	333.41410	Detected	Detected	Detected	Detected
19	A_24_P67898		Hs.500842		Bifunctional protein NCOAT (Nuclear cy	3014.84080	2208.58230	1384.95850	798.38180	Detected	Detected	Detected	Detected
20	A_24_P28657	AHCTF1	Hs.300887	AT hook containing transcription factor	Homo sapiens AT hook containing trans	1268.80160	1246.25400	1193.54550	1022.65520	Detected	Detected	Detected	Detected
21	A_33_P3344292	LOC644925	Hs.680372	hypothetical LOC644925	PREDICTED: Homo sapiens hypothetica	33.93909	57.96402	4.79489	23.41600	Detected	Detected	Compromised	Detected
22	A_33_P3317618	SYN2	Hs.445503	synapsin II	Homo sapiens synapsin II (SYN2), trans	113.04173	38.31403	74.20045	20.90509	Detected	Detected	Detected	Detected
23	A_23_P2873	KLC1	Hs.20107	kinesin light chain 1	Homo sapiens kinesin light chain 1 (KLC	4327.02200	8586.78600	3702.86300	6769.10160	Detected	Detected	Detected	Detected
24	A_23_P171095	USP27X	Hs.143587	ubiquitin specific peptidase 27, X-linked	Homo sapiens ubiquitin specific peptida	39.39882	18.38253	13.24209	5.01283	Detected	Compromised	Compromised	Compromised
25	A_33_P3223631		Hs.721481		PREDICTED: Homo sapiens similar to h	722.89703	1068.55480	307.83838	323.97760	Detected	Detected	Detected	Detected
26	A_23_P213014	SLC2A9	Hs.656895	solute carrier family 2 (facilitated gluco	Homo sapiens solute carrier family 2 (fa	55.15659	20.40927	38.86599	13.71653	Detected	Detected	Detected	Compromised
27	A_23_P256455	RPA3	Hs.487540	replication protein A3, 14kDa	Homo sapiens replication protein A3, 14	6412.85640	10356.60450	5242.56150	7045.41400	Detected	Detected	Detected	Detected
28	A_33_P3217009	LOC154860	Hs.722224	hypothetical protein LOC154860	PREDICTED: Homo sapiens hypothetica	274.85696	301.52615	110.00894	120.64180	Detected	Detected	Detected	Detected
29	A_23_P112846	MTHFD2L	Hs.479954	methylenetetrahydrofolate dehydrogena	Homo sapiens methylenetetrahydrofolat	61.77427	31.95139	27.87302	13.79247	Detected	Detected	Detected	Detected
30	A_23_P31135	ACAT2	Hs.571037	acetyl-Coenzyme A acetyltransferase 2	Homo sapiens acetyl-Coenzyme A acet	9296.85800	9503.29700	6892.36770	5979.56600	Detected	Detected	Detected	Detected
31	A_23_P332509	NBAS	Hs.467759	neuroblastoma amplified sequence	Homo sapiens neuroblastoma amplified	1744.19300	1523.23890	1591.78500	1235.15490	Detected	Detected	Detected	Detected
32	A_23_P110266	C4orf31	Hs.709520	chromosome 4 open reading frame 31	Homo sapiens chromosome 4 open read	21.75164	93.39444	8.78769	43.81885	Detected	Detected	Compromised	Detected
33	A_33_P3403053					114.81238	4.12402	101.79661	3.72467	Detected	Compromised	Detected	Compromised
34	A_23_P105138	CAT	Hs.502302	catalase	Homo sapiens catalase (CAT), mRNA [N	2159.42330	1586.89650	2039.66270	1263.32320	Detected	Detected	Detected	Detected
35	A_23_P144264	TOMM70A	Hs.227253	translocase of outer mitochondrial mem	Homo sapiens translocase of outer mitc	762.01780	833.20685	574.03700	496.85934	Detected	Detected	Detected	Detected
36	A_23_P100344	ORC6L	Hs.49760	origin recognition complex, subunit 6 lik	Homo sapiens origin recognition comple	193.56079	2001.85140	157.03879	1461.40050	Detected	Detected	Detected	Detected
37	A_23_P162486	PTPN6	Hs.63489	protein tyrosine phosphatase, non-rece	Homo sapiens protein tyrosine phospha	26.10123	32.68503	17.01707	17.01764	Detected	Detected	Detected	Detected
38	A_23_P103104	MFNG	Hs.517603	MFNG O-fucosylpeptide 3-beta-N-ace	Homo sapiens MFNG O-fucosylpeptide	93.21178	93.62312	25.68426	19.19042	Detected	Detected	Detected	Detected
39	A_23_P1170	ANUBL1	Hs.89029	AN1, ubiquitin-like, homolog (Xenopus la	Homo sapiens AN1, ubiquitin-like, homo	104.87665	72.50406	77.12078	42.08402	Detected	Detected	Detected	Detected
40	A_23_P169017	DEFB103A	Hs.283082	defensin, beta 103A	Homo sapiens defensin, beta 103A (DEF	13.66321	33.46262	3.78317	17.31846	Compromised	Detected	Compromised	Compromised
41	A_32_P815507	LOC100130920	Hs.683904	hypothetical protein LOC100130920	Homo sapiens cDNA FLJ36320 fis, clon	24.00464	37.24003	31.49851	33.63567	Detected	Detected	Detected	Detected
42	A_33_P3785051		Hs.719432		EF-hand domain-containing protein LO	36.41463	62.84164	5.81634	14.29968	Detected	Detected	Compromised	Compromised
43	A_23_P102950	RSPH1	Hs.661069	radial spoke head 1 homolog (Chlamydo	Homo sapiens radial spoke head 1 homo	39.55026	21.19944	22.31084	3.52550	Detected	Detected	Detected	Compromised
44	A_23_P102517	PDE6D	Hs.516808	phosphodiesterase 6D, cGMP-specific,	Homo sapiens phosphodiesterase 6D, c	1507.63870	1051.19810	1090.53710	657.78280	Detected	Detected	Detected	Detected
45	A_24_P630490	DFNB59	Hs.87734	deafness, autosomal recessive 59	Homo sapiens deafness, autosomal rece	308.57205	66.14830	299.01940	43.24982	Detected	Detected	Detected	Detected
46	A_33_P3288969	LOC100128507	Hs.711892	similar to 6.8 kDa mitochondrial proteoli	PREDICTED: Homo sapiens similar to 6	44.12194	39.21046	3.79028	3.82034	Detected	Detected	Compromised	Compromised
47	A_32_P196263	ADAMTS9	Hs.656071	ADAM metallopeptidase with thrombosp	Homo sapiens ADAM metallopeptidase	202.86755	29.63640	131.94072	10.67120	Detected	Detected	Detected	Compromised
48	A_33_P3225690	ZNF516	Hs.709890	zinc finger protein 516	Homo sapiens zinc finger protein 516 (Z	897.14800	1970.54610	671.33636	1281.59000	Detected	Detected	Detected	Detected
49	A_24_P277934	COL1A2	Hs.489142	collagen, type I, alpha 2	Homo sapiens collagen, type I, alpha 2 (	68040.57000	199252.80000	52074.42000	132084.42000	Detected	Detected	Detected	Detected
50	A_23_P13914	DHX37	Hs.107382	DEAH (Asp-Glu-Ala-His) box polypepti	Homo sapiens DEAH (Asp-Glu-Ala-His)	1697.20800	1725.99320	1878.34580	1787.53610	Detected	Detected	Detected	Detected
51	A_23_P131089	KANK3	Hs.322473	KN motif and ankyrin repeat domains 3	Homo sapiens KN motif and ankyrin rep	620.36920	129.67040	651.26260	108.81519	Detected	Detected	Detected	Detected
52	A_23_P101521	C19orf41	Hs.414175	chromosome 19 open reading frame 41	Homo sapiens chromosome 19 open rea	9.57179	21.59066	4.23034	3.88139	Compromised	Detected	Compromised	Compromised
53	A_33_P3695548	POLE	Hs.524871	polymerase (DNA directed), epsilon	Homo sapiens polymerase (DNA directe	212.75995	407.65518	161.05402	320.00740	Detected	Detected	Detected	Detected
54	A_33_P3266889					366.57760	124.83607	120.72449	32.51704	Detected	Detected	Detected	Detected
55	A_33_P3775741	RPL32P3	Hs.652423	ribosomal protein L32 pseudogene 3	Homo sapiens ribosomal protein L32 ps	19171.25200	14369.90000	15244.39550	10034.64400	Detected	Detected	Detected	Detected
56	A_23_P163113	PRPF39	Hs.274337	PRP39 pre-mRNA processing factor 39	Homo sapiens PRP39 pre-mRNA proce	1314.23050	789.49660	1085.79750	577.80560	Detected	Detected	Detected	Detected
57	A_23_P51587	RGS7	Hs.655739	regulator of G-protein signaling 7	Homo sapiens regulator of G-protein si	103.42441	3.77266	79.21559	3.38741	Detected	Compromised	Detected	Compromised
58	A_33_P3338788					25.11048	25.96995	12.98912	10.11608	Detected	Detected	Compromised	Compromised
59	A_23_P120557	TASP1	Hs.348297	taspase, threonine aspartase, 1	Homo sapiens taspase, threonine aspar	553.07965	448.48712	391.89948	273.81747	Detected	Detected	Detected	Detected
60	A_24_P413669	PFKFB2	Hs.282702	6-phosphofructo-2-kinase/fructose-2,6	Homo sapiens 6-phosphofructo-2-kinas	30.80143	8.49458	15.60664	3.40284	Detected	Compromised	Compromised	Compromised
61	A_33_P3367615	KLF6	Hs.4055	Kruppel-like factor 6	Homo sapiens Kruppel-like factor 6 (KL	30.12388	37.92067	15.58878	18.18893	Detected	Detected	Compromised	Detected
62	A_33_P3379886	FGF2	Hs.284244	fibroblast growth factor 2 (basic)	Homo sapiens fibroblast growth factor 2	1263.73830	1800.41600	242.74430	190.57625	Detected	Detected	Detected	Detected
63	A_24_P915007	NACC1	Hs.531614	nucleus accumbens associated 1, BEN	Homo sapiens nucleus accumbens asso	6437.30760	15972.20500	4971.10300	9740.92900	Detected	Detected	Detected	Detected
64	A_23_P53057	ZNF215	Hs.523457	zinc finger protein 215	Homo sapiens zinc finger protein 215 (Z	593.00116	687.10645	454.91840	438.72006	Detected	Detected	Detected	Detected
65	A_23_P49338	TNFRSF12A	Hs.355899	tumor necrosis factor receptor superfa	Homo sapiens tumor necrosis factor re	18841.79500	62919.28000	19337.05500	57211.10000	Detected	Detected	Detected	Detected
66	A_23_P371266	DNM3	Hs.654775	dynamin 3	Homo sapiens dynamin 3 (DNM3), trans	382.21167	188.32277	300.72693	107.03173	Detected	Detected	Detected	Detected
67	A_23_P200874	CCDC21	Hs.63795	coiled-coil domain containing 21	Homo sapiens coiled-coil domain contai	161.21939	199.32555	154.29295	182.56364	Detected	Detected	Detected	Detected
68	A_23_P11262	F8A1	Hs.533543	coagulation factor VIII-associated (intr	Homo sapiens coagulation factor VIII-as	10490.38600	11562.22500	9664.66800	9812.93800	Detected	Detected	Detected	Detected
69	A_23_P103511	C1orf226	Hs.447011	chromosome 1 open reading frame 226	Homo sapiens chromosome 1 open read	73.44186	81.18276	30.47147	21.94553	Detected	Detected	Detected	Detected
70	A_24_P89080	DCK	Hs.709	deoxycytidine kinase	Homo sapiens deoxycytidine kinase (DC	255.18234	600.77160	151.52339	257.75850	Detected	Detected	Detected	Detected
71	A_23_P157607	INTS10	Hs.512627	integrator complex subunit 10	Homo sapiens integrator complex subur	13873.02200	5951.35100	13738.12300	5040.64200	Detected	Detected	Detected	Detected

72	A_23_P145777	NDUFA4	Hs.50098	NADH dehydrogenase (ubiquinone) 1 alp	Homo sapiens NADH dehydrogenase (ub	43067.13000	40436.36700	42536.23400	33783.27700	Detected	Detected	Detected	Detected
73	A_23_P107693	ZNF586	Hs.705652	zinc finger protein 586	Homo sapiens zinc finger protein 586 (Z	62.73046	112.97427	70.56735	99.87886	Detected	Detected	Detected	Detected
74	A_23_P160828	C1orf159	Hs.235095	chromosome 1 open reading frame 159	Homo sapiens chromosome 1 open read	334.55417	338.89276	328.08383	311.91174	Detected	Detected	Detected	Detected
75	A_23_P120237	STARD7	Hs.469331	StAR-related lipid transfer (START) do	Homo sapiens StAR-related lipid transf	11558.66300	17915.84200	7934.47170	10781.01900	Detected	Detected	Detected	Detected
76	A_23_P99360	TRIM13	Hs.436922	tripartite motif-containing 13	Homo sapiens tripartite motif-containin	1300.43030	358.35098	1649.46260	294.07470	Detected	Detected	Detected	Detected
77	A_33_P3329339	LOC644990	Hs.631912	hypothetical LOC644990	PREDICTED: Homo sapiens hypothetica	123.95170	22.94157	113.72761	10.44829	Detected	Detected	Detected	Compromised
78	A_32_P34876	WDR93	Hs.177557	WD repeat domain 93	Homo sapiens WD repeat domain 93 (W	1152.25180	1058.03150	365.50134	275.53113	Detected	Detected	Detected	Detected
79	A_23_P112220	INSL4	Hs.418506	insulin-like 4 (placenta)	Homo sapiens insulin-like 4 (placenta) (	2.84537	4.55091	3.25192	14.49325	Compromised	Compromised	Compromised	Detected
80	A_32_P210642	EGFL7	Hs.91481	EGF-like-domain, multiple 7	Homo sapiens EGF-like-domain, multipl	1151.68600	634.69070	1178.89650	485.46405	Detected	Detected	Detected	Detected
81	A_32_P50123	SRGAP2	Hs.497575	SLIT-ROBO Rho GTPase activating pro	Homo sapiens SLIT-ROBO Rho GTPase	26.73210	35.15131	18.43379	25.60389	Detected	Detected	Detected	Detected
82	A_23_P114134	SSX3	Hs.558445	synovial sarcoma, X breakpoint 3	Homo sapiens synovial sarcoma, X brea	14.53879	20.26415	7.83031	6.17704	Detected	Detected	Compromised	Compromised
83	A_23_P10995	RBMS3	Hs.696468	RNA binding motif, single stranded inter	Homo sapiens RNA binding motif, single	355.86197	350.62730	50.01029	46.77221	Detected	Detected	Detected	Detected
84	A_33_P3315314	MT1H	Hs.438462	metallothionein 1H	Homo sapiens metallothionein 1H (MT1H	87825.70000	140101.16000	133314.58000	167274.08000	Detected	Detected	Detected	Detected
85	A_23_P120414	YWHAB	Hs.643544	tyrosine 3-monooxygenase/tryptophan	Homo sapiens tyrosine 3-monooxygena	29735.98800	33212.41400	20501.37300	18813.03100	Detected	Detected	Detected	Detected
86	A_23_P9280	LOC389765	Hs.601256	kinesin family member 27 pseudogene	Homo sapiens kinesin family member 27	371.47488	95.04108	216.14198	39.41015	Detected	Detected	Detected	Detected
87	A_32_P223210	C7orf45	Hs.351816	chromosome 7 open reading frame 45	Homo sapiens chromosome 7 open read	25.53161	6.63309	30.59643	3.72938	Detected	Compromised	Detected	Compromised
88	A_23_P104146	ZMYM4	Hs.269211	zinc finger, MYM-type 4	Homo sapiens zinc finger, MYM-type 4	752.12450	736.23334	499.56845	389.13090	Detected	Detected	Detected	Detected
89	A_23_P122228	NDUFS6	Hs.408257	NADH dehydrogenase (ubiquinone) Fe-S	Homo sapiens NADH dehydrogenase (ub	14056.52700	15592.48300	11564.07400	11178.60100	Detected	Detected	Detected	Detected
90	A_23_P213518	CAST	Hs.436186	calpastatin	Homo sapiens calpastatin (CAST), trans	32890.22300	23140.53500	22608.11100	13006.82000	Detected	Detected	Detected	Detected
91	A_33_P3406939	KIF24	Hs.710246	kinesin family member 24	Homo sapiens kinesin family member 24	25.42756	268.34540	5.73247	114.85307	Detected	Detected	Compromised	Detected
92	A_23_P77160	RNF111	Hs.404423	ring finger protein 111	Homo sapiens ring finger protein 111 (R	1565.72340	1271.72050	1131.48890	766.77260	Detected	Detected	Detected	Detected
93	A_33_P3388745	LOC100132207	Hs.633570	hypothetical LOC100132207	Homo sapiens cDNA FLJ41345 fis, clon	163.23900	243.72630	71.90201	72.90302	Detected	Detected	Detected	Detected
94	A_23_P331598	IPO7	Hs.523470	importin 7	Homo sapiens importin 7 (IPO7), mRNA	1556.99700	1661.80240	1673.05380	1310.42820	Detected	Detected	Detected	Detected
95	A_33_P3372297	ABR	Hs.159306	active BCR-related gene	Homo sapiens active BCR-related gene	101.10068	47.51266	92.58960	40.05791	Detected	Detected	Detected	Detected
96	A_33_P3284586	FBXL8	Hs.710714	F-box and leucine-rich repeat protein 8	Homo sapiens F-box and leucine-rich re	1109.19290	834.88230	1046.96030	723.60890	Detected	Detected	Detected	Detected
97	A_23_P118493	TOM1L1	Hs.153504	target of myb1 (chicken)-like 1	Homo sapiens target of myb1 (chicken)-	671.71770	482.24512	550.84920	313.44376	Detected	Detected	Detected	Detected
98	A_23_P100203	HSBP1	Hs.250899	heat shock factor binding protein 1	Homo sapiens heat shock factor binding	6321.13130	6116.29200	4707.03700	3773.30440	Detected	Detected	Detected	Detected
99	A_33_P3343106	ETV2	Hs.194061	ets variant 2	Homo sapiens ets variant 2 (ETV2), mR	561.07680	546.80730	492.48250	403.02860	Detected	Detected	Detected	Detected
100	A_23_P355455	TBC1D5	Hs.475629	TBC1 domain family, member 5	Homo sapiens TBC1 domain family, mem	449.66266	285.69348	437.17240	229.63182	Detected	Detected	Detected	Detected
101	A_23_P109345	PTTG1IP	Hs.474010	pituitary tumor-transforming 1 interacti	Homo sapiens pituitary tumor-transform	10179.49500	22835.09000	9968.43750	19840.63700	Detected	Detected	Detected	Detected
102	A_24_P381136	PACSIN3	Hs.334639	protein kinase C and casein kinase subs	Homo sapiens protein kinase C and cas	1187.30600	484.53012	1297.02200	560.73750	Detected	Detected	Detected	Detected
103	A_23_P106405	NDN	Hs.50130	necdin homolog (mouse)	Homo sapiens necdin homolog (mouse)	4478.53120	1223.63540	3049.49000	681.19244	Detected	Detected	Detected	Detected
104	A_23_P370097	ALS2CR4	Hs.12319	amyotrophic lateral sclerosis 2 (juvenile	Homo sapiens amyotrophic lateral scler	1122.66320	2501.33840	1174.52500	2031.89250	Detected	Detected	Detected	Detected
105	A_23_P118254	FOXF1	Hs.155591	forkhead box F1	Homo sapiens forkhead box F1 (FOXF1)	79.43672	504.11752	46.29013	276.16678	Detected	Detected	Detected	Detected
106	A_23_P70998		Hs.652371		cDNA FLJ54628HCG19535Hypothetical	1315.94730	784.14720	1103.89990	498.49240	Detected	Detected	Detected	Detected
107	A_23_P109055	TP53RK	Hs.440263	TP53 regulating kinase	Homo sapiens TP53 regulating kinase (T	138.54453	120.36256	97.41026	79.36237	Detected	Detected	Detected	Detected
108	A_23_P214211	RARS2	Hs.485910	arginyl-tRNA synthetase 2, mitochondri	Homo sapiens arginyl-tRNA synthetase	26120.35400	27882.78300	23415.64800	21330.49000	Detected	Detected	Detected	Detected
109	A_23_P106505	LCMT2	Hs.720397	leucine carboxyl methyltransferase 2	Homo sapiens leucine carboxyl methyltr	603.38043	1181.67160	429.01193	655.74920	Detected	Detected	Detected	Detected
110	A_24_P158089	SERPINE1	Hs.414795	serpin peptidase inhibitor, clade E (nexi	Homo sapiens serpin peptidase inhibitor	3734.19600	26512.47300	3013.17300	18273.82000	Detected	Detected	Detected	Detected
111	A_23_P56787	CNTNAP5	Hs.660653	contactin associated protein-like 5	Homo sapiens contactin associated pro	84.01149	146.23239	34.74711	43.58251	Detected	Detected	Detected	Detected
112	A_23_P120048	BAZ2B	Hs.470369	bromodomain adjacent to zinc finger do	Homo sapiens bromodomain adjacent to	282.03073	203.38553	170.08627	101.64353	Detected	Detected	Detected	Detected
113	A_33_P3301445					1331.43480	1365.16420	1261.86930	1087.59190	Detected	Detected	Detected	Detected
114	A_23_P215214	LMBR1	Hs.209989	limb region 1 homolog (mouse)	Homo sapiens limb region 1 homolog (m	425.36660	437.77880	240.91410	192.56967	Detected	Detected	Detected	Detected
115	A_23_P45365	COL4A5	Hs.369089	collagen, type IV, alpha 5	Homo sapiens collagen, type IV, alpha 5	848.75995	146.56627	580.25070	102.26360	Detected	Detected	Detected	Detected
116	A_23_P250800	ST3GAL6	Hs.148716	ST3 beta-galactoside alpha-2,3-sialyltr	Homo sapiens ST3 beta-galactoside alp	51.48545	18.63228	39.71555	7.25721	Detected	Detected	Detected	Compromised
117	A_23_P321160	ZNF594	Hs.658402	zinc finger protein 594	Homo sapiens zinc finger protein 594 (Z	127.00730	131.66238	106.28617	72.91481	Detected	Detected	Detected	Detected
118	A_23_P134454	CAV1	Hs.74034	caveolin 1, caveolae protein, 22kDa	Homo sapiens caveolin 1, caveolae prot	20129.15400	97452.82000	13978.40500	56212.50800	Detected	Detected	Detected	Detected
119	A_23_P217475	IDS	Hs.460960	iduronate 2-sulfatase	Homo sapiens iduronate 2-sulfatase (ID	12132.33300	4314.56640	9169.97200	2594.18120	Detected	Detected	Detected	Detected
120	A_23_P89509	SPAG5	Hs.514033	sperm associated antigen 5	Homo sapiens sperm associated antigen	536.76495	15264.78500	436.38416	11749.24800	Detected	Detected	Detected	Detected
121	A_23_P253524	CENPE	Hs.75573	centromere protein E, 312kDa	Homo sapiens centromere protein E, 31	281.99063	3350.53000	274.09690	3089.85570	Detected	Detected	Detected	Detected
122	A_23_P114405	MORF4L2	Hs.326387	mortality factor 4 like 2	Homo sapiens mortality factor 4 like 2 (	8575.57100	18026.60700	6524.99700	11338.49600	Detected	Detected	Detected	Detected
123	A_33_P3409508	MAPK11	Hs.57732	mitogen-activated protein kinase 11	Homo sapiens mitogen-activated protei	400.08008	369.72815	332.67657	245.05434	Detected	Detected	Detected	Detected
124	A_23_P254353	NOXA1	Hs.495554	NADPH oxidase activator 1	Homo sapiens NADPH oxidase activato	131.27791	24.45296	184.75035	43.78117	Detected	Detected	Detected	Detected
125	A_23_P102391	SLC40A1	Hs.643005	solute carrier family 40 (iron-regulated	Homo sapiens solute carrier family 40 (i	163.22023	4.39595	118.72677	3.50902	Detected	Compromised	Detected	Compromised
126	A_23_P56810	SLC4A1AP	Hs.306000	solute carrier family 4 (anion exchanger	Homo sapiens solute carrier family 4 (an	2439.86300	2737.26780	2339.96200	2116.62520	Detected	Detected	Detected	Detected
127	A_23_P124164	ISY1	Hs.512661	ISY1 splicing factor homolog (S. cerevis	Homo sapiens ISY1 splicing factor homo	974.44450	1066.23200	620.53040	595.75543	Detected	Detected	Detected	Detected
128	A_23_P98431	HMBS	Hs.82609	hydroxymethylbilane synthase	Homo sapiens hydroxymethylbilane synt	13358.95600	17343.03100	14205.19700	16156.63700	Detected	Detected	Detected	Detected
129	A_23_P51397	ENAH	Hs.497893	enabled homolog (Drosophila)	Homo sapiens enabled homolog (Drosop	5975.50800	11088.65800	3879.18380	5857.63200	Detected	Detected	Detected	Detected
130	A_23_P108554	DDX1	Hs.440599	DEAD (Asp-Glu-Ala-Asp) box polypept	Homo sapiens DEAD (Asp-Glu-Ala-Asp	29074.92800	31296.07000	23857.32600	22628.62500	Detected	Detected	Detected	Detected
131	A_33_P3372788	NBPF15	Hs.515947	neuroblastoma breakpoint family, memb	Homo sapiens neuroblastoma breakpoin	354.08286	353.82617	213.44414	180.16720	Detected	Detected	Detected	Detected
132	A_24_P122732	SLC41A1	Hs.20274	solute carrier family 41, member 1	Homo sapiens solute carrier family 41, r	1284.98910	1474.70600	1220.56400	1058.08950	Detected	Detected	Detected	Detected
133	A_33_P3269864	LOC284009	Hs.632244	hypothetical protein LOC284009	Homo sapiens hypothetical protein LOC	28.50096	26.99078	13.21819	11.56046	Detected	Detected	Compromised	Compromised
134	A_23_P211504	KDELR3	Hs.720434	KDEL (Lys-Asp-Glu-Leu) endoplasmic	Homo sapiens KDEL (Lys-Asp-Glu-Leu	3830.99760	7122.68950	3529.61350	4744.64300	Detected	Detected	Detected	Detected
135	A_23_P114095	MBTPS2	Hs.443490	membrane-bound transcription factor p	Homo sapiens membrane-bound transcr	52.78520	96.02151	39.97866	64.93119	Detected	Detected	Detected	Detected
136	A_33_P3424577		Hs.722995		Homo sapiens clone J3N.4 T cell recept	200.56895	251.46222	67.85310	71.86373	Detected	Detected	Detected	Detected
137	A_33_P3362567					667.92620	670.64980	261.00507	221.80353	Detected	Detected	Detected	Detected
138	A_33_P3323607				Coiled-coil domain-containing protein 1	200.76161	240.81848	97.26218	92.90617	Detected	Detected	Detected	Detected
139	A_23_P374902	CLDND2	Hs.720536	claudin domain containing 2	Homo sapiens claudin domain containing	20.31547	41.67067	8.64128	31.12904	Detected	Detected	Compromised	Detected
140	A_33_P3291877	ARID1B	Hs.291587	AT rich interactive domain 1B (SWI1-lik	Homo sapiens AT rich interactive doma	842.11340	1086.42930	407.97570	405.93338	Detected	Detected	Detected	Detected
141	A_23_P24997	CDK4	Hs.95577	cyclin-dependent kinase 4	Homo sapiens cyclin-dependent kinase	6099.91940	5577.28000	6660.33840	5247.21800	Detected	Detected	Detected	Detected
142	A_23_P48088	CD27	Hs.355307	CD27 molecule	Homo sapiens CD27 molecule (CD27), r	42.75664	65.42853	45.26162	58.67195	Detected	Detected	Detected	Detected
143	A_23_P103149	ACO2	Hs.643610	aconitase 2, mitochondrial	Homo sapiens aconitase 2, mitochondria	7714.12160	9364.40200	6106.24950	6582.92530	Detected	Detected	Detected	Detected
144	A_23_P110492	MARCH6	Hs.432862	membrane-associated ring finger (C3HC	Homo sapiens membrane-associated rin	2207.17380	1049.18980	1223.10000	476.82025	Detected	Detected	Detected	Detected
145	A_23_P20876	PTPDC1	Hs.437943	protein tyrosine phosphatase domain co	Homo sapiens protein tyrosine phospha	661.28930	658.09546	540.68726	458.52167	Detected	Detected	Detected	Detected
146	A_33_P3234222	TSPO2	Hs.357392	translocator protein 2	Homo sapiens translocator protein 2 (T	1853.08690	10103.76000	609.54254	3096.64820	Detected	Detected	Detected	Detected

147	A_33_P3354569	GPD2	Hs.512382	glycerol-3-phosphate dehydrogenase 2	Homo sapiens glycerol-3-phosphate de	4125.64100	1630.68430	2954.59500	1007.78326	Detected	Detected	Detected	Detected
148	A_24_P132470	ESYT2	Hs.490795	extended synaptotagmin-like protein 2	Homo sapiens extended synaptotagmin-	5697.10100	3609.18460	4821.05200	2594.07300	Detected	Detected	Detected	Detected
149	A_33_P3248629		Hs.6385		DENN domain-containing protein 2A [S	30.60560	27.98837	17.24825	12.70303	Detected	Detected	Compromised	Compromised
150	A_24_P281988	PLCL2	Hs.202010	phospholipase C-like 2	Homo sapiens phospholipase C-like 2 (F	715.88900	194.89008	537.98160	117.16563	Detected	Detected	Detected	Detected
151	A_24_P410086	SSBP4	Hs.515259	single stranded DNA binding protein 4	Homo sapiens single stranded DNA binc	8295.20900	5512.23970	6073.36430	3159.68820	Detected	Detected	Detected	Detected
152	A_33_P3235454					11711.36900	7201.85940	508.86542	196.84091	Detected	Detected	Detected	Detected
153	A_23_P37778	FHOD1	Hs.95231	formin homology 2 domain containing 1	Homo sapiens formin homology 2 domai	3308.83620	5935.24300	3857.19100	5984.48100	Detected	Detected	Detected	Detected
154	A_33_P3409513	MAPK11	Hs.57732	mitogen-activated protein kinase 11	Homo sapiens mitogen-activated protei	1659.31380	911.79050	2082.68240	1132.84200	Detected	Detected	Detected	Detected
155	A_24_P200162	HIGD1A	Hs.719119	HIG1 hypoxia inducible domain family, m	Homo sapiens HIG1 hypoxia inducible d	15586.65000	23699.48200	15793.98100	20435.83200	Detected	Detected	Detected	Detected
156	A_23_P5983	PLTP	Hs.439312	phospholipid transfer protein	Homo sapiens phospholipid transfer pro	3554.11060	451.75687	4003.48320	401.05980	Detected	Detected	Detected	Detected
157	A_23_P386254	NKX3-2	Hs.590927	NK3 homeobox 2	Homo sapiens NK3 homeobox 2 (NKX3-	16.44924	3.77345	3.33947	3.38622	Detected	Compromised	Compromised	Compromised
158	A_23_P409516	FAM122A	Hs.655572	family with sequence similarity 122A	Homo sapiens family with sequence sim	1227.72780	1594.31240	934.25650	948.24080	Detected	Detected	Detected	Detected
159	A_33_P3270404	ATG9A	Hs.323363	ATG9 autophagy related 9 homolog A (S	Homo sapiens ATG9 autophagy related	2303.43000	2346.89720	2375.85100	2323.57540	Detected	Detected	Detected	Detected
160	A_24_P882732		Hs.579266		cDNA FLJ61294, highly similar to Kerat	2.76101	42.33516	3.36381	43.83626	Compromised	Detected	Compromised	Detected
161	A_23_P128613	KDELC1	Hs.408629	KDEL (Lys-Asp-Glu-Leu) containing 1	Homo sapiens KDEL (Lys-Asp-Glu-Leu	1403.32980	1808.61680	895.15700	934.23790	Detected	Detected	Detected	Detected
162	A_33_P3328883	HGC6.3	Hs.448059	similar to HGC6.3	Homo sapiens similar to HGC6.3 (HGC6	2.76951	30.61433	3.37365	10.52219	Compromised	Detected	Compromised	Compromised
163	A_33_P3349384					157.14082	75.30514	4.31198	4.31844	Detected	Detected	Compromised	Compromised
164	A_23_P114656	HDAC1	Hs.88556	histone deacetylase 1	Homo sapiens histone deacetylase 1 (H	8041.79000	8951.84800	7358.90300	7247.70200	Detected	Detected	Detected	Detected
165	A_33_P3253365		Hs.658328		Contactin-associated protein-like 3B P	94.49448	125.05528	68.27509	63.55217	Detected	Detected	Detected	Detected
166	A_33_P3230478	C1S	Hs.458355	complement component 1, s subcompor	Homo sapiens complement component	3915.60230	528.34470	3311.75100	412.44962	Detected	Detected	Detected	Detected
167	A_23_P102202	MSH6	Hs.445052	mutS homolog 6 (E. coli)	Homo sapiens mutS homolog 6 (E. coli)	13264.38000	9478.57800	9780.13100	6114.48540	Detected	Detected	Detected	Detected
168	A_33_P3349036	LOC100133612	Hs.307652	similar to hCG1815312	Homo sapiens similar to hCG1815312 (L	33.16634	20.79912	6.94871	3.45970	Detected	Detected	Compromised	Compromised
169	A_33_P3388618	TNK1	Hs.203420	tyrosine kinase, non-receptor, 1	Homo sapiens tyrosine kinase, non-rece	233.16905	302.00510	135.85812	73.86742	Detected	Detected	Detected	Detected
170	A_23_P36397	CYP27B1	Hs.524528	cytochrome P450, family 27, subfamily B	Homo sapiens cytochrome P450, family	36.47665	50.42074	32.60569	40.25559	Detected	Detected	Detected	Detected
171	A_23_P8083	LY6G6C	Hs.241586	lymphocyte antigen 6 complex, locus G6	Homo sapiens lymphocyte antigen 6 cor	131.99780	168.14409	29.68471	37.61633	Detected	Detected	Detected	Detected
172	A_32_P212471	C1orf230	Hs.297967	chromosome 1 open reading frame 230	Homo sapiens chromosome 1 open reac	76.72168	198.61569	59.04790	129.42210	Detected	Detected	Detected	Detected
173	A_33_P3215768	GALNT6	Hs.505575	UDP-N-acetyl-alpha-D-galactosamine:	Homo sapiens UDP-N-acetyl-alpha-D-	3431.40820	2265.09250	3150.09900	1895.68570	Detected	Detected	Detected	Detected
174	A_23_P206441	FANCA	Hs.719210	Fanconi anemia, complementation group	Homo sapiens Fanconi anemia, complen	193.04425	527.31616	146.39120	336.32297	Detected	Detected	Detected	Detected
175	A_23_P153037	ZNF624	Hs.128078	zinc finger protein 624	Homo sapiens zinc finger protein 624 (Z	207.14868	140.14551	202.71689	104.31908	Detected	Detected	Detected	Detected
176	A_33_P3310552		Hs.654630		Formin-like protein 2 (Formin homology	141.73060	52.16145	128.23239	39.52482	Detected	Detected	Detected	Detected
177	A_24_P151498	PRMT2	Hs.154163	protein arginine methyltransferase 2	Homo sapiens protein arginine methyltra	1086.87110	3484.48410	1271.60610	3690.02500	Detected	Detected	Detected	Detected
178	A_23_P113777	ITGBL1	Hs.696554	integrin, beta-like 1 (with EGF-like repe	Homo sapiens integrin, beta-like 1 (with	965.24610	6529.00930	656.64970	3803.54860	Detected	Detected	Detected	Detected
179	A_33_P3237977	LARP6	Hs.416755	La ribonucleoprotein domain family, mer	Homo sapiens La ribonucleoprotein dom	17275.53500	20470.02300	13098.91100	13030.03900	Detected	Detected	Detected	Detected
180	A_23_P1102	ACTA1	Hs.1288	actin, alpha 1, skeletal muscle	Homo sapiens actin, alpha 1, skeletal mu	15.13551	16.06805	13.77614	7.70394	Detected	Detected	Detected	Compromised
181	A_24_P381975	TMEM231	Hs.156784	transmembrane protein 231	Homo sapiens transmembrane protein 2	63.02515	66.42916	55.11193	46.86706	Detected	Detected	Detected	Detected
182	A_23_P102412	PLEKHA3	Hs.41086	pleckstrin homology domain containing,	Homo sapiens pleckstrin homology dom	3413.59180	3440.11570	1728.53140	1411.13640	Detected	Detected	Detected	Detected
183	A_23_P134109	EPB41L2	Hs.486470	erythrocyte membrane protein band 4.1	Homo sapiens erythrocyte membrane pr	804.57196	986.05493	792.51260	867.01404	Detected	Detected	Detected	Detected
184	A_33_P3392580	AK2	Hs.470907	adenylate kinase 2	Homo sapiens adenylate kinase 2 (AK2)	1788.56320	1058.02170	1625.52920	854.95860	Detected	Detected	Detected	Detected
185	A_23_P145761	ARL4A	Hs.245540	ADP-ribosylation factor-like 4A	Homo sapiens ADP-ribosylation factor-	283.53006	799.90686	201.56413	414.50912	Detected	Detected	Detected	Detected
186	A_23_P104372	DNAJC9	Hs.654694	DnaJ (Hsp40) homolog, subfamily C, me	Homo sapiens DnaJ (Hsp40) homolog, s	6473.46830	25225.11000	4526.09900	14234.18700	Detected	Detected	Detected	Detected
187	A_33_P3309741		Hs.147762		Coiled-coil domain-containing protein 1	41.62708	55.00295	18.52189	13.27050	Detected	Detected	Detected	Compromised
188	A_23_P321320	STK11IP	Hs.22410	serine/threonine kinase 11 interacting p	Homo sapiens serine/threonine kinase	5699.07470	4148.94530	5535.94970	3559.17240	Detected	Detected	Detected	Detected
189	A_23_P209519	DNAJB2	Hs.77768	DnaJ (Hsp40) homolog, subfamily B, me	Homo sapiens DnaJ (Hsp40) homolog, s	337.27072	278.45636	393.12726	327.52283	Detected	Detected	Detected	Detected
190	A_23_P108948	MREG	Hs.707104	melanoregulin	Homo sapiens melanoregulin (MREG), m	749.38556	596.01500	803.56104	566.95105	Detected	Detected	Detected	Detected
191	A_23_P160406	KCTD3	Hs.335139	potassium channel tetramerisation doma	Homo sapiens potassium channel tetrar	12058.04800	12011.32600	9081.25500	6663.44300	Detected	Detected	Detected	Detected
192	A_23_P111635	STYXL1	Hs.11615	serine/threonine/tyrosine interacting-li	Homo sapiens serine/threonine/tyrosin	548.70245	949.65027	447.03120	670.71985	Detected	Detected	Detected	Detected
193	A_24_P170753		Hs.122115		EP400 N-terminal-like protein [Source	458.37195	1132.04410	363.75073	802.60810	Detected	Detected	Detected	Detected
194	A_23_P212196	OGG1	Hs.380271	8-oxoguanine DNA glycosylase	Homo sapiens 8-oxoguanine DNA glyco	611.45870	686.14060	424.40240	437.90790	Detected	Detected	Detected	Detected
195	A_24_P406754	LOXL4	Hs.306814	lysyl oxidase-like 4	Homo sapiens lysyl oxidase-like 4 (LOX	978.69100	671.81537	783.33640	455.31372	Detected	Detected	Detected	Detected
196	A_24_P400172	hCG_1817306	Hs.661872	hypothetical LOC100130691	Homo sapiens hypothetical LOC100130	93.65880	117.72070	64.20893	71.47038	Detected	Detected	Detected	Detected
197	A_33_P3243544	ESRRA	Hs.110849	estrogen-related receptor alpha	Homo sapiens estrogen-related recepto	2363.66200	1414.96980	2491.88350	1707.42680	Detected	Detected	Detected	Detected
198	A_24_P7790	WFDC10B	Hs.237392	WAP four-disulfide core domain 10B	Homo sapiens WAP four-disulfide core	31.88236	4.23759	18.86051	3.82794	Detected	Compromised	Detected	Compromised
199	A_23_P304682	EMP2	Hs.531561	epithelial membrane protein 2	Homo sapiens epithelial membrane prot	6902.67700	10349.43300	5260.07100	7201.19300	Detected	Detected	Detected	Detected
200	A_33_P3358295	ABTB1	Hs.107812	ankyrin repeat and BTB (POZ) domain c	Homo sapiens ankyrin repeat and BTB (	3334.50220	1942.98740	2751.95260	1371.70250	Detected	Detected	Detected	Detected
201	A_23_P204423	PPP1CC	Hs.79081	protein phosphatase 1, catalytic subunit	Homo sapiens protein phosphatase 1, ca	7347.22750	6043.89200	5665.36670	3842.53740	Detected	Detected	Detected	Detected
202	A_23_P104025	TSEN15	Hs.548197	tRNA splicing endonuclease 15 homolog	Homo sapiens tRNA splicing endonuclea	868.15820	815.03230	721.75060	561.78860	Detected	Detected	Detected	Detected
203	A_23_P101131	GRP	Hs.153444	gastrin-releasing peptide	Homo sapiens gastrin-releasing peptide	11.93863	20.13042	14.82036	15.22815	Detected	Detected	Detected	Detected
204	A_23_P110846	CNOT8	Hs.26703	CCR4-NOT transcription complex, subu	Homo sapiens CCR4-NOT transcription	2545.82420	1890.11240	1629.64770	998.00420	Detected	Detected	Detected	Detected
205	A_23_P252335	C21orf45	Hs.190518	chromosome 21 open reading frame 45	Homo sapiens chromosome 21 open rea	1400.17850	2261.20300	1301.46480	1724.18910	Detected	Detected	Detected	Detected
206	A_23_P425752	TRIM14	Hs.575631	tripartite motif-containing 14	Homo sapiens tripartite motif-containin	60.63707	80.22573	56.75068	69.42812	Detected	Detected	Detected	Detected
207	A_23_P117506	DHRS7	Hs.59719	dehydrogenase/reductase (SDR family)	Homo sapiens dehydrogenase/reductas	10933.61500	3901.65010	9161.44700	2814.46850	Detected	Detected	Detected	Detected
208	A_23_P316741	TSPAN4	Hs.654836	tetraspanin 4	Homo sapiens tetraspanin 4 (TSPAN4),	21434.40800	28842.15400	19914.49600	24260.99200	Detected	Detected	Detected	Detected
209	A_33_P3398111		Hs.120392		Putative uncharacterized protein C16or	40.53354	11.77204	40.46209	5.94287	Detected	Compromised	Detected	Compromised
210	A_23_P3592	HSF4	Hs.512156	heat shock transcription factor 4	Homo sapiens heat shock transcription	158.30745	56.71010	151.64409	41.83772	Detected	Detected	Detected	Detected
211	A_33_P3364060	HR	Hs.272367	hairless homolog (mouse)	Homo sapiens hairless homolog (mouse)	3319.86740	1613.31260	2510.93040	621.47906	Detected	Detected	Detected	Detected
212	A_23_P434919	RAB42	Hs.652321	RAB42, member RAS oncogene family	Homo sapiens RAB42, member RAS onc	1132.36660	599.87000	965.10126	471.32614	Detected	Detected	Detected	Detected
213	A_33_P3231878	PIK3C2A	Hs.175343	phosphoinositide-3-kinase, class 2, alph	Homo sapiens phosphoinositide-3-kinas	2599.24150	2050.63900	1927.46750	1253.36320	Detected	Detected	Detected	Detected
214	A_24_P19810	PPCS	Hs.710166	phosphopantothenoylcysteine syntheta:	Homo sapiens phosphopantothenoylcys	2071.16100	1686.28770	1797.19960	1228.90110	Detected	Detected	Detected	Detected
215	A_33_P3256565					23.22006	24.76438	14.80642	12.67118	Detected	Detected	Compromised	Compromised
216	A_23_P122001	MSH3	Hs.280987	mutS homolog 3 (E. coli)	Homo sapiens mutS homolog 3 (E. coli)	531.97830	950.65710	277.10013	394.15330	Detected	Detected	Detected	Detected
217	A_23_P399078	TIMP3	Hs.644633	TIMP metallopeptidase inhibitor 3	Homo sapiens TIMP metallopeptidase ir	11521.52800	45790.97700	10837.78900	34576.11000	Detected	Detected	Detected	Detected
218	A_33_P3419696	FGF2	Hs.284244	fibroblast growth factor 2 (basic)	Homo sapiens fibroblast growth factor 2	1274.61220	1775.48770	447.46106	400.32004	Detected	Detected	Detected	Detected
219	A_24_P58620	GRAMD1B	Hs.144725	GRAM domain containing 1B	Homo sapiens GRAM domain containing	58.04118	159.23955	46.23160	115.79726	Detected	Detected	Detected	Detected
220	A_33_P3405459	C20orf195	Hs.197755	chromosome 20 open reading frame 195	Homo sapiens chromosome 20 open rea	1157.32290	107.52171	954.09510	73.09479	Detected	Detected	Detected	Detected
221	A_23_P8416	GALNT11	Hs.647109	UDP-N-acetyl-alpha-D-galactosamine:	Homo sapiens UDP-N-acetyl-alpha-D-	635.57654	351.05240	440.22430	232.91263	Detected	Detected	Detected	Detected

222	A_23_P355623	ANKIB1	Hs.83293	ankyrin repeat and IBR domain containi	Homo sapiens ankyrin repeat and IBR d	776.54380	571.94055	603.15155	367.13754	Detected	Detected	Detected	Detected
223	A_23_P102770	NAT5	Hs.368783	N-acetyltransferase 5 (GCN5-related, p	Homo sapiens N-acetyltransferase 5 (G	9954.30000	13623.68700	6973.06000	7627.51500	Detected	Detected	Detected	Detected
224	A_23_P205575	GPR135	Hs.647573	G protein-coupled receptor 135	Homo sapiens G protein-coupled recep	353.31830	187.05194	293.12643	155.80132	Detected	Detected	Detected	Detected
225	A_23_P112026	IDO1	Hs.840	indoleamine 2,3-dioxygenase 1	Homo sapiens indoleamine 2,3-dioxyger	3.61674	23.76277	15.77175	7.35935	Compromised	Not Detected	Detected	Compromised
226	A_33_P3315410	COIL	Hs.532795	coilin	Homo sapiens coilin (COIL), mRNA [NM	2380.99680	2454.38770	2316.39820	2093.25500	Detected	Detected	Detected	Detected
227	A_23_P250118	HSPBAP1	Hs.29169	HSPB (heat shock 27kDa) associated p	Homo sapiens HSPB (heat shock 27kDa	1811.91720	940.86930	1575.67640	767.08435	Detected	Detected	Detected	Detected
228	A_33_P3663105	LOC284263	Hs.135642	hypothetical protein LOC284263	Homo sapiens hypothetical protein LOC	22.20245	18.62885	3.63211	6.28394	Detected	Compromised	Compromised	Compromised
229	A_23_P120442	NCOA3	Hs.592142	nuclear receptor coactivator 3	Homo sapiens nuclear receptor coactiva	303.95483	174.07848	180.05148	81.03122	Detected	Detected	Detected	Detected
230	A_23_P113471	FAAH2	Hs.720593	fatty acid amide hydrolase 2	Homo sapiens fatty acid amide hydrolas	17.73367	3.96608	22.46531	3.74537	Not Detected	Compromised	Detected	Compromised
231	A_23_P10206	HAS2	Hs.159226	hyaluronan synthase 2	Homo sapiens hyaluronan synthase 2 (H	50.60540	2683.23930	28.46118	1519.01870	Detected	Detected	Detected	Detected
232	A_24_P48723	PTGIS	Hs.302085	prostaglandin I2 (prostacyclin) synthase	Homo sapiens prostaglandin I2 (prostac	225.35703	1336.00780	113.96143	595.67750	Detected	Detected	Detected	Detected
233	A_23_P412321	CCR5	Hs.450802	chemokine (C-C motif) receptor 5	Homo sapiens chemokine (C-C motif) r	82.19899	111.65822	61.21126	74.92043	Detected	Detected	Detected	Detected
234	A_33_P3222019	WSCD2	Hs.143591	WSC domain containing 2	Homo sapiens WSC domain containing 2	123.71006	169.38098	184.69356	243.45780	Detected	Detected	Detected	Detected
235	A_33_P3619819		Hs.719003		Homo sapiens cDNA FLJ42959 fis, clon	59452.91400	61749.37500	34553.65000	28384.22700	Detected	Detected	Detected	Detected
236	A_23_P369815	FASLG	Hs.2007	Fas ligand (TNF superfamily, member 6)	Homo sapiens Fas ligand (TNF superfan	12.81226	18.83764	6.46779	16.50493	Detected	Detected	Compromised	Detected
237	A_33_P3219682		Hs.575390		yx87g09.r1 Soares melanocyte 2NbHM	858.86600	902.93870	423.82294	365.87024	Detected	Detected	Detected	Detected
238	A_33_P3252612	CYP2W1	Hs.272795	cytochrome P450, family 2, subfamily W	Homo sapiens cytochrome P450, family	225337.62000	224232.40000	157891.27000	137522.42000	Detected	Detected	Detected	Detected
239	A_23_P115645	CUGBP2	Hs.309288	CUG triplet repeat, RNA binding protein	Homo sapiens CUG triplet repeat, RNA	775.10376	87.93366	587.70910	55.62444	Detected	Detected	Detected	Detected
240	A_24_P191664	GOLIM4	Hs.143600	golgi integral membrane protein 4	Homo sapiens golgi integral membrane p	639.12190	955.71106	353.65198	403.77768	Detected	Detected	Detected	Detected
241	A_33_P3318653	OR5H6	Hs.554712	olfactory receptor, family 5, subfamily H	Homo sapiens olfactory receptor, family	16.90426	13.87496	7.20105	11.21199	Detected	Compromised	Compromised	Compromised
242	A_23_P218807	ZC3H7B	Hs.592188	zinc finger CCCH-type containing 7B	Homo sapiens zinc finger CCCH-type c	1114.82280	2116.88840	228.78206	340.64600	Detected	Detected	Detected	Detected
243	A_23_P404730	SNX26	Hs.515364	sorting nexin 26	Homo sapiens sorting nexin 26 (SNX26)	39.82050	115.73998	30.90159	102.81837	Detected	Detected	Detected	Detected
244	A_23_P14543	ALKBH1	Hs.94542	alkB, alkylation repair homolog 1 (E. coli	Homo sapiens alkB, alkylation repair ho	361.32070	398.79077	319.53897	308.20898	Detected	Detected	Detected	Detected
245	A_33_P3210647	COL16A1	Hs.368921	collagen, type XVI, alpha 1	Homo sapiens collagen, type XVI, alpha	1963.33250	3717.65700	1019.17760	1897.83470	Detected	Detected	Detected	Detected
246	A_33_P3341105	PDE6G	Hs.654482	phosphodiesterase 6G, cGMP-specific,	Homo sapiens phosphodiesterase 6G, c	338.99582	224.22704	254.72655	137.45850	Detected	Detected	Detected	Detected
247	A_23_P50096	TYMS	Hs.592338	thymidylate synthetase	Homo sapiens thymidylate synthetase (	1101.61240	29064.81800	973.80450	20754.01200	Detected	Detected	Detected	Detected
248	A_23_P386384	C1orf87	Hs.47385	chromosome 1 open reading frame 87	Homo sapiens chromosome 1 open read	55.31427	4.23366	48.83391	3.81855	Detected	Compromised	Detected	Compromised
249	A_33_P3264121	TBCA	Hs.291212	tubulin folding cofactor A	Homo sapiens tubulin folding cofactor A	45063.80500	53427.74000	37295.40600	34584.91000	Detected	Detected	Detected	Detected
250	A_32_P142128	BMS1P1	Hs.652450	BMS1 pseudogene 1	Homo sapiens BMS1 pseudogene 1 (BM	24.76301	40.38594	13.92202	18.75528	Detected	Detected	Compromised	Compromised
251	A_32_P3534	LOC145814	Hs.592021	pyroglutamyl-peptidase 1-like	Homo sapiens pyroglutamyl-peptidase 1	27.79167	14.08358	18.44430	10.86374	Detected	Compromised	Compromised	Compromised
252	A_24_P384569	EWSR1	Hs.374477	Ewing sarcoma breakpoint region 1	Homo sapiens Ewing sarcoma breakpoir	51.32769	118.83717	49.81579	87.31752	Detected	Detected	Detected	Detected
253	A_23_P50276	ANGPTL6	Hs.712850	angiopoietin-like 6	Homo sapiens angiopoietin-like 6 (ANGI	120.57459	80.33311	151.01143	65.18100	Detected	Detected	Detected	Detected
254	A_23_P123905	EXOSC3	Hs.602571	exosome component 3	Homo sapiens exosome component 3 (E	2815.01930	3297.03300	2036.63540	1985.85880	Detected	Detected	Detected	Detected
255	A_23_P501010	COL17A1	Hs.117938	collagen, type XVII, alpha 1	Homo sapiens collagen, type XVII, alpha	13.42431	18.95098	6.02284	21.58603	Compromised	Detected	Compromised	Detected
256	A_23_P105862	FRY	Hs.507669	furry homolog (Drosophila)	Homo sapiens furry homolog (Drosophila	101.94138	225.31250	67.99375	121.28308	Detected	Detected	Detected	Detected
257	A_23_P107432		Hs.375092		Putative uncharacterized protein ENSP	49.10269	29.39653	38.28626	17.35869	Detected	Detected	Detected	Detected
258	A_33_P3281816	CAP1	Hs.370581	CAP, adenylate cyclase-associated pro	Homo sapiens CAP, adenylate cyclase-	1056.21950	1509.93750	945.93440	1280.13020	Detected	Detected	Detected	Detected
259	A_23_P115202	CRNN	Hs.242057	cornulin	Homo sapiens cornulin (CRNN), mRNA	20.98559	29.08763	15.42283	15.11236	Detected	Detected	Detected	Detected
260	A_33_P3257528	ZNF451	Hs.485628	zinc finger protein 451	Homo sapiens zinc finger protein 451 (Z	139.52263	155.66980	122.41516	100.97221	Detected	Detected	Detected	Detected
261	A_23_P115167	LRRC8B	Hs.482017	leucine rich repeat containing 8 family,	Homo sapiens leucine rich repeat conta	269.97700	148.65495	187.93321	88.66384	Detected	Detected	Detected	Detected
262	A_32_P131143	CECR4		cat eye syndrome chromosome region,	Homo sapiens cat eye syndrome chrom	177.56955	28.14981	201.00829	27.25383	Detected	Detected	Detected	Detected
263	A_23_P88404	TGFB3	Hs.592317	transforming growth factor, beta 3	Homo sapiens transforming growth fact	161.79344	125.09430	213.41719	135.19325	Detected	Detected	Detected	Detected
264	A_23_P106741	PSMD7	Hs.440604	proteasome (prosome, macropain) 26S	Homo sapiens proteasome (prosome, m	22510.25600	29541.36000	20076.54500	23491.57000	Detected	Detected	Detected	Detected
265	A_23_P3221	SQRDL	Hs.511251	sulfide quinone reductase-like (yeast)	Homo sapiens sulfide quinone reductase	121426.74000	51801.71500	116282.48400	41195.93800	Detected	Detected	Detected	Detected
266	A_23_P74526	PPIE	Hs.524690	peptidylprolyl isomerase E (cyclophilin E	Homo sapiens peptidylprolyl isomerase	1228.71350	1486.56860	1804.83680	1756.05220	Detected	Detected	Detected	Detected
267	A_33_P3264116	NHLRC1	Hs.348351	NHL repeat containing 1	Homo sapiens NHL repeat containing 1	86.61855	136.53958	87.76751	130.91841	Detected	Detected	Detected	Detected
268	A_23_P117274	MIPEP	Hs.507498	mitochondrial intermediate peptidase	Homo sapiens mitochondrial intermediat	635.09106	331.85750	700.65360	323.96625	Detected	Detected	Detected	Detected
269	A_33_P3211804	RUNX1	Hs.149261	runt-related transcription factor 1	Homo sapiens runt-related transcriptior	1593.63700	8768.98400	1287.10510	6070.55100	Detected	Detected	Detected	Detected
270	A_33_P3335682	PPP1R14A	Hs.631569	protein phosphatase 1, regulatory (inhib	Homo sapiens protein phosphatase 1, re	16419.85500	22724.33600	4031.65010	3129.40230	Detected	Detected	Detected	Detected
271	A_23_P105625	COQ5	Hs.17250	coenzyme Q5 homolog, methyltransfera	Homo sapiens coenzyme Q5 homolog, m	4231.96400	2243.18430	3402.94200	1501.22910	Detected	Detected	Detected	Detected
272	A_23_P388812	CKAP2L	Hs.434250	cytoskeleton associated protein 2-like	Homo sapiens cytoskeleton associated	54.82716	1337.86650	66.33037	1574.61960	Detected	Detected	Detected	Detected
273	A_23_P133284	RIOK2	Hs.27021	RIO kinase 2 (yeast)	Homo sapiens RIO kinase 2 (yeast) (RIC	2428.73730	3257.57420	1881.22250	2111.18950	Detected	Detected	Detected	Detected
274	A_23_P30736	HLA-DOB	Hs.1802	major histocompatibility complex, class	Homo sapiens major histocompatibility c	129.15878	92.17059	106.30138	56.80027	Detected	Detected	Detected	Detected
275	A_23_P122068	C1QTNF3	Hs.171929	C1q and tumor necrosis factor related p	Homo sapiens C1q and tumor necrosis	90.51125	136.13124	51.33919	78.78398	Detected	Detected	Detected	Detected
276	A_23_P53137	HBG1	Hs.712539	hemoglobin, gamma A	Homo sapiens hemoglobin, gamma A (HI	4.29004	4.69585	13.45721	3.71014	Compromised	Compromised	Not Detected	Compromised
277	A_24_P91094	RUNDC3A	Hs.500197	RUN domain containing 3A	Homo sapiens RUN domain containing 3	75.60793	29.53190	72.62940	21.66069	Detected	Detected	Detected	Detected
278	A_33_P3215239	KIF20B	Hs.240	kinesin family member 20B	Homo sapiens kinesin family member 20	101.26609	612.50684	79.09248	440.94220	Detected	Detected	Detected	Detected
279	A_23_P115492	VASH2	Hs.96885	vasohibin 2	Homo sapiens vasohibin 2 (VASH2), tra	77.50459	79.64204	30.76549	24.86110	Detected	Detected	Detected	Detected
280	A_32_P169574	OTUD5	Hs.496098	OTU domain containing 5	Homo sapiens OTU domain containing 5	374.53850	378.15796	248.63083	258.51820	Detected	Detected	Detected	Detected
281	A_33_P3242418	LOC100128348	Hs.637572	hypothetical protein LOC100128348	Homo sapiens cDNA FLJ46249 fis, clon	9129.76300	12173.44000	3653.20400	4121.34000	Detected	Detected	Detected	Detected
282	A_23_P308021	CABP4	Hs.143036	calcium binding protein 4	Homo sapiens calcium binding protein 4	117.42122	157.50574	74.60456	99.35929	Detected	Detected	Detected	Detected
283	A_23_P139722	TNFRSF1A	Hs.279594	tumor necrosis factor receptor superfa	Homo sapiens tumor necrosis factor re	4079.99410	4821.05500	1582.50730	1455.37230	Detected	Detected	Detected	Detected
284	A_23_P120428	TP53TG5	Hs.401982	TP53 target 5	Homo sapiens TP53 target 5 (TP53TG5	122.44292	23.50081	8.32186	6.37444	Not Detected	Not Detected	Compromised	Compromised
285	A_33_P3218872	METTL6	Hs.149487	methyltransferase like 6	Homo sapiens methyltransferase like 6	25.61977	23.89393	3.99296	9.37413	Detected	Detected	Compromised	Compromised
286	A_24_P393470	LOC729991	Hs.153629	hypothetical protein LOC729991	Homo sapiens hypothetical protein LOC	497.70776	821.57970	372.96472	496.94934	Detected	Detected	Detected	Detected
287	A_23_P54636	ATP6V0D1	Hs.106876	ATPase, H+ transporting, lysosomal 38k	Homo sapiens ATPase, H+ transporting,	6112.02000	7560.09000	5474.66600	6177.42000	Detected	Detected	Detected	Detected
288	A_24_P22050	RAB20	Hs.508720	RAB20, member RAS oncogene family	Homo sapiens RAB20, member RAS on	343.59024	56.89898	403.56980	73.81477	Detected	Detected	Detected	Detected
289	A_23_P24444	DHCR7	Hs.503134	7-dehydrocholesterol reductase	Homo sapiens 7-dehydrocholesterol rec	3646.61740	3849.10910	3704.85600	3528.59890	Detected	Detected	Detected	Detected
290	A_23_P107724	ZFP112	Hs.48589	zinc finger protein 112 homolog (mouse	Homo sapiens zinc finger protein 112 ho	460.45038	357.49283	319.14294	212.32805	Detected	Detected	Detected	Detected
291	A_23_P23669	PALMD	Hs.483993	palmdelphin	Homo sapiens palmdelphin (PALMD), ml	26.10146	165.71666	21.52662	110.18900	Detected	Detected	Detected	Detected
292	A_24_P302584	SOX11	Hs.432638	SRY (sex determining region Y)-box 11	Homo sapiens SRY (sex determining reg	42.08003	76.51428	22.66728	47.86318	Detected	Detected	Detected	Detected
293	A_33_P3386181	ZFP64	Hs.473082	zinc finger protein 64 homolog (mouse)	Homo sapiens zinc finger protein 64 hor	216.98482	364.86720	194.47139	315.03677	Detected	Detected	Detected	Detected
294	A_23_P395524	PWP1	Hs.506652	PWP1 homolog (S. cerevisiae)	Homo sapiens PWP1 homolog (S. cerevi	24743.15400	42737.33200	23856.58600	37206.39000	Detected	Detected	Detected	Detected
295	A_24_P398092	RAD23B	Hs.521640	RAD23 homolog B (S. cerevisiae)	Homo sapiens RAD23 homolog B (S. ce	4573.98200	7736.58100	3685.97560	4956.23600	Detected	Detected	Detected	Detected
296	A_23_P316531	GPR25	Hs.534316	G protein-coupled receptor 25	Homo sapiens G protein-coupled recep	281.48184	406.64346	412.33267	610.58660	Detected	Detected	Detected	Detected

297	A_33_P3320772	ATXN1	Hs.434961	ataxin 1	Homo sapiens ataxin 1 (ATXN1), transc	45.63403	109.63612	48.18290	89.65896	Detected	Detected	Detected	Detected
298	A_23_P37441	B2M	Hs.534255	beta-2-microglobulin	Homo sapiens beta-2-microglobulin (B2	149971.44000	80471.71000	135857.52000	62127.36300	Detected	Detected	Detected	Detected
299	A_23_P137035	PIR	Hs.495728	pirin (iron-binding nuclear protein)	Homo sapiens pirin (iron-binding nuclea	695.31350	408.51624	599.87396	333.87613	Detected	Detected	Detected	Detected
300	A_23_P169092	CYP7B1	Hs.667720	cytochrome P450, family 7, subfamily B	Homo sapiens cytochrome P450, family	101.53169	57.15080	121.74761	48.86221	Detected	Detected	Detected	Detected
301	A_24_P405002	PDIK1L	Hs.468801	PDLIM1 interacting kinase 1 like	Homo sapiens PDLIM1 interacting kinas	454.64120	481.61118	327.76474	273.01913	Detected	Detected	Detected	Detected
302	A_23_P139509	ERGIC2	Hs.339453	ERGIC and golgi 2	Homo sapiens ERGIC and golgi 2 (ERGI	2598.45460	2400.87160	2582.30740	2226.90580	Detected	Detected	Detected	Detected
303	A_23_P114314	CDX4	Hs.553488	caudal type homeobox 4	Homo sapiens caudal type homeobox 4	3.31210	21.95581	3.10158	3.54689	Compromised	Detected	Compromised	Compromised
304	A_23_P87664	SART3	Hs.584842	squamous cell carcinoma antigen recog	Homo sapiens squamous cell carcinoma	985.43980	944.87695	1060.36510	907.13495	Detected	Detected	Detected	Detected
305	A_23_P79794	TGIF2	Hs.632264	TGFB-induced factor homeobox 2	Homo sapiens TGFB-induced factor ho	98.62981	55.71090	99.85552	48.37042	Detected	Detected	Detected	Detected
306	A_23_P430842	HAPLN4	Hs.367829	hyaluronan and proteoglycan link protei	Homo sapiens hyaluronan and proteogly	13.55808	8.40414	18.40147	16.80085	Detected	Compromised	Detected	Detected
307	A_23_P150583	SCGB1A1	Hs.523732	secretoglobin, family 1A, member 1 (ute	Homo sapiens secretoglobin, family 1A,	157.05810	226.35764	107.83568	101.54572	Detected	Detected	Detected	Detected
308	A_33_P3296372	EIF2B3	Hs.533549	eukaryotic translation initiation factor 2	Homo sapiens eukaryotic translation ini	4506.29150	6898.31100	4255.32230	5221.31250	Detected	Detected	Detected	Detected
309	A_33_P3259092	FKTN	Hs.55777	fukutin	Homo sapiens fukutin (FKTN), transcrip	3.36455	21.16682	6.40561	16.49477	Compromised	Detected	Compromised	Compromised
310	A_23_P110879	TRAF3IP2	Hs.561514	TRAF3 interacting protein 2	Homo sapiens TRAF3 interacting protei	739.56140	923.42444	496.79102	561.38950	Detected	Detected	Detected	Detected
311	A_23_P310350	SHPK	Hs.579217	sedoheptulokinase	Homo sapiens sedoheptulokinase (SHPK	2539.90100	2974.08250	2610.13700	2539.28300	Detected	Detected	Detected	Detected
312	A_23_P416314	HRASLS5	Hs.410316	HRAS-like suppressor family, member 5	Homo sapiens HRAS-like suppressor fa	31345.61500	19749.08000	3636.43260	1124.74460	Detected	Detected	Detected	Detected
313	A_23_P122624	LTV1	Hs.185675	LTV1 homolog (S. cerevisiae)	Homo sapiens LTV1 homolog (S. cerevi	3926.17970	4362.75100	3339.67500	3076.06700	Detected	Detected	Detected	Detected
314	A_23_P83976	CCDC46	Hs.408676	coiled-coil domain containing 46	Homo sapiens coiled-coil domain contai	274.39510	319.97630	147.49680	133.32112	Detected	Detected	Detected	Detected
315	A_33_P3253427	LRRC37B2	Hs.558901	leucine rich repeat containing 37, memb	Homo sapiens leucine rich repeat conta	622.48596	867.76690	437.09494	511.48547	Detected	Detected	Detected	Detected
316	A_33_P3396646	BEND3	Hs.418045	BEN domain containing 3	Homo sapiens BEN domain containing 3	68.74713	64.38129	45.42617	35.27922	Detected	Detected	Detected	Detected
317	A_23_P58321	CCNA2	Hs.58974	cyclin A2	Homo sapiens cyclin A2 (CCNA2), mRN	59.81775	2093.80300	43.77638	1529.33180	Detected	Detected	Detected	Detected
318	A_23_P118002	UQCRC2	Hs.528803	ubiquinol-cytochrome c reductase core	Homo sapiens ubiquinol-cytochrome c r	15652.18400	14814.78100	7772.06800	5959.72460	Detected	Detected	Detected	Detected
319	A_23_P214139	REV3L	Hs.232021	REV3-like, catalytic subunit of DNA pol	Homo sapiens REV3-like, catalytic subu	7977.15970	2187.70480	5887.04440	1352.96420	Detected	Detected	Detected	Detected
320	A_24_P218001	ZNF273	Hs.520889	zinc finger protein 273	Homo sapiens zinc finger protein 273 (Z	124.61050	143.51869	74.85217	84.67571	Detected	Detected	Detected	Detected
321	A_32_P94087	RPL21P44	Hs.452943	ribosomal protein L21 pseudogene 44	Homo sapiens ribosomal protein L21 ps	62548.83600	52866.22700	45729.50800	32182.30300	Detected	Detected	Detected	Detected
322	A_23_P102420	CCT4	Hs.421509	chaperonin containing TCP1, subunit 4	Homo sapiens chaperonin containing TC	78166.71000	119635.51000	55808.93400	71288.97000	Detected	Detected	Detected	Detected
323	A_23_P101208	CYB5A	Hs.465413	cytochrome b5 type A (microsomal)	Homo sapiens cytochrome b5 type A (m	9334.73600	6390.06500	8355.93900	4999.05030	Detected	Detected	Detected	Detected
324	A_24_P86868	METTL10	Hs.706064	methyltransferase like 10	Homo sapiens methyltransferase like 10	1593.50320	2468.69380	1403.61560	1876.67290	Detected	Detected	Detected	Detected
325	A_33_P3387463	ATP6V1D	Hs.272630	ATPase, H+ transporting, lysosomal 34k	Homo sapiens ATPase, H+ transporting,	3396.11500	5674.24200	2501.98500	3592.13300	Detected	Detected	Detected	Detected
326	A_23_P103256	CFHR3	Hs.709217	complement factor H-related 3	Homo sapiens complement factor H-rel	308.11078	335.20874	239.71861	240.44920	Detected	Detected	Detected	Detected
327	A_23_P201845	ZMYM6	Hs.675613	zinc finger, MYM-type 6	Homo sapiens zinc finger, MYM-type 6	226.64526	286.72876	216.44167	269.52548	Detected	Detected	Detected	Detected
328	A_23_P110777	LECT2	Hs.512580	leukocyte cell-derived chemotaxin 2	Homo sapiens leukocyte cell-derived ch	94.45591	10.05742	74.50124	14.12698	Detected	Compromised	Detected	Detected
329	A_23_P125078	SLC26A11	Hs.4866	solute carrier family 26, member 11	Homo sapiens solute carrier family 26, r	8433.02600	7986.48300	6357.56350	5192.93260	Detected	Detected	Detected	Detected
330	A_23_P103414	YTHDF2	Hs.532286	YTH domain family, member 2	Homo sapiens YTH domain family, mem	7164.74560	9044.09400	4628.73730	4894.52540	Detected	Detected	Detected	Detected
331	A_23_P121533	SPON2	Hs.720064	spondin 2, extracellular matrix protein	Homo sapiens spondin 2, extracellular n	57357.42000	187252.52000	38992.57400	113375.89000	Detected	Detected	Detected	Detected
332	A_23_P257538	RBBP9	Hs.69330	retinoblastoma binding protein 9	Homo sapiens retinoblastoma binding pr	829.24950	721.11010	655.72450	443.12494	Detected	Detected	Detected	Detected
333	A_23_P110196	HERC5	Hs.26663	hect domain and RLD 5	Homo sapiens hect domain and RLD 5 (	71.27563	27.46673	40.60429	8.38442	Detected	Detected	Detected	Compromised
334	A_23_P63521	LCE2C	Hs.553713	late cornified envelope 2C	Homo sapiens late cornified envelope 2(	19.64014	85.49013	11.85671	62.79385	Detected	Detected	Compromised	Detected
335	A_23_P109026	KCNK15	Hs.528664	potassium channel, subfamily K, membe	Homo sapiens potassium channel, subfa	3014.55370	4097.82500	1568.25820	1670.86220	Detected	Detected	Detected	Detected
336	A_23_P381449	SP2	Hs.514276	Sp2 transcription factor	Homo sapiens Sp2 transcription factor (	165.98477	396.32965	87.86260	214.51236	Detected	Detected	Detected	Detected
337	A_23_P19938	KDELR2	Hs.654552	KDEL (Lys-Asp-Glu-Leu) endoplasmic	Homo sapiens KDEL (Lys-Asp-Glu-Leu	8688.83300	34607.48400	5689.05470	18571.34800	Detected	Detected	Detected	Detected
338	A_24_P913339	C2orf18	Hs.516034	chromosome 2 open reading frame 18	Homo sapiens chromosome 2 open read	2498.72070	2490.83130	2130.52950	2001.48500	Detected	Detected	Detected	Detected
339	A_23_P102320	NUP35	Hs.180591	nucleoporin 35kDa	Homo sapiens nucleoporin 35kDa (NUP3	2596.14530	2448.12000	1839.92750	1494.98540	Detected	Detected	Detected	Detected
340	A_23_P120270	MCFD2	Hs.293689	multiple coagulation factor deficiency 2	Homo sapiens multiple coagulation facto	4643.62600	9721.23200	3787.29080	6507.01800	Detected	Detected	Detected	Detected
341	A_23_P110569	TRIM36	Hs.519514	tripartite motif-containing 36	Homo sapiens tripartite motif-containin	26.10351	30.04322	15.36885	16.31843	Detected	Detected	Detected	Detected
342	A_23_P46844	TRIM8	Hs.336810	tripartite motif-containing 8	Homo sapiens tripartite motif-containin	8150.90970	8717.70800	5471.93460	5464.39450	Detected	Detected	Detected	Detected
343	A_33_P3310371	LOC283788	Hs.657131	FSHD region gene 1 pseudogene	Homo sapiens FSHD region gene 1 pseu	704.92440	620.07935	161.95700	187.10178	Detected	Detected	Detected	Detected
344	A_23_P151820	RIN3	Hs.326822	Ras and Rab interactor 3	Homo sapiens Ras and Rab interactor 3	505.42352	1419.34660	440.24030	1275.22400	Detected	Detected	Detected	Detected
345	A_23_P105495	TAS2R14	Hs.679406	taste receptor, type 2, member 14	Homo sapiens taste receptor, type 2, m	12.99995	16.93549	16.37124	17.46031	Detected	Not Detected	Detected	Detected
346	A_23_P33196	COL5A2	Hs.445827	collagen, type V, alpha 2	Homo sapiens collagen, type V, alpha 2	9524.81800	23557.50400	6864.69040	15168.54300	Detected	Detected	Detected	Detected
347	A_23_P162589	VDR	Hs.524368	vitamin D (1,25- dihydroxyvitamin D3) r	Homo sapiens vitamin D (1,25- dihydrox	3232.79960	3227.61080	2402.65920	2044.08980	Detected	Detected	Detected	Detected
348	A_33_P3264346					2655.98200	5074.66650	624.97015	688.74220	Detected	Detected	Detected	Detected
349	A_23_P125253	LDB2	Hs.714330	LIM domain binding 2	Homo sapiens LIM domain binding 2 (LD	75.30250	21.90350	74.57307	18.55379	Detected	Detected	Detected	Compromised
350	A_24_P369898				Putative myosin-XVB (Unconventional	97.56057	62.52729	73.18980	26.31119	Detected	Detected	Detected	Detected
351	A_33_P3238461					136511.10000	120043.01000	99692.67000	73010.53000	Detected	Detected	Detected	Detected
352	A_23_P123539	PPP2R2A	Hs.146339	protein phosphatase 2 (formerly 2A), re	Homo sapiens protein phosphatase 2 (fo	1834.07930	2599.33080	1328.67320	1678.44520	Detected	Detected	Detected	Detected
353	A_23_P119143	ICAM5	Hs.465862	intercellular adhesion molecule 5, telenc	Homo sapiens intercellular adhesion mo	462.23810	125.53117	346.64862	87.21031	Detected	Detected	Detected	Detected
354	A_23_P120125	COLEC11	Hs.32603	collectin sub-family member 11	Homo sapiens collectin sub-family mem	22.98119	65.55999	18.79908	42.55752	Detected	Detected	Detected	Detected
355	A_23_P206369	TMEM208	Hs.433203	transmembrane protein 208	Homo sapiens transmembrane protein 2	15504.13400	25457.01400	12792.29300	17291.75800	Detected	Detected	Detected	Detected
356	A_23_P251767	BANP	Hs.461705	BTG3 associated nuclear protein	Homo sapiens BTG3 associated nuclear	199.67494	131.42691	265.06744	169.94950	Detected	Detected	Detected	Detected
357	A_33_P3260307	CARHSP1	Hs.632184	calcium regulated heat stable protein 1,	Homo sapiens calcium regulated heat st	5669.90770	10804.94800	6200.62100	10058.36500	Detected	Detected	Detected	Detected
358	A_23_P10182	ACOX2	Hs.444959	acyl-Coenzyme A oxidase 2, branched	Homo sapiens acyl-Coenzyme A oxidas	1395.57300	768.96844	1035.01250	509.30225	Detected	Detected	Detected	Detected
359	A_23_P11025	ZNF185	Hs.16622	zinc finger protein 185 (LIM domain)	Homo sapiens zinc finger protein 185 (L	440.92180	1209.13490	299.68650	683.45850	Detected	Detected	Detected	Detected
360	A_24_P940921	SETX	Hs.460317	senataxin	Homo sapiens senataxin (SETX), mRNA	1957.37800	1546.56160	1344.58860	856.37160	Detected	Detected	Detected	Detected
361	A_23_P114689	ASAP3	Hs.437379	ArfGAP with SH3 domain, ankyrin repea	Homo sapiens ArfGAP with SH3 domain	886.52000	202.27348	882.92330	190.50250	Detected	Detected	Detected	Detected
362	A_23_P252748	ZNF195	Hs.386294	zinc finger protein 195	Homo sapiens zinc finger protein 195 (Z	475.62274	646.39290	330.21173	364.93378	Detected	Detected	Detected	Detected
363	A_23_P120467	ZFP64	Hs.473082	zinc finger protein 64 homolog (mouse)	Homo sapiens zinc finger protein 64 hor	469.24078	393.61874	282.28943	198.15991	Detected	Detected	Detected	Detected
364	A_23_P422044	PCDHGA2	Hs.368160	protocadherin gamma subfamily A, 2	Homo sapiens protocadherin gamma sul	146.91820	193.10628	124.41732	131.54303	Detected	Detected	Detected	Detected
365	A_33_P3335902	ARMCX6	Hs.83530	armadillo repeat containing, X-linked 6	Homo sapiens armadillo repeat containi	392.38705	211.33449	364.32803	167.21849	Detected	Detected	Detected	Detected
366	A_33_P3349637	PCDH1	Hs.79769	protocadherin 1	Homo sapiens protocadherin 1 (PCDH1)	167.96523	242.64388	107.24281	114.77361	Detected	Detected	Detected	Detected
367	A_23_P169470	CDK9	Hs.150423	cyclin-dependent kinase 9	Homo sapiens cyclin-dependent kinase	4120.88230	3437.60840	2113.75600	1530.85110	Detected	Detected	Detected	Detected
368	A_23_P112159	EIF2C2	Hs.449415	eukaryotic translation initiation factor 2	Homo sapiens eukaryotic translation ini	838.95300	2109.58810	715.51850	1632.06570	Detected	Detected	Detected	Detected
369	A_23_P109442	HPS4	Hs.474436	Hermansky-Pudlak syndrome 4	Homo sapiens Hermansky-Pudlak syndr	3728.68070	3283.25500	2029.65330	1500.19350	Detected	Detected	Detected	Detected
370	A_24_P9671	DNAJA1	Hs.445203	DnaJ (Hsp40) homolog, subfamily A, me	Homo sapiens DnaJ (Hsp40) homolog, s	13155.57600	9336.36600	10455.37600	6627.38230	Detected	Detected	Detected	Detected
371	A_33_P3346826	IL32	Hs.943	interleukin 32	Homo sapiens interleukin 32 (IL32), tran	60.08030	260.67145	55.96246	228.39061	Detected	Detected	Detected	Detected

372	A_23_P168080	SKIV2L	Hs.89864	superkiller viralicidic activity 2-like (S. c	Homo sapiens superkiller viralicidic acti	1220.77540	1266.02930	1535.42790	1617.40660	Detected	Detected	Detected	Detected
373	A_23_P112289	TMOD1	Hs.494595	tropomodulin 1	Homo sapiens tropomodulin 1 (TMOD1)	19.13916	9.23398	18.46538	13.04287	Detected	Compromised	Detected	Detected
374	A_33_P3284584					2609.08300	2609.62100	152.47360	98.99217	Detected	Detected	Detected	Detected
375	A_24_P729905	AGAP7	Hs.528346	ArfGAP with GTPase domain, ankyrin re	Homo sapiens ArfGAP with GTPase dom	237.48666	246.74965	165.51729	155.20789	Detected	Detected	Detected	Detected
376	A_33_P3359590	DAZAP1	Hs.222510	DAZ associated protein 1	Homo sapiens DAZ associated protein 1	24.70894	35.79172	16.65136	28.49818	Detected	Detected	Compromised	Detected
377	A_33_P3377005	C11orf35	Hs.669395	chromosome 11 open reading frame 35	Homo sapiens chromosome 11 open rea	1069.36820	170.96336	860.87420	115.02079	Detected	Detected	Detected	Detected
378	A_24_P270769	VPS35	Hs.454528	vacuolar protein sorting 35 homolog (S.	Homo sapiens vacuolar protein sorting 3	19062.54900	17573.06200	15632.11300	13082.24500	Detected	Detected	Detected	Detected
379	A_23_P123563	RPS6	Hs.408073	ribosomal protein S6	Homo sapiens ribosomal protein S6 (RP	269200.66000	233727.64000	227650.20000	178508.70000	Detected	Detected	Detected	Detected
380	A_33_P3330643	KIF6	Hs.588202	kinesin family member 6	Homo sapiens kinesin family member 6	116.06253	11.36079	136.87100	9.05671	Detected	Compromised	Detected	Compromised
381	A_33_P3241696				Membrane-associated transporter prot	102.95721	219.36009	8.28345	11.20790	Detected	Detected	Compromised	Compromised
382	A_23_P105562	VWF	Hs.440848	von Willebrand factor	Homo sapiens von Willebrand factor (VW	82.05867	46.74668	78.85735	29.87193	Detected	Detected	Detected	Detected
383	A_23_P115356	AKR7A2	Hs.571886	aldo-keto reductase family 7, member A	Homo sapiens aldo-keto reductase fam	4485.53200	8933.47500	3727.86720	6377.00500	Detected	Detected	Detected	Detected
384	A_23_P112634	C4orf34	Hs.576320	chromosome 4 open reading frame 34	Homo sapiens chromosome 4 open read	4578.44100	3537.48050	2977.51320	1852.29080	Detected	Detected	Detected	Detected
385	A_33_P3354851				Putative uncharacterized protein ENSP	206.86480	291.83203	101.89591	114.14252	Detected	Detected	Detected	Detected
386	A_24_P71973	KDR	Hs.479756	kinase insert domain receptor (a type II	Homo sapiens kinase insert domain rece	140.01378	443.84348	116.16223	352.02094	Detected	Detected	Detected	Detected
387	A_32_P180265	LOC100287241	Hs.555508	similar to hCG2038619	PREDICTED: Homo sapiens similar to h	133.42670	111.54578	116.78499	117.97673	Detected	Detected	Detected	Detected
388	A_33_P3251252					56.31438	48.18000	32.73143	24.27336	Detected	Detected	Detected	Detected
389	A_24_P93741	RFT1	Hs.720810	RFT1 homolog (S. cerevisiae)	Homo sapiens RFT1 homolog (S. cerevi	480.47437	509.22583	391.31546	346.99045	Detected	Detected	Detected	Detected
390	A_23_P102037	COQ10B	Hs.715692	coenzyme Q10 homolog B (S. cerevisiae	Homo sapiens coenzyme Q10 homolog B	703.40600	882.88245	495.24536	549.98690	Detected	Detected	Detected	Detected
391	A_23_P122863	GRB10	Hs.164060	growth factor receptor-bound protein 1	Homo sapiens growth factor receptor-b	4064.03760	4854.24560	3641.15670	3728.88530	Detected	Detected	Detected	Detected
392	A_23_P120710	TTC3	Hs.368214	tetratricopeptide repeat domain 3	Homo sapiens tetratricopeptide repeat	2404.25440	2191.18730	1237.26980	923.30054	Detected	Detected	Detected	Detected
393	A_23_P82299	C7orf47	Hs.720433	chromosome 7 open reading frame 47	Homo sapiens chromosome 7 open read	21068.07800	17029.45000	16573.68000	13041.56800	Detected	Detected	Detected	Detected
394	A_33_P3878964	C12orf30	Hs.530941	chromosome 12 open reading frame 30	Homo sapiens chromosome 12 open rea	1297.23460	1406.71060	883.78810	759.71674	Detected	Detected	Detected	Detected
395	A_24_P934800	ERI2	Hs.248437	ERI1 exoribonuclease family member 2	Homo sapiens ERI1 exoribonuclease far	275.09033	642.22955	255.73276	503.32370	Detected	Detected	Detected	Detected
396	A_23_P74290	GBP5	Hs.513726	guanylate binding protein 5	Homo sapiens guanylate binding protein	652.39667	124.62351	675.21375	120.90954	Detected	Detected	Detected	Detected
397	A_23_P113825	NACC2	Hs.112895	NACC family member 2, BEN and BTB	Homo sapiens NACC family member 2,	2208.61430	9783.18900	1584.71720	5834.90040	Detected	Detected	Detected	Detected
398	A_23_P102364	NGEF	Hs.97316	neuronal guanine nucleotide exchange f	Homo sapiens neuronal guanine nucleot	1081.15360	283.86688	750.09326	169.72530	Detected	Detected	Detected	Detected
399	A_23_P63178	TAF12	Hs.530251	TAF12 RNA polymerase II, TATA box bi	Homo sapiens TAF12 RNA polymerase	9835.53100	6441.71340	7070.82300	3998.03540	Detected	Detected	Detected	Detected
400	A_23_P106727	RAB11FIP3	Hs.531642	RAB11 family interacting protein 3 (clas	Homo sapiens RAB11 family interacting	5390.14940	4227.49560	4191.91800	2938.39380	Detected	Detected	Detected	Detected
401	A_23_P148150	COBRA1	Hs.655043	cofactor of BRCA1	Homo sapiens cofactor of BRCA1 (COB	5209.36400	5375.83450	6809.52440	7226.24660	Detected	Detected	Detected	Detected
402	A_24_P149713	ZBTB43	Hs.355581	zinc finger and BTB domain containing	Homo sapiens zinc finger and BTB dom	231.43903	436.21545	171.70480	246.80568	Detected	Detected	Detected	Detected
403	A_23_P110052	FOXL2	Hs.289292	forkhead box L2	Homo sapiens forkhead box L2 (FOXL2)	5660.33200	3505.89040	4011.26600	2236.26560	Detected	Detected	Detected	Detected
404	A_23_P141208	DHRS7B	Hs.386989	dehydrogenase/reductase (SDR family)	Homo sapiens dehydrogenase/reductas	1355.88750	1536.24820	1522.84630	1568.45720	Detected	Detected	Detected	Detected
405	A_33_P3404899	LPHN2	Hs.24212	latrophilin 2	Homo sapiens latrophilin 2 (LPHN2), mR	49.21060	15.41660	32.42074	12.87143	Detected	Compromised	Detected	Compromised
406	A_33_P3222598					704.82654	975.41060	3.27752	3.34219	Detected	Detected	Compromised	Compromised
407	A_23_P80778	DIRC2	Hs.477346	disrupted in renal carcinoma 2	Homo sapiens disrupted in renal carcino	1945.11680	2075.99680	1625.09400	1349.37720	Detected	Detected	Detected	Detected
408	A_24_P49597					7348.04350	12254.92300	4111.52250	5201.08840	Detected	Detected	Detected	Detected
409	A_23_P101185	NARS	Hs.465224	asparaginyl-tRNA synthetase	Homo sapiens asparaginyl-tRNA synthe	8322.22100	5775.84100	5426.73440	3071.07080	Detected	Detected	Detected	Detected
410	A_23_P121064	PTX3	Hs.591286	pentraxin-related gene, rapidly induced	Homo sapiens pentraxin-related gene, r	972.81750	12487.65800	677.31525	7262.37300	Detected	Detected	Detected	Detected
411	A_33_P3291160	LOC100133655		hypothetical protein LOC100133655	PREDICTED: Homo sapiens hypothetica	38.35226	35.12378	19.17463	22.41189	Detected	Detected	Detected	Detected
412	A_23_P403955	TARDBP	Hs.300624	TAR DNA binding protein	Homo sapiens TAR DNA binding protein	962.93650	797.10980	640.02240	449.55597	Detected	Detected	Detected	Detected
413	A_24_P135921					797.51355	1553.49220	828.41840	1455.13380	Detected	Detected	Detected	Detected
414	A_24_P271014	PPP4R1	Hs.464595	protein phosphatase 4, regulatory subur	Homo sapiens protein phosphatase 4, re	1366.32350	1687.43410	775.86090	840.35120	Detected	Detected	Detected	Detected
415	A_23_P111621	GTF2IRD1	Hs.647056	GTF2I repeat domain containing 1	Homo sapiens GTF2I repeat domain cor	4791.10250	5151.34400	2759.35000	2577.99220	Detected	Detected	Detected	Detected
416	A_23_P118571	SOST	Hs.349204	sclerosteosis	Homo sapiens sclerosteosis (SOST), ml	21.52464	18.19920	18.17187	4.95681	Detected	Not Detected	Detected	Compromised
417	A_23_P108501	EPHA4	Hs.371218	EPH receptor A4	Homo sapiens EPH receptor A4 (EPHA4	311.36337	195.28638	215.34747	109.55516	Detected	Detected	Detected	Detected
418	A_32_P66222	ANKRD20A2	Hs.632663	ankyrin repeat domain 20 family, membe	Homo sapiens ankyrin repeat domain 20	16.25730	3.78598	9.40287	5.87389	Detected	Compromised	Compromised	Compromised
419	A_23_P112016	DLC1	Hs.720057	deleted in liver cancer 1	Homo sapiens deleted in liver cancer 1	23.31969	48.45659	18.30392	25.70453	Detected	Detected	Detected	Detected
420	A_33_P3784283	JAK1	Hs.207538	Janus kinase 1	Homo sapiens Janus kinase 1 (JAK1), n	2056.43950	2267.06400	1432.49270	1375.41710	Detected	Detected	Detected	Detected
421	A_23_P163278	PARP16	Hs.30634	poly (ADP-ribose) polymerase family, m	Homo sapiens poly (ADP-ribose) polym	524.44025	334.70282	451.18000	245.59143	Detected	Detected	Detected	Detected
422	A_24_P93736	FLJ40292	Hs.721002	hypothetical LOC643210	Homo sapiens hypothetical LOC643210	27.95281	35.10012	9.61531	9.12931	Detected	Detected	Compromised	Compromised
423	A_23_P203439	KCNC1	Hs.552896	potassium voltage-gated channel, Shaw	Homo sapiens potassium voltage-gated	23.78365	5.25829	20.46592	3.46164	Detected	Compromised	Detected	Compromised
424	A_33_P3338036	ZNF470	Hs.204449	zinc finger protein 470	Homo sapiens zinc finger protein 470 (Z	258.70642	290.87598	172.82852	194.84976	Detected	Detected	Detected	Detected
425	A_23_P106661	CMTM1	Hs.698621	CKLF-like MARVEL transmembrane dor	Homo sapiens CKLF-like MARVEL tran	799.86930	190.37970	729.16110	136.20396	Detected	Detected	Detected	Detected
426	A_23_P120316	MTHFD2	Hs.469030	methylenetetrahydrofolate dehydrogena	Homo sapiens methylenetetrahydrofolat	3658.45780	5712.04900	2372.16160	2918.96070	Detected	Detected	Detected	Detected
427	A_24_P383076	AASDH	Hs.104347	aminoadipate-semialdehyde dehydroger	Homo sapiens aminoadipate-semialdehy	274.10180	183.49289	190.30495	135.00415	Detected	Detected	Detected	Detected
428	A_23_P112311	TRIM32	Hs.591910	tripartite motif-containing 32	Homo sapiens tripartite motif-containin	618.11210	688.30066	397.91050	386.45822	Detected	Detected	Detected	Detected
429	A_23_P156708	TNXB	Hs.485104	tenascin XB	Homo sapiens tenascin XB (TNXB), trar	5805.15700	626.72424	5762.05130	530.25757	Detected	Detected	Detected	Detected
430	A_32_P48466	LCA5L	Hs.517284	Leber congenital amaurosis 5-like	Homo sapiens Leber congenital amauro	97.81629	25.66273	83.47168	17.75692	Detected	Detected	Detected	Compromised
431	A_23_P142950	GIGYF2	Hs.565319	GRB10 interacting GYF protein 2	Homo sapiens GRB10 interacting GYF p	3670.29800	3285.05470	2477.30220	1970.15670	Detected	Detected	Detected	Detected
432	A_33_P3315263	KRT79	Hs.711471	keratin 79	Homo sapiens keratin 79 (KRT79), mRN	31.77119	8.53439	4.45110	6.53466	Detected	Compromised	Compromised	Compromised
433	A_23_P344421	ROBO4	Hs.524121	roundabout homolog 4, magic roundabou	Homo sapiens roundabout homolog 4, m	154.22581	17.96187	107.01122	19.90135	Detected	Compromised	Detected	Detected
434	A_33_P3854217	KIAA0319L	Hs.456507	KIAA0319-like	Homo sapiens KIAA0319-like (KIAA031	673.28690	369.54465	568.93300	270.92648	Detected	Detected	Detected	Detected
435	A_23_P163992	GRB7	Hs.86859	growth factor receptor-bound protein 7	Homo sapiens growth factor receptor-b	167.09584	217.06401	71.56998	70.84439	Detected	Detected	Detected	Detected
436	A_23_P12363	ROR1	Hs.654491	receptor tyrosine kinase-like orphan re	Homo sapiens receptor tyrosine kinase-	373.42627	1419.42630	264.36820	937.33710	Detected	Detected	Detected	Detected
437	A_33_P3272165	C8orf38	Hs.435500	chromosome 8 open reading frame 38	Homo sapiens chromosome 8 open read	1474.27620	2041.52280	1328.04270	1615.01780	Detected	Detected	Detected	Detected
438	A_33_P3414799	SH3BP2	Hs.167679	SH3-domain binding protein 2	Homo sapiens SH3-domain binding prot	21.16889	27.04291	20.21460	34.00600	Detected	Detected	Detected	Detected
439	A_23_P330561	C19orf59	Hs.709539	chromosome 19 open reading frame 59	Homo sapiens chromosome 19 open rea	24.56215	41.32567	10.94997	15.00771	Detected	Detected	Compromised	Compromised
440	A_24_P108738	SCARF2	Hs.474251	scavenger receptor class F, member 2	Homo sapiens scavenger receptor class	5266.88700	6749.67400	3122.49850	2933.21800	Detected	Detected	Detected	Detected
441	A_23_P102731	SMOX	Hs.433337	spermine oxidase	Homo sapiens spermine oxidase (SMOX	4814.96530	6881.27340	3350.60740	4212.73540	Detected	Detected	Detected	Detected
442	A_24_P268893	THAP6	Hs.479971	THAP domain containing 6	Homo sapiens THAP domain containing	686.15100	344.40820	573.78060	265.35130	Detected	Detected	Detected	Detected
443	A_33_P3381827	OSBPL2	Hs.473254	oxysterol binding protein-like 2	Homo sapiens oxysterol binding protein	310.29044	395.82773	256.32172	351.00160	Detected	Detected	Detected	Detected
444	A_33_P3434927	PR47	Hs.683780	platelet receptor for type III collagen, 47	Homo sapiens platelet receptor for type	26.88312	10.90445	17.25789	4.95021	Detected	Compromised	Compromised	Compromised
445	A_23_P100074	AVEN	Hs.555966	apoptosis, caspase activation inhibitor	Homo sapiens apoptosis, caspase activa	2960.03540	8326.81000	2313.94360	5605.15100	Detected	Detected	Detected	Detected
446	A_23_P5785	BBS5	Hs.233398	Bardet-Biedl syndrome 5	Homo sapiens Bardet-Biedl syndrome 5	77.82369	267.76580	65.52163	187.85620	Detected	Detected	Detected	Detected

447	A_33_P3309481	PTPRV	Hs.716275	protein tyrosine phosphatase, receptor	Homo sapiens protein tyrosine phospha	70.28989	53.99856	45.89647	34.42334	Detected	Detected	Detected	Detected
448	A_23_P339098	SLC35F2	Hs.524014	solute carrier family 35, member F2	Homo sapiens solute carrier family 35,	4183.43650	4599.09800	4278.52730	3963.07890	Detected	Detected	Detected	Detected
449	A_23_P113634	CBFB	Hs.460988	core-binding factor, beta subunit	Homo sapiens core-binding factor, beta	5911.96440	10033.42300	3801.44460	4988.38200	Detected	Detected	Detected	Detected
450	A_23_P35645	RBM17	Hs.498548	RNA binding motif protein 17	Homo sapiens RNA binding motif protei	5679.22170	3098.40920	5591.55100	2361.70200	Detected	Detected	Detected	Detected
451	A_24_P282237	PRIM2	Hs.654580	primase, DNA, polypeptide 2 (58kDa)	Homo sapiens primase, DNA, polypeptid	109.93461	390.04382	64.25757	191.68808	Detected	Detected	Detected	Detected
452	A_33_P3250394					163.24138	164.92937	103.84569	102.32317	Detected	Detected	Detected	Detected
453	A_24_P364236	NDUFC2	Hs.407860	NADH dehydrogenase (ubiquinone) 1, su	Homo sapiens NADH dehydrogenase (ul	550.65735	812.72644	398.45640	436.34090	Detected	Detected	Detected	Detected
454	A_24_P712562	C17orf67	Hs.658949	chromosome 17 open reading frame 67	Homo sapiens chromosome 17 open rea	70.44097	41.53251	45.73420	19.83071	Detected	Detected	Detected	Detected
455	A_32_P74955	ARID2	Hs.317304	AT rich interactive domain 2 (ARID, RF	Homo sapiens AT rich interactive doma	675.48157	403.44656	535.30756	260.05220	Detected	Detected	Detected	Detected
456	A_23_P49220	TCF25	Hs.415342	transcription factor 25 (basic helix-loop	Homo sapiens transcription factor 25 (b	2700.51220	3692.43550	3702.93870	4723.45400	Detected	Detected	Detected	Detected
457	A_24_P586390	LOC100133331	Hs.646112	hypothetical LOC100133331	Homo sapiens hypothetical LOC100133	128.08100	51.90045	89.97714	30.76644	Detected	Detected	Detected	Detected
458	A_23_P165840	ODC1	Hs.467701	ornithine decarboxylase 1	Homo sapiens ornithine decarboxylase	13138.87200	31721.78000	10350.43200	21221.34000	Detected	Detected	Detected	Detected
459	A_33_P3441639	LOC145694	Hs.147229	hypothetical protein LOC145694	Homo sapiens cDNA FLJ32231 fis, clon	96.26368	1003.94870	101.53353	778.87370	Detected	Detected	Detected	Detected
460	A_23_P34915	ATF3	Hs.460	activating transcription factor 3	Homo sapiens activating transcription f	1185.92400	3447.05420	912.66490	2292.97800	Detected	Detected	Detected	Detected
461	A_23_P324538	LCE3B	Hs.553715	late cornified envelope 3B	Homo sapiens late cornified envelope 3	152.03499	259.96110	109.32539	132.37140	Detected	Detected	Detected	Detected
462	A_23_P122924	INHBA	Hs.583348	inhibin, beta A	Homo sapiens inhibin, beta A (INHBA),	8814.29800	45767.67600	7573.83060	31416.12300	Detected	Detected	Detected	Detected
463	A_33_P3289338	LOC100131346	Hs.640125	hypothetical protein LOC100131346	Homo sapiens cDNA FLJ45704 fis, clon	17.68627	9.85156	14.18271	5.43988	Detected	Compromised	Compromised	Compromised
464	A_24_P366122	ACBD4	Hs.110298	acyl-Coenzyme A binding domain conta	Homo sapiens acyl-Coenzyme A binding	9043.65100	11064.89700	2639.03560	2192.78690	Detected	Detected	Detected	Detected
465	A_23_P121396	DNAJC19	Hs.230601	DnaJ (Hsp40) homolog, subfamily C, me	Homo sapiens DnaJ (Hsp40) homolog, s	455.95102	400.94160	376.59137	251.43497	Detected	Detected	Detected	Detected
466	A_23_P110851	TERT	Hs.492203	telomerase reverse transcriptase	Homo sapiens telomerase reverse trans	95.27364	96.42165	41.57469	35.98146	Detected	Detected	Detected	Detected
467	A_33_P3273173	USP11	Hs.171501	ubiquitin specific peptidase 11	Homo sapiens ubiquitin specific peptida	113.59079	112.60268	96.29980	98.54799	Detected	Detected	Detected	Detected
468	A_24_P883629	C2orf49	Hs.549577	chromosome 2 open reading frame 49	Ashwin [Source:UniProtKB/Swiss-Pro	274.46460	163.03271	163.04430	92.83398	Detected	Detected	Detected	Detected
469	A_23_P11995	PRDX1	Hs.180909	peroxiredoxin 1	Homo sapiens peroxiredoxin 1 (PRDX1)	51651.16400	81395.00000	47045.33000	64838.53000	Detected	Detected	Detected	Detected
470	A_24_P409661					1393.76150	5690.68700	1100.15770	3885.01930	Detected	Detected	Detected	Detected
471	A_33_P3238148		Hs.417077		Leucine-rich repeat and death domain-	3.32305	40.26442	8.40743	13.30925	Compromised	Detected	Compromised	Compromised
472	A_23_P109677					7804.94600	10478.82500	6941.59380	8374.55300	Detected	Detected	Detected	Detected
473	A_33_P3420695				Putative uncharacterized protein ENSP	197.82020	319.30438	59.39119	57.17635	Detected	Detected	Detected	Detected
474	A_23_P218317	NARF	Hs.256526	nuclear prelamin A recognition factor	Homo sapiens nuclear prelamin A recog	5891.88000	3355.73660	5824.31450	2939.64500	Detected	Detected	Detected	Detected
475	A_33_P3242919	HNRNPM	Hs.465808	heterogeneous nuclear ribonucleoprotei	Homo sapiens heterogeneous nuclear ri	13352.18500	25247.18000	6494.33300	9672.36200	Detected	Detected	Detected	Detected
476	A_23_P122144	UGT3A1	Hs.254699	UDP glycosyltransferase 3 family, polyp	Homo sapiens UDP glycosyltransferase	34.34445	51.26796	18.59548	20.39441	Detected	Detected	Detected	Detected
477	A_32_P176594	KIAA1614	Hs.647760	KIAA1614	Homo sapiens KIAA1614 (KIAA1614), m	165.76558	121.86799	130.42130	108.96890	Detected	Detected	Detected	Detected
478	A_23_P321855	ARHGEF7	Hs.508738	Rho guanine nucleotide exchange facto	Homo sapiens Rho guanine nucleotide e	2596.92970	1771.47950	2269.37280	1461.14840	Detected	Detected	Detected	Detected
479	A_33_P3662000	LOC401037	Hs.447665	hypothetical gene supported by AK0562	Homo sapiens cDNA FLJ31684 fis, clon	16.56085	12.42196	18.23892	10.96318	Detected	Compromised	Detected	Compromised
480	A_23_P103433	OSCP1	Hs.202207	organic solute carrier partner 1	Homo sapiens organic solute carrier pa	2944.67630	2217.56900	2202.44000	1442.82290	Detected	Detected	Detected	Detected
481	A_23_P51996	STXBP3	Hs.530436	syntaxin binding protein 3	Homo sapiens syntaxin binding protein	6450.99000	5923.81740	4364.42430	3318.70680	Detected	Detected	Detected	Detected
482	A_23_P345799	FAM129C	Hs.434133	family with sequence similarity 129, mer	Homo sapiens family with sequence sim	31.00659	64.92902	10.12871	12.42890	Detected	Detected	Compromised	Compromised
483	A_24_P23921	C17orf98	Hs.250182	chromosome 17 open reading frame 98	Homo sapiens chromosome 17 open rea	25.02121	4.11445	33.07570	3.69507	Detected	Compromised	Detected	Compromised
484	A_23_P134237	RARRES2	Hs.647064	retinoic acid receptor responder (tazaro	Homo sapiens retinoic acid receptor res	37.86021	27.04658	45.04725	39.21018	Detected	Detected	Detected	Detected
485	A_23_P117225	ERCC5	Hs.258429	excision repair cross-complementing ro	Homo sapiens excision repair cross-com	6527.45600	6276.25100	5398.29400	4665.99560	Detected	Detected	Detected	Detected
486	A_33_P3379962	HLA-A	Hs.181244	major histocompatibility complex, class	Homo sapiens major histocompatibility	188684.40000	127831.20000	164110.97000	108709.16000	Detected	Detected	Detected	Detected
487	A_24_P289404	RPS26	Hs.567235	ribosomal protein S26	Homo sapiens ribosomal protein S26 (R	20058.80900	34060.54300	13056.27500	17572.66800	Detected	Detected	Detected	Detected
488	A_33_P3298062	ABCC5	Hs.606340	ATP-binding cassette, sub-family C (CF	Homo sapiens ATP-binding cassette, su	1481.47520	515.40530	1004.20960	299.34830	Detected	Detected	Detected	Detected
489	A_33_P3409277		Hs.722546		1008982 Human Fat Cell 5'-Stretch Plu	91.91484	118.79000	41.39313	37.90961	Detected	Detected	Detected	Detected
490	A_23_P34018	RPL39	Hs.558387	ribosomal protein L39	Homo sapiens ribosomal protein L39 (R	133620.56000	78743.73000	95512.11000	46191.97700	Detected	Detected	Detected	Detected
491	A_23_P70328	CENPQ	Hs.88663	centromere protein Q	Homo sapiens centromere protein Q (C	2065.80640	2638.72880	1646.84900	1887.64270	Detected	Detected	Detected	Detected
492	A_33_P3334205	TMEM184A	Hs.592174	transmembrane protein 184A	Homo sapiens transmembrane protein	35.72694	36.32193	31.63289	31.21107	Detected	Detected	Detected	Detected
493	A_24_P358164	RPSA	Hs.449909	ribosomal protein SA	Homo sapiens ribosomal protein SA (RF	37964.79700	74424.34000	37845.13000	63703.35000	Detected	Detected	Detected	Detected
494	A_23_P134614	COG5	Hs.239631	component of oligomeric golgi complex	Homo sapiens component of oligomeric	1950.13530	999.23535	1546.08230	693.82635	Detected	Detected	Detected	Detected
495	A_32_P396186	TRIM66	Hs.130836	tripartite motif-containing 66	Homo sapiens tripartite motif-containin	524.36084	93.73052	239.64003	42.93500	Detected	Detected	Detected	Detected
496	A_23_P49155	CDH3	Hs.191842	cadherin 3, type 1, P-cadherin (placenta	Homo sapiens cadherin 3, type 1, P-cad	71.58225	47.52973	42.08263	20.29404	Detected	Detected	Detected	Detected
497	A_32_P54137	UQCRH	Hs.481571	ubiquinol-cytochrome c reductase hinge	Homo sapiens ubiquinol-cytochrome c	52509.30500	51392.63000	49620.84000	42008.44500	Detected	Detected	Detected	Detected
498	A_23_P58489	FASTKD3	Hs.653162	FAST kinase domains 3	Homo sapiens FAST kinase domains 3 (	1143.32920	1209.82570	1047.28280	939.93310	Detected	Detected	Detected	Detected
499	A_33_P3215128	C1orf69	Hs.237017	chromosome 1 open reading frame 69	Homo sapiens chromosome 1 open reac	322.23030	439.73044	273.49493	340.89896	Detected	Detected	Detected	Detected
500	A_23_P142022	PRPF31	Hs.515598	PRP31 pre-mRNA processing factor 31	Homo sapiens PRP31 pre-mRNA proce	3544.00150	3713.83030	3969.38160	3992.47070	Detected	Detected	Detected	Detected
501	A_23_P167595	UBE2B	Hs.644421	ubiquitin-conjugating enzyme E2B (RAD	Homo sapiens ubiquitin-conjugating enz	788.76215	945.44550	603.47980	570.30850	Detected	Detected	Detected	Detected
502	A_23_P57137	C20orf29	Hs.104806	chromosome 20 open reading frame 29	Homo sapiens chromosome 20 open rea	1521.25850	3173.40280	1408.08370	2458.87480	Detected	Detected	Detected	Detected
503	A_33_P3326733	LOC729668		golgi autoantigen, golgin subfamily a, 6 p	Homo sapiens golgi autoantigen, golgin	2555.53440	1891.58980	1125.10230	435.33310	Detected	Detected	Detected	Detected
504	A_23_P52311	TAF5	Hs.96103	TAF5 RNA polymerase II, TATA box bin	Homo sapiens TAF5 RNA polymerase II	97.32083	247.51009	66.78814	135.44788	Detected	Detected	Detected	Detected
505	A_33_P3318459	SUMO1	Hs.81424	SMT3 suppressor of mif two 3 homolog	Homo sapiens SMT3 suppressor of mif	4798.09400	3307.26830	3531.74440	2099.82400	Detected	Detected	Detected	Detected
506	A_33_P3332215	MUC1	Hs.89603	mucin 1, cell surface associated	Homo sapiens mucin 1, cell surface ass	26.86598	54.17699	24.09920	24.29657	Detected	Detected	Detected	Detected
507	A_33_P3354646	PNLIPRP1	Hs.73923	pancreatic lipase-related protein 1	Homo sapiens pancreatic lipase-related	157.59848	241.16473	80.17725	96.68468	Detected	Detected	Detected	Detected
508	A_23_P212968	UGT2B11	Hs.339811	UDP glucuronosyltransferase 2 family, p	Homo sapiens UDP glucuronosyltransfe	20.71307	3.76288	8.85763	3.34683	Detected	Compromised	Compromised	Compromised
509	A_23_P360626	PLD6	Hs.31652	phospholipase D family, member 6	Homo sapiens phospholipase D family, r	333.45640	469.60974	278.91672	312.61190	Detected	Detected	Detected	Detected
510	A_33_P3240931	C5orf43	Hs.508479	chromosome 5 open reading frame 43	Homo sapiens chromosome 5 open reac	2863.35330	4652.38870	738.63556	1266.43620	Detected	Detected	Detected	Detected
511	A_23_P101342	ATG4D	Hs.512799	ATG4 autophagy related 4 homolog D (S	Homo sapiens ATG4 autophagy related	241.77296	440.57715	155.77104	255.05907	Detected	Detected	Detected	Detected
512	A_33_P3564409					44.01296	42.97599	57.49984	30.01708	Detected	Detected	Detected	Detected
513	A_24_P256243	SSX2	Hs.661107	synovial sarcoma, X breakpoint 2	Homo sapiens synovial sarcoma, X brea	291.61084	388.59827	208.03545	190.01822	Detected	Detected	Detected	Detected
514	A_32_P32391	OR7E156P	Hs.363054	olfactory receptor, family 7, subfamily E	Homo sapiens olfactory receptor, family	184.90160	271.77994	150.32948	261.29785	Detected	Detected	Detected	Detected
515	A_24_P66312	KRTAP12-1	Hs.688628	keratin associated protein 12-1	Homo sapiens keratin associated protei	18.01324	14.91404	11.52317	11.93854	Detected	Compromised	Compromised	Compromised
516	A_33_P3347055	LOC100130966		similar to SMT3 suppressor of mif two 3	PREDICTED: Homo sapiens similar to S	1145.85110	1609.48650	774.37290	956.31390	Detected	Detected	Detected	Detected
517	A_33_P3407266	GTF3C4	Hs.656646	general transcription factor IIIC, polype	Homo sapiens general transcription fact	100.29210	94.40801	84.82456	61.30132	Detected	Detected	Detected	Detected
518	A_23_P115366	CMPK1	Hs.11463	cytidine monophosphate (UMP-CMP) ki	Homo sapiens cytidine monophosphate	4502.36700	3976.84330	2631.66670	1870.89950	Detected	Detected	Detected	Detected
519	A_23_P58132	RHOH	Hs.654594	ras homolog gene family, member H	Homo sapiens ras homolog gene family,	45.47711	53.54306	41.48669	44.61155	Detected	Detected	Detected	Detected
520	A_23_P121082	GBE1	Hs.436062	glucan (1,4-alpha-), branching enzyme	Homo sapiens glucan (1,4-alpha-), bran	50205.56200	10494.25600	36161.38700	6626.56300	Detected	Detected	Detected	Detected
521	A_23_P209288	CUL3	Hs.372286	cullin 3	Homo sapiens cullin 3 (CUL3), mRNA [N	84.66550	11.01301	75.32278	6.89077	Detected	Compromised	Detected	Compromised

522	A_33_P3782469	PYGO1	Hs.87194	pygopus homolog 1 (Drosophila)	Pygopus homolog 1 [Source:UniProtKB	451.14478	126.00648	285.68118	73.55151	Detected	Detected	Detected	Detected
523	A_24_P296808	PNMAL1	Hs.8395	PNMA-like 1	Homo sapiens PNMA-like 1 (PNMAL1),	4609.22360	3879.35030	3649.76930	2731.47660	Detected	Detected	Detected	Detected
524	A_23_P34835	LMNA	Hs.594444	lamin A/C	Homo sapiens lamin A/C (LMNA), trans	82125.27000	119083.51600	76971.39000	96952.92000	Detected	Detected	Detected	Detected
525	A_33_P3227375	THBS2	Hs.371147	thrombospondin 2	Homo sapiens thrombospondin 2 (THBS	10447.23700	6864.01860	8009.26900	4667.91750	Detected	Detected	Detected	Detected
526	A_32_P222695	FLJ41603	Hs.256206	FLJ41603 protein	Homo sapiens FLJ41603 protein (FLJ41	71.09638	13.58448	64.62899	16.88072	Detected	Compromised	Detected	Compromised
527	A_23_P102842	NFS1	Hs.194692	NFS1 nitrogen fixation 1 homolog (S. ce	Homo sapiens NFS1 nitrogen fixation 1	2517.09600	1225.12290	2233.55620	950.70550	Detected	Detected	Detected	Detected
528	A_23_P138849	KAT5	Hs.397010	K(lysine) acetyltransferase 5	Homo sapiens K(lysine) acetyltransferas	1961.10020	2119.69170	2179.33330	2066.40040	Detected	Detected	Detected	Detected
529	A_33_P3297642	C10orf84	Hs.372309	chromosome 10 open reading frame 84	Homo sapiens chromosome 10 open rea	5176.25930	6594.56300	4891.89900	5007.58000	Detected	Detected	Detected	Detected
530	A_23_P122531	C6orf48	Hs.640836	chromosome 6 open reading frame 48	Homo sapiens chromosome 6 open read	26326.96700	38390.00400	23046.74200	29493.80900	Detected	Detected	Detected	Detected
531	A_24_P925040	CAV2	Hs.212332	caveolin 2	Homo sapiens caveolin 2 (CAV2), trans	837.27875	1938.00010	452.67847	818.64246	Detected	Detected	Detected	Detected
532	A_24_P197284	C2orf85	Hs.376307	chromosome 2 open reading frame 85	Homo sapiens chromosome 2 open read	22.90743	32.43810	18.27090	20.06642	Detected	Detected	Detected	Detected
533	A_33_P3416376	LOC342918	Hs.531650	similar to mCG134545	PREDICTED: Homo sapiens hypothetica	72.99901	78.94990	43.47972	49.12862	Detected	Detected	Detected	Detected
534	A_33_P3619171	PMAIP1	Hs.96	phorbol-12-myristate-13-acetate-indu	Homo sapiens phorbol-12-myristate-13	1125.90720	3819.66650	894.84045	2481.20780	Detected	Detected	Detected	Detected
535	A_33_P3391429	ZNF672	Hs.521151	zinc finger protein 672	Homo sapiens zinc finger protein 672 (Z	1253.25550	1612.77840	1183.80210	1568.27580	Detected	Detected	Detected	Detected
536	A_23_P119886	GCKR	Hs.89771	glucokinase (hexokinase 4) regulator	Homo sapiens glucokinase (hexokinase	43.61835	32.54997	43.68837	29.34973	Detected	Detected	Detected	Detected
537	A_33_P3343503	LOC645602	Hs.632349	hypothetical LOC645602	PREDICTED: Homo sapiens hypothetica	38.72920	52.33947	46.83429	55.70081	Detected	Detected	Detected	Detected
538	A_23_P111929	OTUD6B	Hs.30532	OTU domain containing 6B	Homo sapiens OTU domain containing 6	34.15876	30.68196	30.99400	19.88240	Detected	Detected	Detected	Detected
539	A_23_P72853	MFSD5	Hs.654660	major facilitator superfamily domain cor	Homo sapiens major facilitator superfan	2736.92240	4272.29000	3102.27860	4416.58350	Detected	Detected	Detected	Detected
540	A_33_P3312030					3769.64700	4887.22170	1643.83840	1712.62430	Detected	Detected	Detected	Detected
541	A_33_P3408762	LMNA	Hs.594444	lamin A/C	Homo sapiens lamin A/C (LMNA), trans	39074.83200	55807.83000	43306.87500	58758.24000	Detected	Detected	Detected	Detected
542	A_33_P3364289	HSH2D	Hs.631617	hematopoietic SH2 domain containing	Homo sapiens hematopoietic SH2 doma	255.53252	304.12082	129.84470	127.55432	Detected	Detected	Detected	Detected
543	A_33_P3221980					79.16579	71.99860	37.09886	30.41040	Detected	Detected	Detected	Detected
544	A_23_P417148	RAI1	Hs.655395	retinoic acid induced 1	Homo sapiens retinoic acid induced 1 (F	3418.94380	4218.88870	2697.05470	3461.09840	Detected	Detected	Detected	Detected
545	A_23_P105276	ZNF84	Hs.445019	zinc finger protein 84	Homo sapiens zinc finger protein 84 (ZN	647.78520	667.70105	503.03665	449.85443	Detected	Detected	Detected	Detected
546	A_23_P101093	COPZ2	Hs.408434	coatomer protein complex, subunit zeta	Homo sapiens coatomer protein comple	3416.54250	10621.14600	2415.69400	6519.27150	Detected	Detected	Detected	Detected
547	A_32_P12994	LOC440900	Hs.662205	hypothetical LOC440900	PREDICTED: Homo sapiens hypothetica	31.67730	147.45746	24.28995	105.17532	Detected	Detected	Detected	Detected
548	A_23_P115922	EIF4EBP2	Hs.643279	eukaryotic translation initiation factor 4	Homo sapiens eukaryotic translation ini	5135.72100	6858.30400	3538.56100	4002.73170	Detected	Detected	Detected	Detected
549	A_23_P110606	MAML1	Hs.631951	mastermind-like 1 (Drosophila)	Homo sapiens mastermind-like 1 (Droso	464.30182	459.84567	339.40842	301.97934	Detected	Detected	Detected	Detected
550	A_24_P645765	KLHDC5	Hs.505104	kelch domain containing 5	Homo sapiens kelch domain containing	1070.57620	840.94965	895.11200	585.02060	Detected	Detected	Detected	Detected
551	A_23_P114929	BRP44	Hs.517768	brain protein 44	Homo sapiens brain protein 44 (BRP44)	9805.58500	16610.62000	7739.89060	10964.49100	Detected	Detected	Detected	Detected
552	A_23_P168288	IL22RA2	Hs.126891	interleukin 22 receptor, alpha 2	Homo sapiens interleukin 22 receptor, a	15.58777	12.99581	3.22367	3.30128	Detected	Compromised	Compromised	Compromised
553	A_23_P113613	CDCP1	Hs.476093	CUB domain containing protein 1	Homo sapiens CUB domain containing p	1550.28330	209.81682	1471.51890	163.31450	Detected	Detected	Detected	Detected
554	A_24_P64362	C10orf26	Hs.500897	chromosome 10 open reading frame 26	Homo sapiens chromosome 10 open rea	3184.24560	3028.40920	2103.93380	1742.25930	Detected	Detected	Detected	Detected
555	A_23_P382188	STAP2	Hs.194385	signal transducing adaptor family memb	Homo sapiens signal transducing adapto	117.96211	116.99578	133.46160	119.55611	Detected	Detected	Detected	Detected
556	A_23_P10291	CTSE	Hs.644082	cathepsin E	Homo sapiens cathepsin E (CTSE), tran	119.44481	139.52678	34.36173	34.16397	Detected	Detected	Detected	Detected
557	A_33_P3382560	RPL23A	Hs.419463	ribosomal protein L23a	Homo sapiens ribosomal protein L23a (F	76901.72000	58674.52000	50323.05000	33195.49200	Detected	Detected	Detected	Detected
558	A_23_P122508	DPCR1	Hs.631993	diffuse panbronchiolitis critical region 1	Homo sapiens diffuse panbronchiolitis c	16.06864	17.75563	4.49426	4.74671	Detected	Detected	Compromised	Compromised
559	A_23_P218597	NPAS2	Hs.156832	neuronal PAS domain protein 2	Homo sapiens neuronal PAS domain pro	320.64136	131.45767	348.08838	131.70540	Detected	Detected	Detected	Detected
560	A_23_P204016	CACNB3	Hs.250712	calcium channel, voltage-dependent, be	Homo sapiens calcium channel, voltage-	908.01807	1077.30690	1120.19620	1288.64430	Detected	Detected	Detected	Detected
561	A_23_P115636	TRDMT1	Hs.351665	tRNA aspartic acid methyltransferase 1	Homo sapiens tRNA aspartic acid meth	139.66856	222.86473	141.89554	182.38548	Detected	Detected	Detected	Detected
562	A_33_P3275826				Putative uncharacterized protein ENSP	231437.16000	274513.38000	277021.30000	261155.02000	Detected	Detected	Detected	Detected
563	A_23_P126197	SFRS4	Hs.469970	splicing factor, arginine/serine-rich 4	Homo sapiens splicing factor, arginine/s	5993.66200	5739.44400	3333.30980	2708.10080	Detected	Detected	Detected	Detected
564	A_23_P109322	PCP4	Hs.80296	Purkinje cell protein 4	Homo sapiens Purkinje cell protein 4 (P	91.05113	126.43391	22.19374	27.54967	Detected	Detected	Detected	Detected
565	A_23_P109593	TBC1D22A	Hs.435044	TBC1 domain family, member 22A	Homo sapiens TBC1 domain family, mer	580.68810	1016.92523	384.67285	693.61310	Detected	Detected	Detected	Detected
566	A_32_P129950	NHLRC3	Hs.507783	NHL repeat containing 3	Homo sapiens NHL repeat containing 3	554.27313	309.39760	505.92157	259.48620	Detected	Detected	Detected	Detected
567	A_33_P3233784	TMEM211	Hs.329040	transmembrane protein 211	Homo sapiens transmembrane protein 2	36.71519	32.12401	31.91140	27.29348	Detected	Detected	Detected	Detected
568	A_23_P30024	NFKB1	Hs.654408	nuclear factor of kappa light polypeptide	Homo sapiens nuclear factor of kappa li	3279.40040	2524.93500	2859.08450	1980.95400	Detected	Detected	Detected	Detected
569	A_33_P3220475	FLJ23867		hypothetical protein FLJ23867	Homo sapiens hypothetical protein FLJ	128.25473	113.27683	90.34032	62.17117	Detected	Detected	Detected	Detected
570	A_23_P101351	ZNF426	Hs.594011	zinc finger protein 426	Homo sapiens zinc finger protein 426 (Z	333.71152	384.36987	190.94237	175.56686	Detected	Detected	Detected	Detected
571	A_23_P127662	OR8D1	Hs.504211	olfactory receptor, family 8, subfamily D	Homo sapiens olfactory receptor, family	17.64634	16.57016	7.52918	6.72273	Detected	Compromised	Compromised	Compromised
572	A_23_P212339	FYCO1	Hs.200227	FYVE and coiled-coil domain containing	Homo sapiens FYVE and coiled-coil dor	2703.36990	1146.07980	2130.31270	777.25970	Detected	Detected	Detected	Detected
573	A_33_P3239884	NCAM2	Hs.473450	neural cell adhesion molecule 2	Homo sapiens neural cell adhesion mole	110.57481	127.28230	44.65458	39.79016	Detected	Detected	Detected	Detected
574	A_24_P203315	CNIH2	Hs.437072	cornichon homolog 2 (Drosophila)	Homo sapiens cornichon homolog 2 (Dr	368.88104	349.34906	107.68288	87.83997	Detected	Detected	Detected	Detected
575	A_33_P3333054	GOLSYN	Hs.390738	Golgi-localized protein	Homo sapiens Golgi-localized protein (C	160.25647	61.39557	145.88925	50.37893	Detected	Detected	Detected	Detected
576	A_23_P62709	SPRR3	Hs.139322	small proline-rich protein 3	Homo sapiens small proline-rich protein	185.35520	165.89163	123.19068	113.54950	Detected	Detected	Detected	Detected
577	A_23_P55731	CIC	Hs.388236	capicua homolog (Drosophila)	Homo sapiens capicua homolog (Drosop	2379.00830	1313.16410	2311.80900	1237.64610	Detected	Detected	Detected	Detected
578	A_33_P3276207	FAM55C	Hs.595933	family with sequence similarity 55, mem	Homo sapiens family with sequence sim	150.68631	84.34351	189.42834	62.05583	Detected	Detected	Detected	Detected
579	A_24_P177353	THOC2	Hs.149991	THO complex 2	Homo sapiens THO complex 2 (THOC2)	3286.80320	2130.17040	2759.17330	1501.06150	Detected	Detected	Detected	Detected
580	A_23_P12343	GSTM3	Hs.2006	glutathione S-transferase mu 3 (brain)	Homo sapiens glutathione S-transferase	20186.01200	13198.71100	17799.95500	10416.67500	Detected	Detected	Detected	Detected
581	A_23_P115885	MINPP1	Hs.121260	multiple inositol polyphosphate histidine	Homo sapiens multiple inositol polyphos	352.60062	399.24127	254.67418	245.12270	Detected	Detected	Detected	Detected
582	A_24_P345846	ANTXR2	Hs.162963	anthrax toxin receptor 2	Homo sapiens anthrax toxin receptor 2	62.50034	117.67095	43.03818	81.11925	Detected	Detected	Detected	Detected
583	A_23_P12272	PPP1R8	Hs.533474	protein phosphatase 1, regulatory (inhib	Homo sapiens protein phosphatase 1, re	6763.98440	7641.24200	4949.47850	4617.51000	Detected	Detected	Detected	Detected
584	A_23_P302134	hCG_2008140	Hs.720157	hypothetical LOC729614	Homo sapiens hypothetical LOC729614	323.08963	285.07584	323.08878	261.70392	Detected	Detected	Detected	Detected
585	A_24_P225635	HNRPLL		heterogeneous nuclear ribonucleoprotei	Heterogeneous nuclear ribonucleoprote	217.07750	104.29340	109.40680	40.54174	Detected	Detected	Detected	Detected
586	A_24_P375683					28928.53500	23246.96900	21483.06200	16287.89300	Detected	Detected	Detected	Detected
587	A_23_P115785	FANK1	Hs.352591	fibronectin type III and ankyrin repeat d	Homo sapiens fibronectin type III and ar	158.81055	124.65037	128.17247	86.81207	Detected	Detected	Detected	Detected
588	A_32_P10396	WDFY3	Hs.480116	WD repeat and FYVE domain containing	Homo sapiens WD repeat and FYVE dor	749.05920	376.18732	524.47840	249.70060	Detected	Detected	Detected	Detected
589	A_23_P114947	RGS2	Hs.78944	regulator of G-protein signaling 2, 24kD	Homo sapiens regulator of G-protein sig	6021.32500	1853.08690	4250.02930	1129.09350	Detected	Detected	Detected	Detected
590	A_33_P3279847	RAET1E	Hs.511818	retinoic acid early transcript 1E	Homo sapiens retinoic acid early transc	42.68399	27.87403	25.55145	19.68227	Detected	Detected	Detected	Detected
591	A_23_P373687	PUS10	Hs.368348	pseudouridylate synthase 10	Homo sapiens pseudouridylate synthase	38.42065	39.55555	28.98142	34.06977	Detected	Detected	Detected	Detected
592	A_24_P206344	ZNF746	Hs.24643	zinc finger protein 746	Homo sapiens zinc finger protein 746 (Z	7831.64450	8952.54600	5953.24760	5651.20600	Detected	Detected	Detected	Detected
593	A_23_P218096	RBM26	Hs.558528	RNA binding motif protein 26	Homo sapiens RNA binding motif protei	377.70380	465.29886	313.27826	321.04410	Detected	Detected	Detected	Detected
594	A_23_P216915	NR5A1	Hs.495108	nuclear receptor subfamily 5, group A, r	Homo sapiens nuclear receptor subfami	20.04108	16.33543	9.64472	12.47020	Detected	Compromised	Compromised	Compromised
595	A_33_P3369393	NCF1	Hs.647047	neutrophil cytosolic factor 1	Homo sapiens neutrophil cytosolic facto	387.89532	650.55520	96.50538	109.68501	Detected	Detected	Detected	Detected
596	A_33_P3228751	LOC388152	Hs.710289	hypothetical LOC388152	Homo sapiens hypothetical LOC388152	202.87541	253.90549	105.97163	112.92820	Detected	Detected	Detected	Detected

597	A_23_P136986	APOOL	Hs.706885	apolipoprotein O-like	Homo sapiens apolipoprotein O-like (AP	69.09710	191.59320	72.28335	161.63518	Detected	Detected	Detected	Detected
598	A_23_P110184	SC4MOL	Hs.105269	sterol-C4-methyl oxidase-like	Homo sapiens sterol-C4-methyl oxidas	463.43927	337.96414	334.59177	202.95488	Detected	Detected	Detected	Detected
599	A_33_P3388016	CCDC72	Hs.356440	coiled-coil domain containing 72	Homo sapiens coiled-coil domain conta	12795.48900	21338.77000	10159.26800	14504.97100	Detected	Detected	Detected	Detected
600	A_23_P117580	ENTPD5	Hs.720540	ectonucleoside triphosphate diphosphol	Homo sapiens ectonucleoside triphosph	38.29009	38.32716	22.03913	19.32851	Detected	Detected	Detected	Detected
601	A_32_P108826	ZBTB41	Hs.529439	zinc finger and BTB domain containing	Homo sapiens zinc finger and BTB dom	542.62000	276.23087	391.21097	131.42178	Detected	Detected	Detected	Detected
602	A_24_P280868	FAM86B2	Hs.458413	family with sequence similarity 86, mem	Homo sapiens family with sequence sim	2240.04600	3051.66580	1430.15940	1562.05140	Detected	Detected	Detected	Detected
603	A_23_P123330	RPL30	Hs.400295	ribosomal protein L30	Homo sapiens ribosomal protein L30 (R	89299.20000	55853.04000	60343.14500	31797.50400	Detected	Detected	Detected	Detected
604	A_23_P368101	SAP30L	Hs.592566	SAP30-like	Homo sapiens SAP30-like (SAP30L), tr	5523.52150	5149.56740	5653.78030	4519.51900	Detected	Detected	Detected	Detected
605	A_23_P303155	TMEM87B	Hs.656298	transmembrane protein 87B	Homo sapiens transmembrane protein 8	153.72748	156.25978	102.31258	83.96524	Detected	Detected	Detected	Detected
606	A_23_P133058	MRFAP1L1	Hs.593159	Morf4 family associated protein 1-like 1	Homo sapiens Morf4 family associated	5684.83300	5242.79740	4623.90870	3590.05350	Detected	Detected	Detected	Detected
607	A_23_P114221	RBBP7	Hs.495755	retinoblastoma binding protein 7	Homo sapiens retinoblastoma binding pr	14197.65000	27995.94300	10492.53500	17264.20900	Detected	Detected	Detected	Detected
608	A_24_P125881	DGCR2	Hs.517357	DiGeorge syndrome critical region gene	Homo sapiens DiGeorge syndrome critic	15976.79900	11970.69500	11235.80800	7628.78600	Detected	Detected	Detected	Detected
609	A_24_P286935	ARL3	Hs.182215	ADP-ribosylation factor-like 3	Homo sapiens ADP-ribosylation factor-	565.07880	243.85716	517.30304	188.26495	Detected	Detected	Detected	Detected
610	A_33_P3336242	USP17L7		ubiquitin specific peptidase 17-like 7	Inactive ubiquitin carboxyl-terminal hyd	19.14826	28.64423	12.86442	9.50037	Detected	Detected	Compromised	Compromised
611	A_23_P83045	VCP	Hs.529782	valosin-containing protein	Homo sapiens valosin-containing protei	38942.91000	76720.75000	49085.22300	80375.27000	Detected	Detected	Detected	Detected
612	A_23_P119857	TTC32	Hs.591547	tetratricopeptide repeat domain 32	Homo sapiens tetratricopeptide repeat	4784.26300	2179.57100	3509.95750	1292.61660	Detected	Detected	Detected	Detected
613	A_23_P314145	UST	Hs.657370	uronyl-2-sulfotransferase	Homo sapiens uronyl-2-sulfotransferas	532.54930	618.21100	387.11557	409.83673	Detected	Detected	Detected	Detected
614	A_23_P59988	AMAC1L2	Hs.458397	acyl-malonyl condensing enzyme 1-like	Homo sapiens acyl-malonyl condensing	61.93527	73.74247	41.42238	38.88979	Detected	Detected	Detected	Detected
615	A_33_P3354881					210.54735	134.89243	280.93030	106.81499	Detected	Detected	Detected	Detected
616	A_33_P3285391					44.26123	41.66835	16.71006	11.28690	Detected	Detected	Detected	Compromised
617	A_23_P200443	SHC1	Hs.433795	SHC (Src homology 2 domain containing	Homo sapiens SHC (Src homology 2 do	13762.14600	13593.58600	12691.51400	10890.12800	Detected	Detected	Detected	Detected
618	A_33_P3215948	MPZL2	Hs.116651	myelin protein zero-like 2	Homo sapiens myelin protein zero-like 2	21.61127	33.56926	8.24838	10.09243	Detected	Detected	Compromised	Compromised
619	A_23_P148969	LRRC40	Hs.147836	leucine rich repeat containing 40	Homo sapiens leucine rich repeat conta	2744.84720	2552.02320	1853.48860	1320.86610	Detected	Detected	Detected	Detected
620	A_32_P194264	CHAC2	Hs.585944	ChaC, cation transport regulator homolo	Homo sapiens ChaC, cation transport re	130.48581	320.81350	114.49580	295.60193	Detected	Detected	Detected	Detected
621	A_24_P379353	CCDC24	Hs.632394	coiled-coil domain containing 24	Homo sapiens coiled-coil domain conta	27.24988	21.03618	39.08530	26.50699	Detected	Detected	Detected	Detected
622	A_23_P114349	XAGE3	Hs.43879	X antigen family, member 3	Homo sapiens X antigen family, member	18.40501	23.22373	14.78686	9.08851	Detected	Detected	Detected	Compromised
623	A_33_P3370424	NOTCH1	Hs.495473	Notch homolog 1, translocation-associa	Homo sapiens Notch homolog 1, translo	2561.36940	3684.28500	843.76440	823.50775	Detected	Detected	Detected	Detected
624	A_23_P100883	SUZ12	Hs.462732	suppressor of zeste 12 homolog (Droso	Homo sapiens suppressor of zeste 12 h	6627.46100	5271.18160	4463.31050	3008.12430	Detected	Detected	Detected	Detected
625	A_32_P120895	LYSMD2	Hs.603629	LysM, putative peptidoglycan-binding, d	Homo sapiens LysM, putative peptidogly	1514.78320	3434.91900	1077.56040	2271.60620	Detected	Detected	Detected	Detected
626	A_33_P3267543				Dolichol phosphate-mannose biosynthe	133.23033	193.15952	62.00203	55.36285	Detected	Detected	Detected	Detected
627	A_23_P120254	DUSP22	Hs.29106	dual specificity phosphatase 22	Homo sapiens dual specificity phosphat	626.15485	481.40110	782.88550	553.35790	Detected	Detected	Detected	Detected
628	A_23_P105313	EIF2B1	Hs.78592	eukaryotic translation initiation factor 2	Homo sapiens eukaryotic translation ini	6750.54400	8012.37800	5599.21500	5385.83640	Detected	Detected	Detected	Detected
629	A_23_P104438	MYPN	Hs.55205	myopalladin	Homo sapiens myopalladin (MYPN), mR	61.96690	372.07748	46.03132	230.61809	Detected	Detected	Detected	Detected
630	A_24_P510377		Hs.647667		Homo sapiens cDNA clone IMAGE:5268	26.63440	198.12343	21.52508	148.47331	Detected	Detected	Detected	Detected
631	A_23_P252062	PPARG	Hs.162646	peroxisome proliferator-activated recep	Homo sapiens peroxisome proliferator-a	3062.86800	1086.32960	2929.38920	925.55970	Detected	Detected	Detected	Detected
632	A_33_P3369336	GCG	Hs.516494	glucagon	Homo sapiens glucagon (GCG), mRNA [	84.41979	97.25581	57.20939	49.79559	Detected	Detected	Detected	Detected
633	A_24_P547010					51335.79300	57659.56600	47042.46500	48408.95700	Detected	Detected	Detected	Detected
634	A_23_P121898	PARP8	Hs.369581	poly (ADP-ribose) polymerase family, m	Homo sapiens poly (ADP-ribose) polym	91.81804	44.57198	71.21668	31.90408	Detected	Detected	Detected	Detected
635	A_33_P3401661	LOC100128074	Hs.718995	hypothetical LOC100128074	PREDICTED: Homo sapiens hypothetica	52.29960	61.85313	23.05898	25.70508	Detected	Detected	Detected	Detected
636	A_33_P3246733	MNT	Hs.632239	MAX binding protein	Homo sapiens MAX binding protein (MN	145.78595	208.21559	138.99217	189.45526	Detected	Detected	Detected	Detected
637	A_23_P112113	KIAA0196	Hs.270043	KIAA0196	Homo sapiens KIAA0196 (KIAA0196), m	7237.29440	4490.97950	5179.14450	2683.14180	Detected	Detected	Detected	Detected
638	A_23_P98183	HRAS	Hs.37003	v-Ha-ras Harvey rat sarcoma viral onco	Homo sapiens v-Ha-ras Harvey rat sar	2770.59810	5895.77200	2502.50120	4911.89500	Detected	Detected	Detected	Detected
639	A_24_P671164	PRLR	Hs.368587	prolactin receptor	Homo sapiens prolactin receptor (PRLR	7.58914	23.88566	3.55654	8.99610	Compromised	Detected	Compromised	Compromised
640	A_33_P3246418	MDFI	Hs.520119	MyoD family inhibitor	Homo sapiens MyoD family inhibitor (MD	31.51549	68.85824	26.23564	74.00040	Detected	Detected	Detected	Detected
641	A_33_P3378707	LOC646214		p21-activated kinase 2 pseudogene	Homo sapiens p21-activated kinase 2 p	3064.70300	3275.75850	1083.96810	1009.59690	Detected	Detected	Detected	Detected
642	A_33_P3419098	PLEC1	Hs.434248	plectin 1, intermediate filament binding	Homo sapiens plectin 1, intermediate fil	52.98082	62.25632	38.66954	42.46640	Detected	Detected	Detected	Detected
643	A_24_P69095	ENC1	Hs.104925	ectodermal-neural cortex (with BTB-lik	Homo sapiens ectodermal-neural cortex	223.46048	780.91060	217.75276	650.98410	Detected	Detected	Detected	Detected
644	A_23_P166716	RG9MTD1	Hs.594839	RNA (guanine-9-) methyltransferase do	Homo sapiens RNA (guanine-9-) methy	10560.91700	11402.44400	7546.45900	7175.04930	Detected	Detected	Detected	Detected
645	A_33_P3242014	PHC1	Hs.305985	polyhomeotic homolog 1 (Drosophila)	Homo sapiens polyhomeotic homolog 1	2592.50800	775.88760	1949.52170	483.98657	Detected	Detected	Detected	Detected
646	A_23_P113393	APLN	Hs.303084	apelin	Homo sapiens apelin (APLN), mRNA [N	148.97510	37.50589	118.28753	25.80565	Detected	Detected	Detected	Detected
647	A_33_P3340025	GINS1	Hs.658464	GINS complex subunit 1 (Psf1 homolog)	Homo sapiens GINS complex subunit 1	373.13315	2020.66400	230.54713	1184.58180	Detected	Detected	Detected	Detected
648	A_23_P112412	TEX10	Hs.494648	testis expressed 10	Homo sapiens testis expressed 10 (TEX	6841.68100	6801.10300	5345.42700	4690.64300	Detected	Detected	Detected	Detected
649	A_24_P154948	GARS	Hs.404321	glycyl-tRNA synthetase	Homo sapiens glycyl-tRNA synthetase	4937.86430	10938.27100	2528.00460	4625.42800	Detected	Detected	Detected	Detected
650	A_23_P11237	TAF1	Hs.158560	TAF1 RNA polymerase II, TATA box bin	Homo sapiens TAF1 RNA polymerase II	105.50584	87.33856	67.94047	56.05565	Detected	Detected	Detected	Detected
651	A_23_P15654	TRAF4	Hs.8375	TNF receptor-associated factor 4	Homo sapiens TNF receptor-associated	958.32310	997.67505	1023.51820	978.01120	Detected	Detected	Detected	Detected
652	A_23_P100730	SKAP1	Hs.316931	src kinase associated phosphoprotein 1	Homo sapiens src kinase associated ph	4.63256	17.05091	47.80388	15.15218	Compromised	Detected	Not Detected	Detected
653	A_23_P201086	ARF1	Hs.286221	ADP-ribosylation factor 1	Homo sapiens ADP-ribosylation factor	2060.60740	3560.21000	1938.66640	3327.57250	Detected	Detected	Detected	Detected
654	A_23_P10785	VTI1A	Hs.194554	vesicle transport through interaction wi	Homo sapiens vesicle transport through	1221.82570	1780.20860	896.23580	1142.28670	Detected	Detected	Detected	Detected
655	A_24_P211709	XPNPEP2	Hs.170499	X-prolyl aminopeptidase (aminopeptidas	Homo sapiens X-prolyl aminopeptidase	28.44586	41.76027	16.96006	20.84309	Detected	Detected	Compromised	Detected
656	A_23_P101707	PLIN3	Hs.140452	perilipin 3	Homo sapiens perilipin 3 (PLIN3), trans	9676.57700	13567.12200	9885.42100	13041.19800	Detected	Detected	Detected	Detected
657	A_33_P3411741	SIDT2	Hs.712144	SID1 transmembrane family, member 2	Homo sapiens SID1 transmembrane fam	550.97156	471.44640	595.84050	469.47565	Detected	Detected	Detected	Detected
658	A_23_P100441	FAM192A	Hs.396740	family with sequence similarity 192, mer	Homo sapiens family with sequence sim	2551.51370	2153.64260	2031.70580	1462.20840	Detected	Detected	Detected	Detected
659	A_32_P6832	C5orf51	Hs.29899	chromosome 5 open reading frame 51	Homo sapiens chromosome 5 open reac	2628.23680	1772.78210	2348.98170	1562.34570	Detected	Detected	Detected	Detected
660	A_33_P3323041				KN motif and ankyrin repeat domain-co	63.28421	135.18053	53.49307	116.73660	Detected	Detected	Detected	Detected
661	A_23_P101319	ZNF285A	Hs.709428	zinc finger protein 285A	Homo sapiens zinc finger protein 285A	100.44051	70.37079	72.02496	40.00580	Detected	Detected	Detected	Detected
662	A_23_P306890	POLI	Hs.438533	polymerase (DNA directed) iota	Homo sapiens polymerase (DNA directe	3923.29980	1271.07150	2167.64380	581.05750	Detected	Detected	Detected	Detected
663	A_23_P43049	DCTN6	Hs.158427	dynactin 6	Homo sapiens dynactin 6 (DCTN6), mR	4808.91550	5009.49700	5081.23240	4643.57670	Detected	Detected	Detected	Detected
664	A_33_P3351371	CYP19A1	Hs.260074	cytochrome P450, family 19, subfamily A	Homo sapiens cytochrome P450, family	801.56730	87.40097	997.23000	94.50887	Detected	Detected	Detected	Detected
665	A_23_P23765	ITGB3BP	Hs.166539	integrin beta 3 binding protein (beta3-e	Homo sapiens integrin beta 3 binding pr	1443.40380	1475.64510	1252.50670	1102.72500	Detected	Detected	Detected	Detected
666	A_23_P23850	DAB1	Hs.477370	disabled homolog 1 (Drosophila)	Homo sapiens disabled homolog 1 (Dros	307.81174	539.99677	55.11408	67.45129	Detected	Detected	Detected	Detected
667	A_33_P3227793	CGREF1	Hs.159525	cell growth regulator with EF-hand dom	Homo sapiens cell growth regulator with	65.74155	153.84291	65.87000	159.67647	Detected	Detected	Detected	Detected
668	A_23_P254801	PLCG1	Hs.268177	phospholipase C, gamma 1	Homo sapiens phospholipase C, gamma	625.58720	754.19510	545.65760	646.18950	Detected	Detected	Detected	Detected
669	A_33_P3280066	PTRF	Hs.437191	polymerase I and transcript release fact	Homo sapiens polymerase I and transcr	173520.55000	185939.12000	76379.59000	80102.85000	Detected	Detected	Detected	Detected
670	A_23_P252653	STK25	Hs.516807	serine/threonine kinase 25 (STE20 hom	Homo sapiens serine/threonine kinase 2	6867.71300	8575.86100	6795.01000	8395.77600	Detected	Detected	Detected	Detected
671	A_23_P113462	KIF21A	Hs.374201	kinesin family member 21A	Homo sapiens kinesin family member 21	16.29826	23.13297	14.32434	4.07560	Detected	Not Detected	Not Detected	Compromised

672	A_24_P220485	OLFML2A	Hs.357004	olfactomedin-like 2A	Homo sapiens olfactomedin-like 2A (OL	7593.59700	246.87650	4915.97800	126.51431	Detected	Detected	Detected	Detected
673	A_23_P110288	GSTCD	Hs.161429	glutathione S-transferase, C-terminal d	Homo sapiens glutathione S-transferas	54.87114	47.93187	40.79844	34.81185	Detected	Detected	Detected	Detected
674	A_23_P12082	CHI3L2	Hs.514840	chitinase 3-like 2	Homo sapiens chitinase 3-like 2 (CHI3L	495.44020	28.41073	384.22250	19.65017	Detected	Detected	Detected	Detected
675	A_23_P142421	MLL4	Hs.92236	myeloid/lymphoid or mixed-lineage leuk	Homo sapiens myeloid/lymphoid or mixe	882.47284	664.09247	727.60443	580.30360	Detected	Detected	Detected	Detected
676	A_23_P103775	LRRC8C	Hs.412836	leucine rich repeat containing 8 family,	Homo sapiens leucine rich repeat conta	153.54979	282.94592	122.95410	180.92285	Detected	Detected	Detected	Detected
677	A_33_P3278303					219.21985	198.13292	137.18141	95.58826	Detected	Detected	Detected	Detected
678	A_33_P3365995				Microtubule-associated serine/threonin	51.11112	60.46194	29.27356	46.07600	Detected	Detected	Detected	Detected
679	A_24_P266285	IQCE	Hs.520627	IQ motif containing E	Homo sapiens IQ motif containing E (IQ	138.20790	388.02097	174.45781	470.15268	Detected	Detected	Detected	Detected
680	A_23_P54041	THTPA	Hs.655179	thiamine triphosphatase	Homo sapiens thiamine triphosphatase (	513.70490	377.18457	387.13885	260.78070	Detected	Detected	Detected	Detected
681	A_33_P3267431	LOC642648	Hs.631363	hypothetical LOC642648	PREDICTED: Homo sapiens hypothetica	48.51016	57.81953	57.78690	51.45633	Detected	Detected	Detected	Detected
682	A_23_P69537	NMU	Hs.418367	neuromedin U	Homo sapiens neuromedin U (NMU), mR	7.94597	35.37153	3.35202	25.01012	Compromised	Detected	Compromised	Detected
683	A_24_P202329	LOC146880	Hs.117853	hypothetical LOC146880	Homo sapiens hypothetical LOC146880	21.80720	24.81102	22.96783	18.99092	Detected	Detected	Detected	Detected
684	A_23_P250122	FAM20C	Hs.134742	family with sequence similarity 20, mem	Homo sapiens family with sequence sim	25324.03300	9058.07600	25336.24000	8199.23200	Detected	Detected	Detected	Detected
685	A_24_P316489	NDUFA10	Hs.277677	NADH dehydrogenase (ubiquinone) 1 alp	Homo sapiens NADH dehydrogenase (ul	709.25116	747.37950	712.75270	720.48737	Detected	Detected	Detected	Detected
686	A_23_P393034	HAS3	Hs.592069	hyaluronan synthase 3	Homo sapiens hyaluronan synthase 3 (H	32.47226	20.63026	23.07009	20.87432	Detected	Detected	Detected	Detected
687	A_23_P11862	C1orf112	Hs.443551	chromosome 1 open reading frame 112	Homo sapiens chromosome 1 open read	1192.95790	880.54230	935.87100	588.97034	Detected	Detected	Detected	Detected
688	A_24_P246963					1061.89000	1116.39140	828.91470	866.75214	Detected	Detected	Detected	Detected
689	A_23_P104054	C1orf9	Hs.204559	chromosome 1 open reading frame 9	Homo sapiens chromosome 1 open read	4377.34800	5940.77800	2870.05220	3254.35450	Detected	Detected	Detected	Detected
690	A_33_P3346573	MAP4	Hs.517949	microtubule-associated protein 4	Homo sapiens microtubule-associated p	141.03023	283.97310	126.82512	234.45319	Detected	Detected	Detected	Detected
691	A_24_P63019	IL1R2	Hs.25333	interleukin 1 receptor, type II	Homo sapiens interleukin 1 receptor, ty	53.48637	18.04451	26.17889	10.67185	Detected	Compromised	Detected	Compromised
692	A_33_P3387646	LOC643201	Hs.390285	hypothetical protein LOC643201	Homo sapiens cDNA clone IMAGE:5268	11.94922	22.33660	14.27600	22.04235	Compromised	Detected	Compromised	Detected
693	A_23_P134100	ULBP3	Hs.326556	UL16 binding protein 3	Homo sapiens UL16 binding protein 3 (U	14.20116	30.60357	9.70166	33.61372	Compromised	Detected	Compromised	Detected
694	A_24_P7600	FBXL7	Hs.433057	F-box and leucine-rich repeat protein 7	Homo sapiens F-box and leucine-rich r	18.32358	22.50575	23.62901	19.66648	Detected	Detected	Detected	Detected
695	A_33_P3334384	LOC100132774	Hs.655569	hypothetical LOC100132774	Homo sapiens cDNA clone IMAGE:5485	27.57604	45.30392	21.36378	40.99035	Detected	Detected	Detected	Detected
696	A_33_P3400823	C1orf190	Hs.568642	chromosome 1 open reading frame 190	Homo sapiens chromosome 1 open read	249.94577	301.84503	197.39060	226.07076	Detected	Detected	Detected	Detected
697	A_23_P346405	CCDC109A	Hs.591366	coiled-coil domain containing 109A	Homo sapiens coiled-coil domain contai	220.53255	393.13403	167.03670	264.13867	Detected	Detected	Detected	Detected
698	A_23_P106145	ERO1L	Hs.592304	ERO1-like (S. cerevisiae)	Homo sapiens ERO1-like (S. cerevisiae)	6061.08400	6190.61570	4804.23540	4049.94460	Detected	Detected	Detected	Detected
699	A_23_P105619	TMEM116	Hs.506815	transmembrane protein 116	Homo sapiens transmembrane protein 1	427.42422	117.45231	390.12170	92.39768	Detected	Detected	Detected	Detected
700	A_23_P411102	DLG4	Hs.463928	discs, large homolog 4 (Drosophila)	Homo sapiens discs, large homolog 4 (D	1640.11000	1123.88840	668.63700	380.23520	Detected	Detected	Detected	Detected
701	A_23_P89665	KRT33B	Hs.32950	keratin 33B	Homo sapiens keratin 33B (KRT33B), m	29.46528	45.02346	19.65440	46.59269	Detected	Detected	Detected	Detected
702	A_33_P3221708					10577.33500	16500.32800	9534.87300	12531.63500	Detected	Detected	Detected	Detected
703	A_23_P117558	FKBP3	Hs.509226	FK506 binding protein 3, 25kDa	Homo sapiens FK506 binding protein 3,	8074.10640	12383.76900	7154.01660	9740.45900	Detected	Detected	Detected	Detected
704	A_23_P123848	DAB2IP	Hs.522378	DAB2 interacting protein	Homo sapiens DAB2 interacting protein	889.43964	1226.94480	692.77780	912.62810	Detected	Detected	Detected	Detected
705	A_33_P3345812	GPER	Hs.20961	G protein-coupled estrogen receptor 1	Homo sapiens G protein-coupled estrog	164.18349	258.35828	162.74919	294.15286	Detected	Detected	Detected	Detected
706	A_23_P100292	LOC645937	Hs.645336	similar to zinc finger protein 598	PREDICTED: Homo sapiens similar to z	40788.85500	29697.97700	30198.21000	19865.67800	Detected	Detected	Detected	Detected
707	A_23_P52826	BUD13	Hs.437341	BUD13 homolog (S. cerevisiae)	Homo sapiens BUD13 homolog (S. cere	4147.89500	5649.12200	3219.12620	3860.18800	Detected	Detected	Detected	Detected
708	A_23_P150064	MMRN2	Hs.524479	multimerin 2	Homo sapiens multimerin 2 (MMRN2), m	208.78930	351.19653	111.04121	232.94820	Detected	Detected	Detected	Detected
709	A_23_P212458	SEC61A1	Hs.518236	Sec61 alpha 1 subunit (S. cerevisiae)	Homo sapiens Sec61 alpha 1 subunit (S	3684.43120	5775.75300	9014.04200	13310.43200	Detected	Detected	Detected	Detected
710	A_33_P3231156				Metallothionein [Source:UniProtKB/TrE	25760.56800	40388.28000	16730.61500	21671.22300	Detected	Detected	Detected	Detected
711	A_33_P3381666	ABLIM2	Hs.233404	actin binding LIM protein family, membe	Homo sapiens actin binding LIM protein	281.47607	4.24681	265.85660	8.69907	Detected	Compromised	Detected	Compromised
712	A_33_P3418942	NUDT21	Hs.528834	nudix (nucleoside diphosphate linked mo	Homo sapiens nudix (nucleoside diphosp	767.24207	1391.43800	952.91205	1570.44600	Detected	Detected	Detected	Detected
713	A_23_P201918	ABCB10	Hs.17614	ATP-binding cassette, sub-family B (MI	Homo sapiens ATP-binding cassette, su	1337.01940	2094.30540	873.85767	1097.39770	Detected	Detected	Detected	Detected
714	A_23_P26511	GDPD3	Hs.289015	glycerophosphodiester phosphodiestera	Homo sapiens glycerophosphodiester ph	41.87480	154.05829	46.70894	166.94452	Detected	Detected	Detected	Detected
715	A_24_P15821					11034.00100	14754.88100	12394.26000	14962.13200	Detected	Detected	Detected	Detected
716	A_23_P212002	NKTR	Hs.529509	natural killer-tumor recognition sequen	Homo sapiens natural killer-tumor reco	3304.30300	2375.53540	2398.50950	1436.91410	Detected	Detected	Detected	Detected
717	A_33_P3293760	KRTAP10-8	Hs.528021	keratin associated protein 10-8	Homo sapiens keratin associated protei	409.00125	603.77510	204.15688	199.56200	Detected	Detected	Detected	Detected
718	A_33_P3324765	CSNK1D	Hs.631725	casein kinase 1, delta	Homo sapiens casein kinase 1, delta (CS	2329.12550	2849.07000	3109.18070	3568.94730	Detected	Detected	Detected	Detected
719	A_24_P413470	TP73	Hs.697294	tumor protein p73	Homo sapiens tumor protein p73 (TP73	83.05737	99.66113	35.42877	32.54079	Detected	Detected	Detected	Detected
720	A_23_P258418	TNIP2	Hs.719161	TNFAIP3 interacting protein 2	Homo sapiens TNFAIP3 interacting prot	3071.04030	2527.55350	3004.71970	2468.12600	Detected	Detected	Detected	Detected
721	A_23_P103503	MDM4	Hs.497492	Mdm4 p53 binding protein homolog (mo	Homo sapiens Mdm4 p53 binding protei	756.61145	339.57065	546.32025	206.08778	Detected	Detected	Detected	Detected
722	A_32_P106732	FANCM	Hs.509229	Fanconi anemia, complementation group	Homo sapiens Fanconi anemia, complen	1007.73400	1624.75780	695.71210	937.61660	Detected	Detected	Detected	Detected
723	A_24_P406830	MTMR2	Hs.181326	myotubularin related protein 2	Homo sapiens myotubularin related prot	734.43384	1194.71060	723.73190	1043.69190	Detected	Detected	Detected	Detected
724	A_24_P351466	KIAA1524	Hs.591308	KIAA1524	Homo sapiens KIAA1524 (KIAA1524), m	2.70181	22.10339	3.26288	3.33902	Compromised	Detected	Compromised	Compromised
725	A_33_P3266928	LMTK3	Hs.207426	lemur tyrosine kinase 3	Homo sapiens lemur tyrosine kinase 3 (	382.56100	135.08199	376.16867	127.52279	Detected	Detected	Detected	Detected
726	A_23_P81880	CTDSP2	Hs.524530	CTD (carboxy-terminal domain, RNA po	Homo sapiens CTD (carboxy-terminal d	43913.25800	45320.18000	38237.46000	34645.13700	Detected	Detected	Detected	Detected
727	A_32_P137920	RSPH4A	Hs.160380	radial spoke head 4 homolog A (Chlamy	Homo sapiens radial spoke head 4 homo	39.85558	32.32417	27.89310	26.30266	Detected	Detected	Detected	Detected
728	A_23_P136817	KNTC1	Hs.300559	kinetochore associated 1	Homo sapiens kinetochore associated 1	281.64804	774.93317	195.76562	449.83630	Detected	Detected	Detected	Detected
729	A_23_P214244	ENPP5	Hs.35198	ectonucleotide pyrophosphatase/phosp	Homo sapiens ectonucleotide pyrophos	65.70152	43.67677	62.89889	35.75092	Detected	Detected	Detected	Detected
730	A_23_P10385	DTL	Hs.656473	denticleless homolog (Drosophila)	Homo sapiens denticleless homolog (Dr	81.02996	875.81525	61.50004	548.71590	Detected	Detected	Detected	Detected
731	A_24_P98555	FAM45A	Hs.434241	family with sequence similarity 45, mem	Homo sapiens family with sequence sim	915.11160	885.81354	828.18350	697.97520	Detected	Detected	Detected	Detected
732	A_23_P64689	PAN2	Hs.273397	PAN2 poly(A) specific ribonuclease sub	Homo sapiens PAN2 poly(A) specific rib	610.54224	143.33139	390.88320	75.20065	Detected	Detected	Detected	Detected
733	A_23_P106835	BBS2	Hs.333738	Bardet-Biedl syndrome 2	Homo sapiens Bardet-Biedl syndrome 2	5034.76400	1789.01500	3699.98800	1103.07350	Detected	Detected	Detected	Detected
734	A_32_P93036	LOC100129500	Hs.714236	hypothetical LOC100129500	PREDICTED: Homo sapiens hypothetica	711.66700	722.85785	712.50630	445.33762	Detected	Detected	Detected	Detected
735	A_24_P195794		Hs.660042		Homo sapiens cDNA FLJ27420 fis, clon	47155.66800	53583.22000	64870.51600	53414.14000	Detected	Detected	Detected	Detected
736	A_33_P3466016	LOC200830	Hs.691402	hypothetical protein LOC200830	Homo sapiens cDNA FLJ30391 fis, clon	749.51190	478.02423	221.05556	151.96028	Detected	Detected	Detected	Detected
737	A_23_P25019	PRIM1	Hs.534339	primase, DNA, polypeptide 1 (49kDa)	Homo sapiens primase, DNA, polypeptid	295.59567	739.43870	311.15802	671.58673	Detected	Detected	Detected	Detected
738	A_24_P280113	IL13RA1	Hs.496646	interleukin 13 receptor, alpha 1	Homo sapiens interleukin 13 receptor, a	48419.90200	23484.04000	33455.41400	14316.92700	Detected	Detected	Detected	Detected
739	A_24_P50818				General transcription factor 3C polypep	31.87553	46.13782	16.62914	28.94454	Detected	Detected	Compromised	Detected
740	A_23_P357207	MRAP2	Hs.370055	melanocortin 2 receptor accessory prot	Homo sapiens melanocortin 2 receptor	52.16884	253.25250	48.21416	177.32857	Detected	Detected	Detected	Detected
741	A_33_P3411612	TMEM221	Hs.711548	transmembrane protein 221	Transmembrane protein 221 [Source:U	90.60114	117.85038	49.20109	52.88782	Detected	Detected	Detected	Detected
742	A_33_P3361584	LOC100130930	Hs.661664	hypothetical LOC100130930	Homo sapiens cDNA FLJ44616 fis, clon	23.60213	31.25521	26.81547	30.97587	Detected	Detected	Detected	Detected
743	A_24_P29277	COL4A3BP	Hs.270437	collagen, type IV, alpha 3 (Goodpasture	Homo sapiens collagen, type IV, alpha 3	3519.91140	1579.87960	2687.12300	1070.97900	Detected	Detected	Detected	Detected
744	A_23_P373568	FUZ	Hs.288800	fuzzy homolog (Drosophila)	Homo sapiens fuzzy homolog (Drosophil	803.96216	457.86320	538.50930	277.23240	Detected	Detected	Detected	Detected
745	A_23_P213255	SMARCAD1	Hs.410406	SWI/SNF-related, matrix-associated ac	Homo sapiens SWI/SNF-related, matrix	2295.46070	1995.36720	1735.48560	1337.48700	Detected	Detected	Detected	Detected
746	A_24_P73290	ATP2A2	Hs.506759	ATPase, Ca++ transporting, cardiac mus	Homo sapiens ATPase, Ca++ transporti	3221.21660	2632.95970	1398.58560	929.96550	Detected	Detected	Detected	Detected

747	A_23_P117851	CPLX3	Hs.187694	complexin 3	Homo sapiens complexin 3 (CPLX3), mR	7.70515	20.45369	18.53984	17.21188	Compromised	Detected	Detected	Detected
748	A_23_P29939	SNCA	Hs.21374	synuclein, alpha (non A4 component of	Homo sapiens synuclein, alpha (non A4	532.17030	63.69193	519.76685	50.48723	Detected	Detected	Detected	Detected
749	A_23_P391164	ZNF512	Hs.529178	zinc finger protein 512	Homo sapiens zinc finger protein 512 (Z	346.99000	289.52050	258.12800	185.19420	Detected	Detected	Detected	Detected
750	A_23_P121141	ASTE1	Hs.100878	asteroid homolog 1 (Drosophila)	Homo sapiens asteroid homolog 1 (Dros	226.97954	190.24930	185.08803	145.97945	Detected	Detected	Detected	Detected
751	A_24_P120346	NIT2	Hs.439152	nitrilase family, member 2	Homo sapiens nitrilase family, member 2	17897.08000	11102.23400	11867.25400	6563.69780	Detected	Detected	Detected	Detected
752	A_33_P3421209	FMN2	Hs.24889	formin 2	Homo sapiens formin 2 (FMN2), mRNA	93.08854	124.78079	94.81374	112.85839	Detected	Detected	Detected	Detected
753	A_23_P77669	ZNF821	Hs.643531	zinc finger protein 821	Homo sapiens zinc finger protein 821 (Z	450.44153	256.86860	512.62280	240.60709	Detected	Detected	Detected	Detected
754	A_23_P33613	DCTPP1	Hs.632191	dCTP pyrophosphatase 1	Homo sapiens dCTP pyrophosphatase 1	5713.63130	8800.09300	6367.72070	8494.34000	Detected	Detected	Detected	Detected
755	A_23_P113351	SPARCL1	Hs.62886	SPARC-like 1 (hevin)	Homo sapiens SPARC-like 1 (hevin) (SF	12.89796	4.60063	4.49258	2.91204	Detected	Compromised	Compromised	Compromised
756	A_33_P3378659	TARP	Hs.534032	TCR gamma alternate reading frame pro	Homo sapiens TCR gamma alternate re	1008.97046	1386.28380	332.66052	345.37372	Detected	Detected	Detected	Detected
757	A_33_P3330546	ZNF746	Hs.24643	zinc finger protein 746	Homo sapiens zinc finger protein 746 (Z	20.90990	21.06484	21.36938	24.99513	Detected	Detected	Detected	Detected
758	A_24_P263910	C14orf182	Hs.660867	chromosome 14 open reading frame 182	Homo sapiens mRNA for FLJ00328 prot	71.69605	54.85810	36.04732	22.88400	Detected	Detected	Detected	Detected
759	A_23_P121527	KLHL5	Hs.272251	kelch-like 5 (Drosophila)	Homo sapiens kelch-like 5 (Drosophila)	1230.58370	553.58220	987.60876	356.11777	Detected	Detected	Detected	Detected
760	A_33_P3474319	SLC37A2	Hs.352661	solute carrier family 37 (glycerol-3-pho	Homo sapiens solute carrier family 37 (	217.26886	225.50157	221.93185	195.34640	Detected	Detected	Detected	Detected
761	A_33_P3335984				Uncharacterized protein FLJ39653 [So	134.82810	55.87217	124.29179	48.66838	Detected	Detected	Detected	Detected
762	A_33_P3212630	FAM90A10	Hs.715411	family with sequence similarity 90, mem	Homo sapiens family with sequence sim	94.24470	22.06375	100.14470	22.22345	Detected	Detected	Detected	Detected
763	A_33_P3381429					214455.89000	214543.52000	245327.97000	208773.81000	Detected	Detected	Detected	Detected
764	A_24_P84508					75396.27000	63594.42000	63095.25400	44838.09800	Detected	Detected	Detected	Detected
765	A_24_P416346	ETV4	Hs.434059	ets variant 4	Homo sapiens ets variant 4 (ETV4), tra	896.55160	472.22210	957.19240	502.19308	Detected	Detected	Detected	Detected
766	A_23_P36700	TAPBPL	Hs.504597	TAP binding protein-like	Homo sapiens TAP binding protein-like	151.25780	179.09170	166.48140	158.50708	Detected	Detected	Detected	Detected
767	A_24_P264166	GOLGA8F	Hs.510812	golgi autoantigen, golgin subfamily a, 8F	Homo sapiens golgi autoantigen, golgin s	216.56589	39.10727	95.13943	8.32781	Detected	Detected	Detected	Compromised
768	A_23_P100499	ROGDI	Hs.459795	rogdi homolog (Drosophila)	Homo sapiens rogdi homolog (Drosophil	145.75377	113.26849	157.12710	145.46260	Detected	Detected	Detected	Detected
769	A_24_P245246	PIP4K2B	Hs.260603	phosphatidylinositol-5-phosphate 4-kin	Homo sapiens phosphatidylinositol-5-ph	5946.80100	5582.46200	4800.20070	3609.71900	Detected	Detected	Detected	Detected
770	A_23_P56736	TUBA3D	Hs.503749	tubulin, alpha 3d	Homo sapiens tubulin, alpha 3d (TUBA3	1093.81200	1178.75160	1179.49580	935.72500	Detected	Detected	Detected	Detected
771	A_24_P159648	BAIAP2	Hs.128316	BAI1-associated protein 2	Homo sapiens BAI1-associated protein	402.59990	537.75800	385.03970	512.57740	Detected	Detected	Detected	Detected
772	A_24_P945283	DLG3	Hs.649747	discs, large homolog 3 (Drosophila)	Homo sapiens discs, large homolog 3 (D	2060.24240	537.21936	1754.79200	427.33580	Detected	Detected	Detected	Detected
773	A_23_P204550	SCYL2	Hs.506481	SCY1-like 2 (S. cerevisiae)	Homo sapiens SCY1-like 2 (S. cerevisia	3399.29300	3571.06180	3172.04170	2845.50170	Detected	Detected	Detected	Detected
774	A_33_P3802146		Hs.255729		Homo sapiens cDNA FLJ16301 fis, clon	4248.87740	3704.80200	2333.14580	1557.63100	Detected	Detected	Detected	Detected
775	A_23_P167159	SCRG1	Hs.7122	stimulator of chondrogenesis 1	Homo sapiens stimulator of chondrogen	2.66140	108.40463	3.20510	70.48447	Compromised	Detected	Compromised	Detected
776	A_33_P3246010				Protein FAM38A (Membrane protein ind	6925.48340	9493.57700	3438.11990	3468.98700	Detected	Detected	Detected	Detected
777	A_24_P6305	RPL10	Hs.534404	ribosomal protein L10	Homo sapiens ribosomal protein L10 (R	12.14226	49.30301	7.22529	38.90824	Compromised	Detected	Compromised	Detected
778	A_24_P291426	REV1	Hs.443077	REV1 homolog (S. cerevisiae)	Homo sapiens REV1 homolog (S. cerevi	413.74673	314.12512	271.70178	175.29237	Detected	Detected	Detected	Detected
779	A_23_P210131	LOC400987	Hs.646318	similar to ankyrin repeat domain 36	UPF0634 protein C (Protein immuno-re	164.48074	288.26126	121.74620	217.12500	Detected	Detected	Detected	Detected
780	A_23_P104109	RPS6KC1	Hs.591416	ribosomal protein S6 kinase, 52kDa, poly	Homo sapiens ribosomal protein S6 kina	874.70087	912.95760	562.27080	497.66763	Detected	Detected	Detected	Detected
781	A_33_P3366241		Hs.591833		Scavenger receptor class A member 5 (	89.78728	77.27410	28.74745	18.74963	Detected	Detected	Detected	Detected
782	A_33_P3242099	CAMSAP1L1	Hs.23585	calmodulin regulated spectrin-associate	Homo sapiens calmodulin regulated spe	515.76447	706.94590	273.98910	264.07060	Detected	Detected	Detected	Detected
783	A_23_P113572	CD19	Hs.652262	CD19 molecule	Homo sapiens CD19 molecule (CD19), r	67.70406	45.31294	54.63095	22.15318	Detected	Detected	Detected	Detected
784	A_23_P117662	HDC	Hs.1481	histidine decarboxylase	Homo sapiens histidine decarboxylase (	34.44180	85.00227	20.95400	17.39580	Detected	Detected	Detected	Detected
785	A_23_P12113	FLVCR1	Hs.7055	feline leukemia virus subgroup C cellula	Homo sapiens feline leukemia virus sub	565.82150	757.94760	389.51718	446.94342	Detected	Detected	Detected	Detected
786	A_33_P3228593	WDR45	Hs.632807	WD repeat domain 45	Homo sapiens WD repeat domain 45, ml	338.74493	232.49922	178.60486	59.88124	Detected	Detected	Detected	Detected
787	A_23_P55190	EFTUD2	Hs.151787	elongation factor Tu GTP binding domai	Homo sapiens elongation factor Tu GTP	19182.44500	24448.64600	16764.96900	19980.94100	Detected	Detected	Detected	Detected
788	A_23_P354798	COQ10A	Hs.4096	coenzyme Q10 homolog A (S. cerevisiae	Homo sapiens coenzyme Q10 homolog	838.65967	720.69650	697.33880	482.50946	Detected	Detected	Detected	Detected
789	A_23_P117782	LARP6	Hs.416755	La ribonucleoprotein domain family, mer	Homo sapiens La ribonucleoprotein dom	12857.91100	9605.35300	10348.49500	6805.23630	Detected	Detected	Detected	Detected
790	A_23_P90233	ZSCAN5A	Hs.177688	zinc finger and SCAN domain containing	Homo sapiens zinc finger and SCAN do	657.89404	886.05710	609.64970	747.15674	Detected	Detected	Detected	Detected
791	A_23_P50907	ITGAV	Hs.436873	integrin, alpha V (vitronectin receptor, a	Homo sapiens integrin, alpha V (vitrone	10187.13500	18803.59600	7656.25900	11967.71200	Detected	Detected	Detected	Detected
792	A_24_P376129		Hs.93836		Whirlin (Autosomal recessive deafness	149.40216	195.34177	108.05399	109.94192	Detected	Detected	Detected	Detected
793	A_33_P3374589				Ataxin-10 (Spinocerebellar ataxia type	242.74606	284.62710	800.80360	613.87490	Detected	Detected	Detected	Detected
794	A_23_P99632	RNF31	Hs.375217	ring finger protein 31	Homo sapiens ring finger protein 31 (RN	1967.10380	1799.35580	2547.03800	2069.33670	Detected	Detected	Detected	Detected
795	A_33_P3282000	LOC643072	Hs.632541	hypothetical LOC643072	PREDICTED: Homo sapiens hypothetica	52.51284	77.33141	33.34234	36.06546	Detected	Detected	Detected	Detected
796	A_23_P112531	FAM102A	Hs.568044	family with sequence similarity 102, mem	Homo sapiens family with sequence sim	13182.29200	18441.71000	8252.18300	9882.72950	Detected	Detected	Detected	Detected
797	A_23_P106481	TMEM85	Hs.250905	transmembrane protein 85	Homo sapiens transmembrane protein 8	14902.77700	14191.36300	13433.92700	11195.32100	Detected	Detected	Detected	Detected
798	A_33_P3239790	ZNF646	Hs.119273	zinc finger protein 646	Homo sapiens zinc finger protein 646 (Z	41.30814	34.89105	31.33370	32.94060	Detected	Detected	Detected	Detected
799	A_24_P53282	CPD	Hs.446079	carboxypeptidase D	Homo sapiens carboxypeptidase D (CPD	2291.85200	2063.93240	1452.83280	1080.53490	Detected	Detected	Detected	Detected
800	A_23_P115726	SLC16A9	Hs.499709	solute carrier family 16, member 9 (mon	Homo sapiens solute carrier family 16, r	16.86954	28.06795	20.92413	21.46313	Detected	Detected	Detected	Detected
801	A_23_P153480	KLK5	Hs.50915	kallikrein-related peptidase 5	Homo sapiens kallikrein-related peptida	9.42779	13.84925	18.81193	15.23162	Compromised	Compromised	Detected	Compromised
802	A_23_P118615	ABCA8	Hs.58351	ATP-binding cassette, sub-family A (AB	Homo sapiens ATP-binding cassette, su	65.97136	22.06179	66.81093	27.47368	Detected	Detected	Detected	Detected
803	A_23_P101960	ZFP36L2	Hs.503093	zinc finger protein 36, C3H type-like 2	Homo sapiens zinc finger protein 36, C3	32716.27100	29788.91200	26003.61700	19739.86500	Detected	Detected	Detected	Detected
804	A_23_P209694	PAPOLG	Hs.387471	poly(A) polymerase gamma	Homo sapiens poly(A) polymerase gamm	585.47600	436.38013	500.87690	325.39178	Detected	Detected	Detected	Detected
805	A_23_P110062	EIF2B5	Hs.283551	eukaryotic translation initiation factor 2	Homo sapiens eukaryotic translation ini	1865.55580	1719.04740	1897.12980	1505.83190	Detected	Detected	Detected	Detected
806	A_23_P97283	PAQR6	Hs.235873	progestin and adipoQ receptor family m	Homo sapiens progestin and adipoQ rec	1708.25320	340.16060	1398.29350	232.12390	Detected	Detected	Detected	Detected
807	A_23_P149084	GRIK3	Hs.128848	glutamate receptor, ionotropic, kainate	Homo sapiens glutamate receptor, ionot	28.24760	31.61923	21.51933	27.10223	Detected	Detected	Detected	Detected
808	A_23_P112596	ADK	Hs.656586	adenosine kinase	Homo sapiens adenosine kinase (ADK),	4213.28400	7458.65300	3540.32760	5101.83000	Detected	Detected	Detected	Detected
809	A_24_P481783	LOC390595	Hs.659256	similar to ubiquitin-associated protein 1	Homo sapiens similar to ubiquitin-assoc	679.22797	215.25311	585.92430	166.13730	Detected	Detected	Detected	Detected
810	A_33_P3294881	C3orf19	Hs.517820	chromosome 3 open reading frame 19	Homo sapiens chromosome 3 open read	1844.13210	2587.24020	1322.00090	1689.61280	Detected	Detected	Detected	Detected
811	A_24_P343233	HLA-DRB1	Hs.701691	major histocompatibility complex, class	Homo sapiens major histocompatibility	980.47363	1216.81230	342.29175	420.99658	Detected	Detected	Detected	Detected
812	A_33_P3252196	EZH2	Hs.444082	enhancer of zeste homolog 2 (Drosophil	Homo sapiens enhancer of zeste homol	1197.51430	2797.64090	813.46760	1642.11160	Detected	Detected	Detected	Detected
813	A_23_P107744	S1PR5	Hs.501561	sphingosine-1-phosphate receptor 5	Homo sapiens sphingosine-1-phosphate	49.65671	89.81369	66.94942	100.21469	Detected	Detected	Detected	Detected
814	A_23_P115919	PHYH	Hs.498732	phytanoyl-CoA 2-hydroxylase	Homo sapiens phytanoyl-CoA 2-hydrox	2403.89840	1363.31740	1775.63440	806.07904	Detected	Detected	Detected	Detected
815	A_24_P14634	EMID1	Hs.289106	EMI domain containing 1	Homo sapiens EMI domain containing 1	57.34748	10.74492	64.32759	9.78679	Detected	Compromised	Detected	Compromised
816	A_23_P145024	ADRB2	Hs.2551	adrenergic, beta-2-, receptor, surface	Homo sapiens adrenergic, beta-2-, rece	608.74084	1181.05000	417.65040	715.37616	Detected	Detected	Detected	Detected
817	A_23_P122304	HDAC2	Hs.3352	histone deacetylase 2	Homo sapiens histone deacetylase 2 (H	5731.90670	5244.99500	6187.29350	4808.73100	Detected	Detected	Detected	Detected
818	A_33_P3406245	TAF1A	Hs.153088	TATA box binding protein (TBP)-associ	Homo sapiens TATA box binding protein	36.54511	24.16068	21.13162	15.74168	Detected	Detected	Detected	Compromised
819	A_23_P93258	HIST1H3B	Hs.533292	histone cluster 1, H3b	Homo sapiens histone cluster 1, H3b (H	50.12993	418.89240	40.98689	356.53467	Detected	Detected	Detected	Detected
820	A_23_P11685	PLA2G4A	Hs.497200	phospholipase A2, group IVA (cytosolic,	Homo sapiens phospholipase A2, group	931.89905	110.55888	821.69480	79.81390	Detected	Detected	Detected	Detected
821	A_33_P3325009					289271.97000	204436.67000	143496.23000	88029.02000	Detected	Detected	Detected	Detected

822	A_23_P122615	SFRS18	Hs.520287	splicing factor, arginine/serine-rich 18	Homo sapiens splicing factor, arginine/s	1306.21810	678.13330	754.11930	327.59340	Detected	Detected	Detected	Detected
823	A_23_P49539	BAHCC1	Hs.514580	BAH domain and coiled-coil containing	Homo sapiens BAH domain and coiled-c	269.70300	282.12338	267.24590	244.58356	Detected	Detected	Detected	Detected
824	A_33_P3289162		Hs.674050		Putative uncharacterized protein FLJ46	29.28445	40.55767	24.34878	29.31738	Detected	Detected	Detected	Detected
825	A_33_P3358943	GRM2	Hs.121510	glutamate receptor, metabotropic 2	Homo sapiens glutamate receptor, meta	20.29636	9.00768	9.50821	3.80904	Detected	Compromised	Compromised	Compromised
826	A_33_P3315504	ERI1	Hs.20000	exoribonuclease 1	Homo sapiens exoribonuclease 1 (ERI1)	3123.13450	1752.84310	2723.85130	1316.30490	Detected	Detected	Detected	Detected
827	A_33_P3885807	LOC399875	Hs.434602	hypothetical gene supported by BC0402	Homo sapiens cDNA clone IMAGE:4822	26.32151	22.64927	12.74148	3.80494	Detected	Detected	Compromised	Compromised
828	A_23_P322845	PPAPDC1B	Hs.567619	phosphatidic acid phosphatase type 2 d	Homo sapiens phosphatidic acid phosph	1207.62920	1175.58450	1001.25464	781.65790	Detected	Detected	Detected	Detected
829	A_33_P3299309	RPL28	Hs.652114	ribosomal protein L28	Homo sapiens ribosomal protein L28 (R	19.13248	13.05522	9.35214	12.42895	Detected	Compromised	Compromised	Compromised
830	A_24_P103264	UGT8	Hs.144197	UDP glycosyltransferase 8	Homo sapiens UDP glycosyltransferase	53.82041	86.44183	11.27970	17.96323	Detected	Detected	Compromised	Compromised
831	A_23_P213863	FAM193B	Hs.484289	family with sequence similarity 193, mer	Homo sapiens family with sequence sim	377.52182	205.80489	471.43020	272.31800	Detected	Detected	Detected	Detected
832	A_23_P121196	TMEM43	Hs.517817	transmembrane protein 43	Homo sapiens transmembrane protein 4	2758.04080	4197.61100	2762.73050	3762.57930	Detected	Detected	Detected	Detected
833	A_23_P414713	ZNF528	Hs.662043	zinc finger protein 528	Homo sapiens zinc finger protein 528 (Z	162.09383	131.19931	79.88126	51.73975	Detected	Detected	Detected	Detected
834	A_23_P115482	UBE2T	Hs.5199	ubiquitin-conjugating enzyme E2T (puta	Homo sapiens ubiquitin-conjugating enz	1200.05410	7390.82900	825.05634	4432.43850	Detected	Detected	Detected	Detected
835	A_23_P8497	GHRHR	Hs.767	growth hormone releasing hormone rece	Homo sapiens growth hormone releasin	15.00459	7.72648	3.23663	8.49851	Detected	Compromised	Compromised	Compromised
836	A_23_P138881	ACTN3	Hs.654432	actinin, alpha 3	Homo sapiens actinin, alpha 3 (ACTN3),	864.84406	1461.38600	385.14532	521.59040	Detected	Detected	Detected	Detected
837	A_33_P3233070	DEXI	Hs.659355	Dexi homolog (mouse)	Homo sapiens cDNA FLJ30967 fis, clon	35.96233	53.28534	25.19099	36.85331	Detected	Detected	Detected	Detected
838	A_33_P3239654	HCG4P6	Hs.181244	HLA complex group 4 pseudogene 6	Homo sapiens cDNA FLJ32165 fis, clon	15.79415	8.72445	3.27319	3.34995	Detected	Compromised	Compromised	Compromised
839	A_23_P17826	SLC5A1	Hs.1964	solute carrier family 5 (sodium/glucose	Homo sapiens solute carrier family 5 (so	22.35986	37.61562	12.73977	14.72557	Detected	Detected	Compromised	Compromised
840	A_33_P3355688				Putative uncharacterized protein FLJ42	52.49930	53.78156	12.63947	18.52653	Detected	Detected	Compromised	Detected
841	A_33_P3253598				Putative uncharacterized protein GYG2	111.10660	103.27957	75.14176	71.32240	Detected	Detected	Detected	Detected
842	A_33_P3319580		Hs.713360		Putative uncharacterized protein FLJ40	21.22710	41.22523	9.03324	15.67992	Detected	Detected	Compromised	Compromised
843	A_23_P216630	SLC44A1	Hs.573495	solute carrier family 44, member 1	Homo sapiens solute carrier family 44, r	60259.83600	23629.21500	53149.87500	18428.78000	Detected	Detected	Detected	Detected
844	A_23_P11372	HPRT1	Hs.412707	hypoxanthine phosphoribosyltransferase	Homo sapiens hypoxanthine phosphorib	3732.00700	7673.89160	2640.50850	4332.57470	Detected	Detected	Detected	Detected
845	A_23_P388433	C4orf3	Hs.173705	chromosome 4 open reading frame 3	Homo sapiens chromosome 4 open read	13789.96700	9726.65700	12059.19500	7433.07300	Detected	Detected	Detected	Detected
846	A_23_P133923	BAT4	Hs.247478	HLA-B associated transcript 4	Homo sapiens HLA-B associated transc	19504.91000	14031.98100	19676.62900	12785.13300	Detected	Detected	Detected	Detected
847	A_23_P23966	ZNF488	Hs.27788	zinc finger protein 488	Homo sapiens zinc finger protein 488 (Z	39.08716	49.83999	19.38133	14.93819	Detected	Detected	Detected	Compromised
848	A_23_P122563	PFDN6	Hs.446374	prefoldin subunit 6	Homo sapiens prefoldin subunit 6 (PFDN	3595.97000	1557.39730	5077.81050	2999.26270	Detected	Detected	Detected	Detected
849	A_23_P206901	NDE1	Hs.655378	nudE nuclear distribution gene E homolo	Homo sapiens nudE nuclear distribution	5214.57280	11269.88400	3789.06130	7210.97200	Detected	Detected	Detected	Detected
850	A_23_P102965	BCL2L13	Hs.631672	BCL2-like 13 (apoptosis facilitator)	Homo sapiens BCL2-like 13 (apoptosis	1393.70430	1625.07140	1013.11750	1047.64710	Detected	Detected	Detected	Detected
851	A_33_P3246935	CSMD2	Hs.656915	CUB and Sushi multiple domains 2	Homo sapiens CUB and Sushi multiple c	55.48075	104.03577	42.46431	90.06294	Detected	Detected	Detected	Detected
852	A_23_P122775	RTN4IP1	Hs.155839	reticulon 4 interacting protein 1	Homo sapiens reticulon 4 interacting pr	3084.30300	3006.01700	2730.10470	2344.78980	Detected	Detected	Detected	Detected
853	A_23_P12173	CRTC2	Hs.406392	CREB regulated transcription coactivat	Homo sapiens CREB regulated transcrip	13492.45100	11754.39800	11857.30300	9190.95100	Detected	Detected	Detected	Detected
854	A_24_P177604	PPP1R3F	Hs.433652	protein phosphatase 1, regulatory (inhib	Homo sapiens protein phosphatase 1, re	405.06677	139.60307	405.28558	125.95813	Detected	Detected	Detected	Detected
855	A_24_P171268	RASSF5	Hs.497579	Ras association (RalGDS/AF-6) domain	Homo sapiens Ras association (RalGDS	93.80930	66.94930	64.86975	40.81809	Detected	Detected	Detected	Detected
856	A_23_P105747	APPL2	Hs.506603	adaptor protein, phosphotyrosine intera	Homo sapiens adaptor protein, phospho	1149.97520	2381.23460	711.80720	1178.22800	Detected	Detected	Detected	Detected
857	A_33_P3393801	PDZK1IP1	Hs.431099	PDZK1 interacting protein 1	Homo sapiens PDZK1 interacting protei	17.71410	25.68504	9.92471	8.20522	Detected	Detected	Compromised	Compromised
858	A_23_P124003	P2RX2	Hs.258580	purinergic receptor P2X, ligand-gated ic	Homo sapiens purinergic receptor P2X,	2700.56760	2457.33900	456.51350	382.18410	Detected	Detected	Detected	Detected
859	A_23_P100455	MTHFSD	Hs.343627	methenyltetrahydrofolate synthetase do	Homo sapiens methenyltetrahydrofolate	265.32147	186.13228	194.84628	111.83366	Detected	Detected	Detected	Detected
860	A_33_P3295333	CLEC1A	Hs.29549	C-type lectin domain family 1, member	Homo sapiens C-type lectin domain fam	2.85839	82.62470	3.45768	53.87229	Compromised	Detected	Compromised	Detected
861	A_24_P42501	ACOT9	Hs.298885	acyl-CoA thioesterase 9	Homo sapiens acyl-CoA thioesterase 9	1966.19370	4432.07230	1740.85940	3519.77420	Detected	Detected	Detected	Detected
862	A_23_P205046	ANKRD10	Hs.525163	ankyrin repeat domain 10	Homo sapiens ankyrin repeat domain 10	2511.67580	653.72064	1506.67720	310.83487	Detected	Detected	Detected	Detected
863	A_23_P420417	TLCD1	Hs.499952	TLC domain containing 1	Homo sapiens TLC domain containing 1	766.87550	572.63470	941.96704	680.16160	Detected	Detected	Detected	Detected
864	A_23_P104318	DDIT4	Hs.719970	DNA-damage-inducible transcript 4	Homo sapiens DNA-damage-inducible t	8787.71100	3317.90600	6731.84130	2367.15360	Detected	Detected	Detected	Detected
865	A_23_P135132	FRMD3	Hs.709357	FERM domain containing 3	Homo sapiens FERM domain containing	43.15263	64.83310	38.39076	61.09072	Detected	Detected	Detected	Detected
866	A_24_P40529	TMLHE	Hs.133321	trimethyllysine hydroxylase, epsilon	Homo sapiens trimethyllysine hydroxyla	18.33834	18.40200	10.58942	6.69326	Detected	Compromised	Compromised	Compromised
867	A_23_P206532	PHKB	Hs.78060	phosphorylase kinase, beta	Homo sapiens phosphorylase kinase, be	1954.04770	4543.39840	1644.12210	3356.88820	Detected	Detected	Detected	Detected
868	A_24_P80500	BDH2	Hs.124696	3-hydroxybutyrate dehydrogenase, type	Homo sapiens 3-hydroxybutyrate dehyd	159.72878	44.33409	138.96709	36.07628	Detected	Detected	Detected	Detected
869	A_23_P115215	VPS72	Hs.2430	vacuolar protein sorting 72 homolog (S.	Homo sapiens vacuolar protein sorting 7	3411.09470	3226.27560	2600.53400	2174.32130	Detected	Detected	Detected	Detected
870	A_33_P3268726					15.40797	4.15374	18.31080	5.09554	Detected	Compromised	Detected	Compromised
871	A_23_P44207	ACOT12	Hs.591756	acyl-CoA thioesterase 12	Homo sapiens acyl-CoA thioesterase 1	24.87373	9.61117	5.72850	8.40575	Detected	Compromised	Compromised	Compromised
872	A_23_P372096		Hs.516978		Uncharacterized protein C20orf112 [Sc	82.57635	226.54326	67.42421	167.67569	Detected	Detected	Detected	Detected
873	A_33_P3217238	ATAD2	Hs.370834	ATPase family, AAA domain containing	Homo sapiens ATPase family, AAA dom	1075.89710	4066.99100	892.74066	2598.76220	Detected	Detected	Detected	Detected
874	A_23_P106056	DAD1	Hs.82890	defender against cell death 1	Homo sapiens defender against cell dea	34131.63000	34000.36700	30198.70900	25798.94700	Detected	Detected	Detected	Detected
875	A_23_P120062	C2orf3	Hs.303808	chromosome 2 open reading frame 3	Homo sapiens chromosome 2 open read	2689.47950	847.44360	1702.17190	458.84640	Detected	Detected	Detected	Detected
876	A_23_P323094	PHC1	Hs.305985	polyhomeotic homolog 1 (Drosophila)	Homo sapiens polyhomeotic homolog 1	188.86420	44.03547	144.88390	26.05678	Detected	Detected	Detected	Detected
877	A_23_P104651	CDCA5	Hs.434886	cell division cycle associated 5	Homo sapiens cell division cycle associ	479.77963	8467.47000	399.53090	6184.82370	Detected	Detected	Detected	Detected
878	A_24_P400324	THSD7A	Hs.120855	thrombospondin, type I, domain containi	Homo sapiens thrombospondin, type I, d	1179.51700	4.24710	781.19403	3.80622	Detected	Compromised	Detected	Compromised
879	A_32_P91250	UBE2L3	Hs.108104	ubiquitin-conjugating enzyme E2L 3	Homo sapiens ubiquitin-conjugating enz	4454.00000	8115.27050	5123.72070	7709.00900	Detected	Detected	Detected	Detected
880	A_33_P3404824	ZNF300	Hs.134885	zinc finger protein 300	Homo sapiens zinc finger protein 300 (Z	42.79029	58.70283	44.71042	46.97850	Detected	Detected	Detected	Detected
881	A_33_P3419180	LOC283547	Hs.675941	hypothetical protein LOC283547	PREDICTED: Homo sapiens hypothetica	3316.73300	2570.39450	3762.58180	2854.06350	Detected	Detected	Detected	Detected
882	A_23_P17330	UCKL1	Hs.504998	uridine-cytidine kinase 1-like 1	Homo sapiens uridine-cytidine kinase 1-	967.77420	808.32385	868.40670	731.00320	Detected	Detected	Detected	Detected
883	A_33_P3329644	ALG1L2	Hs.722197	asparagine-linked glycosylation 1-like 2	Homo sapiens asparagine-linked glycosy	1476.82500	1672.26110	1811.91720	1804.80100	Detected	Detected	Detected	Detected
884	A_23_P117437	IL25	Hs.302036	interleukin 25	Homo sapiens interleukin 25 (IL25), tran	2.70902	22.95530	2.86861	2.99638	Compromised	Detected	Compromised	Compromised
885	A_23_P136635	AP3B1	Hs.532091	adaptor-related protein complex 3, beta	Homo sapiens adaptor-related protein c	5589.33740	7223.62940	5301.03700	6181.70900	Detected	Detected	Detected	Detected
886	A_32_P177955	LOC441461	Hs.163155	hypothetical LOC441461	PREDICTED: Homo sapiens hypothetica	78.00308	38.96744	51.08580	24.88862	Detected	Detected	Detected	Detected
887	A_23_P56798	ACP1	Hs.558296	acid phosphatase 1, soluble	Homo sapiens acid phosphatase 1, solul	7364.26030	6920.75100	6636.55300	5434.15430	Detected	Detected	Detected	Detected
888	A_23_P97584	DNTTIP2	Hs.85769	deoxynucleotidyltransferase, terminal, in	Homo sapiens deoxynucleotidyltransfera	9163.24600	9501.05500	7547.98100	6728.12200	Detected	Detected	Detected	Detected
889	A_23_P111672	TES	Hs.592286	testis derived transcript (3 LIM domains	Homo sapiens testis derived transcript	65.47565	339.87695	36.94475	176.14014	Detected	Detected	Detected	Detected
890	A_23_P117157	SUCLA2	Hs.546323	succinate-CoA ligase, ADP-forming, be	Homo sapiens succinate-CoA ligase, AD	2733.37500	3474.49490	582.17474	444.36484	Detected	Detected	Detected	Detected
891	A_24_P129417	BMP1	Hs.1274	bone morphogenetic protein 1	Homo sapiens bone morphogenetic prot	821.00366	1121.99110	621.16364	947.58780	Detected	Detected	Detected	Detected
892	A_23_P135769	ACTB	Hs.520640	actin, beta	Homo sapiens actin, beta (ACTB), mRN	50621.32400	79707.12500	44023.97000	58563.92600	Detected	Detected	Detected	Detected
893	A_23_P101992	MARCO	Hs.67726	macrophage receptor with collagenous s	Homo sapiens macrophage receptor wit	73.30414	49.33618	66.39297	41.21670	Detected	Detected	Detected	Detected
894	A_33_P3412125					47057.33200	30728.11000	25329.86000	13813.44100	Detected	Detected	Detected	Detected
895	A_23_P152272	RNPS1	Hs.355643	RNA binding protein S1, serine-rich dom	Homo sapiens RNA binding protein S1, s	7054.14260	10391.25600	7280.04150	10942.55300	Detected	Detected	Detected	Detected
896	A_23_P15414	SCARF1	Hs.647430	scavenger receptor class F, member 1	Homo sapiens scavenger receptor class	135.00023	145.67589	51.13325	52.72356	Detected	Detected	Detected	Detected

897	A_24_P175783	ARHGEF12	Hs.24598	Rho guanine nucleotide exchange facto	Homo sapiens Rho guanine nucleotide e	96.08659	77.47396	72.83769	61.28416	Detected	Detected	Detected	Detected
898	A_23_P340922	ZNF414	Hs.515114	zinc finger protein 414	Homo sapiens zinc finger protein 414 (Z	149.18155	273.24230	140.72543	250.11923	Detected	Detected	Detected	Detected
899	A_24_P6825	ZCCHC18	Hs.648338	zinc finger, CCHC domain containing 18	Homo sapiens zinc finger, CCHC domai	14.43170	3.77993	6.43068	3.38089	Detected	Compromised	Compromised	Compromised
900	A_24_P230176	CCDC137	Hs.405942	coiled-coil domain containing 137	Homo sapiens coiled-coil domain conta	2201.38920	3319.32760	1849.63940	2553.57570	Detected	Detected	Detected	Detected
901	A_23_P113283	ZMAT3	Hs.386299	zinc finger, matrin type 3	Homo sapiens zinc finger, matrin type 3	246.85732	351.26898	185.98137	209.28821	Detected	Detected	Detected	Detected
902	A_23_P354027	KCTD11	Hs.592112	potassium channel tetramerisation dom	Homo sapiens potassium channel tetra	2641.23300	2607.35350	2546.66430	2322.06900	Detected	Detected	Detected	Detected
903	A_23_P158277	TMCO4	Hs.656313	transmembrane and coiled-coil domains	Homo sapiens transmembrane and coile	1554.97270	565.47040	1804.99320	646.17500	Detected	Detected	Detected	Detected
904	A_33_P3831566		Hs.667154		Homo sapiens HSFE-1 mRNA, partial c	204.75128	48.97406	115.23281	26.47856	Detected	Detected	Detected	Detected
905	A_33_P3328958					6.46318	28.64680	3.34601	11.09791	Compromised	Detected	Compromised	Compromised
906	A_23_P35168	ALG6	Hs.258501	asparagine-linked glycosylation 6, alpha	Homo sapiens asparagine-linked glycos	1362.14810	768.55990	1410.17130	684.49930	Detected	Detected	Detected	Detected
907	A_23_P75083	ZMYND11	Hs.292265	zinc finger, MYND domain containing 11	Homo sapiens zinc finger, MYND domai	241.85088	329.96298	242.00433	270.78824	Detected	Detected	Detected	Detected
908	A_23_P161004	NR5A2	Hs.33446	nuclear receptor subfamily 5, group A, r	Homo sapiens nuclear receptor subfami	16.15895	19.02849	19.29856	11.36839	Detected	Compromised	Detected	Compromised
909	A_23_P411296	CEBPB	Hs.517106	CCAAT/enhancer binding protein (C/E	Homo sapiens CCAAT/enhancer bindin	110539.60000	75886.49000	88849.47000	54427.82000	Detected	Detected	Detected	Detected
910	A_23_P429581	TMEM67	Hs.116240	transmembrane protein 67	Homo sapiens transmembrane protein 6	47.64972	30.65307	37.87642	13.54631	Detected	Detected	Detected	Compromised
911	A_23_P354791	NAV2	Hs.502116	neuron navigator 2	Homo sapiens neuron navigator 2 (NAV	110.14399	196.71123	52.70222	107.80849	Detected	Detected	Detected	Detected
912	A_24_P87490	FAM18B2	Hs.647422	family with sequence similarity 18, mem	Homo sapiens family with sequence sim	2314.70850	1998.54200	2193.86550	1862.89770	Detected	Detected	Detected	Detected
913	A_23_P45087	ZNF107	Hs.50216	zinc finger protein 107	Homo sapiens zinc finger protein 107 (Z	124.11240	290.92810	83.24802	173.91727	Detected	Detected	Detected	Detected
914	A_23_P142294	ETHE1	Hs.7486	ethylmalonic encephalopathy 1	Homo sapiens ethylmalonic encephalop	4396.12400	4394.90670	4132.14260	3541.24050	Detected	Detected	Detected	Detected
915	A_23_P130429	ROCK1	Hs.306307	Rho-associated, coiled-coil containing	Homo sapiens Rho-associated, coiled-c	174.70016	267.23355	120.72138	155.43172	Detected	Detected	Detected	Detected
916	A_33_P3345931					33.42170	52.65413	21.52689	25.33718	Detected	Detected	Detected	Detected
917	A_23_P120973	FAM118A	Hs.265018	family with sequence similarity 118, me	Homo sapiens family with sequence sim	98.75579	154.21587	100.52882	120.08604	Detected	Detected	Detected	Detected
918	A_33_P3254121		Hs.529989		Ribonuclease T2 Precursor (EC 3.1.27.-	550.78510	982.96890	111.17313	94.66509	Detected	Detected	Detected	Detected
919	A_32_P117313	C8orf83	Hs.440643	chromosome 8 open reading frame 83	Homo sapiens chromosome 8 open rea	1836.87610	787.87990	1397.76030	484.38730	Detected	Detected	Detected	Detected
920	A_24_P920188	ZNF24	Hs.514802	zinc finger protein 24	Homo sapiens zinc finger protein 24 (Z	1605.34140	1214.36710	869.89307	410.52300	Detected	Detected	Detected	Detected
921	A_23_P372638	CHD9	Hs.59159	chromodomain helicase DNA binding pr	Homo sapiens chromodomain helicase D	1119.43960	998.27800	825.58110	670.56110	Detected	Detected	Detected	Detected
922	A_23_P111481	SRRT	Hs.111801	serrate RNA effector molecule homolog	Homo sapiens serrate RNA effector mo	9605.46700	20241.77100	5611.90230	10462.61400	Detected	Detected	Detected	Detected
923	A_23_P10442	OSBPL1A	Hs.370725	oxysterol binding protein-like 1A	Homo sapiens oxysterol binding protein	2378.80370	1028.76560	2031.50440	769.83510	Detected	Detected	Detected	Detected
924	A_23_P108376	THADA	Hs.369592	thyroid adenoma associated	Homo sapiens thyroid adenoma associa	2281.72850	1042.67750	1880.75370	739.17820	Detected	Detected	Detected	Detected
925	A_23_P1691	MMP1	Hs.83169	matrix metallopeptidase 1 (interstitial c	Homo sapiens matrix metallopeptidase	468847.25000	164139.19000	383133.40000	121058.71000	Detected	Detected	Detected	Detected
926	A_33_P3416814	LOC100130663		hypothetical LOC100130663	PREDICTED: Homo sapiens hypothetica	34.55475	48.90920	35.04152	38.71647	Detected	Detected	Detected	Detected
927	A_23_P114299	CXCR3	Hs.198252	chemokine (C-X-C motif) receptor 3	Homo sapiens chemokine (C-X-C moti	332.59213	377.59020	75.89556	74.98702	Detected	Detected	Detected	Detected
928	A_23_P329198	OBFC2A	Hs.591610	oligonucleotide/oligosaccharide-binding	Homo sapiens oligonucleotide/oligosac	2407.04660	2013.57970	2340.53880	1691.42980	Detected	Detected	Detected	Detected
929	A_33_P3260322					127.35831	45.28754	69.63222	23.03556	Detected	Detected	Detected	Detected
930	A_23_P23584	CTNNBIP1	Hs.463759	catenin, beta interacting protein 1	Homo sapiens catenin, beta interacting	9324.21600	5147.47750	8523.47100	4188.43070	Detected	Detected	Detected	Detected
931	A_33_P3246927		Hs.656915		CUB and sushi domain-containing prote	24.92211	51.59345	27.24453	49.53201	Detected	Detected	Detected	Detected
932	A_23_P30634	BACH2	Hs.269764	BTB and CNC homology 1, basic leucin	Homo sapiens BTB and CNC homology	42.06140	33.36184	31.40398	8.00621	Detected	Detected	Detected	Compromised
933	A_23_P11915	GDAP2	Hs.594430	ganglioside induced differentiation asso	Homo sapiens ganglioside induced diffe	146.21446	121.97743	129.75288	99.17205	Detected	Detected	Detected	Detected
934	A_23_P117494	MTHFD1	Hs.652308	methylenetetrahydrofolate dehydrogena	Homo sapiens methylenetetrahydrofolat	3109.31840	10099.13300	2295.80800	6513.62400	Detected	Detected	Detected	Detected
935	A_33_P3405491	HCRP1	Hs.675399	hepatocellular carcinoma-related HCRP	Homo sapiens hepatocellular carcinoma	16.17973	19.64408	27.70888	22.26854	Detected	Compromised	Detected	Detected
936	A_23_P151544	DDX24	Hs.510328	DEAD (Asp-Glu-Ala-Asp) box polypept	Homo sapiens DEAD (Asp-Glu-Ala-Asp	1313.96550	1505.00200	1050.22510	1068.01820	Detected	Detected	Detected	Detected
937	A_24_P350649	PDPR	Hs.461183	pyruvate dehydrogenase phosphatase r	Homo sapiens pyruvate dehydrogenase	1512.96690	1994.75270	1226.60600	1420.08700	Detected	Detected	Detected	Detected
938	A_23_P54929	LYRM1	Hs.185489	LYR motif containing 1	Homo sapiens LYR motif containing 1 (L	445.42770	771.23330	481.29895	739.91350	Detected	Detected	Detected	Detected
939	A_33_P3244369	LOC100132672	Hs.648146	similar to glycosyltransferase 8 domain	PREDICTED: Homo sapiens similar to g	39.96475	83.11401	16.81464	31.26050	Detected	Detected	Detected	Detected
940	A_23_P80902	KIF15	Hs.658939	kinesin family member 15	Homo sapiens kinesin family member 15	54.24877	982.69727	51.76542	837.83075	Detected	Detected	Detected	Detected
941	A_33_P3251984	MRGPRF	Hs.118513	MAS-related GPR, member F	Homo sapiens MAS-related GPR, memb	461.34885	301.96497	356.53256	217.04994	Detected	Detected	Detected	Detected
942	A_33_P3236340		Hs.543740		Putative uncharacterized protein [Sour	189.53017	304.81860	77.89249	108.37360	Detected	Detected	Detected	Detected
943	A_23_P119254	ASF1B	Hs.26516	ASF1 anti-silencing function 1 homolog	Homo sapiens ASF1 anti-silencing func	230.42084	1846.07390	193.23960	1382.33170	Detected	Detected	Detected	Detected
944	A_23_P148475	KIF4A	Hs.648326	kinesin family member 4A	Homo sapiens kinesin family member 4A	83.02992	3551.12330	51.60595	2410.07670	Detected	Detected	Detected	Detected
945	A_32_P135902	EIF4A1	Hs.129673	eukaryotic translation initiation factor 4	Homo sapiens eukaryotic translation ini	9153.44400	13653.88900	6795.60700	9284.42800	Detected	Detected	Detected	Detected
946	A_33_P3298661	ZNF148	Hs.592591	zinc finger protein 148	Homo sapiens zinc finger protein 148 (Z	520.55880	550.59326	354.94525	337.80310	Detected	Detected	Detected	Detected
947	A_24_P322908	USP27X	Hs.143587	ubiquitin specific peptidase 27, X-linked	Homo sapiens ubiquitin specific peptida	383.09546	215.11493	293.19424	158.60970	Detected	Detected	Detected	Detected
948	A_23_P96331	WAS	Hs.2157	Wiskott-Aldrich syndrome (eczema-thr	Homo sapiens Wiskott-Aldrich syndrom	76.57919	82.62203	43.02859	45.96906	Detected	Detected	Detected	Detected
949	A_23_P112825	LCMT1	Hs.337730	leucine carboxyl methyltransferase 1	Homo sapiens leucine carboxyl methylt	4812.69970	4456.08800	4216.41600	3508.12060	Detected	Detected	Detected	Detected
950	A_23_P383132	HIC2	Hs.632767	hypermethylated in cancer 2	Homo sapiens hypermethylated in canc	1825.66530	1454.89140	1437.14780	1142.44980	Detected	Detected	Detected	Detected
951	A_23_P149259	TMEM79	Hs.347408	transmembrane protein 79	Homo sapiens transmembrane protein 7	326.71344	191.22145	316.16418	188.05704	Detected	Detected	Detected	Detected
952	A_23_P117797	CLN6	Hs.584921	ceroid-lipofuscinosis, neuronal 6, late in	Homo sapiens ceroid-lipofuscinosis, ne	626.44965	1220.16940	569.31480	1025.03000	Detected	Detected	Detected	Detected
953	A_23_P114282	MCTS1	Hs.102696	malignant T cell amplified sequence 1	Homo sapiens malignant T cell amplifie	6873.20400	11185.67500	5856.50930	7748.36300	Detected	Detected	Detected	Detected
954	A_33_P3361817					4309.69780	6474.46100	4677.08800	6422.20200	Detected	Detected	Detected	Detected
955	A_23_P156890	TCF21	Hs.720025	transcription factor 21	Homo sapiens transcription factor 21 (T	144.70180	6.63889	168.67722	9.13705	Detected	Compromised	Detected	Compromised
956	A_23_P80382	PRR5	Hs.102336	proline rich 5 (renal)	Homo sapiens proline rich 5 (renal) (PR	1187.05260	1254.68700	1193.89590	1305.39550	Detected	Detected	Detected	Detected
957	A_33_P3283206					14.24555	19.06428	10.46900	18.67058	Detected	Compromised	Compromised	Detected
958	A_33_P3328609	PKP4	Hs.407580	plakophilin 4	Homo sapiens plakophilin 4 (PKP4), tra	121.33360	129.52515	97.70614	83.43006	Detected	Detected	Detected	Detected
959	A_23_P394836	INF2	Hs.24956	inverted formin, FH2 and WH2 domain c	Homo sapiens inverted formin, FH2 and	1143.37760	2281.43750	1008.98180	2120.44580	Detected	Detected	Detected	Detected
960	A_32_P122940	LOC642852	Hs.710382	hypothetical LOC642852	Homo sapiens hypothetical LOC642852	766.01980	346.21237	723.54240	238.27888	Detected	Detected	Detected	Detected
961	A_23_P119923	CNNM4	Hs.175043	cyclin M4	Homo sapiens cyclin M4 (CNNM4), mRN	3875.92330	5146.29700	2512.88650	2898.48320	Detected	Detected	Detected	Detected
962	A_23_P156531	PSMB1	Hs.352768	proteasome (prosome, macropain) subu	Homo sapiens proteasome (prosome, m	38424.28000	42807.50800	35999.84400	34173.05500	Detected	Detected	Detected	Detected
963	A_23_P106241	TRIP11	Hs.632339	thyroid hormone receptor interactor 11	Homo sapiens thyroid hormone recepto	3542.68500	2292.74000	2468.05900	1419.25370	Detected	Detected	Detected	Detected
964	A_33_P3268522					17.18368	19.07467	12.39664	8.40921	Detected	Compromised	Compromised	Compromised
965	A_23_P102582	C20orf24	Hs.584985	chromosome 20 open reading frame 24	Homo sapiens chromosome 20 open rea	23381.59000	39843.08000	17774.79300	25271.00600	Detected	Detected	Detected	Detected
966	A_23_P163737	OR2C1	Hs.258574	olfactory receptor, family 2, subfamily C	Homo sapiens olfactory receptor, family	15.57999	21.81591	13.46416	17.30349	Detected	Detected	Compromised	Compromised
967	A_24_P227585	KIAA1704	Hs.720091	KIAA1704	Homo sapiens KIAA1704 (KIAA1704), m	251.19757	262.96585	307.11320	268.58286	Detected	Detected	Detected	Detected
968	A_33_P3247534	LOC389834	Hs.720653	ankyrin repeat domain 57 pseudogene	Homo sapiens ankyrin repeat domain 57	9864.57100	12377.28700	2654.34250	2184.78420	Detected	Detected	Detected	Detected
969	A_24_P180680	LAPTM4B	Hs.492314	lysosomal protein transmembrane 4 bet	Homo sapiens lysosomal protein transm	19713.35500	11354.88500	17256.25800	8290.77400	Detected	Detected	Detected	Detected
970	A_33_P3280927	SNHG7		small nucleolar RNA host gene 7 (non-p	Homo sapiens small nucleolar RNA host	2252.31760	4994.27100	1330.15040	2375.85570	Detected	Detected	Detected	Detected
971	A_24_P110141	C15orf51	Hs.567763	dynamin 1 pseudogene	Homo sapiens chromosome 15 open rea	121.87061	24.71523	120.24781	24.10910	Detected	Detected	Detected	Detected

972	A_23_P76034	PVRL1	Hs.334846	poliovirus receptor-related 1 (herpesvir	Homo sapiens poliovirus receptor-relate	152.19275	241.37956	157.33118	246.24338	Detected	Detected	Detected	Detected
973	A_23_P316472	DNHD1	Hs.720080	dynein heavy chain domain 1	Homo sapiens dynein heavy chain doma	84.83690	23.23619	117.27170	21.06353	Detected	Detected	Detected	Detected
974	A_24_P940135	CTBS	Hs.513557	chitobiase, di-N-acetyl-	Homo sapiens chitobiase, di-N-acetyl-	1472.48220	866.09520	1095.50600	450.05728	Detected	Detected	Detected	Detected
975	A_33_P3374623	ABCA7	Hs.134514	ATP-binding cassette, sub-family A (AE	Homo sapiens ATP-binding cassette, su	312.98970	25.74829	378.68980	26.24146	Detected	Detected	Detected	Detected
976	A_32_P395879	C9orf93	Hs.17267	chromosome 9 open reading frame 93	Homo sapiens chromosome 9 open read	49.18223	33.21718	31.63214	18.10078	Detected	Detected	Detected	Compromised
977	A_33_P3415551	GPAT2	Hs.348629	glycerol-3-phosphate acyltransferase 2	Homo sapiens glycerol-3-phosphate ac	348.43805	2224.45750	329.21090	1910.69310	Detected	Detected	Detected	Detected
978	A_23_P407565	CX3CR1	Hs.78913	chemokine (C-X3-C motif) receptor 1	Homo sapiens chemokine (C-X3-C mot	21.83493	4.24515	16.45748	5.78100	Detected	Compromised	Compromised	Compromised
979	A_23_P34637	REN	Hs.3210	renin	Homo sapiens renin (REN), mRNA [NM_	17.67091	27.84867	34.59341	45.88975	Detected	Detected	Detected	Detected
980	A_23_P212545	RAB5A	Hs.475663	RAB5A, member RAS oncogene family	Homo sapiens RAB5A, member RAS on	6276.54150	7416.65100	5831.69630	5737.93400	Detected	Detected	Detected	Detected
981	A_33_P3337019	LOC728975	Hs.647238	hypothetical protein LOC728975	PREDICTED: Homo sapiens hypothetica	365.74182	809.42960	255.08148	435.13834	Detected	Detected	Detected	Detected
982	A_23_P41424	SLC39A8	Hs.288034	solute carrier family 39 (zinc transporte	Homo sapiens solute carrier family 39 (	8019.47270	573.18195	7354.79350	448.96796	Detected	Detected	Detected	Detected
983	A_24_P940776	BDP1	Hs.258272	B double prime 1, subunit of RNA polym	Homo sapiens B double prime 1, subuni	506.45828	543.96747	345.96230	318.59512	Detected	Detected	Detected	Detected
984	A_24_P253003	WNT11	Hs.108219	wingless-type MMTV integration site fa	Homo sapiens wingless-type MMTV inte	18.52752	50.63562	12.98368	37.67300	Detected	Detected	Compromised	Detected
985	A_24_P204675	NPIPL3	Hs.720286	nuclear pore complex interacting protei	Homo sapiens nuclear pore complex int	15571.78000	13666.20600	14614.62300	10661.85450	Detected	Detected	Detected	Detected
986	A_33_P3382309	PRDM16	Hs.99500	PR domain containing 16	Homo sapiens PR domain containing 16	62.11982	42.02167	45.19642	20.55967	Detected	Detected	Detected	Detected
987	A_33_P3253234	IQSEC2		IQ motif and Sec7 domain 2	Homo sapiens IQ motif and Sec7 domai	1183.53300	964.14480	860.86110	574.49480	Detected	Detected	Detected	Detected
988	A_33_P3252588	LOC100130093		hypothetical LOC100130093	Homo sapiens hypothetical LOC100130	133.93530	20.74734	118.36622	23.01815	Detected	Detected	Detected	Detected
989	A_23_P136238	TMEM203	Hs.231029	transmembrane protein 203	Homo sapiens transmembrane protein 2	8166.44240	9924.04400	6804.01270	7249.45100	Detected	Detected	Detected	Detected
990	A_33_P3278684		Hs.636424		PREDICTED: Homo sapiens hypothetica	100.33367	93.63979	126.25039	95.48750	Detected	Detected	Detected	Detected
991	A_33_P3294921	PRUNE2	Hs.262857	prune homolog 2 (Drosophila)	Homo sapiens prune homolog 2 (Drosop	54.46020	29.22584	41.94334	20.58717	Detected	Detected	Detected	Detected
992	A_23_P111804	PARP12	Hs.12646	poly (ADP-ribose) polymerase family, m	Homo sapiens poly (ADP-ribose) polym	778.31067	373.78537	599.01886	245.47343	Detected	Detected	Detected	Detected
993	A_23_P112201	KDM4C	Hs.709425	lysine (K)-specific demethylase 4C	Homo sapiens lysine (K)-specific demet	525.39620	560.55590	365.96770	307.90738	Detected	Detected	Detected	Detected
994	A_23_P118306	DNAJA3	Hs.459779	DnaJ (Hsp40) homolog, subfamily A, me	Homo sapiens DnaJ (Hsp40) homolog, s	5877.90300	4280.84400	4363.44040	2892.00300	Detected	Detected	Detected	Detected
995	A_23_P56213	GRAMD1A	Hs.515351	GRAM domain containing 1A	Homo sapiens GRAM domain containing	201.12883	138.04698	211.36124	132.26538	Detected	Detected	Detected	Detected
996	A_24_P221092		Hs.684473		Homo sapiens clone pp9953 unknown m	23.14664	41.50464	18.44238	27.85264	Detected	Detected	Detected	Detected
997	A_23_P116414	PLA2G16	Hs.502775	phospholipase A2, group XVI	Homo sapiens phospholipase A2, group	1151.05590	3987.13000	813.60315	2673.35130	Detected	Detected	Detected	Detected
998	A_23_P217704	GYG2	Hs.567381	glycogenin 2	Homo sapiens glycogenin 2 (GYG2), tra	113.63329	32.52123	115.74461	34.68008	Detected	Detected	Detected	Detected
999	A_24_P941148	HAUS6	Hs.533468	HAUS augmin-like complex, subunit 6	Homo sapiens HAUS augmin-like compl	3444.16600	4110.05470	2320.84160	2411.36430	Detected	Detected	Detected	Detected
1000	A_24_P379693	DNPEP	Hs.258551	aspartyl aminopeptidase	Homo sapiens aspartyl aminopeptidase	402.50412	204.77686	245.53162	137.09543	Detected	Detected	Detected	Detected
1001	A_33_P3280549	LOC100132108		similar to hCG1644176	PREDICTED: Homo sapiens similar to h	4094.57890	6638.18100	3929.26930	5654.97660	Detected	Detected	Detected	Detected
1002	A_23_P115932	KCNK18	Hs.449650	potassium channel, subfamily K, membe	Homo sapiens potassium channel, subfa	2.51963	3.63207	2.83199	13.91203	Compromised	Compromised	Compromised	Not Detected
1003	A_23_P60101	ZNF696	Hs.512740	zinc finger protein 696	Homo sapiens zinc finger protein 696 (Z	444.67780	411.38577	373.39320	359.20430	Detected	Detected	Detected	Detected
1004	A_33_P3695899	FLJ31104	Hs.482141	hypothetical gene supported by AK0556	Homo sapiens cDNA clone IMAGE:5267	154.56404	186.93686	111.81731	100.41240	Detected	Detected	Detected	Detected
1005	A_33_P3273369	SH3BP4	Hs.516777	SH3-domain binding protein 4	Homo sapiens SH3-domain binding prot	79.81108	199.41457	107.49062	214.42757	Detected	Detected	Detected	Detected
1006	A_23_P120982	PKDREJ	Hs.241383	polycystic kidney disease (polycystin) a	Homo sapiens polycystic kidney disease	15.70648	19.38140	3.47697	4.88679	Not Detected	Detected	Compromised	Compromised
1007	A_33_P3303430					187.15070	267.88904	101.02499	81.97353	Detected	Detected	Detected	Detected
1008	A_33_P3318624					36.94327	31.92149	36.75251	40.67007	Detected	Detected	Detected	Detected
1009	A_23_P14174	TNFSF13B	Hs.525157	tumor necrosis factor (ligand) superfam	Homo sapiens tumor necrosis factor (lig	110.16267	158.45831	120.07958	160.02422	Detected	Detected	Detected	Detected
1010	A_23_P218841	NGLY1	Hs.368960	N-glycanase 1	Homo sapiens N-glycanase 1 (NGLY1),	8867.49100	4665.23700	7657.73700	3549.51100	Detected	Detected	Detected	Detected
1011	A_33_P3330283	SP1	Hs.649191	Sp1 transcription factor	Homo sapiens Sp1 transcription factor	2189.53930	1660.19700	1589.94570	1027.00100	Detected	Detected	Detected	Detected
1012	A_23_P12053	SPEN	Hs.558463	spen homolog, transcriptional regulator	Homo sapiens spen homolog, transcripti	614.72190	700.91675	325.58997	301.30716	Detected	Detected	Detected	Detected
1013	A_33_P3313555	PSPC1	Hs.213198	paraspeckle component 1	Homo sapiens paraspeckle component	1369.64400	1360.97990	1130.04630	956.85333	Detected	Detected	Detected	Detected
1014	A_23_P100196	USP10	Hs.136778	ubiquitin specific peptidase 10	Homo sapiens ubiquitin specific peptida	616.16534	1313.40710	481.25696	813.84680	Detected	Detected	Detected	Detected
1015	A_33_P3646051	LOC284408	Hs.570010	hypothetical protein LOC284408	Homo sapiens cDNA FLJ37005 fis, clon	16.41377	15.99440	17.52248	19.20894	Detected	Compromised	Detected	Detected
1016	A_23_P6546	TUBGCP6	Hs.336431	tubulin, gamma complex associated prot	Homo sapiens tubulin, gamma complex	474.61615	374.46050	477.21850	378.08224	Detected	Detected	Detected	Detected
1017	A_23_P115137	HBXIP	Hs.439815	hepatitis B virus x interacting protein	Homo sapiens hepatitis B virus x intera	16115.11300	16749.19500	17060.75000	14610.52700	Detected	Detected	Detected	Detected
1018	A_23_P37068	TRAF3	Hs.510528	TNF receptor-associated factor 3	Homo sapiens TNF receptor-associated	380.67532	482.47820	150.20293	146.16513	Detected	Detected	Detected	Detected
1019	A_33_P3280325	LOC651986		similar to Forkhead box protein L1 (Forl	PREDICTED: Homo sapiens similar to fo	30.28803	42.25825	24.93146	23.89955	Detected	Detected	Detected	Detected
1020	A_33_P3365735	THBS2	Hs.371147	thrombospondin 2	Homo sapiens thrombospondin 2 (THBS	2514.45000	1897.65530	1717.43330	1078.26370	Detected	Detected	Detected	Detected
1021	A_23_P118061	CKLF	Hs.15159	chemokine-like factor	Homo sapiens chemokine-like factor (C	3879.94800	2342.53250	2915.17720	1382.74200	Detected	Detected	Detected	Detected
1022	A_33_P3213204	MAGEF1	Hs.306123	melanoma antigen family F, 1	Homo sapiens melanoma antigen family	1272.64800	1212.61190	1449.58560	1330.15750	Detected	Detected	Detected	Detected
1023	A_23_P129433	SLC9A5	Hs.439650	solute carrier family 9 (sodium/hydroge	Homo sapiens solute carrier family 9 (s	2510.48900	2439.88720	2162.48440	1705.72330	Detected	Detected	Detected	Detected
1024	A_23_P157495	PPP3CC	Hs.655661	protein phosphatase 3 (formerly 2B), ca	Homo sapiens protein phosphatase 3 (fo	8280.85450	10478.02100	6472.01370	6409.93650	Detected	Detected	Detected	Detected
1025	A_33_P3224331	DDX3Y	Hs.99120	DEAD (Asp-Glu-Ala-Asp) box polypept	Homo sapiens DEAD (Asp-Glu-Ala-Asp	4161.63000	4.15513	2590.18330	3.71233	Detected	Compromised	Detected	Compromised
1026	A_33_P3289227	DNALI1	Hs.406050	dynein, axonemal, light intermediate cha	Homo sapiens dynein, axonemal, light in	762.18000	112.13401	532.49790	71.13265	Detected	Detected	Detected	Detected
1027	A_23_P305550	CAPN10	Hs.112218	calpain 10	Homo sapiens calpain 10 (CAPN10), tra	34.96281	37.07065	27.39984	21.48525	Detected	Detected	Detected	Detected
1028	A_23_P119337	ATF5	Hs.9754	activating transcription factor 5	Homo sapiens activating transcription fa	835.30680	1101.17270	882.94080	1156.89300	Detected	Detected	Detected	Detected
1029	A_33_P3400374	PRIC285	Hs.517180	peroxisomal proliferator-activated rece	Homo sapiens peroxisomal proliferator-	3772.68850	2164.46800	3282.21120	1666.56130	Detected	Detected	Detected	Detected
1030	A_24_P256307	ARF3	Hs.119177	ADP-ribosylation factor 3	Homo sapiens ADP-ribosylation factor	10019.33200	8564.49600	7717.38100	6101.62550	Detected	Detected	Detected	Detected
1031	A_23_P423864	PHC2	Hs.524271	polyhomeotic homolog 2 (Drosophila)	Homo sapiens polyhomeotic homolog 2	107648.39000	54306.70700	100621.29000	55595.08000	Detected	Detected	Detected	Detected
1032	A_32_P139229	ZNF543	Hs.202544	zinc finger protein 543	Homo sapiens zinc finger protein 543 (Z	124.42460	75.77698	151.30970	79.48805	Detected	Detected	Detected	Detected
1033	A_23_P76705	TUBGCP3	Hs.224152	tubulin, gamma complex associated prot	Homo sapiens tubulin, gamma complex	231.96722	851.01166	190.08424	609.94920	Detected	Detected	Detected	Detected
1034	A_23_P132121	SIK1	Hs.282113	salt-inducible kinase 1	Homo sapiens salt-inducible kinase 1 (S	321.55768	429.35240	266.49475	247.65419	Detected	Detected	Detected	Detected
1035	A_23_P120921	ANKRD54	Hs.135259	ankyrin repeat domain 54	Homo sapiens ankyrin repeat domain 54	350.43057	364.74057	409.75897	391.38843	Detected	Detected	Detected	Detected
1036	A_23_P424734	EXOC3L	Hs.647356	exocyst complex component 3-like	Homo sapiens exocyst complex compor	105.88271	191.08612	92.17638	74.04449	Detected	Detected	Detected	Detected
1037	A_24_P135406	KCTD9	Hs.72071	potassium channel tetramerisation dom	Homo sapiens potassium channel tetrar	311.59170	630.27980	266.09270	450.29892	Detected	Detected	Detected	Detected
1038	A_23_P104471	DUSP13	Hs.720464	dual specificity phosphatase 13	Homo sapiens dual specificity phosphat	25.30144	10.66538	15.89626	4.91083	Detected	Compromised	Detected	Compromised
1039	A_23_P253068	ANAPC2	Hs.533262	anaphase promoting complex subunit 2	Homo sapiens anaphase promoting com	108.76617	99.64976	130.34860	105.09522	Detected	Detected	Detected	Detected
1040	A_33_P3224426		Hs.148365		Putative uncharacterized protein ENSP	55.33249	68.99858	10.08675	5.54596	Detected	Detected	Compromised	Compromised
1041	A_24_P458479		Hs.567050		Protein FAM27E1 [Source:UniProtKB/	45.32588	17.00646	41.43237	8.99442	Detected	Compromised	Detected	Compromised
1042	A_23_P26810	TP53	Hs.654481	tumor protein p53	Homo sapiens tumor protein p53 (TP53	39.54759	24.71022	30.21457	18.04590	Detected	Detected	Detected	Detected
1043	A_23_P207879	CARD14	Hs.696253	caspase recruitment domain family, mer	Homo sapiens caspase recruitment dom	31.13783	18.12910	34.46156	26.35273	Detected	Compromised	Detected	Detected
1044	A_24_P204214	NCOR1	Hs.462323	nuclear receptor co-repressor 1	Homo sapiens nuclear receptor co-repr	1397.95890	1352.89890	960.29090	798.29150	Detected	Detected	Detected	Detected
1045	A_23_P122197	CCNB1	Hs.23960	cyclin B1	Homo sapiens cyclin B1 (CCNB1), mRN	517.57600	22906.70500	392.00906	15501.72600	Detected	Detected	Detected	Detected
1046	A_23_P324327	GPRC5B	Hs.148685	G protein-coupled receptor, family C, gr	Homo sapiens G protein-coupled recep	144.73958	31.29900	145.35898	22.17813	Detected	Detected	Detected	Detected

1047	A_23_P52761	MMP7	Hs.2256	matrix metallopeptidase 7 (matrilysin, ut	Homo sapiens matrix metallopeptidase 7	15.54122	47.14092	13.93855	38.65622	Detected	Detected	Compromised	Detected
1048	A_23_P168868	PTDSS1	Hs.292579	phosphatidylserine synthase 1	Homo sapiens phosphatidylserine synth	28533.48400	34998.33000	24853.82200	26698.06800	Detected	Detected	Detected	Detected
1049	A_23_P170978	ZNF692	Hs.377705	zinc finger protein 692	Homo sapiens zinc finger protein 692 (Z	1319.79170	376.90143	1346.99580	381.12326	Detected	Detected	Detected	Detected
1050	A_24_P681011	HIPK2	Hs.397465	homeodomain interacting protein kinase	Homo sapiens homeodomain interacting	1389.61160	1398.65390	716.10100	568.13960	Detected	Detected	Detected	Detected
1051	A_33_P3383955	DDB2	Hs.700338	damage-specific DNA binding protein 2,	Homo sapiens damage-specific DNA bir	642.84674	1090.26040	605.19720	983.92190	Detected	Detected	Detected	Detected
1052	A_24_P128312	ZNF79	Hs.522399	zinc finger protein 79	Homo sapiens zinc finger protein 79 (ZN	264.09763	343.99750	178.55257	209.47679	Detected	Detected	Detected	Detected
1053	A_33_P3244872	CEP350	Hs.413045	centrosomal protein 350kDa	Homo sapiens centrosomal protein 350k	1985.11600	1374.58360	806.07800	493.07245	Detected	Detected	Detected	Detected
1054	A_23_P96542	VMA21	Hs.58633	VMA21 vacuolar H+-ATPase homolog (	Homo sapiens VMA21 vacuolar H+-ATP	677.05560	1066.72690	682.82086	941.36890	Detected	Detected	Detected	Detected
1055	A_24_P343377	ATP5J2	Hs.521056	ATP synthase, H+ transporting, mitocho	Homo sapiens ATP synthase, H+ transp	10680.23000	15133.90900	9751.87500	11919.07900	Detected	Detected	Detected	Detected
1056	A_33_P3396522	POLR2G	Hs.14839	polymerase (RNA) II (DNA directed) pol	Homo sapiens polymerase (RNA) II (DNA	22535.61100	23312.40200	16342.39900	14768.12200	Detected	Detected	Detected	Detected
1057	A_23_P121956	THG1L	Hs.353090	tRNA-histidine guanylyltransferase 1-lik	Homo sapiens tRNA-histidine guanylyltr	805.62200	985.55190	753.12897	852.28960	Detected	Detected	Detected	Detected
1058	A_32_P182156	ZNF511	Hs.422113	zinc finger protein 511	Homo sapiens zinc finger protein 511 (Z	3762.44240	3706.43580	3743.18700	3438.77660	Detected	Detected	Detected	Detected
1059	A_23_P110234	CSN1S1	Hs.3155	casein alpha s1	Homo sapiens casein alpha s1 (CSN1S1	69.34351	21.73105	53.49363	17.72514	Detected	Detected	Detected	Detected
1060	A_23_P329361	COG7	Hs.185807	component of oligomeric golgi complex	Homo sapiens component of oligomeric	888.58813	1556.24780	939.73926	1512.33500	Detected	Detected	Detected	Detected
1061	A_24_P32887	BRD3	Hs.522472	bromodomain containing 3	Homo sapiens bromodomain containing	527.41200	221.69505	704.65830	353.01645	Detected	Detected	Detected	Detected
1062	A_24_P397817	LEP	Hs.194236	leptin	Homo sapiens leptin (LEP), mRNA [NM_	79.96177	4.49224	34.25934	2.98008	Detected	Compromised	Detected	Compromised
1063	A_33_P3269408	FOXI2	Hs.449674	forkhead box I2	Homo sapiens forkhead box I2 (FOXI2),	103115.52000	74460.16400	17097.23800	7869.91850	Detected	Detected	Detected	Detected
1064	A_33_P3209167	LOC100131053	Hs.697104	hypothetical LOC100131053	Homo sapiens cDNA FLJ38245 fis, clon	10.51555	3.77343	17.46403	3.37121	Compromised	Compromised	Detected	Compromised
1065	A_23_P101737	DOT1L	Hs.713641	DOT1-like, histone H3 methyltransferas	Homo sapiens DOT1-like, histone H3 m	353.71066	338.35980	307.84326	293.77356	Detected	Detected	Detected	Detected
1066	A_23_P99515	C13orf33	Hs.147880	chromosome 13 open reading frame 33	Homo sapiens chromosome 13 open rea	32043.70700	7032.33940	30612.00000	5781.85740	Detected	Detected	Detected	Detected
1067	A_23_P167040	PDIA5	Hs.477352	protein disulfide isomerase family A, me	Homo sapiens protein disulfide isomeras	6394.74700	7966.40430	7214.01860	7364.71800	Detected	Detected	Detected	Detected
1068	A_24_P306720	LOC400236	Hs.585207	hypothetical LOC400236	PREDICTED: Homo sapiens hypothetica	131.54169	337.58365	137.39941	309.83398	Detected	Detected	Detected	Detected
1069	A_23_P2041	MICALCL	Hs.128196	MICAL C-terminal like	Homo sapiens MICAL C-terminal like (M	113.37088	287.02362	94.48461	188.78000	Detected	Detected	Detected	Detected
1070	A_23_P58102	EXOSC7	Hs.719958	exosome component 7	Homo sapiens exosome component 7 (E	2801.30500	3428.37670	2971.41800	3134.51600	Detected	Detected	Detected	Detected
1071	A_33_P3288294		Hs.458713		Alpha-(1,3)-fucosyltransferase 10 (EC 2	105.85919	143.51761	79.41782	131.30737	Detected	Detected	Detected	Detected
1072	A_33_P3310430	FAM86B2	Hs.458413	family with sequence similarity 86, mem	Homo sapiens family with sequence sim	2098.41140	2894.38010	2144.90800	2591.80200	Detected	Detected	Detected	Detected
1073	A_23_P761	PSMB4	Hs.89545	proteasome (prosome, macropain) subu	Homo sapiens proteasome (prosome, m	55704.33600	53719.33600	50162.77000	41264.26600	Detected	Detected	Detected	Detected
1074	A_23_P110212	ACSL1	Hs.406678	acyl-CoA synthetase long-chain family	Homo sapiens acyl-CoA synthetase lon	1497.37680	1385.02810	1425.64200	1155.93590	Detected	Detected	Detected	Detected
1075	A_23_P117599	AHSA1	Hs.204041	AHA1, activator of heat shock 90kDa pr	Homo sapiens AHA1, activator of heat s	4891.29400	6012.76560	4340.18260	4906.94240	Detected	Detected	Detected	Detected
1076	A_33_P3212823	LOC100132247	Hs.720286	similar to Uncharacterized protein KIAA	Homo sapiens similar to Uncharacterize	109743.09000	95008.97000	56435.83000	42851.42000	Detected	Detected	Detected	Detected
1077	A_23_P161399	MXI1	Hs.501023	MAX interactor 1	Homo sapiens MAX interactor 1 (MXI1),	3046.89670	674.31670	1828.73330	314.20944	Detected	Detected	Detected	Detected
1078	A_23_P104362	ECHS1	Hs.76394	enoyl Coenzyme A hydratase, short cha	Homo sapiens enoyl Coenzyme A hydra	11691.68400	16766.20000	10524.81000	13050.08900	Detected	Detected	Detected	Detected
1079	A_33_P3718734	LOC729083	Hs.713395	hypothetical protein LOC729083	Homo sapiens, clone IMAGE:4779414, m	49.48377	32.90549	46.55763	35.17748	Detected	Detected	Detected	Detected
1080	A_23_P374288	CASC3	Hs.592129	cancer susceptibility candidate 3	Homo sapiens cancer susceptibility can	5648.86430	5969.70800	4457.38130	4097.70360	Detected	Detected	Detected	Detected
1081	A_23_P11214	NKRF	Hs.437084	NFKB repressing factor	Homo sapiens NFKB repressing factor (	816.86940	699.53827	621.61220	432.13828	Detected	Detected	Detected	Detected
1082	A_23_P87560	BTG1	Hs.255935	B-cell translocation gene 1, anti-prolife	Homo sapiens B-cell translocation gene	9403.50100	5875.42800	8000.91360	4154.16800	Detected	Detected	Detected	Detected
1083	A_24_P135193	UEVLD	Hs.407991	UEV and lactate/malate dehyrogenase	Homo sapiens UEV and lactate/malate	69.81501	66.13709	60.04185	49.72934	Detected	Detected	Detected	Detected
1084	A_23_P25994	LGMN	Hs.719135	legumain	Homo sapiens legumain (LGMN), transc	778.30190	1661.62000	802.65110	1362.90100	Detected	Detected	Detected	Detected
1085	A_23_P104734	STT3A	Hs.504237	STT3, subunit of the oligosaccharyltran	Homo sapiens STT3, subunit of the olig	2761.03980	4923.57500	1893.25370	2995.04270	Detected	Detected	Detected	Detected
1086	A_23_P206759	KIAA0174	Hs.232194	KIAA0174	Homo sapiens KIAA0174 (KIAA0174), m	1380.61290	1270.28450	938.91595	818.33950	Detected	Detected	Detected	Detected
1087	A_23_P68669	CHODL	Hs.283725	chondrolectin	Homo sapiens chondrolectin (CHODL),	43.11492	54.68632	29.61943	27.12751	Detected	Detected	Detected	Detected
1088	A_33_P3684897					25.23290	27.62695	31.93717	25.43645	Detected	Detected	Detected	Detected
1089	A_23_P68486	C20orf108	Hs.143736	chromosome 20 open reading frame 108	Homo sapiens chromosome 20 open rea	12347.75300	4728.08700	12067.50500	3971.38430	Detected	Detected	Detected	Detected
1090	A_33_P3336282	TAF4B	Hs.369519	TAF4b RNA polymerase II, TATA box bi	Homo sapiens TAF4b RNA polymerase	19.03182	5.04400	12.72849	5.76888	Detected	Compromised	Compromised	Compromised
1091	A_33_P3358686	LOC728627	Hs.432735	similar to leucine rich repeat containing	PREDICTED: Homo sapiens similar to le	1520.79500	1828.33680	218.49570	183.29323	Detected	Detected	Detected	Detected
1092	A_23_P121596	PPBP	Hs.2164	pro-platelet basic protein (chemokine ((	Homo sapiens pro-platelet basic protein	33.30248	18.48150	4.80041	4.81174	Not Detected	Detected	Compromised	Compromised
1093	A_23_P117873	CHRM5	Hs.584747	cholinergic receptor, muscarinic 5	Homo sapiens cholinergic receptor, mus	16.31043	18.92067	15.12908	6.17398	Detected	Detected	Not Detected	Compromised
1094	A_23_P123265	SUMF2	Hs.279696	sulfatase modifying factor 2	Homo sapiens sulfatase modifying facto	774.89680	1563.08530	646.99730	1148.97450	Detected	Detected	Detected	Detected
1095	A_33_P3282202		Hs.689588		Homo sapiens cDNA FLJ42047 fis, clon	132.45810	128.65862	15.78532	12.64083	Detected	Detected	Compromised	Compromised
1096	A_23_P40194	DDX27	Hs.65234	DEAD (Asp-Glu-Ala-Asp) box polypept	Homo sapiens DEAD (Asp-Glu-Ala-Asp	14452.35900	10422.06100	12575.66600	8029.22750	Detected	Detected	Detected	Detected
1097	A_23_P12241	MCOLN3	Hs.535239	mucolipin 3	Homo sapiens mucolipin 3 (MCOLN3), m	28.86564	9.20777	13.98728	4.69957	Detected	Compromised	Detected	Compromised
1098	A_33_P3361348	ALOX15	Hs.73809	arachidonate 15-lipoxygenase	Homo sapiens arachidonate 15-lipoxyge	42.91057	30.87564	26.41250	19.49341	Detected	Detected	Detected	Detected
1099	A_23_P106708	RPS2	Hs.356366	ribosomal protein S2	Homo sapiens ribosomal protein S2 (RP	503202.22000	529424.94000	431637.16000	396972.25000	Detected	Detected	Detected	Detected
1100	A_33_P3331267				Sestrin-1 (p53-regulated protein PA26)	36.57106	29.55504	12.14836	19.90220	Detected	Detected	Compromised	Detected
1101	A_33_P3345608	C10orf137	Hs.468688	chromosome 10 open reading frame 137	Homo sapiens chromosome 10 open rea	69.99095	151.92462	73.56426	156.50908	Detected	Detected	Detected	Detected
1102	A_24_P58727					8325.50000	11084.72300	4997.14400	6067.89500	Detected	Detected	Detected	Detected
1103	A_23_P361419	DEPDC1B	Hs.482233	DEP domain containing 1B	Homo sapiens DEP domain containing 1	59.24345	996.99400	38.78222	578.17737	Detected	Detected	Detected	Detected
1104	A_32_P738377	LOC284837	Hs.313689	hypothetical LOC284837	Homo sapiens hypothetical LOC284837	52.52415	52.55147	43.46619	30.46356	Detected	Detected	Detected	Detected
1105	A_23_P154256	ELMOD3	Hs.269990	ELMO/CED-12 domain containing 3	Homo sapiens ELMO/CED-12 domain c	2430.25830	1309.93710	2140.59900	1026.58840	Detected	Detected	Detected	Detected
1106	A_24_P23522	THAP5	Hs.707689	THAP domain containing 5	Homo sapiens THAP domain containing	76.20138	83.73710	67.81885	52.27008	Detected	Detected	Detected	Detected
1107	A_33_P3246997		Hs.130293		Cisplatin resistance-associated overexp	618.27190	104.25731	299.26996	41.28412	Detected	Detected	Detected	Detected
1108	A_33_P3815935	LOC654779	Hs.655101	hypothetical gene LOC654779	RST40456 Athersys RAGE Library Hom	34.86784	31.89333	6.57514	5.21903	Detected	Detected	Compromised	Compromised
1109	A_33_P3277259	NPB	Hs.585089	neuropeptide B	Homo sapiens cDNA clone IMAGE:5019	1224.02120	1331.82470	1274.08140	1217.07210	Detected	Detected	Detected	Detected
1110	A_23_P52266	IFIT1	Hs.20315	interferon-induced protein with tetratric	Homo sapiens interferon-induced prote	295.90360	253.49805	307.41766	201.44151	Detected	Detected	Detected	Detected
1111	A_33_P3285047	ZNF75A	Hs.513292	zinc finger protein 75a	Homo sapiens zinc finger protein 75a (Z	2506.41160	1984.87840	1737.32090	1177.53820	Detected	Detected	Detected	Detected
1112	A_24_P195037	C1orf52	Hs.26226	chromosome 1 open reading frame 52	Homo sapiens chromosome 1 open read	1218.89090	1583.23860	1284.81580	1545.31500	Detected	Detected	Detected	Detected
1113	A_23_P77440	NFATC3	Hs.632209	nuclear factor of activated T-cells, cyto	Homo sapiens nuclear factor of activate	428.89597	363.94797	360.07750	291.73810	Detected	Detected	Detected	Detected
1114	A_33_P3330877					21.20288	24.61056	16.61713	13.88007	Detected	Detected	Detected	Compromised
1115	A_33_P3277883	LOC100129931	Hs.707753	hypothetical LOC100129931	Homo sapiens cDNA FLJ44077 fis, clon	54.26789	43.70100	20.98677	15.30473	Detected	Detected	Detected	Compromised
1116	A_24_P140827	PCMT1	Hs.279257	protein-L-isoaspartate (D-aspartate) O	Homo sapiens protein-L-isoaspartate (I	10260.21800	8471.45600	8965.55100	6642.92630	Detected	Detected	Detected	Detected
1117	A_23_P323783	LSM14B	Hs.105379	LSM14B, SCD6 homolog B (S. cerevisia	Homo sapiens LSM14B, SCD6 homolog	99.16212	174.08797	90.72172	157.62470	Detected	Detected	Detected	Detected
1118	A_23_P101392	TMEM38A	Hs.436068	transmembrane protein 38A	Homo sapiens transmembrane protein 3	215.79321	75.42906	191.55850	37.95522	Detected	Detected	Detected	Detected
1119	A_33_P3286218	DLEU2L	Hs.547964	deleted in lymphocytic leukemia 2-like	Homo sapiens deleted in lymphocytic le	99.98975	218.26775	66.52727	128.85132	Detected	Detected	Detected	Detected
1120	A_33_P3221999	GSDMB	Hs.306777	gasdermin B	Homo sapiens gasdermin B (GSDMB), tr	827.25714	207.68341	649.26166	135.29141	Detected	Detected	Detected	Detected
1121	A_33_P3313929	CCR6	Hs.46468	chemokine (C-C motif) receptor 6	Homo sapiens chemokine (C-C motif) r	678.15704	731.41644	189.17307	167.04211	Detected	Detected	Detected	Detected

1122	A_33_P3390456				YY1-associated factor 2 [Source:UniPr	284.10360	485.32736	234.41127	299.32516	Detected	Detected	Detected	Detected
1123	A_23_P75707	OR8H1	Hs.553628	olfactory receptor, family 8, subfamily H	Homo sapiens olfactory receptor, family	57.23581	99.41369	14.16778	20.54213	Detected	Detected	Compromised	Detected
1124	A_33_P3355407	RNU105A		RNA, U105A small nucleolar	Homo sapiens RNA, U105A small nucleo	131.75168	111.69402	226.84213	113.87223	Detected	Detected	Detected	Detected
1125	A_23_P90079	ZNF799	Hs.546637	zinc finger protein 799	Homo sapiens zinc finger protein 799 (Z	119.87309	176.37514	40.42624	56.51087	Detected	Detected	Detected	Detected
1126	A_33_P3267745					130.86570	154.96768	82.57242	71.03182	Detected	Detected	Detected	Detected
1127	A_23_P115597	ANKRD26	Hs.361041	ankyrin repeat domain 26	Homo sapiens ankyrin repeat domain 26	317.10880	129.73405	268.57310	94.80158	Detected	Detected	Detected	Detected
1128	A_33_P3374365	LOC100129702	Hs.709968	similar to RAB40A, member RAS oncog	PREDICTED: Homo sapiens similar to R	3051.86550	4594.54000	1681.76260	1693.40780	Detected	Detected	Detected	Detected
1129	A_33_P3338698	IHH	Hs.654504	Indian hedgehog homolog (Drosophila)	Homo sapiens Indian hedgehog homolog	43.55446	44.52634	33.75560	27.85471	Detected	Detected	Detected	Detected
1130	A_23_P109774	ZBTB11	Hs.655286	zinc finger and BTB domain containing	Homo sapiens zinc finger and BTB dom	1051.91830	849.61206	585.14940	382.05453	Detected	Detected	Detected	Detected
1131	A_32_P128391	LOC728431	Hs.380738	hypothetical LOC728431	PREDICTED: Homo sapiens hypothetica	685.19830	146.84780	866.53270	173.30140	Detected	Detected	Detected	Detected
1132	A_24_P399980	HEPH	Hs.31720	hephaestin	Homo sapiens hephaestin (HEPH), trans	300.36337	425.74133	288.16733	391.67596	Detected	Detected	Detected	Detected
1133	A_23_P12405	ESPN	Hs.719151	espin	Homo sapiens espin (ESPN), mRNA [NM	40.61718	52.73604	20.68593	19.25098	Detected	Detected	Detected	Detected
1134	A_23_P118246	GINS2	Hs.433180	GINS complex subunit 2 (Psf2 homolog)	Homo sapiens GINS complex subunit 2	185.42964	3909.97680	134.80475	2417.23240	Detected	Detected	Detected	Detected
1135	A_33_P3365845					21.12058	44.81330	3.49761	11.21828	Detected	Detected	Compromised	Compromised
1136	A_23_P101461	CCDC130	Hs.24998	coiled-coil domain containing 130	Homo sapiens coiled-coil domain contai	11920.14300	9282.03500	8000.56450	5591.59900	Detected	Detected	Detected	Detected
1137	A_23_P103897	SPATA1	Hs.584956	spermatogenesis associated 1	Homo sapiens spermatogenesis associa	39.48523	44.21411	18.85486	16.81137	Detected	Detected	Detected	Detected
1138	A_23_P11160	MAP7D3		MAP7 domain containing 3	MAP7 domain-containing protein 3 [So	193.71370	329.89755	198.68195	284.80328	Detected	Detected	Detected	Detected
1139	A_24_P926760	NKTR	Hs.529509	natural killer-tumor recognition sequen	Homo sapiens natural killer-tumor reco	811.99130	610.15650	979.72534	666.01404	Detected	Detected	Detected	Detected
1140	A_23_P86283	LAPTM5	Hs.371021	lysosomal protein transmembrane 5	Homo sapiens lysosomal protein transm	66.41994	17.39035	59.10638	24.13623	Detected	Compromised	Detected	Detected
1141	A_33_P3356752	GPER	Hs.20961	G protein-coupled estrogen receptor 1	Homo sapiens G protein-coupled estrog	135.51518	204.46745	116.29504	198.31061	Detected	Detected	Detected	Detected
1142	A_33_P3210069	LOC401847		similar to hCG1793095	PREDICTED: Homo sapiens similar to h	591.52560	484.41003	393.88030	265.55008	Detected	Detected	Detected	Detected
1143	A_23_P31143	TPD52L1	Hs.591347	tumor protein D52-like 1	Homo sapiens tumor protein D52-like 1	250.78758	222.02745	236.62260	171.95338	Detected	Detected	Detected	Detected
1144	A_33_P3376026		Hs.666223		Putative uncharacterized protein FLJ46	128.00317	155.24487	81.46289	86.69218	Detected	Detected	Detected	Detected
1145	A_23_P1145	MTPAP	Hs.173946	mitochondrial poly(A) polymerase	Homo sapiens mitochondrial poly(A) pol	489.28143	570.52870	466.81244	469.79385	Detected	Detected	Detected	Detected
1146	A_23_P103476	UBIAD1	Hs.522933	UbiA prenyltransferase domain containi	Homo sapiens UbiA prenyltransferase d	1134.52890	1292.73360	934.05084	910.62744	Detected	Detected	Detected	Detected
1147	A_33_P3285893	SCRN1	Hs.520740	secernin 1	Homo sapiens secernin 1 (SCRN1), tran	62.18074	14.34843	25.57776	27.42809	Detected	Compromised	Detected	Detected
1148	A_23_P402287	LNX2	Hs.132359	ligand of numb-protein X 2	Homo sapiens ligand of numb-protein X	605.68146	560.51660	500.37952	410.71190	Detected	Detected	Detected	Detected
1149	A_33_P3354256	MTSS1	Hs.336994	metastasis suppressor 1	Homo sapiens metastasis suppressor 1	25.01372	60.63431	21.30042	33.06904	Detected	Detected	Detected	Detected
1150	A_23_P112061	HGSNAT	Hs.600384	heparan-alpha-glucosaminide N-acetylt	Homo sapiens heparan-alpha-glucosam	11915.31300	4483.33250	9373.46300	3065.41820	Detected	Detected	Detected	Detected
1151	A_33_P3389634	CAPG	Hs.516155	capping protein (actin filament), gelsolin	Homo sapiens capping protein (actin fila	9270.95400	12605.97000	11140.07200	13071.25000	Detected	Detected	Detected	Detected
1152	A_23_P352266	BCL2	Hs.150749	B-cell CLL/lymphoma 2	Homo sapiens B-cell CLL/lymphoma 2	70.87732	56.72204	45.99906	35.74418	Detected	Detected	Detected	Detected
1153	A_24_P410610	DPYD	Hs.335034	dihydropyrimidine dehydrogenase	Homo sapiens dihydropyrimidine dehydr	18.79734	13.85666	20.31011	7.97539	Detected	Compromised	Detected	Compromised
1154	A_23_P136077	PARK2	Hs.132954	Parkinson disease (autosomal recessive	Homo sapiens Parkinson disease (autos	71.08962	4.18808	42.12795	3.72791	Detected	Compromised	Detected	Compromised
1155	A_23_P162874	HSP90AA1	Hs.525600	heat shock protein 90kDa alpha (cytoso	Homo sapiens heat shock protein 90kDa	18301.20300	23803.94000	9934.07900	10942.35000	Detected	Detected	Detected	Detected
1156	A_23_P205531	RNASE4	Hs.283749	ribonuclease, RNase A family, 4	Homo sapiens ribonuclease, RNase A fa	2726.94820	1125.98460	2920.86770	1065.41130	Detected	Detected	Detected	Detected
1157	A_32_P141612	ORC4L	Hs.558364	origin recognition complex, subunit 4-lik	Homo sapiens origin recognition comple	76.64851	39.91639	67.14749	30.65461	Detected	Detected	Detected	Detected
1158	A_33_P3257027	FGF7	Hs.567268	fibroblast growth factor 7 (keratinocyte	Homo sapiens fibroblast growth factor 7	2284.72600	1349.23100	1994.99540	1000.02826	Detected	Detected	Detected	Detected
1159	A_24_P11506	KYNU	Hs.470126	kynureninase (L-kynurenine hydrolase)	Homo sapiens kynureninase (L-kynuren	3616.03830	10.54684	3028.44920	4.51670	Detected	Compromised	Detected	Compromised
1160	A_24_P541919	DENND5B	Hs.118166	DENN/MADD domain containing 5B	Homo sapiens DENN/MADD domain co	760.60920	254.02534	527.85670	146.79733	Detected	Detected	Detected	Detected
1161	A_23_P148541	CTAG1A	Hs.534310	cancer/testis antigen 1A	Homo sapiens cancer/testis antigen 1A	93.85477	24223.07800	35.34504	17983.58200	Detected	Detected	Detected	Detected
1162	A_32_P97169	GPC6	Hs.444329	glypican 6	Homo sapiens glypican 6 (GPC6), mRNA	2322.60500	1689.52120	1850.85140	1190.10970	Detected	Detected	Detected	Detected
1163	A_23_P107513	CEP76	Hs.236940	centrosomal protein 76kDa	Homo sapiens centrosomal protein 76kD	333.83356	377.48862	276.35306	259.34473	Detected	Detected	Detected	Detected
1164	A_33_P3355296				D-2-hydroxyglutarate dehydrogenase, r	336.28473	319.24612	218.69438	202.74362	Detected	Detected	Detected	Detected
1165	A_23_P55632	SERPINB3	Hs.227948	serpin peptidase inhibitor, clade B (oval	Homo sapiens serpin peptidase inhibitor	162.06169	3.75615	155.98383	3.32972	Detected	Compromised	Detected	Compromised
1166	A_23_P156683	LTA	Hs.36	lymphotoxin alpha (TNF superfamily, me	Homo sapiens lymphotoxin alpha (TNF s	29.26082	44.43180	11.79450	19.84348	Detected	Detected	Compromised	Detected
1167	A_23_P216845	GFI1B	Hs.553160	growth factor independent 1B transcrip	Homo sapiens growth factor independen	41.19049	45.82921	34.28896	38.09025	Detected	Detected	Detected	Detected
1168	A_23_P79518	IL1B	Hs.126256	interleukin 1, beta	Homo sapiens interleukin 1, beta (IL1B)	1613.51530	226.39677	1139.80290	127.00109	Detected	Detected	Detected	Detected
1169	A_33_P3319905					110.68692	17.70433	97.31604	5.92417	Detected	Compromised	Detected	Compromised
1170	A_23_P118749	DERL2	Hs.286131	Der1-like domain family, member 2	Homo sapiens Der1-like domain family,	3715.04320	5618.57280	3157.38530	4083.48300	Detected	Detected	Detected	Detected
1171	A_23_P53267	RSRC2	Hs.432996	arginine/serine-rich coiled-coil 2	Homo sapiens arginine/serine-rich coile	12012.49300	9679.45600	9184.55600	6858.98400	Detected	Detected	Detected	Detected
1172	A_23_P123596	GLDC	Hs.584238	glycine dehydrogenase (decarboxylating	Homo sapiens glycine dehydrogenase (d	165.26158	133.90130	122.23808	46.02000	Detected	Detected	Detected	Detected
1173	A_33_P3405531	TMF1	Hs.267632	TATA element modulatory factor 1	Homo sapiens TATA element modulator	1749.60880	1385.68190	1007.32306	667.53424	Detected	Detected	Detected	Detected
1174	A_33_P3406072	FRMD3	Hs.709357	FERM domain containing 3	Homo sapiens FERM domain containing	267.67484	383.73087	248.43747	367.96290	Detected	Detected	Detected	Detected
1175	A_23_P17471	FAM113A	Hs.29341	family with sequence similarity 113, mer	Homo sapiens family with sequence sim	209.01004	156.94104	189.54771	90.23242	Detected	Detected	Detected	Detected
1176	A_23_P82941	ARFGEF1	Hs.656902	ADP-ribosylation factor guanine nucleo	Homo sapiens ADP-ribosylation factor g	2906.18630	2436.70870	1714.88770	1123.58010	Detected	Detected	Detected	Detected
1177	A_33_P3340847	CARD6	Hs.200242	caspase recruitment domain family, mer	Homo sapiens caspase recruitment dom	411.14246	279.66260	370.43762	254.77638	Detected	Detected	Detected	Detected
1178	A_33_P3677061	LOC283887	Hs.652665	hypothetical protein LOC283887	Homo sapiens mRNA; cDNA DKFZp547	20.13222	11.09121	13.05424	6.16848	Detected	Compromised	Compromised	Compromised
1179	A_33_P3358347				ADP-ribosylation factor GTPase-activa	595.84880	657.24510	493.94250	547.58826	Detected	Detected	Detected	Detected
1180	A_33_P3350575	OC90	Hs.653174	otoconin 90	Homo sapiens otoconin 90 (OC90), mRN	39.92285	64.85728	19.15262	25.42303	Detected	Detected	Detected	Detected
1181	A_24_P413126	PMEPA1	Hs.517155	prostate transmembrane protein, andro	Homo sapiens prostate transmembrane	5515.95000	16015.94400	3364.78610	8744.47700	Detected	Detected	Detected	Detected
1182	A_33_P3344264	DUS3L	Hs.284297	dihydrouridine synthase 3-like (S. cerev	Homo sapiens dihydrouridine synthase 3	3164.11080	6252.64300	2771.42800	5154.11400	Detected	Detected	Detected	Detected
1183	A_24_P169343	C9orf21	Hs.44640	chromosome 9 open reading frame 21	Homo sapiens chromosome 9 open read	1090.84220	1059.86760	1177.50780	990.43380	Detected	Detected	Detected	Detected
1184	A_32_P109922	ZFAT	Hs.446172	zinc finger and AT hook domain contain	Homo sapiens zinc finger and AT hook	1672.81050	917.40650	1429.76810	740.57556	Detected	Detected	Detected	Detected
1185	A_33_P3308706		Hs.577256		Ubiquitin carboxyl-terminal hydrolase 4	33.29884	41.63071	31.43056	41.26685	Detected	Detected	Detected	Detected
1186	A_23_P111724	RUNDC3B	Hs.411488	RUN domain containing 3B	Homo sapiens RUN domain containing 3	39.68838	71.04257	26.29304	41.38032	Detected	Detected	Detected	Detected
1187	A_32_P167122	RCOR3	Hs.356399	REST corepressor 3	Homo sapiens REST corepressor 3 (RC	1114.93120	632.10440	696.51276	334.31827	Detected	Detected	Detected	Detected
1188	A_23_P133665	FRK	Hs.89426	fyn-related kinase	Homo sapiens fyn-related kinase (FRK)	15.92773	15.71352	3.49821	11.18789	Detected	Compromised	Compromised	Compromised
1189	A_23_P17393	CSE1L	Hs.90073	CSE1 chromosome segregation 1-like (	Homo sapiens CSE1 chromosome segre	9280.00400	21881.56000	8756.91900	17633.64300	Detected	Detected	Detected	Detected
1190	A_24_P109554	CEP110	Hs.653263	centrosomal protein 110kDa	Homo sapiens centrosomal protein 110k	103.42161	128.13693	56.01720	71.63020	Detected	Detected	Detected	Detected
1191	A_33_P3311863	AP2A2	Hs.19121	adaptor-related protein complex 2, alph	Homo sapiens adaptor-related protein c	243.34645	136.62134	174.20705	122.23148	Detected	Detected	Detected	Detected
1192	A_33_P3261937	RNASE13	Hs.666729	ribonuclease, RNase A family, 13 (non-a	Homo sapiens ribonuclease, RNase A fa	1131.54870	1810.15140	110.88670	85.66677	Detected	Detected	Detected	Detected
1193	A_33_P3250845	NIPA2	Hs.591003	non imprinted in Prader-Willi/Angelman	Homo sapiens non imprinted in Prader-	17696.08000	23129.02000	13520.78100	15308.56100	Detected	Detected	Detected	Detected
1194	A_23_P110699	ZNF300	Hs.134885	zinc finger protein 300	Homo sapiens zinc finger protein 300 (Z	81.74606	90.61826	56.37897	53.56196	Detected	Detected	Detected	Detected
1195	A_23_P112950	ATF7IP	Hs.714407	activating transcription factor 7 interac	Homo sapiens activating transcription fa	241.04623	386.73883	139.26628	212.72437	Detected	Detected	Detected	Detected
1196	A_23_P104942	TMX2	Hs.654965	thioredoxin-related transmembrane pro	Homo sapiens thioredoxin-related trans	2514.71920	4271.43700	1970.45240	2931.57420	Detected	Detected	Detected	Detected

1197	A_23_P162734	RNF6	Hs.136885	ring finger protein (C3H2C3 type) 6	Homo sapiens ring finger protein (C3H2	2248.30880	3217.64100	1995.19060	2207.40720	Detected	Detected	Detected	Detected
1198	A_33_P3349619					71.34329	54.91906	51.25352	38.12842	Detected	Detected	Detected	Detected
1199	A_33_P3318627					16.29344	21.78499	14.45190	18.49300	Detected	Detected	Compromised	Compromised
1200	A_33_P3245784	ZNF620	Hs.581541	zinc finger protein 620	Homo sapiens zinc finger protein 620 (Z	24.95015	19.83372	12.24821	8.99479	Detected	Compromised	Compromised	Compromised
1201	A_23_P170186	OPLAH	Hs.305882	5-oxoprolinase (ATP-hydrolysing)	Homo sapiens 5-oxoprolinase (ATP-hyd	1979.40030	579.93390	1974.36650	562.37270	Detected	Detected	Detected	Detected
1202	A_33_P3303146	ATG4C	Hs.7353	ATG4 autophagy related 4 homolog C (S	Homo sapiens ATG4 autophagy related	513.85535	237.94833	121.32635	44.28156	Detected	Detected	Detected	Detected
1203	A_24_P418044	HLA-J	Hs.720762	major histocompatibility complex, class	Homo sapiens major histocompatibility c	109189.96000	41243.02300	75351.33600	21124.85000	Detected	Detected	Detected	Detected
1204	A_23_P82523	ABCB1	Hs.489033	ATP-binding cassette, sub-family B (MI	Homo sapiens ATP-binding cassette, su	6.32705	20.28619	7.86219	14.13803	Compromised	Detected	Compromised	Detected
1205	A_23_P121806	ENOPH1	Hs.18442	enolase-phosphatase 1	Homo sapiens enolase-phosphatase 1 (	2477.38600	3859.11910	1883.21450	2450.61700	Detected	Detected	Detected	Detected
1206	A_23_P325501	MORC3	Hs.421150	MORC family CW-type zinc finger 3	Homo sapiens MORC family CW-type zi	2874.07710	2487.69170	2155.77780	1528.01210	Detected	Detected	Detected	Detected
1207	A_33_P3297611		Hs.580542		wn53g02.x1 NCI_CGAP_Lu19 Homo sapi	241.22452	300.87827	138.20270	132.00226	Detected	Detected	Detected	Detected
1208	A_23_P76961	RPS29	Hs.156367	ribosomal protein S29	Homo sapiens ribosomal protein S29 (R	193487.40000	133141.44000	130470.52000	76793.01000	Detected	Detected	Detected	Detected
1209	A_23_P98085	PTEN	Hs.500466	phosphatase and tensin homolog	Homo sapiens phosphatase and tensin h	1352.29900	1087.48990	936.97900	635.78076	Detected	Detected	Detected	Detected
1210	A_23_P117068	SNRPF	Hs.105465	small nuclear ribonucleoprotein polypep	Homo sapiens small nuclear ribonucleop	13093.72600	28375.03500	12874.63300	22540.66200	Detected	Detected	Detected	Detected
1211	A_33_P3457052	LOC221272	Hs.586272	hypothetical LOC221272	Homo sapiens cDNA: FLJ21659 fis, clon	228.27719	172.30862	106.66448	72.61296	Detected	Detected	Detected	Detected
1212	A_33_P3788772	LOC200609	Hs.635880	hypothetical protein LOC200609	Homo sapiens mRNA; cDNA DKFZp434I	706.29150	344.48900	501.61115	236.71703	Detected	Detected	Detected	Detected
1213	A_23_P156327	TGFBI	Hs.369397	transforming growth factor, beta-induce	Homo sapiens transforming growth fact	73874.69000	432881.84000	54878.18400	231352.42000	Detected	Detected	Detected	Detected
1214	A_33_P3369646					1309.30350	1420.63720	599.49700	729.81780	Detected	Detected	Detected	Detected
1215	A_33_P3215452	LOC100129361		similar to mCG115122	PREDICTED: Homo sapiens similar to m	153.70390	163.53958	127.18259	115.92141	Detected	Detected	Detected	Detected
1216	A_33_P3334590	MTMR10	Hs.30141	myotubularin related protein 10	Homo sapiens myotubularin related prot	62.34150	62.50912	52.67447	43.19898	Detected	Detected	Detected	Detected
1217	A_24_P333019	RNF24	Hs.589884	ring finger protein 24	Homo sapiens ring finger protein 24 (RN	2224.85330	1379.01950	2323.50340	1229.29680	Detected	Detected	Detected	Detected
1218	A_23_P92895	RBM22	Hs.713564	RNA binding motif protein 22	Homo sapiens RNA binding motif protei	562.68870	562.20950	494.79184	398.38553	Detected	Detected	Detected	Detected
1219	A_23_P256021	LAS1L	Hs.522675	LAS1-like (S. cerevisiae)	Homo sapiens LAS1-like (S. cerevisiae)	10599.82400	11259.13200	12083.48500	11294.63000	Detected	Detected	Detected	Detected
1220	A_23_P75299	LHPP	Hs.527748	phospholysine phosphohistidine inorgani	Homo sapiens phospholysine phosphohi	2222.17530	2329.37650	2173.22780	2016.21150	Detected	Detected	Detected	Detected
1221	A_23_P502930	FASTK	Hs.647094	Fas-activated serine/threonine kinase	Homo sapiens Fas-activated serine/thr	29514.96900	24381.89500	23591.83600	18157.22700	Detected	Detected	Detected	Detected
1222	A_23_P123172	OR2A9P	Hs.591830	olfactory receptor, family 2, subfamily A	Homo sapiens olfactory receptor, family	151.84846	197.62030	114.53770	139.56890	Detected	Detected	Detected	Detected
1223	A_23_P106389	SEMA7A	Hs.24640	semaphorin 7A, GPI membrane anchor (	Homo sapiens semaphorin 7A, GPI mem	156.63383	2496.80880	101.89106	1686.83400	Detected	Detected	Detected	Detected
1224	A_23_P109171	BFSP1	Hs.129702	beaded filament structural protein 1, file	Homo sapiens beaded filament structura	2199.23070	397.20908	1914.57340	312.66193	Detected	Detected	Detected	Detected
1225	A_23_P501007	EFEMP1	Hs.76224	EGF-containing fibulin-like extracellular	Homo sapiens EGF-containing fibulin-lik	25235.10000	5012.92900	19172.07600	3261.20040	Detected	Detected	Detected	Detected
1226	A_33_P3323722	ARL4C	Hs.111554	ADP-ribosylation factor-like 4C	Homo sapiens ADP-ribosylation factor-	2411.62400	5263.33700	2570.67480	4611.01000	Detected	Detected	Detected	Detected
1227	A_33_P3363316	FLJ35390	Hs.661253	hypothetical LOC255031	Homo sapiens hypothetical LOC255031	24.81452	26.40394	7.21911	11.67594	Detected	Detected	Compromised	Compromised
1228	A_33_P3349334	SOCS4	Hs.532610	suppressor of cytokine signaling 4	Homo sapiens suppressor of cytokine s	4678.84130	3311.07890	3273.22360	2010.28270	Detected	Detected	Detected	Detected
1229	A_23_P500364	BCL7B	Hs.647051	B-cell CLL/lymphoma 7B	Homo sapiens B-cell CLL/lymphoma 7B	1892.94700	2883.19000	849.28640	1080.99000	Detected	Detected	Detected	Detected
1230	A_24_P316305	AQR	Hs.510958	aquarius homolog (mouse)	Intron-binding protein aquarius (Intron-	685.74927	494.41846	389.98428	225.67471	Detected	Detected	Detected	Detected
1231	A_23_P381351	CSH1	Hs.654390	chorionic somatomammotropin hormone	Homo sapiens chorionic somatomammo	44.78872	60.22410	19.97027	21.73015	Detected	Detected	Detected	Detected
1232	A_33_P3365408	UNQ2963	Hs.246046	hypothetical LOC283314	Homo sapiens hypothetical LOC283314	51.41370	12.50075	25.30603	5.23378	Detected	Compromised	Detected	Compromised
1233	A_23_P360209		Hs.723065		F1262 Human cultured dermal papilla ce	8614.19400	4426.06740	4911.44700	1876.16430	Detected	Detected	Detected	Detected
1234	A_23_P251043	TMEM90B	Hs.124638	transmembrane protein 90B	Homo sapiens transmembrane protein 9	4.93395	64.12730	6.77633	60.33364	Compromised	Detected	Compromised	Detected
1235	A_23_P404685	LCE1A	Hs.534645	late cornified envelope 1A	Homo sapiens late cornified envelope 1A	24550.25400	42273.81000	9751.17900	12024.40200	Detected	Detected	Detected	Detected
1236	A_23_P100420	ZCCHC14	Hs.156231	zinc finger, CCHC domain containing 14	Homo sapiens zinc finger, CCHC domai	5807.85940	4601.27540	4370.19800	2992.23660	Detected	Detected	Detected	Detected
1237	A_24_P260443	THBS4	Hs.211426	thrombospondin 4	Homo sapiens thrombospondin 4 (THBS	118.17413	81.80875	113.10646	52.12481	Detected	Detected	Detected	Detected
1238	A_23_P17053	IL1F9	Hs.211238	interleukin 1 family, member 9	Homo sapiens interleukin 1 family, mem	9.21036	28.50419	3.31594	4.46103	Compromised	Detected	Compromised	Compromised
1239	A_23_P54477	NOP10	Hs.14317	NOP10 ribonucleoprotein homolog (yeas	Homo sapiens NOP10 ribonucleoprotein	34107.95000	47893.29000	35008.88000	39743.83200	Detected	Detected	Detected	Detected
1240	A_23_P108244	COX6B1	Hs.431668	cytochrome c oxidase subunit Vib polyp	Homo sapiens cytochrome c oxidase su	14171.42400	12925.03700	18452.97900	14562.10450	Detected	Detected	Detected	Detected
1241	A_23_P51565	TNNI1	Hs.320890	troponin I type 1 (skeletal, slow)	Homo sapiens troponin I type 1 (skeleta	42.60733	45.29801	42.67028	34.21759	Detected	Detected	Detected	Detected
1242	A_23_P11081	AKAP4	Hs.97633	A kinase (PRKA) anchor protein 4	Homo sapiens A kinase (PRKA) anchor	2.52634	18.64227	3.51346	2.85797	Compromised	Not Detected	Compromised	Compromised
1243	A_23_P204640	NANOG	Hs.661360	Nanog homeobox	Homo sapiens Nanog homeobox (NANO	129.54599	15.85863	67.55973	8.73794	Detected	Compromised	Detected	Compromised
1244	A_24_P341535	LOC388152	Hs.160329	hypothetical LOC388152	Homo sapiens hypothetical LOC388152	267.81448	431.97690	141.98308	177.06250	Detected	Detected	Detected	Detected
1245	A_23_P111961	MAK16	Hs.583805	MAK16 homolog (S. cerevisiae)	Homo sapiens MAK16 homolog (S. cere	1645.21030	2001.95600	1271.93650	1384.67920	Detected	Detected	Detected	Detected
1246	A_33_P3384322	LOC100132006	Hs.593666	hypothetical protein LOC100132006	Homo sapiens mRNA; cDNA DKFZp686I	82.13948	66.29800	60.10638	34.13068	Detected	Detected	Detected	Detected
1247	A_33_P3403576	FCGR2A	Hs.352642	Fc fragment of IgG, low affinity IIa, rece	Homo sapiens Fc fragment of IgG, low a	88.30619	4.36164	74.44190	13.94122	Detected	Compromised	Detected	Compromised
1248	A_23_P23728	SV2A	Hs.516153	synaptic vesicle glycoprotein 2A	Homo sapiens synaptic vesicle glycopro	567.12164	624.66986	288.74530	188.70152	Detected	Detected	Detected	Detected
1249	A_23_P217277	SLITRK2	Hs.320368	SLIT and NTRK-like family, member 2	Homo sapiens SLIT and NTRK-like fami	296.35090	3.94263	232.92703	3.50736	Detected	Compromised	Detected	Compromised
1250	A_23_P32029	SLC35D2	Hs.494556	solute carrier family 35, member D2	Homo sapiens solute carrier family 35, r	1114.01320	984.95593	878.00525	675.90190	Detected	Detected	Detected	Detected
1251	A_33_P3268368	FZR1	Hs.413133	fizzy/cell division cycle 20 related 1 (Dr	Homo sapiens fizzy/cell division cycle 2	1736.16490	3006.47800	1179.43660	2043.69260	Detected	Detected	Detected	Detected
1252	A_33_P3231162	ZNF776	Hs.720254	zinc finger protein 776	Homo sapiens zinc finger protein 776 (Z	178.87135	133.56560	177.02359	116.59901	Detected	Detected	Detected	Detected
1253	A_33_P3330608	PRAM1	Hs.465812	PML-RARA regulated adaptor molecule	Homo sapiens PML-RARA regulated ad	33.96907	33.20594	36.75217	31.96862	Detected	Detected	Detected	Detected
1254	A_23_P160618	SH2D2A	Hs.103527	SH2 domain protein 2A	Homo sapiens SH2 domain protein 2A (S	369.07227	478.97284	213.88580	168.80705	Detected	Detected	Detected	Detected
1255	A_23_P114155	GPKOW	Hs.503666	G patch domain and KOW motifs	Homo sapiens G patch domain and KOW	4567.77600	5500.89750	4349.76700	4684.14840	Detected	Detected	Detected	Detected
1256	A_23_P167401	PCDHB11	Hs.283084	protocadherin beta 11	Homo sapiens protocadherin beta 11 (P	197.84271	135.77548	210.51680	117.30520	Detected	Detected	Detected	Detected
1257	A_33_P3350227	MED22	Hs.78354	mediator complex subunit 22	Homo sapiens mediator complex subuni	584.31030	813.12354	228.15575	251.20561	Detected	Detected	Detected	Detected
1258	A_33_P3341568	LOC390834	Hs.647590	similar to fem-1 homolog a	PREDICTED: Homo sapiens similar to fe	8669.50400	14087.58700	416.66815	501.85560	Detected	Detected	Detected	Detected
1259	A_23_P31721	E2F5	Hs.445758	E2F transcription factor 5, p130-binding	Homo sapiens E2F transcription factor	99.44153	23.11706	67.42403	17.87401	Detected	Detected	Detected	Compromised
1260	A_24_P74329	ZNF493	Hs.656558	zinc finger protein 493	Homo sapiens zinc finger protein 493 (Z	483.14844	666.46857	306.12915	352.22440	Detected	Detected	Detected	Detected
1261	A_33_P3384932	NUDT8	Hs.433329	nudix (nucleoside diphosphate linked mo	Homo sapiens nudix (nucleoside diphosp	3954.42200	2581.80710	2022.27690	1615.97140	Detected	Detected	Detected	Detected
1262	A_23_P106822	NOB1	Hs.271695	NIN1/RPN12 binding protein 1 homolog	Homo sapiens NIN1/RPN12 binding prot	3269.60800	3230.87700	3323.37180	2835.45240	Detected	Detected	Detected	Detected
1263	A_33_P3398897	QDPR	Hs.75438	quinoid dihydropteridine reductase	Homo sapiens quinoid dihydropteridine r	1297.34520	1657.98390	855.34357	936.62726	Detected	Detected	Detected	Detected
1264	A_33_P3262022					141.69720	72.54900	3.66193	4.69882	Detected	Detected	Compromised	Compromised
1265	A_23_P156852	PECI	Hs.15250	peroxisomal D3,D2-enoyl-CoA isomeras	Homo sapiens peroxisomal D3,D2-enoyl	14820.39400	19657.77700	13296.07000	15156.52500	Detected	Detected	Detected	Detected
1266	A_33_P3334155	LOC730495	Hs.369771	hypothetical protein LOC730495	Homo sapiens cDNA FLJ45934 fis, clon	127.75951	41.26251	52.35310	13.17601	Detected	Detected	Detected	Compromised
1267	A_23_P390722	TOR1AIP2	Hs.716051	torsin A interacting protein 2	Homo sapiens torsin A interacting prote	43.38502	36.63496	30.15589	22.33517	Detected	Detected	Detected	Detected
1268	A_33_P3282836	RPS28	Hs.719103	ribosomal protein S28	Homo sapiens ribosomal protein S28 (R	248414.19000	242912.56000	228401.75000	176766.12000	Detected	Detected	Detected	Detected
1269	A_33_P3538104	LOC283854	Hs.266768	hypothetical protein LOC283854	Homo sapiens cDNA FLJ35892 fis, clon	66.21852	81.15754	25.54128	21.87931	Detected	Detected	Detected	Detected
1270	A_33_P3422240	KIAA1530	Hs.380475	KIAA1530	Homo sapiens KIAA1530 (KIAA1530), m	60.49444	17.94405	52.01551	23.59981	Detected	Compromised	Detected	Detected
1271	A_23_P13907	IGF1	Hs.160562	insulin-like growth factor 1 (somatomed	Homo sapiens insulin-like growth factor	3.25768	4.82735	25.69300	6.02610	Compromised	Compromised	Not Detected	Compromised

1272	A_23_P133536	CAPSL	Hs.55150	calcyphosine-like	Homo sapiens calcyphosine-like (CAPS	20.24168	35.92854	14.76967	23.13265	Detected	Detected	Compromised	Detected
1273	A_23_P105066	ILK	Hs.706355	integrin-linked kinase	Homo sapiens integrin-linked kinase (IL	2925.66380	5225.79100	2693.26880	4283.79400	Detected	Detected	Detected	Detected
1274	A_33_P3260747	NHSL2	Hs.397836	NHS-like 2	NHS-like protein 2 [Source:UniProtKB	41.10994	63.04778	27.37388	21.39308	Detected	Detected	Detected	Detected
1275	A_32_P506600	RAN	Hs.10842	RAN, member RAS oncogene family	Homo sapiens RAN, member RAS onco	58519.81200	144522.44000	42442.34000	82300.92000	Detected	Detected	Detected	Detected
1276	A_33_P3795649	LOC100288303	Hs.386685	similar to C21orf105 protein	PREDICTED: Homo sapiens similar to C	24.07138	24.38616	12.06645	12.40069	Detected	Detected	Compromised	Compromised
1277	A_33_P3227944	C17orf69	Hs.128813	chromosome 17 open reading frame 69	Homo sapiens chromosome 17 open rea	1349.81790	92.10795	1015.02660	66.06757	Detected	Detected	Detected	Detected
1278	A_33_P3839897	RNU4ATAC	Hs.689638	RNA, U4atac small nuclear (U12-depen	HHAGE001732 Human liver regeneratio	36.73500	175.55450	7.73602	116.03821	Detected	Detected	Compromised	Detected
1279	A_23_P117298	F7	Hs.36989	coagulation factor VII (serum prothromt	Homo sapiens coagulation factor VII (se	31.84604	20.85692	39.49864	20.37916	Detected	Detected	Detected	Detected
1280	A_23_P10497	ATP9B	Hs.465475	ATPase, class II, type 9B	Homo sapiens ATPase, class II, type 9B	722.73553	608.65030	351.83972	264.49930	Detected	Detected	Detected	Detected
1281	A_23_P362759	PRDM5	Hs.666782	PR domain containing 5	Homo sapiens PR domain containing 5 (	77.84949	63.27969	71.62949	41.98037	Detected	Detected	Detected	Detected
1282	A_23_P301896	PRDM2	Hs.371823	PR domain containing 2, with ZNF doma	Homo sapiens PR domain containing 2,	701.29865	719.74760	458.36438	432.44202	Detected	Detected	Detected	Detected
1283	A_32_P233938	FAM133B	Hs.489105	family with sequence similarity 133, mer	Homo sapiens family with sequence sim	751.93610	529.90576	694.12450	471.05640	Detected	Detected	Detected	Detected
1284	A_24_P248606	ACSL3	Hs.655772	acyl-CoA synthetase long-chain family	Homo sapiens acyl-CoA synthetase lon	566.23047	368.98130	299.86423	159.84781	Detected	Detected	Detected	Detected
1285	A_23_P70213	APC	Hs.158932	adenomatous polyposis coli	Homo sapiens adenomatous polyposis c	49.14510	48.56448	35.24165	28.34723	Detected	Detected	Detected	Detected
1286	A_23_P121825	C5orf44	Hs.591760	chromosome 5 open reading frame 44	Homo sapiens chromosome 5 open read	395.10098	316.63898	307.10587	199.98814	Detected	Detected	Detected	Detected
1287	A_23_P252403	COMMD10	Hs.483136	COMM domain containing 10	Homo sapiens COMM domain containing	2457.55200	2594.02000	1807.47550	1672.79960	Detected	Detected	Detected	Detected
1288	A_33_P3304162	COL19A1	Hs.444842	collagen, type XIX, alpha 1	Homo sapiens collagen, type XIX, alpha	19.40791	10.57739	5.98452	6.28884	Detected	Compromised	Compromised	Compromised
1289	A_33_P3544856	LOC100292680	Hs.391695	hypothetical LOC100292680	Homo sapiens cDNA FLJ33297 fis, clon	8.87600	41.85446	4.52442	29.30852	Compromised	Detected	Compromised	Detected
1290	A_23_P122532	C6orf47	Hs.247323	chromosome 6 open reading frame 47	Homo sapiens chromosome 6 open read	1396.16910	1513.48300	1268.83940	1210.67440	Detected	Detected	Detected	Detected
1291	A_23_P31477	NUDCD3	Hs.488171	NudC domain containing 3	Homo sapiens NudC domain containing	3393.02050	3141.76830	2889.03740	2414.43630	Detected	Detected	Detected	Detected
1292	A_33_P3403778	ZNF579	Hs.112529	zinc finger protein 579	Homo sapiens zinc finger protein 579 (Z	14054.19500	14810.14900	12281.37700	11564.99900	Detected	Detected	Detected	Detected
1293	A_23_P202837	CCND1	Hs.523852	cyclin D1	Homo sapiens cyclin D1 (CCND1), mRN	8636.83100	14990.51500	6060.25050	9930.45500	Detected	Detected	Detected	Detected
1294	A_33_P3299982	USF2	Hs.454534	upstream transcription factor 2, c-fos i	Homo sapiens upstream transcription fa	27718.82200	17720.41600	14469.88200	8476.00400	Detected	Detected	Detected	Detected
1295	A_33_P3399380					232.16020	240.63023	5.15137	4.28847	Detected	Detected	Compromised	Compromised
1296	A_23_P420326	FNDC5	Hs.524234	fibronectin type III domain containing 5	Homo sapiens fibronectin type III domai	3.62574	31.66074	3.32071	27.44941	Compromised	Detected	Compromised	Detected
1297	A_32_P168886	EIF4H	Hs.520943	eukaryotic translation initiation factor 4	Homo sapiens eukaryotic translation ini	34138.78000	50353.49600	27030.35500	33757.62500	Detected	Detected	Detected	Detected
1298	A_24_P262738	DDA1	Hs.466154	DET1 and DDB1 associated 1	Homo sapiens DET1 and DDB1 associat	478.37700	988.37490	533.19495	1022.36725	Detected	Detected	Detected	Detected
1299	A_33_P3255499	MCART1	Hs.645492	mitochondrial carrier triple repeat 1	Homo sapiens mitochondrial carrier trip	2120.72050	1766.96040	648.97030	478.17910	Detected	Detected	Detected	Detected
1300	A_33_P3281171	LOC100130987	Hs.664585	similar to hCG1815675	Homo sapiens similar to hCG1815675 (L	45.38583	47.55063	16.41747	19.63251	Detected	Detected	Compromised	Detected
1301	A_23_P120931	APOBEC3C	Hs.441124	apolipoprotein B mRNA editing enzyme,	Homo sapiens apolipoprotein B mRNA e	279.26300	638.70630	260.96160	604.37366	Detected	Detected	Detected	Detected
1302	A_23_P23303	EXO1	Hs.498248	exonuclease 1	Homo sapiens exonuclease 1 (EXO1), tr	58.60251	689.63330	55.82058	537.35110	Detected	Detected	Detected	Detected
1303	A_23_P121345	TBCCD1	Hs.518469	TBCC domain containing 1	Homo sapiens TBCC domain containing	219.66518	270.72327	218.71477	222.13330	Detected	Detected	Detected	Detected
1304	A_24_P43092	SEPT2	Hs.712994	septin 2	Homo sapiens septin 2 (SEPT2), transc	230.83337	509.85736	197.36574	386.27970	Detected	Detected	Detected	Detected
1305	A_33_P3292844	NEUROG3	Hs.532682	neurogenin 3	Homo sapiens neurogenin 3 (NEUROG3	1658.53970	2234.26660	778.11260	769.27856	Detected	Detected	Detected	Detected
1306	A_23_P142849	RND3	Hs.6838	Rho family GTPase 3	Homo sapiens Rho family GTPase 3 (RN	65578.12500	39188.36300	49797.89000	24620.23400	Detected	Detected	Detected	Detected
1307	A_23_P102242	MARCH7	Hs.529272	membrane-associated ring finger (C3HC	Homo sapiens membrane-associated rir	413.04852	570.14905	325.88107	371.84240	Detected	Detected	Detected	Detected
1308	A_33_P3356811	LCE1E	Hs.250236	late cornified envelope 1E	Homo sapiens late cornified envelope 1I	5849.91600	3581.37840	1063.45800	589.23630	Detected	Detected	Detected	Detected
1309	A_33_P3762733	D21S2088E	Hs.145622	D21S2088E	Homo sapiens D21S2088E mRNA seque	643.01970	380.87115	235.03299	103.53149	Detected	Detected	Detected	Detected
1310	A_33_P3209960	RASGRP2	Hs.99491	RAS guanyl releasing protein 2 (calcium	Homo sapiens RAS guanyl releasing pro	1899.03060	126.37660	2270.63480	110.87439	Detected	Detected	Detected	Detected
1311	A_33_P3364508	C7orf43	Hs.533139	chromosome 7 open reading frame 43	Homo sapiens chromosome 7 open read	635.65216	754.77070	410.23056	437.51150	Detected	Detected	Detected	Detected
1312	A_33_P3212769		Hs.650455		LOC100132686 protein [Source:UniPro	18.54182	6.95867	3.66883	3.76111	Detected	Compromised	Compromised	Compromised
1313	A_24_P12401	VEGFA	Hs.73793	vascular endothelial growth factor A	Homo sapiens vascular endothelial grow	2253.19630	1254.58670	1277.76650	647.88710	Detected	Detected	Detected	Detected
1314	A_33_P3355608	PCDHA7	Hs.199343	protocadherin alpha 7	Homo sapiens protocadherin alpha 7 (P	763.23224	1091.86220	229.73680	226.72116	Detected	Detected	Detected	Detected
1315	A_23_P48717	NPC2	Hs.433222	Niemann-Pick disease, type C2	Homo sapiens Niemann-Pick disease, ty	67055.57000	53708.36300	64834.20300	40389.27000	Detected	Detected	Detected	Detected
1316	A_33_P3349414	ZBTB43	Hs.355581	zinc finger and BTB domain containing	Homo sapiens zinc finger and BTB dom	2545.08060	3190.27560	1338.61740	1473.41500	Detected	Detected	Detected	Detected
1317	A_24_P265856	SENP7	Hs.529551	SUMO1/sentrin specific peptidase 7	Homo sapiens SUMO1/sentrin specific	1720.99050	733.43250	890.90430	307.47903	Detected	Detected	Detected	Detected
1318	A_33_P3305058				Cullin-9 (CUL-9)(p53-associated parkir	17.86639	36.34711	22.42768	29.13753	Detected	Detected	Detected	Detected
1319	A_33_P3329784	ANKLE2	Hs.654628	ankyrin repeat and LEM domain contain	Homo sapiens ankyrin repeat and LEM	541.18536	825.35630	327.34662	445.57200	Detected	Detected	Detected	Detected
1320	A_23_P320897		Hs.657779		DENN domain-containing protein 1B (P	329.95166	313.39072	201.54220	150.21832	Detected	Detected	Detected	Detected
1321	A_33_P3224505	LOC100131099	Hs.664462	hypothetical protein LOC100131099	Homo sapiens cDNA FLJ43943 fis, clon	20.03097	51.34262	11.63518	18.35186	Detected	Detected	Compromised	Compromised
1322	A_33_P3349546	DCAF17	Hs.659439	DDB1 and CUL4 associated factor 17	Homo sapiens DDB1 and CUL4 associat	1806.56520	1090.73400	1220.79800	580.99990	Detected	Detected	Detected	Detected
1323	A_33_P3399935	RPS6KA2	Hs.719131	ribosomal protein S6 kinase, 90kDa, pol	Homo sapiens ribosomal protein S6 kina	133.67040	120.18027	118.15440	92.70250	Detected	Detected	Detected	Detected
1324	A_33_P3388806				Fucose-1-phosphate guanylyltransferas	33.63691	11.98844	19.23536	3.69258	Detected	Compromised	Detected	Compromised
1325	A_23_P122375	ZFAND3	Hs.36959	zinc finger, AN1-type domain 3	Homo sapiens zinc finger, AN1-type do	841.67820	768.39374	736.24207	585.74000	Detected	Detected	Detected	Detected
1326	A_23_P102542	AFTPH	Hs.655167	aftiphilin	Homo sapiens aftiphilin (AFTPH), transc	1324.83870	908.24380	924.63446	499.84000	Detected	Detected	Detected	Detected
1327	A_23_P7313	SPP1	Hs.313	secreted phosphoprotein 1	Homo sapiens secreted phosphoprotein	5.62395	990.71533	4.06797	708.43726	Compromised	Detected	Compromised	Detected
1328	A_24_P831309	C1orf229	Hs.456511	chromosome 1 open reading frame 229	Homo sapiens chromosome 1 open read	3464.21360	5808.17040	8647.27300	8591.66300	Detected	Detected	Detected	Detected
1329	A_24_P411186	BCL11A	Hs.370549	B-cell CLL/lymphoma 11A (zinc finger	Homo sapiens B-cell CLL/lymphoma 11	116.56587	76.76495	80.73995	33.13287	Detected	Detected	Detected	Detected
1330	A_23_P345692	IL17D	Hs.655142	interleukin 17D	Homo sapiens interleukin 17D (IL17D),	1784.27080	226.63747	1443.01650	165.38304	Detected	Detected	Detected	Detected
1331	A_23_P500861	SYNE1	Hs.12967	spectrin repeat containing, nuclear enve	Homo sapiens spectrin repeat containin	1374.86990	1049.78780	1096.03190	662.50000	Detected	Detected	Detected	Detected
1332	A_23_P76435	GATC	Hs.369624	glutamyl-tRNA(Gln) amidotransferase, s	Homo sapiens glutamyl-tRNA(Gln) amid	52.15594	190.68456	48.54683	177.14010	Detected	Detected	Detected	Detected
1333	A_33_P3241646	PANK3	Hs.591729	pantothenate kinase 3	Homo sapiens pantothenate kinase 3 (P	152.77860	186.20274	163.96028	177.76521	Detected	Detected	Detected	Detected
1334	A_33_P3245489	ADAMTSL5	Hs.371674	ADAMTS-like 5	Homo sapiens ADAMTS-like 5 (ADAMT	52474.88300	70349.98400	46557.81600	42679.76600	Detected	Detected	Detected	Detected
1335	A_33_P3234031	LOC645435	Hs.568209	hypothetical protein LOC645435	Homo sapiens cDNA FLJ43993 fis, clon	103.81680	121.30714	45.63228	39.94216	Detected	Detected	Detected	Detected
1336	A_33_P3371727				Diamine acetyltransferase 1 (EC 2.3.1.5	154.69963	115.51297	156.33298	89.99480	Detected	Detected	Detected	Detected
1337	A_23_P416434	PHF15	Hs.483419	PHD finger protein 15	Homo sapiens PHD finger protein 15 (P	370.91223	457.02466	106.32700	88.43488	Detected	Detected	Detected	Detected
1338	A_23_P100711	PMP22	Hs.372031	peripheral myelin protein 22	Homo sapiens peripheral myelin protein	36953.60500	44365.45300	31256.39500	32973.70700	Detected	Detected	Detected	Detected
1339	A_23_P79108	ATP8B3	Hs.306212	ATPase, class I, type 8B, member 3	Homo sapiens ATPase, class I, type 8B	72.12391	10.26929	50.27422	12.43389	Detected	Compromised	Detected	Compromised
1340	A_23_P66766	RPAIN	Hs.462086	RPA interacting protein	Homo sapiens RPA interacting protein (	5565.69700	2109.98140	5454.36200	1860.09700	Detected	Detected	Detected	Detected
1341	A_24_P315862				Putative uncharacterized protein ENSP	126.43747	142.31577	98.75804	94.25829	Detected	Detected	Detected	Detected
1342	A_23_P35796	PPP2R5B	Hs.75199	protein phosphatase 2, regulatory subur	Homo sapiens protein phosphatase 2, re	1481.05520	1488.84420	1719.47130	1684.11990	Detected	Detected	Detected	Detected
1343	A_23_P381102	CCDC74B	Hs.29383	coiled-coil domain containing 74B	Homo sapiens coiled-coil domain conta	3369.48930	1675.57400	2140.37550	928.76940	Detected	Detected	Detected	Detected
1344	A_33_P3574055	LOC401504	Hs.446271	hypothetical gene supported by AK0917	Homo sapiens cDNA FLJ13634 fis, clon	1641.24340	1040.92420	733.38885	363.00250	Detected	Detected	Detected	Detected
1345	A_23_P120588	CST11	Hs.128100	cystatin 11	Homo sapiens cystatin 11 (CST11), trar	17.50763	5.14770	3.36184	3.45022	Not Detected	Compromised	Compromised	Compromised
1346	A_33_P3357626	LOC390998		similar to hCG1644589	PREDICTED: Homo sapiens similar to h	16017.90300	14177.74900	2449.56250	1655.01970	Detected	Detected	Detected	Detected

1347	A_23_P435657	ALMS1P	Hs.278983	Alstrom syndrome 1 pseudogene	Homo sapiens Alstrom syndrome 1 pseu	99.82863	182.63486	56.84922	77.53744	Detected	Detected	Detected	Detected
1348	A_23_P95923	ZNF562	Hs.371107	zinc finger protein 562	Homo sapiens zinc finger protein 562 (Z	370.13776	420.19640	182.21515	178.19995	Detected	Detected	Detected	Detected
1349	A_23_P98844	GEFT	Hs.61581	RhoA/RAC/CDC42 exchange factor	Homo sapiens RhoA/RAC/CDC42 exch	327.26917	69.79605	640.58704	150.07327	Detected	Detected	Detected	Detected
1350	A_23_P111240	PHACTR2	Hs.102471	phosphatase and actin regulator 2	Homo sapiens phosphatase and actin re	765.06433	1829.64010	506.04733	1048.49230	Detected	Detected	Detected	Detected
1351	A_32_P204019	RPL29	Hs.425125	ribosomal protein L29	Homo sapiens ribosomal protein L29 (R	49998.96500	38493.93800	55379.29700	37761.36300	Detected	Detected	Detected	Detected
1352	A_23_P208210	ZNF432	Hs.655934	zinc finger protein 432	Homo sapiens zinc finger protein 432 (Z	265.32236	141.27548	185.45544	83.63014	Detected	Detected	Detected	Detected
1353	A_23_P126393	SETDB1	Hs.643565	SET domain, bifurcated 1	Homo sapiens SET domain, bifurcated 1	4084.57900	3291.13840	3064.40300	2186.63350	Detected	Detected	Detected	Detected
1354	A_33_P3412233	POMZP3	Hs.488877	POM (POM121 homolog, rat) and ZP3 f	Homo sapiens POM (POM121 homolog,	149.12431	149.00986	94.80813	78.56072	Detected	Detected	Detected	Detected
1355	A_32_P153812	TMEM132B	Hs.524838	transmembrane protein 132B	Homo sapiens transmembrane protein 1	2151.39010	41.31860	1649.45290	25.46683	Detected	Detected	Detected	Detected
1356	A_33_P3370940				Laminin subunit beta-1 Precursor (Lam	16.94696	30.07958	7.85147	26.07350	Detected	Detected	Compromised	Detected
1357	A_33_P3409506	C9orf85	Hs.534190	chromosome 9 open reading frame 85	Homo sapiens chromosome 9 open read	173.58333	293.69473	131.91826	217.21364	Detected	Detected	Detected	Detected
1358	A_33_P3209591	AQP3	Hs.234642	aquaporin 3 (Gill blood group)	Homo sapiens aquaporin 3 (Gill blood gr	24.58905	12.98493	19.79410	11.77383	Detected	Compromised	Detected	Compromised
1359	A_24_P74932	PLP2	Hs.77422	proteolipid protein 2 (colonic epithelium	Homo sapiens proteolipid protein 2 (col	4629.26700	9578.67800	4482.98600	7878.46000	Detected	Detected	Detected	Detected
1360	A_33_P3423551	IER3	Hs.591785	immediate early response 3	Homo sapiens immediate early response	23844.66600	7017.26600	27244.95900	7280.40700	Detected	Detected	Detected	Detected
1361	A_24_P108291	IMPACT	Hs.515317	Impact homolog (mouse)	Homo sapiens Impact homolog (mouse)	840.16064	465.10696	525.59143	216.08942	Detected	Detected	Detected	Detected
1362	A_33_P3375368	GPR35	Hs.239891	G protein-coupled receptor 35	Homo sapiens G protein-coupled recept	155.04700	159.02985	56.77490	67.20444	Detected	Detected	Detected	Detected
1363	A_23_P380857	APOL4	Hs.115099	apolipoprotein L, 4	Homo sapiens apolipoprotein L, 4 (APO	203.77370	149.26875	59.21015	28.06168	Detected	Detected	Detected	Detected
1364	A_33_P3211604	RAP1B	Hs.369920	RAP1B, member of RAS oncogene fami	Homo sapiens RAP1B, member of RAS	23063.08000	31383.82600	14644.62100	15693.05500	Detected	Detected	Detected	Detected
1365	A_24_P133253	KITLG	Hs.1048	KIT ligand	Homo sapiens KIT ligand (KITLG), trans	2280.19500	6386.40400	1364.39650	2841.64620	Detected	Detected	Detected	Detected
1366	A_33_P3405168	MMAA	Hs.452864	methylmalonic aciduria (cobalamin defic	Homo sapiens methylmalonic aciduria (c	256.49360	134.21950	178.96904	64.64983	Detected	Detected	Detected	Detected
1367	A_32_P88120	YPEL1	Hs.517436	yippee-like 1 (Drosophila)	Homo sapiens yippee-like 1 (Drosophila	30.11133	19.30241	35.47582	23.11827	Detected	Compromised	Detected	Detected
1368	A_33_P3598363	LOC400662	Hs.531297	hypothetical gene supported by AK0554	Homo sapiens cDNA FLJ30849 fis, clon	111.03230	162.75550	55.21700	52.89266	Detected	Detected	Detected	Detected
1369	A_23_P80377		Hs.162877		Protein kinase C and casein kinase sub	825.75195	1414.22460	823.20135	1265.77660	Detected	Detected	Detected	Detected
1370	A_23_P62764	CCDC28B	Hs.718470	coiled-coil domain containing 28B	Homo sapiens coiled-coil domain contai	580.21155	495.23816	649.81506	467.04086	Detected	Detected	Detected	Detected
1371	A_24_P398432	ARAP1	Hs.503165	ArfGAP with RhoGAP domain, ankyrin r	Homo sapiens ArfGAP with RhoGAP do	2897.54080	1644.57420	2424.01540	1276.79710	Detected	Detected	Detected	Detected
1372	A_33_P3284552		Hs.10326		Putative uncharacterized protein ENSP	34.22800	46.78733	23.34998	22.68800	Detected	Detected	Detected	Detected
1373	A_23_P110243	METTL14	Hs.657806	methyltransferase like 14	Homo sapiens methyltransferase like 14	894.08124	581.96200	629.05940	342.81436	Detected	Detected	Detected	Detected
1374	A_23_P102000	CXCR4	Hs.593413	chemokine (C-X-C motif) receptor 4	Homo sapiens chemokine (C-X-C motif	1326.76600	20.77413	994.89056	4.50236	Detected	Not Detected	Detected	Compromised
1375	A_33_P3233105	MLLT4	Hs.614974	myeloid/lymphoid or mixed-lineage leuk	Homo sapiens myeloid/lymphoid or mixe	23.20151	33.69999	20.15449	16.02622	Detected	Detected	Detected	Compromised
1376	A_33_P3349496	LOC100130413		hypothetical protein LOC100130413	PREDICTED: Homo sapiens hypothetica	309.46512	237.03355	229.35382	144.46582	Detected	Detected	Detected	Detected
1377	A_33_P3213169					9.98022	9.84647	18.63347	5.30973	Compromised	Compromised	Detected	Compromised
1378	A_23_P125772	SRPK3	Hs.104865	SFRS protein kinase 3	Homo sapiens SFRS protein kinase 3 (S	107.63232	155.42406	66.21594	103.65099	Detected	Detected	Detected	Detected
1379	A_23_P115703	PCGF6	Hs.335808	polycomb group ring finger 6	Homo sapiens polycomb group ring finge	979.81400	1555.94640	602.85380	759.05865	Detected	Detected	Detected	Detected
1380	A_33_P3211054	ZNF684	Hs.524767	zinc finger protein 684	Homo sapiens zinc finger protein 684 (Z	191.38623	335.92392	210.38206	226.82031	Detected	Detected	Detected	Detected
1381	A_33_P3371493	TOP1	Hs.472737	topoisomerase (DNA) I	Homo sapiens topoisomerase (DNA) I (T	55.12286	76.47514	67.71658	67.03650	Detected	Detected	Detected	Detected
1382	A_24_P389994	NADK	Hs.654792	NAD kinase	Homo sapiens NAD kinase (NADK), mRI	318.39236	510.51297	353.86594	536.19760	Detected	Detected	Detected	Detected
1383	A_23_P113184	FTO	Hs.528833	fat mass and obesity associated	Homo sapiens fat mass and obesity ass	970.41925	1656.47970	1123.68120	1635.79640	Detected	Detected	Detected	Detected
1384	A_32_P224525	COL6A6	Hs.591282	collagen, type VI, alpha 6	Homo sapiens collagen type VI alpha 6	2.68150	40.72498	3.22207	29.38497	Compromised	Detected	Compromised	Detected
1385	A_24_P100351	GNL3L	Hs.654677	guanine nucleotide binding protein-like	Homo sapiens guanine nucleotide bindin	2031.89510	1946.93320	1777.03760	1636.37280	Detected	Detected	Detected	Detected
1386	A_23_P12874	GTPBP4	Hs.215766	GTP binding protein 4	Homo sapiens GTP binding protein 4 (G	12689.88700	18496.39300	8487.18400	11050.84100	Detected	Detected	Detected	Detected
1387	A_33_P3241582	RPS15A	Hs.370504	ribosomal protein S15a	Homo sapiens ribosomal protein S15a (	98742.85000	81270.05500	100366.99000	77143.05000	Detected	Detected	Detected	Detected
1388	A_23_P114061	TRPM3	Hs.47288	transient receptor potential cation char	Homo sapiens transient receptor poten	49.71554	23.39952	35.30045	15.48263	Detected	Detected	Detected	Detected
1389	A_23_P100127	CASC5	Hs.181855	cancer susceptibility candidate 5	Homo sapiens cancer susceptibility can	13.65018	603.53723	7.98926	346.84253	Detected	Detected	Compromised	Detected
1390	A_33_P3229869	LOC100128714	Hs.587854	hypothetical protein LOC100128714	cDNA FLJ42943 fis, clone BRSTN2003	66.62164	74.25617	53.69245	47.77965	Detected	Detected	Detected	Detected
1391	A_23_P108437	FZD5	Hs.17631	frizzled homolog 5 (Drosophila)	Homo sapiens frizzled homolog 5 (Dros	80.81711	159.70993	70.44272	111.47355	Detected	Detected	Detected	Detected
1392	A_33_P3336632	LOC100008589	Hs.552217	28S ribosomal RNA	Homo sapiens 28S ribosomal RNA (LOC	190020.95000	238526.58000	89073.63000	76744.29000	Detected	Detected	Detected	Detected
1393	A_33_P3300152		Hs.675410		Homo sapiens cDNA FLJ20034 fis, clon	34.37806	42.97366	23.52529	24.21975	Detected	Detected	Detected	Detected
1394	A_23_P217958	HK1	Hs.370365	hexokinase 1	Homo sapiens hexokinase 1 (HK1), nucl	11271.24000	15459.91200	8325.77000	10490.95400	Detected	Detected	Detected	Detected
1395	A_33_P3230798				Muscle, skeletal receptor tyrosine prote	157.04108	336.03894	68.46333	86.59948	Detected	Detected	Detected	Detected
1396	A_24_P320665	FAM126A	Hs.85603	family with sequence similarity 126, mer	Homo sapiens family with sequence sim	144.50880	246.58496	97.41837	123.67940	Detected	Detected	Detected	Detected
1397	A_33_P3259615	TTC36	Hs.653449	tetratricopeptide repeat domain 36	Homo sapiens tetratricopeptide repeat	332.40332	477.62088	44.74777	60.27825	Detected	Detected	Detected	Detected
1398	A_24_P57170	C19orf55	Hs.527982	chromosome 19 open reading frame 55	Homo sapiens chromosome 19 open rea	59.65129	55.55150	61.68535	55.05029	Detected	Detected	Detected	Detected
1399	A_33_P3251369	GAB3	Hs.496982	GRB2-associated binding protein 3	Homo sapiens GRB2-associated binding	34.36458	15.22294	24.17799	16.24427	Detected	Compromised	Detected	Compromised
1400	A_23_P109877	WDR82	Hs.194110	WD repeat domain 82	Homo sapiens WD repeat domain 82 (W	1701.03120	2080.65400	1023.92960	922.04770	Detected	Detected	Detected	Detected
1401	A_33_P3371999	TPPP	Hs.481466	tubulin polymerization promoting protei	Homo sapiens tubulin polymerization pr	50.96421	82.69587	25.10345	45.10500	Detected	Detected	Detected	Detected
1402	A_33_P3256848	ADAM12	Hs.594537	ADAM metallopeptidase domain 12	Homo sapiens ADAM metallopeptidase	194.18146	263.96640	170.42188	260.28848	Detected	Detected	Detected	Detected
1403	A_24_P88031	C8orf33	Hs.169615	chromosome 8 open reading frame 33	Homo sapiens chromosome 8 open read	1976.40870	1734.14270	1639.44450	1324.03250	Detected	Detected	Detected	Detected
1404	A_23_P79441	C2orf42	Hs.720049	chromosome 2 open reading frame 42	Homo sapiens chromosome 2 open read	383.63702	294.04926	240.07771	164.83968	Detected	Detected	Detected	Detected
1405	A_33_P3407945	TMEM19	Hs.688627	transmembrane protein 19	Homo sapiens transmembrane protein 1	1822.60610	2195.63700	1360.42660	1405.53200	Detected	Detected	Detected	Detected
1406	A_23_P58443	ANKHD1-EIF4EBP3	Hs.594084	ANKHD1-EIF4EBP3 readthrough transc	Homo sapiens ANKHD1-EIF4EBP3 read	1667.69200	469.45703	1574.97050	377.53854	Detected	Detected	Detected	Detected
1407	A_24_P6911	PMS2L11	Hs.675888	postmeiotic segregation increased 2-lik	Homo sapiens postmeiotic segregation i	440.54773	632.18964	496.39807	636.63030	Detected	Detected	Detected	Detected
1408	A_33_P3705275	DPY19L3	Hs.194392	dpy-19-like 3 (C. elegans)	Homo sapiens dpy-19-like 3 (C. elegans	35.89215	39.94192	33.40836	24.47237	Detected	Detected	Detected	Detected
1409	A_23_P6802	RRP9	Hs.153768	ribosomal RNA processing 9, small subu	Homo sapiens ribosomal RNA processin	1235.94400	1973.07870	1583.85950	2185.41550	Detected	Detected	Detected	Detected
1410	A_23_P203305	IMMP1L	Hs.502223	IMP1 inner mitochondrial membrane pep	Homo sapiens IMP1 inner mitochondrial	137.06902	230.09960	125.57180	167.09932	Detected	Detected	Detected	Detected
1411	A_24_P84880	LOC148709	Hs.162880	actin pseudogene	Homo sapiens actin pseudogene (LOC1	5217.83840	6675.51860	2851.92480	3043.07230	Detected	Detected	Detected	Detected
1412	A_32_P167791	ST13	Hs.712713	suppression of tumorigenicity 13 (colon	Homo sapiens suppression of tumorigen	11059.44200	7864.59300	4741.60800	3018.88720	Detected	Detected	Detected	Detected
1413	A_23_P102508	SLC5A6	Hs.435735	solute carrier family 5 (sodium-depende	Homo sapiens solute carrier family 5 (s	1082.19970	709.30650	716.89100	420.26614	Detected	Detected	Detected	Detected
1414	A_33_P3254695	RNU105A		RNA, U105A small nucleolar	Homo sapiens RNA, U105A small nucleo	394.56046	421.93604	387.02576	272.38280	Detected	Detected	Detected	Detected
1415	A_23_P111381	ATG5	Hs.486063	ATG5 autophagy related 5 homolog (S.	Homo sapiens ATG5 autophagy related	3381.44900	4442.00050	2078.45460	2268.21220	Detected	Detected	Detected	Detected
1416	A_33_P3378545		Hs.589247		DB092099 TESTI4 Homo sapiens cDNA	116.72753	118.04409	50.40474	41.43196	Detected	Detected	Detected	Detected
1417	A_23_P18413	C3orf1	Hs.477287	chromosome 3 open reading frame 1	Homo sapiens chromosome 3 open read	14160.86100	11884.25800	14295.84700	10549.56400	Detected	Detected	Detected	Detected
1418	A_32_P142818	DLX1	Hs.407015	distal-less homeobox 1	Homo sapiens distal-less homeobox 1 (	120.70478	243.43849	110.87640	182.45392	Detected	Detected	Detected	Detected
1419	A_33_P3294986	LIPE	Hs.656980	lipase, hormone-sensitive	Homo sapiens lipase, hormone-sensitive	6094.75630	3470.40900	4906.05370	2717.12740	Detected	Detected	Detected	Detected
1420	A_32_P26330	USP10	Hs.136778	ubiquitin specific peptidase 10	Homo sapiens ubiquitin specific peptida	31.46402	61.47191	25.84444	42.81350	Detected	Detected	Detected	Detected
1421	A_33_P3364582	TNXB	Hs.485104	tenascin XB	Homo sapiens tenascin XB (TNXB), trar	3423.97400	4046.63960	1731.73250	1584.03540	Detected	Detected	Detected	Detected

1422	A_33_P3247988	PCSK4	Hs.46884	proprotein convertase subtilisin/kexin t	Homo sapiens proprotein convertase su	1169.35850	238.50572	1092.87670	125.03481	Detected	Detected	Detected	Detected
1423	A_33_P3254996	DSCR9	Hs.720778	Down syndrome critical region gene 9 (r	Homo sapiens Down syndrome critical r	180.91150	229.31175	71.90726	72.82404	Detected	Detected	Detected	Detected
1424	A_32_P62211		Hs.408455		Homo sapiens mRNA; cDNA DKFZp686	47.43689	27.99337	53.72361	19.56152	Detected	Detected	Detected	Detected
1425	A_23_P119943	IGFBP2	Hs.438102	insulin-like growth factor binding protei	Homo sapiens insulin-like growth factor	98704.35000	1367.89500	85870.87000	1037.92020	Detected	Detected	Detected	Detected
1426	A_23_P149813	RSU1	Hs.524161	Ras suppressor protein 1	Homo sapiens Ras suppressor protein 1	256.64980	249.31813	135.48799	93.19151	Detected	Detected	Detected	Detected
1427	A_23_P101871	FBXO17	Hs.531770	F-box protein 17	Homo sapiens F-box protein 17 (FBXO	1277.89390	781.65580	887.95460	422.08250	Detected	Detected	Detected	Detected
1428	A_33_P3397865	TNNT1	Hs.631558	troponin T type 1 (skeletal, slow)	Homo sapiens troponin T type 1 (skelet	4095.03610	318.22552	4024.74560	305.09824	Detected	Detected	Detected	Detected
1429	A_23_P27709	CGB2	Hs.567650	chorionic gonadotropin, beta polypeptid	Homo sapiens chorionic gonadotropin, b	122.72106	95.47541	36.08270	28.25173	Detected	Detected	Detected	Detected
1430	A_24_P158065	FAM133B	Hs.489105	family with sequence similarity 133, mer	Homo sapiens family with sequence sim	4594.48830	3654.72830	2863.19630	1813.41500	Detected	Detected	Detected	Detected
1431	A_33_P3214943	SPOCK2	Hs.523009	sparc/osteonectin, cwcv and kazal-like	Homo sapiens sparc/osteonectin, cwcv	774.65760	977.61820	333.75565	286.71054	Detected	Detected	Detected	Detected
1432	A_23_P10121	SFRP1	Hs.213424	secreted frizzled-related protein 1	Homo sapiens secreted frizzled-related	2333.26930	2285.01600	1946.71180	1642.90530	Detected	Detected	Detected	Detected
1433	A_33_P3331952	OR2M3	Hs.553581	olfactory receptor, family 2, subfamily M	Homo sapiens olfactory receptor, family	12.92117	25.72506	7.69173	8.74693	Compromised	Detected	Compromised	Compromised
1434	A_33_P3410981	PCDHGB7	Hs.368160	protocadherin gamma subfamily B, 7	Homo sapiens protocadherin gamma sul	48.51154	56.71970	53.70390	40.11918	Detected	Detected	Detected	Detected
1435	A_33_P3308872	POM121L1P	Hs.318898	POM121 membrane glycoprotein-like 1	Homo sapiens POM121 membrane glyco	284.25183	65.39221	257.48587	47.06519	Detected	Detected	Detected	Detected
1436	A_23_P500873	RAD51L3	Hs.631757	RAD51-like 3 (S. cerevisiae)	Homo sapiens RAD51-like 3 (S. cerevis	201.67795	219.39998	267.48434	254.78581	Detected	Detected	Detected	Detected
1437	A_23_P119763	ABCG5	Hs.132992	ATP-binding cassette, sub-family G (WI	Homo sapiens ATP-binding cassette, su	29.16080	25.74877	15.77105	15.20898	Detected	Detected	Detected	Detected
1438	A_23_P169003	SH2D4A	Hs.303208	SH2 domain containing 4A	Homo sapiens SH2 domain containing 4	143.82076	373.77426	142.13403	291.44107	Detected	Detected	Detected	Detected
1439	A_23_P142634	METTL5	Hs.470553	methyltransferase like 5	Homo sapiens methyltransferase like 5	12526.64100	7409.58940	9786.95400	4857.16650	Detected	Detected	Detected	Detected
1440	A_33_P3364989	C20orf134	Hs.592151	chromosome 20 open reading frame 134	Homo sapiens chromosome 20 open rea	184.42924	540.29890	169.56700	474.28494	Detected	Detected	Detected	Detected
1441	A_33_P3316068					34.50556	73.38557	42.27893	86.91682	Detected	Detected	Detected	Detected
1442	A_24_P415601	RNH1	Hs.530687	ribonuclease/angiogenin inhibitor 1	Homo sapiens ribonuclease/angiogenin	19107.75200	21706.70700	23768.13900	28762.91000	Detected	Detected	Detected	Detected
1443	A_23_P250564	PRKCE	Hs.580351	protein kinase C, epsilon	Homo sapiens protein kinase C, epsilon	520.60250	556.97160	409.88116	339.80396	Detected	Detected	Detected	Detected
1444	A_33_P3386508					186.89473	180.46962	153.39260	111.73231	Detected	Detected	Detected	Detected
1445	A_32_P157208	LOC572558	Hs.571626	hypothetical locus LOC572558	Homo sapiens hypothetical locus LOC5	185.77200	121.89828	166.45547	89.38618	Detected	Detected	Detected	Detected
1446	A_23_P357860	SEC62	Hs.592561	SEC62 homolog (S. cerevisiae)	Homo sapiens SEC62 homolog (S. cerev	83.24557	46.76672	64.36334	34.09317	Detected	Detected	Detected	Detected
1447	A_23_P327426	TIPRL	Hs.209431	TIP41, TOR signaling pathway regulator	Homo sapiens TIP41, TOR signaling pat	5750.25930	7246.96000	5140.28600	4954.95200	Detected	Detected	Detected	Detected
1448	A_33_P3254666					554.82190	742.17224	259.50748	277.45220	Detected	Detected	Detected	Detected
1449	A_33_P3324298	hCG_1817208	Hs.58456	hCG1817208	PREDICTED: Homo sapiens hCG181720	319.80267	570.82404	40.00533	85.99321	Detected	Detected	Detected	Detected
1450	A_23_P501634	BTN2A1	Hs.159028	butyrophilin, subfamily 2, member A1	Homo sapiens butyrophilin, subfamily 2,	1172.42200	1905.87070	329.89060	382.34787	Detected	Detected	Detected	Detected
1451	A_24_P315444	LOC644422	Hs.647418	similar to arginine/serine-rich splicing f	PREDICTED: Homo sapiens similar to a	19.13226	145.01631	12.38194	70.36520	Detected	Detected	Compromised	Detected
1452	A_23_P127781	SCGB1D1	Hs.202686	secretoglobin, family 1D, member 1	Homo sapiens secretoglobin, family 1D,	23.60077	3.85707	12.36649	3.42654	Detected	Compromised	Compromised	Compromised
1453	A_23_P209962	SMC6	Hs.526728	structural maintenance of chromosomes	Homo sapiens structural maintenance o	2053.50660	2288.18400	1441.62550	1265.86700	Detected	Detected	Detected	Detected
1454	A_23_P259632	TMEM120A	Hs.488835	transmembrane protein 120A	Homo sapiens transmembrane protein 1	1751.52980	1968.61790	2073.16360	2012.36880	Detected	Detected	Detected	Detected
1455	A_32_P183367	BRUNOL4	Hs.435976	bruno-like 4, RNA binding protein (Dros	Homo sapiens bruno-like 4, RNA binding	15.16039	14.86654	7.34466	6.52383	Detected	Compromised	Compromised	Compromised
1456	A_23_P10815	PUM1	Hs.281707	pumilio homolog 1 (Drosophila)	Homo sapiens pumilio homolog 1 (Droso	7505.51560	6965.80860	4116.42800	3275.39670	Detected	Detected	Detected	Detected
1457	A_24_P299318	FAM101B	Hs.719324	family with sequence similarity 101, mer	Homo sapiens family with sequence sim	14576.42600	29270.37100	10349.98600	17372.44000	Detected	Detected	Detected	Detected
1458	A_23_P123672	TDRD7	Hs.193842	tudor domain containing 7	Homo sapiens tudor domain containing	1311.08060	1408.20300	946.58050	847.23914	Detected	Detected	Detected	Detected
1459	A_23_P435521	TMEM106A	Hs.536474	transmembrane protein 106A	Homo sapiens transmembrane protein 1	13.62472	39.65275	10.31649	29.32615	Compromised	Detected	Compromised	Detected
1460	A_33_P3407195	BOD1L	Hs.444517	biorientation of chromosomes in cell div	Homo sapiens biorientation of chromoso	2902.34280	1920.83570	2009.96120	1088.11620	Detected	Detected	Detected	Detected
1461	A_24_P400997	SMCHD1	Hs.8118	structural maintenance of chromosomes	Homo sapiens structural maintenance o	158.06845	106.83659	97.22261	35.72718	Detected	Detected	Detected	Detected
1462	A_33_P3354176				Myoferlin (Fer-1-like protein 3) [Source	92.09124	116.95058	103.49047	104.89010	Detected	Detected	Detected	Detected
1463	A_23_P102890	MRPS6	Hs.302742	mitochondrial ribosomal protein S6	Homo sapiens mitochondrial ribosomal p	31434.26000	27257.13700	23957.03100	17149.48800	Detected	Detected	Detected	Detected
1464	A_24_P175187	SAMD9	Hs.65641	sterile alpha motif domain containing 9	Homo sapiens sterile alpha motif domai	121.21391	143.88033	87.98939	78.98187	Detected	Detected	Detected	Detected
1465	A_23_P400984	PIKFYVE	Hs.720192	phosphoinositide kinase, FYVE finger co	Homo sapiens phosphoinositide kinase,	56.06999	47.55398	37.68149	18.23771	Detected	Detected	Detected	Compromised
1466	A_33_P3419790		Hs.126409		Uncharacterized protein C6orf105 [Sou	10.74763	54.72655	8.15376	52.31204	Compromised	Detected	Compromised	Detected
1467	A_33_P3309924	HDAC2	Hs.3352	histone deacetylase 2	Homo sapiens histone deacetylase 2 (H	103.30002	44.81296	89.82162	32.10235	Detected	Detected	Detected	Detected
1468	A_33_P3328292	PLAGL2	Hs.154104	pleiomorphic adenoma gene-like 2	Homo sapiens pleiomorphic adenoma ge	112.56729	162.65842	60.82809	59.48638	Detected	Detected	Detected	Detected
1469	A_23_P123308	TEX15	Hs.458316	testis expressed 15	Homo sapiens testis expressed 15 (TEX	2.57303	3.67624	18.22511	3.50653	Compromised	Compromised	Not Detected	Compromised
1470	A_33_P3223467	VCPIP1	Hs.632066	valosin containing protein (p97)/p47 co	Homo sapiens valosin containing protein	2544.17240	1774.30270	1583.92640	889.29620	Detected	Detected	Detected	Detected
1471	A_23_P40059	PMS1	Hs.111749	PMS1 postmeiotic segregation increase	Homo sapiens PMS1 postmeiotic segreg	1165.18870	401.17540	851.95557	238.35237	Detected	Detected	Detected	Detected
1472	A_33_P3296852	LOC286434	Hs.657302	hypothetical protein LOC286434	Homo sapiens cDNA FLJ20463 fis, clon	323612.40000	336114.00000	241442.31000	203740.53000	Detected	Detected	Detected	Detected
1473	A_33_P3357748	PVT1	Hs.133107	Pvt1 oncogene (non-protein coding)	Homo sapiens Pvt1 oncogene (non-prot	628.20830	588.11330	414.41187	268.08743	Detected	Detected	Detected	Detected
1474	A_32_P228268	DNAH10	Hs.622654	dynein, axonemal, heavy chain 10	Homo sapiens dynein, axonemal, heavy	26.91915	41.06598	37.82899	39.73002	Detected	Detected	Detected	Detected
1475	A_23_P209195	C19orf12	Hs.529094	chromosome 19 open reading frame 12	Homo sapiens chromosome 19 open rea	670.02010	682.22980	577.05400	471.37817	Detected	Detected	Detected	Detected
1476	A_23_P216501	TPM2	Hs.300772	tropomyosin 2 (beta)	Homo sapiens tropomyosin 2 (beta) (TP	91498.38000	204131.75000	97944.29000	176771.50000	Detected	Detected	Detected	Detected
1477	A_33_P3267305	ANKRD52	Hs.524506	ankyrin repeat domain 52	Homo sapiens ankyrin repeat domain 52	14725.62000	16381.56300	9919.46600	10406.61900	Detected	Detected	Detected	Detected
1478	A_24_P111996	HFE	Hs.233325	hemochromatosis	Homo sapiens hemochromatosis (HFE),	14.01389	93.43751	12.81305	69.76872	Compromised	Detected	Compromised	Detected
1479	A_23_P253301	PFN2	Hs.91747	profilin 2	Homo sapiens profilin 2 (PFN2), transcr	2241.87670	1711.02990	2216.45210	1305.25830	Detected	Detected	Detected	Detected
1480	A_33_P3260974		Hs.584210		DA734158 NT2RP2 Homo sapiens cDN/	17.56684	23.92119	16.33876	9.34583	Detected	Detected	Compromised	Compromised
1481	A_23_P116168	KIAA1826	Hs.266782	KIAA1826	Homo sapiens KIAA1826 (KIAA1826), m	934.48254	849.48346	648.57874	499.00870	Detected	Detected	Detected	Detected
1482	A_23_P404005	FAM58A	Hs.496943	family with sequence similarity 58, mem	Homo sapiens family with sequence sim	9164.68200	14141.03900	7311.95950	9305.36700	Detected	Detected	Detected	Detected
1483	A_23_P143274	NRSN2	Hs.416024	neurensin 2	Homo sapiens neurensin 2 (NRSN2), mF	359.99515	256.57416	407.06976	259.40320	Detected	Detected	Detected	Detected
1484	A_23_P124024	MED10	Hs.13885	mediator complex subunit 10	Homo sapiens mediator complex subuni	1929.77260	3893.56790	1289.26030	2327.91550	Detected	Detected	Detected	Detected
1485	A_23_P48997	PSTPIP1	Hs.129758	proline-serine-threonine phosphatase ir	Homo sapiens proline-serine-threonine	61.06587	80.89352	31.05070	44.59847	Detected	Detected	Detected	Detected
1486	A_33_P3279940	TM2D1	Hs.656790	TM2 domain containing 1	Homo sapiens TM2 domain containing 1	4367.93650	3406.99560	4111.33200	2865.58200	Detected	Detected	Detected	Detected
1487	A_23_P106874	PMFBP1	Hs.714939	polyamine modulated factor 1 binding pr	Homo sapiens polyamine modulated fac	35.17079	33.76305	21.88135	17.20816	Detected	Detected	Detected	Detected
1488	A_33_P3311498	LOC283392	Hs.363603	hypothetical LOC283392	Homo sapiens hypothetical LOC283392	5.94969	255.63634	4.39060	157.43460	Compromised	Detected	Compromised	Detected
1489	A_23_P367628	KIAA0528	Hs.271014	KIAA0528	Homo sapiens KIAA0528 (KIAA0528), m	1700.39940	985.47370	1159.81420	518.01685	Detected	Detected	Detected	Detected
1490	A_33_P3299892	UQCRH	Hs.481571	ubiquinol-cytochrome c reductase hinge	Homo sapiens ubiquinol-cytochrome c r	92919.30500	88909.04000	66696.16400	52603.80000	Detected	Detected	Detected	Detected
1491	A_33_P3424826	tcag7.1213		hypothetical LOC393076	PREDICTED: Homo sapiens hypothetica	31.73826	66.27889	21.93320	34.30376	Detected	Detected	Detected	Detected
1492	A_33_P3223660	C9orf130	Hs.494549	chromosome 9 open reading frame 130	Homo sapiens chromosome 9 open read	508.27173	405.16693	452.88528	346.05197	Detected	Detected	Detected	Detected
1493	A_32_P331052	C20orf151	Hs.180374	chromosome 20 open reading frame 151	Homo sapiens chromosome 20 open rea	116.86658	78.50308	19.45052	10.65391	Detected	Detected	Detected	Compromised
1494	A_33_P3414683	KIFC2	Hs.528713	kinesin family member C2	Homo sapiens kinesin family member C2	87.21989	56.85388	47.76035	13.43961	Detected	Detected	Detected	Compromised
1495	A_23_P161615	POLA2	Hs.201897	polymerase (DNA directed), alpha 2 (70	Homo sapiens polymerase (DNA directe	415.58380	1982.39360	433.80743	1894.37920	Detected	Detected	Detected	Detected
1496	A_24_P395317	DIS3	Hs.643464	DIS3 mitotic control homolog (S. cerevi	Homo sapiens DIS3 mitotic control hom	27.36654	16.22963	17.17850	9.41461	Detected	Compromised	Detected	Compromised

1497	A_33_P3388815	FPGT	Hs.480085	fucose-1-phosphate guanylyltransferas	Homo sapiens fucose-1-phosphate gua	268.93604	185.13918	178.08177	88.65531	Detected	Detected	Detected	Detected
1498	A_23_P121795	SORBS2	Hs.655143	sorbin and SH3 domain containing 2	Homo sapiens sorbin and SH3 domain c	13.70106	552.91187	14.33083	429.31635	Detected	Detected	Detected	Detected
1499	A_33_P3329592	BLOC1S3	Hs.103902	biogenesis of lysosomal organelles com	Homo sapiens biogenesis of lysosomal c	44.02268	83.33167	53.91716	97.17870	Detected	Detected	Detected	Detected
1500	A_33_P3384362					17.98289	3.82858	13.12855	3.40118	Detected	Compromised	Compromised	Compromised
1501	A_33_P3231602	ZNF569	Hs.511848	zinc finger protein 569	Homo sapiens zinc finger protein 569 (Z	24.17414	44.95028	6.35017	13.59800	Detected	Detected	Compromised	Compromised
1502	A_33_P3316348					1365.42690	2162.93630	271.55170	332.11100	Detected	Detected	Detected	Detected
1503	A_24_P216087	PAF1	Hs.466714	Paf1, RNA polymerase II associated fac	Homo sapiens Paf1, RNA polymerase II	3296.37480	4485.94730	2863.57100	3238.95360	Detected	Detected	Detected	Detected
1504	A_23_P301336	C10orf28	Hs.419800	chromosome 10 open reading frame 28	Homo sapiens chromosome 10 open rea	265.71112	237.56477	245.63810	201.27533	Detected	Detected	Detected	Detected
1505	A_23_P28105	TSN	Hs.75066	translin	Homo sapiens translin (TSN), mRNA [N	2349.64620	5643.04930	2457.87350	4846.15000	Detected	Detected	Detected	Detected
1506	A_33_P3355717	C14orf181	Hs.655774	chromosome 14 open reading frame 181	Homo sapiens chromosome 14 open rea	7992.43500	12899.11500	8061.49900	7632.19600	Detected	Detected	Detected	Detected
1507	A_23_P36345	STARD10	Hs.188606	StAR-related lipid transfer (START) do	Homo sapiens StAR-related lipid transf	758.61220	345.12747	833.33075	331.67526	Detected	Detected	Detected	Detected
1508	A_23_P117852	KIAA0101	Hs.81892	KIAA0101	Homo sapiens KIAA0101 (KIAA0101), tr	540.27780	20635.85700	324.31445	10278.34300	Detected	Detected	Detected	Detected
1509	A_23_P52531	FAM24B	Hs.114648	family with sequence similarity 24, mem	Homo sapiens family with sequence sim	1736.34280	823.09310	1515.78900	604.79690	Detected	Detected	Detected	Detected
1510	A_23_P211584	C22orf32	Hs.306083	chromosome 22 open reading frame 32	Homo sapiens chromosome 22 open rea	60.46630	52.91518	61.99287	45.00499	Detected	Detected	Detected	Detected
1511	A_23_P210675	SYCP2	Hs.202676	synaptonemal complex protein 2	Homo sapiens synaptonemal complex p	55.64732	27.42587	28.83688	14.44981	Detected	Detected	Detected	Compromised
1512	A_23_P204751	ACCN2	Hs.274361	amiloride-sensitive cation channel 2, ne	Homo sapiens amiloride-sensitive catio	396.58017	225.94466	317.09705	169.89159	Detected	Detected	Detected	Detected
1513	A_23_P429092	HBS1L	Hs.378532	HBS1-like (S. cerevisiae)	Homo sapiens HBS1-like (S. cerevisiae)	16.76979	32.11503	7.99807	13.05882	Detected	Detected	Compromised	Compromised
1514	A_23_P28886	PCNA	Hs.147433	proliferating cell nuclear antigen	Homo sapiens proliferating cell nuclear	4267.33940	17406.92600	3028.38300	9776.35600	Detected	Detected	Detected	Detected
1515	A_23_P54230	ZC3H14	Hs.325846	zinc finger CCCH-type containing 14	Homo sapiens zinc finger CCCH-type c	484.19778	716.08777	493.66534	648.45654	Detected	Detected	Detected	Detected
1516	A_32_P85999	CDH13	Hs.654386	cadherin 13, H-cadherin (heart)	Homo sapiens cadherin 13, H-cadherin	10328.62200	3312.05960	7102.83840	1896.80870	Detected	Detected	Detected	Detected
1517	A_23_P40174	MMP9	Hs.297413	matrix metallopeptidase 9 (gelatinase B	Homo sapiens matrix metallopeptidase	579.02760	177.94675	464.11280	87.39334	Detected	Detected	Detected	Detected
1518	A_33_P3353672	RAB28	Hs.656060	RAB28, member RAS oncogene family	Homo sapiens RAB28, member RAS on	6175.09670	5197.61000	4696.03800	3278.33200	Detected	Detected	Detected	Detected
1519	A_33_P3246920		Hs.656915		CUB and sushi domain-containing prote	16.57642	201.57332	19.46281	156.52893	Detected	Detected	Detected	Detected
1520	A_24_P328231	CPSF3L	Hs.6449	cleavage and polyadenylation specific fa	Homo sapiens cleavage and polyadenyla	94.55737	112.28199	144.88925	156.04600	Detected	Detected	Detected	Detected
1521	A_23_P311201	SFRS13A		splicing factor, arginine/serine-rich 13A	FUS-interacting serine-arginine-rich pr	994.05835	1815.84170	159.88829	132.46672	Detected	Detected	Detected	Detected
1522	A_33_P3267198		Hs.568869		cDNA FLJ43696 fis, clone TBAES2007	33.84724	45.15588	45.67319	53.21255	Detected	Detected	Detected	Detected
1523	A_23_P56553	METTL8	Hs.135146	methyltransferase like 8	Homo sapiens methyltransferase like 8	780.41570	1373.99220	821.13824	1005.84720	Detected	Detected	Detected	Detected
1524	A_33_P3344204	ZDHHC11	Hs.654874	zinc finger, DHHC-type containing 11	Homo sapiens zinc finger, DHHC-type c	297.60416	71.81793	211.07822	56.64791	Detected	Detected	Detected	Detected
1525	A_23_P210900	ACSS2	Hs.517034	acyl-CoA synthetase short-chain family	Homo sapiens acyl-CoA synthetase sho	1462.09910	1160.16460	1409.60930	933.66990	Detected	Detected	Detected	Detected
1526	A_23_P101013	TMC6	Hs.632227	transmembrane channel-like 6	Homo sapiens transmembrane channel-	15.35553	5.68979	19.99438	40.52969	Not Detected	Compromised	Detected	Not Detected
1527	A_23_P107211	RAB5C	Hs.650382	RAB5C, member RAS oncogene family	Homo sapiens RAB5C, member RAS on	1340.25370	2068.53250	1122.98610	1412.47380	Detected	Detected	Detected	Detected
1528	A_23_P112482	AQP3	Hs.234642	aquaporin 3 (Gill blood group)	Homo sapiens aquaporin 3 (Gill blood gr	56.82177	25.70020	53.52462	24.72588	Detected	Detected	Detected	Detected
1529	A_23_P121665	SORCS2	Hs.479099	sortilin-related VPS10 domain containin	Homo sapiens sortilin-related VPS10 d	15.44346	45.79244	13.65241	25.88040	Detected	Detected	Detected	Detected
1530	A_33_P3382086	FAM195A	Hs.417710	family with sequence similarity 195, mem	Homo sapiens family with sequence sim	1709.89700	2007.93070	1893.43260	1748.01110	Detected	Detected	Detected	Detected
1531	A_23_P11800	CAMK2N1	Hs.197922	calcium/calmodulin-dependent protein	Homo sapiens calcium/calmodulin-depe	19919.65600	21986.54900	13438.27900	12159.31700	Detected	Detected	Detected	Detected
1532	A_23_P30307	MED7	Hs.279902	mediator complex subunit 7	Homo sapiens mediator complex subuni	1530.87540	2311.17650	1033.11010	1261.07710	Detected	Detected	Detected	Detected
1533	A_23_P2967	AP1G2	Hs.343244	adaptor-related protein complex 1, gam	Homo sapiens adaptor-related protein c	87.11292	105.00568	76.98847	83.96058	Detected	Detected	Detected	Detected
1534	A_33_P3259557	LOC440104	Hs.616500	hypothetical LOC440104	Homo sapiens cDNA FLJ32617 fis, clon	5470.93260	2859.35100	3361.15800	1706.98780	Detected	Detected	Detected	Detected
1535	A_32_P130577					287.03616	326.30377	212.44296	201.17677	Detected	Detected	Detected	Detected
1536	A_33_P3312658				Kynureninase (EC 3.7.1.3)(L-kynurenine	92.85991	3.74943	60.55794	3.30727	Detected	Compromised	Detected	Compromised
1537	A_24_P126471	SLC12A9	Hs.521087	solute carrier family 12 (potassium/chlo	Homo sapiens solute carrier family 12 (	152.54280	220.08669	189.83868	276.11786	Detected	Detected	Detected	Detected
1538	A_24_P13381	TRPV4	Hs.506713	transient receptor potential cation chan	Homo sapiens transient receptor poten	46.06806	82.32536	41.89491	81.96199	Detected	Detected	Detected	Detected
1539	A_23_P89410	BECN1	Hs.716464	beclin 1, autophagy related	Homo sapiens beclin 1, autophagy relat	5827.58450	5296.85800	5457.34770	4338.72800	Detected	Detected	Detected	Detected
1540	A_23_P145694	ASNS	Hs.489207	asparagine synthetase	Homo sapiens asparagine synthetase (A	6760.25630	21077.17400	5756.45500	15705.99600	Detected	Detected	Detected	Detected
1541	A_23_P101380	B3GNT8	Hs.441681	UDP-GlcNAc:betaGal beta-1,3-N-acety	Homo sapiens UDP-GlcNAc:betaGal be	541.66956	1878.11120	432.96918	1351.37850	Detected	Detected	Detected	Detected
1542	A_24_P248185	C12orf53	Hs.44067	chromosome 12 open reading frame 53	Homo sapiens chromosome 12 open rea	1478.97810	122.60394	787.68616	59.89415	Detected	Detected	Detected	Detected
1543	A_33_P3332438	C6orf72	Hs.438872	chromosome 6 open reading frame 72	Homo sapiens chromosome 6 open read	2816.24950	2491.54270	2548.24220	1880.22290	Detected	Detected	Detected	Detected
1544	A_33_P3406408	MANBAL	Hs.6126	mannosidase, beta A, lysosomal-like	Homo sapiens mannosidase, beta A, lys	5531.33350	5196.19140	5596.37300	4814.59600	Detected	Detected	Detected	Detected
1545	A_33_P3306659	LOC728254	Hs.646684	hypothetical protein LOC728254	PREDICTED: Homo sapiens hypothetica	15.31099	18.10641	11.70692	10.56453	Detected	Compromised	Compromised	Compromised
1546	A_24_P269895	HNRNPA3	Hs.516539	heterogeneous nuclear ribonucleoprotei	Homo sapiens heterogeneous nuclear ri	445.74786	628.62830	272.55356	353.27368	Detected	Detected	Detected	Detected
1547	A_33_P3613516	LOC254057	Hs.586109	hypothetical protein LOC254057	Homo sapiens cDNA: FLJ21000 fis, clon	440.99380	193.84851	289.53830	112.08910	Detected	Detected	Detected	Detected
1548	A_33_P3479999	LOC494150	Hs.662179	prohibitin pseudogene	Homo sapiens prohibitin pseudogene, m	127039.69500	127755.34000	59330.18800	45032.65600	Detected	Detected	Detected	Detected
1549	A_24_P64100	SLC25A37	Hs.596025	solute carrier family 25, member 37	Homo sapiens FP15737 mRNA, complet	1194.87170	497.07680	1073.22690	355.58615	Detected	Detected	Detected	Detected
1550	A_23_P153855	ZNF492	Hs.232108	zinc finger protein 492	Homo sapiens zinc finger protein 492 (Z	215.54400	156.38942	32.11036	5.65291	Detected	Detected	Detected	Compromised
1551	A_23_P46781	ITGA8	Hs.171311	integrin, alpha 8	Homo sapiens integrin, alpha 8 (ITGA8),	9.06729	44.01004	7.85035	19.64209	Compromised	Detected	Compromised	Detected
1552	A_24_P535219	PHF10	Hs.435933	PHD finger protein 10	Homo sapiens mRNA full length insert c	347.87400	140.74320	225.31897	70.24012	Detected	Detected	Detected	Detected
1553	A_24_P38290	TAC1	Hs.2563	tachykinin, precursor 1	Homo sapiens tachykinin, precursor 1 (T	2521.29250	3.80366	1705.67370	3.38048	Detected	Compromised	Detected	Compromised
1554	A_33_P3240295					42.65594	53.26815	50.35004	44.95982	Detected	Detected	Detected	Detected
1555	A_33_P3379506	MITD1	Hs.14222	MIT, microtubule interacting and transp	Homo sapiens MIT, microtubule interact	6443.39100	3675.02500	5589.66260	2734.82280	Detected	Detected	Detected	Detected
1556	A_33_P3272539	PLEKHG5	Hs.284232	pleckstrin homology domain containing,	Homo sapiens pleckstrin homology dom	14225.76800	18601.89300	8412.25400	7521.18500	Detected	Detected	Detected	Detected
1557	A_33_P3365392		Hs.640138		Homo sapiens cDNA FLJ45328 fis, clon	42.40881	66.40528	42.84626	46.60346	Detected	Detected	Detected	Detected
1558	A_23_P116387	INCENP	Hs.142179	inner centromere protein antigens 135/	Homo sapiens inner centromere protein	468.88020	3715.00630	230.12834	1518.62410	Detected	Detected	Detected	Detected
1559	A_33_P3216357	KDM5C	Hs.631768	lysine (K)-specific demethylase 5C	Homo sapiens lysine (K)-specific demet	6.20700	13.14827	22.60662	17.71990	Compromised	Compromised	Detected	Detected
1560	A_33_P3288484	LYRM2	Hs.177275	LYR motif containing 2	Homo sapiens LYR motif containing 2 (L	84.48849	78.87724	83.77087	64.87953	Detected	Detected	Detected	Detected
1561	A_23_P256375	STX4	Hs.83734	syntaxin 4	Homo sapiens syntaxin 4 (STX4), mRNA	7710.11960	6955.63530	10647.77500	8302.26400	Detected	Detected	Detected	Detected
1562	A_33_P3229818	TBPL2	Hs.528278	TATA box binding protein like 2	Homo sapiens TATA box binding protei	20.33875	3.95317	12.92671	3.50444	Detected	Compromised	Compromised	Compromised
1563	A_24_P56388	HIF1A	Hs.597216	hypoxia inducible factor 1, alpha subuni	Homo sapiens hypoxia inducible factor	15846.90900	14017.76900	8832.70600	5921.42900	Detected	Detected	Detected	Detected
1564	A_32_P12104	ANAPC1	Hs.436527	anaphase promoting complex subunit 1	Homo sapiens anaphase promoting com	59.23720	113.74076	36.30728	65.42297	Detected	Detected	Detected	Detected
1565	A_33_P3645465	LOC282997	Hs.599931	hypothetical protein LOC282997	Homo sapiens hypothetical protein LOC	477.09296	406.35114	303.90018	216.80345	Detected	Detected	Detected	Detected
1566	A_23_P114626	SERPINC1	Hs.75599	serpin peptidase inhibitor, clade C (anti	Homo sapiens serpin peptidase inhibitor	15.28680	21.79922	14.58882	6.38597	Detected	Detected	Not Detected	Compromised
1567	A_23_P114164	UBQLN2	Hs.179309	ubiquilin 2	Homo sapiens ubiquilin 2 (UBQLN2), mR	1690.80600	1754.44060	1070.60880	896.21620	Detected	Detected	Detected	Detected
1568	A_23_P117261	RXFP2	Hs.680763	relaxin/insulin-like family peptide recep	Homo sapiens relaxin/insulin-like family	2.99940	4.11840	2.95612	30.42671	Compromised	Compromised	Compromised	Not Detected
1569	A_23_P1331	COL13A1	Hs.695934	collagen, type XIII, alpha 1	Homo sapiens collagen, type XIII, alpha	1187.77160	4264.45170	736.68463	2372.27610	Detected	Detected	Detected	Detected
1570	A_32_P90080	ARMC10	Hs.287412	armadillo repeat containing 10	Homo sapiens armadillo repeat containi	1174.49230	1112.68100	841.67320	623.00140	Detected	Detected	Detected	Detected
1571	A_33_P3302280	PDIK1L	Hs.468801	PDLIM1 interacting kinase 1 like	Homo sapiens PDLIM1 interacting kinas	19.96028	18.14612	23.01782	13.89694	Detected	Compromised	Detected	Compromised

1572	A_23_P312174	ALMS1	Hs.184720	Alstrom syndrome 1	Homo sapiens Alstrom syndrome 1 (ALI	1091.14500	1884.48600	634.09770	862.41600	Detected	Detected	Detected	Detected
1573	A_32_P57854	LOC100128731	Hs.502948	dolichyl-diphosphooligosaccharide--pro	Homo sapiens dolichyl-diphosphooligosa	11769.59100	21569.52100	10493.54100	14428.34300	Detected	Detected	Detected	Detected
1574	A_33_P3277527	LAMC3	Hs.201805	laminin, gamma 3	Homo sapiens laminin, gamma 3 (LAMC	127.50391	8.27836	150.00008	11.06315	Detected	Compromised	Detected	Compromised
1575	A_33_P3414058	MEN1	Hs.423348	multiple endocrine neoplasia I	Homo sapiens multiple endocrine neopla	649.50200	480.71487	664.00160	482.12592	Detected	Detected	Detected	Detected
1576	A_32_P76035	PNPT1	Hs.388733	polyribonucleotide nucleotidyltransferas	Homo sapiens polyribonucleotide nucleo	187.26505	186.38432	133.55179	122.49246	Detected	Detected	Detected	Detected
1577	A_33_P3296482	PTPRK	Hs.155919	protein tyrosine phosphatase, receptor	Homo sapiens protein tyrosine phospha	117.16093	132.82806	111.91042	119.51261	Detected	Detected	Detected	Detected
1578	A_33_P3238573	DENND4B	Hs.716414	DENN/MADD domain containing 4B	Homo sapiens DENN/MADD domain co	12138.57300	6520.68500	7285.13000	3425.69920	Detected	Detected	Detected	Detected
1579	A_33_P3371910	HNRPLL	Hs.445497	heterogeneous nuclear ribonucleoprotei	Homo sapiens heterogeneous nuclear ri	655.36350	522.98760	655.50260	435.95080	Detected	Detected	Detected	Detected
1580	A_23_P122815	CALU	Hs.718425	calumenin	Homo sapiens calumenin (CALU), trans	3573.08300	12901.30700	2560.03200	7356.63130	Detected	Detected	Detected	Detected
1581	A_23_P200801	PDE4DIP	Hs.568247	phosphodiesterase 4D interacting prote	Homo sapiens phosphodiesterase 4D in	917.42650	1051.03760	594.07980	624.11640	Detected	Detected	Detected	Detected
1582	A_23_P111701	GNG11	Hs.83381	guanine nucleotide binding protein (G pr	Homo sapiens guanine nucleotide bindin	102376.44000	89298.96000	68053.80000	46760.98400	Detected	Detected	Detected	Detected
1583	A_33_P3403048				S-adenosylmethionine synthetase isofo	242.40956	207.61128	194.62140	136.34305	Detected	Detected	Detected	Detected
1584	A_23_P94762	ZNF354B	Hs.591734	zinc finger protein 354B	Homo sapiens zinc finger protein 354B	684.70250	346.64633	510.58620	244.14685	Detected	Detected	Detected	Detected
1585	A_33_P3267250	TDRD10	Hs.387671	tudor domain containing 10	Homo sapiens tudor domain containing	41.44941	72.21324	20.91515	34.10947	Detected	Detected	Detected	Detected
1586	A_23_P64888	TAS2R10	Hs.533756	taste receptor, type 2, member 10	Homo sapiens taste receptor, type 2, m	80.36944	34.71766	70.04536	25.72558	Detected	Detected	Detected	Detected
1587	A_33_P3244141	NDEL1	Hs.372123	nudE nuclear distribution gene E homol	Homo sapiens nudE nuclear distribution	14763.74900	19034.99800	12273.49600	14540.77000	Detected	Detected	Detected	Detected
1588	A_33_P3239084	FAM86A	Hs.406461	family with sequence similarity 86, mem	Homo sapiens family with sequence sim	467.96210	411.81677	472.91843	347.02970	Detected	Detected	Detected	Detected
1589	A_23_P106602	CRISPLD2	Hs.513779	cysteine-rich secretory protein LCCL d	Homo sapiens cysteine-rich secretory	491.89820	1029.92790	504.73540	885.13806	Detected	Detected	Detected	Detected
1590	A_33_P3259253	CD28	Hs.591629	CD28 molecule	Homo sapiens CD28 molecule (CD28), r	45.70366	58.73283	34.81178	24.49695	Detected	Detected	Detected	Detected
1591	A_23_P90589	MRPL44	Hs.203559	mitochondrial ribosomal protein L44	Homo sapiens mitochondrial ribosomal	1758.11160	2135.37130	1947.75320	1890.86980	Detected	Detected	Detected	Detected
1592	A_23_P56140	CSNK1G2	Hs.651905	casein kinase 1, gamma 2	Homo sapiens casein kinase 1, gamma 2	2427.47100	1562.27040	1824.13680	1144.63450	Detected	Detected	Detected	Detected
1593	A_33_P3360326	LOC100131326	Hs.710649	hypothetical LOC100131326	PREDICTED: Homo sapiens hypothetica	277.99344	329.54490	142.27560	119.58520	Detected	Detected	Detected	Detected
1594	A_23_P103361	LCK	Hs.470627	lymphocyte-specific protein tyrosine ki	Homo sapiens lymphocyte-specific prot	19.34507	25.67285	15.80272	13.89970	Detected	Detected	Detected	Detected
1595	A_32_P133072	SPON1	Hs.643864	spondin 1, extracellular matrix protein	Homo sapiens spondin 1, extracellular m	7575.24760	266.24368	5809.44530	194.11876	Detected	Detected	Detected	Detected
1596	A_33_P3423425	ZNF770	Hs.718498	zinc finger protein 770	Homo sapiens zinc finger protein 770 (Z	10513.67700	7165.08200	3304.90800	2117.71400	Detected	Detected	Detected	Detected
1597	A_33_P3215611					231.60783	68.73375	23.10415	8.08869	Detected	Detected	Detected	Compromised
1598	A_24_P300302	LOC134466	Hs.661876	zinc finger protein 300 pseudogene	Homo sapiens zinc finger protein 300 ps	85.26833	126.18031	49.83664	76.99819	Detected	Detected	Detected	Detected
1599	A_23_P819	ISG15	Hs.458485	ISG15 ubiquitin-like modifier	Homo sapiens ISG15 ubiquitin-like modi	9293.83500	20410.92400	6646.26900	9472.83400	Detected	Detected	Detected	Detected
1600	A_23_P4611	SLC27A5	Hs.292177	solute carrier family 27 (fatty acid trans	Homo sapiens solute carrier family 27 (	2012.86770	1026.13040	2095.60720	897.74304	Detected	Detected	Detected	Detected
1601	A_23_P164638	ZNF419	Hs.710285	zinc finger protein 419	Homo sapiens zinc finger protein 419 (Z	123.72057	124.61153	81.65655	71.61351	Detected	Detected	Detected	Detected
1602	A_33_P3367077					22.19438	48.15757	14.06983	21.24636	Detected	Detected	Compromised	Detected
1603	A_33_P3252719	TIRAP	Hs.537126	toll-interleukin 1 receptor (TIR) domain	Homo sapiens toll-interleukin 1 recepto	38.74030	30.32185	28.06706	19.25303	Detected	Detected	Detected	Detected
1604	A_23_P207981	SOCS6	Hs.44439	suppressor of cytokine signaling 6	Homo sapiens suppressor of cytokine s	527.53125	359.70923	245.63250	138.11967	Detected	Detected	Detected	Detected
1605	A_23_P119040	GREB1L	Hs.149020	growth regulation by estrogen in breast	Homo sapiens growth regulation by estr	13.50212	65.36356	12.12886	35.16392	Detected	Detected	Not Detected	Detected
1606	A_32_P215179	NPIP	Hs.676266	nuclear pore complex interacting protei	Homo sapiens nuclear pore complex int	724.42830	634.37480	503.65588	360.86362	Detected	Detected	Detected	Detected
1607	A_24_P319354	SUMO1	Hs.81424	SMT3 suppressor of mif two 3 homolog	Homo sapiens SMT3 suppressor of mif	1376.75790	1371.01640	1278.53610	1170.52170	Detected	Detected	Detected	Detected
1608	A_32_P30710	RPL23	Hs.406300	ribosomal protein L23	Homo sapiens ribosomal protein L23 (R	128030.27000	100495.39000	71010.82000	45528.08600	Detected	Detected	Detected	Detected
1609	A_33_P3390207	TMEM155	Hs.27524	transmembrane protein 155	Homo sapiens transmembrane protein 1	26.57940	8.09377	25.20557	3.79520	Detected	Compromised	Detected	Compromised
1610	A_24_P324712	NPC1L1	Hs.567486	NPC1 (Niemann-Pick disease, type C1,	Homo sapiens NPC1 (Niemann-Pick dis	49.30521	47.25186	38.93060	35.08763	Detected	Detected	Detected	Detected
1611	A_23_P116640	OR51B2	Hs.680163	olfactory receptor, family 51, subfamily	Homo sapiens olfactory receptor, family	4.53532	18.46321	2.79938	2.90751	Compromised	Not Detected	Compromised	Compromised
1612	A_23_P216468	SLC1A1	Hs.444915	solute carrier family 1 (neuronal/epithel	Homo sapiens solute carrier family 1 (n	1603.62430	1579.17160	1014.07360	814.91920	Detected	Detected	Detected	Detected
1613	A_23_P107036	TMEM11	Hs.719186	transmembrane protein 11	Homo sapiens transmembrane protein 1	4338.75100	6483.16800	3626.57100	4817.22900	Detected	Detected	Detected	Detected
1614	A_33_P3591810	EPS15L2		epidermal growth factor receptor pathw	Homo sapiens PRO1866 mRNA, comple	648.98700	641.67370	11.36640	8.81306	Detected	Detected	Compromised	Compromised
1615	A_33_P3367850	CHRM4	Hs.248100	cholinergic receptor, muscarinic 4	Homo sapiens cholinergic receptor, mus	75.79919	42.00768	54.94397	24.36362	Detected	Detected	Detected	Detected
1616	A_32_P208120	CAMK1D	Hs.659517	calcium/calmodulin-dependent protein	Homo sapiens calcium/calmodulin-depe	93.56437	53.62555	73.33310	38.01377	Detected	Detected	Detected	Detected
1617	A_23_P122174	XRCC4	Hs.567359	X-ray repair complementing defective r	Homo sapiens X-ray repair complement	870.97595	910.95856	763.26904	698.32640	Detected	Detected	Detected	Detected
1618	A_33_P3395403					221.57733	197.83590	141.47281	101.43029	Detected	Detected	Detected	Detected
1619	A_33_P3243023	ADRA1D	Hs.557	adrenergic, alpha-1D-, receptor	Homo sapiens adrenergic, alpha-1D-, re	8.43609	28.22494	11.79499	30.68854	Compromised	Detected	Compromised	Detected
1620	A_33_P3405743	CRYZ	Hs.83114	crystallin, zeta (quinone reductase)	Homo sapiens crystallin, zeta (quinone	2869.40330	1227.79110	2323.81670	811.88556	Detected	Detected	Detected	Detected
1621	A_23_P210210	EPAS1	Hs.468410	endothelial PAS domain protein 1	Homo sapiens endothelial PAS domain	32220.51200	19718.33400	24703.52300	12904.91500	Detected	Detected	Detected	Detected
1622	A_23_P47517	VPS37C	Hs.523715	vacuolar protein sorting 37 homolog C (	Homo sapiens vacuolar protein sorting	7006.21630	8211.45800	6464.73600	5928.29900	Detected	Detected	Detected	Detected
1623	A_23_P146198	CYP7A1	Hs.1644	cytochrome P450, family 7, subfamily A	Homo sapiens cytochrome P450, family	33.43415	34.69432	24.50140	8.63669	Detected	Detected	Detected	Compromised
1624	A_23_P8432	COPS6	Hs.15591	COP9 constitutive photomorphogenic h	Homo sapiens COP9 constitutive photo	21012.47000	30056.03700	23286.25000	29902.41200	Detected	Detected	Detected	Detected
1625	A_23_P300056	CDC42	Hs.467637	cell division cycle 42 (GTP binding prot	Homo sapiens cell division cycle 42 (GT	109.17782	492.77332	60.26164	205.13826	Detected	Detected	Detected	Detected
1626	A_23_P117602	GZMB	Hs.1051	granzyme B (granzyme 2, cytotoxic T-l	Homo sapiens granzyme B (granzyme 2	241.45609	125.65916	154.19038	84.17164	Detected	Detected	Detected	Detected
1627	A_33_P3271430	MAP3K3	Hs.29282	mitogen-activated protein kinase kinase	Homo sapiens mitogen-activated protei	9840.01200	18672.09000	8844.63700	14882.23200	Detected	Detected	Detected	Detected
1628	A_23_P341503	C3orf35	Hs.475945	chromosome 3 open reading frame 35	Homo sapiens chromosome 3 open read	161.50237	80.87975	84.12185	34.64769	Detected	Detected	Detected	Detected
1629	A_23_P205686	PSEN1	Hs.3260	presenilin 1	Homo sapiens presenilin 1 (PSEN1), tra	2267.96310	2432.34600	2007.49540	1892.51920	Detected	Detected	Detected	Detected
1630	A_23_P110504	CLPTM1L	Hs.444673	CLPTM1-like	Homo sapiens CLPTM1-like (CLPTM1L	7167.67770	9916.29300	6148.10500	7863.70950	Detected	Detected	Detected	Detected
1631	A_23_P89249	ERBB2	Hs.446352	v-erb-b2 erythroblastic leukemia viral o	Homo sapiens v-erb-b2 erythroblastic l	3886.74300	3671.79030	3512.54100	3049.01220	Detected	Detected	Detected	Detected
1632	A_23_P108922	MOBKL1B		MOB1, Mps One Binder kinase activato	Mps one binder kinase activator-like 1B	240.97191	191.85687	220.68436	174.04527	Detected	Detected	Detected	Detected
1633	A_23_P355471	TBXA2R	Hs.442530	thromboxane A2 receptor	Homo sapiens thromboxane A2 recepto	1980.90810	1093.80540	303.74994	225.90576	Detected	Detected	Detected	Detected
1634	A_24_P942730	ZSCAN29	Hs.418287	zinc finger and SCAN domain containing	Homo sapiens zinc finger and SCAN do	512.43610	350.54874	344.65735	179.23756	Detected	Detected	Detected	Detected
1635	A_33_P3436316	ASXL1	Hs.374043	additional sex combs like 1 (Drosophila)	Homo sapiens additional sex combs like	251.65645	1136.73380	204.08989	851.96740	Detected	Detected	Detected	Detected
1636	A_23_P146417	C9orf5	Hs.308074	chromosome 9 open reading frame 5	Homo sapiens chromosome 9 open read	2401.84620	2062.08030	1926.11230	1465.28140	Detected	Detected	Detected	Detected
1637	A_23_P14915	CSNK2A2	Hs.82201	casein kinase 2, alpha prime polypeptide	Homo sapiens casein kinase 2, alpha pri	880.97894	809.72076	644.04193	507.81635	Detected	Detected	Detected	Detected
1638	A_32_P25253	ISCA1	Hs.449291	iron-sulfur cluster assembly 1 homolog	Homo sapiens iron-sulfur cluster assem	1237.06290	1522.72640	997.72845	1064.46730	Detected	Detected	Detected	Detected
1639	A_33_P3264505	FCF1	Hs.579828	FCF1 small subunit (SSU) processome	Homo sapiens FCF1 small subunit (SSU	826.28980	887.55023	443.30014	411.28380	Detected	Detected	Detected	Detected
1640	A_23_P114670	ARHGEF16	Hs.87435	Rho guanine exchange factor (GEF) 16	Homo sapiens Rho guanine exchange fa	103.10102	27.70688	115.26511	27.38974	Detected	Detected	Detected	Detected
1641	A_33_P3369956	ATXN2L	Hs.460499	ataxin 2-like	Homo sapiens ataxin 2-like (ATXN2L), t	1268.98360	1953.08310	585.60680	898.11040	Detected	Detected	Detected	Detected
1642	A_33_P3514487	VSTM1	Hs.444431	V-set and transmembrane domain cont	Homo sapiens V-set and transmembran	24.35807	25.41314	27.65239	20.97433	Detected	Detected	Detected	Detected
1643	A_24_P247536	FAM133B	Hs.489105	family with sequence similarity 133, me	Homo sapiens family with sequence sim	890.01605	687.55760	267.99954	164.67973	Detected	Detected	Detected	Detected
1644	A_24_P59111		Hs.703229		Dipeptidyl aminopeptidase-like protein 6	16.71814	16.15555	13.58506	12.32821	Detected	Compromised	Compromised	Compromised
1645	A_33_P3330498	ALDH7A1	Hs.587300	aldehyde dehydrogenase 7 family, memb	Alpha-aminoadipic semialdehyde dehydr	102.58849	139.42578	62.27552	91.02733	Detected	Detected	Detected	Detected
1646	A_32_P79115	LOC100133075	Hs.271249	hypothetical LOC100133075	PREDICTED: Homo sapiens hypothetica	398.46480	400.39862	140.73302	101.72339	Detected	Detected	Detected	Detected

1647	A_23_P101615	ZNF565	Hs.651111	zinc finger protein 565	Homo sapiens zinc finger protein 565 (Z	371.54782	383.42203	244.97173	217.45020	Detected	Detected	Detected	Detected
1648	A_23_P87580	ANP32D	Hs.532658	acidic (leucine-rich) nuclear phosphopro	Homo sapiens acidic (leucine-rich) nucl	90.10606	151.59602	94.84433	113.32031	Detected	Detected	Detected	Detected
1649	A_33_P3752697	LOC100288106		similar to putative p150	PREDICTED: Homo sapiens similar to p	5267.18300	4647.13870	2513.09570	1728.44790	Detected	Detected	Detected	Detected
1650	A_23_P128828	PABPN1	Hs.707712	poly(A) binding protein, nuclear 1	Homo sapiens poly(A) binding protein, n	10086.51700	11374.64300	8570.25600	8335.06400	Detected	Detected	Detected	Detected
1651	A_23_P140563	TTC23	Hs.513195	tetratricopeptide repeat domain 23	Homo sapiens tetratricopeptide repeat	467.90765	474.82513	392.12440	346.86548	Detected	Detected	Detected	Detected
1652	A_32_P153725	KIAA1033	Hs.12144	KIAA1033	Homo sapiens KIAA1033 (KIAA1033), m	7993.92500	7968.63700	6815.41000	5698.93900	Detected	Detected	Detected	Detected
1653	A_23_P303810	CRYBG3	Hs.714457	beta-gamma crystallin domain containin	Homo sapiens beta-gamma crystallin do	1989.93900	1350.12220	1254.43160	656.11300	Detected	Detected	Detected	Detected
1654	A_32_P157945	DSP	Hs.519873	desmoplakin	Homo sapiens desmoplakin (DSP), trans	7540.03660	42567.88000	4960.26400	25296.22700	Detected	Detected	Detected	Detected
1655	A_33_P3238415	GSG1	Hs.240053	germ cell associated 1	Homo sapiens germ cell associated 1 (G	91.68673	248.81383	79.88535	172.54080	Detected	Detected	Detected	Detected
1656	A_23_P96990	NVL	Hs.497867	nuclear VCP-like	Homo sapiens nuclear VCP-like (NVL),	2755.77370	3515.64430	2677.53760	3142.48070	Detected	Detected	Detected	Detected
1657	A_33_P3287997	C9orf106	Hs.449434	chromosome 9 open reading frame 106	Homo sapiens chromosome 9 open read	34.47268	33.47616	7.66638	5.56889	Detected	Detected	Compromised	Compromised
1658	A_32_P8251	PHRF1	Hs.325838	PHD and ring finger domains 1	Homo sapiens PHD and ring finger doma	26494.37500	31285.95500	27220.15200	30284.19000	Detected	Detected	Detected	Detected
1659	A_23_P115407	GSTM1	Hs.301961	glutathione S-transferase mu 1	Homo sapiens glutathione S-transferase	716.28140	470.72583	600.76370	348.15417	Detected	Detected	Detected	Detected
1660	A_23_P145197	BYSL	Hs.106880	bystin-like	Homo sapiens bystin-like (BYSL), mRNA	2883.05660	4069.19070	3290.30320	4125.01950	Detected	Detected	Detected	Detected
1661	A_23_P39375	KLHL26	Hs.250632	kelch-like 26 (Drosophila)	Homo sapiens kelch-like 26 (Drosophila	230.81697	191.01521	233.63391	188.13867	Detected	Detected	Detected	Detected
1662	A_23_P10647	CYTL1	Hs.13872	cytokine-like 1	Homo sapiens cytokine-like 1 (CYTL1),	4.79888	4.02784	24.12216	3.06760	Compromised	Compromised	Not Detected	Compromised
1663	A_33_P3337727					328.45944	286.63550	113.51089	60.71406	Detected	Detected	Detected	Detected
1664	A_23_P213699	NRG2	Hs.408515	neuregulin 2	Homo sapiens neuregulin 2 (NRG2), tran	185.20956	30.91628	118.61247	18.29059	Detected	Detected	Detected	Detected
1665	A_23_P114862	ANGPTL7	Hs.146559	angiopoietin-like 7	Homo sapiens angiopoietin-like 7 (ANGP	2.51769	25.25676	2.79030	17.78848	Compromised	Not Detected	Compromised	Not Detected
1666	A_33_P3263523	C1orf35	Hs.715607	chromosome 1 open reading frame 35	Homo sapiens chromosome 1 open read	1650.03520	1468.12510	1941.83830	1309.09140	Detected	Detected	Detected	Detected
1667	A_33_P3280729	SHISA9	Hs.130661	shisa homolog 9 (Xenopus laevis)	Homo sapiens shisa homolog 9 (Xenopu	4.85130	35.57634	3.31543	19.21895	Compromised	Detected	Compromised	Detected
1668	A_23_P53736	FBXO21	Hs.159699	F-box protein 21	Homo sapiens F-box protein 21 (FBXO2	7180.80960	5212.21600	6342.91360	4098.76270	Detected	Detected	Detected	Detected
1669	A_33_P3357580	MRTO4	Hs.463797	mRNA turnover 4 homolog (S. cerevisia	Homo sapiens mRNA turnover 4 homolo	661.24520	1791.30600	442.33154	899.29380	Detected	Detected	Detected	Detected
1670	A_33_P3266410	MAZ	Hs.23650	MYC-associated zinc finger protein (pu	Homo sapiens MYC-associated zinc fin	66.34839	87.09406	115.82513	147.82540	Detected	Detected	Detected	Detected
1671	A_23_P118815	BIRC5	Hs.514527	baculoviral IAP repeat-containing 5	Homo sapiens baculoviral IAP repeat-co	897.69434	78166.19500	630.66320	55196.27000	Detected	Detected	Detected	Detected
1672	A_33_P3738458	TNS1	Hs.471381	tensin 1	Homo sapiens tensin 1 (TNS1), mRNA [	2177.32030	3073.85330	2013.70870	2385.21700	Detected	Detected	Detected	Detected
1673	A_23_P204503	PRKAB1	Hs.6061	protein kinase, AMP-activated, beta 1 n	Homo sapiens protein kinase, AMP-acti	766.38947	1487.00100	563.26590	906.64636	Detected	Detected	Detected	Detected
1674	A_24_P66001	UCRC	Hs.284292	ubiquinol-cytochrome c reductase com	Homo sapiens ubiquinol-cytochrome c r	229.82939	460.31040	235.11147	384.20322	Detected	Detected	Detected	Detected
1675	A_33_P3375762		Hs.689633		Putative UPF0607 protein ENSP000003	95.67955	104.60849	45.33667	40.52943	Detected	Detected	Detected	Detected
1676	A_32_P220307	RPL39	Hs.558387	ribosomal protein L39	Homo sapiens ribosomal protein L39 (R	138685.66000	79859.76000	98103.56000	48981.69500	Detected	Detected	Detected	Detected
1677	A_23_P120594	ACSS1	Hs.529353	acyl-CoA synthetase short-chain family	Homo sapiens acyl-CoA synthetase sho	783.54300	155.04270	575.39484	111.56105	Detected	Detected	Detected	Detected
1678	A_23_P205216	UTP14A	Hs.458598	UTP14, U3 small nucleolar ribonucleopr	Homo sapiens UTP14, U3 small nucleola	263.37430	447.24518	262.71704	397.93810	Detected	Detected	Detected	Detected
1679	A_23_P106859	COX4NB	Hs.173162	COX4 neighbor	Homo sapiens COX4 neighbor (COX4NB	14585.67900	22396.75600	10623.54100	13770.81800	Detected	Detected	Detected	Detected
1680	A_24_P411899	RNF19A	Hs.292882	ring finger protein 19A	Homo sapiens ring finger protein 19A (R	716.78253	744.73220	599.04620	555.96405	Detected	Detected	Detected	Detected
1681	A_23_P12189	DFFA	Hs.484782	DNA fragmentation factor, 45kDa, alpha	Homo sapiens DNA fragmentation facto	1463.61930	1767.88590	1013.82965	1079.72020	Detected	Detected	Detected	Detected
1682	A_33_P3287310					1061.45070	1513.36900	280.81240	327.29340	Detected	Detected	Detected	Detected
1683	A_24_P208909	TRIM2	Hs.435711	tripartite motif-containing 2	Homo sapiens tripartite motif-containin	1864.69650	766.46344	1235.39750	423.85010	Detected	Detected	Detected	Detected
1684	A_33_P3236563	ALDH3B1	Hs.523841	aldehyde dehydrogenase 3 family, memb	Homo sapiens aldehyde dehydrogenase	352.97235	250.86435	234.14691	142.13718	Detected	Detected	Detected	Detected
1685	A_24_P49383	C11orf67	Hs.503357	chromosome 11 open reading frame 67	Homo sapiens chromosome 11 open rea	3478.12100	4427.55130	3718.72140	4072.84250	Detected	Detected	Detected	Detected
1686	A_33_P3314301	SV2C	Hs.663229	synaptic vesicle glycoprotein 2C	Homo sapiens synaptic vesicle glycopro	33.85528	46.48112	17.70220	16.62194	Detected	Detected	Compromised	Compromised
1687	A_33_P3218694	PNN	Hs.409965	pinin, desmosome associated protein	Homo sapiens pinin, desmosome associ	141.42307	190.54810	86.77242	105.94929	Detected	Detected	Detected	Detected
1688	A_23_P302094	IMP4	Hs.91579	IMP4, U3 small nucleolar ribonucleoprot	Homo sapiens IMP4, U3 small nucleolar	4155.39840	7470.37200	4048.50700	6327.84130	Detected	Detected	Detected	Detected
1689	A_33_P3220663	STAC3	Hs.417595	SH3 and cysteine rich domain 3	Homo sapiens SH3 and cysteine rich do	568.72205	159.42508	479.86017	118.89889	Detected	Detected	Detected	Detected
1690	A_23_P14948	MBTPS1	Hs.75890	membrane-bound transcription factor p	Homo sapiens membrane-bound transc	3960.48100	3781.88200	2928.96310	2309.60500	Detected	Detected	Detected	Detected
1691	A_23_P164826	RNASEH2A	Hs.532851	ribonuclease H2, subunit A	Homo sapiens ribonuclease H2, subunit	2053.90160	7131.15040	2164.36350	7455.89750	Detected	Detected	Detected	Detected
1692	A_33_P3245454	LOC389834	Hs.720653	ankyrin repeat domain 57 pseudogene	Homo sapiens ankyrin repeat domain 57	88.69790	32.12179	62.19928	15.83307	Detected	Detected	Detected	Compromised
1693	A_23_P329152	ILF3	Hs.465885	interleukin enhancer binding factor 3, 90	Homo sapiens interleukin enhancer bind	2767.79200	3939.67020	3037.30860	4111.25900	Detected	Detected	Detected	Detected
1694	A_23_P94009	LSM8	Hs.657737	LSM8 homolog, U6 small nuclear RNA a	Homo sapiens LSM8 homolog, U6 small	15666.69600	16646.45500	7999.42240	6573.92800	Detected	Detected	Detected	Detected
1695	A_23_P36226	SLC25A33	Hs.568613	solute carrier family 25, member 33	Homo sapiens solute carrier family 25, r	2805.08130	4177.27700	2708.41750	3464.55710	Detected	Detected	Detected	Detected
1696	A_23_P13438	BTBD10	Hs.332382	BTB (POZ) domain containing 10	Homo sapiens BTB (POZ) domain conta	1027.94100	1301.44580	742.97870	913.51056	Detected	Detected	Detected	Detected
1697	A_23_P166051	RBCK1	Hs.247280	RanBP-type and C3HC4-type zinc finge	Homo sapiens RanBP-type and C3HC4-	1304.87100	1048.06880	1234.65540	974.62300	Detected	Detected	Detected	Detected
1698	A_24_P124672	DYNLL1	Hs.5120	dynein, light chain, LC8-type 1	Homo sapiens dynein, light chain, LC8-t	21378.99400	23400.90200	20025.14500	18353.95100	Detected	Detected	Detected	Detected
1699	A_33_P3228375	XPO5	Hs.203206	exportin 5	Homo sapiens exportin 5 (XPO5), mRNA	159.03217	164.81837	82.56895	65.96793	Detected	Detected	Detected	Detected
1700	A_23_P100795	STAT3	Hs.463059	signal transducer and activator of trans	Homo sapiens signal transducer and act	636.58484	218.01482	335.40182	106.81764	Detected	Detected	Detected	Detected
1701	A_33_P3411848	CNFN	Hs.148590	cornifelin	Homo sapiens cornifelin (CNFN), mRNA	521.93460	686.39056	439.68018	398.46008	Detected	Detected	Detected	Detected
1702	A_33_P3803639	TRAF2	Hs.522506	TNF receptor-associated factor 2	Homo sapiens TNF receptor-associated	8730.62000	11871.33400	7542.26030	9004.57300	Detected	Detected	Detected	Detected
1703	A_24_P187614	LOC100129648	Hs.711285	hypothetical LOC100129648	Homo sapiens cDNA FLJ40318 fis, clon	141.13306	144.38715	67.21690	66.08736	Detected	Detected	Detected	Detected
1704	A_23_P154037	AOX1	Hs.406238	aldehyde oxidase 1	Homo sapiens aldehyde oxidase 1 (AOX	8622.15300	3673.86620	8402.61700	3224.74680	Detected	Detected	Detected	Detected
1705	A_32_P176550	JMY	Hs.482605	junction mediating and regulatory protei	Homo sapiens junction mediating and re	408.93503	455.20930	307.45200	282.20230	Detected	Detected	Detected	Detected
1706	A_23_P108823	OSBPL6	Hs.318775	oxysterol binding protein-like 6	Homo sapiens oxysterol binding protein-	1639.29920	2233.13840	1310.90120	1549.11450	Detected	Detected	Detected	Detected
1707	A_23_P114185	TSPAN7	Hs.441664	tetraspanin 7	Homo sapiens tetraspanin 7 (TSPAN7),	13.22887	3.42315	12.42338	2.91472	Detected	Compromised	Not Detected	Compromised
1708	A_23_P1956	TMEM223	Hs.530463	transmembrane protein 223	Homo sapiens transmembrane protein 2	2577.05520	1983.51700	2269.14040	1495.42610	Detected	Detected	Detected	Detected
1709	A_33_P3377274	LOC729519	Hs.667466	hypothetical LOC729519	PREDICTED: Homo sapiens hypothetica	189.72252	188.40936	84.44486	79.24128	Detected	Detected	Detected	Detected
1710	A_33_P3277407	MLL5	Hs.592262	myeloid/lymphoid or mixed-lineage leuk	Homo sapiens myeloid/lymphoid or mixe	40.25471	30.86736	38.18246	23.11229	Detected	Detected	Detected	Detected
1711	A_33_P3312754					1152.63200	1848.00290	414.99603	531.00010	Detected	Detected	Detected	Detected
1712	A_33_P3212640	NOTCH2NL	Hs.709526	Notch homolog 2 (Drosophila) N-termin	Homo sapiens Notch homolog 2 (Drosop	217.71080	65.81836	154.98172	54.57623	Detected	Detected	Detected	Detected
1713	A_23_P346800	AVPR2	Hs.567240	arginine vasopressin receptor 2	Homo sapiens arginine vasopressin rece	33.12175	43.50823	7.85220	11.03678	Detected	Detected	Compromised	Compromised
1714	A_32_P74409	LOC387763	Hs.714890	hypothetical protein LOC387763	Homo sapiens hypothetical protein LOC	46052.15600	2774.76980	33126.72700	1771.72070	Detected	Detected	Detected	Detected
1715	A_23_P107612	RAB27B	Hs.25318	RAB27B, member RAS oncogene family	Homo sapiens RAB27B, member RAS o	14.14487	20.75849	20.66783	19.26638	Detected	Detected	Detected	Detected
1716	A_33_P3233160	CCDC104	Hs.264208	coiled-coil domain containing 104	Homo sapiens coiled-coil domain conta	1497.15900	1294.38050	1541.53280	1014.76090	Detected	Detected	Detected	Detected
1717	A_23_P32233	KLF4	Hs.376206	Kruppel-like factor 4 (gut)	Homo sapiens Kruppel-like factor 4 (gut	652.56530	691.54694	832.71185	735.51825	Detected	Detected	Detected	Detected
1718	A_33_P3396886	C11orf52	Hs.97013	chromosome 11 open reading frame 52	Homo sapiens chromosome 11 open rea	164.95445	151.87357	197.77066	140.38347	Detected	Detected	Detected	Detected
1719	A_24_P96474	LDOC1L	Hs.715637	leucine zipper, down-regulated in cance	Homo sapiens leucine zipper, down-regu	1674.59850	1868.35850	1222.94000	1128.63010	Detected	Detected	Detected	Detected
1720	A_24_P156576	GEMIN8	Hs.592237	gem (nuclear organelle) associated prot	Homo sapiens gem (nuclear organelle) a	505.82794	272.70330	490.19534	228.91942	Detected	Detected	Detected	Detected
1721	A_24_P56194	CREBL2	Hs.591156	cAMP responsive element binding prote	Homo sapiens cAMP responsive elemen	1328.08920	827.08930	876.45120	447.61102	Detected	Detected	Detected	Detected

1722	A_24_P233960	SNX27	Hs.192326	sorting nexin family member 27	Homo sapiens sorting nexin family mem	2915.12920	1332.64110	2500.64580	982.70184	Detected	Detected	Detected	Detected
1723	A_33_P3337901	PDE4DIP	Hs.568247	phosphodiesterase 4D interacting prote	Homo sapiens phosphodiesterase 4D in	78.79833	45.21901	54.48180	39.11085	Detected	Detected	Detected	Detected
1724	A_23_P120243	HOXD1	Hs.83465	homeobox D1	Homo sapiens homeobox D1 (HOXD1),	75.71083	47.70395	56.18820	33.16987	Detected	Detected	Detected	Detected
1725	A_33_P3249731	LOC100294070	Hs.720272	hypothetical protein LOC100294070	PREDICTED: Homo sapiens hypothetica	53.68424	46.09525	31.20835	23.68081	Detected	Detected	Detected	Detected
1726	A_23_P123393	KCNQ3	Hs.374023	potassium voltage-gated channel, KQT-	Homo sapiens potassium voltage-gated	77.21819	85.11265	68.56007	59.21242	Detected	Detected	Detected	Detected
1727	A_23_P45304	XK	Hs.78919	X-linked Kx blood group (McLeod syndr	Homo sapiens X-linked Kx blood group	20.49775	3.88278	10.38769	3.43803	Detected	Compromised	Compromised	Compromised
1728	A_33_P3372124	TRAFD1	Hs.5148	TRAF-type zinc finger domain containir	Homo sapiens TRAF-type zinc finger do	4731.92000	4450.08900	4050.21750	3016.38380	Detected	Detected	Detected	Detected
1729	A_23_P61580	NSUN6	Hs.396175	NOL1/NOP2/Sun domain family, memb	Homo sapiens NOL1/NOP2/Sun domai	493.22990	114.96678	437.74265	97.29054	Detected	Detected	Detected	Detected
1730	A_23_P1014	C1orf97		chromosome 1 open reading frame 97	Homo sapiens chromosome 1 open reac	3694.27400	1353.47310	3149.08100	1054.03220	Detected	Detected	Detected	Detected
1731	A_33_P3577671	DPP8	Hs.591106	dipeptidyl-peptidase 8	Homo sapiens dipeptidyl-peptidase 8 (D	9430.87300	9225.01300	6170.71040	5444.32960	Detected	Detected	Detected	Detected
1732	A_23_P11664	SFRS11	Hs.479693	splicing factor, arginine/serine-rich 11	Homo sapiens splicing factor, arginine/s	10049.33400	5854.79830	7084.94400	3484.89180	Detected	Detected	Detected	Detected
1733	A_23_P106463	OAZ2	Hs.74563	ornithine decarboxylase antizyme 2	Homo sapiens ornithine decarboxylase a	10424.67300	10277.43300	8014.59200	7491.36400	Detected	Detected	Detected	Detected
1734	A_33_P3258392	EDN1	Hs.511899	endothelin 1	Homo sapiens endothelin 1 (EDN1), mR	33.26720	221.06725	39.43983	221.70598	Detected	Detected	Detected	Detected
1735	A_32_P42925	MYL12B	Hs.464472	myosin, light chain 12B, regulatory	Homo sapiens myosin, light chain 12B, r	57813.52700	93345.69500	51319.20000	69218.99000	Detected	Detected	Detected	Detected
1736	A_23_P123866	UBAP1	Hs.268963	ubiquitin associated protein 1	Homo sapiens ubiquitin associated prot	1611.48900	1893.00090	1309.18230	1369.37240	Detected	Detected	Detected	Detected
1737	A_23_P121716	ANXA3	Hs.480042	annexin A3	Homo sapiens annexin A3 (ANXA3), mR	76.53193	59.86021	66.82349	41.76126	Detected	Detected	Detected	Detected
1738	A_23_P105691	GPRC5D	Hs.644599	G protein-coupled receptor, family C, g	Homo sapiens G protein-coupled recep	7.44875	8.56666	15.14021	15.03066	Compromised	Compromised	Detected	Detected
1739	A_24_P936010		Hs.548018		Homo sapiens clone DNA172120 micror	60.46618	98.50328	26.99496	24.70843	Detected	Detected	Detected	Detected
1740	A_23_P106633	DDX28	Hs.458313	DEAD (Asp-Glu-Ala-Asp) box polypept	Homo sapiens DEAD (Asp-Glu-Ala-Asp	5512.37100	7094.15300	4877.47170	5492.44970	Detected	Detected	Detected	Detected
1741	A_33_P3223825	SRRM3	Hs.511025	serine/arginine repetitive matrix 3	Homo sapiens serine/arginine repetitive	58.25058	91.83710	64.90981	61.64842	Detected	Detected	Detected	Detected
1742	A_23_P19987	IGF2BP3	Hs.700696	insulin-like growth factor 2 mRNA bindi	Homo sapiens insulin-like growth factor	4443.59230	5163.01030	2792.80900	2793.88750	Detected	Detected	Detected	Detected
1743	A_23_P11859	HSD17B7	Hs.492925	hydroxysteroid (17-beta) dehydrogenas	Homo sapiens hydroxysteroid (17-beta)	303.81080	130.54123	229.04300	78.31182	Detected	Detected	Detected	Detected
1744	A_32_P212897	LOC643406	Hs.431161	hypothetical LOC643406	Homo sapiens hypothetical LOC643406	8.04846	49.98111	4.84814	41.39180	Compromised	Detected	Compromised	Detected
1745	A_23_P27606	IL27RA	Hs.132781	interleukin 27 receptor, alpha	Homo sapiens interleukin 27 receptor, a	653.52360	271.32578	627.74506	230.69022	Detected	Detected	Detected	Detected
1746	A_24_P414952	TMEM168	Hs.709689	transmembrane protein 168	Homo sapiens transmembrane protein 1	778.63480	463.52106	514.32500	244.08109	Detected	Detected	Detected	Detected
1747	A_23_P87257	MRPL17	Hs.523456	mitochondrial ribosomal protein L17	Homo sapiens mitochondrial ribosomal p	2604.27700	3840.16580	3445.32060	4387.62060	Detected	Detected	Detected	Detected
1748	A_33_P3214803	PRDM13	Hs.287386	PR domain containing 13	Homo sapiens PR domain containing 13	123.74760	119.01479	79.93701	79.39349	Detected	Detected	Detected	Detected
1749	A_33_P3258801	SLURP1	Hs.676358	secreted LY6/PLAUR domain containin	Homo sapiens secreted LY6/PLAUR do	208.87248	212.20233	109.14376	103.84219	Detected	Detected	Detected	Detected
1750	A_23_P137578	FBXO28	Hs.64691	F-box protein 28	Homo sapiens F-box protein 28 (FBXO2	895.64526	1459.91360	647.66700	909.39720	Detected	Detected	Detected	Detected
1751	A_23_P113701	PDGFA	Hs.535898	platelet-derived growth factor alpha pol	Homo sapiens platelet-derived growth f	60.29870	54.92622	41.38086	36.37785	Detected	Detected	Detected	Detected
1752	A_23_P108785	ACTR3	Hs.433512	ARP3 actin-related protein 3 homolog (	Homo sapiens ARP3 actin-related prote	8371.43700	15905.82700	7452.10300	11663.35400	Detected	Detected	Detected	Detected
1753	A_33_P3394710	SLC35A1	Hs.423163	solute carrier family 35 (CMP-sialic aci	Homo sapiens solute carrier family 35 (	409.90256	119.69861	561.53460	144.98631	Detected	Detected	Detected	Detected
1754	A_33_P3365010	ELK4	Hs.497520	ELK4, ETS-domain protein (SRF access	Homo sapiens ELK4, ETS-domain prote	48.34406	61.15321	79.14658	63.98995	Detected	Detected	Detected	Detected
1755	A_23_P386764	DOLPP1	Hs.21701	dolichyl pyrophosphate phosphatase 1	Homo sapiens dolichyl pyrophosphate p	1068.53370	2535.95170	742.20510	1506.54080	Detected	Detected	Detected	Detected
1756	A_23_P129486	SEPX1	Hs.655346	selenoprotein X, 1	Homo sapiens selenoprotein X, 1 (SEPX	2499.52860	2475.55570	3384.92000	3133.87380	Detected	Detected	Detected	Detected
1757	A_33_P3237760					66.44135	117.72787	38.19702	46.58601	Detected	Detected	Detected	Detected
1758	A_24_P80204	MALL	Hs.720026	mal, T-cell differentiation protein-like	Homo sapiens mal, T-cell differentiatior	60.29674	725.18097	64.13989	737.17810	Detected	Detected	Detected	Detected
1759	A_33_P3416009				Protein downstream neighbor of Son (B	2.66017	22.10548	3.10114	4.88934	Compromised	Detected	Compromised	Compromised
1760	A_24_P375322	GGT8P	Hs.650223	gamma-glutamyltransferase 8 pseudoge	Homo sapiens gamma-glutamyltransfera	16.26524	3.74969	15.05574	5.26612	Detected	Compromised	Compromised	Compromised
1761	A_23_P111487	SRRT	Hs.111801	serrate RNA effector molecule homolog	Homo sapiens serrate RNA effector mo	358.79090	760.16690	277.13052	546.87274	Detected	Detected	Detected	Detected
1762	A_23_P89310	EPN2	Hs.309467	epsin 2	Homo sapiens epsin 2 (EPN2), transcrip	6935.94730	6899.27800	7704.01100	6890.99600	Detected	Detected	Detected	Detected
1763	A_23_P388900	SLC22A15	Hs.125482	solute carrier family 22, member 15	Homo sapiens solute carrier family 22, r	1648.67770	926.16740	1235.05210	575.61470	Detected	Detected	Detected	Detected
1764	A_24_P206121	KCNMB1	Hs.484099	potassium large conductance calcium-a	Homo sapiens potassium large conducta	4028.32280	3542.66380	717.24414	482.36597	Detected	Detected	Detected	Detected
1765	A_23_P396353	NIN	Hs.310429	ninein (GSK3B interacting protein)	Homo sapiens ninein (GSK3B interactin	787.49250	1055.79500	648.08246	740.06660	Detected	Detected	Detected	Detected
1766	A_33_P3229246	HIST2H2BE	Hs.2178	histone cluster 2, H2be	Homo sapiens histone cluster 2, H2be (	5.17327	29.13987	11.10427	32.70495	Compromised	Detected	Compromised	Detected
1767	A_24_P943613	TBC1D1	Hs.176503	TBC1 (tre-2/USP6, BUB2, cdc16) dom	Homo sapiens TBC1 (tre-2/USP6, BUB	737.70166	2433.79830	498.70430	1396.91500	Detected	Detected	Detected	Detected
1768	A_23_P89431	CCL2	Hs.303649	chemokine (C-C motif) ligand 2	Homo sapiens chemokine (C-C motif) li	1433.46640	353.18146	1353.83640	280.19830	Detected	Detected	Detected	Detected
1769	A_33_P3416583	ARAP1	Hs.503165	ArfGAP with RhoGAP domain, ankyrin r	Homo sapiens ArfGAP with RhoGAP do	63.31400	39.45239	48.04392	26.73786	Detected	Detected	Detected	Detected
1770	A_23_P121602	SAP30	Hs.591715	Sin3A-associated protein, 30kDa	Homo sapiens Sin3A-associated protei	5298.16400	7484.37000	3468.27400	4104.94500	Detected	Detected	Detected	Detected
1771	A_23_P45294	CXorf39	Hs.592223	chromosome X open reading frame 39	Homo sapiens chromosome X open reac	1169.99410	1977.72080	1155.70670	1555.75510	Detected	Detected	Detected	Detected
1772	A_23_P368691	ABCC9	Hs.446050	ATP-binding cassette, sub-family C (CF	Homo sapiens ATP-binding cassette, su	24.10232	44.17260	23.20503	32.13445	Detected	Detected	Detected	Detected
1773	A_23_P47034	HHEX	Hs.118651	hematopoietically expressed homeobox	Homo sapiens hematopoietically expres	123.42711	103.26540	105.80071	79.94048	Detected	Detected	Detected	Detected
1774	A_24_P709377	LOC654433		hypothetical LOC654433	Homo sapiens hypothetical LOC654433	661.29675	434.76140	728.01140	405.76007	Detected	Detected	Detected	Detected
1775	A_33_P3244279		Hs.694999		PRO0756 [Source:UniProtKB/TrEMBL	38.84963	50.13576	32.41941	22.35877	Detected	Detected	Detected	Detected
1776	A_33_P3618429	PRKXP1	Hs.12250	protein kinase, X-linked, pseudogene 1	Homo sapiens cDNA FLJ38474 fis, clon	1432.22000	402.04300	817.58410	210.05856	Detected	Detected	Detected	Detected
1777	A_24_P58177				UPF0317 protein C14orf159, mitochond	80.71615	146.09052	80.97205	130.34671	Detected	Detected	Detected	Detected
1778	A_24_P143127	GRIA3	Hs.377070	glutamate receptor, ionotrophic, AMPA	Homo sapiens glutamate receptor, ionot	48.16047	77.54987	30.73405	46.68222	Detected	Detected	Detected	Detected
1779	A_24_P16124	IFITM4P		interferon induced transmembrane prot	Homo sapiens interferon induced transr	19027.96500	5757.29600	17262.69100	4370.86570	Detected	Detected	Detected	Detected
1780	A_33_P3255814	MRS2	Hs.719256	MRS2 magnesium homeostasis factor h	Homo sapiens MRS2 magnesium homeo	593.03450	754.31630	341.60638	354.92087	Detected	Detected	Detected	Detected
1781	A_23_P82065	ELOVL4	Hs.101915	elongation of very long chain fatty acids	Homo sapiens elongation of very long c	378.43780	258.95245	231.27539	129.06938	Detected	Detected	Detected	Detected
1782	A_23_P92672	OCLN	Hs.592605	occludin	Homo sapiens occludin (OCLN), mRNA	16.91504	4.58487	9.13593	3.34762	Detected	Compromised	Compromised	Compromised
1783	A_33_P3269803	CLSTN3	Hs.535378	calsyntenin 3	Homo sapiens calsyntenin 3 (CLSTN3),	3730.11550	619.33580	4428.73100	965.65900	Detected	Detected	Detected	Detected
1784	A_24_P341187	GBA2	Hs.443134	glucosidase, beta (bile acid) 2	Homo sapiens glucosidase, beta (bile ac	299.52270	156.41878	207.38434	120.17201	Detected	Detected	Detected	Detected
1785	A_33_P3288384	PSMA1	Hs.102798	proteasome (prosome, macropain) subu	Homo sapiens proteasome (prosome, m	377.71854	207.49356	327.07062	151.30500	Detected	Detected	Detected	Detected
1786	A_24_P407930	SEH1L	Hs.301048	SEH1-like (S. cerevisiae)	Homo sapiens SEH1-like (S. cerevisiae)	3112.92970	3209.36520	2603.84520	2303.70730	Detected	Detected	Detected	Detected
1787	A_32_P48559	C16orf92	Hs.651588	chromosome 16 open reading frame 92	Homo sapiens chromosome 16 open rea	17.07506	23.19780	8.87119	16.52107	Detected	Detected	Compromised	Compromised
1788	A_23_P203743	GAB2	Hs.429434	GRB2-associated binding protein 2	Homo sapiens GRB2-associated binding	2877.33900	2769.32900	1908.85380	1669.17760	Detected	Detected	Detected	Detected
1789	A_33_P3274768	LOC100132790	Hs.632969	hypothetical protein LOC100132790	Homo sapiens cDNA FLJ44869 fis, clon	318.47467	471.37628	191.67287	233.16905	Detected	Detected	Detected	Detected
1790	A_24_P353103	ZNF599	Hs.590961	zinc finger protein 599	Homo sapiens zinc finger protein 599 (Z	76.48120	28.26169	51.81128	16.24329	Detected	Detected	Detected	Compromised
1791	A_23_P94494	TLE4	Hs.444213	transducin-like enhancer of split 4 (E(sp	Homo sapiens transducin-like enhancer	118.18448	168.53250	73.70315	93.24374	Detected	Detected	Detected	Detected
1792	A_23_P5018	LOC80054	Hs.287521	hypothetical LOC80054	Homo sapiens hypothetical LOC80054 (	29.94474	3.92553	33.00670	5.84772	Detected	Compromised	Detected	Compromised
1793	A_24_P79054	TGFB1	Hs.645227	transforming growth factor, beta 1	Homo sapiens transforming growth fact	1290.80900	2370.60330	1484.78210	2797.66110	Detected	Detected	Detected	Detected
1794	A_23_P124619	S100A14	Hs.288998	S100 calcium binding protein A14	Homo sapiens S100 calcium binding pro	55.32674	25.46544	33.82554	18.56610	Detected	Detected	Detected	Detected
1795	A_33_P3389178				Rap guanine nucleotide exchange factor	1578.32640	1274.01830	510.92993	434.40662	Detected	Detected	Detected	Detected
1796	A_33_P3244728	LRP2	Hs.657729	low density lipoprotein-related protein 2	Homo sapiens low density lipoprotein-re	583.19740	2571.84080	190.76208	992.82770	Detected	Detected	Detected	Detected

1797	A_23_P10873	TLR1	Hs.654532	toll-like receptor 1	Homo sapiens toll-like receptor 1 (TLR	35.10439	63.92487	20.99570	34.76000	Detected	Detected	Detected	Detected
1798	A_24_P108863	SCML1	Hs.109655	sex comb on midleg-like 1 (Drosophila)	Homo sapiens sex comb on midleg-like	693.87770	863.99550	510.25116	525.26110	Detected	Detected	Detected	Detected
1799	A_33_P3246068	ROBLD3	Hs.632483	roadblock domain containing 3	Homo sapiens roadblock domain contai	12241.79000	13985.44800	9603.08000	9322.13900	Detected	Detected	Detected	Detected
1800	A_23_P5551	NCL	Hs.79110	nucleolin	Homo sapiens nucleolin (NCL), mRNA [	51256.07400	76580.09000	39422.88000	51306.00400	Detected	Detected	Detected	Detected
1801	A_33_P3385101	TOLLIP	Hs.719949	toll interacting protein	Homo sapiens toll interacting protein (T	11121.61100	19188.19000	11112.55000	16994.37500	Detected	Detected	Detected	Detected
1802	A_33_P3254650	LOC728353	Hs.621210	hypothetical protein LOC728353	PREDICTED: Homo sapiens hypothetica	24.96817	99.68270	19.35469	65.61013	Detected	Detected	Detected	Detected
1803	A_24_P780052	RPSA	Hs.449909	ribosomal protein SA	Homo sapiens ribosomal protein SA (RF	58413.18000	115889.75000	40555.27300	64196.50800	Detected	Detected	Detected	Detected
1804	A_23_P76690	UCHL3	Hs.162241	ubiquitin carboxyl-terminal esterase L3	Homo sapiens ubiquitin carboxyl-termin	3284.78100	8253.27900	2586.29100	5182.47700	Detected	Detected	Detected	Detected
1805	A_24_P721699	NCRNA00181		non-protein coding RNA 181	Homo sapiens non-protein coding RNA	493.78482	1057.26450	583.48004	1120.73240	Detected	Detected	Detected	Detected
1806	A_33_P3389336	LOC100129531	Hs.712924	hypothetical LOC100129531	PREDICTED: Homo sapiens hypothetica	15.02970	14.23063	19.59397	9.55707	Compromised	Compromised	Detected	Compromised
1807	A_23_P211627	NUP50	Hs.475103	nucleoporin 50kDa	Homo sapiens nucleoporin 50kDa (NUP	189.14912	496.11038	194.19499	407.95926	Detected	Detected	Detected	Detected
1808	A_23_P144165	DZIP3	Hs.409210	DAZ interacting protein 3, zinc finger	Homo sapiens DAZ interacting protein 3	935.79486	542.39910	703.36180	319.88430	Detected	Detected	Detected	Detected
1809	A_24_P169092	MAML2	Hs.428214	mastermind-like 2 (Drosophila)	Homo sapiens mastermind-like 2 (Droso	101.07880	102.58917	91.69740	86.83594	Detected	Detected	Detected	Detected
1810	A_33_P3379116					51.51944	70.92008	25.49731	36.73567	Detected	Detected	Detected	Detected
1811	A_33_P3351092	KRTAP20-2	Hs.553698	keratin associated protein 20-2	Homo sapiens keratin associated protei	905.42334	1318.05320	305.45540	332.66733	Detected	Detected	Detected	Detected
1812	A_23_P20852	AUH	Hs.175905	AU RNA binding protein/enoyl-Coenzyr	Homo sapiens AU RNA binding protein/	2142.00050	830.17700	1548.35180	526.14795	Detected	Detected	Detected	Detected
1813	A_33_P3413168				Collagen alpha-1(XXVII) chain Precurso	22.28613	26.80419	7.90837	9.78146	Detected	Detected	Compromised	Compromised
1814	A_23_P10463	NDUFS5	Hs.632385	NADH dehydrogenase (ubiquinone) Fe-S	Homo sapiens NADH dehydrogenase (ul	103601.30500	70526.55000	84266.80000	49350.83600	Detected	Detected	Detected	Detected
1815	A_23_P250571	DMXL1	Hs.181042	Dmx-like 1	Homo sapiens Dmx-like 1 (DMXL1), mR	637.77050	489.72086	418.60010	261.58597	Detected	Detected	Detected	Detected
1816	A_23_P10081	ARL13B	Hs.533086	ADP-ribosylation factor-like 13B	Homo sapiens ADP-ribosylation factor-	286.93124	266.37692	213.24135	167.49606	Detected	Detected	Detected	Detected
1817	A_23_P201264	NMNAT1	Hs.633762	nicotinamide nucleotide adenylyltransfe	Homo sapiens nicotinamide nucleotide a	248.75208	360.21700	190.87448	234.58682	Detected	Detected	Detected	Detected
1818	A_23_P31725	BLK	Hs.146591	B lymphoid tyrosine kinase	Homo sapiens B lymphoid tyrosine kina	10.29856	36.02604	11.48014	38.27507	Compromised	Detected	Compromised	Detected
1819	A_33_P3327470				Putative uncharacterized protein NTF6	45.08403	66.85687	18.76889	26.62400	Detected	Detected	Detected	Detected
1820	A_23_P204472	RPLP0	Hs.546285	ribosomal protein, large, P0	Homo sapiens ribosomal protein, large,	212989.14000	157549.30000	159896.98000	103470.17000	Detected	Detected	Detected	Detected
1821	A_24_P551028	SPOPL	Hs.333297	speckle-type POZ protein-like	Homo sapiens speckle-type POZ protei	1246.57290	779.68300	952.15515	495.50864	Detected	Detected	Detected	Detected
1822	A_23_P125643	ASB9	Hs.19404	ankyrin repeat and SOCS box-containin	Homo sapiens ankyrin repeat and SOCS	135.25587	63.36802	88.68623	34.92737	Detected	Detected	Detected	Detected
1823	A_33_P3215134	ZNF777	Hs.38512	zinc finger protein 777	Homo sapiens zinc finger protein 777 (Z	699.60910	1089.39070	363.30570	514.01263	Detected	Detected	Detected	Detected
1824	A_33_P3283350					23.06857	30.64400	14.71290	17.99553	Detected	Detected	Compromised	Detected
1825	A_33_P3334404		Hs.100322		Carbonic anhydrase 6 Precursor (EC 4.	14.74625	13.58789	7.52860	5.19435	Detected	Compromised	Compromised	Compromised
1826	A_24_P244162	PI4K2A	Hs.25300	phosphatidylinositol 4-kinase type 2 alp	Homo sapiens phosphatidylinositol 4-ki	2617.14450	3328.04350	3102.62520	3703.96220	Detected	Detected	Detected	Detected
1827	A_24_P345002	NUDT11	Hs.200016	nudix (nucleoside diphosphate linked m	Homo sapiens nudix (nucleoside diphos	258.90982	204.88261	203.38452	140.89168	Detected	Detected	Detected	Detected
1828	A_33_P3388855	LRPPRC	Hs.368084	leucine-rich PPR-motif containing	Homo sapiens leucine-rich PPR-motif c	139.12960	114.20241	94.59354	75.48550	Detected	Detected	Detected	Detected
1829	A_24_P415327	HPS1	Hs.404568	Hermansky-Pudlak syndrome 1	Homo sapiens Hermansky-Pudlak syndr	3080.81250	1971.10380	2522.89280	1539.03420	Detected	Detected	Detected	Detected
1830	A_23_P156826	C6orf105	Hs.126409	chromosome 6 open reading frame 105	Homo sapiens chromosome 6 open rea	330.42178	1415.80820	223.17432	781.10060	Detected	Detected	Detected	Detected
1831	A_33_P3305536		Hs.243326		Putative S-adenosyl-L-methionine-dep	126.98888	76.77132	115.93800	51.57267	Detected	Detected	Detected	Detected
1832	A_33_P3284029	CSE1L	Hs.90073	CSE1 chromosome segregation 1-like (	Homo sapiens CSE1 chromosome segre	4353.30220	10337.05800	4588.51500	8636.16900	Detected	Detected	Detected	Detected
1833	A_23_P209477	PPM1G	Hs.643951	protein phosphatase 1G (formerly 2C),	Homo sapiens protein phosphatase 1G	5933.71730	7617.88100	4877.57200	5835.15870	Detected	Detected	Detected	Detected
1834	A_32_P56143	LOC100133109	Hs.719492	hypothetical LOC100133109	PREDICTED: Homo sapiens hypothetica	23.87510	20.11610	21.28090	13.06065	Detected	Detected	Detected	Compromised
1835	A_33_P3315554	ANKMY1	Hs.656615	ankyrin repeat and MYND domain conta	Homo sapiens ankyrin repeat and MYND	498.42090	131.41740	478.09375	112.27869	Detected	Detected	Detected	Detected
1836	A_23_P102404	CCT7	Hs.368149	chaperonin containing TCP1, subunit 7	Homo sapiens chaperonin containing T	15536.79000	26748.78500	13378.39000	20490.86000	Detected	Detected	Detected	Detected
1837	A_32_P164246	FOXQ1	Hs.591352	forkhead box Q1	Homo sapiens forkhead box Q1 (FOXQ1	2959.93200	6949.03120	2403.73460	4792.34700	Detected	Detected	Detected	Detected
1838	A_23_P27346	SMAD4	Hs.75862	SMAD family member 4	Homo sapiens SMAD family member 4 (	355.12833	351.53937	250.68706	186.94841	Detected	Detected	Detected	Detected
1839	A_33_P3346331				Putative uncharacterized protein ENSP	37.21508	567.70900	38.32880	418.44406	Detected	Detected	Detected	Detected
1840	A_23_P132669	GLT8D1	Hs.297304	glycosyltransferase 8 domain containing	Homo sapiens glycosyltransferase 8 do	5895.04700	5652.34900	6451.01560	5191.43600	Detected	Detected	Detected	Detected
1841	A_33_P3400688	SLC24A5	Hs.710240	solute carrier family 24, member 5	Homo sapiens solute carrier family 24,	38.08117	36.04252	5.79815	5.52586	Detected	Detected	Compromised	Compromised
1842	A_32_P32653	SENP5	Hs.240770	SUMO1/sentrin specific peptidase 5	Homo sapiens SUMO1/sentrin specific	4112.91700	2561.58250	3105.13230	1615.16520	Detected	Detected	Detected	Detected
1843	A_23_P432545	EFCAB4A	Hs.660936	EF-hand calcium binding domain 4A	Homo sapiens EF-hand calcium binding	18437.73600	10854.79600	4819.89200	2329.23780	Detected	Detected	Detected	Detected
1844	A_23_P101655	ACTN4	Hs.270291	actinin, alpha 4	Homo sapiens actinin, alpha 4 (ACTN4),	1384.12010	2922.57890	1210.31430	2468.40500	Detected	Detected	Detected	Detected
1845	A_23_P67748	RAB11B	Hs.626404	RAB11B, member RAS oncogene family	Homo sapiens RAB11B, member RAS o	102.79363	114.53642	111.22652	120.16251	Detected	Detected	Detected	Detected
1846	A_23_P202245	RET	Hs.350321	ret proto-oncogene	Homo sapiens ret proto-oncogene (RET	21.89975	4.04819	14.71816	3.58042	Detected	Compromised	Compromised	Compromised
1847	A_23_P426021	SEL1L3	Hs.479384	sel-1 suppressor of lin-12-like 3 (C. ele	Homo sapiens sel-1 suppressor of lin-1	551.32385	330.48514	333.30170	172.53539	Detected	Detected	Detected	Detected
1848	A_23_P118392	RASD1	Hs.25829	RAS, dexamethasone-induced 1	Homo sapiens RAS, dexamethasone-in	29303.37900	5485.62060	15891.10400	2739.84570	Detected	Detected	Detected	Detected
1849	A_23_P410725	ZNF491	Hs.631634	zinc finger protein 491	Homo sapiens zinc finger protein 491 (Z	27.32645	33.94445	9.86071	10.13777	Detected	Detected	Compromised	Compromised
1850	A_32_P233304	LIN9	Hs.120817	lin-9 homolog (C. elegans)	Homo sapiens lin-9 homolog (C. elegans	1239.48410	2969.53880	950.16266	1871.15430	Detected	Detected	Detected	Detected
1851	A_23_P83149	FAM108B1	Hs.380389	family with sequence similarity 108, me	Homo sapiens family with sequence sim	48.55465	73.25330	41.86822	59.20858	Detected	Detected	Detected	Detected
1852	A_24_P93896	CNNM2	Hs.719266	cyclin M2	Homo sapiens cyclin M2 (CNNM2), tran	119.05457	93.89989	66.21830	39.98264	Detected	Detected	Detected	Detected
1853	A_23_P29495	CTNNB1	Hs.476018	catenin (cadherin-associated protein), b	Homo sapiens catenin (cadherin-associ	12186.83800	9199.78300	7340.79500	4663.45750	Detected	Detected	Detected	Detected
1854	A_33_P3252394	GADD45G	Hs.9701	growth arrest and DNA-damage-inducib	Homo sapiens growth arrest and DNA-	1010.35004	231.38281	808.46600	134.47029	Detected	Detected	Detected	Detected
1855	A_33_P3332871		Hs.676335		601279876F1 NIH_MGC_39 Homo sapie	28.20323	26.37890	18.10532	12.37204	Detected	Detected	Detected	Compromised
1856	A_33_P3285334	LOC100128838	Hs.200644	hypothetical LOC100128838	PREDICTED: Homo sapiens hypothetica	34.93885	28.42328	16.56649	18.71813	Detected	Detected	Compromised	Detected
1857	A_23_P250212	PRAGMIN	Hs.657673	homolog of rat pragma of Rnd2	Homo sapiens homolog of rat pragma o	697.69700	1733.92370	574.19714	1258.92910	Detected	Detected	Detected	Detected
1858	A_23_P155830	TBC1D14	Hs.518611	TBC1 domain family, member 14	Homo sapiens TBC1 domain family, me	2703.01250	2223.94020	2979.68900	2274.47000	Detected	Detected	Detected	Detected
1859	A_33_P3230528				Myosin phosphatase Rho-interacting pr	1367.61740	1455.95840	952.71870	810.84290	Detected	Detected	Detected	Detected
1860	A_33_P3308432				MORN repeat-containing protein 1 [So	70.05751	135.03882	71.07686	124.02789	Detected	Detected	Detected	Detected
1861	A_23_P334608	GUSB	Hs.255230	glucuronidase, beta	Homo sapiens glucuronidase, beta (GUS	18418.63500	12044.09900	12580.59700	7244.47850	Detected	Detected	Detected	Detected
1862	A_23_P415558	ZNF212	Hs.490510	zinc finger protein 212	Homo sapiens zinc finger protein 212 (Z	1271.72460	1453.36880	901.79170	969.18646	Detected	Detected	Detected	Detected
1863	A_23_P110412	TMEM150C	Hs.720835	transmembrane protein 150C	Homo sapiens transmembrane protein 1	68.57480	23.15794	55.91653	8.58880	Detected	Not Detected	Detected	Compromised
1864	A_32_P226009	C5orf42	Hs.586199	chromosome 5 open reading frame 42	Homo sapiens chromosome 5 open rea	152.86406	287.68713	94.25620	176.49504	Detected	Detected	Detected	Detected
1865	A_33_P3383524	LOC91948	Hs.130423	hypothetical LOC91948	Homo sapiens hypothetical LOC91948 (	668.35910	558.51935	70.63525	38.09009	Detected	Detected	Detected	Detected
1866	A_23_P212800	FGF5	Hs.37055	fibroblast growth factor 5	Homo sapiens fibroblast growth factor 5	371.24854	1640.06020	315.40643	1135.95400	Detected	Detected	Detected	Detected
1867	A_32_P184518	RPL21	Hs.381123	ribosomal protein L21	Homo sapiens ribosomal protein L21 (R	150991.64000	138306.45000	116270.50000	83589.39000	Detected	Detected	Detected	Detected
1868	A_23_P49041	TMEM62	Hs.511175	transmembrane protein 62	Homo sapiens transmembrane protein 6	409.19543	596.05960	435.29540	589.90200	Detected	Detected	Detected	Detected
1869	A_23_P253921	ZNF167	Hs.529512	zinc finger protein 167	Homo sapiens zinc finger protein 167 (Z	175.08075	163.88220	132.21608	115.90413	Detected	Detected	Detected	Detected
1870	A_33_P3230290	ACAP3	Hs.535257	ArfGAP with coiled-coil, ankyrin repeat	Homo sapiens ArfGAP with coiled-coil,	9415.53000	9779.57500	5090.08740	3898.65190	Detected	Detected	Detected	Detected
1871	A_23_P115105	C1orf144	Hs.252967	chromosome 1 open reading frame 144	Homo sapiens chromosome 1 open rea	3118.76680	10141.88900	2434.52700	6805.19600	Detected	Detected	Detected	Detected

1872	A_23_P74653	NUDC	Hs.263812	nuclear distribution gene C homolog (A.	Homo sapiens nuclear distribution gene	8950.19600	14143.09600	10148.82300	14332.95800	Detected	Detected	Detected	Detected
1873	A_33_P3345309	LOC646960	Hs.570310	similar to hCG1818432	PREDICTED: Homo sapiens similar to h	35.36649	21.87631	10.50267	7.54128	Detected	Detected	Compromised	Compromised
1874	A_23_P16722	DOCK10	Hs.46578	dedicator of cytokinesis 10	Homo sapiens dedicator of cytokinesis	659.40796	1582.60460	579.55835	1217.26280	Detected	Detected	Detected	Detected
1875	A_23_P93464	BCKDHB	Hs.654441	branched chain keto acid dehydrogenas	Homo sapiens branched chain keto acid	1122.86800	927.35960	947.06555	684.23830	Detected	Detected	Detected	Detected
1876	A_23_P128408	TRIAP1	Hs.69499	TP53 regulated inhibitor of apoptosis 1	Homo sapiens TP53 regulated inhibitor	2188.28200	3030.79030	2803.24070	3238.21920	Detected	Detected	Detected	Detected
1877	A_33_P3252177	EID2B	Hs.135181	EP300 interacting inhibitor of differentia	Homo sapiens EP300 interacting inhibito	91.24942	137.98302	125.26244	167.92438	Detected	Detected	Detected	Detected
1878	A_33_P3322814	NTRK3	Hs.410969	neurotrophic tyrosine kinase, receptor,	Homo sapiens neurotrophic tyrosine kin	287.61640	57.43557	213.15840	36.75718	Detected	Detected	Detected	Detected
1879	A_33_P3304252	MRPL32	Hs.50252	mitochondrial ribosomal protein L32	Homo sapiens mitochondrial ribosomal p	6190.57470	5038.18000	3675.80960	2587.28120	Detected	Detected	Detected	Detected
1880	A_33_P3269069	HS2ST1	Hs.719144	heparan sulfate 2-O-sulfotransferase 1	Homo sapiens heparan sulfate 2-O-sulf	165.29790	324.51065	113.02361	165.68768	Detected	Detected	Detected	Detected
1881	A_23_P107963	FUT1	Hs.69747	fucosyltransferase 1 (galactoside 2-alph	Homo sapiens fucosyltransferase 1 (gal	5.72921	7.71060	7.45843	18.88900	Compromised	Compromised	Compromised	Detected
1882	A_23_P3849	TRAP1	Hs.30345	TNF receptor-associated protein 1	Homo sapiens TNF receptor-associated	2429.43900	2359.10400	2157.28170	2197.77600	Detected	Detected	Detected	Detected
1883	A_33_P3306413	INCA1	Hs.658926	inhibitor of CDK, cyclin A1 interacting p	Homo sapiens inhibitor of CDK, cyclin A	77.87960	25.40955	107.72173	41.96820	Detected	Detected	Detected	Detected
1884	A_33_P3256957	LOC100128402	Hs.689574	hypothetical protein LOC100128402	Homo sapiens cDNA FLJ42583 fis, clon	13.65084	5.72396	7.38090	3.76501	Detected	Compromised	Compromised	Compromised
1885	A_23_P112652	CNOT10	Hs.444851	CCR4-NOT transcription complex, subu	Homo sapiens CCR4-NOT transcription	1792.86150	1658.67990	1263.33830	1003.52673	Detected	Detected	Detected	Detected
1886	A_23_P415401	KLF9	Hs.150557	Kruppel-like factor 9	Homo sapiens Kruppel-like factor 9 (KL	29898.14500	13526.10800	27647.41600	10676.27050	Detected	Detected	Detected	Detected
1887	A_33_P3369133	LOC100134230	Hs.568247	hypothetical protein LOC100134230	Myomegalin (Phosphodiesterase 4D-int	66.51432	123.54601	64.09679	101.55032	Detected	Detected	Detected	Detected
1888	A_23_P316239	CXorf40B	Hs.545950	chromosome X open reading frame 40B	Homo sapiens chromosome X open rea	5192.52400	6195.09400	4948.14060	5398.71500	Detected	Detected	Detected	Detected
1889	A_24_P391586	OAF	Hs.445081	OAF homolog (Drosophila)	Homo sapiens OAF homolog (Drosophila	2569.19170	3642.67100	2056.79960	2662.14450	Detected	Detected	Detected	Detected
1890	A_33_P3222783	SURF4	Hs.512465	surfeit 4	Homo sapiens surfeit 4 (SURF4), mRNA	1676.78830	4457.88800	1317.89900	3451.89280	Detected	Detected	Detected	Detected
1891	A_23_P101950	MDH1	Hs.526521	malate dehydrogenase 1, NAD (soluble)	Homo sapiens malate dehydrogenase 1,	31279.00800	43145.31600	22061.52700	24245.81000	Detected	Detected	Detected	Detected
1892	A_33_P3321993					18.73614	6.55114	10.18288	4.82046	Detected	Compromised	Compromised	Compromised
1893	A_23_P113789	USP19	Hs.255596	ubiquitin specific peptidase 19	Homo sapiens ubiquitin specific peptida	802.36460	424.79114	505.96472	274.97726	Detected	Detected	Detected	Detected
1894	A_33_P3412538	ANKRD17	Hs.719092	ankyrin repeat domain 17	Homo sapiens ankyrin repeat domain 17	3277.98100	2488.35450	2686.37720	1602.67050	Detected	Detected	Detected	Detected
1895	A_32_P167076	CAPN14	Hs.468059	calpain 14	Homo sapiens calpain 14 (CAPN14), mR	14.86231	7.20775	4.10426	5.65117	Detected	Compromised	Compromised	Compromised
1896	A_23_P215956	MYC	Hs.202453	v-myc myelocytomatosis viral oncogene	Homo sapiens v-myc myelocytomatosis	14814.03700	11366.35300	13425.91900	8388.20400	Detected	Detected	Detected	Detected
1897	A_23_P10518	TFDP3	Hs.142908	transcription factor Dp family, member	Homo sapiens transcription factor Dp fa	67.03096	126.81033	14.77768	20.58058	Detected	Detected	Detected	Detected
1898	A_23_P344884	ATPGD1	Hs.502982	ATP-grasp domain containing 1	Homo sapiens ATP-grasp domain conta	98.13254	18.85325	80.57062	14.82761	Detected	Compromised	Detected	Compromised
1899	A_23_P64204	ZW10	Hs.503886	ZW10, kinetochore associated, homolog	Homo sapiens ZW10, kinetochore assoc	1119.24730	1731.89160	817.83057	1104.12040	Detected	Detected	Detected	Detected
1900	A_33_P3793702	LOC157381	Hs.651593	hypothetical LOC157381	Homo sapiens hypothetical LOC157381	374.19897	429.59030	144.66296	138.78416	Detected	Detected	Detected	Detected
1901	A_33_P3223121	HIPK2	Hs.397465	homeodomain interacting protein kinase	Homo sapiens homeodomain interacting	147.78836	221.40230	149.28952	168.73683	Detected	Detected	Detected	Detected
1902	A_24_P339071	CDR2	Hs.513430	cerebellar degeneration-related protein	Homo sapiens cerebellar degeneration-	805.32600	1190.62830	748.46027	946.43410	Detected	Detected	Detected	Detected
1903	A_23_P82738	RAD54B	Hs.30561	RAD54 homolog B (S. cerevisiae)	Homo sapiens RAD54 homolog B (S. ce	142.36089	552.97473	109.51458	363.11770	Detected	Detected	Detected	Detected
1904	A_23_P70409	POLR1C	Hs.584839	polymerase (RNA) I polypeptide C, 30kD	Homo sapiens polymerase (RNA) I polyp	3287.22270	2949.68500	2881.96850	2365.28120	Detected	Detected	Detected	Detected
1905	A_24_P4816	GABARAPL1	Hs.524250	GABA(A) receptor-associated protein l	Homo sapiens GABA(A) receptor-assoc	3703.68300	2591.34640	2862.17630	1867.02030	Detected	Detected	Detected	Detected
1906	A_23_P119789	TMEM185B	Hs.376722	transmembrane protein 185B (pseudoge	Homo sapiens transmembrane protein 1	385.83105	606.92430	350.41550	522.78230	Detected	Detected	Detected	Detected
1907	A_23_P26037	FRMD5	Hs.578544	FERM domain containing 5	Homo sapiens FERM domain containing	30.18117	219.72450	20.69545	93.19758	Detected	Detected	Detected	Detected
1908	A_23_P142289	GNA11	Hs.650575	guanine nucleotide binding protein (G pr	Homo sapiens guanine nucleotide bindin	2633.38260	3929.70310	3200.77200	4509.65870	Detected	Detected	Detected	Detected
1909	A_23_P97195	FAM54B	Hs.323396	family with sequence similarity 54, mem	Homo sapiens family with sequence sim	1505.84580	1288.03610	1391.49130	1002.03394	Detected	Detected	Detected	Detected
1910	A_23_P56228	GMIP	Hs.49427	GEM interacting protein	Homo sapiens GEM interacting protein (	821.48900	1005.88116	862.09570	1076.90470	Detected	Detected	Detected	Detected
1911	A_24_P945113	ACVRL1	Hs.591026	activin A receptor type II-like 1	Homo sapiens activin A receptor type II	13233.51700	5614.27700	15768.31900	6260.98140	Detected	Detected	Detected	Detected
1912	A_24_P273043					169.45938	201.29596	3.54343	3.63374	Detected	Detected	Compromised	Compromised
1913	A_24_P822704	TMEM198	Hs.446664	transmembrane protein 198	Homo sapiens transmembrane protein 1	457.51315	120.12550	441.14774	110.83108	Detected	Detected	Detected	Detected
1914	A_33_P3216090	SHC3	Hs.656806	SHC (Src homology 2 domain containin	Homo sapiens SHC (Src homology 2 do	606.78467	25.71041	739.49786	27.65789	Detected	Detected	Detected	Detected
1915	A_23_P202658	GSTP1	Hs.523836	glutathione S-transferase pi 1	Homo sapiens glutathione S-transferas	90616.33600	133079.84000	92509.90000	117240.64000	Detected	Detected	Detected	Detected
1916	A_24_P108451	GPI	Hs.466471	glucose phosphate isomerase	Homo sapiens glucose phosphate isome	29175.50400	52518.47000	16594.79500	31945.63500	Detected	Detected	Detected	Detected
1917	A_23_P49412	PRR14	Hs.293629	proline rich 14	Homo sapiens proline rich 14 (PRR14),	1741.34280	1900.04650	1960.01550	1867.75220	Detected	Detected	Detected	Detected
1918	A_33_P3399571	VNN1	Hs.12114	vanin 1	Homo sapiens vanin 1 (VNN1), mRNA [N	96.48717	28.80472	64.80814	18.78009	Detected	Detected	Detected	Detected
1919	A_23_P89931	ZNF574	Hs.13323	zinc finger protein 574	Homo sapiens zinc finger protein 574 (Z	846.95020	889.16455	907.88420	914.58000	Detected	Detected	Detected	Detected
1920	A_23_P359430	NBPF14	Hs.607640	neuroblastoma breakpoint family, memb	Homo sapiens neuroblastoma breakpoin	3577.44580	3867.38550	2835.49000	2685.52120	Detected	Detected	Detected	Detected
1921	A_23_P212617	TFRC	Hs.529618	transferrin receptor (p90, CD71)	Homo sapiens transferrin receptor (p90	6975.37000	11855.03000	5845.83800	9051.54000	Detected	Detected	Detected	Detected
1922	A_24_P842006	C16orf93	Hs.65735	chromosome 16 open reading frame 93	Homo sapiens chromosome 16 open rea	65.16528	107.06187	65.70547	90.33307	Detected	Detected	Detected	Detected
1923	A_33_P3240912	MAGT1	Hs.323562	magnesium transporter 1	Homo sapiens magnesium transporter 1	253.86395	360.44280	267.33145	314.34512	Detected	Detected	Detected	Detected
1924	A_33_P3759737	LOC100287050	Hs.652076	hypothetical protein LOC100287050	PREDICTED: Homo sapiens hypothetica	34.33199	36.51983	10.15560	7.92077	Detected	Detected	Compromised	Compromised
1925	A_33_P3358601				CD225 family protein FLJ76511 [Sourc	1804.15690	1070.00800	1519.03380	793.92940	Detected	Detected	Detected	Detected
1926	A_33_P3401084					2237.34350	3358.59060	1767.64620	1838.10770	Detected	Detected	Detected	Detected
1927	A_23_P93032	ZBED3	Hs.584988	zinc finger, BED-type containing 3	Homo sapiens zinc finger, BED-type co	749.30880	498.80190	651.48220	367.26620	Detected	Detected	Detected	Detected
1928	A_23_P118353	SRP68	Hs.514495	signal recognition particle 68kDa	Homo sapiens signal recognition particle	2275.71680	3002.14670	1726.02280	2061.44920	Detected	Detected	Detected	Detected
1929	A_24_P210569	SCG3	Hs.232618	secretogranin III	Homo sapiens secretogranin III (SCG3),	24.87489	22.84964	9.41887	9.28441	Detected	Detected	Compromised	Compromised
1930	A_23_P108028	ZNF146	Hs.643436	zinc finger protein 146	Homo sapiens zinc finger protein 146 (Z	753.25650	580.34235	588.61847	348.60324	Detected	Detected	Detected	Detected
1931	A_33_P3343467	FLJ35390		hypothetical LOC255031	Homo sapiens hypothetical LOC255031	121.47746	72.06722	97.39436	35.84551	Detected	Detected	Detected	Detected
1932	A_33_P3219105					13725.72400	12710.51400	6157.56100	4353.25050	Detected	Detected	Detected	Detected
1933	A_33_P3214884	KPNA3	Hs.527919	karyopherin alpha 3 (importin alpha 4)	Homo sapiens karyopherin alpha 3 (imp	12107.22500	15522.00800	8228.29700	8617.64100	Detected	Detected	Detected	Detected
1934	A_33_P3246133	HPCA	Hs.632391	hippocalcin	Homo sapiens hippocalcin (HPCA), mRN	17.34007	7.15411	15.09058	6.85082	Detected	Compromised	Compromised	Compromised
1935	A_24_P363896	COL27A1	Hs.494892	collagen, type XXVII, alpha 1	Homo sapiens collagen, type XXVII, alph	1393.91710	2196.72300	713.99994	981.78436	Detected	Detected	Detected	Detected
1936	A_23_P209778	POLR2D	Hs.554831	polymerase (RNA) II (DNA directed) pol	Homo sapiens polymerase (RNA) II (DN	1183.40390	3806.24490	1018.74790	3001.87700	Detected	Detected	Detected	Detected
1937	A_33_P3418400	ATG10	Hs.713698	ATG10 autophagy related 10 homolog (	Homo sapiens ATG10 autophagy relate	22.00236	19.53854	11.40840	14.57910	Detected	Detected	Compromised	Compromised
1938	A_33_P3383611	KCP	Hs.371746	kielin/chordin-like protein	Homo sapiens kielin/chordin-like protei	485.41943	509.76233	150.06776	109.23431	Detected	Detected	Detected	Detected
1939	A_23_P83159	KLHL9	Hs.522029	kelch-like 9 (Drosophila)	Homo sapiens kelch-like 9 (Drosophila)	5352.83840	6235.31200	4220.41460	4306.38000	Detected	Detected	Detected	Detected
1940	A_33_P3323564	LOC646324	Hs.655713	hypothetical LOC646324	Homo sapiens cDNA FLJ42907 fis, clon	27.79241	143.91875	21.90435	132.54730	Detected	Detected	Detected	Detected
1941	A_23_P105227	ME3	Hs.199743	malic enzyme 3, NADP(+)-dependent, m	Homo sapiens malic enzyme 3, NADP(+	66.25468	74.92918	63.00586	62.41366	Detected	Detected	Detected	Detected
1942	A_23_P128940	C14orf138	Hs.558541	chromosome 14 open reading frame 138	Homo sapiens chromosome 14 open rea	606.02870	633.60410	521.82450	535.17170	Detected	Detected	Detected	Detected
1943	A_23_P219197	RGS3	Hs.494875	regulator of G-protein signaling 3	Homo sapiens regulator of G-protein si	4962.77340	16170.66600	4110.55220	11551.05600	Detected	Detected	Detected	Detected
1944	A_24_P56052	ZFP91	Hs.524920	zinc finger protein 91 homolog (mouse)	Homo sapiens zinc finger protein 91 ho	2729.56150	3426.40800	1953.51050	2101.70430	Detected	Detected	Detected	Detected
1945	A_23_P34827	HCN3	Hs.706960	hyperpolarization activated cyclic nucle	Homo sapiens hyperpolarization activat	159.80170	89.35528	113.56823	38.83109	Detected	Detected	Detected	Detected
1946	A_23_P12079	KCNC4	Hs.153521	potassium voltage-gated channel, Shaw	Homo sapiens potassium voltage-gated	46.86871	243.07242	44.27237	230.51590	Detected	Detected	Detected	Detected

1947	A_33_P3378284	COX6B2	Hs.550544	cytochrome c oxidase subunit VIb polyp	Homo sapiens cytochrome c oxidase su	11661.04100	20412.81200	8021.78960	10407.10800	Detected	Detected	Detected	Detected
1948	A_24_P250922	PTGS2	Hs.196384	prostaglandin-endoperoxide synthase 2	Homo sapiens prostaglandin-endoperox	1656.07690	209.32854	1191.17150	119.12826	Detected	Detected	Detected	Detected
1949	A_23_P119141	KEAP1	Hs.465870	kelch-like ECH-associated protein 1	Homo sapiens kelch-like ECH-associate	6995.20200	7442.51300	5038.00340	4935.88000	Detected	Detected	Detected	Detected
1950	A_23_P210425	MYL9	Hs.504687	myosin, light chain 9, regulatory	Homo sapiens myosin, light chain 9, reg	2269.80050	7691.10200	2354.34470	7738.79100	Detected	Detected	Detected	Detected
1951	A_23_P71981	ERAL1	Hs.3426	Era G-protein-like 1 (E. coli)	Homo sapiens Era G-protein-like 1 (E.	446.10397	784.46550	564.83980	939.99524	Detected	Detected	Detected	Detected
1952	A_23_P34527	FLAD1	Hs.118666	FAD1 flavin adenine dinucleotide synthe	Homo sapiens FAD1 flavin adenine dinu	1229.99830	1440.91560	1158.48330	1230.69850	Detected	Detected	Detected	Detected
1953	A_32_P136402	THOC7	Hs.288151	THO complex 7 homolog (Drosophila)	Homo sapiens THO complex 7 homolog	9992.63000	11214.15600	7977.62350	7496.72900	Detected	Detected	Detected	Detected
1954	A_33_P3593774	PIK3R3	Hs.655387	phosphoinositide-3-kinase, regulatory s	Homo sapiens phosphoinositide-3-kinas	32.12064	38.71846	15.92576	26.66664	Detected	Detected	Compromised	Detected
1955	A_24_P282210	SOLH	Hs.632219	small optic lobes homolog (Drosophila)	Homo sapiens small optic lobes homolo	5616.20360	7545.59130	4847.92200	5431.04440	Detected	Detected	Detected	Detected
1956	A_23_P56380	ZC3H15	Hs.719934	zinc finger CCCH-type containing 15	Homo sapiens zinc finger CCCH-type c	9943.81000	10347.44900	7799.12840	6634.42200	Detected	Detected	Detected	Detected
1957	A_24_P915806	HNMT	Hs.42151	histamine N-methyltransferase	Homo sapiens histamine N-methyltrans	259.10245	176.05247	245.48390	131.50739	Detected	Detected	Detected	Detected
1958	A_33_P3383233	NDRG2	Hs.525205	NDRG family member 2	Homo sapiens NDRG family member 2 (	534.21844	104.13041	450.45462	81.04186	Detected	Detected	Detected	Detected
1959	A_23_P114353	NXF5	Hs.307077	nuclear RNA export factor 5	Homo sapiens nuclear RNA export facto	14.83041	14.75797	4.77193	3.98723	Detected	Detected	Compromised	Compromised
1960	A_23_P50678	MATK	Hs.631845	megakaryocyte-associated tyrosine kin	Homo sapiens megakaryocyte-associate	20.40160	12.65557	4.19567	3.68624	Detected	Compromised	Compromised	Compromised
1961	A_23_P104876	SPA17	Hs.286233	sperm autoantigenic protein 17	Homo sapiens sperm autoantigenic prot	1185.80800	1804.85830	1327.59240	1693.90940	Detected	Detected	Detected	Detected
1962	A_33_P3326025				KN motif and ankyrin repeat domain-co	2930.47360	6972.94040	830.19950	1096.46080	Detected	Detected	Detected	Detected
1963	A_23_P104563	CPT1A	Hs.503043	carnitine palmitoyltransferase 1A (liver)	Homo sapiens carnitine palmitoyltransfe	587.23490	526.27840	461.07507	388.78710	Detected	Detected	Detected	Detected
1964	A_33_P3314810	LOC730978	Hs.722585	similar to capicua-like protein/double h	PREDICTED: Homo sapiens similar to c	63.03229	78.74628	45.24146	52.64378	Detected	Detected	Detected	Detected
1965	A_32_P52018	PHACTR1	Hs.436996	phosphatase and actin regulator 1	Homo sapiens phosphatase and actin re	52.35361	16.76431	84.57846	29.54536	Detected	Compromised	Detected	Detected
1966	A_24_P28811	C2orf14	Hs.710729	chromosome 2 open reading frame 14	Homo sapiens chromosome 2 open read	6129.57200	8890.72300	1586.54930	1339.67180	Detected	Detected	Detected	Detected
1967	A_23_P363406	ANGEL1	Hs.497448	angel homolog 1 (Drosophila)	Homo sapiens angel homolog 1 (Drosop	246.57306	104.61752	177.48225	65.28661	Detected	Detected	Detected	Detected
1968	A_23_P360167	DCTN2	Hs.289123	dynactin 2 (p50)	Homo sapiens dynactin 2 (p50) (DCTN2	17731.94500	17777.31800	17868.42600	16383.39400	Detected	Detected	Detected	Detected
1969	A_23_P112554	COL15A1	Hs.409034	collagen, type XV, alpha 1	Homo sapiens collagen, type XV, alpha	12.94998	203.46391	12.76627	101.67959	Detected	Detected	Not Detected	Detected
1970	A_23_P396666	TBC1D2B	Hs.567426	TBC1 domain family, member 2B	Homo sapiens TBC1 domain family, mer	3765.41820	1994.96770	2854.69950	1383.64530	Detected	Detected	Detected	Detected
1971	A_33_P3329098	WTAP	Hs.446091	Wilms tumor 1 associated protein	Homo sapiens Wilms tumor 1 associated	1902.59160	1052.83310	1477.75170	769.04266	Detected	Detected	Detected	Detected
1972	A_23_P43726	NUP160	Hs.643526	nucleoporin 160kDa	Homo sapiens nucleoporin 160kDa (NUF	1028.64110	905.57720	937.93710	706.49786	Detected	Detected	Detected	Detected
1973	A_33_P3236686				Homo sapiens FP15331 mRNA, complet	85.34432	69.44849	59.79829	50.89603	Detected	Detected	Detected	Detected
1974	A_33_P3220929	JOSD2	Hs.467151	Josephin domain containing 2	Homo sapiens Josephin domain contain	86.48673	71.43179	93.55015	90.90904	Detected	Detected	Detected	Detected
1975	A_24_P915196	C9orf91	Hs.522357	chromosome 9 open reading frame 91	Homo sapiens chromosome 9 open read	228.09113	164.94801	233.81213	134.28069	Detected	Detected	Detected	Detected
1976	A_33_P3395971	CTU1	Hs.148425	cytosolic thiouridylase subunit 1 homol	Homo sapiens cytosolic thiouridylase su	51.39080	53.67150	48.07098	36.81426	Detected	Detected	Detected	Detected
1977	A_23_P109333	C21orf33	Hs.413482	chromosome 21 open reading frame 33	Homo sapiens chromosome 21 open rea	4830.25800	4294.48600	4166.80500	3019.68550	Detected	Detected	Detected	Detected
1978	A_23_P210886	BCL2L1	Hs.516966	BCL2-like 1	Homo sapiens BCL2-like 1 (BCL2L1), n	787.23956	1246.14440	566.96906	852.23944	Detected	Detected	Detected	Detected
1979	A_23_P96087	H1FX	Hs.75307	H1 histone family, member X	Homo sapiens H1 histone family, memb	1756.28210	1683.17050	1995.04020	1868.68840	Detected	Detected	Detected	Detected
1980	A_23_P502142	FYN	Hs.390567	FYN oncogene related to SRC, FGR, YE	Homo sapiens FYN oncogene related to	6777.12000	9046.24600	4823.74560	5648.94300	Detected	Detected	Detected	Detected
1981	A_33_P3209581		Hs.722301		IQ motif and SEC7 domain-containing p	2862.86130	3894.95780	7820.72900	7651.79600	Detected	Detected	Detected	Detected
1982	A_33_P3396200	LRCH4	Hs.719669	leucine-rich repeats and calponin homo	Homo sapiens leucine-rich repeats and	7892.68500	6339.12400	2770.80320	2489.31370	Detected	Detected	Detected	Detected
1983	A_23_P106675	PLCG2	Hs.413111	phospholipase C, gamma 2 (phosphatidy	Homo sapiens phospholipase C, gamma	36.12347	44.12079	17.32239	26.71643	Detected	Detected	Detected	Detected
1984	A_33_P3309643					100.51939	108.25504	43.70604	49.34143	Detected	Detected	Detected	Detected
1985	A_24_P35891	ZNF219	Hs.250493	zinc finger protein 219	Homo sapiens zinc finger protein 219 (Z	3963.87260	2230.98560	2922.19070	1366.88540	Detected	Detected	Detected	Detected
1986	A_23_P388146		Hs.642598		Ubiquitin UBF-fl [Source:UniProtKB/T	176.48915	3300.55700	151.90073	2018.59190	Detected	Detected	Detected	Detected
1987	A_24_P294124	SERTAD2	Hs.591569	SERTA domain containing 2	Homo sapiens SERTA domain containin	2714.52420	3607.30740	1793.57140	2010.87550	Detected	Detected	Detected	Detected
1988	A_33_P3311473	ATP10D	Hs.437241	ATPase, class V, type 10D	Homo sapiens ATPase, class V, type 10	15.70906	23.65361	14.37604	19.84956	Detected	Compromised	Detected	Detected
1989	A_23_P66260	ZNF267	Hs.460645	zinc finger protein 267	Homo sapiens zinc finger protein 267 (Z	608.45276	473.27870	501.63070	363.33524	Detected	Detected	Detected	Detected
1990	A_23_P26777	NUP85	Hs.362817	nucleoporin 85kDa	Homo sapiens nucleoporin 85kDa (NUPI	4499.04100	6794.49300	3595.71100	4949.99560	Detected	Detected	Detected	Detected
1991	A_33_P3211965		Hs.537262		Putative uncharacterized protein ENSP	634.00340	984.61420	456.20276	521.85626	Detected	Detected	Detected	Detected
1992	A_24_P185036	FTSJD1	Hs.72782	FtsJ methyltransferase domain contain	Homo sapiens FtsJ methyltransferase c	605.53015	506.05435	441.07748	327.08813	Detected	Detected	Detected	Detected
1993	A_23_P34433	ZCCHC11	Hs.655407	zinc finger, CCHC domain containing 11	Homo sapiens zinc finger, CCHC domai	1511.03780	819.90106	689.37213	295.61053	Detected	Detected	Detected	Detected
1994	A_32_P228501	CDC26	Hs.530284	cell division cycle 26 homolog (S. cerev	Homo sapiens cell division cycle 26 hon	6722.74700	4353.44600	7653.01500	3923.90670	Detected	Detected	Detected	Detected
1995	A_23_P123732	C9orf103	Hs.530261	chromosome 9 open reading frame 103	Homo sapiens chromosome 9 open read	648.50714	675.39185	469.29755	394.34510	Detected	Detected	Detected	Detected
1996	A_23_P21425	GPR119	Hs.496762	G protein-coupled receptor 119	Homo sapiens G protein-coupled recep	569.79144	518.60140	243.46826	192.88939	Detected	Detected	Detected	Detected
1997	A_23_P321452	PDZD8	Hs.501149	PDZ domain containing 8	Homo sapiens PDZ domain containing 8	542.08080	663.41590	318.04843	307.43350	Detected	Detected	Detected	Detected
1998	A_23_P378690	TMEM64	Hs.567759	transmembrane protein 64	Homo sapiens transmembrane protein 6	695.12920	541.06586	586.86430	399.08298	Detected	Detected	Detected	Detected
1999	A_23_P63896	FAS	Hs.244139	Fas (TNF receptor superfamily, member	Homo sapiens Fas (TNF receptor super	1993.31590	2821.30270	1330.60950	1565.69960	Detected	Detected	Detected	Detected
2000	A_24_P82155	ANKDD1A	Hs.207157	ankyrin repeat and death domain contai	Homo sapiens ankyrin repeat and death	89.05508	35.46917	52.62431	23.53967	Detected	Detected	Detected	Detected
2001	A_33_P3317431					80021.46000	73622.74000	42769.43800	32638.20500	Detected	Detected	Detected	Detected
2002	A_23_P205255	WDR20	Hs.36859	WD repeat domain 20	Homo sapiens WD repeat domain 20 (W	189.98690	258.56827	171.43738	201.37036	Detected	Detected	Detected	Detected
2003	A_23_P266	PIN1L	Hs.654711	peptidylprolyl cis/trans isomerase, NIM/	Homo sapiens peptidylprolyl cis/trans is	855.62750	1317.85290	534.58655	845.52765	Detected	Detected	Detected	Detected
2004	A_23_P216307	RUNX1T1	Hs.368431	runt-related transcription factor 1; tran	Homo sapiens runt-related transcriptior	1616.90950	1357.23790	1146.37990	854.24510	Detected	Detected	Detected	Detected
2005	A_33_P3266614					1442.81430	2080.54960	977.77466	1019.51385	Detected	Detected	Detected	Detected
2006	A_23_P11841	ATP2B4	Hs.343522	ATPase, Ca++ transporting, plasma mer	Homo sapiens ATPase, Ca++ transporti	46.20483	153.91339	48.57132	128.59906	Detected	Detected	Detected	Detected
2007	A_32_P22401	MAP7D1	Hs.700595	MAP7 domain containing 1	Homo sapiens MAP7 domain containing	21747.75600	30397.99000	17310.03300	26164.35200	Detected	Detected	Detected	Detected
2008	A_33_P3367924	LOC390748	Hs.690706	similar to poly(A)binding protein nuclear	Homo sapiens similar to poly(A)binding	18.89989	16.50422	13.22419	8.83519	Detected	Compromised	Compromised	Compromised
2009	A_23_P24104	PLAU	Hs.77274	plasminogen activator, urokinase	Homo sapiens plasminogen activator, ur	7172.39840	2974.28520	6783.54300	2416.34740	Detected	Detected	Detected	Detected
2010	A_23_P99253	LIN7A	Hs.144333	lin-7 homolog A (C. elegans)	Homo sapiens lin-7 homolog A (C. elega	103.33338	514.76074	102.15040	377.80142	Detected	Detected	Detected	Detected
2011	A_24_P135444	AMFR	Hs.295137	autocrine motility factor receptor	Homo sapiens autocrine motility factor	6671.69800	4252.06350	5083.24460	2950.21750	Detected	Detected	Detected	Detected
2012	A_23_P57413	PPM1F	Hs.112728	protein phosphatase 1F (PP2C domain	Homo sapiens protein phosphatase 1F (	55.52918	473.33124	46.82087	492.54144	Detected	Detected	Detected	Detected
2013	A_23_P159476	SSNA1	Hs.530314	Sjogren syndrome nuclear autoantigen	Homo sapiens Sjogren syndrome nuclea	3723.35300	5181.68200	5011.52100	6457.04830	Detected	Detected	Detected	Detected
2014	A_23_P208400	ADCK4	Hs.130712	aarF domain containing kinase 4	Homo sapiens aarF domain containing k	177.98210	41.72846	171.90611	47.21402	Detected	Detected	Detected	Detected
2015	A_23_P214533	ZNF323	Hs.656413	zinc finger protein 323	Homo sapiens zinc finger protein 323 (Z	16.14028	4.10265	10.10185	3.62558	Detected	Compromised	Compromised	Compromised
2016	A_23_P141520	C17orf49	Hs.511801	chromosome 17 open reading frame 49	Homo sapiens chromosome 17 open rea	5741.79300	4265.04400	6837.83840	4175.03000	Detected	Detected	Detected	Detected
2017	A_23_P10858	ANKLE2	Hs.654628	ankyrin repeat and LEM domain contain	Homo sapiens ankyrin repeat and LEM	1122.94820	2158.12480	669.61584	1057.51400	Detected	Detected	Detected	Detected
2018	A_23_P169503	CEL	Hs.533258	carboxyl ester lipase (bile salt-stimulat	Homo sapiens carboxyl ester lipase (bile	45.40538	56.17001	13.03276	7.63225	Detected	Detected	Compromised	Compromised
2019	A_23_P115792	PLEKHA1	Hs.643512	pleckstrin homology domain containing,	Homo sapiens pleckstrin homology dom	1977.07780	907.05220	1293.16190	556.70435	Detected	Detected	Detected	Detected
2020	A_23_P11697	HMGB4	Hs.568628	high-mobility group box 4	Homo sapiens high-mobility group box 4	14.98157	20.38981	14.27565	24.52206	Detected	Detected	Detected	Not Detected
2021	A_23_P24215	TBC1D12	Hs.500598	TBC1 domain family, member 12	Homo sapiens TBC1 domain family, mer	1022.44904	655.65515	590.71750	297.21173	Detected	Detected	Detected	Detected

2022	A_23_P162322	WNT10B	Hs.91985	wingless-type MMTV integration site fa	Homo sapiens wingless-type MMTV inte	46.94071	50.73909	49.08708	47.39446	Detected	Detected	Detected	Detected
2023	A_23_P47377	HSD17B12	Hs.132513	hydroxysteroid (17-beta) dehydrogenas	Homo sapiens hydroxysteroid (17-beta)	6045.05470	9620.99800	3601.20580	4766.99850	Detected	Detected	Detected	Detected
2024	A_23_P14105	RCBTB2	Hs.652712	regulator of chromosome condensation	Homo sapiens regulator of chromosome	403.74670	348.20480	335.06055	223.68468	Detected	Detected	Detected	Detected
2025	A_24_P256404		Hs.722379		Putative uncharacterized protein FLJ35	40.51621	32.29931	24.66402	18.61077	Detected	Detected	Detected	Detected
2026	A_23_P202501	RNLS	Hs.149849	renalase, FAD-dependent amine oxidase	Homo sapiens renalase, FAD-dependent	104.61561	120.14777	97.82945	107.95711	Detected	Detected	Detected	Detected
2027	A_33_P3334535	GIN1	Hs.24088	gypsy retrotransposon integrase 1	Homo sapiens gypsy retrotransposon in	478.44754	604.08450	123.92626	128.30739	Detected	Detected	Detected	Detected
2028	A_23_P204609	VEZT	Hs.24135	vezatin, adherens junctions transmembr	Homo sapiens vezatin, adherens junctio	1280.34520	1927.72360	1111.58500	1456.37710	Detected	Detected	Detected	Detected
2029	A_23_P123727	ZCCHC6	Hs.655162	zinc finger, CCHC domain containing 6	Homo sapiens zinc finger, CCHC domain	1885.87790	864.86346	1123.71010	407.25546	Detected	Detected	Detected	Detected
2030	A_33_P3420486	LOC731419	Hs.612899	hypothetical protein LOC731419	PREDICTED: Homo sapiens hypothetica	44.13735	49.48304	36.74349	32.64608	Detected	Detected	Detected	Detected
2031	A_23_P56703	C2orf89	Hs.469134	chromosome 2 open reading frame 89	Homo sapiens chromosome 2 open read	325.68726	284.88123	338.62897	253.56818	Detected	Detected	Detected	Detected
2032	A_33_P3344603	ZNF280D	Hs.511477	zinc finger protein 280D	Homo sapiens zinc finger protein 280D	41.02706	220.05440	25.73241	136.92195	Detected	Detected	Detected	Detected
2033	A_24_P187086	LOC646891		similar to Serologically defined colon ca	PREDICTED: Homo sapiens similar to S	85.79000	37.85763	3.11074	5.42802	Detected	Detected	Compromised	Compromised
2034	A_33_P3351197	MGC21881	Hs.721511	hypothetical locus MGC21881	Homo sapiens hypothetical locus MGC2	215.36563	74.94025	193.17574	76.02843	Detected	Detected	Detected	Detected
2035	A_32_P122793	C22orf27	Hs.592202	chromosome 22 open reading frame 27	Homo sapiens chromosome 22 open rea	518.49274	358.59103	525.16254	380.37573	Detected	Detected	Detected	Detected
2036	A_33_P3259203					390.24680	508.66498	316.07706	420.88000	Detected	Detected	Detected	Detected
2037	A_23_P15466	SCO1	Hs.14511	SCO cytochrome oxidase deficient hom	Homo sapiens SCO cytochrome oxidase	3749.74240	4253.87400	2989.79470	3083.74630	Detected	Detected	Detected	Detected
2038	A_23_P9883	NLRP3	Hs.159483	NLR family, pyrin domain containing 3	Homo sapiens NLR family, pyrin domain	133.15118	58.13710	97.48431	34.48034	Detected	Detected	Detected	Detected
2039	A_23_P56978	PTK6	Hs.51133	PTK6 protein tyrosine kinase 6	Homo sapiens PTK6 protein tyrosine kin	19.58452	8.94460	13.74731	16.40214	Detected	Compromised	Compromised	Compromised
2040	A_24_P140608	HBEGF	Hs.799	heparin-binding EGF-like growth factor	Homo sapiens heparin-binding EGF-like	99.59885	238.62132	81.78620	183.89543	Detected	Detected	Detected	Detected
2041	A_32_P88719	RAB40AL	Hs.449517	RAB40A, member RAS oncogene family	Homo sapiens RAB40A, member RAS o	229.21521	203.74333	250.87065	169.82468	Detected	Detected	Detected	Detected
2042	A_23_P100517	TMEM170A	Hs.487510	transmembrane protein 170A	Homo sapiens transmembrane protein 1	309.90710	309.43628	230.79180	202.51738	Detected	Detected	Detected	Detected
2043	A_24_P374516	TMSB4X	Hs.522584	thymosin beta 4, X-linked	Homo sapiens thymosin beta 4, X-linked	178017.31000	102741.44500	100446.77000	53055.38000	Detected	Detected	Detected	Detected
2044	A_23_P354288	FAM71A	Hs.129293	family with sequence similarity 71, mem	Homo sapiens family with sequence sim	12.50436	20.79723	9.88659	4.82139	Compromised	Detected	Compromised	Compromised
2045	A_33_P3235420	RASSF6	Hs.590920	Ras association (RalGDS/AF-6) domain	Homo sapiens Ras association (RalGDS	199.80830	199.05127	37.66149	33.89800	Detected	Detected	Detected	Detected
2046	A_32_P197524	EIF2A	Hs.655782	eukaryotic translation initiation factor 2	Homo sapiens eukaryotic translation ini	15.03625	84.49441	6.46038	46.50312	Detected	Detected	Compromised	Detected
2047	A_23_P108404	AGAP1	Hs.435039	ArfGAP with GTPase domain, ankyrin re	Homo sapiens ArfGAP with GTPase dor	978.68774	773.64026	663.44180	448.14810	Detected	Detected	Detected	Detected
2048	A_33_P3232273					18.38986	8.29829	7.48527	10.53737	Detected	Compromised	Compromised	Compromised
2049	A_33_P3299872	HINT3	Hs.72325	histidine triad nucleotide binding protein	Homo sapiens histidine triad nucleotide	1605.20240	1621.44350	1182.48440	877.93150	Detected	Detected	Detected	Detected
2050	A_23_P154235	NMI	Hs.54483	N-myc (and STAT) interactor	Homo sapiens N-myc (and STAT) intera	1422.65300	1184.83330	886.40280	626.62400	Detected	Detected	Detected	Detected
2051	A_33_P3418838	LOC100133047	Hs.309921	hypothetical LOC100133047	PREDICTED: Homo sapiens hypothetica	37.52659	35.61948	19.99468	12.05247	Detected	Detected	Detected	Compromised
2052	A_33_P3401301	RPL39	Hs.558387	ribosomal protein L39	Homo sapiens ribosomal protein L39 (R	26409.97000	16773.41400	24366.05500	12281.06800	Detected	Detected	Detected	Detected
2053	A_23_P16022	ZNF256	Hs.596242	zinc finger protein 256	Homo sapiens zinc finger protein 256 (Z	1052.22360	950.22736	868.29706	672.74730	Detected	Detected	Detected	Detected
2054	A_23_P377141	VPS13A	Hs.459790	vacuolar protein sorting 13 homolog A (	Homo sapiens vacuolar protein sorting	339.35254	114.01082	156.21278	47.08122	Detected	Detected	Detected	Detected
2055	A_33_P3316691					411.41678	537.65880	216.21480	240.04503	Detected	Detected	Detected	Detected
2056	A_23_P45799	ORC1L	Hs.17908	origin recognition complex, subunit 1-lik	Homo sapiens origin recognition comple	30.27161	143.02261	23.49678	141.74663	Detected	Detected	Detected	Detected
2057	A_23_P410613	C12orf23	Hs.257664	chromosome 12 open reading frame 23	Homo sapiens chromosome 12 open rea	13925.71600	17375.08000	11023.64800	12562.93700	Detected	Detected	Detected	Detected
2058	A_33_P3313456	CXorf30	Hs.632791	chromosome X open reading frame 30	Homo sapiens chromosome X open read	189.91864	96.06388	25.48234	21.92592	Detected	Detected	Detected	Detected
2059	A_24_P309645	TPCN1	Hs.524763	two pore segment channel 1	Homo sapiens two pore segment chann	991.42030	215.45064	881.98350	188.44418	Detected	Detected	Detected	Detected
2060	A_23_P115743	C10orf84	Hs.372309	chromosome 10 open reading frame 84	Homo sapiens chromosome 10 open rea	825.18176	836.95500	586.23970	523.40955	Detected	Detected	Detected	Detected
2061	A_23_P331253	XPNPEP1	Hs.390623	X-prolyl aminopeptidase (aminopeptidas	Homo sapiens X-prolyl aminopeptidase	1853.95560	1485.77480	1948.75630	1362.77670	Detected	Detected	Detected	Detected
2062	A_23_P109928	PSMD6	Hs.152536	proteasome (prosome, macropain) 26S	Homo sapiens proteasome (prosome, m	8920.20900	14306.30300	7745.36700	10278.92100	Detected	Detected	Detected	Detected
2063	A_24_P751074	ETS1	Hs.369438	v-ets erythroblastosis virus E26 oncoge	Homo sapiens v-ets erythroblastosis vi	806.30820	1018.91760	685.88530	735.99680	Detected	Detected	Detected	Detected
2064	A_33_P3264577	DCTN1	Hs.516111	dynactin 1 (p150, glued homolog, Droso	Homo sapiens dynactin 1 (p150, glued h	3069.88940	3969.03000	3029.34620	4039.42100	Detected	Detected	Detected	Detected
2065	A_33_P3360525	LOC399804	Hs.621182	similar to nucleophosmin 1	PREDICTED: Homo sapiens similar to n	24432.37700	35848.17000	2529.18300	2667.64230	Detected	Detected	Detected	Detected
2066	A_23_P404965	GNL1	Hs.654655	guanine nucleotide binding protein-like	Guanine nucleotide-binding protein-like	6723.96040	1848.74950	4459.11430	1037.00330	Detected	Detected	Detected	Detected
2067	A_33_P3273664	ARSK	Hs.585051	arylsulfatase family, member K	Homo sapiens arylsulfatase family, mem	59.62927	81.82692	39.12861	51.66271	Detected	Detected	Detected	Detected
2068	A_23_P118158	HS3ST2	Hs.720257	heparan sulfate (glucosamine) 3-O-sulf	Homo sapiens heparan sulfate (glucosar	611.00050	1744.84790	512.09920	1283.14380	Detected	Detected	Detected	Detected
2069	A_23_P156861	RGS17	Hs.166313	regulator of G-protein signaling 17	Homo sapiens regulator of G-protein sig	574.71590	183.13281	349.17980	89.02399	Detected	Detected	Detected	Detected
2070	A_23_P79628	PSME4	Hs.413801	proteasome (prosome, macropain) activ	Homo sapiens proteasome (prosome, m	3024.55600	4444.99070	2659.45500	3159.52100	Detected	Detected	Detected	Detected
2071	A_23_P396981	CCDC66	Hs.476399	coiled-coil domain containing 66	Homo sapiens coiled-coil domain contai	272.79346	103.07387	106.65109	42.05142	Detected	Detected	Detected	Detected
2072	A_23_P208302	APOC2	Hs.75615	apolipoprotein C-II	Homo sapiens apolipoprotein C-II (APO	43.14624	6.56667	38.91995	5.00136	Detected	Compromised	Detected	Compromised
2073	A_23_P11543	FUCA1	Hs.370858	fucosidase, alpha-L- 1, tissue	Homo sapiens fucosidase, alpha-L- 1, t	58.85664	304.33360	56.24582	262.91977	Detected	Detected	Detected	Detected
2074	A_23_P26854	RICH2	Hs.499758	Rho-type GTPase-activating protein RI	Homo sapiens Rho-type GTPase-activa	319.55734	374.78345	368.78036	350.03073	Detected	Detected	Detected	Detected
2075	A_33_P3258046	TAC4	Hs.652130	tachykinin 4 (hemokinin)	Homo sapiens tachykinin 4 (hemokinin)	90.48984	77.63732	10.82015	5.73683	Detected	Detected	Compromised	Compromised
2076	A_23_P372860	HIST1H2AC	Hs.484950	histone cluster 1, H2ac	Homo sapiens histone cluster 1, H2ac (	21.07598	29.73556	12.23563	14.91814	Detected	Detected	Compromised	Compromised
2077	A_33_P3325643	FLJ42418	Hs.446195	FLJ42418 protein	PREDICTED: Homo sapiens FLJ42418 p	23.09349	27.86834	29.09650	30.47577	Detected	Detected	Detected	Detected
2078	A_24_P214598	PPM1K	Hs.291000	protein phosphatase 1K (PP2C domain	Homo sapiens protein phosphatase 1K (	141.68091	139.21492	96.40479	93.49657	Detected	Detected	Detected	Detected
2079	A_23_P16157	KHSRP	Hs.91142	KH-type splicing regulatory protein	Homo sapiens KH-type splicing regulato	13133.37600	22287.11100	10961.67300	17851.63700	Detected	Detected	Detected	Detected
2080	A_33_P3404601	C2	Hs.408903	complement component 2	Homo sapiens complement component	57.17582	9.17417	55.39499	9.21821	Detected	Compromised	Detected	Compromised
2081	A_23_P103703	HSPB7	Hs.502612	heat shock 27kDa protein family, memb	Homo sapiens heat shock 27kDa protein	286.49078	6085.28370	206.45137	4225.50930	Detected	Detected	Detected	Detected
2082	A_23_P120744	MCM3AP	Hs.389037	minichromosome maintenance complex	Homo sapiens minichromosome mainter	5711.35000	5220.64650	4943.70400	4119.31900	Detected	Detected	Detected	Detected
2083	A_33_P3708413	MFAP5	Hs.512842	microfibrillar associated protein 5	Homo sapiens microfibrillar associated	356.31840	14618.14000	262.57050	12498.96400	Detected	Detected	Detected	Detected
2084	A_33_P3411025	ARHGAP19	Hs.80305	Rho GTPase activating protein 19	Homo sapiens Rho GTPase activating p	61.69612	157.64058	45.44716	101.90420	Detected	Detected	Detected	Detected
2085	A_33_P3299435	SART1	Hs.502883	squamous cell carcinoma antigen recog	Homo sapiens squamous cell carcinoma	130.41031	268.56528	89.54284	217.92377	Detected	Detected	Detected	Detected
2086	A_23_P9255	SYK	Hs.371720	spleen tyrosine kinase	Homo sapiens spleen tyrosine kinase (S	16.32016	24.60377	4.99654	6.15717	Not Detected	Not Detected	Compromised	Compromised
2087	A_24_P21056	PHF14	Hs.655688	PHD finger protein 14	Homo sapiens PHD finger protein 14 (P	654.59370	743.09360	610.13995	566.44010	Detected	Detected	Detected	Detected
2088	A_33_P3390753					817.70734	477.31125	177.66390	72.75214	Detected	Detected	Detected	Detected
2089	A_33_P3489675	PRR5-ARHGAP8	Hs.720401	PRR5-ARHGAP8 readthrough	Homo sapiens PRR5-ARHGAP8 fusion	35.05180	4.83539	33.34568	3.22881	Detected	Compromised	Detected	Compromised
2090	A_33_P3222307	LOC100129380	Hs.169054	hypothetical protein LOC100129380	Homo sapiens cDNA FLJ43532 fis, clon	22.31362	21.88772	10.05497	13.43023	Detected	Detected	Compromised	Compromised
2091	A_23_P37736	TNFRSF17	Hs.2556	tumor necrosis factor receptor superfar	Homo sapiens tumor necrosis factor rec	62.76551	10.38595	42.85702	4.93273	Detected	Compromised	Detected	Compromised
2092	A_23_P385206	STX12	Hs.523855	syntaxin 12	Homo sapiens syntaxin 12 (STX12), mR	3980.24900	2800.06080	3522.62800	2246.15190	Detected	Detected	Detected	Detected
2093	A_23_P105571	CHPT1	Hs.293077	choline phosphotransferase 1	Homo sapiens choline phosphotransfera	1878.62150	1539.57250	1250.16170	883.31030	Detected	Detected	Detected	Detected
2094	A_33_P3275500	LOC400743	Hs.522935	FLJ45933 protein	Homo sapiens cDNA FLJ45933 fis, clon	14.36889	100.37981	9.80482	75.24503	Detected	Detected	Compromised	Detected
2095	A_23_P67725	LMNB2	Hs.538286	lamin B2	Homo sapiens lamin B2 (LMNB2), mRNA	2246.70730	6377.94900	1009.52484	2312.76440	Detected	Detected	Detected	Detected
2096	A_33_P3328511	ARL17	Hs.720602	ADP-ribosylation factor-like 17	Homo sapiens ADP-ribosylation factor-	229.91320	295.11655	190.11816	235.79294	Detected	Detected	Detected	Detected

2097	A_23_P254079	STBD1	Hs.109590	starch binding domain 1	Homo sapiens starch binding domain 1 (	462.90378	582.47040	333.78480	394.29868	Detected	Detected	Detected	Detected
2098	A_33_P3392823	USE1	Hs.16187	unconventional SNARE in the ER 1 hom	Homo sapiens unconventional SNARE ir	2155.26500	3246.86600	2276.20430	2954.05420	Detected	Detected	Detected	Detected
2099	A_33_P3422030				FXYD domain-containing ion transport r	35.22102	43.29158	37.07488	43.94627	Detected	Detected	Detected	Detected
2100	A_33_P3353866	MOGAT2	Hs.288568	monoacylglycerol O-acyltransferase 2	Homo sapiens monoacylglycerol O-acyl	2.67834	6.06573	9.13182	23.85420	Compromised	Compromised	Compromised	Detected
2101	A_33_P3222565	TSPAN14	Hs.633177	tetraspanin 14	Homo sapiens tetraspanin 14 (TSPAN14	98.85284	75.72607	83.25223	54.48145	Detected	Detected	Detected	Detected
2102	A_24_P116805	STAT3	Hs.463059	signal transducer and activator of trans	Homo sapiens signal transducer and act	1370.47530	624.47090	847.36410	421.76620	Detected	Detected	Detected	Detected
2103	A_33_P3250398					837.41600	1395.32950	570.09550	912.69750	Detected	Detected	Detected	Detected
2104	A_33_P3343616				PHD finger protein 12 (PHD factor 1)(P	75.29411	37.98934	59.39712	31.26974	Detected	Detected	Detected	Detected
2105	A_33_P3275199	JRK	Hs.535903	jerky homolog (mouse)	Homo sapiens jerky homolog (mouse) (J	604.57904	775.92650	358.87677	374.94205	Detected	Detected	Detected	Detected
2106	A_23_P123071	CAV2	Hs.212332	caveolin 2	Homo sapiens caveolin 2 (CAV2), trans	2633.48170	8755.60450	2972.14090	8906.31600	Detected	Detected	Detected	Detected
2107	A_23_P110253	KIT	Hs.479754	v-kit Hardy-Zuckerman 4 feline sarcom	Homo sapiens v-kit Hardy-Zuckerman 4	26.55960	58.28436	17.32201	30.12346	Detected	Detected	Detected	Detected
2108	A_33_P3316555	MGST3	Hs.191734	microsomal glutathione S-transferase 3	Homo sapiens microsomal glutathione S	34184.85000	22583.88500	30647.19000	17225.57600	Detected	Detected	Detected	Detected
2109	A_32_P177539	IRX6	Hs.369907	iroquois homeobox 6	Homo sapiens iroquois homeobox 6 (IRX	16.56641	7.78626	13.75806	5.66619	Detected	Compromised	Compromised	Compromised
2110	A_23_P40096	PROC	Hs.224698	protein C (inactivator of coagulation fac	Homo sapiens protein C (inactivator of	13.99061	3.77206	6.58614	4.75081	Detected	Compromised	Compromised	Compromised
2111	A_23_P153256	ZNF773	Hs.579576	zinc finger protein 773	Homo sapiens zinc finger protein 773 (Z	312.17990	556.87070	265.36813	325.81967	Detected	Detected	Detected	Detected
2112	A_33_P3406066	ZNF776	Hs.720254	zinc finger protein 776	Homo sapiens zinc finger protein 776 (Z	859.00793	646.85876	725.23660	458.67627	Detected	Detected	Detected	Detected
2113	A_33_P3351982	C2orf64	Hs.596537	chromosome 2 open reading frame 64	Homo sapiens chromosome 2 open read	2605.54610	909.83320	2168.25000	599.80060	Detected	Detected	Detected	Detected
2114	A_33_P3411315	KRTAP3-3	Hs.662759	keratin associated protein 3-3	Homo sapiens keratin associated protei	1908.41720	2459.68920	839.63710	923.41406	Detected	Detected	Detected	Detected
2115	A_23_P109488	PIK3IP1	Hs.26670	phosphoinositide-3-kinase interacting p	Homo sapiens phosphoinositide-3-kinas	144.37561	342.56560	148.12619	321.43335	Detected	Detected	Detected	Detected
2116	A_32_P94	LHFPL4	Hs.56782	lipoma HMGIC fusion partner-like 4	Homo sapiens lipoma HMGIC fusion par	54.96972	49.90353	61.03211	42.19609	Detected	Detected	Detected	Detected
2117	A_23_P125829	PGK1	Hs.78771	phosphoglycerate kinase 1	Homo sapiens phosphoglycerate kinase	42181.88700	64944.57000	44744.44500	59624.55000	Detected	Detected	Detected	Detected
2118	A_24_P17453	TTC30A	Hs.128384	tetratricopeptide repeat domain 30A	Homo sapiens tetratricopeptide repeat	29.87690	25.83341	8.65536	5.28123	Detected	Detected	Compromised	Compromised
2119	A_23_P152038	TUBGCP5	Hs.533898	tubulin, gamma complex associated prot	Homo sapiens tubulin, gamma complex a	463.74417	502.87573	399.21490	390.82635	Detected	Detected	Detected	Detected
2120	A_33_P3298251					76.79231	97.01284	58.41750	70.55638	Detected	Detected	Detected	Detected
2121	A_33_P3255176	LOC100133116	Hs.375067	similar to uromodulin-like	PREDICTED: Homo sapiens similar to u	29.09106	27.12194	14.94152	17.80434	Detected	Detected	Compromised	Compromised
2122	A_23_P110345	CHIC2	Hs.335393	cysteine-rich hydrophobic domain 2	Homo sapiens cysteine-rich hydrophobi	1642.19300	2070.13280	1077.58530	1142.54350	Detected	Detected	Detected	Detected
2123	A_23_P380965	JKAMP	Hs.446850	JNK1/MAPK8-associated membrane pr	Homo sapiens JNK1/MAPK8-associate	9974.64000	8694.26800	7330.43700	5582.07470	Detected	Detected	Detected	Detected
2124	A_24_P234116	C1orf56	Hs.549171	chromosome 1 open reading frame 56	Homo sapiens chromosome 1 open read	3078.98220	4097.19140	2045.24150	2254.99020	Detected	Detected	Detected	Detected
2125	A_33_P3319953				Transcriptional regulator ATRX (EC 3.6.	16.34311	14.25762	12.73777	7.88158	Detected	Compromised	Compromised	Compromised
2126	A_32_P85813	RAP1GDS1	Hs.132858	RAP1, GTP-GDP dissociation stimulato	Homo sapiens RAP1, GTP-GDP dissoci	3975.38960	3210.15010	2795.36700	1948.32870	Detected	Detected	Detected	Detected
2127	A_23_P206310	KIAA0513	Hs.301658	KIAA0513	Homo sapiens KIAA0513 (KIAA0513), m	2250.17480	1620.00790	1759.12880	1178.83800	Detected	Detected	Detected	Detected
2128	A_23_P208013	ZNF407	Hs.536490	zinc finger protein 407	Homo sapiens zinc finger protein 407 (Z	241.36224	316.82740	154.29580	190.27094	Detected	Detected	Detected	Detected
2129	A_23_P1199	NUDT5	Hs.555956	nudix (nucleoside diphosphate linked mo	Homo sapiens nudix (nucleoside diphosp	5104.63670	7231.18750	4895.88130	5981.09570	Detected	Detected	Detected	Detected
2130	A_24_P129632	DLG5	Hs.652690	discs, large homolog 5 (Drosophila)	Homo sapiens discs, large homolog 5 (D	5040.84200	3467.51300	3203.48970	1869.15900	Detected	Detected	Detected	Detected
2131	A_33_P3349843					43.40028	37.87083	32.37069	30.04557	Detected	Detected	Detected	Detected
2132	A_33_P3324890	HSP90AB2P		heat shock protein 90kDa alpha (cytoso	Homo sapiens heat shock protein 90kDa	3985.18000	6600.01860	2586.82130	3979.49660	Detected	Detected	Detected	Detected
2133	A_33_P3215277	TTBK2	Hs.659846	tau tubulin kinase 2	Homo sapiens tau tubulin kinase 2 (TTE	1342.48820	523.28950	877.88160	314.32162	Detected	Detected	Detected	Detected
2134	A_23_P327483	SPATA6	Hs.538103	spermatogenesis associated 6	Homo sapiens spermatogenesis associa	407.93903	100.96921	373.56552	80.11219	Detected	Detected	Detected	Detected
2135	A_23_P17287	IAH1	Hs.570189	isoamyl acetate-hydrolyzing esterase 1	Homo sapiens isoamyl acetate-hydrolyz	15594.24600	14720.15000	15403.40900	13342.25900	Detected	Detected	Detected	Detected
2136	A_23_P122233	MRPL22	Hs.483924	mitochondrial ribosomal protein L22	Homo sapiens mitochondrial ribosomal p	15281.73600	14491.84100	11627.41100	9268.47300	Detected	Detected	Detected	Detected
2137	A_23_P357546	PNKD	Hs.98475	paroxysmal nonkinesigenic dyskinesia	Homo sapiens paroxysmal nonkinesigeni	74.47138	46.38449	66.78574	20.94506	Detected	Detected	Detected	Detected
2138	A_23_P73345	MITF	Hs.166017	microphthalmia-associated transcription	Homo sapiens microphthalmia-associate	76.27582	15.01550	75.48770	6.73453	Detected	Compromised	Detected	Compromised
2139	A_33_P3313221	ZNF691	Hs.20879	zinc finger protein 691	Homo sapiens zinc finger protein 691 (Z	81.16470	132.67113	59.32092	85.76550	Detected	Detected	Detected	Detected
2140	A_23_P71415	WDYHV1	Hs.18029	WDYHV motif containing 1	Homo sapiens WDYHV motif containing	1073.72860	1926.77200	1060.15000	1605.51440	Detected	Detected	Detected	Detected
2141	A_24_P100742	ADD1	Hs.183706	adducin 1 (alpha)	Homo sapiens adducin 1 (alpha) (ADD1)	419.41248	576.48706	252.18817	327.34274	Detected	Detected	Detected	Detected
2142	A_33_P3295550	TET1	Hs.567594	tet oncogene 1	Homo sapiens tet oncogene 1 (TET1), n	386.89430	152.28192	346.90192	118.27197	Detected	Detected	Detected	Detected
2143	A_23_P251421	CDCA7	Hs.470654	cell division cycle associated 7	Homo sapiens cell division cycle associ	738.63850	895.73470	584.07320	594.63180	Detected	Detected	Detected	Detected
2144	A_33_P3294961	LAT2		linker for activation of T cells family, me	Linker for activation of T-cells family m	9568.57700	7590.82200	1616.10280	1136.18000	Detected	Detected	Detected	Detected
2145	A_33_P3286846	TP53TG3	Hs.592038	TP53 target 3	Homo sapiens TP53 target 3 (TP53TG3	41.54462	30.56686	24.25711	19.88500	Detected	Detected	Detected	Detected
2146	A_33_P3267160	LOC100132652	Hs.650662	similar to hCG2038304	PREDICTED: Homo sapiens similar to h	145.90959	27.01693	51.06142	6.01555	Detected	Detected	Detected	Compromised
2147	A_33_P3283485		Hs.683875		Hypothetical LOC440337cDNA FLJ370	84.29483	120.65002	76.91571	93.91841	Detected	Detected	Detected	Detected
2148	A_23_P256084	ARSE	Hs.386975	arylsulfatase E (chondrodysplasia punct	Homo sapiens arylsulfatase E (chondroc	50.32349	27.66060	58.17704	32.45991	Detected	Detected	Detected	Detected
2149	A_32_P226646	LOC100129781		hypothetical protein LOC100129781	PREDICTED: Homo sapiens hypothetica	63.69746	120.96450	53.89512	81.25420	Detected	Detected	Detected	Detected
2150	A_23_P73097	RGS20	Hs.368733	regulator of G-protein signaling 20	Homo sapiens regulator of G-protein sig	1431.64160	1727.12650	1357.82030	1455.30020	Detected	Detected	Detected	Detected
2151	A_23_P322116	TFAP2E	Hs.567844	transcription factor AP-2 epsilon (activ	Homo sapiens transcription factor AP-2	178.71380	96.92868	97.19186	44.28960	Detected	Detected	Detected	Detected
2152	A_33_P3356587	LOC100134040	Hs.552781	hypothetical LOC100134040	Homo sapiens mRNA; cDNA DKFZp434	149.45530	20.37862	85.51138	12.53385	Detected	Detected	Detected	Compromised
2153	A_33_P3421219	LOC647070	Hs.660884	hypothetical LOC647070	PREDICTED: Homo sapiens hypothetica	8720.20900	7727.40200	1836.07240	1470.98300	Detected	Detected	Detected	Detected
2154	A_23_P98898	CDK2	Hs.19192	cyclin-dependent kinase 2	Homo sapiens cyclin-dependent kinase	86.59659	193.96509	74.40871	167.57513	Detected	Detected	Detected	Detected
2155	A_33_P3321417	DMAP1	Hs.8008	DNA methyltransferase 1 associated pr	Homo sapiens DNA methyltransferase 1	321.52026	492.20490	305.56003	457.69614	Detected	Detected	Detected	Detected
2156	A_33_P3223097	C1orf97	Hs.523932	chromosome 1 open reading frame 97	Homo sapiens chromosome 1 open read	798.95514	370.15735	1010.64660	354.80050	Detected	Detected	Detected	Detected
2157	A_33_P3296074	ZNF131	Hs.535804	zinc finger protein 131	Homo sapiens zinc finger protein 131 (Z	7660.87060	7271.48540	6455.40100	5072.38570	Detected	Detected	Detected	Detected
2158	A_23_P70249	CDC25C	Hs.656	cell division cycle 25 homolog C (S. pom	Homo sapiens cell division cycle 25 hom	30.77745	1855.70400	18.09614	1125.36250	Detected	Detected	Detected	Detected
2159	A_33_P3406636	ZFP62	Hs.509227	zinc finger protein 62 homolog (mouse)	Homo sapiens zinc finger protein 62 hom	30.76229	25.87432	22.76644	13.32350	Detected	Detected	Detected	Compromised
2160	A_23_P28120	SIX2	Hs.101937	SIX homeobox 2	Homo sapiens SIX homeobox 2 (SIX2), r	64.34388	63.19782	56.07661	57.46753	Detected	Detected	Detected	Detected
2161	A_23_P356152	MAPK8	Hs.138211	mitogen-activated protein kinase 8	Homo sapiens mitogen-activated protei	29.49658	33.55302	32.26333	34.59254	Detected	Detected	Detected	Detected
2162	A_23_P90444	RBM42	Hs.5086	RNA binding motif protein 42	Homo sapiens RNA binding motif protei	5058.90770	6732.50900	5283.52730	6230.69300	Detected	Detected	Detected	Detected
2163	A_33_P3299791					24.16588	14.36726	9.19799	6.49271	Detected	Compromised	Compromised	Compromised
2164	A_33_P3312222				Putative TAF11-like protein ENSP0000	16.40478	7.81293	3.52555	7.61020	Detected	Compromised	Compromised	Compromised
2165	A_33_P3397433					603.29710	626.84030	44.88273	36.20022	Detected	Detected	Detected	Detected
2166	A_23_P256735	PGCP	Hs.156178	plasma glutamate carboxypeptidase	Homo sapiens plasma glutamate carbox	2672.74540	2849.21700	2260.57960	2124.31230	Detected	Detected	Detected	Detected
2167	A_23_P106773	SULT1A2	Hs.546304	sulfotransferase family, cytosolic, 1A, pl	Homo sapiens sulfotransferase family, c	2024.97100	637.23364	622.47650	173.98610	Detected	Detected	Detected	Detected
2168	A_23_P143446	MRPL39	Hs.420696	mitochondrial ribosomal protein L39	Homo sapiens mitochondrial ribosomal p	4360.02100	5785.40900	3645.77120	4162.60940	Detected	Detected	Detected	Detected
2169	A_23_P45488	FAM3A	Hs.289108	family with sequence similarity 3, memb	Homo sapiens family with sequence sim	532.11470	430.76670	485.73850	387.15192	Detected	Detected	Detected	Detected
2170	A_23_P410717	C1orf51	Hs.54680	chromosome 1 open reading frame 51	Homo sapiens chromosome 1 open read	135.76960	680.90520	159.60750	692.22840	Detected	Detected	Detected	Detected
2171	A_24_P59667	JAK3	Hs.515247	Janus kinase 3	Homo sapiens Janus kinase 3 (JAK3), m	5083.65430	518.39813	4898.01760	492.29178	Detected	Detected	Detected	Detected

2172	A_24_P296698	MAP2K3	Hs.514012	mitogen-activated protein kinase kinase	Homo sapiens mitogen-activated protei	28513.53500	47226.51600	27793.79100	41260.28500	Detected	Detected	Detected	Detected
2173	A_33_P3344229	HIST1H4A	Hs.248178	histone cluster 1, H4a	Homo sapiens histone cluster 1, H4a (H	168.59038	588.18440	47.36457	116.33629	Detected	Detected	Detected	Detected
2174	A_33_P3305023	WWOX	Hs.461453	WW domain containing oxidoreductase	Homo sapiens WW domain containing ox	143.47217	200.21758	92.02263	169.71037	Detected	Detected	Detected	Detected
2175	A_23_P44724	CSRP2	Hs.530904	cysteine and glycine-rich protein 2	Homo sapiens cysteine and glycine-rich	3936.21310	4890.64450	3245.25660	3531.87520	Detected	Detected	Detected	Detected
2176	A_33_P3309039	CPSF7	Hs.720314	cleavage and polyadenylation specific fa	Homo sapiens cleavage and polyadenyla	7384.22200	9523.13100	4383.47500	4625.43400	Detected	Detected	Detected	Detected
2177	A_33_P3357651	KRTAP10-12	Hs.297526	keratin associated protein 10-12	Homo sapiens keratin associated protei	4149.05220	5206.43070	769.85034	594.25415	Detected	Detected	Detected	Detected
2178	A_33_P3389376	MDM4	Hs.497492	Mdm4 p53 binding protein homolog (mou	Homo sapiens Mdm4 p53 binding protei	139.39375	40.00084	116.69451	33.00345	Detected	Detected	Detected	Detected
2179	A_23_P12816	HELLS	Hs.655830	helicase, lymphoid-specific	Homo sapiens helicase, lymphoid-specif	90.17854	352.28516	60.02296	240.76237	Detected	Detected	Detected	Detected
2180	A_23_P153320	ICAM1	Hs.643447	intercellular adhesion molecule 1	Homo sapiens intercellular adhesion mo	3194.19000	1615.29640	2412.40380	1155.25070	Detected	Detected	Detected	Detected
2181	A_33_P3211739	CREB3L2	Hs.490273	cAMP responsive element binding prote	Homo sapiens cAMP responsive elemer	14823.48900	28261.16600	8724.36600	14111.46600	Detected	Detected	Detected	Detected
2182	A_24_P93452					39954.99000	30688.05900	7547.80600	4776.35700	Detected	Detected	Detected	Detected
2183	A_23_P344281	ZIK1	Hs.454830	zinc finger protein interacting with K pr	Homo sapiens zinc finger protein intera	429.73486	588.41700	390.22230	438.00925	Detected	Detected	Detected	Detected
2184	A_23_P250102	CAND2	Hs.343664	cullin-associated and neddylation-disso	Homo sapiens cullin-associated and ne	253.27393	230.94250	146.74785	116.05359	Detected	Detected	Detected	Detected
2185	A_23_P133424	SKP1	Hs.171626	S-phase kinase-associated protein 1	Homo sapiens S-phase kinase-associat	50433.71500	57696.20700	32667.36700	30591.62900	Detected	Detected	Detected	Detected
2186	A_33_P3358342	SIAH2	Hs.477959	seven in absentia homolog 2 (Drosophila	Homo sapiens seven in absentia homolo	11107.50500	11433.97200	6966.80800	6147.84100	Detected	Detected	Detected	Detected
2187	A_33_P3362148	LOC284900		hypothetical LOC284900	Homo sapiens hypothetical LOC284900	236.40208	50.62920	235.46126	35.74389	Detected	Detected	Detected	Detected
2188	A_33_P3298930	LOC389300	Hs.254516	similar to hCG1654959	PREDICTED: Homo sapiens similar to h	8.30819	106.49459	4.20289	41.41038	Compromised	Detected	Compromised	Detected
2189	A_33_P3288871		Hs.719858		DC378344 PEBLM2 Homo sapiens cDN	22.87634	28.15365	23.43205	26.95001	Detected	Detected	Detected	Detected
2190	A_23_P120227	LBH	Hs.567598	limb bud and heart development homolo	Homo sapiens limb bud and heart devel	903.55070	4713.00240	603.57950	2757.51220	Detected	Detected	Detected	Detected
2191	A_23_P202448	CXCL12	Hs.522891	chemokine (C-X-C motif) ligand 12 (str	Homo sapiens chemokine (C-X-C motif	8794.89400	19656.62100	7958.87740	15016.15100	Detected	Detected	Detected	Detected
2192	A_23_P417331	RPS6KA3	Hs.445387	ribosomal protein S6 kinase, 90kDa, poly	Homo sapiens ribosomal protein S6 kina	1742.10270	1059.73170	1637.30760	842.18463	Detected	Detected	Detected	Detected
2193	A_23_P126939	RAB7L1	Hs.115325	RAB7, member RAS oncogene family-li	Homo sapiens RAB7, member RAS onc	1428.78080	1202.10570	1253.90190	803.31780	Detected	Detected	Detected	Detected
2194	A_24_P329795	C10orf10	Hs.93675	chromosome 10 open reading frame 10	Homo sapiens chromosome 10 open rea	11099.41600	1655.16410	5629.94530	748.05273	Detected	Detected	Detected	Detected
2195	A_23_P103310	S100A7	Hs.112408	S100 calcium binding protein A7	Homo sapiens S100 calcium binding pro	100.88084	87.61566	30.71276	21.59525	Detected	Detected	Detected	Detected
2196	A_33_P3262028					113.34728	117.98805	49.00671	47.59304	Detected	Detected	Detected	Detected
2197	A_32_P187599	SERBP1	Hs.719078	SERPINE1 mRNA binding protein 1	Homo sapiens SERPINE1 mRNA binding	10189.00100	15505.64100	7315.48900	8935.55400	Detected	Detected	Detected	Detected
2198	A_33_P3389271	LOC100129058	Hs.628892	similar to hCG2038656	PREDICTED: Homo sapiens similar to h	16.90221	19.96329	8.11624	10.04934	Detected	Compromised	Compromised	Compromised
2199	A_33_P3260223	CYorf15B	Hs.592254	chromosome Y open reading frame 15B	Homo sapiens chromosome Y open read	756.09600	100.16287	471.55392	17.26516	Detected	Detected	Detected	Compromised
2200	A_23_P254179	ADNP	Hs.570355	activity-dependent neuroprotector hom	Homo sapiens activity-dependent neur	2665.11840	2565.74460	1925.34220	1620.22530	Detected	Detected	Detected	Detected
2201	A_23_P155514	AHSG	Hs.324746	alpha-2-HS-glycoprotein	Homo sapiens alpha-2-HS-glycoprotein	23.08028	28.02454	20.88123	24.60150	Detected	Detected	Detected	Detected
2202	A_33_P3229402	PECAM1	Hs.514412	platelet/endothelial cell adhesion molec	Homo sapiens platelet/endothelial cell a	42.67268	11.65248	42.51489	3.55187	Detected	Compromised	Detected	Compromised
2203	A_23_P107132	RCVRN	Hs.80539	recoverin	Homo sapiens recoverin (RCVRN), mRN	2.55484	3.49403	12.88450	3.02340	Compromised	Compromised	Detected	Compromised
2204	A_24_P129277	NOD1	Hs.405153	nucleotide-binding oligomerization doma	Homo sapiens nucleotide-binding oligom	544.28796	197.63246	301.43228	94.44206	Detected	Detected	Detected	Detected
2205	A_23_P413923	DMRTA1	Hs.371976	DMRT-like family A1	Homo sapiens DMRT-like family A1 (DM	21.53626	11.97686	23.54715	6.21829	Detected	Compromised	Detected	Compromised
2206	A_33_P3358069	IDH3B	Hs.436405	isocitrate dehydrogenase 3 (NAD+) beta	Homo sapiens isocitrate dehydrogenase	6254.59400	6370.98400	4995.89900	4495.41400	Detected	Detected	Detected	Detected
2207	A_23_P84070	LARP7	Hs.713663	La ribonucleoprotein domain family, mer	Homo sapiens La ribonucleoprotein dom	3639.43140	3407.23220	3055.93950	2514.66110	Detected	Detected	Detected	Detected
2208	A_23_P99138	MRPL51	Hs.55847	mitochondrial ribosomal protein L51	Homo sapiens mitochondrial ribosomal p	35343.50800	44817.11700	28397.49600	31078.77700	Detected	Detected	Detected	Detected
2209	A_23_P427622	UNQ1887	Hs.507087	signal peptide peptidase 3	Homo sapiens signal peptide peptidase	850.40826	1059.72250	681.38116	782.64075	Detected	Detected	Detected	Detected
2210	A_33_P3548860	SEC1		secretory blood group 1	Homo sapiens secretory blood group 1	50.33355	24.58209	31.65206	18.54473	Detected	Detected	Detected	Detected
2211	A_24_P72479	ARPC1A	Hs.124126	actin related protein 2/3 complex, subu	Homo sapiens actin related protein 2/3	7425.28470	16909.78100	7775.52100	16076.08600	Detected	Detected	Detected	Detected
2212	A_33_P3209229	RAB26	Hs.3797	RAB26, member RAS oncogene family	Homo sapiens RAB26, member RAS on	237.89832	130.20642	191.36688	87.62357	Detected	Detected	Detected	Detected
2213	A_33_P3262124	MMP25	Hs.654979	matrix metallopeptidase 25	Homo sapiens matrix metallopeptidase 2	49.90495	63.90376	15.99097	28.49379	Detected	Detected	Detected	Detected
2214	A_23_P105118	OR51G1	Hs.554541	olfactory receptor, family 51, subfamily	Homo sapiens olfactory receptor, family	16.03785	18.54282	5.06194	5.92097	Detected	Detected	Compromised	Compromised
2215	A_24_P912048	MGC50722	Hs.530383	hypothetical MGC50722	Homo sapiens hypothetical MGC50722	14.09464	12.12891	3.17680	3.28900	Detected	Compromised	Compromised	Compromised
2216	A_23_P117582	JDP2	Hs.196482	Jun dimerization protein 2	Homo sapiens Jun dimerization protein	14049.43900	18113.37500	11026.28700	13086.18100	Detected	Detected	Detected	Detected
2217	A_23_P114232	PRDX4	Hs.83383	peroxiredoxin 4	Homo sapiens peroxiredoxin 4 (PRDX4)	43424.86700	60481.24000	33372.80500	38828.88300	Detected	Detected	Detected	Detected
2218	A_23_P351138	CLDN9	Hs.296949	claudin 9	Homo sapiens claudin 9 (CLDN9), mRN	152.69121	130.89291	52.91251	49.96745	Detected	Detected	Detected	Detected
2219	A_33_P3277953	LOC100128811	Hs.700407	hypothetical protein LOC100128811	Homo sapiens hypothetical protein LOC	89.69562	60.53955	19.76451	15.33971	Detected	Detected	Detected	Compromised
2220	A_33_P3235217	CTPS	Hs.473087	CTP synthase	Homo sapiens CTP synthase (CTPS), m	4127.44730	9287.70000	3256.01560	6842.28760	Detected	Detected	Detected	Detected
2221	A_23_P100868	MYO19	Hs.302051	myosin XIX	Homo sapiens myosin XIX (MYO19), tra	907.86860	2868.36600	831.71230	2396.28930	Detected	Detected	Detected	Detected
2222	A_23_P203013	NPAT	Hs.171061	nuclear protein, ataxia-telangiectasia lo	Homo sapiens nuclear protein, ataxia-te	234.98949	200.56412	118.76802	89.26743	Detected	Detected	Detected	Detected
2223	A_23_P130444	ZNF701	Hs.710468	zinc finger protein 701	Homo sapiens zinc finger protein 701 (Z	225.70730	216.00700	126.91888	100.40543	Detected	Detected	Detected	Detected
2224	A_23_P384056	CCDC14	Hs.645028	coiled-coil domain containing 14	Homo sapiens coiled-coil domain contai	4390.66750	2669.29350	3534.41630	1830.88380	Detected	Detected	Detected	Detected
2225	A_23_P100539	ABCC6	Hs.442182	ATP-binding cassette, sub-family C (CF	Homo sapiens ATP-binding cassette, su	36.33172	25.74478	21.94041	32.76024	Detected	Detected	Detected	Not Detected
2226	A_33_P3277437	ERI2	Hs.248437	ERI1 exoribonuclease family member 2	Homo sapiens ERI1 exoribonuclease fa	61.34220	146.50820	41.35730	90.73431	Detected	Detected	Detected	Detected
2227	A_33_P3265304				Uncharacterized protein C20orf4 [Sour	18.69924	19.75395	8.43305	19.66085	Detected	Detected	Compromised	Detected
2228	A_23_P301138	IP6K2	Hs.595983	inositol hexakisphosphate kinase 2	Homo sapiens inositol hexakisphosphate	863.15290	1375.04430	706.62585	965.75920	Detected	Detected	Detected	Detected
2229	A_33_P3279949	LOC100128170	Hs.26608	hypothetical protein LOC100128170	Homo sapiens cDNA FLJ42176 fis, clon	21.82879	18.54460	8.16503	9.31322	Detected	Compromised	Compromised	Compromised
2230	A_23_P253221	ARHGEF4	Hs.469935	Rho guanine nucleotide exchange facto	Homo sapiens Rho guanine nucleotide e	269.74258	629.06146	230.65292	542.83954	Detected	Detected	Detected	Detected
2231	A_23_P141505	CLEC10A	Hs.54403	C-type lectin domain family 10, member	Homo sapiens C-type lectin domain fan	41.10267	65.10747	12.77112	15.40458	Detected	Detected	Compromised	Compromised
2232	A_33_P3260563	SOX30	Hs.716685	SRY (sex determining region Y)-box 30	Homo sapiens SRY (sex determining reg	24.12229	18.30031	10.51070	6.75890	Detected	Compromised	Compromised	Compromised
2233	A_23_P15603	MRM1	Hs.194864	mitochondrial rRNA methyltransferase 1	Homo sapiens mitochondrial rRNA meth	172.99522	217.42647	233.54960	280.97116	Detected	Detected	Detected	Detected
2234	A_24_P636974	LOC100289383	Hs.652458	hypothetical protein LOC100289383	PREDICTED: Homo sapiens hypothetica	59.58913	38.06313	41.48145	21.26539	Detected	Detected	Detected	Detected
2235	A_23_P121637	PRSS12	Hs.654823	protease, serine, 12 (neurotrypsin, motc	Homo sapiens protease, serine, 12 (neu	566.19640	661.61200	361.07407	367.82794	Detected	Detected	Detected	Detected
2236	A_24_P332230	LARP7	Hs.713663	La ribonucleoprotein domain family, mer	Homo sapiens La ribonucleoprotein dom	3040.30370	2972.47300	2034.76760	1538.20730	Detected	Detected	Detected	Detected
2237	A_23_P157007	TMEM176B	Hs.647090	transmembrane protein 176B	Homo sapiens transmembrane protein 1	234.20009	36.85263	218.94563	8.39808	Detected	Detected	Detected	Compromised
2238	A_33_P3409086	S100A1	Hs.515715	S100 calcium binding protein A1	Homo sapiens S100 calcium binding pro	211.72447	93.30845	33.45704	17.77634	Detected	Detected	Detected	Detected
2239	A_23_P115011	ADAMTSL4	Hs.719991	ADAMTS-like 4	Homo sapiens ADAMTS-like 4 (ADAMT	84.49645	43.40765	69.13534	26.89241	Detected	Detected	Detected	Detected
2240	A_23_P122976	GNAI1	Hs.134587	guanine nucleotide binding protein (G pr	Homo sapiens guanine nucleotide bindin	594.19720	660.33220	425.53143	366.53497	Detected	Detected	Detected	Detected
2241	A_23_P401024	C16orf81	Hs.375664	chromosome 16 open reading frame 81	Homo sapiens chromosome 16 open rea	15.11145	4.01311	3.42210	3.53370	Detected	Compromised	Compromised	Compromised
2242	A_33_P3298771				Putative olfactory receptor 52L2 (Olfac	74.45934	69.03259	30.15762	24.54872	Detected	Detected	Detected	Detected
2243	A_33_P3229156	SLC17A9	Hs.512686	solute carrier family 17, member 9	Homo sapiens solute carrier family 17, r	2997.91280	6665.39450	1711.73800	3596.43820	Detected	Detected	Detected	Detected
2244	A_24_P649624	KIF1B	Hs.97858	kinesin family member 1B	Homo sapiens kinesin family member 1E	2085.14140	4076.48410	1365.62900	2145.11770	Detected	Detected	Detected	Detected
2245	A_23_P354074	LYST	Hs.532411	lysosomal trafficking regulator	Homo sapiens lysosomal trafficking regu	1707.49670	529.70337	914.33340	230.33540	Detected	Detected	Detected	Detected
2246	A_32_P56525	FAM115A	Hs.406492	family with sequence similarity 115, mer	Homo sapiens family with sequence sim	1477.64530	1387.72330	409.13538	335.47876	Detected	Detected	Detected	Detected

2247	A_33_P3381338	TNXB	Hs.485104	tenascin XB	Homo sapiens tenascin XB (TNXB), trar	1737.41860	245.88000	1497.45800	176.52000	Detected	Detected	Detected	Detected
2248	A_23_P105028	ATL3	Hs.356719	atlastin GTPase 3	Homo sapiens atlastin GTPase 3 (ATL3	3835.88820	6868.41700	2637.57250	4000.02600	Detected	Detected	Detected	Detected
2249	A_23_P420236	PCDH9	Hs.654709	protocadherin 9	Homo sapiens protocadherin 9 (PCDH9	17.59978	4.98287	9.23901	6.23751	Detected	Compromised	Compromised	Compromised
2250	A_23_P114952	TMEM9	Hs.181444	transmembrane protein 9	Homo sapiens transmembrane protein 9	2419.35700	2150.95360	2236.89400	1807.28310	Detected	Detected	Detected	Detected
2251	A_23_P66110	TSC2	Hs.90303	tuberous sclerosis 2	Homo sapiens tuberous sclerosis 2 (TS	1141.37870	940.88300	1206.94290	935.42130	Detected	Detected	Detected	Detected
2252	A_33_P3237784	PORCN	Hs.386453	porcupine homolog (Drosophila)	Homo sapiens porcupine homolog (Dros	8853.98400	9558.10800	9307.83500	8737.75000	Detected	Detected	Detected	Detected
2253	A_23_P215913	CLU	Hs.436657	clusterin	Homo sapiens clusterin (CLU), transcrip	556.66350	1041.31640	430.91937	827.94867	Detected	Detected	Detected	Detected
2254	A_33_P3252191				Protein kinase C epsilon type (EC 2.7.1	45.22749	81.90081	24.73911	41.82303	Detected	Detected	Detected	Detected
2255	A_33_P3362232					29.78399	30.27191	36.29910	19.74011	Detected	Detected	Detected	Detected
2256	A_23_P105002	ROM1	Hs.281564	retinal outer segment membrane protei	Homo sapiens retinal outer segment me	5963.19240	2177.42240	4960.14600	1572.18270	Detected	Detected	Detected	Detected
2257	A_23_P203888	MMP19	Hs.591033	matrix metallopeptidase 19	Homo sapiens matrix metallopeptidase	6055.67720	5066.75930	4615.29740	2811.35000	Detected	Detected	Detected	Detected
2258	A_23_P411833	WDR90	Hs.511903	WD repeat domain 90	Homo sapiens WD repeat domain 90 (W	6949.26070	3522.06450	5872.54300	2686.40550	Detected	Detected	Detected	Detected
2259	A_33_P3214314	FAM190B	Hs.461988	family with sequence similarity 190, mer	Homo sapiens family with sequence sim	600.41350	534.51690	438.94980	372.31244	Detected	Detected	Detected	Detected
2260	A_33_P3363485	SOBP	Hs.445244	sine oculis binding protein homolog (Dro	Homo sapiens sine oculis binding protei	39.57497	13.00156	25.01229	3.99202	Detected	Compromised	Detected	Compromised
2261	A_23_P103919	B4GALT3	Hs.321231	UDP-Gal:betaGlcNAc beta 1,4- galacto	Homo sapiens UDP-Gal:betaGlcNAc be	2553.43900	4191.12000	1927.71470	2816.99600	Detected	Detected	Detected	Detected
2262	A_33_P3389291	TEX9	Hs.511476	testis expressed 9	Homo sapiens testis expressed 9 (TEX9	13.78618	3.73410	6.28083	3.20531	Detected	Compromised	Compromised	Compromised
2263	A_23_P148807	CDC7	Hs.533573	cell division cycle 7 homolog (S. cerevis	Homo sapiens cell division cycle 7 homo	603.62463	534.75793	404.50137	334.83093	Detected	Detected	Detected	Detected
2264	A_33_P3380751	ST8SIA1	Hs.408614	ST8 alpha-N-acetyl-neuraminide alpha-	Homo sapiens ST8 alpha-N-acetyl-neu	105.47878	71.12400	39.71795	25.37727	Detected	Detected	Detected	Detected
2265	A_33_P3240144					97.15759	28.22687	91.06741	23.14658	Detected	Detected	Detected	Detected
2266	A_23_P35684	INPP5F	Hs.369755	inositol polyphosphate-5-phosphatase	Homo sapiens inositol polyphosphate-5	521.02110	556.69257	453.20694	435.05463	Detected	Detected	Detected	Detected
2267	A_23_P98252	ARL2	Hs.502836	ADP-ribosylation factor-like 2	Homo sapiens ADP-ribosylation factor-	5361.07500	3142.39550	8310.67600	4274.89750	Detected	Detected	Detected	Detected
2268	A_23_P30200	PCDHB16	Hs.147674	protocadherin beta 16	Homo sapiens protocadherin beta 16 (P	48.44599	58.20495	26.67815	30.42681	Detected	Detected	Detected	Detected
2269	A_23_P165691	PSMD14	Hs.567410	proteasome (prosome, macropain) 26S	Homo sapiens proteasome (prosome, m	16447.11100	32044.76600	13285.49300	23190.53300	Detected	Detected	Detected	Detected
2270	A_33_P3308903	MIB2	Hs.135805	mindbomb homolog 2 (Drosophila)	Homo sapiens mindbomb homolog 2 (Dr	832.52200	298.79830	1062.63570	411.41678	Detected	Detected	Detected	Detected
2271	A_33_P3252099	ANKLE1	Hs.721610	ankyrin repeat and LEM domain contain	Homo sapiens ankyrin repeat and LEM	57.83297	21.29430	51.61974	10.38640	Detected	Detected	Detected	Compromised
2272	A_33_P3255509	FCHO1	Hs.96485	FCH domain only 1	Homo sapiens FCH domain only 1 (FCH	2036.98400	666.85504	1496.57320	418.47750	Detected	Detected	Detected	Detected
2273	A_33_P3391455	SCN5A	Hs.517898	sodium channel, voltage-gated, type V,	Homo sapiens sodium channel, voltage-	34.67255	29.28114	23.75503	17.45392	Detected	Detected	Detected	Detected
2274	A_24_P301547	C17orf80	Hs.12929	chromosome 17 open reading frame 80	Homo sapiens chromosome 17 open rea	356.60373	264.80038	302.94946	210.37581	Detected	Detected	Detected	Detected
2275	A_24_P92174		Hs.720867		Putative uncharacterized protein ENSP	14.42619	11.46667	8.97610	14.98599	Detected	Compromised	Compromised	Compromised
2276	A_33_P3297921	MRPL37	Hs.584908	mitochondrial ribosomal protein L37	Homo sapiens mitochondrial ribosomal	3307.95040	7516.95500	4147.50050	8528.30100	Detected	Detected	Detected	Detected
2277	A_23_P58993	MOCS1	Hs.718492	molybdenum cofactor synthesis 1	Homo sapiens molybdenum cofactor sy	309.97635	351.87833	265.80054	301.78418	Detected	Detected	Detected	Detected
2278	A_33_P3346866	C12orf41	Hs.720554	chromosome 12 open reading frame 41	Homo sapiens chromosome 12 open rea	18073.75000	18410.57600	14212.39300	12396.09500	Detected	Detected	Detected	Detected
2279	A_23_P84910	ZNF157	Hs.89897	zinc finger protein 157	Homo sapiens zinc finger protein 157 (Z	171.63087	108.57980	118.51070	70.23446	Detected	Detected	Detected	Detected
2280	A_23_P257795	NDUFA2	Hs.534333	NADH dehydrogenase (ubiquinone) 1 alp	Homo sapiens NADH dehydrogenase (u	11323.34400	10749.16000	12575.50800	10224.40900	Detected	Detected	Detected	Detected
2281	A_24_P661641	LOC400027	Hs.597122	hypothetical protein LOC400027	Homo sapiens hypothetical protein LOC	852.02484	804.05835	540.11926	403.35138	Detected	Detected	Detected	Detected
2282	A_24_P45446	GBP4	Hs.409925	guanylate binding protein 4	Homo sapiens guanylate binding protein	92.93600	99.17304	53.70445	52.06817	Detected	Detected	Detected	Detected
2283	A_33_P3391578	RPL23AP64		ribosomal protein L23a pseudogene 64	Homo sapiens ribosomal protein L23a p	582.32294	929.96430	76.10249	102.24319	Detected	Detected	Detected	Detected
2284	A_23_P397376	MAF	Hs.134859	v-maf musculoaponeurotic fibrosarcoma	Homo sapiens v-maf musculoaponeurot	118.95010	90.76361	65.83588	32.35656	Detected	Detected	Detected	Detected
2285	A_23_P110167	MGST2	Hs.81874	microsomal glutathione S-transferase 2	Homo sapiens microsomal glutathione S	667.10150	748.03790	505.60230	478.87340	Detected	Detected	Detected	Detected
2286	A_23_P53467	IKBIP	Hs.252543	IKBKB interacting protein	Homo sapiens IKBKB interacting protein	5008.28200	9636.49600	3932.61350	5971.43260	Detected	Detected	Detected	Detected
2287	A_23_P121095	TOP2B	Hs.475733	topoisomerase (DNA) II beta 180kDa	Homo sapiens topoisomerase (DNA) II b	12027.82000	9583.20600	9226.52000	6139.55200	Detected	Detected	Detected	Detected
2288	A_23_P146187	RRS1	Hs.71827	RRS1 ribosome biogenesis regulator ho	Homo sapiens RRS1 ribosome biogenes	3865.25400	6045.78600	2811.62900	3729.31420	Detected	Detected	Detected	Detected
2289	A_24_P743672	LOC652147		similar to U5 snRNP-specific protein, 20	PREDICTED: Homo sapiens similar to U	701.98830	925.50100	209.33595	212.03127	Detected	Detected	Detected	Detected
2290	A_33_P3246995					22157.05000	16175.30600	5471.50800	3010.00780	Detected	Detected	Detected	Detected
2291	A_33_P3270966					61974.77700	70569.26600	48626.15000	48461.72300	Detected	Detected	Detected	Detected
2292	A_23_P115824	HNRNPH3	Hs.643472	heterogeneous nuclear ribonucleoprotei	Homo sapiens heterogeneous nuclear ri	1164.53650	2008.83870	879.66960	1258.73270	Detected	Detected	Detected	Detected
2293	A_23_P100654	ZBTB4	Hs.35096	zinc finger and BTB domain containing	Homo sapiens zinc finger and BTB dom	5847.12100	5767.18000	3201.71780	2691.91800	Detected	Detected	Detected	Detected
2294	A_33_P3216150	PRB4	Hs.528651	proline-rich protein BstNI subfamily 4	Homo sapiens proline-rich protein BstN	64.38258	154.75209	65.93984	137.54308	Detected	Detected	Detected	Detected
2295	A_23_P84334	TMEM39A	Hs.594171	transmembrane protein 39A	Homo sapiens transmembrane protein 3	680.84674	569.36450	460.59680	312.88730	Detected	Detected	Detected	Detected
2296	A_23_P109254	RBM39	Hs.282901	RNA binding motif protein 39	Homo sapiens RNA binding motif protei	8229.49900	6103.79150	6423.87060	3963.60800	Detected	Detected	Detected	Detected
2297	A_23_P113803	KATNA1	Hs.450175	katanin p60 (ATPase-containing) subun	Homo sapiens katanin p60 (ATPase-co	5619.92500	5718.28600	3773.20460	3247.87700	Detected	Detected	Detected	Detected
2298	A_23_P104972	MS4A5	Hs.178066	membrane-spanning 4-domains, subfam	Homo sapiens membrane-spanning 4-d	16.78607	21.26718	4.91905	27.14591	Detected	Detected	Compromised	Not Detected
2299	A_23_P35916	ATM	Hs.367437	ataxia telangiectasia mutated	Homo sapiens ataxia telangiectasia mut	84.30158	35.41534	68.13844	21.70727	Detected	Detected	Detected	Detected
2300	A_23_P157117	CREB5	Hs.437075	cAMP responsive element binding prote	Homo sapiens cAMP responsive elemer	293.64404	183.01367	229.37944	108.97150	Detected	Detected	Detected	Detected
2301	A_24_P330518	CA12	Hs.210995	carbonic anhydrase XII	Homo sapiens carbonic anhydrase XII (	4428.40000	1643.03880	2400.52400	860.14606	Detected	Detected	Detected	Detected
2302	A_33_P3414880	LOC339192	Hs.560498	hypothetical protein LOC339192	PREDICTED: Homo sapiens hypothetica	160.01030	40.00394	170.36528	20.03998	Detected	Detected	Detected	Detected
2303	A_33_P3212221	DEPDC5	Hs.435022	DEP domain containing 5	Homo sapiens DEP domain containing 5	31.63203	56.65369	17.75788	27.06622	Detected	Detected	Detected	Detected
2304	A_24_P147398	VPS53	Hs.461819	vacuolar protein sorting 53 homolog (S.	Homo sapiens vacuolar protein sorting	26.40779	65.23292	10.51792	33.12555	Detected	Detected	Compromised	Detected
2305	A_33_P3444555	LOC158960	Hs.553131	hypothetical protein BC009467	full-length cDNA clone CS0DI017YH09	35.71218	36.64232	31.67026	24.38922	Detected	Detected	Detected	Detected
2306	A_23_P119778	SLC39A10	Hs.650158	solute carrier family 39 (zinc transporte	Homo sapiens solute carrier family 39 (	287.13446	339.94570	209.61642	236.98593	Detected	Detected	Detected	Detected
2307	A_23_P380881	ANKRD13B	Hs.662164	ankyrin repeat domain 13B	Homo sapiens ankyrin repeat domain 1	75.89824	36.72879	80.31434	30.77660	Detected	Detected	Detected	Detected
2308	A_24_P45476	XCL1	Hs.546295	chemokine (C motif) ligand 1	Homo sapiens chemokine (C motif) ligar	17.33644	4.15300	5.57219	3.66432	Detected	Compromised	Compromised	Compromised
2309	A_33_P3220919	ADRBK2	Hs.657494	adrenergic, beta, receptor kinase 2	Homo sapiens adrenergic, beta, recepto	1639.76640	83.74319	1101.26150	49.08750	Detected	Detected	Detected	Detected
2310	A_32_P182439	POLD3	Hs.82502	polymerase (DNA-directed), delta 3, acc	Homo sapiens polymerase (DNA-directe	3067.75340	3528.29100	2416.95300	2339.55350	Detected	Detected	Detected	Detected
2311	A_33_P3311956	FEZ2	Hs.258563	fasciculation and elongation protein zet	Homo sapiens fasciculation and elongat	2765.81840	5453.91460	1905.21060	3373.72170	Detected	Detected	Detected	Detected
2312	A_23_P342600	STK35	Hs.100057	serine/threonine kinase 35	Homo sapiens serine/threonine kinase	19.39942	38.39412	30.40301	42.59630	Detected	Detected	Detected	Detected
2313	A_23_P113034	C10orf11	Hs.118161	chromosome 10 open reading frame 11	Homo sapiens chromosome 10 open rea	42.39873	6.20188	36.82369	5.09725	Detected	Compromised	Detected	Compromised
2314	A_33_P3367830	EFEMP2	Hs.719933	EGF-containing fibulin-like extracellular	Homo sapiens EGF-containing fibulin-li	18855.47500	36876.76600	16793.77300	29906.32600	Detected	Detected	Detected	Detected
2315	A_33_P3388357	WDR87	Hs.664194	WD repeat domain 87	Homo sapiens WD repeat domain 87 (W	23.53359	29.67230	13.95388	15.82918	Detected	Detected	Compromised	Compromised
2316	A_23_P115955	MRPL21	Hs.503047	mitochondrial ribosomal protein L21	Homo sapiens mitochondrial ribosomal	11540.94200	12961.13400	12064.25900	11277.12800	Detected	Detected	Detected	Detected
2317	A_23_P122674	PAK1IP1	Hs.310231	PAK1 interacting protein 1	Homo sapiens PAK1 interacting protein	2134.96780	2399.96070	1804.00430	1632.94310	Detected	Detected	Detected	Detected
2318	A_24_P419120	BICD2	Hs.436939	bicaudal D homolog 2 (Drosophila)	Homo sapiens bicaudal D homolog 2 (Dr	3104.85130	4019.31640	2326.99700	2546.62900	Detected	Detected	Detected	Detected
2319	A_24_P212481	MCTP1	Hs.655087	multiple C2 domains, transmembrane 1	Homo sapiens multiple C2 domains, trar	317.66730	77.89672	247.94930	62.20525	Detected	Detected	Detected	Detected
2320	A_32_P783	RPL37A	Hs.433701	ribosomal protein L37a	Homo sapiens ribosomal protein L37a (I	159747.81000	154882.25000	145008.95000	122386.18000	Detected	Detected	Detected	Detected
2321	A_24_P153043	RPL23AP7	Hs.588209	ribosomal protein L23a pseudogene 7	Homo sapiens ribosomal protein L23a p	89181.10000	80730.66000	47693.24200	40672.43400	Detected	Detected	Detected	Detected

2322	A_23_P120860	NIPSNAP1	Hs.173878	nipsnap homolog 1 (C. elegans)	Homo sapiens nipsnap homolog 1 (C. el	1474.94010	628.72100	971.49370	368.09573	Detected	Detected	Detected	Detected
2323	A_33_P3265075	PAAF1	Hs.525017	proteasomal ATPase-associated factor	Homo sapiens proteasomal ATPase-ass	5229.20500	3931.80000	4117.52500	2869.96300	Detected	Detected	Detected	Detected
2324	A_33_P3298980					96.71539	125.59820	64.30115	82.61039	Detected	Detected	Detected	Detected
2325	A_24_P273413	EML4	Hs.712760	echinoderm microtubule associated pro	Homo sapiens echinoderm microtubule	3847.96170	3082.88620	3131.32280	2203.41280	Detected	Detected	Detected	Detected
2326	A_23_P114826	MRPS15	Hs.352839	mitochondrial ribosomal protein S15	Homo sapiens mitochondrial ribosomal p	3457.69580	5077.08900	3611.84080	4459.99560	Detected	Detected	Detected	Detected
2327	A_23_P209799	MYO7B	Hs.154578	myosin VIIB	Homo sapiens myosin VIIB (MYO7B), m	13.81875	9.45723	3.54276	10.70073	Detected	Compromised	Compromised	Compromised
2328	A_33_P3281435				Putative uncharacterized protein ENSP	13.69518	29.49984	5.20377	14.68489	Detected	Detected	Compromised	Compromised
2329	A_23_P217737	ATP7A	Hs.496414	ATPase, Cu++ transporting, alpha polyp	Homo sapiens ATPase, Cu++ transporti	603.74194	304.36680	388.37433	176.14241	Detected	Detected	Detected	Detected
2330	A_33_P3794109	LOC253805	Hs.667492	hypothetical protein LOC253805	Homo sapiens hypothetical protein LOC	18.54796	14.31542	8.67144	15.58153	Detected	Compromised	Compromised	Compromised
2331	A_23_P153853	ECH1	Hs.196176	enoyl Coenzyme A hydratase 1, peroxis	Homo sapiens enoyl Coenzyme A hydra	3190.86820	1564.21000	3705.49270	1721.73010	Detected	Detected	Detected	Detected
2332	A_23_P150857	SUOX	Hs.558403	sulfite oxidase	Homo sapiens sulfite oxidase (SUOX), r	231.26935	215.29532	193.78323	196.90390	Detected	Detected	Detected	Detected
2333	A_33_P3416221	LOC732160	Hs.277677	similar to NADH dehydrogenase (ubiqui	PREDICTED: Homo sapiens similar to N	543.85850	570.05520	531.46090	429.83090	Detected	Detected	Detected	Detected
2334	A_23_P103110	MAFF	Hs.517617	v-maf musculoaponeurotic fibrosarcoma	Homo sapiens v-maf musculoaponeurot	2639.66720	3748.21100	1903.97560	2423.26700	Detected	Detected	Detected	Detected
2335	A_23_P55749	COL5A3	Hs.235368	collagen, type V, alpha 3	Homo sapiens collagen, type V, alpha 3	296.58588	557.20105	334.47590	573.40480	Detected	Detected	Detected	Detected
2336	A_33_P3389394	RPPH1		ribonuclease P RNA component H1	Homo sapiens ribonuclease P RNA com	373.21750	376.75820	369.51047	298.12772	Detected	Detected	Detected	Detected
2337	A_23_P407206	CLN8	Hs.127675	ceroid-lipofuscinosis, neuronal 8 (epilep	Homo sapiens ceroid-lipofuscinosis, neu	572.85394	360.61893	214.27405	124.31838	Detected	Detected	Detected	Detected
2338	A_23_P10743	PDE6B	Hs.654544	phosphodiesterase 6B, cGMP-specific,	Homo sapiens phosphodiesterase 6B, c	15.77939	15.68814	9.42206	6.51224	Detected	Compromised	Compromised	Compromised
2339	A_33_P3321836	PLXNB2	Hs.3989	plexin B2	Homo sapiens plexin B2 (PLXNB2), mRI	1604.13850	1513.96840	1136.73830	1142.26940	Detected	Detected	Detected	Detected
2340	A_33_P3385266	ABCC6	Hs.442182	ATP-binding cassette, sub-family C (CF	Homo sapiens ATP-binding cassette, su	214.72296	65.14271	185.94418	37.55487	Detected	Detected	Detected	Detected
2341	A_23_P110882	TSPYL4	Hs.284141	TSPY-like 4	Homo sapiens TSPY-like 4 (TSPYL4), n	3644.14970	3217.60940	2466.76070	1785.07780	Detected	Detected	Detected	Detected
2342	A_23_P138507	CDC2	Hs.334562	cell division cycle 2, G1 to S and G2 to	Homo sapiens cell division cycle 2, G1 t	119.53158	7760.52200	82.25016	4933.13040	Detected	Detected	Detected	Detected
2343	A_23_P7761	C5orf22	Hs.519246	chromosome 5 open reading frame 22	Homo sapiens chromosome 5 open read	395.45505	359.42400	306.30807	254.85413	Detected	Detected	Detected	Detected
2344	A_23_P320021	TULP1	Hs.485208	tubby like protein 1	Homo sapiens tubby like protein 1 (TUL	19.21633	13.03723	12.16025	5.70135	Detected	Compromised	Compromised	Compromised
2345	A_23_P306655	AMIGO3	Hs.567903	adhesion molecule with Ig-like domain 3	Homo sapiens adhesion molecule with I	125.28960	224.97696	121.32731	209.57895	Detected	Detected	Detected	Detected
2346	A_23_P115861	ZNF485	Hs.147440	zinc finger protein 485	Homo sapiens zinc finger protein 485 (Z	99.89243	100.61312	72.14692	53.58863	Detected	Detected	Detected	Detected
2347	A_33_P3279470	AGRP	Hs.104633	agouti related protein homolog (mouse)	Homo sapiens agouti related protein ho	82.88689	116.86708	38.09244	36.50859	Detected	Detected	Detected	Detected
2348	A_33_P3252228				Neurensin-1 (Vesicular membrane prote	34.89465	38.97252	18.90318	19.43680	Detected	Detected	Detected	Detected
2349	A_23_P335239	GAB1	Hs.80720	GRB2-associated binding protein 1	Homo sapiens GRB2-associated binding	16.29989	19.07008	5.39382	9.05337	Detected	Detected	Compromised	Compromised
2350	A_33_P3214720	ZC3H12A	Hs.656294	zinc finger CCCH-type containing 12A	Homo sapiens zinc finger CCCH-type c	1008.76904	626.99316	948.49530	575.10090	Detected	Detected	Detected	Detected
2351	A_23_P121851	PCDHB15	Hs.130757	protocadherin beta 15	Homo sapiens protocadherin beta 15 (P	3.14201	29.38744	2.94792	3.18877	Compromised	Not Detected	Compromised	Compromised
2352	A_24_P278367	ACTR1A	Hs.153961	ARP1 actin-related protein 1 homolog A	Homo sapiens ARP1 actin-related prote	4456.57400	6114.48100	3862.00780	5159.05600	Detected	Detected	Detected	Detected
2353	A_33_P3268567	NCK2	Hs.529244	NCK adaptor protein 2	Homo sapiens NCK adaptor protein 2 (N	13815.28600	15406.67900	13849.74900	14606.33600	Detected	Detected	Detected	Detected
2354	A_33_P3296193	PSIMCT-1	Hs.373741	MCTS1 pseudogene	Homo sapiens MCTS1 pseudogene (PSI	19.62921	16.89328	3.36859	4.04464	Detected	Compromised	Compromised	Compromised
2355	A_23_P66664	TADA2A	Hs.500066	transcriptional adaptor 2A	Homo sapiens transcriptional adaptor 2A	80.79347	138.64108	84.55923	121.70480	Detected	Detected	Detected	Detected
2356	A_23_P250485	TRIM62	Hs.656006	tripartite motif-containing 62	Homo sapiens tripartite motif-containin	123.15246	93.19906	65.55616	42.27545	Detected	Detected	Detected	Detected
2357	A_32_P53558					952.29114	868.94590	417.28198	317.28284	Detected	Detected	Detected	Detected
2358	A_33_P3420247	LOC340515	Hs.721129	hypothetical protein LOC340515	Homo sapiens cDNA clone IMAGE:5744	230.81766	141.04561	45.55514	20.18376	Detected	Detected	Detected	Detected
2359	A_23_P91552	FTCD	Hs.415846	formiminotransferase cyclodeaminase	Homo sapiens formiminotransferase cyc	24.26622	8.80801	24.03099	12.58043	Detected	Compromised	Detected	Compromised
2360	A_33_P3235029		Hs.556125		MGC3.5.1.2.1.H09.F.1 NIH_MGC_331 Hor	437.55576	439.25427	99.84553	89.06879	Detected	Detected	Detected	Detected
2361	A_23_P117037	LETMD1	Hs.655272	LETM1 domain containing 1	Homo sapiens LETM1 domain containin	1744.80190	1077.71440	1428.65520	729.49756	Detected	Detected	Detected	Detected
2362	A_33_P3254012	REXO1L1	Hs.373854	REX1, RNA exonuclease 1 homolog (S.	Homo sapiens REX1, RNA exonuclease	16.37702	20.68464	16.72645	21.46636	Detected	Detected	Compromised	Detected
2363	A_23_P11331	TCEAL8	Hs.389734	transcription elongation factor A (SII)-li	Homo sapiens transcription elongation f	12893.59100	10549.60600	10301.72200	7293.87100	Detected	Detected	Detected	Detected
2364	A_24_P252973	CLP1	Hs.523687	CLP1, cleavage and polyadenylation fac	Homo sapiens CLP1, cleavage and poly	1337.91860	1651.32930	1315.01070	1455.39640	Detected	Detected	Detected	Detected
2365	A_33_P3345036	POMC	Hs.1897	proopiomelanocortin	Homo sapiens proopiomelanocortin (PO	32.64461	54.94627	27.84700	39.46231	Detected	Detected	Detected	Detected
2366	A_23_P141146	FBXL20	Hs.462946	F-box and leucine-rich repeat protein 2	Homo sapiens F-box and leucine-rich r	106.12477	86.72531	116.26141	75.29050	Detected	Detected	Detected	Detected
2367	A_24_P48069	DOK4	Hs.279832	docking protein 4	Homo sapiens docking protein 4 (DOK4	1631.55110	1693.31930	1569.19860	1483.70290	Detected	Detected	Detected	Detected
2368	A_33_P3424794					284.04596	259.29382	98.61629	61.33932	Detected	Detected	Detected	Detected
2369	A_33_P3227686	FLJ43860	Hs.689547	FLJ43860 protein	Homo sapiens FLJ43860 protein (FLJ43	1396.64640	1927.15660	307.66913	251.40380	Detected	Detected	Detected	Detected
2370	A_23_P105900	BTF3L1	Hs.567241	basic transcription factor 3, like 1 pseu	Homo sapiens basic transcription factor	15126.01900	13230.79600	14303.48400	10592.33700	Detected	Detected	Detected	Detected
2371	A_23_P103486	CYP2J2	Hs.152096	cytochrome P450, family 2, subfamily J,	Homo sapiens cytochrome P450, family	122.54324	66.94190	69.79430	30.03850	Detected	Detected	Detected	Detected
2372	A_24_P34545	ING5	Hs.529172	inhibitor of growth family, member 5	Homo sapiens inhibitor of growth family	109.24323	278.54086	106.71159	276.43106	Detected	Detected	Detected	Detected
2373	A_33_P3386581	ANKRD40	Hs.463426	ankyrin repeat domain 40	Homo sapiens ankyrin repeat domain 40	7260.40500	8378.59500	5046.94340	5176.97460	Detected	Detected	Detected	Detected
2374	A_33_P3370015					315.73290	301.19565	137.75090	106.63371	Detected	Detected	Detected	Detected
2375	A_23_P106887	FUS	Hs.513522	fusion (involved in t(12;16) in malignant	Homo sapiens fusion (involved in t(12;1	6003.02340	9299.84600	4938.03370	7377.67430	Detected	Detected	Detected	Detected
2376	A_23_P208880	UHRF1	Hs.108106	ubiquitin-like with PHD and ring finger d	Homo sapiens ubiquitin-like with PHD a	543.58500	4040.31570	392.17184	2484.55930	Detected	Detected	Detected	Detected
2377	A_23_P64954	USP5	Hs.631661	ubiquitin specific peptidase 5 (isopeptid	Homo sapiens ubiquitin specific peptida	976.78754	1078.16750	800.31040	960.81220	Detected	Detected	Detected	Detected
2378	A_23_P204702	TMBIM6	Hs.708025	transmembrane BAX inhibitor motif con	Homo sapiens transmembrane BAX inhi	4795.31350	22768.22700	4798.39200	19181.36700	Detected	Detected	Detected	Detected
2379	A_23_P4679	ERF	Hs.655969	Ets2 repressor factor	Homo sapiens Ets2 repressor factor (EI	638.18970	706.96130	648.12100	651.82935	Detected	Detected	Detected	Detected
2380	A_23_P353035	IGFBP7	Hs.479808	insulin-like growth factor binding protei	Homo sapiens insulin-like growth factor	79215.16400	86658.94000	54895.63700	50116.73000	Detected	Detected	Detected	Detected
2381	A_32_P13795	DNAJC27	Hs.434993	DnaJ (Hsp40) homolog, subfamily C, me	Homo sapiens DnaJ (Hsp40) homolog, s	438.46200	353.58817	361.72600	252.60002	Detected	Detected	Detected	Detected
2382	A_23_P149892	CSGALNACT2	Hs.657569	chondroitin sulfate N-acetylgalactosam	Homo sapiens chondroitin sulfate N-ace	2310.70920	2849.39600	1682.21620	1818.00350	Detected	Detected	Detected	Detected
2383	A_33_P3347707	NOVA2	Hs.375439	neuro-oncological ventral antigen 2	Homo sapiens neuro-oncological ventra	32.60312	37.35228	13.70408	10.13262	Detected	Detected	Compromised	Compromised
2384	A_33_P3236591	RLTPR	Hs.611432	RGD motif, leucine rich repeats, tropom	Homo sapiens RGD motif, leucine rich r	2991.15280	3607.02830	1656.70750	1162.47530	Detected	Detected	Detected	Detected
2385	A_23_P397969	FOXK2	Hs.591140	forkhead box K2	Homo sapiens forkhead box K2 (FOXK2	3674.08230	3505.60990	2720.64280	2568.99300	Detected	Detected	Detected	Detected
2386	A_23_P71530	TNFRSF11B	Hs.81791	tumor necrosis factor receptor superfar	Homo sapiens tumor necrosis factor rec	826.93080	126.02147	664.49290	93.16795	Detected	Detected	Detected	Detected
2387	A_32_P84728	LOC100131727	Hs.563183	hypothetical LOC100131727	PREDICTED: Homo sapiens hypothetica	255.97444	104.44083	145.09705	50.93578	Detected	Detected	Detected	Detected
2388	A_32_P230547	DOCK7	Hs.406156	dedicator of cytokinesis 7	Homo sapiens dedicator of cytokinesis	2683.76070	2515.16480	1729.90760	1484.14820	Detected	Detected	Detected	Detected
2389	A_24_P56130	MYL6	Hs.632717	myosin, light chain 6, alkali, smooth mus	Homo sapiens myosin, light chain 6, alka	54984.75800	117847.17000	49092.33000	84939.58000	Detected	Detected	Detected	Detected
2390	A_23_P80940	PPAT	Hs.331420	phosphoribosyl pyrophosphate amidotra	Homo sapiens phosphoribosyl pyrophos	84.15266	98.54565	46.21906	55.32512	Detected	Detected	Detected	Detected
2391	A_33_P3403867	PMEPA1	Hs.517155	prostate transmembrane protein, andro	Homo sapiens prostate transmembrane	776.55720	2647.57470	671.12210	2054.87800	Detected	Detected	Detected	Detected
2392	A_24_P251688	ABCF3	Hs.361323	ATP-binding cassette, sub-family F (GC	Homo sapiens ATP-binding cassette, su	3906.01120	3670.72750	3223.85160	2901.86100	Detected	Detected	Detected	Detected
2393	A_23_P24234	OPN4	Hs.283922	opsin 4	Homo sapiens opsin 4 (OPN4), transcrip	111.43553	126.84243	79.82829	81.82214	Detected	Detected	Detected	Detected
2394	A_33_P3410194	H3F3B	Hs.180877	H3 histone, family 3B (H3.3B)	Homo sapiens H3 histone, family 3B (H3	4220.37840	2920.95850	3268.74370	1906.95950	Detected	Detected	Detected	Detected
2395	A_23_P96633	CXorf48	Hs.272804	chromosome X open reading frame 48	Homo sapiens chromosome X open read	54.30847	54.51199	31.63998	31.87781	Detected	Detected	Detected	Detected
2396	A_23_P83683	MORC1	Hs.591296	MORC family CW-type zinc finger 1	Homo sapiens MORC family CW-type zi	16.72377	24.94928	13.50091	15.25153	Detected	Detected	Compromised	Compromised

2397	A_23_P74737	EYA3	Hs.185774	eyes absent homolog 3 (Drosophila)	Homo sapiens eyes absent homolog 3 (I	89.79858	219.09164	89.88936	186.38850	Detected	Detected	Detected	Detected
2398	A_23_P5325	ERCC3	Hs.469872	excision repair cross-complementing ro	Homo sapiens excision repair cross-cor	2368.44340	2102.64090	2241.78780	1736.60680	Detected	Detected	Detected	Detected
2399	A_33_P3343972	RSPH1	Hs.661069	radial spoke head 1 homolog (Chlamydo	Homo sapiens radial spoke head 1 homo	25.61244	9.93981	16.24456	6.57159	Detected	Compromised	Detected	Compromised
2400	A_33_P3280801	LMO7	Hs.207631	LIM domain 7	Homo sapiens LIM domain 7 (LMO7), tra	226.85376	1123.61880	254.31744	1067.32710	Detected	Detected	Detected	Detected
2401	A_24_P174793	PCSK1	Hs.78977	proprotein convertase subtilisin/kexin t	Homo sapiens proprotein convertase su	2139.17900	31.84399	1470.16550	15.20441	Detected	Detected	Detected	Compromised
2402	A_23_P45871	IFI44L	Hs.389724	interferon-induced protein 44-like	Homo sapiens interferon-induced prote	100.15311	108.04471	78.70588	59.92763	Detected	Detected	Detected	Detected
2403	A_23_P132526	PIK3R4	Hs.149032	phosphoinositide-3-kinase, regulatory s	Homo sapiens phosphoinositide-3-kinas	1242.12610	1134.72310	889.77606	691.41895	Detected	Detected	Detected	Detected
2404	A_33_P3272698	DUSP23	Hs.425801	dual specificity phosphatase 23	Homo sapiens dual specificity phosphat	11618.40700	7114.15330	11984.11500	6283.75240	Detected	Detected	Detected	Detected
2405	A_23_P127684	OR5F1	Hs.553702	olfactory receptor, family 5, subfamily F	Homo sapiens olfactory receptor, family	39.46109	73.76115	24.93589	25.86773	Detected	Detected	Detected	Detected
2406	A_23_P208706	BAX	Hs.624291	BCL2-associated X protein	Homo sapiens BCL2-associated X prot	12109.02500	16313.72100	9929.89700	13850.42600	Detected	Detected	Detected	Detected
2407	A_33_P3336878	TMEM180	Hs.309069	transmembrane protein 180	Homo sapiens transmembrane protein 1	29.07715	11.84825	34.67252	18.70655	Detected	Compromised	Detected	Detected
2408	A_23_P380951	ZNF420	Hs.444992	zinc finger protein 420	Homo sapiens zinc finger protein 420 (Z	62.95886	46.31055	28.12083	16.25842	Detected	Detected	Detected	Compromised
2409	A_33_P3267185	ATP2B1	Hs.506276	ATPase, Ca++ transporting, plasma mer	Homo sapiens ATPase, Ca++ transporti	37.99635	38.57431	21.02027	22.89940	Detected	Detected	Detected	Detected
2410	A_23_P129801	RAB40B	Hs.484068	RAB40B, member RAS oncogene family	Homo sapiens RAB40B, member RAS o	1055.59060	886.05835	1047.19780	749.53270	Detected	Detected	Detected	Detected
2411	A_23_P121222	RAD18	Hs.375684	RAD18 homolog (S. cerevisiae)	Homo sapiens RAD18 homolog (S. cere	264.78900	864.88180	207.24443	512.35315	Detected	Detected	Detected	Detected
2412	A_23_P337270		Hs.506115		Serine/threonine-protein kinase Nek10	12.39888	53.23028	13.00825	45.76311	Compromised	Detected	Compromised	Detected
2413	A_23_P102060	SSFA2	Hs.591602	sperm specific antigen 2	Homo sapiens sperm specific antigen 2	3846.70300	3128.98460	2842.69290	1736.31290	Detected	Detected	Detected	Detected
2414	A_23_P375549	FUNDC1	Hs.7549	FUN14 domain containing 1	Homo sapiens FUN14 domain containing	1173.74800	1309.30520	1315.93520	1224.60400	Detected	Detected	Detected	Detected
2415	A_33_P3239839	FBXL18	Hs.623974	F-box and leucine-rich repeat protein 1	Homo sapiens F-box and leucine-rich r	3362.71580	2259.13350	2089.31080	1149.76460	Detected	Detected	Detected	Detected
2416	A_23_P83438	UBE2Z	Hs.514297	ubiquitin-conjugating enzyme E2Z	Homo sapiens ubiquitin-conjugating enz	19816.02100	22181.08400	13425.04100	12382.95000	Detected	Detected	Detected	Detected
2417	A_33_P3252491					4420.25800	3583.11160	185.99545	100.86658	Detected	Detected	Detected	Detected
2418	A_23_P110430	MSX1	Hs.424414	msh homeobox 1	Homo sapiens msh homeobox 1 (MSX1)	2068.21660	1613.19750	2373.48850	1667.24560	Detected	Detected	Detected	Detected
2419	A_33_P3212704	LOC100130710		hypothetical LOC100130710	PREDICTED: Homo sapiens hypothetica	501.58530	785.03980	109.54839	114.62341	Detected	Detected	Detected	Detected
2420	A_33_P3221489	KIRREL	Hs.657006	kin of IRRE like (Drosophila)	Homo sapiens kin of IRRE like (Drosoph	187.96786	201.18196	119.49950	131.30708	Detected	Detected	Detected	Detected
2421	A_23_P148047	PTGER4	Hs.199248	prostaglandin E receptor 4 (subtype EP	Homo sapiens prostaglandin E receptor	677.30450	753.29030	514.46850	432.76917	Detected	Detected	Detected	Detected
2422	A_23_P90099	TMEM205	Hs.8036	transmembrane protein 205	Homo sapiens transmembrane protein 2	47074.19500	15654.46000	50075.70000	14435.72700	Detected	Detected	Detected	Detected
2423	A_33_P3317593	AP1B1	Hs.368794	adaptor-related protein complex 1, beta	Homo sapiens adaptor-related protein c	47170.51000	50623.16400	34277.31000	33920.81000	Detected	Detected	Detected	Detected
2424	A_23_P49279	C16orf87	Hs.406551	chromosome 16 open reading frame 87	Homo sapiens chromosome 16 open rea	552.93854	725.95320	643.57120	726.62006	Detected	Detected	Detected	Detected
2425	A_33_P3346806		Hs.552561		BP396343 Homo sapiens pancreatic isle	25.76447	3.99473	3.38534	3.47442	Detected	Compromised	Compromised	Compromised
2426	A_23_P160809	COG2	Hs.211800	component of oligomeric golgi complex	Homo sapiens component of oligomeric	4603.18260	2610.04200	4006.61520	1957.44020	Detected	Detected	Detected	Detected
2427	A_23_P256956	KIF20A	Hs.718626	kinesin family member 20A	Homo sapiens kinesin family member 20	18.31738	2359.94800	16.03709	1555.57620	Detected	Detected	Detected	Detected
2428	A_23_P256948	MSC	Hs.442619	musculin (activated B-cell factor-1)	Homo sapiens musculin (activated B-ce	134.59822	130.85430	98.25531	99.57071	Detected	Detected	Detected	Detected
2429	A_24_P246467	ATF2	Hs.592510	activating transcription factor 2	Homo sapiens activating transcription fa	155.83351	197.48267	138.42122	192.55917	Detected	Detected	Detected	Detected
2430	A_24_P187948	BID	Hs.591054	BH3 interacting domain death agonist	Homo sapiens BH3 interacting domain d	6439.20560	9515.05800	5418.35350	7209.19900	Detected	Detected	Detected	Detected
2431	A_23_P56127	C19orf61	Hs.466875	chromosome 19 open reading frame 61	Homo sapiens chromosome 19 open rea	605.10480	1127.77470	523.98490	1046.25180	Detected	Detected	Detected	Detected
2432	A_33_P3394828	NOC4L	Hs.558536	nucleolar complex associated 4 homolog	Homo sapiens nucleolar complex associ	2854.19560	2777.62900	3577.19040	3059.59800	Detected	Detected	Detected	Detected
2433	A_23_P12329	APH1A	Hs.108408	anterior pharynx defective 1 homolog A	Homo sapiens anterior pharynx defectiv	1296.92500	1349.87230	623.07965	591.06670	Detected	Detected	Detected	Detected
2434	A_23_P105833	BIVM	Hs.288809	basic, immunoglobulin-like variable moti	Homo sapiens basic, immunoglobulin-lik	1253.26040	624.37010	914.37384	385.69950	Detected	Detected	Detected	Detected
2435	A_24_P314681		Hs.454452		BX281397 Soares_NFL_T_GBC_S1 Homo	65.38776	166.24452	42.82311	72.70578	Detected	Detected	Detected	Detected
2436	A_33_P3370944	RAX	Hs.278957	retina and anterior neural fold homeobo	Homo sapiens retina and anterior neura	15.12022	20.41091	8.78668	12.07128	Detected	Detected	Compromised	Compromised
2437	A_23_P117734	LYSMD4	Hs.562568	LysM, putative peptidoglycan-binding, d	Homo sapiens LysM, putative peptidogly	519.51337	689.87310	444.42300	507.08038	Detected	Detected	Detected	Detected
2438	A_32_P170003	TRAF3IP1	Hs.631898	TNF receptor-associated factor 3 inter	Homo sapiens TNF receptor-associated	2357.54980	1851.15000	2033.18100	1456.53020	Detected	Detected	Detected	Detected
2439	A_23_P120883	HMOX1	Hs.517581	heme oxygenase (decycling) 1	Homo sapiens heme oxygenase (decycli	45352.93400	166194.17000	33572.63000	109559.42000	Detected	Detected	Detected	Detected
2440	A_23_P336565	FAM185A	Hs.202543	family with sequence similarity 185, mer	Homo sapiens family with sequence sim	88.75517	107.82499	60.95045	87.29969	Detected	Detected	Detected	Detected
2441	A_33_P3257367	TRIM33	Hs.26837	tripartite motif-containing 33	Homo sapiens tripartite motif-containin	2396.15620	1128.11770	1001.41016	369.51633	Detected	Detected	Detected	Detected
2442	A_23_P340251	RAB2A	Hs.369017	RAB2A, member RAS oncogene family	Homo sapiens RAB2A, member RAS on	8632.79500	11757.19600	8282.38200	9813.23600	Detected	Detected	Detected	Detected
2443	A_23_P18579	PTTG2	Hs.668806	pituitary tumor-transforming 2	Homo sapiens pituitary tumor-transforn	712.10800	10411.25700	735.17993	8998.83700	Detected	Detected	Detected	Detected
2444	A_33_P3330353	MOSPD1	Hs.590789	motile sperm domain containing 1	Homo sapiens motile sperm domain con	280.09866	372.61750	226.23326	243.50061	Detected	Detected	Detected	Detected
2445	A_33_P3263614	ASAP3	Hs.437379	ArfGAP with SH3 domain, ankyrin repea	Homo sapiens ArfGAP with SH3 domain	144.78566	110.72907	58.39502	32.63666	Detected	Detected	Detected	Detected
2446	A_33_P3221343	HECTD2	Hs.596096	HECT domain containing 2	Homo sapiens HECT domain containing	191.55583	128.51650	79.62141	48.59214	Detected	Detected	Detected	Detected
2447	A_23_P208325	ZNF235	Hs.298089	zinc finger protein 235	Homo sapiens zinc finger protein 235 (Z	104.96338	71.91000	104.37112	61.23324	Detected	Detected	Detected	Detected
2448	A_24_P143574	SIRPB1	Hs.709487	signal-regulatory protein beta 1	Homo sapiens signal-regulatory protein	27.46608	63.22277	24.31192	63.23936	Detected	Detected	Detected	Detected
2449	A_33_P3271470	GREB1L	Hs.149020	growth regulation by estrogen in breast	Homo sapiens growth regulation by estr	4.37835	25.57806	3.25463	21.54242	Compromised	Detected	Compromised	Detected
2450	A_23_P122052	GPX8	Hs.289044	glutathione peroxidase 8 (putative)	Homo sapiens glutathione peroxidase 8	2764.64300	6416.41400	2030.27200	3958.92100	Detected	Detected	Detected	Detected
2451	A_32_P50066	MAP9	Hs.61271	microtubule-associated protein 9	Homo sapiens microtubule-associated p	2113.23780	1453.46680	1568.55770	920.09454	Detected	Detected	Detected	Detected
2452	A_33_P3365097	ODF2L	Hs.149360	outer dense fiber of sperm tails 2-like	Homo sapiens outer dense fiber of sper	345.89944	551.21234	108.13820	141.44990	Detected	Detected	Detected	Detected
2453	A_23_P215744	CTTNBP2	Hs.592285	cortactin binding protein 2	Homo sapiens cortactin binding protein	167.83832	19.23782	116.03054	9.54176	Detected	Compromised	Detected	Compromised
2454	A_24_P203056	BCL7A	Hs.530970	B-cell CLL/lymphoma 7A	Homo sapiens B-cell CLL/lymphoma 7A	432.36743	985.49976	329.79092	647.01917	Detected	Detected	Detected	Detected
2455	A_23_P218608	EIF5B	Hs.158688	eukaryotic translation initiation factor 5	Homo sapiens eukaryotic translation ini	1652.22460	1290.14270	1042.88840	676.49950	Detected	Detected	Detected	Detected
2456	A_23_P116624	SLC6A5	Hs.136557	solute carrier family 6 (neurotransmitte	Homo sapiens solute carrier family 6 (ne	75.74562	80.57264	33.01852	29.52816	Detected	Detected	Detected	Detected
2457	A_23_P120845	XBP1	Hs.437638	X-box binding protein 1	Homo sapiens X-box binding protein 1 (	12351.69600	19454.02100	11409.97800	15626.80300	Detected	Detected	Detected	Detected
2458	A_32_P224522	SLC25A23	Hs.356231	solute carrier family 25 (mitochondrial c	Homo sapiens solute carrier family 25 (	1127.95140	294.58700	944.26320	186.42442	Detected	Detected	Detected	Detected
2459	A_23_P214977	SEC63	Hs.26904	SEC63 homolog (S. cerevisiae)	Homo sapiens SEC63 homolog (S. cerev	11263.08400	9699.56200	8930.12100	6097.55270	Detected	Detected	Detected	Detected
2460	A_33_P3347193	VSTM2A	Hs.335933	V-set and transmembrane domain conta	Homo sapiens V-set and transmembran	101.42349	161.79912	19.80123	32.34695	Detected	Detected	Detected	Detected
2461	A_33_P3327592	RNF13	Hs.12333	ring finger protein 13	Homo sapiens ring finger protein 13 (RN	15010.57400	6845.23240	7842.13000	3139.07570	Detected	Detected	Detected	Detected
2462	A_24_P163537	TMED4	Hs.598832	transmembrane emp24 protein transpor	Homo sapiens transmembrane emp24 p	641.75214	804.91830	968.07874	934.76550	Detected	Detected	Detected	Detected
2463	A_32_P182511	KIAA1609	Hs.288274	KIAA1609	Homo sapiens KIAA1609 (KIAA1609), m	187.88048	413.53125	180.67159	366.00787	Detected	Detected	Detected	Detected
2464	A_33_P3257628	COG4	Hs.208680	component of oligomeric golgi complex	Homo sapiens component of oligomeric	248.26903	253.74776	175.55260	180.53190	Detected	Detected	Detected	Detected
2465	A_23_P18739	TMEM184C	Hs.203896	transmembrane protein 184C	Homo sapiens transmembrane protein 1	334.89493	347.85742	245.71078	206.88013	Detected	Detected	Detected	Detected
2466	A_33_P3403153	C1QTNF2	Hs.110062	C1q and tumor necrosis factor related p	Homo sapiens C1q and tumor necrosis	20.84400	46.66761	9.08190	27.17382	Detected	Detected	Compromised	Detected
2467	A_24_P386622	ARRB1	Hs.503284	arrestin, beta 1	Homo sapiens arrestin, beta 1 (ARRB1)	243.61226	99.66040	184.05457	81.61316	Detected	Detected	Detected	Detected
2468	A_33_P3362631	GUF1	Hs.546419	GUF1 GTPase homolog (S. cerevisiae)	Homo sapiens GUF1 GTPase homolog (	1217.56700	900.21750	825.63464	460.36517	Detected	Detected	Detected	Detected
2469	A_24_P382630	GTPBP5	Hs.340636	GTP binding protein 5 (putative)	Homo sapiens GTP binding protein 5 (pu	618.16974	363.90417	477.07626	235.29338	Detected	Detected	Detected	Detected
2470	A_33_P3270315	MGC16384	Hs.661642	hypothetical LOC114130	Homo sapiens hypothetical LOC114130	114.28989	111.84341	109.25196	89.18120	Detected	Detected	Detected	Detected
2471	A_33_P3642217	C21orf135	Hs.382029	chromosome 21 open reading frame 135	601489274F1 NIH_MGC_69 Homo sapier	21.38478	25.40276	9.70549	3.68434	Detected	Detected	Compromised	Compromised

2472	A_33_P3307955	ABCD3	Hs.700576	ATP-binding cassette, sub-family D (AL	Homo sapiens ATP-binding cassette, su	3209.77880	5199.61700	2954.90160	3574.02320	Detected	Detected	Detected	Detected
2473	A_33_P3876591	SNORA76	Hs.405444	small nucleolar RNA, H/ACA box 76	AGENCOURT_13972169 NIH_MGC_173	20.91671	29.15334	11.88931	10.20867	Detected	Detected	Compromised	Compromised
2474	A_23_P101237	C18orf21	Hs.37883	chromosome 18 open reading frame 21	Homo sapiens chromosome 18 open rea	3475.02100	3296.73880	3286.23600	2800.24220	Detected	Detected	Detected	Detected
2475	A_23_P106933	ACSM1	Hs.306812	acyl-CoA synthetase medium-chain fan	Homo sapiens acyl-CoA synthetase me	14.40413	5.32441	3.14038	3.09112	Not Detected	Compromised	Compromised	Compromised
2476	A_24_P389608	C10orf47	Hs.435775	chromosome 10 open reading frame 47	Homo sapiens chromosome 10 open rea	11.33406	20.16173	7.88111	17.98927	Compromised	Compromised	Compromised	Detected
2477	A_33_P3388958	SNX32	Hs.591950	sorting nexin 32	Homo sapiens sorting nexin 32 (SNX32)	402.90420	214.68896	303.47098	85.31072	Detected	Detected	Detected	Detected
2478	A_23_P78750	SLC17A7	Hs.375616	solute carrier family 17 (sodium-depend	Homo sapiens solute carrier family 17 (	364.00906	552.78140	151.25479	135.33789	Detected	Detected	Detected	Detected
2479	A_33_P3422265	LOC729305	Hs.148365	hypothetical protein LOC729305	Homo sapiens cDNA FLJ43050 fis, clon	127.50803	202.60837	101.81568	127.94379	Detected	Detected	Detected	Detected
2480	A_23_P29036	IFNGR2	Hs.634632	interferon gamma receptor 2 (interferon	Homo sapiens interferon gamma recept	2805.84900	2510.35130	2984.45950	2285.64600	Detected	Detected	Detected	Detected
2481	A_24_P58187	LOC120364		similar to heterogeneous nuclear ribonu	PREDICTED: Homo sapiens similar to h	3792.75000	5938.75930	1004.10004	1216.10060	Detected	Detected	Detected	Detected
2482	A_23_P503127	SEPT3	Hs.120483	septin 3	Homo sapiens septin 3 (SEPT3), transc	21.07546	17.76925	6.91864	7.93567	Detected	Compromised	Compromised	Compromised
2483	A_33_P3413038	PLXNB3	Hs.632833	plexin B3	Homo sapiens plexin B3 (PLXNB3), tran	149.88683	266.96060	109.43118	179.14671	Detected	Detected	Detected	Detected
2484	A_24_P928604					27459.32400	24355.58800	30454.75000	24742.09200	Detected	Detected	Detected	Detected
2485	A_33_P3255794	POLE	Hs.524871	polymerase (DNA directed), epsilon	Homo sapiens polymerase (DNA directe	13.06518	37.48183	14.74840	27.08976	Compromised	Detected	Compromised	Detected
2486	A_23_P150325	TMEM133	Hs.44004	transmembrane protein 133	Homo sapiens transmembrane protein 1	86.32243	52.28108	59.60612	25.82756	Detected	Detected	Detected	Detected
2487	A_33_P3216483	LOC644277	Hs.577348	hypothetical LOC644277	PREDICTED: Homo sapiens hypothetica	551.08594	838.60980	168.02975	175.51529	Detected	Detected	Detected	Detected
2488	A_24_P218074	ZNF467	Hs.112158	zinc finger protein 467	Homo sapiens zinc finger protein 467 (Z	43077.75000	48524.74200	85585.14000	73460.27000	Detected	Detected	Detected	Detected
2489	A_32_P149492	RP11-94I2.2	Hs.636561	neuroblastoma breakpoint family, memb	Homo sapiens neuroblastoma breakpoin	2723.53100	1785.70790	1273.07960	632.08514	Detected	Detected	Detected	Detected
2490	A_33_P3357879	LOC100133981		hypothetical protein LOC100133981	PREDICTED: Homo sapiens hypothetica	169.91360	208.55591	52.41670	61.62074	Detected	Detected	Detected	Detected
2491	A_23_P257649	RBP1	Hs.529571	retinol binding protein 1, cellular	Homo sapiens retinol binding protein 1,	16.89369	34.26114	5.87006	21.57604	Detected	Detected	Compromised	Detected
2492	A_33_P3249489	WDR89	Hs.655666	WD repeat domain 89	Homo sapiens WD repeat domain 89 (W	162.84148	210.30309	96.22245	112.48071	Detected	Detected	Detected	Detected
2493	A_33_P3299599	NKX2-5	Hs.54473	NK2 transcription factor related, locus 5	Homo sapiens NK2 transcription factor	83453.80000	96445.89000	106123.38000	101573.06000	Detected	Detected	Detected	Detected
2494	A_33_P3357753	LOC728190		hypothetical LOC728190	Homo sapiens hypothetical LOC728190	1708.67430	3726.58860	1189.76120	2226.01250	Detected	Detected	Detected	Detected
2495	A_24_P386323	RABEPK	Hs.19012	Rab9 effector protein with kelch motifs	Homo sapiens Rab9 effector protein wit	4342.58150	4792.30600	3516.86230	3409.84130	Detected	Detected	Detected	Detected
2496	A_23_P30956	KIAA0776	Hs.149367	KIAA0776	Homo sapiens KIAA0776 (KIAA0776), m	3649.79100	3236.55000	2831.96630	1957.75720	Detected	Detected	Detected	Detected
2497	A_33_P3659876	NCAPG2	Hs.18616	non-SMC condensin II complex, subunit	Homo sapiens non-SMC condensin II co	270.82816	1406.82780	178.78595	780.55347	Detected	Detected	Detected	Detected
2498	A_23_P103349	OCLM	Hs.679230	oculomedin	Homo sapiens oculomedin (OCLM), mR	4.55822	5.50744	29.01499	3.28640	Compromised	Compromised	Not Detected	Compromised
2499	A_33_P3384442				Laminin subunit alpha-5 Precursor [So	90648.93000	80070.03000	28888.48200	20182.39600	Detected	Detected	Detected	Detected
2500	A_33_P3325231	LOC728684	Hs.640184	hypothetical protein LOC728684	Homo sapiens cDNA FLJ44019 fis, clon	14.44007	10.39048	7.39506	8.88628	Detected	Compromised	Compromised	Compromised
2501	A_23_P140527	FOXB1	Hs.160375	forkhead box B1	Homo sapiens forkhead box B1 (FOXB1	3133.43680	4631.23830	621.78644	496.82385	Detected	Detected	Detected	Detected
2502	A_24_P182764	ATG4B	Hs.283610	ATG4 autophagy related 4 homolog B (S	Homo sapiens ATG4 autophagy related	2273.42100	1521.87920	1328.95340	730.32166	Detected	Detected	Detected	Detected
2503	A_33_P3702055	SLC11A1	Hs.591607	solute carrier family 11 (proton-coupled	Homo sapiens solute carrier family 11 (	376.03240	506.79922	180.69786	181.30525	Detected	Detected	Detected	Detected
2504	A_33_P3400918	DISC1	Hs.13318	disrupted in schizophrenia 1	Homo sapiens disrupted in schizophreni	24.57733	29.88065	3.32910	3.44367	Detected	Detected	Compromised	Compromised
2505	A_24_P210513	BTBD7	Hs.525549	BTB (POZ) domain containing 7	Homo sapiens BTB (POZ) domain conta	72.39625	144.23480	75.92795	149.35088	Detected	Detected	Detected	Detected
2506	A_23_P341443	MNT	Hs.632239	MAX binding protein	Homo sapiens MAX binding protein (MN	4980.52000	5364.44630	3814.65530	3453.83280	Detected	Detected	Detected	Detected
2507	A_24_P102315	KIAA1755	Hs.472690	KIAA1755	Homo sapiens KIAA1755 (KIAA1755), m	58.82764	30.63051	37.34959	17.43736	Detected	Detected	Detected	Compromised
2508	A_33_P3262376	OTUD7A	Hs.355236	OTU domain containing 7A	Homo sapiens OTU domain containing 7	120.16871	25.89410	106.65578	13.77191	Detected	Detected	Detected	Compromised
2509	A_23_P43690	BNC2	Hs.656581	basonuclin 2	Homo sapiens basonuclin 2 (BNC2), mR	72.26465	93.12322	37.88060	37.42574	Detected	Detected	Detected	Detected
2510	A_33_P3371785	DTNA	Hs.643454	dystrobrevin, alpha	Homo sapiens dystrobrevin, alpha (DTN	2106.61990	2022.83970	932.42470	628.27140	Detected	Detected	Detected	Detected
2511	A_23_P42829	SND1	Hs.122523	staphylococcal nuclease and tudor dom	Homo sapiens staphylococcal nuclease	40462.57000	52079.62000	27529.62300	30556.86700	Detected	Detected	Detected	Detected
2512	A_24_P117029	LDLR	Hs.213289	low density lipoprotein receptor	Homo sapiens low density lipoprotein re	12457.53100	10873.77700	8925.06300	6665.58840	Detected	Detected	Detected	Detected
2513	A_23_P44466	CCDC102B	Hs.280781	coiled-coil domain containing 102B	Homo sapiens coiled-coil domain conta	64.58260	7.83565	48.19966	3.66847	Detected	Compromised	Detected	Compromised
2514	A_23_P122464	ZNF193	Hs.100921	zinc finger protein 193	Homo sapiens zinc finger protein 193 (Z	548.89620	335.15600	468.79218	247.91217	Detected	Detected	Detected	Detected
2515	A_23_P90130	NAPSA	Hs.512843	napsin A aspartic peptidase	Homo sapiens napsin A aspartic peptida	56.11298	57.19011	62.00703	35.47931	Detected	Detected	Detected	Detected
2516	A_33_P3362611	ELTD1	Hs.132314	EGF, latrophilin and seven transmembra	Homo sapiens EGF, latrophilin and seve	1397.97550	1692.93660	1170.89390	1152.98790	Detected	Detected	Detected	Detected
2517	A_33_P3424566	FLJ38717	Hs.656652	FLJ38717 protein	PREDICTED: Homo sapiens FLJ38717 p	24.02079	30.92277	7.67367	13.26477	Detected	Detected	Compromised	Compromised
2518	A_23_P209538	KIAA1310	Hs.516341	KIAA1310	Homo sapiens KIAA1310 (KIAA1310), tr	554.21050	881.44210	486.41614	628.71310	Detected	Detected	Detected	Detected
2519	A_32_P22078	RPS9	Hs.546288	ribosomal protein S9	Homo sapiens ribosomal protein S9 (RP	28149.29700	24184.10400	34883.16400	24220.45100	Detected	Detected	Detected	Detected
2520	A_33_P3340385	ZNF43	Hs.534365	zinc finger protein 43	Homo sapiens zinc finger protein 43 (ZN	32.05516	50.93849	5.97747	3.67648	Detected	Detected	Compromised	Compromised
2521	A_23_P82950	PCM1	Hs.491148	pericentriolar material 1	Homo sapiens pericentriolar material 1	5109.62650	6645.30270	3897.92240	4429.80220	Detected	Detected	Detected	Detected
2522	A_33_P3407549	ANKRD43	Hs.13308	ankyrin repeat domain 43	Homo sapiens ankyrin repeat domain 43	95.91531	102.98928	29.67011	28.46346	Detected	Detected	Detected	Detected
2523	A_33_P3357653		Hs.664097		Putative uncharacterized protein ENSP	171.40490	101.37302	162.95212	87.08159	Detected	Detected	Detected	Detected
2524	A_32_P111996				MGC39584 protein [Source:UniProtKB	25.51359	13.45420	3.52179	3.77732	Detected	Compromised	Compromised	Compromised
2525	A_23_P52668	NAALADL1	Hs.13967	N-acetylated alpha-linked acidic dipept	Homo sapiens N-acetylated alpha-linke	239.52084	336.39166	207.91397	296.36343	Detected	Detected	Detected	Detected
2526	A_33_P3279181	NBPF8	Hs.534675	neuroblastoma breakpoint family, memb	PREDICTED: Homo sapiens neuroblasto	36.98255	31.34727	15.20551	11.17400	Detected	Detected	Compromised	Compromised
2527	A_24_P127159	LOC346329	Hs.666838	similar to guanine nucleotide binding pro	PREDICTED: Homo sapiens similar to g	683.09357	968.18820	605.53570	808.53815	Detected	Detected	Detected	Detected
2528	A_23_P45496	GDI1	Hs.74576	GDP dissociation inhibitor 1	Homo sapiens GDP dissociation inhibito	2984.25800	2520.58420	2346.68630	1747.68190	Detected	Detected	Detected	Detected
2529	A_24_P374973					479.16348	537.82270	71.13698	54.01461	Detected	Detected	Detected	Detected
2530	A_33_P3256033	CBL	Hs.504096	Cas-Br-M (murine) ecotropic retroviral	Homo sapiens Cas-Br-M (murine) ecot	682.40485	759.58120	446.76263	435.34980	Detected	Detected	Detected	Detected
2531	A_33_P3241433	ZNF347	Hs.467239	zinc finger protein 347	Homo sapiens zinc finger protein 347 (Z	366.87286	231.03809	242.35990	129.06613	Detected	Detected	Detected	Detected
2532	A_33_P3366102	KLHL7	Hs.654817	kelch-like 7 (Drosophila)	Homo sapiens kelch-like 7 (Drosophila)	994.87480	1830.64180	812.86830	1354.08350	Detected	Detected	Detected	Detected
2533	A_33_P3317103	CCDC157	Hs.505597	coiled-coil domain containing 157	Homo sapiens coiled-coil domain conta	17.62233	7.17910	12.80742	11.67708	Detected	Compromised	Compromised	Compromised
2534	A_23_P31109	C6orf72	Hs.438872	chromosome 6 open reading frame 72	Homo sapiens chromosome 6 open read	9151.92600	7710.33900	7418.92100	5644.80300	Detected	Detected	Detected	Detected
2535	A_23_P102694	DEFB129	Hs.112087	defensin, beta 129	Homo sapiens defensin, beta 129 (DEFE	16.63207	23.15651	43.31644	7.64730	Detected	Detected	Not Detected	Compromised
2536	A_23_P131240	UBXN2A	Hs.591576	UBX domain protein 2A	Homo sapiens UBX domain protein 2A (	420.17947	634.39290	344.10280	473.84247	Detected	Detected	Detected	Detected
2537	A_23_P48056	CKAP4	Hs.74368	cytoskeleton-associated protein 4	Homo sapiens cytoskeleton-associated	5516.12900	15862.84000	2588.54370	6443.81300	Detected	Detected	Detected	Detected
2538	A_23_P110433	PPWD1	Hs.121432	peptidylprolyl isomerase domain and WD	Homo sapiens peptidylprolyl isomerase	3553.96020	2656.88260	2399.56420	1497.78470	Detected	Detected	Detected	Detected
2539	A_23_P1280	ASCC1	Hs.500007	activating signal cointegrator 1 complex	Homo sapiens activating signal cointegr	1004.22815	710.44495	823.00110	523.44900	Detected	Detected	Detected	Detected
2540	A_23_P105545	VAMP1	Hs.20021	vesicle-associated membrane protein 1	Homo sapiens vesicle-associated memb	141.68227	73.21196	135.64742	58.04005	Detected	Detected	Detected	Detected
2541	A_23_P14804	TSPAN3	Hs.716484	tetraspanin 3	Homo sapiens tetraspanin 3 (TSPAN3),	13588.87900	13731.28600	8887.98400	7613.89360	Detected	Detected	Detected	Detected
2542	A_33_P3294133	EIF4B	Hs.648394	eukaryotic translation initiation factor 4	Homo sapiens eukaryotic translation ini	6080.00440	8205.99900	3994.66970	4732.04200	Detected	Detected	Detected	Detected
2543	A_23_P122041	PPP2CA	Hs.105818	protein phosphatase 2 (formerly 2A), ca	Homo sapiens protein phosphatase 2 (fo	3276.90400	5568.56740	2551.75000	3571.94000	Detected	Detected	Detected	Detected
2544	A_33_P3310751					52.92713	49.00340	60.90427	48.08644	Detected	Detected	Detected	Detected
2545	A_23_P122796	HDDC2	Hs.32826	HD domain containing 2	Homo sapiens HD domain containing 2 (	403.69067	89.76413	352.45148	62.35508	Detected	Detected	Detected	Detected
2546	A_23_P327022	MDFIC	Hs.427236	MyoD family inhibitor domain containing	Homo sapiens MyoD family inhibitor dor	3386.20460	3955.48880	1683.15610	1488.66600	Detected	Detected	Detected	Detected

2547	A_23_P92320	NUP54	Hs.430435	nucleoporin 54kDa	Homo sapiens nucleoporin 54kDa (NUP5	5980.13500	7815.05470	4149.99850	4406.19340	Detected	Detected	Detected	Detected
2548	A_33_P3768196	LOC284551	Hs.722371	hypothetical LOC284551	Homo sapiens hypothetical LOC284551	14.35302	11.13055	3.17168	4.23459	Detected	Compromised	Compromised	Compromised
2549	A_23_P100660	SERPINF1	Hs.532768	serpin peptidase inhibitor, clade F (alpha	Homo sapiens serpin peptidase inhibitor	18300.59800	2946.54570	17098.51600	2496.02500	Detected	Detected	Detected	Detected
2550	A_33_P3417313	PYCRL	Hs.165186	pyrroline-5-carboxylate reductase-like	Homo sapiens pyrroline-5-carboxylate r	35.44446	48.72376	58.63870	75.13699	Detected	Detected	Detected	Detected
2551	A_24_P110558	C5orf53	Hs.696360	chromosome 5 open reading frame 53	Homo sapiens chromosome 5 open read	873.86580	283.04617	522.34670	123.11062	Detected	Detected	Detected	Detected
2552	A_23_P103617	ANXA9	Hs.720072	annexin A9	Homo sapiens annexin A9 (ANXA9), mR	17.42940	4.76261	29.12772	3.69587	Not Detected	Compromised	Not Detected	Compromised
2553	A_24_P67806	MTR	Hs.498187	5-methyltetrahydrofolate-homocysteine	Homo sapiens 5-methyltetrahydrofolate	404.45297	192.03223	244.24532	99.37378	Detected	Detected	Detected	Detected
2554	A_24_P248863	ZCCHC4	Hs.278945	zinc finger, CCHC domain containing 4	Homo sapiens zinc finger, CCHC domain	1285.49890	609.39910	614.19390	232.48978	Detected	Detected	Detected	Detected
2555	A_23_P318300	ZAK	Hs.444451	sterile alpha motif and leucine zipper co	Homo sapiens sterile alpha motif and le	7616.00800	8080.61000	5640.58060	4870.09400	Detected	Detected	Detected	Detected
2556	A_23_P368645	AGPS	Hs.516543	alkylglycerone phosphate synthase	Homo sapiens alkylglycerone phosphate	341.21887	560.90730	301.90510	422.88962	Detected	Detected	Detected	Detected
2557	A_33_P3314857	KCNJ13	Hs.467338	potassium inwardly-rectifying channel, s	Homo sapiens potassium inwardly-recti	21.59785	16.73457	11.03369	3.37248	Detected	Compromised	Compromised	Compromised
2558	A_24_P83738	ASTN2	Hs.601562	astrotactin 2	Homo sapiens astrotactin 2 (ASTN2), tr	10812.09300	18954.29900	1110.44140	1692.36380	Detected	Detected	Detected	Detected
2559	A_33_P3209356	RFWD2	Hs.523744	ring finger and WD repeat domain 2	Homo sapiens ring finger and WD repea	1573.10580	1949.28210	1362.55790	1337.27050	Detected	Detected	Detected	Detected
2560	A_33_P3277323					166273.72000	149382.34000	58362.73400	43341.64500	Detected	Detected	Detected	Detected
2561	A_32_P16315	RP11-631M21.2	Hs.532659	tubulin, beta 8	Homo sapiens tubulin, beta 8 (TUBB8),	74.24895	34.94233	29.85961	11.41146	Detected	Detected	Detected	Compromised
2562	A_23_P416395	STC2	Hs.233160	stanniocalcin 2	Homo sapiens stanniocalcin 2 (STC2), r	760.69970	5591.76900	383.56146	2438.42300	Detected	Detected	Detected	Detected
2563	A_23_P55174	G6PC3	Hs.294005	glucose 6 phosphatase, catalytic, 3	Homo sapiens glucose 6 phosphatase, c	234.77444	242.53418	206.85661	202.19615	Detected	Detected	Detected	Detected
2564	A_23_P44505	KLF11	Hs.12229	Kruppel-like factor 11	Homo sapiens Kruppel-like factor 11 (K	968.34564	1171.47530	686.28760	681.79660	Detected	Detected	Detected	Detected
2565	A_33_P3254606	WDR62	Hs.116244	WD repeat domain 62	Homo sapiens WD repeat domain 62 (W	1323.76900	10077.61200	303.36768	5412.51860	Detected	Detected	Detected	Detected
2566	A_23_P256903	LOC100128416	Hs.432355	RAB28, member RAS oncogene family p	PREDICTED: Homo sapiens RAB28, me	499.14627	455.17828	365.29483	279.83383	Detected	Detected	Detected	Detected
2567	A_24_P122337	SYTL4	Hs.592224	synaptotagmin-like 4	Homo sapiens synaptotagmin-like 4 (SY	192.51208	93.25483	137.57982	59.14547	Detected	Detected	Detected	Detected
2568	A_23_P95165	SEMA4B	Hs.474935	sema domain, immunoglobulin domain (Ig	Homo sapiens sema domain, immunoglo	532.27234	824.94290	243.19698	287.64720	Detected	Detected	Detected	Detected
2569	A_23_P147431	LYN	Hs.699154	v-yes-1 Yamaguchi sarcoma viral relate	Homo sapiens v-yes-1 Yamaguchi sarc	416.26483	545.23680	272.71304	288.71590	Detected	Detected	Detected	Detected
2570	A_33_P3404489	MAMLD1	Hs.20136	mastermind-like domain containing 1	Homo sapiens mastermind-like domain c	17.55753	8.98434	15.65806	12.87312	Detected	Compromised	Compromised	Compromised
2571	A_23_P108932	RPL23AP32	Hs.657366	ribosomal protein L23a pseudogene 32	Homo sapiens ribosomal protein L23a p	288.02164	372.92060	144.64647	148.53084	Detected	Detected	Detected	Detected
2572	A_23_P215406	RAC1	Hs.413812	ras-related C3 botulinum toxin substrat	Homo sapiens ras-related C3 botulinum	20449.83200	29429.57800	11517.67500	14962.25000	Detected	Detected	Detected	Detected
2573	A_33_P3389478				snRNA-activating protein complex subu	20.68666	24.31269	21.24471	13.52110	Detected	Detected	Detected	Compromised
2574	A_23_P102471	MSH2	Hs.597656	mutS homolog 2, colon cancer, nonpolyp	Homo sapiens mutS homolog 2, colon c	2155.33330	2370.67870	1507.89280	1420.97530	Detected	Detected	Detected	Detected
2575	A_24_P414256		Hs.356440		Coiled-coil domain-containing protein 7	28808.75000	43731.77300	23635.77500	32002.19500	Detected	Detected	Detected	Detected
2576	A_23_P35576	ZNF518A	Hs.600823	zinc finger protein 518A	Homo sapiens zinc finger protein 518A	44.57802	38.52347	37.84677	20.15976	Detected	Detected	Detected	Detected
2577	A_23_P71316	RBPMS	Hs.334587	RNA binding protein with multiple splicir	Homo sapiens RNA binding protein with	2291.84450	2843.52170	1785.76220	1965.84800	Detected	Detected	Detected	Detected
2578	A_23_P393051	C1orf172	Hs.188881	chromosome 1 open reading frame 172	Homo sapiens chromosome 1 open read	39.43598	44.29857	18.03458	11.87048	Detected	Detected	Detected	Compromised
2579	A_33_P3413483	SORD	Hs.633539	sorbitol dehydrogenase	Homo sapiens cDNA FLJ33419 fis, clon	762.24854	153.75491	459.68338	75.08749	Detected	Detected	Detected	Detected
2580	A_23_P502590	KIR2DS4	Hs.654608	killer cell immunoglobulin-like receptor,	Homo sapiens killer cell immunoglobulin	36.19452	14.72299	8.09144	4.00474	Detected	Compromised	Compromised	Compromised
2581	A_24_P364838	SLC9A3R2	Hs.440896	solute carrier family 9 (sodium/hydroge	Homo sapiens solute carrier family 9 (s	3499.85380	4528.10450	3629.71300	4125.87300	Detected	Detected	Detected	Detected
2582	A_33_P3245922	MAP3K4	Hs.390428	mitogen-activated protein kinase kinase	Homo sapiens mitogen-activated protei	974.31770	724.54470	526.75210	337.53366	Detected	Detected	Detected	Detected
2583	A_33_P3263277					266934.97000	272440.22000	166385.00000	134832.58000	Detected	Detected	Detected	Detected
2584	A_23_P104201	YME1L1	Hs.499145	YME1-like 1 (S. cerevisiae)	Homo sapiens YME1-like 1 (S. cerevisia	8989.46100	8856.21500	6140.21730	5007.22950	Detected	Detected	Detected	Detected
2585	A_33_P3397801					802.38635	1177.72030	243.86433	278.05942	Detected	Detected	Detected	Detected
2586	A_33_P3228600	CPA5	Hs.567642	carboxypeptidase A5	Homo sapiens carboxypeptidase A5 (CF	15.05960	14.77897	6.55037	3.51765	Detected	Compromised	Compromised	Compromised
2587	A_33_P3306342	OR52N5	Hs.690207	olfactory receptor, family 52, subfamily	Homo sapiens olfactory receptor, family	71.23083	143.73502	17.41715	27.36123	Detected	Detected	Detected	Detected
2588	A_23_P114445	MAGEE1	Hs.8453	melanoma antigen family E, 1	Homo sapiens melanoma antigen family	168.53578	179.07437	72.66856	67.82385	Detected	Detected	Detected	Detected
2589	A_23_P351837	KLHL35	Hs.292451	kelch-like 35 (Drosophila)	Homo sapiens kelch-like 35 (Drosophila	77.51599	140.63997	55.38683	126.39268	Detected	Detected	Detected	Detected
2590	A_23_P11397	TTTY13	Hs.615432	testis-specific transcript, Y-linked 13 (r	Homo sapiens testis-specific transcript	15.07728	19.53622	18.07214	15.02274	Detected	Detected	Detected	Detected
2591	A_23_P300847	DIO3OS	Hs.525597	DIO3 opposite strand (non-protein codi	Homo sapiens deiodinase, iodothyronine	22.91060	27.20717	9.88653	8.96729	Detected	Detected	Compromised	Compromised
2592	A_33_P3293237					40.04447	51.22209	16.15358	19.69040	Detected	Detected	Detected	Detected
2593	A_33_P3386775	ADAM11	Hs.6088	ADAM metallopeptidase domain 11	Homo sapiens ADAM metallopeptidase	3776.89800	4175.11000	3242.51200	3031.03880	Detected	Detected	Detected	Detected
2594	A_23_P40782	COMMD2	Hs.591315	COMM domain containing 2	Homo sapiens COMM domain containing	339.17470	602.09430	267.65820	435.15674	Detected	Detected	Detected	Detected
2595	A_33_P3410296	DPYSL4	Hs.100058	dihydropyrimidinase-like 4	Homo sapiens dihydropyrimidinase-like	1981.48830	2696.62870	1528.12620	1650.84250	Detected	Detected	Detected	Detected
2596	A_33_P3671378	CERCAM	Hs.495230	cerebral endothelial cell adhesion molec	Homo sapiens cerebral endothelial cell a	341.46594	661.74976	222.19014	469.07330	Detected	Detected	Detected	Detected
2597	A_33_P3245699		Hs.683855		cDNA FLJ38166 fis, clone DFNES20055	86.28027	86.56045	25.36723	33.19969	Detected	Detected	Detected	Detected
2598	A_33_P3345504	RC3H2	Hs.533499	ring finger and CCCH-type zinc finger d	Homo sapiens ring finger and CCCH-typ	152.99765	157.66324	138.74922	132.04358	Detected	Detected	Detected	Detected
2599	A_23_P157147	ZDHHC4	Hs.5268	zinc finger, DHHC-type containing 4	Homo sapiens zinc finger, DHHC-type c	12214.25900	11157.29500	10460.15200	8471.14700	Detected	Detected	Detected	Detected
2600	A_24_P20746	PRR16	Hs.720992	proline rich 16	Homo sapiens proline rich 16 (PRR16),	373.83182	223.61482	357.53964	180.98027	Detected	Detected	Detected	Detected
2601	A_24_P135483	TSPAN14	Hs.633177	tetraspanin 14	Homo sapiens tetraspanin 14 (TSPAN14	244.19914	159.22461	176.60217	114.32568	Detected	Detected	Detected	Detected
2602	A_33_P3409282	LOC100132515	Hs.626442	similar to hCG2045429	PREDICTED: Homo sapiens similar to h	23.64616	26.19959	20.83384	10.64577	Detected	Detected	Detected	Compromised
2603	A_23_P312610		Hs.172847		DnaJ homolog subfamily C member 4 (N	230.22108	98.75668	236.02367	89.16381	Detected	Detected	Detected	Detected
2604	A_23_P59677	RINT1	Hs.531388	RAD50 interactor 1	Homo sapiens RAD50 interactor 1 (RIN	3706.70300	4043.62870	2351.66630	2056.61330	Detected	Detected	Detected	Detected
2605	A_24_P216681	LSM10	Hs.720083	LSM10, U7 small nuclear RNA associate	Homo sapiens LSM10, U7 small nuclear	4184.44040	4772.61100	5641.49800	5959.75200	Detected	Detected	Detected	Detected
2606	A_32_P8551	NRN1L	Hs.435464	neuritin 1-like	Homo sapiens neuritin 1-like (NRN1L),	142.99129	36.05116	113.14838	25.11734	Detected	Detected	Detected	Detected
2607	A_23_P106798	SIAH1	Hs.706828	seven in absentia homolog 1 (Drosophila	Homo sapiens seven in absentia homolo	552.40590	440.01978	602.05210	412.43652	Detected	Detected	Detected	Detected
2608	A_33_P3244834					14.09311	17.34514	5.45699	6.18087	Detected	Compromised	Compromised	Compromised
2609	A_33_P3413741	OXTR	Hs.2820	oxytocin receptor	Homo sapiens oxytocin receptor (OXTR	122.57455	1894.74850	71.96461	945.67554	Detected	Detected	Detected	Detected
2610	A_23_P79069	RASAL3	Hs.136979	RAS protein activator like 3	Homo sapiens RAS protein activator lik	211.35180	322.58536	151.29540	115.45328	Detected	Detected	Detected	Detected
2611	A_33_P3361925	PHF8	Hs.133352	PHD finger protein 8	Homo sapiens PHD finger protein 8 (PH	21.54451	35.37641	12.55942	8.91782	Detected	Detected	Compromised	Compromised
2612	A_33_P3398513	hCG_1645220	Hs.602756	hCG1645220	Homo sapiens hCG1645220 (LOC72881	47.31722	105.15989	28.41791	56.79264	Detected	Detected	Detected	Detected
2613	A_33_P3221284	CCDC80	Hs.477128	coiled-coil domain containing 80	Homo sapiens coiled-coil domain conta	50.18488	824.81036	31.70135	486.89645	Detected	Detected	Detected	Detected
2614	A_23_P354175	TMEM129	Hs.518562	transmembrane protein 129	Homo sapiens transmembrane protein 1	9994.84400	5457.23240	4389.00150	2106.25630	Detected	Detected	Detected	Detected
2615	A_23_P40108	COL9A3	Hs.716639	collagen, type IX, alpha 3	Homo sapiens collagen, type IX, alpha 3	1469.43380	132.05458	1365.08750	107.78027	Detected	Detected	Detected	Detected
2616	A_24_P274270	STAT1	Hs.642990	signal transducer and activator of trans	Homo sapiens signal transducer and act	3114.68140	1557.75050	2593.71140	1166.62460	Detected	Detected	Detected	Detected
2617	A_23_P303803	C19orf18	Hs.134209	chromosome 19 open reading frame 18	Homo sapiens chromosome 19 open rea	275.15582	64.08912	216.96654	33.88465	Detected	Detected	Detected	Detected
2618	A_33_P3242416					394.90176	467.17148	159.51173	193.43263	Detected	Detected	Detected	Detected
2619	A_23_P427502	ZNRF3	Hs.655242	zinc and ring finger 3	Homo sapiens zinc and ring finger 3 (ZN	134.04137	170.31961	120.04362	152.20357	Detected	Detected	Detected	Detected
2620	A_33_P3347511	LOC100132630	Hs.722585	similar to capicua-like protein/double h	PREDICTED: Homo sapiens similar to c	16.89980	13.24396	14.15179	16.05165	Detected	Compromised	Compromised	Compromised
2621	A_23_P256473	SEMA3C	Hs.269109	sema domain, immunoglobulin domain (Ig	Homo sapiens sema domain, immunoglo	443.03766	1307.77930	348.35953	789.58325	Detected	Detected	Detected	Detected

2622	A_23_P40956	GHRL	Hs.590080	ghrelin/obestatin prepropeptide	Homo sapiens ghrelin/obestatin prepro	18.52518	36.70391	19.82644	26.36577	Detected	Detected	Detected	Detected
2623	A_23_P53788	MTIF3	Hs.534582	mitochondrial translational initiation fac	Homo sapiens mitochondrial translation	4353.64450	3524.47270	4376.23140	2826.81540	Detected	Detected	Detected	Detected
2624	A_33_P3409294					60.58967	65.56145	48.09064	61.23172	Detected	Detected	Detected	Detected
2625	A_33_P3377399					429.88174	376.17240	163.92874	123.99534	Detected	Detected	Detected	Detected
2626	A_24_P701030		Hs.586271		Novel proteinHCG2030731 ; [Source:Un	26.68885	14.34424	27.86946	12.40766	Detected	Compromised	Detected	Compromised
2627	A_33_P3372722	LOC100129203	Hs.635299	similar to mCG50504	PREDICTED: Homo sapiens similar to m	29.55850	33.98525	3.51683	3.63988	Detected	Detected	Compromised	Compromised
2628	A_33_P3416797	OVOS2	Hs.524331	ovostatin 2	Homo sapiens mRNA; cDNA DKFZp434	29.84448	61.26599	12.50534	9.97156	Detected	Detected	Compromised	Compromised
2629	A_33_P3373745	BRD4	Hs.187763	bromodomain containing 4	Homo sapiens bromodomain containing	117.31850	160.43465	96.93913	122.65691	Detected	Detected	Detected	Detected
2630	A_23_P47148	NOX4	Hs.371036	NADPH oxidase 4	Homo sapiens NADPH oxidase 4 (NOX4	332.40770	969.71070	271.24277	650.84640	Detected	Detected	Detected	Detected
2631	A_32_P846696	LOC349196	Hs.722381	hypothetical LOC349196	Homo sapiens hypothetical LOC349196	577.95800	169.51305	376.68848	99.03562	Detected	Detected	Detected	Detected
2632	A_23_P84230	OTP	Hs.202247	orthopedia homeobox	Homo sapiens orthopedia homeobox (O	50.15221	39.31325	6.32570	3.67585	Detected	Detected	Compromised	Compromised
2633	A_32_P120604				Sorting nexin-29 [Source:UniProtKB/S	254.42490	227.27817	152.22304	100.64140	Detected	Detected	Detected	Detected
2634	A_23_P126757	INTS3	Hs.516522	integrator complex subunit 3	Homo sapiens integrator complex subur	426.53683	322.09420	327.94354	226.82152	Detected	Detected	Detected	Detected
2635	A_23_P55376	KIAA1267	Hs.648744	KIAA1267	Homo sapiens KIAA1267 (KIAA1267), m	2373.86820	1770.26320	1699.04550	995.61300	Detected	Detected	Detected	Detected
2636	A_33_P3233754	ZNF154	Hs.646378	zinc finger protein 154	Homo sapiens zinc finger protein 154 (Z	29.79250	13.06857	17.96855	8.13489	Detected	Compromised	Detected	Compromised
2637	A_33_P3238295	FAM66C	Hs.512257	family with sequence similarity 66, mem	Homo sapiens family with sequence sim	187.90637	18.43527	81.00075	5.82873	Detected	Compromised	Detected	Compromised
2638	A_33_P3291559		Hs.649979		Homo sapiens cDNA FLJ12279 fis, clon	77.34202	84.09570	21.22963	12.92340	Detected	Detected	Detected	Compromised
2639	A_24_P128205	PSMD1	Hs.3887	proteasome (prosome, macropain) 26S	Homo sapiens proteasome (prosome, m	2443.08280	3404.98950	2114.21310	2642.76270	Detected	Detected	Detected	Detected
2640	A_33_P3406255					2079.29150	2515.54740	986.95230	1123.57790	Detected	Detected	Detected	Detected
2641	A_33_P3414312	USP6	Hs.448851	ubiquitin specific peptidase 6 (Tre-2 on	Homo sapiens ubiquitin specific peptida	1098.65620	696.51640	874.72170	517.47815	Detected	Detected	Detected	Detected
2642	A_32_P211248	LOC100131138	Hs.152129	similar to hCG2040918	Putative uncharacterized protein [Sour	3913.73930	2384.57230	145.52238	93.10965	Detected	Detected	Detected	Detected
2643	A_23_P164047	MMD	Hs.463483	monocyte to macrophage differentiatior	Homo sapiens monocyte to macrophage	6297.08400	3940.43300	5078.56600	2941.54610	Detected	Detected	Detected	Detected
2644	A_33_P3366053	ADPRH	Hs.99884	ADP-ribosylarginine hydrolase	Homo sapiens ADP-ribosylarginine hydr	206.51852	431.53024	117.29849	217.23776	Detected	Detected	Detected	Detected
2645	A_33_P3372090					193.16321	325.77985	59.44298	82.00323	Detected	Detected	Detected	Detected
2646	A_33_P3300273	C1orf52	Hs.26226	chromosome 1 open reading frame 52	Homo sapiens chromosome 1 open reac	558.85876	328.41885	442.55410	219.34103	Detected	Detected	Detected	Detected
2647	A_33_P3269718	IVD	Hs.513646	isovaleryl Coenzyme A dehydrogenase	Homo sapiens isovaleryl Coenzyme A d	655.92220	1604.88100	590.08170	1195.45640	Detected	Detected	Detected	Detected
2648	A_33_P3408624	IQSEC3	Hs.720133	IQ motif and Sec7 domain 3	Homo sapiens IQ motif and Sec7 domai	13.84207	22.27090	3.14173	3.28306	Detected	Detected	Compromised	Compromised
2649	A_23_P1186	C1QL3	Hs.676792	complement component 1, q subcompor	Homo sapiens complement component	2.53652	3.72837	2.99657	27.45563	Compromised	Compromised	Compromised	Not Detected
2650	A_23_P126716	ATPIF1	Hs.590908	ATPase inhibitory factor 1	Homo sapiens ATPase inhibitory factor	34016.53000	52342.61000	37756.57000	48264.94500	Detected	Detected	Detected	Detected
2651	A_33_P3360753				Potassium voltage-gated channel subfa	259.27360	226.45035	55.81136	61.78664	Detected	Detected	Detected	Detected
2652	A_23_P329573	ITGB2	Hs.375957	integrin, beta 2 (complement componen	Homo sapiens integrin, beta 2 (complem	36.38856	37.07243	18.86575	17.56248	Detected	Detected	Detected	Detected
2653	A_24_P196499				Homo sapiens mRNA for RBP1-like pro	8.55729	19.85654	3.14798	9.23774	Compromised	Detected	Compromised	Compromised
2654	A_23_P103276	KTI12	Hs.655082	KTI12 homolog, chromatin associated (S	Homo sapiens KTI12 homolog, chromati	496.96246	851.51920	354.53630	525.78010	Detected	Detected	Detected	Detected
2655	A_23_P111303	RBM16	Hs.591329	RNA binding motif protein 16	Homo sapiens RNA binding motif protei	1733.97770	1602.58260	1295.05150	926.41296	Detected	Detected	Detected	Detected
2656	A_33_P3327799	LOC441806		similar to hCG1644617	PREDICTED: Homo sapiens similar to h	12607.72900	19121.48600	13011.00900	17379.62900	Detected	Detected	Detected	Detected
2657	A_33_P3545065	SH3GLP1	Hs.672460	SH3-domain GRB2-like pseudogene 1	H.sapiens SH3GLP1 pseudogene, 3' end	1666.42580	1910.45700	1543.05590	1664.14380	Detected	Detected	Detected	Detected
2658	A_24_P194420	CCDC134	Hs.474991	coiled-coil domain containing 134	Homo sapiens coiled-coil domain contai	44.92689	110.85669	30.18556	106.05341	Detected	Detected	Detected	Detected
2659	A_24_P389916	LRRC32	Hs.151641	leucine rich repeat containing 32	Homo sapiens leucine rich repeat conta	1187.20250	2407.40160	1214.05880	2141.77400	Detected	Detected	Detected	Detected
2660	A_23_P118888	PAFAH1B1	Hs.77318	platelet-activating factor acetylhydrolas	Homo sapiens platelet-activating factor	2956.88100	7023.48440	1849.35200	3767.28050	Detected	Detected	Detected	Detected
2661	A_32_P75299	TOMM5	Hs.130774	translocase of outer mitochondrial mem	Homo sapiens translocase of outer mitc	18464.38700	28659.93000	16377.53500	20848.13300	Detected	Detected	Detected	Detected
2662	A_23_P138680	IL15RA	Hs.524117	interleukin 15 receptor, alpha	Homo sapiens interleukin 15 receptor, a	582.63480	322.13593	626.89410	291.36710	Detected	Detected	Detected	Detected
2663	A_23_P166823	TNNC1	Hs.118845	troponin C type 1 (slow)	Homo sapiens troponin C type 1 (slow)	104.09418	61.46121	109.14289	35.63929	Detected	Detected	Detected	Detected
2664	A_33_P3287105				Putative uncharacterized protein ENSP	1942.74980	3567.09570	940.55590	1298.04430	Detected	Detected	Detected	Detected
2665	A_33_P3228300	RCL1	Hs.194121	RNA terminal phosphate cyclase-like 1	Homo sapiens RNA terminal phosphate	924.96460	2952.72850	880.01800	2295.25730	Detected	Detected	Detected	Detected
2666	A_23_P146050	ZFAND1	Hs.655453	zinc finger, AN1-type domain 1	Homo sapiens zinc finger, AN1-type do	2157.42290	1592.06180	2093.41000	1396.77560	Detected	Detected	Detected	Detected
2667	A_23_P72059	VSIG10	Hs.187624	V-set and immunoglobulin domain conta	Homo sapiens V-set and immunoglobuli	527.21540	534.65405	314.69086	245.84604	Detected	Detected	Detected	Detected
2668	A_23_P309739	ESR1	Hs.208124	estrogen receptor 1	Homo sapiens estrogen receptor 1 (ESF	19.05133	20.91436	14.20425	6.83851	Detected	Detected	Detected	Compromised
2669	A_23_P112452	GGTA1	Hs.97469	glycoprotein, alpha-galactosyltransferas	Homo sapiens glycoprotein, alpha-galac	137.28374	220.00660	18.06272	13.91303	Detected	Detected	Not Detected	Not Detected
2670	A_33_P3342156		Hs.121492		HADV36S1 Fragment [Source:UniProtK	18.63246	19.61933	10.67534	10.36764	Detected	Compromised	Compromised	Compromised
2671	A_23_P122947	AVL9	Hs.128056	AVL9 homolog (S. cerevisiase)	Homo sapiens AVL9 homolog (S. cerevi	272.98288	285.17892	189.15399	165.80157	Detected	Detected	Detected	Detected
2672	A_33_P3305810	KIAA1161	Hs.522083	KIAA1161	Homo sapiens KIAA1161 (KIAA1161), m	55.48276	107.51659	16.92374	17.42081	Detected	Detected	Detected	Compromised
2673	A_23_P80832	TMEM41A	Hs.707060	transmembrane protein 41A	Homo sapiens transmembrane protein 4	309.82764	510.87927	203.24112	281.41280	Detected	Detected	Detected	Detected
2674	A_33_P3378334	SMC2	Hs.119023	structural maintenance of chromosome	Homo sapiens structural maintenance o	358.75272	775.16370	309.67610	500.75143	Detected	Detected	Detected	Detected
2675	A_23_P163467	C15orf52	Hs.32433	chromosome 15 open reading frame 52	Homo sapiens chromosome 15 open rea	3356.67940	6306.08540	3103.62820	5068.40100	Detected	Detected	Detected	Detected
2676	A_33_P3245841	LOC729907	Hs.712224	hypothetical LOC729907	PREDICTED: Homo sapiens hypothetica	53.03219	103.75052	31.53245	27.74506	Detected	Detected	Detected	Detected
2677	A_23_P59179	RXRB	Hs.388034	retinoid X receptor, beta	Homo sapiens retinoid X receptor, beta	1867.02640	676.76480	1553.91430	514.78520	Detected	Detected	Detected	Detected
2678	A_33_P3363082	SCARNA5	Hs.663619	small Cajal body-specific RNA 5	Homo sapiens small Cajal body-specific	14.19011	22.22615	14.72785	9.85950	Compromised	Detected	Compromised	Compromised
2679	A_23_P120194	ORMDL1	Hs.700632	ORM1-like 1 (S. cerevisiae)	Homo sapiens ORM1-like 1 (S. cerevisi	7985.87550	6491.77050	8114.30500	5942.87500	Detected	Detected	Detected	Detected
2680	A_32_P203515	CYTSA	Hs.474384	cytospin A	Homo sapiens cytospin A (CYTSA), tra	4557.59030	6000.67500	3577.10060	4235.16260	Detected	Detected	Detected	Detected
2681	A_33_P3225011	LOC100287322	Hs.723016	hypothetical protein LOC100287322	PREDICTED: Homo sapiens hypothetica	56.14383	41.68602	23.69406	15.63740	Detected	Detected	Detected	Compromised
2682	A_23_P103720	AGMAT	Hs.567583	agmatine ureohydrolase (agmatinase)	Homo sapiens agmatine ureohydrolase (	65.23018	489.61954	54.77769	338.37344	Detected	Detected	Detected	Detected
2683	A_23_P14157	DZIP1	Hs.656580	DAZ interacting protein 1	Homo sapiens DAZ interacting protein 1	2718.34550	1284.03550	2402.41630	989.07355	Detected	Detected	Detected	Detected
2684	A_23_P130020	UTP18	Hs.709327	UTP18, small subunit (SSU) processom	Homo sapiens UTP18, small subunit (SS	4774.61770	3651.15200	3159.28930	1998.73750	Detected	Detected	Detected	Detected
2685	A_33_P3225696	ZNF526	Hs.137282	zinc finger protein 526	Homo sapiens zinc finger protein 526 (Z	43750.33600	48493.80000	21599.51400	18698.35000	Detected	Detected	Detected	Detected
2686	A_33_P3300308	MAP1LC3A	Hs.632273	microtubule-associated protein 1 light c	Homo sapiens microtubule-associated p	650.49115	436.21313	732.80054	420.89307	Detected	Detected	Detected	Detected
2687	A_33_P3308050	EIF4E2	Hs.292026	eukaryotic translation initiation factor 4	Homo sapiens eukaryotic translation ini	5805.62940	14714.00200	6912.24270	14652.37800	Detected	Detected	Detected	Detected
2688	A_23_P255714	IFT74	Hs.145402	intraflagellar transport 74 homolog (Chla	Homo sapiens intraflagellar transport 74	1169.65310	503.55734	774.35040	274.85605	Detected	Detected	Detected	Detected
2689	A_33_P3327058	ARHGEF2	Hs.720178	Rho/Rac guanine nucleotide exchange f	Homo sapiens Rho/Rac guanine nucleo	100.12960	95.43900	94.35572	81.11054	Detected	Detected	Detected	Detected
2690	A_33_P3280044	ANKRD11	Hs.335003	ankyrin repeat domain 11	Homo sapiens ankyrin repeat domain 11	4785.98730	8118.00150	2722.82520	3804.70780	Detected	Detected	Detected	Detected
2691	A_23_P213661	HISPPD1	Hs.212046	histidine acid phosphatase domain cont	Homo sapiens histidine acid phosphatas	5689.35940	5770.43700	2630.91360	2122.48750	Detected	Detected	Detected	Detected
2692	A_24_P522631	TMEM201	Hs.632365	transmembrane protein 201	Homo sapiens transmembrane protein 2	10622.69800	6494.24700	1706.45970	727.10960	Detected	Detected	Detected	Detected
2693	A_23_P398491	MRPL42P5		mitochondrial ribosomal protein L42 pse	Homo sapiens mitochondrial ribosomal p	254.70819	272.73070	42.34442	21.60514	Detected	Detected	Detected	Detected
2694	A_24_P213643	TSPAN10	Hs.208219	tetraspanin 10	Homo sapiens tetraspanin 10 (TSPAN10	541.93787	755.98456	567.44714	690.74380	Detected	Detected	Detected	Detected
2695	A_23_P121926	SEPP1	Hs.275775	selenoprotein P, plasma, 1	Homo sapiens selenoprotein P, plasma,	2963.17580	482.79280	1896.74230	237.60953	Detected	Detected	Detected	Detected
2696	A_33_P3404744	KIAA1958	Hs.656619	KIAA1958	Homo sapiens KIAA1958 (KIAA1958), m	24.62376	18.11491	5.14977	3.25715	Detected	Compromised	Compromised	Compromised

2697	A_24_P324405	ANKRD11	Hs.335003	ankyrin repeat domain 11	Homo sapiens ankyrin repeat domain 11	1350.21330	2166.63820	287.37277	331.23290	Detected	Detected	Detected	Detected
2698	A_32_P66035	TMEM86A	Hs.502100	transmembrane protein 86A	Homo sapiens transmembrane protein 8	47.58443	9.25724	36.57175	3.25898	Detected	Compromised	Detected	Compromised
2699	A_23_P93009		Hs.637001		U2 small nuclear ribonucleoprotein auxi	344.49790	318.66232	246.15160	218.81360	Detected	Detected	Detected	Detected
2700	A_33_P3320301	LOC388789	Hs.349092	hypothetical LOC388789	Homo sapiens hypothetical LOC388789	17698.09200	18110.63300	17671.55500	14969.00700	Detected	Detected	Detected	Detected
2701	A_24_P315014					14.24914	7.28014	3.14312	3.25834	Detected	Compromised	Compromised	Compromised
2702	A_23_P111865	ZSCAN21	Hs.632294	zinc finger and SCAN domain containing	Homo sapiens zinc finger and SCAN do	591.77590	357.66113	615.91736	312.04993	Detected	Detected	Detected	Detected
2703	A_23_P17914	PNPLA3	Hs.654800	patatin-like phospholipase domain conta	Homo sapiens patatin-like phospholipas	1106.68360	937.08923	728.67487	546.23035	Detected	Detected	Detected	Detected
2704	A_23_P5757	TPRKB	Hs.157401	TP53RK binding protein	Homo sapiens TP53RK binding protein (	5068.18950	7285.01460	3835.60670	4678.47950	Detected	Detected	Detected	Detected
2705	A_23_P130585	CIB3	Hs.255432	calcium and integrin binding family mem	Homo sapiens calcium and integrin bind	74.22844	90.96667	30.30788	27.19747	Detected	Detected	Detected	Detected
2706	A_33_P3286709	LOC647281	Hs.690294	hypothetical LOC647281	PREDICTED: Homo sapiens hypothetica	295.31170	259.93893	16.90780	9.97010	Detected	Detected	Detected	Compromised
2707	A_23_P100189	PRM1	Hs.2909	protamine 1	Homo sapiens protamine 1 (PRM1), mR	47.14488	54.83314	25.05262	23.01687	Detected	Detected	Detected	Detected
2708	A_33_P3239195					1843.97850	2450.66720	1240.83280	1252.54980	Detected	Detected	Detected	Detected
2709	A_33_P3357087	MPV17	Hs.75659	MpV17 mitochondrial inner membrane p	Homo sapiens MpV17 mitochondrial inn	16365.35000	8532.82300	13228.92500	5721.03120	Detected	Detected	Detected	Detected
2710	A_33_P3322654	RNPEPL1	Hs.5345	arginyl aminopeptidase (aminopeptidase	Homo sapiens arginyl aminopeptidase (a	21856.09000	20348.49400	24390.10000	19184.61300	Detected	Detected	Detected	Detected
2711	A_23_P125624	ACOT9	Hs.298885	acyl-CoA thioesterase 9	Homo sapiens acyl-CoA thioesterase 9	11937.86900	30433.82000	7223.85840	14855.45900	Detected	Detected	Detected	Detected
2712	A_33_P3393106	FAM188A	Hs.158870	family with sequence similarity 188, mer	Homo sapiens family with sequence sim	180.37521	164.88599	146.45358	117.71366	Detected	Detected	Detected	Detected
2713	A_23_P108492	MARS2	Hs.116602	methionyl-tRNA synthetase 2, mitochor	Homo sapiens methionyl-tRNA syntheta	28.15889	34.33653	15.80295	15.58445	Detected	Detected	Detected	Detected
2714	A_33_P3224281					17.98154	21.54088	3.17647	3.28779	Detected	Detected	Compromised	Compromised
2715	A_32_P201496	POLR3H	Hs.712617	polymerase (RNA) III (DNA directed) po	Homo sapiens polymerase (RNA) III (DN	506.32710	1706.74510	597.65393	1723.28660	Detected	Detected	Detected	Detected
2716	A_23_P253484	AADAT	Hs.529735	aminoadipate aminotransferase	Homo sapiens aminoadipate aminotrans	526.44160	759.75290	336.91574	411.90280	Detected	Detected	Detected	Detected
2717	A_33_P3386995	TXNDC11	Hs.313847	thioredoxin domain containing 11	Homo sapiens thioredoxin domain conta	35.63405	67.97138	28.06592	55.39988	Detected	Detected	Detected	Detected
2718	A_33_P3354539	CHURC1	Hs.325531	churchill domain containing 1	Homo sapiens churchill domain containi	471.26663	200.14810	396.32178	135.30496	Detected	Detected	Detected	Detected
2719	A_32_P107493	C16orf55	Hs.655171	chromosome 16 open reading frame 55	Homo sapiens chromosome 16 open rea	134.84169	64.69071	99.47999	37.06722	Detected	Detected	Detected	Detected
2720	A_23_P118834	TOP2A	Hs.156346	topoisomerase (DNA) II alpha 170kDa	Homo sapiens topoisomerase (DNA) II a	227.44055	4578.82500	159.51663	2723.89280	Detected	Detected	Detected	Detected
2721	A_23_P164258	PIPOX	Hs.462585	pipecolic acid oxidase	Homo sapiens pipecolic acid oxidase (P	158.51918	156.34952	115.86682	67.75285	Detected	Detected	Detected	Detected
2722	A_33_P3277328	LOC100131700	Hs.149540	similar to Quinone oxidoreductase-like (	PREDICTED: Homo sapiens similar to m	24.28950	54.61445	49.68618	53.69992	Detected	Detected	Detected	Detected
2723	A_23_P109821	TADA3	Hs.386390	transcriptional adaptor 3	Homo sapiens transcriptional adaptor 3	27600.15800	22132.96900	19858.11500	14513.10400	Detected	Detected	Detected	Detected
2724	A_33_P3407434		Hs.117259		BX117604 Soares_fetal_liver_spleen_1NF	16.92752	66.39913	7.46430	26.33849	Detected	Detected	Compromised	Detected
2725	A_23_P308603	SRC	Hs.195659	v-src sarcoma (Schmidt-Ruppin A-2) v	Homo sapiens v-src sarcoma (Schmidt-	549.33280	953.43750	363.34170	572.43790	Detected	Detected	Detected	Detected
2726	A_32_P51084	NUP205	Hs.632299	nucleoporin 205kDa	Homo sapiens nucleoporin 205kDa (NUF	5877.96880	8722.75800	4171.18700	5438.95560	Detected	Detected	Detected	Detected
2727	A_32_P129288	RAB1B	Hs.300816	RAB1B, member RAS oncogene family	Homo sapiens RAB1B, member RAS on	3906.05000	5443.91200	3457.13820	4380.41360	Detected	Detected	Detected	Detected
2728	A_23_P39931	DYSF	Hs.252180	dysferlin, limb girdle muscular dystrophy	Homo sapiens dysferlin, limb girdle mus	34.48962	33.86914	8.57504	4.33912	Detected	Detected	Compromised	Compromised
2729	A_23_P121702	OCIAD2	Hs.95835	OCIA domain containing 2	Homo sapiens OCIA domain containing	7259.35840	8286.70800	6629.06450	6234.98200	Detected	Detected	Detected	Detected
2730	A_33_P3360456	ZNF154	Hs.646378	zinc finger protein 154	Homo sapiens zinc finger protein 154 (Z	63.60870	68.93355	4.43116	8.59125	Detected	Detected	Compromised	Compromised
2731	A_23_P105307	DGKA	Hs.524488	diacylglycerol kinase, alpha 80kDa	Homo sapiens diacylglycerol kinase, alp	670.70780	362.89142	615.08450	298.68555	Detected	Detected	Detected	Detected
2732	A_24_P103686	AGPAT6	Hs.355753	1-acylglycerol-3-phosphate O-acyltran	Homo sapiens 1-acylglycerol-3-phosph	2390.94580	3816.69820	1971.90260	3036.94920	Detected	Detected	Detected	Detected
2733	A_23_P43273	EXT1	Hs.492618	exostoses (multiple) 1	Homo sapiens exostoses (multiple) 1 (E	4294.26170	6585.80220	3667.81700	4842.87400	Detected	Detected	Detected	Detected
2734	A_33_P3264731				Putative uncharacterized protein ENSP	174.00450	208.08716	53.84684	44.93541	Detected	Detected	Detected	Detected
2735	A_24_P942354	PITPNA	Hs.429819	phosphatidylinositol transfer protein, alp	Homo sapiens phosphatidylinositol trans	24695.42400	23367.76000	16227.22700	13208.53000	Detected	Detected	Detected	Detected
2736	A_23_P106158	L2HGDH	Hs.256034	L-2-hydroxyglutarate dehydrogenase	Homo sapiens L-2-hydroxyglutarate de	162.64810	113.38887	193.53706	112.97513	Detected	Detected	Detected	Detected
2737	A_23_P77653	SLC12A3	Hs.669115	solute carrier family 12 (sodium/chlorid	Homo sapiens solute carrier family 12 (	18.52985	24.49734	18.03696	19.97282	Detected	Detected	Detected	Detected
2738	A_23_P408095	DSTN	Hs.304192	destrin (actin depolymerizing factor)	Homo sapiens destrin (actin depolymeri	58503.95300	65757.66400	34799.33200	32527.90600	Detected	Detected	Detected	Detected
2739	A_33_P3238323	RBCK1	Hs.247280	RanBP-type and C3HC4-type zinc fing	Homo sapiens RanBP-type and C3HC4	658.18115	549.60925	528.77690	445.77026	Detected	Detected	Detected	Detected
2740	A_33_P3358750	MYBPHL	Hs.443402	myosin binding protein H-like	Homo sapiens myosin binding protein H	15.63080	22.37740	8.16558	6.27531	Detected	Detected	Compromised	Compromised
2741	A_23_P214821	EDN1	Hs.511899	endothelin 1	Homo sapiens endothelin 1 (EDN1), mR	152.43022	1179.52870	146.98103	928.41644	Detected	Detected	Detected	Detected
2742	A_23_P138168	CNN3	Hs.483454	calponin 3, acidic	Homo sapiens calponin 3, acidic (CNN3)	1373.70430	3430.09280	1432.24460	3086.94630	Detected	Detected	Detected	Detected
2743	A_33_P3259890	FLJ35946	Hs.586188	hypothetical protein FLJ35946	PREDICTED: Homo sapiens hypothetica	175.96004	163.55261	138.26282	116.03529	Detected	Detected	Detected	Detected
2744	A_23_P209337	FAM119A	Hs.664764	family with sequence similarity 119, mer	Homo sapiens family with sequence sim	752.80000	587.16583	718.86510	478.67877	Detected	Detected	Detected	Detected
2745	A_33_P3297150					26.94191	31.09520	6.89276	7.02391	Detected	Detected	Compromised	Compromised
2746	A_24_P235305	ZNF706	Hs.374485	zinc finger protein 706	Homo sapiens zinc finger protein 706 (Z	188.55315	102.88902	130.50926	66.26334	Detected	Detected	Detected	Detected
2747	A_23_P385105	PLCD4	Hs.632528	phospholipase C, delta 4	Homo sapiens phospholipase C, delta 4	97.12318	64.96831	95.35312	47.99252	Detected	Detected	Detected	Detected
2748	A_33_P3392517	LBX2	Hs.722114	ladybird homeobox 2	Homo sapiens ladybird homeobox 2 (LB	89.78796	70.39221	66.96814	36.58361	Detected	Detected	Detected	Detected
2749	A_23_P208835	MAP2K2	Hs.465627	mitogen-activated protein kinase kinase	Homo sapiens mitogen-activated protei	7839.35700	7754.65530	9135.57600	8301.92300	Detected	Detected	Detected	Detected
2750	A_33_P3229452	FLJ44477	Hs.531403	FLJ44477 protein	PREDICTED: Homo sapiens FLJ44477 p	548.64154	651.51620	630.36810	588.60486	Detected	Detected	Detected	Detected
2751	A_32_P230148	LOC728836		hypothetical LOC728836	PREDICTED: Homo sapiens hypothetica	27.09776	32.15066	28.04604	23.93599	Detected	Detected	Detected	Detected
2752	A_23_P133691	RRAGD	Hs.31712	Ras-related GTP binding D	Homo sapiens Ras-related GTP binding	72.32405	25.43640	45.94708	7.52638	Detected	Detected	Detected	Compromised
2753	A_33_P3297818					20.36287	23.87266	3.43492	4.82557	Detected	Detected	Compromised	Compromised
2754	A_24_P194931	SMEK2	Hs.516182	SMEK homolog 2, suppressor of mek1 (	Homo sapiens SMEK homolog 2, suppre	1073.46400	376.61180	706.49554	194.15303	Detected	Detected	Detected	Detected
2755	A_23_P315836	BAIAP2	Hs.128316	BAI1-associated protein 2	Homo sapiens BAI1-associated protein	13.31569	90.66317	7.23191	85.38717	Detected	Detected	Compromised	Detected
2756	A_33_P3377199	PRDX1	Hs.180909	peroxiredoxin 1	Homo sapiens peroxiredoxin 1 (PRDX1)	66918.39000	94932.51000	45110.80500	52421.38700	Detected	Detected	Detected	Detected
2757	A_23_P398637	RG9MTD2	Hs.380412	RNA (guanine-9-) methyltransferase do	Homo sapiens RNA (guanine-9-) methy	294.73407	236.87567	205.08308	137.09221	Detected	Detected	Detected	Detected
2758	A_24_P153576	SHPRH	Hs.124537	SNF2 histone linker PHD RING helicase	Homo sapiens SNF2 histone linker PHD	516.04860	259.77438	360.71646	137.26318	Detected	Detected	Detected	Detected
2759	A_33_P3238521	LOC100294070	Hs.720272	hypothetical protein LOC100294070	PREDICTED: Homo sapiens hypothetica	7156.80760	8781.05600	6029.86100	5715.83350	Detected	Detected	Detected	Detected
2760	A_23_P59630		Hs.659871		Postmeiotic segregation increased 2-lik	558.30084	682.96260	486.74005	518.31177	Detected	Detected	Detected	Detected
2761	A_23_P380998	R3HDM1	Hs.412462	R3H domain containing 1	Homo sapiens R3H domain containing 1	1125.76440	1471.89150	758.69900	907.57400	Detected	Detected	Detected	Detected
2762	A_33_P3388651	ABLIM1	Hs.438236	actin binding LIM protein 1	Homo sapiens actin binding LIM protein	85.66856	12.79598	40.75651	3.25387	Detected	Compromised	Detected	Compromised
2763	A_23_P115261	AGT	Hs.19383	angiotensinogen (serpin peptidase inhibi	Homo sapiens angiotensinogen (serpin p	1720.13070	67.72418	1436.19140	47.87763	Detected	Detected	Detected	Detected
2764	A_33_P3303602	DGKZ	Hs.502461	diacylglycerol kinase, zeta 104kDa	Homo sapiens cDNA FLJ41384 fis, clon	68.80434	74.12510	38.07715	33.64038	Detected	Detected	Detected	Detected
2765	A_33_P3386019	LOC402509		similar to solute carrier family 29 (nucle	PREDICTED: Homo sapiens similar to s	19.64319	16.35257	19.22674	8.59874	Detected	Compromised	Detected	Compromised
2766	A_33_P3246885	DMKN	Hs.417795	dermokine	Homo sapiens dermokine (DMKN), trans	4826.60350	405.97660	5578.00240	197.20377	Detected	Detected	Detected	Detected
2767	A_24_P15765	RPS7P5	Hs.677723	ribosomal protein S7 pseudogene 5	Homo sapiens cDNA FLJ25739 fis, clon	62122.35000	55091.03000	34550.70300	24862.92600	Detected	Detected	Detected	Detected
2768	A_23_P156907	SOBP	Hs.445244	sine oculis binding protein homolog (Dro	Homo sapiens sine oculis binding protei	289.61200	10.47626	219.32841	4.09201	Detected	Compromised	Detected	Compromised
2769	A_23_P53276	TIMELESS	Hs.118631	timeless homolog (Drosophila)	Homo sapiens timeless homolog (Droso	1799.55690	10329.36600	1222.47600	5792.52700	Detected	Detected	Detected	Detected
2770	A_32_P213881	C20orf202	Hs.148503	chromosome 20 open reading frame 202	Homo sapiens chromosome 20 open rea	14.37132	3.71874	9.80482	3.27050	Detected	Compromised	Compromised	Compromised
2771	A_24_P910060					597.50380	431.21686	15.21303	6.84455	Detected	Detected	Compromised	Compromised

2772	A_33_P3390042	LOC143188	Hs.287723	hypothetical LOC143188	Homo sapiens hypothetical LOC143188	26.32727	41.04678	14.52489	22.67739	Detected	Detected	Compromised	Detected
2773	A_24_P251962	PCDHB2	Hs.533023	protocadherin beta 2	Homo sapiens protocadherin beta 2 (PC	96.02367	66.89636	86.81425	57.09324	Detected	Detected	Detected	Detected
2774	A_33_P3291510	VCY	Hs.632284	variable charge, Y-linked	Homo sapiens variable charge, Y-linked	35.01013	40.09716	12.52725	11.51151	Detected	Detected	Compromised	Compromised
2775	A_33_P3318343	CTAG2	Hs.87225	cancer/testis antigen 2	Homo sapiens cancer/testis antigen 2 (	62.47739	6749.38800	50.49297	6441.90500	Detected	Detected	Detected	Detected
2776	A_33_P3604069	RPS2P45	Hs.661121	ribosomal protein S2 pseudogene 45	Homo sapiens cDNA FLJ12331 fis, clon	17.65326	13.01531	3.19952	5.06044	Detected	Compromised	Compromised	Compromised
2777	A_24_P139620	USP21	Hs.8015	ubiquitin specific peptidase 21	Homo sapiens ubiquitin specific peptida	130.86545	83.65043	97.21465	48.88602	Detected	Detected	Detected	Detected
2778	A_33_P3384255					15.78322	17.01820	8.42453	6.76089	Detected	Compromised	Compromised	Compromised
2779	A_33_P3365200				Novel protein [Source:UniProtKB/TrEM	39.09910	35.96704	15.31337	14.58285	Detected	Detected	Compromised	Compromised
2780	A_33_P3243519	RAPGEF6	Hs.483329	Rap guanine nucleotide exchange factor	Homo sapiens Rap guanine nucleotide e	37.49061	28.38590	23.32370	17.36894	Detected	Detected	Detected	Detected
2781	A_33_P3357573	MAML3	Hs.586165	mastermind-like 3 (Drosophila)	Homo sapiens mastermind-like 3 (Droso	32.93113	29.80530	16.76047	10.99099	Detected	Detected	Detected	Compromised
2782	A_24_P173823	PBX1	Hs.557097	pre-B-cell leukemia homeobox 1	Homo sapiens pre-B-cell leukemia hom	12832.71800	6878.65800	8602.51300	3953.62740	Detected	Detected	Detected	Detected
2783	A_33_P3404286	KIAA0368	Hs.368255	KIAA0368	Homo sapiens KIAA0368 (KIAA0368), m	3339.64840	3252.17380	2887.94900	2401.73340	Detected	Detected	Detected	Detected
2784	A_33_P3335498	KLF8	Hs.646614	Kruppel-like factor 8	Homo sapiens Kruppel-like factor 8 (KL	32.17826	28.36698	6.29733	10.22252	Detected	Detected	Compromised	Compromised
2785	A_23_P100912	GIT1	Hs.514051	G protein-coupled receptor kinase inter	Homo sapiens G protein-coupled recep	186.26768	164.31030	191.09220	167.52086	Detected	Detected	Detected	Detected
2786	A_33_P3211078	HMGA2	Hs.505924	high mobility group AT-hook 2	Homo sapiens high mobility group AT-h	10150.24500	13649.88200	3386.57620	3074.66630	Detected	Detected	Detected	Detected
2787	A_24_P161293		Hs.445080		Novel protein [Source:UniProtKB/TrEM	9167.03200	11324.76600	5923.69200	5409.65000	Detected	Detected	Detected	Detected
2788	A_23_P400235	MUT	Hs.485527	methylmalonyl Coenzyme A mutase	Homo sapiens methylmalonyl Coenzyme	4239.01370	2333.09960	2433.77560	1122.37610	Detected	Detected	Detected	Detected
2789	A_24_P330822	HNF1B	Hs.191144	HNF1 homeobox B	Homo sapiens HNF1 homeobox B (HNF	180.08803	137.50880	61.29490	37.81079	Detected	Detected	Detected	Detected
2790	A_33_P3304107	KLHL28	Hs.550906	kelch-like 28 (Drosophila)	Homo sapiens kelch-like 28 (Drosophila	1203.87110	626.96580	531.03940	222.59912	Detected	Detected	Detected	Detected
2791	A_23_P164284	CLDN7	Hs.513915	claudin 7	Homo sapiens claudin 7 (CLDN7), mRNA	615.63446	1362.54350	560.19880	1087.43530	Detected	Detected	Detected	Detected
2792	A_33_P3714482	LRP5L	Hs.634058	low density lipoprotein receptor-related	Homo sapiens low density lipoprotein re	31.52933	34.09250	26.98733	33.56067	Detected	Detected	Detected	Detected
2793	A_23_P11598	ZNF684	Hs.524767	zinc finger protein 684	Homo sapiens zinc finger protein 684 (Z	71.22820	54.68333	68.63631	48.34488	Detected	Detected	Detected	Detected
2794	A_32_P227317	MGC13005	Hs.712940	hypothetical LOC84771	Homo sapiens hypothetical LOC84771 (	87.43752	173.86382	60.82715	113.23599	Detected	Detected	Detected	Detected
2795	A_24_P88801	NPHP1	Hs.280388	nephronophthisis 1 (juvenile)	Homo sapiens nephronophthisis 1 (juver	1405.62020	405.17764	1274.20360	331.71980	Detected	Detected	Detected	Detected
2796	A_23_P139864	GSG1	Hs.240053	germ cell associated 1	Homo sapiens germ cell associated 1 (C	139.54771	320.14725	110.96970	213.56856	Detected	Detected	Detected	Detected
2797	A_33_P3393734				40S ribosomal protein S27 (Metallopan-	166.93758	77.59078	143.55637	51.91401	Detected	Detected	Detected	Detected
2798	A_33_P3228435	FXYD1	Hs.442498	FXYD domain containing ion transport r	Homo sapiens FXYD domain containing	263.68960	150.87663	271.38390	137.26332	Detected	Detected	Detected	Detected
2799	A_33_P3409077	C6orf170	Hs.121396	chromosome 6 open reading frame 170	Homo sapiens chromosome 6 open read	22.31720	9.35191	20.28304	3.64940	Detected	Compromised	Detected	Compromised
2800	A_23_P105535	WDR51B	Hs.25130	WD repeat domain 51B	Homo sapiens WD repeat domain 51B (W	408.51650	389.79250	297.65866	233.02498	Detected	Detected	Detected	Detected
2801	A_33_P3238325	LOC650621		similar to Anaphase promoting complex	PREDICTED: Homo sapiens similar to a	82.57561	90.02976	26.85707	18.74593	Detected	Detected	Detected	Detected
2802	A_33_P3294153				Neuron-specific vesicular protein calcy	18.84023	34.76728	11.90012	16.91790	Detected	Detected	Compromised	Compromised
2803	A_24_P365679	RRP7B	Hs.534041	ribosomal RNA processing 7 homolog B	Homo sapiens cDNA FLJ40882 fis, clon	70.84905	4.09944	69.48125	7.51962	Detected	Compromised	Detected	Compromised
2804	A_33_P3279851	LOC100286979	Hs.436527	similar to anaphase promoting complex	PREDICTED: Homo sapiens similar to a	18.86121	24.65449	8.00881	12.87818	Detected	Detected	Compromised	Compromised
2805	A_23_P102262	MRPL19	Hs.44024	mitochondrial ribosomal protein L19	Homo sapiens mitochondrial ribosomal p	5107.14800	8845.05200	4585.37700	6152.63800	Detected	Detected	Detected	Detected
2806	A_23_P118741	SLC16A13	Hs.448010	solute carrier family 16, member 13 (mo	Homo sapiens solute carrier family 16, r	55.09461	39.05560	25.32491	20.22998	Detected	Detected	Detected	Detected
2807	A_33_P3362371	RTN3	Hs.473761	reticulon 3	Homo sapiens reticulon 3 (RTN3), trans	299.06390	428.35650	229.89693	246.60175	Detected	Detected	Detected	Detected
2808	A_24_P349274	OR4X2	Hs.553565	olfactory receptor, family 4, subfamily X	Homo sapiens olfactory receptor, family	42.54639	68.14776	29.60827	33.67575	Detected	Detected	Detected	Detected
2809	A_23_P146367	C9orf89	Hs.434213	chromosome 9 open reading frame 89	Homo sapiens chromosome 9 open read	63335.78000	28680.07600	54753.35500	23475.03700	Detected	Detected	Detected	Detected
2810	A_24_P141019	ST7	Hs.368131	suppression of tumorigenicity 7	Homo sapiens suppression of tumorigen	99.77786	129.05449	96.81867	126.98039	Detected	Detected	Detected	Detected
2811	A_23_P112135	TRAM1	Hs.491988	translocation associated membrane pro	Homo sapiens translocation associated	33250.02000	24418.49200	24622.53000	15410.67100	Detected	Detected	Detected	Detected
2812	A_23_P114466	TBL1Y	Hs.664560	transducin (beta)-like 1Y-linked	Homo sapiens transducin (beta)-like 1Y	392.38086	277.96220	310.11160	206.64552	Detected	Detected	Detected	Detected
2813	A_23_P428219	EZH1	Hs.194669	enhancer of zeste homolog 1 (Drosophil	Homo sapiens enhancer of zeste homol	783.29550	695.14667	692.25037	654.52234	Detected	Detected	Detected	Detected
2814	A_23_P168443	EPHB4	Hs.437008	EPH receptor B4	Homo sapiens EPH receptor B4 (EPHB4	272.39542	160.83267	271.83670	150.66590	Detected	Detected	Detected	Detected
2815	A_23_P116512	PRR5L	Hs.19987	proline rich 5 like	Homo sapiens proline rich 5 like (PRR5L	29.67471	56.71708	20.91380	29.39013	Detected	Detected	Detected	Detected
2816	A_33_P3222867	THBS3	Hs.658188	thrombospondin 3	Homo sapiens mRNA; cDNA DKFZp686	21.09000	24.41338	6.63418	7.74577	Detected	Detected	Compromised	Compromised
2817	A_23_P51217	CLCA1	Hs.194659	chloride channel accessory 1	Homo sapiens chloride channel accesso	18.43954	27.19888	3.13235	3.25123	Detected	Detected	Compromised	Compromised
2818	A_23_P502750	MDM2	Hs.484551	Mdm2 p53 binding protein homolog (mou	Homo sapiens Mdm2 p53 binding protei	77.31155	87.69299	46.55076	44.56888	Detected	Detected	Detected	Detected
2819	A_33_P3241150					59.92439	21.48062	30.79430	8.49506	Detected	Detected	Detected	Compromised
2820	A_23_P406385	FBXL16	Hs.513244	F-box and leucine-rich repeat protein 1	Homo sapiens F-box and leucine-rich r	1099.33190	517.77140	675.88135	268.29184	Detected	Detected	Detected	Detected
2821	A_23_P67529	KCNN4	Hs.10082	potassium intermediate/small conducta	Homo sapiens potassium intermediate/s	1354.62780	911.87070	1146.44510	726.71277	Detected	Detected	Detected	Detected
2822	A_33_P3270489	C6orf97	Hs.660044	chromosome 6 open reading frame 97	Homo sapiens chromosome 6 open read	145.51329	26.38336	87.39236	9.37675	Detected	Detected	Detected	Compromised
2823	A_33_P3243028	DSPP	Hs.678914	dentin sialophosphoprotein	Homo sapiens dentin sialophosphoprote	14.52444	26.88087	3.14244	8.63025	Detected	Detected	Compromised	Compromised
2824	A_33_P3381613					51.26918	50.63003	22.56755	13.95304	Detected	Detected	Detected	Compromised
2825	A_23_P93658	ACCN3	Hs.647113	amiloride-sensitive cation channel 3	Homo sapiens amiloride-sensitive catio	59.50608	9.34186	54.43339	6.68384	Detected	Compromised	Detected	Compromised
2826	A_33_P3266923	LMTK2	Hs.444179	lemur tyrosine kinase 2	Homo sapiens lemur tyrosine kinase 2 (	285.02402	749.49160	184.87233	404.97170	Detected	Detected	Detected	Detected
2827	A_23_P70148	NNT	Hs.482043	nicotinamide nucleotide transhydrogena	Homo sapiens nicotinamide nucleotide t	293.24970	660.18340	212.52597	404.57920	Detected	Detected	Detected	Detected
2828	A_33_P3317211	MECP2	Hs.200716	methyl CpG binding protein 2 (Rett synd	Homo sapiens methyl CpG binding prote	84.84977	109.43400	59.83625	70.18552	Detected	Detected	Detected	Detected
2829	A_23_P65262	N4BP2L2	Hs.507680	NEDD4 binding protein 2-like 2	Homo sapiens NEDD4 binding protein 2-	1810.32970	905.08950	1708.08480	715.91310	Detected	Detected	Detected	Detected
2830	A_23_P364544	C12orf60	Hs.659951	chromosome 12 open reading frame 60	Homo sapiens chromosome 12 open rea	192.05450	100.44659	146.07057	74.77097	Detected	Detected	Detected	Detected
2831	A_23_P75288	ATAD1	Hs.435948	ATPase family, AAA domain containing	Homo sapiens ATPase family, AAA dom	4394.68000	3961.34500	2863.74900	2047.48960	Detected	Detected	Detected	Detected
2832	A_23_P384857				GB	128.09091	142.85141	20.97969	24.66324	Detected	Detected	Detected	Detected
2833	A_23_P110571	MAST4	Hs.595458	microtubule associated serine/threonin	Homo sapiens microtubule associated s	1270.99010	1351.25710	835.07275	884.26140	Detected	Detected	Detected	Detected
2834	A_33_P3253596	KIF4A	Hs.648326	kinesin family member 4A	Homo sapiens kinesin family member 4A	31.42260	757.36957	16.23617	561.93823	Detected	Detected	Detected	Detected
2835	A_23_P401106	PDE2A	Hs.503163	phosphodiesterase 2A, cGMP-stimulate	Homo sapiens phosphodiesterase 2A, c	500.68225	467.43744	389.42963	114.73867	Detected	Detected	Detected	Detected
2836	A_23_P67702	ZNF85	Hs.37138	zinc finger protein 85	Homo sapiens zinc finger protein 85 (ZN	866.42334	1158.91980	142.81577	176.33183	Detected	Detected	Detected	Detected
2837	A_24_P274615	ARRDC3	Hs.24684	arrestin domain containing 3	Homo sapiens arrestin domain containin	5402.46340	2600.69530	2439.44380	943.23130	Detected	Detected	Detected	Detected
2838	A_23_P118289	BCL7C	Hs.658547	B-cell CLL/lymphoma 7C	Homo sapiens B-cell CLL/lymphoma 7C	1313.86120	1907.40830	1401.26650	1959.81340	Detected	Detected	Detected	Detected
2839	A_23_P35444	INA	Hs.500916	internexin neuronal intermediate filamen	Homo sapiens internexin neuronal intern	159.99069	225.32866	108.65849	89.18188	Detected	Detected	Detected	Detected
2840	A_23_P2920	SERPINA3	Hs.534293	serpin peptidase inhibitor, clade A (alph	Homo sapiens serpin peptidase inhibitor	209.56696	48.50884	232.33983	38.81937	Detected	Detected	Detected	Detected
2841	A_33_P3773168	LOC285768	Hs.209463	hypothetical LOC285768	Homo sapiens hypothetical LOC285768	14.21312	3.93935	6.81712	3.50364	Detected	Compromised	Compromised	Compromised
2842	A_24_P106357	WDR36	Hs.533237	WD repeat domain 36	Homo sapiens WD repeat domain 36 (W	1120.71530	788.99316	719.46650	431.31320	Detected	Detected	Detected	Detected
2843	A_33_P3397150	FLJ22184	Hs.288540	hypothetical protein FLJ22184	PREDICTED: Homo sapiens hypothetica	6243.39550	8240.93200	2121.48460	1725.32240	Detected	Detected	Detected	Detected
2844	A_23_P3312	ISLR	Hs.699822	immunoglobulin superfamily containing l	Homo sapiens immunoglobulin superfam	13129.58200	39519.65600	12272.19800	34666.41800	Detected	Detected	Detected	Detected
2845	A_33_P3238171	ZDHHC8	Hs.63128	zinc finger, DHHC-type containing 8	Probable palmitoyltransferase ZDHHC8	575.46747	323.26180	428.31686	201.77210	Detected	Detected	Detected	Detected
2846	A_23_P29257	H1F0	Hs.715673	H1 histone family, member 0	Homo sapiens H1 histone family, membe	2684.51150	2304.30710	2654.72500	2257.14380	Detected	Detected	Detected	Detected

2847	A_33_P3353742					18.74858	29.12946	15.97016	9.81940	Detected	Detected	Compromised	Compromised
2848	A_23_P38677	SLMO1	Hs.514718	slowmo homolog 1 (Drosophila)	Homo sapiens slowmo homolog 1 (Dros	1760.95780	4612.03370	1383.03630	3261.94650	Detected	Detected	Detected	Detected
2849	A_33_P3734384	LOC285957	Hs.298296	hypothetical protein LOC285957	Homo sapiens cDNA FLJ40207 fis, clon	16.24046	19.44561	3.88189	4.05785	Detected	Compromised	Compromised	Compromised
2850	A_33_P3224423	POLM	Hs.596982	polymerase (DNA directed), mu	Homo sapiens polymerase (DNA directe	274.86510	285.08280	186.66920	164.39601	Detected	Detected	Detected	Detected
2851	A_23_P103601	MAN1C1	Hs.197043	mannosidase, alpha, class 1C, member	Homo sapiens mannosidase, alpha, clas	534.25380	246.82867	354.27810	128.56140	Detected	Detected	Detected	Detected
2852	A_33_P3299130	LOC100133001		similar to hCG30082	PREDICTED: Homo sapiens similar to h	210.23741	270.80040	326.42654	333.53644	Detected	Detected	Detected	Detected
2853	A_23_P369237	ADIPOQ	Hs.80485	adiponectin, C1Q and collagen domain c	Homo sapiens adiponectin, C1Q and col	16.04537	5.47203	3.89835	16.94979	Not Detected	Compromised	Compromised	Not Detected
2854	A_23_P6943	GPR15	Hs.563128	G protein-coupled receptor 15	Homo sapiens G protein-coupled recep	52.97058	50.08783	24.38557	17.83667	Detected	Detected	Detected	Compromised
2855	A_23_P67771	BARD1	Hs.591642	BRCA1 associated RING domain 1	Homo sapiens BRCA1 associated RING	460.69882	2007.54970	412.85117	1560.29810	Detected	Detected	Detected	Detected
2856	A_33_P3385453				Lys-63-specific deubiquitinase BRCC3	22.35689	15.54965	8.19251	5.36261	Detected	Compromised	Compromised	Compromised
2857	A_33_P3362696	RIC8B	Hs.131306	resistance to inhibitors of cholinesteras	Homo sapiens resistance to inhibitors o	1215.74050	624.55835	806.94110	343.99728	Detected	Detected	Detected	Detected
2858	A_23_P5441	ABCB6	Hs.107911	ATP-binding cassette, sub-family B (MI	Homo sapiens ATP-binding cassette, su	1620.53700	651.17773	1270.89180	481.20670	Detected	Detected	Detected	Detected
2859	A_24_P41149					39132.11300	32187.32400	1505.99010	979.25916	Detected	Detected	Detected	Detected
2860	A_32_P133670	ANP32A	Hs.458747	acidic (leucine-rich) nuclear phosphopr	Homo sapiens acidic (leucine-rich) nucl	154.50836	243.79220	137.53545	123.77321	Detected	Detected	Detected	Detected
2861	A_32_P88231					221.12338	15.52549	132.96002	9.35926	Detected	Compromised	Detected	Compromised
2862	A_23_P27947	PDCD2L	Hs.515344	programmed cell death 2-like	Homo sapiens programmed cell death 2	1834.34730	1510.40770	1469.51840	983.74630	Detected	Detected	Detected	Detected
2863	A_23_P103442	MEAF6	Hs.17118	MYST/Esa1-associated factor 6	Homo sapiens MYST/Esa1-associated	5796.74800	6411.39400	4830.60300	4352.62000	Detected	Detected	Detected	Detected
2864	A_24_P898945	C18orf19	Hs.13034	chromosome 18 open reading frame 19	Homo sapiens chromosome 18 open rea	566.15970	604.10520	256.85992	211.31493	Detected	Detected	Detected	Detected
2865	A_24_P281009	AQP7P2	Hs.639769	aquaporin 7 pseudogene 2	Homo sapiens aquaporin 7 pseudogene	13.64391	8.62802	3.09043	3.22576	Detected	Compromised	Compromised	Compromised
2866	A_33_P3380897	ATP6V1C1	Hs.86905	ATPase, H+ transporting, lysosomal 42k	Homo sapiens ATPase, H+ transporting,	18029.63500	11175.79300	14049.16100	8068.57370	Detected	Detected	Detected	Detected
2867	A_33_P3388822	ZNF233	Hs.466891	zinc finger protein 233	Homo sapiens zinc finger protein 233 (Z	77.82809	72.29757	10.80178	9.15387	Detected	Detected	Compromised	Compromised
2868	A_33_P3255209	LOC151162	Hs.4988	hypothetical LOC151162	Homo sapiens hypothetical LOC151162	8390.40000	11415.79400	7128.75630	8668.56900	Detected	Detected	Detected	Detected
2869	A_33_P3376836				Putative uncharacterized protein ENSP	9216.72950	6704.11300	602.10547	277.60320	Detected	Detected	Detected	Detected
2870	A_33_P3404336		Hs.676976		CDNA FLJ27256 fis, clone SYN09689	58.86171	173.88405	28.69825	142.82162	Detected	Detected	Detected	Detected
2871	A_23_P6836	IP6K2	Hs.595983	inositol hexakisphosphate kinase 2	Homo sapiens inositol hexakisphosphate	677.74880	548.71840	519.66140	400.61520	Detected	Detected	Detected	Detected
2872	A_23_P421423	TNFAIP2	Hs.525607	tumor necrosis factor, alpha-induced pr	Homo sapiens tumor necrosis factor, al	19523.68000	9441.80800	15985.91600	7304.73730	Detected	Detected	Detected	Detected
2873	A_23_P107173	MEOX1	Hs.438	mesenchyme homeobox 1	Homo sapiens mesenchyme homeobox	32.32433	5.24817	27.00598	3.49333	Detected	Compromised	Detected	Compromised
2874	A_23_P153050	ALKBH5	Hs.462392	alkB, alkylation repair homolog 5 (E. coli	Homo sapiens alkB, alkylation repair ho	2527.97120	2602.21220	1783.88990	1635.33030	Detected	Detected	Detected	Detected
2875	A_23_P395585	C3orf22	Hs.178210	chromosome 3 open reading frame 22	Homo sapiens chromosome 3 open read	176.11516	223.36626	57.58423	51.02618	Detected	Detected	Detected	Detected
2876	A_23_P65174	PHF11	Hs.369039	PHD finger protein 11	Homo sapiens PHD finger protein 11 (P	1056.69090	1268.98030	778.17175	817.35065	Detected	Detected	Detected	Detected
2877	A_23_P117163	RCBTB1	Hs.508021	regulator of chromosome condensation	Homo sapiens regulator of chromosome	1136.60970	1192.70400	778.53910	708.29260	Detected	Detected	Detected	Detected
2878	A_23_P114423	RGN	Hs.77854	regucalcin (senescence marker protein-	Homo sapiens regucalcin (senescence m	299.85828	573.17474	209.09510	330.70740	Detected	Detected	Detected	Detected
2879	A_23_P363174	HIST1H2AL	Hs.233568	histone cluster 1, H2al	Homo sapiens histone cluster 1, H2al (H	7.46676	52.65493	3.13748	37.79842	Compromised	Detected	Compromised	Detected
2880	A_33_P3353906	LOC100132848		hypothetical LOC100132848	PREDICTED: Homo sapiens hypothetica	1300.58410	1590.67000	9065.45000	8050.60300	Detected	Detected	Detected	Detected
2881	A_23_P105873	TRPC4	Hs.262960	transient receptor potential cation char	Homo sapiens transient receptor poten	86.88139	242.86981	78.24825	172.86086	Detected	Detected	Detected	Detected
2882	A_24_P81841	CDKN1B	Hs.238990	cyclin-dependent kinase inhibitor 1B (p	Homo sapiens cyclin-dependent kinase	1204.32870	889.14105	1015.86096	582.99115	Detected	Detected	Detected	Detected
2883	A_33_P3307068					156.86734	231.12020	74.18463	78.41783	Detected	Detected	Detected	Detected
2884	A_23_P257478	CYP21A2	Hs.654479	cytochrome P450, family 21, subfamily A	Homo sapiens cytochrome P450, family	71.24484	54.06644	54.56234	33.44042	Detected	Detected	Detected	Detected
2885	A_23_P350555	TCP10L	Hs.42034	t-complex 10 (mouse)-like	Homo sapiens t-complex 10 (mouse)-lik	361.97480	154.98264	269.50946	67.82232	Detected	Detected	Detected	Detected
2886	A_23_P31873	RAB11FIP1	Hs.191179	RAB11 family interacting protein 1 (clas	Homo sapiens RAB11 family interacting	222.77083	387.61133	207.03404	342.05945	Detected	Detected	Detected	Detected
2887	A_23_P310956	COL6A2	Hs.420269	collagen, type VI, alpha 2	Homo sapiens collagen, type VI, alpha 2	3361.17140	5325.57700	3940.72240	6329.13800	Detected	Detected	Detected	Detected
2888	A_24_P808100	MCTS1	Hs.102696	malignant T cell amplified sequence 1	Homo sapiens malignant T cell amplified	205.85536	63.73870	123.76613	33.47527	Detected	Detected	Detected	Detected
2889	A_33_P3345782				Coiled-coil domain-containing protein 8	9.68236	24.46997	4.07507	3.82765	Compromised	Detected	Compromised	Compromised
2890	A_23_P408353	HLA-A	Hs.181244	major histocompatibility complex, class	Homo sapiens major histocompatibility	34335.16000	14197.12400	34391.88000	15095.70200	Detected	Detected	Detected	Detected
2891	A_33_P3287636		Hs.697089		V-alpha FR1 Precursor; Fragment [Sou	33.53708	35.92987	55.65504	41.16341	Detected	Detected	Detected	Detected
2892	A_32_P334325	RIMBP2	Hs.657441	RIMS binding protein 2	Homo sapiens RIMS binding protein 2 (F	60.01526	77.58448	30.54478	34.07240	Detected	Detected	Detected	Detected
2893	A_33_P3251054	RNF38	Hs.333503	ring finger protein 38	Homo sapiens ring finger protein 38 (RN	1586.93850	1735.63820	1069.54700	996.58610	Detected	Detected	Detected	Detected
2894	A_23_P2705	LPAR6	Hs.123464	lysophosphatidic acid receptor 6	Homo sapiens lysophosphatidic acid rec	325.91467	301.55743	228.45978	160.60649	Detected	Detected	Detected	Detected
2895	A_33_P3358856					2933.70480	2978.67070	2657.23930	2249.54270	Detected	Detected	Detected	Detected
2896	A_33_P3243502	LOC389634	Hs.700070	hypothetical LOC389634	Homo sapiens hypothetical LOC389634	789.77740	300.24545	655.50130	121.70493	Detected	Detected	Detected	Detected
2897	A_33_P3262292	UBXN7	Hs.518524	UBX domain protein 7	Homo sapiens UBX domain protein 7 (U	18.97112	23.04177	17.42876	19.28250	Detected	Detected	Detected	Detected
2898	A_33_P3260066	BEAN	Hs.97805	brain expressed, associated with Nedd4	Homo sapiens brain expressed, associat	362.66074	234.56635	279.90543	99.85780	Detected	Detected	Detected	Detected
2899	A_33_P3267003	LOC100132588	Hs.689599	hypothetical protein LOC100132588	PREDICTED: Homo sapiens hypothetica	5.90499	110.03313	3.37833	76.64602	Compromised	Detected	Compromised	Detected
2900	A_23_P35848	NADSYN1	Hs.556986	NAD synthetase 1	Homo sapiens NAD synthetase 1 (NADS	1444.04990	1501.82870	1634.45070	1547.05350	Detected	Detected	Detected	Detected
2901	A_23_P118633	SPATA20	Hs.103147	spermatogenesis associated 20	Homo sapiens spermatogenesis associa	3119.87620	1974.70450	2515.85670	1370.32780	Detected	Detected	Detected	Detected
2902	A_32_P806841	ARL4A	Hs.245540	ADP-ribosylation factor-like 4A	Homo sapiens ADP-ribosylation factor-	214.72679	750.11957	118.97802	297.10507	Detected	Detected	Detected	Detected
2903	A_23_P121564	GUCY1B3	Hs.77890	guanylate cyclase 1, soluble, beta 3	Homo sapiens guanylate cyclase 1, solu	175.75934	53.47599	111.30045	25.53968	Detected	Detected	Detected	Detected
2904	A_23_P110531	FST	Hs.9914	follistatin	Homo sapiens follistatin (FST), transcri	3438.59720	6330.69870	2510.01440	4199.99500	Detected	Detected	Detected	Detected
2905	A_33_P3364607	LOC100130051	Hs.710155	hypothetical protein LOC100130051	Homo sapiens cDNA FLJ45363 fis, clon	30.55104	20.03159	12.03151	7.26857	Detected	Compromised	Compromised	Compromised
2906	A_24_P918032	WHAMML1	Hs.212670	WAS protein homolog associated with a	Homo sapiens WAS protein homolog as	62.91232	52.45760	15.75114	8.92253	Detected	Detected	Compromised	Compromised
2907	A_33_P3326992	PDE4DIP	Hs.568247	phosphodiesterase 4D interacting prote	Homo sapiens phosphodiesterase 4D in	2937.65380	1921.73610	2444.14230	1331.17600	Detected	Detected	Detected	Detected
2908	A_33_P3704280	FLJ38109	Hs.652083	hypothetical protein FLJ38109	Homo sapiens cDNA FLJ38109 fis, clon	30.08203	22.86355	16.16808	5.06588	Detected	Detected	Compromised	Compromised
2909	A_33_P3336484	NPIP	Hs.676266	nuclear pore complex interacting protei	Homo sapiens nuclear pore complex int	1324.64260	1153.08310	749.12060	544.23650	Detected	Detected	Detected	Detected
2910	A_24_P19544	STK39	Hs.276271	serine threonine kinase 39 (STE20/SPS	Homo sapiens serine threonine kinase 3	1238.39000	1550.81020	619.47955	619.70970	Detected	Detected	Detected	Detected
2911	A_23_P165891	TAF1B	Hs.584833	TATA box binding protein (TBP)-associ	Homo sapiens TATA box binding protein	689.25320	845.50920	711.23760	755.25480	Detected	Detected	Detected	Detected
2912	A_23_P58293	UBE2D3	Hs.518773	ubiquitin-conjugating enzyme E2D 3 (UE	Homo sapiens ubiquitin-conjugating enz	5784.67100	7460.28500	4910.66100	5412.63670	Detected	Detected	Detected	Detected
2913	A_33_P3345011	ACTL9	Hs.209206	actin-like 9	Homo sapiens actin-like 9 (ACTL9), mR	80.60034	103.30712	8.14517	7.63540	Detected	Detected	Compromised	Compromised
2914	A_33_P3361771	TPR	Hs.279640	translocated promoter region (to activa	Homo sapiens translocated promoter re	73.96522	46.03376	35.52308	23.07175	Detected	Detected	Detected	Detected
2915	A_23_P303238	VN1R5	Hs.553686	vomeronasal 1 receptor 5 (gene/pseudo	Homo sapiens vomeronasal 1 receptor	13.41614	31.37005	19.72023	20.61628	Compromised	Detected	Detected	Detected
2916	A_24_P281439	OR2T5	Hs.553808	olfactory receptor, family 2, subfamily T	Homo sapiens olfactory receptor, family	14.86529	6.02743	8.09551	4.47396	Detected	Compromised	Compromised	Compromised
2917	A_33_P3312790	MEGF11	Hs.712886	multiple EGF-like-domains 11	Homo sapiens multiple EGF-like-domair	472.74650	538.40640	180.55128	170.68767	Detected	Detected	Detected	Detected
2918	A_23_P398854	DOK7	Hs.122110	docking protein 7	Homo sapiens docking protein 7 (DOK7	2.55532	164.43954	3.03850	92.52573	Compromised	Detected	Compromised	Detected
2919	A_33_P3246635	RP11-756A22.3		transmembrane phosphoinositide 3-pho	Homo sapiens transmembrane phospho	24.46156	21.67962	19.83631	7.16346	Detected	Detected	Detected	Compromised
2920	A_33_P3336642	THOC3	Hs.484227	THO complex 3	Homo sapiens THO complex 3 (THOC3	349.59370	645.93670	524.94037	910.52270	Detected	Detected	Detected	Detected
2921	A_23_P10374	R3HDM2	Hs.443673	R3H domain containing 2	Homo sapiens R3H domain containing 2	2265.61380	1174.94350	1553.63180	679.66675	Detected	Detected	Detected	Detected

2922	A_33_P3437283	LOC643551	Hs.513081	hypothetical LOC643551	Homo sapiens cDNA FLJ46123 fis, clon	132.71692	48.14031	61.36118	15.44709	Detected	Detected	Detected	Compromised
2923	A_23_P60387	NOTCH1	Hs.495473	Notch homolog 1, translocation-associa	Homo sapiens Notch homolog 1, translo	66.15070	130.14928	41.77545	79.15676	Detected	Detected	Detected	Detected
2924	A_23_P124044	DEAF1	Hs.243994	deformed epidermal autoregulatory fact	Homo sapiens deformed epidermal auto	7695.43500	2089.42290	5654.64000	1439.57040	Detected	Detected	Detected	Detected
2925	A_23_P55127	C17orf48	Hs.47668	chromosome 17 open reading frame 48	Homo sapiens chromosome 17 open rea	774.45795	554.56964	628.33710	432.53693	Detected	Detected	Detected	Detected
2926	A_33_P3265965	KIAA1430	Hs.535734	KIAA1430	Homo sapiens KIAA1430 (KIAA1430), m	247.18414	282.48038	251.29987	237.75517	Detected	Detected	Detected	Detected
2927	A_23_P157593	CHRNA6	Hs.103128	cholinergic receptor, nicotinic, alpha 6	Homo sapiens cholinergic receptor, nico	57.73914	46.76027	13.57455	14.11165	Detected	Detected	Compromised	Compromised
2928	A_33_P3399181					2184.57470	2943.13700	776.37134	576.14040	Detected	Detected	Detected	Detected
2929	A_33_P3789382	LOC84989	Hs.659950	hypothetical LOC84989	Homo sapiens hypothetical LOC84989 (	253.59265	348.79733	192.15678	244.50224	Detected	Detected	Detected	Detected
2930	A_23_P204364	NOP2	Hs.534334	NOP2 nucleolar protein homolog (yeast	Homo sapiens NOP2 nucleolar protein h	1651.06840	1570.87060	1652.41800	1511.32060	Detected	Detected	Detected	Detected
2931	A_23_P202496	NOC3L	Hs.74899	nucleolar complex associated 3 homolo	Homo sapiens nucleolar complex associ	2909.44630	923.51540	2156.93950	582.20013	Detected	Detected	Detected	Detected
2932	A_24_P282416	ABL1	Hs.431048	c-abl oncogene 1, receptor tyrosine kin	Homo sapiens c-abl oncogene 1, recept	30.29367	72.76679	34.40602	74.16642	Detected	Detected	Detected	Detected
2933	A_23_P120335	MTX2	Hs.470728	metaxin 2	Homo sapiens metaxin 2 (MTX2), nucle	5514.16000	4226.14300	4077.92600	2796.39280	Detected	Detected	Detected	Detected
2934	A_23_P205768	ARPP19	Hs.512908	cAMP-regulated phosphoprotein, 19kDa	Homo sapiens cAMP-regulated phospho	4252.83100	4705.35400	3378.89500	3057.70530	Detected	Detected	Detected	Detected
2935	A_33_P3212274	F2R	Hs.679786	coagulation factor II (thrombin) recepto	602291527F1 NIH_MGC_86 Homo sapien	469.04385	1024.72180	433.64343	792.59644	Detected	Detected	Detected	Detected
2936	A_23_P91019	PRKRA	Hs.570274	protein kinase, interferon-inducible dou	Homo sapiens protein kinase, interferon	8062.65530	6395.53300	4476.10400	2984.77510	Detected	Detected	Detected	Detected
2937	A_33_P3326662	LOC100128400	Hs.681117	hypothetical protein LOC100128400	Homo sapiens cDNA FLJ42244 fis, clon	26.76864	26.52134	19.26665	19.01369	Detected	Detected	Detected	Detected
2938	A_33_P3423755	NCRNA00200	Hs.721191	non-protein coding RNA 200	Homo sapiens non-protein coding RNA	2.68139	19.30973	3.14298	10.48575	Compromised	Detected	Compromised	Compromised
2939	A_23_P75149	SFXN4	Hs.655168	sideroflexin 4	Homo sapiens sideroflexin 4 (SFXN4), m	1297.27150	1406.74130	1066.58450	1069.58950	Detected	Detected	Detected	Detected
2940	A_33_P3376541	LOC285033	Hs.58648	hypothetical protein LOC285033	Homo sapiens hypothetical protein LOC	53.95388	52.22937	33.04043	34.63057	Detected	Detected	Detected	Detected
2941	A_33_P3327462	TMEM108	Hs.191616	transmembrane protein 108	Homo sapiens transmembrane protein 1	21.54784	18.52993	13.26466	11.15108	Detected	Compromised	Compromised	Compromised
2942	A_33_P3339256		Hs.679155		Putative uncharacterized protein ENSP	66.91878	69.07703	13.39121	11.07139	Detected	Detected	Compromised	Compromised
2943	A_24_P101391	YBX1	Hs.473583	Y box binding protein 1	Homo sapiens Y box binding protein 1 (	10333.94800	13176.17700	9977.88500	10156.44000	Detected	Detected	Detected	Detected
2944	A_33_P3382849	ACR	Hs.370870	acrosin	Homo sapiens acrosin (ACR), mRNA [N	78.17465	100.50620	58.51514	47.83707	Detected	Detected	Detected	Detected
2945	A_23_P127584	NNMT	Hs.503911	nicotinamide N-methyltransferase	Homo sapiens nicotinamide N-methyltra	163610.11000	45981.79700	119643.73400	34721.07000	Detected	Detected	Detected	Detected
2946	A_23_P26954	VAT1	Hs.514199	vesicle amine transport protein 1 homo	Homo sapiens vesicle amine transport p	22410.89800	32064.76800	28296.30900	37273.47300	Detected	Detected	Detected	Detected
2947	A_23_P27075	GABARAP	Hs.647421	GABA(A) receptor-associated protein	Homo sapiens GABA(A) receptor-assoc	12075.41300	9864.25200	11516.13700	8548.56300	Detected	Detected	Detected	Detected
2948	A_23_P314120	CHKB	Hs.654827	choline kinase beta	Homo sapiens choline kinase beta (CHK	1945.75070	801.29610	1746.35270	579.85000	Detected	Detected	Detected	Detected
2949	A_33_P3748714	FUT10	Hs.458713	fucosyltransferase 10 (alpha (1,3) fucos	Homo sapiens fucosyltransferase 10 (al	134.30150	270.65903	99.82431	152.53035	Detected	Detected	Detected	Detected
2950	A_23_P137238	KDM5D	Hs.80358	lysine (K)-specific demethylase 5D	Homo sapiens lysine (K)-specific demet	282.81165	103.24251	234.84654	32.68670	Detected	Detected	Detected	Detected
2951	A_23_P100632	HN1	Hs.532803	hematological and neurological express	Homo sapiens hematological and neurol	15509.49500	130478.28000	14559.64100	92464.78000	Detected	Detected	Detected	Detected
2952	A_33_P3370787	EPHB2	Hs.523329	EPH receptor B2	Homo sapiens EPH receptor B2 (EPHB2	179.79773	332.58774	117.39219	211.87056	Detected	Detected	Detected	Detected
2953	A_33_P3279353	AZU1	Hs.72885	azurocidin 1	Homo sapiens azurocidin 1 (AZU1), mR	10.55162	24.80081	14.15482	13.24163	Compromised	Detected	Compromised	Compromised
2954	A_23_P104046	BPNT1	Hs.406134	3'(2'), 5'-bisphosphate nucleotidase 1	Homo sapiens 3'(2'), 5'-bisphosphate nu	476.57318	411.04544	392.77347	292.02866	Detected	Detected	Detected	Detected
2955	A_23_P320739	MEF2C	Hs.649965	myocyte enhancer factor 2C	Homo sapiens myocyte enhancer facto	189.57979	384.67180	155.37459	242.54076	Detected	Detected	Detected	Detected
2956	A_24_P481824	DPY19L4	Hs.567828	dpy-19-like 4 (C. elegans)	Homo sapiens dpy-19-like 4 (C. elegans	596.10016	249.25774	308.51492	89.62986	Detected	Detected	Detected	Detected
2957	A_33_P3210079	SF3A2	Hs.115232	splicing factor 3a, subunit 2, 66kDa	Homo sapiens splicing factor 3a, subuni	2768.93550	3736.50220	3088.03000	3233.11160	Detected	Detected	Detected	Detected
2958	A_24_P391604	KRTAP6-3	Hs.553691	keratin associated protein 6-3	Homo sapiens keratin associated protei	207.55200	316.82993	67.37071	68.19248	Detected	Detected	Detected	Detected
2959	A_23_P401709	C20orf196	Hs.529340	chromosome 20 open reading frame 196	Homo sapiens chromosome 20 open rea	35.92156	36.22750	43.53861	28.26889	Detected	Detected	Detected	Detected
2960	A_33_P3364389		Hs.161031		Synaptotagmin-8 (Synaptotagmin VIII)(	692.78143	779.12350	223.93057	294.52197	Detected	Detected	Detected	Detected
2961	A_23_P20882	ATP6V1G1	Hs.388654	ATPase, H+ transporting, lysosomal 13k	Homo sapiens ATPase, H+ transporting,	15300.09300	12785.42900	10507.75000	7361.61300	Detected	Detected	Detected	Detected
2962	A_23_P83073	HIATL1	Hs.555996	hippocampus abundant transcript-like 1	Homo sapiens hippocampus abundant tr	2258.60350	2236.23600	1751.02380	1483.18500	Detected	Detected	Detected	Detected
2963	A_23_P79161	PRELID1	Hs.279529	PRELI domain containing 1	Homo sapiens PRELI domain containing	13642.80100	23967.89600	16204.87600	23875.23200	Detected	Detected	Detected	Detected
2964	A_23_P391926	LPHN1	Hs.94229	latrophilin 1	Homo sapiens latrophilin 1 (LPHN1), tra	320.01184	147.60540	257.91754	44.31344	Detected	Detected	Detected	Detected
2965	A_23_P138693	NMT2	Hs.60339	N-myristoyltransferase 2	Homo sapiens N-myristoyltransferase 2	370.33966	681.16540	264.68590	382.64624	Detected	Detected	Detected	Detected
2966	A_24_P181101	TMEM135	Hs.188591	transmembrane protein 135	Homo sapiens transmembrane protein 1	541.27264	356.76855	399.69995	207.75241	Detected	Detected	Detected	Detected
2967	A_23_P102058	MATN3	Hs.656199	matrilin 3	Homo sapiens matrilin 3 (MATN3), mRN	15.85547	103.94723	5.30359	25.89163	Detected	Detected	Compromised	Detected
2968	A_23_P102937	SUMO3	Hs.474005	SMT3 suppressor of mif two 3 homolog	Homo sapiens SMT3 suppressor of mif	5769.31930	7374.43650	3609.80500	3806.76830	Detected	Detected	Detected	Detected
2969	A_23_P250982	ISOC1	Hs.483296	isochorismatase domain containing 1	Homo sapiens isochorismatase domain	1969.87540	717.06067	1481.04830	446.45620	Detected	Detected	Detected	Detected
2970	A_32_P97862	C1orf223	Hs.443299	chromosome 1 open reading frame 223	Homo sapiens chromosome 1 open read	15.39718	19.23098	12.23762	10.11781	Detected	Detected	Compromised	Compromised
2971	A_33_P3281303					21.66663	40.03494	3.10209	5.12204	Detected	Detected	Compromised	Compromised
2972	A_33_P3260455					1470.25780	1461.32520	977.37854	920.44855	Detected	Detected	Detected	Detected
2973	A_33_P3311308	LOC727918		hypothetical LOC727918	PREDICTED: Homo sapiens hypothetica	20.52316	40.60236	3.10923	5.51865	Detected	Detected	Compromised	Compromised
2974	A_23_P211738	UBP1	Hs.716083	upstream binding protein 1 (LBP-1a)	Homo sapiens upstream binding protein	4176.83500	4092.70070	2567.03250	2018.31450	Detected	Detected	Detected	Detected
2975	A_33_P3280009					43.28673	88.88355	35.08458	62.61284	Detected	Detected	Detected	Detected
2976	A_23_P32143	ALDOB	Hs.530274	aldolase B, fructose-bisphosphate	Homo sapiens aldolase B, fructose-bisp	14.90303	34.16979	3.94471	5.92935	Detected	Detected	Compromised	Compromised
2977	A_33_P3221613	LOC100129169	Hs.570084	hypothetical LOC100129169	PREDICTED: Homo sapiens hypothetica	9.90726	18.92704	11.29978	11.53894	Compromised	Detected	Compromised	Compromised
2978	A_33_P3543133	LOC283624	Hs.525210	hypothetical protein LOC283624	Homo sapiens cDNA clone IMAGE:5264	69.29273	179.49455	50.96552	92.30598	Detected	Detected	Detected	Detected
2979	A_23_P105794	EPSTI1	Hs.546467	epithelial stromal interaction 1 (breast)	Homo sapiens epithelial stromal interac	27.88620	19.69243	21.73393	15.84323	Detected	Detected	Detected	Detected
2980	A_23_P116902	ART4	Hs.655792	ADP-ribosyltransferase 4 (Dombrock bl	Homo sapiens ADP-ribosyltransferase 4	5.56126	21.47685	3.28363	4.47768	Compromised	Detected	Compromised	Compromised
2981	A_23_P416747	CD3E	Hs.3003	CD3e molecule, epsilon (CD3-TCR com	Homo sapiens CD3e molecule, epsilon (	45.75336	61.93827	15.87961	21.43873	Detected	Detected	Detected	Detected
2982	A_23_P364517	SELK	Hs.58471	selenoprotein K	Homo sapiens selenoprotein K (SELK),	16225.59600	11475.95200	20474.92400	12889.91800	Detected	Detected	Detected	Detected
2983	A_23_P100386	IL34	Hs.461214	interleukin 34	Homo sapiens interleukin 34 (IL34), mR	129.99280	47.83975	83.53969	22.83147	Detected	Detected	Detected	Detected
2984	A_24_P185709	EPB41L1	Hs.704100	erythrocyte membrane protein band 4.1	Homo sapiens erythrocyte membrane p	405.51980	70.45564	326.80048	48.09829	Detected	Detected	Detected	Detected
2985	A_23_P256223	VBP1	Hs.436803	von Hippel-Lindau binding protein 1	Homo sapiens von Hippel-Lindau bindin	520.42730	928.95730	431.00882	590.56604	Detected	Detected	Detected	Detected
2986	A_23_P115200	FCRL4	Hs.120260	Fc receptor-like 4	Homo sapiens Fc receptor-like 4 (FCRL	3.74802	21.95841	3.44157	25.87869	Compromised	Not Detected	Compromised	Not Detected
2987	A_23_P250054	RNF123	Hs.553723	ring finger protein 123	Homo sapiens ring finger protein 123 (R	498.46010	282.36838	423.47763	242.90096	Detected	Detected	Detected	Detected
2988	A_23_P218282	ZNF434	Hs.592078	zinc finger protein 434	Homo sapiens zinc finger protein 434 (Z	973.78970	591.80524	848.31177	462.82330	Detected	Detected	Detected	Detected
2989	A_33_P3273424					14.67248	17.52165	10.74501	8.79425	Detected	Compromised	Compromised	Compromised
2990	A_24_P414999	LAPTM4B	Hs.492314	lysosomal protein transmembrane 4 bet	Homo sapiens lysosomal protein transm	2096.64500	1165.74660	2164.54250	1043.16000	Detected	Detected	Detected	Detected
2991	A_23_P158925	GPR125	Hs.99195	G protein-coupled receptor 125	Homo sapiens G protein-coupled recep	9099.14900	2883.05470	6871.06450	1767.50840	Detected	Detected	Detected	Detected
2992	A_23_P217015	SET	Hs.436687	SET nuclear oncogene	Homo sapiens SET nuclear oncogene (S	661.65137	908.59510	724.61456	767.37396	Detected	Detected	Detected	Detected
2993	A_23_P15876	ALPK2	Hs.628152	alpha-kinase 2	Homo sapiens alpha-kinase 2 (ALPK2),	440.79620	2103.52780	288.80008	1218.03710	Detected	Detected	Detected	Detected
2994	A_24_P383523	SAMD4A	Hs.98259	sterile alpha motif domain containing 4A	Homo sapiens sterile alpha motif domai	443.15744	1403.81980	512.88617	1464.62200	Detected	Detected	Detected	Detected
2995	A_23_P157620	FAM160B2	Hs.491223	family with sequence similarity 160, me	Homo sapiens family with sequence sim	1581.39150	1891.87120	1651.06630	1933.38220	Detected	Detected	Detected	Detected
2996	A_23_P142424	TMEM149	Hs.352548	transmembrane protein 149	Homo sapiens transmembrane protein 1	2362.54690	1637.27090	2242.28600	1302.70510	Detected	Detected	Detected	Detected

2997	A_23_P73526	CITED1	Hs.40403	Cbp/p300-interacting transactivator, w	Homo sapiens Cbp/p300-interacting tra	52.00576	20.65315	57.90118	19.17595	Detected	Detected	Detected	Detected
2998	A_23_P126735	IL10	Hs.193717	interleukin 10	Homo sapiens interleukin 10 (IL10), mR	13.83336	12.29018	14.93680	14.99750	Detected	Compromised	Detected	Detected
2999	A_24_P77947	CCDC132	Hs.222282	coiled-coil domain containing 132	Homo sapiens coiled-coil domain conta	22.23203	34.59441	11.70854	21.26237	Detected	Detected	Compromised	Detected
3000	A_33_P3358208	PADI1	Hs.412941	peptidyl arginine deiminase, type I	Homo sapiens peptidyl arginine deimina	4.16737	86.65952	3.39096	70.57236	Compromised	Detected	Compromised	Detected
3001	A_23_P110961	BRP44L	Hs.172755	brain protein 44-like	Homo sapiens brain protein 44-like (BR	6826.11430	6816.97360	5493.21200	4460.49370	Detected	Detected	Detected	Detected
3002	A_33_P3339138	RPRML	Hs.367999	reprimo-like	Homo sapiens reprimo-like (RPRML), m	27.14795	38.24723	4.82195	10.04672	Detected	Detected	Compromised	Compromised
3003	A_24_P162454	PABPC1L2B	Hs.567854	poly(A) binding protein, cytoplasmic 1-li	Homo sapiens poly(A) binding protein, c	47.85794	61.77092	14.14711	17.14573	Detected	Detected	Compromised	Compromised
3004	A_24_P517901	HNRNPA1	Hs.546261	heterogeneous nuclear ribonucleoprotei	Homo sapiens heterogeneous nuclear ri	3313.05150	5380.16460	1625.99340	2107.12400	Detected	Detected	Detected	Detected
3005	A_23_P122906	AUTS2	Hs.21631	autism susceptibility candidate 2	Homo sapiens autism susceptibility can	2278.23660	1249.48250	1322.46140	599.09607	Detected	Detected	Detected	Detected
3006	A_33_P3380793	C22orf34	Hs.133159	chromosome 22 open reading frame 34	Homo sapiens chromosome 22 open rea	15.31921	16.93539	3.49842	6.19314	Detected	Compromised	Compromised	Compromised
3007	A_33_P3379941	HMGXB3	Hs.719189	HMG box domain containing 3	Homo sapiens HMG box domain contain	9613.45100	9315.88000	5704.52830	4825.51200	Detected	Detected	Detected	Detected
3008	A_23_P110362	MAPKSP1	Hs.433332	MAPK scaffold protein 1	Homo sapiens MAPK scaffold protein 1	1768.97410	1390.24650	1569.91460	1122.55790	Detected	Detected	Detected	Detected
3009	A_33_P3382118					2016.32210	3568.70900	1397.49780	1992.18570	Detected	Detected	Detected	Detected
3010	A_33_P3212356	FAM163B	Hs.709706	family with sequence similarity 163, mer	Homo sapiens family with sequence sim	23.31815	33.52815	19.00357	8.11484	Detected	Detected	Detected	Compromised
3011	A_33_P3331451	TGFBR1	Hs.494622	transforming growth factor, beta recept	Homo sapiens transforming growth fact	739.15530	891.72620	453.95830	446.39984	Detected	Detected	Detected	Detected
3012	A_23_P43086	BIN3	Hs.718405	bridging integrator 3	Homo sapiens bridging integrator 3 (BIN	3499.14140	3093.96780	3407.20970	2648.73900	Detected	Detected	Detected	Detected
3013	A_23_P313031		Hs.653239		cDNA FLJ44566 fis, clone UTERU30095	254.77554	294.78820	132.45030	119.32182	Detected	Detected	Detected	Detected
3014	A_23_P306956	ZNF566	Hs.533939	zinc finger protein 566	Homo sapiens zinc finger protein 566 (Z	24.33081	7.51235	18.33152	4.90817	Detected	Compromised	Detected	Compromised
3015	A_23_P55421	CBX8	Hs.387258	chromobox homolog 8 (Pc class homolo	Homo sapiens chromobox homolog 8 (P	234.37013	139.30983	203.12682	107.16369	Detected	Detected	Detected	Detected
3016	A_23_P169838	UNC84A	Hs.438072	unc-84 homolog A (C. elegans)	Homo sapiens unc-84 homolog A (C. el	4176.07280	3579.74300	3232.50460	2123.51050	Detected	Detected	Detected	Detected
3017	A_23_P201863	PCTK3	Hs.445402	PCTAIRE protein kinase 3	Homo sapiens PCTAIRE protein kinase	27.15768	36.94061	6.50868	3.49201	Detected	Detected	Compromised	Compromised
3018	A_33_P3389298	ZNF30	Hs.657402	zinc finger protein 30	Homo sapiens zinc finger protein 30 (ZN	300.46106	189.13799	201.10470	118.07948	Detected	Detected	Detected	Detected
3019	A_23_P359245	MET	Hs.132966	met proto-oncogene (hepatocyte growt	Homo sapiens met proto-oncogene (he	3089.59200	7596.94630	1885.15870	3993.20430	Detected	Detected	Detected	Detected
3020	A_33_P3301297	LOC100128086	Hs.600666	similar to APAF1 interacting protein	PREDICTED: Homo sapiens similar to A	300.00280	420.57407	50.55911	47.80298	Detected	Detected	Detected	Detected
3021	A_32_P163147	VSIG1	Hs.177164	V-set and immunoglobulin domain conta	Homo sapiens V-set and immunoglobuli	43.43386	31.15377	32.87548	12.16334	Detected	Detected	Detected	Compromised
3022	A_33_P3248231	LOC283028	Hs.646699	hypothetical protein LOC283028	PREDICTED: Homo sapiens hypothetica	43.56344	20.50653	22.81017	6.28551	Detected	Detected	Detected	Compromised
3023	A_33_P3257330	DCBLD1	Hs.658304	discoidin, CUB and LCCL domain conta	Homo sapiens discoidin, CUB and LCCL	67.11993	312.76157	32.62805	130.40501	Detected	Detected	Detected	Detected
3024	A_33_P3282649	LOC100134147		hypothetical protein LOC100134147	PREDICTED: Homo sapiens hypothetica	36.68533	42.31262	14.72927	16.73238	Detected	Detected	Compromised	Detected
3025	A_24_P317907	SORBS1	Hs.595068	sorbin and SH3 domain containing 1	Homo sapiens sorbin and SH3 domain c	22.94284	25.02874	5.94142	8.12431	Detected	Detected	Compromised	Compromised
3026	A_33_P3411090	ADAL	Hs.533913	adenosine deaminase-like	Homo sapiens adenosine deaminase-like	794.97076	368.83545	638.23950	237.62712	Detected	Detected	Detected	Detected
3027	A_23_P46025	ETNK2	Hs.497469	ethanolamine kinase 2	Homo sapiens ethanolamine kinase 2 (E	236.97293	26.13535	141.08897	15.05522	Detected	Detected	Detected	Compromised
3028	A_24_P40721	SPTLC3	Hs.425023	serine palmitoyltransferase, long chain b	Homo sapiens serine palmitoyltransfera	14.24299	16.07324	6.33990	3.50840	Detected	Compromised	Compromised	Compromised
3029	A_33_P3293474	OPN3	Hs.409081	opsin 3	Homo sapiens opsin 3 (OPN3), mRNA [N	349.21283	591.52325	184.46278	201.42813	Detected	Detected	Detected	Detected
3030	A_23_P94932	MMADHC	Hs.5324	methylmalonic aciduria (cobalamin defic	Homo sapiens methylmalonic aciduria (c	15922.80700	21400.95300	13318.90700	15166.54800	Detected	Detected	Detected	Detected
3031	A_33_P3240333	PITX1	Hs.84136	paired-like homeodomain 1	Homo sapiens paired-like homeodomain	91.91876	283.69223	42.20107	214.13847	Detected	Detected	Detected	Detected
3032	A_24_P21985	FOXJ2	Hs.120844	forkhead box J2	Homo sapiens forkhead box J2 (FOXJ2)	274.66696	411.36893	252.22777	331.20312	Detected	Detected	Detected	Detected
3033	A_23_P132444	TCEA1	Hs.491745	transcription elongation factor A (SII), 1	Homo sapiens transcription elongation f	9819.31100	9267.63900	9486.70200	7714.81000	Detected	Detected	Detected	Detected
3034	A_23_P323166	SRRM2	Hs.719123	serine/arginine repetitive matrix 2	Homo sapiens serine/arginine repetitive	14454.04100	16999.74600	11823.37300	13327.79400	Detected	Detected	Detected	Detected
3035	A_33_P3272568	LOC100292021		similar to thioredoxin peroxidase	Peroxiredoxin-4 (EC 1.11.1.15)(Prx-IV)(	12.50706	39.39356	3.35681	11.00656	Compromised	Detected	Compromised	Compromised
3036	A_23_P350689	ZDHHC23	Hs.21902	zinc finger, DHHC-type containing 23	Homo sapiens zinc finger, DHHC-type c	173.66672	140.52121	138.09210	91.22174	Detected	Detected	Detected	Detected
3037	A_23_P106682	EMP2	Hs.531561	epithelial membrane protein 2	Homo sapiens epithelial membrane prot	15984.47900	31226.22300	13185.94300	22670.80300	Detected	Detected	Detected	Detected
3038	A_23_P86021	SELENBP1	Hs.632460	selenium binding protein 1	Homo sapiens selenium binding protein	4541.43460	1796.47970	3642.94560	1288.19710	Detected	Detected	Detected	Detected
3039	A_24_P183150	CXCL3	Hs.89690	chemokine (C-X-C motif) ligand 3	Homo sapiens chemokine (C-X-C motif	186.66193	687.75720	146.50864	473.22174	Detected	Detected	Detected	Detected
3040	A_33_P3367855	PIK3R2	Hs.371344	phosphoinositide-3-kinase, regulatory s	Homo sapiens phosphoinositide-3-kinas	29275.82800	30662.83400	18262.39800	17653.13000	Detected	Detected	Detected	Detected
3041	A_33_P3243832	ZEB2	Hs.34871	zinc finger E-box binding homeobox 2	Homo sapiens zinc finger E-box binding	91.71288	110.38741	84.16265	81.20136	Detected	Detected	Detected	Detected
3042	A_24_P411561	HAVCR2	Hs.710500	hepatitis A virus cellular receptor 2	Homo sapiens hepatitis A virus cellular	26.30078	9.16842	17.67664	3.24993	Detected	Compromised	Detected	Compromised
3043	A_33_P3293768	KRTAP10-6	Hs.689666	keratin associated protein 10-6	Homo sapiens keratin associated protei	102.79489	86.62193	40.03988	33.11286	Detected	Detected	Detected	Detected
3044	A_23_P112341	RMI1	Hs.284137	RMI1, RecQ mediated genome instability	Homo sapiens RMI1, RecQ mediated ge	823.71080	1466.41610	661.06335	974.01560	Detected	Detected	Detected	Detected
3045	A_24_P119259	BLZF1	Hs.130746	basic leucine zipper nuclear factor 1	Homo sapiens basic leucine zipper nucl	75.06079	106.34366	35.35048	41.74528	Detected	Detected	Detected	Detected
3046	A_33_P3391803					12887.02100	12087.52050	833.25476	595.55225	Detected	Detected	Detected	Detected
3047	A_24_P130792	NSUN4	Hs.163424	NOL1/NOP2/Sun domain family, memb	Homo sapiens NOL1/NOP2/Sun domai	251.29846	306.07895	229.84906	244.98938	Detected	Detected	Detected	Detected
3048	A_23_P166526	RIBC2	Hs.475110	RIB43A domain with coiled-coils 2	Homo sapiens RIB43A domain with coile	19.78241	75.39253	15.19062	47.66806	Detected	Detected	Compromised	Detected
3049	A_24_P291133	UHMK1	Hs.127310	U2AF homology motif (UHM) kinase 1	Homo sapiens U2AF homology motif (U	44.21506	54.72244	39.17293	43.19856	Detected	Detected	Detected	Detected
3050	A_32_P104746	ZFYVE28	Hs.292056	zinc finger, FYVE domain containing 28	Homo sapiens zinc finger, FYVE domain	491.07004	213.55408	471.85046	170.66832	Detected	Detected	Detected	Detected
3051	A_24_P370970	NSL1	Hs.497692	NSL1, MIND kinetochore complex comp	Homo sapiens NSL1, MIND kinetochore	135.07368	155.99278	94.92791	96.44428	Detected	Detected	Detected	Detected
3052	A_33_P3280905				GB	16.74024	14.20966	3.22600	3.34548	Detected	Compromised	Compromised	Compromised
3053	A_33_P3322909	CUL4A	Hs.339735	cullin 4A	Homo sapiens cullin 4A (CUL4A), transc	914.60840	793.38635	709.50720	563.17210	Detected	Detected	Detected	Detected
3054	A_24_P135628	LOC728449	Hs.463110	hypothetical protein LOC728449	Putative uncharacterized protein ENSP	8904.53800	11832.83400	5908.41260	6061.05570	Detected	Detected	Detected	Detected
3055	A_33_P3310475	SEPT11	Hs.128199	septin 11	Homo sapiens septin 11 (SEPT11), mRN	19772.34600	48314.36000	14137.60900	30575.15200	Detected	Detected	Detected	Detected
3056	A_23_P338505	C19orf40	Hs.579899	chromosome 19 open reading frame 40	Homo sapiens chromosome 19 open rea	83.04067	113.78890	43.63382	54.03855	Detected	Detected	Detected	Detected
3057	A_33_P3884179	LOC150759	Hs.651352	hypothetical protein LOC150759	Homo sapiens cDNA FLJ33034 fis, clon	224.85059	568.56340	186.91480	351.75833	Detected	Detected	Detected	Detected
3058	A_23_P107994	TMEM160	Hs.105606	transmembrane protein 160	Homo sapiens transmembrane protein 1	2724.86160	2883.94480	3683.97560	3347.94630	Detected	Detected	Detected	Detected
3059	A_23_P83266	ENDOG	Hs.591905	endonuclease G	Homo sapiens endonuclease G (ENDOG	3068.38620	4191.50540	3350.00300	3932.96730	Detected	Detected	Detected	Detected
3060	A_23_P101476	ZNF442	Hs.253193	zinc finger protein 442	Homo sapiens zinc finger protein 442 (Z	89.26200	267.71005	40.17199	105.85170	Detected	Detected	Detected	Detected
3061	A_23_P157449	POLR2K	Hs.351475	polymerase (RNA) II (DNA directed) pol	Homo sapiens polymerase (RNA) II (DNA	4693.97400	4396.13870	3182.46950	2679.75600	Detected	Detected	Detected	Detected
3062	A_24_P201404	C11orf54	Hs.8360	chromosome 11 open reading frame 54	Homo sapiens chromosome 11 open rea	869.39404	406.12610	753.98724	277.08200	Detected	Detected	Detected	Detected
3063	A_23_P62953	PBX1	Hs.557097	pre-B-cell leukemia homeobox 1	Homo sapiens pre-B-cell leukemia hom	90.33609	80.83762	82.23103	49.54766	Detected	Detected	Detected	Detected
3064	A_23_P2129	TMEM126B	Hs.525063	transmembrane protein 126B	Homo sapiens transmembrane protein 1	5569.11900	3603.77300	4605.36200	2370.63800	Detected	Detected	Detected	Detected
3065	A_33_P3262416	ATPBD4	Hs.107196	ATP binding domain 4	Homo sapiens ATP binding domain 4 (A	10.49456	26.87047	3.42838	8.04942	Compromised	Detected	Compromised	Compromised
3066	A_33_P3327500	UFSP1	Hs.534845	UFM1-specific peptidase 1 (non-functio	Homo sapiens UFM1-specific peptidase	102.69580	208.36705	113.16232	226.13315	Detected	Detected	Detected	Detected
3067	A_23_P202156	NFKB2	Hs.73090	nuclear factor of kappa light polypeptide	Homo sapiens nuclear factor of kappa li	374.10846	409.83456	320.63245	311.31130	Detected	Detected	Detected	Detected
3068	A_23_P355824	MGRN1	Hs.526494	mahogunin, ring finger 1	Homo sapiens mahogunin, ring finger 1 (	16686.91000	18952.35700	13368.12600	12744.31000	Detected	Detected	Detected	Detected
3069	A_33_P3391791	MOG	Hs.141308	myelin oligodendrocyte glycoprotein	Homo sapiens myelin oligodendrocyte g	19.41591	7.95996	11.50257	3.94605	Detected	Compromised	Compromised	Compromised
3070	A_33_P3285277	CACNB2	Hs.59093	calcium channel, voltage-dependent, be	Homo sapiens calcium channel, voltage-	48.26880	34.32385	32.54459	6.75993	Detected	Detected	Detected	Compromised
3071	A_23_P25121	FKBP11	Hs.655103	FK506 binding protein 11, 19 kDa	Homo sapiens FK506 binding protein 11	2337.28120	16227.85900	2160.92260	13026.02500	Detected	Detected	Detected	Detected

3072	A_32_P165933	IQCF2	Hs.412294	IQ motif containing F2	Homo sapiens IQ motif containing F2 (IC	18.58750	20.30776	6.84623	5.64536	Detected	Compromised	Compromised	Compromised
3073	A_23_P10559	AATK	Hs.514575	apoptosis-associated tyrosine kinase	Homo sapiens apoptosis-associated tyr	535.70970	329.16020	317.69630	197.93263	Detected	Detected	Detected	Detected
3074	A_23_P29747	RPL23A	Hs.419463	ribosomal protein L23a	Homo sapiens ribosomal protein L23a (F	222381.05000	173402.22000	165839.48000	109881.01600	Detected	Detected	Detected	Detected
3075	A_23_P131417	HDLBP	Hs.471851	high density lipoprotein binding protein	Homo sapiens high density lipoprotein b	11885.66400	20071.90000	10749.56500	16638.80900	Detected	Detected	Detected	Detected
3076	A_33_P3343250	GPR1	Hs.184907	G protein-coupled receptor 1	Homo sapiens G protein-coupled recep	26.06809	303.07584	3.05885	52.40424	Detected	Detected	Compromised	Detected
3077	A_23_P127565	LAYN	Hs.503831	layilin	Homo sapiens layilin (LAYN), mRNA [NM	5772.36000	9591.44400	5807.38700	8644.21600	Detected	Detected	Detected	Detected
3078	A_33_P3321442	ZSCAN5C		zinc finger and SCAN domain containing	Zinc finger and SCAN domain-containin	195.99463	247.41586	88.87888	110.14541	Detected	Detected	Detected	Detected
3079	A_24_P93948		Hs.151219		Brain my050 protein [Source:UniProtKI	26.21840	15.84463	17.36172	3.22371	Detected	Compromised	Detected	Compromised
3080	A_32_P230868	FAM65A	Hs.152717	family with sequence similarity 65, mem	Homo sapiens family with sequence sim	20376.79000	24717.29300	10746.14100	12272.26300	Detected	Detected	Detected	Detected
3081	A_33_P3618561	LOC730961	Hs.597933	hypothetical protein LOC730961	Homo sapiens cDNA clone IMAGE:4826	21.85534	120.93172	17.06258	72.73414	Detected	Detected	Detected	Detected
3082	A_33_P3332081	KHDRBS3	Hs.444558	KH domain containing, RNA binding, sign	Homo sapiens KH domain containing, RI	821.22240	1309.14250	592.00750	797.87103	Detected	Detected	Detected	Detected
3083	A_23_P120146	TGFBRAP1	Hs.446350	transforming growth factor, beta recept	Homo sapiens transforming growth fact	1303.21670	1114.86110	817.96740	630.41310	Detected	Detected	Detected	Detected
3084	A_23_P40821	HPS3	Hs.591311	Hermansky-Pudlak syndrome 3	Homo sapiens Hermansky-Pudlak syndr	722.28080	458.49863	514.98060	250.60852	Detected	Detected	Detected	Detected
3085	A_23_P89981	CYP2F1	Hs.558318	cytochrome P450, family 2, subfamily F,	Homo sapiens cytochrome P450, family	5.51846	15.22285	20.69836	23.23175	Compromised	Compromised	Detected	Detected
3086	A_33_P3287785	NEFL	Hs.521461	neurofilament, light polypeptide	Homo sapiens neurofilament, light polyp	14.31782	32.24802	12.74158	22.89769	Detected	Detected	Compromised	Detected
3087	A_23_P135857	EIF2AK3	Hs.591589	eukaryotic translation initiation factor 2	Homo sapiens eukaryotic translation ini	600.82840	418.09677	266.62903	154.22830	Detected	Detected	Detected	Detected
3088	A_33_P3307363	LPHN2	Hs.24212	latrophilin 2	Homo sapiens latrophilin 2 (LPHN2), mF	3728.10380	1235.86060	2523.89720	717.68335	Detected	Detected	Detected	Detected
3089	A_32_P159400	UBR5	Hs.591856	ubiquitin protein ligase E3 component n	Homo sapiens ubiquitin protein ligase E3	669.16800	673.02980	500.22240	403.14877	Detected	Detected	Detected	Detected
3090	A_33_P3371752				Epidermal growth factor receptor subst	1185.38320	1019.77405	693.80250	449.71796	Detected	Detected	Detected	Detected
3091	A_23_P218434	ZNF226	Hs.145956	zinc finger protein 226	Homo sapiens zinc finger protein 226 (Z	2238.56710	2685.78930	1749.69560	1798.24890	Detected	Detected	Detected	Detected
3092	A_23_P106720	TBL3	Hs.513267	transducin (beta)-like 3	Homo sapiens transducin (beta)-like 3 (	336.07160	319.12817	327.70062	279.02090	Detected	Detected	Detected	Detected
3093	A_23_P27677	IRF3	Hs.75254	interferon regulatory factor 3	Homo sapiens interferon regulatory fact	708.77360	657.82446	722.93384	674.92365	Detected	Detected	Detected	Detected
3094	A_23_P85765	CACNA1S	Hs.1294	calcium channel, voltage-dependent, L t	Homo sapiens calcium channel, voltage-	20.75914	28.57916	12.76248	20.02464	Detected	Detected	Compromised	Detected
3095	A_33_P3219197	TBC1D10B	Hs.632182	TBC1 domain family, member 10B	Homo sapiens TBC1 domain family, mer	151.60205	162.52250	98.08707	113.11139	Detected	Detected	Detected	Detected
3096	A_24_P306726	TPT1	Hs.374596	tumor protein, translationally-controlled	Homo sapiens tumor protein, translatior	130960.45000	74925.08000	80014.90000	44686.18400	Detected	Detected	Detected	Detected
3097	A_24_P93371	COMMD4	Hs.351327	COMM domain containing 4	Homo sapiens COMM domain containing	1352.33910	2182.70560	1553.27340	2243.32670	Detected	Detected	Detected	Detected
3098	A_23_P49429	NUDT16L1	Hs.513315	nudix (nucleoside diphosphate linked mo	Homo sapiens nudix (nucleoside diphosp	3519.95800	4583.21900	3626.57100	4075.09130	Detected	Detected	Detected	Detected
3099	A_23_P435002	SRFBP1	Hs.107622	serum response factor binding protein 1	Homo sapiens serum response factor bi	767.13000	1080.44200	645.93370	725.87494	Detected	Detected	Detected	Detected
3100	A_23_P201079	PRDM2	Hs.371823	PR domain containing 2, with ZNF doma	Homo sapiens PR domain containing 2,	209.07913	125.02217	137.77217	65.31496	Detected	Detected	Detected	Detected
3101	A_33_P3241482	ZNF346	Hs.484259	zinc finger protein 346	Homo sapiens zinc finger protein 346 (Z	2440.60990	4134.60700	2026.71720	2685.24980	Detected	Detected	Detected	Detected
3102	A_23_P117146	PRDM4	Hs.506655	PR domain containing 4	Homo sapiens PR domain containing 4 (	1464.19600	1715.17910	976.66614	1044.17830	Detected	Detected	Detected	Detected
3103	A_33_P3346348	WDR67	Hs.492716	WD repeat domain 67	Homo sapiens WD repeat domain 67 (W	6.36736	21.94600	4.71907	9.77026	Compromised	Detected	Compromised	Compromised
3104	A_23_P19313	TBP	Hs.590872	TATA box binding protein	Homo sapiens TATA box binding proteir	890.72290	724.48570	583.76980	345.25046	Detected	Detected	Detected	Detected
3105	A_33_P3297651	LOC100130540	Hs.689563	hypothetical protein LOC100130540	Homo sapiens cDNA FLJ43078 fis, clon	30.75827	46.75160	15.15630	12.66219	Detected	Detected	Compromised	Compromised
3106	A_32_P173058	TMEM41B	Hs.594563	transmembrane protein 41B	Homo sapiens transmembrane protein 4	3709.04130	1894.72600	3209.68770	1410.74560	Detected	Detected	Detected	Detected
3107	A_33_P3300495				Plasminogen-related protein B Precurs	355.92218	70.03501	153.10292	29.83661	Detected	Detected	Detected	Detected
3108	A_33_P3338968	LOC100133737		similar to hCG1809973	PREDICTED: Homo sapiens similar to A	2047.54250	2589.45580	972.55320	854.24400	Detected	Detected	Detected	Detected
3109	A_33_P3235761	LOC284023	Hs.354493	hypothetical protein LOC284023	Homo sapiens hypothetical protein LOC	17.51339	13.35032	12.45074	10.90192	Detected	Compromised	Compromised	Compromised
3110	A_33_P3218925	STEAP2	Hs.489051	six transmembrane epithelial antigen of	Homo sapiens six transmembrane epith	101.28952	14.25143	69.37874	7.40381	Detected	Compromised	Detected	Compromised
3111	A_24_P24685	LOC729595		similar to high mobility group box 3	PREDICTED: Homo sapiens similar to h	108.68327	533.83470	77.04117	397.01743	Detected	Detected	Detected	Detected
3112	A_23_P121253	TNFSF10	Hs.478275	tumor necrosis factor (ligand) superfam	Homo sapiens tumor necrosis factor (lig	28.49304	34.54272	23.17142	22.84033	Detected	Detected	Detected	Detected
3113	A_24_P941322	QKI	Hs.510324	quaking homolog, KH domain RNA bindin	Homo sapiens quaking homolog, KH don	937.94080	870.95170	267.76544	162.03030	Detected	Detected	Detected	Detected
3114	A_24_P235049	MTHFD1L	Hs.591343	methylenetetrahydrofolate dehydrogena	Homo sapiens methylenetetrahydrofolat	4391.28000	5274.08840	3006.85180	3152.57180	Detected	Detected	Detected	Detected
3115	A_24_P142118	THBS1	Hs.164226	thrombospondin 1	Homo sapiens thrombospondin 1 (THBS	8381.52000	87372.07000	6690.92400	66152.31000	Detected	Detected	Detected	Detected
3116	A_33_P3401317	P704P	Hs.654289	prostate-specific P704P	Homo sapiens prostate-specific P704P	16.94385	13.44935	3.77322	3.62428	Detected	Compromised	Compromised	Compromised
3117	A_33_P3243069	KIAA0040	Hs.518138	KIAA0040	Homo sapiens KIAA0040 (KIAA0040), tr	74.05147	115.29102	32.95936	43.86429	Detected	Detected	Detected	Detected
3118	A_23_P142174	FOXA3	Hs.36137	forkhead box A3	Homo sapiens forkhead box A3 (FOXA3	41.94216	40.78092	19.32692	12.72244	Detected	Detected	Detected	Compromised
3119	A_32_P9963	HSF2	Hs.158195	heat shock transcription factor 2	Homo sapiens heat shock transcription	3190.83230	3167.81300	3366.93970	2162.78470	Not Detected	Detected	Detected	Detected
3120	A_33_P3302613				Centromere protein M (CENP-M)(Interp	33.45667	49.21133	21.84188	18.29244	Detected	Detected	Detected	Detected
3121	A_24_P3415	PPP2R2C	Hs.479069	protein phosphatase 2 (formerly 2A), re	Homo sapiens protein phosphatase 2 (fo	60.90144	34.68731	46.74001	36.99606	Detected	Detected	Detected	Detected
3122	A_33_P3378101	AGBL4	Hs.679809	ATP/GTP binding protein-like 4	Homo sapiens ATP/GTP binding proteir	157.49080	165.40103	65.12114	46.01537	Detected	Detected	Detected	Detected
3123	A_23_P28434	VAMP8	Hs.714302	vesicle-associated membrane protein 8	Homo sapiens vesicle-associated memb	271.03610	158.86841	250.83228	101.82161	Detected	Detected	Detected	Detected
3124	A_33_P3354067					83.21247	68.74693	42.16253	32.10800	Detected	Detected	Detected	Detected
3125	A_23_P406616	FLJ36031	Hs.29692	hypothetical protein FLJ36031	Homo sapiens hypothetical protein FLJ	7461.30100	15200.69300	6030.00830	9462.94500	Detected	Detected	Detected	Detected
3126	A_24_P65121	TTC23	Hs.513195	tetratricopeptide repeat domain 23	Homo sapiens tetratricopeptide repeat	14.83880	43.70466	10.54224	15.29494	Detected	Detected	Compromised	Compromised
3127	A_24_P475814	OPA3	Hs.719374	optic atrophy 3 (autosomal recessive, w	Homo sapiens optic atrophy 3 (autosom	2259.48460	3736.11700	1747.25790	2525.11040	Detected	Detected	Detected	Detected
3128	A_23_P112296	DBH	Hs.591890	dopamine beta-hydroxylase (dopamine	Homo sapiens dopamine beta-hydroxyla	2.96786	4.67724	47.96599	5.28065	Compromised	Compromised	Not Detected	Compromised
3129	A_24_P1255	BCCIP	Hs.715543	BRCA2 and CDKN1A interacting protein	Homo sapiens BRCA2 and CDKN1A inte	65.14663	149.67029	47.64932	100.29369	Detected	Detected	Detected	Detected
3130	A_23_P430558	CHRND	Hs.156289	cholinergic receptor, nicotinic, delta	Homo sapiens cholinergic receptor, nico	14.38336	19.72271	16.32973	19.60557	Detected	Detected	Detected	Detected
3131	A_33_P3214501	AEN	Hs.436102	apoptosis enhancing nuclease	Homo sapiens apoptosis enhancing nucl	537.58026	880.52660	388.66943	795.90460	Detected	Detected	Detected	Detected
3132	A_23_P207614	KIAA0100	Hs.151761	KIAA0100	Homo sapiens KIAA0100 (KIAA0100), m	6754.72600	9127.49300	6253.68900	8020.73440	Detected	Detected	Detected	Detected
3133	A_33_P3351087				cGMP-dependent protein kinase 1, beta	26.90071	116.53132	14.61953	37.33798	Detected	Detected	Compromised	Detected
3134	A_23_P408913	TTC30B	Hs.447659	tetratricopeptide repeat domain 30B	Homo sapiens tetratricopeptide repeat	299.44745	234.69664	239.10187	170.14660	Detected	Detected	Detected	Detected
3135	A_23_P100141	UNKL	Hs.643536	unkempt homolog (Drosophila)-like	Homo sapiens unkempt homolog (Droso	785.27700	455.40402	631.70294	329.52856	Detected	Detected	Detected	Detected
3136	A_23_P29773	LAMP3	Hs.518448	lysosomal-associated membrane protei	Homo sapiens lysosomal-associated me	30.17534	17.05645	17.42260	4.18152	Detected	Compromised	Detected	Compromised
3137	A_32_P118847	FLJ42709	Hs.457407	hypothetical LOC441094	Homo sapiens hypothetical LOC441094	1437.20470	2582.76660	955.20575	1340.74460	Detected	Detected	Detected	Detected
3138	A_24_P286527	RABL5	Hs.389104	RAB, member RAS oncogene family-like	Homo sapiens RAB, member RAS oncog	840.21010	807.20470	778.01900	614.22350	Detected	Detected	Detected	Detected
3139	A_23_P254797	BPIL1	Hs.257045	bactericidal/permeability-increasing pro	Homo sapiens bactericidal/permeability	488.12620	1121.78270	89.05166	160.70000	Detected	Detected	Detected	Detected
3140	A_33_P3236906	C6orf217	Hs.510098	chromosome 6 open reading frame 217	Homo sapiens chromosome 6 open reac	77.77840	14.99797	42.23470	3.22162	Detected	Compromised	Detected	Compromised
3141	A_33_P3245927	OGFOD2	Hs.524817	2-oxoglutarate and iron-dependent oxy	Homo sapiens 2-oxoglutarate and iron-	1781.05370	1566.75770	1981.52100	1627.01050	Detected	Detected	Detected	Detected
3142	A_33_P3214105	ATF3	Hs.460	activating transcription factor 3	Homo sapiens activating transcription fa	173.78299	237.98262	74.29054	125.73639	Detected	Detected	Detected	Detected
3143	A_23_P129101	HEXA	Hs.604479	hexosaminidase A (alpha polypeptide)	Homo sapiens hexosaminidase A (alpha	3983.53120	2579.49580	4123.24200	2601.36080	Detected	Detected	Detected	Detected
3144	A_33_P3289466					41.28385	51.01352	47.09954	51.17204	Detected	Detected	Detected	Detected
3145	A_23_P349127	CDAN1	Hs.599232	congenital dyserythropoietic anemia, typ	Homo sapiens congenital dyserythropoi	117.17371	140.08739	71.87343	77.67474	Detected	Detected	Detected	Detected
3146	A_33_P3297245	RRAS2	Hs.502004	related RAS viral (r-ras) oncogene hom	Homo sapiens related RAS viral (r-ras)	8672.16900	11464.14600	6275.08940	7000.85500	Detected	Detected	Detected	Detected

3147	A_23_P93543	PEX7	Hs.280932	peroxisomal biogenesis factor 7	Homo sapiens peroxisomal biogenesis fa	1978.64930	1949.24480	1617.83970	1297.89500	Detected	Detected	Detected	Detected
3148	A_24_P114438	OLA1	Hs.157351	Obg-like ATPase 1	Homo sapiens Obg-like ATPase 1 (OLA	163.09319	141.97679	119.00890	82.25976	Detected	Detected	Detected	Detected
3149	A_24_P179504	WDR66	Hs.709837	WD repeat domain 66	Homo sapiens WD repeat domain 66 (W	210.04163	209.28503	189.05806	148.92577	Detected	Detected	Detected	Detected
3150	A_24_P419309	SNRNP40	Hs.33962	small nuclear ribonucleoprotein 40kDa (	Homo sapiens small nuclear ribonucleop	858.53450	1075.39090	822.14140	845.08570	Detected	Detected	Detected	Detected
3151	A_23_P201002	RNF220	Hs.456557	ring finger protein 220	Homo sapiens ring finger protein 220 (R	18538.66000	16614.59600	13730.63300	10953.22000	Detected	Detected	Detected	Detected
3152	A_33_P3327140					2529.34030	1274.67200	201.80990	57.85199	Detected	Detected	Detected	Detected
3153	A_33_P3456341	LARP4	Hs.26613	La ribonucleoprotein domain family, mer	Homo sapiens La ribonucleoprotein dom	335.86618	526.44610	313.17346	465.19244	Detected	Detected	Detected	Detected
3154	A_23_P148798	UCHL5	Hs.591458	ubiquitin carboxyl-terminal hydrolase L5	Homo sapiens ubiquitin carboxyl-termin	2581.25780	2746.10670	2441.48600	2221.02400	Detected	Detected	Detected	Detected
3155	A_33_P3277826		Hs.643437		Zinc finger and SCAN domain-containin	172.35043	149.09845	92.17474	66.44051	Detected	Detected	Detected	Detected
3156	A_24_P357169	EPPK1	Hs.200412	epiplakin 1	Homo sapiens epiplakin 1 (EPPK1), mRN	25.68030	33.53632	13.77992	14.32146	Detected	Detected	Compromised	Compromised
3157	A_33_P3265872	LOC100128994	Hs.453788	hypothetical LOC100128994	PREDICTED: Homo sapiens hypothetica	139.54039	515.27820	164.21724	530.07150	Detected	Detected	Detected	Detected
3158	A_33_P3389028					31.42353	40.22412	5.88388	5.93404	Detected	Detected	Compromised	Compromised
3159	A_33_P3216994	HERC4	Hs.51891	hect domain and RLD 4	Homo sapiens hect domain and RLD 4 (	63.56657	131.76300	61.54115	109.95721	Detected	Detected	Detected	Detected
3160	A_23_P161488	TIAL1	Hs.501203	TIA1 cytotoxic granule-associated RNA	Homo sapiens TIA1 cytotoxic granule-a	1938.61130	1790.57560	1653.31380	1361.48730	Detected	Detected	Detected	Detected
3161	A_33_P3394366		Hs.632180		C-type lectin domain-containing protein	53.25347	49.33812	13.54198	18.88700	Detected	Detected	Compromised	Detected
3162	A_33_P3393941	MBTPS1	Hs.75890	membrane-bound transcription factor p	Homo sapiens membrane-bound transcr	5430.20800	5501.80270	4451.38330	3647.19120	Detected	Detected	Detected	Detected
3163	A_23_P63908	TRUB1	Hs.21187	TruB pseudouridine (psi) synthase homo	Homo sapiens TruB pseudouridine (psi)	2588.81400	2036.23820	1279.47800	798.15485	Detected	Detected	Detected	Detected
3164	A_33_P3224996					506.24808	498.06155	256.75740	196.32748	Detected	Detected	Detected	Detected
3165	A_23_P302787	LOC375295	Hs.659070	hypothetical protein LOC375295	PREDICTED: Homo sapiens hypothetica	1065.05640	2848.41670	593.11487	1198.39260	Detected	Detected	Detected	Detected
3166	A_33_P3781394	LOC728769	Hs.586193	hypothetical protein LOC728769	PREDICTED: Homo sapiens hypothetica	1370.96100	2497.48560	1159.97130	2014.93640	Detected	Detected	Detected	Detected
3167	A_33_P3256282	KRTAP10-2	Hs.664660	keratin associated protein 10-2	Homo sapiens keratin associated protei	168.44139	211.16809	58.81296	65.59853	Detected	Detected	Detected	Detected
3168	A_23_P258393	PPM1M	Hs.373560	protein phosphatase 1M (PP2C domain	Homo sapiens protein phosphatase 1M	380.01584	338.38843	385.34473	287.29205	Detected	Detected	Detected	Detected
3169	A_33_P3299766				UPF0518 protein FAM160B1 [Source:U	166.51425	182.25357	117.15552	88.18571	Detected	Detected	Detected	Detected
3170	A_33_P3231267	LOC257396	Hs.12326	hypothetical protein LOC257396	Homo sapiens hypothetical protein LOC	117.53079	141.76976	84.58897	67.39201	Detected	Detected	Detected	Detected
3171	A_24_P20630	LEF1	Hs.555947	lymphoid enhancer-binding factor 1	Homo sapiens lymphoid enhancer-bindi	162.12495	391.73346	121.71104	206.98984	Detected	Detected	Detected	Detected
3172	A_33_P3358253	INPPL1	Hs.523875	inositol polyphosphate phosphatase-like	Homo sapiens inositol polyphosphate ph	1972.96260	1570.40760	2166.59100	1626.84060	Detected	Detected	Detected	Detected
3173	A_23_P55880	ZNF564	Hs.655615	zinc finger protein 564	Homo sapiens zinc finger protein 564 (Z	321.58190	304.50565	228.02826	180.95610	Detected	Detected	Detected	Detected
3174	A_23_P163682	RHBDF1	Hs.57988	rhomboid 5 homolog 1 (Drosophila)	Homo sapiens rhomboid 5 homolog 1 (D	7799.57200	5169.94000	8953.68200	5069.35250	Detected	Detected	Detected	Detected
3175	A_32_P178499	C16orf86	Hs.632208	chromosome 16 open reading frame 86	Homo sapiens chromosome 16 open rea	667.11255	175.98761	835.20966	196.82967	Detected	Detected	Detected	Detected
3176	A_33_P3360814	NF-E4		transcription factor NF-E4	Homo sapiens transcription factor NF-E	13.99187	19.80841	5.74212	4.83242	Compromised	Detected	Compromised	Compromised
3177	A_23_P58009	C3orf52	Hs.434247	chromosome 3 open reading frame 52	Homo sapiens chromosome 3 open read	161.91531	979.21770	117.84353	585.36920	Detected	Detected	Detected	Detected
3178	A_33_P3388865	LRRC10	Hs.448708	leucine rich repeat containing 10	Homo sapiens leucine rich repeat conta	47.85720	79.06751	33.88496	30.87973	Detected	Detected	Detected	Detected
3179	A_23_P116280	WT1	Hs.591980	Wilms tumor 1	Homo sapiens Wilms tumor 1 (WT1), tra	2.62647	3.61221	20.80758	2.73972	Compromised	Compromised	Not Detected	Compromised
3180	A_23_P68401	CTNNBL1	Hs.472667	catenin, beta like 1	Homo sapiens catenin, beta like 1 (CTN	2584.22680	1842.42630	2238.57150	1388.42710	Detected	Detected	Detected	Detected
3181	A_24_P704446	TMEM72	Hs.292788	transmembrane protein 72	Homo sapiens transmembrane protein 7	35.98392	42.96879	3.37527	9.83861	Detected	Detected	Compromised	Compromised
3182	A_24_P200427	PAICS	Hs.518774	phosphoribosylaminoimidazole carboxyla	Homo sapiens phosphoribosylaminoimid	2941.30500	4938.14800	2382.64200	3076.68100	Detected	Detected	Detected	Detected
3183	A_33_P3263569	AKNAD1	Hs.720879	AKNA domain containing 1	Homo sapiens AKNA domain containing	113.92490	45.93173	52.07269	14.97472	Detected	Detected	Detected	Compromised
3184	A_33_P3367293	MT1IP		metallothionein 1I (pseudogene)	Homo sapiens metallothionein 1I (pseud	68.19785	99.39256	28.73135	26.58406	Detected	Detected	Detected	Detected
3185	A_23_P39910	C2orf64	Hs.596537	chromosome 2 open reading frame 64	Homo sapiens chromosome 2 open read	2596.91940	690.71954	2323.07030	488.38410	Detected	Detected	Detected	Detected
3186	A_23_P155989	CENPK	Hs.529778	centromere protein K	Homo sapiens centromere protein K (C	282.01190	1032.24870	118.56199	302.66720	Detected	Detected	Detected	Detected
3187	A_33_P3356525	FLJ45482	Hs.644332	hypothetical LOC645566	Homo sapiens cDNA FLJ45482 fis, clon	160.85086	175.16705	127.91288	151.72410	Detected	Detected	Detected	Detected
3188	A_33_P3271800	SIRT2	Hs.466693	sirtuin (silent mating type information r	Homo sapiens sirtuin (silent mating type	457.84534	460.77680	365.93045	368.33792	Detected	Detected	Detected	Detected
3189	A_33_P3346048	POM121L1P	Hs.546584	POM121 membrane glycoprotein-like 1	Homo sapiens POM121 membrane glyco	145.73503	112.56190	51.86681	27.70785	Detected	Detected	Detected	Detected
3190	A_24_P37253	LYPD6	Hs.21929	LY6/PLAUR domain containing 6	Homo sapiens LY6/PLAUR domain con	2.63021	169.18219	3.09921	88.08434	Compromised	Detected	Compromised	Detected
3191	A_23_P41588	HARS2	Hs.432560	histidyl-tRNA synthetase 2, mitochondr	Homo sapiens histidyl-tRNA synthetase	3145.77300	4383.41750	3211.24320	4086.34250	Detected	Detected	Detected	Detected
3192	A_33_P3280779	C10orf57	Hs.169982	chromosome 10 open reading frame 57	Homo sapiens chromosome 10 open rea	57.41723	63.80368	43.78356	37.82629	Detected	Detected	Detected	Detected
3193	A_23_P206454	CENPBD1	Hs.513832	CENPB DNA-binding domains containin	Homo sapiens CENPB DNA-binding dor	1486.97550	1386.79610	1482.20890	1222.63600	Detected	Detected	Detected	Detected
3194	A_33_P3216898	LOC286367	Hs.720355	FP944	Homo sapiens FP944 (LOC286367), nor	305.39200	88.20250	214.72481	47.20954	Detected	Detected	Detected	Detected
3195	A_23_P123228	SLC26A3	Hs.1650	solute carrier family 26, member 3	Homo sapiens solute carrier family 26, r	5.49373	17.42438	3.16996	3.92206	Compromised	Detected	Compromised	Compromised
3196	A_33_P3276323	C8orf81	Hs.706174	chromosome 8 open reading frame 81	PREDICTED: Homo sapiens chromosom	26.26470	44.91344	14.40895	15.90481	Detected	Detected	Compromised	Compromised
3197	A_23_P139396	C11orf73	Hs.283322	chromosome 11 open reading frame 73	Homo sapiens chromosome 11 open rea	10175.53900	8686.94900	10735.03600	7912.59900	Detected	Detected	Detected	Detected
3198	A_33_P3210605					13.61354	23.76384	3.09964	4.53219	Detected	Detected	Compromised	Compromised
3199	A_23_P119964	CEBPZ	Hs.135406	CCAAT/enhancer binding protein (C/EI	Homo sapiens CCAAT/enhancer bindin	4706.30900	2705.39720	3506.29600	1656.97050	Detected	Detected	Detected	Detected
3200	A_33_P3666797	LOC144874	Hs.439363	hypothetical protein LOC144874	Homo sapiens mRNA; cDNA DKFZp667I	299.72510	237.34000	222.33508	139.73288	Detected	Detected	Detected	Detected
3201	A_32_P416583	NLRC5	Hs.528836	NLR family, CARD domain containing 5	Homo sapiens NLR family, CARD domai	393.71683	390.12866	203.25247	303.30032	Detected	Detected	Detected	Detected
3202	A_23_P355776		Hs.722814		Putative uncharacterized protein C20or	13.01365	20.36121	4.74201	10.44705	Compromised	Detected	Compromised	Compromised
3203	A_33_P3211513	CLK1	Hs.433732	CDC-like kinase 1	Homo sapiens CDC-like kinase 1 (CLK1	329.33148	244.17421	372.13577	236.74652	Detected	Detected	Detected	Detected
3204	A_24_P246943	PPIA	Hs.356331	peptidylprolyl isomerase A (cyclophilin A	Homo sapiens peptidylprolyl isomerase	11810.19500	15636.50600	9172.99700	10844.46800	Detected	Detected	Detected	Detected
3205	A_24_P732099	HBBP1		hemoglobin, beta pseudogene 1	Homo sapiens hemoglobin, beta pseudo	21.56074	19.19583	5.78945	8.82303	Detected	Detected	Compromised	Compromised
3206	A_23_P41267	LOC401127	Hs.383197	WD repeat domain 5 pseudogene	Homo sapiens WD repeat domain 5 pseu	334.72890	936.46070	336.61120	850.78670	Detected	Detected	Detected	Detected
3207	A_33_P3249349				Melanoma antigen preferentially expres	332.26910	319.19254	124.85340	109.35373	Detected	Detected	Detected	Detected
3208	A_24_P172768	GMFB	Hs.151413	glia maturation factor, beta	Homo sapiens glia maturation factor, be	4351.40040	6027.15900	2816.40970	3235.92970	Detected	Detected	Detected	Detected
3209	A_24_P30194	IFIT5	Hs.252839	interferon-induced protein with tetratric	Homo sapiens interferon-induced prote	2236.36180	1535.31820	1511.74930	798.31840	Detected	Detected	Detected	Detected
3210	A_23_P367405	PCBD1	Hs.3192	pterin-4 alpha-carbinolamine dehydrata	Homo sapiens pterin-4 alpha-carbinolar	1268.28000	1834.45280	1001.51013	1329.18880	Detected	Detected	Detected	Detected
3211	A_23_P784	LZIC	Hs.327252	leucine zipper and CTNNBIP1 domain c	Homo sapiens leucine zipper and CTNN	1426.21410	3157.31640	1448.92740	2602.15900	Detected	Detected	Detected	Detected
3212	A_23_P148484	RLIM	Hs.653288	ring finger protein, LIM domain interacti	Homo sapiens ring finger protein, LIM d	847.53937	1695.58420	627.69446	994.11945	Detected	Detected	Detected	Detected
3213	A_33_P3719214		Hs.313507		602662785F1 NIH_MGC_21 Homo sapier	411.41324	743.01800	155.99521	211.01517	Detected	Detected	Detected	Detected
3214	A_23_P144843	ESM1	Hs.129944	endothelial cell-specific molecule 1	Homo sapiens endothelial cell-specific	40.72820	457.42970	25.56133	249.28648	Detected	Detected	Detected	Detected
3215	A_23_P397019	DNAJC25	Hs.534196	DnaJ (Hsp40) homolog, subfamily C , m	Homo sapiens DnaJ (Hsp40) homolog, s	578.44840	330.71658	314.16522	160.87558	Detected	Detected	Detected	Detected
3216	A_33_P3227857	COX18	Hs.356697	COX18 cytochrome c oxidase assembly	Homo sapiens COX18 cytochrome c ox	65.72430	33.20455	59.11636	27.78757	Detected	Detected	Detected	Detected
3217	A_23_P435636	DAND5	Hs.331981	DAN domain family, member 5	Homo sapiens DAN domain family, mem	28.79373	80.30779	12.55294	50.76249	Detected	Detected	Compromised	Detected
3218	A_23_P117694	CORO2B	Hs.551213	coronin, actin binding protein, 2B	Homo sapiens coronin, actin binding pro	706.12760	382.64218	545.60140	265.31656	Detected	Detected	Detected	Detected
3219	A_33_P3281196	MRPS36	Hs.631971	mitochondrial ribosomal protein S36	Homo sapiens mitochondrial ribosomal p	1121.63880	1714.36400	463.04056	552.33610	Detected	Detected	Detected	Detected
3220	A_33_P3421733	EIF3CL	Hs.535464	eukaryotic translation initiation factor 3	Homo sapiens eukaryotic translation ini	1940.45240	1671.35270	1456.71120	1063.79760	Detected	Detected	Detected	Detected
3221	A_24_P206047	SLC25A4	Hs.246506	solute carrier family 25 (mitochondrial c	Homo sapiens solute carrier family 25 (	590.48170	1820.15840	651.65790	1827.50550	Detected	Detected	Detected	Detected

3222	A_24_P117323	KLHL22	Hs.517419	kelch-like 22 (Drosophila)	Homo sapiens kelch-like 22 (Drosophila	676.04670	497.19083	500.68555	388.32760	Detected	Detected	Detected	Detected
3223	A_23_P108265	OR7C2	Hs.589620	olfactory receptor, family 7, subfamily C	Homo sapiens olfactory receptor, family	95.64929	104.16111	33.38456	28.57242	Detected	Detected	Detected	Detected
3224	A_23_P60537	PRPF4	Hs.374973	PRP4 pre-mRNA processing factor 4 ho	Homo sapiens PRP4 pre-mRNA process	2610.59940	3870.30350	1768.46440	2199.05760	Detected	Detected	Detected	Detected
3225	A_24_P100190	C21orf93	Hs.171428	chromosome 21 open reading frame 93	PREDICTED: Homo sapiens chromosom	344.75534	483.62552	213.24600	183.33904	Detected	Detected	Detected	Detected
3226	A_23_P40847	CHST2	Hs.8786	carbohydrate (N-acetylglucosamine-6-(	Homo sapiens carbohydrate (N-acetylgl	14784.90300	12185.58900	8727.42400	5883.54300	Detected	Detected	Detected	Detected
3227	A_33_P3383149	ZCWPW2	Hs.659030	zinc finger, CW type with PWWP domain	Homo sapiens zinc finger, CW type with	54.03496	38.97273	47.10782	20.21014	Detected	Detected	Detected	Detected
3228	A_33_P3350374	C10orf58	Hs.500333	chromosome 10 open reading frame 58	Homo sapiens chromosome 10 open rea	298.76492	14.03263	361.52570	9.18739	Detected	Compromised	Detected	Compromised
3229	A_23_P429998	FOSB	Hs.590958	FBJ murine osteosarcoma viral oncogen	Homo sapiens FBJ murine osteosarcom	246.31786	836.67290	154.63303	547.23505	Detected	Detected	Detected	Detected
3230	A_24_P156267	SOX12	Hs.43627	SRY (sex determining region Y)-box 12	Homo sapiens SRY (sex determining reg	72.84500	22.40542	55.37658	10.15128	Detected	Detected	Detected	Compromised
3231	A_33_P3357964				Putative uncharacterized protein PRO0	36.00971	57.26234	33.49946	34.98207	Detected	Detected	Detected	Detected
3232	A_23_P111811	ING3	Hs.489811	inhibitor of growth family, member 3	Homo sapiens inhibitor of growth family	979.74084	1187.90750	673.10810	690.82640	Detected	Detected	Detected	Detected
3233	A_33_P3256880	LOC642980	Hs.458216	hypothetical LOC642980	Homo sapiens cDNA FLJ16523 fis, clon	88.35986	116.52319	39.89599	39.13820	Detected	Detected	Detected	Detected
3234	A_23_P100315	DECR2	Hs.628831	2,4-dienoyl CoA reductase 2, peroxisom	Homo sapiens 2,4-dienoyl CoA reducta	6110.64650	5102.55860	6173.11330	4611.67430	Detected	Detected	Detected	Detected
3235	A_23_P359854	BEND3	Hs.418045	BEN domain containing 3	Homo sapiens BEN domain containing 3	947.11380	747.11774	617.76447	387.41510	Detected	Detected	Detected	Detected
3236	A_23_P140069	FBXL3	Hs.508284	F-box and leucine-rich repeat protein 3	Homo sapiens F-box and leucine-rich r	4509.58400	4271.88330	3925.80860	3126.81960	Detected	Detected	Detected	Detected
3237	A_33_P3406449	SLC15A4	Hs.699387	solute carrier family 15, member 4	Homo sapiens solute carrier family 15, r	881.13446	928.85230	983.43353	871.52594	Detected	Detected	Detected	Detected
3238	A_33_P3214293	PPP3R1	Hs.280604	protein phosphatase 3 (formerly 2B), re	Homo sapiens protein phosphatase 3 (fo	28.79519	65.36010	35.55488	29.72365	Detected	Detected	Detected	Detected
3239	A_33_P3352562	C19orf28	Hs.656901	chromosome 19 open reading frame 28	Homo sapiens chromosome 19 open rea	2634.89920	1803.37120	2593.97880	1817.98670	Detected	Detected	Detected	Detected
3240	A_24_P298174	CBX1	Hs.77254	chromobox homolog 1 (HP1 beta homol	Homo sapiens chromobox homolog 1 (H	583.26910	1080.55700	451.16656	710.47170	Detected	Detected	Detected	Detected
3241	A_23_P21086	LGI3	Hs.33470	leucine-rich repeat LGI family, member	Homo sapiens leucine-rich repeat LGI f	257.62445	281.29733	107.91883	79.79578	Detected	Detected	Detected	Detected
3242	A_23_P431569		Hs.655804		LOC100049716 protein Fragment [Sou	402.96573	215.25511	256.92444	107.23760	Detected	Detected	Detected	Detected
3243	A_33_P3215565				Putative uncharacterized protein ENSP	35.60484	63.71058	27.36239	43.52412	Detected	Detected	Detected	Detected
3244	A_23_P383278	PYCRL	Hs.165186	pyrroline-5-carboxylate reductase-like	Homo sapiens pyrroline-5-carboxylate r	1056.31800	1601.78090	1015.81730	1606.99460	Detected	Detected	Detected	Detected
3245	A_23_P313828	CENPV	Hs.433422	centromere protein V	Homo sapiens centromere protein V (C	122.02570	112.76178	129.92398	94.37787	Detected	Detected	Detected	Detected
3246	A_33_P3372742				Keratin-associated protein 5-11 (Kerati	3349.73700	4869.41500	2140.20730	1929.29160	Detected	Detected	Detected	Detected
3247	A_23_P104892	EIF4G2	Hs.183684	eukaryotic translation initiation factor 4	Homo sapiens eukaryotic translation ini	27663.57800	50411.58200	25294.73800	40611.88000	Detected	Detected	Detected	Detected
3248	A_23_P61202	C21orf70	Hs.410830	chromosome 21 open reading frame 70	Homo sapiens chromosome 21 open rea	1024.18000	1959.67350	1213.43230	2118.58080	Detected	Detected	Detected	Detected
3249	A_23_P105730	MDM1	Hs.655702	Mdm1 nuclear protein homolog (mouse)	Homo sapiens Mdm1 nuclear protein ho	7.61416	16.89531	8.14518	13.78905	Compromised	Detected	Compromised	Detected
3250	A_33_P3308105	GGH	Hs.78619	gamma-glutamyl hydrolase (conjugase,	Homo sapiens gamma-glutamyl hydrolas	14639.66600	27526.20300	13622.64200	20745.76800	Detected	Detected	Detected	Detected
3251	A_33_P3266530	DUS1L	Hs.514599	dihydrouridine synthase 1-like (S. cerev	Homo sapiens dihydrouridine synthase	10563.50700	12418.46900	9100.11100	9961.29700	Detected	Detected	Detected	Detected
3252	A_33_P3393694	GLTSCR2	Hs.421907	glioma tumor suppressor candidate regi	Homo sapiens glioma tumor suppressor	84522.08000	30313.23000	81016.13000	28724.60500	Detected	Detected	Detected	Detected
3253	A_33_P3244347	PC	Hs.89890	pyruvate carboxylase	Homo sapiens pyruvate carboxylase (PC	2079.25850	1716.75700	1551.88490	895.78620	Detected	Detected	Detected	Detected
3254	A_23_P115346	LGTN	Hs.497581	ligatin	Homo sapiens ligatin (LGTN), mRNA [N	1377.62280	1586.47070	1132.12260	1058.66250	Detected	Detected	Detected	Detected
3255	A_23_P87575	CCNT1	Hs.279906	cyclin T1	Homo sapiens cyclin T1 (CCNT1), mRN	13.42048	9.88063	17.53400	13.91847	Compromised	Compromised	Detected	Compromised
3256	A_33_P3418496					717.04790	925.30950	358.62280	371.89084	Detected	Detected	Detected	Detected
3257	A_33_P3408152	NEFH	Hs.198760	neurofilament, heavy polypeptide	Homo sapiens neurofilament, heavy poly	21.57550	32.48154	26.54647	34.18483	Detected	Detected	Detected	Detected
3258	A_23_P119478	EBI3	Hs.501452	Epstein-Barr virus induced 3	Homo sapiens Epstein-Barr virus induc	18.83064	23.11770	21.47668	20.05334	Detected	Detected	Detected	Detected
3259	A_23_P146004	CBLL1	Hs.592271	Cas-Br-M (murine) ecotropic retroviral	Homo sapiens Cas-Br-M (murine) ecot	248.29808	459.02023	167.86006	268.55338	Detected	Detected	Detected	Detected
3260	A_23_P78762	HSD17B14	Hs.18788	hydroxysteroid (17-beta) dehydrogenas	Homo sapiens hydroxysteroid (17-beta)	293.95422	345.34402	278.46057	305.66670	Detected	Detected	Detected	Detected
3261	A_23_P58538	C5orf26		chromosome 5 open reading frame 26	Homo sapiens chromosome 5 open read	45263.95300	24616.44100	39921.01000	17717.98000	Detected	Detected	Detected	Detected
3262	A_32_P181638	BVES	Hs.221660	blood vessel epicardial substance	Homo sapiens blood vessel epicardial su	539.69850	544.49490	407.86407	344.65598	Detected	Detected	Detected	Detected
3263	A_33_P3327986					37296.29300	29387.23800	10525.35200	7307.03600	Detected	Detected	Detected	Detected
3264	A_24_P544882	SSR1	Hs.114033	signal sequence receptor, alpha	Homo sapiens signal sequence receptor	1227.70510	1722.56120	647.05676	742.71160	Detected	Detected	Detected	Detected
3265	A_23_P45166	PROSC	Hs.304792	proline synthetase co-transcribed homo	Homo sapiens proline synthetase co-tra	889.13983	970.68616	821.21860	752.10460	Detected	Detected	Detected	Detected
3266	A_33_P3286621	SCARNA16	Hs.722642	small Cajal body-specific RNA 16	Homo sapiens small Cajal body-specific	792.75360	256.14117	563.69430	169.05106	Detected	Detected	Detected	Detected
3267	A_23_P116840	C12orf44	Hs.9911	chromosome 12 open reading frame 44	Homo sapiens chromosome 12 open rea	2843.58370	4064.01340	2971.80500	3853.17200	Detected	Detected	Detected	Detected
3268	A_23_P43107	TM7SF4	Hs.652230	transmembrane 7 superfamily member 4	Homo sapiens transmembrane 7 superfa	66.72916	47.41620	58.16419	30.13075	Detected	Detected	Detected	Detected
3269	A_23_P117515	ARID4A	Hs.161000	AT rich interactive domain 4A (RBP1-li	Homo sapiens AT rich interactive doma	94.40005	67.61522	78.13593	42.20647	Detected	Detected	Detected	Detected
3270	A_33_P3368706	NFYC	Hs.720048	nuclear transcription factor Y, gamma	Homo sapiens nuclear transcription fact	2564.36450	3001.66260	1583.75480	1488.73890	Detected	Detected	Detected	Detected
3271	A_33_P3287745	MYST1	Hs.533803	MYST histone acetyltransferase 1	Homo sapiens MYST histone acetyltran	7788.97360	4520.74700	7518.14300	3566.72560	Detected	Detected	Detected	Detected
3272	A_23_P13753	NFE2	Hs.75643	nuclear factor (erythroid-derived 2), 45	Homo sapiens nuclear factor (erythroid	199.92067	15.10880	194.15591	9.36264	Detected	Compromised	Detected	Compromised
3273	A_23_P120153	RNF149	Hs.142074	ring finger protein 149	Homo sapiens ring finger protein 149 (R	12486.95600	13394.14000	9946.07800	9027.53700	Detected	Detected	Detected	Detected
3274	A_33_P3219286	LOC100130262	Hs.721956	hypothetical LOC100130262	PREDICTED: Homo sapiens hypothetica	91.27609	124.60016	39.48027	44.33758	Detected	Detected	Detected	Detected
3275	A_24_P397515	HMGN4	Hs.236774	high mobility group nucleosomal binding	Homo sapiens high mobility group nucle	2766.81320	3995.44600	2841.06980	3487.89010	Detected	Detected	Detected	Detected
3276	A_23_P348298	SAC3D1	Hs.23642	SAC3 domain containing 1	Homo sapiens SAC3 domain containing	2461.94870	6078.92720	2271.81500	4768.10740	Detected	Detected	Detected	Detected
3277	A_33_P3415086				Putative uncharacterized protein ENSP	74.68657	83.49936	79.28328	68.23444	Detected	Detected	Detected	Detected
3278	A_23_P116797	C12orf10	Hs.655988	chromosome 12 open reading frame 10	Homo sapiens chromosome 12 open rea	2922.05350	2980.24240	3367.29150	3032.82060	Detected	Detected	Detected	Detected
3279	A_33_P3217700	USP9Y	Hs.598540	ubiquitin specific peptidase 9, Y-linked	Homo sapiens ubiquitin specific peptida	836.06976	6.32727	605.62220	3.20250	Detected	Compromised	Detected	Compromised
3280	A_23_P400078	MTHFR	Hs.214142	5,10-methylenetetrahydrofolate reducta	Homo sapiens 5,10-methylenetetrahydr	239.93740	157.21594	170.53581	88.83180	Detected	Detected	Detected	Detected
3281	A_24_P399362	PSMG2	Hs.464652	proteasome (prosome, macropain) asse	Homo sapiens proteasome (prosome, m	3367.83900	4289.66550	3039.88160	3381.93630	Detected	Detected	Detected	Detected
3282	A_24_P117147	KIR3DL1	Hs.645228	killer cell immunoglobulin-like receptor,	Homo sapiens killer cell immunoglobulin	35.51101	45.42644	26.13291	18.84281	Detected	Detected	Detected	Detected
3283	A_33_P3303572	EDA	Hs.105407	ectodysplasin A	Homo sapiens ectodysplasin A (EDA), t	49.93486	38.67900	39.52055	20.93252	Detected	Detected	Detected	Detected
3284	A_33_P3391275	LOC284749	Hs.299080	hypothetical LOC284749	Homo sapiens hypothetical LOC284749	176.99046	253.04358	77.07339	69.82050	Detected	Detected	Detected	Detected
3285	A_23_P214487	HIST1H4C	Hs.46423	histone cluster 1, H4c	Homo sapiens histone cluster 1, H4c (H	21490.90200	82005.71000	16907.02000	53649.42600	Detected	Detected	Detected	Detected
3286	A_23_P36183	GTF2H1	Hs.577202	general transcription factor IIH, polypep	Homo sapiens general transcription fact	4870.39650	3241.78100	3714.91800	2115.58180	Detected	Detected	Detected	Detected
3287	A_33_P3269233					15.79573	25.97099	19.44780	16.61274	Detected	Detected	Detected	Detected
3288	A_32_P160561	DOK6	Hs.278285	docking protein 6	Homo sapiens docking protein 6 (DOK6)	11.81122	56.51425	23.61119	54.97334	Compromised	Detected	Detected	Detected
3289	A_24_P196704	MLL5	Hs.592262	myeloid/lymphoid or mixed-lineage leuk	Homo sapiens myeloid/lymphoid or mixe	1860.90260	1187.41280	1422.65580	745.31870	Detected	Detected	Detected	Detected
3290	A_23_P106898	ORAI3	Hs.720562	ORAI calcium release-activated calciun	Homo sapiens ORAI calcium release-ac	1296.18460	1013.60315	1172.10900	860.44860	Detected	Detected	Detected	Detected
3291	A_23_P51202	ZNF436	Hs.293798	zinc finger protein 436	Homo sapiens zinc finger protein 436 (Z	1182.31080	674.35190	788.27167	390.12946	Detected	Detected	Detected	Detected
3292	A_33_P3732466	DKFZp547G183	Hs.585502	hypothetical LOC55525	Homo sapiens mRNA; cDNA DKFZp547	144.26490	106.88159	125.34370	75.52788	Detected	Detected	Detected	Detected
3293	A_32_P170749	STAG3L2	Hs.709564	stromal antigen 3-like 2	Homo sapiens stromal antigen 3-like 2	629.89700	761.31090	637.42993	790.41770	Detected	Detected	Detected	Detected
3294	A_23_P8961	IL7	Hs.591873	interleukin 7	Homo sapiens interleukin 7 (IL7), mRNA	3.94324	77.80329	3.08973	28.16472	Compromised	Detected	Compromised	Detected
3295	A_33_P3665777	HSP90AA1	Hs.525600	heat shock protein 90kDa alpha (cytoso	Homo sapiens heat shock protein 90kDa	97113.15000	125822.51000	65895.86000	67951.04000	Detected	Detected	Detected	Detected
3296	A_33_P3325257	LOC442421	Hs.591892	hypothetical LOC442421	Homo sapiens hypothetical LOC442421	68.73027	94.23741	43.45919	43.14025	Detected	Detected	Detected	Detected

3297	A_23_P134125	MAP3K5	Hs.186486	mitogen-activated protein kinase kinase	Homo sapiens mitogen-activated protei	5312.23050	1519.55100	3606.47780	854.43660	Detected	Detected	Detected	Detected
3298	A_23_P161152	PDSS1	Hs.558468	prenyl (decaprenyl) diphosphate synthas	Homo sapiens prenyl (decaprenyl) dipho	638.59270	1370.11760	623.96783	1154.05310	Detected	Detected	Detected	Detected
3299	A_33_P3255716	LOC285095	Hs.85890	hypothetical LOC285095	Putative uncharacterized protein ENSP	584.67346	827.31080	178.96786	192.59622	Detected	Detected	Detected	Detected
3300	A_33_P3229370	ID4	Hs.519601	inhibitor of DNA binding 4, dominant ne	Homo sapiens inhibitor of DNA binding	1238.90590	310.18090	990.65704	191.32608	Detected	Detected	Detected	Detected
3301	A_23_P256784	MUC2	Hs.315	mucin 2, oligomeric mucus/gel-forming	Homo sapiens mucin 2, oligomeric mucu	274.86078	226.11156	62.60069	60.04185	Detected	Detected	Detected	Detected
3302	A_33_P3316522	DEFB124	Hs.381373	defensin, beta 124	Homo sapiens defensin, beta 124 (DEFE	205.09602	649.11870	58.34496	90.76322	Detected	Detected	Detected	Detected
3303	A_33_P3228499					32.73024	13.68750	25.74217	5.94535	Detected	Compromised	Detected	Compromised
3304	A_33_P3277753	ANKRD23	Hs.643430	ankyrin repeat domain 23	Homo sapiens ankyrin repeat domain 23	461.73390	264.05835	305.22894	159.33832	Detected	Detected	Detected	Detected
3305	A_33_P3257538	AGPHD1	Hs.307962	aminoglycoside phosphotransferase dom	Homo sapiens aminoglycoside phosphot	148.06862	198.82173	90.18886	100.40490	Detected	Detected	Detected	Detected
3306	A_23_P416894	PION	Hs.186649	pigeon homolog (Drosophila)	Homo sapiens pigeon homolog (Drosoph	233.35948	75.13628	219.79060	50.83225	Detected	Detected	Detected	Detected
3307	A_23_P166807	PCBP4	Hs.20930	poly(rC) binding protein 4	Homo sapiens poly(rC) binding protein 4	12760.91700	12659.18800	13378.46900	12034.16700	Detected	Detected	Detected	Detected
3308	A_33_P3416772	C1orf159	Hs.235095	chromosome 1 open reading frame 159	Homo sapiens chromosome 1 open read	780.75780	889.64430	697.00280	762.34680	Detected	Detected	Detected	Detected
3309	A_33_P3287907	LOC728723	Hs.161338	hypothetical LOC728723	Homo sapiens hypothetical LOC728723	31.82555	12.53240	8.27923	3.36843	Detected	Compromised	Compromised	Compromised
3310	A_23_P206018	TPM1	Hs.133892	tropomyosin 1 (alpha)	Homo sapiens tropomyosin 1 (alpha) (T	12588.56500	64961.57400	11102.85700	49353.77300	Detected	Detected	Detected	Detected
3311	A_33_P3293169	AMBRA1	Hs.654644	autophagy/beclin-1 regulator 1	Homo sapiens autophagy/beclin-1 regu	24.29869	23.03964	24.17879	20.20831	Detected	Detected	Detected	Detected
3312	A_23_P94174	MRPL15	Hs.18349	mitochondrial ribosomal protein L15	Homo sapiens mitochondrial ribosomal p	9538.90100	18874.40400	9467.80100	15226.56000	Detected	Detected	Detected	Detected
3313	A_23_P118	EXTL2	Hs.357637	exostoses (multiple)-like 2	Homo sapiens exostoses (multiple)-like	367.50420	238.57668	232.88947	136.09335	Detected	Detected	Detected	Detected
3314	A_23_P5731	FAHD2A	Hs.546387	fumarylacetoacetate hydrolase domain	Homo sapiens fumarylacetoacetate hyd	527.77190	908.42930	528.48060	865.76610	Detected	Detected	Detected	Detected
3315	A_23_P406928					1899.18380	1104.11230	1541.99550	773.14590	Detected	Detected	Detected	Detected
3316	A_33_P3365985		Hs.721481		Putative uncharacterized protein FLJ4	704.49023	562.05530	537.07733	351.67603	Detected	Detected	Detected	Detected
3317	A_23_P130182	AURKB	Hs.442658	aurora kinase B	Homo sapiens aurora kinase B (AURKB	24.55805	1091.36500	20.76012	1019.13385	Detected	Detected	Detected	Detected
3318	A_33_P3273298	ZNF813	Hs.710780	zinc finger protein 813	Homo sapiens zinc finger protein 813 (Z	86.08008	117.72826	54.54988	69.76330	Detected	Detected	Detected	Detected
3319	A_33_P3399061	ADARB1		adenosine deaminase, RNA-specific, B1	Homo sapiens adenosine deaminase, RN	33.89433	170.17253	48.96199	164.17480	Detected	Detected	Detected	Detected
3320	A_33_P3665553	FLJ44253	Hs.595418	hypothetical gene supported by AK0969	Homo sapiens cDNA FLJ44253 fis, clon	47.49411	15.96725	29.68685	4.74835	Detected	Compromised	Detected	Compromised
3321	A_23_P125286	OR52A1	Hs.553520	olfactory receptor, family 52, subfamily	Homo sapiens olfactory receptor, family	84.83070	80.56767	22.20584	17.92940	Detected	Detected	Detected	Detected
3322	A_33_P3386770	LOC642574	Hs.449407	IQ motif and ankyrin repeat domain-con	IQ motif and ankyrin repeat domain-con	77.79485	112.52953	44.57018	43.06647	Detected	Detected	Detected	Detected
3323	A_33_P3229161	UTP14C	Hs.512963	UTP14, U3 small nucleolar ribonucleopr	Homo sapiens UTP14, U3 small nucleola	251.51950	223.85109	245.82613	199.18237	Detected	Detected	Detected	Detected
3324	A_23_P61569	VAMP4	Hs.6651	vesicle-associated membrane protein 4	Homo sapiens vesicle-associated memb	337.09064	508.25897	231.90842	292.08040	Detected	Detected	Detected	Detected
3325	A_32_P231617	TM4SF1	Hs.351316	transmembrane 4 L six family member 1	Homo sapiens transmembrane 4 L six fa	17096.97900	5196.66900	16455.67000	4709.62650	Detected	Detected	Detected	Detected
3326	A_23_P401	CENPF	Hs.497741	centromere protein F, 350/400ka (mitos	Homo sapiens centromere protein F, 35	1128.54430	34518.81200	808.48630	21192.85400	Detected	Detected	Detected	Detected
3327	A_23_P377245		Hs.533986		Homo sapiens zinc finger, MYM-type 6,	397.33510	183.59372	168.12486	53.48137	Detected	Detected	Detected	Detected
3328	A_24_P417352				Immunoglobulin heavy variable 3-6 [Sou	13.85318	30.37977	24.16655	11.46511	Detected	Detected	Detected	Compromised
3329	A_33_P3334313	ACTR3B	Hs.647117	ARP3 actin-related protein 3 homolog E	Homo sapiens ARP3 actin-related prote	759.95886	262.32153	628.76086	178.04646	Detected	Detected	Detected	Detected
3330	A_23_P29422	GYG1	Hs.477892	glycogenin 1	Homo sapiens glycogenin 1 (GYG1), mR	669.44680	921.87590	615.70734	710.30450	Detected	Detected	Detected	Detected
3331	A_33_P3329467	STYXL1	Hs.11615	serine/threonine/tyrosine interacting-li	Homo sapiens serine/threonine/tyrosin	4222.46000	7494.99460	3906.78340	5912.52700	Detected	Detected	Detected	Detected
3332	A_24_P876522	GPX8	Hs.289044	glutathione peroxidase 8 (putative)	Homo sapiens glutathione peroxidase 8	2686.06450	7460.49200	2674.32350	6270.64840	Detected	Detected	Detected	Detected
3333	A_33_P3333587	LOC90110	Hs.595347	hypothetical protein LOC90110	Homo sapiens hypothetical protein LOC	2276.77900	1730.56070	1700.57030	1124.00950	Detected	Detected	Detected	Detected
3334	A_24_P225878	KIF9	Hs.373947	kinesin family member 9	Homo sapiens kinesin family member 9	62.38912	26.94854	48.96149	12.45222	Detected	Detected	Detected	Compromised
3335	A_24_P136124	LOC100131642	Hs.680766	PP5241	Homo sapiens PP5241 mRNA, complete	19.30365	22.78182	3.48262	7.52374	Detected	Detected	Compromised	Compromised
3336	A_33_P3722568	NCRNA00107	Hs.575741	non-protein coding RNA 107	Homo sapiens non-protein coding RNA	228.31711	153.72742	115.00258	66.75295	Detected	Detected	Detected	Detected
3337	A_23_P140035	WBP4	Hs.411300	WW domain binding protein 4 (formin bir	Homo sapiens WW domain binding prote	3760.66200	4357.30400	3261.61650	3141.53980	Detected	Detected	Detected	Detected
3338	A_33_P3238157	MYO18A	Hs.462590	myosin XVIIIA	Homo sapiens myosin XVIIIA (MYO18A)	752.54920	535.76105	570.16710	349.87506	Detected	Detected	Detected	Detected
3339	A_33_P3210168	PIN1	Hs.465849	peptidylprolyl cis/trans isomerase, NIM	Homo sapiens peptidylprolyl cis/trans is	9642.41600	15489.20900	10399.96300	15217.38300	Detected	Detected	Detected	Detected
3340	A_33_P3330149	PAX6	Hs.270303	paired box 6	Homo sapiens paired box 6 (PAX6), tran	2.62064	126.24968	3.08544	82.59633	Compromised	Detected	Compromised	Detected
3341	A_33_P3346801	BMF	Hs.591104	Bcl2 modifying factor	Homo sapiens Bcl2 modifying factor (BM	7.11277	18.42336	3.08540	4.59273	Compromised	Detected	Compromised	Compromised
3342	A_33_P3278510	LOC100128402	Hs.689574	hypothetical protein LOC100128402	Homo sapiens cDNA FLJ42583 fis, clon	17.72130	15.42700	13.08799	7.02277	Detected	Compromised	Compromised	Compromised
3343	A_23_P126908	TNFRSF14	Hs.512898	tumor necrosis factor receptor superfar	Homo sapiens tumor necrosis factor rec	3789.02860	1347.75830	2289.28250	675.68300	Detected	Detected	Detected	Detected
3344	A_33_P3369829	tcag7.1307		hypothetical LOC154822	Homo sapiens hypothetical LOC154822	6.03031	18.28381	3.08385	3.20701	Compromised	Detected	Compromised	Compromised
3345	A_23_P106016	PRKD1	Hs.508999	protein kinase D1	Homo sapiens protein kinase D1 (PRKD	739.77120	1074.18090	387.87326	473.36160	Detected	Detected	Detected	Detected
3346	A_33_P3369039	PRPF31	Hs.515598	PRP31 pre-mRNA processing factor 31	Homo sapiens PRP31 pre-mRNA proce	13526.49300	13869.76800	14964.02050	13178.16200	Detected	Detected	Detected	Detected
3347	A_33_P3318971	C12orf72	Hs.585084	chromosome 12 open reading frame 72	UPF0516 protein C12orf72 [Source:Un	82.15444	38.04686	56.51485	12.66644	Detected	Detected	Detected	Compromised
3348	A_23_P364478	FAM175B	Hs.280695	family with sequence similarity 175, mer	Homo sapiens family with sequence sim	626.39124	827.56020	345.64413	325.74173	Detected	Detected	Detected	Detected
3349	A_23_P356965		Hs.631724		Coiled-coil domain-containing protein 5	487.53995	488.84790	215.22160	164.22739	Detected	Detected	Detected	Detected
3350	A_33_P3252954				Protein naked cuticle homolog 2 (Naked	18.14649	17.71265	4.82994	3.20644	Detected	Compromised	Compromised	Compromised
3351	A_23_P101281	ZNF587	Hs.719987	zinc finger protein 587	Homo sapiens zinc finger protein 587 (Z	384.41992	475.31442	331.09872	343.82970	Detected	Detected	Detected	Detected
3352	A_24_P147461	SERPINB8	Hs.368077	serpin peptidase inhibitor, clade B (ova	Homo sapiens serpin peptidase inhibitor	317.75300	638.06360	351.91882	579.22590	Detected	Detected	Detected	Detected
3353	A_33_P3398526	BCL2L11	Hs.469658	BCL2-like 11 (apoptosis facilitator)	Homo sapiens BCL2-like 11 (apoptosis	483.31775	537.67975	173.81056	127.45330	Detected	Detected	Detected	Detected
3354	A_33_P3308626	MON1B	Hs.513743	MON1 homolog B (yeast)	Homo sapiens MON1 homolog B (yeast)	10185.95000	13764.43000	5790.85740	5447.79640	Detected	Detected	Detected	Detected
3355	A_33_P3386429	SENP6	Hs.485784	SUMO1/sentrin specific peptidase 6	Homo sapiens SUMO1/sentrin specific	40.14382	11.40541	27.55107	11.72163	Detected	Compromised	Detected	Compromised
3356	A_23_P104199	ITGB1	Hs.643813	integrin, beta 1 (fibronectin receptor, be	Homo sapiens integrin, beta 1 (fibronect	7670.34130	12386.62600	6220.86130	8549.88000	Detected	Detected	Detected	Detected
3357	A_23_P206280	GPR56	Hs.513633	G protein-coupled receptor 56	Homo sapiens G protein-coupled recep	10218.61800	778.46450	7705.78900	567.35785	Detected	Detected	Detected	Detected
3358	A_23_P312851	SILV	Hs.95972	silver homolog (mouse)	Homo sapiens silver homolog (mouse) (	70.37856	22.44931	81.25911	28.98480	Detected	Detected	Detected	Detected
3359	A_23_P158053	C9orf16	Hs.522412	chromosome 9 open reading frame 16	Homo sapiens chromosome 9 open read	1194.02610	494.19592	1403.99050	554.98114	Detected	Detected	Detected	Detected
3360	A_23_P104762	YAP1	Hs.503692	Yes-associated protein 1, 65kDa	Homo sapiens Yes-associated protein 1	933.40027	1764.13610	880.37524	1514.33870	Detected	Detected	Detected	Detected
3361	A_33_P3248052	NLGN4Y	Hs.439199	neuroligin 4, Y-linked	Homo sapiens neuroligin 4, Y-linked (NL	114.60700	3.77633	98.18084	3.28599	Detected	Compromised	Detected	Compromised
3362	A_33_P3228460	FXYD3	Hs.301350	FXYD domain containing ion transport r	Homo sapiens FXYD domain containing	93.05353	26.39510	87.39114	24.51946	Detected	Detected	Detected	Detected
3363	A_33_P3362781	KLHL36	Hs.578546	kelch-like 36 (Drosophila)	Homo sapiens kelch-like 36 (Drosophila	298.40080	468.60284	388.04688	603.33936	Detected	Detected	Detected	Detected
3364	A_23_P209805	NAB1	Hs.107474	NGFI-A binding protein 1 (EGR1 binding	Homo sapiens NGFI-A binding protein 1	970.44940	1447.08960	827.34796	1128.39280	Detected	Detected	Detected	Detected
3365	A_33_P3376071					97667.05500	77142.74000	77256.65000	52874.82400	Detected	Detected	Detected	Detected
3366	A_23_P56146	RAX2	Hs.532691	retina and anterior neural fold homeobo	Homo sapiens retina and anterior neura	88.09255	105.39271	15.01838	17.82400	Detected	Detected	Compromised	Detected
3367	A_23_P254978	TATDN1	Hs.170568	TatD DNase domain containing 1	Homo sapiens TatD DNase domain cont	1236.48490	1088.31040	921.30444	639.78380	Detected	Detected	Detected	Detected
3368	A_33_P3225278	LOC100128084	Hs.623860	hypothetical LOC100128084	PREDICTED: Homo sapiens hypothetica	911.63200	1278.86240	202.20236	141.80650	Detected	Detected	Detected	Detected
3369	A_23_P114057	SEMA4C	Hs.516220	sema domain, immunoglobulin domain (I	Homo sapiens sema domain, immunoglo	19549.52100	11693.64600	14257.72000	7603.88430	Detected	Detected	Detected	Detected
3370	A_23_P213369	KIAA0141	Hs.210532	KIAA0141	Homo sapiens KIAA0141 (KIAA0141), tr	505.12457	601.57590	472.68870	477.12357	Detected	Detected	Detected	Detected
3371	A_33_P3421365	ZNF169	Hs.387623	zinc finger protein 169	Homo sapiens zinc finger protein 169 (Z	192.72867	175.94590	175.67043	134.88185	Detected	Detected	Detected	Detected

3372	A_24_P281636		Hs.653188		Putative tripartite motif-containing prot	129.33751	141.14247	89.90645	77.91230	Detected	Detected	Detected	Detected
3373	A_24_P277673	HIST1H4G	Hs.553507	histone cluster 1, H4g	Homo sapiens histone cluster 1, H4g (H	11.78839	21.68811	3.34728	4.52056	Compromised	Detected	Compromised	Compromised
3374	A_33_P3424108					151.38853	128.34819	60.32774	43.66964	Detected	Detected	Detected	Detected
3375	A_24_P416257	GGA2	Hs.460336	golgi associated, gamma adaptin ear co	Homo sapiens golgi associated, gamma	1215.45600	774.58594	1041.11340	588.70180	Detected	Detected	Detected	Detected
3376	A_23_P251412	SCGN	Hs.116428	secretagogin, EF-hand calcium binding	Homo sapiens secretagogin, EF-hand ca	56.60117	25.91132	36.47100	7.15872	Detected	Detected	Detected	Compromised
3377	A_33_P3364373	KRTAP5-2	Hs.673618	keratin associated protein 5-2	Homo sapiens keratin associated protei	2289.78800	2685.83000	256.03503	224.74805	Detected	Detected	Detected	Detected
3378	A_33_P3398187	LOC100128909	Hs.535950	similar to hCG1817494	PREDICTED: Homo sapiens similar to h	25.27664	26.87233	11.23930	7.26110	Detected	Detected	Compromised	Compromised
3379	A_33_P3374035					23.43846	37.77541	6.63125	7.12557	Detected	Detected	Compromised	Compromised
3380	A_23_P104224	A1CF	Hs.499643	APOBEC1 complementation factor	Homo sapiens APOBEC1 complementat	15.27172	5.76507	27.42098	16.50308	Detected	Compromised	Detected	Detected
3381	A_32_P12639	PSMD12	Hs.592689	proteasome (prosome, macropain) 26S	Homo sapiens proteasome (prosome, m	4192.44730	8981.93000	3842.61870	6355.00500	Detected	Detected	Detected	Detected
3382	A_23_P338233	BCDIN3D	Hs.142736	BCDIN3 domain containing	Homo sapiens BCDIN3 domain containi	1794.80730	1414.93800	1642.34920	988.33700	Detected	Detected	Detected	Detected
3383	A_33_P3379939	HLA-F	Hs.519972	major histocompatibility complex, class	Homo sapiens major histocompatibility	99214.07000	38676.38300	94341.70000	31188.24400	Detected	Detected	Detected	Detected
3384	A_23_P69362	CCDC51	Hs.187657	coiled-coil domain containing 51	Homo sapiens coiled-coil domain conta	1917.84640	1632.29240	1964.34510	1352.84340	Detected	Detected	Detected	Detected
3385	A_33_P3232995	TDRD1	Hs.333132	tudor domain containing 1	Homo sapiens tudor domain containing	52.20979	12.63799	37.70708	3.55891	Detected	Compromised	Detected	Compromised
3386	A_23_P53126	LMO2	Hs.34560	LIM domain only 2 (rhombotin-like 1)	Homo sapiens LIM domain only 2 (rhom	93.84444	357.18740	61.91695	213.24445	Detected	Detected	Detected	Detected
3387	A_24_P334456	ADAMTS6	Hs.482291	ADAM metallopeptidase with thrombosp	Homo sapiens ADAM metallopeptidase	9.31852	31.09095	5.58943	23.14423	Compromised	Detected	Compromised	Detected
3388	A_23_P68730	PDXK	Hs.284491	pyridoxal (pyridoxine, vitamin B6) kinase	Homo sapiens pyridoxal (pyridoxine, vita	8882.55700	5475.35740	8375.37800	4664.09570	Detected	Detected	Detected	Detected
3389	A_33_P3317580	GFRA2	Hs.441202	GDNF family receptor alpha 2	Homo sapiens GDNF family receptor alp	114.49471	129.94649	74.30718	66.79653	Detected	Detected	Detected	Detected
3390	A_24_P119577	PHACTR4	Hs.225641	phosphatase and actin regulator 4	Homo sapiens phosphatase and actin re	664.88050	766.37130	449.49982	415.14392	Detected	Detected	Detected	Detected
3391	A_33_P3227703					90.41065	123.35370	71.73908	66.81243	Detected	Detected	Detected	Detected
3392	A_23_P48481	RAD51L1	Hs.172587	RAD51-like 1 (S. cerevisiae)	Homo sapiens RAD51-like 1 (S. cerevis	126.26617	140.90807	106.33621	106.55779	Detected	Detected	Detected	Detected
3393	A_33_P3342780	ADAMTS13	Hs.131433	ADAM metallopeptidase with thrombosp	Homo sapiens ADAM metallopeptidase	48.36455	45.73002	12.59919	7.96360	Detected	Detected	Compromised	Compromised
3394	A_24_P372932	PEX5	Hs.567327	peroxisomal biogenesis factor 5	Homo sapiens peroxisomal biogenesis fa	425.88570	222.11120	371.94073	203.65620	Detected	Detected	Detected	Detected
3395	A_23_P111088	OR2B2	Hs.307111	olfactory receptor, family 2, subfamily B	Homo sapiens olfactory receptor, family	34.00925	47.58770	18.24934	22.87111	Detected	Detected	Detected	Detected
3396	A_33_P3249354	CCDC99	Hs.368710	coiled-coil domain containing 99	Homo sapiens coiled-coil domain conta	682.93640	1673.94430	550.39636	1170.66170	Detected	Detected	Detected	Detected
3397	A_23_P402936	PPFIA2	Hs.506216	protein tyrosine phosphatase, receptor	Homo sapiens protein tyrosine phospha	70.73332	7.06043	71.99606	3.66959	Detected	Compromised	Detected	Compromised
3398	A_24_P178175	GGT1	Hs.645535	gamma-glutamyltransferase 1	Homo sapiens gamma-glutamyltransfera	1105.55540	1329.60970	435.68405	410.06192	Detected	Detected	Detected	Detected
3399	A_32_P80089	LOC283788	Hs.657131	FSHD region gene 1 pseudogene	Homo sapiens FSHD region gene 1 pseu	6973.88100	5000.89800	4368.16800	3034.30640	Detected	Detected	Detected	Detected
3400	A_23_P20980	CYC1	Hs.289271	cytochrome c-1	Homo sapiens cytochrome c-1 (CYC1),	5709.61770	7831.41700	6874.61230	8559.44200	Detected	Detected	Detected	Detected
3401	A_33_P3372859	DDX54	Hs.506861	DEAD (Asp-Glu-Ala-Asp) box polypept	Homo sapiens DEAD (Asp-Glu-Ala-Asp	411.68338	554.87695	243.27489	318.55300	Detected	Detected	Detected	Detected
3402	A_24_P350622	KIR2DL4	Hs.661219	killer cell immunoglobulin-like receptor,	Homo sapiens killer cell immunoglobulin	39.58674	54.48058	36.17704	32.20427	Detected	Detected	Detected	Detected
3403	A_33_P3378654		Hs.706372		Putative uncharacterized protein FLJ31	384.45193	392.77972	168.61680	164.84212	Detected	Detected	Detected	Detected
3404	A_23_P99027	PTPN11	Hs.506852	protein tyrosine phosphatase, non-rece	Homo sapiens protein tyrosine phospha	8076.47100	5978.89800	5998.12900	3890.84060	Detected	Detected	Detected	Detected
3405	A_23_P59418	NRF1	Hs.654363	nuclear respiratory factor 1	Homo sapiens nuclear respiratory facto	94.79867	193.44331	80.19270	157.55557	Detected	Detected	Detected	Detected
3406	A_33_P3229122	HIST1H2BF	Hs.182137	histone cluster 1, H2bf	Homo sapiens histone cluster 1, H2bf (H	3.49279	18.47677	3.07647	15.05870	Compromised	Detected	Compromised	Compromised
3407	A_33_P3388222	hCG_1999814	Hs.666511	hCG1999814	Homo sapiens cDNA FLJ32758 fis, clon	13.43120	16.45344	5.80789	6.51248	Detected	Compromised	Compromised	Compromised
3408	A_33_P3298634	NFS1	Hs.194692	NFS1 nitrogen fixation 1 homolog (S. ce	Homo sapiens NFS1 nitrogen fixation 1	107.45822	53.27097	100.38499	51.49176	Detected	Detected	Detected	Detected
3409	A_33_P3394352	VPS53	Hs.461819	vacuolar protein sorting 53 homolog (S.	Homo sapiens vacuolar protein sorting	114.93372	161.78960	25.34364	30.17225	Detected	Detected	Detected	Detected
3410	A_23_P253464	FAM175A	Hs.334772	family with sequence similarity 175, me	Homo sapiens family with sequence sim	545.28550	526.08325	376.20422	293.98180	Detected	Detected	Detected	Detected
3411	A_24_P281395	LOC642521		similar to nuclear DNA-binding protein	PREDICTED: Homo sapiens similar to n	359.40550	853.44025	195.81113	296.01550	Detected	Detected	Detected	Detected
3412	A_33_P3304824					39.37814	68.44783	31.48504	49.80347	Detected	Detected	Detected	Detected
3413	A_23_P13740	NAV3	Hs.655301	neuron navigator 3	Homo sapiens neuron navigator 3 (NAV	1286.13290	2973.10570	1098.14370	2310.58080	Detected	Detected	Detected	Detected
3414	A_33_P3320403	LOC100128528	Hs.711978	hypothetical protein LOC100128528	CDNA FLJ26814 fis, clone PRS06344	547.24160	684.28845	115.94540	161.82500	Detected	Detected	Detected	Detected
3415	A_33_P3319140	OVOS2	Hs.524331	ovostatin 2	Homo sapiens ovostatin 2, mRNA (cDN	73.18561	9.42977	53.39385	9.92642	Detected	Compromised	Detected	Compromised
3416	A_33_P3288334					18.95079	20.14965	12.23504	12.17700	Detected	Detected	Compromised	Compromised
3417	A_32_P207243	LOC100134253	Hs.689001	hypothetical protein LOC100134253	PREDICTED: Homo sapiens hypothetica	309.98822	348.03568	183.95647	160.75210	Detected	Detected	Detected	Detected
3418	A_23_P431789	ARF4	Hs.652183	ADP-ribosylation factor 4	Homo sapiens ADP-ribosylation factor	21377.59600	46357.74000	12916.14550	21784.02700	Detected	Detected	Detected	Detected
3419	A_33_P3334220	ACACB	Hs.234898	acetyl-Coenzyme A carboxylase beta	Homo sapiens acetyl-Coenzyme A carb	564.99210	65.09295	436.61673	45.58112	Detected	Detected	Detected	Detected
3420	A_23_P130064	SNF8	Hs.127249	SNF8, ESCRT-II complex subunit, homo	Homo sapiens SNF8, ESCRT-II complex	16228.34200	19845.70100	21351.93400	21775.72000	Detected	Detected	Detected	Detected
3421	A_33_P3255631	ZNF780A	Hs.115140	zinc finger protein 780A	Homo sapiens zinc finger protein 780A	166.03130	141.88387	186.30585	135.74242	Detected	Detected	Detected	Detected
3422	A_33_P3264269	MOV10	Hs.514941	Mov10, Moloney leukemia virus 10, hom	Homo sapiens Mov10, Moloney leukemia	58.01872	59.27392	45.00548	43.66569	Detected	Detected	Detected	Detected
3423	A_23_P402733	SON	Hs.517262	SON DNA binding protein	Homo sapiens SON DNA binding protein	504.47020	632.52405	468.58057	498.64038	Detected	Detected	Detected	Detected
3424	A_24_P286898	MAPK8	Hs.522924	mitogen-activated protein kinase 8	Mitogen-activated protein kinase 8 (EC	183.55780	155.06883	102.30268	67.93449	Detected	Detected	Detected	Detected
3425	A_32_P52816	TM7SF3	Hs.438641	transmembrane 7 superfamily member 3	Homo sapiens transmembrane 7 superfa	213.72850	439.85895	131.82759	251.26300	Detected	Detected	Detected	Detected
3426	A_23_P313512	DCP1B	Hs.130934	DCP1 decapping enzyme homolog B (S.	Homo sapiens DCP1 decapping enzyme	5258.56540	4461.10450	5133.18260	3679.13280	Detected	Detected	Detected	Detected
3427	A_23_P65983	CCDC102A	Hs.644611	coiled-coil domain containing 102A	Homo sapiens coiled-coil domain conta	2185.53400	880.05290	2180.18200	800.56860	Detected	Detected	Detected	Detected
3428	A_23_P71790	MAMDC4	Hs.376780	MAM domain containing 4	Homo sapiens MAM domain containing	102.62872	76.34545	63.96878	42.00723	Detected	Detected	Detected	Detected
3429	A_24_P284893	PSMB2	Hs.471441	proteasome (prosome, macropain) subu	Homo sapiens proteasome (prosome, m	4480.11800	8321.88500	4509.67970	6714.56640	Detected	Detected	Detected	Detected
3430	A_24_P302998	ATP5I	Hs.85539	ATP synthase, H+ transporting, mitocho	Homo sapiens ATP synthase, H+ transp	22608.88900	25095.34800	21405.43800	18460.96900	Detected	Detected	Detected	Detected
3431	A_24_P16815	ZMYM3	Hs.522684	zinc finger, MYM-type 3	Homo sapiens zinc finger, MYM-type 3	1083.84410	852.69250	1199.98670	828.02690	Detected	Detected	Detected	Detected
3432	A_24_P71649	CNTN3	Hs.12723	contactin 3 (plasmacytoma associated)	Homo sapiens contactin 3 (plasmacyto	169.10806	166.17046	125.36839	95.48235	Detected	Detected	Detected	Detected
3433	A_23_P35309	TAF5L	Hs.270621	TAF5-like RNA polymerase II, p300/CB	Homo sapiens TAF5-like RNA polymera	369.04202	478.94434	284.77768	305.28363	Detected	Detected	Detected	Detected
3434	A_33_P3400171	AKT2	Hs.631535	v-akt murine thymoma viral oncogene h	Homo sapiens v-akt murine thymoma v	121.02948	179.78935	149.86840	207.85956	Detected	Detected	Detected	Detected
3435	A_23_P115022	TMEM125	Hs.104476	transmembrane protein 125	Homo sapiens transmembrane protein 1	22.70938	34.21439	17.14432	17.91027	Detected	Detected	Detected	Detected
3436	A_24_P152404		Hs.16004		UPF0668 protein C10orf76 [Source:Un	1521.51310	1661.90340	1368.24950	1310.38660	Detected	Detected	Detected	Detected
3437	A_24_P945228	CYP4V2	Hs.720053	cytochrome P450, family 4, subfamily V	Homo sapiens cytochrome P450, family	67.38983	64.00000	42.90029	30.50212	Detected	Detected	Detected	Detected
3438	A_33_P3323699				Atrial natriuretic factor Precursor (ANF	447.33210	559.54706	210.15106	142.74720	Detected	Detected	Detected	Detected
3439	A_23_P112478	NUDT2	Hs.493767	nudix (nucleoside diphosphate linked m	Homo sapiens nudix (nucleoside diphos	2336.83180	2538.80640	2194.20200	2064.61330	Detected	Detected	Detected	Detected
3440	A_23_P209904	GPC1	Hs.328232	glypican 1	Homo sapiens glypican 1 (GPC1), mRNA	9630.09700	31134.83200	9392.59200	31278.53100	Detected	Detected	Detected	Detected
3441	A_23_P166566	CCDC48	Hs.134807	coiled-coil domain containing 48	Homo sapiens coiled-coil domain conta	54.66911	56.04485	18.39978	3.16809	Detected	Detected	Detected	Compromised
3442	A_23_P131375	PQLC3	Hs.274415	PQ loop repeat containing 3	Homo sapiens PQ loop repeat containin	2300.94340	1120.47680	1956.06770	771.18690	Detected	Detected	Detected	Detected
3443	A_33_P3271652	HHIPL1	Hs.288522	HHIP-like 1	Homo sapiens HHIP-like 1 (HHIPL1), tr	6.16106	41.05143	6.16175	32.20711	Compromised	Detected	Compromised	Detected
3444	A_33_P3301559	CLUAP1	Hs.155995	clusterin associated protein 1	Homo sapiens clusterin associated prot	1067.74990	933.85016	863.12610	639.26980	Detected	Detected	Detected	Detected
3445	A_24_P319923	MYLK	Hs.477375	myosin light chain kinase	Homo sapiens myosin light chain kinase	1076.15450	3712.11720	786.66473	2435.52510	Detected	Detected	Detected	Detected
3446	A_23_P7732	CETN3	Hs.591767	centrin, EF-hand protein, 3 (CDC31 hor	Homo sapiens centrin, EF-hand protein,	1152.42180	1076.25210	751.49930	579.53270	Detected	Detected	Detected	Detected

3447	A_23_P107116	RNF112	Hs.189482	ring finger protein 112	Homo sapiens ring finger protein 112 (R	3522.35550	346.45276	2656.85990	223.03967	Detected	Detected	Detected	Detected
3448	A_33_P3224730		Hs.32194		cDNA FLJ23816 fis, clone HSI02685HC	180.25728	120.34463	110.04931	47.16255	Detected	Detected	Detected	Detected
3449	A_33_P3407414	LOC92249	Hs.708316	hypothetical LOC92249	Homo sapiens hypothetical LOC92249 (	91.92116	118.36113	83.40850	89.86390	Detected	Detected	Detected	Detected
3450	A_33_P3388183		Hs.529517		Homo sapiens cDNA FLJ44249 fis, clon	29.81422	35.55904	3.07594	3.20558	Detected	Detected	Compromised	Compromised
3451	A_24_P246777	LOC100132658	Hs.711160	similar to TGF beta-inducible nuclear p	PREDICTED: Homo sapiens similar to T	337.65665	278.32108	6.82318	9.27809	Detected	Detected	Compromised	Compromised
3452	A_33_P3239152	RABEP2	Hs.555978	rabaptin, RAB GTPase binding effector	Homo sapiens rabaptin, RAB GTPase bi	4558.49760	11647.59800	3990.33620	10127.57200	Detected	Detected	Detected	Detected
3453	A_33_P3237393	ZNF140	Hs.181552	zinc finger protein 140	Homo sapiens zinc finger protein 140 (Z	4.56436	18.67969	3.07390	3.38087	Compromised	Detected	Compromised	Compromised
3454	A_33_P3245011	DAK	Hs.6278	dihydroxyacetone kinase 2 homolog (S.	Homo sapiens dihydroxyacetone kinase	4872.65100	3661.47000	4633.94530	3313.76340	Detected	Detected	Detected	Detected
3455	A_24_P101101					15139.13300	23590.97700	9848.14000	13118.33900	Detected	Detected	Detected	Detected
3456	A_32_P201979	HECTD2	Hs.596096	HECT domain containing 2	Homo sapiens HECT domain containing	674.49774	336.14290	453.95470	185.15290	Detected	Detected	Detected	Detected
3457	A_33_P3223577	LOC283104		hypothetical LOC283104	PREDICTED: Homo sapiens hypothetica	21.12083	33.30550	6.39395	10.25923	Detected	Detected	Compromised	Compromised
3458	A_23_P102681		Hs.280135		HCG1785561MGC4294 protein ; [Sourc	90.13903	135.04851	94.29453	119.63454	Detected	Detected	Detected	Detected
3459	A_33_P3209950		Hs.99491		RAS guanyl-releasing protein 2 (Calciu	110.22422	197.17998	114.24246	109.78338	Detected	Detected	Detected	Detected
3460	A_33_P3415820	THBS1	Hs.164226	thrombospondin 1	Homo sapiens thrombospondin 1 (THBS	415.86053	3910.35400	451.28854	4125.84330	Detected	Detected	Detected	Detected
3461	A_23_P133359	ZFP2	Hs.654533	zinc finger protein 2 homolog (mouse)	Homo sapiens zinc finger protein 2 hom	196.07133	32.65834	163.67316	17.92992	Detected	Detected	Detected	Detected
3462	A_33_P3273290	ZNF28	Hs.554778	zinc finger protein 28	Homo sapiens zinc finger protein 28, mF	635.26740	423.01794	355.53867	173.96896	Detected	Detected	Detected	Detected
3463	A_33_P3673310					39.38107	36.44084	23.86037	17.20401	Detected	Detected	Detected	Detected
3464	A_23_P161125	MOV10	Hs.514941	Mov10, Moloney leukemia virus 10, hom	Homo sapiens Mov10, Moloney leukemia	588.64453	635.90770	507.96338	499.72370	Detected	Detected	Detected	Detected
3465	A_33_P3245679		Hs.685856		Putative uncharacterized protein ENSP	237.14319	279.56168	178.50826	178.35480	Detected	Detected	Detected	Detected
3466	A_23_P72912	SLC4A8	Hs.370636	solute carrier family 4, sodium bicarbon	Homo sapiens solute carrier family 4, so	15.49214	15.80152	9.01963	8.30706	Detected	Compromised	Compromised	Compromised
3467	A_33_P3334248		Hs.247186		Probable fibrosin-1 long transcript prot	51.17283	50.44029	43.92687	44.44549	Detected	Detected	Detected	Detected
3468	A_23_P344408	CSHL1	Hs.655225	chorionic somatomammotropin hormone	Homo sapiens chorionic somatomammo	154.33319	236.36668	40.67241	47.58835	Detected	Detected	Detected	Detected
3469	A_24_P144439	EFHB	Hs.670883	EF-hand domain family, member B	Homo sapiens EF-hand domain family, r	16.02420	3.73103	15.17499	3.24624	Detected	Compromised	Compromised	Compromised
3470	A_23_P216596	SVEP1	Hs.522334	sushi, von Willebrand factor type A, EGF	Homo sapiens sushi, von Willebrand fact	904.60315	641.37897	747.00165	416.55716	Detected	Detected	Detected	Detected
3471	A_33_P3420747	YTHDC2	Hs.231942	YTH domain containing 2	Homo sapiens YTH domain containing 2	1229.02540	677.20380	740.49870	331.33847	Detected	Detected	Detected	Detected
3472	A_23_P73142	TACO1	Hs.174134	translational activator of mitochondrially	Homo sapiens translational activator of	1302.77750	2209.53150	1157.97020	1680.29910	Detected	Detected	Detected	Detected
3473	A_23_P406438	SRPK2	Hs.285197	SFRS protein kinase 2	Homo sapiens SFRS protein kinase 2 (S	1486.52930	1755.12040	831.47864	781.24460	Detected	Detected	Detected	Detected
3474	A_23_P91930	QTRTD1	Hs.477162	queuine tRNA-ribosyltransferase domai	Homo sapiens queuine tRNA-ribosyltrar	1213.52440	1791.00050	727.22504	931.01056	Detected	Detected	Detected	Detected
3475	A_23_P112103	GSDMD	Hs.118983	gasdermin D	Homo sapiens gasdermin D (GSDMD), t	257.10727	153.29889	337.75775	205.04655	Detected	Detected	Detected	Detected
3476	A_33_P3312730	BHLHA15	Hs.674510	basic helix-loop-helix family, member a	Homo sapiens basic helix-loop-helix far	36.44653	27.90533	25.44523	21.47941	Detected	Detected	Detected	Detected
3477	A_24_P208567	IL18R1	Hs.469521	interleukin 18 receptor 1	Homo sapiens interleukin 18 receptor 1	2480.25510	66.95126	1807.45400	35.27838	Detected	Detected	Detected	Detected
3478	A_23_P215778	DUS4L	Hs.97627	dihydrouridine synthase 4-like (S. cerev	Homo sapiens dihydrouridine synthase 4	367.48016	268.62726	293.28845	188.74582	Detected	Detected	Detected	Detected
3479	A_33_P3436646	LOC151657	Hs.587187	hypothetical protein LOC151657	Homo sapiens cDNA FLJ33795 fis, clon	35.25608	8.76835	19.69265	8.93090	Detected	Compromised	Detected	Compromised
3480	A_33_P3421626	KIAA1147	Hs.521240	KIAA1147	Homo sapiens KIAA1147 (KIAA1147), m	926.03400	260.06198	502.30463	109.59837	Detected	Detected	Detected	Detected
3481	A_23_P395172	ABHD2	Hs.122337	abhydrolase domain containing 2	Homo sapiens abhydrolase domain cont	1163.03260	1412.93970	901.18445	957.46387	Detected	Detected	Detected	Detected
3482	A_24_P475864	LOC653390	Hs.720348	RRN3 RNA polymerase I transcription fa	Homo sapiens RRN3 RNA polymerase I	18.60097	24.95473	19.54440	17.88165	Detected	Detected	Detected	Detected
3483	A_33_P3270445	BAI2	Hs.524138	brain-specific angiogenesis inhibitor 2	Homo sapiens brain-specific angiogenes	472.03265	100.92283	417.83597	88.56529	Detected	Detected	Detected	Detected
3484	A_23_P59877	FABP5	Hs.408061	fatty acid binding protein 5 (psoriasis-a	Homo sapiens fatty acid binding protein	6713.36400	11506.07800	4670.83450	6433.40670	Detected	Detected	Detected	Detected
3485	A_23_P34375	TCEA3	Hs.446354	transcription elongation factor A (SII), 3	Homo sapiens transcription elongation f	944.67584	740.93353	906.01130	556.42290	Detected	Detected	Detected	Detected
3486	A_33_P3284711	NCRNA00095	Hs.711535	non-protein coding RNA 95	Homo sapiens non-protein coding RNA	329.44165	375.82343	218.62044	212.80095	Detected	Detected	Detected	Detected
3487	A_24_P260639	HIST1H1D	Hs.136857	histone cluster 1, H1d	Homo sapiens histone cluster 1, H1d (H	408.88016	1345.15490	419.31656	1024.28980	Detected	Detected	Detected	Detected
3488	A_23_P252052	FILIP1L	Hs.104672	filamin A interacting protein 1-like	Homo sapiens filamin A interacting prot	564.97160	727.73140	473.65646	560.57666	Detected	Detected	Detected	Detected
3489	A_33_P3343210	SLC25A37	Hs.716436	solute carrier family 25, member 37	Homo sapiens solute carrier family 25, r	1401.65730	903.73413	1073.36670	468.68280	Detected	Detected	Detected	Detected
3490	A_33_P3254375	LOC730651	Hs.662603	similar to hCG2040227	PREDICTED: Homo sapiens similar to h	34.79230	38.54320	18.54798	8.73496	Detected	Detected	Detected	Compromised
3491	A_24_P237511	EIF1AY	Hs.461178	eukaryotic translation initiation factor 1	Homo sapiens eukaryotic translation ini	297.78046	4.11110	239.38290	3.58643	Detected	Compromised	Detected	Compromised
3492	A_23_P151297	TENC1	Hs.6147	tensin like C1 domain containing phosph	Homo sapiens tensin like C1 domain co	2127.41650	1216.80580	1970.89390	1000.07850	Detected	Detected	Detected	Detected
3493	A_33_P3321342	INSIG2	Hs.7089	insulin induced gene 2	Homo sapiens insulin induced gene 2 (IN	1174.80690	749.01660	1075.99300	582.46040	Detected	Detected	Detected	Detected
3494	A_23_P66827	FAM106A	Hs.674403	family with sequence similarity 106, mer	Homo sapiens family with sequence sim	17.14978	4.11035	12.76762	4.42336	Detected	Compromised	Compromised	Compromised
3495	A_33_P3251148	TSPO	Hs.202	translocator protein (18kDa)	Homo sapiens translocator protein (18k	97258.73000	148750.72000	87694.83000	109202.23400	Detected	Detected	Detected	Detected
3496	A_33_P3263603	LOC100130600	Hs.721358	similar to hCG2014367	PREDICTED: Homo sapiens similar to h	512.12420	355.00555	187.72015	131.91634	Detected	Detected	Detected	Detected
3497	A_32_P75425	LOC100133161	Hs.531400	hypothetical LOC100133161	Homo sapiens hypothetical LOC100133	725.76940	510.82220	450.82550	290.07587	Detected	Detected	Detected	Detected
3498	A_33_P3335401	LOC100128598	Hs.713353	hypothetical LOC100128598	PREDICTED: Homo sapiens hypothetica	20.18642	27.17982	20.56330	15.99071	Detected	Detected	Detected	Compromised
3499	A_33_P3230017	AURKAPS1		aurora kinase A pseudogene 1	Homo sapiens aurora kinase A pseudog	151.15030	2322.68630	104.20519	1115.05490	Detected	Detected	Detected	Detected
3500	A_33_P3254831					95.88637	104.69943	52.71114	36.98898	Detected	Detected	Detected	Detected
3501	A_23_P30474	WDR70	Hs.213690	WD repeat domain 70	Homo sapiens WD repeat domain 70 (W	3132.16720	2754.72100	2592.58180	1915.53300	Detected	Detected	Detected	Detected
3502	A_23_P375147	RC3H2	Hs.709775	ring finger and CCCH-type zinc finger d	Ring finger and CCCH-type zinc finger	2160.22700	1124.74230	1513.71020	630.32227	Detected	Detected	Detected	Detected
3503	A_33_P3306018		Hs.689480		Putative uncharacterized protein FLJ45	2.55258	19.84448	3.02145	3.16950	Compromised	Detected	Compromised	Compromised
3504	A_33_P3265270	SLC17A5	Hs.597422	solute carrier family 17 (anion/sugar tra	Homo sapiens solute carrier family 17 (a	163.57640	316.66010	151.33180	209.44745	Detected	Detected	Detected	Detected
3505	A_33_P3405946	RBM39	Hs.282901	RNA binding motif protein 39	Homo sapiens RNA binding motif protei	7483.25200	5473.94240	6450.27830	4231.19140	Detected	Detected	Detected	Detected
3506	A_23_P74042	LPHN2	Hs.24212	latrophilin 2	Homo sapiens latrophilin 2 (LPHN2), mR	530.42377	270.98343	513.01330	204.61084	Detected	Detected	Detected	Detected
3507	A_24_P929369	AP4E1	Hs.413366	adaptor-related protein complex 4, epsi	Homo sapiens adaptor-related protein c	2598.95120	3831.92190	2034.79580	2587.42300	Detected	Detected	Detected	Detected
3508	A_23_P39517	SRBD1	Hs.14229	S1 RNA binding domain 1	Homo sapiens S1 RNA binding domain 1	259.55588	196.86063	187.93115	137.39600	Detected	Detected	Detected	Detected
3509	A_32_P2738	TCTN3	Hs.438991	tectonic family member 3	Homo sapiens tectonic family member 3	9325.55300	7698.07100	7522.01500	5490.71040	Detected	Detected	Detected	Detected
3510	A_33_P3273552	KRT83	Hs.720768	keratin 83	Homo sapiens keratin 83 (KRT83), mRN	36.98336	93.52235	25.46344	70.26061	Detected	Detected	Detected	Detected
3511	A_24_P116017	PSMD9	Hs.131151	proteasome (prosome, macropain) 26S s	Homo sapiens proteasome (prosome, m	157.24770	337.60420	154.82465	287.15590	Detected	Detected	Detected	Detected
3512	A_23_P414273	C5orf62	Hs.29444	chromosome 5 open reading frame 62	Homo sapiens chromosome 5 open read	36171.25800	49710.96000	21015.23600	24255.84600	Detected	Detected	Detected	Detected
3513	A_23_P67162	PIN1	Hs.465849	peptidylprolyl cis/trans isomerase, NIMA	Homo sapiens peptidylprolyl cis/trans is	4332.78660	6734.06640	3687.86180	5386.82600	Detected	Detected	Detected	Detected
3514	A_33_P3345108	LOC647055	Hs.710912	similar to hCG1790759	PREDICTED: Homo sapiens similar to h	33.77455	56.80375	27.56506	33.70466	Detected	Detected	Detected	Detected
3515	A_23_P202104	PPIF	Hs.381072	peptidylprolyl isomerase F	Homo sapiens peptidylprolyl isomerase	17602.96500	34932.34000	14306.02800	25539.40200	Detected	Detected	Detected	Detected
3516	A_33_P3305467	LOC400406	Hs.714167	hypothetical gene supported by NM_014	PREDICTED: Homo sapiens similar to C	1006.31080	821.77850	1071.97410	804.04720	Detected	Detected	Detected	Detected
3517	A_33_P3365750	EML5	Hs.558671	echinoderm microtubule associated pro	Homo sapiens echinoderm microtubule	42.74799	3.63289	22.00829	3.18511	Detected	Compromised	Detected	Compromised
3518	A_32_P219279	ELFN2	Hs.660812	extracellular leucine-rich repeat and fib	Homo sapiens extracellular leucine-rich	137.20078	105.24229	107.57069	73.41871	Detected	Detected	Detected	Detected
3519	A_23_P33856	STAG2	Hs.496710	stromal antigen 2	Homo sapiens stromal antigen 2 (STAG	2688.09670	2431.27860	1580.13960	1103.11060	Detected	Detected	Detected	Detected
3520	A_24_P374652	NUCKS1	Hs.719942	nuclear casein kinase and cyclin-depen	Homo sapiens nuclear casein kinase an	1595.00800	2085.41140	1026.20970	1084.02260	Detected	Detected	Detected	Detected
3521	A_33_P3409886	VAMP2	Hs.25348	vesicle-associated membrane protein 2	Homo sapiens vesicle-associated memb	934.78400	1231.55540	674.70374	643.99170	Detected	Detected	Detected	Detected

3522	A_23_P121459	THPO	Hs.1166	thrombopoietin	Homo sapiens thrombopoietin (THPO),	4.58897	3.31517	4.46823	29.13180	Compromised	Compromised	Compromised	Not Detected
3523	A_33_P3310512				Putative uncharacterized protein ENSP	177.15059	319.79126	60.62751	68.67412	Detected	Detected	Detected	Detected
3524	A_24_P390070	SFRS9	Hs.706889	splicing factor, arginine/serine-rich 9	Homo sapiens splicing factor, arginine/s	5376.21100	7944.55900	5688.13700	7367.19140	Detected	Detected	Detected	Detected
3525	A_24_P113287	ZNF229	Hs.709348	zinc finger protein 229	Homo sapiens zinc finger protein 229 (Z	13.97551	8.44306	3.12314	3.24453	Detected	Compromised	Compromised	Compromised
3526	A_33_P3396562					24.54587	35.85000	10.42205	15.46231	Detected	Detected	Compromised	Compromised
3527	A_23_P82249	ABCB8	Hs.647118	ATP-binding cassette, sub-family B (MI	Homo sapiens ATP-binding cassette, su	51.70463	85.81496	69.09673	105.34900	Detected	Detected	Detected	Detected
3528	A_23_P983	PRDX6	Hs.120	peroxiredoxin 6	Homo sapiens peroxiredoxin 6 (PRDX6)	50095.90000	53265.45300	38368.16000	33791.13700	Detected	Detected	Detected	Detected
3529	A_33_P3386237	LOC100132529	Hs.710296	hypothetical LOC100132529	PREDICTED: Homo sapiens hypothetica	130.81581	62.78484	116.78588	44.14994	Detected	Detected	Detected	Detected
3530	A_24_P641130	BAT2	Hs.436093	HLA-B associated transcript 2	Homo sapiens HLA-B associated trans	519.94147	639.66670	427.22195	506.11860	Detected	Detected	Detected	Detected
3531	A_33_P3330179	LOC100131372	Hs.704272	hypothetical LOC100131372	PREDICTED: Homo sapiens hypothetica	20.77679	18.24654	3.21512	3.34173	Detected	Compromised	Compromised	Compromised
3532	A_33_P3327587	LOC100127904	Hs.632266	hypothetical LOC100127904	PREDICTED: Homo sapiens hypothetica	317.74900	173.73337	55.11639	18.77859	Detected	Detected	Detected	Detected
3533	A_33_P3235034		Hs.675838		Putative uncharacterized protein [Sour	31.18600	121.61682	40.24796	95.73933	Detected	Detected	Detected	Detected
3534	A_23_P47614	PHLDA2	Hs.154036	pleckstrin homology-like domain, family	Homo sapiens pleckstrin homology-like	13875.65400	16594.16800	10634.79000	9816.64900	Detected	Detected	Detected	Detected
3535	A_23_P71037	IL6	Hs.654458	interleukin 6 (interferon, beta 2)	Homo sapiens interleukin 6 (interferon,	2241.15360	956.33417	1901.17970	704.10190	Detected	Detected	Detected	Detected
3536	A_23_P376088	LIME1	Hs.233220	Lck interacting transmembrane adaptor	Homo sapiens Lck interacting transmem	477.59943	422.48110	282.52316	195.22855	Detected	Detected	Detected	Detected
3537	A_23_P106617	WFDC1	Hs.36688	WAP four-disulfide core domain 1	Homo sapiens WAP four-disulfide core	14.54028	211.41685	13.83263	162.06807	Detected	Detected	Detected	Detected
3538	A_33_P3362498	ATR	Hs.271791	ataxia telangiectasia and Rad3 related	Homo sapiens ataxia telangiectasia and	251.81055	198.54736	240.35036	179.96730	Detected	Detected	Detected	Detected
3539	A_33_P3355040	MGC21881	Hs.459704	hypothetical locus MGC21881	zx56g04.r1 Soares_fetal_liver_spleen_1NF	286.07500	72.10458	183.35406	33.13226	Detected	Detected	Detected	Detected
3540	A_33_P3263027		Hs.611445		Novel protein [Source:UniProtKB/TrEM	91.03259	106.63671	51.93793	50.90483	Detected	Detected	Detected	Detected
3541	A_33_P3393582	CCNDBP1		cyclin D-type binding-protein 1	Homo sapiens cDNA FLJ46628 fis, clon	64.24725	57.33639	53.55763	38.84863	Detected	Detected	Detected	Detected
3542	A_32_P119197	TPM3	Hs.644306	tropomyosin 3	Homo sapiens tropomyosin 3 (TPM3), tr	138.41902	310.49130	96.61615	190.53928	Detected	Detected	Detected	Detected
3543	A_24_P78556	RASSF8	Hs.696433	Ras association (RalGDS/AF-6) domain	Homo sapiens Ras association (RalGDS	75.75031	58.82087	82.08059	42.07400	Detected	Detected	Detected	Detected
3544	A_33_P3247190	CC2D1B	Hs.591451	coiled-coil and C2 domain containing 1E	Homo sapiens coiled-coil and C2 domai	548.46783	524.10236	617.49950	564.16030	Detected	Detected	Detected	Detected
3545	A_33_P3288942	FAM107B	Hs.446315	family with sequence similarity 107, mer	Homo sapiens family with sequence sim	253.25677	527.55010	328.20877	566.63745	Detected	Detected	Detected	Detected
3546	A_24_P392060	C5orf25	Hs.519783	chromosome 5 open reading frame 25	Homo sapiens chromosome 5 open read	531.61230	726.07556	303.13934	365.03003	Detected	Detected	Detected	Detected
3547	A_24_P132039	RNF14	Hs.483616	ring finger protein 14	Homo sapiens ring finger protein 14 (RN	837.76320	1082.35000	709.15270	813.07196	Detected	Detected	Detected	Detected
3548	A_33_P3325306				10 kDa heat shock protein, mitochondri	25162.37300	28160.28000	19756.68000	18619.70500	Detected	Detected	Detected	Detected
3549	A_23_P62659	PPT1	Hs.3873	palmitoyl-protein thioesterase 1	Homo sapiens palmitoyl-protein thioest	16286.41700	11046.15900	12479.26600	7047.27730	Detected	Detected	Detected	Detected
3550	A_23_P35609	UROS	Hs.501376	uroporphyrinogen III synthase	Homo sapiens uroporphyrinogen III synt	923.29140	1521.20530	927.26090	1299.31320	Detected	Detected	Detected	Detected
3551	A_32_P67623	FAM120C	Hs.86045	family with sequence similarity 120C	Homo sapiens family with sequence sim	2931.53340	890.44270	2161.20830	540.29315	Detected	Detected	Detected	Detected
3552	A_23_P122662	GFOD1	Hs.484686	glucose-fructose oxidoreductase domai	Homo sapiens glucose-fructose oxidore	513.81150	773.06210	394.23328	532.53595	Detected	Detected	Detected	Detected
3553	A_23_P165676	FAM128B	Hs.655067	family with sequence similarity 128, mer	Homo sapiens family with sequence sim	195.37779	226.13129	132.80458	158.08199	Detected	Detected	Detected	Detected
3554	A_23_P43326	SPTLC1	Hs.90458	serine palmitoyltransferase, long chain b	Homo sapiens serine palmitoyltransfera	4071.00150	4460.12550	2813.97560	2430.13940	Detected	Detected	Detected	Detected
3555	A_33_P3332112	FAS	Hs.244139	Fas (TNF receptor superfamily, member	Homo sapiens Fas (TNF receptor super	714.53820	1000.09770	447.47670	495.85630	Detected	Detected	Detected	Detected
3556	A_33_P3292337	KIF5B	Hs.327736	kinesin family member 5B	Homo sapiens kinesin family member 5E	302.04883	749.28845	271.56247	491.68005	Detected	Detected	Detected	Detected
3557	A_33_P3357813	HTR1D		5-hydroxytryptamine (serotonin) recept	5-hydroxytryptamine receptor 1D (5-HT	3.18393	23.03002	6.45261	5.29949	Compromised	Detected	Compromised	Compromised
3558	A_23_P114210	POU3F4	Hs.2229	POU class 3 homeobox 4	Homo sapiens POU class 3 homeobox 4	26.27575	29.72889	15.43023	10.52291	Detected	Detected	Detected	Compromised
3559	A_33_P3274134	TMEM151B	Hs.632851	transmembrane protein 151B	Homo sapiens transmembrane protein 1	2473.76930	3263.78610	2593.39400	1762.63830	Detected	Detected	Detected	Detected
3560	A_23_P422083	TMEM55A	Hs.202517	transmembrane protein 55A	Homo sapiens transmembrane protein 5	5012.87940	2928.71530	4015.05710	1970.59300	Detected	Detected	Detected	Detected
3561	A_33_P3797820	FAM22B	Hs.662805	family with sequence similarity 22, mem	PREDICTED: Homo sapiens family with	36.15212	173.19661	13.93794	90.44382	Detected	Detected	Compromised	Detected
3562	A_23_P216355	NFKBIL2	Hs.675285	nuclear factor of kappa light polypeptide	Homo sapiens nuclear factor of kappa li	146.05565	245.68253	125.42364	222.08603	Detected	Detected	Detected	Detected
3563	A_24_P244410	C11orf51	Hs.38044	chromosome 11 open reading frame 51	Homo sapiens chromosome 11 open rea	618.88560	1101.15450	728.44030	1220.92540	Detected	Detected	Detected	Detected
3564	A_23_P218646	TNFRSF6B	Hs.434878	tumor necrosis factor receptor superfa	Homo sapiens tumor necrosis factor re	3738.91600	592.36180	4989.08060	702.60580	Detected	Detected	Detected	Detected
3565	A_23_P418199	LOC730098	Hs.459590	similar to chemokine (C-C motif) ligand	PREDICTED: Homo sapiens similar to c	1388.05730	723.22970	1438.59350	634.19116	Detected	Detected	Detected	Detected
3566	A_23_P429491	C11orf82	Hs.165607	chromosome 11 open reading frame 82	Homo sapiens chromosome 11 open rea	158.07306	1334.83810	123.22599	829.54780	Detected	Detected	Detected	Detected
3567	A_23_P218086	TPCN1	Hs.524763	two pore segment channel 1	Homo sapiens two pore segment chann	24734.60200	3410.49950	15893.71900	2041.51070	Detected	Detected	Detected	Detected
3568	A_24_P409250				Elongation factor 1-gamma (EF-1-gam	24.14008	116.48773	18.54161	98.04096	Detected	Detected	Detected	Detected
3569	A_33_P3554053	NCRNA00106	Hs.495569	non-protein coding RNA 106	UI-H-ED1-axv-n-20-0-UI.s1 NCI_CGA	453.63464	653.58716	305.75177	381.21146	Detected	Detected	Detected	Detected
3570	A_23_P152353	EARS2	Hs.620541	glutamyl-tRNA synthetase 2, mitochond	Homo sapiens glutamyl-tRNA synthetas	532.26526	311.60883	553.44977	259.21466	Detected	Detected	Detected	Detected
3571	A_33_P3232552	GUCY1B3	Hs.77890	guanylate cyclase 1, soluble, beta 3	Homo sapiens guanylate cyclase 1, solu	62.12696	28.53018	36.41146	8.98200	Detected	Detected	Detected	Compromised
3572	A_33_P3215392	EXOC3L2	Hs.721742	exocyst complex component 3-like 2	Homo sapiens exocyst complex compor	7.95078	18.28802	8.84290	9.41375	Compromised	Detected	Compromised	Compromised
3573	A_23_P216402	UCK1	Hs.9597	uridine-cytidine kinase 1	Homo sapiens uridine-cytidine kinase 1	2050.88280	2389.31050	1790.23460	1824.36960	Detected	Detected	Detected	Detected
3574	A_33_P3377649	PRSS16	Hs.274407	protease, serine, 16 (thymus)	Homo sapiens protease, serine, 16 (thyr	71.87200	76.22425	11.43345	5.72382	Detected	Detected	Compromised	Compromised
3575	A_33_P3374504				Thioredoxin reductase 2, mitochondrial	362.18732	152.57301	504.50092	151.95255	Detected	Detected	Detected	Detected
3576	A_23_P107322	CORO6	Hs.143046	coronin 6	Homo sapiens coronin 6 (CORO6), mRN	1165.93920	505.81223	827.40485	280.04460	Detected	Detected	Detected	Detected
3577	A_24_P22746	ZADH2	Hs.465433	zinc binding alcohol dehydrogenase dom	Homo sapiens zinc binding alcohol dehy	834.33780	664.48240	553.46765	354.11002	Detected	Detected	Detected	Detected
3578	A_23_P111888	CTHRC1	Hs.405614	collagen triple helix repeat containing 1	Homo sapiens collagen triple helix repea	7924.49660	20901.31200	6844.78900	15267.00500	Detected	Detected	Detected	Detected
3579	A_33_P3270852	RPS29	Hs.156367	ribosomal protein S29	Homo sapiens ribosomal protein S29 (R	95066.90000	64055.52000	67156.09000	39647.29300	Detected	Detected	Detected	Detected
3580	A_24_P134816	BCL9L	Hs.414740	B-cell CLL/lymphoma 9-like	Homo sapiens B-cell CLL/lymphoma 9-	363.65674	495.27023	360.63992	452.21130	Detected	Detected	Detected	Detected
3581	A_33_P3352382	ARG1	Hs.440934	arginase, liver	Homo sapiens arginase, liver (ARG1), m	24.63676	3.70057	16.70089	3.21929	Detected	Compromised	Detected	Compromised
3582	A_24_P941930	EAF1	Hs.716733	ELL associated factor 1	Homo sapiens ELL associated factor 1	600.74660	707.98130	371.59620	347.94666	Detected	Detected	Detected	Detected
3583	A_23_P109143	PRNP	Hs.472010	prion protein	Homo sapiens prion protein (PRNP), tra	18712.97700	17937.51600	10890.53000	8359.13900	Detected	Detected	Detected	Detected
3584	A_33_P3364864	NAMPT	Hs.489615	nicotinamide phosphoribosyltransferase	Homo sapiens nicotinamide phosphoribo	2450.68820	188.60071	2941.43900	189.21902	Detected	Detected	Detected	Detected
3585	A_24_P355626	ABCG4	Hs.126378	ATP-binding cassette, sub-family G (W	Homo sapiens ATP-binding cassette, su	59.73692	30.84316	33.37523	17.72617	Detected	Detected	Detected	Detected
3586	A_33_P3313635		Hs.569588		601344576F1 NIH_MGC_8 Homo sapiens	294.16180	503.93762	179.27982	225.11836	Detected	Detected	Detected	Detected
3587	A_23_P103631	EBNA1BP2	Hs.346868	EBNA1 binding protein 2	Homo sapiens EBNA1 binding protein 2	36351.32000	70968.87000	27304.31200	46107.08000	Detected	Detected	Detected	Detected
3588	A_33_P3275235	AKT1	Hs.525622	v-akt murine thymoma viral oncogene h	Homo sapiens v-akt murine thymoma v	5285.09230	7743.84670	3720.40300	4864.10640	Detected	Detected	Detected	Detected
3589	A_33_P3392123	LOC100233209	Hs.657722	hypothetical LOC100233209	Homo sapiens hypothetical LOC100233	2271.25200	2232.75170	309.28290	234.32675	Detected	Detected	Detected	Detected
3590	A_23_P428640	PPP1R3E	Hs.601513	protein phosphatase 1, regulatory (inhib	Homo sapiens protein phosphatase 1, re	84.25430	47.05807	92.37248	30.56352	Detected	Detected	Detected	Detected
3591	A_33_P3374215					78.33289	103.52770	72.51947	61.74706	Detected	Detected	Detected	Detected
3592	A_23_P116614	ME3	Hs.199743	malic enzyme 3, NADP(+)-dependent, m	Homo sapiens malic enzyme 3, NADP(+	1146.45480	929.24347	1429.12940	900.25385	Detected	Detected	Detected	Detected
3593	A_23_P53193	SYTL2	Hs.369520	synaptotagmin-like 2	Homo sapiens synaptotagmin-like 2 (SY	141.18517	233.95282	104.26830	112.89551	Detected	Detected	Detected	Detected
3594	A_23_P47077	BAG3	Hs.523309	BCL2-associated athanogene 3	Homo sapiens BCL2-associated athano	1576.09840	3658.70460	1042.70170	2137.00930	Detected	Detected	Detected	Detected
3595	A_23_P105144	SCUBE2	Hs.523468	signal peptide, CUB domain, EGF-like 2	Homo sapiens signal peptide, CUB doma	38.50871	207.59084	22.64691	92.23489	Detected	Detected	Detected	Detected
3596	A_23_P108699	FSHR	Hs.1428	follicle stimulating hormone receptor	Homo sapiens follicle stimulating hormo	17.53664	23.62109	6.83166	15.64501	Detected	Detected	Compromised	Detected

3597	A_24_P148094	LEPROT	Hs.23581	leptin receptor overlapping transcript	Homo sapiens leptin receptor overlappir	5808.13600	1977.00980	3804.80350	988.05740	Detected	Detected	Detected	Detected
3598	A_33_P3228271	CST3	Hs.304682	cystatin C	Cystatin-C Precursor (Cystatin-3)(Neu	841.97760	775.87836	377.11030	280.07940	Detected	Detected	Detected	Detected
3599	A_33_P3389779				WD repeat-containing protein 33 (WD r	19.68360	28.80119	10.64071	6.88706	Detected	Detected	Compromised	Compromised
3600	A_23_P299314	RPL28	Hs.652114	ribosomal protein L28	Homo sapiens ribosomal protein L28 (R	2502.22830	2729.71480	2164.29350	2104.29000	Detected	Detected	Detected	Detected
3601	A_23_P408930	OR7E5P	Hs.350816	olfactory receptor, family 7, subfamily E	Homo sapiens olfactory receptor, family	49.24318	159.57928	18.91004	34.87352	Detected	Detected	Detected	Detected
3602	A_33_P3352004					343.90973	454.89462	106.78580	101.58218	Detected	Detected	Detected	Detected
3603	A_23_P332260	PBOV1	Hs.302016	prostate and breast cancer overexpress	Homo sapiens prostate and breast canc	34.77721	36.69527	16.32275	15.59543	Detected	Detected	Compromised	Compromised
3604	A_23_P61498	KRT76	Hs.654392	keratin 76	Homo sapiens keratin 76 (KRT76), mRN	24.73173	29.19605	15.71926	19.94016	Detected	Detected	Compromised	Detected
3605	A_23_P200216	MAGOH	Hs.421576	mago-nashi homolog, proliferation-asso	Homo sapiens mago-nashi homolog, pro	1605.56640	1942.24900	1236.21360	1351.94410	Detected	Detected	Detected	Detected
3606	A_23_P31840	EEF1D	Hs.333388	eukaryotic translation elongation factor	Homo sapiens eukaryotic translation elo	41525.56000	57598.91800	45897.58600	57214.35500	Detected	Detected	Detected	Detected
3607	A_23_P207445	MAP2K6	Hs.463978	mitogen-activated protein kinase kinase	Homo sapiens mitogen-activated protei	28.65283	32.18968	14.98219	11.29621	Detected	Detected	Compromised	Compromised
3608	A_23_P336929	MED29	Hs.611541	mediator complex subunit 29	Homo sapiens mediator complex subuni	546.58466	579.62160	86.28284	53.63518	Detected	Detected	Detected	Detected
3609	A_33_P3308672		Hs.130423		hypothetical LOC91948 (LOC91948), tr	8.62934	26.48964	11.70268	8.00043	Compromised	Detected	Compromised	Compromised
3610	A_33_P3407742	PPM1B	Hs.416769	protein phosphatase 1B (formerly 2C), r	Homo sapiens protein phosphatase 1B (	325.24040	390.15060	286.53662	249.41992	Detected	Detected	Detected	Detected
3611	A_33_P3331345	DUSP11	Hs.14611	dual specificity phosphatase 11 (RNA/R	Homo sapiens dual specificity phosphat	325.75680	435.71370	278.09305	311.83734	Detected	Detected	Detected	Detected
3612	A_23_P209320	PER2	Hs.58756	period homolog 2 (Drosophila)	Homo sapiens period homolog 2 (Drosop	598.52686	860.80870	444.70132	531.58136	Detected	Detected	Detected	Detected
3613	A_24_P366535	LOC100128760	Hs.106861	hypothetical LOC100128760	PREDICTED: Homo sapiens similar to p	4470.04250	6541.75830	5853.83450	7173.22850	Detected	Detected	Detected	Detected
3614	A_33_P3293114	SFRS11	Hs.479693	splicing factor, arginine/serine-rich 11	Homo sapiens splicing factor, arginine/s	7999.56000	5443.28600	4743.44800	2708.84900	Detected	Detected	Detected	Detected
3615	A_33_P3251289	SELS	Hs.32148	selenoprotein S	Homo sapiens selenoprotein S (SELS),	4867.29400	8679.40600	4924.01100	7468.93500	Detected	Detected	Detected	Detected
3616	A_23_P29723	SGOL1	Hs.105153	shugoshin-like 1 (S. pombe)	Homo sapiens shugoshin-like 1 (S. pom	9.41642	139.59583	4.46658	89.90624	Compromised	Detected	Compromised	Detected
3617	A_23_P104741	KIRREL3	Hs.657065	kin of IRRE like 3 (Drosophila)	Homo sapiens kin of IRRE like 3 (Droso	273.28130	2270.66630	296.64136	2178.04860	Detected	Detected	Detected	Detected
3618	A_32_P186027	ANP32A	Hs.458747	acidic (leucine-rich) nuclear phosphopro	Homo sapiens acidic (leucine-rich) nucl	1031.71170	960.40010	852.38310	650.12830	Detected	Detected	Detected	Detected
3619	A_33_P3242234	F8A2	Hs.593687	coagulation factor VIII-associated (intro	Homo sapiens coagulation factor VIII-as	2371.12200	2948.92100	2105.83030	2632.56640	Detected	Detected	Detected	Detected
3620	A_23_P16652	ZNF555	Hs.47712	zinc finger protein 555	Homo sapiens zinc finger protein 555 (Z	1115.28050	946.47230	840.06620	652.01465	Detected	Detected	Detected	Detected
3621	A_24_P704878					117.40407	70.44379	3.30701	6.51560	Detected	Detected	Compromised	Compromised
3622	A_23_P87603	TARBP2	Hs.326	TAR (HIV-1) RNA binding protein 2	Homo sapiens TAR (HIV-1) RNA binding	1426.60930	1819.32120	1716.07280	1997.32030	Detected	Detected	Detected	Detected
3623	A_33_P3305458	ATF6B	Hs.42853	activating transcription factor 6 beta	Homo sapiens activating transcription fa	533.55920	664.87256	116.51588	128.75821	Detected	Detected	Detected	Detected
3624	A_23_P31671	UQCRB	Hs.131255	ubiquinol-cytochrome c reductase bindi	Homo sapiens ubiquinol-cytochrome c r	9800.39500	12976.53900	7567.90000	7947.80500	Detected	Detected	Detected	Detected
3625	A_23_P86182	MRPS21	Hs.405880	mitochondrial ribosomal protein S21	Homo sapiens mitochondrial ribosomal p	13507.64800	17554.30000	14052.59800	15256.51300	Detected	Detected	Detected	Detected
3626	A_23_P121875	C5orf28	Hs.558531	chromosome 5 open reading frame 28	Homo sapiens chromosome 5 open read	951.70026	920.52890	898.63420	655.70760	Detected	Detected	Detected	Detected
3627	A_23_P24345	SLC39A13	Hs.523664	solute carrier family 39 (zinc transporte	Homo sapiens solute carrier family 39 (	62497.66800	69122.88000	52904.15600	51693.34800	Detected	Detected	Detected	Detected
3628	A_23_P116829	UBE2N	Hs.524630	ubiquitin-conjugating enzyme E2N (UBC	Homo sapiens ubiquitin-conjugating enz	639.73773	851.75510	425.97552	476.19626	Detected	Detected	Detected	Detected
3629	A_23_P99891	MESDC1	Hs.513071	mesoderm development candidate 1	Homo sapiens mesoderm development	2197.71780	4282.91360	1645.08740	2505.46900	Detected	Detected	Detected	Detected
3630	A_24_P126417	USP34	Hs.644708	ubiquitin specific peptidase 34	Homo sapiens ubiquitin specific peptida	2161.53120	1444.90450	1823.26280	1005.10876	Detected	Detected	Detected	Detected
3631	A_23_P406330	SMAP2	Hs.15200	small ArfGAP2	Homo sapiens small ArfGAP2 (SMAP2),	350.60040	428.31885	248.06897	242.95700	Detected	Detected	Detected	Detected
3632	A_23_P4190	ACSF2	Hs.288959	acyl-CoA synthetase family member 2	Homo sapiens acyl-CoA synthetase fan	864.44560	250.68234	731.38354	182.81906	Detected	Detected	Detected	Detected
3633	A_23_P170518	DYM	Hs.162996	dymeclin	Homo sapiens dymeclin (DYM), mRNA [	5898.32500	7114.96240	4294.13400	4041.23970	Detected	Detected	Detected	Detected
3634	A_24_P261383	TAF1D	Hs.355750	TATA box binding protein (TBP)-associ	Homo sapiens TATA box binding protein	2751.02320	2815.48270	1965.21920	1598.93930	Detected	Detected	Detected	Detected
3635	A_23_P48676	PYGL	Hs.282417	phosphorylase, glycogen, liver	Homo sapiens phosphorylase, glycogen,	1820.91170	1321.06870	1630.84060	937.35443	Detected	Detected	Detected	Detected
3636	A_33_P3370634					357.75098	434.35608	336.57138	339.07740	Detected	Detected	Detected	Detected
3637	A_24_P341731	LOC100133224		similar to hCG2041320	PREDICTED: Homo sapiens similar to h	974.11930	927.64230	385.40050	266.90042	Detected	Detected	Detected	Detected
3638	A_33_P3232508	SMARCB1	Hs.534350	SWI/SNF related, matrix associated, ac	Homo sapiens SWI/SNF related, matrix	6441.28300	14711.79400	3524.96660	6114.16200	Detected	Detected	Detected	Detected
3639	A_24_P664995	CBX5	Hs.349283	chromobox homolog 5 (HP1 alpha homo	Homo sapiens chromobox homolog 5 (H	2187.83500	2994.10420	1614.58860	1649.30790	Detected	Detected	Detected	Detected
3640	A_23_P400515	KIF17	Hs.130411	kinesin family member 17	Homo sapiens kinesin family member 17	100.96758	108.01096	70.97641	71.02161	Detected	Detected	Detected	Detected
3641	A_24_P92952	ARID1A	Hs.468972	AT rich interactive domain 1A (SWI-like	Homo sapiens AT rich interactive doma	407.74222	539.60750	261.42416	323.17380	Detected	Detected	Detected	Detected
3642	A_33_P3422260		Hs.148365		Putative uncharacterized protein ENSP	1450.88650	708.40076	180.22879	88.66742	Detected	Detected	Detected	Detected
3643	A_33_P3247644	C7orf41	Hs.200100	chromosome 7 open reading frame 41	Homo sapiens chromosome 7 open read	406.84433	520.19794	404.88446	461.61150	Detected	Detected	Detected	Detected
3644	A_23_P107412	P4HB	Hs.464336	prolyl 4-hydroxylase, beta polypeptide	Homo sapiens prolyl 4-hydroxylase, bet	28360.87700	70007.99000	20931.53700	49110.79700	Detected	Detected	Detected	Detected
3645	A_33_P3269899	RECK	Hs.388918	reversion-inducing-cysteine-rich protei	Homo sapiens reversion-inducing-cyste	473.27023	1214.84310	366.85492	747.34644	Detected	Detected	Detected	Detected
3646	A_33_P3360565	ZNF474	Hs.646680	zinc finger protein 474	Homo sapiens zinc finger protein 474 (Z	38.41990	39.78241	34.95677	28.26808	Detected	Detected	Detected	Detected
3647	A_23_P215060	PODXL	Hs.16426	podocalyxin-like	Homo sapiens podocalyxin-like (PODXL	8632.86900	51856.15600	7474.39750	35253.09000	Detected	Detected	Detected	Detected
3648	A_33_P3411397	SLC25A6	Hs.350927	solute carrier family 25 (mitochondrial c	Homo sapiens solute carrier family 25 (	149325.88000	172816.78000	99440.98400	92776.05500	Detected	Detected	Detected	Detected
3649	A_23_P205830	BTBD1	Hs.459149	BTB (POZ) domain containing 1	Homo sapiens BTB (POZ) domain conta	16612.59600	12453.71100	12012.27900	7275.40400	Detected	Detected	Detected	Detected
3650	A_33_P3352970	IRAK2	Hs.449207	interleukin-1 receptor-associated kinas	Homo sapiens interleukin-1 receptor-as	3700.22020	1778.29380	2705.62260	1045.05110	Detected	Detected	Detected	Detected
3651	A_23_P101246	VSIG10L	Hs.293236	V-set and immunoglobulin domain conta	Homo sapiens V-set and immunoglobuli	351.07907	254.02040	307.29330	205.44781	Detected	Detected	Detected	Detected
3652	A_23_P132294	GGA1	Hs.499158	golgi associated, gamma adaptin ear cor	Homo sapiens golgi associated, gamma	17857.46900	14405.41300	16675.32000	12695.65000	Detected	Detected	Detected	Detected
3653	A_33_P3350643	LMX1B	Hs.129133	LIM homeobox transcription factor 1, be	Homo sapiens LIM homeobox transcript	101.04950	107.19359	129.91948	141.25864	Detected	Detected	Detected	Detected
3654	A_23_P94517	DBC1	Hs.532316	deleted in bladder cancer 1	Homo sapiens deleted in bladder cance	377.23993	37.11812	273.39642	19.46183	Detected	Detected	Detected	Detected
3655	A_23_P214587	TRIM26	Hs.720054	tripartite motif-containing 26	Homo sapiens tripartite motif-containin	871.49365	909.80670	671.62976	695.70380	Detected	Detected	Detected	Detected
3656	A_23_P163711	FAM57B	Hs.558560	family with sequence similarity 57, mem	Homo sapiens family with sequence sim	141.43677	159.02170	60.21539	59.08681	Detected	Detected	Detected	Detected
3657	A_24_P943566		Hs.436996		Phosphatase and actin regulator 1 [So	51.18278	4.83447	14.03618	3.55113	Detected	Compromised	Compromised	Compromised
3658	A_33_P3368014	HVCN1	Hs.334637	hydrogen voltage-gated channel 1	Homo sapiens hydrogen voltage-gated	69.30987	75.37782	70.63410	62.69996	Detected	Detected	Detected	Detected
3659	A_23_P15450	TMEM100	Hs.173233	transmembrane protein 100	Homo sapiens transmembrane protein 1	225.62056	16.14026	227.26352	4.50800	Detected	Compromised	Detected	Compromised
3660	A_24_P185205	TP53I13	Hs.514050	tumor protein p53 inducible protein 13	Homo sapiens tumor protein p53 inducil	760.68360	789.40894	907.07556	1060.74300	Detected	Detected	Detected	Detected
3661	A_23_P376599	RALBP1	Hs.719181	ralA binding protein 1	Homo sapiens ralA binding protein 1 (R	14422.56300	15588.50600	13274.77400	11683.74100	Detected	Detected	Detected	Detected
3662	A_23_P433229	PHYHIP	Hs.334688	phytanoyl-CoA 2-hydroxylase interactir	Homo sapiens phytanoyl-CoA 2-hydrox	56.49025	28.38153	57.59419	27.73176	Detected	Detected	Detected	Detected
3663	A_33_P3259861	LOC388079	Hs.451294	hypothetical LOC388079	PREDICTED: Homo sapiens similar to h	4898.53560	5426.50440	625.79280	611.48370	Detected	Detected	Detected	Detected
3664	A_33_P3253804	CEBPD	Hs.440829	CCAAT/enhancer binding protein (C/EI	Homo sapiens CCAAT/enhancer binding	45074.29300	42913.78500	58385.61700	42687.97700	Detected	Detected	Detected	Detected
3665	A_23_P216149	TERF1	Hs.442707	telomeric repeat binding factor (NIMA-i	Homo sapiens telomeric repeat binding	1073.33760	1078.89690	798.06520	605.61700	Detected	Detected	Detected	Detected
3666	A_33_P3243929					79.17230	38.47890	5.46459	3.51118	Detected	Detected	Compromised	Compromised
3667	A_23_P113111	AR	Hs.496240	androgen receptor	Homo sapiens androgen receptor (AR),	2104.53610	820.86910	983.63250	310.67914	Detected	Detected	Detected	Detected
3668	A_23_P160546	FAM63A	Hs.3346	family with sequence similarity 63, mem	Homo sapiens family with sequence sim	1415.32710	365.88910	852.81430	205.48088	Detected	Detected	Detected	Detected
3669	A_23_P106998	MRPS23	Hs.5836	mitochondrial ribosomal protein S23	Homo sapiens mitochondrial ribosomal p	2250.86960	2514.08280	2009.38990	1920.24790	Detected	Detected	Detected	Detected
3670	A_33_P3222367	SNORA74B	Hs.692720	small nucleolar RNA, H/ACA box 74B	Homo sapiens small nucleolar RNA, H/A	40.57015	57.63852	20.48475	33.61913	Detected	Detected	Detected	Detected
3671	A_23_P111919	PURG	Hs.373778	purine-rich element binding protein G	Homo sapiens purine-rich element bindi	50.30246	20.43952	22.47478	4.42312	Detected	Not Detected	Detected	Compromised

3672	A_33_P3354374				Putative uncharacterized protein ENSP	104.87907	31.12331	76.15112	9.84085	Detected	Detected	Detected	Compromised
3673	A_33_P3215729					23.72802	24.23484	3.02510	3.17167	Detected	Detected	Compromised	Compromised
3674	A_23_P349416	ERBB3	Hs.118681	v-erb-b2 erythroblastic leukemia viral o	Homo sapiens v-erb-b2 erythroblastic l	10.23193	23.82036	12.30037	8.10503	Compromised	Detected	Compromised	Compromised
3675	A_33_P3346791	SCAMP3	Hs.200600	secretory carrier membrane protein 3	Homo sapiens secretory carrier membra	24454.40000	25223.47500	23000.91600	20572.45500	Detected	Detected	Detected	Detected
3676	A_23_P135742	C1orf170	Hs.271462	chromosome 1 open reading frame 170	Homo sapiens chromosome 1 open read	35.56104	17.32753	20.51798	3.86375	Detected	Compromised	Detected	Compromised
3677	A_33_P3327097				Myotubularin-related protein 1 (EC 3.1.3	2.58600	38.17262	4.57051	16.43150	Compromised	Detected	Compromised	Detected
3678	A_23_P52806	BACE1	Hs.504003	beta-site APP-cleaving enzyme 1	Homo sapiens beta-site APP-cleaving e	17168.89600	20922.85000	13782.10200	13983.32200	Detected	Detected	Detected	Detected
3679	A_23_P56833	ASB3	Hs.40763	ankyrin repeat and SOCS box-containin	Homo sapiens ankyrin repeat and SOCS	2002.55960	1523.49770	1559.18040	1061.47390	Detected	Detected	Detected	Detected
3680	A_23_P45970	C1orf109	Hs.272673	chromosome 1 open reading frame 109	Homo sapiens chromosome 1 open read	757.02820	1112.84070	751.32745	951.36310	Detected	Detected	Detected	Detected
3681	A_33_P3243878	GOLGA6L9	Hs.630181	golgi autoantigen, golgin subfamily a, 6-	Homo sapiens golgi autoantigen, golgin s	5707.50830	5452.51600	3649.25540	2176.95900	Detected	Detected	Detected	Detected
3682	A_33_P3211679	TSPAN11	Hs.505141	tetraspanin 11	Homo sapiens tetraspanin 11 (TSPAN11	708.99475	1099.06300	323.01297	371.60135	Detected	Detected	Detected	Detected
3683	A_33_P3424339					4175.54300	4630.06900	312.28870	346.01380	Detected	Detected	Detected	Detected
3684	A_23_P93938	NACAD	Hs.96633	NAC alpha domain containing	Homo sapiens NAC alpha domain conta	275.69363	354.65866	284.45050	387.97174	Detected	Detected	Detected	Detected
3685	A_24_P317719	WHSC1L1	Hs.608111	Wolf-Hirschhorn syndrome candidate 1-	Homo sapiens Wolf-Hirschhorn syndrom	781.41350	579.85610	600.99920	402.56186	Detected	Detected	Detected	Detected
3686	A_23_P314672	C11orf40	Hs.350556	chromosome 11 open reading frame 40	Homo sapiens chromosome 11 open rea	10.75375	19.04333	3.04881	3.17011	Compromised	Detected	Compromised	Compromised
3687	A_33_P3312212	PRKAA1	Hs.43322	protein kinase, AMP-activated, alpha 1	Homo sapiens protein kinase, AMP-acti	212.14041	208.20355	266.75650	199.32469	Detected	Detected	Detected	Detected
3688	A_33_P3270228				Chloride transport protein 6 (ClC-6) [So	22.68033	43.62730	14.24216	24.35965	Detected	Detected	Compromised	Detected
3689	A_32_P155091	ATXN2L	Hs.460499	ataxin 2-like	Homo sapiens ataxin 2-like (ATXN2L), t	21321.59600	29509.62500	18978.86100	21572.50200	Detected	Detected	Detected	Detected
3690	A_33_P3236133	SHQ1	Hs.606584	SHQ1 homolog (S. cerevisiae)	Homo sapiens SHQ1 homolog (S. cerevi	441.95570	368.36227	201.05984	149.04489	Detected	Detected	Detected	Detected
3691	A_33_P3394972	OSBPL5	Hs.436166	oxysterol binding protein-like 5	Homo sapiens oxysterol binding protein	156.49019	68.01246	140.49767	47.83389	Detected	Detected	Detected	Detected
3692	A_24_P100517	C9orf140	Hs.19322	chromosome 9 open reading frame 140	Homo sapiens chromosome 9 open read	68.86566	931.86950	70.17153	990.95636	Detected	Detected	Detected	Detected
3693	A_23_P142708	LMAN2L	Hs.655743	lectin, mannose-binding 2-like	Homo sapiens lectin, mannose-binding 2	1044.31980	1751.98830	932.27057	1353.51530	Detected	Detected	Detected	Detected
3694	A_33_P3326210	ESCO2	Hs.99480	establishment of cohesion 1 homolog 2	Homo sapiens establishment of cohesio	2.66392	98.89648	3.07945	70.35697	Compromised	Detected	Compromised	Detected
3695	A_33_P3422581	C2orf86	Hs.414952	chromosome 2 open reading frame 86	Homo sapiens chromosome 2 open read	134.61362	54.39821	122.18694	47.30804	Detected	Detected	Detected	Detected
3696	A_33_P3406899	TRAK1	Hs.535711	trafficking protein, kinesin binding 1	Homo sapiens trafficking protein, kinesi	1165.44210	3535.48170	1013.55820	2402.76250	Detected	Detected	Detected	Detected
3697	A_23_P427299	LOC729915	Hs.546584	similar to Putative POM121-like protein	PREDICTED: Homo sapiens similar to P	24.40855	23.68303	26.24596	13.30729	Detected	Detected	Detected	Compromised
3698	A_23_P142688	FAM134A	Hs.516707	family with sequence similarity 134, mer	Homo sapiens family with sequence sim	3550.44260	1986.39050	2596.85770	1405.32950	Detected	Detected	Detected	Detected
3699	A_23_P163380	MTHFS	Hs.459049	5,10-methenyltetrahydrofolate syntheta	Homo sapiens 5,10-methenyltetrahydro	1028.02870	4096.51170	1047.30640	3436.86550	Detected	Detected	Detected	Detected
3700	A_23_P403424	JMJD7-PLA2G4B	Hs.198161	JMJD7-PLA2G4B readthrough transcri	Homo sapiens JMJD7-PLA2G4B readth	1220.54770	407.94740	1173.06000	294.16785	Detected	Detected	Detected	Detected
3701	A_23_P5460	FEV	Hs.234759	FEV (ETS oncogene family)	Homo sapiens FEV (ETS oncogene fam	9.71678	12.55692	15.05101	19.44387	Compromised	Compromised	Compromised	Detected
3702	A_23_P41716	GNB2L1	Hs.5662	guanine nucleotide binding protein (G pr	Homo sapiens guanine nucleotide bindin	57664.10000	50391.82400	56208.79300	44123.28500	Detected	Detected	Detected	Detected
3703	A_33_P3325087	CCDC55	Hs.462663	coiled-coil domain containing 55	Homo sapiens coiled-coil domain conta	918.15330	846.39557	794.68176	610.43207	Detected	Detected	Detected	Detected
3704	A_33_P3313519	CHRFAM7A	Hs.510853	CHRNA7 (cholinergic receptor, nicotinic	Homo sapiens CHRNA7 (cholinergic rec	42.21831	67.76662	49.33638	41.90054	Detected	Detected	Detected	Detected
3705	A_24_P49687					73130.51600	75141.13000	81227.08600	71977.47000	Detected	Detected	Detected	Detected
3706	A_24_P76018	C3orf71	Hs.720727	chromosome 3 open reading frame 71	Homo sapiens chromosome 3 open read	141.55972	153.45310	134.83083	123.07050	Detected	Detected	Detected	Detected
3707	A_24_P687594	LIX1L	Hs.644778	Lix1 homolog (mouse)-like	LIX1-like protein [Source:UniProtKB/S	1777.24440	1418.64210	1400.81820	898.02320	Detected	Detected	Detected	Detected
3708	A_23_P88909	SYNGR3	Hs.435277	synaptogyrin 3	Homo sapiens synaptogyrin 3 (SYNGR3	172.61182	91.56157	183.05075	79.45996	Detected	Detected	Detected	Detected
3709	A_32_P89310	PLEKHM3	Hs.159188	pleckstrin homology domain containing,	Homo sapiens pleckstrin homology dom	794.11884	759.25940	599.37335	443.49506	Detected	Detected	Detected	Detected
3710	A_33_P3284858	LOC729867	Hs.674191	hypothetical protein LOC729867	Homo sapiens cDNA FLJ35980 fis, clon	101.47379	87.24214	89.08826	70.79285	Detected	Detected	Detected	Detected
3711	A_33_P3544887	TTC28	Hs.387856	tetratricopeptide repeat domain 28	Homo sapiens tetratricopeptide repeat	961.18760	1678.76150	693.63050	822.83356	Detected	Detected	Detected	Detected
3712	A_23_P78152	MIS12	Hs.267194	MIS12, MIND kinetochore complex com	Homo sapiens MIS12, MIND kinetochore	791.99830	769.23280	642.05770	469.66064	Detected	Detected	Detected	Detected
3713	A_23_P326009	ZNF471	Hs.710590	zinc finger protein 471	Homo sapiens zinc finger protein 471 (Z	24.82238	42.05902	20.00964	23.85708	Detected	Detected	Detected	Detected
3714	A_24_P288915		Hs.649499		Coiled-coil domain-containing protein 1	66.31436	6.91733	58.88777	5.83075	Detected	Compromised	Detected	Compromised
3715	A_23_P403745	MTSS1L	Hs.432387	metastasis suppressor 1-like	Homo sapiens metastasis suppressor 1-	447.17126	322.58280	376.47140	213.15073	Detected	Detected	Detected	Detected
3716	A_23_P152047	SCAMP5	Hs.374180	secretory carrier membrane protein 5	Homo sapiens secretory carrier membra	246.35417	60.74674	203.43346	44.34990	Detected	Detected	Detected	Detected
3717	A_23_P314811	SLC10A4	Hs.132591	solute carrier family 10 (sodium/bile aci	Homo sapiens solute carrier family 10 (	17.40044	17.30580	10.02643	11.08551	Detected	Compromised	Compromised	Compromised
3718	A_23_P102832	CEP250	Hs.443976	centrosomal protein 250kDa	Homo sapiens centrosomal protein 250k	562.88740	1141.28900	523.92190	1084.91150	Detected	Detected	Detected	Detected
3719	A_33_P3507270	EFCAB5	Hs.662411	EF-hand calcium binding domain 5	Homo sapiens EF-hand calcium binding	27.19609	9.85468	15.27364	6.05592	Detected	Compromised	Compromised	Compromised
3720	A_24_P213478	SEMA6A	Hs.156967	sema domain, transmembrane domain (T	Homo sapiens sema domain, transmemb	14.88906	3.99834	3.47972	3.45424	Detected	Compromised	Compromised	Compromised
3721	A_33_P3421363	ZNF169	Hs.387623	zinc finger protein 169	Homo sapiens zinc finger protein 169 (Z	459.00717	635.31490	278.13956	263.30032	Detected	Detected	Detected	Detected
3722	A_24_P156388	TTC38	Hs.435832	tetratricopeptide repeat domain 38	Homo sapiens tetratricopeptide repeat	1459.37130	1486.80740	916.80930	862.72960	Detected	Detected	Detected	Detected
3723	A_33_P3355926		Hs.678899		cDNA FLJ37701 fis, clone BRHIP20169	10.11982	18.26346	14.58379	12.70461	Compromised	Detected	Compromised	Compromised
3724	A_23_P353614	C8orf46	Hs.268869	chromosome 8 open reading frame 46	Homo sapiens chromosome 8 open read	13.99843	55.68430	7.76348	36.09570	Detected	Detected	Compromised	Detected
3725	A_23_P145984	TSPAN12	Hs.16529	tetraspanin 12	Homo sapiens tetraspanin 12 (TSPAN12	164.56580	53.34979	87.04568	18.13400	Detected	Detected	Detected	Detected
3726	A_33_P3257150	CDC42	Hs.467637	cell division cycle 42 (GTP binding prote	Homo sapiens cell division cycle 42 (GT	2800.02000	8537.06000	2134.20300	5654.62500	Detected	Detected	Detected	Detected
3727	A_23_P16275	TSKS	Hs.515858	testis-specific serine kinase substrate	Homo sapiens testis-specific serine kin	16.27254	11.17215	15.94602	7.21021	Detected	Compromised	Detected	Compromised
3728	A_32_P46238	LOC339240	Hs.420616	keratin pseudogene	Homo sapiens keratin pseudogene (LOC	22.46601	30.31690	21.42847	20.35729	Detected	Detected	Detected	Detected
3729	A_33_P3291959	LOC729204	Hs.638320	similar to hCG2045644	PREDICTED: Homo sapiens similar to h	14.42846	21.33093	15.11563	15.12980	Detected	Detected	Compromised	Compromised
3730	A_23_P148463	CUL4B	Hs.102914	cullin 4B	Homo sapiens cullin 4B (CUL4B), transc	2989.83030	3253.13280	2319.21140	2035.24910	Detected	Detected	Detected	Detected
3731	A_23_P323751	FAM83D	Hs.472716	family with sequence similarity 83, mem	Homo sapiens family with sequence sim	68.57602	2546.37650	38.00895	1276.28080	Detected	Detected	Detected	Detected
3732	A_32_P207428	ZNF845	Hs.720672	zinc finger protein 845	Homo sapiens zinc finger protein 845 (Z	805.16520	894.53410	798.42710	721.85223	Detected	Detected	Detected	Detected
3733	A_33_P3417765	KLHDC7A	Hs.406913	kelch domain containing 7A	Homo sapiens kelch domain containing	18.06164	28.05654	17.98172	13.66685	Detected	Detected	Detected	Compromised
3734	A_23_P216568	C9orf6	Hs.29276	chromosome 9 open reading frame 6	Homo sapiens chromosome 9 open read	1100.51270	1439.57320	777.26280	837.41766	Detected	Detected	Detected	Detected
3735	A_33_P3241334		Hs.709192		Pregnancy-specific beta-1-glycoprotei	7.45382	212.25935	3.04265	3.16443	Compromised	Detected	Compromised	Compromised
3736	A_23_P62634	RHCE	Hs.449968	Rh blood group, CcEe antigens	Homo sapiens Rh blood group, CcEe an	128.85187	95.65226	142.75809	77.98186	Detected	Detected	Detected	Detected
3737	A_23_P337033	GMEB2	Hs.473286	glucocorticoid modulatory element bindi	Homo sapiens glucocorticoid modulator	341.90277	415.66705	255.08073	274.81970	Detected	Detected	Detected	Detected
3738	A_33_P3289045	GSTT2B	Hs.656498	glutathione S-transferase theta 2B (ger	Homo sapiens glutathione S-transferase	450.79840	1265.97580	691.95807	1545.40380	Detected	Detected	Detected	Detected
3739	A_33_P3250750	LOC100130711	Hs.719173	similar to hCG1778032	PREDICTED: Homo sapiens similar to h	3303.07130	2119.09200	749.35730	311.95670	Detected	Detected	Detected	Detected
3740	A_24_P930418	SDHALP2	Hs.334587	succinate dehydrogenase complex, subu	Homo sapiens cDNA FLJ32971 fis, clon	58.54857	92.38989	40.63631	62.66066	Detected	Detected	Detected	Detected
3741	A_23_P159741	BCOR	Hs.659681	BCL6 co-repressor	Homo sapiens BCL6 co-repressor (BCO	542.97310	569.53650	410.22433	364.15253	Detected	Detected	Detected	Detected
3742	A_23_P2114	C11orf59	Hs.530753	chromosome 11 open reading frame 59	Homo sapiens chromosome 11 open rea	8339.12100	8659.14300	8874.87400	8326.59000	Detected	Detected	Detected	Detected
3743	A_23_P101054	KRT34	Hs.296942	keratin 34	Homo sapiens keratin 34 (KRT34), mRN	149.22154	942.93024	145.92633	817.49384	Detected	Detected	Detected	Detected
3744	A_33_P3405680		Hs.549631		BX090838 Soares_NFL_T_GBC_S1 Hom	57.89622	62.36929	21.33504	23.50965	Detected	Detected	Detected	Detected
3745	A_33_P3296067	CDC14C	Hs.567757	CDC14 cell division cycle 14 homolog C	Homo sapiens CDC14 cell division cycle	443.61030	632.87573	796.84796	807.80896	Detected	Detected	Detected	Detected
3746	A_33_P3306153	KIAA1841	Hs.468653	KIAA1841	Homo sapiens KIAA1841 (KIAA1841), tr	44.34510	83.78438	60.54691	63.45842	Detected	Detected	Detected	Detected

3747	A_23_P92025	CIDEC	Hs.567562	cell death-inducing DFFA-like effector	Homo sapiens cell death-inducing DFFA	179.63165	335.00630	245.74489	399.44507	Detected	Detected	Detected	Detected
3748	A_23_P161698	MMP3	Hs.375129	matrix metallopeptidase 3 (stromelysin	Homo sapiens matrix metallopeptidase 3	74141.90000	39074.59800	76679.69000	35777.27000	Detected	Detected	Detected	Detected
3749	A_33_P3218564					43623.12500	58027.59400	34324.38000	33067.13000	Detected	Detected	Detected	Detected
3750	A_32_P4364	BCAP31	Hs.522817	B-cell receptor-associated protein 31	Homo sapiens B-cell receptor-associat	43482.20000	52129.80500	42452.30000	41695.77000	Detected	Detected	Detected	Detected
3751	A_23_P171336	NXF3	Hs.60386	nuclear RNA export factor 3	Homo sapiens nuclear RNA export facto	348.48297	374.09190	150.54353	115.19428	Detected	Detected	Detected	Detected
3752	A_24_P141688	PCBP2	Hs.546271	poly(rC) binding protein 2	Homo sapiens poly(rC) binding protein 2	829.47760	1855.49790	760.82690	1483.51060	Detected	Detected	Detected	Detected
3753	A_23_P122579	DAXX	Hs.336916	death-domain associated protein	Homo sapiens death-domain associated	2411.26540	2188.64380	2323.36960	1879.68240	Detected	Detected	Detected	Detected
3754	A_33_P3334225	ACACA	Hs.160556	acetyl-Coenzyme A carboxylase alpha	Homo sapiens acetyl-Coenzyme A carb	5003.60940	4399.63040	4044.71140	3229.02370	Detected	Detected	Detected	Detected
3755	A_23_P137381	ID3	Hs.76884	inhibitor of DNA binding 3, dominant neg	Homo sapiens inhibitor of DNA binding 3	1231.34620	1069.48340	1302.13850	1066.99210	Detected	Detected	Detected	Detected
3756	A_23_P96853	FAF1	Hs.530402	Fas (TNFRSF6) associated factor 1	Homo sapiens Fas (TNFRSF6) associat	4890.55370	7132.62600	4786.74660	5693.56600	Detected	Detected	Detected	Detected
3757	A_33_P3400147	TLE6	Hs.334507	transducin-like enhancer of split 6 (E(sp	Homo sapiens transducin-like enhancer	54.14709	6.74904	53.50415	8.00051	Detected	Compromised	Detected	Compromised
3758	A_33_P3370226	RPL7	Hs.571841	ribosomal protein L7	Homo sapiens ribosomal protein L7 (RP	149018.22000	146905.60000	117125.84000	94787.16000	Detected	Detected	Detected	Detected
3759	A_23_P112801	CHP	Hs.406234	calcium binding protein P22	Homo sapiens calcium binding protein P	40959.08000	28076.30000	27497.57800	16345.74800	Detected	Detected	Detected	Detected
3760	A_33_P3350094	PATL2	Hs.11594	protein associated with topoisomerase I	Homo sapiens protein associated with t	41.72835	29.93844	33.88764	16.82487	Detected	Detected	Detected	Compromised
3761	A_33_P3328026	CDC2L6	Hs.584867	cell division cycle 2-like 6 (CDK8-like)	Homo sapiens cell division cycle 2-like	5819.26560	5259.28860	4551.96000	3334.98270	Detected	Detected	Detected	Detected
3762	A_24_P678104	STMN3	Hs.639609	stathmin-like 3	Homo sapiens stathmin-like 3 (STMN3)	957.99506	186.15714	914.94160	162.76710	Detected	Detected	Detected	Detected
3763	A_23_P217120	EHMT1	Hs.495511	euchromatic histone-lysine N-methyltra	Homo sapiens euchromatic histone-lysi	1111.75540	1009.11520	788.95404	563.31805	Detected	Detected	Detected	Detected
3764	A_33_P3302662				Transducin-like enhancer protein 4 [So	24.19757	18.69328	13.72011	8.83070	Detected	Compromised	Compromised	Compromised
3765	A_23_P108751	FHL2	Hs.443687	four and a half LIM domains 2	Homo sapiens four and a half LIM doma	56261.91400	116901.68000	46107.32000	84241.62000	Detected	Detected	Detected	Detected
3766	A_24_P258955	ZNF317	Hs.465829	zinc finger protein 317	Homo sapiens zinc finger protein 317 (Z	1087.61930	1335.87540	801.70667	903.41340	Detected	Detected	Detected	Detected
3767	A_23_P128554	EBPL	Hs.433278	emopamil binding protein-like	Homo sapiens emopamil binding protein	30852.97900	18309.35700	25701.34400	12516.23600	Detected	Detected	Detected	Detected
3768	A_23_P371145	ADPRHL1	Hs.98669	ADP-ribosylhydrolase like 1	Homo sapiens ADP-ribosylhydrolase lik	87.93218	178.60947	61.74085	121.37371	Detected	Detected	Detected	Detected
3769	A_33_P3238976	TRMT5	Hs.380159	TRM5 tRNA methyltransferase 5 homol	Homo sapiens TRM5 tRNA methyltransf	915.45886	3528.32350	1061.80580	3684.14840	Detected	Detected	Detected	Detected
3770	A_24_P261724	RNF10	Hs.442798	ring finger protein 10	Homo sapiens ring finger protein 10 (RN	4691.08640	4705.65870	4139.19870	3600.37500	Detected	Detected	Detected	Detected
3771	A_23_P59388	DST	Hs.604915	dystonin	Homo sapiens dystonin (DST), transcrip	79.74980	155.51573	78.83347	103.75947	Detected	Detected	Detected	Detected
3772	A_33_P3249066					42.95923	24.86806	29.13458	5.51760	Detected	Detected	Detected	Compromised
3773	A_33_P3250463	PMS1	Hs.111749	PMS1 postmeiotic segregation increase	Homo sapiens PMS1 postmeiotic segreg	132.50740	159.03421	136.77449	117.70429	Detected	Detected	Detected	Detected
3774	A_32_P183904	SHF	Hs.310399	Src homology 2 domain containing F	Homo sapiens Src homology 2 domain c	381.23312	70.37430	315.02243	44.27502	Detected	Detected	Detected	Detected
3775	A_33_P3226367					17.93448	29.81625	13.53667	9.25846	Detected	Detected	Compromised	Compromised
3776	A_33_P3395237	LOC134505		similar to hCG39609	PREDICTED: Homo sapiens similar to h	2899.80570	2980.74880	844.12256	554.69354	Detected	Detected	Detected	Detected
3777	A_23_P171237	ACRC	Hs.135167	acidic repeat containing	Homo sapiens acidic repeat containing (	333.81458	273.99762	313.19025	220.29556	Detected	Detected	Detected	Detected
3778	A_23_P254025	ZFP37	Hs.150406	zinc finger protein 37 homolog (mouse)	Homo sapiens zinc finger protein 37 hor	97.20121	67.52898	57.83863	27.84483	Detected	Detected	Detected	Detected
3779	A_33_P3348220	ENGASE	Hs.29288	endo-beta-N-acetylglucosaminidase	Homo sapiens endo-beta-N-acetylgluc	42.04091	50.78405	17.97124	38.26683	Detected	Detected	Detected	Detected
3780	A_33_P3277367	LOC732146		hypothetical protein LOC732146	PREDICTED: Homo sapiens hypothetica	52.68789	42.79372	40.03375	7.68341	Detected	Detected	Detected	Compromised
3781	A_24_P97770	ERGIC1	Hs.509163	endoplasmic reticulum-golgi intermediat	Homo sapiens endoplasmic reticulum-g	324.16870	432.68850	394.06314	445.13217	Detected	Detected	Detected	Detected
3782	A_23_P200404	AK2	Hs.470907	adenylate kinase 2	Homo sapiens adenylate kinase 2 (AK2)	4669.96040	7942.01200	3457.46950	4844.39450	Detected	Detected	Detected	Detected
3783	A_23_P73702	MED12	Hs.409226	mediator complex subunit 12	Homo sapiens mediator complex subuni	247.44940	147.17899	187.15262	111.09232	Detected	Detected	Detected	Detected
3784	A_23_P12680	PSAP	Hs.523004	prosaposin	Homo sapiens prosaposin (PSAP), trans	23059.81800	9800.14200	22953.10400	8796.32300	Detected	Detected	Detected	Detected
3785	A_33_P3278191					14.13377	31.59641	5.08957	4.29597	Detected	Detected	Compromised	Compromised
3786	A_23_P77818	ATP5H	Hs.514465	ATP synthase, H+ transporting, mitocho	Homo sapiens ATP synthase, H+ transp	53992.21500	59022.88000	50337.06000	44865.70000	Detected	Detected	Detected	Detected
3787	A_33_P3235521					3.00789	22.03484	7.52112	6.54470	Compromised	Detected	Compromised	Compromised
3788	A_23_P360964	DACT3	Hs.515490	dapper, antagonist of beta-catenin, hom	Homo sapiens dapper, antagonist of bet	10025.61900	8877.49100	7774.19000	5798.79440	Detected	Detected	Detected	Detected
3789	A_23_P92730	HSPB3	Hs.41707	heat shock 27kDa protein 3	Homo sapiens heat shock 27kDa protei	272.07672	3458.43400	274.89764	3066.69500	Detected	Detected	Detected	Detected
3790	A_23_P40354	MAPRE1	Hs.472437	microtubule-associated protein, RP/EB	Homo sapiens microtubule-associated p	1271.74990	2813.00660	1364.57420	2656.99150	Detected	Detected	Detected	Detected
3791	A_33_P3368375	RANBP3	Hs.531752	RAN binding protein 3	Homo sapiens RAN binding protein 3 (R	150.75530	284.13327	98.06442	173.43108	Detected	Detected	Detected	Detected
3792	A_23_P115316	TOR3A	Hs.584957	torsin family 3, member A	Homo sapiens torsin family 3, member A	3151.59860	3680.75510	3254.72660	3448.13000	Detected	Detected	Detected	Detected
3793	A_23_P104996	BEST1	Hs.712676	bestrophin 1	Homo sapiens bestrophin 1 (BEST1), tra	51.46502	38.26264	46.59691	33.25069	Detected	Detected	Detected	Detected
3794	A_23_P216689	BRD3	Hs.522472	bromodomain containing 3	Homo sapiens bromodomain containing	689.19855	286.09677	1261.16800	596.15686	Detected	Detected	Detected	Detected
3795	A_33_P3231653	GOLM1	Hs.494337	golgi membrane protein 1	Homo sapiens golgi membrane protein 1	808.54297	974.63007	549.38080	556.11630	Detected	Detected	Detected	Detected
3796	A_33_P3404097	PGM5P2	Hs.434753	phosphoglucomutase 5 pseudogene 2	Homo sapiens phosphoglucomutase 5 p	11343.49600	8963.81400	4745.75050	2850.37940	Detected	Detected	Detected	Detected
3797	A_23_P253571	PHF5A	Hs.474980	PHD finger protein 5A	Homo sapiens PHD finger protein 5A (P	1325.79900	1486.49490	978.17285	927.66430	Detected	Detected	Detected	Detected
3798	A_23_P41365	SMR3A	Hs.701334	submaxillary gland androgen regulated p	Homo sapiens submaxillary gland androg	133.07468	172.85017	89.11476	90.91073	Detected	Detected	Detected	Detected
3799	A_23_P217208	SLC35A2	Hs.21899	solute carrier family 35 (UDP-galactose	Homo sapiens solute carrier family 35 (	146.66997	253.11224	107.45531	168.53590	Detected	Detected	Detected	Detected
3800	A_23_P26094	SLTM	Hs.512932	SAFB-like, transcription modulator	Homo sapiens SAFB-like, transcription	5253.10740	3734.68240	3408.75730	2042.82020	Detected	Detected	Detected	Detected
3801	A_33_P3370019		Hs.510635		Ig delta chain C region [Source:UniProt	25.79987	24.29970	14.44361	13.91375	Detected	Detected	Compromised	Compromised
3802	A_33_P3385477	ATAD3B	Hs.23413	ATPase family, AAA domain containing	Homo sapiens ATPase family, AAA dom	11669.33000	26354.74000	10203.61300	19975.23600	Detected	Detected	Detected	Detected
3803	A_23_P31315	CBX3	Hs.381189	chromobox homolog 3 (HP1 gamma hom	Homo sapiens chromobox homolog 3 (H	29921.15800	56158.20700	23938.11300	38225.61000	Detected	Detected	Detected	Detected
3804	A_23_P103877	LRRC38	Hs.657356	leucine rich repeat containing 38	Leucine-rich repeat-containing protein	13.63118	31.24960	4.15942	19.09707	Detected	Detected	Compromised	Detected
3805	A_24_P285501	UBR3	Hs.379548	ubiquitin protein ligase E3 component n	Homo sapiens ubiquitin protein ligase E3	509.62958	501.96774	237.11073	164.49988	Detected	Detected	Detected	Detected
3806	A_24_P419132	CENPI	Hs.348920	centromere protein I	Homo sapiens centromere protein I (CE	2.85082	84.95754	3.28953	66.62502	Compromised	Detected	Compromised	Detected
3807	A_32_P184367	RPL10	Hs.534404	ribosomal protein L10	Homo sapiens ribosomal protein L10 (R	172393.48000	170910.00000	165875.88000	137830.94000	Detected	Detected	Detected	Detected
3808	A_33_P3358804	LOC100133998		hypothetical protein LOC100133998	PREDICTED: Homo sapiens similar to C	37.81796	58.13389	22.78910	26.19341	Detected	Detected	Detected	Detected
3809	A_33_P3328726	CCDC33	Hs.383206	coiled-coil domain containing 33	Homo sapiens coiled-coil domain contai	91.68973	109.70891	40.09355	34.26024	Detected	Detected	Detected	Detected
3810	A_23_P205997	APH1B	Hs.511703	anterior pharynx defective 1 homolog B	Homo sapiens anterior pharynx defectiv	1692.42420	1583.33290	2017.79690	1530.65270	Detected	Detected	Detected	Detected
3811	A_33_P3400728	RIPK4	Hs.517310	receptor-interacting serine-threonine k	Homo sapiens receptor-interacting seri	91.76295	118.07086	47.60225	50.67316	Detected	Detected	Detected	Detected
3812	A_33_P3280950	LOC144571	Hs.592432	hypothetical protein LOC144571	Homo sapiens hypothetical protein LOC	258.87560	302.92102	180.42882	179.38246	Detected	Detected	Detected	Detected
3813	A_33_P3311403	MRVI1	Hs.501898	murine retrovirus integration site 1 hom	Homo sapiens murine retrovirus integra	252.12675	280.32870	196.64082	168.07985	Detected	Detected	Detected	Detected
3814	A_23_P206371	NOL3	Hs.513667	nucleolar protein 3 (apoptosis repressor	Homo sapiens nucleolar protein 3 (apop	7650.59230	5952.66900	6637.75540	4278.46830	Detected	Detected	Detected	Detected
3815	A_23_P398172	FAM135A	Hs.211700	family with sequence similarity 135, mer	Homo sapiens family with sequence sim	504.74140	151.19731	308.59796	65.19569	Detected	Detected	Detected	Detected
3816	A_23_P259054	SNX14	Hs.485871	sorting nexin 14	Homo sapiens sorting nexin 14 (SNX14)	9668.93700	6206.73140	9162.05300	4673.25240	Detected	Detected	Detected	Detected
3817	A_33_P3274618	LOC731275	Hs.611274	hypothetical LOC731275	Homo sapiens hypothetical LOC731275	118.20213	158.07321	131.25813	115.39918	Detected	Detected	Detected	Detected
3818	A_23_P115161	DARC	Hs.153381	Duffy blood group, chemokine receptor	Homo sapiens Duffy blood group, chemo	13.07614	40.66947	7.34868	4.94004	Detected	Not Detected	Compromised	Compromised
3819	A_33_P3372332	CYBA	Hs.513803	cytochrome b-245, alpha polypeptide	Homo sapiens cytochrome b-245, alpha	4230.31900	3350.41700	4931.71530	3159.81980	Detected	Detected	Detected	Detected
3820	A_23_P127233	SMNDC1	Hs.632093	survival motor neuron domain containin	Homo sapiens survival motor neuron do	7019.14000	8039.19630	4724.80900	4330.39000	Detected	Detected	Detected	Detected
3821	A_33_P3380642				Extracellular matrix protein FRAS1 Pre	21.02560	20.54025	14.45131	8.54465	Detected	Detected	Compromised	Compromised

3822	A_23_P123256	PDAP1	Hs.632296	PDGFA associated protein 1	Homo sapiens PDGFA associated prote	1374.45830	1747.62520	2261.85470	3556.88160	Detected	Detected	Detected	Detected
3823	A_32_P725218	METTL2A	Hs.381204	methyltransferase like 2A	Homo sapiens methyltransferase like 2A	232.97476	467.58237	202.91196	376.22040	Detected	Detected	Detected	Detected
3824	A_33_P3415032	KCNA3	Hs.169948	potassium voltage-gated channel, shake	Homo sapiens potassium voltage-gated	62.31734	41.84573	37.05456	4.91855	Detected	Detected	Detected	Compromised
3825	A_33_P3386262	CDT1	Hs.122908	chromatin licensing and DNA replication	Homo sapiens chromatin licensing and D	320.29288	4330.27540	284.40067	3326.46400	Detected	Detected	Detected	Detected
3826	A_23_P106433	RCCD1	Hs.655895	RCC1 domain containing 1	Homo sapiens RCC1 domain containing	1549.01110	2062.03540	1076.09840	1247.56210	Detected	Detected	Detected	Detected
3827	A_33_P3266744	SYTL1	Hs.469175	synaptotagmin-like 1	Homo sapiens synaptotagmin-like 1 (SY	764.47894	711.04395	427.26205	280.40340	Detected	Detected	Detected	Detected
3828	A_24_P683861		Hs.719733		Ig heavy chain V-I region V35 Precurso	92.97750	66.86392	28.62893	21.24211	Detected	Detected	Detected	Detected
3829	A_24_P759674	OBFC1	Hs.62314	oligonucleotide/oligosaccharide-binding	Homo sapiens oligonucleotide/oligosacc	1293.16530	141.00398	787.50500	71.85342	Detected	Detected	Detected	Detected
3830	A_23_P310274	PRSS2	Hs.622865	protease, serine, 2 (trypsin 2)	Homo sapiens protease, serine, 2 (tryps	4365.01560	4358.27500	4047.71780	3435.20300	Detected	Detected	Detected	Detected
3831	A_24_P30557	TBX5	Hs.381715	T-box 5	Homo sapiens T-box 5 (TBX5), transcri	4.14714	193.41812	3.04195	100.91200	Compromised	Detected	Compromised	Detected
3832	A_33_P3412463	C10orf81	Hs.228320	chromosome 10 open reading frame 81	Homo sapiens chromosome 10 open rea	65.60441	52.96472	16.16549	14.36548	Detected	Detected	Detected	Compromised
3833	A_33_P3385837	C10orf68	Hs.585464	chromosome 10 open reading frame 68	Homo sapiens chromosome 10 open rea	20.04126	17.67061	19.81384	5.51836	Detected	Compromised	Detected	Compromised
3834	A_23_P12062	TRIT1	Hs.356554	tRNA isopentenyltransferase 1	Homo sapiens tRNA isopentenyltransfer	1874.70510	1268.29170	1313.05290	710.30030	Detected	Detected	Detected	Detected
3835	A_32_P45009	IDH1	Hs.593422	isocitrate dehydrogenase 1 (NADP+), so	Homo sapiens isocitrate dehydrogenase	3496.21880	2437.56250	2445.42720	1425.16150	Detected	Detected	Detected	Detected
3836	A_23_P119344	TEAD2	Hs.515534	TEA domain family member 2	Homo sapiens TEA domain family memb	226.92830	201.53775	202.22047	140.56775	Detected	Detected	Detected	Detected
3837	A_23_P119627	NDUFA13	Hs.534453	NADH dehydrogenase (ubiquinone) 1 alp	Homo sapiens NADH dehydrogenase (ub	14446.12200	12769.41400	20009.55700	14620.35300	Detected	Detected	Detected	Detected
3838	A_23_P256682	APEX2	Hs.659558	APEX nuclease (apurinic/apyrimidinic e	Homo sapiens APEX nuclease (apurinic/	757.12683	1398.24820	963.39874	1425.31510	Detected	Detected	Detected	Detected
3839	A_33_P3213089					38.11620	41.39959	15.49532	15.41689	Detected	Detected	Detected	Compromised
3840	A_32_P207124	CT47A11	Hs.522775	cancer/testis antigen family 47, membe	Homo sapiens cancer/testis antigen fan	17773.47700	18012.43200	3681.93550	2278.43820	Detected	Detected	Detected	Detected
3841	A_24_P658427	NFIB	Hs.644095	nuclear factor I/B	Homo sapiens nuclear factor I/B (NFIB)	3430.19040	321.76053	1965.67790	128.21251	Detected	Detected	Detected	Detected
3842	A_24_P350546	LOC646976	Hs.632485	hypothetical LOC646976	PREDICTED: Homo sapiens hypothetica	15.87192	17.24732	12.96652	4.46093	Detected	Compromised	Compromised	Compromised
3843	A_33_P3340926	KIR3DX1		killer cell immunoglobulin-like receptor,	Homo sapiens killer cell immunoglobulin	46.94402	54.00395	20.83592	21.61592	Detected	Detected	Detected	Detected
3844	A_24_P76300	ZNF154	Hs.649031	zinc finger protein 154	Homo sapiens zinc finger protein 154, m	79.83522	61.76200	60.17498	42.59740	Detected	Detected	Detected	Detected
3845	A_23_P163942	SUPT4H1	Hs.439481	suppressor of Ty 4 homolog 1 (S. cerev	Homo sapiens suppressor of Ty 4 homo	21693.85400	26003.63500	20434.81800	20564.78700	Detected	Detected	Detected	Detected
3846	A_33_P3233659	RPP30	Hs.139120	ribonuclease P/MRP 30kDa subunit	Homo sapiens ribonuclease P/MRP 30k	3612.18240	5490.90670	1996.92030	2503.48560	Detected	Detected	Detected	Detected
3847	A_33_P3313421	UHRF1BP1	Hs.700656	UHRF1 binding protein 1	Homo sapiens UHRF1 binding protein 1	1486.08220	1714.85940	974.07940	999.57227	Detected	Detected	Detected	Detected
3848	A_24_P406301	NDUFB2	Hs.655788	NADH dehydrogenase (ubiquinone) 1 be	Homo sapiens NADH dehydrogenase (ub	29002.63700	58575.93000	28703.13900	38020.22700	Detected	Detected	Detected	Detected
3849	A_24_P346807	HERC4	Hs.51891	hect domain and RLD 4	Homo sapiens hect domain and RLD 4 (	2769.73020	2901.61180	1927.61400	1545.84520	Detected	Detected	Detected	Detected
3850	A_23_P374689	GAD1	Hs.420036	glutamate decarboxylase 1 (brain, 67kDa	Homo sapiens glutamate decarboxylase	12.30295	18.67002	4.31243	9.06273	Compromised	Detected	Compromised	Compromised
3851	A_33_P3308585	UNQ2963	Hs.246046	hypothetical LOC283314	Homo sapiens hypothetical LOC283314	13.81035	3.75938	8.53236	7.45404	Detected	Compromised	Compromised	Compromised
3852	A_23_P250404	RAD50	Hs.655835	RAD50 homolog (S. cerevisiae)	Homo sapiens RAD50 homolog (S. cerev	1642.65870	1104.59780	1264.38260	727.92865	Detected	Detected	Detected	Detected
3853	A_33_P3683076	SNORA11	Hs.679783	small nucleolar RNA, H/ACA box 11	602630895F1 NCI_CGAP_Skn3 Homo sa	354.33246	590.16910	477.53440	721.38950	Detected	Detected	Detected	Detected
3854	A_33_P3241204	CALHM3	Hs.364624	calcium homeostasis modulator 3	Homo sapiens calcium homeostasis mod	17.89732	34.24387	14.93891	20.12687	Detected	Detected	Compromised	Detected
3855	A_23_P422212	SLC35F3	Hs.158748	solute carrier family 35, member F3	Homo sapiens solute carrier family 35, r	199.96461	299.60764	17.90004	25.06656	Detected	Detected	Detected	Detected
3856	A_23_P130466	ZNF8	Hs.590941	zinc finger protein 8	Homo sapiens zinc finger protein 8 (ZNF	480.55570	358.28775	448.52716	265.82687	Detected	Detected	Detected	Detected
3857	A_33_P3398597	EPS8L1	Hs.438862	EPS8-like 1	Homo sapiens EPS8-like 1 (EPS8L1), tr	48.29141	9.39267	56.12793	6.74249	Detected	Compromised	Detected	Compromised
3858	A_33_P3210278	SYNE2	Hs.525392	spectrin repeat containing, nuclear enve	Homo sapiens spectrin repeat containin	372.47934	849.52950	220.72856	402.64276	Detected	Detected	Detected	Detected
3859	A_23_P211039	ADAMTS1	Hs.643357	ADAM metallopeptidase with thrombosp	Homo sapiens ADAM metallopeptidase	558.26170	2319.09940	480.39910	1676.57230	Detected	Detected	Detected	Detected
3860	A_23_P102109	TUBA4A	Hs.75318	tubulin, alpha 4a	Homo sapiens tubulin, alpha 4a (TUBA4	8879.71000	13729.91600	5819.99660	2818.92400	Detected	Detected	Detected	Detected
3861	A_23_P104617	GYLTL1B	Hs.86543	glycosyltransferase-like 1B	Homo sapiens glycosyltransferase-like	270.88940	284.88200	326.73007	285.12200	Detected	Detected	Detected	Detected
3862	A_23_P212522	ATP11B	Hs.478429	ATPase, class VI, type 11B	Homo sapiens ATPase, class VI, type 1	909.43390	588.77060	699.78570	401.74652	Detected	Detected	Detected	Detected
3863	A_24_P407717	GRB2	Hs.444356	growth factor receptor-bound protein 2	Homo sapiens growth factor receptor-b	2003.66780	2370.21310	2045.87020	2088.23800	Detected	Detected	Detected	Detected
3864	A_23_P36825	GPRC5A	Hs.631733	G protein-coupled receptor, family C, gr	Homo sapiens G protein-coupled recep	33.24828	44.87690	25.53676	30.84180	Detected	Detected	Detected	Detected
3865	A_23_P119130	RPS19	Hs.438429	ribosomal protein S19	Homo sapiens ribosomal protein S19 (R	36187.76000	30937.51000	43179.27000	31443.54900	Detected	Detected	Detected	Detected
3866	A_23_P52082	INTS7	Hs.369285	integrator complex subunit 7	Homo sapiens integrator complex subur	239.75371	358.81012	177.63460	221.07990	Detected	Detected	Detected	Detected
3867	A_23_P108761	C2orf29	Hs.593973	chromosome 2 open reading frame 29	Homo sapiens chromosome 2 open read	919.64820	1276.46630	708.34314	814.23530	Detected	Detected	Detected	Detected
3868	A_33_P3381305	LOC100132614		similar to rCG63049	PREDICTED: Homo sapiens hypothetica	375.71738	415.34540	211.16426	152.17194	Detected	Detected	Detected	Detected
3869	A_32_P228414	GAPVD1	Hs.495134	GTPase activating protein and VPS9 do	Homo sapiens GTPase activating protei	435.83527	710.24490	288.95322	410.99410	Detected	Detected	Detected	Detected
3870	A_33_P3397443	PKMYT1	Hs.77783	protein kinase, membrane associated ty	Homo sapiens protein kinase, membran	845.12085	16746.18400	606.99930	16077.28700	Detected	Detected	Detected	Detected
3871	A_33_P3303772	SLC6A3	Hs.406	solute carrier family 6 (neurotransmitte	Homo sapiens solute carrier family 6 (n	203.10928	309.99643	164.00641	513.39600	Detected	Detected	Detected	Detected
3872	A_24_P418637	MACF1	Hs.580782	microtubule-actin crosslinking factor 1	Homo sapiens microtubule-actin crossli	79.23869	87.39732	93.03868	75.99187	Detected	Detected	Detected	Detected
3873	A_24_P191790	TMEM33	Hs.31082	transmembrane protein 33	Homo sapiens transmembrane protein 3	256.50513	214.86708	126.89879	70.54521	Detected	Detected	Detected	Detected
3874	A_33_P3380932	ZNF800	Hs.159006	zinc finger protein 800	Homo sapiens zinc finger protein 800 (Z	182.25768	174.62679	167.26779	137.08710	Detected	Detected	Detected	Detected
3875	A_23_P404091	GRPEL2	Hs.511816	GrpE-like 2, mitochondrial (E. coli)	Homo sapiens GrpE-like 2, mitochondri	1735.98010	1904.21720	1297.84020	1184.56070	Detected	Detected	Detected	Detected
3876	A_33_P3272553	NCAPH2	Hs.180903	non-SMC condensin II complex, subunit	Homo sapiens non-SMC condensin II co	735.60675	935.34406	421.16583	428.15778	Detected	Detected	Detected	Detected
3877	A_23_P73457	RUFY1	Hs.306769	RUN and FYVE domain containing 1	Homo sapiens RUN and FYVE domain c	8030.82000	6109.43900	8088.04350	5278.23540	Detected	Detected	Detected	Detected
3878	A_24_P178963				Putative uncharacterized protein UNC9	181.24228	156.39587	205.47151	169.82753	Detected	Detected	Detected	Detected
3879	A_23_P126540	ADORA3	Hs.281342	adenosine A3 receptor	Homo sapiens adenosine A3 receptor (A	12.99843	23.58610	7.99999	5.89696	Detected	Detected	Compromised	Compromised
3880	A_23_P131626	SNRNP200	Hs.246112	small nuclear ribonucleoprotein 200kDa	Homo sapiens small nuclear ribonucleop	16933.55000	13258.26100	12600.96300	8495.87300	Detected	Detected	Detected	Detected
3881	A_33_P3401058				Homo sapiens cDNA FLJ12078 fis, clon	50.67567	42.69764	34.75398	22.38289	Detected	Detected	Detected	Detected
3882	A_24_P586523	ALG1	Hs.592086	asparagine-linked glycosylation 1, beta-	Homo sapiens asparagine-linked glycos	1792.14100	2065.39670	1761.32320	1739.43420	Detected	Detected	Detected	Detected
3883	A_23_P74162	IQCC	Hs.274356	IQ motif containing C	Homo sapiens IQ motif containing C (IQ	119.39271	161.26091	103.99912	125.02411	Detected	Detected	Detected	Detected
3884	A_23_P501933	CACNG6	Hs.631560	calcium channel, voltage-dependent, ga	Homo sapiens calcium channel, voltage-	358.90784	30.24085	334.77650	13.17023	Detected	Detected	Detected	Compromised
3885	A_23_P397347	MCM9	Hs.279008	minichromosome maintenance complex	Homo sapiens minichromosome mainter	102.70622	72.83482	75.63982	36.07800	Detected	Detected	Detected	Detected
3886	A_23_P325661	ZNF134	Hs.469694	zinc finger protein 134	Homo sapiens zinc finger protein 134 (Z	396.48477	523.75464	314.62140	326.87330	Detected	Detected	Detected	Detected
3887	A_33_P3365860	MAGEB5	Hs.224079	melanoma antigen family B, 5	Melanoma-associated antigen B5 (MAG	13.80851	18.76720	19.99039	9.41478	Detected	Detected	Detected	Compromised
3888	A_33_P3278714				Protein FAM179A [Source:UniProtKB/	83.67554	98.57907	50.98960	38.56059	Detected	Detected	Detected	Detected
3889	A_33_P3284596	ARL5C	Hs.568007	ADP-ribosylation factor-like 5C	Homo sapiens ADP-ribosylation factor-	121.38956	217.71642	84.00455	108.48237	Detected	Detected	Detected	Detected
3890	A_33_P3371055	RAB1A	Hs.310645	RAB1A, member RAS oncogene family	Homo sapiens RAB1A, member RAS on	32326.67000	39941.23000	24067.34800	23104.87500	Detected	Detected	Detected	Detected
3891	A_32_P193646	RBMX	Hs.380118	RNA binding motif protein, X-linked	Homo sapiens RNA binding motif protei	767.20660	2467.91720	511.97495	1229.86240	Detected	Detected	Detected	Detected
3892	A_33_P3289780	C14orf2	Hs.109052	chromosome 14 open reading frame 2	Homo sapiens chromosome 14 open rea	3620.91800	3907.33100	3041.83740	2751.24150	Detected	Detected	Detected	Detected
3893	A_23_P330616	WIPF1	Hs.128067	WAS/WASL interacting protein family, r	Homo sapiens WAS/WASL interacting p	90.88859	223.28610	85.63651	194.16342	Detected	Detected	Detected	Detected
3894	A_33_P3408918	SAA2	Hs.1955	serum amyloid A2	Homo sapiens serum amyloid A2 (SAA2	19.70596	59.47710	16.43667	35.36048	Detected	Detected	Detected	Detected
3895	A_33_P3214874	KPNA6	Hs.470588	karyopherin alpha 6 (importin alpha 7)	Homo sapiens karyopherin alpha 6 (impo	9249.64550	13457.43700	7204.99200	9154.33400	Detected	Detected	Detected	Detected
3896	A_23_P358662	KIAA1712	Hs.555989	KIAA1712	Homo sapiens KIAA1712 (KIAA1712), tr	699.72003	288.33597	444.88538	133.92720	Detected	Detected	Detected	Detected

3897	A_23_P47208	BANF1	Hs.433759	barrier to autointegration factor 1	Homo sapiens barrier to autointegration	3579.11570	6888.07230	3131.86300	5176.65300	Detected	Detected	Detected	Detected
3898	A_32_P42197	HNRNPA1	Hs.546261	heterogeneous nuclear ribonucleoprotei	Homo sapiens heterogeneous nuclear ri	67173.85000	95084.74000	55051.60500	64303.28000	Detected	Detected	Detected	Detected
3899	A_33_P3244361					2952.66200	4891.24900	1649.27730	1986.11220	Detected	Detected	Detected	Detected
3900	A_23_P21376	MAGI2	Hs.603842	membrane associated guanylate kinase,	Homo sapiens membrane associated gu	293.67280	129.99911	174.71713	52.72998	Detected	Detected	Detected	Detected
3901	A_23_P433791	C1orf124	Hs.554892	chromosome 1 open reading frame 124	Homo sapiens chromosome 1 open read	229.79886	209.40321	180.91520	134.68419	Detected	Detected	Detected	Detected
3902	A_33_P3412061				Tetratricopeptide repeat protein 32 (TP	63.82623	19.38232	3.18377	5.35303	Detected	Detected	Compromised	Compromised
3903	A_23_P144311	POLR2B	Hs.602757	polymerase (RNA) II (DNA directed) pol	Homo sapiens polymerase (RNA) II (DNA	7208.86100	6326.45650	5652.89500	4178.67970	Detected	Detected	Detected	Detected
3904	A_33_P3374723	ZEB1	Hs.124503	zinc finger E-box binding homeobox 1	Homo sapiens zinc finger E-box binding	15555.12400	9915.04100	10596.94500	5717.97600	Detected	Detected	Detected	Detected
3905	A_33_P3368144	LOC652614		similar to HLA class I histocompatibility	PREDICTED: Homo sapiens similar to M	26.42275	26.86960	9.56121	4.55289	Detected	Detected	Compromised	Compromised
3906	A_33_P3333360	C16orf5	Hs.654653	chromosome 16 open reading frame 5	Homo sapiens chromosome 16 open rea	1612.44260	1810.82400	700.81200	629.55980	Detected	Detected	Detected	Detected
3907	A_33_P3337134	ABLIM2	Hs.233404	actin binding LIM protein family, membe	Homo sapiens actin binding LIM protein	118.96076	6.37250	97.44179	3.38190	Detected	Compromised	Detected	Compromised
3908	A_33_P3389599		Hs.721707		chromosome 21 open reading frame 81	20.18729	33.23436	9.55626	19.04255	Detected	Detected	Compromised	Detected
3909	A_33_P3347147	SUPT3H	Hs.368325	suppressor of Ty 3 homolog (S. cerevisi	Homo sapiens suppressor of Ty 3 homo	37.27906	21.22792	22.91353	11.84544	Detected	Detected	Detected	Compromised
3910	A_33_P3272957	LOC100133311	Hs.658061	similar to hCG1644697	PREDICTED: Homo sapiens similar to h	2.87419	103.57362	3.31359	69.26411	Compromised	Detected	Compromised	Detected
3911	A_24_P7157	RIMKLB	Hs.504670	ribosomal modification protein rimK-like	Homo sapiens ribosomal modification pr	113.03825	69.40007	108.01521	58.24527	Detected	Detected	Detected	Detected
3912	A_23_P48936	SMAD3	Hs.36915	SMAD family member 3	Homo sapiens SMAD family member 3 (	942.04120	906.65060	566.50190	471.78156	Detected	Detected	Detected	Detected
3913	A_23_P25403	HCFC2	Hs.506558	host cell factor C2	Homo sapiens host cell factor C2 (HCF	947.89484	632.53700	547.71857	263.27823	Detected	Detected	Detected	Detected
3914	A_33_P3400263	CHRAC1	Hs.279704	chromatin accessibility complex 1	Homo sapiens chromatin accessibility c	1333.28340	1012.32220	976.90440	631.07400	Detected	Detected	Detected	Detected
3915	A_23_P118300	FAHD1	Hs.513265	fumarylacetoacetate hydrolase domain	Homo sapiens fumarylacetoacetate hyd	745.33990	1933.49340	669.80225	1451.84580	Detected	Detected	Detected	Detected
3916	A_23_P88865	CMTM3	Hs.298198	CKLF-like MARVEL transmembrane do	Homo sapiens CKLF-like MARVEL tran	24190.06600	32721.91600	19071.97700	21633.39300	Detected	Detected	Detected	Detected
3917	A_23_P71908	SNAPC3	Hs.546299	small nuclear RNA activating complex, p	Homo sapiens small nuclear RNA activa	214.81737	285.06378	194.63728	206.01587	Detected	Detected	Detected	Detected
3918	A_33_P3262118		Hs.257900		Leucine zipper protein 1 [Source:UniPr	17.28480	13.35092	10.92846	4.13293	Detected	Compromised	Compromised	Compromised
3919	A_33_P3369844	CD24	Hs.644105	CD24 molecule	Homo sapiens CD24 molecule (CD24), r	27.24082	49.88758	12.99351	33.96960	Detected	Detected	Compromised	Detected
3920	A_23_P120472	TFAP2C	Hs.473152	transcription factor AP-2 gamma (activ	Homo sapiens transcription factor AP-2	870.85790	778.95557	686.40870	528.58470	Detected	Detected	Detected	Detected
3921	A_32_P92281	LOC100132832	Hs.720896	postmeiotic segregation increased 2-lik	Homo sapiens postmeiotic segregation	44.97765	96.33431	37.44844	64.73505	Detected	Detected	Detected	Detected
3922	A_33_P3250944	C1orf189	Hs.104967	chromosome 1 open reading frame 189	Homo sapiens chromosome 1 open read	37.76807	24.75015	20.02984	12.23289	Detected	Detected	Detected	Compromised
3923	A_33_P3249534	NEFM	Hs.719965	neurofilament, medium polypeptide	Homo sapiens neurofilament, medium po	215.03410	235.17209	179.29338	186.94902	Detected	Detected	Detected	Detected
3924	A_33_P3288605	LOC100133287	Hs.684477	hypothetical protein LOC100133287	Homo sapiens clone pp10199 unknown	23.53306	31.81386	16.70908	12.79776	Detected	Detected	Detected	Compromised
3925	A_33_P3281478					71.83350	106.70172	62.93208	64.00671	Detected	Detected	Detected	Detected
3926	A_24_P782308	NEDD4L	Hs.185677	neural precursor cell expressed, develo	Homo sapiens neural precursor cell exp	31.40292	29.50642	23.71002	16.06863	Detected	Detected	Detected	Detected
3927	A_33_P3816266	ZNF253	Hs.659321	zinc finger protein 253	Homo sapiens cDNA FLJ90276 fis, clon	177.91998	110.15317	85.65245	39.84946	Detected	Detected	Detected	Detected
3928	A_24_P184388	LRTOMT	Hs.317243	leucine rich transmembrane and O-meth	Homo sapiens leucine rich transmembra	129.65210	167.72247	100.85617	124.06918	Detected	Detected	Detected	Detected
3929	A_23_P304524	DCLK2	Hs.591683	doublecortin-like kinase 2	Homo sapiens doublecortin-like kinase	97.21934	163.32892	107.30568	169.67258	Detected	Detected	Detected	Detected
3930	A_33_P3263170	LOC100130932	Hs.631639	small nuclear ribonucleoprotein G-like p	Homo sapiens small nuclear ribonucleop	33110.85000	45337.34800	27167.37500	31680.74800	Detected	Detected	Detected	Detected
3931	A_24_P36890	RAP1GAP	Hs.148178	RAP1 GTPase activating protein	Homo sapiens RAP1 GTPase activating	28.80279	33.46730	8.39505	21.23230	Detected	Detected	Compromised	Detected
3932	A_33_P3396872	C7orf58	Hs.189652	chromosome 7 open reading frame 58	Homo sapiens chromosome 7 open read	146.09843	293.31445	116.89478	191.46861	Detected	Detected	Detected	Detected
3933	A_23_P257895	MAPK1	Hs.431850	mitogen-activated protein kinase 1	Homo sapiens mitogen-activated protei	896.05273	1108.75660	720.65840	835.80396	Detected	Detected	Detected	Detected
3934	A_32_P58614	KIAA1377	Hs.156352	KIAA1377	Homo sapiens KIAA1377 (KIAA1377), m	128.32167	20.41560	48.60483	3.16196	Detected	Detected	Detected	Compromised
3935	A_23_P153616	MADCAM1	Hs.102598	mucosal vascular addressin cell adhesio	Homo sapiens mucosal vascular addres	52.81176	21.21942	18.55060	3.15962	Detected	Detected	Detected	Compromised
3936	A_33_P3231437					13.25268	18.77207	3.85055	3.15706	Detected	Detected	Compromised	Compromised
3937	A_33_P3354203				Isocitrate dehydrogenase 3 (NAD+) gam	229.42615	306.93400	65.83530	74.99915	Detected	Detected	Detected	Detected
3938	A_33_P3353259					112283.53000	106797.71000	84578.19500	68049.21000	Detected	Detected	Detected	Detected
3939	A_33_P3221373		Hs.571415		AGENCOURT_13445090 NIH_MGC_187	89.88357	83.12487	20.85138	14.97488	Detected	Detected	Detected	Compromised
3940	A_23_P348227	ZNF135	Hs.85863	zinc finger protein 135	Homo sapiens zinc finger protein 135 (Z	162.47015	141.67937	113.63348	79.81692	Detected	Detected	Detected	Detected
3941	A_33_P3264910	NKX6-2	Hs.134013	NK6 homeobox 2	Homo sapiens NK6 homeobox 2 (NKX6-	8.81191	23.23373	13.50177	12.21949	Compromised	Detected	Compromised	Compromised
3942	A_33_P3386932	FAM86A	Hs.406461	family with sequence similarity 86, mem	Homo sapiens family with sequence sim	2017.74350	2887.82100	1870.85250	2165.57150	Detected	Detected	Detected	Detected
3943	A_33_P3240078					2084.64230	2276.76250	2330.76420	1913.14640	Detected	Detected	Detected	Detected
3944	A_33_P3307307	MLL	Hs.258855	myeloid/lymphoid or mixed-lineage leuk	Homo sapiens myeloid/lymphoid or mixe	166.79413	151.84702	95.89465	76.93249	Detected	Detected	Detected	Detected
3945	A_33_P3405434				von Willebrand factor A domain-contain	28.88396	39.33635	13.58323	11.34456	Detected	Detected	Compromised	Compromised
3946	A_24_P354689	SPOCK1	Hs.596136	sparc/osteonectin, cwcv and kazal-like	Homo sapiens sparc/osteonectin, cwcv	5326.84130	6716.79500	3079.24050	3332.21260	Detected	Detected	Detected	Detected
3947	A_24_P186124	MTERFD2	Hs.159556	MTERF domain containing 2	Homo sapiens MTERF domain containin	3697.10060	3815.33620	2939.60990	2517.28880	Detected	Detected	Detected	Detected
3948	A_24_P373126	TRMT61A	Hs.525610	tRNA methyltransferase 61 homolog A (	Homo sapiens tRNA methyltransferase	327.33063	401.79166	194.56350	276.03427	Detected	Detected	Detected	Detected
3949	A_33_P3375269					27.24106	17.34370	12.55356	9.73318	Detected	Compromised	Compromised	Compromised
3950	A_23_P501372	SMCR7	Hs.655555	Smith-Magenis syndrome chromosome	Homo sapiens Smith-Magenis syndrome	1080.95140	1477.44400	1112.19640	1449.29710	Detected	Detected	Detected	Detected
3951	A_23_P393777	PTGDR	Hs.306831	prostaglandin D2 receptor (DP)	Homo sapiens prostaglandin D2 recepto	497.72388	177.86531	466.53850	123.26009	Detected	Detected	Detected	Detected
3952	A_23_P26522	AQP8	Hs.176658	aquaporin 8	Homo sapiens aquaporin 8 (AQP8), mRI	74.23408	188.35146	48.04609	92.22843	Detected	Detected	Detected	Detected
3953	A_23_P502047	CHRD	Hs.166186	chordin	Homo sapiens chordin (CHRD), mRNA [	525.83710	246.49597	627.15780	401.91970	Detected	Detected	Detected	Detected
3954	A_33_P3407424	CDC42EP1	Hs.225356	CDC42 effector protein (Rho GTPase b	Homo sapiens CDC42 effector protein (	20032.55900	37547.47700	16783.97500	28921.36500	Detected	Detected	Detected	Detected
3955	A_23_P29769	WWTR1	Hs.477921	WW domain containing transcription reg	Homo sapiens WW domain containing tr	300.61365	777.64980	292.62402	632.50226	Detected	Detected	Detected	Detected
3956	A_23_P57306	CHAF1B	Hs.75238	chromatin assembly factor 1, subunit B	Homo sapiens chromatin assembly fact	1814.68620	3942.04600	1618.94170	3021.62840	Detected	Detected	Detected	Detected
3957	A_33_P3628481	MGC27345	Hs.552129	hypothetical protein MGC27345	PREDICTED: Homo sapiens hypothetica	582.44460	813.99365	364.11954	430.14810	Detected	Detected	Detected	Detected
3958	A_23_P210379	ARFGAP1	Hs.25584	ADP-ribosylation factor GTPase activa	Homo sapiens ADP-ribosylation factor	7257.94340	8297.95100	8661.71300	8981.43700	Detected	Detected	Detected	Detected
3959	A_23_P150549	PGA3	Hs.601055	pepsinogen 3, group I (pepsinogen A)	Homo sapiens pepsinogen 3, group I (pe	12.50874	26.69241	7.56412	14.18200	Compromised	Detected	Compromised	Compromised
3960	A_33_P3217028	TM9SF1	Hs.91586	transmembrane 9 superfamily member	Homo sapiens transmembrane 9 superfa	1561.51150	1993.63480	1406.48100	1537.87790	Detected	Detected	Detected	Detected
3961	A_23_P413641	PREX1	Hs.153310	phosphatidylinositol-3,4,5-trisphosphate	Homo sapiens phosphatidylinositol-3,4,5	1698.27860	711.58984	1520.15480	504.29385	Detected	Detected	Detected	Detected
3962	A_23_P51761	OR6K2	Hs.554489	olfactory receptor, family 6, subfamily K	Homo sapiens olfactory receptor, family	39.14969	59.96766	39.63957	35.74550	Detected	Detected	Detected	Detected
3963	A_33_P3447304	OXR1	Hs.148778	oxidation resistance 1	Homo sapiens oxidation resistance 1 (O	598.05347	546.13794	464.97632	367.48914	Detected	Detected	Detected	Detected
3964	A_23_P253074	PIWIL2	Hs.614809	piwi-like 2 (Drosophila)	Homo sapiens piwi-like 2 (Drosophila) (F	27.68733	56.00263	17.64961	38.35213	Detected	Detected	Detected	Detected
3965	A_33_P3356325	ETV6	Hs.504765	ets variant 6	Homo sapiens ets variant 6 (ETV6), mR	66.02714	85.80685	81.01636	79.86774	Detected	Detected	Detected	Detected
3966	A_33_P3266132		Hs.657920		Grainyhead-like protein 3 homolog (Sist	20.16297	14.29657	9.76947	9.41192	Detected	Compromised	Compromised	Compromised
3967	A_23_P108641	FAM136A	Hs.516077	family with sequence similarity 136, mer	Homo sapiens family with sequence sim	8433.96900	10528.52500	6289.18800	6594.29250	Detected	Detected	Detected	Detected
3968	A_23_P168882	TP53INP1	Hs.492261	tumor protein p53 inducible nuclear pro	Homo sapiens tumor protein p53 induci	3393.81700	4473.63700	2763.79700	2946.39000	Detected	Detected	Detected	Detected
3969	A_33_P3302652		Hs.671099		Chromosome 1 open reading frame 132	17.85942	11.72815	23.36105	11.04579	Detected	Compromised	Detected	Compromised
3970	A_33_P3416124	LOC100130872		hypothetical LOC100130872	Homo sapiens hypothetical LOC100130	89.03939	141.48703	63.49580	82.65270	Detected	Detected	Detected	Detected
3971	A_33_P3363560	TMEM51	Hs.465305	transmembrane protein 51	Homo sapiens transmembrane protein 5	428.72476	1590.30940	403.14370	1230.38610	Detected	Detected	Detected	Detected

3972	A_23_P257578	SRA1	Hs.653135	steroid receptor RNA activator 1	Homo sapiens steroid receptor RNA act	2375.07200	5082.40770	1864.97270	3317.11230	Detected	Detected	Detected	Detected
3973	A_23_P110837	IRX4	Hs.196927	iroquois homeobox 4	Homo sapiens iroquois homeobox 4 (IRX	1106.68080	21.10374	989.28080	17.46779	Detected	Not Detected	Detected	Detected
3974	A_24_P65098	TMEM87A	Hs.597178	transmembrane protein 87A	Homo sapiens transmembrane protein 8	11.75098	99.89788	19.55616	54.35128	Compromised	Detected	Detected	Detected
3975	A_33_P3772150	LOC100130729	Hs.586663	hypothetical protein LOC100130729	Homo sapiens cDNA FLJ45419 fis, clon	17.02328	26.59152	17.32205	12.19539	Detected	Detected	Detected	Compromised
3976	A_32_P142440	PCSK9	Hs.18844	proprotein convertase subtilisin/kexin t	Homo sapiens proprotein convertase su	2.92709	40.81410	5.23401	20.72815	Compromised	Detected	Compromised	Detected
3977	A_33_P3406706	EXOG	Hs.517897	endo/exonuclease (5'-3'), endonuclease	Homo sapiens endo/exonuclease (5'-3')	167.71399	175.26822	245.14507	214.34737	Detected	Detected	Detected	Detected
3978	A_33_P3341494	ZNF658	Hs.522147	zinc finger protein 658	Homo sapiens zinc finger protein 658 (Z	180.87292	98.37334	152.45934	56.24946	Detected	Detected	Detected	Detected
3979	A_33_P3335920	SYNE1	Hs.12967	spectrin repeat containing, nuclear enve	Homo sapiens spectrin repeat containin	18716.52700	22608.32000	4655.76660	3580.85030	Detected	Detected	Detected	Detected
3980	A_23_P157361	WDR60	Hs.389945	WD repeat domain 60	Homo sapiens WD repeat domain 60 (W	1735.24350	668.35730	1064.59530	331.62662	Detected	Detected	Detected	Detected
3981	A_33_P3423285		Hs.598958		Putative uncharacterized protein FLJ46	52.85229	46.05763	18.54220	19.49658	Detected	Detected	Detected	Detected
3982	A_23_P216023	ANGPT1	Hs.369675	angiopoietin 1	Homo sapiens angiopoietin 1 (ANGPT1)	130.44817	1093.00240	114.93729	860.66705	Detected	Detected	Detected	Detected
3983	A_23_P211207	ADARB1	Hs.474018	adenosine deaminase, RNA-specific, B1	Homo sapiens adenosine deaminase, RN	2904.15820	2061.81320	2197.38090	1334.76940	Detected	Detected	Detected	Detected
3984	A_23_P10870	DOLK	Hs.531563	dolichol kinase	Homo sapiens dolichol kinase (DOLK), n	2677.24730	2794.90140	2209.02610	2150.18040	Detected	Detected	Detected	Detected
3985	A_24_P941708	RUFY2	Hs.653144	RUN and FYVE domain containing 2	Homo sapiens RUN and FYVE domain c	65.07817	64.97730	40.49668	29.73896	Detected	Detected	Detected	Detected
3986	A_33_P3272117	ASPSCR1	Hs.298351	alveolar soft part sarcoma chromosome	Homo sapiens alveolar soft part sarcom	903.18410	568.65470	819.75910	515.16614	Detected	Detected	Detected	Detected
3987	A_23_P393080	CALML3	Hs.239600	calmodulin-like 3	Homo sapiens calmodulin-like 3 (CALMI	961.73480	2002.00890	465.37082	976.64355	Detected	Detected	Detected	Detected
3988	A_33_P3392320	SLC6A15	Hs.44424	solute carrier family 6 (neutral amino ac	Homo sapiens solute carrier family 6 (ne	118.91280	33.07696	119.35487	20.97982	Detected	Detected	Detected	Detected
3989	A_23_P13713	PRPH	Hs.37044	peripherin	Homo sapiens peripherin (PRPH), mRNA	129.22130	225.89209	31.20463	33.15455	Detected	Detected	Detected	Detected
3990	A_23_P122852	SMARCD3	Hs.647067	SWI/SNF related, matrix associated, ac	Homo sapiens SWI/SNF related, matrix	10948.12300	5085.48970	13912.39300	5254.73970	Detected	Detected	Detected	Detected
3991	A_33_P3291294	EXTL3	Hs.491354	exostoses (multiple)-like 3	Homo sapiens exostoses (multiple)-like	13706.74600	17959.53700	9945.05900	9930.09500	Detected	Detected	Detected	Detected
3992	A_24_P287189	TOLLIP	Hs.719949	toll interacting protein	Homo sapiens toll interacting protein (T	504.90024	228.13170	225.51454	36.42691	Detected	Detected	Detected	Detected
3993	A_23_P34788	KIF2C	Hs.720061	kinesin family member 2C	Homo sapiens kinesin family member 2C	273.92862	16853.63900	207.39778	12643.64200	Detected	Detected	Detected	Detected
3994	A_33_P3257944					62080.15200	43551.44000	13335.32800	7604.43750	Detected	Detected	Detected	Detected
3995	A_23_P215296	CDC2L5	Hs.233552	cell division cycle 2-like 5 (cholinestera	Homo sapiens cell division cycle 2-like	601.40020	574.21655	521.64870	406.02167	Detected	Detected	Detected	Detected
3996	A_33_P3320619	HDAC6	Hs.6764	histone deacetylase 6	Homo sapiens histone deacetylase 6 (H	40.98766	55.52511	36.62278	34.69270	Detected	Detected	Detected	Detected
3997	A_33_P3259042	MYOCD	Hs.567641	myocardin	Homo sapiens myocardin (MYOCD), tra	7.10948	18.20330	6.43418	10.72063	Compromised	Detected	Compromised	Compromised
3998	A_23_P110661	SKIV2L2	Hs.274531	superkiller viralicidic activity 2-like 2 (S	Homo sapiens superkiller viralicidic acti	4296.19240	4004.39100	3512.60130	2701.86910	Detected	Detected	Detected	Detected
3999	A_33_P3317277	DPF1	Hs.631576	D4, zinc and double PHD fingers family	Homo sapiens D4, zinc and double PHD	698.30610	944.74960	246.31444	423.70612	Detected	Detected	Detected	Detected
4000	A_23_P158775	UMOD	Hs.654425	uromodulin	Homo sapiens uromodulin (UMOD), tran	190.55829	283.40020	114.23948	119.60761	Detected	Detected	Detected	Detected
4001	A_23_P107454	KRTAP3-1	Hs.307027	keratin associated protein 3-1	Homo sapiens keratin associated protei	23.65426	30.50023	18.04009	20.53068	Detected	Detected	Detected	Detected
4002	A_24_P238143	LRRC37A2	Hs.646568	leucine rich repeat containing 37, memb	Homo sapiens leucine rich repeat conta	374.14432	265.15332	288.77182	181.29416	Detected	Detected	Detected	Detected
4003	A_24_P374382	TOP1P2	Hs.381281	topoisomerase (DNA) I pseudogene 2	Homo sapiens topoisomerase (DNA) I p	863.34640	1013.73846	538.07837	516.89150	Detected	Detected	Detected	Detected
4004	A_24_P306136	KIAA1432	Hs.211520	KIAA1432	Homo sapiens KIAA1432 (KIAA1432), tr	56.45766	53.62382	37.38542	34.56426	Detected	Detected	Detected	Detected
4005	A_33_P3221055					32.18138	14.68908	14.12771	7.35045	Detected	Compromised	Compromised	Compromised
4006	A_33_P3310706		Hs.576915		DB043695 TESTI2 Homo sapiens cDNA	149.40631	179.22057	100.55471	91.08478	Detected	Detected	Detected	Detected
4007	A_24_P212819				CDNA FLJ25155 fis, clone CBR07976	32538.81000	44001.36300	33174.87000	33013.27000	Detected	Detected	Detected	Detected
4008	A_33_P3382856	DCN	Hs.156316	decorin	Homo sapiens decorin (DCN), transcript	51018.80500	26404.82000	45476.50000	23155.08800	Detected	Detected	Detected	Detected
4009	A_23_P115149	WDR77	Hs.204773	WD repeat domain 77	Homo sapiens WD repeat domain 77 (W	586.81230	1510.84510	483.81512	1161.36390	Detected	Detected	Detected	Detected
4010	A_33_P3393543		Hs.590968		Putative uncharacterized protein LOC4	57.80022	20.65415	23.89595	9.53139	Detected	Detected	Detected	Compromised
4011	A_33_P3407691					45.75657	51.76244	26.44276	30.27413	Detected	Detected	Detected	Detected
4012	A_23_P54758	GDE1	Hs.512607	glycerophosphodiester phosphodiestera	Homo sapiens glycerophosphodiester ph	4758.81250	2824.72900	2658.71680	1293.66800	Detected	Detected	Detected	Detected
4013	A_23_P168165	C6orf208	Hs.677182	chromosome 6 open reading frame 208	Homo sapiens chromosome 6 open read	47.70738	70.10933	15.88053	27.48226	Detected	Detected	Detected	Detected
4014	A_23_P206501	CLEC18B	Hs.556045	C-type lectin domain family 18, member	Homo sapiens C-type lectin domain fam	37.56757	62.01484	35.35198	58.10139	Detected	Detected	Detected	Detected
4015	A_33_P3315874	DHX30	Hs.517948	DEAH (Asp-Glu-Ala-His) box polypepti	Homo sapiens DEAH (Asp-Glu-Ala-His)	31035.76800	34466.43800	19162.04100	18418.50200	Detected	Detected	Detected	Detected
4016	A_24_P350759	SLC1A2	Hs.502338	solute carrier family 1 (glial high affinity	Homo sapiens solute carrier family 1 (gl	111.41322	82.19964	69.13790	40.48160	Detected	Detected	Detected	Detected
4017	A_33_P3727762	SET	Hs.436687	SET nuclear oncogene	Homo sapiens SET nuclear oncogene (S	16120.79200	21444.01800	11911.97400	12154.76100	Detected	Detected	Detected	Detected
4018	A_23_P59022	TRERF1	Hs.485392	transcriptional regulating factor 1	Homo sapiens transcriptional regulating	467.61020	601.93756	399.52480	449.37445	Detected	Detected	Detected	Detected
4019	A_23_P310560	NUDT16	Hs.591313	nudix (nucleoside diphosphate linked mo	Homo sapiens nudix (nucleoside diphosp	1184.33910	1642.19190	1075.31120	1281.76780	Detected	Detected	Detected	Detected
4020	A_33_P3308045				Eukaryotic translation initiation factor 4	22.88191	21.31399	9.85554	16.64638	Detected	Detected	Compromised	Compromised
4021	A_32_P175739	HK2	Hs.591588	hexokinase 2	Homo sapiens hexokinase 2 (HK2), mRN	4148.66000	6282.76000	3199.02880	3973.48220	Detected	Detected	Detected	Detected
4022	A_33_P3261914		Hs.512544		Homo sapiens cDNA clone IMAGE:5267	310.97482	316.74720	123.07250	112.98742	Detected	Detected	Detected	Detected
4023	A_33_P3423859	PPP1R3D	Hs.42215	protein phosphatase 1, regulatory (inhib	Homo sapiens protein phosphatase 1, re	1121.56690	1551.33410	938.71796	1112.71720	Detected	Detected	Detected	Detected
4024	A_23_P19182	REEP2	Hs.416090	receptor accessory protein 2	Homo sapiens receptor accessory prote	10293.22000	2173.38450	9871.66900	1857.93050	Detected	Detected	Detected	Detected
4025	A_23_P426472	ZNF45	Hs.381285	zinc finger protein 45	Homo sapiens zinc finger protein 45 (ZN	410.57483	425.96457	380.47433	331.92444	Detected	Detected	Detected	Detected
4026	A_23_P127948	ADM	Hs.441047	adrenomedullin	Homo sapiens adrenomedullin (ADM), m	103370.63000	141143.77000	69297.46000	80770.05000	Detected	Detected	Detected	Detected
4027	A_33_P3217743	LOC728417	Hs.646112	hypothetical LOC728417	PREDICTED: Homo sapiens hypothetica	23.13366	15.67215	14.08668	11.89035	Detected	Compromised	Compromised	Compromised
4028	A_33_P3330413					2320.49340	5225.58740	2252.40400	4130.44400	Detected	Detected	Detected	Detected
4029	A_33_P3235568	CAPZB	Hs.432760	capping protein (actin filament) muscle	Homo sapiens capping protein (actin fila	7198.66550	10172.07500	5844.39260	6797.31900	Detected	Detected	Detected	Detected
4030	A_33_P3369317	DNAJB5	Hs.237506	DnaJ (Hsp40) homolog, subfamily B, me	Homo sapiens DnaJ (Hsp40) homolog, s	515.74880	582.42570	534.97470	500.81036	Detected	Detected	Detected	Detected
4031	A_23_P60458	PPP2R4	Hs.400740	protein phosphatase 2A activator, regul	Homo sapiens protein phosphatase 2A a	1319.33390	1380.15040	1228.92630	1275.79400	Detected	Detected	Detected	Detected
4032	A_33_P3861385	PGM5P1	Hs.650460	phosphoglucomutase 5 pseudogene 1	Homo sapiens cDNA FLJ38840 fis, clon	20.53688	4.03941	12.81287	3.49722	Detected	Compromised	Compromised	Compromised
4033	A_32_P351936	PMS2L5	Hs.720896	postmeiotic segregation increased 2-lik	Homo sapiens postmeiotic segregation i	427.01303	583.32500	483.35577	545.40015	Detected	Detected	Detected	Detected
4034	A_24_P197537	PDE8B	Hs.584830	phosphodiesterase 8B	Homo sapiens phosphodiesterase 8B (P	20.28899	17.49742	21.65499	13.43312	Detected	Compromised	Detected	Compromised
4035	A_32_P74712	SENP2	Hs.401388	SUMO1/sentrin/SMT3 specific peptidas	Homo sapiens SUMO1/sentrin/SMT3 s	3267.37820	3423.80420	2663.39430	2482.02800	Detected	Detected	Detected	Detected
4036	A_33_P3396326					18.35821	39.49315	5.45150	4.56597	Detected	Detected	Compromised	Compromised
4037	A_33_P3293432	LRRC37A3	Hs.551962	leucine rich repeat containing 37, memb	Homo sapiens leucine rich repeat conta	404.13922	227.25758	268.15497	121.07182	Detected	Detected	Detected	Detected
4038	A_32_P62997	PBK	Hs.104741	PDZ binding kinase	Homo sapiens PDZ binding kinase (PBK	272.00460	9988.02700	235.21033	7407.88530	Detected	Detected	Detected	Detected
4039	A_33_P3375234	FAM82A2	Hs.511067	family with sequence similarity 82, mem	Homo sapiens family with sequence sim	1026.78110	1236.98610	1112.51050	1215.60070	Detected	Detected	Detected	Detected
4040	A_33_P3371514	IPPK	Hs.459896	inositol 1,3,4,5,6-pentakisphosphate 2-k	Homo sapiens inositol 1,3,4,5,6-pentakis	231.16832	478.99847	223.43190	372.31122	Detected	Detected	Detected	Detected
4041	A_23_P373724	PPFIBP1	Hs.172445	PTPRF interacting protein, binding prot	Homo sapiens PTPRF interacting protei	5138.70600	8257.43750	2758.75560	3369.77880	Detected	Detected	Detected	Detected
4042	A_23_P130435	LIM2	Hs.162754	lens intrinsic membrane protein 2, 19kD	Homo sapiens lens intrinsic membrane p	16.04314	21.31989	5.11823	3.38470	Detected	Detected	Compromised	Compromised
4043	A_23_P161998	HPX	Hs.426485	hemopexin	Homo sapiens hemopexin (HPX), mRNA	50.10967	59.73994	31.18187	33.19838	Detected	Detected	Detected	Detected
4044	A_24_P181585	LRRC59	Hs.370927	leucine rich repeat containing 59	Homo sapiens leucine rich repeat conta	9999.81900	36114.66000	6029.58600	17237.49200	Detected	Detected	Detected	Detected
4045	A_33_P3258383					55.43835	24.61155	63.49353	18.54869	Detected	Detected	Detected	Detected
4046	A_33_P3267532	KCNJ15	Hs.411299	potassium inwardly-rectifying channel, s	Homo sapiens potassium inwardly-recti	127.23847	147.24852	137.68167	138.45737	Detected	Detected	Detected	Detected

4047	A_33_P3259428	WDR55		WD repeat domain 55	WD repeat-containing protein 55 [Sour	1531.73670	1792.51890	891.70496	830.69354	Detected	Detected	Detected	Detected
4048	A_33_P3272209	MFSD6	Hs.715029	major facilitator superfamily domain cor	Homo sapiens major facilitator superfan	459.31890	705.91670	382.04720	472.71683	Detected	Detected	Detected	Detected
4049	A_33_P3298617	LOC645781	Hs.646773	similar to mCG141871	PREDICTED: Homo sapiens similar to m	1312.70500	1438.75890	4360.91750	4281.17800	Detected	Detected	Detected	Detected
4050	A_24_P277955	FIS1	Hs.423968	fission 1 (mitochondrial outer membrane	Homo sapiens fission 1 (mitochondrial c	4003.53880	5189.56640	5440.10600	6309.90530	Detected	Detected	Detected	Detected
4051	A_33_P3222917	CD276	Hs.77873	CD276 molecule	Homo sapiens CD276 molecule (CD276)	66174.44500	70087.08000	45271.19500	43922.43400	Detected	Detected	Detected	Detected
4052	A_23_P111132	HSPA1A	Hs.274402	heat shock 70kDa protein 1A	Homo sapiens heat shock 70kDa proteir	16387.78000	12141.62300	10518.42500	6770.46200	Detected	Detected	Detected	Detected
4053	A_33_P3336810	NUDT17	Hs.585066	nudix (nucleoside diphosphate linked mc	Homo sapiens nudix (nucleoside diphosp	62.48917	123.01572	60.41163	74.60288	Detected	Detected	Detected	Detected
4054	A_33_P3357431		Hs.683806		Novel protein (FLJ40547) [Source:UniF	55.71569	58.22394	16.22490	18.54182	Detected	Detected	Detected	Detected
4055	A_23_P9614	NDUFA4L2	Hs.720062	NADH dehydrogenase (ubiquinone) 1 alp	Homo sapiens NADH dehydrogenase (ul	22.48559	180.38762	19.47534	125.40830	Detected	Detected	Detected	Detected
4056	A_23_P301372	TAPT1	Hs.479223	transmembrane anterior posterior trans	Homo sapiens transmembrane anterior	444.77274	328.69165	321.39640	190.61322	Detected	Detected	Detected	Detected
4057	A_24_P737939	C6orf154	Hs.445552	chromosome 6 open reading frame 154	Homo sapiens chromosome 6 open reac	931.62370	418.55360	930.20715	324.06244	Detected	Detected	Detected	Detected
4058	A_23_P55123	COX10	Hs.462278	COX10 homolog, cytochrome c oxidase	Homo sapiens COX10 homolog, cytochr	240.69203	280.82077	184.92244	188.54710	Detected	Detected	Detected	Detected
4059	A_24_P64329	STK32C	Hs.469002	serine/threonine kinase 32C	Homo sapiens serine/threonine kinase :	1142.86300	764.59820	1284.06600	952.67206	Detected	Detected	Detected	Detected
4060	A_32_P119616	ETFA	Hs.39925	electron-transfer-flavoprotein, alpha pc	Homo sapiens electron-transfer-flavopr	1714.96810	2215.05930	2135.96700	2273.95300	Detected	Detected	Detected	Detected
4061	A_24_P49106	TCEAL7	Hs.21861	transcription elongation factor A (SII)-li	Homo sapiens transcription elongation f	177.47421	131.67636	197.40952	130.70862	Detected	Detected	Detected	Detected
4062	A_24_P941643	PLCB1	Hs.431173	phospholipase C, beta 1 (phosphoinositi	Homo sapiens phospholipase C, beta 1	457.83414	372.31094	335.87350	206.63540	Detected	Detected	Detected	Detected
4063	A_24_P309594	SLC48A1	Hs.438867	solute carrier family 48 (heme transport	Homo sapiens solute carrier family 48 (	5426.91700	2108.20650	4635.96730	1677.90490	Detected	Detected	Detected	Detected
4064	A_23_P53891	KLF5	Hs.508234	Kruppel-like factor 5 (intestinal)	Homo sapiens Kruppel-like factor 5 (int	446.72632	557.84784	370.85806	381.01846	Detected	Detected	Detected	Detected
4065	A_24_P408424	MYH9	Hs.474751	myosin, heavy chain 9, non-muscle	Homo sapiens myosin, heavy chain 9, nc	15862.03200	22816.04700	11337.22200	14443.10450	Detected	Detected	Detected	Detected
4066	A_23_P208937	TLE6	Hs.334507	transducin-like enhancer of split 6 (E(sp	Homo sapiens transducin-like enhancer	71.28606	37.15451	74.84522	25.68150	Detected	Detected	Detected	Detected
4067	A_23_P70231	ALDH7A1	Hs.483239	aldehyde dehydrogenase 7 family, memb	Homo sapiens aldehyde dehydrogenase	3064.00800	5183.31300	2720.77780	4086.59380	Detected	Detected	Detected	Detected
4068	A_23_P110712	DUSP1	Hs.171695	dual specificity phosphatase 1	Homo sapiens dual specificity phosphata	17122.89000	12835.65300	12634.75200	7788.88570	Detected	Detected	Detected	Detected
4069	A_33_P3366575	NXT2	Hs.25010	nuclear transport factor 2-like export fa	Homo sapiens nuclear transport factor	63.05940	37.72027	67.03584	48.16822	Detected	Detected	Detected	Detected
4070	A_23_P66241	MT1M	Hs.647370	metallothionein 1M	Homo sapiens metallothionein 1M (MT1	43360.70000	60967.18400	21808.89600	21617.15400	Detected	Detected	Detected	Detected
4071	A_23_P119593	EPHX3	Hs.156457	epoxide hydrolase 3	Homo sapiens epoxide hydrolase 3 (EPH	28191.90400	33377.52700	4673.91360	6081.91360	Detected	Detected	Detected	Detected
4072	A_24_P348203	LRRC8E	Hs.501511	leucine rich repeat containing 8 family,	Homo sapiens leucine rich repeat conta	1149.84220	1660.16640	1056.95350	1519.02380	Detected	Detected	Detected	Detected
4073	A_33_P3325914	TAPBP	Hs.370937	TAP binding protein (tapasin)	Homo sapiens TAP binding protein (tapa	363.99850	510.99040	299.18195	375.90320	Detected	Detected	Detected	Detected
4074	A_33_P3221748	RUNX3	Hs.170019	runt-related transcription factor 3	Homo sapiens runt-related transcriptior	4805.45170	4154.45600	3406.64000	2584.98200	Detected	Detected	Detected	Detected
4075	A_24_P194881	SHANK3	Hs.149035	SH3 and multiple ankyrin repeat domain	Homo sapiens SH3 and multiple ankyrin	1950.59170	664.26416	1894.82500	587.44226	Detected	Detected	Detected	Detected
4076	A_23_P93431	AIG1	Hs.567501	androgen-induced 1	Homo sapiens androgen-induced 1 (AIG	1887.26220	1289.83830	1559.55970	852.40510	Detected	Detected	Detected	Detected
4077	A_23_P211797	OPA1	Hs.594504	optic atrophy 1 (autosomal dominant)	Homo sapiens optic atrophy 1 (autosom	2776.12920	2421.42400	2173.66600	1562.37460	Detected	Detected	Detected	Detected
4078	A_33_P3314673	SYCE1L	Hs.635157	synaptonemal complex central element	Homo sapiens synaptonemal complex c	57.95960	67.52575	42.79836	40.47538	Detected	Detected	Detected	Detected
4079	A_32_P42574	C1orf198	Hs.520494	chromosome 1 open reading frame 198	Homo sapiens chromosome 1 open reac	39845.16000	17961.08000	34561.94000	13225.13900	Detected	Detected	Detected	Detected
4080	A_23_P146284	SQLE	Hs.71465	squalene epoxidase	Homo sapiens squalene epoxidase (SQL	4667.05370	6049.06800	2792.91970	2609.91770	Detected	Detected	Detected	Detected
4081	A_33_P3362548					100.01103	126.26704	135.97070	105.73009	Detected	Detected	Detected	Detected
4082	A_24_P920125	PPIA	Hs.356331	peptidylprolyl isomerase A (cyclophilin /	Homo sapiens peptidylprolyl isomerase	2083.08980	1501.45540	1461.21120	872.80990	Detected	Detected	Detected	Detected
4083	A_23_P6151	PLAGL2	Hs.154104	pleiomorphic adenoma gene-like 2	Homo sapiens pleiomorphic adenoma ge	596.66660	503.51138	360.28226	256.60806	Detected	Detected	Detected	Detected
4084	A_33_P3344086	HIST1H2AJ	Hs.406691	histone cluster 1, H2aj	Homo sapiens histone cluster 1, H2aj (H	13088.87100	14806.35200	14338.90900	12739.95500	Detected	Detected	Detected	Detected
4085	A_23_P23947	MAP3K8	Hs.432453	mitogen-activated protein kinase kinase	Homo sapiens mitogen-activated protei	384.24410	110.77798	360.49335	83.20309	Detected	Detected	Detected	Detected
4086	A_23_P57547	SLC25A17	Hs.474938	solute carrier family 25 (mitochondrial c	Homo sapiens solute carrier family 25 (	1811.95790	2198.18850	1837.89150	1885.71960	Detected	Detected	Detected	Detected
4087	A_33_P3364498	LOC728701		hypothetical LOC728701	PREDICTED: Homo sapiens hypothetica	2770.55960	1567.38550	2997.72660	1388.10130	Detected	Detected	Detected	Detected
4088	A_33_P3317523	STMN1	Hs.209983	stathmin 1	Homo sapiens stathmin 1 (STMN1), trar	8335.58300	39894.79000	6252.74660	25663.01000	Detected	Detected	Detected	Detected
4089	A_24_P242132	NRBP2	Hs.521926	nuclear receptor binding protein 2	Homo sapiens nuclear receptor binding	326.01510	236.98921	226.33887	162.32689	Detected	Detected	Detected	Detected
4090	A_33_P3230269	GRHL1	Hs.418493	grainyhead-like 1 (Drosophila)	Homo sapiens grainyhead-like 1 (Droso	81.55605	141.99350	51.90007	64.28283	Detected	Detected	Detected	Detected
4091	A_24_P50554					81970.80500	80209.25000	56297.70300	46876.82400	Detected	Detected	Detected	Detected
4092	A_23_P12336	PRMT6	Hs.26006	protein arginine methyltransferase 6	Homo sapiens protein arginine methyltr	1135.03750	1204.91560	898.01130	811.99664	Detected	Detected	Detected	Detected
4093	A_23_P339954	LOC728741	Hs.719525	hypothetical LOC728741	Nuclear pore complex-interacting prote	16523.86100	14409.86100	20135.14300	15752.12900	Detected	Detected	Detected	Detected
4094	A_23_P356425	C1orf125	Hs.658505	chromosome 1 open reading frame 125	Homo sapiens chromosome 1 open reac	51.96343	7.16536	26.74605	3.35532	Detected	Compromised	Detected	Compromised
4095	A_33_P3318581	PLOD2	Hs.477866	procollagen-lysine, 2-oxoglutarate 5-dic	Homo sapiens procollagen-lysine, 2-oxc	4076.25510	12681.68500	2914.74760	7324.13100	Detected	Detected	Detected	Detected
4096	A_33_P3304888	USP6	Hs.448851	ubiquitin specific peptidase 6 (Tre-2 on	Homo sapiens ubiquitin specific peptida	900.00390	619.17290	449.98917	260.76898	Detected	Detected	Detected	Detected
4097	A_23_P105664	CCDC59	Hs.582627	coiled-coil domain containing 59	Homo sapiens coiled-coil domain conta	5002.88870	5963.61430	4559.77830	4655.08350	Detected	Detected	Detected	Detected
4098	A_33_P3685572	LOC157562	Hs.27371	hypothetical protein LOC157562	Homo sapiens mRNA; cDNA DKFZp686	682.59430	131.98306	415.00290	53.48939	Detected	Detected	Detected	Detected
4099	A_24_P167377	OR10P1	Hs.554551	olfactory receptor, family 10, subfamily	Homo sapiens olfactory receptor, family	38.37795	50.67060	20.70620	19.26529	Detected	Detected	Detected	Detected
4100	A_24_P178093	TOMM40	Hs.655909	translocase of outer mitochondrial mem	Homo sapiens translocase of outer mitc	341.55830	626.79010	306.30847	532.82080	Detected	Detected	Detected	Detected
4101	A_33_P3560679	LOC651721	Hs.598754	hypothetical protein LOC651721	Homo sapiens hypothetical protein LOC	31.79598	152.30840	17.21336	120.68978	Detected	Detected	Detected	Detected
4102	A_23_P211355	DGCR8	Hs.643452	DiGeorge syndrome critical region gene	Homo sapiens DiGeorge syndrome critic	415.87152	715.91170	476.12512	704.57776	Detected	Detected	Detected	Detected
4103	A_33_P3306894	C11orf41	Hs.502266	chromosome 11 open reading frame 41	Homo sapiens chromosome 11 open rea	26.13967	186.36116	14.15435	116.18719	Detected	Detected	Compromised	Detected
4104	A_24_P60845	ACHE	Hs.154495	acetylcholinesterase (Yt blood group)	Homo sapiens acetylcholinesterase (Yt	203.27812	415.82645	116.45770	203.37140	Detected	Detected	Detected	Detected
4105	A_23_P76799	BAZ1A	Hs.509140	bromodomain adjacent to zinc finger do	Homo sapiens bromodomain adjacent tc	1135.47730	1601.05790	799.36926	914.10913	Detected	Detected	Detected	Detected
4106	A_33_P3388527					395.48773	554.60450	307.67400	296.46198	Detected	Detected	Detected	Detected
4107	A_23_P52727	NAV2	Hs.502116	neuron navigator 2	Homo sapiens neuron navigator 2 (NAV	5073.67400	7030.07130	3656.82100	3989.69260	Detected	Detected	Detected	Detected
4108	A_33_P3322428	MARK4	Hs.34314	MAP/microtubule affinity-regulating kin	Homo sapiens MAP/microtubule affinity	63.33602	65.59636	62.98912	49.76494	Detected	Detected	Detected	Detected
4109	A_33_P3279059	RIPK1	Hs.519842	receptor (TNFRSF)-interacting serine-t	Homo sapiens receptor (TNFRSF)-inter	6095.99300	8266.54200	3639.34960	4156.56050	Detected	Detected	Detected	Detected
4110	A_33_P3349536	CHEK1	Hs.24529	CHK1 checkpoint homolog (S. pombe)	Homo sapiens CHK1 checkpoint homolo	393.43924	1467.11610	390.91937	1139.11670	Detected	Detected	Detected	Detected
4111	A_23_P213319	ADAMTS6	Hs.482291	ADAM metallopeptidase with thrombosp	Homo sapiens ADAM metallopeptidase	354.72382	691.42950	218.51137	348.44107	Detected	Detected	Detected	Detected
4112	A_23_P130642	GBP6	Hs.254338	guanylate binding protein family, membe	Homo sapiens guanylate binding protein	6358.55960	9855.01000	4927.98050	5417.21830	Detected	Detected	Detected	Detected
4113	A_33_P3257187	PRKAA1	Hs.43322	protein kinase, AMP-activated, alpha 1	Homo sapiens protein kinase, AMP-acti	1046.12800	347.84064	661.96720	172.13160	Detected	Detected	Detected	Detected
4114	A_23_P80839	MAP6D1	Hs.478465	MAP6 domain containing 1	Homo sapiens MAP6 domain containing	679.18260	1825.89820	636.15155	1379.28930	Detected	Detected	Detected	Detected
4115	A_33_P3218951	RPA1	Hs.461925	replication protein A1, 70kDa	Homo sapiens replication protein A1, 7C	378.90866	113.34091	339.45755	80.22252	Detected	Detected	Detected	Detected
4116	A_33_P3284152		Hs.121670		Putative uncharacterized protein ENSP	77.05721	91.59116	54.52567	53.68060	Detected	Detected	Detected	Detected
4117	A_24_P165595	ARL5A	Hs.470233	ADP-ribosylation factor-like 5A	Homo sapiens ADP-ribosylation factor-	883.23846	856.44120	761.70050	529.37256	Detected	Detected	Detected	Detected
4118	A_23_P353679	LENEP	Hs.272399	lens epithelial protein	Homo sapiens lens epithelial protein (LE	15.46737	13.79059	3.12024	5.06773	Detected	Compromised	Compromised	Compromised
4119	A_33_P3282354	LOC732443		hypothetical protein LOC732443	PREDICTED: Homo sapiens hypothetica	17.52188	12.03231	9.50568	5.28655	Detected	Compromised	Compromised	Compromised
4120	A_24_P122403	TCEB3	Hs.15535	transcription elongation factor B (SIII),	Homo sapiens transcription elongation f	900.98254	1754.09730	656.95667	1050.71530	Detected	Detected	Detected	Detected
4121	A_32_P85676	STK32B	Hs.133062	serine/threonine kinase 32B	Homo sapiens serine/threonine kinase :	30.18532	17.34413	24.92646	11.51208	Detected	Compromised	Detected	Compromised

4122	A_33_P3233981	NASP	Hs.319334	nuclear autoantigenic sperm protein (his	Homo sapiens nuclear autoantigenic spe	22191.73000	40916.29300	11169.84000	15702.63200	Detected	Detected	Detected	Detected
4123	A_24_P98613	TSPAN14	Hs.633177	tetraspanin 14	Homo sapiens tetraspanin 14 (TSPAN14	1398.45800	682.62366	1216.28870	495.56497	Detected	Detected	Detected	Detected
4124	A_32_P26969	ERICH1	Hs.655310	glutamate-rich 1	Homo sapiens glutamate-rich 1 (ERICH	1083.98020	1364.73290	802.27820	792.82697	Detected	Detected	Detected	Detected
4125	A_23_P116602	USP35	Hs.531249	ubiquitin specific peptidase 35	Homo sapiens ubiquitin specific peptida	3133.00170	5785.89100	2705.99270	4449.28270	Detected	Detected	Detected	Detected
4126	A_23_P205646	MAP4K5	Hs.130491	mitogen-activated protein kinase kinase	Homo sapiens mitogen-activated protei	2019.24060	1727.01570	1355.51280	962.70166	Detected	Detected	Detected	Detected
4127	A_33_P3423017					15.28729	10.99508	10.52227	8.78331	Detected	Compromised	Compromised	Compromised
4128	A_23_P115223	HAX1	Hs.199625	HCLS1 associated protein X-1	Homo sapiens HCLS1 associated prote	6975.28200	8141.67500	8841.51500	8354.68700	Detected	Detected	Detected	Detected
4129	A_33_P3283906	NIP7	Hs.719308	nuclear import 7 homolog (S. cerevisiae	Homo sapiens nuclear import 7 homolog	27965.92400	36006.60500	7881.68950	6818.64600	Detected	Detected	Detected	Detected
4130	A_23_P90014	GPR108	Hs.167641	G protein-coupled receptor 108	Homo sapiens G protein-coupled recep	1019.08670	719.45130	1202.73120	693.16570	Detected	Detected	Detected	Detected
4131	A_23_P131449	C2orf81	Hs.445377	chromosome 2 open reading frame 81	Homo sapiens chromosome 2 open read	360.63060	364.82380	288.20200	244.65591	Detected	Detected	Detected	Detected
4132	A_23_P127394	CRY2	Hs.532491	cryptochrome 2 (photolyase-like)	Homo sapiens cryptochrome 2 (photoly	2859.68360	2501.63100	2147.32520	1734.74460	Detected	Detected	Detected	Detected
4133	A_33_P3420496	SNORA53	Hs.693250	small nucleolar RNA, H/ACA box 53	Homo sapiens small nucleolar RNA, H/A	27.10165	18.61462	30.54386	12.70118	Detected	Compromised	Detected	Compromised
4134	A_24_P115621	EIF4EBP2	Hs.643279	eukaryotic translation initiation factor 4	Homo sapiens eukaryotic translation ini	7310.94000	8487.36900	3878.21700	3647.40100	Detected	Detected	Detected	Detected
4135	A_23_P30666	TNFRSF21	Hs.443577	tumor necrosis factor receptor superfa	Homo sapiens tumor necrosis factor re	7500.66460	981.13820	6455.31150	733.37820	Detected	Detected	Detected	Detected
4136	A_33_P3224858	EIF2AK2	Hs.131431	eukaryotic translation initiation factor 2	Homo sapiens eukaryotic translation ini	66.36637	76.24574	59.93886	57.65025	Detected	Detected	Detected	Detected
4137	A_23_P110811	COX7C	Hs.430075	cytochrome c oxidase subunit VIIc	Homo sapiens cytochrome c oxidase su	37337.19500	25066.54900	30320.86700	17099.20500	Detected	Detected	Detected	Detected
4138	A_23_P383819	TBX3	Hs.714737	T-box 3	Homo sapiens T-box 3 (TBX3), transcri	1834.99980	5889.47700	854.32250	2400.74930	Detected	Detected	Detected	Detected
4139	A_23_P345710	C3orf21	Hs.478741	chromosome 3 open reading frame 21	Homo sapiens chromosome 3 open read	857.91614	894.22784	712.62260	652.29706	Detected	Detected	Detected	Detected
4140	A_33_P3390032	EXOC7	Hs.514496	exocyst complex component 7	Homo sapiens exocyst complex compor	4419.69400	4415.48900	5603.25800	4620.77700	Detected	Detected	Detected	Detected
4141	A_33_P3461633	LOC284454	Hs.436426	hypothetical protein LOC284454	Homo sapiens mRNA; cDNA DKFZp686	1574.56130	2490.42290	669.43304	780.08716	Detected	Detected	Detected	Detected
4142	A_23_P202458	ZNF22	Hs.462693	zinc finger protein 22 (KOX 15)	Homo sapiens zinc finger protein 22 (K	5688.99070	1950.14040	3826.23440	1148.88560	Detected	Detected	Detected	Detected
4143	A_23_P43248	TAF2	Hs.122752	TAF2 RNA polymerase II, TATA box bin	Homo sapiens TAF2 RNA polymerase II	860.76324	752.94330	679.59310	548.35144	Detected	Detected	Detected	Detected
4144	A_23_P62731	MRPS14	Hs.702192	mitochondrial ribosomal protein S14	Homo sapiens mitochondrial ribosomal p	2075.72360	2690.52150	2052.45750	2239.16500	Detected	Detected	Detected	Detected
4145	A_24_P258235	OR5L2	Hs.528356	olfactory receptor, family 5, subfamily L	Homo sapiens olfactory receptor, family	181.33305	172.26490	47.45421	32.21701	Detected	Detected	Detected	Detected
4146	A_23_P217938	SPHAR	Hs.716553	S-phase response (cyclin related)	Homo sapiens S-phase response (cycli	1314.79170	1277.62370	1098.40860	874.99410	Detected	Detected	Detected	Detected
4147	A_33_P3376781					906.36835	1071.61990	734.14215	733.83887	Detected	Detected	Detected	Detected
4148	A_23_P32115	LCN12	Hs.440519	lipocalin 12	Homo sapiens lipocalin 12 (LCN12), mR	14.73142	17.74704	11.70949	17.15006	Detected	Compromised	Compromised	Detected
4149	A_23_P53162	STIM1	Hs.501735	stromal interaction molecule 1	Homo sapiens stromal interaction mole	462.14734	444.13226	413.06436	335.12634	Detected	Detected	Detected	Detected
4150	A_24_P415280	SEC61A2	Hs.112955	Sec61 alpha 2 subunit (S. cerevisiae)	Homo sapiens Sec61 alpha 2 subunit (S	88.62353	188.42038	72.33949	104.85079	Detected	Detected	Detected	Detected
4151	A_23_P69030	COL8A1	Hs.654548	collagen, type VIII, alpha 1	Homo sapiens collagen, type VIII, alpha	392.14618	9558.00700	288.84400	7201.69240	Detected	Detected	Detected	Detected
4152	A_23_P420692	PPFIA4	Hs.153648	protein tyrosine phosphatase, receptor	Homo sapiens protein tyrosine phospha	113.75821	68.69614	97.69784	40.99103	Detected	Detected	Detected	Detected
4153	A_33_P3218491					48.33289	76.11459	19.21467	19.93933	Detected	Detected	Detected	Detected
4154	A_32_P182388	ZNF77	Hs.38004	zinc finger protein 77	Homo sapiens zinc finger protein 77 (ZN	1181.33120	1092.18700	1006.94690	751.80440	Detected	Detected	Detected	Detected
4155	A_33_P3275330	NADK	Hs.654792	NAD kinase	Homo sapiens NAD kinase (NADK), mR	190.21509	261.70444	186.10078	222.13249	Detected	Detected	Detected	Detected
4156	A_23_P18123	NLGN1	Hs.478289	neuroligin 1	Homo sapiens neuroligin 1 (NLGN1), mR	152.01715	201.98314	105.62311	101.06690	Detected	Detected	Detected	Detected
4157	A_23_P114414	LONRF3	Hs.144266	LON peptidase N-terminal domain and r	Homo sapiens LON peptidase N-termin	148.35696	137.54636	74.79376	49.62935	Detected	Detected	Detected	Detected
4158	A_33_P3394272	C6orf176		chromosome 6 open reading frame 176	Homo sapiens chromosome 6 open read	17.97071	3.60232	8.32217	3.12638	Detected	Compromised	Compromised	Compromised
4159	A_33_P3391970					89.06776	149.92369	3.00688	6.91307	Detected	Detected	Compromised	Compromised
4160	A_23_P10911	PLBD2	Hs.115896	phospholipase B domain containing 2	Homo sapiens phospholipase B domain	766.39813	344.97806	771.40710	231.68225	Detected	Detected	Detected	Detected
4161	A_23_P111662	ABCB5	Hs.658821	ATP-binding cassette, sub-family B (MI	Homo sapiens ATP-binding cassette, su	2.69704	3.41312	21.30628	3.21978	Compromised	Compromised	Not Detected	Compromised
4162	A_23_P79221	ACVR1	Hs.470316	activin A receptor, type I	Homo sapiens activin A receptor, type I	2150.21440	2045.68360	2114.50540	1617.45800	Detected	Detected	Detected	Detected
4163	A_33_P3816042	FKSG73		ARP3 actin-related protein 3 homolog E	Homo sapiens ARP3 actin-related prote	19.02494	26.16369	14.67790	14.62436	Detected	Detected	Compromised	Compromised
4164	A_33_P3325708	hCG_2045614	Hs.126452	hCG2045614	PREDICTED: Homo sapiens hCG204561	25.09461	30.77162	25.82048	17.92616	Detected	Detected	Detected	Detected
4165	A_33_P3237005	ZFATAS		ZFAT antisense RNA (non-protein codi	Homo sapiens ZFAT antisense RNA (no	22.64752	29.64640	3.04335	6.77730	Detected	Detected	Compromised	Compromised
4166	A_23_P119611	FXYD7	Hs.134729	FXYD domain containing ion transport r	Homo sapiens FXYD domain containing	48.43345	41.77168	19.73008	9.22760	Detected	Detected	Detected	Compromised
4167	A_33_P3213134					4537.48300	3724.21460	1346.11290	775.81116	Detected	Detected	Detected	Detected
4168	A_24_P75220	MAGI1	Hs.651939	membrane associated guanylate kinase,	Homo sapiens membrane associated gu	65.90208	97.34602	40.63495	69.31344	Detected	Detected	Detected	Detected
4169	A_23_P71440	GOLGA7	Hs.654773	golgi autoantigen, golgin subfamily a, 7	Homo sapiens golgi autoantigen, golgin s	10298.96400	13360.52000	9627.94500	9859.14100	Detected	Detected	Detected	Detected
4170	A_23_P68922	MICALL1	Hs.517610	MICAL-like 1	Homo sapiens MICAL-like 1 (MICALL1)	231.45456	706.22190	179.47130	488.38086	Detected	Detected	Detected	Detected
4171	A_23_P137705	TBX19	Hs.716656	T-box 19	Homo sapiens T-box 19 (TBX19), mRNA	643.15106	460.01474	326.19530	108.16612	Detected	Detected	Detected	Detected
4172	A_33_P3342295	VSX1	Hs.274264	visual system homeobox 1	Homo sapiens visual system homeobox	49732.78500	61092.20700	57651.29300	49938.38300	Detected	Detected	Detected	Detected
4173	A_33_P3359354		Hs.107101		Uncharacterized protein C1orf86 [Sour	468.89536	394.65436	499.35680	361.34436	Detected	Detected	Detected	Detected
4174	A_24_P101201	PDIA3	Hs.591095	protein disulfide isomerase family A, me	Homo sapiens protein disulfide isomera	5067.45950	10883.03900	4364.11870	7823.91940	Detected	Detected	Detected	Detected
4175	A_23_P118150	ARL6IP1	Hs.634882	ADP-ribosylation factor-like 6 interacti	Homo sapiens ADP-ribosylation factor-	2155.11380	5321.04830	1496.81860	3065.61280	Detected	Detected	Detected	Detected
4176	A_33_P3383531	LOC729312		hypothetical LOC729312	PREDICTED: Homo sapiens hypothetica	22.45556	27.81657	15.41627	14.11455	Detected	Detected	Compromised	Compromised
4177	A_23_P303210	IKBIP	Hs.252543	IKBKB interacting protein	Homo sapiens IKBKB interacting protei	480.37527	2250.26400	400.22476	1724.82410	Detected	Detected	Detected	Detected
4178	A_33_P3317850	RIOK3	Hs.445511	RIO kinase 3 (yeast)	Homo sapiens RIO kinase 3 (yeast) (RIO	18089.18000	34253.52300	13357.20800	21594.27300	Detected	Detected	Detected	Detected
4179	A_32_P191262	ACR	Hs.370870	acrosin	Homo sapiens acrosin (ACR), mRNA [N	170.28033	159.96260	105.09692	67.17253	Detected	Detected	Detected	Detected
4180	A_23_P317056					1200.32230	1509.89370	1381.85010	1242.63940	Detected	Detected	Detected	Detected
4181	A_33_P3357949	ETV1	Hs.22634	ets variant 1	Homo sapiens ets variant 1 (ETV1), trar	2218.69500	683.17706	1149.69780	312.82526	Detected	Detected	Detected	Detected
4182	A_23_P301925	LOC100288578	Hs.557644	similar to cytochrome c oxidase subunit	PREDICTED: Homo sapiens similar to c	35900.15200	37491.47300	33137.48000	31729.60200	Detected	Detected	Detected	Detected
4183	A_33_P3334423	SPRR2G	Hs.490253	small proline-rich protein 2G	Homo sapiens small proline-rich protein	11.63138	17.00212	20.56595	18.39780	Compromised	Compromised	Detected	Detected
4184	A_33_P3221253	LPP	Hs.444362	LIM domain containing preferred translo	Homo sapiens LIM domain containing pr	31.37648	47.17257	20.45422	27.85953	Detected	Detected	Detected	Detected
4185	A_33_P3336567					27.94412	49.35439	42.73656	38.12025	Detected	Detected	Detected	Detected
4186	A_33_P3213707	PDCD1LG2	Hs.532279	programmed cell death 1 ligand 2	Homo sapiens programmed cell death 1	109.27439	180.27533	94.65931	139.64590	Detected	Detected	Detected	Detected
4187	A_33_P3334803					16.34723	30.93641	11.56318	11.52816	Detected	Detected	Compromised	Compromised
4188	A_23_P171117	MORC4	Hs.496544	MORC family CW-type zinc finger 4	Homo sapiens MORC family CW-type z	2137.66200	2620.00240	1671.23780	1575.63170	Detected	Detected	Detected	Detected
4189	A_23_P256244		Hs.148778		Oxidation resistance protein 1 [Source	65.98434	178.56082	55.19980	130.15552	Detected	Detected	Detected	Detected
4190	A_24_P185854	DMD	Hs.495912	dystrophin	Homo sapiens dystrophin (DMD), transc	40.66435	62.85912	26.73364	26.18836	Detected	Detected	Detected	Detected
4191	A_23_P148959	TM2D1	Hs.656790	TM2 domain containing 1	Homo sapiens TM2 domain containing 1	10756.28200	9233.37700	7790.00830	5498.35250	Detected	Detected	Detected	Detected
4192	A_33_P3296687	KRT33A	Hs.512579	keratin 33A	Homo sapiens keratin 33A (KRT33A), m	103.06110	124.95802	34.29577	22.59015	Detected	Detected	Detected	Detected
4193	A_23_P145247	ZNF187	Hs.157883	zinc finger protein 187	Homo sapiens zinc finger protein 187 (Z	698.82745	564.56055	720.52020	425.90880	Detected	Detected	Detected	Detected
4194	A_23_P1519	PITPNM1	Hs.372295	phosphatidylinositol transfer protein, me	Homo sapiens phosphatidylinositol trans	562.36414	250.78635	575.59830	291.75583	Detected	Detected	Detected	Detected
4195	A_33_P3273255				Zinc finger protein 761 [Source:UniPro	30.57966	83.88039	20.52007	38.46174	Detected	Detected	Detected	Detected
4196	A_23_P40588	HSCB	Hs.632780	HscB iron-sulfur cluster co-chaperone	Homo sapiens HscB iron-sulfur cluster	504.58762	802.41820	427.76650	493.10860	Detected	Detected	Detected	Detected

4197	A_33_P3335845	WDR33	Hs.620490	WD repeat domain 33	Homo sapiens WD repeat domain 33 (W	3150.26150	2247.66380	2627.13380	1554.71470	Detected	Detected	Detected	Detected
4198	A_24_P763243	EEF1A1	Hs.535192	eukaryotic translation elongation factor	Homo sapiens eukaryotic translation elo	272603.06000	271318.38000	249340.42000	187743.83000	Detected	Detected	Detected	Detected
4199	A_33_P3316928	PELI1	Hs.719982	pellino homolog 1 (Drosophila)	Homo sapiens pellino homolog 1 (Droso	1707.78690	1255.88350	997.04410	594.00476	Detected	Detected	Detected	Detected
4200	A_23_P257091	NUP214	Hs.654530	nucleoporin 214kDa	Homo sapiens nucleoporin 214kDa (NUF	1167.23550	1957.22400	1177.35290	1749.64230	Detected	Detected	Detected	Detected
4201	A_23_P24135	TACR2	Hs.88372	tachykinin receptor 2	Homo sapiens tachykinin receptor 2 (T/	622.43580	470.40823	424.69147	249.33502	Detected	Detected	Detected	Detected
4202	A_23_P85004	DIAPH2	Hs.226483	diaphanous homolog 2 (Drosophila)	Homo sapiens diaphanous homolog 2 (D	301.84780	487.49255	227.15042	267.35352	Detected	Detected	Detected	Detected
4203	A_33_P3758973	DKFZP761C1711	Hs.675490	hypothetical protein DKFZp761C1711	Homo sapiens mRNA; cDNA DKFZp761	14.63438	15.40041	13.39886	11.41851	Detected	Compromised	Compromised	Compromised
4204	A_24_P310756	FBXO28	Hs.64691	F-box protein 28	Homo sapiens F-box protein 28 (FBXO	1797.21500	1631.73020	979.03390	682.29974	Detected	Detected	Detected	Detected
4205	A_33_P3251144	CDCA7L	Hs.520245	cell division cycle associated 7-like	Homo sapiens cell division cycle associ	44.69637	59.66919	39.23323	51.35245	Detected	Detected	Detected	Detected
4206	A_33_P3242798	CPS1	Hs.149252	carbamoyl-phosphate synthetase 1, mit	Homo sapiens carbamoyl-phosphate sy	124.52889	306.77658	78.26087	158.00630	Detected	Detected	Detected	Detected
4207	A_33_P3366667	EYA1	Hs.491997	eyes absent homolog 1 (Drosophila)	Homo sapiens eyes absent homolog 1 (	182.09389	376.90344	46.16349	35.22107	Detected	Detected	Detected	Detected
4208	A_23_P27215	UBB	Hs.356190	ubiquitin B	Homo sapiens ubiquitin B (UBB), mRNA	114096.75000	134812.62000	91248.44500	85741.43000	Detected	Detected	Detected	Detected
4209	A_23_P139704	DUSP6	Hs.298654	dual specificity phosphatase 6	Homo sapiens dual specificity phosphat	10497.58600	11434.61400	6363.48800	5269.09130	Detected	Detected	Detected	Detected
4210	A_23_P501080	ZNF92	Hs.9521	zinc finger protein 92	Homo sapiens zinc finger protein 92 (ZN	702.89655	696.10770	552.30800	359.12500	Detected	Detected	Detected	Detected
4211	A_23_P215331	CRHR2	Hs.546246	corticotropin releasing hormone recepto	Homo sapiens corticotropin releasing ho	48.77800	71.16409	42.33597	36.30618	Detected	Detected	Detected	Detected
4212	A_23_P106362	AQP9	Hs.104624	aquaporin 9	Homo sapiens aquaporin 9 (AQP9), mRN	1583.02420	3273.91190	1304.57510	2258.61550	Detected	Detected	Detected	Detected
4213	A_24_P379104	PIM2	Hs.719294	pim-2 oncogene	Homo sapiens pim-2 oncogene (PIM2),	434.67892	340.69464	377.98328	258.27768	Detected	Detected	Detected	Detected
4214	A_23_P156788	STX11	Hs.118958	syntaxin 11	Homo sapiens syntaxin 11 (STX11), mR	94.07705	84.41670	104.83389	91.17074	Detected	Detected	Detected	Detected
4215	A_23_P40952	RAF1	Hs.159130	v-raf-1 murine leukemia viral oncogene	Homo sapiens v-raf-1 murine leukemia	4205.22900	5214.92430	4102.21880	4562.10600	Detected	Detected	Detected	Detected
4216	A_23_P114713	CYP4B1	Hs.436317	cytochrome P450, family 4, subfamily B	Homo sapiens cytochrome P450, family	2.75396	3.89423	21.91268	3.82290	Compromised	Compromised	Not Detected	Compromised
4217	A_24_P196117	GTF2H5	Hs.356224	general transcription factor IIH, polypep	Homo sapiens general transcription fact	5021.63200	4867.96700	4907.58800	3686.23630	Detected	Detected	Detected	Detected
4218	A_23_P99452	BRCA2	Hs.34012	breast cancer 2, early onset	Homo sapiens breast cancer 2, early on	17.06263	54.49803	8.90761	31.79808	Detected	Detected	Compromised	Detected
4219	A_33_P3403132	NR2F6	Hs.466148	nuclear receptor subfamily 2, group F, m	Homo sapiens nuclear receptor subfami	26472.45700	27967.79100	21227.69500	18667.17800	Detected	Detected	Detected	Detected
4220	A_33_P3646133	NFATC2IP	Hs.513470	nuclear factor of activated T-cells, cyto	Homo sapiens nuclear factor of activate	385.39084	378.47833	193.66504	182.92659	Detected	Detected	Detected	Detected
4221	A_33_P3357669					15736.89300	14070.97500	14136.54400	10460.27050	Detected	Detected	Detected	Detected
4222	A_23_P152087	FAM82A2	Hs.511067	family with sequence similarity 82, mem	Homo sapiens family with sequence sim	4778.75100	5699.22500	4251.29050	4139.80600	Detected	Detected	Detected	Detected
4223	A_33_P3250253	LOC100131150	Hs.124147	hypothetical protein LOC100131150	Homo sapiens cDNA FLJ38875 fis, clon	137.59420	201.45170	43.64181	47.17813	Detected	Detected	Detected	Detected
4224	A_33_P3358099	CD300E	Hs.158954	CD300e molecule	CMRF35-like molecule 2 Precursor (CL	521.10724	861.07733	260.00630	313.44666	Detected	Detected	Detected	Detected
4225	A_24_P929754	MKNK2	Hs.515032	MAP kinase interacting serine/threonin	Homo sapiens MAP kinase interacting s	590.69250	822.81450	495.38223	601.23600	Detected	Detected	Detected	Detected
4226	A_24_P410017	POTEE	Hs.686146	POTE ankyrin domain family, member E	Homo sapiens POTE ankyrin domain far	47714.97000	67909.09000	33693.95300	43022.82400	Detected	Detected	Detected	Detected
4227	A_32_P119248	FOXD4	Hs.584759	forkhead box D4	Homo sapiens forkhead box D4 (FOXD4	22.48050	18.03764	18.57067	7.40590	Detected	Compromised	Detected	Compromised
4228	A_23_P389102	MYO1D	Hs.658000	myosin ID	Homo sapiens myosin ID (MYO1D), mRN	7918.14000	2571.38530	4539.04900	1222.18100	Detected	Detected	Detected	Detected
4229	A_24_P71468	QPCT	Hs.79033	glutaminyl-peptide cyclotransferase	Homo sapiens glutaminyl-peptide cyclot	6975.57760	2538.72240	5833.95560	1623.20230	Detected	Detected	Detected	Detected
4230	A_23_P144458	CAMK2D	Hs.144114	calcium/calmodulin-dependent protein	Homo sapiens calcium/calmodulin-depe	736.71720	738.75665	991.55005	755.96454	Detected	Detected	Detected	Detected
4231	A_33_P3383326	LPAR1	Hs.126667	lysophosphatidic acid receptor 1	Homo sapiens lysophosphatidic acid rec	12336.42100	5438.08200	6781.10840	2426.59640	Detected	Detected	Detected	Detected
4232	A_23_P384329	DENND4A	Hs.654567	DENN/MADD domain containing 4A	Homo sapiens DENN/MADD domain co	41.46604	23.16443	43.07253	16.08686	Detected	Detected	Detected	Compromised
4233	A_24_P787897	XYLT1	Hs.22907	xylosyltransferase I	Homo sapiens xylosyltransferase I (XYL	900.12350	1584.97600	572.19617	784.51013	Detected	Detected	Detected	Detected
4234	A_33_P3244249				SH3 domain and tetratricopeptide repea	6.70742	42.88037	5.22224	38.38015	Compromised	Detected	Compromised	Detected
4235	A_33_P3417880	NKAPL	Hs.239181	NFKB activating protein-like	Homo sapiens NFKB activating protein-	1192.53760	1417.58750	471.78372	421.97147	Detected	Detected	Detected	Detected
4236	A_24_P135902	RPS2	Hs.356366	ribosomal protein S2	Homo sapiens ribosomal protein S2 (RP	281185.72000	270182.25000	294061.06000	245132.47000	Detected	Detected	Detected	Detected
4237	A_23_P21162	TCTEX1D2	Hs.135997	Tctex1 domain containing 2	Homo sapiens Tctex1 domain containin	4039.42900	4026.33700	2215.59810	1752.99270	Detected	Detected	Detected	Detected
4238	A_23_P376686	BTNL2	Hs.534471	butyrophilin-like 2 (MHC class II associ	Homo sapiens butyrophilin-like 2 (MHC	84.17316	104.87907	50.14227	45.30479	Detected	Detected	Detected	Detected
4239	A_23_P395075	KDM3A	Hs.557425	lysine (K)-specific demethylase 3A	Homo sapiens lysine (K)-specific demet	916.69180	527.98755	395.48248	185.72774	Detected	Detected	Detected	Detected
4240	A_24_P916656	CRCP	Hs.300684	CGRP receptor component	Homo sapiens CGRP receptor compone	84.35514	170.22462	87.76881	133.15062	Detected	Detected	Detected	Detected
4241	A_23_P39402	ALKBH6	Hs.71941	alkB, alkylation repair homolog 6 (E. coli	Homo sapiens alkB, alkylation repair ho	446.28653	593.40430	441.05298	528.08390	Detected	Detected	Detected	Detected
4242	A_33_P3331366	TRIM25	Hs.528952	tripartite motif-containing 25	Homo sapiens tripartite motif-containin	16180.23600	8772.28400	14129.56400	6948.50730	Detected	Detected	Detected	Detected
4243	A_23_P99604	G2E3	Hs.509008	G2/M-phase specific E3 ubiquitin ligase	Homo sapiens G2/M-phase specific E3	216.62044	370.52258	166.81833	250.21315	Detected	Detected	Detected	Detected
4244	A_33_P3327762				Dedicator of cytokinesis protein 8 [Sou	39.62342	90.07700	19.49684	24.84941	Detected	Detected	Detected	Detected
4245	A_23_P151368	N6AMT2	Hs.26674	N-6 adenine-specific DNA methyltransf	Homo sapiens N-6 adenine-specific DN	1368.30100	842.06274	1475.91250	753.39886	Detected	Detected	Detected	Detected
4246	A_33_P3531857	DARS	Hs.503787	aspartyl-tRNA synthetase	Homo sapiens aspartyl-tRNA synthetas	16495.83600	12112.66800	14075.37500	8826.04000	Detected	Detected	Detected	Detected
4247	A_23_P81898	UBD	Hs.44532	ubiquitin D	Homo sapiens ubiquitin D (UBD), mRNA	121.69443	49.74078	72.73988	3.13442	Detected	Detected	Detected	Compromised
4248	A_33_P3237719	LOC100129652		hypothetical protein LOC100129652	PREDICTED: Homo sapiens hypothetica	6.13799	31.92258	8.55560	14.14132	Compromised	Detected	Compromised	Compromised
4249	A_23_P118690	ZNF207	Hs.500775	zinc finger protein 207	Homo sapiens zinc finger protein 207 (Z	8248.67600	7840.93260	5381.65140	4035.82570	Detected	Detected	Detected	Detected
4250	A_23_P110791	CSF1R	Hs.586219	colony stimulating factor 1 receptor	Homo sapiens colony stimulating factor	613.51870	846.10077	470.15640	565.98380	Detected	Detected	Detected	Detected
4251	A_33_P3220939	PCID2	Hs.508769	PCI domain containing 2	Homo sapiens PCI domain containing 2	2978.63920	2523.43330	1310.37270	894.69910	Detected	Detected	Detected	Detected
4252	A_33_P3245439	CD40	Hs.472860	CD40 molecule, TNF receptor superfam	Homo sapiens CD40 molecule, TNF rec	223.80263	94.80080	281.99493	110.60050	Detected	Detected	Detected	Detected
4253	A_23_P59637	DOCK4	Hs.654652	dedicator of cytokinesis 4	Homo sapiens dedicator of cytokinesis	922.59220	175.08434	492.09235	65.27118	Detected	Detected	Detected	Detected
4254	A_23_P430181	ZBTB3	Hs.147554	zinc finger and BTB domain containing	Homo sapiens zinc finger and BTB dom	455.60938	1033.87870	369.34000	763.32860	Detected	Detected	Detected	Detected
4255	A_33_P3256952	EGLN3	Hs.135507	egl nine homolog 3 (C. elegans)	Homo sapiens egl nine homolog 3 (C. el	32.55422	7.80420	25.16468	3.10849	Detected	Compromised	Detected	Compromised
4256	A_24_P186216	PDS5A	Hs.331431	PDS5, regulator of cohesion maintenanc	Homo sapiens PDS5, regulator of cohes	313.74120	920.00510	192.64104	464.73816	Detected	Detected	Detected	Detected
4257	A_23_P137765	PAFAH2	Hs.477083	platelet-activating factor acetylhydrolas	Homo sapiens platelet-activating factor	74.24947	80.33927	41.69019	32.93355	Detected	Detected	Detected	Detected
4258	A_33_P3311551		Hs.647659		Integral membrane protein GPR177 Pre	276.73438	242.35085	213.09134	186.10583	Detected	Detected	Detected	Detected
4259	A_33_P3424364	CCDC152	Hs.718521	coiled-coil domain containing 152	Homo sapiens coiled-coil domain contai	105.69899	42.48478	105.29570	31.82538	Detected	Detected	Detected	Detected
4260	A_33_P3213512	LOC100131988	Hs.710099	hypothetical LOC100131988	PREDICTED: Homo sapiens hypothetica	37.03846	47.37972	20.48719	30.73992	Detected	Detected	Detected	Detected
4261	A_24_P404033	C15orf38	Hs.6734	chromosome 15 open reading frame 38	Homo sapiens chromosome 15 open rea	173.78426	525.45180	138.88650	359.11148	Detected	Detected	Detected	Detected
4262	A_33_P3699445	LOC644613		hypothetical protein LOC644613	PREDICTED: Homo sapiens hypothetica	492.41644	394.61707	569.79410	426.86374	Detected	Detected	Detected	Detected
4263	A_23_P130547	OR7D4	Hs.446910	olfactory receptor, family 7, subfamily D	Homo sapiens olfactory receptor, family	14.69497	13.32654	4.42048	7.38775	Detected	Compromised	Compromised	Compromised
4264	A_23_P401568	MAT2A	Hs.516157	methionine adenosyltransferase II, alpha	Homo sapiens methionine adenosyltrans	8966.58200	8740.76600	6912.72700	5817.71140	Detected	Detected	Detected	Detected
4265	A_33_P3325606					16.38692	15.37391	7.30161	9.63747	Detected	Compromised	Compromised	Compromised
4266	A_23_P28969	CHMP4B	Hs.472471	chromatin modifying protein 4B	Homo sapiens chromatin modifying prot	3434.68500	2589.71780	6636.46140	5348.43100	Detected	Detected	Detected	Detected
4267	A_23_P80032	E2F1	Hs.654393	E2F transcription factor 1	Homo sapiens E2F transcription factor	512.26470	4651.27250	357.43234	3045.51730	Detected	Detected	Detected	Detected
4268	A_24_P406006	LPCAT1	Hs.368853	lysophosphatidylcholine acyltransferase	Homo sapiens lysophosphatidylcholine a	972.44910	1145.97300	873.20340	861.65015	Detected	Detected	Detected	Detected
4269	A_33_P3401647	PPP1R14A	Hs.631569	protein phosphatase 1, regulatory (inhib	Homo sapiens protein phosphatase 1, re	35.51206	99.07933	28.80223	48.56027	Detected	Detected	Detected	Detected
4270	A_24_P16340				GB	67613.91400	47257.18000	86629.84000	50440.28000	Detected	Detected	Detected	Detected
4271	A_24_P361457	FLJ35220	Hs.389678	hypothetical protein FLJ35220	Homo sapiens hypothetical protein FLJ3	180.23248	93.54668	145.01985	41.39437	Detected	Detected	Detected	Detected

4272	A_24_P400473	SLC25A44	Hs.532375	solute carrier family 25, member 44	Homo sapiens solute carrier family 25, r	3187.86400	3213.27950	2661.61300	2458.06200	Detected	Detected	Detected	Detected
4273	A_24_P244356	NLRX1	Hs.524082	NLR family member X1	Homo sapiens NLR family member X1 (N	1917.20510	2871.98800	2116.97660	2724.95000	Detected	Detected	Detected	Detected
4274	A_24_P264909	NDFIP2	Hs.525093	Nedd4 family interacting protein 2	Homo sapiens Nedd4 family interacting	1830.10220	1167.60600	1006.04803	519.23870	Detected	Detected	Detected	Detected
4275	A_23_P14184	THSD1	Hs.325667	thrombospondin, type I, domain containi	Homo sapiens thrombospondin, type I, d	1572.18900	2119.85840	999.30730	1279.09910	Detected	Detected	Detected	Detected
4276	A_33_P3310409					28.41065	35.58866	15.30090	17.17907	Detected	Detected	Compromised	Compromised
4277	A_23_P36464	C12orf11	Hs.505077	chromosome 12 open reading frame 11	Homo sapiens chromosome 12 open rea	1606.96910	1202.92440	963.26373	569.21850	Detected	Detected	Detected	Detected
4278	A_23_P253421	AUP1	Hs.411480	ancient ubiquitous protein 1	Homo sapiens ancient ubiquitous protei	7017.58600	7171.87700	6488.16260	6349.60900	Detected	Detected	Detected	Detected
4279	A_24_P366415	LOC643997		similar to peptidylprolyl isomerase A	PREDICTED: Homo sapiens similar to T	11012.17800	14104.15400	7619.55300	8667.79000	Detected	Detected	Detected	Detected
4280	A_33_P3318946	HAPLN2	Hs.410719	hyaluronan and proteoglycan link protei	Homo sapiens hyaluronan and proteogly	297.76727	179.99692	181.40578	36.26995	Detected	Detected	Detected	Detected
4281	A_23_P217054	DCAF10	Hs.118394	DDB1 and CUL4 associated factor 10	Homo sapiens DDB1 and CUL4 associa	881.60870	945.62177	744.12150	678.16830	Detected	Detected	Detected	Detected
4282	A_23_P110643	CDKL3	Hs.719926	cyclin-dependent kinase-like 3	Homo sapiens cyclin-dependent kinase-	673.81500	381.36040	554.11450	263.12146	Detected	Detected	Detected	Detected
4283	A_24_P185986	UPRT	Hs.91612	uracil phosphoribosyltransferase (FUR1	Homo sapiens uracil phosphoribosyltran	1335.53720	1720.78340	1133.43530	1100.98270	Detected	Detected	Detected	Detected
4284	A_23_P120170	TIGD1	Hs.211823	tigger transposable element derived 1	Homo sapiens tigger transposable eleme	319.26385	326.37906	251.50487	213.02255	Detected	Detected	Detected	Detected
4285	A_23_P170574	SNAI3	Hs.673548	snail homolog 3 (Drosophila)	Homo sapiens snail homolog 3 (Drosoph	22.02261	20.02051	10.15327	3.52310	Detected	Detected	Compromised	Compromised
4286	A_24_P166311	C5orf54	Hs.529464	chromosome 5 open reading frame 54	Homo sapiens chromosome 5 open read	97.99213	74.37478	79.25645	37.18189	Detected	Detected	Detected	Detected
4287	A_33_P3230264	GPC3	Hs.644108	glypican 3	Homo sapiens glypican 3 (GPC3), transc	212.16965	275.46957	161.67290	160.97860	Detected	Detected	Detected	Detected
4288	A_23_P32454	TG	Hs.654591	thyroglobulin	Homo sapiens thyroglobulin (TG), mRNA	82.45951	169.68474	61.02495	126.60231	Detected	Detected	Detected	Detected
4289	A_24_P110983	AKT3		v-akt murine thymoma viral oncogene h	RAC-gamma serine/threonine-protein k	1409.38480	1944.27480	1011.86190	1129.86710	Detected	Detected	Detected	Detected
4290	A_24_P401637		Hs.634471		cDNA FLJ38408 fis, clone FEBRA2009(	329.60916	244.35207	295.99496	179.77213	Detected	Detected	Detected	Detected
4291	A_33_P3289236	HPR	Hs.655361	haptoglobin-related protein	Homo sapiens haptoglobin-related prote	35.02179	148.30803	14.83061	11.01715	Detected	Detected	Compromised	Compromised
4292	A_23_P46215	TRIM46	Hs.287735	tripartite motif-containing 46	Homo sapiens tripartite motif-containin	27.92592	27.77200	30.91382	13.44092	Detected	Detected	Detected	Compromised
4293	A_23_P254733	MLF1IP	Hs.575032	MLF1 interacting protein	Homo sapiens MLF1 interacting protein	491.06420	10777.37800	376.09010	6348.35400	Detected	Detected	Detected	Detected
4294	A_23_P103661	YY1AP1	Hs.584927	YY1 associated protein 1	Homo sapiens YY1 associated protein 1	3056.26270	3920.14750	2201.77760	2538.33640	Detected	Detected	Detected	Detected
4295	A_32_P144596	TNKS	Hs.370267	tankyrase, TRF1-interacting ankyrin-re	Homo sapiens tankyrase, TRF1-interact	627.73220	306.14856	523.99320	193.74545	Detected	Detected	Detected	Detected
4296	A_23_P42884	MRPS24	Hs.284286	mitochondrial ribosomal protein S24	Homo sapiens mitochondrial ribosomal p	49056.02700	54125.93400	46711.02700	43166.06000	Detected	Detected	Detected	Detected
4297	A_23_P101374	CYP2S1	Hs.98370	cytochrome P450, family 2, subfamily S,	Homo sapiens cytochrome P450, family	18.00857	22.97875	5.43182	4.63717	Not Detected	Not Detected	Compromised	Compromised
4298	A_23_P420269	INTS2	Hs.279646	integrator complex subunit 2	Homo sapiens integrator complex subur	144.23180	154.63951	88.11593	55.93204	Detected	Detected	Detected	Detected
4299	A_33_P3212716		Hs.640952		DB028998 TESTI2 Homo sapiens cDNA	656.72656	93.20602	610.00446	61.89534	Detected	Detected	Detected	Detected
4300	A_33_P3297580	CACNA1C	Hs.118262	calcium channel, voltage-dependent, L t	Homo sapiens calcium channel, voltage-	75.81902	126.05113	64.92164	82.31429	Detected	Detected	Detected	Detected
4301	A_33_P3358307	OTUD7B	Hs.98322	OTU domain containing 7B	Homo sapiens OTU domain containing 7	836.68510	729.29650	519.10693	348.30450	Detected	Detected	Detected	Detected
4302	A_33_P3321275	MLL3	Hs.647120	myeloid/lymphoid or mixed-lineage leuk	Homo sapiens myeloid/lymphoid or mixe	104.68749	86.11656	51.41141	24.71013	Detected	Detected	Detected	Detected
4303	A_23_P117727	MTFMT	Hs.531615	mitochondrial methionyl-tRNA formyltra	Homo sapiens mitochondrial methionyl-	1154.50020	1177.91810	1108.41590	937.07750	Detected	Detected	Detected	Detected
4304	A_23_P139740	PRMT8	Hs.504530	protein arginine methyltransferase 8	Homo sapiens protein arginine methyltra	13.36769	13.41034	7.77173	3.77225	Detected	Compromised	Compromised	Compromised
4305	A_33_P3215635	CHRNB2	Hs.2306	cholinergic receptor, nicotinic, beta 2 (n	Homo sapiens cholinergic receptor, nico	18.44157	3.83412	15.58569	5.71129	Detected	Compromised	Detected	Compromised
4306	A_33_P3303136	SERPINB6	Hs.719963	serpin peptidase inhibitor, clade B (ovall	Homo sapiens serpin peptidase inhibitor	9976.87500	12993.28600	13200.74400	14763.28000	Detected	Detected	Detected	Detected
4307	A_23_P140057	TNFRSF19	Hs.149168	tumor necrosis factor receptor superfar	Homo sapiens tumor necrosis factor rec	6.51908	22.59849	4.57305	11.08194	Compromised	Detected	Compromised	Compromised
4308	A_23_P140511	CLPX	Hs.113823	ClpX caseinolytic peptidase X homolog	Homo sapiens ClpX caseinolytic peptida	3527.00830	3646.06540	2912.66360	2488.55700	Detected	Detected	Detected	Detected
4309	A_23_P121686	AIMP1	Hs.591680	aminoacyl tRNA synthetase complex-in	Homo sapiens aminoacyl tRNA syntheta	10271.95800	9609.52500	7181.59770	5893.54700	Detected	Detected	Detected	Detected
4310	A_24_P373844	KCTD15	Hs.221873	potassium channel tetramerisation dom	Homo sapiens potassium channel tetran	387.38590	141.41602	241.98073	87.01706	Detected	Detected	Detected	Detected
4311	A_33_P3221019	ZAN	Hs.307004	zonadhesin	Homo sapiens zonadhesin (ZAN), transc	72.18246	125.84303	72.66040	81.27568	Detected	Detected	Detected	Detected
4312	A_23_P329962	SUNC1	Hs.406741	Sad1 and UNC84 domain containing 1	Homo sapiens Sad1 and UNC84 domain	19.86798	20.11431	20.65978	17.21868	Detected	Detected	Detected	Detected
4313	A_23_P83094	TLE4	Hs.444213	transducin-like enhancer of split 4 (E(sp	Homo sapiens transducin-like enhancer	911.45550	1323.76820	797.79350	831.02370	Detected	Detected	Detected	Detected
4314	A_32_P182662	AIDA	Hs.534965	axin interactor, dorsalization associated	Homo sapiens axin interactor, dorsalizat	591.93085	1040.96200	580.09610	823.17523	Detected	Detected	Detected	Detected
4315	A_32_P113114	ZNF561	Hs.720081	zinc finger protein 561	Homo sapiens zinc finger protein 561 (Z	529.17630	392.14694	476.12164	297.42145	Detected	Detected	Detected	Detected
4316	A_33_P3249214	CNOT6L	Hs.592519	CCR4-NOT transcription complex, subu	Homo sapiens CCR4-NOT transcription	1006.46180	736.34770	775.90040	481.65414	Detected	Detected	Detected	Detected
4317	A_33_P3394769	TMEM8C	Hs.512467	transmembrane protein 8C	Homo sapiens transmembrane protein 8	43756.38300	54462.15200	48608.95300	46087.21000	Detected	Detected	Detected	Detected
4318	A_23_P77562	TMEM219	Hs.460574	transmembrane protein 219	Homo sapiens transmembrane protein 2	2180.17770	2090.20600	2937.66750	2473.46500	Detected	Detected	Detected	Detected
4319	A_33_P3301689	ZNF326	Hs.306221	zinc finger protein 326	Homo sapiens zinc finger protein 326 (Z	347.23392	304.56866	186.65637	137.35341	Detected	Detected	Detected	Detected
4320	A_33_P3372318				Echinoderm microtubule-associated pro	62.42786	87.81080	32.92608	26.41738	Detected	Detected	Detected	Detected
4321	A_24_P921366	CALD1	Hs.490203	caldesmon 1	Homo sapiens caldesmon 1 (CALD1), tr	31079.19000	30390.89800	19238.24400	15616.76600	Detected	Detected	Detected	Detected
4322	A_33_P3243717	ZNF248	Hs.528423	zinc finger protein 248	Homo sapiens zinc finger protein 248 (Z	88.83656	55.67775	57.79451	44.85306	Detected	Detected	Detected	Detected
4323	A_32_P49350					24783.89800	30094.78500	22554.98600	23816.40600	Detected	Detected	Detected	Detected
4324	A_23_P103099	RBM9	Hs.282998	RNA binding motif protein 9	Homo sapiens RNA binding motif protei	942.13025	1037.25700	687.22640	687.89950	Detected	Detected	Detected	Detected
4325	A_23_P122007	C5orf30	Hs.482976	chromosome 5 open reading frame 30	Homo sapiens chromosome 5 open read	4282.01030	8207.12800	2607.82080	4277.33400	Detected	Detected	Detected	Detected
4326	A_24_P96762	TRNAU1AP	Hs.533626	tRNA selenocysteine 1 associated prote	Homo sapiens tRNA selenocysteine 1 a	959.55990	421.37997	779.50775	281.41022	Detected	Detected	Detected	Detected
4327	A_33_P3502305	LOC100132101	Hs.721803	similar to hCG2006901	PREDICTED: Homo sapiens similar to h	18.48218	21.28582	17.37473	11.20551	Detected	Detected	Detected	Compromised
4328	A_23_P107644	SNRPD1	Hs.464734	small nuclear ribonucleoprotein D1 poly	Homo sapiens small nuclear ribonucleop	20698.20900	34129.96000	15867.58000	22479.66800	Detected	Detected	Detected	Detected
4329	A_33_P3224780				Voltage-dependent calcium channel gar	17.73284	28.29773	16.29657	17.30338	Detected	Detected	Detected	Detected
4330	A_33_P3278826				Leukocyte tyrosine kinase receptor Pre	39.45715	35.08118	27.98605	28.41069	Detected	Detected	Detected	Detected
4331	A_23_P111395	SLC22A2	Hs.436385	solute carrier family 22 (organic cation	Homo sapiens solute carrier family 22 (	3.69828	6.28326	4.19068	42.74104	Compromised	Compromised	Compromised	Not Detected
4332	A_33_P3231428		Hs.677538		Putative uncharacterized protein ENSP	67.45341	89.78813	24.74580	16.25784	Detected	Detected	Detected	Detected
4333	A_33_P3364884	PDHB	Hs.161357	pyruvate dehydrogenase (lipoamide) bet	Homo sapiens pyruvate dehydrogenase	6648.58940	9112.37400	4148.88530	5247.89100	Detected	Detected	Detected	Detected
4334	A_33_P3270863	XDH	Hs.250	xanthine dehydrogenase	Homo sapiens xanthine dehydrogenase	633.99250	768.71783	548.02750	610.38245	Detected	Detected	Detected	Detected
4335	A_23_P91640	ASPHD2	Hs.567547	aspartate beta-hydroxylase domain con	Homo sapiens aspartate beta-hydroxyla	315.80950	149.65936	423.05765	93.03819	Detected	Detected	Detected	Detected
4336	A_23_P252306	ID1	Hs.504609	inhibitor of DNA binding 1, dominant neg	Homo sapiens inhibitor of DNA binding	18107.00600	16030.09500	17078.64600	11803.24900	Detected	Detected	Detected	Detected
4337	A_24_P778906	LOC100289026	Hs.609146	similar to hCG1744891	PREDICTED: Homo sapiens similar to h	16.55136	21.02830	13.64320	10.59634	Detected	Detected	Compromised	Compromised
4338	A_33_P3248384	HCG2P7	Hs.720720	HLA complex group 2 pseudogene 7	Homo sapiens HLA complex group 2 ps	917.06210	1467.72140	309.44180	365.22336	Detected	Detected	Detected	Detected
4339	A_33_P3401394					55.04575	53.96940	3.22829	3.12462	Detected	Detected	Compromised	Compromised
4340	A_24_P66125	STAG2	Hs.496710	stromal antigen 2	Homo sapiens stromal antigen 2 (STAG	4314.27250	3860.06800	3149.33280	2242.53340	Detected	Detected	Detected	Detected
4341	A_24_P325176	KIAA1109	Hs.408142	KIAA1109	Homo sapiens KIAA1109 (KIAA1109), m	974.44650	516.67834	564.38150	231.37180	Detected	Detected	Detected	Detected
4342	A_33_P3351027	PXMP3	Hs.437966	peroxisomal membrane protein 3, 35kDa	Homo sapiens peroxisomal membrane p	75.50127	25.79222	60.17407	8.30736	Detected	Detected	Detected	Compromised
4343	A_23_P159125	SLC16A5	Hs.592095	solute carrier family 16, member 5 (mon	Homo sapiens solute carrier family 16, r	13275.01100	6323.04930	11959.81200	4757.49760	Detected	Detected	Detected	Detected
4344	A_23_P214727	GPR63	Hs.632612	G protein-coupled receptor 63	Homo sapiens G protein-coupled recep	17.71935	29.34414	24.30586	12.09202	Detected	Detected	Detected	Compromised
4345	A_33_P3319815		Hs.187816		Putative uncharacterized protein CXorf	37.43110	56.72214	34.94214	29.18210	Detected	Detected	Detected	Detected
4346	A_23_P160336	LEFTY1	Hs.720965	left-right determination factor 1	Homo sapiens left-right determination f	94.15096	3.57629	80.80880	3.62055	Detected	Compromised	Detected	Compromised

4347	A_24_P51080	FLJ42709		hypothetical LOC441094	Homo sapiens hypothetical LOC441094	40.22015	35.70239	25.03539	22.97747	Detected	Detected	Detected	Detected
4348	A_24_P175176	PHTF2	Hs.203965	putative homeodomain transcription fac	Homo sapiens putative homeodomain tr	1704.90040	1842.20210	903.90204	803.52014	Detected	Detected	Detected	Detected
4349	A_33_P3831730	MGC4859	Hs.571301	hypothetical LOC79150	Homo sapiens hypothetical protein MG(	1495.80080	1472.96120	673.39514	604.23065	Detected	Detected	Detected	Detected
4350	A_33_P3304528	SENP1	Hs.371957	SUMO1/sentrin specific peptidase 1	Homo sapiens SUMO1/sentrin specific	1525.55360	1683.22510	1133.55520	953.15080	Detected	Detected	Detected	Detected
4351	A_23_P37910	MAPK3	Hs.861	mitogen-activated protein kinase 3	Homo sapiens mitogen-activated protei	903.69170	672.52410	835.93713	696.31850	Detected	Detected	Detected	Detected
4352	A_23_P47466	PRG3	Hs.251386	proteoglycan 3	Homo sapiens proteoglycan 3 (PRG3), r	51.21634	94.27433	19.91306	25.97524	Detected	Detected	Detected	Detected
4353	A_24_P388570	MINA	Hs.607776	MYC induced nuclear antigen	Homo sapiens MYC induced nuclear ant	482.91183	516.39734	548.25580	512.37480	Detected	Detected	Detected	Detected
4354	A_24_P381199	TRIM6	Hs.125300	tripartite motif-containing 6	Homo sapiens tripartite motif-containin	90.76167	585.51120	57.89333	267.60248	Detected	Detected	Detected	Detected
4355	A_23_P79426	CAB39	Hs.603930	calcium binding protein 39	Homo sapiens calcium binding protein 3	2082.11400	1993.26840	1772.68730	1363.36960	Detected	Detected	Detected	Detected
4356	A_33_P3416301	E2F6	Hs.603093	E2F transcription factor 6	Homo sapiens E2F transcription factor	69.80108	47.48319	100.99050	68.28635	Detected	Detected	Detected	Detected
4357	A_24_P412156	CXCL12	Hs.522891	chemokine (C-X-C motif) ligand 12 (str	Homo sapiens chemokine (C-X-C motif	727.78530	963.79047	288.09020	312.46317	Detected	Detected	Detected	Detected
4358	A_23_P218331	CYB561	Hs.355264	cytochrome b-561	Homo sapiens cytochrome b-561 (CYB!	1036.01660	1154.08220	837.70890	850.51110	Detected	Detected	Detected	Detected
4359	A_23_P82868	PLAT	Hs.491582	plasminogen activator, tissue	Homo sapiens plasminogen activator, tis	3778.89090	6668.21100	2556.87280	3799.02050	Detected	Detected	Detected	Detected
4360	A_33_P3591761	FAM91A1	Hs.459174	family with sequence similarity 91, mem	Homo sapiens family with sequence sim	342.13995	208.26123	184.80269	89.05307	Detected	Detected	Detected	Detected
4361	A_23_P31798	NAT2	Hs.2	N-acetyltransferase 2 (arylamine N-ace	Homo sapiens N-acetyltransferase 2 (a	52.63606	63.30480	25.98324	24.83295	Detected	Detected	Detected	Detected
4362	A_23_P344125	ISM2	Hs.29742	isthmin 2 homolog (zebrafish)	Homo sapiens isthmin 2 homolog (zebra	19.71674	32.83685	15.42175	8.17032	Detected	Detected	Compromised	Compromised
4363	A_33_P3422330	KLHL17	Hs.109212	kelch-like 17 (Drosophila)	Homo sapiens kelch-like 17 (Drosophila	5069.48970	2793.53470	4916.35840	2426.11060	Detected	Detected	Detected	Detected
4364	A_23_P96688	SUV420H1	Hs.632120	suppressor of variegation 4-20 homolog	Homo sapiens suppressor of variegatior	65.34137	139.47772	43.25069	76.82851	Detected	Detected	Detected	Detected
4365	A_23_P203891	NCOR2	Hs.137510	nuclear receptor co-repressor 2	Homo sapiens nuclear receptor co-repr	46171.67600	50683.74000	38945.68800	43142.95000	Detected	Detected	Detected	Detected
4366	A_23_P29029	C21orf119	Hs.58149	chromosome 21 open reading frame 119	Homo sapiens chromosome 21 open rea	968.08130	430.20126	1164.46030	469.91345	Detected	Detected	Detected	Detected
4367	A_24_P109069	SYT15	Hs.696346	synaptotagmin XV	Homo sapiens synaptotagmin XV (SYT1	77.25349	118.94649	80.53534	109.76219	Detected	Detected	Detected	Detected
4368	A_33_P3368646	CNKSR3	Hs.16064	CNKSR family member 3	Homo sapiens CNKSR family member 3	39.53285	30.44714	32.53808	15.23065	Detected	Detected	Detected	Compromised
4369	A_23_P320829	C14orf145	Hs.162889	chromosome 14 open reading frame 145	Homo sapiens chromosome 14 open rea	12.44311	49.52603	12.80256	26.22872	Compromised	Detected	Compromised	Detected
4370	A_23_P84448	TUBA4A	Hs.75318	tubulin, alpha 4a	Homo sapiens tubulin, alpha 4a (TUBA4	3445.81030	2193.02880	4222.82280	1707.34200	Detected	Detected	Detected	Detected
4371	A_23_P167856	TMEM63B	Hs.414473	transmembrane protein 63B	Homo sapiens transmembrane protein 6	562.40985	603.90717	491.71094	540.74976	Detected	Detected	Detected	Detected
4372	A_23_P108657	WDSUB1	Hs.20848	WD repeat, sterile alpha motif and U-bo	Homo sapiens WD repeat, sterile alpha	274.33182	274.18660	219.12119	187.65694	Detected	Detected	Detected	Detected
4373	A_33_P3379176					18.54990	18.89701	28.33592	20.32524	Detected	Compromised	Detected	Detected
4374	A_33_P3282740		Hs.660463		hypothetical LOC728855 (LOC728855),	13.03383	10.85138	17.60691	6.82808	Compromised	Compromised	Detected	Compromised
4375	A_33_P3214869	KPNA7	Hs.253050	karyopherin alpha 7 (importin alpha 8)	Homo sapiens karyopherin alpha 7 (impo	23.92537	17.75214	13.91151	5.99858	Detected	Compromised	Compromised	Compromised
4376	A_32_P182473	ZNF625	Hs.512823	zinc finger protein 625	Homo sapiens zinc finger protein 625 (Z	779.23883	987.75305	774.53674	721.64276	Detected	Detected	Detected	Detected
4377	A_33_P3385957	TTLL1	Hs.660298	tubulin tyrosine ligase-like family, memb	Homo sapiens tubulin tyrosine ligase-lik	481.88663	296.21555	558.20380	265.77470	Detected	Detected	Detected	Detected
4378	A_33_P3221528	IREB2	Hs.436031	iron-responsive element binding protein	Homo sapiens iron-responsive element	6193.97200	6529.19900	3894.28080	3401.11130	Detected	Detected	Detected	Detected
4379	A_24_P587443	LOC731852	Hs.136941	hypothetical LOC731852	PREDICTED: Homo sapiens hypothetica	5.10486	22.76509	9.15973	4.46807	Compromised	Detected	Compromised	Compromised
4380	A_33_P3378920	RBM4	Hs.533712	RNA binding motif protein 4	Homo sapiens RNA binding motif protei	21258.78500	23805.48200	19427.66400	18365.61500	Detected	Detected	Detected	Detected
4381	A_33_P3423445	ZNF730	Hs.720743	zinc finger protein 730	Putative zinc finger protein 730 [Sourc	4.77951	31.74057	5.36864	14.41995	Compromised	Detected	Compromised	Compromised
4382	A_23_P103628	HEATR1	Hs.708114	HEAT repeat containing 1	Homo sapiens HEAT repeat containing	2491.69920	2913.46850	1509.67500	1477.19890	Detected	Detected	Detected	Detected
4383	A_33_P3241369	UCN3	Hs.511775	urocortin 3 (stresscopin)	Homo sapiens urocortin 3 (stresscopin)	296.64313	269.08417	85.21415	59.14236	Detected	Detected	Detected	Detected
4384	A_23_P252322	ATP5E	Hs.177530	ATP synthase, H+ transporting, mitocho	Homo sapiens ATP synthase, H+ transp	132323.70000	142143.17000	103618.57000	84416.77000	Detected	Detected	Detected	Detected
4385	A_33_P3395581	TRIM53	Hs.719886	tripartite motif-containing 53	Homo sapiens tripartite motif-containin	13.57604	23.48080	19.18556	14.81500	Detected	Detected	Detected	Compromised
4386	A_33_P3212679	LOC100130932	Hs.631639	small nuclear ribonucleoprotein G-like p	Homo sapiens small nuclear ribonucleop	24054.91000	33227.65200	18377.65000	19120.86700	Detected	Detected	Detected	Detected
4387	A_33_P3307133	GRIK2	Hs.98262	glutamate receptor, ionotropic, kainate	Homo sapiens glutamate receptor, ionot	11.48295	34.16568	7.92374	22.56036	Compromised	Detected	Compromised	Detected
4388	A_23_P1452	NPFFR1	Hs.302026	neuropeptide FF receptor 1	Homo sapiens neuropeptide FF recepto	22.75856	32.89338	25.03903	18.49329	Detected	Detected	Detected	Detected
4389	A_33_P3391476	CRYZ	Hs.83114	crystallin, zeta (quinone reductase)	Homo sapiens crystallin, zeta (quinone	403.97095	306.26578	379.44830	243.44154	Detected	Detected	Detected	Detected
4390	A_23_P138025	GPN2	Hs.14333	GPN-loop GTPase 2	Homo sapiens GPN-loop GTPase 2 (GF	937.90730	2280.83890	1277.48940	2575.95830	Detected	Detected	Detected	Detected
4391	A_24_P26897	INPP5A	Hs.523360	inositol polyphosphate-5-phosphatase,	Homo sapiens inositol polyphosphate-5	1352.38020	1856.58620	1077.25420	1241.99390	Detected	Detected	Detected	Detected
4392	A_23_P256933	RPL24	Hs.477028	ribosomal protein L24	Homo sapiens ribosomal protein L24 (R	147968.50000	136053.27000	137699.62000	98539.28000	Detected	Detected	Detected	Detected
4393	A_24_P469641	RNF216	Hs.487458	ring finger protein 216	Homo sapiens ring finger protein 216 (R	507.54886	893.48456	424.08690	626.69196	Detected	Detected	Detected	Detected
4394	A_33_P3306452	LOC653510	Hs.635227	similar to COBW domain containing 1	COBW domain-containing protein 3 (Co	86.54411	58.95406	78.83817	34.49773	Detected	Detected	Detected	Detected
4395	A_33_P3232493		Hs.660492		Homo sapiens cDNA FLJ27284 fis, clon	28.22407	24.49258	9.57194	6.76678	Detected	Detected	Compromised	Compromised
4396	A_33_P3402783					1140.97740	723.07240	1270.30640	627.35016	Detected	Detected	Detected	Detected
4397	A_32_P186474	RACGAP1	Hs.505469	Rac GTPase activating protein 1	Homo sapiens Rac GTPase activating p	1142.04660	10445.47700	657.50240	4478.19970	Detected	Detected	Detected	Detected
4398	A_33_P3261520	FLJ41309	Hs.364045	hypothetical protein LOC645079	Homo sapiens cDNA FLJ41309 fis, clon	76.15928	22.99678	47.47089	6.07652	Detected	Detected	Detected	Compromised
4399	A_23_P12463	QSOX1	Hs.518374	quiescin Q6 sulfhydryl oxidase 1	Homo sapiens quiescin Q6 sulfhydryl ox	40045.81600	43156.10000	37465.93000	34670.70300	Detected	Detected	Detected	Detected
4400	A_33_P3404116					50.12833	40.05836	20.90065	12.68644	Detected	Detected	Detected	Compromised
4401	A_24_P178065		Hs.477114		Pleckstrin homology-like domain family	386.86642	934.64746	17.73886	20.80199	Detected	Detected	Detected	Detected
4402	A_32_P358887	SLC4A4	Hs.5462	solute carrier family 4, sodium bicarbon	Homo sapiens solute carrier family 4, so	19.60631	346.53430	13.04699	218.66792	Detected	Detected	Compromised	Detected
4403	A_23_P83818	COL5A1	Hs.210283	collagen, type V, alpha 1	Homo sapiens collagen, type V, alpha 1	233.04053	3570.03980	226.51959	3340.94240	Detected	Detected	Detected	Detected
4404	A_23_P152995	SLC6A4	Hs.134662	solute carrier family 6 (neurotransmitte	Homo sapiens solute carrier family 6 (n	16.18967	23.18904	16.19665	6.74260	Detected	Detected	Detected	Compromised
4405	A_32_P140898	FOXN2	Hs.468478	forkhead box N2	Homo sapiens forkhead box N2 (FOXN2	487.44810	562.57640	449.89697	320.54770	Detected	Detected	Detected	Detected
4406	A_23_P5903	SLCO4A1	Hs.235782	solute carrier organic anion transporter	Homo sapiens solute carrier organic ani	328.30510	9.52320	306.34595	6.31691	Detected	Compromised	Detected	Compromised
4407	A_33_P3274622	LOC731275	Hs.722924	hypothetical LOC731275	Homo sapiens hypothetical LOC731275	63.40539	39.05120	44.17804	22.00750	Detected	Detected	Detected	Detected
4408	A_33_P3339915	REXO1L1	Hs.373854	REX1, RNA exonuclease 1 homolog (S.	Homo sapiens REX1, RNA exonuclease	268.37238	356.57687	75.70846	65.47316	Detected	Detected	Detected	Detected
4409	A_23_P69573	GUCY1A3	Hs.24258	guanylate cyclase 1, soluble, alpha 3	Homo sapiens guanylate cyclase 1, solu	135.01192	20.75872	107.33898	10.15567	Detected	Detected	Detected	Compromised
4410	A_23_P421306	SYT12	Hs.287636	synaptotagmin XII	Homo sapiens synaptotagmin XII (SYT1	141.13390	12.71280	106.00036	3.39201	Detected	Compromised	Detected	Compromised
4411	A_23_P124427	NEK1	Hs.481181	NIMA (never in mitosis gene a)-related	Homo sapiens NIMA (never in mitosis g	149.27620	111.45911	132.93541	89.89560	Detected	Detected	Detected	Detected
4412	A_33_P3326817					2219.67070	2616.41630	592.22906	501.55580	Detected	Detected	Detected	Detected
4413	A_23_P11224	MMGT1	Hs.110702	membrane magnesium transporter 1	Homo sapiens membrane magnesium tra	5969.53800	6749.91900	4405.78860	4382.58600	Detected	Detected	Detected	Detected
4414	A_33_P3353552	SLC48A1	Hs.438867	solute carrier family 48 (heme transport	Homo sapiens solute carrier family 48 (	678.56647	668.38640	499.30930	464.01830	Detected	Detected	Detected	Detected
4415	A_33_P3233081	MSH5	Hs.647011	mutS homolog 5 (E. coli)	Homo sapiens mutS homolog 5 (E. coli)	419.27610	376.40274	329.73730	257.50900	Detected	Detected	Detected	Detected
4416	A_33_P3340468		Hs.348920		Centromere protein I (CENP-I)(Interpha	9.16946	159.74335	10.91147	170.30363	Compromised	Detected	Compromised	Detected
4417	A_33_P3247919					119.16449	151.84792	59.94217	53.96574	Detected	Detected	Detected	Detected
4418	A_33_P3250887	C22orf41	Hs.535660	chromosome 22 open reading frame 41	Homo sapiens chromosome 22 open rea	34.68342	16.15800	32.61946	11.84706	Detected	Compromised	Detected	Compromised
4419	A_33_P3293753	KRTAP10-9	Hs.474000	keratin associated protein 10-9	Homo sapiens keratin associated protei	607.18310	637.95390	81.19594	72.72286	Detected	Detected	Detected	Detected
4420	A_33_P3388810		Hs.480085		Fucose-1-phosphate guanylyltransferas	363.03560	89.09296	360.86264	76.59300	Detected	Detected	Detected	Detected
4421	A_23_P259328	PEX3	Hs.7277	peroxisomal biogenesis factor 3	Homo sapiens peroxisomal biogenesis fa	849.51324	1136.41520	685.72930	746.88810	Detected	Detected	Detected	Detected

4422	A_33_P3219295					12.34007	14.89735	18.38269	11.58272	Compromised	Compromised	Detected	Compromised
4423	A_23_P70318	ENPP4	Hs.643497	ectonucleotide pyrophosphatase/phosp	Homo sapiens ectonucleotide pyrophos	303.78067	816.39435	200.24220	525.50420	Detected	Detected	Detected	Detected
4424	A_32_P82111	LRFN2	Hs.250015	leucine rich repeat and fibronectin type	Homo sapiens leucine rich repeat and fi	14.57191	10.45277	10.38243	3.11754	Detected	Compromised	Compromised	Compromised
4425	A_33_P3263284	LOC100130238		hypothetical LOC100130238	Homo sapiens hypothetical LOC100130	11795.29400	16828.17800	4967.69340	3048.58300	Detected	Detected	Detected	Detected
4426	A_23_P44581	NOMO1	Hs.209113	NODAL modulator 1	Homo sapiens NODAL modulator 1 (NO	4125.51860	5222.16160	3295.87800	3732.13040	Detected	Detected	Detected	Detected
4427	A_23_P319874	C3orf23	Hs.55131	chromosome 3 open reading frame 23	Homo sapiens chromosome 3 open read	36.61928	42.74885	25.92867	27.02862	Detected	Detected	Detected	Detected
4428	A_33_P3289352	PYGO2	Hs.533597	pygopus homolog 2 (Drosophila)	Homo sapiens pygopus homolog 2 (Dros	238.12944	211.53170	111.11340	109.76139	Detected	Detected	Detected	Detected
4429	A_23_P397341	PAQR4	Hs.351474	progestin and adipoQ receptor family m	Homo sapiens progestin and adipoQ rec	425.20610	400.01080	713.32490	639.44684	Detected	Detected	Detected	Detected
4430	A_23_P140170	SEC23A	Hs.272927	Sec23 homolog A (S. cerevisiae)	Homo sapiens Sec23 homolog A (S. cer	7745.81800	12517.15400	5542.72750	6762.65670	Detected	Detected	Detected	Detected
4431	A_24_P409126	FNDC3A	Hs.508010	fibronectin type III domain containing 3A	Homo sapiens cDNA FLJ31509 fis, clon	39.30961	13.14010	21.45673	3.13000	Detected	Compromised	Detected	Compromised
4432	A_23_P105963	AK7	Hs.667462	adenylate kinase 7	Homo sapiens adenylate kinase 7 (AK7)	44.38477	5.77970	41.50077	5.30490	Detected	Compromised	Detected	Compromised
4433	A_23_P10305	CTPS2	Hs.227049	CTP synthase II	Homo sapiens CTP synthase II (CTPS2	54.16229	230.71088	44.44841	170.04990	Detected	Detected	Detected	Detected
4434	A_23_P213883	NIPBL	Hs.481927	Nipped-B homolog (Drosophila)	Homo sapiens Nipped-B homolog (Dros	905.82806	608.17930	782.72770	421.56635	Detected	Detected	Detected	Detected
4435	A_33_P3788618	ACER2	Hs.41379	alkaline ceramidase 2	Homo sapiens alkaline ceramidase 2 (A	67.23721	100.92090	53.08019	46.52017	Detected	Detected	Detected	Detected
4436	A_23_P5912	YTHDF1	Hs.11747	YTH domain family, member 1	Homo sapiens YTH domain family, mem	12533.58100	13767.00500	13595.41000	12860.64800	Detected	Detected	Detected	Detected
4437	A_23_P7582	TCF7	Hs.573153	transcription factor 7 (T-cell specific, H	Homo sapiens transcription factor 7 (T-	784.26800	1424.91370	987.40643	1583.39670	Detected	Detected	Detected	Detected
4438	A_33_P3364741	MRC2	Hs.7835	mannose receptor, C type 2	Homo sapiens mannose receptor, C typ	81524.23400	96052.31000	75307.31000	73682.53000	Detected	Detected	Detected	Detected
4439	A_33_P3613358	FLJ39824		hypothetical LOC441173	Homo sapiens cDNA FLJ39824 fis, clon	1228.11250	2077.57840	485.73560	575.33777	Detected	Detected	Detected	Detected
4440	A_24_P323941	C20orf106	Hs.504907	chromosome 20 open reading frame 106	Homo sapiens chromosome 20 open rea	628.41350	576.70660	454.91418	351.14896	Detected	Detected	Detected	Detected
4441	A_23_P38537	KRT16	Hs.655160	keratin 16	Homo sapiens keratin 16 (KRT16), mRN	8.27474	33.26911	9.40091	7.37636	Compromised	Detected	Compromised	Compromised
4442	A_24_P185314	NAPG	Hs.464622	N-ethylmaleimide-sensitive factor attac	Homo sapiens N-ethylmaleimide-sensit	4196.61470	4818.15600	3187.61740	2813.23540	Detected	Detected	Detected	Detected
4443	A_33_P3556532	DNAH17	Hs.713207	dynein, axonemal, heavy chain 17	full-length cDNA clone CS0DJ002YI14	2.94526	21.85653	2.96272	10.43644	Compromised	Detected	Compromised	Compromised
4444	A_32_P1533	LOC202181		hypothetical LOC202181	Homo sapiens hypothetical protein LOC	62.41500	54.10782	40.55858	21.61473	Detected	Detected	Detected	Detected
4445	A_33_P3398448	PARP10	Hs.348609	poly (ADP-ribose) polymerase family, m	Homo sapiens poly (ADP-ribose) polym	3033.73490	1849.76650	2369.03220	1307.30910	Detected	Detected	Detected	Detected
4446	A_32_P69930	CCDC96	Hs.656757	coiled-coil domain containing 96	Homo sapiens coiled-coil domain contai	2166.84000	2912.83100	1978.84160	2385.74220	Detected	Detected	Detected	Detected
4447	A_32_P11450	BMS1	Hs.10848	BMS1 homolog, ribosome assembly prot	Homo sapiens BMS1 homolog, ribosome	1022.28210	918.93840	765.00520	578.99316	Detected	Detected	Detected	Detected
4448	A_33_P3422085	SPANXN2	Hs.535083	SPANX family, member N2	Homo sapiens SPANX family, member N	16.05144	24.81165	17.79046	12.27117	Detected	Detected	Detected	Compromised
4449	A_33_P3499692	LOC645261		PP565	Homo sapiens PP565 mRNA, complete	3342.08890	4256.25400	1324.74600	951.29080	Detected	Detected	Detected	Detected
4450	A_23_P424316	TCF20	Hs.475018	transcription factor 20 (AR1)	Homo sapiens transcription factor 20 (A	1201.44150	687.88310	1037.61010	644.21436	Detected	Detected	Detected	Detected
4451	A_32_P71788	FKBP4	Hs.524183	FK506 binding protein 4, 59kDa	Homo sapiens FK506 binding protein 4,	241.10233	199.68669	191.62993	125.86545	Detected	Detected	Detected	Detected
4452	A_23_P125383	TMEFF2	Hs.144513	transmembrane protein with EGF-like a	Homo sapiens transmembrane protein w	264.64325	197.55017	184.06143	105.23708	Detected	Detected	Detected	Detected
4453	A_24_P413437	NONO	Hs.533282	non-POU domain containing, octamer-b	Homo sapiens non-POU domain contair	3584.90500	2897.99660	4188.03500	2726.94650	Detected	Detected	Detected	Detected
4454	A_23_P256504	AMBP	Hs.436911	alpha-1-microglobulin/bikunin precurso	Homo sapiens alpha-1-microglobulin/bi	2.89774	7.50736	7.41574	17.73235	Compromised	Compromised	Compromised	Detected
4455	A_33_P3413114	ADAMTSL1	Hs.522019	ADAMTS-like 1	Homo sapiens ADAMTS-like 1 (ADAMT	168.77023	402.40380	134.27980	304.90690	Detected	Detected	Detected	Detected
4456	A_23_P35591	EXOSC1	Hs.632089	exosome component 1	Homo sapiens exosome component 1 (E	3825.69580	7000.82700	4041.60570	6297.39600	Detected	Detected	Detected	Detected
4457	A_33_P3359748		Hs.531176		Seryl-tRNA synthetase, cytoplasmic (E	342.14764	439.59384	180.36574	275.85970	Detected	Detected	Detected	Detected
4458	A_33_P3288659	ACTL8	Hs.2149	actin-like 8	Homo sapiens actin-like 8 (ACTL8), mR	79.80515	101.35298	37.87872	33.49907	Detected	Detected	Detected	Detected
4459	A_23_P83498	IGF2BP1	Hs.144936	insulin-like growth factor 2 mRNA bindi	Homo sapiens insulin-like growth factor	590.03534	854.25640	450.22778	505.83730	Detected	Detected	Detected	Detected
4460	A_23_P57521	EIF3L	Hs.446852	eukaryotic translation initiation factor 3	Homo sapiens eukaryotic translation ini	10645.51700	7880.98630	11570.13800	7523.62160	Detected	Detected	Detected	Detected
4461	A_33_P3421490	KIAA1024	Hs.301654	KIAA1024	Homo sapiens KIAA1024 (KIAA1024), m	561.25073	350.30713	328.40560	199.19237	Detected	Detected	Detected	Detected
4462	A_33_P3376551	PTGFR	Hs.654365	prostaglandin F receptor (FP)	Homo sapiens prostaglandin F receptor	5007.08060	955.31060	4037.26120	603.33060	Detected	Detected	Detected	Detected
4463	A_23_P36513	PRKAG1	Hs.530862	protein kinase, AMP-activated, gamma	Homo sapiens protein kinase, AMP-acti	1995.70230	1069.84400	2210.92530	1228.59400	Detected	Detected	Detected	Detected
4464	A_33_P3240767	CIDEC	Hs.567562	cell death-inducing DFFA-like effector	Homo sapiens cell death-inducing DFFA	917.97370	1779.78740	547.95290	928.74670	Detected	Detected	Detected	Detected
4465	A_33_P3363720	C1orf213	Hs.61884	chromosome 1 open reading frame 213	Homo sapiens chromosome 1 open read	193.36092	66.57955	248.85594	90.39758	Detected	Detected	Detected	Detected
4466	A_23_P209246	GLI2	Hs.111867	GLI family zinc finger 2	Homo sapiens GLI family zinc finger 2 (	327.69476	549.52936	328.82028	483.19540	Detected	Detected	Detected	Detected
4467	A_33_P3253960				Dehydrogenase/reductase SDR family r	228.60367	227.46364	183.26282	175.88899	Detected	Detected	Detected	Detected
4468	A_33_P3346688					51.22588	64.69074	41.54768	41.82814	Detected	Detected	Detected	Detected
4469	A_33_P3398697	PICK1	Hs.180871	protein interacting with PRKCA 1	Homo sapiens protein interacting with P	1503.77170	2836.98220	1226.94900	2185.60180	Detected	Detected	Detected	Detected
4470	A_33_P3361746	TRPS1	Hs.657018	trichorhinophalangeal syndrome I	Homo sapiens trichorhinophalangeal syr	577.37760	297.25360	557.56650	213.42545	Detected	Detected	Detected	Detected
4471	A_33_P3405429	VWA5B1	Hs.205178	von Willebrand factor A domain containi	Homo sapiens cDNA FLJ43845 fis, clon	25.43535	20.18617	7.24739	9.85145	Detected	Detected	Compromised	Compromised
4472	A_23_P110473	NAIP	Hs.654500	NLR family, apoptosis inhibitory protein	Homo sapiens NLR family, apoptosis inh	370.56180	227.53679	193.30666	92.32456	Detected	Detected	Detected	Detected
4473	A_24_P167614	INTS6	Hs.439440	integrator complex subunit 6	Homo sapiens integrator complex subur	766.97687	590.60974	579.60740	365.34290	Detected	Detected	Detected	Detected
4474	A_23_P106973	SEPT9	Hs.440932	septin 9	Homo sapiens septin 9 (SEPT9), transc	109112.47000	161606.53000	113287.19000	153434.20000	Detected	Detected	Detected	Detected
4475	A_23_P402787	FAM100A	Hs.513313	family with sequence similarity 100, mer	Homo sapiens family with sequence sim	1244.01110	1022.46594	1287.81140	925.26117	Detected	Detected	Detected	Detected
4476	A_24_P56689	ZNF205	Hs.592088	zinc finger protein 205	Homo sapiens zinc finger protein 205 (Z	3434.14940	3608.23270	996.68500	774.96900	Detected	Detected	Detected	Detected
4477	A_23_P398836	TXNDC16	Hs.532609	thioredoxin domain containing 16	Homo sapiens thioredoxin domain conta	161.43700	164.72322	108.41789	95.62779	Detected	Detected	Detected	Detected
4478	A_33_P3402454	HAS3	Hs.592069	hyaluronan synthase 3	Homo sapiens hyaluronan synthase 3 (H	81.60000	57.46177	64.51149	48.60440	Detected	Detected	Detected	Detected
4479	A_23_P351342	PLEKHM2	Hs.646775	pleckstrin homology domain containing,	Homo sapiens pleckstrin homology dom	2168.08230	1651.28670	2224.72530	1811.12330	Detected	Detected	Detected	Detected
4480	A_23_P61524	CCDC71	Hs.211472	coiled-coil domain containing 71	Homo sapiens coiled-coil domain conta	202.04503	210.99368	252.19210	254.62360	Detected	Detected	Detected	Detected
4481	A_33_P3400699	SLC26A5	Hs.585146	solute carrier family 26, member 5 (pres	Homo sapiens solute carrier family 26, r	26.87829	18.86993	27.91637	9.37458	Detected	Compromised	Detected	Compromised
4482	A_33_P3256168	SNX30	Hs.522350	sorting nexin family member 30	Homo sapiens sorting nexin family mem	33.79947	30.05612	36.89750	37.31852	Detected	Detected	Detected	Detected
4483	A_23_P350512	ADAM12	Hs.594537	ADAM metallopeptidase domain 12	Homo sapiens ADAM metallopeptidase	295.06018	397.58392	212.32173	244.96371	Detected	Detected	Detected	Detected
4484	A_33_P3236921	PARP1	Hs.177766	poly (ADP-ribose) polymerase 1	Homo sapiens poly (ADP-ribose) polym	1283.46280	1854.55980	880.63544	916.50880	Detected	Detected	Detected	Detected
4485	A_23_P259071	AREG	Hs.270833	amphiregulin	Homo sapiens amphiregulin (AREG), mR	51166.50000	2510.09900	45443.98400	1748.41210	Detected	Detected	Detected	Detected
4486	A_33_P3362770	RRN3	Hs.460078	RRN3 RNA polymerase I transcription f	Homo sapiens RRN3 RNA polymerase I	124.72530	106.66970	115.78911	80.03819	Detected	Detected	Detected	Detected
4487	A_33_P3305625	LOC100131015	Hs.591633	hypothetical LOC100131015	Homo sapiens cDNA FLJ42518 fis, clon	40.58105	42.45229	15.54411	12.36063	Detected	Detected	Detected	Compromised
4488	A_24_P356453	CCDC56	Hs.16059	coiled-coil domain containing 56	Homo sapiens coiled-coil domain conta	7684.95300	9312.32100	8081.30400	8342.87600	Detected	Detected	Detected	Detected
4489	A_24_P169843					58.25988	77.65492	48.03464	41.55749	Detected	Detected	Detected	Detected
4490	A_23_P156667	PPP1R10	Hs.106019	protein phosphatase 1, regulatory (inhib	Homo sapiens protein phosphatase 1, re	1925.88200	2937.26150	1681.01230	2224.25170	Detected	Detected	Detected	Detected
4491	A_24_P77676	HSPA9	Hs.184233	heat shock 70kDa protein 9 (mortalin)	Homo sapiens heat shock 70kDa protei	8030.23100	12366.66300	5174.53500	6148.33250	Detected	Detected	Detected	Detected
4492	A_32_P150086	LOC284232	Hs.633153	ankyrin repeat domain 20 family, membe	Homo sapiens ankyrin repeat domain 20	6.08420	20.91937	7.41386	7.58294	Compromised	Detected	Compromised	Compromised
4493	A_23_P369746				HSPC254 Fragment [Source:UniProtKE	359.53333	277.86996	356.36432	229.73355	Detected	Detected	Detected	Detected
4494	A_23_P132175	RTN4R	Hs.30868	reticulon 4 receptor	Homo sapiens reticulon 4 receptor (RTI	43.72976	79.50623	20.94223	40.65098	Detected	Detected	Detected	Detected
4495	A_23_P123974	DTYMK	Hs.471873	deoxythymidylate kinase (thymidylate ki	Homo sapiens deoxythymidylate kinase	10900.91700	46200.00800	7156.72700	25024.23200	Detected	Detected	Detected	Detected
4496	A_24_P134789	KRTAP10-10	Hs.474001	keratin associated protein 10-10	Homo sapiens keratin associated protei	526.06620	582.60170	184.23685	162.79375	Detected	Detected	Detected	Detected

4497	A_23_P83939	SYAP1	Hs.605178	synapse associated protein 1, SAP47 h	Homo sapiens synapse associated prote	9587.89500	16061.91600	6249.47800	8277.97600	Detected	Detected	Detected	Detected
4498	A_33_P3216869	CRABP1	Hs.346950	cellular retinoic acid binding protein 1	Homo sapiens cellular retinoic acid bind	126.71162	145.04492	101.62167	73.44978	Detected	Detected	Detected	Detected
4499	A_33_P3242878	EFCAB6	Hs.658996	EF-hand calcium binding domain 6	Homo sapiens EF-hand calcium binding	29.95172	8.09910	26.79373	3.08033	Detected	Compromised	Detected	Compromised
4500	A_23_P101811	ZNF14	Hs.659932	zinc finger protein 14	Homo sapiens zinc finger protein 14 (ZN	163.44724	157.43646	110.12983	86.14726	Detected	Detected	Detected	Detected
4501	A_23_P163148	C14orf133	Hs.16157	chromosome 14 open reading frame 133	Homo sapiens chromosome 14 open rea	1620.65270	1294.15640	1302.34350	905.33360	Detected	Detected	Detected	Detected
4502	A_32_P319858	NKPD1	Hs.299256	NTPase, KAP family P-loop domain con	Homo sapiens NTPase, KAP family P-lo	22.74639	10.03057	11.89693	8.90446	Detected	Compromised	Compromised	Compromised
4503	A_24_P196534	PKNOX1	Hs.431043	PBX/knotted 1 homeobox 1	Homo sapiens PBX/knotted 1 homeobo	1917.38000	1033.50900	1360.55700	525.32640	Detected	Detected	Detected	Detected
4504	A_23_P26336	ZC3H7A	Hs.371856	zinc finger CCCH-type containing 7A	Homo sapiens zinc finger CCCH-type c	4398.45850	7321.99100	3273.46390	4435.10840	Detected	Detected	Detected	Detected
4505	A_23_P53614	BRAP	Hs.530940	BRCA1 associated protein	Homo sapiens BRCA1 associated prote	345.11264	585.18270	296.19324	410.76730	Detected	Detected	Detected	Detected
4506	A_32_P112894	FAM76A	Hs.469359	family with sequence similarity 76, mem	Homo sapiens family with sequence sim	26.06089	30.24686	17.27253	13.63008	Detected	Detected	Detected	Compromised
4507	A_23_P164253	PPY2	Hs.20588	pancreatic polypeptide 2	Homo sapiens pancreatic polypeptide 2	13.34145	14.06733	12.89715	12.76937	Detected	Compromised	Compromised	Compromised
4508	A_23_P130653	RTBDN	Hs.21162	retbindin	Homo sapiens retbindin (RTBDN), trans	1121.39270	1623.84400	310.63663	283.68716	Detected	Detected	Detected	Detected
4509	A_33_P3296831	TP73	Hs.697294	tumor protein p73	Homo sapiens tumor protein p73 (TP73	26.14122	24.84367	6.96058	6.27512	Detected	Detected	Compromised	Compromised
4510	A_24_P290286	P4HA3	Hs.660541	prolyl 4-hydroxylase, alpha polypeptide	Homo sapiens prolyl 4-hydroxylase, alph	387.73460	1022.77420	428.14023	1011.86660	Detected	Detected	Detected	Detected
4511	A_32_P149546	CROCC	Hs.309403	ciliary rootlet coiled-coil, rootletin	Homo sapiens ciliary rootlet coiled-coil,	50.55953	14.55234	43.12153	12.66369	Detected	Compromised	Detected	Compromised
4512	A_23_P61398	PIM3	Hs.530381	pim-3 oncogene	Homo sapiens pim-3 oncogene (PIM3),	25620.33000	15180.08900	21473.50800	11775.08500	Detected	Detected	Detected	Detected
4513	A_33_P3270800	CPSF4L	Hs.534707	cleavage and polyadenylation specific fa	Homo sapiens cleavage and polyadenyla	30.39167	26.03968	24.25326	21.11570	Detected	Detected	Detected	Detected
4514	A_23_P63681	IDE	Hs.500546	insulin-degrading enzyme	Homo sapiens insulin-degrading enzyme	506.26483	633.81060	448.78280	481.08743	Detected	Detected	Detected	Detected
4515	A_32_P393316	RAPGEF3	Hs.8578	Rap guanine nucleotide exchange factor	Homo sapiens Rap guanine nucleotide e	687.12010	906.56490	184.69014	158.98244	Detected	Detected	Detected	Detected
4516	A_33_P3319006	C8G	Hs.1285	complement component 8, gamma polyp	Homo sapiens complement component	47.04166	63.36031	38.60202	39.38100	Detected	Detected	Detected	Detected
4517	A_33_P3305617		Hs.369104		C3orf57 proteincDNA FLJ41354 fis, clo	20.83025	19.47741	11.67638	6.93752	Detected	Detected	Compromised	Compromised
4518	A_33_P3243093	RGS5	Hs.24950	regulator of G-protein signaling 5	Homo sapiens regulator of G-protein si	164.69100	48.89890	119.81332	27.49131	Detected	Detected	Detected	Detected
4519	A_33_P3294911	C12orf66		chromosome 12 open reading frame 66	UPF0536 protein C12orf66 [Source:Un	35.35727	12.44580	34.55707	12.63295	Detected	Compromised	Detected	Compromised
4520	A_33_P3322460	LOC100128256		hypothetical LOC100128256	PREDICTED: Homo sapiens hypothetica	359.50574	382.93317	66.70797	59.17765	Detected	Detected	Detected	Detected
4521	A_23_P163492	BAIAP3	Hs.458427	BAI1-associated protein 3	Homo sapiens BAI1-associated protein	41.67684	3.89145	33.76196	3.35231	Detected	Compromised	Detected	Compromised
4522	A_32_P40375					184.96738	181.51228	184.45168	148.18314	Detected	Detected	Detected	Detected
4523	A_23_P114084	PHEX	Hs.495834	phosphate regulating endopeptidase hor	Homo sapiens phosphate regulating end	61.35286	61.51650	40.60182	38.53147	Detected	Detected	Detected	Detected
4524	A_23_P315206	CCBL1	Hs.495250	cysteine conjugate-beta lyase, cytoplas	Homo sapiens cysteine conjugate-beta	414.71167	316.10460	278.36728	231.25490	Detected	Detected	Detected	Detected
4525	A_24_P162287	CEP250	Hs.443976	centrosomal protein 250kDa	Homo sapiens centrosomal protein 250k	36.43417	88.11762	29.00269	56.36321	Detected	Detected	Detected	Detected
4526	A_23_P360874	LRWD1	Hs.274135	leucine-rich repeats and WD repeat dor	Homo sapiens leucine-rich repeats and	6001.27000	8832.05800	7046.31300	9269.61200	Detected	Detected	Detected	Detected
4527	A_33_P3209030	ZNF623	Hs.694018	zinc finger protein 623	Homo sapiens zinc finger protein 623 (Z	212.39221	184.63040	140.65135	111.38420	Detected	Detected	Detected	Detected
4528	A_24_P121956	TMEM86B	Hs.135215	transmembrane protein 86B	Homo sapiens transmembrane protein 8	336.35297	318.52606	252.58232	199.79457	Detected	Detected	Detected	Detected
4529	A_33_P3235611	SLC46A1	Hs.446689	solute carrier family 46 (folate transpor	Homo sapiens solute carrier family 46 (	337.85980	277.92783	266.22202	186.97238	Detected	Detected	Detected	Detected
4530	A_33_P3270451	TXNDC5	Hs.150837	thioredoxin domain containing 5 (endopl	Homo sapiens thioredoxin domain conta	1888.26670	3893.40040	1849.99650	3368.90580	Detected	Detected	Detected	Detected
4531	A_32_P177040	SPDYE5	Hs.632298	speedy homolog E5 (Xenopus laevis)	Homo sapiens speedy homolog E5 (Xen	41.18334	52.17546	23.65478	23.45130	Detected	Detected	Detected	Detected
4532	A_23_P17134	MAL	Hs.80395	mal, T-cell differentiation protein	Homo sapiens mal, T-cell differentiation	30.36956	35.90467	17.36510	12.44694	Detected	Detected	Detected	Compromised
4533	A_23_P42241	OR5V1	Hs.666316	olfactory receptor, family 5, subfamily V	Homo sapiens olfactory receptor, family	281.84528	555.73444	87.10194	119.93051	Detected	Detected	Detected	Detected
4534	A_24_P85619				Putative uncharacterized protein ENSP	682.99740	397.58392	542.30660	282.35492	Detected	Detected	Detected	Detected
4535	A_23_P502464	NOS2	Hs.709191	nitric oxide synthase 2, inducible	Homo sapiens nitric oxide synthase 2, i	41.90181	7.56190	27.47658	4.34394	Detected	Compromised	Detected	Compromised
4536	A_23_P104323	MGMT	Hs.501522	O-6-methylguanine-DNA methyltransfe	Homo sapiens O-6-methylguanine-DNA	3721.63060	2115.60700	4502.93260	2330.07250	Detected	Detected	Detected	Detected
4537	A_24_P832426	B3GALTL	Hs.13205	beta 1,3-galactosyltransferase-like	Homo sapiens beta 1,3-galactosyltransf	1158.94510	1950.60190	748.35540	1081.99730	Detected	Detected	Detected	Detected
4538	A_23_P43317	PRDM12	Hs.495311	PR domain containing 12	Homo sapiens PR domain containing 12	77.02335	24.48676	66.33418	22.18207	Detected	Detected	Detected	Detected
4539	A_33_P3397348	LOC149134		hypothetical LOC149134	Homo sapiens hypothetical LOC149134	140.50378	196.27084	93.79817	97.46291	Detected	Detected	Detected	Detected
4540	A_23_P65068	EID3	Hs.659857	EP300 interacting inhibitor of differentia	Homo sapiens EP300 interacting inhibito	477.25540	238.81890	523.64280	217.94588	Detected	Detected	Detected	Detected
4541	A_33_P3413355	CHST1	Hs.104576	carbohydrate (keratan sulfate Gal-6) su	Homo sapiens carbohydrate (keratan su	703.24930	64.68276	387.51987	27.96841	Detected	Detected	Detected	Detected
4542	A_32_P722809		Hs.449621		Putative uncharacterized protein ENSP	7.79047	19.08362	10.07553	3.12289	Compromised	Detected	Compromised	Compromised
4543	A_33_P3375451	NYNRIN	Hs.288348	NYN domain and retroviral integrase co	Homo sapiens NYN domain and retrovir	27.04986	65.63056	22.58610	26.19789	Detected	Detected	Detected	Detected
4544	A_33_P3423949	CBX2	Hs.368410	chromobox homolog 2 (Pc class homolo	Homo sapiens chromobox homolog 2 (P	2502.97920	382.37213	1845.42590	279.15244	Detected	Detected	Detected	Detected
4545	A_33_P3411805					242.13771	501.22433	123.65410	156.41588	Detected	Detected	Detected	Detected
4546	A_33_P3305105	VWA5A	Hs.152944	von Willebrand factor A domain containi	Homo sapiens von Willebrand factor A d	207.28683	134.44350	210.79391	42.47305	Detected	Detected	Detected	Detected
4547	A_23_P129085	SPESP1	Hs.657932	sperm equatorial segment protein 1	Homo sapiens sperm equatorial segmen	453.54560	186.85470	337.27563	113.08470	Detected	Detected	Detected	Detected
4548	A_23_P102117	WNT10A	Hs.121540	wingless-type MMTV integration site fa	Homo sapiens wingless-type MMTV inte	14.83481	23.59448	19.29561	20.52696	Detected	Detected	Detected	Not Detected
4549	A_23_P59452	ABP1	Hs.647097	amiloride binding protein 1 (amine oxida	Homo sapiens amiloride binding protein	36.19832	64.70358	10.87545	5.18399	Detected	Detected	Compromised	Compromised
4550	A_23_P156809	FAM119A	Hs.664764	family with sequence similarity 119, mer	Homo sapiens family with sequence sim	1706.47890	1973.72180	1040.20810	967.45245	Detected	Detected	Detected	Detected
4551	A_24_P337657	SRF	Hs.520140	serum response factor (c-fos serum res	Homo sapiens serum response factor (c	629.78080	448.82108	346.52032	221.79056	Detected	Detected	Detected	Detected
4552	A_23_P203255	API5	Hs.435771	apoptosis inhibitor 5	Homo sapiens apoptosis inhibitor 5 (AP	198.85117	550.17194	205.20730	450.15073	Detected	Detected	Detected	Detected
4553	A_33_P3222630	FBXO43	Hs.339577	F-box protein 43	Homo sapiens F-box protein 43 (FBXO4	24.23979	150.14178	24.66990	100.70612	Detected	Detected	Detected	Detected
4554	A_24_P823684	HSP90AB1	Hs.509736	heat shock protein 90kDa alpha (cytoso	Homo sapiens heat shock protein 90kDa	4251.95400	7093.95100	2821.44650	3881.43850	Detected	Detected	Detected	Detected
4555	A_33_P3305472		Hs.288215		small nucleolar RNA, C/D box 1C (SNO	39.18271	124.96590	18.12705	68.69997	Detected	Detected	Detected	Detected
4556	A_32_P749354	EIF1AD	Hs.425178	eukaryotic translation initiation factor 1	Homo sapiens eukaryotic translation ini	1217.43260	1393.32020	953.00525	833.73960	Detected	Detected	Detected	Detected
4557	A_23_P207967	KIAA0427	Hs.145230	KIAA0427	Homo sapiens KIAA0427 (KIAA0427), tr	1708.68910	1424.69040	1837.75290	1354.05040	Detected	Detected	Detected	Detected
4558	A_33_P3358957	PAPL	Hs.448934	iron/zinc purple acid phosphatase-like	Homo sapiens iron/zinc purple acid pho	4344.26700	6337.13800	303.95563	248.38164	Detected	Detected	Detected	Detected
4559	A_33_P3357773					32.74730	45.27027	27.12211	16.61863	Detected	Detected	Detected	Detected
4560	A_24_P397150	GAK	Hs.369607	cyclin G associated kinase	Homo sapiens cyclin G associated kinas	4263.65430	6096.01760	5575.23730	6286.39060	Detected	Detected	Detected	Detected
4561	A_33_P3237699	RMND5A	Hs.75277	required for meiotic nuclear division 5 h	Homo sapiens required for meiotic nucl	283.08792	409.97058	241.28520	304.61920	Detected	Detected	Detected	Detected
4562	A_24_P60680	OR10C1	Hs.631997	olfactory receptor, family 10, subfamily	Homo sapiens olfactory receptor, family	57.45465	216.49855	42.17722	79.56069	Detected	Detected	Detected	Detected
4563	A_33_P3280461	THRAP3	Hs.160211	thyroid hormone receptor associated pr	Homo sapiens thyroid hormone recepto	423.29123	610.32470	360.57650	422.68030	Detected	Detected	Detected	Detected
4564	A_24_P295379	GPR107	Hs.512461	G protein-coupled receptor 107	Homo sapiens G protein-coupled recep	6447.26400	5561.00050	4708.57130	3382.92240	Detected	Detected	Detected	Detected
4565	A_33_P3238690	TET2	Hs.367639	tet oncogene family member 2	Homo sapiens tet oncogene family mem	51.40903	47.97693	49.24601	34.64314	Detected	Detected	Detected	Detected
4566	A_23_P122805	TMEM209	Hs.267245	transmembrane protein 209	Homo sapiens transmembrane protein 2	1871.50070	1408.43850	1200.52150	814.45330	Detected	Detected	Detected	Detected
4567	A_33_P3363420	FRMD3	Hs.709357	FERM domain containing 3	Homo sapiens FERM domain containing	3200.97300	5672.41200	2551.47000	3788.90600	Detected	Detected	Detected	Detected
4568	A_23_P121506	FAM193A	Hs.652364	family with sequence similarity 193, mer	Homo sapiens family with sequence sim	536.26350	491.90210	439.51047	377.03824	Detected	Detected	Detected	Detected
4569	A_23_P116207	C11orf60	Hs.533738	chromosome 11 open reading frame 60	Homo sapiens chromosome 11 open rea	1507.50010	1335.90890	1579.11840	1226.76560	Detected	Detected	Detected	Detected
4570	A_33_P3227212	LOC391073	Hs.647684	similar to glyceraldehyde-3-phosphate	PREDICTED: Homo sapiens similar to g	28.80946	44.30026	24.41609	22.84948	Detected	Detected	Detected	Detected
4571	A_33_P3276455	KDM5B	Hs.443650	lysine (K)-specific demethylase 5B	Homo sapiens lysine (K)-specific demet	522.43304	605.57654	239.36820	217.51495	Detected	Detected	Detected	Detected

4572	A_23_P384698	LOC100293611	Hs.666816	hypothetical protein LOC100293611	PREDICTED: Homo sapiens hypothetica	101.52199	236.37146	69.98246	142.15442	Detected	Detected	Detected	Detected
4573	A_33_P3390708	TMEM2	Hs.494146	transmembrane protein 2	Homo sapiens transmembrane protein 2	45.28901	61.29089	35.53810	40.14062	Detected	Detected	Detected	Detected
4574	A_23_P52639	COX8A	Hs.433901	cytochrome c oxidase subunit 8A (ubiqu	Homo sapiens cytochrome c oxidase su	25614.62300	47355.68400	26046.81600	41242.28500	Detected	Detected	Detected	Detected
4575	A_33_P3250068	SNX17	Hs.278569	sorting nexin 17	Homo sapiens sorting nexin 17 (SNX17)	12733.66600	16554.79100	10505.34000	11734.40000	Detected	Detected	Detected	Detected
4576	A_23_P48977	MRPS11	Hs.111286	mitochondrial ribosomal protein S11	Homo sapiens mitochondrial ribosomal p	2840.63200	6643.24270	3574.40360	7227.88040	Detected	Detected	Detected	Detected
4577	A_24_P376294	HNRNPUL1	Hs.155218	heterogeneous nuclear ribonucleoprotei	Homo sapiens heterogeneous nuclear ri	558.53784	717.20483	522.12060	549.76117	Detected	Detected	Detected	Detected
4578	A_33_P3218960	CACNA1H	Hs.459642	calcium channel, voltage-dependent, T	Homo sapiens calcium channel, voltage-	81.47275	32.67845	47.59345	14.64669	Detected	Detected	Detected	Compromised
4579	A_23_P120863	GAL3ST1	Hs.17958	galactose-3-O-sulfotransferase 1	Homo sapiens galactose-3-O-sulfotran	28.98957	6.37298	22.76339	6.69659	Detected	Compromised	Detected	Compromised
4580	A_24_P366777		Hs.709526		Notch homolog 2 N-terminal-like protei	714.23175	412.19614	313.25195	180.96335	Detected	Detected	Detected	Detected
4581	A_33_P3288594	LOC100129055	Hs.148265	cyclin Y-like pseudogene	Homo sapiens cyclin Y-like pseudogene	82.89698	4.85968	35.88683	3.40206	Detected	Compromised	Detected	Compromised
4582	A_33_P3215288	LOC284757	Hs.534781	hypothetical protein LOC284757	Homo sapiens cDNA FLJ46426 fis, clon	136.87941	160.42653	61.92843	60.83615	Detected	Detected	Detected	Detected
4583	A_23_P115842	CCAR1	Hs.49853	cell division cycle and apoptosis regulat	Homo sapiens cell division cycle and ap	3517.78080	3732.60910	2123.52170	1789.64310	Detected	Detected	Detected	Detected
4584	A_33_P3344276	HS1BP3	Hs.531785	HCLS1 binding protein 3	Homo sapiens HCLS1 binding protein 3	71.70268	194.48598	60.07006	192.18785	Detected	Detected	Detected	Detected
4585	A_24_P252846	C11orf74	Hs.159376	chromosome 11 open reading frame 74	Homo sapiens chromosome 11 open rea	1465.06450	1366.06600	971.25420	690.46760	Detected	Detected	Detected	Detected
4586	A_24_P254346	DSCR3	Hs.369488	Down syndrome critical region gene 3	Homo sapiens Down syndrome critical r	4910.87550	4114.01500	4018.91000	3135.38040	Detected	Detected	Detected	Detected
4587	A_23_P74828	GJA9	Hs.632402	gap junction protein, alpha 9, 59kDa	Homo sapiens gap junction protein, alph	16.10347	12.43168	2.97665	3.11790	Detected	Compromised	Compromised	Compromised
4588	A_23_P358470	CCDC111	Hs.481307	coiled-coil domain containing 111	Homo sapiens coiled-coil domain conta	380.15830	283.18150	317.95554	171.14870	Detected	Detected	Detected	Detected
4589	A_24_P35478	PARD3	Hs.131489	par-3 partitioning defective 3 homolog (	Homo sapiens par-3 partitioning defecti	729.39966	415.39540	728.43210	329.85330	Detected	Detected	Detected	Detected
4590	A_33_P3272080	QRFP	Hs.660704	pyroglutamylated RFamide peptide	oe35d02.y1 Human keratoconus cornea	269.47882	279.86250	254.40463	191.80995	Detected	Detected	Detected	Detected
4591	A_33_P3217819	CCNE2	Hs.567387	cyclin E2	Homo sapiens cyclin E2 (CCNE2), mRN	7.36852	49.24119	3.61515	28.81312	Compromised	Detected	Compromised	Detected
4592	A_23_P204269	USP15	Hs.434951	ubiquitin specific peptidase 15	Homo sapiens ubiquitin specific peptida	930.65186	371.98550	711.45020	207.81890	Detected	Detected	Detected	Detected
4593	A_33_P3337405	LOC440993	Hs.709411	hypothetical LOC440993	PREDICTED: Homo sapiens hypothetical	61.94769	37.05709	39.55944	19.53496	Detected	Detected	Detected	Detected
4594	A_23_P118406	C17orf62	Hs.163113	chromosome 17 open reading frame 62	Homo sapiens chromosome 17 open rea	1521.51600	795.79254	1609.12270	754.80960	Detected	Detected	Detected	Detected
4595	A_23_P155907	METAP1	Hs.480364	methionyl aminopeptidase 1	Homo sapiens methionyl aminopeptidase	5828.70170	6636.99660	4738.21140	4449.87700	Detected	Detected	Detected	Detected
4596	A_33_P3304282	RPS15A	Hs.370504	ribosomal protein S15a	Homo sapiens ribosomal protein S15a (	89804.03000	78164.54000	45987.24000	33403.57000	Detected	Detected	Detected	Detected
4597	A_23_P215454	ELN	Hs.647061	elastin	Homo sapiens elastin (ELN), transcript	51.50940	2019.68640	45.34365	2097.67200	Detected	Detected	Detected	Detected
4598	A_32_P223059	SLC45A1	Hs.463036	solute carrier family 45, member 1	Homo sapiens solute carrier family 45, r	104.31739	23.56400	96.16173	15.26625	Detected	Detected	Detected	Compromised
4599	A_23_P83278	CHMP5	Hs.635313	chromatin modifying protein 5	Homo sapiens chromatin modifying prot	12882.14100	10393.76400	10629.77900	6927.77730	Detected	Detected	Detected	Detected
4600	A_23_P359588	PCDHGB4	Hs.368160	protocadherin gamma subfamily B, 4	Homo sapiens protocadherin gamma sul	76.29316	168.89574	70.24512	144.16612	Detected	Detected	Detected	Detected
4601	A_33_P3296772					28.53963	87.97130	32.48265	62.06285	Detected	Detected	Detected	Detected
4602	A_33_P3233489					4.16698	86.30691	6.32585	54.17547	Compromised	Detected	Compromised	Detected
4603	A_23_P11774	UTP11L	Hs.472038	UTP11-like, U3 small nucleolar ribonucl	Homo sapiens UTP11-like, U3 small nuc	6170.73500	7107.71830	3949.13750	3765.96340	Detected	Detected	Detected	Detected
4604	A_33_P3265606	GCLM	Hs.315562	glutamate-cysteine ligase, modifier subu	Homo sapiens glutamate-cysteine ligase	17763.60000	16573.21900	4533.71730	3904.24050	Detected	Detected	Detected	Detected
4605	A_33_P3258362	HBA2	Hs.654744	hemoglobin, alpha 2	Homo sapiens hemoglobin, alpha 2 (HBA	28.27968	105.17101	11.54820	15.40257	Detected	Detected	Compromised	Compromised
4606	A_33_P3372266		Hs.719643		V3-4 protein Fragment [Source:UniPro	17.73159	8.93256	6.95933	3.15801	Detected	Compromised	Compromised	Compromised
4607	A_23_P6771	LMCD1	Hs.475353	LIM and cysteine-rich domains 1	Homo sapiens LIM and cysteine-rich do	4452.59570	17044.63000	4454.90100	15431.52800	Detected	Detected	Detected	Detected
4608	A_24_P49997	PFN3	Hs.130196	profilin 3	Homo sapiens profilin 3 (PFN3), mRNA	34.00441	24.84094	28.81979	15.11779	Detected	Detected	Detected	Compromised
4609	A_23_P85015	MAOB	Hs.654473	monoamine oxidase B	Homo sapiens monoamine oxidase B (M	33.09285	12.71895	21.01397	3.68081	Detected	Compromised	Detected	Compromised
4610	A_33_P3284197	POMGNT1	Hs.525134	protein O-linked mannose beta1,2-N-ac	Homo sapiens protein O-linked mannos	7354.26800	10595.83100	7922.54300	9835.57000	Detected	Detected	Detected	Detected
4611	A_33_P3231888	C5orf15	Hs.355177	chromosome 5 open reading frame 15	Homo sapiens chromosome 5 open read	1397.69370	1459.53430	1314.35200	1128.03420	Detected	Detected	Detected	Detected
4612	A_33_P3401422	LIPM	Hs.55118	lipase, family member M	Homo sapiens lipase, family member M	93.81883	91.57192	38.40746	28.39243	Detected	Detected	Detected	Detected
4613	A_33_P3386558	FRMD4A	Hs.330463	FERM domain containing 4A	Homo sapiens FERM domain containing	14.37203	27.09540	14.83714	21.94632	Detected	Detected	Compromised	Detected
4614	A_32_P121085	DOK3	Hs.720849	docking protein 3	Homo sapiens docking protein 3 (DOK3	31.97475	59.59426	27.16032	31.85868	Detected	Detected	Detected	Detected
4615	A_23_P321703	BCL2A1	Hs.227817	BCL2-related protein A1	Homo sapiens BCL2-related protein A1	2655.44290	1675.59840	2077.53700	1131.85550	Detected	Detected	Detected	Detected
4616	A_24_P251841	SETD7	Hs.480792	SET domain containing (lysine methyltra	Homo sapiens SET domain containing (	1716.86930	2650.46750	874.13617	1064.82820	Detected	Detected	Detected	Detected
4617	A_33_P3228722		Hs.464391		Tubulin-specific chaperone D (Tubulin-	354.42593	290.81870	131.69006	63.83908	Detected	Detected	Detected	Detected
4618	A_23_P57036	CD40	Hs.472860	CD40 molecule, TNF receptor superfam	Homo sapiens CD40 molecule, TNF rec	399.17374	157.06107	486.50168	200.41289	Detected	Detected	Detected	Detected
4619	A_23_P122724	VNN2	Hs.293130	vanin 2	Homo sapiens vanin 2 (VNN2), transcrip	68.66112	73.77902	56.53732	42.21378	Detected	Detected	Detected	Detected
4620	A_23_P126291	SNRPE	Hs.334612	small nuclear ribonucleoprotein polypep	Homo sapiens small nuclear ribonucleop	9283.42800	11104.17200	7371.78030	7012.72170	Detected	Detected	Detected	Detected
4621	A_24_P497226	RPS6KB1	Hs.463642	ribosomal protein S6 kinase, 70kDa, pol	Homo sapiens ribosomal protein S6 kina	1754.81410	1214.22250	1145.17480	648.52045	Detected	Detected	Detected	Detected
4622	A_23_P39237	ZFP36	Hs.534052	zinc finger protein 36, C3H type, homolo	Homo sapiens zinc finger protein 36, C3	6868.32600	3952.55350	7251.86230	3699.29600	Detected	Detected	Detected	Detected
4623	A_23_P204850	RB1	Hs.408528	retinoblastoma 1	Homo sapiens retinoblastoma 1 (RB1), r	5617.05760	4242.45300	3652.02600	2234.07450	Detected	Detected	Detected	Detected
4624	A_33_P3227920	SLC16A4	Hs.351306	solute carrier family 16, member 4 (mon	Homo sapiens solute carrier family 16, r	601.84880	747.61127	478.24515	443.87310	Detected	Detected	Detected	Detected
4625	A_23_P372660	GAPVD1	Hs.495134	GTPase activating protein and VPS9 do	Homo sapiens GTPase activating protei	1042.66710	1153.99510	629.04480	567.87604	Detected	Detected	Detected	Detected
4626	A_23_P343808	SOS1	Hs.709893	son of sevenless homolog 1 (Drosophila	Homo sapiens son of sevenless homolo	43.98110	36.89191	42.32370	34.88517	Detected	Detected	Detected	Detected
4627	A_23_P165722	EIF4E2	Hs.292026	eukaryotic translation initiation factor 4	Homo sapiens eukaryotic translation ini	14696.79600	34794.29300	15347.95500	31430.66400	Detected	Detected	Detected	Detected
4628	A_32_P196115	ZNF498	Hs.446297	zinc finger protein 498	Homo sapiens zinc finger protein 498 (Z	191.17874	186.48630	182.45670	191.40483	Detected	Detected	Detected	Detected
4629	A_33_P3305487	REM2	Hs.444911	RAS (RAD and GEM)-like GTP binding	Homo sapiens RAS (RAD and GEM)-like	518.81020	153.13045	456.49930	113.12332	Detected	Detected	Detected	Detected
4630	A_33_P3393091	LOC100130819	Hs.712140	hypothetical LOC100130819	PREDICTED: Homo sapiens hypothetica	259.97192	318.19397	217.08719	244.29207	Detected	Detected	Detected	Detected
4631	A_24_P166443	HLA-DPB1	Hs.485130	major histocompatibility complex, class	Homo sapiens major histocompatibility	1104.11010	1835.47290	1344.35670	1927.49170	Detected	Detected	Detected	Detected
4632	A_33_P3399291	GUK1	Hs.376933	guanylate kinase 1	Homo sapiens guanylate kinase 1 (GUK	5542.12700	7786.59000	8888.80700	10635.82300	Detected	Detected	Detected	Detected
4633	A_24_P918266	TMTC3	Hs.331268	transmembrane and tetratricopeptide re	Homo sapiens transmembrane and tetra	1852.95090	3127.82450	1563.90900	2112.06880	Detected	Detected	Detected	Detected
4634	A_33_P3234939	ZNF266	Hs.656185	zinc finger protein 266	Homo sapiens zinc finger protein 266 (Z	509.40662	280.32110	423.23526	192.53282	Detected	Detected	Detected	Detected
4635	A_23_P365189	CAMTA2	Hs.632242	calmodulin binding transcription activato	Homo sapiens calmodulin binding transc	139.01982	111.36270	81.50205	82.57277	Detected	Detected	Detected	Detected
4636	A_33_P3252322	LOC286434	Hs.657302	hypothetical protein LOC286434	Homo sapiens cDNA FLJ20463 fis, clon	117.63922	60.32607	64.32689	13.82207	Detected	Detected	Detected	Compromised
4637	A_32_P209094	FGGY	Hs.444301	FGGY carbohydrate kinase domain cont	Homo sapiens FGGY carbohydrate kina	648.58900	997.71265	630.08984	826.82416	Detected	Detected	Detected	Detected
4638	A_23_P258340	PPIA	Hs.356331	peptidylprolyl isomerase A (cyclophilin A	Homo sapiens peptidylprolyl isomerase	23565.47900	35695.72300	26648.06400	37455.06000	Detected	Detected	Detected	Detected
4639	A_33_P3222648	STK4	Hs.472838	serine/threonine kinase 4	Homo sapiens serine/threonine kinase	2036.75780	2639.55200	1327.22390	1287.22170	Detected	Detected	Detected	Detected
4640	A_33_P3365037	ERCC1	Hs.435981	excision repair cross-complementing ro	Homo sapiens excision repair cross-cor	4248.75100	3561.51880	4743.32570	3297.91360	Detected	Detected	Detected	Detected
4641	A_23_P310331	RANBP3	Hs.531752	RAN binding protein 3	Homo sapiens RAN binding protein 3 (R	12179.12200	10494.10300	7420.09330	5132.03900	Detected	Detected	Detected	Detected
4642	A_33_P3225685	B3GAT3	Hs.502759	beta-1,3-glucuronyltransferase 3 (glucu	Homo sapiens beta-1,3-glucuronyltrans	2476.46400	1968.04930	2871.85500	2049.69560	Detected	Detected	Detected	Detected
4643	A_24_P377225	USP46	Hs.7966	ubiquitin specific peptidase 46	Homo sapiens ubiquitin specific peptida	333.52628	506.78516	301.23648	359.96994	Detected	Detected	Detected	Detected
4644	A_33_P3342792					1523.09500	2380.57200	864.33997	1002.10680	Detected	Detected	Detected	Detected
4645	A_24_P179351	TPT1	Hs.374596	tumor protein, translationally-controlled	Homo sapiens tumor protein, translation	184830.08000	118478.14000	124236.00000	68620.54000	Detected	Detected	Detected	Detected
4646	A_24_P317450	OSTCL	Hs.487035	oligosaccharyltransferase complex subu	Homo sapiens oligosaccharyltransferase	4424.02900	11232.77800	1530.06500	2154.99980	Detected	Detected	Detected	Detected

4647	A_23_P60166	DEPDC6	Hs.112981	DEP domain containing 6	Homo sapiens DEP domain containing 6	75.83478	160.22548	52.52521	116.59759	Detected	Detected	Detected	Detected
4648	A_24_P388786	DNAH5	Hs.212360	dynein, axonemal, heavy chain 5	Homo sapiens dynein, axonemal, heavy	18.42734	34.69323	17.13323	6.04542	Detected	Detected	Detected	Compromised
4649	A_33_P3402868	GRIN2D	Hs.445015	glutamate receptor, ionotropic, N-methy	Homo sapiens glutamate receptor, ionot	660.89470	785.68823	613.10350	665.75700	Detected	Detected	Detected	Detected
4650	A_33_P3324980	ARID5B	Hs.535297	AT rich interactive domain 5B (MRF1-li	Homo sapiens AT rich interactive doma	72.51736	77.51679	91.70085	74.04576	Detected	Detected	Detected	Detected
4651	A_24_P116351	SIVA1	Hs.112058	SIVA1, apoptosis-inducing factor	Homo sapiens SIVA1, apoptosis-inducir	14334.61600	16182.68600	19646.08600	17320.51800	Detected	Detected	Detected	Detected
4652	A_23_P203920	SSPN	Hs.183428	sarcospan (Kras oncogene-associated g	Homo sapiens sarcospan (Kras oncoger	1142.13650	936.11847	873.26230	641.20557	Detected	Detected	Detected	Detected
4653	A_23_P252541	RAB7B	Hs.534612	RAB7B, member RAS oncogene family	Homo sapiens RAB7B, member RAS on	1235.91280	1084.59240	1165.29350	830.67926	Detected	Detected	Detected	Detected
4654	A_33_P3346552	LOC100008589	Hs.690421	28S ribosomal RNA	Homo sapiens 28S ribosomal RNA (LOC	428004.30000	464142.80000	381827.25000	370363.12000	Detected	Detected	Detected	Detected
4655	A_33_P3250953	SLC35B4	Hs.490181	solute carrier family 35, member B4	Homo sapiens solute carrier family 35, r	2078.29660	895.79510	1360.52200	474.43580	Detected	Detected	Detected	Detected
4656	A_24_P391431	TAF9B	Hs.592248	TAF9B RNA polymerase II, TATA box b	Homo sapiens TAF9B RNA polymerase	358.28467	617.81590	275.55807	342.96054	Detected	Detected	Detected	Detected
4657	A_23_P62868	EXOSC10	Hs.632368	exosome component 10	Homo sapiens exosome component 10 (	5480.58250	7652.98500	4787.55960	5762.97700	Detected	Detected	Detected	Detected
4658	A_24_P376422	LOC100286937	Hs.649203	similar to HSPC047 protein	HSPC047 protein [Source:UniProtKB/T	379.28152	194.57547	393.49594	160.19900	Detected	Detected	Detected	Detected
4659	A_23_P503233	EDARADD	Hs.352224	EDAR-associated death domain	Homo sapiens EDAR-associated death	36804.70300	58512.77000	20518.72700	28547.97500	Detected	Detected	Detected	Detected
4660	A_32_P215113	CCDC58	Hs.220594	coiled-coil domain containing 58	Homo sapiens coiled-coil domain contai	833.64100	1567.70460	608.71027	953.09660	Detected	Detected	Detected	Detected
4661	A_33_P3219138				Kalirin (EC 2.7.11.1)(Huntingtin-associat	92.74611	120.53419	41.39759	33.05323	Detected	Detected	Detected	Detected
4662	A_33_P3295148	LOC100133994		similar to hCG2040250	PREDICTED: Homo sapiens similar to h	3539.84330	4613.77200	2312.29830	1986.64140	Detected	Detected	Detected	Detected
4663	A_33_P3247022	CCNE2	Hs.567387	cyclin E2	Homo sapiens cyclin E2 (CCNE2), mRN/	73.33092	514.74200	45.47872	267.49158	Detected	Detected	Detected	Detected
4664	A_23_P209394	CFLAR	Hs.390736	CASP8 and FADD-like apoptosis regula	Homo sapiens CASP8 and FADD-like ap	733.48920	3548.11280	626.20910	2602.93480	Detected	Detected	Detected	Detected
4665	A_23_P101480	MAST1	Hs.227489	microtubule associated serine/threonin	Homo sapiens microtubule associated s	433.96893	417.83072	226.37985	131.47919	Detected	Detected	Detected	Detected
4666	A_23_P19624	BMP6	Hs.285671	bone morphogenetic protein 6	Homo sapiens bone morphogenetic prot	1000.64856	3530.09420	923.59540	2744.45730	Detected	Detected	Detected	Detected
4667	A_33_P3213857	RNASEH2B	Hs.306291	ribonuclease H2, subunit B	Homo sapiens ribonuclease H2, subunit	1516.20080	2284.47390	1371.25650	1701.19650	Detected	Detected	Detected	Detected
4668	A_33_P3241190	XRCC2	Hs.647093	X-ray repair complementing defective r	Homo sapiens X-ray repair complement	155.07538	120.99213	25.57997	28.57226	Detected	Detected	Detected	Detected
4669	A_24_P83183	WHSC2	Hs.21771	Wolf-Hirschhorn syndrome candidate 2	Homo sapiens Wolf-Hirschhorn syndrom	857.24380	986.33984	499.83173	465.16724	Detected	Detected	Detected	Detected
4670	A_32_P114284	IKZF2	Hs.604950	IKAROS family zinc finger 2 (Helios)	Homo sapiens IKAROS family zinc finge	183.10570	862.85565	154.60490	566.77640	Detected	Detected	Detected	Detected
4671	A_33_P3385681	LOC100130768	Hs.640088	hypothetical LOC100130768	Homo sapiens cDNA FLJ46357 fis, clon	41.70779	43.13681	25.07678	15.64639	Detected	Detected	Detected	Compromised
4672	A_33_P3256894	FAM21C	Hs.365286	family with sequence similarity 21, mem	Homo sapiens family with sequence sim	636.24115	635.18340	573.17250	424.71338	Detected	Detected	Detected	Detected
4673	A_33_P3372389	NPHP4	Hs.462348	nephronophthisis 4	Homo sapiens nephronophthisis 4 (NPH	66.20605	58.99488	62.54359	41.80791	Detected	Detected	Detected	Detected
4674	A_33_P3290830	C21orf99	Hs.579474	cancer-testis SP-1	Homo sapiens chromosome 21 open rea	14.39386	8.92663	6.50822	3.57075	Detected	Compromised	Compromised	Compromised
4675	A_32_P865708	FAM24A	Hs.369829	family with sequence similarity 24, mem	Homo sapiens family with sequence sim	22.97281	14.80768	7.18573	3.42798	Detected	Compromised	Compromised	Compromised
4676	A_23_P328022	SLC5A10	Hs.462418	solute carrier family 5 (sodium/glucose	Homo sapiens solute carrier family 5 (so	26.34082	28.83185	34.86835	28.32267	Detected	Detected	Detected	Detected
4677	A_23_P95029	SNTB1	Hs.46701	syntrophin, beta 1 (dystrophin-associat	Homo sapiens syntrophin, beta 1 (dystro	507.26074	346.87418	451.34464	267.65378	Detected	Detected	Detected	Detected
4678	A_33_P3413815	LAMB2L		laminin, beta 2-like	Homo sapiens laminin, beta 2-like (LAM	815.41060	128.73285	530.09143	78.80706	Detected	Detected	Detected	Detected
4679	A_33_P3314151	EFCAB4B	Hs.504534	EF-hand calcium binding domain 4B	Homo sapiens EF-hand calcium binding	78.77635	90.07497	56.69815	51.14805	Detected	Detected	Detected	Detected
4680	A_23_P7535	HRH2	Hs.247885	histamine receptor H2	Homo sapiens histamine receptor H2 (H	124.16550	177.46770	53.43567	54.12347	Detected	Detected	Detected	Detected
4681	A_23_P421011	KAZALD1	Hs.632079	Kazal-type serine peptidase inhibitor do	Homo sapiens Kazal-type serine peptida	652.39233	750.73663	559.57776	545.15070	Detected	Detected	Detected	Detected
4682	A_33_P3413053	NUBP1	Hs.81469	nucleotide binding protein 1 (MinD homo	Homo sapiens nucleotide binding protei	3786.39200	4809.30300	3775.66480	4144.82280	Detected	Detected	Detected	Detected
4683	A_23_P8558	ABHD11	Hs.647045	abhydrolase domain containing 11	Homo sapiens abhydrolase domain cont	340.11093	491.08334	430.28120	555.80756	Detected	Detected	Detected	Detected
4684	A_23_P13183	EXT2	Hs.368404	exostoses (multiple) 2	Homo sapiens exostoses (multiple) 2 (E	7216.40800	10451.22300	6185.53500	8298.04400	Detected	Detected	Detected	Detected
4685	A_24_P250227	NR1D1	Hs.592130	nuclear receptor subfamily 1, group D, r	Homo sapiens nuclear receptor subfami	907.86500	1912.61010	1091.90900	2175.28610	Detected	Detected	Detected	Detected
4686	A_33_P3373765	DRD4	Hs.99922	dopamine receptor D4	Homo sapiens dopamine receptor D4 (D	181.08372	82.87054	133.36740	49.99016	Detected	Detected	Detected	Detected
4687	A_24_P542375	PTMA	Hs.459927	prothymosin, alpha	Homo sapiens prothymosin, alpha (PTM	63568.15200	69032.28000	32955.90200	28629.10400	Detected	Detected	Detected	Detected
4688	A_33_P3370763	SARM1	Hs.719354	sterile alpha and TIR motif containing 1	Homo sapiens sterile alpha and TIR mot	12.13934	24.57278	13.23847	13.36773	Compromised	Detected	Compromised	Compromised
4689	A_24_P192434	TERF1	Hs.442707	telomeric repeat binding factor (NIMA-i	Homo sapiens telomeric repeat binding	637.66345	473.13165	461.31775	269.89220	Detected	Detected	Detected	Detected
4690	A_33_P3642648	ZNF460	Hs.99971	zinc finger protein 460	Homo sapiens zinc finger protein 460 (Z	22.90077	29.07365	18.23157	11.13262	Detected	Detected	Detected	Compromised
4691	A_33_P3282075	SP6	Hs.253603	Sp6 transcription factor	Homo sapiens Sp6 transcription factor (	252.77016	263.01782	258.30246	201.51984	Detected	Detected	Detected	Detected
4692	A_33_P3380618	HSPG2	Hs.562227	heparan sulfate proteoglycan 2	Homo sapiens heparan sulfate proteogly	35.65439	72.96546	36.04528	59.30328	Detected	Detected	Detected	Detected
4693	A_23_P17706	IL17RA	Hs.129751	interleukin 17 receptor A	Homo sapiens interleukin 17 receptor A	2531.31740	2544.80300	2313.47100	2046.39900	Detected	Detected	Detected	Detected
4694	A_23_P201368	CTBS	Hs.513557	chitobiase, di-N-acetyl-	Homo sapiens chitobiase, di-N-acetyl-	2628.13260	1062.23830	2341.48600	771.18690	Detected	Detected	Detected	Detected
4695	A_23_P6344	SDF2L1	Hs.303116	stromal cell-derived factor 2-like 1	Homo sapiens stromal cell-derived fact	34137.43000	42818.40600	35945.50800	39642.00000	Detected	Detected	Detected	Detected
4696	A_33_P3326588	TNFRSF10D	Hs.213467	tumor necrosis factor receptor superfa	Homo sapiens tumor necrosis factor re	33785.08600	29402.64800	25602.97700	18409.77300	Detected	Detected	Detected	Detected
4697	A_24_P282309	MYOF	Hs.602086	myoferlin	Homo sapiens myoferlin (MYOF), transc	156.00873	424.63310	152.63959	391.52920	Detected	Detected	Detected	Detected
4698	A_23_P71727	CKS2	Hs.83758	CDC28 protein kinase regulatory subun	Homo sapiens CDC28 protein kinase re	5413.06600	33660.00400	4014.77030	19306.26000	Detected	Detected	Detected	Detected
4699	A_23_P83798	ALX1	Hs.41683	ALX homeobox 1	Homo sapiens ALX homeobox 1 (ALX1)	289.79892	105.18860	255.48160	80.85695	Detected	Detected	Detected	Detected
4700	A_33_P3419557	OR5K3	Hs.554709	olfactory receptor, family 5, subfamily K	Homo sapiens olfactory receptor, family	42.47395	36.92362	21.73170	15.28226	Detected	Detected	Detected	Detected
4701	A_33_P3375528	MIER3	Hs.657594	mesoderm induction early response 1, f	Homo sapiens mesoderm induction early	56.20774	35.14603	65.18869	42.56298	Detected	Detected	Detected	Detected
4702	A_24_P193592	CCNF	Hs.1973	cyclin F	Homo sapiens cyclin F (CCNF), mRNA [	27.45196	219.67622	19.30103	168.89502	Detected	Detected	Detected	Detected
4703	A_23_P55468	TIMM22	Hs.592108	translocase of inner mitochondrial mem	Homo sapiens translocase of inner mito	1544.34070	3332.86250	1298.62280	2676.19260	Detected	Detected	Detected	Detected
4704	A_33_P3227774	LOC80154	Hs.710289	hypothetical LOC80154	Homo sapiens hypothetical LOC80154 (	255.74612	328.29578	223.31644	297.01297	Detected	Detected	Detected	Detected
4705	A_23_P57474	OSBP2	Hs.517546	oxysterol binding protein 2	Homo sapiens oxysterol binding protein	106.95686	13.63679	84.62169	8.07980	Detected	Compromised	Detected	Compromised
4706	A_23_P121356	BBX	Hs.124366	bobby sox homolog (Drosophila)	Homo sapiens bobby sox homolog (Dros	4651.13700	2668.55420	2599.99930	1261.46900	Detected	Detected	Detected	Detected
4707	A_23_P60793	NCRNA00105	Hs.521856	non-protein coding RNA 105	Homo sapiens non-protein coding RNA	80.64959	80.63637	63.97473	51.13976	Detected	Detected	Detected	Detected
4708	A_33_P3355266	TINAGL1	Hs.199368	tubulointerstitial nephritis antigen-like 1	Homo sapiens tubulointerstitial nephritis	741.25977	996.57825	568.88780	491.48215	Detected	Detected	Detected	Detected
4709	A_33_P3394465	PROP1	Hs.158301	PROP paired-like homeobox 1	Homo sapiens PROP paired-like homeo	1069.86800	1820.17760	332.42795	129.41072	Not Detected	Not Detected	Not Detected	Detected
4710	A_23_P411612	SPRYD4	Hs.128676	SPRY domain containing 4	Homo sapiens SPRY domain containing	843.38855	1154.27210	714.88360	775.44403	Detected	Detected	Detected	Detected
4711	A_24_P400507	C3orf64	Hs.518059	chromosome 3 open reading frame 64	Homo sapiens chromosome 3 open read	153.99496	149.04262	59.71325	44.68838	Detected	Detected	Detected	Detected
4712	A_23_P257593	LOH12CR1	Hs.720779	loss of heterozygosity, 12, chromosoma	Homo sapiens loss of heterozygosity, 12	644.20886	780.73303	558.13184	580.49390	Detected	Detected	Detected	Detected
4713	A_33_P3297626				Uncharacterized protein KIAA0090 Pred	29.33166	29.07937	13.77224	16.47643	Detected	Detected	Compromised	Detected
4714	A_23_P61371	TMEM173	Hs.379754	transmembrane protein 173	Homo sapiens transmembrane protein 1	4192.79100	10186.41400	3677.53520	8016.83840	Detected	Detected	Detected	Detected
4715	A_23_P130735	SLC6A16	Hs.130949	solute carrier family 6, member 16	Homo sapiens solute carrier family 6, m	429.04285	135.33570	239.76053	51.86281	Detected	Detected	Detected	Detected
4716	A_33_P3291650		Hs.541909		Putative uncharacterized protein ENSP	35.43890	43.52216	23.02638	21.18078	Detected	Detected	Detected	Detected
4717	A_33_P3246248	LOC729879	Hs.589661	hypothetical protein LOC729879	PREDICTED: Homo sapiens hypothetica	29.97668	46.69782	22.41873	19.97442	Detected	Detected	Detected	Detected
4718	A_23_P66767	GGT6	Hs.130749	gamma-glutamyltransferase 6	Homo sapiens gamma-glutamyltransfera	20.83229	22.99856	6.29064	9.81083	Detected	Detected	Compromised	Compromised
4719	A_33_P3225086	PEX16	Hs.100915	peroxisomal biogenesis factor 16	Homo sapiens peroxisomal biogenesis fa	4495.47950	4415.70170	4560.52300	4360.19970	Detected	Detected	Detected	Detected
4720	A_33_P3271594	TRIM54	Hs.516036	tripartite motif-containing 54	Homo sapiens tripartite motif-containin	177.99298	248.06987	51.97665	63.53077	Detected	Detected	Detected	Detected
4721	A_23_P1492	AVPI1	Hs.23918	arginine vasopressin-induced 1	Homo sapiens arginine vasopressin-indu	3427.74900	1568.93100	3907.66160	1649.20530	Detected	Detected	Detected	Detected

4722	A_33_P3590279					31835.17200	23812.32800	1074.91600	595.93070	Detected	Detected	Detected	Detected
4723	A_23_P217564	ACSL4	Hs.268785	acyl-CoA synthetase long-chain family	Homo sapiens acyl-CoA synthetase lon	1710.32420	1511.72330	1110.22860	846.43414	Detected	Detected	Detected	Detected
4724	A_33_P3300877	LOC644193		similar to hCG1744883	PREDICTED: Homo sapiens similar to h	138.15387	198.16946	77.45043	69.37791	Detected	Detected	Detected	Detected
4725	A_33_P3210223				Zinc finger CCHC domain-containing pr	16.06959	6.95193	11.13752	7.25885	Detected	Compromised	Compromised	Compromised
4726	A_33_P3299754	RAB18	Hs.406799	RAB18, member RAS oncogene family	Homo sapiens RAB18, member RAS onc	726.82947	838.90533	540.28815	495.29840	Detected	Detected	Detected	Detected
4727	A_33_P3335248	SDCCAG3	Hs.94300	serologically defined colon cancer antig	Homo sapiens serologically defined colo	2759.71680	2592.02300	3119.96480	2939.00170	Detected	Detected	Detected	Detected
4728	A_24_P62659	TSPAN2	Hs.310458	tetraspanin 2	Homo sapiens tetraspanin 2 (TSPAN2),	2.87176	55.06810	8.26957	34.80544	Compromised	Detected	Compromised	Detected
4729	A_23_P161481	KIAA1274	Hs.202351	KIAA1274	Homo sapiens KIAA1274 (KIAA1274), m	40.79353	45.49888	21.52618	24.76312	Detected	Detected	Detected	Detected
4730	A_23_P89799	ACAA2	Hs.200136	acetyl-Coenzyme A acyltransferase 2	Homo sapiens acetyl-Coenzyme A acyl	10141.85500	9198.58500	5313.23140	4017.66400	Detected	Detected	Detected	Detected
4731	A_32_P155631	SDHD	Hs.356270	succinate dehydrogenase complex, subu	Homo sapiens succinate dehydrogenase	4577.39100	3695.41700	3328.83760	2190.86280	Detected	Detected	Detected	Detected
4732	A_33_P3378790	CASC1	Hs.407771	cancer susceptibility candidate 1	Homo sapiens cancer susceptibility can	48.57039	3.59331	33.91716	3.08308	Detected	Compromised	Detected	Compromised
4733	A_33_P3351207	MGC21881	Hs.721511	hypothetical locus MGC21881	Homo sapiens hypothetical locus MGC2	2234.59600	1097.26930	1592.76810	677.52260	Detected	Detected	Detected	Detected
4734	A_23_P354208	WDR81	Hs.234572	WD repeat domain 81	Homo sapiens WD repeat domain 81 (W	4783.15000	5089.03100	5467.79440	5205.43260	Detected	Detected	Detected	Detected
4735	A_24_P315184					27837.29500	15342.45700	28761.80900	14026.24000	Detected	Detected	Detected	Detected
4736	A_23_P251705	ARHGEF9	Hs.54697	Cdc42 guanine nucleotide exchange fac	Homo sapiens Cdc42 guanine nucleotide	179.08679	164.78410	182.58560	123.30850	Detected	Detected	Detected	Detected
4737	A_33_P3394599	HMG20B	Hs.406534	high-mobility group 20B	Homo sapiens high-mobility group 20B (	24819.28000	30048.74600	13407.58700	8584.86800	Detected	Detected	Detected	Detected
4738	A_33_P3268466	MPDU1	Hs.246381	mannose-P-dolichol utilization defect 1	Homo sapiens mannose-P-dolichol utili	371.66498	688.71985	333.83618	557.46670	Detected	Detected	Detected	Detected
4739	A_23_P10458	C22orf26	Hs.567529	chromosome 22 open reading frame 26	Homo sapiens chromosome 22 open rea	43.77982	53.02567	39.97013	29.79784	Detected	Detected	Detected	Detected
4740	A_23_P96369	CXorf57	Hs.274267	chromosome X open reading frame 57	Homo sapiens chromosome X open read	1024.83020	375.42640	806.11050	242.34772	Detected	Detected	Detected	Detected
4741	A_33_P3286349	C19orf51	Hs.351582	chromosome 19 open reading frame 51	Homo sapiens chromosome 19 open rea	50.80563	24.36252	60.23245	9.37581	Detected	Detected	Detected	Compromised
4742	A_33_P3366987	GPR125	Hs.99195	G protein-coupled receptor 125	Homo sapiens G protein-coupled recep	280.18810	113.83508	231.64964	71.41628	Detected	Detected	Detected	Detected
4743	A_33_P3398091	ZNF562	Hs.371107	zinc finger protein 562	Homo sapiens zinc finger protein 562 (Z	483.70096	629.71300	390.72305	477.69287	Detected	Detected	Detected	Detected
4744	A_33_P3234010	FLJ41856	Hs.446909	hypothetical LOC388550	Homo sapiens hypothetical LOC388550	8.41708	24.72885	14.31565	10.77357	Compromised	Detected	Compromised	Compromised
4745	A_33_P3422747	DIS3L2	Hs.471637	DIS3 mitotic control homolog (S. cerevi	Homo sapiens DIS3 mitotic control hom	61.28582	113.32222	75.18996	107.82231	Detected	Detected	Detected	Detected
4746	A_33_P3341189	UBE2A	Hs.379466	ubiquitin-conjugating enzyme E2A (RAD	Homo sapiens ubiquitin-conjugating enz	3969.53640	6208.10600	3752.31860	4830.74300	Detected	Detected	Detected	Detected
4747	A_23_P88880	ZNF423	Hs.530930	zinc finger protein 423	Homo sapiens zinc finger protein 423 (Z	95.23167	83.70467	39.43089	14.11618	Detected	Detected	Detected	Compromised
4748	A_23_P55682	ZSCAN18	Hs.235390	zinc finger and SCAN domain containing	Homo sapiens zinc finger and SCAN do	3288.82450	2393.18500	2877.55980	1908.38200	Detected	Detected	Detected	Detected
4749	A_23_P105368	MGC16384	Hs.661642	hypothetical LOC114130	Homo sapiens hypothetical LOC114130	36.50864	40.18161	17.76127	17.23869	Detected	Detected	Detected	Detected
4750	A_33_P3294504	FCRL1	Hs.656112	Fc receptor-like 1	Homo sapiens Fc receptor-like 1 (FCRL	62.39751	62.20136	27.37092	25.19044	Detected	Detected	Detected	Detected
4751	A_33_P3427239	LOC100134937	Hs.659762	hypothetical LOC100134937	Homo sapiens cDNA clone IMAGE:5263	2536.55520	817.61170	1584.55690	420.85272	Detected	Detected	Detected	Detected
4752	A_33_P3287228		Hs.568276		IQ domain-containing protein D [Sourc	37.31191	11.58055	27.00180	3.19626	Detected	Compromised	Detected	Compromised
4753	A_33_P3384045		Hs.623961		Nervous system abundant protein 11 [	9.43825	16.16872	20.42660	20.52279	Compromised	Compromised	Detected	Detected
4754	A_24_P694760	ERC1	Hs.601216	ELKS/RAB6-interacting/CAST family m	Homo sapiens ELKS/RAB6-interacting/	1477.50940	2254.17500	715.83026	865.54120	Detected	Detected	Detected	Detected
4755	A_32_P159612	ACCN1	Hs.368417	amiloride-sensitive cation channel 1, ne	Homo sapiens amiloride-sensitive catio	94.85637	114.84726	42.46571	34.81399	Detected	Detected	Detected	Detected
4756	A_33_P3259722	TMEM222	Hs.469171	transmembrane protein 222	Homo sapiens transmembrane protein 2	172.97212	177.62668	134.04181	144.43178	Detected	Detected	Detected	Detected
4757	A_33_P3307990	MASP2	Hs.655645	mannan-binding lectin serine peptidase	Homo sapiens mannan-binding lectin se	43.35148	16.55871	10.38281	4.33183	Detected	Compromised	Compromised	Compromised
4758	A_24_P161463	C20orf199	Hs.356766	chromosome 20 open reading frame 199	Homo sapiens chromosome 20 open rea	62689.36700	28832.90800	40148.71000	14684.11300	Detected	Detected	Detected	Detected
4759	A_24_P151834	ANKFY1	Hs.696087	ankyrin repeat and FYVE domain contai	Homo sapiens ankyrin repeat and FYVE	219.80928	206.38669	110.00400	85.01939	Detected	Detected	Detected	Detected
4760	A_23_P150741	C2CD3	Hs.557938	C2 calcium-dependent domain containin	Homo sapiens mRNA; cDNA DKFZp586	646.93100	648.24740	469.35153	394.96677	Detected	Detected	Detected	Detected
4761	A_33_P3394605	HMG20B	Hs.406534	high-mobility group 20B	Homo sapiens high-mobility group 20B (	5069.70070	4630.23100	3992.64750	3257.66260	Detected	Detected	Detected	Detected
4762	A_33_P3220670	LOC644649	Hs.721083	apolipoprotein O pseudogene	BX108667 Soares_NFL_T_GBC_S1 Homo	61.51376	88.42042	12.12348	15.77099	Detected	Detected	Compromised	Compromised
4763	A_23_P11629	TMEM61	Hs.663950	transmembrane protein 61	Homo sapiens transmembrane protein 6	62.79213	34.00399	53.69297	30.73929	Detected	Detected	Detected	Detected
4764	A_24_P338145	STOML2	Hs.3439	stomatin (EPB72)-like 2	Homo sapiens stomatin (EPB72)-like 2	3961.26900	4770.10000	4503.47700	4784.70600	Detected	Detected	Detected	Detected
4765	A_33_P3291394	GPN1	Hs.18259	GPN-loop GTPase 1	Homo sapiens GPN-loop GTPase 1 (GP	3526.63530	6002.76950	2990.70210	4458.10000	Detected	Detected	Detected	Detected
4766	A_23_P55706	RELB	Hs.654402	v-rel reticuloendotheliosis viral oncogen	Homo sapiens v-rel reticuloendotheliosi	19956.70500	18088.55900	13627.10900	11365.70600	Detected	Detected	Detected	Detected
4767	A_33_P3297946	LOC283693	Hs.721080	actin, gamma pseudogene	Homo sapiens actin, gamma pseudogene	48.52438	53.11452	38.34666	24.94997	Detected	Detected	Detected	Detected
4768	A_23_P39131	GLTSCR2	Hs.421907	glioma tumor suppressor candidate regi	Homo sapiens glioma tumor suppressor	7135.15400	2232.04170	9520.51300	2941.48780	Detected	Detected	Detected	Detected
4769	A_23_P150281	TP53I11	Hs.554791	tumor protein p53 inducible protein 11	Homo sapiens tumor protein p53 inducib	804.52466	467.72240	659.26587	375.71713	Detected	Detected	Detected	Detected
4770	A_24_P250650	RABL2A	Hs.446425	RAB, member of RAS oncogene family-	Homo sapiens RAB, member of RAS on	8050.58200	3779.81270	6484.52640	2671.33060	Detected	Detected	Detected	Detected
4771	A_23_P138253	C1orf77	Hs.611057	chromosome 1 open reading frame 77	Homo sapiens chromosome 1 open read	1507.04870	2689.96830	999.90204	1420.58840	Detected	Detected	Detected	Detected
4772	A_33_P3221971	C15orf58	Hs.304673	chromosome 15 open reading frame 58	Homo sapiens chromosome 15 open rea	99.97488	119.26571	57.11032	65.05092	Detected	Detected	Detected	Detected
4773	A_33_P3851513	LIN54	Hs.96952	lin-54 homolog (C. elegans)	Homo sapiens lin-54 homolog (C. elegar	570.52300	897.84393	329.74930	448.92758	Detected	Detected	Detected	Detected
4774	A_23_P98310	CSTF3	Hs.44402	cleavage stimulation factor, 3' pre-RNA	Homo sapiens cleavage stimulation fact	2804.16000	2380.21480	2062.45920	1447.92880	Detected	Detected	Detected	Detected
4775	A_23_P27167	RNASEH1	Hs.568006	ribonuclease H1	Homo sapiens ribonuclease H1 (RNASE	1934.40230	3470.16580	1477.40490	2327.76950	Detected	Detected	Detected	Detected
4776	A_23_P2601	HSP90B1	Hs.192374	heat shock protein 90kDa beta (Grp94),	Homo sapiens heat shock protein 90kDa	74395.12500	150189.70000	53818.43000	96205.08000	Detected	Detected	Detected	Detected
4777	A_24_P177553					40.16356	27.08609	32.70244	24.00318	Detected	Detected	Detected	Detected
4778	A_23_P68327	CCDC104	Hs.264208	coiled-coil domain containing 104	Homo sapiens coiled-coil domain contai	6438.52050	5179.37060	4772.94200	3638.36080	Detected	Detected	Detected	Detected
4779	A_33_P3407746	PPM1B	Hs.416769	protein phosphatase 1B (formerly 2C), r	Homo sapiens protein phosphatase 1B (	17.91791	5.95311	11.92083	5.39601	Detected	Compromised	Compromised	Compromised
4780	A_23_P207058	SOCS3	Hs.527973	suppressor of cytokine signaling 3	Homo sapiens suppressor of cytokine s	5844.79830	2763.73320	5653.16400	2525.30400	Detected	Detected	Detected	Detected
4781	A_33_P3284557	ZAK	Hs.444451	sterile alpha motif and leucine zipper co	Homo sapiens sterile alpha motif and le	5493.61130	4900.65700	3374.44200	2528.54960	Detected	Detected	Detected	Detected
4782	A_33_P3248024					219.85164	324.38605	138.04309	136.51973	Detected	Detected	Detected	Detected
4783	A_24_P121271	CPSF6	Hs.369606	cleavage and polyadenylation specific fa	Homo sapiens cleavage and polyadenyla	2102.59230	3415.57130	2082.50760	2904.02200	Detected	Detected	Detected	Detected
4784	A_33_P3255924	ACCSL	Hs.558851	1-aminocyclopropane-1-carboxylate sy	Homo sapiens 1-aminocyclopropane-1-	52.51191	74.72166	15.81474	20.49462	Detected	Detected	Detected	Detected
4785	A_33_P3326275	LOC100131554	Hs.287726	hypothetical LOC100131554	CDNA: FLJ23120 fis, clone LNG07989H	66.55672	89.65102	55.58537	16.45411	Detected	Detected	Detected	Detected
4786	A_33_P3404651	TTC7A	Hs.370603	tetratricopeptide repeat domain 7A	Homo sapiens tetratricopeptide repeat	634.87573	1639.03380	613.56366	1557.47860	Detected	Detected	Detected	Detected
4787	A_24_P12413	TRAM2	Hs.520182	translocation associated membrane pro	Homo sapiens translocation associated	4790.47200	13572.64700	3906.67240	8861.51500	Detected	Detected	Detected	Detected
4788	A_32_P15421	RNF185	Hs.517553	ring finger protein 185	Homo sapiens ring finger protein 185 (R	752.35126	711.36414	416.02770	333.53555	Detected	Detected	Detected	Detected
4789	A_24_P92163					91.78154	229.82756	70.62071	161.90894	Detected	Detected	Detected	Detected
4790	A_24_P55295	GJA1	Hs.74471	gap junction protein, alpha 1, 43kDa	Homo sapiens gap junction protein, alph	4381.10250	6285.47270	3163.03170	3659.37940	Detected	Detected	Detected	Detected
4791	A_24_P109082	USP37	Hs.166068	ubiquitin specific peptidase 37	Homo sapiens ubiquitin specific peptida	559.25420	505.61970	318.67917	234.20898	Detected	Detected	Detected	Detected
4792	A_24_P330773	CALCOCO2	Hs.514920	calcium binding and coiled-coil domain 2	Homo sapiens calcium binding and coile	1003.85583	598.26340	682.68410	330.01840	Detected	Detected	Detected	Detected
4793	A_23_P77000	VASH1	Hs.525479	vasohibin 1	Homo sapiens vasohibin 1 (VASH1), mR	1781.85220	355.59600	1720.44460	289.88130	Detected	Detected	Detected	Detected
4794	A_24_P631848	LOC100288891	Hs.308060	hypothetical protein LOC100288891	PREDICTED: Homo sapiens hypothetica	1261.28310	1863.86340	575.30084	305.01938	Not Detected	Detected	Detected	Detected
4795	A_33_P3258467	SPTBN1	Hs.503178	spectrin, beta, non-erythrocytic 1	Homo sapiens spectrin, beta, non-eryth	5281.16700	5972.81840	3537.98800	3623.55660	Detected	Detected	Detected	Detected
4796	A_24_P28977	TRPC1	Hs.250687	transient receptor potential cation char	Homo sapiens transient receptor poten	1778.47960	1148.60930	1230.25930	650.43650	Detected	Detected	Detected	Detected

4797	A_33_P3215193	NMNAT1	Hs.633762	nicotinamide nucleotide adenylyltransfe	Homo sapiens nicotinamide nucleotide a	245.97560	368.65290	197.96320	242.69247	Detected	Detected	Detected	Detected
4798	A_32_P34444	FHOD3	Hs.719944	formin homology 2 domain containing 3	Homo sapiens formin homology 2 domai	2108.41280	3620.82200	1521.93960	2325.96830	Detected	Detected	Detected	Detected
4799	A_23_P34126	BGN	Hs.821	biglycan	Homo sapiens biglycan (BGN), mRNA [N	383.01090	847.96470	440.74258	994.82680	Detected	Detected	Detected	Detected
4800	A_23_P102122	ARPC2	Hs.529303	actin related protein 2/3 complex, subu	Homo sapiens actin related protein 2/3	54669.52000	97316.34000	46999.60000	68880.94500	Detected	Detected	Detected	Detected
4801	A_23_P103398	PSEN2	Hs.25363	presenilin 2 (Alzheimer disease 4)	Homo sapiens presenilin 2 (Alzheimer d	9182.99600	2334.02200	6278.15140	1493.17300	Detected	Detected	Detected	Detected
4802	A_23_P251927	CHCHD3	Hs.655010	coiled-coil-helix-coiled-coil-helix doma	Homo sapiens coiled-coil-helix-coiled-c	3306.30000	4239.59380	2800.51250	3092.68380	Detected	Detected	Detected	Detected
4803	A_24_P228130	CCL3L3	Hs.512304	chemokine (C-C motif) ligand 3-like 3	Homo sapiens chemokine (C-C motif) li	18.60374	9.22518	26.48538	5.78994	Detected	Compromised	Detected	Compromised
4804	A_23_P69840	SLC35A4	Hs.406840	solute carrier family 35, member A4	Homo sapiens solute carrier family 35, r	894.47675	907.58480	1159.53210	1051.61380	Detected	Detected	Detected	Detected
4805	A_23_P152782	IFI35	Hs.632258	interferon-induced protein 35	Homo sapiens interferon-induced prote	422.75378	891.37555	361.37277	676.92896	Detected	Detected	Detected	Detected
4806	A_23_P215634	IGFBP3	Hs.450230	insulin-like growth factor binding protei	Homo sapiens insulin-like growth factor	1824.75790	127283.97000	1505.56410	87855.55500	Detected	Detected	Detected	Detected
4807	A_23_P206324	HSDL1	Hs.555992	hydroxysteroid dehydrogenase like 1	Homo sapiens hydroxysteroid dehydrog	204.11276	234.33604	133.05528	114.39587	Detected	Detected	Detected	Detected
4808	A_23_P152620	TNFSF13	Hs.54673	tumor necrosis factor (ligand) superfam	Homo sapiens tumor necrosis factor (lig	241.37310	75.93219	250.86080	65.42909	Detected	Detected	Detected	Detected
4809	A_33_P3229335	HIST3H2BB	Hs.376691	histone cluster 3, H2bb	Homo sapiens histone cluster 3, H2bb (	25.78246	57.22399	26.66236	26.72782	Detected	Detected	Detected	Detected
4810	A_23_P4592	SIGLEC6	Hs.720304	sialic acid binding Ig-like lectin 6	Homo sapiens sialic acid binding Ig-like	94.00835	100.19449	15.11246	10.93310	Detected	Detected	Compromised	Compromised
4811	A_33_P3361636	MGP	Hs.365706	matrix Gla protein	Homo sapiens matrix Gla protein (MGP)	556.05340	1454.91060	381.32965	785.03357	Detected	Detected	Detected	Detected
4812	A_32_P94001	FAM92A3	Hs.652187	family with sequence similarity 92, mem	Homo sapiens family with sequence sim	295.24496	396.31577	78.16783	87.88522	Detected	Detected	Detected	Detected
4813	A_23_P394166	CNOT7	Hs.598387	CCR4-NOT transcription complex, subu	Homo sapiens CCR4-NOT transcription	4884.57030	5159.08000	3300.69210	2795.71830	Detected	Detected	Detected	Detected
4814	A_33_P3410454				General transcription factor II-I repeat	16.13193	10.13086	4.57190	3.30872	Detected	Compromised	Compromised	Compromised
4815	A_24_P922631	C5orf58	Hs.519749	chromosome 5 open reading frame 58	Homo sapiens chromosome 5 open read	960.30610	787.18230	212.55211	117.30151	Detected	Detected	Detected	Detected
4816	A_23_P26557	C16orf59	Hs.534491	chromosome 16 open reading frame 59	Homo sapiens chromosome 16 open rea	397.71536	1391.35700	445.41122	1414.42750	Detected	Detected	Detected	Detected
4817	A_33_P3214239	GNASAS	Hs.122718	GNAS antisense RNA (non-protein cod	Homo sapiens GNAS antisense RNA (no	75.65220	95.73440	58.45865	64.76164	Detected	Detected	Detected	Detected
4818	A_23_P106024	JAG2	Hs.433445	jagged 2	Homo sapiens jagged 2 (JAG2), transcri	46.37534	7.42493	29.68383	4.38479	Detected	Compromised	Detected	Compromised
4819	A_23_P104073	S100A3	Hs.557609	S100 calcium binding protein A3	Homo sapiens S100 calcium binding pro	2292.10960	1639.03810	2062.71560	1279.89670	Detected	Detected	Detected	Detected
4820	A_33_P3232993	MARK2	Hs.567261	MAP/microtubule affinity-regulating kin	Homo sapiens MAP/microtubule affinity	296.72235	336.72740	292.21463	329.89690	Detected	Detected	Detected	Detected
4821	A_23_P12147	C1orf74	Hs.497642	chromosome 1 open reading frame 74	Homo sapiens chromosome 1 open read	224.21222	276.06348	220.26468	236.31950	Detected	Detected	Detected	Detected
4822	A_23_P107247	CACNA1G	Hs.591169	calcium channel, voltage-dependent, T	Homo sapiens calcium channel, voltage-	1343.89110	84.34154	1270.26770	68.78381	Detected	Detected	Detected	Detected
4823	A_23_P113161	C1orf21	Hs.719188	chromosome 1 open reading frame 21	Homo sapiens chromosome 1 open read	460.13150	274.04202	269.09485	126.44144	Detected	Detected	Detected	Detected
4824	A_23_P28375	GMCL1	Hs.293971	germ cell-less homolog 1 (Drosophila)	Homo sapiens germ cell-less homolog 1	304.80573	202.88070	250.71034	145.80070	Detected	Detected	Detected	Detected
4825	A_23_P120694	KCNE2	Hs.551521	potassium voltage-gated channel, Isk-re	Homo sapiens potassium voltage-gated	16.62007	24.21027	15.05628	4.31332	Detected	Not Detected	Detected	Compromised
4826	A_23_P124837	LRP1	Hs.162757	low density lipoprotein-related protein	Homo sapiens low density lipoprotein-re	2513.14230	1481.99730	1732.08630	942.97943	Detected	Detected	Detected	Detected
4827	A_23_P62605	RPL11	Hs.719951	ribosomal protein L11	Homo sapiens ribosomal protein L11 (R	252854.92000	207995.53000	187285.19000	122377.44000	Detected	Detected	Detected	Detected
4828	A_24_P297551	FAM19A2	Hs.269745	family with sequence similarity 19 (chem	Homo sapiens family with sequence sim	23.30094	13.53786	19.91161	9.81893	Detected	Compromised	Detected	Compromised
4829	A_24_P175989	VPS29	Hs.600114	vacuolar protein sorting 29 homolog (S.	Homo sapiens vacuolar protein sorting 2	4625.28760	7462.95850	4158.42900	5441.19900	Detected	Detected	Detected	Detected
4830	A_23_P338519	NKIRAS1	Hs.173202	NFKB inhibitor interacting Ras-like 1	Homo sapiens NFKB inhibitor interactin	550.06396	733.12470	471.44766	520.05865	Detected	Detected	Detected	Detected
4831	A_33_P3407364	NXF5	Hs.307077	nuclear RNA export factor 5	Homo sapiens nuclear RNA export facto	32.55703	40.32529	26.88809	23.95960	Detected	Detected	Detected	Detected
4832	A_33_P3284108	NSF	Hs.431279	N-ethylmaleimide-sensitive factor	Homo sapiens N-ethylmaleimide-sensiti	64.13476	72.18134	70.84371	48.90011	Detected	Detected	Detected	Detected
4833	A_23_P252740	DSCC1	Hs.315167	defective in sister chromatid cohesion 1	Homo sapiens defective in sister chrom	48.38689	308.93390	35.22436	195.17061	Detected	Detected	Detected	Detected
4834	A_23_P113972	EXOC1	Hs.269665	exocyst complex component 1	Homo sapiens exocyst complex compor	1166.11180	843.86870	834.53800	525.08790	Detected	Detected	Detected	Detected
4835	A_33_P3320503	RAI1	Hs.655395	retinoic acid induced 1	Homo sapiens retinoic acid induced 1 (R	105.88050	127.05701	55.28748	50.06311	Detected	Detected	Detected	Detected
4836	A_32_P135348	TANC1	Hs.61590	tetratricopeptide repeat, ankyrin repeat	Homo sapiens tetratricopeptide repeat,	1245.65440	1533.90710	968.92896	940.69330	Detected	Detected	Detected	Detected
4837	A_33_P3382553				HCG1773879PRO1438 ; [Source:UniPro	29.98128	56.29163	10.92608	9.77008	Detected	Detected	Compromised	Compromised
4838	A_23_P105392	CCT2	Hs.189772	chaperonin containing TCP1, subunit 2	Homo sapiens chaperonin containing TC	47756.71500	60058.89000	32201.15600	35337.15200	Detected	Detected	Detected	Detected
4839	A_33_P3328716	CCDC13	Hs.709716	coiled-coil domain containing 13	Homo sapiens coiled-coil domain conta	17.92289	26.79277	7.45691	3.05113	Detected	Detected	Compromised	Compromised
4840	A_23_P113317	P4HTM	Hs.654944	prolyl 4-hydroxylase, transmembrane (e	Homo sapiens prolyl 4-hydroxylase, tran	1087.97670	578.58720	800.59560	379.07925	Detected	Detected	Detected	Detected
4841	A_33_P3231833				Acyl-coenzyme A thioesterase 8 (EC 3.	95.53854	368.98618	91.19238	270.55136	Detected	Detected	Detected	Detected
4842	A_23_P54376	STOML1	Hs.194816	stomatin (EPB72)-like 1	Homo sapiens stomatin (EPB72)-like 1	7298.84380	8003.92400	6180.72460	6227.76100	Detected	Detected	Detected	Detected
4843	A_24_P323104	DIAPH1	Hs.529451	diaphanous homolog 1 (Drosophila)	Homo sapiens diaphanous homolog 1 (D	31052.10200	37284.21500	23520.33200	23999.70100	Detected	Detected	Detected	Detected
4844	A_33_P3305482	EIF2B3	Hs.533549	eukaryotic translation initiation factor 2	Homo sapiens eukaryotic translation ini	2997.61160	4383.57670	2386.29100	2871.25510	Detected	Detected	Detected	Detected
4845	A_33_P3358243	KIAA1161	Hs.522083	KIAA1161	Homo sapiens KIAA1161 (KIAA1161), m	1652.06520	1656.20600	696.22140	654.96510	Detected	Detected	Detected	Detected
4846	A_33_P3408034	TDGF2		teratocarcinoma-derived growth factor	PREDICTED: Homo sapiens similar to h	33.76842	48.98740	21.18830	21.92804	Detected	Detected	Detected	Detected
4847	A_33_P3322430				Cystinosin [Source:UniProtKB/Swiss-P	19376.86100	14107.53900	3633.50830	1952.97850	Detected	Detected	Detected	Detected
4848	A_24_P394940	CYP2E1	Hs.12907	cytochrome P450, family 2, subfamily E,	Homo sapiens cytochrome P450, family	15.95504	11.51997	9.55558	10.03151	Detected	Compromised	Compromised	Compromised
4849	A_33_P3330731				Rhomboid domain-containing protein 3	21.96300	22.61615	16.40047	13.41242	Detected	Detected	Detected	Compromised
4850	A_24_P131222	ATP13A2	Hs.128866	ATPase type 13A2	Homo sapiens ATPase type 13A2 (ATP	207.87927	456.09995	188.49626	427.67830	Detected	Detected	Detected	Detected
4851	A_24_P518369					86.05388	95.20278	58.15544	48.38419	Detected	Detected	Detected	Detected
4852	A_32_P751535					6508.47660	5071.20560	4327.63430	2785.21680	Detected	Detected	Detected	Detected
4853	A_24_P185394	GSK3A	Hs.466828	glycogen synthase kinase 3 alpha	Homo sapiens glycogen synthase kinase	12302.68900	15735.36300	10897.65200	12440.06200	Detected	Detected	Detected	Detected
4854	A_33_P3502311	HERC2P7	Hs.722631	hect domain and RLD 2 pseudogene 7	Homo sapiens cDNA FLJ43661 fis, clon	185.14413	117.79369	152.24180	55.73543	Detected	Detected	Detected	Detected
4855	A_33_P3291349		Hs.668462		Regulation of nuclear pre-mRNA domai	726.13696	1346.07820	574.30760	943.46643	Detected	Detected	Detected	Detected
4856	A_23_P90369	C19orf6	Hs.515003	chromosome 19 open reading frame 6	Homo sapiens chromosome 19 open rea	27920.50200	20032.13500	15783.75300	9657.83400	Detected	Detected	Detected	Detected
4857	A_23_P64611	P2RY6	Hs.16362	pyrimidinergic receptor P2Y, G-protein	Homo sapiens pyrimidinergic receptor P	4.36511	48.17956	3.08048	50.93531	Compromised	Detected	Compromised	Detected
4858	A_33_P3264940	CXorf25	Hs.562045	chromosome X open reading frame 25	PREDICTED: Homo sapiens chromosom	21.20039	19.37977	7.55514	3.22647	Detected	Detected	Compromised	Compromised
4859	A_33_P3416762	LYPLA1	Hs.435850	lysophospholipase I	Homo sapiens lysophospholipase I (LYP	5626.30300	5606.26660	3808.84740	2920.43330	Detected	Detected	Detected	Detected
4860	A_23_P206107	ULK3	Hs.513034	unc-51-like kinase 3 (C. elegans)	Homo sapiens unc-51-like kinase 3 (C.	1459.37130	647.63745	1221.96300	534.68976	Detected	Detected	Detected	Detected
4861	A_24_P288298	KIR2DL4	Hs.661219	killer cell immunoglobulin-like receptor,	Homo sapiens killer cell immunoglobulin	19.15971	25.68978	15.07396	10.06525	Detected	Detected	Compromised	Compromised
4862	A_24_P313993	CAPS	Hs.584744	calcyphosine	Homo sapiens calcyphosine (CAPS), tra	447.17984	164.24948	454.80234	175.16560	Detected	Detected	Detected	Detected
4863	A_23_P210619	C20orf43	Hs.517134	chromosome 20 open reading frame 43	Homo sapiens chromosome 20 open rea	5528.72300	6321.11870	5458.29700	5394.70850	Detected	Detected	Detected	Detected
4864	A_33_P3417820	NOL12	Hs.632778	nucleolar protein 12	Homo sapiens nucleolar protein 12 (NO	4406.23730	6306.49400	3384.07420	4466.91460	Detected	Detected	Detected	Detected
4865	A_33_P3393311	MCHR1	Hs.248122	melanin-concentrating hormone recepto	Homo sapiens melanin-concentrating ho	39.34761	35.61929	29.88549	24.10449	Detected	Detected	Detected	Detected
4866	A_33_P3327245	SETDB2	Hs.631789	SET domain, bifurcated 2	Homo sapiens SET domain, bifurcated 2	350.25525	520.46790	339.68088	434.28027	Detected	Detected	Detected	Detected
4867	A_33_P3369266		Hs.682246		CR997556 RZPD no.9016 Homo sapiens	10.64243	9.91824	22.49788	14.76679	Compromised	Compromised	Detected	Compromised
4868	A_33_P3307073		Hs.578228		DA455367 CTONG2 Homo sapiens cDN	19.28507	28.28487	19.01172	18.54028	Detected	Detected	Detected	Detected
4869	A_24_P359117	GPATCH8	Hs.463129	G patch domain containing 8	Homo sapiens G patch domain containir	1263.67070	982.83435	739.85380	478.81137	Detected	Detected	Detected	Detected
4870	A_23_P502343	ADAM33	Hs.173716	ADAM metallopeptidase domain 33	Homo sapiens ADAM metallopeptidase	3425.10600	744.04626	3382.75460	541.68854	Detected	Detected	Detected	Detected
4871	A_23_P117546	SOS2	Hs.291533	son of sevenless homolog 2 (Drosophila	Homo sapiens son of sevenless homolog	741.64500	633.58276	486.53482	322.04822	Detected	Detected	Detected	Detected

4872	A_24_P69379	CASD1	Hs.260041	CAS1 domain containing 1	Homo sapiens CAS1 domain containing	234.01291	158.00260	229.30751	111.03010	Detected	Detected	Detected	Detected
4873	A_33_P3214690	NLGN1	Hs.478289	neuroligin 1	Homo sapiens neuroligin 1 (NLGN1), mR	20.49768	19.52077	10.14666	11.73548	Detected	Compromised	Compromised	Compromised
4874	A_24_P353619	ALPL	Hs.75431	alkaline phosphatase, liver/bone/kidney	Homo sapiens alkaline phosphatase, live	22.69063	37.91129	12.90409	22.35175	Detected	Detected	Compromised	Detected
4875	A_33_P3358740	OSBPL7	Hs.463320	oxysterol binding protein-like 7	Homo sapiens oxysterol binding protein-	2041.52950	869.38776	1520.55480	613.15094	Detected	Detected	Detected	Detected
4876	A_23_P151907	PCSK6	Hs.498494	proprotein convertase subtilisin/kexin t	Homo sapiens proprotein convertase su	18.13198	15.36286	15.80287	7.14207	Detected	Compromised	Compromised	Compromised
4877	A_24_P303454	TIAM2	Hs.586279	T-cell lymphoma invasion and metastas	Homo sapiens T-cell lymphoma invasior	1092.43250	1830.30180	733.11500	1107.15120	Detected	Detected	Detected	Detected
4878	A_23_P24709	OSBP	Hs.597091	oxysterol binding protein	Homo sapiens oxysterol binding protein	1890.16250	1639.85200	2056.24780	1590.50390	Detected	Detected	Detected	Detected
4879	A_33_P3254510				Putative uncharacterized protein ENSP	715.79430	984.16260	579.50010	774.17847	Detected	Detected	Detected	Detected
4880	A_23_P35316	ZNF695	Hs.669893	zinc finger protein 695	Homo sapiens zinc finger protein 695 (Z	26.60894	11.13454	18.38606	6.49864	Detected	Compromised	Detected	Compromised
4881	A_23_P11279	ALG13	Hs.443061	asparagine-linked glycosylation 13 hom	Homo sapiens asparagine-linked glycos	1308.89840	2238.24300	1227.71400	1842.79900	Detected	Detected	Detected	Detected
4882	A_33_P3455785	LOC401312	Hs.680441	hypothetical LOC401312	Homo sapiens cDNA clone IMAGE:5221	29.38309	25.65504	18.76679	18.24544	Detected	Detected	Detected	Detected
4883	A_23_P312150	EDN2	Hs.1407	endothelin 2	Homo sapiens endothelin 2 (EDN2), mR	21.60421	9.21082	12.45530	5.13787	Detected	Compromised	Compromised	Compromised
4884	A_23_P101671	ATCAY	Hs.418055	ataxia, cerebellar, Cayman type	Homo sapiens ataxia, cerebellar, Cayma	2.46193	37.86528	2.68531	3.42527	Compromised	Not Detected	Compromised	Compromised
4885	A_23_P123096	GNGT1	Hs.702084	guanine nucleotide binding protein (G pr	Homo sapiens guanine nucleotide bindir	18.43834	25.70851	6.22313	3.08832	Detected	Not Detected	Compromised	Compromised
4886	A_23_P119362	EMP3	Hs.9999	epithelial membrane protein 3	Homo sapiens epithelial membrane prot	6815.04100	18346.03100	10205.37400	25049.20700	Detected	Detected	Detected	Detected
4887	A_23_P26468	RHBDL1	Hs.137572	rhomboid, veinlet-like 1 (Drosophila)	Homo sapiens rhomboid, veinlet-like 1 (	37842.42600	49895.02700	21560.67000	17728.10200	Detected	Detected	Detected	Detected
4888	A_33_P3339825	C19orf63	Hs.515550	chromosome 19 open reading frame 63	Homo sapiens chromosome 19 open rea	127.78632	179.25285	167.12108	200.85779	Detected	Detected	Detected	Detected
4889	A_23_P71649	MUSK	Hs.521653	muscle, skeletal, receptor tyrosine kina	Homo sapiens muscle, skeletal, recepto	73.36720	86.00475	50.51124	59.64523	Detected	Detected	Detected	Detected
4890	A_32_P114574	CACYBP	Hs.508524	calcyclin binding protein	Homo sapiens calcyclin binding protein	5418.16750	11163.06400	3934.83180	6347.07000	Detected	Detected	Detected	Detected
4891	A_33_P3380502	GPR85	Hs.152009	G protein-coupled receptor 85	Homo sapiens G protein-coupled recep	2.54855	18.29177	2.94568	5.17329	Compromised	Detected	Compromised	Compromised
4892	A_33_P3265309	CEACAM4	Hs.12	carcinoembryonic antigen-related cell a	Homo sapiens carcinoembryonic antiger	29.97047	47.50848	16.34149	19.08776	Detected	Detected	Detected	Detected
4893	A_24_P134195	MYADM	Hs.380906	myeloid-associated differentiation mark	Homo sapiens myeloid-associated differ	649.05865	758.90360	598.63010	752.35270	Detected	Detected	Detected	Detected
4894	A_23_P46455	DNAJC8	Hs.433540	DnaJ (Hsp40) homolog, subfamily C, me	Homo sapiens DnaJ (Hsp40) homolog, s	1058.26160	1342.08890	969.09485	970.56744	Detected	Detected	Detected	Detected
4895	A_24_P849801	RPL22	Hs.515329	ribosomal protein L22	Homo sapiens ribosomal protein L22 (R	2896.40300	2962.30420	2539.58350	2426.42650	Detected	Detected	Detected	Detected
4896	A_24_P289383	CHD7	Hs.20395	chromodomain helicase DNA binding pr	Homo sapiens chromodomain helicase D	202.66945	106.89371	127.86469	47.08938	Detected	Detected	Detected	Detected
4897	A_23_P109907	ILDR1	Hs.98484	immunoglobulin-like domain containing	Homo sapiens immunoglobulin-like dom	91778.49000	75997.94000	15600.71100	11111.77900	Detected	Detected	Detected	Detected
4898	A_33_P3281333	SNTB2	Hs.461117	syntrophin, beta 2 (dystrophin-associat	Homo sapiens syntrophin, beta 2 (dystr	2727.68920	1997.91980	2224.00630	1327.26430	Detected	Detected	Detected	Detected
4899	A_33_P3348091	LTBP4	Hs.466766	latent transforming growth factor beta	Homo sapiens latent transforming growt	579.85077	764.27430	292.44476	281.00090	Detected	Detected	Detected	Detected
4900	A_23_P215669	POLR2J2	Hs.610255	polymerase (RNA) II (DNA directed) pol	Homo sapiens polymerase (RNA) II (DN	1237.66740	2088.79980	919.49805	1307.77230	Detected	Detected	Detected	Detected
4901	A_23_P9465	FPGS	Hs.335084	folylpolyglutamate synthase	Homo sapiens folylpolyglutamate syntha	3040.84840	3212.07350	2688.73100	2686.29540	Detected	Detected	Detected	Detected
4902	A_23_P65768	RSL24D1	Hs.274772	ribosomal L24 domain containing 1	Homo sapiens ribosomal L24 domain co	15356.90600	8564.67200	10925.86300	5246.89550	Detected	Detected	Detected	Detected
4903	A_33_P3229328	NBPF6	Hs.712226	neuroblastoma breakpoint family, memb	Homo sapiens neuroblastoma breakpoin	126.80471	154.17133	53.86775	48.57469	Detected	Detected	Detected	Detected
4904	A_24_P399942	ATP11C	Hs.88252	ATPase, class VI, type 11C	Homo sapiens ATPase, class VI, type 1	1141.16900	857.70746	799.50030	504.29020	Detected	Detected	Detected	Detected
4905	A_33_P3408137	LOC100130248	Hs.656333	hypothetical protein LOC100130248	Homo sapiens cDNA FLJ42222 fis, clon	16.73500	4.27684	8.58857	6.03917	Detected	Compromised	Compromised	Compromised
4906	A_33_P3413810	LOC100128155	Hs.678824	hypothetical protein LOC100128155	Homo sapiens cDNA FLJ26965 fis, clon	40.85985	49.92973	8.82337	11.42858	Detected	Detected	Compromised	Compromised
4907	A_33_P3336592	LOC283711	Hs.585100	hypothetical protein LOC283711	Homo sapiens cDNA FLJ45109 fis, clon	212.14960	38.71746	182.26791	23.71974	Detected	Detected	Detected	Detected
4908	A_33_P3280003	HERC2	Hs.434890	hect domain and RLD 2	Homo sapiens hect domain and RLD 2 (	42.99153	35.88403	14.17319	15.16178	Detected	Detected	Compromised	Compromised
4909	A_23_P2661	RAP1B	Hs.369920	RAP1B, member of RAS oncogene fami	Homo sapiens RAP1B, member of RAS	28784.30900	39344.70000	19844.04700	20545.22000	Detected	Detected	Detected	Detected
4910	A_23_P129786	SREBF1	Hs.592123	sterol regulatory element binding transc	Homo sapiens sterol regulatory element	26699.83400	15046.79600	21071.93600	10172.88900	Detected	Detected	Detected	Detected
4911	A_24_P665504	LOC100133331	Hs.649644	hypothetical LOC100133331	Homo sapiens hypothetical LOC100133	395.70060	298.88010	289.02084	198.52013	Detected	Detected	Detected	Detected
4912	A_23_P63798	KLF6	Hs.4055	Kruppel-like factor 6	Homo sapiens Kruppel-like factor 6 (KL	39329.15000	81547.52000	24657.90600	40467.89000	Detected	Detected	Detected	Detected
4913	A_23_P427703	MT1L	Hs.647358	metallothionein 1L (gene/pseudogene)	Homo sapiens metallothionein 1L (gene	215425.19000	315728.25000	166826.40000	187586.52000	Detected	Detected	Detected	Detected
4914	A_33_P3322874	LOC100128131	Hs.638438	hypothetical LOC100128131	PREDICTED: Homo sapiens hypothetica	19.10009	11.00650	25.92960	14.20215	Detected	Compromised	Detected	Compromised
4915	A_23_P114983	TRIM63	Hs.279709	tripartite motif-containing 63	Homo sapiens tripartite motif-containin	47.12495	182.49371	25.88433	128.28160	Detected	Detected	Detected	Detected
4916	A_33_P3375673		Hs.634966		tg48b03.x1 Soares_NFL_T_GBC_S1 Hom	58227.47700	59818.38700	42556.31600	37760.26600	Detected	Detected	Detected	Detected
4917	A_32_P8813	LOC283663	Hs.181297	hypothetical LOC283663	Homo sapiens hypothetical LOC283663	677.82940	432.65533	574.22296	334.17355	Detected	Detected	Detected	Detected
4918	A_33_P3419938	VHL	Hs.517792	von Hippel-Lindau tumor suppressor	Homo sapiens von Hippel-Lindau tumor	30678.75000	23164.53300	9296.97500	5460.45500	Detected	Detected	Detected	Detected
4919	A_23_P80694	ACTR8	Hs.412186	ARP8 actin-related protein 8 homolog (	Homo sapiens ARP8 actin-related prote	423.30887	344.22177	297.61792	213.42065	Detected	Detected	Detected	Detected
4920	A_33_P3281552	RPGR	Hs.61438	retinitis pigmentosa GTPase regulator	Homo sapiens retinitis pigmentosa GTP	32.21437	30.41781	14.74636	14.24778	Detected	Detected	Compromised	Compromised
4921	A_24_P71439	PUM2	Hs.467824	pumilio homolog 2 (Drosophila)	Homo sapiens pumilio homolog 2 (Droso	490.22708	286.23410	402.15198	209.41443	Detected	Detected	Detected	Detected
4922	A_33_P3412556					115.12538	183.93202	13.90517	22.89599	Detected	Detected	Compromised	Detected
4923	A_33_P3296169	CDCA7	Hs.470654	cell division cycle associated 7	Homo sapiens cell division cycle associ	265.19644	353.14575	155.85725	162.46570	Detected	Detected	Detected	Detected
4924	A_33_P3357082	METTL8	Hs.135146	methyltransferase like 8	Homo sapiens methyltransferase like 8	266.51978	392.15213	261.82452	334.39720	Detected	Detected	Detected	Detected
4925	A_23_P94857	MAN1B1	Hs.279881	mannosidase, alpha, class 1B, member 1	Homo sapiens mannosidase, alpha, clas	2963.65770	3347.26250	3106.57370	3590.50930	Detected	Detected	Detected	Detected
4926	A_33_P3245631	TTC39A	Hs.112949	tetratricopeptide repeat domain 39A	Homo sapiens tetratricopeptide repeat	14.85822	3.95722	19.78834	10.74111	Detected	Compromised	Detected	Compromised
4927	A_23_P353667	C19orf30	Hs.326728	chromosome 19 open reading frame 30	Homo sapiens chromosome 19 open rea	35.70073	47.58844	13.44074	9.75340	Detected	Detected	Compromised	Compromised
4928	A_33_P3398877					207.28950	259.50406	114.98547	88.74558	Detected	Detected	Detected	Detected
4929	A_33_P3293265					15.54563	20.24695	11.25091	8.65161	Detected	Detected	Compromised	Compromised
4930	A_33_P3247072	LOC100131098		hypothetical protein LOC100131098	PREDICTED: Homo sapiens hypothetica	167.46754	214.92460	114.64269	147.61662	Detected	Detected	Detected	Detected
4931	A_23_P86731	ZNF239	Hs.25040	zinc finger protein 239	Homo sapiens zinc finger protein 239 (Z	714.44470	777.64105	435.43182	452.44482	Detected	Detected	Detected	Detected
4932	A_23_P90484	SARS2	Hs.709416	seryl-tRNA synthetase 2, mitochondrial	Homo sapiens seryl-tRNA synthetase 2	403.97308	785.40830	448.76560	863.45935	Detected	Detected	Detected	Detected
4933	A_23_P303072	GRIA1	Hs.519693	glutamate receptor, ionotropic, AMPA 1	Homo sapiens glutamate receptor, ionot	3.36312	54.18872	2.92022	43.71250	Compromised	Detected	Compromised	Detected
4934	A_33_P3396434					250016.12000	301707.12000	364240.80000	336172.22000	Detected	Detected	Detected	Detected
4935	A_33_P3240532	RGL1	Hs.497148	ral guanine nucleotide dissociation stim	Homo sapiens ral guanine nucleotide dis	5280.38530	4688.96300	4443.66600	3518.06230	Detected	Detected	Detected	Detected
4936	A_33_P3262515	RASA3	Hs.593075	RAS p21 protein activator 3	Homo sapiens RAS p21 protein activato	20208.05500	16082.20800	19063.04100	13632.63200	Detected	Detected	Detected	Detected
4937	A_33_P3372397				CDNA FLJ26601 fis, clone LVR00207 [	24.28226	25.45065	8.35216	8.28802	Detected	Detected	Compromised	Compromised
4938	A_24_P184305	BBS1	Hs.502915	Bardet-Biedl syndrome 1	Homo sapiens Bardet-Biedl syndrome 1	240.09296	87.54874	168.84958	43.20460	Detected	Detected	Detected	Detected
4939	A_33_P3396591	FAM113A	Hs.29341	family with sequence similarity 113, me	Homo sapiens family with sequence sim	85.48875	83.91150	95.74873	52.80249	Detected	Detected	Detected	Detected
4940	A_23_P81717	FRMD1	Hs.266746	FERM domain containing 1	Homo sapiens FERM domain containing	154.45868	186.93544	69.48184	57.70215	Detected	Detected	Detected	Detected
4941	A_33_P3304678	RP5-1022P6.6	Hs.123609	hypothetical LOC149837	Homo sapiens hypothetical LOC149837	2.82983	29.57470	2.94014	8.22998	Compromised	Detected	Compromised	Compromised
4942	A_23_P58862	FAM135A	Hs.211700	family with sequence similarity 135, me	Homo sapiens family with sequence sim	47.05703	27.13497	36.56421	9.37284	Detected	Detected	Detected	Compromised
4943	A_33_P3238685	TET2	Hs.367639	tet oncogene family member 2	Homo sapiens tet oncogene family mem	213.31656	193.17886	153.49661	121.19629	Detected	Detected	Detected	Detected
4944	A_23_P394567	KIAA1467	Hs.132660	KIAA1467	Homo sapiens KIAA1467 (KIAA1467), m	82.85770	65.56017	69.87397	34.68608	Detected	Detected	Detected	Detected
4945	A_33_P3338853	LOC401233	Hs.582928	similar to HIV TAT specific factor 1; co	PREDICTED: Homo sapiens similar to H	40.62010	51.47076	20.67147	21.19159	Detected	Detected	Detected	Detected
4946	A_23_P120822	UPB1	Hs.474388	ureidopropionase, beta	Homo sapiens ureidopropionase, beta (U	43.50107	49.61540	18.45366	6.57452	Detected	Detected	Detected	Compromised

4947	A_33_P3426943	LOC151484	Hs.559309	hypothetical protein LOC151484	Homo sapiens hypothetical protein LOC	8.63074	29.61856	18.66737	16.50391	Compromised	Detected	Detected	Detected
4948	A_23_P111947	CDH17	Hs.591853	cadherin 17, LI cadherin (liver-intestine	Homo sapiens cadherin 17, LI cadherin	2.76736	19.01676	2.73337	3.14125	Compromised	Not Detected	Compromised	Compromised
4949	A_24_P90900	CTRL	Hs.654546	chymotrypsin-like	Homo sapiens chymotrypsin-like (CTRL	53.40529	65.74533	68.65685	66.62008	Detected	Detected	Detected	Detected
4950	A_33_P3396951	BNIP3L	Hs.131226	BCL2/adenovirus E1B 19kDa interactin	Homo sapiens BCL2/adenovirus E1B 19	1410.98060	823.53500	1359.38560	685.29974	Detected	Detected	Detected	Detected
4951	A_23_P123424	CHRNB3	Hs.654576	cholinergic receptor, nicotinic, beta 3	Homo sapiens cholinergic receptor, nico	103.14775	115.53944	23.01159	20.14128	Detected	Detected	Detected	Detected
4952	A_33_P3291796	CAMKV	Hs.145156	CaM kinase-like vesicle-associated	Homo sapiens CaM kinase-like vesicle-	25.37171	34.78996	11.77637	9.78383	Detected	Detected	Compromised	Compromised
4953	A_23_P347528	TFCP2	Hs.48849	transcription factor CP2	Homo sapiens transcription factor CP2	1164.65520	1475.28430	719.38324	712.01380	Detected	Detected	Detected	Detected
4954	A_23_P145397	CCNC	Hs.430646	cyclin C	Homo sapiens cyclin C (CCNC), transcr	1748.27700	1815.54400	1701.77700	1439.74460	Detected	Detected	Detected	Detected
4955	A_23_P408108	MTERF	Hs.532216	mitochondrial transcription termination	Homo sapiens mitochondrial transcriptio	496.56744	453.67123	265.20154	200.22102	Detected	Detected	Detected	Detected
4956	A_33_P3286372	C2orf48	Hs.676025	chromosome 2 open reading frame 48	Homo sapiens chromosome 2 open read	2.56037	66.59853	3.76110	62.23827	Compromised	Detected	Compromised	Detected
4957	A_32_P150391	C17orf100	Hs.262480	chromosome 17 open reading frame 100	Homo sapiens chromosome 17 open rea	304.92603	137.40367	273.53470	109.54278	Detected	Detected	Detected	Detected
4958	A_23_P357351	MPV17L	Hs.720673	MPV17 mitochondrial membrane protein	Homo sapiens MPV17 mitochondrial me	16.45169	28.54130	3.09284	6.60109	Detected	Detected	Compromised	Compromised
4959	A_33_P3347937	HSF1	Hs.530227	heat shock transcription factor 1	Homo sapiens heat shock transcription	26.97747	41.78755	28.18115	50.05398	Detected	Detected	Detected	Detected
4960	A_23_P215790	EGFR	Hs.488293	epidermal growth factor receptor (eryth	Homo sapiens epidermal growth factor	1418.99660	1567.06430	859.47314	808.94446	Detected	Detected	Detected	Detected
4961	A_23_P107684	ZNF324	Hs.515660	zinc finger protein 324	Homo sapiens zinc finger protein 324 (Z	918.96640	721.33940	1013.40550	717.73740	Detected	Detected	Detected	Detected
4962	A_23_P385771	PAOX	Hs.532469	polyamine oxidase (exo-N4-amino)	Homo sapiens polyamine oxidase (exo-N	3074.66020	1574.31770	3012.40230	1390.10220	Detected	Detected	Detected	Detected
4963	A_24_P161581	SHISA7	Hs.6664	shisa homolog 7 (Xenopus laevis)	Homo sapiens shisa homolog 7 (Xenopu	2598.57670	3733.99850	1058.94460	994.54600	Detected	Detected	Detected	Detected
4964	A_33_P3220192	C6orf41		chromosome 6 open reading frame 41	Homo sapiens chromosome 6 open read	27.61332	22.76671	15.81933	9.02185	Detected	Detected	Detected	Compromised
4965	A_23_P371254	TAS2R43	Hs.688195	taste receptor, type 2, member 43	Homo sapiens taste receptor, type 2, m	13.46229	12.26503	13.24256	9.02798	Detected	Compromised	Compromised	Compromised
4966	A_33_P3274001					482.83493	297.13257	94.02902	64.09547	Detected	Detected	Detected	Detected
4967	A_33_P3380529	PRTFDC1	Hs.405619	phosphoribosyl transferase domain cont	Homo sapiens phosphoribosyl transfera	237.89384	213.53221	205.76605	162.26895	Detected	Detected	Detected	Detected
4968	A_23_P200780	TGFBR3	Hs.482390	transforming growth factor, beta recept	Homo sapiens transforming growth fact	1267.44950	577.22150	1232.22560	485.14740	Detected	Detected	Detected	Detected
4969	A_33_P3214284					42.97879	57.91702	19.95411	26.62967	Detected	Detected	Detected	Detected
4970	A_33_P3298206	PPP4R4	Hs.259599	protein phosphatase 4, regulatory subur	Homo sapiens protein phosphatase 4, re	41.84698	9.89384	35.81535	3.17325	Detected	Compromised	Detected	Compromised
4971	A_23_P33364	SH3D19	Hs.720175	SH3 domain containing 19	Homo sapiens SH3 domain containing 1	2602.65770	2891.54220	2076.89840	1867.35230	Detected	Detected	Detected	Detected
4972	A_33_P3724750	LOC151878	Hs.680377	hypothetical protein LOC151878	Homo sapiens hypothetical protein LOC	33.98023	52.53129	24.72521	35.60107	Detected	Detected	Detected	Detected
4973	A_23_P107073	RPA1	Hs.461925	replication protein A1, 70kDa	Homo sapiens replication protein A1, 70	10540.53000	11147.29100	8482.63500	7782.01030	Detected	Detected	Detected	Detected
4974	A_33_P3244956	NRIP3	Hs.523467	nuclear receptor interacting protein 3	Homo sapiens nuclear receptor interact	243.32732	371.34695	163.67212	201.86328	Detected	Detected	Detected	Detected
4975	A_23_P219144	CLTA	Hs.522114	clathrin, light chain (Lca)	Homo sapiens clathrin, light chain (Lca)	31326.16400	56615.78000	22761.81600	35641.30500	Detected	Detected	Detected	Detected
4976	A_23_P89921	ZNF180	Hs.22305	zinc finger protein 180	Homo sapiens zinc finger protein 180 (Z	251.20047	288.10390	194.87520	178.88400	Detected	Detected	Detected	Detected
4977	A_24_P291978	ADCK2	Hs.534141	aarF domain containing kinase 2	Homo sapiens aarF domain containing k	211.50813	192.90788	196.97206	167.27940	Detected	Detected	Detected	Detected
4978	A_33_P3422213	NEK10	Hs.506115	NIMA (never in mitosis gene a)- related	Homo sapiens NIMA (never in mitosis g	22.79477	23.67672	6.72997	13.13697	Detected	Detected	Compromised	Compromised
4979	A_33_P3333708					1954.52190	2573.28120	2095.47700	2700.32300	Detected	Detected	Detected	Detected
4980	A_32_P213349	LOC100130897	Hs.666764	hypothetical LOC100130897	PREDICTED: Homo sapiens hypothetica	30.04039	37.13551	23.55560	19.90707	Detected	Detected	Detected	Detected
4981	A_23_P92499	TLR2	Hs.519033	toll-like receptor 2	Homo sapiens toll-like receptor 2 (TLR2	3031.82840	1062.31350	2123.45430	634.39325	Detected	Detected	Detected	Detected
4982	A_33_P3319581	FIGNL2	Hs.648218	fidgetin-like 2	Homo sapiens fidgetin-like 2 (FIGNL2),	1539.77300	2085.05440	1264.33500	1491.67440	Detected	Detected	Detected	Detected
4983	A_24_P414269	ALG3	Hs.478481	asparagine-linked glycosylation 3, alpha	Homo sapiens asparagine-linked glycosy	4393.43700	5342.65400	5466.29100	6092.38770	Detected	Detected	Detected	Detected
4984	A_33_P3264399					36.83421	26.94198	14.12249	9.27332	Detected	Detected	Compromised	Compromised
4985	A_24_P294703	PRRC1	Hs.483259	proline-rich coiled-coil 1	Homo sapiens proline-rich coiled-coil 1	1117.00110	1373.03700	1145.85490	1473.58540	Detected	Detected	Detected	Detected
4986	A_23_P202269	ANK3	Hs.499725	ankyrin 3, node of Ranvier (ankyrin G)	Homo sapiens ankyrin 3, node of Ranvie	60.79306	39.22450	33.28387	17.83108	Detected	Detected	Detected	Detected
4987	A_32_P9575	MRPL45	Hs.462913	mitochondrial ribosomal protein L45	Homo sapiens mitochondrial ribosomal p	665.59460	1782.52950	734.00040	1687.52660	Detected	Detected	Detected	Detected
4988	A_23_P49181	LRRC29	Hs.461000	leucine rich repeat containing 29	Homo sapiens leucine rich repeat conta	24.56881	17.65986	33.11531	26.06244	Detected	Compromised	Detected	Detected
4989	A_33_P3397579		Hs.434693		T1560 [Source:UniProtKB/TrEMBL;Ac	343.03128	272.73215	174.94632	119.77448	Detected	Detected	Detected	Detected
4990	A_23_P29735	BSN	Hs.194684	bassoon (presynaptic cytomatrix protei	Homo sapiens bassoon (presynaptic cyt	28.83126	21.78337	20.53968	16.38342	Detected	Detected	Detected	Compromised
4991	A_23_P70566	FKBPL	Hs.520042	FK506 binding protein like	Homo sapiens FK506 binding protein lik	240.52400	253.10054	220.63301	206.93546	Detected	Detected	Detected	Detected
4992	A_33_P3316983	NCRNA00081	Hs.232543	non-protein coding RNA 81	Homo sapiens non-protein coding RNA	1275.06210	1346.29440	1099.82400	989.49146	Detected	Detected	Detected	Detected
4993	A_23_P416608	LAMP2	Hs.496684	lysosomal-associated membrane protei	Homo sapiens lysosomal-associated me	4057.94650	4388.27640	2776.43950	2576.44700	Detected	Detected	Detected	Detected
4994	A_33_P3571901	DKFZP586K1520	Hs.516864	DKFZP586K1520 protein	Homo sapiens mRNA; cDNA DKFZp586	6149.94630	2602.38940	6742.88600	2603.93630	Detected	Detected	Detected	Detected
4995	A_33_P3218704		Hs.526500		Dynein heavy chain 3, axonemal (Axone	16.87268	3.84459	3.16312	3.27394	Detected	Compromised	Compromised	Compromised
4996	A_24_P15391	FOXI3	Hs.716722	forkhead box I3	Homo sapiens forkhead box I3 (FOXI3),	38.30276	43.14490	21.06836	14.51134	Detected	Detected	Detected	Compromised
4997	A_23_P107653	ETFB	Hs.348531	electron-transfer-flavoprotein, beta pol	Homo sapiens electron-transfer-flavopr	27485.69300	20765.29100	28718.45500	19851.98400	Detected	Detected	Detected	Detected
4998	A_33_P3408450	C14orf176	Hs.540107	chromosome 14 open reading frame 176	Homo sapiens chromosome 14 open rea	34.51291	25.11194	35.73367	24.02192	Detected	Detected	Detected	Detected
4999	A_23_P56356	PLB1	Hs.444933	phospholipase B1	Homo sapiens phospholipase B1 (PLB1)	77.66901	87.89800	45.28247	31.80753	Detected	Detected	Detected	Detected
5000	A_24_P416289	KIAA0195	Hs.514474	KIAA0195	Homo sapiens KIAA0195 (KIAA0195), m	428.97366	236.28577	368.72964	196.68054	Detected	Detected	Detected	Detected
5001	A_23_P47790	METTL1	Hs.42957	methyltransferase like 1	Homo sapiens methyltransferase like 1	911.14220	2300.69380	1117.72270	2555.30640	Detected	Detected	Detected	Detected
5002	A_23_P218751	GNB1L	Hs.105642	guanine nucleotide binding protein (G pr	Homo sapiens guanine nucleotide bindir	3083.46310	2589.71950	2977.46800	2380.20390	Detected	Detected	Detected	Detected
5003	A_24_P392958	TBC1D20	Hs.590876	TBC1 domain family, member 20	Homo sapiens TBC1 domain family, mer	910.49260	667.48883	887.78790	600.10400	Detected	Detected	Detected	Detected
5004	A_23_P201386	DDAH1	Hs.719968	dimethylarginine dimethylaminohydrolas	Homo sapiens dimethylarginine dimethy	7626.14400	16781.25600	6153.71200	11571.33100	Detected	Detected	Detected	Detected
5005	A_23_P87545	IFITM3	Hs.374650	interferon induced transmembrane prot	Homo sapiens interferon induced transr	54624.94500	20234.01400	64728.22000	21708.58800	Detected	Detected	Detected	Detected
5006	A_33_P3298473		Hs.547855		Putative lung carcinoma-associated pro	17.84077	37.97535	2.93890	3.07612	Detected	Detected	Compromised	Compromised
5007	A_33_P3268129		Hs.575858		Putative uncharacterized protein C1orf	82.94727	39.21912	63.63493	18.73886	Detected	Detected	Detected	Detected
5008	A_33_P3383184	SP9		Sp9 transcription factor homolog (mous	Sp9 transcription factor homolog [Sour	1256.24300	565.27045	1066.03760	396.33698	Detected	Detected	Detected	Detected
5009	A_23_P69310	CCRL2	Hs.535713	chemokine (C-C motif) receptor-like 2	Homo sapiens chemokine (C-C motif) r	35.68720	36.72383	35.79386	11.58241	Detected	Detected	Detected	Compromised
5010	A_33_P3294985	LOC554203	Hs.648316	alanyl-tRNA synthetase domain contain	Homo sapiens alanyl-tRNA synthetase	66.83788	174.39539	46.88966	99.00875	Detected	Detected	Detected	Detected
5011	A_23_P49060	SPINT1	Hs.233950	serine peptidase inhibitor, Kunitz type 1	Homo sapiens serine peptidase inhibitor	2.74321	19.61894	2.91923	4.09215	Compromised	Detected	Compromised	Compromised
5012	A_24_P16249	ZNF479	Hs.616660	zinc finger protein 479	Homo sapiens zinc finger protein 479 (Z	201.65749	121.85429	7.31414	3.04421	Detected	Detected	Compromised	Compromised
5013	A_23_P360797	NTF3	Hs.99171	neurotrophin 3	Homo sapiens neurotrophin 3 (NTF3), tr	31.01014	65.81131	18.65075	31.76139	Detected	Detected	Detected	Detected
5014	A_33_P3388397	GJB4	Hs.351203	gap junction protein, beta 4, 30.3kDa	Gap junction beta-4 protein (Connexin-	142.57292	176.98210	82.04473	72.60053	Detected	Detected	Detected	Detected
5015	A_23_P1043	C1orf106	Hs.518997	chromosome 1 open reading frame 106	Homo sapiens chromosome 1 open read	2.46684	4.04864	3.11176	61.19597	Compromised	Compromised	Compromised	Not Detected
5016	A_33_P3258013		Hs.374067		Ubiquitin-protein ligase E3B (EC 6.3.2.-	3590.30520	2956.21660	3689.66870	2517.22240	Detected	Detected	Detected	Detected
5017	A_23_P118607	AARSD1	Hs.317403	alanyl-tRNA synthetase domain contain	Homo sapiens alanyl-tRNA synthetase	1167.61900	2304.33940	1067.39360	1803.58220	Detected	Detected	Detected	Detected
5018	A_23_P64499	HCCA2	Hs.370360	HCCA2 protein	Homo sapiens HCCA2 protein (HCCA2)	3876.40260	3103.82300	3717.35990	2995.34350	Detected	Detected	Detected	Detected
5019	A_23_P29836	TMEM42	Hs.646856	transmembrane protein 42	Homo sapiens transmembrane protein 4	13661.94200	5728.52000	12193.45300	4467.91260	Detected	Detected	Detected	Detected
5020	A_33_P3375185	TCTEX1D4	Hs.585137	Tctex1 domain containing 4	Homo sapiens Tctex1 domain containin	383.92572	288.41070	233.78339	125.64628	Detected	Detected	Detected	Detected
5021	A_23_P112406	GTF3C5	Hs.495417	general transcription factor IIIC, polype	Homo sapiens general transcription fact	3609.12960	4965.34900	4930.28760	5982.05270	Detected	Detected	Detected	Detected

5022	A_33_P3334575					11926.73100	14811.95100	10473.64500	10406.03500	Detected	Detected	Detected	Detected
5023	A_23_P101623	ZNF667	Hs.676605	zinc finger protein 667	Homo sapiens zinc finger protein 667 (Z	156.87020	112.19800	53.37379	21.23962	Detected	Detected	Detected	Detected
5024	A_24_P203964	LOC653391	Hs.652536	glucuronidase, beta pseudogene	Homo sapiens glucuronidase, beta pseu	513.60880	411.30066	196.21727	92.01597	Detected	Detected	Detected	Detected
5025	A_23_P41948	CCDC99	Hs.368710	coiled-coil domain containing 99	Homo sapiens coiled-coil domain conta	1656.22790	4432.48830	1145.57760	2463.52300	Detected	Detected	Detected	Detected
5026	A_33_P3235562		Hs.432760		F-actin-capping protein subunit beta (C	24.00909	64.12550	23.93008	48.70724	Detected	Detected	Detected	Detected
5027	A_33_P3395636	LOC100130721	Hs.711443	similar to ribosomal protein L24-like	PREDICTED: Homo sapiens similar to ri	1500.44350	823.42610	283.53400	146.45688	Detected	Detected	Detected	Detected
5028	A_23_P137248	PRKY	Hs.632287	protein kinase, Y-linked	Homo sapiens protein kinase, Y-linked (	601.05920	124.56294	412.87408	71.10033	Detected	Detected	Detected	Detected
5029	A_32_P19806	DNAH11	Hs.655326	dynein, axonemal, heavy chain 11	Homo sapiens dynein, axonemal, heavy	133.03981	118.20471	125.34071	95.33714	Detected	Detected	Detected	Detected
5030	A_33_P3391796	NOG	Hs.248201	noggin	Homo sapiens noggin (NOG), mRNA [NM	306.61194	120.02342	232.86661	72.25366	Detected	Detected	Detected	Detected
5031	A_33_P3527721	LOC284219	Hs.657510	hypothetical protein LOC284219	Homo sapiens cDNA FLJ37117 fis, clon	1370.09680	622.46790	962.72327	360.99387	Detected	Detected	Detected	Detected
5032	A_24_P935682	UNQ6228	Hs.661972	hypothetical LOC100131541	Homo sapiens clone DNA166629 MRSS	74.27256	60.09716	39.07020	16.77013	Detected	Detected	Detected	Detected
5033	A_23_P77593	VAC14	Hs.445061	Vac14 homolog (S. cerevisiae)	Homo sapiens Vac14 homolog (S. cerev	347.03656	276.56247	441.65106	359.04050	Detected	Detected	Detected	Detected
5034	A_33_P3881812	IRGM	Hs.519680	immunity-related GTPase family, M	Homo sapiens immunity-related GTPas	239.72581	140.45160	197.61958	97.45438	Detected	Detected	Detected	Detected
5035	A_33_P3401826	CMPK2	Hs.7155	cytidine monophosphate (UMP-CMP) ki	Homo sapiens cytidine monophosphate	825.16974	290.70990	571.33026	184.58140	Detected	Detected	Detected	Detected
5036	A_23_P162300	IRAK3	Hs.369265	interleukin-1 receptor-associated kinas	Homo sapiens interleukin-1 receptor-as	573.99450	275.87173	467.30026	213.84021	Detected	Detected	Detected	Detected
5037	A_24_P49199	GLDN	Hs.526441	gliomedin	Homo sapiens gliomedin (GLDN), mRNA	2829.93600	551.73000	1863.59850	315.72330	Detected	Detected	Detected	Detected
5038	A_33_P3404257	LOC729885	Hs.685640	similar to pheromone receptor	PREDICTED: Homo sapiens similar to p	58.53431	5.69532	54.92576	5.33778	Detected	Compromised	Detected	Compromised
5039	A_24_P337334	DCTD	Hs.183850	dCMP deaminase	Homo sapiens dCMP deaminase (DCTD	4680.84570	3706.20800	4012.44900	2968.28470	Detected	Detected	Detected	Detected
5040	A_33_P3370094	MME	Hs.307734	membrane metallo-endopeptidase	Homo sapiens membrane metallo-endop	7869.74170	1397.77590	6427.11570	1060.21730	Detected	Detected	Detected	Detected
5041	A_32_P69849	BEX5	Hs.47209	brain expressed, X-linked 5	Homo sapiens brain expressed, X-linked	500.22473	24.78589	406.50775	18.12798	Detected	Detected	Detected	Detected
5042	A_33_P3345936	CCDC9	Hs.227782	coiled-coil domain containing 9	Homo sapiens coiled-coil domain conta	58668.54700	35589.77000	14873.05700	6259.69000	Detected	Detected	Detected	Detected
5043	A_24_P181295	C14orf37	Hs.535420	chromosome 14 open reading frame 37	Homo sapiens chromosome 14 open rea	62.33652	360.57507	47.69210	273.30246	Detected	Detected	Detected	Detected
5044	A_23_P212508	TF	Hs.518267	transferrin	Homo sapiens transferrin (TF), mRNA [	36.56189	3.57096	17.42358	3.06163	Detected	Compromised	Detected	Compromised
5045	A_33_P3666346	AHCTF1	Hs.300887	AT hook containing transcription factor	Homo sapiens AT hook containing trans	34.75244	52.15048	27.25175	34.40731	Detected	Detected	Detected	Detected
5046	A_33_P3353502	PLCB4	Hs.472101	phospholipase C, beta 4	Homo sapiens phospholipase C, beta 4	98.23375	200.93408	66.35543	128.04353	Detected	Detected	Detected	Detected
5047	A_23_P70719	LAMA2	Hs.200841	laminin, alpha 2	Homo sapiens laminin, alpha 2 (LAMA2)	1168.06070	2194.87080	909.87530	1524.15040	Detected	Detected	Detected	Detected
5048	A_33_P3369505	MANEAL	Hs.534562	mannosidase, endo-alpha-like	Homo sapiens mannosidase, endo-alpha	345.29907	238.84146	161.11382	106.68349	Detected	Detected	Detected	Detected
5049	A_33_P3422298		Hs.547600		Putative uncharacterized protein ENSP	75.83568	30.93161	49.64283	19.68213	Detected	Detected	Detected	Detected
5050	A_23_P338952	S100PBP	Hs.440880	S100P binding protein	Homo sapiens S100P binding protein (S	2314.40400	1609.17910	1875.60130	1241.40950	Detected	Detected	Detected	Detected
5051	A_23_P78170	MYBBP1A	Hs.22824	MYB binding protein (P160) 1a	Homo sapiens MYB binding protein (P16	1344.80290	2033.30070	1434.31100	2192.28830	Detected	Detected	Detected	Detected
5052	A_24_P127192	LOC646936	Hs.646817	similar to C-terminal-binding protein 2	PREDICTED: Homo sapiens similar to C	344.78336	413.40826	121.62772	120.10133	Detected	Detected	Detected	Detected
5053	A_33_P3268124	ZNF654	Hs.591650	zinc finger protein 654	Homo sapiens zinc finger protein 654 (Z	735.39624	557.41504	507.84387	288.08774	Detected	Detected	Detected	Detected
5054	A_23_P88643	OR4N4	Hs.719028	olfactory receptor, family 4, subfamily N	Homo sapiens olfactory receptor, family	54.95746	85.87885	20.40236	23.89045	Detected	Detected	Detected	Detected
5055	A_23_P138125	FAIM3	Hs.58831	Fas apoptotic inhibitory molecule 3	Homo sapiens Fas apoptotic inhibitory r	308.68770	379.90860	256.60434	154.70898	Detected	Detected	Detected	Detected
5056	A_23_P36928	POLR1D	Hs.711105	polymerase (RNA) I polypeptide D, 16kD	Homo sapiens polymerase (RNA) I polyp	2566.08400	3750.63720	2818.70800	3492.77470	Detected	Detected	Detected	Detected
5057	A_24_P50091	C15orf51	Hs.585319	dynamin 1 pseudogene	Homo sapiens chromosome 15 open rea	31.72077	23.94946	18.69385	8.01825	Detected	Detected	Detected	Compromised
5058	A_23_P151805	FBLN5	Hs.719986	fibulin 5	Homo sapiens fibulin 5 (FBLN5), mRNA	13289.91100	49615.20000	11493.29200	35871.78000	Detected	Detected	Detected	Detected
5059	A_23_P349966	TMEM130	Hs.270753	transmembrane protein 130	Homo sapiens transmembrane protein 1	98.66630	107.85889	80.59113	62.06765	Detected	Detected	Detected	Detected
5060	A_24_P331704	KRT80	Hs.140978	keratin 80	Homo sapiens keratin 80 (KRT80), trans	41.88565	36.73359	33.41480	8.30090	Detected	Detected	Detected	Compromised
5061	A_32_P73452	ANO8	Hs.590990	anoctamin 8	Homo sapiens anoctamin 8 (ANO8), mR	595.59674	276.43344	133.82800	48.66736	Detected	Detected	Detected	Detected
5062	A_33_P3386955	MRPS5	Hs.655259	mitochondrial ribosomal protein S5	Homo sapiens mitochondrial ribosomal p	29125.88500	36479.19000	20229.84000	21532.68200	Detected	Detected	Detected	Detected
5063	A_33_P3312564					21213.92000	15053.38200	4841.28960	2365.62840	Detected	Detected	Detected	Detected
5064	A_23_P146637	SIGMAR1	Hs.522087	sigma non-opioid intracellular receptor	Homo sapiens sigma non-opioid intrace	3960.10640	6314.10700	3959.09350	5598.26000	Detected	Detected	Detected	Detected
5065	A_23_P134542	FOXP2	Hs.656280	forkhead box P2	Homo sapiens forkhead box P2 (FOXP2	34.95438	10.39367	19.81325	3.74891	Detected	Compromised	Detected	Compromised
5066	A_23_P103070	YWHAH	Hs.226755	tyrosine 3-monooxygenase/tryptophan	Homo sapiens tyrosine 3-monooxygena	2437.30960	5595.00800	1686.43150	3207.72560	Detected	Detected	Detected	Detected
5067	A_24_P32520	RNF214	Hs.591934	ring finger protein 214	Homo sapiens ring finger protein 214 (R	621.45276	285.67230	294.99738	110.63473	Detected	Detected	Detected	Detected
5068	A_33_P3337485	CD248	Hs.195727	CD248 molecule, endosialin	Homo sapiens CD248 molecule, endosia	11370.38100	8967.82700	10807.22000	7869.27340	Detected	Detected	Detected	Detected
5069	A_33_P3278560	ZIK1	Hs.454830	zinc finger protein interacting with K pr	Homo sapiens zinc finger protein intera	383.54040	416.07117	223.13316	242.66055	Detected	Detected	Detected	Detected
5070	A_23_P74843	IARS2	Hs.262823	isoleucyl-tRNA synthetase 2, mitochon	Homo sapiens isoleucyl-tRNA syntheta	10194.81400	8489.01300	9374.78300	6416.57960	Detected	Detected	Detected	Detected
5071	A_24_P385280	ALDH9A1	Hs.2533	aldehyde dehydrogenase 9 family, memb	Homo sapiens aldehyde dehydrogenase	2141.08030	3255.56600	908.78546	1218.57590	Detected	Detected	Detected	Detected
5072	A_33_P3334923	LOC338758		hypothetical LOC338758	Homo sapiens hypothetical LOC338758	78.34594	21.80352	63.77973	18.58729	Detected	Detected	Detected	Detected
5073	A_24_P228875	ARHGEF6	Hs.522795	Rac/Cdc42 guanine nucleotide exchang	Homo sapiens Rac/Cdc42 guanine nucl	195.52030	121.51288	122.24757	63.69351	Detected	Detected	Detected	Detected
5074	A_23_P139260	SLC22A18	Hs.50868	solute carrier family 22, member 18	Homo sapiens solute carrier family 22, r	8157.17330	1835.17080	8984.94600	1459.10610	Detected	Detected	Detected	Detected
5075	A_33_P3332547	SCHIP1	Hs.134665	schwannomin interacting protein 1	Homo sapiens schwannomin interacting	174.20377	373.82390	177.53787	303.44028	Detected	Detected	Detected	Detected
5076	A_24_P16214					464.31473	504.99136	277.33250	239.27975	Detected	Detected	Detected	Detected
5077	A_33_P3279831	PNRC1	Hs.75969	proline-rich nuclear receptor coactivato	Homo sapiens proline-rich nuclear rece	4833.97270	1574.92870	4292.03120	1066.88490	Detected	Detected	Detected	Detected
5078	A_33_P3230738				Tetratricopeptide repeat protein 19 (TP	343505.72000	333431.44000	208680.83000	176153.03000	Detected	Detected	Detected	Detected
5079	A_24_P85200	C14orf43	Hs.594157	chromosome 14 open reading frame 43	Homo sapiens chromosome 14 open rea	693.50946	589.27450	504.94930	374.43353	Detected	Detected	Detected	Detected
5080	A_32_P310503	OR2M2	Hs.534728	olfactory receptor, family 2, subfamily M	Homo sapiens olfactory receptor, family	14.94847	29.72208	3.02851	7.80161	Detected	Detected	Compromised	Compromised
5081	A_33_P3407105	YTHDF1	Hs.11747	YTH domain family, member 1	Homo sapiens YTH domain family, mem	1159.55430	1342.34440	296.36282	290.70914	Detected	Detected	Detected	Detected
5082	A_33_P3235098	FAM22D	Hs.701044	family with sequence similarity 22, mem	Homo sapiens family with sequence sim	161.52900	212.78287	92.62523	105.74659	Detected	Detected	Detected	Detected
5083	A_23_P103131	SMCR7L	Hs.148677	Smith-Magenis syndrome chromosome	Homo sapiens Smith-Magenis syndrome	7327.67100	7484.19200	4121.71040	3591.20000	Detected	Detected	Detected	Detected
5084	A_33_P3520693		Hs.632559		LOC646903 protein Fragment [Source:	116.73042	114.75741	75.21176	56.59399	Detected	Detected	Detected	Detected
5085	A_24_P16559	FOXP2	Hs.656280	forkhead box P2	Homo sapiens forkhead box P2 (FOXP2	18.74792	3.81385	8.85488	3.26559	Detected	Compromised	Compromised	Compromised
5086	A_23_P69468	NDUFB4	Hs.304613	NADH dehydrogenase (ubiquinone) 1 be	Homo sapiens NADH dehydrogenase (u	30373.30500	25005.21700	22355.65000	16339.31000	Detected	Detected	Detected	Detected
5087	A_24_P827	SUPT3H	Hs.368325	suppressor of Ty 3 homolog (S. cerevisi	Homo sapiens suppressor of Ty 3 homo	43.96681	85.74927	19.85378	50.06265	Detected	Detected	Detected	Detected
5088	A_23_P93046	EXOC3	Hs.481464	exocyst complex component 3	Homo sapiens exocyst complex compor	437.09640	317.92800	227.47266	167.34897	Detected	Detected	Detected	Detected
5089	A_23_P101564	FGF21	Hs.283015	fibroblast growth factor 21	Homo sapiens fibroblast growth factor 2	15.88445	12.61913	8.80544	11.03211	Detected	Compromised	Compromised	Compromised
5090	A_33_P3306843	ZNF836	Hs.631584	zinc finger protein 836	Homo sapiens zinc finger protein 836 (Z	38.65924	30.38854	32.32166	18.05452	Detected	Detected	Detected	Detected
5091	A_33_P3263994					6.42548	9.59848	17.05752	11.09258	Compromised	Compromised	Detected	Compromised
5092	A_32_P78491	ETV1	Hs.22634	ets variant 1	Homo sapiens ets variant 1 (ETV1), tra	1174.53590	335.42465	818.79990	193.00845	Detected	Detected	Detected	Detected
5093	A_23_P215505	RAPGEF5	Hs.174768	Rap guanine nucleotide exchange factor	Homo sapiens Rap guanine nucleotide e	30.84725	15.90043	12.38912	3.94355	Detected	Compromised	Compromised	Compromised
5094	A_33_P3342081	PRDM1	Hs.436023	PR domain containing 1, with ZNF doma	Homo sapiens PR domain containing 1,	7129.81640	4964.54150	4413.17770	2746.75500	Detected	Detected	Detected	Detected
5095	A_33_P3290622	PDZD11	Hs.11042	PDZ domain containing 11	Homo sapiens PDZ domain containing 1	637.31410	1200.71910	512.23460	820.09030	Detected	Detected	Detected	Detected
5096	A_33_P3303355					647.15985	792.87573	331.45980	314.59250	Detected	Detected	Detected	Detected

5097	A_33_P3374338	LOC100292758	Hs.414183	hypothetical protein LOC100292758	PREDICTED: Homo sapiens hypothetica	11.39641	7.39258	15.88357	4.38671	Compromised	Compromised	Detected	Compromised
5098	A_23_P123402	TRIM55	Hs.85524	tripartite motif-containing 55	Homo sapiens tripartite motif-containin	2.81697	3.53010	18.90220	20.74781	Compromised	Compromised	Not Detected	Detected
5099	A_33_P3256680	MFHAS1	Hs.379414	malignant fibrous histiocytoma amplified	Homo sapiens malignant fibrous histiocy	164.68953	150.31691	135.28731	104.29340	Detected	Detected	Detected	Detected
5100	A_33_P3333642		Hs.533590		Putative uncharacterized protein ENSP	3546.72920	3324.54740	1805.57910	1393.18950	Detected	Detected	Detected	Detected
5101	A_33_P3283237	YY2	Hs.673601	YY2 transcription factor	Homo sapiens YY2 transcription factor	2830.02000	2254.20040	78.11636	50.12974	Detected	Detected	Detected	Detected
5102	A_23_P204847	LCP1	Hs.381099	lymphocyte cytosolic protein 1 (L-plast	Homo sapiens lymphocyte cytosolic pro	2142.38280	92.58511	1806.19030	60.63090	Detected	Detected	Detected	Detected
5103	A_32_P131050	ZNF148	Hs.592591	zinc finger protein 148	Homo sapiens zinc finger protein 148 (Z	711.98510	246.83333	613.58154	187.49352	Detected	Detected	Detected	Detected
5104	A_24_P362572	BZW1	Hs.355983	basic leucine zipper and W2 domains 1	Homo sapiens basic leucine zipper and	13008.83300	13294.66500	11341.97500	10148.70200	Detected	Detected	Detected	Detected
5105	A_23_P357316	UPP2	Hs.128427	uridine phosphorylase 2	Homo sapiens uridine phosphorylase 2 (	7.80181	21.87900	7.28632	7.34486	Compromised	Detected	Compromised	Compromised
5106	A_33_P3305250				26S proteasome non-ATPase regulator	3.62711	41.73700	2.91894	15.48627	Compromised	Detected	Compromised	Detected
5107	A_33_P3461039	LOC728705	Hs.721313	hypothetical protein LOC728705	Homo sapiens cDNA clone IMAGE:4821	232.26527	238.50856	207.36310	195.70657	Detected	Detected	Detected	Detected
5108	A_33_P3232544					475.95047	594.08484	430.70350	556.12506	Detected	Detected	Detected	Detected
5109	A_24_P781757	LOC148413	Hs.702346	hypothetical LOC148413	Homo sapiens hypothetical LOC148413	898.78630	590.06573	1112.43030	705.88870	Detected	Detected	Detected	Detected
5110	A_33_P3386414	DCAF4L2	Hs.371738	DDB1 and CUL4 associated factor 4-lik	Homo sapiens DDB1 and CUL4 associa	8.81621	26.96727	10.21778	7.80952	Compromised	Detected	Compromised	Compromised
5111	A_23_P88893	DEF8	Hs.62771	differentially expressed in FDCP 8 hom	Homo sapiens differentially expressed i	22075.35500	27901.55300	21822.10500	23989.91400	Detected	Detected	Detected	Detected
5112	A_23_P37702	TPSAB1	Hs.405479	tryptase alpha/beta 1	Homo sapiens tryptase alpha/beta 1 (T	5.43328	17.97537	2.89450	3.01986	Compromised	Detected	Compromised	Compromised
5113	A_33_P3229527	LOC100134387		similar to DEAH (Asp-Glu-Ala-His) box	PREDICTED: Homo sapiens similar to D	265.18997	148.51180	229.39333	95.61038	Detected	Detected	Detected	Detected
5114	A_24_P174775	NNT		nicotinamide nucleotide transhydrogena	NAD(P) transhydrogenase, mitochondri	31.88774	33.24893	9.82550	5.55617	Detected	Detected	Compromised	Compromised
5115	A_24_P409402					10592.78500	8918.29900	1095.45960	599.00616	Detected	Detected	Detected	Detected
5116	A_24_P289636		Hs.568066		Homo sapiens cDNA FLJ43380 fis, clon	1514.98140	1227.80160	649.98004	387.22280	Detected	Detected	Detected	Detected
5117	A_24_P168925	CHRDL1	Hs.496587	chordin-like 1	Homo sapiens chordin-like 1 (CHRDL1)	18.17714	3.49960	12.29744	3.01156	Detected	Compromised	Compromised	Compromised
5118	A_32_P150142	DKFZp434L192	Hs.568108	hypothetical protein DKFZp434L192	Homo sapiens hypothetical protein DKF	14.44668	15.39076	15.04854	7.00347	Detected	Compromised	Detected	Compromised
5119	A_24_P333326	CTAGE5	Hs.540038	CTAGE family, member 5	Homo sapiens CTAGE family, member 5	19.69100	18.33876	22.50626	10.85294	Detected	Detected	Detected	Compromised
5120	A_32_P171061	ASCL2	Hs.152475	achaete-scute complex homolog 2 (Dro	Homo sapiens achaete-scute complex	12.75135	23.35045	2.89812	8.90905	Compromised	Detected	Compromised	Compromised
5121	A_24_P332623	RP11-159J2.1	Hs.567920	CTAGE family, member 5 pseudogene	Homo sapiens CTAGE family, member 5	356.37186	343.73315	425.58880	356.64430	Detected	Detected	Detected	Detected
5122	A_23_P158041	AQP7	Hs.455323	aquaporin 7	Homo sapiens aquaporin 7 (AQP7), mR	20.26492	20.53089	13.91236	8.72563	Detected	Detected	Compromised	Compromised
5123	A_23_P417113				RNA-binding protein 33 (RNA-binding	128.72472	772.42560	100.11581	443.26965	Detected	Detected	Detected	Detected
5124	A_33_P3277890		Hs.498519		Putative uncharacterized protein ENSP	48.19155	29.70886	27.87934	24.97707	Detected	Detected	Detected	Detected
5125	A_33_P3251332	HEATR5A	Hs.370299	HEAT repeat containing 5A	Homo sapiens HEAT repeat containing	465.10144	281.60318	296.21204	161.78447	Detected	Detected	Detected	Detected
5126	A_23_P378526	RTEL1	Hs.434878	regulator of telomere elongation helicas	Homo sapiens regulator of telomere elo	990.81683	1004.77400	1164.66280	1043.59940	Detected	Detected	Detected	Detected
5127	A_33_P3305775					45.03801	58.29038	29.72970	36.35414	Detected	Detected	Detected	Detected
5128	A_32_P210202	E2F7	Hs.416375	E2F transcription factor 7	Homo sapiens E2F transcription factor	412.02400	5993.82800	307.05228	3496.70870	Detected	Detected	Detected	Detected
5129	A_33_P3291594		Hs.709187		Lipoamide acyltransferase component	26.10882	36.65236	16.05820	19.36006	Detected	Detected	Detected	Detected
5130	A_33_P3270623	FAM181B	Hs.448218	family with sequence similarity 181, me	Homo sapiens family with sequence sim	16.17003	20.20001	15.35311	16.28932	Detected	Detected	Detected	Detected
5131	A_23_P366125	EME2	Hs.7247	essential meiotic endonuclease 1 homo	Probable crossover junction endonuclea	98.71200	84.05460	81.55718	74.04900	Detected	Detected	Detected	Detected
5132	A_33_P3274422	LOC402679		similar to Serine/threonine-protein kina	PREDICTED: Homo sapiens similar to S	24.07808	30.17051	17.46489	17.20066	Detected	Detected	Detected	Detected
5133	A_23_P42257	IER3	Hs.591785	immediate early response 3	Homo sapiens immediate early response	67134.43000	19357.94000	53813.27300	14346.06700	Detected	Detected	Detected	Detected
5134	A_23_P64828	OAS1	Hs.524760	2',5'-oligoadenylate synthetase 1, 40/46	Homo sapiens 2',5'-oligoadenylate synth	18.03274	3.81481	8.80052	3.26537	Detected	Compromised	Compromised	Compromised
5135	A_24_P29733	PFTK1	Hs.430742	PFTAIRE protein kinase 1	Homo sapiens PFTAIRE protein kinase	613.25880	1026.76070	489.48360	707.90220	Detected	Detected	Detected	Detected
5136	A_33_P3318766					23.63883	20.73374	11.82860	7.47757	Detected	Detected	Compromised	Compromised
5137	A_23_P206733	CES1	Hs.558865	carboxylesterase 1 (monocyte/macroph	Homo sapiens carboxylesterase 1 (mon	3190.61300	480.54108	3727.48050	406.42984	Detected	Detected	Detected	Detected
5138	A_33_P3309034	CDC10L	Hs.568390	CDC10 cell division cycle 10 homolog (	Homo sapiens CDC10 cell division cycle	256.38614	230.20937	89.82842	62.81056	Detected	Detected	Detected	Detected
5139	A_24_P225468	ANP32E	Hs.656466	acidic (leucine-rich) nuclear phosphopr	Homo sapiens acidic (leucine-rich) nucl	866.63640	3032.59400	541.87620	1568.29440	Detected	Detected	Detected	Detected
5140	A_23_P50241	CLPTM1	Hs.444441	cleft lip and palate associated transmer	Homo sapiens cleft lip and palate assoc	525.89484	574.23096	527.08890	607.48865	Detected	Detected	Detected	Detected
5141	A_23_P121374	SEMA5B	Hs.210870	sema domain, seven thrombospondin re	Homo sapiens sema domain, seven thro	27.78108	48.67250	6.09376	7.10086	Detected	Detected	Compromised	Compromised
5142	A_33_P3232011	RAB17	Hs.44278	RAB17, member RAS oncogene family	Homo sapiens RAB17, member RAS on	109.76988	81.97602	73.13536	59.01947	Detected	Detected	Detected	Detected
5143	A_33_P3231407		Hs.710248		Pyrin and HIN domain-containing protei	51.24419	56.45499	41.54337	39.69608	Detected	Detected	Detected	Detected
5144	A_33_P3229380	LOC731275	Hs.655981	hypothetical LOC731275	Homo sapiens hypothetical LOC731275	219.00687	216.72342	155.56549	133.32906	Detected	Detected	Detected	Detected
5145	A_23_P149529	TACSTD2	Hs.23582	tumor-associated calcium signal transd	Homo sapiens tumor-associated calciu	615.56080	128.47751	409.90692	75.57325	Detected	Detected	Detected	Detected
5146	A_32_P85591	H2BFXP	Hs.590799	H2B histone family, member X, pseudog	Homo sapiens H2B histone family, mem	114.21988	62.12004	93.00495	47.65176	Detected	Detected	Detected	Detected
5147	A_23_P119196	KLF2	Hs.107740	Kruppel-like factor 2 (lung)	Homo sapiens Kruppel-like factor 2 (lu	6568.12060	1902.35140	5248.78960	1335.64230	Detected	Detected	Detected	Detected
5148	A_23_P167308	RNF4	Hs.66394	ring finger protein 4	Homo sapiens ring finger protein 4 (RN	10109.53200	15570.48300	5806.07470	7782.81300	Detected	Detected	Detected	Detected
5149	A_33_P3854953	LOC440117	Hs.127100	hypothetical gene supported by BC037	Homo sapiens hypothetical gene suppor	91.32793	6.06833	26.25056	9.41983	Detected	Compromised	Detected	Compromised
5150	A_24_P295245	ASPH	Hs.591874	aspartate beta-hydroxylase	Homo sapiens aspartate beta-hydroxyla	4673.23100	5825.19240	3891.50420	4302.53860	Detected	Detected	Detected	Detected
5151	A_23_P136909	HMGN5	Hs.282204	high-mobility group nucleosome binding	Homo sapiens high-mobility group nucle	521.52590	241.71542	423.56525	166.20770	Detected	Detected	Detected	Detected
5152	A_33_P3318966	C12orf72	Hs.585084	chromosome 12 open reading frame 72	Homo sapiens chromosome 12 open rea	115.25025	114.32318	83.90834	72.03936	Detected	Detected	Detected	Detected
5153	A_23_P379020	GNRHR2		gonadotropin-releasing hormone (type 2	Homo sapiens gonadotropin-releasing h	17.56091	21.44272	24.52408	18.92038	Detected	Detected	Detected	Detected
5154	A_32_P108474	ABCE1	Hs.12013	ATP-binding cassette, sub-family E (O	Homo sapiens ATP-binding cassette, su	814.75330	730.88290	415.50497	292.87503	Detected	Detected	Detected	Detected
5155	A_33_P3560878	LOC283587	Hs.525513	hypothetical protein LOC283587	Homo sapiens cDNA clone IMAGE:4734	106.59161	29.83018	51.57369	3.05249	Detected	Detected	Detected	Compromised
5156	A_33_P3297883				LP2570 [Source:UniProtKB/TrEMBL;A	14.73939	37.17885	7.16560	5.27109	Detected	Detected	Compromised	Compromised
5157	A_33_P3372526				Putative malate dehydrogenase 1B (EC	720.69410	605.82200	303.75256	172.28836	Detected	Detected	Detected	Detected
5158	A_33_P3236316	LOC729218	Hs.646112	hypothetical LOC729218	PREDICTED: Homo sapiens hypothetica	371.08832	420.46704	120.84358	98.87719	Detected	Detected	Detected	Detected
5159	A_33_P3398922	MBD1	Hs.405610	methyl-CpG binding domain protein 1	Homo sapiens methyl-CpG binding dom	7958.07230	6479.38500	5313.44900	3889.51100	Detected	Detected	Detected	Detected
5160	A_33_P3404749	FMN1	Hs.657649	formin 1	Homo sapiens formin 1 (FMN1), mRNA	119.75382	151.43674	65.97489	58.97061	Detected	Detected	Detected	Detected
5161	A_33_P3258612	PCNA	Hs.147433	proliferating cell nuclear antigen	Homo sapiens proliferating cell nuclear	3013.90010	11773.21000	2210.71340	7265.10000	Detected	Detected	Detected	Detected
5162	A_23_P319719	PGAM5	Hs.102558	phosphoglycerate mutase family membe	Homo sapiens phosphoglycerate mutas	160.68784	443.35846	205.26537	570.38275	Detected	Detected	Detected	Detected
5163	A_23_P164011	SOX15	Hs.95582	SRY (sex determining region Y)-box 15	Homo sapiens SRY (sex determining re	22.50150	19.71123	2.88492	3.00808	Detected	Detected	Compromised	Compromised
5164	A_24_P251661	KCNMB3	Hs.591285	potassium large conductance calcium-a	Homo sapiens potassium large conduct	58.10039	72.37022	50.63598	53.77029	Detected	Detected	Detected	Detected
5165	A_23_P115762	ECD	Hs.631822	ecdysoneless homolog (Drosophila)	Homo sapiens ecdysoneless homolog (D	3319.90920	5429.90970	2658.19680	3520.94480	Detected	Detected	Detected	Detected
5166	A_24_P9090	HNRPDL	Hs.527105	heterogeneous nuclear ribonucleoprote	Homo sapiens heterogeneous nuclear ri	3392.65500	5904.58640	1691.68480	2400.84770	Detected	Detected	Detected	Detected
5167	A_23_P339773	TPRG1L	Hs.20529	tumor protein p63 regulated 1-like	Homo sapiens tumor protein p63 regula	2457.28880	3289.55500	2493.66480	2375.33600	Detected	Detected	Detected	Detected
5168	A_33_P3299119	HNF1A	Hs.654455	HNF1 homeobox A	Homo sapiens HNF1 homeobox A (HNF	18.47665	14.10118	11.20857	11.84060	Detected	Compromised	Compromised	Compromised
5169	A_23_P39602	NCOA1	Hs.596314	nuclear receptor coactivator 1	Homo sapiens nuclear receptor coactiv	1028.27330	662.72906	1016.14240	584.49340	Detected	Detected	Detected	Detected
5170	A_23_P29555	SEC13	Hs.166924	SEC13 homolog (S. cerevisiae)	Homo sapiens SEC13 homolog (S. cere	57397.55000	119134.54000	38548.10000	63294.68400	Detected	Detected	Detected	Detected
5171	A_33_P3210561	LOC391359		similar to hCG1784039	PREDICTED: Homo sapiens similar to h	8469.39550	16457.47500	7195.47300	12367.27600	Detected	Detected	Detected	Detected

5172	A_23_P254181	MGC5566	Hs.255479	hypothetical protein MGC5566	Homo sapiens hypothetical protein MG(	188.44904	24.47981	212.33977	21.52325	Detected	Detected	Detected	Detected
5173	A_23_P325080	PTOV1	Hs.587979	prostate tumor overexpressed 1	Homo sapiens prostate tumor overexpr	1085.92250	864.80457	1238.83370	1041.12370	Detected	Detected	Detected	Detected
5174	A_32_P415151	WDR27	Hs.131903	WD repeat domain 27	Homo sapiens WD repeat domain 27 (W	38.49739	14.24524	38.15366	22.04081	Detected	Compromised	Detected	Detected
5175	A_32_P64096	ZKSCAN2	Hs.513451	zinc finger with KRAB and SCAN domai	Homo sapiens zinc finger with KRAB an	1072.93690	682.49680	1041.48100	623.43850	Detected	Detected	Detected	Detected
5176	A_33_P3409740	LOC649294	Hs.588395	hypothetical LOC649294	Homo sapiens cDNA FLJ33940 fis, clon	139.81313	213.54780	45.70821	70.75050	Detected	Detected	Detected	Detected
5177	A_32_P95729	FANCI	Hs.513126	Fanconi anemia, complementation group	Homo sapiens Fanconi anemia, complen	763.60620	3251.58900	795.07324	2562.31250	Detected	Detected	Detected	Detected
5178	A_33_P3409625	SORBS3	Hs.528572	sorbin and SH3 domain containing 3	Homo sapiens sorbin and SH3 domain c	81724.74000	103183.98400	53576.17000	57970.73400	Detected	Detected	Detected	Detected
5179	A_23_P109964	CCDC54	Hs.164799	coiled-coil domain containing 54	Homo sapiens coiled-coil domain contai	5.12998	5.03178	14.19253	26.08669	Compromised	Compromised	Not Detected	Not Detected
5180	A_33_P3317168	EPS8L3	Hs.485352	EPS8-like 3	Homo sapiens EPS8-like 3 (EPS8L3), tr	145.47395	172.66837	89.88609	102.26840	Detected	Detected	Detected	Detected
5181	A_24_P150580	RASL12	Hs.27018	RAS-like, family 12	Homo sapiens RAS-like, family 12 (RAS	1016.60394	6.04048	965.77515	8.78895	Detected	Compromised	Detected	Compromised
5182	A_33_P3371889	NUAK1	Hs.524692	NUAK family, SNF1-like kinase, 1	Homo sapiens NUAK family, SNF1-like I	24.93593	12.13341	12.90229	7.45943	Detected	Compromised	Compromised	Compromised
5183	A_32_P194779	ZBTB34	Hs.177633	zinc finger and BTB domain containing	Homo sapiens zinc finger and BTB dom	1488.94850	1041.48570	1207.64900	710.67460	Detected	Detected	Detected	Detected
5184	A_23_P256603	MLLT4	Hs.614974	myeloid/lymphoid or mixed-lineage leuk	Homo sapiens myeloid/lymphoid or mixe	29.40695	32.16655	28.89231	28.78011	Detected	Detected	Detected	Detected
5185	A_23_P373079	PATL1	Hs.591960	protein associated with topoisomerase I	Homo sapiens protein associated with t	899.68930	1026.92770	791.42000	870.93620	Detected	Detected	Detected	Detected
5186	A_33_P3252539	HIGD1C	Hs.702849	HIG1 hypoxia inducible domain family, m	Homo sapiens HIG1 hypoxia inducible d	21.88695	3.85550	10.45727	3.30319	Detected	Compromised	Compromised	Compromised
5187	A_23_P368259	EID2B	Hs.135181	EP300 interacting inhibitor of differentia	Homo sapiens EP300 interacting inhibit	706.57355	927.43870	436.09060	535.17914	Detected	Detected	Detected	Detected
5188	A_23_P11507	ZZZ3	Hs.480506	zinc finger, ZZ-type containing 3	Homo sapiens zinc finger, ZZ-type cont	1150.23360	1278.17610	707.78520	604.62860	Detected	Detected	Detected	Detected
5189	A_23_P65089	DDX47	Hs.719938	DEAD (Asp-Glu-Ala-Asp) box polypept	Homo sapiens DEAD (Asp-Glu-Ala-Asp	14868.37800	16866.24400	12394.97300	12508.32900	Detected	Detected	Detected	Detected
5190	A_32_P151800	FAM72D	Hs.535577	family with sequence similarity 72, mem	Homo sapiens family with sequence sim	99.80597	753.53080	78.22633	538.95370	Detected	Detected	Detected	Detected
5191	A_24_P264664	CHD2	Hs.220864	chromodomain helicase DNA binding pro	Homo sapiens chromodomain helicase D	3162.00950	1981.56950	2400.23200	1329.72060	Detected	Detected	Detected	Detected
5192	A_33_P3366039	RPL29	Hs.425125	ribosomal protein L29	Homo sapiens ribosomal protein L29 (R	6653.90500	5184.33060	5629.16400	3804.23000	Detected	Detected	Detected	Detected
5193	A_23_P104151	TMEM69	Hs.436502	transmembrane protein 69	Homo sapiens transmembrane protein 6	391.02536	579.10800	394.38028	461.61517	Detected	Detected	Detected	Detected
5194	A_32_P61857	KIAA1468	Hs.465323	KIAA1468	Homo sapiens KIAA1468 (KIAA1468), m	724.93317	447.15027	708.73800	359.73395	Detected	Detected	Detected	Detected
5195	A_23_P99642	SLC7A7	Hs.513147	solute carrier family 7 (cationic amino a	Homo sapiens solute carrier family 7 (c	765.40125	210.32936	527.87520	118.94975	Detected	Detected	Detected	Detected
5196	A_23_P103837	UBAP2L	Hs.490551	ubiquitin associated protein 2-like	Homo sapiens ubiquitin associated prot	2259.04100	1991.24830	1866.64640	1439.51050	Detected	Detected	Detected	Detected
5197	A_33_P3253175				WD repeat-containing protein 37 [Sour	32.21724	101.34390	24.57428	68.55726	Detected	Detected	Detected	Detected
5198	A_33_P3356492		Hs.645542		Uncharacterized protein DKFZP434B06	147.98764	97.60637	36.75357	22.19642	Detected	Detected	Detected	Detected
5199	A_33_P3410831	LOC729609	Hs.554726	hypothetical protein LOC729609	Homo sapiens hypothetical protein LOC	2574.86520	2715.01780	986.53090	1008.15310	Detected	Detected	Detected	Detected
5200	A_33_P3381127	FAS	Hs.244139	Fas (TNF receptor superfamily, member	Homo sapiens Fas (TNF receptor super	1151.46390	1600.47490	772.68195	933.99650	Detected	Detected	Detected	Detected
5201	A_33_P3396239	SNRPD3	Hs.356549	small nuclear ribonucleoprotein D3 poly	Homo sapiens small nuclear ribonucleop	7568.29050	15022.67500	4705.99460	7889.33100	Detected	Detected	Detected	Detected
5202	A_23_P327777	GATS	Hs.556063	GATS, stromal antigen 3 opposite stran	Homo sapiens GATS, stromal antigen 3	374.94104	284.57687	258.13757	177.28842	Detected	Detected	Detected	Detected
5203	A_23_P416212	HSPB9	Hs.620611	heat shock protein, alpha-crystallin-rela	Homo sapiens heat shock protein, alpha	474.05896	586.19086	1058.51980	1064.29700	Detected	Detected	Detected	Detected
5204	A_33_P3395196		Hs.577850		DA380124 BRTHA2 Homo sapiens cDN	41.37176	48.82461	34.07310	31.36341	Detected	Detected	Detected	Detected
5205	A_24_P652700	CEP152	Hs.597323	centrosomal protein 152kDa	Homo sapiens centrosomal protein 152k	157.53043	294.75940	122.03239	215.22125	Detected	Detected	Detected	Detected
5206	A_33_P3403459	C14orf167	Hs.569361	chromosome 14 open reading frame 167	Homo sapiens chromosome 14 open rea	1126.53710	1076.67400	527.61880	317.94586	Detected	Detected	Detected	Detected
5207	A_23_P124108	ITGAM	Hs.172631	integrin, alpha M (complement compone	Homo sapiens integrin, alpha M (comple	478.17276	383.45270	42.75130	27.67741	Detected	Detected	Detected	Detected
5208	A_33_P3380913	LOC100287245	Hs.650098	similar to nudix-type motif 15	PREDICTED: Homo sapiens similar to n	43.25538	134.81104	20.64441	45.96591	Detected	Detected	Detected	Detected
5209	A_33_P3317073	MOBKL1A	Hs.700445	MOB1, Mps One Binder kinase activato	Homo sapiens MOB1, Mps One Binder k	12.22344	38.60724	21.09019	21.19990	Compromised	Detected	Detected	Detected
5210	A_33_P3399318	GNG12	Hs.431101	guanine nucleotide binding protein (G pr	Homo sapiens guanine nucleotide bindir	13127.72600	17913.10500	9963.61500	11894.17600	Detected	Detected	Detected	Detected
5211	A_33_P3626709	LOC100129280	Hs.678032	hypothetical protein LOC100129280	Homo sapiens cDNA FLJ44650 fis, clon	72.66760	47.69230	34.98270	9.99633	Detected	Detected	Detected	Compromised
5212	A_33_P3309799	TRIM10	Hs.709483	tripartite motif-containing 10	Homo sapiens tripartite motif-containin	386.35095	229.75761	139.27586	84.00935	Detected	Detected	Detected	Detected
5213	A_33_P3421203	hCG_1990547		family with sequence similarity 86, mem	Homo sapiens family with sequence sim	272.97638	218.30693	197.47588	128.14320	Detected	Detected	Detected	Detected
5214	A_23_P205336	C14orf129	Hs.4104	chromosome 14 open reading frame 129	Homo sapiens chromosome 14 open rea	4698.39500	8083.60160	3916.63260	5254.91000	Detected	Detected	Detected	Detected
5215	A_23_P37347	SNW1	Hs.445498	SNW domain containing 1	Homo sapiens SNW domain containing 1	7844.92970	7490.05660	7368.96900	5684.17200	Detected	Detected	Detected	Detected
5216	A_23_P31444	ERVWE1		endogenous retroviral family W, env(C7)	Homo sapiens endogenous retroviral fa	6.86740	17.35525	2.88383	3.00881	Compromised	Detected	Compromised	Compromised
5217	A_33_P3414242	MOG	Hs.141308	myelin oligodendrocyte glycoprotein	Homo sapiens myelin oligodendrocyte g	1145.83290	1704.82930	312.28806	279.36400	Detected	Detected	Detected	Detected
5218	A_33_P3354631		Hs.578624		Putative uncharacterized protein FLJ45	18.84815	20.56081	16.08715	4.14308	Detected	Detected	Detected	Compromised
5219	A_33_P3429864	EPN2	Hs.309467	epsin 2	Homo sapiens epsin 2 (EPN2), transcrip	48.60666	66.64263	41.27185	45.28219	Detected	Detected	Detected	Detected
5220	A_23_P143734	CYP2D6	Hs.648256	cytochrome P450, family 2, subfamily D	Homo sapiens cytochrome P450, family	55.66696	32.25240	47.58781	28.39000	Detected	Detected	Detected	Detected
5221	A_24_P272917	USP17	Hs.631527	ubiquitin specific peptidase 17	Homo sapiens ubiquitin specific peptida	317.92294	386.33154	114.86563	110.58007	Detected	Detected	Detected	Detected
5222	A_33_P3339625	IL17C	Hs.278911	interleukin 17C	Homo sapiens interleukin 17C (IL17C), r	18.78623	14.20546	10.78854	7.61870	Detected	Compromised	Compromised	Compromised
5223	A_23_P94819	RPH3AL	Hs.651925	rabphilin 3A-like (without C2 domains)	Homo sapiens rabphilin 3A-like (without	51.62018	53.24840	38.01583	35.84042	Detected	Detected	Detected	Detected
5224	A_32_P221452	RABL3	Hs.444360	RAB, member of RAS oncogene family-	Homo sapiens RAB, member of RAS on	202.82385	168.40298	136.87418	101.56169	Detected	Detected	Detected	Detected
5225	A_33_P3351474	SMARCAD1	Hs.410406	SWI/SNF-related, matrix-associated ac	Homo sapiens SWI/SNF-related, matrix	488.73157	467.67790	353.78340	257.46375	Detected	Detected	Detected	Detected
5226	A_33_P3317317	KRTAP5-4	Hs.539087	keratin associated protein 5-4	Homo sapiens keratin associated protei	8081.89700	12592.15300	818.63165	874.07605	Detected	Detected	Detected	Detected
5227	A_23_P202219	CALHM2	Hs.241545	calcium homeostasis modulator 2	Homo sapiens calcium homeostasis mo	2681.11700	3231.49410	2775.52370	2981.87400	Detected	Detected	Detected	Detected
5228	A_33_P3369311	BBX	Hs.124366	bobby sox homolog (Drosophila)	Homo sapiens bobby sox homolog (Dros	2973.32760	1688.40700	1882.33580	943.24817	Detected	Detected	Detected	Detected
5229	A_23_P344037	CHFR	Hs.656770	checkpoint with forkhead and ring finger	Homo sapiens checkpoint with forkhead	397.38500	568.75287	291.53240	400.90424	Detected	Detected	Detected	Detected
5230	A_23_P29803	POLR2H	Hs.432574	polymerase (RNA) II (DNA directed) pol	Homo sapiens polymerase (RNA) II (DN	10815.73100	9629.61900	9382.27800	6998.43500	Detected	Detected	Detected	Detected
5231	A_23_P309779	N4BP2	Hs.391463	NEDD4 binding protein 2	Homo sapiens NEDD4 binding protein 2	292.96060	292.88782	253.67447	178.52365	Detected	Detected	Detected	Detected
5232	A_33_P3283744	LOC339742	Hs.98178	hypothetical protein LOC339742	PREDICTED: Homo sapiens hypothetica	223.84058	42.55518	177.21689	14.56343	Detected	Detected	Detected	Compromised
5233	A_33_P3325280					1296.70310	1214.85610	1386.81640	1217.46400	Detected	Detected	Detected	Detected
5234	A_33_P3234794	C20orf95		chromosome 20 open reading frame 95	Homo sapiens chromosome 20 open rea	49.75720	92.42056	19.82646	25.71120	Detected	Detected	Detected	Detected
5235	A_24_P225010	RPS15	Hs.406683	ribosomal protein S15	Homo sapiens ribosomal protein S15 (R	99190.85000	98548.14000	91772.30500	81329.17000	Detected	Detected	Detected	Detected
5236	A_33_P3223869	LOC647302	Hs.646813	LSM3 homolog, U6 small nuclear RNA a	PREDICTED: Homo sapiens similar to L	3051.32080	5570.34200	2106.94020	3155.80350	Detected	Detected	Detected	Detected
5237	A_32_P35800	KIAA0947	Hs.449296	KIAA0947	Homo sapiens KIAA0947 (KIAA0947), m	1288.45010	1136.46700	972.27234	666.51570	Detected	Detected	Detected	Detected
5238	A_24_P51061	DCBLD2	Hs.203691	discoidin, CUB and LCCL domain conta	Homo sapiens discoidin, CUB and LCCL	746.05270	977.70230	533.85803	661.11316	Detected	Detected	Detected	Detected
5239	A_33_P3243332	FLJ32810	Hs.269837	Rho-type GTPase-activating protein FL	Homo sapiens Rho-type GTPase-activa	25.30957	15.12208	13.05708	3.79721	Detected	Compromised	Compromised	Compromised
5240	A_24_P43588	MAD2L1BP	Hs.122346	MAD2L1 binding protein	Homo sapiens MAD2L1 binding protein	3858.14200	4028.51460	4640.91600	4469.18900	Detected	Detected	Detected	Detected
5241	A_33_P3290588	ASPSCR1	Hs.298351	alveolar soft part sarcoma chromosome	Homo sapiens cDNA FLJ45020 fis, clon	23.59190	24.67499	13.80515	12.51469	Detected	Detected	Compromised	Compromised
5242	A_23_P251945	DCTN4	Hs.675564	dynactin 4 (p62)	Homo sapiens dynactin 4 (p62) (DCTN4	3847.80400	4104.48100	3221.28470	3031.08280	Detected	Detected	Detected	Detected
5243	A_24_P252043	PTP4A1	Hs.227777	protein tyrosine phosphatase type IVA,	Homo sapiens protein tyrosine phospha	263.18008	569.86340	179.55948	294.43008	Detected	Detected	Detected	Detected
5244	A_24_P190168	TMEM97	Hs.199695	transmembrane protein 97	Homo sapiens transmembrane protein 9	771.30664	1515.49730	659.35610	1104.69120	Detected	Detected	Detected	Detected
5245	A_33_P3336657	PLEKHM3	Hs.159188	pleckstrin homology domain containing,	Homo sapiens pleckstrin homology dom	10.90549	14.16208	16.35710	25.28051	Compromised	Compromised	Detected	Detected
5246	A_23_P46903	CAMK2G	Hs.523045	calcium/calmodulin-dependent protein I	Homo sapiens calcium/calmodulin-depe	199.94995	129.17949	199.37674	104.89079	Detected	Detected	Detected	Detected

5247	A_33_P3361157				Olfactory receptor 52E5 [Source:UniPr	173.92854	197.57211	92.25581	78.04766	Detected	Detected	Detected	Detected
5248	A_33_P3238058	LOC84931	Hs.334519	hypothetical LOC84931	Homo sapiens hypothetical LOC84931 (	1625.27340	872.10455	752.19310	341.18414	Detected	Detected	Detected	Detected
5249	A_23_P22096	PTK2	Hs.395482	PTK2 protein tyrosine kinase 2	Homo sapiens PTK2 protein tyrosine ki	411.25906	435.86926	514.67700	474.42290	Detected	Detected	Detected	Detected
5250	A_33_P3349693	LCLAT1	Hs.468048	lysocardiolipin acyltransferase 1	Homo sapiens lysocardiolipin acyltransf	18.55192	25.08752	15.34319	23.64169	Detected	Detected	Compromised	Detected
5251	A_32_P37592	SCARNA17	Hs.668351	small Cajal body-specific RNA 17	Homo sapiens small Cajal body-specific	4342.96630	810.94310	4166.35060	696.86414	Detected	Detected	Detected	Detected
5252	A_32_P234935	TARDBP	Hs.300624	TAR DNA binding protein	Homo sapiens TAR DNA binding protein	34148.49000	41172.41800	21341.37900	22075.04900	Detected	Detected	Detected	Detected
5253	A_33_P3322769	OR51G2	Hs.542302	olfactory receptor, family 51, subfamily	Homo sapiens olfactory receptor, family	27.77811	32.89664	9.75365	12.73143	Detected	Detected	Compromised	Compromised
5254	A_33_P3397073		Hs.549204		Proline-rich transmembrane protein 1	562.12177	179.89493	548.24457	156.35257	Detected	Detected	Detected	Detected
5255	A_23_P333228	MARCH4	Hs.170388	membrane-associated ring finger (C3H	Homo sapiens membrane-associated ri	263.17224	1798.02190	121.30525	717.65485	Detected	Detected	Detected	Detected
5256	A_33_P3296991	FLJ42393	Hs.679026	hypothetical LOC401105	Homo sapiens hypothetical LOC401105	297.83658	372.68646	132.03398	125.97741	Detected	Detected	Detected	Detected
5257	A_24_P182122					3565.58940	2762.02200	2390.19070	1559.69370	Detected	Detected	Detected	Detected
5258	A_23_P355311	PTCH2	Hs.591497	patched homolog 2 (Drosophila)	Homo sapiens patched homolog 2 (Dros	118.65012	195.11786	74.55373	92.86921	Detected	Detected	Detected	Detected
5259	A_33_P3229177	POLE4	Hs.469060	polymerase (DNA-directed), epsilon 4 (p	Homo sapiens polymerase (DNA-directe	8131.34770	8390.96100	9198.99900	8159.80000	Detected	Detected	Detected	Detected
5260	A_33_P3216913	ABHD15	Hs.106510	abhydrolase domain containing 15	Homo sapiens abhydrolase domain cont	727.00540	681.67535	372.40870	261.15472	Detected	Detected	Detected	Detected
5261	A_33_P3365553	LOC389634	Hs.647958	hypothetical LOC389634	Homo sapiens hypothetical LOC389634	72.88973	48.05279	122.04389	28.38252	Detected	Detected	Detected	Detected
5262	A_23_P502312	CD97	Hs.466039	CD97 molecule	Homo sapiens CD97 molecule (CD97), t	12405.13700	27000.37300	11619.81250	24869.30300	Detected	Detected	Detected	Detected
5263	A_24_P51588	CCNL2	Hs.515704	cyclin L2	Homo sapiens cyclin L2 (CCNL2), trans	3750.09420	2246.91200	3172.61000	1713.34670	Detected	Detected	Detected	Detected
5264	A_33_P3350828	EPS15L1	Hs.654639	epidermal growth factor receptor pathw	Homo sapiens epidermal growth factor	569.54670	861.71850	436.32764	600.83875	Detected	Detected	Detected	Detected
5265	A_33_P3265376	HOMER3	Hs.720208	homer homolog 3 (Drosophila)	Homo sapiens homer homolog 3 (Droso	3487.56270	1628.55350	5318.75200	3398.12040	Detected	Detected	Detected	Detected
5266	A_23_P301053	PAIP2B	Hs.416735	poly(A) binding protein interacting prote	Homo sapiens poly(A) binding protein in	21.88923	26.67883	12.76020	10.45579	Detected	Detected	Compromised	Compromised
5267	A_24_P419300	LOC25845	Hs.719132	hypothetical LOC25845	Homo sapiens hypothetical LOC25845 (	2375.37520	1709.13530	1798.55320	1105.94210	Detected	Detected	Detected	Detected
5268	A_23_P91317	RAD21L1	Hs.713451	RAD21-like 1 (S. pombe)	Homo sapiens RAD21-like 1 (S. pombe)	6.79083	21.41805	2.88247	3.00859	Compromised	Detected	Compromised	Compromised
5269	A_23_P165201	PRODH2	Hs.515366	proline dehydrogenase (oxidase) 2	Homo sapiens proline dehydrogenase (o	8.20413	29.89708	2.87968	4.39094	Compromised	Detected	Compromised	Compromised
5270	A_33_P3666884	PNPLA7	Hs.294147	patatin-like phospholipase domain conta	Homo sapiens patatin-like phospholipas	3373.47400	776.34210	2216.74830	195.84381	Detected	Detected	Detected	Detected
5271	A_33_P3418130	UPRT	Hs.91612	uracil phosphoribosyltransferase (FUR1	Homo sapiens uracil phosphoribosyltran	26.78055	30.62606	34.20039	31.76866	Detected	Detected	Detected	Detected
5272	A_23_P218817	CPT1B	Hs.439777	carnitine palmitoyltransferase 1B (musc	Homo sapiens carnitine palmitoyltransfe	496.39972	108.84767	452.31473	86.38345	Detected	Detected	Detected	Detected
5273	A_23_P161634	MUS81	Hs.288798	MUS81 endonuclease homolog (S. cerev	Homo sapiens MUS81 endonuclease ho	3137.17360	1746.81210	2432.71260	1208.39580	Detected	Detected	Detected	Detected
5274	A_33_P3211423	PANK3	Hs.388400	pantothenate kinase 3	Pantothenate kinase 3 (EC 2.7.1.33)(Pa	4869.78800	4600.15330	3408.18500	2752.17110	Detected	Detected	Detected	Detected
5275	A_24_P320033	CD80	Hs.838	CD80 molecule	Homo sapiens CD80 molecule (CD80), r	16.07032	16.90339	21.01261	18.58429	Detected	Compromised	Detected	Detected
5276	A_24_P287233	ARL6IP4	Hs.103561	ADP-ribosylation-like factor 6 interacti	Homo sapiens ADP-ribosylation-like fa	56090.24200	60438.55500	60106.19000	57624.01600	Detected	Detected	Detected	Detected
5277	A_23_P68031	STAT4	Hs.80642	signal transducer and activator of trans	Homo sapiens signal transducer and act	1149.00940	66.64448	871.92413	57.28394	Detected	Detected	Detected	Detected
5278	A_33_P3292694	LOC643355	Hs.647680	hypothetical LOC643355	PREDICTED: Homo sapiens hypothetica	57.55818	81.64613	56.81684	58.53087	Detected	Detected	Detected	Detected
5279	A_33_P3347413	PBXIP1	Hs.505806	pre-B-cell leukemia homeobox interact	Homo sapiens pre-B-cell leukemia hom	270.04980	124.36762	226.69194	98.13870	Detected	Detected	Detected	Detected
5280	A_33_P3215999	AFMID	Hs.558614	arylformamidase	Homo sapiens arylformamidase (AFMID)	233.59670	335.90060	292.09296	383.93713	Detected	Detected	Detected	Detected
5281	A_33_P3339066	RNPC3	Hs.632423	RNA-binding region (RNP1, RRM) conta	Homo sapiens RNA-binding region (RNF	598.81380	775.50400	444.94684	301.22852	Detected	Detected	Detected	Detected
5282	A_33_P3318322	STEAP4	Hs.521008	STEAP family member 4	Homo sapiens STEAP family member 4	26.60883	30.36606	16.70850	11.32813	Detected	Detected	Detected	Compromised
5283	A_23_P141126	GALK1	Hs.407966	galactokinase 1	Homo sapiens galactokinase 1 (GALK1)	1694.06160	1224.68580	2177.11870	1484.12850	Detected	Detected	Detected	Detected
5284	A_33_P3222210	FAM108A1	Hs.465542	family with sequence similarity 108, me	Homo sapiens family with sequence sim	2678.46500	2550.96900	2650.47830	2752.50760	Detected	Detected	Detected	Detected
5285	A_24_P131589	CD86	Hs.171182	CD86 molecule	Homo sapiens CD86 molecule (CD86), t	1226.64230	373.84344	46.24458	23.60967	Detected	Detected	Detected	Detected
5286	A_33_P3340869	LOC643719	Hs.722749	hypothetical LOC643719	Homo sapiens hypothetical LOC643719	16.31489	15.17138	12.21478	13.94589	Detected	Compromised	Compromised	Compromised
5287	A_23_P416686	GPR137	Hs.523763	G protein-coupled receptor 137	Homo sapiens G protein-coupled recep	295.33280	154.37222	280.28387	141.39438	Detected	Detected	Detected	Detected
5288	A_23_P111288	TMEM14C	Hs.519557	transmembrane protein 14C	Homo sapiens transmembrane protein 1	23766.70900	32276.62000	15694.26900	17589.69300	Detected	Detected	Detected	Detected
5289	A_23_P94365	ADCK5	Hs.283374	aarF domain containing kinase 5	Homo sapiens aarF domain containing k	972.78534	695.81240	974.69836	670.82135	Detected	Detected	Detected	Detected
5290	A_32_P80255	DDX6	Hs.654366	DEAD (Asp-Glu-Ala-Asp) box polypept	Homo sapiens DEAD (Asp-Glu-Ala-Asp	2405.39260	1905.71700	1058.40220	902.89874	Detected	Detected	Detected	Detected
5291	A_24_P12065	CCNG2	Hs.13291	cyclin G2	Homo sapiens cyclin G2 (CCNG2), mRN	133.78284	91.74135	104.49297	65.95150	Detected	Detected	Detected	Detected
5292	A_24_P314515	C12orf27	Hs.612351	chromosome 12 open reading frame 27	Homo sapiens chromosome 12 open rea	19.85997	20.77937	3.79779	10.45722	Detected	Detected	Compromised	Compromised
5293	A_33_P3405369	KRTAP5-4	Hs.539087	keratin associated protein 5-4	Homo sapiens keratin associated protei	22.80168	17.61503	8.89320	3.26397	Detected	Compromised	Compromised	Compromised
5294	A_33_P3380126	MORN3	Hs.434154	MORN repeat containing 3	Homo sapiens MORN repeat containing	43.32909	15.51238	48.84450	5.76834	Detected	Compromised	Detected	Compromised
5295	A_23_P137361	WDR8	Hs.31714	WD repeat domain 8	Homo sapiens WD repeat domain 8 (WD	1197.39160	636.00270	1059.74990	543.81720	Detected	Detected	Detected	Detected
5296	A_24_P56270	DYRK2	Hs.173135	dual-specificity tyrosine-(Y)-phosphory	Homo sapiens dual-specificity tyrosine-	1087.97020	1685.22690	909.02160	1276.74480	Detected	Detected	Detected	Detected
5297	A_24_P106794	NUDCD1	Hs.380291	NudC domain containing 1	Homo sapiens NudC domain containing	347.13248	81.47108	192.71810	38.18600	Detected	Detected	Detected	Detected
5298	A_33_P3304247	LOC644075	Hs.673368	hypothetical LOC644075	Homo sapiens cDNA FLJ26757 fis, clon	109.69459	138.65286	43.55159	42.41294	Detected	Detected	Detected	Detected
5299	A_33_P3293958	LOC100133479		hypothetical protein LOC100133479	PREDICTED: Homo sapiens hypothetica	29.32807	128.49760	33.80766	97.82661	Detected	Detected	Detected	Detected
5300	A_23_P394259	MPP2	Hs.514208	membrane protein, palmitoylated 2 (MA	Homo sapiens membrane protein, palmit	82.90224	64.16913	69.63572	65.70638	Detected	Detected	Detected	Detected
5301	A_23_P61426	MSRA	Hs.490981	methionine sulfoxide reductase A	Homo sapiens methionine sulfoxide redu	5740.26760	6684.18200	4973.00300	5425.46100	Detected	Detected	Detected	Detected
5302	A_23_P142590	ILKAP	Hs.92033	integrin-linked kinase-associated serine	Homo sapiens integrin-linked kinase-as	749.15280	1024.23690	593.34235	796.60205	Detected	Detected	Detected	Detected
5303	A_23_P4353	WSB1	Hs.446017	WD repeat and SOCS box-containing 1	Homo sapiens WD repeat and SOCS bo	4795.02150	3917.78220	2385.02780	1650.54680	Detected	Detected	Detected	Detected
5304	A_33_P3231086					76.65481	94.91850	69.60194	62.18270	Detected	Detected	Detected	Detected
5305	A_33_P3253214				Histone H2A type 2-A (H2A.2)(H2A/o)	5751.40770	17396.60700	3995.19530	10910.20200	Detected	Detected	Detected	Detected
5306	A_23_P259207	THNSL2	Hs.516179	threonine synthase-like 2 (S. cerevisiae	Homo sapiens threonine synthase-like 2	977.65640	359.19340	969.96200	357.16638	Detected	Detected	Detected	Detected
5307	A_33_P3314471					33.84198	28.20792	41.40944	37.16726	Detected	Detected	Detected	Detected
5308	A_24_P146683	MSMB	Hs.255462	microseminoprotein, beta-	Homo sapiens microseminoprotein, beta	24.91783	9.90244	18.36275	5.21215	Detected	Compromised	Detected	Compromised
5309	A_24_P341674	DIO2	Hs.202354	deiodinase, iodothyronine, type II	Homo sapiens deiodinase, iodothyronine	72.66090	10.60158	68.62457	3.49052	Detected	Compromised	Detected	Compromised
5310	A_33_P3294608	MVP	Hs.632177	major vault protein	Homo sapiens major vault protein (MVP	86812.02000	74481.14000	95525.37000	79368.21000	Detected	Detected	Detected	Detected
5311	A_23_P303181	SPHK2	Hs.528006	sphingosine kinase 2	Homo sapiens sphingosine kinase 2 (SP	4320.42000	5208.24800	4286.02340	4870.32570	Detected	Detected	Detected	Detected
5312	A_33_P3399101				Cathepsin L1 Precursor (EC 3.4.22.15)(	1029.46080	525.76100	467.65253	173.54770	Detected	Detected	Detected	Detected
5313	A_32_P75264	TMEM26	Hs.623955	transmembrane protein 26	Homo sapiens transmembrane protein 2	91.53088	99.22815	61.76375	50.12204	Detected	Detected	Detected	Detected
5314	A_23_P421221	R3HCC1	Hs.458644	R3H domain and coiled-coil containing	Homo sapiens R3H domain and coiled-c	3322.93680	3337.12620	4044.39330	3677.23360	Detected	Detected	Detected	Detected
5315	A_23_P132159	USP18	Hs.38260	ubiquitin specific peptidase 18	Homo sapiens ubiquitin specific peptida	109.29862	234.59573	80.87825	162.80795	Detected	Detected	Detected	Detected
5316	A_23_P326204	SGMS2	Hs.595423	sphingomyelin synthase 2	Homo sapiens sphingomyelin synthase 2	87.11810	104.82089	83.86012	77.00710	Detected	Detected	Detected	Detected
5317	A_23_P65618	TGM1	Hs.508950	transglutaminase 1 (K polypeptide epide	Homo sapiens transglutaminase 1 (K po	62.67423	66.74986	81.72791	43.98004	Detected	Detected	Detected	Detected
5318	A_23_P322008	TRHR	Hs.3022	thyrotropin-releasing hormone receptor	Homo sapiens thyrotropin-releasing hor	11.40994	25.28057	13.53037	11.61394	Compromised	Detected	Compromised	Compromised
5319	A_24_P4877	ZCRB1	Hs.496279	zinc finger CCHC-type and RNA binding	Homo sapiens zinc finger CCHC-type a	2561.57930	1578.09890	1349.87060	572.89330	Detected	Detected	Detected	Detected
5320	A_23_P11005	ADAMTS7	Hs.16441	ADAM metallopeptidase with thrombos	Homo sapiens ADAM metallopeptidase	24701.12300	30931.54000	25382.35500	22608.96300	Detected	Detected	Detected	Detected
5321	A_23_P350005	TRIML2	Hs.276429	tripartite motif family-like 2	Homo sapiens tripartite motif family-like	15.98617	61.85148	5.70030	13.68122	Detected	Detected	Compromised	Compromised

5322	A_24_P920646		Hs.260074		Cytochrome P450 19A1 (EC 1.14.14.1)(	35.04839	16.98896	20.52118	3.01328	Detected	Compromised	Detected	Compromised
5323	A_23_P41075	MON1A	Hs.655014	MON1 homolog A (yeast)	Homo sapiens MON1 homolog A (yeast)	2929.47630	3220.98440	3165.95210	3201.05760	Detected	Detected	Detected	Detected
5324	A_33_P3240392	FOXO3	Hs.220950	forkhead box O3	Homo sapiens forkhead box O3 (FOXO3	19983.25200	20457.16000	12750.63000	11242.26500	Detected	Detected	Detected	Detected
5325	A_23_P218579	GLB1L	Hs.181173	galactosidase, beta 1-like	Homo sapiens galactosidase, beta 1-like	270.00214	225.14209	256.80762	182.75490	Detected	Detected	Detected	Detected
5326	A_33_P3372667					219.88689	202.51760	202.77618	160.55617	Detected	Detected	Detected	Detected
5327	A_23_P67424	ZNF461	Hs.590972	zinc finger protein 461	Homo sapiens zinc finger protein 461 (Z	241.45352	260.33047	155.96010	140.16936	Detected	Detected	Detected	Detected
5328	A_23_P56894	CYP20A1	Hs.446065	cytochrome P450, family 20, subfamily A	Homo sapiens cytochrome P450, family	439.43726	354.94785	414.41132	288.39087	Detected	Detected	Detected	Detected
5329	A_23_P307544	PLXNA2	Hs.497626	plexin A2	Homo sapiens plexin A2 (PLXNA2), mRN	33.63466	126.85350	20.70332	92.53270	Detected	Detected	Detected	Detected
5330	A_23_P134650	PTCD1	Hs.632313	pentatricopeptide repeat domain 1	Homo sapiens pentatricopeptide repeat	5051.18550	5751.73100	5684.26200	5950.62700	Detected	Detected	Detected	Detected
5331	A_24_P145911		Hs.533122		Transformer-2 protein homolog beta (T	1133.12510	1281.83980	524.48737	401.93298	Detected	Detected	Detected	Detected
5332	A_23_P251505	NSUN5	Hs.647060	NOL1/NOP2/Sun domain family, memb	Homo sapiens NOL1/NOP2/Sun domain	5153.25900	5018.92040	4362.31600	4612.69240	Detected	Detected	Detected	Detected
5333	A_33_P3395605	TMEM119	Hs.720826	transmembrane protein 119	Homo sapiens transmembrane protein 1	7731.99500	4388.73800	9315.91700	4732.60840	Detected	Detected	Detected	Detected
5334	A_23_P140698	DNAJC17	Hs.511069	DnaJ (Hsp40) homolog, subfamily C, me	Homo sapiens DnaJ (Hsp40) homolog, s	258.07120	422.62665	185.86966	299.02435	Detected	Detected	Detected	Detected
5335	A_23_P155257	FOXP1	Hs.431498	forkhead box P1	Homo sapiens forkhead box P1 (FOXP1	13483.95400	22135.26800	12792.32700	18178.96500	Detected	Detected	Detected	Detected
5336	A_24_P296457	PHKG2	Hs.196177	phosphorylase kinase, gamma 2 (testis)	Homo sapiens phosphorylase kinase, ga	216.17506	234.80588	204.83142	206.45170	Detected	Detected	Detected	Detected
5337	A_23_P50052		Hs.334493		Uncharacterized protein C18orf12 (Prot	1540.88590	2012.69200	631.24170	528.65430	Detected	Detected	Detected	Detected
5338	A_24_P228228	B4GALT6	Hs.591063	UDP-Gal:betaGlcNAc beta 1,4- galacto	Homo sapiens UDP-Gal:betaGlcNAc be	51.59884	34.94069	20.00821	13.82293	Detected	Detected	Detected	Compromised
5339	A_23_P168993	ADRB3	Hs.2549	adrenergic, beta-3-, receptor	Homo sapiens adrenergic, beta-3-, rece	42.92918	37.99310	33.85064	30.60424	Detected	Detected	Detected	Detected
5340	A_33_P3316587	SLC22A18AS	Hs.300076	solute carrier family 22 (organic cation	Homo sapiens solute carrier family 22 (	109.80609	91.91815	88.64005	63.37541	Detected	Detected	Detected	Detected
5341	A_24_P319942	SSR3	Hs.518346	signal sequence receptor, gamma (trans	Homo sapiens signal sequence receptor	99.63514	437.34814	109.68317	546.20980	Detected	Detected	Detected	Detected
5342	A_23_P361085	SNHG5	Hs.292457	small nucleolar RNA host gene 5 (non-p	Homo sapiens small nucleolar RNA host	93611.03000	84802.01600	80833.82000	61193.33200	Detected	Detected	Detected	Detected
5343	A_33_P3309626		Hs.513200		A disintegrin and metalloproteinase with	16.62743	14.59630	13.18001	5.67692	Detected	Compromised	Compromised	Compromised
5344	A_33_P3360077	LOC100130745	Hs.634078	hypothetical LOC100130745	Homo sapiens cDNA FLJ46338 fis, clon	86.47552	92.77601	66.50394	61.14061	Detected	Detected	Detected	Detected
5345	A_24_P12281	AP4S1	Hs.293411	adaptor-related protein complex 4, sigm	Homo sapiens adaptor-related protein c	74.46210	162.58118	53.91050	100.26260	Detected	Detected	Detected	Detected
5346	A_33_P3287477	C10orf120	Hs.363649	chromosome 10 open reading frame 120	Homo sapiens chromosome 10 open rea	24.08344	20.03894	22.14956	15.83803	Detected	Detected	Detected	Compromised
5347	A_33_P3359683	IL16	Hs.459095	interleukin 16 (lymphocyte chemoattrac	Homo sapiens interleukin 16 (lymphocyt	77.12784	77.96263	33.80088	34.68241	Detected	Detected	Detected	Detected
5348	A_23_P1361	ALDH18A1	Hs.500645	aldehyde dehydrogenase 18 family, mem	Homo sapiens aldehyde dehydrogenase	1561.68700	2794.98100	1757.20070	2865.47400	Detected	Detected	Detected	Detected
5349	A_23_P365226	FAM161B	Hs.660789	family with sequence similarity 161, mer	Homo sapiens family with sequence sim	58.36326	55.65416	42.20603	42.97092	Detected	Detected	Detected	Detected
5350	A_33_P3391865	INF2	Hs.24956	inverted formin, FH2 and WH2 domain c	Homo sapiens inverted formin, FH2 and	409.83350	708.78534	488.57556	854.95074	Detected	Detected	Detected	Detected
5351	A_23_P145408	FUCA2	Hs.591332	fucosidase, alpha-L- 2, plasma	Homo sapiens fucosidase, alpha-L- 2, p	5669.56640	8035.52500	4899.49400	6373.12350	Detected	Detected	Detected	Detected
5352	A_33_P3251547	LOC100128370	Hs.587424	hypothetical LOC100128370	PREDICTED: Homo sapiens hypothetica	360.08084	264.42100	288.21990	169.13959	Detected	Detected	Detected	Detected
5353	A_23_P360769	MAN2A1	Hs.432822	mannosidase, alpha, class 2A, member 1	Homo sapiens mannosidase, alpha, clas	4536.43600	4092.63300	1625.53990	1290.49570	Detected	Detected	Detected	Detected
5354	A_23_P147805		Hs.488240		Uridine phosphorylase 1 (UrdPase 1)(UF	9353.82300	6314.79540	10163.78800	5885.52300	Detected	Detected	Detected	Detected
5355	A_33_P3380837	AMZ1	Hs.42221	archaelysin family metallopeptidase 1	Homo sapiens archaelysin family metall	19433.93200	15516.64300	4362.01600	2954.69460	Detected	Detected	Detected	Detected
5356	A_23_P258071	RNF113A	Hs.458365	ring finger protein 113A	Homo sapiens ring finger protein 113A (	8366.65100	8256.77100	7758.35740	6655.73930	Detected	Detected	Detected	Detected
5357	A_23_P156408	C6orf155		chromosome 6 open reading frame 155	Homo sapiens chromosome 6 open read	36.54325	27.12920	22.50295	10.92795	Detected	Detected	Detected	Compromised
5358	A_33_P3376828	CMTM7	Hs.440494	CKLF-like MARVEL transmembrane do	Homo sapiens CKLF-like MARVEL tran	578.14870	561.38390	562.96070	486.10373	Detected	Detected	Detected	Detected
5359	A_23_P146512	GOLM1	Hs.494337	golgi membrane protein 1	Homo sapiens golgi membrane protein 1	1283.05210	1506.72200	982.46800	963.41974	Detected	Detected	Detected	Detected
5360	A_33_P3249072				Dihydrodipicolinate synthase-like, mitoc	63.17305	37.50060	39.94757	19.12858	Detected	Detected	Detected	Detected
5361	A_33_P3403107	PRR19	Hs.585901	proline rich 19	Homo sapiens proline rich 19 (PRR19),	182.84052	142.19545	178.15436	120.88952	Detected	Detected	Detected	Detected
5362	A_23_P66328	ACSM2B	Hs.567879	acyl-CoA synthetase medium-chain far	Homo sapiens acyl-CoA synthetase me	27.67321	36.17820	26.00691	24.01582	Detected	Detected	Detected	Detected
5363	A_33_P3324786		Hs.126248		Transcription factor-like 5 protein (Cha	843.28375	440.60013	707.53870	338.81348	Detected	Detected	Detected	Detected
5364	A_32_P148710	CFL1	Hs.170622	cofilin 1 (non-muscle)	Homo sapiens cofilin 1 (non-muscle) (C	6964.90620	13352.33500	5818.79640	10290.87300	Detected	Detected	Detected	Detected
5365	A_23_P203737	INTS4	Hs.533723	integrator complex subunit 4	Homo sapiens integrator complex subur	1722.82650	1362.90280	1575.13700	1061.41080	Detected	Detected	Detected	Detected
5366	A_33_P3208995		Hs.712133		Zinc finger protein 663 [Source:UniPro	17.85014	16.47715	10.59702	12.19167	Detected	Compromised	Compromised	Compromised
5367	A_32_P22501	LOC645212	Hs.698054	hypothetical LOC645212	LOC645212 protein Fragment [Source:	660.20874	263.45877	714.31200	264.87723	Detected	Detected	Detected	Detected
5368	A_32_P87697	HLA-DRA	Hs.520048	major histocompatibility complex, class	Homo sapiens major histocompatibility	2.51772	17.49646	5.39050	17.34696	Compromised	Compromised	Compromised	Detected
5369	A_32_P86763	TGM2	Hs.517033	transglutaminase 2 (C polypeptide, prot	Homo sapiens transglutaminase 2 (C po	1059.96140	2759.12200	1114.69900	2899.76730	Detected	Detected	Detected	Detected
5370	A_23_P15516	TMEM101	Hs.514211	transmembrane protein 101	Homo sapiens transmembrane protein 1	2380.36100	1454.53940	1639.85960	930.09280	Detected	Detected	Detected	Detected
5371	A_33_P3258324	LOC729645	Hs.164360	similar to C-type lectin domain family 4	PREDICTED: Homo sapiens similar to C	413.40500	490.27863	241.50134	254.40866	Detected	Detected	Detected	Detected
5372	A_23_P200325	RABGAP1L	Hs.585378	RAB GTPase activating protein 1-like	Homo sapiens RAB GTPase activating p	1663.08030	1587.65520	1318.84380	946.19390	Detected	Detected	Detected	Detected
5373	A_33_P3386970		Hs.53565		Homo sapiens cDNA clone IMAGE:5302	620.36470	533.48410	417.65674	305.70670	Detected	Detected	Detected	Detected
5374	A_23_P3038	GPX2	Hs.2704	glutathione peroxidase 2 (gastrointestin	Homo sapiens glutathione peroxidase 2	46.93354	73.39539	30.13962	36.93401	Detected	Detected	Detected	Detected
5375	A_23_P150394	FXYD6	Hs.713034	FXYD domain containing ion transport r	Homo sapiens FXYD domain containing	61.55625	97.52746	36.80216	31.25339	Detected	Detected	Detected	Detected
5376	A_24_P655268	LOC729082		hypothetical protein LOC729082	Homo sapiens hypothetical protein LOC	134.68200	163.77538	121.54441	109.53359	Detected	Detected	Detected	Detected
5377	A_33_P3364211	LOC100129282	Hs.711998	hypothetical LOC100129282	PREDICTED: Homo sapiens hypothetica	39.13612	73.50092	49.38351	60.72636	Detected	Detected	Detected	Detected
5378	A_33_P3282634	ALDH1L2	Hs.42572	aldehyde dehydrogenase 1 family, memb	Homo sapiens aldehyde dehydrogenase	27.16264	145.18670	23.58183	108.95201	Detected	Detected	Detected	Detected
5379	A_23_P201342	DVL1	Hs.74375	dishevelled, dsh homolog 1 (Drosophila)	Homo sapiens dishevelled, dsh homolog	54778.62000	49015.93400	43924.20300	38006.45300	Detected	Detected	Detected	Detected
5380	A_23_P128372	FKBP4	Hs.524183	FK506 binding protein 4, 59kDa	Homo sapiens FK506 binding protein 4,	3536.48220	3807.22360	1698.37850	1296.64560	Detected	Detected	Detected	Detected
5381	A_33_P3270509	SIAH1	Hs.706828	seven in absentia homolog 1 (Drosophila	Homo sapiens seven in absentia homolo	848.47550	678.61710	911.53503	657.07740	Detected	Detected	Detected	Detected
5382	A_23_P395595	FNBP4	Hs.6834	formin binding protein 4	Homo sapiens formin binding protein 4 (	3798.40770	3320.33940	2445.84770	1726.11290	Detected	Detected	Detected	Detected
5383	A_24_P281643					12.93323	18.51986	8.86967	9.43997	Detected	Detected	Compromised	Compromised
5384	A_33_P3347108	TTC5	Hs.98553	tetratricopeptide repeat domain 5	Homo sapiens tetratricopeptide repeat	4159.75700	3410.34620	4146.88100	2905.30440	Detected	Detected	Detected	Detected
5385	A_23_P37569	MAN2A2	Hs.116459	mannosidase, alpha, class 2A, member 2	Homo sapiens mannosidase, alpha, clas	303.45290	232.02872	252.54585	179.60179	Detected	Detected	Detected	Detected
5386	A_23_P419150	VENTX	Hs.125231	VENT homeobox homolog (Xenopus lae	Homo sapiens VENT homeobox homolo	56.10853	3.47086	35.47286	2.98157	Detected	Compromised	Detected	Compromised
5387	A_24_P128163				A disintegrin and metalloproteinase with	286.90964	211.37654	271.51794	183.29560	Detected	Detected	Detected	Detected
5388	A_33_P3266780	PODXL2	Hs.591290	podocalyxin-like 2	Homo sapiens podocalyxin-like 2 (PODX	24.97647	3.49030	11.10032	3.40549	Detected	Compromised	Compromised	Compromised
5389	A_24_P18802	VPS18	Hs.23876	vacuolar protein sorting 18 homolog (S.	Homo sapiens vacuolar protein sorting	7455.87260	10059.12300	5720.02540	9182.89800	Detected	Detected	Detected	Detected
5390	A_23_P104583	CORO1B	Hs.6191	coronin, actin binding protein, 1B	Homo sapiens coronin, actin binding pro	741.19360	746.21844	715.77920	777.15340	Detected	Detected	Detected	Detected
5391	A_33_P3377994	WNK4	Hs.105448	WNK lysine deficient protein kinase 4	Homo sapiens WNK lysine deficient prot	187.89755	244.00809	108.60898	106.49941	Detected	Detected	Detected	Detected
5392	A_23_P310582	ST7L	Hs.201921	suppression of tumorigenicity 7 like	Homo sapiens suppression of tumoriger	296.81592	167.10920	265.94006	121.23379	Detected	Detected	Detected	Detected
5393	A_33_P3331346	TRIM65	Hs.189823	tripartite motif-containing 65	Homo sapiens tripartite motif-containin	3313.69780	1983.78440	3317.20650	1566.97220	Detected	Detected	Detected	Detected
5394	A_23_P416191	TAS2R31	Hs.688197	taste receptor, type 2, member 31	Homo sapiens taste receptor, type 2, m	122.95931	121.72298	58.35227	55.51266	Detected	Detected	Detected	Detected
5395	A_23_P141779	CXXC1	Hs.180933	CXXC finger 1 (PHD domain)	Homo sapiens CXXC finger 1 (PHD dom	1422.63230	984.82245	1535.47450	1041.99120	Detected	Detected	Detected	Detected
5396	A_24_P220771	ANKRD17	Hs.719092	ankyrin repeat domain 17	Homo sapiens ankyrin repeat domain 17	33.52263	29.32722	10.61715	10.83920	Detected	Detected	Compromised	Compromised

5397	A_23_P166	MOBKL2C	Hs.632401	MOB1, Mps One Binder kinase activato	Homo sapiens MOB1, Mps One Binder k	893.87800	595.52313	757.14580	472.21024	Detected	Detected	Detected	Detected
5398	A_33_P3217153	GRIA4	Hs.503743	glutamate receptor, ionotrophic, AMPA	Homo sapiens glutamate receptor, ionot	303.32556	927.42770	37.94178	52.59076	Detected	Detected	Detected	Detected
5399	A_33_P3418209	ITGBL1	Hs.696554	integrin, beta-like 1 (with EGF-like repe	Homo sapiens integrin, beta-like 1 (with	1171.59300	7821.42140	952.68840	5142.42720	Detected	Detected	Detected	Detected
5400	A_23_P134684	HMBOX1	Hs.591836	homeobox containing 1	Homo sapiens homeobox containing 1 (I	2018.79080	821.52050	1518.69540	571.78630	Detected	Detected	Detected	Detected
5401	A_23_P250813	WRN	Hs.632050	Werner syndrome, RecQ helicase-like	Homo sapiens Werner syndrome, RecQ	4016.17460	2295.43950	2896.86080	1410.88270	Detected	Detected	Detected	Detected
5402	A_24_P76675	MFAP3L	Hs.593942	microfibrillar-associated protein 3-like	Homo sapiens microfibrillar-associated	51.72767	63.88748	48.34835	43.08481	Detected	Detected	Detected	Detected
5403	A_23_P144054	PRKCD	Hs.155342	protein kinase C, delta	Homo sapiens protein kinase C, delta (F	1719.02700	3505.25780	1530.27330	2905.06840	Detected	Detected	Detected	Detected
5404	A_23_P145218				Butyrophilin subfamily 2 member A3 Pre	1040.93730	1259.17130	598.80110	641.68510	Detected	Detected	Detected	Detected
5405	A_24_P399622	COPE	Hs.10326	coatomer protein complex, subunit epsi	Homo sapiens coatomer protein comple	687.02924	777.42004	842.55273	943.92993	Detected	Detected	Detected	Detected
5406	A_33_P3238866	SBDS	Hs.110445	Shwachman-Bodian-Diamond syndrome	Homo sapiens Shwachman-Bodian-Diar	1777.72730	2426.95260	1884.81480	2307.31760	Detected	Detected	Detected	Detected
5407	A_33_P3387856	CENPN	Hs.55028	centromere protein N	Homo sapiens centromere protein N (CI	18.99727	115.05539	20.83892	92.46666	Detected	Detected	Detected	Detected
5408	A_24_P392925	GLTPD2	Hs.131035	glycolipid transfer protein domain conta	Homo sapiens glycolipid transfer protein	87.35011	87.37558	48.11359	35.56260	Detected	Detected	Detected	Detected
5409	A_33_P3610123	POLR1A	Hs.531818	polymerase (RNA) I polypeptide A, 194k	Homo sapiens polymerase (RNA) I polyp	660.38070	445.55316	619.43120	335.04750	Detected	Detected	Detected	Detected
5410	A_33_P3298139	CRLF2	Hs.287729	cytokine receptor-like factor 2	Homo sapiens cytokine receptor-like fa	27.53067	29.60868	14.98864	12.87802	Detected	Detected	Compromised	Compromised
5411	A_33_P3261084	IDH3B	Hs.436405	isocitrate dehydrogenase 3 (NAD+) beta	Homo sapiens isocitrate dehydrogenase	1079.02230	1063.73990	1057.04160	950.39450	Detected	Detected	Detected	Detected
5412	A_23_P12128	TSHB	Hs.406687	thyroid stimulating hormone, beta	Homo sapiens thyroid stimulating hormo	115.26675	195.11760	38.68305	56.57420	Detected	Detected	Detected	Detected
5413	A_32_P360193	DNHD1	Hs.720080	dynein heavy chain domain 1	Homo sapiens dynein heavy chain doma	49.19489	31.26731	30.70627	19.47881	Detected	Detected	Detected	Detected
5414	A_33_P3299055	FER1L5	Hs.534025	fer-1-like 5 (C. elegans)	Homo sapiens fer-1-like 5 (C. elegans)	17.99474	17.09232	7.26615	12.89321	Detected	Compromised	Compromised	Compromised
5415	A_23_P133470	PJA2	Hs.483036	praja ring finger 2	Homo sapiens praja ring finger 2 (PJA2)	3839.56150	2197.71780	2832.12550	1367.33630	Detected	Detected	Detected	Detected
5416	A_32_P7823	MAPK1IP1L	Hs.594338	mitogen-activated protein kinase 1 inte	Homo sapiens mitogen-activated protei	1339.95870	2545.35820	947.66675	1635.64390	Detected	Detected	Detected	Detected
5417	A_23_P78608	DENND1C	Hs.236449	DENN/MADD domain containing 1C	Homo sapiens DENN/MADD domain co	25.70837	27.09400	20.01991	20.82499	Detected	Detected	Detected	Detected
5418	A_24_P818010	LOC100133161	Hs.356758	hypothetical LOC100133161	Homo sapiens hypothetical LOC100133	468.77637	292.00920	221.88931	103.47029	Detected	Detected	Detected	Detected
5419	A_33_P3311245	KRTAP19-7	Hs.553696	keratin associated protein 19-7	Homo sapiens keratin associated protei	258.95758	664.50745	72.93908	78.64144	Detected	Detected	Detected	Detected
5420	A_23_P70746	AHI1	Hs.386684	Abelson helper integration site 1	Homo sapiens Abelson helper integratio	465.91443	115.33756	303.56808	65.54598	Detected	Detected	Detected	Detected
5421	A_23_P11843	LRRN2	Hs.26312	leucine rich repeat neuronal 2	Homo sapiens leucine rich repeat neuro	300.77744	561.27450	162.47525	211.03888	Detected	Detected	Detected	Detected
5422	A_33_P3392330				Ubiquinone biosynthesis protein COQ4	22.16212	22.62187	5.12009	9.28082	Detected	Detected	Compromised	Compromised
5423	A_33_P3355886	OOSP1		oocyte-secreted protein 1 homolog	PREDICTED: Homo sapiens oocyte-sec	18.98476	15.00651	8.96612	17.67520	Detected	Compromised	Compromised	Detected
5424	A_23_P153251	ZNF274	Hs.83761	zinc finger protein 274	Homo sapiens zinc finger protein 274 (Z	696.44324	486.62360	443.45694	246.98105	Detected	Detected	Detected	Detected
5425	A_23_P251342	GSK3B	Hs.445733	glycogen synthase kinase 3 beta	Homo sapiens glycogen synthase kinase	1153.02600	1536.16080	1369.24660	1583.24290	Detected	Detected	Detected	Detected
5426	A_24_P360763	CREM	Hs.200250	cAMP responsive element modulator	Homo sapiens cAMP responsive elemer	342.63538	273.04135	395.32278	316.63416	Detected	Detected	Detected	Detected
5427	A_33_P3424067	SUV420H1	Hs.632120	suppressor of variegation 4-20 homolog	Homo sapiens suppressor of variegation	92.04596	25.18086	47.91030	7.75972	Detected	Detected	Detected	Compromised
5428	A_23_P200560	CDC42	Hs.467637	cell division cycle 42 (GTP binding prot	Homo sapiens cell division cycle 42 (GT	31280.08200	40758.10500	14271.35450	15551.29900	Detected	Detected	Detected	Detected
5429	A_33_P3220674		Hs.636923		Novel proteinHCG1981372, isoform CRA	95.78862	63.41810	126.44069	47.22224	Detected	Detected	Detected	Detected
5430	A_23_P133974	C6orf106	Hs.643498	chromosome 6 open reading frame 106	Homo sapiens chromosome 6 open read	804.73846	977.01355	675.16690	732.94780	Detected	Detected	Detected	Detected
5431	A_23_P141706	TCEB3B	Hs.375035	transcription elongation factor B polype	Homo sapiens transcription elongation f	3.09817	19.24957	2.87890	3.00752	Compromised	Detected	Compromised	Compromised
5432	A_23_P122216	LOX	Hs.102267	lysyl oxidase	Homo sapiens lysyl oxidase (LOX), mRN	6056.68600	34951.09400	5062.68160	25198.01200	Detected	Detected	Detected	Detected
5433	A_23_P117928	BAHD1	Hs.22109	bromo adjacent homology domain conta	Homo sapiens bromo adjacent homology	3482.08250	4260.36900	2951.22780	3239.94290	Detected	Detected	Detected	Detected
5434	A_33_P3215059	NSMCE1	Hs.284295	non-SMC element 1 homolog (S. cerevi	Homo sapiens non-SMC element 1 hom	1243.74800	1650.17480	1028.10960	1119.13040	Detected	Detected	Detected	Detected
5435	A_33_P3224307	SCARNA12	Hs.689636	small Cajal body-specific RNA 12	Homo sapiens small Cajal body-specific	859.88800	284.50446	787.28845	245.86115	Detected	Detected	Detected	Detected
5436	A_32_P96036	MEX3A	Hs.591496	mex-3 homolog A (C. elegans)	Homo sapiens mex-3 homolog A (C. ele	725.40920	98.61645	455.00530	56.20992	Detected	Detected	Detected	Detected
5437	A_33_P3410935	C17orf89	Hs.356545	chromosome 17 open reading frame 89	Homo sapiens chromosome 17 open rea	13013.77000	21689.41000	6858.24500	9005.85900	Detected	Detected	Detected	Detected
5438	A_33_P3281532	RMND5A	Hs.75277	required for meiotic nuclear division 5 h	Homo sapiens required for meiotic nucle	204.80109	144.67807	193.01387	118.77752	Detected	Detected	Detected	Detected
5439	A_33_P3392787	CCDC147	Hs.253576	coiled-coil domain containing 147	Homo sapiens coiled-coil domain contai	86.56288	20.19100	61.93649	24.79793	Detected	Detected	Detected	Detected
5440	A_23_P38328	DBF4B	Hs.720187	DBF4 homolog B (S. cerevisiae)	Homo sapiens DBF4 homolog B (S. cere	121.82361	217.55673	74.05666	114.74262	Detected	Detected	Detected	Detected
5441	A_23_P89824	C18orf45	Hs.137562	chromosome 18 open reading frame 45	Homo sapiens chromosome 18 open rea	94.39742	128.22467	89.75909	103.49543	Detected	Detected	Detected	Detected
5442	A_33_P3259148	ZNF286B	Hs.534279	zinc finger protein 286B	Homo sapiens zinc finger protein 286B (	3198.02470	2905.64260	2071.84420	1472.38390	Detected	Detected	Detected	Detected
5443	A_23_P6762	JAGN1	Hs.24054	jagunal homolog 1 (Drosophila)	Homo sapiens jagunal homolog 1 (Droso	3555.61910	5090.47070	2613.38450	4193.10700	Detected	Detected	Detected	Detected
5444	A_33_P3276505	ATP11C	Hs.88252	ATPase, class VI, type 11C	Homo sapiens ATPase, class VI, type 1	365.67462	496.44763	248.35380	255.23439	Detected	Detected	Detected	Detected
5445	A_23_P211212	COL18A1	Hs.517356	collagen, type XVIII, alpha 1	Homo sapiens collagen, type XVIII, alpha	30172.25200	26947.32600	21074.22000	16227.93500	Detected	Detected	Detected	Detected
5446	A_23_P407684	ZNF598	Hs.343828	zinc finger protein 598	Homo sapiens zinc finger protein 598 (Z	2917.09550	2172.09470	3581.80930	2522.28100	Detected	Detected	Detected	Detected
5447	A_33_P3321602				Phosphatase and actin regulator 4 [So	72.64707	99.21579	39.24189	48.44576	Detected	Detected	Detected	Detected
5448	A_23_P47728	MAP6	Hs.585540	microtubule-associated protein 6	Homo sapiens microtubule-associated p	287.22748	229.57945	231.21034	180.16156	Detected	Detected	Detected	Detected
5449	A_33_P3382399	KRTAP3-1	Hs.307027	keratin associated protein 3-1	Homo sapiens keratin associated protei	132.96547	132.67358	97.45367	86.08364	Detected	Detected	Detected	Detected
5450	A_23_P111228	COQ3	Hs.713623	coenzyme Q3 homolog, methyltransfera	Homo sapiens coenzyme Q3 homolog, m	463.54100	943.97610	403.75525	663.24927	Detected	Detected	Detected	Detected
5451	A_33_P3297030	LOC100129974	Hs.461819	hypothetical protein LOC100129974	Homo sapiens cDNA FLJ46583 fis, clon	332.94760	570.88934	151.73819	202.61526	Detected	Detected	Detected	Detected
5452	A_23_P145711	C7orf10	Hs.586313	chromosome 7 open reading frame 10	Homo sapiens chromosome 7 open read	233.82372	175.50177	222.20960	138.19010	Detected	Detected	Detected	Detected
5453	A_33_P3222942	GPR78	Hs.350588	G protein-coupled receptor 78	Homo sapiens G protein-coupled recept	29.40975	26.54613	9.57384	10.61654	Detected	Detected	Compromised	Compromised
5454	A_33_P3312182	C10orf47	Hs.435775	chromosome 10 open reading frame 47	Homo sapiens chromosome 10 open rea	29.93525	49.74223	23.45097	46.07514	Detected	Detected	Detected	Detected
5455	A_24_P125690	MRPL34	Hs.515242	mitochondrial ribosomal protein L34	Homo sapiens mitochondrial ribosomal p	16794.92800	11431.45500	15114.86300	9297.28800	Detected	Detected	Detected	Detected
5456	A_33_P3392677					45.51341	55.76545	33.07849	32.56044	Detected	Detected	Detected	Detected
5457	A_33_P3352843					197.12999	179.27228	37.33619	26.43093	Detected	Detected	Detected	Detected
5458	A_32_P481377	KRTAP11-1	Hs.407654	keratin associated protein 11-1	Homo sapiens keratin associated protei	135.74843	186.60764	30.75040	33.48414	Detected	Detected	Detected	Detected
5459	A_23_P502224	CYB5R3	Hs.700572	cytochrome b5 reductase 3	Homo sapiens cytochrome b5 reductase	214156.70000	348125.60000	159182.38000	239561.90000	Detected	Detected	Detected	Detected
5460	A_24_P183864	IMPA1	Hs.656694	inositol(myo)-1(or 4)-monophosphatase	Homo sapiens inositol(myo)-1(or 4)-mo	2698.62230	4650.13200	2212.81420	3310.33030	Detected	Detected	Detected	Detected
5461	A_23_P139682	PZP	Hs.707491	pregnancy-zone protein	Homo sapiens pregnancy-zone protein (	338.51840	39.38823	5.94839	3.34267	Detected	Detected	Compromised	Compromised
5462	A_23_P380614	ATP9A	Hs.700437	ATPase, class II, type 9A	Homo sapiens ATPase, class II, type 9A	7356.82100	6574.57570	6272.33600	4899.93160	Detected	Detected	Detected	Detected
5463	A_24_P151727	NONO	Hs.533282	non-POU domain containing, octamer-b	Homo sapiens non-POU domain contair	5850.64700	6931.58840	4844.31150	5008.51030	Detected	Detected	Detected	Detected
5464	A_33_P3268900	LOC100129791	Hs.646497	hypothetical protein LOC100129791	PREDICTED: Homo sapiens hypothetica	3986.31300	3596.31230	2434.71220	1812.04050	Detected	Detected	Detected	Detected
5465	A_23_P300076	IQCG	Hs.591675	IQ motif containing G	Homo sapiens IQ motif containing G (IQ	100.42143	45.71759	89.66157	32.14840	Detected	Detected	Detected	Detected
5466	A_23_P106737	LUC7L	Hs.16803	LUC7-like (S. cerevisiae)	Homo sapiens LUC7-like (S. cerevisiae)	916.90875	424.43497	614.36890	251.21625	Detected	Detected	Detected	Detected
5467	A_23_P13934	MGC14436	Hs.675961	hypothetical LOC84983	Homo sapiens hypothetical LOC84983 (	28.18038	47.07571	29.16535	19.68297	Detected	Detected	Detected	Detected
5468	A_23_P368225	EME1	Hs.514330	essential meiotic endonuclease 1 homol	Homo sapiens essential meiotic endonu	138.05186	103.90790	91.36069	49.91707	Detected	Detected	Detected	Detected
5469	A_23_P1505	LRP5	Hs.6347	low density lipoprotein receptor-related	Homo sapiens low density lipoprotein re	42.15891	54.82018	38.47614	45.36396	Detected	Detected	Detected	Detected
5470	A_23_P62351	ARMCX6	Hs.83530	armadillo repeat containing, X-linked 6	Homo sapiens armadillo repeat containi	3017.32700	4130.28270	3227.95430	3990.53400	Detected	Detected	Detected	Detected
5471	A_23_P8763	PTPN12	Hs.61812	protein tyrosine phosphatase, non-rece	Homo sapiens protein tyrosine phospha	14496.05700	12935.97800	10067.53200	6898.87700	Detected	Detected	Detected	Detected

5472	A_23_P143906	MLF1	Hs.85195	myeloid leukemia factor 1	Homo sapiens myeloid leukemia factor	2573.09060	2333.12570	2778.91820	2139.37700	Detected	Detected	Detected	Detected
5473	A_24_P130363	C18orf1	Hs.149363	chromosome 18 open reading frame 1	Homo sapiens chromosome 18 open rea	19.02401	15.26673	9.79324	19.83447	Detected	Compromised	Compromised	Detected
5474	A_33_P3264746					4994.05660	6278.59960	3248.19040	3464.28700	Detected	Detected	Detected	Detected
5475	A_33_P3353622	RBAK	Hs.396178	RB-associated KRAB zinc finger	Homo sapiens RB-associated KRAB zir	16.30989	22.11372	12.69832	15.91313	Detected	Detected	Compromised	Detected
5476	A_33_P3245434					30.37122	34.52234	7.49803	8.21883	Detected	Detected	Compromised	Compromised
5477	A_23_P66421	NAT9	Hs.144058	N-acetyltransferase 9 (GCN5-related, p	Homo sapiens N-acetyltransferase 9 (G	4256.32670	5114.62100	5326.42600	5866.83540	Detected	Detected	Detected	Detected
5478	A_24_P558365	FABP12	Hs.586370	fatty acid binding protein 12	Homo sapiens fatty acid binding protein	5.22616	18.98084	3.55921	3.02390	Compromised	Detected	Compromised	Compromised
5479	A_32_P23010	SDHAF1	Hs.356460	succinate dehydrogenase complex asse	Homo sapiens succinate dehydrogenase	3474.29520	3757.80440	3470.96660	3392.58540	Detected	Detected	Detected	Detected
5480	A_33_P3288246	MCC	Hs.593171	mutated in colorectal cancers	Homo sapiens mutated in colorectal car	15.38564	28.98046	17.24503	13.42756	Detected	Detected	Detected	Compromised
5481	A_33_P3318272	OR5M11	Hs.553632	olfactory receptor, family 5, subfamily M	Homo sapiens olfactory receptor, family	12.18461	28.87194	3.84092	3.00481	Compromised	Detected	Compromised	Compromised
5482	A_33_P3398143				Integrin beta-2 Precursor (Cell surface	212.15640	385.90350	97.38693	151.88742	Detected	Detected	Detected	Detected
5483	A_23_P144622	GNPDA1	Hs.633853	glucosamine-6-phosphate deaminase 1	Homo sapiens glucosamine-6-phosphat	1547.27750	742.03830	1540.01110	625.95374	Detected	Detected	Detected	Detected
5484	A_33_P3380076	LOC100131673		similar to hCG1995779	PREDICTED: Homo sapiens hypothetica	5.85481	19.26212	2.86511	2.99137	Compromised	Detected	Compromised	Compromised
5485	A_24_P576174	DCP1A	Hs.476353	DCP1 decapping enzyme homolog A (S.	Homo sapiens DCP1 decapping enzyme	2264.46970	1933.93570	1996.33030	1560.38570	Detected	Detected	Detected	Detected
5486	A_33_P3422289					1174.75870	105.52359	924.01980	58.35923	Detected	Detected	Detected	Detected
5487	A_23_P133629	CDC23	Hs.73625	cell division cycle 23 homolog (S. cerev	Homo sapiens cell division cycle 23 hon	1105.57590	2331.65940	1090.60280	1792.62670	Detected	Detected	Detected	Detected
5488	A_23_P118174	PLK1	Hs.592049	polo-like kinase 1 (Drosophila)	Homo sapiens polo-like kinase 1 (Droso	27.14974	2558.50440	23.68674	2743.66100	Detected	Detected	Detected	Detected
5489	A_33_P3290149	LOC100133554		hypothetical protein LOC100133554	PREDICTED: Homo sapiens hypothetica	33.47741	28.52071	31.22284	18.66937	Detected	Detected	Detected	Detected
5490	A_32_P4228		Hs.560990		PREDICTED: Homo sapiens hypothetica	112.21799	99.85021	79.47427	52.95938	Detected	Detected	Detected	Detected
5491	A_24_P366526	SYNGR2	Hs.464210	synaptogyrin 2	Homo sapiens synaptogyrin 2, mRNA (c	6876.00340	6002.01950	5986.38430	4624.31840	Detected	Detected	Detected	Detected
5492	A_33_P3555009	LOC284373	Hs.188680	hypothetical protein LOC284373	Homo sapiens mRNA; cDNA DKFZp686	7702.36670	5908.25700	3372.48630	1840.56740	Detected	Detected	Detected	Detected
5493	A_33_P3210218	GALNT1	Hs.514806	UDP-N-acetyl-alpha-D-galactosamine:	Homo sapiens UDP-N-acetyl-alpha-D-	833.38367	1196.95170	502.76605	538.55980	Detected	Detected	Detected	Detected
5494	A_23_P119535	EFNA2	Hs.532655	ephrin-A2	Homo sapiens ephrin-A2 (EFNA2), mRN	30.66620	30.13324	17.19171	24.11985	Detected	Detected	Detected	Detected
5495	A_32_P34387	SPATA7	Hs.525518	spermatogenesis associated 7	Homo sapiens spermatogenesis associa	647.01404	390.14750	474.73035	237.10486	Detected	Detected	Detected	Detected
5496	A_23_P255805	ZNF7	Hs.493218	zinc finger protein 7	Homo sapiens zinc finger protein 7 (ZNI	1546.10570	1180.51620	1490.73900	991.11950	Detected	Detected	Detected	Detected
5497	A_24_P935782	ZNF121	Hs.501537	zinc finger protein 121	Homo sapiens zinc finger protein 121 (Z	133.72600	92.17784	83.85738	53.67476	Detected	Detected	Detected	Detected
5498	A_24_P295806	CHID1	Hs.144468	chitinase domain containing 1	Homo sapiens chitinase domain contain	914.96820	1400.71550	881.03200	1197.92440	Detected	Detected	Detected	Detected
5499	A_33_P3417990	VRK2	Hs.631890	vaccinia related kinase 2	Homo sapiens vaccinia related kinase 2	447.63736	378.21747	350.95890	245.26842	Detected	Detected	Detected	Detected
5500	A_23_P96777	LRRC47	Hs.268488	leucine rich repeat containing 47	Homo sapiens leucine rich repeat conta	4826.25900	7080.73500	2946.37620	3810.39600	Detected	Detected	Detected	Detected
5501	A_33_P3280421	CDC14C	Hs.567757	CDC14 cell division cycle 14 homolog C	Homo sapiens CDC14 cell division cycle	195.47249	263.63560	113.26616	137.17440	Detected	Detected	Detected	Detected
5502	A_23_P254472	C6orf211	Hs.15929	chromosome 6 open reading frame 211	Homo sapiens chromosome 6 open read	1634.93690	2596.89040	1407.60450	1842.46770	Detected	Detected	Detected	Detected
5503	A_32_P203528	SYCE2	Hs.655173	synaptonemal complex central element	Homo sapiens synaptonemal complex c	82.35737	67.45087	36.16878	32.65242	Detected	Detected	Detected	Detected
5504	A_32_P44775	C9orf85	Hs.534190	chromosome 9 open reading frame 85	Homo sapiens chromosome 9 open read	268.70148	444.08588	254.72300	365.37740	Detected	Detected	Detected	Detected
5505	A_32_P18824	BRD7	Hs.437894	bromodomain containing 7	Homo sapiens bromodomain containing	5829.98900	5259.16700	4659.71040	3731.54830	Detected	Detected	Detected	Detected
5506	A_23_P255126	GAB3	Hs.496982	GRB2-associated binding protein 3	Homo sapiens GRB2-associated binding	66.51953	64.96368	41.43200	34.28501	Detected	Detected	Detected	Detected
5507	A_23_P161644	RBM14	Hs.714949	RNA binding motif protein 14	Homo sapiens RNA binding motif protei	34.10689	33.83769	18.92158	17.96596	Detected	Detected	Detected	Detected
5508	A_33_P3263061	AK3	Hs.493362	adenylate kinase 3	Homo sapiens adenylate kinase 3 (AK3)	19065.64800	11752.82900	15474.45100	8521.96100	Detected	Detected	Detected	Detected
5509	A_24_P129341	AKR1B10	Hs.116724	aldo-keto reductase family 1, member B	Homo sapiens aldo-keto reductase fam	85.13577	92.97130	69.71199	46.67335	Detected	Detected	Detected	Detected
5510	A_23_P58529	DIMT1L	Hs.719162	DIM1 dimethyladenosine transferase 1-	Homo sapiens DIM1 dimethyladenosine	6072.07200	7200.79300	5962.39200	6135.80700	Detected	Detected	Detected	Detected
5511	A_23_P27404	ZNF841	Hs.665751	zinc finger protein 841	Homo sapiens zinc finger protein 841 (Z	114.31970	85.69257	96.44208	58.55387	Detected	Detected	Detected	Detected
5512	A_33_P3337733		Hs.446616		Putative uncharacterized protein FLJ45	239.83502	260.26096	18.84962	14.15563	Detected	Detected	Detected	Compromised
5513	A_23_P79962	MKKS	Hs.472119	McKusick-Kaufman syndrome	Homo sapiens McKusick-Kaufman synd	2796.82180	1542.70410	2645.62230	1325.62280	Detected	Detected	Detected	Detected
5514	A_33_P3255131	KCTD19	Hs.299127	potassium channel tetramerisation dom	Homo sapiens potassium channel tetrar	10769.89900	13928.78100	3844.79100	3652.18500	Detected	Detected	Detected	Detected
5515	A_23_P75255	VPS26A	Hs.499925	vacuolar protein sorting 26 homolog A (	Homo sapiens vacuolar protein sorting	6872.99000	6805.08800	5060.10840	4596.09800	Detected	Detected	Detected	Detected
5516	A_23_P353005	RNF217	Hs.368639	ring finger protein 217	Homo sapiens ring finger protein 217 (R	256.13880	236.80045	193.38597	173.36642	Detected	Detected	Detected	Detected
5517	A_33_P3315801	CRKRS	Hs.416108	Cdc2-related kinase, arginine/serine-ric	Homo sapiens Cdc2-related kinase, arg	50.88180	108.52128	52.21736	108.88412	Detected	Detected	Detected	Detected
5518	A_23_P217384	AP1S2	Hs.653504	adaptor-related protein complex 1, sigm	Homo sapiens adaptor-related protein c	3533.00780	4473.56050	2490.89200	2815.79150	Detected	Detected	Detected	Detected
5519	A_33_P3292387	LRRIQ3	Hs.644625	leucine-rich repeats and IQ motif conta	Homo sapiens leucine-rich repeats and	198.47008	93.93043	155.73573	58.51874	Detected	Detected	Detected	Detected
5520	A_23_P24485	NFRKB	Hs.530539	nuclear factor related to kappaB bindin	Homo sapiens nuclear factor related to	204.57182	174.41705	165.38545	152.06161	Detected	Detected	Detected	Detected
5521	A_23_P379649	BMF	Hs.591104	Bcl2 modifying factor	Homo sapiens Bcl2 modifying factor (BI	713.95844	389.59290	665.32544	310.53934	Detected	Detected	Detected	Detected
5522	A_33_P3324505					41.20751	16.22265	24.68537	12.05809	Detected	Compromised	Detected	Compromised
5523	A_24_P942904	BMPER	Hs.660998	BMP binding endothelial regulator	BMP-binding endothelial regulator prote	20.73415	44.44391	14.69899	39.62135	Detected	Detected	Detected	Detected
5524	A_24_P234838	PCDH1	Hs.79769	protocadherin 1	Homo sapiens protocadherin 1 (PCDH1)	21.69634	30.67941	7.75148	10.57935	Detected	Detected	Compromised	Compromised
5525	A_33_P3333752				Protein FAM183A [Source:UniProtKB/	34.79124	47.38303	26.10190	36.10160	Detected	Detected	Detected	Detected
5526	A_33_P3311165		Hs.451549		cDNA FLJ44313 fis, clone TRACH2025	123.72891	33.06974	66.33850	12.07507	Detected	Detected	Detected	Compromised
5527	A_24_P117942	TOMM20L	Hs.592307	translocase of outer mitochondrial mem	Homo sapiens translocase of outer mito	749.40436	828.13530	267.17140	243.83145	Detected	Detected	Detected	Detected
5528	A_23_P349928	SP100	Hs.369056	SP100 nuclear antigen	Homo sapiens SP100 nuclear antigen (S	39478.00800	87444.76000	25006.67200	44652.16800	Detected	Detected	Detected	Detected
5529	A_23_P4489	NETO1	Hs.465407	neuropilin (NRP) and tolloid (TLL)-like 1	Homo sapiens neuropilin (NRP) and tollo	145.75449	66.45059	157.37918	35.47916	Detected	Detected	Detected	Detected
5530	A_33_P3409392	FZD6	Hs.591863	frizzled homolog 6 (Drosophila)	Homo sapiens frizzled homolog 6 (Droso	743.55010	2558.26250	432.54324	1166.24600	Detected	Detected	Detected	Detected
5531	A_33_P3324086	CCDC90A	Hs.214043	coiled-coil domain containing 90A	Homo sapiens coiled-coil domain contai	11784.60800	20534.11300	9573.51200	13953.47000	Detected	Detected	Detected	Detected
5532	A_33_P3327108	NLK	Hs.208759	nemo-like kinase	Homo sapiens nemo-like kinase (NLK),	1429.46950	1238.50710	1202.25590	743.94836	Detected	Detected	Detected	Detected
5533	A_24_P362646	TXNDC9	Hs.536122	thioredoxin domain containing 9	Homo sapiens thioredoxin domain conta	2755.16160	4165.68700	2723.06100	3452.13920	Detected	Detected	Detected	Detected
5534	A_23_P211417	RFPL1	Hs.648249	ret finger protein-like 1	Homo sapiens ret finger protein-like 1 (	16.74429	28.92757	3.48728	2.99728	Detected	Detected	Compromised	Compromised
5535	A_33_P3584591	HIST2H2BA	Hs.585427	histone cluster 2, H2ba	AGENCOURT_8230913 Lupski_dorsal_ro	4.37379	25.88455	2.86167	2.98817	Compromised	Detected	Compromised	Compromised
5536	A_23_P404494	IL7R	Hs.591742	interleukin 7 receptor	Homo sapiens interleukin 7 receptor (IL	85.23578	953.81573	54.39650	675.06730	Detected	Detected	Detected	Detected
5537	A_23_P64121	C11orf41	Hs.502266	chromosome 11 open reading frame 41	Homo sapiens chromosome 11 open rea	587.89716	938.60815	329.49720	414.68400	Detected	Detected	Detected	Detected
5538	A_23_P217098	VPS13A	Hs.459790	vacuolar protein sorting 13 homolog A (	Homo sapiens vacuolar protein sorting	1091.27700	348.49725	638.02350	172.92480	Detected	Detected	Detected	Detected
5539	A_33_P3356857					17.21906	25.62079	2.84268	2.96825	Detected	Detected	Compromised	Compromised
5540	A_23_P208288	ZNF304	Hs.287374	zinc finger protein 304	Homo sapiens zinc finger protein 304 (Z	427.01530	410.82135	293.58950	213.96245	Detected	Detected	Detected	Detected
5541	A_23_P75867	OR10A4	Hs.448685	olfactory receptor, family 10, subfamily	Homo sapiens olfactory receptor, family	22.31735	26.65768	10.30060	14.09949	Detected	Detected	Compromised	Compromised
5542	A_33_P3416366	CHAF1A	Hs.79018	chromatin assembly factor 1, subunit A	Homo sapiens chromatin assembly facto	151.97786	394.63272	149.46780	414.52173	Detected	Detected	Detected	Detected
5543	A_33_P3297562	IRX2	Hs.282089	iroquois homeobox 2	Homo sapiens iroquois homeobox 2 (IR)	13259.07600	10770.64700	9207.19900	6661.55800	Detected	Detected	Detected	Detected
5544	A_23_P398275	LOC388152	Hs.607498	hypothetical LOC388152	Homo sapiens hypothetical LOC388152	526.11285	363.52167	469.39777	316.54333	Detected	Detected	Detected	Detected
5545	A_24_P933418	ABI2	Hs.471156	abl-interactor 2	Homo sapiens abl-interactor 2 (ABI2), r	558.16284	275.72900	280.35745	122.81622	Detected	Detected	Detected	Detected
5546	A_23_P111657	SHH	Hs.164537	sonic hedgehog homolog (Drosophila)	Homo sapiens sonic hedgehog homolog	28.48942	45.29599	18.93315	18.72265	Detected	Detected	Detected	Detected

5547	A_33_P3288159	ASPM	Hs.121028	asp (abnormal spindle) homolog, microce	Homo sapiens asp (abnormal spindle) ho	38.55512	2860.60820	11.57788	1466.15250	Detected	Detected	Compromised	Detected
5548	A_23_P167585	GDF9	Hs.25022	growth differentiation factor 9	Homo sapiens growth differentiation fac	22.61592	3.53515	21.61738	3.01030	Detected	Compromised	Detected	Compromised
5549	A_23_P94552	TMEM2	Hs.494146	transmembrane protein 2	Homo sapiens transmembrane protein 2	2018.77610	2541.22400	1839.65150	1966.44590	Detected	Detected	Detected	Detected
5550	A_33_P3227320	MIER3	Hs.657594	mesoderm induction early response 1, f	Homo sapiens mesoderm induction early	1826.57670	1255.05530	1055.44060	554.99760	Detected	Detected	Detected	Detected
5551	A_33_P3651994	GNL3LP	Hs.435237	guanine nucleotide binding protein-like 3	Homo sapiens cDNA FLJ12595 fis, clon	200.66165	259.56840	161.31145	171.86595	Detected	Detected	Detected	Detected
5552	A_33_P3394213	GRIN3B	Hs.660378	glutamate receptor, ionotropic, N-methy	Homo sapiens glutamate receptor, ionot	512.19226	195.24643	480.25845	156.54953	Detected	Detected	Detected	Detected
5553	A_23_P121614	MUC7	Hs.631946	mucin 7, secreted	Homo sapiens mucin 7, secreted (MUC7	15.28755	8.31787	7.59696	7.08379	Not Detected	Compromised	Compromised	Compromised
5554	A_23_P66881	RGS9	Hs.664380	regulator of G-protein signaling 9	Homo sapiens regulator of G-protein sig	273.88373	28.98886	265.73007	15.58286	Detected	Detected	Detected	Detected
5555	A_23_P255317	RNF14	Hs.483616	ring finger protein 14	Homo sapiens ring finger protein 14 (RN	2357.57320	3326.47500	1793.07360	2290.38750	Detected	Detected	Detected	Detected
5556	A_33_P3266719	TET2	Hs.367639	tet oncogene family member 2	Homo sapiens tet oncogene family mem	108.72416	33.90730	75.47787	20.75903	Detected	Detected	Detected	Detected
5557	A_33_P3401156	ETV1	Hs.22634	ets variant 1	Homo sapiens ets variant 1 (ETV1), tran	874.94086	238.44322	798.51160	182.56226	Detected	Detected	Detected	Detected
5558	A_33_P3215913				Transmembrane protein 184B (Putative	27.12199	40.42902	3.07372	3.20847	Detected	Detected	Compromised	Compromised
5559	A_32_P96692	POLH	Hs.655467	polymerase (DNA directed), eta	Homo sapiens polymerase (DNA directe	433.96088	536.48930	355.26215	375.83087	Detected	Detected	Detected	Detected
5560	A_32_P1445	PTPN2	Hs.654527	protein tyrosine phosphatase, non-rece	Homo sapiens protein tyrosine phospha	417.98320	459.43823	371.74362	336.61210	Detected	Detected	Detected	Detected
5561	A_33_P3336552	LOC100130701	Hs.711483	similar to hCG1657343	PREDICTED: Homo sapiens similar to h	13.29634	10.34963	21.47342	5.16019	Compromised	Compromised	Detected	Compromised
5562	A_23_P1594	VEGFB	Hs.78781	vascular endothelial growth factor B	Homo sapiens vascular endothelial grow	24183.35200	13999.79400	20032.82400	10150.33900	Detected	Detected	Detected	Detected
5563	A_33_P3691758	OR4C1P	Hs.550274	olfactory receptor, family 4, subfamily C	H.sapiens mRNA HTPCRX11 for olfacto	71.38695	63.41304	25.35875	19.18739	Detected	Detected	Detected	Detected
5564	A_32_P3998	ZNF813	Hs.710780	zinc finger protein 813	Homo sapiens zinc finger protein 813 (Z	562.86430	463.06476	333.65417	259.59790	Detected	Detected	Detected	Detected
5565	A_23_P154708	HAO1	Hs.193640	hydroxyacid oxidase (glycolate oxidase)	Homo sapiens hydroxyacid oxidase (glyc	11.00218	20.20710	3.86506	8.06122	Compromised	Detected	Compromised	Compromised
5566	A_23_P133807	TAF8	Hs.520122	TAF8 RNA polymerase II, TATA box bin	Homo sapiens TAF8 RNA polymerase II	31.61265	34.24954	30.08978	20.06069	Detected	Detected	Detected	Detected
5567	A_33_P3294031	KCNQ1OT1		KCNQ1 overlapping transcript 1 (non-p	Homo sapiens KCNQ1 overlapping trans	181.08061	172.93630	111.05428	85.25322	Detected	Detected	Detected	Detected
5568	A_23_P360179	NEURL4	Hs.654794	neuralized homolog 4 (Drosophila)	Homo sapiens neuralized homolog 4 (Dr	1403.54700	644.19730	1120.43620	519.05646	Detected	Detected	Detected	Detected
5569	A_32_P137966	DEPDC4	Hs.653118	DEP domain containing 4	Homo sapiens DEP domain containing 4	25.50711	49.60483	31.14655	36.57092	Detected	Detected	Detected	Detected
5570	A_33_P3210349		Hs.592071		cDNA FLJ44904 fis, clone BRAMY3005	99.92869	56.86625	22.63895	11.96547	Detected	Detected	Detected	Compromised
5571	A_33_P3401119	FAM22F	Hs.648183	family with sequence similarity 22, mem	Homo sapiens family with sequence sim	9.26051	9.21345	20.19276	12.63372	Compromised	Compromised	Detected	Compromised
5572	A_23_P116091	DPP3	Hs.502914	dipeptidyl-peptidase 3	Homo sapiens dipeptidyl-peptidase 3 (D	3595.66330	4714.32670	3317.01560	4575.81500	Detected	Detected	Detected	Detected
5573	A_23_P71864	FAM73B	Hs.632693	family with sequence similarity 73, mem	Homo sapiens family with sequence sim	594.21484	596.01306	567.96650	573.39800	Detected	Detected	Detected	Detected
5574	A_33_P3385662				Putative uncharacterized protein ENSP	63.14967	65.16907	54.89994	46.64375	Detected	Detected	Detected	Detected
5575	A_24_P910733	CCDC50	Hs.478682	coiled-coil domain containing 50	Homo sapiens coiled-coil domain conta	1218.81520	1422.80730	960.87250	1030.85890	Detected	Detected	Detected	Detected
5576	A_33_P3252499	WDR52	Hs.584936	WD repeat domain 52	Homo sapiens WD repeat domain 52 (W	17.42707	5.13524	8.96346	5.57949	Detected	Compromised	Compromised	Compromised
5577	A_33_P3339197	LOC647343		similar to T-cell receptor beta chain V	PREDICTED: Homo sapiens similar to h	18.00927	19.54380	5.24416	16.57096	Detected	Detected	Compromised	Detected
5578	A_23_P151459	MYCBP2	Hs.591221	MYC binding protein 2	Homo sapiens MYC binding protein 2 (M	5996.61800	3681.19070	3114.95730	1557.71240	Detected	Detected	Detected	Detected
5579	A_32_P103633	MCM2	Hs.477481	minichromosome maintenance complex	Homo sapiens minichromosome mainter	1604.82690	3026.15380	1683.70690	3129.58450	Detected	Detected	Detected	Detected
5580	A_33_P3308219					110.40868	78.96509	112.92981	40.14286	Detected	Detected	Detected	Detected
5581	A_23_P6914	OSBPL11	Hs.477440	oxysterol binding protein-like 11	Homo sapiens oxysterol binding protein-	1463.72080	1625.93320	1233.08450	1220.76490	Detected	Detected	Detected	Detected
5582	A_33_P3247753	TSIX	Hs.696585	XIST antisense RNA (non-protein codin	Homo sapiens XIST antisense RNA (nor	227.46875	194.22896	97.77937	59.88827	Detected	Detected	Detected	Detected
5583	A_33_P3579336	LOC285000	Hs.295012	hypothetical protein LOC285000	Homo sapiens hypothetical protein LOC	3.66306	25.47837	2.87368	3.00469	Compromised	Detected	Compromised	Compromised
5584	A_24_P203622	DOCK7	Hs.406156	dedicator of cytokinesis 7	Homo sapiens dedicator of cytokinesis 7	55.18329	91.49967	51.89207	45.03661	Detected	Detected	Detected	Detected
5585	A_33_P3335137	ATG4B	Hs.283610	ATG4 autophagy related 4 homolog B (S	Homo sapiens ATG4 autophagy related	3526.15750	3731.07710	6096.79100	4652.16700	Detected	Detected	Detected	Detected
5586	A_33_P3303577	TMEM110	Hs.705605	transmembrane protein 110	Homo sapiens transmembrane protein 1	541.11060	981.58386	404.63214	756.19946	Detected	Detected	Detected	Detected
5587	A_33_P3387365	PXMP4	Hs.654857	peroxisomal membrane protein 4, 24kDa	Homo sapiens peroxisomal membrane p	730.81330	348.55026	583.65326	216.55357	Detected	Detected	Detected	Detected
5588	A_23_P202594	C10orf119	Hs.124246	chromosome 10 open reading frame 119	Homo sapiens chromosome 10 open rea	3218.39200	3427.46140	1958.93650	1696.33870	Detected	Detected	Detected	Detected
5589	A_33_P3318960	POLR2F	Hs.436578	polymerase (RNA) II (DNA directed) pol	Homo sapiens polymerase (RNA) II (DNA	12467.40000	15066.28300	13435.79200	13418.52800	Detected	Detected	Detected	Detected
5590	A_23_P69249	ACTL6A	Hs.435326	actin-like 6A	Homo sapiens actin-like 6A (ACTL6A),	6663.68550	7997.35500	6483.44240	6870.00150	Detected	Detected	Detected	Detected
5591	A_33_P3306823	ZNF846	Hs.665717	zinc finger protein 846	Homo sapiens zinc finger protein 846 (Z	14.85290	12.73272	11.89291	2.96350	Detected	Compromised	Compromised	Compromised
5592	A_33_P3245606	BHLHA9		basic helix-loop-helix family, member a9	Homo sapiens basic helix-loop-helix far	17.86018	9.38772	11.63444	2.96133	Detected	Compromised	Compromised	Compromised
5593	A_33_P3409210		Hs.710582		cDNA FLJ44654 fis, clone BRACE2047	104.31122	285.45505	88.91026	249.75607	Detected	Detected	Detected	Detected
5594	A_23_P317691	UIMC1	Hs.232721	ubiquitin interaction motif containing 1	Homo sapiens ubiquitin interaction moti	8816.63500	9070.09000	6967.82130	6115.14800	Detected	Detected	Detected	Detected
5595	A_24_P48177	ST3GAL2	Hs.368611	ST3 beta-galactoside alpha-2,3-sialyltr	Homo sapiens ST3 beta-galactoside alp	2835.86670	3390.91720	1736.91330	1844.87340	Detected	Detected	Detected	Detected
5596	A_23_P47839	DDX55	Hs.286173	DEAD (Asp-Glu-Ala-Asp) box polypept	Homo sapiens DEAD (Asp-Glu-Ala-Asp	474.73130	687.94820	418.31350	504.59930	Detected	Detected	Detected	Detected
5597	A_23_P136172	TMEM68	Hs.420076	transmembrane protein 68	Homo sapiens transmembrane protein 6	192.17203	170.22990	198.86491	124.27351	Detected	Detected	Detected	Detected
5598	A_23_P89621	CBX4	Hs.405046	chromobox homolog 4 (Pc class homolo	Homo sapiens chromobox homolog 4 (P	314.12805	210.89420	353.96585	225.51857	Detected	Detected	Detected	Detected
5599	A_23_P201628	LAMC1	Hs.609663	laminin, gamma 1 (formerly LAMB2)	Homo sapiens laminin, gamma 1 (former	9360.96800	15083.04400	7472.10550	9555.25300	Detected	Detected	Detected	Detected
5600	A_32_P231086	GOLGA6L9	Hs.630181	golgi autoantigen, golgin subfamily a, 6-l	Homo sapiens golgi autoantigen, golgin s	944.64900	615.83270	681.87950	374.21010	Detected	Detected	Detected	Detected
5601	A_23_P122134	NMUR2	Hs.283093	neuromedin U receptor 2	Homo sapiens neuromedin U receptor 2	16.49329	26.14739	15.23018	17.99435	Detected	Detected	Detected	Detected
5602	A_23_P110957	FOXF2	Hs.484423	forkhead box F2	Homo sapiens forkhead box F2 (FOXF2)	2461.97950	3972.21660	1652.06630	2298.30130	Detected	Detected	Detected	Detected
5603	A_33_P3290707	MME	Hs.307734	membrane metallo-endopeptidase	Homo sapiens membrane metallo-endop	2849.60500	1039.20120	3660.71480	662.04380	Detected	Detected	Detected	Detected
5604	A_23_P65041	RACGAP1P	Hs.650500	Rac GTPase activating protein 1 pseudo	Homo sapiens Rac GTPase activating p	26.28015	305.61334	32.67522	296.10828	Detected	Detected	Detected	Detected
5605	A_23_P115124	TRIM11	Hs.13543	tripartite motif-containing 11	Homo sapiens tripartite motif-containin	1008.93910	1224.39600	1206.67000	1337.71960	Detected	Detected	Detected	Detected
5606	A_23_P25293	NCAPD2	Hs.5719	non-SMC condensin I complex, subunit	Homo sapiens non-SMC condensin I co	659.44366	2117.33470	729.57230	2049.88940	Detected	Detected	Detected	Detected
5607	A_33_P3287562	BCAP29	Hs.303787	B-cell receptor-associated protein 29	Homo sapiens B-cell receptor-associat	19291.99800	11598.00300	12150.72900	6467.84380	Detected	Detected	Detected	Detected
5608	A_32_P24140	GAS2	Hs.632151	growth arrest-specific 2	Homo sapiens growth arrest-specific 2	92.59290	5.81353	78.18802	3.07793	Detected	Compromised	Detected	Compromised
5609	A_23_P427217	JMJD1C	Hs.413416	jumonji domain containing 1C	Homo sapiens jumonji domain containing	1213.90230	832.74430	850.74560	483.36280	Detected	Detected	Detected	Detected
5610	A_23_P125837	CXorf26	Hs.370100	chromosome X open reading frame 26	Homo sapiens chromosome X open read	552.09120	824.77386	454.18222	591.12024	Detected	Detected	Detected	Detected
5611	A_23_P215549	PON3	Hs.440967	paraoxonase 3	Homo sapiens paraoxonase 3 (PON3), m	45.28404	95.44412	24.34313	50.41994	Detected	Detected	Detected	Detected
5612	A_23_P379026	GTPBP2	Hs.485449	GTP binding protein 2	Homo sapiens GTP binding protein 2 (G	2585.45200	2772.50170	2043.30490	1699.44760	Detected	Detected	Detected	Detected
5613	A_23_P31489	URG4	Hs.709317	up-regulated gene 4	Homo sapiens up-regulated gene 4 (UR	1704.49580	1745.50900	1833.12300	1971.73180	Detected	Detected	Detected	Detected
5614	A_24_P196384	LOC100129502	Hs.6734	hypothetical protein LOC100129502	Homo sapiens cDNA FLJ30690 fis, clon	51.84105	53.41185	26.70035	29.36077	Detected	Detected	Detected	Detected
5615	A_23_P110802	CENPH	Hs.631967	centromere protein H	Homo sapiens centromere protein H (C	794.73270	1885.61240	521.67830	1021.24670	Detected	Detected	Detected	Detected
5616	A_33_P3417150	P2RY1	Hs.654526	purinergic receptor P2Y, G-protein cou	Homo sapiens purinergic receptor P2Y,	40.09369	29.97777	5.69890	3.84224	Detected	Detected	Compromised	Compromised
5617	A_23_P420256	C2orf43	Hs.187823	chromosome 2 open reading frame 43	Homo sapiens chromosome 2 open read	868.80460	378.47183	775.47220	317.08510	Detected	Detected	Detected	Detected
5618	A_33_P3232038	LOC389458	Hs.720724	hypothetical LOC389458	Homo sapiens hypothetical LOC389458	10451.78200	13640.95200	6156.15300	5585.20460	Detected	Detected	Detected	Detected
5619	A_23_P112004	LRRC6	Hs.591865	leucine rich repeat containing 6	Homo sapiens leucine rich repeat conta	45.89535	38.72318	36.20078	25.28111	Detected	Detected	Detected	Detected
5620	A_24_P323997	TPRX1	Hs.629812	tetra-peptide repeat homeobox 1	Homo sapiens tetra-peptide repeat hom	51.05117	78.56464	27.92441	36.49838	Detected	Detected	Detected	Detected
5621	A_23_P3355	POLG	Hs.706868	polymerase (DNA directed), gamma	Homo sapiens polymerase (DNA directe	3369.80540	3060.13000	2562.53780	2100.18500	Detected	Detected	Detected	Detected

5622	A_33_P3259708	CMA1	Hs.135626	chymase 1, mast cell	Homo sapiens chymase 1, mast cell (CM	41.18157	42.40707	17.00753	17.73487	Detected	Detected	Detected	Detected
5623	A_23_P391228	MANEAL	Hs.534562	mannosidase, endo-alpha-like	Homo sapiens mannosidase, endo-alpha	67.91811	26.81895	52.51436	17.25199	Detected	Detected	Detected	Detected
5624	A_23_P101829	LPAR2	Hs.122575	lysophosphatidic acid receptor 2	Homo sapiens lysophosphatidic acid rec	148.92572	75.36151	117.43903	51.37141	Detected	Detected	Detected	Detected
5625	A_23_P17844	PVALB	Hs.295449	parvalbumin	Homo sapiens parvalbumin (PVALB), m	70.34963	81.02230	24.42361	22.61610	Detected	Detected	Detected	Detected
5626	A_23_P380766	CDC42BPB	Hs.654634	CDC42 binding protein kinase beta (DM	Homo sapiens CDC42 binding protein ki	6645.30270	7606.32760	5020.61960	5120.39900	Detected	Detected	Detected	Detected
5627	A_23_P133279	FAM173B	Hs.481569	family with sequence similarity 173, mer	Homo sapiens family with sequence sim	385.68190	448.54170	322.80505	301.25060	Detected	Detected	Detected	Detected
5628	A_23_P259580	TAPBP	Hs.370937	TAP binding protein (tapasin)	Homo sapiens TAP binding protein (tapa	73.96967	89.79683	79.61233	89.20175	Detected	Detected	Detected	Detected
5629	A_23_P141479	SUPT6H	Hs.250429	suppressor of Ty 6 homolog (S. cerevisi	Homo sapiens suppressor of Ty 6 homo	3831.37740	3122.48630	3591.82200	2656.20140	Detected	Detected	Detected	Detected
5630	A_23_P50081	IMPA2	Hs.720058	inositol(myo)-1(or 4)-monophosphatase	Homo sapiens inositol(myo)-1(or 4)-mo	7392.97460	2044.49670	6137.30760	1524.36000	Detected	Detected	Detected	Detected
5631	A_23_P145357	BAK1	Hs.485139	BCL2-antagonist/killer 1	Homo sapiens BCL2-antagonist/killer 1	1202.95950	2899.68100	1258.46310	2945.30710	Detected	Detected	Detected	Detected
5632	A_33_P3272330	DNMT3A	Hs.515840	DNA (cytosine-5-)-methyltransferase 3	Homo sapiens DNA (cytosine-5-)-meth	92.53301	74.09419	76.99387	50.08971	Detected	Detected	Detected	Detected
5633	A_23_P16242	ZNF20	Hs.709334	zinc finger protein 20	Homo sapiens zinc finger protein 20 (ZN	168.86382	194.58119	105.17442	103.50449	Detected	Detected	Detected	Detected
5634	A_24_P323967	ZNF880	Hs.502314	zinc finger protein 880	Homo sapiens zinc finger protein 880 (Z	280.67398	256.89847	46.93728	32.33081	Detected	Detected	Detected	Detected
5635	A_23_P84000	CACNG2	Hs.670146	calcium channel, voltage-dependent, ga	Homo sapiens calcium channel, voltage-	17.05386	42.54961	7.66197	6.78929	Detected	Detected	Compromised	Compromised
5636	A_32_P231446	HIPK1	Hs.532363	homeodomain interacting protein kinase	Homo sapiens homeodomain interacting	5878.03100	4153.26460	4217.96040	2367.08000	Detected	Detected	Detected	Detected
5637	A_23_P70307	SMOC2	Hs.487200	SPARC related modular calcium binding	Homo sapiens SPARC related modular	5.30047	34.32664	2.85926	3.97113	Compromised	Detected	Compromised	Compromised
5638	A_33_P3283083	INPP4B	Hs.658245	inositol polyphosphate-4-phosphatase,	Homo sapiens inositol polyphosphate-4	7.55689	38.36041	6.10817	6.81470	Compromised	Detected	Compromised	Compromised
5639	A_23_P29365	RYBP	Hs.7910	RING1 and YY1 binding protein	Homo sapiens RING1 and YY1 binding p	684.88763	982.09330	577.87054	684.64954	Detected	Detected	Detected	Detected
5640	A_24_P282751	SUPT7L	Hs.6232	suppressor of Ty 7 (S. cerevisiae)-like	Homo sapiens suppressor of Ty 7 (S. ce	31.70395	34.27554	21.37351	16.94335	Detected	Detected	Detected	Detected
5641	A_33_P3387561	OSCAR	Hs.347655	osteoclast associated, immunoglobulin-	Homo sapiens osteoclast associated, im	10021.31250	12034.86700	7754.51170	6255.91360	Detected	Detected	Detected	Detected
5642	A_23_P154306	TANK	Hs.132257	TRAF family member-associated NFKB	Homo sapiens TRAF family member-ass	6199.75200	3649.15400	7168.13700	3787.89160	Detected	Detected	Detected	Detected
5643	A_24_P169896	LOC100289922		hypothetical protein LOC100289922	cDNA FLJ32655 fis, clone TESTI10000	17.60120	8.19474	11.43444	6.23901	Detected	Compromised	Compromised	Compromised
5644	A_23_P54891	C16orf58	Hs.9003	chromosome 16 open reading frame 58	Homo sapiens chromosome 16 open rea	2231.13300	1028.13610	2074.00370	912.18120	Detected	Detected	Detected	Detected
5645	A_33_P3437907	FAM171A2	Hs.154001	family with sequence similarity 171, mer	Homo sapiens family with sequence sim	100.71490	95.86520	86.74512	76.35674	Detected	Detected	Detected	Detected
5646	A_23_P149042	PYCR2	Hs.654718	pyrroline-5-carboxylate reductase fami	Homo sapiens pyrroline-5-carboxylate	826.31000	673.85690	1074.27880	767.19900	Detected	Detected	Detected	Detected
5647	A_23_P390097	TTC39B	Hs.563630	tetratricopeptide repeat domain 39B	Homo sapiens tetratricopeptide repeat	219.73584	265.13983	182.11460	183.62060	Detected	Detected	Detected	Detected
5648	A_33_P3319406				Rho GTPase-activating protein 21 (Rho	33.15133	55.20000	18.83115	26.41073	Detected	Detected	Detected	Detected
5649	A_33_P3220857				UPF0553 protein C9orf64 [Source:UniF	56.42101	128.00050	69.20654	127.65440	Detected	Detected	Detected	Detected
5650	A_33_P3399268				Interleukin-15 receptor subunit alpha P	185.00815	173.01930	205.45522	160.77092	Detected	Detected	Detected	Detected
5651	A_33_P3333092	LOC729242	Hs.680206	similar to keratin 17	PREDICTED: Homo sapiens similar to k	15.23123	16.37261	7.84885	10.04400	Detected	Compromised	Compromised	Compromised
5652	A_33_P3389540					58160.42600	53914.00000	45952.75000	36811.37000	Detected	Detected	Detected	Detected
5653	A_33_P3828101	CCDC112	Hs.436121	coiled-coil domain containing 112	Homo sapiens coiled-coil domain conta	484.40170	541.24475	309.75903	279.36050	Detected	Detected	Detected	Detected
5654	A_33_P3352073	hCG_1806964		hypothetical LOC401093	Homo sapiens hypothetical LOC401093	17.47629	35.09377	13.65129	21.34896	Detected	Detected	Compromised	Detected
5655	A_23_P320530	ZNF780A	Hs.115140	zinc finger protein 780A	Homo sapiens zinc finger protein 780A	49.56512	26.23464	15.63957	8.20350	Detected	Detected	Detected	Compromised
5656	A_23_P255672	ABLIM2	Hs.233404	actin binding LIM protein family, membe	Homo sapiens actin binding LIM protein	493.69733	10.30636	355.38593	5.65312	Detected	Compromised	Detected	Compromised
5657	A_23_P368740	HDAC10	Hs.26593	histone deacetylase 10	Homo sapiens histone deacetylase 10 (	118.17491	65.73660	98.99884	55.44788	Detected	Detected	Detected	Detected
5658	A_24_P185158	FAM134C	Hs.632262	family with sequence similarity 134, mer	Homo sapiens family with sequence sim	2799.82930	2516.00460	2009.60240	1726.60600	Detected	Detected	Detected	Detected
5659	A_33_P3396050	LOC645307	Hs.558726	hypothetical LOC645307	PREDICTED: Homo sapiens hypothetica	10.94363	20.22203	5.55514	14.64144	Compromised	Detected	Compromised	Compromised
5660	A_33_P3420167				GB	14253.26300	13824.26600	15185.73900	12614.48900	Detected	Detected	Detected	Detected
5661	A_33_P3318534	ACPL2	Hs.657887	acid phosphatase-like 2	Homo sapiens acid phosphatase-like 2	369.05618	172.81972	212.95341	130.85135	Detected	Detected	Detected	Detected
5662	A_33_P3818787	LOC344967	Hs.534633	acyl-CoA thioesterase 7 pseudogene	Homo sapiens acyl-CoA thioesterase 7	217.93270	828.47534	61.97788	199.51576	Detected	Detected	Detected	Detected
5663	A_33_P3399373	TPRA1	Hs.6418	transmembrane protein, adipocyte asso	Homo sapiens transmembrane protein,	7209.92800	9424.73400	3651.77700	4401.41360	Detected	Detected	Detected	Detected
5664	A_23_P146922	GAS6	Hs.646346	growth arrest-specific 6	Homo sapiens growth arrest-specific 6	6633.65870	10849.22950	7201.36430	12030.05500	Detected	Detected	Detected	Detected
5665	A_33_P3379763	LOC100130320	Hs.722605	similar to succinate dehydrogenase con	PREDICTED: Homo sapiens similar to s	554.30470	467.99300	466.01514	337.29590	Detected	Detected	Detected	Detected
5666	A_23_P150238	C11orf68	Hs.433573	chromosome 11 open reading frame 68	Homo sapiens chromosome 11 open rea	2264.84250	3437.62430	2540.79900	3619.29790	Detected	Detected	Detected	Detected
5667	A_23_P141802	SERPINB7	Hs.138202	serpin peptidase inhibitor, clade B (oval	Homo sapiens serpin peptidase inhibitor	66.62235	485.98645	74.93073	467.97940	Detected	Detected	Detected	Detected
5668	A_33_P3312544				Gamma-aminobutyric acid receptor sub	435.19385	486.96533	107.91630	91.30059	Detected	Detected	Detected	Detected
5669	A_33_P3609431	FLJ45949		FLJ45949 protein	Homo sapiens cDNA FLJ45949 fis, clon	14045.90100	11728.73900	2191.07710	1560.36720	Detected	Detected	Detected	Detected
5670	A_33_P3272563	NMT2	Hs.60339	N-myristoyltransferase 2	Homo sapiens N-myristoyltransferase 2	586.75415	537.84753	748.54690	666.20294	Detected	Detected	Detected	Detected
5671	A_32_P28223		Hs.656851		Homo sapiens full length insert cDNA Y	219.19884	88.04734	159.30766	57.96906	Detected	Detected	Detected	Detected
5672	A_33_P3401093	FBXO36	Hs.140666	F-box protein 36	Homo sapiens F-box protein 36 (FBXO3	136.39389	100.65643	162.62283	90.35278	Detected	Detected	Detected	Detected
5673	A_24_P357709	VPS8	Hs.269263	vacuolar protein sorting 8 homolog (S. c	Homo sapiens vacuolar protein sorting	381.04138	274.27762	280.31186	172.04788	Detected	Detected	Detected	Detected
5674	A_23_P84565	POLR3D	Hs.148342	polymerase (RNA) III (DNA directed) po	Homo sapiens polymerase (RNA) III (DN	561.50195	706.60767	477.01538	526.65290	Detected	Detected	Detected	Detected
5675	A_24_P215882	ARHGDIG	Hs.647388	Rho GDP dissociation inhibitor (GDI) ga	Homo sapiens Rho GDP dissociation inh	22.81186	24.74208	16.64939	14.51193	Detected	Detected	Detected	Compromised
5676	A_23_P201636	LAMC2	Hs.591484	laminin, gamma 2	Homo sapiens laminin, gamma 2 (LAMC	800.69934	2835.61180	720.53546	2281.80700	Detected	Detected	Detected	Detected
5677	A_33_P3259022	SLC6A2	Hs.78036	solute carrier family 6 (neurotransmitte	Homo sapiens solute carrier family 6 (n	282.29932	399.59216	86.66883	83.16106	Detected	Detected	Detected	Detected
5678	A_24_P339664	NCLN	Hs.657032	nicalin homolog (zebrafish)	Homo sapiens nicalin homolog (zebrafis	14882.93100	29634.85200	15295.79500	28107.92600	Detected	Detected	Detected	Detected
5679	A_23_P429689	BOD1L	Hs.444517	biorientation of chromosomes in cell div	Homo sapiens biorientation of chromoso	1550.95260	1148.60690	796.01825	518.38060	Detected	Detected	Detected	Detected
5680	A_24_P338648	ARL1	Hs.372616	ADP-ribosylation factor-like 1	Homo sapiens ADP-ribosylation factor-	5757.15870	6706.92720	4163.30860	4352.08200	Detected	Detected	Detected	Detected
5681	A_23_P31945	IL33	Hs.348390	interleukin 33	Homo sapiens interleukin 33 (IL33), mR	409.89960	170.21780	387.54675	135.73192	Detected	Detected	Detected	Detected
5682	A_33_P3377719	LOC100129112	Hs.659741	hypothetical protein LOC100129112	Homo sapiens cDNA FLJ41845 fis, clon	15.71753	20.73640	6.61073	3.97431	Detected	Detected	Compromised	Compromised
5683	A_33_P3360087	BBS9	Hs.372360	Bardet-Biedl syndrome 9	Homo sapiens Bardet-Biedl syndrome 9	66.83823	35.29454	59.93847	18.89915	Detected	Detected	Detected	Detected
5684	A_23_P135257	PRSS3	Hs.654513	protease, serine, 3	Homo sapiens protease, serine, 3 (PRS	1935.98730	2030.39670	2333.39580	2031.09350	Detected	Detected	Detected	Detected
5685	A_23_P62133	MTM1	Hs.655056	myotubularin 1	Homo sapiens myotubularin 1 (MTM1),	283.96298	234.16107	202.72165	166.84280	Detected	Detected	Detected	Detected
5686	A_24_P935345		Hs.676497		NINP6167 [Source:UniProtKB/TrEMBL	85.10198	118.83929	33.82225	42.79303	Detected	Detected	Detected	Detected
5687	A_24_P204819	SPRY3	Hs.381912	sprouty homolog 3 (Drosophila)	Homo sapiens sprouty homolog 3 (Dros	19.56641	15.61483	3.74689	10.54934	Detected	Compromised	Compromised	Compromised
5688	A_33_P3269789		Hs.534420		Putative uncharacterized protein ENSP	13.86681	7.18439	2.87422	5.12644	Detected	Compromised	Compromised	Compromised
5689	A_33_P3389728	NR5A2	Hs.33446	nuclear receptor subfamily 5, group A, r	Homo sapiens nuclear receptor subfami	298.60205	658.35345	206.43166	446.82407	Detected	Detected	Detected	Detected
5690	A_23_P145935	EPHB6	Hs.380089	EPH receptor B6	Homo sapiens EPH receptor B6 (EPHB	376.65836	92.19390	474.44800	111.21818	Detected	Detected	Detected	Detected
5691	A_32_P62571	RBM8A	Hs.719989	RNA binding motif protein 8A	Homo sapiens RNA binding motif protei	638.53534	1017.88763	433.62164	523.64417	Detected	Detected	Detected	Detected
5692	A_33_P3249936	C3orf67	Hs.368434	chromosome 3 open reading frame 67	Homo sapiens chromosome 3 open read	485.95013	125.25383	328.04327	66.87203	Detected	Detected	Detected	Detected
5693	A_23_P137143	DKC1	Hs.4747	dyskeratosis congenita 1, dyskerin	Homo sapiens dyskeratosis congenita 1	6394.51030	8949.11400	5512.26500	6778.71970	Detected	Detected	Detected	Detected
5694	A_33_P3407636					119.22460	659.51215	83.65967	384.33353	Detected	Detected	Detected	Detected
5695	A_33_P3343957	EHBP1	Hs.271667	EH domain binding protein 1	Homo sapiens EH domain binding protei	569.20870	392.51263	494.07180	308.24707	Detected	Detected	Detected	Detected
5696	A_23_P128166	RAB21	Hs.524590	RAB21, member RAS oncogene family	Homo sapiens RAB21, member RAS on	6364.22400	8958.98700	5936.95800	7245.59000	Detected	Detected	Detected	Detected

5697	A_33_P3283992	ZNF671	Hs.180402	zinc finger protein 671	Homo sapiens zinc finger protein 671 (Z	108.08907	158.55666	85.17768	90.88654	Detected	Detected	Detected	Detected
5698	A_33_P3230594				GB	868.46870	290.24560	547.20870	133.41669	Detected	Detected	Detected	Detected
5699	A_24_P200219	UPK1B	Hs.271580	uroplakin 1B	Homo sapiens uroplakin 1B (UPK1B), m	2.51242	31.97251	2.86027	2.98971	Compromised	Detected	Compromised	Compromised
5700	A_23_P304287	PSMC2	Hs.437366	proteasome (prosome, macropain) 26S	Homo sapiens proteasome (prosome, m	12096.13700	15560.74900	10789.37400	13045.49100	Detected	Detected	Detected	Detected
5701	A_33_P3404418	MAP3K6	Hs.194694	mitogen-activated protein kinase kinase	Homo sapiens mitogen-activated protei	480.47977	648.71340	525.54486	835.41230	Detected	Detected	Detected	Detected
5702	A_33_P3258478	KIR3DP1	Hs.690615	killer-cell Ig-like receptor	Homo sapiens killer-cell Ig-like recepto	23.74017	91.47687	14.91342	41.54417	Detected	Detected	Detected	Detected
5703	A_32_P118568	RFPL1S	Hs.167750	RFPL1 antisense RNA (non-protein coc	Homo sapiens RFPL1 antisense RNA (n	43.10982	10.27129	31.32931	2.95532	Detected	Compromised	Detected	Compromised
5704	A_23_P75402	NDUFS3	Hs.502528	NADH dehydrogenase (ubiquinone) Fe-S	Homo sapiens NADH dehydrogenase (ul	13561.59000	11754.28200	15640.37200	11489.32600	Detected	Detected	Detected	Detected
5705	A_33_P3271810	PHLPP2	Hs.709458	PH domain and leucine rich repeat prot	Homo sapiens PH domain and leucine ri	13.01441	21.56240	13.82625	20.53851	Detected	Detected	Compromised	Detected
5706	A_33_P3233055		Hs.720773		UI-H-BW1-anr-f-12-0-UI.s1 NCI_CGAI	84.45535	110.37121	122.66299	123.20510	Detected	Detected	Detected	Detected
5707	A_24_P606663					45488.65000	32950.61000	16224.71700	9852.69700	Detected	Detected	Detected	Detected
5708	A_33_P3362526	MED31	Hs.567493	mediator complex subunit 31	Homo sapiens mediator complex subuni	166.61902	142.04663	140.05766	89.53911	Detected	Detected	Detected	Detected
5709	A_32_P41375	tcag7.907	Hs.534942	hypothetical LOC402483	Homo sapiens hypothetical LOC402483	390.31982	230.08089	319.57340	175.33040	Detected	Detected	Detected	Detected
5710	A_33_P3378702				Putative uncharacterized protein ENSP	106.32911	143.67831	102.49580	92.95548	Detected	Detected	Detected	Detected
5711	A_33_P3352407	LOC729009	Hs.449153	similar to ferritin, heavy polypeptide 1	PREDICTED: Homo sapiens similar to F	235018.52000	203625.28000	224159.80000	182277.70000	Detected	Detected	Detected	Detected
5712	A_23_P314835	C20orf85	Hs.43977	chromosome 20 open reading frame 85	Homo sapiens chromosome 20 open rea	13.93233	16.59781	11.76394	14.97931	Detected	Compromised	Compromised	Detected
5713	A_33_P3324495	ZFP41	Hs.668016	zinc finger protein 41 homolog (mouse)	Homo sapiens zinc finger protein 41 hor	2808.01270	1307.90380	2471.86500	932.15410	Detected	Detected	Detected	Detected
5714	A_23_P115838	C10orf12	Hs.427927	chromosome 10 open reading frame 12	Homo sapiens chromosome 10 open rea	58.12010	111.44312	37.21150	66.51118	Detected	Detected	Detected	Detected
5715	A_33_P3273324	ZNF880	Hs.502314	zinc finger protein 880	Homo sapiens zinc finger protein LOC4(	1218.62020	1098.03910	1026.89250	699.99390	Detected	Detected	Detected	Detected
5716	A_32_P87013	IL8	Hs.624	interleukin 8	Homo sapiens interleukin 8 (IL8), mRNA	3709.52930	4145.03100	1420.30100	1049.12320	Detected	Detected	Detected	Detected
5717	A_33_P3337034	PRPF4B	Hs.159014	PRP4 pre-mRNA processing factor 4 h	Homo sapiens PRP4 pre-mRNA proces	365.07370	260.97470	282.29538	187.29816	Detected	Detected	Detected	Detected
5718	A_23_P301247	HIST2H2AC	Hs.408067	histone cluster 2, H2ac	Homo sapiens histone cluster 2, H2ac (	559.30225	976.56415	420.23474	606.72210	Detected	Detected	Detected	Detected
5719	A_23_P29282	NPTXR	Hs.91622	neuronal pentraxin receptor	Homo sapiens neuronal pentraxin recep	74.91033	107.40868	67.77159	91.25624	Detected	Detected	Detected	Detected
5720	A_23_P411162	PER2	Hs.58756	period homolog 2 (Drosophila)	Homo sapiens period homolog 2 (Drosop	30.52989	41.60015	15.66415	31.48260	Detected	Detected	Detected	Detected
5721	A_33_P3327158	LOC100131432	Hs.640116	hypothetical LOC100131432	Homo sapiens cDNA FLJ45862 fis, clon	467.04950	529.11194	72.83270	64.97926	Detected	Detected	Detected	Detected
5722	A_23_P17593	CDH4	Hs.473231	cadherin 4, type 1, R-cadherin (retinal)	Homo sapiens cadherin 4, type 1, R-cac	134.48900	793.39480	119.07429	668.90670	Detected	Detected	Detected	Detected
5723	A_33_P3235990	PTPN21	Hs.437040	protein tyrosine phosphatase, non-rece	Homo sapiens protein tyrosine phospha	2559.63260	2128.66580	2203.11430	1489.15220	Detected	Detected	Detected	Detected
5724	A_33_P3242614	LOC606724		coronin, actin binding protein, 1A pseud	Homo sapiens coronin, actin binding pro	46.88162	37.96114	28.65409	16.73704	Detected	Detected	Detected	Detected
5725	A_33_P3312246	PHIP	Hs.511817	pleckstrin homology domain interacting	Homo sapiens pleckstrin homology dom	2338.89650	1363.41650	1752.59880	883.00385	Detected	Detected	Detected	Detected
5726	A_23_P139123	SERPING1	Hs.384598	serpin peptidase inhibitor, clade G (C1 i	Homo sapiens serpin peptidase inhibitor	2228.46390	1016.62680	1566.13440	685.07510	Detected	Detected	Detected	Detected
5727	A_32_P8529	C17orf86	Hs.720923	chromosome 17 open reading frame 86	Homo sapiens chromosome 17 open rea	32.35884	52.02356	56.04335	67.02494	Detected	Detected	Detected	Detected
5728	A_23_P388670	LTA4H	Hs.524648	leukotriene A4 hydrolase	Homo sapiens leukotriene A4 hydrolase	9068.13000	6750.59300	9762.78100	6423.21700	Detected	Detected	Detected	Detected
5729	A_33_P3423631	WHSC1	Hs.113876	Wolf-Hirschhorn syndrome candidate 1	Homo sapiens Wolf-Hirschhorn syndron	85.14700	489.86136	123.90369	529.43170	Detected	Detected	Detected	Detected
5730	A_33_P3378126	FBXO32	Hs.403933	F-box protein 32	Homo sapiens F-box protein 32 (FBXO3	14752.09100	6356.78170	10623.82200	3797.98850	Detected	Detected	Detected	Detected
5731	A_33_P3331726	LOC647979	Hs.401218	hypothetical LOC647979	Homo sapiens hypothetical LOC647979	795.83250	1384.07980	314.78345	454.12010	Detected	Detected	Detected	Detected
5732	A_24_P74571	CBY1	Hs.720074	chibby homolog 1 (Drosophila)	Homo sapiens chibby homolog 1 (Droso	558.35065	442.82568	536.22130	428.11984	Detected	Detected	Detected	Detected
5733	A_24_P383478	ESR1	Hs.208124	estrogen receptor 1	Homo sapiens estrogen receptor 1 (ESF	77.27553	81.85938	30.49788	31.63243	Detected	Detected	Detected	Detected
5734	A_33_P3219434	MOSC2	Hs.369042	MOCO sulphurase C-terminal domain c	Homo sapiens MOCO sulphurase C-ter	123.46970	63.81842	102.22758	46.69906	Detected	Detected	Detected	Detected
5735	A_33_P3349501	LOC100132413		similar to hCG1782458	PREDICTED: Homo sapiens similar to h	198.30530	210.64862	57.37755	55.56039	Detected	Detected	Detected	Detected
5736	A_23_P72025	SLC25A20	Hs.13845	solute carrier family 25 (carnitine/acylc	Homo sapiens solute carrier family 25 (	955.46027	672.74066	964.17980	582.78656	Detected	Detected	Detected	Detected
5737	A_23_P128215	SOCS2	Hs.485572	suppressor of cytokine signaling 2	Homo sapiens suppressor of cytokine s	4416.62450	3973.29050	3729.67400	2885.34450	Detected	Detected	Detected	Detected
5738	A_23_P101073	OR1E1	Hs.278485	olfactory receptor, family 1, subfamily E	Homo sapiens olfactory receptor, family	33.42365	38.88860	14.90000	15.19916	Detected	Detected	Compromised	Compromised
5739	A_33_P3339860	PLEKHA2	Hs.369123	pleckstrin homology domain containing,	Homo sapiens pleckstrin homology dom	4697.16200	9201.64500	3373.01680	5708.09670	Detected	Detected	Detected	Detected
5740	A_23_P38732	CDH2	Hs.464829	cadherin 2, type 1, N-cadherin (neurona	Homo sapiens cadherin 2, type 1, N-cac	213.39350	1515.36610	191.76831	1222.59730	Detected	Detected	Detected	Detected
5741	A_33_P3218955	KDM6A	Hs.522616	lysine (K)-specific demethylase 6A	Homo sapiens lysine (K)-specific demet	142.93520	121.76021	100.01070	75.71861	Detected	Detected	Detected	Detected
5742	A_23_P255653	TNFRSF10A	Hs.591834	tumor necrosis factor receptor superfar	Homo sapiens tumor necrosis factor rec	88.61099	352.94740	91.94637	333.06207	Detected	Detected	Detected	Detected
5743	A_33_P3351175	WNK2	Hs.654856	WNK lysine deficient protein kinase 2	Homo sapiens WNK lysine deficient prot	28.41998	3.51085	20.12625	3.16905	Detected	Compromised	Detected	Compromised
5744	A_23_P73982	TMEM48	Hs.476525	transmembrane protein 48	Homo sapiens transmembrane protein 4	1190.70300	2734.19200	962.20795	1913.63450	Detected	Detected	Detected	Detected
5745	A_33_P3347132	EXOSC2	Hs.654643	exosome component 2	Homo sapiens exosome component 2 (E	545.13306	1500.72020	468.58923	1151.55290	Detected	Detected	Detected	Detected
5746	A_33_P3363690	LOC391636	Hs.646338	similar to chromosome 9 open reading f	PREDICTED: Homo sapiens similar to c	339.06396	412.82385	28.59783	35.90949	Detected	Detected	Detected	Detected
5747	A_33_P3332970	CLEC2B	Hs.85201	C-type lectin domain family 2, member	Homo sapiens C-type lectin domain fam	6559.26900	1694.38760	6492.38000	1562.61010	Detected	Detected	Detected	Detected
5748	A_33_P3257703	C9orf131	Hs.148250	chromosome 9 open reading frame 131	Homo sapiens chromosome 9 open reac	36.07060	43.76042	25.58928	25.32075	Detected	Detected	Detected	Detected
5749	A_24_P406245	PMS2L2	Hs.720746	postmeiotic segregation increased 2-lik	Homo sapiens cDNA clone IMAGE:3456	124.00018	311.03650	101.31708	227.92752	Detected	Detected	Detected	Detected
5750	A_33_P3280965	SNHG9		small nucleolar RNA host gene 9 (non-p	Homo sapiens small nucleolar RNA host	11961.74800	15751.84900	11344.44100	12824.22100	Detected	Detected	Detected	Detected
5751	A_23_P390700	CNTN1	Hs.143434	contactin 1	Homo sapiens contactin 1 (CNTN1), tra	4.43926	18.51506	2.86166	2.99279	Compromised	Detected	Compromised	Compromised
5752	A_33_P3278362	ANKRD2	Hs.73708	ankyrin repeat domain 2 (stretch respor	Homo sapiens ankyrin repeat domain 2	53.20981	373.87830	53.70923	338.05417	Detected	Detected	Detected	Detected
5753	A_24_P216014	GH2	Hs.406754	growth hormone 2	Homo sapiens growth hormone 2 (GH2)	2.64374	30.76777	2.85337	2.98188	Compromised	Detected	Compromised	Compromised
5754	A_24_P40094	ATCAY	Hs.418055	ataxia, cerebellar, Cayman type	Homo sapiens ataxia, cerebellar, Cayma	9.59126	43.86983	6.63278	5.96894	Compromised	Detected	Compromised	Compromised
5755	A_24_P280497	FBRSL1	Hs.411138	fibrosin-like 1	Homo sapiens fibrosin-like 1 (FBRSL1),	24491.41400	29965.19700	11340.71500	9594.08900	Detected	Detected	Detected	Detected
5756	A_23_P15045	E4F1	Hs.513268	E4F transcription factor 1	Homo sapiens E4F transcription factor	967.32710	1195.96230	1525.55460	1847.34700	Detected	Detected	Detected	Detected
5757	A_33_P3214056	LOC728875	Hs.551637	hypothetical LOC728875	Homo sapiens hypothetical LOC728875	4032.15920	2652.40100	2852.15670	1687.99070	Detected	Detected	Detected	Detected
5758	A_24_P329229	KCTD7	Hs.546627	potassium channel tetramerisation dom	Homo sapiens potassium channel tetrar	11.91641	22.49742	2.83102	9.76708	Compromised	Detected	Compromised	Compromised
5759	A_24_P339944	PDGFB	Hs.1976	platelet-derived growth factor beta poly	Homo sapiens platelet-derived growth f	6.85375	21.64097	7.13727	15.15994	Compromised	Detected	Compromised	Detected
5760	A_33_P3284939	TMEM189	Hs.719899	transmembrane protein 189	Homo sapiens transmembrane protein 1	5037.79740	7133.37100	4024.94500	5029.90770	Detected	Detected	Detected	Detected
5761	A_23_P164022	MYO1C	Hs.286226	myosin IC	Homo sapiens myosin IC (MYO1C), tran	55075.33600	55216.96000	30580.12500	26876.38000	Detected	Detected	Detected	Detected
5762	A_23_P212383	SACM1L	Hs.156509	SAC1 suppressor of actin mutations 1-	Homo sapiens SAC1 suppressor of acti	1608.77170	1520.79910	1160.97630	951.51843	Detected	Detected	Detected	Detected
5763	A_33_P3391626				Putative uncharacterized protein ENSP	97024.60000	93668.97000	61926.20700	58686.11700	Detected	Detected	Detected	Detected
5764	A_33_P3318671	LYPLA2	Hs.533479	lysophospholipase II	Homo sapiens lysophospholipase II (LYF	21554.25600	18375.02500	13017.71000	9976.41400	Detected	Detected	Detected	Detected
5765	A_24_P233944	CEPT1	Hs.363572	choline/ethanolamine phosphotransfera	Homo sapiens choline/ethanolamine ph	156.97133	97.02003	168.87173	73.59670	Detected	Detected	Detected	Detected
5766	A_33_P3439713	LOC283713	Hs.4986	hypothetical protein LOC283713	Homo sapiens cDNA FLJ37663 fis, clon	74.07384	7.02089	50.07333	6.10264	Detected	Compromised	Detected	Compromised
5767	A_33_P3239455	GTF2IRD2	Hs.647017	GTF2I repeat domain containing 2	Homo sapiens GTF2I repeat domain cor	58.94321	24.01915	39.85822	10.43398	Detected	Detected	Detected	Compromised
5768	A_23_P215088	ZC3HC1	Hs.194157	zinc finger, C3HC-type containing 1	Homo sapiens zinc finger, C3HC-type c	1400.14230	3035.71300	1256.30870	2345.43950	Detected	Detected	Detected	Detected
5769	A_33_P3290829	LOC100128703	Hs.640061	hypothetical protein LOC100128703	PREDICTED: Homo sapiens hypothetica	38.91952	48.17169	23.45034	18.07244	Detected	Detected	Detected	Detected
5770	A_33_P3341901	TSR2	Hs.522662	TSR2, 20S rRNA accumulation, homolog	Homo sapiens TSR2, 20S rRNA accumu	3092.72050	6419.79540	2268.96200	4215.22000	Detected	Detected	Detected	Detected
5771	A_33_P3293913	BICC1	Hs.158745	bicaudal C homolog 1 (Drosophila)	Homo sapiens bicaudal C homolog 1 (Dr	75.19399	20.12886	15.77041	3.37592	Detected	Detected	Detected	Compromised

5772	A_23_P119907	ANKZF1	Hs.437647	ankyrin repeat and zinc finger domain c	Homo sapiens ankyrin repeat and zinc f	736.00660	268.21878	589.90080	202.64246	Detected	Detected	Detected	Detected
5773	A_33_P3306264	LYPD3	Hs.631594	LY6/PLAUR domain containing 3	Homo sapiens LY6/PLAUR domain con	73.82488	55.35597	71.92293	59.57527	Detected	Detected	Detected	Detected
5774	A_23_P389987	TLX2	Hs.168586	T-cell leukemia homeobox 2	Homo sapiens T-cell leukemia homeobo	44.07448	208.12553	40.50041	175.57297	Detected	Detected	Detected	Detected
5775	A_33_P3342569		Hs.712333		Putative UPF0607 protein ENSP000003	34.03972	29.32715	19.92758	19.61238	Detected	Detected	Detected	Detected
5776	A_33_P3404954		Hs.200738		Probable sodium-coupled neutral amino	4971.10300	4320.70850	3507.76340	2573.98560	Detected	Detected	Detected	Detected
5777	A_23_P48455	AMN	Hs.534494	amnionless homolog (mouse)	Homo sapiens amnionless homolog (mou	2412.86620	3015.34300	1930.24960	1844.19670	Detected	Detected	Detected	Detected
5778	A_23_P400945	ETV3	Hs.105636	ets variant 3	Homo sapiens ets variant 3 (ETV3), tra	65.07116	120.54582	50.15067	71.13472	Detected	Detected	Detected	Detected
5779	A_33_P3339202	LOC149086	Hs.675936	hypothetical protein LOC149086	PREDICTED: Homo sapiens hypothetica	418.17804	499.45850	178.99040	172.14610	Detected	Detected	Detected	Detected
5780	A_23_P141974	TPM4	Hs.631618	tropomyosin 4	Homo sapiens tropomyosin 4 (TPM4), tr	20339.79300	23133.95900	16948.51200	17744.41000	Detected	Detected	Detected	Detected
5781	A_33_P3387493					44.48738	51.28348	27.55856	27.79760	Detected	Detected	Detected	Detected
5782	A_33_P3264875	GRK6	Hs.235116	G protein-coupled receptor kinase 6	Homo sapiens G protein-coupled recep	1920.78110	2289.14480	1449.82700	2194.67770	Detected	Detected	Detected	Detected
5783	A_23_P23296	PKP1	Hs.497350	plakophilin 1 (ectodermal dysplasia/skin	Homo sapiens plakophilin 1 (ectodermal	10.54824	13.40185	29.98664	32.24558	Compromised	Compromised	Detected	Detected
5784	A_33_P3213892	PRDM15	Hs.720605	PR domain containing 15	Homo sapiens PR domain containing 15	38.32715	82.74328	27.97446	56.43785	Detected	Detected	Detected	Detected
5785	A_23_P500271	IRF5	Hs.521181	interferon regulatory factor 5	Homo sapiens interferon regulatory fact	64.92951	37.42121	50.05281	16.13107	Detected	Detected	Detected	Compromised
5786	A_33_P3332744	CYTH1	Hs.191215	cytohesin 1	Homo sapiens cytohesin 1 (CYTH1), tra	139.39150	113.92640	131.82677	93.22398	Detected	Detected	Detected	Detected
5787	A_24_P280762	KHDC1	Hs.715645	KH homology domain containing 1	Homo sapiens KH homology domain cor	489.36295	342.35870	690.68530	403.80542	Detected	Detected	Detected	Detected
5788	A_33_P3245133					188.40027	262.64984	92.31041	99.29255	Detected	Detected	Detected	Detected
5789	A_33_P3764802	SIRT5	Hs.567431	sirtuin (silent mating type information re	Homo sapiens sirtuin (silent mating type	440.43008	223.59177	401.69473	166.59550	Detected	Detected	Detected	Detected
5790	A_23_P383422	NFKBID	Hs.466531	nuclear factor of kappa light polypeptide	Homo sapiens nuclear factor of kappa li	58.46615	57.98586	35.99563	29.49577	Detected	Detected	Detected	Detected
5791	A_23_P213431	GFM2	Hs.277154	G elongation factor, mitochondrial 2	Homo sapiens G elongation factor, mito	6431.79100	4319.87740	4353.08940	2508.51660	Detected	Detected	Detected	Detected
5792	A_33_P3414122	ZNF260	Hs.18103	zinc finger protein 260	Homo sapiens zinc finger protein 260 (Z	1307.85790	713.17970	1060.55500	513.23785	Detected	Detected	Detected	Detected
5793	A_33_P3250612	MOCS1	Hs.718492	molybdenum cofactor synthesis 1	Homo sapiens molybdenum cofactor sy	257.32745	346.29640	401.19266	467.57960	Detected	Detected	Detected	Detected
5794	A_23_P417282	IGF1R	Hs.643120	insulin-like growth factor 1 receptor	Homo sapiens insulin-like growth factor	3364.82150	1713.07500	3176.18550	1371.22660	Detected	Detected	Detected	Detected
5795	A_33_P3318027	CSNK2B	Hs.73527	casein kinase 2, beta polypeptide	Homo sapiens casein kinase 2, beta pol	9364.18600	11634.11600	12693.88000	14366.03400	Detected	Detected	Detected	Detected
5796	A_23_P143143	ID2	Hs.180919	inhibitor of DNA binding 2, dominant neg	Homo sapiens inhibitor of DNA binding 2	3452.06150	1841.29150	2542.77000	1259.71240	Detected	Detected	Detected	Detected
5797	A_23_P170587	SMYD2	Hs.66170	SET and MYND domain containing 2	Homo sapiens SET and MYND domain c	4418.50800	7534.69530	3829.11650	5710.25200	Detected	Detected	Detected	Detected
5798	A_23_P111797	DKFZp434F142	Hs.588334	hypothetical DKFZp434F142	Homo sapiens mRNA; cDNA DKFZp434	1763.88540	1025.49840	836.85380	444.04507	Detected	Detected	Detected	Detected
5799	A_33_P3376463		Hs.555973		Nuclear receptor subfamily 2 group C m	89.85175	114.21388	61.88114	61.66886	Detected	Detected	Detected	Detected
5800	A_33_P3653912	FAHD2A	Hs.531247	fumarylacetoacetate hydrolase domain (	Fumarylacetoacetate hydrolase domain	56.49834	57.70201	80.30524	42.59773	Detected	Detected	Detected	Detected
5801	A_33_P3420224	ENTPD8	Hs.512562	ectonucleoside triphosphate diphosphoh	Homo sapiens ectonucleoside triphosph	21.82459	41.33719	8.25697	2.97995	Detected	Detected	Compromised	Compromised
5802	A_24_P296508	SLC43A2	Hs.160550	solute carrier family 43, member 2	Homo sapiens solute carrier family 43, r	956.45996	2364.62480	368.64752	815.95087	Detected	Detected	Detected	Detected
5803	A_23_P24633	THYN1	Hs.13645	thymocyte nuclear protein 1	Homo sapiens thymocyte nuclear prote	4286.59000	2536.94580	4111.75340	2241.89010	Detected	Detected	Detected	Detected
5804	A_33_P3258617				Adipophilin (Adipose differentiation-rela	2671.99440	394.87200	2063.65530	231.06764	Detected	Detected	Detected	Detected
5805	A_33_P3325135	ZSCAN23	Hs.721841	zinc finger and SCAN domain containing	Homo sapiens zinc finger and SCAN do	2.50484	23.86334	2.83106	2.95572	Compromised	Detected	Compromised	Compromised
5806	A_23_P160869	LRIG2	Hs.448972	leucine-rich repeats and immunoglobulin	Homo sapiens leucine-rich repeats and	479.45750	587.72460	448.19223	433.41418	Detected	Detected	Detected	Detected
5807	A_33_P3270203	DIS3L2	Hs.471637	DIS3 mitotic control homolog (S. cerevi	Homo sapiens DIS3 mitotic control hom	177.71317	197.20865	71.52568	59.87196	Detected	Detected	Detected	Detected
5808	A_24_P267293	SARM1	Hs.719354	sterile alpha and TIR motif containing 1	Homo sapiens sterile alpha and TIR mot	159.31409	81.26252	190.04422	93.65626	Detected	Detected	Detected	Detected
5809	A_23_P24755	STX5	Hs.654602	syntaxin 5	Homo sapiens syntaxin 5 (STX5), mRNA	2456.51100	1971.93260	2776.58420	2026.80030	Detected	Detected	Detected	Detected
5810	A_33_P3403392	AACS	Hs.656073	acetoacetyl-CoA synthetase	Homo sapiens acetoacetyl-CoA synthe	130.18047	240.73357	118.50428	205.28864	Detected	Detected	Detected	Detected
5811	A_23_P12989	PRDX5	Hs.502823	peroxiredoxin 5	Homo sapiens peroxiredoxin 5 (PRDX5)	22140.56400	15217.51400	21456.42400	14153.84200	Detected	Detected	Detected	Detected
5812	A_33_P3214209	LOC728411		glucuronidase, beta pseudogene	Homo sapiens glucuronidase, beta pseu	556.02580	879.48300	176.50804	274.67133	Detected	Detected	Detected	Detected
5813	A_33_P3270519	FAM40A	Hs.584996	family with sequence similarity 40, mem	Homo sapiens family with sequence sim	70.13436	85.50539	64.04029	55.86905	Detected	Detected	Detected	Detected
5814	A_33_P3305958	TECPR2	Hs.195667	tectonin beta-propeller repeat containin	Homo sapiens tectonin beta-propeller r	18.03383	30.57272	17.63189	18.87126	Detected	Detected	Detected	Detected
5815	A_33_P3219245	C9orf130		chromosome 9 open reading frame 130	Homo sapiens chromosome 9 open reac	150.53383	92.46513	98.71474	44.62233	Detected	Detected	Detected	Detected
5816	A_23_P136870	MAGEA6	Hs.441113	melanoma antigen family A, 6	Homo sapiens melanoma antigen family	23.16131	29.72754	19.80973	20.57198	Detected	Detected	Detected	Detected
5817	A_32_P86905	KGFLP2		keratinocyte growth factor-like protein	Homo sapiens keratinocyte growth fact	628.43054	300.84265	366.38525	149.61860	Detected	Detected	Detected	Detected
5818	A_24_P93855	FOXP4	Hs.131436	forkhead box P4	Homo sapiens forkhead box P4 (FOXP4	111.39562	135.11418	100.46989	117.99206	Detected	Detected	Detected	Detected
5819	A_24_P615822	BMS1	Hs.10848	BMS1 homolog, ribosome assembly prot	Homo sapiens BMS1 homolog, ribosome	301.50860	522.50590	235.93265	352.02676	Detected	Detected	Detected	Detected
5820	A_24_P678418	FLJ45244		hypothetical locus FLJ45244	Homo sapiens hypothetical locus FLJ45	502.15625	136.62286	423.51877	100.34090	Detected	Detected	Detected	Detected
5821	A_23_P131208	NR4A2	Hs.563344	nuclear receptor subfamily 4, group A, r	Homo sapiens nuclear receptor subfami	1141.15920	37.51196	476.82245	7.83885	Detected	Detected	Detected	Compromised
5822	A_32_P86289	CYP19A1	Hs.260074	cytochrome P450, family 19, subfamily A	Homo sapiens cytochrome P450, family	25.81845	4.16865	20.56400	3.00817	Detected	Compromised	Detected	Compromised
5823	A_33_P3218301		Hs.567723		Homo sapiens solute carrier family 26 (	43.05892	51.16461	15.63027	19.48603	Detected	Detected	Detected	Detected
5824	A_33_P3216621	LOC728792		hypothetical LOC728792	PREDICTED: Homo sapiens hypothetica	115.77419	105.41789	13.30099	13.62044	Detected	Detected	Compromised	Compromised
5825	A_23_P82651	NPTX2	Hs.3281	neuronal pentraxin II	Homo sapiens neuronal pentraxin II (NP	3557.59400	14453.35300	2555.26250	9103.91400	Detected	Detected	Detected	Detected
5826	A_33_P3307500	STRA6	Hs.24553	stimulated by retinoic acid gene 6 homo	Homo sapiens stimulated by retinoic ac	9.30964	110.74777	4.63203	88.60502	Compromised	Detected	Compromised	Detected
5827	A_33_P3365352	LOC100130476	Hs.598050	similar to hCG2036711	PREDICTED: Homo sapiens similar to h	143.06839	176.33078	86.03423	93.50977	Detected	Detected	Detected	Detected
5828	A_23_P22915	SLC30A7	Hs.533903	solute carrier family 30 (zinc transporte	Homo sapiens solute carrier family 30 (	174.44300	311.16626	141.59663	221.48910	Detected	Detected	Detected	Detected
5829	A_23_P56578	VIT	Hs.137415	vitrin	Homo sapiens vitrin (VIT), mRNA [NM_0	75.32980	363.38544	66.07529	284.43450	Detected	Detected	Detected	Detected
5830	A_23_P307536	C1orf113	Hs.524496	chromosome 1 open reading frame 113	SH3 domain-containing protein C1orf11	279.54670	94.15263	189.11418	51.26257	Detected	Detected	Detected	Detected
5831	A_23_P141738	SS18	Hs.404263	synovial sarcoma translocation, chromo	Homo sapiens synovial sarcoma translo	1870.47410	2073.45400	1461.99870	1353.05270	Detected	Detected	Detected	Detected
5832	A_33_P3841819	LOC440944	Hs.598958	hypothetical LOC440944	Homo sapiens hypothetical LOC440944	348.56567	227.83728	356.80840	201.04960	Detected	Detected	Detected	Detected
5833	A_24_P186944					44984.19000	29744.69700	43171.68000	31701.50800	Detected	Detected	Detected	Detected
5834	A_23_P33583	DNAH7	Hs.97403	dynein, axonemal, heavy chain 7	Homo sapiens dynein, axonemal, heavy	53.21900	12.81922	45.18938	9.75539	Detected	Compromised	Detected	Compromised
5835	A_33_P3373358	GJC1	Hs.532593	gap junction protein, gamma 1, 45kDa	Homo sapiens gap junction protein, gam	4257.75340	5572.50000	4091.99460	4721.51200	Detected	Detected	Detected	Detected
5836	A_23_P83234	ZBTB6	Hs.654596	zinc finger and BTB domain containing	Homo sapiens zinc finger and BTB dom	159.29951	120.51148	137.39201	113.22540	Detected	Detected	Detected	Detected
5837	A_33_P3228366	XPO5	Hs.203206	exportin 5	Homo sapiens exportin 5 (XPO5), mRNA	1031.91360	1127.33040	743.75903	775.29440	Detected	Detected	Detected	Detected
5838	A_32_P32856	PCDHGC5	Hs.368160	protocadherin gamma subfamily C, 5	Homo sapiens protocadherin gamma su	26.16206	34.38536	29.87371	33.16810	Detected	Detected	Detected	Detected
5839	A_33_P3253124	LOC100129700	Hs.711048	similar to hCG1805148	PREDICTED: Homo sapiens similar to h	22.91832	26.51579	13.04718	7.10808	Detected	Detected	Compromised	Compromised
5840	A_23_P63980	LRFN4	Hs.209979	leucine rich repeat and fibronectin type	Homo sapiens leucine rich repeat and fi	4045.50020	8240.48400	3829.94400	7453.76860	Detected	Detected	Detected	Detected
5841	A_32_P28939	ALKBH2	Hs.374458	alkB, alkylation repair homolog 2 (E. coli	Homo sapiens alkB, alkylation repair ho	7601.20070	3133.63350	9921.27000	3769.93870	Detected	Detected	Detected	Detected
5842	A_33_P3246950		Hs.127078		BX117927 Soares_NFL_T_GBC_S1 Hom	231.29510	492.69644	49.73011	59.12139	Detected	Detected	Detected	Detected
5843	A_23_P46369	RAB13	Hs.151536	RAB13, member RAS oncogene family	Homo sapiens RAB13, member RAS onc	8092.15600	4127.13600	5463.58540	2278.25900	Detected	Detected	Detected	Detected
5844	A_33_P3422301	PCDHGC4	Hs.368160	protocadherin gamma subfamily C, 4	Homo sapiens protocadherin gamma su	14.74863	21.34185	15.53642	13.21635	Detected	Detected	Detected	Compromised
5845	A_24_P322635	ELMO2	Hs.210469	engulfment and cell motility 2	Homo sapiens engulfment and cell moti	2669.55640	2451.57280	1102.85330	963.86180	Detected	Detected	Detected	Detected
5846	A_24_P626850	H6PD	Hs.463511	hexose-6-phosphate dehydrogenase (gl	Homo sapiens hexose-6-phosphate deh	606.41656	89.92889	516.40515	59.54870	Detected	Detected	Detected	Detected

5847	A_33_P3315763				Putative uncharacterized protein ENSP	190015.94000	194421.80000	128265.60000	110709.26000	Detected	Detected	Detected	Detected
5848	A_33_P3289870		Hs.722282		K-EST0009229 S5SNU484 Homo sapier	23.36645	25.72798	19.87618	15.72364	Detected	Detected	Detected	Detected
5849	A_33_P3395804	C20orf26	Hs.176013	chromosome 20 open reading frame 26	Homo sapiens chromosome 20 open rea	38.45551	42.34431	9.48799	15.98397	Detected	Detected	Compromised	Detected
5850	A_33_P3503937				Putative uncharacterized protein FLJ37	435.16193	493.94183	180.49103	182.05838	Detected	Detected	Detected	Detected
5851	A_23_P98057	ZNF32	Hs.522885	zinc finger protein 32	Homo sapiens zinc finger protein 32 (ZN	944.19574	545.96576	1099.51800	524.65140	Detected	Detected	Detected	Detected
5852	A_23_P88781	CIAPIN1	Hs.4900	cytokine induced apoptosis inhibitor 1	Homo sapiens cytokine induced apoptos	3668.03270	4836.55500	3818.55800	4368.72200	Detected	Detected	Detected	Detected
5853	A_33_P3226761	SOX18	Hs.8619	SRY (sex determining region Y)-box 18	Homo sapiens SRY (sex determining reg	2.50588	22.10151	3.48455	4.24819	Compromised	Detected	Compromised	Compromised
5854	A_23_P170213	NKAIN2	Hs.656604	Na+/K+ transporting ATPase interactin	Homo sapiens Na+/K+ transporting ATP	5.56543	32.45965	4.27244	5.04132	Compromised	Detected	Compromised	Compromised
5855	A_23_P58396	PDGFC	Hs.570855	platelet derived growth factor C	Homo sapiens platelet derived growth fa	3267.70020	7061.98100	2618.98750	4978.69630	Detected	Detected	Detected	Detected
5856	A_23_P92948	PRRC1	Hs.483259	proline-rich coiled-coil 1	Homo sapiens proline-rich coiled-coil 1	392.12130	1073.58180	429.81100	965.67050	Detected	Detected	Detected	Detected
5857	A_33_P3338937	LOC731414		similar to S100 calcium-binding protein	PREDICTED: Homo sapiens similar to S	23.76253	39.54381	4.02666	2.94832	Detected	Detected	Compromised	Compromised
5858	A_33_P3417865	ZFP36L1	Hs.85155	zinc finger protein 36, C3H type-like 1	Homo sapiens zinc finger protein 36, C3	11346.23300	13901.73700	9242.81250	9050.06250	Detected	Detected	Detected	Detected
5859	A_33_P3280721	GPR177	Hs.647659	G protein-coupled receptor 177	Homo sapiens G protein-coupled recep	12352.93750	4442.12400	10153.52700	3136.23850	Detected	Detected	Detected	Detected
5860	A_24_P414371	PPP3CA	Hs.435512	protein phosphatase 3 (formerly 2B), ca	Homo sapiens protein phosphatase 3 (fo	3973.42970	5360.68160	3532.61550	4180.77700	Detected	Detected	Detected	Detected
5861	A_23_P88222	PLD4	Hs.407101	phospholipase D family, member 4	Homo sapiens phospholipase D family, r	7.57155	21.41245	4.13943	5.29950	Compromised	Detected	Compromised	Compromised
5862	A_23_P203900	SCARB1	Hs.709216	scavenger receptor class B, member 1	Homo sapiens scavenger receptor class	9151.40500	3461.51270	8312.78500	2883.49880	Detected	Detected	Detected	Detected
5863	A_33_P3394297				Threonine synthase-like 2 (TSH2)(EC 4	149.06760	31.63256	140.17593	23.33377	Detected	Detected	Detected	Detected
5864	A_33_P3265593		Hs.719950		cDNA FLJ44869 fis, clone BRAMY2015	1047.54380	1347.67810	1083.27690	1287.47440	Detected	Detected	Detected	Detected
5865	A_23_P30649	SPACA1	Hs.161241	sperm acrosome associated 1	Homo sapiens sperm acrosome associa	85.94672	89.24778	28.31570	31.53657	Detected	Detected	Detected	Detected
5866	A_24_P262407	THRA	Hs.724	thyroid hormone receptor, alpha (erythr	Homo sapiens thyroid hormone recepto	399.32680	434.92093	386.86234	436.40262	Detected	Detected	Detected	Detected
5867	A_33_P3290343	CYP1B1	Hs.154654	cytochrome P450, family 1, subfamily B	Homo sapiens cytochrome P450, family	1449.83090	762.80600	1832.40580	841.76720	Detected	Detected	Detected	Detected
5868	A_24_P83678	C6orf167	Hs.444292	chromosome 6 open reading frame 167	Homo sapiens chromosome 6 open read	132.22234	161.78789	112.08473	119.14389	Detected	Detected	Detected	Detected
5869	A_23_P160240	ACP6	Hs.562154	acid phosphatase 6, lysophosphatidic	Homo sapiens acid phosphatase 6, lysop	285.65872	80.91502	333.53930	90.21769	Detected	Detected	Detected	Detected
5870	A_33_P3359253				Mitotic spindle assembly checkpoint pro	34.93909	26.69670	34.44181	24.31870	Detected	Detected	Detected	Detected
5871	A_24_P342096	FAM27A	Hs.651534	family with sequence similarity 27, mem	Homo sapiens family with sequence sim	471.75630	472.84964	453.11975	363.51300	Detected	Detected	Detected	Detected
5872	A_33_P3235766	QSER1	Hs.369368	glutamine and serine rich 1	Homo sapiens glutamine and serine rich	3412.55180	3072.78880	2410.76340	1691.02620	Detected	Detected	Detected	Detected
5873	A_23_P392962	GRAP	Hs.567416	GRB2-related adaptor protein	Homo sapiens GRB2-related adaptor pr	16.12816	26.51250	9.95103	6.17531	Detected	Detected	Compromised	Compromised
5874	A_33_P3329991					8462.55700	8583.96000	1211.62070	949.08044	Detected	Detected	Detected	Detected
5875	A_23_P217899	CCNL2	Hs.515704	cyclin L2	Homo sapiens cyclin L2 (CCNL2), trans	1514.91940	800.29663	1558.05220	792.40625	Detected	Detected	Detected	Detected
5876	A_33_P3354713		Hs.588854		Alpha-(1,3)-fucosyltransferase 11 (EC 2	31.74972	41.28693	23.40314	23.62636	Detected	Detected	Detected	Detected
5877	A_33_P3354553	LOC390638		similar to hCG32587	PREDICTED: Homo sapiens similar to h	21.65480	29.71254	17.51552	23.09614	Detected	Detected	Detected	Detected
5878	A_23_P116743	LOC338799	Hs.524804	hypothetical LOC338799	Homo sapiens hypothetical LOC338799	1627.24170	2930.58030	1529.91720	2489.07670	Detected	Detected	Detected	Detected
5879	A_33_P3342260	GCK	Hs.1270	glucokinase (hexokinase 4)	Homo sapiens glucokinase (hexokinase	588.88470	466.31436	417.36694	201.04588	Detected	Detected	Detected	Detected
5880	A_24_P124349	PDGFD	Hs.352298	platelet derived growth factor D	Homo sapiens platelet derived growth fa	16.62358	58.07933	13.00552	44.15033	Detected	Detected	Compromised	Detected
5881	A_32_P125338	FAM43B	Hs.429179	family with sequence similarity 43, mem	Homo sapiens family with sequence sim	159.30914	21.03658	120.04231	16.56563	Detected	Detected	Detected	Detected
5882	A_33_P3319845	OR51B6	Hs.553730	olfactory receptor, family 51, subfamily	Homo sapiens olfactory receptor, family	15.92864	16.43621	13.20379	9.93790	Detected	Compromised	Compromised	Compromised
5883	A_23_P150092	SEPHS1	Hs.124027	selenophosphate synthetase 1	Homo sapiens selenophosphate synthet	7028.26800	7645.98140	5986.17800	5929.83100	Detected	Detected	Detected	Detected
5884	A_23_P87310	LMO1	Hs.654426	LIM domain only 1 (rhombotin 1)	Homo sapiens LIM domain only 1 (rhom	53.27324	73.33632	31.88286	30.52608	Detected	Detected	Detected	Detected
5885	A_23_P27353	SLC14A2	Hs.710927	solute carrier family 14 (urea transporte	Homo sapiens solute carrier family 14 (	132.60072	180.68071	34.47023	44.53359	Detected	Detected	Detected	Detected
5886	A_23_P19852	IQCE	Hs.520627	IQ motif containing E	Homo sapiens IQ motif containing E (IQ	3871.89260	2219.74680	3153.63900	1634.75430	Detected	Detected	Detected	Detected
5887	A_33_P3378531	AS3MT	Hs.720370	arsenic (+3 oxidation state) methyltrans	Homo sapiens arsenic (+3 oxidation sta	270159.75000	289076.22000	173337.58000	155839.00000	Detected	Detected	Detected	Detected
5888	A_33_P3346526					15.70367	21.96430	3.19217	3.00284	Detected	Detected	Compromised	Compromised
5889	A_33_P3379456	SH3YL1	Hs.515951	SH3 domain containing, Ysc84-like 1 (S	Homo sapiens SH3 domain containing, Y	3618.67100	1701.22800	3006.76250	1191.93950	Detected	Detected	Detected	Detected
5890	A_33_P3388376	TBC1D3B	Hs.454713	TBC1 domain family, member 3B	Homo sapiens TBC1 domain family, mer	18.65774	5.80775	13.53422	4.50287	Detected	Compromised	Compromised	Compromised
5891	A_24_P303589	CUL1	Hs.146806	cullin 1	Homo sapiens cullin 1 (CUL1), mRNA [N	3192.36620	2665.68920	2726.99340	2012.45520	Detected	Detected	Detected	Detected
5892	A_33_P3645805	LOC149401	Hs.675489	hypothetical protein LOC149401	Homo sapiens mRNA; cDNA DKFZp434	12946.15700	11059.20800	8234.59300	6879.48600	Detected	Detected	Detected	Detected
5893	A_23_P28466	WDR69	Hs.424594	WD repeat domain 69	Homo sapiens WD repeat domain 69 (W	436.32706	71.78383	418.93643	56.42390	Detected	Detected	Detected	Detected
5894	A_33_P3215864	FAM3A	Hs.289108	family with sequence similarity 3, memb	Homo sapiens family with sequence sim	273.25476	239.53842	209.82368	180.97095	Detected	Detected	Detected	Detected
5895	A_23_P136405	PDCD1	Hs.158297	programmed cell death 1	Homo sapiens programmed cell death 1	89.82444	125.92113	65.67442	67.66341	Detected	Detected	Detected	Detected
5896	A_24_P276888	CENPO	Hs.718431	centromere protein O	Centromere protein O (CENP-O)(Interp	406.66095	2227.50800	332.06950	1795.17630	Detected	Detected	Detected	Detected
5897	A_23_P73023	TBC1D1	Hs.176503	TBC1 (tre-2/USP6, BUB2, cdc16) doma	Homo sapiens TBC1 (tre-2/USP6, BUB	2078.15380	2788.51440	1944.96770	2253.23950	Detected	Detected	Detected	Detected
5898	A_24_P349002	POM121	Hs.655217	POM121 membrane glycoprotein (rat)	Homo sapiens POM121 membrane glyco	1031.76280	1600.15840	523.81555	666.18800	Detected	Detected	Detected	Detected
5899	A_24_P712350	CHML	Hs.654545	choroideremia-like (Rab escort protein	Homo sapiens choroideremia-like (Rab	4630.38100	2314.75590	3044.36400	1317.63160	Detected	Detected	Detected	Detected
5900	A_24_P701776	ARHGEF5L	Hs.534621	Rho guanine nucleotide exchange facto	Homo sapiens Rho guanine nucleotide e	22.13001	68.41540	24.18276	53.80462	Detected	Detected	Detected	Detected
5901	A_33_P3280508	LOC100128613	Hs.120493	hypothetical LOC100128613	PREDICTED: Homo sapiens hypothetica	15.56274	36.81731	10.73147	7.90042	Detected	Detected	Compromised	Compromised
5902	A_33_P3281616	C22orf40	Hs.592209	chromosome 22 open reading frame 40	Homo sapiens chromosome 22 open rea	1833.26230	2201.22750	1991.49320	2092.39840	Detected	Detected	Detected	Detected
5903	A_23_P325562	SLC1A7	Hs.104637	solute carrier family 1 (glutamate transp	Homo sapiens solute carrier family 1 (gl	126.28503	169.08437	71.48184	50.23654	Detected	Detected	Detected	Detected
5904	A_23_P412427	NS3BP	Hs.711081	NS3BP	PREDICTED: Homo sapiens NS3BP (NS	80.67822	56.96281	103.66239	42.69446	Detected	Detected	Detected	Detected
5905	A_33_P3399560	RABL4	Hs.415172	RAB, member of RAS oncogene family-	Homo sapiens RAB, member of RAS on	1056.22020	637.47253	1154.57130	533.09344	Detected	Detected	Detected	Detected
5906	A_23_P99930	TIPIN	Hs.572318	TIMELESS interacting protein	Homo sapiens TIMELESS interacting pr	2584.49320	4141.85350	2146.64000	3479.64360	Detected	Detected	Detected	Detected
5907	A_33_P3341601	WDR86	Hs.647083	WD repeat domain 86	Homo sapiens WD repeat domain 86 (W	166.58890	9.71910	155.19218	2.94105	Detected	Compromised	Detected	Compromised
5908	A_33_P3355055	ARL6IP4	Hs.103561	ADP-ribosylation-like factor 6 interacti	Homo sapiens ADP-ribosylation-like fac	2486.40280	2590.57900	4087.89600	4132.08800	Detected	Detected	Detected	Detected
5909	A_33_P3533001	UBE2E4P	Hs.567831	ubiquitin-conjugating enzyme E2E 4 pse	AL568675 Homo sapiens PLACENTA H	72.16199	122.13699	69.58280	81.29784	Detected	Detected	Detected	Detected
5910	A_32_P150300	LOC100131646	Hs.713783	hypothetical LOC100131646	PREDICTED: Homo sapiens hypothetica	162.19115	266.18472	88.10043	193.03639	Detected	Detected	Detected	Detected
5911	A_23_P13772	TBX3	Hs.714737	T-box 3	Homo sapiens T-box 3 (TBX3), transcri	513.15137	991.43207	578.74900	1010.74884	Detected	Detected	Detected	Detected
5912	A_24_P192197	WRNIP1	Hs.236828	Werner helicase interacting protein 1	Homo sapiens Werner helicase interacti	1487.11010	1291.94170	1484.32390	1169.36780	Detected	Detected	Detected	Detected
5913	A_23_P421401	PDGFRB	Hs.509067	platelet-derived growth factor receptor	Homo sapiens platelet-derived growth f	52989.03000	20623.27000	35322.79000	11988.06800	Detected	Detected	Detected	Detected
5914	A_32_P183609	ASB1	Hs.516788	ankyrin repeat and SOCS box-containir	Homo sapiens ankyrin repeat and SOCS	2493.51000	2214.45600	2144.04740	1678.59280	Detected	Detected	Detected	Detected
5915	A_23_P147900	POU6F2	Hs.137106	POU class 6 homeobox 2	Homo sapiens POU class 6 homeobox 2	12.93213	17.72429	13.42442	11.63290	Detected	Detected	Compromised	Compromised
5916	A_33_P3259135	D4S234E	Hs.518595	DNA segment on chromosome 4 (uniqu	Homo sapiens DNA segment on chromo	15.34606	63.16761	15.54790	35.92776	Detected	Detected	Detected	Detected
5917	A_32_P76811	RP3-377H14.5		hypothetical LOC285830	Homo sapiens hypothetical LOC285830	33509.90600	25547.98000	23071.20700	15831.19300	Detected	Detected	Detected	Detected
5918	A_33_P3281010	FAM95B1	Hs.584289	family with sequence similarity 95, mem	Homo sapiens cDNA FLJ43862 fis, clon	116.87856	156.77959	17.99843	27.93453	Detected	Detected	Detected	Detected
5919	A_24_P65722	LILRB4	Hs.67846	leukocyte immunoglobulin-like receptor,	Leukocyte immunoglobulin-like recepto	46.46482	39.61328	24.75706	16.88066	Detected	Detected	Detected	Detected
5920	A_33_P3311621	LOC338739	Hs.423476	hypothetical protein LOC338739	Homo sapiens hypothetical protein LOC	84.93229	89.33570	23.42293	20.30010	Detected	Detected	Detected	Detected
5921	A_33_P3827416	RP11-144G6.7	Hs.652837	hypothetical LOC399753	Homo sapiens cDNA FLJ14094 fis, clon	105843.71000	98661.30000	35077.51600	25769.12000	Detected	Detected	Detected	Detected

5922	A_24_P324011	KCTD2	Hs.514468	potassium channel tetramerisation dom	Homo sapiens potassium channel tetrar	776.73890	797.74976	1205.53710	1167.36360	Detected	Detected	Detected	Detected
5923	A_24_P398323	TRIM34	Hs.125300	tripartite motif-containing 34	Homo sapiens tripartite motif-containin	90.12923	180.35649	72.25922	122.21331	Detected	Detected	Detected	Detected
5924	A_24_P69439	SLC25A32	Hs.607819	solute carrier family 25, member 32	Homo sapiens solute carrier family 25, r	5389.05960	9833.72400	4059.25300	6379.76270	Detected	Detected	Detected	Detected
5925	A_33_P3249574	SCAMP1	Hs.482587	secretory carrier membrane protein 1	Homo sapiens secretory carrier membra	152.01710	109.17439	144.94218	96.91926	Detected	Detected	Detected	Detected
5926	A_23_P152655	ICAM2	Hs.431460	intercellular adhesion molecule 2	Homo sapiens intercellular adhesion mo	108.77898	339.28143	103.91580	318.78473	Detected	Detected	Detected	Detected
5927	A_23_P171249	IGBP1	Hs.496267	immunoglobulin (CD79A) binding protein	Homo sapiens immunoglobulin (CD79A)	7036.20360	4880.77800	5072.40900	3051.91400	Detected	Detected	Detected	Detected
5928	A_23_P125748	ZMAT1	Hs.496512	zinc finger, matrin type 1	Homo sapiens zinc finger, matrin type 1	88.71359	10.02164	49.78720	6.58169	Detected	Compromised	Detected	Compromised
5929	A_23_P372144	C19orf29	Hs.720039	chromosome 19 open reading frame 29	Homo sapiens chromosome 19 open rea	9847.99600	10234.91000	4634.29440	3657.59230	Detected	Detected	Detected	Detected
5930	A_23_P418413	OXSR1	Hs.475970	oxidative-stress responsive 1	Homo sapiens oxidative-stress respons	6390.46240	8246.46800	4468.00440	4564.98100	Detected	Detected	Detected	Detected
5931	A_24_P399083	TXNL4B	Hs.134406	thioredoxin-like 4B	Homo sapiens thioredoxin-like 4B (TXN	171.77344	336.35430	140.21793	242.57306	Detected	Detected	Detected	Detected
5932	A_33_P3676515	C21orf122	Hs.309203	chromosome 21 open reading frame 122	Homo sapiens chromosome 21 open rea	984.71893	333.67800	696.38110	233.26941	Detected	Detected	Detected	Detected
5933	A_33_P3381092	OR10W1	Hs.531507	olfactory receptor, family 10, subfamily	Homo sapiens olfactory receptor, family	25.47415	18.19081	23.83231	12.76905	Detected	Compromised	Detected	Compromised
5934	A_23_P208674	C19orf63	Hs.515550	chromosome 19 open reading frame 63	Homo sapiens chromosome 19 open rea	5546.60900	5862.57470	5754.34330	5752.30860	Detected	Detected	Detected	Detected
5935	A_23_P62890	GBP1	Hs.62661	guanylate binding protein 1, interferon-i	Homo sapiens guanylate binding protein	375.55002	60.98749	337.86630	48.50555	Detected	Detected	Detected	Detected
5936	A_23_P258124	ZNF346	Hs.484259	zinc finger protein 346	Homo sapiens zinc finger protein 346 (Z	421.02747	277.41260	294.37015	145.59012	Detected	Detected	Detected	Detected
5937	A_33_P3285444	TERF1	Hs.442707	telomeric repeat binding factor (NIMA-i	Homo sapiens telomeric repeat binding	35.72988	29.09573	9.03980	4.70955	Detected	Detected	Compromised	Compromised
5938	A_23_P154411	PPIG	Hs.470544	peptidylprolyl isomerase G (cyclophilin	Homo sapiens peptidylprolyl isomerase	3519.93240	2928.36300	2087.49270	1433.93850	Detected	Detected	Detected	Detected
5939	A_33_P3252286	CRLF1	Hs.114948	cytokine receptor-like factor 1	Homo sapiens cytokine receptor-like fa	13849.45600	52525.27300	12980.70000	43780.62500	Detected	Detected	Detected	Detected
5940	A_33_P3424222	HLA-DQB1	Hs.409934	major histocompatibility complex, class	Homo sapiens major histocompatibility	397.15393	547.00757	113.31918	120.30589	Detected	Detected	Detected	Detected
5941	A_24_P372562	PTPMT1	Hs.700746	protein tyrosine phosphatase, mitochon	Homo sapiens protein tyrosine phospha	7437.53120	9533.43600	7588.94040	8569.39000	Detected	Detected	Detected	Detected
5942	A_23_P92362	NDUFC1	Hs.84549	NADH dehydrogenase (ubiquinone) 1, su	Homo sapiens NADH dehydrogenase (u	25419.88500	24949.75600	21440.82800	18361.82800	Detected	Detected	Detected	Detected
5943	A_23_P334021	IGF2R	Hs.487062	insulin-like growth factor 2 receptor	Homo sapiens insulin-like growth factor	4491.60900	3106.07470	3354.05570	2260.54370	Detected	Detected	Detected	Detected
5944	A_23_P209032	ZNF302	Hs.436350	zinc finger protein 302	Homo sapiens zinc finger protein 302 (Z	969.15250	627.36053	654.24720	347.08612	Detected	Detected	Detected	Detected
5945	A_33_P3375636	FAM196B	Hs.721917	family with sequence similarity 196, mer	Homo sapiens family with sequence sim	38.61979	88.53218	20.46457	50.21685	Detected	Detected	Detected	Detected
5946	A_33_P3373308	LOC440700	Hs.568703	carbonic anhydrase XIV (CA14) pseudo	Homo sapiens carbonic anhydrase XIV	12.38166	3.50185	2.85286	3.00658	Detected	Compromised	Compromised	Compromised
5947	A_33_P3351351				Rho guanine nucleotide exchange facto	33.99439	41.64095	24.37367	21.64848	Detected	Detected	Detected	Detected
5948	A_23_P252082	TMEM176A	Hs.647116	transmembrane protein 176A	Homo sapiens transmembrane protein 1	256.53650	35.33626	304.49338	27.87198	Detected	Detected	Detected	Detected
5949	A_23_P73721	RRAGB	Hs.50282	Ras-related GTP binding B	Homo sapiens Ras-related GTP binding	130.89249	143.60277	111.78572	101.70540	Detected	Detected	Detected	Detected
5950	A_23_P16166	PNPLA6	Hs.631863	patatin-like phospholipase domain conta	Homo sapiens patatin-like phospholipas	519.66830	555.65070	474.35770	538.41000	Detected	Detected	Detected	Detected
5951	A_23_P30069	DDX60L	Hs.535011	DEAD (Asp-Glu-Ala-Asp) box polypept	Homo sapiens DEAD (Asp-Glu-Ala-Asp	330.91187	198.18997	241.56097	129.61928	Detected	Detected	Detected	Detected
5952	A_23_P215566	AHR	Hs.171189	aryl hydrocarbon receptor	Homo sapiens aryl hydrocarbon recepto	2162.68300	4747.77400	1764.58170	3271.41110	Detected	Detected	Detected	Detected
5953	A_24_P190472	SLPI	Hs.517070	secretory leukocyte peptidase inhibitor	Homo sapiens secretory leukocyte pept	10.90877	21.73602	9.50408	12.19293	Compromised	Detected	Compromised	Compromised
5954	A_23_P56734	HNMT	Hs.42151	histamine N-methyltransferase	Homo sapiens histamine N-methyltrans	427.34088	141.07480	414.78076	94.21686	Detected	Detected	Detected	Detected
5955	A_24_P301186	WDR89	Hs.655666	WD repeat domain 89	Homo sapiens WD repeat domain 89 (W	101.10229	112.87070	55.57903	35.47010	Detected	Detected	Detected	Detected
5956	A_23_P213228	PHOX2B	Hs.87202	paired-like homeobox 2b	Homo sapiens paired-like homeobox 2b	3.41934	20.91289	2.83012	2.95728	Compromised	Detected	Compromised	Compromised
5957	A_23_P200551	USP48	Hs.467524	ubiquitin specific peptidase 48	Homo sapiens ubiquitin specific peptida	3469.49400	3671.26370	2538.10160	2522.60940	Detected	Detected	Detected	Detected
5958	A_23_P85922	BMP8A	Hs.472497	bone morphogenetic protein 8a	Homo sapiens bone morphogenetic prot	10.17271	37.67982	2.81777	9.18504	Compromised	Detected	Compromised	Compromised
5959	A_23_P119095	PPP1R13L	Hs.466937	protein phosphatase 1, regulatory (inhib	Homo sapiens protein phosphatase 1, re	1323.23700	914.18170	1356.62070	879.05115	Detected	Detected	Detected	Detected
5960	A_33_P3358462	GLI2	Hs.111867	GLI family zinc finger 2	Homo sapiens GLI family zinc finger 2 (	30.97022	52.34563	61.41718	70.25842	Detected	Detected	Detected	Detected
5961	A_23_P152858	C17orf79	Hs.462729	chromosome 17 open reading frame 79	Homo sapiens chromosome 17 open rea	32723.82000	56553.50000	35003.09400	48420.54300	Detected	Detected	Detected	Detected
5962	A_33_P3265394	WDR74	Hs.655372	WD repeat domain 74	Homo sapiens cDNA FLJ78749 complet	163.39317	67.58331	141.28226	34.20799	Detected	Detected	Detected	Detected
5963	A_32_P118397	HEPACAM2	Hs.443169	HEPACAM family member 2	Homo sapiens HEPACAM family membe	17.98209	15.04626	10.17763	2.92118	Detected	Compromised	Compromised	Compromised
5964	A_33_P3368188	SEPT9	Hs.440932	septin 9	Homo sapiens septin 9 (SEPT9), transc	110.41970	202.64581	126.44424	192.74944	Detected	Detected	Detected	Detected
5965	A_33_P3404032	HIST3H2A	Hs.26331	histone cluster 3, H2a	Homo sapiens histone cluster 3, H2a (H	47.73618	62.14280	20.87698	41.31546	Detected	Detected	Detected	Detected
5966	A_23_P301877	MED27	Hs.374262	mediator complex subunit 27	Homo sapiens mediator complex subuni	698.88610	1278.70940	849.76710	1448.37110	Detected	Detected	Detected	Detected
5967	A_23_P11744	WASH1	Hs.709408	WAS protein family homolog 1	Homo sapiens WAS protein family homo	13523.88300	7425.36330	16478.50800	8361.72500	Detected	Detected	Detected	Detected
5968	A_33_P3244882	EIF2C3	Hs.657659	eukaryotic translation initiation factor 2	Homo sapiens eukaryotic translation ini	358.69272	1248.60660	335.00763	1040.26930	Detected	Detected	Detected	Detected
5969	A_33_P3399028	TLK2	Hs.445078	tousled-like kinase 2	Homo sapiens tousled-like kinase 2 (TL	130.07281	178.57965	160.46376	187.79083	Detected	Detected	Detected	Detected
5970	A_32_P89679	ALG10B	Hs.259305	asparagine-linked glycosylation 10, alph	Homo sapiens asparagine-linked glycos	520.20650	368.48575	453.52910	297.68560	Detected	Detected	Detected	Detected
5971	A_23_P97795	ACBD5	Hs.530597	acyl-Coenzyme A binding domain conta	Homo sapiens acyl-Coenzyme A bindin	761.39044	1091.42350	541.27155	709.50490	Detected	Detected	Detected	Detected
5972	A_33_P3419165	TFPT	Hs.590939	TCF3 (E2A) fusion partner (in childhood	Homo sapiens TCF3 (E2A) fusion partn	297.54730	644.95154	260.60270	515.48395	Detected	Detected	Detected	Detected
5973	A_33_P3381132					19.05364	20.98166	5.06695	7.59985	Detected	Detected	Compromised	Compromised
5974	A_24_P346886	NDUFB8	Hs.523215	NADH dehydrogenase (ubiquinone) 1 be	Homo sapiens NADH dehydrogenase (u	23414.27100	33651.75000	22061.76000	25791.75000	Detected	Detected	Detected	Detected
5975	A_23_P46606	LPGAT1	Hs.497674	lysophosphatidylglycerol acyltransferase	Homo sapiens lysophosphatidylglycerol	416.38260	964.15940	588.63600	862.05580	Detected	Detected	Detected	Detected
5976	A_23_P152055	EFTUD1	Hs.459114	elongation factor Tu GTP binding domai	Homo sapiens elongation factor Tu GTF	5623.25800	7780.65230	3688.33000	4478.01600	Detected	Detected	Detected	Detected
5977	A_23_P141680	BCAS3	Hs.655028	breast carcinoma amplified sequence 3	Homo sapiens breast carcinoma amplifi	278.07153	120.79601	203.22055	87.85716	Detected	Detected	Detected	Detected
5978	A_33_P3337144	ZNF704	Hs.632067	zinc finger protein 704	Homo sapiens zinc finger protein 704 (Z	10.67958	8.32803	13.68748	19.31438	Compromised	Compromised	Compromised	Detected
5979	A_24_P244952	SMG1	Hs.460179	SMG1 homolog, phosphatidylinositol 3-k	Homo sapiens SMG1 homolog, phosphat	1153.86710	1284.95250	525.51430	499.98538	Detected	Detected	Detected	Detected
5980	A_23_P80321	POLR2F	Hs.436578	polymerase (RNA) II (DNA directed) pol	Homo sapiens polymerase (RNA) II (DN	28380.04300	34959.80500	34208.95000	37068.15600	Detected	Detected	Detected	Detected
5981	A_33_P3310296				cAMP-dependent protein kinase inhibit	117.58830	153.36345	58.80870	67.14562	Detected	Detected	Detected	Detected
5982	A_23_P141248	SRR	Hs.461954	serine racemase	Homo sapiens serine racemase (SRR), r	253.25903	563.57654	218.80774	389.12677	Detected	Detected	Detected	Detected
5983	A_33_P3221438	C3orf21	Hs.478741	chromosome 3 open reading frame 21	Homo sapiens chromosome 3 open read	2475.15480	2519.04000	2293.29640	2075.62500	Detected	Detected	Detected	Detected
5984	A_33_P3281151	FUBP1	Hs.567380	far upstream element (FUSE) binding pr	Homo sapiens far upstream element (FU	730.42500	1597.18580	665.80850	1325.14360	Detected	Detected	Detected	Detected
5985	A_33_P3274189					118.87154	153.99806	91.17574	93.86771	Detected	Detected	Detected	Detected
5986	A_23_P79999	ENTPD6	Hs.719917	ectonucleoside triphosphate diphosphol	Homo sapiens ectonucleoside triphosph	3661.23270	6317.03300	3560.96800	5870.55570	Detected	Detected	Detected	Detected
5987	A_24_P213161	NLRP2	Hs.369279	NLR family, pyrin domain containing 2	Homo sapiens NLR family, pyrin domain	25.49520	26.48509	7.43641	10.22462	Detected	Detected	Compromised	Compromised
5988	A_33_P3275873	SFTPB	Hs.512690	surfactant protein B	Homo sapiens surfactant protein B (SFT	11.17468	3.79850	15.54381	3.23739	Compromised	Compromised	Detected	Compromised
5989	A_23_P69863	PCDHB5	Hs.119693	protocadherin beta 5	Homo sapiens protocadherin beta 5 (PC	28.55352	25.64111	20.77180	8.22323	Detected	Detected	Detected	Compromised
5990	A_23_P328237	SPTY2D1	Hs.268668	SPT2, Suppressor of Ty, domain contai	Homo sapiens SPT2, Suppressor of Ty,	154.97214	192.55542	99.12499	93.86823	Detected	Detected	Detected	Detected
5991	A_33_P3460043	C8orf56	Hs.459124	chromosome 8 open reading frame 56	Homo sapiens chromosome 8 open read	630.95340	912.46040	525.02330	527.11993	Detected	Detected	Detected	Detected
5992	A_23_P11256	MAGEE2	Hs.356869	melanoma antigen family E, 2	Homo sapiens melanoma antigen family	14.61824	18.07728	5.42245	6.74504	Detected	Detected	Compromised	Compromised
5993	A_33_P3292130					4494.18070	6218.38600	1898.20480	1751.11650	Detected	Detected	Detected	Detected
5994	A_33_P3368109					53.28788	80.21735	109.69919	127.36958	Detected	Detected	Detected	Detected
5995	A_32_P70203	FAM41C	Hs.449006	family with sequence similarity 41, mem	Homo sapiens family with sequence sim	35.19427	99.83477	3.14977	3.27428	Detected	Detected	Compromised	Compromised
5996	A_23_P376449	LOC642413	Hs.558776	similar to Putative cathepsin L-like prot	PREDICTED: Homo sapiens similar to P	18216.83400	4980.42770	19787.43400	4436.98830	Detected	Detected	Detected	Detected

5997	A_33_P3342822	LOC100132672	Hs.648146	similar to glycosyltransferase 8 domain	PREDICTED: Homo sapiens similar to g	34.62092	72.19582	24.67194	28.75968	Detected	Detected	Detected	Detected
5998	A_23_P407132	KIRREL3	Hs.657065	kin of IRRE like 3 (Drosophila)	Homo sapiens kin of IRRE like 3 (Droso	7.88733	43.75179	8.01686	23.30890	Compromised	Detected	Compromised	Detected
5999	A_33_P3228637		Hs.639846		Zinc finger protein 584 [Source:UniPro	52.10278	41.79255	10.02209	6.57738	Detected	Detected	Compromised	Compromised
6000	A_33_P3216694	HIVEP3	Hs.648369	human immunodeficiency virus type I en	Homo sapiens human immunodeficiency	32.32219	43.98869	19.62098	16.21435	Detected	Detected	Detected	Detected
6001	A_33_P3220822	LOC441687	Hs.685242	similar to testis expressed gene 21	PREDICTED: Homo sapiens similar to te	100.22500	19.95661	79.45029	17.95789	Detected	Detected	Detected	Detected
6002	A_24_P734953	TRNP1	Hs.355747	TMF1-regulated nuclear protein 1	Homo sapiens TMF1-regulated nuclear	4016.02050	15697.93100	4386.20260	15838.56250	Detected	Detected	Detected	Detected
6003	A_23_P102988	TXNRD2	Hs.443430	thioredoxin reductase 2	Homo sapiens thioredoxin reductase 2 (	188.09993	156.13211	182.62020	169.56673	Detected	Detected	Detected	Detected
6004	A_23_P352358	KIAA2013	Hs.520094	KIAA2013	Homo sapiens KIAA2013 (KIAA2013), m	7847.19100	8435.82600	9065.41400	9315.05500	Detected	Detected	Detected	Detected
6005	A_23_P115444	TNFSF18	Hs.248197	tumor necrosis factor (ligand) superfam	Homo sapiens tumor necrosis factor (lig	3.02406	4.03485	31.69821	3.21525	Compromised	Compromised	Not Detected	Compromised
6006	A_33_P3363799	NCAM1	Hs.503878	neural cell adhesion molecule 1	Homo sapiens neural cell adhesion mole	100.27211	235.10501	69.17639	139.70822	Detected	Detected	Detected	Detected
6007	A_23_P91512	CLDN14	Hs.660278	claudin 14	Homo sapiens claudin 14 (CLDN14), tra	23.47915	81.99339	10.71079	40.05732	Detected	Detected	Compromised	Detected
6008	A_23_P311144	CCDC138	Hs.362702	coiled-coil domain containing 138	Homo sapiens coiled-coil domain contai	140.03790	154.76581	121.08686	99.22025	Detected	Detected	Detected	Detected
6009	A_23_P66694	EVI2B	Hs.5509	ecotropic viral integration site 2B	Homo sapiens ecotropic viral integratio	22.86298	50.51148	15.00267	28.17931	Detected	Detected	Detected	Detected
6010	A_23_P72503	KLHL2	Hs.388668	kelch-like 2, Mayven (Drosophila)	Homo sapiens kelch-like 2, Mayven (Dro	860.52313	447.61667	676.17850	267.63327	Detected	Detected	Detected	Detected
6011	A_24_P93206	TMEM179	Hs.655004	transmembrane protein 179	Homo sapiens transmembrane protein 1	21.24773	40.60987	13.40708	15.05815	Detected	Detected	Compromised	Detected
6012	A_33_P3219697	ANKRD45	Hs.130054	ankyrin repeat domain 45	Homo sapiens ankyrin repeat domain 45	15729.71600	13515.48100	4182.22800	3074.27200	Detected	Detected	Detected	Detected
6013	A_23_P414964	ZNF584	Hs.439551	zinc finger protein 584	Homo sapiens zinc finger protein 584 (Z	1294.82980	1287.77650	1401.23030	1384.00840	Detected	Detected	Detected	Detected
6014	A_24_P332341	POLR2J4	Hs.657028	polymerase (RNA) II (DNA directed) pol	Homo sapiens polymerase (RNA) II (DNA	582.94495	771.24347	662.25300	740.13510	Detected	Detected	Detected	Detected
6015	A_33_P3241657	C2orf34	Hs.468349	chromosome 2 open reading frame 34	Homo sapiens chromosome 2 open read	322.12274	175.30391	285.39280	137.29391	Detected	Detected	Detected	Detected
6016	A_33_P3213767				Coiled-coil domain-containing protein 1	296.55390	323.64110	302.04760	252.86975	Detected	Detected	Detected	Detected
6017	A_23_P2181	CYB5R2	Hs.414362	cytochrome b5 reductase 2	Homo sapiens cytochrome b5 reductase	16733.46900	14155.74100	15368.69700	11483.80700	Detected	Detected	Detected	Detected
6018	A_24_P221968	LOC643802	Hs.451336	similar to M-phase phosphoprotein 10 (	PREDICTED: Homo sapiens similar to M	171.82118	162.14398	29.13746	7.91467	Detected	Detected	Detected	Compromised
6019	A_24_P25326	ZMYM6	Hs.675613	zinc finger, MYM-type 6	Homo sapiens zinc finger, MYM-type 6	206.39207	256.21942	180.39555	185.57832	Detected	Detected	Detected	Detected
6020	A_24_P242688	HADHA	Hs.516032	hydroxyacyl-Coenzyme A dehydrogenas	Homo sapiens hydroxyacyl-Coenzyme A	10539.15800	9882.42200	6097.31100	5031.29100	Detected	Detected	Detected	Detected
6021	A_33_P3243439	GPR162	Hs.631654	G protein-coupled receptor 162	Homo sapiens G protein-coupled recep	1031.80580	232.76956	1050.26260	186.54080	Detected	Detected	Detected	Detected
6022	A_23_P30275	PCYOX1L	Hs.644397	prenylcysteine oxidase 1 like	Homo sapiens prenylcysteine oxidase 1	836.70000	830.21540	784.58276	635.89720	Detected	Detected	Detected	Detected
6023	A_23_P370494	PCDHGB7	Hs.368160	protocadherin gamma subfamily B, 7	Homo sapiens protocadherin gamma sub	55.03197	63.35861	44.08073	23.06486	Detected	Detected	Detected	Detected
6024	A_23_P129209	IDH2	Hs.596461	isocitrate dehydrogenase 2 (NADP+), m	Homo sapiens isocitrate dehydrogenase	1915.64940	4030.49900	1751.16740	3414.44730	Detected	Detected	Detected	Detected
6025	A_33_P3351944	EGFR	Hs.488293	epidermal growth factor receptor (eryth	Homo sapiens epidermal growth factor r	683.43460	387.50806	500.50488	233.68805	Detected	Detected	Detected	Detected
6026	A_23_P132784	FXR1	Hs.478407	fragile X mental retardation, autosomal	Homo sapiens fragile X mental retardati	12772.05700	10219.30100	10654.42200	7270.27440	Detected	Detected	Detected	Detected
6027	A_33_P3235390	UBA5	Hs.170737	ubiquitin-like modifier activating enzyme	Homo sapiens ubiquitin-like modifier ac	821.08673	956.43090	615.28050	672.09125	Detected	Detected	Detected	Detected
6028	A_23_P92860	CCNO	Hs.3041	cyclin O	Homo sapiens cyclin O (CCNO), mRNA	82.25579	187.02054	74.60291	151.63116	Detected	Detected	Detected	Detected
6029	A_23_P15073	JMJD8	Hs.533771	jumonji domain containing 8	Homo sapiens jumonji domain containing	782.96160	754.66144	608.27060	616.91690	Detected	Detected	Detected	Detected
6030	A_23_P92410	CASP3	Hs.141125	caspase 3, apoptosis-related cysteine p	Homo sapiens caspase 3, apoptosis-rel	11994.05400	17404.87700	11425.78900	13977.43600	Detected	Detected	Detected	Detected
6031	A_24_P23445	RNU12	Hs.574857	RNA, U12 small nuclear	Homo sapiens RNA, U12 small nuclear (	38.04002	22.96338	43.03594	22.11578	Detected	Detected	Detected	Detected
6032	A_33_P3293346	KCTD3	Hs.335139	potassium channel tetramerisation dom	Homo sapiens potassium channel tetran	202.85971	167.19362	174.50644	138.48642	Detected	Detected	Detected	Detected
6033	A_23_P69226	TMEM111	Hs.475392	transmembrane protein 111	Homo sapiens transmembrane protein 1	5669.83640	5496.75730	4741.13770	4096.42530	Detected	Detected	Detected	Detected
6034	A_33_P3215640	PI16	Hs.720179	peptidase inhibitor 16	Homo sapiens peptidase inhibitor 16 (PI	75.91495	1205.29880	80.89175	1189.84070	Detected	Detected	Detected	Detected
6035	A_33_P3354604	CCL4L1	Hs.661942	chemokine (C-C motif) ligand 4-like 1	Homo sapiens CC chemokine ligand 4L2	39.36432	35.37931	24.78358	15.27299	Detected	Detected	Detected	Compromised
6036	A_23_P383009	IGFBP5	Hs.607212	insulin-like growth factor binding protei	Homo sapiens insulin-like growth factor	131.08830	19.28795	80.21664	7.93594	Detected	Detected	Detected	Compromised
6037	A_23_P23346	MLLT11	Hs.75823	myeloid/lymphoid or mixed-lineage leuk	Homo sapiens myeloid/lymphoid or mixe	2799.99780	2949.02220	2350.16040	2171.16870	Detected	Detected	Detected	Detected
6038	A_33_P3258846					2381.17800	3710.85600	1102.58720	1378.15650	Detected	Detected	Detected	Detected
6039	A_24_P355267	SLC25A25	Hs.5476	solute carrier family 25 (mitochondrial c	Homo sapiens solute carrier family 25 (	2961.32130	3764.69140	1526.02160	1604.95740	Detected	Detected	Detected	Detected
6040	A_23_P8640	GPER	Hs.20961	G protein-coupled estrogen receptor 1	Homo sapiens G protein-coupled estrog	1097.87290	2446.29050	1032.70390	2207.35620	Detected	Detected	Detected	Detected
6041	A_33_P3275070	LOC730456	Hs.587281	hypothetical LOC730456	PREDICTED: Homo sapiens hypothetica	21.11728	14.72634	10.94888	9.42728	Detected	Compromised	Compromised	Compromised
6042	A_23_P202071	CUGBP2	Hs.309288	CUG triplet repeat, RNA binding protein	Homo sapiens CUG triplet repeat, RNA	11394.29200	1292.52170	7654.13800	737.34140	Detected	Detected	Detected	Detected
6043	A_33_P3420053	ZNF415	Hs.147765	zinc finger protein 415	Homo sapiens zinc finger protein 415 (Z	533.91943	436.99470	441.21628	330.82742	Detected	Detected	Detected	Detected
6044	A_33_P3310274				Leucine-rich repeat-containing G-prote	13.79512	40.35233	13.31105	31.34366	Detected	Detected	Compromised	Detected
6045	A_23_P92042	ITPR1	Hs.567295	inositol 1,4,5-triphosphate receptor, typ	Homo sapiens inositol 1,4,5-triphosphat	470.72705	93.28436	436.75528	77.49809	Detected	Detected	Detected	Detected
6046	A_23_P91919	SERP1	Hs.518326	stress-associated endoplasmic reticulu	Homo sapiens stress-associated endop	7329.45850	8902.43200	4629.35250	4764.05600	Detected	Detected	Detected	Detected
6047	A_23_P63050	UROD	Hs.78601	uroporphyrinogen decarboxylase	Homo sapiens uroporphyrinogen decarb	13115.06200	19247.49000	13467.10100	17501.87100	Detected	Detected	Detected	Detected
6048	A_23_P113748	ZNF385D	Hs.21026	zinc finger protein 385D	Homo sapiens zinc finger protein 385D (	369.35660	453.36664	302.50680	338.48776	Detected	Detected	Detected	Detected
6049	A_24_P207150	UBE3A	Hs.598862	ubiquitin protein ligase E3A	Homo sapiens ubiquitin protein ligase E3	1612.03340	1452.96530	1098.89530	808.17267	Detected	Detected	Detected	Detected
6050	A_32_P221822	RRP15	Hs.660109	ribosomal RNA processing 15 homolog (	Homo sapiens ribosomal RNA processin	485.38190	408.43200	257.94208	182.10226	Detected	Detected	Detected	Detected
6051	A_23_P213718	UQCRQ	Hs.146602	ubiquinol-cytochrome c reductase, com	Homo sapiens ubiquinol-cytochrome c r	74889.33000	74489.12000	64585.20700	56949.20300	Detected	Detected	Detected	Detected
6052	A_23_P20035	GPR146	Hs.718624	G protein-coupled receptor 146	Homo sapiens G protein-coupled recep	66.73664	69.38261	57.11290	35.89881	Detected	Detected	Detected	Detected
6053	A_24_P178300	PRR21	Hs.693479	proline rich 21	Homo sapiens proline rich 21 (PRR21),	82.14011	165.37993	49.23406	43.76566	Detected	Detected	Detected	Detected
6054	A_23_P389532	AMAC1	Hs.514814	acyl-malonyl condensing enzyme 1	Homo sapiens acyl-malonyl condensing	2.49248	19.83752	2.81184	2.93700	Compromised	Detected	Compromised	Compromised
6055	A_33_P3246990	XIAP	Hs.356076	X-linked inhibitor of apoptosis	Homo sapiens X-linked inhibitor of apop	5372.37400	5593.05200	4107.96340	3345.49730	Detected	Detected	Detected	Detected
6056	A_33_P3307408					11.33737	25.64879	9.59873	9.54254	Compromised	Detected	Compromised	Compromised
6057	A_23_P405148	MFF	Hs.471528	mitochondrial fission factor	Homo sapiens mitochondrial fission fact	37531.86700	25869.01400	32278.15600	19894.45700	Detected	Detected	Detected	Detected
6058	A_33_P3210139	PCF11	Hs.128959	PCF11, cleavage and polyadenylation fa	Homo sapiens PCF11, cleavage and poly	2276.14870	1451.59830	1432.56750	762.07117	Detected	Detected	Detected	Detected
6059	A_23_P18292	RPL14	Hs.446522	ribosomal protein L14	Homo sapiens ribosomal protein L14 (R	256127.60000	248365.27000	200190.16000	172578.88000	Detected	Detected	Detected	Detected
6060	A_23_P165727	MSTN	Hs.41565	myostatin	Homo sapiens myostatin (MSTN), mRNA	12.96214	3.40375	2.78607	2.90602	Detected	Compromised	Compromised	Compromised
6061	A_24_P301837	HRH3	Hs.251399	histamine receptor H3	Homo sapiens histamine receptor H3 (H	98.00059	127.20858	53.33311	68.67786	Detected	Detected	Detected	Detected
6062	A_23_P98022	SIRT1	Hs.369779	sirtuin (silent mating type information re	Homo sapiens sirtuin (silent mating type	976.05690	961.76790	860.72050	711.48400	Detected	Detected	Detected	Detected
6063	A_33_P3361337	TMEM184A	Hs.592174	transmembrane protein 184A	Homo sapiens transmembrane protein 1	15.19493	22.34018	9.65746	16.50362	Detected	Detected	Compromised	Detected
6064	A_23_P376870	C14orf79	Hs.27183	chromosome 14 open reading frame 79	Homo sapiens chromosome 14 open rea	304.97360	160.23915	263.16113	128.56511	Detected	Detected	Detected	Detected
6065	A_23_P131935	FERMT1	Hs.472054	fermitin family homolog 1 (Drosophila)	Homo sapiens fermitin family homolog 1	101.83689	661.18050	72.09717	407.88727	Detected	Detected	Detected	Detected
6066	A_23_P116898	A2M	Hs.212838	alpha-2-macroglobulin	Homo sapiens alpha-2-macroglobulin (A	8472.32900	948.47900	5920.55600	590.06384	Detected	Detected	Detected	Detected
6067	A_23_P354805	KLF12	Hs.373857	Kruppel-like factor 12	Homo sapiens Kruppel-like factor 12 (K	2844.92600	3256.43530	2003.26000	1836.77770	Detected	Detected	Detected	Detected
6068	A_23_P133068	ANK2	Hs.620557	ankyrin 2, neuronal	Homo sapiens ankyrin 2, neuronal (ANK	49.01297	42.11720	26.57825	24.70576	Detected	Detected	Detected	Detected
6069	A_24_P373768	GIPR	Hs.658534	gastric inhibitory polypeptide receptor	Homo sapiens gastric inhibitory polypep	400.45210	523.95580	226.34663	216.39640	Detected	Detected	Detected	Detected
6070	A_33_P3360675	PRKAR1B	Hs.520851	protein kinase, cAMP-dependent, regula	Homo sapiens protein kinase, cAMP-de	1440.59850	562.78510	1115.41690	381.24750	Detected	Detected	Detected	Detected
6071	A_33_P3269862					6.95258	58.60853	3.46605	3.01131	Compromised	Detected	Compromised	Compromised

6072	A_23_P200710	PIK3C2B	Hs.497487	phosphoinositide-3-kinase, class 2, beta	Homo sapiens phosphoinositide-3-kinas	60.94561	20.65533	50.10544	11.52305	Detected	Detected	Detected	Compromised
6073	A_23_P18325	PDCD10	Hs.478150	programmed cell death 10	Homo sapiens programmed cell death 1(	9298.38900	9049.55400	6644.38000	5573.41360	Detected	Detected	Detected	Detected
6074	A_33_P3372189	LOC729255	Hs.647666	hypothetical LOC729255	PREDICTED: Homo sapiens hypothetica	95828.94500	104348.69000	61819.29300	59578.65000	Detected	Detected	Detected	Detected
6075	A_32_P72394	PRKAR1B	Hs.520851	protein kinase, cAMP-dependent, regula	Homo sapiens protein kinase, cAMP-de	248.34608	137.14401	230.35916	111.81469	Detected	Detected	Detected	Detected
6076	A_23_P334751	B3GALNT2	Hs.716571	beta-1,3-N-acetylgalactosaminyltransfe	Homo sapiens beta-1,3-N-acetylgalacto	408.10437	428.54086	356.44394	317.36190	Detected	Detected	Detected	Detected
6077	A_33_P3346538	LOC100132364	Hs.606475	hypothetical LOC100132364	PREDICTED: Homo sapiens hypothetica	70.00687	64.78051	45.66395	38.84081	Detected	Detected	Detected	Detected
6078	A_33_P3321055	TNFRSF19	Hs.149168	tumor necrosis factor receptor superfar	Homo sapiens tumor necrosis factor re	51.96604	54.20837	48.86760	46.29742	Detected	Detected	Detected	Detected
6079	A_23_P164237	UTP6	Hs.589862	UTP6, small subunit (SSU) processome	Homo sapiens UTP6, small subunit (SSU	4039.39430	3789.40430	3642.52660	3082.49500	Detected	Detected	Detected	Detected
6080	A_24_P942694	C10orf118	Hs.159066	chromosome 10 open reading frame 118	Homo sapiens chromosome 10 open rea	491.19470	309.82720	271.44186	146.87886	Detected	Detected	Detected	Detected
6081	A_23_P500400	ABCA6	Hs.709514	ATP-binding cassette, sub-family A (AE	Homo sapiens ATP-binding cassette, su	3165.52730	535.03450	2400.01460	331.76846	Detected	Detected	Detected	Detected
6082	A_33_P3371320	BRE	Hs.258314	brain and reproductive organ-expressed	Homo sapiens brain and reproductive or	855.69420	532.53485	828.61444	453.15180	Detected	Detected	Detected	Detected
6083	A_23_P78209	MAFG	Hs.252229	v-maf musculoaponeurotic fibrosarcoma	Homo sapiens v-maf musculoaponeurot	4105.38430	3800.87940	2748.91280	2207.23070	Detected	Detected	Detected	Detected
6084	A_23_P22994	NFYC	Hs.720048	nuclear transcription factor Y, gamma	Homo sapiens nuclear transcription fact	527.80505	669.23615	564.09760	590.04663	Detected	Detected	Detected	Detected
6085	A_33_P3242080	DDX19A	Hs.656037	DEAD (Asp-Glu-Ala-As) box polypeptide	Homo sapiens DEAD (Asp-Glu-Ala-As)	3918.62400	4044.47070	2707.71950	2289.78050	Detected	Detected	Detected	Detected
6086	A_33_P3365755	SYT2	Hs.25422	synaptotagmin II	Homo sapiens synaptotagmin II (SYT2),	39.76437	48.70399	17.06175	16.31250	Detected	Detected	Detected	Detected
6087	A_24_P918175	LOC100128392	Hs.607889	hypothetical LOC100128392	PREDICTED: Homo sapiens hypothetica	15.43492	6.55301	22.91605	9.50450	Detected	Compromised	Detected	Compromised
6088	A_24_P194688	EFHA2	Hs.403594	EF-hand domain family, member A2	Homo sapiens EF-hand domain family, r	494.04797	292.72580	379.82123	160.96153	Detected	Detected	Detected	Detected
6089	A_23_P81241	NDFIP1	Hs.9788	Nedd4 family interacting protein 1	Homo sapiens Nedd4 family interacting	4196.38200	5602.36500	2775.78050	3238.77170	Detected	Detected	Detected	Detected
6090	A_33_P3272921	ARID3A	Hs.501296	AT rich interactive domain 3A (BRIGHT	Homo sapiens AT rich interactive doma	2254.22270	2012.68810	1556.20870	1181.51160	Detected	Detected	Detected	Detected
6091	A_23_P369479	MSI2	Hs.658922	musashi homolog 2 (Drosophila)	Homo sapiens musashi homolog 2 (Dros	120.25652	63.27131	118.42922	64.64677	Detected	Detected	Detected	Detected
6092	A_24_P234856	CAMLG	Hs.529846	calcium modulating ligand	Homo sapiens calcium modulating ligand	508.52646	254.10118	264.69626	104.66673	Detected	Detected	Detected	Detected
6093	A_33_P3216714	DNAJC6	Hs.647643	DnaJ (Hsp40) homolog, subfamily C, me	Homo sapiens DnaJ (Hsp40) homolog, s	931.92190	737.44520	672.63480	434.43506	Detected	Detected	Detected	Detected
6094	A_33_P3302632	HIST1H2BE	Hs.534369	histone cluster 1, H2be	Homo sapiens histone cluster 1, H2be (	37.06138	62.74258	25.99282	42.13002	Detected	Detected	Detected	Detected
6095	A_33_P3246543	MAT2B	Hs.54642	methionine adenosyltransferase II, beta	Homo sapiens methionine adenosyltrans	432.44430	367.79290	416.36746	307.36462	Detected	Detected	Detected	Detected
6096	A_24_P159094	UBR7	Hs.648806	ubiquitin protein ligase E3 component n	Homo sapiens ubiquitin protein ligase E	1783.59390	3037.03340	1581.87880	2224.44730	Detected	Detected	Detected	Detected
6097	A_23_P35148	TAF13	Hs.632426	TAF13 RNA polymerase II, TATA box bi	Homo sapiens TAF13 RNA polymerase	36.37207	118.08963	40.37082	115.04269	Detected	Detected	Detected	Detected
6098	A_24_P914513	BCKDHB	Hs.654441	branched chain keto acid dehydrogenas	Homo sapiens branched chain keto acid	33.29901	83.51744	10.99819	27.32194	Detected	Detected	Compromised	Detected
6099	A_24_P520767	LOC149351	Hs.546492	hypothetical protein LOC149351	Homo sapiens cDNA FLJ12836 fis, clon	304.43420	73.70721	288.58423	44.07230	Detected	Detected	Detected	Detected
6100	A_24_P187921	MANEA	Hs.533323	mannosidase, endo-alpha	Homo sapiens mannosidase, endo-alpha	60.42229	81.61797	47.42405	54.23855	Detected	Detected	Detected	Detected
6101	A_33_P3210521				UPF0554 protein C2orf43 [Source:UniF	1110.28590	576.78250	710.00220	342.92450	Detected	Detected	Detected	Detected
6102	A_23_P419239	ETNK1	Hs.29464	ethanolamine kinase 1	Homo sapiens ethanolamine kinase 1 (E	430.27980	341.26038	293.04236	194.73927	Detected	Detected	Detected	Detected
6103	A_23_P303718	DST	Hs.604915	dystonin	Homo sapiens dystonin (DST), transcrip	1218.26260	1413.25920	749.08100	799.01215	Detected	Detected	Detected	Detected
6104	A_24_P250765	PRKG1	Hs.654556	protein kinase, cGMP-dependent, type I	Homo sapiens protein kinase, cGMP-de	33.04324	79.37972	25.01828	47.91743	Detected	Detected	Detected	Detected
6105	A_33_P3251940	LOC100131608	Hs.650715	hypothetical LOC100131608	PREDICTED: Homo sapiens hypothetica	6.93965	20.41564	4.59221	5.53787	Compromised	Detected	Compromised	Compromised
6106	A_23_P357284	GPR4	Hs.17170	G protein-coupled receptor 4	Homo sapiens G protein-coupled recep	1731.05040	167.70346	1539.17870	139.04767	Detected	Detected	Detected	Detected
6107	A_23_P2645	SDS	Hs.439023	serine dehydratase	Homo sapiens serine dehydratase (SDS	25.36711	32.42664	29.59186	31.76617	Detected	Detected	Detected	Detected
6108	A_23_P363313	SLC16A11	Hs.336564	solute carrier family 16, member 11 (mo	Homo sapiens solute carrier family 16, r	205.39194	214.39548	172.85349	111.92669	Detected	Detected	Detected	Detected
6109	A_33_P3404411	MIPEP	Hs.507498	mitochondrial intermediate peptidase	Homo sapiens mitochondrial intermediat	3132.45310	1696.75480	3144.68750	1473.17930	Detected	Detected	Detected	Detected
6110	A_33_P3306442	QRICH2	Hs.252739	glutamine rich 2	Homo sapiens glutamine rich 2 (QRICH2	12.63070	19.02203	27.19263	31.99103	Detected	Detected	Detected	Detected
6111	A_24_P258051	MASTL	Hs.276905	microtubule associated serine/threonine	Homo sapiens microtubule associated s	235.41441	817.08545	204.54713	565.30480	Detected	Detected	Detected	Detected
6112	A_33_P3219517	LOC100131416	Hs.714984	hypothetical LOC100131416	PREDICTED: Homo sapiens hypothetica	91.62378	62.55540	92.91283	57.66104	Detected	Detected	Detected	Detected
6113	A_24_P370887	VAMP3	Hs.66708	vesicle-associated membrane protein 3	Homo sapiens vesicle-associated memb	4615.84330	6369.11040	3614.13840	4146.14060	Detected	Detected	Detected	Detected
6114	A_23_P357248	ZNF343	Hs.516846	zinc finger protein 343	Homo sapiens zinc finger protein 343 (Z	115.82501	114.26319	90.03348	97.46172	Detected	Detected	Detected	Detected
6115	A_33_P3362193	RAD23A	Hs.643267	RAD23 homolog A (S. cerevisiae)	Homo sapiens RAD23 homolog A (S. ce	44305.37000	56558.02300	34922.48000	38219.11700	Detected	Detected	Detected	Detected
6116	A_33_P3302881	KLHL31	Hs.131064	kelch-like 31 (Drosophila)	Homo sapiens kelch-like 31 (Drosophila	9.86326	9.46690	14.23532	11.35470	Compromised	Compromised	Detected	Compromised
6117	A_33_P3402943	LOC100128591	Hs.640065	hypothetical protein LOC100128591	Homo sapiens cDNA FLJ46872 fis, clon	15.14350	20.50326	3.74296	3.29517	Detected	Detected	Compromised	Compromised
6118	A_32_P857658	RPLP1	Hs.356502	ribosomal protein, large, P1	Homo sapiens ribosomal protein, large, I	329317.28000	329158.40000	214744.14000	182892.05000	Detected	Detected	Detected	Detected
6119	A_23_P111297	RPP40	Hs.511756	ribonuclease P/MRP 40kDa subunit	Homo sapiens ribonuclease P/MRP 40k	2138.36740	3311.84940	1781.59890	2181.44200	Detected	Detected	Detected	Detected
6120	A_23_P140375	NGB	Hs.274363	neuroglobin	Homo sapiens neuroglobin (NGB), mRNA	65.61896	60.70142	17.84150	20.17035	Detected	Detected	Detected	Detected
6121	A_33_P3279379	LOC171220	Hs.661103	destrin-2 pseudogene	Human destrin-2 pseudogene mRNA, cc	1034.38660	1132.92520	93.51190	91.41077	Detected	Detected	Detected	Detected
6122	A_33_P3414574	DNAJA2	Hs.368078	DnaJ (Hsp40) homolog, subfamily A, me	Homo sapiens DnaJ (Hsp40) homolog, s	2166.57670	2689.45260	1599.11490	1707.38720	Detected	Detected	Detected	Detected
6123	A_23_P140362	C14orf115	Hs.578167	chromosome 14 open reading frame 115	Homo sapiens chromosome 14 open rea	16.59598	10.87119	3.48857	2.99905	Detected	Compromised	Compromised	Compromised
6124	A_33_P3319765				Fanconi anemia group C protein (Protei	19.41148	100.42371	12.26209	58.58151	Detected	Detected	Compromised	Detected
6125	A_23_P416965	FAM149A	Hs.357025	family with sequence similarity 149, mer	Homo sapiens family with sequence sim	35.50081	35.45934	37.86006	36.63333	Detected	Detected	Detected	Detected
6126	A_33_P3239765	LOC100129936	Hs.708523	hypothetical LOC100129936	Homo sapiens cDNA FLJ42830 fis, clon	13.56317	14.57097	6.66419	6.68751	Detected	Compromised	Compromised	Compromised
6127	A_33_P3280385	COL6A3	Hs.233240	collagen, type VI, alpha 3	Homo sapiens collagen, type VI, alpha 3	2893.73340	1960.85520	1934.56860	1210.03520	Detected	Detected	Detected	Detected
6128	A_23_P119418	C19orf2	Hs.466391	chromosome 19 open reading frame 2	Homo sapiens chromosome 19 open rea	1243.72250	1840.80710	993.63525	1341.76950	Detected	Detected	Detected	Detected
6129	A_33_P3326099	FUBP3	Hs.98751	far upstream element (FUSE) binding pr	Homo sapiens far upstream element (FL	57.12774	57.77520	64.07346	65.66797	Detected	Detected	Detected	Detected
6130	A_24_P135501					22859.31400	20113.30000	32927.33200	24741.50200	Detected	Detected	Detected	Detected
6131	A_23_P300600	NEFH	Hs.198760	neurofilament, heavy polypeptide	Homo sapiens neurofilament, heavy poly	529.61720	916.18896	532.23114	786.63873	Detected	Detected	Detected	Detected
6132	A_23_P395464	ZNF582	Hs.244391	zinc finger protein 582	Homo sapiens zinc finger protein 582 (Z	28.25690	19.83436	20.92675	14.46911	Detected	Detected	Detected	Compromised
6133	A_23_P14986	HSD11B2	Hs.1376	hydroxysteroid (11-beta) dehydrogenas	Homo sapiens hydroxysteroid (11-beta)	82.54202	48.12079	63.27200	16.43854	Detected	Detected	Detected	Detected
6134	A_33_P3254380	SLC9A7	Hs.620643	solute carrier family 9 (sodium/hydroge	Sodium/hydrogen exchanger 7 (Na(+)/H	151.78690	396.23560	124.76785	253.95550	Detected	Detected	Detected	Detected
6135	A_23_P52885	C11orf61	Hs.146079	chromosome 11 open reading frame 61	Homo sapiens chromosome 11 open rea	303.12372	308.22730	261.37665	207.55893	Detected	Detected	Detected	Detected
6136	A_32_P81676	LOC157627		hypothetical LOC157627	Homo sapiens hypothetical LOC157627	18.35619	19.15088	11.61488	7.81506	Detected	Detected	Compromised	Compromised
6137	A_23_P206684	WWP2	Hs.408458	WW domain containing E3 ubiquitin prot	Homo sapiens WW domain containing E3	670.59125	760.26790	631.46204	678.15170	Detected	Detected	Detected	Detected
6138	A_23_P423926	SS18L1	Hs.154429	synovial sarcoma translocation gene on	Homo sapiens synovial sarcoma translo	501.48053	291.99432	342.10037	169.53731	Detected	Detected	Detected	Detected
6139	A_23_P330070	TFPI	Hs.516578	tissue factor pathway inhibitor (lipoprot	Homo sapiens tissue factor pathway inh	8663.04800	4366.39800	7773.44970	3043.41240	Detected	Detected	Detected	Detected
6140	A_33_P3299811	LQK1	Hs.552649	hypothetical LOC642946	Homo sapiens hypothetical LOC642946	85.05660	40.98655	86.16750	32.78058	Detected	Detected	Detected	Detected
6141	A_23_P22129	HAP1	Hs.158300	huntingtin-associated protein 1	Homo sapiens huntingtin-associated pr	88.69928	93.74313	94.32015	82.10154	Detected	Detected	Detected	Detected
6142	A_23_P391506	IVNS1ABP	Hs.497183	influenza virus NS1A binding protein	Homo sapiens influenza virus NS1A bin	1752.86060	1084.46190	1379.26750	720.28880	Detected	Detected	Detected	Detected
6143	A_23_P308058	TUSC5	Hs.146059	tumor suppressor candidate 5	Homo sapiens tumor suppressor candid	46.46771	43.36250	19.14707	11.94587	Detected	Detected	Detected	Compromised
6144	A_33_P3779229	GK5	Hs.135904	glycerol kinase 5 (putative)	Homo sapiens glycerol kinase 5 (putativ	201.06330	95.43150	131.95859	52.71764	Detected	Detected	Detected	Detected
6145	A_24_P237878	FAM195B	Hs.514632	family with sequence similarity 195, mer	Homo sapiens family with sequence sim	31443.44500	33628.86700	32209.41800	31522.36100	Detected	Detected	Detected	Detected
6146	A_23_P344568	FAM124A	Hs.71913	family with sequence similarity 124A	Homo sapiens family with sequence sim	52.94270	17.49849	40.19567	16.46163	Detected	Compromised	Detected	Detected

6147	A_33_P3293734					744.85034	1566.40660	738.13770	1397.23830	Detected	Detected	Detected	Detected
6148	A_23_P361544	CES8	Hs.346947	carboxylesterase 8 (putative)	Homo sapiens carboxylesterase 8 (puta	17.99902	15.52796	12.00290	8.30374	Detected	Compromised	Compromised	Compromised
6149	A_33_P3350703	ERO1LB	Hs.558519	ERO1-like beta (S. cerevisiae)	Homo sapiens ERO1-like beta (S. cerev	228.95142	130.40518	175.89957	68.43462	Detected	Detected	Detected	Detected
6150	A_23_P422245	C5orf36	Hs.425123	chromosome 5 open reading frame 36	Homo sapiens chromosome 5 open read	12.64150	3.47892	7.01882	2.99558	Detected	Compromised	Compromised	Compromised
6151	A_24_P192262	RALA	Hs.6906	v-ral simian leukemia viral oncogene ho	Homo sapiens v-ral simian leukemia vira	9308.37600	12158.33700	6730.54200	7217.91100	Detected	Detected	Detected	Detected
6152	A_23_P317347	ESCO1	Hs.464733	establishment of cohesion 1 homolog 1	Homo sapiens establishment of cohesio	393.30505	243.33568	291.76105	146.19257	Detected	Detected	Detected	Detected
6153	A_23_P161727	HSPB2	Hs.709660	heat shock 27kDa protein 2	Homo sapiens heat shock 27kDa protei	4778.94700	16545.60200	3946.50700	11562.66000	Detected	Detected	Detected	Detected
6154	A_33_P3248654	SARS	Hs.531176	seryl-tRNA synthetase	Homo sapiens seryl-tRNA synthetase (	19308.66000	30031.37100	14908.81800	19968.39600	Detected	Detected	Detected	Detected
6155	A_33_P3287158	NLGN4X	Hs.21107	neuroligin 4, X-linked	Homo sapiens neuroligin 4, X-linked (NL	30.49761	8.33449	2.83332	2.96557	Detected	Compromised	Compromised	Compromised
6156	A_23_P79818	C20orf111	Hs.75798	chromosome 20 open reading frame 111	Homo sapiens chromosome 20 open rea	9274.77200	8480.03900	6795.23440	5338.93650	Detected	Detected	Detected	Detected
6157	A_24_P160413	MTMR9L	Hs.471067	myotubularin related protein 9-like	Homo sapiens myotubularin related prot	533.08640	156.50012	389.90180	94.65812	Detected	Detected	Detected	Detected
6158	A_24_P309415	TMEM123	Hs.503709	transmembrane protein 123	Homo sapiens transmembrane protein 1	12135.52400	13403.74100	8292.79100	7571.58300	Detected	Detected	Detected	Detected
6159	A_23_P88119	HSPH1	Hs.36927	heat shock 105kDa/110kDa protein 1	Homo sapiens heat shock 105kDa/110k	9927.14700	10553.59600	7371.12740	6609.30900	Detected	Detected	Detected	Detected
6160	A_23_P138760	CLCF1	Hs.502977	cardiotrophin-like cytokine factor 1	Homo sapiens cardiotrophin-like cytoki	558.07245	537.10070	470.60727	381.94974	Detected	Detected	Detected	Detected
6161	A_32_P88880					261.83160	14.98536	222.54968	3.10922	Detected	Compromised	Detected	Compromised
6162	A_23_P110624	CTNND2	Hs.314543	catenin (cadherin-associated protein), d	Homo sapiens catenin (cadherin-associ	61.01253	36.02285	42.13758	17.41351	Detected	Detected	Detected	Detected
6163	A_33_P3252635	ZNF254	Hs.434406	zinc finger protein 254	Homo sapiens zinc finger protein 254 (Z	331.66016	327.91556	250.22101	191.14581	Detected	Detected	Detected	Detected
6164	A_33_P3345210	TLCD2		TLC domain containing 2	Homo sapiens TLC domain containing 2	21.23530	21.39751	22.70827	17.40658	Detected	Detected	Detected	Detected
6165	A_23_P212595	VPRBP	Hs.716623	Vpr (HIV-1) binding protein	Homo sapiens Vpr (HIV-1) binding prote	193.84294	149.11372	163.66120	116.69632	Detected	Detected	Detected	Detected
6166	A_23_P160567	ZMYND12	Hs.294009	zinc finger, MYND-type containing 12	Homo sapiens zinc finger, MYND-type c	26.71981	8.87254	27.62315	7.41927	Detected	Compromised	Detected	Compromised
6167	A_33_P3224891		Hs.713417		cDNA FLJ26048 fis, clone PRS02384 [	297.05500	270.01392	340.36688	242.75833	Detected	Detected	Detected	Detected
6168	A_24_P917178		Hs.306978		Hypothetical gene supported by BC001	11.55057	22.94474	12.83736	16.54653	Compromised	Detected	Compromised	Detected
6169	A_33_P3791552	LOC284950	Hs.570227	hypothetical protein LOC284950	Homo sapiens cDNA FLJ37719 fis, clon	171.15028	218.05590	128.63069	160.01703	Detected	Detected	Detected	Detected
6170	A_24_P122636	BPNT1	Hs.406134	3'(2'), 5'-bisphosphate nucleotidase 1	Homo sapiens 3'(2'), 5'-bisphosphate nu	389.81262	292.87988	31.60121	16.72446	Detected	Detected	Detected	Detected
6171	A_24_P941359	FAM65B	Hs.559459	family with sequence similarity 65, mem	Homo sapiens family with sequence sim	284.77618	67.61446	188.60900	33.29909	Detected	Detected	Detected	Detected
6172	A_23_P356004	KCNIP3	Hs.437376	Kv channel interacting protein 3, calsen	Homo sapiens Kv channel interacting pr	8600.85700	3932.01320	7498.34470	3309.68290	Detected	Detected	Detected	Detected
6173	A_33_P3718352	LOC170425	Hs.434131	hypothetical protein LOC170425	Homo sapiens cDNA FLJ31999 fis, clon	351.60860	254.80586	8.55359	9.37767	Detected	Detected	Compromised	Compromised
6174	A_32_P138042		Hs.715338		naf71f09.x1 NCI_CGAP_Brn66 Homo sa	40.09229	55.37050	19.97757	17.48777	Detected	Detected	Detected	Detected
6175	A_33_P3380462	GPR88	Hs.170053	G protein-coupled receptor 88	Homo sapiens G protein-coupled recep	642.84330	938.00340	409.21356	441.62510	Detected	Detected	Detected	Detected
6176	A_24_P243396	TCOF1	Hs.519672	Treacher Collins-Franceschetti syndror	Homo sapiens Treacher Collins-France	117.56401	527.05860	107.63834	409.97464	Detected	Detected	Detected	Detected
6177	A_24_P401090					661.18530	1565.13150	139.63788	254.35855	Detected	Detected	Detected	Detected
6178	A_23_P160934	ANP32E	Hs.656466	acidic (leucine-rich) nuclear phosphopro	Homo sapiens acidic (leucine-rich) nucl	189.92499	764.61285	129.73839	403.92905	Detected	Detected	Detected	Detected
6179	A_33_P3390580	CCDC144A	Hs.721149	coiled-coil domain containing 144A	Homo sapiens coiled-coil domain conta	88.15624	9.56912	58.62548	6.37955	Detected	Compromised	Detected	Compromised
6180	A_33_P3286312				Tripartite motif-containing protein ENS	28.04098	25.27024	15.33565	11.35236	Detected	Detected	Detected	Compromised
6181	A_23_P132417	LSG1	Hs.720060	large subunit GTPase 1 homolog (S. cer	Homo sapiens large subunit GTPase 1 h	1646.39720	1591.28960	1482.45480	1217.28300	Detected	Detected	Detected	Detected
6182	A_33_P3235360					79.17709	154.24925	57.41756	50.83376	Detected	Detected	Detected	Detected
6183	A_33_P3370615				Putative uncharacterized protein ENSP	42.83526	80.41865	21.36199	39.00223	Detected	Detected	Detected	Detected
6184	A_23_P315571	RFTN1	Hs.98910	raftlin, lipid raft linker 1	Homo sapiens raftlin, lipid raft linker 1 (	6515.51460	15186.57200	3921.42260	7757.10100	Detected	Detected	Detected	Detected
6185	A_33_P3410821	LOC100134360		hypothetical protein LOC100134360	PREDICTED: Homo sapiens hypothetica	24.44852	28.30274	29.39100	33.88952	Detected	Detected	Detected	Detected
6186	A_23_P37654	MGA	Hs.187569	MAX gene associated	Homo sapiens MAX gene associated (M	935.90314	796.80426	834.23303	674.87994	Detected	Detected	Detected	Detected
6187	A_24_P159181	PDCD7	Hs.458596	programmed cell death 7	Homo sapiens programmed cell death 7	3605.92460	3067.30320	2740.08130	1986.42990	Detected	Detected	Detected	Detected
6188	A_33_P3844650	ANGPTL2	Hs.653262	angiopoietin-like 2	Homo sapiens angiopoietin-like 2 (ANG	16207.33000	2729.26030	17331.30700	2866.21850	Detected	Detected	Detected	Detected
6189	A_23_P63243	C1orf43	Hs.287471	chromosome 1 open reading frame 43	Homo sapiens chromosome 1 open read	18155.90600	10173.84400	19141.00000	9432.86800	Detected	Detected	Detected	Detected
6190	A_24_P8109	ANO9	Hs.501622	anoctamin 9	Homo sapiens anoctamin 9 (ANO9), mR	13.91276	14.44905	23.82850	18.72803	Detected	Compromised	Detected	Detected
6191	A_33_P3362143	LOC284900		hypothetical LOC284900	Homo sapiens hypothetical LOC284900	277.78660	122.03377	168.28268	57.01615	Detected	Detected	Detected	Detected
6192	A_33_P3328426	ANO10	Hs.656657	anoctamin 10	Homo sapiens anoctamin 10 (ANO10), r	17378.81400	19146.69500	14397.61600	13367.26900	Detected	Detected	Detected	Detected
6193	A_33_P3760937	LOC497256	Hs.584108	hypothetical LOC497256	Homo sapiens cDNA FLJ37669 fis, clon	4636.93900	7632.76070	222.87187	132.00056	Detected	Detected	Detected	Detected
6194	A_23_P356139	FAM178A	Hs.447458	family with sequence similarity 178, mer	Homo sapiens family with sequence sim	304.18620	135.61546	203.93115	90.49079	Detected	Detected	Detected	Detected
6195	A_23_P204947	GJB2	Hs.524894	gap junction protein, beta 2, 26kDa	Homo sapiens gap junction protein, beta	19.86098	3.33521	17.26534	2.97960	Detected	Compromised	Detected	Compromised
6196	A_24_P400604	RBMY1B	Hs.536001	RNA binding motif protein, Y-linked, fan	Homo sapiens RNA binding motif protei	16.17409	3.81763	12.24152	3.24992	Detected	Compromised	Compromised	Compromised
6197	A_23_P217228	TRO	Hs.633653	trophinin	Homo sapiens trophinin (TRO), transcri	1584.30650	879.05810	1415.78580	686.35925	Detected	Detected	Detected	Detected
6198	A_24_P259276	ZDHHC24	Hs.659933	zinc finger, DHHC-type containing 24	Homo sapiens zinc finger, DHHC-type c	3317.01560	3680.00100	3054.81640	2859.86130	Detected	Detected	Detected	Detected
6199	A_33_P3240353	SLC39A4	Hs.521934	solute carrier family 39 (zinc transporte	Homo sapiens solute carrier family 39 (	1489.50180	1316.32140	1662.42090	1207.04490	Detected	Detected	Detected	Detected
6200	A_33_P3238177	ZNF37A	Hs.292575	zinc finger protein 37A	Homo sapiens zinc finger protein 37A (Z	1460.34690	1762.95870	973.62616	1046.00430	Detected	Detected	Detected	Detected
6201	A_23_P57155	CHGB	Hs.516874	chromogranin B (secretogranin 1)	Homo sapiens chromogranin B (secreto	100.27039	7.79014	101.51112	9.16573	Detected	Compromised	Detected	Compromised
6202	A_24_P337239	FGF5	Hs.37055	fibroblast growth factor 5	Homo sapiens fibroblast growth factor 5	5.87650	27.82625	2.83158	2.98551	Compromised	Detected	Compromised	Compromised
6203	A_33_P3234641					92264.15000	62020.67600	72366.19500	41280.20000	Detected	Detected	Detected	Detected
6204	A_23_P170088	EXD3	Hs.495553	exonuclease 3'-5' domain containing 3	Homo sapiens exonuclease 3'-5' domai	97.32549	98.72785	87.81820	87.78399	Detected	Detected	Detected	Detected
6205	A_23_P361584	TMEM154	Hs.518900	transmembrane protein 154	Homo sapiens transmembrane protein 1	290.38380	464.55510	198.06155	269.30072	Detected	Detected	Detected	Detected
6206	A_23_P259863	CD177	Hs.232165	CD177 molecule	Homo sapiens CD177 molecule (CD177	20.33878	13.02086	23.40525	15.17513	Detected	Compromised	Detected	Detected
6207	A_33_P3379956				Doublecortin domain-containing protein	17.95586	26.13642	9.70055	5.49559	Detected	Detected	Compromised	Compromised
6208	A_23_P259586	TTK	Hs.169840	TTK protein kinase	Homo sapiens TTK protein kinase (TTK	25.58659	1487.64700	12.98803	840.60840	Detected	Detected	Compromised	Detected
6209	A_24_P231829	BHMT2	Hs.114172	betaine-homocysteine methyltransferas	Homo sapiens betaine-homocysteine m	87.09492	281.01630	62.57184	187.41867	Detected	Detected	Detected	Detected
6210	A_24_P101047	KIAA1586	Hs.709521	KIAA1586	Homo sapiens KIAA1586 (KIAA1586), m	46.68339	63.94198	26.17948	20.34057	Detected	Detected	Detected	Detected
6211	A_23_P394323	FAM98B	Hs.6799	family with sequence similarity 98, mem	Protein FAM98B [Source:UniProtKB/S	6.30244	24.21579	3.28111	7.92631	Compromised	Detected	Compromised	Compromised
6212	A_24_P238118	LOC100192379	Hs.607691	hypothetical LOC100192379	Homo sapiens hypothetical LOC100192	45.46611	54.10691	13.41028	13.19325	Detected	Detected	Compromised	Compromised
6213	A_33_P3295423	ZXDC	Hs.440049	ZXD family zinc finger C	Homo sapiens ZXD family zinc finger C	86.53945	69.00472	59.36798	26.09438	Detected	Detected	Detected	Detected
6214	A_23_P118462	OVCA2	Hs.513856	ovarian tumor suppressor candidate 2	Homo sapiens ovarian tumor suppresso	17604.42000	26920.78000	13450.97100	17854.95300	Detected	Detected	Detected	Detected
6215	A_23_P151778	CMA1	Hs.135626	chymase 1, mast cell	Homo sapiens chymase 1, mast cell (CM	2.64003	30.26581	4.74813	5.76386	Compromised	Detected	Compromised	Compromised
6216	A_24_P417935	hCG_2014417	Hs.656080	hypothetical LOC100130776	Homo sapiens hypothetical LOC100130	361.34985	858.60657	438.65830	1050.98410	Detected	Detected	Detected	Detected
6217	A_33_P3343432	THEM4	Hs.164070	thioesterase superfamily member 4	Homo sapiens thioesterase superfamily	189.81818	401.09772	152.20322	270.06497	Detected	Detected	Detected	Detected
6218	A_33_P3213792	KCNK4	Hs.647233	potassium channel, subfamily K, membe	Homo sapiens potassium channel, subfa	114.83621	31.29369	102.70381	28.24970	Detected	Detected	Detected	Detected
6219	A_33_P3385351	LOC100133402		similar to hCG2012269	PREDICTED: Homo sapiens hypothetica	79.53516	113.96285	24.36357	25.05223	Detected	Detected	Detected	Detected
6220	A_24_P111054	SLC2A5	Hs.530003	solute carrier family 2 (facilitated gluco	Solute carrier family 2, facilitated gluco	174.85977	317.97256	49.17836	65.20573	Detected	Detected	Detected	Detected
6221	A_33_P3312039	RAD23B	Hs.521640	RAD23 homolog B (S. cerevisiae)	Homo sapiens RAD23 homolog B (S. ce	251.55632	336.34253	154.54642	208.96335	Detected	Detected	Detected	Detected

6222	A_33_P3246883	DMKN	Hs.417795	dermokine	Homo sapiens dermokine (DMKN), trans	4352.25200	167.08998	4441.97700	188.62196	Detected	Detected	Detected	Detected
6223	A_33_P3248004		Hs.559970		Uncharacterized protein FLJ41423 [So	28.50503	28.88320	17.06128	5.37426	Detected	Detected	Detected	Compromised
6224	A_33_P3429576	AP3S1	Hs.406191	adaptor-related protein complex 3, sigm	Homo sapiens adaptor-related protein c	3801.00240	3462.37300	2901.48700	2238.42200	Detected	Detected	Detected	Detected
6225	A_23_P65830	HDDC3	Hs.349979	HD domain containing 3	Homo sapiens HD domain containing 3 (	2116.90380	1563.12880	2198.16240	1370.86950	Detected	Detected	Detected	Detected
6226	A_24_P941505	FAM120A	Hs.372003	family with sequence similarity 120A	Homo sapiens family with sequence sim	2524.76320	3569.80620	1648.04360	1732.74820	Detected	Detected	Detected	Detected
6227	A_32_P202703	LOC389831	Hs.389638	hypothetical gene supported by AL7137	Homo sapiens mRNA; cDNA DKFZp667I	649.24615	631.44830	574.54114	481.12115	Detected	Detected	Detected	Detected
6228	A_23_P423108	TDRD10	Hs.387671	tudor domain containing 10	Homo sapiens tudor domain containing	16.91662	19.13191	9.94240	11.44878	Detected	Detected	Compromised	Compromised
6229	A_23_P11353	ATP6AP2	Hs.495960	ATPase, H+ transporting, lysosomal acc	Homo sapiens ATPase, H+ transporting,	9215.62700	7517.02500	7185.91900	4641.75700	Detected	Detected	Detected	Detected
6230	A_23_P354827	ZNF550		zinc finger protein 550	zinc finger protein 550 [Source:RefSeq	1192.79700	1040.58910	865.99677	603.51886	Detected	Detected	Detected	Detected
6231	A_24_P943922	CACHD1	Hs.443891	cache domain containing 1	Homo sapiens cache domain containing	65.52362	74.32055	53.95264	38.59877	Detected	Detected	Detected	Detected
6232	A_24_P405054	C1orf144	Hs.252967	chromosome 1 open reading frame 144	Homo sapiens chromosome 1 open read	261.73074	1011.78700	347.85260	1234.69530	Detected	Detected	Detected	Detected
6233	A_24_P339416	ARSG	Hs.437249	arylsulfatase G	Homo sapiens arylsulfatase G (ARSG),	608.62090	127.90578	719.41080	103.42311	Detected	Detected	Detected	Detected
6234	A_33_P3240679				Bromodomain-containing protein 3 (RIN	46.04787	49.77331	7.25211	5.32390	Detected	Detected	Compromised	Compromised
6235	A_33_P3288364	SPATA2L	Hs.374556	spermatogenesis associated 2-like	Homo sapiens spermatogenesis associa	3867.92000	3285.10450	4005.44210	3047.90000	Detected	Detected	Detected	Detected
6236	A_23_P429478	CCDC140	Hs.350729	coiled-coil domain containing 140	Homo sapiens coiled-coil domain conta	120.96010	161.89568	70.82580	84.75140	Detected	Detected	Detected	Detected
6237	A_24_P338992	EDC3	Hs.96852	enhancer of mRNA decapping 3 homolo	Homo sapiens enhancer of mRNA decap	663.15940	761.00903	703.77060	771.22510	Detected	Detected	Detected	Detected
6238	A_23_P259692	PSAT1	Hs.494261	phosphoserine aminotransferase 1	Homo sapiens phosphoserine aminotran	2168.18140	7507.80270	1129.59960	3180.38770	Detected	Detected	Detected	Detected
6239	A_33_P3394378					630.98260	532.59080	202.44391	141.80680	Detected	Detected	Detected	Detected
6240	A_33_P3365002	TUBB2A	Hs.654543	tubulin, beta 2A	Homo sapiens tubulin, beta 2A (TUBB2A	2988.80080	7557.59670	3583.82470	8636.80300	Detected	Detected	Detected	Detected
6241	A_23_P27035	TMEM98	Hs.3447	transmembrane protein 98	Homo sapiens transmembrane protein 9	9816.85600	14946.25600	10022.12100	12658.62800	Detected	Detected	Detected	Detected
6242	A_24_P384755	NOL10	Hs.222494	nucleolar protein 10	Homo sapiens nucleolar protein 10 (NO	146.77098	210.26791	117.26907	144.97600	Detected	Detected	Detected	Detected
6243	A_24_P167825	VPS39	Hs.88025	vacuolar protein sorting 39 homolog (S.	Homo sapiens vacuolar protein sorting	1624.34110	1113.95200	1618.67090	1098.30700	Detected	Detected	Detected	Detected
6244	A_33_P3343479	C1orf167	Hs.585415	chromosome 1 open reading frame 167	PREDICTED: Homo sapiens hypothetica	28.72789	35.30093	12.40097	10.50338	Detected	Detected	Compromised	Compromised
6245	A_23_P101551	BCAT2	Hs.512670	branched chain aminotransferase 2, mit	Homo sapiens branched chain aminotra	1476.31640	2626.71500	1530.86520	2452.77400	Detected	Detected	Detected	Detected
6246	A_23_P379692	C14orf178	Hs.375834	chromosome 14 open reading frame 178	Homo sapiens chromosome 14 open rea	15.07650	23.22352	13.09773	12.02010	Detected	Detected	Compromised	Compromised
6247	A_23_P371885	ARL17P1	Hs.720602	ADP-ribosylation factor-like 17 pseudo	Homo sapiens ADP-ribosylation factor-	191.93216	226.30235	101.24246	94.71812	Detected	Detected	Detected	Detected
6248	A_32_P166693	HEG1	Hs.477420	HEG homolog 1 (zebrafish)	Homo sapiens HEG homolog 1 (zebrafis	4540.74760	7407.39060	2397.68360	3142.46830	Detected	Detected	Detected	Detected
6249	A_23_P54116	DAAM1	Hs.654934	dishevelled associated activator of mor	Homo sapiens dishevelled associated ac	133.25739	173.78000	138.76381	149.22263	Detected	Detected	Detected	Detected
6250	A_24_P332471					29345.05700	17050.41200	21535.25800	10431.40000	Detected	Detected	Detected	Detected
6251	A_23_P141100	ADAT1	Hs.188661	adenosine deaminase, tRNA-specific 1	Homo sapiens adenosine deaminase, tR	657.34937	713.07263	511.48715	521.57210	Detected	Detected	Detected	Detected
6252	A_23_P408232	MGC23270	Hs.352220	hypothetical LOC196872	Homo sapiens hypothetical LOC196872	16.35067	12.70547	12.59482	4.11783	Detected	Compromised	Compromised	Compromised
6253	A_33_P3218252	SMG1	Hs.460179	SMG1 homolog, phosphatidylinositol 3-k	Homo sapiens SMG1 homolog, phospha	2817.35740	2924.73560	2044.10740	1789.07410	Detected	Detected	Detected	Detected
6254	A_33_P3379391	H2AFJ	Hs.524280	H2A histone family, member J	Homo sapiens H2A histone family, mem	20.29649	17.93600	30.41344	22.92387	Detected	Compromised	Detected	Detected
6255	A_23_P152262	DPEP1	Hs.109	dipeptidase 1 (renal)	Homo sapiens dipeptidase 1 (renal) (DP	7.82363	35.07455	7.00571	26.06125	Compromised	Detected	Compromised	Detected
6256	A_23_P210253	DGKD	Hs.471675	diacylglycerol kinase, delta 130kDa	Homo sapiens diacylglycerol kinase, del	2855.76400	1724.38060	2594.21880	1537.49160	Detected	Detected	Detected	Detected
6257	A_33_P3329795				Solute carrier family 22 member 20 (Or	51.53378	63.23092	25.67969	23.95257	Detected	Detected	Detected	Detected
6258	A_33_P3333982	PDE4DIP	Hs.568247	phosphodiesterase 4D interacting prote	Homo sapiens phosphodiesterase 4D in	4658.19240	7176.07370	3756.73400	5204.09770	Detected	Detected	Detected	Detected
6259	A_33_P3359308	KLHL29		kelch-like 29 (Drosophila)	Homo sapiens kelch-like 29 (Drosophila	562.27900	627.15360	232.62428	241.08487	Detected	Detected	Detected	Detected
6260	A_24_P912372	KIAA0754	Hs.658760	KIAA0754	Homo sapiens KIAA0754 (KIAA0754), m	76.23433	103.28858	49.46975	54.29508	Detected	Detected	Detected	Detected
6261	A_33_P3502640	DTX2	Hs.187058	deltex homolog 2 (Drosophila)	Homo sapiens deltex homolog 2 (Drosop	426.94247	482.03925	475.61047	495.72620	Detected	Detected	Detected	Detected
6262	A_33_P3263307	RANGAP1	Hs.183800	Ran GTPase activating protein 1	Homo sapiens Ran GTPase activating p	35303.34400	76605.72000	31365.24400	61598.69000	Detected	Detected	Detected	Detected
6263	A_23_P64083	SF3B2	Hs.406423	splicing factor 3b, subunit 2, 145kDa	Homo sapiens splicing factor 3b, subuni	26462.20500	25092.41200	23349.03100	20058.72900	Detected	Detected	Detected	Detected
6264	A_32_P2103					2132.57700	1803.77300	1480.91910	1072.70500	Detected	Detected	Detected	Detected
6265	A_33_P3291092	THSD1	Hs.325667	thrombospondin, type I, domain contain	Homo sapiens thrombospondin, type I, d	9.17102	42.92023	12.13744	22.10692	Compromised	Detected	Compromised	Detected
6266	A_23_P73239	NCKAP1	Hs.603732	NCK-associated protein 1	Homo sapiens NCK-associated protein	7042.31800	8038.03200	3525.73100	2935.42140	Detected	Detected	Detected	Detected
6267	A_23_P3982	C17orf37	Hs.333526	chromosome 17 open reading frame 37	Homo sapiens chromosome 17 open rea	7512.61870	10307.95500	8754.29100	10259.05900	Detected	Detected	Detected	Detected
6268	A_23_P16143	GTF2F1	Hs.68257	general transcription factor IIF, polypep	Homo sapiens general transcription fact	4905.12650	5249.03560	5143.32130	5255.81700	Detected	Detected	Detected	Detected
6269	A_33_P3256272	KRTAP10-5	Hs.688629	keratin associated protein 10-5	Homo sapiens keratin associated protei	113.36560	88.46947	30.77415	24.05520	Detected	Detected	Detected	Detected
6270	A_33_P3288684					111.34014	47.56698	60.89954	13.00236	Detected	Detected	Detected	Compromised
6271	A_33_P3305433	LOC100131581	Hs.711569	hypothetical LOC100131381	PREDICTED: Homo sapiens hypothetica	53.83004	71.17228	30.92132	30.47660	Detected	Detected	Detected	Detected
6272	A_23_P204286	MGP	Hs.365706	matrix Gla protein	Homo sapiens matrix Gla protein (MGP)	306.85180	782.39820	254.50783	547.47314	Detected	Detected	Detected	Detected
6273	A_23_P211106	SETD4	Hs.606200	SET domain containing 4	Homo sapiens SET domain containing 4	474.19968	264.90927	420.81350	194.64738	Detected	Detected	Detected	Detected
6274	A_23_P55281	HOXB7	Hs.436181	homeobox B7	Homo sapiens homeobox B7 (HOXB7),	2.50469	256.59653	2.77442	108.12709	Compromised	Detected	Compromised	Detected
6275	A_23_P20255	COMMD5	Hs.631856	COMM domain containing 5	Homo sapiens COMM domain containing	4230.12900	7664.45700	4949.23000	7622.00150	Detected	Detected	Detected	Detected
6276	A_33_P3232280	C12orf11	Hs.505077	chromosome 12 open reading frame 11	Homo sapiens chromosome 12 open rea	331.96000	260.07660	273.30103	172.20934	Detected	Detected	Detected	Detected
6277	A_23_P103864	TTC13	Hs.424788	tetratricopeptide repeat domain 13	Homo sapiens tetratricopeptide repeat	478.16202	241.88254	318.28357	135.88731	Detected	Detected	Detected	Detected
6278	A_24_P113686	ZNHIT6	Hs.5111	zinc finger, HIT type 6	Homo sapiens zinc finger, HIT type 6 (Z	761.71910	1044.12180	561.87213	658.30865	Detected	Detected	Detected	Detected
6279	A_24_P296070	COG3	Hs.507948	component of oligomeric golgi complex	Homo sapiens component of oligomeric	1998.07180	1731.28380	1458.23730	1102.16460	Detected	Detected	Detected	Detected
6280	A_23_P65208	ZMYM5	Hs.530988	zinc finger, MYM-type 5	Homo sapiens zinc finger, MYM-type 5	762.29944	1049.58310	497.91574	588.27893	Detected	Detected	Detected	Detected
6281	A_23_P68240	GPAT2	Hs.348629	glycerol-3-phosphate acyltransferase 2	Homo sapiens glycerol-3-phosphate ac	756.55396	2871.61400	505.43155	2199.24500	Detected	Detected	Detected	Detected
6282	A_33_P3233819				Putative uncharacterized protein ENSP	12.90386	14.03247	18.22324	18.88724	Compromised	Compromised	Detected	Detected
6283	A_32_P132477					317.78912	521.56384	300.96460	420.72420	Detected	Detected	Detected	Detected
6284	A_33_P3331085	SEC24A	Hs.595540	SEC24 family, member A (S. cerevisiae)	Homo sapiens SEC24 family, member A	425.63522	686.71820	317.00170	436.10130	Detected	Detected	Detected	Detected
6285	A_23_P110076	WDR53	Hs.385865	WD repeat domain 53	Homo sapiens WD repeat domain 53 (W	1350.79170	1529.00540	1072.36180	1010.36870	Detected	Detected	Detected	Detected
6286	A_24_P168574				Guanine nucleotide-binding protein G(s	27.38589	87.09503	33.84385	69.39768	Detected	Detected	Detected	Detected
6287	A_24_P339514	CYP2B6	Hs.1360	cytochrome P450, family 2, subfamily B	Homo sapiens cytochrome P450, family	186.35962	253.96213	79.11897	32.17953	Detected	Detected	Detected	Detected
6288	A_33_P3236272				Protein BANP (Btg3-associated nuclea	50.39393	42.95858	55.49107	39.78638	Detected	Detected	Detected	Detected
6289	A_23_P128574	ENOX1	Hs.128258	ecto-NOX disulfide-thiol exchanger 1	Homo sapiens ecto-NOX disulfide-thiol	750.96000	303.53700	588.78780	208.28291	Detected	Detected	Detected	Detected
6290	A_23_P55107	ULK2	Hs.168762	unc-51-like kinase 2 (C. elegans)	Homo sapiens unc-51-like kinase 2 (C.	1796.20540	1457.15320	1185.20210	865.87085	Detected	Detected	Detected	Detected
6291	A_33_P3333712	SCYL2	Hs.506481	SCY1-like 2 (S. cerevisiae)	Homo sapiens SCY1-like 2 (S. cerevisia	51.38754	55.04919	55.08820	45.49254	Detected	Detected	Detected	Detected
6292	A_23_P383915	BTBD16	Hs.422466	BTB (POZ) domain containing 16	Homo sapiens BTB (POZ) domain conta	14.33021	14.65037	15.36431	13.43181	Detected	Compromised	Compromised	Compromised
6293	A_33_P3777584	SERAC1	Hs.154706	serine active site containing 1	Homo sapiens serine active site contain	255.24070	270.32254	145.92368	122.39631	Detected	Detected	Detected	Detected
6294	A_23_P36724	FBXL14	Hs.367956	F-box and leucine-rich repeat protein 1	Homo sapiens F-box and leucine-rich r	513.62646	199.29932	423.21848	95.50861	Detected	Detected	Detected	Detected
6295	A_23_P15123	UBFD1	Hs.3459	ubiquitin family domain containing 1	Homo sapiens ubiquitin family domain c	417.62885	1624.61600	408.02830	1200.18350	Detected	Detected	Detected	Detected
6296	A_33_P3420083	hCG_1647286	Hs.711769	hypothetical protein LOC728690	Homo sapiens cDNA FLJ37323 fis, clon	14.13333	8.64488	9.83191	6.74361	Detected	Compromised	Compromised	Compromised

6297	A_32_P49848	RHOQ	Hs.709193	ras homolog gene family, member Q	Homo sapiens ras homolog gene family,	1485.46140	1210.30480	1523.25200	1017.79070	Detected	Detected	Detected	Detected
6298	A_23_P145006	SCGB3A2	Hs.483765	secretoglobin, family 3A, member 2	Homo sapiens secretoglobin, family 3A,	6955.71340	11079.89500	920.89404	1198.67700	Detected	Detected	Detected	Detected
6299	A_33_P3373119					33.17876	33.98977	34.10426	35.66327	Detected	Detected	Detected	Detected
6300	A_33_P3843285	LOC151877	Hs.664969	hypothetical protein LOC151877	Homo sapiens cDNA FLJ32304 fis, clon	12.32328	14.74664	16.19919	16.89697	Compromised	Compromised	Detected	Detected
6301	A_33_P3217517	C5orf55	Hs.446702	chromosome 5 open reading frame 55	Homo sapiens chromosome 5 open read	734.75530	433.67786	786.75885	424.63422	Detected	Detected	Detected	Detected
6302	A_23_P98483	ZBED5	Hs.655066	zinc finger, BED-type containing 5	Homo sapiens zinc finger, BED-type co	9474.90000	5046.24600	8972.18600	4084.14700	Detected	Detected	Detected	Detected
6303	A_24_P346587	MTFR1	Hs.584788	mitochondrial fission regulator 1	Homo sapiens mitochondrial fission regu	866.11350	1932.37280	793.81550	1421.52310	Detected	Detected	Detected	Detected
6304	A_24_P827491	PA2G4	Hs.524498	proliferation-associated 2G4, 38kDa	Homo sapiens proliferation-associated	1970.64650	3901.98300	530.25090	817.70624	Detected	Detected	Detected	Detected
6305	A_23_P384499	RPTOR	Hs.133044	regulatory associated protein of MTOR,	Homo sapiens regulatory associated pro	3050.85300	4294.84670	2511.81860	3244.37940	Detected	Detected	Detected	Detected
6306	A_24_P935881	SERF1B	Hs.658079	small EDRK-rich factor 1B (centromeric	Homo sapiens small EDRK-rich factor 1	2128.36330	3704.30500	2760.92480	3884.07450	Detected	Detected	Detected	Detected
6307	A_33_P3292218					588.15840	1142.21570	451.24768	843.43820	Detected	Detected	Detected	Detected
6308	A_33_P3262253				Histone-lysine N-methyltransferase SU	15.48363	21.58634	12.20534	12.10699	Detected	Detected	Compromised	Compromised
6309	A_33_P3418000	RELL1	Hs.283378	RELT-like 1	Homo sapiens RELT-like 1 (RELL1), tra	216.84558	155.74020	198.77771	135.45760	Detected	Detected	Detected	Detected
6310	A_33_P3350634	KIF22	Hs.612151	kinesin family member 22	Homo sapiens kinesin family member 22	549.70230	4080.30710	545.88920	3827.57760	Detected	Detected	Detected	Detected
6311	A_24_P246293	FDXACB1	Hs.697132	ferredoxin-fold anticodon binding domai	Homo sapiens ferredoxin-fold anticodon	17.75206	32.89586	18.93753	22.46475	Detected	Detected	Detected	Detected
6312	A_33_P3410849	C8orf58	Hs.553539	chromosome 8 open reading frame 58	Homo sapiens chromosome 8 open read	13343.50300	17883.09000	3280.14840	3456.56000	Detected	Detected	Detected	Detected
6313	A_23_P406025	PRUNE2	Hs.262857	prune homolog 2 (Drosophila)	Homo sapiens prune homolog 2 (Drosop	1853.21060	1676.99790	1151.88780	823.77783	Detected	Detected	Detected	Detected
6314	A_23_P310972	PCDHGB1	Hs.368160	protocadherin gamma subfamily B, 1	Homo sapiens protocadherin gamma sul	8.79056	21.13807	6.73388	16.42243	Compromised	Detected	Compromised	Detected
6315	A_33_P3227217	SNORA81	Hs.688848	small nucleolar RNA, H/ACA box 81	Homo sapiens small nucleolar RNA, H/A	67.18250	73.10549	42.86226	23.00446	Detected	Detected	Detected	Detected
6316	A_33_P3215363				Transcription factor 23 [Source:UniPro	22.91709	32.34838	25.81775	18.80683	Detected	Detected	Detected	Detected
6317	A_23_P121945	SNCB	Hs.90297	synuclein, beta	Homo sapiens synuclein, beta (SNCB), t	248.18805	241.04878	84.12805	57.67044	Detected	Detected	Detected	Detected
6318	A_23_P59602	MIOS	Hs.520215	missing oocyte, meiosis regulator, homo	Homo sapiens missing oocyte, meiosis r	1743.50350	6199.34230	1785.36450	5455.04700	Detected	Detected	Detected	Detected
6319	A_32_P65533	ZC3H12D	Hs.632618	zinc finger CCCH-type containing 12D	Homo sapiens zinc finger CCCH-type c	39.60955	49.21735	11.91900	10.40319	Detected	Detected	Compromised	Compromised
6320	A_24_P944040	RRP1B	Hs.654727	ribosomal RNA processing 1 homolog B	Homo sapiens ribosomal RNA processin	1071.15730	1632.17800	958.99677	1244.99410	Detected	Detected	Detected	Detected
6321	A_24_P892472	EMX2OS	Hs.312592	EMX2 opposite strand (non-protein cod	Homo sapiens EMX2 opposite strand (n	4735.68750	1292.17140	3871.12230	899.16900	Detected	Detected	Detected	Detected
6322	A_23_P18372	B3GNT5	Hs.718506	UDP-GlcNAc:betaGal beta-1,3-N-acety	Homo sapiens UDP-GlcNAc:betaGal be	8222.96300	4491.50200	5923.81740	2759.88840	Detected	Detected	Detected	Detected
6323	A_23_P78479	ZIM2	Hs.719209	zinc finger, imprinted 2	Homo sapiens zinc finger, imprinted 2 (	21.30275	27.93983	23.20063	25.87556	Detected	Detected	Detected	Detected
6324	A_23_P128084	ITGA7	Hs.524484	integrin, alpha 7	Homo sapiens integrin, alpha 7 (ITGA7),	9235.27000	1862.41870	11556.64600	2033.64880	Detected	Detected	Detected	Detected
6325	A_33_P3262789	REEP6	Hs.76277	receptor accessory protein 6	Homo sapiens receptor accessory prot	1256.40090	460.89743	1352.45360	444.56552	Detected	Detected	Detected	Detected
6326	A_32_P205241	GJA3	Hs.130313	gap junction protein, alpha 3, 46kDa	Homo sapiens gap junction protein, alph	81.18858	122.36888	66.71558	79.37804	Detected	Detected	Detected	Detected
6327	A_33_P3309289		Hs.591947		CMP-N-acetylneuraminate-beta-galact	24.08888	70.02098	21.26356	49.96451	Detected	Detected	Detected	Detected
6328	A_23_P209625	CYP1B1	Hs.154654	cytochrome P450, family 1, subfamily B	Homo sapiens cytochrome P450, family	9671.18000	4649.27440	6000.36500	2641.90530	Detected	Detected	Detected	Detected
6329	A_23_P55136	RANGRF	Hs.408233	RAN guanine nucleotide release factor	Homo sapiens RAN guanine nucleotide	2279.56800	2895.59900	2365.92870	2869.30100	Detected	Detected	Detected	Detected
6330	A_24_P367211	SOCS7	Hs.632261	suppressor of cytokine signaling 7	Homo sapiens suppressor of cytokine s	11.68562	28.51689	11.77062	24.30900	Compromised	Detected	Compromised	Detected
6331	A_33_P3274304	WIPF2	Hs.421622	WAS/WASL interacting protein family, r	Homo sapiens WAS/WASL interacting p	2737.45070	3313.12160	2499.77150	2555.50240	Detected	Detected	Detected	Detected
6332	A_33_P3344399	LRRC49	Hs.12692	leucine rich repeat containing 49	Homo sapiens leucine rich repeat conta	489.76294	426.24200	399.98700	290.18652	Detected	Detected	Detected	Detected
6333	A_24_P941557	FAM125B	Hs.162659	family with sequence similarity 125, mer	Homo sapiens family with sequence sim	14.19450	128.63310	4.88828	67.83497	Detected	Detected	Compromised	Detected
6334	A_33_P3268649	SLC2A11	Hs.632772	solute carrier family 2 (facilitated glucos	Homo sapiens solute carrier family 2 (fa	60.38124	66.80468	74.03867	85.37899	Detected	Detected	Detected	Detected
6335	A_23_P15202	DHODH	Hs.654427	dihydroorotate dehydrogenase	Homo sapiens dihydroorotate dehydroge	880.76180	1265.41860	698.91110	856.31160	Detected	Detected	Detected	Detected
6336	A_23_P313550	SLC25A41	Hs.375135	solute carrier family 25, member 41	Homo sapiens solute carrier family 25, r	73.00770	11.61971	126.56374	12.62074	Detected	Compromised	Detected	Compromised
6337	A_33_P3313704		Hs.683855		cDNA FLJ38166 fis, clone DFNES20055	95.28104	159.09567	68.42284	66.98496	Detected	Detected	Detected	Detected
6338	A_23_P153236	CHMP2A	Hs.12107	chromatin modifying protein 2A	Homo sapiens chromatin modifying prot	19765.36700	12070.20100	25308.15200	13387.55500	Detected	Detected	Detected	Detected
6339	A_23_P124252	CAMK1D	Hs.659517	calcium/calmodulin-dependent protein	Homo sapiens calcium/calmodulin-depe	21.32434	20.95844	16.09867	11.95264	Detected	Detected	Detected	Compromised
6340	A_33_P3222653	LOC100131366	Hs.147881	hypothetical protein LOC100131366	Homo sapiens cDNA clone IMAGE:4826	15.31397	25.57720	8.91013	23.01011	Detected	Detected	Compromised	Detected
6341	A_33_P3382867	LOC389834	Hs.718898	ankyrin repeat domain 57 pseudogene	Homo sapiens ankyrin repeat domain 57	25.33643	3.80128	3.10763	3.23581	Detected	Compromised	Compromised	Compromised
6342	A_23_P207336	PPY	Hs.558368	pancreatic polypeptide	Homo sapiens pancreatic polypeptide (F	69.69177	71.62412	68.94918	62.02329	Detected	Detected	Detected	Detected
6343	A_24_P417596	FLYWCH2	Hs.534525	FLYWCH family member 2	Homo sapiens FLYWCH family member	666.34960	635.11163	184.54974	137.73920	Detected	Detected	Detected	Detected
6344	A_33_P3376762	LOC388152	Hs.719728	hypothetical LOC388152	Homo sapiens hypothetical LOC388152	7092.10350	9056.37600	4887.69040	5156.91500	Detected	Detected	Detected	Detected
6345	A_23_P83110	CDK5RAP2	Hs.269560	CDK5 regulatory subunit associated pro	Homo sapiens CDK5 regulatory subunit	3057.19020	3548.44820	2583.22920	2615.17480	Detected	Detected	Detected	Detected
6346	A_33_P3286754	SEC14L2	Hs.335614	SEC14-like 2 (S. cerevisiae)	Homo sapiens SEC14-like 2 (S. cerevisi	76.59461	165.34949	46.08950	83.73217	Detected	Detected	Detected	Detected
6347	A_23_P91619	MIF	Hs.407995	macrophage migration inhibitory factor (	Homo sapiens macrophage migration inh	182776.50000	193847.14000	168360.55000	154611.98000	Detected	Detected	Detected	Detected
6348	A_23_P212792	CEP135	Hs.518767	centrosomal protein 135kDa	Homo sapiens centrosomal protein 135	799.89100	1512.75790	568.91266	900.83010	Detected	Detected	Detected	Detected
6349	A_24_P79712	FAM36A	Hs.657313	family with sequence similarity 36, mem	Homo sapiens family with sequence sim	2555.82350	3004.92040	1926.34530	1821.51980	Detected	Detected	Detected	Detected
6350	A_24_P330112	MAGOHB	Hs.104650	mago-nashi homolog B (Drosophila)	Homo sapiens mago-nashi homolog B (D	56.82209	146.83475	45.77235	97.29925	Detected	Detected	Detected	Detected
6351	A_33_P3281323					241.84512	445.29257	44.27757	63.88731	Detected	Detected	Detected	Detected
6352	A_23_P27584	MYADM	Hs.380906	myeloid-associated differentiation mark	Homo sapiens myeloid-associated differ	21905.52700	26001.04000	19822.41600	21269.97500	Detected	Detected	Detected	Detected
6353	A_32_P358115	LOC284648	Hs.253475	hypothetical protein LOC284648	PREDICTED: Homo sapiens hypothetica	14.08319	7.59700	8.64931	2.95278	Detected	Compromised	Compromised	Compromised
6354	A_23_P420942				Metallothionein-1E (MT-1E)(Metallothio	275.97333	406.68784	290.55698	372.45618	Detected	Detected	Detected	Detected
6355	A_23_P47777	MARCH9	Hs.632709	membrane-associated ring finger (C3HC	Homo sapiens membrane-associated rir	1206.71860	996.08887	920.76135	326.21796	Detected	Detected	Detected	Detected
6356	A_33_P3363425	FRMD3		FERM domain containing 3	FERM domain-containing protein 3 (Bar	3318.99410	5746.63800	2116.44480	3089.91480	Detected	Detected	Detected	Detected
6357	A_33_P3229012	LOC652586		similar to double homeobox 4c	PREDICTED: Homo sapiens similar to fa	451.40997	404.33884	88.93345	66.63168	Detected	Detected	Detected	Detected
6358	A_23_P44257	COMMD8	Hs.23956	COMM domain containing 8	Homo sapiens COMM domain containing	11132.48200	10546.39200	8405.73900	6734.10200	Detected	Detected	Detected	Detected
6359	A_23_P388220	RALYL	Hs.121663	RALY RNA binding protein-like	Homo sapiens RALY RNA binding prote	16.93804	19.41648	7.56515	6.56719	Detected	Detected	Compromised	Compromised
6360	A_33_P3338961					1742.68200	1552.66270	436.91095	290.10693	Detected	Detected	Detected	Detected
6361	A_23_P75380	AIP	Hs.412433	aryl hydrocarbon receptor interacting p	Homo sapiens aryl hydrocarbon recepto	4037.50680	2402.62260	4683.06500	2428.76950	Detected	Detected	Detected	Detected
6362	A_23_P129425	TSNAXIP1	Hs.632212	translin-associated factor X interacting	Homo sapiens translin-associated facto	134.33679	21.33831	129.42801	28.76905	Detected	Detected	Detected	Detected
6363	A_33_P3382105					3138.07800	2960.60100	1301.24450	971.30237	Detected	Detected	Detected	Detected
6364	A_24_P156748	SLC30A2	Hs.143545	solute carrier family 30 (zinc transporte	Homo sapiens solute carrier family 30 (	125.83151	167.20699	79.72057	84.04671	Detected	Detected	Detected	Detected
6365	A_33_P3417670	SLC24A1	Hs.173092	solute carrier family 24 (sodium/potass	Homo sapiens solute carrier family 24 (	13.76604	12.80795	4.20096	12.97939	Detected	Compromised	Compromised	Compromised
6366	A_23_P69586	FAT1	Hs.481371	FAT tumor suppressor homolog 1 (Dros	Homo sapiens FAT tumor suppressor h	1207.71370	5964.42630	889.89900	3619.16140	Detected	Detected	Detected	Detected
6367	A_32_P29615	ZNF468	Hs.467223	zinc finger protein 468	Homo sapiens zinc finger protein 468 (Z	55.62089	57.12334	67.77298	70.45299	Detected	Detected	Detected	Detected
6368	A_23_P55356	VMO1	Hs.122561	vitelline membrane outer layer 1 homolo	Homo sapiens vitelline membrane outer	150.18779	36.97193	222.73726	59.43932	Detected	Detected	Detected	Detected
6369	A_24_P96593	EVI5	Hs.594434	ecotropic viral integration site 5	Homo sapiens ecotropic viral integratior	1688.19710	1960.39770	1216.55810	1104.85910	Detected	Detected	Detected	Detected
6370	A_24_P205589	ACOT7	Hs.126137	acyl-CoA thioesterase 7	Homo sapiens acyl-CoA thioesterase 7	1732.32790	6763.89500	2208.02030	8665.93900	Detected	Detected	Detected	Detected
6371	A_23_P113237	CRLF2	Hs.287729	cytokine receptor-like factor 2	Homo sapiens cytokine receptor-like fa	32.24876	31.79553	16.73864	16.79141	Detected	Detected	Detected	Detected

6372	A_24_P127075	TMEM135	Hs.188591	transmembrane protein 135	Homo sapiens transmembrane protein 1	35.41678	12.07530	21.68153	7.50305	Detected	Compromised	Detected	Compromised
6373	A_24_P876408	C11orf95	Hs.191073	chromosome 11 open reading frame 95	Homo sapiens chromosome 11 open rea	1573.90580	1045.59700	1539.75560	954.87750	Detected	Detected	Detected	Detected
6374	A_23_P76015	ARHGEF17	Hs.533719	Rho guanine nucleotide exchange facto	Homo sapiens Rho guanine nucleotide e	4615.08700	5152.31500	3446.45260	3475.63900	Detected	Detected	Detected	Detected
6375	A_33_P3313825				TGF-beta receptor type-2 Precursor (E	20.30415	23.96305	9.87174	15.42405	Detected	Detected	Compromised	Detected
6376	A_23_P141092	TFAP4	Hs.513305	transcription factor AP-4 (activating en	Homo sapiens transcription factor AP-4	20.41479	10.08173	12.45214	10.34755	Detected	Compromised	Compromised	Compromised
6377	A_33_P3449397	METTL3	Hs.168799	methyltransferase like 3	Homo sapiens methyltransferase like 3	3052.45070	2755.83570	2339.14230	1817.40390	Detected	Detected	Detected	Detected
6378	A_23_P18142	RPL32	Hs.265174	ribosomal protein L32	Homo sapiens ribosomal protein L32 (R	181567.70000	155990.90000	157821.98000	115206.13000	Detected	Detected	Detected	Detected
6379	A_23_P131596	C2orf7	Hs.61268	chromosome 2 open reading frame 7	Homo sapiens chromosome 2 open read	1205.43820	2230.72310	1618.85820	2546.91330	Detected	Detected	Detected	Detected
6380	A_23_P211136	BRWD1	Hs.654740	bromodomain and WD repeat domain co	Homo sapiens bromodomain and WD rep	413.68164	166.69190	310.11346	107.27631	Detected	Detected	Detected	Detected
6381	A_33_P3326423	POLL	Hs.523230	polymerase (DNA directed), lambda	Homo sapiens polymerase (DNA directe	2389.97560	1837.27920	2790.89700	1885.00420	Detected	Detected	Detected	Detected
6382	A_24_P204204					791.07530	1149.43410	189.06686	225.83670	Detected	Detected	Detected	Detected
6383	A_33_P3218148	C5orf42	Hs.586199	chromosome 5 open reading frame 42	Homo sapiens chromosome 5 open read	53.84967	53.76146	33.84839	30.20574	Detected	Detected	Detected	Detected
6384	A_33_P3281567	CMAH	Hs.484918	cytidine monophosphate-N-acetylneura	Homo sapiens cytidine monophosphate-	100.16287	132.39262	96.74695	100.01103	Detected	Detected	Detected	Detected
6385	A_33_P3405349		Hs.533139		Uncharacterized protein C7orf43 [Sour	424.78918	622.91570	142.48097	147.56902	Detected	Detected	Detected	Detected
6386	A_33_P3250133	VSIG10	Hs.187624	V-set and immunoglobulin domain conta	Homo sapiens V-set and immunoglobuli	92.17714	194.15508	89.54816	148.84123	Detected	Detected	Detected	Detected
6387	A_23_P118854	KRT37	Hs.673852	keratin 37	Homo sapiens keratin 37 (KRT37), mRN	12.05978	3.45631	4.58338	2.92615	Not Detected	Compromised	Compromised	Compromised
6388	A_33_P3236102	IER5L	Hs.529857	immediate early response 5-like	Homo sapiens immediate early response	2244.03100	883.62274	1469.55140	501.11768	Detected	Detected	Detected	Detected
6389	A_33_P3364869					7328.43100	497.73868	5648.88670	280.28220	Detected	Detected	Detected	Detected
6390	A_33_P3324894				Huntingtin-interacting protein 1-related	127.99480	135.37860	40.56386	41.35929	Detected	Detected	Detected	Detected
6391	A_33_P3405474	LOC100130943	Hs.609218	hypothetical LOC100130943	PREDICTED: Homo sapiens hypothetica	472.21555	503.85333	333.17725	340.88544	Detected	Detected	Detected	Detected
6392	A_33_P3352349				Putative keratin-associated protein 9-2	4429.05270	5091.49700	1629.47200	1551.32690	Detected	Detected	Detected	Detected
6393	A_23_P164341	VAMP2	Hs.25348	vesicle-associated membrane protein 2	Homo sapiens vesicle-associated memb	755.60986	746.89500	677.82270	605.81080	Detected	Detected	Detected	Detected
6394	A_33_P3264163	DDR1	Hs.631988	discoidin domain receptor tyrosine kina	Homo sapiens discoidin domain recepto	15.23726	4.03931	14.01807	7.31668	Detected	Compromised	Compromised	Compromised
6395	A_33_P3355743	GPATCH2	Hs.420757	G patch domain containing 2	Homo sapiens G patch domain containi	161.93388	200.45218	137.29909	130.77435	Detected	Detected	Detected	Detected
6396	A_33_P3376454	UNQ1870	Hs.679984	GALI1870	Homo sapiens clone DNA62312 GALI18	248.58417	500.63162	138.24632	199.25658	Detected	Detected	Detected	Detected
6397	A_23_P314712	CABYR	Hs.511983	calcium binding tyrosine-(Y)-phosphory	Homo sapiens calcium binding tyrosine-	564.90960	1122.71600	623.13030	1009.10986	Detected	Detected	Detected	Detected
6398	A_33_P3215605	LOC100131746	Hs.693955	hypothetical LOC100131746	PREDICTED: Homo sapiens hypothetica	9.95920	14.53936	8.29647	14.92691	Compromised	Compromised	Compromised	Detected
6399	A_32_P9842	MTMR14	Hs.475382	myotubularin related protein 14	Homo sapiens myotubularin related prot	22079.77700	21112.80000	19267.94700	15459.73800	Detected	Detected	Detected	Detected
6400	A_24_P106591	MUTED	Hs.719272	muted homolog (mouse)	Homo sapiens muted homolog (mouse)	4484.45900	3545.65110	3958.12800	2512.39650	Detected	Detected	Detected	Detected
6401	A_23_P214360	IRF4	Hs.401013	interferon regulatory factor 4	Homo sapiens interferon regulatory fact	19.71884	3.44710	4.15737	2.97435	Detected	Compromised	Compromised	Compromised
6402	A_32_P151875	ZNF398	Hs.654721	zinc finger protein 398	Homo sapiens zinc finger protein 398 (Z	2753.78340	3764.76370	2001.88590	2395.99320	Detected	Detected	Detected	Detected
6403	A_23_P103588	HMGCS2	Hs.59889	3-hydroxy-3-methylglutaryl-Coenzyme	Homo sapiens 3-hydroxy-3-methylgluta	8.86007	6.84490	3.68416	15.88497	Compromised	Compromised	Compromised	Not Detected
6404	A_23_P200685	MOSC2	Hs.369042	MOCO sulphurase C-terminal domain c	Homo sapiens MOCO sulphurase C-ter	352.78665	192.34685	311.45230	139.06496	Detected	Detected	Detected	Detected
6405	A_33_P3272160	REXO4	Hs.438409	REX4, RNA exonuclease 4 homolog (S.	Homo sapiens REX4, RNA exonuclease	5060.11500	5012.20650	5497.77500	5011.71300	Detected	Detected	Detected	Detected
6406	A_33_P3382471	CYP4A22	Hs.720099	cytochrome P450, family 4, subfamily A	Homo sapiens cytochrome P450, family	14.67549	37.66693	2.99643	5.31290	Detected	Detected	Compromised	Compromised
6407	A_33_P3306085	RAD51AP2	Hs.722353	RAD51 associated protein 2	Homo sapiens RAD51 associated protei	18.74500	25.83727	11.80497	7.80183	Detected	Detected	Compromised	Compromised
6408	A_24_P235266	GRB10	Hs.164060	growth factor receptor-bound protein 1	Homo sapiens growth factor receptor-b	4017.86330	2444.75980	2410.29960	1292.85240	Detected	Detected	Detected	Detected
6409	A_23_P309224	AGK	Hs.720082	acylglycerol kinase	Homo sapiens acylglycerol kinase (AGK	381.74478	463.85977	341.54633	349.97614	Detected	Detected	Detected	Detected
6410	A_33_P3471712	LOC284600	Hs.551839	hypothetical protein LOC284600	Homo sapiens cDNA FLJ31924 fis, clon	270585.47000	274654.22000	158394.10000	124383.42000	Detected	Detected	Detected	Detected
6411	A_23_P80643	SETMAR	Hs.716054	SET domain and mariner transposase fu	Homo sapiens SET domain and mariner	1164.46800	1346.09860	1012.21910	1038.08960	Detected	Detected	Detected	Detected
6412	A_33_P3241269	CES1	Hs.558865	carboxylesterase 1 (monocyte/macroph	Homo sapiens carboxylesterase 1 (mon	50269.65600	5297.46040	33968.60000	3212.68500	Detected	Detected	Detected	Detected
6413	A_23_P393531	INPP4A	Hs.469386	inositol polyphosphate-4-phosphatase,	Homo sapiens inositol polyphosphate-4	128.00453	252.20502	110.78728	176.90128	Detected	Detected	Detected	Detected
6414	A_33_P3418170	DDX58	Hs.190622	DEAD (Asp-Glu-Ala-Asp) box polypept	Homo sapiens DEAD (Asp-Glu-Ala-Asp	242.55197	140.69469	154.42091	58.08788	Detected	Detected	Detected	Detected
6415	A_33_P3227360				Homeobox protein DLX-1 [Source:UniP	2.47228	17.90130	7.70356	13.10687	Compromised	Detected	Compromised	Compromised
6416	A_33_P3314531	LOC646853		similar to high-mobility group nucleosor	PREDICTED: Homo sapiens similar to h	37.07333	142.77480	12.77107	30.84141	Detected	Detected	Compromised	Detected
6417	A_33_P3294669	LOC100129626		similar to translocase of outer mitochor	PREDICTED: Homo sapiens similar to tr	554.30835	902.54730	376.20523	479.92267	Detected	Detected	Detected	Detected
6418	A_23_P111995	LOXL2	Hs.626637	lysyl oxidase-like 2	Homo sapiens lysyl oxidase-like 2 (LOX	9818.66100	63102.42600	6884.45360	40602.16400	Detected	Detected	Detected	Detected
6419	A_23_P8906	LRP12	Hs.600630	low density lipoprotein-related protein	Homo sapiens low density lipoprotein-r	1024.92910	1280.45600	705.47910	719.89890	Detected	Detected	Detected	Detected
6420	A_32_P524014	UTRN	Hs.133135	utrophin	Homo sapiens utrophin (UTRN), mRNA	1662.83960	2604.58890	1136.67360	1543.30900	Detected	Detected	Detected	Detected
6421	A_33_P3242355				Putative uncharacterized protein LOC3	2357.57000	3136.61160	1793.01550	1499.51140	Detected	Detected	Detected	Detected
6422	A_33_P3318911	INPP5K	Hs.632238	inositol polyphosphate-5-phosphatase	Homo sapiens inositol polyphosphate-5	3647.33330	3084.62500	3729.13670	2898.55030	Detected	Detected	Detected	Detected
6423	A_23_P26704	MFSD11	Hs.73965	major facilitator superfamily domain cor	Homo sapiens major facilitator superfan	2674.15920	2837.27100	2380.80640	2174.00240	Detected	Detected	Detected	Detected
6424	A_32_P113508	ATG16L1	Hs.529322	ATG16 autophagy related 16-like 1 (S.	Homo sapiens ATG16 autophagy relate	2018.65600	2172.21680	2007.09190	1926.78980	Detected	Detected	Detected	Detected
6425	A_23_P390116	SPATA13	Hs.595391	spermatogenesis associated 13	Homo sapiens spermatogenesis associa	425.47320	177.63371	308.29355	126.02992	Detected	Detected	Detected	Detected
6426	A_24_P212860	C9orf156	Hs.9196	chromosome 9 open reading frame 156	Homo sapiens chromosome 9 open read	151.57072	191.50665	154.07750	180.69296	Detected	Detected	Detected	Detected
6427	A_23_P11652	USP1	Hs.35086	ubiquitin specific peptidase 1	Homo sapiens ubiquitin specific peptida	5417.05700	8553.71300	3321.74220	4348.25200	Detected	Detected	Detected	Detected
6428	A_24_P456723	LOC728537	Hs.469369	hypothetical protein LOC728537	PREDICTED: Homo sapiens hypothetica	346.23230	153.71710	304.51190	120.75785	Detected	Detected	Detected	Detected
6429	A_24_P319364	F11R	Hs.517293	F11 receptor	Homo sapiens F11 receptor (F11R), mR	265.14560	98.61254	255.14026	88.32552	Detected	Detected	Detected	Detected
6430	A_23_P40049	CAD	Hs.377010	carbamoyl-phosphate synthetase 2, asp	Homo sapiens carbamoyl-phosphate sy	2509.19650	2445.65190	2358.95120	2411.02470	Detected	Detected	Detected	Detected
6431	A_23_P146084	GSR	Hs.271510	glutathione reductase	Homo sapiens glutathione reductase (G	791.02620	1009.30066	811.27124	933.10110	Detected	Detected	Detected	Detected
6432	A_33_P3242388	PIGX	Hs.223296	phosphatidylinositol glycan anchor biosy	Homo sapiens phosphatidylinositol glyca	2317.69400	1827.86180	2100.18070	1407.14030	Detected	Detected	Detected	Detected
6433	A_23_P154058	EIF2B4	Hs.169474	eukaryotic translation initiation factor 2	Homo sapiens eukaryotic translation ini	1791.09640	1389.45450	1682.03050	1309.13720	Detected	Detected	Detected	Detected
6434	A_23_P215832	ATP5J2	Hs.521056	ATP synthase, H+ transporting, mitocho	Homo sapiens ATP synthase, H+ transp	8887.27300	12567.52100	8419.71400	10286.90700	Detected	Detected	Detected	Detected
6435	A_33_P3374324	OR51I1	Hs.553732	olfactory receptor, family 51, subfamily	Homo sapiens olfactory receptor, family	16.18175	11.81096	8.57046	7.77733	Detected	Compromised	Compromised	Compromised
6436	A_33_P3246448	KCNE4	Hs.348522	potassium voltage-gated channel, Isk-re	Homo sapiens potassium voltage-gated	309.30887	354.52878	224.22740	214.48747	Detected	Detected	Detected	Detected
6437	A_33_P3330503	ALDH7A1	Hs.483239	aldehyde dehydrogenase 7 family, memb	Homo sapiens aldehyde dehydrogenase	1016.87146	452.72336	675.88760	222.91962	Detected	Detected	Detected	Detected
6438	A_33_P3211924	RCOR1	Hs.510521	REST corepressor 1	Homo sapiens REST corepressor 1 (RC	2418.42160	2500.56150	1758.16610	1366.16630	Detected	Detected	Detected	Detected
6439	A_23_P204436	GIT2	Hs.434996	G protein-coupled receptor kinase inter	Homo sapiens G protein-coupled recep	134.20543	266.86300	127.55027	231.19789	Detected	Detected	Detected	Detected
6440	A_24_P914940	LOC729991	Hs.153629	hypothetical protein LOC729991	Homo sapiens hypothetical protein LOC	701.38995	1519.78100	756.97920	1643.44840	Detected	Detected	Detected	Detected
6441	A_24_P320328	SUB1	Hs.229641	SUB1 homolog (S. cerevisiae)	Homo sapiens SUB1 homolog (S. cerevi	7648.75200	6879.35840	6261.95400	4610.79350	Detected	Detected	Detected	Detected
6442	A_32_P159192		Hs.652919		Ig kappa chain V-III region VH Precurso	63.74764	51.93294	34.09478	31.29619	Detected	Detected	Detected	Detected
6443	A_23_P318581	KIAA1430	Hs.535734	KIAA1430	Homo sapiens KIAA1430 (KIAA1430), m	12426.27800	9342.86700	8459.93400	5392.49800	Detected	Detected	Detected	Detected
6444	A_33_P3410892	LOC100289383	Hs.652458	hypothetical protein LOC100289383	PREDICTED: Homo sapiens hypothetica	319.39578	487.91006	153.86508	180.11993	Detected	Detected	Detected	Detected
6445	A_24_P130962	TOR3A	Hs.584957	torsin family 3, member A	Homo sapiens torsin family 3, member A	617.75305	748.84780	557.97430	608.06555	Detected	Detected	Detected	Detected
6446	A_23_P68577	HSPC072	Hs.584899	hypothetical LOC29075	Homo sapiens hypothetical LOC29075 (	18.36211	26.34152	21.97964	17.21508	Detected	Detected	Detected	Detected

6447	A_23_P381461	LRRC45	Hs.143774	leucine rich repeat containing 45	Homo sapiens leucine rich repeat conta	730.02190	662.58450	1044.35170	917.92694	Detected	Detected	Detected	Detected
6448	A_23_P143047	ATP6V1E2	Hs.659656	ATPase, H+ transporting, lysosomal 31k	Homo sapiens ATPase, H+ transporting,	328.10248	195.53981	249.58823	136.07869	Detected	Detected	Detected	Detected
6449	A_24_P170395					209.59521	209.97595	19.51209	18.19290	Detected	Detected	Detected	Detected
6450	A_33_P3261565	ZRANB1	Hs.595158	zinc finger, RAN-binding domain contair	Homo sapiens zinc finger, RAN-binding	307.94952	216.74413	264.10934	157.49564	Detected	Detected	Detected	Detected
6451	A_33_P3373298	STAG3L4	Hs.632013	stromal antigen 3-like 4	Homo sapiens stromal antigen 3-like 4 (	2127.71900	2297.51460	1447.43490	1349.92760	Detected	Detected	Detected	Detected
6452	A_32_P107746	ENSA	Hs.632456	endosulfine alpha	Homo sapiens endosulfine alpha (ENSA)	665.74774	539.82830	1100.42690	1165.46220	Detected	Detected	Detected	Detected
6453	A_33_P3421794	DHX34	Hs.151706	DEAH (Asp-Glu-Ala-His) box polypepti	Homo sapiens DEAH (Asp-Glu-Ala-His)	4.13520	5.81505	22.46474	18.13274	Compromised	Compromised	Detected	Detected
6454	A_23_P129133	OCA2	Hs.654411	oculocutaneous albinism II	Homo sapiens oculocutaneous albinism	136.87029	13.61720	102.98220	4.23454	Detected	Compromised	Detected	Compromised
6455	A_23_P151337	DLEU1	Hs.591229	deleted in lymphocytic leukemia 1 (non-	Homo sapiens deleted in lymphocytic le	777.18884	1197.90310	510.15506	710.02710	Detected	Detected	Detected	Detected
6456	A_24_P153763		Hs.617072		Putative uncharacterized protein Fragm	25.44260	18.47607	11.51968	8.61940	Detected	Detected	Compromised	Compromised
6457	A_23_P212830	FGFR3	Hs.1420	fibroblast growth factor receptor 3	Homo sapiens fibroblast growth factor r	14.93448	20.82092	18.11966	11.56241	Detected	Detected	Detected	Compromised
6458	A_33_P3343573	FAM108A1	Hs.653679	family with sequence similarity 108, mer	Homo sapiens mRNA for FLJ00358 prot	126.09513	77.48026	123.11147	58.69955	Detected	Detected	Detected	Detected
6459	A_23_P110276	LOC84740	Hs.663029	hypothetical LOC84740	Homo sapiens hypothetical LOC84740 (	2.92835	4.46657	3.12752	17.04352	Compromised	Compromised	Compromised	Not Detected
6460	A_33_P3233947	FAM36A	Hs.657313	family with sequence similarity 36, mem	Homo sapiens family with sequence sim	2443.26680	6594.82400	1404.97840	3326.71260	Detected	Detected	Detected	Detected
6461	A_23_P214144	COL10A1	Hs.520339	collagen, type X, alpha 1	Homo sapiens collagen, type X, alpha 1	850.93290	1260.63710	675.20746	820.24150	Detected	Detected	Detected	Detected
6462	A_33_P3338047	PNPLA7	Hs.294147	patatin-like phospholipase domain conta	Homo sapiens patatin-like phospholipas	13.32527	15.45485	6.46789	2.93813	Detected	Compromised	Compromised	Compromised
6463	A_33_P3229067	HIST1H2BN	Hs.673851	histone cluster 1, H2bn	Homo sapiens histone cluster 1, H2bn (	808.81934	1059.14970	307.33902	338.87372	Detected	Detected	Detected	Detected
6464	A_23_P121447	ARPM1	Hs.135411	actin related protein M1	Homo sapiens actin related protein M1	145.67104	191.46855	127.63533	152.08946	Detected	Detected	Detected	Detected
6465	A_23_P113005	EFNA1	Hs.516664	ephrin-A1	Homo sapiens ephrin-A1 (EFNA1), trans	124.18421	91.84532	124.05991	67.88595	Detected	Detected	Detected	Detected
6466	A_24_P291973	SEPT7	Hs.191346	septin 7	Homo sapiens septin 7 (SEPT7), transc	1569.73080	1431.69000	1285.82090	1090.61500	Detected	Detected	Detected	Detected
6467	A_23_P207089	SHBG	Hs.632235	sex hormone-binding globulin	Homo sapiens sex hormone-binding glob	43.24232	29.60692	50.52682	22.73223	Detected	Detected	Detected	Detected
6468	A_33_P3277097				lymphocyte antigen 6 complex, locus G	132.29988	472.43472	151.45505	463.66724	Detected	Detected	Detected	Detected
6469	A_33_P3417547					45.70442	35.88381	19.43917	4.57107	Detected	Detected	Detected	Compromised
6470	A_32_P73821	CSDE1	Hs.69855	cold shock domain containing E1, RNA-	Homo sapiens cold shock domain conta	6958.06350	10330.27400	3939.39670	4706.51400	Detected	Detected	Detected	Detected
6471	A_24_P58242					8565.44700	7201.95900	280.89355	146.08331	Detected	Detected	Detected	Detected
6472	A_23_P424900	C1orf88	Hs.172510	chromosome 1 open reading frame 88	Homo sapiens chromosome 1 open read	38.69124	48.52680	32.89849	17.57398	Detected	Detected	Detected	Detected
6473	A_33_P3265030	SEPT5	Hs.283743	septin 5	Homo sapiens septin 5 (SEPT5), mRNA	7704.13400	4785.02540	6248.41200	2627.45500	Detected	Detected	Detected	Detected
6474	A_23_P157736	PPAPDC3	Hs.134292	phosphatidic acid phosphatase type 2 d	Homo sapiens phosphatidic acid phosph	1563.39950	4056.99170	1377.32860	3110.83980	Detected	Detected	Detected	Detected
6475	A_33_P3235335	MTMR3	Hs.474536	myotubularin related protein 3	Homo sapiens myotubularin related prot	230.76305	263.04460	204.00592	193.57806	Detected	Detected	Detected	Detected
6476	A_23_P208516	MBOAT7	Hs.467279	membrane bound O-acyltransferase do	Homo sapiens membrane bound O-acyl	6030.00050	9333.98300	6212.46600	9239.27000	Detected	Detected	Detected	Detected
6477	A_23_P128543	MED4	Hs.181112	mediator complex subunit 4	Homo sapiens mediator complex subuni	20267.90800	15553.22200	19506.00600	12755.28300	Detected	Detected	Detected	Detected
6478	A_24_P667968	LOC26102	Hs.561893	hypothetical LOC26102	Homo sapiens hypothetical LOC26102 (	19.97752	21.44401	33.66542	23.42827	Detected	Detected	Detected	Detected
6479	A_33_P3254555		Hs.721800		DNA replication licensing factor MCM9	16.83345	51.05166	20.76773	32.66146	Detected	Detected	Detected	Detected
6480	A_33_P3261695	LOC647310	Hs.710431	similar to testis expressed gene 22	PREDICTED: Homo sapiens similar to t	13.55802	23.17667	18.95246	8.00012	Detected	Detected	Detected	Compromised
6481	A_32_P190151	FAM86D	Hs.591652	family with sequence similarity 86, mem	Homo sapiens family with sequence sim	63.71791	60.00253	29.30447	26.53322	Detected	Detected	Detected	Detected
6482	A_33_P3813684	FLJ31662	Hs.514123	hypothetical gene supported by AK0562	Homo sapiens cDNA FLJ31662 fis, clon	2522.07760	3383.21310	1235.01450	1094.49180	Detected	Detected	Detected	Detected
6483	A_33_P3397506	LOC653653	Hs.631741	adaptor-related protein complex 1, sigm	PREDICTED: Homo sapiens adaptor-rel	26.20936	31.66154	17.96899	27.80703	Detected	Detected	Detected	Detected
6484	A_24_P376120	MYPN	Hs.55205	myopalladin	Homo sapiens myopalladin (MYPN), mR	6.69271	29.09572	2.78283	15.59751	Compromised	Detected	Compromised	Detected
6485	A_23_P104116	RBM8A	Hs.719989	RNA binding motif protein 8A	Homo sapiens RNA binding motif protei	37569.32400	66899.81000	35255.63000	53977.33200	Detected	Detected	Detected	Detected
6486	A_33_P3367037					31.67678	29.59793	17.73357	22.87837	Detected	Detected	Detected	Detected
6487	A_23_P109636	LRIG1	Hs.518055	leucine-rich repeats and immunoglobuli	Homo sapiens leucine-rich repeats and	4087.11800	2158.40720	3662.97830	1685.18680	Detected	Detected	Detected	Detected
6488	A_33_P3250383	CNP	Hs.273621	2',3'-cyclic nucleotide 3' phosphodieste	Homo sapiens 2',3'-cyclic nucleotide 3'	950.65460	609.37854	714.49146	444.44415	Detected	Detected	Detected	Detected
6489	A_23_P132956	UCHL1	Hs.518731	ubiquitin carboxyl-terminal esterase L1	Homo sapiens ubiquitin carboxyl-termin	30940.37300	44227.83000	23813.13000	30990.46000	Detected	Detected	Detected	Detected
6490	A_23_P151746	ISCA2	Hs.291079	iron-sulfur cluster assembly 2 homolog	Homo sapiens iron-sulfur cluster assem	11907.14100	11627.44200	9394.45400	8160.72200	Detected	Detected	Detected	Detected
6491	A_33_P3212570					14.75470	30.90664	19.12933	34.52834	Detected	Detected	Detected	Detected
6492	A_33_P3337009				Phosphopantothenate--cysteine ligase	116.38690	102.75790	122.38510	92.93501	Detected	Detected	Detected	Detected
6493	A_23_P130158	WNT3	Hs.445884	wingless-type MMTV integration site fa	Homo sapiens wingless-type MMTV inte	719.23870	220.16235	544.41500	130.95697	Detected	Detected	Detected	Detected
6494	A_32_P138628				Novel protein [Source:UniProtKB/TrEM	25.30155	34.00605	15.82622	15.89120	Detected	Detected	Detected	Detected
6495	A_32_P34552	POLB	Hs.654484	polymerase (DNA directed), beta	Homo sapiens polymerase (DNA directe	290.09350	142.25322	186.35555	67.33865	Detected	Detected	Detected	Detected
6496	A_33_P3250268	LOC442132	Hs.589351	similar to hypothetical protein FLJ3614	PREDICTED: Homo sapiens similar to h	104.29919	126.32230	118.54581	130.06306	Detected	Detected	Detected	Detected
6497	A_33_P3229467	STK17A	Hs.709489	serine/threonine kinase 17a	Homo sapiens serine/threonine kinase	101.97670	103.96190	63.92939	52.45011	Detected	Detected	Detected	Detected
6498	A_23_P360245	NUDCD2	Hs.140443	NudC domain containing 2	Homo sapiens NudC domain containing	12503.50900	11633.03900	9028.49200	6667.60250	Detected	Detected	Detected	Detected
6499	A_23_P165624	TNFAIP6	Hs.437322	tumor necrosis factor, alpha-induced pr	Homo sapiens tumor necrosis factor, al	3267.00240	450.51390	3085.84670	365.31656	Detected	Detected	Detected	Detected
6500	A_23_P58729	SLC34A1	Hs.936	solute carrier family 34 (sodium phosph	Homo sapiens solute carrier family 34 (	20.13468	24.34481	18.27083	18.21198	Detected	Detected	Detected	Detected
6501	A_23_P256148	AKIRIN1	Hs.293563	akirin 1	Homo sapiens akirin 1 (AKIRIN1), transc	1148.16520	962.82385	831.95700	650.42535	Detected	Detected	Detected	Detected
6502	A_24_P87114	USP30	Hs.486434	ubiquitin specific peptidase 30	Homo sapiens ubiquitin specific peptida	59.71055	75.14018	57.91970	68.38856	Detected	Detected	Detected	Detected
6503	A_33_P3503279	BOD1P	Hs.127882	biorientation of chromosomes in cell div	AGENCOURT_6565391 NIH_MGC_119 H	32.87357	42.56589	19.97179	15.91796	Detected	Detected	Detected	Compromised
6504	A_33_P3387861	CENPN	Hs.55028	centromere protein N	Homo sapiens centromere protein N (C	319.12270	3035.08890	254.22676	2292.62040	Detected	Detected	Detected	Detected
6505	A_33_P3343428	TRAF3IP2	Hs.561514	TRAF3 interacting protein 2	Homo sapiens TRAF3 interacting protei	3719.15400	4912.77830	3477.52340	4009.06600	Detected	Detected	Detected	Detected
6506	A_24_P41850	MASP1	Hs.89983	mannan-binding lectin serine peptidase	Homo sapiens mannan-binding lectin se	49.45198	354.75385	39.38162	281.03452	Detected	Detected	Detected	Detected
6507	A_23_P37870	STUB1	Hs.592081	STIP1 homology and U-box containing	Homo sapiens STIP1 homology and U-b	26819.39500	27281.95900	24845.57600	23532.71000	Detected	Detected	Detected	Detected
6508	A_33_P3388312	SKAP2	Hs.200770	src kinase associated phosphoprotein 2	Homo sapiens src kinase associated ph	1254.76500	4270.05660	924.84290	2581.05130	Detected	Detected	Detected	Detected
6509	A_23_P106761	CORO1A	Hs.415067	coronin, actin binding protein, 1A	Homo sapiens coronin, actin binding pro	55.34289	176.16493	66.37285	164.54393	Detected	Detected	Detected	Detected
6510	A_23_P135730	ZNF627	Hs.526665	zinc finger protein 627	Homo sapiens zinc finger protein 627 (Z	721.13873	629.96110	636.11540	468.58520	Detected	Detected	Detected	Detected
6511	A_23_P16992	PKP4	Hs.407580	plakophilin 4	Homo sapiens plakophilin 4 (PKP4), tran	314.79218	318.05643	269.54990	234.94212	Detected	Detected	Detected	Detected
6512	A_33_P3325634	C19orf69	Hs.649452	chromosome 19 open reading frame 69	Homo sapiens chromosome 19 open rea	1125.82690	1334.43410	863.51874	736.81510	Detected	Detected	Detected	Detected
6513	A_33_P3224680		Hs.440371		tRNA guanosine-2'-O-methyltransferas	121.83609	146.71481	107.68072	116.49870	Detected	Detected	Detected	Detected
6514	A_33_P3369079	LOC100144603		hypothetical transcript	Homo sapiens hypothetical transcript (L	110.42521	167.81668	91.98720	116.40137	Detected	Detected	Detected	Detected
6515	A_33_P3263423	TLR5	Hs.604542	toll-like receptor 5	Homo sapiens toll-like receptor 5 (TLR	250.20778	290.88540	136.57831	138.97954	Detected	Detected	Detected	Detected
6516	A_23_P38457	TAOK1	Hs.631758	TAO kinase 1	Homo sapiens TAO kinase 1 (TAOK1), r	1424.14720	1328.93050	649.64970	535.32560	Detected	Detected	Detected	Detected
6517	A_24_P400457	CYB5RL	Hs.591426	cytochrome b5 reductase-like	Homo sapiens cytochrome b5 reductase	76.25545	19.40226	35.40360	6.28445	Detected	Detected	Detected	Compromised
6518	A_23_P61633	TNK2	Hs.518513	tyrosine kinase, non-receptor, 2	Homo sapiens tyrosine kinase, non-rece	7032.19040	4152.13600	7456.59230	4226.85840	Detected	Detected	Detected	Detected
6519	A_33_P3319150					23.30779	20.32439	9.22504	6.49276	Detected	Detected	Compromised	Compromised
6520	A_33_P3234697	LXN	Hs.478067	latexin	Homo sapiens latexin (LXN), mRNA [NM	204.84123	940.08720	173.78930	716.05554	Detected	Detected	Detected	Detected
6521	A_24_P297537	MAMSTR	Hs.191815	MEF2 activating motif and SAP domain	Homo sapiens MEF2 activating motif an	312.28497	441.18536	140.74226	121.06049	Detected	Detected	Detected	Detected

6522	A_23_P321501	DHRS2	Hs.272499	dehydrogenase/reductase (SDR family)	Homo sapiens dehydrogenase/reductas	65.39507	616.19836	33.44551	527.96630	Detected	Detected	Detected	Detected
6523	A_23_P73780	IRAK1	Hs.522819	interleukin-1 receptor-associated kinas	Homo sapiens interleukin-1 receptor-as	14526.07000	10735.07900	15599.88600	11837.35600	Detected	Detected	Detected	Detected
6524	A_23_P110005	NICN1	Hs.191460	nicolin 1	Homo sapiens nicolin 1 (NICN1), mRNA	574.93210	608.23480	663.46545	605.80760	Detected	Detected	Detected	Detected
6525	A_23_P2431	C3AR1	Hs.591148	complement component 3a receptor 1	Homo sapiens complement component	2079.10450	2232.45800	432.45260	391.52997	Detected	Detected	Detected	Detected
6526	A_24_P557479	XAF1	Hs.441975	XIAP associated factor 1	Homo sapiens XIAP associated factor 1	151.64720	152.21579	97.60082	102.60622	Detected	Detected	Detected	Detected
6527	A_23_P116037	TM7SF2	Hs.31130	transmembrane 7 superfamily member 2	Homo sapiens transmembrane 7 superfa	56.69408	26.48223	41.57246	18.79347	Detected	Detected	Detected	Detected
6528	A_23_P135616	STX18	Hs.584913	syntaxin 18	Homo sapiens syntaxin 18 (STX18), mR	2726.83100	3530.72440	2676.05640	2935.26250	Detected	Detected	Detected	Detected
6529	A_23_P382199	VAPA	Hs.699980	VAMP (vesicle-associated membrane p	Homo sapiens VAMP (vesicle-associate	4959.73970	5758.76100	4451.37160	4090.29370	Detected	Detected	Detected	Detected
6530	A_23_P82296	GNB2	Hs.185172	guanine nucleotide binding protein (G pr	Homo sapiens guanine nucleotide bindin	7534.34200	9487.45400	7254.71500	9090.79900	Detected	Detected	Detected	Detected
6531	A_23_P106002	NFKBIA	Hs.81328	nuclear factor of kappa light polypeptide	Homo sapiens nuclear factor of kappa li	33393.74600	17720.60400	25451.31000	11735.43200	Detected	Detected	Detected	Detected
6532	A_23_P408955	E2F2	Hs.194333	E2F transcription factor 2	Homo sapiens E2F transcription factor	36.62947	516.58480	39.07835	438.20502	Detected	Detected	Detected	Detected
6533	A_33_P3316505	SNORA73A		small nucleolar RNA, H/ACA box 73A	Homo sapiens small nucleolar RNA, H/A	71.74272	64.78045	121.28834	59.37486	Detected	Detected	Detected	Detected
6534	A_23_P215024	LRRC1	Hs.657695	leucine rich repeat containing 1	Homo sapiens leucine rich repeat conta	85.10286	81.80512	74.31735	65.06821	Detected	Detected	Detected	Detected
6535	A_23_P255968	TAAR5	Hs.248198	trace amine associated receptor 5	Homo sapiens trace amine associated r	1819.87680	3161.86100	220.82707	223.19063	Detected	Detected	Detected	Detected
6536	A_33_P3369834	REEP6	Hs.76277	receptor accessory protein 6	Homo sapiens receptor accessory prote	324.65936	283.27810	211.49254	153.14320	Detected	Detected	Detected	Detected
6537	A_33_P3211929	RCOR2	Hs.98788	REST corepressor 2	Homo sapiens REST corepressor 2 (RC	2262.04470	760.26434	1265.19950	385.63880	Detected	Detected	Detected	Detected
6538	A_23_P66719	DHRS13	Hs.631760	dehydrogenase/reductase (SDR family)	Homo sapiens dehydrogenase/reductas	1320.20620	296.66312	1130.45040	256.21637	Detected	Detected	Detected	Detected
6539	A_33_P3474328	PPP1R11	Hs.82887	protein phosphatase 1, regulatory (inhib	Homo sapiens protein phosphatase 1, re	501.28232	489.51016	392.35828	360.54553	Detected	Detected	Detected	Detected
6540	A_33_P3231858	LOC100133991	Hs.656430	similar to hCG1995169	Homo sapiens similar to hCG1995169 (L	34.61082	28.79249	37.25903	23.76066	Detected	Detected	Detected	Detected
6541	A_23_P111402	RSPO3	Hs.135254	R-spondin 3 homolog (Xenopus laevis)	Homo sapiens R-spondin 3 homolog (Xe	69.72252	36.68558	37.23166	16.31046	Detected	Detected	Detected	Detected
6542	A_33_P3351259	LOC100130560	Hs.711988	hypothetical protein LOC100130560	Homo sapiens cDNA FLJ45554 fis, clon	538.23773	640.59717	189.52522	221.10196	Detected	Detected	Detected	Detected
6543	A_33_P3308115	LOC728416	Hs.544999	hypothetical LOC728416	PREDICTED: Homo sapiens hypothetica	17673.06200	19177.13300	13832.03500	12276.71800	Detected	Detected	Detected	Detected
6544	A_23_P98763	LRTOMT	Hs.317243	leucine rich transmembrane and 0-meth	Homo sapiens leucine rich transmembra	40.74731	71.12833	51.26942	69.04110	Detected	Detected	Detected	Detected
6545	A_24_P192988	CCDC89	Hs.376241	coiled-coil domain containing 89	Homo sapiens coiled-coil domain conta	44.48689	147.48987	38.00570	137.13028	Detected	Detected	Detected	Detected
6546	A_24_P143076	FAM109A	Hs.173088	family with sequence similarity 109, mer	Homo sapiens family with sequence sim	777.23505	971.20480	661.91907	799.16003	Detected	Detected	Detected	Detected
6547	A_24_P109432	NBEAL1	Hs.648846	neurobeachin-like 1	Homo sapiens neurobeachin-like 1 (NBE	502.23790	445.42328	461.38547	358.25836	Detected	Detected	Detected	Detected
6548	A_23_P141656	DRG2	Hs.78582	developmentally regulated GTP binding	Homo sapiens developmentally regulate	1570.86440	1787.10020	1831.71670	1977.99550	Detected	Detected	Detected	Detected
6549	A_23_P333498	EEPD1	Hs.487994	endonuclease/exonuclease/phosphatas	Homo sapiens endonuclease/exonuclea	376.90717	174.96413	331.80774	133.61930	Detected	Detected	Detected	Detected
6550	A_33_P3311076	CYB5A	Hs.465413	cytochrome b5 type A (microsomal)	Homo sapiens cytochrome b5 type A (m	4640.51400	3038.76050	5685.11130	3363.89430	Detected	Detected	Detected	Detected
6551	A_23_P346048	HERC2P2	Hs.531509	hect domain and RLD 2 pseudogene 2	Homo sapiens hect domain and RLD 2 p	1363.26680	1132.49010	979.12130	676.43830	Detected	Detected	Detected	Detected
6552	A_23_P163161	SDR39U1	Hs.643552	short chain dehydrogenase/reductase f	Homo sapiens short chain dehydrogena	2174.32420	1081.63180	2497.01170	1165.71640	Detected	Detected	Detected	Detected
6553	A_23_P72117	SMPDL3A	Hs.486357	sphingomyelin phosphodiesterase, acid-	Homo sapiens sphingomyelin phosphodi	1580.62340	2144.09280	1126.19840	1203.56400	Detected	Detected	Detected	Detected
6554	A_33_P3423585	UNC13C	Hs.657273	unc-13 homolog C (C. elegans)	Homo sapiens unc-13 homolog C (C. el	2.73679	94.13646	3.03744	51.94047	Compromised	Detected	Compromised	Detected
6555	A_33_P3317198	GTF2F2	Hs.654582	general transcription factor IIF, polypep	Homo sapiens general transcription fact	6025.90430	6033.00240	5218.97300	4319.23500	Detected	Detected	Detected	Detected
6556	A_33_P3484806	FLJ21408	Hs.434412	hypothetical gene supported by AK0250	Homo sapiens cDNA: FLJ21408 fis, clor	30.50491	23.74432	3.76673	4.26996	Detected	Detected	Compromised	Compromised
6557	A_33_P3306973					583.08020	775.24160	516.45294	557.71730	Detected	Detected	Detected	Detected
6558	A_33_P3257513	FAT3	Hs.98523	FAT tumor suppressor homolog 3 (Dros	Homo sapiens FAT tumor suppressor ho	1108.94870	2121.84380	956.10850	1515.80900	Detected	Detected	Detected	Detected
6559	A_33_P3271316	RPP25	Hs.8562	ribonuclease P/MRP 25kDa subunit	Homo sapiens ribonuclease P/MRP 25k	239.43782	200.38977	199.89325	131.86581	Detected	Detected	Detected	Detected
6560	A_23_P67896	SCN3A	Hs.435274	sodium channel, voltage-gated, type III,	Homo sapiens sodium channel, voltage-	17.74976	22.81960	19.06968	14.68090	Detected	Detected	Detected	Compromised
6561	A_24_P402836	ZNF141	Hs.654355	zinc finger protein 141	Homo sapiens zinc finger protein 141 (Z	271.41315	257.97482	88.91840	61.47218	Detected	Detected	Detected	Detected
6562	A_33_P3259620	TTC37	Hs.482868	tetratricopeptide repeat domain 37	Homo sapiens tetratricopeptide repeat	3154.09770	2436.72970	2177.07570	1285.24480	Detected	Detected	Detected	Detected
6563	A_33_P3310780	CTTN	Hs.596164	cortactin	Homo sapiens cortactin (CTTN), transc	75103.98400	62880.99000	52012.49600	38050.77300	Detected	Detected	Detected	Detected
6564	A_23_P409541	POLR1D	Hs.711105	polymerase (RNA) I polypeptide D, 16kD	Homo sapiens polymerase (RNA) I polyp	357.51175	299.90090	304.46460	200.47060	Detected	Detected	Detected	Detected
6565	A_33_P3226565					608.57350	1067.92920	50.83133	54.82324	Detected	Detected	Detected	Detected
6566	A_33_P3229918	PTCRA	Hs.169002	pre T-cell antigen receptor alpha	Homo sapiens pre T-cell antigen recept	6.28337	24.45166	9.99613	23.39850	Compromised	Detected	Compromised	Detected
6567	A_24_P1054	NFKBIL2	Hs.675285	nuclear factor of kappa light polypeptide	Homo sapiens nuclear factor of kappa li	264.16330	308.65692	158.56204	156.39500	Detected	Detected	Detected	Detected
6568	A_24_P921321	PTPRJ	Hs.318547	protein tyrosine phosphatase, receptor	Homo sapiens protein tyrosine phospha	331.86783	1477.81910	230.61170	844.48310	Detected	Detected	Detected	Detected
6569	A_23_P165061	AES	Hs.515053	amino-terminal enhancer of split	Homo sapiens amino-terminal enhancer	26070.26000	19674.60000	22289.20300	13870.44500	Detected	Detected	Detected	Detected
6570	A_33_P3254670		Hs.585418		UI-E-EJ1-ajb-m-22-0-UI.r1 UI-E-EJ1	21.23292	32.88799	19.55916	17.01909	Detected	Detected	Detected	Detected
6571	A_23_P15146	IL32	Hs.943	interleukin 32	Homo sapiens interleukin 32 (IL32), tran	75.85977	579.07544	91.34475	600.76404	Detected	Detected	Detected	Detected
6572	A_23_P140760	GPR97	Hs.383403	G protein-coupled receptor 97	Homo sapiens G protein-coupled recep	49.36665	64.93378	24.69367	24.66895	Detected	Detected	Detected	Detected
6573	A_24_P3005	SCN9A	Hs.439145	sodium channel, voltage-gated, type IX,	Homo sapiens sodium channel, voltage-	17.04645	94.26747	8.24805	62.02753	Detected	Detected	Compromised	Detected
6574	A_33_P3233700	LOC100128675	Hs.633989	hypothetical LOC100128675	Homo sapiens hypothetical LOC100128	55.48637	78.10996	40.78633	35.27318	Detected	Detected	Detected	Detected
6575	A_24_P933908		Hs.190495		Transmembrane glycoprotein NMB Prec	149.47705	112.74397	72.38379	45.26667	Detected	Detected	Detected	Detected
6576	A_33_P3389827	PROM2	Hs.469313	prominin 2	Homo sapiens prominin 2 (PROM2), tra	68.96548	41.59382	13.52840	9.75264	Detected	Detected	Compromised	Compromised
6577	A_23_P38041	NAGPA	Hs.21334	N-acetylglucosamine-1-phosphodiester	Homo sapiens N-acetylglucosamine-1-	3300.93000	4056.07980	3814.63770	4227.92330	Detected	Detected	Detected	Detected
6578	A_23_P87742	IFFO1	Hs.15243	intermediate filament family orphan 1	Homo sapiens intermediate filament fan	8097.47460	11658.26700	7706.05370	9742.44800	Detected	Detected	Detected	Detected
6579	A_33_P3421325	ZNF782	Hs.592420	zinc finger protein 782	Homo sapiens zinc finger protein 782 (Z	137.54971	120.14309	71.58379	43.72523	Detected	Detected	Detected	Detected
6580	A_33_P3243907	CTSD	Hs.654447	cathepsin D	Homo sapiens cathepsin D (CTSD), mR	5605.72900	3074.80030	5381.04640	2967.97460	Detected	Detected	Detected	Detected
6581	A_23_P69908	GLRX	Hs.28988	glutaredoxin (thioltransferase)	Homo sapiens glutaredoxin (thioltransfe	27475.53700	10545.77100	32001.03100	9363.66000	Detected	Detected	Detected	Detected
6582	A_33_P3365582	RASGRP3	Hs.143674	RAS guanyl releasing protein 3 (calcium	Homo sapiens RAS guanyl releasing pro	7.75243	6.77454	10.92380	14.27821	Compromised	Compromised	Compromised	Detected
6583	A_33_P3312549	LOC100131154	Hs.546565	similar to hCG1818167	PREDICTED: Homo sapiens similar to c	94.68296	103.95892	38.45997	44.32685	Detected	Detected	Detected	Detected
6584	A_33_P3284394		Hs.88778		Carbonyl reductase [NADPH] 1 (EC 1.1	13.52372	17.84357	10.93210	9.49761	Detected	Detected	Compromised	Compromised
6585	A_33_P3301782	LOC220930	Hs.372654	hypothetical LOC220930	Homo sapiens hypothetical LOC220930	168.16254	61.78086	105.26550	32.35434	Detected	Detected	Detected	Detected
6586	A_23_P43226	KCTD9	Hs.72071	potassium channel tetramerisation dom	Homo sapiens potassium channel tetrar	5839.02150	11108.34200	4955.06350	8348.41500	Detected	Detected	Detected	Detected
6587	A_33_P3212092	PDCD4	Hs.711490	programmed cell death 4 (neoplastic tra	Homo sapiens programmed cell death 4	1272.39900	684.91210	1191.02820	637.71150	Detected	Detected	Detected	Detected
6588	A_33_P3406806				Putative keratin-associated protein 9-2	35.50654	39.39400	20.50166	20.40923	Detected	Detected	Detected	Detected
6589	A_23_P83931	NET1	Hs.25155	neuroepithelial cell transforming 1	Homo sapiens neuroepithelial cell transf	2421.09130	7461.27150	1569.30760	4392.47600	Detected	Detected	Detected	Detected
6590	A_23_P108673	FAM176A	Hs.302346	family with sequence similarity 176, mer	Homo sapiens family with sequence sim	1181.12110	3040.98500	926.00890	1898.60890	Detected	Detected	Detected	Detected
6591	A_24_P334529	PCDHGA3	Hs.368160	protocadherin gamma subfamily A, 3	Homo sapiens protocadherin gamma sul	33.13506	42.01219	35.88997	39.67756	Detected	Detected	Detected	Detected
6592	A_23_P301360	ZNF572	Hs.175350	zinc finger protein 572	Homo sapiens zinc finger protein 572 (Z	168.22330	103.98273	148.82903	65.81583	Detected	Detected	Detected	Detected
6593	A_33_P3256510	KCNK12	Hs.591586	potassium channel, subfamily K, membe	Homo sapiens potassium channel, subfa	195.18300	119.07240	167.58870	83.80016	Detected	Detected	Detected	Detected
6594	A_23_P126605	TMOD4	Hs.709681	tropomodulin 4 (muscle)	Homo sapiens tropomodulin 4 (muscle)	16.43727	14.02111	10.66863	9.28506	Detected	Compromised	Compromised	Compromised
6595	A_33_P3379199	UCRC	Hs.284292	ubiquinol-cytochrome c reductase com	Homo sapiens ubiquinol-cytochrome c	1660.53520	2723.89650	2352.54320	3138.47400	Detected	Detected	Detected	Detected
6596	A_23_P208636	SHANK1	Hs.274255	SH3 and multiple ankyrin repeat domain	Homo sapiens SH3 and multiple ankyrin	20.94702	20.47164	15.52573	7.03252	Detected	Detected	Detected	Compromised

6597	A_24_P375761		Hs.517850		STGC3 [Source:UniProtKB/TrEMBL;A	117.88248	93.63335	107.67897	97.94447	Detected	Detected	Detected	Detected
6598	A_33_P3238375	LOC730081	Hs.534953	hypothetical protein LOC730081	PREDICTED: Homo sapiens hypothetica	11.35201	13.38420	15.07361	12.08532	Compromised	Compromised	Detected	Compromised
6599	A_33_P3404126				Polycomb group RING finger protein 3 (	2306.61450	1619.72630	1665.65820	949.46826	Detected	Detected	Detected	Detected
6600	A_33_P3234859	UTRN	Hs.133135	utrophin	Homo sapiens utrophin (UTRN), mRNA	32.83000	56.02164	24.45905	37.72967	Detected	Detected	Detected	Detected
6601	A_24_P218970	EIF4B	Hs.648394	eukaryotic translation initiation factor 4	Homo sapiens eukaryotic translation ini	3691.98900	2746.94920	2353.57450	1504.47180	Detected	Detected	Detected	Detected
6602	A_23_P12849	FBXO18	Hs.498543	F-box protein, helicase, 18	Homo sapiens F-box protein, helicase,	1635.16500	1229.45850	1278.14900	901.33100	Detected	Detected	Detected	Detected
6603	A_33_P3383606	KCP	Hs.371746	kielin/chordin-like protein	Homo sapiens kielin/chordin-like protei	8868.67500	12198.82300	4594.81640	3972.42920	Detected	Detected	Detected	Detected
6604	A_33_P3331831	DKFZp451A211	Hs.646609	DKFZp451A211 protein	PREDICTED: Homo sapiens DKFZp451A	2893.88840	5461.77250	2180.19630	3594.30180	Detected	Detected	Detected	Detected
6605	A_33_P3339531	CHADL	Hs.344488	chondroadherin-like	Homo sapiens chondroadherin-like (CHA	77.06607	232.80196	68.10331	206.32674	Detected	Detected	Detected	Detected
6606	A_23_P133338	PCDH24	Hs.4205	protocadherin 24	Homo sapiens protocadherin 24 (PCDH	202.05286	390.53894	50.15382	72.07687	Detected	Detected	Detected	Detected
6607	A_24_P383850	EIF4G3	Hs.467084	eukaryotic translation initiation factor 4	Homo sapiens eukaryotic translation ini	104.79061	175.37120	70.13005	118.36000	Detected	Detected	Detected	Detected
6608	A_23_P48198	GLT8D2	Hs.631650	glycosyltransferase 8 domain containing	Homo sapiens glycosyltransferase 8 do	1705.91380	2042.76480	1343.10510	1343.89990	Detected	Detected	Detected	Detected
6609	A_33_P3310548		Hs.689628		AHPA9419 [Source:UniProtKB/TrEMB	39.34523	46.27998	25.56127	26.99758	Detected	Detected	Detected	Detected
6610	A_23_P44932	EIF2A	Hs.655782	eukaryotic translation initiation factor 2	Homo sapiens eukaryotic translation ini	1683.71810	1880.14090	1287.44370	1262.07030	Detected	Detected	Detected	Detected
6611	A_23_P68628	NECAB3	Hs.516986	N-terminal EF-hand calcium binding pro	Homo sapiens N-terminal EF-hand calc	1617.55740	867.97800	1523.95000	761.64610	Detected	Detected	Detected	Detected
6612	A_24_P200000	STEAP3	Hs.647822	STEAP family member 3	Homo sapiens STEAP family member 3	400.57280	359.79794	501.87018	464.70038	Detected	Detected	Detected	Detected
6613	A_23_P405942	LARP4B	Hs.631814	La ribonucleoprotein domain family, mer	Homo sapiens La ribonucleoprotein dom	4168.91000	4352.70360	5080.44300	4890.28860	Detected	Detected	Detected	Detected
6614	A_23_P48029	CLEC4A	Hs.504657	C-type lectin domain family 4, member	Homo sapiens C-type lectin domain fan	261.87177	77.51788	182.94298	42.44064	Detected	Detected	Detected	Detected
6615	A_33_P3362891	HNRNPA2B1	Hs.487774	heterogeneous nuclear ribonucleoprote	Homo sapiens heterogeneous nuclear ri	1017.28290	2419.08840	1197.04100	2707.92360	Detected	Detected	Detected	Detected
6616	A_33_P3227934				Putative uncharacterized protein ENSP	112.77117	170.55458	53.14667	56.43244	Detected	Detected	Detected	Detected
6617	A_23_P111273	TBC1D7	Hs.484678	TBC1 domain family, member 7	Homo sapiens TBC1 domain family, mer	2135.51250	2953.47220	1706.99790	2030.69070	Detected	Detected	Detected	Detected
6618	A_32_P43812	DCUN1D4	Hs.605388	DCN1, defective in cullin neddylation 1,	Homo sapiens DCN1, defective in cullin	1983.56290	1211.40700	1133.76120	604.90125	Detected	Detected	Detected	Detected
6619	A_23_P50674	C19orf36	Hs.424049	chromosome 19 open reading frame 36	Homo sapiens chromosome 19 open rea	67.30064	43.12028	101.04199	67.42194	Detected	Detected	Detected	Detected
6620	A_33_P3403117	NR2F1	Hs.519445	nuclear receptor subfamily 2, group F, m	Homo sapiens nuclear receptor subfami	2577.96340	1472.55140	1576.52250	750.07196	Detected	Detected	Detected	Detected
6621	A_23_P424878	KIAA1688	Hs.148988	KIAA1688 protein	Homo sapiens KIAA1688 protein (KIAA1	169.93938	146.32808	138.49388	89.68646	Detected	Detected	Detected	Detected
6622	A_23_P46539	PSRC1	Hs.405925	proline/serine-rich coiled-coil 1	Homo sapiens proline/serine-rich coilec	656.59110	2672.18530	577.50890	2059.57100	Detected	Detected	Detected	Detected
6623	A_24_P252130	PPARD	Hs.696032	peroxisome proliferator-activated recep	Homo sapiens peroxisome proliferator-a	5541.29150	5043.60160	4728.47400	3969.72530	Detected	Detected	Detected	Detected
6624	A_23_P11295	MTCP1NB	Hs.6917	mature T-cell proliferation 1 neighbor	Homo sapiens mature T-cell proliferatio	3961.08840	2397.64230	3216.33540	1668.04700	Detected	Detected	Detected	Detected
6625	A_24_P390495	CX3CL1	Hs.531668	chemokine (C-X3-C motif) ligand 1	Homo sapiens chemokine (C-X3-C mot	11.09669	12.82816	15.47971	10.34474	Compromised	Compromised	Detected	Compromised
6626	A_33_P3404588	FGD4	Hs.117835	FYVE, RhoGEF and PH domain containi	Homo sapiens FYVE, RhoGEF and PH d	118.37510	161.24721	112.71491	138.32420	Detected	Detected	Detected	Detected
6627	A_33_P3399768	LOC100129269	Hs.689728	hypothetical protein LOC100129269	full-length cDNA clone CS0DI076YF22	203.35172	250.64075	107.85119	123.99661	Detected	Detected	Detected	Detected
6628	A_24_P633902	RNF115	Hs.523550	ring finger protein 115	Homo sapiens ring finger protein 115 (R	1403.37160	2251.93500	1232.41870	1724.45130	Detected	Detected	Detected	Detected
6629	A_23_P49849	SEZ6	Hs.21837	seizure related 6 homolog (mouse)	Homo sapiens seizure related 6 homolo	9.76059	5.94369	14.03453	10.39091	Compromised	Compromised	Detected	Compromised
6630	A_33_P3383189	SP9	Hs.516603	Sp9 transcription factor homolog (mous	Homo sapiens Sp9 transcription factor	180.59290	161.69160	320.35260	297.88187	Detected	Detected	Detected	Detected
6631	A_23_P126031	OR10J5	Hs.553587	olfactory receptor, family 10, subfamily	Homo sapiens olfactory receptor, family	30.28610	39.40596	44.35135	37.46638	Detected	Detected	Detected	Detected
6632	A_23_P12746	MRC1L1	Hs.461247	mannose receptor, C type 1-like 1	Homo sapiens mannose receptor, C typ	199.85626	344.04935	11.58383	15.75160	Detected	Detected	Compromised	Detected
6633	A_33_P3382303	FMNL1	Hs.720131	formin-like 1	Homo sapiens formin-like 1 (FMNL1), m	127.98245	39.09370	129.19661	30.33828	Detected	Detected	Detected	Detected
6634	A_33_P3284838		Hs.132442		Putative uncharacterized protein C10or	30.72678	3.40712	32.55511	9.96175	Detected	Compromised	Detected	Compromised
6635	A_23_P43504	ABCA2	Hs.421202	ATP-binding cassette, sub-family A (AE	Homo sapiens ATP-binding cassette, su	226.15760	119.89203	252.81828	128.91930	Detected	Detected	Detected	Detected
6636	A_33_P3289391	LOC100132168	Hs.629558	hypothetical LOC100132168	PREDICTED: Homo sapiens hypothetica	1314.38850	1373.52720	592.20400	408.09412	Detected	Detected	Detected	Detected
6637	A_32_P88310	LOC730183	Hs.620916	hypothetical protein LOC730183	PREDICTED: Homo sapiens hypothetica	653.70250	296.91150	602.06885	258.31354	Detected	Detected	Detected	Detected
6638	A_23_P56938	REL	Hs.631886	v-rel reticuloendotheliosis viral oncoger	Homo sapiens v-rel reticuloendotheliosi	155.10855	85.92960	104.58041	48.77384	Detected	Detected	Detected	Detected
6639	A_33_P3399363	GPR155	Hs.516604	G protein-coupled receptor 155	Homo sapiens G protein-coupled recept	159.63731	48.76824	123.96223	29.96816	Detected	Detected	Detected	Detected
6640	A_33_P3888568	SNORA70B	Hs.675825	small nucleolar RNA, H/ACA box 70B (r	UI-H-DT0-aub-m-04-0-UI.s1 NCI_CGA	35.99945	24.82054	23.59237	17.79532	Detected	Detected	Detected	Detected
6641	A_33_P3360863	LOC644794	Hs.552124	hypothetical LOC644794	PREDICTED: Homo sapiens hypothetica	27.22332	17.03954	25.74767	20.84037	Detected	Compromised	Detected	Detected
6642	A_33_P3257222	COMTD1	Hs.355333	catechol-O-methyltransferase domain	Homo sapiens catechol-O-methyltransf	1169.52390	1266.84390	1272.79690	1252.71700	Detected	Detected	Detected	Detected
6643	A_23_P84995	MTMR8	Hs.442892	myotubularin related protein 8	Homo sapiens myotubularin related prot	15.14692	24.12569	7.05277	4.71819	Detected	Detected	Compromised	Compromised
6644	A_23_P256172	NDUFAF3	Hs.31387	NADH dehydrogenase (ubiquinone) 1 alp	Homo sapiens NADH dehydrogenase (ul	14982.36100	27660.59800	17418.40000	28586.85500	Detected	Detected	Detected	Detected
6645	A_33_P3255766	NIF3L1	Hs.145284	NIF3 NGG1 interacting factor 3-like 1 (	Homo sapiens NIF3 NGG1 interacting fa	1766.82970	1334.09720	1726.76340	1202.08830	Detected	Detected	Detected	Detected
6646	A_33_P3273272	ZNF701	Hs.710468	zinc finger protein 701	Homo sapiens zinc finger protein 701 (Z	47.06821	72.51446	31.71472	38.18873	Detected	Detected	Detected	Detected
6647	A_24_P95822	NPTN	Hs.187866	neuroplastin	Homo sapiens neuroplastin (NPTN), trar	434.33542	991.63390	572.98460	1172.15540	Detected	Detected	Detected	Detected
6648	A_33_P3271455	PXDN	Hs.332197	peroxidasin homolog (Drosophila)	Homo sapiens peroxidasin homolog (Drc	16410.87100	61953.02700	9350.12000	29403.44500	Detected	Detected	Detected	Detected
6649	A_24_P255303					12058.96500	16435.86100	9288.94000	10863.38800	Detected	Detected	Detected	Detected
6650	A_33_P3278931		Hs.631578		Putative uncharacterized protein ENSP	7.47072	5.31832	15.31747	13.03836	Compromised	Compromised	Detected	Compromised
6651	A_33_P3251538	MAPKAP1	Hs.495138	mitogen-activated protein kinase assoc	Homo sapiens mitogen-activated protei	795.83887	1328.71260	704.33330	975.63950	Detected	Detected	Detected	Detected
6652	A_23_P147665	OLFML1	Hs.503500	olfactomedin-like 1	Homo sapiens olfactomedin-like 1 (OLF	52.02829	76.18189	44.76571	41.37922	Detected	Detected	Detected	Detected
6653	A_33_P3375566		Hs.126160		BX093647 Soares_testis_NHT Homo sap	109.08609	3.70836	92.31986	5.59381	Detected	Compromised	Detected	Compromised
6654	A_24_P6030		Hs.656195		Protein FAM165B [Source:UniProtKB/	2048.03400	460.08408	1352.40710	227.97784	Detected	Detected	Detected	Detected
6655	A_24_P53353	RAB12	Hs.270074	RAB12, member RAS oncogene family	Homo sapiens RAB12, member RAS onc	95.92825	108.97388	94.90438	92.00389	Detected	Detected	Detected	Detected
6656	A_32_P86245	EFHC1	Hs.403171	EF-hand domain (C-terminal) containing	Homo sapiens EF-hand domain (C-term	25.92156	6.79409	25.66364	6.43415	Detected	Compromised	Detected	Compromised
6657	A_24_P164894	DSCR3	Hs.369488	Down syndrome critical region gene 3	Homo sapiens Down syndrome critical r	112.01707	212.95384	128.10370	181.99083	Detected	Detected	Detected	Detected
6658	A_23_P72138	MRPS22	Hs.720211	mitochondrial ribosomal protein S22	Homo sapiens mitochondrial ribosomal p	6391.99170	8029.66260	6159.93900	6933.06400	Detected	Detected	Detected	Detected
6659	A_23_P34741	ZNF593	Hs.477273	zinc finger protein 593	Homo sapiens zinc finger protein 593 (Z	5590.03200	5409.48900	6186.45500	5464.96880	Detected	Detected	Detected	Detected
6660	A_33_P3286208	PPIL5	Hs.451090	peptidylprolyl isomerase (cyclophilin)-lik	Homo sapiens peptidylprolyl isomerase	191.65773	446.28595	263.79022	541.30340	Detected	Detected	Detected	Detected
6661	A_23_P89550	NLRP1	Hs.652273	NLR family, pyrin domain containing 1	Homo sapiens NLR family, pyrin domain	365.20910	402.58127	336.01608	304.45816	Detected	Detected	Detected	Detected
6662	A_33_P3258472	SPTBN1	Hs.503178	spectrin, beta, non-erythrocytic 1	Homo sapiens spectrin, beta, non-eryth	18.54857	43.12497	10.72287	25.24206	Detected	Detected	Compromised	Detected
6663	A_24_P923102	KLHL23	Hs.574626	kelch-like 23 (Drosophila)	Kelch-like protein 23 [Source:UniProtK	40.36454	3.72764	41.62518	3.15200	Detected	Compromised	Detected	Compromised
6664	A_24_P303420	LOC221442	Hs.374076	adenylate cyclase 10 pseudogene	Homo sapiens adenylate cyclase 10 pse	41.43341	22.97343	32.37032	19.32091	Detected	Detected	Detected	Detected
6665	A_33_P3392187	CCDC85A	Hs.117136	coiled-coil domain containing 85A	Homo sapiens coiled-coil domain conta	11.20793	190.14186	11.74969	142.92550	Compromised	Detected	Compromised	Detected
6666	A_24_P5550	PHF23	Hs.647432	PHD finger protein 23	Homo sapiens PHD finger protein 23 (P	800.56010	733.66910	891.72620	913.78410	Detected	Detected	Detected	Detected
6667	A_23_P205746	EML1	Hs.12451	echinoderm microtubule associated pro	Homo sapiens echinoderm microtubule	5322.68800	3411.83890	4448.97700	2529.83520	Detected	Detected	Detected	Detected
6668	A_33_P3296682	NDUFB5	Hs.518424	NADH dehydrogenase (ubiquinone) 1 be	Homo sapiens NADH dehydrogenase (ul	9899.78100	8736.11600	5729.45950	4478.49850	Detected	Detected	Detected	Detected
6669	A_23_P6381	MN1	Hs.268515	meningioma (disrupted in balanced tran	Homo sapiens meningioma (disrupted in	47.89284	84.14571	36.50382	84.29112	Detected	Detected	Detected	Detected
6670	A_33_P3567512	EEF1DP3	Hs.507667	eukaryotic translation elongation factor	Homo sapiens eukaryotic translation elo	2274.34230	2449.10420	1174.48460	1108.38000	Detected	Detected	Detected	Detected
6671	A_32_P104518	C9orf130	Hs.494549	chromosome 9 open reading frame 130	Homo sapiens chromosome 9 open read	33.04272	56.40272	52.64962	55.20076	Detected	Detected	Detected	Detected

6672	A_23_P213375	PCDHB2	Hs.533023	protocadherin beta 2	Homo sapiens protocadherin beta 2 (PC	80.61588	47.47965	65.58167	36.85380	Detected	Detected	Detected	Detected
6673	A_33_P3318661	E2F7	Hs.416375	E2F transcription factor 7	Homo sapiens E2F transcription factor	2.46618	54.81842	2.76984	46.04974	Compromised	Detected	Compromised	Detected
6674	A_33_P3235880	TBC1D28	Hs.434123	TBC1 domain family, member 28	Homo sapiens TBC1 domain family, mer	10.10242	21.10721	11.13678	13.01092	Compromised	Detected	Compromised	Compromised
6675	A_23_P14083	AMIGO2	Hs.121520	adhesion molecule with Ig-like domain 2	Homo sapiens adhesion molecule with I	126.29672	1310.28090	76.09612	663.57996	Detected	Detected	Detected	Detected
6676	A_24_P79070	GNG8	Hs.283961	guanine nucleotide binding protein (G pr	Homo sapiens guanine nucleotide bindir	124.93972	133.51846	52.69640	39.89711	Detected	Detected	Detected	Detected
6677	A_24_P88800	COX7A2L	Hs.339639	cytochrome c oxidase subunit VIIa poly	Homo sapiens cytochrome c oxidase su	3583.39100	3212.59350	4713.06500	3620.83400	Detected	Detected	Detected	Detected
6678	A_23_P39386	HCST	Hs.117339	hematopoietic cell signal transducer	Homo sapiens hematopoietic cell signal	633.13310	421.56775	625.73080	331.92947	Detected	Detected	Detected	Detected
6679	A_33_P3239267	C3orf54	Hs.86674	chromosome 3 open reading frame 54	Homo sapiens chromosome 3 open read	20902.62500	15271.44600	14165.83200	7721.47360	Detected	Detected	Detected	Detected
6680	A_33_P3272291	AKR1C4	Hs.567245	aldo-keto reductase family 1, member C	Homo sapiens aldo-keto reductase fam	1686.14060	594.76227	312.36713	76.25301	Detected	Detected	Detected	Detected
6681	A_33_P3289848	CDX1	Hs.1545	caudal type homeobox 1	Homo sapiens caudal type homeobox 1	1210.86570	1668.95360	542.44070	539.71270	Detected	Detected	Detected	Detected
6682	A_23_P146077	ZNF395	Hs.435535	zinc finger protein 395	Homo sapiens zinc finger protein 395 (Z	10988.08700	4006.48800	11013.39400	3623.41280	Detected	Detected	Detected	Detected
6683	A_23_P406105	GCN1L1	Hs.298716	GCN1 general control of amino-acid sy	Homo sapiens GCN1 general control of	23946.78700	23217.97500	16430.28000	13999.68400	Detected	Detected	Detected	Detected
6684	A_24_P280833	LOC100129138	Hs.514487	similar to THAP domain containing, apo	PREDICTED: Homo sapiens similar to T	432.71368	496.92930	452.27768	414.69083	Detected	Detected	Detected	Detected
6685	A_23_P91283	CASS4	Hs.473144	Cas scaffolding protein family member 4	Homo sapiens Cas scaffolding protein f	13.53693	17.16136	19.78437	22.02164	Detected	Detected	Detected	Detected
6686	A_33_P3244224	LOC100129048	Hs.666120	hypothetical LOC100129048	Homo sapiens cDNA FLJ46195 fis, clon	30.24559	65.47630	13.54188	16.17644	Detected	Detected	Compromised	Detected
6687	A_33_P3334748	LOC100129200	Hs.576209	hypothetical LOC100129200	PREDICTED: Homo sapiens hypothetica	11.93054	19.52262	14.24737	10.92662	Compromised	Detected	Detected	Compromised
6688	A_23_P135778	PPP2R2D	Hs.380372	protein phosphatase 2, regulatory subur	Homo sapiens protein phosphatase 2, r	3314.16200	3283.48700	2725.09770	2581.85670	Detected	Detected	Detected	Detected
6689	A_23_P159027	ZNF521	Hs.116935	zinc finger protein 521	Homo sapiens zinc finger protein 521 (Z	4290.11900	800.27075	3140.01590	531.77400	Detected	Detected	Detected	Detected
6690	A_23_P48628	ZBTB25	Hs.654571	zinc finger and BTB domain containing	Homo sapiens zinc finger and BTB dom	161.78307	116.78009	171.69324	103.01178	Detected	Detected	Detected	Detected
6691	A_33_P3296352	PPM1B	Hs.416769	protein phosphatase 1B (formerly 2C), r	Homo sapiens protein phosphatase 1B	499.72534	666.06555	533.10754	619.16230	Detected	Detected	Detected	Detected
6692	A_33_P3382031	FAM23A	Hs.564139	family with sequence similarity 23, mem	Homo sapiens family with sequence sim	19.18336	22.67436	17.20304	21.43304	Detected	Detected	Detected	Detected
6693	A_23_P39840	VAMP5	Hs.534373	vesicle-associated membrane protein 5	Homo sapiens vesicle-associated memb	5666.53560	4137.00500	7508.14060	4944.68750	Detected	Detected	Detected	Detected
6694	A_32_P93328	LOC100134713		hypothetical protein LOC100134713	Homo sapiens hypothetical protein LOC	398.90363	425.18164	162.98961	181.44025	Detected	Detected	Detected	Detected
6695	A_32_P134290	ZCCHC2	Hs.114191	zinc finger, CCHC domain containing 2	Homo sapiens zinc finger, CCHC domai	1173.92880	375.93920	901.53064	254.99518	Detected	Detected	Detected	Detected
6696	A_23_P75786	SLC15A3	Hs.237856	solute carrier family 15, member 3	Homo sapiens solute carrier family 15,	470.82385	250.32880	600.90784	339.85830	Detected	Detected	Detected	Detected
6697	A_33_P3660204	LOC340073	Hs.434422	hypothetical protein LOC340073	Homo sapiens hypothetical protein LOC	23.49136	33.23942	26.60815	20.36184	Detected	Detected	Detected	Detected
6698	A_32_P216872	LOC729602	Hs.446876	NPIP-like protein ENSP00000283050	PREDICTED: Homo sapiens NPIP-like p	679.08026	372.94473	448.45334	201.12059	Detected	Detected	Detected	Detected
6699	A_23_P9061	EIF3H	Hs.492599	eukaryotic translation initiation factor 3	Homo sapiens eukaryotic translation ini	111258.02000	84484.20000	78547.52000	50084.47300	Detected	Detected	Detected	Detected
6700	A_33_P3360785	LOC100128371	Hs.689386	hypothetical protein LOC100128371	Homo sapiens cDNA FLJ26611 fis, clon	7.57186	22.02423	10.44184	6.64215	Compromised	Detected	Compromised	Compromised
6701	A_24_P171983	NDUFAF4	Hs.512144	NADH dehydrogenase (ubiquinone) 1 al	Homo sapiens NADH dehydrogenase (u	2882.32300	3616.39920	2302.08450	2328.42970	Detected	Detected	Detected	Detected
6702	A_24_P350589	RNF150	Hs.659104	ring finger protein 150	Homo sapiens ring finger protein 150 (R	35.77187	71.07806	29.37649	39.08086	Detected	Detected	Detected	Detected
6703	A_33_P3277110	SLC5A3	Hs.302742	solute carrier family 5 (sodium/myo-ino	Homo sapiens solute carrier family 5 (s	3635.91820	1584.72970	2275.12870	740.41650	Detected	Detected	Detected	Detected
6704	A_23_P86801	RAPSN	Hs.81218	receptor-associated protein of the syna	Homo sapiens receptor-associated prot	133.10600	127.68208	72.50660	64.34332	Detected	Detected	Detected	Detected
6705	A_23_P118722	ASGR1	Hs.12056	asialoglycoprotein receptor 1	Homo sapiens asialoglycoprotein recept	1028.25290	449.41156	843.52520	320.22787	Detected	Detected	Detected	Detected
6706	A_23_P114774	PMVK	Hs.30954	phosphomevalonate kinase	Homo sapiens phosphomevalonate kina	3190.68680	3461.26100	3484.58760	3503.55500	Detected	Detected	Detected	Detected
6707	A_23_P55948	PRR12	Hs.590971	proline rich 12	Homo sapiens proline rich 12 (PRR12),	669.98553	936.03925	621.49060	751.69507	Detected	Detected	Detected	Detected
6708	A_33_P3339575	LOC647983	Hs.677472	hypothetical protein LOC647983	Homo sapiens cDNA FLJ44880 fis, clon	9.89043	9.63630	15.08671	18.22092	Compromised	Compromised	Compromised	Detected
6709	A_33_P3403356	TIMM23B		translocase of inner mitochondrial mem	PREDICTED: Homo sapiens translocase	1131.37150	2022.72070	1739.01570	2685.82840	Detected	Detected	Detected	Detected
6710	A_24_P314554	GOLGA2LY1	Hs.459129	golgi autoantigen, golgin subfamily a, 2-	Homo sapiens golgi autoantigen, golgin	179.00868	166.58977	93.61541	61.54752	Detected	Detected	Detected	Detected
6711	A_24_P406334	STEAP1	Hs.61635	six transmembrane epithelial antigen of	Homo sapiens six transmembrane epith	12670.62400	11554.66800	10526.70200	8482.51200	Detected	Detected	Detected	Detected
6712	A_23_P88381	NUMB	Hs.654609	numb homolog (Drosophila)	Homo sapiens numb homolog (Drosophi	2925.15330	3136.03520	2618.31600	2320.54860	Detected	Detected	Detected	Detected
6713	A_33_P3279730				D-dopachrome decarboxylase (EC 4.1.1	50.48250	233.81213	68.81451	250.95956	Detected	Detected	Detected	Detected
6714	A_23_P50799	OR10H2	Hs.247694	olfactory receptor, family 10, subfamily	Homo sapiens olfactory receptor, family	5423.79000	7653.40000	722.07416	573.28580	Detected	Detected	Detected	Detected
6715	A_32_P16854	ANAPC13	Hs.106909	anaphase promoting complex subunit 13	Homo sapiens anaphase promoting com	3831.62060	2858.22100	3584.60420	2424.64200	Detected	Detected	Detected	Detected
6716	A_23_P29663	ZMYND10	Hs.526735	zinc finger, MYND-type containing 10	Homo sapiens zinc finger, MYND-type c	112.50269	34.78461	114.30830	32.73550	Detected	Detected	Detected	Detected
6717	A_23_P152949	LRRC46	Hs.130767	leucine rich repeat containing 46	Homo sapiens leucine rich repeat conta	72.03764	102.51291	76.12808	71.82327	Detected	Detected	Detected	Detected
6718	A_33_P3357007	RP11-45B20.2	Hs.642624	C1q and tumor necrosis factor related	Homo sapiens C1q and tumor necrosis	11.09452	10.89134	21.68560	23.71134	Compromised	Compromised	Detected	Detected
6719	A_24_P280029	PDXP	Hs.632762	pyridoxal (pyridoxine, vitamin B6) phosp	Homo sapiens pyridoxal (pyridoxine, vita	568.25000	545.35724	677.24450	717.21520	Detected	Detected	Detected	Detected
6720	A_23_P430902	MORN4	Hs.217409	MORN repeat containing 4	Homo sapiens MORN repeat containing	271.67020	103.43839	264.17780	99.70356	Detected	Detected	Detected	Detected
6721	A_33_P3373165	DUX4	Hs.553518	double homeobox, 4	Homo sapiens double homeobox, 4 (DU	18.05265	20.03039	17.96594	13.23557	Detected	Detected	Detected	Compromised
6722	A_33_P3362267	DGCR10		DiGeorge syndrome critical region gene	Homo sapiens DiGeorge syndrome critic	5.04222	14.06518	14.02855	7.03962	Compromised	Compromised	Detected	Compromised
6723	A_33_P3232504	C9orf169	Hs.512469	chromosome 9 open reading frame 169	Homo sapiens chromosome 9 open read	743.68480	373.79996	670.78723	281.03043	Detected	Detected	Detected	Detected
6724	A_33_P3313796	CCDC34	Hs.143733	coiled-coil domain containing 34	Homo sapiens coiled-coil domain conta	2715.68200	4855.38500	1903.26830	2853.34300	Detected	Detected	Detected	Detected
6725	A_23_P64404	FADS3	Hs.21765	fatty acid desaturase 3	Homo sapiens fatty acid desaturase 3 (	3775.63720	2169.08000	4023.19480	2488.61280	Detected	Detected	Detected	Detected
6726	A_24_P29117	SLC26A1	Hs.658244	solute carrier family 26 (sulfate transpo	Homo sapiens solute carrier family 26 (	56.04364	23.62315	56.65553	18.51136	Detected	Detected	Detected	Detected
6727	A_33_P3381458	LOC100128842	Hs.679154	hypothetical protein LOC100128842	Homo sapiens hypothetical protein LOC	12.43582	21.60807	19.11869	18.87158	Detected	Detected	Detected	Detected
6728	A_33_P3338724	LENG9	Hs.590976	leukocyte receptor cluster (LRC) memb	Homo sapiens leukocyte receptor clust	411.04980	200.95248	176.01780	106.31888	Detected	Detected	Detected	Detected
6729	A_33_P3402635	C16orf3	Hs.633032	chromosome 16 open reading frame 3	Homo sapiens chromosome 16 open rea	34.31527	36.09451	21.25353	13.15106	Detected	Detected	Detected	Compromised
6730	A_23_P88278	RPGRIP1	Hs.126035	retinitis pigmentosa GTPase regulator i	Homo sapiens retinitis pigmentosa GTP	16.46728	3.33241	16.91269	2.84551	Detected	Compromised	Detected	Compromised
6731	A_24_P282762	TLK1	Hs.596942	tousled-like kinase 1	Homo sapiens tousled-like kinase 1 (TL	38.37436	48.36628	33.89228	29.58465	Detected	Detected	Detected	Detected
6732	A_23_P90845	TIA1	Hs.413123	TIA1 cytotoxic granule-associated RNA	Homo sapiens TIA1 cytotoxic granule-a	405.72787	322.33554	351.75995	244.49190	Detected	Detected	Detected	Detected
6733	A_33_P3231908	POU3F3	Hs.673855	POU class 3 homeobox 3	Homo sapiens POU class 3 homeobox 3	11.94260	7.51123	20.20154	5.07508	Compromised	Compromised	Detected	Compromised
6734	A_33_P3304819					47.37251	95.08643	26.85074	38.69822	Detected	Detected	Detected	Detected
6735	A_33_P3211968		Hs.633538		Full-length cDNA clone CS0DG006YJ1	2.46478	3.34388	14.69812	4.02894	Compromised	Compromised	Detected	Compromised
6736	A_23_P150903	FAR2	Hs.719237	fatty acyl CoA reductase 2	Homo sapiens fatty acyl CoA reductase	259.52362	207.78778	314.22772	230.57016	Detected	Detected	Detected	Detected
6737	A_23_P77310	ZFP106	Hs.511143	zinc finger protein 106 homolog (mouse	Homo sapiens zinc finger protein 106 h	2690.56420	2586.54420	2541.89620	2027.72770	Detected	Detected	Detected	Detected
6738	A_23_P62901	BTG2	Hs.519162	BTG family, member 2	Homo sapiens BTG family, member 2 (B	227.29897	401.58005	207.92484	318.85764	Detected	Detected	Detected	Detected
6739	A_24_P418809	GNAS	Hs.125898	GNAS complex locus	Homo sapiens GNAS complex locus (GN	36395.63700	47970.31600	31430.06200	38584.83600	Detected	Detected	Detected	Detected
6740	A_33_P3832852	HAUS2	Hs.14347	HAUS augmin-like complex, subunit 2	Homo sapiens HAUS augmin-like compl	909.97150	995.85780	846.88860	836.62976	Detected	Detected	Detected	Detected
6741	A_23_P89710	CEP192	Hs.100914	centrosomal protein 192kDa	Homo sapiens centrosomal protein 192k	1228.28310	1756.42370	849.97720	959.95764	Detected	Detected	Detected	Detected
6742	A_33_P3398156	CYS1	Hs.27092	cystin 1	Homo sapiens cystin 1 (CYS1), mRNA [	526.39430	381.98890	588.24470	369.97510	Detected	Detected	Detected	Detected
6743	A_23_P20894	EHMT1	Hs.495511	euchromatic histone-lysine N-methyltra	Homo sapiens euchromatic histone-lysi	179.13368	137.89375	160.61844	136.63240	Detected	Detected	Detected	Detected
6744	A_23_P144202	EEFSEC	Hs.477498	eukaryotic elongation factor, selenocyst	Homo sapiens eukaryotic elongation fac	174.81938	160.90680	95.05682	85.21790	Detected	Detected	Detected	Detected
6745	A_33_P3258747	C21orf63	Hs.208358	chromosome 21 open reading frame 63	Homo sapiens chromosome 21 open rea	244.18236	219.76736	258.13364	175.90556	Detected	Detected	Detected	Detected
6746	A_23_P105465	CMKLR1	Hs.197143	chemokine-like receptor 1	Homo sapiens chemokine-like receptor	3.31700	8.22242	21.60531	8.23685	Compromised	Compromised	Not Detected	Compromised

6747	A_23_P93629	TRIM24	Hs.490287	tripartite motif-containing 24	Homo sapiens tripartite motif-containin	4312.56740	4893.42870	4137.02400	3937.88650	Detected	Detected	Detected	Detected
6748	A_33_P3405763	TMPPE	Hs.443031	transmembrane protein with metallopho	Homo sapiens transmembrane protein w	103.08480	143.04001	86.84564	101.07983	Detected	Detected	Detected	Detected
6749	A_23_P21230	TRIM37	Hs.579079	tripartite motif-containing 37	Homo sapiens tripartite motif-containin	728.13320	867.30980	661.35270	717.86554	Detected	Detected	Detected	Detected
6750	A_33_P3263193	C12orf32	Hs.198853	chromosome 12 open reading frame 32	Homo sapiens chromosome 12 open rea	809.03326	3123.21900	438.11462	1328.13400	Detected	Detected	Detected	Detected
6751	A_33_P3285540	CLDN5	Hs.505337	claudin 5	Homo sapiens claudin 5 (CLDN5), trans	19.81551	6.05243	11.14288	7.47590	Detected	Compromised	Compromised	Compromised
6752	A_24_P363745	LRRC14	Hs.459391	leucine rich repeat containing 14	Homo sapiens leucine rich repeat conta	4742.42630	3086.68530	2598.27950	1559.64430	Detected	Detected	Detected	Detected
6753	A_24_P49447	KIAA1432	Hs.211520	KIAA1432	Homo sapiens KIAA1432 (KIAA1432), tr	1627.85830	1216.99730	1140.98340	757.18240	Detected	Detected	Detected	Detected
6754	A_33_P3383861	LOC646471	Hs.720666	hypothetical LOC646471	Homo sapiens hypothetical LOC646471	14.41845	16.66343	9.99940	8.26018	Detected	Compromised	Compromised	Compromised
6755	A_32_P64475	C1orf55	Hs.520192	chromosome 1 open reading frame 55	Homo sapiens chromosome 1 open read	251.00652	505.93094	194.22960	359.37054	Detected	Detected	Detected	Detected
6756	A_23_P341065	RP3-402G11.5	Hs.365405	selenoprotein O	Homo sapiens selenoprotein O (SELO),	1349.77060	919.82025	1824.82310	1279.05350	Detected	Detected	Detected	Detected
6757	A_33_P3239148	ZNF548	Hs.657353	zinc finger protein 548	Homo sapiens zinc finger protein 548 (Z	81.77966	62.15253	89.29434	52.88560	Detected	Detected	Detected	Detected
6758	A_33_P3259560					191.56944	211.86777	49.33508	58.02342	Detected	Detected	Detected	Detected
6759	A_33_P3411325					133.18213	201.96638	86.60177	87.98531	Detected	Detected	Detected	Detected
6760	A_33_P3260572	CLIP4	Hs.122927	CAP-GLY domain containing linker prot	Homo sapiens CAP-GLY domain contai	428.92914	217.78299	343.21387	129.47177	Detected	Detected	Detected	Detected
6761	A_23_P83436	PEPD	Hs.36473	peptidase D	Homo sapiens peptidase D (PEPD), tran	20695.40400	11280.37800	15673.66800	7263.46730	Detected	Detected	Detected	Detected
6762	A_33_P3798739	LOC286382	Hs.668691	hypothetical protein LOC286382	Homo sapiens cDNA FLJ37037 fis, clon	342.10920	297.78046	141.07527	119.41169	Detected	Detected	Detected	Detected
6763	A_23_P156061	LNPEP	Hs.656905	leucyl/cystinyl aminopeptidase	Homo sapiens leucyl/cystinyl aminopep	87.57545	291.26157	71.33482	204.11552	Detected	Detected	Detected	Detected
6764	A_24_P7629	RPL32P3		ribosomal protein L32 pseudogene 3	Homo sapiens ribosomal protein L32 ps	24.22241	11.32208	16.68334	7.07140	Detected	Compromised	Detected	Compromised
6765	A_33_P3222600	LOC100132588		hypothetical protein LOC100132588	PREDICTED: Homo sapiens hypothetica	2.46339	76.51893	2.80229	57.04184	Compromised	Detected	Compromised	Detected
6766	A_33_P3234571	MAP2K3	Hs.514012	mitogen-activated protein kinase kinase	Homo sapiens mitogen-activated protei	436.02576	726.23444	363.91140	607.48663	Detected	Detected	Detected	Detected
6767	A_23_P256470	NPY	Hs.1832	neuropeptide Y	Homo sapiens neuropeptide Y (NPY), m	19.79165	49.41306	22.13791	30.32477	Detected	Detected	Detected	Detected
6768	A_33_P3276142	C15orf51	Hs.585319	dynamin 1 pseudogene	Homo sapiens chromosome 15 open rea	161.35826	163.71670	117.19219	99.82210	Detected	Detected	Detected	Detected
6769	A_33_P3374117	LOC645634	Hs.162595	hypothetical LOC645634	PREDICTED: Homo sapiens hypothetica	126.00081	125.87260	54.57761	39.74834	Detected	Detected	Detected	Detected
6770	A_33_P3355371	TTC9C	Hs.31704	tetratricopeptide repeat domain 9C	Homo sapiens clone pp8376 unknown m	94.17227	63.42287	76.46660	43.55389	Detected	Detected	Detected	Detected
6771	A_23_P147383	GPAA1	Hs.627962	glycosylphosphatidylinositol anchor atta	Homo sapiens glycosylphosphatidylinosi	1158.65250	1911.25660	1133.55970	1997.85780	Detected	Detected	Detected	Detected
6772	A_33_P3234487	BOLA2B	Hs.647333	bolA homolog 2B (E. coli)	Homo sapiens bolA homolog 2B (E. coli)	20181.62300	28509.25800	22324.94700	26404.26400	Detected	Detected	Detected	Detected
6773	A_33_P3325871	hCG_1993592		hypothetical LOC644903	Homo sapiens hypothetical LOC644903	119.94800	88.69506	72.95523	35.29963	Detected	Detected	Detected	Detected
6774	A_33_P3212555	PROS1	Hs.64016	protein S (alpha)	Homo sapiens protein S (alpha) (PROS1	589.21760	251.68166	386.64860	122.92542	Detected	Detected	Detected	Detected
6775	A_24_P626951		Hs.705191		Putative uncharacterized protein ENSP	25.69199	51.40555	23.91397	41.17196	Detected	Detected	Detected	Detected
6776	A_33_P3304382	C8orf38	Hs.435500	chromosome 8 open reading frame 38	Homo sapiens chromosome 8 open read	442.75864	655.11490	307.51868	360.48404	Detected	Detected	Detected	Detected
6777	A_33_P3364696	STAMBP	Hs.469018	STAM binding protein	Homo sapiens STAM binding protein (ST	676.54870	273.05200	626.55610	219.32414	Detected	Detected	Detected	Detected
6778	A_23_P418597	TNKS1BP1	Hs.530730	tankyrase 1 binding protein 1, 182kDa	Homo sapiens tankyrase 1 binding prote	1058.11120	665.47850	1141.68200	692.92065	Detected	Detected	Detected	Detected
6779	A_33_P3315634	NKX1-1		NK1 homeobox 1	PREDICTED: Homo sapiens NK1 homeo	68.75529	118.83009	28.70459	55.28974	Detected	Detected	Detected	Detected
6780	A_23_P77980	SLC4A1	Hs.443948	solute carrier family 4, anion exchanger,	Homo sapiens solute carrier family 4, ar	470.94586	741.86320	87.11764	155.75940	Detected	Detected	Detected	Detected
6781	A_33_P3391387	LOC100268168		hypothetical LOC100268168	Homo sapiens hypothetical LOC100268	164.30878	206.63959	98.69891	117.82612	Detected	Detected	Detected	Detected
6782	A_23_P41599	PCDHB8	Hs.287793	protocadherin beta 8	Homo sapiens protocadherin beta 8 (PC	49.69988	35.64441	33.24172	19.41358	Detected	Detected	Detected	Detected
6783	A_24_P15292	C1orf124	Hs.554892	chromosome 1 open reading frame 124	Homo sapiens chromosome 1 open read	216.20444	315.50592	260.11185	375.08954	Detected	Detected	Detected	Detected
6784	A_33_P3243897	RPAP2	Hs.444421	RNA polymerase II associated protein 2	Homo sapiens RNA polymerase II assoc	294.59167	182.21550	217.55457	123.31942	Detected	Detected	Detected	Detected
6785	A_24_P34611	SIX3	Hs.567336	SIX homeobox 3	Homo sapiens SIX homeobox 3 (SIX3),	16.22232	13.27408	12.08702	7.50414	Detected	Compromised	Compromised	Compromised
6786	A_23_P147388	KIF13B	Hs.444767	kinesin family member 13B	Homo sapiens kinesin family member 13	1769.67240	926.88340	1317.79370	674.76306	Detected	Detected	Detected	Detected
6787	A_33_P3492750	MGC27382	Hs.352665	hypothetical MGC27382	Homo sapiens cDNA FLJ34438 fis, clon	394.44180	252.56697	73.48261	32.74221	Detected	Detected	Detected	Detected
6788	A_33_P3454968	LOC645553	Hs.613068	hypothetical LOC645553	Homo sapiens cDNA clone IMAGE:5478	9457.46100	9485.70500	6124.20950	4484.18300	Detected	Detected	Detected	Detected
6789	A_32_P98927	STAG3L4	Hs.632013	stromal antigen 3-like 4	Homo sapiens stromal antigen 3-like 4 (	62.99878	87.02034	76.07897	75.50060	Detected	Detected	Detected	Detected
6790	A_23_P201939	PPM1J	Hs.655231	protein phosphatase 1J (PP2C domain c	Homo sapiens protein phosphatase 1J (	578.89020	171.99289	745.83720	191.68364	Detected	Detected	Detected	Detected
6791	A_23_P82959	FOXH1	Hs.708365	forkhead box H1	Homo sapiens forkhead box H1 (FOXH1	135.31789	336.25873	94.61042	202.14550	Detected	Detected	Detected	Detected
6792	A_23_P99693	ZBTB1	Hs.655536	zinc finger and BTB domain containing	Homo sapiens zinc finger and BTB dom	353.84653	254.87940	202.12251	147.56131	Detected	Detected	Detected	Detected
6793	A_23_P215491	CCL24	Hs.247838	chemokine (C-C motif) ligand 24	Homo sapiens chemokine (C-C motif) li	17104.04500	21974.24200	2844.07060	2655.00760	Detected	Detected	Detected	Detected
6794	A_33_P3330079	LOC100128242	Hs.651710	similar to GRAM domain containing 1B	PREDICTED: Homo sapiens similar to G	119.79380	725.48114	97.99329	580.30554	Detected	Detected	Detected	Detected
6795	A_33_P3666817	C10orf55	Hs.585453	chromosome 10 open reading frame 55	Homo sapiens chromosome 10 open rea	69.61294	37.46403	49.68577	23.01921	Detected	Detected	Detected	Detected
6796	A_23_P19084	HNRNPAB	Hs.591731	heterogeneous nuclear ribonucleoprotei	Homo sapiens heterogeneous nuclear ri	2991.29320	5220.43950	2200.37650	3743.79320	Detected	Detected	Detected	Detected
6797	A_24_P195081	C9orf93	Hs.17267	chromosome 9 open reading frame 93	Homo sapiens chromosome 9 open read	20.74102	14.66326	10.46549	6.04877	Detected	Compromised	Compromised	Compromised
6798	A_33_P3352632	LOC728147	Hs.653119	hypothetical protein LOC728147	PREDICTED: Homo sapiens hypothetica	30.49795	24.90463	19.22646	9.37207	Detected	Detected	Detected	Compromised
6799	A_23_P500998	HOXA9	Hs.659350	homeobox A9	Homo sapiens homeobox A9 (HOXA9),	3.55414	2179.96880	3.05108	1328.80490	Compromised	Detected	Compromised	Detected
6800	A_23_P337934	FBLIM1	Hs.530101	filamin binding LIM protein 1	Homo sapiens filamin binding LIM protei	4791.84670	4560.45950	3383.35620	3239.44820	Detected	Detected	Detected	Detected
6801	A_33_P3288039	ATP9B	Hs.465475	ATPase, class II, type 9B	Homo sapiens ATPase, class II, type 9B	214.85764	160.29631	224.67177	134.45117	Detected	Detected	Detected	Detected
6802	A_33_P3389230	CXCR3	Hs.198252	chemokine (C-X-C motif) receptor 3	Homo sapiens chemokine (C-X-C motif	424.86023	363.47986	318.81674	236.88153	Detected	Detected	Detected	Detected
6803	A_32_P16258	EXOC6B	Hs.303454	exocyst complex component 6B	Exocyst complex component 6B (Exocy	354.08075	181.18274	262.20260	107.58481	Detected	Detected	Detected	Detected
6804	A_32_P412313	ASB6	Hs.125037	ankyrin repeat and SOCS box-containin	Homo sapiens ankyrin repeat and SOCS	540.58390	593.39606	370.39697	392.78440	Detected	Detected	Detected	Detected
6805	A_33_P3335596	C17orf62	Hs.163113	chromosome 17 open reading frame 62	Homo sapiens chromosome 17 open rea	653.86370	340.96793	586.04790	301.98013	Detected	Detected	Detected	Detected
6806	A_24_P363583	AGFG2	Hs.521083	ArfGAP with FG repeats 2	Homo sapiens ArfGAP with FG repeats	15.11284	10.18519	20.96355	9.79876	Detected	Compromised	Detected	Compromised
6807	A_33_P3417113	NPSR1	Hs.652373	neuropeptide S receptor 1	Homo sapiens neuropeptide S receptor	59.34942	49.39188	24.51761	11.01552	Detected	Detected	Detected	Compromised
6808	A_33_P3315899					1427.36570	1648.19070	3345.78250	3234.98440	Detected	Detected	Detected	Detected
6809	A_23_P16063	GRIK5	Hs.367799	glutamate receptor, ionotropic, kainate	Homo sapiens glutamate receptor, ionot	106.83709	146.49759	45.06868	46.85510	Detected	Detected	Detected	Detected
6810	A_23_P24774	ABCC8	Hs.54470	ATP-binding cassette, sub-family C (CF	Homo sapiens ATP-binding cassette, su	36.93746	37.40045	19.76947	16.70522	Detected	Detected	Detected	Detected
6811	A_23_P8452	LFNG	Hs.159142	LFNG O-fucosylpeptide 3-beta-N-acet	Homo sapiens LFNG O-fucosylpeptide	321.72757	376.39078	162.72972	155.38507	Detected	Detected	Detected	Detected
6812	A_33_P3295838	C17orf53	Hs.437059	chromosome 17 open reading frame 53	Homo sapiens chromosome 17 open rea	127.08948	182.91316	101.21867	121.47128	Detected	Detected	Detected	Detected
6813	A_33_P3329765					7.99235	23.88538	8.95986	7.16805	Compromised	Detected	Compromised	Compromised
6814	A_33_P3331080		Hs.595540		Protein transport protein Sec24A (SEC	27.42535	33.74467	19.32281	19.93037	Detected	Detected	Detected	Detected
6815	A_23_P119916	WNT6	Hs.29764	wingless-type MMTV integration site fa	Homo sapiens wingless-type MMTV inte	107.32568	71.96708	65.05959	37.48494	Detected	Detected	Detected	Detected
6816	A_32_P178966	TMEM170B	Hs.146317	transmembrane protein 170B	Homo sapiens transmembrane protein 1	331.79984	73.22176	260.85242	42.92730	Detected	Detected	Detected	Detected
6817	A_33_P3272140	NUP35	Hs.180591	nucleoporin 35kDa	Homo sapiens nucleoporin 35kDa (NUP3	1328.15330	1318.37740	1247.31380	1001.73790	Detected	Detected	Detected	Detected
6818	A_33_P3223958	NAT8B	Hs.458287	N-acetyltransferase 8B (GCN5-related,	Homo sapiens N-acetyltransferase 8B (	14.65505	13.47936	11.89937	6.81652	Detected	Compromised	Compromised	Compromised
6819	A_23_P368718	C13orf1	Hs.44235	chromosome 13 open reading frame 1	Homo sapiens chromosome 13 open rea	188.06512	336.10226	122.12657	177.77610	Detected	Detected	Detected	Detected
6820	A_24_P328969	YIPF5	Hs.372050	Yip1 domain family, member 5	Homo sapiens Yip1 domain family, meml	1254.01950	1596.70020	985.32190	1019.45190	Detected	Detected	Detected	Detected
6821	A_23_P141651	NCOR1	Hs.462323	nuclear receptor co-repressor 1	Homo sapiens nuclear receptor co-repr	462.32242	475.83218	383.30115	344.84240	Detected	Detected	Detected	Detected

6822	A_33_P3260511	LOC729061	Hs.722585	similar to capicua-like protein/double h	PREDICTED: Homo sapiens similar to c	16.06148	9.77163	13.09416	13.54351	Detected	Compromised	Compromised	Compromised
6823	A_23_P214168	COL12A1	Hs.101302	collagen, type XII, alpha 1	Homo sapiens collagen, type XII, alpha 1	1915.52160	11180.96600	1511.68230	7038.72950	Detected	Detected	Detected	Detected
6824	A_33_P3233135				Kinesin-like protein KIF16B (Sorting ne:	116.29769	215.13675	93.26060	143.10074	Detected	Detected	Detected	Detected
6825	A_33_P3342345	LOC100288426	Hs.187134	hypothetical protein LOC100288426	cDNA FLJ45512 fis, clone BRTHA2021	284.01480	231.11745	272.80518	159.34901	Detected	Detected	Detected	Detected
6826	A_23_P99186	UBE3B	Hs.374067	ubiquitin protein ligase E3B	Homo sapiens ubiquitin protein ligase E3	299.63715	200.04904	158.35062	88.61723	Detected	Detected	Detected	Detected
6827	A_23_P8269	TAAR8	Hs.350571	trace amine associated receptor 8	Homo sapiens trace amine associated r	20.64247	27.28824	18.36937	18.43497	Detected	Detected	Detected	Detected
6828	A_33_P3277965	NFIC	Hs.170131	nuclear factor I/C (CCAAT-binding tran	Homo sapiens nuclear factor I/C (CCAA	88.74127	97.99955	77.66933	67.39917	Detected	Detected	Detected	Detected
6829	A_23_P352870	PVRL2	Hs.655455	poliovirus receptor-related 2 (herpesvir	Homo sapiens poliovirus receptor-relate	896.38983	1339.05820	1255.78480	1942.58410	Detected	Detected	Detected	Detected
6830	A_23_P156319	LARP1	Hs.292078	La ribonucleoprotein domain family, mer	Homo sapiens La ribonucleoprotein dom	10684.67200	13710.81500	12096.26500	14845.85700	Detected	Detected	Detected	Detected
6831	A_23_P124733	COQ2	Hs.144304	coenzyme Q2 homolog, prenyltransferas	Homo sapiens coenzyme Q2 homolog, p	346.70132	398.39320	401.31415	477.90417	Detected	Detected	Detected	Detected
6832	A_33_P3332627					34246.32400	38165.56600	4225.67400	5369.65430	Detected	Detected	Detected	Detected
6833	A_32_P34589	RSRC1	Hs.591283	arginine/serine-rich coiled-coil 1	Homo sapiens arginine/serine-rich coile	1175.87510	1384.32690	1062.94700	1137.06900	Detected	Detected	Detected	Detected
6834	A_32_P116840	CTAGE5	Hs.540038	CTAGE family, member 5	Homo sapiens CTAGE family, member 5	309.39413	297.39825	116.19917	95.48839	Detected	Detected	Detected	Detected
6835	A_23_P321201	DENND5A	Hs.501857	DENN/MADD domain containing 5A	Homo sapiens DENN/MADD domain co	4877.94600	5913.21500	4410.69870	4892.97900	Detected	Detected	Detected	Detected
6836	A_32_P64200	GUCA1B	Hs.446529	guanylate cyclase activator 1B (retina)	Homo sapiens guanylate cyclase activat	265.88593	49.43856	201.63210	35.59463	Detected	Detected	Detected	Detected
6837	A_23_P69141	DYNC1LI1	Hs.529495	dynein, cytoplasmic 1, light intermediate	Homo sapiens dynein, cytoplasmic 1, lig	1136.13430	981.67596	1050.19310	766.25930	Detected	Detected	Detected	Detected
6838	A_33_P3380850	PAX2	Hs.155644	paired box 2	Homo sapiens paired box 2 (PAX2), trar	15.10595	20.26371	7.19476	11.52944	Detected	Detected	Compromised	Compromised
6839	A_23_P50897	MKI67IP	Hs.367842	MKI67 (FHA domain) interacting nucleol	Homo sapiens MKI67 (FHA domain) inte	4139.82500	4569.45200	4059.33350	3912.20530	Detected	Detected	Detected	Detected
6840	A_33_P3409904	VAMP4	Hs.6651	vesicle-associated membrane protein 4	Homo sapiens vesicle-associated memb	241.98033	185.74222	231.09277	133.21748	Detected	Detected	Detected	Detected
6841	A_33_P3253440	PQLC3	Hs.274415	PQ loop repeat containing 3	Homo sapiens PQ loop repeat containin	442.58044	274.25024	379.07123	177.04002	Detected	Detected	Detected	Detected
6842	A_33_P3702281	LOC284014	Hs.131035	hypothetical protein LOC284014	Homo sapiens cDNA FLJ38248 fis, clon	4473.92430	3268.18820	1721.50260	897.55020	Detected	Detected	Detected	Detected
6843	A_23_P69791	AP1AR	Hs.435991	adaptor-related protein complex 1 asso	Homo sapiens adaptor-related protein c	676.41860	436.57086	742.30273	414.42117	Detected	Detected	Detected	Detected
6844	A_23_P383679	HYDIN	Hs.461229	hydrocephalus inducing homolog (mouse	Homo sapiens hydrocephalus inducing h	27.99303	5.45842	35.48910	5.58931	Detected	Compromised	Detected	Compromised
6845	A_23_P112173	SLC24A2	Hs.283014	solute carrier family 24 (sodium/potass	Homo sapiens solute carrier family 24 (s	5.61595	5.09030	16.44186	15.32906	Compromised	Compromised	Detected	Detected
6846	A_33_P3319880	EVC2	Hs.87306	Ellis van Creveld syndrome 2	Homo sapiens Ellis van Creveld syndror	613.05164	310.63870	533.17560	266.45636	Detected	Detected	Detected	Detected
6847	A_33_P3217356	LOC388630	Hs.576171	UPF0632 protein A	PREDICTED: Homo sapiens UPF0632 p	35.69952	35.04402	32.72874	28.48659	Detected	Detected	Detected	Detected
6848	A_23_P213093	OCIAD1	Hs.518750	OCIA domain containing 1	Homo sapiens OCIA domain containing	15073.11700	15828.65000	12234.61400	11959.05300	Detected	Detected	Detected	Detected
6849	A_23_P202988	AASDHPPT	Hs.524009	aminoadipate-semialdehyde dehydroger	Homo sapiens aminoadipate-semialdehy	2650.90200	2440.01300	1947.18540	1727.71800	Detected	Detected	Detected	Detected
6850	A_32_P389118	HEATR5B	Hs.468186	HEAT repeat containing 5B	Homo sapiens HEAT repeat containing	446.54416	320.86612	197.72340	113.94199	Detected	Detected	Detected	Detected
6851	A_24_P146892	ORAI1	Hs.55148	ORAI calcium release-activated calcium	Homo sapiens ORAI calcium release-ac	1139.99800	1070.87130	848.44635	793.03720	Detected	Detected	Detected	Detected
6852	A_32_P205624	SHC2	Hs.30965	SHC (Src homology 2 domain containing	Homo sapiens SHC (Src homology 2 do	135.27054	22.52849	158.03465	12.30763	Detected	Detected	Detected	Compromised
6853	A_33_P3334895	GRIN2A	Hs.411472	glutamate receptor, ionotropic, N-methy	Homo sapiens glutamate receptor, ionot	145.15820	140.97650	78.86428	66.99258	Detected	Detected	Detected	Detected
6854	A_33_P3291567	TSNARE1	Hs.370931	t-SNARE domain containing 1	Homo sapiens t-SNARE domain contair	305.61234	311.84250	146.73994	107.54250	Detected	Detected	Detected	Detected
6855	A_33_P3347049					168.68887	134.61311	81.03452	46.77826	Detected	Detected	Detected	Detected
6856	A_23_P43779	COPS4	Hs.190384	COP9 constitutive photomorphogenic h	Homo sapiens COP9 constitutive photo	7944.86400	7780.16900	5695.51950	4537.09000	Detected	Detected	Detected	Detected
6857	A_23_P209426	TRAK2	Hs.152774	trafficking protein, kinesin binding 2	Homo sapiens trafficking protein, kinesi	1764.44750	1784.53970	1018.84960	911.14703	Detected	Detected	Detected	Detected
6858	A_33_P3386463					16.32165	15.49783	15.10796	12.14548	Detected	Compromised	Detected	Compromised
6859	A_23_P47857	TM7SF3	Hs.438641	transmembrane 7 superfamily member 3	Homo sapiens transmembrane 7 superfa	1652.83640	3447.23410	1171.13850	2244.50440	Detected	Detected	Detected	Detected
6860	A_33_P3388870	BLOC1S1	Hs.94672	biogenesis of lysosomal organelles com	Homo sapiens biogenesis of lysosomal c	932.95984	919.63055	1690.32670	1496.61880	Detected	Detected	Detected	Detected
6861	A_24_P195669					56.77738	3.35538	52.61725	6.58083	Detected	Compromised	Detected	Compromised
6862	A_33_P3235162					11.63942	30.99974	3.21604	6.73552	Compromised	Detected	Compromised	Compromised
6863	A_33_P3347291	INMT	Hs.632629	indolethylamine N-methyltransferase	Homo sapiens indolethylamine N-methy	1179.10220	26.21479	955.78310	4.32800	Detected	Detected	Detected	Compromised
6864	A_24_P693321	LOC100190986	Hs.676485	hypothetical LOC100190986	Homo sapiens hypothetical LOC100190	75.42542	70.69460	43.55101	25.20663	Detected	Detected	Detected	Detected
6865	A_33_P3224345				GB	22.41678	32.15904	8.94222	8.73134	Detected	Detected	Compromised	Compromised
6866	A_23_P215419	ICA1	Hs.487561	islet cell autoantigen 1, 69kDa	Homo sapiens islet cell autoantigen 1, 6	27.63704	33.14576	31.48160	25.64964	Detected	Detected	Detected	Detected
6867	A_23_P211056	C21orf66	Hs.644004	chromosome 21 open reading frame 66	Homo sapiens chromosome 21 open rea	160.50645	102.81490	135.28986	72.06739	Detected	Detected	Detected	Detected
6868	A_33_P3349947	TCP1	Hs.363137	t-complex 1	Homo sapiens t-complex 1 (TCP1), trar	1152.68920	1612.23490	818.67660	1029.16760	Detected	Detected	Detected	Detected
6869	A_33_P3327842	ZNF436	Hs.293798	zinc finger protein 436	Homo sapiens zinc finger protein 436 (Z	18.07686	3.98808	17.51654	7.61163	Detected	Compromised	Detected	Compromised
6870	A_33_P3336557		Hs.721131		full-length cDNA clone CS0DL001YG15	8.35874	18.32567	9.71695	9.07582	Compromised	Detected	Compromised	Compromised
6871	A_33_P3382538	JRKL	Hs.105940	jerky homolog-like (mouse)	Homo sapiens jerky homolog-like (mous	58.89602	49.08956	39.02926	29.70244	Detected	Detected	Detected	Detected
6872	A_33_P3407324	GULP1	Hs.470887	GULP, engulfment adaptor PTB domain	Homo sapiens GULP, engulfment adapto	361.97550	188.17294	387.11840	174.20872	Detected	Detected	Detected	Detected
6873	A_33_P3360867					489.32315	248.97968	224.25668	126.47250	Detected	Detected	Detected	Detected
6874	A_23_P163238	STRC	Hs.657395	stereocilin	Homo sapiens stereocilin (STRC), mRN/	70.51856	3.32356	72.56361	7.61694	Detected	Compromised	Detected	Compromised
6875	A_33_P3351811				DNA-directed RNA polymerase II subur	18.54673	12.21828	16.49707	15.80009	Detected	Compromised	Detected	Detected
6876	A_33_P3419339	GNAI3	Hs.73799	guanine nucleotide binding protein (G pr	Homo sapiens guanine nucleotide bindin	1102.33510	1029.85080	634.33374	500.88120	Detected	Detected	Detected	Detected
6877	A_33_P3216008	SKA3	Hs.88523	spindle and kinetochore associated com	Homo sapiens spindle and kinetochore a	12.93789	682.42560	8.85430	378.06476	Detected	Detected	Compromised	Detected
6878	A_23_P78037	CCL7	Hs.251526	chemokine (C-C motif) ligand 7	Homo sapiens chemokine (C-C motif) li	243.96648	17.14749	236.21362	13.62833	Detected	Detected	Detected	Compromised
6879	A_23_P300087	FKSG83	Hs.326753	FKSG83	Homo sapiens FKSG83 (FKSG83), mRN	33.12524	33.67173	18.92976	23.31157	Detected	Detected	Detected	Detected
6880	A_24_P311771	ZFR	Hs.435231	zinc finger RNA binding protein	Homo sapiens zinc finger RNA binding p	711.28090	320.39453	563.86346	268.39987	Detected	Detected	Detected	Detected
6881	A_33_P3403936	SERINC4	Hs.604985	serine incorporator 4	Homo sapiens serine incorporator 4 (SE	16.18715	16.53342	8.07762	8.78011	Detected	Compromised	Compromised	Compromised
6882	A_23_P169909	DGKI	Hs.242947	diacylglycerol kinase, iota	Homo sapiens diacylglycerol kinase, iota	127.27599	279.06445	119.32119	246.25339	Detected	Detected	Detected	Detected
6883	A_32_P46191	ZNF727	Hs.640774	zinc finger protein 727	Homo sapiens zinc finger protein 727 (Z	2736.90230	3167.04880	1302.92300	1397.24840	Detected	Detected	Detected	Detected
6884	A_33_P3314974	LOC100130522		hypothetical LOC100130522	Homo sapiens hypothetical LOC100130	1037.72670	626.75080	205.15019	137.82004	Detected	Detected	Detected	Detected
6885	A_32_P174083	CYCS	Hs.437060	cytochrome c, somatic	Homo sapiens cytochrome c, somatic (C	596.46630	232.61952	557.13140	191.20439	Detected	Detected	Detected	Detected
6886	A_32_P203786	FAM7A1	Hs.574255	family with sequence similarity 7, memb	Homo sapiens family with sequence sim	10.91427	24.29497	9.81501	18.18439	Compromised	Detected	Compromised	Detected
6887	A_33_P3315824					59.53817	78.91189	26.28234	24.02014	Detected	Detected	Detected	Detected
6888	A_23_P54179	ZNF410	Hs.270869	zinc finger protein 410	Homo sapiens zinc finger protein 410 (Z	4545.47460	6468.77150	4203.05570	5324.48600	Detected	Detected	Detected	Detected
6889	A_33_P3242543	MAOA	Hs.183109	monoamine oxidase A	Homo sapiens monoamine oxidase A (M	8552.10600	968.99040	5194.00730	496.81860	Detected	Detected	Detected	Detected
6890	A_33_P3294654	C1orf89	Hs.546430	chromosome 1 open reading frame 89	Homo sapiens chromosome 1 open reac	268.28192	182.48491	225.47058	159.05229	Detected	Detected	Detected	Detected
6891	A_23_P20275	PLEKHF2	Hs.29724	pleckstrin homology domain containing,	Homo sapiens pleckstrin homology dom	2327.13700	2930.83200	1586.47390	1628.35660	Detected	Detected	Detected	Detected
6892	A_23_P431853					2877.09180	1731.38110	3432.76270	1713.53920	Detected	Detected	Detected	Detected
6893	A_33_P3240053	UFSP1	Hs.534845	UFM1-specific peptidase 1 (non-functic	Homo sapiens UFM1-specific peptidase	154.89793	314.54092	215.01030	397.48960	Detected	Detected	Detected	Detected
6894	A_23_P130553	SHKBP1	Hs.26506	SH3KBP1 binding protein 1	Homo sapiens SH3KBP1 binding proteir	951.98334	595.25510	741.25000	493.68884	Detected	Detected	Detected	Detected
6895	A_33_P3407999					20.32329	16.85931	19.88308	16.82268	Detected	Compromised	Detected	Detected
6896	A_33_P3278152					1096.90730	818.20910	424.33368	284.54040	Detected	Detected	Detected	Detected

6897	A_23_P173	CASQ1	Hs.632476	calsequestrin 1 (fast-twitch, skeletal mu	Homo sapiens calsequestrin 1 (fast-twi	43.71622	77.02342	32.45422	33.01180	Detected	Detected	Detected	Detected
6898	A_33_P3269678	LOC541471		hypothetical LOC541471	Homo sapiens hypothetical LOC541471	764.95360	1227.30100	541.22687	725.51135	Detected	Detected	Detected	Detected
6899	A_23_P205098	PDS5B	Hs.646051	PDS5, regulator of cohesion maintenanc	Homo sapiens PDS5, regulator of cohes	964.64075	665.34650	973.59980	565.21890	Detected	Detected	Detected	Detected
6900	A_23_P316085					22.11897	25.49272	9.04807	7.22993	Detected	Detected	Compromised	Compromised
6901	A_23_P93704	C7orf61	Hs.632306	chromosome 7 open reading frame 61	Homo sapiens chromosome 7 open read	121.51906	56.73119	95.19649	36.24519	Detected	Detected	Detected	Detected
6902	A_23_P32064	NELF	Hs.455336	nasal embryonic LHRH factor	Homo sapiens nasal embryonic LHRH fa	1130.45420	596.67730	819.25366	489.23648	Detected	Detected	Detected	Detected
6903	A_33_P3288110	PKP4	Hs.407580	plakophilin 4	Homo sapiens plakophilin 4 (PKP4), tran	149.94510	132.92021	114.21317	102.71040	Detected	Detected	Detected	Detected
6904	A_23_P164179	TOB1	Hs.531550	transducer of ERBB2, 1	Homo sapiens transducer of ERBB2, 1 (	575.46173	871.05710	508.81330	763.66830	Detected	Detected	Detected	Detected
6905	A_33_P3298750				Homo sapiens cDNA clone IMAGE:3463	75.39227	70.45631	39.58123	22.48010	Detected	Detected	Detected	Detected
6906	A_23_P56746	FAP	Hs.654370	fibroblast activation protein, alpha	Homo sapiens fibroblast activation prot	22461.62100	29330.14300	15746.09100	17162.83600	Detected	Detected	Detected	Detected
6907	A_33_P3251163	LOC100131869	Hs.529989	hypothetical protein LOC100131869	Homo sapiens cDNA FLJ42372 fis, clon	18.87657	8.51767	10.86844	8.51985	Detected	Compromised	Compromised	Compromised
6908	A_23_P78458	ZNF350	Hs.407694	zinc finger protein 350	Homo sapiens zinc finger protein 350 (Z	1198.48610	796.64680	898.47740	520.76770	Detected	Detected	Detected	Detected
6909	A_24_P250335	SNRPA	Hs.466775	small nuclear ribonucleoprotein polypep	Homo sapiens small nuclear ribonucleop	3799.67850	6747.24700	3046.85640	5567.06540	Detected	Detected	Detected	Detected
6910	A_33_P3360887	PRIC285	Hs.517180	peroxisomal proliferator-activated recep	Homo sapiens peroxisomal proliferator-	4330.39600	3417.50240	2000.29050	1307.55200	Detected	Detected	Detected	Detected
6911	A_33_P3280355	LOC100132857	Hs.711080	similar to heat shock 90kDa protein 1, b	PREDICTED: Homo sapiens similar to h	40549.75400	66451.05000	23202.49400	32281.59200	Detected	Detected	Detected	Detected
6912	A_33_P3384958	LPPR4	Hs.13245	lipid phosphate phosphatase-related pro	Homo sapiens lipid phosphate phosphat	47.06005	57.26815	29.14679	27.99749	Detected	Detected	Detected	Detected
6913	A_23_P39955	ACTG2	Hs.516105	actin, gamma 2, smooth muscle, enteric	Homo sapiens actin, gamma 2, smooth r	146.85382	90.75291	164.08328	94.83800	Detected	Detected	Detected	Detected
6914	A_33_P3342628	HES4	Hs.154029	hairy and enhancer of split 4 (Drosophil	Homo sapiens hairy and enhancer of sp	5532.15620	13677.19500	5212.37060	10895.11700	Detected	Detected	Detected	Detected
6915	A_33_P3336437	TTLL5	Hs.709609	tubulin tyrosine ligase-like family, memb	Homo sapiens tubulin tyrosine ligase-lik	35.93230	178.68008	31.67737	116.47976	Detected	Detected	Detected	Detected
6916	A_24_P103469	LST1	Hs.436066	leukocyte specific transcript 1	Homo sapiens leukocyte specific transc	12.89567	16.31936	18.81442	9.95439	Detected	Compromised	Detected	Compromised
6917	A_23_P135357	RNMTL1	Hs.182729	RNA methyltransferase like 1	Homo sapiens RNA methyltransferase l	1764.58980	2149.92720	1563.85950	1670.08590	Detected	Detected	Detected	Detected
6918	A_23_P152807	RSAD1	Hs.8033	radical S-adenosyl methionine domain c	Homo sapiens radical S-adenosyl methi	3902.36450	2200.44340	4661.97200	2418.64700	Detected	Detected	Detected	Detected
6919	A_24_P304051	GSTO1	Hs.190028	glutathione S-transferase omega 1	Homo sapiens glutathione S-transferase	64521.79700	88532.17000	55261.94500	62071.04300	Detected	Detected	Detected	Detected
6920	A_33_P3299565	LEKR1	Hs.478048	leucine, glutamate and lysine rich 1	Homo sapiens leucine, glutamate and ly	7.97407	3.31318	16.51427	2.82525	Compromised	Compromised	Detected	Compromised
6921	A_33_P3216664	LOC151174	Hs.424165	hypothetical LOC151174	Homo sapiens hypothetical LOC151174	795.59160	739.98200	758.18460	578.39070	Detected	Detected	Detected	Detected
6922	A_33_P3390202	C20orf165	Hs.128235	chromosome 20 open reading frame 165	Homo sapiens chromosome 20 open rea	111.46843	99.27480	85.71889	55.54049	Detected	Detected	Detected	Detected
6923	A_24_P54131	DCLRE1B	Hs.591412	DNA cross-link repair 1B (PSO2 homol	Homo sapiens DNA cross-link repair 1B	217.30606	637.52435	154.04399	325.44750	Detected	Detected	Detected	Detected
6924	A_23_P251132	SNTG2	Hs.595069	syntrophin, gamma 2	Homo sapiens syntrophin, gamma 2 (SN	2.65865	31.16880	6.89224	25.28930	Compromised	Detected	Compromised	Detected
6925	A_33_P3275846	CACNA1A	Hs.501632	calcium channel, voltage-dependent, P/	Homo sapiens calcium channel, voltage-	1924.42220	1033.68400	1601.78830	732.06510	Detected	Detected	Detected	Detected
6926	A_23_P141894	PVR	Hs.171844	poliovirus receptor	Homo sapiens poliovirus receptor (PVR	97.11194	214.48697	77.75445	176.88145	Detected	Detected	Detected	Detected
6927	A_23_P386561	TWISTNB	Hs.353035	TWIST neighbor	Homo sapiens TWIST neighbor (TWISTN	1720.42860	1531.23770	1153.27670	842.83514	Detected	Detected	Detected	Detected
6928	A_33_P3680462	LOC339539	Hs.434301	hypothetical protein LOC339539	Homo sapiens cDNA clone IMAGE:5170	20.55779	3.34328	9.58752	3.37255	Detected	Compromised	Compromised	Compromised
6929	A_23_P60146	PDGFRL	Hs.458573	platelet-derived growth factor receptor	Homo sapiens platelet-derived growth f	2403.17380	130.73070	1754.59250	79.94280	Detected	Detected	Detected	Detected
6930	A_23_P30805	HIST1H4J	Hs.278483	histone cluster 1, H4j	Homo sapiens histone cluster 1, H4j (HI	363.35350	914.25300	387.43097	867.31960	Detected	Detected	Detected	Detected
6931	A_32_P471485		Hs.58559		Rhotekin-2 (Pleckstrin homology domai	2.49951	25.42164	2.73433	19.44717	Compromised	Detected	Compromised	Detected
6932	A_23_P363826	FBXO3	Hs.406787	F-box protein 3	Homo sapiens F-box protein 3 (FBXO3)	362.55023	333.59024	334.51460	283.28183	Detected	Detected	Detected	Detected
6933	A_24_P118884	LOC100289079		similar to ribosomal protein L36	Putative uncharacterized protein ENSP	112.87630	97.89113	13.85419	9.52537	Detected	Detected	Detected	Compromised
6934	A_23_P111583	CD36	Hs.120949	CD36 molecule (thrombospondin recept	Homo sapiens CD36 molecule (thrombo	13.91738	20.65486	30.18742	14.32074	Not Detected	Detected	Not Detected	Detected
6935	A_23_P22499	GNL3L	Hs.654677	guanine nucleotide binding protein-like 3	Homo sapiens guanine nucleotide bindin	165.58722	154.61937	166.86040	160.16614	Detected	Detected	Detected	Detected
6936	A_32_P115050	LOC646576	Hs.632595	hypothetical LOC646576	PREDICTED: Homo sapiens hypothetica	114.55550	76.93150	79.01563	52.74386	Detected	Detected	Detected	Detected
6937	A_33_P3300610	OR7G2	Hs.553774	olfactory receptor, family 7, subfamily G	Homo sapiens olfactory receptor, family	107.58154	37.73705	10.55803	7.50182	Detected	Detected	Compromised	Compromised
6938	A_33_P3252989	KIAA0913	Hs.65135	KIAA0913	Homo sapiens KIAA0913 (KIAA0913), m	17915.74600	9175.89600	13435.71200	6677.46900	Detected	Detected	Detected	Detected
6939	A_33_P3599591	PAPPA	Hs.643599	pregnancy-associated plasma protein A	Homo sapiens pregnancy-associated pla	4090.38280	3035.05080	3259.29320	2155.01680	Detected	Detected	Detected	Detected
6940	A_32_P309404	SLC22A3	Hs.567337	solute carrier family 22 (extraneuronal r	Homo sapiens solute carrier family 22 (	14.26344	3.52431	7.30801	2.96508	Detected	Compromised	Compromised	Compromised
6941	A_23_P25873	WDHD1	Hs.385998	WD repeat and HMG-box DNA binding p	Homo sapiens WD repeat and HMG-box	888.45184	1719.96240	765.12650	1264.04580	Detected	Detected	Detected	Detected
6942	A_33_P3411313	LOC100131276		hypothetical LOC100131276	PREDICTED: Homo sapiens hypothetica	123.95256	121.11320	20.40745	18.36416	Detected	Detected	Detected	Detected
6943	A_23_P50775	LRFN3	Hs.143792	leucine rich repeat and fibronectin type	Homo sapiens leucine rich repeat and fi	5403.69600	5684.93070	3746.86080	3623.47500	Detected	Detected	Detected	Detected
6944	A_33_P3269423	C13orf35	Hs.591213	chromosome 13 open reading frame 35	Homo sapiens chromosome 13 open rea	11.66988	13.62025	18.71600	14.13346	Compromised	Compromised	Detected	Compromised
6945	A_23_P393015	MYL10	Hs.247831	myosin, light chain 10, regulatory	Homo sapiens myosin, light chain 10, re	2.67354	75.12500	4.47056	69.86723	Compromised	Detected	Compromised	Detected
6946	A_33_P3276713	HGF	Hs.396530	hepatocyte growth factor (hepapoietin A	Homo sapiens hepatocyte growth facto	1101.39330	27.81671	1136.79310	24.93893	Detected	Detected	Detected	Detected
6947	A_24_P367242	KRTAP19-1	Hs.61552	keratin associated protein 19-1	Homo sapiens keratin associated protei	71.88564	117.95221	14.35190	10.77261	Detected	Detected	Compromised	Compromised
6948	A_33_P3327454	RSPRY1	Hs.460885	ring finger and SPRY domain containing	Homo sapiens ring finger and SPRY dom	10184.66400	10766.53300	9861.82600	9179.83400	Detected	Detected	Detected	Detected
6949	A_23_P35995	ASAM	Hs.591949	adipocyte-specific adhesion molecule	Homo sapiens adipocyte-specific adhes	5353.99900	3617.44900	10875.86100	7347.99950	Detected	Detected	Detected	Detected
6950	A_33_P3216467	FAM83E	Hs.165803	family with sequence similarity 83, mem	Homo sapiens family with sequence sim	45.21943	52.31142	19.96738	20.99705	Detected	Detected	Detected	Detected
6951	A_24_P80338	NEK4	Hs.631921	NIMA (never in mitosis gene a)-related	Homo sapiens NIMA (never in mitosis g	1578.50890	1320.52310	1039.97500	710.55620	Detected	Detected	Detected	Detected
6952	A_23_P155969	PLK4	Hs.172052	polo-like kinase 4 (Drosophila)	Homo sapiens polo-like kinase 4 (Droso	26.50033	568.24250	9.90814	352.40582	Detected	Detected	Compromised	Detected
6953	A_23_P129659	ZNF689	Hs.454685	zinc finger protein 689	Homo sapiens zinc finger protein 689 (Z	549.74300	596.42926	556.60580	549.25226	Detected	Detected	Detected	Detected
6954	A_33_P3368895					3199.80710	10030.20000	1203.02150	3286.40550	Detected	Detected	Detected	Detected
6955	A_23_P168551	SLC29A4	Hs.4302	solute carrier family 29 (nucleoside tran	Homo sapiens solute carrier family 29 (	120.09771	34.78423	130.02846	24.18835	Detected	Detected	Detected	Detected
6956	A_33_P3282988	LOC100131582	Hs.671136	hypothetical protein LOC100131582	PREDICTED: Homo sapiens hypothetica	66.58831	14.14516	70.79940	21.56137	Detected	Compromised	Detected	Detected
6957	A_23_P100220	ESRP2	Hs.592053	epithelial splicing regulatory protein 2	Homo sapiens epithelial splicing regulato	161.46059	158.63821	71.86554	58.61312	Detected	Detected	Detected	Detected
6958	A_32_P54018	KIAA1143	Hs.476082	KIAA1143	Homo sapiens KIAA1143 (KIAA1143), m	1033.28770	1125.35220	828.74590	691.79250	Detected	Detected	Detected	Detected
6959	A_23_P156471	CDC5L	Hs.485471	CDC5 cell division cycle 5-like (S. pomb	Homo sapiens CDC5 cell division cycle	4109.84030	3661.72660	3386.56030	2525.82980	Detected	Detected	Detected	Detected
6960	A_33_P3392740				Protein atonal homolog 8 (Helix-loop-he	57.65628	71.84376	41.81544	27.60621	Detected	Detected	Detected	Detected
6961	A_24_P101402	NOP56	Hs.376064	NOP56 ribonucleoprotein homolog (yeas	Homo sapiens NOP56 ribonucleoprotein	19707.88300	31939.97700	14514.62000	21609.85400	Detected	Detected	Detected	Detected
6962	A_33_P3245006	DAK	Hs.6278	dihydroxyacetone kinase 2 homolog (S.	Homo sapiens dihydroxyacetone kinase	1026.40990	478.42350	742.99786	200.24557	Detected	Detected	Detected	Detected
6963	A_24_P152094	RP11-431O22.2	Hs.643463	similar to peptidyl-Pro cis trans isomera	PREDICTED: Homo sapiens similar to p	5828.94040	8044.57100	4096.57960	5198.57900	Detected	Detected	Detected	Detected
6964	A_33_P3412658	LOC100289509	Hs.720874	hypothetical protein LOC100289509	PREDICTED: Homo sapiens hypothetica	12.41569	8.90556	12.30640	12.27123	Detected	Compromised	Compromised	Compromised
6965	A_33_P3351920	LOC649941		similar to double homeobox 4c	PREDICTED: Homo sapiens similar to d	57.95241	65.20539	24.82075	23.12932	Detected	Detected	Detected	Detected
6966	A_33_P3379775		Hs.710058		Transducin-like enhancer protein 1 (En	983.76904	1859.22180	309.81020	424.13290	Detected	Detected	Detected	Detected
6967	A_33_P3367361	FAM107B	Hs.446315	family with sequence similarity 107, mer	Homo sapiens family with sequence sim	1916.15220	2967.23120	1246.30260	1563.46460	Detected	Detected	Detected	Detected
6968	A_33_P3226542	SNORD3B-1	Hs.658815	small nucleolar RNA, C/D box 3B-1	Homo sapiens small nucleolar RNA, C/D	7005.36870	1829.83480	5235.83250	1326.55810	Detected	Detected	Detected	Detected
6969	A_23_P152420	KIAA0182	Hs.461647	KIAA0182	Homo sapiens KIAA0182 (KIAA0182), tr	365.27430	569.88600	244.72313	309.84912	Detected	Detected	Detected	Detected
6970	A_24_P316234	LOC643475	Hs.675960	FLJ00409 protein	Homo sapiens mRNA for FLJ00409 prot	14.60104	36.26165	8.77799	15.68298	Detected	Detected	Compromised	Detected
6971	A_32_P352697	YTHDC1	Hs.175955	YTH domain containing 1	Homo sapiens YTH domain containing 1	91.65198	110.68381	97.02182	85.47855	Detected	Detected	Detected	Detected

6972	A_33_P3268652				Solute carrier family 2, facilitated glucos	25.21025	20.50139	20.48054	9.13682	Detected	Detected	Detected	Compromised
6973	A_33_P3251901	APBB2	Hs.479602	amyloid beta (A4) precursor protein-bin	Homo sapiens amyloid beta (A4) precur	2106.27170	2949.37330	661.13940	796.37354	Detected	Detected	Detected	Detected
6974	A_33_P3389942	NHP2	Hs.720093	NHP2 ribonucleoprotein homolog (yeast	Homo sapiens NHP2 ribonucleoprotein	46258.47000	54943.95700	35583.95700	38018.66800	Detected	Detected	Detected	Detected
6975	A_23_P133739	HUS1B	Hs.669039	HUS1 checkpoint homolog b (S. pombe)	Homo sapiens HUS1 checkpoint homolo	119.46708	159.41743	97.09272	117.78037	Detected	Detected	Detected	Detected
6976	A_33_P3292083		Hs.22495		601590766F1 NIH_MGC_7 Homo sapiens	20.46576	17.47292	6.00174	8.80073	Detected	Detected	Compromised	Compromised
6977	A_23_P94422	MELK	Hs.184339	maternal embryonic leucine zipper kinas	Homo sapiens maternal embryonic leuc	675.75670	5955.62900	609.38257	4352.22600	Detected	Detected	Detected	Detected
6978	A_23_P87769	C12orf48	Hs.330663	chromosome 12 open reading frame 48	Homo sapiens chromosome 12 open rea	690.75930	2965.82520	521.31670	1843.18150	Detected	Detected	Detected	Detected
6979	A_24_P920979	ASB7	Hs.31845	ankyrin repeat and SOCS box-containin	Homo sapiens ankyrin repeat and SOCS	486.75647	466.44693	332.26822	254.66551	Detected	Detected	Detected	Detected
6980	A_33_P3772937	KRT8P12	Hs.595594	keratin 8 pseudogene 12	Homo sapiens mRNA; cDNA DKFZp434	339.42993	285.31525	397.73035	304.52900	Detected	Detected	Detected	Detected
6981	A_24_P408457	NAGA	Hs.75372	N-acetylgalactosaminidase, alpha-	Homo sapiens N-acetylgalactosaminida	312.78287	372.53473	223.11583	236.88702	Detected	Detected	Detected	Detected
6982	A_23_P145863	S100A11	Hs.417004	S100 calcium binding protein A11	Homo sapiens S100 calcium binding pro	7555.38400	12863.45400	9942.90900	15218.03600	Detected	Detected	Detected	Detected
6983	A_33_P3325723	CHN1	Hs.654534	chimerin (chimaerin) 1	Homo sapiens chimerin (chimaerin) 1 (C	778.31165	2566.08400	529.56190	1533.36880	Detected	Detected	Detected	Detected
6984	A_24_P212531	B3GALNT2	Hs.716571	beta-1,3-N-acetylgalactosaminyltransfe	Homo sapiens beta-1,3-N-acetylgalact	82.23638	116.83912	87.36694	96.41241	Detected	Detected	Detected	Detected
6985	A_24_P181422	CATSPER2	Hs.662284	cation channel, sperm associated 2	Homo sapiens cation channel, sperm as	152.04112	82.77786	98.98254	54.61385	Detected	Detected	Detected	Detected
6986	A_24_P99046	STK38L	Hs.184523	serine/threonine kinase 38 like	Homo sapiens serine/threonine kinase	125.80090	21.57544	73.38590	15.28585	Detected	Detected	Detected	Detected
6987	A_33_P3217230	ZNF91	Hs.654471	zinc finger protein 91	Homo sapiens zinc finger protein 91 (Z	56.68010	85.57460	39.74873	49.21378	Detected	Detected	Detected	Detected
6988	A_24_P373174	RAB27A	Hs.654978	RAB27A, member RAS oncogene family	Homo sapiens RAB27A, member RAS o	899.51200	432.68878	749.47030	298.36588	Detected	Detected	Detected	Detected
6989	A_33_P3403494	LOC150527	Hs.125706	hypothetical LOC150527	Homo sapiens hypothetical LOC150527	447.29367	533.12800	192.27000	183.96576	Detected	Detected	Detected	Detected
6990	A_23_P121182	HACL1	Hs.63290	2-hydroxyacyl-CoA lyase 1	Homo sapiens 2-hydroxyacyl-CoA lyas	9963.52900	6400.67200	6544.92870	3616.13400	Detected	Detected	Detected	Detected
6991	A_24_P267522	ZNF585A	Hs.390568	zinc finger protein 585A	Homo sapiens zinc finger protein 585A	19.30548	10.26894	17.28775	5.74043	Detected	Compromised	Detected	Compromised
6992	A_33_P3275741		Hs.333383		Ficolin-3 Precursor (Collagen/fibrinoge	152.06343	215.63985	77.02734	79.78758	Detected	Detected	Detected	Detected
6993	A_24_P174613	FBXW7	Hs.679796	F-box and WD repeat domain containing	Homo sapiens F-box and WD repeat do	2207.98220	1977.64500	1302.27810	1005.91240	Detected	Detected	Detected	Detected
6994	A_33_P3261640	LOC100134365		hypothetical protein LOC100134365	PREDICTED: Homo sapiens similar to a	40.21115	164.37189	19.78440	52.07305	Detected	Detected	Detected	Detected
6995	A_33_P3350858					15.39112	17.82533	4.23733	3.02987	Detected	Compromised	Compromised	Compromised
6996	A_23_P206856	WDR59	Hs.280951	WD repeat domain 59	Homo sapiens WD repeat domain 59 (W	1246.26880	926.37010	1319.21250	845.89940	Detected	Detected	Detected	Detected
6997	A_23_P140928	TMC7	Hs.187377	transmembrane channel-like 7	Homo sapiens transmembrane channel-	50.38262	68.38400	37.83168	35.26643	Detected	Detected	Detected	Detected
6998	A_32_P69465	MORN2	Hs.720519	MORN repeat containing 2	Homo sapiens MORN repeat containing	4612.97900	4144.88300	3500.90900	2710.72630	Detected	Detected	Detected	Detected
6999	A_33_P3219720					6942.37840	5964.91100	2481.45560	1818.81500	Detected	Detected	Detected	Detected
7000	A_23_P413761	SFRS3	Hs.405144	splicing factor, arginine/serine-rich 3	Homo sapiens splicing factor, arginine/s	31272.47500	82299.51000	22369.75200	49963.74200	Detected	Detected	Detected	Detected
7001	A_24_P68991	NKX6-1	Hs.546270	NK6 homeobox 1	Homo sapiens NK6 homeobox 1 (NKX6-	26.09088	51.14008	22.97795	43.24320	Detected	Detected	Detected	Detected
7002	A_33_P3299590	SRRT	Hs.111801	serrate RNA effector molecule homolog	Homo sapiens serrate RNA effector mo	115.51160	211.95819	117.85178	222.10907	Detected	Detected	Detected	Detected
7003	A_23_P64980	SLC6A13	Hs.504398	solute carrier family 6 (neurotransmitte	Homo sapiens solute carrier family 6 (n	11.44664	22.15135	13.40283	15.14615	Compromised	Detected	Compromised	Compromised
7004	A_33_P3409266	GAFA2	Hs.675726	FGF-2 activity-associated protein 2	FGF-2 activity-associated protein 2HC	35.09311	55.94032	19.29671	26.79140	Detected	Detected	Detected	Detected
7005	A_33_P3353345	SULT4A1	Hs.189810	sulfotransferase family 4A, member 1	Homo sapiens sulfotransferase family 4	174.02548	47.31189	143.57425	38.34769	Detected	Detected	Detected	Detected
7006	A_33_P3884230	NFIX	Hs.257970	nuclear factor I/X (CCAAT-binding tran	Homo sapiens nuclear factor I/X (CCA	220.09076	210.23825	223.17978	228.36533	Detected	Detected	Detected	Detected
7007	A_23_P409093	ANO4	Hs.58785	anoctamin 4	Homo sapiens anoctamin 4 (ANO4), mR	90.61234	304.33844	52.78136	194.40788	Detected	Detected	Detected	Detected
7008	A_33_P3266010	RIN1	Hs.1030	Ras and Rab interactor 1	Homo sapiens Ras and Rab interactor 1	12453.02800	18813.20700	9602.96600	14406.99400	Detected	Detected	Detected	Detected
7009	A_33_P3821660	C9orf86	Hs.370555	chromosome 9 open reading frame 86	Homo sapiens chromosome 9 open rea	188.06755	321.45142	175.11009	363.08963	Detected	Detected	Detected	Detected
7010	A_33_P3300346	DCUN1D2	Hs.708150	DCN1, defective in cullin neddylation 1,	Homo sapiens DCN1, defective in cullin	70.37476	92.25849	76.21800	78.43066	Detected	Detected	Detected	Detected
7011	A_33_P3253304	LOC100270710		hypothetical LOC100270710	Homo sapiens hypothetical LOC100270	44.21044	62.37588	47.30441	47.20962	Detected	Detected	Detected	Detected
7012	A_23_P163567	SMPD3	Hs.368421	sphingomyelin phosphodiesterase 3, neu	Homo sapiens sphingomyelin phosphodi	18.40829	939.38586	2.77939	62.48648	Detected	Detected	Compromised	Detected
7013	A_33_P3354796	FLJ42627		hypothetical LOC645644	Homo sapiens hypothetical LOC645644	343.68497	419.51367	183.93665	184.82516	Detected	Detected	Detected	Detected
7014	A_23_P215175	ABCF2	Hs.654958	ATP-binding cassette, sub-family F (GC	Homo sapiens ATP-binding cassette, su	2458.73340	3572.31080	2049.19850	3121.55350	Detected	Detected	Detected	Detected
7015	A_23_P422178	CUL4B	Hs.102914	cullin 4B	Homo sapiens cullin 4B (CUL4B), trans	4208.70200	6050.21600	4061.50500	4911.61570	Detected	Detected	Detected	Detected
7016	A_33_P3324552					77.35424	67.51477	31.01563	21.94588	Detected	Detected	Detected	Detected
7017	A_33_P3319134		Hs.675915		Homo sapiens clone pp9372 unknown m	95.34293	66.41017	57.35764	31.60656	Detected	Detected	Detected	Detected
7018	A_33_P3219010				Inorganic pyrophosphatase 2, mitochon	15731.43200	18455.73400	28626.41600	27195.49400	Detected	Detected	Detected	Detected
7019	A_33_P3271196	AMOTL1	Hs.503594	angiomotin like 1	Homo sapiens angiomotin like 1 (AMOT	725.06540	681.31720	631.49460	544.80560	Detected	Detected	Detected	Detected
7020	A_32_P104063	CRNDE	Hs.237396	colorectal neoplasia differentially expres	PREDICTED: Homo sapiens hCG181549	24081.86100	9364.43400	17524.34200	5336.36040	Detected	Detected	Detected	Detected
7021	A_33_P3211956	RGS19	Hs.422336	regulator of G-protein signaling 19	Homo sapiens regulator of G-protein si	3226.06230	3285.25660	2773.08080	2190.42970	Detected	Detected	Detected	Detected
7022	A_33_P3380056		Hs.530272		UPF0439 protein C9orf30 [Source:UniP	497.94208	1080.03500	399.72693	785.58435	Detected	Detected	Detected	Detected
7023	A_33_P3303474	CYP4A11	Hs.1645	cytochrome P450, family 4, subfamily A	Homo sapiens cytochrome P450, family	84.81509	100.67703	28.24759	16.38229	Detected	Detected	Detected	Detected
7024	A_33_P3273233		Hs.678171		cDNA FLJ45526 fis, clone BRTHA2027	108.92349	115.48026	81.13523	72.55364	Detected	Detected	Detected	Detected
7025	A_23_P10077	PNPLA2	Hs.654697	patatin-like phospholipase domain conta	Homo sapiens patatin-like phospholipas	759.58970	633.97614	1099.53320	864.51996	Detected	Detected	Detected	Detected
7026	A_33_P3329750					32.80483	8.02918	33.68646	10.10606	Detected	Compromised	Detected	Compromised
7027	A_33_P3304452	LOC100128028	Hs.675276	hypothetical protein LOC100128028	Homo sapiens cDNA FLJ41161 fis, clon	133.21858	108.55209	14.33388	9.70562	Detected	Detected	Detected	Compromised
7028	A_32_P142881	SMG1	Hs.460179	SMG1 homolog, phosphatidylinositol 3-k	Homo sapiens SMG1 homolog, phospha	124.93031	110.15280	91.61254	64.83753	Detected	Detected	Detected	Detected
7029	A_32_P145153	RPL31	Hs.469473	ribosomal protein L31	Homo sapiens ribosomal protein L31 (R	170425.12000	128868.33000	109084.20000	72961.91000	Detected	Detected	Detected	Detected
7030	A_23_P46936	EGR2	Hs.1395	early growth response 2	Homo sapiens early growth response 2	286.86203	664.37650	210.38345	448.26780	Detected	Detected	Detected	Detected
7031	A_33_P3287314					295.47086	419.20874	124.03989	130.15552	Detected	Detected	Detected	Detected
7032	A_23_P11286	HNRNPH2	Hs.632828	heterogeneous nuclear ribonucleoprotei	Homo sapiens heterogeneous nuclear ri	5948.57800	4484.15970	4094.76560	2550.29470	Detected	Detected	Detected	Detected
7033	A_23_P426663	MITF	Hs.166017	microphthalmia-associated transcriptio	Homo sapiens microphthalmia-associat	549.13640	44.41470	367.20917	20.05947	Detected	Detected	Detected	Detected
7034	A_33_P3423979	PALLD	Hs.151220	palladin, cytoskeletal associated protein	Homo sapiens palladin, cytoskeletal ass	872.69570	1666.84890	485.42233	866.10380	Detected	Detected	Detected	Detected
7035	A_33_P3225882	BET1L	Hs.414418	blocked early in transport 1 homolog (S	Homo sapiens blocked early in transpor	315.61606	428.80554	393.82407	505.41318	Detected	Detected	Detected	Detected
7036	A_33_P3395310	LOC100130982	Hs.717171	hypothetical LOC100130982	PREDICTED: Homo sapiens hypothetica	50.35426	62.87877	39.82287	37.88488	Detected	Detected	Detected	Detected
7037	A_33_P3309929	HDAC3	Hs.519632	histone deacetylase 3	Homo sapiens histone deacetylase 3 (H	31182.26800	18828.72000	24186.54900	13129.47000	Detected	Detected	Detected	Detected
7038	A_33_P3407863					42.69673	41.81358	33.25561	26.29837	Detected	Detected	Detected	Detected
7039	A_33_P3293254	GPSM1	Hs.239370	G-protein signaling modulator 1 (AGS3-	Homo sapiens G-protein signaling modu	24.38764	54.51491	36.98367	60.55776	Detected	Detected	Detected	Detected
7040	A_23_P323272	OSR1	Hs.123933	odd-skipped related 1 (Drosophila)	Homo sapiens odd-skipped related 1 (D	234.41684	385.79688	183.19632	273.60236	Detected	Detected	Detected	Detected
7041	A_23_P393749	CATSPER3	Hs.631804	cation channel, sperm associated 3	Homo sapiens cation channel, sperm as	28.93592	35.55150	34.05087	19.73938	Detected	Detected	Detected	Detected
7042	A_33_P3256677	CCDC101	Hs.655476	coiled-coil domain containing 101	Homo sapiens coiled-coil domain conta	271.35233	255.93713	412.31604	334.83228	Detected	Detected	Detected	Detected
7043	A_23_P207940	PTPN2	Hs.654527	protein tyrosine phosphatase, non-rece	Homo sapiens protein tyrosine phospha	4398.26950	3993.61100	3191.89140	2506.48600	Detected	Detected	Detected	Detected
7044	A_33_P3399208		Hs.77961		HLA class I histocompatibility antigen, E	13922.18100	2168.60550	17484.75600	2802.14580	Detected	Detected	Detected	Detected
7045	A_24_P185604	NDRG3	Hs.437338	NDRG family member 3	Homo sapiens NDRG family member 3 (	1279.07890	672.11300	800.05970	350.60504	Detected	Detected	Detected	Detected
7046	A_33_P3238250	F11R	Hs.517293	F11 receptor	Homo sapiens F11 receptor (F11R), mR	26.57830	13.16831	16.69701	10.16820	Detected	Compromised	Detected	Compromised

7047	A_33_P3298430	FAM171A1	Hs.66762	family with sequence similarity 171, mer	Homo sapiens family with sequence sim	287.76627	461.84683	250.12212	395.17648	Detected	Detected	Detected	Detected
7048	A_24_P291658	ADH1A	Hs.654433	alcohol dehydrogenase 1A (class I), alph	Homo sapiens alcohol dehydrogenase 1.	5846.36330	10.59284	3907.21220	4.09455	Detected	Compromised	Detected	Compromised
7049	A_24_P112160	UPK3B	Hs.488861	uroplakin 3B	Homo sapiens uroplakin 3B (UPK3B), tr	189.72208	242.92023	180.06923	190.00409	Detected	Detected	Detected	Detected
7050	A_33_P3221384					218.55946	94.95828	209.27245	165.65850	Detected	Detected	Detected	Detected
7051	A_23_P415411	HIST1H4E	Hs.662174	histone cluster 1, H4e	Homo sapiens histone cluster 1, H4e (H	151.07298	325.15463	163.74376	283.37810	Detected	Detected	Detected	Detected
7052	A_33_P3250348	PCDH18	Hs.591691	protocadherin 18	Homo sapiens protocadherin 18 (PCDH	1938.88170	1215.51390	1628.31670	875.24054	Detected	Detected	Detected	Detected
7053	A_23_P70384	RNF8	Hs.485278	ring finger protein 8	Homo sapiens ring finger protein 8 (RNF	1184.84810	1776.19560	899.30510	1144.81600	Detected	Detected	Detected	Detected
7054	A_23_P50535	DMPK	Hs.631596	dystrophia myotonica-protein kinase	Homo sapiens dystrophia myotonica-pr	1900.48610	1282.00170	1794.22240	1025.86580	Detected	Detected	Detected	Detected
7055	A_33_P3248272	UBXN8	Hs.153678	UBX domain protein 8	Homo sapiens UBX domain protein 8 (U	239.31630	508.45520	251.12311	501.30320	Detected	Detected	Detected	Detected
7056	A_33_P3399064	LOC100008587	Hs.613975	5.8S ribosomal RNA	Homo sapiens 5.8S ribosomal RNA (LOC	2935.65230	2615.90260	2408.74020	1631.52320	Detected	Detected	Detected	Detected
7057	A_33_P3418055	ZNF235	Hs.298089	zinc finger protein 235	Homo sapiens zinc finger protein 235 (Z	36.01980	28.03680	37.67404	23.53239	Detected	Detected	Detected	Detected
7058	A_33_P3236846	GPR17	Hs.46453	G protein-coupled receptor 17	Homo sapiens G protein-coupled recep	15.88805	13.08389	17.01021	12.76600	Detected	Compromised	Detected	Compromised
7059	A_33_P3307475	LOC100133817	Hs.666068	hypothetical protein LOC100133817	PREDICTED: Homo sapiens hypothetica	12.22731	10.56407	12.81183	11.28990	Detected	Compromised	Compromised	Compromised
7060	A_23_P103282	TMEM59	Hs.523262	transmembrane protein 59	Homo sapiens transmembrane protein 5	23186.79300	23277.75400	21416.94500	18021.56600	Detected	Detected	Detected	Detected
7061	A_32_P31182	RPL7	Hs.571841	ribosomal protein L7	Homo sapiens ribosomal protein L7 (RP	7055.97070	6956.14550	6541.55100	5076.58540	Detected	Detected	Detected	Detected
7062	A_33_P3241756		Hs.597664		Inhibitor of nuclear factor kappa-B kina	12.59193	4.66137	14.43501	7.45312	Detected	Compromised	Detected	Compromised
7063	A_33_P3229002	C17orf50	Hs.127194	chromosome 17 open reading frame 50	Homo sapiens chromosome 17 open rea	166.00243	179.77853	53.49820	58.30288	Detected	Detected	Detected	Detected
7064	A_33_P3293446	KIAA1462	Hs.533953	KIAA1462	Homo sapiens KIAA1462 (KIAA1462), m	854.72015	3423.94460	757.26495	2633.29900	Detected	Detected	Detected	Detected
7065	A_33_P3399638	ZNF257	Hs.283900	zinc finger protein 257	Homo sapiens zinc finger protein 257 (Z	32.16019	73.80660	17.58391	46.13667	Detected	Detected	Detected	Detected
7066	A_24_P67534	LOC644538	Hs.42239	hypothetical protein LOC644538	Homo sapiens hypothetical protein LOC	109.90656	118.81612	109.65453	108.22759	Detected	Detected	Detected	Detected
7067	A_33_P3287567	BCAP29	Hs.303787	B-cell receptor-associated protein 29	Homo sapiens B-cell receptor-associat	4934.54600	4265.16200	3500.39280	2659.20560	Detected	Detected	Detected	Detected
7068	A_23_P64173	CARD16	Hs.348365	caspase recruitment domain family, mer	Homo sapiens caspase recruitment dom	191.75708	160.90855	141.23277	100.46581	Detected	Detected	Detected	Detected
7069	A_33_P3395733		Hs.534482		603027139F1 NIH_MGC_114 Homo sapie	17.43463	20.70727	19.18392	21.40829	Detected	Detected	Detected	Detected
7070	A_23_P96209	REEP4	Hs.289063	receptor accessory protein 4	Homo sapiens receptor accessory prote	356.71173	447.36550	410.49097	554.40436	Detected	Detected	Detected	Detected
7071	A_23_P390704	STK38	Hs.409578	serine/threonine kinase 38	Homo sapiens serine/threonine kinase 3	455.23877	446.80370	451.55743	380.58800	Detected	Detected	Detected	Detected
7072	A_33_P3268304	LIMS2	Hs.469881	LIM and senescent cell antigen-like dor	Homo sapiens LIM and senescent cell a	4083.51540	8671.07400	3601.13430	6526.89500	Detected	Detected	Detected	Detected
7073	A_33_P3248354	OLFM1	Hs.522484	olfactomedin 1	Homo sapiens olfactomedin 1 (OLFM1),	197.62769	246.58072	219.39012	240.00037	Detected	Detected	Detected	Detected
7074	A_33_P3317253	PTER	Hs.444321	phosphotriesterase related	Homo sapiens phosphotriesterase relate	94.17305	496.90990	67.41232	271.67593	Detected	Detected	Detected	Detected
7075	A_33_P3292417	WDR5	Hs.397638	WD repeat domain 5	Homo sapiens WD repeat domain 5 (WD	24693.79100	19880.45500	21391.84400	15893.14200	Detected	Detected	Detected	Detected
7076	A_33_P3287770	TELO2	Hs.271044	TEL2, telomere maintenance 2, homolog	Homo sapiens TEL2, telomere maintena	15913.46200	23040.29900	13760.86300	19817.98600	Detected	Detected	Detected	Detected
7077	A_24_P96961	SPSB1	Hs.8261	splA/ryanodine receptor domain and SO	Homo sapiens splA/ryanodine receptor	10291.22000	8407.30100	7532.75830	5976.32130	Detected	Detected	Detected	Detected
7078	A_24_P299355	GRAPL	Hs.677674	GRB2-related adaptor protein-like	Homo sapiens GRB2-related adaptor pr	24.56451	26.85251	6.94361	9.93207	Detected	Detected	Compromised	Compromised
7079	A_24_P335358	PUS1	Hs.592004	pseudouridylate synthase 1	Homo sapiens pseudouridylate synthase	1385.03810	1704.73360	1721.35570	2417.76780	Detected	Detected	Detected	Detected
7080	A_23_P151075	ARHGDIB	Hs.504877	Rho GDP dissociation inhibitor (GDI) be	Homo sapiens Rho GDP dissociation inh	850.53980	966.77290	727.40770	729.22034	Detected	Detected	Detected	Detected
7081	A_33_P3219840	NCRNA00171	Hs.653168	non-protein coding RNA 171	Homo sapiens non-protein coding RNA	105.55917	13.91801	89.23410	14.06738	Detected	Compromised	Detected	Compromised
7082	A_24_P182494	DUSP10	Hs.497822	dual specificity phosphatase 10	Homo sapiens dual specificity phosphat	638.47455	1152.40500	734.03580	1258.45140	Detected	Detected	Detected	Detected
7083	A_24_P109887		Hs.375105		LOC554207 protein [Source:UniProtKB	16.97433	11.23112	12.54624	4.99649	Detected	Compromised	Compromised	Compromised
7084	A_23_P400298	PRSS22	Hs.459709	protease, serine, 22	Homo sapiens protease, serine, 22 (PRS	35.85894	41.53428	30.71303	24.55147	Detected	Detected	Detected	Detected
7085	A_23_P501795	MED22	Hs.78354	mediator complex subunit 22	Homo sapiens mediator complex subuni	1766.15360	2015.20830	1908.43620	2191.15970	Detected	Detected	Detected	Detected
7086	A_23_P150950	ZFC3H1	Hs.527874	zinc finger, C3H1-type containing	Homo sapiens zinc finger, C3H1-type c	1582.86830	803.79480	1354.26700	635.91440	Detected	Detected	Detected	Detected
7087	A_23_P70060	PPAP2A	Hs.696231	phosphatidic acid phosphatase type 2A	Homo sapiens phosphatidic acid phosph	3647.43460	4521.67330	4041.74460	4408.61470	Detected	Detected	Detected	Detected
7088	A_24_P56330	C13orf16	Hs.210677	chromosome 13 open reading frame 16	Homo sapiens chromosome 13 open rea	647.93164	106.83939	615.80820	46.82224	Detected	Detected	Detected	Detected
7089	A_33_P3620488	CATSPERG	Hs.324335	cation channel, sperm-associated, gamr	Homo sapiens cation channel, sperm-as	533.60290	777.14410	217.84232	215.70215	Detected	Detected	Detected	Detected
7090	A_33_P3232204	LOC100128343	Hs.675262	hypothetical protein LOC100128343	Homo sapiens clone pp9378 unknown m	41.89986	50.68631	37.31885	48.48700	Detected	Detected	Detected	Detected
7091	A_33_P3364268	LBH	Hs.567598	limb bud and heart development homolo	Homo sapiens limb bud and heart devel	77.30021	705.02090	80.12980	549.08673	Detected	Detected	Detected	Detected
7092	A_33_P3347035	LOC100130401	Hs.655345	similar to PRO0641	Homo sapiens cDNA clone IMAGE:7262	17.02849	25.22709	21.78922	29.72016	Detected	Detected	Detected	Detected
7093	A_33_P3374833	PLDN	Hs.7037	pallidin homolog (mouse)	Homo sapiens pallidin homolog (mouse)	15418.04800	11978.27800	11049.03700	7375.98730	Detected	Detected	Detected	Detected
7094	A_33_P3401753	FFAR3	Hs.248055	free fatty acid receptor 3	Homo sapiens free fatty acid receptor 3	11.12367	8.48280	21.19775	18.60925	Compromised	Compromised	Detected	Detected
7095	A_23_P365086	ANKRD5	Hs.70903	ankyrin repeat domain 5	Homo sapiens ankyrin repeat domain 5	17.12206	7.97144	15.47770	3.13802	Detected	Compromised	Detected	Compromised
7096	A_32_P228775	PPP4R2	Hs.431092	protein phosphatase 4, regulatory subur	Homo sapiens protein phosphatase 4, re	396.05150	478.06943	336.94983	334.26614	Detected	Detected	Detected	Detected
7097	A_23_P324994	KLHL7	Hs.654817	kelch-like 7 (Drosophila)	Homo sapiens kelch-like 7 (Drosophila)	1577.45230	1154.36060	914.03970	555.09520	Detected	Detected	Detected	Detected
7098	A_23_P111188	ZBTB22	Hs.206770	zinc finger and BTB domain containing 2	Homo sapiens zinc finger and BTB dom	276.10490	276.49430	263.83572	229.52028	Detected	Detected	Detected	Detected
7099	A_23_P252642	BBS5	Hs.233398	Bardet-Biedl syndrome 5	Homo sapiens Bardet-Biedl syndrome 5	18413.91200	13275.42400	12924.90000	7730.23730	Detected	Detected	Detected	Detected
7100	A_24_P673063	FABP5	Hs.408061	fatty acid binding protein 5 (psoriasis-a	Homo sapiens fatty acid binding protein	2132.69120	3695.79080	2070.55440	3028.94360	Detected	Detected	Detected	Detected
7101	A_33_P3397066		Hs.640480		CR736467 Homo sapiens library (Ebert	16.12217	18.04307	17.12843	21.34982	Detected	Compromised	Detected	Detected
7102	A_33_P3408844	LOC100133091	Hs.712837	hypothetical LOC100133091	Homo sapiens hypothetical LOC100133	296.24002	346.12177	240.70334	230.83887	Detected	Detected	Detected	Detected
7103	A_33_P3230414	ZNF774	Hs.55307	zinc finger protein 774	Homo sapiens zinc finger protein 774 (Z	21.12307	35.95339	15.04888	21.24899	Detected	Detected	Detected	Detected
7104	A_33_P3424207	RABEP2	Hs.555978	rabaptin, RAB GTPase binding effector	Homo sapiens rabaptin, RAB GTPase bi	3258.06100	8377.60900	3548.11280	8863.38300	Detected	Detected	Detected	Detected
7105	A_23_P84836	NPEPPS	Hs.443837	aminopeptidase puromycin sensitive	Homo sapiens aminopeptidase puromyc	1511.69730	786.41150	1139.63110	547.38885	Detected	Detected	Detected	Detected
7106	A_33_P3399988	HCP5P10	Hs.662200	HLA complex P5 pseudogene 10	Homo sapiens HLA complex P5 pseudog	19.09507	7.72116	23.92854	8.52175	Detected	Compromised	Detected	Compromised
7107	A_23_P230	TTC4	Hs.720251	tetratricopeptide repeat domain 4	Homo sapiens tetratricopeptide repeat	858.17990	1483.58420	815.21497	1380.97900	Detected	Detected	Detected	Detected
7108	A_23_P150080	RPP38	Hs.94986	ribonuclease P/MRP 38kDa subunit	Homo sapiens ribonuclease P/MRP 38k	2528.62300	2737.97900	2414.36600	2335.73970	Detected	Detected	Detected	Detected
7109	A_33_P3275600	PLA2G4E	Hs.668060	phospholipase A2, group IVE	Homo sapiens phospholipase A2, group	7.66141	8.73622	21.15447	17.08173	Compromised	Compromised	Detected	Detected
7110	A_33_P3339375	ARHGAP11B	Hs.659621	Rho GTPase activating protein 11B	Homo sapiens Rho GTPase activating p	19.36673	445.88050	16.37650	297.87656	Detected	Detected	Detected	Detected
7111	A_24_P169634	MBL1P1	Hs.102310	mannose-binding lectin (protein A) 1, ps	Homo sapiens mannose-binding lectin (	3.97766	26.64194	8.55314	37.48676	Compromised	Detected	Compromised	Detected
7112	A_33_P3260445		Hs.445999		Dynein light chain Tctex-type 1 (T-com	171.99512	126.06567	144.85722	86.73609	Detected	Detected	Detected	Detected
7113	A_23_P368154	PODN	Hs.586141	podocan	Homo sapiens podocan (PODN), mRNA	6.83837	56.34272	7.41624	66.12500	Compromised	Detected	Compromised	Detected
7114	A_23_P385427		Hs.721709		Putative zinc finger protein 56 (Putativ	428.57513	583.30770	155.56775	165.80333	Detected	Detected	Detected	Detected
7115	A_23_P16573	DDX49	Hs.143187	DEAD (Asp-Glu-Ala-Asp) box polypept	Homo sapiens DEAD (Asp-Glu-Ala-Asp	1189.94850	1578.40470	1047.55080	1314.78920	Detected	Detected	Detected	Detected
7116	A_23_P352389	C17orf46	Hs.412719	chromosome 17 open reading frame 46	Homo sapiens chromosome 17 open rea	24.13543	35.58738	25.55820	23.20413	Detected	Detected	Detected	Detected
7117	A_23_P17955	FBXL2	Hs.475872	F-box and leucine-rich repeat protein 2	Homo sapiens F-box and leucine-rich r	159.25587	176.19977	137.37338	114.60170	Detected	Detected	Detected	Detected
7118	A_33_P3351851	HIST1H4L	Hs.533295	histone cluster 1, H4l	Homo sapiens histone cluster 1, H4l (HI	6.44134	39.27768	5.11999	26.48525	Compromised	Detected	Compromised	Detected
7119	A_23_P4919	KDELR1	Hs.515515	KDEL (Lys-Asp-Glu-Leu) endoplasmic	Homo sapiens KDEL (Lys-Asp-Glu-Leu	3369.74760	7017.56300	2952.00830	4946.59030	Detected	Detected	Detected	Detected
7120	A_23_P131050	ACSBG2	Hs.465720	acyl-CoA synthetase bubblegum family	Homo sapiens acyl-CoA synthetase bub	9.52741	15.23515	13.78467	11.67883	Compromised	Compromised	Detected	Compromised
7121	A_23_P41942	POLR3G	Hs.282387	polymerase (RNA) III (DNA directed) po	Homo sapiens polymerase (RNA) III (DN	39.33811	56.78059	51.69717	47.28400	Detected	Detected	Detected	Detected

7122	A_24_P204238	tcag7.1239	Hs.632023	similar to Huntingtin interacting protein	PREDICTED: Homo sapiens similar to H	2735.34330	3307.40380	151.12178	114.04500	Detected	Detected	Detected	Detected
7123	A_23_P162945	SRP54	Hs.167535	signal recognition particle 54kDa	Homo sapiens signal recognition particle	2750.59960	3264.13350	1728.69590	1698.51770	Detected	Detected	Detected	Detected
7124	A_23_P161171	ASAH2	Hs.512645	N-acylsphingosine amidohydrolase (non	Homo sapiens N-acylsphingosine amido	53.73758	62.81629	32.54276	42.32240	Detected	Detected	Detected	Detected
7125	A_33_P3362727				Heterogeneous nuclear ribonucleoprote	30.63105	24.01363	20.22239	10.47958	Detected	Detected	Detected	Compromised
7126	A_33_P3235891				Homo sapiens cDNA FLJ46681 fis, clon	653.71765	624.92180	136.08562	110.65773	Detected	Detected	Detected	Detected
7127	A_23_P62741	ELTD1	Hs.132314	EGF, latrophilin and seven transmembra	Homo sapiens EGF, latrophilin and seve	828.61940	975.35516	750.67460	775.44460	Detected	Detected	Detected	Detected
7128	A_23_P14886	GNPTG	Hs.241575	N-acetylglucosamine-1-phosphate tran	Homo sapiens N-acetylglucosamine-1-p	10074.59600	9155.39900	11073.65600	9304.39000	Detected	Detected	Detected	Detected
7129	A_33_P3283718	LOC285696	Hs.646924	hypothetical LOC285696	Homo sapiens cDNA FLJ43202 fis, clon	16.90122	9.86679	4.64049	8.06870	Detected	Compromised	Compromised	Compromised
7130	A_23_P19657	LRP11	Hs.408355	low density lipoprotein receptor-related	Homo sapiens low density lipoprotein re	622.68680	365.06815	551.68990	293.34393	Detected	Detected	Detected	Detected
7131	A_24_P108301	RTTN	Hs.654809	rotatin	Homo sapiens rotatin (RTTN), mRNA [N	1398.65950	881.92930	876.07870	483.74893	Detected	Detected	Detected	Detected
7132	A_33_P3329187	DNMT1	Hs.202672	DNA (cytosine-5-)-methyltransferase 1	Homo sapiens DNA (cytosine-5-)-meth	3086.66480	3865.63720	1025.23880	990.61120	Detected	Detected	Detected	Detected
7133	A_23_P28084	STX10		syntaxin 10	Syntaxin-10 (Syn10) [Source:UniProtKE	6344.75900	4088.60400	8228.66100	4741.56740	Detected	Detected	Detected	Detected
7134	A_23_P121106	HESX1	Hs.171980	HESX homeobox 1	Homo sapiens HESX homeobox 1 (HESX	68.82225	16.86980	65.66126	6.92329	Detected	Detected	Detected	Compromised
7135	A_33_P3232627		Hs.303454		Exocyst complex component 6B (Exocy	44.66393	61.00870	26.30289	15.65480	Detected	Detected	Detected	Detected
7136	A_24_P105391	ERMAP	Hs.439437	erythroblast membrane-associated prot	Homo sapiens erythroblast membrane-a	207.08577	119.55983	193.45450	101.98321	Detected	Detected	Detected	Detected
7137	A_24_P941376	ZNF473	Hs.440553	zinc finger protein 473	Homo sapiens zinc finger protein 473 (Z	208.07450	145.32579	136.56580	95.64018	Detected	Detected	Detected	Detected
7138	A_23_P97394	BCAR3	Hs.36958	breast cancer anti-estrogen resistance	Homo sapiens breast cancer anti-estro	3851.13230	9854.52800	2828.53880	6070.65530	Detected	Detected	Detected	Detected
7139	A_33_P3316639	VPS24	Hs.591582	vacuolar protein sorting 24 homolog (S.	Homo sapiens vacuolar protein sorting 2	1130.55430	2237.12770	1200.52310	2128.80790	Detected	Detected	Detected	Detected
7140	A_23_P502627	ZXDA	Hs.591214	zinc finger, X-linked, duplicated A	Homo sapiens zinc finger, X-linked, dup	19.93386	21.46882	24.31918	26.57486	Detected	Detected	Detected	Detected
7141	A_23_P310	MARCKSL1	Hs.75061	MARCKS-like 1	Homo sapiens MARCKS-like 1 (MARCK	542.26430	1166.36080	595.13000	1240.79990	Detected	Detected	Detected	Detected
7142	A_23_P61447	ETFDH	Hs.155729	electron-transferring-flavoprotein dehy	Homo sapiens electron-transferring-fla	3135.77830	2937.97630	2671.51760	2115.63090	Detected	Detected	Detected	Detected
7143	A_33_P3302796	U2AF2	Hs.528007	U2 small nuclear RNA auxiliary factor 2	Homo sapiens U2 small nuclear RNA au	15374.16300	19828.99400	15247.44900	19327.67400	Detected	Detected	Detected	Detected
7144	A_23_P211047	BACH1	Hs.154276	BTB and CNC homology 1, basic leucin	Homo sapiens BTB and CNC homology	4200.22560	2965.89580	3766.77760	2183.56420	Detected	Detected	Detected	Detected
7145	A_33_P3310047	SHROOM4	Hs.420541	shroom family member 4	Homo sapiens shroom family member 4	876.08680	795.94354	264.50226	186.49153	Detected	Detected	Detected	Detected
7146	A_23_P30630		Hs.279008		Homo sapiens cDNA FLJ20170 fis, clon	29.71254	78.18810	22.37025	61.05659	Detected	Detected	Detected	Detected
7147	A_24_P41408		Hs.683855		cDNA FLJ38166 fis, clone DFNES2005	58.79561	50.43039	31.07081	28.63034	Detected	Detected	Detected	Detected
7148	A_24_P166663	CDK6	Hs.119882	cyclin-dependent kinase 6	Homo sapiens cyclin-dependent kinase	3692.00600	12714.48800	2710.45950	7249.11570	Detected	Detected	Detected	Detected
7149	A_23_P352717	TMEM31	Hs.98843	transmembrane protein 31	Homo sapiens transmembrane protein 3	52.78613	18.91105	40.44062	19.16273	Detected	Detected	Detected	Detected
7150	A_33_P3368900					334.99655	479.17804	373.60083	456.16780	Detected	Detected	Detected	Detected
7151	A_23_P307844	PHYHD1	Hs.709447	phytanoyl-CoA dioxygenase domain cor	Homo sapiens phytanoyl-CoA dioxygena	705.89100	160.91031	482.79248	100.34256	Detected	Detected	Detected	Detected
7152	A_32_P192474	PRRT1	Hs.699821	proline-rich transmembrane protein 1	Homo sapiens proline-rich transmembra	437.59653	393.31990	229.55495	106.46890	Detected	Detected	Detected	Detected
7153	A_23_P71752	ZFAND5	Hs.406096	zinc finger, AN1-type domain 5	Homo sapiens zinc finger, AN1-type do	31059.06200	18865.42400	26120.52700	14234.47900	Detected	Detected	Detected	Detected
7154	A_33_P3352053	LOC100132716	Hs.672618	hypothetical LOC100132716	Homo sapiens cDNA FLJ43840 fis, clon	194.22113	310.96042	105.03439	143.18400	Detected	Detected	Detected	Detected
7155	A_32_P26376	KTELC1	Hs.231750	KTEL (Lys-Tyr-Glu-Leu) containing 1	Homo sapiens KTEL (Lys-Tyr-Glu-Leu)	1412.56250	1088.44000	915.94250	565.77850	Detected	Detected	Detected	Detected
7156	A_33_P3316572	LOC100128007		similar to Spi-C transcription factor (Sp	PREDICTED: Homo sapiens similar to S	2.44336	22.30797	6.27096	2.91598	Compromised	Detected	Compromised	Compromised
7157	A_24_P345679	MLF1	Hs.85195	myeloid leukemia factor 1	Homo sapiens myeloid leukemia factor 1	294.87690	198.59514	260.44220	155.80977	Detected	Detected	Detected	Detected
7158	A_24_P648176	LYRM5	Hs.209151	LYR motif containing 5	Homo sapiens LYR motif containing 5 (L	395.81570	342.08362	255.97403	187.22215	Detected	Detected	Detected	Detected
7159	A_33_P3341224	PCTK2	Hs.506415	PCTAIRE protein kinase 2	Homo sapiens PCTAIRE protein kinase	744.30054	353.57413	535.92790	206.98094	Detected	Detected	Detected	Detected
7160	A_33_P3277140	C1orf151	Hs.466662	chromosome 1 open reading frame 151	Homo sapiens chromosome 1 open read	41365.20700	65175.13300	42862.66800	56333.74000	Detected	Detected	Detected	Detected
7161	A_33_P3251093	SLC36A1	Hs.269004	solute carrier family 36 (proton/amino a	Homo sapiens solute carrier family 36 (	3973.72920	4247.65870	2727.70920	2480.02220	Detected	Detected	Detected	Detected
7162	A_23_P79836	SERINC3	Hs.272168	serine incorporator 3	Homo sapiens serine incorporator 3 (SE	9931.27000	9074.21600	7122.52440	5788.47300	Detected	Detected	Detected	Detected
7163	A_33_P3306207	KLRG2	Hs.17572	killer cell lectin-like receptor subfamily	Homo sapiens killer cell lectin-like rece	471.64215	588.55350	332.63720	327.70538	Detected	Detected	Detected	Detected
7164	A_32_P155666	ECEL1	Hs.26880	endothelin converting enzyme-like 1	Homo sapiens endothelin converting en	2.43232	3.28965	20.10550	2.79834	Compromised	Compromised	Detected	Compromised
7165	A_23_P68211	SPR	Hs.301540	sepiapterin reductase (7,8-dihydrobiopt	Homo sapiens sepiapterin reductase (7,	1078.17680	1054.49770	1156.08500	961.18695	Detected	Detected	Detected	Detected
7166	A_33_P3372288		Hs.114246		Zinc finger protein 81 (HFZ20) [Source:	31.95927	50.54675	19.33180	18.25527	Detected	Detected	Detected	Detected
7167	A_23_P59798	MKRN1	Hs.490347	makorin ring finger protein 1	Homo sapiens makorin ring finger protei	12908.59200	13708.78500	11459.97200	10018.60400	Detected	Detected	Detected	Detected
7168	A_33_P3308158					1864.71250	1751.77510	753.45166	544.66406	Detected	Detected	Detected	Detected
7169	A_32_P130788	SAMD13	Hs.591445	sterile alpha motif domain containing 13	Homo sapiens sterile alpha motif domai	141.98491	30.69155	121.84958	14.76097	Detected	Detected	Detected	Detected
7170	A_24_P933011	TMEM200C	Hs.346566	transmembrane protein 200C	Homo sapiens transmembrane protein 2	30.19460	37.66693	22.83115	17.63998	Detected	Detected	Detected	Detected
7171	A_24_P941038	C10orf72	Hs.522928	chromosome 10 open reading frame 72	Homo sapiens chromosome 10 open rea	190.67654	106.95199	195.41124	97.64970	Detected	Detected	Detected	Detected
7172	A_33_P3512350	LOC339807	Hs.434736	hypothetical protein LOC339807	Homo sapiens hypothetical protein LOC	69.03314	101.11742	50.43181	68.42209	Detected	Detected	Detected	Detected
7173	A_23_P102258	MRPL53	Hs.534527	mitochondrial ribosomal protein L53	Homo sapiens mitochondrial ribosomal p	11810.27300	15248.48700	8999.43200	10418.64500	Detected	Detected	Detected	Detected
7174	A_23_P169154	ERMP1	Hs.591078	endoplasmic reticulum metallopeptidase	Homo sapiens endoplasmic reticulum m	72.55721	51.35657	64.78037	47.70816	Detected	Detected	Detected	Detected
7175	A_33_P3243917	OBP2B	Hs.449629	odorant binding protein 2B	Homo sapiens odorant binding protein 2	21.09048	30.95299	17.69152	22.54322	Detected	Detected	Detected	Detected
7176	A_24_P336931	ANKRD36	Hs.532921	ankyrin repeat domain 36	Homo sapiens ankyrin repeat domain 36	410.46304	461.10800	309.31888	312.97665	Detected	Detected	Detected	Detected
7177	A_24_P832156	FAM120AOS	Hs.350364	family with sequence similarity 120A op	Homo sapiens family with sequence sim	4302.30800	4940.08450	3055.62230	3082.82710	Detected	Detected	Detected	Detected
7178	A_24_P374427	ZDHHC21	Hs.649522	zinc finger, DHHC-type containing 21	Homo sapiens zinc finger, DHHC-type c	22.71465	18.79182	19.06180	11.97489	Detected	Detected	Detected	Compromised
7179	A_33_P3258699	DUX4	Hs.553518	double homeobox, 4	Homo sapiens double homeobox, 4 (DUX	197.70680	262.75836	136.26894	134.49197	Detected	Detected	Detected	Detected
7180	A_33_P3217119	OSCP1	Hs.202207	organic solute carrier partner 1	Homo sapiens organic solute carrier par	1401.55460	1387.94070	1034.99390	880.90674	Detected	Detected	Detected	Detected
7181	A_33_P3229288	ACE	Hs.654434	angiotensin I converting enzyme (peptid	Homo sapiens angiotensin I converting e	463.38382	637.25134	208.11343	223.95470	Detected	Detected	Detected	Detected
7182	A_33_P3375348					42.12613	55.32987	48.87037	62.07386	Detected	Detected	Detected	Detected
7183	A_33_P3244998	TMEM212	Hs.580330	transmembrane protein 212	Homo sapiens transmembrane protein 2	15255.12100	12959.05300	8795.99800	6486.00540	Detected	Detected	Detected	Detected
7184	A_23_P121885	ROPN1L	Hs.381089	ropporin 1-like	Homo sapiens ropporin 1-like (ROPN1L	189.76573	74.45407	171.42032	50.53081	Detected	Detected	Detected	Detected
7185	A_23_P132644	NCEH1	Hs.444099	neutral cholesterol ester hydrolase 1	Homo sapiens neutral cholesterol ester	1132.31040	7846.91100	800.06177	4653.32030	Detected	Detected	Detected	Detected
7186	A_23_P207927	C17orf80	Hs.12929	chromosome 17 open reading frame 80	Homo sapiens chromosome 17 open rea	1196.74600	691.02190	1224.52620	633.45880	Detected	Detected	Detected	Detected
7187	A_23_P434301	PTMA	Hs.459927	prothymosin, alpha	Homo sapiens prothymosin, alpha (PTM	31704.12700	33875.70000	34860.62000	33903.81600	Detected	Detected	Detected	Detected
7188	A_32_P7204	RAB28	Hs.656060	RAB28, member RAS oncogene family	Homo sapiens RAB28, member RAS onc	226.60443	239.27350	160.86638	129.46022	Detected	Detected	Detected	Detected
7189	A_24_P110799	LSS	Hs.596543	lanosterol synthase (2,3-oxidosqualene-	Homo sapiens lanosterol synthase (2,3-	4679.29600	5875.56400	3953.14940	4710.44530	Detected	Detected	Detected	Detected
7190	A_23_P85800	CD52	Hs.276770	CD52 molecule	Homo sapiens CD52 molecule (CD52), r	507.29092	17.52693	446.61680	8.51103	Detected	Compromised	Detected	Compromised
7191	A_32_P28402	LRRC37B2		leucine rich repeat containing 37, memb	Homo sapiens leucine rich repeat conta	712.66974	748.60870	408.49762	355.03278	Detected	Detected	Detected	Detected
7192	A_33_P3305731	NINL	Hs.631508	ninein-like	Homo sapiens ninein-like (NINL), mRNA	159.46797	37.16459	126.57566	32.12083	Detected	Detected	Detected	Detected
7193	A_23_P251377	C3orf37	Hs.458320	chromosome 3 open reading frame 37	Homo sapiens chromosome 3 open read	1336.72610	1354.65550	1300.60730	1233.10000	Detected	Detected	Detected	Detected
7194	A_33_P3298455		Hs.648259		Em:AC005003.4 protein [Source:UniPro	43.11263	16.02244	37.16148	14.84233	Detected	Compromised	Detected	Compromised
7195	A_23_P346912	SPATA17	Hs.171130	spermatogenesis associated 17	Homo sapiens spermatogenesis associa	81.48010	23.98258	70.66014	16.43363	Detected	Detected	Detected	Detected
7196	A_32_P34920	FOXD1	Hs.519385	forkhead box D1	Homo sapiens forkhead box D1 (FOXD1	5160.05900	17783.90000	4136.47700	11259.78700	Detected	Detected	Detected	Detected

7197	A_24_P170234	RNF148	Hs.675191	ring finger protein 148	Homo sapiens ring finger protein 148 (R	15.48232	11.32320	8.13730	12.47276	Detected	Compromised	Compromised	Compromised
7198	A_33_P3270369	LOC100128342	Hs.672522	hypothetical protein LOC100128342	Homo sapiens cDNA FLJ26896 fis, clon	32.05883	47.14226	23.86891	27.11267	Detected	Detected	Detected	Detected
7199	A_23_P149221	HECTD3	Hs.525084	HECT domain containing 3	Homo sapiens HECT domain containing	192.83005	168.07808	204.73761	187.70190	Detected	Detected	Detected	Detected
7200	A_24_P84008	LOC441722	Hs.535106	U2 small nuclear RNA auxiliary factor 1	PREDICTED: Homo sapiens similar to U	1260.04470	1672.82600	1462.32140	2004.13810	Detected	Detected	Detected	Detected
7201	A_32_P215938	GPSM1	Hs.239370	G-protein signaling modulator 1 (AGS3-	Homo sapiens G-protein signaling modu	47622.83200	49791.43800	37611.16800	35777.03500	Detected	Detected	Detected	Detected
7202	A_33_P3236632	MIA3	Hs.118474	melanoma inhibitory activity family, men	Homo sapiens melanoma inhibitory activ	463.77084	391.00520	544.06384	345.31345	Detected	Detected	Detected	Detected
7203	A_23_P210581	KCNG1	Hs.118695	potassium voltage-gated channel, subfa	Homo sapiens potassium voltage-gated	1568.94460	3360.65140	1314.69450	2682.33840	Detected	Detected	Detected	Detected
7204	A_23_P384635	DZIP1L	Hs.351403	DAZ interacting protein 1-like	Homo sapiens DAZ interacting protein	546.14264	714.13590	240.86362	234.96844	Detected	Detected	Detected	Detected
7205	A_33_P3273143		Hs.660384		Solute carrier family 35 member E4 [S	16.36342	4.04610	2.77077	11.46634	Detected	Compromised	Compromised	Compromised
7206	A_23_P13852	STRAP	Hs.504895	serine/threonine kinase receptor assoc	Homo sapiens serine/threonine kinase	69986.71000	89258.01000	48125.59800	52432.96000	Detected	Detected	Detected	Detected
7207	A_33_P3228837	CD8A	Hs.85258	CD8a molecule	Homo sapiens CD8a molecule (CD8A),	366.02917	334.23764	142.47390	110.11389	Detected	Detected	Detected	Detected
7208	A_23_P218637	RGPD5	Hs.469630	RANBP2-like and GRIP domain contain	Homo sapiens RANBP2-like and GRIP	4617.48200	4440.71800	3321.61250	2725.76780	Detected	Detected	Detected	Detected
7209	A_33_P3492042	D21S2091E	Hs.561028	D21S2091E	Homo sapiens D21S2091E mRNA seque	2.44662	3.33544	8.69196	15.57333	Compromised	Compromised	Compromised	Detected
7210	A_23_P25475	SOAT2	Hs.656544	sterol O-acyltransferase 2	Homo sapiens sterol O-acyltransferase	75.64575	96.40365	41.17838	58.23792	Detected	Detected	Detected	Detected
7211	A_24_P73943	COX11	Hs.591171	COX11 homolog, cytochrome c oxidase	Homo sapiens COX11 homolog, cytochr	562.36334	373.78513	381.43933	198.45866	Detected	Detected	Detected	Detected
7212	A_23_P52227	GDF10	Hs.2171	growth differentiation factor 10	Homo sapiens growth differentiation fac	53.35391	12.38138	45.57743	2.83467	Detected	Compromised	Detected	Compromised
7213	A_33_P3344406	MEF2B	Hs.153629	myocyte enhancer factor 2B	Homo sapiens myocyte enhancer factor	820.90704	551.74970	900.80860	571.50250	Detected	Detected	Detected	Detected
7214	A_23_P163639	ANKRD11	Hs.335003	ankyrin repeat domain 11	Homo sapiens ankyrin repeat domain 11	1913.34620	3426.52340	2290.59960	3571.82200	Detected	Detected	Detected	Detected
7215	A_32_P173298	SF3B3	Hs.514435	splicing factor 3b, subunit 3, 130kDa	Homo sapiens splicing factor 3b, subuni	7.87145	11.70448	15.29840	10.84230	Compromised	Compromised	Detected	Compromised
7216	A_23_P68899	TXN2	Hs.211929	thioredoxin 2	Homo sapiens thioredoxin 2 (TXN2), nu	3946.07400	3828.02800	3819.06570	3382.13300	Detected	Detected	Detected	Detected
7217	A_33_P3211229	SLC35F2	Hs.524014	solute carrier family 35, member F2	Homo sapiens solute carrier family 35,	70.30839	113.03859	77.75462	81.31470	Detected	Detected	Detected	Detected
7218	A_33_P3230990	SCUBE1	Hs.133995	signal peptide, CUB domain, EGF-like 1	Homo sapiens signal peptide, CUB dom	92.77337	155.27393	67.71291	71.06904	Detected	Detected	Detected	Detected
7219	A_33_P3399150	FLJ42258	Hs.712208	FLJ42258 protein	Homo sapiens FLJ42258 protein, mRNA	15.07984	22.66677	6.89596	8.14443	Detected	Detected	Compromised	Compromised
7220	A_33_P3281572	CMAH	Hs.484918	cytidine monophosphate-N-acetylneura	Homo sapiens cytidine monophosphate-	53.72680	63.07251	46.21258	40.76316	Detected	Detected	Detected	Detected
7221	A_23_P137103	EIF4A1	Hs.129673	eukaryotic translation initiation factor 4	Homo sapiens eukaryotic translation initi	44825.11700	67825.29000	46201.01600	58483.96000	Detected	Detected	Detected	Detected
7222	A_33_P3266080	TAF6L	Hs.714400	TAF6-like RNA polymerase II, p300/CB	Homo sapiens TAF6-like RNA polymera	2236.53470	2599.58860	2314.95480	2393.37480	Detected	Detected	Detected	Detected
7223	A_23_P12477	SCMH1	Hs.571874	sex comb on midleg homolog 1 (Drosop	Homo sapiens sex comb on midleg hom	495.29874	582.09470	586.24005	667.49230	Detected	Detected	Detected	Detected
7224	A_24_P66932	LOC730107	Hs.646666	similar to Glycine cleavage system H pr	PREDICTED: Homo sapiens similar to G	737.61290	747.88460	114.69530	96.84631	Detected	Detected	Detected	Detected
7225	A_32_P226149	YWHAZ	Hs.492407	tyrosine 3-monooxygenase/tryptophan	Homo sapiens tyrosine 3-monooxygena	7637.93400	13365.02400	6600.12300	10275.85600	Detected	Detected	Detected	Detected
7226	A_23_P108342	ZNF571	Hs.590944	zinc finger protein 571	Homo sapiens zinc finger protein 571 (Z	89.53955	60.47379	61.38794	35.71553	Detected	Detected	Detected	Detected
7227	A_23_P409951	OAZ1	Hs.446427	ornithine decarboxylase antizyme 1	Homo sapiens ornithine decarboxylase	309426.80000	275334.10000	222538.98000	178776.05000	Detected	Detected	Detected	Detected
7228	A_23_P7144	CXCL1	Hs.789	chemokine (C-X-C motif) ligand 1 (mela	Homo sapiens chemokine (C-X-C motif	25784.42000	24184.34200	19118.27500	15976.75700	Detected	Detected	Detected	Detected
7229	A_24_P152356					14701.10100	11239.86400	11353.36900	8092.91000	Detected	Detected	Detected	Detected
7230	A_23_P333129				D4S2463 homeobox-like Fragment [So	61898.99200	69272.45000	101156.69000	101019.43000	Detected	Detected	Detected	Detected
7231	A_23_P252362	MRPS30	Hs.124165	mitochondrial ribosomal protein S30	Homo sapiens mitochondrial ribosomal	695.43665	879.04820	615.68940	623.23160	Detected	Detected	Detected	Detected
7232	A_23_P34733	FH	Hs.592490	fumarate hydratase	Homo sapiens fumarate hydratase (FH),	6526.12300	8027.43900	8464.56500	9122.70400	Detected	Detected	Detected	Detected
7233	A_24_P305223	CTAGE1	Hs.406709	cutaneous T-cell lymphoma-associated	Homo sapiens cutaneous T-cell lympho	95.17258	93.91565	69.96144	57.75960	Detected	Detected	Detected	Detected
7234	A_33_P3401452	CHD6	Hs.720576	chromodomain helicase DNA binding pr	Homo sapiens chromodomain helicase D	32.38490	19.20777	34.23957	19.80135	Detected	Detected	Detected	Detected
7235	A_23_P115872	CEP55	Hs.14559	centrosomal protein 55kDa	Homo sapiens centrosomal protein 55kD	178.33205	10675.41700	124.08308	6484.93360	Detected	Detected	Detected	Detected
7236	A_33_P3404959	C2orf68	Hs.516159	chromosome 2 open reading frame 68	Homo sapiens chromosome 2 open read	935.58890	1188.44230	909.39606	1057.50560	Detected	Detected	Detected	Detected
7237	A_23_P452	DDR2	Hs.593833	discoidin domain receptor tyrosine kina	Homo sapiens discoidin domain recepto	641.39040	827.21610	682.29070	779.49800	Detected	Detected	Detected	Detected
7238	A_23_P397543	NCRNA00174	Hs.50755	non-protein coding RNA 174	Homo sapiens non-protein coding RNA	118.46078	24.11794	60.70594	16.50932	Detected	Detected	Detected	Detected
7239	A_33_P3352887	LOC388692	Hs.723057	hypothetical LOC388692	Homo sapiens hypothetical LOC388692	151.87473	104.34677	145.78387	83.03050	Detected	Detected	Detected	Detected
7240	A_23_P152002	BCL2A1	Hs.227817	BCL2-related protein A1	Homo sapiens BCL2-related protein A1	167.29521	20.48223	149.31548	11.12467	Detected	Detected	Detected	Compromised
7241	A_23_P45345	HTATSF1	Hs.204475	HIV-1 Tat specific factor 1	Homo sapiens HIV-1 Tat specific factor	12348.44700	12596.63200	8921.45400	7401.65400	Detected	Detected	Detected	Detected
7242	A_33_P3258339	C17orf76	Hs.25425	chromosome 17 open reading frame 76	Homo sapiens chromosome 17 open rea	47.81069	666.62280	34.30236	160.46663	Detected	Detected	Detected	Detected
7243	A_33_P3306217	TRIM39	Hs.413493	tripartite motif-containing 39	Homo sapiens tripartite motif-containin	181.60272	205.32507	135.75327	128.92390	Detected	Detected	Detected	Detected
7244	A_24_P118472	LOC100131785	Hs.601625	similar to ring finger protein 181	PREDICTED: Homo sapiens similar to r	2962.53320	3054.43870	2200.61650	1831.97830	Detected	Detected	Detected	Detected
7245	A_23_P358195	GOLGA6L10	Hs.715226	golgi autoantigen, golgin subfamily a, 6-	Homo sapiens golgi autoantigen, golgin	1384.57210	1400.95800	513.61800	332.49606	Detected	Detected	Detected	Detected
7246	A_23_P161190	VIM	Hs.628678	vimentin	Homo sapiens vimentin (VIM), mRNA [N	346050.56000	405479.28000	215145.40000	245570.70000	Detected	Detected	Detected	Detected
7247	A_24_P401645	AP4S1	Hs.293411	adaptor-related protein complex 4, sigm	Homo sapiens adaptor-related protein	56.60312	107.36290	35.65637	46.09366	Detected	Detected	Detected	Detected
7248	A_33_P3265260					16.87539	3.68333	10.96474	3.50885	Detected	Compromised	Compromised	Compromised
7249	A_24_P196592	MMP28	Hs.380710	matrix metallopeptidase 28	Homo sapiens matrix metallopeptidase	559.88600	735.02880	291.23770	329.13583	Detected	Detected	Detected	Detected
7250	A_33_P3287379	COG8	Hs.130849	component of oligomeric golgi complex	Homo sapiens component of oligomeric	1791.98580	1631.42930	1461.52120	1200.40950	Detected	Detected	Detected	Detected
7251	A_33_P3229722	PSMF1	Hs.471917	proteasome (prosome, macropain) inhib	Homo sapiens proteasome (prosome, m	743.67596	749.41580	627.87330	585.37225	Detected	Detected	Detected	Detected
7252	A_33_P3404331	MAP3K7IP2	Hs.269775	mitogen-activated protein kinase kinase	Homo sapiens mitogen-activated protei	99.83236	64.77137	106.18350	74.72247	Detected	Detected	Detected	Detected
7253	A_33_P3275687	TBC1D3B	Hs.454713	TBC1 domain family, member 3B	Homo sapiens TBC1 domain family, mer	51.13411	5.99428	44.80544	6.24887	Detected	Compromised	Detected	Compromised
7254	A_23_P369987	KIAA0317	Hs.497417	KIAA0317	Homo sapiens KIAA0317 (KIAA0317), m	2408.83900	2618.79700	1777.58150	1812.80370	Detected	Detected	Detected	Detected
7255	A_33_P3368471	NT5DC1	Hs.719198	5'-nucleotidase domain containing 1	Homo sapiens 5'-nucleotidase domain c	304.37524	251.09662	366.51364	245.80313	Detected	Detected	Detected	Detected
7256	A_24_P203298	IQUB	Hs.159650	IQ motif and ubiquitin domain containing	Homo sapiens IQ motif and ubiquitin do	32.16048	3.37267	26.01299	5.08923	Detected	Compromised	Detected	Compromised
7257	A_24_P83075		Hs.11637		Uncharacterized protein C21orf86 [Sou	40.55607	47.94800	57.62795	52.25444	Detected	Detected	Detected	Detected
7258	A_24_P67585	VGLL3	Hs.720159	vestigial like 3 (Drosophila)	Homo sapiens vestigial like 3 (Drosophil	919.85370	864.03546	377.92180	328.50330	Detected	Detected	Detected	Detected
7259	A_23_P161156	ZNF438	Hs.660642	zinc finger protein 438	Homo sapiens zinc finger protein 438 (Z	8951.29700	2822.42580	6589.07230	1904.39220	Detected	Detected	Detected	Detected
7260	A_23_P47058	CUZD1	Hs.647182	CUB and zona pellucida-like domains 1	Homo sapiens CUB and zona pellucida-	175.23486	96.28971	145.48958	68.92304	Detected	Detected	Detected	Detected
7261	A_23_P63032	GUCA2B	Hs.32966	guanylate cyclase activator 2B (urogua	Homo sapiens guanylate cyclase activat	33.48439	30.49013	26.07258	13.85506	Detected	Detected	Detected	Compromised
7262	A_32_P199767	ZNF630	Hs.650883	zinc finger protein 630	Homo sapiens zinc finger protein 630 (Z	65.58949	39.26794	50.60721	17.71032	Detected	Detected	Detected	Detected
7263	A_33_P3331711	LOC100130463	Hs.684304	hypothetical protein LOC100130463	Homo sapiens cDNA FLJ42307 fis, clon	132.39993	69.55470	100.10621	56.46957	Detected	Detected	Detected	Detected
7264	A_33_P3812815	PKD1	Hs.75813	polycystic kidney disease 1 (autosomal	Homo sapiens polycystic kidney disease	1301.07340	1586.64370	642.39514	607.09924	Detected	Detected	Detected	Detected
7265	A_23_P168092	LY6G6E		lymphocyte antigen 6 complex, locus G	Homo sapiens lymphocyte antigen 6 co	2.42745	6.68363	23.04215	6.60671	Compromised	Compromised	Detected	Compromised
7266	A_33_P3225512	OAS2	Hs.414332	2'-5'-oligoadenylate synthetase 2, 69/7	Homo sapiens 2'-5'-oligoadenylate synt	49.05518	70.99319	36.69605	35.93460	Detected	Detected	Detected	Detected
7267	A_33_P3227252	LOC100294070	Hs.720272	hypothetical protein LOC100294070	PREDICTED: Homo sapiens hypothetica	3424.90670	4116.22700	2533.42820	1981.96910	Detected	Detected	Detected	Detected
7268	A_23_P1998	APBB1	Hs.372840	amyloid beta (A4) precursor protein-bin	Homo sapiens amyloid beta (A4) precur	1155.40580	1278.10350	1101.44420	1145.39140	Detected	Detected	Detected	Detected
7269	A_23_P155755	CXCL6	Hs.164021	chemokine (C-X-C motif) ligand 6 (gran	Homo sapiens chemokine (C-X-C motif	186.20544	70.81077	261.69284	96.14695	Detected	Detected	Detected	Detected
7270	A_33_P3344648	LOC440461	Hs.661522	SH3 domain containing 20 pseudogene	Homo sapiens SH3 domain containing 2	155.58560	184.37470	61.08816	47.08899	Detected	Detected	Detected	Detected
7271	A_24_P187774		Hs.522334		Sushi, von Willebrand factor type A, EG	36.56668	25.71072	31.84260	20.34277	Detected	Detected	Detected	Detected

7272	A_24_P202319	ATP2A3	Hs.513870	ATPase, Ca++ transporting, ubiquitous	Homo sapiens ATPase, Ca++ transporti	24.27437	3.29360	32.57851	8.10773	Detected	Compromised	Detected	Compromised
7273	A_23_P317207	ATXN7L2	Hs.118248	ataxin 7-like 2	Homo sapiens ataxin 7-like 2 (ATXN7L2	362.00204	551.68840	241.92812	216.25189	Detected	Detected	Detected	Detected
7274	A_23_P160226	C1orf175	Hs.412482	chromosome 1 open reading frame 175	Homo sapiens chromosome 1 open reac	96.97963	109.06806	66.86774	49.74494	Detected	Detected	Detected	Detected
7275	A_24_P225604	DNAJC10	Hs.516632	DnaJ (Hsp40) homolog, subfamily C, me	Homo sapiens DnaJ (Hsp40) homolog, s	4019.78420	5579.14160	3270.02510	3967.28560	Detected	Detected	Detected	Detected
7276	A_33_P3274930				Peripheral myelin protein 22 (PMP-22)(	128.39932	163.55905	101.41521	121.72270	Detected	Detected	Detected	Detected
7277	A_33_P3280796				LIM domain only protein 7 (LOMP)(F-bo	28.58457	104.78590	20.30353	68.03269	Detected	Detected	Detected	Detected
7278	A_33_P3225572	LOC100129463	Hs.687800	hypothetical LOC100129463	PREDICTED: Homo sapiens hypothetica	3127.31790	4735.08640	2273.34720	2546.59700	Detected	Detected	Detected	Detected
7279	A_33_P3378318	LOC100128934		similar to FXYD domain-containing ion t	PREDICTED: Homo sapiens similar to F	20.50046	21.33798	21.19454	19.17075	Detected	Detected	Detected	Detected
7280	A_32_P139894	ABL2	Hs.159472	v-abl Abelson murine leukemia viral onc	Homo sapiens v-abl Abelson murine leu	2367.07400	5087.65200	1638.53110	3087.82400	Detected	Detected	Detected	Detected
7281	A_23_P337658	ALPI	Hs.37009	alkaline phosphatase, intestinal	Homo sapiens alkaline phosphatase, inte	34.92049	37.39041	33.46057	34.01130	Detected	Detected	Detected	Detected
7282	A_33_P3285809	SSX2IP	Hs.22587	synovial sarcoma, X breakpoint 2 intera	Homo sapiens synovial sarcoma, X brea	323.74340	505.85770	347.19006	468.82440	Detected	Detected	Detected	Detected
7283	A_33_P3310929	ADAM12	Hs.594537	ADAM metallopeptidase domain 12	Homo sapiens ADAM metallopeptidase	707.72110	1396.58180	501.25450	867.91430	Detected	Detected	Detected	Detected
7284	A_33_P3369286	IPO8	Hs.505136	importin 8	Homo sapiens importin 8 (IPO8), mRNA	3891.00000	3759.95120	1822.93140	1405.12790	Detected	Detected	Detected	Detected
7285	A_33_P3251647	PCBP3OT	Hs.474049	poly(rC) binding protein 3 overlapping tr	Homo sapiens cDNA FLJ44028 fis, clon	53.53653	80.07542	57.75162	52.64162	Detected	Detected	Detected	Detected
7286	A_33_P3385775	ZNF703	Hs.288042	zinc finger protein 703	Homo sapiens zinc finger protein 703 (Z	101.85399	233.29138	77.79172	163.93622	Detected	Detected	Detected	Detected
7287	A_33_P3250273	LOC442132	Hs.453260	similar to hypothetical protein FLJ3614	PREDICTED: Homo sapiens similar to h	87.98531	120.43957	13.12055	10.53666	Detected	Detected	Compromised	Compromised
7288	A_23_P144476	SPRY1	Hs.436944	sprouty homolog 1, antagonist of FGF s	Homo sapiens sprouty homolog 1, antag	403.17890	77.50677	255.63626	41.05924	Detected	Detected	Detected	Detected
7289	A_33_P3317752	KRTAP4-7	Hs.632746	keratin associated protein 4-7	Homo sapiens keratin associated protei	541.68964	412.48670	130.70340	105.13451	Detected	Detected	Detected	Detected
7290	A_33_P3289777	LOC728417	Hs.646112	hypothetical LOC728417	PREDICTED: Homo sapiens hypothetica	8.47184	17.44263	17.80640	18.16713	Compromised	Compromised	Detected	Detected
7291	A_23_P106391	THAP10	Hs.591123	THAP domain containing 10	Homo sapiens THAP domain containing	340.66200	367.62836	186.81900	163.05573	Detected	Detected	Detected	Detected
7292	A_32_P60223	ING5	Hs.529172	inhibitor of growth family, member 5	Homo sapiens inhibitor of growth family	1388.01780	743.54620	1009.03720	479.77548	Detected	Detected	Detected	Detected
7293	A_33_P3333232				Coiled-coil domain-containing protein C	41.58016	20.80196	27.30990	10.62436	Detected	Detected	Detected	Compromised
7294	A_24_P173325	UBA52	Hs.5308	ubiquitin A-52 residue ribosomal protein	Homo sapiens ubiquitin A-52 residue rib	62085.49000	61823.17600	76696.72000	67106.39000	Detected	Detected	Detected	Detected
7295	A_23_P149121	DIRAS3	Hs.194695	DIRAS family, GTP-binding RAS-like 3	Homo sapiens DIRAS family, GTP-bindi	62.51156	650.01830	54.66158	477.63540	Detected	Detected	Detected	Detected
7296	A_32_P11894	C12orf65	Hs.319128	chromosome 12 open reading frame 65	Homo sapiens chromosome 12 open rea	851.21310	1071.17930	604.23100	582.07086	Detected	Detected	Detected	Detected
7297	A_33_P3230876	ALG11	Hs.512963	asparagine-linked glycosylation 11, alph	Homo sapiens asparagine-linked glycos	806.26825	789.74506	327.41394	266.35403	Detected	Detected	Detected	Detected
7298	A_23_P207319	MAP3K14	Hs.404183	mitogen-activated protein kinase kinase	Homo sapiens mitogen-activated protei	659.59280	296.26627	780.44305	363.24063	Detected	Detected	Detected	Detected
7299	A_24_P347480	NEK9	Hs.719118	NIMA (never in mitosis gene a)- related	Homo sapiens NIMA (never in mitosis g	5006.76950	2997.72460	3060.57900	1666.85000	Detected	Detected	Detected	Detected
7300	A_33_P3403179	LOC728411	Hs.88181	glucuronidase, beta pseudogene	Homo sapiens, clone IMAGE:4590099, m	156.15637	103.88448	80.80130	42.36783	Detected	Detected	Detected	Detected
7301	A_23_P165333	BIN1	Hs.193163	bridging integrator 1	Homo sapiens bridging integrator 1 (BIN	5002.38900	2916.18730	3867.84330	2184.79420	Detected	Detected	Detected	Detected
7302	A_23_P52336	UNC5B	Hs.522997	unc-5 homolog B (C. elegans)	Homo sapiens unc-5 homolog B (C. ele	3769.13130	4889.60350	2077.60030	2309.58060	Detected	Detected	Detected	Detected
7303	A_33_P3372666	PDGFA	Hs.535898	platelet-derived growth factor alpha po	Homo sapiens platelet-derived growth f	161.11732	284.29560	65.50455	59.47228	Detected	Detected	Detected	Detected
7304	A_23_P152136	GINS3	Hs.47125	GINS complex subunit 3 (Psf3 homolog)	Homo sapiens GINS complex subunit 3	596.59766	4031.30100	504.73474	2948.92680	Detected	Detected	Detected	Detected
7305	A_24_P340941	LOC100131149	Hs.606533	similar to HSPC125	PREDICTED: Homo sapiens similar to h	149.51999	205.24828	8.77985	12.04498	Detected	Detected	Compromised	Compromised
7306	A_33_P3333985					747.79760	965.20330	684.13513	784.11870	Detected	Detected	Detected	Detected
7307	A_23_P110445	APBB3	Hs.529449	amyloid beta (A4) precursor protein-bin	Homo sapiens amyloid beta (A4) precur	477.55396	297.80942	394.38757	170.78038	Detected	Detected	Detected	Detected
7308	A_33_P3238484	DIP2C	Hs.432397	DIP2 disco-interacting protein 2 homolo	Homo sapiens DIP2 disco-interacting p	28.52858	30.23777	38.88112	45.74978	Detected	Detected	Detected	Detected
7309	A_24_P90878	THUMPD1	Hs.460232	THUMP domain containing 1	Homo sapiens THUMP domain containir	845.68530	722.14430	452.17123	319.80100	Detected	Detected	Detected	Detected
7310	A_33_P3421178	LOC100131830	Hs.628359	hypothetical LOC100131830	PREDICTED: Homo sapiens hypothetica	373.99197	374.98044	302.09494	274.06265	Detected	Detected	Detected	Detected
7311	A_23_P202392	SUV39H2	Hs.554883	suppressor of variegation 3-9 homolog	Homo sapiens suppressor of variegatior	688.45540	1625.57310	622.60736	1337.73020	Detected	Detected	Detected	Detected
7312	A_23_P79931	ATRN	Hs.276252	attractin	Homo sapiens attractin (ATRN), transcr	5378.37200	1789.61820	5075.10940	1572.27420	Detected	Detected	Detected	Detected
7313	A_24_P605190	PGGT1B	Hs.254006	protein geranylgeranyltransferase type	Homo sapiens protein geranylgeranyltra	414.04138	655.99243	337.19160	452.54202	Detected	Detected	Detected	Detected
7314	A_23_P502425	MRPL47	Hs.283734	mitochondrial ribosomal protein L47	Homo sapiens mitochondrial ribosomal p	17479.28000	23199.71700	15315.02800	17736.54100	Detected	Detected	Detected	Detected
7315	A_24_P161764		Hs.721648		Putative uncharacterized protein ENSP	3.99826	40.36176	2.69015	4.28084	Compromised	Detected	Compromised	Compromised
7316	A_23_P376661	NIT1	Hs.517342	nitrilase 1	Homo sapiens nitrilase 1 (NIT1), mRNA	885.99680	493.29837	963.96124	525.25760	Detected	Detected	Detected	Detected
7317	A_32_P21579	TRDMT1	Hs.351665	tRNA aspartic acid methyltransferase 1	Homo sapiens tRNA aspartic acid meth	10.66561	19.54670	5.97023	8.92450	Compromised	Detected	Compromised	Compromised
7318	A_33_P3319261		Hs.495553		Probable exonuclease mut-7 homolog (	137.25108	110.33546	184.72147	141.82639	Detected	Detected	Detected	Detected
7319	A_23_P252962	ITSN1	Hs.160324	intersectin 1 (SH3 domain protein)	Homo sapiens intersectin 1 (SH3 domai	729.79803	826.10520	505.68484	532.74300	Detected	Detected	Detected	Detected
7320	A_24_P365180	DSEL	Hs.124673	dermatan sulfate epimerase-like	Homo sapiens dermatan sulfate epimera	1420.88230	628.65360	675.55530	236.99023	Detected	Detected	Detected	Detected
7321	A_24_P284805	INPP5J	Hs.517549	inositol polyphosphate-5-phosphatase	Homo sapiens inositol polyphosphate-5	9.15968	12.24386	16.28513	12.56611	Compromised	Compromised	Detected	Compromised
7322	A_33_P3352340				Putative keratin-associated protein 9-2	480.12766	453.78220	177.35535	144.20573	Detected	Detected	Detected	Detected
7323	A_23_P69958	AP3S1	Hs.406191	adaptor-related protein complex 3, sigm	Homo sapiens adaptor-related protein	33010.47700	31055.03700	26994.85700	21183.37100	Detected	Detected	Detected	Detected
7324	A_23_P155596	FMO3	Hs.445350	flavin containing monooxygenase 3	Homo sapiens flavin containing monoox	22.59917	3.56980	23.85950	3.00388	Detected	Compromised	Detected	Compromised
7325	A_23_P162807	MRPS31	Hs.596607	mitochondrial ribosomal protein S31	Homo sapiens mitochondrial ribosomal p	4299.19530	3217.43290	4200.66700	2734.10500	Detected	Detected	Detected	Detected
7326	A_24_P142305	HBA2	Hs.654744	hemoglobin, alpha 2	Homo sapiens hemoglobin, alpha 2 (HBA	30.57024	46.10620	42.05200	54.29128	Detected	Detected	Detected	Detected
7327	A_23_P46964	HIF1AN	Hs.500788	hypoxia inducible factor 1, alpha subunit	Homo sapiens hypoxia inducible factor	167.62420	167.68794	218.70276	205.31340	Detected	Detected	Detected	Detected
7328	A_33_P3255404	CLDN11	Hs.31595	claudin 11	Homo sapiens claudin 11 (CLDN11), mR	9547.06300	22035.67000	6177.75500	11584.47200	Detected	Detected	Detected	Detected
7329	A_23_P305210	ZNF680	Hs.520886	zinc finger protein 680	Homo sapiens zinc finger protein 680 (Z	39.72292	48.27413	4.54943	3.09810	Detected	Detected	Compromised	Compromised
7330	A_23_P80122	WRB	Hs.198308	tryptophan rich basic protein	Homo sapiens tryptophan rich basic pro	2798.10700	2703.62380	2708.68100	2216.28080	Detected	Detected	Detected	Detected
7331	A_24_P3804	C6orf26	Hs.371225	chromosome 6 open reading frame 26	Homo sapiens chromosome 6 open reac	690.42510	471.68020	476.91104	303.78186	Detected	Detected	Detected	Detected
7332	A_32_P134968	SPTB	Hs.417303	spectrin, beta, erythrocytic	Homo sapiens spectrin, beta, erythrocyt	13.31615	20.24308	9.99919	15.80825	Compromised	Detected	Compromised	Detected
7333	A_23_P323143	ZNF767	Hs.520785	zinc finger family member 767	Homo sapiens zinc finger family membe	160.72661	123.01032	179.83708	120.66537	Detected	Detected	Detected	Detected
7334	A_33_P3441060	C6orf35	Hs.157212	chromosome 6 open reading frame 35	Homo sapiens chromosome 6 open reac	1122.75000	1131.99680	823.65314	773.96606	Detected	Detected	Detected	Detected
7335	A_23_P138706	ADRA2A	Hs.249159	adrenergic, alpha-2A-, receptor	Homo sapiens adrenergic, alpha-2A-, re	9.42170	41.24627	3.56301	29.71798	Compromised	Detected	Compromised	Detected
7336	A_23_P149375	THEM4	Hs.164070	thioesterase superfamily member 4	Homo sapiens thioesterase superfamily	386.38263	722.74316	365.64830	593.15060	Detected	Detected	Detected	Detected
7337	A_24_P288685	IL13RA1	Hs.496646	interleukin 13 receptor, alpha 1	Homo sapiens interleukin 13 receptor, a	631.16200	463.94287	643.34753	442.81485	Detected	Detected	Detected	Detected
7338	A_24_P216313	ERGIC3	Hs.472558	ERGIC and golgi 3	Homo sapiens ERGIC and golgi 3 (ERGI	5619.74600	4261.84500	6294.41200	4218.63230	Detected	Detected	Detected	Detected
7339	A_33_P3393931					75.95828	71.16974	31.84436	31.51705	Detected	Detected	Detected	Detected
7340	A_23_P204801	SLC41A2	Hs.577463	solute carrier family 41, member 2	Homo sapiens solute carrier family 41, r	117.18940	316.04675	100.55089	229.87710	Detected	Detected	Detected	Detected
7341	A_23_P88626	ANPEP	Hs.1239	alanyl (membrane) aminopeptidase	Homo sapiens alanyl (membrane) amino	7205.97360	20283.25400	7611.53860	20139.64300	Detected	Detected	Detected	Detected
7342	A_33_P3281066	ZNF295	Hs.434947	zinc finger protein 295	Homo sapiens zinc finger protein 295 (Z	806.83390	734.50520	795.43176	648.62460	Detected	Detected	Detected	Detected
7343	A_33_P3377786				Homo sapiens ELK2, member of ETS or	1070.93230	1434.41250	694.47375	722.93190	Detected	Detected	Detected	Detected
7344	A_33_P3415300	CPLX2	Hs.193235	complexin 2	Homo sapiens complexin 2 (CPLX2), tra	26.60775	33.34330	22.51742	16.73911	Detected	Detected	Detected	Detected
7345	A_23_P125082	IRF2	Hs.654566	interferon regulatory factor 2	Homo sapiens interferon regulatory fact	21.86831	24.07956	24.68289	17.51266	Detected	Detected	Detected	Detected
7346	A_24_P372134	TMEM140	Hs.521213	transmembrane protein 140	Homo sapiens transmembrane protein 1	147.30157	116.76160	169.34549	95.47737	Detected	Detected	Detected	Detected

7347	A_23_P311020	MITD1	Hs.14222	MIT, microtubule interacting and transp	Homo sapiens MIT, microtubule interact	5288.85640	3299.47490	4268.47900	2190.28930	Detected	Detected	Detected	Detected
7348	A_23_P86411	MYO3A	Hs.662630	myosin IIIA	Homo sapiens myosin IIIA (MYO3A), mR	8.32692	13.15172	17.11780	11.95125	Compromised	Compromised	Detected	Compromised
7349	A_33_P3216568		Hs.721515		Mucin-5B Precursor (MUC-5B)(Mucin-	15.05963	24.54194	31.66838	19.30190	Detected	Detected	Detected	Detected
7350	A_23_P121276	NEK4	Hs.631921	NIMA (never in mitosis gene a)-related	Homo sapiens NIMA (never in mitosis g	324.40128	274.75160	224.01111	164.54800	Detected	Detected	Detected	Detected
7351	A_23_P90357	TBXA2R	Hs.442530	thromboxane A2 receptor	Homo sapiens thromboxane A2 recepto	1026.52530	1958.74230	690.44160	896.53080	Detected	Detected	Detected	Detected
7352	A_23_P157170	DNAJC30	Hs.647046	DnaJ (Hsp40) homolog, subfamily C, me	Homo sapiens DnaJ (Hsp40) homolog, s	598.98004	1544.45200	877.11010	2031.80380	Detected	Detected	Detected	Detected
7353	A_33_P3265956		Hs.153934		Protein CBFA2T2 (MTG8-related prote	412.26645	400.58157	399.86010	351.05658	Detected	Detected	Detected	Detected
7354	A_33_P3362321	FHL2	Hs.443687	four and a half LIM domains 2	Homo sapiens four and a half LIM doma	6334.86800	13891.23800	7268.36670	14552.13600	Detected	Detected	Detected	Detected
7355	A_24_P24790					122068.32000	103804.08000	95913.87000	69750.51600	Detected	Detected	Detected	Detected
7356	A_23_P165442	NRBP1	Hs.515876	nuclear receptor binding protein 1	Homo sapiens nuclear receptor binding	8100.25340	10022.29300	7829.51700	9545.11400	Detected	Detected	Detected	Detected
7357	A_33_P3415560	ZFP82	Hs.558734	zinc finger protein 82 homolog (mouse)	Homo sapiens zinc finger protein 82 hor	177.52466	164.23325	172.92635	139.91391	Detected	Detected	Detected	Detected
7358	A_23_P131771	TPD52L2	Hs.473296	tumor protein D52-like 2	Homo sapiens tumor protein D52-like 2	1554.62430	1644.89930	1315.14470	1313.61020	Detected	Detected	Detected	Detected
7359	A_24_P104091	ORAOV1	Hs.667996	oral cancer overexpressed 1	Homo sapiens oral cancer overexpresse	2999.85160	4322.28800	1734.57840	2016.92870	Detected	Detected	Detected	Detected
7360	A_23_P251095	PDHA1	Hs.530331	pyruvate dehydrogenase (lipoamide) alp	Homo sapiens pyruvate dehydrogenase	11650.59300	14916.92100	8149.46040	8817.07100	Detected	Detected	Detected	Detected
7361	A_33_P3262580				Ectonucleoside triphosphate diphosphol	480.21368	740.68670	223.03134	225.39360	Detected	Detected	Detected	Detected
7362	A_33_P3359012	DUSP8	Hs.41688	dual specificity phosphatase 8	Homo sapiens dual specificity phosphat	24503.54300	19829.03300	8854.32300	4372.69500	Detected	Detected	Detected	Detected
7363	A_24_P315638	MRGPRE	Hs.706565	MAS-related GPR, member E	Homo sapiens MAS-related GPR, memb	93.89787	107.49999	57.62416	54.82940	Detected	Detected	Detected	Detected
7364	A_23_P211428	SMTN	Hs.149098	smoothelin	Homo sapiens smoothelin (SMTN), trans	1545.50290	3637.10080	2150.85840	5014.38700	Detected	Detected	Detected	Detected
7365	A_33_P3367447	ALDH3B1	Hs.523841	aldehyde dehydrogenase 3 family, memb	Homo sapiens aldehyde dehydrogenase	1011.63520	291.53010	771.86926	236.44507	Detected	Detected	Detected	Detected
7366	A_33_P3391167				Putative uncharacterized protein ENSP	936.09680	1198.53360	2251.24150	2258.59000	Detected	Detected	Detected	Detected
7367	A_23_P68740	AIRE	Hs.129829	autoimmune regulator	Homo sapiens autoimmune regulator (A	71.34989	55.67081	55.89057	36.54223	Detected	Detected	Detected	Detected
7368	A_23_P25638	RNF219	Hs.567576	ring finger protein 219	Homo sapiens ring finger protein 219 (R	2554.69500	1709.46070	1897.86990	1119.28430	Detected	Detected	Detected	Detected
7369	A_33_P3301316	ANUBL1	Hs.89029	AN1, ubiquitin-like, homolog (Xenopus la	Homo sapiens AN1, ubiquitin-like, homo	18.41830	36.84430	23.16426	34.88789	Detected	Detected	Detected	Detected
7370	A_23_P36647	TMUB2	Hs.181391	transmembrane and ubiquitin-like doma	Homo sapiens transmembrane and ubiqu	2038.87820	2063.74680	1897.85470	1973.38390	Detected	Detected	Detected	Detected
7371	A_33_P3355877				NF-X1-type zinc finger protein NFXL1	35.26935	40.77150	41.25526	35.29543	Detected	Detected	Detected	Detected
7372	A_23_P66715	PIGS	Hs.462550	phosphatidylinositol glycan anchor biosy	Homo sapiens phosphatidylinositol glyca	510.69860	1029.38060	489.18472	925.62335	Detected	Detected	Detected	Detected
7373	A_33_P3287631	CTSB	Hs.520898	cathepsin B	Homo sapiens cathepsin B (CTSB), tran	43379.19000	10985.90100	35090.57400	8282.38800	Detected	Detected	Detected	Detected
7374	A_23_P210920	GSS	Hs.82327	glutathione synthetase	Homo sapiens glutathione synthetase ((	4579.67600	8388.02100	4859.38230	7140.07470	Detected	Detected	Detected	Detected
7375	A_24_P4661	EI24	Hs.643514	etoposide induced 2.4 mRNA	Homo sapiens etoposide induced 2.4 mF	3896.47970	5435.02000	4372.02440	5499.24660	Detected	Detected	Detected	Detected
7376	A_32_P199884	HORMAD1	Hs.298312	HORMA domain containing 1	Homo sapiens HORMA domain containi	14.39697	3.65606	16.56413	3.08841	Detected	Compromised	Detected	Compromised
7377	A_23_P16058	ZNF296	Hs.192237	zinc finger protein 296	Homo sapiens zinc finger protein 296 (Z	85.84721	102.77403	57.33899	76.02686	Detected	Detected	Detected	Detected
7378	A_33_P3300212	BLCAP	Hs.718404	bladder cancer associated protein	Homo sapiens bladder cancer associate	429.28088	811.47670	411.65836	697.63850	Detected	Detected	Detected	Detected
7379	A_23_P353014	CACNA2D4	Hs.13768	calcium channel, voltage-dependent, alp	Homo sapiens calcium channel, voltage-	31.74249	8.11009	27.10278	7.78559	Detected	Compromised	Detected	Compromised
7380	A_33_P3271651	HLA-DPB1	Hs.485130	major histocompatibility complex, class	Homo sapiens major histocompatibility	596.61420	1073.61510	802.04224	1245.04930	Detected	Detected	Detected	Detected
7381	A_33_P3340254	MLL2	Hs.120228	myeloid/lymphoid or mixed-lineage leuk	Homo sapiens myeloid/lymphoid or mixe	1583.40610	1624.61500	1030.70420	915.97880	Detected	Detected	Detected	Detected
7382	A_33_P3358019	LOC642515		similar to NG5 protein	PREDICTED: Homo sapiens similar to N	235.49979	320.47375	77.23488	85.90253	Detected	Detected	Detected	Detected
7383	A_33_P3341540				Putative uncharacterized protein ENSP	350.73254	601.80270	185.09488	225.25024	Detected	Detected	Detected	Detected
7384	A_23_P380298	ProSAPiP1	Hs.90232	ProSAPiP1 protein	Homo sapiens ProSAPiP1 protein (ProS	272.87408	170.44722	200.26338	134.14100	Detected	Detected	Detected	Detected
7385	A_33_P3292679	SNX6	Hs.356647	sorting nexin 6	Homo sapiens sorting nexin 6 (SNX6), t	5421.39550	6182.00340	4078.59470	4227.19400	Detected	Detected	Detected	Detected
7386	A_33_P3365524	SETD3	Hs.510407	SET domain containing 3	Homo sapiens SET domain containing 3	192.84349	346.74008	151.78775	206.59683	Detected	Detected	Detected	Detected
7387	A_24_P198820	CRKRS	Hs.416108	Cdc2-related kinase, arginine/serine-ric	Homo sapiens Cdc2-related kinase, arg	339.86148	292.26100	262.78824	178.78712	Detected	Detected	Detected	Detected
7388	A_23_P65386	OTUB2	Hs.278815	OTU domain, ubiquitin aldehyde binding	Homo sapiens OTU domain, ubiquitin al	35.39555	48.31780	30.62669	44.54240	Detected	Detected	Detected	Detected
7389	A_23_P102706	SNPH	Hs.323833	syntaphilin	Homo sapiens syntaphilin (SNPH), mRN	1118.95210	777.18524	1126.04050	847.10364	Detected	Detected	Detected	Detected
7390	A_33_P3262927	PCBP4	Hs.20930	poly(rC) binding protein 4	Homo sapiens poly(rC) binding protein 4	796.98390	780.31250	708.44196	796.63200	Detected	Detected	Detected	Detected
7391	A_33_P3257728	C1RL	Hs.631730	complement component 1, r subcompor	Homo sapiens complement component	13.10563	7.29867	24.47788	14.73210	Detected	Compromised	Detected	Detected
7392	A_24_P13041	RTKN2	Hs.58559	rhotekin 2	Homo sapiens rhotekin 2 (RTKN2), mRN	2.44072	5.30510	2.74950	14.67723	Compromised	Compromised	Compromised	Detected
7393	A_23_P133227	HARS	Hs.528050	histidyl-tRNA synthetase	Homo sapiens histidyl-tRNA synthetase	7972.73140	14342.45500	5572.70260	9098.70400	Detected	Detected	Detected	Detected
7394	A_24_P364042	PPYR1	Hs.524719	pancreatic polypeptide receptor 1	Homo sapiens pancreatic polypeptide re	100.42408	112.63152	51.90744	47.67221	Detected	Detected	Detected	Detected
7395	A_33_P3630129	FLJ46875	Hs.641142	hypothetical LOC440918	Homo sapiens cDNA FLJ46875 fis, clon	287.96625	61.31950	194.59586	26.24131	Detected	Detected	Detected	Detected
7396	A_23_P379794	PIGW	Hs.378885	phosphatidylinositol glycan anchor biosy	Homo sapiens phosphatidylinositol glyca	378.89062	627.17060	340.73315	429.81924	Detected	Detected	Detected	Detected
7397	A_32_P514599					1494.44380	2202.52000	713.75836	819.16864	Detected	Detected	Detected	Detected
7398	A_23_P26629	PYCARD	Hs.499094	PYD and CARD domain containing	Homo sapiens PYD and CARD domain c	2689.52400	1422.83450	3430.62350	1733.60510	Detected	Detected	Detected	Detected
7399	A_24_P66838					838.00525	828.22125	18.86874	13.80111	Detected	Detected	Detected	Compromised
7400	A_23_P4133	AOC2	Hs.143102	amine oxidase, copper containing 2 (reti	Homo sapiens amine oxidase, copper co	20.93111	15.89842	19.20649	12.18158	Detected	Compromised	Detected	Compromised
7401	A_23_P118105	NDUFB10	Hs.513266	NADH dehydrogenase (ubiquinone) 1 be	Homo sapiens NADH dehydrogenase (u	19744.52500	17233.00200	23478.11000	18122.33400	Detected	Detected	Detected	Detected
7402	A_24_P393844	DPH2	Hs.632398	DPH2 homolog (S. cerevisiae)	Homo sapiens DPH2 homolog (S. cerevi	1841.92580	2518.30420	1596.01540	1902.37150	Detected	Detected	Detected	Detected
7403	A_33_P3296975	LOC390251	Hs.712234	hypothetical LOC390251	PREDICTED: Homo sapiens hypothetica	66.53839	91.66979	71.61285	71.36381	Detected	Detected	Detected	Detected
7404	A_23_P379746	DENND5B	Hs.118166	DENN/MADD domain containing 5B	Homo sapiens DENN/MADD domain co	380.99630	211.78563	318.52963	140.38838	Detected	Detected	Detected	Detected
7405	A_33_P3299705	NOMO1		NODAL modulator 1	Nodal modulator 1 Precursor (pM5) [So	28408.83600	32054.89300	16680.27000	16067.59800	Detected	Detected	Detected	Detected
7406	A_33_P3289561				Putative uncharacterized protein C1orf	640.44855	296.04270	630.01904	241.99345	Detected	Detected	Detected	Detected
7407	A_24_P116242	KLHDC2	Hs.509264	kelch domain containing 2	Homo sapiens kelch domain containing	13675.94700	8878.94000	10555.45200	5849.67630	Detected	Detected	Detected	Detected
7408	A_24_P167654	SLC8A3	Hs.337696	solute carrier family 8 (sodium/calcium	Homo sapiens solute carrier family 8 (s	20.04742	3.28530	21.81239	7.77320	Detected	Compromised	Detected	Compromised
7409	A_33_P3298810	FFAR3	Hs.248055	free fatty acid receptor 3	Homo sapiens free fatty acid receptor 3	22.33439	5.23169	31.04200	9.37250	Detected	Compromised	Detected	Compromised
7410	A_24_P366457					29097.53700	16436.86100	8525.39700	3905.32960	Detected	Detected	Detected	Detected
7411	A_24_P344711	AGPAT3	Hs.248785	1-acylglycerol-3-phosphate O-acyltran	Homo sapiens 1-acylglycerol-3-phosph	2197.12940	2231.76730	1668.56750	1541.70300	Detected	Detected	Detected	Detected
7412	A_24_P163405	TRIM27	Hs.440382	tripartite motif-containing 27	Homo sapiens tripartite motif-containin	2241.66480	2939.32420	2125.51760	2807.42070	Detected	Detected	Detected	Detected
7413	A_24_P273143	NCRNA00152		non-protein coding RNA 152	Homo sapiens non-protein coding RNA	10698.47500	17549.95000	9769.95400	14270.98600	Detected	Detected	Detected	Detected
7414	A_33_P3300092	NDST1	Hs.222055	N-deacetylase/N-sulfotransferase (hep	Homo sapiens N-deacetylase/N-sulfotr	11444.12000	14841.51100	12358.35300	22950.90200	Detected	Detected	Detected	Detected
7415	A_24_P348861	TTTY15	Hs.433656	testis-specific transcript, Y-linked 15 (r	Homo sapiens testis-specific transcript	65.23033	3.46278	32.06854	2.90244	Detected	Compromised	Detected	Compromised
7416	A_33_P3239864		Hs.207157		Ankyrin repeat and death domain-conta	271.25800	315.55765	182.38422	174.88521	Detected	Detected	Detected	Detected
7417	A_23_P502575	CSNK2A1	Hs.644056	casein kinase 2, alpha 1 polypeptide	Homo sapiens casein kinase 2, alpha 1 p	1319.85720	1421.66770	1108.39030	1024.09550	Detected	Detected	Detected	Detected
7418	A_33_P3354394	KRTAP12-4	Hs.689664	keratin associated protein 12-4	Homo sapiens keratin associated protei	18.77581	23.72819	17.12035	11.08279	Detected	Detected	Detected	Compromised
7419	A_33_P3311917					202.12732	41.29287	151.32675	25.85368	Detected	Detected	Detected	Detected
7420	A_33_P3253460	LOC100130654	Hs.636509	hypothetical protein LOC100130654	Homo sapiens cDNA FLJ37924 fis, clon	10699.09000	17635.22700	1237.49650	1675.05460	Detected	Detected	Detected	Detected
7421	A_33_P3378031	TMEM18	Hs.43899	transmembrane protein 18	Homo sapiens transmembrane protein 1	1058.48830	2291.71120	1132.64060	1970.15150	Detected	Detected	Detected	Detected

7422	A_23_P93881	SYPL1	Hs.80919	synaptophysin-like 1	Homo sapiens synaptophysin-like 1 (SY	11815.79500	14012.73800	9218.81900	8731.73400	Detected	Detected	Detected	Detected
7423	A_33_P3232980	MARK4	Hs.34314	MAP/microtubule affinity-regulating kin	Homo sapiens MAP/microtubule affinity	230.11807	189.32893	123.35819	86.15833	Detected	Detected	Detected	Detected
7424	A_23_P24433	CTSF	Hs.11590	cathepsin F	Homo sapiens cathepsin F (CTSF), mRN	30439.95700	6693.87900	28025.80900	5727.43360	Detected	Detected	Detected	Detected
7425	A_24_P357100	RASD2	Hs.474711	RASD family, member 2	Homo sapiens RASD family, member 2 (	77.09639	117.12098	63.27604	80.97433	Detected	Detected	Detected	Detected
7426	A_33_P3213235	CLEC4G	Hs.220649	C-type lectin domain family 4, member	Homo sapiens C-type lectin domain fam	112.60416	180.46263	47.39768	69.60403	Detected	Detected	Detected	Detected
7427	A_24_P40978	CYHR1	Hs.459379	cysteine/histidine-rich 1	Homo sapiens cysteine/histidine-rich 1	164.65154	419.78998	162.61520	469.84760	Detected	Detected	Detected	Detected
7428	A_24_P101812	LOC341378		hypothetical LOC341378	PREDICTED: Homo sapiens hypothetica	192.23543	111.79382	5.24014	4.94177	Detected	Detected	Compromised	Compromised
7429	A_24_P151	KCNAB2	Hs.440497	potassium voltage-gated channel, shake	Homo sapiens potassium voltage-gated	50.67333	32.04783	33.13292	23.48307	Detected	Detected	Detected	Detected
7430	A_32_P68746	SMU1	Hs.655351	smu-1 suppressor of mec-8 and unc-52	Homo sapiens smu-1 suppressor of me	767.93570	1132.91540	707.83765	967.63666	Detected	Detected	Detected	Detected
7431	A_24_P213950	HEPACAM	Hs.159863	hepatocyte cell adhesion molecule	Homo sapiens hepatocyte cell adhesion	32.86781	47.47872	15.55268	19.07591	Detected	Detected	Detected	Detected
7432	A_33_P3224324	NOX4	Hs.371036	NADPH oxidase 4	Homo sapiens NADPH oxidase 4 (NOX4	22.75373	181.93068	26.26116	166.38739	Detected	Detected	Detected	Detected
7433	A_33_P3259801	TMEM189	Hs.719899	transmembrane protein 189	Homo sapiens transmembrane protein 1	2324.55250	3022.72310	1047.86730	1152.63810	Detected	Detected	Detected	Detected
7434	A_33_P3349552	CASKIN1	Hs.643537	CASK interacting protein 1	Homo sapiens CASK interacting protein	30093.45100	37421.84800	45418.34000	42542.90200	Detected	Detected	Detected	Detected
7435	A_33_P3380762	LOC729467		hypothetical LOC729467	Homo sapiens hypothetical LOC729467	80.16272	12.68284	72.74611	20.96267	Detected	Compromised	Detected	Detected
7436	A_23_P205900	NTRK3	Hs.410969	neurotrophic tyrosine kinase, receptor,	Homo sapiens neurotrophic tyrosine kin	58.84325	6.16370	57.21378	17.27734	Detected	Compromised	Detected	Detected
7437	A_33_P3298024	ABCC3	Hs.463421	ATP-binding cassette, sub-family C (CF	Homo sapiens ATP-binding cassette, su	1651.23110	2363.76050	2042.30430	2859.36230	Detected	Detected	Detected	Detected
7438	A_23_P31116	ACOT13	Hs.9676	acyl-CoA thioesterase 13	Homo sapiens acyl-CoA thioesterase 1	8893.26200	4211.64400	8939.50000	3929.49020	Detected	Detected	Detected	Detected
7439	A_24_P322741	IL10RB	Hs.654593	interleukin 10 receptor, beta	Homo sapiens interleukin 10 receptor, b	2206.98850	1485.59400	1323.32100	864.25653	Detected	Detected	Detected	Detected
7440	A_23_P98963	LOC144438	Hs.92308	hypothetical LOC144438	Homo sapiens hypothetical LOC144438	708.42510	640.96106	489.04022	437.82340	Detected	Detected	Detected	Detected
7441	A_24_P333567	hCG_2022304	Hs.593896	similar to hCG2022304	Homo sapiens similar to hCG2022304 (L	975.81305	264.06116	689.26090	167.28897	Detected	Detected	Detected	Detected
7442	A_23_P7684	CCNJL	Hs.14070	cyclin J-like	Homo sapiens cyclin J-like (CCNJL), m	12.07663	3.32529	20.28200	5.48395	Compromised	Compromised	Detected	Compromised
7443	A_23_P403521	C7orf36	Hs.83313	chromosome 7 open reading frame 36	Homo sapiens chromosome 7 open read	936.43225	935.95080	975.10820	860.31836	Detected	Detected	Detected	Detected
7444	A_33_P3298785	CUX1	Hs.654389	cut-like homeobox 1	Homo sapiens cut-like homeobox 1 (CU	78.37483	86.05070	78.91773	71.19913	Detected	Detected	Detected	Detected
7445	A_32_P140706	PMS2	Hs.632637	PMS2 postmeiotic segregation increase	Homo sapiens PMS2 postmeiotic segreg	185.32014	176.22481	189.27287	177.86030	Detected	Detected	Detected	Detected
7446	A_33_P3371219	SDC2	Hs.1501	syndecan 2	Homo sapiens syndecan 2 (SDC2), mRN	44.12290	91.60685	23.41175	43.63336	Detected	Detected	Detected	Detected
7447	A_23_P162165	KCTD14	Hs.709780	potassium channel tetramerisation dom	Homo sapiens potassium channel tetran	41.20672	33.64582	35.83420	30.64113	Detected	Detected	Detected	Detected
7448	A_23_P47304	CASP5	Hs.213327	caspase 5, apoptosis-related cysteine p	Homo sapiens caspase 5, apoptosis-rel	7891.73050	4523.79000	6843.58540	3634.29150	Detected	Detected	Detected	Detected
7449	A_33_P3423565	LOC100291714		hypothetical protein LOC100291714	PREDICTED: Homo sapiens hypothetica	12.10876	44.27559	20.60895	41.56035	Detected	Detected	Detected	Detected
7450	A_23_P303260	STX7	Hs.593148	syntaxin 7	Homo sapiens syntaxin 7 (STX7), mRNA	402.38920	358.86206	280.52853	210.20955	Detected	Detected	Detected	Detected
7451	A_23_P166663	APPL1	Hs.476415	adaptor protein, phosphotyrosine intera	Homo sapiens adaptor protein, phospho	3237.63060	2669.31980	1617.13200	1008.93774	Detected	Detected	Detected	Detected
7452	A_33_P3411260	LOC100288884	Hs.613108	hypothetical LOC100288884	Homo sapiens cDNA FLJ42128 fis, clon	17.05435	14.65683	20.49250	9.73666	Detected	Compromised	Detected	Compromised
7453	A_24_P335305	OAS3	Hs.528634	2'-5'-oligoadenylate synthetase 3, 100k	Homo sapiens 2'-5'-oligoadenylate synt	45.46606	65.03587	29.00628	38.11857	Detected	Detected	Detected	Detected
7454	A_33_P3363061	ERLIN2	Hs.705490	ER lipid raft associated 2	Homo sapiens ER lipid raft associated 2	116.49632	266.05243	138.86328	252.88371	Detected	Detected	Detected	Detected
7455	A_33_P3367126	RGNEF	Hs.482521	Rho-guanine nucleotide exchange facto	Homo sapiens Rho-guanine nucleotide e	55.18639	74.52091	48.22378	49.21029	Detected	Detected	Detected	Detected
7456	A_33_P3272823	MAG	Hs.643440	myelin associated glycoprotein	Homo sapiens myelin associated glycop	230.29848	321.09464	103.51093	94.74059	Detected	Detected	Detected	Detected
7457	A_23_P203445	UEVLD	Hs.407991	UEV and lactate/malate dehyrogenase	Homo sapiens UEV and lactate/malate	844.94380	711.53390	639.79565	437.57745	Detected	Detected	Detected	Detected
7458	A_23_P312358	BEND7	Hs.498740	BEN domain containing 7	Homo sapiens BEN domain containing 7	60.32234	148.71849	47.69342	98.63226	Detected	Detected	Detected	Detected
7459	A_23_P30464	PRR7	Hs.534492	proline rich 7 (synaptic)	Homo sapiens proline rich 7 (synaptic) (	4407.65000	2733.79050	6055.13670	3508.54250	Detected	Detected	Detected	Detected
7460	A_33_P3278590	AKAP14	Hs.592245	A kinase (PRKA) anchor protein 14	Homo sapiens A kinase (PRKA) anchor	12.13000	8.62628	14.27733	13.31178	Compromised	Compromised	Detected	Compromised
7461	A_33_P3220095	ZNF341	Hs.516989	zinc finger protein 341	Homo sapiens zinc finger protein 341 (Z	960.50290	1123.90170	777.37740	871.26843	Detected	Detected	Detected	Detected
7462	A_33_P3390950	hCG_1995004	Hs.493171	hypothetical LOC100128338	Homo sapiens cDNA FLJ43864 fis, clon	358.66403	440.14050	188.22581	196.17317	Detected	Detected	Detected	Detected
7463	A_23_P250619	ZDHHC14	Hs.187459	zinc finger, DHHC-type containing 14	Homo sapiens zinc finger, DHHC-type c	1812.47670	411.83936	1712.24170	362.29483	Detected	Detected	Detected	Detected
7464	A_23_P214300	GSTA2	Hs.94107	glutathione S-transferase alpha 2	Homo sapiens glutathione S-transferase	39.77264	118.55846	21.59130	75.28162	Detected	Detected	Detected	Detected
7465	A_23_P162547	MYL2	Hs.75535	myosin, light chain 2, regulatory, cardiac	Homo sapiens myosin, light chain 2, reg	73.80911	241.00575	36.16277	90.54007	Detected	Detected	Detected	Detected
7466	A_23_P27627	ASNA1	Hs.465985	arsA arsenite transporter, ATP-binding,	Homo sapiens arsA arsenite transporter	3804.86870	3212.82080	3799.89700	3356.86820	Detected	Detected	Detected	Detected
7467	A_23_P1523	RHOD	Hs.15114	ras homolog gene family, member D	Homo sapiens ras homolog gene family,	102.22119	149.31651	117.78808	164.94530	Detected	Detected	Detected	Detected
7468	A_23_P156218	GZMK	Hs.277937	granzyme K (granzyme 3; tryptase II)	Homo sapiens granzyme K (granzyme 3;	5.09379	49.06254	4.55026	46.81402	Compromised	Detected	Compromised	Detected
7469	A_33_P3230548	KIF14	Hs.3104	kinesin family member 14	Homo sapiens kinesin family member 14	107.26451	1188.64820	61.23342	511.77700	Detected	Detected	Detected	Detected
7470	A_24_P136758	SAMD4B	Hs.612332	sterile alpha motif domain containing 4B	Homo sapiens sterile alpha motif domai	500.57535	625.52435	304.55075	327.61398	Detected	Detected	Detected	Detected
7471	A_23_P421379	IGF2	Hs.272259	insulin-like growth factor 2 (somatomed	Homo sapiens insulin-like growth factor	97.98582	16.39024	134.85498	12.48476	Detected	Compromised	Detected	Compromised
7472	A_33_P3269539	COL6A2	Hs.420269	collagen, type VI, alpha 2	Homo sapiens collagen, type VI, alpha 2	19445.99800	13529.90000	23290.93000	17159.20700	Detected	Detected	Detected	Detected
7473	A_23_P76291	PRR4	Hs.408153	proline rich 4 (lacrimal)	Homo sapiens proline rich 4 (lacrimal) (P	376.08115	284.34204	500.11660	278.98290	Detected	Detected	Detected	Detected
7474	A_33_P3424867					495.41367	261.72086	274.30426	119.41446	Detected	Detected	Detected	Detected
7475	A_23_P119448	SAPS1	Hs.515610	SAPS domain family, member 1	Homo sapiens SAPS domain family, mer	591.53930	444.97684	155.73788	141.72452	Detected	Detected	Detected	Detected
7476	A_33_P3355708	C14orf180	Hs.153827	chromosome 14 open reading frame 180	Homo sapiens chromosome 14 open rea	14.75952	17.50868	13.67158	8.88500	Detected	Compromised	Compromised	Compromised
7477	A_33_P3276638	HMBOX1	Hs.591836	homeobox containing 1	Homo sapiens homeobox containing 1 (H	1221.12480	834.30580	488.73386	224.74908	Detected	Detected	Detected	Detected
7478	A_23_P125668	SLC25A43	Hs.496658	solute carrier family 25, member 43	Homo sapiens solute carrier family 25, r	1131.52030	1480.13510	970.69710	1030.39300	Detected	Detected	Detected	Detected
7479	A_24_P67552				Putative uncharacterized protein ENSP	1810.68030	1369.62950	1062.98700	654.52750	Detected	Detected	Detected	Detected
7480	A_32_P217543		Hs.211561		DB326574 OCBBF2 Homo sapiens cDN	14.64974	8.55058	11.42161	16.15855	Detected	Compromised	Compromised	Detected
7481	A_33_P3233219	NPBWR1	Hs.248117	neuropeptides B/W receptor 1	Homo sapiens neuropeptides B/W rece	14.42512	17.84905	17.80584	17.77708	Detected	Compromised	Detected	Detected
7482	A_23_P17144	GPN1	Hs.18259	GPN-loop GTPase 1	Homo sapiens GPN-loop GTPase 1 (GP	9203.29300	15664.65600	8399.59600	12903.84000	Detected	Detected	Detected	Detected
7483	A_33_P3411279	CYP21A2	Hs.654479	cytochrome P450, family 21, subfamily A	Homo sapiens cytochrome P450, family	25.25434	31.23885	26.09656	25.71828	Detected	Detected	Detected	Detected
7484	A_23_P373799	CWC22	Hs.311363	CWC22 spliceosome-associated protein	Homo sapiens CWC22 spliceosome-ass	614.47815	394.05322	494.58680	289.24924	Detected	Detected	Detected	Detected
7485	A_32_P60065	F2RL2	Hs.42502	coagulation factor II (thrombin) recepto	Homo sapiens coagulation factor II (thro	408.44794	268.11633	266.87677	144.42500	Detected	Detected	Detected	Detected
7486	A_23_P379327	FAM63B	Hs.591122	family with sequence similarity 63, mem	Homo sapiens family with sequence sim	748.50880	641.52313	486.69820	383.39417	Detected	Detected	Detected	Detected
7487	A_23_P76918	TIMM9	Hs.440525	translocase of inner mitochondrial mem	Homo sapiens translocase of inner mito	8586.77000	8852.48000	7690.44630	6443.92400	Detected	Detected	Detected	Detected
7488	A_23_P423197	RXRA	Hs.590886	retinoid X receptor, alpha	Homo sapiens retinoid X receptor, alpha	3900.16750	2918.38960	3079.95920	1968.67770	Detected	Detected	Detected	Detected
7489	A_33_P3242659	KIF13A	Hs.189915	kinesin family member 13A	Homo sapiens kinesin family member 13	204.78369	471.56890	226.06859	441.67792	Detected	Detected	Detected	Detected
7490	A_33_P3424612		Hs.705093		Ig kappa chain V-III region VG Precurso	125.43209	102.98696	112.27461	78.12534	Detected	Detected	Detected	Detected
7491	A_23_P302681	FIGNL1	Hs.137516	fidgetin-like 1	Homo sapiens fidgetin-like 1 (FIGNL1),	68.84042	127.59539	52.61412	83.14224	Detected	Detected	Detected	Detected
7492	A_33_P3263287	LOC100129111	Hs.627496	hypothetical LOC100129111	Homo sapiens cDNA FLJ44947 fis, clon	77.55720	103.16460	48.23406	42.85287	Detected	Detected	Detected	Detected
7493	A_23_P30254	PLK2	Hs.398157	polo-like kinase 2 (Drosophila)	Homo sapiens polo-like kinase 2 (Droso	957.32150	3466.32810	654.82007	2071.33740	Detected	Detected	Detected	Detected
7494	A_23_P112187	FIBCD1	Hs.133205	fibrinogen C domain containing 1	Homo sapiens fibrinogen C domain cont	26.56942	60.07597	31.03868	54.87181	Detected	Detected	Detected	Detected
7495	A_23_P4551	SETBP1	Hs.435458	SET binding protein 1	Homo sapiens SET binding protein 1 (SE	2580.92680	2639.90280	1874.49820	1729.12790	Detected	Detected	Detected	Detected
7496	A_23_P377339	C4orf36	Hs.339646	chromosome 4 open reading frame 36	Homo sapiens chromosome 4 open read	19.89038	9.32149	23.73599	17.67168	Detected	Compromised	Detected	Detected

7497	A_23_P24044	CNNM2	Hs.719266	cyclin M2	Homo sapiens cyclin M2 (CNNM2), tran	52.26291	34.40937	45.17306	23.66960	Detected	Detected	Detected	Detected
7498	A_33_P3271001	NIPSNAP3B	Hs.720820	nipsnap homolog 3B (C. elegans)	Homo sapiens nipsnap homolog 3B (C. e	31.68244	3.26301	33.86998	4.59980	Detected	Compromised	Detected	Compromised
7499	A_33_P3364641		Hs.711405		Putative uncharacterized protein FLJ42	73.69876	49.03242	50.92661	36.59575	Detected	Detected	Detected	Detected
7500	A_23_P251316	CTNND1	Hs.166011	catenin (cadherin-associated protein), d	Homo sapiens catenin (cadherin-associ	541.68964	496.88922	381.88083	299.87890	Detected	Detected	Detected	Detected
7501	A_24_P101786	THADA	Hs.369592	thyroid adenoma associated	Homo sapiens thyroid adenoma associa	117.56770	199.20015	98.43018	139.85834	Detected	Detected	Detected	Detected
7502	A_23_P93082	NUS1	Hs.289008	nuclear undecaprenyl pyrophosphate sy	Homo sapiens nuclear undecaprenyl pyr	333.56155	581.45090	429.55252	624.74664	Detected	Detected	Detected	Detected
7503	A_23_P144639	TMCO6	Hs.712585	transmembrane and coiled-coil domains	Homo sapiens transmembrane and coile	449.45020	312.18137	387.72995	223.60810	Detected	Detected	Detected	Detected
7504	A_23_P25503	FNDC3A	Hs.508010	fibronectin type III domain containing 3A	Homo sapiens fibronectin type III domai	17337.04700	3379.41110	13145.40500	2221.50980	Detected	Detected	Detected	Detected
7505	A_33_P3248245		Hs.689016		fs26e07.y1 Human Lens cDNA (Normali	2.42446	3.27085	20.44195	3.55606	Compromised	Compromised	Detected	Compromised
7506	A_23_P49674	ARHGEF15	Hs.443109	Rho guanine nucleotide exchange facto	Homo sapiens Rho guanine nucleotide e	4193.38130	2162.64300	425.91107	155.02490	Detected	Detected	Detected	Detected
7507	A_33_P3256054	MLST8	Hs.29203	MTOR associated protein, LST8 homolo	Homo sapiens MTOR associated protei	3591.76980	2624.73120	4483.00100	3168.92110	Detected	Detected	Detected	Detected
7508	A_23_P31747	C8orf76	Hs.521800	chromosome 8 open reading frame 76	Homo sapiens chromosome 8 open rea	3014.48600	3527.29980	2706.20750	2695.23880	Detected	Detected	Detected	Detected
7509	A_33_P3370163				WUGSC:H_DJ1086D14.1 protein Fragme	37.70301	35.24078	25.91581	26.42673	Detected	Detected	Detected	Detected
7510	A_24_P398064		Hs.719327		Retrograde Golgi transport protein RGP	4085.67800	2767.53400	2993.49490	1967.36000	Detected	Detected	Detected	Detected
7511	A_23_P377616	CIRBP	Hs.618145	cold inducible RNA binding protein	Homo sapiens cold inducible RNA bindi	710.36460	109.50278	620.83110	104.94281	Detected	Detected	Detected	Detected
7512	A_23_P402319	GAPT	Hs.547697	GRB2-binding adaptor protein, transme	Homo sapiens GRB2-binding adaptor pr	5.68826	8.52619	13.94601	11.50902	Compromised	Compromised	Detected	Compromised
7513	A_33_P3276369	HSD17B7	Hs.492925	hydroxysteroid (17-beta) dehydrogenas	Homo sapiens hydroxysteroid (17-beta)	111.05358	56.42326	118.17542	61.71288	Detected	Detected	Detected	Detected
7514	A_33_P3290955	KIAA1875	Hs.686343	KIAA1875	Homo sapiens KIAA1875 (KIAA1875), n	6647.96500	10235.60100	5198.73050	6524.47500	Detected	Detected	Detected	Detected
7515	A_33_P3410659	CLEC12B	Hs.127937	C-type lectin domain family 12, membe	Homo sapiens C-type lectin domain fam	17.86912	22.87861	10.76614	21.60335	Detected	Detected	Compromised	Detected
7516	A_24_P253251	SLC7A1	Hs.14846	solute carrier family 7 (cationic amino a	Homo sapiens solute carrier family 7 (c	2154.40160	5707.24400	1371.49130	3077.87300	Detected	Detected	Detected	Detected
7517	A_23_P142345	PRTN3	Hs.928	proteinase 3	Homo sapiens proteinase 3 (PRTN3), m	20.14180	19.75725	30.12624	19.58725	Detected	Detected	Detected	Detected
7518	A_32_P49867	LOC645277	Hs.282811	Uncharacterized protein LOC645277	PREDICTED: Homo sapiens Uncharacte	10.94258	12.97560	13.72785	12.44866	Compromised	Compromised	Detected	Compromised
7519	A_23_P20463	RAD21	Hs.81848	RAD21 homolog (S. pombe)	Homo sapiens RAD21 homolog (S. pomb	14911.00600	19125.73400	12972.62900	13017.82200	Detected	Detected	Detected	Detected
7520	A_23_P134953	PLIN2	Hs.3416	perilipin 2	Homo sapiens perilipin 2 (PLIN2), mRNA	10996.10900	4124.06540	10729.60200	3637.36550	Detected	Detected	Detected	Detected
7521	A_23_P154522	MTA3	Hs.435413	metastasis associated 1 family, member	Homo sapiens metastasis associated 1	1156.69420	1038.78560	884.54846	687.82310	Detected	Detected	Detected	Detected
7522	A_23_P103465	PLA2G5	Hs.319438	phospholipase A2, group V	Homo sapiens phospholipase A2, group	34.07610	44.77688	38.82804	35.59290	Detected	Detected	Detected	Detected
7523	A_23_P162288	MYO1A	Hs.5394	myosin IA	Homo sapiens myosin IA (MYO1A), mRN	27.45301	32.21744	12.39413	13.78953	Detected	Detected	Compromised	Compromised
7524	A_23_P3602	NUDT7	Hs.282665	nudix (nucleoside diphosphate linked mo	Homo sapiens nudix (nucleoside diphos	970.30634	183.98637	668.95490	92.74550	Detected	Detected	Detected	Detected
7525	A_33_P3424112	DHX40	Hs.29403	DEAH (Asp-Glu-Ala-His) box polypepti	Homo sapiens DEAH (Asp-Glu-Ala-His)	198.12599	102.55642	187.77078	70.25285	Detected	Detected	Detected	Detected
7526	A_23_P119042	NKG7	Hs.10306	natural killer cell group 7 sequence	Homo sapiens natural killer cell group 7	260.82780	365.95538	55.96083	54.42605	Detected	Detected	Detected	Detected
7527	A_23_P155463	LRRC2	Hs.657345	leucine rich repeat containing 2	Homo sapiens leucine rich repeat conta	287.88080	154.58295	23.03155	45.79820	Detected	Detected	Detected	Detected
7528	A_32_P100430	LOC100128737	Hs.721809	hypothetical LOC100128737	PREDICTED: Homo sapiens hypothetica	210.49495	950.18970	234.54007	988.67285	Detected	Detected	Detected	Detected
7529	A_33_P3421571	RAPH1	Hs.471162	Ras association (RalGDS/AF-6) and ple	Homo sapiens Ras association (RalGDS	2869.31980	4053.23600	3371.02860	3641.93680	Detected	Detected	Detected	Detected
7530	A_23_P334263	SENP8	Hs.513002	SUMO/sentrin specific peptidase family	Homo sapiens SUMO/sentrin specific p	83.91871	106.58368	99.76184	103.00511	Detected	Detected	Detected	Detected
7531	A_24_P649388	C21orf63	Hs.208358	chromosome 21 open reading frame 63	Homo sapiens chromosome 21 open rea	180.68794	91.11498	229.37073	104.87658	Detected	Detected	Detected	Detected
7532	A_23_P129118	PDCD7	Hs.458596	programmed cell death 7	Homo sapiens programmed cell death 7	1120.67090	987.71716	875.31920	691.32250	Detected	Detected	Detected	Detected
7533	A_23_P212213	THUMPD3	Hs.443081	THUMP domain containing 3	Homo sapiens THUMP domain containi	3399.14480	3604.44000	2999.43700	2738.52220	Detected	Detected	Detected	Detected
7534	A_23_P137046	NYX	Hs.302019	nyctalopin	Homo sapiens nyctalopin (NYX), mRNA	929.00946	1363.44620	499.78513	503.72880	Detected	Detected	Detected	Detected
7535	A_23_P54953	SAP30BP	Hs.655088	SAP30 binding protein	Homo sapiens SAP30 binding protein (S	1919.56760	2541.11330	1384.28210	1621.07590	Detected	Detected	Detected	Detected
7536	A_33_P3298043	ABCC6	Hs.442182	ATP-binding cassette, sub-family C (CF	Homo sapiens ATP-binding cassette, su	93.21674	48.85289	92.33732	33.13603	Detected	Detected	Detected	Detected
7537	A_33_P3767773	UBR4	Hs.148078	ubiquitin protein ligase E3 component n	Homo sapiens ubiquitin protein ligase E	17330.98600	19693.97500	13200.59600	13572.52100	Detected	Detected	Detected	Detected
7538	A_24_P415624	MLXIP	Hs.721711	MLX interacting protein	Homo sapiens MLX interacting protein (	179.00946	239.61943	149.77159	181.67278	Detected	Detected	Detected	Detected
7539	A_33_P3228190	KCNAB2	Hs.440497	potassium voltage-gated channel, shake	Homo sapiens potassium voltage-gated	26.95176	17.10450	32.10412	18.15509	Detected	Compromised	Detected	Detected
7540	A_33_P3258274				Tissue factor pathway inhibitor Precurs	10121.89000	6367.16100	6264.05700	3287.29200	Detected	Detected	Detected	Detected
7541	A_23_P67042	MOCOS	Hs.405028	molybdenum cofactor sulfurase	Homo sapiens molybdenum cofactor su	831.65510	695.35803	692.07104	501.97272	Detected	Detected	Detected	Detected
7542	A_23_P217088	AK1	Hs.175473	adenylate kinase 1	Homo sapiens adenylate kinase 1 (AK1)	5124.55700	3963.11000	6966.73440	5131.61800	Detected	Detected	Detected	Detected
7543	A_32_P221958	AGAP7	Hs.528346	ArfGAP with GTPase domain, ankyrin re	Homo sapiens ArfGAP with GTPase do	933.28120	1025.91810	305.86618	282.04248	Detected	Detected	Detected	Detected
7544	A_33_P3287223	DPP4	Hs.368912	dipeptidyl-peptidase 4	Homo sapiens dipeptidyl-peptidase 4 (D	5817.76170	828.92180	5390.58060	697.43190	Detected	Detected	Detected	Detected
7545	A_23_P3823	BCKDK	Hs.513520	branched chain ketoacid dehydrogenase	Homo sapiens branched chain ketoacid	5135.71800	6493.46140	5654.47560	6483.04350	Detected	Detected	Detected	Detected
7546	A_33_P3407895	RINL	Hs.446933	Ras and Rab interactor-like	Homo sapiens Ras and Rab interactor-l	780.31100	168.07335	897.40247	183.72337	Detected	Detected	Detected	Detected
7547	A_23_P387031	COL23A1	Hs.660026	collagen, type XXIII, alpha 1	Homo sapiens collagen, type XXIII, alph	99.96572	72.74659	100.65170	69.98785	Detected	Detected	Detected	Detected
7548	A_23_P320242	KIAA1324L	Hs.208093	KIAA1324-like	Homo sapiens KIAA1324-like (KIAA132	535.53830	149.16720	405.43512	95.24467	Detected	Detected	Detected	Detected
7549	A_33_P3229577	LOC100129035	Hs.716185	hypothetical LOC100129035	PREDICTED: Homo sapiens hypothetica	184.27924	225.72581	137.13345	109.44329	Detected	Detected	Detected	Detected
7550	A_33_P3436732	DSCR10	Hs.147695	Down syndrome critical region gene 10	Homo sapiens Down syndrome critical r	638.00160	473.40260	74.11694	59.34852	Detected	Detected	Detected	Detected
7551	A_33_P3306624	HCRT	Hs.158348	hypocretin (orexin) neuropeptide precur	Homo sapiens hypocretin (orexin) neuro	14.03532	13.87179	15.16734	17.90422	Detected	Compromised	Detected	Detected
7552	A_23_P147296	NFU1	Hs.430439	NFU1 iron-sulfur cluster scaffold homol	Homo sapiens NFU1 iron-sulfur cluster	5670.81500	6298.69100	4367.52400	4038.21680	Detected	Detected	Detected	Detected
7553	A_23_P131074	THEG	Hs.250002	Theg homolog (mouse)	Homo sapiens Theg homolog (mouse) (T	25.77035	23.94946	20.50564	17.43448	Detected	Detected	Detected	Detected
7554	A_33_P3374903					236.53323	233.71794	44.05866	30.15449	Detected	Detected	Detected	Detected
7555	A_33_P3278475	FOXN2	Hs.468478	forkhead box N2	Homo sapiens forkhead box N2 (FOXN2	3587.07640	3241.88400	3707.25440	2608.79150	Detected	Detected	Detected	Detected
7556	A_32_P183970	C15orf62	Hs.631715	chromosome 15 open reading frame 62	Homo sapiens chromosome 15 open rea	16.14035	3.25167	16.50773	6.08134	Detected	Compromised	Detected	Compromised
7557	A_33_P3250438				UPF0608 protein C19orf42 Precursor [	152.76082	961.47170	159.04251	747.25653	Detected	Detected	Detected	Detected
7558	A_23_P84922	HDAC8	Hs.310536	histone deacetylase 8	Homo sapiens histone deacetylase 8 (H	212.73463	155.03398	285.10486	186.78116	Detected	Detected	Detected	Detected
7559	A_23_P83579	ARNT2	Hs.459070	aryl-hydrocarbon receptor nuclear tran	Homo sapiens aryl-hydrocarbon recept	4552.67630	3743.09030	3370.80130	2297.29130	Detected	Detected	Detected	Detected
7560	A_23_P314760	PRKAG2	Hs.647072	protein kinase, AMP-activated, gamma	Homo sapiens protein kinase, AMP-acti	1269.43250	1713.13940	1220.52200	1365.48280	Detected	Detected	Detected	Detected
7561	A_24_P47547	RAN	Hs.10842	RAN, member RAS oncogene family	Homo sapiens RAN, member RAS onco	7879.93400	21563.35000	6987.78000	16778.05000	Detected	Detected	Detected	Detected
7562	A_24_P160104	TUBA8	Hs.137400	tubulin, alpha 8	Homo sapiens tubulin, alpha 8 (TUBA8),	1005.29680	920.13890	363.45703	293.64230	Detected	Detected	Detected	Detected
7563	A_33_P3423401	ZNF790	Hs.282067	zinc finger protein 790	Homo sapiens zinc finger protein 790 (Z	204.36522	174.43540	150.36705	107.12930	Detected	Detected	Detected	Detected
7564	A_23_P376188	BEND7	Hs.498740	BEN domain containing 7	Homo sapiens BEN domain containing 7	177.55084	236.30428	159.83562	191.21362	Detected	Detected	Detected	Detected
7565	A_23_P93641	AKR1B10	Hs.116724	aldo-keto reductase family 1, member B	Homo sapiens aldo-keto reductase fam	102.68280	96.25636	85.36409	66.39071	Detected	Detected	Detected	Detected
7566	A_33_P3227400	COL4A4	Hs.591645	collagen, type IV, alpha 4	Homo sapiens collagen, type IV, alpha 4	254.18837	45.21323	213.89528	29.64439	Detected	Detected	Detected	Detected
7567	A_33_P3294149				Neuron-specific vesicular protein calcy	16.49631	17.84572	22.38862	15.61942	Detected	Detected	Detected	Detected
7568	A_23_P367013	TAS2R60	Hs.688222	taste receptor, type 2, member 60	Homo sapiens taste receptor, type 2, m	99.18818	109.26608	34.79522	31.71020	Detected	Detected	Detected	Detected
7569	A_33_P3260100	C6orf129	Hs.284207	chromosome 6 open reading frame 129	Homo sapiens chromosome 6 open rea	6067.84670	9549.75300	6574.63600	9329.24000	Detected	Detected	Detected	Detected
7570	A_23_P18465	RFC1	Hs.507475	replication factor C (activator 1) 1, 145	Homo sapiens replication factor C (acti	1653.96000	1650.78140	1540.61500	1244.44370	Detected	Detected	Detected	Detected
7571	A_23_P258190	AKR1B1	Hs.521212	aldo-keto reductase family 1, member B	Homo sapiens aldo-keto reductase fam	59975.41800	14000.75700	68133.56000	15516.85800	Detected	Detected	Detected	Detected

7572	A_33_P3217213	PDLIM7	Hs.533040	PDZ and LIM domain 7 (enigma)	Homo sapiens PDZ and LIM domain 7 (e	1046.65440	810.54280	1116.80250	889.94135	Detected	Detected	Detected	Detected
7573	A_33_P3368771	C3orf47	Hs.636552	chromosome 3 open reading frame 47	Homo sapiens chromosome 3 open read	174.39711	191.03238	165.19571	168.41818	Detected	Detected	Detected	Detected
7574	A_24_P301846	GART	Hs.473648	phosphoribosylglycinamide formyltransfe	Homo sapiens phosphoribosylglycinamid	481.07535	677.20330	478.70187	563.48566	Detected	Detected	Detected	Detected
7575	A_24_P751247		Hs.722533		BX117289 Soares_NFL_T_GBC_S1 Homo	12.93751	9.19231	11.58147	8.84860	Detected	Compromised	Compromised	Compromised
7576	A_33_P3399755					7594.20460	4935.99400	5587.33150	2337.04500	Detected	Detected	Detected	Detected
7577	A_33_P3338275		Hs.498322		Putative uncharacterized protein FLJ43	114.45502	157.85388	49.02289	40.41557	Detected	Detected	Detected	Detected
7578	A_33_P3211203		Hs.467500		Homo sapiens cDNA FLJ43576 fis, clon	733.57940	720.66930	360.69308	305.07382	Detected	Detected	Detected	Detected
7579	A_32_P703	LOC646626	Hs.653214	hypothetical LOC646626	PREDICTED: Homo sapiens hypothetica	561.56134	622.41270	550.06830	558.35840	Detected	Detected	Detected	Detected
7580	A_23_P127824	TNNT3	Hs.73454	troponin T type 3 (skeletal, fast)	Homo sapiens troponin T type 3 (skelet	24.94082	33.35895	19.51799	23.45023	Detected	Detected	Detected	Detected
7581	A_33_P3280805	LMO7	Hs.207631	LIM domain 7	Homo sapiens LIM domain 7 (LMO7), tr	41.05179	180.78215	52.77922	240.14589	Detected	Detected	Detected	Detected
7582	A_23_P68087	ATIC	Hs.90280	5-aminoimidazole-4-carboxamide ribon	Homo sapiens 5-aminoimidazole-4-carb	6453.78400	7354.13670	6410.65230	6473.89600	Detected	Detected	Detected	Detected
7583	A_23_P375566	STXBP4	Hs.35199	syntaxin binding protein 4	Homo sapiens syntaxin binding protein 4	85.29376	84.67739	69.36487	60.62491	Detected	Detected	Detected	Detected
7584	A_33_P3636590	SUCLG2	Hs.655250	succinate-CoA ligase, GDP-forming, be	Homo sapiens succinate-CoA ligase, GD	2633.36870	2712.22120	2362.52950	1963.89060	Detected	Detected	Detected	Detected
7585	A_23_P207811	PAIP1	Hs.482038	poly(A) binding protein interacting prote	Homo sapiens poly(A) binding protein in	2219.31250	5289.07300	1768.01090	3476.45240	Detected	Detected	Detected	Detected
7586	A_23_P79496	GTF2A1L	Hs.44385	general transcription factor IIA, 1-like	Homo sapiens general transcription fact	62.05046	8.96835	48.18505	6.71037	Detected	Compromised	Detected	Compromised
7587	A_33_P3691168	IL31RA	Hs.55378	interleukin 31 receptor A	Homo sapiens interleukin 31 receptor A	16.87226	20.58691	16.93884	20.84505	Detected	Detected	Detected	Detected
7588	A_23_P357929	SLC35D1	Hs.213642	solute carrier family 35 (UDP-glucuroni	Homo sapiens solute carrier family 35 (	68.23728	310.42870	53.08312	205.62512	Detected	Detected	Detected	Detected
7589	A_23_P252711	LACTB2	Hs.118554	lactamase, beta 2	Homo sapiens lactamase, beta 2 (LACT	1623.86990	1352.03700	1099.99700	733.46106	Detected	Detected	Detected	Detected
7590	A_33_P3322288	AZI2	Hs.706676	5-azacytidine induced 2	Homo sapiens 5-azacytidine induced 2	1683.16830	1418.54360	1329.51050	894.53705	Detected	Detected	Detected	Detected
7591	A_33_P3256344				Putative uncharacterized protein ENSP	4842.56000	6846.11960	3170.52610	3606.79470	Detected	Detected	Detected	Detected
7592	A_23_P217712	ARSD	Hs.528631	arylsulfatase D	Homo sapiens arylsulfatase D (ARSD), t	160.65842	216.27990	100.57462	108.18443	Detected	Detected	Detected	Detected
7593	A_24_P187799	C7orf58	Hs.189652	chromosome 7 open reading frame 58	Homo sapiens chromosome 7 open read	53.83073	109.20142	29.21242	57.89966	Detected	Detected	Detected	Detected
7594	A_23_P39561	UBE2F	Hs.471785	ubiquitin-conjugating enzyme E2F (puta	Homo sapiens ubiquitin-conjugating enz	1101.75010	2587.61470	898.92413	1925.05080	Detected	Detected	Detected	Detected
7595	A_33_P3415923	FMNL3	Hs.179838	formin-like 3	Homo sapiens formin-like 3 (FMNL3), tr	391.72076	405.23093	500.34048	471.48163	Detected	Detected	Detected	Detected
7596	A_33_P3389286	SFN	Hs.523718	stratifin	Homo sapiens stratifin (SFN), mRNA [N	196.03201	196.59235	156.40228	261.41016	Detected	Detected	Detected	Detected
7597	A_33_P3287879	HIST1H3H	Hs.591778	histone cluster 1, H3h	Homo sapiens histone cluster 1, H3h (H	690.24536	731.72350	139.45822	210.52835	Detected	Detected	Detected	Detected
7598	A_33_P3395028	LOC152225	Hs.376768	hypothetical LOC152225	Homo sapiens hypothetical LOC152225	28.37306	18.96412	24.47730	11.02921	Detected	Detected	Detected	Compromised
7599	A_33_P3226546					147.78470	138.35072	5.73645	5.26898	Detected	Detected	Compromised	Compromised
7600	A_24_P940086	NXPH3	Hs.55069	neurexophilin 3	Homo sapiens neurexophilin 3 (NXPH3),	187.19920	38.63708	184.58086	39.30105	Detected	Detected	Detected	Detected
7601	A_33_P3357199	SH2D6	Hs.209542	SH2 domain containing 6	Homo sapiens SH2 domain containing 6	28.19188	20.14340	31.75531	11.23727	Detected	Detected	Detected	Compromised
7602	A_23_P116942	LAG3	Hs.409523	lymphocyte-activation gene 3	Homo sapiens lymphocyte-activation ge	92.78269	55.56650	118.59660	62.05795	Detected	Detected	Detected	Detected
7603	A_23_P62967	DISC1	Hs.13318	disrupted in schizophrenia 1	Homo sapiens disrupted in schizophreni	494.50803	225.94347	386.41428	145.27892	Detected	Detected	Detected	Detected
7604	A_33_P3295077					128.60727	123.60188	89.57892	81.25828	Detected	Detected	Detected	Detected
7605	A_24_P341476	LOC729313	Hs.647367	hypothetical LOC729313	PREDICTED: Homo sapiens similar to T	11328.74100	15199.99000	15761.75400	17999.98200	Detected	Detected	Detected	Detected
7606	A_33_P3332885	BTN2A1	Hs.159028	butyrophilin, subfamily 2, member A1	Homo sapiens butyrophilin, subfamily 2,	189.05968	305.91693	171.75908	217.55493	Detected	Detected	Detected	Detected
7607	A_23_P204696	CDKN1B	Hs.238990	cyclin-dependent kinase inhibitor 1B (p	Homo sapiens cyclin-dependent kinase	77.79624	70.33453	85.73209	68.91789	Detected	Detected	Detected	Detected
7608	A_23_P161624	FOSL1	Hs.283565	FOS-like antigen 1	Homo sapiens FOS-like antigen 1 (FOS	1134.81770	2069.64450	954.96710	1607.21020	Detected	Detected	Detected	Detected
7609	A_32_P220472	ZFAND6	Hs.596679	zinc finger, AN1-type domain 6	Homo sapiens zinc finger, AN1-type do	16902.74000	12500.03800	15481.15300	9277.23100	Detected	Detected	Detected	Detected
7610	A_24_P277155	HLTF	Hs.3068	helicase-like transcription factor	Homo sapiens helicase-like transcriptio	1323.09450	1091.59080	1153.76560	778.57050	Detected	Detected	Detected	Detected
7611	A_33_P3251518		Hs.654718		Pyrroline-5-carboxylate reductase 2 (P	4.62888	9.17621	14.26402	9.05835	Compromised	Compromised	Detected	Compromised
7612	A_33_P3213064	STAT2	Hs.530595	signal transducer and activator of trans	Homo sapiens signal transducer and act	15311.22200	10262.19900	13402.12000	7433.21100	Detected	Detected	Detected	Detected
7613	A_33_P3211734	SIKE1	Hs.709277	suppressor of IKBKE 1	Homo sapiens suppressor of IKBKE 1 (S	1718.34190	2819.40870	1286.78170	1812.30180	Detected	Detected	Detected	Detected
7614	A_23_P143512	HSF2BP	Hs.406157	heat shock transcription factor 2 bindin	Homo sapiens heat shock transcription	66.72583	12.73651	76.42082	18.83087	Detected	Compromised	Detected	Detected
7615	A_23_P58228	ODAM	Hs.143811	odontogenic, ameloblast asssociated	Homo sapiens odontogenic, ameloblast	2.98308	4.88931	17.86589	16.04250	Compromised	Compromised	Detected	Detected
7616	A_23_P63219	POGZ	Hs.489873	pogo transposable element with ZNF do	Homo sapiens pogo transposable eleme	2126.76680	1672.36390	1521.76650	1054.73060	Detected	Detected	Detected	Detected
7617	A_23_P254212	RPA4	Hs.659349	replication protein A4, 34kDa	Homo sapiens replication protein A4, 34	438.31510	252.75871	173.31750	129.25136	Detected	Detected	Detected	Detected
7618	A_24_P307808	KCTD16	Hs.661870	potassium channel tetramerisation dom	Homo sapiens potassium channel tetran	7.27348	6.47645	17.73487	11.61274	Compromised	Compromised	Detected	Compromised
7619	A_33_P3314978	LOC100130522		hypothetical LOC100130522	Homo sapiens hypothetical LOC100130	89.28635	104.88119	60.88968	54.75899	Detected	Detected	Detected	Detected
7620	A_24_P940666	MED1	Hs.643754	mediator complex subunit 1	Homo sapiens mediator complex subuni	246.42535	344.35492	183.97148	193.33370	Detected	Detected	Detected	Detected
7621	A_23_P59202	TAF11	Hs.112444	TAF11 RNA polymerase II, TATA box bi	Homo sapiens TAF11 RNA polymerase	1834.09630	2973.66970	1857.16800	2538.89700	Detected	Detected	Detected	Detected
7622	A_24_P687582	TADA2B	Hs.518614	transcriptional adaptor 2B	Homo sapiens transcriptional adaptor 2	143.48213	178.99371	117.01102	128.20078	Detected	Detected	Detected	Detected
7623	A_23_P258018	MYL5	Hs.410970	myosin, light chain 5, regulatory	Homo sapiens myosin, light chain 5, reg	803.04440	928.80010	720.27970	602.60280	Detected	Detected	Detected	Detected
7624	A_23_P218190	CAPN3	Hs.143261	calpain 3, (p94)	Homo sapiens calpain 3, (p94) (CAPN3)	81.76717	73.07670	95.23557	72.63447	Detected	Detected	Detected	Detected
7625	A_33_P3231923		Hs.570209		Putative uncharacterized protein ENSP	3553.63350	1648.25710	359.68760	110.79108	Detected	Detected	Detected	Detected
7626	A_23_P63289	SSU72	Hs.657061	SSU72 RNA polymerase II CTD phospha	Homo sapiens SSU72 RNA polymerase	17074.95500	8196.37100	12517.89900	5295.89160	Detected	Detected	Detected	Detected
7627	A_33_P3360271	EVX2	Hs.452341	even-skipped homeobox 2	Homo sapiens even-skipped homeobox	27.25938	39.67777	43.56790	50.39588	Detected	Detected	Detected	Detected
7628	A_23_P2492	C1S	Hs.458355	complement component 1, s subcompor	Homo sapiens complement component	99613.09000	15813.12000	66059.58600	9950.22800	Detected	Detected	Detected	Detected
7629	A_24_P212072	ANKRD32	Hs.657315	ankyrin repeat domain 32	Homo sapiens ankyrin repeat domain 32	136.44412	134.06170	148.43343	120.00668	Detected	Detected	Detected	Detected
7630	A_24_P117410	KLHDC7B	Hs.137007	kelch domain containing 7B	Homo sapiens kelch domain containing	55.47781	64.58825	42.77114	42.85590	Detected	Detected	Detected	Detected
7631	A_33_P3320152	SNRPE	Hs.334612	small nuclear ribonucleoprotein polypep	Homo sapiens small nuclear ribonucleop	2888.59450	3485.75120	1370.72630	1230.35680	Detected	Detected	Detected	Detected
7632	A_33_P3382835	NYNRIN	Hs.288348	NYN domain and retroviral integrase co	Homo sapiens NYN domain and retrovir	289.93054	360.69977	110.58340	121.36601	Detected	Detected	Detected	Detected
7633	A_23_P500886	CLDN15	Hs.38738	claudin 15	Homo sapiens claudin 15 (CLDN15), mR	151.00240	64.38749	120.14507	45.05403	Detected	Detected	Detected	Detected
7634	A_33_P3351615					60103.97000	29431.48600	36331.47700	17706.60000	Detected	Detected	Detected	Detected
7635	A_23_P22660	CYSLTR1	Hs.201300	cysteinyl leukotriene receptor 1	Homo sapiens cysteinyl leukotriene rec	32.25481	37.92138	32.05790	37.12317	Detected	Detected	Detected	Detected
7636	A_33_P3387781	OR2L1P	Hs.684887	olfactory receptor, family 2, subfamily L	Homo sapiens olfactory receptor, family	25.91796	35.39547	32.51806	31.40518	Detected	Detected	Detected	Detected
7637	A_33_P3260175	LOC100288891	Hs.308060	hypothetical protein LOC100288891	PREDICTED: Homo sapiens hypothetica	30.98264	52.06646	24.63136	24.59014	Detected	Detected	Detected	Detected
7638	A_24_P382119	MTMR4	Hs.514373	myotubularin related protein 4	Homo sapiens myotubularin related prot	11533.94900	6159.33600	9733.28200	4450.34770	Detected	Detected	Detected	Detected
7639	A_33_P3889862	LOC151475	Hs.528154	hypothetical protein LOC151475	Homo sapiens cDNA FLJ30454 fis, clon	25.53617	22.03210	19.62162	8.15393	Detected	Detected	Detected	Compromised
7640	A_23_P8848	INTS9	Hs.162397	integrator complex subunit 9	Homo sapiens integrator complex subur	3455.63700	2752.37840	3057.92160	2349.48780	Detected	Detected	Detected	Detected
7641	A_23_P165186	LIN37	Hs.529100	lin-37 homolog (C. elegans)	Homo sapiens lin-37 homolog (C. elegar	2444.27800	3587.47970	2228.86600	2895.85180	Detected	Detected	Detected	Detected
7642	A_33_P3391418	TMEM40	Hs.475502	transmembrane protein 40	Homo sapiens transmembrane protein 4	82.03069	85.63739	30.73828	29.50971	Detected	Detected	Detected	Detected
7643	A_33_P3298775	PRSSL1	Hs.245146	protease, serine-like 1	Homo sapiens protease, serine-like 1 (F	16.09948	5.82953	21.15192	4.60282	Detected	Compromised	Detected	Compromised
7644	A_33_P3409649	ADAMTS2	Hs.591725	ADAM metallopeptidase with thrombosp	Homo sapiens ADAM metallopeptidase	311.91174	358.95102	229.79218	263.64185	Detected	Detected	Detected	Detected
7645	A_23_P77073	SPPL2A	Hs.401537	signal peptide peptidase-like 2A	Homo sapiens signal peptide peptidase-	2504.74370	5601.07670	1908.45760	3725.30540	Detected	Detected	Detected	Detected
7646	A_33_P3217649	C9orf116	Hs.414028	chromosome 9 open reading frame 116	Homo sapiens chromosome 9 open read	784.76636	247.42477	753.50745	230.75587	Detected	Detected	Detected	Detected

7647	A_33_P3400578	HLF	Hs.196952	hepatic leukemia factor	Homo sapiens hepatic leukemia factor (	156.09073	3.36660	100.69540	2.88222	Detected	Compromised	Detected	Compromised
7648	A_33_P3419632				Beta-galactosidase-1-like protein 3 (E(	17.97596	20.90284	9.66675	14.01524	Detected	Detected	Compromised	Compromised
7649	A_23_P138461	C10orf2	Hs.22678	chromosome 10 open reading frame 2	Homo sapiens chromosome 10 open rea	381.26538	423.59550	364.46365	343.84064	Detected	Detected	Detected	Detected
7650	A_24_P276628	PPT1	Hs.3873	palmitoyl-protein thioesterase 1	Homo sapiens palmitoyl-protein thioest	969.53440	830.05190	936.04236	748.59040	Detected	Detected	Detected	Detected
7651	A_33_P3283064					574.55330	713.05524	338.09283	321.17444	Detected	Detected	Detected	Detected
7652	A_24_P42136	KRT18	Hs.406013	keratin 18	Homo sapiens keratin 18 (KRT18), trans	101.46272	121.99409	130.38747	155.47878	Detected	Detected	Detected	Detected
7653	A_23_P14072	KRT8	Hs.533782	keratin 8	Homo sapiens keratin 8 (KRT8), mRNA	1151.19740	1257.03900	877.05620	773.95435	Detected	Detected	Detected	Detected
7654	A_32_P72181	LOC647946	Hs.649350	hypothetical protein LOC647946	Homo sapiens hypothetical protein LOC	28.65982	38.15098	28.12687	28.97585	Detected	Detected	Detected	Detected
7655	A_23_P18490	MAEA	Hs.139896	macrophage erythroblast attacher	Homo sapiens macrophage erythroblast	2207.14450	1999.49210	1476.64250	1305.27810	Detected	Detected	Detected	Detected
7656	A_33_P3350452				Ribosomal protein L15 [Source:UniProt	2436.19500	2695.22630	856.00653	771.38980	Detected	Detected	Detected	Detected
7657	A_23_P91702	EIF3D	Hs.55682	eukaryotic translation initiation factor 3	Homo sapiens eukaryotic translation ini	32309.90000	44701.31200	37027.45300	47182.39000	Detected	Detected	Detected	Detected
7658	A_24_P348989	LILRA1	Hs.710507	leukocyte immunoglobulin-like receptor,	Homo sapiens leukocyte immunoglobulin	61.95285	91.59196	44.95077	42.51800	Detected	Detected	Detected	Detected
7659	A_33_P3258782	AP1S2	Hs.653504	adaptor-related protein complex 1, sigm	Homo sapiens adaptor-related protein c	663.96344	879.91620	677.63050	769.18805	Detected	Detected	Detected	Detected
7660	A_33_P3353630					28.57620	29.31804	20.50711	21.25972	Detected	Detected	Detected	Detected
7661	A_33_P3306818		Hs.302963		cDNA FLJ34460 fis, clone HLUNG2002	13.76019	16.03510	34.08410	42.32504	Detected	Compromised	Detected	Detected
7662	A_24_P94054	STK4	Hs.472838	serine/threonine kinase 4	Homo sapiens serine/threonine kinase	398.67430	402.87570	472.16467	444.18423	Detected	Detected	Detected	Detected
7663	A_24_P40907	PPAPDC2	Hs.107510	phosphatidic acid phosphatase type 2 d	Homo sapiens phosphatidic acid phosph	1407.93860	829.34490	1009.37006	457.72974	Detected	Detected	Detected	Detected
7664	A_33_P3297606		Hs.177968		Phospholipid scramblase 2 (PL scrambl	15.15626	3.26780	22.53095	3.90642	Detected	Compromised	Detected	Compromised
7665	A_24_P945147	RABEP1	Hs.592121	rabaptin, RAB GTPase binding effector	Homo sapiens rabaptin, RAB GTPase bi	1402.62960	1241.46030	910.85800	665.19040	Detected	Detected	Detected	Detected
7666	A_24_P202558	SIPA1L3	Hs.655502	signal-induced proliferation-associated	Homo sapiens signal-induced proliferati	288.77527	528.62600	161.87292	193.47784	Detected	Detected	Detected	Detected
7667	A_33_P3395146	LOC646762	Hs.30579	hypothetical LOC646762	Homo sapiens hypothetical LOC646762	1188.48000	728.65466	1007.88324	589.14050	Detected	Detected	Detected	Detected
7668	A_24_P195272				GB	28.47838	39.18382	26.14722	27.02454	Detected	Detected	Detected	Detected
7669	A_23_P64019	MTMR2	Hs.181326	myotubularin related protein 2	Homo sapiens myotubularin related prot	1320.09110	1991.40360	836.24000	1143.55680	Detected	Detected	Detected	Detected
7670	A_23_P15348	MPRIP	Hs.462341	myosin phosphatase Rho interacting pr	Homo sapiens myosin phosphatase Rho	6380.43300	22459.92800	5414.05370	17805.38000	Detected	Detected	Detected	Detected
7671	A_33_P3413927	VEZT	Hs.24135	vezatin, adherens junctions transmembr	Homo sapiens vezatin, adherens junctio	173.16278	284.47345	186.31201	275.31340	Detected	Detected	Detected	Detected
7672	A_33_P3220090	ZNF391	Hs.651176	zinc finger protein 391	Homo sapiens zinc finger protein 391 (Z	28.47206	28.03139	31.23404	30.53726	Detected	Detected	Detected	Detected
7673	A_23_P356646	LOC100132288	Hs.487562	hypothetical protein LOC100132288	Homo sapiens hypothetical protein LOC	181.26780	89.09624	142.53410	53.75972	Detected	Detected	Detected	Detected
7674	A_23_P117971	LOC100287593	Hs.654375	hypothetical protein LOC100287593	PREDICTED: Homo sapiens hypothetica	26849.37100	21084.76800	6612.70000	4519.79350	Detected	Detected	Detected	Detected
7675	A_33_P3408305	LASS3	Hs.662371	LAG1 homolog, ceramide synthase 3	Homo sapiens LAG1 homolog, ceramide	4550.42870	7325.92240	471.17410	680.17914	Detected	Detected	Detected	Detected
7676	A_32_P20691	CLK3	Hs.584748	CDC-like kinase 3	Homo sapiens CDC-like kinase 3 (CLK3	8012.86500	6749.12060	4052.11100	3070.20000	Detected	Detected	Detected	Detected
7677	A_24_P6903	ACTBL2	Hs.482167	actin, beta-like 2	Homo sapiens actin, beta-like 2 (ACTB	2719.90920	4652.42530	1949.98320	3233.96660	Detected	Detected	Detected	Detected
7678	A_23_P151634	SUPT16H	Hs.213724	suppressor of Ty 16 homolog (S. cerevi	Homo sapiens suppressor of Ty 16 hom	1210.32240	1761.39660	984.39940	1239.80870	Detected	Detected	Detected	Detected
7679	A_33_P3846177	B4GALNT1	Hs.591019	beta-1,4-N-acetyl-galactosaminyl trans	Homo sapiens beta-1,4-N-acetyl-galac	66.17393	487.64984	53.93387	442.10440	Detected	Detected	Detected	Detected
7680	A_24_P323114	ANXA2P3	Hs.591361	annexin A2 pseudogene 3	Homo sapiens annexin A2 pseudogene	5581.56900	18179.59000	1035.35520	2701.13900	Detected	Detected	Detected	Detected
7681	A_24_P336957	HTRA2	Hs.469045	HtrA serine peptidase 2	Homo sapiens HtrA serine peptidase 2	5201.23300	4970.74800	4582.23200	4017.93770	Detected	Detected	Detected	Detected
7682	A_24_P306443	LOC100132288	Hs.487562	hypothetical protein LOC100132288	Homo sapiens hypothetical protein LOC	97.59769	75.83553	99.58904	67.91160	Detected	Detected	Detected	Detected
7683	A_24_P322369	VPS4B	Hs.126550	vacuolar protein sorting 4 homolog B (S	Homo sapiens vacuolar protein sorting	7389.76400	8447.11400	5855.04200	5426.31450	Detected	Detected	Detected	Detected
7684	A_33_P3326682	SDHC	Hs.444472	succinate dehydrogenase complex, subu	Homo sapiens succinate dehydrogenase	196.10432	241.84673	175.90830	191.29889	Detected	Detected	Detected	Detected
7685	A_32_P919718	TMEM105	Hs.364191	transmembrane protein 105	Homo sapiens transmembrane protein	712.08580	474.05520	375.73648	209.94493	Detected	Detected	Detected	Detected
7686	A_23_P35343	ZNF248	Hs.528423	zinc finger protein 248	Homo sapiens zinc finger protein 248 (Z	244.93214	72.53056	176.16220	44.62397	Detected	Detected	Detected	Detected
7687	A_23_P350551	C12orf57	Hs.591045	chromosome 12 open reading frame 57	Homo sapiens chromosome 12 open rea	26888.30500	10159.26800	33555.77000	10974.73700	Detected	Detected	Detected	Detected
7688	A_33_P3389658	PDE4D	Hs.117545	phosphodiesterase 4D, cAMP-specific (	Homo sapiens phosphodiesterase 4D, c	45.17578	6.64635	58.90025	13.12352	Detected	Compromised	Detected	Compromised
7689	A_24_P24724					6063.17140	6091.81200	1555.66560	1302.78770	Detected	Detected	Detected	Detected
7690	A_33_P3529860	KDM2B	Hs.524800	lysine (K)-specific demethylase 2B	Homo sapiens lysine (K)-specific demet	165.74470	233.50507	164.48352	213.47110	Detected	Detected	Detected	Detected
7691	A_33_P3647427	ZNF29	Hs.684886	zinc finger protein 29 pseudogene	Human Kox26 mRNA for zinc finger pro	122.96751	140.79391	15.33226	9.89934	Detected	Detected	Detected	Compromised
7692	A_23_P64721	GPR109B	Hs.458425	G protein-coupled receptor 109B	Homo sapiens G protein-coupled recep	12.12387	6.43179	6.24587	6.68591	Detected	Compromised	Compromised	Compromised
7693	A_23_P165521	STRN	Hs.656726	striatin, calmodulin binding protein	Homo sapiens striatin, calmodulin bindi	34.30307	97.31465	42.95839	90.07593	Detected	Detected	Detected	Detected
7694	A_23_P214681	PPARD	Hs.696032	peroxisome proliferator-activated rece	Homo sapiens peroxisome proliferator-	99.55288	95.33679	72.35202	60.26026	Detected	Detected	Detected	Detected
7695	A_33_P3381796	tcag7.1023	Hs.632008	hypothetical LOC393078	PREDICTED: Homo sapiens hypothetica	66.02609	79.81090	34.94209	40.52845	Detected	Detected	Detected	Detected
7696	A_24_P927189	OXNAD1	Hs.655449	oxidoreductase NAD-binding domain co	Homo sapiens oxidoreductase NAD-bin	455.81630	269.58250	277.11478	148.35628	Detected	Detected	Detected	Detected
7697	A_33_P3381410	ORC2L	Hs.444870	origin recognition complex, subunit 2-lik	Homo sapiens origin recognition comple	59.28096	40.54231	45.44872	26.54941	Detected	Detected	Detected	Detected
7698	A_24_P43876	C9orf40	Hs.532296	chromosome 9 open reading frame 40	Homo sapiens chromosome 9 open read	636.22050	1341.18250	414.11804	704.79440	Detected	Detected	Detected	Detected
7699	A_23_P140256	NP	Hs.75514	nucleoside phosphorylase	Homo sapiens nucleoside phosphorylase	11246.79100	30999.82400	11443.06100	27516.10500	Detected	Detected	Detected	Detected
7700	A_33_P3273599	INTU	Hs.391481	inturned planar cell polarity effector ho	Homo sapiens inturned planar cell polar	34.06942	43.25365	39.20428	33.47139	Detected	Detected	Detected	Detected
7701	A_23_P430201	C14orf145	Hs.162889	chromosome 14 open reading frame 145	Homo sapiens chromosome 14 open rea	260.05440	419.90125	196.09389	266.92105	Detected	Detected	Detected	Detected
7702	A_33_P3353051	C6orf48	Hs.640836	chromosome 6 open reading frame 48	Homo sapiens chromosome 6 open read	11731.18900	15420.30100	13815.25800	15926.49000	Detected	Detected	Detected	Detected
7703	A_24_P63030	SFXN5	Hs.368171	sideroflexin 5	Homo sapiens sideroflexin 5 (SFXN5), m	868.24255	1503.09110	839.49554	1344.75390	Detected	Detected	Detected	Detected
7704	A_33_P3412716					7676.15430	8576.59200	2941.13960	2681.85960	Detected	Detected	Detected	Detected
7705	A_33_P3371564	hCG_1990547	Hs.656318	family with sequence similarity 86, mem	Homo sapiens family with sequence sim	354.36642	505.84265	541.36176	669.99350	Detected	Detected	Detected	Detected
7706	A_33_P3248982	FAIM2	Hs.567424	Fas apoptotic inhibitory molecule 2	Homo sapiens Fas apoptotic inhibitory	1640.81920	366.23972	939.99090	102.68311	Detected	Detected	Detected	Detected
7707	A_23_P79927	NOP56	Hs.376064	NOP56 ribonucleoprotein homolog (yea	Homo sapiens NOP56 ribonucleoprotei	4248.59900	5976.59400	3360.04050	3891.15160	Detected	Detected	Detected	Detected
7708	A_24_P322354	SKA1	Hs.134726	spindle and kinetochore associated com	Homo sapiens spindle and kinetochore	33.14546	1021.33580	43.43451	865.45593	Detected	Detected	Detected	Detected
7709	A_33_P3259443		Hs.635230		Putative uncharacterized protein ENSP	102.07733	25.88511	15.45255	4.35843	Detected	Detected	Detected	Compromised
7710	A_23_P145569	SLC22A1	Hs.117367	solute carrier family 22 (organic cation	Homo sapiens solute carrier family 22 (	93.72649	137.16232	39.35754	38.69991	Detected	Detected	Detected	Detected
7711	A_23_P14716	ADPGK	Hs.654636	ADP-dependent glucokinase	Homo sapiens ADP-dependent glucoki	2213.92480	2158.41720	1496.76220	1256.04800	Detected	Detected	Detected	Detected
7712	A_32_P3572					12.95191	43.15993	5.57263	25.04007	Detected	Detected	Compromised	Detected
7713	A_33_P3257808	FANCD2	Hs.208388	Fanconi anemia, complementation group	Homo sapiens Fanconi anemia, complen	45.18990	186.04999	45.28367	157.79900	Detected	Detected	Detected	Detected
7714	A_32_P83845	HEY1	Hs.234434	hairy/enhancer-of-split related with YR	Homo sapiens hairy/enhancer-of-split	1826.20000	921.02435	1203.56470	509.18073	Detected	Detected	Detected	Detected
7715	A_33_P3308497	BMS1P4	Hs.559827	BMS1 pseudogene 4	Homo sapiens BMS1 pseudogene 4 (BM	87.57261	65.57517	84.58654	53.18325	Detected	Detected	Detected	Detected
7716	A_23_P94030	LAMB1	Hs.650585	laminin, beta 1	Homo sapiens laminin, beta 1 (LAMB1),	47138.31000	26356.39500	37413.19000	19638.25600	Detected	Detected	Detected	Detected
7717	A_23_P211141	DSCAM	Hs.397800	Down syndrome cell adhesion molecule	Homo sapiens Down syndrome cell adhe	13.38958	9.20786	13.82505	19.75375	Detected	Compromised	Detected	Detected
7718	A_23_P141044	ZNF688	Hs.301463	zinc finger protein 688	Homo sapiens zinc finger protein 688 (Z	3797.02470	2152.66200	3010.26250	1580.80730	Detected	Detected	Detected	Detected
7719	A_24_P31003	GFER	Hs.27184	growth factor, augmenter of liver regen	Homo sapiens growth factor, augmente	84.68369	151.27320	99.21130	163.21753	Detected	Detected	Detected	Detected
7720	A_33_P3297907	FGF22	Hs.248087	fibroblast growth factor 22	Homo sapiens fibroblast growth factor	52.45741	27.51495	71.89887	25.07756	Detected	Detected	Detected	Detected
7721	A_33_P3285235	ZNF507	Hs.205392	zinc finger protein 507	Homo sapiens zinc finger protein 507 (Z	78.23895	64.53561	62.35719	38.39958	Detected	Detected	Detected	Detected

7722	A_33_P3314441	FBXL17	Hs.721914	F-box and leucine-rich repeat protein 1	Homo sapiens F-box and leucine-rich r	156.44655	79.49745	108.74220	57.61035	Detected	Detected	Detected	Detected
7723	A_23_P134274	POP7	Hs.416994	processing of precursor 7, ribonuclease	Homo sapiens processing of precursor	30276.30300	60661.82000	24823.47900	45258.02700	Detected	Detected	Detected	Detected
7724	A_23_P402610	PFAS	Hs.573976	phosphoribosylformylglycinamidine synt	Homo sapiens phosphoribosylformylglyc	203.91296	288.88544	181.66342	246.50371	Detected	Detected	Detected	Detected
7725	A_33_P3290082	HPS4	Hs.474436	Hermansky-Pudlak syndrome 4	Homo sapiens Hermansky-Pudlak syndr	268.57610	187.94632	228.34239	179.31537	Detected	Detected	Detected	Detected
7726	A_33_P3215166					66.12214	44.16578	2.94606	3.48844	Detected	Detected	Compromised	Compromised
7727	A_23_P303317	ZXDC	Hs.440049	ZXD family zinc finger C	Homo sapiens ZXD family zinc finger C	373.88050	694.16260	336.02630	548.43304	Detected	Detected	Detected	Detected
7728	A_33_P3236986					161.91590	445.28610	95.32117	157.53099	Detected	Detected	Detected	Detected
7729	A_23_P155487	SLC38A3	Hs.76460	solute carrier family 38, member 3	Homo sapiens solute carrier family 38, r	23.50630	20.72906	29.79906	21.46368	Detected	Detected	Detected	Detected
7730	A_33_P3239849	GPX1	Hs.76686	glutathione peroxidase 1	Homo sapiens glutathione peroxidase 1	27937.28300	28164.97000	36077.10000	29881.94100	Detected	Detected	Detected	Detected
7731	A_32_P41496	LOC100132831		A20-binding inhibitor of NF-kappaB act	Homo sapiens A20-binding inhibitor of N	4334.85200	3576.18330	4164.19780	3402.69680	Detected	Detected	Detected	Detected
7732	A_23_P251785	NARG1L	Hs.512914	NMDA receptor regulated 1-like	Homo sapiens NMDA receptor regulated	1403.58780	1071.91030	885.20950	578.75055	Detected	Detected	Detected	Detected
7733	A_23_P60079	ANGPT2	Hs.583870	angiopoietin 2	Homo sapiens angiopoietin 2 (ANGPT2)	87.30627	66.88567	76.66309	59.11623	Detected	Detected	Detected	Detected
7734	A_23_P4522	TXNL1	Hs.114412	thioredoxin-like 1	Homo sapiens thioredoxin-like 1 (TXNL	25216.19000	28647.14500	20228.58400	18907.58400	Detected	Detected	Detected	Detected
7735	A_33_P3362668		Hs.721583		602504137F1 NIH_MGC_77 Homo sapier	1010.75750	927.82440	808.20630	550.23740	Detected	Detected	Detected	Detected
7736	A_23_P66525	HS3ST3A1	Hs.462270	heparan sulfate (glucosamine) 3-O-sulf	Homo sapiens heparan sulfate (glucosar	2479.67800	2838.01950	1128.02890	972.51404	Detected	Detected	Detected	Detected
7737	A_23_P3186	CCNK	Hs.510409	cyclin K	Homo sapiens cyclin K (CCNK), transcr	514.48620	861.42633	555.65950	782.99270	Detected	Detected	Detected	Detected
7738	A_33_P3322879					53.39572	101.07960	16.46955	25.32829	Detected	Detected	Detected	Detected
7739	A_24_P328872	CCT5	Hs.720003	chaperonin containing TCP1, subunit 5 (	Homo sapiens chaperonin containing TC	187.38608	156.89140	118.31107	78.14844	Detected	Detected	Detected	Detected
7740	A_23_P334709	FKBP9	Hs.103934	FK506 binding protein 9, 63 kDa	Homo sapiens FK506 binding protein 9,	22018.50400	25520.65200	18588.62900	19283.26400	Detected	Detected	Detected	Detected
7741	A_33_P3361422	CYP27A1	Hs.516700	cytochrome P450, family 27, subfamily A	Homo sapiens cytochrome P450, family	4265.50050	2024.37540	4144.21140	1765.92360	Detected	Detected	Detected	Detected
7742	A_24_P337058	CCNL1	Hs.4859	cyclin L1	Homo sapiens cyclin L1 (CCNL1), mRNA	2211.41480	1626.26100	1921.58750	1223.10410	Detected	Detected	Detected	Detected
7743	A_33_P3257155				Stromal membrane-associated protein	73.84318	88.48795	70.71357	75.32826	Detected	Detected	Detected	Detected
7744	A_24_P274814	TBXAS1	Hs.520757	thromboxane A synthase 1 (platelet)	Homo sapiens thromboxane A synthase	449.25684	110.91463	419.25920	44.69256	Detected	Detected	Detected	Detected
7745	A_33_P3235690	THOC2	Hs.149991	THO complex 2	Homo sapiens THO complex 2 (THOC2)	420.04367	311.37840	272.55573	156.88580	Detected	Detected	Detected	Detected
7746	A_23_P129188	CALML4	Hs.709550	calmodulin-like 4	Homo sapiens calmodulin-like 4 (CALMI	570.59920	880.52075	489.57162	561.51050	Detected	Detected	Detected	Detected
7747	A_33_P3522511	KIAA0485	Hs.604754	hypothetical LOC57235	Homo sapiens mRNA, chromosome 1 sp	143.48929	66.88791	100.67370	33.68170	Detected	Detected	Detected	Detected
7748	A_33_P3379841	CG030	Hs.658751	hypothetical CG030	Homo sapiens hypothetical CG030 (CG(	126.17167	63.97669	81.37260	28.94234	Detected	Detected	Detected	Detected
7749	A_33_P3419419				Phosphatidylinositol-4-phosphate 3-kin	231.53038	312.89640	113.74599	110.19621	Detected	Detected	Detected	Detected
7750	A_23_P3527	POLR2C	Hs.79402	polymerase (RNA) II (DNA directed) pol	Homo sapiens polymerase (RNA) II (DNA	2003.83080	2319.76780	2867.36300	3206.39870	Detected	Detected	Detected	Detected
7751	A_33_P3311046	OR2M7	Hs.530570	olfactory receptor, family 2, subfamily M	Homo sapiens olfactory receptor, family	8.05155	4.74752	15.10618	12.92337	Compromised	Compromised	Detected	Compromised
7752	A_23_P401675	MARVELD2	Hs.657687	MARVEL domain containing 2	Homo sapiens MARVEL domain contain	171.31157	435.13257	128.06538	243.22343	Detected	Detected	Detected	Detected
7753	A_33_P3370714	PGC	Hs.1867	progastricsin (pepsinogen C)	Homo sapiens progastricsin (pepsinoger	57.64784	56.05222	34.08705	38.84194	Detected	Detected	Detected	Detected
7754	A_33_P3343715				Homeobox protein EMX1 (Empty spiracl	82.78330	160.75221	51.10543	57.41027	Detected	Detected	Detected	Detected
7755	A_33_P3398077	FLJ31813	Hs.712902	hypothetical protein FLJ31813	Homo sapiens cDNA FLJ31813 fis, clon	95.47680	182.30792	123.14550	188.65962	Detected	Detected	Detected	Detected
7756	A_23_P169117	RRAGA	Hs.98943	Ras-related GTP binding A	Homo sapiens Ras-related GTP binding	28584.26800	36582.16000	19592.48400	20426.81000	Detected	Detected	Detected	Detected
7757	A_33_P3291118	LRRC37B	Hs.514071	leucine rich repeat containing 37B	Homo sapiens leucine rich repeat conta	84.38462	89.53289	79.33048	64.57655	Detected	Detected	Detected	Detected
7758	A_23_P340376	MSTP9	Hs.655432	macrophage stimulating, pseudogene 9	Homo sapiens macrophage stimulating,	16.01123	3.37798	21.15229	3.94749	Detected	Compromised	Detected	Compromised
7759	A_24_P54174	TNFRSF1B	Hs.256278	tumor necrosis factor receptor superfar	Homo sapiens tumor necrosis factor rec	322.86844	279.34260	278.61084	245.47270	Detected	Detected	Detected	Detected
7760	A_23_P372308	RGMA	Hs.271277	RGM domain family, member A	Homo sapiens RGM domain family, mem	1537.38490	33.29382	837.68400	20.15876	Detected	Detected	Detected	Detected
7761	A_33_P3409854	LHX1	Hs.443727	LIM homeobox 1	Homo sapiens LIM homeobox 1 (LHX1),	25.23806	20.74613	10.85923	8.38698	Detected	Detected	Compromised	Compromised
7762	A_33_P3833211	DNA2	Hs.532446	DNA replication helicase 2 homolog (yea	Homo sapiens DNA replication helicase	41.13052	109.72628	31.38795	75.56111	Detected	Detected	Detected	Detected
7763	A_23_P70448	HIST1H1A	Hs.150206	histone cluster 1, H1a	Homo sapiens histone cluster 1, H1a (H	165.76979	416.41428	141.29810	355.91653	Detected	Detected	Detected	Detected
7764	A_33_P3316379					20908.54100	11019.62700	8496.86100	3686.64330	Detected	Detected	Detected	Detected
7765	A_24_P169544	ZNF17	Hs.710278	zinc finger protein 17	Homo sapiens zinc finger protein 17 (ZN	430.10913	531.02230	366.74000	412.47060	Detected	Detected	Detected	Detected
7766	A_33_P3215906	NBPF10	Hs.607640	neuroblastoma breakpoint family, memb	Homo sapiens neuroblastoma breakpoin	396.19815	210.88520	268.23883	123.48669	Detected	Detected	Detected	Detected
7767	A_33_P3278313	MSRB3	Hs.339024	methionine sulfoxide reductase B3	Homo sapiens methionine sulfoxide redu	2159.45040	4420.27540	1664.44410	2929.11430	Detected	Detected	Detected	Detected
7768	A_32_P76853		Hs.525905		Golgin subfamily A member 8-like prote	828.08330	238.07475	713.12400	161.48187	Detected	Detected	Detected	Detected
7769	A_33_P3380693	ADAM15	Hs.312098	ADAM metallopeptidase domain 15	Homo sapiens ADAM metallopeptidase	42563.49200	20087.97500	48000.73400	25477.31600	Detected	Detected	Detected	Detected
7770	A_23_P50137	MEX3C	Hs.465144	mex-3 homolog C (C. elegans)	Homo sapiens mex-3 homolog C (C. ele	4375.68750	3524.03000	4021.30700	2687.09810	Detected	Detected	Detected	Detected
7771	A_33_P3241596					21598.84200	16033.79400	6710.64800	4365.29000	Detected	Detected	Detected	Detected
7772	A_33_P3319572	FAM71E2	Hs.528319	family with sequence similarity 71, mem	Homo sapiens family with sequence sim	755.34970	601.07666	223.28920	191.01717	Detected	Detected	Detected	Detected
7773	A_24_P177279	CSDC2	Hs.310893	cold shock domain containing C2, RNA	Homo sapiens cold shock domain conta	44.47540	9.90647	43.42648	13.81576	Detected	Compromised	Detected	Compromised
7774	A_33_P3263664	KRTAP6-2	Hs.553690	keratin associated protein 6-2	Homo sapiens keratin associated protei	79.18224	73.20400	38.61773	33.32576	Detected	Detected	Detected	Detected
7775	A_23_P147605	C11orf49	Hs.368296	chromosome 11 open reading frame 49	Homo sapiens chromosome 11 open rea	1416.35860	1396.93530	1542.23700	1468.07170	Detected	Detected	Detected	Detected
7776	A_23_P202587	KIAA1598	Hs.501140	KIAA1598	Homo sapiens KIAA1598 (KIAA1598), tr	407.81717	489.11197	215.15810	197.36508	Detected	Detected	Detected	Detected
7777	A_33_P3306964	PPP1R2	Hs.535731	protein phosphatase 1, regulatory (inhib	Homo sapiens protein phosphatase 1, re	330.89655	345.18655	257.67743	210.22588	Detected	Detected	Detected	Detected
7778	A_23_P410224	C6orf52	Hs.61389	chromosome 6 open reading frame 52	Homo sapiens chromosome 6 open read	59.84782	40.98766	28.22509	13.67274	Detected	Detected	Detected	Compromised
7779	A_24_P68247	TRIM4	Hs.50749	tripartite motif-containing 4	Homo sapiens tripartite motif-containin	83.70671	91.74648	66.85080	72.90652	Detected	Detected	Detected	Detected
7780	A_33_P3231572	LOC100130456	Hs.683861	hypothetical LOC100130456	Homo sapiens cDNA FLJ37693 fis, clon	2895.75420	3714.24020	921.92460	1121.25260	Detected	Detected	Detected	Detected
7781	A_23_P215051	ECHDC1	Hs.486410	enoyl Coenzyme A hydratase domain co	Homo sapiens enoyl Coenzyme A hydra	8559.62300	8004.85500	7535.89100	5826.58300	Detected	Detected	Detected	Detected
7782	A_33_P3259775	DOCK5	Hs.195403	dedicator of cytokinesis 5	Homo sapiens dedicator of cytokinesis	61.65580	223.65318	41.67636	119.28570	Detected	Detected	Detected	Detected
7783	A_24_P366082	MICAL3	Hs.528024	microtubule associated monoxygenase,	Homo sapiens microtubule associated n	361.05950	469.45860	324.40472	398.87885	Detected	Detected	Detected	Detected
7784	A_33_P3361067	ABCG2	Hs.480218	ATP-binding cassette, sub-family G (WI	Homo sapiens ATP-binding cassette, su	22.36160	46.82163	18.32889	26.93418	Detected	Detected	Detected	Detected
7785	A_32_P196854	FAM76A	Hs.469359	family with sequence similarity 76, mem	Homo sapiens family with sequence sim	687.28500	556.55975	590.50433	402.91006	Detected	Detected	Detected	Detected
7786	A_33_P3518572	LOC400019	Hs.355210	hypothetical LOC400019	Homo sapiens cDNA clone IMAGE:6023	1885.46780	2724.87260	834.33340	908.56200	Detected	Detected	Detected	Detected
7787	A_23_P46673	LOC730167		similar to protein tyrosine phosphatase	PREDICTED: Homo sapiens similar to p	130.69073	217.30994	39.69554	59.87980	Detected	Detected	Detected	Detected
7788	A_33_P3326382	STX1B	Hs.542230	syntaxin 1B	Homo sapiens syntaxin 1B (STX1B), mF	52.41391	38.92402	198.25195	237.60985	Detected	Detected	Detected	Detected
7789	A_23_P87827	UNC119B	Hs.127610	unc-119 homolog B (C. elegans)	Homo sapiens unc-119 homolog B (C. e	2936.06000	2447.30960	2404.59130	1782.32460	Detected	Detected	Detected	Detected
7790	A_23_P259797	ACSF3	Hs.461727	acyl-CoA synthetase family member 3	Homo sapiens acyl-CoA synthetase fan	434.88470	349.74164	377.20825	347.52716	Detected	Detected	Detected	Detected
7791	A_24_P360078	LRBA	Hs.480938	LPS-responsive vesicle trafficking, bea	Homo sapiens LPS-responsive vesicle t	1375.25160	883.75183	973.38806	531.76483	Detected	Detected	Detected	Detected
7792	A_33_P3354574					15.71410	21.43181	27.23060	27.27198	Detected	Detected	Detected	Detected
7793	A_33_P3303865	FAM120C	Hs.86045	family with sequence similarity 120C	Homo sapiens family with sequence sim	1568.30580	2548.42070	751.80835	1205.81520	Detected	Detected	Detected	Detected
7794	A_33_P3419945	LOC100128562	Hs.667110	hypothetical protein LOC100128562	Homo sapiens cDNA FLJ27214 fis, clon	1637.61610	788.50560	119.97112	31.70260	Detected	Detected	Detected	Detected
7795	A_23_P322756	TET1	Hs.567594	tet oncogene 1	Homo sapiens tet oncogene 1 (TET1), n	24.59942	16.83509	11.71336	7.06170	Detected	Detected	Compromised	Compromised
7796	A_24_P126651	TCEA1	Hs.491745	transcription elongation factor A (SII), 1	Homo sapiens transcription elongation f	142.60747	139.13037	216.30264	172.53282	Detected	Detected	Detected	Detected

7797	A_23_P49842	UNC119	Hs.410455	unc-119 homolog (C. elegans)	Homo sapiens unc-119 homolog (C. ele	903.04680	904.36100	1052.00730	1051.71600	Detected	Detected	Detected	Detected
7798	A_33_P3368879	LMOD1	Hs.519075	leiomodin 1 (smooth muscle)	Homo sapiens leiomodin 1 (smooth mus	37.84213	12.28481	32.98058	7.68402	Detected	Compromised	Detected	Compromised
7799	A_23_P25587	LECT1	Hs.421391	leukocyte cell derived chemotaxin 1	Homo sapiens leukocyte cell derived ch	32.14131	28.27386	17.32542	11.85575	Detected	Detected	Detected	Compromised
7800	A_24_P219920	FEM1A	Hs.515082	fem-1 homolog a (C. elegans)	Homo sapiens fem-1 homolog a (C. ele	331.11620	664.48370	288.21838	533.87775	Detected	Detected	Detected	Detected
7801	A_32_P138004	FAM45A	Hs.434241	family with sequence similarity 45, mem	Homo sapiens family with sequence sim	4099.45100	3927.14530	4571.66360	4031.03980	Detected	Detected	Detected	Detected
7802	A_33_P3221177				CDNA FLJ25952 fis, clone SYN00911	126.03088	215.07100	76.04722	88.33106	Detected	Detected	Detected	Detected
7803	A_33_P3807062	HJURP	Hs.532968	Holliday junction recognition protein	Homo sapiens Holliday junction recognit	274.61868	16112.07700	196.39200	9754.37600	Detected	Detected	Detected	Detected
7804	A_23_P12733	H2AFY2	Hs.499953	H2A histone family, member Y2	Homo sapiens H2A histone family, mem	117.90220	79.90233	112.27256	61.37737	Detected	Detected	Detected	Detected
7805	A_23_P159907	MAGED4B	Hs.522650	melanoma antigen family D, 4B	Homo sapiens melanoma antigen family	1584.74850	888.10720	2422.93730	1676.14340	Detected	Detected	Detected	Detected
7806	A_33_P3345743	C1orf152	Hs.657186	profilin 1 pseudogene	Homo sapiens chromosome 1 open read	211.92126	190.27080	103.88348	79.11128	Detected	Detected	Detected	Detected
7807	A_23_P146811	FBXO38	Hs.483772	F-box protein 38	Homo sapiens F-box protein 38 (FBXO	1834.00540	1254.24000	1590.25890	968.62350	Detected	Detected	Detected	Detected
7808	A_24_P134319	ADNP	Hs.720215	activity-dependent neuroprotector hom	Activity-dependent neuroprotector hon	3763.17630	2991.00460	2984.22070	2151.20120	Detected	Detected	Detected	Detected
7809	A_24_P272061	RPL13A	Hs.523185	ribosomal protein L13a	Homo sapiens ribosomal protein L13a (F	103572.55000	85778.62000	93769.08600	72996.93000	Detected	Detected	Detected	Detected
7810	A_33_P3302741		Hs.596302		Zinc finger protein 397OS (Zinc finger p	36.43682	36.95010	34.23615	30.81822	Detected	Detected	Detected	Detected
7811	A_32_P75902	C16orf73	Hs.662737	chromosome 16 open reading frame 73	Homo sapiens chromosome 16 open rea	237.07697	45.10858	204.68510	32.40978	Detected	Detected	Detected	Detected
7812	A_33_P3385561				cDNA FLJ27422 fis, clone WMC08087P	15809.21100	15287.73800	2434.32760	2093.19950	Detected	Detected	Detected	Detected
7813	A_33_P3239143	ZNF497	Hs.447840	zinc finger protein 497	Homo sapiens zinc finger protein 497 (Z	750.16223	872.03424	210.69012	194.36922	Detected	Detected	Detected	Detected
7814	A_33_P3390057	TM4SF1	Hs.351316	transmembrane 4 L six family member 1	Homo sapiens transmembrane 4 L six fa	87213.04000	24191.88900	55772.64500	15690.85500	Detected	Detected	Detected	Detected
7815	A_23_P8311	TTRAP	Hs.716397	TRAF and TNF receptor associated pro	Homo sapiens TRAF and TNF receptor	11949.38800	11415.53700	8418.52300	7186.11870	Detected	Detected	Detected	Detected
7816	A_33_P3228252	LOC730413	Hs.581653	similar to cholinergic receptor, muscarin	PREDICTED: Homo sapiens hypothetica	412.64570	390.11243	315.42917	283.23220	Detected	Detected	Detected	Detected
7817	A_33_P3234809	PAX8	Hs.469728	paired box 8	Homo sapiens paired box 8 (PAX8), trar	152.59767	136.82722	149.82407	110.77219	Detected	Detected	Detected	Detected
7818	A_23_P129476	C16orf7	Hs.164410	chromosome 16 open reading frame 7	Homo sapiens chromosome 16 open rea	2444.93580	1453.48510	2627.96040	1598.84220	Detected	Detected	Detected	Detected
7819	A_33_P3377239	LOC100270804	Hs.598768	hypothetical LOC100270804	Homo sapiens hypothetical LOC100270	342.58307	116.56322	321.20905	84.44455	Detected	Detected	Detected	Detected
7820	A_33_P3565787	KIAA1908	Hs.436146	hypothetical LOC114796	Homo sapiens hypothetical LOC114796	83.71654	76.41473	80.96729	70.37865	Detected	Detected	Detected	Detected
7821	A_23_P424080	YIPF4	Hs.468099	Yip1 domain family, member 4	Homo sapiens Yip1 domain family, meml	691.06805	641.00470	575.29175	446.54947	Detected	Detected	Detected	Detected
7822	A_33_P3269428		Hs.720142		Complement C1q tumor necrosis factor	86.59393	102.85399	72.90408	45.16439	Detected	Detected	Detected	Detected
7823	A_23_P423237	SGCG	Hs.37167	sarcoglycan, gamma (35kDa dystrophin-	Homo sapiens sarcoglycan, gamma (35k	2.61723	136.12785	8.27940	96.48359	Compromised	Detected	Compromised	Detected
7824	A_23_P391396	EBF3	Hs.591374	early B-cell factor 3	Homo sapiens early B-cell factor 3 (EB	1128.68080	556.69556	885.29020	360.84976	Detected	Detected	Detected	Detected
7825	A_33_P3389872		Hs.187636		Leucine-rich repeat serine/threonine-p	28.54237	5.29163	32.58225	7.56011	Detected	Compromised	Detected	Compromised
7826	A_23_P126752	CAPZB	Hs.432760	capping protein (actin filament) muscle	Homo sapiens capping protein (actin fila	6387.07960	8923.81250	5166.93650	6432.38200	Detected	Detected	Detected	Detected
7827	A_23_P50504	FTL	Hs.433670	ferritin, light polypeptide	Homo sapiens ferritin, light polypeptide	76797.67000	58842.35000	73301.94500	54527.24600	Detected	Detected	Detected	Detected
7828	A_24_P20777	RPL37	Hs.80545	ribosomal protein L37	Homo sapiens ribosomal protein L37 (R	83714.69500	51277.86300	61829.84000	34640.14500	Detected	Detected	Detected	Detected
7829	A_33_P3220242	C9orf82	Hs.178357	chromosome 9 open reading frame 82	Homo sapiens chromosome 9 open read	1376.02820	1029.58810	1003.16724	621.56287	Detected	Detected	Detected	Detected
7830	A_23_P15705	PSMB6	Hs.77060	proteasome (prosome, macropain) subu	Homo sapiens proteasome (prosome, m	22273.35500	35341.47300	28374.17200	36496.68000	Detected	Detected	Detected	Detected
7831	A_23_P114883	FMOD	Hs.705207	fibromodulin	Homo sapiens fibromodulin (FMOD), mR	2032.07520	3134.35450	1793.92360	2462.73320	Detected	Detected	Detected	Detected
7832	A_23_P54055	JUB	Hs.655832	jub, ajuba homolog (Xenopus laevis)	Homo sapiens jub, ajuba homolog (Xeno	950.61310	1280.62110	695.77277	713.89760	Detected	Detected	Detected	Detected
7833	A_24_P918384	NSD1	Hs.106861	nuclear receptor binding SET domain pr	Homo sapiens nuclear receptor binding	38.93200	31.13560	11.45811	8.97608	Detected	Detected	Compromised	Compromised
7834	A_23_P99614	BTBD6	Hs.7367	BTB (POZ) domain containing 6	Homo sapiens BTB (POZ) domain conta	33802.08600	53703.53500	33041.78000	46944.74000	Detected	Detected	Detected	Detected
7835	A_33_P3382100	IGFN1	Hs.519024	immunoglobulin-like and fibronectin typ	Homo sapiens immunoglobulin-like and	35.01601	33.64971	13.78444	15.16071	Detected	Detected	Compromised	Detected
7836	A_24_P123347	PPAT	Hs.331420	phosphoribosyl pyrophosphate amidotra	Homo sapiens phosphoribosyl pyrophos	735.95450	843.90280	821.96265	823.76910	Detected	Detected	Detected	Detected
7837	A_33_P3429575	LOC643454	Hs.662086	adaptor-related protein complex 3, sigm	Homo sapiens adaptor-related protein c	12601.86200	10827.35500	9376.56800	7620.93800	Detected	Detected	Detected	Detected
7838	A_23_P127579	PTS	Hs.503860	6-pyruvoyltetrahydropterin synthase	Homo sapiens 6-pyruvoyltetrahydropter	3490.42500	10840.21800	3003.05790	7926.05800	Detected	Detected	Detected	Detected
7839	A_23_P169576	EXOC6	Hs.655657	exocyst complex component 6	Homo sapiens exocyst complex compor	183.48518	281.40186	133.85370	179.32349	Detected	Detected	Detected	Detected
7840	A_33_P3465703	SNORA60	Hs.706159	small nucleolar RNA, H/ACA box 60	601888042F1 NIH_MGC_17 Homo sapier	16.27865	16.28063	2.72731	2.86663	Detected	Compromised	Compromised	Compromised
7841	A_33_P3330549	SLC44A2	Hs.534560	solute carrier family 44, member 2	Homo sapiens solute carrier family 44, r	827.57385	1135.77310	726.75696	937.52856	Detected	Detected	Detected	Detected
7842	A_33_P3407049					184.34400	92.01822	135.83720	58.13566	Detected	Detected	Detected	Detected
7843	A_23_P343594	USP40	Hs.96513	ubiquitin specific peptidase 40	Homo sapiens ubiquitin specific peptida	227.19330	208.17307	174.74475	154.68132	Detected	Detected	Detected	Detected
7844	A_23_P212639	TRA2B	Hs.533122	transformer 2 beta homolog (Drosophila	Homo sapiens transformer 2 beta homo	12622.10450	19599.19500	8915.34600	12142.41800	Detected	Detected	Detected	Detected
7845	A_23_P422981	FBXO36	Hs.140666	F-box protein 36	Homo sapiens F-box protein 36 (FBXO	735.79645	471.39218	546.87240	304.26660	Detected	Detected	Detected	Detected
7846	A_33_P3251347		Hs.188256		Mitogen-activated protein kinase kinase	6.65649	16.94770	8.53700	11.20479	Compromised	Detected	Compromised	Compromised
7847	A_24_P696761	LEMD1	Hs.655520	LEM domain containing 1	Homo sapiens LEM domain containing 1	29.16211	5.92217	26.10342	5.54261	Detected	Compromised	Detected	Compromised
7848	A_33_P3348469	ANKRD13C	Hs.105016	ankyrin repeat domain 13C	Homo sapiens ankyrin repeat domain 13	164.14230	165.32675	128.20700	120.01002	Detected	Detected	Detected	Detected
7849	A_23_P118038	NUTF2	Hs.356630	nuclear transport factor 2	Homo sapiens nuclear transport factor	3111.92360	6977.74950	3522.37650	7170.55900	Detected	Detected	Detected	Detected
7850	A_24_P5750	KLK2	Hs.515560	kallikrein-related peptidase 2	Homo sapiens kallikrein-related peptida	59.13968	76.25777	60.70266	51.11041	Detected	Detected	Detected	Detected
7851	A_24_P150874	GNA13	Hs.515018	guanine nucleotide binding protein (G pr	Homo sapiens guanine nucleotide bindin	640.42865	686.61975	452.07710	425.75848	Detected	Detected	Detected	Detected
7852	A_24_P74896	GPR144	Hs.454099	G protein-coupled receptor 144	Homo sapiens G protein-coupled recep	110.73702	120.97154	44.26140	39.15278	Detected	Detected	Detected	Detected
7853	A_23_P389692	KIAA1328	Hs.4045	KIAA1328	Homo sapiens KIAA1328 (KIAA1328), m	226.51743	242.46797	180.08124	151.95969	Detected	Detected	Detected	Detected
7854	A_33_P3311543					12.96915	13.98911	27.34616	22.15227	Detected	Compromised	Detected	Detected
7855	A_23_P154526	GRB14	Hs.411881	growth factor receptor-bound protein 1	Homo sapiens growth factor receptor-b	82.34106	1923.31840	55.05007	1148.67370	Detected	Detected	Detected	Detected
7856	A_33_P3276100	LOC100130015	Hs.719553	hypothetical LOC100130015	Homo sapiens hypothetical LOC100130	294.14316	61.81912	201.16812	43.27463	Detected	Detected	Detected	Detected
7857	A_23_P255876	DNAI1	Hs.112667	dynein, axonemal, intermediate chain 1	Homo sapiens dynein, axonemal, interm	14.00250	11.15324	12.01216	9.76156	Detected	Compromised	Compromised	Compromised
7858	A_23_P501887	DHPS	Hs.79064	deoxyhypusine synthase	Homo sapiens deoxyhypusine synthase	730.79443	1056.63570	978.83390	1248.02480	Detected	Detected	Detected	Detected
7859	A_24_P406132	MAPK13	Hs.178695	mitogen-activated protein kinase 13	Homo sapiens mitogen-activated protei	798.85740	612.28940	914.94824	629.24530	Detected	Detected	Detected	Detected
7860	A_23_P59099	OR11A1	Hs.676010	olfactory receptor, family 11, subfamily	Homo sapiens olfactory receptor, family	636.94135	1079.31910	148.70232	189.22260	Detected	Detected	Detected	Detected
7861	A_32_P158746	RPL17	Hs.374588	ribosomal protein L17	Homo sapiens ribosomal protein L17 (R	58691.90000	58831.69500	30909.07600	26784.95100	Detected	Detected	Detected	Detected
7862	A_33_P3373560	SPTAN1	Hs.372331	spectrin, alpha, non-erythrocytic 1 (alph	Homo sapiens spectrin, alpha, non-eryt	380.25858	340.41210	242.10610	245.47385	Detected	Detected	Detected	Detected
7863	A_33_P3539345	MYO6	Hs.149387	myosin VI	Homo sapiens myosin VI (MYO6), mRNA	2130.87040	1210.11190	1239.24260	610.12830	Detected	Detected	Detected	Detected
7864	A_33_P3349651	SWAP70	Hs.153026	SWAP switching B-cell complex 70kDa	Homo sapiens SWAP switching B-cell c	1497.31640	1575.78060	1101.91910	905.24260	Detected	Detected	Detected	Detected
7865	A_33_P3381943	DMTF1	Hs.196129	cyclin D binding myb-like transcription	Homo sapiens cyclin D binding myb-like	107.38522	53.44023	103.71880	46.91865	Detected	Detected	Detected	Detected
7866	A_24_P56837	ASB16	Hs.534517	ankyrin repeat and SOCS box-containir	Homo sapiens ankyrin repeat and SOCS	1186.53020	1113.98070	235.42702	173.36992	Detected	Detected	Detected	Detected
7867	A_23_P58353	HNRNPD	Hs.480073	heterogeneous nuclear ribonucleoprotei	Homo sapiens heterogeneous nuclear ri	28547.50200	50210.64000	20246.83000	31249.86700	Detected	Detected	Detected	Detected
7868	A_24_P9321	HIST1H3I	Hs.132854	histone cluster 1, H3i	Homo sapiens histone cluster 1, H3i (HI	1582.77510	2020.76150	433.61823	466.35196	Detected	Detected	Detected	Detected
7869	A_23_P30315	TRIM7	Hs.487412	tripartite motif-containing 7	Homo sapiens tripartite motif-containin	2742.09060	2532.72490	2856.62100	2375.55570	Detected	Detected	Detected	Detected
7870	A_33_P3418668	PAR-SN	Hs.564847	paternally expressed transcript PAR-SN	Homo sapiens paternally expressed trar	480.06006	91.39077	658.66907	137.96678	Detected	Detected	Detected	Detected
7871	A_33_P3605352	LOC440983	Hs.535002	hypothetical gene supported by BC0669	Homo sapiens hypothetical gene suppor	17.84400	5.46695	15.09054	7.94811	Detected	Compromised	Detected	Compromised

7872	A_24_P294982	VTA1	Hs.431367	Vps20-associated 1 homolog (S. cerevis	Homo sapiens Vps20-associated 1 hom	570.84973	683.55927	442.59537	479.21160	Detected	Detected	Detected	Detected
7873	A_33_P3415052	NIPAL4	Hs.4285	NIPA-like domain containing 4	Homo sapiens NIPA-like domain contair	150.43858	69.08849	129.04839	51.57492	Detected	Detected	Detected	Detected
7874	A_23_P137814	ATP6V0B	Hs.596514	ATPase, H+ transporting, lysosomal 21k	Homo sapiens ATPase, H+ transporting,	10289.98800	14172.50100	10977.28400	13451.78800	Detected	Detected	Detected	Detected
7875	A_33_P3289356	CD58	Hs.34341	CD58 molecule	Homo sapiens CD58 molecule (CD58), t	1320.87490	816.56760	1134.60390	599.36820	Detected	Detected	Detected	Detected
7876	A_33_P3381513	CD274	Hs.521989	CD274 molecule	Homo sapiens CD274 molecule (CD274	18.79324	30.91879	19.23845	32.76265	Detected	Detected	Detected	Detected
7877	A_23_P423331	NTNG2	Hs.163642	netrin G2	Homo sapiens netrin G2 (NTNG2), mRN	215.40080	211.42119	127.73459	116.48076	Detected	Detected	Detected	Detected
7878	A_33_P3281273	S1PR4	Hs.662006	sphingosine-1-phosphate receptor 4	Homo sapiens sphingosine-1-phosphate	18.20723	21.87316	15.59199	12.07503	Detected	Detected	Detected	Compromised
7879	A_23_P340263	RNF175	Hs.388364	ring finger protein 175	Homo sapiens ring finger protein 175 (R	1145.89500	75.80601	1278.16940	76.20176	Detected	Detected	Detected	Detected
7880	A_23_P73632	NR0B1	Hs.268490	nuclear receptor subfamily 0, group B, r	Homo sapiens nuclear receptor subfami	257.53506	40.78642	306.05145	38.58382	Detected	Detected	Detected	Detected
7881	A_33_P3237379	KCNC1	Hs.552896	potassium voltage-gated channel, Shaw	Homo sapiens potassium voltage-gated	29.11208	3.53352	27.44197	2.96705	Detected	Compromised	Detected	Compromised
7882	A_23_P144490	DCHS2	Hs.655664	dachsous 2 (Drosophila)	Homo sapiens dachsous 2 (Drosophila)	20.32828	25.36023	11.81477	12.70044	Detected	Detected	Compromised	Compromised
7883	A_33_P3433156	TRMU	Hs.439524	tRNA 5-methylaminomethyl-2-thiouridy	Homo sapiens tRNA 5-methylaminomet	752.11360	992.80800	655.23010	807.76086	Detected	Detected	Detected	Detected
7884	A_33_P3390177	FLJ34208	Hs.407087	hypothetical protein LOC401106	Homo sapiens cDNA FLJ34208 fis, clon	26.27153	29.60819	32.22845	25.16800	Detected	Detected	Detected	Detected
7885	A_23_P311640	AGFG2	Hs.521083	ArfGAP with FG repeats 2	Homo sapiens ArfGAP with FG repeats	361.49246	405.26523	254.44398	231.78305	Detected	Detected	Detected	Detected
7886	A_23_P70359	C6orf59	Hs.664873	chromosome 6 open reading frame 59	Homo sapiens chromosome 6 open reac	682.47473	392.06195	466.21110	216.64150	Detected	Detected	Detected	Detected
7887	A_23_P84140	IQCB1	Hs.604110	IQ motif containing B1	Homo sapiens IQ motif containing B1 (I	1539.95720	1880.29630	995.21155	982.17900	Detected	Detected	Detected	Detected
7888	A_23_P367676	SIN3A	Hs.513039	SIN3 homolog A, transcription regulator	Homo sapiens SIN3 homolog A, transcri	1193.71200	812.02080	1105.25000	693.91070	Detected	Detected	Detected	Detected
7889	A_33_P3360204					60.89751	91.80007	51.55786	76.51737	Detected	Detected	Detected	Detected
7890	A_33_P3213997	DHRS4L2	Hs.528385	dehydrogenase/reductase (SDR family)	Homo sapiens dehydrogenase/reductas	14244.94500	12781.65100	10005.05000	7824.38500	Detected	Detected	Detected	Detected
7891	A_24_P139094	SH3GL1	Hs.97616	SH3-domain GRB2-like 1	Homo sapiens SH3-domain GRB2-like	15038.63200	18618.89300	15285.55600	17786.19300	Detected	Detected	Detected	Detected
7892	A_24_P66679	NAT12	Hs.165465	N-acetyltransferase 12 (GCN5-related,	Homo sapiens N-acetyltransferase 12 (	137.58409	128.53630	90.22625	66.92271	Detected	Detected	Detected	Detected
7893	A_33_P3268507	CEACAM1	Hs.512682	carcinoembryonic antigen-related cell a	Homo sapiens carcinoembryonic antiger	11.93268	18.87370	10.90321	15.28419	Compromised	Detected	Compromised	Detected
7894	A_24_P55250	HDDC2	Hs.32826	HD domain containing 2	Homo sapiens HD domain containing 2 (	4876.42100	3440.35450	3615.37380	2352.88380	Detected	Detected	Detected	Detected
7895	A_32_P144920	NECAP1	Hs.555927	NECAP endocytosis associated 1	Homo sapiens NECAP endocytosis asso	309.62085	308.71140	204.87150	167.52728	Detected	Detected	Detected	Detected
7896	A_33_P3531206	PCOTH	Hs.721128	prostate collagen triple helix	Homo sapiens prostate collagen triple h	6.09393	107.66082	10.07329	101.67017	Compromised	Detected	Compromised	Detected
7897	A_33_P3709525	LOC100128233	Hs.497323	hypothetical protein LOC100128233	human full-length cDNA clone CS0DI00	24.20766	7.06216	9.10767	15.00228	Detected	Compromised	Compromised	Detected
7898	A_33_P3253501	HIST2H2BF	Hs.632451	histone cluster 2, H2bf	Homo sapiens histone cluster 2, H2bf (H	2812.03980	2290.12100	494.44034	846.60820	Detected	Detected	Detected	Detected
7899	A_32_P129419	NARG2	Hs.200943	NMDA receptor regulated 2	Homo sapiens NMDA receptor regulate	127.11490	29.81319	60.29860	14.07613	Detected	Detected	Detected	Compromised
7900	A_33_P3728698	FLJ45248	Hs.224506	FLJ45248 protein	PREDICTED: Homo sapiens FLJ45248 p	819.40530	1073.28020	506.02325	567.93910	Detected	Detected	Detected	Detected
7901	A_24_P119678	LOC100129366	Hs.668296	hypothetical protein LOC100129366	Homo sapiens mRNA for hypothetical p	84.07090	176.33452	17.98316	10.58485	Detected	Detected	Detected	Compromised
7902	A_24_P315921					948.86220	235.61275	1090.01460	226.69583	Detected	Detected	Detected	Detected
7903	A_23_P319859	EYA2	Hs.472877	eyes absent homolog 2 (Drosophila)	Homo sapiens eyes absent homolog 2 (I	31.42043	188.71936	22.63615	84.95781	Detected	Detected	Detected	Detected
7904	A_33_P3307495	STRA6	Hs.24553	stimulated by retinoic acid gene 6 homo	Homo sapiens stimulated by retinoic ac	24.68651	2093.29790	17.08419	1411.16990	Detected	Detected	Detected	Detected
7905	A_24_P93754	KDM4C	Hs.709425	lysine (K)-specific demethylase 4C	Homo sapiens lysine (K)-specific demet	41.91743	44.25726	20.72341	16.01356	Detected	Detected	Detected	Detected
7906	A_33_P3267280	CTDSP2	Hs.524530	CTD (carboxy-terminal domain, RNA po	Homo sapiens CTD (carboxy-terminal d	1143.84400	1545.64390	1244.47580	1640.79860	Detected	Detected	Detected	Detected
7907	A_33_P3405360	PDCL	Hs.271749	phosducin-like	Homo sapiens phosducin-like (PDCL), n	217.13211	395.32306	217.35225	324.63490	Detected	Detected	Detected	Detected
7908	A_23_P207400	BRCA1	Hs.194143	breast cancer 1, early onset	Homo sapiens breast cancer 1, early on	177.70929	983.38610	122.69441	584.51580	Detected	Detected	Detected	Detected
7909	A_23_P203191	APOA1	Hs.93194	apolipoprotein A-I	Homo sapiens apolipoprotein A-I (APOA	6.45793	27.28085	4.96122	17.07688	Compromised	Detected	Compromised	Detected
7910	A_23_P21316	PRUNE	Hs.78524	prune homolog (Drosophila)	Homo sapiens prune homolog (Drosophi	100.79349	71.06373	89.84543	50.07617	Detected	Detected	Detected	Detected
7911	A_33_P3422991	SLC36A4	Hs.148766	solute carrier family 36 (proton/amino a	Homo sapiens solute carrier family 36 (	1662.41760	673.82120	1322.35400	456.94946	Detected	Detected	Detected	Detected
7912	A_32_P41526	AGAP7	Hs.528346	ArfGAP with GTPase domain, ankyrin re	Homo sapiens ArfGAP with GTPase dor	387.61746	410.69778	268.36847	240.62856	Detected	Detected	Detected	Detected
7913	A_23_P92424	UTP3	Hs.322901	UTP3, small subunit (SSU) processome	Homo sapiens UTP3, small subunit (SSU	11473.35100	16700.22900	9053.26400	10674.35800	Detected	Detected	Detected	Detected
7914	A_23_P13701	TMBIM4	Hs.505934	transmembrane BAX inhibitor motif con	Homo sapiens transmembrane BAX inhi	3494.01930	3399.34470	3924.19480	3357.41630	Detected	Detected	Detected	Detected
7915	A_33_P3277611	TMEM8C	Hs.512467	transmembrane protein 8C	Homo sapiens transmembrane protein 8	6072.12000	4121.84400	207.93837	109.60482	Detected	Detected	Detected	Detected
7916	A_33_P3243128	COQ5	Hs.17250	coenzyme Q5 homolog, methyltransfera	Homo sapiens coenzyme Q5 homolog, n	2591.78150	1455.52620	3572.26830	1721.33640	Detected	Detected	Detected	Detected
7917	A_23_P92687	DAP	Hs.75189	death-associated protein	Homo sapiens death-associated protein	9265.94200	14111.66200	8047.53300	10463.20200	Detected	Detected	Detected	Detected
7918	A_33_P3320832	NMS	Hs.567676	neuromedin S	Homo sapiens neuromedin S (NMS), mR	9.34217	10.01763	9.06217	25.06055	Compromised	Compromised	Compromised	Detected
7919	A_33_P3393537	PTAFR		platelet-activating factor receptor	Homo sapiens platelet-activating factor	2186.71170	2803.14430	1103.96340	1267.00220	Detected	Detected	Detected	Detected
7920	A_33_P3390122	GIPC2	Hs.659356	GIPC PDZ domain containing family, me	Homo sapiens GIPC PDZ domain contai	9.10933	47.82937	10.15155	60.15094	Compromised	Detected	Compromised	Detected
7921	A_33_P3370881	LOC390937	Hs.590950	similar to hCG2040171	PREDICTED: Homo sapiens similar to h	129.51163	187.39699	83.74814	75.17326	Detected	Detected	Detected	Detected
7922	A_23_P64214	PCSK7	Hs.648612	proprotein convertase subtilisin/kexin t	Homo sapiens proprotein convertase su	233.92607	334.12076	223.99333	341.78370	Detected	Detected	Detected	Detected
7923	A_33_P3307163	RBM20	Hs.116630	RNA binding motif protein 20	Homo sapiens RNA binding motif protei	115.40898	126.75250	92.27990	72.30983	Detected	Detected	Detected	Detected
7924	A_33_P3360555	ZNF444	Hs.24545	zinc finger protein 444	Homo sapiens zinc finger protein 444 (Z	7745.48000	5095.78030	7805.07470	4565.98300	Detected	Detected	Detected	Detected
7925	A_24_P598406	RNF216L	Hs.520636	ring finger protein 216-like	Homo sapiens ring finger protein 216-lik	4408.47460	11070.47300	4525.04350	9950.18200	Detected	Detected	Detected	Detected
7926	A_24_P148026	LOC100294391	Hs.638348	hypothetical protein LOC100294391	PREDICTED: Homo sapiens hypothetica	131.99005	34.39092	123.85309	32.94152	Detected	Detected	Detected	Detected
7927	A_24_P98277	GOLGA7	Hs.654773	golgi autoantigen, golgin subfamily a, 7	Homo sapiens golgi autoantigen, golgin	953.79560	1478.77670	818.05710	1082.15890	Detected	Detected	Detected	Detected
7928	A_24_P284324	TMEM104	Hs.370262	transmembrane protein 104	Homo sapiens transmembrane protein 1	3256.63770	3753.06790	2978.04080	3371.05080	Detected	Detected	Detected	Detected
7929	A_24_P40551	BEX4	Hs.184736	brain expressed, X-linked 4	Homo sapiens brain expressed, X-linked	2022.77820	531.20306	1583.26700	385.22784	Detected	Detected	Detected	Detected
7930	A_32_P2392	GOLGA8A	Hs.720151	golgi autoantigen, golgin subfamily a, 8A	Homo sapiens golgi autoantigen, golgin	467.74530	69.26858	314.52844	31.68479	Detected	Detected	Detected	Detected
7931	A_33_P3209351	IARS	Hs.445403	isoleucyl-tRNA synthetase	Homo sapiens isoleucyl-tRNA syntheta	2279.55150	3841.11520	1603.00850	2270.51930	Detected	Detected	Detected	Detected
7932	A_33_P3423365	GSN	Hs.522373	gelsolin (amyloidosis, Finnish type)	Homo sapiens gelsolin (amyloidosis, Finn	17059.72500	10324.17800	14310.98400	7172.23240	Detected	Detected	Detected	Detected
7933	A_33_P3244863	CEP350	Hs.413045	centrosomal protein 350kDa	Homo sapiens centrosomal protein 350k	85.80878	51.36941	53.79936	27.32346	Detected	Detected	Detected	Detected
7934	A_33_P3374643	PAAF1	Hs.525017	proteasomal ATPase-associated factor	Homo sapiens proteasomal ATPase-ass	5838.40770	4425.02900	4978.43300	3313.55100	Detected	Detected	Detected	Detected
7935	A_23_P374389	PWWP2B	Hs.527751	PWWP domain containing 2B	Homo sapiens PWWP domain containing	1746.44040	2616.45260	1814.20030	1852.32870	Detected	Detected	Detected	Detected
7936	A_33_P3301351	RBM44	Hs.720233	RNA binding motif protein 44	Homo sapiens RNA binding motif protei	87.92421	22.29676	69.63701	15.58324	Detected	Detected	Detected	Detected
7937	A_33_P3360412	PIGQ	Hs.644153	phosphatidylinositol glycan anchor biosy	Homo sapiens phosphatidylinositol glyca	19.55603	24.25235	25.88672	29.35451	Detected	Detected	Detected	Detected
7938	A_33_P3286151	LOC100132891	Hs.137674	hypothetical protein LOC100132891	PREDICTED: Homo sapiens hypothetica	111.11372	92.55371	124.54651	94.02408	Detected	Detected	Detected	Detected
7939	A_23_P69100	SGEF	Hs.240845	Src homology 3 domain-containing guar	Homo sapiens Src homology 3 domain-c	106.25249	37.29717	100.07902	31.18078	Detected	Detected	Detected	Detected
7940	A_23_P142918	C2orf69	Hs.471040	chromosome 2 open reading frame 69	Homo sapiens chromosome 2 open reac	695.57550	1025.04500	570.57886	614.58093	Detected	Detected	Detected	Detected
7941	A_24_P212234	SLC6A18	Hs.213284	solute carrier family 6, member 18	Homo sapiens solute carrier family 6, m	142.68526	120.95918	184.71152	190.21346	Detected	Detected	Detected	Detected
7942	A_24_P674924	LRPPRC	Hs.368084	leucine-rich PPR-motif containing	Homo sapiens leucine-rich PPR-motif c	275.77475	212.24014	247.59477	201.34846	Detected	Detected	Detected	Detected
7943	A_33_P3341424	MYO10	Hs.481720	myosin X	Homo sapiens myosin X (MYO10), mRN	812.23444	2867.81620	695.86430	1902.00800	Detected	Detected	Detected	Detected
7944	A_23_P40315	C20orf12	Hs.472225	chromosome 20 open reading frame 12	Homo sapiens chromosome 20 open rea	276.58075	220.27720	220.30757	163.32228	Detected	Detected	Detected	Detected
7945	A_33_P3316239	LOC100130419	Hs.651773	hypothetical protein LOC100130419	Homo sapiens cDNA FLJ44830 fis, clon	21.46429	17.12181	15.41429	21.36423	Detected	Detected	Detected	Detected
7946	A_33_P3348802	LOC729570	Hs.598194	hypothetical LOC729570	PREDICTED: Homo sapiens hypothetica	1354.99320	1757.80250	1176.02980	1320.13820	Detected	Detected	Detected	Detected

7947	A_24_P391568	ARNT	Hs.632446	aryl hydrocarbon receptor nuclear trans	Homo sapiens aryl hydrocarbon recepto	678.76117	433.83984	682.94543	366.87674	Detected	Detected	Detected	Detected
7948	A_23_P218706	ZNF343	Hs.516846	zinc finger protein 343	Homo sapiens zinc finger protein 343 (Z	457.35410	439.96362	302.78370	269.12170	Detected	Detected	Detected	Detected
7949	A_24_P83922	SNRPC	Hs.1063	small nuclear ribonucleoprotein polypep	Homo sapiens small nuclear ribonucleop	325.55615	504.63464	28.93313	39.35492	Detected	Detected	Detected	Detected
7950	A_24_P63347	PF4V1	Hs.72933	platelet factor 4 variant 1	Homo sapiens platelet factor 4 variant	73.49332	45.69723	54.37374	23.74132	Detected	Detected	Detected	Detected
7951	A_33_P3281250		Hs.573317		Putative uncharacterized protein ENSP	20.44692	25.39373	29.88739	18.93258	Detected	Detected	Detected	Detected
7952	A_33_P3355821	ZNF273	Hs.520889	zinc finger protein 273	Homo sapiens zinc finger protein 273 (Z	63.10246	111.73818	22.57808	33.18304	Detected	Detected	Detected	Detected
7953	A_33_P3292230	LOC402160	Hs.558938	similar to hCG18094	PREDICTED: Homo sapiens similar to h	2.81535	3.23919	8.37266	27.86561	Compromised	Compromised	Compromised	Detected
7954	A_33_P3308456	C17orf93		chromosome 17 open reading frame 93	Homo sapiens chromosome 17 open rea	348.30264	526.51434	254.99037	267.80563	Detected	Detected	Detected	Detected
7955	A_24_P79403	PF4	Hs.81564	platelet factor 4	Homo sapiens platelet factor 4 (PF4), m	244.66570	1905.09730	185.89767	1319.96450	Detected	Detected	Detected	Detected
7956	A_24_P135769	VWA5A	Hs.152944	von Willebrand factor A domain contain	Homo sapiens von Willebrand factor A d	110.08601	17.71646	107.84480	12.60848	Detected	Detected	Detected	Compromised
7957	A_33_P3311992	MAGI2	Hs.603842	membrane associated guanylate kinase,	Homo sapiens membrane associated gu	10.81799	3.67816	8.41682	13.92058	Compromised	Compromised	Compromised	Detected
7958	A_33_P3366221	NTNG1	Hs.657434	netrin G1	Homo sapiens netrin G1 (NTNG1), trans	671.97620	285.23227	582.32910	228.33453	Detected	Detected	Detected	Detected
7959	A_23_P53567	CCDC53	Hs.405692	coiled-coil domain containing 53	Homo sapiens coiled-coil domain conta	3280.85550	3891.46040	2558.06470	2549.50070	Detected	Detected	Detected	Detected
7960	A_33_P3313622	MIR17HG	Hs.24115	MIR17 host gene (non-protein coding)	Homo sapiens MIR17 host gene (non-pr	54.61401	41.82233	58.29137	29.57836	Detected	Detected	Detected	Detected
7961	A_33_P3364338				Putative uncharacterized protein ENSP	146.01912	240.89346	5.80885	4.77786	Detected	Detected	Compromised	Compromised
7962	A_23_P19115	GSPT2	Hs.59523	G1 to S phase transition 2	Homo sapiens G1 to S phase transition	1145.05900	1476.44910	837.53394	646.49500	Detected	Detected	Detected	Detected
7963	A_32_P113584	ZNF292	Hs.485892	zinc finger protein 292	Homo sapiens zinc finger protein 292 (Z	455.88202	214.24437	276.92285	122.87330	Detected	Detected	Detected	Detected
7964	A_33_P3357843	SGCB	Hs.438953	sarcoglycan, beta (43kDa dystrophin-as	Homo sapiens sarcoglycan, beta (43kDa	2546.41020	3378.82570	2312.05220	2336.51340	Detected	Detected	Detected	Detected
7965	A_33_P3376075					293.55905	300.06192	137.11433	176.33340	Detected	Detected	Detected	Detected
7966	A_33_P3366758	ST8SIA6	Hs.677766	ST8 alpha-N-acetyl-neuraminide alpha-	Homo sapiens ST8 alpha-N-acetyl-neu	2.59020	3.24374	6.13663	17.25095	Compromised	Compromised	Compromised	Detected
7967	A_33_P3348362	C14orf128	Hs.496755	chromosome 14 open reading frame 128	Homo sapiens chromosome 14 open rea	235.08652	185.59813	281.91888	182.12856	Detected	Detected	Detected	Detected
7968	A_24_P66337				Lysocardiolipin acyltransferase 1 (EC 2.	4.45771	5.40923	8.04887	17.29719	Compromised	Compromised	Compromised	Detected
7969	A_33_P3415895	NRBF2	Hs.449628	nuclear receptor binding factor 2	Homo sapiens nuclear receptor binding	954.44270	1013.46110	1113.28590	977.79987	Detected	Detected	Detected	Detected
7970	A_23_P88069	LHFP	Hs.507798	lipoma HMGIC fusion partner	Homo sapiens lipoma HMGIC fusion par	10321.17600	21598.88500	7784.03700	13096.95500	Detected	Detected	Detected	Detected
7971	A_24_P268662	ABHD10	Hs.708240	abhydrolase domain containing 10	Homo sapiens abhydrolase domain cont	20.74780	24.96027	2.62673	2.73716	Detected	Detected	Compromised	Compromised
7972	A_24_P307289	TMEM95	Hs.259432	transmembrane protein 95	Homo sapiens transmembrane protein 9	58.86835	100.15261	35.53265	26.98069	Detected	Detected	Detected	Detected
7973	A_33_P3289456					123.94580	195.80356	70.95399	81.59620	Detected	Detected	Detected	Detected
7974	A_23_P428729		Hs.675613		Zinc finger MYM-type protein 6 (Zinc fi	34.15274	73.41062	21.86367	55.45189	Detected	Detected	Detected	Detected
7975	A_33_P3367102	C1orf152	Hs.632440	profilin 1 pseudogene	Homo sapiens chromosome 1 open read	4978.56800	10377.78100	5833.32700	11505.60600	Detected	Detected	Detected	Detected
7976	A_33_P3399090	DIXDC1	Hs.655626	DIX domain containing 1	Homo sapiens DIX domain containing 1	3898.04100	8198.46900	3456.51660	6555.65800	Detected	Detected	Detected	Detected
7977	A_23_P111753	MRPL32	Hs.50252	mitochondrial ribosomal protein L32	Homo sapiens mitochondrial ribosomal	12374.99600	10131.70300	11828.00100	8427.20900	Detected	Detected	Detected	Detected
7978	A_33_P3225096	MTMR10	Hs.30141	myotubularin related protein 10	Homo sapiens myotubularin related prot	164.98499	367.85712	173.26881	303.80237	Detected	Detected	Detected	Detected
7979	A_23_P205697	DLST	Hs.525459	dihydrolipoamide S-succinyltransferase	Homo sapiens dihydrolipoamide S-succ	2091.50240	1686.75490	5604.11400	5313.71240	Detected	Detected	Detected	Detected
7980	A_23_P159956	MID2	Hs.12256	midline 2	Homo sapiens midline 2 (MID2), transcri	205.57144	692.05960	135.97160	437.05478	Detected	Detected	Detected	Detected
7981	A_33_P3378212	MED25	Hs.656639	mediator complex subunit 25	Homo sapiens mediator complex subuni	2687.24900	3843.38090	2878.27120	3671.45560	Detected	Detected	Detected	Detected
7982	A_24_P20873	HIST1H4I	Hs.248172	histone cluster 1, H4i	Homo sapiens histone cluster 1, H4i (HI	70.15601	207.04198	65.08009	160.63701	Detected	Detected	Detected	Detected
7983	A_33_P3263890	PRRX1	Hs.283416	paired related homeobox 1	Homo sapiens paired related homeobox	83364.58600	61739.94500	50134.75800	32801.78500	Detected	Detected	Detected	Detected
7984	A_32_P173662	CRISP2	Hs.2042	cysteine-rich secretory protein 2	Homo sapiens cysteine-rich secretory	15.80311	25.67292	7.54247	8.48123	Detected	Detected	Compromised	Compromised
7985	A_23_P502797	WDFY1	Hs.368359	WD repeat and FYVE domain containing	Homo sapiens WD repeat and FYVE dor	1137.01930	1354.58840	803.24133	794.73694	Detected	Detected	Detected	Detected
7986	A_32_P146659	LOC401431	Hs.556998	hypothetical LOC401431	Homo sapiens hypothetical LOC401431	208.09108	127.36542	155.13531	86.60884	Detected	Detected	Detected	Detected
7987	A_24_P246692					29392.93200	32561.31600	17453.02300	16068.87200	Detected	Detected	Detected	Detected
7988	A_24_P394533	NEU1	Hs.520037	sialidase 1 (lysosomal sialidase)	Homo sapiens sialidase 1 (lysosomal sia	6906.65200	4446.64840	5670.29740	3258.54790	Detected	Detected	Detected	Detected
7989	A_24_P293120	ZNF576	Hs.11110	zinc finger protein 576	Homo sapiens zinc finger protein 576 (Z	364.62512	483.12863	502.12802	587.05524	Detected	Detected	Detected	Detected
7990	A_33_P3390873					108310.63000	84180.50000	55663.66000	35826.23400	Detected	Detected	Detected	Detected
7991	A_33_P3284646	FBXL6	Hs.12271	F-box and leucine-rich repeat protein 6	Homo sapiens F-box and leucine-rich r	9450.47500	12703.00400	5514.00340	5139.36870	Detected	Detected	Detected	Detected
7992	A_33_P3370060	ARHGAP23	Hs.374446	Rho GTPase activating protein 23	Homo sapiens Rho GTPase activating p	124.66121	224.52858	80.99135	148.91937	Detected	Detected	Detected	Detected
7993	A_33_P3343605					4603.13100	4270.46040	18.13498	13.75652	Detected	Detected	Detected	Compromised
7994	A_23_P348253	FLJ23834	Hs.150120	hypothetical protein FLJ23834	Homo sapiens hypothetical protein FLJ	60.15961	31.61555	38.46408	21.89460	Detected	Detected	Detected	Detected
7995	A_23_P86917	FADD	Hs.86131	Fas (TNFRSF6)-associated via death d	Homo sapiens Fas (TNFRSF6)-associat	14174.62800	16299.41700	12436.94500	13709.35600	Detected	Detected	Detected	Detected
7996	A_23_P128930	PSMC6	Hs.156171	proteasome (prosome, macropain) 26S	Homo sapiens proteasome (prosome, m	6857.13000	6581.98970	5772.08900	4627.14360	Detected	Detected	Detected	Detected
7997	A_24_P327499	KIAA0406	Hs.655481	KIAA0406	Homo sapiens KIAA0406 (KIAA0406), m	185.27997	171.40100	134.27904	114.82221	Detected	Detected	Detected	Detected
7998	A_23_P46045	RGS5	Hs.24950	regulator of G-protein signaling 5	Homo sapiens regulator of G-protein si	287.01130	64.67562	199.01865	40.10272	Detected	Detected	Detected	Detected
7999	A_23_P57370	CECR5	Hs.26890	cat eye syndrome chromosome region,	Homo sapiens cat eye syndrome chrom	5834.00200	3734.53660	5363.18070	3290.43800	Detected	Detected	Detected	Detected
8000	A_23_P341349	CAPN10	Hs.112218	calpain 10	Homo sapiens calpain 10 (CAPN10), tra	1878.20300	1437.53540	1040.44810	680.37070	Detected	Detected	Detected	Detected
8001	A_23_P171143	TSPAN6	Hs.43233	tetraspanin 6	Homo sapiens tetraspanin 6 (TSPAN6),	3673.38800	1995.79720	3705.95070	1868.27950	Detected	Detected	Detected	Detected
8002	A_24_P126628	TMEM194A	Hs.591040	transmembrane protein 194A	Homo sapiens transmembrane protein 1	706.11880	764.80290	455.42780	466.14053	Detected	Detected	Detected	Detected
8003	A_23_P133123	MND1	Hs.294088	meiotic nuclear divisions 1 homolog (S.	Homo sapiens meiotic nuclear divisions	190.46000	2352.91330	132.15727	1461.11170	Detected	Detected	Detected	Detected
8004	A_23_P76901	PLEKHG3	Hs.509637	pleckstrin homology domain containing,	Homo sapiens pleckstrin homology dom	1481.36760	1636.69740	1154.51010	1196.91050	Detected	Detected	Detected	Detected
8005	A_33_P3331588	ATAD3B	Hs.23413	ATPase family, AAA domain containing	Homo sapiens ATPase family, AAA dom	10746.49600	23536.00800	13221.15200	27393.22300	Detected	Detected	Detected	Detected
8006	A_33_P3720195	LOC375196	Hs.659035	hypothetical protein LOC375196	Homo sapiens hypothetical protein LOC	109.24542	150.01767	95.28227	116.24812	Detected	Detected	Detected	Detected
8007	A_33_P3376080	OR1J1	Hs.632692	olfactory receptor, family 1, subfamily J	Homo sapiens olfactory receptor, family	36.85961	32.68249	31.64087	14.08681	Detected	Detected	Detected	Detected
8008	A_24_P416370	HOXB4	Hs.664706	homeobox B4	Homo sapiens homeobox B4 (HOXB4),	21.78193	1104.94100	13.86268	924.07230	Detected	Detected	Detected	Detected
8009	A_23_P14769	FES	Hs.7636	feline sarcoma oncogene	Homo sapiens feline sarcoma oncogene	465.49112	437.56473	375.07687	440.79620	Detected	Detected	Detected	Detected
8010	A_33_P3334791	C1orf85	Hs.202522	chromosome 1 open reading frame 85	Homo sapiens chromosome 1 open read	644.51420	578.62200	431.63522	400.09543	Detected	Detected	Detected	Detected
8011	A_23_P115046	EIF2B3	Hs.533549	eukaryotic translation initiation factor 2	Homo sapiens eukaryotic translation ini	616.64530	903.38470	491.59750	622.31116	Detected	Detected	Detected	Detected
8012	A_24_P22800	PSG11	Hs.646353	pregnancy specific beta-1-glycoprotein	Homo sapiens pregnancy specific beta-	8.48527	25.53615	11.59793	28.18888	Compromised	Detected	Compromised	Detected
8013	A_23_P372255	ITPKB	Hs.528087	inositol 1,4,5-trisphosphate 3-kinase B	Homo sapiens inositol 1,4,5-trisphospha	318.83636	127.08860	277.03710	88.62856	Detected	Detected	Detected	Detected
8014	A_23_P433111	C5orf35	Hs.85950	chromosome 5 open reading frame 35	Homo sapiens chromosome 5 open read	437.07703	546.59010	400.91590	401.45184	Detected	Detected	Detected	Detected
8015	A_23_P344578	FAM154A	Hs.720871	family with sequence similarity 154, mer	Homo sapiens family with sequence sim	9.94275	3.21106	15.86384	10.53082	Compromised	Compromised	Detected	Compromised
8016	A_23_P156180	SLC22A4	Hs.310591	solute carrier family 22 (organic cation/	Homo sapiens solute carrier family 22 (	2129.00070	1979.92240	1581.09990	1271.43040	Detected	Detected	Detected	Detected
8017	A_23_P45955	TEKT2	Hs.127111	tektin 2 (testicular)	Homo sapiens tektin 2 (testicular) (TEK	60.55796	21.97670	58.01646	17.79255	Detected	Detected	Detected	Detected
8018	A_24_P391230	CYYR1	Hs.37445	cysteine/tyrosine-rich 1	Homo sapiens cysteine/tyrosine-rich 1	48.30590	23.49316	44.55092	14.79356	Detected	Detected	Detected	Detected
8019	A_23_P59294	RREB1	Hs.298248	ras responsive element binding protein	Homo sapiens ras responsive element b	57.91427	149.64166	58.44019	149.05257	Detected	Detected	Detected	Detected
8020	A_33_P3380883	XPA	Hs.654364	xeroderma pigmentosum, complementat	Homo sapiens xeroderma pigmentosum,	119.18170	60.74545	115.25208	55.38242	Detected	Detected	Detected	Detected
8021	A_32_P222961	SPIN4	Hs.612782	spindlin family, member 4	Homo sapiens spindlin family, member 4	380.52060	489.63830	199.07207	186.71560	Detected	Detected	Detected	Detected

8022	A_23_P119102	VASP	Hs.515469	vasodilator-stimulated phosphoprotein	Homo sapiens vasodilator-stimulated ph	3410.79930	4733.45950	2446.03030	2855.13530	Detected	Detected	Detected	Detected
8023	A_23_P22263	BANP	Hs.461705	BTG3 associated nuclear protein	Homo sapiens BTG3 associated nuclear	11003.70800	9539.26700	12659.99100	10114.40500	Detected	Detected	Detected	Detected
8024	A_33_P3405022	LETM1	Hs.120165	leucine zipper-EF-hand containing trans	Homo sapiens leucine zipper-EF-hand c	450.01385	690.49176	360.62872	472.36072	Detected	Detected	Detected	Detected
8025	A_23_P3204	MAPK6	Hs.411847	mitogen-activated protein kinase 6	Homo sapiens mitogen-activated protei	5530.90600	10660.41700	3458.50950	5121.77700	Detected	Detected	Detected	Detected
8026	A_33_P3318069	LOC100129295	Hs.134183	hypothetical LOC100129295	PREDICTED: Homo sapiens similar to ri	43.36105	22.36383	36.83081	18.42329	Detected	Detected	Detected	Detected
8027	A_23_P205913	SLC24A1	Hs.173092	solute carrier family 24 (sodium/potass	Homo sapiens solute carrier family 24 (	121.70661	71.95690	82.71143	52.95803	Detected	Detected	Detected	Detected
8028	A_23_P420293	C11orf45	Hs.351133	chromosome 11 open reading frame 45	Homo sapiens chromosome 11 open rea	18.41250	30.78590	15.60334	18.84498	Detected	Detected	Detected	Detected
8029	A_33_P3388491	PQLC2	Hs.647620	PQ loop repeat containing 2	Homo sapiens PQ loop repeat containin	341373.38000	351646.22000	262552.94000	232448.78000	Detected	Detected	Detected	Detected
8030	A_23_P15394	CD68	Hs.647419	CD68 molecule	Homo sapiens CD68 molecule (CD68), t	8877.93100	3832.47680	10008.60800	4214.55200	Detected	Detected	Detected	Detected
8031	A_33_P3285260				ES1 protein homolog, mitochondrial Pre	310.96844	1996.87410	265.62155	1654.15450	Detected	Detected	Detected	Detected
8032	A_23_P142310	MKNK2	Hs.515032	MAP kinase interacting serine/threonin	Homo sapiens MAP kinase interacting s	16913.94000	28954.89800	14866.50000	21822.00400	Detected	Detected	Detected	Detected
8033	A_33_P3343206		Hs.520384			352.74258	545.50793	142.68352	129.50438	Detected	Detected	Detected	Detected
8034	A_33_P3278144	ANKRD42	Hs.503438	ankyrin repeat domain 42	Homo sapiens ankyrin repeat domain 42	30760.93200	23608.21500	21901.09600	13557.47600	Detected	Detected	Detected	Detected
8035	A_23_P93898	FSCN3	Hs.712692	fascin homolog 3, actin-bundling protein	Homo sapiens fascin homolog 3, actin-b	25.55307	17.17876	14.17952	9.49881	Detected	Compromised	Compromised	Compromised
8036	A_23_P159406	SPRR1B	Hs.1076	small proline-rich protein 1B (cornifin)	Homo sapiens small proline-rich protein	15.26595	17.29326	31.94412	37.69524	Detected	Compromised	Detected	Detected
8037	A_24_P127928	RAB3GAP2	Hs.654849	RAB3 GTPase activating protein subuni	Homo sapiens RAB3 GTPase activating	4870.53500	3996.72730	3763.04200	2426.50340	Detected	Detected	Detected	Detected
8038	A_33_P3281033		Hs.661576		MACRO domain-containing protein 2 [	72.67815	55.19303	45.03738	26.86372	Detected	Detected	Detected	Detected
8039	A_23_P30435	TNIP1	Hs.355141	TNFAIP3 interacting protein 1	Homo sapiens TNFAIP3 interacting prot	5760.61900	8937.84600	6546.58600	8828.43800	Detected	Detected	Detected	Detected
8040	A_23_P40307	SNRPB2	Hs.280378	small nuclear ribonucleoprotein polypep	Homo sapiens small nuclear ribonucleop	10376.82000	12612.75500	6329.74070	5981.91400	Detected	Detected	Detected	Detected
8041	A_24_P74070	PARD6G	Hs.654920	par-6 partitioning defective 6 homolog g	Homo sapiens par-6 partitioning defecti	220.93913	98.64500	162.84933	63.88125	Detected	Detected	Detected	Detected
8042	A_23_P46755	GDF2	Hs.279463	growth differentiation factor 2	Homo sapiens growth differentiation fac	11.35572	10.67908	10.20144	19.97336	Compromised	Compromised	Compromised	Detected
8043	A_33_P3416767	MAFG	Hs.252229	v-maf musculoaponeurotic fibrosarcoma	Homo sapiens v-maf musculoaponeurot	816.44240	807.72565	859.50780	778.51697	Detected	Detected	Detected	Detected
8044	A_33_P3420235				Actin-related protein 2/3 complex subu	773.52110	2027.77590	666.90090	1765.52090	Detected	Detected	Detected	Detected
8045	A_33_P3230073					17028.28000	13320.45700	13492.70100	9225.23200	Detected	Detected	Detected	Detected
8046	A_33_P3374971	TRIM53	Hs.719886	tripartite motif-containing 53	Homo sapiens tripartite motif-containin	17.26868	16.86829	13.44140	11.83960	Detected	Compromised	Compromised	Compromised
8047	A_23_P130040	PHB	Hs.514303	prohibitin	Homo sapiens prohibitin (PHB), mRNA [	8605.47000	8014.32700	6741.52250	5497.98540	Detected	Detected	Detected	Detected
8048	A_24_P932594	ULK2	Hs.168762	unc-51-like kinase 2 (C. elegans)	Homo sapiens unc-51-like kinase 2 (C.	13.08518	6.91976	3.11491	6.61303	Detected	Compromised	Compromised	Compromised
8049	A_33_P3303951	NUPL2	Hs.408241	nucleoporin like 2	Homo sapiens nucleoporin like 2 (NUPL	379.88900	596.18800	406.46664	549.16840	Detected	Detected	Detected	Detected
8050	A_33_P3409675		Hs.453260		cDNA FLJ42124 fis, clone TESTI20094	2392.86230	2218.22880	1150.35380	800.12160	Detected	Detected	Detected	Detected
8051	A_23_P99853	KIAA1370	Hs.152385	KIAA1370	Homo sapiens KIAA1370 (KIAA1370), m	4360.22270	3279.26600	2964.28470	2027.53330	Detected	Detected	Detected	Detected
8052	A_23_P160729	AP4B1	Hs.515048	adaptor-related protein complex 4, beta	Homo sapiens adaptor-related protein c	678.45840	1296.77760	741.39890	1203.75010	Detected	Detected	Detected	Detected
8053	A_23_P95823	NSMCE1	Hs.284295	non-SMC element 1 homolog (S. cerevi	Homo sapiens non-SMC element 1 hom	2114.46340	2884.12400	1884.62290	2238.77700	Detected	Detected	Detected	Detected
8054	A_33_P3336587	LOC283710	Hs.591097	hypothetical LOC283710	PREDICTED: Homo sapiens hypothetica	112.04903	51.81383	12.65105	6.18818	Detected	Detected	Compromised	Compromised
8055	A_23_P128993	GZMH	Hs.348264	granzyme H (cathepsin G-like 2, protein	Homo sapiens granzyme H (cathepsin G	13.15494	12.61988	8.06504	20.63083	Detected	Compromised	Compromised	Detected
8056	A_23_P167367	PITX2	Hs.643588	paired-like homeodomain 2	Homo sapiens paired-like homeodomain	1018.36414	1991.34300	868.77185	1427.44030	Detected	Detected	Detected	Detected
8057	A_33_P3395389	ZNF420	Hs.444992	zinc finger protein 420	Homo sapiens zinc finger protein 420 (Z	269.64453	287.19860	32.28725	23.72687	Detected	Detected	Detected	Detected
8058	A_33_P3340565	POL3S	Hs.569575	polyserase 3	Homo sapiens polyserase 3 (POL3S), m	1517.99180	1289.91160	1368.73250	1051.51990	Detected	Detected	Detected	Detected
8059	A_33_P3245480		Hs.656830		hypothetical LOC728855 (LOC728855),	17.46534	9.73013	25.42032	6.30999	Detected	Compromised	Detected	Compromised
8060	A_24_P12904	C9orf5	Hs.308074	chromosome 9 open reading frame 5	Homo sapiens chromosome 9 open read	130.29497	262.40826	95.86076	191.04622	Detected	Detected	Detected	Detected
8061	A_23_P42897	MGAM	Hs.122785	maltase-glucoamylase (alpha-glucosida	Homo sapiens maltase-glucoamylase (a	2.39401	4.91066	13.77754	5.76475	Compromised	Compromised	Detected	Compromised
8062	A_23_P137209	UBA1	Hs.533273	ubiquitin-like modifier activating enzyme	Homo sapiens ubiquitin-like modifier ac	2374.20100	4398.04250	2051.07130	3402.61350	Detected	Detected	Detected	Detected
8063	A_33_P3312187	LOC100131209		hypothetical protein LOC100131209	PREDICTED: Homo sapiens hypothetica	29.23642	34.23098	48.36091	43.22566	Detected	Detected	Detected	Detected
8064	A_33_P3313596	CDC2L1	Hs.709182	cell division cycle 2-like 1 (PITSLRE pr	Homo sapiens cell division cycle 2-like	1171.23220	904.30600	432.53150	261.58130	Detected	Detected	Detected	Detected
8065	A_23_P100602	TBCD	Hs.464391	tubulin folding cofactor D	Homo sapiens tubulin folding cofactor D	3271.69430	2778.22020	1739.73080	1343.24450	Detected	Detected	Detected	Detected
8066	A_33_P3286953	ADAMTS6	Hs.482291	ADAM metallopeptidase with thrombosp	Homo sapiens ADAM metallopeptidase	181.99226	742.56824	146.56259	489.50787	Detected	Detected	Detected	Detected
8067	A_24_P37589	ACPP	Hs.433060	acid phosphatase, prostate	Homo sapiens acid phosphatase, prosta	32.75102	3.21556	23.29688	5.25906	Detected	Compromised	Detected	Compromised
8068	A_23_P377434	TNRC18	Hs.520638	trinucleotide repeat containing 18	Homo sapiens trinucleotide repeat cont	7156.42430	9088.85700	4070.31520	3987.66500	Detected	Detected	Detected	Detected
8069	A_24_P81947	CORO1C	Hs.330384	coronin, actin binding protein, 1C	Homo sapiens coronin, actin binding pro	7013.74600	14709.49900	4976.76800	7926.98000	Detected	Detected	Detected	Detected
8070	A_23_P7791	OGFRL1	Hs.656091	opioid growth factor receptor-like 1	Homo sapiens opioid growth factor rece	5912.58540	4496.87000	4176.67200	2502.62700	Detected	Detected	Detected	Detected
8071	A_32_P144342	PARP4	Hs.117825	poly (ADP-ribose) polymerase family, m	Homo sapiens poly (ADP-ribose) polym	1175.28060	757.64900	137.02680	60.25371	Detected	Detected	Detected	Detected
8072	A_24_P246636	LOC100130383	Hs.711098	similar to HIG1 domain family, member	PREDICTED: Homo sapiens similar to H	329.21070	443.53052	22.83668	14.75135	Detected	Detected	Detected	Detected
8073	A_24_P85557	GPR37	Hs.719904	G protein-coupled receptor 37 (endothe	Homo sapiens G protein-coupled recep	2.45177	17.05505	2.63798	2.74898	Compromised	Detected	Compromised	Compromised
8074	A_24_P14260	CARD8	Hs.446146	caspase recruitment domain family, mer	Homo sapiens caspase recruitment dom	216.15434	52.50522	189.69969	42.44869	Detected	Detected	Detected	Detected
8075	A_33_P3371588	FUNDC2	Hs.356050	FUN14 domain containing 2	Homo sapiens FUN14 domain containing	66.66740	48.98645	54.30255	38.29719	Detected	Detected	Detected	Detected
8076	A_32_P14894	RPS10	Hs.645317	ribosomal protein S10	Homo sapiens ribosomal protein S10 (R	91747.07000	78800.90000	92485.32000	70754.23400	Detected	Detected	Detected	Detected
8077	A_24_P412512	NIN	Hs.310429	ninein (GSK3B interacting protein)	Homo sapiens ninein (GSK3B interactin	167.24850	365.98755	115.64679	207.11404	Detected	Detected	Detected	Detected
8078	A_23_P137984	S100A10	Hs.143873	S100 calcium binding protein A10	Homo sapiens S100 calcium binding pro	39216.01600	50362.22000	22041.26600	20311.83400	Detected	Detected	Detected	Detected
8079	A_32_P203430	ZNF30	Hs.657402	zinc finger protein 30	Homo sapiens zinc finger protein 30 (ZN	502.12170	316.34103	280.56100	149.18263	Detected	Detected	Detected	Detected
8080	A_23_P165952	ACTR5	Hs.371585	ARP5 actin-related protein 5 homolog (	Homo sapiens ARP5 actin-related prote	410.99734	329.84500	480.35400	317.57135	Detected	Detected	Detected	Detected
8081	A_32_P447001	FLJ27352	Hs.97300	hypothetical LOC145788	RST28817 Athersys RAGE Library Hom	63.44371	166.19188	48.17236	91.12878	Detected	Detected	Detected	Detected
8082	A_33_P3357049	RPS15AP10		ribosomal protein S15a pseudogene 10	Homo sapiens ribosomal protein S15a p	129.20280	128.72060	74.78327	58.62544	Detected	Detected	Detected	Detected
8083	A_24_P82032	RHOJ	Hs.656339	ras homolog gene family, member J	Homo sapiens ras homolog gene family,	15.60592	3.52146	23.39463	2.95482	Detected	Compromised	Detected	Compromised
8084	A_33_P3408047	LOC100131368	Hs.589889	similar to hCG1783157	PREDICTED: Homo sapiens similar to h	16.78262	15.96891	7.30570	9.10776	Detected	Compromised	Compromised	Compromised
8085	A_23_P48237	MCRS1	Hs.25313	microspherule protein 1	Homo sapiens microspherule protein 1 (	985.46200	1876.40110	1065.20500	1848.97800	Detected	Detected	Detected	Detected
8086	A_23_P413193	C10orf78	Hs.93667	chromosome 10 open reading frame 78	Homo sapiens chromosome 10 open rea	1084.01970	1448.46580	791.61096	867.31726	Detected	Detected	Detected	Detected
8087	A_32_P155247	FTL	Hs.433670	ferritin, light polypeptide	Homo sapiens ferritin, light polypeptide	92411.98400	68669.91000	86937.33000	57931.54700	Detected	Detected	Detected	Detected
8088	A_23_P218770	RAC2	Hs.517601	ras-related C3 botulinum toxin substrat	Homo sapiens ras-related C3 botulinum	3425.68330	5751.48340	3119.67630	4825.10740	Detected	Detected	Detected	Detected
8089	A_33_P3273258	ZNF701	Hs.710468	zinc finger protein 701	Homo sapiens zinc finger protein 701 (Z	1144.37410	1094.71900	886.64075	801.69770	Detected	Detected	Detected	Detected
8090	A_23_P67589	C19orf60	Hs.655317	chromosome 19 open reading frame 60	Homo sapiens chromosome 19 open rea	6135.35250	4802.97800	7433.54000	4933.09770	Detected	Detected	Detected	Detected
8091	A_24_P176493	ATMIN	Hs.16349	ATM interactor	Homo sapiens ATM interactor (ATMIN),	919.52356	644.13776	383.85290	212.82310	Detected	Detected	Detected	Detected
8092	A_24_P409800	KCTD16	Hs.661870	potassium channel tetramerisation dom	Homo sapiens potassium channel tetran	14.80215	3.59848	15.21657	7.54938	Detected	Compromised	Detected	Compromised
8093	A_23_P133236	PCDHB14	Hs.658497	protocadherin beta 14	Homo sapiens protocadherin beta 14 (P	80.37313	33.25761	61.64074	17.11332	Detected	Detected	Detected	Detected
8094	A_33_P3252889	PHF12	Hs.444173	PHD finger protein 12	Homo sapiens PHD finger protein 12 (P	537.13556	711.36743	123.22811	119.52790	Detected	Detected	Detected	Detected
8095	A_23_P212329	PTPN23	Hs.25524	protein tyrosine phosphatase, non-rece	Homo sapiens protein tyrosine phospha	625.62900	787.87990	784.73680	909.73630	Detected	Detected	Detected	Detected
8096	A_33_P3406004	C21orf57	Hs.474066	chromosome 21 open reading frame 57	Homo sapiens chromosome 21 open rea	1938.44850	1145.31190	1821.19230	979.76090	Detected	Detected	Detected	Detected

8097	A_23_P91657	EWSR1	Hs.374477	Ewing sarcoma breakpoint region 1	Homo sapiens Ewing sarcoma breakpoir	1331.60730	2905.99020	1147.41320	2547.61380	Detected	Detected	Detected	Detected
8098	A_33_P3510837		Hs.710496		Homo sapiens cDNA FLJ39124 fis, clon	176.25370	173.23670	57.32010	28.92053	Detected	Detected	Detected	Detected
8099	A_33_P3315169				Seven transmembrane helix receptor [	2.41658	3.29486	19.20797	7.80545	Compromised	Compromised	Detected	Compromised
8100	A_33_P3358885	LOC100130913	Hs.649020	hypothetical LOC100130913	Homo sapiens cDNA FLJ46498 fis, clon	33.17669	39.28299	26.08009	27.47108	Detected	Detected	Detected	Detected
8101	A_24_P322709	SNTA1	Hs.31121	syntrophin, alpha 1 (dystrophin-associa	Homo sapiens syntrophin, alpha 1 (dyst	883.29630	659.19135	1146.79900	844.67630	Detected	Detected	Detected	Detected
8102	A_24_P418418	RPS17	Hs.433427	ribosomal protein S17	Homo sapiens ribosomal protein S17 (R	293900.94000	242499.92000	238560.95000	178060.16000	Detected	Detected	Detected	Detected
8103	A_33_P3318377					97.93223	153.34404	98.50585	122.64280	Detected	Detected	Detected	Detected
8104	A_24_P53976	GLUL	Hs.518525	glutamate-ammonia ligase (glutamine sy	Homo sapiens glutamate-ammonia ligas	11734.61700	11349.53400	10139.85100	8226.87200	Detected	Detected	Detected	Detected
8105	A_33_P3236416	GPR179	Hs.462915	G protein-coupled receptor 179	Homo sapiens G protein-coupled recep	76.96792	5.25644	3.83964	6.05667	Detected	Compromised	Compromised	Compromised
8106	A_33_P3336038					136808.66000	137466.98000	54895.78000	41783.95000	Detected	Detected	Detected	Detected
8107	A_33_P3284933	IL27	Hs.528111	interleukin 27	Homo sapiens interleukin 27 (IL27), mR	51.34401	122.46668	55.38432	128.74553	Detected	Detected	Detected	Detected
8108	A_23_P311358	ZNF282	Hs.657701	zinc finger protein 282	Homo sapiens zinc finger protein 282 (Z	297.79620	448.30010	173.42712	240.02028	Detected	Detected	Detected	Detected
8109	A_23_P159237	GPR20	Hs.188859	G protein-coupled receptor 20	Homo sapiens G protein-coupled recep	13.82131	10.75055	29.28503	13.27689	Detected	Compromised	Detected	Compromised
8110	A_33_P3265866					61.30286	75.18638	85.42723	79.60028	Detected	Detected	Detected	Detected
8111	A_23_P60962	MAPK15	Hs.493169	mitogen-activated protein kinase 15	Homo sapiens mitogen-activated protei	41.62768	57.26900	29.59609	30.06472	Detected	Detected	Detected	Detected
8112	A_23_P146551	MOBKL2B	Hs.369022	MOB1, Mps One Binder kinase activato	Homo sapiens MOB1, Mps One Binder k	122.35542	3.20207	98.99301	3.32278	Detected	Compromised	Detected	Compromised
8113	A_23_P142776	EIF3F	Hs.516023	eukaryotic translation initiation factor 3	Homo sapiens eukaryotic translation ini	9693.57500	7992.70400	8369.19000	5820.55030	Detected	Detected	Detected	Detected
8114	A_24_P272088	SPTB	Hs.417303	spectrin, beta, erythrocytic	Homo sapiens spectrin, beta, erythrocy	2.39486	3.21654	14.64953	5.76771	Compromised	Compromised	Detected	Compromised
8115	A_23_P97573	PRPF3	Hs.11776	PRP3 pre-mRNA processing factor 3 h	Homo sapiens PRP3 pre-mRNA proces	555.30220	770.30540	519.16220	575.08220	Detected	Detected	Detected	Detected
8116	A_24_P217848	HIST1H2AK	Hs.558421	histone cluster 1, H2ak	Homo sapiens histone cluster 1, H2ak (	9341.51500	11164.45900	10342.59400	10092.12500	Detected	Detected	Detected	Detected
8117	A_23_P119266	DNASE2	Hs.720012	deoxyribonuclease II, lysosomal	Homo sapiens deoxyribonuclease II, lys	1900.57410	1204.51270	1713.00460	1039.32420	Detected	Detected	Detected	Detected
8118	A_33_P3369436	LOC100130111	Hs.525720	hypothetical protein LOC100130111	PREDICTED: Homo sapiens hypothetica	315.63858	372.04648	252.04044	243.06447	Detected	Detected	Detected	Detected
8119	A_23_P218997	PDCD6	Hs.50823	programmed cell death 6	Homo sapiens programmed cell death 6	26340.26600	22583.97500	20728.16800	14966.01900	Detected	Detected	Detected	Detected
8120	A_33_P3376321	ANK1	Hs.654438	ankyrin 1, erythrocytic	Homo sapiens ankyrin 1, erythrocytic (A	40.78545	10.66018	34.80586	5.85430	Detected	Compromised	Detected	Compromised
8121	A_32_P36046	C2orf68	Hs.516159	chromosome 2 open reading frame 68	Homo sapiens chromosome 2 open rea	921.02435	429.53690	761.71660	334.84244	Detected	Detected	Detected	Detected
8122	A_33_P3285077	COMMD8	Hs.23956	COMM domain containing 8	Homo sapiens COMM domain containin	1074.03880	968.81880	1137.51100	887.78320	Detected	Detected	Detected	Detected
8123	A_23_P103226	FAAH	Hs.720143	fatty acid amide hydrolase	Homo sapiens fatty acid amide hydrolas	27.16059	17.32744	24.44943	12.17342	Detected	Detected	Detected	Compromised
8124	A_23_P332713	ZFR2	Hs.65750	zinc finger RNA binding protein 2	Homo sapiens zinc finger RNA binding p	13.42054	13.49560	6.82540	9.76644	Detected	Compromised	Compromised	Compromised
8125	A_23_P315964	UMODL1	Hs.242520	uromodulin-like 1	Homo sapiens uromodulin-like 1 (UMOD	13.40429	22.36356	2.70668	9.77041	Detected	Detected	Compromised	Compromised
8126	A_33_P3295458	LOC339674		hypothetical LOC339674	Homo sapiens hypothetical LOC339674	49.31111	46.41822	11.41999	22.01907	Detected	Detected	Compromised	Detected
8127	A_23_P335905	STK36	Hs.471404	serine/threonine kinase 36, fused homo	Homo sapiens serine/threonine kinase	439.50842	296.60860	317.20105	212.97755	Detected	Detected	Detected	Detected
8128	A_23_P14493	C14orf104	Hs.231761	chromosome 14 open reading frame 104	Homo sapiens chromosome 14 open rea	2810.37480	1324.50600	2195.48900	872.38245	Detected	Detected	Detected	Detected
8129	A_33_P3234546	CD8B	Hs.405667	CD8b molecule	Homo sapiens CD8b molecule (CD8B),	18.64010	21.46886	11.70636	13.16171	Detected	Detected	Compromised	Compromised
8130	A_33_P3335257	TMEM146	Hs.631842	transmembrane protein 146	Homo sapiens transmembrane protein 1	273.63074	328.12177	177.86707	173.89462	Detected	Detected	Detected	Detected
8131	A_23_P326760	MYRIP	Hs.594535	myosin VIIA and Rab interacting protein	Homo sapiens myosin VIIA and Rab inte	12.64973	42.15561	8.14695	22.42786	Compromised	Detected	Compromised	Detected
8132	A_32_P50417	LOC100130107	Hs.710718	hypothetical LOC100130107	PREDICTED: Homo sapiens hypothetica	2097.10100	819.90430	1340.98130	490.56195	Detected	Detected	Detected	Detected
8133	A_23_P325606	ESX1	Hs.223782	ESX homeobox 1	Homo sapiens ESX homeobox 1 (ESX1)	9.89546	18.46791	8.96940	6.12556	Compromised	Detected	Compromised	Compromised
8134	A_33_P3260733	GHR	Hs.125180	growth hormone receptor	Homo sapiens growth hormone recepto	29.98511	9.97161	21.49529	9.62162	Detected	Compromised	Detected	Compromised
8135	A_23_P140301	PSMA3	Hs.558799	proteasome (prosome, macropain) subu	Homo sapiens proteasome (prosome, m	13855.54500	16041.44800	11561.48300	10977.26300	Detected	Detected	Detected	Detected
8136	A_23_P159952	BEX1	Hs.334370	brain expressed, X-linked 1	Homo sapiens brain expressed, X-linke	8086.93100	1801.44100	4423.39400	797.31903	Detected	Detected	Detected	Detected
8137	A_32_P40744					202.00531	160.23311	124.98420	81.34921	Detected	Detected	Detected	Detected
8138	A_32_P100109	REPS2	Hs.186810	RALBP1 associated Eps domain contai	Homo sapiens RALBP1 associated Eps	133.74800	3.58485	77.76267	2.99581	Detected	Compromised	Detected	Compromised
8139	A_33_P3840630	FRYL	Hs.595553	FRY-like	Homo sapiens FRY-like (FRYL), mRNA	338.88425	379.20657	213.99702	210.65337	Detected	Detected	Detected	Detected
8140	A_33_P3224265				60S ribosomal protein L23 (Ribosomal p	1130.36010	1526.71370	1033.75500	1229.69090	Detected	Detected	Detected	Detected
8141	A_33_P3416568				Androgen-induced protein 1 (AIG-1) [S	899.29860	752.39703	650.39870	487.67017	Detected	Detected	Detected	Detected
8142	A_23_P207742	THRA	Hs.724	thyroid hormone receptor, alpha (erythr	Homo sapiens thyroid hormone recepto	6269.96040	7144.05470	5300.12260	5452.70360	Detected	Detected	Detected	Detected
8143	A_33_P3221403		Hs.710766		Putative uncharacterized protein ENSP	8.22852	3.38524	17.73204	13.22099	Compromised	Compromised	Detected	Compromised
8144	A_33_P3287529	SDF4	Hs.42806	stromal cell derived factor 4	Homo sapiens stromal cell derived fact	3173.07980	3556.50300	2157.36160	2578.46100	Detected	Detected	Detected	Detected
8145	A_23_P24515	ACAT1	Hs.232375	acetyl-Coenzyme A acetyltransferase 1	Homo sapiens acetyl-Coenzyme A acet	16206.85800	18579.05900	15675.54300	15081.70800	Detected	Detected	Detected	Detected
8146	A_32_P840463		Hs.672807		full-length cDNA clone CS0DN005YK16	517.39540	754.85800	434.37100	474.73288	Detected	Detected	Detected	Detected
8147	A_32_P342064	FTH1	Hs.524910	ferritin, heavy polypeptide 1	Homo sapiens ferritin, heavy polypeptid	164886.28000	173206.31000	82085.22000	69598.52000	Detected	Detected	Detected	Detected
8148	A_23_P164958	CCDC8	Hs.97876	coiled-coil domain containing 8	Homo sapiens coiled-coil domain conta	242.36613	2629.01510	243.71422	2500.55980	Detected	Detected	Detected	Detected
8149	A_33_P3374563	SPATA7	Hs.525518	spermatogenesis associated 7	Homo sapiens spermatogenesis associa	2561.65040	1528.03540	2037.38540	1041.27930	Detected	Detected	Detected	Detected
8150	A_23_P1552	CTSC	Hs.128065	cathepsin C	Homo sapiens cathepsin C (CTSC), tran	1044.99180	1523.85930	1243.45700	1595.02710	Detected	Detected	Detected	Detected
8151	A_33_P3386364	FANCF	Hs.713574	Fanconi anemia, complementation group	Homo sapiens Fanconi anemia, complen	605.39450	979.88210	636.97590	893.99023	Detected	Detected	Detected	Detected
8152	A_33_P3264089	ACTR3C	Hs.647117	ARP3 actin-related protein 3 homolog C	Homo sapiens ARP3 actin-related prote	53.51188	24.62030	32.46536	19.41249	Detected	Detected	Detected	Detected
8153	A_33_P3800664		Hs.601293		Homo sapiens cDNA FLJ33330 fis, clon	2664.27120	1999.59520	1299.94290	895.55350	Detected	Detected	Detected	Detected
8154	A_33_P3356821	LCE1C	Hs.516429	late cornified envelope 1C	Homo sapiens late cornified envelope 1	47.40436	41.91419	45.04185	25.84189	Detected	Detected	Detected	Detected
8155	A_33_P3417650	KLK10	Hs.275464	kallikrein-related peptidase 10	Homo sapiens kallikrein-related peptida	16.51674	18.07455	14.66304	4.30398	Detected	Detected	Detected	Compromised
8156	A_33_P3404697	PSTK	Hs.281004	phosphoseryl-tRNA kinase	Homo sapiens phosphoseryl-tRNA kina	733.59200	1056.23560	568.09490	651.89570	Detected	Detected	Detected	Detected
8157	A_24_P336577	C1orf183	Hs.657857	chromosome 1 open reading frame 183	Homo sapiens chromosome 1 open rea	872.66565	867.37573	575.47850	386.80940	Detected	Detected	Detected	Detected
8158	A_23_P433016	FBLN1	Hs.24601	fibulin 1	Homo sapiens fibulin 1 (FBLN1), transc	21080.49000	4119.26460	18950.06400	4037.08520	Detected	Detected	Detected	Detected
8159	A_33_P3216438	SPATA21	Hs.705501	spermatogenesis associated 21	Homo sapiens spermatogenesis associa	4375.73100	2566.30620	304.48593	147.98520	Detected	Detected	Detected	Detected
8160	A_33_P3400653	BNIP2	Hs.592515	BCL2/adenovirus E1B 19kDa interactin	BCL2/adenovirus E1B 19 kDa protein-i	14051.62200	8695.03800	10709.58400	5185.67770	Detected	Detected	Detected	Detected
8161	A_33_P3307903	CDKN3	Hs.84113	cyclin-dependent kinase inhibitor 3	Homo sapiens cyclin-dependent kinase	74.23734	1541.01530	98.50087	1436.78020	Detected	Detected	Detected	Detected
8162	A_24_P336718	RABIF	Hs.90875	RAB interacting factor	Homo sapiens RAB interacting factor (F	46.98939	53.34866	32.49504	28.45789	Detected	Detected	Detected	Detected
8163	A_33_P3317548					9.50813	24.48768	11.18813	24.89634	Compromised	Detected	Compromised	Detected
8164	A_23_P365149	BCKDK	Hs.513520	branched chain ketoacid dehydrogenase	Homo sapiens branched chain ketoacid	1687.19310	1981.51590	1920.21740	2133.77690	Detected	Detected	Detected	Detected
8165	A_23_P73530	MTMR1	Hs.347187	myotubularin related protein 1	Homo sapiens myotubularin related prot	903.27905	815.98270	758.72253	584.26160	Detected	Detected	Detected	Detected
8166	A_33_P3355230	LAIR1	Hs.572535	leukocyte-associated immunoglobulin-li	Homo sapiens leukocyte-associated im	142.02522	169.21373	74.42757	73.36568	Detected	Detected	Detected	Detected
8167	A_23_P117424	DCAF11	Hs.525251	DDB1 and CUL4 associated factor 11	Homo sapiens DDB1 and CUL4 associa	788.63910	1033.32180	582.42340	737.04895	Detected	Detected	Detected	Detected
8168	A_23_P380318	EGR4	Hs.3052	early growth response 4	Homo sapiens early growth response 4	24.95375	384.39703	20.23956	328.71967	Detected	Detected	Detected	Detected
8169	A_23_P630	MSTO1	Hs.656547	misato homolog 1 (Drosophila)	Homo sapiens misato homolog 1 (Droso	251.57030	391.95752	257.35040	463.06137	Detected	Detected	Detected	Detected
8170	A_33_P3297452					341.79703	260.89847	54.18988	42.03466	Detected	Detected	Detected	Detected
8171	A_23_P46982	MMS19	Hs.500721	MMS19 nucleotide excision repair homo	Homo sapiens MMS19 nucleotide excisi	6549.82370	4651.08800	3375.88100	1929.93840	Detected	Detected	Detected	Detected

8172	A_23_P104188	ELF3	Hs.67928	E74-like factor 3 (ets domain transcript	Homo sapiens E74-like factor 3 (ets do	16.28275	3.42071	13.55224	3.09708	Detected	Compromised	Compromised	Compromised
8173	A_33_P3283824	SLC39A8	Hs.288034	solute carrier family 39 (zinc transporte	Homo sapiens solute carrier family 39 (	50.50548	3.44698	36.34823	3.53954	Detected	Compromised	Detected	Compromised
8174	A_23_P209360	KLHL29		kelch-like 29 (Drosophila)	Homo sapiens kelch-like 29 (Drosophila	2601.47780	1803.82300	2330.92750	1417.74300	Detected	Detected	Detected	Detected
8175	A_23_P152970	RAPGEFL1	Hs.632254	Rap guanine nucleotide exchange factor	Homo sapiens Rap guanine nucleotide e	75.32838	14.38494	65.30569	13.38655	Detected	Compromised	Detected	Compromised
8176	A_33_P3303956	LOC100133311	Hs.658061	similar to hCG1644697	PREDICTED: Homo sapiens similar to h	2.59771	115.96621	3.62127	85.19312	Compromised	Detected	Compromised	Detected
8177	A_23_P50008	TTC19	Hs.462316	tetratricopeptide repeat domain 19	Homo sapiens tetratricopeptide repeat	4576.45300	3969.28700	3372.79400	2471.45830	Detected	Detected	Detected	Detected
8178	A_23_P50942	RAB3GAP1	Hs.306327	RAB3 GTPase activating protein subuni	Homo sapiens RAB3 GTPase activating	5500.90100	6676.90430	2683.74850	2703.31450	Detected	Detected	Detected	Detected
8179	A_24_P39454	INO80	Hs.292949	INO80 homolog (S. cerevisiae)	Homo sapiens INO80 homolog (S. cerev	265.13492	250.77092	339.42545	266.10132	Detected	Detected	Detected	Detected
8180	A_23_P118842	KRTAP1-5	Hs.534499	keratin associated protein 1-5	Homo sapiens keratin associated protei	69.21690	5934.33300	38.83360	3691.36670	Detected	Detected	Detected	Detected
8181	A_24_P66528	SRP9	Hs.511425	signal recognition particle 9kDa	Homo sapiens signal recognition particle	3239.46120	4498.60940	3143.77300	3442.35860	Detected	Detected	Detected	Detected
8182	A_23_P10232	BANK1	Hs.480400	B-cell scaffold protein with ankyrin rep	Homo sapiens B-cell scaffold protein w	17.54608	39.42660	8.68094	20.09248	Detected	Detected	Compromised	Detected
8183	A_24_P218814	RDH5	Hs.600940	retinol dehydrogenase 5 (11-cis/9-cis)	Homo sapiens retinol dehydrogenase 5	426.10538	244.67978	484.42606	278.59370	Detected	Detected	Detected	Detected
8184	A_24_P201491	MRPL49	Hs.719977	mitochondrial ribosomal protein L49	Homo sapiens mitochondrial ribosomal p	5411.83150	5905.57400	5209.83540	5264.30400	Detected	Detected	Detected	Detected
8185	A_32_P86118	PM20D2	Hs.356247	peptidase M20 domain containing 2	Homo sapiens peptidase M20 domain co	400.22160	202.58540	294.15616	122.53121	Detected	Detected	Detected	Detected
8186	A_23_P94095	ANKRD46	Hs.530199	ankyrin repeat domain 46	Homo sapiens ankyrin repeat domain 46	554.30210	269.30377	352.58594	131.45080	Detected	Detected	Detected	Detected
8187	A_23_P77731	CRYM	Hs.924	crystallin, mu	Homo sapiens crystallin, mu (CRYM), tr	2952.99980	3972.17970	2508.74540	2918.19480	Detected	Detected	Detected	Detected
8188	A_23_P358542	KIFC2	Hs.528713	kinesin family member C2	Homo sapiens kinesin family member C2	556.58040	147.40990	577.44290	141.83932	Detected	Detected	Detected	Detected
8189	A_24_P196528	CRB1	Hs.126135	crumbs homolog 1 (Drosophila)	Homo sapiens crumbs homolog 1 (Dros	16.09217	3.33709	2.70257	2.84536	Detected	Compromised	Compromised	Compromised
8190	A_33_P3846114	LOC286442	Hs.638478	hypothetical protein LOC286442	Homo sapiens cDNA FLJ36359 fis, clon	30.74355	4.59157	2.70537	6.09687	Detected	Compromised	Compromised	Compromised
8191	A_33_P3347417	SPEN	Hs.558463	spen homolog, transcriptional regulator	Homo sapiens spen homolog, transcripti	555.16270	282.87933	79.45810	69.25946	Detected	Detected	Detected	Detected
8192	A_33_P3255290	JAKMIP2	Hs.184323	janus kinase and microtubule interacting	Homo sapiens janus kinase and microtu	11.50306	3.30861	13.51270	2.83406	Compromised	Compromised	Detected	Compromised
8193	A_33_P3219279	JPH4	Hs.597841	junctophilin 4	Homo sapiens junctophilin 4 (JPH4), tra	28.55239	3.29497	32.48565	13.17107	Detected	Compromised	Detected	Compromised
8194	A_23_P24375	OTUB1	Hs.473788	OTU domain, ubiquitin aldehyde binding	Homo sapiens OTU domain, ubiquitin al	1453.71370	2272.71460	2044.26700	2969.91580	Detected	Detected	Detected	Detected
8195	A_23_P140384	CTSG	Hs.421724	cathepsin G	Homo sapiens cathepsin G (CTSG), mR	25.83420	32.85134	15.25849	14.85059	Detected	Detected	Detected	Detected
8196	A_33_P3231120	CASD1	Hs.260041	CAS1 domain containing 1	Homo sapiens CAS1 domain containing	48.75614	32.28587	37.57118	17.41883	Detected	Detected	Detected	Detected
8197	A_33_P3264612	TPCN2	Hs.131851	two pore segment channel 2	Homo sapiens two pore segment chann	1338.94750	866.93610	906.31800	519.20640	Detected	Detected	Detected	Detected
8198	A_23_P22926	GNB1	Hs.430425	guanine nucleotide binding protein (G pr	Homo sapiens guanine nucleotide bindin	6019.29930	9869.29400	4171.03800	5468.84300	Detected	Detected	Detected	Detected
8199	A_33_P3251617	LOC388948	Hs.720073	hypothetical protein LOC388948	Putative uncharacterized protein ENSP	168.14642	167.50298	156.43724	115.16443	Detected	Detected	Detected	Detected
8200	A_33_P3390387	NCRNA00159		non-protein coding RNA 159	Homo sapiens non-protein coding RNA	2.39077	3.21296	2.61593	13.92859	Compromised	Compromised	Compromised	Detected
8201	A_32_P165340	SRP9	Hs.511425	signal recognition particle 9kDa	Homo sapiens signal recognition particle	8618.24400	11424.27100	2527.96950	2461.21800	Detected	Detected	Detected	Detected
8202	A_33_P3250289	MAPK8	Hs.138211	mitogen-activated protein kinase 8	Homo sapiens mitogen-activated protei	28.71922	29.81456	26.13865	30.86146	Detected	Detected	Detected	Detected
8203	A_23_P205875	SCAND2	Hs.658114	SCAN domain containing 2 pseudogene	Homo sapiens SCAN domain containing	157.82024	162.87834	169.02988	149.51514	Detected	Detected	Detected	Detected
8204	A_32_P163125	SGMS1	Hs.654698	sphingomyelin synthase 1	Homo sapiens sphingomyelin synthase	916.70450	1618.81640	668.27423	891.98267	Detected	Detected	Detected	Detected
8205	A_24_P298027	AXIN2	Hs.156527	axin 2	Homo sapiens axin 2 (AXIN2), mRNA [N	29.58959	18.98939	27.46104	14.02410	Detected	Detected	Detected	Detected
8206	A_23_P89270	SERPINF2	Hs.159509	serpin peptidase inhibitor, clade F (alpha	Homo sapiens serpin peptidase inhibitor	5.85210	6.19239	15.79068	12.16312	Compromised	Compromised	Detected	Compromised
8207	A_23_P79251	EHD3	Hs.368808	EH-domain containing 3	Homo sapiens EH-domain containing 3	1814.89000	1206.01300	1656.66480	1077.35390	Detected	Detected	Detected	Detected
8208	A_24_P161973	ATP11A	Hs.29189	ATPase, class VI, type 11A	Homo sapiens ATPase, class VI, type 1	613.94020	474.58853	411.95862	245.46458	Detected	Detected	Detected	Detected
8209	A_24_P21044	PSMG3	Hs.446311	proteasome (prosome, macropain) asse	Homo sapiens proteasome (prosome, m	4064.77900	5410.45100	4667.87100	5471.12060	Detected	Detected	Detected	Detected
8210	A_23_P141730	DSG2	Hs.412597	desmoglein 2	Homo sapiens desmoglein 2 (DSG2), mR	7.26556	7.30651	14.53189	21.61447	Compromised	Compromised	Detected	Detected
8211	A_24_P418816	GPX7	Hs.43728	glutathione peroxidase 7	Homo sapiens glutathione peroxidase 7	426.21863	751.34330	394.81720	574.53660	Detected	Detected	Detected	Detected
8212	A_24_P76854	KRTAP2-1	Hs.528921	keratin associated protein 2-1	Homo sapiens keratin associated protei	255.66904	357.02783	146.82295	163.14154	Detected	Detected	Detected	Detected
8213	A_33_P3773374	LOC286063	Hs.21550	hypothetical protein LOC286063	Homo sapiens cDNA FLJ33573 fis, clon	118.21983	46.79547	43.67109	11.28020	Detected	Detected	Detected	Compromised
8214	A_23_P166633	ITGB5	Hs.536663	integrin, beta 5	Homo sapiens integrin, beta 5 (ITGB5),	32168.37100	51416.21000	30380.49400	45037.72000	Detected	Detected	Detected	Detected
8215	A_23_P218358	FBXW10	Hs.592128	F-box and WD repeat domain containing	Homo sapiens F-box and WD repeat do	15.65503	3.26885	16.96856	4.55499	Detected	Compromised	Detected	Compromised
8216	A_33_P3346333					50.39791	51.59510	45.53624	35.51970	Detected	Detected	Detected	Detected
8217	A_32_P160972	C6orf115	Hs.600861	chromosome 6 open reading frame 115	Homo sapiens chromosome 6 open read	7132.70170	9961.67900	5400.67430	6441.29600	Detected	Detected	Detected	Detected
8218	A_23_P18223	ITIH1	Hs.420257	inter-alpha (globulin) inhibitor H1	Homo sapiens inter-alpha (globulin) inhi	20.22944	18.55426	22.62479	10.84261	Detected	Detected	Detected	Compromised
8219	A_23_P124642	RASGRP1	Hs.591127	RAS guanyl releasing protein 1 (calcium	Homo sapiens RAS guanyl releasing pro	3.27837	57.53051	2.68507	36.80635	Compromised	Detected	Compromised	Detected
8220	A_24_P940166	PAPSS2	Hs.524491	3'-phosphoadenosine 5'-phosphosulfate	Homo sapiens 3'-phosphoadenosine 5'-	1346.17250	2566.37230	1398.37390	2285.20920	Detected	Detected	Detected	Detected
8221	A_23_P83917	KDM2A	Hs.124147	lysine (K)-specific demethylase 2A	Homo sapiens lysine (K)-specific demet	572.10880	766.01720	604.60980	699.51056	Detected	Detected	Detected	Detected
8222	A_23_P305723	MIER1	Hs.605432	mesoderm induction early response 1 h	Homo sapiens mesoderm induction early	3609.69290	2017.65420	3098.10200	1414.23490	Detected	Detected	Detected	Detected
8223	A_33_P3252414	TH	Hs.435609	tyrosine hydroxylase	Homo sapiens tyrosine hydroxylase (TH	569.78770	837.53340	260.93488	389.59520	Detected	Detected	Detected	Detected
8224	A_32_P141238	ANO2	Hs.148970	anoctamin 2	Homo sapiens anoctamin 2 (ANO2), mR	20.42992	22.74553	18.47583	12.76885	Detected	Detected	Detected	Compromised
8225	A_33_P3301095					1091.96180	765.59607	82.04286	27.54778	Detected	Detected	Detected	Detected
8226	A_32_P153773	CACNA1B	Hs.495522	calcium channel, voltage-dependent, N	Homo sapiens calcium channel, voltage-	26.21260	28.15770	15.58989	13.33385	Detected	Detected	Detected	Compromised
8227	A_24_P84808					82291.01600	78380.19500	42459.06600	32675.27100	Detected	Detected	Detected	Detected
8228	A_33_P3247933	GCC2	Hs.436505	GRIP and coiled-coil domain containing	Homo sapiens GRIP and coiled-coil dom	790.95140	539.80260	530.86290	282.94254	Detected	Detected	Detected	Detected
8229	A_33_P3223544	TMBIM4	Hs.505934	transmembrane BAX inhibitor motif con	Homo sapiens transmembrane BAX inhi	14144.49800	12586.48500	13085.67900	9750.08300	Detected	Detected	Detected	Detected
8230	A_33_P3329301	LOC646014	Hs.657259	hypothetical protein LOC646014	Homo sapiens cDNA FLJ44593 fis, clon	263.70425	269.48932	92.25822	75.78161	Detected	Detected	Detected	Detected
8231	A_33_P3829391	LOC641510	Hs.669824	hypothetical LOC641510	Homo sapiens, mRNA (cDNA clone IMA	242.65270	399.23100	224.51418	307.02914	Detected	Detected	Detected	Detected
8232	A_33_P3238410	SBF1	Hs.589924	SET binding factor 1	Homo sapiens SET binding factor 1 (SB	221.32578	215.90622	101.77201	86.07272	Detected	Detected	Detected	Detected
8233	A_23_P351275	UPP1	Hs.488240	uridine phosphorylase 1	Homo sapiens uridine phosphorylase 1 (	802.09260	1323.23540	688.88654	1040.28310	Detected	Detected	Detected	Detected
8234	A_33_P3349265	C6orf140	Hs.585172	chromosome 6 open reading frame 140	Homo sapiens chromosome 6 open read	30.87686	31.78021	18.60187	22.34012	Detected	Detected	Detected	Detected
8235	A_33_P3290667	SELT	Hs.369052	selenoprotein T	Homo sapiens selenoprotein T (SELT),	1044.63610	1929.50460	848.76556	1339.87100	Detected	Detected	Detected	Detected
8236	A_33_P3272580	FUCA2	Hs.591332	fucosidase, alpha-L- 2, plasma	Homo sapiens fucosidase, alpha-L- 2, p	900.08484	1389.86050	853.53394	1168.49390	Detected	Detected	Detected	Detected
8237	A_33_P3413463	BRF1	Hs.424484	BRF1 homolog, subunit of RNA polymer	Homo sapiens BRF1 homolog, subunit o	7350.92430	5723.42530	4613.53120	3607.68900	Detected	Detected	Detected	Detected
8238	A_23_P207666	USP6	Hs.448851	ubiquitin specific peptidase 6 (Tre-2 on	Homo sapiens ubiquitin specific peptida	829.12240	500.44797	501.90634	251.28807	Detected	Detected	Detected	Detected
8239	A_33_P3352019	SCARA3	Hs.128856	scavenger receptor class A, member 3	Homo sapiens scavenger receptor class	168.41946	600.53260	182.38518	580.32930	Detected	Detected	Detected	Detected
8240	A_24_P406601	OLFM1	Hs.522484	olfactomedin 1	Homo sapiens olfactomedin 1 (OLFM1),	277.42746	267.87115	165.24770	141.56908	Detected	Detected	Detected	Detected
8241	A_33_P3310104	SERPINB5	Hs.55279	serpin peptidase inhibitor, clade B (oval	Homo sapiens serpin peptidase inhibitor	16.09594	13.40774	4.14146	14.16912	Detected	Compromised	Compromised	Detected
8242	A_23_P382506	OR1F2P	Hs.651203	olfactory receptor, family 1, subfamily F	Homo sapiens olfactory receptor, family	42.81683	53.29217	9.00552	13.94097	Detected	Detected	Compromised	Compromised
8243	A_23_P31389	TRA2A	Hs.445652	transformer 2 alpha homolog (Drosophil	Homo sapiens transformer 2 alpha hom	286.51240	381.23087	230.91907	267.37085	Detected	Detected	Detected	Detected
8244	A_23_P374782	SH3KBP1	Hs.444770	SH3-domain kinase binding protein 1	Homo sapiens SH3-domain kinase bindi	6134.74850	6320.31200	5045.70900	4403.82300	Detected	Detected	Detected	Detected
8245	A_32_P225816	PRDM16	Hs.99500	PR domain containing 16	Homo sapiens PR domain containing 16	54.21896	3.30866	62.82385	2.83294	Detected	Compromised	Detected	Compromised
8246	A_24_P723735				Putative uncharacterized protein ZNF72	40.68850	37.97491	2.67892	2.80355	Detected	Detected	Compromised	Compromised

8247	A_32_P420563	RNF215	Hs.592194	ring finger protein 215	Homo sapiens ring finger protein 215 (R	799.20917	320.00253	362.33005	134.04292	Detected	Detected	Detected	Detected
8248	A_23_P18384	ARMC8	Hs.622438	armadillo repeat containing 8	Homo sapiens armadillo repeat containi	754.17720	1502.01730	663.29970	1029.96180	Detected	Detected	Detected	Detected
8249	A_23_P36305	ATG16L2	Hs.653186	ATG16 autophagy related 16-like 2 (S.	Homo sapiens ATG16 autophagy relate	1355.87670	1278.65780	1168.28450	1033.72690	Detected	Detected	Detected	Detected
8250	A_23_P131954	SNX5	Hs.316890	sorting nexin 5	Homo sapiens sorting nexin 5 (SNX5), t	17601.02300	20804.78000	13746.89800	14203.39500	Detected	Detected	Detected	Detected
8251	A_32_P47107	LOC100129104	Hs.598335	similar to hydroxyproline-rich glycoprot	PREDICTED: Homo sapiens similar to h	1907.93040	2167.48200	1386.18660	1383.97920	Detected	Detected	Detected	Detected
8252	A_24_P149124	C5orf13	Hs.36053	chromosome 5 open reading frame 13	Homo sapiens chromosome 5 open read	3553.91550	25115.22900	2497.92970	15254.72900	Detected	Detected	Detected	Detected
8253	A_24_P79153	SCAMP4	Hs.144980	secretory carrier membrane protein 4	Homo sapiens secretory carrier membra	163.47977	229.41463	169.56386	231.91140	Detected	Detected	Detected	Detected
8254	A_23_P342934	TLE3	Hs.287362	transducin-like enhancer of split 3 (E(sp	Homo sapiens transducin-like enhancer	386.39820	352.55750	311.37592	261.18994	Detected	Detected	Detected	Detected
8255	A_23_P98830	UBQLN3	Hs.189184	ubiquilin 3	Homo sapiens ubiquilin 3 (UBQLN3), mF	31.21750	34.54750	16.10843	12.70807	Detected	Detected	Detected	Compromised
8256	A_23_P209987	POLR1B	Hs.86337	polymerase (RNA) I polypeptide B, 128k	Homo sapiens polymerase (RNA) I polyp	708.78625	666.03125	500.18207	394.81772	Detected	Detected	Detected	Detected
8257	A_33_P3417950	AKAP8	Hs.594496	A kinase (PRKA) anchor protein 8	Homo sapiens A kinase (PRKA) anchor	6430.09470	7619.89060	3727.88940	3611.01730	Detected	Detected	Detected	Detected
8258	A_23_P304897	BDKRB2	Hs.654542	bradykinin receptor B2	Homo sapiens bradykinin receptor B2 (E	7759.26500	1071.63960	7206.35000	907.77020	Detected	Detected	Detected	Detected
8259	A_23_P46907	CBARA1	Hs.524367	calcium binding atopy-related autoantig	Homo sapiens calcium binding atopy-re	795.81940	1292.46100	567.66700	766.23910	Detected	Detected	Detected	Detected
8260	A_23_P127613	TRAPPC4	Hs.132760	trafficking protein particle complex 4	Homo sapiens trafficking protein particl	7264.27900	6684.67720	5149.75800	3947.62900	Detected	Detected	Detected	Detected
8261	A_33_P3408983	APC	Hs.158932	adenomatous polyposis coli	Homo sapiens adenomatous polyposis c	216.42950	226.41450	159.83714	142.23761	Detected	Detected	Detected	Detected
8262	A_23_P28834	PHACTR3	Hs.473218	phosphatase and actin regulator 3	Homo sapiens phosphatase and actin re	21.47659	3.24186	15.20828	2.70012	Detected	Compromised	Detected	Compromised
8263	A_23_P419107	TCP11L2	Hs.696047	t-complex 11 (mouse)-like 2	Homo sapiens t-complex 11 (mouse)-lik	155.06088	37.11098	147.00056	40.69178	Detected	Detected	Detected	Detected
8264	A_33_P3354688	LOC730236	Hs.444913	hypothetical LOC730236	PREDICTED: Homo sapiens hypothetica	243.22270	122.55770	176.38902	80.35494	Detected	Detected	Detected	Detected
8265	A_23_P127385	MYBPC3	Hs.524906	myosin binding protein C, cardiac	Homo sapiens myosin binding protein C	67.41184	73.86156	51.32774	46.10299	Detected	Detected	Detected	Detected
8266	A_33_P3388070		Hs.577832		DB226000 TRACH3 Homo sapiens cDN	35.40722	25.92164	20.84122	21.09744	Detected	Detected	Detected	Detected
8267	A_33_P3362991					54.91648	38.31458	32.75600	19.88372	Detected	Detected	Detected	Detected
8268	A_33_P3286302		Hs.512898		Tumor necrosis factor receptor superfa	559.21020	592.27250	339.44028	249.09236	Detected	Detected	Detected	Detected
8269	A_23_P213840	ATP6V0E1	Hs.484188	ATPase, H+ transporting, lysosomal 9kD	Homo sapiens ATPase, H+ transporting	18432.02100	27235.54100	18783.35700	25370.95000	Detected	Detected	Detected	Detected
8270	A_23_P3663	FAM195A	Hs.417710	family with sequence similarity 195, mer	Homo sapiens family with sequence sim	4158.91300	4235.52340	4641.79350	4402.26030	Detected	Detected	Detected	Detected
8271	A_24_P95439	CARS	Hs.274873	cysteinyl-tRNA synthetase	Homo sapiens cysteinyl-tRNA syntheta	1302.59740	1350.56760	824.34270	763.56980	Detected	Detected	Detected	Detected
8272	A_24_P382401	ZNF791	Hs.720677	zinc finger protein 791	Homo sapiens zinc finger protein 791 (Z	91.46251	77.55145	32.85729	37.26100	Detected	Detected	Detected	Detected
8273	A_23_P340722	XPOT	Hs.85951	exportin, tRNA (nuclear export receptor	Homo sapiens exportin, tRNA (nuclear e	3430.51460	3013.23300	3387.17820	2497.57960	Detected	Detected	Detected	Detected
8274	A_33_P3323392	LOC731932	Hs.539723	hypothetical LOC731932	PREDICTED: Homo sapiens hypothetica	165.01149	112.12060	99.40015	53.84625	Detected	Detected	Detected	Detected
8275	A_33_P3244165	LOC100008589	Hs.552217	28S ribosomal RNA	Homo sapiens 28S ribosomal RNA (LOC	339958.72000	365216.30000	291796.30000	279405.50000	Detected	Detected	Detected	Detected
8276	A_33_P3253420					32.84102	38.11917	11.24177	10.58572	Detected	Detected	Compromised	Compromised
8277	A_33_P3266444	MRPS25	Hs.715617	mitochondrial ribosomal protein S25	Homo sapiens mitochondrial ribosomal p	1602.99580	492.88116	1236.05750	343.64975	Detected	Detected	Detected	Detected
8278	A_23_P411335	SGOL2	Hs.655182	shugoshin-like 2 (S. pombe)	Homo sapiens shugoshin-like 2 (S. pom	176.75575	1365.24820	146.04740	907.11280	Detected	Detected	Detected	Detected
8279	A_24_P115971	RIC8A	Hs.592292	resistance to inhibitors of cholinesteras	Homo sapiens resistance to inhibitors o	3516.56960	5266.46600	3560.97730	5085.65770	Detected	Detected	Detected	Detected
8280	A_33_P3253179					4.88307	6.01397	5.94761	15.33472	Compromised	Compromised	Compromised	Detected
8281	A_23_P254404	SNX33	Hs.8705	sorting nexin 33	Homo sapiens sorting nexin 33 (SNX33)	689.01996	813.76556	547.48584	625.59717	Detected	Detected	Detected	Detected
8282	A_33_P3264179	LCE3E	Hs.490208	late cornified envelope 3E	Homo sapiens late cornified envelope 3	15.29184	26.82595	32.07620	24.14248	Detected	Detected	Detected	Detected
8283	A_24_P261005	C8orf40	Hs.655320	chromosome 8 open reading frame 40	Homo sapiens chromosome 8 open read	4703.88600	1666.62620	3282.40430	968.91550	Detected	Detected	Detected	Detected
8284	A_23_P376557	MMP25	Hs.654979	matrix metallopeptidase 25	Homo sapiens matrix metallopeptidase	86.06284	156.53525	52.92314	74.92806	Detected	Detected	Detected	Detected
8285	A_23_P23748	WDR47	Hs.570055	WD repeat domain 47	Homo sapiens WD repeat domain 47 (W	375.04886	454.47385	194.92159	179.10788	Detected	Detected	Detected	Detected
8286	A_23_P78664	DDX39	Hs.311609	DEAD (Asp-Glu-Ala-Asp) box polypept	Homo sapiens DEAD (Asp-Glu-Ala-Asp	9388.38300	29449.62300	9159.12800	24791.05900	Detected	Detected	Detected	Detected
8287	A_33_P3357535	CYB5B	Hs.461131	cytochrome b5 type B (outer mitochond	Homo sapiens cytochrome b5 type B (o	513.16693	281.02747	311.78640	144.45837	Detected	Detected	Detected	Detected
8288	A_32_P25357	CDH15	Hs.148090	cadherin 15, type 1, M-cadherin (myotu	Homo sapiens cadherin 15, type 1, M-ca	22.61115	100.12960	20.82790	60.94959	Detected	Detected	Detected	Detected
8289	A_33_P3317937	KRTAP20-1	Hs.553697	keratin associated protein 20-1	Homo sapiens keratin associated protei	82.45542	80.21542	27.30302	24.76463	Detected	Detected	Detected	Detected
8290	A_33_P3420879	LOC646482	Hs.713256	hypothetical LOC646482	PREDICTED: Homo sapiens hypothetica	35.70228	54.52293	23.84327	33.96371	Detected	Detected	Detected	Detected
8291	A_33_P3374085	LRRFIP1	Hs.471779	leucine rich repeat (in FLII) interacting	Homo sapiens leucine rich repeat (in FL	1196.17020	1976.48970	915.25616	1337.44020	Detected	Detected	Detected	Detected
8292	A_24_P365327	LSM14A	Hs.407368	LSM14A, SCD6 homolog A (S. cerevisia	Homo sapiens LSM14A, SCD6 homolog	4781.98700	5957.62900	3257.55050	3146.65480	Detected	Detected	Detected	Detected
8293	A_23_P315892	ST6GALNAC6	Hs.109672	ST6 (alpha-N-acetyl-neuraminyl-2,3-be	Homo sapiens ST6 (alpha-N-acetyl-neu	16830.12500	6805.05200	12706.80900	4891.80500	Detected	Detected	Detected	Detected
8294	A_32_P160563	OPCML	Hs.4817	opioid binding protein/cell adhesion mol	Homo sapiens opioid binding protein/ce	2.37851	14.34993	2.56447	15.97528	Compromised	Compromised	Compromised	Detected
8295	A_33_P3519683	ZBTB8OS	Hs.655921	zinc finger and BTB domain containing	Homo sapiens zinc finger and BTB dom	1488.26560	2752.35300	1409.02420	2197.57980	Detected	Detected	Detected	Detected
8296	A_33_P3239087		Hs.568078		Protein FAM87B [Source:UniProtKB/S	330.75024	548.54220	289.54672	375.36115	Detected	Detected	Detected	Detected
8297	A_33_P3414422	GPHN	Hs.208765	gephyrin	Homo sapiens gephyrin (GPHN), transcr	1523.54410	967.46906	1147.64760	619.36285	Detected	Detected	Detected	Detected
8298	A_23_P45976	RAP1GAP	Hs.148178	RAP1 GTPase activating protein	Homo sapiens RAP1 GTPase activating	31.99902	39.09724	26.92430	29.39153	Detected	Detected	Detected	Detected
8299	A_23_P367071	UBE2DNL	Hs.460809	ubiquitin-conjugating enzyme E2D N-te	Homo sapiens ubiquitin-conjugating enz	58.49555	63.11289	44.64999	33.04923	Detected	Detected	Detected	Detected
8300	A_23_P34496	TMEM39B	Hs.25544	transmembrane protein 39B	Homo sapiens transmembrane protein 3	1757.76420	1367.03090	1811.45370	1263.52030	Detected	Detected	Detected	Detected
8301	A_23_P121011	CSRNP1	Hs.370950	cysteine-serine-rich nuclear protein 1	Homo sapiens cysteine-serine-rich nuc	1127.76200	855.19210	842.68866	697.62190	Detected	Detected	Detected	Detected
8302	A_24_P30567	UNQ1887	Hs.507087	signal peptide peptidase 3	Homo sapiens signal peptide peptidase	3050.43970	1843.08400	2803.81880	1512.54400	Detected	Detected	Detected	Detected
8303	A_24_P256552	CSTF3	Hs.44402	cleavage stimulation factor, 3' pre-RNA	Homo sapiens cleavage stimulation fact	426.02032	866.94980	621.37430	1139.19060	Detected	Detected	Detected	Detected
8304	A_33_P3257861	SARDH	Hs.198003	sarcosine dehydrogenase	Homo sapiens sarcosine dehydrogenase	79.09638	38.99924	82.24799	51.46558	Detected	Detected	Detected	Detected
8305	A_33_P3258146	LOC100130539	Hs.694626	hypothetical protein LOC100130539	PREDICTED: Homo sapiens hypothetica	183.81381	225.05550	36.41563	43.58212	Detected	Detected	Detected	Detected
8306	A_23_P130764	KCNJ14	Hs.590945	potassium inwardly-rectifying channel, s	Homo sapiens potassium inwardly-recti	2137.91650	1645.79760	1850.08810	1175.14090	Detected	Detected	Detected	Detected
8307	A_33_P3248953	FLJ44606		glutaredoxin-like protein YDR286C hom	Homo sapiens glutaredoxin-like protein	19.34342	20.17139	14.55258	24.57988	Detected	Detected	Detected	Detected
8308	A_32_P104478	FGD6	Hs.506381	FYVE, RhoGEF and PH domain containi	Homo sapiens FYVE, RhoGEF and PH d	176.04987	167.80869	130.42677	108.34420	Detected	Detected	Detected	Detected
8309	A_33_P3248644	QARS	Hs.79322	glutaminyl-tRNA synthetase	Homo sapiens glutaminyl-tRNA synthet	38662.22300	23428.80900	33537.60500	18352.25400	Detected	Detected	Detected	Detected
8310	A_33_P3289541	MLLT1	Hs.10095	myeloid/lymphoid or mixed-lineage leuk	Homo sapiens myeloid/lymphoid or mixe	1757.04740	1429.60460	1599.41720	1430.00920	Detected	Detected	Detected	Detected
8311	A_23_P53530	MTERFD3	Hs.5009	MTERF domain containing 3	Homo sapiens MTERF domain containin	292.31818	153.34459	257.52298	106.52303	Detected	Detected	Detected	Detected
8312	A_23_P136777	APOD	Hs.522555	apolipoprotein D	Homo sapiens apolipoprotein D (APOD)	1574.38330	20.92940	1158.70530	19.86256	Detected	Detected	Detected	Detected
8313	A_23_P152984	THOC4	Hs.534385	THO complex 4	Homo sapiens THO complex 4 (THOC4)	7230.83350	22502.32600	5037.84030	12113.68500	Detected	Detected	Detected	Detected
8314	A_23_P215883	NCALD	Hs.492427	neurocalcin delta	Homo sapiens neurocalcin delta (NCALI	325.54132	271.13843	304.71506	243.65850	Detected	Detected	Detected	Detected
8315	A_23_P153797	NCAN	Hs.169047	neurocan	Homo sapiens neurocan (NCAN), mRNA	29.78795	26.25062	12.28963	12.70647	Detected	Detected	Compromised	Compromised
8316	A_23_P41327	LYAR	Hs.425427	Ly1 antibody reactive homolog (mouse)	Homo sapiens Ly1 antibody reactive ho	2374.15060	3193.51440	1903.94910	2188.09100	Detected	Detected	Detected	Detected
8317	A_23_P160849	FCER1G	Hs.433300	Fc fragment of IgE, high affinity I, recep	Homo sapiens Fc fragment of IgE, high	3777.93400	6224.21340	962.47766	1028.99820	Detected	Detected	Detected	Detected
8318	A_33_P3384229					40.90521	69.79791	15.08623	21.30942	Detected	Detected	Detected	Detected
8319	A_33_P3413987	SERPING1	Hs.384598	serpin peptidase inhibitor, clade G (C1 i	Homo sapiens serpin peptidase inhibitor	1265.57670	1081.22940	1184.16920	871.88260	Detected	Detected	Detected	Detected
8320	A_23_P64837	SMAGP	Hs.652389	small cell adhesion glycoprotein	Homo sapiens small cell adhesion glyco	937.69220	2307.16500	951.29140	2094.36350	Detected	Detected	Detected	Detected
8321	A_33_P3279136	LOC286177	Hs.385655	hypothetical protein LOC286177	PREDICTED: Homo sapiens hypothetica	13.81028	3.32017	2.69614	3.23999	Detected	Compromised	Compromised	Compromised

8322	A_24_P409519	PTPN5	Hs.79092	protein tyrosine phosphatase, non-rece	Homo sapiens protein tyrosine phospha	23.47920	17.22315	27.75398	30.89535	Detected	Detected	Detected	Detected
8323	A_33_P3244753	DRP2	Hs.159291	dystrophin related protein 2	Homo sapiens dystrophin related protei	4615.62060	6946.21600	592.89575	675.52893	Detected	Detected	Detected	Detected
8324	A_24_P141629	FAM111A	Hs.150651	family with sequence similarity 111, mer	Homo sapiens family with sequence sim	740.32740	900.88780	604.01140	643.51294	Detected	Detected	Detected	Detected
8325	A_23_P69188	DPH3	Hs.388087	DPH3, KTI11 homolog (S. cerevisiae)	Homo sapiens DPH3, KTI11 homolog (S	2719.44360	5643.43000	2013.99770	3348.81180	Detected	Detected	Detected	Detected
8326	A_33_P3328445		Hs.422967		Putative uncharacterized protein ENSP	237.86066	237.40300	151.60335	114.49459	Detected	Detected	Detected	Detected
8327	A_24_P15062	ZNF490	Hs.655860	zinc finger protein 490	Homo sapiens zinc finger protein 490 (Z	93.13335	77.65667	71.13625	58.09058	Detected	Detected	Detected	Detected
8328	A_23_P376172	C1orf64	Hs.29190	chromosome 1 open reading frame 64	Homo sapiens chromosome 1 open reac	21.81780	28.24578	11.03333	11.76700	Detected	Detected	Compromised	Compromised
8329	A_23_P413585	FOXD2	Hs.166188	forkhead box D2	Homo sapiens forkhead box D2 (FOXD2	71.13244	63.12175	35.13295	15.32175	Detected	Detected	Detected	Detected
8330	A_33_P3218649	GPM6B	Hs.495710	glycoprotein M6B	Homo sapiens glycoprotein M6B (GPM6	28022.99400	2133.90670	22387.32600	1514.62400	Detected	Detected	Detected	Detected
8331	A_33_P3253682	PPIL3	Hs.121076	peptidylprolyl isomerase (cyclophilin)-lik	Homo sapiens peptidylprolyl isomerase	5125.17720	3840.62520	3905.58280	2536.10570	Detected	Detected	Detected	Detected
8332	A_32_P91042	LOC100129034	Hs.654980	hypothetical protein LOC100129034	Homo sapiens hypothetical protein LOC	233.00272	369.98584	193.69595	279.37784	Detected	Detected	Detected	Detected
8333	A_33_P3294053	ANKRD39	Hs.709507	ankyrin repeat domain 39	Homo sapiens ankyrin repeat domain 39	3466.96730	7768.21530	2636.70100	4876.42720	Detected	Detected	Detected	Detected
8334	A_23_P71855	C5	Hs.494997	complement component 5	Homo sapiens complement component	78.24587	72.63454	80.97328	61.17237	Detected	Detected	Detected	Detected
8335	A_23_P390148	RALGPS1	Hs.432842	Ral GEF with PH domain and SH3 bindir	Homo sapiens Ral GEF with PH domain	55.35303	5.47720	52.88453	4.40901	Detected	Compromised	Detected	Compromised
8336	A_23_P168812	C7orf38	Hs.596587	chromosome 7 open reading frame 38	Homo sapiens chromosome 7 open reac	294.88390	210.19913	126.97289	75.54845	Detected	Detected	Detected	Detected
8337	A_23_P216655	TRIM14	Hs.575631	tripartite motif-containing 14	Homo sapiens tripartite motif-containin	125.05213	173.69788	136.55240	142.31177	Detected	Detected	Detected	Detected
8338	A_33_P3363515					138233.33000	133330.10000	58149.77700	48687.83600	Detected	Detected	Detected	Detected
8339	A_33_P3344332	RPL39	Hs.558387	ribosomal protein L39	Homo sapiens ribosomal protein L39 (R	20812.19300	11325.64900	12545.81600	5659.10900	Detected	Detected	Detected	Detected
8340	A_23_P115573	SHISA4	Hs.632471	shisa homolog 4 (Xenopus laevis)	Homo sapiens shisa homolog 4 (Xenopu	18879.84600	17968.39500	15934.48300	10608.97950	Detected	Detected	Detected	Detected
8341	A_23_P336015	NOC2L	Hs.405987	nucleolar complex associated 2 homolog	Homo sapiens nucleolar complex associ	791.33264	1011.23535	877.53930	1042.31910	Detected	Detected	Detected	Detected
8342	A_33_P3331188	ARHGAP23	Hs.374446	Rho GTPase activating protein 23	Homo sapiens Rho GTPase activating p	131.22339	422.91113	122.70849	358.96243	Detected	Detected	Detected	Detected
8343	A_33_P3332052	SLC26A11	Hs.4866	solute carrier family 26, member 11	Homo sapiens solute carrier family 26, r	57.56595	56.72519	48.10790	43.00554	Detected	Detected	Detected	Detected
8344	A_33_P3209802	OR2C3	Hs.23491	olfactory receptor, family 2, subfamily C	Homo sapiens olfactory receptor, family	14.62285	19.39216	8.94000	8.21735	Detected	Detected	Compromised	Compromised
8345	A_24_P18621	AEBP2	Hs.126497	AE binding protein 2	Homo sapiens AE binding protein 2 (AE	2984.69020	2839.30860	2411.90300	1765.40770	Detected	Detected	Detected	Detected
8346	A_23_P133075	TBCK	Hs.292986	TBC1 domain containing kinase	Homo sapiens TBC1 domain containing	123.43331	158.86400	135.79290	146.62402	Detected	Detected	Detected	Detected
8347	A_33_P3383422	DHRS4	Hs.719241	dehydrogenase/reductase (SDR family)	Homo sapiens dehydrogenase/reductas	1387.42880	1086.65990	1768.74380	1260.12460	Detected	Detected	Detected	Detected
8348	A_23_P160167	TSPAN1	Hs.38972	tetraspanin 1	Homo sapiens tetraspanin 1 (TSPAN1),	296.39868	269.83070	187.61116	141.66531	Detected	Detected	Detected	Detected
8349	A_23_P133133	ALPK1	Hs.652825	alpha-kinase 1	Homo sapiens alpha-kinase 1 (ALPK1),	913.01610	387.61670	574.33080	197.97858	Detected	Detected	Detected	Detected
8350	A_33_P3226810	TNFSF10	Hs.478275	tumor necrosis factor (ligand) superfam	Homo sapiens tumor necrosis factor (lig	11.61518	20.91834	8.39425	16.17728	Compromised	Detected	Compromised	Detected
8351	A_23_P252201	EAF2	Hs.477325	ELL associated factor 2	Homo sapiens ELL associated factor 2	98.59631	62.60365	78.49319	60.41434	Detected	Detected	Detected	Detected
8352	A_23_P252211	MTRR	Hs.481551	5-methyltetrahydrofolate-homocysteine	Homo sapiens 5-methyltetrahydrofolate	492.25890	342.64940	523.73627	325.45117	Detected	Detected	Detected	Detected
8353	A_33_P3348670					15.87302	12.28053	21.04924	20.64105	Detected	Compromised	Detected	Detected
8354	A_33_P3399911					16.02123	17.16905	13.87946	21.61728	Detected	Detected	Detected	Detected
8355	A_33_P3212645	NOTCH2NL	Hs.709526	Notch homolog 2 (Drosophila) N-termin	Homo sapiens Notch homolog 2 (Drosop	24244.47900	29757.83400	16946.11500	18021.07800	Detected	Detected	Detected	Detected
8356	A_24_P254949	PGM5	Hs.307835	phosphoglucomutase 5	Homo sapiens phosphoglucomutase 5 (F	102.83608	165.75945	37.31512	46.29348	Detected	Detected	Detected	Detected
8357	A_33_P3846653		Hs.527883		Keratin 19 Fragment [Source:UniProtK	11.36448	119.09602	22.22349	145.86003	Compromised	Detected	Detected	Detected
8358	A_33_P3423954	CBX2	Hs.368410	chromobox homolog 2 (Pc class homolo	Homo sapiens chromobox homolog 2 (P	133.34175	167.96712	44.71868	45.19058	Detected	Detected	Detected	Detected
8359	A_23_P65674	TMOD3	Hs.4998	tropomodulin 3 (ubiquitous)	Homo sapiens tropomodulin 3 (ubiquitou	339.26416	516.43146	368.86667	460.40960	Detected	Detected	Detected	Detected
8360	A_24_P270033	MPZL3	Hs.15396	myelin protein zero-like 3	Myelin protein zero-like protein 3 Precu	3.56797	34.92887	2.78325	24.03949	Compromised	Detected	Compromised	Detected
8361	A_24_P333663	MAPK6	Hs.411847	mitogen-activated protein kinase 6	Homo sapiens mitogen-activated protei	164.40840	327.16296	129.40807	231.70255	Detected	Detected	Detected	Detected
8362	A_23_P69497	CLEC3B	Hs.476092	C-type lectin domain family 3, member	Homo sapiens C-type lectin domain fan	1889.17930	6469.37450	1760.17680	5920.17300	Detected	Detected	Detected	Detected
8363	A_33_P3253634	LOC400419	Hs.569516	hypothetical gene supported by AK131C	PREDICTED: Homo sapiens similar to C	2094.32350	2827.16110	1053.40670	1051.82510	Detected	Detected	Detected	Detected
8364	A_33_P3329686	SEPT5	Hs.283743	septin 5	Homo sapiens septin 5 (SEPT5), mRNA	93.61037	45.18395	101.79849	55.51631	Detected	Detected	Detected	Detected
8365	A_24_P16143	CDH29	Hs.363312	cadherin-like 29	Homo sapiens cadherin-like 29 (CDH29	17.71332	13.57830	10.97245	10.83565	Detected	Compromised	Compromised	Compromised
8366	A_24_P144620	C6orf35	Hs.157212	chromosome 6 open reading frame 35	Homo sapiens chromosome 6 open reac	346.15015	457.31268	252.60136	282.21777	Detected	Detected	Detected	Detected
8367	A_23_P4628	ZNF606	Hs.654967	zinc finger protein 606	Homo sapiens zinc finger protein 606 (Z	205.93724	98.07031	166.96725	65.70073	Detected	Detected	Detected	Detected
8368	A_24_P161914					44859.79700	45390.12500	2549.14840	1965.60120	Detected	Detected	Detected	Detected
8369	A_33_P3355252				Novel proteinPutative uncharacterized	108.92868	31.46297	132.45271	27.05674	Detected	Detected	Detected	Detected
8370	A_33_P3285629	DNAI2	Hs.147472	dynein, axonemal, intermediate chain 2	Homo sapiens dynein, axonemal, interm	18.57869	14.74952	15.54828	8.46465	Detected	Compromised	Detected	Compromised
8371	A_33_P3346508				60 kDa lysophospholipase (EC 3.1.1.5) [	297.58900	359.19458	142.92404	128.96819	Detected	Detected	Detected	Detected
8372	A_33_P3413958		Hs.524431		Homo sapiens olfactory receptor, family	206.07681	160.34140	92.11054	97.17355	Detected	Detected	Detected	Detected
8373	A_33_P3260575	CERCAM	Hs.495230	cerebral endothelial cell adhesion molec	Homo sapiens cerebral endothelial cell	5316.37940	20793.72500	5428.62840	18855.91000	Detected	Detected	Detected	Detected
8374	A_33_P3249394	CACNA1C	Hs.118262	calcium channel, voltage-dependent, L t	Homo sapiens calcium channel, voltage-	12.34892	16.90813	13.64180	15.09873	Compromised	Compromised	Compromised	Detected
8375	A_33_P3247659	TMEM87A	Hs.597178	transmembrane protein 87A	Homo sapiens transmembrane protein 8	118.92278	206.08723	74.21664	142.31610	Detected	Detected	Detected	Detected
8376	A_33_P3283828	CCDC154	Hs.355232	coiled-coil domain containing 154	Homo sapiens coiled-coil domain contai	835.36926	728.71630	330.98163	241.54740	Detected	Detected	Detected	Detected
8377	A_33_P3260722	UVRAG	Hs.719175	UV radiation resistance associated gen	Homo sapiens UV radiation resistance a	339.56033	116.50182	243.02728	78.88307	Detected	Detected	Detected	Detected
8378	A_33_P3388588	LOC400558	Hs.513818	FLJ27068 protein	cDNA FLJ27068 fis, clone SPL01475 [	26538.65200	33221.74600	18194.14800	14824.59700	Detected	Detected	Detected	Detected
8379	A_33_P3342111	ZNF169	Hs.387623	zinc finger protein 169	Homo sapiens zinc finger protein 169 (Z	440.11954	127.94099	306.36032	88.60101	Detected	Detected	Detected	Detected
8380	A_33_P3263412	LOC642278	Hs.689380	hypothetical LOC642278	PREDICTED: Homo sapiens hypothetica	132.00972	207.62082	24.38257	27.52512	Detected	Detected	Detected	Detected
8381	A_32_P51894	HNRNPU	Hs.106212	heterogeneous nuclear ribonucleoprotei	Homo sapiens heterogeneous nuclear ri	905.94244	842.59620	763.37200	602.89720	Detected	Detected	Detected	Detected
8382	A_24_P49517	ZNF251	Hs.534516	zinc finger protein 251	Homo sapiens zinc finger protein 251 (Z	575.11150	578.09630	430.86353	337.12607	Detected	Detected	Detected	Detected
8383	A_33_P3373750	BRD4	Hs.187763	bromodomain containing 4	Homo sapiens bromodomain containing	1794.57710	3181.56080	1277.57810	1962.40340	Detected	Detected	Detected	Detected
8384	A_24_P409346	NPEPPS	Hs.443837	aminopeptidase puromycin sensitive	Homo sapiens aminopeptidase puromyc	192.56935	309.01233	108.06799	166.56984	Detected	Detected	Detected	Detected
8385	A_23_P144827	FBXL7	Hs.433057	F-box and leucine-rich repeat protein 7	Homo sapiens F-box and leucine-rich r	2638.93100	1872.94010	2629.57470	1705.04920	Detected	Detected	Detected	Detected
8386	A_32_P120043	FAM26E	Hs.660142	family with sequence similarity 26, mem	Homo sapiens family with sequence sim	46.13914	147.80028	27.43779	83.16188	Detected	Detected	Detected	Detected
8387	A_23_P397899	C9orf80	Hs.658575	chromosome 9 open reading frame 80	Homo sapiens chromosome 9 open reac	1083.68000	925.95807	874.54880	642.57184	Detected	Detected	Detected	Detected
8388	A_23_P65609	GTF2A1	Hs.592334	general transcription factor IIA, 1, 19/3	Homo sapiens general transcription fact	253.45187	251.01108	148.35721	115.73328	Detected	Detected	Detected	Detected
8389	A_33_P3304998	LOC100128960		similar to Putative paraneoplastic antige	PREDICTED: Homo sapiens similar to p	2.39021	12.39096	17.92675	25.38967	Compromised	Compromised	Detected	Detected
8390	A_23_P39813	TTC31	Hs.557709	tetratricopeptide repeat domain 31	Homo sapiens tetratricopeptide repeat	357.75620	409.90527	346.76392	369.57300	Detected	Detected	Detected	Detected
8391	A_24_P357847		Hs.652919		Ig kappa chain V-III region CLL Precurs	9.59219	19.23479	13.52577	18.96227	Compromised	Detected	Detected	Detected
8392	A_33_P3302255	ITM2B	Hs.643683	integral membrane protein 2B	Homo sapiens integral membrane protei	14659.67300	13003.42500	11683.47100	8238.07100	Detected	Detected	Detected	Detected
8393	A_23_P140146	IFI27L2	Hs.94695	interferon, alpha-inducible protein 27-lil	Homo sapiens interferon, alpha-inducibl	14792.18600	21267.78100	15322.08600	19295.55700	Detected	Detected	Detected	Detected
8394	A_24_P82880	TPM4	Hs.631618	tropomyosin 4	Homo sapiens tropomyosin 4 (TPM4), tr	24105.43400	19156.72000	17362.12000	14275.28900	Detected	Detected	Detected	Detected
8395	A_23_P429082	SPTY2D1	Hs.268668	SPT2, Suppressor of Ty, domain contai	Homo sapiens SPT2, Suppressor of Ty,	481.18950	570.48267	224.33252	175.41380	Detected	Detected	Detected	Detected
8396	A_33_P3224045	DAPK3	Hs.631844	death-associated protein kinase 3	Homo sapiens death-associated protein	29001.98400	27115.91800	34180.62500	27004.74600	Detected	Detected	Detected	Detected

8397	A_33_P3365441				LIM/homeobox protein Lhx9 (LIM home	11.28066	13.92198	12.88380	11.92726	Compromised	Compromised	Detected	Compromised
8398	A_33_P3323448	HM13	Hs.373741	histocompatibility (minor) 13	Homo sapiens histocompatibility (minor)	30.93633	31.95709	21.41938	20.22015	Detected	Detected	Detected	Detected
8399	A_33_P3283167					7511.16360	11067.28200	6480.34000	8117.84100	Detected	Detected	Detected	Detected
8400	A_23_P145485	ULBP2	Hs.656778	UL16 binding protein 2	Homo sapiens UL16 binding protein 2 (U	248.94669	292.64453	334.24625	336.03230	Detected	Detected	Detected	Detected
8401	A_23_P24157	PYROXD2	Hs.238303	pyridine nucleotide-disulphide oxidored	Homo sapiens pyridine nucleotide-disul	166.88841	103.82750	174.61743	104.32739	Detected	Detected	Detected	Detected
8402	A_33_P3292829	SCYL2	Hs.506481	SCY1-like 2 (S. cerevisiae)	Homo sapiens SCY1-like 2 (S. cerevisia	64.80842	65.46470	57.34429	53.78643	Detected	Detected	Detected	Detected
8403	A_23_P115780	LRIT1	Hs.226000	leucine-rich repeat, immunoglobulin-like	Homo sapiens leucine-rich repeat, immu	17.58559	16.64137	4.10877	6.30124	Detected	Detected	Compromised	Compromised
8404	A_23_P156284	DBN1	Hs.130316	drebrin 1	Homo sapiens drebrin 1 (DBN1), transc	25180.02700	35160.79000	20872.94000	24585.12100	Detected	Detected	Detected	Detected
8405	A_23_P255569	DUS1L	Hs.514599	dihydrouridine synthase 1-like (S. cerev	Homo sapiens dihydrouridine synthase	2069.93870	2664.63200	2408.83280	2935.70870	Detected	Detected	Detected	Detected
8406	A_24_P100650	C9orf11	Hs.163070	chromosome 9 open reading frame 11	Homo sapiens chromosome 9 open read	5.76220	3.23111	4.32713	13.46868	Compromised	Compromised	Compromised	Detected
8407	A_24_P382253	RPRD1A	Hs.464912	regulation of nuclear pre-mRNA domain	Homo sapiens regulation of nuclear pre	3233.75070	1385.87510	2306.38130	903.13635	Detected	Detected	Detected	Detected
8408	A_32_P171181	NBPF10	Hs.607640	neuroblastoma breakpoint family, memb	Homo sapiens neuroblastoma breakpoin	640.11676	544.21277	375.63467	275.32210	Detected	Detected	Detected	Detected
8409	A_23_P25720	SERPINA4	Hs.719893	serpin peptidase inhibitor, clade A (alph	Homo sapiens serpin peptidase inhibitor	2.43472	3.26754	8.30253	16.94575	Compromised	Compromised	Compromised	Detected
8410	A_33_P3335629	TMEM208	Hs.433203	transmembrane protein 208	Homo sapiens transmembrane protein 2	2590.94560	3865.42020	3352.99830	4932.49100	Detected	Detected	Detected	Detected
8411	A_33_P3307144	GRIK2	Hs.98262	glutamate receptor, ionotropic, kainate	Homo sapiens glutamate receptor, ionot	15.54582	34.91761	16.67583	29.16857	Detected	Detected	Detected	Detected
8412	A_33_P3386099	ELK1	Hs.181128	ELK1, member of ETS oncogene family	Homo sapiens ELK1, member of ETS or	22.63811	21.43820	23.99842	26.37944	Detected	Detected	Detected	Detected
8413	A_33_P3230090	LUZP6	Hs.602015	leucine zipper protein 6	Homo sapiens leucine zipper protein 6 (	237.74574	340.28455	202.83934	264.30127	Detected	Detected	Detected	Detected
8414	A_33_P3248610	ASXL2	Hs.119815	additional sex combs like 2 (Drosophila)	Homo sapiens additional sex combs like	13.32497	13.59800	9.98684	11.78278	Detected	Compromised	Compromised	Compromised
8415	A_33_P3387272	EEF1A1	Hs.535192	eukaryotic translation elongation factor	Homo sapiens eukaryotic translation elo	12288.92900	12538.56200	10059.82100	8915.72400	Detected	Detected	Detected	Detected
8416	A_33_P3412468	SLC25A27	Hs.40510	solute carrier family 25, member 27	Homo sapiens solute carrier family 25, r	1077.63880	179.11522	857.74146	125.09339	Detected	Detected	Detected	Detected
8417	A_23_P24616	SIAE	Hs.10056	sialic acid acetylesterase	Homo sapiens sialic acid acetylesterase	116.93633	255.85408	98.95667	169.38042	Detected	Detected	Detected	Detected
8418	A_33_P3385750	ZNF793	Hs.568010	zinc finger protein 793	Homo sapiens zinc finger protein 793 (Z	36.01196	41.94919	32.27803	32.63802	Detected	Detected	Detected	Detected
8419	A_33_P3227896	LIMS3-LOC440895	Hs.535619	LIMS3-LOC440895 read-through	Homo sapiens LIMS3-LOC440895 read-	11.80766	16.47960	20.53888	19.43414	Compromised	Compromised	Detected	Detected
8420	A_24_P120537	SH3RF2	Hs.443728	SH3 domain containing ring finger 2	Homo sapiens SH3 domain containing ri	14.97374	13.40656	10.68649	10.41543	Detected	Compromised	Compromised	Compromised
8421	A_33_P3255194	OSBPL6	Hs.318775	oxysterol binding protein-like 6	Homo sapiens oxysterol binding protein	57.76999	97.23200	33.39087	50.19142	Detected	Detected	Detected	Detected
8422	A_23_P310532	C19orf52	Hs.164026	chromosome 19 open reading frame 52	Homo sapiens chromosome 19 open rea	1270.71290	3150.41970	1056.75020	2329.95850	Detected	Detected	Detected	Detected
8423	A_33_P3210059	SF3A1	Hs.406277	splicing factor 3a, subunit 1, 120kDa	Homo sapiens splicing factor 3a, subuni	1844.62590	2035.37830	1555.02310	1368.44400	Detected	Detected	Detected	Detected
8424	A_33_P3215422	LYL1	Hs.46446	lymphoblastic leukemia derived sequenc	Homo sapiens lymphoblastic leukemia d	37.80429	81.34155	47.13634	88.36628	Detected	Detected	Detected	Detected
8425	A_33_P3396553	LOC732419		similar to AAA-ATPase TOB3	PREDICTED: Homo sapiens similar to b	807.77313	1860.59510	286.89597	666.25714	Detected	Detected	Detected	Detected
8426	A_23_P109420	BMS1	Hs.10848	BMS1 homolog, ribosome assembly prot	Homo sapiens BMS1 homolog, ribosome	3710.25510	4188.24270	3447.12940	3360.56930	Detected	Detected	Detected	Detected
8427	A_23_P34142	WBP5	Hs.533287	WW domain binding protein 5	Homo sapiens WW domain binding prote	19942.49000	13991.59800	13499.77500	7814.36230	Detected	Detected	Detected	Detected
8428	A_33_P3213086		Hs.721707		BX457161 Homo sapiens THYMUS Hom	39.28583	37.40937	14.27432	8.45479	Detected	Detected	Detected	Compromised
8429	A_33_P3280681				Leukocyte receptor cluster member 8	242.16692	290.36688	154.80832	139.04726	Detected	Detected	Detected	Detected
8430	A_23_P159390	TOPBP1	Hs.593379	topoisomerase (DNA) II binding protein	Homo sapiens topoisomerase (DNA) II b	494.12994	733.20514	246.90938	312.91193	Detected	Detected	Detected	Detected
8431	A_24_P775249	LOC400657	Hs.61508	hypothetical LOC400657	Homo sapiens hypothetical LOC400657	224.57162	235.58348	137.24251	101.92989	Detected	Detected	Detected	Detected
8432	A_23_P312999	C4orf22	Hs.527104	chromosome 4 open reading frame 22	Homo sapiens chromosome 4 open read	64.34728	91.39397	59.20103	51.48421	Detected	Detected	Detected	Detected
8433	A_23_P209619	ATL2	Hs.190440	atlastin GTPase 2	Homo sapiens atlastin GTPase 2 (ATL2	2387.53120	2132.23300	1829.10680	1303.00920	Detected	Detected	Detected	Detected
8434	A_23_P24004	IFIT2	Hs.437609	interferon-induced protein with tetratri	Homo sapiens interferon-induced prote	400.81253	196.78328	391.67624	177.57138	Detected	Detected	Detected	Detected
8435	A_23_P359457		Hs.367649		Dynein heavy chain 2, axonemal (Axone	670.97260	1236.66720	42.58586	66.11750	Detected	Detected	Detected	Detected
8436	A_23_P424269		Hs.432364		Putative DNA repair and recombination	577.83044	439.00696	453.89140	309.32297	Detected	Detected	Detected	Detected
8437	A_23_P133375	SLC25A46	Hs.75639	solute carrier family 25, member 46	Homo sapiens solute carrier family 25, r	3843.09380	3083.77690	2493.76540	1476.99880	Detected	Detected	Detected	Detected
8438	A_33_P3330453	PBRM1	Hs.189920	polybromo 1	Homo sapiens polybromo 1 (PBRM1), tr	3648.85740	5530.88000	2366.20240	2825.78050	Detected	Detected	Detected	Detected
8439	A_33_P3283599	ANKRD20B	Hs.722109	ankyrin repeat domain 20B	Homo sapiens ankyrin repeat domain 20	15.35937	13.94276	14.02557	25.22953	Detected	Compromised	Detected	Detected
8440	A_23_P163195	LRFN5	Hs.136893	leucine rich repeat and fibronectin type	Homo sapiens leucine rich repeat and fi	11.98265	97.03247	14.42112	87.54005	Detected	Detected	Detected	Detected
8441	A_33_P3334015	C17orf103	Hs.654713	chromosome 17 open reading frame 103	Homo sapiens chromosome 17 open rea	11.09434	14.15645	15.46274	13.53533	Compromised	Compromised	Detected	Detected
8442	A_33_P3360382	MEG8		maternally expressed 8 (non-protein co	Homo sapiens maternally expressed 8 (	35.93178	104.55823	33.20224	59.95642	Detected	Detected	Detected	Detected
8443	A_33_P3313855	UNQ6484	Hs.555530	ISPF6484	ISPF6484 [Source:UniProtKB/TrEMBL	5.71818	4.26639	15.05253	9.37569	Compromised	Compromised	Detected	Compromised
8444	A_23_P1641	RCE1	Hs.654972	RCE1 homolog, prenyl protein peptidase	Homo sapiens RCE1 homolog, prenyl pr	217.35973	244.88115	220.93219	196.44458	Detected	Detected	Detected	Detected
8445	A_33_P3321533	LOC100129514	Hs.591827	hypothetical LOC100129514	PREDICTED: Homo sapiens hypothetica	34.51815	50.16054	16.06262	23.66650	Detected	Detected	Detected	Detected
8446	A_33_P3276758					56.54942	59.58638	48.98322	56.13404	Detected	Detected	Detected	Detected
8447	A_33_P3366493	LOC100133990	Hs.685977	hypothetical LOC100133990	PREDICTED: Homo sapiens similar to h	47.01513	55.99833	42.04421	37.16980	Detected	Detected	Detected	Detected
8448	A_33_P3411414	FAM156B	Hs.6451	family with sequence similarity 156, mem	Homo sapiens family with sequence sim	601.87470	699.72740	732.68720	790.78200	Detected	Detected	Detected	Detected
8449	A_33_P3361027	TFIP11	Hs.20225	tuftelin interacting protein 11	Homo sapiens tuftelin interacting prote	8518.91300	10896.31300	10108.08200	12437.56200	Detected	Detected	Detected	Detected
8450	A_23_P24365	ANKRD49	Hs.29052	ankyrin repeat domain 49	Homo sapiens ankyrin repeat domain 49	2061.33760	1412.93790	1597.53740	990.87970	Detected	Detected	Detected	Detected
8451	A_23_P23564	PIP5K1A	Hs.655131	phosphatidylinositol-4-phosphate 5-kin	Homo sapiens phosphatidylinositol-4-pl	45.12754	47.37390	7.98699	7.40308	Detected	Detected	Compromised	Compromised
8452	A_23_P127079	PPRC1	Hs.533551	peroxisome proliferator-activated recep	Homo sapiens peroxisome proliferator-a	2888.71850	4347.59670	2582.67770	3367.79000	Detected	Detected	Detected	Detected
8453	A_33_P3271126					6635.96600	7559.58540	5057.40670	5151.93360	Detected	Detected	Detected	Detected
8454	A_23_P25989	SETD3	Hs.510407	SET domain containing 3	Homo sapiens SET domain containing 3	957.04120	1362.12830	702.61920	881.73926	Detected	Detected	Detected	Detected
8455	A_33_P3325753	TRIOBP	Hs.533030	TRIO and F-actin binding protein	Homo sapiens TRIO and F-actin binding	875.52290	1611.90660	459.96637	756.63050	Detected	Detected	Detected	Detected
8456	A_23_P81408	MAT2B	Hs.54642	methionine adenosyltransferase II, beta	Homo sapiens methionine adenosyltrans	3144.83720	2668.54370	3007.29900	2254.82030	Detected	Detected	Detected	Detected
8457	A_24_P133488	CDCA4	Hs.34045	cell division cycle associated 4	Homo sapiens cell division cycle associ	153.17271	411.84833	111.15553	258.92590	Detected	Detected	Detected	Detected
8458	A_23_P401076	SUSD3	Hs.88417	sushi domain containing 3	Homo sapiens sushi domain containing 3	848.85254	326.99390	680.45526	152.96039	Detected	Detected	Detected	Detected
8459	A_33_P3419204					134.10513	76.78716	14.21153	10.75671	Detected	Detected	Detected	Compromised
8460	A_23_P359540	HIST1H4F	Hs.247816	histone cluster 1, H4f	Homo sapiens histone cluster 1, H4f (HI	42.68813	177.87689	55.66727	161.37617	Detected	Detected	Detected	Detected
8461	A_33_P3386965	PIGM	Hs.552810	phosphatidylinositol glycan anchor biosy	Homo sapiens phosphatidylinositol glyca	71.60859	86.78091	45.09698	48.83105	Detected	Detected	Detected	Detected
8462	A_33_P3296769					34.20839	39.77549	20.80552	24.46855	Detected	Detected	Detected	Detected
8463	A_33_P3221414	NTNG1		netrin G1	Netrin-G1 Precursor (Laminet-1) [Sour	71.04034	18.83402	53.79359	16.72850	Detected	Detected	Detected	Detected
8464	A_32_P50223	PLK5P	Hs.195352	polo-like kinase 5 pseudogene	Homo sapiens polo-like kinase 5 pseudo	167.70512	13.19038	121.56341	9.46378	Detected	Compromised	Detected	Compromised
8465	A_23_P382065	EMCN	Hs.152913	endomucin	Homo sapiens endomucin (EMCN), trans	1237.75990	21.41273	994.08000	11.00449	Detected	Detected	Detected	Compromised
8466	A_32_P44512	ST13	Hs.712713	suppression of tumorigenicity 13 (colon	Homo sapiens suppression of tumoriger	8425.43750	7106.67530	6331.13400	4440.24200	Detected	Detected	Detected	Detected
8467	A_33_P3347012	LOC728857	Hs.647778	hypothetical protein LOC728857	PREDICTED: Homo sapiens hypothetica	212.39705	138.27905	14.00433	17.53993	Detected	Detected	Compromised	Detected
8468	A_23_P167129	HHIP	Hs.507991	hedgehog interacting protein	Homo sapiens hedgehog interacting pro	68.96924	377.16910	54.35737	299.02258	Detected	Detected	Detected	Detected
8469	A_23_P362228	C1orf213	Hs.61884	chromosome 1 open reading frame 213	Homo sapiens chromosome 1 open read	2012.35950	735.29560	1519.62040	569.51544	Detected	Detected	Detected	Detected
8470	A_33_P3410895				NADPH--cytochrome P450 reductase (	20568.60700	23314.80700	43353.79300	28208.99800	Detected	Detected	Detected	Detected
8471	A_24_P361643	C19orf55	Hs.527982	chromosome 19 open reading frame 55	Homo sapiens chromosome 19 open rea	36.28454	41.33006	40.81302	37.63675	Detected	Detected	Detected	Detected

8472	A_23_P61406	SHC3	Hs.656806	SHC (Src homology 2 domain containing	Homo sapiens SHC (Src homology 2 do	1888.98830	52.79705	1255.19140	35.17455	Detected	Detected	Detected	Detected
8473	A_33_P3292840	C11orf49	Hs.368296	chromosome 11 open reading frame 49	Homo sapiens chromosome 11 open rea	211.55281	302.29490	231.08292	254.06087	Detected	Detected	Detected	Detected
8474	A_23_P27649	ZNF433	Hs.667776	zinc finger protein 433	Homo sapiens zinc finger protein 433 (Z	451.62280	484.15490	186.97029	207.00140	Detected	Detected	Detected	Detected
8475	A_23_P380815	KIAA1279	Hs.279580	KIAA1279	Homo sapiens KIAA1279 (KIAA1279), m	1119.70530	2042.05600	680.97680	1099.23600	Detected	Detected	Detected	Detected
8476	A_33_P3340229	LOC100129081		hypothetical LOC100129081	PREDICTED: Homo sapiens hypothetica	29.18718	45.60054	23.24080	24.10243	Detected	Detected	Detected	Detected
8477	A_33_P3306828	ZNF806	Hs.433138	zinc finger protein 806	PREDICTED: Homo sapiens zinc finger	15.40863	15.13881	27.95872	17.64334	Detected	Compromised	Detected	Detected
8478	A_33_P3216938					67.45725	91.61239	46.81474	37.84532	Detected	Detected	Detected	Detected
8479	A_33_P3225111				Solute carrier family 12 member 5 (Elec	21.43166	21.71707	25.16504	22.20831	Detected	Detected	Detected	Detected
8480	A_33_P3393573	PCMT1	Hs.279257	protein-L-isoaspartate (D-aspartate) O	Homo sapiens protein-L-isoaspartate (	5565.51660	7064.58150	7787.95900	8507.87800	Detected	Detected	Detected	Detected
8481	A_33_P3309734	CCDC108	Hs.147762	coiled-coil domain containing 108	Homo sapiens coiled-coil domain conta	3.41628	8.81907	17.17422	15.38123	Compromised	Compromised	Detected	Detected
8482	A_33_P3592015	HABP4	Hs.494567	hyaluronan binding protein 4	Homo sapiens hyaluronan binding protei	9324.41300	7395.58940	8325.48800	5931.43070	Detected	Detected	Detected	Detected
8483	A_33_P3269000	C8orf48	Hs.104941	chromosome 8 open reading frame 48	Homo sapiens chromosome 8 open read	303.74854	372.03885	193.71286	188.16747	Detected	Detected	Detected	Detected
8484	A_23_P205428	FOXG1	Hs.695962	forkhead box G1	Homo sapiens forkhead box G1 (FOXG1	30.33493	57.40498	23.34911	40.58195	Detected	Detected	Detected	Detected
8485	A_24_P6428	C20orf177	Hs.504920	chromosome 20 open reading frame 177	Homo sapiens chromosome 20 open rea	54.87759	57.69280	34.23815	23.87836	Detected	Detected	Detected	Detected
8486	A_23_P36157	WDR74	Hs.655372	WD repeat domain 74	Homo sapiens WD repeat domain 74 (W	4385.60250	2388.49270	5074.28760	2473.79050	Detected	Detected	Detected	Detected
8487	A_24_P41021	FBXO33	Hs.324342	F-box protein 33	Homo sapiens F-box protein 33 (FBXO3	1754.58910	2202.00050	1177.98030	1165.63170	Detected	Detected	Detected	Detected
8488	A_33_P3281028	MACROD2	Hs.661576	MACRO domain containing 2	Homo sapiens MACRO domain containi	14.55479	3.16885	13.68711	2.66121	Detected	Compromised	Detected	Compromised
8489	A_23_P17103	TSGA10	Hs.120267	testis specific, 10	Homo sapiens testis specific, 10 (TSGA	114.22592	82.73174	96.75443	58.46405	Detected	Detected	Detected	Detected
8490	A_33_P3349676		Hs.712083		PREDICTED: Homo sapiens hypothetica	36.87775	52.44677	21.95910	12.08709	Detected	Detected	Detected	Compromised
8491	A_23_P106042	CMTM5	Hs.99272	CKLF-like MARVEL transmembrane do	Homo sapiens CKLF-like MARVEL tran	9.49007	5.24031	10.03364	16.76071	Compromised	Compromised	Compromised	Detected
8492	A_23_P146830	SLC25A10	Hs.548187	solute carrier family 25 (mitochondrial c	Homo sapiens solute carrier family 25 (	236.91623	270.10748	295.46478	341.38696	Detected	Detected	Detected	Detected
8493	A_24_P95038	PPIA	Hs.356331	peptidylprolyl isomerase A (cyclophilin A	Homo sapiens peptidylprolyl isomerase	99037.41000	135681.97000	69132.57000	83609.06000	Detected	Detected	Detected	Detected
8494	A_24_P652502		Hs.454579		Putative uncharacterized protein ENSP	2.38410	3.19392	2.55437	13.25412	Compromised	Compromised	Compromised	Detected
8495	A_33_P3407445				GB	116.28970	96.45605	109.22051	68.34638	Detected	Detected	Detected	Detected
8496	A_23_P393607	SNAP47	Hs.325081	synaptosomal-associated protein, 47kD	Homo sapiens synaptosomal-associated	8000.08350	8102.91500	7157.73000	6503.45750	Detected	Detected	Detected	Detected
8497	A_24_P341504	RNPC3	Hs.632423	RNA-binding region (RNP1, RRM) conta	Homo sapiens RNA-binding region (RNP	617.57500	1153.69080	505.20105	799.87510	Detected	Detected	Detected	Detected
8498	A_23_P301340	C17orf56	Hs.631761	chromosome 17 open reading frame 56	Homo sapiens chromosome 17 open rea	7637.72200	6684.61500	7494.25630	6921.63870	Detected	Detected	Detected	Detected
8499	A_23_P251916	FAM82B	Hs.145386	family with sequence similarity 82, mem	Homo sapiens family with sequence sim	8983.55000	3721.92820	8850.39700	3400.47300	Detected	Detected	Detected	Detected
8500	A_23_P401014	ARIH1	Hs.268787	ariadne homolog, ubiquitin-conjugating e	Homo sapiens ariadne homolog, ubiquiti	183.61307	260.28076	201.40762	271.39377	Detected	Detected	Detected	Detected
8501	A_33_P3352687	LOC100133161	Hs.710409	hypothetical LOC100133161	Homo sapiens hypothetical LOC100133	30789.43400	37312.29700	31682.88300	29791.57000	Detected	Detected	Detected	Detected
8502	A_23_P369994	DCLK1	Hs.507755	doublecortin-like kinase 1	Homo sapiens doublecortin-like kinase	97.92042	136.03506	84.35487	99.46201	Detected	Detected	Detected	Detected
8503	A_33_P3369565	APTX	Hs.20158	aprataxin	Homo sapiens aprataxin (APTX), transc	308.69077	277.62265	287.00958	242.79324	Detected	Detected	Detected	Detected
8504	A_23_P62999	EXTL1	Hs.150956	exostoses (multiple)-like 1	Homo sapiens exostoses (multiple)-like	64.44457	132.66600	58.16955	100.35732	Detected	Detected	Detected	Detected
8505	A_23_P64770	DDX23	Hs.130098	DEAD (Asp-Glu-Ala-Asp) box polypept	Homo sapiens DEAD (Asp-Glu-Ala-Asp	7643.50540	8364.18000	6546.14450	6521.66800	Detected	Detected	Detected	Detected
8506	A_23_P61466	CD163L1	Hs.631727	CD163 molecule-like 1	Homo sapiens CD163 molecule-like 1 (	1274.04190	284.51294	1365.30410	240.30660	Detected	Detected	Detected	Detected
8507	A_33_P3308862	FAM22A	Hs.710565	family with sequence similarity 22, mem	Homo sapiens family with sequence sim	40.06661	53.26067	44.31945	32.39283	Detected	Detected	Detected	Detected
8508	A_32_P29806	CRADD	Hs.591016	CASP2 and RIPK1 domain containing ad	Homo sapiens CASP2 and RIPK1 domai	4098.17770	2420.15040	4274.41700	2103.18430	Detected	Detected	Detected	Detected
8509	A_32_P95223	FDPSL2A	Hs.609978	MGC44478	Homo sapiens MGC44478 (FDPSL2A), r	5907.67500	6190.58700	6270.60300	5989.31740	Detected	Detected	Detected	Detected
8510	A_33_P3341429	NEXN	Hs.612385	nexilin (F actin binding protein)	Homo sapiens nexilin (F actin binding pr	500.97128	2531.24020	220.64992	895.68616	Detected	Detected	Detected	Detected
8511	A_23_P91001	ASNSD1	Hs.101364	asparagine synthetase domain containin	Homo sapiens asparagine synthetase do	9475.51500	8611.01800	8617.33300	6067.28500	Detected	Detected	Detected	Detected
8512	A_33_P3330872	MSL3	Hs.655288	male-specific lethal 3 homolog (Drosoph	Homo sapiens male-specific lethal 3 ho	80.15284	79.45319	63.35889	46.31295	Detected	Detected	Detected	Detected
8513	A_33_P3395738	LOC100131601	Hs.479747	similar to hCG1980470	Homo sapiens cDNA FLJ45059 fis, clon	331.75270	306.33157	250.69708	209.90976	Detected	Detected	Detected	Detected
8514	A_33_P3379454	UHRF1	Hs.108106	ubiquitin-like with PHD and ring finger d	Homo sapiens ubiquitin-like with PHD a	37.88173	136.46721	33.17343	134.19444	Detected	Detected	Detected	Detected
8515	A_32_P475513	MYO15B	Hs.390817	myosin XVB pseudogene	Homo sapiens myosin XVB pseudogene	20.01581	10.67407	11.82322	5.09658	Detected	Compromised	Compromised	Compromised
8516	A_24_P21447	SURF6	Hs.274430	surfeit 6	Homo sapiens surfeit 6 (SURF6), mRNA	231.85654	689.03680	172.62419	417.87628	Detected	Detected	Detected	Detected
8517	A_23_P15511	DDX5	Hs.279806	DEAD (Asp-Glu-Ala-Asp) box polypept	Homo sapiens DEAD (Asp-Glu-Ala-Asp	51258.41000	51430.56000	41566.45300	36930.82400	Detected	Detected	Detected	Detected
8518	A_32_P189790	DKFZp779M0652	Hs.632142	hypothetical DKFZp779M0652	Homo sapiens hypothetical DKFZp779M	40.87058	38.86438	31.21527	33.04925	Detected	Detected	Detected	Detected
8519	A_33_P3234864	UTRN	Hs.133135	utrophin	Homo sapiens utrophin (UTRN), mRNA	51.75586	105.60370	62.83224	85.39883	Detected	Detected	Detected	Detected
8520	A_24_P258846	NFATC1	Hs.534074	nuclear factor of activated T-cells, cyto	Homo sapiens nuclear factor of activate	968.79190	414.02853	1147.36010	489.98282	Detected	Detected	Detected	Detected
8521	A_33_P3332066	METTL10	Hs.706064	methyltransferase like 10	Homo sapiens methyltransferase like 10	599.99414	905.99274	652.96440	858.94434	Detected	Detected	Detected	Detected
8522	A_23_P375281	TRPV1	Hs.655380	transient receptor potential cation char	Homo sapiens transient receptor potent	87.34578	92.49539	81.20133	78.78432	Detected	Detected	Detected	Detected
8523	A_32_P923011	FAM138A	Hs.640260	family with sequence similarity 138, mer	Homo sapiens family with sequence sim	248.87569	347.83743	465.03873	445.49660	Detected	Detected	Detected	Detected
8524	A_33_P3215412	C2CD4C	Hs.223770	C2 calcium-dependent domain containin	Homo sapiens C2 calcium-dependent d	2791.00400	3979.55180	982.72720	849.38745	Detected	Detected	Detected	Detected
8525	A_33_P3315764	TP53	Hs.654481	tumor protein p53	Homo sapiens tumor protein p53 (TP53	25.66715	17.18451	24.48101	16.23080	Detected	Detected	Detected	Detected
8526	A_23_P23279	RCSD1	Hs.493867	RCSD domain containing 1	Homo sapiens RCSD domain containing	69.52772	37.49831	62.32583	22.57387	Detected	Detected	Detected	Detected
8527	A_33_P3220565	UBTD2	Hs.131570	ubiquitin domain containing 2	Homo sapiens ubiquitin domain containi	1833.72050	1597.36320	965.77900	614.93730	Detected	Detected	Detected	Detected
8528	A_23_P85218	SOX3	Hs.157429	SRY (sex determining region Y)-box 3	Homo sapiens SRY (sex determining reg	16.56520	25.06779	12.51989	14.78570	Detected	Detected	Compromised	Detected
8529	A_23_P250283	RAB1A	Hs.310645	RAB1A, member RAS oncogene family	Homo sapiens RAB1A, member RAS on	3862.60000	3824.97750	3347.17630	2808.54350	Detected	Detected	Detected	Detected
8530	A_33_P3343442	ZNF316	Hs.720748	zinc finger protein 316	PREDICTED: Homo sapiens zinc finger	52.71945	53.92333	39.49667	33.42044	Detected	Detected	Detected	Detected
8531	A_23_P44569	ABCC2	Hs.368243	ATP-binding cassette, sub-family C (CF	Homo sapiens ATP-binding cassette, su	70.51619	104.22889	51.46082	63.64042	Detected	Detected	Detected	Detected
8532	A_33_P3410409	LAMP2	Hs.496684	lysosomal-associated membrane protei	Homo sapiens lysosomal-associated me	1713.55630	6367.96900	1486.29540	4905.14600	Detected	Detected	Detected	Detected
8533	A_32_P326819	KRR1	Hs.645517	KRR1, small subunit (SSU) processome	Homo sapiens KRR1, small subunit (SSU	240.27141	220.97266	212.43700	157.05359	Detected	Detected	Detected	Detected
8534	A_24_P254177	C19orf42	Hs.356467	chromosome 19 open reading frame 42	Homo sapiens chromosome 19 open rea	2217.77420	5193.44780	1798.23690	3581.02080	Detected	Detected	Detected	Detected
8535	A_33_P3298356	SH3GLB1	Hs.136309	SH3-domain GRB2-like endophilin B1	Homo sapiens SH3-domain GRB2-like e	13819.63400	21126.19000	10658.87400	13698.52100	Detected	Detected	Detected	Detected
8536	A_33_P3317988	CMPK1	Hs.11463	cytidine monophosphate (UMP-CMP) ki	Homo sapiens cytidine monophosphate	407.99000	620.30360	362.67680	446.66342	Detected	Detected	Detected	Detected
8537	A_24_P324520	MTCP1	Hs.6917	mature T-cell proliferation 1	Homo sapiens mature T-cell proliferatio	28.51474	35.62500	56.36462	37.06551	Detected	Detected	Detected	Detected
8538	A_33_P3311083				Pleckstrin homology-like domain family	8.04312	3.16474	16.88327	8.44929	Compromised	Compromised	Detected	Compromised
8539	A_33_P3273459		Hs.705501		Spermatogenesis-associated protein 21	115.42710	116.99501	107.05218	76.40730	Detected	Detected	Detected	Detected
8540	A_24_P281801	ZNF732	Hs.698668	zinc finger protein 732	Homo sapiens zinc finger protein 732 (Z	580.47314	758.25620	82.74880	83.91952	Detected	Detected	Detected	Detected
8541	A_24_P253827	AP2B1	Hs.514819	adaptor-related protein complex 2, beta	Homo sapiens adaptor-related protein c	3223.03340	2310.13480	2636.57890	1728.38620	Detected	Detected	Detected	Detected
8542	A_33_P3263989					178.63408	267.20566	147.44649	175.94125	Detected	Detected	Detected	Detected
8543	A_33_P3364801	LOC401180	Hs.534795	similar to hCG1745223	PREDICTED: Homo sapiens similar to h	5.12246	3.17755	18.41112	9.28092	Compromised	Compromised	Detected	Compromised
8544	A_23_P26759	CANT1	Hs.8859	calcium activated nucleotidase 1	Homo sapiens calcium activated nucleo	665.51500	976.58740	520.48990	675.82460	Detected	Detected	Detected	Detected
8545	A_23_P116430	USH1C	Hs.502072	Usher syndrome 1C (autosomal recessi	Homo sapiens Usher syndrome 1C (aut	8.52403	12.07954	9.81525	15.72409	Compromised	Compromised	Compromised	Detected
8546	A_23_P117380	FAM181A	Hs.525550	family with sequence similarity 181, mer	Homo sapiens family with sequence sim	348.93777	528.50400	87.06603	123.39912	Detected	Detected	Detected	Detected

8547	A_23_P45699	FUBP1	Hs.567380	far upstream element (FUSE) binding pr	Homo sapiens far upstream element (FU	1492.37570	3518.52950	917.64606	1720.44570	Detected	Detected	Detected	Detected
8548	A_33_P3224867	GSG1	Hs.240053	germ cell associated 1	Homo sapiens germ cell associated 1 (G	88.75209	126.97913	84.39614	73.55129	Detected	Detected	Detected	Detected
8549	A_24_P943781	C7orf58	Hs.189652	chromosome 7 open reading frame 58	Homo sapiens chromosome 7 open read	254.33778	206.51572	211.53806	133.24773	Detected	Detected	Detected	Detected
8550	A_23_P252536	MRPS26	Hs.18946	mitochondrial ribosomal protein S26	Homo sapiens mitochondrial ribosomal p	7714.25930	7395.36470	9763.99500	8732.59400	Detected	Detected	Detected	Detected
8551	A_33_P3233378		Hs.648234		Uncharacterized protein DKFZP434B06	20.04970	19.20263	11.73260	15.18335	Detected	Detected	Compromised	Detected
8552	A_33_P3281905	LOC100144604	Hs.245476	hypothetical LOC100144604	Homo sapiens hypothetical LOC100144	2244.68380	1405.63800	1575.67850	422.49340	Detected	Detected	Detected	Detected
8553	A_33_P3227551					13.10493	5.71870	10.40672	8.84863	Detected	Compromised	Compromised	Compromised
8554	A_23_P253597	LRRC3	Hs.326579	leucine rich repeat containing 3	Homo sapiens leucine rich repeat conta	19.49851	21.32076	20.84424	23.96807	Detected	Detected	Detected	Detected
8555	A_33_P3381044	LOC646709	Hs.688894	similar to NADH dehydrogenase (ubiqui	PREDICTED: Homo sapiens similar to N	755.61084	774.32684	51.44575	46.43938	Detected	Detected	Detected	Detected
8556	A_23_P300033	PDGFRA	Hs.74615	platelet-derived growth factor receptor	Homo sapiens platelet-derived growth f	31031.85000	27632.85500	20934.84600	16762.46300	Detected	Detected	Detected	Detected
8557	A_23_P56288	LENG8	Hs.720354	leukocyte receptor cluster (LRC) memb	Homo sapiens leukocyte receptor clust	620.83320	790.16490	149.60680	147.44595	Detected	Detected	Detected	Detected
8558	A_23_P307392	DPF3	Hs.162868	D4, zinc and double PHD fingers, family	Homo sapiens cDNA FLJ14079 fis, clon	31.95336	40.55076	29.41871	39.12954	Detected	Detected	Detected	Detected
8559	A_24_P49747	LOC646993	Hs.591080	similar to high mobility group box 3	PREDICTED: Homo sapiens similar to h	160.52905	741.94855	37.17392	155.11348	Detected	Detected	Detected	Detected
8560	A_23_P329945	HIPK4	Hs.79363	homeodomain interacting protein kinase	Homo sapiens homeodomain interacting	20.12576	16.63349	16.86052	13.64664	Detected	Compromised	Detected	Compromised
8561	A_24_P393565		Hs.351005		Zinc finger protein 396 (Zinc finger and	24.32697	5.47728	28.87855	7.91667	Detected	Compromised	Detected	Compromised
8562	A_33_P3235491					96.14616	109.46069	94.34156	101.15710	Detected	Detected	Detected	Detected
8563	A_33_P3306146	PLAU	Hs.77274	plasminogen activator, urokinase	Homo sapiens plasminogen activator, ur	380.63810	151.09871	501.72690	186.48705	Detected	Detected	Detected	Detected
8564	A_23_P193	DEM1	Hs.59584	defects in morphology 1 homolog (S. ce	Homo sapiens defects in morphology 1	1131.33480	1503.19240	1063.98180	1322.54460	Detected	Detected	Detected	Detected
8565	A_33_P3424132	LOC158376		hypothetical LOC158376	Homo sapiens hypothetical LOC158376	25.63182	31.84499	27.96895	34.29906	Detected	Detected	Detected	Detected
8566	A_23_P129334	CLCN7	Hs.459649	chloride channel 7	Homo sapiens chloride channel 7 (CLCN	814.10236	599.36224	904.78020	692.19135	Detected	Detected	Detected	Detected
8567	A_23_P124122	PXMP2	Hs.430299	peroxisomal membrane protein 2, 22kDa	Homo sapiens peroxisomal membrane p	966.07970	3781.88450	720.31880	2381.51000	Detected	Detected	Detected	Detected
8568	A_24_P128442	TBX15	Hs.146196	T-box 15	Homo sapiens T-box 15 (TBX15), mRNA	6124.38000	3946.45750	2667.79600	1543.38040	Detected	Detected	Detected	Detected
8569	A_23_P300728	LMBR1	Hs.209989	limb region 1 homolog (mouse)	Homo sapiens limb region 1 homolog (m	298.62198	304.46298	247.95306	212.40962	Detected	Detected	Detected	Detected
8570	A_33_P3356711	ING3	Hs.489811	inhibitor of growth family, member 3	Homo sapiens inhibitor of growth family	344.33603	1170.11010	309.24940	969.20440	Detected	Detected	Detected	Detected
8571	A_33_P3221353	KIAA0564	Hs.368282	KIAA0564	Homo sapiens KIAA0564 (KIAA0564), tr	25.69818	53.36924	29.13800	38.06493	Detected	Detected	Detected	Detected
8572	A_23_P354297	CHTF18	Hs.153850	CTF18, chromosome transmission fideli	Homo sapiens CTF18, chromosome tran	1519.76300	3925.16500	1825.87410	4579.13430	Detected	Detected	Detected	Detected
8573	A_23_P411806	SLC44A1	Hs.573495	solute carrier family 44, member 1	Homo sapiens solute carrier family 44, r	2725.93000	629.90826	2734.24050	570.81310	Detected	Detected	Detected	Detected
8574	A_33_P3301219	FAM27A	Hs.555508	family with sequence similarity 27, mem	Homo sapiens family with sequence sim	45.39132	39.28957	51.08906	45.63453	Detected	Detected	Detected	Detected
8575	A_24_P100368	DYNLT3	Hs.446392	dynein, light chain, Tctex-type 3	Homo sapiens dynein, light chain, Tctex	442.63780	377.44672	550.86774	388.68073	Detected	Detected	Detected	Detected
8576	A_33_P3320212					8.15047	12.77767	25.06290	18.23862	Compromised	Compromised	Detected	Detected
8577	A_33_P3375710					558.00085	98.50436	544.37286	75.57945	Detected	Detected	Detected	Detected
8578	A_23_P428842	TMEM44	Hs.478729	transmembrane protein 44	Homo sapiens transmembrane protein 4	1968.31350	1572.40100	2228.74500	1573.13600	Detected	Detected	Detected	Detected
8579	A_24_P116871	TXNL4A	Hs.465498	thioredoxin-like 4A	Homo sapiens thioredoxin-like 4A (TXN	3482.01590	7831.30300	2410.92260	4378.30030	Detected	Detected	Detected	Detected
8580	A_24_P371758	MRPL36	Hs.719465	mitochondrial ribosomal protein L36	Homo sapiens mitochondrial ribosomal p	4317.33940	6450.12900	4919.89840	6551.32600	Detected	Detected	Detected	Detected
8581	A_23_P303548		Hs.516978		Uncharacterized protein C20orf112 [So	2.39316	18.05723	5.46754	18.42136	Compromised	Detected	Compromised	Detected
8582	A_23_P391725	DR1	Hs.348418	down-regulator of transcription 1, TBP-	Homo sapiens down-regulator of transc	1734.64370	4886.12000	1154.77270	2596.41820	Detected	Detected	Detected	Detected
8583	A_23_P161098	ELK4	Hs.497520	ELK4, ETS-domain protein (SRF access	Homo sapiens ELK4, ETS-domain prote	19.07014	40.04675	22.83272	30.93284	Detected	Detected	Detected	Detected
8584	A_33_P3376873					44.25631	44.86863	33.67215	31.09192	Detected	Detected	Detected	Detected
8585	A_33_P3259393	HAPLN3	Hs.447530	hyaluronan and proteoglycan link protei	Homo sapiens hyaluronan and proteogly	2059.75600	2920.14180	1395.38950	1952.54870	Detected	Detected	Detected	Detected
8586	A_23_P425750	ARMC6	Hs.77876	armadillo repeat containing 6	Homo sapiens armadillo repeat containi	4306.53660	7247.84130	1153.67640	2042.33280	Detected	Detected	Detected	Detected
8587	A_33_P3247838	ZNF844	Hs.646391	zinc finger protein 844	Homo sapiens zinc finger protein 844 (Z	650.25380	517.47300	337.54663	219.93784	Detected	Detected	Detected	Detected
8588	A_33_P3260342	NFASC	Hs.13349	neurofascin homolog (chicken)	Homo sapiens neurofascin homolog (chi	58.91195	189.01350	61.74173	149.11308	Detected	Detected	Detected	Detected
8589	A_23_P109768	RNF7	Hs.134623	ring finger protein 7	Homo sapiens ring finger protein 7 (RNF	25788.49400	32988.22700	19780.53000	20995.65800	Detected	Detected	Detected	Detected
8590	A_23_P54692				Putative uncharacterized protein ENSP	72.36359	28.01513	58.64199	39.82821	Detected	Detected	Detected	Detected
8591	A_33_P3316313	MTERFD2	Hs.159556	MTERF domain containing 2	Homo sapiens MTERF domain containin	1758.71250	1894.96640	1562.78880	1398.25560	Detected	Detected	Detected	Detected
8592	A_33_P3267410	KILLIN	Hs.559820	killin protein	Homo sapiens killin protein (KILLIN), mF	147.49318	103.13899	107.65868	63.90482	Detected	Detected	Detected	Detected
8593	A_23_P24796	FAM160A2	Hs.501793	family with sequence similarity 160, mer	Homo sapiens family with sequence sim	2536.70950	2781.00200	2458.04900	2550.47170	Detected	Detected	Detected	Detected
8594	A_23_P436476	RBM34	Hs.535224	RNA binding motif protein 34	Homo sapiens RNA binding motif protei	6607.65770	5687.60700	5516.03100	4125.45300	Detected	Detected	Detected	Detected
8595	A_33_P3211327		Hs.712317		Putative uncharacterized protein FLJ42	137.84400	146.36682	94.12603	95.46257	Detected	Detected	Detected	Detected
8596	A_33_P3420931	LOC100129509	Hs.712168	hypothetical LOC100129509	PREDICTED: Homo sapiens hypothetica	10.57340	11.56119	11.96726	19.89779	Compromised	Compromised	Compromised	Detected
8597	A_33_P3262625	CECR7		cat eye syndrome chromosome region,	Homo sapiens cat eye syndrome chrom	95.35472	213.16524	52.40698	113.12040	Detected	Detected	Detected	Detected
8598	A_33_P3233869		Hs.446446		Homo sapiens, clone IMAGE:6155889, m	107.14988	86.60008	96.18245	76.14614	Detected	Detected	Detected	Detected
8599	A_24_P540057	CA5A	Hs.177446	carbonic anhydrase VA, mitochondrial	Homo sapiens carbonic anhydrase VA, r	52.43564	17.68948	57.16416	20.57550	Detected	Detected	Detected	Detected
8600	A_23_P40025	DAZAP2	Hs.369761	DAZ associated protein 2	Homo sapiens DAZ associated protein 2	33913.20700	32176.60200	24551.08200	20998.58600	Detected	Detected	Detected	Detected
8601	A_23_P41765	IRF1	Hs.436061	interferon regulatory factor 1	Homo sapiens interferon regulatory fact	641.23610	623.04050	773.89750	726.93616	Detected	Detected	Detected	Detected
8602	A_33_P3325229		Hs.535995		Novel proteinHCG1981372, isoform CRA	86.48792	177.74472	79.53285	173.94199	Detected	Detected	Detected	Detected
8603	A_23_P154447	NOP58	Hs.471104	NOP58 ribonucleoprotein homolog (yeas	Homo sapiens NOP58 ribonucleoprotein	10888.45700	19738.80900	7442.48900	10993.39100	Detected	Detected	Detected	Detected
8604	A_23_P326157	GAL3ST2	Hs.632556	galactose-3-O-sulfotransferase 2	Homo sapiens galactose-3-O-sulfotran	10.77746	10.89831	17.40399	12.92415	Compromised	Compromised	Detected	Compromised
8605	A_33_P3230436	ZNF714	Hs.466291	zinc finger protein 714	Homo sapiens zinc finger protein 714 (Z	350.28467	743.44640	199.11089	401.88437	Detected	Detected	Detected	Detected
8606	A_23_P37676	GPR176	Hs.37196	G protein-coupled receptor 176	Homo sapiens G protein-coupled recep	194.99400	335.04282	231.96599	352.59480	Detected	Detected	Detected	Detected
8607	A_33_P3395428	LOC100131594	Hs.689599	hypothetical protein LOC100131594	PREDICTED: Homo sapiens hypothetica	2.57165	62.51181	4.25822	43.29396	Compromised	Detected	Compromised	Detected
8608	A_33_P3407103	YTHDF1	Hs.11747	YTH domain family, member 1	Homo sapiens YTH domain family, meml	32.37426	87.22479	37.54954	85.27808	Detected	Detected	Detected	Detected
8609	A_33_P3349325	RAB28	Hs.656060	RAB28, member RAS oncogene family	Homo sapiens RAB28, member RAS onc	242.89421	216.84860	219.96088	176.11528	Detected	Detected	Detected	Detected
8610	A_33_P3270742	LOC100288406		hypothetical protein LOC100288406	PREDICTED: Homo sapiens hypothetica	8.66535	14.38479	16.32550	14.30160	Compromised	Compromised	Detected	Compromised
8611	A_23_P81492	RPS14	Hs.381126	ribosomal protein S14	Homo sapiens ribosomal protein S14 (R	324378.72000	245812.03000	316709.88000	215317.84000	Detected	Detected	Detected	Detected
8612	A_23_P303978	LOC285943	Hs.714774	hypothetical protein LOC285943	PREDICTED: Homo sapiens hypothetica	2.60220	20.56742	2.86364	14.06456	Compromised	Detected	Compromised	Compromised
8613	A_24_P23034	ZNFX1	Hs.371794	zinc finger, NFX1-type containing 1	Homo sapiens zinc finger, NFX1-type c	925.92320	824.25440	760.32513	706.08000	Detected	Detected	Detected	Detected
8614	A_33_P3324909	JUND	Hs.2780	jun D proto-oncogene	Homo sapiens jun D proto-oncogene (J	468.22137	263.42044	380.57092	237.04076	Detected	Detected	Detected	Detected
8615	A_33_P3321793		Hs.681925		UI-CF-EC1-abx-d-10-0-UI.s1 UI-CF-E	378.10450	321.01060	147.85408	118.12059	Detected	Detected	Detected	Detected
8616	A_23_P253200	RPL15	Hs.381219	ribosomal protein L15	Homo sapiens ribosomal protein L15 (R	20534.48000	14435.58400	13260.42600	8525.15500	Detected	Detected	Detected	Detected
8617	A_32_P185628					33.00386	20.40342	29.25992	22.78651	Detected	Detected	Detected	Detected
8618	A_23_P38446	TNFAIP1	Hs.76090	tumor necrosis factor, alpha-induced pr	Homo sapiens tumor necrosis factor, al	8111.06050	10790.35800	8027.15300	9851.06250	Detected	Detected	Detected	Detected
8619	A_33_P3273364	YIPF2	Hs.720136	Yip1 domain family, member 2	Homo sapiens Yip1 domain family, meml	2246.61820	5022.52150	1760.52000	3586.41970	Detected	Detected	Detected	Detected
8620	A_24_P330385	SLC22A17	Hs.373498	solute carrier family 22, member 17	Homo sapiens solute carrier family 22, r	388.71234	276.17026	333.33630	246.68050	Detected	Detected	Detected	Detected
8621	A_23_P151614	PSME1	Hs.75348	proteasome (prosome, macropain) activ	Homo sapiens proteasome (prosome, m	1137.43270	526.30414	1033.87520	426.86627	Detected	Detected	Detected	Detected

8622	A_33_P3396370	ACRBP	Hs.123239	acrosin binding protein	Homo sapiens acrosin binding protein (A	83.33027	93.76968	59.49383	44.30151	Detected	Detected	Detected	Detected
8623	A_24_P649282	LUZP2	Hs.144138	leucine zipper protein 2	Homo sapiens leucine zipper protein 2 (	2.39659	3.24033	2.64730	14.74644	Compromised	Compromised	Compromised	Detected
8624	A_33_P3271410	THAP9	Hs.582050	THAP domain containing 9	Homo sapiens THAP domain containing	239.02696	162.48561	161.21976	99.44616	Detected	Detected	Detected	Detected
8625	A_24_P74371	CTSA	Hs.609336	cathepsin A	Homo sapiens cathepsin A (CTSA), tran	12460.35600	19783.54900	11073.72950	16434.24400	Detected	Detected	Detected	Detected
8626	A_33_P3217609		Hs.435044		TBC1 domain family member 22A [Sou	91.85586	30.40470	73.24652	21.56771	Detected	Detected	Detected	Detected
8627	A_23_P91221	PKIG	Hs.472831	protein kinase (cAMP-dependent, cataly	Homo sapiens protein kinase (cAMP-de	4716.73340	4534.67630	5941.35900	5660.48540	Detected	Detected	Detected	Detected
8628	A_23_P50146	SIGLEC15	Hs.287692	sialic acid binding Ig-like lectin 15	Homo sapiens sialic acid binding Ig-like	338.35800	564.59564	198.95682	249.87787	Detected	Detected	Detected	Detected
8629	A_33_P3248602	DUX4	Hs.553518	double homeobox, 4	Homo sapiens double homeobox, 4 (DU	183.98851	254.98320	125.73967	123.95891	Detected	Detected	Detected	Detected
8630	A_23_P131149	ORC4L	Hs.558364	origin recognition complex, subunit 4-lik	Homo sapiens origin recognition comple	2041.52820	1600.10240	1804.61010	1151.38090	Detected	Detected	Detected	Detected
8631	A_33_P3288725					45.76602	54.69024	20.42202	24.51126	Detected	Detected	Detected	Detected
8632	A_24_P416131	COTL1	Hs.289092	coactosin-like 1 (Dictyostelium)	Homo sapiens coactosin-like 1 (Dictyos	71617.43000	116964.29000	40629.06600	55215.75400	Detected	Detected	Detected	Detected
8633	A_23_P255827	FKSG2	Hs.651853	apoptosis inhibitor	apoptosis inhibitor [Source:RefSeq pep	20285.65400	12203.95200	649.54065	311.00360	Detected	Detected	Detected	Detected
8634	A_23_P339480	HAT1	Hs.632532	histone acetyltransferase 1	Homo sapiens histone acetyltransferase	8124.14840	14868.91800	7339.43650	11700.34300	Detected	Detected	Detected	Detected
8635	A_33_P3421476		Hs.575906		DA798455 OCBBF3 Homo sapiens cDN	22.08542	27.87972	20.59552	20.38250	Detected	Detected	Detected	Detected
8636	A_33_P3249439	NCAPH2	Hs.180903	non-SMC condensin II complex, subunit	Homo sapiens non-SMC condensin II co	9450.85000	26838.54700	8418.79000	21410.91400	Detected	Detected	Detected	Detected
8637	A_23_P15108	YPEL3	Hs.513491	yippee-like 3 (Drosophila)	Homo sapiens yippee-like 3 (Drosophila	1058.32870	566.32855	972.06415	495.68097	Detected	Detected	Detected	Detected
8638	A_23_P159839	C1GALT1C1	Hs.643920	C1GALT1-specific chaperone 1	Homo sapiens C1GALT1-specific chape	8159.21730	8525.00900	7807.87160	6983.78100	Detected	Detected	Detected	Detected
8639	A_33_P3230161	GRIA2	Hs.32763	glutamate receptor, ionotropic, AMPA 2	Homo sapiens glutamate receptor, ionot	7.92119	19.64796	6.78169	8.58173	Compromised	Detected	Compromised	Compromised
8640	A_33_P3213317				Eppin Precursor (Epididymal protease in	59.88384	23.28406	20.80347	7.42930	Detected	Detected	Detected	Compromised
8641	A_23_P165698	C2orf49	Hs.549577	chromosome 2 open reading frame 49	Homo sapiens chromosome 2 open read	342.50992	352.16058	281.25717	254.45550	Detected	Detected	Detected	Detected
8642	A_33_P3312365	LONP1	Hs.350265	lon peptidase 1, mitochondrial	Homo sapiens lon peptidase 1, mitochon	4241.49400	3264.35600	5283.84500	4096.08100	Detected	Detected	Detected	Detected
8643	A_24_P368943	EVX1	Hs.369879	even-skipped homeobox 1	Homo sapiens even-skipped homeobox	9136.09600	11696.48300	4037.17600	3631.08800	Detected	Detected	Detected	Detected
8644	A_23_P258689	HEATR2	Hs.535896	HEAT repeat containing 2	Homo sapiens HEAT repeat containing	1168.78200	1058.77800	950.02515	746.40840	Detected	Detected	Detected	Detected
8645	A_23_P73667	RIBC1	Hs.351743	RIB43A domain with coiled-coils 1	Homo sapiens RIB43A domain with coile	82.43041	111.39580	74.07132	80.61561	Detected	Detected	Detected	Detected
8646	A_23_P393099	TFF3	Hs.82961	trefoil factor 3 (intestinal)	Homo sapiens trefoil factor 3 (intestinal	51.87015	73.89201	25.50851	27.09465	Detected	Detected	Detected	Detected
8647	A_23_P128094	ABCB9	Hs.511951	ATP-binding cassette, sub-family B (MD	Homo sapiens ATP-binding cassette, su	99.89072	90.61781	90.14338	84.03085	Detected	Detected	Detected	Detected
8648	A_33_P3230558	EML5	Hs.558671	echinoderm microtubule associated pro	Homo sapiens echinoderm microtubule	54.52297	34.73977	8.25367	4.43160	Detected	Detected	Compromised	Compromised
8649	A_23_P312344	RBM19	Hs.7482	RNA binding motif protein 19	Homo sapiens RNA binding motif protei	1812.68270	1901.68250	1891.93000	1988.57410	Detected	Detected	Detected	Detected
8650	A_33_P3349269	PIAS2	Hs.658013	protein inhibitor of activated STAT, 2	Homo sapiens protein inhibitor of activa	1026.33940	2642.21950	931.23150	2033.50620	Detected	Detected	Detected	Detected
8651	A_33_P3239123		Hs.256639		Peptidyl-prolyl cis-trans isomerase H (	67.51627	76.52073	57.94709	66.82513	Detected	Detected	Detected	Detected
8652	A_23_P319640	hCG_1811732	Hs.516124	hypothetical LOC151534	Homo sapiens hypothetical LOC151534	696.26000	626.63600	371.69470	309.95874	Detected	Detected	Detected	Detected
8653	A_23_P41987	GFRA3	Hs.58042	GDNF family receptor alpha 3	Homo sapiens GDNF family receptor alp	94.00839	98.13052	39.24457	34.30808	Detected	Detected	Detected	Detected
8654	A_33_P3264965					18.50807	14.23110	18.55289	11.09507	Detected	Compromised	Detected	Compromised
8655	A_33_P3396612					12546.38900	13713.21700	11068.82600	9828.48600	Detected	Detected	Detected	Detected
8656	A_24_P231173	GIGYF1	Hs.414396	GRB10 interacting GYF protein 1	Homo sapiens GRB10 interacting GYF p	129.70178	93.51731	148.62787	96.27335	Detected	Detected	Detected	Detected
8657	A_33_P3262485	ZNF563	Hs.663510	zinc finger protein 563	Homo sapiens zinc finger protein 563 (Z	179.81883	271.14163	95.83659	112.02862	Detected	Detected	Detected	Detected
8658	A_33_P3501900	BC036928	Hs.549564	hypothetical protein BC036928	Homo sapiens hypothetical protein BC0	1134.69690	984.86610	892.67750	678.76746	Detected	Detected	Detected	Detected
8659	A_33_P3313779	CCDC64	Hs.369763	coiled-coil domain containing 64	Homo sapiens coiled-coil domain conta	36.16052	5.69717	22.14104	8.03587	Detected	Compromised	Detected	Compromised
8660	A_24_P326491	MKX	Hs.128193	mohawk homeobox	Homo sapiens mohawk homeobox (MKX	155.40741	4.84315	110.28508	4.25191	Detected	Compromised	Detected	Compromised
8661	A_24_P203689	KIF7	Hs.513134	kinesin family member 7	Homo sapiens kinesin family member 7	484.34473	667.95800	430.15607	565.58850	Detected	Detected	Detected	Detected
8662	A_24_P217758	MFAP3	Hs.432818	microfibrillar-associated protein 3	Homo sapiens microfibrillar-associated	722.63043	755.83060	654.70450	610.48370	Detected	Detected	Detected	Detected
8663	A_23_P214678	CUTA	Hs.520070	cutA divalent cation tolerance homolog	Homo sapiens cutA divalent cation toler	25475.78300	37522.46500	27983.93600	36429.96000	Detected	Detected	Detected	Detected
8664	A_32_P193583	C4orf39	Hs.178648	chromosome 4 open reading frame 39	Homo sapiens chromosome 4 open read	24.00664	41.83076	16.72947	42.67223	Detected	Detected	Detected	Detected
8665	A_23_P345887	GFPT1	Hs.580300	glutamine-fructose-6-phosphate transa	Homo sapiens glutamine-fructose-6-ph	395.02316	353.93707	274.35233	229.33624	Detected	Detected	Detected	Detected
8666	A_24_P298013	GTPBP2	Hs.485449	GTP binding protein 2	Homo sapiens GTP binding protein 2 (G	159.07237	187.29840	168.44507	206.93771	Detected	Detected	Detected	Detected
8667	A_33_P3577120		Hs.620382		LOC283868 protein Fragment [Source:	1171.50320	1252.29240	435.65180	490.55290	Detected	Detected	Detected	Detected
8668	A_23_P155556	CLDND1	Hs.531371	claudin domain containing 1	Homo sapiens claudin domain containin	4438.17900	2958.34940	3826.22440	2291.57800	Detected	Detected	Detected	Detected
8669	A_23_P133596	RNASEN	Hs.97997	ribonuclease type III, nuclear	Homo sapiens ribonuclease type III, nuc	6350.16300	7410.38770	5050.77500	5272.16800	Detected	Detected	Detected	Detected
8670	A_23_P322013		Hs.683919		Putative uncharacterized protein C8orf	18.22963	14.27582	13.59192	11.66763	Detected	Compromised	Detected	Compromised
8671	A_23_P387943	CASP2	Hs.368982	caspase 2, apoptosis-related cysteine p	Homo sapiens caspase 2, apoptosis-rel	574.16680	603.57750	262.65018	221.13646	Detected	Detected	Detected	Detected
8672	A_33_P3323463					164.71669	109.41042	131.20392	76.20448	Detected	Detected	Detected	Detected
8673	A_33_P3382380	UBQLN4	Hs.283739	ubiquilin 4	Homo sapiens ubiquilin 4 (UBQLN4), mR	375.00200	325.54776	329.16064	253.56616	Detected	Detected	Detected	Detected
8674	A_33_P3372580	COG1	Hs.283109	component of oligomeric golgi complex	Homo sapiens component of oligomeric	7210.86230	7102.58000	5809.00800	5240.16400	Detected	Detected	Detected	Detected
8675	A_23_P170498	NTAN1	Hs.592045	N-terminal asparagine amidase	Homo sapiens N-terminal asparagine an	5918.95200	6768.77930	5258.26000	5343.10250	Detected	Detected	Detected	Detected
8676	A_24_P219378	CASKIN1	Hs.643537	CASK interacting protein 1	Homo sapiens CASK interacting protein	353.57742	187.10376	274.32166	129.45628	Detected	Detected	Detected	Detected
8677	A_24_P261259	PFKFB3	Hs.195471	6-phosphofructo-2-kinase/fructose-2,6	Homo sapiens 6-phosphofructo-2-kinas	4227.55700	1377.31410	3518.72730	1123.16580	Detected	Detected	Detected	Detected
8678	A_23_P250413	PARVG	Hs.658995	parvin, gamma	Homo sapiens parvin, gamma (PARVG),	9.06001	8.02399	13.44084	17.31711	Compromised	Compromised	Detected	Detected
8679	A_23_P14515	ACOT4	Hs.49433	acyl-CoA thioesterase 4	Homo sapiens acyl-CoA thioesterase 4	53.32838	64.14956	55.05061	55.76027	Detected	Detected	Detected	Detected
8680	A_33_P3348797	C5orf24	Hs.708536	chromosome 5 open reading frame 24	Homo sapiens chromosome 5 open read	1370.67920	1009.88794	818.99860	485.07782	Detected	Detected	Detected	Detected
8681	A_23_P91468	PSMA7	Hs.233952	proteasome (prosome, macropain) subu	Homo sapiens proteasome (prosome, m	16513.16000	24392.32400	14207.85500	17585.04300	Detected	Detected	Detected	Detected
8682	A_33_P3420680	NEUROG1	Hs.248149	neurogenin 1	Homo sapiens neurogenin 1 (NEUROG1	14.55265	11.78375	2.53522	3.48444	Detected	Compromised	Compromised	Compromised
8683	A_23_P94204	OXR1	Hs.148778	oxidation resistance 1	Homo sapiens oxidation resistance 1 (O	1053.32520	792.21246	895.26587	597.06433	Detected	Detected	Detected	Detected
8684	A_23_P347059	MOBKL1A	Hs.700445	MOB1, Mps One Binder kinase activato	Homo sapiens MOB1, Mps One Binder k	2808.61600	3839.73930	1876.70630	2084.30900	Detected	Detected	Detected	Detected
8685	A_33_P3329104					79.16471	106.72920	37.50547	36.80174	Detected	Detected	Detected	Detected
8686	A_24_P393864	PHTF1	Hs.655824	putative homeodomain transcription fac	Homo sapiens putative homeodomain tr	675.54990	846.91327	529.41876	522.78955	Detected	Detected	Detected	Detected
8687	A_33_P3374049	KIAA2013	Hs.520094	KIAA2013	Homo sapiens KIAA2013 (KIAA2013), m	3065.52560	3168.80790	3087.34420	3166.44380	Detected	Detected	Detected	Detected
8688	A_23_P502269	POLDIP3	Hs.505802	polymerase (DNA-directed), delta intera	Homo sapiens polymerase (DNA-directe	4706.68200	4091.36180	5122.16300	4106.51370	Detected	Detected	Detected	Detected
8689	A_23_P336198	GLCCI1	Hs.131673	glucocorticoid induced transcript 1	Homo sapiens glucocorticoid induced tr	1249.86100	1401.58790	858.17930	799.21130	Detected	Detected	Detected	Detected
8690	A_23_P323227	PPP1CA	Hs.183994	protein phosphatase 1, catalytic subunit	Homo sapiens protein phosphatase 1, c	12131.15300	18578.52000	13229.21300	18473.36000	Detected	Detected	Detected	Detected
8691	A_33_P3479449	LOC151438	Hs.516245	hypothetical protein LOC151438	Homo sapiens cDNA FLJ31315 fis, clon	98.46256	3.27998	71.40328	3.28433	Detected	Compromised	Detected	Compromised
8692	A_33_P3244669	TMEM30A	Hs.108530	transmembrane protein 30A	Homo sapiens transmembrane protein 3	20158.39600	32139.67800	11072.34600	13379.21200	Detected	Detected	Detected	Detected
8693	A_23_P54605	RSL1D1	Hs.401842	ribosomal L1 domain containing 1	Homo sapiens ribosomal L1 domain con	3208.61840	3083.01050	2617.27420	2128.82320	Detected	Detected	Detected	Detected
8694	A_32_P204676	FABP5	Hs.408061	fatty acid binding protein 5 (psoriasis-a	Homo sapiens fatty acid binding protein	1739.61820	3041.28660	2006.04810	2893.63010	Detected	Detected	Detected	Detected
8695	A_24_P217572	EDNRA	Hs.183713	endothelin receptor type A	Homo sapiens endothelin receptor type	7132.73440	821.12360	5827.34900	545.83330	Detected	Detected	Detected	Detected
8696	A_23_P15272	ABCC6	Hs.442182	ATP-binding cassette, sub-family C (CF	Homo sapiens ATP-binding cassette, su	742.62714	306.52844	403.74457	103.95039	Detected	Detected	Detected	Detected

8697	A_23_P357811	MBNL1	Hs.478000	muscleblind-like (Drosophila)	Homo sapiens muscleblind-like (Drosop	6798.65330	9383.61800	6593.77400	7993.40200	Detected	Detected	Detected	Detected
8698	A_24_P331560	STS	Hs.522578	steroid sulfatase (microsomal), isozyme	Homo sapiens steroid sulfatase (micros	141.84367	232.12114	135.34769	175.54690	Detected	Detected	Detected	Detected
8699	A_23_P126706	ANGPTL1	Hs.591474	angiopoietin-like 1	Homo sapiens angiopoietin-like 1 (ANG	42.62610	31.19981	30.49238	21.73756	Detected	Detected	Detected	Detected
8700	A_33_P3237096	INPP5F		inositol polyphosphate-5-phosphatase	Homo sapiens inositol polyphosphate-5	251.82303	759.62585	194.63162	506.29930	Detected	Detected	Detected	Detected
8701	A_33_P3305840	SFRS7	Hs.309090	splicing factor, arginine/serine-rich 7, 3	Homo sapiens splicing factor, arginine/s	5269.89600	18269.95700	4822.97700	13807.04200	Detected	Detected	Detected	Detected
8702	A_23_P157051	AP4M1	Hs.632317	adaptor-related protein complex 4, mu	Homo sapiens adaptor-related protein c	613.35730	629.27106	664.18115	643.01544	Detected	Detected	Detected	Detected
8703	A_23_P95930	HMGA2	Hs.505924	high mobility group AT-hook 2	Homo sapiens high mobility group AT-h	6805.54200	10396.39550	4631.40620	5724.05370	Detected	Detected	Detected	Detected
8704	A_32_P389770		Hs.376015		Proline-rich protein 10 [Source:UniPro	16.53882	24.94761	10.33532	11.60618	Detected	Detected	Compromised	Compromised
8705	A_33_P3386572					52.69799	64.79812	38.51343	49.28180	Detected	Detected	Detected	Detected
8706	A_24_P36097	RPAIN	Hs.462086	RPA interacting protein	Homo sapiens RPA interacting protein (	3953.25630	1700.89640	3774.77640	1327.43000	Detected	Detected	Detected	Detected
8707	A_33_P3398719				V4-3 protein Fragment [Source:UniPro	1109.33000	1599.52610	476.54453	504.94397	Detected	Detected	Detected	Detected
8708	A_23_P166248	RCAN1	Hs.282326	regulator of calcineurin 1	Homo sapiens regulator of calcineurin 1	2715.13090	2849.63900	1860.74770	1491.98410	Detected	Detected	Detected	Detected
8709	A_23_P24275	C10orf110	Hs.644603	chromosome 10 open reading frame 110	Homo sapiens chromosome 10 open rea	75.80736	78.50741	61.10500	53.19846	Detected	Detected	Detected	Detected
8710	A_23_P51711	CELA2B	Hs.631871	chymotrypsin-like elastase family, mem	Homo sapiens chymotrypsin-like elasta	41.12223	37.58899	61.44664	73.82766	Detected	Detected	Detected	Detected
8711	A_33_P3261902	ENOSF1	Hs.369762	enolase superfamily member 1	Homo sapiens enolase superfamily mem	857.75620	775.91270	796.31354	632.06550	Detected	Detected	Detected	Detected
8712	A_33_P3417260	C9orf95	Hs.494186	chromosome 9 open reading frame 95	Homo sapiens chromosome 9 open reac	1337.90980	695.46606	860.40200	374.69897	Detected	Detected	Detected	Detected
8713	A_32_P743407	GLYR1	Hs.387255	glyoxylate reductase 1 homolog (Arabid	Homo sapiens glyoxylate reductase 1 h	214.83896	138.42464	223.99919	138.60837	Detected	Detected	Detected	Detected
8714	A_23_P212310	C3orf75	Hs.311100	chromosome 3 open reading frame 75	Homo sapiens chromosome 3 open reac	2206.94340	2594.81230	1903.65970	1857.71440	Detected	Detected	Detected	Detected
8715	A_33_P3308686	CC2D2A	Hs.590928	coiled-coil and C2 domain containing 2A	Homo sapiens coiled-coil and C2 domai	151.60490	545.58950	102.56459	266.28204	Detected	Detected	Detected	Detected
8716	A_23_P83028	RECK	Hs.388918	reversion-inducing-cysteine-rich protei	Homo sapiens reversion-inducing-cyste	2598.48400	3196.82400	2077.19650	2183.97700	Detected	Detected	Detected	Detected
8717	A_33_P3230541				Myosin phosphatase Rho-interacting pr	102.01978	214.62845	27.06718	49.06304	Detected	Detected	Detected	Detected
8718	A_32_P77502	HNRNPA3	Hs.516539	heterogeneous nuclear ribonucleoprotei	Homo sapiens heterogeneous nuclear ri	18019.06600	26923.14600	11752.76000	15159.21100	Detected	Detected	Detected	Detected
8719	A_33_P3424462	CNST	Hs.368353	consortin, connexin sorting protein	Homo sapiens consortin, connexin sorti	196.21461	437.82250	104.84539	214.01508	Detected	Detected	Detected	Detected
8720	A_23_P113623	MRPL45	Hs.462913	mitochondrial ribosomal protein L45	Homo sapiens mitochondrial ribosomal p	8750.66300	8529.09600	7446.60250	6089.83500	Detected	Detected	Detected	Detected
8721	A_33_P3211628	PRKRIR	Hs.503315	protein-kinase, interferon-inducible dou	Homo sapiens protein-kinase, interferon	1603.52040	1259.04470	1309.56570	897.07210	Detected	Detected	Detected	Detected
8722	A_33_P3305243				26S proteasome non-ATPase regulator	57.37618	68.17216	50.95158	47.42955	Detected	Detected	Detected	Detected
8723	A_33_P3421318					27.45886	36.12769	16.03678	3.14683	Detected	Detected	Detected	Compromised
8724	A_23_P253896	NPNT	Hs.518921	nephronectin	Homo sapiens nephronectin (NPNT), mR	38.67376	21.20606	18.45088	12.14906	Detected	Detected	Detected	Compromised
8725	A_23_P331700	SRRM3	Hs.511025	serine/arginine repetitive matrix 3	Homo sapiens serine/arginine repetitive	760.71930	431.03644	759.76650	465.31238	Detected	Detected	Detected	Detected
8726	A_23_P215658	TBRG4	Hs.231411	transforming growth factor beta regulat	Homo sapiens transforming growth fact	3409.42650	3364.23440	3606.37040	3521.74780	Detected	Detected	Detected	Detected
8727	A_33_P3248137	OLFML2A	Hs.357004	olfactomedin-like 2A	Homo sapiens olfactomedin-like 2A (OL	116.03997	120.34908	86.96171	75.93922	Detected	Detected	Detected	Detected
8728	A_24_P72750	SPIN1	Hs.146804	spindlin 1	Homo sapiens spindlin 1 (SPIN1), mRNA	2616.28500	3129.90920	1867.53740	1656.13160	Detected	Detected	Detected	Detected
8729	A_24_P236251	DLK1	Hs.533717	delta-like 1 homolog (Drosophila)	Homo sapiens delta-like 1 homolog (Dro	72.87358	14.29931	20.77113	28.10795	Detected	Compromised	Detected	Detected
8730	A_24_P924862	RAPH1	Hs.471162	Ras association (RalGDS/AF-6) and ple	Homo sapiens Ras association (RalGDS	6308.97900	5497.55300	3892.63350	2815.95530	Detected	Detected	Detected	Detected
8731	A_33_P3252834	PHLDA3	Hs.268557	pleckstrin homology-like domain, family	Homo sapiens pleckstrin homology-like	2909.99440	4788.82280	3309.15550	5518.73340	Detected	Detected	Detected	Detected
8732	A_33_P3324394	CIITA	Hs.701991	class II, major histocompatibility comple	Homo sapiens class II, major histocomp	22.70656	23.22531	18.80866	17.99700	Detected	Detected	Detected	Detected
8733	A_23_P372368	C21orf87	Hs.190548	chromosome 21 open reading frame 87	PREDICTED: Homo sapiens chromosom	108.74378	129.69243	36.82956	36.05229	Detected	Detected	Detected	Detected
8734	A_32_P211117	LOC401021	Hs.591636	hypothetical gene supported by BC047	Homo sapiens hypothetical gene suppor	3.46688	36.92355	9.09632	21.02701	Compromised	Detected	Compromised	Detected
8735	A_23_P153390	CLEC4G	Hs.220649	C-type lectin domain family 4, member	Homo sapiens C-type lectin domain fam	6.70164	3.94823	12.75499	10.24008	Compromised	Compromised	Detected	Compromised
8736	A_23_P84189	PITPNC1	Hs.591185	phosphatidylinositol transfer protein, cy	Homo sapiens phosphatidylinositol trans	363.66635	258.89374	350.46414	198.38986	Detected	Detected	Detected	Detected
8737	A_24_P142228	RPL13	Hs.410817	ribosomal protein L13	Homo sapiens ribosomal protein L13 (R	275875.16000	244626.12000	275552.94000	220674.47000	Detected	Detected	Detected	Detected
8738	A_33_P3349252		Hs.567600		Putative uncharacterized protein DKFZ	44019.31200	52589.44000	8607.60400	6532.04440	Detected	Detected	Detected	Detected
8739	A_24_P245108	USP7	Hs.386939	ubiquitin specific peptidase 7 (herpes vi	Homo sapiens ubiquitin specific peptida	1247.66600	1139.10100	1093.91970	825.29140	Detected	Detected	Detected	Detected
8740	A_33_P3409159	SLC22A23	Hs.713588	solute carrier family 22, member 23	Homo sapiens solute carrier family 22, r	1429.05850	715.81090	642.83990	256.06380	Detected	Detected	Detected	Detected
8741	A_33_P3243168	MZF1	Hs.399810	myeloid zinc finger 1	Homo sapiens myeloid zinc finger 1 (MZ	3705.25020	1740.01380	3333.14450	1429.68490	Detected	Detected	Detected	Detected
8742	A_32_P70135	C13orf23	Hs.318526	chromosome 13 open reading frame 23	Homo sapiens chromosome 13 open rea	2691.51220	2653.01800	1912.19800	1550.89110	Detected	Detected	Detected	Detected
8743	A_32_P101844					473.03253	470.42130	314.76285	244.69417	Detected	Detected	Detected	Detected
8744	A_24_P367329	LOC440292	Hs.326276	similar to COMM domain containing 4	PREDICTED: Homo sapiens similar to C	545.90150	924.56300	863.76990	1322.13890	Detected	Detected	Detected	Detected
8745	A_33_P3369146	ATP2B3	Hs.533956	ATPase, Ca++ transporting, plasma mer	Homo sapiens ATPase, Ca++ transporti	132.53421	155.62149	68.44340	72.01627	Detected	Detected	Detected	Detected
8746	A_33_P3295261	LMOD1	Hs.519075	leiomodin 1 (smooth muscle)	Homo sapiens leiomodin 1 (smooth mus	9.36807	3.40340	16.82253	9.93805	Compromised	Compromised	Detected	Compromised
8747	A_33_P3381483	ZNF331	Hs.185674	zinc finger protein 331	Homo sapiens zinc finger protein 331 (Z	416.52823	610.24646	275.23657	333.91058	Detected	Detected	Detected	Detected
8748	A_33_P3348927					12.59241	10.61988	8.60903	9.32853	Detected	Compromised	Compromised	Compromised
8749	A_23_P39799	LOXL3	Hs.720335	lysyl oxidase-like 3	Homo sapiens lysyl oxidase-like 3 (LOX	61.53202	1167.07190	58.72586	725.99590	Detected	Detected	Detected	Detected
8750	A_24_P77904	HOXA10	Hs.110637	homeobox A10	Homo sapiens homeobox A10 (HOXA10	2.51297	553.14400	2.69428	242.11569	Compromised	Detected	Compromised	Detected
8751	A_33_P3228807	SPEG	Hs.21639	SPEG complex locus	Homo sapiens SPEG complex locus (SF	578.77430	448.07434	453.54318	316.82110	Detected	Detected	Detected	Detected
8752	A_33_P3712341	CXCL12	Hs.522891	chemokine (C-X-C motif) ligand 12 (str	Homo sapiens chemokine (C-X-C motif	372.28757	855.56920	208.04623	432.06543	Detected	Detected	Detected	Detected
8753	A_33_P3315060	ZC3H11A	Hs.532399	zinc finger CCCH-type containing 11A	Homo sapiens mRNA; cDNA DKFZp781	84.48402	59.95505	64.69343	28.48030	Detected	Detected	Detected	Detected
8754	A_33_P3344243	C4orf46	Hs.380920	chromosome 4 open reading frame 46	Homo sapiens chromosome 4 open reac	2867.72140	7097.15900	2252.21660	4710.57000	Detected	Detected	Detected	Detected
8755	A_23_P159974	KLHL13	Hs.348262	kelch-like 13 (Drosophila)	Homo sapiens kelch-like 13 (Drosophila	66.84304	23.64190	63.65384	18.73332	Detected	Detected	Detected	Detected
8756	A_23_P353744	LARP1B	Hs.657067	La ribonucleoprotein domain family, mer	Homo sapiens La ribonucleoprotein dom	4242.28200	4594.60060	3573.53880	3360.88260	Detected	Detected	Detected	Detected
8757	A_24_P343559	NTRK2	Hs.494312	neurotrophic tyrosine kinase, receptor,	Homo sapiens neurotrophic tyrosine kin	48.63181	70.04300	14.64395	21.85574	Detected	Detected	Detected	Detected
8758	A_33_P3333282	FGF11	Hs.655193	fibroblast growth factor 11	Homo sapiens fibroblast growth factor 1	113.39652	14.29735	91.81173	9.15862	Detected	Compromised	Detected	Compromised
8759	A_23_P6624	UMPS	Hs.2057	uridine monophosphate synthetase	Homo sapiens uridine monophosphate s	5179.10700	8383.64700	3816.45870	4797.47270	Detected	Detected	Detected	Detected
8760	A_33_P3251108	SLC33A1	Hs.478031	solute carrier family 33 (acetyl-CoA tra	Homo sapiens solute carrier family 33 (	151.49580	149.41402	106.72782	82.00347	Detected	Detected	Detected	Detected
8761	A_23_P5831	HPCAL1	Hs.580427	hippocalcin-like 1	Homo sapiens hippocalcin-like 1 (HPCA	3280.34570	2176.33890	3014.64750	1948.97550	Detected	Detected	Detected	Detected
8762	A_23_P97906	NT5C2	Hs.97439	5'-nucleotidase, cytosolic II	Homo sapiens 5'-nucleotidase, cytosoli	1466.40150	1608.08910	1196.87010	1147.60820	Detected	Detected	Detected	Detected
8763	A_23_P41166	B3GALNT1	Hs.418062	beta-1,3-N-acetylgalactosaminyltransfe	Homo sapiens beta-1,3-N-acetylgalact	431.74652	1200.68570	358.65360	939.74740	Detected	Detected	Detected	Detected
8764	A_23_P68121	PSD4	Hs.516306	pleckstrin and Sec7 domain containing	Homo sapiens pleckstrin and Sec7 dom	126.41027	159.63450	117.19708	141.09512	Detected	Detected	Detected	Detected
8765	A_33_P3399870	LASS6	Hs.506829	LAG1 homolog, ceramide synthase 6	Homo sapiens LAG1 homolog, ceramide	354.63333	638.92120	254.09563	440.07388	Detected	Detected	Detected	Detected
8766	A_33_P3519223					2816.99600	1854.98280	62.79829	35.80115	Detected	Detected	Detected	Detected
8767	A_23_P201764	DHDDS	Hs.369385	dehydrodolichyl diphosphate synthase	Homo sapiens dehydrodolichyl diphosph	154.46388	259.83070	178.39572	260.01470	Detected	Detected	Detected	Detected
8768	A_33_P3439765	ZC3H14	Hs.325846	zinc finger CCCH-type containing 14	Homo sapiens zinc finger CCCH-type c	8320.41600	7305.47600	6185.50630	4814.45600	Detected	Detected	Detected	Detected
8769	A_23_P104607	PSMC3	Hs.250758	proteasome (prosome, macropain) 26S	Homo sapiens proteasome (prosome, m	5250.62900	13366.92400	5814.00930	14044.76000	Detected	Detected	Detected	Detected
8770	A_23_P55342	DVL2	Hs.118640	dishevelled, dsh homolog 2 (Drosophila)	Homo sapiens dishevelled, dsh homolog	1958.61410	1035.20000	1404.91150	698.07166	Detected	Detected	Detected	Detected
8771	A_33_P3670415	NAT8L	Hs.318529	N-acetyltransferase 8-like (GCN5-relat	Homo sapiens N-acetyltransferase 8-lik	4745.12600	3055.38000	417.95834	203.91876	Detected	Detected	Detected	Detected

8772	A_23_P42507	PPIL4	Hs.593411	peptidylprolyl isomerase (cyclophilin)-lik	Homo sapiens peptidylprolyl isomerase	431.45610	279.86545	308.06598	184.15161	Detected	Detected	Detected	Detected
8773	A_33_P3377763	FLJ36000	Hs.310247	hypothetical FLJ36000	Homo sapiens hypothetical FLJ36000 (F	117.66270	166.91754	105.42566	111.00001	Detected	Detected	Detected	Detected
8774	A_24_P365954	THAP11	Hs.632200	THAP domain containing 11	Homo sapiens THAP domain containing	977.30035	556.18610	1044.10310	514.89070	Detected	Detected	Detected	Detected
8775	A_33_P3398564	ACAP3	Hs.535257	ArfGAP with coiled-coil, ankyrin repeat	Homo sapiens ArfGAP with coiled-coil,	8382.18000	4898.39000	8382.16800	4719.70850	Detected	Detected	Detected	Detected
8776	A_23_P43425	C9orf40	Hs.532296	chromosome 9 open reading frame 40	Homo sapiens chromosome 9 open read	756.24646	980.49115	567.59120	613.64966	Detected	Detected	Detected	Detected
8777	A_23_P74716	DEDD	Hs.146406	death effector domain containing	Homo sapiens death effector domain co	749.94110	1141.00300	679.14264	840.56790	Detected	Detected	Detected	Detected
8778	A_23_P323924	ZFYVE27	Hs.523194	zinc finger, FYVE domain containing 27	Homo sapiens zinc finger, FYVE domain	13890.18200	10599.85300	12250.41900	9248.54500	Detected	Detected	Detected	Detected
8779	A_23_P356565	RRP8	Hs.652255	ribosomal RNA processing 8, methyltran	Homo sapiens ribosomal RNA processin	5633.02050	7745.32130	5765.06900	6706.68360	Detected	Detected	Detected	Detected
8780	A_24_P181055	ST3GAL4	Hs.591947	ST3 beta-galactoside alpha-2,3-sialyltr	Homo sapiens ST3 beta-galactoside alp	1618.86780	1873.74870	1865.25500	2356.06050	Detected	Detected	Detected	Detected
8781	A_23_P69329	HYAL1	Hs.75619	hyaluronoglucosaminidase 1	Homo sapiens hyaluronoglucosaminidas	19.17929	11.33319	28.40954	13.64313	Detected	Compromised	Detected	Detected
8782	A_33_P3416387	RNU105C		RNA, U105C small nucleolar	Homo sapiens RNA, U105C small nucleo	15.84825	16.45923	6.87379	6.38315	Detected	Detected	Compromised	Compromised
8783	A_32_P178945	YOD1	Hs.567533	YOD1 OTU deubiquinating enzyme 1 ho	Homo sapiens YOD1 OTU deubiquinatin	1774.71800	2550.12100	1593.43050	2019.67180	Detected	Detected	Detected	Detected
8784	A_23_P44112	LAT	Hs.632179	linker for activation of T cells	Homo sapiens linker for activation of T	701.32086	768.30690	368.20743	405.17175	Detected	Detected	Detected	Detected
8785	A_23_P415061	FAM104B	Hs.415414	family with sequence similarity 104, mer	Homo sapiens family with sequence sim	28.31433	15.30461	22.84185	12.75686	Detected	Compromised	Detected	Compromised
8786	A_23_P71270	AZGP1	Hs.546239	alpha-2-glycoprotein 1, zinc-binding	Homo sapiens alpha-2-glycoprotein 1, z	30.34832	21.84959	41.64492	23.79109	Detected	Detected	Detected	Detected
8787	A_23_P143692	SH3BP1	Hs.601143	SH3-domain binding protein 1	Homo sapiens SH3-domain binding prot	176.61816	143.73254	162.72682	105.64273	Detected	Detected	Detected	Detected
8788	A_24_P252996	FOLR3	Hs.352	folate receptor 3 (gamma)	Homo sapiens folate receptor 3 (gamma	220.06894	263.27560	276.11093	270.27410	Detected	Detected	Detected	Detected
8789	A_23_P205959	ALDH1A3	Hs.459538	aldehyde dehydrogenase 1 family, memb	Homo sapiens aldehyde dehydrogenase	208.54344	939.00590	173.49884	598.22186	Detected	Detected	Detected	Detected
8790	A_33_P3328485	IKZF2	Hs.604950	IKAROS family zinc finger 2 (Helios)	Homo sapiens IKAROS family zinc finge	23.65237	42.62778	22.21364	33.17990	Detected	Detected	Detected	Detected
8791	A_23_P153676	TLE2	Hs.332173	transducin-like enhancer of split 2 (E(sp	Homo sapiens transducin-like enhancer	645.62850	218.96901	633.44830	188.39542	Detected	Detected	Detected	Detected
8792	A_33_P3372699	SEC14L4	Hs.517541	SEC14-like 4 (S. cerevisiae)	Homo sapiens SEC14-like 4 (S. cerevisi	93.03751	79.95025	38.20171	30.32473	Detected	Detected	Detected	Detected
8793	A_33_P3244553	LOC100131968	Hs.694468	hypothetical LOC100131968	PREDICTED: Homo sapiens hypothetica	20.12982	25.07792	26.31074	23.94398	Detected	Detected	Detected	Detected
8794	A_24_P204244	ANXA2P1	Hs.546235	annexin A2 pseudogene 1	Homo sapiens annexin A2 pseudogene 1	26224.94000	75800.86000	26965.82200	68880.94500	Detected	Detected	Detected	Detected
8795	A_24_P29723	POR	Hs.354056	P450 (cytochrome) oxidoreductase	Homo sapiens P450 (cytochrome) oxido	2619.65600	1803.17690	2699.65720	1876.44090	Detected	Detected	Detected	Detected
8796	A_33_P3367681					17.34834	14.64378	10.89874	16.83083	Detected	Compromised	Compromised	Detected
8797	A_33_P3358731	PCSK5	Hs.368542	proprotein convertase subtilisin/kexin t	Homo sapiens proprotein convertase su	299.60547	109.63779	261.85168	96.90212	Detected	Detected	Detected	Detected
8798	A_23_P144465	PAPSS1	Hs.368610	3'-phosphoadenosine 5'-phosphosulfate	Homo sapiens 3'-phosphoadenosine 5'-	2618.16380	1641.28560	2349.50660	1208.90390	Detected	Detected	Detected	Detected
8799	A_33_P3333187					14.85899	125.10724	16.00103	45.11354	Detected	Detected	Detected	Detected
8800	A_23_P399146	ZNF549	Hs.720803	zinc finger protein 549	Homo sapiens zinc finger protein 549 (Z	129.18760	125.28935	80.37693	71.55656	Detected	Detected	Detected	Detected
8801	A_24_P42681	PSMD2	Hs.518464	proteasome (prosome, macropain) 26S s	Homo sapiens proteasome (prosome, ma	15062.32000	34039.36300	12046.03400	24416.29700	Detected	Detected	Detected	Detected
8802	A_33_P3364051	TOP3B	Hs.436401	topoisomerase (DNA) III beta	Homo sapiens topoisomerase (DNA) III	210.33006	265.97876	188.43515	234.73775	Detected	Detected	Detected	Detected
8803	A_33_P3344765					304.64478	546.33620	194.59097	263.12650	Detected	Detected	Detected	Detected
8804	A_33_P3294089					21.35827	9.79993	25.85777	8.50999	Detected	Compromised	Detected	Compromised
8805	A_23_P142969	ZNF2	Hs.590916	zinc finger protein 2	Homo sapiens zinc finger protein 2 (ZNF	136.30421	136.23817	166.42500	139.16768	Detected	Detected	Detected	Detected
8806	A_24_P52004	PDS5A	Hs.331431	PDS5, regulator of cohesion maintenanc	Homo sapiens PDS5, regulator of cohes	1305.76400	1020.43490	1403.36150	882.10364	Detected	Detected	Detected	Detected
8807	A_23_P166686	AMOTL2	Hs.426312	angiomotin like 2	Homo sapiens angiomotin like 2 (AMOT	12692.48700	28411.76600	13985.12500	26509.26600	Detected	Detected	Detected	Detected
8808	A_24_P350008					18963.91200	15546.43750	10451.65800	7350.53100	Detected	Detected	Detected	Detected
8809	A_24_P104407	SYNM	Hs.207106	synemin, intermediate filament protein	Homo sapiens synemin, intermediate fila	72.96240	117.40582	49.98889	68.12206	Detected	Detected	Detected	Detected
8810	A_33_P3386760	CHEK2	Hs.291363	CHK2 checkpoint homolog (S. pombe)	Homo sapiens CHK2 checkpoint homolo	37.22844	27.85218	20.71462	28.38907	Detected	Detected	Detected	Detected
8811	A_24_P208436	PDE1A	Hs.191046	phosphodiesterase 1A, calmodulin-depe	Homo sapiens phosphodiesterase 1A, ca	25.68504	16.99416	26.51155	15.96192	Detected	Detected	Compromised	Detected
8812	A_24_P82466	GAS7	Hs.462214	growth arrest-specific 7	Homo sapiens growth arrest-specific 7	123.92180	24.22800	73.06556	8.60148	Detected	Detected	Detected	Compromised
8813	A_32_P47554	HINT1	Hs.483305	histidine triad nucleotide binding protein	Homo sapiens histidine triad nucleotide	88408.77000	123001.44000	62256.12000	70905.09000	Detected	Detected	Detected	Detected
8814	A_23_P76245	SCN8A	Hs.710638	sodium channel, voltage gated, type VIII	Homo sapiens sodium channel, voltage g	98.86392	115.32510	113.33042	114.55716	Detected	Detected	Detected	Detected
8815	A_24_P357726	PSG8	Hs.466843	pregnancy specific beta-1-glycoprotein	Homo sapiens pregnancy specific beta-	95.66386	353.83860	97.68231	116.63563	Detected	Detected	Detected	Detected
8816	A_33_P3362636	CISD3	Hs.713595	CDGSH iron sulfur domain 3	Homo sapiens CDGSH iron sulfur domai	19171.12500	22462.58600	43751.29000	42492.11300	Detected	Detected	Detected	Detected
8817	A_33_P3404546	OBSCN	Hs.656999	obscurin, cytoskeletal calmodulin and tit	Homo sapiens obscurin, cytoskeletal ca	416.93677	277.47037	76.41319	58.29656	Detected	Detected	Detected	Detected
8818	A_23_P399156	IL12RB1	Hs.567294	interleukin 12 receptor, beta 1	Homo sapiens interleukin 12 receptor, b	91.38950	107.16681	39.69604	33.34669	Detected	Detected	Detected	Detected
8819	A_33_P3265314	FLJ44054	Hs.680435	hypothetical protein LOC643365	Homo sapiens hypothetical protein LOC	36.99557	30.69725	6.58495	14.53638	Detected	Detected	Compromised	Detected
8820	A_32_P18159	LYRM7	Hs.115467	Lyrm7 homolog (mouse)	Homo sapiens Lyrm7 homolog (mouse) (	182.86311	144.54425	148.62512	83.05701	Detected	Detected	Detected	Detected
8821	A_23_P135184	RALGDS	Hs.106185	ral guanine nucleotide dissociation stimu	Homo sapiens ral guanine nucleotide dis	5785.60800	2433.13720	3049.38300	1355.49570	Detected	Detected	Detected	Detected
8822	A_32_P132589	ZYG11A	Hs.658458	zyg-11 homolog A (C. elegans)	Homo sapiens zyg-11 homolog A (C. ele	63.34996	65.61015	55.62574	61.20653	Detected	Detected	Detected	Detected
8823	A_33_P3265494	C6orf138	Hs.659409	chromosome 6 open reading frame 138	Homo sapiens chromosome 6 open read	19.64230	8.65778	22.74603	8.93176	Detected	Compromised	Detected	Compromised
8824	A_33_P3328087				PRO2532 [Source:UniProtKB/TrEMBL	25.92933	20.83626	9.56369	2.69727	Detected	Detected	Compromised	Compromised
8825	A_23_P218456	ILF3	Hs.465885	interleukin enhancer binding factor 3, 90	Homo sapiens interleukin enhancer bind	1930.40800	5176.58450	1607.09020	3760.96300	Detected	Detected	Detected	Detected
8826	A_23_P413803	C5orf37	Hs.432726	chromosome 5 open reading frame 37	Homo sapiens chromosome 5 open read	434.48474	351.44806	409.28415	281.03600	Detected	Detected	Detected	Detected
8827	A_23_P4294	ZNF232	Hs.279914	zinc finger protein 232	Homo sapiens zinc finger protein 232 (Z	468.45697	428.88235	406.92260	355.11633	Detected	Detected	Detected	Detected
8828	A_32_P94160	PRKAA2	Hs.437039	protein kinase, AMP-activated, alpha 2	Homo sapiens protein kinase, AMP-acti	53.61281	4.69636	27.89293	2.91112	Detected	Compromised	Detected	Compromised
8829	A_23_P51085	SPC25	Hs.421956	SPC25, NDC80 kinetochore complex co	Homo sapiens SPC25, NDC80 kinetoch	98.13633	11337.70200	62.00933	7774.36960	Detected	Detected	Detected	Detected
8830	A_33_P3391339	LOC100287820	Hs.648247	hypothetical protein LOC100287820	Hypothetical gene supported by BC066	96.22102	37.60498	68.31778	22.60620	Detected	Detected	Detected	Detected
8831	A_32_P103945	B4GALT4	Hs.13225	UDP-Gal:betaGlcNAc beta 1,4- galacto	Homo sapiens UDP-Gal:betaGlcNAc be	1727.02710	2590.91480	1107.91990	1362.22640	Detected	Detected	Detected	Detected
8832	A_33_P3346966	SPAG16	Hs.602792	sperm associated antigen 16	Homo sapiens sperm associated antigen	23.50736	20.42728	55.72788	22.23059	Detected	Detected	Detected	Detected
8833	A_33_P3619221	ZMYND8	Hs.446240	zinc finger, MYND-type containing 8	Homo sapiens zinc finger, MYND-type c	7359.05800	4876.55960	6802.94700	4036.72490	Detected	Detected	Detected	Detected
8834	A_23_P132856	LRRC33	Hs.709536	leucine rich repeat containing 33	Homo sapiens leucine rich repeat conta	49.25140	50.93151	46.79756	51.23081	Detected	Detected	Detected	Detected
8835	A_33_P3280811	TTC16	Hs.642748	tetratricopeptide repeat domain 16	Homo sapiens tetratricopeptide repeat	983.85425	948.38116	260.98782	221.50133	Detected	Detected	Detected	Detected
8836	A_33_P3312258	TUBB	Hs.636480	tubulin, beta	Homo sapiens tubulin, beta (TUBB), mR	6120.86300	21747.45300	5897.70560	18783.91600	Detected	Detected	Detected	Detected
8837	A_23_P10685	HSPBP1	Hs.53066	HSPA (heat shock 70kDa) binding prote	Homo sapiens HSPA (heat shock 70kDa	6246.09960	6976.39840	6265.20400	6284.28860	Detected	Detected	Detected	Detected
8838	A_33_P3303464	C19orf39	Hs.631619	chromosome 19 open reading frame 39	Homo sapiens chromosome 19 open rea	73.43026	29.42701	16.18536	8.19766	Detected	Detected	Detected	Compromised
8839	A_33_P3214491	PDE4DIP	Hs.568247	phosphodiesterase 4D interacting prote	Homo sapiens phosphodiesterase 4D in	56.40102	38.27688	40.23848	31.72549	Detected	Detected	Detected	Detected
8840	A_23_P129058	USP8	Hs.644563	ubiquitin specific peptidase 8	Homo sapiens ubiquitin specific peptida	1076.95800	1100.76300	885.45233	725.56604	Detected	Detected	Detected	Detected
8841	A_33_P3285132	ZNF599	Hs.590961	zinc finger protein 599	Homo sapiens zinc finger protein 599 (Z	34.52332	10.94009	25.91915	14.59877	Detected	Compromised	Detected	Detected
8842	A_33_P3234025				Putative uncharacterized protein C1orf	306.80780	3.22771	297.64487	6.47243	Detected	Compromised	Detected	Compromised
8843	A_33_P3345796		Hs.12102		Sorting nexin-3 (Protein SDP3) [Source	36.14097	23.98878	42.07123	33.04477	Detected	Detected	Detected	Detected
8844	A_24_P371281	TMEM22	Hs.477692	transmembrane protein 22	Homo sapiens transmembrane protein 2	1625.91810	2113.03540	1323.53630	1533.32620	Detected	Detected	Detected	Detected
8845	A_33_P3344831	TMEM45A	Hs.658956	transmembrane protein 45A	Homo sapiens transmembrane protein 4	5340.63770	7123.95600	4120.91500	4871.53960	Detected	Detected	Detected	Detected
8846	A_23_P153084	RALBP1	Hs.719181	ralA binding protein 1	Homo sapiens ralA binding protein 1 (RA	1161.90060	1708.23060	1011.58435	1319.92180	Detected	Detected	Detected	Detected

8847	A_24_P272073					1264.36340	2650.83740	426.88068	656.94714	Detected	Detected	Detected	Detected
8848	A_33_P3296366	PODXL2	Hs.591290	podocalyxin-like 2	Homo sapiens podocalyxin-like 2 (PODX	68.53091	46.82324	70.02786	40.65199	Detected	Detected	Detected	Detected
8849	A_33_P3384710	C17orf63	Hs.564533	chromosome 17 open reading frame 63	Homo sapiens chromosome 17 open rea	8480.22950	11847.52500	2840.81800	1996.32120	Detected	Detected	Detected	Detected
8850	A_33_P3217704	KIAA1539	Hs.301696	KIAA1539	Homo sapiens KIAA1539 (KIAA1539), m	2048.75150	2569.67920	1464.32960	1519.43960	Detected	Detected	Detected	Detected
8851	A_23_P88904	NTHL1	Hs.66196	nth endonuclease III-like 1 (E. coli)	Homo sapiens nth endonuclease III-like	2725.71920	1475.36620	3353.30860	1599.66560	Detected	Detected	Detected	Detected
8852	A_33_P3304212	PLEKHG3	Hs.509637	pleckstrin homology domain containing,	Homo sapiens pleckstrin homology dom	1155.61280	1273.95180	891.12690	885.41780	Detected	Detected	Detected	Detected
8853	A_32_P234738	RPL21	Hs.381123	ribosomal protein L21	Homo sapiens ribosomal protein L21 (R	67232.76000	56611.74000	40246.84000	28541.76600	Detected	Detected	Detected	Detected
8854	A_24_P54178	TMED5	Hs.482873	transmembrane emp24 protein transpor	Homo sapiens transmembrane emp24 p	7314.03860	7448.17330	4152.57000	3377.83200	Detected	Detected	Detected	Detected
8855	A_24_P158385	ZMYND19	Hs.128096	zinc finger, MYND-type containing 19	Homo sapiens zinc finger, MYND-type c	1152.16500	1110.08250	1212.28420	1106.39550	Detected	Detected	Detected	Detected
8856	A_23_P215070	TSGA14	Hs.368315	testis specific, 14	Homo sapiens testis specific, 14 (TSGA	272.22620	427.99533	188.16031	231.37357	Detected	Detected	Detected	Detected
8857	A_33_P3462155	LOC401188	Hs.449289	hypothetical gene supported by AK0910	Homo sapiens cDNA FLJ33694 fis, clon	22.20522	17.16740	9.38955	6.35230	Detected	Compromised	Compromised	Compromised
8858	A_23_P417942	FNBP1L	Hs.134060	formin binding protein 1-like	Homo sapiens formin binding protein 1-	2283.83960	663.13574	1493.06930	335.81310	Detected	Detected	Detected	Detected
8859	A_33_P3377691	C4orf46	Hs.380920	chromosome 4 open reading frame 46	Homo sapiens chromosome 4 open read	291.06967	978.94006	223.33296	638.08420	Detected	Detected	Detected	Detected
8860	A_33_P3339361	ARHGAP11A	Hs.591130	Rho GTPase activating protein 11A	Homo sapiens Rho GTPase activating p	46.56337	979.22546	27.35365	413.81128	Detected	Detected	Detected	Detected
8861	A_33_P3354935	CSF1	Hs.591402	colony stimulating factor 1 (macrophage	Homo sapiens colony stimulating factor	1645.12980	4191.43900	475.55264	841.62700	Detected	Detected	Detected	Detected
8862	A_33_P3239101	GOLGA6L10	Hs.630181	golgi autoantigen, golgin subfamily a, 6-l	Homo sapiens golgi autoantigen, golgin s	578.43463	479.66070	204.69377	117.94792	Detected	Detected	Detected	Detected
8863	A_24_P93309	CDNF	Hs.559067	cerebral dopamine neurotrophic factor	Homo sapiens cerebral dopamine neuro	106.76208	40.32684	70.83563	23.11818	Detected	Detected	Detected	Detected
8864	A_23_P356484	RPS10	Hs.645317	ribosomal protein S10	Homo sapiens ribosomal protein S10 (R	316466.70000	295991.78000	196194.67000	145493.27000	Detected	Detected	Detected	Detected
8865	A_33_P3384988	SLC30A5	Hs.631975	solute carrier family 30 (zinc transporte	Homo sapiens solute carrier family 30 (	44.18130	60.82819	6.21703	13.76596	Detected	Detected	Compromised	Compromised
8866	A_33_P3291831	CEP55	Hs.14559	centrosomal protein 55kDa	Homo sapiens centrosomal protein 55kD	19.57336	1378.80080	10.99036	891.50810	Detected	Detected	Compromised	Detected
8867	A_23_P144807	SEPT8	Hs.522057	septin 8	Homo sapiens septin 8 (SEPT8), transc	614.05994	127.69660	509.69327	114.39742	Detected	Detected	Detected	Detected
8868	A_33_P3564394	SH3GLP2	Hs.684860	SH3-domain GRB2-like pseudogene 2	H.sapiens SH3GLP2 pseudogene, 5' end	109.85579	116.84947	110.11520	116.81472	Detected	Detected	Detected	Detected
8869	A_33_P3322945	SPDYC	Hs.450642	speedy homolog C (Xenopus laevis)	Homo sapiens speedy homolog C (Xeno	90.99760	114.45842	60.56106	53.71029	Detected	Detected	Detected	Detected
8870	A_24_P138022	FAM120A	Hs.372003	family with sequence similarity 120A	Homo sapiens family with sequence sim	834.48175	1089.01560	880.14370	1010.67930	Detected	Detected	Detected	Detected
8871	A_24_P64393	MARCH5	Hs.573490	membrane-associated ring finger (C3HC	Homo sapiens membrane-associated rin	691.86790	703.59900	602.56537	499.67844	Detected	Detected	Detected	Detected
8872	A_24_P219114	SEL1L	Hs.181300	sel-1 suppressor of lin-12-like (C. elega	Protein sel-1 homolog 1 Precursor (Sup	43.78898	70.33588	20.66688	23.67973	Detected	Detected	Detected	Detected
8873	A_23_P115683	HPS6	Hs.125133	Hermansky-Pudlak syndrome 6	Homo sapiens Hermansky-Pudlak syndr	16725.06400	22194.16600	14506.46700	18743.20900	Detected	Detected	Detected	Detected
8874	A_33_P3808996	SPATS2	Hs.654826	spermatogenesis associated, serine-ricl	Homo sapiens spermatogenesis associa	588.90686	552.98456	551.70996	475.85358	Detected	Detected	Detected	Detected
8875	A_33_P3378634					34.05927	6.89628	29.58238	4.72503	Detected	Compromised	Detected	Compromised
8876	A_33_P3401008	TMEM150B	Hs.382625	transmembrane protein 150B	Homo sapiens transmembrane protein 1	21.45020	42.13478	21.50138	41.14356	Detected	Detected	Detected	Detected
8877	A_24_P419028	MOP-1	Hs.679418	MOP-1	Homo sapiens mRNA for MOP-1, compl	6510.58060	9190.93900	2092.91600	1627.08900	Detected	Detected	Detected	Detected
8878	A_23_P251795	GPC2	Hs.211701	glypican 2	Homo sapiens glypican 2 (GPC2), mRNA	424.61820	247.67146	306.92038	137.03505	Detected	Detected	Detected	Detected
8879	A_33_P3371144					6883.32960	9022.40000	7285.25050	5935.90230	Detected	Detected	Detected	Detected
8880	A_33_P3239879	LSMD1	Hs.565094	LSM domain containing 1	Homo sapiens LSM domain containing 1	54745.72000	52897.47700	52041.79700	43901.59000	Detected	Detected	Detected	Detected
8881	A_23_P51213	MYOM3	Hs.523413	myomesin family, member 3	Homo sapiens myomesin family, membe	2.35949	19.18428	11.41953	17.48877	Compromised	Detected	Compromised	Detected
8882	A_33_P3640690	ZEB1	Hs.124503	zinc finger E-box binding homeobox 1	Homo sapiens zinc finger E-box binding	2018.62800	1399.52510	913.16156	550.92490	Detected	Detected	Detected	Detected
8883	A_24_P355876	TMBIM6	Hs.708025	transmembrane BAX inhibitor motif con	Homo sapiens transmembrane BAX inhi	102465.80500	125910.87000	63300.04000	65248.37000	Detected	Detected	Detected	Detected
8884	A_33_P3321781	FLJ44342	Hs.710026	hypothetical LOC645460	PREDICTED: Homo sapiens hypothetica	7158.82760	4065.86200	4283.61570	2162.51170	Detected	Detected	Detected	Detected
8885	A_33_P3255824					4.28859	137.94330	7.36967	60.84053	Compromised	Detected	Compromised	Detected
8886	A_33_P3268310	LIMS3	Hs.535619	LIM and senescent cell antigen-like dor	Homo sapiens LIM and senescent cell a	293.52295	727.04333	319.16278	737.34863	Detected	Detected	Detected	Detected
8887	A_33_P3224675		Hs.440371		tRNA guanosine-2'-O-methyltransferas	41.67744	53.01451	35.00890	27.54780	Detected	Detected	Detected	Detected
8888	A_23_P8561	RHBDD2	Hs.488827	rhomboid domain containing 2	Homo sapiens rhomboid domain contain	3251.82350	1958.55580	3340.44340	1952.87130	Detected	Detected	Detected	Detected
8889	A_33_P3221443	ZNF254	Hs.434406	zinc finger protein 254	Homo sapiens zinc finger protein 254 (Z	411.61700	189.41751	339.35052	128.72488	Detected	Detected	Detected	Detected
8890	A_24_P16378	HGSNAT	Hs.600384	heparan-alpha-glucosaminide N-acetylt	Homo sapiens heparan-alpha-glucosam	54.65733	41.16343	43.88644	41.41850	Detected	Detected	Detected	Detected
8891	A_32_P118586	FAM116A	Hs.91085	family with sequence similarity 116, mer	Homo sapiens family with sequence sim	2799.50730	3546.60740	2151.00630	2169.15600	Detected	Detected	Detected	Detected
8892	A_23_P76882	CCNB1IP1	Hs.107003	cyclin B1 interacting protein 1	Homo sapiens cyclin B1 interacting prot	5434.20460	4261.96630	6240.19400	3836.06840	Detected	Detected	Detected	Detected
8893	A_33_P3241046					12.58404	15.51610	4.29955	19.82121	Detected	Compromised	Compromised	Detected
8894	A_24_P229638	C9orf117	Hs.652519	chromosome 9 open reading frame 117	Homo sapiens chromosome 9 open read	2.38750	3.19144	6.93065	13.64372	Compromised	Compromised	Compromised	Detected
8895	A_33_P3262758	UNC93B1	Hs.502989	unc-93 homolog B1 (C. elegans)	Homo sapiens unc-93 homolog B1 (C. e	385.88974	337.30640	395.20650	337.20030	Detected	Detected	Detected	Detected
8896	A_33_P3418466	LOC391578	Hs.633991	similar to MAF1 homolog (S. cerevisiae)	PREDICTED: Homo sapiens similar to M	121.14138	119.19880	67.48407	54.98649	Detected	Detected	Detected	Detected
8897	A_33_P3358745	SEPP1	Hs.275775	selenoprotein P, plasma, 1	Homo sapiens selenoprotein P, plasma,	2137.99700	467.51993	1985.97560	281.98600	Detected	Detected	Detected	Detected
8898	A_23_P410859	ZSWIM6	Hs.720283	zinc finger, SWIM-type containing 6	Homo sapiens zinc finger, SWIM-type c	1551.73100	928.61470	1345.32920	716.03380	Detected	Detected	Detected	Detected
8899	A_33_P3354678	TRIMP1	Hs.709401	tripartite motif-containing pseudogene	Homo sapiens tripartite motif-containin	33.58771	53.77036	32.62042	31.71368	Detected	Detected	Detected	Detected
8900	A_23_P91636	POM121L9P	Hs.318898	POM121 membrane glycoprotein-like 9	Homo sapiens POM121 membrane glyco	257.61490	67.68008	229.35724	50.95446	Detected	Detected	Detected	Detected
8901	A_33_P3319502					33038.22300	11561.68200	31721.59000	10837.98900	Detected	Detected	Detected	Detected
8902	A_33_P3340105	LOC646201	Hs.646643	brain abundant, membrane attached sig	PREDICTED: Homo sapiens brain abund	194.30806	291.38095	231.19063	356.85345	Detected	Detected	Detected	Detected
8903	A_23_P18205	RAD54L2	Hs.105399	RAD54-like 2 (S. cerevisiae)	Homo sapiens RAD54-like 2 (S. cerevis	1954.86170	2025.54660	1222.80980	1114.21940	Detected	Detected	Detected	Detected
8904	A_33_P3268798	ADAT2	Hs.709561	adenosine deaminase, tRNA-specific 2,	Homo sapiens adenosine deaminase, tR	288.07535	165.87480	205.15196	104.84958	Detected	Detected	Detected	Detected
8905	A_32_P229132	FMN2	Hs.24889	formin 2	Homo sapiens formin 2 (FMN2), mRNA	533.05120	1047.60960	430.86496	672.96080	Detected	Detected	Detected	Detected
8906	A_23_P1948	C11orf48	Hs.9061	chromosome 11 open reading frame 48	Homo sapiens chromosome 11 open rea	6465.42800	9541.08300	5397.54100	6609.41400	Detected	Detected	Detected	Detected
8907	A_33_P3285877				Chromosome 9 open reading frame 146	36.17161	39.83929	13.95089	13.22918	Detected	Detected	Detected	Compromised
8908	A_24_P408736	GALNT5	Hs.269027	UDP-N-acetyl-alpha-D-galactosamine:	Homo sapiens UDP-N-acetyl-alpha-D-	230.25905	604.21027	148.92079	399.62228	Detected	Detected	Detected	Detected
8909	A_33_P3404729	LOC642350	Hs.632647	similar to Coagulation factor V precurso	PREDICTED: Homo sapiens similar to C	14.27679	67.87863	8.64606	46.02578	Detected	Detected	Compromised	Detected
8910	A_33_P3389758					21.07979	23.56560	17.62566	10.80712	Detected	Detected	Detected	Compromised
8911	A_33_P3363804	NCAM1	Hs.503878	neural cell adhesion molecule 1	Homo sapiens neural cell adhesion mole	26.83234	24.75264	18.45216	13.70385	Detected	Detected	Detected	Compromised
8912	A_33_P3289025	FAM176A	Hs.302346	family with sequence similarity 176, mer	Homo sapiens family with sequence sim	284.20447	895.92834	429.49830	1077.21010	Detected	Detected	Detected	Detected
8913	A_24_P273865	MED20	Hs.278434	mediator complex subunit 20	Homo sapiens mediator complex subuni	557.14280	752.72437	405.82230	436.22525	Detected	Detected	Detected	Detected
8914	A_23_P134935	DUSP4	Hs.417962	dual specificity phosphatase 4	Homo sapiens dual specificity phosphat	563.96454	1482.05210	312.40347	927.73850	Detected	Detected	Detected	Detected
8915	A_33_P3351249	CXCL16	Hs.513885	chemokine (C-X-C motif) ligand 16	Homo sapiens chemokine (C-X-C motif	214.68492	38.27278	215.68155	37.75723	Detected	Detected	Detected	Detected
8916	A_23_P39542	C2orf76	Hs.99488	chromosome 2 open reading frame 76	Homo sapiens chromosome 2 open read	834.34607	486.34897	739.48126	365.89755	Detected	Detected	Detected	Detected
8917	A_23_P202978	CASP1	Hs.2490	caspase 1, apoptosis-related cysteine p	Homo sapiens caspase 1, apoptosis-rela	730.93840	465.22165	649.85590	324.10486	Detected	Detected	Detected	Detected
8918	A_23_P55256	ZNF652	Hs.463375	zinc finger protein 652	Homo sapiens zinc finger protein 652 (Z	435.97415	255.73598	431.87240	289.31580	Detected	Detected	Detected	Detected
8919	A_24_P128524	ICMT	Hs.515688	isoprenylcysteine carboxyl methyltransf	Homo sapiens isoprenylcysteine carbox	2295.48490	3307.07600	468.72522	552.92426	Detected	Detected	Detected	Detected
8920	A_33_P3247473	KRTAP23-1	Hs.553688	keratin associated protein 23-1	Homo sapiens keratin associated protei	53.59001	86.29829	68.74181	64.72795	Detected	Detected	Detected	Detected
8921	A_33_P3420704	LOC100130131		hypothetical LOC100130131	PREDICTED: Homo sapiens hypothetica	26.65978	47.71196	26.77594	53.58603	Detected	Detected	Detected	Detected

8922	A_33_P3270599	TPM2	Hs.300772	tropomyosin 2 (beta)	Homo sapiens tropomyosin 2 (beta) (TP	69898.86000	154134.22000	63249.09400	121393.99000	Detected	Detected	Detected	Detected
8923	A_33_P3364062	FLJ31356	Hs.562970	hypothetical protein FLJ31356	Homo sapiens cDNA FLJ31356 fis, clon	22.31790	3.28466	16.45729	2.79828	Detected	Compromised	Detected	Compromised
8924	A_33_P3278801	LRCH3	Hs.518414	leucine-rich repeats and calponin homo	Homo sapiens cDNA FLJ44976 fis, clon	38.73059	25.80026	20.39835	13.29018	Detected	Detected	Detected	Compromised
8925	A_23_P83328	ENG	Hs.76753	endoglin	Homo sapiens endoglin (ENG), transcrip	1537.30470	3593.05200	1367.05350	3361.78030	Detected	Detected	Detected	Detected
8926	A_33_P3356406	SOS1	Hs.709893	son of sevenless homolog 1 (Drosophila	Homo sapiens son of sevenless homolog	178.81662	221.97778	108.32948	114.66599	Detected	Detected	Detected	Detected
8927	A_24_P221883	PARL	Hs.478469	presenilin associated, rhomboid-like	Homo sapiens presenilin associated, rho	3142.60550	4033.07890	3304.55860	3758.93000	Detected	Detected	Detected	Detected
8928	A_24_P120934	GADD45G	Hs.9701	growth arrest and DNA-damage-inducib	Homo sapiens growth arrest and DNA-c	115.22611	42.43369	148.78069	38.89514	Detected	Detected	Detected	Detected
8929	A_33_P3345132	ZNF578	Hs.157287	zinc finger protein 578	Homo sapiens zinc finger protein 578 (Z	36.03907	48.12175	36.40131	34.79262	Detected	Detected	Detected	Detected
8930	A_24_P60972	TSTD2	Hs.404289	thiosulfate sulfurtransferase (rhodanese	Homo sapiens thiosulfate sulfurtransfer	485.32993	407.55710	341.69513	255.15408	Detected	Detected	Detected	Detected
8931	A_32_P133518	SUMO1	Hs.596171	SMT3 suppressor of mif two 3 homolog	AL565221 Homo sapiens FETAL BRAIN	109.69662	116.71059	92.59168	74.53316	Detected	Detected	Detected	Detected
8932	A_33_P3347787	LOC652022		similar to skeletrophin	PREDICTED: Homo sapiens similar to m	30.83417	36.75079	19.20950	18.17481	Detected	Detected	Detected	Detected
8933	A_24_P254833		Hs.546371		Homo sapiens mRNA for T cell recepto	7.71964	10.30891	15.66659	2.69870	Compromised	Compromised	Detected	Compromised
8934	A_23_P96833	SLAMF9	Hs.661712	SLAM family member 9	Homo sapiens SLAM family member 9 (	439.70224	967.30347	492.34583	1105.86170	Detected	Detected	Detected	Detected
8935	A_32_P100830	KIF19	Hs.372773	kinesin family member 19	Homo sapiens kinesin family member 19	22.98185	25.42072	19.44624	13.00870	Detected	Detected	Detected	Compromised
8936	A_33_P3438201		Hs.721489		603032464F1 NIH_MGC_115 Homo sapie	124.25044	166.30183	64.63117	67.23479	Detected	Detected	Detected	Detected
8937	A_23_P13359	NXF1	Hs.523739	nuclear RNA export factor 1	Homo sapiens nuclear RNA export facto	3361.44680	3478.05660	2526.62650	2516.32900	Detected	Detected	Detected	Detected
8938	A_23_P2283	TAC3	Hs.9730	tachykinin 3	Homo sapiens tachykinin 3 (TAC3), mRI	17.97314	9.01501	20.42554	11.53785	Detected	Compromised	Detected	Compromised
8939	A_33_P3272231	MFSD2A	Hs.655177	major facilitator superfamily domain cor	Homo sapiens major facilitator superfan	2591.77800	2055.87800	1279.69120	855.73267	Detected	Detected	Detected	Detected
8940	A_23_P422018	SPRR4	Hs.433629	small proline-rich protein 4	Homo sapiens small proline-rich protein	2.34971	3.13589	25.88413	8.40341	Compromised	Compromised	Detected	Compromised
8941	A_24_P330796	MKS1	Hs.408843	Meckel syndrome, type 1	Homo sapiens Meckel syndrome, type 1	147.06973	100.64389	176.77188	120.43264	Detected	Detected	Detected	Detected
8942	A_23_P107801	C19orf44	Hs.631627	chromosome 19 open reading frame 44	Homo sapiens chromosome 19 open rea	796.50670	586.84534	746.11140	400.19833	Detected	Detected	Detected	Detected
8943	A_33_P3250669					14.95861	11.42217	13.03236	7.82995	Detected	Compromised	Detected	Compromised
8944	A_33_P3354214					20.14956	11.79106	8.11381	8.50556	Detected	Compromised	Compromised	Compromised
8945	A_23_P141345	MPP3	Hs.396566	membrane protein, palmitoylated 3 (MA(	Homo sapiens membrane protein, palmit	2040.09960	4084.95970	1409.60930	2240.49600	Detected	Detected	Detected	Detected
8946	A_33_P3350259	FAM129C	Hs.434133	family with sequence similarity 129, mer	Homo sapiens family with sequence sim	21.30033	19.01962	15.61474	13.95435	Detected	Detected	Detected	Detected
8947	A_23_P128855	SLC39A2	Hs.175783	solute carrier family 39 (zinc transporte	Homo sapiens solute carrier family 39 (:	27.09677	28.76322	20.30783	16.82905	Detected	Detected	Detected	Detected
8948	A_33_P3252444	LOC651581		similar to deleted in malignant brain tum	PREDICTED: Homo sapiens hypothetica	3.99780	3.14716	12.62833	9.45446	Compromised	Compromised	Detected	Compromised
8949	A_23_P23141	H3F3A	Hs.533624	H3 histone, family 3A	Homo sapiens H3 histone, family 3A (H3	78691.17000	63170.79000	79881.62000	55693.29000	Detected	Detected	Detected	Detected
8950	A_23_P137097	SLC16A2	Hs.75317	solute carrier family 16, member 2 (mon	Homo sapiens solute carrier family 16, r	2224.80620	7456.44900	1511.55740	4380.56700	Detected	Detected	Detected	Detected
8951	A_33_P3388061	KRTAP21-2	Hs.553699	keratin associated protein 21-2	Homo sapiens keratin associated protei	14.36426	6.83358	20.55763	4.39867	Detected	Compromised	Detected	Compromised
8952	A_33_P3407529	PRRT4	Hs.381096	proline-rich transmembrane protein 4	Homo sapiens proline-rich transmembra	63.65750	21.60231	45.81823	10.73814	Detected	Detected	Detected	Compromised
8953	A_33_P3318646	CALY	Hs.148680	calcyon neuron-specific vesicular prote	Homo sapiens calcyon neuron-specific	128143.23000	140992.50000	129435.21000	122436.51000	Detected	Detected	Detected	Detected
8954	A_33_P3368506	SLC38A8	Hs.461575	solute carrier family 38, member 8	Homo sapiens solute carrier family 38, r	4.39715	3.64497	9.36415	20.30222	Compromised	Compromised	Compromised	Detected
8955	A_24_P18270	UCMA	Hs.112998	upper zone of growth plate and cartilag	Homo sapiens upper zone of growth pla	14.95715	11.58529	17.03761	12.54113	Detected	Compromised	Detected	Compromised
8956	A_23_P167298	C4orf17	Hs.97501	chromosome 4 open reading frame 17	Homo sapiens chromosome 4 open read	5.27288	10.43850	14.93718	18.07043	Compromised	Compromised	Detected	Detected
8957	A_33_P3338360	SCARNA13	Hs.722636	small Cajal body-specific RNA 13	Homo sapiens small Cajal body-specific	767.83624	432.57953	558.74646	269.43445	Detected	Detected	Detected	Detected
8958	A_33_P3376527	CHST10	Hs.516370	carbohydrate sulfotransferase 10	Homo sapiens carbohydrate sulfotransfe	36.19944	105.37380	34.63421	94.32127	Detected	Detected	Detected	Detected
8959	A_23_P372467	AHSA2	Hs.655602	AHA1, activator of heat shock 90kDa pı	Homo sapiens AHA1, activator of heat s	5222.73140	1781.54480	4575.80270	1330.98340	Detected	Detected	Detected	Detected
8960	A_33_P3345663	KLRAQ1	Hs.654619	KLRAQ motif containing 1	Homo sapiens KLRAQ motif containing	335.16312	214.70146	309.87125	144.13191	Detected	Detected	Detected	Detected
8961	A_33_P3281686	TSC2	Hs.90303	tuberous sclerosis 2	Homo sapiens tuberous sclerosis 2 (TS(	36.34605	30.70144	37.39202	22.98943	Detected	Detected	Detected	Detected
8962	A_23_P44264	EMX2	Hs.202095	empty spiracles homeobox 2	Homo sapiens empty spiracles homeobo	2820.60720	2387.52500	2058.72020	1614.14050	Detected	Detected	Detected	Detected
8963	A_33_P3340007	LOC220429	Hs.646637	CTAGE family, member 5 pseudogene	Homo sapiens CTAGE family, member 5	109.19511	103.41053	75.34980	58.92254	Detected	Detected	Detected	Detected
8964	A_32_P24372	TBC1D3B	Hs.454713	TBC1 domain family, member 3B	Homo sapiens TBC1 domain family, mer	217.75290	34.82607	40.65864	6.02993	Detected	Detected	Detected	Compromised
8965	A_33_P3240543	AGAP3	Hs.647075	ArfGAP with GTPase domain, ankyrin re	Homo sapiens ArfGAP with GTPase dor	8167.95400	13878.78200	7197.81840	10711.35400	Detected	Detected	Detected	Detected
8966	A_33_P3542801	LOC729291	Hs.129278	hypothetical protein LOC729291	PREDICTED: Homo sapiens hypothetica	151.06679	129.46368	82.86876	46.90983	Detected	Detected	Detected	Detected
8967	A_33_P3383287	LOC440839	Hs.722241	tigger transposable element derived 1 p	Homo sapiens tigger transposable elem	448.47348	414.20535	189.99982	181.86641	Detected	Detected	Detected	Detected
8968	A_33_P3419970	NPB	Hs.708916	neuropeptide B	Homo sapiens neuropeptide B (NPB), m	18705.38300	22628.29700	6762.08000	5111.40040	Detected	Detected	Detected	Detected
8969	A_23_P139099	OR4A15	Hs.554529	olfactory receptor, family 4, subfamily A	Homo sapiens olfactory receptor, family	25.80156	39.64533	23.01840	27.90012	Detected	Detected	Detected	Detected
8970	A_33_P3551349	LOC439911		hypothetical gene supported by NM_194	Homo sapiens cDNA FLJ45542 fis, clon	324.43988	444.04330	236.78119	217.92348	Detected	Detected	Detected	Detected
8971	A_33_P3239527	LOC100128517	Hs.484706	hypothetical protein LOC100128517	Homo sapiens cDNA FLJ44820 fis, clon	25.89623	50.22429	24.27096	49.82145	Detected	Detected	Detected	Detected
8972	A_33_P3237879		Hs.532673			6.70618	11.35149	14.88185	7.26131	Compromised	Compromised	Detected	Compromised
8973	A_23_P162782	ARGLU1	Hs.508644	arginine and glutamate rich 1	Homo sapiens arginine and glutamate ri	1610.52530	1009.30800	1323.00010	760.01460	Detected	Detected	Detected	Detected
8974	A_33_P3231297	CREG1	Hs.5710	cellular repressor of E1A-stimulated ge	Homo sapiens cellular repressor of E1A	18655.48400	6743.64400	12225.44600	3982.93630	Detected	Detected	Detected	Detected
8975	A_23_P329375	POU6F1	Hs.555886	POU class 6 homeobox 1	Homo sapiens POU class 6 homeobox 1	469.63860	171.95833	342.09560	115.01604	Detected	Detected	Detected	Detected
8976	A_23_P157136	SCIN	Hs.655515	scinderin	Homo sapiens scinderin (SCIN), transcr	2.38629	88.41367	5.01626	34.16774	Compromised	Detected	Compromised	Detected
8977	A_32_P36694	JAZF1	Hs.368944	JAZF zinc finger 1	Homo sapiens JAZF zinc finger 1 (JAZF	1159.16720	876.90720	635.96360	367.53262	Detected	Detected	Detected	Detected
8978	A_33_P3287253	LOC100128401	Hs.567547	hypothetical protein LOC100128401	Homo sapiens cDNA FLJ42506 fis, clon	2.38991	3.23787	43.84176	5.57333	Compromised	Compromised	Detected	Compromised
8979	A_23_P391857	ESRRB	Hs.435845	estrogen-related receptor beta	Homo sapiens estrogen-related recepto	50.07010	60.34140	34.50986	37.32019	Detected	Detected	Detected	Detected
8980	A_23_P155103	ADSL	Hs.75527	adenylosuccinate lyase	Homo sapiens adenylosuccinate lyase (/	1015.10710	3486.59470	1118.49280	3363.38940	Detected	Detected	Detected	Detected
8981	A_33_P3276250	TMEM191C	Hs.645547	transmembrane protein 191C	Homo sapiens cDNA FLJ36990 fis, clon	12.25225	17.59095	12.07616	11.29773	Detected	Detected	Compromised	Compromised
8982	A_33_P3255001	LOC152024		hypothetical LOC152024	Homo sapiens hypothetical LOC152024	15.11448	14.04020	7.74070	11.14301	Detected	Compromised	Compromised	Compromised
8983	A_24_P940517	PCYOX1	Hs.567502	prenylcysteine oxidase 1	Homo sapiens prenylcysteine oxidase 1	1528.57690	1852.48060	990.74550	1073.69810	Detected	Detected	Detected	Detected
8984	A_23_P151895	CILP	Hs.720636	cartilage intermediate layer protein, nuc	Homo sapiens cartilage intermediate lay	76.52758	95.91911	103.25919	63.44126	Detected	Detected	Detected	Detected
8985	A_33_P3211809	RUNX1	Hs.149261	runt-related transcription factor 1	Homo sapiens runt-related transcriptior	82.81162	146.88211	72.93238	126.22569	Detected	Detected	Detected	Detected
8986	A_23_P309515	KCTD18	Hs.605775	potassium channel tetramerisation dom	Homo sapiens potassium channel tetrar	649.83570	362.33770	684.28620	314.66300	Detected	Detected	Detected	Detected
8987	A_33_P3321130	DENND3		DENN/MADD domain containing 3	Homo sapiens cDNA FLJ43517 fis, clon	264.49597	235.78024	176.16785	131.37354	Detected	Detected	Detected	Detected
8988	A_33_P3226080	C17orf87	Hs.462080	chromosome 17 open reading frame 87	Homo sapiens chromosome 17 open rea	16.37831	22.38042	17.63978	16.15610	Detected	Detected	Detected	Detected
8989	A_33_P3365932	WASH1	Hs.709408	WAS protein family homolog 1	Homo sapiens WAS protein family homo	1048.55810	782.25440	822.56820	484.78290	Detected	Detected	Detected	Detected
8990	A_33_P3452003	LOC143286	Hs.705563	hypothetical protein LOC143286	Homo sapiens mRNA; cDNA DKFZp586I	236.77313	385.19373	142.60289	163.57360	Detected	Detected	Detected	Detected
8991	A_33_P3270514	NBLA00301	Hs.61435	Nbla00301	Homo sapiens Nbla00301 (NBLA00301),	4.17646	130.49452	10.87418	80.82700	Compromised	Detected	Compromised	Detected
8992	A_33_P3414689				Homo sapiens FP15529 mRNA, complet	139.76915	111.89791	91.51395	72.28605	Detected	Detected	Detected	Detected
8993	A_24_P66545	KIAA0226	Hs.478868	KIAA0226	Homo sapiens KIAA0226 (KIAA0226), tr	8.19863	27.75651	11.03095	27.44350	Compromised	Detected	Compromised	Detected
8994	A_24_P23245	NDUFA6	Hs.274416	NADH dehydrogenase (ubiquinone) 1 alp	Homo sapiens NADH dehydrogenase (ul	13940.26800	21299.63300	9799.63700	11330.94300	Detected	Detected	Detected	Detected
8995	A_33_P3319937	LOC100291206		hypothetical protein LOC100291206	PREDICTED: Homo sapiens hypothetica	21443.11000	24464.71700	54543.43400	52855.67000	Detected	Detected	Detected	Detected
8996	A_23_P160559	ECM1	Hs.81071	extracellular matrix protein 1	Homo sapiens extracellular matrix prote	5502.98900	5784.90800	5280.28370	5071.20900	Detected	Detected	Detected	Detected

8997	A_33_P3287502	MSH2	Hs.597656	mutS homolog 2, colon cancer, nonpoly	Homo sapiens mutS homolog 2, colon c	1300.38990	1381.21080	1066.53590	1007.42830	Detected	Detected	Detected	Detected
8998	A_33_P3214466	MESP1	Hs.447531	mesoderm posterior 1 homolog (mouse)	Homo sapiens mesoderm posterior 1 ho	189.52153	102.79931	156.76414	65.50794	Detected	Detected	Detected	Detected
8999	A_33_P3220105	LOC644246	Hs.644600	hypothetical protein LOC644246	LOC644246 protein Fragment [Source:	153.64711	67.84344	235.44266	70.49780	Detected	Detected	Detected	Detected
9000	A_33_P3362503	ISPD	Hs.636502	isoprenoid synthase domain containing	Homo sapiens isoprenoid synthase dom	4.70097	30.35291	4.87257	30.23124	Compromised	Detected	Compromised	Detected
9001	A_23_P70509	C6orf134	Hs.654798	chromosome 6 open reading frame 134	Homo sapiens chromosome 6 open read	13.50238	17.10863	10.89710	12.83652	Detected	Detected	Compromised	Compromised
9002	A_23_P164797	ZNF580	Hs.631551	zinc finger protein 580	Homo sapiens zinc finger protein 580 (Z	4439.21800	3549.48050	4746.80400	3243.26440	Detected	Detected	Detected	Detected
9003	A_23_P356616	ABTB2	Hs.23361	ankyrin repeat and BTB (POZ) domain c	Homo sapiens ankyrin repeat and BTB (	436.00067	1741.04930	367.96410	1164.68520	Detected	Detected	Detected	Detected
9004	A_32_P82623	AGBL3	Hs.648616	ATP/GTP binding protein-like 3	Homo sapiens ATP/GTP binding protein	1040.30300	1240.64400	598.02423	485.06850	Detected	Detected	Detected	Detected
9005	A_33_P3218178	MCART6	Hs.660022	mitochondrial carrier triple repeat 6	Homo sapiens mitochondrial carrier trip	22.99856	20.72115	2.72509	2.83277	Detected	Detected	Compromised	Compromised
9006	A_23_P41304	GYPE	Hs.709199	glycophorin E	Homo sapiens glycophorin E (GYPE), tra	52.15321	75.18969	41.18375	25.95681	Detected	Detected	Detected	Detected
9007	A_24_P191417	NAB1	Hs.107474	NGFI-A binding protein 1 (EGR1 binding	Homo sapiens NGFI-A binding protein 1	2311.19800	677.54900	1538.06290	365.98004	Detected	Detected	Detected	Detected
9008	A_24_P690924	SEC23B	Hs.369373	Sec23 homolog B (S. cerevisiae)	Homo sapiens Sec23 homolog B (S. cer	1316.43190	1351.96100	1051.68120	983.30290	Detected	Detected	Detected	Detected
9009	A_33_P3228315	CARD14	Hs.696253	caspase recruitment domain family, mer	Homo sapiens caspase recruitment dom	1683.69690	1925.18190	653.17080	651.96340	Detected	Detected	Detected	Detected
9010	A_33_P3317473					3637.81250	2633.71170	176.60439	94.79046	Detected	Detected	Detected	Detected
9011	A_33_P3341474	ZBTB38	Hs.518301	zinc finger and BTB domain containing	Homo sapiens zinc finger and BTB dom	223.92850	134.39746	168.57712	89.57682	Detected	Detected	Detected	Detected
9012	A_23_P408285	PRICKLE1	Hs.720221	prickle homolog 1 (Drosophila)	Homo sapiens prickle homolog 1 (Droso	443.67276	378.36502	366.94904	230.78912	Detected	Detected	Detected	Detected
9013	A_33_P3237552	FIBCD1	Hs.133205	fibrinogen C domain containing 1	Homo sapiens fibrinogen C domain cont	208.78088	264.33865	105.37532	114.83402	Detected	Detected	Detected	Detected
9014	A_32_P54242	LCORL	Hs.446201	ligand dependent nuclear receptor core	Homo sapiens ligand dependent nuclear	88.31808	52.12592	71.13213	38.50362	Detected	Detected	Detected	Detected
9015	A_24_P73669	GSPT1	Hs.528780	G1 to S phase transition 1	Homo sapiens G1 to S phase transition	807.27190	2167.50200	718.55110	1692.36490	Detected	Detected	Detected	Detected
9016	A_24_P915692	PHLDA1	Hs.602085	pleckstrin homology-like domain, family	Homo sapiens pleckstrin homology-like	7209.12740	7980.37160	4503.84600	4485.15600	Detected	Detected	Detected	Detected
9017	A_32_P93149	DDX10	Hs.591931	DEAD (Asp-Glu-Ala-Asp) box polypept	Homo sapiens DEAD (Asp-Glu-Ala-Asp	95.29058	119.31736	22.62365	28.31091	Detected	Detected	Detected	Detected
9018	A_23_P5200	NPHS1	Hs.122186	nephrosis 1, congenital, Finnish type (ne	Homo sapiens nephrosis 1, congenital, F	28.92750	45.77320	21.24429	52.33985	Detected	Detected	Detected	Detected
9019	A_33_P3279475				PRO2007 [Source:UniProtKB/TrEMBL	27.20094	23.41501	19.54409	17.09517	Detected	Detected	Detected	Detected
9020	A_23_P2223	MYL6B	Hs.632731	myosin, light chain 6B, alkali, smooth mu	Homo sapiens myosin, light chain 6B, al	12724.35100	7507.69870	18266.10400	9448.38800	Detected	Detected	Detected	Detected
9021	A_24_P13475	SPATA13	Hs.595391	spermatogenesis associated 13	Homo sapiens spermatogenesis associa	367.62497	159.54680	343.94590	141.71303	Detected	Detected	Detected	Detected
9022	A_33_P3261927	ZNF789	Hs.440384	zinc finger protein 789	Homo sapiens zinc finger protein 789 (Z	133.35242	220.98602	111.51962	179.44449	Detected	Detected	Detected	Detected
9023	A_33_P3437273	MGC15705	Hs.652898	hypothetical protein MGC15705	Homo sapiens hypothetical protein MGC	19.73402	25.43211	41.13441	16.92933	Detected	Detected	Detected	Detected
9024	A_23_P47879	STAT6	Hs.524518	signal transducer and activator of trans	Homo sapiens signal transducer and act	8510.07700	8617.16800	6537.66000	5803.63040	Detected	Detected	Detected	Detected
9025	A_23_P66306	ALG1	Hs.592086	asparagine-linked glycosylation 1, beta-	Homo sapiens asparagine-linked glycosy	3420.41330	4800.15300	2566.20950	3407.20970	Detected	Detected	Detected	Detected
9026	A_23_P428887	KLHL34	Hs.448572	kelch-like 34 (Drosophila)	Homo sapiens kelch-like 34 (Drosophila	18.63266	19.62713	10.56031	9.36225	Detected	Detected	Compromised	Compromised
9027	A_23_P156310	SKP2	Hs.23348	S-phase kinase-associated protein 2 (p	Homo sapiens S-phase kinase-associat	266.01180	797.66907	227.98456	623.54360	Detected	Detected	Detected	Detected
9028	A_24_P41570	H2AFZ	Hs.119192	H2A histone family, member Z	Homo sapiens H2A histone family, mem	16723.64800	51055.71500	17300.33400	42940.54300	Detected	Detected	Detected	Detected
9029	A_33_P3266091	LOC100133839		hypothetical protein LOC100133839	PREDICTED: Homo sapiens hypothetica	16.39120	25.87657	13.38062	5.26575	Detected	Detected	Detected	Compromised
9030	A_33_P3282973				Brain and acute leukemia cytoplasmic p	127.08301	322.33957	113.78412	352.37973	Detected	Detected	Detected	Detected
9031	A_24_P35400	SARDH	Hs.198003	sarcosine dehydrogenase	Homo sapiens sarcosine dehydrogenase	10.49168	6.42893	21.96643	11.60515	Compromised	Compromised	Detected	Compromised
9032	A_33_P3680789	LOC219731	Hs.585473	hypothetical protein LOC219731	Homo sapiens cDNA FLJ32141 fis, clon	4.30552	9.25785	20.15212	3.89611	Compromised	Compromised	Detected	Compromised
9033	A_33_P3364348	JPH1	Hs.657367	junctophilin 1	Homo sapiens junctophilin 1 (JPH1), mR	131.98358	14.71776	77.05670	10.21930	Detected	Compromised	Detected	Compromised
9034	A_33_P3416687				GB	361.41077	267.26694	119.65700	43.85080	Detected	Detected	Detected	Detected
9035	A_33_P3321462	OR5B21	Hs.553647	olfactory receptor, family 5, subfamily B	Homo sapiens olfactory receptor, family	7.70297	12.43901	13.03679	6.69399	Compromised	Compromised	Detected	Compromised
9036	A_24_P144303	LOC442459	Hs.706599	X-ray repair complementing defective r	Homo sapiens X-ray repair complement	634.41003	870.16160	19.13367	13.59370	Detected	Detected	Detected	Detected
9037	A_33_P3350508	FTO	Hs.528833	fat mass and obesity associated	Homo sapiens fat mass and obesity ass	64.18758	96.45519	59.61289	103.48853	Detected	Detected	Detected	Detected
9038	A_23_P421811	C20orf132	Hs.349125	chromosome 20 open reading frame 132	Homo sapiens chromosome 20 open rea	269.55347	21.72731	216.83226	20.70338	Detected	Detected	Detected	Detected
9039	A_23_P116850	KRT2	Hs.707	keratin 2	Homo sapiens keratin 2 (KRT2), mRNA	2.40478	3.21113	2.69493	20.31255	Compromised	Compromised	Compromised	Not Detected
9040	A_33_P3417305	LOC645225		similar to ATP synthase, H+ transportin	PREDICTED: Homo sapiens similar to A	433.88000	94.69554	309.63763	10.96050	Detected	Detected	Detected	Compromised
9041	A_23_P201711	S100A6	Hs.275243	S100 calcium binding protein A6	Homo sapiens S100 calcium binding pro	450185.16000	334624.22000	305397.22000	214127.81000	Detected	Detected	Detected	Detected
9042	A_23_P92727	RAI14	Hs.431400	retinoic acid induced 14	Homo sapiens retinoic acid induced 14 (	4647.59030	7224.56050	4662.52050	5978.60160	Detected	Detected	Detected	Detected
9043	A_24_P307135	TNXB	Hs.485104	tenascin XB	Homo sapiens tenascin XB (TNXB), tran	141.70418	134.83028	86.74541	52.29593	Detected	Detected	Detected	Detected
9044	A_32_P113566	BTBD9	Hs.603858	BTB (POZ) domain containing 9	Homo sapiens BTB (POZ) domain conta	9.86783	23.35382	6.20850	9.95000	Compromised	Detected	Compromised	Compromised
9045	A_23_P143190	MYBL2	Hs.179718	v-myb myeloblastosis viral oncogene ho	Homo sapiens v-myb myeloblastosis vir	247.45096	2326.74340	103.16248	2345.65940	Detected	Detected	Detected	Detected
9046	A_33_P3325547	C10orf53	Hs.131287	chromosome 10 open reading frame 53	Homo sapiens chromosome 10 open rea	22.71774	25.46579	17.36243	22.74655	Detected	Detected	Detected	Detected
9047	A_23_P89727	CTDP1	Hs.465490	CTD (carboxy-terminal domain, RNA po	Homo sapiens CTD (carboxy-terminal d	231.29977	227.02936	227.58139	274.22867	Detected	Detected	Detected	Detected
9048	A_23_P101905	APC2	Hs.446376	adenomatosis polyposis coli 2	Homo sapiens adenomatosis polyposis c	341.53775	175.93759	353.33115	173.60617	Detected	Detected	Detected	Detected
9049	A_33_P3221418	NTNG1	Hs.657434	netrin G1	Homo sapiens netrin G1 (NTNG1), trans	12.31479	3.32254	16.20202	6.56387	Compromised	Compromised	Detected	Compromised
9050	A_33_P3365870	BSDC1	Hs.353454	BSD domain containing 1	Homo sapiens BSD domain containing 1	63.55568	58.12148	92.44508	62.30201	Detected	Detected	Detected	Detected
9051	A_33_P3421923	CADM3	Hs.365689	cell adhesion molecule 3	Homo sapiens cell adhesion molecule 3	65.71242	9.95383	92.42572	6.77069	Detected	Compromised	Detected	Compromised
9052	A_23_P20022	C7orf68	Hs.710088	chromosome 7 open reading frame 68	Homo sapiens chromosome 7 open read	7732.06150	3822.25300	7576.07370	3159.98900	Detected	Detected	Detected	Detected
9053	A_33_P3260634	PLCB4	Hs.472101	phospholipase C, beta 4	Homo sapiens phospholipase C, beta 4 (	40.01251	388.80792	39.97358	237.73419	Detected	Detected	Detected	Detected
9054	A_33_P3268167					1554.52360	1859.32870	762.62050	783.40270	Detected	Detected	Detected	Detected
9055	A_33_P3377269					24.98017	35.97260	15.34014	26.28217	Detected	Detected	Detected	Detected
9056	A_33_P3394031	UBXN11	Hs.145061	UBX domain protein 11	Homo sapiens UBX domain protein 11 (	76.68469	76.31135	57.51881	44.16782	Detected	Detected	Detected	Detected
9057	A_23_P313734	KIAA1731	Hs.458418	KIAA1731	Homo sapiens KIAA1731 (KIAA1731), m	717.84090	1025.11890	521.85350	556.12506	Detected	Detected	Detected	Detected
9058	A_33_P3322363	HMSD	Hs.721604	histocompatibility (minor) serpin domain	Homo sapiens histocompatibility (minor)	14.86305	224.39763	25.78065	175.33514	Detected	Detected	Detected	Detected
9059	A_24_P134235	KHSRP	Hs.91142	KH-type splicing regulatory protein	Homo sapiens KH-type splicing regulato	72.53574	184.08406	94.75519	186.12355	Detected	Detected	Detected	Detected
9060	A_33_P3346533					410.29510	2772.01980	435.57750	2440.13090	Detected	Detected	Detected	Detected
9061	A_24_P194819		Hs.648184		Putative serine protease LOC138652 P	23.10347	23.10598	26.71047	19.10516	Detected	Detected	Detected	Detected
9062	A_33_P3229022	LOC100129966	Hs.639219	similar to mitochondrial carrier homolog	PREDICTED: Homo sapiens similar to m	7949.50240	13101.01600	5895.64360	9377.91300	Detected	Detected	Detected	Detected
9063	A_24_P238131	SUSD5	Hs.196647	sushi domain containing 5	Homo sapiens sushi domain containing 5	64.05328	131.39621	70.96616	137.00189	Detected	Detected	Detected	Detected
9064	A_23_P325040	TMPO	Hs.11355	thymopoietin	Homo sapiens thymopoietin (TMPO), tra	102.42580	450.28763	80.07113	294.42773	Detected	Detected	Detected	Detected
9065	A_33_P3299898	LOC644021	Hs.690144	similar to Cathepsin L1	PREDICTED: Homo sapiens similar to C	10856.85500	3897.18550	11138.44400	2643.16650	Detected	Detected	Detected	Detected
9066	A_23_P94296	ADAM7	Hs.116147	ADAM metallopeptidase domain 7	Homo sapiens ADAM metallopeptidase (	20.62238	34.80878	9.01879	9.59626	Detected	Detected	Compromised	Compromised
9067	A_33_P3397603	LOC100130278	Hs.638469	hypothetical protein LOC100130278	Homo sapiens cDNA FLJ36795 fis, clon	9.62989	7.37632	13.63893	4.85706	Compromised	Compromised	Detected	Compromised
9068	A_33_P3240972	LOC100133758	Hs.664651	hypothetical protein LOC100133758	PREDICTED: Homo sapiens hypothetica	29.21190	41.66560	15.82651	16.72642	Detected	Detected	Detected	Detected
9069	A_23_P108835	YPEL5	Hs.515890	yippee-like 5 (Drosophila)	Homo sapiens yippee-like 5 (Drosophila	8477.79200	7451.84230	6408.62740	4777.51560	Detected	Detected	Detected	Detected
9070	A_33_P3288754	C19orf48	Hs.256301	chromosome 19 open reading frame 48	Homo sapiens chromosome 19 open rea	2742.41870	5076.99170	3405.42630	5766.19300	Detected	Detected	Detected	Detected
9071	A_23_P56298	ZNF430	Hs.466289	zinc finger protein 430	Homo sapiens zinc finger protein 430 (Z	58.18762	98.31570	38.52749	49.57049	Detected	Detected	Detected	Detected

9072	A_33_P3351836				Uncharacterized protein C2orf73 [Sour	201.41922	212.85526	135.34827	104.32022	Detected	Detected	Detected	Detected
9073	A_24_P332647	SSH1	Hs.199763	slingshot homolog 1 (Drosophila)	Homo sapiens slingshot homolog 1 (Dro	206.51122	857.84296	149.85355	600.52185	Detected	Detected	Detected	Detected
9074	A_23_P74928	MR1	Hs.101840	major histocompatibility complex, class	Homo sapiens major histocompatibility	433.40643	380.20130	354.98703	331.41687	Detected	Detected	Detected	Detected
9075	A_33_P3365856		Hs.721471		fs15d03.y2 Human Lens cDNA (Normali	210.62537	186.26225	169.03082	157.34128	Detected	Detected	Detected	Detected
9076	A_33_P3787904	LOC400604	Hs.641441	hypothetical gene supported by BC039	Homo sapiens cDNA FLJ41720 fis, clon	71886.36000	69127.81000	30333.71700	25561.91600	Detected	Detected	Detected	Detected
9077	A_33_P3266873	MSL1	Hs.532786	male-specific lethal 1 homolog (Drosop	Homo sapiens male-specific lethal 1 ho	3968.63650	2846.41600	2997.03700	1754.70040	Detected	Detected	Detected	Detected
9078	A_23_P48121	ADIPOR2	Hs.371642	adiponectin receptor 2	Homo sapiens adiponectin receptor 2 (A	5957.26270	3836.15450	4456.26170	2540.51700	Detected	Detected	Detected	Detected
9079	A_32_P143000	FAM189A1	Hs.383564	family with sequence similarity 189, mer	Homo sapiens family with sequence sim	25.96095	15.73206	36.01842	14.14829	Detected	Detected	Detected	Detected
9080	A_33_P3283684	C7orf31	Hs.122055	chromosome 7 open reading frame 31	Homo sapiens chromosome 7 open read	13.55404	3.12612	7.41918	4.79336	Detected	Compromised	Compromised	Compromised
9081	A_33_P3326904	LOC728650	Hs.696467	hypothetical LOC728650	PREDICTED: Homo sapiens hypothetica	352.75470	61.48365	252.71835	32.68150	Detected	Detected	Detected	Detected
9082	A_33_P3355732				Uromodulin-like 1 Precursor (Olfactorin	30.68053	23.85505	12.08243	15.70561	Detected	Detected	Compromised	Detected
9083	A_23_P250294	ABHD5	Hs.19385	abhydrolase domain containing 5	Homo sapiens abhydrolase domain cont	2585.13060	3378.58000	2546.48070	2730.74000	Detected	Detected	Detected	Detected
9084	A_33_P3285754	LOC729537		hypothetical LOC729537	PREDICTED: Homo sapiens hypothetica	108.53728	123.63289	82.92921	75.20331	Detected	Detected	Detected	Detected
9085	A_33_P3278515	LOC642340	Hs.655951	hypothetical LOC642340	PREDICTED: Homo sapiens hypothetica	42.69819	23.88781	35.24102	14.81118	Detected	Detected	Detected	Detected
9086	A_33_P3290139	ASB15	Hs.83916	ankyrin repeat and SOCS box-containi	Homo sapiens ankyrin repeat and SOCS	3.03536	3.12663	9.98927	21.34644	Compromised	Compromised	Compromised	Detected
9087	A_23_P31335	ZNF12	Hs.431471	zinc finger protein 12	Homo sapiens zinc finger protein 12 (ZN	1897.42460	1183.05500	1311.76370	634.44570	Detected	Detected	Detected	Detected
9088	A_23_P4850	ZNF625	Hs.512823	zinc finger protein 625	Homo sapiens zinc finger protein 625 (Z	117.76243	147.86551	13.17973	23.81399	Detected	Detected	Detected	Detected
9089	A_33_P3226610	PSIP1	Hs.658434	PC4 and SFRS1 interacting protein 1	Homo sapiens PC4 and SFRS1 interact	442.40875	431.57217	90.72591	61.42552	Detected	Detected	Detected	Detected
9090	A_32_P210872	HEPN1	Hs.159863	HEPACAM opposite strand 1	Homo sapiens HEPACAM opposite stra	16.85573	3.14658	7.97256	17.64964	Detected	Compromised	Compromised	Detected
9091	A_33_P3238290	FAM65C	Hs.372578	family with sequence similarity 65, mem	Homo sapiens family with sequence sim	5840.50340	1355.56650	4975.03120	1005.00250	Detected	Detected	Detected	Detected
9092	A_23_P24149	SLC39A12	Hs.350895	solute carrier family 39 (zinc transporte	Homo sapiens solute carrier family 39 (	5.27535	4.77092	7.32855	15.66440	Compromised	Compromised	Compromised	Detected
9093	A_33_P3376971	CHAC1	Hs.155569	ChaC, cation transport regulator homol	Homo sapiens ChaC, cation transport re	500.00160	1797.34330	502.52450	1944.32100	Detected	Detected	Detected	Detected
9094	A_24_P77364	BOLA3	Hs.61472	bolA homolog 3 (E. coli)	Homo sapiens bolA homolog 3 (E. coli) (	11556.58500	25143.62000	10180.66600	17948.35700	Detected	Detected	Detected	Detected
9095	A_24_P134727	TFAM	Hs.642966	transcription factor A, mitochondrial	Homo sapiens transcription factor A, mi	366.86197	385.40793	143.72775	125.06367	Detected	Detected	Detected	Detected
9096	A_23_P21324	TWIST2	Hs.422585	twist homolog 2 (Drosophila)	Homo sapiens twist homolog 2 (Drosoph	43909.63300	31395.34200	38007.36000	24190.46700	Detected	Detected	Detected	Detected
9097	A_33_P3416037	FAM96A	Hs.439548	family with sequence similarity 96, mem	Homo sapiens family with sequence sim	8405.55000	6327.61500	8221.69100	5318.17200	Detected	Detected	Detected	Detected
9098	A_23_P42368	VTA1	Hs.431367	Vps20-associated 1 homolog (S. cerevis	Homo sapiens Vps20-associated 1 hom	777.55524	1019.57790	936.55360	1063.70410	Detected	Detected	Detected	Detected
9099	A_33_P3244122	HAAO	Hs.368805	3-hydroxyanthranilate 3,4-dioxygenase	Homo sapiens 3-hydroxyanthranilate 3,4	14.96962	26.18989	23.31793	29.76039	Detected	Detected	Detected	Detected
9100	A_32_P186731	ISM1	Hs.559353	isthmin 1 homolog (zebrafish)	Homo sapiens isthmin 1 homolog (zebra	29.81801	53.95092	24.44836	40.76854	Detected	Detected	Detected	Detected
9101	A_23_P99741	CDKL1	Hs.679430	cyclin-dependent kinase-like 1 (CDC2-	Homo sapiens cyclin-dependent kinase-	147.03264	107.98370	118.82751	98.97499	Detected	Detected	Detected	Detected
9102	A_33_P3494748	TMEM65	Hs.187646	transmembrane protein 65	Homo sapiens transmembrane protein 6	1151.17540	879.05520	606.31720	429.54855	Detected	Detected	Detected	Detected
9103	A_33_P3360823	C10orf88	Hs.501286	chromosome 10 open reading frame 88	Homo sapiens chromosome 10 open rea	1577.61290	1841.17100	1336.99290	1266.61610	Detected	Detected	Detected	Detected
9104	A_33_P3359647	OPRL1	Hs.2859	opiate receptor-like 1	Homo sapiens opiate receptor-like 1 (O	29.15271	26.57490	15.65875	11.48654	Detected	Detected	Detected	Compromised
9105	A_32_P76627		Hs.546903		full-length cDNA clone CS0DI013YN06	17.77038	36.36503	17.56453	37.98599	Detected	Detected	Detected	Detected
9106	A_24_P213370		Hs.720324		cDNA FLJ26472 fis, clone KDN04506 [	796.91327	171.49223	388.07460	61.06018	Detected	Detected	Detected	Detected
9107	A_24_P344295	RNF167	Hs.7158	ring finger protein 167	Homo sapiens ring finger protein 167 (R	102.76479	134.43309	112.61890	121.83540	Detected	Detected	Detected	Detected
9108	A_24_P99838	ZNF223	Hs.720813	zinc finger protein 223	Homo sapiens zinc finger protein 223 (Z	639.09440	471.00003	444.55200	244.36797	Detected	Detected	Detected	Detected
9109	A_23_P109547	SMCR7L	Hs.148677	Smith-Magenis syndrome chromosome	Homo sapiens Smith-Magenis syndrome	1963.01840	2070.95830	1567.41550	1400.44870	Detected	Detected	Detected	Detected
9110	A_33_P3248424	FAM105B	Hs.406335	family with sequence similarity 105, me	Homo sapiens family with sequence sim	854.46710	640.41940	623.96783	396.90277	Detected	Detected	Detected	Detected
9111	A_23_P141770	NAPG	Hs.464622	N-ethylmaleimide-sensitive factor atta	Homo sapiens N-ethylmaleimide-sensiti	896.25653	1569.76820	805.67206	1067.47960	Detected	Detected	Detected	Detected
9112	A_32_P486620	IGSF22	Hs.434152	immunoglobulin superfamily, member 22	Homo sapiens immunoglobulin superfam	20.89318	9.93411	17.45664	7.42735	Detected	Compromised	Detected	Compromised
9113	A_23_P343963	FAM83F	Hs.197680	family with sequence similarity 83, mem	Homo sapiens family with sequence sim	14.70678	10.20593	4.68212	5.36104	Detected	Compromised	Compromised	Compromised
9114	A_33_P3319920	KDM4B	Hs.654816	lysine (K)-specific demethylase 4B	Homo sapiens lysine (K)-specific demet	8208.53800	5382.73970	7335.76000	4860.81840	Detected	Detected	Detected	Detected
9115	A_24_P50908		Hs.599464		Tripartite motif-containing protein 11 (E	205.40375	225.12030	177.48471	181.75345	Detected	Detected	Detected	Detected
9116	A_33_P3355014	TMEM229B	Hs.509707	transmembrane protein 229B	Homo sapiens transmembrane protein 2	339.57043	683.53170	45.22252	41.62141	Detected	Detected	Detected	Detected
9117	A_33_P3254141	LOC285696		hypothetical LOC285696	Homo sapiens hypothetical LOC285696	6.31693	3.28759	13.74076	2.78757	Compromised	Compromised	Detected	Compromised
9118	A_33_P3374758	DYNC1LI2	Hs.369068	dynein, cytoplasmic 1, light intermediate	Homo sapiens dynein, cytoplasmic 1, lig	473.05692	565.92210	255.39868	246.98145	Detected	Detected	Detected	Detected
9119	A_33_P3371663	LTK	Hs.434481	leukocyte receptor tyrosine kinase	Homo sapiens leukocyte receptor tyros	954.10770	1338.91210	620.05270	634.57320	Detected	Detected	Detected	Detected
9120	A_24_P935986	BCAT1	Hs.438993	branched chain aminotransferase 1, cyt	Homo sapiens branched chain aminotra	13809.91700	11137.07500	8108.33200	5313.96530	Detected	Detected	Detected	Detected
9121	A_23_P55064	WRAP53	Hs.437460	WD repeat containing, antisense to TP5	Homo sapiens WD repeat containing, an	224.18324	472.92374	265.14490	532.91907	Detected	Detected	Detected	Detected
9122	A_24_P272873	RPL13AP3	Hs.663461	ribosomal protein L13a pseudogene 3	Homo sapiens ribosomal protein L13a p	158639.92000	128078.36000	62475.81600	41029.50800	Detected	Detected	Detected	Detected
9123	A_33_P3359250				Mitotic spindle assembly checkpoint pro	135.80188	156.47302	97.37624	73.67570	Detected	Detected	Detected	Detected
9124	A_24_P133584	MFGE8	Hs.3745	milk fat globule-EGF factor 8 protein	Homo sapiens milk fat globule-EGF fact	689.48517	2460.04860	638.23950	2401.75730	Detected	Detected	Detected	Detected
9125	A_23_P131096	POLRMT	Hs.254113	polymerase (RNA) mitochondrial (DNA	Homo sapiens polymerase (RNA) mitoch	5832.69800	3398.01680	7070.87000	4074.88920	Detected	Detected	Detected	Detected
9126	A_33_P3283601	LOC389033		placenta-specific 9 pseudogene	Homo sapiens placenta-specific 9 pseu	15027.16400	28109.15200	3204.23540	8850.04600	Detected	Detected	Detected	Detected
9127	A_23_P401904	PHF19	Hs.460124	PHD finger protein 19	Homo sapiens PHD finger protein 19 (P	1946.19320	8152.69870	2070.30960	7671.90330	Detected	Detected	Detected	Detected
9128	A_32_P95914	C6orf167	Hs.444292	chromosome 6 open reading frame 167	Homo sapiens chromosome 6 open read	393.69860	292.99740	268.21524	146.59999	Detected	Detected	Detected	Detected
9129	A_33_P3283626					11.67043	8.12470	34.41100	2.66090	Compromised	Compromised	Detected	Compromised
9130	A_33_P3663974	THRB	Hs.187861	thyroid hormone receptor, beta (erythr	Homo sapiens thyroid hormone recepto	106.54867	29.69543	96.40772	21.56174	Detected	Detected	Detected	Detected
9131	A_23_P26457	HBA2	Hs.654744	hemoglobin, alpha 2	Homo sapiens hemoglobin, alpha 2 (HBA	35.58730	146.37220	24.14582	20.74697	Detected	Detected	Detected	Detected
9132	A_24_P160202	PANX2	Hs.440092	pannexin 2	Homo sapiens pannexin 2 (PANX2), tran	257.88684	353.07596	97.28342	79.50246	Detected	Detected	Detected	Detected
9133	A_33_P3331021	LOC100129148	Hs.370475	hypothetical protein LOC100129148	PREDICTED: Homo sapiens hypothetica	13.94933	19.26838	8.18611	5.41508	Detected	Detected	Compromised	Compromised
9134	A_33_P3293573	VPS37D	Hs.647037	vacuolar protein sorting 37 homolog D (	Homo sapiens vacuolar protein sorting 3	3974.66460	3144.88500	3496.43820	2507.34940	Detected	Detected	Detected	Detected
9135	A_23_P45517	PPP2R3B	Hs.124942	protein phosphatase 2 (formerly 2A), re	Homo sapiens protein phosphatase 2 (f	964.68030	836.31240	1241.52840	1049.93550	Detected	Detected	Detected	Detected
9136	A_24_P109176	ZC3H12D	Hs.632618	zinc finger CCCH-type containing 12D	Homo sapiens zinc finger CCCH-type c	18.39056	43.29374	15.52489	19.70140	Detected	Detected	Detected	Detected
9137	A_23_P410507	PSPN	Hs.248159	persephin	Homo sapiens persephin (PSPN), mRNA	170.05270	176.72188	77.50720	66.37892	Detected	Detected	Detected	Detected
9138	A_33_P3210904				Protein SEC13 homolog (SEC13-relate	28.90517	43.20622	44.64623	43.07351	Detected	Detected	Detected	Detected
9139	A_23_P133543	KLHL3	Hs.655084	kelch-like 3 (Drosophila)	Homo sapiens kelch-like 3 (Drosophila)	58.87436	31.57631	44.67199	40.35296	Detected	Detected	Detected	Detected
9140	A_33_P3421832	LOC100131706	Hs.705836	hypothetical LOC100131706	PREDICTED: Homo sapiens hypothetica	98.44811	80.99057	110.14927	80.56181	Detected	Detected	Detected	Detected
9141	A_23_P28507	MGAT4A	Hs.177576	mannosyl (alpha-1,3-)-glycoprotein bet	Homo sapiens mannosyl (alpha-1,3-)-gl	15.95063	11.06094	11.94294	6.17342	Detected	Compromised	Compromised	Compromised
9142	A_23_P42306	HLA-DMA	Hs.351279	major histocompatibility complex, class	Homo sapiens major histocompatibility	147.08130	131.16602	194.24063	170.88230	Detected	Detected	Detected	Detected
9143	A_33_P3249135	NBEAL1	Hs.648846	neurobeachin-like 1	Homo sapiens neurobeachin-like 1 (NBE	23.40156	46.73758	24.73073	35.01914	Detected	Detected	Detected	Detected
9144	A_33_P3276997	MAP6	Hs.585540	microtubule-associated protein 6	Homo sapiens microtubule-associated p	572.71610	604.77966	454.14890	437.10623	Detected	Detected	Detected	Detected
9145	A_33_P3311750	CCDC144A	Hs.721149	coiled-coil domain containing 144A	Homo sapiens coiled-coil domain conta	99.35824	7.53528	44.80317	5.65477	Detected	Compromised	Detected	Compromised
9146	A_24_P54808	KLHL8	Hs.106601	kelch-like 8 (Drosophila)	Homo sapiens kelch-like 8 (Drosophila)	709.46124	549.67865	522.74360	363.89456	Detected	Detected	Detected	Detected

9147	A_23_P29153	RTDR1	Hs.526920	rhabdoid tumor deletion region gene 1	Homo sapiens rhabdoid tumor deletion r	120.39317	30.01149	124.28970	20.76825	Detected	Detected	Detected	Detected
9148	A_23_P73540	CCDC120	Hs.522643	coiled-coil domain containing 120	Homo sapiens coiled-coil domain contai	325.07297	198.90875	222.40262	118.72060	Detected	Detected	Detected	Detected
9149	A_33_P3424591		Hs.510635		Ig gamma-1 chain C region [Source:Un	148.26181	165.21138	121.23158	135.28815	Detected	Detected	Detected	Detected
9150	A_24_P336759	MCL1	Hs.632486	myeloid cell leukemia sequence 1 (BCL2	Homo sapiens myeloid cell leukemia seq	14453.68750	5746.49000	9370.38400	3255.05370	Detected	Detected	Detected	Detected
9151	A_23_P336644	TOR1AIP2	Hs.655655	torsin A interacting protein 2	Homo sapiens torsin A interacting prote	108.62951	335.77072	81.21252	208.09396	Detected	Detected	Detected	Detected
9152	A_24_P115700	COX15	Hs.28326	COX15 homolog, cytochrome c oxidase	Homo sapiens COX15 homolog, cytochr	145.35582	41.96235	99.63257	29.80107	Detected	Detected	Detected	Detected
9153	A_23_P67847	GALNT14	Hs.468058	UDP-N-acetyl-alpha-D-galactosamine:	Homo sapiens UDP-N-acetyl-alpha-D-	1565.03740	1461.47110	1496.66240	1217.21130	Detected	Detected	Detected	Detected
9154	A_23_P76488	EMP1	Hs.436298	epithelial membrane protein 1	Homo sapiens epithelial membrane prot	142269.70000	186369.08000	92752.26000	99039.76600	Detected	Detected	Detected	Detected
9155	A_33_P3576797	LOC158863	Hs.138411	hypothetical protein LOC158863	Homo sapiens mRNA; cDNA DKFZp586	26.75259	7.25151	27.86678	10.90243	Detected	Compromised	Detected	Compromised
9156	A_23_P348264	LETM2	Hs.696457	leucine zipper-EF-hand containing trans	Homo sapiens leucine zipper-EF-hand c	109.61449	123.43307	111.40651	94.42108	Detected	Detected	Detected	Detected
9157	A_24_P200942	TSC22D4	Hs.719125	TSC22 domain family, member 4	Homo sapiens TSC22 domain family, me	987.59247	1107.28220	930.76320	1000.67960	Detected	Detected	Detected	Detected
9158	A_33_P3225298	XKR9	Hs.458938	XK, Kell blood group complex subunit-re	Homo sapiens XK, Kell blood group com	216.93993	271.59476	141.04407	113.91740	Detected	Detected	Detected	Detected
9159	A_23_P163027	PARP2	Hs.409412	poly (ADP-ribose) polymerase 2	Homo sapiens poly (ADP-ribose) polym	2529.19650	2723.10600	2258.56900	1917.87060	Detected	Detected	Detected	Detected
9160	A_33_P3336992	MRPS18B	Hs.655329	mitochondrial ribosomal protein S18B	Homo sapiens mitochondrial ribosomal p	369.59818	251.25320	379.75820	205.56471	Detected	Detected	Detected	Detected
9161	A_33_P3362806	GRIPAP1	Hs.109929	GRIP1 associated protein 1	Homo sapiens GRIP1 associated protei	32.09176	33.11180	27.15097	25.28083	Detected	Detected	Detected	Detected
9162	A_24_P321068	SLC31A1	Hs.532315	solute carrier family 31 (copper transpo	Homo sapiens solute carrier family 31 (	419.91013	428.75677	243.66550	243.92407	Detected	Detected	Detected	Detected
9163	A_24_P276490	LYPLA2	Hs.533479	lysophospholipase II	Homo sapiens lysophospholipase II (LYF	157.93220	114.78559	151.17223	117.35433	Detected	Detected	Detected	Detected
9164	A_24_P411815	C7orf11	Hs.654989	chromosome 7 open reading frame 11	Homo sapiens chromosome 7 open reac	3281.62770	2271.09720	2279.29100	1367.74850	Detected	Detected	Detected	Detected
9165	A_33_P3400389	SPATS2L	Hs.120323	spermatogenesis associated, serine-ricl	Homo sapiens spermatogenesis associa	5087.46700	9243.30700	3300.45430	5414.62650	Detected	Detected	Detected	Detected
9166	A_33_P3279581	UNQ353	Hs.81892	GKGM353	Homo sapiens clone DNA48309 GKGM3	217.01515	291.14838	146.04715	157.84653	Detected	Detected	Detected	Detected
9167	A_33_P3342190	LOC100133580		hypothetical protein LOC100133580	PREDICTED: Homo sapiens hypothetica	2.37495	3.27387	22.94387	5.38038	Compromised	Compromised	Detected	Compromised
9168	A_33_P3356926	APBB3	Hs.529449	amyloid beta (A4) precursor protein-bin	Homo sapiens amyloid beta (A4) precur	60.46646	43.73781	56.44490	45.53855	Detected	Detected	Detected	Detected
9169	A_33_P3388948	SNX30	Hs.522350	sorting nexin family member 30	Homo sapiens sorting nexin family mem	5292.00730	5104.46140	4070.29900	3475.20240	Detected	Detected	Detected	Detected
9170	A_23_P2203	TAOK3	Hs.644420	TAO kinase 3	Homo sapiens TAO kinase 3 (TAOK3), r	521.94116	522.11990	409.28552	340.88544	Detected	Detected	Detected	Detected
9171	A_24_P349547					5768.00000	17136.63700	3106.61060	6649.32760	Detected	Detected	Detected	Detected
9172	A_23_P141302	ZNF18	Hs.370473	zinc finger protein 18	Homo sapiens zinc finger protein 18 (ZN	1768.11020	965.36860	1553.62570	788.21796	Detected	Detected	Detected	Detected
9173	A_23_P353514	MSL3	Hs.655288	male-specific lethal 3 homolog (Drosopl	Homo sapiens male-specific lethal 3 ho	111.04089	117.12551	60.53143	52.94499	Detected	Detected	Detected	Detected
9174	A_32_P89730	LOH12CR2		loss of heterozygosity, 12, chromosoma	Homo sapiens loss of heterozygosity, 12	383.75647	215.96574	301.46057	139.70444	Detected	Detected	Detected	Detected
9175	A_33_P3256620	LOC100134162		similar to Solute carrier family 22, mem	PREDICTED: Homo sapiens similar to s	69.59493	74.74999	37.96536	34.25653	Detected	Detected	Detected	Detected
9176	A_33_P3283420	RBM12B	Hs.606916	RNA binding motif protein 12B	Homo sapiens RNA binding motif protei	166.82896	113.53948	74.18709	57.19763	Detected	Detected	Detected	Detected
9177	A_33_P3278033	TUSC1	Hs.26268	tumor suppressor candidate 1	Homo sapiens tumor suppressor candid	3878.21700	4575.03760	1866.20700	1319.11400	Detected	Detected	Detected	Detected
9178	A_24_P386334	SH3GLB2	Hs.460238	SH3-domain GRB2-like endophilin B2	Homo sapiens SH3-domain GRB2-like e	656.86810	454.60516	594.98840	400.15018	Detected	Detected	Detected	Detected
9179	A_23_P206396	CKLF	Hs.15159	chemokine-like factor	Homo sapiens chemokine-like factor (C	3829.53900	4240.84900	3513.76030	3348.79400	Detected	Detected	Detected	Detected
9180	A_23_P205529	METT11D1	Hs.512693	methyltransferase 11 domain containing	Homo sapiens methyltransferase 11 dor	1521.90830	1491.09080	1604.61790	1460.70700	Detected	Detected	Detected	Detected
9181	A_33_P3220723	KIAA0922	Hs.205572	KIAA0922	Homo sapiens KIAA0922 (KIAA0922), tr	1148.55220	791.53406	725.72577	411.03730	Detected	Detected	Detected	Detected
9182	A_33_P3381781				Regulation of nuclear pre-mRNA domai	30.57419	18.01776	20.30864	2.87295	Detected	Detected	Detected	Compromised
9183	A_33_P3423300	TNPO2	Hs.416049	transportin 2	Homo sapiens transportin 2 (TNPO2), tr	13764.51200	12713.39550	8966.97950	7444.10250	Detected	Detected	Detected	Detected
9184	A_33_P3230254	NCAPG	Hs.567567	non-SMC condensin I complex, subunit	Homo sapiens non-SMC condensin I co	8.17935	227.49911	12.68985	153.34033	Compromised	Detected	Detected	Detected
9185	A_33_P3221408	NTNG1	Hs.657434	netrin G1	Homo sapiens netrin G1 (NTNG1), trans	3468.41600	957.74940	2579.15820	610.85266	Detected	Detected	Detected	Detected
9186	A_33_P3344277				HCLS1-binding protein 3 (HS1-binding	60.86503	221.19077	41.65284	149.43239	Detected	Detected	Detected	Detected
9187	A_33_P3503537	LOC285178	Hs.586683	hypothetical protein LOC285178	Homo sapiens cDNA FLJ34252 fis, clon	191.77565	258.27563	117.48857	136.25809	Detected	Detected	Detected	Detected
9188	A_33_P3375145	C9orf150	Hs.445356	chromosome 9 open reading frame 150	Homo sapiens chromosome 9 open reac	938.29425	600.82684	556.08540	285.75030	Detected	Detected	Detected	Detected
9189	A_23_P109269	LAMA5	Hs.473256	laminin, alpha 5	Homo sapiens laminin, alpha 5 (LAMA5)	701.03300	70.64972	769.16620	68.26530	Detected	Detected	Detected	Detected
9190	A_33_P3400763	PLIN4	Hs.591387	perilipin 4	Homo sapiens perilipin 4 (PLIN4), mRNA	363.90515	11.41286	283.48096	7.29991	Detected	Compromised	Detected	Compromised
9191	A_23_P167168	IGJ	Hs.643431	immunoglobulin J polypeptide, linker pro	Homo sapiens immunoglobulin J polypep	69.15413	100.27921	46.59057	55.10980	Detected	Detected	Detected	Detected
9192	A_23_P207245	TEKT3	Hs.414648	tektin 3	Homo sapiens tektin 3 (TEKT3), mRNA	20.63918	3.13850	17.57672	7.79037	Detected	Compromised	Detected	Compromised
9193	A_23_P9415	ACO1	Hs.567229	aconitase 1, soluble	Homo sapiens aconitase 1, soluble (ACC	5992.25540	4799.20750	3645.24120	2377.41400	Detected	Detected	Detected	Detected
9194	A_23_P129513	MRPL28	Hs.513230	mitochondrial ribosomal protein L28	Homo sapiens mitochondrial ribosomal p	6954.77100	6275.29300	9078.57100	7345.62500	Detected	Detected	Detected	Detected
9195	A_24_P255314					13760.86300	15699.53900	6000.85740	5384.32700	Detected	Detected	Detected	Detected
9196	A_32_P117422	PMS2L4	Hs.720896	postmeiotic segregation increased 2-lik	Homo sapiens postmeiotic segregation i	784.08655	1156.76680	635.60760	774.83430	Detected	Detected	Detected	Detected
9197	A_24_P153456	ZDHHC11	Hs.654874	zinc finger, DHHC-type containing 11	Homo sapiens zinc finger, DHHC-type c	101.79839	34.77900	99.72676	33.49789	Detected	Detected	Detected	Detected
9198	A_23_P64650	TSFM	Hs.632704	Ts translation elongation factor, mitoch	Homo sapiens Ts translation elongation	11540.68400	16283.88800	11868.35200	14330.36200	Detected	Detected	Detected	Detected
9199	A_23_P93690	MCM7	Hs.438720	minichromosome maintenance complex	Homo sapiens minichromosome mainter	6916.26460	32294.97500	8675.46000	35834.95300	Detected	Detected	Detected	Detected
9200	A_32_P98975	C15orf57	Hs.720139	chromosome 15 open reading frame 57	Homo sapiens chromosome 15 open rea	664.86554	703.44037	672.46940	674.86523	Detected	Detected	Detected	Detected
9201	A_24_P280926	LOC391358		similar to CG12975	PREDICTED: Homo sapiens similar to C	7738.54540	7578.66360	8965.72900	7278.15530	Detected	Detected	Detected	Detected
9202	A_33_P3372941	TINF2	Hs.496191	TERF1 (TRF1)-interacting nuclear facto	Homo sapiens TERF1 (TRF1)-interactir	8515.85600	7353.04800	7825.04700	5763.94800	Detected	Detected	Detected	Detected
9203	A_33_P3360728	BLVRB	Hs.515785	biliverdin reductase B (flavin reductase	Homo sapiens biliverdin reductase B (fla	14791.13000	7317.26860	14110.56100	5560.36470	Detected	Detected	Detected	Detected
9204	A_33_P3329419	DNM1	Hs.522413	dynamin 1	Homo sapiens dynamin 1 (DNM1), trans	122229.33000	144585.12000	129047.69000	119180.23000	Detected	Detected	Detected	Detected
9205	A_24_P183264	BTF3	Hs.591768	basic transcription factor 3	Homo sapiens basic transcription factor	12859.45800	10420.05700	12824.16100	8442.71600	Detected	Detected	Detected	Detected
9206	A_23_P320250	MYO19	Hs.302051	myosin XIX	Homo sapiens myosin XIX (MYO19), tra	4015.17920	4560.32370	3302.74580	3012.64140	Detected	Detected	Detected	Detected
9207	A_23_P17880	DNAL4	Hs.632766	dynein, axonemal, light chain 4	Homo sapiens dynein, axonemal, light ch	424.15475	200.54045	446.51758	185.32689	Detected	Detected	Detected	Detected
9208	A_33_P3261197	NCBP1	Hs.595669	nuclear cap binding protein subunit 1, 8	Homo sapiens nuclear cap binding prote	23.51609	35.22049	30.59248	26.45947	Detected	Detected	Detected	Detected
9209	A_24_P370471	ATG10	Hs.713698	ATG10 autophagy related 10 homolog (S	Homo sapiens ATG10 autophagy relatec	109.99349	125.40167	103.44920	99.43982	Detected	Detected	Detected	Detected
9210	A_33_P3338166	NUAK2	Hs.497512	NUAK family, SNF1-like kinase, 2	Homo sapiens NUAK family, SNF1-like k	363.11435	665.04140	289.94644	466.75537	Detected	Detected	Detected	Detected
9211	A_23_P395426	DIDO1	Hs.517172	death inducer-obliterator 1	Homo sapiens death inducer-obliterator	763.52290	548.43700	599.55960	355.37183	Detected	Detected	Detected	Detected
9212	A_33_P3390731	AKTIP	Hs.380897	AKT interacting protein	Homo sapiens AKT interacting protein (	865.33580	1001.71470	614.55940	547.15370	Detected	Detected	Detected	Detected
9213	A_33_P3372161					118811.30500	98402.58600	43414.24600	28166.77500	Detected	Detected	Detected	Detected
9214	A_33_P3239854	ALKBH5	Hs.462392	alkB, alkylation repair homolog 5 (E. coli	Homo sapiens alkB, alkylation repair hor	727.74290	770.16900	370.16223	376.54233	Detected	Detected	Detected	Detected
9215	A_33_P3310696	UBE2J2	Hs.191987	ubiquitin-conjugating enzyme E2, J2 (UI	Homo sapiens ubiquitin-conjugating enz	8488.52400	10103.23900	8010.77300	9759.06500	Detected	Detected	Detected	Detected
9216	A_24_P352637	TSSK4	Hs.314432	testis-specific serine kinase 4	Homo sapiens testis-specific serine kin	10.93978	4.15106	21.11306	3.85474	Compromised	Compromised	Detected	Compromised
9217	A_33_P3345474				hypothetical LOC400931 (LOC400931),	365.18280	561.00850	382.67102	531.80774	Detected	Detected	Detected	Detected
9218	A_23_P213085	SLC10A7	Hs.659209	solute carrier family 10 (sodium/bile aci	Homo sapiens solute carrier family 10 (	94.28904	68.60733	81.08459	39.72110	Detected	Detected	Detected	Detected
9219	A_23_P127088	CUEDC2	Hs.500874	CUE domain containing 2	Homo sapiens CUE domain containing 2	5541.58840	3772.27200	6133.30470	3750.50830	Detected	Detected	Detected	Detected
9220	A_23_P386268	C4orf26	Hs.24510	chromosome 4 open reading frame 26	Homo sapiens chromosome 4 open reac	20.56818	8.49630	23.73701	14.29418	Detected	Compromised	Detected	Detected
9221	A_33_P3246193	C10orf92		chromosome 10 open reading frame 92	Uncharacterized protein C10orf92 [Sou	27.31672	42.13725	17.03149	17.87975	Detected	Detected	Detected	Detected

9222	A_23_P211196	C21orf67	Hs.534504	chromosome 21 open reading frame 67	Homo sapiens chromosome 21 open rea	43.40780	58.68576	80.73630	104.10020	Detected	Detected	Detected	Detected
9223	A_33_P3415430	HSPA1B	Hs.719966	heat shock 70kDa protein 1B	Homo sapiens heat shock 70kDa protei	2588.05440	2947.68730	2434.48700	2282.21260	Detected	Detected	Detected	Detected
9224	A_23_P33326	ADRA1B	Hs.368632	adrenergic, alpha-1B-, receptor	Homo sapiens adrenergic, alpha-1B-, re	374.43750	1373.74700	347.30530	1216.00570	Detected	Detected	Detected	Detected
9225	A_33_P3386467	RPL23AP7	Hs.406135	ribosomal protein L23a pseudogene 7	Homo sapiens ribosomal protein L23a p	1648.42050	3921.47200	1426.96480	2821.46310	Detected	Detected	Detected	Detected
9226	A_23_P217755	SHROOM2	Hs.567236	shroom family member 2	Homo sapiens shroom family member 2	2.36952	18.85009	2.58399	2.69607	Compromised	Detected	Compromised	Compromised
9227	A_32_P108156	MIR155HG	Hs.697120	MIR155 host gene (non-protein coding)	Homo sapiens MIR155 host gene (non-p	320.03870	62.22223	318.92530	51.03583	Detected	Detected	Detected	Detected
9228	A_33_P3217218	AGPAT5	Hs.624002	1-acylglycerol-3-phosphate O-acyltran	Homo sapiens 1-acylglycerol-3-phosph	508.60077	234.64754	417.62830	151.36873	Detected	Detected	Detected	Detected
9229	A_23_P90533	POP4	Hs.412870	processing of precursor 4, ribonuclease	Homo sapiens processing of precursor	8069.77900	9618.94300	8094.24200	8533.52200	Detected	Detected	Detected	Detected
9230	A_24_P148590	TACR1	Hs.633301	tachykinin receptor 1	Homo sapiens tachykinin receptor 1 (TA	2.35437	5.78303	12.88781	2.65430	Compromised	Compromised	Detected	Compromised
9231	A_23_P24903	P2RY2	Hs.339	purinergic receptor P2Y, G-protein cou	Homo sapiens purinergic receptor P2Y,	4.72663	20.12577	2.54075	13.71020	Compromised	Detected	Compromised	Detected
9232	A_23_P7074	C4orf14	Hs.8715	chromosome 4 open reading frame 14	Homo sapiens chromosome 4 open read	5849.96240	4512.83450	4043.84250	2502.13550	Detected	Detected	Detected	Detected
9233	A_24_P105564	PRKAB2	Hs.50732	protein kinase, AMP-activated, beta 2 r	Homo sapiens protein kinase, AMP-acti	2936.35300	3304.53000	1824.10190	1615.25050	Detected	Detected	Detected	Detected
9234	A_33_P3366471					26.53721	5.94164	7.48528	4.89754	Detected	Compromised	Compromised	Compromised
9235	A_33_P3390027				Exocyst complex component 7 (Exocys	462.14673	483.14650	393.32610	365.37912	Detected	Detected	Detected	Detected
9236	A_24_P476086	KPNA5		karyopherin alpha 5 (importin alpha 6)	Importin subunit alpha-6 (Karyopherin s	389.28653	148.20027	247.94650	72.18704	Detected	Detected	Detected	Detected
9237	A_33_P3877728	SMCR6	Hs.660306	Smith-Magenis syndrome chromosome	Homo sapiens mRNA; cDNA DKFZp686	277.92984	223.06584	278.42047	178.06665	Detected	Detected	Detected	Detected
9238	A_32_P140489	GDF6	Hs.492277	growth differentiation factor 6	Homo sapiens growth differentiation fac	3.73760	134.75726	6.48856	53.49357	Compromised	Detected	Compromised	Detected
9239	A_33_P3325978				Tribbles homolog 2 (TRB-2) [Source:Ur	1105.56640	1979.47740	920.60920	1367.65720	Detected	Detected	Detected	Detected
9240	A_23_P46639	C8A	Hs.93210	complement component 8, alpha polype	Homo sapiens complement component	21.89501	23.08410	17.32473	9.27754	Detected	Detected	Detected	Compromised
9241	A_23_P394377	KCNRG	Hs.660161	potassium channel regulator	Homo sapiens potassium channel regula	47.57196	24.10659	37.27490	16.76990	Detected	Detected	Detected	Detected
9242	A_23_P37415	SECISBP2L	Hs.9997	SECIS binding protein 2-like	Homo sapiens SECIS binding protein 2-	93.71813	165.66020	98.59586	130.94931	Detected	Detected	Detected	Detected
9243	A_33_P3318384	LOC644893		similar to hCG201263	PREDICTED: Homo sapiens similar to h	154.28990	102.38406	26.85972	40.54791	Detected	Detected	Detected	Detected
9244	A_33_P3339231	UBN2	Hs.153458	ubinuclein 2	Homo sapiens ubinuclein 2 (UBN2), mR	1532.54300	902.52936	1133.31380	520.60930	Detected	Detected	Detected	Detected
9245	A_23_P152462	STX1B	Hs.542230	syntaxin 1B	Homo sapiens syntaxin 1B (STX1B), mR	14.67962	9.81346	17.68655	5.72746	Detected	Compromised	Detected	Compromised
9246	A_24_P515319	FAM90A7	Hs.715411	family with sequence similarity 90, mem	Homo sapiens family with sequence sim	335.25464	28.92349	316.86030	16.19896	Detected	Detected	Detected	Detected
9247	A_33_P3311979	ZFPM1	Hs.632218	zinc finger protein, multitype 1	Homo sapiens zinc finger protein, multit	1069.92250	755.68080	1154.95210	671.14470	Detected	Detected	Detected	Detected
9248	A_24_P130041	CYP51A1	Hs.417077	cytochrome P450, family 51, subfamily A	Homo sapiens cytochrome P450, family	4300.37200	5391.36470	3301.79420	3145.15750	Detected	Detected	Detected	Detected
9249	A_33_P3244610	SIRPD	Hs.664861	signal-regulatory protein delta	Homo sapiens signal-regulatory protein	26.55673	6.12310	34.94795	2.63196	Detected	Compromised	Detected	Compromised
9250	A_24_P359067					46.27107	25.28401	26.12363	16.62979	Detected	Detected	Detected	Detected
9251	A_24_P256018	LOC729189	Hs.647699	hypothetical LOC729189	PREDICTED: Homo sapiens hypothetica	79.59483	100.87571	28.26825	32.55504	Detected	Detected	Detected	Detected
9252	A_33_P3700794	METTL1	Hs.42957	methyltransferase like 1	Homo sapiens methyltransferase like 1	839.50720	1891.00350	857.11346	1803.62630	Detected	Detected	Detected	Detected
9253	A_33_P3225397					2.42159	34.38652	6.62371	33.28554	Compromised	Detected	Compromised	Detected
9254	A_33_P3215317		Hs.616610		cAMP-dependent protein kinase catalyt	12.65387	13.69425	20.93862	21.06009	Detected	Compromised	Detected	Detected
9255	A_33_P3286724	PLD2	Hs.104519	phospholipase D2	Homo sapiens phospholipase D2 (PLD2)	12001.40500	5159.67000	10477.38400	4156.60700	Detected	Detected	Detected	Detected
9256	A_33_P3390868	SYNPO2	Hs.655519	synaptopodin 2	Homo sapiens synaptopodin 2 (SYNPO2	25.57292	16.19988	34.62911	14.61900	Detected	Compromised	Detected	Detected
9257	A_33_P3394203				Protein YIPF3 (YIP1 family member 3)(	213.55733	226.45499	97.04010	95.33021	Detected	Detected	Detected	Detected
9258	A_33_P3271171				CCR4-NOT transcription complex subu	294.22482	339.94775	285.29470	250.17900	Detected	Detected	Detected	Detected
9259	A_24_P330971	EIF3K	Hs.314359	eukaryotic translation initiation factor 3	Homo sapiens eukaryotic translation ini	10665.41500	11386.24300	13591.84300	12236.87900	Detected	Detected	Detected	Detected
9260	A_24_P13715	FBXO22	Hs.591115	F-box protein 22	Homo sapiens F-box protein 22 (FBXO2	94.76907	69.56827	37.06923	29.59467	Detected	Detected	Detected	Detected
9261	A_33_P3314231	CRYBA2	Hs.415790	crystallin, beta A2	Homo sapiens crystallin, beta A2 (CRYE	188.97790	274.06412	96.07983	113.12287	Detected	Detected	Detected	Detected
9262	A_33_P3282898		Hs.471014		Talin-1 [Source:UniProtKB/Swiss-Pro	325.30707	360.15845	91.74026	85.67770	Detected	Detected	Detected	Detected
9263	A_33_P3417980		Hs.632208		Uncharacterized protein C16orf86 [Sou	36.60969	30.38393	46.11882	27.93048	Detected	Detected	Detected	Detected
9264	A_24_P824592	RBMX	Hs.380118	RNA binding motif protein, X-linked	Homo sapiens RNA binding motif protei	142.08969	409.53586	72.17301	144.17990	Detected	Detected	Detected	Detected
9265	A_24_P302802	PCCB	Hs.63788	propionyl Coenzyme A carboxylase, bet	Homo sapiens propionyl Coenzyme A ca	1545.16670	1676.80830	1352.55730	1356.22350	Detected	Detected	Detected	Detected
9266	A_33_P3212804	C22orf42	Hs.426551	chromosome 22 open reading frame 42	Homo sapiens chromosome 22 open rea	16.78405	45.06262	10.48611	13.76863	Detected	Detected	Compromised	Compromised
9267	A_24_P87931	APOL1	Hs.114309	apolipoprotein L, 1	Homo sapiens apolipoprotein L, 1 (APO	4937.09380	6741.40670	1716.83750	1586.16240	Detected	Detected	Detected	Detected
9268	A_33_P3346108	RUFY4	Hs.570069	RUN and FYVE domain containing 4	Homo sapiens RUN and FYVE domain c	202.51250	258.38324	84.77543	91.76355	Detected	Detected	Detected	Detected
9269	A_33_P3221568	ARMC5	Hs.720682	armadillo repeat containing 5	Homo sapiens armadillo repeat containi	2462.78690	2287.35550	865.38560	625.80930	Detected	Detected	Detected	Detected
9270	A_23_P78685	FARSA	Hs.23111	phenylalanyl-tRNA synthetase, alpha su	Homo sapiens phenylalanyl-tRNA synth	2675.45680	2740.29320	2341.14700	2487.44340	Detected	Detected	Detected	Detected
9271	A_24_P41975	TDRKH	Hs.144439	tudor and KH domain containing	Homo sapiens tudor and KH domain cor	65.60009	32.30399	52.24258	28.08325	Detected	Detected	Detected	Detected
9272	A_23_P139669	SLC2A3	Hs.419240	solute carrier family 2 (facilitated glucos	Homo sapiens solute carrier family 2 (fa	305.25296	148.14822	183.16533	78.58719	Detected	Detected	Detected	Detected
9273	A_24_P48587	PSENEN	Hs.534465	presenilin enhancer 2 homolog (C. elega	Homo sapiens presenilin enhancer 2 hor	1152.91860	1087.03990	1268.20460	1065.00500	Detected	Detected	Detected	Detected
9274	A_23_P58647	CTNNA1	Hs.534797	catenin (cadherin-associated protein), a	Homo sapiens catenin (cadherin-associ	7889.29500	9711.28300	6839.84100	7306.45170	Detected	Detected	Detected	Detected
9275	A_23_P321473	FAM76A	Hs.469359	family with sequence similarity 76, mem	Homo sapiens family with sequence sim	629.73300	531.77680	440.64703	367.97046	Detected	Detected	Detected	Detected
9276	A_24_P143440	DYNLRB1	Hs.593920	dynein, light chain, roadblock-type 1	Homo sapiens dynein, light chain, roadb	2971.31960	3963.13100	3947.72020	4561.11100	Detected	Detected	Detected	Detected
9277	A_33_P3244112	UOX	Hs.647646	urate oxidase (pseudogene)	Homo sapiens urate oxidase (pseudoger	20.60939	8.20991	18.55851	3.86548	Detected	Compromised	Detected	Compromised
9278	A_23_P337729	TMEM180	Hs.309069	transmembrane protein 180	Homo sapiens transmembrane protein 1	623.67960	122.83911	692.44214	130.13388	Detected	Detected	Detected	Detected
9279	A_33_P3262205	CPNE1	Hs.246413	copine I	Homo sapiens copine I (CPNE1), transc	3230.38570	4323.16500	2743.03340	3401.25540	Detected	Detected	Detected	Detected
9280	A_33_P3230399	ZNF784	Hs.53996	zinc finger protein 784	Homo sapiens zinc finger protein 784 (Z	973.69635	1637.95600	1109.79710	1878.40050	Detected	Detected	Detected	Detected
9281	A_23_P398770	PWWP2A	Hs.438851	PWWP domain containing 2A	Homo sapiens PWWP domain containing	1081.32150	1277.02590	818.16095	774.15796	Detected	Detected	Detected	Detected
9282	A_33_P3703637	LOC285300	Hs.638436	hypothetical protein LOC285300	Homo sapiens cDNA FLJ38336 fis, clon	284.20280	785.07820	179.70844	366.34530	Detected	Detected	Detected	Detected
9283	A_33_P3391005	NEDD4L	Hs.185677	neural precursor cell expressed, develo	Homo sapiens neural precursor cell exp	51.34712	77.57996	46.33381	49.06812	Detected	Detected	Detected	Detected
9284	A_33_P3222139	SREBF1	Hs.592123	sterol regulatory element binding transc	Homo sapiens sterol regulatory element	541.43365	778.97516	360.40850	411.90823	Detected	Detected	Detected	Detected
9285	A_24_P39211	ING1	Hs.46700	inhibitor of growth family, member 1	Homo sapiens inhibitor of growth family	914.31104	1056.38500	1088.87600	1154.65060	Detected	Detected	Detected	Detected
9286	A_23_P382240	TMEM26	Hs.623955	transmembrane protein 26	Homo sapiens transmembrane protein 2	24.65031	31.13321	21.20381	24.56933	Detected	Detected	Detected	Detected
9287	A_24_P372553	C10orf137	Hs.468688	chromosome 10 open reading frame 137	Homo sapiens chromosome 10 open rea	256.63013	143.91724	231.74957	125.78681	Detected	Detected	Detected	Detected
9288	A_33_P3405114	ENHO	Hs.522085	energy homeostasis associated	Homo sapiens energy homeostasis asso	39.49242	39.49656	24.39947	21.92454	Detected	Detected	Detected	Detected
9289	A_33_P3221828	LOC100128253	Hs.447497	hypothetical protein LOC100128253	PREDICTED: Homo sapiens hypothetica	312.87717	364.28638	185.43147	154.49520	Detected	Detected	Detected	Detected
9290	A_32_P221305	LOC389634		hypothetical LOC389634	Homo sapiens hypothetical LOC389634	22.70916	23.01786	24.73434	20.26225	Detected	Detected	Detected	Detected
9291	A_23_P256773	TSSC1	Hs.502770	tumor suppressing subtransferable canc	Homo sapiens tumor suppressing subtra	2439.29540	5501.24660	2752.17460	5317.79250	Detected	Detected	Detected	Detected
9292	A_33_P3365047	RBBP9	Hs.69330	retinoblastoma binding protein 9	Homo sapiens retinoblastoma binding pr	28.07087	38.38120	30.85762	37.87996	Detected	Detected	Detected	Detected
9293	A_23_P169278	AGTPBP1	Hs.719980	ATP/GTP binding protein 1	Homo sapiens ATP/GTP binding protei	1521.22640	520.86270	1091.82900	308.00854	Detected	Detected	Detected	Detected
9294	A_33_P3252093	TMED10	Hs.74137	transmembrane emp24-like trafficking p	Homo sapiens transmembrane emp24-li	27503.90400	30030.21900	17843.54500	16259.19100	Detected	Detected	Detected	Detected
9295	A_33_P3368049	LOC643988	Hs.528816	hypothetical LOC643988	PREDICTED: Homo sapiens hypothetica	2736.58400	2101.00270	2690.82900	1860.89280	Detected	Detected	Detected	Detected
9296	A_24_P191656	ACAD9	Hs.567482	acyl-Coenzyme A dehydrogenase family	Homo sapiens acyl-Coenzyme A dehydr	1227.31480	1693.96200	1178.16030	1612.94910	Detected	Detected	Detected	Detected

9297	A_24_P289178	C16orf74	Hs.461655	chromosome 16 open reading frame 74	Homo sapiens chromosome 16 open rea	364.91302	622.32550	532.76794	764.88434	Detected	Detected	Detected	Detected
9298	A_24_P102920	WDR33		WD repeat domain 33	WD repeat-containing protein 33 (WD re	56.17299	15.98086	41.71609	10.62620	Detected	Compromised	Detected	Compromised
9299	A_33_P3267651	DZIP3	Hs.409210	DAZ interacting protein 3, zinc finger	Homo sapiens DAZ interacting protein 3	27.80087	18.69374	28.04115	14.30463	Detected	Detected	Detected	Detected
9300	A_33_P3397613					48.83320	52.75506	15.42836	27.87464	Detected	Detected	Detected	Detected
9301	A_23_P384748	PLEKHH2	Hs.164162	pleckstrin homology domain containing,	Homo sapiens pleckstrin homology dom	401.65756	79.17293	250.45103	41.84094	Detected	Detected	Detected	Detected
9302	A_33_P3346032	LOC727808	Hs.589350	hypothetical protein LOC727808	PREDICTED: Homo sapiens hypothetica	48.89111	45.93119	41.41632	39.94535	Detected	Detected	Detected	Detected
9303	A_32_P120791	LOC727916	Hs.706850	hypothetical protein LOC727916	PREDICTED: Homo sapiens hypothetica	53.48153	254.30273	43.69270	160.62193	Detected	Detected	Detected	Detected
9304	A_24_P232696	SMARCD1	Hs.79335	SWI/SNF related, matrix associated, ac	Homo sapiens SWI/SNF related, matrix	1644.11880	2314.58150	1644.95690	1921.69040	Detected	Detected	Detected	Detected
9305	A_23_P4662	BCL3	Hs.31210	B-cell CLL/lymphoma 3	Homo sapiens B-cell CLL/lymphoma 3	10567.56400	5512.40000	10185.99700	4650.01500	Detected	Detected	Detected	Detected
9306	A_33_P3304764					66.87077	95.26183	46.61299	48.42633	Detected	Detected	Detected	Detected
9307	A_33_P3422479	CRELD1	Hs.9383	cysteine-rich with EGF-like domains 1	Homo sapiens cysteine-rich with EGF-l	37.26279	43.53835	37.34510	48.07254	Detected	Detected	Detected	Detected
9308	A_33_P3246153				Seven transmembrane helix receptor [S	13.36519	11.58240	37.90176	17.91634	Detected	Compromised	Detected	Detected
9309	A_33_P3318444	RNF222	Hs.526550	ring finger protein 222	Homo sapiens ring finger protein 222 (R	2445.50500	2845.77610	1005.96027	1367.04710	Detected	Detected	Detected	Detected
9310	A_33_P3288824	H2AFB3	Hs.632841	H2A histone family, member B3	Homo sapiens H2A histone family, mem	18.84365	15.46096	12.05762	11.67718	Detected	Compromised	Compromised	Compromised
9311	A_23_P119936	REG3A	Hs.567312	regenerating islet-derived 3 alpha	Homo sapiens regenerating islet-derive	10.81568	17.26954	12.79340	9.68179	Compromised	Detected	Compromised	Compromised
9312	A_24_P136551		Hs.464333		Nuclear protein localization protein 4 ho	13.81564	15.19224	9.66120	9.76195	Detected	Compromised	Compromised	Compromised
9313	A_33_P3319943	ATRX	Hs.533526	alpha thalassemia/mental retardation sy	Homo sapiens alpha thalassemia/menta	1880.34350	1241.34140	1565.96670	790.58810	Detected	Detected	Detected	Detected
9314	A_23_P115036	IPO13	Hs.158497	importin 13	Homo sapiens importin 13 (IPO13), mRN	487.53670	349.19160	470.70340	346.99274	Detected	Detected	Detected	Detected
9315	A_24_P273253	AHNAK2	Hs.441783	AHNAK nucleoprotein 2	Homo sapiens AHNAK nucleoprotein 2 (	827.14780	543.38490	584.55020	363.75748	Detected	Detected	Detected	Detected
9316	A_24_P9883	DKFZp761E198	Hs.591957	DKFZp761E198 protein	Homo sapiens DKFZp761E198 protein (	69.86681	29.94687	185.50381	82.60452	Detected	Detected	Detected	Detected
9317	A_23_P501822	JUP	Hs.514174	junction plakoglobin	Homo sapiens junction plakoglobin (JUP	216.02950	134.52110	285.81058	121.13493	Detected	Detected	Detected	Detected
9318	A_23_P255896	C2orf89	Hs.469134	chromosome 2 open reading frame 89	Homo sapiens chromosome 2 open read	49.68837	56.52045	48.20181	64.57096	Detected	Detected	Detected	Detected
9319	A_33_P3338674	C1orf231	Hs.461300	chromosome 1 open reading frame 231	Homo sapiens chromosome 1 open read	13.66467	15.33024	12.72578	16.24959	Detected	Compromised	Compromised	Detected
9320	A_33_P3220545	SLC6A19	Hs.481478	solute carrier family 6 (neutral amino ac	Homo sapiens solute carrier family 6 (ne	56.35831	79.48962	31.00996	40.39283	Detected	Detected	Detected	Detected
9321	A_33_P3266848				Seven transmembrane helix receptor [S	310.90103	426.10678	145.68277	166.53165	Detected	Detected	Detected	Detected
9322	A_33_P3321225					96.91126	124.33658	79.00492	76.11039	Detected	Detected	Detected	Detected
9323	A_33_P3299796		Hs.279881		Endoplasmic reticulum mannosyl-oligosa	51.23203	23.60925	40.11793	24.23261	Detected	Detected	Detected	Detected
9324	A_33_P3349469	ATAD3A	Hs.640083	ATPase family, AAA domain containing	Homo sapiens ATPase family, AAA dom	14890.58700	27680.51600	16320.12800	27627.19300	Detected	Detected	Detected	Detected
9325	A_33_P3329088	PRSS8	Hs.75799	protease, serine, 8	Homo sapiens protease, serine, 8 (PRSS	25.31643	18.08651	17.63175	9.58715	Detected	Detected	Detected	Compromised
9326	A_33_P3219965	TCIRG1	Hs.495985	T-cell, immune regulator 1, ATPase, H+	Homo sapiens T-cell, immune regulator	4701.54540	7432.36100	6318.92100	8590.87400	Detected	Detected	Detected	Detected
9327	A_23_P166408	OSM	Hs.248156	oncostatin M	Homo sapiens oncostatin M (OSM), mR	10.87188	10.60837	14.07833	7.37423	Compromised	Compromised	Detected	Compromised
9328	A_23_P140830	ELMO3	Hs.377416	engulfment and cell motility 3	Homo sapiens engulfment and cell motil	114.54050	42.27829	132.72249	40.26522	Detected	Detected	Detected	Detected
9329	A_32_P212095	C17orf105	Hs.252707	chromosome 17 open reading frame 105	Homo sapiens chromosome 17 open rea	12.72164	7.12114	14.19570	14.06859	Detected	Compromised	Detected	Detected
9330	A_33_P3294297	RNF126	Hs.69554	ring finger protein 126	Homo sapiens ring finger protein 126 (R	4841.84960	10572.48300	4770.55400	9459.70600	Detected	Detected	Detected	Detected
9331	A_33_P3523583	LOC642924	Hs.434882	hypothetical protein LOC642924	Homo sapiens cDNA clone IMAGE:5277	59.20582	3.17288	47.81736	2.62575	Detected	Compromised	Detected	Compromised
9332	A_33_P3325933	PQBP1	Hs.534384	polyglutamine binding protein 1	Homo sapiens polyglutamine binding pro	461.57642	597.92420	495.56204	587.91077	Detected	Detected	Detected	Detected
9333	A_23_P411353	ZCCHC5	Hs.134873	zinc finger, CCHC domain containing 5	Homo sapiens zinc finger, CCHC domain	3.32482	22.25131	14.20888	16.69952	Compromised	Detected	Detected	Detected
9334	A_33_P3248539	LCN1	Hs.530311	lipocalin 1 (tear prealbumin)	Homo sapiens lipocalin 1 (tear prealbum	8.69893	13.68588	14.41197	7.65503	Compromised	Compromised	Detected	Compromised
9335	A_23_P300905	CCNY	Hs.14745	cyclin Y	Homo sapiens cyclin Y (CCNY), transcr	1582.50210	1229.48940	1300.59450	731.66740	Detected	Detected	Detected	Detected
9336	A_33_P3270360	OR1S1	Hs.553645	olfactory receptor, family 1, subfamily S	Homo sapiens olfactory receptor, family	48.61759	62.34711	36.73217	35.04812	Detected	Detected	Detected	Detected
9337	A_33_P3395230		Hs.185235		Homo sapiens, Similar to nitric oxide sy	12.59049	5.55958	13.11570	11.78038	Detected	Compromised	Detected	Compromised
9338	A_33_P3257568		Hs.653263		Centriolin (110 kDa centrosomal protein	13.55334	24.62427	11.56696	15.61266	Detected	Detected	Compromised	Detected
9339	A_23_P216429	ASPN	Hs.435655	asporin	Homo sapiens asporin (ASPN), mRNA [	6.12644	44.50567	12.62557	62.93216	Compromised	Detected	Detected	Detected
9340	A_32_P75094	AIFM2	Hs.655377	apoptosis-inducing factor, mitochondria	Homo sapiens apoptosis-inducing facto	3826.26730	3599.52080	3781.40970	2977.42070	Detected	Detected	Detected	Detected
9341	A_24_P389491	COQ4	Hs.98541	coenzyme Q4 homolog (S. cerevisiae)	Homo sapiens coenzyme Q4 homolog (S	1058.41270	735.22220	1437.73220	973.25230	Detected	Detected	Detected	Detected
9342	A_33_P3264013				PRO2176 [Source:UniProtKB/TrEMBL	19.98295	45.57377	23.41162	34.11706	Detected	Detected	Detected	Detected
9343	A_33_P3382714	LOC144742	Hs.524782	hypothetical LOC144742	Homo sapiens hypothetical LOC144742	2.35740	5.36833	5.36713	33.31650	Compromised	Compromised	Compromised	Detected
9344	A_33_P3268035	LYZL1	Hs.558572	lysozyme-like 1	Homo sapiens lysozyme-like 1 (LYZL1),	21.65354	17.96827	45.20168	32.06171	Detected	Detected	Detected	Detected
9345	A_33_P3413845	TIMM13	Hs.75056	translocase of inner mitochondrial mem	Homo sapiens translocase of inner mito	240.81690	287.45370	278.56958	348.79642	Detected	Detected	Detected	Detected
9346	A_24_P380536	CD164	Hs.520313	CD164 molecule, sialomucin	Homo sapiens CD164 molecule, sialomu	923.72420	1798.87780	855.42786	1430.53960	Detected	Detected	Detected	Detected
9347	A_33_P3215720	PPP1R2	Hs.535731	protein phosphatase 1, regulatory (inhib	Homo sapiens protein phosphatase 1, re	171.68279	181.98848	85.03074	87.36842	Detected	Detected	Detected	Detected
9348	A_23_P66137	SOX8	Hs.243678	SRY (sex determining region Y)-box 8	Homo sapiens SRY (sex determining reg	4125.94400	3671.28320	1753.01230	309.91550	Detected	Detected	Detected	Detected
9349	A_24_P252497	TRIB1	Hs.444947	tribbles homolog 1 (Drosophila)	Homo sapiens tribbles homolog 1 (Dros	11923.41300	15057.33300	9613.55300	10623.38700	Detected	Detected	Detected	Detected
9350	A_23_P28012	ZNF682	Hs.306298	zinc finger protein 682	Homo sapiens zinc finger protein 682 (Z	62.88941	72.07880	42.97356	53.36730	Detected	Detected	Detected	Detected
9351	A_23_P148308	RBM3	Hs.301404	RNA binding motif (RNP1, RRM) protein	Homo sapiens RNA binding motif (RNP1	41690.28500	47137.93800	28512.33000	26734.99600	Detected	Detected	Detected	Detected
9352	A_33_P3315190		Hs.501928		Protein MICAL-2 [Source:UniProtKB/S	332.73572	408.90747	226.34910	287.76987	Detected	Detected	Detected	Detected
9353	A_33_P3254756	LOC550112	Hs.479879	hypothetical LOC550112	Homo sapiens hypothetical LOC550112	690.30786	732.30950	724.93890	638.19520	Detected	Detected	Detected	Detected
9354	A_33_P3256773	C9orf80	Hs.658575	chromosome 9 open reading frame 80	Homo sapiens chromosome 9 open read	880.01746	758.21564	771.18585	588.70917	Detected	Detected	Detected	Detected
9355	A_23_P47565	LDHA	Hs.2795	lactate dehydrogenase A	Homo sapiens lactate dehydrogenase A	76183.23400	182746.06000	70225.93000	149686.80000	Detected	Detected	Detected	Detected
9356	A_24_P113824	TMEM50A	Hs.523054	transmembrane protein 50A	Homo sapiens transmembrane protein 5	4551.43950	7553.36600	3966.91850	5786.75540	Detected	Detected	Detected	Detected
9357	A_33_P3266609					20.90933	22.66924	7.52382	8.34500	Detected	Detected	Compromised	Compromised
9358	A_33_P3344282				HCLS1-binding protein 3 (HS1-binding	41.84630	258.80066	30.82096	162.26150	Detected	Detected	Detected	Detected
9359	A_33_P3653330	MAP2K4	Hs.514681	mitogen-activated protein kinase kinase	Homo sapiens mitogen-activated protei	1675.24390	2667.77300	1286.60060	1947.01940	Detected	Detected	Detected	Detected
9360	A_33_P3245160				GB	337.61868	557.20140	171.36470	222.19153	Detected	Detected	Detected	Detected
9361	A_23_P161446	GDI2	Hs.299055	GDP dissociation inhibitor 2	Homo sapiens GDP dissociation inhibito	7448.15800	11842.68700	6629.82280	9029.80500	Detected	Detected	Detected	Detected
9362	A_23_P54006	HECTD1	Hs.708017	HECT domain containing 1	Homo sapiens HECT domain containing	9532.00500	7908.95400	9055.29300	6657.85600	Detected	Detected	Detected	Detected
9363	A_33_P3274164				Histone-lysine N-methyltransferase, H3	416.38867	537.77010	295.62207	305.49457	Detected	Detected	Detected	Detected
9364	A_33_P3357227					28.79807	48.30300	2.80054	2.88436	Detected	Detected	Compromised	Compromised
9365	A_23_P101699	HDGF2	Hs.43071	hepatoma-derived growth factor-relate	Homo sapiens hepatoma-derived growth	844.71370	1161.42300	848.37740	1006.67535	Detected	Detected	Detected	Detected
9366	A_32_P220739	ZNF658	Hs.522147	zinc finger protein 658	Homo sapiens zinc finger protein 658 (Z	228.95164	126.47270	232.63544	125.83072	Detected	Detected	Detected	Detected
9367	A_23_P151975	RHCG	Hs.459284	Rh family, C glycoprotein	Homo sapiens Rh family, C glycoprotein	20.51694	35.32925	36.33695	55.59158	Detected	Detected	Detected	Detected
9368	A_33_P3240637					15.19428	21.82345	29.92669	21.52530	Detected	Detected	Detected	Detected
9369	A_23_P78734	MYH14	Hs.467142	myosin, heavy chain 14	Homo sapiens myosin, heavy chain 14 (	11531.57800	16951.63900	4967.78860	4807.88570	Detected	Detected	Detected	Detected
9370	A_24_P309360	TM9SF3	Hs.500674	transmembrane 9 superfamily member 3	Homo sapiens transmembrane 9 superfa	5199.47270	6607.27340	3977.30320	3992.54470	Detected	Detected	Detected	Detected
9371	A_33_P3344477	FERMT2	Hs.509343	fermitin family homolog 2 (Drosophila)	Homo sapiens fermitin family homolog 2	11354.79500	10249.18300	8216.32300	6299.08200	Detected	Detected	Detected	Detected

9372	A_33_P3407524	SUV39H1	Hs.522639	suppressor of variegation 3-9 homolog	Homo sapiens suppressor of variegatior	28.29967	114.60712	21.92879	87.53416	Detected	Detected	Detected	Detected
9373	A_33_P3340454				UPF0594 protein C13orf38 [Source:Un	32.82027	3.18273	22.20318	6.07510	Detected	Compromised	Detected	Compromised
9374	A_23_P27917	OR10H1	Hs.533925	olfactory receptor, family 10, subfamily	Homo sapiens olfactory receptor, family	21.78593	38.99443	11.40156	15.94139	Detected	Detected	Compromised	Detected
9375	A_32_P138617	KIAA2018	Hs.632570	KIAA2018	Homo sapiens KIAA2018 (KIAA2018), m	622.09235	519.59406	451.24023	335.89816	Detected	Detected	Detected	Detected
9376	A_33_P3333224	GLIS2	Hs.592087	GLIS family zinc finger 2	Homo sapiens GLIS family zinc finger 2	3349.71500	2146.23270	1214.32060	689.30194	Detected	Detected	Detected	Detected
9377	A_33_P3233437	LOC100133176		similar to zinc finger protein 195	PREDICTED: Homo sapiens similar to z	16.30072	15.88478	17.76827	13.22943	Detected	Detected	Detected	Detected
9378	A_23_P253752	FAM54A	Hs.121536	family with sequence similarity 54, mem	Homo sapiens family with sequence sim	213.55846	3529.86800	163.92441	2358.63160	Detected	Detected	Detected	Detected
9379	A_33_P3249305	CHRD	Hs.166186	chordin	Homo sapiens chordin (CHRD), mRNA [	62.49342	80.74168	17.61906	17.34270	Detected	Detected	Detected	Detected
9380	A_24_P196372	AGBL5	Hs.138207	ATP/GTP binding protein-like 5	Homo sapiens ATP/GTP binding protei	643.01843	326.04742	471.79900	258.02905	Detected	Detected	Detected	Detected
9381	A_23_P59481	UBE3C	Hs.118351	ubiquitin protein ligase E3C	Homo sapiens ubiquitin protein ligase E	1959.11390	3159.75930	1778.95580	2475.97140	Detected	Detected	Detected	Detected
9382	A_23_P16976	ANXA4	Hs.422986	annexin A4	Homo sapiens annexin A4 (ANXA4), mR	3802.01270	7346.18800	3979.70170	6152.65040	Detected	Detected	Detected	Detected
9383	A_33_P3304603	ZMIZ1	Hs.193118	zinc finger, MIZ-type containing 1	Homo sapiens zinc finger, MIZ-type cor	413.51486	764.51733	324.06027	387.78534	Detected	Detected	Detected	Detected
9384	A_23_P170839	SEPT2	Hs.712994	septin 2	Homo sapiens septin 2 (SEPT2), transc	7847.15500	8448.66700	6177.95070	5067.42900	Detected	Detected	Detected	Detected
9385	A_33_P3315331	CNTD2	Hs.631603	cyclin N-terminal domain containing 2	Homo sapiens cyclin N-terminal domain	148.48862	102.16238	61.28627	41.14066	Detected	Detected	Detected	Detected
9386	A_33_P3279241	ZNF608	Hs.266616	zinc finger protein 608	Homo sapiens zinc finger protein 608 (Z	70.12565	66.23899	65.12096	49.45082	Detected	Detected	Detected	Detected
9387	A_33_P3342957	STK17B	Hs.88297	serine/threonine kinase 17b	Homo sapiens serine/threonine kinase	604.75690	1413.37980	547.88440	1010.85110	Detected	Detected	Detected	Detected
9388	A_23_P77135	ATPBD4	Hs.107196	ATP binding domain 4	Homo sapiens ATP binding domain 4 (A	52.67199	12.97613	49.46977	14.83178	Detected	Compromised	Detected	Detected
9389	A_33_P3297005	EME2	Hs.7247	essential meiotic endonuclease 1 homol	Homo sapiens essential meiotic endonu	17.52211	13.67831	18.60528	14.32471	Detected	Compromised	Detected	Detected
9390	A_23_P207632	ATP2A3	Hs.513870	ATPase, Ca++ transporting, ubiquitous	Homo sapiens ATPase, Ca++ transporti	582.04010	42.80889	392.92307	31.21948	Detected	Detected	Detected	Detected
9391	A_33_P3329241		Hs.149545		DA734585 NT2RP2 Homo sapiens cDN/	11.45954	15.68588	14.31880	10.45150	Compromised	Compromised	Detected	Compromised
9392	A_24_P336417	XPR1	Hs.227656	xenotropic and polytropic retrovirus rec	Homo sapiens xenotropic and polytropi	679.44980	445.58230	642.81910	371.47806	Detected	Detected	Detected	Detected
9393	A_23_P111517	WBSCR17	Hs.488591	Williams-Beuren syndrome chromosome	Homo sapiens Williams-Beuren syndrom	14.73622	11.31541	14.37721	17.17336	Detected	Compromised	Detected	Detected
9394	A_33_P3363630	LOC100131993	Hs.25447	similar to hCG2020760	Homo sapiens cDNA FLJ43180 fis, clon	22.50280	18.94530	24.62602	15.27020	Detected	Detected	Detected	Detected
9395	A_33_P3389188	TFAM	Hs.642966	transcription factor A, mitochondrial	Homo sapiens transcription factor A, m	1019.93520	1052.94170	1085.37990	891.41560	Detected	Detected	Detected	Detected
9396	A_24_P38081	FKBP5	Hs.407190	FK506 binding protein 5	Homo sapiens FK506 binding protein 5 (	2246.99240	2074.29700	1518.33010	1170.60680	Detected	Detected	Detected	Detected
9397	A_33_P3264771	NOTO	Hs.694384	notochord homeobox	Homo sapiens notochord homeobox (NC	5.54969	3.31843	15.09831	13.23901	Compromised	Compromised	Detected	Compromised
9398	A_33_P3382730	LARP4B	Hs.631814	La ribonucleoprotein domain family, mer	Homo sapiens La ribonucleoprotein dom	59.41051	61.64251	51.14074	54.32513	Detected	Detected	Detected	Detected
9399	A_33_P3416668	VWA1	Hs.449009	von Willebrand factor A domain contain	Homo sapiens von Willebrand factor A c	73.37101	80.32998	72.69727	71.83455	Detected	Detected	Detected	Detected
9400	A_33_P3311974				Prospero homeobox protein 2 (Homeob	5.13720	16.82082	4.75180	8.52713	Compromised	Detected	Compromised	Compromised
9401	A_23_P200396	C1orf91	Hs.272299	chromosome 1 open reading frame 91	Homo sapiens chromosome 1 open rea	441.30350	625.23460	564.72253	738.01575	Detected	Detected	Detected	Detected
9402	A_24_P203502	LOC284288		similar to ribosomal protein L24-like	PREDICTED: Homo sapiens similar to ri	548.47546	327.64905	309.81064	145.71663	Detected	Detected	Detected	Detected
9403	A_32_P72940	RPL35	Hs.182825	ribosomal protein L35	Homo sapiens ribosomal protein L35 (R	25908.53000	25311.43200	34952.36300	33092.27700	Detected	Detected	Detected	Detected
9404	A_23_P9523	RBKS	Hs.11916	ribokinase	Homo sapiens ribokinase (RBKS), mRN/	855.15027	493.90494	795.45490	411.99646	Detected	Detected	Detected	Detected
9405	A_24_P65910	UQCC	Hs.714359	ubiquinol-cytochrome c reductase com	Homo sapiens ubiquinol-cytochrome c	3565.35840	2576.54420	2765.10550	1648.48380	Detected	Detected	Detected	Detected
9406	A_23_P251268	C3orf42	Hs.302131	chromosome 3 open reading frame 42	Homo sapiens chromosome 3 open rea	76.11200	42.10264	72.73938	38.57610	Detected	Detected	Detected	Detected
9407	A_24_P152983					66188.88000	63246.16800	40444.57400	33436.42000	Detected	Detected	Detected	Detected
9408	A_23_P154832	ATP5J	Hs.246310	ATP synthase, H+ transporting, mitoch	Homo sapiens ATP synthase, H+ transp	22305.75600	23244.02000	24964.30700	22933.69000	Detected	Detected	Detected	Detected
9409	A_33_P3319940	ATRX	Hs.533526	alpha thalassemia/mental retardation s	Homo sapiens alpha thalassemia/menta	261.56070	170.83430	134.05821	85.21494	Detected	Detected	Detected	Detected
9410	A_33_P3399474					27.58037	32.78729	33.86806	29.68038	Detected	Detected	Detected	Detected
9411	A_33_P3413335				Homo sapiens cervical cancer suppress	397.34274	222.17104	319.24002	147.95386	Detected	Detected	Detected	Detected
9412	A_23_P99927	PTPLAD1	Hs.512973	protein tyrosine phosphatase-like A dor	Homo sapiens protein tyrosine phospha	6861.10640	8610.20400	5073.48600	5375.40100	Detected	Detected	Detected	Detected
9413	A_23_P47226	YIF1A	Hs.446445	Yip1 interacting factor homolog A (S. ce	Homo sapiens Yip1 interacting factor h	20624.28000	29446.35400	21034.88900	25433.53300	Detected	Detected	Detected	Detected
9414	A_33_P3244433				UPF0526 protein C7orf67 (Protein FAM	26.80921	28.90692	24.22409	20.51914	Detected	Detected	Detected	Detected
9415	A_24_P347704	CTCF	Hs.368367	CCCTC-binding factor (zinc finger prot	Homo sapiens CCCTC-binding factor (z	739.46857	782.82700	644.21014	597.98114	Detected	Detected	Detected	Detected
9416	A_33_P3414107	LOC729040	Hs.678658	hypothetical LOC729040	PREDICTED: Homo sapiens hypothetica	53.62075	10.64894	47.26759	15.96916	Detected	Compromised	Detected	Detected
9417	A_33_P3228023	SCPEP1	Hs.514950	serine carboxypeptidase 1	Homo sapiens serine carboxypeptidase	180.83676	85.41187	231.55954	121.63503	Detected	Detected	Detected	Detected
9418	A_33_P3358938	CLOCK	Hs.436975	clock homolog (mouse)	Homo sapiens clock homolog (mouse) (	395.27783	438.87784	263.83347	278.07336	Detected	Detected	Detected	Detected
9419	A_23_P125815	RBM10	Hs.401509	RNA binding motif protein 10	Homo sapiens RNA binding motif protei	5122.28470	6383.51270	5396.80960	6333.93000	Detected	Detected	Detected	Detected
9420	A_24_P942604	SMC1A	Hs.211602	structural maintenance of chromosome	Homo sapiens structural maintenance o	730.95050	1205.97390	665.64215	974.45610	Detected	Detected	Detected	Detected
9421	A_33_P3223239	ST7L	Hs.201921	suppression of tumorigenicity 7 like	Homo sapiens suppression of tumoriger	199.28976	130.11260	250.20456	130.91676	Detected	Detected	Detected	Detected
9422	A_33_P3411427	ZNF837	Hs.222236	zinc finger protein 837	Homo sapiens zinc finger protein 837 (Z	2316.46260	1989.17220	2344.21950	1866.42540	Detected	Detected	Detected	Detected
9423	A_33_P3390823					2317.59300	767.38970	1903.72630	714.42535	Detected	Detected	Detected	Detected
9424	A_23_P99292	RAD51AP1	Hs.504550	RAD51 associated protein 1	Homo sapiens RAD51 associated prote	124.78508	1082.54600	114.96819	974.53090	Detected	Detected	Detected	Detected
9425	A_33_P3320062	PLD1	Hs.382865	phospholipase D1, phosphatidylcholine-	Homo sapiens phospholipase D1, phosp	522.73880	34.48394	370.27994	33.87997	Detected	Detected	Detected	Detected
9426	A_23_P109452	CHEK2	Hs.291363	CHK2 checkpoint homolog (S. pombe)	Homo sapiens CHK2 checkpoint homol	646.76385	479.17426	594.31580	395.81150	Detected	Detected	Detected	Detected
9427	A_33_P3285038	LASS5	Hs.270525	LAG1 homolog, ceramide synthase 5	Homo sapiens LAG1 homolog, ceramide	4804.18460	6555.92720	5595.28500	6498.71340	Detected	Detected	Detected	Detected
9428	A_33_P3445679	FLJ42392	Hs.577960	hypothetical gene supported by AK1243	Homo sapiens cDNA FLJ42392 fis, clon	137.62662	115.28111	72.17735	36.68576	Detected	Detected	Detected	Detected
9429	A_24_P143843				Putative uncharacterized protein ENSP	268.29398	522.70557	123.14941	191.45291	Detected	Detected	Detected	Detected
9430	A_24_P17870	HCP5	Hs.130838	HLA complex P5	Homo sapiens HLA complex P5 (HCP5)	70.11137	46.63548	46.62469	28.13505	Detected	Detected	Detected	Detected
9431	A_33_P3384260					7329.14840	5415.43160	5829.98100	3514.06900	Detected	Detected	Detected	Detected
9432	A_33_P3255782					57058.63300	49595.06600	69425.66400	48879.65000	Detected	Detected	Detected	Detected
9433	A_23_P324384	RPS4Y2	Hs.367761	ribosomal protein S4, Y-linked 2	Homo sapiens ribosomal protein S4, Y-	44035.61000	11.21212	40853.48400	2.57502	Detected	Compromised	Detected	Compromised
9434	A_33_P3253628				Alanine--glyoxylate aminotransferase 2	19.48911	15.66556	14.57309	10.87678	Detected	Detected	Detected	Compromised
9435	A_23_P146209	RNF170	Hs.491626	ring finger protein 170	Homo sapiens ring finger protein 170 (R	577.13947	475.61804	640.86150	379.27075	Detected	Detected	Detected	Detected
9436	A_24_P333802	MAGT1	Hs.323562	magnesium transporter 1	Homo sapiens magnesium transporter 1	16298.45800	17572.61000	10302.18500	9107.32100	Detected	Detected	Detected	Detected
9437	A_23_P355447	ZDHHC22	Hs.525485	zinc finger, DHHC-type containing 22	Homo sapiens zinc finger, DHHC-type c	16.79664	18.80100	4.86688	8.85396	Detected	Detected	Compromised	Compromised
9438	A_33_P3361758	C15orf33	Hs.712959	chromosome 15 open reading frame 33	Homo sapiens chromosome 15 open rea	11.72691	3.20045	12.89860	3.23806	Compromised	Compromised	Detected	Compromised
9439	A_23_P107307	SLC13A2	Hs.102307	solute carrier family 13 (sodium-depend	Homo sapiens solute carrier family 13 (	348.07020	336.33496	231.05275	210.02219	Detected	Detected	Detected	Detected
9440	A_23_P373119	HMGB3L1	Hs.558624	high-mobility group box 3-like 1	Homo sapiens high-mobility group box 3	1296.64650	6755.07030	837.34796	3629.98630	Detected	Detected	Detected	Detected
9441	A_23_P93988	ARHGEF5	Hs.334	Rho guanine nucleotide exchange facto	Homo sapiens Rho guanine nucleotide e	404.30008	671.26404	355.22974	549.69210	Detected	Detected	Detected	Detected
9442	A_33_P3399718	LOC128322		similar to nuclear transport factor 2	PREDICTED: Homo sapiens similar to n	478.85886	1162.41920	627.62683	1275.69620	Detected	Detected	Detected	Detected
9443	A_23_P127195	PRPF18	Hs.161181	PRP18 pre-mRNA processing factor 18	Homo sapiens PRP18 pre-mRNA proce	4879.83250	5388.43260	3621.04960	3576.35570	Detected	Detected	Detected	Detected
9444	A_23_P82975	VPS13B	Hs.191540	vacuolar protein sorting 13 homolog B (	Homo sapiens vacuolar protein sorting	21.07999	23.99711	15.86801	15.70500	Detected	Detected	Detected	Detected
9445	A_33_P3304576	KRTAP5-5	Hs.653467	keratin associated protein 5-5	Homo sapiens keratin associated protei	73.34900	83.61591	29.69916	21.91049	Detected	Detected	Detected	Detected
9446	A_23_P328034	C20orf96	Hs.348112	chromosome 20 open reading frame 96	Homo sapiens chromosome 20 open rea	75.09042	26.61464	82.52980	19.61817	Detected	Detected	Detected	Detected

9447	A_24_P58037	LOC392288	Hs.568035	similar to microtubule-associated prote	PREDICTED: Homo sapiens similar to m	656.83984	1083.31260	569.28094	782.83480	Detected	Detected	Detected	Detected
9448	A_33_P3390673					27.70171	20.99465	23.37118	11.73920	Detected	Detected	Detected	Compromised
9449	A_23_P131348	THUMPD2	Hs.468254	THUMP domain containing 2	Homo sapiens THUMP domain containir	791.67950	821.49060	820.79530	724.79280	Detected	Detected	Detected	Detected
9450	A_32_P49199	PGR	Hs.32405	progesterone receptor	Homo sapiens progesterone receptor (F	122.83887	48.87093	63.82708	11.00331	Detected	Detected	Detected	Compromised
9451	A_24_P190541	BRWD1	Hs.654740	bromodomain and WD repeat domain co	Homo sapiens bromodomain and WD rep	581.29224	243.44540	402.90714	121.31480	Detected	Detected	Detected	Detected
9452	A_24_P314571		Hs.621849		Kinetochore protein Spc24 (hSpc24) [S	88.55961	2153.04350	98.14356	2331.25270	Detected	Detected	Detected	Detected
9453	A_33_P3331242	G3BP1	Hs.587054	GTPase activating protein (SH3 domain	Homo sapiens GTPase activating protei	6825.17140	12953.73300	5657.29500	9366.89200	Detected	Detected	Detected	Detected
9454	A_33_P3316456	NPL	Hs.496969	N-acetylneuraminate pyruvate lyase (di	Homo sapiens N-acetylneuraminate pyr	66.36359	19.71821	54.51500	10.09606	Detected	Detected	Detected	Compromised
9455	A_23_P330999	MED23	Hs.29679	mediator complex subunit 23	Homo sapiens mediator complex subuni	514.33044	687.94180	418.83395	449.45880	Detected	Detected	Detected	Detected
9456	A_33_P3267502	ANKRD16	Hs.289828	ankyrin repeat domain 16	Homo sapiens ankyrin repeat domain 16	2312.42820	4809.49700	2076.30960	3909.34080	Detected	Detected	Detected	Detected
9457	A_32_P74366	VCPIP1	Hs.632066	valosin containing protein (p97)/p47 co	Deubiquitinating protein VCIP135 (EC 3	1071.66090	1211.89150	697.28020	719.02480	Detected	Detected	Detected	Detected
9458	A_24_P131173	C1orf115	Hs.519839	chromosome 1 open reading frame 115	Homo sapiens chromosome 1 open read	145.61519	36.02716	116.87350	38.50933	Detected	Detected	Detected	Detected
9459	A_23_P321377	C1orf216	Hs.112023	chromosome 1 open reading frame 216	Homo sapiens chromosome 1 open read	969.89530	754.02014	553.30084	379.92215	Detected	Detected	Detected	Detected
9460	A_33_P3350420	SATL1	Hs.640783	spermidine/spermine N1-acetyl transfe	Homo sapiens spermidine/spermine N1-	2.59471	4.05907	9.19833	14.00228	Compromised	Compromised	Compromised	Detected
9461	A_33_P3333960	RP11-374F3.4		hypothetical LOC100188949	Homo sapiens hypothetical LOC100188	34.91412	16.07896	2.63576	8.87851	Detected	Compromised	Compromised	Compromised
9462	A_23_P99811	MDP1	Hs.220963	magnesium-dependent phosphatase 1	Homo sapiens magnesium-dependent pl	5811.06640	2784.75500	4632.14750	1797.97310	Detected	Detected	Detected	Detected
9463	A_23_P338603	CHCHD7	Hs.436913	coiled-coil-helix-coiled-coil-helix doma	Homo sapiens coiled-coil-helix-coiled-c	816.74250	1683.39090	625.68690	1102.89990	Detected	Detected	Detected	Detected
9464	A_24_P175427	MRRF	Hs.368011	mitochondrial ribosome recycling factor	Homo sapiens mitochondrial ribosome r	146.02003	144.55963	100.77071	76.88953	Detected	Detected	Detected	Detected
9465	A_33_P3373364	CLIC4	Hs.440544	chloride intracellular channel 4	Homo sapiens chloride intracellular cha	903.82074	4146.40100	833.44090	3049.46780	Detected	Detected	Detected	Detected
9466	A_33_P3297155	SIP1	Hs.652307	survival of motor neuron protein interac	Homo sapiens survival of motor neuron	40.23253	59.16676	21.92046	32.08240	Detected	Detected	Detected	Detected
9467	A_24_P382489	SLC27A1	Hs.363138	solute carrier family 27 (fatty acid trans	Homo sapiens solute carrier family 27 (	4379.76800	3599.70390	3778.92580	2756.70850	Detected	Detected	Detected	Detected
9468	A_33_P3354945	CSF1	Hs.591402	colony stimulating factor 1 (macrophage	Homo sapiens colony stimulating factor	12.74747	13.18349	23.48087	13.27571	Detected	Compromised	Detected	Compromised
9469	A_24_P144601	POU5F1	Hs.249184	POU class 5 homeobox 1	Homo sapiens POU class 5 homeobox 1	34.96261	15.51868	40.08314	18.15659	Detected	Compromised	Detected	Detected
9470	A_24_P472007	UNC13A	Hs.164502	unc-13 homolog A (C. elegans)	Homo sapiens unc-13 homolog A (C. ele	32.32014	4.03884	17.77806	2.67663	Detected	Compromised	Detected	Compromised
9471	A_23_P319005	HMP19	Hs.559412	HMP19 protein	Homo sapiens HMP19 protein (HMP19),	11.85455	5.46846	11.21257	11.86947	Detected	Compromised	Compromised	Compromised
9472	A_33_P3251989	CRIPT	Hs.133998	cysteine-rich PDZ-binding protein	Homo sapiens cysteine-rich PDZ-bindir	890.44270	1322.24560	792.02450	972.23444	Detected	Detected	Detected	Detected
9473	A_33_P3335571	LOC100133315		transient receptor potential cation char	Homo sapiens transient receptor potent	39.83188	59.45031	30.81570	32.34291	Detected	Detected	Detected	Detected
9474	A_23_P90223	ZNF585B	Hs.631552	zinc finger protein 585B	Homo sapiens zinc finger protein 585B (	382.38754	285.71765	320.46082	200.29059	Detected	Detected	Detected	Detected
9475	A_33_P3542911	SNAR-C4	Hs.560634	small ILF3/NF90-associated RNA C4	602076150F1 NIH_MGC_62 Homo sapier	204.65102	257.29224	63.76453	53.62031	Detected	Detected	Detected	Detected
9476	A_32_P52785	DAAM2	Hs.357128	dishevelled associated activator of mor	Homo sapiens dishevelled associated ac	1866.95240	1201.48840	1880.28140	1045.53970	Detected	Detected	Detected	Detected
9477	A_33_P3281785	NKX3-1	Hs.55999	NK3 homeobox 1	Homo sapiens NK3 homeobox 1 (NKX3-	2.32629	10.03570	4.50406	26.20068	Compromised	Compromised	Compromised	Detected
9478	A_24_P374319	RAP2C	Hs.119889	RAP2C, member of RAS oncogene fami	Homo sapiens RAP2C, member of RAS	1260.81790	1154.05160	949.85500	659.97003	Detected	Detected	Detected	Detected
9479	A_23_P427114	GORASP1	Hs.613614	golgi reassembly stacking protein 1, 65k	Homo sapiens golgi reassembly stacking	152.75032	228.97790	103.29022	140.26330	Detected	Detected	Detected	Detected
9480	A_33_P3285470	BAI1	Hs.194654	brain-specific angiogenesis inhibitor 1	Homo sapiens brain-specific angiogenes	539.04380	305.51315	440.41640	212.13655	Detected	Detected	Detected	Detected
9481	A_23_P48835	KIF23	Hs.270845	kinesin family member 23	Homo sapiens kinesin family member 23	474.74826	13523.92800	324.82614	9163.31800	Detected	Detected	Detected	Detected
9482	A_33_P3329607	RAB1B	Hs.300816	RAB1B, member RAS oncogene family	Homo sapiens RAB1B, member RAS on	296.85733	406.42957	236.24829	347.61490	Detected	Detected	Detected	Detected
9483	A_23_P200073	C1orf128	Hs.31819	chromosome 1 open reading frame 128	Homo sapiens chromosome 1 open read	1167.67310	1389.03480	1015.33997	1113.62220	Detected	Detected	Detected	Detected
9484	A_23_P149189	ZP4	Hs.136241	zona pellucida glycoprotein 4	Homo sapiens zona pellucida glycoprote	196.96684	15.15949	187.11588	10.81741	Detected	Compromised	Detected	Compromised
9485	A_23_P201605	DENND1B	Hs.657779	DENN/MADD domain containing 1B	Homo sapiens DENN/MADD domain co	38.32481	51.30813	38.03793	47.23921	Detected	Detected	Detected	Detected
9486	A_33_P3270009	NCOA1	Hs.596314	nuclear receptor coactivator 1	Homo sapiens nuclear receptor coactiva	60.72897	38.73277	24.86862	12.73731	Detected	Detected	Detected	Compromised
9487	A_23_P157109	ADCYAP1R1	Hs.377783	adenylate cyclase activating polypeptid	Homo sapiens adenylate cyclase activa	62.45704	64.98663	19.80857	16.36342	Detected	Detected	Detected	Detected
9488	A_24_P120734	MGC16075	Hs.488236	hypothetical protein MGC16075	Homo sapiens hypothetical protein MGC	40.85338	47.25901	31.79608	30.84505	Detected	Detected	Detected	Detected
9489	A_23_P203819	GOLGA3	Hs.507333	golgi autoantigen, golgin subfamily a, 3	Homo sapiens golgi autoantigen, golgin s	9845.87400	16178.79200	8272.50000	12134.41800	Detected	Detected	Detected	Detected
9490	A_23_P61149	INPP5D	Hs.601911	inositol polyphosphate-5-phosphatase,	Homo sapiens inositol polyphosphate-5	16.12785	19.56152	11.20683	9.95311	Detected	Detected	Compromised	Compromised
9491	A_33_P3423610	SEP15	Hs.362728	15 kDa selenoprotein	Homo sapiens 15 kDa selenoprotein (SE	12934.13100	11179.39800	10732.19100	7846.24700	Detected	Detected	Detected	Detected
9492	A_33_P3229181	SLC12A9	Hs.521087	solute carrier family 12 (potassium/chlo	Homo sapiens solute carrier family 12 (	17652.17400	13648.05100	9404.31500	7102.65040	Detected	Detected	Detected	Detected
9493	A_23_P151529	C14orf132	Hs.6434	chromosome 14 open reading frame 132	Homo sapiens chromosome 14 open rea	3387.01050	1142.49960	2517.54030	780.52510	Detected	Detected	Detected	Detected
9494	A_33_P3404922	KIAA1217	Hs.445885	KIAA1217	Homo sapiens KIAA1217 (KIAA1217), tr	6.60209	26.79005	11.82033	18.64976	Compromised	Detected	Compromised	Detected
9495	A_23_P23996	MAT1A	Hs.282670	methionine adenosyltransferase I, alpha	Homo sapiens methionine adenosyltrans	21.76002	30.27417	7.81787	6.66790	Detected	Detected	Compromised	Compromised
9496	A_23_P207194	GH1	Hs.655229	growth hormone 1	Homo sapiens growth hormone 1 (GH1)	37.02459	46.93849	24.58865	22.72495	Detected	Detected	Detected	Detected
9497	A_23_P56347	PSG3	Hs.654413	pregnancy specific beta-1-glycoprotein	Homo sapiens pregnancy specific beta-	93.47945	4149.07400	45.92394	1780.67700	Detected	Detected	Detected	Detected
9498	A_23_P69637	OTUD4	Hs.270851	OTU domain containing 4	Homo sapiens OTU domain containing 4	44.85463	44.98289	25.24869	22.21102	Detected	Detected	Detected	Detected
9499	A_23_P305245	LOC401097	Hs.710781	Similar to LOC166075	LOC401097 protein Fragment [Source:	3.18542	18.17814	2.79508	8.42875	Compromised	Detected	Compromised	Compromised
9500	A_33_P3261132	CYCSP52		cytochrome c, somatic pseudogene 52	Homo sapiens cytochrome c, somatic p	102.00030	240.88272	5.73450	8.72894	Detected	Detected	Compromised	Compromised
9501	A_33_P3317761	DAD1L		defender against cell death 1-like	Homo sapiens defender against cell dea	101.66131	196.95636	38.42505	75.80004	Detected	Detected	Detected	Detected
9502	A_33_P3414477	LOC729739		hypothetical LOC729739	PREDICTED: Homo sapiens hypothetica	26.10718	17.11563	11.63925	3.34427	Detected	Detected	Compromised	Compromised
9503	A_33_P3374947	KLF14	Hs.125240	Kruppel-like factor 14	Homo sapiens Kruppel-like factor 14 (K	10.67169	16.18280	22.82804	23.48467	Compromised	Detected	Detected	Detected
9504	A_24_P83899	GRM5	Hs.147361	glutamate receptor, metabotropic 5	Homo sapiens glutamate receptor, meta	32.92325	40.76404	13.56192	12.00511	Detected	Detected	Detected	Compromised
9505	A_23_P362637	UBR2	Hs.529925	ubiquitin protein ligase E3 component n	Homo sapiens ubiquitin protein ligase E3	809.17500	1030.01830	676.42890	739.97906	Detected	Detected	Detected	Detected
9506	A_33_P3407675	TIPRL	Hs.209431	TIP41, TOR signaling pathway regulator	Homo sapiens TIP41, TOR signaling pat	1583.88560	2277.89450	1641.56020	1876.68520	Detected	Detected	Detected	Detected
9507	A_33_P3260614	PLCB2	Hs.355888	phospholipase C, beta 2	Homo sapiens phospholipase C, beta 2 (	94.67899	197.52680	94.11113	161.41405	Detected	Detected	Detected	Detected
9508	A_33_P3368925					82.07124	97.87454	42.78486	61.72170	Detected	Detected	Detected	Detected
9509	A_23_P211007	NRIP1	Hs.155017	nuclear receptor interacting protein 1	Homo sapiens nuclear receptor interact	4351.59030	3620.12080	2969.90190	1993.18290	Detected	Detected	Detected	Detected
9510	A_23_P109881	ITIH4	Hs.709406	inter-alpha (globulin) inhibitor H4 (plasm	Homo sapiens inter-alpha (globulin) inhi	198.11977	121.08946	163.03540	77.40247	Detected	Detected	Detected	Detected
9511	A_33_P3282394	MLLT1	Hs.10095	myeloid/lymphoid or mixed-lineage leuk	Homo sapiens myeloid/lymphoid or mixe	12815.54300	11482.97200	9331.44200	7339.99400	Detected	Detected	Detected	Detected
9512	A_23_P134663	BAALC	Hs.533446	brain and acute leukemia, cytoplasmic	Homo sapiens brain and acute leukemia	581.92426	6014.82100	391.14270	3431.36500	Detected	Detected	Detected	Detected
9513	A_23_P140309	MAPK1IP1L	Hs.594338	mitogen-activated protein kinase 1 inte	Homo sapiens mitogen-activated protei	5111.90700	8294.84200	3568.89060	4765.02930	Detected	Detected	Detected	Detected
9514	A_24_P254965	HDAC8	Hs.310536	histone deacetylase 8	Homo sapiens histone deacetylase 8 (H	240.58586	429.12598	232.14386	354.79090	Detected	Detected	Detected	Detected
9515	A_33_P3333627	PHACTR1	Hs.436996	phosphatase and actin regulator 1	Homo sapiens phosphatase and actin re	33.95574	19.08847	49.32464	10.49799	Detected	Detected	Detected	Compromised
9516	A_24_P302685	ARHGEF4	Hs.469935	Rho guanine nucleotide exchange facto	Homo sapiens Rho guanine nucleotide e	3.91432	10.98175	8.35678	13.62465	Compromised	Compromised	Compromised	Detected
9517	A_23_P217178	MAGEC1	Hs.132194	melanoma antigen family C, 1	Homo sapiens melanoma antigen family	7.00699	12.93369	13.97026	7.98694	Compromised	Compromised	Detected	Compromised
9518	A_33_P3256828	LOC100127910	Hs.647752	hypothetical protein LOC100127910	Homo sapiens cDNA FLJ27201 fis, clon	61.60994	78.21748	30.83247	36.61612	Detected	Detected	Detected	Detected
9519	A_33_P3424347	LOC100131271	Hs.624900	hypothetical protein LOC100131271	PREDICTED: Homo sapiens hypothetica	44.51840	25.36332	39.87806	10.71824	Detected	Detected	Detected	Compromised
9520	A_23_P319133	DNAJC10	Hs.516632	DnaJ (Hsp40) homolog, subfamily C, me	Homo sapiens DnaJ (Hsp40) homolog, s	10515.25400	7926.26200	7057.58500	4156.60700	Detected	Detected	Detected	Detected
9521	A_24_P99795	ISOC2	Hs.467306	isochorismatase domain containing 2	Homo sapiens isochorismatase domain (	2043.00510	2412.35740	2685.65450	2837.33860	Detected	Detected	Detected	Detected

9522	A_23_P23411	PRCC	Hs.516948	papillary renal cell carcinoma (transloca	Homo sapiens papillary renal cell carcin	477.06207	638.71344	442.34380	535.56560	Detected	Detected	Detected	Detected
9523	A_33_P3364205	SBDSP		Shwachman-Bodian-Diamond syndrome	Homo sapiens Shwachman-Bodian-Diar	3308.11670	5028.36800	1859.58810	2285.67460	Detected	Detected	Detected	Detected
9524	A_23_P111361	PPIL6	Hs.32234	peptidylprolyl isomerase (cyclophilin)-lik	Homo sapiens peptidylprolyl isomerase	286.86790	18.43625	183.14062	5.95100	Detected	Detected	Detected	Compromised
9525	A_23_P18649	FAT4	Hs.563205	FAT tumor suppressor homolog 4 (Dros	Homo sapiens FAT tumor suppressor h	389.20110	269.91754	323.02023	168.90138	Detected	Detected	Detected	Detected
9526	A_33_P3593546	MARK3	Hs.35828	MAP/microtubule affinity-regulating kin	Homo sapiens MAP/microtubule affinity	11624.33800	11319.10700	7974.10160	6719.25500	Detected	Detected	Detected	Detected
9527	A_24_P213783	RPL31	Hs.469473	ribosomal protein L31	Homo sapiens ribosomal protein L31 (R	159320.28000	141054.40000	115843.64000	92459.77000	Detected	Detected	Detected	Detected
9528	A_33_P3411296	PURA	Hs.443121	purine-rich element binding protein A	Homo sapiens purine-rich element bindi	5895.86570	3583.08080	3489.22780	1723.08170	Detected	Detected	Detected	Detected
9529	A_24_P194017	NOSIP	Hs.7236	nitric oxide synthase interacting protein	Homo sapiens nitric oxide synthase inte	1838.11010	2322.74630	1981.36910	2415.04270	Detected	Detected	Detected	Detected
9530	A_33_P3267320	RFT1	Hs.720810	RFT1 homolog (S. cerevisiae)	Homo sapiens RFT1 homolog (S. cerevi	37.89586	95.22031	44.33249	93.78940	Detected	Detected	Detected	Detected
9531	A_23_P353574	NEK7	Hs.720168	NIMA (never in mitosis gene a)-related	Homo sapiens NIMA (never in mitosis g	67.85575	61.15735	30.79610	57.69431	Detected	Detected	Detected	Detected
9532	A_23_P212728	TBC1D23	Hs.477003	TBC1 domain family, member 23	Homo sapiens TBC1 domain family, mer	4759.04830	3480.42550	3869.82520	2450.71400	Detected	Detected	Detected	Detected
9533	A_32_P218228	FAM109B	Hs.368312	family with sequence similarity 109, mer	Homo sapiens family with sequence sim	903.30650	962.07630	686.63430	750.20087	Detected	Detected	Detected	Detected
9534	A_33_P3365267					34.89776	43.42908	19.42755	24.24906	Detected	Detected	Detected	Detected
9535	A_33_P3398927	MBD1	Hs.405610	methyl-CpG binding domain protein 1	Homo sapiens methyl-CpG binding dom	1148.46800	800.68396	1285.99520	884.50750	Detected	Detected	Detected	Detected
9536	A_33_P3274397	CHM	Hs.496449	choroideremia (Rab escort protein 1)	Homo sapiens choroideremia (Rab esco	124.25421	434.80970	88.05138	253.39459	Detected	Detected	Detected	Detected
9537	A_23_P137173	TMSB15A	Hs.56145	thymosin beta 15a	Homo sapiens thymosin beta 15a (TMSI	349.33080	492.43533	262.78320	351.17728	Detected	Detected	Detected	Detected
9538	A_23_P78383	C18orf8	Hs.464779	chromosome 18 open reading frame 8	Homo sapiens chromosome 18 open rea	899.66425	1056.23900	840.36615	871.89417	Detected	Detected	Detected	Detected
9539	A_23_P202720	SLC35C1	Hs.12211	solute carrier family 35, member C1	Homo sapiens solute carrier family 35, r	2121.63600	3655.39750	2029.07390	3460.35280	Detected	Detected	Detected	Detected
9540	A_23_P134113	C6orf192	Hs.347144	chromosome 6 open reading frame 192	Homo sapiens chromosome 6 open reac	1408.01120	807.50300	1216.93210	598.33856	Detected	Detected	Detected	Detected
9541	A_24_P318593	SCRN2	Hs.239718	secernin 2	Homo sapiens secernin 2 (SCRN2), trar	1683.83830	1665.23320	1742.15800	1771.69150	Detected	Detected	Detected	Detected
9542	A_24_P21752	TBRG1	Hs.436410	transforming growth factor beta regulat	Homo sapiens transforming growth fact	2503.80350	2440.47600	2196.02880	1882.92570	Detected	Detected	Detected	Detected
9543	A_33_P3311458	LOC283398	Hs.567794	similar to sucb	PREDICTED: Homo sapiens similar to s	1260.21220	1236.86760	684.39660	643.03710	Detected	Detected	Detected	Detected
9544	A_33_P3361811	NARG1L	Hs.512914	NMDA receptor regulated 1-like	Homo sapiens NMDA receptor regulate	208.91832	159.08658	164.72144	93.13486	Detected	Detected	Detected	Detected
9545	A_33_P3526315	MGC12488	Hs.659351	hypothetical protein MGC12488	Homo sapiens, clone IMAGE:3932794, m	305.95898	175.62537	200.76447	89.48689	Detected	Detected	Detected	Detected
9546	A_33_P3304293	STAG2	Hs.496710	stromal antigen 2	Homo sapiens stromal antigen 2 (STAG	74.53111	58.89986	38.39740	27.82972	Detected	Detected	Detected	Detected
9547	A_23_P301846	CALCA	Hs.37058	calcitonin-related polypeptide alpha	Homo sapiens calcitonin-related polype	20.72523	12.87869	6.63551	3.92429	Detected	Compromised	Compromised	Compromised
9548	A_24_P292470	UCP3	Hs.101337	uncoupling protein 3 (mitochondrial, pro	Homo sapiens uncoupling protein 3 (mit	811.94403	1081.10000	416.88028	392.55026	Detected	Detected	Detected	Detected
9549	A_33_P3428642	DMWD	Hs.515474	dystrophia myotonica, WD repeat conta	Homo sapiens dystrophia myotonica, WI	376.32410	513.35864	397.74140	442.11902	Detected	Detected	Detected	Detected
9550	A_23_P75310	ARHGAP22	Hs.655672	Rho GTPase activating protein 22	Homo sapiens Rho GTPase activating p	15794.79600	36165.71000	16419.27000	34851.61300	Detected	Detected	Detected	Detected
9551	A_23_P107795	ATP5SL	Hs.351099	ATP5S-like	Homo sapiens ATP5S-like (ATP5SL), m	11261.68500	15007.05300	8581.14100	9816.53900	Detected	Detected	Detected	Detected
9552	A_23_P34568	ADPRHL2	Hs.18021	ADP-ribosylhydrolase like 2	Homo sapiens ADP-ribosylhydrolase lik	1911.91990	2381.65650	2113.03660	2462.34420	Detected	Detected	Detected	Detected
9553	A_33_P3418234	BECN1L1		Beclin-1-like protein 1	PREDICTED: Homo sapiens Beclin-1-li	8.42086	12.83084	4.18006	28.58665	Compromised	Compromised	Compromised	Detected
9554	A_33_P3270657	FAM111B	Hs.186579	family with sequence similarity 111, mer	Homo sapiens family with sequence sim	67.20326	159.71347	25.12072	71.83579	Detected	Detected	Detected	Detected
9555	A_23_P96041	TMEM164	Hs.659535	transmembrane protein 164	Homo sapiens transmembrane protein 1	1648.67440	2827.82670	1118.24890	1535.23290	Detected	Detected	Detected	Detected
9556	A_33_P3324904					51.24548	50.96988	46.29549	32.73806	Detected	Detected	Detected	Detected
9557	A_33_P3407985	TMEM99	Hs.353163	transmembrane protein 99	Homo sapiens transmembrane protein 9	69.67983	129.40752	47.70123	72.81222	Detected	Detected	Detected	Detected
9558	A_23_P50786	CLIP3	Hs.466539	CAP-GLY domain containing linker prot	Homo sapiens CAP-GLY domain contai	2509.25120	1669.63220	2582.68970	1422.00150	Detected	Detected	Detected	Detected
9559	A_33_P3308949	DBT	Hs.709187	dihydrolipoamide branched chain transa	Homo sapiens dihydrolipoamide branche	906.63916	203.39856	724.56714	127.14599	Detected	Detected	Detected	Detected
9560	A_32_P174365	SATB2	Hs.719907	SATB homeobox 2	Homo sapiens SATB homeobox 2 (SATI	909.45795	958.10650	533.09380	477.80720	Detected	Detected	Detected	Detected
9561	A_33_P3328123	LOC100127919		similar to developmental pluripotency-a	PREDICTED: Homo sapiens similar to d	360.67712	515.73140	202.27101	222.31612	Detected	Detected	Detected	Detected
9562	A_33_P3404123	KRTAP9L1		keratin associated protein 9-like 1	Putative keratin-associated protein 9-2	49.64193	59.00927	45.08349	27.68379	Detected	Detected	Detected	Detected
9563	A_33_P3336384	PAOX	Hs.532469	polyamine oxidase (exo-N4-amino)	Homo sapiens polyamine oxidase (exo-N	177.26082	116.37140	216.51872	124.95628	Detected	Detected	Detected	Detected
9564	A_33_P3337931					2262.30790	1310.95320	1918.06580	862.85565	Detected	Detected	Detected	Detected
9565	A_33_P3422233	KIAA1530	Hs.380475	KIAA1530	Homo sapiens KIAA1530 (KIAA1530), m	1540.61290	632.95105	1014.71936	343.99203	Detected	Detected	Detected	Detected
9566	A_24_P942068	TANC2	Hs.410889	tetratricopeptide repeat, ankyrin repeat	Homo sapiens tetratricopeptide repeat,	3517.52950	1776.97780	2238.01370	907.26086	Detected	Detected	Detected	Detected
9567	A_23_P204417	ITFG2	Hs.446098	integrin alpha FG-GAP repeat containin	Homo sapiens integrin alpha FG-GAP r	236.39012	943.59430	187.88979	759.10077	Detected	Detected	Detected	Detected
9568	A_23_P79803	VSTM2L	Hs.517029	V-set and transmembrane domain conta	Homo sapiens V-set and transmembrar	44.37958	368.32720	49.42203	299.55457	Detected	Detected	Detected	Detected
9569	A_23_P79703	HADHB	Hs.515848	hydroxyacyl-Coenzyme A dehydrogenas	Homo sapiens hydroxyacyl-Coenzyme A	24970.72700	23713.07000	18070.26200	15057.56200	Detected	Detected	Detected	Detected
9570	A_23_P26124	RORA	Hs.560343	RAR-related orphan receptor A	Homo sapiens RAR-related orphan rece	1093.90810	1254.70100	482.83844	477.63510	Detected	Detected	Detected	Detected
9571	A_33_P3297141	WDR48	Hs.109778	WD repeat domain 48	Homo sapiens WD repeat domain 48 (W	1815.35560	976.97156	1568.11510	763.19840	Detected	Detected	Detected	Detected
9572	A_33_P3682006	NCRNA00118	Hs.223858	non-protein coding RNA 118	Homo sapiens clone BPR-4 mRNA sequ	117.44932	106.10337	33.87792	24.56609	Detected	Detected	Detected	Detected
9573	A_33_P3228612	CACNA1E	Hs.437444	calcium channel, voltage-dependent, R	Homo sapiens calcium channel, voltage-	7.61690	20.76307	2.54779	10.40906	Compromised	Detected	Compromised	Compromised
9574	A_23_P80048	FER1L4	Hs.72222	fer-1-like 4 (C. elegans)	Homo sapiens fer-1-like 4 (C. elegans)	858.55945	193.70550	722.50960	109.77960	Detected	Detected	Detected	Detected
9575	A_33_P3408212	HSP90B1	Hs.192374	heat shock protein 90kDa beta (Grp94),	Homo sapiens heat shock protein 90kD	2519.94290	6079.15230	1664.37380	3266.82540	Detected	Detected	Detected	Detected
9576	A_23_P70201	CHD1	Hs.643465	chromodomain helicase DNA binding pro	Homo sapiens chromodomain helicase D	798.43290	550.63910	555.13810	318.70190	Detected	Detected	Detected	Detected
9577	A_23_P48669	CDKN3	Hs.84113	cyclin-dependent kinase inhibitor 3	Homo sapiens cyclin-dependent kinase	540.94354	9767.11300	474.77963	7360.56150	Detected	Detected	Detected	Detected
9578	A_33_P3218772	UPF2	Hs.370689	UPF2 regulator of nonsense transcripts	Homo sapiens UPF2 regulator of nonse	76.96952	83.42615	51.99729	52.77638	Detected	Detected	Detected	Detected
9579	A_33_P3577142	LOC283861	Hs.192155	hypothetical locus LOC283861	Homo sapiens cDNA FLJ40824 fis, clon	821.93005	425.69067	671.15760	282.49030	Detected	Detected	Detected	Detected
9580	A_33_P3217357	LOC100132501	Hs.634003	hypothetical protein LOC100132501	Homo sapiens cDNA FLJ45888 fis, clon	12.91508	15.93846	10.60558	10.74908	Detected	Compromised	Compromised	Compromised
9581	A_33_P3369667					216.22887	270.27680	102.10142	96.75267	Detected	Detected	Detected	Detected
9582	A_33_P3237634	TSC22D3	Hs.522074	TSC22 domain family, member 3	Homo sapiens TSC22 domain family, me	22364.50400	19566.41800	15667.52400	12556.38600	Detected	Detected	Detected	Detected
9583	A_33_P3263417	FLJ43663		hypothetical LOC378805	Homo sapiens hypothetical LOC378805	788.43994	468.48517	729.08490	398.02017	Detected	Detected	Detected	Detected
9584	A_32_P205637	PARD6B	Hs.589848	par-6 partitioning defective 6 homolog b	Homo sapiens par-6 partitioning defecti	31.55992	21.09397	10.64454	12.34000	Detected	Detected	Compromised	Compromised
9585	A_33_P3228959	MRPL11	Hs.418450	mitochondrial ribosomal protein L11	Homo sapiens mitochondrial ribosomal p	2669.35160	4107.21140	3447.58960	4773.31100	Detected	Detected	Detected	Detected
9586	A_23_P3502	ZC3H18	Hs.93670	zinc finger CCCH-type containing 18	Homo sapiens zinc finger CCCH-type c	685.66455	547.23505	543.56525	406.68594	Detected	Detected	Detected	Detected
9587	A_33_P3367396	FAM177B	Hs.697608	family with sequence similarity 177, mer	Homo sapiens family with sequence sim	164.37129	147.66893	80.57211	59.37061	Detected	Detected	Detected	Detected
9588	A_33_P3389153	STK10	Hs.719134	serine/threonine kinase 10	Homo sapiens serine/threonine kinase	1499.50340	1454.02990	1182.30690	1055.92420	Detected	Detected	Detected	Detected
9589	A_33_P3289461	C20orf135	Hs.266774	chromosome 20 open reading frame 135	Homo sapiens chromosome 20 open rea	23.20330	19.88924	17.62624	14.94376	Detected	Detected	Detected	Detected
9590	A_33_P3306192	hCG_1645727	Hs.705349	hCG1645727	Homo sapiens hCG1645727 (LOC39059	1739.92070	2687.21200	1021.40330	1199.80820	Detected	Detected	Detected	Detected
9591	A_23_P149613	FMO1	Hs.1424	flavin containing monooxygenase 1	Homo sapiens flavin containing monoox	191.70462	101.83814	120.43762	20.92966	Detected	Detected	Detected	Detected
9592	A_23_P104098	SYT2	Hs.25422	synaptotagmin II	Homo sapiens synaptotagmin II (SYT2),	38.53122	52.37784	50.63830	48.10413	Detected	Detected	Detected	Detected
9593	A_23_P23206	MAD2L2	Hs.19400	MAD2 mitotic arrest deficient-like 2 (ye	Homo sapiens MAD2 mitotic arrest defi	3183.72780	3263.60060	5445.73340	5057.86130	Detected	Detected	Detected	Detected
9594	A_33_P3298612					9.03694	25.70428	8.93457	15.30335	Compromised	Detected	Compromised	Detected
9595	A_33_P3210468	MATN4	Hs.278489	matrilin 4	Homo sapiens matrilin 4 (MATN4), trans	10.97351	5.00817	15.16150	9.01856	Compromised	Compromised	Detected	Compromised
9596	A_23_P111037	HIST1H3A	Hs.546315	histone cluster 1, H3a	Homo sapiens histone cluster 1, H3a (H	9893.66500	7567.75000	10007.17300	6621.70260	Detected	Detected	Detected	Detected

9597	A_33_P3373850	WDR24	Hs.459632	WD repeat domain 24	Homo sapiens WD repeat domain 24 (W	1765.68900	1055.93190	2208.49320	1186.51600	Detected	Detected	Detected	Detected
9598	A_33_P3245575	GLT25D1	Hs.418795	glycosyltransferase 25 domain containir	Homo sapiens glycosyltransferase 25 do	183.56787	341.60275	164.88654	321.38086	Detected	Detected	Detected	Detected
9599	A_33_P3287646	HSPB1	Hs.520973	heat shock 27kDa protein 1	Homo sapiens heat shock 27kDa protei	28385.48400	72761.05500	15268.13600	33376.15600	Detected	Detected	Detected	Detected
9600	A_33_P3407469	MYH7B	Hs.414122	myosin, heavy chain 7B, cardiac muscle	Homo sapiens myosin, heavy chain 7B,	150.09232	24.66338	149.09140	22.26326	Detected	Detected	Detected	Detected
9601	A_32_P50431	ST13	Hs.712713	suppression of tumorigenicity 13 (colon	Homo sapiens suppression of tumoriger	339.62048	222.29459	102.91751	73.43933	Detected	Detected	Detected	Detected
9602	A_23_P425516	TSPAN17	Hs.532129	tetraspanin 17	Homo sapiens tetraspanin 17 (TSPAN17	966.71790	1312.52100	652.31600	908.22640	Detected	Detected	Detected	Detected
9603	A_23_P93750	LSM5	Hs.424908	LSM5 homolog, U6 small nuclear RNA a	Homo sapiens LSM5 homolog, U6 small	6294.29150	11592.05600	4713.16750	6894.67770	Detected	Detected	Detected	Detected
9604	A_33_P3392250	FAM178A	Hs.447458	family with sequence similarity 178, mer	Homo sapiens family with sequence sim	497.74954	502.22662	435.12020	379.67587	Detected	Detected	Detected	Detected
9605	A_33_P3263938	LCN15	Hs.413902	lipocalin 15	Homo sapiens lipocalin 15 (LCN15), mR	41.06605	49.30457	20.15180	12.07109	Detected	Detected	Detected	Compromised
9606	A_24_P655849	SMAD9	Hs.586812	SMAD family member 9	Mothers against decapentaplegic homol	511.62480	652.91565	365.36026	351.94300	Detected	Detected	Detected	Detected
9607	A_33_P3344599				Ubiquitin conjugation factor E4 B (Ubiqu	13.25875	11.37392	29.66088	24.73285	Detected	Compromised	Detected	Detected
9608	A_33_P3309665	ZFP3	Hs.48832	zinc finger protein 3 homolog (mouse)	Homo sapiens zinc finger protein 3 hom	185.02907	212.16719	110.36658	121.80074	Detected	Detected	Detected	Detected
9609	A_23_P145330	CCHCR1	Hs.485075	coiled-coil alpha-helical rod protein 1	Homo sapiens coiled-coil alpha-helical	884.15970	882.78845	1217.96300	1163.11870	Detected	Detected	Detected	Detected
9610	A_33_P3245066	RP11-345P4.4	Hs.655255	similar to solute carrier family 35, memb	Homo sapiens similar to solute carrier f	1376.75430	1293.10020	871.39980	758.18054	Detected	Detected	Detected	Detected
9611	A_23_P33216	ATP5B	Hs.406510	ATP synthase, H+ transporting, mitocho	Homo sapiens ATP synthase, H+ transp	24075.73400	33906.57000	19854.42600	24394.79100	Detected	Detected	Detected	Detected
9612	A_33_P3329444	MAMSTR	Hs.191815	MEF2 activating motif and SAP domain	Homo sapiens MEF2 activating motif an	763.00270	529.83680	604.36847	442.42627	Detected	Detected	Detected	Detected
9613	A_23_P336060					14.12134	13.36552	14.21292	13.78602	Detected	Compromised	Detected	Detected
9614	A_23_P166775	IL17RC	Hs.129959	interleukin 17 receptor C	Homo sapiens interleukin 17 receptor C	1252.31970	1405.56920	1308.64060	1495.87300	Detected	Detected	Detected	Detected
9615	A_23_P136683	HLA-DQB1	Hs.409934	major histocompatibility complex, class	Homo sapiens major histocompatibility	10.89868	3.85394	20.64814	2.71657	Compromised	Compromised	Detected	Compromised
9616	A_33_P3210343	ETV6	Hs.504765	ets variant 6	Homo sapiens ets variant 6 (ETV6), mR	933.38916	926.27640	708.72020	610.26340	Detected	Detected	Detected	Detected
9617	A_23_P99540	ZFP36L1	Hs.85155	zinc finger protein 36, C3H type-like 1	Homo sapiens zinc finger protein 36, C3	34178.86300	41685.90200	27216.62700	29790.78300	Detected	Detected	Detected	Detected
9618	A_33_P3221341	HECTD2	Hs.596096	HECT domain containing 2	Homo sapiens HECT domain containing	44.76840	31.93737	29.30558	17.49333	Detected	Detected	Detected	Detected
9619	A_23_P201979	CREM	Hs.200250	cAMP responsive element modulator	Homo sapiens cAMP responsive elemer	1330.59720	664.93280	1169.48070	472.16810	Detected	Detected	Detected	Detected
9620	A_23_P134601	WNT16	Hs.272375	wingless-type MMTV integration site fa	Homo sapiens wingless-type MMTV inte	36.36884	133.63458	23.00234	65.64746	Detected	Detected	Detected	Detected
9621	A_33_P3394517	SMG1	Hs.460179	SMG1 homolog, phosphatidylinositol 3-k	Homo sapiens SMG1 homolog, phosphat	64.38178	62.04640	64.39609	64.91850	Detected	Detected	Detected	Detected
9622	A_23_P371682	GPC6	Hs.444329	glypican 6	Homo sapiens glypican 6 (GPC6), mRNA	25.45998	29.53196	34.39009	32.15201	Detected	Detected	Detected	Detected
9623	A_33_P3396972	LOC100129113	Hs.677516	hypothetical protein LOC100129113	Homo sapiens cDNA FLJ37158 fis, clon	60.41572	66.00887	65.57156	58.59077	Detected	Detected	Detected	Detected
9624	A_23_P200143	DCAF8	Hs.632447	DDB1 and CUL4 associated factor 8	Homo sapiens DDB1 and CUL4 associa	1555.59780	573.33276	1056.15730	374.70392	Detected	Detected	Detected	Detected
9625	A_23_P434430	ZNF439	Hs.528731	zinc finger protein 439	Homo sapiens zinc finger protein 439 (Z	478.76898	149.58860	287.89963	82.74487	Detected	Detected	Detected	Detected
9626	A_23_P69431	RPL4	Hs.644628	ribosomal protein L4	Homo sapiens ribosomal protein L4 (RP	127354.16000	110119.66400	118263.58600	102019.47000	Detected	Detected	Detected	Detected
9627	A_33_P3347932	HSF1	Hs.530227	heat shock transcription factor 1	Homo sapiens heat shock transcription	15217.22200	24756.53700	11597.00700	17060.77300	Detected	Detected	Detected	Detected
9628	A_23_P155288	GFM1	Hs.518355	G elongation factor, mitochondrial 1	Homo sapiens G elongation factor, mito	2762.89840	3116.62770	1868.35360	1776.72050	Detected	Detected	Detected	Detected
9629	A_33_P3221978					23.21033	25.55956	16.71027	7.00376	Detected	Detected	Detected	Compromised
9630	A_33_P3258056	PALM3	Hs.444298	Paralemmin-3	Homo sapiens Paralemmin-3 (PALM3),	12.02710	5.40438	4.18000	6.97328	Detected	Compromised	Compromised	Compromised
9631	A_33_P3319760	TAGLN2	Hs.517168	transgelin 2	Homo sapiens transgelin 2 (TAGLN2), m	8985.56900	13617.23900	10701.50200	15788.99200	Detected	Detected	Detected	Detected
9632	A_23_P7655	BNIP1	Hs.145726	BCL2/adenovirus E1B 19kDa interactin	Homo sapiens BCL2/adenovirus E1B 19	540.90314	1374.73800	613.63184	1370.54330	Detected	Detected	Detected	Detected
9633	A_23_P97328	C1orf35	Hs.715607	chromosome 1 open reading frame 35	Homo sapiens chromosome 1 open reac	5880.53760	4929.58000	7089.46000	5605.29930	Detected	Detected	Detected	Detected
9634	A_33_P3405204	LOC729911	Hs.513931	hypothetical LOC729911	PREDICTED: Homo sapiens hypothetica	8.25306	23.61732	2.60537	12.58916	Compromised	Detected	Compromised	Compromised
9635	A_23_P29318	SAMM50	Hs.505824	sorting and assembly machinery compo	Homo sapiens sorting and assembly ma	11699.81600	14814.88900	10572.17700	12107.05600	Detected	Detected	Detected	Detected
9636	A_33_P3343981					999.45530	1494.57420	434.99770	492.80005	Detected	Detected	Detected	Detected
9637	A_23_P71513	EFR3A	Hs.204564	EFR3 homolog A (S. cerevisiae)	Homo sapiens EFR3 homolog A (S. cere	4633.88400	3858.44040	2780.90820	1874.07320	Detected	Detected	Detected	Detected
9638	A_33_P3287119	FAM178B	Hs.107922	family with sequence similarity 178, mer	Homo sapiens family with sequence sim	3080.74500	3914.08060	626.13086	490.12112	Detected	Detected	Detected	Detected
9639	A_24_P222890	C2orf16	Hs.131021	chromosome 2 open reading frame 16	Homo sapiens chromosome 2 open reac	58.06050	58.48320	43.97315	43.29131	Detected	Detected	Detected	Detected
9640	A_23_P8139	ZNF76	Hs.388024	zinc finger protein 76 (expressed in tes	Homo sapiens zinc finger protein 76 (ex	486.79153	286.12930	409.51123	254.66417	Detected	Detected	Detected	Detected
9641	A_23_P309837	STON2	Hs.14248	stonin 2	Homo sapiens stonin 2 (STON2), mRNA	3.92606	4.57075	2.54048	46.88997	Compromised	Compromised	Compromised	Detected
9642	A_33_P3227731					2581.49320	3973.45340	2259.98950	3074.17630	Detected	Detected	Detected	Detected
9643	A_33_P3210735	LRRC55	Hs.199853	leucine rich repeat containing 55	Homo sapiens leucine rich repeat conta	2.45378	6.50794	22.17749	5.54944	Compromised	Compromised	Detected	Compromised
9644	A_33_P3243997	PSME4	Hs.413801	proteasome (prosome, macropain) activ	Homo sapiens proteasome (prosome, m	1645.58980	896.43540	1130.85630	547.36490	Detected	Detected	Detected	Detected
9645	A_32_P407245	DNAJC22	Hs.659300	DnaJ (Hsp40) homolog, subfamily C, me	Homo sapiens DnaJ (Hsp40) homolog, s	53.27324	90.28615	52.03591	69.52351	Detected	Detected	Detected	Detected
9646	A_33_P3278455	ANKRD6	Hs.702213	ankyrin repeat domain 6	Homo sapiens ankyrin repeat domain 6	62.13267	11.20253	52.52755	5.68066	Detected	Compromised	Detected	Compromised
9647	A_23_P315122	EMX1	Hs.516090	empty spiracles homeobox 1	Homo sapiens empty spiracles homeobo	1214.71950	2071.31420	626.63890	777.54030	Detected	Detected	Detected	Detected
9648	A_33_P3292525	TMEM201	Hs.632365	transmembrane protein 201	Homo sapiens transmembrane protein 2	3055.46680	5581.89360	2467.04300	4075.11550	Detected	Detected	Detected	Detected
9649	A_23_P302654	CEP72	Hs.591741	centrosomal protein 72kDa	Homo sapiens centrosomal protein 72kD	236.12088	578.41974	233.85294	491.51138	Detected	Detected	Detected	Detected
9650	A_33_P3234984	IFT81	Hs.528382	intraflagellar transport 81 homolog (Chla	Homo sapiens intraflagellar transport 81	68.08637	76.09655	53.64220	71.37858	Detected	Detected	Detected	Detected
9651	A_23_P300100	PLA2G2D	Hs.189507	phospholipase A2, group IID	Homo sapiens phospholipase A2, group	19.22845	18.80504	5.19380	13.68924	Detected	Detected	Compromised	Detected
9652	A_32_P128258	SIGLECP3	Hs.132045	sialic acid binding Ig-like lectin, pseudog	Homo sapiens sialic acid binding Ig-like	5.45634	3.07938	2.46544	17.35080	Compromised	Compromised	Compromised	Detected
9653	A_24_P201381	FGFBP3	Hs.466120	fibroblast growth factor binding protein	Homo sapiens fibroblast growth factor b	158.75125	81.18778	104.17248	53.45922	Detected	Detected	Detected	Detected
9654	A_32_P192376	ENPP1	Hs.527295	ectonucleotide pyrophosphatase/phosph	Homo sapiens ectonucleotide pyrophos	774.22350	2538.88010	495.65933	1301.34010	Detected	Detected	Detected	Detected
9655	A_33_P3236813	GPR19	Hs.657862	G protein-coupled receptor 19	Homo sapiens G protein-coupled recep	44.41781	71.98067	22.49988	45.33276	Detected	Detected	Detected	Detected
9656	A_33_P3363188	FLJ43315	Hs.632789	similar to Asparagine synthetase [glutar	Homo sapiens similar to Asparagine syn	58.08824	190.22081	40.36947	110.34768	Detected	Detected	Detected	Detected
9657	A_33_P3333030	SUFU	Hs.404089	suppressor of fused homolog (Drosophil	Homo sapiens suppressor of fused hom	24.32019	76.66287	29.24629	70.19083	Detected	Detected	Detected	Detected
9658	A_23_P23815	SLC30A1	Hs.519469	solute carrier family 30 (zinc transporte	Homo sapiens solute carrier family 30 (	686.80270	1362.41020	718.42380	1261.54820	Detected	Detected	Detected	Detected
9659	A_33_P3228322	IL18BP	Hs.591967	interleukin 18 binding protein	Homo sapiens interleukin 18 binding pro	20.84341	29.84125	16.81322	25.03914	Detected	Detected	Detected	Detected
9660	A_32_P163089	C12orf75	Hs.368938	chromosome 12 open reading frame 75	Homo sapiens chromosome 12 open rea	5469.39200	25568.35500	4796.12800	18685.19000	Detected	Detected	Detected	Detected
9661	A_23_P16915	QPCT	Hs.79033	glutaminyl-peptide cyclotransferase	Homo sapiens glutaminyl-peptide cyclot	4553.40200	1703.89990	4829.67400	1581.85250	Detected	Detected	Detected	Detected
9662	A_33_P3337771	TGS1	Hs.335068	trimethylguanosine synthase homolog (S	Homo sapiens trimethylguanosine synth	3696.75850	3212.03930	2946.87870	2072.11250	Detected	Detected	Detected	Detected
9663	A_33_P3382959	CASP8AP2	Hs.558218	caspase 8 associated protein 2	Homo sapiens caspase 8 associated pro	167.21141	147.22287	139.72905	101.05297	Detected	Detected	Detected	Detected
9664	A_33_P3327539	TBC1D9	Hs.480819	TBC1 domain family, member 9 (with Gl	Homo sapiens TBC1 domain family, mer	337.59860	374.67940	476.81015	488.85500	Detected	Detected	Detected	Detected
9665	A_33_P3418576				Protein SERAC1 [Source:UniProtKB/S	105.76183	123.97038	68.92027	77.88120	Detected	Detected	Detected	Detected
9666	A_33_P3415491	WDR90	Hs.511903	WD repeat domain 90	Homo sapiens WD repeat domain 90 (W	772.95580	1170.84050	497.96643	681.81550	Detected	Detected	Detected	Detected
9667	A_23_P211244	PRMT2	Hs.154163	protein arginine methyltransferase 2	Homo sapiens protein arginine methyltr	1390.70790	546.90234	1171.91060	418.56772	Detected	Detected	Detected	Detected
9668	A_32_P114003	LOC100192378	Hs.596420	hypothetical LOC100192378	Homo sapiens hypothetical LOC100192	13.69514	3.26477	3.77483	2.70599	Detected	Compromised	Compromised	Compromised
9669	A_24_P392632	TRPM3	Hs.47288	transient receptor potential cation char	Homo sapiens transient receptor poten	3.19016	3.27700	19.58200	4.80367	Compromised	Compromised	Detected	Compromised
9670	A_24_P89835	MAPK15	Hs.493169	mitogen-activated protein kinase 15	Homo sapiens mitogen-activated protei	13.10841	16.79522	9.74651	9.77365	Detected	Detected	Compromised	Compromised
9671	A_23_P128246	FICD	Hs.661891	FIC domain containing	Homo sapiens FIC domain containing (F	289.04837	650.01400	336.16672	692.98250	Detected	Detected	Detected	Detected

9672	A_32_P52911	ADI1	Hs.502773	acireductone dioxygenase 1	Homo sapiens acireductone dioxygenas	1000.77290	2789.11720	714.53820	1659.61220	Detected	Detected	Detected	Detected
9673	A_24_P942017	hCG_2039148	Hs.649468	hypothetical protein LOC100132356	PREDICTED: Homo sapiens hypothetica	51.54080	52.49022	33.77681	30.77309	Detected	Detected	Detected	Detected
9674	A_33_P3315425	METTL4	Hs.126888	methyltransferase like 4	Homo sapiens methyltransferase like 4	259.61627	351.61768	165.76733	169.39570	Detected	Detected	Detected	Detected
9675	A_23_P89056	NUP93	Hs.276878	nucleoporin 93kDa	Homo sapiens nucleoporin 93kDa (NUP	4297.42530	6046.09770	3353.14670	4377.48400	Detected	Detected	Detected	Detected
9676	A_32_P226205		Hs.525247		Zinc finger homeobox protein 2 (Zinc fir	20.33369	43.37669	23.41800	26.42780	Detected	Detected	Detected	Detected
9677	A_23_P204277	H2AFJ	Hs.524280	H2A histone family, member J	Homo sapiens H2A histone family, mem	31158.84000	35787.82000	38974.45700	40565.94500	Detected	Detected	Detected	Detected
9678	A_33_P3287883	LOC100133331	Hs.531193	hypothetical LOC100133331	Homo sapiens hypothetical LOC100133	242.45316	296.94427	104.78691	187.40894	Detected	Detected	Detected	Detected
9679	A_24_P324563	FAM108A1	Hs.465542	family with sequence similarity 108, mer	Homo sapiens family with sequence sim	5494.64650	5133.70500	6080.31400	6116.41360	Detected	Detected	Detected	Detected
9680	A_33_P3270776	HTRA3	Hs.479119	HtrA serine peptidase 3	Homo sapiens HtrA serine peptidase 3 (	237.17336	119.17733	199.94624	112.86816	Detected	Detected	Detected	Detected
9681	A_23_P156993	CNOT4	Hs.490224	CCR4-NOT transcription complex, subu	Homo sapiens CCR4-NOT transcription	41.63683	27.68796	39.73552	16.15536	Detected	Detected	Detected	Detected
9682	A_23_P62907	ATF6	Hs.492740	activating transcription factor 6	Homo sapiens activating transcription fa	1513.13700	3432.69240	1265.10240	2515.40260	Detected	Detected	Detected	Detected
9683	A_23_P131299	ZFAND2B	Hs.534540	zinc finger, AN1-type domain 2B	Homo sapiens zinc finger, AN1-type do	1630.23380	1643.05300	1647.03400	1703.63750	Detected	Detected	Detected	Detected
9684	A_23_P510	PLD5	Hs.672452	phospholipase D family, member 5	Homo sapiens phospholipase D family, r	5.17320	20.71751	5.18547	19.16083	Compromised	Detected	Compromised	Detected
9685	A_23_P330578	LRRC39	Hs.44277	leucine rich repeat containing 39	Homo sapiens leucine rich repeat conta	294.63920	118.04792	196.84312	76.13449	Detected	Detected	Detected	Detected
9686	A_23_P252825	SCAP	Hs.531789	SREBF chaperone	Homo sapiens SREBF chaperone (SCAI	5989.63770	4614.15040	5536.71500	4301.15100	Detected	Detected	Detected	Detected
9687	A_24_P550924	BTBD19	Hs.632400	BTB (POZ) domain containing 19	Homo sapiens BTB (POZ) domain conta	5.75307	11.67084	10.35721	15.83431	Compromised	Compromised	Compromised	Detected
9688	A_33_P3273399					300.83710	388.19235	201.11162	189.72847	Detected	Detected	Detected	Detected
9689	A_33_P3220152	SBNO2	Hs.408708	strawberry notch homolog 2 (Drosophila	Homo sapiens strawberry notch homolo	3177.29860	3410.81520	4573.80960	4885.38400	Detected	Detected	Detected	Detected
9690	A_33_P3376954					56.59811	68.75227	36.11568	75.01189	Detected	Detected	Detected	Detected
9691	A_33_P3389261	TMEM194B	Hs.659824	transmembrane protein 194B	Homo sapiens transmembrane protein 1	26.23440	25.05034	60.52998	16.70513	Detected	Detected	Detected	Detected
9692	A_33_P3383656	DUSP28	Hs.369297	dual specificity phosphatase 28	Homo sapiens dual specificity phosphat	1676.95130	1440.25950	1220.45580	896.07050	Detected	Detected	Detected	Detected
9693	A_33_P3233318	LOC442366		hypothetical LOC442366	PREDICTED: Homo sapiens hypothetica	13.24727	21.54850	17.71499	19.56027	Detected	Detected	Detected	Detected
9694	A_33_P3605969	FOXO6	Hs.559677	forkhead box O6	601582007F1 NIH_MGC_7 Homo sapiens	539.91364	802.01150	315.18112	352.29330	Detected	Detected	Detected	Detected
9695	A_33_P3272628				High affinity immunoglobulin epsilon rec	29.45701	46.30628	7.63257	20.47408	Detected	Detected	Compromised	Detected
9696	A_33_P3383065	LOC100130705	Hs.641532	hypothetical LOC100130705	PREDICTED: Homo sapiens hypothetica	31.00382	18.42895	38.12509	43.65508	Detected	Detected	Detected	Detected
9697	A_33_P3342096	ZHX3	Hs.380133	zinc fingers and homeoboxes 3	Homo sapiens zinc fingers and homeob	421.80720	594.89680	273.00922	349.33542	Detected	Detected	Detected	Detected
9698	A_23_P386320	MFI2	Hs.184727	antigen p97 (melanoma associated) ider	Homo sapiens antigen p97 (melanoma a	273.43402	269.35930	194.76233	158.34361	Detected	Detected	Detected	Detected
9699	A_23_P60225	GRHPR	Hs.713140	glyoxylate reductase/hydroxypyruvate r	Homo sapiens glyoxylate reductase/hyd	3124.14820	4284.23600	3724.42870	5046.55660	Detected	Detected	Detected	Detected
9700	A_32_P161762	RUNX2	Hs.535845	runt-related transcription factor 2	Homo sapiens runt-related transcriptior	862.62700	1652.41580	642.16800	1074.87490	Detected	Detected	Detected	Detected
9701	A_23_P57941	RBM6	Hs.696735	RNA binding motif protein 6	Homo sapiens RNA binding motif protei	1244.05380	1040.56910	1349.03650	954.03394	Detected	Detected	Detected	Detected
9702	A_33_P3219601	ABL2	Hs.159472	v-abl Abelson murine leukemia viral onc	Homo sapiens v-abl Abelson murine leu	2092.76510	1401.37570	1609.67700	914.45370	Detected	Detected	Detected	Detected
9703	A_23_P133332	MRPS27	Hs.482491	mitochondrial ribosomal protein S27	Homo sapiens mitochondrial ribosomal p	1469.60490	1794.67920	1713.38750	1820.53260	Detected	Detected	Detected	Detected
9704	A_24_P32215				Putative uncharacterized protein ARL4I	87.32372	216.90236	11.01740	12.97082	Detected	Detected	Compromised	Detected
9705	A_33_P3369520	MAGI3	Hs.486189	membrane associated guanylate kinase,	Homo sapiens membrane associated gu	698.87317	483.67667	490.72476	268.23050	Detected	Detected	Detected	Detected
9706	A_23_P253932	FAIM	Hs.173438	Fas apoptotic inhibitory molecule	Homo sapiens Fas apoptotic inhibitory r	2035.11330	3996.37870	1741.80680	2886.12840	Detected	Detected	Detected	Detected
9707	A_33_P3378272		Hs.647036		Putative uncharacterized protein WUGS	20.57214	21.97258	11.73670	12.53150	Detected	Detected	Compromised	Compromised
9708	A_33_P3235262	PIP5KL1	Hs.445486	phosphatidylinositol-4-phosphate 5-kin	Homo sapiens phosphatidylinositol-4-pl	406.55316	179.81961	350.87190	131.45598	Detected	Detected	Detected	Detected
9709	A_32_P100641	LOC100128139	Hs.550683	hypothetical LOC100128139	PREDICTED: Homo sapiens hypothetica	36.41717	45.77239	43.83915	74.56945	Detected	Detected	Detected	Detected
9710	A_33_P3269203	SERPINH1	Hs.596449	serpin peptidase inhibitor, clade H (heat	Homo sapiens serpin peptidase inhibitor	29063.24400	64655.47700	23792.29900	48866.95000	Detected	Detected	Detected	Detected
9711	A_23_P111311	AKAP12	Hs.371240	A kinase (PRKA) anchor protein 12	Homo sapiens A kinase (PRKA) anchor	1617.15970	1318.57700	1230.26990	822.39960	Detected	Detected	Detected	Detected
9712	A_33_P3280405	OR7E37P	Hs.507862	olfactory receptor, family 7, subfamily E	Homo sapiens olfactory receptor, family	196.59637	1006.62880	163.57385	716.54565	Detected	Detected	Detected	Detected
9713	A_23_P78782	CA11	Hs.428446	carbonic anhydrase XI	Homo sapiens carbonic anhydrase XI (C	252.82680	11.81024	288.16467	7.37579	Detected	Compromised	Detected	Compromised
9714	A_33_P3220347	CNPY1	Hs.146751	canopy 1 homolog (zebrafish)	Homo sapiens canopy 1 homolog (zebra	71.05870	89.28761	21.56042	28.59382	Detected	Detected	Detected	Detected
9715	A_33_P3255647	HNRNPH2	Hs.632828	heterogeneous nuclear ribonucleoprotei	Homo sapiens heterogeneous nuclear ri	292.24457	259.64185	218.76103	175.77927	Detected	Detected	Detected	Detected
9716	A_23_P67355	PRRG2	Hs.35101	proline rich Gla (G-carboxyglutamic aci	Homo sapiens proline rich Gla (G-carbo	40.77185	42.88954	14.49991	6.23848	Detected	Detected	Detected	Compromised
9717	A_23_P34946	C1orf107	Hs.194754	chromosome 1 open reading frame 107	Homo sapiens chromosome 1 open read	233.51271	284.03018	200.06451	243.49240	Detected	Detected	Detected	Detected
9718	A_33_P3412035	UBA6	Hs.719086	ubiquitin-like modifier activating enzym	Homo sapiens ubiquitin-like modifier ac	168.37427	205.17848	141.52318	150.16670	Detected	Detected	Detected	Detected
9719	A_23_P8185	DYNLT1	Hs.445999	dynein, light chain, Tctex-type 1	Homo sapiens dynein, light chain, Tctex	20676.87500	31588.00400	19260.43600	24442.53700	Detected	Detected	Detected	Detected
9720	A_23_P428184	HIST1H2AD	Hs.679229	histone cluster 1, H2ad	Homo sapiens histone cluster 1, H2ad (	2691.97850	6105.12300	1796.40610	4640.54800	Detected	Detected	Detected	Detected
9721	A_33_P3292851				SUMO-conjugating enzyme UBC9 (EC 6	113.75261	115.35891	74.00735	71.73288	Detected	Detected	Detected	Detected
9722	A_23_P128663	SACS	Hs.159492	spastic ataxia of Charlevoix-Saguenay (	Homo sapiens spastic ataxia of Charlev	9825.57600	19148.34000	7116.64650	12521.35800	Detected	Detected	Detected	Detected
9723	A_33_P3345414				40S ribosomal protein S4, X isoform (Si	54.73739	59.70083	31.76308	38.22566	Detected	Detected	Detected	Detected
9724	A_33_P3368238					12.01981	12.75119	18.31735	25.91798	Detected	Compromised	Detected	Detected
9725	A_23_P119617	SF4	Hs.515274	splicing factor 4	Homo sapiens splicing factor 4 (SF4), m	2383.87900	1725.73540	2379.62480	1606.32540	Detected	Detected	Detected	Detected
9726	A_33_P3391656	TP53RK	Hs.440263	TP53 regulating kinase	Homo sapiens TP53 regulating kinase (T	1906.34690	1030.19000	1481.55750	722.83250	Detected	Detected	Detected	Detected
[truncated: 3,339,775 more chars]
