# Supplementary material for: Lactic Acid Bacteria Convert Human Fibroblasts to Multipotent Cells
Source: PLoS One. 2012 Dec 26;7(12):e51866. doi: 10.1371/journal.pone.0051866 (PMC3530539; doi:10.1371/journal.pone.0051866)
Supplement: Table S5 — List of 50 genes that is decreased by a factor of fifteen or more at least between LAB-incorporated cell clusters-1 vs. HDF-1 and LAB-incorporated cell clusters-2 vs. HDF-2 and has a GO term [development]. (PDF) [file pone.0051866.s014.pdf]

Table S5: List of 50 genes that is decreased by a factor of fifteen or more at least between LAB-incorporated cell clusters-1 vs HDF-1 or LAB-incorporated cell clusters-2 vs HDF-2 and has a GO term [development」

no	ProbeName	GeneSymbol	UniGeneID	GeneName	Description	Fold change [LAB-inc. cells-1] vs [HDF-1]	Regulation [LAB-inc. cells-1] vs [HDF-1]	Fold change [LAB-inc. cells-2] vs [HDF-2]	Regulation [LAB-inc. cells-2] vs [HDF-2]
1	A_23_P307310	ACAN	Hs.2159	aggrecan	Homo sapiens aggrecan (ACAN), transcript variant 2, mRNA [NM_013227]	1.17244	down	37.48496	down
2	A_23_P27013	HOXB9	Hs.463350	homeobox B9	Homo sapiens homeobox B9 (HOXB9), mRNA [NM_024017]	6.62605	down	30.84917	down
3	A_33_P3377519	HOXA6	Hs.679517	homeobox A6	Homo sapiens homeobox A6 (HOXA6), mRNA [NM_024014]	7.97321	down	34.13733	down
4	A_23_P301942	NPPC	Hs.247916	natriuretic peptide precursor C	Homo sapiens natriuretic peptide precursor C (NPPC), mRNA [NM_024409]	11.58496	down	42.84185	down
5	A_23_P93772	HOXA5	Hs.655218	homeobox A5	Homo sapiens homeobox A5 (HOXA5), mRNA [NM_019102]	19.32321	down	31.22134	down
6	A_23_P155969	PLK4	Hs.172052	polo-like kinase 4 (Drosophila)	Homo sapiens polo-like kinase 4 (Drosophila) (PLK4), mRNA [NM_014264]	20.70854	down	40.33532	down
7	A_23_P46039	FCRLA	Hs.266331	Fc receptor-like A	Homo sapiens Fc receptor-like A (FCRLA), mRNA [NM_032738]	20.82261	down	36.28435	down
8	A_23_P213336	FGF1	Hs.483635	fibroblast growth factor 1 (acidic)	Homo sapiens fibroblast growth factor 1 (acidic) (FGF1), transcript variant 1, mRNA [NM_000800]	21.22867	down	42.34012	down
9	A_23_P389897	NGFR	Hs.415768	nerve growth factor receptor (TNFR superfamily, member 16)	Homo sapiens nerve growth factor receptor (TNFR superfamily, member 16) (NGFR), mRNA [NM_002507]	23.03998	down	30.64351	down
10	A_23_P96158	KRT17	Hs.2785	keratin 17	Homo sapiens keratin 17 (KRT17), mRNA [NM_000422]	30.95973	down	18.17780	down
11	A_23_P107283	HOXB2	Hs.514289	homeobox B2	Homo sapiens homeobox B2 (HOXB2), mRNA [NM_002145]	31.19959	down	31.77469	down
12	A_23_P501538	HOXA3	Hs.659337	homeobox A3	Homo sapiens homeobox A3 (HOXA3), transcript variant 2, mRNA [NM_153631]	33.35369	down	18.09414	down
13	A_23_P58321	CCNA2	Hs.58974	cyclin A2	Homo sapiens cyclin A2 (CCNA2), mRNA [NM_001237]	33.80436	down	39.61835	down
14	A_23_P157136	SCIN	Hs.655515	scinderin	Homo sapiens scinderin (SCIN), transcript variant 2, mRNA [NM_033128]	35.78193	down	7.72451	down
15	A_23_P215454	ELN	Hs.647061	elastin	Homo sapiens elastin (ELN), transcript variant 1, mRNA [NM_000501]	37.86731	down	52.46331	down
16	A_23_P167159	SCRG1	Hs.7122	stimulator of chondrogenesis 1	Homo sapiens stimulator of chondrogenesis 1 (SCRG1), mRNA [NM_007281]	39.33724	down	24.93944	down
17	A_23_P373521	HAND2	Hs.388245	heart and neural crest derivatives expressed 2	Homo sapiens heart and neural crest derivatives expressed 2 (HAND2), mRNA [NM_021973]	41.60090	down	32.86138	down
18	A_23_P100127	CASC5	Hs.181855	cancer susceptibility candidate 5	Homo sapiens cancer susceptibility candidate 5 (CASC5), transcript variant 1, mRNA [NM_170589]	42.70049	down	49.23346	down
19	A_23_P122197	CCNB1	Hs.23960	cyclin B1	Homo sapiens cyclin B1 (CCNB1), mRNA [NM_031966]	42.74203	down	44.84546	down
20	A_23_P146274	STMN2	Hs.521651	stathmin-like 2	Homo sapiens stathmin-like 2 (STMN2), mRNA [NM_007029]	43.26243	down	38.34229	down
21	A_24_P30557	TBX5	Hs.381715	T-box 5	Homo sapiens T-box 5 (TBX5), transcript variant 1, mRNA [NM_000192]	45.04174	down	37.62053	down
22	A_33_P3330149	PAX6	Hs.270303	paired box 6	Homo sapiens paired box 6 (PAX6), transcript variant 1, mRNA [NM_000280]	46.52534	down	30.35840	down
23	A_24_P416370	HOXB4	Hs.664706	homeobox B4	Homo sapiens homeobox B4 (HOXB4), mRNA [NM_024015]	48.99025	down	75.59501	down
24	A_23_P163567	SMPD3	Hs.368421	sphingomyelin phosphodiesterase 3, neutral membrane (neutral sphingomyelinase II)	Homo sapiens sphingomyelin phosphodiesterase 3, neutral membrane (neutral sphingomyelinase II) (SMPD3), mRNA [NM_018667]	49.28304	down	25.49599	down
25	A_23_P24129	DKK1	Hs.40499	dickkopf homolog 1 (Xenopus laevis)	Homo sapiens dickkopf homolog 1 (Xenopus laevis) (DKK1), mRNA [NM_012242]	49.39177	down	47.39847	down
26	A_23_P423237	SGCG	Hs.37167	sarcoglycan, gamma (35kDa dystrophin-associated glycoprotein)	Homo sapiens sarcoglycan, gamma (35kDa dystrophin-associated glycoprotein) (SGCG), mRNA [NM_000231]	50.23103	down	13.21567	down
27	A_23_P210176	ITGA6	Hs.133397	integrin, alpha 6	Homo sapiens integrin, alpha 6 (ITGA6), transcript variant 2, mRNA [NM_000210]	53.34168	down	45.93769	down
28	A_23_P212844	TACC3	Hs.104019	transforming, acidic coiled-coil containing protein 3	Homo sapiens transforming, acidic coiled-coil containing protein 3 (TACC3), mRNA [NM_006342]	53.95437	down	50.58793	down
29	A_33_P3377529	HOXA4	Hs.533357	homeobox A4	Homo sapiens homeobox A4 (HOXA4), mRNA [NM_002141]	57.78450	down	98.33543	down
30	A_23_P52017	ASPM	Hs.121028	asp (abnormal spindle) homolog, microcephaly associated (Drosophila)	Homo sapiens asp (abnormal spindle) homolog, microcephaly associated (Drosophila) (ASPM), mRNA [NM_018136]	65.81075	down	70.01885	down
31	A_23_P316511	HOXB3	Hs.654560	homeobox B3	Homo sapiens homeobox B3 (HOXB3), mRNA [NM_002146]	65.99564	down	33.93036	down
32	A_23_P215634	IGFBP3	Hs.450230	insulin-like growth factor binding protein 3	Homo sapiens insulin-like growth factor binding protein 3 (IGFBP3), transcript variant 1, mRNA [NM_001013398]	67.36516	down	66.17660	down
33	A_23_P65757	CCNB2	Hs.194698	cyclin B2	Homo sapiens cyclin B2 (CCNB2), mRNA [NM_004701]	71.74918	down	79.26724	down
34	A_23_P370588	HOXB8	Hs.514292	homeobox B8	Homo sapiens homeobox B8 (HOXB8), mRNA [NM_024016]	76.56639	down	23.89782	down
35	A_33_P3421243	AFP	Hs.518808	alpha-fetoprotein	Homo sapiens alpha-fetoprotein (AFP), mRNA [NM_001134]	91.34399	down	55.39948	down
36	A_23_P55281	HOXB7	Hs.436181	homeobox B7	Homo sapiens homeobox B7 (HOXB7), mRNA [NM_004502]	98.93823	down	44.19750	down
37	A_24_P218805	HOXC10	Hs.44276	homeobox C10	Homo sapiens homeobox C10 (HOXC10), mRNA [NM_017409]	116.49500	down	100.85654	down
38	A_23_P25150	HOXC9	Hs.658823	homeobox C9	Homo sapiens homeobox C9 (HOXC9), mRNA [NM_006897]	122.49983	down	222.03098	down
39	A_24_P264943	COMP	Hs.1584	cartilage oligomeric matrix protein	Homo sapiens cartilage oligomeric matrix protein (COMP), mRNA [NM_000095]	126.96592	down	201.15356	down
40	A_33_P3300975	HOXC4	Hs.549040	homeobox C4	Homo sapiens homeobox C4 (HOXC4), transcript variant 1, mRNA [NM_014620]	131.16129	down	304.63330	down
41	A_33_P3264528	HOXA11	Hs.249171	homeobox A11	Homo sapiens homeobox A11 (HOXA11), mRNA [NM_005523]	135.72766	down	157.62318	down
42	A_23_P374695	TEK	Hs.89640	TEK tyrosine kinase, endothelial	Homo sapiens TEK tyrosine kinase, endothelial (TEK), mRNA [NM_000459]	168.16338	down	99.82339	down
43	A_23_P7313	SPP1	Hs.313	secreted phosphoprotein 1	Homo sapiens secreted phosphoprotein 1 (SPP1), transcript variant 1, mRNA [NM_001040058]	170.12749	down	197.49605	down
44	A_24_P77904	HOXA10	Hs.110637	homeobox A10	Homo sapiens homeobox A10 (HOXA10), transcript variant 1, mRNA [NM_018951]	212.57760	down	101.90968	down
45	A_33_P3300965	HOXC6	Hs.549040	homeobox C6	Homo sapiens homeobox C6 (HOXC6), transcript variant 2, mRNA [NM_153693]	247.46118	down	651.67040	down
46	A_23_P66682	HOXB6	Hs.98428	homeobox B6	Homo sapiens homeobox B6 (HOXB6), mRNA [NM_018952]	385.91574	down	1998.17210	down
47	A_23_P363316	HOXB5	Hs.654456	homeobox B5	Homo sapiens homeobox B5 (HOXB5), mRNA [NM_002147]	489.89343	down	455.47385	down
48	A_23_P70968	HOXA7	Hs.660918	homeobox A7	Homo sapiens homeobox A7 (HOXA7), mRNA [NM_006896]	547.44600	down	274.53156	down
49	A_23_P500998	HOXA9	Hs.659350	homeobox A9	Homo sapiens homeobox A9 (HOXA9), mRNA [NM_152739]	592.35516	down	493.90430	down
50	A_24_P124558	HOXC8	Hs.664500	homeobox C8	Homo sapiens homeobox C8 (HOXC8), mRNA [NM_022658]	594.18225	down	534.99910	down
